# SRSF1-mediated alternative splicing is required for spermatogenesis

**DOI:** 10.7150/ijbs.83474

**Published:** 2023-09-11

**Authors:** Wen-Long Lei, Yuan-Yuan Li, Zongchang Du, Ruibao Su, Tie-Gang Meng, Yan Ning, Guanmei Hou, Heide Schatten, Zhen-Bo Wang, Zhiming Han, Fei Sun, Wei-Ping Qian, Chenli Liu, Qing-Yuan Sun

**Affiliations:** 1 Guangdong and Shenzhen Key Laboratory of Reproductive Medicine and Genetics, The Center of Reproductive Medicine, Peking University Shenzhen Hospital, Shenzhen 518000, China.; 2 Department of Urology & Andrology, Sir Run Run Shaw Hospital, Zhejiang University School of Medicine, Hangzhou, 310016, China.; 3 State Key Laboratory of Stem Cell and Reproductive Biology, Institute of Zoology, Chinese Academy of Sciences, Beijing, 100101, China.; 4 School of Artificial Intelligence, University of Chinese Academy of Sciences, Beijing, 100049, China.; 5 Guangzhou Key Laboratory of Metabolic Diseases and Reproductive Health, Guangdong-Hongkong Metabolism & Reproduction Joint Laboratory, Reproductive Medicine Center, Guangdong Second Provincial General Hospital, Guangzhou, 510317, China.; 6 Department of Veterinary Pathobiology, University of Missouri, Columbia, MO 65211, USA.; 7 Beijing Institute for Stem Cell and Regenerative Medicine, Beijing, 100101, China.; 8 CAS Key Laboratory of Quantitative Engineering Biology, Shenzhen Institute of Synthetic Biology, Shenzhen Institutes of Advanced Technology, Chinese Academy of Sciences, Shenzhen, 518055, China.

**Keywords:** SRSF1, male infertility, alternative splicing, spermatogenesis

## Abstract

Alternative splicing (AS) plays significant roles in a multitude of fundamental biological activities. AS is prevalent in the testis, but the regulations of AS in spermatogenesis is only little explored. Here, we report that Serine/arginine-rich splicing factor 1 (SRSF1) plays critical roles in alternative splicing and male reproduction. Male germ cell-specific deletion of *Srsf1* led to complete infertility by affecting spermatogenesis. Mechanistically, by combining RNA-seq data with LACE-seq data, we showed that SRSF1 affected the AS of *Stra8* in a direct manner and *Dazl*, *Dmc1*, *Mre11a*, *Syce2* and *Rif1* in an indirect manner. Our findings demonstrate that SRSF1 has crucial functions in spermatogenesis and male fertility by regulating alternative splicing.

## Introduction

In mammalian species, the male germline stem cells are initiated to enter meiosis, continuously producing haploid male gametes after puberty 1. Spermatogenesis consists of three sequential processes: mitosis, meiosis and spermiogenesis 2. In the first phase of spermatogenesis, spermatogonia stem cells undergo mitotic self-renewal and differentiation into primary spermatocytes. Then, spermatocytes commence two constitutive meiotic divisions without an interval of DNA synthesis to yield haploid round spermatids in the second phase of spermatogenesis. Finally, before being released into seminiferous tubular lumen as spermatozoa, haploid round spermatids undergo a series of stepwise morphogenetic changes 2. Dysfunction in any of these processes can cause the failure of spermatogenesis, further resulting in severe consequences including infertility 3.

It is well known that alternative splicing (AS) is one of the most important transcriptional and post-transcriptional regulatory mechanisms to enrich the amount of mRNA and protein isoforms from a single gene, and these different protein isoforms always have different structural characteristics and functions 4-6. Generally, AS occurs in about 95% of human genes and in about 60% of mouse genes, and AS is most prevalent in the testis and brain when compared to other tissues 7, 8. There are numerous AS events during many developmental processes. Recently, it has been shown that several RNA binding proteins have functions in AS events during spermatogenesis 9-11, indicating the importance of AS events during spermatogenesis. However, understanding the splicing factors that control the AS of key genes in spermatogenesis remains ambiguous.

The critical regulators of alternative splicing are members of the serine/arginine (SR)-rich splicing factor family, which bind to splicing enhancer sequences to promote alternative splicing 12, 13. In humans, the SR family has at least 12 members that share a conserved SR domain 14. It has been reported that these SR splicing factors are critical for development. The SR splicing factors can identify the splicing components of precursor RNA, then recruit and assemble spliceosomes to promote or inhibit the occurrence of alternative splicing events 15. There are large amounts of studies suggesting that SR splicing factors have functions in nearly every step of spliceosome assembly, mRNA export, mRNA stability and genomic stability 16, 17. Serine/arginine-rich splicing factor 1 (SRSF1), also known as SF2/ASF, is a prototype member of the SR protein family 18. So far, several studies have suggested that SRSF1 regulates post-transcriptional gene expression via pre-mRNA alternative splicing, mRNA stability, and translation 19, 20. While SRSF1 is an important regulator of gene expression, very little is known of its functions in the male germ cells.

In this study, by crossing *Srsf1^Floxed/Floxed^* (*Srsf1^F/F^*) mice with* Stra8-Cre* mice to generate mutant mice with specific deletion of the *Srsf1* gene in male germ cells, we found that SRSF1 knockout caused complete infertility and germ cells were drastically lost during spermatogenesis. We further verified that deletion of the *Srsf1* gene in germ cells led to meiotic arrest at the pachytene stage. By combining advanced linear amplification of complementary DNA ends and sequencing (LACE-seq) and RNA-seq with bioinformatics analysis, we unbiasedly mapped the binding sites of SRSF1 at single-nucleotide resolution and revealed the changes of the transcriptome and transcripts splicing in SRSF1-null testes. Our data suggest that SRSF1-mediated alternative splicing is required for spermatogenesis.

## Results

### Germ cell-specific *Srsf1* knockout results in complete male infertility

**Figure 1:**
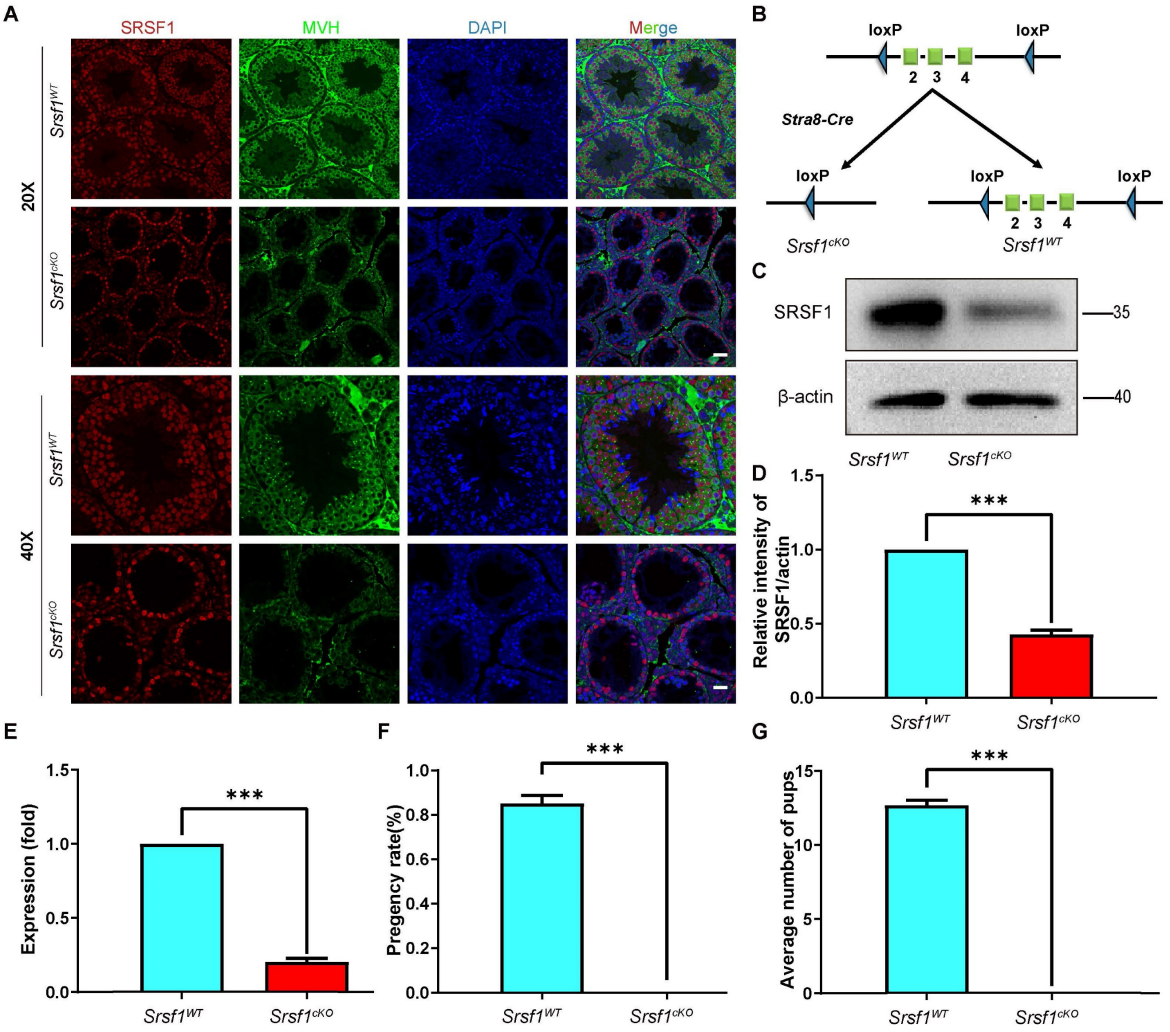
**Germ cell-specific *Srsf1* knockout results in complete male infertility.** (A) Representative images of localization of SRSF1 in the control and *Srsf1^cKO^* testes of 8-week-old mice. The DNA was stained with DAPI, and SRSF1 (red) and MVH (green) were stained with corresponding antibodies. Scale bar: (top) 50 μm; (bottom) 20 μm. (B) Schematic diagram of deletion of *Srsf1* exons 2, 3 and 4 and generation of *Srsf1* Δ allele by *Stra8- Cre*-mediated recombination in male germ cells. (C) Western blotting analysis of SRSF1 protein in *Srsf1^WT^* and *Srsf1^cKO^* total testes of 8-week-old mice. β-actin was detected as an internal control. (D) The relative intensity of SRSF1/actin. β-actin was detected as an internal control. (E) Quantitative RT-PCR analyses showing *Srsf1* mRNA level was decreased. β-actin was used as the internal control. Data are presented as the mean ± SEM.* P*<0.05(*), 0.01(**) or 0.001(***). (F) Pregnancy rates (%) of plugged wild-type females after mating with *Srsf1^WT^* and *Srsf1^cKO^* 8-week-old males. (G) Average litter size of plugged wild-type females after mating with *Srsf1^WT^* and *Srsf1^cKO^* 8-week-old males. For this part, at least 3 mice (8-week-old) of each genotype were used for the analysis. Data are presented as the mean ± SEM.* P*<0.05(*), 0.01(**) or 0.001(***).

To explore the function of SRSF1 in spermatogenesis, we first analyzed the expression of SRSF1 in the testis by using the anti-SRSF1 antibody. As a well-known splicing factor, co-staining of cross-sections of seminiferous tubules in the adult mouse testis showed that SRSF1 was expressed in both germ cells and somatic cells of the testis (Fig. 1A), implying its potential role in spermatogenesis.

Then, we generated *Srsf1* conditional knockout mice (referred to as *Srsf1^cKO^*) by crossing *Srsf1^Floxed/Floxed^* (*Srsf1^F/F^*) mice in which the exons 2-4 were floxed by loxP sites 21, and *Stra8-Cre* mice in which cre activity is initiated at 3 days after birth 22. *Srsf1* was specifically deleted (Fig. 1B), and the knockout efficiency of SRSF1 was confirmed by using quantitative RT-PCR and Western blotting. The mRNA and protein level of *Srsf1* gene was significantly decreased in testes of *Srsf1^cKO^* mice (Fig. 1C,1D and Fig. 1E). Also, SRSF1 expression was barely detected in Mouse Vasa Homologue (MVH)-positive germ cells of *Srsf1^cKO^* mice (Fig. 1A). Thus, we successfully generated male germ cell-specific knockout mice for SRSF1. Next, we analyzed the phenotypes and found that compared to their littermate controls, the *Srsf1^cKO^* male mice were completely infertile (Fig. 1F and Fig. 1G). Although copulatory plugs were routinely observed, no pups were obtained when adult *Srsf1^cKO^* males were mated with normal fertile females.

### *Srsf1* knockout leads to severe defects in spermatogenesis

**Figure 2:**
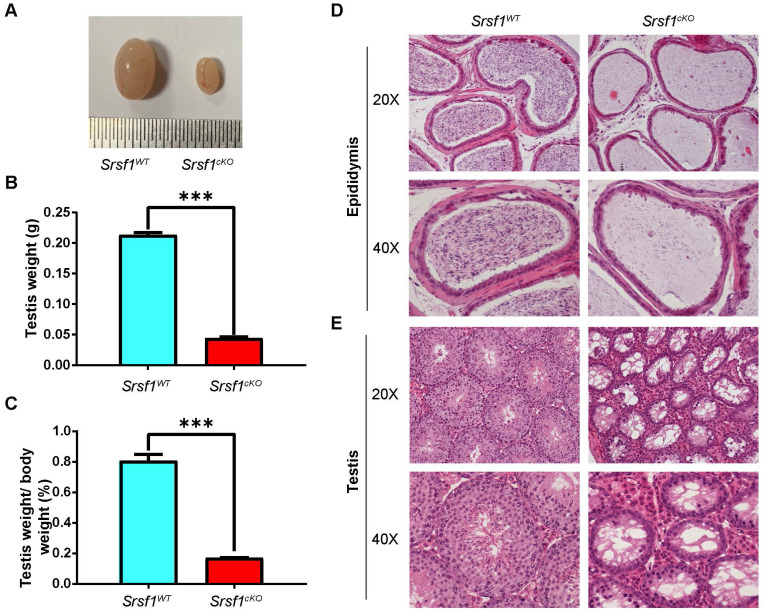
**SRSF1 is required for spermatogenesis.** (A) The testes of *Srsf1^cKO^* were smaller than those of the control (8-week-old, the same as below). (B) Testis weight of *Srsf1^WT^* and *Srsf1^cKO^* 8-week-old male mice (n=3). (C) Testis weight to body weight ratio of *Srsf1^WT^* and *Srsf1^cKO^* 8-week-old male mice (n=3). Data are presented as the mean ± SEM.* P*<0.05(*), 0.01(**) or 0.001(***). (D) Histological analysis of the caudal epididymes of the *Srsf1^WT^* and *Srsf1^cKO^* mice. Scale bar: (top) 100 μm; (bottom) 50 μm. (E) Histological analysis of the seminiferous tubules of the *Srsf1^WT^* and *Srsf1^cKO^* mice. Scale bar: (top) 100 μm; (bottom) 50 μm. For this part, at least 3 mice (8-week-old) of each genotype were used for the analysis.

To determine the reasons of infertility in *Srsf1^cKO^* male mice, we firstly analyzed the phenotypes. Compared with controls, *Srsf1^cKO^* male mice had much smaller testes (Fig. 2A). The testis weight and the testis weight to body weight ratio of* Srsf1^cKO^* mice were significantly lower (Fig. 2B and Fig. 2C). Then we analyzed the histology of the epididymes and testes by Hematoxylin and Eosin (H&E) staining. Consistent with this phenotype, hematoxylin staining results showed that no mature spermatozoa were found in the epididymal lumen of* Srsf1^cKO^* mice (Fig. 2D). The seminiferous tubules of *Srsf1^WT^* testes contained a basal population of spermatogonia, several types of spermatocytes and spermatids. However, germ cells were severely reduced in number, almost no spermatids were present in the seminiferous tubules of *Srsf1^cKO^* testes (Fig. 2E). These findings collectively suggested that germ cell-specific *Srsf1* knockout results in spermatogenesis failure and thus male infertility.

### *Srsf1*-deficient male germ cells arrest at the pachytene stage

**Figure 3:**
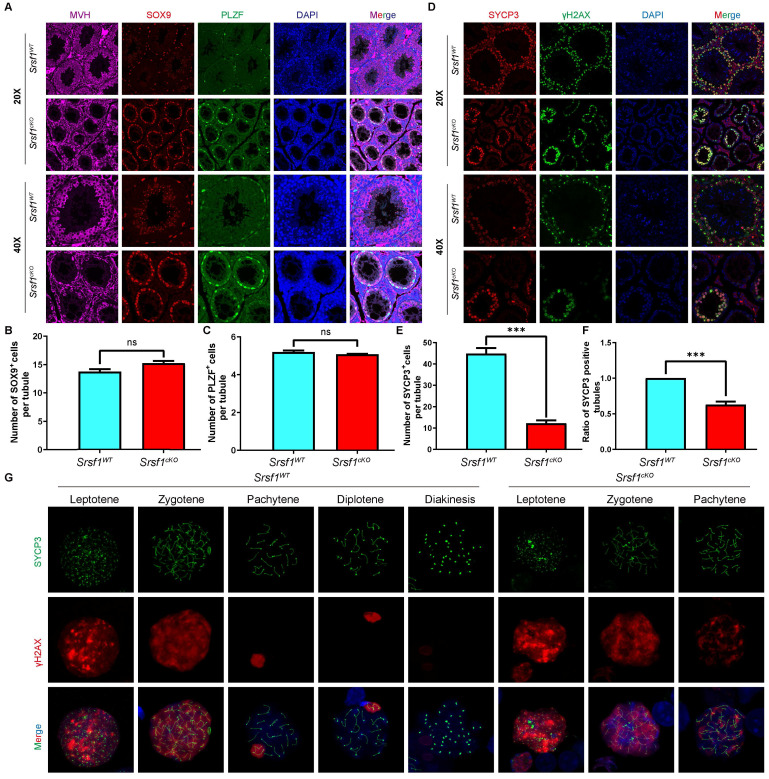
***Srsf1* deficiency causes the loss of germ cells.** (A) MVH (a marker of germ cells, purple), SOX9 (a marker of Sertoli cells, red) and PLZF (a marker of undifferentiated spermatogonia, green), immunofluorescence analysis of the *Srsf1^WT^* and *Srsf1^cKO^* 8-week-old male mice. Scale bar: (top) 50 μm; (bottom) 20 μm. (B) Quantification of the SOX9 positive signal cells in the seminiferous tubules of the *Srsf1^WT^* and *Srsf1^cKO^* mice. (C) Quantification of the PLZF positive signal cells in the seminiferous tubules of the *Srsf1^WT^* and *Srsf1^cKO^* mice. (D) SYCP3 (red) and γH2AX (green) immunofluorescence analysis of the *Srsf1^WT^* and *Srsf1^cKO^* 8-week-old male mice. Scale bar: (top) 50 μm; (bottom) 20 μm. (E) Quantification of the SYCP3 positive signal cells in the seminiferous tubules of the *Srsf1^WT^* and *Srsf1^cKO^* mice. (F) Statistics of the ratio of SYCP3-positive tubule cross-sections in *Srsf1^WT^* and *Srsf1^cKO^* mice. (G) *Srsf1^WT^* and *Srsf1^cKO^* mice chromosome spreads of spermatocytes were immunostained with antibodies against SYCP3 (green) and γH2AX (red). γH2AX marked DSB in spermatocytes(8-weekold). Scale bar: 10 μm. In this part, at least 3 mice of each genotype were used for the analysis.

To validate the above results, we performed immunofluorescent co-staining by using antibodies against MVH, SOX9 and PLZF, markers for the germ cells, Sertoli cells, and undifferentiated spermatogonia, respectively. Immunofluorescence results indicated that the number of MVH positive signals was significantly reduced in cKO testicular sections compared with those in the control (Fig. 3A). Sertoli cells marker SOX9 staining and undifferentiated spermatogonia marker PLZF did not show an obvious change (Fig. 3B and 3C).

Meiosis is a unique process for the differentiation of germ cells. Meiotic recombination and homologous chromosome synapsis are two pivotal events in meiotic progression. Next, we examined meiotic progression by immunostaining the axial element component of the synaptonemal complex with SYCP3 and double-strand break (DSB) marker γH2AX. Similarly, immunofluorescence results indicated that SYCP3 andγH2AX double-positive cells were significantly decreased in *Srsf1^cKO^* mice compared with controls (Fig. 3D, 3E and 3F). To further evaluate the detailed phenotype, we observed spermatocytes using mouse germ cell surface spreading by co-immunostaining with antibodies against synaptonemal complex protein 3 (SYCP3) and phosphorylated histone H2AX (γH2AX) (Fig. 3G). The result indicated that loss of SRSF1 led to meiotic arrest at the pachytene stage.

**Figure 4:**
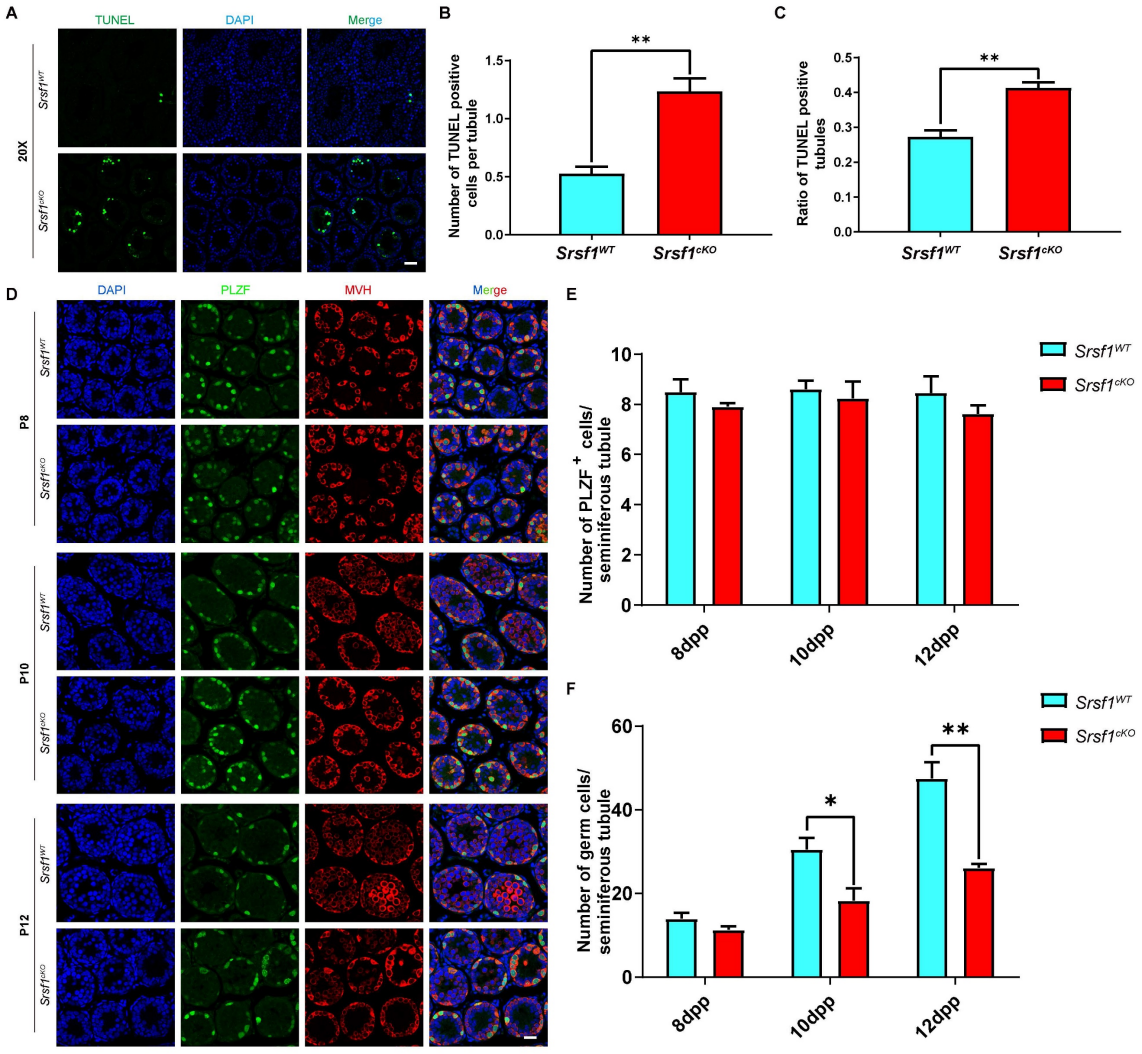
**SRSF1 depletion results in the apoptotic death of germ cells.** (A) TUNEL immunofluorescence staining of the testes of *Srsf1^WT^* and *Srsf1^cKO^*. (Scale bar: 50 μm) Green: TUNEL positive signal; Blue: DAPI. At least 3 mice (8-week-old) of each genotype were used for analysis. (B) Number of TUNEL positive cells per tubule section. (C) Quantification of TUNEL positive seminiferous tubules (8-week-old). (D) PLZF (green) and MVH (red) immunofluorescence analysis of the *Srsf1^WT^* and *Srsf1^cKO^* male mice at P8, P10 and P12. Scale bar, 20 μm. In this part, at least 3 mice of each genotype were used for the analysis. (E) Quantification of the PLZF positive signal cells in the seminiferous tubules of the juvenile *Srsf1^WT^* and *Srsf1^cKO^* mice. For each time point, at least 3 mice of each genotype were used for the analysis. Data are presented as the mean ± SEM.* P*<0.05(*), 0.01(**) or 0.001(***). (F) Quantification of the germ cells in the seminiferous tubules of the juvenile *Srsf1^WT^* and *Srsf1^cKO^* mice. For each time point, at least 3 mice of each genotype were used for the analysis. Data are presented as the mean ± SEM.* P*<0.05(*), 0.01(**) or 0.001(***).

The number of germ cells was sharply decreased in *Srsf1^cKO^* mice, probably because the germ cells were undergoing apoptosis. To test this possibility, TUNEL assay was performed, and the results showed that germ cells underwent apoptosis in the *Srsf1^cKO^* mice (Fig. 4A, 4B and 4C). Then, to further identify which stage of spermatogenesis was firstly impaired in SRSF1-deficient mice, we performed immunofluorescence staining of the PLZF protein and the germ cell marker MVH to characterize the first wave of spermatogenesis in mice at postnatal day 8 (P8), P10, and P12. Undifferentiated spermatogonia marker PLZF staining showed that the number and location of undifferentiated spermatogonia did not display an obvious change (Fig. 4D and Fig. 4E). However, the results revealed that the numbers of germ cells of *Srsf1^cKO^* mice were similar to *Srsf1^WT^* mice at P8, but these numbers started to decrease at P10 and sharply dropped at P12 (Fig. 4Dand Fig. 4F). Altogether, these results again supported that *Srsf1* has functions during spermatogenesis.

### Genome-wide analysis of SRSF1-binding genes in mouse testes

**Figure 5:**
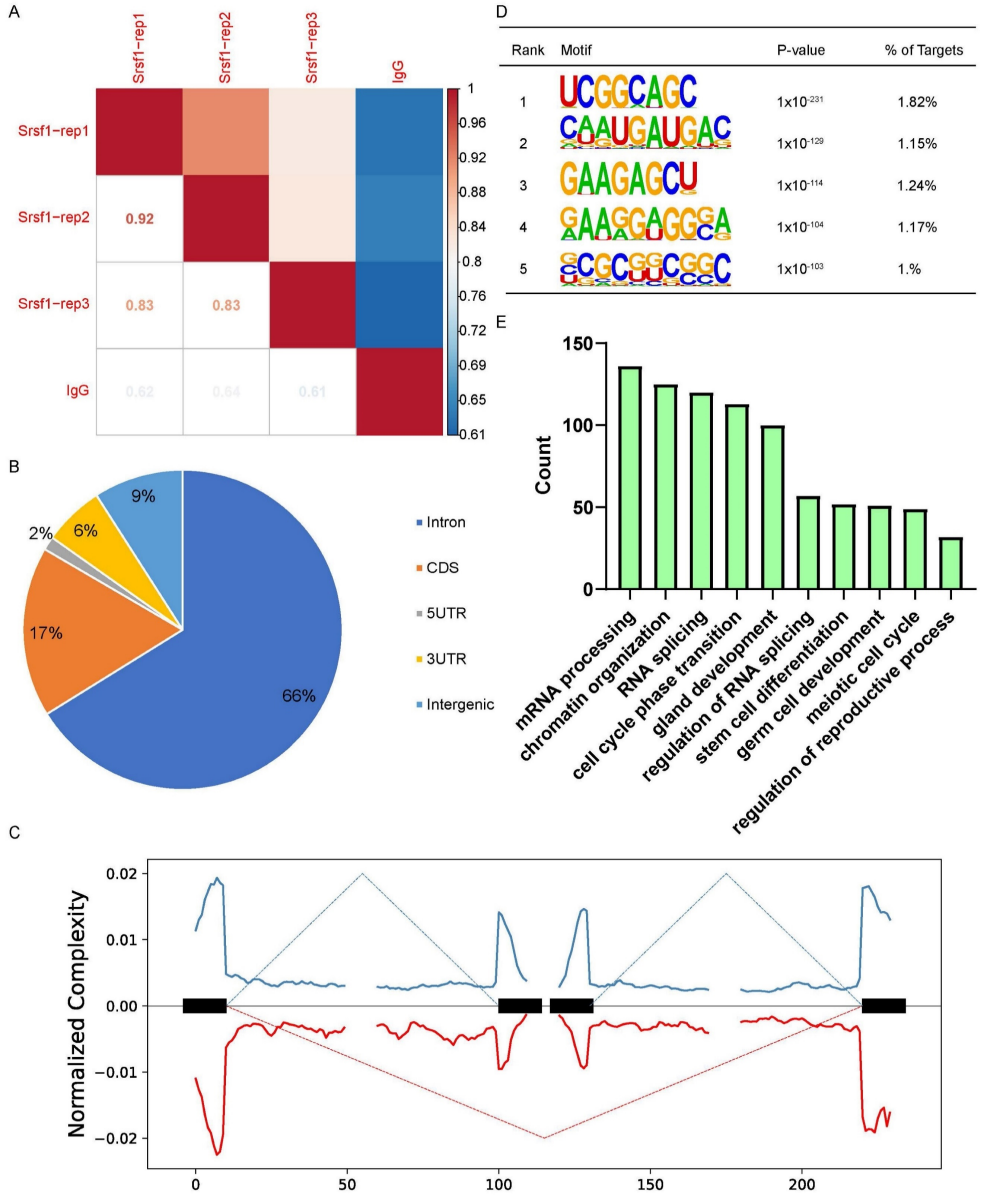
**Global landscape of SRSF1-binding sites in mouse testes as revealed by using LACE-seq.** (A) The reproducibility of the LACE-seq data. (B) Genomic distribution of SRSF1 binding sites in testes. CDS, coding sequence. UTR3, 3′ untranslated region. UTR5, 5′ untranslated region. (C) Schematic analysis showing the distribution of SRSF1-binding sites in the vicinity of the 5′ exon-intron and the 3′ intron-exon boundaries. (D) Enriched hexamer motifs bound by SRSF1. The top five enriched motifs are shown. (E) GO enrichment map of SRSF1-binding genes.

To further investigate the molecular mechanisms by which SRSF1 causes the failure of spermatogenesis, we performed LACE-seq analysis of testes collected at P10 to profile SRSF1-binding sites. Three independent replicates with a high correlation in read counts were pooled together for the subsequent analysis (Fig. 5A). Among these SRSF1 clusters, more than half of them were derived from intergenic regions, while others were aligned to intron, CDS (coding sequence), UTR3 (3′ untranslated region), and UTR5 (5′ untranslated region) (Fig. 5B). We also found that SRSF1 “preferentially” bound to exons and enriched between 0 and 50 nt of the 5′ and 3′ exonic sequences flanking the constitutive splice sites as revealed by analyzing the distributions of SRSF1-binding peaks within 200 nucleotides (nt) upstream or downstream of the constitutive splice site (Fig. 5C). Among these SRSF1 peaks, most of them were significantly enriched for UC-rich consensus motif (Fig. 5D). GO analysis showed that these SRSF1-binding genes were involved in the regulation of mRNA processing, RNA splicing, germ cell development, stem cell differentiation, meiotic cell cycle, and regulation of the reproductive process (Fig. 5E). Together, these analyses suggested that SRSF1 functions as a crucial regulator by directly binding its targets in the reproductive development.

### Changes in transcriptome and splicing of transcripts in SRSF1-null testes

**Figure 6:**
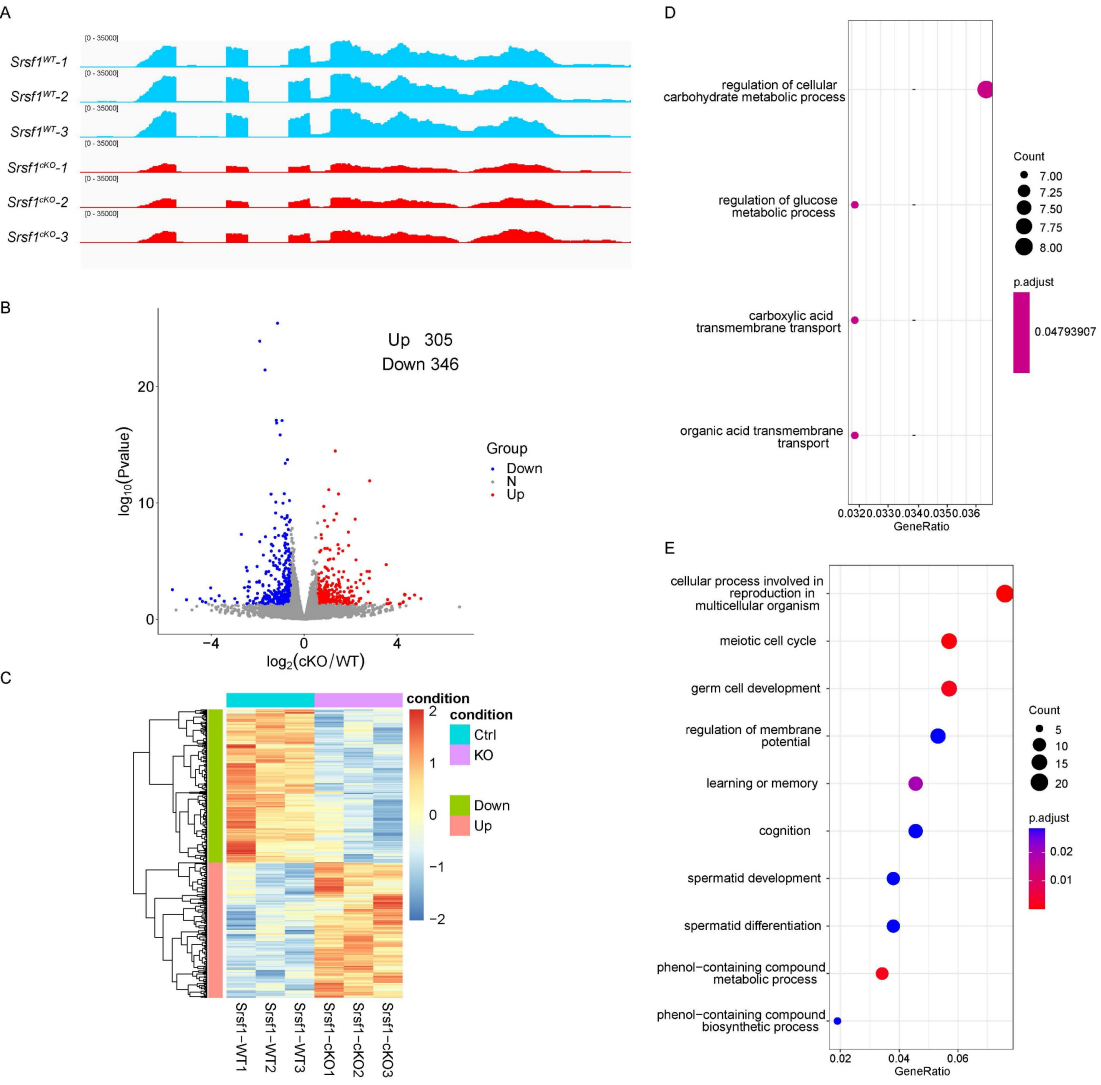
** Transcriptome changes in SRSF1-null testes.** (A) RNA-seq results showing the reduction of *Srsf1* RNA in *Srsf1^cKO^* mice testes. Three independent RNA-seq experiments are shown. (B) Volcano plot showing transcriptome difference between *Srsf1^WT^* and *Srsf1^cKO^* testes. (C) Heatmap showing hierarchical clustering of differential expression genes of* Srsf1^WT^* and *Srsf1^cKO^* male mice testes. (D) GO term enrichment analysis of upregulated genes. (E) GO term enrichment analysis of downregulated genes.

According to the above-presented data, SRSF1 cKO mice displayed defects in spermatogenesis. To explore a comprehensive perspective of the mechanisms of SRSF1 deletion in male germ cells, we isolated mRNA from *Srsf1^WT^* and *Srsf1^cKO^* testes at P10 and then performed RNA sequencing (RNA-seq). RNA-seq results firstly showed the reduction of *Srsf1* RNA in *Srsf1^cKO^* mice testes (Fig. 6A). A total of 305 genes were upregulated, and 346 genes were downregulated in *Srsf1^cKO^* testes (P value of <0.05, |log2FoldChange| ≥ 0.58) (Fig. 6B). Heatmap analysis showed hierarchical clustering of differential expression genes (DEGs) of* Srsf1^WT^* and *Srsf1^cKO^* testes (Fig. 6C). To obtain more comprehensive information, we then performed Gene Ontology (GO) annotation. GO analysis showed that these upregulated genes were involved in carbohydrate metabolic processes and carboxylic acid transmembrane transport, (Fig. 6D). Meiotic cell cycle, germ cell development, and cellular processes involved in reproduction in multicellular organisms were significantly enriched among these downregulated genes (Fig. 6E). In short, these differential expression genes may account for the SRSF1-null phenotypes in spermatogenesis.

**Figure 7:**
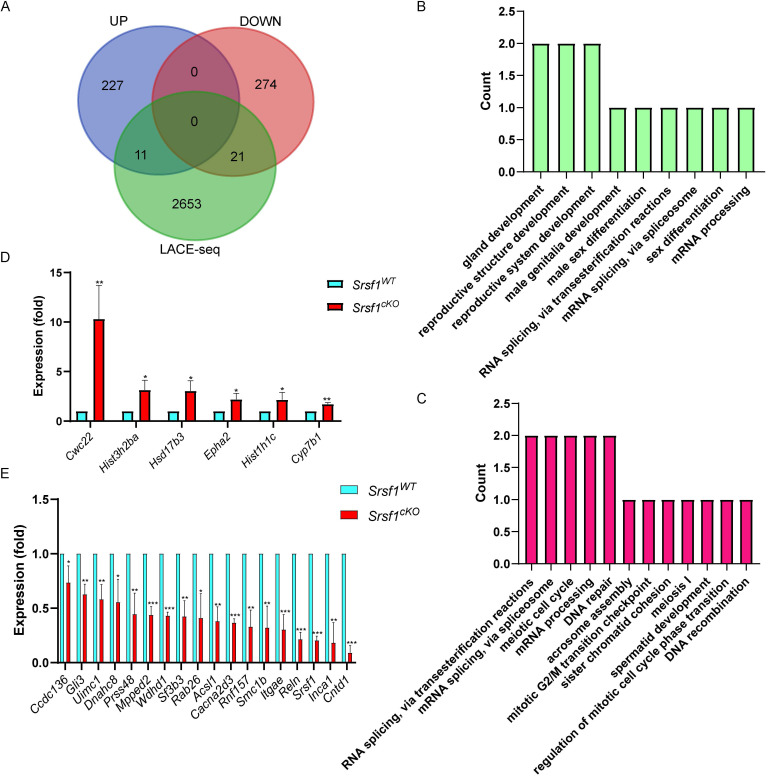
** The expressions of key SRSF1-binding genes involved in the spermatogenesis change after *Srsf1* KO.** (A) Venn diagram showing the overlapped genes between differentially expressed genes and SRSF1-binding genes. (B) Correlation analysis between the RNA-seq and LACE-seq. GO analysis of the significantly upregulated genes and SRSF1-binding genes. (C) Correlation analysis between the RNA-seq and LACE-seq. GO analysis of the significantly downregulated genes and SRSF1-binding genes. (D) Quantitative RT-PCR validation of the expression of genes involved in (B). β-actin was used as the internal control. Data are presented as the mean ± SEM.* P*<0.05(*), 0.01(**) or 0.001(***). (E) Quantitative RT-PCR validation of the expression of genes involved in (C). β-actin was used as the internal control. Data are presented as the mean ± SEM.* P*<0.05(*), 0.01(**) or 0.001(***).

Furthermore, by combining RNA-seq data with LACE-seq identified peaks, we only identified 21 downregulated, and 11 upregulated transcripts as direct targets of SRSF1 in testes (Fig. 7A). To obtain more comprehensive information, similarly, we then performed GO annotation. GO analysis showed that both significantly upregulated genes and SRSF1-binding genes were involved in reproductive development, male sex differentiation, mRNA processing and mRNA splicing (Fig. 7B). And RNA splicing, meiotic cell cycle, meiosis I, spermatid development, DNA repair, and DNA recombination were significantly enriched among both of these significantly downregulated genes and SRSF1-binding genes (Fig. 7C). We next validated both of these significantly DEGs and SRSF1-binding genes which were involved in spermatogenesis by using quantitative polymerase chain reaction (qPCR) to check the mRNA abundance (Fig. 7D and Fig. 7E). These data reflected that deletion of SRSF1 directly affects the expression levels of critical genes involved in spermatogenesis. Nevertheless, most of the changes in gene expression might be regulated indirectly by SRSF1.

**Figure 8:**
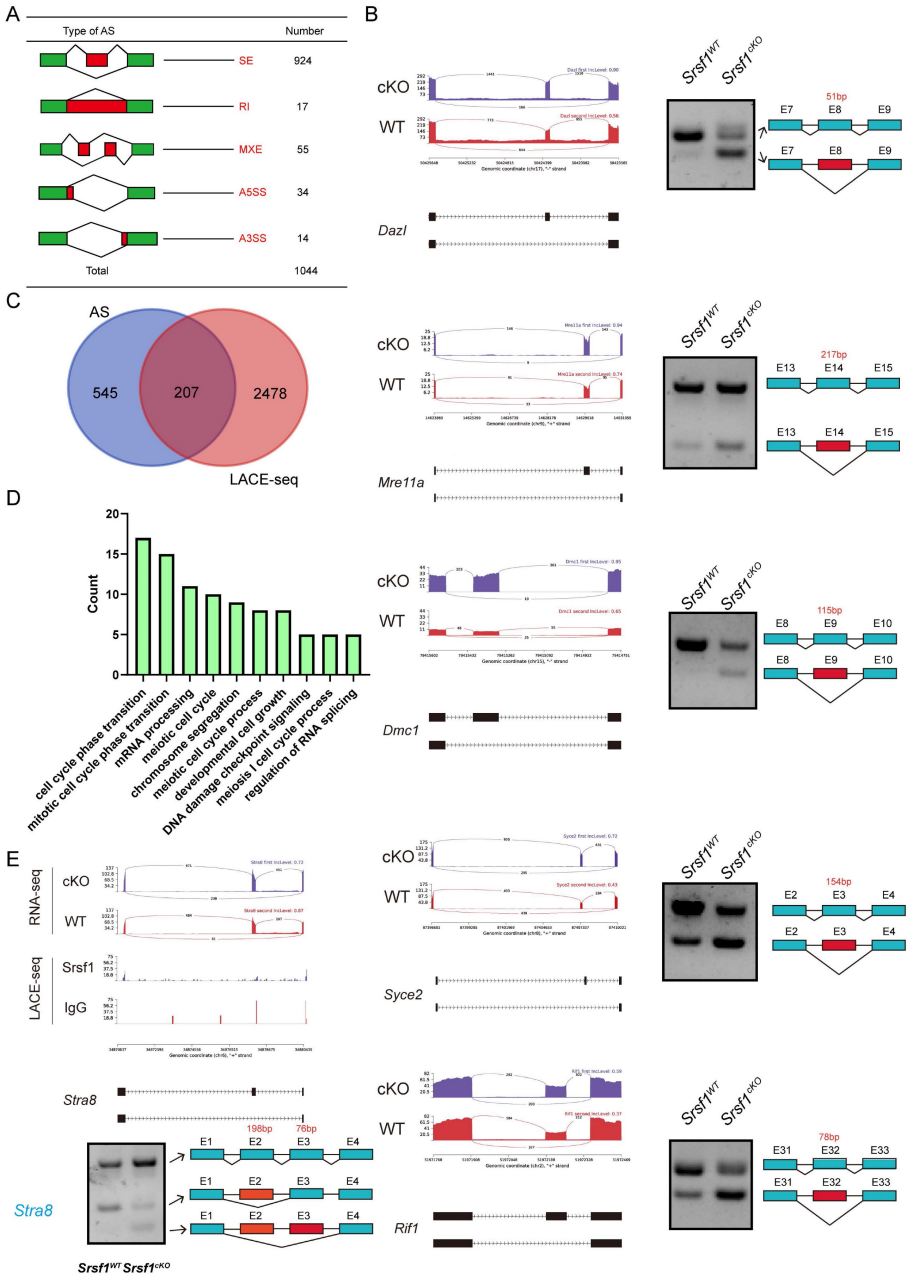
***Srsf1* is required for normal splicing of functional genes during spermatogenesis.** (A) The five different types of alternative splicing (AS) events. The numbers of abnormal AS events were counted between* Srsf1^WT^* and *Srsf1^cKO^* testes by rMATS software. (B) A magnified view showing RNA-seq signals of the selected candidate genes. IgG, immunoglobulin G. Semiquantitative RT-PCR analysis of AS patterns of the changed spliced genes in *Srsf1^WT^* and *Srsf1^cKO^* testes at P10. (C) Venn diagram shows the correlation between SRSF1-binding genes and AS genes. (D) GO analysis of the AS genes and SRSF1-binding genes. (E) A magnified view showing RNA-seq and LACE-seq signals of the *Stra8* genes. IgG, immunoglobulin G. Semiquantitative RT-PCR analysis of AS patterns of the changed spliced genes in *Srsf1^WT^* and *Srsf1^cKO^* testes at P10. PCR primers are listed in Table S1.

Because SRSF1 is an SR protein and played critical roles in AS, we then analyzed the five different types of AS events between *Srsf1^WT^* and *Srsf1^cKO^* testes by using the rMATS computational tool. Compared with the *Srsf1^WT^* group, a total of 1044 AS events were identified as significantly changed in the *Srsf1^cKO^* group (|Diff| > 0.05, FDR < 0.001). Among these 1044 changed AS events, most (924) of AS events were skipped exons (SE). Moreover, there were 14 alternative 3′ splice sites (A3SS), 34 alternative 5′ splice sites (A5SS), 55 mutually exclusive exons (MXE), and 17 retained introns (RI) (Fig. 8A and Fig. S1). Importantly, we successfully verified that loss of SRSF1 in testes resulted in differential splicing in *Dazl*, *Dmc1*, *Mre11*a, *Syce2* and *Rif1* (Fig. 8B), which were critical for spermatogenesis.

We then asked how these differentially spliced genes were regulated by SRSF1. Venn diagram showed that among these differentially spliced genes, 207 genes were bound by SRSF1 as identified in LACE-seq (Fig. 8C), indicating that these genes are probably the direct targets of SRSF1. GO analysis showed that both AS genes and SRSF1-binding genes were involved in meiotic cell cycle, mRNA processing, chromosome segregation and developmental cell growth (Fig. 8D). Importantly, the data showed that the splicing of *Stra8* mRNA was changed after SRSF1 cKO (Fig. 8E). We also performed semiquantitative reverse transcription PCR to confirm the above results (Fig. 8E). Together, these results suggest that SRSF1 is essential for RNA splicing during spermatogenesis.

## Discussion

In higher eukaryotes, most genes express primary transcripts that undergo AS regulation in the nucleus to generate different functional mRNAs 23-25. It is important for spermatogenesis to regulate AS processes, and splicing factors and AS is regulated in a stage-specific manner during spermatogenesis 26, 27. The SR proteins are well-known splicing factors that are critical for the regulation of AS. As members of the SR protein family, SRs which include 12 members in mammalian systems (SRSF1-12) are well-known for their regulatory function of splicing 28. The first SRs identified were SRSF1 (previously known as SF2/ASF) 14. Like other SR splicing factors, several studies in recent years have shown that SRSF1, as a crucial RNA-binding protein (RBP), has well-documented functions as posttranscriptional regulator for mRNA splicing and translation during a multitude of physiological processes and multiple cancer genesis events 29-31.

Recently, it also has been found that RBPs have important functions during germline and early embryo development. As an RBP, SRSF1 is also expressed in testis, however, its functions in male germ cells are still unknown. In this study, by crossing *Srsf1^F/F^* mice with *Stra8-Cre* mice to generate mutant mice, we found that SRSF1 is essential for spermatogenesis and fertility in males.

Our findings showed that the number of germ cells was sharply decreased in *Srsf1^cKO^* mice, and *Srsf1*-deficient male germ cells arrest at the pachytene stage, probably because the pachytene checkpoint-arrested spermatocytes and other abnormal germ cells were undergoing apoptosis. To test this possibility, TUNEL assay was performed, and the results showed that abnormal germ cells underwent apoptosis in the *Srsf1^cKO^* mice. To gain a comprehensive perspective of the mechanisms of SRSF1 depletion in male germ cells, we isolated testes from wildtype mouse at P10 and systematically profiled binding landscapes of SRSF1 proteins by using LACE-seq. The results showed that SRSF1 proteins could bind numerous genes in a direct manner. Then, our analysis showed that these SRSF1-binding genes were closely involved in the regulation of mRNA processing, RNA splicing, germ cell development, stem cell differentiation, meiotic cell cycle, and regulation of reproductive processes. In addition, RNA-seq analysis further showed that transcriptome and splicing of transcripts change in SRSF1-null testes. By combining RNA-seq and LACE-seq data, we found that deletion of SRSF1 directly or indirectly affects the expression levels of critical genes involved in spermatogenesis.

Spermatogonial stem cell differentiation and the mitosis-meiosis transition are important steps for spermatogenesis. Retinoic acid (RA) is a crucial factor of spermatogenesis, with functions on spermatogonial stem cell differentiation and subsequent initiation of meiosis 32, 33. RA has two certain targets, *Stra8* and *Kit*. There is already some evidence suggesting that *Stra8* has two different functions in spermatogenesis. On one hand, under the influence of RA, *Stra8* functions as a transcriptional repressor of the pluripotency program during differentiation of spermatogonia. When differentiating spermatogonia are near the end of their mitotic phase, *Stra8* switches to the second role and acts as a transcription activator of genes involved in meiosis initiation 34-36. In addition to RA signaling, *Dazl* is a key intrinsic factor for initiating meiosis 37. The switch from mitosis to meiosis is a critical step of germ cell development that requires *Dazl*. In meiosis I, the Mre11 complex is essential for meiotic recombination. During the meiotic prophase, numerous DNA double-strand breaks (DSB) are formed in the genome in order to initiate recombination between homologous chromosomes. The conserved Mre11 complex has functions in mitotic cells for sensing and repairing DSB 38, 39. In most eukaryotic cells, two recombinases, Rad51 and Dmc1, are responsible for the homologous recombination process. Dmc1, which is essential for both mitotic and meiotic recombination, is present only in meiosis while Rad51 is expressed in both meiotic and mitotic cells 40-42. Also, *Syce2* is required for synaptonemal complex assembly, double strand break repair, and homologous recombination 43. The surveys indicated that *Rif1* had crucial functions in DNA double-strand repair by its interaction with TP53BP1 (tumor protein p53 binding protein) 44.Here, we crossed *Stra8-Cre* mice with *Srsf1^flox/flox^* (*Srsf1^cKO^*) mice to study the functions of *Srsf1* in spermatogenesis. We found that the number of c-KIT^+^ cells was obviously reduced in cKO testes compared with that in the control. SYCP3 and γH2AX positive cells were also significantly decreased in *Srsf1^cKO^* testes compared with controls. These results indicated that *Srsf1* had critical roles during spermatogenesis. Of particular note, one of our critical findings was that we identified the binding motif and splicing targets of *Srsf1* during spermatogenesis through combined analysis of RNA-seq and LACE-seq data. The two omics data indicated that SRSF1 affects the AS of *Stra8* in a direct manner and *Dazl*, *Dmc1*, *Mre11a*, *Syce2* and *Rif1* in an indirect manner. These above genes are critical for the male germ cell developmental process. However, limitation of the study is that transcriptome and GO data provided here may only due to a lack of germ cells in testis from SRSF1 cKO mice.

In summary, our findings suggest that SRSF1 is critical for male fertility and spermatogenesis. Mechanistic analyses indicate that SRSF1 has functions in posttranscriptional regulation by specifically adjusting the gene expression and AS in direct or indirect manners during spermatogenesis. Specifically, we verified *Stra8* as one of the splicing targets of SRSF1. Our investigation suggests that SRSF1-mediated AS is important for spermatogenesis.

## Methods

### Mice

Mice lacking *Srsf1* in male germ cells (referred to as *Srsf1^cKO^*) were generated by crossing *Srsf1^Floxed/Floxed^* (*Srsf1^F/F^*) mice with* Stra8-Cre* mice. All transgenic mouse lines had C57BL/6J genomic background. Genotyping PCR for *Srsf1* was performed using the following primers: forward: GGGACTAATGTGGGAAGAATG, and reverse: AACCTAAACTATTGCTCCCATCTG. The PCR conditions were as follows: 94 °C for 5 min; 35 rounds of 94 °C for 30 sec, 60 °C for 30 sec, and 72 °C for 30 sec; and 72 °C for 5 min. Genotyping PCR for *Stra8-Cre* was performed using the following primers: forward: ACTCCAAGCACTGGGCAGAA, wildtype reverse: GCCACCATAGCAGCATCAAA and reverse: CGTTTACGTCGCCGTCCAG. The PCR conditions were as follows: 94 °C for 5 min; 35 rounds of 94 °C for 30 sec, 60 °C for 30 sec, and 72 °C for 30 sec; and 72 °C for 5 min. Four genotypes in the progeny, including *Srsf1^F/+^*,* Srsf1^F/-^*, *Srsf1^F/+^; Stra8-Cre* and *Srsf1^F/-^; Stra8-Cre* were identified. The *Srsf1^F/+^* male mice were used as control group.

The mice were maintained under specific-pathogen-free (SPF) conditions and housed under controlled environmental conditions with free access to water and food. All animal operations were approved by the Animal Care and Use Committee of the Institute of Zoology, Chinese Academy of Sciences (CAS). The assigned accreditation number of the laboratory was IOZ20160033.

### Antibodies

β-actin antibody (mouse, sc-47778; Santa Cruz); SYCP3 (mouse, sc-74569; Santa Cruz); γH2AX (rabbit, 9718; Cell Signaling Technology, Inc.); MVH (mouse, ab27591; Abcam); SOX9 antibody (rabbit, AB5535, Sigma-Aldrich); PLZF antibody (goat, AF2944, R&D Systems); SFRS1 polyclonal antibody (rabbit, 12929-2-AP, Proteintech); c-Kit antibody (goat, AF1356, R&D Systems). Horseradish peroxidase-conjugated secondary antibodies were purchased from Zhongshan Golden Bridge Biotechnology Co, LTD (Beijing). Alexa Fluor 488-conjugated antibody, 594-conjugated antibody and Alexa Fluor 647-conjugated antibody were purchased from Life Technologies.

### Breeding assay

Males of different genotypes (8 weeks) were used for the breeding assay. Each male mouse was caged with two wild-type ICR (Institute of Cancer Research) females (7 weeks), and their vaginal plugs were checked every morning. The number of pups in each cage was counted within a week of birth. Each male underwent at least eight cycles of the above breeding assay.

### Immunoblotting

To prepare protein extracts, testes were homogenized in RIPA lysis buffer supplemented with protease and phosphatase inhibitor cocktail (Roche Diagnostics). After transient ultrasound treatment, the testis lysates were incubated on ice for 30 min and then centrifuged at 4 ℃, 12000 rpm for 20 min. The supernatant was transferred to a new tube and quantified using a BCA reagent kit (Beyotime, P0012-1). Then equal volume loading buffer was added. After being boiled at 95 ℃ for 10 min, the protein lysates were used for immunoblotting analysis. Immunoblotting was performed as described previously 45. Briefly, the separated proteins in SDS-PAGE were electrically transferred to a polyvinylidene fluoride membrane. After incubation with primary and secondary antibodies, the membranes were scanned with Bio-Rad ChemiDoc XRS+.

### Tissue collection and histological analysis

For histological analysis, at least three adult mice for each genotype were analyzed. Testes and caudal epididymides were dissected immediately following euthanasia. The tissues were then fixed in Bouin's fixative (saturated picric acid: 37% formaldehyde: glacial acetic acid= 15: 5: 1) overnight at room temperature, dehydrated in an ethanol series, and embedded in paraffin wax. Then, 5μm sections were cut with a microtome. After 48 °C overnight drying, the sections were deparaffinized in xylene, hydrated by a graded alcohol series and stained with Hematoxylin and Eosin for histological analysis. Images were collected with a Nikon inverted microscope with a charge coupled device (CCD) (Nikon, Eclipse Ti-S, Tokyo, Japan).

### Immunofluorescence

Testes used for immunostaining were fixed in 4% paraformaldehyde (pH 7.4) overnight at 4 °C, dehydrated, and embedded in paraffin. Paraffin-embedded testes were cut into sections of 5μm thickness. Then, the sections were deparaffinized, immersed in sodium citrate buffer (pH 6.0) and heated for 15 min in a microwave for antigen retrieval. After blocking with 5% donkey serum albumin, sections were incubated with primary antibodies at 4 °C overnight. Then the sections were incubated with an appropriate FITC-conjugated secondary antibody. The nuclei were stained with DAPI. Images were captured using a laser scanning confocal microscope LSM880 (Carl Zeiss, Germany).

### TUNEL assay

TUNEL assay was carried out in accordance with the DeadEnd^TM^ Fluorometric TUNEL System (Promega BioSciences, Madison, WI, USA). Images were captured using a laser scanning confocal microscope LSM880 (Carl Zeiss, Germany).

### RNA extraction and gene expression analysis

Total RNA was extracted from whole testes using TRNzol Universal Reagent (cat. # DP424, Tiangen, China) according to the manufacturer's instructions. Then reverse transcription (RT) was performed using the 5X All-In-One RT MasterMix (cat. # G490, Abm, Canada). RT-PCR was performed using the UltraSYBR Mixture (cat. # CW0957, Cowin Bio, China) on a LightCycler 480 instrument (Roche). The results were analyzed based on the 2^-ΔΔCt^ method to calculate the fold changes. *β-actin* was used as an internal control. At least three independent experiments were analyzed. All primer sequences are listed in Table S1.

Semiquantitative PCR experiment was carried out with primers (listed in Table S1) amplifying endogenous transcripts. Then the PCR products were detected on 2% agarose gels. *Gapdh* was used as an internal control.

### RNA sequencing and data analysis

Total testes samples were used from P10 *Srsf1^WT^* and *Srsf1^cKO^* male mice according to three individual collections. One Total RNA was extracted from whole testes using TRNzol Universal Reagent (cat. # DP424, Tiangen, China) according to the manufacturer's instructions. The quality of RNA samples was examined by NanoDrop 2000&8000 and Agilent 2100 Bioanalyzer, Agilent RNA 6000 Nano Kit. High-quality RNAs were used to prepare the libraries, followed by high-throughput sequencing on an Illumina NovaSeq 6000. The RNA sequencing experiment was supported by Annoroad BioLabs.

After trimming adaptor sequence and rRNA, the retained reads from *Srsf1* control and cKO samples were aligned to the mouse genome (mm9) using HISAT2 with default parameters. Only non-RCR duplicate and uniquely mapped reads were used for subsequent analysis. Significantly changed genes were screened using DESeq2 with |log2FC| > 0.58 and p<0.05. Alternative splicing events were identified by rMATS with default parameters. Only events with FDR<0.001 and splicing difference > 0.05 were regarded as significant.

### LACE-sequencing and data analysis

Total testes samples were used from P10 WT male mice for LACE-seq. LACE-seq method was performed as described recently by us 46. Briefly, the samples were firstly irradiated twice with UV-C light on ice at 400 mJ. Then RNA immunoprecipitation of the samples was performed. The immunoprecipitated RNAs were then fragmented by MNase and dephosphorylated. Then a series of steps was performed to include, reverse transcription, first-strand cDNA capture by streptavidin beads, poly(A) tailing, pre-PCR, IVT, RNA purification, RT, PCR barcoding and deep sequencing.

The adapter sequences and poly(A) tails at the 3′ end of raw reads were removed using Cutadapt (v.1.15) with two parameters: -f fastq -q 30,0 -a ATCTCGTATGCCGTCTTCTGCTT -m 18 --max-n 0.25 --trim-n., and -f fastq -a A -m 18 -n 2. Clean reads were first aligned to mouse pre-rRNA using Bowtie, and the remaining unmapped reads were then aligned to the mouse (mm9) reference genome. For LACE-seq data mapping, two mismatches were allowed (Bowtie parameters: -v 2 -m 10 --best -strata; -v 2 -k 10 --best -strata). Peaks were identified by Piranha with parameters: -s -b 20 -p 0.01. Peaks without IgG signal were selected for further usage. For motif analysis, LACE-seq peaks/clusters were first extended 30 nt to 5′ upstream, and overrepresented hexamers in the extended sequences were identified as previously described 47. The consensus motifs were generated from the top-10 enriched hexamers using WebLogo.

### Statistical analysis

All of the experiments were performed at least three times independently. Paired two-tailed Student's t-test was used for statistical analysis. Data analyses were carried out via GraphPad Prism 8.00 (GraphPad Software, Inc.) and presented as mean ± SEM and *P*<0.05(*), 0.01(**) or 0.001(***) was considered statistically significant.

## Supplementary Material

Supplementary figures and tables.
