# Supplementary material for: SRSF1-mediated alternative splicing is required for spermatogenesis
Source: Int J Biol Sci. 2023 Sep 11;19(15):4883–97. doi: 10.7150/ijbs.83474 (PMC10539708; doi:10.7150/ijbs.83474)
Supplement: Supplementary file 1 — Supplementary figures and tables. [file ijbsv19p4883s1.zip › Supplementary materials/Table 1 Primer List.pdf]

RT-PCR

Gene	Forward primer	Reverse primer
β-actin	GGCTGTATTCCCCTCCATCG	CCAGTTGGTAACAATGCCATGT
Srsf1	GTGGTTGTCTCTGGACTGCC	GTTGCTTCTGCTACGGCTTC
Cwc22	CGGAAAGGCTATCGAAGGAAC	ATTTGAGACCACACTCTTTGAGG
Epha2	GCACAGGGAAAGGAAGTTGTT	CATGTAGATAGGCATGTCGTCC
Hsd17b3	TTAGTCGGACACTGGAAAAGC	ATTCTGGCTCTCACCGGAAGT
Ccdc136	AGCAGAAGTATTCACCAAGCAG	TTGGGCCAAATGCAACTCCT
Cntd1	AACAAGTTCATCCTCCGATTGG	CAGGACTGTAATGTTGCTGACA
Dnahc8	ACAGGGTGGTTGGGTGTTAC	TCAGTGGTTATCCACACTCTGAA
Smc1b	CATGAGGGAAAACGTCAGCAG	TGACACAGATCAAGCAGTCTTC
Uimc1	AGTCTTCCCAAGGTCTGTTTGT	TCTATTGAGGTCAAGGGGTTCA
Unc5d	TCAGCCTGCAATGTTCGCT	GACTGACCATTCACTCCACAC
a	GTGCTTCTTCACCGTCCACA	GCCACGATAGAAACAGAGGAGA
Rab26	GGAGCGGTTCCGAAGTGTC	GCCTGAATGTTGTCGAAGGAAT
Trim2	TGGACAGTTCAAAAGTCGTTTCG	AATGCTAACCCACTTGTTGTCAT
Hist3h2ba	CAAGGAGAGCTACTCCATCTAC	GACGAACGAGTTCATGATGCC
Reln	GAATTTCATGGCTACTGCAACAC	CAGCAAGGTGCGAGTAAGCA
Hist1h4j-	TAAAAAGACGGCGCTCAGGCTC	GATGTTGTCGCGCAGCACTTTG
Cacna2d3	AAGAAATCGACGGTCTCCAAC	GGTCATTGGGGGCTAAGATGAA
Hist1h1d	GTAAAGCTTAGAACATGTCCG	GTTGTTCTTCTCCACATCGTAC
Mpped2-	AACAGATGGTATCCAGATGCCT	GGCAGGTTTCCTAACCAGTCAT
Rnf157	CTGGACCGTGAAGTCTACCCT	CCACCTTGGAATCTTGTGTGTT
Inca1	ATGCCTCAGCCGTATGGAGAT	GCCCTCAGAATTGGTGGAATGTA
Col22a1	GTGACGCCCTGCGTTATATCA	ACCTGCTTGAAGGCACGATTC
Prss48	CACCTTCTCATCGGTCATTCTG	TTGGAGGGTAGAGGGGTAGTG
Itgae	CCTGTGCAGCATGTAAAAGAATG	CAAGGATCGGCAGTTCAGATAC
Acsl1	ACCAGCCCTATGAGTGGATTT	CAAGGCTTGAACCCCTTCTG
Gli3	CACAGCTCTACGGCGACTG	CTGCATAGTGATTGCGTTTCTTC
Sf3b3	TCCTTCAGGACAGTTGAATGAGT	GTCTGATGGGTCGAGGGAGAT
Trim17	CTTGCCAGACGGTTACAAGAG	CTCAGCCACTTTTGTCAGGAG
Cyp7b1	GGAGCCACGACCCTAGATG	GCCATGCCAAGATAAGGAAGC
Hist1h4k	GTGCTGAAGGTGTTCCTGGAGAA	GCCCTGAAAAGGGCCTTTTGT
Hist1h1c	AACCCCAGGCTAAGAAGGC	TGGCTTTACGGCTTTAGACGC
Wdhd1	AAGGTACTCCCTGTTACGTGG	TGCAGTGCTCTCTTATGTTGC

Semiquantitative PCR

Gene	Forward primer	Reverse primer
Stra8	TCCTGCGTGTTCCACAAGTG	GCTGTTGGGATTCCCATCTTGC
Dazl	TCCTCCTTATCCAAGTTCACCA	TCAGCTCCTGGATCAACTTCAC
Rif1	GCGTTGCTCTGTTGTTAGATC	CACAGTTCACCAAAGCTGGGTA
Dmc1	TGAAGCAGTACTGGACAACG	TCTTCCCTTGCGCAAGCTTA
Mre11a	GTTTCCGAGAAAGCAGACAGAG	GCTGTTGTCGGGTAGATCTGA
Syce2	TGGAGTTAAAGGACCAGGAGCC	GGTCCTTGTACACCGTTTCCAC
Gapdh	ACCACAGTCCATGCCATCAC	TCCACCACCCTGTTGCTGTA
Appl1	GGCTGGATACCTAAATGCTAG	GAAAGATGGGGAAGGAGTGACA
Cenpf	CAGTTCCAGCTGGACTCTCT	GAACTCGGTGTGAGTGGAGTTG
Dmrt1	GAACTGGGTATCAGCCACCCAATT	GACCAAGCCAGAATCTTGAC
Ezh2	CAAGGAGTCCAGTATCATAGCAC	GTGTTGCATTGTGCTTTGCAC
Fn1	CAGAACCGGAACGGAGAAAG	GAACACTGGGTGCTATCCAC
Nek1	GGATCAGATTAGGTTCCTGAAG	GAGCCATCGACCATAGATTC
Picalm	CTACCCCTAGTTCTTCTAACAG	CAAAGTCAACATTAAGGCCAGC
Prkdc	GCAGGATGATATTGACAGAGC	GCATGCAACGAACATCTTCC
Rcc1	CACAACACAGAACCAGGCTTG	GCACTTGAACAGGTACCATGC
Stxbp4	GACTGCCAATTGCGAAAATCAG	CTTGATTCCATCTGCTGTGTAAG
