# Supplementary material for: SRSF1-mediated alternative splicing is required for spermatogenesis
Source: Int J Biol Sci. 2023 Sep 11;19(15):4883–97. doi: 10.7150/ijbs.83474 (PMC10539708; doi:10.7150/ijbs.83474)
Supplement: Supplementary file 1 — Supplementary figures and tables. [file ijbsv19p4883s1.zip › Supplementary materials/Table 2 motif_Srsf1_ - Homer de novo Motif Results.pdf]

Homer *de novo* Motif Results (motif_Srsf1/)

Known Motif Enrichment Results
Gene Ontology Enrichment Results
If Homer is having trouble matching a motif to a known motif, try copy/pasting the matrix file into STAMP
More information on motif finding results: HOMER | Description of Results | Tips
Total target sequences = 7826
Total background sequences = 6000
* - possible false positive

Rank	Motif	P-value	log P-pvalue	% of Targets	% of Background	STD(Bg STD)	Best Match/Details	Motif File
1	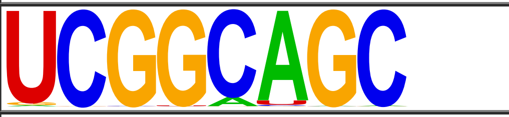	1e-231	-5.336e+02	1.82%	0.01%	12.2bp (0.0bp)	hsa-miR-22 MIMAT0000077 Homo sapiens miR-22 Targets (miRBase)(0.656) More Information | Similar Motifs Found	motif file (matrix)
2	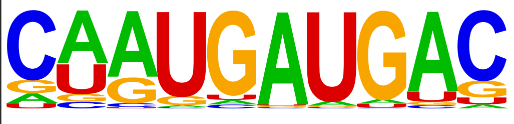	1e-129	-2.984e+02	1.15%	0.03%	8.2bp (2.6bp)	hsa-miR-598 MIMAT0003266 Homo sapiens miR-598 Targets (miRBase)(0.670) More Information | Similar Motifs Found	motif file (matrix)
3	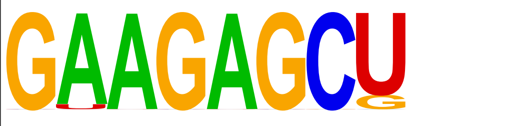	1e-114	-2.626e+02	1.24%	0.05%	7.3bp (8.1bp)	hsa-miR-3194-3p MIMAT0019218 Homo sapiens miR-3194-3p Targets (miRBase)(0.711) More Information | Similar Motifs Found	motif file (matrix)
4	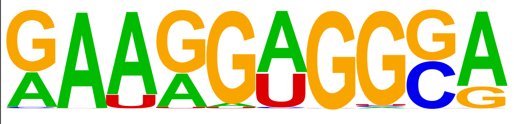	1e-104	-2.408e+02	1.17%	0.04%	9.2bp (0.6bp)	hsa-miR-4667-3p MIMAT0019744 Homo sapiens miR-4667-3p Targets (miRBase)(0.774) More Information | Similar Motifs Found	motif file (matrix)
5	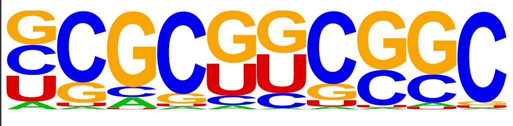	1e-103	-2.392e+02	1.61%	0.11%	10.4bp (3.5bp)	hsa-miR-3960 MIMAT0019337 Homo sapiens miR-3960 Targets (miRBase)(0.609) More Information | Similar Motifs Found	motif file (matrix)
6	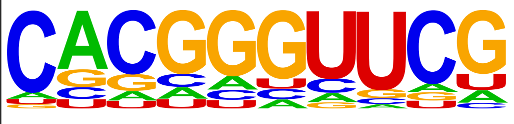	1e-90	-2.089e+02	1.05%	0.04%	8.3bp (6.0bp)	hsa-miR-1273d MIMAT0015090 Homo sapiens miR-1273d Targets (miRBase)(0.634) More Information | Similar Motifs Found	motif file (matrix)
7	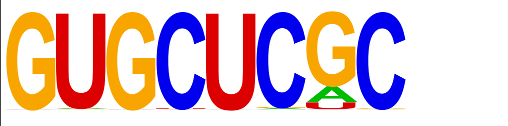	1e-81	-1.874e+02	0.81%	0.03%	4.8bp (1.9bp)	hsa-miR-3663-3p MIMAT0018085 Homo sapiens miR-3663-3p Targets (miRBase)(0.702) More Information | Similar Motifs Found	motif file (matrix)
8	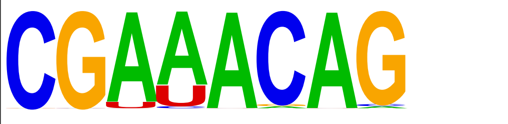	1e-76	-1.757e+02	0.77%	0.00%	9.7bp (0.0bp)	hsa-miR-4676-3p MIMAT0019759 Homo sapiens miR-4676-3p Targets (miRBase)(0.703) More Information | Similar Motifs Found	motif file (matrix)
9	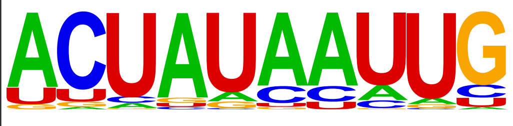	1e-70	-1.613e+02	0.87%	0.04%	4.0bp (6.6bp)	hsa-miR-944 MIMAT0004987 Homo sapiens miR-944 Targets (miRBase)(0.647) More Information | Similar Motifs Found	motif file (matrix)
10	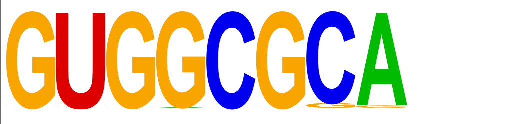	1e-69	-1.594e+02	1.31%	0.13%	9.4bp (9.3bp)	hsa-miR-4787-3p MIMAT0019957 Homo sapiens miR-4787-3p Targets (miRBase)(0.707) More Information | Similar Motifs Found	motif file (matrix)
11	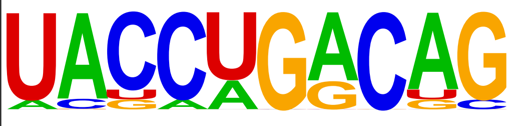	1e-68	-1.566e+02	0.70%	0.03%	6.5bp (4.2bp)	hsa-miR-626 MIMAT0003295 Homo sapiens miR-626 Targets (miRBase)(0.602) More Information | Similar Motifs Found	motif file (matrix)
12	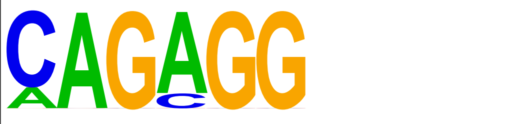	1e-53	-1.231e+02	8.50%	4.46%	11.2bp (14.5bp)	hsa-miR-4757-5p MIMAT0019901 Homo sapiens miR-4757-5p Targets (miRBase)(0.720) More Information | Similar Motifs Found	motif file (matrix)
13	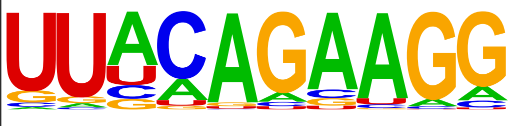	1e-52	-1.219e+02	0.90%	0.08%	6.0bp (5.3bp)	hsa-miR-3182 MIMAT0015062 Homo sapiens miR-3182 Targets (miRBase)(0.748) More Information | Similar Motifs Found	motif file (matrix)
14	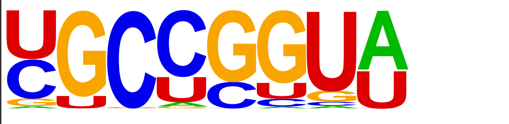	1e-50	-1.166e+02	0.69%	0.05%	8.0bp (4.5bp)	hsa-miR-634 MIMAT0003304 Homo sapiens miR-634 Targets (miRBase)(0.687) More Information | Similar Motifs Found	motif file (matrix)
15	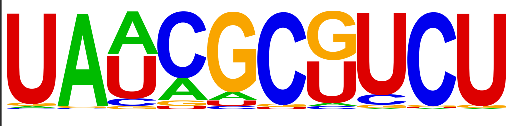	1e-48	-1.126e+02	0.55%	0.03%	8.4bp (5.9bp)	hsa-miR-139-3p MIMAT0004552 Homo sapiens miR-139-3p Targets (miRBase)(0.592) More Information | Similar Motifs Found	motif file (matrix)
16	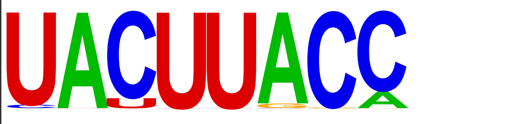	1e-46	-1.075e+02	0.65%	0.05%	6.7bp (5.5bp)	hsa-miR-555 MIMAT0003219 Homo sapiens miR-555 Targets (miRBase)(0.651) More Information | Similar Motifs Found	motif file (matrix)
17	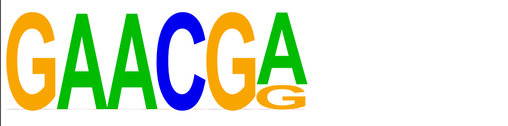	1e-46	-1.064e+02	1.37%	0.23%	8.7bp (11.9bp)	hsa-miR-130b* MIMAT0004680 Homo sapiens miR-130b* Targets (miRBase)(0.607) More Information | Similar Motifs Found	motif file (matrix)
18	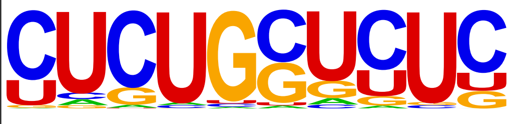	1e-44	-1.015e+02	0.63%	0.04%	9.1bp (5.3bp)	hsa-miR-4468 MIMAT0018995 Homo sapiens miR-4468 Targets (miRBase)(0.757) More Information | Similar Motifs Found	motif file (matrix)
19	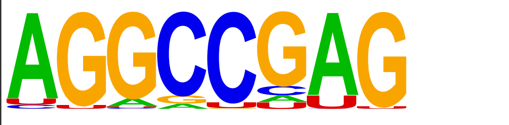	1e-42	-9.788e+01	1.82%	0.44%	9.9bp (10.8bp)	hsa-miR-1234 MIMAT0005589 Homo sapiens miR-1234 Targets (miRBase)(0.721) More Information | Similar Motifs Found	motif file (matrix)
20	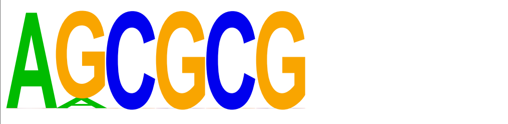	1e-41	-9.519e+01	0.49%	0.00%	12.4bp (0.0bp)	hsa-miR-937 MIMAT0004980 Homo sapiens miR-937 Targets (miRBase)(0.715) More Information | Similar Motifs Found	motif file (matrix)
21	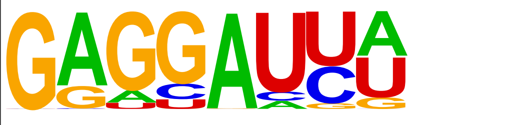	1e-39	-9.098e+01	0.81%	0.09%	8.7bp (8.2bp)	hsa-miR-4326 MIMAT0016888 Homo sapiens miR-4326 Targets (miRBase)(0.641) More Information | Similar Motifs Found	motif file (matrix)
22	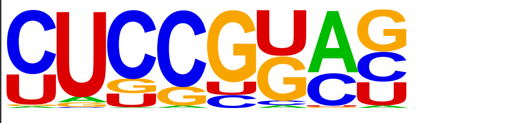	1e-32	-7.461e+01	0.65%	0.08%	10.1bp (8.3bp)	hsa-miR-1203 MIMAT0005866 Homo sapiens miR-1203 Targets (miRBase)(0.645) More Information | Similar Motifs Found	motif file (matrix)
23	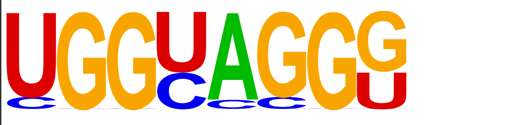	1e-31	-7.274e+01	0.58%	0.06%	8.3bp (8.5bp)	hsa-miR-4722-3p MIMAT0019837 Homo sapiens miR-4722-3p Targets (miRBase)(0.772) More Information | Similar Motifs Found	motif file (matrix)
24	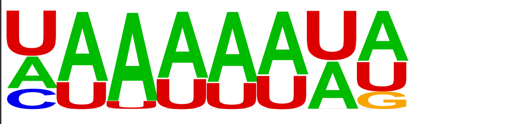	1e-27	-6.221e+01	9.34%	6.18%	5.4bp (12.1bp)	hsa-miR-590-3p MIMAT0004801 Homo sapiens miR-590-3p Targets (miRBase)(0.665) More Information | Similar Motifs Found	motif file (matrix)
25	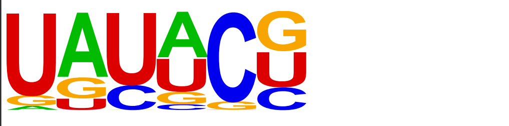	1e-25	-5.927e+01	1.83%	0.66%	7.1bp (9.2bp)	hsa-miR-3669 MIMAT0018092 Homo sapiens miR-3669 Targets (miRBase)(0.606) More Information | Similar Motifs Found	motif file (matrix)
26	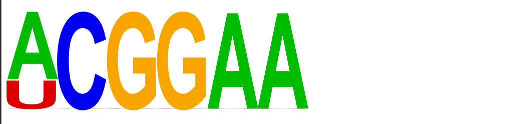	1e-24	-5.634e+01	1.77%	0.64%	7.7bp (14.6bp)	hsa-miR-718 MIMAT0012735 Homo sapiens miR-718 Targets (miRBase)(0.657) More Information | Similar Motifs Found	motif file (matrix)
27	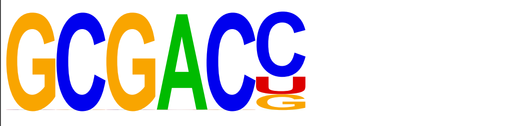	1e-21	-4.911e+01	0.88%	0.22%	9.9bp (16.1bp)	hsa-miR-1181 MIMAT0005826 Homo sapiens miR-1181 Targets (miRBase)(0.695) More Information | Similar Motifs Found	motif file (matrix)
28	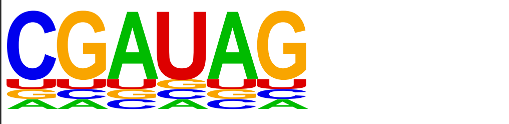	1e-13	-3.213e+01	0.35%	0.05%	7.7bp (12.4bp)	hsa-miR-454* MIMAT0003884 Homo sapiens miR-454* Targets (miRBase)(0.647) More Information | Similar Motifs Found	motif file (matrix)
