# Supplementary material for: SRSF1-mediated alternative splicing is required for spermatogenesis
Source: Int J Biol Sci. 2023 Sep 11;19(15):4883–97. doi: 10.7150/ijbs.83474 (PMC10539708; doi:10.7150/ijbs.83474)
Supplement: Supplementary file 1 — Supplementary figures and tables. [file ijbsv19p4883s1.zip › Supplementary materials/Table 3 The list of SRSF1-binding & differentially expressed genes.pdf]

Names	total	elements
LACE-seq &UP	11	Cwc22
		Trim17
		Hist1h4j
		Hsd17b3
		Hist1h1c
		Cyp7b1
		Hist1h4k
		Hist1h1d
		Trim2
		Epha2
		Hist3h2ba
LACE-seq&DOWN	21	Cntd1
		Reln
		Cacna2d3
		Mpped2
		Wdhd1
		Unc5d
		Dnahc8
		a
		Rnf157
		Col22a1
		Itgae
		Smc1b
		Ccdc136
		Gli3
		Rab26
		Inca1
		Uimc1
		Prss48
		Acsl1
		Srsf1
		Sf3b3
