# Supplementary material for: SRSF1-mediated alternative splicing is required for spermatogenesis
Source: Int J Biol Sci. 2023 Sep 11;19(15):4883–97. doi: 10.7150/ijbs.83474 (PMC10539708; doi:10.7150/ijbs.83474)
Supplement: Supplementary file 1 — Supplementary figures and tables. [file ijbsv19p4883s1.zip › Supplementary materials/Table 4 The list of SRSF1-binding & differentially spliced genes.pdf]

Names	total	elements
AS&LACE-seq	207	Wdr33
		Tom1l2
		Ppp1r12b
		Mpzl1
		Fam40b
		Orc3
		Chd3
		Glyr1
		Icmt
		Bptf
		Gpbp1
		Braf
		Arvcf
		Cep63
		Cadm1
		Whsc1
		Nop56
		Vps13c
		Nab2
		Gak
		Prkci
		Suv420h1
		Tex14
		Prrc2b
		Gnb1l
		Eif4g3
		Erg
		Ubr4
		Csnk1g3
		Ece1
		Bclaf1
		Traf7
		Fam178a
		Dcp1b
		Mycbp2
		4931406P16Rik
		Cbx5
		Mllt4
		Csde1
		Nktr
		Fip1l1
		Myef2
		Kdm4b
		Fyttd1
		Pphln1
		Uhrf1
		Kdm5a
		Hps3
		Paip2
		Eri3
		Prkdc
		Ensa
		Dmrt1
		Fn1
		Zmym2
		Cenpi
		Ezh2

Usp3
Dhx30
Mapk8
Cacna1c
Stxbp4
Ccdc91
Epn2
Arhgap12
Stard9
Usp34
Heatr7a
Gpsm2
Nek1
Slc30a9
Adprh
Pbxip1
Hnrnpa2b1
Rnf157
Rcc1
Dicer1
Stk25
Mov10
Picalm
Ext2
Sestd1
Atg10
Stag1
Ell
Spata5
Ubac1
Wapal
Prrc2c
Kctd3
Appl1
Arhgef18
Hnrpdl
Nsfl1c
Cit
Hnrnph3
Zfp644
Ep400
Bcas3
Birc6
Stx16
Smarce1
Ewsr1
Zbtb20
Dtl
Sipa1l1
Rnf220
Crtc1
Senp6
Chic2
Epn1
Mapk8ip3
Chka
Tnrc6c
Sycp1

Papola
Hells
Zfp384
Pikfyve
Herc1
Smg6
Mll3
Fubp1
Smc1b
Arfip1
Lrrc16a
Ptbp2
Ccdc136
Fgfr1op2
Arhgef11
Amhr2
Rbfox2
Pcnt
Ap1m1
Zfp407
Ccdc88c
Dab1
Thrap3
App
Numa1
Sbf2
Ccdc57
Fam188a
Syt7
Ddx4
Rab26
Eif5
Raly
Hnrnpd
Mrps18c
U2af1
Stra8
Stk11
Nap1l4
Zfp655
2410089E03Rik
Cdk8
Wdfy3
Capn7
Herc2
Abcc1
Casp8ap2
Cdc42bpa
Myh10
Mprip
Akap9
Ivns1abp
Nvl
Prpsap1
2810422J05Rik
Cep110
Cenpf
Pcgf3

Lrrfip2
Mdn1
Uimc1
Ttc13
Ireb2
Wac
Gtdc1
Tatdn2
Gsk3b
Clasp2
Setd5
Dgkd
Pcm1
Huwe1
Zfml
Fam13b
Zmym4
Ddi2
Card10
Ube2e2
Rps24
Atpbd4
Sfrs18
Atf6b
Ncor1
Usp31
Tsga14
Brwd1
Gnl3
Hnrnpa1
Enah
Cdc42se2
Bahcc1
Bcor
