# Supplementary material for: SRSF1-mediated alternative splicing is required for spermatogenesis
Source: Int J Biol Sci. 2023 Sep 11;19(15):4883–97. doi: 10.7150/ijbs.83474 (PMC10539708; doi:10.7150/ijbs.83474)
Supplement: Supplementary file 1 — Supplementary figures and tables. [file ijbsv19p4883s1.zip › Supplementary materials/Table 6 The list of SRSF1-binding genes.pdf]

chr1	4519076	4519097	peak_1	0	+	.	
chr1	4775710	4775731	peak_4	0	-	ENSMUSG	protein_coding
chr1	5092593	5092615	peak_5	0	+	ENSMUSG	protein_coding
chr1	6224066	6224087	peak_6	0	+	ENSMUSG	protein_coding
chr1	6259057	6259079	peak_7	0	+	ENSMUSG	protein_coding
chr1	9735684	9735705	peak_8	0	-	ENSMUSG	protein_coding
chr1	10128816	10128838	peak_10	0	-	ENSMUSG	protein_coding
chr1	12711170	12711191	peak_11	0	+	ENSMUSG	protein_coding
chr1	13132269	13132290	peak_12	0	-	ENSMUSG	protein_coding
chr1	13273119	13273145	peak_13	0	-	ENSMUSG	protein_coding
chr1	13316573	13316594	peak_14	0	-	ENSMUSG	protein_coding
chr1	13358476	13358497	peak_15	0	-	ENSMUSG	protein_coding
chr1	13361772	13361794	peak_16	0	-	ENSMUSG	protein_coding
chr1	13556446	13556467	peak_17	0	-	ENSMUSG	protein_coding
chr1	13617497	13617520	peak_18	0	-	ENSMUSG	protein_coding
chr1	15404850	15404871	peak_19	0	+	ENSMUSG	protein_coding
chr1	16636723	16636744	peak_20	0	-	ENSMUSG	protein_coding
chr1	21777326	21777347	peak_22	0	-	ENSMUSG	protein_coding
chr1	21801995	21802018	peak_23	0	-	ENSMUSG	protein_coding
chr1	24019252	24019273	peak_24	0	-	ENSMUSG	protein_coding
chr1	25336369	25336391	peak_25	0	-	ENSMUSG	protein_coding
chr1	30860839	30860881	peak_26	0	-	ENSMUSG	protein_coding
chr1	34077935	34077956	peak_27	0	+	ENSMUSG	protein_coding
chr1	34191986	34192007	peak_28	0	+	ENSMUSG	protein_coding
chr1	34221028	34221049	peak_29	0	+	ENSMUSG	protein_coding
chr1	34221056	34221077	peak_30	0	+	ENSMUSG	protein_coding
chr1	34221171	34221192	peak_31	0	+	ENSMUSG	protein_coding
chr1	34224695	34224716	peak_32	0	+	ENSMUSG	protein_coding
chr1	34224802	34224843	peak_33	0	+	ENSMUSG	protein_coding
chr1	34242809	34242830	peak_34	0	+	ENSMUSG	protein_coding
chr1	34243742	34243764	peak_35	0	+	ENSMUSG	protein_coding
chr1	34268070	34268091	peak_36	0	+	ENSMUSG	protein_coding
chr1	34278795	34278816	peak_37	0	+	ENSMUSG	protein_coding
chr1	34301332	34301354	peak_38	0	+	ENSMUSG	protein_coding
chr1	34304524	34304545	peak_39	0	+	ENSMUSG	protein_coding
chr1	34318852	34318873	peak_40	0	+	ENSMUSG	protein_coding
chr1	34318911	34318932	peak_41	0	+	ENSMUSG	protein_coding
chr1	34319002	34319023	peak_42	0	+	ENSMUSG	protein_coding
chr1	34320118	34320139	peak_43	0	+	ENSMUSG	protein_coding
chr1	34321655	34321676	peak_44	0	+	ENSMUSG	protein_coding
chr1	34323713	34323738	peak_45	0	+	ENSMUSG	protein_coding
chr1	34323742	34323763	peak_46	0	+	ENSMUSG	protein_coding
chr1	34347296	34347317	peak_47	0	+	ENSMUSG	protein_coding
chr1	34352121	34352143	peak_48	0	+	ENSMUSG	protein_coding
chr1	34352298	34352319	peak_49	0	+	ENSMUSG	protein_coding
chr1	34814995	34815016	peak_51	0	+	ENSMUSG	protein_coding
chr1	36411334	36411355	peak_52	0	-	ENSMUSG	protein_coding
chr1	36411665	36411686	peak_53	0	-	ENSMUSG	protein_coding
chr1	36414916	36414950	peak_54	0	-	ENSMUSG	protein_coding
chr1	36415000	36415021	peak_55	0	-	ENSMUSG	protein_coding
chr1	36424085	36424106	peak_56	0	-	ENSMUSG	protein_coding
chr1	36868392	36868418	peak_58	0	-	ENSMUSG	protein_coding
chr1	38076309	38076330	peak_59	0	+	ENSMUSG	protein_coding
chr1	38304994	38305015	peak_60	0	-	ENSMUSG	protein_coding
chr1	38373802	38373823	peak_61	0	-	ENSMUSG	protein_coding
chr1	38429040	38429061	peak_62	0	-	ENSMUSG	protein_coding
chr1	38553685	38553706	peak_63	0	-	ENSMUSG	protein_coding
chr1	38602760	38602781	peak_64	0	-	ENSMUSG	protein_coding

chr1	38652949	38652971	peak_65	0	-	ENSMUSG	protein_coding
chr1	39048113	39048134	peak_66	0	+	ENSMUSG	protein_coding
chr1	39051747	39051768	peak_67	0	+	ENSMUSG	protein_coding
chr1	39427869	39427890	peak_68	0	+	ENSMUSG	protein_coding
chr1	39962019	39962041	peak_71	0	+	ENSMUSG	protein_coding
chr1	39980492	39980513	peak_72	0	+	ENSMUSG	protein_coding
chr1	39997611	39997633	peak_73	0	+	ENSMUSG	protein_coding
chr1	40022122	40022143	peak_74	0	+	ENSMUSG	protein_coding
chr1	40033565	40033586	peak_75	0	+	ENSMUSG	protein_coding
chr1	40043592	40043613	peak_76	0	+	ENSMUSG	protein_coding
chr1	40082073	40082094	peak_77	0	+	ENSMUSG	protein_coding
chr1	42941440	42941461	peak_78	0	+	ENSMUSG	protein_coding
chr1	42950771	42950792	peak_79	0	+	ENSMUSG	protein_coding
chr1	43593879	43593900	peak_81	0	+	ENSMUSG	protein_coding
chr1	43829172	43829193	peak_82	0	-	ENSMUSG	protein_coding
chr1	43835771	43835794	peak_83	0	-	ENSMUSG	protein_coding
chr1	43864199	43864220	peak_84	0	-	ENSMUSG	protein_coding
chr1	44003615	44003636	peak_85	0	+	ENSMUSG	protein_coding
chr1	44012188	44012209	peak_86	0	+	ENSMUSG	protein_coding
chr1	45368453	45368474	peak_87	0	+	ENSMUSG	protein_coding
chr1	45368567	45368588	peak_88	0	+	ENSMUSG	protein_coding
chr1	45391132	45391153	peak_89	0	+	ENSMUSG	protein_coding
chr1	45402889	45402910	peak_90	0	+	ENSMUSG	protein_coding
chr1	45405427	45405448	peak_91	0	+	ENSMUSG	protein_coding
chr1	51530738	51530759	peak_93	0	-	ENSMUSG	protein_coding
chr1	52208556	52208577	peak_94	0	+	ENSMUSG	protein_coding
chr1	52268278	52268299	peak_95	0	-	ENSMUSG	protein_coding
chr1	54916243	54916264	peak_96	0	-	ENSMUSG	protein_coding
chr1	55058227	55058248	peak_97	0	-	ENSMUSG	protein_coding
chr1	55058385	55058408	peak_98	0	-	ENSMUSG	protein_coding
chr1	55084240	55084261	peak_99	0	-	ENSMUSG	protein_coding
chr1	58451041	58451062	peak_100	0	+	ENSMUSG	protein_coding
chr1	58473125	58473146	peak_101	0	-	ENSMUSG	protein_coding
chr1	58477106	58477127	peak_102	0	-	ENSMUSG	protein_coding
chr1	58622721	58622742	peak_103	0	-	ENSMUSG	protein_coding
chr1	59003151	59003173	peak_104	0	-	ENSMUSG	protein_coding
chr1	59268172	59268195	peak_105	0	-	ENSMUSG	protein_coding
chr1	59748764	59748785	peak_106	0	+	ENSMUSG	protein_coding
chr1	59749339	59749361	peak_107	0	+	ENSMUSG	protein_coding
chr1	59761083	59761105	peak_108	0	+	ENSMUSG	protein_coding
chr1	59761614	59761653	peak_109	0	+	ENSMUSG	protein_coding
chr1	59767323	59767345	peak_110	0	+	ENSMUSG	protein_coding
chr1	59925431	59925452	peak_111	0	+	ENSMUSG	protein_coding
chr1	60466578	60466599	peak_112	0	+	ENSMUSG	protein_coding
chr1	60572287	60572308	peak_113	0	-	ENSMUSG	protein_coding
chr1	61827090	61827111	peak_114	0	+	ENSMUSG	protein_coding
chr1	61881318	61881339	peak_115	0	+	ENSMUSG	protein_coding
chr1	61922050	61922071	peak_116	0	+	ENSMUSG	protein_coding
chr1	63193893	63193914	peak_117	0	+	ENSMUSG	protein_coding
chr1	63223688	63223709	peak_118	0	+	ENSMUSG	protein_coding
chr1	63225555	63225576	peak_119	0	+	ENSMUSG	protein_coding
chr1	63225650	63225671	peak_120	0	+	ENSMUSG	protein_coding
chr1	65218406	65218427	peak_121	0	-	ENSMUSG	protein_coding
chr1	65237044	65237065	peak_122	0	+	ENSMUSG	protein_coding
chr1	65302142	65302163	peak_123	0	+	ENSMUSG	protein_coding
chr1	66272353	66272376	peak_124	0	+	ENSMUSG	protein_coding
chr1	66799893	66799916	peak_125	0	+	ENSMUSG	protein_coding
chr1	68374715	68374737	peak_126	0	-	ENSMUSG	protein_coding

chr1	71643891	71643916	peak_128	0	-	ENSMUSG	protein_coding
chr1	72632856	72632890	peak_134	0	+	ENSMUSG	protein_coding
chr1	72758773	72758794	peak_135	0	+	ENSMUSG	protein_coding
chr1	74022464	74022485	peak_136	0	-	ENSMUSG	protein_coding
chr1	74331249	74331270	peak_137	0	-	ENSMUSG	protein_coding
chr1	74442858	74442880	peak_138	0	+	ENSMUSG	protein_coding
chr1	74742964	74742995	peak_139	0	+	ENSMUSG	protein_coding
chr1	74745101	74745126	peak_140	0	+	ENSMUSG	protein_coding
chr1	74745149	74745170	peak_141	0	+	ENSMUSG	protein_coding
chr1	74825881	74825902	peak_142	0	+	ENSMUSG	protein_coding
chr1	74825945	74825967	peak_143	0	+	ENSMUSG	protein_coding
chr1	75206352	75206374	peak_144	0	-	ENSMUSG	protein_coding
chr1	75408971	75408992	peak_145	0	+	ENSMUSG	protein_coding
chr1	75493722	75493743	peak_146	0	-	ENSMUSG	protein_coding
chr1	75496548	75496569	peak_147	0	-	ENSMUSG	protein_coding
chr1	75500037	75500058	peak_148	0	-	ENSMUSG	protein_coding
chr1	75500411	75500433	peak_149	0	-	ENSMUSG	protein_coding
chr1	78661718	78661739	peak_150	0	+	ENSMUSG	protein_coding
chr1	78661718	78661739	peak_150	0	+	ENSMUSG	protein_coding
chr1	79795893	79795916	peak_151	0	-	ENSMUSG	protein_coding
chr1	80286694	80286715	peak_153	0	-	ENSMUSG	protein_coding
chr1	80317854	80317875	peak_154	0	-	ENSMUSG	protein_coding
chr1	82447355	82447378	peak_156	0	-	ENSMUSG	protein_coding
chr1	82453290	82453311	peak_157	0	-	ENSMUSG	protein_coding
chr1	82524448	82524469	peak_158	0	-	ENSMUSG	protein_coding
chr1	82659900	82659921	peak_159	0	+	ENSMUSG	protein_coding
chr1	84724519	84724546	peak_160	0	-	ENSMUSG	protein_coding
chr1	84754085	84754106	peak_161	0	-	ENSMUSG	protein_coding
chr1	84755730	84755752	peak_162	0	-	ENSMUSG	protein_coding
chr1	84757361	84757382	peak_163	0	-	ENSMUSG	protein_coding
chr1	84774266	84774288	peak_164	0	-	ENSMUSG	protein_coding
chr1	85254863	85254884	peak_165	0	+	ENSMUSG	protein_coding
chr1	87801804	87801825	peak_166	0	+	ENSMUSG	protein_coding
chr1	88247871	88247892	peak_167	0	-	ENSMUSG	protein_coding
chr1	88248662	88248683	peak_168	0	-	ENSMUSG	protein_coding
chr1	88250386	88250407	peak_169	0	-	ENSMUSG	protein_coding
chr1	88252902	88252925	peak_170	0	-	ENSMUSG	protein_coding
chr1	88423500	88423521	peak_171	0	+	ENSMUSG	protein_coding
chr1	88425722	88425743	peak_172	0	+	ENSMUSG	protein_coding
chr1	89188852	89188874	peak_173	0	+	ENSMUSG	protein_coding
chr1	89250988	89251009	peak_174	0	+	ENSMUSG	protein_coding
chr1	89256096	89256117	peak_175	0	+	ENSMUSG	protein_coding
chr1	89261410	89261431	peak_176	0	+	ENSMUSG	protein_coding
chr1	89262122	89262157	peak_177	0	+	ENSMUSG	protein_coding
chr1	89321620	89321641	peak_178	0	+	ENSMUSG	protein_coding
chr1	89757416	89757437	peak_179	0	+	ENSMUSG	protein_coding
chr1	89790975	89790996	peak_180	0	+	ENSMUSG	protein_coding
chr1	89797178	89797199	peak_181	0	+	ENSMUSG	protein_coding
chr1	91389130	91389151	peak_182	0	+	ENSMUSG	protein_coding
chr1	91453412	91453433	peak_183	0	+	ENSMUSG	protein_coding
chr1	91482251	91482272	peak_184	0	+	ENSMUSG	protein_coding
chr1	91523149	91523170	peak_185	0	+	ENSMUSG	protein_coding
chr1	91546657	91546679	peak_186	0	+	ENSMUSG	protein_coding
chr1	91669807	91669828	peak_187	0	+	ENSMUSG	protein_coding
chr1	91685367	91685389	peak_188	0	+	ENSMUSG	protein_coding
chr1	91692981	91693004	peak_189	0	+	ENSMUSG	protein_coding
chr1	91716335	91716356	peak_190	0	+	ENSMUSG	protein_coding
chr1	91762023	91762045	peak_191	0	+	ENSMUSG	protein_coding

chr1	91870437	91870458	peak_192	0	-	ENSMUSG	protein_coding
chr1	92700440	92700461	peak_193	0	-	ENSMUSG	protein_coding
chr1	92707929	92707950	peak_194	0	-	ENSMUSG	protein_coding
chr1	93974896	93974917	peak_196	0	-	ENSMUSG	protein_coding
chr1	94033258	94033280	peak_197	0	-	ENSMUSG	protein_coding
chr1	94036667	94036688	peak_198	0	-	ENSMUSG	protein_coding
chr1	94963657	94963678	peak_199	0	-	ENSMUSG	protein_coding
chr1	95317953	95317978	peak_201	0	-	ENSMUSG	protein_coding
chr1	95392205	95392226	peak_202	0	+	ENSMUSG	protein_coding
chr1	95525719	95525741	peak_203	0	-	ENSMUSG	protein_coding
chr1	95659371	95659393	peak_204	0	+	ENSMUSG	protein_coding
chr1	95708383	95708404	peak_205	0	+	ENSMUSG	protein_coding
chr1	1.07E+08	1.07E+08	peak_209	0	-	ENSMUSG	protein_coding
chr1	1.2E+08	1.2E+08	peak_212	0	-	ENSMUSG	protein_coding
chr1	1.2E+08	1.2E+08	peak_213	0	+	ENSMUSG	protein_coding
chr1	1.21E+08	1.21E+08	peak_214	0	-	ENSMUSG	protein_coding
chr1	1.21E+08	1.21E+08	peak_215	0	-	ENSMUSG	protein_coding
chr1	1.21E+08	1.21E+08	peak_216	0	-	ENSMUSG	protein_coding
chr1	1.21E+08	1.21E+08	peak_217	0	-	ENSMUSG	protein_coding
chr1	1.22E+08	1.22E+08	peak_218	0	-	ENSMUSG	protein_coding
chr1	1.27E+08	1.27E+08	peak_221	0	-	ENSMUSG	protein_coding
chr1	1.27E+08	1.27E+08	peak_222	0	-	ENSMUSG	protein_coding
chr1	1.28E+08	1.28E+08	peak_223	0	-	ENSMUSG	protein_coding
chr1	1.28E+08	1.28E+08	peak_224	0	-	ENSMUSG	protein_coding
chr1	1.28E+08	1.28E+08	peak_225	0	-	ENSMUSG	protein_coding
chr1	1.29E+08	1.29E+08	peak_226	0	+	ENSMUSG	protein_coding
chr1	1.3E+08	1.3E+08	peak_227	0	-	ENSMUSG	protein_coding
chr1	1.3E+08	1.3E+08	peak_229	0	-	ENSMUSG	protein_coding
chr1	1.33E+08	1.33E+08	peak_230	0	-	ENSMUSG	protein_coding
chr1	1.33E+08	1.33E+08	peak_231	0	-	ENSMUSG	protein_coding
chr1	1.33E+08	1.33E+08	peak_232	0	-	ENSMUSG	protein_coding
chr1	1.34E+08	1.34E+08	peak_233	0	+	ENSMUSG	protein_coding
chr1	1.34E+08	1.34E+08	peak_234	0	+	ENSMUSG	protein_coding
chr1	1.34E+08	1.34E+08	peak_235	0	-	ENSMUSG	protein_coding
chr1	1.34E+08	1.34E+08	peak_236	0	+	ENSMUSG	protein_coding
chr1	1.34E+08	1.34E+08	peak_237	0	+	ENSMUSG	protein_coding
chr1	1.35E+08	1.35E+08	peak_238	0	-	ENSMUSG	protein_coding
chr1	1.35E+08	1.35E+08	peak_239	0	-	ENSMUSG	protein_coding
chr1	1.36E+08	1.36E+08	peak_242	0	-	ENSMUSG	protein_coding
chr1	1.36E+08	1.36E+08	peak_243	0	-	ENSMUSG	protein_coding
chr1	1.36E+08	1.36E+08	peak_244	0	-	ENSMUSG	protein_coding
chr1	1.36E+08	1.36E+08	peak_245	0	-	ENSMUSG	protein_coding
chr1	1.36E+08	1.36E+08	peak_246	0	-	ENSMUSG	protein_coding
chr1	1.36E+08	1.36E+08	peak_247	0	-	ENSMUSG	protein_coding
chr1	1.36E+08	1.36E+08	peak_248	0	-	ENSMUSG	protein_coding
chr1	1.36E+08	1.36E+08	peak_249	0	-	ENSMUSG	protein_coding
chr1	1.36E+08	1.36E+08	peak_250	0	-	ENSMUSG	protein_coding
chr1	1.36E+08	1.36E+08	peak_251	0	-	ENSMUSG	protein_coding
chr1	1.36E+08	1.36E+08	peak_252	0	+	ENSMUSG	protein_coding
chr1	1.36E+08	1.36E+08	peak_253	0	+	ENSMUSG	protein_coding
chr1	1.36E+08	1.36E+08	peak_254	0	+	ENSMUSG	protein_coding
chr1	1.37E+08	1.37E+08	peak_255	0	+	ENSMUSG	protein_coding
chr1	1.37E+08	1.37E+08	peak_256	0	+	ENSMUSG	protein_coding
chr1	1.37E+08	1.37E+08	peak_257	0	-	ENSMUSG	protein_coding
chr1	1.37E+08	1.37E+08	peak_258	0	-	ENSMUSG	protein_coding
chr1	1.37E+08	1.37E+08	peak_259	0	-	ENSMUSG	protein_coding
chr1	1.37E+08	1.37E+08	peak_260	0	-	ENSMUSG	protein_coding
chr1	1.37E+08	1.37E+08	peak_262	0	+	ENSMUSG	protein_coding

chr1	1.37E+08	1.37E+08	peak_263	0	-	ENSMUSG	protein_coding
chr1	1.37E+08	1.37E+08	peak_264	0	-	ENSMUSG	protein_coding
chr1	1.38E+08	1.38E+08	peak_265	0	-	ENSMUSG	protein_coding
chr1	1.41E+08	1.41E+08	peak_267	0	+	ENSMUSG	protein_coding
chr1	1.41E+08	1.41E+08	peak_268	0	+	ENSMUSG	protein_coding
chr1	1.41E+08	1.41E+08	peak_269	0	+	ENSMUSG	protein_coding
chr1	1.46E+08	1.46E+08	peak_270	0	-	ENSMUSG	protein_coding
chr1	1.53E+08	1.53E+08	peak_272	0	-	ENSMUSG	protein_coding
chr1	1.53E+08	1.53E+08	peak_273	0	+	ENSMUSG	protein_coding
chr1	1.53E+08	1.53E+08	peak_274	0	+	ENSMUSG	protein_coding
chr1	1.53E+08	1.53E+08	peak_275	0	-	ENSMUSG	protein_coding
chr1	1.53E+08	1.53E+08	peak_276	0	-	ENSMUSG	protein_coding
chr1	1.54E+08	1.54E+08	peak_277	0	-	ENSMUSG	protein_coding
chr1	1.54E+08	1.54E+08	peak_278	0	+	ENSMUSG	protein_coding
chr1	1.54E+08	1.54E+08	peak_281	0	-	ENSMUSG	protein_coding
chr1	1.54E+08	1.54E+08	peak_282	0	-	ENSMUSG	protein_coding
chr1	1.55E+08	1.55E+08	peak_283	0	+	ENSMUSG	protein_coding
chr1	1.55E+08	1.55E+08	peak_284	0	-	ENSMUSG	protein_coding
chr1	1.55E+08	1.55E+08	peak_285	0	-	ENSMUSG	protein_coding
chr1	1.55E+08	1.55E+08	peak_286	0	-	ENSMUSG	protein_coding
chr1	1.55E+08	1.55E+08	peak_287	0	-	ENSMUSG	protein_coding
chr1	1.57E+08	1.57E+08	peak_288	0	-	ENSMUSG	protein_coding
chr1	1.57E+08	1.57E+08	peak_289	0	-	ENSMUSG	protein_coding
chr1	1.57E+08	1.57E+08	peak_290	0	-	ENSMUSG	protein_coding
chr1	1.58E+08	1.58E+08	peak_291	0	-	ENSMUSG	protein_coding
chr1	1.58E+08	1.58E+08	peak_292	0	-	ENSMUSG	protein_coding
chr1	1.58E+08	1.58E+08	peak_293	0	-	ENSMUSG	protein_coding
chr1	1.58E+08	1.58E+08	peak_294	0	+	ENSMUSG	protein_coding
chr1	1.58E+08	1.58E+08	peak_294	0	+	ENSMUSG	protein_coding
chr1	1.61E+08	1.61E+08	peak_296	0	+	ENSMUSG	protein_coding
chr1	1.62E+08	1.62E+08	peak_297	0	+	ENSMUSG	protein_coding
chr1	1.62E+08	1.62E+08	peak_298	0	-	ENSMUSG	protein_coding
chr1	1.63E+08	1.63E+08	peak_299	0	+	ENSMUSG	protein_coding
chr1	1.63E+08	1.63E+08	peak_300	0	+	ENSMUSG	protein_coding
chr1	1.63E+08	1.63E+08	peak_301	0	+	ENSMUSG	protein_coding
chr1	1.63E+08	1.63E+08	peak_302	0	-	ENSMUSG	protein_coding
chr1	1.64E+08	1.64E+08	peak_311	0	-	ENSMUSG	protein_coding
chr1	1.65E+08	1.65E+08	peak_312	0	-	ENSMUSG	protein_coding
chr1	1.65E+08	1.65E+08	peak_313	0	-	ENSMUSG	protein_coding
chr1	1.65E+08	1.65E+08	peak_314	0	-	ENSMUSG	protein_coding
chr1	1.65E+08	1.65E+08	peak_315	0	-	ENSMUSG	protein_coding
chr1	1.65E+08	1.65E+08	peak_316	0	-	ENSMUSG	protein_coding
chr1	1.65E+08	1.65E+08	peak_317	0	-	ENSMUSG	protein_coding
chr1	1.65E+08	1.65E+08	peak_318	0	-	ENSMUSG	protein_coding
chr1	1.65E+08	1.65E+08	peak_319	0	-	ENSMUSG	protein_coding
chr1	1.65E+08	1.65E+08	peak_320	0	-	ENSMUSG	protein_coding
chr1	1.65E+08	1.65E+08	peak_321	0	-	ENSMUSG	protein_coding
chr1	1.66E+08	1.66E+08	peak_322	0	+	ENSMUSG	protein_coding
chr1	1.66E+08	1.66E+08	peak_323	0	+	ENSMUSG	protein_coding
chr1	1.66E+08	1.66E+08	peak_324	0	+	ENSMUSG	protein_coding
chr1	1.67E+08	1.67E+08	peak_325	0	-	ENSMUSG	protein_coding
chr1	1.68E+08	1.68E+08	peak_326	0	-	ENSMUSG	protein_coding
chr1	1.68E+08	1.68E+08	peak_327	0	-	ENSMUSG	protein_coding
chr1	1.7E+08	1.7E+08	peak_329	0	-	ENSMUSG	protein_coding
chr1	1.7E+08	1.7E+08	peak_330	0	-	ENSMUSG	protein_coding
chr1	1.7E+08	1.7E+08	peak_331	0	-	ENSMUSG	protein_coding
chr1	1.7E+08	1.7E+08	peak_332	0	-	ENSMUSG	protein_coding
chr1	1.7E+08	1.7E+08	peak_333	0	-	ENSMUSG	protein_coding

chr1	1.7E+08	1.7E+08	peak_334	0	-	ENSMUSG	protein_coding
chr1	1.7E+08	1.7E+08	peak_335	0	-	ENSMUSG	protein_coding
chr1	1.7E+08	1.7E+08	peak_336	0	-	ENSMUSG	protein_coding
chr1	1.7E+08	1.7E+08	peak_337	0	-	ENSMUSG	protein_coding
chr1	1.7E+08	1.7E+08	peak_338	0	-	ENSMUSG	protein_coding
chr1	1.72E+08	1.72E+08	peak_339	0	-	ENSMUSG	protein_coding
chr1	1.72E+08	1.72E+08	peak_340	0	+	ENSMUSG	protein_coding
chr1	1.73E+08	1.73E+08	peak_341	0	-	ENSMUSG	protein_coding
chr1	1.73E+08	1.73E+08	peak_348	0	+	ENSMUSG	protein_coding
chr1	1.73E+08	1.73E+08	peak_349	0	-	ENSMUSG	protein_coding
chr1	1.74E+08	1.74E+08	peak_350	0	+	ENSMUSG	protein_coding
chr1	1.74E+08	1.74E+08	peak_351	0	+	ENSMUSG	protein_coding
chr1	1.79E+08	1.79E+08	peak_353	0	-	ENSMUSG	protein_coding
chr1	1.8E+08	1.8E+08	peak_354	0	-	ENSMUSG	protein_coding
chr1	1.8E+08	1.8E+08	peak_355	0	-	ENSMUSG	protein_coding
chr1	1.8E+08	1.8E+08	peak_355	0	-	ENSMUSG	protein_coding
chr1	1.8E+08	1.8E+08	peak_356	0	+	ENSMUSG	protein_coding
chr1	1.8E+08	1.8E+08	peak_357	0	-	ENSMUSG	protein_coding
chr1	1.81E+08	1.81E+08	peak_358	0	+	ENSMUSG	protein_coding
chr1	1.81E+08	1.81E+08	peak_359	0	-	ENSMUSG	protein_coding
chr1	1.81E+08	1.81E+08	peak_360	0	-	ENSMUSG	protein_coding
chr1	1.82E+08	1.82E+08	peak_361	0	-	ENSMUSG	protein_coding
chr1	1.82E+08	1.82E+08	peak_362	0	+	ENSMUSG	protein_coding
chr1	1.82E+08	1.82E+08	peak_363	0	-	ENSMUSG	protein_coding
chr1	1.82E+08	1.82E+08	peak_364	0	-	ENSMUSG	protein_coding
chr1	1.82E+08	1.82E+08	peak_365	0	+	ENSMUSG	protein_coding
chr1	1.83E+08	1.83E+08	peak_366	0	+	ENSMUSG	protein_coding
chr1	1.83E+08	1.83E+08	peak_367	0	-	ENSMUSG	protein_coding
chr1	1.83E+08	1.83E+08	peak_368	0	-	ENSMUSG	protein_coding
chr1	1.83E+08	1.83E+08	peak_369	0	-	ENSMUSG	protein_coding
chr1	1.83E+08	1.83E+08	peak_370	0	-	ENSMUSG	protein_coding
chr1	1.83E+08	1.83E+08	peak_371	0	-	ENSMUSG	protein_coding
chr1	1.84E+08	1.84E+08	peak_373	0	-	ENSMUSG	protein_coding
chr1	1.84E+08	1.84E+08	peak_374	0	-	ENSMUSG	protein_coding
chr1	1.87E+08	1.87E+08	peak_375	0	-	ENSMUSG	protein_coding
chr1	1.87E+08	1.87E+08	peak_376	0	+	ENSMUSG	protein_coding
chr1	1.87E+08	1.87E+08	peak_377	0	+	ENSMUSG	protein_coding
chr1	1.89E+08	1.89E+08	peak_379	0	+	ENSMUSG	protein_coding
chr1	1.89E+08	1.89E+08	peak_380	0	+	ENSMUSG	protein_coding
chr1	1.91E+08	1.91E+08	peak_381	0	-	ENSMUSG	protein_coding
chr1	1.91E+08	1.91E+08	peak_382	0	-	ENSMUSG	protein_coding
chr1	1.91E+08	1.91E+08	peak_383	0	-	ENSMUSG	protein_coding
chr1	1.91E+08	1.91E+08	peak_385	0	-	ENSMUSG	protein_coding
chr1	1.92E+08	1.92E+08	peak_386	0	-	ENSMUSG	protein_coding
chr1	1.92E+08	1.92E+08	peak_387	0	-	ENSMUSG	protein_coding
chr1	1.93E+08	1.93E+08	peak_388	0	-	ENSMUSG	protein_coding
chr1	1.93E+08	1.93E+08	peak_389	0	-	ENSMUSG	protein_coding
chr1	1.95E+08	1.95E+08	peak_390	0	+	ENSMUSG	protein_coding
chr1	1.97E+08	1.97E+08	peak_391	0	+	ENSMUSG	protein_coding
chr10	3142199	3142220	peak_393	0	+	ENSMUSG	protein_coding
chr10	3202592	3202613	peak_394	0	+	ENSMUSG	protein_coding
chr10	5991293	5991315	peak_395	0	-	ENSMUSG	protein_coding
chr10	6009201	6009223	peak_396	0	-	ENSMUSG	protein_coding
chr10	6080176	6080199	peak_397	0	-	ENSMUSG	protein_coding
chr10	6394809	6394830	peak_398	0	-	ENSMUSG	protein_coding
chr10	7452824	7452845	peak_399	0	+	ENSMUSG	protein_coding
chr10	7453156	7453177	peak_400	0	+	ENSMUSG	protein_coding
chr10	8190569	8190590	peak_401	0	-	ENSMUSG	protein_coding

chr10	8553632	8553653	peak_402	0	-	ENSMUSG	protein_coding
chr10	12356509	12356530	peak_403	0	-	ENSMUSG	protein_coding
chr10	12434044	12434065	peak_404	0	-	ENSMUSG	protein_coding
chr10	12454937	12454958	peak_405	0	-	ENSMUSG	protein_coding
chr10	12459994	12460015	peak_406	0	-	ENSMUSG	protein_coding
chr10	12951571	12951598	peak_408	0	-	ENSMUSG	protein_coding
chr10	12965284	12965305	peak_409	0	-	ENSMUSG	protein_coding
chr10	13499693	13499722	peak_410	0	-	ENSMUSG	protein_coding
chr10	17822535	17822557	peak_411	0	+	ENSMUSG	protein_coding
chr10	18244457	18244481	peak_412	0	+	ENSMUSG	protein_coding
chr10	19869875	19869901	peak_415	0	+	ENSMUSG	protein_coding
chr10	20043603	20043625	peak_416	0	+	ENSMUSG	protein_coding
chr10	23505437	23505458	peak_417	0	-	ENSMUSG	protein_coding
chr10	23505617	23505648	peak_418	0	-	ENSMUSG	protein_coding
chr10	23506347	23506369	peak_419	0	-	ENSMUSG	protein_coding
chr10	24315823	24315844	peak_421	0	+	ENSMUSG	protein_coding
chr10	24317364	24317385	peak_422	0	+	ENSMUSG	protein_coding
chr10	24613764	24613785	peak_423	0	+	ENSMUSG	protein_coding
chr10	25096685	25096707	peak_424	0	+	ENSMUSG	protein_coding
chr10	25100044	25100065	peak_425	0	+	ENSMUSG	protein_coding
chr10	25175373	25175395	peak_426	0	+	ENSMUSG	protein_coding
chr10	25221631	25221652	peak_427	0	+	ENSMUSG	protein_coding
chr10	25221861	25221882	peak_428	0	+	ENSMUSG	protein_coding
chr10	27309255	27309276	peak_429	0	-	ENSMUSG	protein_coding
chr10	27824560	27824587	peak_430	0	+	ENSMUSG	protein_coding
chr10	27868949	27868974	peak_431	0	+	ENSMUSG	protein_coding
chr10	30392751	30392772	peak_434	0	-	ENSMUSG	protein_coding
chr10	30488675	30488696	peak_435	0	-	ENSMUSG	protein_coding
chr10	31099113	31099135	peak_437	0	-	ENSMUSG	protein_coding
chr10	36695977	36695998	peak_438	0	+	ENSMUSG	protein_coding
chr10	36711564	36711585	peak_439	0	+	ENSMUSG	protein_coding
chr10	39152788	39152809	peak_440	0	+	ENSMUSG	protein_coding
chr10	39176611	39176632	peak_441	0	+	ENSMUSG	protein_coding
chr10	39206447	39206468	peak_442	0	+	ENSMUSG	protein_coding
chr10	39235443	39235465	peak_443	0	+	ENSMUSG	protein_coding
chr10	39245263	39245284	peak_444	0	+	ENSMUSG	protein_coding
chr10	39283592	39283629	peak_445	0	+	ENSMUSG	protein_coding
chr10	39459532	39459553	peak_446	0	+	ENSMUSG	protein_coding
chr10	39544738	39544759	peak_447	0	+	ENSMUSG	protein_coding
chr10	39569056	39569079	peak_448	0	+	ENSMUSG	protein_coding
chr10	40619865	40619886	peak_450	0	+	ENSMUSG	protein_coding
chr10	40939298	40939319	peak_451	0	-	ENSMUSG	protein_coding
chr10	41521590	41521632	peak_452	0	-	ENSMUSG	protein_coding
chr10	41537494	41537515	peak_453	0	+	ENSMUSG	protein_coding
chr10	41944108	41944130	peak_454	0	-	ENSMUSG	protein_coding
chr10	41968793	41968814	peak_455	0	-	ENSMUSG	protein_coding
chr10	41978513	41978534	peak_456	0	-	ENSMUSG	protein_coding
chr10	41979549	41979578	peak_457	0	-	ENSMUSG	protein_coding
chr10	41979901	41979924	peak_458	0	-	ENSMUSG	protein_coding
chr10	41983374	41983396	peak_459	0	-	ENSMUSG	protein_coding
chr10	41985118	41985139	peak_460	0	-	ENSMUSG	protein_coding
chr10	42514561	42514582	peak_461	0	+	ENSMUSG	protein_coding
chr10	42538327	42538350	peak_462	0	+	ENSMUSG	protein_coding
chr10	42751949	42751970	peak_463	0	-	ENSMUSG	protein_coding
chr10	42779559	42779580	peak_464	0	-	ENSMUSG	protein_coding
chr10	42819048	42819071	peak_465	0	-	ENSMUSG	protein_coding
chr10	42826940	42826961	peak_466	0	-	ENSMUSG	protein_coding
chr10	42827786	42827808	peak_467	0	-	ENSMUSG	protein_coding

chr10	42838036	42838057	peak_468	0	-	ENSMUSG	protein_coding
chr10	42873062	42873083	peak_469	0	-	ENSMUSG	protein_coding
chr10	43176346	43176367	peak_470	0	-	ENSMUSG	protein_coding
chr10	44810842	44810866	peak_472	0	+	ENSMUSG	protein_coding
chr10	44850388	44850409	peak_473	0	+	ENSMUSG	protein_coding
chr10	45372386	45372407	peak_474	0	+	ENSMUSG	protein_coding
chr10	50338009	50338030	peak_475	0	+	ENSMUSG	protein_coding
chr10	50474365	50474386	peak_476	0	+	ENSMUSG	protein_coding
chr10	52096506	52096527	peak_477	0	-	ENSMUSG	protein_coding
chr10	56104636	56104658	peak_478	0	+	ENSMUSG	protein_coding
chr10	57807229	57807251	peak_479	0	+	ENSMUSG	protein_coding
chr10	57821808	57821829	peak_480	0	+	ENSMUSG	protein_coding
chr10	59584211	59584232	peak_482	0	+	ENSMUSG	protein_coding
chr10	59594715	59594736	peak_483	0	+	ENSMUSG	protein_coding
chr10	59767873	59767894	peak_484	0	-	ENSMUSG	protein_coding
chr10	59881570	59881591	peak_485	0	-	ENSMUSG	protein_coding
chr10	59952096	59952117	peak_486	0	-	ENSMUSG	protein_coding
chr10	60796044	60796065	peak_487	0	-	ENSMUSG	protein_coding
chr10	60837399	60837420	peak_488	0	-	ENSMUSG	protein_coding
chr10	60904702	60904723	peak_489	0	-	ENSMUSG	protein_coding
chr10	62047461	62047482	peak_490	0	-	ENSMUSG	protein_coding
chr10	62064924	62064945	peak_491	0	-	ENSMUSG	protein_coding
chr10	62113831	62113853	peak_492	0	-	ENSMUSG	protein_coding
chr10	62226278	62226299	peak_493	0	-	ENSMUSG	protein_coding
chr10	62234700	62234721	peak_494	0	-	ENSMUSG	protein_coding
chr10	62478435	62478456	peak_496	0	-	ENSMUSG	protein_coding
chr10	62478435	62478456	peak_496	0	-	ENSMUSG	protein_coding
chr10	62480319	62480340	peak_497	0	-	ENSMUSG	protein_coding
chr10	62480319	62480340	peak_497	0	-	ENSMUSG	protein_coding
chr10	62481529	62481550	peak_498	0	-	ENSMUSG	protein_coding
chr10	62753285	62753306	peak_499	0	+	ENSMUSG	protein_coding
chr10	62755931	62755952	peak_500	0	+	ENSMUSG	protein_coding
chr10	62775978	62775999	peak_501	0	+	ENSMUSG	protein_coding
chr10	62788072	62788093	peak_502	0	-	ENSMUSG	protein_coding
chr10	63000395	63000416	peak_503	0	+	ENSMUSG	protein_coding
chr10	63263014	63263037	peak_504	0	+	ENSMUSG	protein_coding
chr10	63417019	63417040	peak_505	0	+	ENSMUSG	protein_coding
chr10	63417019	63417040	peak_505	0	+	ENSMUSG	protein_coding
chr10	63470504	63470525	peak_506	0	+	ENSMUSG	protein_coding
chr10	63470504	63470525	peak_506	0	+	ENSMUSG	protein_coding
chr10	63473983	63474004	peak_507	0	+	ENSMUSG	protein_coding
chr10	63473983	63474004	peak_507	0	+	ENSMUSG	protein_coding
chr10	63494171	63494194	peak_508	0	+	ENSMUSG	protein_coding
chr10	63494171	63494194	peak_508	0	+	ENSMUSG	protein_coding
chr10	63747560	63747581	peak_509	0	+	ENSMUSG	protein_coding
chr10	66600821	66600842	peak_510	0	+	ENSMUSG	protein_coding
chr10	66640551	66640572	peak_511	0	+	ENSMUSG	protein_coding
chr10	66648594	66648618	peak_512	0	+	ENSMUSG	protein_coding
chr10	66696220	66696241	peak_513	0	+	ENSMUSG	protein_coding
chr10	67737930	67737951	peak_514	0	-	ENSMUSG	protein_coding
chr10	69673099	69673127	peak_516	0	+	ENSMUSG	protein_coding
chr10	70621552	70621577	peak_517	0	-	ENSMUSG	protein_coding
chr10	72117683	72117705	peak_518	0	+	ENSMUSG	protein_coding
chr10	72120848	72120870	peak_519	0	+	ENSMUSG	protein_coding
chr10	72124901	72124923	peak_520	0	+	ENSMUSG	protein_coding
chr10	74598411	74598432	peak_521	0	+	ENSMUSG	protein_coding
chr10	74721745	74721766	peak_522	0	+	ENSMUSG	protein_coding
chr10	75123290	75123311	peak_524	0	-	ENSMUSG	protein_coding

chr10	75166376	75166415	peak_525	0	-	ENSMUSG	protein_coding
chr10	75168072	75168093	peak_526	0	-	ENSMUSG	protein_coding
chr10	75757654	75757675	peak_527	0	-	ENSMUSG	protein_coding
chr10	75757707	75757728	peak_528	0	-	ENSMUSG	protein_coding
chr10	75866615	75866636	peak_529	0	-	ENSMUSG	protein_coding
chr10	75882953	75882974	peak_530	0	-	ENSMUSG	protein_coding
chr10	75883911	75883932	peak_531	0	-	ENSMUSG	protein_coding
chr10	75885610	75885633	peak_532	0	-	ENSMUSG	protein_coding
chr10	75973856	75973878	peak_533	0	+	ENSMUSG	protein_coding
chr10	76521545	76521576	peak_534	0	-	ENSMUSG	protein_coding
chr10	76521545	76521576	peak_534	0	-	ENSMUSG	protein_coding
chr10	77068947	77068968	peak_535	0	+	ENSMUSG	protein_coding
chr10	79172810	79172831	peak_539	0	+	ENSMUSG	protein_coding
chr10	79521471	79521493	peak_541	0	-	ENSMUSG	protein_coding
chr10	79590814	79590835	peak_542	0	+	ENSMUSG	protein_coding
chr10	79612912	79612936	peak_543	0	+	ENSMUSG	protein_coding
chr10	79619974	79619995	peak_544	0	+	ENSMUSG	protein_coding
chr10	79711891	79711912	peak_545	0	+	ENSMUSG	protein_coding
chr10	79755193	79755214	peak_546	0	+	ENSMUSG	protein_coding
chr10	80263971	80263992	peak_547	0	+	ENSMUSG	protein_coding
chr10	80639368	80639389	peak_548	0	+	ENSMUSG	protein_coding
chr10	80640598	80640619	peak_549	0	+	ENSMUSG	protein_coding
chr10	80640895	80640917	peak_550	0	+	ENSMUSG	protein_coding
chr10	80641671	80641698	peak_551	0	+	ENSMUSG	protein_coding
chr10	80641957	80641978	peak_552	0	+	ENSMUSG	protein_coding
chr10	80642309	80642330	peak_553	0	+	ENSMUSG	protein_coding
chr10	80642757	80642778	peak_554	0	+	ENSMUSG	protein_coding
chr10	80643318	80643339	peak_555	0	+	ENSMUSG	protein_coding
chr10	80644116	80644137	peak_556	0	+	ENSMUSG	protein_coding
chr10	80644941	80644962	peak_557	0	+	ENSMUSG	protein_coding
chr10	80711248	80711269	peak_558	0	+	ENSMUSG	protein_coding
chr10	81612943	81612969	peak_559	0	+	ENSMUSG	protein_coding
chr10	82215299	82215321	peak_562	0	-	ENSMUSG	protein_coding
chr10	82219136	82219159	peak_563	0	-	ENSMUSG	protein_coding
chr10	82460343	82460364	peak_565	0	+	ENSMUSG	protein_coding
chr10	83063648	83063669	peak_568	0	-	ENSMUSG	protein_coding
chr10	85772653	85772674	peak_569	0	+	ENSMUSG	protein_coding
chr10	85772653	85772674	peak_569	0	+	ENSMUSG	protein_coding
chr10	86154446	86154467	peak_570	0	-	ENSMUSG	protein_coding
chr10	86158635	86158656	peak_571	0	-	ENSMUSG	protein_coding
chr10	86167111	86167132	peak_572	0	-	ENSMUSG	protein_coding
chr10	86168056	86168081	peak_573	0	-	ENSMUSG	protein_coding
chr10	86242181	86242202	peak_574	0	+	ENSMUSG	protein_coding
chr10	88231415	88231437	peak_575	0	-	ENSMUSG	protein_coding
chr10	88257094	88257115	peak_576	0	-	ENSMUSG	protein_coding
chr10	90484938	90484959	peak_577	0	+	ENSMUSG	protein_coding
chr10	90581125	90581148	peak_578	0	-	ENSMUSG	protein_coding
chr10	90610539	90610560	peak_579	0	-	ENSMUSG	protein_coding
chr10	90612478	90612499	peak_580	0	-	ENSMUSG	protein_coding
chr10	92181840	92181861	peak_585	0	-	ENSMUSG	protein_coding
chr10	93349790	93349811	peak_587	0	-	ENSMUSG	protein_coding
chr10	93679089	93679110	peak_588	0	+	ENSMUSG	protein_coding
chr10	93679119	93679140	peak_589	0	+	ENSMUSG	protein_coding
chr10	93980022	93980062	peak_590	0	+	ENSMUSG	protein_coding
chr10	94306556	94306579	peak_591	0	-	ENSMUSG	protein_coding
chr10	94357316	94357338	peak_592	0	-	ENSMUSG	protein_coding
chr10	95015127	95015148	peak_593	0	-	ENSMUSG	protein_coding
chr10	95439809	95439830	peak_594	0	+	ENSMUSG	protein_coding

chr10	95445523	95445545	peak_595	0	+	ENSMUSG	protein_coding
chr10	95492210	95492234	peak_596	0	+	ENSMUSG	protein_coding
chr10	98389960	98389981	peak_599	0	+	ENSMUSG	protein_coding
chr10	99535150	99535171	peak_600	0	+	ENSMUSG	protein_coding
chr10	1.05E+08	1.05E+08	peak_602	0	-	ENSMUSG	protein_coding
chr10	1.05E+08	1.05E+08	peak_603	0	-	ENSMUSG	protein_coding
chr10	1.05E+08	1.05E+08	peak_604	0	-	ENSMUSG	protein_coding
chr10	1.08E+08	1.08E+08	peak_605	0	+	ENSMUSG	protein_coding
chr10	1.11E+08	1.11E+08	peak_606	0	+	ENSMUSG	protein_coding
chr10	1.12E+08	1.12E+08	peak_607	0	+	ENSMUSG	protein_coding
chr10	1.16E+08	1.16E+08	peak_608	0	-	ENSMUSG	protein_coding
chr10	1.16E+08	1.16E+08	peak_609	0	-	ENSMUSG	protein_coding
chr10	1.16E+08	1.16E+08	peak_610	0	-	ENSMUSG	protein_coding
chr10	1.17E+08	1.17E+08	peak_611	0	-	ENSMUSG	protein_coding
chr10	1.17E+08	1.17E+08	peak_613	0	-	ENSMUSG	protein_coding
chr10	1.19E+08	1.19E+08	peak_614	0	+	ENSMUSG	protein_coding
chr10	1.19E+08	1.19E+08	peak_615	0	+	ENSMUSG	protein_coding
chr10	1.19E+08	1.19E+08	peak_616	0	+	ENSMUSG	protein_coding
chr10	1.19E+08	1.19E+08	peak_617	0	+	ENSMUSG	protein_coding
chr10	1.19E+08	1.19E+08	peak_618	0	+	ENSMUSG	protein_coding
chr10	1.2E+08	1.2E+08	peak_619	0	+	ENSMUSG	protein_coding
chr10	1.2E+08	1.2E+08	peak_620	0	-	ENSMUSG	protein_coding
chr10	1.2E+08	1.2E+08	peak_621	0	-	ENSMUSG	protein_coding
chr10	1.21E+08	1.21E+08	peak_622	0	-	ENSMUSG	protein_coding
chr10	1.23E+08	1.23E+08	peak_623	0	-	ENSMUSG	protein_coding
chr10	1.27E+08	1.27E+08	peak_625	0	-	ENSMUSG	protein_coding
chr10	1.27E+08	1.27E+08	peak_626	0	+	ENSMUSG	protein_coding
chr10	1.27E+08	1.27E+08	peak_627	0	-	ENSMUSG	protein_coding
chr10	1.27E+08	1.27E+08	peak_628	0	-	ENSMUSG	protein_coding
chr10	1.27E+08	1.27E+08	peak_629	0	+	ENSMUSG	protein_coding
chr10	1.27E+08	1.27E+08	peak_630	0	-	ENSMUSG	protein_coding
chr10	1.27E+08	1.27E+08	peak_631	0	-	ENSMUSG	protein_coding
chr10	1.28E+08	1.28E+08	peak_632	0	+	ENSMUSG	protein_coding
chr10	1.28E+08	1.28E+08	peak_632	0	+	ENSMUSG	protein_coding
chr10	1.28E+08	1.28E+08	peak_637	0	+	ENSMUSG	protein_coding
chr10	1.28E+08	1.28E+08	peak_638	0	+	ENSMUSG	protein_coding
chr10	1.28E+08	1.28E+08	peak_639	0	+	ENSMUSG	protein_coding
chr10	1.28E+08	1.28E+08	peak_642	0	-	ENSMUSG	protein_coding
chr10	1.28E+08	1.28E+08	peak_643	0	-	ENSMUSG	protein_coding
chr10	1.28E+08	1.28E+08	peak_644	0	-	ENSMUSG	protein_coding
chr10	1.28E+08	1.28E+08	peak_645	0	+	ENSMUSG	protein_coding
chr11	3876096	3876118	peak_646	0	+	ENSMUSG	protein_coding
chr11	3876096	3876118	peak_646	0	+	ENSMUSG	protein_coding
chr11	4583818	4583839	peak_648	0	+	ENSMUSG	protein_coding
chr11	4793813	4793834	peak_649	0	+	ENSMUSG	protein_coding
chr11	4919763	4919784	peak_650	0	+	ENSMUSG	protein_coding
chr11	4987801	4987822	peak_651	0	-	ENSMUSG	protein_coding
chr11	4996259	4996280	peak_652	0	-	ENSMUSG	protein_coding
chr11	4999046	4999067	peak_653	0	-	ENSMUSG	protein_coding
chr11	4999046	4999067	peak_653	0	-	ENSMUSG	protein_coding
chr11	5217704	5217725	peak_654	0	-	ENSMUSG	protein_coding
chr11	5334401	5334425	peak_655	0	-	ENSMUSG	protein_coding
chr11	5340407	5340428	peak_656	0	-	ENSMUSG	protein_coding
chr11	5762209	5762230	peak_657	0	+	ENSMUSG	protein_coding
chr11	5855910	5855931	peak_658	0	+	ENSMUSG	protein_coding
chr11	6205060	6205088	peak_659	0	+	ENSMUSG	protein_coding
chr11	6476686	6476707	peak_662	0	+	ENSMUSG	protein_coding
chr11	8351071	8351092	peak_663	0	-	ENSMUSG	protein_coding

chr11	8434695	8434716	peak_664	0	-	ENSMUSG	protein_coding
chr11	8544224	8544245	peak_665	0	-	ENSMUSG	protein_coding
chr11	11211215	11211236	peak_666	0	-	ENSMUSG	protein_coding
chr11	18816450	18816471	peak_668	0	-	ENSMUSG	protein_coding
chr11	19883642	19883663	peak_669	0	+	ENSMUSG	protein_coding
chr11	22051044	22051065	peak_672	0	-	ENSMUSG	protein_coding
chr11	23174502	23174531	peak_674	0	+	ENSMUSG	protein_coding
chr11	23189395	23189417	peak_675	0	+	ENSMUSG	protein_coding
chr11	23233584	23233605	peak_676	0	+	ENSMUSG	protein_coding
chr11	23288736	23288757	peak_677	0	+	ENSMUSG	protein_coding
chr11	23301537	23301558	peak_678	0	+	ENSMUSG	protein_coding
chr11	23346593	23346615	peak_679	0	+	ENSMUSG	protein_coding
chr11	23354517	23354538	peak_680	0	+	ENSMUSG	protein_coding
chr11	23377812	23377833	peak_681	0	+	ENSMUSG	protein_coding
chr11	29072990	29073011	peak_682	0	+	ENSMUSG	protein_coding
chr11	29093949	29093991	peak_683	0	+	ENSMUSG	protein_coding
chr11	29447704	29447725	peak_684	0	-	ENSMUSG	protein_coding
chr11	29447861	29447883	peak_685	0	-	ENSMUSG	protein_coding
chr11	30000130	30000153	peak_686	0	-	ENSMUSG	protein_coding
chr11	30003271	30003292	peak_687	0	-	ENSMUSG	protein_coding
chr11	30004536	30004557	peak_688	0	-	ENSMUSG	protein_coding
chr11	30009953	30009974	peak_689	0	-	ENSMUSG	protein_coding
chr11	30013906	30013927	peak_690	0	-	ENSMUSG	protein_coding
chr11	30017778	30017804	peak_691	0	-	ENSMUSG	protein_coding
chr11	30020553	30020574	peak_692	0	-	ENSMUSG	protein_coding
chr11	30021525	30021546	peak_693	0	-	ENSMUSG	protein_coding
chr11	30021702	30021724	peak_694	0	-	ENSMUSG	protein_coding
chr11	30023928	30023949	peak_695	0	-	ENSMUSG	protein_coding
chr11	30023986	30024007	peak_696	0	-	ENSMUSG	protein_coding
chr11	30024963	30024984	peak_697	0	-	ENSMUSG	protein_coding
chr11	30037155	30037176	peak_698	0	-	ENSMUSG	protein_coding
chr11	30037186	30037207	peak_699	0	-	ENSMUSG	protein_coding
chr11	30038627	30038648	peak_700	0	-	ENSMUSG	protein_coding
chr11	30038820	30038841	peak_701	0	-	ENSMUSG	protein_coding
chr11	30039163	30039184	peak_702	0	-	ENSMUSG	protein_coding
chr11	30079651	30079673	peak_703	0	-	ENSMUSG	protein_coding
chr11	30147195	30147219	peak_704	0	-	ENSMUSG	protein_coding
chr11	30157597	30157618	peak_705	0	-	ENSMUSG	protein_coding
chr11	30163073	30163095	peak_706	0	-	ENSMUSG	protein_coding
chr11	31267052	31267073	peak_707	0	-	ENSMUSG	protein_coding
chr11	32317116	32317137	peak_708	0	+	ENSMUSG	protein_coding
chr11	32317182	32317203	peak_709	0	+	ENSMUSG	protein_coding
chr11	32385381	32385402	peak_710	0	+	ENSMUSG	protein_coding
chr11	32456992	32457014	peak_711	0	+	ENSMUSG	protein_coding
chr11	33060830	33060870	peak_712	0	-	ENSMUSG	protein_coding
chr11	33874711	33874732	peak_713	0	-	ENSMUSG	protein_coding
chr11	34949790	34949811	peak_714	0	+	ENSMUSG	protein_coding
chr11	34994592	34994613	peak_715	0	+	ENSMUSG	protein_coding
chr11	35007443	35007465	peak_716	0	+	ENSMUSG	protein_coding
chr11	35050243	35050265	peak_717	0	+	ENSMUSG	protein_coding
chr11	35063154	35063175	peak_718	0	+	ENSMUSG	protein_coding
chr11	35063843	35063864	peak_719	0	+	ENSMUSG	protein_coding
chr11	35066230	35066252	peak_720	0	+	ENSMUSG	protein_coding
chr11	35069851	35069872	peak_721	0	+	ENSMUSG	protein_coding
chr11	35099029	35099050	peak_722	0	+	ENSMUSG	protein_coding
chr11	35129738	35129759	peak_723	0	+	ENSMUSG	protein_coding
chr11	35158103	35158124	peak_724	0	+	ENSMUSG	protein_coding
chr11	35168660	35168681	peak_725	0	+	ENSMUSG	protein_coding

chr11	35168870	35168891	peak_726	0	+	ENSMUSG	protein_coding
chr11	35175092	35175113	peak_727	0	+	ENSMUSG	protein_coding
chr11	35192971	35192993	peak_728	0	+	ENSMUSG	protein_coding
chr11	35232281	35232302	peak_729	0	+	ENSMUSG	protein_coding
chr11	35749839	35749862	peak_730	0	-	ENSMUSG	protein_coding
chr11	40565728	40565749	peak_731	0	-	ENSMUSG	protein_coding
chr11	43368766	43368787	peak_733	0	+	ENSMUSG	protein_coding
chr11	44350503	44350525	peak_734	0	+	ENSMUSG	protein_coding
chr11	44468495	44468519	peak_735	0	+	ENSMUSG	protein_coding
chr11	44481412	44481433	peak_736	0	+	ENSMUSG	protein_coding
chr11	44495331	44495352	peak_737	0	+	ENSMUSG	protein_coding
chr11	44540292	44540313	peak_738	0	+	ENSMUSG	protein_coding
chr11	44553880	44553901	peak_739	0	+	ENSMUSG	protein_coding
chr11	44553922	44553943	peak_740	0	+	ENSMUSG	protein_coding
chr11	44561827	44561848	peak_741	0	+	ENSMUSG	protein_coding
chr11	44625629	44625651	peak_742	0	+	ENSMUSG	protein_coding
chr11	45704130	45704151	peak_743	0	+	ENSMUSG	protein_coding
chr11	45704182	45704203	peak_744	0	+	ENSMUSG	protein_coding
chr11	48614346	48614367	peak_745	0	+	ENSMUSG	protein_coding
chr11	48615514	48615536	peak_746	0	+	ENSMUSG	protein_coding
chr11	48616620	48616642	peak_747	0	+	ENSMUSG	protein_coding
chr11	48647457	48647485	peak_752	0	-	ENSMUSG	protein_coding
chr11	48658625	48658646	peak_753	0	+	ENSMUSG	protein_coding
chr11	48676585	48676607	peak_755	0	+	ENSMUSG	protein_coding
chr11	49020000	49020021	peak_756	0	+	ENSMUSG	protein_coding
chr11	49524920	49524941	peak_757	0	-	ENSMUSG	protein_coding
chr11	49674693	49674715	peak_758	0	+	ENSMUSG	protein_coding
chr11	49694715	49694736	peak_759	0	+	ENSMUSG	protein_coding
chr11	50126962	50126983	peak_761	0	-	ENSMUSG	protein_coding
chr11	50196477	50196498	peak_762	0	+	ENSMUSG	protein_coding
chr11	50196791	50196812	peak_763	0	+	ENSMUSG	protein_coding
chr11	50455829	50455850	peak_764	0	+	ENSMUSG	protein_coding
chr11	51419896	51419917	peak_765	0	-	ENSMUSG	protein_coding
chr11	51940664	51940685	peak_766	0	+	ENSMUSG	protein_coding
chr11	52216800	52216821	peak_767	0	+	ENSMUSG	protein_coding
chr11	53086359	53086380	peak_768	0	-	ENSMUSG	protein_coding
chr11	53140896	53140917	peak_769	0	+	ENSMUSG	protein_coding
chr11	53213186	53213207	peak_770	0	+	ENSMUSG	protein_coding
chr11	54537177	54537203	peak_772	0	-	ENSMUSG	protein_coding
chr11	54544660	54544681	peak_773	0	-	ENSMUSG	protein_coding
chr11	54553759	54553781	peak_774	0	-	ENSMUSG	protein_coding
chr11	54748055	54748076	peak_775	0	-	ENSMUSG	protein_coding
chr11	54923494	54923515	peak_776	0	+	ENSMUSG	protein_coding
chr11	57344769	57344791	peak_778	0	+	ENSMUSG	protein_coding
chr11	57838670	57838691	peak_779	0	+	ENSMUSG	protein_coding
chr11	57854988	57855021	peak_780	0	+	ENSMUSG	protein_coding
chr11	57870112	57870133	peak_781	0	+	ENSMUSG	protein_coding
chr11	57927534	57927555	peak_782	0	+	ENSMUSG	protein_coding
chr11	58464160	58464197	peak_786	0	+	ENSMUSG	protein_coding
chr11	58762784	58762805	peak_787	0	+	ENSMUSG	protein_coding
chr11	58768336	58768357	peak_788	0	+	ENSMUSG	protein_coding
chr11	58768336	58768357	peak_788	0	+	ENSMUSG	protein_coding
chr11	58768551	58768572	peak_789	0	+	ENSMUSG	protein_coding
chr11	58768551	58768572	peak_789	0	+	ENSMUSG	protein_coding
chr11	58775252	58775273	peak_790	0	+	ENSMUSG	protein_coding
chr11	58775681	58775722	peak_791	0	+	ENSMUSG	protein_coding
chr11	59557287	59557316	peak_792	0	+	ENSMUSG	protein_coding
chr11	59578005	59578027	peak_793	0	+	ENSMUSG	protein_coding

chr11	59590165	59590186	peak_794	0	+	ENSMUSG	protein_coding
chr11	59963124	59963166	peak_795	0	+	ENSMUSG	protein_coding
chr11	59997765	59997788	peak_796	0	+	ENSMUSG	protein_coding
chr11	60009271	60009292	peak_797	0	+	ENSMUSG	protein_coding
chr11	60121716	60121737	peak_799	0	-	ENSMUSG	protein_coding
chr11	60245874	60245896	peak_800	0	+	ENSMUSG	protein_coding
chr11	61361410	61361433	peak_802	0	-	ENSMUSG	protein_coding
chr11	61723710	61723731	peak_803	0	-	ENSMUSG	protein_coding
chr11	61740167	61740188	peak_804	0	-	ENSMUSG	protein_coding
chr11	62131513	62131534	peak_805	0	-	ENSMUSG	protein_coding
chr11	62131513	62131534	peak_805	0	-	ENSMUSG	protein_coding
chr11	62137826	62137847	peak_806	0	+	ENSMUSG	protein_coding
chr11	62137826	62137847	peak_806	0	+	ENSMUSG	protein_coding
chr11	62247872	62247896	peak_807	0	-	ENSMUSG	protein_coding
chr11	62261610	62261631	peak_808	0	-	ENSMUSG	protein_coding
chr11	62365070	62365091	peak_809	0	+	ENSMUSG	protein_coding
chr11	64825576	64825597	peak_814	0	-	ENSMUSG	protein_coding
chr11	64966143	64966164	peak_815	0	-	ENSMUSG	protein_coding
chr11	64966143	64966164	peak_815	0	-	ENSMUSG	protein_coding
chr11	67489868	67489889	peak_816	0	+	ENSMUSG	protein_coding
chr11	67489868	67489889	peak_816	0	+	ENSMUSG	protein_coding
chr11	68000231	68000252	peak_817	0	+	ENSMUSG	protein_coding
chr11	68534918	68534939	peak_818	0	+	ENSMUSG	protein_coding
chr11	68605393	68605445	peak_819	0	+	ENSMUSG	protein_coding
chr11	68618314	68618335	peak_820	0	+	ENSMUSG	protein_coding
chr11	68626548	68626569	peak_821	0	+	ENSMUSG	protein_coding
chr11	68627898	68627919	peak_822	0	+	ENSMUSG	protein_coding
chr11	68715893	68715914	peak_823	0	+	ENSMUSG	protein_coding
chr11	68717939	68717961	peak_824	0	+	ENSMUSG	protein_coding
chr11	68937074	68937095	peak_836	0	-	ENSMUSG	protein_coding
chr11	69164006	69164043	peak_838	0	-	ENSMUSG	protein_coding
chr11	69173779	69173800	peak_839	0	-	ENSMUSG	protein_coding
chr11	69174251	69174272	peak_840	0	-	ENSMUSG	protein_coding
chr11	69213792	69213813	peak_841	0	-	ENSMUSG	protein_coding
chr11	69418864	69418889	peak_842	0	-	ENSMUSG	protein_coding
chr11	69435730	69435751	peak_845	0	+	ENSMUSG	protein_coding
chr11	69481206	69481227	peak_846	0	-	ENSMUSG	protein_coding
chr11	69481432	69481453	peak_847	0	-	ENSMUSG	protein_coding
chr11	69482399	69482421	peak_848	0	-	ENSMUSG	protein_coding
chr11	69482497	69482518	peak_849	0	-	ENSMUSG	protein_coding
chr11	69483230	69483251	peak_850	0	-	ENSMUSG	protein_coding
chr11	69483281	69483302	peak_851	0	-	ENSMUSG	protein_coding
chr11	69555425	69555450	peak_852	0	-	ENSMUSG	protein_coding
chr11	69572136	69572157	peak_853	0	-	ENSMUSG	protein_coding
chr11	69715928	69715949	peak_854	0	+	ENSMUSG	protein_coding
chr11	69812263	69812285	peak_855	0	+	ENSMUSG	protein_coding
chr11	69812353	69812374	peak_856	0	+	ENSMUSG	protein_coding
chr11	69855404	69855427	peak_857	0	+	ENSMUSG	protein_coding
chr11	69855532	69855553	peak_858	0	+	ENSMUSG	protein_coding
chr11	70417389	70417410	peak_859	0	+	ENSMUSG	protein_coding
chr11	70419594	70419619	peak_860	0	+	ENSMUSG	protein_coding
chr11	70466360	70466382	peak_861	0	-	ENSMUSG	protein_coding
chr11	70511269	70511290	peak_862	0	-	ENSMUSG	protein_coding
chr11	70524033	70524054	peak_863	0	+	ENSMUSG	protein_coding
chr11	71977494	71977515	peak_864	0	-	ENSMUSG	protein_coding
chr11	72219632	72219653	peak_865	0	-	ENSMUSG	protein_coding
chr11	72357387	72357408	peak_866	0	+	ENSMUSG	protein_coding
chr11	72446386	72446410	peak_867	0	+	ENSMUSG	protein_coding

chr11	72628561	72628583	peak_868	0	+	ENSMUSG	protein_coding
chr11	72950847	72950868	peak_869	0	-	ENSMUSG	protein_coding
chr11	72950847	72950868	peak_869	0	-	ENSMUSG	protein_coding
chr11	74448850	74448873	peak_870	0	-	ENSMUSG	protein_coding
chr11	74449037	74449058	peak_871	0	-	ENSMUSG	protein_coding
chr11	74494958	74494979	peak_872	0	-	ENSMUSG	protein_coding
chr11	74504046	74504067	peak_873	0	-	ENSMUSG	protein_coding
chr11	74527801	74527822	peak_874	0	-	ENSMUSG	protein_coding
chr11	74718910	74718931	peak_875	0	+	ENSMUSG	protein_coding
chr11	74896205	74896228	peak_876	0	+	ENSMUSG	protein_coding
chr11	74946649	74946670	peak_877	0	+	ENSMUSG	protein_coding
chr11	75011176	75011198	peak_878	0	+	ENSMUSG	protein_coding
chr11	75546415	75546436	peak_879	0	+	ENSMUSG	protein_coding
chr11	75546566	75546587	peak_880	0	+	ENSMUSG	protein_coding
chr11	75565376	75565397	peak_881	0	+	ENSMUSG	protein_coding
chr11	75565449	75565470	peak_882	0	+	ENSMUSG	protein_coding
chr11	75565494	75565515	peak_883	0	+	ENSMUSG	protein_coding
chr11	76270120	76270141	peak_884	0	-	ENSMUSG	protein_coding
chr11	76721565	76721586	peak_885	0	-	ENSMUSG	protein_coding
chr11	76864150	76864171	peak_886	0	-	ENSMUSG	protein_coding
chr11	77032308	77032329	peak_887	0	+	ENSMUSG	protein_coding
chr11	77227447	77227468	peak_888	0	+	ENSMUSG	protein_coding
chr11	77353619	77353641	peak_889	0	-	ENSMUSG	protein_coding
chr11	77373793	77373814	peak_890	0	-	ENSMUSG	protein_coding
chr11	77614787	77614808	peak_892	0	+	ENSMUSG	protein_coding
chr11	77614811	77614832	peak_893	0	+	ENSMUSG	protein_coding
chr11	77628317	77628338	peak_894	0	+	ENSMUSG	protein_coding
chr11	77655705	77655726	peak_895	0	+	ENSMUSG	protein_coding
chr11	77655944	77655966	peak_896	0	+	ENSMUSG	protein_coding
chr11	77658158	77658179	peak_897	0	+	ENSMUSG	protein_coding
chr11	77659902	77659923	peak_898	0	+	ENSMUSG	protein_coding
chr11	77961222	77961243	peak_899	0	+	ENSMUSG	protein_coding
chr11	77994863	77994885	peak_900	0	-	ENSMUSG	protein_coding
chr11	77994863	77994885	peak_900	0	-	ENSMUSG	protein_coding
chr11	77994955	77994976	peak_901	0	-	ENSMUSG	protein_coding
chr11	77994955	77994976	peak_901	0	-	ENSMUSG	protein_coding
chr11	77995239	77995278	peak_902	0	-	ENSMUSG	protein_coding
chr11	77995239	77995278	peak_902	0	-	ENSMUSG	protein_coding
chr11	77996620	77996646	peak_903	0	-	ENSMUSG	protein_coding
chr11	78026979	78027000	peak_904	0	-	ENSMUSG	protein_coding
chr11	78029785	78029806	peak_905	0	-	ENSMUSG	protein_coding
chr11	78034454	78034475	peak_906	0	-	ENSMUSG	protein_coding
chr11	78431217	78431238	peak_907	0	-	ENSMUSG	protein_coding
chr11	78449247	78449269	peak_908	0	-	ENSMUSG	protein_coding
chr11	78505513	78505534	peak_909	0	-	ENSMUSG	protein_coding
chr11	79201239	79201260	peak_910	0	+	ENSMUSG	protein_coding
chr11	79296974	79296996	peak_911	0	+	ENSMUSG	protein_coding
chr11	79314586	79314608	peak_912	0	+	ENSMUSG	protein_coding
chr11	79314586	79314608	peak_912	0	+	ENSMUSG	protein_coding
chr11	80200087	80200110	peak_913	0	+	ENSMUSG	protein_coding
chr11	80260212	80260233	peak_914	0	+	ENSMUSG	protein_coding
chr11	80364246	80364267	peak_915	0	-	ENSMUSG	protein_coding
chr11	82699977	82699998	peak_916	0	-	ENSMUSG	protein_coding
chr11	83797025	83797046	peak_918	0	+	ENSMUSG	protein_coding
chr11	84074233	84074258	peak_919	0	+	ENSMUSG	protein_coding
chr11	85038212	85038233	peak_920	0	-	ENSMUSG	protein_coding
chr11	85551404	85551425	peak_921	0	+	ENSMUSG	protein_coding
chr11	86083278	86083299	peak_922	0	-	ENSMUSG	protein_coding

chr11	87085312	87085336	peak_923	0	+	ENSMUSG	protein_coding
chr11	87091164	87091185	peak_924	0	-	ENSMUSG	protein_coding
chr11	87236347	87236368	peak_925	0	+	ENSMUSG	protein_coding
chr11	87240196	87240217	peak_926	0	+	ENSMUSG	protein_coding
chr11	87240265	87240286	peak_927	0	+	ENSMUSG	protein_coding
chr11	87256717	87256738	peak_928	0	+	ENSMUSG	protein_coding
chr11	87256840	87256861	peak_929	0	+	ENSMUSG	protein_coding
chr11	87258052	87258073	peak_930	0	-	ENSMUSG	protein_coding
chr11	87262107	87262128	peak_931	0	+	ENSMUSG	protein_coding
chr11	87262240	87262261	peak_932	0	+	ENSMUSG	protein_coding
chr11	87262293	87262314	peak_933	0	+	ENSMUSG	protein_coding
chr11	87269421	87269442	peak_934	0	+	ENSMUSG	protein_coding
chr11	87275766	87275787	peak_935	0	+	ENSMUSG	protein_coding
chr11	87327704	87327725	peak_936	0	+	ENSMUSG	protein_coding
chr11	87341862	87341883	peak_937	0	+	ENSMUSG	protein_coding
chr11	87801008	87801029	peak_938	0	-	ENSMUSG	protein_coding
chr11	87864685	87864707	peak_939	0	+	ENSMUSG	protein_coding
chr11	88166928	88166949	peak_941	0	-	ENSMUSG	protein_coding
chr11	88168607	88168628	peak_942	0	-	ENSMUSG	protein_coding
chr11	88185767	88185792	peak_943	0	-	ENSMUSG	protein_coding
chr11	88268102	88268123	peak_944	0	-	ENSMUSG	protein_coding
chr11	88270843	88270864	peak_945	0	-	ENSMUSG	protein_coding
chr11	88285694	88285715	peak_946	0	-	ENSMUSG	protein_coding
chr11	88347062	88347083	peak_947	0	-	ENSMUSG	protein_coding
chr11	88407139	88407160	peak_948	0	-	ENSMUSG	protein_coding
chr11	88510454	88510480	peak_949	0	-	ENSMUSG	protein_coding
chr11	88531857	88531879	peak_950	0	-	ENSMUSG	protein_coding
chr11	88536973	88536994	peak_951	0	-	ENSMUSG	protein_coding
chr11	88556630	88556651	peak_952	0	-	ENSMUSG	protein_coding
chr11	88558915	88558937	peak_953	0	-	ENSMUSG	protein_coding
chr11	88811410	88811432	peak_954	0	-	ENSMUSG	protein_coding
chr11	88903953	88903975	peak_955	0	-	ENSMUSG	protein_coding
chr11	90493151	90493172	peak_956	0	-	ENSMUSG	protein_coding
chr11	93748590	93748611	peak_957	0	+	ENSMUSG	protein_coding
chr11	93749129	93749150	peak_958	0	+	ENSMUSG	protein_coding
chr11	93805641	93805662	peak_959	0	+	ENSMUSG	protein_coding
chr11	93875024	93875045	peak_960	0	+	ENSMUSG	protein_coding
chr11	93944150	93944171	peak_961	0	+	ENSMUSG	protein_coding
chr11	94152903	94152924	peak_962	0	-	ENSMUSG	protein_coding
chr11	94157333	94157363	peak_963	0	-	ENSMUSG	protein_coding
chr11	94157397	94157418	peak_964	0	-	ENSMUSG	protein_coding
chr11	94158224	94158245	peak_965	0	-	ENSMUSG	protein_coding
chr11	94161363	94161384	peak_966	0	-	ENSMUSG	protein_coding
chr11	94183071	94183092	peak_967	0	-	ENSMUSG	protein_coding
chr11	94797546	94797567	peak_968	0	+	ENSMUSG	protein_coding
chr11	94811825	94811846	peak_969	0	+	ENSMUSG	protein_coding
chr11	94812540	94812561	peak_970	0	+	ENSMUSG	protein_coding
chr11	94826217	94826238	peak_971	0	+	ENSMUSG	protein_coding
chr11	95158607	95158636	peak_972	0	-	ENSMUSG	protein_coding
chr11	95171154	95171175	peak_973	0	-	ENSMUSG	protein_coding
chr11	95288312	95288333	peak_974	0	+	ENSMUSG	protein_coding
chr11	95290692	95290713	peak_975	0	+	ENSMUSG	protein_coding
chr11	95330330	95330356	peak_976	0	+	ENSMUSG	protein_coding
chr11	95610700	95610721	peak_977	0	+	ENSMUSG	protein_coding
chr11	96117496	96117517	peak_979	0	+	ENSMUSG	protein_coding
chr11	96662138	96662162	peak_980	0	+	ENSMUSG	protein_coding
chr11	96668429	96668450	peak_981	0	-	ENSMUSG	protein_coding
chr11	96680227	96680248	peak_982	0	-	ENSMUSG	protein_coding

chr11	96683561	96683582	peak_983	0	-	ENSMUSG	protein_coding
chr11	97337403	97337424	peak_984	0	+	ENSMUSG	protein_coding
chr11	97525375	97525396	peak_987	0	+	ENSMUSG	protein_coding
chr11	97538795	97538816	peak_988	0	+	ENSMUSG	protein_coding
chr11	97543209	97543242	peak_989	0	+	ENSMUSG	protein_coding
chr11	97545452	97545473	peak_990	0	+	ENSMUSG	protein_coding
chr11	97581746	97581767	peak_992	0	-	ENSMUSG	protein_coding
chr11	97643091	97643113	peak_993	0	-	ENSMUSG	protein_coding
chr11	97888253	97888274	peak_999	0	+	ENSMUSG	protein_coding
chr11	97889214	97889235	peak_1000	0	+	ENSMUSG	protein_coding
chr11	97889724	97889745	peak_1001	0	+	ENSMUSG	protein_coding
chr11	97891164	97891193	peak_1002	0	+	ENSMUSG	protein_coding
chr11	98064932	98064953	peak_1003	0	+	ENSMUSG	protein_coding
chr11	98625024	98625046	peak_1005	0	+	ENSMUSG	protein_coding
chr11	98625078	98625099	peak_1006	0	+	ENSMUSG	protein_coding
chr11	98625547	98625568	peak_1007	0	+	ENSMUSG	protein_coding
chr11	98626445	98626466	peak_1008	0	+	ENSMUSG	protein_coding
chr11	98629083	98629104	peak_1009	0	+	ENSMUSG	protein_coding
chr11	98660099	98660120	peak_1010	0	+	ENSMUSG	protein_coding
chr11	98862350	98862371	peak_1011	0	-	ENSMUSG	protein_coding
chr11	98872307	98872328	peak_1012	0	-	ENSMUSG	protein_coding
chr11	99070734	99070759	peak_1013	0	-	ENSMUSG	protein_coding
chr11	1E+08	1E+08	peak_1014	0	+	ENSMUSG	protein_coding
chr11	1E+08	1E+08	peak_1016	0	-	ENSMUSG	protein_coding
chr11	1E+08	1E+08	peak_1017	0	-	ENSMUSG	protein_coding
chr11	1E+08	1E+08	peak_1018	0	-	ENSMUSG	protein_coding
chr11	1E+08	1E+08	peak_1019	0	-	ENSMUSG	protein_coding
chr11	1E+08	1E+08	peak_1020	0	-	ENSMUSG	protein_coding
chr11	1E+08	1E+08	peak_1021	0	-	ENSMUSG	protein_coding
chr11	1.01E+08	1.01E+08	peak_1022	0	+	ENSMUSG	protein_coding
chr11	1.01E+08	1.01E+08	peak_1023	0	-	ENSMUSG	protein_coding
chr11	1.01E+08	1.01E+08	peak_1024	0	-	ENSMUSG	protein_coding
chr11	1.01E+08	1.01E+08	peak_1024	0	-	ENSMUSG	protein_coding
chr11	1.01E+08	1.01E+08	peak_1025	0	+	ENSMUSG	protein_coding
chr11	1.02E+08	1.02E+08	peak_1028	0	-	ENSMUSG	protein_coding
chr11	1.02E+08	1.02E+08	peak_1029	0	-	ENSMUSG	protein_coding
chr11	1.02E+08	1.02E+08	peak_1030	0	-	ENSMUSG	protein_coding
chr11	1.02E+08	1.02E+08	peak_1031	0	-	ENSMUSG	protein_coding
chr11	1.02E+08	1.02E+08	peak_1032	0	-	ENSMUSG	protein_coding
chr11	1.02E+08	1.02E+08	peak_1033	0	-	ENSMUSG	protein_coding
chr11	1.02E+08	1.02E+08	peak_1034	0	-	ENSMUSG	protein_coding
chr11	1.03E+08	1.03E+08	peak_1036	0	-	ENSMUSG	protein_coding
chr11	1.04E+08	1.04E+08	peak_1039	0	-	ENSMUSG	protein_coding
chr11	1.04E+08	1.04E+08	peak_1040	0	-	ENSMUSG	protein_coding
chr11	1.04E+08	1.04E+08	peak_1041	0	-	ENSMUSG	protein_coding
chr11	1.04E+08	1.04E+08	peak_1042	0	-	ENSMUSG	protein_coding
chr11	1.04E+08	1.04E+08	peak_1043	0	-	ENSMUSG	protein_coding
chr11	1.06E+08	1.06E+08	peak_1044	0	+	ENSMUSG	protein_coding
chr11	1.06E+08	1.06E+08	peak_1045	0	+	ENSMUSG	protein_coding
chr11	1.06E+08	1.06E+08	peak_1046	0	-	ENSMUSG	protein_coding
chr11	1.07E+08	1.07E+08	peak_1049	0	-	ENSMUSG	protein_coding
chr11	1.07E+08	1.07E+08	peak_1050	0	-	ENSMUSG	protein_coding
chr11	1.07E+08	1.07E+08	peak_1051	0	-	ENSMUSG	protein_coding
chr11	1.07E+08	1.07E+08	peak_1052	0	-	ENSMUSG	protein_coding
chr11	1.07E+08	1.07E+08	peak_1053	0	-	ENSMUSG	protein_coding
chr11	1.07E+08	1.07E+08	peak_1057	0	-	ENSMUSG	protein_coding
chr11	1.07E+08	1.07E+08	peak_1058	0	-	ENSMUSG	protein_coding
chr11	1.07E+08	1.07E+08	peak_1059	0	-	ENSMUSG	protein_coding

chr11	1.07E+08	1.07E+08	peak_1060	0	-	ENSMUSG	protein_coding
chr11	1.07E+08	1.07E+08	peak_1061	0	-	ENSMUSG	protein_coding
chr11	1.07E+08	1.07E+08	peak_1062	0	+	ENSMUSG	protein_coding
chr11	1.08E+08	1.08E+08	peak_1063	0	+	ENSMUSG	protein_coding
chr11	1.08E+08	1.08E+08	peak_1064	0	-	ENSMUSG	protein_coding
chr11	1.09E+08	1.09E+08	peak_1065	0	+	ENSMUSG	protein_coding
chr11	1.1E+08	1.1E+08	peak_1067	0	+	ENSMUSG	protein_coding
chr11	1.13E+08	1.13E+08	peak_1068	0	+	ENSMUSG	protein_coding
chr11	1.14E+08	1.14E+08	peak_1074	0	+	ENSMUSG	protein_coding
chr11	1.14E+08	1.14E+08	peak_1075	0	-	ENSMUSG	protein_coding
chr11	1.15E+08	1.15E+08	peak_1076	0	+	ENSMUSG	protein_coding
chr11	1.15E+08	1.15E+08	peak_1077	0	+	ENSMUSG	protein_coding
chr11	1.15E+08	1.15E+08	peak_1078	0	+	ENSMUSG	protein_coding
chr11	1.15E+08	1.15E+08	peak_1080	0	-	ENSMUSG	protein_coding
chr11	1.15E+08	1.15E+08	peak_1081	0	-	ENSMUSG	protein_coding
chr11	1.16E+08	1.16E+08	peak_1082	0	-	ENSMUSG	protein_coding
chr11	1.16E+08	1.16E+08	peak_1083	0	+	ENSMUSG	protein_coding
chr11	1.16E+08	1.16E+08	peak_1085	0	-	ENSMUSG	protein_coding
chr11	1.16E+08	1.16E+08	peak_1086	0	+	ENSMUSG	protein_coding
chr11	1.16E+08	1.16E+08	peak_1087	0	-	ENSMUSG	protein_coding
chr11	1.16E+08	1.16E+08	peak_1088	0	-	ENSMUSG	protein_coding
chr11	1.16E+08	1.16E+08	peak_1089	0	-	ENSMUSG	protein_coding
chr11	1.16E+08	1.16E+08	peak_1090	0	-	ENSMUSG	protein_coding
chr11	1.17E+08	1.17E+08	peak_1091	0	+	ENSMUSG	protein_coding
chr11	1.17E+08	1.17E+08	peak_1092	0	+	ENSMUSG	protein_coding
chr11	1.17E+08	1.17E+08	peak_1093	0	+	ENSMUSG	protein_coding
chr11	1.17E+08	1.17E+08	peak_1094	0	+	ENSMUSG	protein_coding
chr11	1.17E+08	1.17E+08	peak_1095	0	+	ENSMUSG	protein_coding
chr11	1.17E+08	1.17E+08	peak_1096	0	+	ENSMUSG	protein_coding
chr11	1.17E+08	1.17E+08	peak_1097	0	+	ENSMUSG	protein_coding
chr11	1.17E+08	1.17E+08	peak_1098	0	+	ENSMUSG	protein_coding
chr11	1.17E+08	1.17E+08	peak_1099	0	+	ENSMUSG	protein_coding
chr11	1.17E+08	1.17E+08	peak_1100	0	+	ENSMUSG	protein_coding
chr11	1.18E+08	1.18E+08	peak_1101	0	+	ENSMUSG	protein_coding
chr11	1.18E+08	1.18E+08	peak_1102	0	+	ENSMUSG	protein_coding
chr11	1.18E+08	1.18E+08	peak_1103	0	+	ENSMUSG	protein_coding
chr11	1.18E+08	1.18E+08	peak_1104	0	+	ENSMUSG	protein_coding
chr11	1.18E+08	1.18E+08	peak_1105	0	-	ENSMUSG	protein_coding
chr11	1.18E+08	1.18E+08	peak_1106	0	-	ENSMUSG	protein_coding
chr11	1.18E+08	1.18E+08	peak_1107	0	-	ENSMUSG	protein_coding
chr11	1.19E+08	1.19E+08	peak_1108	0	+	ENSMUSG	protein_coding
chr11	1.19E+08	1.19E+08	peak_1109	0	+	ENSMUSG	protein_coding
chr11	1.19E+08	1.19E+08	peak_1110	0	+	ENSMUSG	protein_coding
chr11	1.2E+08	1.2E+08	peak_1111	0	+	ENSMUSG	protein_coding
chr11	1.2E+08	1.2E+08	peak_1112	0	-	ENSMUSG	protein_coding
chr11	1.2E+08	1.2E+08	peak_1113	0	+	ENSMUSG	protein_coding
chr11	1.2E+08	1.2E+08	peak_1114	0	+	ENSMUSG	protein_coding
chr11	1.2E+08	1.2E+08	peak_1115	0	-	ENSMUSG	protein_coding
chr11	1.2E+08	1.2E+08	peak_1116	0	-	ENSMUSG	protein_coding
chr11	1.21E+08	1.21E+08	peak_1117	0	-	ENSMUSG	protein_coding
chr11	1.21E+08	1.21E+08	peak_1118	0	-	ENSMUSG	protein_coding
chr11	1.21E+08	1.21E+08	peak_1119	0	-	ENSMUSG	protein_coding
chr11	1.21E+08	1.21E+08	peak_1120	0	-	ENSMUSG	protein_coding
chr11	1.21E+08	1.21E+08	peak_1121	0	-	ENSMUSG	protein_coding
chr11	1.21E+08	1.21E+08	peak_1122	0	-	ENSMUSG	protein_coding
chr11	1.21E+08	1.21E+08	peak_1123	0	-	ENSMUSG	protein_coding
chr12	3589171	3589192	peak_1124	0	+	ENSMUSG	protein_coding
chr12	3640305	3640326	peak_1125	0	+	ENSMUSG	protein_coding

chr12	3777145	3777168	peak_1126	0	+	ENSMUSG	protein_coding
chr12	3815901	3815922	peak_1127	0	+	ENSMUSG	protein_coding
chr12	3874230	3874251	peak_1128	0	+	ENSMUSG	protein_coding
chr12	4602978	4602999	peak_1129	0	+	ENSMUSG	protein_coding
chr12	4641521	4641542	peak_1130	0	+	ENSMUSG	protein_coding
chr12	4641670	4641691	peak_1131	0	+	ENSMUSG	protein_coding
chr12	8681528	8681549	peak_1133	0	+	ENSMUSG	protein_coding
chr12	13572024	13572045	peak_1137	0	+	ENSMUSG	protein_coding
chr12	15822274	15822300	peak_1139	0	-	ENSMUSG	protein_coding
chr12	16975447	16975468	peak_1140	0	+	ENSMUSG	protein_coding
chr12	17375216	17375237	peak_1141	0	+	ENSMUSG	protein_coding
chr12	17552928	17552949	peak_1142	0	+	ENSMUSG	protein_coding
chr12	17746685	17746707	peak_1144	0	+	ENSMUSG	protein_coding
chr12	21305929	21305950	peak_1146	0	+	ENSMUSG	protein_coding
chr12	21398930	21398962	peak_1147	0	-	ENSMUSG	protein_coding
chr12	25526259	25526280	peak_1150	0	+	ENSMUSG	protein_coding
chr12	25531142	25531164	peak_1151	0	+	ENSMUSG	protein_coding
chr12	25742629	25742650	peak_1152	0	+	ENSMUSG	protein_coding
chr12	27072574	27072599	peak_1153	0	-	ENSMUSG	protein_coding
chr12	29320316	29320341	peak_1154	0	-	ENSMUSG	protein_coding
chr12	29320484	29320515	peak_1155	0	-	ENSMUSG	protein_coding
chr12	29320738	29320764	peak_1156	0	-	ENSMUSG	protein_coding
chr12	31980306	31980327	peak_1157	0	+	ENSMUSG	protein_coding
chr12	31989612	31989633	peak_1158	0	+	ENSMUSG	protein_coding
chr12	32531061	32531083	peak_1159	0	+	ENSMUSG	protein_coding
chr12	32587534	32587555	peak_1160	0	+	ENSMUSG	protein_coding
chr12	33851239	33851260	peak_1161	0	+	ENSMUSG	protein_coding
chr12	33911675	33911696	peak_1162	0	+	ENSMUSG	protein_coding
chr12	35748304	35748325	peak_1164	0	+	ENSMUSG	protein_coding
chr12	35770218	35770239	peak_1165	0	+	ENSMUSG	protein_coding
chr12	40764480	40764501	peak_1166	0	-	ENSMUSG	protein_coding
chr12	41172328	41172349	peak_1167	0	-	ENSMUSG	protein_coding
chr12	42197435	42197456	peak_1168	0	+	ENSMUSG	protein_coding
chr12	42197435	42197456	peak_1168	0	+	ENSMUSG	protein_coding
chr12	42910183	42910204	peak_1169	0	+	ENSMUSG	protein_coding
chr12	45991215	45991236	peak_1170	0	-	ENSMUSG	protein_coding
chr12	51524271	51524292	peak_1172	0	-	ENSMUSG	protein_coding
chr12	51671426	51671447	peak_1173	0	-	ENSMUSG	protein_coding
chr12	52792477	52792498	peak_1174	0	-	ENSMUSG	protein_coding
chr12	52792477	52792498	peak_1174	0	-	ENSMUSG	protein_coding
chr12	52885998	52886019	peak_1175	0	-	ENSMUSG	protein_coding
chr12	53618205	53618227	peak_1176	0	+	ENSMUSG	protein_coding
chr12	53620598	53620621	peak_1177	0	+	ENSMUSG	protein_coding
chr12	53644168	53644190	peak_1178	0	+	ENSMUSG	protein_coding
chr12	56401009	56401030	peak_1186	0	-	ENSMUSG	protein_coding
chr12	56748900	56748924	peak_1188	0	-	ENSMUSG	protein_coding
chr12	56778110	56778131	peak_1189	0	-	ENSMUSG	protein_coding
chr12	56830978	56830999	peak_1190	0	-	ENSMUSG	protein_coding
chr12	60172463	60172484	peak_1193	0	+	ENSMUSG	protein_coding
chr12	60172733	60172754	peak_1194	0	+	ENSMUSG	protein_coding
chr12	60235123	60235144	peak_1196	0	+	ENSMUSG	protein_coding
chr12	66139107	66139129	peak_1197	0	+	ENSMUSG	protein_coding
chr12	66157541	66157562	peak_1198	0	+	ENSMUSG	protein_coding
chr12	66198426	66198447	peak_1199	0	+	ENSMUSG	protein_coding
chr12	70444621	70444642	peak_1201	0	-	ENSMUSG	protein_coding
chr12	70708268	70708290	peak_1203	0	-	ENSMUSG	protein_coding
chr12	70764978	70764999	peak_1204	0	+	ENSMUSG	protein_coding
chr12	72138329	72138350	peak_1206	0	+	ENSMUSG	protein_coding

chr12	72140805	72140835	peak_1207	0	+	ENSMUSG	protein_coding
chr12	73885093	73885114	peak_1208	0	+	ENSMUSG	protein_coding
chr12	73895541	73895562	peak_1209	0	+	ENSMUSG	protein_coding
chr12	75019662	75019683	peak_1210	0	+	ENSMUSG	protein_coding
chr12	75084922	75084948	peak_1211	0	+	ENSMUSG	protein_coding
chr12	76694271	76694304	peak_1212	0	-	ENSMUSG	protein_coding
chr12	76696624	76696646	peak_1213	0	-	ENSMUSG	protein_coding
chr12	76919314	76919335	peak_1214	0	+	ENSMUSG	protein_coding
chr12	76964540	76964561	peak_1215	0	+	ENSMUSG	protein_coding
chr12	76966539	76966560	peak_1216	0	+	ENSMUSG	protein_coding
chr12	77000246	77000267	peak_1217	0	+	ENSMUSG	protein_coding
chr12	77030803	77030824	peak_1218	0	+	ENSMUSG	protein_coding
chr12	77031533	77031555	peak_1219	0	+	ENSMUSG	protein_coding
chr12	77045466	77045487	peak_1220	0	+	ENSMUSG	protein_coding
chr12	77046057	77046090	peak_1221	0	+	ENSMUSG	protein_coding
chr12	77053931	77053952	peak_1222	0	+	ENSMUSG	protein_coding
chr12	77066723	77066744	peak_1223	0	+	ENSMUSG	protein_coding
chr12	77074421	77074442	peak_1224	0	+	ENSMUSG	protein_coding
chr12	77075120	77075142	peak_1225	0	+	ENSMUSG	protein_coding
chr12	77090182	77090203	peak_1226	0	+	ENSMUSG	protein_coding
chr12	77106352	77106373	peak_1227	0	+	ENSMUSG	protein_coding
chr12	77109678	77109699	peak_1228	0	+	ENSMUSG	protein_coding
chr12	77109838	77109859	peak_1229	0	+	ENSMUSG	protein_coding
chr12	77132613	77132634	peak_1230	0	+	ENSMUSG	protein_coding
chr12	77208545	77208566	peak_1231	0	+	ENSMUSG	protein_coding
chr12	77939156	77939180	peak_1232	0	+	ENSMUSG	protein_coding
chr12	78396044	78396065	peak_1233	0	+	ENSMUSG	protein_coding
chr12	79408889	79408911	peak_1234	0	+	ENSMUSG	protein_coding
chr12	79411453	79411476	peak_1235	0	+	ENSMUSG	protein_coding
chr12	79980596	79980617	peak_1236	0	+	ENSMUSG	protein_coding
chr12	80083378	80083399	peak_1237	0	-	ENSMUSG	protein_coding
chr12	80269227	80269248	peak_1238	0	-	ENSMUSG	protein_coding
chr12	81213998	81214019	peak_1239	0	-	ENSMUSG	protein_coding
chr12	81361042	81361063	peak_1240	0	-	ENSMUSG	protein_coding
chr12	81535770	81535791	peak_1241	0	-	ENSMUSG	protein_coding
chr12	82046498	82046522	peak_1243	0	+	ENSMUSG	protein_coding
chr12	82048470	82048496	peak_1244	0	+	ENSMUSG	protein_coding
chr12	82605299	82605320	peak_1245	0	-	ENSMUSG	protein_coding
chr12	82605299	82605320	peak_1245	0	-	ENSMUSG	protein_coding
chr12	82677443	82677464	peak_1246	0	-	ENSMUSG	protein_coding
chr12	83013675	83013696	peak_1247	0	+	ENSMUSG	protein_coding
chr12	83279139	83279160	peak_1248	0	+	ENSMUSG	protein_coding
chr12	84982716	84982737	peak_1250	0	+	ENSMUSG	protein_coding
chr12	84992234	84992255	peak_1251	0	+	ENSMUSG	protein_coding
chr12	85001605	85001626	peak_1252	0	+	ENSMUSG	protein_coding
chr12	85004947	85004968	peak_1253	0	+	ENSMUSG	protein_coding
chr12	85005105	85005126	peak_1254	0	+	ENSMUSG	protein_coding
chr12	85058190	85058212	peak_1255	0	+	ENSMUSG	protein_coding
chr12	85249627	85249648	peak_1256	0	-	ENSMUSG	protein_coding
chr12	85679934	85679955	peak_1257	0	+	ENSMUSG	protein_coding
chr12	86371182	86371203	peak_1258	0	+	ENSMUSG	protein_coding
chr12	86383146	86383167	peak_1259	0	+	ENSMUSG	protein_coding
chr12	86490159	86490181	peak_1260	0	+	ENSMUSG	protein_coding
chr12	86711179	86711201	peak_1261	0	-	ENSMUSG	protein_coding
chr12	87095789	87095810	peak_1262	0	+	ENSMUSG	protein_coding
chr12	87424870	87424891	peak_1263	0	+	ENSMUSG	protein_coding
chr12	87430364	87430385	peak_1264	0	+	ENSMUSG	protein_coding
chr12	87460482	87460503	peak_1265	0	+	ENSMUSG	protein_coding

chr12	88685658	88685679	peak_1266	0	-	ENSMUSG	protein_coding
chr12	88694843	88694864	peak_1267	0	-	ENSMUSG	protein_coding
chr12	88797842	88797863	peak_1268	0	-	ENSMUSG	protein_coding
chr12	96956510	96956531	peak_1272	0	+	ENSMUSG	protein_coding
chr12	96981224	96981245	peak_1273	0	+	ENSMUSG	protein_coding
chr12	1E+08	1E+08	peak_1275	0	-	ENSMUSG	protein_coding
chr12	1E+08	1E+08	peak_1276	0	-	ENSMUSG	protein_coding
chr12	1.01E+08	1.01E+08	peak_1277	0	-	ENSMUSG	protein_coding
chr12	1.01E+08	1.01E+08	peak_1278	0	-	ENSMUSG	protein_coding
chr12	1.01E+08	1.01E+08	peak_1279	0	-	ENSMUSG	protein_coding
chr12	1.01E+08	1.01E+08	peak_1280	0	-	ENSMUSG	protein_coding
chr12	1.01E+08	1.01E+08	peak_1281	0	+	ENSMUSG	protein_coding
chr12	1.01E+08	1.01E+08	peak_1282	0	+	ENSMUSG	protein_coding
chr12	1.02E+08	1.02E+08	peak_1283	0	-	ENSMUSG	protein_coding
chr12	1.03E+08	1.03E+08	peak_1284	0	-	ENSMUSG	protein_coding
chr12	1.03E+08	1.03E+08	peak_1285	0	-	ENSMUSG	protein_coding
chr12	1.04E+08	1.04E+08	peak_1286	0	-	ENSMUSG	protein_coding
chr12	1.04E+08	1.04E+08	peak_1287	0	-	ENSMUSG	protein_coding
chr12	1.04E+08	1.04E+08	peak_1288	0	-	ENSMUSG	protein_coding
chr12	1.04E+08	1.04E+08	peak_1289	0	-	ENSMUSG	protein_coding
chr12	1.06E+08	1.06E+08	peak_1290	0	-	ENSMUSG	protein_coding
chr12	1.07E+08	1.07E+08	peak_1292	0	-	ENSMUSG	protein_coding
chr12	1.07E+08	1.07E+08	peak_1293	0	+	ENSMUSG	protein_coding
chr12	1.07E+08	1.07E+08	peak_1294	0	+	ENSMUSG	protein_coding
chr12	1.07E+08	1.07E+08	peak_1295	0	+	ENSMUSG	protein_coding
chr12	1.07E+08	1.07E+08	peak_1296	0	+	ENSMUSG	protein_coding
chr12	1.09E+08	1.09E+08	peak_1297	0	+	ENSMUSG	protein_coding
chr12	1.1E+08	1.1E+08	peak_1298	0	+	ENSMUSG	protein_coding
chr12	1.1E+08	1.1E+08	peak_1299	0	+	ENSMUSG	protein_coding
chr12	1.1E+08	1.1E+08	peak_1300	0	+	ENSMUSG	protein_coding
chr12	1.1E+08	1.1E+08	peak_1301	0	+	ENSMUSG	protein_coding
chr12	1.1E+08	1.1E+08	peak_1302	0	+	ENSMUSG	protein_coding
chr12	1.1E+08	1.1E+08	peak_1303	0	+	ENSMUSG	protein_coding
chr12	1.12E+08	1.12E+08	peak_1317	0	+	ENSMUSG	protein_coding
chr12	1.12E+08	1.12E+08	peak_1318	0	+	ENSMUSG	protein_coding
chr12	1.12E+08	1.12E+08	peak_1319	0	+	ENSMUSG	protein_coding
chr12	1.12E+08	1.12E+08	peak_1320	0	+	ENSMUSG	protein_coding
chr12	1.12E+08	1.12E+08	peak_1321	0	+	ENSMUSG	protein_coding
chr12	1.12E+08	1.12E+08	peak_1322	0	+	ENSMUSG	protein_coding
chr12	1.12E+08	1.12E+08	peak_1323	0	+	ENSMUSG	protein_coding
chr12	1.12E+08	1.12E+08	peak_1324	0	+	ENSMUSG	protein_coding
chr12	1.12E+08	1.12E+08	peak_1325	0	+	ENSMUSG	protein_coding
chr12	1.12E+08	1.12E+08	peak_1326	0	+	ENSMUSG	protein_coding
chr12	1.12E+08	1.12E+08	peak_1327	0	-	ENSMUSG	protein_coding
chr12	1.12E+08	1.12E+08	peak_1328	0	-	ENSMUSG	protein_coding
chr12	1.12E+08	1.12E+08	peak_1329	0	-	ENSMUSG	protein_coding
chr12	1.12E+08	1.12E+08	peak_1330	0	-	ENSMUSG	protein_coding
chr12	1.12E+08	1.12E+08	peak_1331	0	+	ENSMUSG	protein_coding
chr12	1.12E+08	1.12E+08	peak_1332	0	+	ENSMUSG	protein_coding
chr12	1.12E+08	1.12E+08	peak_1333	0	+	ENSMUSG	protein_coding
chr12	1.12E+08	1.12E+08	peak_1334	0	+	ENSMUSG	protein_coding
chr12	1.13E+08	1.13E+08	peak_1335	0	-	ENSMUSG	protein_coding
chr12	1.13E+08	1.13E+08	peak_1336	0	-	ENSMUSG	protein_coding
chr12	1.13E+08	1.13E+08	peak_1337	0	+	ENSMUSG	protein_coding
chr12	1.13E+08	1.13E+08	peak_1338	0	+	ENSMUSG	protein_coding
chr12	1.13E+08	1.13E+08	peak_1339	0	+	ENSMUSG	protein_coding
chr13	4606727	4606748	peak_1341	0	-	ENSMUSG	protein_coding
chr13	8870918	8870939	peak_1342	0	-	ENSMUSG	protein_coding

chr13	8979205	8979226	peak_1343	0	-	ENSMUSG	protein_coding
chr13	9124547	9124568	peak_1344	0	+	ENSMUSG	protein_coding
chr13	9329186	9329208	peak_1345	0	+	ENSMUSG	protein_coding
chr13	9335581	9335602	peak_1346	0	+	ENSMUSG	protein_coding
chr13	9349637	9349658	peak_1347	0	+	ENSMUSG	protein_coding
chr13	9726326	9726347	peak_1348	0	-	ENSMUSG	protein_coding
chr13	12526180	12526212	peak_1349	0	+	ENSMUSG	protein_coding
chr13	14705503	14705524	peak_1350	0	+	ENSMUSG	protein_coding
chr13	15716882	15716904	peak_1351	0	+	ENSMUSG	protein_coding
chr13	17849972	17849993	peak_1353	0	-	ENSMUSG	protein_coding
chr13	17864751	17864772	peak_1354	0	-	ENSMUSG	protein_coding
chr13	17895273	17895294	peak_1355	0	-	ENSMUSG	protein_coding
chr13	17985137	17985158	peak_1357	0	-	ENSMUSG	protein_coding
chr13	18307124	18307145	peak_1358	0	-	ENSMUSG	protein_coding
chr13	20251695	20251716	peak_1359	0	+	ENSMUSG	protein_coding
chr13	20375891	20375912	peak_1360	0	+	ENSMUSG	protein_coding
chr13	20569015	20569037	peak_1361	0	+	ENSMUSG	protein_coding
chr13	20577004	20577025	peak_1362	0	+	ENSMUSG	protein_coding
chr13	21808602	21808623	peak_1374	0	+	ENSMUSG	protein_coding
chr13	21809481	21809502	peak_1375	0	+	ENSMUSG	protein_coding
chr13	21809878	21809899	peak_1376	0	+	ENSMUSG	protein_coding
chr13	21814279	21814300	peak_1377	0	+	ENSMUSG	protein_coding
chr13	21827238	21827259	peak_1378	0	+	ENSMUSG	protein_coding
chr13	21842086	21842107	peak_1379	0	-	ENSMUSG	protein_coding
chr13	21845359	21845384	peak_1380	0	-	ENSMUSG	protein_coding
chr13	21845711	21845732	peak_1381	0	-	ENSMUSG	protein_coding
chr13	22127088	22127109	peak_1405	0	-	ENSMUSG	protein_coding
chr13	22128121	22128142	peak_1406	0	+	ENSMUSG	protein_coding
chr13	22134463	22134484	peak_1408	0	-	ENSMUSG	protein_coding
chr13	22134624	22134646	peak_1409	0	-	ENSMUSG	protein_coding
chr13	22135204	22135225	peak_1410	0	+	ENSMUSG	protein_coding
chr13	22135441	22135462	peak_1411	0	+	ENSMUSG	protein_coding
chr13	23636740	23636765	peak_1438	0	+	ENSMUSG	protein_coding
chr13	23643358	23643379	peak_1439	0	-	ENSMUSG	protein_coding
chr13	23647170	23647191	peak_1440	0	+	ENSMUSG	protein_coding
chr13	23653822	23653843	peak_1441	0	-	ENSMUSG	protein_coding
chr13	23663644	23663665	peak_1442	0	+	ENSMUSG	protein_coding
chr13	23665671	23665692	peak_1443	0	-	ENSMUSG	protein_coding
chr13	23673603	23673624	peak_1444	0	+	ENSMUSG	protein_coding
chr13	23677437	23677459	peak_1445	0	-	ENSMUSG	protein_coding
chr13	23775395	23775416	peak_1447	0	-	ENSMUSG	protein_coding
chr13	23790030	23790051	peak_1448	0	-	ENSMUSG	protein_coding
chr13	23830926	23830947	peak_1450	0	+	ENSMUSG	protein_coding
chr13	23831333	23831354	peak_1451	0	+	ENSMUSG	protein_coding
chr13	23838687	23838708	peak_1452	0	+	ENSMUSG	protein_coding
chr13	23855885	23855906	peak_1454	0	+	ENSMUSG	protein_coding
chr13	24025772	24025793	peak_1456	0	-	ENSMUSG	protein_coding
chr13	24179316	24179337	peak_1458	0	-	ENSMUSG	protein_coding
chr13	24330695	24330717	peak_1459	0	-	ENSMUSG	protein_coding
chr13	24372641	24372662	peak_1460	0	-	ENSMUSG	protein_coding
chr13	29044704	29044725	peak_1464	0	-	ENSMUSG	protein_coding
chr13	30067106	30067147	peak_1465	0	-	ENSMUSG	protein_coding
chr13	30074897	30074918	peak_1466	0	-	ENSMUSG	protein_coding
chr13	34269901	34269922	peak_1467	0	+	ENSMUSG	protein_coding
chr13	34975573	34975612	peak_1468	0	+	ENSMUSG	protein_coding
chr13	35854805	35854827	peak_1470	0	+	ENSMUSG	protein_coding
chr13	35895259	35895280	peak_1471	0	+	ENSMUSG	protein_coding
chr13	35900707	35900729	peak_1472	0	+	ENSMUSG	protein_coding

chr13	36452278	36452299	peak_1473	0	+	ENSMUSG	protein_coding
chr13	36601728	36601750	peak_1474	0	+	ENSMUSG	protein_coding
chr13	37919782	37919803	peak_1475	0	+	ENSMUSG	protein_coding
chr13	37963609	37963630	peak_1476	0	+	ENSMUSG	protein_coding
chr13	38151813	38151834	peak_1477	0	+	ENSMUSG	protein_coding
chr13	39032193	39032214	peak_1478	0	-	ENSMUSG	protein_coding
chr13	44827202	44827223	peak_1480	0	+	ENSMUSG	protein_coding
chr13	44829924	44829945	peak_1481	0	+	ENSMUSG	protein_coding
chr13	44837025	44837046	peak_1482	0	+	ENSMUSG	protein_coding
chr13	44849287	44849308	peak_1483	0	+	ENSMUSG	protein_coding
chr13	44980084	44980117	peak_1484	0	+	ENSMUSG	protein_coding
chr13	45015650	45015671	peak_1485	0	+	ENSMUSG	protein_coding
chr13	45019059	45019080	peak_1486	0	+	ENSMUSG	protein_coding
chr13	46822203	46822225	peak_1487	0	-	ENSMUSG	protein_coding
chr13	46903977	46903998	peak_1488	0	-	ENSMUSG	protein_coding
chr13	47193592	47193613	peak_1489	0	-	ENSMUSG	protein_coding
chr13	47218088	47218109	peak_1490	0	+	ENSMUSG	protein_coding
chr13	47227271	47227292	peak_1491	0	+	ENSMUSG	protein_coding
chr13	47279986	47280008	peak_1492	0	+	ENSMUSG	protein_coding
chr13	49062910	49062931	peak_1493	0	-	ENSMUSG	protein_coding
chr13	50526602	50526624	peak_1495	0	+	ENSMUSG	protein_coding
chr13	51197324	51197345	peak_1497	0	+	ENSMUSG	protein_coding
chr13	51233855	51233878	peak_1498	0	+	ENSMUSG	protein_coding
chr13	51751858	51751879	peak_1500	0	+	ENSMUSG	protein_coding
chr13	54480213	54480234	peak_1501	0	+	ENSMUSG	protein_coding
chr13	54605131	54605152	peak_1502	0	+	ENSMUSG	protein_coding
chr13	55136757	55136778	peak_1503	0	-	ENSMUSG	protein_coding
chr13	55209036	55209057	peak_1504	0	+	ENSMUSG	protein_coding
chr13	55335268	55335289	peak_1505	0	+	ENSMUSG	protein_coding
chr13	55356810	55356831	peak_1506	0	+	ENSMUSG	protein_coding
chr13	55397529	55397552	peak_1507	0	+	ENSMUSG	protein_coding
chr13	55399895	55399918	peak_1508	0	+	ENSMUSG	protein_coding
chr13	55577609	55577630	peak_1509	0	-	ENSMUSG	protein_coding
chr13	55636768	55636790	peak_1510	0	-	ENSMUSG	protein_coding
chr13	55636923	55636944	peak_1511	0	-	ENSMUSG	protein_coding
chr13	55739459	55739480	peak_1512	0	+	ENSMUSG	protein_coding
chr13	55741143	55741176	peak_1513	0	+	ENSMUSG	protein_coding
chr13	55851781	55851802	peak_1514	0	+	ENSMUSG	protein_coding
chr13	58373009	58373030	peak_1515	0	-	ENSMUSG	protein_coding
chr13	58374526	58374549	peak_1516	0	-	ENSMUSG	protein_coding
chr13	58494673	58494694	peak_1517	0	-	ENSMUSG	protein_coding
chr13	58496961	58496982	peak_1518	0	-	ENSMUSG	protein_coding
chr13	58500968	58500989	peak_1519	0	-	ENSMUSG	protein_coding
chr13	58501297	58501318	peak_1520	0	-	ENSMUSG	protein_coding
chr13	58501756	58501778	peak_1521	0	-	ENSMUSG	protein_coding
chr13	59749188	59749213	peak_1522	0	-	ENSMUSG	protein_coding
chr13	62253159	62253180	peak_1523	0	+	ENSMUSG	protein_coding
chr13	63349486	63349507	peak_1524	0	+	ENSMUSG	protein_coding
chr13	63491773	63491794	peak_1525	0	-	ENSMUSG	protein_coding
chr13	63639274	63639296	peak_1526	0	-	ENSMUSG	protein_coding
chr13	63648406	63648427	peak_1527	0	-	ENSMUSG	protein_coding
chr13	63657855	63657876	peak_1528	0	-	ENSMUSG	protein_coding
chr13	64162474	64162495	peak_1529	0	-	ENSMUSG	protein_coding
chr13	64162499	64162520	peak_1530	0	-	ENSMUSG	protein_coding
chr13	64470337	64470359	peak_1531	0	-	ENSMUSG	protein_coding
chr13	69755417	69755439	peak_1535	0	+	ENSMUSG	protein_coding
chr13	74075962	74075983	peak_1536	0	+	ENSMUSG	protein_coding
chr13	76009417	76009438	peak_1539	0	-	ENSMUSG	protein_coding

chr13	76013821	76013842	peak_1540	0	-	ENSMUSG	protein_coding
chr13	85337989	85338012	peak_1542	0	+	ENSMUSG	protein_coding
chr13	85383798	85383819	peak_1543	0	-	ENSMUSG	protein_coding
chr13	86184651	86184674	peak_1544	0	-	ENSMUSG	protein_coding
chr13	90233599	90233620	peak_1545	0	+	ENSMUSG	protein_coding
chr13	91062753	91062775	peak_1546	0	+	ENSMUSG	protein_coding
chr13	91063309	91063330	peak_1547	0	+	ENSMUSG	protein_coding
chr13	91063969	91063991	peak_1548	0	+	ENSMUSG	protein_coding
chr13	91223989	91224011	peak_1549	0	-	ENSMUSG	protein_coding
chr13	93410926	93410947	peak_1550	0	+	ENSMUSG	protein_coding
chr13	93470635	93470657	peak_1551	0	+	ENSMUSG	protein_coding
chr13	94223497	94223527	peak_1552	0	-	ENSMUSG	protein_coding
chr13	94223884	94223905	peak_1553	0	-	ENSMUSG	protein_coding
chr13	94269202	94269224	peak_1554	0	-	ENSMUSG	protein_coding
chr13	94900558	94900580	peak_1555	0	+	ENSMUSG	protein_coding
chr13	95210223	95210244	peak_1556	0	+	ENSMUSG	protein_coding
chr13	95225572	95225593	peak_1557	0	+	ENSMUSG	protein_coding
chr13	95602390	95602411	peak_1558	0	+	ENSMUSG	protein_coding
chr13	95814477	95814498	peak_1559	0	-	ENSMUSG	protein_coding
chr13	96005151	96005172	peak_1560	0	-	ENSMUSG	protein_coding
chr13	96134768	96134789	peak_1561	0	-	ENSMUSG	protein_coding
chr13	97864552	97864573	peak_1563	0	+	ENSMUSG	protein_coding
chr13	1E+08	1E+08	peak_1566	0	-	ENSMUSG	protein_coding
chr13	1E+08	1E+08	peak_1567	0	-	ENSMUSG	protein_coding
chr13	1E+08	1E+08	peak_1568	0	-	ENSMUSG	protein_coding
chr13	1E+08	1E+08	peak_1569	0	-	ENSMUSG	protein_coding
chr13	1E+08	1E+08	peak_1570	0	-	ENSMUSG	protein_coding
chr13	1.01E+08	1.01E+08	peak_1571	0	-	ENSMUSG	protein_coding
chr13	1.01E+08	1.01E+08	peak_1572	0	+	ENSMUSG	protein_coding
chr13	1.02E+08	1.02E+08	peak_1574	0	-	ENSMUSG	protein_coding
chr13	1.04E+08	1.04E+08	peak_1575	0	-	ENSMUSG	protein_coding
chr13	1.04E+08	1.04E+08	peak_1576	0	-	ENSMUSG	protein_coding
chr13	1.04E+08	1.04E+08	peak_1577	0	-	ENSMUSG	protein_coding
chr13	1.04E+08	1.04E+08	peak_1578	0	-	ENSMUSG	protein_coding
chr13	1.04E+08	1.04E+08	peak_1579	0	-	ENSMUSG	protein_coding
chr13	1.05E+08	1.05E+08	peak_1580	0	-	ENSMUSG	protein_coding
chr13	1.05E+08	1.05E+08	peak_1581	0	-	ENSMUSG	protein_coding
chr13	1.05E+08	1.05E+08	peak_1583	0	-	ENSMUSG	protein_coding
chr13	1.06E+08	1.06E+08	peak_1584	0	+	ENSMUSG	protein_coding
chr13	1.08E+08	1.08E+08	peak_1585	0	-	ENSMUSG	protein_coding
chr13	1.08E+08	1.08E+08	peak_1586	0	-	ENSMUSG	protein_coding
chr13	1.09E+08	1.09E+08	peak_1587	0	-	ENSMUSG	protein_coding
chr13	1.09E+08	1.09E+08	peak_1588	0	-	ENSMUSG	protein_coding
chr13	1.1E+08	1.1E+08	peak_1589	0	+	ENSMUSG	protein_coding
chr13	1.12E+08	1.12E+08	peak_1590	0	-	ENSMUSG	protein_coding
chr13	1.13E+08	1.13E+08	peak_1591	0	-	ENSMUSG	protein_coding
chr13	1.13E+08	1.13E+08	peak_1592	0	-	ENSMUSG	protein_coding
chr13	1.13E+08	1.13E+08	peak_1593	0	-	ENSMUSG	protein_coding
chr13	1.13E+08	1.13E+08	peak_1594	0	-	ENSMUSG	protein_coding
chr13	1.13E+08	1.13E+08	peak_1595	0	-	ENSMUSG	protein_coding
chr13	1.14E+08	1.14E+08	peak_1597	0	+	ENSMUSG	protein_coding
chr13	1.14E+08	1.14E+08	peak_1598	0	+	ENSMUSG	protein_coding
chr13	1.14E+08	1.14E+08	peak_1599	0	-	ENSMUSG	protein_coding
chr13	1.14E+08	1.14E+08	peak_1600	0	-	ENSMUSG	protein_coding
chr13	1.14E+08	1.14E+08	peak_1601	0	-	ENSMUSG	protein_coding
chr13	1.15E+08	1.15E+08	peak_1602	0	+	ENSMUSG	protein_coding
chr13	1.15E+08	1.15E+08	peak_1603	0	+	ENSMUSG	protein_coding
chr13	1.15E+08	1.15E+08	peak_1604	0	+	ENSMUSG	protein_coding

chr13	1.15E+08	1.15E+08	peak_1605		0	+	ENSMUSG	protein_coding
chr13	1.15E+08	1.15E+08	peak_1606		0	+	ENSMUSG	protein_coding
chr13	1.15E+08	1.15E+08	peak_1607		0	-	ENSMUSG	protein_coding
chr13	1.2E+08	1.2E+08	peak_1608		0	-	ENSMUSG	protein_coding
chr13	1.2E+08	1.2E+08	peak_1609		0	+	ENSMUSG	protein_coding
chr14	8921840	8921861	peak_1610		0	+	ENSMUSG	protein_coding
chr14	9055661	9055682	peak_1611		0	+	ENSMUSG	protein_coding
chr14	12420613	12420634	peak_1612		0	+	ENSMUSG	protein_coding
chr14	12559098	12559119	peak_1613		0	+	ENSMUSG	protein_coding
chr14	12900196	12900217	peak_1614		0	+	ENSMUSG	protein_coding
chr14	12904079	12904100	peak_1615		0	+	ENSMUSG	protein_coding
chr14	14928362	14928387	peak_1616		0	+	ENSMUSG	protein_coding
chr14	17201389	17201415	peak_1617		0	+	ENSMUSG	protein_coding
chr14	17239610	17239631	peak_1618		0	+	ENSMUSG	protein_coding
chr14	19101123	19101150	peak_1619		0	-	ENSMUSG	protein_coding
chr14	19522625	19522646	peak_1620		0	-	ENSMUSG	protein_coding
chr14	19604396	19604417	peak_1621		0	-	ENSMUSG	protein_coding
chr14	19636858	19636880	peak_1622		0	-	ENSMUSG	protein_coding
chr14	19652901	19652925	peak_1623		0	-	ENSMUSG	protein_coding
chr14	21820483	21820504	peak_1625		0	+	ENSMUSG	protein_coding
chr14	21843429	21843450	peak_1626		0	+	ENSMUSG	protein_coding
chr14	21996174	21996195	peak_1627		0	+	ENSMUSG	protein_coding
chr14	21996198	21996219	peak_1628		0	+	ENSMUSG	protein_coding
chr14	22001890	22001924	peak_1629		0	+	ENSMUSG	protein_coding
chr14	22321614	22321635	peak_1630		0	+	ENSMUSG	protein_coding
chr14	22339816	22339837	peak_1631		0	+	ENSMUSG	protein_coding
chr14	22361591	22361612	peak_1632		0	+	ENSMUSG	protein_coding
chr14	24568472	24568493	peak_1633		0	-	ENSMUSG	protein_coding
chr14	24991473	24991495	peak_1634		0	-	ENSMUSG	protein_coding
chr14	25309922	25309949	peak_1636		0	+	ENSMUSG	protein_coding
chr14	26380818	26380847	peak_1639		0	+	ENSMUSG	protein_coding
chr14	27256043	27256064	peak_1640		0	-	ENSMUSG	protein_coding
chr14	27258484	27258505	peak_1641		0	-	ENSMUSG	protein_coding
chr14	27354411	27354432	peak_1642		0	-	ENSMUSG	protein_coding
chr14	27736887	27736908	peak_1643		0	-	ENSMUSG	protein_coding
chr14	27736973	27736994	peak_1644		0	-	ENSMUSG	protein_coding
chr14	28070656	28070678	peak_1648		0	+	ENSMUSG	protein_coding
chr14	28115863	28115885	peak_1649		0	+	ENSMUSG	protein_coding
chr14	28186614	28186635	peak_1650		0	+	ENSMUSG	protein_coding
chr14	28192841	28192862	peak_1651		0	+	ENSMUSG	protein_coding
chr14	28277304	28277325	peak_1652		0	+	ENSMUSG	protein_coding
chr14	30123161	30123183	peak_1653		0	+	ENSMUSG	protein_coding
chr14	31339764	31339785	peak_1654		0	+	ENSMUSG	protein_coding
chr14	31827546	31827567	peak_1655		0	-	ENSMUSG	protein_coding
chr14	31828399	31828421	peak_1656		0	-	ENSMUSG	protein_coding
chr14	31829468	31829489	peak_1657		0	-	ENSMUSG	protein_coding
chr14	31865480	31865501	peak_1658		0	+	ENSMUSG	protein_coding
chr14	31901221	31901242	peak_1659		0	+	ENSMUSG	protein_coding
chr14	31999927	31999950	peak_1660		0	-	ENSMUSG	protein_coding
chr14	31999927	31999950	peak_1660		0	-	ENSMUSG	protein_coding
chr14	32000266	32000287	peak_1661		0	-	ENSMUSG	protein_coding
chr14	32000266	32000287	peak_1661		0	-	ENSMUSG	protein_coding
chr14	32016546	32016567	peak_1662		0	-	ENSMUSG	protein_coding
chr14	32016546	32016567	peak_1662		0	-	ENSMUSG	protein_coding
chr14	32153221	32153242	peak_1663		0	+	ENSMUSG	protein_coding
chr14	32163149	32163170	peak_1664		0	+	ENSMUSG	protein_coding
chr14	32214864	32214885	peak_1665		0	-	ENSMUSG	protein_coding
chr14	32242208	32242229	peak_1666		0	-	ENSMUSG	protein_coding

chr14	33005237	33005260	peak_1667	0	-	ENSMUSG	protein_coding
chr14	33385329	33385354	peak_1668	0	+	ENSMUSG	protein_coding
chr14	34244754	34244775	peak_1669	0	-	ENSMUSG	protein_coding
chr14	35560613	35560634	peak_1670	0	+	ENSMUSG	protein_coding
chr14	41773508	41773534	peak_1671	0	-	ENSMUSG	protein_coding
chr14	45770022	45770050	peak_1672	0	-	ENSMUSG	protein_coding
chr14	46247327	46247349	peak_1674	0	-	ENSMUSG	protein_coding
chr14	47504274	47504295	peak_1675	0	+	ENSMUSG	protein_coding
chr14	47521190	47521212	peak_1676	0	+	ENSMUSG	protein_coding
chr14	47689516	47689537	peak_1677	0	+	ENSMUSG	protein_coding
chr14	47863522	47863543	peak_1678	0	-	ENSMUSG	protein_coding
chr14	48092225	48092246	peak_1679	0	+	ENSMUSG	protein_coding
chr14	48092871	48092892	peak_1680	0	+	ENSMUSG	protein_coding
chr14	48116744	48116765	peak_1681	0	+	ENSMUSG	protein_coding
chr14	48294518	48294540	peak_1682	0	+	ENSMUSG	protein_coding
chr14	48296341	48296366	peak_1683	0	+	ENSMUSG	protein_coding
chr14	48313558	48313579	peak_1684	0	+	ENSMUSG	protein_coding
chr14	48355333	48355354	peak_1685	0	+	ENSMUSG	protein_coding
chr14	48789825	48789846	peak_1686	0	+	ENSMUSG	protein_coding
chr14	52613968	52613989	peak_1696	0	+	ENSMUSG	protein_coding
chr14	52723674	52723695	peak_1697	0	-	ENSMUSG	protein_coding
chr14	52801938	52801960	peak_1698	0	-	ENSMUSG	protein_coding
chr14	52829569	52829591	peak_1699	0	-	ENSMUSG	protein_coding
chr14	52913307	52913328	peak_1700	0	+	ENSMUSG	protein_coding
chr14	55055469	55055490	peak_1701	0	+	ENSMUSG	protein_coding
chr14	55261863	55261884	peak_1704	0	-	ENSMUSG	protein_coding
chr14	55261863	55261884	peak_1704	0	-	ENSMUSG	protein_coding
chr14	55262841	55262863	peak_1705	0	-	ENSMUSG	protein_coding
chr14	55262841	55262863	peak_1705	0	-	ENSMUSG	protein_coding
chr14	55263301	55263322	peak_1706	0	-	ENSMUSG	protein_coding
chr14	55264575	55264596	peak_1707	0	-	ENSMUSG	protein_coding
chr14	55284368	55284389	peak_1708	0	-	ENSMUSG	protein_coding
chr14	55284443	55284464	peak_1709	0	-	ENSMUSG	protein_coding
chr14	55303886	55303911	peak_1710	0	-	ENSMUSG	protein_coding
chr14	55511946	55511971	peak_1711	0	+	ENSMUSG	protein_coding
chr14	55511946	55511971	peak_1711	0	+	ENSMUSG	protein_coding
chr14	55515777	55515798	peak_1712	0	+	ENSMUSG	protein_coding
chr14	55515777	55515798	peak_1712	0	+	ENSMUSG	protein_coding
chr14	55516337	55516358	peak_1713	0	+	ENSMUSG	protein_coding
chr14	55516337	55516358	peak_1713	0	+	ENSMUSG	protein_coding
chr14	55516883	55516905	peak_1714	0	+	ENSMUSG	protein_coding
chr14	55516883	55516905	peak_1714	0	+	ENSMUSG	protein_coding
chr14	55706410	55706431	peak_1715	0	-	ENSMUSG	protein_coding
chr14	56260339	56260361	peak_1716	0	-	ENSMUSG	protein_coding
chr14	57149007	57149034	peak_1718	0	-	ENSMUSG	protein_coding
chr14	57253036	57253057	peak_1719	0	+	ENSMUSG	protein_coding
chr14	57308207	57308228	peak_1720	0	+	ENSMUSG	protein_coding
chr14	57355985	57356006	peak_1721	0	-	ENSMUSG	protein_coding
chr14	57380754	57380776	peak_1722	0	-	ENSMUSG	protein_coding
chr14	57386969	57386996	peak_1723	0	-	ENSMUSG	protein_coding
chr14	57545654	57545676	peak_1724	0	+	ENSMUSG	protein_coding
chr14	57903769	57903790	peak_1725	0	-	ENSMUSG	protein_coding
chr14	58324426	58324447	peak_1726	0	-	ENSMUSG	protein_coding
chr14	58546636	58546657	peak_1727	0	-	ENSMUSG	protein_coding
chr14	62020918	62020939	peak_1728	0	-	ENSMUSG	protein_coding
chr14	62050872	62050893	peak_1729	0	-	ENSMUSG	protein_coding
chr14	63845868	63845889	peak_1731	0	-	ENSMUSG	protein_coding
chr14	64419766	64419787	peak_1733	0	+	ENSMUSG	protein_coding

chr14	65344369	65344390	peak_1734	0	+	ENSMUSG	protein_coding
chr14	65355027	65355049	peak_1735	0	+	ENSMUSG	protein_coding
chr14	66328531	66328552	peak_1736	0	+	ENSMUSG	protein_coding
chr14	66329469	66329490	peak_1737	0	+	ENSMUSG	protein_coding
chr14	66362015	66362036	peak_1738	0	+	ENSMUSG	protein_coding
chr14	66382202	66382223	peak_1739	0	+	ENSMUSG	protein_coding
chr14	66587458	66587479	peak_1740	0	+	ENSMUSG	protein_coding
chr14	66592168	66592190	peak_1741	0	+	ENSMUSG	protein_coding
chr14	66593548	66593569	peak_1742	0	+	ENSMUSG	protein_coding
chr14	66594802	66594823	peak_1743	0	+	ENSMUSG	protein_coding
chr14	66921835	66921856	peak_1744	0	+	ENSMUSG	protein_coding
chr14	66922977	66922998	peak_1745	0	+	ENSMUSG	protein_coding
chr14	67454003	67454024	peak_1746	0	-	ENSMUSG	protein_coding
chr14	67472494	67472515	peak_1747	0	-	ENSMUSG	protein_coding
chr14	68457674	68457695	peak_1748	0	-	ENSMUSG	protein_coding
chr14	68539834	68539855	peak_1749	0	-	ENSMUSG	protein_coding
chr14	70049352	70049374	peak_1750	0	-	ENSMUSG	protein_coding
chr14	70524550	70524572	peak_1751	0	+	ENSMUSG	protein_coding
chr14	70595060	70595081	peak_1752	0	-	ENSMUSG	protein_coding
chr14	73953067	73953088	peak_1753	0	+	ENSMUSG	protein_coding
chr14	75251073	75251094	peak_1755	0	-	ENSMUSG	protein_coding
chr14	75316342	75316363	peak_1756	0	-	ENSMUSG	protein_coding
chr14	75727531	75727552	peak_1757	0	+	ENSMUSG	protein_coding
chr14	75739208	75739229	peak_1758	0	+	ENSMUSG	protein_coding
chr14	76245115	76245142	peak_1759	0	+	ENSMUSG	protein_coding
chr14	76245473	76245494	peak_1760	0	+	ENSMUSG	protein_coding
chr14	76246172	76246193	peak_1761	0	+	ENSMUSG	protein_coding
chr14	76247118	76247139	peak_1762	0	+	ENSMUSG	protein_coding
chr14	76306725	76306747	peak_1763	0	-	ENSMUSG	protein_coding
chr14	76378873	76378894	peak_1764	0	-	ENSMUSG	protein_coding
chr14	76820797	76820818	peak_1766	0	+	ENSMUSG	protein_coding
chr14	76830085	76830106	peak_1767	0	+	ENSMUSG	protein_coding
chr14	76865560	76865582	peak_1768	0	+	ENSMUSG	protein_coding
chr14	76904298	76904319	peak_1769	0	+	ENSMUSG	protein_coding
chr14	76906227	76906248	peak_1770	0	+	ENSMUSG	protein_coding
chr14	76906476	76906497	peak_1771	0	+	ENSMUSG	protein_coding
chr14	76906561	76906582	peak_1772	0	+	ENSMUSG	protein_coding
chr14	76906992	76907013	peak_1773	0	+	ENSMUSG	protein_coding
chr14	78272169	78272190	peak_1774	0	-	ENSMUSG	protein_coding
chr14	78910702	78910723	peak_1775	0	-	ENSMUSG	protein_coding
chr14	79881462	79881483	peak_1776	0	+	ENSMUSG	protein_coding
chr14	79965157	79965178	peak_1777	0	+	ENSMUSG	protein_coding
chr14	80019507	80019529	peak_1778	0	+	ENSMUSG	protein_coding
chr14	87151444	87151465	peak_1780	0	-	ENSMUSG	protein_coding
chr14	87266390	87266412	peak_1781	0	-	ENSMUSG	protein_coding
chr14	87366170	87366191	peak_1782	0	-	ENSMUSG	protein_coding
chr14	87503991	87504012	peak_1783	0	-	ENSMUSG	protein_coding
chr14	98377732	98377753	peak_1784	0	+	ENSMUSG	protein_coding
chr14	99559020	99559041	peak_1785	0	+	ENSMUSG	protein_coding
chr14	1E+08	1E+08	peak_1786	0	-	ENSMUSG	protein_coding
chr14	1E+08	1E+08	peak_1787	0	-	ENSMUSG	protein_coding
chr14	1.02E+08	1.02E+08	peak_1789	0	-	ENSMUSG	protein_coding
chr14	1.03E+08	1.03E+08	peak_1790	0	-	ENSMUSG	protein_coding
chr14	1.04E+08	1.04E+08	peak_1791	0	-	ENSMUSG	protein_coding
chr14	1.04E+08	1.04E+08	peak_1792	0	-	ENSMUSG	protein_coding
chr14	1.04E+08	1.04E+08	peak_1793	0	-	ENSMUSG	protein_coding
chr14	1.16E+08	1.16E+08	peak_1796	0	-	ENSMUSG	protein_coding
chr14	1.21E+08	1.21E+08	peak_1798	0	+	ENSMUSG	protein_coding

chr14	1.21E+08	1.21E+08	peak_1799	0	+	ENSMUSG	protein_coding
chr14	1.22E+08	1.22E+08	peak_1801	0	-	ENSMUSG	protein_coding
chr14	1.22E+08	1.22E+08	peak_1802	0	-	ENSMUSG	protein_coding
chr14	1.22E+08	1.22E+08	peak_1803	0	-	ENSMUSG	protein_coding
chr14	1.22E+08	1.22E+08	peak_1804	0	-	ENSMUSG	protein_coding
chr14	1.22E+08	1.22E+08	peak_1805	0	+	ENSMUSG	protein_coding
chr14	1.22E+08	1.22E+08	peak_1806	0	+	ENSMUSG	protein_coding
chr15	3220978	3220999	peak_1807	0	+	ENSMUSG	protein_coding
chr15	5066632	5066654	peak_1808	0	+	ENSMUSG	protein_coding
chr15	5068387	5068409	peak_1809	0	+	ENSMUSG	protein_coding
chr15	6682186	6682208	peak_1810	0	+	ENSMUSG	protein_coding
chr15	6731468	6731489	peak_1811	0	+	ENSMUSG	protein_coding
chr15	7119065	7119086	peak_1812	0	+	ENSMUSG	protein_coding
chr15	7138131	7138152	peak_1813	0	+	ENSMUSG	protein_coding
chr15	7140105	7140126	peak_1814	0	+	ENSMUSG	protein_coding
chr15	8137386	8137417	peak_1815	0	+	ENSMUSG	protein_coding
chr15	8169167	8169189	peak_1816	0	+	ENSMUSG	protein_coding
chr15	9025712	9025733	peak_1817	0	+	ENSMUSG	protein_coding
chr15	10109474	10109498	peak_1818	0	+	ENSMUSG	protein_coding
chr15	10415558	10415579	peak_1819	0	-	ENSMUSG	protein_coding
chr15	10613095	10613116	peak_1820	0	-	ENSMUSG	protein_coding
chr15	10620196	10620225	peak_1821	0	-	ENSMUSG	protein_coding
chr15	11326980	11327001	peak_1822	0	-	ENSMUSG	protein_coding
chr15	11329273	11329294	peak_1823	0	-	ENSMUSG	protein_coding
chr15	11925761	11925783	peak_1825	0	-	ENSMUSG	protein_coding
chr15	12095991	12096013	peak_1826	0	+	ENSMUSG	protein_coding
chr15	12109457	12109478	peak_1827	0	+	ENSMUSG	protein_coding
chr15	12406017	12406038	peak_1828	0	+	ENSMUSG	protein_coding
chr15	12475624	12475645	peak_1829	0	-	ENSMUSG	protein_coding
chr15	12498131	12498154	peak_1830	0	-	ENSMUSG	protein_coding
chr15	25335755	25335776	peak_1831	0	-	ENSMUSG	protein_coding
chr15	25342834	25342855	peak_1832	0	-	ENSMUSG	protein_coding
chr15	25552266	25552287	peak_1833	0	+	ENSMUSG	protein_coding
chr15	25709464	25709488	peak_1834	0	+	ENSMUSG	protein_coding
chr15	25710966	25710987	peak_1835	0	+	ENSMUSG	protein_coding
chr15	25914856	25914877	peak_1836	0	+	ENSMUSG	protein_coding
chr15	26771853	26771875	peak_1837	0	-	ENSMUSG	protein_coding
chr15	27445425	27445446	peak_1838	0	+	ENSMUSG	protein_coding
chr15	27682495	27682516	peak_1839	0	-	ENSMUSG	protein_coding
chr15	27693797	27693818	peak_1840	0	-	ENSMUSG	protein_coding
chr15	27941051	27941075	peak_1841	0	-	ENSMUSG	protein_coding
chr15	31524181	31524202	peak_1842	0	-	ENSMUSG	protein_coding
chr15	31524278	31524299	peak_1843	0	-	ENSMUSG	protein_coding
chr15	32177357	32177378	peak_1844	0	+	ENSMUSG	protein_coding
chr15	34013073	34013095	peak_1845	0	+	ENSMUSG	protein_coding
chr15	34372492	34372513	peak_1846	0	-	ENSMUSG	protein_coding
chr15	35416267	35416290	peak_1847	0	+	ENSMUSG	protein_coding
chr15	35445416	35445437	peak_1848	0	+	ENSMUSG	protein_coding
chr15	35513115	35513136	peak_1849	0	+	ENSMUSG	protein_coding
chr15	35544432	35544453	peak_1850	0	-	ENSMUSG	protein_coding
chr15	35724347	35724368	peak_1851	0	+	ENSMUSG	protein_coding
chr15	35945463	35945484	peak_1852	0	-	ENSMUSG	protein_coding
chr15	36530716	36530737	peak_1853	0	-	ENSMUSG	protein_coding
chr15	36700618	36700639	peak_1854	0	-	ENSMUSG	protein_coding
chr15	37929719	37929740	peak_1855	0	-	ENSMUSG	protein_coding
chr15	37930376	37930397	peak_1856	0	-	ENSMUSG	protein_coding
chr15	37954563	37954584	peak_1857	0	-	ENSMUSG	protein_coding
chr15	37961555	37961578	peak_1858	0	-	ENSMUSG	protein_coding

chr15	37983626	37983649	peak_1859	0	-	ENSMUSG	protein_coding
chr15	37999055	37999077	peak_1860	0	-	ENSMUSG	protein_coding
chr15	38420157	38420178	peak_1862	0	-	ENSMUSG	protein_coding
chr15	38424092	38424113	peak_1863	0	-	ENSMUSG	protein_coding
chr15	38431171	38431192	peak_1864	0	-	ENSMUSG	protein_coding
chr15	38865503	38865524	peak_1866	0	+	ENSMUSG	protein_coding
chr15	38949324	38949345	peak_1867	0	+	ENSMUSG	protein_coding
chr15	40489816	40489837	peak_1869	0	+	ENSMUSG	protein_coding
chr15	40539991	40540012	peak_1870	0	+	ENSMUSG	protein_coding
chr15	40836905	40836927	peak_1871	0	+	ENSMUSG	protein_coding
chr15	44264149	44264170	peak_1872	0	+	ENSMUSG	protein_coding
chr15	51625379	51625400	peak_1873	0	-	ENSMUSG	protein_coding
chr15	51805769	51805790	peak_1874	0	-	ENSMUSG	protein_coding
chr15	51813812	51813835	peak_1875	0	-	ENSMUSG	protein_coding
chr15	51871678	51871699	peak_1876	0	+	ENSMUSG	protein_coding
chr15	51872447	51872468	peak_1877	0	+	ENSMUSG	protein_coding
chr15	51876379	51876401	peak_1878	0	+	ENSMUSG	protein_coding
chr15	51876454	51876475	peak_1879	0	+	ENSMUSG	protein_coding
chr15	51876612	51876633	peak_1880	0	+	ENSMUSG	protein_coding
chr15	51876716	51876740	peak_1881	0	+	ENSMUSG	protein_coding
chr15	53065876	53065897	peak_1882	0	-	ENSMUSG	protein_coding
chr15	53100930	53100951	peak_1883	0	-	ENSMUSG	protein_coding
chr15	53134212	53134236	peak_1884	0	-	ENSMUSG	protein_coding
chr15	55050964	55050985	peak_1885	0	+	ENSMUSG	protein_coding
chr15	57538378	57538399	peak_1886	0	+	ENSMUSG	protein_coding
chr15	57574252	57574273	peak_1887	0	+	ENSMUSG	protein_coding
chr15	57793714	57793735	peak_1888	0	+	ENSMUSG	protein_coding
chr15	57978147	57978169	peak_1890	0	+	ENSMUSG	protein_coding
chr15	58029018	58029040	peak_1891	0	-	ENSMUSG	protein_coding
chr15	58768946	58768971	peak_1892	0	+	ENSMUSG	protein_coding
chr15	58864335	58864356	peak_1893	0	-	ENSMUSG	protein_coding
chr15	59305507	59305530	peak_1894	0	+	ENSMUSG	protein_coding
chr15	59336241	59336262	peak_1895	0	+	ENSMUSG	protein_coding
chr15	59341513	59341534	peak_1896	0	+	ENSMUSG	protein_coding
chr15	66426439	66426460	peak_1899	0	+	ENSMUSG	protein_coding
chr15	71837076	71837097	peak_1900	0	-	ENSMUSG	protein_coding
chr15	72686049	72686070	peak_1902	0	-	ENSMUSG	protein_coding
chr15	73180396	73180417	peak_1903	0	-	ENSMUSG	protein_coding
chr15	73430918	73430939	peak_1904	0	-	ENSMUSG	protein_coding
chr15	73436239	73436269	peak_1905	0	-	ENSMUSG	protein_coding
chr15	73447795	73447817	peak_1906	0	-	ENSMUSG	protein_coding
chr15	73475529	73475551	peak_1907	0	-	ENSMUSG	protein_coding
chr15	75887911	75887932	peak_1908	0	-	ENSMUSG	protein_coding
chr15	75893209	75893230	peak_1909	0	-	ENSMUSG	protein_coding
chr15	75909909	75909930	peak_1910	0	-	ENSMUSG	protein_coding
chr15	76004191	76004212	peak_1912	0	-	ENSMUSG	protein_coding
chr15	76005569	76005590	peak_1913	0	-	ENSMUSG	protein_coding
chr15	76009187	76009208	peak_1914	0	-	ENSMUSG	protein_coding
chr15	76009898	76009919	peak_1915	0	-	ENSMUSG	protein_coding
chr15	76010151	76010177	peak_1916	0	-	ENSMUSG	protein_coding
chr15	76011380	76011419	peak_1917	0	-	ENSMUSG	protein_coding
chr15	76013604	76013625	peak_1918	0	-	ENSMUSG	protein_coding
chr15	76013795	76013816	peak_1919	0	-	ENSMUSG	protein_coding
chr15	76013984	76014005	peak_1920	0	-	ENSMUSG	protein_coding
chr15	76260702	76260725	peak_1921	0	+	ENSMUSG	protein_coding
chr15	76478576	76478597	peak_1922	0	-	ENSMUSG	protein_coding
chr15	76734492	76734513	peak_1923	0	+	ENSMUSG	protein_coding
chr15	76918367	76918388	peak_1924	0	-	ENSMUSG	protein_coding

chr15	77027499	77027520	peak_1925	0	-	ENSMUSG	protein_coding
chr15	77591769	77591790	peak_1927	0	-	ENSMUSG	protein_coding
chr15	77591978	77591999	peak_1928	0	-	ENSMUSG	protein_coding
chr15	77592409	77592430	peak_1929	0	-	ENSMUSG	protein_coding
chr15	77594771	77594792	peak_1930	0	-	ENSMUSG	protein_coding
chr15	77594992	77595013	peak_1931	0	-	ENSMUSG	protein_coding
chr15	77596240	77596261	peak_1932	0	-	ENSMUSG	protein_coding
chr15	77599653	77599687	peak_1933	0	-	ENSMUSG	protein_coding
chr15	77599936	77599957	peak_1934	0	-	ENSMUSG	protein_coding
chr15	77611921	77611942	peak_1935	0	-	ENSMUSG	protein_coding
chr15	77613841	77613878	peak_1936	0	-	ENSMUSG	protein_coding
chr15	77636961	77636983	peak_1937	0	-	ENSMUSG	protein_coding
chr15	77638415	77638438	peak_1938	0	-	ENSMUSG	protein_coding
chr15	77643475	77643497	peak_1939	0	-	ENSMUSG	protein_coding
chr15	77797826	77797847	peak_1940	0	-	ENSMUSG	protein_coding
chr15	78632071	78632092	peak_1941	0	-	ENSMUSG	protein_coding
chr15	78889456	78889477	peak_1943	0	-	ENSMUSG	protein_coding
chr15	78980593	78980618	peak_1944	0	+	ENSMUSG	protein_coding
chr15	79359667	79359688	peak_1946	0	-	ENSMUSG	protein_coding
chr15	79362616	79362640	peak_1947	0	-	ENSMUSG	protein_coding
chr15	79363460	79363481	peak_1948	0	-	ENSMUSG	protein_coding
chr15	79363686	79363707	peak_1949	0	-	ENSMUSG	protein_coding
chr15	79370129	79370150	peak_1950	0	-	ENSMUSG	protein_coding
chr15	79376981	79377002	peak_1951	0	-	ENSMUSG	protein_coding
chr15	79501356	79501377	peak_1952	0	+	ENSMUSG	protein_coding
chr15	79908445	79908483	peak_1954	0	-	ENSMUSG	protein_coding
chr15	79909018	79909039	peak_1955	0	-	ENSMUSG	protein_coding
chr15	79913349	79913370	peak_1956	0	-	ENSMUSG	protein_coding
chr15	80624542	80624564	peak_1957	0	+	ENSMUSG	protein_coding
chr15	80628999	80629020	peak_1958	0	+	ENSMUSG	protein_coding
chr15	80763625	80763646	peak_1959	0	+	ENSMUSG	protein_coding
chr15	80863732	80863753	peak_1960	0	-	ENSMUSG	protein_coding
chr15	80990768	80990789	peak_1961	0	-	ENSMUSG	protein_coding
chr15	81575726	81575747	peak_1962	0	+	ENSMUSG	protein_coding
chr15	81580225	81580249	peak_1963	0	+	ENSMUSG	protein_coding
chr15	81584755	81584776	peak_1964	0	+	ENSMUSG	protein_coding
chr15	81598365	81598386	peak_1965	0	+	ENSMUSG	protein_coding
chr15	81607509	81607530	peak_1966	0	+	ENSMUSG	protein_coding
chr15	81608316	81608344	peak_1967	0	+	ENSMUSG	protein_coding
chr15	81609560	81609588	peak_1968	0	+	ENSMUSG	protein_coding
chr15	81610866	81610890	peak_1969	0	+	ENSMUSG	protein_coding
chr15	81623830	81623851	peak_1970	0	+	ENSMUSG	protein_coding
chr15	81648169	81648205	peak_1971	0	+	ENSMUSG	protein_coding
chr15	81656703	81656726	peak_1972	0	+	ENSMUSG	protein_coding
chr15	81768659	81768681	peak_1974	0	+	ENSMUSG	protein_coding
chr15	81774356	81774383	peak_1975	0	-	ENSMUSG	protein_coding
chr15	81844678	81844699	peak_1976	0	-	ENSMUSG	protein_coding
chr15	81844678	81844699	peak_1976	0	-	ENSMUSG	protein_coding
chr15	81844703	81844724	peak_1977	0	-	ENSMUSG	protein_coding
chr15	81844703	81844724	peak_1977	0	-	ENSMUSG	protein_coding
chr15	81847559	81847581	peak_1978	0	+	ENSMUSG	protein_coding
chr15	81847559	81847581	peak_1978	0	+	ENSMUSG	protein_coding
chr15	82642726	82642747	peak_1979	0	-	ENSMUSG	protein_coding
chr15	82725639	82725660	peak_1980	0	-	ENSMUSG	protein_coding
chr15	84937359	84937380	peak_1983	0	-	ENSMUSG	protein_coding
chr15	84952118	84952139	peak_1984	0	-	ENSMUSG	protein_coding
chr15	84957938	84957961	peak_1985	0	-	ENSMUSG	protein_coding
chr15	85261220	85261241	peak_1986	0	+	ENSMUSG	protein_coding

chr15	86004280	86004301	peak_1992	0	-	ENSMUSG	protein_coding
chr15	87486220	87486241	peak_1993	0	+	ENSMUSG	protein_coding
chr15	87562329	87562350	peak_1994	0	+	ENSMUSG	protein_coding
chr15	87583828	87583852	peak_1995	0	+	ENSMUSG	protein_coding
chr15	88561056	88561077	peak_1996	0	-	ENSMUSG	protein_coding
chr15	88986307	88986328	peak_1999	0	-	ENSMUSG	protein_coding
chr15	89199024	89199045	peak_2000	0	+	ENSMUSG	protein_coding
chr15	89388582	89388603	peak_2001	0	+	ENSMUSG	protein_coding
chr15	90871352	90871373	peak_2003	0	-	ENSMUSG	protein_coding
chr15	93070904	93070925	peak_2005	0	-	ENSMUSG	protein_coding
chr15	93104397	93104422	peak_2006	0	-	ENSMUSG	protein_coding
chr15	93161975	93161996	peak_2007	0	-	ENSMUSG	protein_coding
chr15	93263851	93263872	peak_2008	0	+	ENSMUSG	protein_coding
chr15	93272572	93272593	peak_2009	0	+	ENSMUSG	protein_coding
chr15	93400350	93400371	peak_2010	0	-	ENSMUSG	protein_coding
chr15	93402953	93402978	peak_2011	0	-	ENSMUSG	protein_coding
chr15	96245534	96245555	peak_2012	0	-	ENSMUSG	protein_coding
chr15	96249292	96249313	peak_2013	0	-	ENSMUSG	protein_coding
chr15	97148791	97148814	peak_2014	0	+	ENSMUSG	protein_coding
chr15	97149264	97149285	peak_2015	0	+	ENSMUSG	protein_coding
chr15	97193066	97193098	peak_2016	0	+	ENSMUSG	protein_coding
chr15	97889408	97889429	peak_2017	0	-	ENSMUSG	protein_coding
chr15	97907875	97907896	peak_2018	0	-	ENSMUSG	protein_coding
chr15	98572444	98572466	peak_2019	0	-	ENSMUSG	protein_coding
chr15	98574826	98574847	peak_2020	0	-	ENSMUSG	protein_coding
chr15	98589586	98589607	peak_2021	0	-	ENSMUSG	protein_coding
chr15	98658950	98658971	peak_2022	0	-	ENSMUSG	protein_coding
chr15	98664102	98664124	peak_2023	0	-	ENSMUSG	protein_coding
chr15	98664235	98664263	peak_2024	0	-	ENSMUSG	protein_coding
chr15	98677230	98677251	peak_2025	0	-	ENSMUSG	protein_coding
chr15	98683542	98683570	peak_2026	0	-	ENSMUSG	protein_coding
chr15	98686038	98686059	peak_2027	0	-	ENSMUSG	protein_coding
chr15	98739053	98739076	peak_2029	0	-	ENSMUSG	protein_coding
chr15	99126192	99126213	peak_2032	0	+	ENSMUSG	protein_coding
chr15	99139513	99139534	peak_2033	0	+	ENSMUSG	protein_coding
chr15	99871149	99871171	peak_2034	0	+	ENSMUSG	protein_coding
chr15	1E+08	1E+08	peak_2035	0	+	ENSMUSG	protein_coding
chr15	1.02E+08	1.02E+08	peak_2036	0	+	ENSMUSG	protein_coding
chr15	1.02E+08	1.02E+08	peak_2037	0	+	ENSMUSG	protein_coding
chr15	1.02E+08	1.02E+08	peak_2038	0	-	ENSMUSG	protein_coding
chr15	1.02E+08	1.02E+08	peak_2039	0	-	ENSMUSG	protein_coding
chr15	1.02E+08	1.02E+08	peak_2043	0	+	ENSMUSG	protein_coding
chr15	1.02E+08	1.02E+08	peak_2044	0	-	ENSMUSG	protein_coding
chr15	1.03E+08	1.03E+08	peak_2046	0	-	ENSMUSG	protein_coding
chr15	1.03E+08	1.03E+08	peak_2047	0	+	ENSMUSG	protein_coding
chr15	1.03E+08	1.03E+08	peak_2048	0	+	ENSMUSG	protein_coding
chr15	1.03E+08	1.03E+08	peak_2049	0	+	ENSMUSG	protein_coding
chr16	3903154	3903175	peak_2050	0	+	ENSMUSG	protein_coding
chr16	3903154	3903175	peak_2050	0	+	ENSMUSG	protein_coding
chr16	3903154	3903175	peak_2050	0	+	ENSMUSG	protein_coding
chr16	3903177	3903198	peak_2051	0	+	ENSMUSG	protein_coding
chr16	3903177	3903198	peak_2051	0	+	ENSMUSG	protein_coding
chr16	3903177	3903198	peak_2051	0	+	ENSMUSG	protein_coding
chr16	4115830	4115851	peak_2052	0	-	ENSMUSG	protein_coding
chr16	4152376	4152398	peak_2053	0	-	ENSMUSG	protein_coding
chr16	4210467	4210488	peak_2054	0	-	ENSMUSG	protein_coding
chr16	4327084	4327105	peak_2055	0	-	ENSMUSG	protein_coding
chr16	4418423	4418444	peak_2056	0	-	ENSMUSG	protein_coding

chr16	4518435	4518456	peak_2057	0	-	ENSMUSG	protein_coding
chr16	5037074	5037095	peak_2058	0	-	ENSMUSG	protein_coding
chr16	8409427	8409448	peak_2059	0	-	ENSMUSG	protein_coding
chr16	8517838	8517859	peak_2060	0	+	ENSMUSG	protein_coding
chr16	8738347	8738368	peak_2061	0	-	ENSMUSG	protein_coding
chr16	8830509	8830530	peak_2062	0	+	ENSMUSG	protein_coding
chr16	10599595	10599616	peak_2063	0	+	ENSMUSG	protein_coding
chr16	11230969	11230990	peak_2064	0	-	ENSMUSG	protein_coding
chr16	11403795	11403816	peak_2065	0	+	ENSMUSG	protein_coding
chr16	11536237	11536258	peak_2066	0	+	ENSMUSG	protein_coding
chr16	13369262	13369283	peak_2067	0	-	ENSMUSG	protein_coding
chr16	14195220	14195241	peak_2069	0	-	ENSMUSG	protein_coding
chr16	14203278	14203299	peak_2070	0	-	ENSMUSG	protein_coding
chr16	14206084	14206105	peak_2071	0	-	ENSMUSG	protein_coding
chr16	14212645	14212666	peak_2072	0	-	ENSMUSG	protein_coding
chr16	14215898	14215919	peak_2073	0	-	ENSMUSG	protein_coding
chr16	14218052	14218074	peak_2074	0	-	ENSMUSG	protein_coding
chr16	14219987	14220008	peak_2075	0	-	ENSMUSG	protein_coding
chr16	14423136	14423157	peak_2076	0	+	ENSMUSG	protein_coding
chr16	15734978	15734999	peak_2078	0	+	ENSMUSG	protein_coding
chr16	16994835	16994856	peak_2079	0	+	ENSMUSG	protein_coding
chr16	17026692	17026713	peak_2080	0	+	ENSMUSG	protein_coding
chr16	17452735	17452756	peak_2082	0	+	ENSMUSG	protein_coding
chr16	18240937	18240958	peak_2083	0	-	ENSMUSG	protein_coding
chr16	18285198	18285220	peak_2084	0	-	ENSMUSG	protein_coding
chr16	18372010	18372031	peak_2085	0	-	ENSMUSG	protein_coding
chr16	18503197	18503218	peak_2086	0	+	ENSMUSG	protein_coding
chr16	18533423	18533446	peak_2087	0	-	ENSMUSG	protein_coding
chr16	20173493	20173514	peak_2089	0	+	ENSMUSG	protein_coding
chr16	20227583	20227604	peak_2090	0	+	ENSMUSG	protein_coding
chr16	20651754	20651779	peak_2091	0	+	ENSMUSG	protein_coding
chr16	20684109	20684130	peak_2092	0	+	ENSMUSG	protein_coding
chr16	20684289	20684310	peak_2093	0	+	ENSMUSG	protein_coding
chr16	20692328	20692349	peak_2094	0	+	ENSMUSG	protein_coding
chr16	22036634	22036655	peak_2095	0	+	ENSMUSG	protein_coding
chr16	22249180	22249201	peak_2097	0	-	ENSMUSG	protein_coding
chr16	22265045	22265066	peak_2098	0	-	ENSMUSG	protein_coding
chr16	22482892	22482913	peak_2099	0	+	ENSMUSG	protein_coding
chr16	23107811	23107832	peak_2100	0	+	ENSMUSG	protein_coding
chr16	23109006	23109028	peak_2101	0	+	ENSMUSG	protein_coding
chr16	23110761	23110784	peak_2102	0	+	ENSMUSG	protein_coding
chr16	23110902	23110923	peak_2103	0	+	ENSMUSG	protein_coding
chr16	23111428	23111449	peak_2104	0	+	ENSMUSG	protein_coding
chr16	23274385	23274407	peak_2105	0	+	ENSMUSG	protein_coding
chr16	23311755	23311776	peak_2106	0	+	ENSMUSG	protein_coding
chr16	23359893	23359914	peak_2107	0	+	ENSMUSG	protein_coding
chr16	24667185	24667207	peak_2108	0	+	ENSMUSG	protein_coding
chr16	24704277	24704298	peak_2109	0	+	ENSMUSG	protein_coding
chr16	24828078	24828099	peak_2110	0	+	ENSMUSG	protein_coding
chr16	27336772	27336794	peak_2111	0	+	ENSMUSG	protein_coding
chr16	28870201	28870222	peak_2112	0	-	ENSMUSG	protein_coding
chr16	28914322	28914344	peak_2113	0	-	ENSMUSG	protein_coding
chr16	29587310	29587331	peak_2114	0	+	ENSMUSG	protein_coding
chr16	30338020	30338041	peak_2115	0	-	ENSMUSG	protein_coding
chr16	30564815	30564836	peak_2116	0	-	ENSMUSG	protein_coding
chr16	30564848	30564869	peak_2117	0	-	ENSMUSG	protein_coding
chr16	31010689	31010712	peak_2118	0	-	ENSMUSG	protein_coding
chr16	32052428	32052469	peak_2119	0	-	ENSMUSG	protein_coding

chr16	32339055	32339077	peak_2120	0	+	ENSMUSG	protein_coding
chr16	32658445	32658466	peak_2121	0	+	ENSMUSG	protein_coding
chr16	32901662	32901683	peak_2122	0	+	ENSMUSG	protein_coding
chr16	32961414	32961435	peak_2123	0	+	ENSMUSG	protein_coding
chr16	32972047	32972069	peak_2124	0	+	ENSMUSG	protein_coding
chr16	33047705	33047728	peak_2125	0	+	ENSMUSG	protein_coding
chr16	33057232	33057254	peak_2126	0	+	ENSMUSG	protein_coding
chr16	33381705	33381726	peak_2128	0	+	ENSMUSG	protein_coding
chr16	33763581	33763603	peak_2129	0	+	ENSMUSG	protein_coding
chr16	33885152	33885174	peak_2130	0	+	ENSMUSG	protein_coding
chr16	34518644	34518665	peak_2131	0	-	ENSMUSG	protein_coding
chr16	34953741	34953762	peak_2132	0	+	ENSMUSG	protein_coding
chr16	34993698	34993719	peak_2133	0	+	ENSMUSG	protein_coding
chr16	34995256	34995277	peak_2134	0	+	ENSMUSG	protein_coding
chr16	35034745	35034767	peak_2135	0	+	ENSMUSG	protein_coding
chr16	35044043	35044064	peak_2136	0	+	ENSMUSG	protein_coding
chr16	35220920	35220941	peak_2137	0	+	ENSMUSG	protein_coding
chr16	35235009	35235030	peak_2138	0	-	ENSMUSG	protein_coding
chr16	35355827	35355868	peak_2139	0	-	ENSMUSG	protein_coding
chr16	35551797	35551820	peak_2140	0	+	ENSMUSG	protein_coding
chr16	35553990	35554011	peak_2141	0	+	ENSMUSG	protein_coding
chr16	35558523	35558544	peak_2142	0	+	ENSMUSG	protein_coding
chr16	35566620	35566642	peak_2143	0	+	ENSMUSG	protein_coding
chr16	35572015	35572036	peak_2144	0	+	ENSMUSG	protein_coding
chr16	35594232	35594253	peak_2145	0	+	ENSMUSG	protein_coding
chr16	35597821	35597842	peak_2146	0	+	ENSMUSG	protein_coding
chr16	35599274	35599295	peak_2147	0	+	ENSMUSG	protein_coding
chr16	35606003	35606025	peak_2148	0	+	ENSMUSG	protein_coding
chr16	35606342	35606363	peak_2149	0	+	ENSMUSG	protein_coding
chr16	35631255	35631285	peak_2150	0	+	ENSMUSG	protein_coding
chr16	35650798	35650819	peak_2151	0	+	ENSMUSG	protein_coding
chr16	35653662	35653683	peak_2152	0	+	ENSMUSG	protein_coding
chr16	36850958	36850981	peak_2154	0	+	ENSMUSG	protein_coding
chr16	36850958	36850981	peak_2154	0	+	ENSMUSG	protein_coding
chr16	36912616	36912637	peak_2155	0	+	ENSMUSG	protein_coding
chr16	36913856	36913878	peak_2156	0	+	ENSMUSG	protein_coding
chr16	36915353	36915374	peak_2157	0	+	ENSMUSG	protein_coding
chr16	36919283	36919305	peak_2158	0	+	ENSMUSG	protein_coding
chr16	36928795	36928816	peak_2159	0	+	ENSMUSG	protein_coding
chr16	37883582	37883603	peak_2160	0	+	ENSMUSG	protein_coding
chr16	37995359	37995380	peak_2161	0	+	ENSMUSG	protein_coding
chr16	38090543	38090564	peak_2163	0	+	ENSMUSG	protein_coding
chr16	38123585	38123606	peak_2164	0	+	ENSMUSG	protein_coding
chr16	38445880	38445901	peak_2165	0	-	ENSMUSG	protein_coding
chr16	38581435	38581456	peak_2166	0	+	ENSMUSG	protein_coding
chr16	42902234	42902255	peak_2168	0	+	ENSMUSG	protein_coding
chr16	42962116	42962137	peak_2169	0	+	ENSMUSG	protein_coding
chr16	43100754	43100775	peak_2170	0	+	ENSMUSG	protein_coding
chr16	43156101	43156123	peak_2171	0	+	ENSMUSG	protein_coding
chr16	43179804	43179827	peak_2172	0	+	ENSMUSG	protein_coding
chr16	43249479	43249520	peak_2173	0	+	ENSMUSG	protein_coding
chr16	43329377	43329399	peak_2174	0	+	ENSMUSG	protein_coding
chr16	43364335	43364356	peak_2175	0	+	ENSMUSG	protein_coding
chr16	43404311	43404333	peak_2176	0	+	ENSMUSG	protein_coding
chr16	43450553	43450574	peak_2177	0	+	ENSMUSG	protein_coding
chr16	43629746	43629767	peak_2178	0	+	ENSMUSG	protein_coding
chr16	43636369	43636391	peak_2179	0	+	ENSMUSG	protein_coding
chr16	43641862	43641883	peak_2180	0	+	ENSMUSG	protein_coding

chr16	43886850	43886871	peak_2181	0	-	ENSMUSG	protein_coding
chr16	43960240	43960261	peak_2182	0	+	ENSMUSG	protein_coding
chr16	44527840	44527870	peak_2183	0	-	ENSMUSG	protein_coding
chr16	45786949	45786970	peak_2184	0	-	ENSMUSG	protein_coding
chr16	48589430	48589466	peak_2185	0	-	ENSMUSG	protein_coding
chr16	52058179	52058200	peak_2186	0	+	ENSMUSG	protein_coding
chr16	52058521	52058542	peak_2187	0	+	ENSMUSG	protein_coding
chr16	52156959	52156981	peak_2188	0	+	ENSMUSG	protein_coding
chr16	52351023	52351044	peak_2189	0	-	ENSMUSG	protein_coding
chr16	57150536	57150557	peak_2190	0	+	ENSMUSG	protein_coding
chr16	57391634	57391655	peak_2191	0	-	ENSMUSG	protein_coding
chr16	57391634	57391655	peak_2191	0	-	ENSMUSG	protein_coding
chr16	57391792	57391813	peak_2192	0	-	ENSMUSG	protein_coding
chr16	57391792	57391813	peak_2192	0	-	ENSMUSG	protein_coding
chr16	57512793	57512814	peak_2193	0	-	ENSMUSG	protein_coding
chr16	57512793	57512814	peak_2193	0	-	ENSMUSG	protein_coding
chr16	57538856	57538880	peak_2194	0	-	ENSMUSG	protein_coding
chr16	57538856	57538880	peak_2194	0	-	ENSMUSG	protein_coding
chr16	57552585	57552606	peak_2195	0	+	ENSMUSG	protein_coding
chr16	57552585	57552606	peak_2195	0	+	ENSMUSG	protein_coding
chr16	57597807	57597829	peak_2196	0	-	ENSMUSG	protein_coding
chr16	76328784	76328806	peak_2200	0	-	ENSMUSG	protein_coding
chr16	77537504	77537525	peak_2201	0	+	ENSMUSG	protein_coding
chr16	77537504	77537525	peak_2201	0	+	ENSMUSG	protein_coding
chr16	77583837	77583864	peak_2202	0	+	ENSMUSG	protein_coding
chr16	77583837	77583864	peak_2202	0	+	ENSMUSG	protein_coding
chr16	77595015	77595036	peak_2203	0	+	ENSMUSG	protein_coding
chr16	77595015	77595036	peak_2203	0	+	ENSMUSG	protein_coding
chr16	77595015	77595036	peak_2203	0	+	ENSMUSG	protein_coding
chr16	77595331	77595352	peak_2204	0	+	ENSMUSG	protein_coding
chr16	77595331	77595352	peak_2204	0	+	ENSMUSG	protein_coding
chr16	77595331	77595352	peak_2204	0	+	ENSMUSG	protein_coding
chr16	77599082	77599103	peak_2205	0	+	ENSMUSG	protein_coding
chr16	77599826	77599847	peak_2206	0	+	ENSMUSG	protein_coding
chr16	84839330	84839352	peak_2212	0	+	ENSMUSG	protein_coding
chr16	84997785	84997808	peak_2213	0	-	ENSMUSG	protein_coding
chr16	85054003	85054024	peak_2214	0	-	ENSMUSG	protein_coding
chr16	85172029	85172050	peak_2215	0	-	ENSMUSG	protein_coding
chr16	87381000	87381021	peak_2217	0	-	ENSMUSG	protein_coding
chr16	89791527	89791548	peak_2218	0	-	ENSMUSG	protein_coding
chr16	89805396	89805417	peak_2219	0	-	ENSMUSG	protein_coding
chr16	89932159	89932180	peak_2220	0	-	ENSMUSG	protein_coding
chr16	89989925	89989946	peak_2221	0	-	ENSMUSG	protein_coding
chr16	90007843	90007866	peak_2222	0	-	ENSMUSG	protein_coding
chr16	90037145	90037166	peak_2223	0	-	ENSMUSG	protein_coding
chr16	90230212	90230250	peak_2224	0	-	ENSMUSG	protein_coding
chr16	91033132	91033153	peak_2225	0	-	ENSMUSG	protein_coding
chr16	91648089	91648110	peak_2226	0	+	ENSMUSG	protein_coding
chr16	91660165	91660188	peak_2227	0	+	ENSMUSG	protein_coding
chr16	91666730	91666751	peak_2228	0	+	ENSMUSG	protein_coding
chr16	91818822	91818844	peak_2229	0	+	ENSMUSG	protein_coding
chr16	91818822	91818844	peak_2229	0	+	ENSMUSG	protein_coding
chr16	91846019	91846040	peak_2230	0	+	ENSMUSG	protein_coding
chr16	91846019	91846040	peak_2230	0	+	ENSMUSG	protein_coding
chr16	91921660	91921681	peak_2231	0	-	ENSMUSG	protein_coding
chr16	92060149	92060170	peak_2232	0	+	ENSMUSG	protein_coding
chr16	92060149	92060170	peak_2232	0	+	ENSMUSG	protein_coding
chr16	92069338	92069359	peak_2233	0	+	ENSMUSG	protein_coding

chr16	92069338	92069359	peak_2233	0	+	ENSMUSG	protein_coding
chr16	92465677	92465698	peak_2234	0	-	ENSMUSG	protein_coding
chr16	93843923	93843944	peak_2235	0	+	ENSMUSG	protein_coding
chr16	93875140	93875161	peak_2236	0	+	ENSMUSG	protein_coding
chr16	94604171	94604193	peak_2237	0	+	ENSMUSG	protein_coding
chr16	94632507	94632528	peak_2238	0	+	ENSMUSG	protein_coding
chr16	94649410	94649432	peak_2239	0	+	ENSMUSG	protein_coding
chr16	94651037	94651064	peak_2240	0	+	ENSMUSG	protein_coding
chr16	94664124	94664145	peak_2241	0	+	ENSMUSG	protein_coding
chr16	94664539	94664560	peak_2242	0	+	ENSMUSG	protein_coding
chr16	94678383	94678404	peak_2243	0	+	ENSMUSG	protein_coding
chr16	94835183	94835204	peak_2244	0	+	ENSMUSG	protein_coding
chr16	95582835	95582856	peak_2245	0	-	ENSMUSG	protein_coding
chr16	96214822	96214843	peak_2246	0	-	ENSMUSG	protein_coding
chr17	5112414	5112435	peak_2247	0	+	ENSMUSG	protein_coding
chr17	5128046	5128084	peak_2248	0	+	ENSMUSG	protein_coding
chr17	5129358	5129379	peak_2249	0	+	ENSMUSG	protein_coding
chr17	5135846	5135867	peak_2250	0	+	ENSMUSG	protein_coding
chr17	5180907	5180928	peak_2251	0	+	ENSMUSG	protein_coding
chr17	5188407	5188428	peak_2252	0	+	ENSMUSG	protein_coding
chr17	5193020	5193041	peak_2253	0	+	ENSMUSG	protein_coding
chr17	5539935	5539956	peak_2254	0	+	ENSMUSG	protein_coding
chr17	5561846	5561867	peak_2255	0	+	ENSMUSG	protein_coding
chr17	5575883	5575904	peak_2256	0	+	ENSMUSG	protein_coding
chr17	5611162	5611183	peak_2257	0	+	ENSMUSG	protein_coding
chr17	5963321	5963342	peak_2258	0	+	ENSMUSG	protein_coding
chr17	7384124	7384145	peak_2259	0	+	ENSMUSG	protein_coding
chr17	7389657	7389678	peak_2260	0	+	ENSMUSG	protein_coding
chr17	7407499	7407520	peak_2261	0	+	ENSMUSG	protein_coding
chr17	7424528	7424549	peak_2262	0	+	ENSMUSG	protein_coding
chr17	7434030	7434051	peak_2263	0	+	ENSMUSG	protein_coding
chr17	7455250	7455271	peak_2264	0	+	ENSMUSG	protein_coding
chr17	7493206	7493227	peak_2265	0	+	ENSMUSG	protein_coding
chr17	8509736	8509757	peak_2266	0	+	ENSMUSG	protein_coding
chr17	10410654	10410675	peak_2267	0	-	ENSMUSG	protein_coding
chr17	10431805	10431826	peak_2268	0	-	ENSMUSG	protein_coding
chr17	10451355	10451376	peak_2269	0	-	ENSMUSG	protein_coding
chr17	10470360	10470381	peak_2270	0	-	ENSMUSG	protein_coding
chr17	12315668	12315689	peak_2271	0	+	ENSMUSG	protein_coding
chr17	12394593	12394631	peak_2272	0	+	ENSMUSG	protein_coding
chr17	12401897	12401918	peak_2273	0	+	ENSMUSG	protein_coding
chr17	12887123	12887144	peak_2274	0	-	ENSMUSG	protein_coding
chr17	12940467	12940488	peak_2275	0	+	ENSMUSG	protein_coding
chr17	13112429	13112450	peak_2277	0	+	ENSMUSG	protein_coding
chr17	13112492	13112513	peak_2278	0	+	ENSMUSG	protein_coding
chr17	13115089	13115110	peak_2279	0	+	ENSMUSG	protein_coding
chr17	13115467	13115488	peak_2280	0	+	ENSMUSG	protein_coding
chr17	13819625	13819646	peak_2281	0	-	ENSMUSG	protein_coding
chr17	14025930	14025951	peak_2282	0	+	ENSMUSG	protein_coding
chr17	14033100	14033125	peak_2283	0	+	ENSMUSG	protein_coding
chr17	15901824	15901845	peak_2284	0	+	ENSMUSG	protein_coding
chr17	17968402	17968423	peak_2287	0	+	ENSMUSG	protein_coding
chr17	17972241	17972263	peak_2288	0	+	ENSMUSG	protein_coding
chr17	21100818	21100839	peak_2290	0	+	ENSMUSG	protein_coding
chr17	23943910	23943931	peak_2299	0	+	ENSMUSG	protein_coding
chr17	23944074	23944095	peak_2300	0	+	ENSMUSG	protein_coding
chr17	23944474	23944495	peak_2301	0	+	ENSMUSG	protein_coding
chr17	23945458	23945479	peak_2302	0	+	ENSMUSG	protein_coding

chr17	23946946	23946983	peak_2303	0	+	ENSMUSG	protein_coding
chr17	23952490	23952511	peak_2304	0	+	ENSMUSG	protein_coding
chr17	23953237	23953258	peak_2305	0	+	ENSMUSG	protein_coding
chr17	23953769	23953791	peak_2306	0	+	ENSMUSG	protein_coding
chr17	23955650	23955672	peak_2307	0	+	ENSMUSG	protein_coding
chr17	23956134	23956155	peak_2308	0	+	ENSMUSG	protein_coding
chr17	23957248	23957269	peak_2309	0	+	ENSMUSG	protein_coding
chr17	24640811	24640832	peak_2311	0	+	ENSMUSG	protein_coding
chr17	24650305	24650326	peak_2312	0	-	ENSMUSG	protein_coding
chr17	24665398	24665420	peak_2313	0	+	ENSMUSG	protein_coding
chr17	24857039	24857061	peak_2314	0	+	ENSMUSG	protein_coding
chr17	25043797	25043818	peak_2315	0	-	ENSMUSG	protein_coding
chr17	25229788	25229815	peak_2316	0	+	ENSMUSG	protein_coding
chr17	25252462	25252484	peak_2317	0	-	ENSMUSG	protein_coding
chr17	25343799	25343820	peak_2318	0	+	ENSMUSG	protein_coding
chr17	26112221	26112242	peak_2320	0	-	ENSMUSG	protein_coding
chr17	26131201	26131222	peak_2321	0	-	ENSMUSG	protein_coding
chr17	26229334	26229355	peak_2322	0	+	ENSMUSG	protein_coding
chr17	26279782	26279803	peak_2323	0	+	ENSMUSG	protein_coding
chr17	26735799	26735820	peak_2324	0	+	ENSMUSG	protein_coding
chr17	26865038	26865061	peak_2325	0	+	ENSMUSG	protein_coding
chr17	27730354	27730375	peak_2326	0	-	ENSMUSG	protein_coding
chr17	27754324	27754345	peak_2327	0	-	ENSMUSG	protein_coding
chr17	27772185	27772207	peak_2328	0	-	ENSMUSG	protein_coding
chr17	27898379	27898400	peak_2329	0	-	ENSMUSG	protein_coding
chr17	27900453	27900475	peak_2330	0	-	ENSMUSG	protein_coding
chr17	27923145	27923166	peak_2331	0	-	ENSMUSG	protein_coding
chr17	28042264	28042286	peak_2333	0	-	ENSMUSG	protein_coding
chr17	28467603	28467624	peak_2334	0	+	ENSMUSG	protein_coding
chr17	28552936	28552958	peak_2335	0	-	ENSMUSG	protein_coding
chr17	28622002	28622023	peak_2336	0	-	ENSMUSG	protein_coding
chr17	28735919	28735940	peak_2337	0	-	ENSMUSG	protein_coding
chr17	28860683	28860705	peak_2338	0	+	ENSMUSG	protein_coding
chr17	28941611	28941632	peak_2339	0	+	ENSMUSG	protein_coding
chr17	29097445	29097466	peak_2340	0	+	ENSMUSG	protein_coding
chr17	29482174	29482195	peak_2342	0	-	ENSMUSG	protein_coding
chr17	29764016	29764037	peak_2343	0	+	ENSMUSG	protein_coding
chr17	29764016	29764037	peak_2343	0	+	ENSMUSG	protein_coding
chr17	29810585	29810606	peak_2344	0	+	ENSMUSG	protein_coding
chr17	29810585	29810606	peak_2344	0	+	ENSMUSG	protein_coding
chr17	29822921	29822942	peak_2345	0	+	ENSMUSG	protein_coding
chr17	29822921	29822942	peak_2345	0	+	ENSMUSG	protein_coding
chr17	30182515	30182536	peak_2346	0	+	ENSMUSG	protein_coding
chr17	30195684	30195705	peak_2347	0	+	ENSMUSG	protein_coding
chr17	30219696	30219717	peak_2348	0	+	ENSMUSG	protein_coding
chr17	30404400	30404421	peak_2349	0	-	ENSMUSG	protein_coding
chr17	30940037	30940058	peak_2350	0	+	ENSMUSG	protein_coding
chr17	31791130	31791151	peak_2351	0	-	ENSMUSG	protein_coding
chr17	32361072	32361093	peak_2352	0	-	ENSMUSG	protein_coding
chr17	32449292	32449313	peak_2353	0	-	ENSMUSG	protein_coding
chr17	33802548	33802569	peak_2354	0	-	ENSMUSG	protein_coding
chr17	33891788	33891809	peak_2355	0	-	ENSMUSG	protein_coding
chr17	33960154	33960176	peak_2356	0	-	ENSMUSG	protein_coding
chr17	33961290	33961312	peak_2357	0	-	ENSMUSG	protein_coding
chr17	33961441	33961462	peak_2358	0	-	ENSMUSG	protein_coding
chr17	34056413	34056434	peak_2359	0	+	ENSMUSG	protein_coding
chr17	34056563	34056584	peak_2360	0	+	ENSMUSG	protein_coding
chr17	34060810	34060831	peak_2361	0	+	ENSMUSG	protein_coding

chr17	34089233	34089254	peak_2362	0	-	ENSMUSG	protein_coding
chr17	34250726	34250752	peak_2363	0	-	ENSMUSG	protein_coding
chr17	34252194	34252215	peak_2364	0	-	ENSMUSG	protein_coding
chr17	34252307	34252328	peak_2365	0	-	ENSMUSG	protein_coding
chr17	34788133	34788154	peak_2367	0	+	ENSMUSG	protein_coding
chr17	34990734	34990755	peak_2368	0	+	ENSMUSG	protein_coding
chr17	34990987	34991008	peak_2369	0	+	ENSMUSG	protein_coding
chr17	35043684	35043705	peak_2370	0	+	ENSMUSG	protein_coding
chr17	35291646	35291667	peak_2374	0	-	ENSMUSG	protein_coding
chr17	35296260	35296296	peak_2375	0	-	ENSMUSG	protein_coding
chr17	35299204	35299225	peak_2376	0	-	ENSMUSG	protein_coding
chr17	35299642	35299663	peak_2377	0	-	ENSMUSG	protein_coding
chr17	35379600	35379622	peak_2378	0	+	ENSMUSG	protein_coding
chr17	35384097	35384136	peak_2379	0	+	ENSMUSG	protein_coding
chr17	35387211	35387232	peak_2380	0	+	ENSMUSG	protein_coding
chr17	35390508	35390529	peak_2381	0	+	ENSMUSG	protein_coding
chr17	35971985	35972006	peak_2382	0	-	ENSMUSG	protein_coding
chr17	35974998	35975019	peak_2383	0	-	ENSMUSG	protein_coding
chr17	36018285	36018306	peak_2384	0	+	ENSMUSG	protein_coding
chr17	36018655	36018676	peak_2385	0	+	ENSMUSG	protein_coding
chr17	36018951	36018972	peak_2386	0	+	ENSMUSG	protein_coding
chr17	36037692	36037713	peak_2387	0	+	ENSMUSG	protein_coding
chr17	36053908	36053930	peak_2388	0	+	ENSMUSG	protein_coding
chr17	36398441	36398462	peak_2390	0	-	ENSMUSG	protein_coding
chr17	36407220	36407241	peak_2391	0	-	ENSMUSG	protein_coding
chr17	36994639	36994660	peak_2392	0	+	ENSMUSG	protein_coding
chr17	36995133	36995155	peak_2393	0	+	ENSMUSG	protein_coding
chr17	45529164	45529185	peak_2407	0	-	ENSMUSG	protein_coding
chr17	45705839	45705860	peak_2409	0	-	ENSMUSG	protein_coding
chr17	45705909	45705930	peak_2410	0	-	ENSMUSG	protein_coding
chr17	45706195	45706216	peak_2411	0	-	ENSMUSG	protein_coding
chr17	45706225	45706246	peak_2412	0	-	ENSMUSG	protein_coding
chr17	45706419	45706440	peak_2413	0	-	ENSMUSG	protein_coding
chr17	45707414	45707435	peak_2414	0	-	ENSMUSG	protein_coding
chr17	45707616	45707637	peak_2415	0	-	ENSMUSG	protein_coding
chr17	45708115	45708136	peak_2416	0	-	ENSMUSG	protein_coding
chr17	45708526	45708547	peak_2417	0	-	ENSMUSG	protein_coding
chr17	45710134	45710155	peak_2418	0	-	ENSMUSG	protein_coding
chr17	45710216	45710237	peak_2419	0	-	ENSMUSG	protein_coding
chr17	46369699	46369720	peak_2421	0	-	ENSMUSG	protein_coding
chr17	46536282	46536303	peak_2422	0	+	ENSMUSG	protein_coding
chr17	46536340	46536362	peak_2423	0	+	ENSMUSG	protein_coding
chr17	46541093	46541114	peak_2424	0	+	ENSMUSG	protein_coding
chr17	46798945	46798966	peak_2425	0	+	ENSMUSG	protein_coding
chr17	47067428	47067450	peak_2426	0	-	ENSMUSG	protein_coding
chr17	47378225	47378246	peak_2427	0	+	ENSMUSG	protein_coding
chr17	47397487	47397508	peak_2428	0	+	ENSMUSG	protein_coding
chr17	47682767	47682789	peak_2429	0	+	ENSMUSG	protein_coding
chr17	47825518	47825539	peak_2430	0	-	ENSMUSG	protein_coding
chr17	48044595	48044616	peak_2431	0	-	ENSMUSG	protein_coding
chr17	49596083	49596104	peak_2432	0	-	ENSMUSG	protein_coding
chr17	49613571	49613592	peak_2433	0	-	ENSMUSG	protein_coding
chr17	49614616	49614640	peak_2434	0	-	ENSMUSG	protein_coding
chr17	51158788	51158809	peak_2435	0	-	ENSMUSG	protein_coding
chr17	56452948	56452969	peak_2436	0	+	ENSMUSG	protein_coding
chr17	56497638	56497659	peak_2437	0	+	ENSMUSG	protein_coding
chr17	56722488	56722509	peak_2438	0	-	ENSMUSG	protein_coding
chr17	56738190	56738211	peak_2439	0	+	ENSMUSG	protein_coding

chr17	56741973	56741994	peak_2440	0	+	ENSMUSG	protein_coding
chr17	56743095	56743116	peak_2441	0	+	ENSMUSG	protein_coding
chr17	56752815	56752837	peak_2442	0	+	ENSMUSG	protein_coding
chr17	63097293	63097315	peak_2444	0	-	ENSMUSG	protein_coding
chr17	63664427	63664448	peak_2445	0	-	ENSMUSG	protein_coding
chr17	63702851	63702872	peak_2446	0	-	ENSMUSG	protein_coding
chr17	63825902	63825924	peak_2447	0	-	ENSMUSG	protein_coding
chr17	64313057	64313078	peak_2448	0	+	ENSMUSG	protein_coding
chr17	64395738	64395759	peak_2449	0	+	ENSMUSG	protein_coding
chr17	64961913	64961934	peak_2450	0	+	ENSMUSG	protein_coding
chr17	65034030	65034051	peak_2451	0	+	ENSMUSG	protein_coding
chr17	65943270	65943291	peak_2452	0	-	ENSMUSG	protein_coding
chr17	65945897	65945919	peak_2453	0	-	ENSMUSG	protein_coding
chr17	65955060	65955081	peak_2454	0	-	ENSMUSG	protein_coding
chr17	66062912	66062933	peak_2455	0	-	ENSMUSG	protein_coding
chr17	66210799	66210820	peak_2456	0	-	ENSMUSG	protein_coding
chr17	66691756	66691777	peak_2457	0	-	ENSMUSG	protein_coding
chr17	66706520	66706541	peak_2458	0	-	ENSMUSG	protein_coding
chr17	66724825	66724846	peak_2459	0	-	ENSMUSG	protein_coding
chr17	66729536	66729557	peak_2460	0	-	ENSMUSG	protein_coding
chr17	66775266	66775289	peak_2461	0	-	ENSMUSG	protein_coding
chr17	67071434	67071455	peak_2462	0	-	ENSMUSG	protein_coding
chr17	67366250	67366272	peak_2463	0	-	ENSMUSG	protein_coding
chr17	67528338	67528359	peak_2464	0	-	ENSMUSG	protein_coding
chr17	67649980	67650001	peak_2465	0	-	ENSMUSG	protein_coding
chr17	67650019	67650040	peak_2466	0	-	ENSMUSG	protein_coding
chr17	67653478	67653499	peak_2467	0	-	ENSMUSG	protein_coding
chr17	68079943	68079969	peak_2468	0	+	ENSMUSG	protein_coding
chr17	69587745	69587766	peak_2469	0	+	ENSMUSG	protein_coding
chr17	71346102	71346124	peak_2471	0	-	ENSMUSG	protein_coding
chr17	71965777	71965798	peak_2472	0	+	ENSMUSG	protein_coding
chr17	71980599	71980620	peak_2473	0	+	ENSMUSG	protein_coding
chr17	71980644	71980665	peak_2474	0	+	ENSMUSG	protein_coding
chr17	71981451	71981472	peak_2475	0	+	ENSMUSG	protein_coding
chr17	71990197	71990218	peak_2476	0	+	ENSMUSG	protein_coding
chr17	71990901	71990923	peak_2477	0	+	ENSMUSG	protein_coding
chr17	71990934	71990955	peak_2478	0	+	ENSMUSG	protein_coding
chr17	73267622	73267645	peak_2479	0	+	ENSMUSG	protein_coding
chr17	74665318	74665340	peak_2481	0	-	ENSMUSG	protein_coding
chr17	74972894	74972915	peak_2482	0	+	ENSMUSG	protein_coding
chr17	75010406	75010427	peak_2483	0	+	ENSMUSG	protein_coding
chr17	75054527	75054548	peak_2484	0	+	ENSMUSG	protein_coding
chr17	75062006	75062027	peak_2485	0	+	ENSMUSG	protein_coding
chr17	79071944	79071965	peak_2486	0	-	ENSMUSG	protein_coding
chr17	79238379	79238400	peak_2487	0	+	ENSMUSG	protein_coding
chr17	79336312	79336333	peak_2488	0	-	ENSMUSG	protein_coding
chr17	80604743	80604764	peak_2489	0	-	ENSMUSG	protein_coding
chr17	80627507	80627528	peak_2491	0	+	ENSMUSG	protein_coding
chr17	80797979	80798000	peak_2492	0	-	ENSMUSG	protein_coding
chr17	80845913	80845935	peak_2493	0	-	ENSMUSG	protein_coding
chr17	80851004	80851025	peak_2494	0	-	ENSMUSG	protein_coding
chr17	81069017	81069038	peak_2495	0	-	ENSMUSG	protein_coding
chr17	81904488	81904509	peak_2496	0	-	ENSMUSG	protein_coding
chr17	81948616	81948637	peak_2497	0	-	ENSMUSG	protein_coding
chr17	83987148	83987169	peak_2498	0	-	ENSMUSG	protein_coding
chr17	84106354	84106375	peak_2499	0	-	ENSMUSG	protein_coding
chr17	84586202	84586223	peak_2501	0	-	ENSMUSG	protein_coding
chr17	84733155	84733177	peak_2502	0	-	ENSMUSG	protein_coding

chr17	85580255	85580276	peak_2503	0	+	ENSMUSG	protein_coding
chr17	86415363	86415384	peak_2504	0	-	ENSMUSG	protein_coding
chr17	86538832	86538853	peak_2505	0	-	ENSMUSG	protein_coding
chr17	86569241	86569264	peak_2506	0	+	ENSMUSG	protein_coding
chr17	86617097	86617118	peak_2507	0	+	ENSMUSG	protein_coding
chr17	86645205	86645226	peak_2508	0	+	ENSMUSG	protein_coding
chr17	86773877	86773898	peak_2509	0	+	ENSMUSG	protein_coding
chr17	86915988	86916009	peak_2510	0	+	ENSMUSG	protein_coding
chr17	87209647	87209668	peak_2511	0	+	ENSMUSG	protein_coding
chr17	87834536	87834557	peak_2512	0	-	ENSMUSG	protein_coding
chr17	87846203	87846225	peak_2513	0	-	ENSMUSG	protein_coding
chr17	88387674	88387695	peak_2514	0	+	ENSMUSG	protein_coding
chr17	88955129	88955152	peak_2515	0	+	ENSMUSG	protein_coding
chr17	89397106	89397127	peak_2516	0	-	ENSMUSG	protein_coding
chr18	4944945	4944975	peak_2518	0	+	ENSMUSG	protein_coding
chr18	4954362	4954383	peak_2519	0	+	ENSMUSG	protein_coding
chr18	4955121	4955142	peak_2520	0	+	ENSMUSG	protein_coding
chr18	5049469	5049490	peak_2521	0	+	ENSMUSG	protein_coding
chr18	5106804	5106825	peak_2522	0	+	ENSMUSG	protein_coding
chr18	5116019	5116040	peak_2523	0	+	ENSMUSG	protein_coding
chr18	5298875	5298896	peak_2524	0	+	ENSMUSG	protein_coding
chr18	5327368	5327390	peak_2525	0	-	ENSMUSG	protein_coding
chr18	5668125	5668146	peak_2526	0	+	ENSMUSG	protein_coding
chr18	5720359	5720380	peak_2527	0	+	ENSMUSG	protein_coding
chr18	5772720	5772741	peak_2528	0	+	ENSMUSG	protein_coding
chr18	6062256	6062277	peak_2529	0	-	ENSMUSG	protein_coding
chr18	6076552	6076573	peak_2530	0	-	ENSMUSG	protein_coding
chr18	6220268	6220300	peak_2531	0	-	ENSMUSG	protein_coding
chr18	6220810	6220831	peak_2532	0	-	ENSMUSG	protein_coding
chr18	6490509	6490530	peak_2533	0	-	ENSMUSG	protein_coding
chr18	7913257	7913278	peak_2535	0	+	ENSMUSG	protein_coding
chr18	10065684	10065706	peak_2536	0	-	ENSMUSG	protein_coding
chr18	10144937	10144958	peak_2537	0	-	ENSMUSG	protein_coding
chr18	10151139	10151160	peak_2538	0	-	ENSMUSG	protein_coding
chr18	10352023	10352045	peak_2539	0	+	ENSMUSG	protein_coding
chr18	10361748	10361769	peak_2540	0	+	ENSMUSG	protein_coding
chr18	10759410	10759431	peak_2541	0	+	ENSMUSG	protein_coding
chr18	12056464	12056485	peak_2542	0	+	ENSMUSG	protein_coding
chr18	12874450	12874471	peak_2543	0	+	ENSMUSG	protein_coding
chr18	12955897	12955918	peak_2544	0	-	ENSMUSG	protein_coding
chr18	14806370	14806391	peak_2545	0	-	ENSMUSG	protein_coding
chr18	14806440	14806463	peak_2546	0	-	ENSMUSG	protein_coding
chr18	14822732	14822753	peak_2547	0	-	ENSMUSG	protein_coding
chr18	15220244	15220265	peak_2548	0	-	ENSMUSG	protein_coding
chr18	16770074	16770096	peak_2549	0	-	ENSMUSG	protein_coding
chr18	21166640	21166662	peak_2552	0	+	ENSMUSG	protein_coding
chr18	21299640	21299661	peak_2554	0	-	ENSMUSG	protein_coding
chr18	23593857	23593878	peak_2560	0	+	ENSMUSG	protein_coding
chr18	23769088	23769109	peak_2561	0	+	ENSMUSG	protein_coding
chr18	23789812	23789833	peak_2562	0	+	ENSMUSG	protein_coding
chr18	24416420	24416441	peak_2563	0	+	ENSMUSG	protein_coding
chr18	24646316	24646340	peak_2564	0	-	ENSMUSG	protein_coding
chr18	24666696	24666717	peak_2565	0	-	ENSMUSG	protein_coding
chr18	31794014	31794035	peak_2566	0	+	ENSMUSG	protein_coding
chr18	31997368	31997389	peak_2568	0	+	ENSMUSG	protein_coding
chr18	32586581	32586602	peak_2569	0	+	ENSMUSG	protein_coding
chr18	33616403	33616424	peak_2570	0	-	ENSMUSG	protein_coding
chr18	34420784	34420805	peak_2571	0	+	ENSMUSG	protein_coding

chr18	34428834	34428855	peak_2572	0	+	ENSMUSG	protein_coding
chr18	34434081	34434102	peak_2573	0	+	ENSMUSG	protein_coding
chr18	34621813	34621834	peak_2574	0	-	ENSMUSG	protein_coding
chr18	34954103	34954124	peak_2575	0	+	ENSMUSG	protein_coding
chr18	34983144	34983165	peak_2576	0	+	ENSMUSG	protein_coding
chr18	34991150	34991173	peak_2577	0	+	ENSMUSG	protein_coding
chr18	35000681	35000704	peak_2578	0	+	ENSMUSG	protein_coding
chr18	35098238	35098259	peak_2579	0	-	ENSMUSG	protein_coding
chr18	35102367	35102388	peak_2580	0	-	ENSMUSG	protein_coding
chr18	35107207	35107228	peak_2581	0	-	ENSMUSG	protein_coding
chr18	35279037	35279058	peak_2582	0	+	ENSMUSG	protein_coding
chr18	35388789	35388810	peak_2583	0	+	ENSMUSG	protein_coding
chr18	35512364	35512390	peak_2584	0	+	ENSMUSG	protein_coding
chr18	35733065	35733086	peak_2586	0	+	ENSMUSG	protein_coding
chr18	35750023	35750044	peak_2587	0	+	ENSMUSG	protein_coding
chr18	35772986	35773007	peak_2588	0	+	ENSMUSG	protein_coding
chr18	35948215	35948236	peak_2589	0	+	ENSMUSG	protein_coding
chr18	36724867	36724889	peak_2590	0	+	ENSMUSG	protein_coding
chr18	36797848	36797869	peak_2591	0	+	ENSMUSG	protein_coding
chr18	36842063	36842084	peak_2592	0	+	ENSMUSG	protein_coding
chr18	36916520	36916541	peak_2593	0	+	ENSMUSG	protein_coding
chr18	36956154	36956175	peak_2594	0	+	ENSMUSG	protein_coding
chr18	37958867	37958888	peak_2596	0	+	ENSMUSG	protein_coding
chr18	38013091	38013112	peak_2597	0	-	ENSMUSG	protein_coding
chr18	38013201	38013222	peak_2598	0	-	ENSMUSG	protein_coding
chr18	39155223	39155255	peak_2600	0	+	ENSMUSG	protein_coding
chr18	39168718	39168739	peak_2601	0	+	ENSMUSG	protein_coding
chr18	39171829	39171850	peak_2602	0	+	ENSMUSG	protein_coding
chr18	39182882	39182904	peak_2603	0	+	ENSMUSG	protein_coding
chr18	39193395	39193421	peak_2604	0	+	ENSMUSG	protein_coding
chr18	39199435	39199456	peak_2605	0	+	ENSMUSG	protein_coding
chr18	39250901	39250922	peak_2606	0	+	ENSMUSG	protein_coding
chr18	39261759	39261780	peak_2607	0	+	ENSMUSG	protein_coding
chr18	39288789	39288811	peak_2608	0	+	ENSMUSG	protein_coding
chr18	39319272	39319293	peak_2609	0	+	ENSMUSG	protein_coding
chr18	39370820	39370841	peak_2610	0	+	ENSMUSG	protein_coding
chr18	39384986	39385008	peak_2611	0	+	ENSMUSG	protein_coding
chr18	39427963	39427984	peak_2612	0	+	ENSMUSG	protein_coding
chr18	39615376	39615397	peak_2613	0	-	ENSMUSG	protein_coding
chr18	42708112	42708133	peak_2615	0	+	ENSMUSG	protein_coding
chr18	42728105	42728126	peak_2616	0	+	ENSMUSG	protein_coding
chr18	43585561	43585582	peak_2617	0	-	ENSMUSG	protein_coding
chr18	44579931	44579961	peak_2618	0	+	ENSMUSG	protein_coding
chr18	46403138	46403159	peak_2619	0	-	ENSMUSG	protein_coding
chr18	46412930	46412952	peak_2620	0	-	ENSMUSG	protein_coding
chr18	46450864	46450887	peak_2621	0	-	ENSMUSG	protein_coding
chr18	46455971	46455992	peak_2622	0	-	ENSMUSG	protein_coding
chr18	47169553	47169574	peak_2625	0	+	ENSMUSG	protein_coding
chr18	47417138	47417168	peak_2626	0	-	ENSMUSG	protein_coding
chr18	50222137	50222158	peak_2627	0	+	ENSMUSG	protein_coding
chr18	53354224	53354245	peak_2628	0	+	ENSMUSG	protein_coding
chr18	53357484	53357505	peak_2629	0	+	ENSMUSG	protein_coding
chr18	54107234	54107255	peak_2631	0	+	ENSMUSG	protein_coding
chr18	56698197	56698219	peak_2632	0	-	ENSMUSG	protein_coding
chr18	56892888	56892913	peak_2634	0	+	ENSMUSG	protein_coding
chr18	56930500	56930524	peak_2635	0	-	ENSMUSG	protein_coding
chr18	57529037	57529058	peak_2636	0	+	ENSMUSG	protein_coding
chr18	60934284	60934305	peak_2638	0	+	ENSMUSG	protein_coding

chr18	60936036	60936057	peak_2639	0	+	ENSMUSG	protein_coding
chr18	60936701	60936722	peak_2640	0	+	ENSMUSG	protein_coding
chr18	61715415	61715437	peak_2641	0	+	ENSMUSG	protein_coding
chr18	61729962	61729983	peak_2642	0	+	ENSMUSG	protein_coding
chr18	62679437	62679458	peak_2647	0	-	ENSMUSG	protein_coding
chr18	64671514	64671536	peak_2648	0	-	ENSMUSG	protein_coding
chr18	66158292	66158315	peak_2649	0	-	ENSMUSG	protein_coding
chr18	67270933	67270954	peak_2650	0	+	ENSMUSG	protein_coding
chr18	67561907	67561928	peak_2651	0	+	ENSMUSG	protein_coding
chr18	67574466	67574487	peak_2652	0	-	ENSMUSG	protein_coding
chr18	67624505	67624526	peak_2653	0	+	ENSMUSG	protein_coding
chr18	69511578	69511600	peak_2654	0	+	ENSMUSG	protein_coding
chr18	69564291	69564312	peak_2655	0	+	ENSMUSG	protein_coding
chr18	69694401	69694422	peak_2656	0	+	ENSMUSG	protein_coding
chr18	69765698	69765719	peak_2657	0	+	ENSMUSG	protein_coding
chr18	69823337	69823358	peak_2658	0	+	ENSMUSG	protein_coding
chr18	73733304	73733325	peak_2659	0	+	ENSMUSG	protein_coding
chr18	73737925	73737946	peak_2660	0	+	ENSMUSG	protein_coding
chr18	73740047	73740069	peak_2661	0	+	ENSMUSG	protein_coding
chr18	73857549	73857570	peak_2662	0	-	ENSMUSG	protein_coding
chr18	73931209	73931230	peak_2663	0	-	ENSMUSG	protein_coding
chr18	74036775	74036796	peak_2664	0	+	ENSMUSG	protein_coding
chr18	74039920	74039941	peak_2665	0	+	ENSMUSG	protein_coding
chr18	74634608	74634630	peak_2666	0	+	ENSMUSG	protein_coding
chr18	74648488	74648509	peak_2667	0	+	ENSMUSG	protein_coding
chr18	74658842	74658863	peak_2668	0	+	ENSMUSG	protein_coding
chr18	74752534	74752555	peak_2669	0	+	ENSMUSG	protein_coding
chr18	75160689	75160711	peak_2672	0	+	ENSMUSG	protein_coding
chr18	75161087	75161108	peak_2673	0	+	ENSMUSG	protein_coding
chr18	75161511	75161533	peak_2674	0	+	ENSMUSG	protein_coding
chr18	75161730	75161751	peak_2675	0	+	ENSMUSG	protein_coding
chr18	75223590	75223611	peak_2676	0	+	ENSMUSG	protein_coding
chr18	75621159	75621181	peak_2677	0	-	ENSMUSG	protein_coding
chr18	75630374	75630396	peak_2678	0	-	ENSMUSG	protein_coding
chr18	75713927	75713948	peak_2679	0	-	ENSMUSG	protein_coding
chr18	76078206	76078227	peak_2680	0	+	ENSMUSG	protein_coding
chr18	77210538	77210560	peak_2681	0	+	ENSMUSG	protein_coding
chr18	80934291	80934312	peak_2685	0	-	ENSMUSG	protein_coding
chr18	80973742	80973777	peak_2686	0	-	ENSMUSG	protein_coding
chr18	83157904	83157926	peak_2688	0	+	ENSMUSG	protein_coding
chr18	84252207	84252228	peak_2689	0	-	ENSMUSG	protein_coding
chr18	84720432	84720475	peak_2690	0	-	ENSMUSG	protein_coding
chr19	3504441	3504466	peak_2691	0	-	ENSMUSG	protein_coding
chr19	3628429	3628450	peak_2693	0	-	ENSMUSG	protein_coding
chr19	3772013	3772034	peak_2694	0	+	ENSMUSG	protein_coding
chr19	3792035	3792056	peak_2695	0	+	ENSMUSG	protein_coding
chr19	3804156	3804177	peak_2696	0	+	ENSMUSG	protein_coding
chr19	3804156	3804177	peak_2696	0	+	ENSMUSG	protein_coding
chr19	3875173	3875194	peak_2697	0	+	ENSMUSG	protein_coding
chr19	4762285	4762306	peak_2699	0	-	ENSMUSG	protein_coding
chr19	4791922	4791944	peak_2700	0	-	ENSMUSG	protein_coding
chr19	4793378	4793400	peak_2701	0	-	ENSMUSG	protein_coding
chr19	4796543	4796564	peak_2702	0	-	ENSMUSG	protein_coding
chr19	5099303	5099324	peak_2706	0	-	ENSMUSG	protein_coding
chr19	5264281	5264302	peak_2707	0	-	ENSMUSG	protein_coding
chr19	5274877	5274898	peak_2708	0	-	ENSMUSG	protein_coding
chr19	5279360	5279381	peak_2709	0	-	ENSMUSG	protein_coding
chr19	5284700	5284721	peak_2710	0	-	ENSMUSG	protein_coding

chr19	5382066	5382087	peak_2711	0	-	ENSMUSG	protein_coding
chr19	5696670	5696691	peak_2712	0	+	ENSMUSG	protein_coding
chr19	5953196	5953218	peak_2735	0	-	ENSMUSG	protein_coding
chr19	6057932	6057953	peak_2736	0	+	ENSMUSG	protein_coding
chr19	6294318	6294339	peak_2737	0	+	ENSMUSG	protein_coding
chr19	6297538	6297559	peak_2738	0	+	ENSMUSG	protein_coding
chr19	6364710	6364731	peak_2739	0	+	ENSMUSG	protein_coding
chr19	6371268	6371289	peak_2740	0	+	ENSMUSG	protein_coding
chr19	6985802	6985823	peak_2741	0	+	ENSMUSG	protein_coding
chr19	7049944	7049965	peak_2742	0	+	ENSMUSG	protein_coding
chr19	7057040	7057061	peak_2743	0	-	ENSMUSG	protein_coding
chr19	7165779	7165800	peak_2744	0	-	ENSMUSG	protein_coding
chr19	7191492	7191514	peak_2745	0	-	ENSMUSG	protein_coding
chr19	7543432	7543453	peak_2746	0	-	ENSMUSG	protein_coding
chr19	7549109	7549135	peak_2747	0	-	ENSMUSG	protein_coding
chr19	7608573	7608595	peak_2748	0	+	ENSMUSG	protein_coding
chr19	7611094	7611115	peak_2749	0	+	ENSMUSG	protein_coding
chr19	8816293	8816314	peak_2758	0	+	ENSMUSG	protein_coding
chr19	8901162	8901183	peak_2759	0	+	ENSMUSG	protein_coding
chr19	9007719	9007740	peak_2760	0	+	ENSMUSG	protein_coding
chr19	9041664	9041687	peak_2761	0	+	ENSMUSG	protein_coding
chr19	9043973	9043994	peak_2762	0	+	ENSMUSG	protein_coding
chr19	9074176	9074197	peak_2763	0	+	ENSMUSG	protein_coding
chr19	9090898	9090920	peak_2764	0	+	ENSMUSG	protein_coding
chr19	9092546	9092567	peak_2765	0	+	ENSMUSG	protein_coding
chr19	9092569	9092590	peak_2766	0	+	ENSMUSG	protein_coding
chr19	10058660	10058682	peak_2769	0	+	ENSMUSG	protein_coding
chr19	10150270	10150291	peak_2770	0	-	ENSMUSG	protein_coding
chr19	10470701	10470729	peak_2771	0	+	ENSMUSG	protein_coding
chr19	10511119	10511140	peak_2772	0	+	ENSMUSG	protein_coding
chr19	10516196	10516217	peak_2773	0	+	ENSMUSG	protein_coding
chr19	10693137	10693158	peak_2774	0	+	ENSMUSG	protein_coding
chr19	12554512	12554533	peak_2777	0	-	ENSMUSG	protein_coding
chr19	14534309	14534330	peak_2778	0	-	ENSMUSG	protein_coding
chr19	14545145	14545166	peak_2779	0	-	ENSMUSG	protein_coding
chr19	14666840	14666861	peak_2780	0	-	ENSMUSG	protein_coding
chr19	16214200	16214221	peak_2782	0	+	ENSMUSG	protein_coding
chr19	16300110	16300131	peak_2783	0	+	ENSMUSG	protein_coding
chr19	16347418	16347439	peak_2784	0	+	ENSMUSG	protein_coding
chr19	16366977	16367003	peak_2785	0	+	ENSMUSG	protein_coding
chr19	16544783	16544805	peak_2786	0	+	ENSMUSG	protein_coding
chr19	16555398	16555419	peak_2787	0	+	ENSMUSG	protein_coding
chr19	17911478	17911499	peak_2788	0	-	ENSMUSG	protein_coding
chr19	18663349	18663370	peak_2789	0	+	ENSMUSG	protein_coding
chr19	20692460	20692481	peak_2792	0	+	ENSMUSG	protein_coding
chr19	23694318	23694339	peak_2794	0	+	ENSMUSG	protein_coding
chr19	23694318	23694339	peak_2794	0	+	ENSMUSG	protein_coding
chr19	24601786	24601807	peak_2795	0	-	ENSMUSG	protein_coding
chr19	24601813	24601834	peak_2796	0	-	ENSMUSG	protein_coding
chr19	25499968	25499990	peak_2797	0	+	ENSMUSG	protein_coding
chr19	25581537	25581560	peak_2798	0	+	ENSMUSG	protein_coding
chr19	25624600	25624621	peak_2799	0	+	ENSMUSG	protein_coding
chr19	25635796	25635817	peak_2800	0	+	ENSMUSG	protein_coding
chr19	25643475	25643496	peak_2801	0	+	ENSMUSG	protein_coding
chr19	25656381	25656402	peak_2802	0	+	ENSMUSG	protein_coding
chr19	26686411	26686433	peak_2803	0	+	ENSMUSG	protein_coding
chr19	26728939	26728960	peak_2804	0	+	ENSMUSG	protein_coding
chr19	26744677	26744699	peak_2805	0	+	ENSMUSG	protein_coding

chr19	26765870	26765891	peak_2806	0	+	ENSMUSG	protein_coding
chr19	26767030	26767051	peak_2807	0	+	ENSMUSG	protein_coding
chr19	26769213	26769234	peak_2808	0	+	ENSMUSG	protein_coding
chr19	26780524	26780545	peak_2809	0	+	ENSMUSG	protein_coding
chr19	26798600	26798621	peak_2810	0	+	ENSMUSG	protein_coding
chr19	26848676	26848697	peak_2811	0	+	ENSMUSG	protein_coding
chr19	29101495	29101516	peak_2812	0	+	ENSMUSG	protein_coding
chr19	30131815	30131836	peak_2813	0	+	ENSMUSG	protein_coding
chr19	32230849	32230870	peak_2814	0	-	ENSMUSG	protein_coding
chr19	32293552	32293573	peak_2815	0	-	ENSMUSG	protein_coding
chr19	32462941	32462962	peak_2816	0	-	ENSMUSG	protein_coding
chr19	34915531	34915553	peak_2819	0	-	ENSMUSG	protein_coding
chr19	36956739	36956760	peak_2822	0	+	ENSMUSG	protein_coding
chr19	37036110	37036135	peak_2823	0	+	ENSMUSG	protein_coding
chr19	37068775	37068797	peak_2824	0	+	ENSMUSG	protein_coding
chr19	37624919	37624951	peak_2825	0	+	ENSMUSG	protein_coding
chr19	37683473	37683494	peak_2826	0	+	ENSMUSG	protein_coding
chr19	38144363	38144384	peak_2827	0	+	ENSMUSG	protein_coding
chr19	38470998	38471019	peak_2828	0	+	ENSMUSG	protein_coding
chr19	38634507	38634529	peak_2829	0	+	ENSMUSG	protein_coding
chr19	38672819	38672841	peak_2830	0	+	ENSMUSG	protein_coding
chr19	38741207	38741228	peak_2831	0	+	ENSMUSG	protein_coding
chr19	38877007	38877028	peak_2832	0	-	ENSMUSG	protein_coding
chr19	38889201	38889222	peak_2833	0	-	ENSMUSG	protein_coding
chr19	39005915	39005936	peak_2834	0	+	ENSMUSG	protein_coding
chr19	40381160	40381183	peak_2835	0	-	ENSMUSG	protein_coding
chr19	41570147	41570170	peak_2836	0	+	ENSMUSG	protein_coding
chr19	41635087	41635108	peak_2837	0	+	ENSMUSG	protein_coding
chr19	41635114	41635135	peak_2838	0	+	ENSMUSG	protein_coding
chr19	44188427	44188462	peak_2839	0	-	ENSMUSG	protein_coding
chr19	44377806	44377827	peak_2840	0	+	ENSMUSG	protein_coding
chr19	44379526	44379547	peak_2841	0	+	ENSMUSG	protein_coding
chr19	45016892	45016913	peak_2844	0	+	ENSMUSG	protein_coding
chr19	45017927	45017948	peak_2845	0	+	ENSMUSG	protein_coding
chr19	45031619	45031640	peak_2846	0	+	ENSMUSG	protein_coding
chr19	45082752	45082773	peak_2847	0	+	ENSMUSG	protein_coding
chr19	45452030	45452053	peak_2848	0	+	ENSMUSG	protein_coding
chr19	45576495	45576516	peak_2849	0	+	ENSMUSG	protein_coding
chr19	45595512	45595533	peak_2850	0	+	ENSMUSG	protein_coding
chr19	45846350	45846372	peak_2851	0	-	ENSMUSG	protein_coding
chr19	46110772	46110793	peak_2852	0	-	ENSMUSG	protein_coding
chr19	46267280	46267302	peak_2853	0	+	ENSMUSG	protein_coding
chr19	46502885	46502906	peak_2854	0	+	ENSMUSG	protein_coding
chr19	46640759	46640780	peak_2855	0	-	ENSMUSG	protein_coding
chr19	46697873	46697894	peak_2856	0	+	ENSMUSG	protein_coding
chr19	46836732	46836753	peak_2857	0	+	ENSMUSG	protein_coding
chr19	46897491	46897512	peak_2858	0	+	ENSMUSG	protein_coding
chr19	47013756	47013777	peak_2859	0	-	ENSMUSG	protein_coding
chr19	47338743	47338764	peak_2861	0	-	ENSMUSG	protein_coding
chr19	47341336	47341358	peak_2862	0	-	ENSMUSG	protein_coding
chr19	47538615	47538636	peak_2863	0	-	ENSMUSG	protein_coding
chr19	53230671	53230693	peak_2864	0	+	ENSMUSG	protein_coding
chr19	53249476	53249497	peak_2865	0	+	ENSMUSG	protein_coding
chr19	53253742	53253763	peak_2866	0	+	ENSMUSG	protein_coding
chr19	53320684	53320705	peak_2867	0	+	ENSMUSG	protein_coding
chr19	53412213	53412234	peak_2869	0	+	ENSMUSG	protein_coding
chr19	54019323	54019344	peak_2870	0	+	ENSMUSG	protein_coding
chr19	55455409	55455431	peak_2871	0	+	ENSMUSG	protein_coding

chr19	55515226	55515247	peak_2872	0	+	ENSMUSG	protein_coding
chr19	55679062	55679083	peak_2873	0	+	ENSMUSG	protein_coding
chr19	55816529	55816550	peak_2874	0	+	ENSMUSG	protein_coding
chr19	55845626	55845648	peak_2875	0	+	ENSMUSG	protein_coding
chr19	55863876	55863897	peak_2876	0	+	ENSMUSG	protein_coding
chr19	55865539	55865560	peak_2877	0	+	ENSMUSG	protein_coding
chr19	55914879	55914902	peak_2878	0	+	ENSMUSG	protein_coding
chr19	56874703	56874724	peak_2879	0	-	ENSMUSG	protein_coding
chr19	56921354	56921375	peak_2880	0	+	ENSMUSG	protein_coding
chr19	57270379	57270400	peak_2881	0	-	ENSMUSG	protein_coding
chr19	57456758	57456783	peak_2882	0	+	ENSMUSG	protein_coding
chr19	57561341	57561363	peak_2883	0	+	ENSMUSG	protein_coding
chr19	58028640	58028665	peak_2884	0	+	ENSMUSG	protein_coding
chr19	58053541	58053562	peak_2885	0	+	ENSMUSG	protein_coding
chr19	58524961	58524982	peak_2886	0	-	ENSMUSG	protein_coding
chr19	59083808	59083836	peak_2887	0	-	ENSMUSG	protein_coding
chr19	60008722	60008762	peak_2888	0	-	ENSMUSG	protein_coding
chr19	60840078	60840099	peak_2890	0	-	ENSMUSG	protein_coding
chr19	60842243	60842264	peak_2891	0	-	ENSMUSG	protein_coding
chr19	60842610	60842632	peak_2892	0	-	ENSMUSG	protein_coding
chr19	60842859	60842880	peak_2893	0	-	ENSMUSG	protein_coding
chr19	60845909	60845935	peak_2894	0	-	ENSMUSG	protein_coding
chr19	60846922	60846952	peak_2895	0	-	ENSMUSG	protein_coding
chr19	60848889	60848910	peak_2896	0	-	ENSMUSG	protein_coding
chr19	60857883	60857904	peak_2897	0	-	ENSMUSG	protein_coding
chr2	3085854	3085875	peak_2898	0	+	ENSMUSG	protein_coding
chr2	3230707	3230728	peak_2899	0	+	ENSMUSG	protein_coding
chr2	4715477	4715498	peak_2900	0	+	ENSMUSG	protein_coding
chr2	4974210	4974231	peak_2901	0	-	ENSMUSG	protein_coding
chr2	5364932	5364953	peak_2902	0	-	ENSMUSG	protein_coding
chr2	5878731	5878752	peak_2903	0	+	ENSMUSG	protein_coding
chr2	5908503	5908525	peak_2904	0	+	ENSMUSG	protein_coding
chr2	5976785	5976806	peak_2905	0	+	ENSMUSG	protein_coding
chr2	6478742	6478764	peak_2906	0	-	ENSMUSG	protein_coding
chr2	6495548	6495572	peak_2907	0	-	ENSMUSG	protein_coding
chr2	6501880	6501901	peak_2908	0	-	ENSMUSG	protein_coding
chr2	6628734	6628755	peak_2909	0	-	ENSMUSG	protein_coding
chr2	6716472	6716493	peak_2910	0	-	ENSMUSG	protein_coding
chr2	6723017	6723038	peak_2911	0	-	ENSMUSG	protein_coding
chr2	6731523	6731544	peak_2912	0	-	ENSMUSG	protein_coding
chr2	6782560	6782582	peak_2913	0	-	ENSMUSG	protein_coding
chr2	6792644	6792665	peak_2914	0	-	ENSMUSG	protein_coding
chr2	6796276	6796297	peak_2915	0	-	ENSMUSG	protein_coding
chr2	6819278	6819299	peak_2916	0	-	ENSMUSG	protein_coding
chr2	6868797	6868818	peak_2917	0	-	ENSMUSG	protein_coding
chr2	6870867	6870888	peak_2918	0	-	ENSMUSG	protein_coding
chr2	6882837	6882858	peak_2919	0	-	ENSMUSG	protein_coding
chr2	6997458	6997479	peak_2920	0	-	ENSMUSG	protein_coding
chr2	9985918	9985939	peak_2922	0	-	ENSMUSG	protein_coding
chr2	10136696	10136718	peak_2923	0	+	ENSMUSG	protein_coding
chr2	10333864	10333885	peak_2924	0	+	ENSMUSG	protein_coding
chr2	12318252	12318273	peak_2925	0	-	ENSMUSG	protein_coding
chr2	13185809	13185830	peak_2926	0	-	ENSMUSG	protein_coding
chr2	13503277	13503298	peak_2928	0	+	ENSMUSG	protein_coding
chr2	14586754	14586775	peak_2929	0	+	ENSMUSG	protein_coding
chr2	14814419	14814441	peak_2930	0	+	ENSMUSG	protein_coding
chr2	18284144	18284165	peak_2931	0	-	ENSMUSG	protein_coding
chr2	20658985	20659006	peak_2934	0	+	ENSMUSG	protein_coding

chr2	20666156	20666177	peak_2935	0	+	ENSMUSG	protein_coding
chr2	20667081	20667106	peak_2936	0	+	ENSMUSG	protein_coding
chr2	20726824	20726845	peak_2937	0	+	ENSMUSG	protein_coding
chr2	22445510	22445531	peak_2938	0	-	ENSMUSG	protein_coding
chr2	22852334	22852355	peak_2940	0	-	ENSMUSG	protein_coding
chr2	23375507	23375528	peak_2942	0	-	ENSMUSG	protein_coding
chr2	24576613	24576634	peak_2943	0	+	ENSMUSG	protein_coding
chr2	24718460	24718483	peak_2944	0	-	ENSMUSG	protein_coding
chr2	25430392	25430413	peak_2945	0	-	ENSMUSG	protein_coding
chr2	25439474	25439496	peak_2946	0	-	ENSMUSG	protein_coding
chr2	25864487	25864508	peak_2947	0	-	ENSMUSG	protein_coding
chr2	25936467	25936504	peak_2948	0	-	ENSMUSG	protein_coding
chr2	26766306	26766327	peak_2950	0	+	ENSMUSG	protein_coding
chr2	26766758	26766779	peak_2951	0	+	ENSMUSG	protein_coding
chr2	26766789	26766810	peak_2952	0	+	ENSMUSG	protein_coding
chr2	26767716	26767737	peak_2953	0	+	ENSMUSG	protein_coding
chr2	26768028	26768068	peak_2954	0	+	ENSMUSG	protein_coding
chr2	26768346	26768368	peak_2955	0	+	ENSMUSG	protein_coding
chr2	27119767	27119788	peak_2956	0	-	ENSMUSG	protein_coding
chr2	27257447	27257478	peak_2957	0	+	ENSMUSG	protein_coding
chr2	27305914	27305935	peak_2958	0	-	ENSMUSG	protein_coding
chr2	27873947	27873968	peak_2960	0	+	ENSMUSG	protein_coding
chr2	29475474	29475495	peak_2961	0	+	ENSMUSG	protein_coding
chr2	29524323	29524344	peak_2962	0	+	ENSMUSG	protein_coding
chr2	29586038	29586059	peak_2963	0	+	ENSMUSG	protein_coding
chr2	29838665	29838686	peak_2964	0	+	ENSMUSG	protein_coding
chr2	29856606	29856627	peak_2965	0	+	ENSMUSG	protein_coding
chr2	29871481	29871502	peak_2966	0	+	ENSMUSG	protein_coding
chr2	29873904	29873925	peak_2967	0	+	ENSMUSG	protein_coding
chr2	29873926	29873951	peak_2968	0	+	ENSMUSG	protein_coding
chr2	29878012	29878033	peak_2969	0	+	ENSMUSG	protein_coding
chr2	29880525	29880546	peak_2970	0	+	ENSMUSG	protein_coding
chr2	29885508	29885529	peak_2971	0	+	ENSMUSG	protein_coding
chr2	29886210	29886236	peak_2972	0	+	ENSMUSG	protein_coding
chr2	30263599	30263620	peak_2974	0	-	ENSMUSG	protein_coding
chr2	30670542	30670564	peak_2975	0	+	ENSMUSG	protein_coding
chr2	30899219	30899243	peak_2976	0	-	ENSMUSG	protein_coding
chr2	31022604	31022626	peak_2977	0	+	ENSMUSG	protein_coding
chr2	31436711	31436732	peak_2978	0	+	ENSMUSG	protein_coding
chr2	31544791	31544815	peak_2979	0	+	ENSMUSG	protein_coding
chr2	31552115	31552139	peak_2980	0	+	ENSMUSG	protein_coding
chr2	31622232	31622253	peak_2981	0	+	ENSMUSG	protein_coding
chr2	31763752	31763773	peak_2982	0	+	ENSMUSG	protein_coding
chr2	31877663	31877685	peak_2983	0	+	ENSMUSG	protein_coding
chr2	32020135	32020156	peak_2984	0	+	ENSMUSG	protein_coding
chr2	32038032	32038053	peak_2985	0	+	ENSMUSG	protein_coding
chr2	32072751	32072772	peak_2986	0	+	ENSMUSG	protein_coding
chr2	32077466	32077487	peak_2987	0	+	ENSMUSG	protein_coding
chr2	32077489	32077510	peak_2988	0	+	ENSMUSG	protein_coding
chr2	32081148	32081170	peak_2989	0	+	ENSMUSG	protein_coding
chr2	32081172	32081193	peak_2990	0	+	ENSMUSG	protein_coding
chr2	32159231	32159252	peak_2991	0	+	ENSMUSG	protein_coding
chr2	32159364	32159385	peak_2992	0	+	ENSMUSG	protein_coding
chr2	32159690	32159712	peak_2993	0	+	ENSMUSG	protein_coding
chr2	32219036	32219057	peak_2994	0	+	ENSMUSG	protein_coding
chr2	32818137	32818180	peak_2995	0	+	ENSMUSG	protein_coding
chr2	32818782	32818804	peak_2996	0	+	ENSMUSG	protein_coding
chr2	32818882	32818905	peak_2997	0	+	ENSMUSG	protein_coding

chr2	32895119	32895140	peak_2998	0	-	ENSMUSG	protein_coding
chr2	32904628	32904649	peak_2999	0	-	ENSMUSG	protein_coding
chr2	32924104	32924125	peak_3000	0	-	ENSMUSG	protein_coding
chr2	32925446	32925467	peak_3001	0	-	ENSMUSG	protein_coding
chr2	34226395	34226416	peak_3003	0	-	ENSMUSG	protein_coding
chr2	34287786	34287808	peak_3004	0	+	ENSMUSG	protein_coding
chr2	34982891	34982912	peak_3005	0	+	ENSMUSG	protein_coding
chr2	35030694	35030715	peak_3006	0	+	ENSMUSG	protein_coding
chr2	35040665	35040686	peak_3007	0	-	ENSMUSG	protein_coding
chr2	35529133	35529154	peak_3008	0	+	ENSMUSG	protein_coding
chr2	37255660	37255682	peak_3010	0	-	ENSMUSG	protein_coding
chr2	37397797	37397819	peak_3011	0	+	ENSMUSG	protein_coding
chr2	37397797	37397819	peak_3011	0	+	ENSMUSG	protein_coding
chr2	37406676	37406697	peak_3012	0	+	ENSMUSG	protein_coding
chr2	37406676	37406697	peak_3012	0	+	ENSMUSG	protein_coding
chr2	37871428	37871449	peak_3013	0	-	ENSMUSG	protein_coding
chr2	37961420	37961441	peak_3014	0	-	ENSMUSG	protein_coding
chr2	38555129	38555150	peak_3016	0	-	ENSMUSG	protein_coding
chr2	38717211	38717232	peak_3017	0	-	ENSMUSG	protein_coding
chr2	38774922	38774943	peak_3018	0	-	ENSMUSG	protein_coding
chr2	38860616	38860637	peak_3019	0	-	ENSMUSG	protein_coding
chr2	38860616	38860637	peak_3019	0	-	ENSMUSG	protein_coding
chr2	38883275	38883302	peak_3020	0	+	ENSMUSG	protein_coding
chr2	38978598	38978619	peak_3021	0	-	ENSMUSG	protein_coding
chr2	41618816	41618839	peak_3022	0	+	ENSMUSG	protein_coding
chr2	41628281	41628302	peak_3023	0	+	ENSMUSG	protein_coding
chr2	44589196	44589217	peak_3024	0	-	ENSMUSG	protein_coding
chr2	44853024	44853045	peak_3025	0	-	ENSMUSG	protein_coding
chr2	44859986	44860007	peak_3026	0	-	ENSMUSG	protein_coding
chr2	44942067	44942088	peak_3027	0	-	ENSMUSG	protein_coding
chr2	44945331	44945353	peak_3028	0	-	ENSMUSG	protein_coding
chr2	44959369	44959390	peak_3029	0	-	ENSMUSG	protein_coding
chr2	48691313	48691334	peak_3030	0	+	ENSMUSG	protein_coding
chr2	52484715	52484736	peak_3031	0	-	ENSMUSG	protein_coding
chr2	52523113	52523134	peak_3032	0	-	ENSMUSG	protein_coding
chr2	52525907	52525930	peak_3033	0	-	ENSMUSG	protein_coding
chr2	52531315	52531336	peak_3034	0	-	ENSMUSG	protein_coding
chr2	52720326	52720351	peak_3035	0	+	ENSMUSG	protein_coding
chr2	52802281	52802302	peak_3036	0	+	ENSMUSG	protein_coding
chr2	52821886	52821908	peak_3037	0	+	ENSMUSG	protein_coding
chr2	52836474	52836504	peak_3038	0	+	ENSMUSG	protein_coding
chr2	53009753	53009774	peak_3039	0	-	ENSMUSG	protein_coding
chr2	53063347	53063369	peak_3040	0	+	ENSMUSG	protein_coding
chr2	59562826	59562847	peak_3043	0	+	ENSMUSG	protein_coding
chr2	59629840	59629861	peak_3044	0	+	ENSMUSG	protein_coding
chr2	59697317	59697338	peak_3045	0	-	ENSMUSG	protein_coding
chr2	59774781	59774802	peak_3046	0	-	ENSMUSG	protein_coding
chr2	59807392	59807413	peak_3047	0	-	ENSMUSG	protein_coding
chr2	60756970	60756994	peak_3048	0	-	ENSMUSG	protein_coding
chr2	65046281	65046304	peak_3049	0	-	ENSMUSG	protein_coding
chr2	65062300	65062322	peak_3050	0	-	ENSMUSG	protein_coding
chr2	68068036	68068058	peak_3052	0	-	ENSMUSG	protein_coding
chr2	68128352	68128373	peak_3053	0	-	ENSMUSG	protein_coding
chr2	68152626	68152647	peak_3054	0	-	ENSMUSG	protein_coding
chr2	68184193	68184215	peak_3055	0	-	ENSMUSG	protein_coding
chr2	68241502	68241523	peak_3056	0	-	ENSMUSG	protein_coding
chr2	68300483	68300504	peak_3057	0	-	ENSMUSG	protein_coding
chr2	68714392	68714413	peak_3058	0	+	ENSMUSG	protein_coding

chr2	68850435	68850456	peak_3059	0	+	ENSMUSG	protein_coding
chr2	68879986	68880010	peak_3060	0	+	ENSMUSG	protein_coding
chr2	68900088	68900109	peak_3061	0	+	ENSMUSG	protein_coding
chr2	69628490	69628511	peak_3062	0	+	ENSMUSG	protein_coding
chr2	69665565	69665586	peak_3063	0	+	ENSMUSG	protein_coding
chr2	69706837	69706859	peak_3064	0	+	ENSMUSG	protein_coding
chr2	69708855	69708876	peak_3065	0	+	ENSMUSG	protein_coding
chr2	69784123	69784144	peak_3066	0	+	ENSMUSG	protein_coding
chr2	69832866	69832887	peak_3067	0	+	ENSMUSG	protein_coding
chr2	70583998	70584019	peak_3068	0	-	ENSMUSG	protein_coding
chr2	70634935	70634956	peak_3069	0	-	ENSMUSG	protein_coding
chr2	70662750	70662772	peak_3070	0	-	ENSMUSG	protein_coding
chr2	71052507	71052528	peak_3071	0	+	ENSMUSG	protein_coding
chr2	71175393	71175414	peak_3072	0	+	ENSMUSG	protein_coding
chr2	71630248	71630269	peak_3073	0	+	ENSMUSG	protein_coding
chr2	71739027	71739048	peak_3074	0	+	ENSMUSG	protein_coding
chr2	72228391	72228413	peak_3075	0	+	ENSMUSG	protein_coding
chr2	73656544	73656565	peak_3076	0	-	ENSMUSG	protein_coding
chr2	73719656	73719677	peak_3077	0	-	ENSMUSG	protein_coding
chr2	76523334	76523358	peak_3078	0	+	ENSMUSG	protein_coding
chr2	76962342	76962363	peak_3079	0	-	ENSMUSG	protein_coding
chr2	77041504	77041525	peak_3080	0	-	ENSMUSG	protein_coding
chr2	77498638	77498659	peak_3081	0	-	ENSMUSG	protein_coding
chr2	77551354	77551375	peak_3082	0	-	ENSMUSG	protein_coding
chr2	77631559	77631580	peak_3083	0	-	ENSMUSG	protein_coding
chr2	77759239	77759260	peak_3084	0	-	ENSMUSG	protein_coding
chr2	78753180	78753201	peak_3085	0	+	ENSMUSG	protein_coding
chr2	79485196	79485217	peak_3086	0	+	ENSMUSG	protein_coding
chr2	84254653	84254674	peak_3087	0	-	ENSMUSG	protein_coding
chr2	84490839	84490860	peak_3088	0	-	ENSMUSG	protein_coding
chr2	84884487	84884521	peak_3090	0	+	ENSMUSG	protein_coding
chr2	84885653	84885674	peak_3091	0	+	ENSMUSG	protein_coding
chr2	90358709	90358730	peak_3093	0	-	ENSMUSG	protein_coding
chr2	90379691	90379712	peak_3094	0	+	ENSMUSG	protein_coding
chr2	90597536	90597557	peak_3095	0	+	ENSMUSG	protein_coding
chr2	90815938	90815959	peak_3096	0	+	ENSMUSG	protein_coding
chr2	91412539	91412565	peak_3097	0	+	ENSMUSG	protein_coding
chr2	91436206	91436228	peak_3098	0	+	ENSMUSG	protein_coding
chr2	91726803	91726824	peak_3099	0	+	ENSMUSG	protein_coding
chr2	91774410	91774431	peak_3100	0	-	ENSMUSG	protein_coding
chr2	92024861	92024882	peak_3101	0	+	ENSMUSG	protein_coding
chr2	92026224	92026245	peak_3102	0	+	ENSMUSG	protein_coding
chr2	92061635	92061656	peak_3103	0	+	ENSMUSG	protein_coding
chr2	92088067	92088088	peak_3104	0	+	ENSMUSG	protein_coding
chr2	92251040	92251061	peak_3105	0	-	ENSMUSG	protein_coding
chr2	92827927	92827948	peak_3106	0	-	ENSMUSG	protein_coding
chr2	93260883	93260905	peak_3107	0	-	ENSMUSG	protein_coding
chr2	93264494	93264515	peak_3108	0	-	ENSMUSG	protein_coding
chr2	93649898	93649919	peak_3109	0	-	ENSMUSG	protein_coding
chr2	93934926	93934948	peak_3110	0	-	ENSMUSG	protein_coding
chr2	94162111	94162133	peak_3111	0	-	ENSMUSG	protein_coding
chr2	94162224	94162245	peak_3112	0	-	ENSMUSG	protein_coding
chr2	1.02E+08	1.02E+08	peak_3113	0	-	ENSMUSG	protein_coding
chr2	1.02E+08	1.02E+08	peak_3114	0	-	ENSMUSG	protein_coding
chr2	1.02E+08	1.02E+08	peak_3115	0	-	ENSMUSG	protein_coding
chr2	1.03E+08	1.03E+08	peak_3116	0	+	ENSMUSG	protein_coding
chr2	1.03E+08	1.03E+08	peak_3117	0	+	ENSMUSG	protein_coding
chr2	1.03E+08	1.03E+08	peak_3118	0	+	ENSMUSG	protein_coding

chr2	1.03E+08	1.03E+08	peak_3119	0	+	ENSMUSG	protein_coding
chr2	1.03E+08	1.03E+08	peak_3120	0	+	ENSMUSG	protein_coding
chr2	1.03E+08	1.03E+08	peak_3121	0	+	ENSMUSG	protein_coding
chr2	1.03E+08	1.03E+08	peak_3122	0	+	ENSMUSG	protein_coding
chr2	1.03E+08	1.03E+08	peak_3123	0	+	ENSMUSG	protein_coding
chr2	1.04E+08	1.04E+08	peak_3124	0	-	ENSMUSG	protein_coding
chr2	1.04E+08	1.04E+08	peak_3125	0	-	ENSMUSG	protein_coding
chr2	1.05E+08	1.05E+08	peak_3127	0	-	ENSMUSG	protein_coding
chr2	1.05E+08	1.05E+08	peak_3131	0	+	ENSMUSG	protein_coding
chr2	1.05E+08	1.05E+08	peak_3132	0	+	ENSMUSG	protein_coding
chr2	1.05E+08	1.05E+08	peak_3133	0	+	ENSMUSG	protein_coding
chr2	1.05E+08	1.05E+08	peak_3134	0	+	ENSMUSG	protein_coding
chr2	1.05E+08	1.05E+08	peak_3135	0	+	ENSMUSG	protein_coding
chr2	1.05E+08	1.05E+08	peak_3136	0	+	ENSMUSG	protein_coding
chr2	1.07E+08	1.07E+08	peak_3137	0	+	ENSMUSG	protein_coding
chr2	1.14E+08	1.14E+08	peak_3140	0	-	ENSMUSG	protein_coding
chr2	1.18E+08	1.18E+08	peak_3141	0	+	ENSMUSG	protein_coding
chr2	1.19E+08	1.19E+08	peak_3142	0	+	ENSMUSG	protein_coding
chr2	1.2E+08	1.2E+08	peak_3146	0	+	ENSMUSG	protein_coding
chr2	1.2E+08	1.2E+08	peak_3147	0	+	ENSMUSG	protein_coding
chr2	1.2E+08	1.2E+08	peak_3148	0	+	ENSMUSG	protein_coding
chr2	1.2E+08	1.2E+08	peak_3149	0	-	ENSMUSG	protein_coding
chr2	1.2E+08	1.2E+08	peak_3149	0	-	ENSMUSG	protein_coding
chr2	1.2E+08	1.2E+08	peak_3150	0	-	ENSMUSG	protein_coding
chr2	1.21E+08	1.21E+08	peak_3151	0	+	ENSMUSG	protein_coding
chr2	1.21E+08	1.21E+08	peak_3152	0	+	ENSMUSG	protein_coding
chr2	1.21E+08	1.21E+08	peak_3153	0	-	ENSMUSG	protein_coding
chr2	1.21E+08	1.21E+08	peak_3153	0	-	ENSMUSG	protein_coding
chr2	1.21E+08	1.21E+08	peak_3154	0	-	ENSMUSG	protein_coding
chr2	1.21E+08	1.21E+08	peak_3155	0	+	ENSMUSG	protein_coding
chr2	1.21E+08	1.21E+08	peak_3156	0	+	ENSMUSG	protein_coding
chr2	1.21E+08	1.21E+08	peak_3157	0	+	ENSMUSG	protein_coding
chr2	1.21E+08	1.21E+08	peak_3157	0	+	ENSMUSG	protein_coding
chr2	1.22E+08	1.22E+08	peak_3158	0	-	ENSMUSG	protein_coding
chr2	1.22E+08	1.22E+08	peak_3159	0	-	ENSMUSG	protein_coding
chr2	1.22E+08	1.22E+08	peak_3160	0	-	ENSMUSG	protein_coding
chr2	1.22E+08	1.22E+08	peak_3161	0	+	ENSMUSG	protein_coding
chr2	1.22E+08	1.22E+08	peak_3166	0	-	ENSMUSG	protein_coding
chr2	1.22E+08	1.22E+08	peak_3167	0	-	ENSMUSG	protein_coding
chr2	1.24E+08	1.24E+08	peak_3168	0	+	ENSMUSG	protein_coding
chr2	1.24E+08	1.24E+08	peak_3169	0	+	ENSMUSG	protein_coding
chr2	1.25E+08	1.25E+08	peak_3170	0	-	ENSMUSG	protein_coding
chr2	1.25E+08	1.25E+08	peak_3171	0	-	ENSMUSG	protein_coding
chr2	1.25E+08	1.25E+08	peak_3172	0	-	ENSMUSG	protein_coding
chr2	1.25E+08	1.25E+08	peak_3173	0	-	ENSMUSG	protein_coding
chr2	1.26E+08	1.26E+08	peak_3174	0	-	ENSMUSG	protein_coding
chr2	1.27E+08	1.27E+08	peak_3175	0	+	ENSMUSG	protein_coding
chr2	1.27E+08	1.27E+08	peak_3175	0	+	ENSMUSG	protein_coding
chr2	1.27E+08	1.27E+08	peak_3176	0	-	ENSMUSG	protein_coding
chr2	1.27E+08	1.27E+08	peak_3177	0	-	ENSMUSG	protein_coding
chr2	1.27E+08	1.27E+08	peak_3178	0	-	ENSMUSG	protein_coding
chr2	1.3E+08	1.3E+08	peak_3179	0	-	ENSMUSG	protein_coding
chr2	1.3E+08	1.3E+08	peak_3180	0	+	ENSMUSG	protein_coding
chr2	1.3E+08	1.3E+08	peak_3181	0	+	ENSMUSG	protein_coding
chr2	1.3E+08	1.3E+08	peak_3182	0	+	ENSMUSG	protein_coding
chr2	1.3E+08	1.3E+08	peak_3183	0	+	ENSMUSG	protein_coding
chr2	1.3E+08	1.3E+08	peak_3184	0	+	ENSMUSG	protein_coding
chr2	1.3E+08	1.3E+08	peak_3185	0	+	ENSMUSG	protein_coding

chr2	1.31E+08	1.31E+08	peak_3186	0	-	ENSMUSG	protein_coding
chr2	1.31E+08	1.31E+08	peak_3187	0	-	ENSMUSG	protein_coding
chr2	1.31E+08	1.31E+08	peak_3188	0	-	ENSMUSG	protein_coding
chr2	1.31E+08	1.31E+08	peak_3189	0	-	ENSMUSG	protein_coding
chr2	1.33E+08	1.33E+08	peak_3190	0	+	ENSMUSG	protein_coding
chr2	1.36E+08	1.36E+08	peak_3191	0	+	ENSMUSG	protein_coding
chr2	1.36E+08	1.36E+08	peak_3192	0	+	ENSMUSG	protein_coding
chr2	1.36E+08	1.36E+08	peak_3193	0	+	ENSMUSG	protein_coding
chr2	1.37E+08	1.37E+08	peak_3194	0	-	ENSMUSG	protein_coding
chr2	1.37E+08	1.37E+08	peak_3195	0	-	ENSMUSG	protein_coding
chr2	1.43E+08	1.43E+08	peak_3197	0	-	ENSMUSG	protein_coding
chr2	1.44E+08	1.44E+08	peak_3198	0	-	ENSMUSG	protein_coding
chr2	1.44E+08	1.44E+08	peak_3199	0	-	ENSMUSG	protein_coding
chr2	1.44E+08	1.44E+08	peak_3200	0	-	ENSMUSG	protein_coding
chr2	1.44E+08	1.44E+08	peak_3201	0	-	ENSMUSG	protein_coding
chr2	1.44E+08	1.44E+08	peak_3202	0	+	ENSMUSG	protein_coding
chr2	1.46E+08	1.46E+08	peak_3203	0	-	ENSMUSG	protein_coding
chr2	1.46E+08	1.46E+08	peak_3204	0	-	ENSMUSG	protein_coding
chr2	1.46E+08	1.46E+08	peak_3205	0	-	ENSMUSG	protein_coding
chr2	1.51E+08	1.51E+08	peak_3206	0	+	ENSMUSG	protein_coding
chr2	1.51E+08	1.51E+08	peak_3207	0	-	ENSMUSG	protein_coding
chr2	1.51E+08	1.51E+08	peak_3208	0	-	ENSMUSG	protein_coding
chr2	1.51E+08	1.51E+08	peak_3209	0	+	ENSMUSG	protein_coding
chr2	1.51E+08	1.51E+08	peak_3210	0	+	ENSMUSG	protein_coding
chr2	1.51E+08	1.51E+08	peak_3211	0	+	ENSMUSG	protein_coding
chr2	1.52E+08	1.52E+08	peak_3212	0	+	ENSMUSG	protein_coding
chr2	1.52E+08	1.52E+08	peak_3213	0	+	ENSMUSG	protein_coding
chr2	1.53E+08	1.53E+08	peak_3214	0	+	ENSMUSG	protein_coding
chr2	1.53E+08	1.53E+08	peak_3215	0	-	ENSMUSG	protein_coding
chr2	1.53E+08	1.53E+08	peak_3216	0	+	ENSMUSG	protein_coding
chr2	1.53E+08	1.53E+08	peak_3217	0	-	ENSMUSG	protein_coding
chr2	1.54E+08	1.54E+08	peak_3218	0	+	ENSMUSG	protein_coding
chr2	1.55E+08	1.55E+08	peak_3219	0	+	ENSMUSG	protein_coding
chr2	1.55E+08	1.55E+08	peak_3220	0	+	ENSMUSG	protein_coding
chr2	1.55E+08	1.55E+08	peak_3220	0	+	ENSMUSG	protein_coding
chr2	1.55E+08	1.55E+08	peak_3221	0	+	ENSMUSG	protein_coding
chr2	1.55E+08	1.55E+08	peak_3221	0	+	ENSMUSG	protein_coding
chr2	1.55E+08	1.55E+08	peak_3222	0	+	ENSMUSG	protein_coding
chr2	1.55E+08	1.55E+08	peak_3222	0	+	ENSMUSG	protein_coding
chr2	1.55E+08	1.55E+08	peak_3223	0	+	ENSMUSG	protein_coding
chr2	1.55E+08	1.55E+08	peak_3223	0	+	ENSMUSG	protein_coding
chr2	1.55E+08	1.55E+08	peak_3224	0	+	ENSMUSG	protein_coding
chr2	1.55E+08	1.55E+08	peak_3225	0	-	ENSMUSG	protein_coding
chr2	1.55E+08	1.55E+08	peak_3226	0	+	ENSMUSG	protein_coding
chr2	1.55E+08	1.55E+08	peak_3226	0	+	ENSMUSG	protein_coding
chr2	1.56E+08	1.56E+08	peak_3227	0	+	ENSMUSG	protein_coding
chr2	1.56E+08	1.56E+08	peak_3227	0	+	ENSMUSG	protein_coding
chr2	1.56E+08	1.56E+08	peak_3228	0	-	ENSMUSG	protein_coding
chr2	1.56E+08	1.56E+08	peak_3229	0	-	ENSMUSG	protein_coding
chr2	1.56E+08	1.56E+08	peak_3230	0	-	ENSMUSG	protein_coding
chr2	1.56E+08	1.56E+08	peak_3231	0	-	ENSMUSG	protein_coding
chr2	1.56E+08	1.56E+08	peak_3232	0	-	ENSMUSG	protein_coding
chr2	1.56E+08	1.56E+08	peak_3233	0	-	ENSMUSG	protein_coding
chr2	1.56E+08	1.56E+08	peak_3234	0	-	ENSMUSG	protein_coding
chr2	1.56E+08	1.56E+08	peak_3235	0	-	ENSMUSG	protein_coding
chr2	1.57E+08	1.57E+08	peak_3237	0	+	ENSMUSG	protein_coding
chr2	1.57E+08	1.57E+08	peak_3238	0	+	ENSMUSG	protein_coding
chr2	1.57E+08	1.57E+08	peak_3239	0	+	ENSMUSG	protein_coding

chr2	1.57E+08	1.57E+08	peak_3240	0	-	ENSMUSG	protein_coding
chr2	1.57E+08	1.57E+08	peak_3241	0	+	ENSMUSG	protein_coding
chr2	1.57E+08	1.57E+08	peak_3242	0	+	ENSMUSG	protein_coding
chr2	1.6E+08	1.6E+08	peak_3245	0	+	ENSMUSG	protein_coding
chr2	1.61E+08	1.61E+08	peak_3246	0	+	ENSMUSG	protein_coding
chr2	1.61E+08	1.61E+08	peak_3247	0	+	ENSMUSG	protein_coding
chr2	1.61E+08	1.61E+08	peak_3248	0	+	ENSMUSG	protein_coding
chr2	1.61E+08	1.61E+08	peak_3249	0	+	ENSMUSG	protein_coding
chr2	1.61E+08	1.61E+08	peak_3249	0	+	ENSMUSG	protein_coding
chr2	1.61E+08	1.61E+08	peak_3250	0	-	ENSMUSG	protein_coding
chr2	1.61E+08	1.61E+08	peak_3251	0	-	ENSMUSG	protein_coding
chr2	1.61E+08	1.61E+08	peak_3252	0	-	ENSMUSG	protein_coding
chr2	1.61E+08	1.61E+08	peak_3253	0	-	ENSMUSG	protein_coding
chr2	1.61E+08	1.61E+08	peak_3254	0	-	ENSMUSG	protein_coding
chr2	1.63E+08	1.63E+08	peak_3255	0	+	ENSMUSG	protein_coding
chr2	1.63E+08	1.63E+08	peak_3256	0	+	ENSMUSG	protein_coding
chr2	1.63E+08	1.63E+08	peak_3257	0	+	ENSMUSG	protein_coding
chr2	1.63E+08	1.63E+08	peak_3258	0	+	ENSMUSG	protein_coding
chr2	1.63E+08	1.63E+08	peak_3259	0	+	ENSMUSG	protein_coding
chr2	1.63E+08	1.63E+08	peak_3260	0	-	ENSMUSG	protein_coding
chr2	1.64E+08	1.64E+08	peak_3261	0	+	ENSMUSG	protein_coding
chr2	1.65E+08	1.65E+08	peak_3262	0	+	ENSMUSG	protein_coding
chr2	1.66E+08	1.66E+08	peak_3263	0	+	ENSMUSG	protein_coding
chr2	1.66E+08	1.66E+08	peak_3264	0	+	ENSMUSG	protein_coding
chr2	1.66E+08	1.66E+08	peak_3265	0	-	ENSMUSG	protein_coding
chr2	1.67E+08	1.67E+08	peak_3266	0	+	ENSMUSG	protein_coding
chr2	1.67E+08	1.67E+08	peak_3267	0	-	ENSMUSG	protein_coding
chr2	1.67E+08	1.67E+08	peak_3268	0	-	ENSMUSG	protein_coding
chr2	1.67E+08	1.67E+08	peak_3269	0	-	ENSMUSG	protein_coding
chr2	1.67E+08	1.67E+08	peak_3270	0	+	ENSMUSG	protein_coding
chr2	1.67E+08	1.67E+08	peak_3271	0	+	ENSMUSG	protein_coding
chr2	1.67E+08	1.67E+08	peak_3272	0	+	ENSMUSG	protein_coding
chr2	1.67E+08	1.67E+08	peak_3273	0	+	ENSMUSG	protein_coding
chr2	1.67E+08	1.67E+08	peak_3274	0	+	ENSMUSG	protein_coding
chr2	1.68E+08	1.68E+08	peak_3275	0	-	ENSMUSG	protein_coding
chr2	1.68E+08	1.68E+08	peak_3275	0	-	ENSMUSG	protein_coding
chr2	1.68E+08	1.68E+08	peak_3276	0	-	ENSMUSG	protein_coding
chr2	1.68E+08	1.68E+08	peak_3276	0	-	ENSMUSG	protein_coding
chr2	1.69E+08	1.69E+08	peak_3278	0	-	ENSMUSG	protein_coding
chr2	1.69E+08	1.69E+08	peak_3280	0	+	ENSMUSG	protein_coding
chr2	1.69E+08	1.69E+08	peak_3281	0	+	ENSMUSG	protein_coding
chr2	1.69E+08	1.69E+08	peak_3282	0	+	ENSMUSG	protein_coding
chr2	1.69E+08	1.69E+08	peak_3283	0	+	ENSMUSG	protein_coding
chr2	1.69E+08	1.69E+08	peak_3284	0	+	ENSMUSG	protein_coding
chr2	1.69E+08	1.69E+08	peak_3285	0	+	ENSMUSG	protein_coding
chr2	1.69E+08	1.69E+08	peak_3286	0	+	ENSMUSG	protein_coding
chr2	1.69E+08	1.69E+08	peak_3287	0	+	ENSMUSG	protein_coding
chr2	1.7E+08	1.7E+08	peak_3288	0	+	ENSMUSG	protein_coding
chr2	1.7E+08	1.7E+08	peak_3289	0	+	ENSMUSG	protein_coding
chr2	1.7E+08	1.7E+08	peak_3290	0	+	ENSMUSG	protein_coding
chr2	1.7E+08	1.7E+08	peak_3291	0	+	ENSMUSG	protein_coding
chr2	1.7E+08	1.7E+08	peak_3292	0	+	ENSMUSG	protein_coding
chr2	1.7E+08	1.7E+08	peak_3293	0	+	ENSMUSG	protein_coding
chr2	1.7E+08	1.7E+08	peak_3294	0	+	ENSMUSG	protein_coding
chr2	1.7E+08	1.7E+08	peak_3295	0	+	ENSMUSG	protein_coding
chr2	1.7E+08	1.7E+08	peak_3296	0	+	ENSMUSG	protein_coding
chr2	1.7E+08	1.7E+08	peak_3297	0	+	ENSMUSG	protein_coding
chr2	1.7E+08	1.7E+08	peak_3298	0	+	ENSMUSG	protein_coding

chr2	1.7E+08	1.7E+08	peak_3299	0	+	ENSMUSG	protein_coding
chr2	1.7E+08	1.7E+08	peak_3300	0	+	ENSMUSG	protein_coding
chr2	1.7E+08	1.7E+08	peak_3301	0	+	ENSMUSG	protein_coding
chr2	1.7E+08	1.7E+08	peak_3302	0	+	ENSMUSG	protein_coding
chr2	1.7E+08	1.7E+08	peak_3303	0	+	ENSMUSG	protein_coding
chr2	1.7E+08	1.7E+08	peak_3304	0	+	ENSMUSG	protein_coding
chr2	1.7E+08	1.7E+08	peak_3305	0	+	ENSMUSG	protein_coding
chr2	1.7E+08	1.7E+08	peak_3306	0	+	ENSMUSG	protein_coding
chr2	1.7E+08	1.7E+08	peak_3307	0	+	ENSMUSG	protein_coding
chr2	1.7E+08	1.7E+08	peak_3308	0	+	ENSMUSG	protein_coding
chr2	1.73E+08	1.73E+08	peak_3309	0	-	ENSMUSG	protein_coding
chr2	1.73E+08	1.73E+08	peak_3310	0	-	ENSMUSG	protein_coding
chr2	1.74E+08	1.74E+08	peak_3313	0	+	ENSMUSG	protein_coding
chr2	1.74E+08	1.74E+08	peak_3314	0	+	ENSMUSG	protein_coding
chr2	1.74E+08	1.74E+08	peak_3314	0	+	ENSMUSG	protein_coding
chr2	1.74E+08	1.74E+08	peak_3315	0	+	ENSMUSG	protein_coding
chr2	1.74E+08	1.74E+08	peak_3315	0	+	ENSMUSG	protein_coding
chr2	1.78E+08	1.78E+08	peak_3316	0	-	ENSMUSG	protein_coding
chr2	1.8E+08	1.8E+08	peak_3317	0	-	ENSMUSG	protein_coding
chr2	1.8E+08	1.8E+08	peak_3318	0	+	ENSMUSG	protein_coding
chr2	1.8E+08	1.8E+08	peak_3319	0	+	ENSMUSG	protein_coding
chr2	1.8E+08	1.8E+08	peak_3320	0	-	ENSMUSG	protein_coding
chr2	1.8E+08	1.8E+08	peak_3321	0	-	ENSMUSG	protein_coding
chr2	1.8E+08	1.8E+08	peak_3322	0	-	ENSMUSG	protein_coding
chr2	1.81E+08	1.81E+08	peak_3323	0	-	ENSMUSG	protein_coding
chr2	1.81E+08	1.81E+08	peak_3324	0	-	ENSMUSG	protein_coding
chr2	1.81E+08	1.81E+08	peak_3325	0	-	ENSMUSG	protein_coding
chr2	1.81E+08	1.81E+08	peak_3326	0	+	ENSMUSG	protein_coding
chr3	5243303	5243324	peak_3327	0	+	ENSMUSG	protein_coding
chr3	8939447	8939468	peak_3328	0	-	ENSMUSG	protein_coding
chr3	9001726	9001749	peak_3329	0	-	ENSMUSG	protein_coding
chr3	9725087	9725108	peak_3331	0	-	ENSMUSG	protein_coding
chr3	16084342	16084363	peak_3332	0	+	ENSMUSG	protein_coding
chr3	16091412	16091434	peak_3333	0	+	ENSMUSG	protein_coding
chr3	18077627	18077648	peak_3335	0	-	ENSMUSG	protein_coding
chr3	19527994	19528017	peak_3336	0	-	ENSMUSG	protein_coding
chr3	19528390	19528411	peak_3337	0	+	ENSMUSG	protein_coding
chr3	19931437	19931458	peak_3340	0	-	ENSMUSG	protein_coding
chr3	25627288	25627309	peak_3344	0	-	ENSMUSG	protein_coding
chr3	27030824	27030845	peak_3345	0	-	ENSMUSG	protein_coding
chr3	27044934	27044955	peak_3346	0	-	ENSMUSG	protein_coding
chr3	27052745	27052766	peak_3347	0	-	ENSMUSG	protein_coding
chr3	27101545	27101566	peak_3348	0	+	ENSMUSG	protein_coding
chr3	27359914	27359935	peak_3349	0	-	ENSMUSG	protein_coding
chr3	27437004	27437026	peak_3350	0	-	ENSMUSG	protein_coding
chr3	27494518	27494539	peak_3351	0	-	ENSMUSG	protein_coding
chr3	27519566	27519588	peak_3352	0	-	ENSMUSG	protein_coding
chr3	27595247	27595268	peak_3353	0	-	ENSMUSG	protein_coding
chr3	27596115	27596139	peak_3354	0	-	ENSMUSG	protein_coding
chr3	28162279	28162300	peak_3355	0	+	ENSMUSG	protein_coding
chr3	28162304	28162325	peak_3356	0	+	ENSMUSG	protein_coding
chr3	28162676	28162697	peak_3357	0	+	ENSMUSG	protein_coding
chr3	28199588	28199609	peak_3358	0	+	ENSMUSG	protein_coding
chr3	28211661	28211683	peak_3359	0	+	ENSMUSG	protein_coding
chr3	28234580	28234601	peak_3360	0	+	ENSMUSG	protein_coding
chr3	28327422	28327443	peak_3361	0	+	ENSMUSG	protein_coding
chr3	28360799	28360821	peak_3362	0	+	ENSMUSG	protein_coding
chr3	28479818	28479843	peak_3363	0	+	ENSMUSG	protein_coding

chr3	28486868	28486890	peak_3364	0	+	ENSMUSG	protein_coding
chr3	30814471	30814492	peak_3365	0	-	ENSMUSG	protein_coding
chr3	30896098	30896119	peak_3366	0	+	ENSMUSG	protein_coding
chr3	32476039	32476060	peak_3367	0	+	ENSMUSG	protein_coding
chr3	33944949	33944970	peak_3368	0	+	ENSMUSG	protein_coding
chr3	35675228	35675250	peak_3369	0	+	ENSMUSG	protein_coding
chr3	35709559	35709580	peak_3370	0	+	ENSMUSG	protein_coding
chr3	35743784	35743805	peak_3371	0	+	ENSMUSG	protein_coding
chr3	36064806	36064827	peak_3372	0	+	ENSMUSG	protein_coding
chr3	37367952	37367973	peak_3373	0	+	ENSMUSG	protein_coding
chr3	37388255	37388276	peak_3374	0	+	ENSMUSG	protein_coding
chr3	37399284	37399305	peak_3375	0	+	ENSMUSG	protein_coding
chr3	40459513	40459534	peak_3376	0	+	ENSMUSG	protein_coding
chr3	41400593	41400614	peak_3377	0	+	ENSMUSG	protein_coding
chr3	41476526	41476547	peak_3378	0	-	ENSMUSG	protein_coding
chr3	49560394	49560416	peak_3380	0	-	ENSMUSG	protein_coding
chr3	51112026	51112047	peak_3381	0	-	ENSMUSG	protein_coding
chr3	51245871	51245892	peak_3386	0	+	ENSMUSG	protein_coding
chr3	51618921	51618942	peak_3387	0	-	ENSMUSG	protein_coding
chr3	51655341	51655362	peak_3388	0	-	ENSMUSG	protein_coding
chr3	51862538	51862559	peak_3389	0	-	ENSMUSG	protein_coding
chr3	51883175	51883196	peak_3390	0	-	ENSMUSG	protein_coding
chr3	52077473	52077494	peak_3391	0	+	ENSMUSG	protein_coding
chr3	52097103	52097124	peak_3392	0	+	ENSMUSG	protein_coding
chr3	55808926	55808947	peak_3396	0	-	ENSMUSG	protein_coding
chr3	55808954	55808975	peak_3397	0	-	ENSMUSG	protein_coding
chr3	55980254	55980278	peak_3398	0	-	ENSMUSG	protein_coding
chr3	57313188	57313209	peak_3399	0	-	ENSMUSG	protein_coding
chr3	58835481	58835502	peak_3401	0	+	ENSMUSG	protein_coding
chr3	58835481	58835502	peak_3401	0	+	ENSMUSG	protein_coding
chr3	60370159	60370180	peak_3402	0	+	ENSMUSG	protein_coding
chr3	63157868	63157889	peak_3403	0	+	ENSMUSG	protein_coding
chr3	68889368	68889389	peak_3404	0	-	ENSMUSG	protein_coding
chr3	69150485	69150506	peak_3405	0	+	ENSMUSG	protein_coding
chr3	69153717	69153738	peak_3406	0	+	ENSMUSG	protein_coding
chr3	69159814	69159835	peak_3407	0	+	ENSMUSG	protein_coding
chr3	69165248	69165271	peak_3408	0	+	ENSMUSG	protein_coding
chr3	69539223	69539248	peak_3410	0	+	ENSMUSG	protein_coding
chr3	75342279	75342300	peak_3411	0	-	ENSMUSG	protein_coding
chr3	78971445	78971466	peak_3412	0	-	ENSMUSG	protein_coding
chr3	79710866	79710889	peak_3414	0	+	ENSMUSG	protein_coding
chr3	79728578	79728599	peak_3415	0	+	ENSMUSG	protein_coding
chr3	84024773	84024794	peak_3416	0	-	ENSMUSG	protein_coding
chr3	84350124	84350145	peak_3417	0	-	ENSMUSG	protein_coding
chr3	84707727	84707750	peak_3418	0	+	ENSMUSG	protein_coding
chr3	84707727	84707750	peak_3418	0	+	ENSMUSG	protein_coding
chr3	85520680	85520703	peak_3419	0	-	ENSMUSG	protein_coding
chr3	85520680	85520703	peak_3419	0	-	ENSMUSG	protein_coding
chr3	85603475	85603496	peak_3420	0	+	ENSMUSG	protein_coding
chr3	85800650	85800671	peak_3422	0	+	ENSMUSG	protein_coding
chr3	85942409	85942430	peak_3423	0	-	ENSMUSG	protein_coding
chr3	85942779	85942801	peak_3424	0	-	ENSMUSG	protein_coding
chr3	85944609	85944632	peak_3425	0	-	ENSMUSG	protein_coding
chr3	85945662	85945686	peak_3426	0	-	ENSMUSG	protein_coding
chr3	86152057	86152080	peak_3427	0	+	ENSMUSG	protein_coding
chr3	86181866	86181888	peak_3428	0	+	ENSMUSG	protein_coding
chr3	87463599	87463620	peak_3429	0	+	ENSMUSG	protein_coding
chr3	87534470	87534491	peak_3430	0	+	ENSMUSG	protein_coding

chr3	87800057	87800078	peak_3431	0	-	ENSMUSG	protein_coding
chr3	88211888	88211909	peak_3433	0	-	ENSMUSG	protein_coding
chr3	88662347	88662368	peak_3434	0	+	ENSMUSG	protein_coding
chr3	88785522	88785549	peak_3435	0	+	ENSMUSG	protein_coding
chr3	88789228	88789249	peak_3436	0	+	ENSMUSG	protein_coding
chr3	88802464	88802485	peak_3437	0	+	ENSMUSG	protein_coding
chr3	88811711	88811733	peak_3438	0	+	ENSMUSG	protein_coding
chr3	88836740	88836761	peak_3439	0	+	ENSMUSG	protein_coding
chr3	88870160	88870181	peak_3440	0	+	ENSMUSG	protein_coding
chr3	89247543	89247564	peak_3441	0	+	ENSMUSG	protein_coding
chr3	89420066	89420087	peak_3442	0	+	ENSMUSG	protein_coding
chr3	89891595	89891616	peak_3443	0	+	ENSMUSG	protein_coding
chr3	89893754	89893775	peak_3444	0	+	ENSMUSG	protein_coding
chr3	90016881	90016902	peak_3446	0	-	ENSMUSG	protein_coding
chr3	94304518	94304539	peak_3450	0	-	ENSMUSG	protein_coding
chr3	94566929	94566950	peak_3451	0	-	ENSMUSG	protein_coding
chr3	94577448	94577469	peak_3452	0	-	ENSMUSG	protein_coding
chr3	94690852	94690873	peak_3453	0	-	ENSMUSG	protein_coding
chr3	94836821	94836842	peak_3454	0	-	ENSMUSG	protein_coding
chr3	94839804	94839825	peak_3455	0	-	ENSMUSG	protein_coding
chr3	94846492	94846513	peak_3456	0	-	ENSMUSG	protein_coding
chr3	94894610	94894652	peak_3457	0	-	ENSMUSG	protein_coding
chr3	94918542	94918564	peak_3458	0	+	ENSMUSG	protein_coding
chr3	94923143	94923166	peak_3459	0	+	ENSMUSG	protein_coding
chr3	94969514	94969535	peak_3460	0	+	ENSMUSG	protein_coding
chr3	95142011	95142032	peak_3461	0	-	ENSMUSG	protein_coding
chr3	95268118	95268139	peak_3462	0	+	ENSMUSG	protein_coding
chr3	95433369	95433390	peak_3463	0	+	ENSMUSG	protein_coding
chr3	95914049	95914070	peak_3464	0	+	ENSMUSG	protein_coding
chr3	95916520	95916541	peak_3465	0	+	ENSMUSG	protein_coding
chr3	96024430	96024455	peak_3466	0	-	ENSMUSG	protein_coding
chr3	96049783	96049804	peak_3467	0	+	ENSMUSG	protein_coding
chr3	96049783	96049804	peak_3467	0	+	ENSMUSG	protein_coding
chr3	96073698	96073719	peak_3468	0	+	ENSMUSG	protein_coding
chr3	96073903	96073924	peak_3469	0	+	ENSMUSG	protein_coding
chr3	96073980	96074001	peak_3470	0	+	ENSMUSG	protein_coding
chr3	96074017	96074053	peak_3471	0	+	ENSMUSG	protein_coding
chr3	96239872	96239893	peak_3482	0	+	ENSMUSG	protein_coding
chr3	96255321	96255342	peak_3483	0	+	ENSMUSG	protein_coding
chr3	96361861	96361882	peak_3491	0	+	ENSMUSG	protein_coding
chr3	96433840	96433862	peak_3492	0	+	ENSMUSG	protein_coding
chr3	96435441	96435467	peak_3493	0	+	ENSMUSG	protein_coding
chr3	97467801	97467822	peak_3495	0	+	ENSMUSG	protein_coding
chr3	97904218	97904243	peak_3496	0	+	ENSMUSG	protein_coding
chr3	97913779	97913800	peak_3497	0	+	ENSMUSG	protein_coding
chr3	97926495	97926516	peak_3498	0	+	ENSMUSG	protein_coding
chr3	97952603	97952624	peak_3499	0	+	ENSMUSG	protein_coding
chr3	99015243	99015264	peak_3500	0	+	ENSMUSG	protein_coding
chr3	1E+08	1E+08	peak_3502	0	-	ENSMUSG	protein_coding
chr3	1.01E+08	1.01E+08	peak_3503	0	-	ENSMUSG	protein_coding
chr3	1.01E+08	1.01E+08	peak_3504	0	-	ENSMUSG	protein_coding
chr3	1.02E+08	1.02E+08	peak_3505	0	+	ENSMUSG	protein_coding
chr3	1.03E+08	1.03E+08	peak_3506	0	-	ENSMUSG	protein_coding
chr3	1.03E+08	1.03E+08	peak_3507	0	+	ENSMUSG	protein_coding
chr3	1.04E+08	1.04E+08	peak_3508	0	-	ENSMUSG	protein_coding
chr3	1.04E+08	1.04E+08	peak_3508	0	-	ENSMUSG	protein_coding
chr3	1.04E+08	1.04E+08	peak_3509	0	-	ENSMUSG	protein_coding
chr3	1.04E+08	1.04E+08	peak_3510	0	-	ENSMUSG	protein_coding

chr3	1.04E+08	1.04E+08	peak_3511	0	-	ENSMUSG	protein_coding
chr3	1.04E+08	1.04E+08	peak_3512	0	-	ENSMUSG	protein_coding
chr3	1.05E+08	1.05E+08	peak_3513	0	-	ENSMUSG	protein_coding
chr3	1.05E+08	1.05E+08	peak_3514	0	-	ENSMUSG	protein_coding
chr3	1.05E+08	1.05E+08	peak_3515	0	-	ENSMUSG	protein_coding
chr3	1.05E+08	1.05E+08	peak_3516	0	+	ENSMUSG	protein_coding
chr3	1.05E+08	1.05E+08	peak_3517	0	+	ENSMUSG	protein_coding
chr3	1.05E+08	1.05E+08	peak_3518	0	+	ENSMUSG	protein_coding
chr3	1.05E+08	1.05E+08	peak_3519	0	+	ENSMUSG	protein_coding
chr3	1.05E+08	1.05E+08	peak_3520	0	+	ENSMUSG	protein_coding
chr3	1.05E+08	1.05E+08	peak_3521	0	+	ENSMUSG	protein_coding
chr3	1.05E+08	1.05E+08	peak_3522	0	+	ENSMUSG	protein_coding
chr3	1.05E+08	1.05E+08	peak_3523	0	+	ENSMUSG	protein_coding
chr3	1.05E+08	1.05E+08	peak_3524	0	+	ENSMUSG	protein_coding
chr3	1.05E+08	1.05E+08	peak_3525	0	+	ENSMUSG	protein_coding
chr3	1.05E+08	1.05E+08	peak_3526	0	+	ENSMUSG	protein_coding
chr3	1.05E+08	1.05E+08	peak_3527	0	+	ENSMUSG	protein_coding
chr3	1.05E+08	1.05E+08	peak_3528	0	+	ENSMUSG	protein_coding
chr3	1.07E+08	1.07E+08	peak_3529	0	-	ENSMUSG	protein_coding
chr3	1.07E+08	1.07E+08	peak_3530	0	-	ENSMUSG	protein_coding
chr3	1.08E+08	1.08E+08	peak_3531	0	-	ENSMUSG	protein_coding
chr3	1.08E+08	1.08E+08	peak_3532	0	-	ENSMUSG	protein_coding
chr3	1.08E+08	1.08E+08	peak_3533	0	-	ENSMUSG	protein_coding
chr3	1.08E+08	1.08E+08	peak_3537	0	+	ENSMUSG	protein_coding
chr3	1.09E+08	1.09E+08	peak_3538	0	-	ENSMUSG	protein_coding
chr3	1.09E+08	1.09E+08	peak_3539	0	-	ENSMUSG	protein_coding
chr3	1.09E+08	1.09E+08	peak_3540	0	-	ENSMUSG	protein_coding
chr3	1.09E+08	1.09E+08	peak_3541	0	-	ENSMUSG	protein_coding
chr3	1.16E+08	1.16E+08	peak_3542	0	+	ENSMUSG	protein_coding
chr3	1.19E+08	1.19E+08	peak_3543	0	+	ENSMUSG	protein_coding
chr3	1.19E+08	1.19E+08	peak_3544	0	-	ENSMUSG	protein_coding
chr3	1.21E+08	1.21E+08	peak_3545	0	+	ENSMUSG	protein_coding
chr3	1.21E+08	1.21E+08	peak_3546	0	+	ENSMUSG	protein_coding
chr3	1.22E+08	1.22E+08	peak_3547	0	+	ENSMUSG	protein_coding
chr3	1.22E+08	1.22E+08	peak_3548	0	+	ENSMUSG	protein_coding
chr3	1.22E+08	1.22E+08	peak_3549	0	+	ENSMUSG	protein_coding
chr3	1.22E+08	1.22E+08	peak_3550	0	+	ENSMUSG	protein_coding
chr3	1.22E+08	1.22E+08	peak_3551	0	+	ENSMUSG	protein_coding
chr3	1.22E+08	1.22E+08	peak_3552	0	+	ENSMUSG	protein_coding
chr3	1.22E+08	1.22E+08	peak_3553	0	+	ENSMUSG	protein_coding
chr3	1.23E+08	1.23E+08	peak_3554	0	+	ENSMUSG	protein_coding
chr3	1.3E+08	1.3E+08	peak_3558	0	+	ENSMUSG	protein_coding
chr3	1.3E+08	1.3E+08	peak_3559	0	-	ENSMUSG	protein_coding
chr3	1.3E+08	1.3E+08	peak_3560	0	-	ENSMUSG	protein_coding
chr3	1.33E+08	1.33E+08	peak_3561	0	-	ENSMUSG	protein_coding
chr3	1.35E+08	1.35E+08	peak_3562	0	+	ENSMUSG	protein_coding
chr3	1.36E+08	1.36E+08	peak_3563	0	+	ENSMUSG	protein_coding
chr3	1.36E+08	1.36E+08	peak_3564	0	+	ENSMUSG	protein_coding
chr3	1.36E+08	1.36E+08	peak_3565	0	+	ENSMUSG	protein_coding
chr3	1.36E+08	1.36E+08	peak_3566	0	+	ENSMUSG	protein_coding
chr3	1.38E+08	1.38E+08	peak_3569	0	+	ENSMUSG	protein_coding
chr3	1.38E+08	1.38E+08	peak_3570	0	+	ENSMUSG	protein_coding
chr3	1.38E+08	1.38E+08	peak_3571	0	+	ENSMUSG	protein_coding
chr3	1.38E+08	1.38E+08	peak_3572	0	+	ENSMUSG	protein_coding
chr3	1.38E+08	1.38E+08	peak_3573	0	+	ENSMUSG	protein_coding
chr3	1.39E+08	1.39E+08	peak_3574	0	-	ENSMUSG	protein_coding
chr3	1.39E+08	1.39E+08	peak_3575	0	-	ENSMUSG	protein_coding
chr3	1.44E+08	1.44E+08	peak_3577	0	-	ENSMUSG	protein_coding

chr3	1.44E+08	1.44E+08	peak_3578	0	-	ENSMUSG	protein_coding
chr3	1.45E+08	1.45E+08	peak_3579	0	+	ENSMUSG	protein_coding
chr3	1.45E+08	1.45E+08	peak_3580	0	+	ENSMUSG	protein_coding
chr3	1.46E+08	1.46E+08	peak_3581	0	+	ENSMUSG	protein_coding
chr3	1.46E+08	1.46E+08	peak_3582	0	-	ENSMUSG	protein_coding
chr3	1.49E+08	1.49E+08	peak_3583	0	-	ENSMUSG	protein_coding
chr3	1.49E+08	1.49E+08	peak_3584	0	-	ENSMUSG	protein_coding
chr3	1.49E+08	1.49E+08	peak_3585	0	-	ENSMUSG	protein_coding
chr3	1.49E+08	1.49E+08	peak_3586	0	-	ENSMUSG	protein_coding
chr3	1.52E+08	1.52E+08	peak_3589	0	+	ENSMUSG	protein_coding
chr3	1.52E+08	1.52E+08	peak_3590	0	+	ENSMUSG	protein_coding
chr3	1.53E+08	1.53E+08	peak_3591	0	-	ENSMUSG	protein_coding
chr3	1.54E+08	1.54E+08	peak_3592	0	-	ENSMUSG	protein_coding
chr3	1.54E+08	1.54E+08	peak_3593	0	-	ENSMUSG	protein_coding
chr3	1.54E+08	1.54E+08	peak_3594	0	-	ENSMUSG	protein_coding
chr3	1.54E+08	1.54E+08	peak_3595	0	-	ENSMUSG	protein_coding
chr3	1.54E+08	1.54E+08	peak_3596	0	-	ENSMUSG	protein_coding
chr3	1.54E+08	1.54E+08	peak_3597	0	-	ENSMUSG	protein_coding
chr4	3762293	3762315	peak_3598	0	-	ENSMUSG	protein_coding
chr4	3762745	3762766	peak_3599	0	-	ENSMUSG	protein_coding
chr4	3900737	3900758	peak_3600	0	-	ENSMUSG	protein_coding
chr4	5681425	5681446	peak_3601	0	+	ENSMUSG	protein_coding
chr4	5692954	5692975	peak_3602	0	+	ENSMUSG	protein_coding
chr4	6828851	6828873	peak_3603	0	-	ENSMUSG	protein_coding
chr4	8511247	8511270	peak_3604	0	+	ENSMUSG	protein_coding
chr4	8753560	8753581	peak_3605	0	+	ENSMUSG	protein_coding
chr4	9565659	9565680	peak_3606	0	-	ENSMUSG	protein_coding
chr4	12097756	12097778	peak_3608	0	-	ENSMUSG	protein_coding
chr4	21786431	21786452	peak_3609	0	+	ENSMUSG	protein_coding
chr4	32051261	32051282	peak_3610	0	+	ENSMUSG	protein_coding
chr4	32357441	32357462	peak_3611	0	+	ENSMUSG	protein_coding
chr4	32727099	32727120	peak_3612	0	+	ENSMUSG	protein_coding
chr4	32754322	32754343	peak_3613	0	+	ENSMUSG	protein_coding
chr4	32759875	32759912	peak_3614	0	+	ENSMUSG	protein_coding
chr4	32785769	32785790	peak_3615	0	+	ENSMUSG	protein_coding
chr4	32794406	32794427	peak_3616	0	+	ENSMUSG	protein_coding
chr4	32803041	32803062	peak_3617	0	+	ENSMUSG	protein_coding
chr4	32821297	32821318	peak_3618	0	+	ENSMUSG	protein_coding
chr4	32825570	32825594	peak_3619	0	+	ENSMUSG	protein_coding
chr4	32825871	32825893	peak_3620	0	+	ENSMUSG	protein_coding
chr4	32833436	32833457	peak_3621	0	+	ENSMUSG	protein_coding
chr4	32847774	32847795	peak_3622	0	+	ENSMUSG	protein_coding
chr4	34525561	34525583	peak_3623	0	-	ENSMUSG	protein_coding
chr4	34752999	34753020	peak_3624	0	-	ENSMUSG	protein_coding
chr4	34829051	34829072	peak_3625	0	-	ENSMUSG	protein_coding
chr4	35052804	35052825	peak_3626	0	-	ENSMUSG	protein_coding
chr4	35165816	35165837	peak_3627	0	-	ENSMUSG	protein_coding
chr4	40932029	40932050	peak_3630	0	+	ENSMUSG	protein_coding
chr4	41110644	41110671	peak_3631	0	+	ENSMUSG	protein_coding
chr4	41150159	41150180	peak_3632	0	-	ENSMUSG	protein_coding
chr4	41158435	41158458	peak_3633	0	-	ENSMUSG	protein_coding
chr4	41158605	41158629	peak_3634	0	-	ENSMUSG	protein_coding
chr4	41160877	41160900	peak_3635	0	-	ENSMUSG	protein_coding
chr4	41330306	41330327	peak_3636	0	-	ENSMUSG	protein_coding
chr4	43085598	43085619	peak_3637	0	+	ENSMUSG	protein_coding
chr4	43100759	43100780	peak_3638	0	+	ENSMUSG	protein_coding
chr4	43150106	43150127	peak_3639	0	+	ENSMUSG	protein_coding
chr4	43183292	43183313	peak_3640	0	+	ENSMUSG	protein_coding

chr4	43183687	43183708	peak_3641	0	+	ENSMUSG	protein_coding
chr4	43184829	43184850	peak_3642	0	+	ENSMUSG	protein_coding
chr4	43216729	43216750	peak_3643	0	+	ENSMUSG	protein_coding
chr4	43227516	43227538	peak_3644	0	+	ENSMUSG	protein_coding
chr4	43505931	43505953	peak_3648	0	-	ENSMUSG	protein_coding
chr4	43576328	43576349	peak_3649	0	+	ENSMUSG	protein_coding
chr4	43893043	43893064	peak_3650	0	+	ENSMUSG	protein_coding
chr4	44024833	44024854	peak_3652	0	+	ENSMUSG	protein_coding
chr4	44141099	44141120	peak_3653	0	-	ENSMUSG	protein_coding
chr4	44192633	44192657	peak_3654	0	-	ENSMUSG	protein_coding
chr4	44781532	44781553	peak_3655	0	+	ENSMUSG	protein_coding
chr4	44784550	44784571	peak_3656	0	+	ENSMUSG	protein_coding
chr4	44796608	44796630	peak_3657	0	+	ENSMUSG	protein_coding
chr4	44800997	44801018	peak_3658	0	+	ENSMUSG	protein_coding
chr4	44827412	44827436	peak_3659	0	+	ENSMUSG	protein_coding
chr4	44833608	44833629	peak_3660	0	+	ENSMUSG	protein_coding
chr4	44859993	44860015	peak_3661	0	+	ENSMUSG	protein_coding
chr4	45242682	45242703	peak_3662	0	+	ENSMUSG	protein_coding
chr4	45366570	45366591	peak_3663	0	+	ENSMUSG	protein_coding
chr4	46482743	46482764	peak_3664	0	+	ENSMUSG	protein_coding
chr4	46484264	46484285	peak_3665	0	+	ENSMUSG	protein_coding
chr4	48603734	48603755	peak_3666	0	+	ENSMUSG	protein_coding
chr4	48628882	48628903	peak_3667	0	+	ENSMUSG	protein_coding
chr4	48641827	48641848	peak_3668	0	+	ENSMUSG	protein_coding
chr4	48649759	48649781	peak_3669	0	+	ENSMUSG	protein_coding
chr4	49302177	49302198	peak_3670	0	+	ENSMUSG	protein_coding
chr4	49669556	49669583	peak_3671	0	+	ENSMUSG	protein_coding
chr4	53074402	53074423	peak_3673	0	-	ENSMUSG	protein_coding
chr4	53531891	53531913	peak_3674	0	+	ENSMUSG	protein_coding
chr4	54960705	54960726	peak_3675	0	+	ENSMUSG	protein_coding
chr4	54965812	54965833	peak_3676	0	+	ENSMUSG	protein_coding
chr4	54977367	54977388	peak_3677	0	+	ENSMUSG	protein_coding
chr4	54981189	54981211	peak_3678	0	+	ENSMUSG	protein_coding
chr4	55010669	55010691	peak_3679	0	+	ENSMUSG	protein_coding
chr4	55023216	55023237	peak_3680	0	+	ENSMUSG	protein_coding
chr4	55036811	55036844	peak_3681	0	+	ENSMUSG	protein_coding
chr4	55363220	55363241	peak_3682	0	+	ENSMUSG	protein_coding
chr4	55397631	55397652	peak_3683	0	+	ENSMUSG	protein_coding
chr4	56883932	56883953	peak_3684	0	-	ENSMUSG	protein_coding
chr4	56910629	56910650	peak_3685	0	-	ENSMUSG	protein_coding
chr4	56925930	56925951	peak_3686	0	-	ENSMUSG	protein_coding
chr4	57631829	57631850	peak_3687	0	+	ENSMUSG	protein_coding
chr4	57631829	57631850	peak_3687	0	+	ENSMUSG	protein_coding
chr4	57736694	57736715	peak_3688	0	+	ENSMUSG	protein_coding
chr4	57736694	57736715	peak_3688	0	+	ENSMUSG	protein_coding
chr4	58862552	58862573	peak_3689	0	-	ENSMUSG	protein_coding
chr4	59771814	59771835	peak_3690	0	+	ENSMUSG	protein_coding
chr4	62026814	62026835	peak_3691	0	+	ENSMUSG	protein_coding
chr4	62068092	62068113	peak_3692	0	-	ENSMUSG	protein_coding
chr4	63089422	63089443	peak_3693	0	-	ENSMUSG	protein_coding
chr4	63096448	63096469	peak_3694	0	-	ENSMUSG	protein_coding
chr4	69964568	69964589	peak_3696	0	-	ENSMUSG	protein_coding
chr4	75786457	75786478	peak_3698	0	-	ENSMUSG	protein_coding
chr4	75868172	75868194	peak_3699	0	-	ENSMUSG	protein_coding
chr4	76007069	76007090	peak_3700	0	-	ENSMUSG	protein_coding
chr4	82016877	82016898	peak_3701	0	-	ENSMUSG	protein_coding
chr4	82105425	82105446	peak_3702	0	-	ENSMUSG	protein_coding
chr4	82641075	82641096	peak_3703	0	-	ENSMUSG	protein_coding

chr4	82964411	82964432	peak_3704	0	+	ENSMUSG	protein_coding
chr4	84163693	84163715	peak_3706	0	-	ENSMUSG	protein_coding
chr4	84225857	84225879	peak_3707	0	-	ENSMUSG	protein_coding
chr4	84307468	84307489	peak_3708	0	-	ENSMUSG	protein_coding
chr4	84692195	84692225	peak_3709	0	+	ENSMUSG	protein_coding
chr4	84894834	84894856	peak_3710	0	+	ENSMUSG	protein_coding
chr4	86124430	86124454	peak_3711	0	+	ENSMUSG	protein_coding
chr4	86257476	86257497	peak_3712	0	-	ENSMUSG	protein_coding
chr4	86501799	86501820	peak_3714	0	-	ENSMUSG	protein_coding
chr4	97444525	97444546	peak_3715	0	+	ENSMUSG	protein_coding
chr4	97444525	97444546	peak_3715	0	+	ENSMUSG	protein_coding
chr4	97450994	97451015	peak_3716	0	+	ENSMUSG	protein_coding
chr4	97461305	97461326	peak_3717	0	+	ENSMUSG	protein_coding
chr4	97483893	97483914	peak_3718	0	+	ENSMUSG	protein_coding
chr4	97490699	97490720	peak_3719	0	+	ENSMUSG	protein_coding
chr4	97500910	97500932	peak_3720	0	+	ENSMUSG	protein_coding
chr4	97506429	97506450	peak_3721	0	+	ENSMUSG	protein_coding
chr4	97545368	97545390	peak_3722	0	+	ENSMUSG	protein_coding
chr4	97570156	97570177	peak_3723	0	+	ENSMUSG	protein_coding
chr4	97586649	97586670	peak_3724	0	+	ENSMUSG	protein_coding
chr4	97732982	97733003	peak_3725	0	+	ENSMUSG	protein_coding
chr4	97776016	97776038	peak_3726	0	+	ENSMUSG	protein_coding
chr4	98613064	98613086	peak_3727	0	-	ENSMUSG	protein_coding
chr4	98625774	98625795	peak_3728	0	-	ENSMUSG	protein_coding
chr4	98627141	98627162	peak_3729	0	-	ENSMUSG	protein_coding
chr4	98672259	98672280	peak_3730	0	-	ENSMUSG	protein_coding
chr4	98730695	98730716	peak_3731	0	-	ENSMUSG	protein_coding
chr4	98765872	98765893	peak_3732	0	-	ENSMUSG	protein_coding
chr4	99571655	99571676	peak_3733	0	+	ENSMUSG	protein_coding
chr4	99571655	99571676	peak_3733	0	+	ENSMUSG	protein_coding
chr4	99640771	99640792	peak_3734	0	+	ENSMUSG	protein_coding
chr4	1.01E+08	1.01E+08	peak_3735	0	-	ENSMUSG	protein_coding
chr4	1.01E+08	1.01E+08	peak_3736	0	-	ENSMUSG	protein_coding
chr4	1.01E+08	1.01E+08	peak_3737	0	+	ENSMUSG	protein_coding
chr4	1.03E+08	1.03E+08	peak_3738	0	+	ENSMUSG	protein_coding
chr4	1.04E+08	1.04E+08	peak_3739	0	+	ENSMUSG	protein_coding
chr4	1.05E+08	1.05E+08	peak_3740	0	+	ENSMUSG	protein_coding
chr4	1.06E+08	1.06E+08	peak_3741	0	+	ENSMUSG	protein_coding
chr4	1.07E+08	1.07E+08	peak_3743	0	+	ENSMUSG	protein_coding
chr4	1.07E+08	1.07E+08	peak_3744	0	+	ENSMUSG	protein_coding
chr4	1.07E+08	1.07E+08	peak_3745	0	+	ENSMUSG	protein_coding
chr4	1.07E+08	1.07E+08	peak_3746	0	+	ENSMUSG	protein_coding
chr4	1.07E+08	1.07E+08	peak_3746	0	+	ENSMUSG	protein_coding
chr4	1.08E+08	1.08E+08	peak_3749	0	+	ENSMUSG	protein_coding
chr4	1.08E+08	1.08E+08	peak_3750	0	+	ENSMUSG	protein_coding
chr4	1.08E+08	1.08E+08	peak_3751	0	-	ENSMUSG	protein_coding
chr4	1.08E+08	1.08E+08	peak_3752	0	+	ENSMUSG	protein_coding
chr4	1.08E+08	1.08E+08	peak_3753	0	+	ENSMUSG	protein_coding
chr4	1.08E+08	1.08E+08	peak_3754	0	+	ENSMUSG	protein_coding
chr4	1.08E+08	1.08E+08	peak_3755	0	-	ENSMUSG	protein_coding
chr4	1.08E+08	1.08E+08	peak_3757	0	-	ENSMUSG	protein_coding
chr4	1.09E+08	1.09E+08	peak_3758	0	+	ENSMUSG	protein_coding
chr4	1.09E+08	1.09E+08	peak_3759	0	+	ENSMUSG	protein_coding
chr4	1.09E+08	1.09E+08	peak_3760	0	-	ENSMUSG	protein_coding
chr4	1.09E+08	1.09E+08	peak_3761	0	-	ENSMUSG	protein_coding
chr4	1.09E+08	1.09E+08	peak_3762	0	+	ENSMUSG	protein_coding
chr4	1.09E+08	1.09E+08	peak_3763	0	+	ENSMUSG	protein_coding
chr4	1.09E+08	1.09E+08	peak_3764	0	+	ENSMUSG	protein_coding

chr4	1.15E+08	1.15E+08	peak_3765	0	+	ENSMUSG	protein_coding
chr4	1.15E+08	1.15E+08	peak_3766	0	+	ENSMUSG	protein_coding
chr4	1.16E+08	1.16E+08	peak_3767	0	+	ENSMUSG	protein_coding
chr4	1.16E+08	1.16E+08	peak_3768	0	-	ENSMUSG	protein_coding
chr4	1.16E+08	1.16E+08	peak_3769	0	-	ENSMUSG	protein_coding
chr4	1.16E+08	1.16E+08	peak_3770	0	-	ENSMUSG	protein_coding
chr4	1.16E+08	1.16E+08	peak_3771	0	-	ENSMUSG	protein_coding
chr4	1.16E+08	1.16E+08	peak_3772	0	+	ENSMUSG	protein_coding
chr4	1.16E+08	1.16E+08	peak_3773	0	+	ENSMUSG	protein_coding
chr4	1.16E+08	1.16E+08	peak_3774	0	+	ENSMUSG	protein_coding
chr4	1.16E+08	1.16E+08	peak_3774	0	+	ENSMUSG	protein_coding
chr4	1.17E+08	1.17E+08	peak_3775	0	-	ENSMUSG	protein_coding
chr4	1.17E+08	1.17E+08	peak_3776	0	-	ENSMUSG	protein_coding
chr4	1.17E+08	1.17E+08	peak_3777	0	-	ENSMUSG	protein_coding
chr4	1.17E+08	1.17E+08	peak_3778	0	-	ENSMUSG	protein_coding
chr4	1.17E+08	1.17E+08	peak_3783	0	-	ENSMUSG	protein_coding
chr4	1.17E+08	1.17E+08	peak_3784	0	+	ENSMUSG	protein_coding
chr4	1.17E+08	1.17E+08	peak_3785	0	+	ENSMUSG	protein_coding
chr4	1.17E+08	1.17E+08	peak_3786	0	+	ENSMUSG	protein_coding
chr4	1.17E+08	1.17E+08	peak_3787	0	+	ENSMUSG	protein_coding
chr4	1.18E+08	1.18E+08	peak_3788	0	-	ENSMUSG	protein_coding
chr4	1.18E+08	1.18E+08	peak_3789	0	-	ENSMUSG	protein_coding
chr4	1.18E+08	1.18E+08	peak_3790	0	-	ENSMUSG	protein_coding
chr4	1.18E+08	1.18E+08	peak_3791	0	-	ENSMUSG	protein_coding
chr4	1.19E+08	1.19E+08	peak_3792	0	-	ENSMUSG	protein_coding
chr4	1.19E+08	1.19E+08	peak_3793	0	-	ENSMUSG	protein_coding
chr4	1.2E+08	1.2E+08	peak_3794	0	+	ENSMUSG	protein_coding
chr4	1.21E+08	1.21E+08	peak_3796	0	-	ENSMUSG	protein_coding
chr4	1.21E+08	1.21E+08	peak_3797	0	-	ENSMUSG	protein_coding
chr4	1.23E+08	1.23E+08	peak_3798	0	-	ENSMUSG	protein_coding
chr4	1.23E+08	1.23E+08	peak_3799	0	-	ENSMUSG	protein_coding
chr4	1.23E+08	1.23E+08	peak_3800	0	-	ENSMUSG	protein_coding
chr4	1.23E+08	1.23E+08	peak_3801	0	-	ENSMUSG	protein_coding
chr4	1.23E+08	1.23E+08	peak_3802	0	-	ENSMUSG	protein_coding
chr4	1.23E+08	1.23E+08	peak_3803	0	-	ENSMUSG	protein_coding
chr4	1.23E+08	1.23E+08	peak_3804	0	-	ENSMUSG	protein_coding
chr4	1.23E+08	1.23E+08	peak_3805	0	-	ENSMUSG	protein_coding
chr4	1.23E+08	1.23E+08	peak_3806	0	-	ENSMUSG	protein_coding
chr4	1.23E+08	1.23E+08	peak_3807	0	-	ENSMUSG	protein_coding
chr4	1.23E+08	1.23E+08	peak_3808	0	-	ENSMUSG	protein_coding
chr4	1.23E+08	1.23E+08	peak_3809	0	-	ENSMUSG	protein_coding
chr4	1.23E+08	1.23E+08	peak_3810	0	-	ENSMUSG	protein_coding
chr4	1.23E+08	1.23E+08	peak_3811	0	-	ENSMUSG	protein_coding
chr4	1.23E+08	1.23E+08	peak_3812	0	-	ENSMUSG	protein_coding
chr4	1.23E+08	1.23E+08	peak_3813	0	-	ENSMUSG	protein_coding
chr4	1.23E+08	1.23E+08	peak_3814	0	-	ENSMUSG	protein_coding
chr4	1.23E+08	1.23E+08	peak_3815	0	-	ENSMUSG	protein_coding
chr4	1.24E+08	1.24E+08	peak_3818	0	+	ENSMUSG	protein_coding
chr4	1.24E+08	1.24E+08	peak_3819	0	+	ENSMUSG	protein_coding
chr4	1.24E+08	1.24E+08	peak_3820	0	+	ENSMUSG	protein_coding
chr4	1.26E+08	1.26E+08	peak_3821	0	+	ENSMUSG	protein_coding
chr4	1.26E+08	1.26E+08	peak_3822	0	-	ENSMUSG	protein_coding
chr4	1.26E+08	1.26E+08	peak_3823	0	-	ENSMUSG	protein_coding
chr4	1.27E+08	1.27E+08	peak_3825	0	+	ENSMUSG	protein_coding
chr4	1.27E+08	1.27E+08	peak_3826	0	-	ENSMUSG	protein_coding
chr4	1.27E+08	1.27E+08	peak_3827	0	+	ENSMUSG	protein_coding
chr4	1.27E+08	1.27E+08	peak_3828	0	+	ENSMUSG	protein_coding
chr4	1.27E+08	1.27E+08	peak_3829	0	+	ENSMUSG	protein_coding

chr4	1.27E+08	1.27E+08	peak_3830	0	+	ENSMUSG	protein_coding
chr4	1.27E+08	1.27E+08	peak_3831	0	+	ENSMUSG	protein_coding
chr4	1.27E+08	1.27E+08	peak_3833	0	+	ENSMUSG	protein_coding
chr4	1.28E+08	1.28E+08	peak_3834	0	+	ENSMUSG	protein_coding
chr4	1.29E+08	1.29E+08	peak_3835	0	-	ENSMUSG	protein_coding
chr4	1.29E+08	1.29E+08	peak_3836	0	-	ENSMUSG	protein_coding
chr4	1.29E+08	1.29E+08	peak_3837	0	+	ENSMUSG	protein_coding
chr4	1.29E+08	1.29E+08	peak_3838	0	-	ENSMUSG	protein_coding
chr4	1.29E+08	1.29E+08	peak_3839	0	-	ENSMUSG	protein_coding
chr4	1.29E+08	1.29E+08	peak_3840	0	-	ENSMUSG	protein_coding
chr4	1.29E+08	1.29E+08	peak_3841	0	-	ENSMUSG	protein_coding
chr4	1.29E+08	1.29E+08	peak_3842	0	-	ENSMUSG	protein_coding
chr4	1.29E+08	1.29E+08	peak_3843	0	+	ENSMUSG	protein_coding
chr4	1.29E+08	1.29E+08	peak_3844	0	-	ENSMUSG	protein_coding
chr4	1.29E+08	1.29E+08	peak_3845	0	-	ENSMUSG	protein_coding
chr4	1.29E+08	1.29E+08	peak_3846	0	-	ENSMUSG	protein_coding
chr4	1.29E+08	1.29E+08	peak_3847	0	-	ENSMUSG	protein_coding
chr4	1.29E+08	1.29E+08	peak_3848	0	-	ENSMUSG	protein_coding
chr4	1.3E+08	1.3E+08	peak_3849	0	+	ENSMUSG	protein_coding
chr4	1.3E+08	1.3E+08	peak_3850	0	+	ENSMUSG	protein_coding
chr4	1.32E+08	1.32E+08	peak_3851	0	-	ENSMUSG	protein_coding
chr4	1.32E+08	1.32E+08	peak_3853	0	+	ENSMUSG	protein_coding
chr4	1.32E+08	1.32E+08	peak_3859	0	-	ENSMUSG	protein_coding
chr4	1.32E+08	1.32E+08	peak_3860	0	-	ENSMUSG	protein_coding
chr4	1.32E+08	1.32E+08	peak_3861	0	-	ENSMUSG	protein_coding
chr4	1.32E+08	1.32E+08	peak_3862	0	-	ENSMUSG	protein_coding
chr4	1.32E+08	1.32E+08	peak_3863	0	-	ENSMUSG	protein_coding
chr4	1.32E+08	1.32E+08	peak_3864	0	-	ENSMUSG	protein_coding
chr4	1.32E+08	1.32E+08	peak_3865	0	+	ENSMUSG	protein_coding
chr4	1.32E+08	1.32E+08	peak_3866	0	-	ENSMUSG	protein_coding
chr4	1.33E+08	1.33E+08	peak_3867	0	+	ENSMUSG	protein_coding
chr4	1.33E+08	1.33E+08	peak_3868	0	+	ENSMUSG	protein_coding
chr4	1.33E+08	1.33E+08	peak_3869	0	+	ENSMUSG	protein_coding
chr4	1.33E+08	1.33E+08	peak_3870	0	+	ENSMUSG	protein_coding
chr4	1.33E+08	1.33E+08	peak_3871	0	+	ENSMUSG	protein_coding
chr4	1.33E+08	1.33E+08	peak_3872	0	-	ENSMUSG	protein_coding
chr4	1.33E+08	1.33E+08	peak_3873	0	-	ENSMUSG	protein_coding
chr4	1.33E+08	1.33E+08	peak_3875	0	-	ENSMUSG	protein_coding
chr4	1.33E+08	1.33E+08	peak_3876	0	-	ENSMUSG	protein_coding
chr4	1.34E+08	1.34E+08	peak_3877	0	-	ENSMUSG	protein_coding
chr4	1.34E+08	1.34E+08	peak_3878	0	+	ENSMUSG	protein_coding
chr4	1.34E+08	1.34E+08	peak_3879	0	+	ENSMUSG	protein_coding
chr4	1.34E+08	1.34E+08	peak_3880	0	+	ENSMUSG	protein_coding
chr4	1.34E+08	1.34E+08	peak_3881	0	+	ENSMUSG	protein_coding
chr4	1.34E+08	1.34E+08	peak_3882	0	+	ENSMUSG	protein_coding
chr4	1.35E+08	1.35E+08	peak_3883	0	-	ENSMUSG	protein_coding
chr4	1.35E+08	1.35E+08	peak_3884	0	-	ENSMUSG	protein_coding
chr4	1.35E+08	1.35E+08	peak_3885	0	-	ENSMUSG	protein_coding
chr4	1.35E+08	1.35E+08	peak_3886	0	+	ENSMUSG	protein_coding
chr4	1.35E+08	1.35E+08	peak_3887	0	+	ENSMUSG	protein_coding
chr4	1.36E+08	1.36E+08	peak_3889	0	+	ENSMUSG	protein_coding
chr4	1.36E+08	1.36E+08	peak_3890	0	-	ENSMUSG	protein_coding
chr4	1.36E+08	1.36E+08	peak_3891	0	-	ENSMUSG	protein_coding
chr4	1.37E+08	1.37E+08	peak_3892	0	-	ENSMUSG	protein_coding
chr4	1.37E+08	1.37E+08	peak_3893	0	-	ENSMUSG	protein_coding
chr4	1.37E+08	1.37E+08	peak_3894	0	+	ENSMUSG	protein_coding
chr4	1.37E+08	1.37E+08	peak_3895	0	+	ENSMUSG	protein_coding
chr4	1.37E+08	1.37E+08	peak_3896	0	+	ENSMUSG	protein_coding

chr4	1.37E+08	1.37E+08	peak_3897	0	+	ENSMUSG	protein_coding
chr4	1.37E+08	1.37E+08	peak_3898	0	+	ENSMUSG	protein_coding
chr4	1.37E+08	1.37E+08	peak_3899	0	+	ENSMUSG	protein_coding
chr4	1.37E+08	1.37E+08	peak_3900	0	+	ENSMUSG	protein_coding
chr4	1.38E+08	1.38E+08	peak_3901	0	+	ENSMUSG	protein_coding
chr4	1.38E+08	1.38E+08	peak_3902	0	+	ENSMUSG	protein_coding
chr4	1.38E+08	1.38E+08	peak_3903	0	-	ENSMUSG	protein_coding
chr4	1.38E+08	1.38E+08	peak_3905	0	+	ENSMUSG	protein_coding
chr4	1.39E+08	1.39E+08	peak_3906	0	+	ENSMUSG	protein_coding
chr4	1.39E+08	1.39E+08	peak_3907	0	+	ENSMUSG	protein_coding
chr4	1.39E+08	1.39E+08	peak_3908	0	+	ENSMUSG	protein_coding
chr4	1.39E+08	1.39E+08	peak_3909	0	+	ENSMUSG	protein_coding
chr4	1.39E+08	1.39E+08	peak_3910	0	+	ENSMUSG	protein_coding
chr4	1.4E+08	1.4E+08	peak_3911	0	+	ENSMUSG	protein_coding
chr4	1.41E+08	1.41E+08	peak_3912	0	-	ENSMUSG	protein_coding
chr4	1.41E+08	1.41E+08	peak_3913	0	+	ENSMUSG	protein_coding
chr4	1.41E+08	1.41E+08	peak_3914	0	+	ENSMUSG	protein_coding
chr4	1.41E+08	1.41E+08	peak_3915	0	+	ENSMUSG	protein_coding
chr4	1.41E+08	1.41E+08	peak_3916	0	-	ENSMUSG	protein_coding
chr4	1.41E+08	1.41E+08	peak_3917	0	-	ENSMUSG	protein_coding
chr4	1.41E+08	1.41E+08	peak_3918	0	-	ENSMUSG	protein_coding
chr4	1.41E+08	1.41E+08	peak_3919	0	-	ENSMUSG	protein_coding
chr4	1.41E+08	1.41E+08	peak_3920	0	-	ENSMUSG	protein_coding
chr4	1.41E+08	1.41E+08	peak_3921	0	-	ENSMUSG	protein_coding
chr4	1.41E+08	1.41E+08	peak_3922	0	-	ENSMUSG	protein_coding
chr4	1.41E+08	1.41E+08	peak_3923	0	-	ENSMUSG	protein_coding
chr4	1.41E+08	1.41E+08	peak_3924	0	-	ENSMUSG	protein_coding
chr4	1.41E+08	1.41E+08	peak_3925	0	-	ENSMUSG	protein_coding
chr4	1.42E+08	1.42E+08	peak_3926	0	-	ENSMUSG	protein_coding
chr4	1.42E+08	1.42E+08	peak_3927	0	-	ENSMUSG	protein_coding
chr4	1.43E+08	1.43E+08	peak_3931	0	-	ENSMUSG	protein_coding
chr4	1.43E+08	1.43E+08	peak_3932	0	-	ENSMUSG	protein_coding
chr4	1.43E+08	1.43E+08	peak_3934	0	-	ENSMUSG	protein_coding
chr4	1.45E+08	1.45E+08	peak_3936	0	-	ENSMUSG	protein_coding
chr4	1.45E+08	1.45E+08	peak_3937	0	-	ENSMUSG	protein_coding
chr4	1.45E+08	1.45E+08	peak_3938	0	-	ENSMUSG	protein_coding
chr4	1.46E+08	1.46E+08	peak_3939	0	-	ENSMUSG	protein_coding
chr4	1.47E+08	1.47E+08	peak_3940	0	+	ENSMUSG	protein_coding
chr4	1.47E+08	1.47E+08	peak_3942	0	-	ENSMUSG	protein_coding
chr4	1.49E+08	1.49E+08	peak_3943	0	-	ENSMUSG	protein_coding
chr4	1.49E+08	1.49E+08	peak_3944	0	+	ENSMUSG	protein_coding
chr4	1.5E+08	1.5E+08	peak_3946	0	+	ENSMUSG	protein_coding
chr4	1.5E+08	1.5E+08	peak_3947	0	+	ENSMUSG	protein_coding
chr4	1.5E+08	1.5E+08	peak_3948	0	+	ENSMUSG	protein_coding
chr4	1.5E+08	1.5E+08	peak_3949	0	+	ENSMUSG	protein_coding
chr4	1.5E+08	1.5E+08	peak_3950	0	+	ENSMUSG	protein_coding
chr4	1.5E+08	1.5E+08	peak_3951	0	+	ENSMUSG	protein_coding
chr4	1.5E+08	1.5E+08	peak_3952	0	+	ENSMUSG	protein_coding
chr4	1.5E+08	1.5E+08	peak_3953	0	+	ENSMUSG	protein_coding
chr4	1.5E+08	1.5E+08	peak_3955	0	+	ENSMUSG	protein_coding
chr4	1.5E+08	1.5E+08	peak_3956	0	-	ENSMUSG	protein_coding
chr4	1.5E+08	1.5E+08	peak_3957	0	-	ENSMUSG	protein_coding
chr4	1.5E+08	1.5E+08	peak_3958	0	-	ENSMUSG	protein_coding
chr4	1.51E+08	1.51E+08	peak_3959	0	-	ENSMUSG	protein_coding
chr4	1.51E+08	1.51E+08	peak_3960	0	-	ENSMUSG	protein_coding
chr4	1.51E+08	1.51E+08	peak_3961	0	+	ENSMUSG	protein_coding
chr4	1.51E+08	1.51E+08	peak_3962	0	+	ENSMUSG	protein_coding
chr4	1.51E+08	1.51E+08	peak_3963	0	+	ENSMUSG	protein_coding

chr4	1.52E+08	1.52E+08	peak_3964	0	+	ENSMUSG	protein_coding
chr4	1.52E+08	1.52E+08	peak_3965	0	+	ENSMUSG	protein_coding
chr4	1.52E+08	1.52E+08	peak_3966	0	+	ENSMUSG	protein_coding
chr4	1.54E+08	1.54E+08	peak_3968	0	+	ENSMUSG	protein_coding
chr4	1.55E+08	1.55E+08	peak_3969	0	-	ENSMUSG	protein_coding
chr4	1.55E+08	1.55E+08	peak_3970	0	-	ENSMUSG	protein_coding
chr4	1.55E+08	1.55E+08	peak_3971	0	+	ENSMUSG	protein_coding
chr4	1.55E+08	1.55E+08	peak_3972	0	+	ENSMUSG	protein_coding
chr4	1.55E+08	1.55E+08	peak_3973	0	-	ENSMUSG	protein_coding
chr4	1.55E+08	1.55E+08	peak_3974	0	-	ENSMUSG	protein_coding
chr4	1.55E+08	1.55E+08	peak_3975	0	+	ENSMUSG	protein_coding
chr4	1.55E+08	1.55E+08	peak_3976	0	+	ENSMUSG	protein_coding
chr4	1.55E+08	1.55E+08	peak_3977	0	+	ENSMUSG	protein_coding
chr4	1.55E+08	1.55E+08	peak_3978	0	+	ENSMUSG	protein_coding
chr5	3492581	3492603	peak_3980	0	+	ENSMUSG	protein_coding
chr5	3520651	3520672	peak_3981	0	+	ENSMUSG	protein_coding
chr5	3757638	3757661	peak_3982	0	-	ENSMUSG	protein_coding
chr5	3968642	3968664	peak_3983	0	+	ENSMUSG	protein_coding
chr5	3989035	3989056	peak_3984	0	+	ENSMUSG	protein_coding
chr5	17208836	17208857	peak_3985	0	+	ENSMUSG	protein_coding
chr5	17772964	17772985	peak_3986	0	-	ENSMUSG	protein_coding
chr5	18750418	18750445	peak_3988	0	+	ENSMUSG	protein_coding
chr5	20300245	20300266	peak_3989	0	-	ENSMUSG	protein_coding
chr5	20451882	20451903	peak_3990	0	-	ENSMUSG	protein_coding
chr5	21157969	21157990	peak_3991	0	+	ENSMUSG	protein_coding
chr5	21524858	21524879	peak_3992	0	-	ENSMUSG	protein_coding
chr5	23069820	23069841	peak_3993	0	-	ENSMUSG	protein_coding
chr5	23296334	23296355	peak_3995	0	+	ENSMUSG	protein_coding
chr5	23471113	23471134	peak_3997	0	-	ENSMUSG	protein_coding
chr5	23836800	23836821	peak_3998	0	-	ENSMUSG	protein_coding
chr5	23972383	23972404	peak_3999	0	+	ENSMUSG	protein_coding
chr5	23994751	23994772	peak_4000	0	+	ENSMUSG	protein_coding
chr5	23998568	23998589	peak_4001	0	+	ENSMUSG	protein_coding
chr5	24549615	24549636	peak_4002	0	-	ENSMUSG	protein_coding
chr5	24835324	24835345	peak_4003	0	-	ENSMUSG	protein_coding
chr5	26329235	26329256	peak_4005	0	+	ENSMUSG	protein_coding
chr5	26330141	26330162	peak_4006	0	+	ENSMUSG	protein_coding
chr5	26344108	26344129	peak_4007	0	+	ENSMUSG	protein_coding
chr5	26348308	26348329	peak_4008	0	+	ENSMUSG	protein_coding
chr5	26413714	26413735	peak_4010	0	+	ENSMUSG	protein_coding
chr5	26426465	26426486	peak_4011	0	+	ENSMUSG	protein_coding
chr5	27827745	27827766	peak_4013	0	+	ENSMUSG	protein_coding
chr5	27827745	27827766	peak_4013	0	+	ENSMUSG	protein_coding
chr5	28099040	28099061	peak_4014	0	-	ENSMUSG	protein_coding
chr5	28099128	28099149	peak_4015	0	-	ENSMUSG	protein_coding
chr5	28709428	28709449	peak_4017	0	+	ENSMUSG	protein_coding
chr5	29761902	29761923	peak_4018	0	+	ENSMUSG	protein_coding
chr5	29762152	29762173	peak_4019	0	+	ENSMUSG	protein_coding
chr5	30062502	30062523	peak_4020	0	+	ENSMUSG	protein_coding
chr5	31257496	31257517	peak_4025	0	-	ENSMUSG	protein_coding
chr5	31363607	31363629	peak_4026	0	+	ENSMUSG	protein_coding
chr5	31364675	31364696	peak_4027	0	+	ENSMUSG	protein_coding
chr5	31550907	31550930	peak_4028	0	+	ENSMUSG	protein_coding
chr5	31585517	31585538	peak_4029	0	-	ENSMUSG	protein_coding
chr5	31800046	31800067	peak_4030	0	+	ENSMUSG	protein_coding
chr5	32761704	32761725	peak_4031	0	+	ENSMUSG	protein_coding
chr5	32934810	32934831	peak_4032	0	+	ENSMUSG	protein_coding
chr5	32947452	32947473	peak_4033	0	+	ENSMUSG	protein_coding

chr5	34011351	34011372	peak_4034	0	+	ENSMUSG	protein_coding
chr5	34168259	34168280	peak_4035	0	+	ENSMUSG	protein_coding
chr5	34225568	34225589	peak_4036	0	+	ENSMUSG	protein_coding
chr5	34692028	34692050	peak_4037	0	+	ENSMUSG	protein_coding
chr5	34723604	34723626	peak_4038	0	+	ENSMUSG	protein_coding
chr5	34820308	34820330	peak_4039	0	+	ENSMUSG	protein_coding
chr5	34962277	34962298	peak_4040	0	+	ENSMUSG	protein_coding
chr5	35371842	35371863	peak_4041	0	+	ENSMUSG	protein_coding
chr5	35915515	35915536	peak_4042	0	-	ENSMUSG	protein_coding
chr5	35916743	35916764	peak_4043	0	-	ENSMUSG	protein_coding
chr5	36003643	36003664	peak_4044	0	-	ENSMUSG	protein_coding
chr5	36006661	36006682	peak_4045	0	-	ENSMUSG	protein_coding
chr5	36405046	36405067	peak_4046	0	-	ENSMUSG	protein_coding
chr5	37186251	37186272	peak_4047	0	-	ENSMUSG	protein_coding
chr5	37186760	37186781	peak_4048	0	-	ENSMUSG	protein_coding
chr5	37187182	37187203	peak_4049	0	-	ENSMUSG	protein_coding
chr5	37187910	37187931	peak_4050	0	-	ENSMUSG	protein_coding
chr5	38931813	38931835	peak_4051	0	-	ENSMUSG	protein_coding
chr5	42198477	42198498	peak_4053	0	-	ENSMUSG	protein_coding
chr5	43907266	43907287	peak_4054	0	+	ENSMUSG	protein_coding
chr5	43928928	43928949	peak_4055	0	+	ENSMUSG	protein_coding
chr5	43968763	43968784	peak_4056	0	+	ENSMUSG	protein_coding
chr5	45113536	45113558	peak_4057	0	-	ENSMUSG	protein_coding
chr5	48427877	48427903	peak_4058	0	+	ENSMUSG	protein_coding
chr5	48779478	48779501	peak_4059	0	+	ENSMUSG	protein_coding
chr5	50414285	50414306	peak_4060	0	-	ENSMUSG	protein_coding
chr5	50417011	50417032	peak_4061	0	-	ENSMUSG	protein_coding
chr5	52548523	52548544	peak_4062	0	-	ENSMUSG	protein_coding
chr5	52550143	52550165	peak_4063	0	-	ENSMUSG	protein_coding
chr5	52581552	52581573	peak_4064	0	-	ENSMUSG	protein_coding
chr5	53138604	53138625	peak_4065	0	+	ENSMUSG	protein_coding
chr5	53180881	53180902	peak_4066	0	+	ENSMUSG	protein_coding
chr5	53547486	53547508	peak_4067	0	-	ENSMUSG	protein_coding
chr5	53985005	53985028	peak_4068	0	+	ENSMUSG	protein_coding
chr5	54463957	54463979	peak_4069	0	+	ENSMUSG	protein_coding
chr5	65364309	65364330	peak_4073	0	+	ENSMUSG	protein_coding
chr5	65547716	65547738	peak_4074	0	+	ENSMUSG	protein_coding
chr5	66081432	66081453	peak_4076	0	-	ENSMUSG	protein_coding
chr5	66156108	66156129	peak_4077	0	+	ENSMUSG	protein_coding
chr5	66162611	66162633	peak_4078	0	+	ENSMUSG	protein_coding
chr5	66900693	66900717	peak_4079	0	-	ENSMUSG	protein_coding
chr5	67746893	67746915	peak_4080	0	+	ENSMUSG	protein_coding
chr5	72810028	72810049	peak_4081	0	-	ENSMUSG	protein_coding
chr5	73456417	73456444	peak_4082	0	-	ENSMUSG	protein_coding
chr5	73474525	73474550	peak_4083	0	-	ENSMUSG	protein_coding
chr5	73559392	73559413	peak_4084	0	-	ENSMUSG	protein_coding
chr5	74867994	74868016	peak_4086	0	-	ENSMUSG	protein_coding
chr5	74935472	74935494	peak_4087	0	+	ENSMUSG	protein_coding
chr5	74946734	74946755	peak_4088	0	+	ENSMUSG	protein_coding
chr5	75431844	75431868	peak_4089	0	-	ENSMUSG	protein_coding
chr5	75548370	75548391	peak_4090	0	+	ENSMUSG	protein_coding
chr5	75559790	75559814	peak_4091	0	+	ENSMUSG	protein_coding
chr5	75562557	75562582	peak_4092	0	+	ENSMUSG	protein_coding
chr5	75588639	75588660	peak_4093	0	+	ENSMUSG	protein_coding
chr5	76017590	76017611	peak_4094	0	+	ENSMUSG	protein_coding
chr5	76669183	76669204	peak_4095	0	-	ENSMUSG	protein_coding
chr5	76669210	76669231	peak_4096	0	-	ENSMUSG	protein_coding
chr5	76691731	76691752	peak_4097	0	-	ENSMUSG	protein_coding

chr5	76698554	76698575	peak_4098	0	-	ENSMUSG	protein_coding
chr5	76714287	76714308	peak_4099	0	-	ENSMUSG	protein_coding
chr5	77059465	77059486	peak_4100	0	+	ENSMUSG	protein_coding
chr5	77813689	77813716	peak_4101	0	-	ENSMUSG	protein_coding
chr5	87243256	87243278	peak_4102	0	+	ENSMUSG	protein_coding
chr5	87246134	87246155	peak_4103	0	+	ENSMUSG	protein_coding
chr5	87264756	87264777	peak_4104	0	+	ENSMUSG	protein_coding
chr5	88983871	88983892	peak_4105	0	+	ENSMUSG	protein_coding
chr5	90712474	90712495	peak_4106	0	-	ENSMUSG	protein_coding
chr5	90729060	90729081	peak_4107	0	-	ENSMUSG	protein_coding
chr5	96565918	96565939	peak_4108	0	-	ENSMUSG	protein_coding
chr5	97254100	97254121	peak_4109	0	+	ENSMUSG	protein_coding
chr5	97473728	97473749	peak_4110	0	+	ENSMUSG	protein_coding
chr5	97505993	97506014	peak_4111	0	+	ENSMUSG	protein_coding
chr5	1E+08	1E+08	peak_4112	0	-	ENSMUSG	protein_coding
chr5	1E+08	1E+08	peak_4113	0	-	ENSMUSG	protein_coding
chr5	1E+08	1E+08	peak_4114	0	-	ENSMUSG	protein_coding
chr5	1E+08	1E+08	peak_4115	0	-	ENSMUSG	protein_coding
chr5	1E+08	1E+08	peak_4117	0	-	ENSMUSG	protein_coding
chr5	1.01E+08	1.01E+08	peak_4120	0	+	ENSMUSG	protein_coding
chr5	1.01E+08	1.01E+08	peak_4121	0	+	ENSMUSG	protein_coding
chr5	1.02E+08	1.02E+08	peak_4122	0	-	ENSMUSG	protein_coding
chr5	1.02E+08	1.02E+08	peak_4123	0	-	ENSMUSG	protein_coding
chr5	1.02E+08	1.02E+08	peak_4124	0	-	ENSMUSG	protein_coding
chr5	1.02E+08	1.02E+08	peak_4125	0	-	ENSMUSG	protein_coding
chr5	1.02E+08	1.02E+08	peak_4126	0	-	ENSMUSG	protein_coding
chr5	1.04E+08	1.04E+08	peak_4127	0	+	ENSMUSG	protein_coding
chr5	1.04E+08	1.04E+08	peak_4128	0	+	ENSMUSG	protein_coding
chr5	1.04E+08	1.04E+08	peak_4129	0	+	ENSMUSG	protein_coding
chr5	1.04E+08	1.04E+08	peak_4130	0	+	ENSMUSG	protein_coding
chr5	1.04E+08	1.04E+08	peak_4131	0	+	ENSMUSG	protein_coding
chr5	1.05E+08	1.05E+08	peak_4132	0	+	ENSMUSG	protein_coding
chr5	1.05E+08	1.05E+08	peak_4133	0	+	ENSMUSG	protein_coding
chr5	1.06E+08	1.06E+08	peak_4134	0	+	ENSMUSG	protein_coding
chr5	1.06E+08	1.06E+08	peak_4135	0	+	ENSMUSG	protein_coding
chr5	1.07E+08	1.07E+08	peak_4136	0	-	ENSMUSG	protein_coding
chr5	1.07E+08	1.07E+08	peak_4137	0	-	ENSMUSG	protein_coding
chr5	1.07E+08	1.07E+08	peak_4138	0	-	ENSMUSG	protein_coding
chr5	1.08E+08	1.08E+08	peak_4139	0	-	ENSMUSG	protein_coding
chr5	1.08E+08	1.08E+08	peak_4140	0	+	ENSMUSG	protein_coding
chr5	1.08E+08	1.08E+08	peak_4141	0	-	ENSMUSG	protein_coding
chr5	1.08E+08	1.08E+08	peak_4142	0	+	ENSMUSG	protein_coding
chr5	1.08E+08	1.08E+08	peak_4143	0	+	ENSMUSG	protein_coding
chr5	1.08E+08	1.08E+08	peak_4144	0	+	ENSMUSG	protein_coding
chr5	1.08E+08	1.08E+08	peak_4145	0	+	ENSMUSG	protein_coding
chr5	1.08E+08	1.08E+08	peak_4146	0	+	ENSMUSG	protein_coding
chr5	1.09E+08	1.09E+08	peak_4147	0	+	ENSMUSG	protein_coding
chr5	1.09E+08	1.09E+08	peak_4149	0	+	ENSMUSG	protein_coding
chr5	1.09E+08	1.09E+08	peak_4150	0	+	ENSMUSG	protein_coding
chr5	1.09E+08	1.09E+08	peak_4151	0	-	ENSMUSG	protein_coding
chr5	1.1E+08	1.1E+08	peak_4155	0	+	ENSMUSG	protein_coding
chr5	1.1E+08	1.1E+08	peak_4156	0	+	ENSMUSG	protein_coding
chr5	1.1E+08	1.1E+08	peak_4157	0	+	ENSMUSG	protein_coding
chr5	1.11E+08	1.11E+08	peak_4158	0	+	ENSMUSG	protein_coding
chr5	1.11E+08	1.11E+08	peak_4159	0	-	ENSMUSG	protein_coding
chr5	1.11E+08	1.11E+08	peak_4160	0	-	ENSMUSG	protein_coding
chr5	1.12E+08	1.12E+08	peak_4163	0	+	ENSMUSG	protein_coding
chr5	1.14E+08	1.14E+08	peak_4164	0	+	ENSMUSG	protein_coding

chr5	1.15E+08	1.15E+08	peak_4165	0	+	ENSMUSG	protein_coding
chr5	1.15E+08	1.15E+08	peak_4166	0	+	ENSMUSG	protein_coding
chr5	1.16E+08	1.16E+08	peak_4167	0	+	ENSMUSG	protein_coding
chr5	1.16E+08	1.16E+08	peak_4168	0	-	ENSMUSG	protein_coding
chr5	1.16E+08	1.16E+08	peak_4169	0	-	ENSMUSG	protein_coding
chr5	1.16E+08	1.16E+08	peak_4170	0	-	ENSMUSG	protein_coding
chr5	1.16E+08	1.16E+08	peak_4171	0	-	ENSMUSG	protein_coding
chr5	1.16E+08	1.16E+08	peak_4172	0	-	ENSMUSG	protein_coding
chr5	1.16E+08	1.16E+08	peak_4173	0	-	ENSMUSG	protein_coding
chr5	1.16E+08	1.16E+08	peak_4174	0	-	ENSMUSG	protein_coding
chr5	1.16E+08	1.16E+08	peak_4175	0	+	ENSMUSG	protein_coding
chr5	1.16E+08	1.16E+08	peak_4176	0	+	ENSMUSG	protein_coding
chr5	1.16E+08	1.16E+08	peak_4179	0	+	ENSMUSG	protein_coding
chr5	1.16E+08	1.16E+08	peak_4180	0	+	ENSMUSG	protein_coding
chr5	1.16E+08	1.16E+08	peak_4181	0	+	ENSMUSG	protein_coding
chr5	1.16E+08	1.16E+08	peak_4182	0	+	ENSMUSG	protein_coding
chr5	1.16E+08	1.16E+08	peak_4183	0	+	ENSMUSG	protein_coding
chr5	1.16E+08	1.16E+08	peak_4184	0	+	ENSMUSG	protein_coding
chr5	1.18E+08	1.18E+08	peak_4185	0	+	ENSMUSG	protein_coding
chr5	1.18E+08	1.18E+08	peak_4186	0	+	ENSMUSG	protein_coding
chr5	1.18E+08	1.18E+08	peak_4187	0	+	ENSMUSG	protein_coding
chr5	1.18E+08	1.18E+08	peak_4188	0	+	ENSMUSG	protein_coding
chr5	1.19E+08	1.19E+08	peak_4189	0	+	ENSMUSG	protein_coding
chr5	1.21E+08	1.21E+08	peak_4190	0	-	ENSMUSG	protein_coding
chr5	1.22E+08	1.22E+08	peak_4191	0	-	ENSMUSG	protein_coding
chr5	1.22E+08	1.22E+08	peak_4192	0	-	ENSMUSG	protein_coding
chr5	1.22E+08	1.22E+08	peak_4193	0	+	ENSMUSG	protein_coding
chr5	1.22E+08	1.22E+08	peak_4194	0	+	ENSMUSG	protein_coding
chr5	1.22E+08	1.22E+08	peak_4195	0	-	ENSMUSG	protein_coding
chr5	1.22E+08	1.22E+08	peak_4196	0	-	ENSMUSG	protein_coding
chr5	1.22E+08	1.22E+08	peak_4197	0	+	ENSMUSG	protein_coding
chr5	1.23E+08	1.23E+08	peak_4198	0	+	ENSMUSG	protein_coding
chr5	1.23E+08	1.23E+08	peak_4199	0	-	ENSMUSG	protein_coding
chr5	1.23E+08	1.23E+08	peak_4200	0	-	ENSMUSG	protein_coding
chr5	1.23E+08	1.23E+08	peak_4201	0	+	ENSMUSG	protein_coding
chr5	1.24E+08	1.24E+08	peak_4202	0	+	ENSMUSG	protein_coding
chr5	1.24E+08	1.24E+08	peak_4203	0	+	ENSMUSG	protein_coding
chr5	1.24E+08	1.24E+08	peak_4204	0	+	ENSMUSG	protein_coding
chr5	1.24E+08	1.24E+08	peak_4205	0	-	ENSMUSG	protein_coding
chr5	1.24E+08	1.24E+08	peak_4206	0	+	ENSMUSG	protein_coding
chr5	1.24E+08	1.24E+08	peak_4207	0	-	ENSMUSG	protein_coding
chr5	1.24E+08	1.24E+08	peak_4208	0	-	ENSMUSG	protein_coding
chr5	1.24E+08	1.24E+08	peak_4209	0	-	ENSMUSG	protein_coding
chr5	1.24E+08	1.24E+08	peak_4210	0	-	ENSMUSG	protein_coding
chr5	1.24E+08	1.24E+08	peak_4211	0	+	ENSMUSG	protein_coding
chr5	1.24E+08	1.24E+08	peak_4212	0	+	ENSMUSG	protein_coding
chr5	1.24E+08	1.24E+08	peak_4213	0	-	ENSMUSG	protein_coding
chr5	1.24E+08	1.24E+08	peak_4214	0	-	ENSMUSG	protein_coding
chr5	1.25E+08	1.25E+08	peak_4215	0	-	ENSMUSG	protein_coding
chr5	1.25E+08	1.25E+08	peak_4216	0	-	ENSMUSG	protein_coding
chr5	1.25E+08	1.25E+08	peak_4217	0	-	ENSMUSG	protein_coding
chr5	1.25E+08	1.25E+08	peak_4218	0	-	ENSMUSG	protein_coding
chr5	1.25E+08	1.25E+08	peak_4219	0	-	ENSMUSG	protein_coding
chr5	1.25E+08	1.25E+08	peak_4220	0	+	ENSMUSG	protein_coding
chr5	1.25E+08	1.25E+08	peak_4221	0	+	ENSMUSG	protein_coding
chr5	1.25E+08	1.25E+08	peak_4221	0	+	ENSMUSG	protein_coding
chr5	1.26E+08	1.26E+08	peak_4222	0	-	ENSMUSG	protein_coding
chr5	1.26E+08	1.26E+08	peak_4223	0	-	ENSMUSG	protein_coding

chr5	1.26E+08	1.26E+08	peak_4224	0	-	ENSMUSG	protein_coding
chr5	1.26E+08	1.26E+08	peak_4225	0	-	ENSMUSG	protein_coding
chr5	1.26E+08	1.26E+08	peak_4226	0	-	ENSMUSG	protein_coding
chr5	1.26E+08	1.26E+08	peak_4227	0	-	ENSMUSG	protein_coding
chr5	1.26E+08	1.26E+08	peak_4228	0	-	ENSMUSG	protein_coding
chr5	1.26E+08	1.26E+08	peak_4229	0	-	ENSMUSG	protein_coding
chr5	1.26E+08	1.26E+08	peak_4230	0	-	ENSMUSG	protein_coding
chr5	1.26E+08	1.26E+08	peak_4231	0	-	ENSMUSG	protein_coding
chr5	1.26E+08	1.26E+08	peak_4232	0	-	ENSMUSG	protein_coding
chr5	1.26E+08	1.26E+08	peak_4233	0	-	ENSMUSG	protein_coding
chr5	1.26E+08	1.26E+08	peak_4234	0	-	ENSMUSG	protein_coding
chr5	1.26E+08	1.26E+08	peak_4235	0	-	ENSMUSG	protein_coding
chr5	1.26E+08	1.26E+08	peak_4236	0	-	ENSMUSG	protein_coding
chr5	1.26E+08	1.26E+08	peak_4236	0	-	ENSMUSG	protein_coding
chr5	1.26E+08	1.26E+08	peak_4237	0	-	ENSMUSG	protein_coding
chr5	1.26E+08	1.26E+08	peak_4237	0	-	ENSMUSG	protein_coding
chr5	1.26E+08	1.26E+08	peak_4239	0	+	ENSMUSG	protein_coding
chr5	1.3E+08	1.3E+08	peak_4241	0	+	ENSMUSG	protein_coding
chr5	1.3E+08	1.3E+08	peak_4242	0	+	ENSMUSG	protein_coding
chr5	1.3E+08	1.3E+08	peak_4243	0	+	ENSMUSG	protein_coding
chr5	1.3E+08	1.3E+08	peak_4244	0	+	ENSMUSG	protein_coding
chr5	1.31E+08	1.31E+08	peak_4245	0	+	ENSMUSG	protein_coding
chr5	1.32E+08	1.32E+08	peak_4246	0	-	ENSMUSG	protein_coding
chr5	1.32E+08	1.32E+08	peak_4247	0	-	ENSMUSG	protein_coding
chr5	1.32E+08	1.32E+08	peak_4248	0	-	ENSMUSG	protein_coding
chr5	1.32E+08	1.32E+08	peak_4249	0	-	ENSMUSG	protein_coding
chr5	1.32E+08	1.32E+08	peak_4250	0	-	ENSMUSG	protein_coding
chr5	1.33E+08	1.33E+08	peak_4251	0	-	ENSMUSG	protein_coding
chr5	1.33E+08	1.33E+08	peak_4252	0	-	ENSMUSG	protein_coding
chr5	1.33E+08	1.33E+08	peak_4253	0	-	ENSMUSG	protein_coding
chr5	1.33E+08	1.33E+08	peak_4254	0	-	ENSMUSG	protein_coding
chr5	1.33E+08	1.33E+08	peak_4255	0	-	ENSMUSG	protein_coding
chr5	1.33E+08	1.33E+08	peak_4256	0	-	ENSMUSG	protein_coding
chr5	1.33E+08	1.33E+08	peak_4257	0	-	ENSMUSG	protein_coding
chr5	1.33E+08	1.33E+08	peak_4258	0	-	ENSMUSG	protein_coding
chr5	1.33E+08	1.33E+08	peak_4259	0	-	ENSMUSG	protein_coding
chr5	1.33E+08	1.33E+08	peak_4260	0	-	ENSMUSG	protein_coding
chr5	1.35E+08	1.35E+08	peak_4261	0	+	ENSMUSG	protein_coding
chr5	1.35E+08	1.35E+08	peak_4262	0	-	ENSMUSG	protein_coding
chr5	1.35E+08	1.35E+08	peak_4263	0	-	ENSMUSG	protein_coding
chr5	1.35E+08	1.35E+08	peak_4264	0	-	ENSMUSG	protein_coding
chr5	1.36E+08	1.36E+08	peak_4265	0	-	ENSMUSG	protein_coding
chr5	1.36E+08	1.36E+08	peak_4266	0	+	ENSMUSG	protein_coding
chr5	1.36E+08	1.36E+08	peak_4267	0	+	ENSMUSG	protein_coding
chr5	1.36E+08	1.36E+08	peak_4268	0	-	ENSMUSG	protein_coding
chr5	1.36E+08	1.36E+08	peak_4269	0	-	ENSMUSG	protein_coding
chr5	1.36E+08	1.36E+08	peak_4270	0	-	ENSMUSG	protein_coding
chr5	1.36E+08	1.36E+08	peak_4271	0	-	ENSMUSG	protein_coding
chr5	1.37E+08	1.37E+08	peak_4275	0	-	ENSMUSG	protein_coding
chr5	1.37E+08	1.37E+08	peak_4276	0	-	ENSMUSG	protein_coding
chr5	1.37E+08	1.37E+08	peak_4277	0	+	ENSMUSG	protein_coding
chr5	1.37E+08	1.37E+08	peak_4278	0	-	ENSMUSG	protein_coding
chr5	1.38E+08	1.38E+08	peak_4280	0	-	ENSMUSG	protein_coding
chr5	1.38E+08	1.38E+08	peak_4281	0	+	ENSMUSG	protein_coding
chr5	1.38E+08	1.38E+08	peak_4281	0	+	ENSMUSG	protein_coding
chr5	1.38E+08	1.38E+08	peak_4282	0	+	ENSMUSG	protein_coding
chr5	1.38E+08	1.38E+08	peak_4282	0	+	ENSMUSG	protein_coding
chr5	1.38E+08	1.38E+08	peak_4283	0	+	ENSMUSG	protein_coding

chr5	1.38E+08	1.38E+08	peak_4283	0	+	ENSMUSG	protein_coding
chr5	1.38E+08	1.38E+08	peak_4284	0	+	ENSMUSG	protein_coding
chr5	1.38E+08	1.38E+08	peak_4285	0	+	ENSMUSG	protein_coding
chr5	1.38E+08	1.38E+08	peak_4286	0	+	ENSMUSG	protein_coding
chr5	1.38E+08	1.38E+08	peak_4287	0	+	ENSMUSG	protein_coding
chr5	1.4E+08	1.4E+08	peak_4290	0	-	ENSMUSG	protein_coding
chr5	1.4E+08	1.4E+08	peak_4291	0	-	ENSMUSG	protein_coding
chr5	1.4E+08	1.4E+08	peak_4292	0	-	ENSMUSG	protein_coding
chr5	1.41E+08	1.41E+08	peak_4294	0	-	ENSMUSG	protein_coding
chr5	1.41E+08	1.41E+08	peak_4296	0	+	ENSMUSG	protein_coding
chr5	1.41E+08	1.41E+08	peak_4297	0	+	ENSMUSG	protein_coding
chr5	1.41E+08	1.41E+08	peak_4298	0	+	ENSMUSG	protein_coding
chr5	1.41E+08	1.41E+08	peak_4299	0	+	ENSMUSG	protein_coding
chr5	1.41E+08	1.41E+08	peak_4300	0	-	ENSMUSG	protein_coding
chr5	1.42E+08	1.42E+08	peak_4301	0	+	ENSMUSG	protein_coding
chr5	1.42E+08	1.42E+08	peak_4302	0	+	ENSMUSG	protein_coding
chr5	1.42E+08	1.42E+08	peak_4303	0	+	ENSMUSG	protein_coding
chr5	1.43E+08	1.43E+08	peak_4304	0	+	ENSMUSG	protein_coding
chr5	1.43E+08	1.43E+08	peak_4305	0	-	ENSMUSG	protein_coding
chr5	1.43E+08	1.43E+08	peak_4306	0	-	ENSMUSG	protein_coding
chr5	1.44E+08	1.44E+08	peak_4307	0	-	ENSMUSG	protein_coding
chr5	1.44E+08	1.44E+08	peak_4308	0	-	ENSMUSG	protein_coding
chr5	1.44E+08	1.44E+08	peak_4309	0	-	ENSMUSG	protein_coding
chr5	1.44E+08	1.44E+08	peak_4310	0	-	ENSMUSG	protein_coding
chr5	1.44E+08	1.44E+08	peak_4311	0	-	ENSMUSG	protein_coding
chr5	1.44E+08	1.44E+08	peak_4312	0	-	ENSMUSG	protein_coding
chr5	1.44E+08	1.44E+08	peak_4314	0	-	ENSMUSG	protein_coding
chr5	1.44E+08	1.44E+08	peak_4315	0	-	ENSMUSG	protein_coding
chr5	1.44E+08	1.44E+08	peak_4316	0	-	ENSMUSG	protein_coding
chr5	1.44E+08	1.44E+08	peak_4317	0	-	ENSMUSG	protein_coding
chr5	1.44E+08	1.44E+08	peak_4318	0	-	ENSMUSG	protein_coding
chr5	1.44E+08	1.44E+08	peak_4319	0	+	ENSMUSG	protein_coding
chr5	1.45E+08	1.45E+08	peak_4320	0	+	ENSMUSG	protein_coding
chr5	1.46E+08	1.46E+08	peak_4321	0	+	ENSMUSG	protein_coding
chr5	1.46E+08	1.46E+08	peak_4322	0	+	ENSMUSG	protein_coding
chr5	1.46E+08	1.46E+08	peak_4323	0	+	ENSMUSG	protein_coding
chr5	1.46E+08	1.46E+08	peak_4324	0	+	ENSMUSG	protein_coding
chr5	1.46E+08	1.46E+08	peak_4325	0	+	ENSMUSG	protein_coding
chr5	1.46E+08	1.46E+08	peak_4326	0	+	ENSMUSG	protein_coding
chr5	1.46E+08	1.46E+08	peak_4327	0	-	ENSMUSG	protein_coding
chr5	1.46E+08	1.46E+08	peak_4328	0	-	ENSMUSG	protein_coding
chr5	1.46E+08	1.46E+08	peak_4329	0	+	ENSMUSG	protein_coding
chr5	1.47E+08	1.47E+08	peak_4330	0	-	ENSMUSG	protein_coding
chr5	1.47E+08	1.47E+08	peak_4331	0	+	ENSMUSG	protein_coding
chr5	1.47E+08	1.47E+08	peak_4332	0	+	ENSMUSG	protein_coding
chr5	1.47E+08	1.47E+08	peak_4333	0	+	ENSMUSG	protein_coding
chr5	1.48E+08	1.48E+08	peak_4334	0	-	ENSMUSG	protein_coding
chr5	1.48E+08	1.48E+08	peak_4335	0	+	ENSMUSG	protein_coding
chr5	1.48E+08	1.48E+08	peak_4337	0	+	ENSMUSG	protein_coding
chr5	1.48E+08	1.48E+08	peak_4338	0	+	ENSMUSG	protein_coding
chr5	1.48E+08	1.48E+08	peak_4339	0	+	ENSMUSG	protein_coding
chr5	1.48E+08	1.48E+08	peak_4340	0	+	ENSMUSG	protein_coding
chr5	1.48E+08	1.48E+08	peak_4341	0	+	ENSMUSG	protein_coding
chr5	1.49E+08	1.49E+08	peak_4342	0	-	ENSMUSG	protein_coding
chr5	1.49E+08	1.49E+08	peak_4343	0	-	ENSMUSG	protein_coding
chr5	1.49E+08	1.49E+08	peak_4344	0	-	ENSMUSG	protein_coding
chr5	1.51E+08	1.51E+08	peak_4348	0	+	ENSMUSG	protein_coding
chr5	1.51E+08	1.51E+08	peak_4349	0	+	ENSMUSG	protein_coding

chr5	1.51E+08	1.51E+08	peak_4350	0	+	ENSMUSG	protein_coding
chr5	1.51E+08	1.51E+08	peak_4351	0	-	ENSMUSG	protein_coding
chr5	1.52E+08	1.52E+08	peak_4352	0	+	ENSMUSG	protein_coding
chr6	4458314	4458335	peak_4355	0	+	ENSMUSG	protein_coding
chr6	4490538	4490559	peak_4356	0	+	ENSMUSG	protein_coding
chr6	4685350	4685371	peak_4357	0	-	ENSMUSG	protein_coding
chr6	4887580	4887601	peak_4358	0	+	ENSMUSG	protein_coding
chr6	4891409	4891430	peak_4359	0	+	ENSMUSG	protein_coding
chr6	4968356	4968377	peak_4360	0	+	ENSMUSG	protein_coding
chr6	5004820	5004841	peak_4361	0	+	ENSMUSG	protein_coding
chr6	5691390	5691411	peak_4362	0	+	ENSMUSG	protein_coding
chr6	5707426	5707447	peak_4363	0	+	ENSMUSG	protein_coding
chr6	5738637	5738658	peak_4364	0	+	ENSMUSG	protein_coding
chr6	5752126	5752147	peak_4365	0	+	ENSMUSG	protein_coding
chr6	5764199	5764220	peak_4366	0	+	ENSMUSG	protein_coding
chr6	5803219	5803240	peak_4367	0	+	ENSMUSG	protein_coding
chr6	5959351	5959372	peak_4368	0	+	ENSMUSG	protein_coding
chr6	6915373	6915394	peak_4370	0	+	ENSMUSG	protein_coding
chr6	6954472	6954497	peak_4371	0	+	ENSMUSG	protein_coding
chr6	8232606	8232635	peak_4372	0	+	ENSMUSG	protein_coding
chr6	17605631	17605652	peak_4373	0	+	ENSMUSG	protein_coding
chr6	17705909	17705930	peak_4374	0	+	ENSMUSG	protein_coding
chr6	22038677	22038698	peak_4375	0	+	ENSMUSG	protein_coding
chr6	22051868	22051889	peak_4376	0	+	ENSMUSG	protein_coding
chr6	22093942	22093963	peak_4377	0	+	ENSMUSG	protein_coding
chr6	22105858	22105879	peak_4378	0	+	ENSMUSG	protein_coding
chr6	22132737	22132759	peak_4379	0	+	ENSMUSG	protein_coding
chr6	22278590	22278611	peak_4380	0	-	ENSMUSG	protein_coding
chr6	28193339	28193360	peak_4381	0	-	ENSMUSG	protein_coding
chr6	28193339	28193360	peak_4381	0	-	ENSMUSG	protein_coding
chr6	28194242	28194264	peak_4382	0	-	ENSMUSG	protein_coding
chr6	28194242	28194264	peak_4382	0	-	ENSMUSG	protein_coding
chr6	28462894	28462915	peak_4383	0	+	ENSMUSG	protein_coding
chr6	28518814	28518836	peak_4384	0	+	ENSMUSG	protein_coding
chr6	28641813	28641834	peak_4385	0	+	ENSMUSG	protein_coding
chr6	28641813	28641834	peak_4385	0	+	ENSMUSG	protein_coding
chr6	28746593	28746615	peak_4386	0	+	ENSMUSG	protein_coding
chr6	28746593	28746615	peak_4386	0	+	ENSMUSG	protein_coding
chr6	28770936	28770957	peak_4387	0	-	ENSMUSG	protein_coding
chr6	28770936	28770957	peak_4387	0	-	ENSMUSG	protein_coding
chr6	29349200	29349224	peak_4388	0	+	ENSMUSG	protein_coding
chr6	29369987	29370009	peak_4389	0	+	ENSMUSG	protein_coding
chr6	29417878	29417899	peak_4390	0	+	ENSMUSG	protein_coding
chr6	29454052	29454073	peak_4391	0	-	ENSMUSG	protein_coding
chr6	29734221	29734243	peak_4392	0	+	ENSMUSG	protein_coding
chr6	29883682	29883703	peak_4393	0	+	ENSMUSG	protein_coding
chr6	29884431	29884452	peak_4394	0	+	ENSMUSG	protein_coding
chr6	30078761	30078782	peak_4395	0	+	ENSMUSG	protein_coding
chr6	30175817	30175839	peak_4396	0	-	ENSMUSG	protein_coding
chr6	30246763	30246784	peak_4397	0	-	ENSMUSG	protein_coding
chr6	30248692	30248713	peak_4398	0	-	ENSMUSG	protein_coding
chr6	30340961	30340982	peak_4399	0	-	ENSMUSG	protein_coding
chr6	30451970	30451993	peak_4400	0	-	ENSMUSG	protein_coding
chr6	30637518	30637541	peak_4401	0	-	ENSMUSG	protein_coding
chr6	30829820	30829842	peak_4402	0	-	ENSMUSG	protein_coding
chr6	32918276	32918297	peak_4403	0	-	ENSMUSG	protein_coding
chr6	33227354	33227375	peak_4404	0	+	ENSMUSG	protein_coding
chr6	33282321	33282342	peak_4405	0	+	ENSMUSG	protein_coding

chr6	33355104	33355126	peak_4406	0	+	ENSMUSG	protein_coding
chr6	33488516	33488537	peak_4407	0	+	ENSMUSG	protein_coding
chr6	33536142	33536163	peak_4408	0	+	ENSMUSG	protein_coding
chr6	33637909	33637931	peak_4409	0	+	ENSMUSG	protein_coding
chr6	33708023	33708045	peak_4410	0	+	ENSMUSG	protein_coding
chr6	33789805	33789826	peak_4411	0	+	ENSMUSG	protein_coding
chr6	33872102	33872123	peak_4412	0	+	ENSMUSG	protein_coding
chr6	34259989	34260010	peak_4413	0	-	ENSMUSG	protein_coding
chr6	34454807	34454828	peak_4414	0	+	ENSMUSG	protein_coding
chr6	34667427	34667448	peak_4415	0	+	ENSMUSG	protein_coding
chr6	34696125	34696146	peak_4416	0	+	ENSMUSG	protein_coding
chr6	34705624	34705645	peak_4417	0	+	ENSMUSG	protein_coding
chr6	34705667	34705688	peak_4418	0	+	ENSMUSG	protein_coding
chr6	34705844	34705865	peak_4419	0	+	ENSMUSG	protein_coding
chr6	34853469	34853490	peak_4420	0	-	ENSMUSG	protein_coding
chr6	34880868	34880889	peak_4421	0	+	ENSMUSG	protein_coding
chr6	35069865	35069886	peak_4422	0	-	ENSMUSG	protein_coding
chr6	35083705	35083726	peak_4423	0	-	ENSMUSG	protein_coding
chr6	37328197	37328220	peak_4424	0	-	ENSMUSG	protein_coding
chr6	37387112	37387141	peak_4425	0	-	ENSMUSG	protein_coding
chr6	37915897	37915918	peak_4426	0	+	ENSMUSG	protein_coding
chr6	38444232	38444253	peak_4427	0	+	ENSMUSG	protein_coding
chr6	38506140	38506162	peak_4428	0	+	ENSMUSG	protein_coding
chr6	38510094	38510115	peak_4429	0	+	ENSMUSG	protein_coding
chr6	38543405	38543426	peak_4430	0	+	ENSMUSG	protein_coding
chr6	38767282	38767303	peak_4431	0	-	ENSMUSG	protein_coding
chr6	38768932	38768968	peak_4432	0	-	ENSMUSG	protein_coding
chr6	39040305	39040326	peak_4433	0	-	ENSMUSG	protein_coding
chr6	39090307	39090328	peak_4434	0	-	ENSMUSG	protein_coding
chr6	39091451	39091472	peak_4435	0	-	ENSMUSG	protein_coding
chr6	39099007	39099028	peak_4436	0	-	ENSMUSG	protein_coding
chr6	39099111	39099132	peak_4437	0	-	ENSMUSG	protein_coding
chr6	39102245	39102266	peak_4438	0	-	ENSMUSG	protein_coding
chr6	39142463	39142485	peak_4439	0	-	ENSMUSG	protein_coding
chr6	39305603	39305624	peak_4440	0	-	ENSMUSG	protein_coding
chr6	39579777	39579801	peak_4441	0	-	ENSMUSG	protein_coding
chr6	42302850	42302871	peak_4444	0	+	ENSMUSG	protein_coding
chr6	42304043	42304064	peak_4445	0	+	ENSMUSG	protein_coding
chr6	43259064	43259085	peak_4447	0	+	ENSMUSG	protein_coding
chr6	47486614	47486635	peak_4450	0	-	ENSMUSG	protein_coding
chr6	47758319	47758340	peak_4461	0	-	ENSMUSG	protein_coding
chr6	48346183	48346207	peak_4464	0	+	ENSMUSG	protein_coding
chr6	51413242	51413263	peak_4465	0	-	ENSMUSG	protein_coding
chr6	51416575	51416596	peak_4466	0	-	ENSMUSG	protein_coding
chr6	51417619	51417644	peak_4467	0	-	ENSMUSG	protein_coding
chr6	51417800	51417822	peak_4468	0	-	ENSMUSG	protein_coding
chr6	52570690	52570711	peak_4469	0	-	ENSMUSG	protein_coding
chr6	52822963	52822985	peak_4470	0	-	ENSMUSG	protein_coding
chr6	52949574	52949595	peak_4471	0	-	ENSMUSG	protein_coding
chr6	52969812	52969835	peak_4472	0	-	ENSMUSG	protein_coding
chr6	53592074	53592095	peak_4473	0	+	ENSMUSG	protein_coding
chr6	54405568	54405590	peak_4474	0	+	ENSMUSG	protein_coding
chr6	54423848	54423869	peak_4475	0	+	ENSMUSG	protein_coding
chr6	54431823	54431844	peak_4476	0	+	ENSMUSG	protein_coding
chr6	54433817	54433838	peak_4477	0	+	ENSMUSG	protein_coding
chr6	54504309	54504330	peak_4478	0	-	ENSMUSG	protein_coding
chr6	56707895	56707919	peak_4479	0	+	ENSMUSG	protein_coding
chr6	56708389	56708410	peak_4480	0	+	ENSMUSG	protein_coding

chr6	57560828	57560850	peak_4481	0	+	ENSMUSG	protein_coding
chr6	66883395	66883416	peak_4483	0	+	ENSMUSG	protein_coding
chr6	66896044	66896066	peak_4484	0	+	ENSMUSG	protein_coding
chr6	67216982	67217005	peak_4485	0	+	ENSMUSG	protein_coding
chr6	67217129	67217150	peak_4486	0	+	ENSMUSG	protein_coding
chr6	67222908	67222929	peak_4487	0	+	ENSMUSG	protein_coding
chr6	71493896	71493917	peak_4489	0	+	ENSMUSG	protein_coding
chr6	71679972	71679994	peak_4490	0	+	ENSMUSG	protein_coding
chr6	71682100	71682121	peak_4491	0	+	ENSMUSG	protein_coding
chr6	71721543	71721566	peak_4492	0	+	ENSMUSG	protein_coding
chr6	71733806	71733827	peak_4493	0	+	ENSMUSG	protein_coding
chr6	71832603	71832624	peak_4495	0	-	ENSMUSG	protein_coding
chr6	71832643	71832664	peak_4496	0	-	ENSMUSG	protein_coding
chr6	71832686	71832707	peak_4497	0	-	ENSMUSG	protein_coding
chr6	71879695	71879732	peak_4498	0	+	ENSMUSG	protein_coding
chr6	72054759	72054780	peak_4499	0	+	ENSMUSG	protein_coding
chr6	72075692	72075713	peak_4500	0	+	ENSMUSG	protein_coding
chr6	72104464	72104485	peak_4501	0	+	ENSMUSG	protein_coding
chr6	72269775	72269796	peak_4503	0	-	ENSMUSG	protein_coding
chr6	72312388	72312409	peak_4504	0	-	ENSMUSG	protein_coding
chr6	72384396	72384417	peak_4505	0	-	ENSMUSG	protein_coding
chr6	72388617	72388639	peak_4506	0	-	ENSMUSG	protein_coding
chr6	72389138	72389159	peak_4507	0	-	ENSMUSG	protein_coding
chr6	72565821	72565842	peak_4508	0	-	ENSMUSG	protein_coding
chr6	76938969	76938990	peak_4513	0	-	ENSMUSG	protein_coding
chr6	77023788	77023809	peak_4514	0	-	ENSMUSG	protein_coding
chr6	77187712	77187733	peak_4515	0	-	ENSMUSG	protein_coding
chr6	77218486	77218507	peak_4516	0	-	ENSMUSG	protein_coding
chr6	77223773	77223797	peak_4517	0	-	ENSMUSG	protein_coding
chr6	77248289	77248310	peak_4518	0	-	ENSMUSG	protein_coding
chr6	77260521	77260542	peak_4519	0	-	ENSMUSG	protein_coding
chr6	77346437	77346458	peak_4520	0	-	ENSMUSG	protein_coding
chr6	77355524	77355545	peak_4521	0	-	ENSMUSG	protein_coding
chr6	77366338	77366360	peak_4522	0	-	ENSMUSG	protein_coding
chr6	77392348	77392369	peak_4523	0	-	ENSMUSG	protein_coding
chr6	77396859	77396881	peak_4524	0	-	ENSMUSG	protein_coding
chr6	77412560	77412581	peak_4525	0	-	ENSMUSG	protein_coding
chr6	77435117	77435138	peak_4526	0	-	ENSMUSG	protein_coding
chr6	77461457	77461478	peak_4527	0	-	ENSMUSG	protein_coding
chr6	77469071	77469094	peak_4528	0	-	ENSMUSG	protein_coding
chr6	77551367	77551388	peak_4529	0	-	ENSMUSG	protein_coding
chr6	77555108	77555129	peak_4530	0	-	ENSMUSG	protein_coding
chr6	77694109	77694130	peak_4531	0	-	ENSMUSG	protein_coding
chr6	77703123	77703144	peak_4532	0	-	ENSMUSG	protein_coding
chr6	77708496	77708517	peak_4533	0	-	ENSMUSG	protein_coding
chr6	82724257	82724278	peak_4534	0	-	ENSMUSG	protein_coding
chr6	83066643	83066664	peak_4535	0	+	ENSMUSG	protein_coding
chr6	83139237	83139258	peak_4536	0	+	ENSMUSG	protein_coding
chr6	83139601	83139627	peak_4537	0	+	ENSMUSG	protein_coding
chr6	83144997	83145018	peak_4538	0	+	ENSMUSG	protein_coding
chr6	83347537	83347558	peak_4539	0	-	ENSMUSG	protein_coding
chr6	83352934	83352956	peak_4540	0	-	ENSMUSG	protein_coding
chr6	83433044	83433065	peak_4541	0	-	ENSMUSG	protein_coding
chr6	83886527	83886549	peak_4542	0	+	ENSMUSG	protein_coding
chr6	83893101	83893122	peak_4543	0	+	ENSMUSG	protein_coding
chr6	84670414	84670435	peak_4545	0	-	ENSMUSG	protein_coding
chr6	84674492	84674513	peak_4546	0	-	ENSMUSG	protein_coding
chr6	84694876	84694910	peak_4547	0	-	ENSMUSG	protein_coding

chr6	84932784	84932805	peak_4548	0	-	ENSMUSG	protein_coding
chr6	85019410	85019431	peak_4549	0	-	ENSMUSG	protein_coding
chr6	85551199	85551220	peak_4550	0	+	ENSMUSG	protein_coding
chr6	85899476	85899498	peak_4551	0	-	ENSMUSG	protein_coding
chr6	85907662	85907684	peak_4552	0	-	ENSMUSG	protein_coding
chr6	86632965	86632986	peak_4556	0	-	ENSMUSG	protein_coding
chr6	86850554	86850576	peak_4558	0	+	ENSMUSG	protein_coding
chr6	87864613	87864634	peak_4560	0	+	ENSMUSG	protein_coding
chr6	88034757	88034778	peak_4561	0	+	ENSMUSG	protein_coding
chr6	88839998	88840019	peak_4562	0	-	ENSMUSG	protein_coding
chr6	89266787	89266808	peak_4563	0	-	ENSMUSG	protein_coding
chr6	89268240	89268262	peak_4564	0	-	ENSMUSG	protein_coding
chr6	90573408	90573429	peak_4565	0	+	ENSMUSG	protein_coding
chr6	90613577	90613599	peak_4566	0	-	ENSMUSG	protein_coding
chr6	90671796	90671817	peak_4567	0	-	ENSMUSG	protein_coding
chr6	91469542	91469563	peak_4568	0	+	ENSMUSG	protein_coding
chr6	92161646	92161668	peak_4569	0	-	ENSMUSG	protein_coding
chr6	92827090	92827112	peak_4570	0	-	ENSMUSG	protein_coding
chr6	92827090	92827112	peak_4570	0	-	ENSMUSG	protein_coding
chr6	92876036	92876057	peak_4571	0	-	ENSMUSG	protein_coding
chr6	92883511	92883533	peak_4572	0	-	ENSMUSG	protein_coding
chr6	93710587	93710609	peak_4573	0	-	ENSMUSG	protein_coding
chr6	94123198	94123219	peak_4574	0	-	ENSMUSG	protein_coding
chr6	94142522	94142552	peak_4575	0	-	ENSMUSG	protein_coding
chr6	94220166	94220187	peak_4576	0	-	ENSMUSG	protein_coding
chr6	94549781	94549803	peak_4577	0	+	ENSMUSG	protein_coding
chr6	95574411	95574434	peak_4579	0	-	ENSMUSG	protein_coding
chr6	97126583	97126609	peak_4580	0	-	ENSMUSG	protein_coding
chr6	97134713	97134734	peak_4581	0	-	ENSMUSG	protein_coding
chr6	97342344	97342366	peak_4582	0	-	ENSMUSG	protein_coding
chr6	99074717	99074738	peak_4585	0	-	ENSMUSG	protein_coding
chr6	99353304	99353325	peak_4586	0	-	ENSMUSG	protein_coding
chr6	99470438	99470459	peak_4587	0	-	ENSMUSG	protein_coding
chr6	1E+08	1E+08	peak_4588	0	-	ENSMUSG	protein_coding
chr6	1.01E+08	1.01E+08	peak_4590	0	+	ENSMUSG	protein_coding
chr6	1.08E+08	1.08E+08	peak_4591	0	+	ENSMUSG	protein_coding
chr6	1.08E+08	1.08E+08	peak_4592	0	+	ENSMUSG	protein_coding
chr6	1.08E+08	1.08E+08	peak_4593	0	+	ENSMUSG	protein_coding
chr6	1.08E+08	1.08E+08	peak_4594	0	+	ENSMUSG	protein_coding
chr6	1.13E+08	1.13E+08	peak_4595	0	-	ENSMUSG	protein_coding
chr6	1.13E+08	1.13E+08	peak_4596	0	-	ENSMUSG	protein_coding
chr6	1.13E+08	1.13E+08	peak_4597	0	-	ENSMUSG	protein_coding
chr6	1.13E+08	1.13E+08	peak_4598	0	-	ENSMUSG	protein_coding
chr6	1.13E+08	1.13E+08	peak_4599	0	-	ENSMUSG	protein_coding
chr6	1.13E+08	1.13E+08	peak_4600	0	+	ENSMUSG	protein_coding
chr6	1.13E+08	1.13E+08	peak_4601	0	+	ENSMUSG	protein_coding
chr6	1.13E+08	1.13E+08	peak_4602	0	+	ENSMUSG	protein_coding
chr6	1.14E+08	1.14E+08	peak_4604	0	+	ENSMUSG	protein_coding
chr6	1.15E+08	1.15E+08	peak_4605	0	-	ENSMUSG	protein_coding
chr6	1.15E+08	1.15E+08	peak_4606	0	-	ENSMUSG	protein_coding
chr6	1.16E+08	1.16E+08	peak_4607	0	-	ENSMUSG	protein_coding
chr6	1.16E+08	1.16E+08	peak_4608	0	-	ENSMUSG	protein_coding
chr6	1.16E+08	1.16E+08	peak_4609	0	-	ENSMUSG	protein_coding
chr6	1.16E+08	1.16E+08	peak_4610	0	-	ENSMUSG	protein_coding
chr6	1.16E+08	1.16E+08	peak_4611	0	-	ENSMUSG	protein_coding
chr6	1.16E+08	1.16E+08	peak_4612	0	+	ENSMUSG	protein_coding
chr6	1.18E+08	1.18E+08	peak_4613	0	-	ENSMUSG	protein_coding
chr6	1.18E+08	1.18E+08	peak_4614	0	-	ENSMUSG	protein_coding

chr6	1.19E+08	1.19E+08	peak_4615	0	-	ENSMUSG	protein_coding
chr6	1.19E+08	1.19E+08	peak_4616	0	-	ENSMUSG	protein_coding
chr6	1.19E+08	1.19E+08	peak_4617	0	-	ENSMUSG	protein_coding
chr6	1.19E+08	1.19E+08	peak_4618	0	-	ENSMUSG	protein_coding
chr6	1.19E+08	1.19E+08	peak_4619	0	-	ENSMUSG	protein_coding
chr6	1.19E+08	1.19E+08	peak_4620	0	+	ENSMUSG	protein_coding
chr6	1.19E+08	1.19E+08	peak_4620	0	+	ENSMUSG	protein_coding
chr6	1.19E+08	1.19E+08	peak_4621	0	-	ENSMUSG	protein_coding
chr6	1.2E+08	1.2E+08	peak_4622	0	-	ENSMUSG	protein_coding
chr6	1.2E+08	1.2E+08	peak_4623	0	-	ENSMUSG	protein_coding
chr6	1.2E+08	1.2E+08	peak_4624	0	-	ENSMUSG	protein_coding
chr6	1.2E+08	1.2E+08	peak_4625	0	+	ENSMUSG	protein_coding
chr6	1.2E+08	1.2E+08	peak_4626	0	+	ENSMUSG	protein_coding
chr6	1.2E+08	1.2E+08	peak_4627	0	+	ENSMUSG	protein_coding
chr6	1.2E+08	1.2E+08	peak_4628	0	+	ENSMUSG	protein_coding
chr6	1.21E+08	1.21E+08	peak_4629	0	+	ENSMUSG	protein_coding
chr6	1.21E+08	1.21E+08	peak_4630	0	+	ENSMUSG	protein_coding
chr6	1.21E+08	1.21E+08	peak_4631	0	+	ENSMUSG	protein_coding
chr6	1.21E+08	1.21E+08	peak_4632	0	-	ENSMUSG	protein_coding
chr6	1.21E+08	1.21E+08	peak_4633	0	-	ENSMUSG	protein_coding
chr6	1.25E+08	1.25E+08	peak_4634	0	+	ENSMUSG	protein_coding
chr6	1.25E+08	1.25E+08	peak_4635	0	-	ENSMUSG	protein_coding
chr6	1.25E+08	1.25E+08	peak_4635	0	-	ENSMUSG	protein_coding
chr6	1.25E+08	1.25E+08	peak_4636	0	-	ENSMUSG	protein_coding
chr6	1.25E+08	1.25E+08	peak_4636	0	-	ENSMUSG	protein_coding
chr6	1.25E+08	1.25E+08	peak_4637	0	+	ENSMUSG	protein_coding
chr6	1.25E+08	1.25E+08	peak_4638	0	+	ENSMUSG	protein_coding
chr6	1.25E+08	1.25E+08	peak_4639	0	+	ENSMUSG	protein_coding
chr6	1.25E+08	1.25E+08	peak_4640	0	+	ENSMUSG	protein_coding
chr6	1.25E+08	1.25E+08	peak_4641	0	+	ENSMUSG	protein_coding
chr6	1.25E+08	1.25E+08	peak_4642	0	+	ENSMUSG	protein_coding
chr6	1.25E+08	1.25E+08	peak_4643	0	+	ENSMUSG	protein_coding
chr6	1.25E+08	1.25E+08	peak_4644	0	+	ENSMUSG	protein_coding
chr6	1.25E+08	1.25E+08	peak_4645	0	+	ENSMUSG	protein_coding
chr6	1.25E+08	1.25E+08	peak_4646	0	+	ENSMUSG	protein_coding
chr6	1.25E+08	1.25E+08	peak_4647	0	+	ENSMUSG	protein_coding
chr6	1.25E+08	1.25E+08	peak_4648	0	-	ENSMUSG	protein_coding
chr6	1.25E+08	1.25E+08	peak_4649	0	-	ENSMUSG	protein_coding
chr6	1.25E+08	1.25E+08	peak_4650	0	-	ENSMUSG	protein_coding
chr6	1.27E+08	1.27E+08	peak_4652	0	-	ENSMUSG	protein_coding
chr6	1.28E+08	1.28E+08	peak_4653	0	-	ENSMUSG	protein_coding
chr6	1.28E+08	1.28E+08	peak_4654	0	-	ENSMUSG	protein_coding
chr6	1.29E+08	1.29E+08	peak_4666	0	-	ENSMUSG	protein_coding
chr6	1.29E+08	1.29E+08	peak_4667	0	+	ENSMUSG	protein_coding
chr6	1.34E+08	1.34E+08	peak_4668	0	+	ENSMUSG	protein_coding
chr6	1.34E+08	1.34E+08	peak_4669	0	+	ENSMUSG	protein_coding
chr6	1.35E+08	1.35E+08	peak_4670	0	-	ENSMUSG	protein_coding
chr6	1.35E+08	1.35E+08	peak_4671	0	-	ENSMUSG	protein_coding
chr6	1.35E+08	1.35E+08	peak_4672	0	-	ENSMUSG	protein_coding
chr6	1.35E+08	1.35E+08	peak_4673	0	+	ENSMUSG	protein_coding
chr6	1.36E+08	1.36E+08	peak_4674	0	+	ENSMUSG	protein_coding
chr6	1.36E+08	1.36E+08	peak_4675	0	+	ENSMUSG	protein_coding
chr6	1.37E+08	1.37E+08	peak_4676	0	+	ENSMUSG	protein_coding
chr6	1.37E+08	1.37E+08	peak_4677	0	+	ENSMUSG	protein_coding
chr6	1.37E+08	1.37E+08	peak_4678	0	+	ENSMUSG	protein_coding
chr6	1.37E+08	1.37E+08	peak_4679	0	-	ENSMUSG	protein_coding
chr6	1.41E+08	1.41E+08	peak_4681	0	-	ENSMUSG	protein_coding
chr6	1.46E+08	1.46E+08	peak_4683	0	+	ENSMUSG	protein_coding

chr6	1.47E+08	1.47E+08	peak_4684	0	+	ENSMUSG	protein_coding
chr6	1.47E+08	1.47E+08	peak_4685	0	-	ENSMUSG	protein_coding
chr6	1.47E+08	1.47E+08	peak_4686	0	+	ENSMUSG	protein_coding
chr6	1.47E+08	1.47E+08	peak_4687	0	+	ENSMUSG	protein_coding
chr6	1.47E+08	1.47E+08	peak_4688	0	+	ENSMUSG	protein_coding
chr7	3655622	3655644	peak_4692	0	+	ENSMUSG	protein_coding
chr7	3658333	3658354	peak_4693	0	+	ENSMUSG	protein_coding
chr7	4085956	4085977	peak_4694	0	+	ENSMUSG	protein_coding
chr7	4097434	4097455	peak_4695	0	+	ENSMUSG	protein_coding
chr7	4433529	4433550	peak_4696	0	-	ENSMUSG	protein_coding
chr7	4433563	4433585	peak_4697	0	-	ENSMUSG	protein_coding
chr7	4583614	4583635	peak_4698	0	-	ENSMUSG	protein_coding
chr7	4587689	4587710	peak_4699	0	-	ENSMUSG	protein_coding
chr7	4745191	4745213	peak_4700	0	+	ENSMUSG	protein_coding
chr7	5013913	5013934	peak_4701	0	+	ENSMUSG	protein_coding
chr7	5016931	5016952	peak_4702	0	+	ENSMUSG	protein_coding
chr7	5018021	5018042	peak_4703	0	+	ENSMUSG	protein_coding
chr7	5042351	5042372	peak_4704	0	+	ENSMUSG	protein_coding
chr7	6093402	6093423	peak_4706	0	-	ENSMUSG	protein_coding
chr7	6340994	6341015	peak_4707	0	+	ENSMUSG	protein_coding
chr7	6660287	6660308	peak_4708	0	-	ENSMUSG	protein_coding
chr7	13353824	13353845	peak_4710	0	-	ENSMUSG	protein_coding
chr7	13507644	13507665	peak_4711	0	+	ENSMUSG	protein_coding
chr7	13611073	13611095	peak_4712	0	+	ENSMUSG	protein_coding
chr7	17057175	17057196	peak_4715	0	+	ENSMUSG	protein_coding
chr7	17123489	17123510	peak_4716	0	-	ENSMUSG	protein_coding
chr7	17149241	17149262	peak_4717	0	-	ENSMUSG	protein_coding
chr7	17502150	17502171	peak_4718	0	-	ENSMUSG	protein_coding
chr7	19484496	19484517	peak_4720	0	-	ENSMUSG	protein_coding
chr7	19492983	19493004	peak_4721	0	-	ENSMUSG	protein_coding
chr7	19627914	19627935	peak_4722	0	+	ENSMUSG	protein_coding
chr7	19941474	19941495	peak_4724	0	-	ENSMUSG	protein_coding
chr7	19941474	19941495	peak_4724	0	-	ENSMUSG	protein_coding
chr7	25669629	25669650	peak_4728	0	+	ENSMUSG	protein_coding
chr7	25670160	25670181	peak_4729	0	+	ENSMUSG	protein_coding
chr7	25670520	25670541	peak_4730	0	+	ENSMUSG	protein_coding
chr7	26063158	26063179	peak_4731	0	+	ENSMUSG	protein_coding
chr7	26511851	26511872	peak_4733	0	-	ENSMUSG	protein_coding
chr7	26539090	26539112	peak_4734	0	-	ENSMUSG	protein_coding
chr7	26551902	26551923	peak_4735	0	-	ENSMUSG	protein_coding
chr7	27943936	27943958	peak_4736	0	-	ENSMUSG	protein_coding
chr7	27976769	27976790	peak_4737	0	-	ENSMUSG	protein_coding
chr7	29183684	29183705	peak_4741	0	+	ENSMUSG	protein_coding
chr7	29516993	29517014	peak_4743	0	+	ENSMUSG	protein_coding
chr7	29598853	29598874	peak_4744	0	+	ENSMUSG	protein_coding
chr7	29600295	29600316	peak_4745	0	+	ENSMUSG	protein_coding
chr7	29680754	29680777	peak_4746	0	+	ENSMUSG	protein_coding
chr7	29703796	29703817	peak_4747	0	-	ENSMUSG	protein_coding
chr7	29716682	29716703	peak_4748	0	-	ENSMUSG	protein_coding
chr7	29746687	29746708	peak_4749	0	-	ENSMUSG	protein_coding
chr7	29766316	29766337	peak_4750	0	-	ENSMUSG	protein_coding
chr7	30680355	30680376	peak_4752	0	-	ENSMUSG	protein_coding
chr7	30680355	30680376	peak_4752	0	-	ENSMUSG	protein_coding
chr7	30951713	30951734	peak_4753	0	-	ENSMUSG	protein_coding
chr7	31371329	31371350	peak_4754	0	-	ENSMUSG	protein_coding
chr7	31754076	31754097	peak_4755	0	-	ENSMUSG	protein_coding
chr7	31754281	31754302	peak_4756	0	-	ENSMUSG	protein_coding
chr7	34906488	34906509	peak_4758	0	-	ENSMUSG	protein_coding

chr7	34926363	34926384	peak_4759	0	-	ENSMUSG	protein_coding
chr7	34998622	34998643	peak_4760	0	-	ENSMUSG	protein_coding
chr7	35068308	35068329	peak_4761	0	+	ENSMUSG	protein_coding
chr7	35426995	35427016	peak_4762	0	-	ENSMUSG	protein_coding
chr7	36428678	36428699	peak_4763	0	-	ENSMUSG	protein_coding
chr7	38265533	38265554	peak_4764	0	-	ENSMUSG	protein_coding
chr7	38265586	38265607	peak_4765	0	-	ENSMUSG	protein_coding
chr7	51502134	51502155	peak_4767	0	+	ENSMUSG	protein_coding
chr7	51503440	51503462	peak_4768	0	+	ENSMUSG	protein_coding
chr7	51503471	51503493	peak_4769	0	+	ENSMUSG	protein_coding
chr7	51505213	51505234	peak_4770	0	+	ENSMUSG	protein_coding
chr7	51505485	51505506	peak_4771	0	+	ENSMUSG	protein_coding
chr7	51505512	51505538	peak_4772	0	+	ENSMUSG	protein_coding
chr7	51505813	51505834	peak_4773	0	+	ENSMUSG	protein_coding
chr7	52178735	52178756	peak_4774	0	-	ENSMUSG	protein_coding
chr7	52291439	52291460	peak_4775	0	-	ENSMUSG	protein_coding
chr7	52378481	52378503	peak_4776	0	-	ENSMUSG	protein_coding
chr7	52379701	52379722	peak_4777	0	-	ENSMUSG	protein_coding
chr7	52381804	52381827	peak_4778	0	-	ENSMUSG	protein_coding
chr7	52382010	52382031	peak_4779	0	-	ENSMUSG	protein_coding
chr7	52382038	52382059	peak_4780	0	-	ENSMUSG	protein_coding
chr7	52382314	52382336	peak_4781	0	-	ENSMUSG	protein_coding
chr7	52382831	52382853	peak_4782	0	-	ENSMUSG	protein_coding
chr7	52383867	52383889	peak_4783	0	-	ENSMUSG	protein_coding
chr7	52384104	52384125	peak_4784	0	-	ENSMUSG	protein_coding
chr7	52637902	52637923	peak_4785	0	-	ENSMUSG	protein_coding
chr7	52642661	52642682	peak_4786	0	-	ENSMUSG	protein_coding
chr7	52714494	52714515	peak_4787	0	-	ENSMUSG	protein_coding
chr7	52719313	52719334	peak_4788	0	-	ENSMUSG	protein_coding
chr7	52784969	52784990	peak_4789	0	+	ENSMUSG	protein_coding
chr7	52973451	52973472	peak_4790	0	+	ENSMUSG	protein_coding
chr7	53326955	53326977	peak_4792	0	+	ENSMUSG	protein_coding
chr7	53705760	53705782	peak_4793	0	+	ENSMUSG	protein_coding
chr7	53957355	53957376	peak_4794	0	-	ENSMUSG	protein_coding
chr7	56502648	56502670	peak_4795	0	+	ENSMUSG	protein_coding
chr7	56516399	56516420	peak_4796	0	+	ENSMUSG	protein_coding
chr7	56731294	56731315	peak_4797	0	+	ENSMUSG	protein_coding
chr7	56786979	56787001	peak_4798	0	+	ENSMUSG	protein_coding
chr7	56820889	56820920	peak_4799	0	+	ENSMUSG	protein_coding
chr7	56835174	56835195	peak_4800	0	+	ENSMUSG	protein_coding
chr7	63259159	63259180	peak_4802	0	-	ENSMUSG	protein_coding
chr7	63390971	63390993	peak_4803	0	+	ENSMUSG	protein_coding
chr7	63419252	63419276	peak_4804	0	+	ENSMUSG	protein_coding
chr7	66057839	66057860	peak_4806	0	+	ENSMUSG	protein_coding
chr7	66509307	66509328	peak_4807	0	+	ENSMUSG	protein_coding
chr7	72487563	72487584	peak_4813	0	-	ENSMUSG	protein_coding
chr7	73093727	73093748	peak_4817	0	+	ENSMUSG	protein_coding
chr7	73294250	73294273	peak_4818	0	+	ENSMUSG	protein_coding
chr7	73306058	73306080	peak_4819	0	+	ENSMUSG	protein_coding
chr7	73505526	73505547	peak_4820	0	-	ENSMUSG	protein_coding
chr7	73517098	73517119	peak_4821	0	-	ENSMUSG	protein_coding
chr7	73517098	73517119	peak_4821	0	-	ENSMUSG	protein_coding
chr7	73518475	73518496	peak_4822	0	-	ENSMUSG	protein_coding
chr7	73518475	73518496	peak_4822	0	-	ENSMUSG	protein_coding
chr7	73814890	73814911	peak_4823	0	-	ENSMUSG	protein_coding
chr7	74880602	74880624	peak_4824	0	-	ENSMUSG	protein_coding
chr7	74881245	74881268	peak_4825	0	-	ENSMUSG	protein_coding
chr7	75102794	75102815	peak_4826	0	+	ENSMUSG	protein_coding

chr7	75152936	75152958	peak_4827	0	+	ENSMUSG	protein_coding
chr7	75169249	75169270	peak_4828	0	+	ENSMUSG	protein_coding
chr7	75169275	75169297	peak_4829	0	+	ENSMUSG	protein_coding
chr7	75175177	75175198	peak_4830	0	+	ENSMUSG	protein_coding
chr7	75204946	75204967	peak_4831	0	+	ENSMUSG	protein_coding
chr7	75232609	75232631	peak_4832	0	+	ENSMUSG	protein_coding
chr7	75258050	75258071	peak_4833	0	+	ENSMUSG	protein_coding
chr7	75365568	75365590	peak_4834	0	+	ENSMUSG	protein_coding
chr7	77501518	77501539	peak_4836	0	-	ENSMUSG	protein_coding
chr7	80574577	80574598	peak_4839	0	-	ENSMUSG	protein_coding
chr7	80614542	80614563	peak_4840	0	-	ENSMUSG	protein_coding
chr7	80646898	80646919	peak_4841	0	-	ENSMUSG	protein_coding
chr7	80675950	80675971	peak_4842	0	-	ENSMUSG	protein_coding
chr7	81693731	81693752	peak_4844	0	-	ENSMUSG	protein_coding
chr7	82681790	82681811	peak_4845	0	+	ENSMUSG	protein_coding
chr7	82724605	82724626	peak_4846	0	+	ENSMUSG	protein_coding
chr7	82754484	82754505	peak_4847	0	+	ENSMUSG	protein_coding
chr7	82754933	82754954	peak_4848	0	+	ENSMUSG	protein_coding
chr7	82800061	82800082	peak_4849	0	+	ENSMUSG	protein_coding
chr7	86442795	86442816	peak_4853	0	+	ENSMUSG	protein_coding
chr7	86892031	86892055	peak_4856	0	-	ENSMUSG	protein_coding
chr7	87184012	87184033	peak_4858	0	+	ENSMUSG	protein_coding
chr7	87208610	87208631	peak_4859	0	+	ENSMUSG	protein_coding
chr7	87768503	87768527	peak_4860	0	-	ENSMUSG	protein_coding
chr7	87883786	87883807	peak_4861	0	-	ENSMUSG	protein_coding
chr7	88069733	88069754	peak_4862	0	-	ENSMUSG	protein_coding
chr7	88490108	88490130	peak_4863	0	-	ENSMUSG	protein_coding
chr7	89042021	89042044	peak_4864	0	-	ENSMUSG	protein_coding
chr7	89340561	89340582	peak_4865	0	+	ENSMUSG	protein_coding
chr7	89920229	89920250	peak_4866	0	+	ENSMUSG	protein_coding
chr7	90016355	90016376	peak_4867	0	+	ENSMUSG	protein_coding
chr7	91122799	91122820	peak_4868	0	+	ENSMUSG	protein_coding
chr7	91126927	91126948	peak_4869	0	+	ENSMUSG	protein_coding
chr7	91129852	91129873	peak_4870	0	+	ENSMUSG	protein_coding
chr7	91787463	91787484	peak_4871	0	-	ENSMUSG	protein_coding
chr7	96382555	96382576	peak_4873	0	-	ENSMUSG	protein_coding
chr7	97315371	97315394	peak_4874	0	+	ENSMUSG	protein_coding
chr7	97596140	97596161	peak_4875	0	+	ENSMUSG	protein_coding
chr7	99396246	99396267	peak_4876	0	+	ENSMUSG	protein_coding
chr7	1.04E+08	1.04E+08	peak_4878	0	+	ENSMUSG	protein_coding
chr7	1.04E+08	1.04E+08	peak_4879	0	+	ENSMUSG	protein_coding
chr7	1.04E+08	1.04E+08	peak_4880	0	+	ENSMUSG	protein_coding
chr7	1.04E+08	1.04E+08	peak_4881	0	+	ENSMUSG	protein_coding
chr7	1.04E+08	1.04E+08	peak_4882	0	+	ENSMUSG	protein_coding
chr7	1.05E+08	1.05E+08	peak_4883	0	+	ENSMUSG	protein_coding
chr7	1.05E+08	1.05E+08	peak_4884	0	+	ENSMUSG	protein_coding
chr7	1.05E+08	1.05E+08	peak_4885	0	-	ENSMUSG	protein_coding
chr7	1.06E+08	1.06E+08	peak_4886	0	-	ENSMUSG	protein_coding
chr7	1.06E+08	1.06E+08	peak_4887	0	-	ENSMUSG	protein_coding
chr7	1.06E+08	1.06E+08	peak_4888	0	+	ENSMUSG	protein_coding
chr7	1.06E+08	1.06E+08	peak_4891	0	-	ENSMUSG	protein_coding
chr7	1.07E+08	1.07E+08	peak_4892	0	-	ENSMUSG	protein_coding
chr7	1.07E+08	1.07E+08	peak_4893	0	-	ENSMUSG	protein_coding
chr7	1.07E+08	1.07E+08	peak_4894	0	-	ENSMUSG	protein_coding
chr7	1.07E+08	1.07E+08	peak_4896	0	-	ENSMUSG	protein_coding
chr7	1.08E+08	1.08E+08	peak_4897	0	+	ENSMUSG	protein_coding
chr7	1.08E+08	1.08E+08	peak_4898	0	+	ENSMUSG	protein_coding
chr7	1.08E+08	1.08E+08	peak_4901	0	+	ENSMUSG	protein_coding

chr7	1.08E+08	1.08E+08	peak_4902	0	+	ENSMUSG	protein_coding
chr7	1.09E+08	1.09E+08	peak_4903	0	+	ENSMUSG	protein_coding
chr7	1.09E+08	1.09E+08	peak_4904	0	+	ENSMUSG	protein_coding
chr7	1.09E+08	1.09E+08	peak_4904	0	+	ENSMUSG	protein_coding
chr7	1.09E+08	1.09E+08	peak_4905	0	+	ENSMUSG	protein_coding
chr7	1.09E+08	1.09E+08	peak_4906	0	+	ENSMUSG	protein_coding
chr7	1.09E+08	1.09E+08	peak_4907	0	+	ENSMUSG	protein_coding
chr7	1.09E+08	1.09E+08	peak_4908	0	+	ENSMUSG	protein_coding
chr7	1.09E+08	1.09E+08	peak_4909	0	+	ENSMUSG	protein_coding
chr7	1.1E+08	1.1E+08	peak_4910	0	+	ENSMUSG	protein_coding
chr7	1.13E+08	1.13E+08	peak_4911	0	-	ENSMUSG	protein_coding
chr7	1.13E+08	1.13E+08	peak_4912	0	-	ENSMUSG	protein_coding
chr7	1.15E+08	1.15E+08	peak_4914	0	+	ENSMUSG	protein_coding
chr7	1.17E+08	1.17E+08	peak_4915	0	+	ENSMUSG	protein_coding
chr7	1.17E+08	1.17E+08	peak_4916	0	+	ENSMUSG	protein_coding
chr7	1.17E+08	1.17E+08	peak_4917	0	-	ENSMUSG	protein_coding
chr7	1.17E+08	1.17E+08	peak_4918	0	-	ENSMUSG	protein_coding
chr7	1.17E+08	1.17E+08	peak_4919	0	-	ENSMUSG	protein_coding
chr7	1.17E+08	1.17E+08	peak_4920	0	-	ENSMUSG	protein_coding
chr7	1.17E+08	1.17E+08	peak_4921	0	-	ENSMUSG	protein_coding
chr7	1.17E+08	1.17E+08	peak_4922	0	-	ENSMUSG	protein_coding
chr7	1.17E+08	1.17E+08	peak_4923	0	+	ENSMUSG	protein_coding
chr7	1.17E+08	1.17E+08	peak_4924	0	-	ENSMUSG	protein_coding
chr7	1.18E+08	1.18E+08	peak_4925	0	-	ENSMUSG	protein_coding
chr7	1.18E+08	1.18E+08	peak_4926	0	+	ENSMUSG	protein_coding
chr7	1.18E+08	1.18E+08	peak_4927	0	-	ENSMUSG	protein_coding
chr7	1.18E+08	1.18E+08	peak_4928	0	-	ENSMUSG	protein_coding
chr7	1.19E+08	1.19E+08	peak_4929	0	+	ENSMUSG	protein_coding
chr7	1.2E+08	1.2E+08	peak_4930	0	+	ENSMUSG	protein_coding
chr7	1.21E+08	1.21E+08	peak_4931	0	-	ENSMUSG	protein_coding
chr7	1.21E+08	1.21E+08	peak_4932	0	-	ENSMUSG	protein_coding
chr7	1.22E+08	1.22E+08	peak_4934	0	+	ENSMUSG	protein_coding
chr7	1.23E+08	1.23E+08	peak_4936	0	-	ENSMUSG	protein_coding
chr7	1.23E+08	1.23E+08	peak_4937	0	-	ENSMUSG	protein_coding
chr7	1.23E+08	1.23E+08	peak_4938	0	-	ENSMUSG	protein_coding
chr7	1.23E+08	1.23E+08	peak_4939	0	-	ENSMUSG	protein_coding
chr7	1.23E+08	1.23E+08	peak_4940	0	-	ENSMUSG	protein_coding
chr7	1.24E+08	1.24E+08	peak_4941	0	-	ENSMUSG	protein_coding
chr7	1.25E+08	1.25E+08	peak_4942	0	+	ENSMUSG	protein_coding
chr7	1.25E+08	1.25E+08	peak_4943	0	-	ENSMUSG	protein_coding
chr7	1.25E+08	1.25E+08	peak_4944	0	-	ENSMUSG	protein_coding
chr7	1.25E+08	1.25E+08	peak_4945	0	-	ENSMUSG	protein_coding
chr7	1.25E+08	1.25E+08	peak_4946	0	-	ENSMUSG	protein_coding
chr7	1.25E+08	1.25E+08	peak_4947	0	-	ENSMUSG	protein_coding
chr7	1.25E+08	1.25E+08	peak_4948	0	-	ENSMUSG	protein_coding
chr7	1.26E+08	1.26E+08	peak_4949	0	-	ENSMUSG	protein_coding
chr7	1.27E+08	1.27E+08	peak_4950	0	-	ENSMUSG	protein_coding
chr7	1.28E+08	1.28E+08	peak_4952	0	-	ENSMUSG	protein_coding
chr7	1.29E+08	1.29E+08	peak_4953	0	-	ENSMUSG	protein_coding
chr7	1.29E+08	1.29E+08	peak_4954	0	-	ENSMUSG	protein_coding
chr7	1.29E+08	1.29E+08	peak_4955	0	+	ENSMUSG	protein_coding
chr7	1.3E+08	1.3E+08	peak_4956	0	+	ENSMUSG	protein_coding
chr7	1.3E+08	1.3E+08	peak_4957	0	+	ENSMUSG	protein_coding
chr7	1.3E+08	1.3E+08	peak_4958	0	+	ENSMUSG	protein_coding
chr7	1.3E+08	1.3E+08	peak_4959	0	+	ENSMUSG	protein_coding
chr7	1.3E+08	1.3E+08	peak_4960	0	+	ENSMUSG	protein_coding
chr7	1.31E+08	1.31E+08	peak_4961	0	-	ENSMUSG	protein_coding
chr7	1.33E+08	1.33E+08	peak_4962	0	-	ENSMUSG	protein_coding

chr7	1.33E+08	1.33E+08	peak_4963	0	-	ENSMUSG	protein_coding
chr7	1.33E+08	1.33E+08	peak_4964	0	-	ENSMUSG	protein_coding
chr7	1.33E+08	1.33E+08	peak_4965	0	+	ENSMUSG	protein_coding
chr7	1.33E+08	1.33E+08	peak_4966	0	+	ENSMUSG	protein_coding
chr7	1.34E+08	1.34E+08	peak_4967	0	-	ENSMUSG	protein_coding
chr7	1.34E+08	1.34E+08	peak_4968	0	-	ENSMUSG	protein_coding
chr7	1.34E+08	1.34E+08	peak_4969	0	-	ENSMUSG	protein_coding
chr7	1.34E+08	1.34E+08	peak_4970	0	-	ENSMUSG	protein_coding
chr7	1.34E+08	1.34E+08	peak_4971	0	-	ENSMUSG	protein_coding
chr7	1.34E+08	1.34E+08	peak_4972	0	-	ENSMUSG	protein_coding
chr7	1.35E+08	1.35E+08	peak_4973	0	+	ENSMUSG	protein_coding
chr7	1.35E+08	1.35E+08	peak_4974	0	+	ENSMUSG	protein_coding
chr7	1.35E+08	1.35E+08	peak_4975	0	+	ENSMUSG	protein_coding
chr7	1.35E+08	1.35E+08	peak_4976	0	+	ENSMUSG	protein_coding
chr7	1.35E+08	1.35E+08	peak_4977	0	+	ENSMUSG	protein_coding
chr7	1.35E+08	1.35E+08	peak_4978	0	+	ENSMUSG	protein_coding
chr7	1.35E+08	1.35E+08	peak_4981	0	+	ENSMUSG	protein_coding
chr7	1.36E+08	1.36E+08	peak_4982	0	-	ENSMUSG	protein_coding
chr7	1.37E+08	1.37E+08	peak_4983	0	+	ENSMUSG	protein_coding
chr7	1.38E+08	1.38E+08	peak_4984	0	-	ENSMUSG	protein_coding
chr7	1.38E+08	1.38E+08	peak_4984	0	-	ENSMUSG	protein_coding
chr7	1.38E+08	1.38E+08	peak_4985	0	+	ENSMUSG	protein_coding
chr7	1.38E+08	1.38E+08	peak_4985	0	+	ENSMUSG	protein_coding
chr7	1.4E+08	1.4E+08	peak_4986	0	-	ENSMUSG	protein_coding
chr7	1.4E+08	1.4E+08	peak_4986	0	-	ENSMUSG	protein_coding
chr7	1.4E+08	1.4E+08	peak_4987	0	-	ENSMUSG	protein_coding
chr7	1.4E+08	1.4E+08	peak_4987	0	-	ENSMUSG	protein_coding
chr7	1.4E+08	1.4E+08	peak_4988	0	-	ENSMUSG	protein_coding
chr7	1.4E+08	1.4E+08	peak_4989	0	+	ENSMUSG	protein_coding
chr7	1.4E+08	1.4E+08	peak_4989	0	+	ENSMUSG	protein_coding
chr7	1.4E+08	1.4E+08	peak_4990	0	+	ENSMUSG	protein_coding
chr7	1.4E+08	1.4E+08	peak_4990	0	+	ENSMUSG	protein_coding
chr7	1.4E+08	1.4E+08	peak_4991	0	-	ENSMUSG	protein_coding
chr7	1.4E+08	1.4E+08	peak_4991	0	-	ENSMUSG	protein_coding
chr7	1.4E+08	1.4E+08	peak_4992	0	-	ENSMUSG	protein_coding
chr7	1.4E+08	1.4E+08	peak_4992	0	-	ENSMUSG	protein_coding
chr7	1.41E+08	1.41E+08	peak_4993	0	-	ENSMUSG	protein_coding
chr7	1.41E+08	1.41E+08	peak_4994	0	-	ENSMUSG	protein_coding
chr7	1.41E+08	1.41E+08	peak_4995	0	-	ENSMUSG	protein_coding
chr7	1.41E+08	1.41E+08	peak_4996	0	-	ENSMUSG	protein_coding
chr7	1.41E+08	1.41E+08	peak_4997	0	-	ENSMUSG	protein_coding
chr7	1.42E+08	1.42E+08	peak_4998	0	+	ENSMUSG	protein_coding
chr7	1.42E+08	1.42E+08	peak_4999	0	+	ENSMUSG	protein_coding
chr7	1.42E+08	1.42E+08	peak_5000	0	+	ENSMUSG	protein_coding
chr7	1.42E+08	1.42E+08	peak_5000	0	+	ENSMUSG	protein_coding
chr7	1.42E+08	1.42E+08	peak_5001	0	+	ENSMUSG	protein_coding
chr7	1.42E+08	1.42E+08	peak_5001	0	+	ENSMUSG	protein_coding
chr7	1.42E+08	1.42E+08	peak_5002	0	+	ENSMUSG	protein_coding
chr7	1.42E+08	1.42E+08	peak_5003	0	+	ENSMUSG	protein_coding
chr7	1.43E+08	1.43E+08	peak_5004	0	+	ENSMUSG	protein_coding
chr7	1.43E+08	1.43E+08	peak_5005	0	-	ENSMUSG	protein_coding
chr7	1.46E+08	1.46E+08	peak_5006	0	+	ENSMUSG	protein_coding
chr7	1.47E+08	1.47E+08	peak_5007	0	+	ENSMUSG	protein_coding
chr7	1.47E+08	1.47E+08	peak_5008	0	+	ENSMUSG	protein_coding
chr7	1.49E+08	1.49E+08	peak_5011	0	+	ENSMUSG	protein_coding
chr7	1.49E+08	1.49E+08	peak_5012	0	-	ENSMUSG	protein_coding
chr7	1.5E+08	1.5E+08	peak_5014	0	-	ENSMUSG	protein_coding
chr7	1.51E+08	1.51E+08	peak_5015	0	-	ENSMUSG	protein_coding

chr7	1.51E+08	1.51E+08	peak_5016	0	-	ENSMUSG	protein_coding
chr7	1.51E+08	1.51E+08	peak_5017	0	-	ENSMUSG	protein_coding
chr7	1.51E+08	1.51E+08	peak_5018	0	+	ENSMUSG	protein_coding
chr7	1.52E+08	1.52E+08	peak_5019	0	+	ENSMUSG	protein_coding
chr7	1.52E+08	1.52E+08	peak_5020	0	-	ENSMUSG	protein_coding
chr7	1.52E+08	1.52E+08	peak_5021	0	-	ENSMUSG	protein_coding
chr8	3152068	3152089	peak_5023	0	-	ENSMUSG	protein_coding
chr8	3432748	3432769	peak_5024	0	+	ENSMUSG	protein_coding
chr8	4324139	4324161	peak_5026	0	-	ENSMUSG	protein_coding
chr8	8681600	8681625	peak_5028	0	-	ENSMUSG	protein_coding
chr8	8682441	8682463	peak_5029	0	-	ENSMUSG	protein_coding
chr8	8690044	8690065	peak_5030	0	-	ENSMUSG	protein_coding
chr8	11236498	11236519	peak_5031	0	-	ENSMUSG	protein_coding
chr8	12846112	12846133	peak_5032	0	+	ENSMUSG	protein_coding
chr8	12972046	12972067	peak_5033	0	+	ENSMUSG	protein_coding
chr8	13131603	13131624	peak_5034	0	+	ENSMUSG	protein_coding
chr8	13167233	13167254	peak_5035	0	+	ENSMUSG	protein_coding
chr8	13292765	13292786	peak_5036	0	+	ENSMUSG	protein_coding
chr8	13876075	13876096	peak_5037	0	+	ENSMUSG	protein_coding
chr8	13974025	13974046	peak_5038	0	-	ENSMUSG	protein_coding
chr8	14979736	14979757	peak_5039	0	+	ENSMUSG	protein_coding
chr8	18690683	18690704	peak_5042	0	+	ENSMUSG	protein_coding
chr8	18690683	18690704	peak_5042	0	+	ENSMUSG	protein_coding
chr8	18754332	18754354	peak_5043	0	+	ENSMUSG	protein_coding
chr8	23255080	23255102	peak_5044	0	-	ENSMUSG	protein_coding
chr8	23694343	23694364	peak_5045	0	-	ENSMUSG	protein_coding
chr8	23813322	23813343	peak_5046	0	-	ENSMUSG	protein_coding
chr8	24042849	24042870	peak_5047	0	+	ENSMUSG	protein_coding
chr8	26271757	26271779	peak_5048	0	-	ENSMUSG	protein_coding
chr8	26279701	26279734	peak_5049	0	-	ENSMUSG	protein_coding
chr8	26294921	26294950	peak_5050	0	-	ENSMUSG	protein_coding
chr8	26643904	26643926	peak_5052	0	+	ENSMUSG	protein_coding
chr8	26746757	26746779	peak_5053	0	+	ENSMUSG	protein_coding
chr8	26751669	26751698	peak_5054	0	+	ENSMUSG	protein_coding
chr8	26783815	26783840	peak_5055	0	+	ENSMUSG	protein_coding
chr8	26846047	26846068	peak_5056	0	-	ENSMUSG	protein_coding
chr8	26846101	26846122	peak_5057	0	-	ENSMUSG	protein_coding
chr8	26946809	26946830	peak_5058	0	-	ENSMUSG	protein_coding
chr8	28209590	28209611	peak_5059	0	-	ENSMUSG	protein_coding
chr8	30246664	30246685	peak_5060	0	-	ENSMUSG	protein_coding
chr8	34802250	34802271	peak_5062	0	+	ENSMUSG	protein_coding
chr8	34808441	34808462	peak_5063	0	+	ENSMUSG	protein_coding
chr8	34909909	34909930	peak_5064	0	-	ENSMUSG	protein_coding
chr8	34915083	34915106	peak_5065	0	-	ENSMUSG	protein_coding
chr8	35901073	35901094	peak_5066	0	-	ENSMUSG	protein_coding
chr8	37705749	37705774	peak_5069	0	-	ENSMUSG	protein_coding
chr8	37731963	37731985	peak_5070	0	-	ENSMUSG	protein_coding
chr8	37755035	37755056	peak_5071	0	-	ENSMUSG	protein_coding
chr8	37758413	37758434	peak_5072	0	-	ENSMUSG	protein_coding
chr8	37758482	37758503	peak_5073	0	-	ENSMUSG	protein_coding
chr8	37773404	37773425	peak_5074	0	-	ENSMUSG	protein_coding
chr8	37821673	37821694	peak_5075	0	-	ENSMUSG	protein_coding
chr8	38000631	38000661	peak_5076	0	-	ENSMUSG	protein_coding
chr8	38001442	38001463	peak_5077	0	-	ENSMUSG	protein_coding
chr8	41551404	41551425	peak_5079	0	+	ENSMUSG	protein_coding
chr8	41596394	41596418	peak_5080	0	-	ENSMUSG	protein_coding
chr8	41976116	41976137	peak_5081	0	+	ENSMUSG	protein_coding
chr8	41994827	41994848	peak_5082	0	+	ENSMUSG	protein_coding

chr8	42129487	42129508	peak_5083	0	-	ENSMUSG	protein_coding
chr8	42189709	42189730	peak_5084	0	-	ENSMUSG	protein_coding
chr8	42344523	42344544	peak_5085	0	+	ENSMUSG	protein_coding
chr8	42361127	42361148	peak_5086	0	+	ENSMUSG	protein_coding
chr8	42361272	42361293	peak_5087	0	+	ENSMUSG	protein_coding
chr8	42437567	42437588	peak_5088	0	-	ENSMUSG	protein_coding
chr8	46035740	46035761	peak_5091	0	+	ENSMUSG	protein_coding
chr8	46116655	46116676	peak_5092	0	+	ENSMUSG	protein_coding
chr8	46133785	46133806	peak_5093	0	+	ENSMUSG	protein_coding
chr8	46753003	46753024	peak_5094	0	+	ENSMUSG	protein_coding
chr8	47576472	47576493	peak_5095	0	+	ENSMUSG	protein_coding
chr8	48594032	48594053	peak_5096	0	-	ENSMUSG	protein_coding
chr8	48610407	48610428	peak_5097	0	-	ENSMUSG	protein_coding
chr8	48616084	48616105	peak_5098	0	-	ENSMUSG	protein_coding
chr8	49066951	49066972	peak_5099	0	-	ENSMUSG	protein_coding
chr8	49703953	49703974	peak_5100	0	-	ENSMUSG	protein_coding
chr8	54694769	54694790	peak_5102	0	-	ENSMUSG	protein_coding
chr8	59990779	59990800	peak_5103	0	+	ENSMUSG	protein_coding
chr8	60112818	60112839	peak_5104	0	-	ENSMUSG	protein_coding
chr8	63531749	63531770	peak_5105	0	+	ENSMUSG	protein_coding
chr8	67123246	67123267	peak_5106	0	-	ENSMUSG	protein_coding
chr8	68526270	68526291	peak_5107	0	-	ENSMUSG	protein_coding
chr8	68526351	68526372	peak_5108	0	-	ENSMUSG	protein_coding
chr8	70432677	70432698	peak_5109	0	-	ENSMUSG	protein_coding
chr8	72329207	72329228	peak_5110	0	+	ENSMUSG	protein_coding
chr8	72932858	72932879	peak_5112	0	-	ENSMUSG	protein_coding
chr8	73034256	73034279	peak_5113	0	-	ENSMUSG	protein_coding
chr8	73034256	73034279	peak_5113	0	-	ENSMUSG	protein_coding
chr8	73105527	73105548	peak_5114	0	+	ENSMUSG	protein_coding
chr8	73421305	73421326	peak_5115	0	-	ENSMUSG	protein_coding
chr8	73891954	73891975	peak_5117	0	+	ENSMUSG	protein_coding
chr8	73893422	73893443	peak_5118	0	+	ENSMUSG	protein_coding
chr8	74103980	74104001	peak_5119	0	+	ENSMUSG	protein_coding
chr8	74136033	74136054	peak_5120	0	+	ENSMUSG	protein_coding
chr8	74743876	74743897	peak_5122	0	+	ENSMUSG	protein_coding
chr8	74774601	74774622	peak_5123	0	+	ENSMUSG	protein_coding
chr8	74905395	74905416	peak_5124	0	-	ENSMUSG	protein_coding
chr8	74906299	74906320	peak_5125	0	-	ENSMUSG	protein_coding
chr8	75057172	75057194	peak_5126	0	-	ENSMUSG	protein_coding
chr8	75247266	75247288	peak_5127	0	+	ENSMUSG	protein_coding
chr8	75249908	75249943	peak_5128	0	+	ENSMUSG	protein_coding
chr8	75856950	75856972	peak_5129	0	-	ENSMUSG	protein_coding
chr8	79800363	79800384	peak_5130	0	-	ENSMUSG	protein_coding
chr8	79806951	79806979	peak_5131	0	-	ENSMUSG	protein_coding
chr8	79896996	79897017	peak_5132	0	-	ENSMUSG	protein_coding
chr8	79932674	79932695	peak_5133	0	-	ENSMUSG	protein_coding
chr8	81083372	81083393	peak_5134	0	+	ENSMUSG	protein_coding
chr8	81131155	81131176	peak_5135	0	+	ENSMUSG	protein_coding
chr8	81597494	81597516	peak_5137	0	+	ENSMUSG	protein_coding
chr8	82560592	82560613	peak_5138	0	-	ENSMUSG	protein_coding
chr8	83324107	83324129	peak_5139	0	-	ENSMUSG	protein_coding
chr8	85295619	85295640	peak_5140	0	-	ENSMUSG	protein_coding
chr8	85794802	85794823	peak_5141	0	+	ENSMUSG	protein_coding
chr8	86431155	86431176	peak_5144	0	+	ENSMUSG	protein_coding
chr8	86432840	86432861	peak_5145	0	+	ENSMUSG	protein_coding
chr8	86434113	86434134	peak_5146	0	+	ENSMUSG	protein_coding
chr8	86448065	86448089	peak_5147	0	+	ENSMUSG	protein_coding
chr8	86462548	86462570	peak_5148	0	+	ENSMUSG	protein_coding

chr8	86462548	86462570	peak_5148	0	+	ENSMUSG	protein_coding
chr8	86498800	86498821	peak_5149	0	+	ENSMUSG	protein_coding
chr8	87200887	87200908	peak_5150	0	-	ENSMUSG	protein_coding
chr8	87272893	87272914	peak_5151	0	-	ENSMUSG	protein_coding
chr8	87296118	87296139	peak_5152	0	-	ENSMUSG	protein_coding
chr8	87307431	87307452	peak_5153	0	-	ENSMUSG	protein_coding
chr8	87368381	87368402	peak_5154	0	-	ENSMUSG	protein_coding
chr8	87368532	87368553	peak_5155	0	-	ENSMUSG	protein_coding
chr8	87370764	87370786	peak_5156	0	-	ENSMUSG	protein_coding
chr8	87493570	87493592	peak_5157	0	+	ENSMUSG	protein_coding
chr8	87493570	87493592	peak_5157	0	+	ENSMUSG	protein_coding
chr8	87577469	87577490	peak_5158	0	+	ENSMUSG	protein_coding
chr8	87635789	87635812	peak_5159	0	-	ENSMUSG	protein_coding
chr8	88444770	88444792	peak_5160	0	+	ENSMUSG	protein_coding
chr8	89177171	89177192	peak_5161	0	+	ENSMUSG	protein_coding
chr8	89264567	89264588	peak_5162	0	-	ENSMUSG	protein_coding
chr8	89384384	89384405	peak_5163	0	-	ENSMUSG	protein_coding
chr8	89402136	89402157	peak_5164	0	-	ENSMUSG	protein_coding
chr8	90481637	90481658	peak_5165	0	-	ENSMUSG	protein_coding
chr8	90767152	90767174	peak_5166	0	+	ENSMUSG	protein_coding
chr8	90769684	90769705	peak_5167	0	+	ENSMUSG	protein_coding
chr8	93378142	93378163	peak_5168	0	+	ENSMUSG	protein_coding
chr8	93914111	93914132	peak_5169	0	+	ENSMUSG	protein_coding
chr8	93976243	93976264	peak_5170	0	+	ENSMUSG	protein_coding
chr8	94009084	94009105	peak_5171	0	+	ENSMUSG	protein_coding
chr8	94055077	94055103	peak_5172	0	+	ENSMUSG	protein_coding
chr8	94107577	94107598	peak_5173	0	+	ENSMUSG	protein_coding
chr8	94155416	94155437	peak_5174	0	+	ENSMUSG	protein_coding
chr8	94164595	94164616	peak_5175	0	+	ENSMUSG	protein_coding
chr8	95363279	95363300	peak_5176	0	-	ENSMUSG	protein_coding
chr8	96496154	96496175	peak_5177	0	-	ENSMUSG	protein_coding
chr8	96836826	96836850	peak_5178	0	+	ENSMUSG	protein_coding
chr8	97101335	97101356	peak_5180	0	-	ENSMUSG	protein_coding
chr8	97139930	97139952	peak_5181	0	+	ENSMUSG	protein_coding
chr8	97183160	97183181	peak_5182	0	+	ENSMUSG	protein_coding
chr8	97770740	97770761	peak_5185	0	+	ENSMUSG	protein_coding
chr8	97997007	97997028	peak_5186	0	-	ENSMUSG	protein_coding
chr8	98268243	98268264	peak_5187	0	-	ENSMUSG	protein_coding
chr8	98316512	98316534	peak_5188	0	-	ENSMUSG	protein_coding
chr8	1.07E+08	1.07E+08	peak_5189	0	+	ENSMUSG	protein_coding
chr8	1.07E+08	1.07E+08	peak_5190	0	+	ENSMUSG	protein_coding
chr8	1.07E+08	1.07E+08	peak_5191	0	-	ENSMUSG	protein_coding
chr8	1.07E+08	1.07E+08	peak_5192	0	-	ENSMUSG	protein_coding
chr8	1.08E+08	1.08E+08	peak_5193	0	+	ENSMUSG	protein_coding
chr8	1.08E+08	1.08E+08	peak_5194	0	+	ENSMUSG	protein_coding
chr8	1.08E+08	1.08E+08	peak_5195	0	+	ENSMUSG	protein_coding
chr8	1.08E+08	1.08E+08	peak_5196	0	-	ENSMUSG	protein_coding
chr8	1.08E+08	1.08E+08	peak_5197	0	+	ENSMUSG	protein_coding
chr8	1.09E+08	1.09E+08	peak_5198	0	-	ENSMUSG	protein_coding
chr8	1.09E+08	1.09E+08	peak_5200	0	+	ENSMUSG	protein_coding
chr8	1.09E+08	1.09E+08	peak_5201	0	+	ENSMUSG	protein_coding
chr8	1.1E+08	1.1E+08	peak_5202	0	+	ENSMUSG	protein_coding
chr8	1.1E+08	1.1E+08	peak_5204	0	+	ENSMUSG	protein_coding
chr8	1.1E+08	1.1E+08	peak_5205	0	+	ENSMUSG	protein_coding
chr8	1.1E+08	1.1E+08	peak_5206	0	+	ENSMUSG	protein_coding
chr8	1.1E+08	1.1E+08	peak_5207	0	+	ENSMUSG	protein_coding
chr8	1.1E+08	1.1E+08	peak_5208	0	+	ENSMUSG	protein_coding
chr8	1.1E+08	1.1E+08	peak_5209	0	+	ENSMUSG	protein_coding

chr8	1.1E+08	1.1E+08	peak_5210	0	+	ENSMUSG	protein_coding
chr8	1.1E+08	1.1E+08	peak_5211	0	+	ENSMUSG	protein_coding
chr8	1.11E+08	1.11E+08	peak_5213	0	+	ENSMUSG	protein_coding
chr8	1.11E+08	1.11E+08	peak_5214	0	+	ENSMUSG	protein_coding
chr8	1.11E+08	1.11E+08	peak_5215	0	+	ENSMUSG	protein_coding
chr8	1.11E+08	1.11E+08	peak_5216	0	+	ENSMUSG	protein_coding
chr8	1.12E+08	1.12E+08	peak_5218	0	-	ENSMUSG	protein_coding
chr8	1.12E+08	1.12E+08	peak_5219	0	+	ENSMUSG	protein_coding
chr8	1.12E+08	1.12E+08	peak_5220	0	+	ENSMUSG	protein_coding
chr8	1.13E+08	1.13E+08	peak_5221	0	+	ENSMUSG	protein_coding
chr8	1.13E+08	1.13E+08	peak_5222	0	-	ENSMUSG	protein_coding
chr8	1.13E+08	1.13E+08	peak_5223	0	-	ENSMUSG	protein_coding
chr8	1.13E+08	1.13E+08	peak_5224	0	-	ENSMUSG	protein_coding
chr8	1.13E+08	1.13E+08	peak_5225	0	+	ENSMUSG	protein_coding
chr8	1.14E+08	1.14E+08	peak_5226	0	+	ENSMUSG	protein_coding
chr8	1.14E+08	1.14E+08	peak_5227	0	-	ENSMUSG	protein_coding
chr8	1.14E+08	1.14E+08	peak_5228	0	-	ENSMUSG	protein_coding
chr8	1.14E+08	1.14E+08	peak_5229	0	-	ENSMUSG	protein_coding
chr8	1.14E+08	1.14E+08	peak_5230	0	-	ENSMUSG	protein_coding
chr8	1.14E+08	1.14E+08	peak_5231	0	-	ENSMUSG	protein_coding
chr8	1.14E+08	1.14E+08	peak_5232	0	+	ENSMUSG	protein_coding
chr8	1.14E+08	1.14E+08	peak_5233	0	+	ENSMUSG	protein_coding
chr8	1.14E+08	1.14E+08	peak_5234	0	+	ENSMUSG	protein_coding
chr8	1.14E+08	1.14E+08	peak_5235	0	-	ENSMUSG	protein_coding
chr8	1.14E+08	1.14E+08	peak_5236	0	-	ENSMUSG	protein_coding
chr8	1.14E+08	1.14E+08	peak_5237	0	-	ENSMUSG	protein_coding
chr8	1.17E+08	1.17E+08	peak_5238	0	+	ENSMUSG	protein_coding
chr8	1.17E+08	1.17E+08	peak_5239	0	+	ENSMUSG	protein_coding
chr8	1.17E+08	1.17E+08	peak_5240	0	+	ENSMUSG	protein_coding
chr8	1.18E+08	1.18E+08	peak_5241	0	-	ENSMUSG	protein_coding
chr8	1.19E+08	1.19E+08	peak_5242	0	-	ENSMUSG	protein_coding
chr8	1.2E+08	1.2E+08	peak_5243	0	+	ENSMUSG	protein_coding
chr8	1.22E+08	1.22E+08	peak_5245	0	+	ENSMUSG	protein_coding
chr8	1.22E+08	1.22E+08	peak_5246	0	+	ENSMUSG	protein_coding
chr8	1.23E+08	1.23E+08	peak_5247	0	+	ENSMUSG	protein_coding
chr8	1.23E+08	1.23E+08	peak_5248	0	+	ENSMUSG	protein_coding
chr8	1.23E+08	1.23E+08	peak_5249	0	+	ENSMUSG	protein_coding
chr8	1.23E+08	1.23E+08	peak_5250	0	-	ENSMUSG	protein_coding
chr8	1.23E+08	1.23E+08	peak_5250	0	-	ENSMUSG	protein_coding
chr8	1.23E+08	1.23E+08	peak_5251	0	+	ENSMUSG	protein_coding
chr8	1.23E+08	1.23E+08	peak_5251	0	+	ENSMUSG	protein_coding
chr8	1.24E+08	1.24E+08	peak_5252	0	-	ENSMUSG	protein_coding
chr8	1.24E+08	1.24E+08	peak_5253	0	-	ENSMUSG	protein_coding
chr8	1.24E+08	1.24E+08	peak_5253	0	-	ENSMUSG	protein_coding
chr8	1.25E+08	1.25E+08	peak_5254	0	+	ENSMUSG	protein_coding
chr8	1.25E+08	1.25E+08	peak_5254	0	+	ENSMUSG	protein_coding
chr8	1.25E+08	1.25E+08	peak_5255	0	+	ENSMUSG	protein_coding
chr8	1.25E+08	1.25E+08	peak_5255	0	+	ENSMUSG	protein_coding
chr8	1.25E+08	1.25E+08	peak_5256	0	+	ENSMUSG	protein_coding
chr8	1.25E+08	1.25E+08	peak_5256	0	+	ENSMUSG	protein_coding
chr8	1.25E+08	1.25E+08	peak_5257	0	+	ENSMUSG	protein_coding
chr8	1.25E+08	1.25E+08	peak_5258	0	-	ENSMUSG	protein_coding
chr8	1.25E+08	1.25E+08	peak_5258	0	-	ENSMUSG	protein_coding
chr8	1.25E+08	1.25E+08	peak_5259	0	-	ENSMUSG	protein_coding
chr8	1.25E+08	1.25E+08	peak_5259	0	-	ENSMUSG	protein_coding
chr8	1.25E+08	1.25E+08	peak_5260	0	-	ENSMUSG	protein_coding
chr8	1.25E+08	1.25E+08	peak_5260	0	-	ENSMUSG	protein_coding
chr8	1.25E+08	1.25E+08	peak_5261	0	-	ENSMUSG	protein_coding

chr8	1.25E+08	1.25E+08	peak_5261	0	-	ENSMUSG	protein_coding
chr8	1.25E+08	1.25E+08	peak_5262	0	-	ENSMUSG	protein_coding
chr8	1.25E+08	1.25E+08	peak_5262	0	-	ENSMUSG	protein_coding
chr8	1.25E+08	1.25E+08	peak_5263	0	-	ENSMUSG	protein_coding
chr8	1.25E+08	1.25E+08	peak_5263	0	-	ENSMUSG	protein_coding
chr8	1.26E+08	1.26E+08	peak_5264	0	+	ENSMUSG	protein_coding
chr8	1.26E+08	1.26E+08	peak_5264	0	+	ENSMUSG	protein_coding
chr8	1.26E+08	1.26E+08	peak_5265	0	+	ENSMUSG	protein_coding
chr8	1.26E+08	1.26E+08	peak_5265	0	+	ENSMUSG	protein_coding
chr8	1.26E+08	1.26E+08	peak_5266	0	+	ENSMUSG	protein_coding
chr8	1.26E+08	1.26E+08	peak_5266	0	+	ENSMUSG	protein_coding
chr8	1.26E+08	1.26E+08	peak_5267	0	-	ENSMUSG	protein_coding
chr8	1.26E+08	1.26E+08	peak_5268	0	-	ENSMUSG	protein_coding
chr8	1.26E+08	1.26E+08	peak_5269	0	+	ENSMUSG	protein_coding
chr8	1.26E+08	1.26E+08	peak_5269	0	+	ENSMUSG	protein_coding
chr8	1.27E+08	1.27E+08	peak_5270	0	+	ENSMUSG	protein_coding
chr8	1.27E+08	1.27E+08	peak_5270	0	+	ENSMUSG	protein_coding
chr8	1.27E+08	1.27E+08	peak_5271	0	+	ENSMUSG	protein_coding
chr8	1.27E+08	1.27E+08	peak_5271	0	+	ENSMUSG	protein_coding
chr8	1.27E+08	1.27E+08	peak_5272	0	+	ENSMUSG	protein_coding
chr8	1.27E+08	1.27E+08	peak_5273	0	-	ENSMUSG	protein_coding
chr8	1.27E+08	1.27E+08	peak_5274	0	+	ENSMUSG	protein_coding
chr8	1.27E+08	1.27E+08	peak_5275	0	-	ENSMUSG	protein_coding
chr8	1.28E+08	1.28E+08	peak_5277	0	+	ENSMUSG	protein_coding
chr8	1.28E+08	1.28E+08	peak_5278	0	+	ENSMUSG	protein_coding
chr8	1.28E+08	1.28E+08	peak_5279	0	+	ENSMUSG	protein_coding
chr8	1.3E+08	1.3E+08	peak_5280	0	+	ENSMUSG	protein_coding
chr8	1.3E+08	1.3E+08	peak_5281	0	+	ENSMUSG	protein_coding
chr8	1.31E+08	1.31E+08	peak_5282	0	+	ENSMUSG	protein_coding
chr8	1.31E+08	1.31E+08	peak_5283	0	+	ENSMUSG	protein_coding
chr9	3404065	3404086	peak_5284	0	+	ENSMUSG	protein_coding
chr9	3454061	3454082	peak_5285	0	+	ENSMUSG	protein_coding
chr9	3454548	3454569	peak_5286	0	+	ENSMUSG	protein_coding
chr9	6202180	6202201	peak_5287	0	+	ENSMUSG	protein_coding
chr9	7821037	7821058	peak_5288	0	-	ENSMUSG	protein_coding
chr9	7821222	7821244	peak_5289	0	-	ENSMUSG	protein_coding
chr9	7824360	7824381	peak_5290	0	-	ENSMUSG	protein_coding
chr9	7973512	7973533	peak_5291	0	-	ENSMUSG	protein_coding
chr9	7979661	7979682	peak_5292	0	-	ENSMUSG	protein_coding
chr9	9238745	9238766	peak_5293	0	-	ENSMUSG	protein_coding
chr9	13636595	13636616	peak_5294	0	+	ENSMUSG	protein_coding
chr9	15070485	15070506	peak_5295	0	-	ENSMUSG	protein_coding
chr9	15110687	15110708	peak_5296	0	+	ENSMUSG	protein_coding
chr9	15118224	15118245	peak_5297	0	+	ENSMUSG	protein_coding
chr9	15118496	15118517	peak_5298	0	+	ENSMUSG	protein_coding
chr9	15119946	15119972	peak_5299	0	+	ENSMUSG	protein_coding
chr9	15130777	15130798	peak_5300	0	-	ENSMUSG	protein_coding
chr9	15130917	15130938	peak_5301	0	-	ENSMUSG	protein_coding
chr9	15334848	15334870	peak_5302	0	+	ENSMUSG	protein_coding
chr9	18139201	18139222	peak_5303	0	-	ENSMUSG	protein_coding
chr9	20322793	20322816	peak_5304	0	-	ENSMUSG	protein_coding
chr9	20693912	20693934	peak_5306	0	+	ENSMUSG	protein_coding
chr9	20695185	20695206	peak_5307	0	+	ENSMUSG	protein_coding
chr9	20702382	20702403	peak_5308	0	-	ENSMUSG	protein_coding
chr9	20731574	20731603	peak_5309	0	-	ENSMUSG	protein_coding
chr9	20757164	20757185	peak_5310	0	-	ENSMUSG	protein_coding
chr9	20946959	20946983	peak_5311	0	-	ENSMUSG	protein_coding
chr9	21200688	21200709	peak_5312	0	+	ENSMUSG	protein_coding

chr9	21234354	21234375	peak_5313	0	+	ENSMUSG	protein_coding
chr9	21393548	21393571	peak_5314	0	+	ENSMUSG	protein_coding
chr9	21393548	21393571	peak_5314	0	+	ENSMUSG	protein_coding
chr9	21443732	21443753	peak_5315	0	+	ENSMUSG	protein_coding
chr9	21490433	21490468	peak_5316	0	+	ENSMUSG	protein_coding
chr9	21504287	21504311	peak_5317	0	+	ENSMUSG	protein_coding
chr9	22622682	22622703	peak_5319	0	+	ENSMUSG	protein_coding
chr9	26883248	26883269	peak_5321	0	+	ENSMUSG	protein_coding
chr9	26943123	26943144	peak_5322	0	-	ENSMUSG	protein_coding
chr9	30850574	30850595	peak_5323	0	+	ENSMUSG	protein_coding
chr9	30963178	30963200	peak_5324	0	-	ENSMUSG	protein_coding
chr9	30978091	30978113	peak_5325	0	-	ENSMUSG	protein_coding
chr9	31107066	31107087	peak_5326	0	+	ENSMUSG	protein_coding
chr9	31167656	31167677	peak_5327	0	+	ENSMUSG	protein_coding
chr9	31216900	31216922	peak_5328	0	+	ENSMUSG	protein_coding
chr9	31961492	31961513	peak_5329	0	+	ENSMUSG	protein_coding
chr9	32004937	32004958	peak_5330	0	+	ENSMUSG	protein_coding
chr9	34854165	34854186	peak_5331	0	-	ENSMUSG	protein_coding
chr9	35021832	35021854	peak_5332	0	+	ENSMUSG	protein_coding
chr9	35022119	35022140	peak_5333	0	+	ENSMUSG	protein_coding
chr9	36773480	36773504	peak_5335	0	-	ENSMUSG	protein_coding
chr9	40020340	40020361	peak_5336	0	+	ENSMUSG	protein_coding
chr9	40144941	40144962	peak_5337	0	-	ENSMUSG	protein_coding
chr9	40196320	40196341	peak_5338	0	-	ENSMUSG	protein_coding
chr9	40198092	40198113	peak_5339	0	-	ENSMUSG	protein_coding
chr9	40211767	40211788	peak_5340	0	-	ENSMUSG	protein_coding
chr9	40609336	40609357	peak_5341	0	+	ENSMUSG	protein_coding
chr9	40610946	40610967	peak_5342	0	+	ENSMUSG	protein_coding
chr9	40611644	40611665	peak_5343	0	+	ENSMUSG	protein_coding
chr9	40611692	40611713	peak_5344	0	+	ENSMUSG	protein_coding
chr9	40612056	40612077	peak_5345	0	+	ENSMUSG	protein_coding
chr9	40612098	40612119	peak_5346	0	+	ENSMUSG	protein_coding
chr9	40612809	40612830	peak_5347	0	+	ENSMUSG	protein_coding
chr9	40612851	40612872	peak_5348	0	+	ENSMUSG	protein_coding
chr9	41389958	41389986	peak_5350	0	+	ENSMUSG	protein_coding
chr9	42254505	42254528	peak_5351	0	-	ENSMUSG	protein_coding
chr9	42358807	42358828	peak_5352	0	-	ENSMUSG	protein_coding
chr9	42780240	42780261	peak_5353	0	-	ENSMUSG	protein_coding
chr9	42805098	42805119	peak_5354	0	-	ENSMUSG	protein_coding
chr9	42808066	42808087	peak_5355	0	-	ENSMUSG	protein_coding
chr9	42828756	42828777	peak_5356	0	-	ENSMUSG	protein_coding
chr9	42910584	42910605	peak_5357	0	-	ENSMUSG	protein_coding
chr9	44142974	44142995	peak_5359	0	+	ENSMUSG	protein_coding
chr9	44425430	44425451	peak_5361	0	+	ENSMUSG	protein_coding
chr9	44443786	44443807	peak_5362	0	+	ENSMUSG	protein_coding
chr9	44524095	44524116	peak_5363	0	-	ENSMUSG	protein_coding
chr9	44614611	44614633	peak_5364	0	-	ENSMUSG	protein_coding
chr9	44655590	44655611	peak_5365	0	-	ENSMUSG	protein_coding
chr9	44656125	44656146	peak_5366	0	-	ENSMUSG	protein_coding
chr9	44957752	44957774	peak_5367	0	-	ENSMUSG	protein_coding
chr9	45665191	45665212	peak_5368	0	-	ENSMUSG	protein_coding
chr9	45665222	45665243	peak_5369	0	-	ENSMUSG	protein_coding
chr9	45739780	45739801	peak_5370	0	-	ENSMUSG	protein_coding
chr9	45827292	45827313	peak_5371	0	+	ENSMUSG	protein_coding
chr9	46020267	46020288	peak_5372	0	+	ENSMUSG	protein_coding
chr9	47358853	47358874	peak_5373	0	+	ENSMUSG	protein_coding
chr9	47369097	47369118	peak_5374	0	+	ENSMUSG	protein_coding
chr9	47369427	47369448	peak_5375	0	+	ENSMUSG	protein_coding

chr9	47382713	47382734	peak_5376	0	+	ENSMUSG	protein_coding
chr9	47394481	47394503	peak_5377	0	+	ENSMUSG	protein_coding
chr9	47412854	47412875	peak_5378	0	+	ENSMUSG	protein_coding
chr9	47533379	47533400	peak_5379	0	+	ENSMUSG	protein_coding
chr9	47539932	47539959	peak_5380	0	+	ENSMUSG	protein_coding
chr9	48464125	48464146	peak_5381	0	-	ENSMUSG	protein_coding
chr9	49311279	49311300	peak_5382	0	-	ENSMUSG	protein_coding
chr9	50415599	50415621	peak_5383	0	-	ENSMUSG	protein_coding
chr9	50638399	50638420	peak_5384	0	+	ENSMUSG	protein_coding
chr9	51881297	51881325	peak_5386	0	+	ENSMUSG	protein_coding
chr9	52916192	52916213	peak_5387	0	-	ENSMUSG	protein_coding
chr9	53012421	53012442	peak_5388	0	-	ENSMUSG	protein_coding
chr9	53021400	53021430	peak_5389	0	-	ENSMUSG	protein_coding
chr9	53031788	53031811	peak_5390	0	-	ENSMUSG	protein_coding
chr9	53358153	53358174	peak_5391	0	+	ENSMUSG	protein_coding
chr9	54264582	54264604	peak_5392	0	-	ENSMUSG	protein_coding
chr9	54293218	54293240	peak_5393	0	-	ENSMUSG	protein_coding
chr9	54448945	54448966	peak_5394	0	+	ENSMUSG	protein_coding
chr9	54451829	54451850	peak_5395	0	+	ENSMUSG	protein_coding
chr9	54470442	54470463	peak_5396	0	-	ENSMUSG	protein_coding
chr9	54508789	54508810	peak_5397	0	-	ENSMUSG	protein_coding
chr9	54732843	54732864	peak_5398	0	+	ENSMUSG	protein_coding
chr9	54748260	54748281	peak_5399	0	+	ENSMUSG	protein_coding
chr9	55527512	55527533	peak_5400	0	-	ENSMUSG	protein_coding
chr9	55610129	55610151	peak_5401	0	-	ENSMUSG	protein_coding
chr9	55705894	55705915	peak_5402	0	-	ENSMUSG	protein_coding
chr9	55756629	55756650	peak_5403	0	-	ENSMUSG	protein_coding
chr9	55995225	55995246	peak_5404	0	-	ENSMUSG	protein_coding
chr9	56071641	56071662	peak_5405	0	-	ENSMUSG	protein_coding
chr9	56106129	56106154	peak_5406	0	-	ENSMUSG	protein_coding
chr9	56257799	56257820	peak_5407	0	-	ENSMUSG	protein_coding
chr9	56773592	56773613	peak_5408	0	-	ENSMUSG	protein_coding
chr9	56955377	56955398	peak_5409	0	+	ENSMUSG	protein_coding
chr9	56956608	56956629	peak_5410	0	+	ENSMUSG	protein_coding
chr9	57004316	57004337	peak_5411	0	-	ENSMUSG	protein_coding
chr9	58139382	58139403	peak_5412	0	-	ENSMUSG	protein_coding
chr9	58781871	58781892	peak_5413	0	-	ENSMUSG	protein_coding
chr9	58782053	58782074	peak_5414	0	-	ENSMUSG	protein_coding
chr9	58787921	58787942	peak_5415	0	-	ENSMUSG	protein_coding
chr9	59238134	59238160	peak_5416	0	-	ENSMUSG	protein_coding
chr9	59319454	59319478	peak_5417	0	-	ENSMUSG	protein_coding
chr9	59319496	59319517	peak_5418	0	-	ENSMUSG	protein_coding
chr9	60718176	60718197	peak_5420	0	+	ENSMUSG	protein_coding
chr9	61255216	61255237	peak_5421	0	+	ENSMUSG	protein_coding
chr9	62212710	62212731	peak_5422	0	+	ENSMUSG	protein_coding
chr9	62658562	62658583	peak_5423	0	-	ENSMUSG	protein_coding
chr9	63179573	63179594	peak_5425	0	-	ENSMUSG	protein_coding
chr9	63447607	63447628	peak_5426	0	-	ENSMUSG	protein_coding
chr9	63486528	63486549	peak_5427	0	+	ENSMUSG	protein_coding
chr9	63577797	63577818	peak_5428	0	-	ENSMUSG	protein_coding
chr9	63588502	63588523	peak_5429	0	-	ENSMUSG	protein_coding
chr9	63590012	63590033	peak_5430	0	-	ENSMUSG	protein_coding
chr9	64022834	64022855	peak_5431	0	+	ENSMUSG	protein_coding
chr9	64023206	64023228	peak_5432	0	+	ENSMUSG	protein_coding
chr9	64023268	64023289	peak_5433	0	+	ENSMUSG	protein_coding
chr9	64023676	64023698	peak_5434	0	+	ENSMUSG	protein_coding
chr9	64024445	64024466	peak_5435	0	+	ENSMUSG	protein_coding
chr9	64024506	64024527	peak_5436	0	+	ENSMUSG	protein_coding

chr9	64024908	64024930	peak_5437	0	+	ENSMUSG	protein_coding
chr9	64026247	64026269	peak_5438	0	+	ENSMUSG	protein_coding
chr9	64078706	64078727	peak_5439	0	-	ENSMUSG	protein_coding
chr9	64906081	64906102	peak_5441	0	+	ENSMUSG	protein_coding
chr9	64965117	64965138	peak_5442	0	-	ENSMUSG	protein_coding
chr9	65334649	65334670	peak_5446	0	+	ENSMUSG	protein_coding
chr9	65706162	65706183	peak_5447	0	-	ENSMUSG	protein_coding
chr9	65950097	65950118	peak_5448	0	-	ENSMUSG	protein_coding
chr9	66309661	66309682	peak_5449	0	+	ENSMUSG	protein_coding
chr9	66324166	66324187	peak_5450	0	+	ENSMUSG	protein_coding
chr9	66358153	66358175	peak_5451	0	+	ENSMUSG	protein_coding
chr9	66390646	66390667	peak_5452	0	-	ENSMUSG	protein_coding
chr9	66432116	66432137	peak_5453	0	-	ENSMUSG	protein_coding
chr9	66697660	66697681	peak_5454	0	-	ENSMUSG	protein_coding
chr9	67696192	67696213	peak_5455	0	+	ENSMUSG	protein_coding
chr9	68547684	68547705	peak_5456	0	+	ENSMUSG	protein_coding
chr9	68575444	68575465	peak_5457	0	+	ENSMUSG	protein_coding
chr9	68597405	68597427	peak_5458	0	+	ENSMUSG	protein_coding
chr9	68651982	68652004	peak_5459	0	+	ENSMUSG	protein_coding
chr9	68679067	68679090	peak_5460	0	+	ENSMUSG	protein_coding
chr9	68685299	68685320	peak_5461	0	+	ENSMUSG	protein_coding
chr9	68711433	68711454	peak_5462	0	+	ENSMUSG	protein_coding
chr9	68712536	68712558	peak_5463	0	+	ENSMUSG	protein_coding
chr9	68729632	68729653	peak_5464	0	+	ENSMUSG	protein_coding
chr9	68745316	68745339	peak_5465	0	+	ENSMUSG	protein_coding
chr9	68780983	68781007	peak_5466	0	+	ENSMUSG	protein_coding
chr9	68791431	68791452	peak_5467	0	+	ENSMUSG	protein_coding
chr9	68792778	68792800	peak_5468	0	+	ENSMUSG	protein_coding
chr9	68936445	68936467	peak_5469	0	+	ENSMUSG	protein_coding
chr9	69264852	69264873	peak_5470	0	+	ENSMUSG	protein_coding
chr9	69316501	69316522	peak_5471	0	+	ENSMUSG	protein_coding
chr9	69316729	69316750	peak_5472	0	+	ENSMUSG	protein_coding
chr9	70402445	70402466	peak_5477	0	+	ENSMUSG	protein_coding
chr9	70402471	70402492	peak_5478	0	+	ENSMUSG	protein_coding
chr9	70428624	70428645	peak_5479	0	+	ENSMUSG	protein_coding
chr9	70429217	70429238	peak_5480	0	+	ENSMUSG	protein_coding
chr9	70431772	70431793	peak_5481	0	+	ENSMUSG	protein_coding
chr9	71331601	71331622	peak_5482	0	-	ENSMUSG	protein_coding
chr9	71331601	71331622	peak_5482	0	-	ENSMUSG	protein_coding
chr9	71528794	71528815	peak_5483	0	-	ENSMUSG	protein_coding
chr9	71543304	71543325	peak_5484	0	-	ENSMUSG	protein_coding
chr9	71564244	71564265	peak_5485	0	-	ENSMUSG	protein_coding
chr9	71741526	71741547	peak_5486	0	-	ENSMUSG	protein_coding
chr9	71856671	71856692	peak_5487	0	-	ENSMUSG	protein_coding
chr9	71910222	71910246	peak_5488	0	-	ENSMUSG	protein_coding
chr9	72162826	72162847	peak_5489	0	+	ENSMUSG	protein_coding
chr9	72512805	72512828	peak_5490	0	+	ENSMUSG	protein_coding
chr9	72523271	72523293	peak_5491	0	+	ENSMUSG	protein_coding
chr9	72537635	72537656	peak_5492	0	+	ENSMUSG	protein_coding
chr9	72541442	72541463	peak_5493	0	+	ENSMUSG	protein_coding
chr9	72560009	72560030	peak_5494	0	+	ENSMUSG	protein_coding
chr9	72566080	72566101	peak_5495	0	+	ENSMUSG	protein_coding
chr9	72591331	72591352	peak_5496	0	+	ENSMUSG	protein_coding
chr9	72799359	72799380	peak_5497	0	+	ENSMUSG	protein_coding
chr9	74861836	74861857	peak_5498	0	+	ENSMUSG	protein_coding
chr9	74885477	74885498	peak_5499	0	+	ENSMUSG	protein_coding
chr9	74885703	74885724	peak_5500	0	+	ENSMUSG	protein_coding
chr9	77329070	77329093	peak_5501	0	-	ENSMUSG	protein_coding

chr9	77814931	77814952	peak_5502	0	+	ENSMUSG	protein_coding
chr9	78326993	78327015	peak_5504	0	-	ENSMUSG	protein_coding
chr9	78327180	78327201	peak_5505	0	-	ENSMUSG	protein_coding
chr9	78327581	78327602	peak_5506	0	-	ENSMUSG	protein_coding
chr9	78327679	78327700	peak_5507	0	-	ENSMUSG	protein_coding
chr9	78328435	78328459	peak_5508	0	-	ENSMUSG	protein_coding
chr9	78329473	78329496	peak_5509	0	-	ENSMUSG	protein_coding
chr9	79665379	79665400	peak_5510	0	-	ENSMUSG	protein_coding
chr9	79924281	79924302	peak_5511	0	+	ENSMUSG	protein_coding
chr9	82800660	82800690	peak_5512	0	-	ENSMUSG	protein_coding
chr9	82802298	82802321	peak_5513	0	-	ENSMUSG	protein_coding
chr9	86533176	86533197	peak_5514	0	-	ENSMUSG	protein_coding
chr9	86556481	86556502	peak_5515	0	-	ENSMUSG	protein_coding
chr9	86929713	86929735	peak_5516	0	+	ENSMUSG	protein_coding
chr9	88357099	88357120	peak_5517	0	-	ENSMUSG	protein_coding
chr9	88359324	88359345	peak_5518	0	-	ENSMUSG	protein_coding
chr9	92467238	92467261	peak_5521	0	+	ENSMUSG	protein_coding
chr9	95372620	95372641	peak_5522	0	-	ENSMUSG	protein_coding
chr9	96133713	96133734	peak_5523	0	+	ENSMUSG	protein_coding
chr9	96144851	96144872	peak_5524	0	+	ENSMUSG	protein_coding
chr9	96616110	96616131	peak_5525	0	-	ENSMUSG	protein_coding
chr9	96983128	96983149	peak_5526	0	-	ENSMUSG	protein_coding
chr9	97763070	97763091	peak_5527	0	-	ENSMUSG	protein_coding
chr9	98288234	98288255	peak_5528	0	+	ENSMUSG	protein_coding
chr9	98470756	98470777	peak_5529	0	+	ENSMUSG	protein_coding
chr9	98893008	98893029	peak_5530	0	+	ENSMUSG	protein_coding
chr9	1E+08	1E+08	peak_5532	0	-	ENSMUSG	protein_coding
chr9	1.01E+08	1.01E+08	peak_5533	0	+	ENSMUSG	protein_coding
chr9	1.01E+08	1.01E+08	peak_5534	0	+	ENSMUSG	protein_coding
chr9	1.01E+08	1.01E+08	peak_5535	0	+	ENSMUSG	protein_coding
chr9	1.03E+08	1.03E+08	peak_5536	0	-	ENSMUSG	protein_coding
chr9	1.03E+08	1.03E+08	peak_5538	0	+	ENSMUSG	protein_coding
chr9	1.03E+08	1.03E+08	peak_5539	0	+	ENSMUSG	protein_coding
chr9	1.03E+08	1.03E+08	peak_5540	0	+	ENSMUSG	protein_coding
chr9	1.08E+08	1.08E+08	peak_5541	0	-	ENSMUSG	protein_coding
chr9	1.08E+08	1.08E+08	peak_5542	0	-	ENSMUSG	protein_coding
chr9	1.08E+08	1.08E+08	peak_5543	0	+	ENSMUSG	protein_coding
chr9	1.08E+08	1.08E+08	peak_5544	0	+	ENSMUSG	protein_coding
chr9	1.08E+08	1.08E+08	peak_5545	0	+	ENSMUSG	protein_coding
chr9	1.09E+08	1.09E+08	peak_5546	0	+	ENSMUSG	protein_coding
chr9	1.1E+08	1.1E+08	peak_5549	0	+	ENSMUSG	protein_coding
chr9	1.1E+08	1.1E+08	peak_5550	0	+	ENSMUSG	protein_coding
chr9	1.1E+08	1.1E+08	peak_5551	0	+	ENSMUSG	protein_coding
chr9	1.1E+08	1.1E+08	peak_5552	0	-	ENSMUSG	protein_coding
chr9	1.1E+08	1.1E+08	peak_5553	0	-	ENSMUSG	protein_coding
chr9	1.1E+08	1.1E+08	peak_5554	0	+	ENSMUSG	protein_coding
chr9	1.1E+08	1.1E+08	peak_5555	0	+	ENSMUSG	protein_coding
chr9	1.1E+08	1.1E+08	peak_5556	0	+	ENSMUSG	protein_coding
chr9	1.1E+08	1.1E+08	peak_5557	0	+	ENSMUSG	protein_coding
chr9	1.1E+08	1.1E+08	peak_5558	0	+	ENSMUSG	protein_coding
chr9	1.1E+08	1.1E+08	peak_5561	0	+	ENSMUSG	protein_coding
chr9	1.1E+08	1.1E+08	peak_5562	0	+	ENSMUSG	protein_coding
chr9	1.1E+08	1.1E+08	peak_5563	0	+	ENSMUSG	protein_coding
chr9	1.11E+08	1.11E+08	peak_5564	0	+	ENSMUSG	protein_coding
chr9	1.11E+08	1.11E+08	peak_5565	0	+	ENSMUSG	protein_coding
chr9	1.11E+08	1.11E+08	peak_5566	0	-	ENSMUSG	protein_coding
chr9	1.11E+08	1.11E+08	peak_5568	0	+	ENSMUSG	protein_coding
chr9	1.11E+08	1.11E+08	peak_5569	0	+	ENSMUSG	protein_coding

chr9	1.11E+08	1.11E+08	peak_5570	0	+	ENSMUSG	protein_coding
chr9	1.11E+08	1.11E+08	peak_5571	0	+	ENSMUSG	protein_coding
chr9	1.11E+08	1.11E+08	peak_5572	0	+	ENSMUSG	protein_coding
chr9	1.11E+08	1.11E+08	peak_5573	0	+	ENSMUSG	protein_coding
chr9	1.11E+08	1.11E+08	peak_5574	0	+	ENSMUSG	protein_coding
chr9	1.11E+08	1.11E+08	peak_5575	0	+	ENSMUSG	protein_coding
chr9	1.11E+08	1.11E+08	peak_5576	0	+	ENSMUSG	protein_coding
chr9	1.14E+08	1.14E+08	peak_5577	0	-	ENSMUSG	protein_coding
chr9	1.14E+08	1.14E+08	peak_5578	0	+	ENSMUSG	protein_coding
chr9	1.14E+08	1.14E+08	peak_5579	0	+	ENSMUSG	protein_coding
chr9	1.14E+08	1.14E+08	peak_5580	0	+	ENSMUSG	protein_coding
chr9	1.14E+08	1.14E+08	peak_5581	0	+	ENSMUSG	protein_coding
chr9	1.14E+08	1.14E+08	peak_5582	0	-	ENSMUSG	protein_coding
chr9	1.15E+08	1.15E+08	peak_5584	0	+	ENSMUSG	protein_coding
chr9	1.15E+08	1.15E+08	peak_5587	0	-	ENSMUSG	protein_coding
chr9	1.18E+08	1.18E+08	peak_5591	0	+	ENSMUSG	protein_coding
chr9	1.18E+08	1.18E+08	peak_5592	0	+	ENSMUSG	protein_coding
chr9	1.19E+08	1.19E+08	peak_5593	0	+	ENSMUSG	protein_coding
chr9	1.19E+08	1.19E+08	peak_5594	0	-	ENSMUSG	protein_coding
chr9	1.2E+08	1.2E+08	peak_5595	0	+	ENSMUSG	protein_coding
chr9	1.2E+08	1.2E+08	peak_5596	0	+	ENSMUSG	protein_coding
chr9	1.2E+08	1.2E+08	peak_5597	0	+	ENSMUSG	protein_coding
chr9	1.2E+08	1.2E+08	peak_5598	0	+	ENSMUSG	protein_coding
chr9	1.21E+08	1.21E+08	peak_5600	0	+	ENSMUSG	protein_coding
chr9	1.21E+08	1.21E+08	peak_5601	0	+	ENSMUSG	protein_coding
chr9	1.21E+08	1.21E+08	peak_5602	0	+	ENSMUSG	protein_coding
chr9	1.21E+08	1.21E+08	peak_5603	0	+	ENSMUSG	protein_coding
chr9	1.21E+08	1.21E+08	peak_5604	0	+	ENSMUSG	protein_coding
chr9	1.21E+08	1.21E+08	peak_5605	0	-	ENSMUSG	protein_coding
chr9	1.21E+08	1.21E+08	peak_5606	0	+	ENSMUSG	protein_coding
chr9	1.21E+08	1.21E+08	peak_5607	0	+	ENSMUSG	protein_coding
chr9	1.21E+08	1.21E+08	peak_5608	0	+	ENSMUSG	protein_coding
chr9	1.21E+08	1.21E+08	peak_5609	0	+	ENSMUSG	protein_coding
chr9	1.21E+08	1.21E+08	peak_5610	0	+	ENSMUSG	protein_coding
chr9	1.21E+08	1.21E+08	peak_5611	0	+	ENSMUSG	protein_coding
chr9	1.21E+08	1.21E+08	peak_5612	0	+	ENSMUSG	protein_coding
chr9	1.22E+08	1.22E+08	peak_5613	0	+	ENSMUSG	protein_coding
chr9	1.23E+08	1.23E+08	peak_5614	0	+	ENSMUSG	protein_coding
chr9	1.23E+08	1.23E+08	peak_5615	0	-	ENSMUSG	protein_coding
chr9	1.23E+08	1.23E+08	peak_5616	0	+	ENSMUSG	protein_coding
chr9	1.23E+08	1.23E+08	peak_5617	0	+	ENSMUSG	protein_coding
chr9	1.23E+08	1.23E+08	peak_5619	0	+	ENSMUSG	protein_coding
chr9	1.24E+08	1.24E+08	peak_5620	0	+	ENSMUSG	protein_coding
chrM	5368	5389	peak_5640	0	+	ENSMUSG	protein_coding
chrM	5525	5547	peak_5641	0	+	ENSMUSG	protein_coding
chrM	5611	5633	peak_5642	0	+	ENSMUSG	protein_coding
chrM	6181	6202	peak_5643	0	+	ENSMUSG	protein_coding
chrM	6305	6326	peak_5644	0	+	ENSMUSG	protein_coding
chrM	6590	6611	peak_5645	0	+	ENSMUSG	protein_coding
chrM	12179	12200	peak_5648	0	+	ENSMUSG	protein_coding
chrM	12992	13015	peak_5649	0	-	ENSMUSG	protein_coding
chrM	14163	14185	peak_5650	0	+	ENSMUSG	protein_coding
chrM	14404	14426	peak_5651	0	+	ENSMUSG	protein_coding
chrM	14632	14653	peak_5652	0	+	ENSMUSG	protein_coding
chrM	15070	15091	peak_5653	0	+	ENSMUSG	protein_coding
chrM	15126	15147	peak_5654	0	+	ENSMUSG	protein_coding
chrM	15280	15321	peak_5655	0	+	ENSMUSG	protein_coding
chrX	5988897	5988918	peak_5659	0	+	ENSMUSG	protein_coding

chrX	7524924	7524945	peak_5661	0	-	ENSMUSG	protein_coding
chrX	7647187	7647208	peak_5662	0	-	ENSMUSG	protein_coding
chrX	7722826	7722847	peak_5663	0	-	ENSMUSG	protein_coding
chrX	10084301	10084324	peak_5665	0	+	ENSMUSG	protein_coding
chrX	10098543	10098564	peak_5666	0	+	ENSMUSG	protein_coding
chrX	10295725	10295746	peak_5667	0	+	ENSMUSG	protein_coding
chrX	11736406	11736427	peak_5668	0	-	ENSMUSG	protein_coding
chrX	12684862	12684884	peak_5671	0	+	ENSMUSG	protein_coding
chrX	12719749	12719770	peak_5672	0	+	ENSMUSG	protein_coding
chrX	12865982	12866003	peak_5673	0	+	ENSMUSG	protein_coding
chrX	20002891	20002912	peak_5675	0	+	ENSMUSG	protein_coding
chrX	20014574	20014596	peak_5676	0	+	ENSMUSG	protein_coding
chrX	34144808	34144829	peak_5679	0	+	ENSMUSG	protein_coding
chrX	34284396	34284417	peak_5680	0	-	ENSMUSG	protein_coding
chrX	34622990	34623011	peak_5681	0	-	ENSMUSG	protein_coding
chrX	34625177	34625198	peak_5682	0	-	ENSMUSG	protein_coding
chrX	35801331	35801352	peak_5683	0	-	ENSMUSG	protein_coding
chrX	39160272	39160293	peak_5685	0	-	ENSMUSG	protein_coding
chrX	39216802	39216824	peak_5686	0	-	ENSMUSG	protein_coding
chrX	45318301	45318322	peak_5687	0	+	ENSMUSG	protein_coding
chrX	45934277	45934298	peak_5688	0	-	ENSMUSG	protein_coding
chrX	45945396	45945417	peak_5689	0	-	ENSMUSG	protein_coding
chrX	48512295	48512316	peak_5691	0	-	ENSMUSG	protein_coding
chrX	49016206	49016232	peak_5692	0	-	ENSMUSG	protein_coding
chrX	54319559	54319580	peak_5696	0	+	ENSMUSG	protein_coding
chrX	54644698	54644719	peak_5697	0	-	ENSMUSG	protein_coding
chrX	57485652	57485673	peak_5699	0	-	ENSMUSG	protein_coding
chrX	67113943	67113964	peak_5704	0	+	ENSMUSG	protein_coding
chrX	68489436	68489458	peak_5705	0	+	ENSMUSG	protein_coding
chrX	70601777	70601798	peak_5706	0	+	ENSMUSG	protein_coding
chrX	71473042	71473063	peak_5707	0	-	ENSMUSG	protein_coding
chrX	71475413	71475434	peak_5708	0	-	ENSMUSG	protein_coding
chrX	74821218	74821239	peak_5710	0	+	ENSMUSG	protein_coding
chrX	80781343	80781366	peak_5711	0	+	ENSMUSG	protein_coding
chrX	82448880	82448902	peak_5712	0	+	ENSMUSG	protein_coding
chrX	89670303	89670324	peak_5714	0	-	ENSMUSG	protein_coding
chrX	89686367	89686388	peak_5715	0	-	ENSMUSG	protein_coding
chrX	91782253	91782274	peak_5716	0	-	ENSMUSG	protein_coding
chrX	91784610	91784636	peak_5717	0	-	ENSMUSG	protein_coding
chrX	91787360	91787381	peak_5718	0	-	ENSMUSG	protein_coding
chrX	91886554	91886575	peak_5719	0	+	ENSMUSG	protein_coding
chrX	94552294	94552316	peak_5720	0	-	ENSMUSG	protein_coding
chrX	95448086	95448108	peak_5721	0	+	ENSMUSG	protein_coding
chrX	95754880	95754901	peak_5722	0	-	ENSMUSG	protein_coding
chrX	98477416	98477437	peak_5725	0	+	ENSMUSG	protein_coding
chrX	98638640	98638662	peak_5726	0	+	ENSMUSG	protein_coding
chrX	98638797	98638818	peak_5727	0	+	ENSMUSG	protein_coding
chrX	99104069	99104090	peak_5729	0	+	ENSMUSG	protein_coding
chrX	99162412	99162435	peak_5730	0	+	ENSMUSG	protein_coding
chrX	99198267	99198288	peak_5731	0	+	ENSMUSG	protein_coding
chrX	99198289	99198310	peak_5732	0	+	ENSMUSG	protein_coding
chrX	99200182	99200203	peak_5733	0	+	ENSMUSG	protein_coding
chrX	99227459	99227480	peak_5734	0	+	ENSMUSG	protein_coding
chrX	99739605	99739627	peak_5735	0	-	ENSMUSG	protein_coding
chrX	1.01E+08	1.01E+08	peak_5737	0	-	ENSMUSG	protein_coding
chrX	1.01E+08	1.01E+08	peak_5738	0	-	ENSMUSG	protein_coding
chrX	1.01E+08	1.01E+08	peak_5739	0	-	ENSMUSG	protein_coding
chrX	1.01E+08	1.01E+08	peak_5741	0	-	ENSMUSG	protein_coding

chrX	1.03E+08	1.03E+08	peak_5742	0	-	ENSMUSG	protein_coding
chrX	1.03E+08	1.03E+08	peak_5743	0	-	ENSMUSG	protein_coding
chrX	1.03E+08	1.03E+08	peak_5744	0	-	ENSMUSG	protein_coding
chrX	1.06E+08	1.06E+08	peak_5745	0	-	ENSMUSG	protein_coding
chrX	1.26E+08	1.26E+08	peak_5746	0	+	ENSMUSG	protein_coding
chrX	1.27E+08	1.27E+08	peak_5747	0	+	ENSMUSG	protein_coding
chrX	1.3E+08	1.3E+08	peak_5748	0	-	ENSMUSG	protein_coding
chrX	1.3E+08	1.3E+08	peak_5749	0	-	ENSMUSG	protein_coding
chrX	1.31E+08	1.31E+08	peak_5750	0	+	ENSMUSG	protein_coding
chrX	1.31E+08	1.31E+08	peak_5751	0	+	ENSMUSG	protein_coding
chrX	1.32E+08	1.32E+08	peak_5752	0	+	ENSMUSG	protein_coding
chrX	1.33E+08	1.33E+08	peak_5753	0	-	ENSMUSG	protein_coding
chrX	1.33E+08	1.33E+08	peak_5754	0	+	ENSMUSG	protein_coding
chrX	1.35E+08	1.35E+08	peak_5760	0	+	ENSMUSG	protein_coding
chrX	1.39E+08	1.39E+08	peak_5764	0	+	ENSMUSG	protein_coding
chrX	1.39E+08	1.39E+08	peak_5765	0	+	ENSMUSG	protein_coding
chrX	1.4E+08	1.4E+08	peak_5766	0	+	ENSMUSG	protein_coding
chrX	1.4E+08	1.4E+08	peak_5767	0	+	ENSMUSG	protein_coding
chrX	1.4E+08	1.4E+08	peak_5768	0	+	ENSMUSG	protein_coding
chrX	1.4E+08	1.4E+08	peak_5769	0	+	ENSMUSG	protein_coding
chrX	1.41E+08	1.41E+08	peak_5770	0	+	ENSMUSG	protein_coding
chrX	1.42E+08	1.42E+08	peak_5771	0	+	ENSMUSG	protein_coding
chrX	1.47E+08	1.47E+08	peak_5772	0	-	ENSMUSG	protein_coding
chrX	1.48E+08	1.48E+08	peak_5775	0	+	ENSMUSG	protein_coding
chrX	1.48E+08	1.48E+08	peak_5776	0	+	ENSMUSG	protein_coding
chrX	1.48E+08	1.48E+08	peak_5777	0	+	ENSMUSG	protein_coding
chrX	1.48E+08	1.48E+08	peak_5778	0	+	ENSMUSG	protein_coding
chrX	1.48E+08	1.48E+08	peak_5779	0	+	ENSMUSG	protein_coding
chrX	1.48E+08	1.48E+08	peak_5780	0	+	ENSMUSG	protein_coding
chrX	1.48E+08	1.48E+08	peak_5781	0	+	ENSMUSG	protein_coding
chrX	1.48E+08	1.48E+08	peak_5782	0	+	ENSMUSG	protein_coding
chrX	1.48E+08	1.48E+08	peak_5783	0	+	ENSMUSG	protein_coding
chrX	1.49E+08	1.49E+08	peak_5784	0	-	ENSMUSG	protein_coding
chrX	1.56E+08	1.56E+08	peak_5785	0	+	ENSMUSG	protein_coding
chrX	1.56E+08	1.56E+08	peak_5786	0	+	ENSMUSG	protein_coding
chrX	1.56E+08	1.56E+08	peak_5787	0	+	ENSMUSG	protein_coding
chrX	1.56E+08	1.56E+08	peak_5788	0	+	ENSMUSG	protein_coding
chrX	1.59E+08	1.59E+08	peak_5790	0	-	ENSMUSG	protein_coding
chrX	1.59E+08	1.59E+08	peak_5791	0	-	ENSMUSG	protein_coding
chrX	1.59E+08	1.59E+08	peak_5792	0	-	ENSMUSG	protein_coding
chrX	1.59E+08	1.59E+08	peak_5793	0	-	ENSMUSG	protein_coding
chrX	1.59E+08	1.59E+08	peak_5794	0	-	ENSMUSG	protein_coding
chrX	1.59E+08	1.59E+08	peak_5795	0	-	ENSMUSG	protein_coding
chrX	1.64E+08	1.64E+08	peak_5796	0	-	ENSMUSG	protein_coding
chrX	1.64E+08	1.64E+08	peak_5797	0	-	ENSMUSG	protein_coding
chrX	1.65E+08	1.65E+08	peak_5798	0	-	ENSMUSG	protein_coding
chrX	1.66E+08	1.66E+08	peak_5799	0	+	ENSMUSG	protein_coding

Mrpl15
Atp6v1h
Rb1cc1
Rb1cc1
Vcpip1
Arfgef1
Sulf1
Ncoa2
Ncoa2
Ncoa2
Ncoa2
Ncoa2
Tram1
Lactb2
Kcnb2
Tceb1
Kcnq5
Kcnq5
Fam135a
Bai3
Phf3
Dst
Dst
Dst
Dst
Dst
Dst
Dst
Dst
Dst
Dst
Dst
Dst
Dst
Dst
Dst
Dst
Dst
Dst
Dst
Dst
Dst
Dst
Dst
Arhgef4
4632411B12Rik
4632411B12Rik
4632411B12Rik
4632411B12Rik
4632411B12Rik
Tmem131
Eif5b
Aff3
Aff3
Aff3
Aff3
Aff3

Aff3
Pdcl3
Pdcl3
Rpl31
Map4k4
Map4k4
Map4k4
Map4k4
Map4k4
Map4k4
Map4k4
Mrps9
Mrps9
Nck2
Uxs1
Uxs1
Uxs1
Tpp2
Tpp2
Col3a1
Col3a1
Col3a1
Col3a1
Col3a1
Obfc2a
Stat1
Gls
Ankrd44
Sf3b1
Sf3b1
Sf3b1
Bzw1
Clk1
Clk1
Fam126b
Trak2
Als2
Nop58
Nop58
Nop58
Nop58
Nop58
Bmpr2
Abi2
Raph1
Pard3b
Pard3b
Pard3b
Ndufs1
Eef1b2
Eef1b2
Eef1b2
Idh1
Pikfyve
Pikfyve
Mtap2
1110028C15Rik
Erbb4

Fn1
Smarcal1
Rpl37a
Tns1
Aamp
Ctdsp1
Ttll4
Ttll4
Ttll4
Wnt6
Wnt6
Glb1l
Speg
Obsl1
Obsl1
Obsl1
Obsl1
Acsl3
Utp14b
Serpine2
Cul3
Cul3
Col4a4
Col4a4
Col4a4
Col4a3
Trip12
Trip12
Trip12
Trip12
Trip12
C130026I21Rik
Itm2c
Ncl
Ncl
Ncl
Ncl
Ptma
Ptma
Efhd1
Gigyf2
Gigyf2
Gigyf2
Gigyf2
Gigyf2
Dgkd
Dgkd
Dgkd
Agap1
Agap1
Agap1
Agap1
Agap1
Agap1
Agap1
Agap1
Agap1
Agap1

Asb18
Col6a3
Col6a3
Hdac4
Hdac4
Hdac4
Kif1a
Hdlbp
2-Sep
Stk25
Atg4b
Ing5
Rnf152
Clasp1
Clasp1
Gli2
Gli2
Gli2
Gli2
Ptpn4
Actr3
Actr3
Nckap5
Nckap5
Nckap5
Mgat5
Tmem163
Dars
Mapkapk2
Srgap2
Srgap2
Slc41a1
Nucks1
Lemd1
Dstyk
Dstyk
Mdm4
Mdm4
Zc3h11a
Zc3h11a
Zc3h11a
Atp2b4
Adora1
Adora1
Adora1
Adora1
Tmem183a
Tmem183a
Adipor1
Adipor1
Kdm5b
Kdm5b
Syt2
Ppp1r12b
Ppp1r12b
Ppp1r12b
Ppp1r12b
Shisa4

Nav1
Nav1
Nav1
Dennd1b
Dennd1b
Aspm
Cdc73
Hmcn1
Ivns1abp
Ivns1abp
1200016B10Rik
Rnf2
Fam129a
Fam129a
1700025G04Rik
Rgl1
Arpc5
Dhx9
Dhx9
Dhx9
Dhx9
BC034090
BC034090
Xpr1
Cep350
Cep350
Cep350
Tor1aip2
Tor1aip2
Rfwd2
Rabgap1l
Rabgap1l
Rc3h1
Rc3h1
Rc3h1
Zbtb37
AI848100
Prrc2c
Prrc2c
Prrc2c
Prrc2c
Prrc2c
Prrc2c
Prrc2c
Prrc2c
Prrc2c
Prrc2c
Kifap3
Slc19a2
Nme7
Dcaf6
Mpzl1
Pou2f1
Pbx1
Pbx1
Pbx1
Pbx1
Pbx1

Pbx1
Pbx1
Pbx1
Pbx1
Pbx1
Nos1ap
Nos1ap
Atf6
Usf1
B930036N10Rik
Copa
Dcaf8
Akt3
Adss
Gm16432
Gm16432
Pppde1
Hnrnpu
Kif26b
Smyd3
Smyd3
Ahctf1
Cdc42bpa
Adck3
Adck3
Itpkb
Acbd3
Lefty1
Nvl
Wdr26
Wdr26
Wdr26
Enah
Enah
C130074G19Rik
Eprs
Eprs
Gpatch2
Esrrg
Kctd3
Kctd3
Kctd3
Cenpf
Cenpf
Cenpf
Rps6kc1
Dtl
Hhat
Plxna2
Cnksr3
Cnksr3
Akap12
Akap12
Akap12
Plekhg1
Katna1
Katna1
Ust

Sash1
Utrn
Utrn
Utrn
Utrn
Phactr2
Phactr2
Aig1
Reps1
Nhsl1
Mtap7
Bclaf1
Rps12
Rps12
Rps12
Ctgf
Ctgf
Med23
Epb41l2
Epb41l2
Epb41l2
Epb41l2
Epb41l2
Lama2
Ptprk
Ptprk
Ncoa7
Ncoa7
Tpd52l1
Hdac2
Hdac2
Fyn
Fyn
Fyn
Fyn
Fyn
Fyn
Rev3l
Rev3l
Rev3l
Wasf1
Fig4
Cep57l1
Sesn1
Foxo3
Foxo3
Foxo3
Foxo3
Foxo3
Foxo3
Foxo3
Sec63
Sec63
Sobp
Sobp
Sobp
Sobp
Sobp

Sobp
Sobp
Pdss2
Prep
Prep
Hace1
Ascc3
Ascc3
Gopc
Gja1
Lims1
Lims1
Spock2
Spock2
Cdh23
Cdh23
Cdh23
X99384
X99384
Eif4ebp2
Ddx21
Ddx21
Ddx50
Ccar1
Ccar1
Hnrnph3
Rufy2
Hnrnph3
Rufy2
Hnrnph3
Herc4
Herc4
Herc4
Sirt1
Ctnna3
Ctnna3
Lrrtm3
Ctnna3
Lrrtm3
Ctnna3
Lrrtm3
Ctnna3
Lrrtm3
Ctnna3
Ctnna3
Jmjd1c
Jmjd1c
Jmjd1c
Jmjd1c
Arid5b
2310015B20Rik
Bicc1
Zwint
Zwint
Zwint
Bcr
Specc1l
Cabin1

Cabin1
Cabin1
Dip2a
Dip2a
Pcnt
Pcnt
Pcnt
Pcnt
Mcm3ap
Slc19a1
Col18a1
Sumo3
Bsg
Sbno2
Stk11
Midn
Midn
Ndufs7
Rps15
Sf3a2
Eef2
Eef2
Eef2
Eef2
Eef2
Eef2
Eef2
Eef2
Eef2
Eef2
Zfr2
AU041133
Nfyb
Nfyb
Chst11
Appl2
Timp3
Syn3
Hsp90b1
Hsp90b1
Hsp90b1
Hsp90b1
Nt5dc3
Utp20
Utp20
Apaf1
Slc25a3
Tmpo
Tmpo
Nedd1
Metap2
Ndufa12
Ndufa12
Tmcc3
Plxnc1
Plxnc1
Nudt4
Eea1

Eea1
Eea1
Atp2b1
Kitl
Tmtc2
Tmtc2
Tmtc2
Ppp1r12a
Nap1l1
Kcnc2
Cnot2
Cnot2
Cct2
Cpsf6
Cpm
Grip1
Grip1
Grip1
Grip1
Grip1
Llph
Msrb3
Msrb3
Tbk1
Usp15
Pip4k2c
R3hdm2
Shmt2
Lrp1
Stat6
Nab2
Nab2
Atp5b
Baz2a
Smarcc2
Smarcc2
Smarcc2
Rps26
Rps26
Rps26
Cd63
4930556J24Rik
Pes1
Ascc2
Nipsnap1
Ap1b1
Ewsr1
Ewsr1
Rhbdd3
Ewsr1
Znrf3
Znrf3
Znrf3
Aebp1
Ykt6
Ogdh
Ccm2
Tns3

Tns3
Tns3
Zpbp
Meis1
Spred2
Ehbp1
Xpo1
Xpo1
Usp34
Usp34
Usp34
Usp34
Usp34
Usp34
Smek2
Smek2
Rps27a
Rps27a
Spnb2
Spnb2
Spnb2
Spnb2
Spnb2
Spnb2
Spnb2
Spnb2
Spnb2
Spnb2
Spnb2
Spnb2
Spnb2
Spnb2
Spnb2
Spnb2
Spnb2
Spnb2
Spnb2
Spnb2
Spnb2
Stc2
Sh3pxd2b
Sh3pxd2b
Ubtd2
Stk10
Npm1
Kcnip1
Slit3
Slit3
Slit3
Slit3
Slit3
Slit3
Slit3
Slit3
Slit3
Slit3
Slit3
Slit3

Slit3
Slit3
Slit3
Slit3
Wwc1
Ccng1
Ccnjl
Rnf145
Ebf1
Ebf1
Ebf1
Ebf1
Ebf1
Ebf1
Ebf1
Ebf1
Clint1
Clint1
Gnb2l1
Gnb2l1
Gnb2l1
Trim7
Trim7
Irgm1
Zfp62
Cnot6
Mapk9
Mapk9
Canx
Hnrnph1
Hnrnph1
Adamts2
Hnrnpab
Ppp2ca
9530068E07Rik
Hspa4
Zcchc10
Aff4
Cdc42se2
Cdc42se2
Cdc42se2
Tnip1
Gm2a
Mfap3
Larp1
Larp1
Larp1
Cnot8
Trim58
Hist3h2ba
Trim17
Hist3h2a
Trim17
Hist3h2a
Trim17
Trim17
Mprip
Mprip

Mprip
Rai1
Rai1
Rai1
Tom1l2
4933439F18Rik
Epn2
Akap10
Akap10
Ttc19
Ncor1
Ttc19
Ncor1
Ncor1
Ncor1
Ubb
Arhgap44
1700086D15Rik
Arhgap44
Gas7
Glp2r
Stx8
Myh10
Myh10
Myh10
Myh10
Myh10
Rpl26
Rpl26
Hes7
Chd3
Chd3
Chd3
Kdm6b
Atp1b2
Sat2
Eif4a1
Eif4a1
Eif4a1
Eif4a1
Eif4a1
Eif4a1
Polr2a
Polr2a
Neurl4
Phf23
Phf23
Dlg4
Dlg4
Mink1
Mink1
Pfn1
Inca1
Kif1c
4933427D14Rik
Smtnl2
Spns3
Ube2g1

Zzef1
Gsg2
Itgae
E130309D14Rik
E130309D14Rik
Pafah1b1
Pafah1b1
Pafah1b1
Tsr1
Smg6
Smg6
Rtn4rl1
Ywhae
Ywhae
Ywhae
Ywhae
Ywhae
Abr
Tmigd1
Ccdc55
Ssh2
Ssh2
Taok1
Taok1
Myo18a
Myo18a
Myo18a
Myo18a
Myo18a
Myo18a
Myo18a
BC017647
Tlcd1
Rpl23a
Tlcd1
Rpl23a
Tlcd1
Rpl23a
Rpl23a
Supt6h
Supt6h
Supt6h
Nlk
Nlk
Nlk
Nf1
Nf1
Omg
Nf1
Zfp207
Psmd11
Myo1d
Rad51l3
Synrg
Acaca
Appbp2
Bcas3
Med13

Ppm1e
Ppm1e
Tex14
Tex14
Tex14
Tex14
Tex14
Tex14
Tex14
Tex14
Tex14
Tex14
Tex14
Tex14
Tex14
Dynll2
Srsf1
Msi2
Msi2
Msi2
Msi2
Msi2
Msi2
Msi2
Msi2
Msi2
Msi2
Msi2
Msi2
Msi2
Scpep1
Dgke
Stxbp4
Mbtd1
Mbtd1
Mbtd1
Spag9
Spag9
Luc7l3
Luc7l3
Luc7l3
Luc7l3
Luc7l3
Luc7l3
Col1a1
Col1a1
Col1a1
Sgca
Myst2
Myst2
Spop
Spop
Spop
Zfp652
Gm53
Cbx1
Cbx1
Nfe2l1

Nfe2l1
Arhgap23
Mllt6
Mllt6
Mllt6
Mllt6
Pip4k2b
Rpl23
Rpl19
Rpl19
Rpl19
Rpl19
Cdk12
Thra
Thra
Thra
Thra
Thra
Msl1
Top2a
Top2a
Smarce1
Eif1
Nt5c3l
Acly
Acly
Acly
Acly
Dnajc7
Atp6v0a1
Plekhh3
Cntd1
Becn1
Rpl27
Hdac5
Ubtf
Ubtf
Gpatch8
Gpatch8
Gpatch8
Gpatch8
Gjc1
Nsf
Nsf
1700081L11Rik
1700081L11Rik
1700081L11Rik
Tanc2
Limd2
Ern1
Ddx5
Ddx5
Ddx5
Ddx5
Ddx5
Bptf
Bptf
Bptf

Bptf
Bptf
Helz
Helz
Prkca
Axin2
Prkar1a
Sox9
D11Wsu47e
Cdc42ep4
Rpl38
Ttyh2
Ict1
Hn1
Gga3
H3f3b
Unk
Srp68
Rnf157
Rnf157
Rnf157
Prpsap1
Rhbdf2
1810032O08Rik
1810032O08Rik
Sec14l1
9-Sep
9-Sep
9-Sep
9-Sep
9-Sep
9-Sep
9-Sep
Tnrc6c
Tnrc6c
Birc5
Pgs1
Dnahc17
Usp36
Cant1
Rnf213
A730011L01Rik
Rptor
Rptor
Slc38a10
Bahcc1
Bahcc1
Nploc4
Arhgdia
Fasn
Fasn
Ccdc57
Ccdc57
Ccdc57
Csnk1d
Tbcd
Dtnb
Dtnb

Dtnb
Dnmt3a
Dnmt3a
Itsn2
Itsn2
Itsn2
Pum2
Nbas
Trib2
Rock2
Nol10
Odc1
Hpcal1
Cpsf3
Ywhaq
Mboat2
Mboat2
Kidins220
Rnf144a
Rps7
Rps7
Rps7
Lamb1
Lamb1
Cog5
Cog5
Atxn7l1
Atxn7l1
Snx13
Snx13
Arl4a
Zfp277
Lrrn3
Immp2l
Immp2l
Stxbp6
Prkd1
Prkd1
Strn3
Ap4s1
Hectd1
Arhgap5
Arhgap5
Arhgap5
Ppp2r3c
Ralgapa1
Ralgapa1
Ralgapa1
Pnn
Pnn
Ctage5
Prpf39
Prpf39
Fancm
Nemf
Sos2
Sos2
Arid4a

Arid4a
Ppm1a
Ppm1a
Hif1a
Snapc1
Ppp2r5e
Ppp2r5e
Syne2
Syne2
Syne2
Syne2
Syne2
Syne2
Syne2
Syne2
Syne2
Syne2
Syne2
Syne2
Syne2
Syne2
Syne2
Syne2
Syne2
Syne2
Fntb
Fut8
Gphn
Gphn
Eif2s1
Tmem229b
Vti1b
Zfp36l1
Actn1
Dcaf5
Srsf5
Srsf5
Synj2bp
Synj2bp
Med6
Pcnx
Sipa1l1
Rbm25
Rbm25
Rbm25
Rbm25
Rbm25
Psen1
Numb
Zfp410
Ylpm1
Ylpm1
Rps6kl1
Tmed10
Mfsd7c
1700019E19Rik
1700019E19Rik
1700019E19Rik

Sptlc2
Sptlc2
Snw1
Flrt2
Flrt2
Foxn3
Foxn3
Foxn3
Foxn3
Foxn3
Foxn3
Kcnk13
Psmc1
Ccdc88c
Trip11
Trip11
Lgmn
Itpk1
Itpk1
Btbd7
Dicer1
Atg2b
Papola
Papola
Papola
Papola
Ccnk
Evl
Evl
Evl
Yy1
Yy1
Yy1
Ppp2r5c
Dync1h1
Dync1h1
Dync1h1
Dync1h1
Dync1h1
Dync1h1
Dync1h1
Dync1h1
Dync1h1
Hsp90aa1
Hsp90aa1
Hsp90aa1
Hsp90aa1
Wdr20a
Zfp839
Tecpr2
Traf3
Cdc42bpb
Cdc42bpb
Eif5
Eif5
Mark3
Akr1e1
Wdr37

Gtpbp4
Larp4b
Dip2c
Dip2c
Dip2c
Zmynd11
Heatr1
Psma2
Gli3
Cdk13
Cdk13
Cdk13
Rala
Pou6f2
Elmo1
Elmo1
Elmo1
Elmo1
Hist1h2ai
Hist1h3h
Hist1h3h
Hist1h2bm
Hist1h4j
Hist1h4k
Hist1h2ak
Hist1h2ak
Hist1h2ah
Hist1h2bk
Hist1h2ag
Hist1h2ag
Hist1h2bj
Hist1h2bj
Hist1h3f
Hist1h4f
Hist1h1d
Hist1h3e
Hist1h2bg
Hist1h2bf
Hist1h4d
Hist1h2be
Hist1h2ac
Hist1h4c
Hist1h1c
Hist1h1c
Hist1h2bb
Hist1h1a
Hist1h2ba
Lrrc16a
Lrrc16a
Lrrc16a
Sox4
E2f3
E2f3
Psmg4
Prpf4b
Cdyl
Cdyl
Cdyl

Fars2
Fars2
Rreb1
Rreb1
Riok1
Slc35b3
Jarid2
Jarid2
Jarid2
Jarid2
Jarid2
Jarid2
Dtnbp1
Nup153
Kif13a
Dek
Rnf144b
Rnf144b
Rnf144b
Fam120a
Fbxw17
Spin1
Spin1
Secisbp2
Cplx2
4732471D19Rik
Uimc1
Zfp346
Nsd1
Nsd1
Nsd1
Nsd1
Dbn1
Ddx41
Ddx41
Ddx46
Ddx46
Pcbd2
Gkap1
Gkap1
Hnrnpk
Hnrnpk
Hnrnpk
Hnrnpk
Hnrnpk
Golm1
Zfp808
2010111I01Rik
Fancc
Ptch1
Ptch1
Ptch1
Hsd17b3
Hsd17b3
Ctsl
Nsun2
Brd9
Rhobtb3

Rhobtb3
Ccnh
Rasa1
Cox7c
Tmem167
Rps23
Rps23
Rps23
Atg10
Serinc5
Serinc5
Jmy
Jmy
Jmy
Lhfpl2
Ap3b1
Ap3b1
Tbca
Pde8b
Pde8b
Aggf1
Fam169a
Mtap1b
Mtap1b
Mtap1b
Mtap1b
Mtap1b
Bdp1
Serf1
Pik3r1
Mast4
Mast4
Mast4
Mast4
Mast4
Srek1
Erbb2ip
Cwc27
Srek1ip1
Ipo11
Kif2a
Zswim6
Ndufaf2
Pde4d
Gpbp1
Map3k1
Map3k1
Map3k1
Map3k1
Ddx4
Slc38a9
Ppap2a
Skiv2l2
Skiv2l2
Skiv2l2
Arl15
Arl15
Arl15

Arl15
Arl15
Ndufs4
Nnt
4833420G17Rik
Rpp14
Kctd6
Ptprg
Ptprg
Ptprg
Ptprg
Atxn7
Top2b
Top2b
Rpl15
Ube2e2
Ube2e2
Ube2e2
Ube2e2
Vcl
Vcl
Adk
Adk
Adk
Myst4
Myst4
Myst4
Kcnma1
Dlg5
Rps24
Zmiz1
Slmap
Slmap
Slmap
Appl1
Appl1
Arhgef3
Arhgef3
Arhgef3
Arhgef3
D14Abb1e
Cacna2d3
Dcp1a
Gnl3
Gnl3
Gnl3
Pbrm1
Pbrm1
Tnnc1
Nisch
Tnnc1
Nisch
Tnnc1
Nisch
Capn7
Capn7
Sh3bp5
Sh3bp5

Timm23
Ercc6
Mapk8
Wapal
Tspan14
Txndc16
Ddhd1
Samd4
Samd4
Samd4
Wdhd1
Fbxo34
Fbxo34
Fbxo34
Ktn1
Ktn1
Ktn1
Ktn1
Peli2
Arhgef40
Hnrnpc
Supt16h
Chd8
Tox4
Mmp14
Gm17606
Acin1
Gm17606
Acin1
Acin1
Acin1
Acin1
Acin1
Acin1
Pabpn1
Gm20521
Pabpn1
Gm20521
Pabpn1
Gm20521
Pabpn1
Gm20521
Zfhx2
Tm9sf1
Cenpj
Parp4
Mphosph8
Pspc1
Pspc1
Pspc1
Zmym2
Cryl1
Lats2
Efha1
Kpna3
Kpna3
Gata4
Xkr6

Kif13b
Kif13b
Scara5
Scara5
Scara5
Scara5
Clu
Clu
Clu
Clu
Trim35
Trim35
Dpysl2
Dpysl2
Dock5
Dock5
Loxl2
Bin3
Sorbs3
Sucla2
Lrch1
Lrch1
Zc3h13
Zc3h13
Tpt1
Tpt1
Tpt1
Tpt1
Gtf2f2
Gtf2f2
Tsc22d1
Tsc22d1
Tsc22d1
Tsc22d1
Tsc22d1
Tsc22d1
Tsc22d1
Tsc22d1
Dnajc15
Akap11
Elf1
Elf1
Sugt1
Diap3
Diap3
Diap3
Diap3
Dach1
Pibf1
Klf12
Klf12
Tbc1d4
Fbxl3
Mycbp2
Mycbp2
Mycbp2
Gpc5
Mbnl2

Mbnl2
Stk24
Stk24
Stk24
Dock9
Ubac2
Ubac2
Sepp1
Rpl37
Rpl37
Rictor
Rictor
Lifr
Lifr
Lifr
2410089E03Rik
2410089E03Rik
1110020G09Rik
Prlr
Brix1
Rai14
Rai14
Tars
Tars
Sub1
Zfr
Zfr
Pdzd2
Pdzd2
Pdzd2
Basp1
Basp1
Myo10
Myo10
Myo10
Zfp622
Fbxl7
Ank
Trio
Trio
Trio
Cct5
Cct5
Sema5a
Mtdh
Rpl30
Vps13b
Vps13b
Vps13b
Vps13b
Vps13b
Rgs22
Pabpc1
Ywhaz
Ubr5
Ubr5
Ubr5
Ubr5

Ubr5
Ubr5
Azin1
Azin1
Azin1
Fzd6
Dcaf13
Zfpm2
Zfpm2
Zfpm2
Eny2
Eif3h
Rad21
Rad21
Aard
Aard
Aard
Aard
Aard
Aard
Ext1
Ext1
Ext1
Deptor
Zhx2
Zhx2
Wdr67
Wdyhv1
Fbxo32
Ndufb9
Mtss1
Nsmce2
Nsmce2
Nsmce2
Phf20l1
Col22a1
Trappc9
Ptk2
Slc45a4
Slc45a4
Slc45a4
Slc45a4
Scrib
Scrib
Puf60
Plec
Plec
Plec
Plec
Plec
Plec
Plec
Plec
Plec
Heatr7a
Cyhr1
Rpl8
Rbfox2

Rbfox2
Myh9
Myh9
Myh9
Myh9
Myh9
Myh9
Myh9
Myh9
Myh9
Myh9
Myh9
Myh9
Myh9
Eif3d
Card10
Ankrd54
Polr2f
Ddx17
Ddx17
Ddx17
Ddx17
Ddx17
Ddx17
Tomm22
Rpl3
Rpl3
Rpl3
Tnrc6b
Tnrc6b
Tnrc6b
Mkl1
Mkl1
Zc3h7b
Zc3h7b
Zc3h7b
Zc3h7b
Zc3h7b
Zc3h7b
Zc3h7b
Zc3h7b
Zc3h7b
Tef
Tef
Csdc2
Csdc2
Xrcc6
Pppde2
Xrcc6
Pppde2
Xrcc6
Pppde2
Tcf20
Tcf20
Smc1b
Smc1b
Smc1b
Atxn10

Cerk
Fam19a5
Fam19a5
Fam19a5
Brd1
Plxnb2
Ncaph2
Shank3
Kif21a
Gxylt1
Gxylt1
Yaf2
Pphln1
Pphln1
Prickle1
Prickle1
Scaf11
Scaf11
Fam113b
Fam113b
Fam113b
Senp1
Senp1
Arf3
Arf3
Arf3
Prkag1
Mll2
Mll2
Mll2
Mll2
Mll2
Lmbr1l
Prpf40b
Prpf40b
Dip2b
Atf1
Eif4b
Eif4b
Spryd3
Spryd3
Amhr2
Tarbp2
Cbx5
Hnrnpa1
Hnrnpa1
Hnrnpa1
Nat15
1700037C18Rik
Cluap1
Nat15
1700037C18Rik
Cluap1
Crebbp
Crebbp
Crebbp
Adcy9
Adcy9

Srl
Glyr1
Cldn26
Abat
Usp7
1810013L24Rik
Clec16a
Gspt1
Snx29
Snx29
Mkl2
Myh11
Myh11
Myh11
Myh11
Myh11
Myh11
Myh11
Abcc1
Prkdc
Mapk1
Mapk1
Crkl
Ranbp1
Dgcr8
Arvcf
Gnb1l
Gnb1l
Yeats2
Yeats2
Psmd2
Eif4g1
Eif4g1
Eif4g1
Senp2
Tra2b
Tra2b
Dgkg
Eif4a2
Eif4a2
Eif4a2
Eif4a2
Eif4a2
St6gal1
St6gal1
St6gal1
Lpp
Lpp
Lpp
Ostn
1600021P15Rik
1600021P15Rik
Opa1
Atp13a3
Lsg1
Lsg1
AI480653
Pak2

Ubxn7
Tnk2
Fyttd1
Lrch3
Lrch3
Iqcg
Rpl35a
Zfp148
Heg1
Itgb5
Kalrn
Mylk
Mylk
Mylk
Ptplb
Ptplb
Adcy5
Adcy5
Sec22a
Sema5b
Sema5b
Sema5b
Sema5b
Sema5b
Sema5b
Sema5b
Sema5b
Sema5b
Sema5b
Sema5b
Sema5b
Sema5b
Eaf2
Iqcb1
Golgb1
Golgb1
Golgb1
Golgb1
Golgb1
Lrrc58
Gpr156
Gsk3b
Gsk3b
Adprh
Tmem39a
Zbtb20
Zbtb20
Zbtb20
Zbtb20
Zbtb20
Zbtb20
Zbtb20
Zbtb20
Zbtb20
Zbtb20
Zbtb20
Zbtb20
Zbtb20

Qtrtd1
2610015P09Rik
Boc
Phldb2
Morc1
Cblb
Cblb
Cblb
Alcam
Tomm70a
2610528E23Rik
Filip1l
2610528E23Rik
Filip1l
2610528E23Rik
Filip1l
2610528E23Rik
Filip1l
2610528E23Rik
Filip1l
2610528E23Rik
Nrip1
2810055G20Rik
2810055G20Rik
2810055G20Rik
2810055G20Rik
2810055G20Rik
2810055G20Rik
Gm11146
2810055G20Rik
2810055G20Rik
Gm11146
Gm11146
Gm11146
Gabpa
App
App
App
Ltn1
Tiam1
Tiam1
Tiam1
Tiam1
Tiam1
Tiam1
Scaf4
Gcfc1
Son
Son
Son
Atp5o
Itsn1
Atp5o
Itsn1
Atp5o
Mrps6
Slc5a3
Mrps6

Slc5a3
Rcan1
Morc3
Morc3
Ttc3
Ttc3
Ttc3
Ttc3
Ttc3
Ttc3
Ttc3
Dyrk1a
Erg
Brwd1
Arid1b
Arid1b
Arid1b
Arid1b
Arid1b
Arid1b
Arid1b
Zdhhc14
Zdhhc14
Zdhhc14
Zdhhc14
Synj2
Rps6ka2
Rps6ka2
Rps6ka2
Rps6ka2
Rps6ka2
Rps6ka2
Rps6ka2
Sft2d1
Qk
Qk
Qk
Qk
Agpat4
Agpat4
Agpat4
Igf2r
Igf2r
Tcp1
Tcp1
Tcp1
Tcp1
Tcte2
Mllt4
Mllt4
Chd1
Ncrna00085
Ncrna00085
Ppp2r1a
Srrm2
Srrm2
Srrm2
Srrm2

Srrm2
Srrm2
Srrm2
Srrm2
Srrm2
Srrm2
Srrm2
Caskin1
Traf7
Rab26
Rps2
Mapk8ip3
Ift140
Telo2
Unkl
Solh
Rab11fip3
Nme4
Axin1
Ergic1
A930001N09Rik
Nudt3
Nudt3
Rps10
D17Wsu92e
D17Wsu92e
D17Wsu92e
Taf11
Rpl10a
Fkbp5
Fkbp5
Srpk1
Mapk14
Brpf3
Kctd20
Mtch1
Ftsjd2
Rnf8
Ftsjd2
Rnf8
Ftsjd2
Rnf8
Zfand3
Zfand3
Zfand3
Btbd9
Dnahc8
U2af1
Brd4
Akap8
Hnrnpm
Rab11b
Rps28
Rps28
Rps28
Tapbp
Tapbp
Tapbp

Rps18
Brd2
Brd2
Brd2
Atf6b
Rdbp
Rdbp
Ehmt2
Prrc2a
Prrc2a
Prrc2a
Prrc2a
Ddx39b
Ddx39b
Ddx39b
Ddx39b
Tubb5
Tubb5
Dhx16
Dhx16
Dhx16
Atat1
Ppp1r10
Trim39
Trim39
Trim26
Trim26
Cdc5l
Hsp90ab1
Hsp90ab1
Hsp90ab1
Hsp90ab1
Hsp90ab1
Hsp90ab1
Hsp90ab1
Hsp90ab1
Hsp90ab1
Hsp90ab1
Hsp90ab1
Xpo5
Zfp318
Zfp318
Zfp318
Cul7
Ubr2
Trerf1
Trerf1
Ccnd3
Prickle4
Foxp4
Daam2
Daam2
Daam2
Tbc1d5
Uhrf1
Kdm4b
Safb2
Safb

Safb
Safb
Rpl36
Efna5
Fbxl17
Fbxl17
Fbxl17
Fer
Fer
Man2a1
Man2a1
Vapa
Vapa
Vapa
Rab31
Ralbp1
1110012J17Rik
1110012J17Rik
1110012J17Rik
1110012J17Rik
1110012J17Rik
Ptprm
Ptprm
Ptprm
Ptprm
Ptprm
Ptprm
Lama1
Epb41l3
2900073G15Rik
Wdr43
Wdr43
Wdr43
Wdr43
Wdr43
Wdr43
Wdr43
Lbh
Memo1
Birc6
Birc6
Birc6
Birc6
Strn
Ccdc75
Cebpz
Srsf7
Gemin6
Sos1
Sos1
Sos1
Map4k3
Slc8a1
Slc8a1
Kcng3
Mta3
Zfp36l2
Thada

1700106N22Rik
Srbd1
Srbd1
Prkce
Prkce
Prkce
Prkce
Prkce
Epas1
Calm2
Calm2
Msh6
Klraq1
Fshr
Svil
Svil
Svil
Svil
Svil
Svil
Zfp438
Zfp438
Zeb1
Zeb1
Zeb1
Arhgap12
Arhgap12
Kif5b
Kif5b
Epc1
Wac
Rock1
Rock1
Rock1
Greb1l
Greb1l
Mib1
Cables1
Ttc39c
Osbpl1a
Ss18
Ss18
Ss18
Kctd1
Cdh2
Rnf138
Fam59a
Dtna
Dtna
Dtna
Galnt1
Rprd1a
Rprd1a
Sap130
Wdr33
Bin1
D0H4S114
Apc

Apc
Apc
Fam13b
Kdm3b
Kdm3b
Kdm3b
Reep2
Hspa9
Hspa9
Hspa9
Ctnna1
Ctnna1
Sil1
Matr3
Matr3
Paip2
Ube2d2
Ankhd1
Ankhd1
Slc35a4
Ik
Zmat2
Pcdhgc5
Diap1
Diap1
Arhgap26
Arhgap26
Arhgap26
Arhgap26
Arhgap26
Arhgap26
Arhgap26
Arhgap26
Arhgap26
Arhgap26
Arhgap26
Arhgap26
Arhgap26
Nr3c1
Tcerg1
Tcerg1
Dpysl3
Dcp2
Pggt1b
Pggt1b
Ccdc112
Ccdc112
Commd10
Sema6a
Tnfaip8
Snx2
Snx2
Csnk1g3
Aldh7a1
Lmnb1
3-Mar
Prrc1
Rps14

Rps14
Rps14
Csnk1a1
Csnk1a1
Fbxo38
Nars
Lman1
Gnal
Tubb6
Afg3l2
Slmo1
Tcf4
Tcf4
Tcf4
Tcf4
Tcf4
Mex3c
Mex3c
Mex3c
Smad4
Me2
Mro
Mro
Myo5b
Myo5b
Myo5b
Myo5b
Rpl17
Rpl17
Rpl17
Rpl17
Dym
Ctif
Ctif
Ctif
Zbtb7c
Hdhd2
Atp9b
Atp9b
Zfp516
Tshz1
Zfp407
Ppp6r3
Lrp5
Suv420h1
Suv420h1
Suv420h1
Gm16066
Chka
Rbm4b
Rbm14
Rbm14
Rbm14
Rab1b
Pacs1
Sf3b2
Sf3b2
Sf3b2

Sart1
Map3k11
Pola2
Fau
Ehd1
Ehd1
Sf1
Sf1
Esrra
Ppp1r14b
Vegfb
Macrod1
Macrod1
Rtn3
Rtn3
Atl3
Atl3
Stx5a
Hnrnpul2
Eml3
Eef1g
Eef1g
Ahnak
Ahnak
Ahnak
Ahnak
Fth1
Fads2
Syt7
Syt7
Syt7
Ddb1
Dtx4
Tle4
Tle4
Tle4
Gnaq
Gnaq
Gnaq
Gnaq
Gna14
Gna14
Pcsk5
Ostf1
Aldh1a1
Gm9493
1700028P14Rik
Pip5k1b
Pip5k1b
Kank1
Dmrt1
Dmrt1
Dmrt1
Dmrt1
Dmrt1
Smarca2
Smarca2
Smarca2

Smarca2
Smarca2
Smarca2
Smarca2
Smarca2
Smarca2
Ak3
Uhrf2
Sgms1
Sgms1
Sgms1
Pank1
Tnks2
Btaf1
Btaf1
Exoc6
Exoc6
Cep55
Tmem20
Plce1
Plce1
Plce1
Noc3l
Noc3l
Hells
Sorbs1
Lcor
Lcor
Lcor
Cwf19l1
Scd2
Scd2
Fam178a
Fam178a
Fam178a
Peo1
Btrc
Btrc
Btrc
Mgea5
Ldb1
Gbf1
Sufu
Arl3
D19Wsu162e
Cnnm2
Cnnm2
Nt5c2
Sh3pxd2a
Sh3pxd2a
Sh3pxd2a
Add3
Add3
Add3
Add3
Mxi1
Shoc2
Vti1a

Vti1a
Vti1a
Tcf7l2
Tcf7l2
Tcf7l2
Tcf7l2
Tcf7l2
A630007B06Rik
Tdrd1
Ablim1
Fam160b1
Trub1
Atrnl1
Atrnl1
Gfra1
4930506M07Rik
Rab11fip2
Eif3a
Eif3a
Eif3a
Eif3a
Eif3a
Eif3a
Eif3a
Eif3a
Fam171a1
Nmt2
Bend7
Optn
Camk1d
Upf2
Upf2
Upf2
Celf2
Celf2
Celf2
Celf2
Celf2
Celf2
Celf2
Celf2
Celf2
Celf2
Celf2
Celf2
Celf2
Celf2
Celf2
Atp5c1
Itih5
Sfmbt2
Fam188a
Rsu1
Vim
Cacnb2
Cacnb2
Dnajc1
Etl4

Etl4
Etl4
Etl4
Myo3a
Abi1
Spopl
Cacna1b
Ehmt1
Phpt1
B230208H17Rik
Ubac1
Nacc2
Rpl7a
Rpl7a
Rpl7a
Rpl7a
Rpl7a
Rpl7a
Vav2
Vav2
Brd3
Col5a1
Rapgef1
Rapgef1
Rapgef1
Spna2
Spna2
Spna2
Spna2
Spna2
Spna2
Spna2
Spna2
Spna2
Crat
Mettl11a
Fnbp1
Gpr107
Fubp3
Abl1
Abl1
Abl1
Lamc3
Nup214
Prrc2b
Prrc2b
Prrc2b
Prrc2b
Prrc2b
Prrc2b
Prrc2b
Golga2
Golga2
Golga2
Ciz1
Rpl12
Rpl12
Rpl12

Garnl3
Garnl3
Garnl3
Garnl3
Pbx3
Mapkap1
Cep110
Cep110
Rab14
Dab2ip
Rc3h2
Rabgap1
Strbp
Rabgap1
Strbp
Dennd1a
Dennd1a
Nr5a1
Nr6a1
Nr6a1
Wdr38
Rpl35
Golga1
Scai
Lrp1b
Lrp1b
Gtdc1
Zeb2
Zeb2
Zeb2
Zeb2
Zeb2
Acvr2a
Cacnb4
Cacnb4
Cacnb4
Cacnb4
Fmnl2
Fmnl2
Fmnl2
Fmnl2
Prpf40a
Arl6ip6
Tanc1
Tanc1
Wdsub1
Baz2b
Baz2b
Rbms1
Cobll1
Cobll1
Stk39
Stk39
Stk39
Stk39
Stk39
Stk39
Lass6

Lass6
Lass6
Lass6
Phospho2
Klhl23
Ssb
Ssb
Ubr3
Ubr3
Tlk1
Tlk1
Tlk1
Dync1i2
Slc25a12
Itga6
Pdk1
B230120H23Rik
Atf2
Atf2
Plekha3
Ccdc141
Sestd1
Zfp385b
Zfp385b
Zfp385b
Cwc22
Ube2e3
Ssfa2
Calcrl
Ctnnd1
Ssrp1
Ssrp1
Ptprj
Ptprj
Fnbp4
Celf1
Ckap5
Ckap5
Ambra1
Dgkz
Phf21a
Phf21a
Phf21a
Phf21a
Cry2
Prdm11
Cd82
Cd82
Ext2
Hsd17b12
Ttc17
Ttc17
Ldlrad3
Ldlrad3
Trim44
Cd44
Cd44
Abtb2

Abtb2
Abtb2
Abtb2
Abtb2
Abtb2
Caprin1
Caprin1
Tcp11l1
Wt1
Wt1
Wt1
Wt1
Wt1
Wt1
Mpped2
Atpbd4
Eif2ak4
1500003O03Rik
Tyro3
Mga
Mga
Ganc
Tmem87a
Zfp106
Stard9
Stard9
Stard9
Cdan1
Ubr1
Pdia3
Pdia3
Serf2
Serinc4
Frmd5
Frmd5
Frmd5
Eif3j
Gatm
Gatm
Sema6d
Sema6d
Myef2
Myef2
Fbn1
Fbn1
Gabpb1
Usp8
Usp50
Trpm7
Trpm7
Ncaph
Snrpb
Nop56
Nop56
Nop56
Nop56
Nop56
Ptpra

4930402H24Rik
4930402H24Rik
4930402H24Rik
4930402H24Rik
Crls1
Plcb4
Plcb4
Plcb4
Jag1
Jag1
Kif16b
Rrbp1
Rrbp1
Rrbp1
Snx5
Csrp2bp
Ralgapa2
Ralgapa2
Ralgapa2
Pygb
Ninl
Ninl
Nsfl1c
Fkbp1a
Fkbp1a
Csnk2a1
Defb36
H13
Bcl2l1
Asxl1
8430427H17Rik
Chmp4b
Chmp4b
Raly
a
Gm14226
a
Gm14226
a
Gm14226
a
Itch
Ncoa6
Acss2
Gss
Cep250
6430550D23Rik
Rbm39
Rbm39
Rbm39
Rbm39
Rbm39
Rbm39
Rbm39
Rbm39
Dlgap4
Dlgap4
Dlgap4

Ndrg3
Rpn2
Src
Top1
Top1
Top1
Top1
Plcg1
Zhx3
Zhx3
Zhx3
Chd6
Chd6
Chd6
Srsf6
Srsf6
Srsf6
Srsf6
Tox2
Serinc3
Ywhab
Dnttip1
Ncoa3
Ncoa3
Sulf2
Ddx27
Znfx1
Znfx1
Znfx1
1500012F01Rik
1500012F01Rik
1500012F01Rik
Slc9a8
Slc9a8
Adnp
Dpm1
Adnp
Dpm1
Zfp64
Tshz2
Tshz2
Tshz2
Tshz2
Tshz2
Tshz2
Tshz2
Tshz2
Tshz2
Tshz2
Tshz2
Tshz2
Tshz2
Tshz2
Tshz2
Tshz2
Tshz2
Tshz2
Tshz2

Tshz2
Tshz2
Tshz2
Tshz2
Tshz2
Tshz2
Tshz2
Tshz2
Tshz2
Pfdn4
Pmepa1
Pmepa1
Stx16
Gnas
AF085738
Gnas
AF085738
Gm14326
Taf4a
Osbpl2
Rps21
Cables2
Dido1
Dido1
Ythdf1
Ythdf1
Zbtb46
Tpd52l2
Zfhx4
Tpd52
Tpd52
Pag1
Ythdf3
Ythdf3
Cyp7b1
1700064H15Rik
1700064H15Rik
Hps3
Nlgn1
Ect2
Ect2
Ect2
Nceh1
Fndc3b
Fndc3b
Fndc3b
Fndc3b
Fndc3b
Fndc3b
Tnik
Tnik
Tnik
Tnik
Tnik
Tnik
Tnik
Tnik
Tnik

Tnik
Phc3
Prkci
Mfn1
Fxr1
Atp11b
Atp11b
Atp11b
D3Ertd254e
Spata5
Spata5
Spata5
Intu
Phf17
Sclt1
Pcdh18
Elf2
Naa15
Maml3
Maml3
Maml3
Maml3
Foxo1
Foxo1
Nbea
Nbea
Nbea
Wwtr1
Gm10071
Med12l
Mbnl1
Mme
Kpna4
Ppm1l
Ppm1l
Ppm1l
Ppm1l
Nmd3
Pdcd10
Rapgef2
Fam198b
Fam198b
Trim2
Arfip1
Fbxw7
Arfip1
Fam160a1
Arfip1
Arfip1
Prss48
Rps3a
Rps3a
Rps3a
Rps3a
Lrba
Lrba
Arhgef11
Arhgef11

Bcan
Pmf1
Gon4l
Ash1l
Ash1l
Ash1l
Ash1l
Ash1l
Ash1l
Pbxip1
Kcnn3
Tpm3
Tpm3
Rps27
Snx27
Cgn
Cgn
Psmb4
Psmd4
Psmd4
Psmd4
Pip5k1a
Vps72
Vps72
Sema6c
Setdb1
Arnt
Ensa
Otud7b
Otud7b
Hist2h2ac
Hist2h2aa1
Hist2h3c1
Hist2h2bb
Hist2h2bb
Hist2h2bb
Hist2h2bb
BC107364
BC107364
Txnip
Rbm8a
Rbm8a
Prkab2
Notch2
Notch2
Notch2
Notch2
Wars2
Man1a2
Atp1a1
Atp1a1
Casq2
Sycp1
Csde1
Gm10964
Hipk1
Hipk1
Hipk1

Hipk1
Lrig2
Mov10
Mov10
Capza1
St7l
St7l
Kcnd3
Kcnd3
Kcnd3
Kcnd3
Kcnd3
Kcnd3
Kcnd3
Kcnd3
Kcnd3
Kcnd3
Kcnd3
Rbm15
Ahcyl1
Gstm2
Gstm1
Gstm1
Taf13
Gpsm2
Stxbp3a
Prpf38b
Prpf38b
Dph5
Dpyd
Ptbp2
Cnn3
Cnn3
Arhgap29
Arhgap29
Arhgap29
Dnttip2
Dnttip2
Bcar3
Bcar3
1810037I17Rik
Col25a1
Rpl34
Rpl34
Gstcd
Ube2d3
Ppp3ca
Ppp3ca
Ppp3ca
Ppp3ca
Eif4e
Tspan5
Tspan5
Tspan5
Tspan5
Tspan5
B930007M17Rik
Hs2st1

Hs2st1
Ddah1
Ddah1
Lpar3
Prkacb
Lphn2
Lphn2
Lphn2
Lphn2
Fubp1
Pigk
St6galnac3
Rabggtb
Rabggtb
Rabggtb
Rabggtb
Rabggtb
Acadm
Rps20
Rps20
Gm11808
Fam110b
Fam110b
Tox
Rab2a
Chd7
Asph
Fam92a
Sfrs18
Map3k7
Bach2
Casp8ap2
Mdn1
Mdn1
Mdn1
Mdn1
Mdn1
Mdn1
Mdn1
Mdn1
Mdn1
Mdn1
Orc3
Zfp292
Zfp292
Mob3b
3110043O21Rik
Nfx1
Ube2r2
Ubap2
Ubap2
Ubap2
Ubap2
Ubap1
Unc13b
Unc13b
Unc13b
Unc13b

Unc13b
Unc13b
Unc13b
Unc13b
Ccdc107
Creb3
Reck
Clta
Rnf38
Rnf38
Zcchc7
Zcchc7
Zcchc7
Zcchc7
Zcchc7
Zcchc7
Zcchc7
Frmpd1
Dcaf10
Anp32b
Anp32b
Tmeff1
Tmeff1
Tmeff1
Tmeff1
E130309F12Rik
Rnf20
Abca1
Slc44a1
Zfp462
Zfp462
Zfp462
Zfp462
Zfp462
Zfp462
Zfp462
Rad23b
Rad23b
D730040F13Rik
D730040F13Rik
D730040F13Rik
Palm2
AF064781
Akap2
AF064781
AI314180
E130308A19Rik
Slc31a1
Cdc26
Whrn
Whrn
Cdk5rap2
Ptprd
Ptprd
Ptprd
Nfib
Nfib
Frem1

Ttc39b
Bnc2
Bnc2
Bnc2
Cntln
Sh3gl2
Fam154a
Haus6
Rps6
E130114P18Rik
Nfia
Nfia
Nfia
Nfia
Nfia
Nfia
Nfia
Nfia
Nfia
Nfia
Nfia
Nfia
Dock7
Dock7
Dock7
Dock7
Dock7
Dock7
Efcab7
Itgb3bp
Pgm2
Jak1
Jak1
Leprot
Mier1
Dab1
Ppap2b
Usp24
Ssbp3
Ssbp3
Ssbp3
Tmem59
2210012G02Rik
Magoh
Scp2
Zcchc11
Zcchc11
Zcchc11
Cc2d1b
Zfyve9
Btf3l4
Nrd1
Osbpl9
Osbpl9
Osbpl9
Eps15
Eps15
Faf1

4732418C07Rik
Atpaf1
Lrrc41
Mast2
Mast2
Mast2
Mast2
Gpbp1l1
Gpbp1l1
Tesk2
Toe1
Rps8
Rps8
Rps8
Rps8
Rnf220
Eri3
Eri3
Eri3
Eri3
St3gal3
Ptprf
Ptprf
Szt2
AU022252
Ybx1
Hivep3
Rlf
Rlf
Macf1
Macf1
Macf1
Macf1
Macf1
Macf1
Macf1
Macf1
Macf1
Macf1
Macf1
Macf1
Macf1
Macf1
Macf1
Macf1
Macf1
Macf1
Sf3a3
Inpp5b
Inpp5b
1700029G01Rik
Thrap3
Thrap3
AU040320
Zmym4
Sfpq
Sfpq
Sfpq

Sfpq
Sfpq
Zmym6
Phc2
S100pbp
S100pbp
Yars
C77080
Sync
Rbbp4
Rbbp4
Rbbp4
Marcksl1
Hdac1
Eif3i
Kpna6
Tmem39b
Khdrbs1
Pum1
Pum1
Ythdf2
Taf12
Rcc1
Rcc1
Rcc1
Rcc1
Phactr4
Phactr4
Eya3
Stx12
Ahdc1
Ahdc1
Ahdc1
Ahdc1
Slc9a1
Nudc
Nudc
Arid1a
Arid1a
Ccdc21
Stmn1
Stmn1
D4Wsu53e
D4Wsu53e
D4Wsu53e
Srrm1
Srrm1
Rcan3
Srsf10
Srsf10
Hnrnpr
Luzp1
Ephb2
Cdc42
Cdc42
Hspg2
Hspg2
Usp48

Usp48
Usp48
Usp48
Ece1
Eif4g3
Eif4g3
Pink1
Pla2g5
Capzb
Ubr4
Ubr4
Ubr4
Iffo2
Padi2
Necap2
Fbxo42
Arhgef19
Epha2
Spen
Spen
Spen
Spen
Spen
Spen
Ddi2
Dnajc16
Efhd2
Efhd2
Kazn
Kazn
Prdm2
Prdm2
Pdpn
Vps13d
Vps13d
Vps13d
Gm13251
Mfn2
2610109H07Rik
Kif1b
Clstn1
Rere
Rere
Rere
Rere
Rere
Rere
Rere
Rere
Errfi1
Per3
Per3
Per3
Camta1
Camta1
Plekhg5
Plekhg5
Plekhg5

Icmt
Rpl22
Rpl22
Pank4
Ski
Ski
Gnb1
Gnb1
Mib2
Atad3a
Dvl1
Dvl1
Cpsf3l
Sdf4
Cdk6
Cdk6
Ankib1
Akap9
Akap9
Sema3c
Gnai1
Magi2
Phtf2
Rsbn1l
Armc10
Reln
Srpk2
Rint1
Fam126a
Kcnh2
Agap3
Agap3
Agap3
Prkag2
Mll3
Gm1979
Gm1979
Gm5862
Gm5862
Gm10471
5031410I06Rik
Speer4b
Dpp6
Paxip1
Paxip1
Rbm33
Nom1
Nom1
Dnajb6
Preb
Cad
Cad
Nrbp1
Ift172
Gpn1
Ppp1cb
Yes1
Yes1

Tacc3
Whsc1
Whsc1
Rnf4
Fam193a
Fam193a
Add1
Rgs12
2310079F23Rik
2310079F23Rik
Htra3
Htra3
Sorcs2
Mrfap1
Mrfap1
Mrfap1
Mrfap1
Wdr1
Bod1l
C1qtnf7
C1qtnf7
C1qtnf7
Ldb2
Slit2
Pacrgl
Gpr125
Gpr125
Dhx15
Dhx15
Dhx15
Pi4k2b
Zcchc4
Sel1l3
Rbpj
Stim2
Fam114a1
Klhl5
Pds5a
N4bp2
N4bp2
Apbb2
Slc30a9
Corin
Fryl
Fryl
Fryl
Scfd2
Fip1l1
Fip1l1
Chic2
Pdgfra
Pdgfra
Pdgfra
Pdgfra
Kit
Clock
Clock
Clock

Clock
Clock
Cep135
Igfbp7
Ythdc1
Ythdc1
Ythdc1
Utp3
Ankrd17
Ankrd17
Cnot6l
Anxa3
Bmp2k
Bmp2k
Hnrnpd
Hnrnpd
Hnrnpd
Hnrnpd
Hnrpdl
Cops4
Mrps18c
Wdfy3
Wdfy3
Wdfy3
Wdfy3
Wdfy3
Ptpn13
Aff1
Aff1
Nudt9
Nudt9
Pkd2
Pkd2
Lrrc8c
Lrrc8d
Zfp644
Zfp644
Zfp644
Tgfbr3
Rpap2
Evi5
Rpl5
Rpl5
Rpl5
Rpl5
Rpl5
Ccdc18
Pcgf3
Pcgf3
Gak
Vmn2r12
Vmn2r12
Gm15446
Pole
Ep400
Ep400
Pitpnb
Sart3

BC057022
Ankrd13a
Sppl3
Mlec
Mlec
Mlec
Rnf10
Dynll1
Dynll1
Dynll1
Msi1
Msi1
Pxn
Rplp0
Rplp0
Gcn1l1
Cit
Cit
Taok3
Taok3
Fbxo21
Fbxo21
Med13l
Dtx1
Ptpn11
Ptpn11
Rpl6
Gm15800
Erp29
Aldh2
Atxn2
Anapc7
Ift81
Ift81
Rnf34
Setd1b
Setd1b
Mlxip
Diablo
Clip1
Clip1
Clip1
Rsrc2
Rsrc2
Denr
Denr
Vps37b
Vps37b
Mphosph9
Sbno1
Sbno1
Sbno1
Sbno1
Setd8
Zfp664
Zfp664
Ncor2
Ncor2

Ncor2
Ncor2
Ncor2
Ncor2
Ncor2
Ncor2
Ncor2
Ncor2
Ncor2
Scarb1
Scarb1
Ubc
Ubc
Gm10382
Ubc
Gm10382
Aacs
Sfswap
Sfswap
Gbas
Gbas
Tyw1
Auts2
Auts2
Auts2
Auts2
Auts2
Auts2
Auts2
Auts2
Auts2
Auts2
Auts2
Auts2
Auts2
Auts2
Auts2
Gtf2ird2
Clip2
Eif4h
Eif4h
Wbscr22
Baz1b
Baz1b
Hip1
Hip1
Hip1
Hip1
Cux1
Cux1
Cux1
Cux1
Srrt
Gigyf1
Epo
Gigyf1
Epo
Gigyf1

Epo
Lrch4
Tsc22d4
Zcwpw1
Zcwpw1
Ints1
Tmem184a
Tmem184a
Mad1l1
Eif3b
Eif3b
Eif3b
Eif3b
Ttyh3
Sdk1
Sdk1
Sdk1
C330006K01Rik
Radil
Radil
Tnrc18
Tnrc18
Tnrc18
Tnrc18
Tnrc18
Tnrc18
Actb
Actb
Actb
Rnf216
Rac1
Cyth3
Bri3
Trrap
Trrap
Trrap
Trrap
Trrap
Trrap
Smurf1
Pdap1
Zfp655
Rnf6
Cdk8
Cdk8
Cdk8
Usp12
Rpl21
Polr1d
Polr1d
Pan3
Pan3
Pan3
Slc7a1
Ubl3
Ubl3
B3galtl
Fry

Fry
N4bp2l2
Pds5b
Col1a2
Col1a2
Sgce
Ppp1r9a
Ppp1r9a
Ppp1r9a
Ppp1r9a
Dync1i1
Dync1i1
Dync1i1
Dync1i1
Dync1i1
Dync1i1
Dync1i1
Acn9
Acn9
Gm16039
Capza2
St7
A430107O13Rik
A430107O13Rik
A430107O13Rik
A430107O13Rik
A430107O13Rik
Fam3c
6530409C15Rik
Zfp800
6530409C15Rik
Zfp800
Snd1
Snd1
Snd1
Lrrc4
Snd1
Lrrc4
Snd1
Lrrc4
Ccdc136
Ccdc136
Atp6v1f
Kcp
Ahcyl2
Fam40b
Fam40b
Nrf1
Ube2h
Ube2h
Ube2h
Zc3hc1
Tmem209
Tsga14
Copg2
Chchd3
Exoc4
Exoc4

Exoc4
Exoc4
Exoc4
Exoc4
Exoc4
Exoc4
Exoc4
Akr1b3
Bpgm
Cald1
Cald1
Cald1
Cald1
Cald1
Wdr91
Stra8
Cnot4
Cnot4
Creb3l2
Creb3l2
Trim24
Ubn2
Luc7l2
Luc7l2
Luc7l2
Hipk2
Hipk2
Parp12
Jhdm1d
Jhdm1d
Jhdm1d
Jhdm1d
Jhdm1d
Jhdm1d
Slc37a3
Braf
Zyx
Zyx
Nobox
Ezh2
Pdia4
Krba1
Hnrnpa2b1
Hnrnpa2b1
Hnrnpa2b1
Hnrnpa2b1
Hibadh
Jazf1
Jazf1
Jazf1
Creb5
Wipf3
Wipf3
Wipf3
Wipf3
Scrn1
Avl9
Avl9

Herc6
Gng12
Gng12
Serbp1
Serbp1
Serbp1
Chmp3
Reep1
Reep1
Reep1
Reep1
Ptcd3
Ptcd3
Ptcd3
Polr1a
St3gal5
St3gal5
St3gal5
Usp39
Rnf181
Mat2a
Mat2a
Mat2a
Tgoln1
Ctnna2
Ctnna2
Ctnna2
Ctnna2
Ctnna2
Ctnna2
Ctnna2
Ctnna2
Ctnna2
Ctnna2
Ctnna2
Ctnna2
Ctnna2
Ctnna2
Ctnna2
Ctnna2
Ctnna2
Ctnna2
Ctnna2
Ctnna2
Ctnna2
Hk2
Mogs
Dctn1
Dctn1
Dctn1
Tet3
Tet3
Dguok
Zfml
Zfml
Exoc6b
Exoc6b
Exoc6b

Exoc6b
Exoc6b
Alms1
Dusp11
Dusp11
Snrnp27
Aak1
8430410A17Rik
Rpn1
Mcm2
Plxna1
Plxna1
Slc41a3
Iqsec1
Iqsec1
Lsm3
Zfyve20
Gm15737
Adamts9
Adamts9
Adamts9
Magi1
Magi1
Magi1
Magi1
Slc25a26
Suclg2
Tmf1
Uba3
Frmd4b
Foxp1
Eif4e3
Eif4e3
Rybp
Pdzrn3
Itpr1
Itpr1
Itpr1
Itpr1
Rad18
Rad18
Srgap3
Srgap3
Srgap3
Setd5
Setd5
Setd5
Tatdn2
Vgll4
Vgll4
Rpl32
Rpl32
Rpl32
Tmcc1
Tmcc1
8-Mar
Bms1
Zfp9

Cacna1c
Cacna1c
Cacna1c
Cacna1c
Cacna1c
Cacna1c
Dcp1b
Wnt5b
Wnk1
Wnk1
Wnk1
Kdm5a
Kdm5a
Kdm5a
Il17ra
Cecr2
Cecr2
Cecr2
Atp6v1e1
Mical3
Phb2
Grcc10
Atn1
Ptms
A230083G16Rik
Zfp384
Zfp384
Ing4
Ing4
Chd4
Chd4
Chd4
Chd4
Chd4
Chd4
Chd4
Ncapd2
Ncapd2
Ncapd2
Ccnd2
Fkbp4
Fkbp4
BC035044
Gabarapl1
Etv6
Etv6
Dusp16
Dusp16
Dusp16
Ddx47
Atf7ip
Atf7ip
Atf7ip
Atf7ip
H2afj
Wbp11
Gm11077
Tuba3b

Fgfr1op2
Tm7sf3
Ppfibp1
Ppfibp1
Ccdc91
Rps9
Rps9
Ttyh1
Leng8
Ppp1r12c
Ppp1r12c
Ppp6r1
Ppp6r1
Rpl28
U2af2
U2af2
U2af2
Epn1
Zfp787
Zfp28
Peg3
Zscan18
Rps5
Trim28
Npas1
Grlf1
Grlf1
Calm3
Ccdc61
Ccdc61
Sympk
Cd3eap
Ercc1
Rps19
Rps19
Rps19
Cic
Hnrnpul1
Hnrnpul1
Axl
Egln2
Snrpa
Paf1
Fbxo17
Hnrnpl
Hnrnpl
Actn4
Actn4
Actn4
Actn4
Eif3k
Zfp27
Zfp420
Zfp146
Wbp7
Lsr
Lsr
Wtip

Uba2
Gpi1
4931406P16Rik
Kctd15
Pdcd5
Zfp536
Zfp536
2410002F23Rik
2410002F23Rik
2410002F23Rik
2410002F23Rik
2410002F23Rik
2410002F23Rik
2410002F23Rik
Ap2a1
Prr12
Rps11
Rps11
Rpl13a
Rpl13a
Rpl13a
Rpl13a
Rpl13a
Rpl13a
Rpl13a
Snrnp70
Snrnp70
Ftl1
Bax
Plekha4
Rpl18
Nomo1
Sergef
Saal1
Nav2
Nav2
Nav2
Nav2
Nav2
Nav2
Nipa1
Herc2
Herc2
Atp10a
Ube3a
Tjp1
Pcsk6
Chsy1
Chsy1
Lrrk1
Gm10974
Lrrk1
Gm10974
Lrrk1
Asb7
Synm
Synm
Igf1r

Igf1r
Igf1r
Igf1r
Igf1r
Igf1r
Igf1r
Igf1r
Igf1r
Nr2f2
Chd2
Chd2
Chd2
Chd2
Slco3a1
Akap13
Akap13
Akap13
Akap13
Akap13
Abhd2
Wdr93
Zfp710
Zfp710
Crtc3
Iqgap1
Sec11a
Rps17
Hdgfrp3
Sh3gl3
Eftud1
Mex3b
9930013L23Rik
9930013L23Rik
9930013L23Rik
Zfand6
Tmem135
Picalm
Crebzf
Dlg2
Gab2
Gab2
Gab2
Gab2
Gab2
Ndufc2
Pak1
Myo7a
2210018M11Rik
2210018M11Rik
Prkrir
Serpinh1
Rps3
Rps3
Rps3
Rnf169
C2cd3
Ucp2
Fchsd2

Fchsd2
Clpb
2400001E08Rik
Tomt
Numa1
Numa1
Numa1
Trpc2
Stim1
Stim1
Fam160a2
Mrpl17
Syt9
Rpl27a
Rpl27a
St5
St5
St5
Nrip3
Dennd5a
AA474408
Wee1
Sbf2
Mrvi1
Ctr9
Eif4g2
Eif4g2
Usp47
Tead1
Rras2
Rras2
Pde3b
Sox6
Sox6
Plekha7
Rps13
Rps13
Pik3c2a
Xylt1
Rps15a
Rps15a
Smg1
Smg1
Smg1
Smg1
Tmc7
Dcun1d3
Polr3e
Usp31
Ears2
Ubfd1
Rbbp6
Rbbp6
Rbbp6
Tnrc6a
Tnrc6a
Arhgap17
Nsmce1

Gtf3c1
Gtf3c1
D430042O09Rik
D430042O09Rik
Atxn2l
Eif3c
Eif3c
Eif3c
Eif3c
Maz
Rnf40
Myst1
Fus
Fus
Fus
Fus
Tgfb1i1
Tial1
Wdr11
Ate1
Fgfr2
Tacc2
Fgfr2
Oat
Fgfr2
Fam53b
Fgfr2
Fgfr2
Fam175b
Fgfr2
Zranb1
Fgfr2
Ctbp2
Fgfr2
Ctbp2
Fgfr2
Dhx32
Dhx32
Adam12
Adam12
Adam12
Dock1
Dock1
Dock1
Fam196a
Dock1
Fam196a
Dock1
Dock1
Ptpre
Mki67
Dpysl4
Inpp5a
Inpp5a
Rplp2
Chid1
Kcnq1
Nap1l4

Nap1l4
Cars
Shank2
Shank2
Cttn
Ppfia1
Insr
Arhgef18
Elavl1
Arglu1
Arglu1
Arglu1
Col4a1
Atp11a
Mcf2l
Cul4a
Lamp1
Tmco3
Zfp828
2610019F03Rik
Arhgef10
Angpt2
Mcph1
Mcph1
Nek3
Vdac3
Ikbkb
Myst3
Tacc1
Tacc1
Tacc1
Fgfr1
Whsc1l1
Whsc1l1
Whsc1l1
Ddhd2
Ddhd2
Ash2l
Gpr124
Unc5d
Gsr
Gsr
Rbpms
Rbpms
Tnks
Dlc1
Dlc1
Dlc1
Dlc1
Dlc1
Dlc1
Dlc1
Dlc1
Dlc1
Zdhhc2
Cnot7
Slc7a2
Slc7a2

Mtus1
Mtus1
Pcm1
Pcm1
Pcm1
Asah1
Fat1
Fat1
Fat1
Sorbs2
Acsl1
D030016E14Rik
D030016E14Rik
D030016E14Rik
Wwc2
Odz3
Neil3
Hmgb2
Galnt7
Nek1
Cpe
1-Mar
1-Mar
Psd3
Atp13a1
Crtc1
Uba52
2810422J05Rik
Ell
Rpl18a
Use1
Use1
Slc27a1
Glt25d1
Fam32a
Ap1m1
Eps15l1
Eps15l1
Med26
Sin3b
Sin3b
Large
Arhgap10
Arhgap10
Arhgap10
Arhgap10
Slc10a7
Slc10a7
Zfp827
Hhip
Gab1
Zfp330
Tbc1d9
Lphn1
Lphn1
Lphn1
Lphn1
Lphn1

Gm10644
Prkaca
Nacc1
Nfix
Nfix
Nfix
Calr
Calr
Calr
Prdx2
Rnaseh2a
Tnpo2
Zfp791
Phkb
Lonp2
Siah1a
N4bp1
N4bp1
Zfp423
Papd5
Papd5
Chd9
Fto
Fto
Fto
Fto
Fto
Fto
Fto
Mmp2
Amfr
Nup93
Fam192a
Rspry1
Rspry1
Cngb1
Csnk2a2
Cnot1
Cnot1
Cdh5
Cklf
Dync1li2
Nae1
Cbfb
Cbfb
D230025D16Rik
4931428F04Rik
Pskh1
Cdh1
Tmco7
Tmco7
Sntb2
Cyb5b
Nfat5
Nfat5
Nfat5
Nfat5
Nfat5

Nfat5
Nfat5
Zfhx3
Zfhx3
Zfhx3
Zfhx3
Dhx38
Ap1g1
Phlpp2
Vac14
Sf3b3
Sf3b3
Sf3b3
St3gal2
Pdpr
Glg1
Glg1
Glg1
Glg1
Glg1
Znrf1
Znrf1
Znrf1
Cfdp1
Cfdp1
Cfdp1
Wwox
Wwox
Wwox
Maf
Cdyl2
4933407C03Rik
Hsbp1
Atp2c2
Gm20388
Gm20388
Gm20388
Cox4nb
Gm20388
Cox4i1
Gm20388
Gm20388
Zcchc14
Gm20388
Banp
Gm20388
Banp
Gm20388
Banp
Gm20388
Gm20388
Ankrd11
Gm20388
Ankrd11
Gm20388
Ankrd11
Gm20388
Ankrd11

Gm20388
Ankrd11
Gm20388
Ankrd11
Gm20388
Rpl13
Gm20388
Rpl13
Gm20388
Tubb3
Gm20388
Gm20388
Gm20388
Rhou
Gm20388
Galnt2
Gm20388
Galnt2
Gm20388
Cog2
Ttc13
Arv1
Egln1
BC021891
BC021891
BC021891
Pard3
Pard3
Nrp1
Nrp1
Cwf19l2
Cwf19l2
Cwf19l2
Pdgfd
Birc2
Birc2
Birc2
Yap1
Yap1
Arhgap42
Fam76b
Med17
Taf1d
Taf1d
Taf1d
Taf1d
5830418K08Rik
5830418K08Rik
BC017612
Naalad2
Zfp266
Ppan
Ppan
Eif3g
Dnmt1
Dnmt1
Cdc37
Ilf3

Dnm2
Yipf2
Carm1
Smarca4
Smarca4
Smarca4
Bbs9
Ncapd3
Jam3
Zbtb44
Aplp2
Aplp2
Prdm10
Prdm10
Nfrkb
Arhgap32
Arhgap32
St3gal4
Srpr
Srpr
Pknox2
Zfp202
Gramd1b
Gramd1b
Gramd1b
Gramd1b
Hspa8
Hspa8
Hspa8
Hspa8
Hspa8
Hspa8
Hspa8
Hspa8
2610203C20Rik
Tbcel
Grik4
Arhgef12
Arhgef12
Arhgef12
Arhgef12
Arhgef12
H2afx
Ddx6
Ddx6
Phldb1
Mll1
Mll1
Mll1
Scn4b
Bace1
Bace1
Tagln
Sik3
Sik3
Cadm1
Cadm1
Cadm1

Cadm1
Cadm1
Cadm1
Cadm1
Cadm1
Zbtb16
Ncam1
AU019823
Alg9
Rdx
Ddx10
Ddx10
Ddx10
Ddx10
Npat
Dmxl2
Dmxl2
Idh3a
Idh3a
Acsbg1
Acsbg1
Ireb2
Ireb2
Scaper
Scaper
Scaper
Scaper
Tspan3
C230081A13Rik
C230081A13Rik
C230081A13Rik
Snx33
Sin3a
Sin3a
Commd4
Loxl1
Neo1
Neo1
Neo1
Arih1
Arih1
Arih1
Uaca
Tle3
Anp32a
Fem1b
Map2k5
Iqch
Aagab
Smad3
Smad3
Smad3
Rpl4
Rpl4
Rpl4
Rpl4
Rpl4
Rpl4

Rpl4
Rpl4
Map2k1
Dpp8
Igdcc4
Spg21
Trip4
Snx1
Herc1
Herc1
Fbxl22
Usp3
Usp3
Rab8b
Vps13c
Rora
Rora
Rora
Rora
Rora
Rora
Rora
Rora
Rora
Rora
Rora
Rora
Rora
Rora
Narg2
Anxa2
Anxa2
Sltm
Sltm
Sltm
Sltm
Sltm
Grinl1a
Grinl1a
Cgnl1
Cgnl1
Cgnl1
Tcf12
Tcf12
Tcf12
Zfp280d
Nedd4
Nedd4
Nedd4
Nedd4
Nedd4
Nedd4
Nedd4
Pygo1
BC031353
Arpp19
Arpp19
Lrrc1

Elovl5
Eef1a1
Eef1a1
Eef1a1
Eef1a1
Eef1a1
Eef1a1
Filip1
Senp6
Phip
Phip
Me1
Me1
Cyb5r4
Syncrip
Syncrip
Plod2
2610101N10Rik
Tfdp2
Tfdp2
Zbtb38
Slc25a36
Clstn2
Nmnat3
Copb2
Faim
Il20rb
Stag1
Stag1
Stag1
Cep63
Ryk
Topbp1
Topbp1
Rbm6
Rbm6
Ip6k1
Usp19
Qrich1
Prkar2a
Mtap4
Mtap4
Mtap4
Dhx30
Dhx30
Smarcc1
Smarcc1
Smarcc1
Smarcc1
Smarcc1
Setd2
Setd2
Setd2
Lrrfip2
Lrrfip2
Mlh1
Trank1
Trank1

Trank1
Trank1
Trank1
Trank1
Trank1
Trank1
Trank1
Pdcd6ip
Clasp2
Clasp2
Ubp1
Ubp1
Trim71
Cmtm6
Stt3b
Golga4
Golga4
Itga9
Itga9
Rpsa
Rpsa
Rpsa
Rpsa
Ctnnb1
Ctnnb1
Ctnnb1
Ctnnb1
Ulk4
Ulk4
Trak1
Trak1
Trak1
Trak1
Trak1
Trak1
Trak1
Nktr
D9Ertd402e
Zfp445
Lars2
Lars2
Sacm1l
Slc6a20b
mt-Co1
mt-Co1
mt-Co1
mt-Co1
mt-Co1
mt-Co1
mt-Nd5
mt-Nd5
mt-Cytb
mt-Cytb
mt-Cytb
mt-Cytb
mt-Cytb
mt-Cytb
Shroom4

Hdac6
Suv39h1
Rbm3
Tspan7
Tspan7
Mid1ip1
Bcor
Usp9x
Usp9x
Ddx3x
Phf16
Phf16
Pgrmc1
Slc25a43
Rpl39
Rpl39
Lamp2
Thoc2
Thoc2
Ocrl
Zfp280c
Zfp280c
Mbnl3
Hs6st2
Htatsf1
Rbmx
Atp11c
Aff2
Mtm1
Zfp275
Flna
Flna
Tbl1x
Dmd
Dmd
Mageb18
Mageb18
Maged1
Maged1
Maged1
Gspt2
Eda2r
Ar
Ophn1
Med12
Nono
Nono
Nhsl2
Nhsl2
Nhsl2
Nhsl2
Nhsl2
Nhsl2
Phka1
Rlim
Rlim
Rlim
C77370

Atrx
Atrx
Atrx
Brwd3
Diap2
Diap2
Sytl4
Sytl4
Cenpi
Rpl36a
Gprasp1
Tceal8
Wbp5
Nrk
Tmem164
Tmem164
Pak3
Pak3
Pak3
Pak3
Alg13
Zcchc16
Maged2
Huwe1
Huwe1
Huwe1
Huwe1
Huwe1
Huwe1
Smc1a
Smc1a
Smc1a
Tspyl2
Rps6ka3
Rps6ka3
A830080D01Rik
Sh3kbp1
Nhs
Nhs
Nhs
Nhs
Txlng
Syap1
Tmsb4x
Tmsb4x
Frmpd4
Arhgap6
