# Supplementary material for: SRSF1-mediated alternative splicing is required for spermatogenesis
Source: Int J Biol Sci. 2023 Sep 11;19(15):4883–97. doi: 10.7150/ijbs.83474 (PMC10539708; doi:10.7150/ijbs.83474)
Supplement: Supplementary file 1 — Supplementary figures and tables. [file ijbsv19p4883s1.zip › Supplementary materials/Table 7 The list of differentially spliced genes.pdf]

chr14	51548680	51548982	ENSMUSG00000035953.7	Tmem55b	4.63E-05	0.141
chr12	74007177	74007269	ENSMUSG00000021098.8	4930447C(	5.12E-05	0.641
chr11	1.16E+08	1.16E+08	ENSMUSG00000057948.6	Unc13d	2.61E-07	-0.725
chr14	35508172	35508291	ENSMUSG00000041408.10	Wapal	1.28E-05	0.136
chr10	79855283	79856025	ENSMUSG00000035478.8	Mbd3	0	0.104
chr3	95007652	95008730	ENSMUSG00000038766.9	Gabpb2	1.85E-09	0.17
chr7	1.27E+08	1.27E+08	ENSMUSG00000030929.9	Eri2	0	0.533
chr3	19930313	19930805	ENSMUSG00000027615.7	Hps3	0.000358	0.114
chr4	1.55E+08	1.55E+08	ENSMUSG00000059939.7	9430015G:	2.22E-05	0.275
chr2	50148328	50148473	ENSMUSG00000026766.10	Mmadhc	1.28E-05	0.078
chr2	1.64E+08	1.64E+08	ENSMUSG00000017721.8	Pigt	4.63E-05	0.054
chr5	33943004	33950876	ENSMUSG00000037339.10	Fam53a	3.86E-12	0.223
chr14	32183887	32185170	ENSMUSG00000021893.7	Capn7	1.86E-09	0.156
chr6	1.19E+08	1.19E+08	ENSMUSG00000041477.8	Dcp1b	6.05E-07	0.163
chrX	70340162	70340421	ENSMUSG00000057836.6	Xlr3a	1.18E-06	-0.18
chr19	4158824	4158919	ENSMUSG00000024830.9	Rps6kb2	0.000228	0.513
chr17	28338205	28339293	ENSMUSG00000024220.7	Zfp523	4.59E-06	0.395
chr17	27696477	27696648	ENSMUSG00000046711.9	Hmga1	2.50E-09	0.17
chr17	24356675	24357255	ENSMUSG00000024118.7	1600002H(	9.24E-05	0.232
chr17	15638117	15638321	ENSMUSG00000014767.10	Tbp	3.69E-10	0.216
chr16	17323080	17323163	ENSMUSG00000041720.6	Pi4ka	1.78E-07	0.304
chr15	12165772	12165903	ENSMUSG00000039458.8	Mtmr12	1.04E-06	0.28
chr11	84654916	84655126	ENSMUSG00000020530.7	Ggnbp2	0.000151	0.138
chr4	1.32E+08	1.32E+08	ENSMUSG00000054405.8	Dnajc8	9.21E-12	0.073
chr2	35982644	35982771	ENSMUSG00000026889.6	Rbm18	7.42E-06	0.076
chr5	1.07E+08	1.07E+08	ENSMUSG00000049606.9	Zfp644	8.23E-05	0.26
chr5	1.07E+08	1.07E+08	ENSMUSG00000049606.9	Zfp644	9.49E-05	-0.418
chr6	29881119	29881829	ENSMUSG00000039629.8	Fam40b	6.41E-05	0.161
chr3	89842736	89842938	ENSMUSG00000042520.9	Ubap2l	0.000793	-0.097
chr15	1.02E+08	1.02E+08	ENSMUSG00000046897.9	Zfp740	1.16E-06	0.122
chr2	1.81E+08	1.81E+08	ENSMUSG00000038671.7	Arfrp1	0.000213	0.206
chr5	31173792	31174228	ENSMUSG00000038828.7	Tmem214	0.000112	0.092
chr1	1.55E+08	1.55E+08	ENSMUSG00000042772.8	Smg7	0	0.294
chr1	1.58E+08	1.58E+08	ENSMUSG00000060985.8	Tdrd5	1.17E-10	0.416
chr7	1.44E+08	1.44E+08	ENSMUSG00000010476.6	Ebf3	0.000406	0.13
chr6	83926195	83927423	ENSMUSG00000030016.7	Zfml	1.88E-07	0.061
chr16	18896546	18896866	ENSMUSG00000022702.9	Hira	4.70E-06	-0.123
chr3	31012329	31012562	ENSMUSG00000027660.9	Skil	4.15E-05	0.196
chr9	45707828	45708339	ENSMUSG00000042790.8	Rnf214	1.54E-06	0.214
chr6	18383819	18385446	ENSMUSG00000000416.9	Cttnbp2	0.000448	0.455
chr5	46082840	46084577	ENSMUSG00000015880.9	Ncapg	1.72E-12	0.27
chr5	46082840	46084577	ENSMUSG00000015880.9	Ncapg	5.57E-05	0.437
chr5	1.16E+08	1.16E+08	ENSMUSG00000029544.9	Cabp1	0.000378	0.629
chr7	1.09E+08	1.09E+08	ENSMUSG00000030649.10	3200002M	9.49E-05	0.062
chr4	1.17E+08	1.17E+08	ENSMUSG00000028677.11	Rnf220	1.41E-09	0.185
chr13	1.12E+08	1.12E+08	ENSMUSG00000032745.10	Gpbp1	0	0.148
chr6	1.13E+08	1.13E+08	ENSMUSG00000001632.9	Brpf1	1.19E-12	0.127
chr11	29053231	29054215	ENSMUSG00000020464.9	Pnpt1	9.44E-05	0.063
chr19	10500788	10501157	ENSMUSG00000024743.7	Syt7	0.000299	-0.355
chr17	50423562	50423676	ENSMUSG00000010592.7	Dazl	0	0.198
chr17	31788590	31788657	ENSMUSG00000061613.6	U2af1	9.21E-06	0.102
chrX	1.58E+08	1.58E+08	ENSMUSG00000000037.9	Scml2	3.15E-09	-0.358
chrX	1.58E+08	1.58E+08	ENSMUSG00000000037.9	Scml2	4.15E-07	-0.28
chr16	17680641	17680854	ENSMUSG00000012114.9	Med15	3.83E-06	-0.22
chr14	60042224	60042321	ENSMUSG00000071350.5	Setdb2	0.000921	0.191
chr12	1E+08	1E+08	ENSMUSG00000021012.9	Zc3h14	3.41E-05	0.059
chr12	85636166	85636285	ENSMUSG00000072946.5	Ptgr2	6.22E-09	0.104
chr12	74009498	74010800	ENSMUSG00000021098.8	4930447C(	0.000357	-0.123

chr16	87474826	87474918	ENSMUSG00000025616.7	Usp16	0.000794	-0.076
chr11	1.05E+08	1.05E+08	ENSMUSG00000020694.9	Tlk2	5.62E-05	-0.143
chr11	1.05E+08	1.05E+08	ENSMUSG00000020694.9	Tlk2	4.46E-06	0.175
chr11	1.05E+08	1.05E+08	ENSMUSG00000020694.9	Tlk2	6.54E-08	-0.194
chr11	82625901	82626312	ENSMUSG00000020696.11	Rffl	4.41E-05	0.213
chr11	69784062	69784244	ENSMUSG00000018565.10	Rai12	0.000996	0.058
chr11	59557272	59557329	ENSMUSG00000005417.10	Mprip	0.000162	0.125
chr11	6333726	6333840	ENSMUSG00000041126.8	H2afv	2.41E-12	-0.142
chr11	3194458	3194550	ENSMUSG00000020453.10	Patz1	0.000409	0.083
chr16	31846919	31847019	ENSMUSG00000022770.9	Dlg1	0	-0.156
chr16	31846922	31847019	ENSMUSG00000022770.9	Dlg1	0	-0.181
chr9	1.01E+08	1.01E+08	ENSMUSG00000037286.8	Stag1	3.28E-07	-0.178
chr9	1.01E+08	1.01E+08	ENSMUSG00000037286.8	Stag1	1.05E-05	0.2
chr2	1.25E+08	1.25E+08	ENSMUSG00000027201.10	Myef2	7.86E-07	-0.188
chr3	95365149	95365202	ENSMUSG00000028109.8	Hormad1	0.000451	-0.081
chr3	95365149	95365202	ENSMUSG00000028109.8	Hormad1	1.19E-06	-0.152
chr7	1.27E+08	1.27E+08	ENSMUSG00000030929.9	Eri2	0	0.272
chr2	1.81E+08	1.81E+08	ENSMUSG00000038671.7	Arfrp1	4.06E-05	0.167
chr2	1.81E+08	1.81E+08	ENSMUSG00000038671.7	Arfrp1	8.54E-05	-0.075
chr9	47621855	47622028	ENSMUSG00000032076.11	Cadm1	0.000134	0.053
chr1	1.93E+08	1.93E+08	ENSMUSG00000026634.9	Angel2	7.55E-06	0.156
chr1	1.93E+08	1.93E+08	ENSMUSG00000026634.9	Angel2	2.57E-08	-0.111
chr15	79414748	79414809	ENSMUSG00000022429.9	Dmc1	0.000313	0.181
chr15	79414748	79414809	ENSMUSG00000022429.9	Dmc1	0.000393	0.221
chr1	1.55E+08	1.55E+08	ENSMUSG00000042772.8	Smg7	1.37E-10	0.091
chr16	18805474	18805530	ENSMUSG00000000028.8	Cdc45	8.62E-05	-0.107
chr2	1.44E+08	1.44E+08	ENSMUSG00000037259.8	6330439K1	3.02E-06	-0.321
chr6	1.19E+08	1.19E+08	ENSMUSG00000051331.7	Cacna1c	0.000189	0.228
chr3	87961955	87962093	ENSMUSG00000001419.10	Mef2d	0	0.177
chr8	74765622	74765837	ENSMUSG00000003033.8	Ap1m1	4.46E-06	-0.074
chr3	1.46E+08	1.46E+08	ENSMUSG00000028188.7	Spata1	0.000423	0.218
chr8	73779545	73779611	ENSMUSG00000035439.7	Haus8	1.58E-08	-0.145
chr5	1E+08	1E+08	ENSMUSG00000029328.8	Hnrpdl	0	0.31
chr10	1.12E+08	1.12E+08	ENSMUSG00000020214.4	Glipr1l2	8.21E-06	-0.368
chr13	1.12E+08	1.12E+08	ENSMUSG00000032745.10	Gpbp1	7.55E-06	0.107
chr6	54565208	54565364	ENSMUSG00000005225.9	Plekha8	9.75E-10	0.191
chr3	37363453	37363580	ENSMUSG00000027722.8	Spata5	3.05E-06	-0.107
chr5	65779800	65779918	ENSMUSG00000047215.8	Rpl9	0.00054	0.083
chr9	59522835	59523002	ENSMUSG00000032294.9	Pkm2	0	0.055
chr9	61768825	61769053	ENSMUSG00000032254.8	Kif23	1.82E-05	0.136
chr15	61938702	61939020	ENSMUSG00000072566.3	Pvt1	7.50E-05	-0.44
chr15	61980793	61980907	ENSMUSG00000072566.3	Pvt1	1.22E-10	1
chr14	57575688	57575940	ENSMUSG00000021945.7	Zmym2	0.00056	0.114
chr13	54660558	54660685	ENSMUSG00000025871.11	4833439L1	0.000556	-0.091
chr13	54663053	54663232	ENSMUSG00000025871.11	4833439L1	1.35E-05	0.134
chrX	70442691	70443724	ENSMUSG00000073125.4	Xlr3b	0.000354	-0.184
chr18	34768071	34769603	ENSMUSG00000003778.8	Brd8	1.46E-05	-0.133
chr15	1.03E+08	1.03E+08	ENSMUSG00000022489.4	Pde1b	0.000336	0.6
chr12	32643340	32645387	ENSMUSG00000002997.9	Prkar2b	9.29E-05	-0.096
chr12	4871942	4873335	ENSMUSG00000037336.8	Mfsd2b	1.02E-05	0.722
chr4	34544556	34546005	ENSMUSG00000040044.5	Orc3	1.99E-05	-0.088
chr7	1.27E+08	1.27E+08	ENSMUSG00000030929.9	Eri2	9.73E-06	-0.144
chr2	1.81E+08	1.81E+08	ENSMUSG00000059540.8	Tcea2	5.12E-05	-0.117
chr7	1.26E+08	1.26E+08	ENSMUSG00000033904.8	6330503K2	4.31E-05	-0.244
chr3	68794663	68798504	ENSMUSG00000027778.8	Ift80	1.86E-06	-0.499
chr3	79399123	79401795	ENSMUSG00000027804.7	Ppid	0.000867	-0.082
chr5	46082840	46084577	ENSMUSG00000015880.9	Ncapg	0.000104	-0.272
chr5	46087271	46087783	ENSMUSG00000015880.9	Ncapg	1.05E-06	-0.162

chr7	1.47E+08	1.47E+08	ENSMUSG00000073795.4	6430531B1	0.000332	-0.305
chr6	90359335	90359644	ENSMUSG00000048794.8	Ccdc37	0.000129	-0.456
chr2	51949022	51950182	ENSMUSG00000036202.8	Rif1	0	-0.232
chr5	67733332	67735808	ENSMUSG00000029221.8	Slc30a9	7.80E-11	-0.111
chrY	2041933	2042022	ENSMUSG00000090652.1	Gm4064	2.36E-11	0.252
chrY	75034	75586	ENSMUSG00000053211.4	Zfy1	4.30E-06	0.35
chrX	1.48E+08	1.48E+08	ENSMUSG00000025261.9	Huwe1	1.76E-06	0.092
chrX	1.48E+08	1.48E+08	ENSMUSG00000025261.9	Huwe1	7.27E-09	0.192
chrX	1.48E+08	1.48E+08	ENSMUSG00000025261.9	Huwe1	3.04E-05	0.073
chrX	1.44E+08	1.44E+08	ENSMUSG00000031290.7	Lrch2	3.19E-06	0.337
chrX	1.37E+08	1.37E+08	ENSMUSG00000072944.4	Nup62cl	0.000274	-0.072
chrX	1.31E+08	1.31E+08	ENSMUSG00000045427.7	Hnrnph2	1.27E-10	0.167
chrX	98613611	98614291	ENSMUSG00000031310.10	Zmym3	3.48E-11	0.132
chrX	98614133	98614291	ENSMUSG00000031310.10	Zmym3	1.90E-13	0.291
chrX	51054686	51055033	ENSMUSG00000054727.2	1700013H	0	0.805
chrX	46210651	46210717	ENSMUSG00000079606.1	Gm595	5.07E-07	0.317
chrX	46210651	46210717	ENSMUSG00000079606.1	Gm595	0.000696	0.411
chrX	45728199	45728365	ENSMUSG00000036959.9	Bcorl1	0.000531	-0.183
chrX	45627977	45628365	ENSMUSG00000063785.6	Utp14a	8.24E-05	0.187
chrX	45630398	45630796	ENSMUSG00000063785.6	Utp14a	0.000406	0.181
chrX	39447543	39448449	ENSMUSG00000025860.8	Xiap	1.15E-05	0.194
chrX	20529333	20529397	ENSMUSG00000001134.3	Uxt	2.14E-05	0.153
chr19	60296956	60297207	ENSMUSG00000057858.6	D19Ertd73	3.76E-05	0.116
chr19	47689685	47689758	ENSMUSG00000025060.8	Slk	0	0.133
chr19	45009422	45010156	ENSMUSG00000036097.6	Fam178a	0	0.107
chr19	39018203	39018431	ENSMUSG00000025001.5	Hells	0	0.096
chr19	36519477	36519567	ENSMUSG00000024805.8	Pcgf5	0	0.113
chr19	36525053	36525113	ENSMUSG00000024805.8	Pcgf5	0	0.699
chr19	36159555	36159611	ENSMUSG00000024800.7	Rpp30	0	0.107
chr19	29365656	29365791	ENSMUSG00000024789.6	Jak2	0.000699	0.126
chr19	5365817	5365956	ENSMUSG00000024844.8	Banf1	6.37E-11	0.051
chr19	5024510	5024592	ENSMUSG00000024891.5	Slc29a2	0.000173	0.159
chr19	4288411	4288507	ENSMUSG00000024858.7	Adrbk1	8.57E-05	0.229
chr18	84727718	84732438	ENSMUSG00000048410.9	Zfp407	1.42E-05	0.134
chr18	80645979	80646987	ENSMUSG00000033323.6	Ctdp1	5.68E-13	0.182
chr18	74377414	74377650	ENSMUSG00000024560.6	Cxxc1	0.000116	0.067
chrX	53391147	53391273	ENSMUSG00000031125.2	3830403N	3.98E-09	0.452
chrX	53391921	53391956	ENSMUSG00000031125.2	3830403N	8.99E-05	0.185
chr18	58071543	58071667	ENSMUSG00000024597.9	Slc12a2	0.000374	0.064
chr18	36829657	36829783	ENSMUSG00000006050.4	Sra1	5.36E-08	0.059
chr18	32062235	32062331	ENSMUSG00000024400.9	Wdr33	0.000627	0.075
chr18	12301444	12301572	ENSMUSG00000024404.6	Riok3	5.25E-05	0.064
chr18	7910513	7910822	ENSMUSG00000024283.8	Wac	5.53E-07	-0.074
chr17	80275141	80275386	ENSMUSG00000059811.5	Atl2	1.37E-06	0.054
chr17	79161339	79161606	ENSMUSG00000039414.8	Heatr5b	0.000158	0.152
chr17	74993703	74993793	ENSMUSG00000024073.6	Birc6	0.000136	0.187
chr17	56564749	56565349	ENSMUSG00000013236.9	Ptprs	9.47E-13	0.363
chr17	56574303	56574437	ENSMUSG00000013236.9	Ptprs	0.000316	0.131
chr17	50424320	50424371	ENSMUSG00000010592.7	Dazl	5.25E-08	0.052
chr17	50424320	50424371	ENSMUSG00000010592.7	Dazl	0	0.341
chr17	46884369	46884493	ENSMUSG00000023973.7	Cnpy3	0.000448	0.096
chr17	35259173	35259405	ENSMUSG00000092417.1	RP23-115(	0.000618	-0.202
chr17	35259173	35259405	ENSMUSG00000092417.1	RP23-115(	1.87E-06	-0.195
chr17	35119585	35119632	ENSMUSG00000007050.10	Lsm2	0.000701	0.118
chr17	34784604	34784684	ENSMUSG00000015461.8	Atf6b	0	0.094
chr17	34167768	34168207	ENSMUSG00000024327.9	Slc39a7	3.71E-06	0.148
chr17	31789446	31789513	ENSMUSG00000061613.6	U2af1	0.000265	0.222
chr17	31791923	31792011	ENSMUSG00000061613.6	U2af1	1.31E-12	0.065

chr17	31639715	31639828	ENSMUSG00000024037.5	Wdr4	3.91E-08	0.264
chr17	28339059	28339293	ENSMUSG00000024220.7	Zfp523	8.59E-06	0.103
chr17	28339379	28339543	ENSMUSG00000024220.7	Zfp523	0.000107	0.061
chr17	27076261	27076313	ENSMUSG00000024194.8	Cuta	0.000775	-0.103
chr17	25918137	25918323	ENSMUSG00000002280.4	Narfl	7.82E-05	0.15
chr17	25918185	25918323	ENSMUSG00000002280.4	Narfl	0.000125	0.077
chr17	24832198	24832395	ENSMUSG00000040888.9	Gfer	2.78E-13	0.182
chr17	24666685	24666762	ENSMUSG00000079657.3	Rab26	0.000117	0.598
chr17	24653449	24653541	ENSMUSG00000052752.7	Traf7	1.20E-12	0.16
chr17	24356675	24357078	ENSMUSG00000024118.7	1600002H(	0.000164	0.181
chr17	15638117	15638321	ENSMUSG00000014767.10	Tbp	1.15E-10	0.062
chr17	15638275	15638321	ENSMUSG00000014767.10	Tbp	4.14E-08	0.209
chr17	13985361	13985382	ENSMUSG00000068036.7	Mllt4	0.000834	-0.235
chr17	7188487	7188540	ENSMUSG00000079724.3	Rnaset2b	7.98E-11	0.24
chr17	7189404	7189462	ENSMUSG00000079724.3	Rnaset2b	8.20E-11	0.431
chr16	96401651	96401727	ENSMUSG00000045275.8	Lca5l	0.000124	-0.265
chr16	96268925	96269054	ENSMUSG00000022914.9	Brwd1	1.87E-11	0.119
chr16	96287631	96287792	ENSMUSG00000022914.9	Brwd1	2.74E-05	0.093
chr16	85043820	85043988	ENSMUSG00000022892.9	App	0.000116	-0.084
chr16	85043824	85043988	ENSMUSG00000022892.9	App	0.000376	-0.102
chr16	49869130	49869193	ENSMUSG00000055447.11	Cd47	3.56E-11	0.164
chr16	38450208	38450563	ENSMUSG00000002844.8	Adprh	1.49E-10	0.055
chr16	38217198	38217237	ENSMUSG00000022812.7	Gsk3b	8.91E-06	0.069
chr16	33963857	33964011	ENSMUSG00000022814.6	Umps	4.36E-12	0.183
chrX	1.58E+08	1.58E+08	ENSMUSG00000000037.9	Scml2	5.63E-07	0.066
chrX	1.58E+08	1.58E+08	ENSMUSG00000000037.9	Scml2	1.09E-07	0.107
chrX	1.58E+08	1.58E+08	ENSMUSG00000000037.9	Scml2	0	0.148
chr16	17680641	17680854	ENSMUSG00000012114.9	Med15	7.56E-09	0.299
chr16	16318537	16318570	ENSMUSG00000022789.7	Dnm1l	5.32E-11	0.256
chr16	15966694	15966945	ENSMUSG00000041974.8	2310008H(	0.000192	0.133
chr16	4764605	4765097	ENSMUSG00000004070.9	Hmox2	0	0.098
chr16	4765762	4765889	ENSMUSG00000004070.9	Hmox2	4.80E-07	0.06
chr16	4621608	4621706	ENSMUSG00000014303.7	Glis2	0.000155	-0.227
chr15	1.03E+08	1.03E+08	ENSMUSG00000022487.8	Gtsf1	4.04E-10	0.104
chr15	1.03E+08	1.03E+08	ENSMUSG00000022487.8	Gtsf1	1.16E-10	0.121
chr15	1.03E+08	1.03E+08	ENSMUSG00000022487.8	Gtsf1	1.47E-05	0.521
chr15	1.03E+08	1.03E+08	ENSMUSG00000022487.8	Gtsf1	0.000188	-0.125
chr15	1.03E+08	1.03E+08	ENSMUSG00000009575.8	Cbx5	8.57E-09	-0.107
chr15	1.03E+08	1.03E+08	ENSMUSG00000009575.8	Cbx5	0.00051	-0.062
chr15	1.02E+08	1.02E+08	ENSMUSG00000023047.4	Amhr2	3.64E-05	-0.594
chr15	1.01E+08	1.01E+08	ENSMUSG00000023032.6	Slc4a8	8.41E-05	-0.241
chr15	99230087	99230302	ENSMUSG00000023010.8	Tmbim6	6.01E-05	-0.436
chr15	93282547	93282604	ENSMUSG00000036167.9	Pphln1	2.42E-11	-0.151
chr18	25498214	25498885	ENSMUSG00000033632.9	AW55491ε	0.000393	-0.172
chr18	25503364	25503463	ENSMUSG00000033632.9	AW55491ε	7.18E-05	-0.234
chr15	88825270	88825455	ENSMUSG00000015365.8	Mov10l1	1.26E-06	0.098
chr15	88825270	88825455	ENSMUSG00000015365.8	Mov10l1	0	0.295
chr15	88826485	88826626	ENSMUSG00000015365.8	Mov10l1	5.07E-14	0.212
chr15	76282234	76282390	ENSMUSG00000022558.9	Heatr7a	8.20E-11	0.126
chr15	75727069	75727170	ENSMUSG00000055762.9	Eef1d	3.60E-13	0.054
chr15	75727655	75727712	ENSMUSG00000055762.9	Eef1d	2.73E-06	0.384
chr15	75669685	75670994	ENSMUSG00000075600.2	Zc3h3	4.94E-07	0.27
chr15	54914959	54915148	ENSMUSG00000022422.7	Dscc1	2.72E-05	0.164
chr15	38912028	38912250	ENSMUSG00000054196.5	Cthrc1	6.35E-05	0.297
chr15	8180214	8180369	ENSMUSG00000039801.6	2410089E(	7.33E-07	0.104
chr14	1.19E+08	1.19E+08	ENSMUSG00000032849.7	Abcc4	1.21E-08	0.264
chr14	1.19E+08	1.19E+08	ENSMUSG00000022131.2	Gpr180	0.000423	0.111
chr16	43577142	43577306	ENSMUSG00000022708.10	Zbtb20	0.00035	-0.235

chr16	56089496	56089577	ENSMUSG00000052917.7	Senp7	0.000168	0.226
chrX	71545588	71545663	ENSMUSG00000019087.7	Atp6ap1	0	0.106
chr14	70795134	70795248	ENSMUSG00000033644.4	Piwil2	2.16E-09	0.088
chr14	60866160	60866303	ENSMUSG00000063895.5	Nupl1	0	0.131
chr14	60056273	60056602	ENSMUSG00000071350.5	Setdb2	0	0.331
chr14	57090127	57090281	ENSMUSG00000000365.7	Rnf17	9.98E-07	0.1
chr14	57094234	57094350	ENSMUSG00000000365.7	Rnf17	4.40E-07	0.114
chr14	57126654	57126780	ENSMUSG00000000365.7	Rnf17	2.54E-07	0.119
chr14	57132872	57133041	ENSMUSG00000000365.7	Rnf17	0.0003	0.084
chr14	51548876	51548982	ENSMUSG00000035953.7	Tmem55b	2.90E-05	0.087
chr18	10593743	10595873	ENSMUSG00000024293.8	Esco1	5.55E-06	0.053
chr18	10595673	10595873	ENSMUSG00000024293.8	Esco1	3.94E-06	0.075
chr17	66391872	66391941	ENSMUSG00000034647.8	Ankrd12	9.43E-09	0.245
chr14	34215809	34215948	ENSMUSG00000021936.7	Mapk8	0.00014	0.052
chr14	33780658	33780838	ENSMUSG00000051506.10	Wdfy4	4.41E-05	0.313
chr14	31411903	31412032	ENSMUSG00000021948.8	Prkcd	2.74E-05	0.102
chr14	21177245	21177362	ENSMUSG00000039599.8	Fam149b	0.000412	-0.348
chr14	19462812	19462945	ENSMUSG00000058317.4	Ube2e2	0.000605	0.061
chr14	19110357	19110615	ENSMUSG00000021772.8	Nkiras1	0.0001	-0.087
chr13	1.09E+08	1.09E+08	ENSMUSG00000021694.11	Ercc8	0.000344	0.164
chr18	34657621	34657813	ENSMUSG00000036501.6	Fam13b	0	0.225
chr13	55573148	55573797	ENSMUSG00000034686.7	Prr7	0.000546	0.115
chr13	55132313	55132377	ENSMUSG00000025878.9	Uimc1	0	0.132
chr13	53440169	53440277	ENSMUSG00000021468.6	Sptlc1	7.38E-12	0.112
chr13	47163832	47164004	ENSMUSG00000038080.9	Kdm1b	0.00054	0.105
chr13	94156299	94156335	ENSMUSG00000007617.10	Homer1	4.29E-05	0.164
chr12	1.18E+08	1.18E+08	ENSMUSG00000042029.6	Ncapg2	1.44E-09	0.385
chr18	42461376	42461670	ENSMUSG00000024491.8	Rbm27	0.000234	0.08
chr12	1.14E+08	1.14E+08	ENSMUSG00000021144.8	Mta1	9.37E-07	0.175
chr12	1.14E+08	1.14E+08	ENSMUSG00000064326.6	Siva1	9.97E-14	0.107
chr12	1.13E+08	1.13E+08	ENSMUSG00000021282.9	Eif5	0	0.053
chr12	1.09E+08	1.09E+08	ENSMUSG00000056770.8	Setd3	0.000853	0.141
chr12	1.02E+08	1.02E+08	ENSMUSG00000021182.7	Ccdc88c	0.000104	0.088
chr12	1E+08	1E+08	ENSMUSG00000021012.9	Zc3h14	0	0.261
chr12	1E+08	1E+08	ENSMUSG00000021012.9	Zc3h14	0	0.251
chr12	99872433	99872529	ENSMUSG00000021007.7	Spata7	0.000255	-0.219
chr12	88610824	88610907	ENSMUSG00000021037.6	Ahsa1	0	0.064
chr12	88061179	88061613	ENSMUSG00000021257.8	Angel1	3.34E-06	0.242
chr12	86603716	86603812	ENSMUSG00000021245.7	Mlh3	3.69E-05	0.172
chr12	85643186	85643357	ENSMUSG00000072946.5	Ptgr2	6.47E-10	0.089
chr12	81743775	81743832	ENSMUSG00000021131.7	Erh	9.88E-06	0.071
chr12	74394685	74394786	ENSMUSG00000044712.8	Slc38a6	0.000795	0.292
chr12	74010890	74011078	ENSMUSG00000021098.8	4930447C(	0.000688	-0.101
chr12	71076954	71077225	ENSMUSG00000021067.7	Sav1	0	0.165
chr12	60118154	60118214	ENSMUSG00000060121.7	Sip1	0	0.131
chr12	25339707	25340641	ENSMUSG00000020653.5	Klf11	2.88E-06	0.094
chr12	11280799	11280917	ENSMUSG00000020608.6	Smc6	0.000656	-0.082
chr12	1.06E+08	1.06E+08	ENSMUSG00000041415.8	Dicer1	2.99E-06	0.051
chr11	1.21E+08	1.21E+08	ENSMUSG00000025144.10	Stra13	1.68E-07	0.093
chr11	1.21E+08	1.21E+08	ENSMUSG00000025144.10	Stra13	9.44E-08	0.096
chr11	1.21E+08	1.21E+08	ENSMUSG00000025144.10	Stra13	1.98E-10	0.148
chr11	1.21E+08	1.21E+08	ENSMUSG00000025142.11	Aspscr1	1.21E-06	0.061
chr11	1.2E+08	1.2E+08	ENSMUSG00000025138.8	Sirt7	4.30E-05	-0.365
chr11	1.2E+08	1.2E+08	ENSMUSG00000025372.10	Baiap2	1.76E-08	-0.143
chr11	1.19E+08	1.19E+08	ENSMUSG00000025579.8	Gaa	8.71E-06	0.056
chr11	1.18E+08	1.18E+08	ENSMUSG00000025572.10	Tmc6	0.000173	0.112
chr11	1.16E+08	1.16E+08	ENSMUSG00000015869.10	Prpsap1	3.61E-06	0.059
chr11	1.16E+08	1.16E+08	ENSMUSG00000020792.9	Exoc7	3.61E-06	0.099

chr14	1.04E+08	1.04E+08	ENSMUSG00000033004.7	Mycbp2	6.00E-10	0.076
chr14	1.04E+08	1.04E+08	ENSMUSG00000033004.7	Mycbp2	3.28E-07	0.053
chr11	1.05E+08	1.05E+08	ENSMUSG00000020694.9	Tlk2	2.44E-12	-0.188
chr11	1.04E+08	1.04E+08	ENSMUSG00000018411.10	Mapt	0.000602	-0.343
chr11	1.04E+08	1.04E+08	ENSMUSG00000020946.7	Gosr2	0.000688	0.196
chr11	1.01E+08	1.01E+08	ENSMUSG00000017119.12	Nbr1	2.66E-05	0.089
chr11	1.01E+08	1.01E+08	ENSMUSG00000017119.12	Nbr1	0.000297	0.089
chr17	78680585	78680706	ENSMUSG00000024074.7	Crim1	0.000361	0.448
chr11	99086034	99086456	ENSMUSG00000037935.9	Smarce1	5.28E-13	0.092
chr11	98703871	98703969	ENSMUSG00000038020.5	Rapgefl1	0.000493	0.194
chr11	97856380	97856441	ENSMUSG00000038352.9	Arl5c	0.000776	0.337
chr11	90396793	90396927	ENSMUSG00000020546.8	Stxbp4	1.60E-05	0.168
chr11	85174525	85174564	ENSMUSG00000059439.8	Bcas3	0.000162	-0.099
chr11	84708979	84709056	ENSMUSG00000020527.8	Myo19	0.000531	-0.393
chr11	84708979	84709056	ENSMUSG00000020527.8	Myo19	0.000889	-0.225
chr11	84667756	84667882	ENSMUSG00000020530.7	Ggnbp2	0	0.066
chr11	83298152	83298204	ENSMUSG00000020680.8	Taf15	0	0.219
chr11	82624529	82624613	ENSMUSG00000020696.11	Rffl	3.15E-06	0.28
chr11	82631917	82632105	ENSMUSG00000020696.11	Rffl	1.21E-05	0.182
chr11	82631917	82632105	ENSMUSG00000020696.11	Rffl	3.40E-08	0.119
chr11	82659253	82659397	ENSMUSG00000020696.11	Rffl	1.86E-06	0.62
chr16	14311139	14311224	ENSMUSG00000022677.8	0610037P(	8.97E-06	0.193
chr16	14313908	14314106	ENSMUSG00000022677.8	0610037P(	3.04E-09	0.212
chr17	83824597	83824771	ENSMUSG00000032624.8	Eml4	0.000474	-0.134
chr17	83824597	83824771	ENSMUSG00000032624.8	Eml4	0.000288	-0.219
chr17	83826562	83826691	ENSMUSG00000032624.8	Eml4	0.000268	-0.14
chr11	78096560	78096660	ENSMUSG00000010277.7	2610507B1	0	0.064
chr11	76934597	76934887	ENSMUSG00000050944.8	Efcab5	0.000786	-0.239
chr13	1.13E+08	1.13E+08	ENSMUSG00000021758.7	Ddx4	0	0.051
chr11	74759508	74759721	ENSMUSG00000038290.9	Smg6	3.02E-05	0.081
chr19	47724537	47724648	ENSMUSG00000025064.8	Col17a1	4.25E-05	0.437
chr11	70758346	70758472	ENSMUSG00000040667.4	Nup88	0	0.121
chr11	70758346	70758487	ENSMUSG00000040667.4	Nup88	0	0.156
chr11	70461909	70462094	ENSMUSG00000040746.9	Rnf167	0.000639	0.288
chr11	70461909	70462094	ENSMUSG00000040746.9	Rnf167	1.38E-06	0.058
chr11	70049197	70049302	ENSMUSG00000020831.10	0610010K1	0.000745	-0.209
chr11	69788541	69788762	ENSMUSG00000018565.10	Rai12	0.00073	0.058
chrX	11632764	11632818	ENSMUSG00000040363.8	Bcor	3.04E-05	0.341
chr13	1.05E+08	1.05E+08	ENSMUSG00000021712.8	Trim23	1.98E-08	-0.814
chr13	1.05E+08	1.05E+08	ENSMUSG00000021712.8	Trim23	0	0.201
chr11	69174046	69174277	ENSMUSG00000018474.10	Chd3	1.16E-11	0.09
chr11	68512711	68513087	ENSMUSG00000020900.9	Myh10	3.04E-05	0.118
chr11	62527166	62528878	ENSMUSG00000005267.6	Zfp287	1.97E-05	0.348
chr11	61359651	61360413	ENSMUSG00000001036.10	Epn2	1.38E-11	0.189
chr11	61361509	61361544	ENSMUSG00000001036.10	Epn2	2.23E-09	0.526
chr11	61361509	61361544	ENSMUSG00000001036.10	Epn2	4.12E-06	0.104
chr11	61367011	61367081	ENSMUSG00000001036.10	Epn2	2.90E-08	-0.466
chr11	61367011	61367081	ENSMUSG00000001036.10	Epn2	8.88E-05	-0.288
chr11	61368970	61369105	ENSMUSG00000001036.10	Epn2	1.64E-05	-0.457
chr11	61379323	61379396	ENSMUSG00000001036.10	Epn2	1.44E-09	0.399
chr11	61379323	61379396	ENSMUSG00000001036.10	Epn2	1.01E-05	0.1
chr11	60046399	60046459	ENSMUSG00000000538.11	Tom1l2	1.80E-06	0.089
chr11	59555492	59555600	ENSMUSG00000005417.10	Mprip	1.46E-13	-0.217
chr11	58123757	58123835	ENSMUSG00000037243.10	Zfp692	2.13E-11	0.337
chr11	57534796	57534899	ENSMUSG00000020520.8	Galnt10	0.000213	0.116
chr16	95598907	95598979	ENSMUSG00000040732.11	Erg	1.27E-06	-0.286
chr11	54569327	54569426	ENSMUSG00000052298.6	Cdc42se2	0.000712	0.108
chr11	54507771	54508074	ENSMUSG00000037533.10	Rapgef6	3.04E-05	0.187

chr11	78332681	78332870	ENSMUSG00000001100.4	Poldip2	5.55E-06	0.051
chr11	49981670	49981727	ENSMUSG00000036644.9	Tbc1d9b	0.000147	0.165
chr11	1.07E+08	1.07E+08	ENSMUSG00000040481.9	Bptf	0	-0.102
chr14	27762627	27762816	ENSMUSG00000040760.4	Appl1	0	0.08
chr11	32155438	32155568	ENSMUSG00000020289.8	Nprl3	0.000654	-0.179
chr11	20596753	20596834	ENSMUSG00000049659.7	Aftph	8.24E-10	0.151
chr11	6333726	6333840	ENSMUSG00000041126.8	H2afv	0	0.123
chr11	4385881	4385992	ENSMUSG00000034354.10	Mtmr3	0.000346	0.097
chr11	3194458	3194550	ENSMUSG00000020453.10	Patz1	0.000626	0.076
chr18	64642987	64643111	ENSMUSG00000024588.7	Fech	9.95E-05	-0.051
chr11	1.01E+08	1.01E+08	ENSMUSG00000010362.3	Rdm1	0.00062	0.078
chr16	31846919	31847023	ENSMUSG00000022770.9	Dlg1	1.52E-09	-0.3
chr16	31846922	31847019	ENSMUSG00000022770.9	Dlg1	2.44E-08	-0.333
chr16	31847721	31847755	ENSMUSG00000022770.9	Dlg1	0.000177	0.054
chr10	1.28E+08	1.28E+08	ENSMUSG00000025353.3	Ormdl2	0.000688	0.063
chr10	1.28E+08	1.28E+08	ENSMUSG00000025357.7	Dgka	0.000293	0.231
chr10	1.28E+08	1.28E+08	ENSMUSG00000005682.7	Pan2	5.90E-06	0.079
chr10	93563146	93563298	ENSMUSG00000020021.4	Fgd6	0.000509	0.066
chr10	87929183	87929301	ENSMUSG00000020059.3	Sycp3	0	0.056
chr10	80072385	80072542	ENSMUSG00000078441.3	Scamp4	0.00028	-0.076
chr10	79873142	79873366	ENSMUSG00000020167.8	Tcf3	5.08E-05	0.196
chr10	79873142	79873369	ENSMUSG00000020167.8	Tcf3	3.64E-07	0.154
chr10	79875571	79875807	ENSMUSG00000020167.8	Tcf3	6.42E-11	0.225
chr10	79855839	79856025	ENSMUSG00000035478.8	Mbd3	0	0.083
chr10	79591135	79592445	ENSMUSG00000003068.8	Stk11	2.23E-06	0.054
chr10	79004258	79004679	ENSMUSG00000042570.8	Mier2	1.42E-07	0.177
chr10	75867166	75867396	ENSMUSG00000001151.8	Pcnt	3.04E-06	0.114
chr10	62481507	62481646	ENSMUSG00000020069.9	Hnrnph3	1.01E-06	0.104
chr15	1.03E+08	1.03E+08	ENSMUSG00000046434.8	Hnrnpa1	4.86E-05	0.074
chr11	83000026	83001066	ENSMUSG00000000204.8	Slfn4	9.89E-07	-0.711
chr4	41063627	41063752	ENSMUSG00000028430.8	Nol6	1.95E-06	0.062
chr4	1.55E+08	1.55E+08	ENSMUSG00000023286.10	Ube2j2	5.07E-14	0.229
chr4	1.55E+08	1.55E+08	ENSMUSG00000051557.8	Pusl1	0.000998	0.22
chr4	1.55E+08	1.55E+08	ENSMUSG00000029068.9	Ccnl2	9.25E-05	0.139
chr1	1.93E+08	1.93E+08	ENSMUSG00000037474.7	Dtl	3.40E-05	0.071
chr4	1.54E+08	1.54E+08	ENSMUSG00000029049.7	Morn1	0.000764	-0.232
chr4	1.54E+08	1.54E+08	ENSMUSG00000029048.3	Rer1	0	0.073
chr4	1.54E+08	1.54E+08	ENSMUSG00000029048.3	Rer1	0	0.108
chr19	5665328	5665627	ENSMUSG00000054874.6	Pcnxl3	3.11E-06	0.063
chr15	99080893	99081013	ENSMUSG00000037570.9	Mcrs1	6.28E-06	0.092
chr15	99080893	99081013	ENSMUSG00000037570.9	Mcrs1	0.00086	0.087
chr17	71439162	71439347	ENSMUSG00000024049.7	Myom1	0.00062	-0.198
chr4	1.41E+08	1.41E+08	ENSMUSG00000078515.3	Ddi2	8.22E-11	-0.222
chr4	1.39E+08	1.39E+08	ENSMUSG00000066036.7	Ubr4	0.000652	0.078
chr4	1.37E+08	1.37E+08	ENSMUSG00000057530.7	Ece1	0.000537	0.342
chr5	42089628	42089717	ENSMUSG00000029128.8	Rab28	1.71E-09	0.093
chr15	99567977	99568084	ENSMUSG00000023021.8	Lass5	4.05E-10	0.054
chr5	92854648	92854836	ENSMUSG00000034826.8	Nup54	1.05E-06	0.094
chr4	1.33E+08	1.33E+08	ENSMUSG00000028860.7	Sytl1	0.000144	0.453
chr15	78632810	78633062	ENSMUSG00000033170.7	Card10	0.000462	0.187
chr11	51012077	51012218	ENSMUSG00000086646.1	5133400J0	7.25E-05	0.614
chr3	30511390	30511474	ENSMUSG00000037730.7	Mynn	8.84E-05	0.209
chr2	1.73E+08	1.73E+08	ENSMUSG00000055897.7	Ppp4r1l-p	5.75E-05	0.738
chr2	1.73E+08	1.73E+08	ENSMUSG00000055897.7	Ppp4r1l-p	0.00045	0.203
chr2	1.73E+08	1.73E+08	ENSMUSG00000055897.7	Ppp4r1l-p	0.000113	0.504
chr9	1.01E+08	1.01E+08	ENSMUSG00000037286.8	Stag1	0	0.31
chr4	1.16E+08	1.16E+08	ENSMUSG00000028689.8	Ccdc163	0.000382	-0.187
chr4	1.16E+08	1.16E+08	ENSMUSG00000028689.8	Ccdc163	4.54E-07	-0.608

chr16	11151958	11152023	ENSMUSG00000037965.8	Zc3h7a	0.000374	0.21
chr1	1.3E+08	1.3E+08	ENSMUSG00000056211.6	R3hdm1	1.68E-05	0.62
chr4	1.17E+08	1.17E+08	ENSMUSG00000033423.9	Eri3	3.63E-06	0.08
chr10	79427670	79427804	ENSMUSG00000035754.7	Wdr18	0.000445	0.066
chr10	79427954	79428096	ENSMUSG00000035754.7	Wdr18	0.000361	0.094
chr9	78300570	78300860	ENSMUSG00000032342.7	Mto1	5.66E-05	0.145
chr7	53996625	53996764	ENSMUSG00000074115.3	Saa1	0.000478	0.332
chr6	83070765	83070867	ENSMUSG00000030035.8	Wbp1	0.000964	0.123
chr6	83070765	83070870	ENSMUSG00000030035.8	Wbp1	0.00056	0.087
chr4	94628393	94628524	ENSMUSG00000062627.3	Mysm1	0	0.369
chr2	1.25E+08	1.25E+08	ENSMUSG00000027201.10	Myef2	2.67E-12	0.171
chr2	1.25E+08	1.25E+08	ENSMUSG00000027201.10	Myef2	1.26E-05	0.115
chr7	52440647	52440699	ENSMUSG00000038292.11	Ccdc155	5.25E-08	0.443
chr7	52450898	52450963	ENSMUSG00000038292.11	Ccdc155	7.66E-05	0.337
chr1	1.72E+08	1.72E+08	ENSMUSG00000026670.9	Uap1	0.000371	-0.181
chr19	7448785	7448935	ENSMUSG00000024970.4	AI846148	2.93E-08	0.078
chrX	9753128	9754007	ENSMUSG00000031174.10	Rpgr	7.98E-06	0.209
chr4	83441103	83441235	ENSMUSG00000052407.7	4930473A(	0.000188	0.179
chr8	1.08E+08	1.08E+08	ENSMUSG00000038000.8	Acd	8.57E-08	0.177
chr17	80752019	80752079	ENSMUSG00000054901.5	Arhgef33	6.04E-05	0.364
chr4	1.16E+08	1.16E+08	ENSMUSG00000028698.7	Pik3r3	1.01E-05	0.069
chr13	38124233	38124493	ENSMUSG00000044566.9	Cage1	0.000285	-0.388
chr4	48196286	48196348	ENSMUSG00000061455.6	Stx17	1.76E-05	0.667
chr14	25310971	25311037	ENSMUSG00000025290.9	Rps24	2.16E-07	0.516
chr2	5725956	5726079	ENSMUSG00000039128.6	Cdc123	1.35E-06	0.092
chr4	40642409	40642451	ENSMUSG00000028411.9	Aptx	7.91E-05	0.072
chr5	1.07E+08	1.07E+08	ENSMUSG00000049606.9	Zfp644	5.98E-06	0.397
chr5	1.07E+08	1.07E+08	ENSMUSG00000049606.9	Zfp644	9.44E-08	-0.722
chr4	32785745	32785887	ENSMUSG00000058006.5	Mdn1	0	0.286
chr17	25045355	25045373	ENSMUSG00000024163.10	Mapk8ip3	0.000551	0.083
chr17	25046094	25046106	ENSMUSG00000024163.10	Mapk8ip3	1.40E-05	0.133
chr9	8023899	8023966	ENSMUSG00000053070.3	9230110C:	0.00064	0.167
chr3	88628271	88628456	ENSMUSG00000078684.5	5830417I1	1.23E-07	0.091
chr4	1.26E+08	1.26E+08	ENSMUSG00000043962.9	Thrap3	0	-0.091
chr4	1.26E+08	1.26E+08	ENSMUSG00000043962.9	Thrap3	1.40E-10	-0.135
chr4	1.26E+08	1.26E+08	ENSMUSG00000043962.9	Thrap3	0	-0.214
chr4	1.26E+08	1.26E+08	ENSMUSG00000043962.9	Thrap3	4.91E-13	-0.131
chr16	87494581	87494672	ENSMUSG00000025613.6	Cct8	0	0.118
chr5	1.07E+08	1.07E+08	ENSMUSG00000043410.9	Hfm1	1.69E-06	0.191
chr3	1.35E+08	1.35E+08	ENSMUSG00000045328.7	Cenpe	1.02E-07	0.099
chr7	1.34E+08	1.34E+08	ENSMUSG00000030714.8	Ccdc101	1.48E-09	0.641
chr13	24114364	24114505	ENSMUSG00000021338.10	Lrrc16a	0.000493	0.14
chr10	84454864	84455009	ENSMUSG00000035620.7	Ric8b	0.000795	0.075
chr4	1.41E+08	1.41E+08	ENSMUSG00000028914.7	Casp9	3.45E-06	0.097
chr4	1.41E+08	1.41E+08	ENSMUSG00000028914.7	Casp9	5.16E-07	0.146
chr16	90776369	90776549	ENSMUSG00000039929.8	Urb1	6.98E-07	0.272
chr3	1.03E+08	1.03E+08	ENSMUSG00000068823.5	Csde1	0.000192	-0.099
chr2	25133843	25134025	ENSMUSG00000026965.6	Anapc2	2.78E-09	0.058
chr11	59278513	59278574	ENSMUSG00000054519.7	Zfp867	4.84E-10	0.194
chr4	32711058	32711141	ENSMUSG00000028282.5	Casp8ap2	9.23E-05	0.156
chr5	8494783	8494905	ENSMUSG00000040570.8	Rundc3b	0.00071	0.088
chr8	98240252	98240394	ENSMUSG00000031671.4	Setd6	2.98E-05	0.157
chr6	29881763	29881829	ENSMUSG00000039629.8	Fam40b	1.23E-05	0.131
chr3	95366401	95366546	ENSMUSG00000028109.8	Hormad1	4.15E-09	0.123
chr3	95374587	95374608	ENSMUSG00000028109.8	Hormad1	0	0.188
chr3	94994474	94994660	ENSMUSG00000038766.9	Gabpb2	0.000239	0.083
chr3	95008562	95008730	ENSMUSG00000038766.9	Gabpb2	4.76E-09	0.12
chr6	1.35E+08	1.35E+08	ENSMUSG00000032641.10	Gpr19	0.000274	-0.514

chr3	30924051	30924141	ENSMUSG00000037643.8	Prkci	0.000232	0.063
chr12	1.07E+08	1.07E+08	ENSMUSG00000021115.8	Vrk1	1.83E-10	0.238
chr18	6031727	6031826	ENSMUSG00000041225.8	Arhgap12	7.18E-05	0.066
chr7	29107021	29107158	ENSMUSG00000003435.8	Supt5h	0	0.09
chr16	32900718	32900793	ENSMUSG00000022800.7	Fyttd1	2.42E-08	0.082
chr2	25860845	25860984	ENSMUSG00000036352.10	Ubac1	0.000153	0.065
chr5	1.02E+08	1.02E+08	ENSMUSG00000029330.8	Cds1	3.70E-07	0.234
chr1	89837943	89838074	ENSMUSG00000070738.4	Dgkd	4.80E-09	0.129
chr15	96528352	96528468	ENSMUSG00000022462.6	Slc38a2	1.78E-05	0.078
chrX	1.51E+08	1.51E+08	ENSMUSG00000079349.3	Magea5	0.000187	0.245
chr6	1.47E+08	1.47E+08	ENSMUSG00000040242.8	Fgfr1op2	0	0.264
chr4	43667777	43667918	ENSMUSG00000028470.4	Hint2	9.62E-08	0.051
chr8	1.13E+08	1.13E+08	ENSMUSG00000044676.8	Zfp612	4.71E-07	0.353
chr3	33706760	33706870	ENSMUSG00000027677.10	Ttc14	0.000158	0.075
chr3	33706775	33706870	ENSMUSG00000027677.10	Ttc14	2.39E-05	0.105
chr3	33706777	33706870	ENSMUSG00000027677.10	Ttc14	7.09E-05	0.087
chr7	1.27E+08	1.27E+08	ENSMUSG00000030929.9	Eri2	0	0.457
chr3	19930558	19930805	ENSMUSG00000027615.7	Hps3	0.000853	0.169
chr3	1.16E+08	1.16E+08	ENSMUSG00000033400.8	Agl	0.000763	0.072
chr2	1.78E+08	1.78E+08	ENSMUSG00000060445.5	Sycp2	0.000293	0.091
chr16	5081986	5082076	ENSMUSG00000039473.6	Ubn1	1.16E-10	-0.237
chrX	51098644	51098679	ENSMUSG00000054626.5	Xlr	3.61E-09	0.175
chrX	51099319	51099397	ENSMUSG00000054626.5	Xlr	0	0.424
chrX	51099319	51099445	ENSMUSG00000054626.5	Xlr	0	0.532
chr16	14447570	14447738	ENSMUSG00000023088.9	Abcc1	0.000832	0.155
chr16	14448492	14448676	ENSMUSG00000023088.9	Abcc1	7.70E-05	0.132
chr3	95430670	95430796	ENSMUSG00000038619.6	Ensa	0	0.124
chr19	25620308	25620592	ENSMUSG00000024837.8	Dmrt1	0	0.052
chr3	84333091	84333187	ENSMUSG00000074513.3	Arfip1	4.83E-05	-0.13
chr3	84333112	84333187	ENSMUSG00000074513.3	Arfip1	4.04E-05	-0.08
chr11	5737291	5737481	ENSMUSG00000020474.5	Polm	1.08E-06	-0.155
chr18	61968585	61968615	ENSMUSG00000032735.8	Ablim3	0.000273	-0.62
chr2	1.65E+08	1.65E+08	ENSMUSG00000017307.9	Acot8	0.000106	-0.182
chr4	56772672	56772786	ENSMUSG00000028431.5	Ikbkap	0	0.146
chr2	1.27E+08	1.27E+08	ENSMUSG00000001999.9	Blvra	0.00013	0.067
chr1	1.53E+08	1.53E+08	ENSMUSG00000023150.8	Ivns1abp	1.71E-07	0.051
chr1	58504477	58504933	ENSMUSG00000026036.10	Nif3l1	3.68E-07	0.075
chr1	1.82E+08	1.82E+08	ENSMUSG00000026490.10	Cdc42bpa	0	0.09
chr11	87298201	87298367	ENSMUSG00000010342.9	Tex14	1.00E-05	0.148
chr11	87362933	87363026	ENSMUSG00000010342.9	Tex14	0	0.14
chr12	78054155	78054263	ENSMUSG00000059436.5	Max	0.000641	0.073
chr17	56451476	56451692	ENSMUSG00000001228.8	Uhrf1	5.07E-14	0.083
chr2	25059717	25059820	ENSMUSG00000013465.11	Cobra1	2.35E-13	0.137
chr2	25060646	25060759	ENSMUSG00000013465.11	Cobra1	1.57E-12	0.143
chr2	1.3E+08	1.3E+08	ENSMUSG00000037885.11	Stk35	3.32E-06	0.272
chr2	1.81E+08	1.81E+08	ENSMUSG00000038671.7	Arfrp1	3.44E-06	0.17
chr2	1.81E+08	1.81E+08	ENSMUSG00000038671.7	Arfrp1	1.06E-05	0.168
chr2	1.81E+08	1.81E+08	ENSMUSG00000038671.7	Arfrp1	6.27E-08	0.102
chr9	47637459	47637492	ENSMUSG00000032076.11	Cadm1	2.09E-05	0.118
chr5	31174093	31174228	ENSMUSG00000038828.7	Tmem214	0.00012	0.058
chr1	1.93E+08	1.93E+08	ENSMUSG00000026634.9	Angel2	5.87E-08	0.099
chr16	31101910	31101972	ENSMUSG00000049076.8	Acap2	2.34E-08	0.116
chr15	79415291	79415406	ENSMUSG00000022429.9	Dmc1	0	0.299
chr9	1.14E+08	1.14E+08	ENSMUSG00000033392.8	Clasp2	1.24E-09	-0.19
chr6	71803166	71803262	ENSMUSG00000052337.8	Immt	0.000837	-0.159
chr12	1.07E+08	1.07E+08	ENSMUSG00000021111.8	Papola	6.96E-07	0.105
chr6	1.16E+08	1.16E+08	ENSMUSG00000030323.7	Ift122	7.25E-05	0.387
chr10	1.22E+08	1.22E+08	ENSMUSG00000034620.10	Tmem5	1.25E-08	0.096

chr1	1.78E+08	1.78E+08	ENSMUSG00000039748.6	Exo1	0	0.294
chr4	1.52E+08	1.52E+08	ENSMUSG00000039662.10	Icmt	2.23E-06	0.183
chr9	1.11E+08	1.11E+08	ENSMUSG00000032497.8	Lrrfip2	3.28E-05	0.556
chr1	1.65E+08	1.65E+08	ENSMUSG00000040225.7	Prrc2c	1.01E-06	0.135
chr9	1.1E+08	1.1E+08	ENSMUSG00000054792.8	Klhl18	0.000112	0.098
chr5	1.36E+08	1.36E+08	ENSMUSG00000019178.10	Styxl1	0.000316	0.584
chr2	66024210	66024278	ENSMUSG00000034848.10	Ttc21b	8.34E-05	0.092
chr1	1.68E+08	1.68E+08	ENSMUSG00000026566.9	Mpzl1	0.000268	0.099
chr1	1.68E+08	1.68E+08	ENSMUSG00000026566.9	Mpzl1	0.00025	0.234
chr1	1.55E+08	1.55E+08	ENSMUSG00000042772.8	Smg7	0	0.161
chr5	3954396	3954450	ENSMUSG00000040407.9	Akap9	5.06E-10	-0.23
chr7	17607763	17608005	ENSMUSG00000003099.5	Ppp5c	7.37E-13	-0.148
chr5	1.11E+08	1.11E+08	ENSMUSG00000029505.9	Ep400	0.000222	0.094
chr3	87536581	87536794	ENSMUSG00000041977.10	Arhgef11	0.000515	0.062
chr3	95685103	95685202	ENSMUSG00000038550.4	Gm129	1.97E-05	0.257
chr3	95685103	95685512	ENSMUSG00000038550.4	Gm129	1.43E-07	0.175
chr5	1.09E+08	1.09E+08	ENSMUSG00000033623.7	Pcgf3	7.94E-09	0.062
chr4	1.38E+08	1.38E+08	ENSMUSG00000028760.9	Eif4g3	7.24E-05	0.203
chr11	1.18E+08	1.18E+08	ENSMUSG00000025571.7	Tnrc6c	0.000133	-0.472
chr1	1.84E+08	1.84E+08	ENSMUSG00000022995.9	Enah	0.000259	-0.07
chr1	1.84E+08	1.84E+08	ENSMUSG00000022995.9	Enah	0.000209	-0.133
chr2	69569547	69569637	ENSMUSG00000042133.10	Ppig	0.000696	0.089
chr2	69569547	69569637	ENSMUSG00000042133.10	Ppig	5.07E-14	0.391
chr2	69569802	69569870	ENSMUSG00000042133.10	Ppig	0	0.263
chr19	3782219	3782279	ENSMUSG00000045098.10	Suv420h1	1.25E-08	0.165
chr1	44148205	44148436	ENSMUSG00000026049.5	1700029F(	0	0.104
chr1	44150108	44150162	ENSMUSG00000026049.5	1700029F(	0	0.35
chr2	12317805	12317885	ENSMUSG00000026767.6	Fam188a	0.00023	0.615
chr2	1.8E+08	1.8E+08	ENSMUSG00000039108.7	Lsm14b	0.000887	0.056
chr1	71113681	71113762	ENSMUSG00000026196.7	Bard1	3.50E-07	0.173
chr16	18808824	18808962	ENSMUSG00000000028.8	Cdc45	2.03E-09	0.171
chr1	51823665	51823752	ENSMUSG00000018417.8	Myo1b	7.69E-11	0.291
chr1	51825493	51825580	ENSMUSG00000018417.8	Myo1b	3.11E-12	0.563
chr4	21786230	21786454	ENSMUSG00000028248.6	Sfrs18	0.000204	0.204
chr1	60274039	60274125	ENSMUSG00000073664.4	Nbeal1	0.00013	0.285
chr1	60285465	60285552	ENSMUSG00000073664.4	Nbeal1	5.96E-10	-0.488
chr16	18405194	18405314	ENSMUSG00000000325.8	Arvcf	0.000169	0.153
chr7	1.51E+08	1.51E+08	ENSMUSG00000059119.7	Nap1l4	3.35E-05	-0.1
chr3	1.09E+08	1.09E+08	ENSMUSG00000027883.8	Gpsm2	3.71E-05	0.187
chr2	1.67E+08	1.67E+08	ENSMUSG00000074582.4	Arfgef2	0.000883	0.219
chr1	39615159	39615256	ENSMUSG00000048234.8	Rnf149	5.47E-08	0.126
chr1	1.8E+08	1.8E+08	ENSMUSG00000026500.6	Fam36a	1.73E-05	0.068
chr17	56528681	56528765	ENSMUSG00000024201.6	Kdm4b	0.000948	0.055
chr15	79596216	79596283	ENSMUSG00000022420.8	Dnalc4	2.60E-09	-0.225
chr3	1.33E+08	1.33E+08	ENSMUSG00000028013.10	Ppa2	3.21E-05	0.053
chr7	63401906	63402099	ENSMUSG00000030451.8	Herc2	1.28E-06	0.073
chr7	63457470	63457578	ENSMUSG00000030451.8	Herc2	0	0.23
chr14	34444092	34444427	ENSMUSG00000021940.8	Ptpn20	1.75E-05	0.51
chr1	1.33E+08	1.33E+08	ENSMUSG00000026427.9	Eif2d	0.000605	-0.056
chr11	87402469	87402587	ENSMUSG00000020486.11	4-Sep	0.000478	0.184
chr2	1.51E+08	1.51E+08	ENSMUSG00000027455.10	Nsfl1c	5.46E-06	-0.136
chr2	93696060	93696137	ENSMUSG00000075023.4	Accsl	4.04E-06	-0.575
chr1	9871840	9871972	ENSMUSG00000025915.7	Sgk3	4.87E-07	0.075
chr18	54108298	54108322	ENSMUSG00000073563.2	Csnk1g3	6.22E-05	-0.165
chr1	46142399	46142547	ENSMUSG00000041144.8	Dnahc7b	0.000546	0.425
chr2	93661766	93661838	ENSMUSG00000027198.9	Ext2	3.52E-06	0.124
chr9	66309506	66309706	ENSMUSG00000038664.9	Herc1	0.000165	0.062
chr9	66309506	66309727	ENSMUSG00000038664.9	Herc1	0.000192	0.057

chr1	1.91E+08	1.91E+08	ENSMUSG00000026608.6	Kctd3	9.37E-07	0.069
chr1	1.91E+08	1.91E+08	ENSMUSG00000026608.6	Kctd3	2.67E-05	0.081
chr4	34580906	34580962	ENSMUSG00000028292.8	Rars2	0.000374	0.125
chr6	83928015	83928081	ENSMUSG00000030016.7	Zfml	4.90E-05	-0.461
chr6	83928015	83928081	ENSMUSG00000030016.7	Zfml	0.000951	-0.294
chr6	84014464	84014557	ENSMUSG00000033788.9	Dysf	0.000541	0.449
chr3	1.52E+08	1.52E+08	ENSMUSG00000028034.8	Fubp1	5.07E-14	0.085
chr17	15369743	15369846	ENSMUSG00000087655.1	Gm5091	2.35E-05	-0.352
chr17	15370892	15370966	ENSMUSG00000087655.1	Gm5091	7.02E-05	-0.333
chr4	1.47E+08	1.47E+08	ENSMUSG00000078498.4	Gm13151	3.21E-05	0.568
chrX	91604784	91604879	ENSMUSG00000043549.7	4932442L(	1.80E-06	-0.266
chr7	51746532	51746619	ENSMUSG00000008140.10	2310044H	1.34E-05	-0.147
chr7	51748559	51748669	ENSMUSG00000008140.10	2310044H	1.90E-13	0.103
chr10	80174856	80174952	ENSMUSG00000020198.7	Ap3d1	0	0.082
chr3	89247022	89247170	ENSMUSG00000042613.3	Pbxip1	2.09E-07	0.216
chr3	89247043	89247170	ENSMUSG00000042613.3	Pbxip1	1.87E-07	0.137
chr7	1.4E+08	1.4E+08	ENSMUSG00000030960.10	Mettl10	0.000543	0.114
chr9	1.07E+08	1.07E+08	ENSMUSG00000032579.8	Hemk1	2.77E-08	0.484
chr9	1.07E+08	1.07E+08	ENSMUSG00000032579.8	Hemk1	0.000155	0.12
chr4	1.55E+08	1.55E+08	ENSMUSG00000059939.7	9430015G	5.09E-07	0.374
chr5	1.16E+08	1.16E+08	ENSMUSG00000060152.8	Pop5	8.22E-07	0.099
chr2	32084782	32084864	ENSMUSG00000039262.9	Prrc2b	3.19E-08	0.224
chr7	1.33E+08	1.33E+08	ENSMUSG00000030752.2	Jmjd5	4.40E-05	0.267
chr7	1.33E+08	1.33E+08	ENSMUSG00000030752.2	Jmjd5	2.09E-06	-0.623
chr2	30249166	30249373	ENSMUSG00000026856.8	Dolpp1	0.000612	0.064
chr2	1.4E+08	1.4E+08	ENSMUSG00000039033.5	Tasp1	3.16E-07	0.214
chr2	1.4E+08	1.4E+08	ENSMUSG00000039033.5	Tasp1	5.07E-14	0.238
chr12	70275378	70276100	ENSMUSG00000034883.7	Lrr1	6.20E-05	0.248
chr2	35016040	35016181	ENSMUSG00000057110.8	Cep110	0.000229	0.103
chr2	35016310	35016437	ENSMUSG00000057110.8	Cep110	9.86E-05	0.114
chr2	35016310	35016437	ENSMUSG00000057110.8	Cep110	4.99E-05	0.098
chr9	1.22E+08	1.22E+08	ENSMUSG00000032525.6	Nktr	0.000186	-0.096
chr2	25228935	25229037	ENSMUSG00000026955.7	2010317E2	3.02E-07	0.41
chr14	59971487	59971619	ENSMUSG00000068245.7	D14Ertd66	0.00055	0.148
chr15	82069722	82069814	ENSMUSG00000068101.4	Cenpm	7.35E-09	0.11
chr1	1.87E+08	1.87E+08	ENSMUSG00000039318.7	Rab3gap2	0.000531	0.084
chr3	1.42E+08	1.42E+08	ENSMUSG00000028273.8	Pdlim5	2.54E-07	0.172
chr5	1.15E+08	1.15E+08	ENSMUSG00000041890.10	Git2	2.98E-05	0.062
chr11	1.2E+08	1.2E+08	ENSMUSG00000039741.9	Bahcc1	2.96E-05	-0.316
chr7	87398464	87398608	ENSMUSG00000045467.7	Ttll13	0.000227	0.535
chr11	78213585	78213707	ENSMUSG00000001095.5	Slc13a2	3.85E-05	0.439
chr9	45707828	45708339	ENSMUSG00000042790.8	Rnf214	0.000322	0.155
chr1	82738392	82738551	ENSMUSG00000026150.7	Mff	8.49E-05	-0.081
chr1	82743657	82743717	ENSMUSG00000026150.7	Mff	1.34E-06	-0.143
chr9	78264469	78264557	ENSMUSG00000070291.3	Ddx43	0.000181	0.167
chr7	1.09E+08	1.09E+08	ENSMUSG00000066306.5	Numa1	0.000445	-0.128
chr19	27997697	27997756	ENSMUSG00000040929.9	Rfx3	9.03E-05	0.499
chr19	28057343	28057436	ENSMUSG00000040929.9	Rfx3	0.000945	-0.412
chr17	50807323	50807433	ENSMUSG00000038910.6	Plcl2	9.15E-05	0.068
chr1	43167842	43168218	ENSMUSG00000010290.7	AI597479	1.24E-05	0.132
chr1	65315271	65315448	ENSMUSG00000025949.9	Pikfyve	1.44E-10	0.109
chr2	26811808	26811883	ENSMUSG00000052406.8	Rexo4	0.000502	0.053
chr4	1.17E+08	1.17E+08	ENSMUSG00000028683.7	Eif2b3	0.000153	0.086
chr2	1.44E+08	1.44E+08	ENSMUSG00000037259.8	6330439K	4.82E-07	-0.559
chr14	19052481	19052570	ENSMUSG00000021775.9	Nr1d2	5.54E-05	0.077
chr8	72924517	72924603	ENSMUSG00000003575.7	Crtc1	1.66E-06	0.272
chr8	72932793	72932910	ENSMUSG00000003575.7	Crtc1	4.74E-05	0.147
chr2	29992640	29992702	ENSMUSG00000039678.6	Tbc1d13	0.000172	0.551

chr2	29992801	29992901	ENSMUSG00000039678.6	Tbc1d13	6.52E-06	0.134
chr9	13588049	13588154	ENSMUSG00000031918.9	Mtmr2	0.000435	-0.131
chr6	30377708	30377795	ENSMUSG00000029775.8	Klhdc10	0	0.291
chr2	76195629	76195658	ENSMUSG00000056436.4	Cyct	0	0.243
chr3	75418375	75418606	ENSMUSG00000027834.9	Serpini1	0.000491	0.093
chr6	30611722	30611855	ENSMUSG00000029790.9	Tsga14	0.000315	-0.09
chr10	30310826	30310908	ENSMUSG00000019792.6	Trmt11	1.41E-11	0.478
chr10	30313920	30313994	ENSMUSG00000019792.6	Trmt11	0.000127	0.282
chr2	33283620	33283740	ENSMUSG00000068966.4	Zbtb34	0.000106	-0.268
chr2	34590070	34590127	ENSMUSG00000026867.11	Gapvd1	0.000203	-0.064
chr16	49013909	49013984	ENSMUSG00000033031.7	C330027C	9.81E-06	0.065
chr8	1.26E+08	1.26E+08	ENSMUSG00000032815.9	Fanca	9.01E-05	0.089
chr11	60689261	60689549	ENSMUSG00000043284.6	Tmem11	4.64E-08	-0.073
chr2	1.14E+08	1.14E+08	ENSMUSG00000057147.7	Atpbd4	2.11E-05	0.198
chr3	1.19E+08	1.19E+08	ENSMUSG00000028134.7	Ptbp2	2.93E-10	0.291
chrY	2397908	2397997	ENSMUSG00000091987.1	Gm3376	0.000174	0.202
chr11	88876846	88876945	ENSMUSG00000000275.9	Trim25	3.68E-07	0.194
chr3	1.33E+08	1.33E+08	ENSMUSG00000040998.12	Npnt	0.000616	0.205
chr9	1.1E+08	1.1E+08	ENSMUSG00000032480.10	Dhx30	0.000419	-0.123
chr5	8403079	8403191	ENSMUSG00000002297.9	Dbf4	5.98E-06	0.082
chr2	60293142	60293288	ENSMUSG00000054580.7	Pla2r1	0.000347	-0.182
chr15	31539320	31539371	ENSMUSG00000039065.9	Fam173b	1.58E-05	-0.071
chr2	70889728	70889790	ENSMUSG00000041975.10	Mettl8	5.36E-08	-0.141
chr2	1.55E+08	1.55E+08	ENSMUSG00000038324.7	Trpc4ap	0	-0.122
chr11	62158736	62158820	ENSMUSG00000018501.10	Ncor1	0.000712	0.069
chr2	73222124	73222249	ENSMUSG00000041762.8	Gpr155	0.000194	0.285
chr8	83511385	83511492	ENSMUSG00000038250.7	Usp38	9.81E-06	0.119
chr2	77079669	77079778	ENSMUSG00000042272.10	Sestd1	3.11E-05	0.102
chr16	16875161	16875248	ENSMUSG00000022779.8	Top3b	4.24E-05	-0.224
chr16	16875164	16875248	ENSMUSG00000022779.8	Top3b	0.000701	-0.204
chr16	16875164	16875248	ENSMUSG00000022779.8	Top3b	4.87E-05	-0.216
chr19	3875734	3875788	ENSMUSG00000024843.9	Chka	0.00082	-0.123
chr18	60991819	60991963	ENSMUSG00000024613.8	Tcof1	3.11E-07	0.275
chr3	1.05E+08	1.05E+08	ENSMUSG00000002227.8	Mov10	0.000891	0.371
chr11	1.16E+08	1.16E+08	ENSMUSG00000020776.12	Fbf1	3.98E-09	0.19
chr10	80538255	80538404	ENSMUSG00000004929.6	Thop1	0.00082	0.105
chr11	87201749	87201829	ENSMUSG00000007646.7	Rad51c	1.13E-07	0.12
chr11	80069363	80069459	ENSMUSG00000017686.10	Rhot1	1.11E-11	0.204
chr11	80069363	80069459	ENSMUSG00000017686.10	Rhot1	0.000164	0.113
chr11	80071015	80071138	ENSMUSG00000017686.10	Rhot1	0.000456	-0.198
chr1	1.37E+08	1.37E+08	ENSMUSG00000073557.4	Ppp1r12b	0.000781	-0.443
chr9	1.09E+08	1.09E+08	ENSMUSG00000032599.6	Ip6k2	0.000149	0.27
chr8	26881550	26881805	ENSMUSG00000037316.8	Bag4	2.32E-11	0.114
chr2	1.56E+08	1.56E+08	ENSMUSG00000027618.10	Nfs1	9.15E-12	0.127
chr3	87961955	87962093	ENSMUSG00000001419.10	Mef2d	0	0.327
chr2	90738540	90738660	ENSMUSG00000005510.3	Ndufs3	0	0.194
chr2	91000473	91000527	ENSMUSG00000040687.9	Madd	0.000565	0.175
chr12	83533739	83533979	ENSMUSG00000042700.8	Sipa1l1	0	0.127
chr8	74765622	74765837	ENSMUSG00000003033.8	Ap1m1	2.75E-06	-0.068
chr6	1.49E+08	1.49E+08	ENSMUSG00000039985.8	Fam60a	0.000373	-0.224
chr3	1.46E+08	1.46E+08	ENSMUSG00000028188.7	Spata1	1.03E-05	0.298
chr2	1.74E+08	1.74E+08	ENSMUSG00000027522.8	Stx16	4.77E-05	0.065
chr11	1.06E+08	1.06E+08	ENSMUSG00000019590.9	Cyb561	1.06E-07	0.197
chr2	50136092	50136178	ENSMUSG00000026766.10	Mmadhc	1.47E-07	-0.059
chr2	1.64E+08	1.64E+08	ENSMUSG00000017721.8	Pigt	1.24E-05	0.077
chr2	1.64E+08	1.64E+08	ENSMUSG00000017721.8	Pigt	7.24E-06	0.07
chr8	73779545	73779611	ENSMUSG00000035439.7	Haus8	0	0.391
chr2	1.21E+08	1.21E+08	ENSMUSG00000033705.9	Stard9	4.28E-06	0.248

chr2	1.21E+08	1.21E+08	ENSMUSG00000033705.9	Stard9	1.20E-05	0.186
chr15	88585189	88585363	ENSMUSG00000034333.9	Zbed4	0.000789	-0.094
chr12	87365169	87365292	ENSMUSG00000012609.10	Ttll5	4.16E-06	0.248
chr4	57089785	57089874	ENSMUSG00000028434.6	Epb4.1l4b	2.11E-05	0.153
chr15	77061816	77061867	ENSMUSG00000033565.9	Rbfox2	2.29E-10	0.225
chr2	1.21E+08	1.21E+08	ENSMUSG00000043909.9	Trp53bp1	6.75E-05	0.062
chr2	1.21E+08	1.21E+08	ENSMUSG00000043909.9	Trp53bp1	0.0003	-0.135
chr4	46002921	46003102	ENSMUSG00000035517.10	Tdrd7	2.43E-05	0.093
chr4	46007178	46007396	ENSMUSG00000035517.10	Tdrd7	0.000909	0.097
chr2	1.27E+08	1.27E+08	ENSMUSG00000046338.3	Gpat2	8.59E-08	0.108
chr4	1.04E+08	1.04E+08	ENSMUSG00000028519.9	Dab1	0.000931	0.464
chr4	1.04E+08	1.04E+08	ENSMUSG00000028519.9	Dab1	6.46E-05	0.465
chr2	1.61E+08	1.61E+08	ENSMUSG00000027412.6	Lpin3	8.13E-05	0.099
chr2	1.3E+08	1.3E+08	ENSMUSG00000027405.10	Nop56	0.000203	-0.056
chr12	29343035	29343160	ENSMUSG00000020630.7	Rnaseh1	6.78E-05	0.159
chr13	59632417	59632564	ENSMUSG00000021557.7	Agtpbp1	9.34E-07	-0.155
chr11	23373169	23373294	ENSMUSG00000056342.9	Usp34	0	0.1
chr6	1.14E+08	1.14E+08	ENSMUSG00000056952.7	Tatdn2	2.61E-07	0.087
chr2	25107240	25107338	ENSMUSG00000006471.10	Ndor1	0.000325	0.123
chr2	25107240	25107338	ENSMUSG00000006471.10	Ndor1	6.07E-05	0.163
chr2	25107250	25107338	ENSMUSG00000006471.10	Ndor1	0.000764	0.279
chr11	1.02E+08	1.02E+08	ENSMUSG00000018677.3	Slc25a39	0	0.072
chr9	7142856	7143012	ENSMUSG00000047193.8	Dync2h1	3.60E-13	0.37
chrX	1.31E+08	1.31E+08	ENSMUSG00000031262.6	Cenpi	0.000145	-0.309
chr4	1.32E+08	1.32E+08	ENSMUSG00000028896.7	Rcc1	0	0.143
chr2	44607566	44607740	ENSMUSG00000036890.6	Gtdc1	0.000374	0.092
chr2	44611787	44611955	ENSMUSG00000036890.6	Gtdc1	0.000471	0.111
chr2	90885882	90885975	ENSMUSG00000086495.1	Gm13778	0.00052	0.425
chr2	1.58E+08	1.58E+08	ENSMUSG00000027652.9	Ralgapb	0.000581	0.079
chr11	1.16E+08	1.16E+08	ENSMUSG00000052949.8	Rnf157	0.000532	-0.174
chr1	57450428	57450538	ENSMUSG00000048495.9	1110034B(	4.32E-07	-0.086
chr3	1.52E+08	1.52E+08	ENSMUSG00000028035.7	Dnajb4	0.000316	-0.523
chr5	1.22E+08	1.22E+08	ENSMUSG00000029454.8	Mapkapk5	1.97E-07	0.089
chr5	1.22E+08	1.22E+08	ENSMUSG00000029454.8	Mapkapk5	9.34E-07	0.101
chr14	42094951	42095133	ENSMUSG00000091110.1	Gm2832	0.000158	-0.43
chr4	1.27E+08	1.27E+08	ENSMUSG00000042446.9	Zmym4	0	0.356
chr3	1.16E+08	1.16E+08	ENSMUSG00000089911.1	Hiat1	2.09E-10	0.086
chr1	58018744	58018839	ENSMUSG00000054770.9	Kctd18	2.48E-05	0.085
chr1	58022194	58022384	ENSMUSG00000054770.9	Kctd18	2.47E-06	0.116
chr5	24871894	24872014	ENSMUSG00000038056.8	Mll3	6.71E-05	0.309
chr7	57053675	57053797	ENSMUSG00000030505.9	Prmt3	0.000117	0.064
chr11	4979487	4979522	ENSMUSG00000009079.10	Ewsr1	0	0.4
chr11	4982286	4982436	ENSMUSG00000009079.10	Ewsr1	0	0.234
chr3	10420196	10420316	ENSMUSG00000027534.9	Snx16	0	0.398
chr5	31846042	31846286	ENSMUSG00000029141.9	Slc4a1ap	1.65E-05	0.108
chr5	31848552	31848639	ENSMUSG00000029141.9	Slc4a1ap	4.85E-05	0.317
chr5	1.47E+08	1.47E+08	ENSMUSG00000029636.7	Wasf3	5.07E-14	0.412
chr4	11970611	11970714	ENSMUSG00000049488.8	Tmem67	0.000349	0.092
chr5	33950112	33950876	ENSMUSG00000037339.10	Fam53a	0	0.137
chr5	33950979	33951138	ENSMUSG00000037339.10	Fam53a	1.46E-13	0.078
chr5	34214055	34214150	ENSMUSG00000057406.8	Whsc1	0.000389	-0.057
chr5	34214055	34214283	ENSMUSG00000057406.8	Whsc1	0.000126	-0.059
chr1	89671387	89671444	ENSMUSG00000026289.8	Atg16l1	3.54E-07	0.129
chr5	46084395	46084577	ENSMUSG00000015880.9	Ncapg	9.97E-14	0.258
chr5	46084395	46084577	ENSMUSG00000015880.9	Ncapg	4.80E-07	0.282
chr5	46084990	46085165	ENSMUSG00000015880.9	Ncapg	7.09E-07	0.21
chr9	4303093	4303215	ENSMUSG00000025894.9	Aasdhppt	2.56E-05	0.089
chr9	4303093	4303215	ENSMUSG00000025894.9	Aasdhppt	9.49E-07	0.178

chr9	20867757	20867840	ENSMUSG00000079681.2	Zglp1	3.14E-06	0.233
chr5	68172226	68172300	ENSMUSG00000037685.9	Atp8a1	0.000386	-0.306
chr5	1.24E+08	1.24E+08	ENSMUSG00000029414.7	Kntc1	5.91E-08	0.312
chr5	74031027	74031219	ENSMUSG00000029156.9	Sgcb	0.00056	0.059
chr5	74981070	74981097	ENSMUSG00000029227.9	Fip1l1	1.29E-05	-0.095
chr5	75407255	75407315	ENSMUSG00000029229.7	Chic2	0	0.065
chr12	74381991	74382643	ENSMUSG00000034442.9	Trmt5	1.80E-07	0.135
chr10	83044001	83044136	ENSMUSG00000034560.5	A230046K(	1.15E-05	0.087
chr5	93063261	93063342	ENSMUSG00000050050.9	Ccdc158	2.89E-12	0.729
chr5	1E+08	1E+08	ENSMUSG00000000568.9	Hnrnpd	1.32E-06	0.13
chr5	1E+08	1E+08	ENSMUSG00000029328.8	Hnrpdl	0	0.344
chr7	51752872	51753003	ENSMUSG00000051113.7	Fam71e1	0.000212	0.188
chr5	1.01E+08	1.01E+08	ENSMUSG00000016833.8	Mrps18c	0	0.086
chr5	1.02E+08	1.02E+08	ENSMUSG00000043940.7	Wdfy3	2.24E-05	-0.094
chr1	1.68E+08	1.68E+08	ENSMUSG00000040596.9	Pogk	6.28E-07	0.315
chr5	1.08E+08	1.08E+08	ENSMUSG00000029276.7	Glmn	0	0.243
chr5	1.09E+08	1.09E+08	ENSMUSG00000029263.9	Pigg	1.87E-05	0.137
chr5	1.09E+08	1.09E+08	ENSMUSG00000029263.9	Pigg	0.000526	0.225
chr5	1.09E+08	1.09E+08	ENSMUSG00000029263.9	Pigg	0.000278	0.13
chr5	1.09E+08	1.09E+08	ENSMUSG00000062234.7	Gak	5.05E-07	0.166
chr5	1.11E+08	1.11E+08	ENSMUSG00000033434.9	Gtpbp6	4.20E-08	0.842
chr5	1.11E+08	1.11E+08	ENSMUSG00000033434.9	Gtpbp6	2.98E-08	0.532
chr5	1.11E+08	1.11E+08	ENSMUSG00000033434.9	Gtpbp6	0.000245	0.077
chr5	1.11E+08	1.11E+08	ENSMUSG00000033434.9	Gtpbp6	3.98E-09	0.209
chr5	1.11E+08	1.11E+08	ENSMUSG00000043510.6	Hscb	2.16E-10	0.202
chr5	1.11E+08	1.11E+08	ENSMUSG00000043510.6	Hscb	5.25E-11	0.13
chr15	82967897	82967984	ENSMUSG00000041815.7	Poldip3	0.000485	0.117
chr5	1.14E+08	1.14E+08	ENSMUSG00000025825.6	Iscu	8.39E-05	-0.11
chr5	1.14E+08	1.14E+08	ENSMUSG00000025825.6	Iscu	5.14E-09	-0.095
chr5	1.16E+08	1.16E+08	ENSMUSG00000029516.11	Cit	0.000725	-0.267
chr7	1.17E+08	1.17E+08	ENSMUSG00000038371.7	Sbf2	1.79E-06	0.16
chr10	1.12E+08	1.12E+08	ENSMUSG00000020214.4	Glipr1l2	0.000551	0.504
chr5	1.23E+08	1.23E+08	ENSMUSG00000029462.12	Vps29	4.90E-08	0.153
chr4	1.07E+08	1.07E+08	ENSMUSG00000028614.8	Tmem48	3.75E-06	0.78
chr4	1.07E+08	1.07E+08	ENSMUSG00000028614.8	Tmem48	2.31E-06	0.058
chr10	20054304	20054451	ENSMUSG00000037608.8	Bclaf1	4.66E-06	0.116
chrX	71438857	71439049	ENSMUSG00000031397.5	Tktl1	3.60E-13	0.091
chr5	1.35E+08	1.35E+08	ENSMUSG00000061979.2	Wbscr16	0.00066	0.08
chr5	1.39E+08	1.39E+08	ENSMUSG00000036928.8	Stag3	6.39E-08	0.275
chr5	1.39E+08	1.39E+08	ENSMUSG00000036928.8	Stag3	4.54E-07	0.218
chr5	1.39E+08	1.39E+08	ENSMUSG00000036928.8	Stag3	8.07E-09	0.055
chrX	1.1E+08	1.1E+08	ENSMUSG00000025531.8	Chm	0.000359	0.104
chr1	36621174	36621327	ENSMUSG00000046337.10	Fam178b	6.35E-05	0.2
chr1	36621174	36621327	ENSMUSG00000046337.10	Fam178b	3.67E-07	0.376
chr1	36621675	36621753	ENSMUSG00000046337.10	Fam178b	7.22E-05	0.343
chr8	42346713	42346830	ENSMUSG00000031592.8	Pcm1	0.00013	0.207
chr14	32183887	32183980	ENSMUSG00000021893.7	Capn7	1.09E-10	0.108
chr5	1.46E+08	1.46E+08	ENSMUSG00000007812.9	Zfp655	3.55E-07	0.063
chr5	1.46E+08	1.46E+08	ENSMUSG00000007812.9	Zfp655	4.45E-05	-0.238
chr5	1.47E+08	1.47E+08	ENSMUSG00000029635.9	Cdk8	4.90E-05	0.117
chr5	1.47E+08	1.47E+08	ENSMUSG00000029635.9	Cdk8	0.000322	0.156
chr11	1.15E+08	1.15E+08	ENSMUSG00000020744.6	Slc25a19	2.47E-05	0.193
chr16	5018932	5019112	ENSMUSG00000022536.7	Glyr1	0	0.197
chr8	13885665	13885881	ENSMUSG00000031458.7	2410022L(	0	0.185
chr3	1.08E+08	1.08E+08	ENSMUSG00000009108.9	Gnat2	0.000959	0.286
chr16	13652737	13652803	ENSMUSG00000022685.8	Parn	5.07E-14	0.152
chr6	29362901	29363087	ENSMUSG00000029769.8	Ccdc136	1.01E-08	0.156
chr6	29364785	29364980	ENSMUSG00000029769.8	Ccdc136	0.000273	0.248

chr6	34877718	34877916	ENSMUSG00000029848.4	Stra8	0	-0.159
chr6	34877718	34877916	ENSMUSG00000029848.4	Stra8	3.64E-06	0.101
chr6	34880360	34880436	ENSMUSG00000029848.4	Stra8	0	0.163
chr6	39090158	39090271	ENSMUSG00000073144.3	4930599N	8.34E-05	-0.667
chr6	39598819	39598855	ENSMUSG00000002413.8	Braf	0.000592	0.153
chr6	40318579	40318674	ENSMUSG00000029916.8	Agk	1.45E-09	0.129
chr6	90967325	90967542	ENSMUSG00000030091.9	Nup210	1.61E-05	0.083
chr6	42312655	42312781	ENSMUSG00000029859.4	Epha1	0.000446	0.154
chr2	25699443	25699520	ENSMUSG00000059625.6	Sohlh1	0.000527	0.19
chr2	25700005	25700192	ENSMUSG00000059625.6	Sohlh1	4.16E-05	0.118
chr13	1.12E+08	1.12E+08	ENSMUSG00000032745.10	Gpbp1	0	0.1
chr6	47491843	47491969	ENSMUSG00000029687.9	Ezh2	3.40E-06	-0.18
chr6	51414426	51414546	ENSMUSG00000004980.9	Hnrnpa2b1	1.13E-09	0.134
chr6	54565208	54565364	ENSMUSG00000005225.9	Plekha8	0	0.48
chr13	14067704	14067901	ENSMUSG00000039242.9	B3galnt2	0.000283	0.45
chr7	1.47E+08	1.47E+08	ENSMUSG00000025474.7	Tubgcp2	0.000639	0.054
chr7	1.2E+08	1.2E+08	ENSMUSG00000038187.8	Btbd10	9.98E-07	0.25
chr7	1.21E+08	1.21E+08	ENSMUSG00000038187.8	Btbd10	2.18E-06	0.204
chr15	88904502	88904628	ENSMUSG00000087331.2	1810021B2	0.000493	-0.684
chr6	82978385	82978526	ENSMUSG00000030041.8	D6Mm5e	0	0.177
chr7	1.29E+08	1.29E+08	ENSMUSG00000063317.6	Usp31	0.000422	0.07
chr5	1.09E+08	1.09E+08	ENSMUSG00000029267.10	Mtf2	0.00064	0.122
chr8	73042263	73042416	ENSMUSG00000055553.10	2810422J0	1.84E-06	0.054
chr6	89417383	89417467	ENSMUSG00000030086.9	Chchd6	3.11E-05	0.068
chr17	71905579	71905829	ENSMUSG00000052525.8	Spdya	0.00028	0.477
chr17	71911842	71911928	ENSMUSG00000052525.8	Spdya	7.98E-06	0.538
chr17	71911842	71911928	ENSMUSG00000052525.8	Spdya	0.000437	0.437
chr2	1.63E+08	1.63E+08	ENSMUSG00000017861.5	Mybl2	1.18E-11	0.159
chr2	1.63E+08	1.63E+08	ENSMUSG00000017861.5	Mybl2	2.22E-07	-0.206
chr9	1.1E+08	1.1E+08	ENSMUSG00000032478.6	Nme6	0.000509	-0.129
chr9	1.1E+08	1.1E+08	ENSMUSG00000032478.6	Nme6	1.56E-06	-0.096
chr5	38497802	38497880	ENSMUSG00000029125.8	Stx18	0.00055	0.075
chr10	19614530	19614608	ENSMUSG00000020003.9	Pex7	1.44E-09	0.175
chr8	1.14E+08	1.14E+08	ENSMUSG00000012519.8	Mlkl	0.000123	0.169
chr1	71650233	71650503	ENSMUSG00000026193.8	Fn1	6.23E-05	0.176
chr6	1.13E+08	1.13E+08	ENSMUSG00000034269.6	Setd5	1.06E-07	-0.225
chr6	1.13E+08	1.13E+08	ENSMUSG00000030269.7	Mtmr14	0.00097	0.17
chr6	1.13E+08	1.13E+08	ENSMUSG00000001632.9	Brpf1	6.65E-12	0.108
chr6	87964579	87964714	ENSMUSG00000079477.3	Rab7	6.49E-06	-0.133
chr6	1.19E+08	1.19E+08	ENSMUSG00000041477.8	Dcp1b	1.69E-07	0.15
chr6	1.2E+08	1.2E+08	ENSMUSG00000030180.9	Kdm5a	1.51E-12	-0.275
chr6	1.21E+08	1.21E+08	ENSMUSG00000067825.5	Pex26	0.00033	0.33
chr6	85390633	85390738	ENSMUSG00000033706.7	Smyd5	1.29E-09	0.129
chr6	1.25E+08	1.25E+08	ENSMUSG00000038346.11	Zfp384	0.00095	0.359
chr6	1.25E+08	1.25E+08	ENSMUSG00000038346.11	Zfp384	5.47E-08	0.152
chr6	1.25E+08	1.25E+08	ENSMUSG00000038346.11	Zfp384	0.000194	-0.148
chr3	37428904	37428941	ENSMUSG00000027722.8	Spata5	1.30E-06	-0.204
chr19	47155030	47155195	ENSMUSG00000025049.5	Taf5	0	0.343
chr11	69264723	69264856	ENSMUSG00000005237.7	Dnahc2	7.87E-08	0.583
chr11	69264723	69264856	ENSMUSG00000005237.7	Dnahc2	0.0002	0.509
chr6	1.42E+08	1.42E+08	ENSMUSG00000030243.10	Recql	2.50E-08	0.148
chr6	1.47E+08	1.47E+08	ENSMUSG00000030301.10	Ccdc91	1.56E-10	0.086
chr6	1.48E+08	1.48E+08	ENSMUSG00000030304.5	Ergic2	0	0.083
chr6	1.49E+08	1.49E+08	ENSMUSG00000030309.9	Caprin2	1.66E-08	0.145
chr8	1.09E+08	1.09E+08	ENSMUSG00000031902.8	Nfatc3	6.13E-07	0.097
chr11	78017566	78017699	ENSMUSG00000044122.8	Proca1	0.000454	0.268
chr19	10784759	10784931	ENSMUSG00000048832.8	Vps37c	1.95E-05	0.111
chr1	1.92E+08	1.92E+08	ENSMUSG00000026605.7	Cenpf	6.48E-08	0.087

chr1	1.92E+08	1.92E+08	ENSMUSG00000026605.7	Cenpf	1.55E-09	-0.102
chr7	28036448	28036661	ENSMUSG00000003762.7	Adck4	0.000255	0.052
chr8	90863307	90863419	ENSMUSG00000031660.7	Brd7	3.87E-08	0.053
chr16	15795120	15795232	ENSMUSG00000022672.7	Prkdc	0.000467	0.107
chr9	1.08E+08	1.08E+08	ENSMUSG00000070283.3	Ndufaf3	1.80E-06	0.131
chr1	58215748	58215863	ENSMUSG00000064294.6	Aox3	0.00013	0.88
chr7	16578428	16578494	ENSMUSG00000070808.3	Gltscr1	0.000462	-0.215
chr7	1.28E+08	1.28E+08	ENSMUSG00000035064.9	Eef2k	0.000305	0.086
chr19	47822331	47822545	ENSMUSG00000044948.9	Wdr96	0.000155	0.59
chr4	1.54E+08	1.54E+08	ENSMUSG00000029029.7	Wdr8	0	0.236
chr10	60183608	60183771	ENSMUSG00000020100.9	Slc29a3	0.00059	0.285
chr10	60186511	60186738	ENSMUSG00000020100.9	Slc29a3	0.000237	0.317
chr2	26771474	26771526	ENSMUSG00000015790.7	Surf1	0.000527	0.256
chr2	70916530	70916636	ENSMUSG00000041966.12	Dcaf17	0.00051	0.124
chr7	31375651	31375720	ENSMUSG00000006310.10	Zbtb32	7.84E-06	0.209
chr7	31376075	31376984	ENSMUSG00000006310.10	Zbtb32	8.98E-05	0.226
chr7	35097107	35097195	ENSMUSG00000066571.6	4931406P1	7.18E-05	-0.574
chr7	35097107	35097195	ENSMUSG00000066571.6	4931406P1	0.000189	-0.871
chr7	35097107	35097195	ENSMUSG00000066571.6	4931406P1	7.84E-06	-0.221
chr1	95524230	95524287	ENSMUSG00000026277.7	Stk25	5.07E-14	0.083
chr1	95524230	95524287	ENSMUSG00000026277.7	Stk25	6.02E-12	0.121
chr1	95525610	95525841	ENSMUSG00000026277.7	Stk25	4.43E-09	0.058
chr7	5033619	5033723	ENSMUSG00000035203.8	Epn1	6.29E-05	0.065
chr2	51972187	51972265	ENSMUSG00000036202.8	Rif1	1.10E-08	0.222
chr7	1.41E+08	1.41E+08	ENSMUSG00000030979.6	Uros	5.00E-05	0.141
chr1	1.35E+08	1.35E+08	ENSMUSG00000026447.10	Pik3c2b	1.31E-05	0.286
chr2	1.4E+08	1.4E+08	ENSMUSG00000027384.5	2310003L2	6.01E-05	0.085
chr17	46815990	46816143	ENSMUSG00000063576.5	Klhdc3	5.07E-14	0.059
chr7	52404670	52405136	ENSMUSG00000007833.7	Aldh16a1	0.000654	0.262
chr7	52405033	52405136	ENSMUSG00000007833.7	Aldh16a1	9.69E-05	0.166
chr7	52773955	52774255	ENSMUSG00000023467.9	Tulp2	0.000677	0.08
chr7	52773955	52774276	ENSMUSG00000023467.9	Tulp2	0.000759	0.081
chr6	87795670	87795787	ENSMUSG00000030057.9	Cnbp	0	0.052
chr6	87799008	87799103	ENSMUSG00000030057.9	Cnbp	1.54E-12	0.665
chr3	1.3E+08	1.3E+08	ENSMUSG00000001052.8	Sec24b	7.75E-07	0.28
chr16	18544203	18544366	ENSMUSG00000000884.9	Gnb1l	0.000639	0.262
chr7	88506471	88506695	ENSMUSG00000025586.9	Cpeb1	1.66E-06	0.099
chr7	89794159	89794339	ENSMUSG00000038570.8	Fam154b	4.42E-05	0.441
chr9	59523374	59523541	ENSMUSG00000032294.9	Pkm2	0	-0.106
chr7	97084236	97084474	ENSMUSG00000062797.5	I7Rn6	7.35E-09	0.18
chr7	97330728	97330878	ENSMUSG00000039361.8	Picalm	0	0.293
chr5	30972064	30972201	ENSMUSG00000029177.3	Cenpa	0.000163	0.117
chr9	1.15E+08	1.15E+08	ENSMUSG00000040875.5	Osbpl10	2.23E-06	-0.146
chr15	84920021	84920175	ENSMUSG00000022432.6	Smc1b	0	0.281
chr15	84952018	84952277	ENSMUSG00000022432.6	Smc1b	0.000465	0.076
chr15	84954150	84954389	ENSMUSG00000022432.6	Smc1b	7.68E-06	-0.526
chr15	84958188	84958301	ENSMUSG00000022432.6	Smc1b	2.13E-10	-0.869
chr1	93061490	93061570	ENSMUSG00000070732.1	Rbm44	0.00025	0.064
chr11	1.21E+08	1.21E+08	ENSMUSG00000048445.6	Ccdc57	1.61E-05	0.628
chr11	64800772	64800831	ENSMUSG00000020549.8	Elac2	0.000177	0.056
chr3	32629002	32629133	ENSMUSG00000037531.7	Mrpl47	6.55E-08	0.088
chr16	37560080	37560195	ENSMUSG00000022827.7	Rabl3	0.000267	-0.141
chrX	53450650	53450768	ENSMUSG00000073177.2	Gm773	3.43E-09	0.149
chrX	53454760	53454795	ENSMUSG00000073177.2	Gm773	3.29E-10	0.334
chrX	53454760	53454795	ENSMUSG00000073177.2	Gm773	1.90E-13	0.155
chr19	3714403	3714625	ENSMUSG00000035372.1	1810055G	0.000523	0.077
chr7	1.35E+08	1.35E+08	ENSMUSG00000070371.5	Prss36	5.31E-06	0.258
chr7	1.35E+08	1.35E+08	ENSMUSG00000070371.5	Prss36	8.63E-06	0.237

chr7	1.35E+08	1.35E+08	ENSMUSG00000070371.5	Prss36	4.90E-05	0.588
chr7	1.35E+08	1.35E+08	ENSMUSG00000070371.5	Prss36	5.98E-06	0.232
chr7	1.35E+08	1.35E+08	ENSMUSG00000070371.5	Prss36	4.05E-06	0.286
chr7	1.35E+08	1.35E+08	ENSMUSG00000070371.5	Prss36	0.000327	0.243
chr7	1.35E+08	1.35E+08	ENSMUSG00000070371.5	Prss36	1.47E-05	0.344
chr7	1.38E+08	1.38E+08	ENSMUSG00000040268.10	Plekha1	1.10E-07	0.092
chr7	1.39E+08	1.39E+08	ENSMUSG00000063179.6	Pstk	5.38E-06	0.103
chr7	1.48E+08	1.48E+08	ENSMUSG00000058886.7	Deaf1	1.09E-05	-0.267
chr7	1.48E+08	1.48E+08	ENSMUSG00000058886.7	Deaf1	2.69E-08	-0.229
chr7	1.49E+08	1.49E+08	ENSMUSG00000058886.7	Deaf1	0.000145	0.08
chr2	26445899	26446015	ENSMUSG00000026921.11	Egfl7	0.000125	0.124
chr2	1.19E+08	1.19E+08	ENSMUSG00000068580.5	Zfyve19	2.29E-10	0.106
chr2	1.19E+08	1.19E+08	ENSMUSG00000068580.5	Zfyve19	0.000397	0.071
chr8	3450295	3450436	ENSMUSG00000004568.6	Arhgef18	7.24E-05	0.059
chr17	27073231	27073336	ENSMUSG00000024193.7	Phf1	0.000872	0.127
chr17	27073456	27073625	ENSMUSG00000024193.7	Phf1	0.000899	0.087
chr17	27073456	27073625	ENSMUSG00000024193.7	Phf1	7.82E-05	0.089
chr2	1.73E+08	1.73E+08	ENSMUSG00000087382.1	1300015D(	0.000268	-0.334
chr9	66390319	66390420	ENSMUSG00000032376.6	Usp3	0	0.351
chr9	66390319	66390433	ENSMUSG00000032376.6	Usp3	0	0.2
chr10	79796710	79796879	ENSMUSG00000035504.10	Reep6	0.000909	0.069
chr2	1.3E+08	1.3E+08	ENSMUSG00000027411.10	Vps16	1.65E-07	0.068
chr15	83194249	83194358	ENSMUSG00000075511.1	1700001L(	0.000479	-0.244
chr15	83195458	83195940	ENSMUSG00000075511.1	1700001L(	1.34E-05	0.311
chr13	21487968	21488443	ENSMUSG00000021327.11	Zkscan3	9.66E-06	0.159
chr13	21487968	21488446	ENSMUSG00000021327.11	Zkscan3	2.32E-06	0.16
chr13	91162174	91162272	ENSMUSG00000021619.5	Atg10	0.000881	0.117
chr9	61771711	61772023	ENSMUSG00000032254.8	Kif23	1.46E-13	0.242
chr9	61781035	61781077	ENSMUSG00000032254.8	Kif23	0.000168	0.206
chr8	26107126	26107322	ENSMUSG00000031555.7	Adam9	6.39E-05	0.067
chr5	89174066	89174175	ENSMUSG00000006262.9	Mob1b	8.33E-05	0.238
chr5	89174081	89174175	ENSMUSG00000006262.9	Mob1b	2.09E-05	0.083
chr8	34627411	34627516	ENSMUSG00000009628.8	Tex15	4.24E-05	0.125
chr8	42282311	42282499	ENSMUSG00000031594.8	Fgl1	8.82E-06	0.272
chr8	63523055	63523130	ENSMUSG00000031644.11	Nek1	4.58E-11	0.136
chr8	71348088	71348166	ENSMUSG00000031864.8	Ints10	0.000164	-0.157
chr8	71348950	71349028	ENSMUSG00000031864.8	Ints10	0.000346	-0.082
chr8	72656310	72656416	ENSMUSG00000071078.5	Nr2c2ap	8.91E-09	-0.19
chr8	73105453	73105719	ENSMUSG00000070002.6	Ell	0.000229	0.062
chr8	73370662	73370671	ENSMUSG00000002908.8	Kcnn1	2.83E-05	0.465
chr8	73860774	73860864	ENSMUSG00000004677.8	Myo9b	0.000633	0.115
chr8	73860774	73860864	ENSMUSG00000004677.8	Myo9b	7.32E-08	0.323
chr8	73862805	73862919	ENSMUSG00000004677.8	Myo9b	2.65E-06	0.313
chr8	73864658	73864698	ENSMUSG00000004677.8	Myo9b	3.32E-06	0.237
chr8	73879136	73879210	ENSMUSG00000004677.8	Myo9b	0.000254	0.285
chr8	73879593	73879786	ENSMUSG00000004677.8	Myo9b	8.59E-08	0.065
chr8	73882514	73882562	ENSMUSG00000004677.8	Myo9b	0.00037	-0.151
chr8	74489692	74489815	ENSMUSG00000052446.10	Zfp961	2.08E-05	0.298
chr8	74489692	74489819	ENSMUSG00000052446.10	Zfp961	6.01E-05	0.243
chr8	74489998	74490059	ENSMUSG00000052446.10	Zfp961	8.18E-06	0.166
chr8	74701518	74701569	ENSMUSG00000003037.9	Rab8a	0.000422	0.069
chr8	80086312	80086415	ENSMUSG00000037134.10	Prmt10	0.000793	0.057
chr8	81343885	81343984	ENSMUSG00000031683.9	Lsm6	0.000561	-0.086
chr8	87396618	87396731	ENSMUSG00000003824.6	Syce2	4.01E-10	0.063
chr8	87407329	87407483	ENSMUSG00000003824.6	Syce2	0	0.124
chr8	87407329	87407483	ENSMUSG00000003824.6	Syce2	8.20E-11	0.287
chr8	87597193	87597315	ENSMUSG00000060038.8	Dhps	0	0.333
chr8	87597401	87597498	ENSMUSG00000060038.8	Dhps	0	0.267

chr14	31827775	31827863	ENSMUSG00000042354.6	Gnl3	0	0.094
chr6	85435914	85436089	ENSMUSG00000047013.9	Fbxo41	5.90E-09	-0.889
chr7	1.35E+08	1.35E+08	ENSMUSG00000030811.7	Fbxl19	0.00056	0.115
chr8	1.08E+08	1.08E+08	ENSMUSG00000036672.4	Cenpt	0.00072	0.264
chr8	1.08E+08	1.08E+08	ENSMUSG00000036672.4	Cenpt	0.000551	0.109
chr13	43761126	43761191	ENSMUSG00000044164.2	Rnf182	0.000794	0.572
chr8	1.27E+08	1.27E+08	ENSMUSG00000037300.10	Ttc13	8.75E-05	-0.112
chrX	1.37E+08	1.37E+08	ENSMUSG00000085146.1	Eif2c5	5.22E-07	0.643
chr9	14608208	14608297	ENSMUSG00000031928.8	Mre11a	4.23E-05	-0.086
chr9	14629584	14629801	ENSMUSG00000031928.8	Mre11a	2.78E-13	0.201
chr18	35759698	35759771	ENSMUSG00000037058.8	Paip2	6.84E-05	-0.38
chr8	28161730	28161873	ENSMUSG00000031485.8	Prosc	0.000192	0.091
chr8	41444763	41444802	ENSMUSG00000039478.8	Efha2	0.000618	0.278
chr9	20395782	20395865	ENSMUSG00000058192.8	Zfp846	0.000311	0.187
chr9	21085485	21085613	ENSMUSG00000035047.2	Kri1	0.000957	0.088
chr9	21862247	21862859	ENSMUSG00000038895.8	Zfp653	0.000349	0.088
chr1	1.83E+08	1.83E+08	ENSMUSG00000026516.7	Nvl	3.20E-07	0.063
chr15	61938702	61939020	ENSMUSG00000072566.3	Pvt1	0.000695	-0.378
chr15	61991551	61991683	ENSMUSG00000072566.3	Pvt1	2.43E-05	-0.617
chr11	4622360	4622514	ENSMUSG00000009076.4	Zmat5	0.000154	-0.07
chr14	57575688	57575940	ENSMUSG00000021945.7	Zmym2	0	0.161
chr3	1.03E+08	1.03E+08	ENSMUSG00000027855.8	Sycp1	0.000274	0.065
chr13	67773934	67774030	ENSMUSG00000048280.10	Zfp738	0.000784	0.238
chr17	80694852	80694989	ENSMUSG00000045257.9	Morn2	3.38E-07	0.102
chr9	53296010	53296175	ENSMUSG00000034218.8	Atm	3.22E-13	0.167
chr4	1.08E+08	1.08E+08	ENSMUSG00000034645.7	Zyg11a	0.0004	0.107
chr9	53430886	53431048	ENSMUSG00000032030.9	Cul5	0	0.109
chr2	1.55E+08	1.55E+08	ENSMUSG00000027593.9	Raly	4.72E-08	-0.084
chr13	54665545	54665633	ENSMUSG00000025871.11	4833439L1	1.77E-06	0.132
chr9	54731703	54731922	ENSMUSG00000032293.7	Ireb2	0.000347	0.054
chr9	55130357	55130423	ENSMUSG00000032311.10	Nrg4	2.60E-06	-0.67
chr9	55343548	55343630	ENSMUSG00000032314.8	Etfa	0.000679	0.522
chr7	52893239	52893387	ENSMUSG00000044562.9	Rasip1	1.70E-08	0.163
chr9	59637842	59637899	ENSMUSG00000039585.9	Myo9a	7.98E-10	0.334
chr9	59723008	59723221	ENSMUSG00000039585.9	Myo9a	4.15E-06	-0.129
chr9	67803473	67803600	ENSMUSG00000035284.8	Vps13c	0.000386	0.061
chr2	29059390	29059528	ENSMUSG00000035513.10	Ntng2	0.000881	0.396
chr9	77942951	77943066	ENSMUSG00000001366.8	Fbxo9	0.000282	0.079
chr10	1.27E+08	1.27E+08	ENSMUSG00000025402.5	Nab2	0.000626	0.058
chr9	78190293	78190404	ENSMUSG00000057933.4	Gsta2	0.000354	-0.262
chr9	79937655	79937713	ENSMUSG00000034252.7	Senp6	4.56E-05	0.116
chr9	83004394	83004481	ENSMUSG00000066456.6	Hmgn3	0.000854	-0.093
chr9	1.03E+08	1.03E+08	ENSMUSG00000032534.9	Cep63	0.000311	0.132
chr17	8334460	8334518	ENSMUSG00000073469.4	Rnaset2a	2.23E-06	0.311
chr17	8335382	8335435	ENSMUSG00000073469.4	Rnaset2a	2.09E-10	0.184
chr9	88610325	88610587	ENSMUSG00000079427.3	Gm2382	0	0.3
chr9	1.05E+08	1.05E+08	ENSMUSG00000032570.8	Atp2c1	0	0.163
chr9	1.08E+08	1.08E+08	ENSMUSG00000047220.4	Ccdc36	0.000347	0.127
chr9	1.08E+08	1.08E+08	ENSMUSG00000047220.4	Ccdc36	1.86E-06	0.231
chr9	1.08E+08	1.08E+08	ENSMUSG00000047220.4	Ccdc36	3.46E-06	0.211
chr9	1.19E+08	1.19E+08	ENSMUSG00000032511.10	Scn5a	0.00017	-0.177
chr10	1.27E+08	1.27E+08	ENSMUSG00000040415.9	Dtx3	0.000122	-0.143
chr9	1.24E+08	1.24E+08	ENSMUSG00000025245.7	Lztfl1	0.000786	0.075
chr9	1.24E+08	1.24E+08	ENSMUSG00000025245.7	Lztfl1	8.60E-06	-0.064
chr2	1.47E+08	1.47E+08	ENSMUSG00000074749.4	Plk1s1	2.08E-05	0.101
chr6	1.35E+08	1.35E+08	ENSMUSG00000032652.7	Crebl2	0.000265	0.137
chr1	1.66E+08	1.66E+08	ENSMUSG00000026577.7	Blzf1	3.50E-06	-0.162
chr1	1.66E+08	1.66E+08	ENSMUSG00000026577.7	Blzf1	1.97E-07	-0.156

A3SS	Up	0	-
A3SS	Up	0	-
A3SS	Down	0	-
A3SS	Up	0	+
A3SS	Up	0	-
A3SS	Up	0	-
A3SS	Up	0	-
A3SS	Up	0	-
A3SS	Up	0	+
A3SS	Up	0	-
A3SS	Up	0	+
A3SS	Up	0	-
A3SS	Up	0	+
A3SS	Up	0	+
A5SS	Down	0	-
A5SS	Up	0	-
A5SS	Up	0	+
A5SS	Up	0	+
A5SS	Up	0	-
A5SS	Up	0	+
A5SS	Up	0	-
A5SS	Up	0	+
A5SS	Up	0	-
A5SS	Up	0	+
A5SS	Up	0	-
A5SS	Up	0	-
A5SS	Down	0	-
A5SS	Up	0	+
A5SS	Down	0	-
A5SS	Up	0	+
A5SS	Up	0	-
A5SS	Up	0	+
A5SS	Up	0	-
A5SS	Up	0	-
A5SS	Up	0	-
A5SS	Up	0	+
A5SS	Down	0	+
A5SS	Up	0	+
A5SS	Up	0	-
A5SS	Up	0	-
A5SS	Up	0	+
A5SS	Up	0	+
A5SS	Up	0	-
A5SS	Up	0	+
A5SS	Up	0	-
A5SS	Up	0	-
A5SS	Up	0	+
A5SS	Up	0	+
MXE	Down	0	+
MXE	Up	0	-
MXE	Up	0	-
MXE	Down	0	+
MXE	Down	0	+
MXE	Down	0	-
MXE	Up	0	-
MXE	Up	0	+
MXE	Up	0	+
MXE	Down	0	-

MXE	Down	0 +
MXE	Down	0 +
MXE	Up	0 +
MXE	Down	0 +
MXE	Up	0 -
MXE	Up	0 -
MXE	Up	0 +
MXE	Down	0 -
MXE	Up	0 +
MXE	Down	0 +
MXE	Down	0 +
MXE	Down	0 +
MXE	Up	0 +
MXE	Down	0 -
MXE	Down	0 +
MXE	Down	0 +
MXE	Up	0 -
MXE	Up	0 -
MXE	Down	0 -
MXE	Up	0 +
MXE	Up	0 +
MXE	Down	0 +
MXE	Up	0 -
MXE	Up	0 -
MXE	Up	0 -
MXE	Down	0 -
MXE	Down	0 -
MXE	Up	0 -
MXE	Up	0 +
MXE	Down	0 +
MXE	Up	0 -
MXE	Down	0 -
MXE	Up	0 -
MXE	Down	0 +
MXE	Up	0 -
MXE	Up	0 +
MXE	Down	0 +
MXE	Up	0 -
MXE	Up	0 +
MXE	Up	0 -
MXE	Down	0 +
MXE	Up	0 +
MXE	Up	0 +
MXE	Down	0 -
MXE	Up	0 -
RI	Down	0 +
RI	Down	0 -
RI	Up	0 +
RI	Down	0 -
RI	Up	0 -
RI	Down	0 -
RI	Down	0 -
RI	Down	0 +
RI	Down	0 +
RI	Down	0 -
RI	Down	0 +
RI	Down	0 +
RI	Down	0 +

RI	Down	0	-
RI	Down	0	-
RI	Down	0	+
RI	Down	0	+
SE	Up	0	-
SE	Up	0	-
SE	Up	0	+
SE	Up	0	+
SE	Up	0	+
SE	Up	0	-
SE	Down	0	-
SE	Up	0	+
SE	Up	0	-
SE	Up	0	-
SE	Up	0	-
SE	Up	0	-
SE	Up	0	-
SE	Down	0	+
SE	Up	0	+
SE	Up	0	+
SE	Up	0	+
SE	Up	0	-
SE	Up	0	-
SE	Up	0	+
SE	Up	0	+
SE	Up	0	+
SE	Up	0	+
SE	Up	0	+
SE	Up	0	+
SE	Up	0	+
SE	Up	0	-
SE	Up	0	+
SE	Up	0	-
SE	Up	0	-
SE	Up	0	-
SE	Up	0	+
SE	Up	0	+
SE	Up	0	+
SE	Up	0	+
SE	Up	0	-
SE	Up	0	+
SE	Up	0	+
SE	Down	0	+
SE	Up	0	-
SE	Up	0	-
SE	Up	0	+
SE	Up	0	-
SE	Up	0	-
SE	Up	0	-
SE	Up	0	-
SE	Up	0	-
SE	Down	0	+
SE	Down	0	+
SE	Up	0	+
SE	Up	0	+
SE	Up	0	-
SE	Up	0	-
SE	Up	0	-

SE	Up	0	-
SE	Up	0	+
SE	Up	0	+
SE	Down	0	-
SE	Up	0	+
SE	Up	0	+
SE	Up	0	-
SE	Up	0	-
SE	Up	0	-
SE	Up	0	-
SE	Up	0	+
SE	Up	0	+
SE	Down	0	+
SE	Up	0	+
SE	Up	0	+
SE	Down	0	-
SE	Up	0	-
SE	Up	0	-
SE	Down	0	-
SE	Down	0	-
SE	Up	0	+
SE	Up	0	-
SE	Up	0	+
SE	Up	0	-
SE	Up	0	+
SE	Up	0	+
SE	Up	0	+
SE	Up	0	-
SE	Up	0	-
SE	Up	0	-
SE	Up	0	+
SE	Up	0	+
SE	Down	0	+
SE	Up	0	-
SE	Up	0	-
SE	Up	0	-
SE	Down	0	-
SE	Down	0	-
SE	Down	0	-
SE	Down	0	+
SE	Down	0	+
SE	Down	0	+
SE	Down	0	+
SE	Down	0	+
SE	Down	0	+
SE	Up	0	+
SE	Up	0	+
SE	Up	0	+
SE	Up	0	+
SE	Up	0	-
SE	Up	0	-
SE	Up	0	-
SE	Up	0	-
SE	Up	0	+
SE	Up	0	+
SE	Up	0	-
SE	Up	0	+
SE	Down	0	+

SE	Up	0 +
SE	Up	0 +
SE	Up	0 -
SE	Up	0 -
SE	Up	0 -
SE	Up	0 +
SE	Up	0 +
SE	Up	0 +
SE	Up	0 +
SE	Up	0 -
SE	Up	0 -
SE	Up	0 -
SE	Up	0 -
SE	Up	0 -
SE	Up	0 -
SE	Up	0 -
SE	Down	0 +
SE	Up	0 -
SE	Down	0 +
SE	Up	0 +
SE	Up	0 -
SE	Up	0 +
SE	Up	0 -
SE	Up	0 -
SE	Up	0 +
SE	Up	0 +
SE	Up	0 +
SE	Up	0 +
SE	Up	0 +
SE	Up	0 +
SE	Up	0 +
SE	Up	0 -
SE	Up	0 -
SE	Up	0 +
SE	Up	0 +
SE	Down	0 +
SE	Up	0 +
SE	Up	0 -
SE	Up	0 -
SE	Up	0 +
SE	Up	0 -
SE	Up	0 +
SE	Down	0 -
SE	Up	0 -
SE	Up	0 +
SE	Up	0 +
SE	Down	0 +
SE	Up	0 -
SE	Up	0 -
SE	Up	0 -
SE	Up	0 -
SE	Up	0 +
SE	Down	0 -
SE	Down	0 +
SE	Up	0 +
SE	Up	0 -
SE	Up	0 -
SE	Up	0 -

SE	Up	0	-
SE	Up	0	-
SE	Down	0	+
SE	Down	0	+
SE	Up	0	-
SE	Up	0	+
SE	Up	0	+
SE	Up	0	+
SE	Up	0	-
SE	Up	0	+
SE	Up	0	-
SE	Up	0	-
SE	Down	0	+
SE	Down	0	+
SE	Down	0	+
SE	Up	0	-
SE	Up	0	+
SE	Up	0	-
SE	Up	0	-
SE	Up	0	-
SE	Up	0	-
SE	Up	0	-
SE	Up	0	-
SE	Down	0	+
SE	Down	0	+
SE	Down	0	+
SE	Up	0	+
SE	Down	0	-
SE	Up	0	-
SE	Up	0	+
SE	Up	0	-
SE	Up	0	-
SE	Up	0	-
SE	Up	0	+
SE	Up	0	+
SE	Down	0	-
SE	Up	0	-
SE	Up	0	-
SE	Down	0	+
SE	Up	0	+
SE	Up	0	-
SE	Up	0	+
SE	Up	0	-
SE	Up	0	-
SE	Up	0	-
SE	Up	0	-
SE	Down	0	-
SE	Down	0	-
SE	Down	0	-
SE	Up	0	-
SE	Up	0	-
SE	Up	0	-
SE	Down	0	+
SE	Up	0	+
SE	Up	0	+
SE	Down	0	-
SE	Up	0	-
SE	Up	0	+

SE	Up	0	+
SE	Up	0	+
SE	Down	0	-
SE	Up	0	-
SE	Down	0	-
SE	Up	0	-
SE	Up	0	-
SE	Up	0	-
SE	Up	0	+
SE	Down	0	-
SE	Up	0	+
SE	Down	0	+
SE	Down	0	+
SE	Up	0	+
SE	Up	0	-
SE	Up	0	-
SE	Up	0	+
SE	Up	0	+
SE	Up	0	+
SE	Down	0	+
SE	Up	0	-
SE	Up	0	-
SE	Up	0	-
SE	Up	0	-
SE	Up	0	+
SE	Up	0	-
SE	Up	0	-
SE	Up	0	-
SE	Up	0	+
SE	Down	0	+
SE	Up	0	-
SE	Up	0	+
SE	Up	0	-
SE	Up	0	+
SE	Up	0	-
SE	Down	0	+
SE	Up	0	-
SE	Up	0	-
SE	Up	0	-
SE	Up	0	-
SE	Up	0	-
SE	Down	0	+
SE	Down	0	-
SE	Up	0	+
SE	Up	0	+
SE	Up	0	-
SE	Up	0	-
SE	Up	0	-
SE	Up	0	-
SE	Up	0	-
SE	Up	0	-
SE	Up	0	+
SE	Up	0	-
SE	Up	0	-
SE	Up	0	-
SE	Up	0	+
SE	Down	0	+
SE	Down	0	+

SE	Up	0	-
SE	Up	0	+
SE	Up	0	+
SE	Up	0	+
SE	Up	0	+
SE	Up	0	+
SE	Up	0	-
SE	Up	0	-
SE	Up	0	-
SE	Up	0	-
SE	Up	0	-
SE	Up	0	-
SE	Up	0	-
SE	Up	0	-
SE	Down	0	-
SE	Up	0	-
SE	Up	0	-
SE	Up	0	+
SE	Up	0	-
SE	Up	0	+
SE	Up	0	+
SE	Down	0	-
SE	Up	0	+
SE	Up	0	+
SE	Up	0	-
SE	Up	0	-
SE	Up	0	-
SE	Down	0	-
SE	Up	0	+
SE	Up	0	-
SE	Up	0	-
SE	Up	0	-
SE	Up	0	+
SE	Down	0	-
SE	Down	0	-
SE	Down	0	-
SE	Down	0	-
SE	Up	0	-
SE	Up	0	-
SE	Up	0	+
SE	Up	0	+
SE	Up	0	-
SE	Up	0	+
SE	Up	0	+
SE	Up	0	+
SE	Up	0	-
SE	Down	0	+
SE	Up	0	+
SE	Up	0	-
SE	Up	0	+
SE	Up	0	-
SE	Up	0	+
SE	Up	0	+
SE	Up	0	+
SE	Up	0	+
SE	Up	0	-
SE	Up	0	-
SE	Down	0	-

SE	Up	0	+
SE	Up	0	+
SE	Up	0	-
SE	Up	0	-
SE	Up	0	+
SE	Up	0	-
SE	Up	0	+
SE	Up	0	+
SE	Up	0	-
SE	Up	0	-
SE	Up	0	+
SE	Up	0	-
SE	Up	0	+
SE	Up	0	+
SE	Up	0	+
SE	Up	0	+
SE	Up	0	-
SE	Up	0	-
SE	Up	0	-
SE	Up	0	-
SE	Down	0	+
SE	Up	0	-
SE	Up	0	-
SE	Up	0	-
SE	Up	0	+
SE	Up	0	+
SE	Up	0	+
SE	Up	0	+
SE	Down	0	-
SE	Down	0	-
SE	Down	0	-
SE	Down	0	-
SE	Down	0	-
SE	Up	0	-
SE	Up	0	+
SE	Up	0	+
SE	Up	0	+
SE	Up	0	+
SE	Up	0	+
SE	Up	0	+
SE	Up	0	-
SE	Up	0	+
SE	Up	0	-
SE	Up	0	-
SE	Up	0	+
SE	Up	0	-
SE	Up	0	-
SE	Up	0	-
SE	Up	0	+
SE	Up	0	+
SE	Up	0	+
SE	Up	0	-
SE	Up	0	-
SE	Down	0	+
SE	Down	0	+
SE	Up	0	+
SE	Up	0	+
SE	Up	0	-

SE	Up	0	+
SE	Up	0	+
SE	Up	0	+
SE	Up	0	-
SE	Up	0	-
SE	Up	0	-
SE	Up	0	-
SE	Up	0	-
SE	Up	0	-
SE	Up	0	-
SE	Down	0	+
SE	Down	0	-
SE	Up	0	-
SE	Up	0	+
SE	Up	0	-
SE	Up	0	-
SE	Up	0	+
SE	Up	0	+
SE	Down	0	+
SE	Down	0	-
SE	Down	0	-
SE	Up	0	+
SE	Up	0	+
SE	Up	0	+
SE	Up	0	+
SE	Up	0	-
SE	Up	0	-
SE	Up	0	-
SE	Up	0	+
SE	Up	0	-
SE	Up	0	-
SE	Up	0	-
SE	Up	0	-
SE	Up	0	+
SE	Up	0	+
SE	Down	0	+
SE	Up	0	+
SE	Down	0	-
SE	Up	0	-
SE	Up	0	+
SE	Up	0	-
SE	Up	0	+
SE	Up	0	+
SE	Down	0	-
SE	Up	0	+
SE	Up	0	+
SE	Up	0	+
SE	Up	0	+
SE	Down	0	+
SE	Up	0	+
SE	Down	0	+
SE	Down	0	-
SE	Up	0	+
SE	Down	0	+
SE	Up	0	+
SE	Up	0	-
SE	Up	0	+
SE	Up	0	+

SE	Up	0	-
SE	Up	0	-
SE	Up	0	+
SE	Down	0	+
SE	Down	0	+
SE	Up	0	+
SE	Up	0	+
SE	Down	0	-
SE	Down	0	-
SE	Up	0	+
SE	Down	0	-
SE	Down	0	-
SE	Up	0	-
SE	Up	0	-
SE	Up	0	+
SE	Up	0	+
SE	Up	0	-
SE	Up	0	-
SE	Up	0	-
SE	Up	0	+
SE	Up	0	+
SE	Up	0	+
SE	Up	0	+
SE	Down	0	+
SE	Up	0	+
SE	Up	0	-
SE	Up	0	-
SE	Up	0	+
SE	Up	0	+
SE	Up	0	+
SE	Up	0	+
SE	Down	0	+
SE	Up	0	+
SE	Up	0	-
SE	Up	0	-
SE	Up	0	+
SE	Up	0	-
SE	Up	0	-
SE	Down	0	+
SE	Up	0	+
SE	Up	0	-
SE	Up	0	-
SE	Down	0	+
SE	Down	0	+
SE	Up	0	+
SE	Down	0	+
SE	Up	0	-
SE	Down	0	-
SE	Up	0	+
SE	Up	0	+
SE	Up	0	+
SE	Up	0	-
SE	Up	0	+
SE	Down	0	-
SE	Up	0	-
SE	Up	0	-
SE	Up	0	-
SE	Up	0	+

SE	Up	0	+
SE	Down	0	+
SE	Up	0	+
SE	Up	0	-
SE	Up	0	+
SE	Down	0	-
SE	Up	0	-
SE	Up	0	-
SE	Down	0	-
SE	Down	0	-
SE	Up	0	+
SE	Up	0	-
SE	Down	0	-
SE	Up	0	-
SE	Up	0	-
SE	Up	0	+
SE	Up	0	+
SE	Up	0	-
SE	Down	0	-
SE	Up	0	-
SE	Down	0	-
SE	Down	0	+
SE	Down	0	-
SE	Down	0	-
SE	Up	0	-
SE	Up	0	-
SE	Up	0	-
SE	Up	0	-
SE	Down	0	+
SE	Down	0	+
SE	Down	0	+
SE	Down	0	+
SE	Up	0	-
SE	Up	0	-
SE	Up	0	-
SE	Up	0	+
SE	Up	0	-
SE	Up	0	+
SE	Up	0	+
SE	Down	0	+
SE	Down	0	-
SE	Up	0	+
SE	Up	0	-
SE	Up	0	-
SE	Up	0	+
SE	Up	0	-
SE	Up	0	-
SE	Up	0	+
SE	Down	0	+
SE	Down	0	-
SE	Up	0	-
SE	Up	0	+
SE	Up	0	-
SE	Down	0	-
SE	Up	0	+
SE	Up	0	+
SE	Up	0	-
SE	Up	0	+

SE	Up	0	+
SE	Down	0	+
SE	Up	0	+
SE	Up	0	-
SE	Up	0	-
SE	Up	0	-
SE	Down	0	-
SE	Up	0	+
SE	Up	0	+
SE	Up	0	+
SE	Up	0	+
SE	Up	0	+
SE	Up	0	+
SE	Down	0	+
SE	Up	0	+
SE	Down	0	-
SE	Up	0	+
SE	Up	0	+
SE	Up	0	-
SE	Up	0	-
SE	Up	0	-
SE	Up	0	-
SE	Up	0	-
SE	Down	0	+
SE	Up	0	-
SE	Up	0	-
SE	Up	0	-
SE	Up	0	-
SE	Up	0	+
SE	Down	0	-
SE	Down	0	-
SE	Down	0	-
SE	Up	0	-
SE	Up	0	-
SE	Down	0	+
SE	Up	0	-
SE	Up	0	-
SE	Up	0	-
SE	Up	0	-
SE	Up	0	-
SE	Up	0	+
SE	Up	0	-
SE	Up	0	-
SE	Up	0	-
SE	Up	0	+
SE	Up	0	+
SE	Up	0	+
SE	Up	0	-
SE	Up	0	-
SE	Up	0	-
SE	Down	0	+
SE	Down	0	+
SE	Up	0	+
SE	Up	0	+
SE	Up	0	+
SE	Up	0	+
SE	Up	0	-
SE	Up	0	-

SE	Up	0	-
SE	Down	0	-
SE	Up	0	+
SE	Up	0	-
SE	Down	0	+
SE	Up	0	-
SE	Up	0	-
SE	Up	0	+
SE	Up	0	-
SE	Up	0	-
SE	Up	0	-
SE	Up	0	+
SE	Up	0	+
SE	Down	0	-
SE	Up	0	-
SE	Up	0	-
SE	Up	0	+
SE	Up	0	+
SE	Up	0	+
SE	Up	0	-
SE	Up	0	-
SE	Up	0	-
SE	Up	0	-
SE	Up	0	-
SE	Up	0	-
SE	Up	0	-
SE	Up	0	-
SE	Down	0	+
SE	Down	0	+
SE	Down	0	+
SE	Up	0	-
SE	Up	0	+
SE	Up	0	+
SE	Up	0	+
SE	Up	0	+
SE	Up	0	+
SE	Up	0	+
SE	Up	0	-
SE	Up	0	+
SE	Up	0	+
SE	Up	0	+
SE	Up	0	-
SE	Up	0	-
SE	Up	0	-
SE	Up	0	-
SE	Up	0	+
SE	Up	0	+
SE	Up	0	+
SE	Down	0	+
SE	Up	0	+
SE	Up	0	+
SE	Up	0	-
SE	Up	0	-
SE	Up	0	-
SE	Up	0	+
SE	Up	0	-
SE	Up	0	+
SE	Up	0	+

SE	Down	0	+
SE	Up	0	+
SE	Up	0	+
SE	Down	0	+
SE	Up	0	-
SE	Up	0	+
SE	Up	0	-
SE	Up	0	-
SE	Up	0	-
SE	Up	0	-
SE	Up	0	-
SE	Down	0	-
SE	Up	0	-
SE	Up	0	+
SE	Up	0	+
SE	Up	0	-
SE	Up	0	-
SE	Up	0	-
SE	Down	0	-
SE	Up	0	+
SE	Up	0	-
SE	Up	0	+
SE	Up	0	-
SE	Up	0	-
SE	Up	0	+
SE	Up	0	+
SE	Up	0	+
SE	Up	0	+
SE	Down	0	+
SE	Down	0	+
SE	Down	0	+
SE	Up	0	+
SE	Up	0	-
SE	Up	0	-
SE	Up	0	-
SE	Down	0	+
SE	Up	0	+
SE	Up	0	+
SE	Down	0	-
SE	Up	0	+
SE	Down	0	+
SE	Up	0	+
SE	Up	0	+
SE	Up	0	+
SE	Up	0	+
SE	Down	0	+
SE	Down	0	+
SE	Up	0	+
SE	Up	0	-
SE	Up	0	-
SE	Up	0	-
SE	Up	0	+
SE	Up	0	-
SE	Up	0	-
SE	Up	0	+
SE	Up	0	+
SE	Up	0	+
SE	Up	0	-

SE	Down	0	-
SE	Up	0	+
SE	Up	0	-
SE	Up	0	+
SE	Up	0	-
SE	Up	0	+
SE	Down	0	-
SE	Up	0	+
SE	Up	0	-
SE	Up	0	+
SE	Up	0	-
SE	Up	0	-
SE	Up	0	-
SE	Up	0	+
SE	Up	0	-
SE	Up	0	-
SE	Down	0	-
SE	Down	0	-
SE	Down	0	-
SE	Up	0	-
SE	Up	0	-
SE	Up	0	-
SE	Up	0	+
SE	Up	0	+
SE	Up	0	-
SE	Up	0	+
SE	Up	0	+
SE	Up	0	-
SE	Up	0	-
SE	Up	0	-
SE	Up	0	+
SE	Up	0	+
SE	Up	0	-
SE	Up	0	-
SE	Up	0	-
SE	Up	0	+
SE	Up	0	-
SE	Up	0	-
SE	Down	0	+
SE	Up	0	-
SE	Up	0	+
SE	Up	0	+
SE	Down	0	+
SE	Up	0	-
SE	Up	0	-
SE	Down	0	-
SE	Down	0	-
SE	Up	0	+
SE	Up	0	-
SE	Up	0	+
SE	Up	0	-
SE	Down	0	+
SE	Up	0	-
SE	Up	0	-
SE	Up	0	-
SE	Up	0	+
SE	Up	0	-
SE	Up	0	-

SE	Up	0	-
SE	Up	0	-
SE	Up	0	-
SE	Up	0	-
SE	Up	0	-
SE	Up	0	+
SE	Up	0	+
SE	Down	0	-
SE	Down	0	-
SE	Up	0	-
SE	Up	0	+
SE	Up	0	+
SE	Up	0	+
SE	Up	0	+
SE	Up	0	+
SE	Up	0	+
SE	Up	0	+
SE	Down	0	+
SE	Up	0	-
SE	Up	0	-
SE	Up	0	+
SE	Up	0	+
SE	Down	0	-
SE	Up	0	-
SE	Up	0	-
SE	Up	0	-
SE	Up	0	-
SE	Up	0	-
SE	Up	0	-
SE	Up	0	-
SE	Up	0	+
SE	Up	0	+
SE	Up	0	+
SE	Up	0	-
SE	Up	0	+
SE	Down	0	+
SE	Down	0	+
SE	Down	0	+
SE	Up	0	+
SE	Up	0	-
SE	Up	0	+
SE	Up	0	+
SE	Up	0	+
SE	Up	0	+
SE	Up	0	+
SE	Up	0	+
SE	Down	0	+
SE	Up	0	+
SE	Up	0	+
SE	Up	0	+
SE	Up	0	+
SE	Up	0	+
SE	Down	0	-
SE	Up	0	+
SE	Up	0	+
SE	Up	0	+
SE	Up	0	+
SE	Up	0	+

SE	Up	0	-
SE	Down	0	-
SE	Up	0	+
SE	Up	0	-
SE	Up	0	-
SE	Up	0	+
SE	Down	0	-
SE	Up	0	+
SE	Down	0	+
SE	Up	0	+
SE	Down	0	+
SE	Up	0	+
SE	Up	0	+
SE	Up	0	+
SE	Up	0	-
SE	Up	0	-
SE	Up	0	-
SE	Down	0	+
SE	Down	0	+
SE	Down	0	+
SE	Up	0	+
SE	Up	0	-
SE	Up	0	-
SE	Up	0	+
SE	Up	0	-
SE	Up	0	-
SE	Up	0	-
SE	Down	0	+
SE	Up	0	-
SE	Up	0	+
SE	Down	0	-
SE	Up	0	-
SE	Up	0	+
SE	Up	0	+
SE	Down	0	+
SE	Up	0	+
SE	Up	0	-
SE	Up	0	-
SE	Up	0	-
SE	Down	0	-
SE	Up	0	+
SE	Down	0	-
SE	Up	0	-
SE	Up	0	-
SE	Up	0	-
SE	Up	0	-
SE	Up	0	-
SE	Up	0	-
SE	Up	0	-
SE	Up	0	-
SE	Down	0	-
SE	Down	0	-
SE	Up	0	-
SE	Down	0	-
SE	Up	0	+
SE	Up	0	+
SE	Down	0	-
SE	Down	0	-
