# Supplementary material for: SRSF1-mediated alternative splicing is required for spermatogenesis
Source: Int J Biol Sci. 2023 Sep 11;19(15):4883–97. doi: 10.7150/ijbs.83474 (PMC10539708; doi:10.7150/ijbs.83474)
Supplement: Supplementary file 1 — Supplementary figures and tables. [file ijbsv19p4883s1.zip › fig legends.pdf]

Fig.S1. SRSF1 regulates mRNA alternative splicing in testes

Five AS events significantly affected by deletion of SRSF1 in the testes at P10.

The different types of alternatively spliced events are shown.

Fig.S2. The original gel pictures in figure 8
