# Supplementary figures and images for: SRSF1-mediated alternative splicing is required for spermatogenesis
Source: Int J Biol Sci. 2023 Sep 11;19(15):4883–97. doi: 10.7150/ijbs.83474 (PMC10539708; doi:10.7150/ijbs.83474)

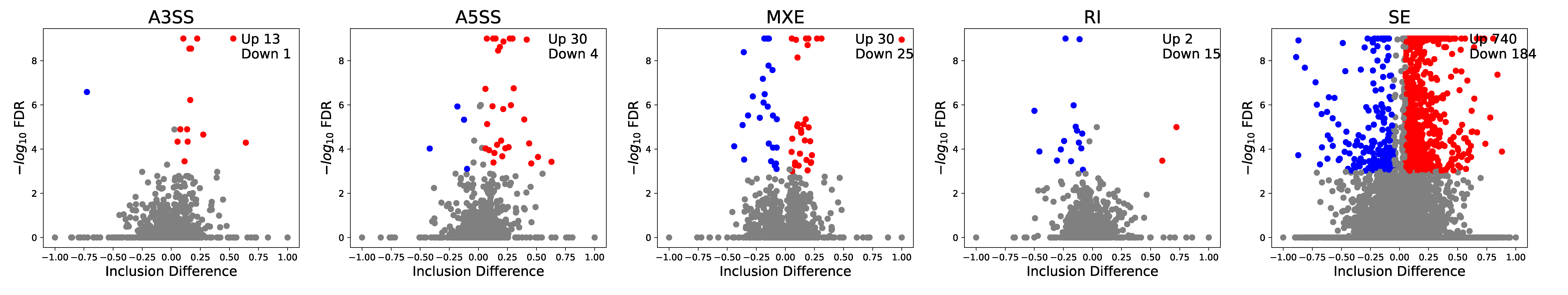

Supplement: Supplementary file 1 — Supplementary figures and tables. [file ijbsv19p4883s1.zip › Supplementary materials/Fig S1.tif]

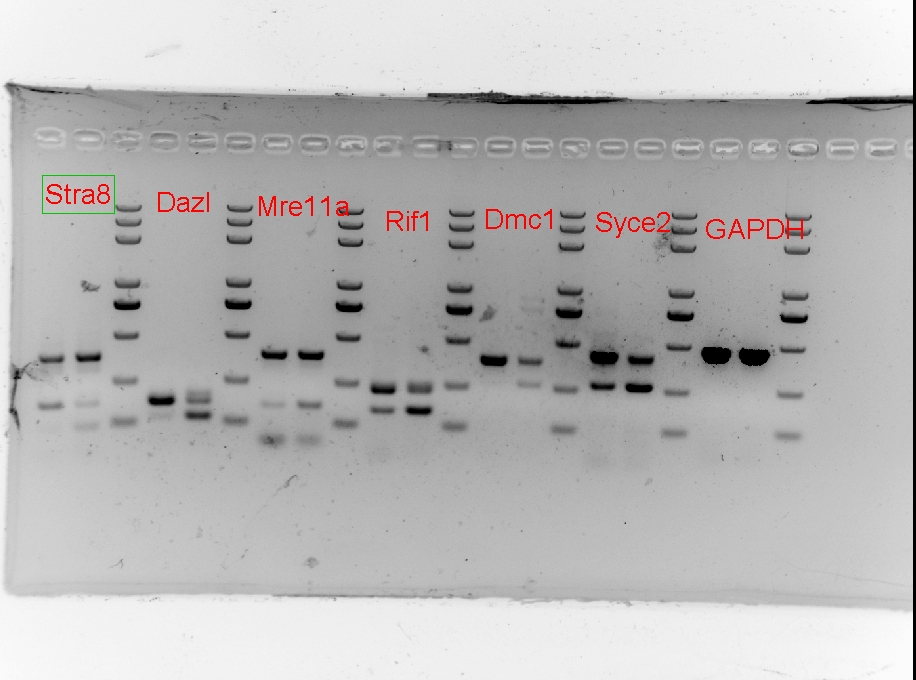

Supplement: Supplementary file 1 — Supplementary figures and tables. [file ijbsv19p4883s1.zip › Supplementary materials/Fig S2.jpg]
