# Supplementary material for: SRSF1-mediated alternative splicing is required for spermatogenesis
Source: Int J Biol Sci. 2023 Sep 11;19(15):4883–97. doi: 10.7150/ijbs.83474 (PMC10539708; doi:10.7150/ijbs.83474)
Supplement: Supplementary file 1 — Supplementary figures and tables. [file Table 5 The list of differentially expressed genes.pdf › Supplementary materials/Table 5 The list of differentially expressed genes.pdf]

| | | | Ctrl | KO | baseMean |
|---|---|---|---|---|---|
| ENSMUSG00000041974 | protein_coding | 2310008H04Rik | 1129.063 | 511.3357 | 820.1994 |
| ENSMUSG00000042453 | protein_coding | Reln | 1343.215 | 358.433 | 850.8238 |
| ENSMUSG00000021257 | protein_coding | Angel1 | 1065.691 | 333.3934 | 699.5421 |
| ENSMUSG00000018379 | protein_coding | Srsf1 | 9926.459 | 4327.811 | 7127.135 |
| ENSMUSG00000038080 | protein_coding | Kdm1b | 1643.876 | 852.5461 | 1248.211 |
| ENSMUSG00000029636 | protein_coding | Wasf3 | 852.5465 | 376.4511 | 614.4988 |
| ENSMUSG00000007833 | protein_coding | Aldh16a1 | 911.8562 | 446.3736 | 679.1149 |
| ENSMUSG00000036181 | protein_coding | Hist1h1c | 367.9019 | 932.9611 | 650.4315 |
| ENSMUSG00000037572 | protein_coding | Wdhd1 | 3731.949 | 2270.721 | 3001.335 |
| ENSMUSG00000001157 | protein_coding | Gmcl1 | 1407.259 | 803.1437 | 1105.201 |
| ENSMUSG00000062007 | protein_coding | Hsh2d | 13.0036 | 91.57175 | 52.28768 |
| ENSMUSG00000042010 | protein_coding | Acacb | 514.6989 | 1074.121 | 794.4098 |
| ENSMUSG00000026944 | protein_coding | Abca2 | 3292.395 | 1821.304 | 2556.849 |
| ENSMUSG00000034384 | protein_coding | Barhl2 | 68.37025 | 190.6703 | 129.5203 |
| ENSMUSG00000063626 | protein_coding | Unc5d | 290.7634 | 108.3386 | 199.551 |
| ENSMUSG00000033732 | protein_coding | Sf3b3 | 5715.099 | 3692.535 | 4703.817 |
| ENSMUSG00000020431 | protein_coding | Adcy1 | 279.3799 | 120.1485 | 199.7642 |
| ENSMUSG00000028683 | protein_coding | Eif2b3 | 1363.526 | 727.5048 | 1045.515 |
| ENSMUSG00000078941 | protein_coding | Taf9 | 376.7828 | 678.6697 | 527.7262 |
| ENSMUSG00000022240 | protein_coding | Ctnnd2 | 280.1465 | 119.666 | 199.9063 |
| ENSMUSG00000030000 | protein_coding | Add2 | 85.81526 | 225.3704 | 155.5928 |
| ENSMUSG00000026709 | protein_coding | Dars2 | 1129.877 | 684.4219 | 907.1496 |
| ENSMUSG00000021747 | protein_coding | 4930452B06Rik | 385.6156 | 197.6626 | 291.6391 |
| ENSMUSG00000028089 | protein_coding | Chd1l | 1762.138 | 1060.52 | 1411.329 |
| ENSMUSG00000052949 | protein_coding | Rnf157 | 1114.655 | 626.4137 | 870.5341 |
| ENSMUSG00000034057 | protein_coding | Gm239 | 19.0789 | 87.23527 | 53.15708 |
| ENSMUSG00000026965 | protein_coding | Anapc2 | 3066.738 | 2029.256 | 2547.997 |
| ENSMUSG00000017692 | protein_coding | Rhbdl3 | 95.57779 | 233.144 | 164.3609 |
| ENSMUSG00000058550 | protein_coding | Dppa4 | 239.4491 | 440.0195 | 339.7343 |
| ENSMUSG00000024789 | protein_coding | Jak2 | 1786.887 | 1150.312 | 1468.6 |
| ENSMUSG00000049932 | protein_coding | H2afx | 1641.801 | 2448.108 | 2044.954 |
| ENSMUSG00000028434 | protein_coding | Epb4.1l4b | 1226.615 | 727.7783 | 977.1969 |
| ENSMUSG00000023169 | protein_coding | Slc38a1 | 170.8635 | 343.4394 | 257.1515 |
| ENSMUSG00000022489 | protein_coding | Pde1b | 200.7438 | 83.79925 | 142.2715 |
| ENSMUSG00000020380 | protein_coding | Rad50 | 2522.419 | 1771.888 | 2147.153 |
| ENSMUSG00000073434 | protein_coding | Wdr90 | 1573.981 | 983.259 | 1278.62 |
| ENSMUSG00000006058 | protein_coding | Snf8 | 1602.036 | 1073.483 | 1337.76 |
| ENSMUSG00000060639 | protein_coding | Hist1h4i | 43.44231 | 163.1093 | 103.2758 |
| ENSMUSG00000041720 | protein_coding | Pi4ka | 3947.552 | 2222.126 | 3084.839 |
| ENSMUSG00000078653 | protein_coding | Cntd1 | 138.5859 | 21.39631 | 79.99108 |
| ENSMUSG00000070732 | protein_coding | Rbm44 | 1720.818 | 1170.761 | 1445.789 |
| ENSMUSG00000004864 | protein_coding | Mapk13 | 428.6421 | 712.918 | 570.78 |
| ENSMUSG00000025878 | protein_coding | Uimc1 | 2287.894 | 1363.84 | 1825.867 |
| ENSMUSG00000030075 | protein_coding | Cntn3 | 221.0737 | 103.0264 | 162.0501 |
| ENSMUSG00000079022 | protein_coding | Col22a1 | 450.3489 | 241.0916 | 345.7203 |
| ENSMUSG00000015880 | protein_coding | Ncapg | 1879.073 | 1070.963 | 1475.018 |
| ENSMUSG00000091219 | lincRNA | Gm17254 | 117.6209 | 37.1187 | 77.3698 |
| ENSMUSG00000006567 | protein_coding | Atp7b | 184.1272 | 77.59367 | 130.8605 |
| ENSMUSG00000031762 | protein_coding | Mt2 | 1754.99 | 2480.405 | 2117.698 |
| ENSMUSG00000024560 | protein_coding | Cxxc1 | 3270.211 | 2267.317 | 2768.764 |
| ENSMUSG00000084911 | processed_transcrip | Gm16185 | 642.6569 | 1082.17 | 862.4133 |
| ENSMUSG00000039176 | protein_coding | Polg | 2680.517 | 1872.222 | 2276.369 |
| ENSMUSG00000024312 | protein_coding | Wdr46 | 1756.808 | 1020.236 | 1388.522 |
| ENSMUSG00000024404 | protein_coding | Riok3 | 2525.934 | 1746.621 | 2136.277 |
| ENSMUSG00000019178 | protein_coding | Styxl1 | 78.02327 | 20.79304 | 49.40815 |
| ENSMUSG00000031660 | protein_coding | Brd7 | 3154.518 | 2285.53 | 2720.024 |
| ENSMUSG00000037621 | protein_coding | Atoh8 | 393.272 | 223.6092 | 308.4406 |

| | | | | | |
|---|---|---|---|---|---|
| ENSMUSG00000036352 | protein_coding | Ubac1 | 2155.467 | 1557.051 | 1856.259 |
| ENSMUSG00000019214 | protein_coding | Chtf18 | 1681.952 | 970.9349 | 1326.443 |
| ENSMUSG00000006517 | protein_coding | Mvd | 840.0138 | 515.1875 | 677.6007 |
| ENSMUSG00000035486 | protein_coding | Plk5 | 211.1073 | 79.51859 | 145.3129 |
| ENSMUSG00000029055 | protein_coding | Plch2 | 77.58405 | 212.4896 | 145.0368 |
| ENSMUSG00000040289 | protein_coding | Hey1 | 441.7511 | 247.0069 | 344.379 |
| ENSMUSG00000031297 | protein_coding | Slc7a3 | 16.79952 | 63.38062 | 40.09007 |
| ENSMUSG00000061601 | protein_coding | Pclo | 301.6413 | 118.8222 | 210.2317 |
| ENSMUSG00000030757 | protein_coding | Zkscan2 | 425.6474 | 245.115 | 335.3812 |
| ENSMUSG00000025236 | protein_coding | Adpgk | 1103.723 | 775.5694 | 939.6462 |
| ENSMUSG00000050345 | protein_coding | 4930486L24Rik | 228.6952 | 456.274 | 342.4846 |
| ENSMUSG00000090430 | lincRNA | Snhg4 | 147.6325 | 273.6734 | 210.653 |
| ENSMUSG00000021318 | protein_coding | Gli3 | 902.3707 | 571.0644 | 736.7176 |
| ENSMUSG00000010760 | protein_coding | Phlda2 | 43.2388 | 122.4744 | 82.85661 |
| ENSMUSG00000021069 | protein_coding | Pygl | 729.9023 | 462.0791 | 595.9907 |
| ENSMUSG00000054405 | protein_coding | Dnajc8 | 3821.75 | 2835.99 | 3328.87 |
| ENSMUSG00000087107 | processed_transcrip | AI662270 | 101.4046 | 219.2395 | 160.3221 |
| ENSMUSG00000021367 | protein_coding | Edn1 | 367.3516 | 606.4254 | 486.8885 |
| ENSMUSG00000048647 | protein_coding | Exd1 | 372.1541 | 177.7763 | 274.9652 |
| ENSMUSG00000036678 | protein_coding | Aaas | 1172.399 | 717.5852 | 944.9919 |
| ENSMUSG00000056895 | protein_coding | Hist3h2ba | 27.06791 | 85.71542 | 56.39167 |
| ENSMUSG00000022236 | protein_coding | Ropn1l | 133.0956 | 50.04729 | 91.57145 |
| ENSMUSG00000038046 | protein_coding | Rnmtl1 | 426.5647 | 250.8378 | 338.7012 |
| ENSMUSG00000035517 | protein_coding | Tdrd7 | 1606.876 | 1012.29 | 1309.583 |
| ENSMUSG00000042211 | protein_coding | Fbxo38 | 2959.939 | 2168.805 | 2564.372 |
| ENSMUSG00000060989 | pseudogene | Gm11847 | 762.4566 | 1213.566 | 988.0111 |
| ENSMUSG00000040860 | protein_coding | Crocc | 1474.451 | 1004.771 | 1239.611 |
| ENSMUSG00000037552 | protein_coding | Plekhg2 | 2220.821 | 1611.753 | 1916.287 |
| ENSMUSG00000032492 | protein_coding | Pth1r | 516.5285 | 293.4417 | 404.9851 |
| ENSMUSG00000005338 | protein_coding | Cadm3 | 180.8283 | 502.434 | 341.6312 |
| ENSMUSG00000031295 | protein_coding | Phka2 | 1216.777 | 812.4892 | 1014.633 |
| ENSMUSG00000024970 | protein_coding | AI846148 | 1060.201 | 717.197 | 888.6988 |
| ENSMUSG00000004798 | protein_coding | Ulk2 | 1871.261 | 1322.475 | 1596.868 |
| ENSMUSG00000034391 | protein_coding | Fbxo15 | 164.532 | 49.06122 | 106.7966 |
| ENSMUSG00000037032 | protein_coding | Apbb1 | 976.1232 | 647.0959 | 811.6095 |
| ENSMUSG00000057265 | protein_coding | 2900006K08Rik | 143.2335 | 58.52946 | 100.8815 |
| ENSMUSG00000030279 | protein_coding | 5730419I09Rik | 1415.468 | 898.8411 | 1157.154 |
| ENSMUSG00000010651 | protein_coding | Acaa1b | 266.425 | 137.0044 | 201.7147 |
| ENSMUSG00000001150 | protein_coding | Mcm3ap | 2539.296 | 1844.692 | 2191.994 |
| ENSMUSG00000026213 | protein_coding | Stk11ip | 1736.76 | 1217.301 | 1477.03 |
| ENSMUSG00000042498 | protein_coding | D330045A20Rik | 877.4802 | 567.9349 | 722.7075 |
| ENSMUSG00000020387 | protein_coding | Phf15 | 1194.521 | 831.1465 | 1012.834 |
| ENSMUSG00000029414 | protein_coding | Kntc1 | 1586.183 | 997.7287 | 1291.956 |
| ENSMUSG00000033105 | protein_coding | Lss | 1406.149 | 1003.999 | 1205.074 |
| ENSMUSG00000038742 | protein_coding | Angptl6 | 121.7879 | 52.43385 | 87.11088 |
| ENSMUSG00000029151 | protein_coding | Slc30a3 | 53.17308 | 128.1988 | 90.68595 |
| ENSMUSG00000043903 | protein_coding | Gm22 | 94.44351 | 204.2144 | 149.329 |
| ENSMUSG00000018654 | protein_coding | Ikzf1 | 127.1685 | 60.76493 | 93.96673 |
| ENSMUSG00000029769 | protein_coding | Ccdc136 | 1226.901 | 751.2843 | 989.0926 |
| ENSMUSG00000045136 | protein_coding | Tubb2b | 112.8237 | 231.9228 | 172.3733 |
| ENSMUSG00000004613 | protein_coding | Lim2 | 11.57196 | 54.62527 | 33.09862 |
| ENSMUSG00000027353 | protein_coding | Mcm8 | 1270.953 | 832.1547 | 1051.554 |
| ENSMUSG00000013833 | protein_coding | Med16 | 1281.617 | 939.6479 | 1110.633 |
| ENSMUSG00000038128 | protein_coding | Camk4 | 313.3997 | 182.7731 | 248.0864 |
| ENSMUSG00000025357 | protein_coding | Dgka | 481.6879 | 312.1338 | 396.9109 |
| ENSMUSG00000040231 | protein_coding | Syngr4 | 191.228 | 69.10866 | 130.1683 |
| ENSMUSG00000024045 | protein_coding | Akap8 | 3807.745 | 5061.05 | 4434.398 |
| ENSMUSG00000033762 | protein_coding | Recql4 | 940.7256 | 635.0656 | 787.8956 |

| | | | | | |
|---|---|---|---|---|---|
| ENSMUSG00000056771 | protein_coding | Gm10010 | 192.422 | 76.86671 | 134.6444 |
| ENSMUSG00000004951 | protein_coding | Hspb1 | 1931.398 | 2814.733 | 2373.065 |
| ENSMUSG00000044216 | protein_coding | Kcnj4 | 224.0589 | 107.7482 | 165.9036 |
| ENSMUSG00000017978 | protein_coding | Cadps2 | 422.5365 | 244.0469 | 333.2917 |
| ENSMUSG00000047171 | protein_coding | Helt | 193.583 | 94.28038 | 143.9317 |
| ENSMUSG00000060499 | protein_coding | Rpl10l | 957.063 | 1321.717 | 1139.39 |
| ENSMUSG00000031583 | protein_coding | Wrn | 1042.658 | 745.4576 | 894.0577 |
| ENSMUSG00000018796 | protein_coding | Acsl1 | 1281.331 | 839.8758 | 1060.603 |
| ENSMUSG00000010505 | protein_coding | Myt1 | 2.287571 | 26.28859 | 14.28808 |
| ENSMUSG00000028391 | protein_coding | Wdr31 | 342.2183 | 190.6543 | 266.4363 |
| ENSMUSG00000015365 | protein_coding | Mov10l1 | 4080.507 | 2216.866 | 3148.686 |
| ENSMUSG00000060985 | lincRNA | Tdrd5 | 977.4717 | 649.6147 | 813.5432 |
| ENSMUSG00000033396 | protein_coding | Spg11 | 2063.584 | 1502.07 | 1782.827 |
| ENSMUSG00000048814 | protein_coding | Lonrf2 | 270.6308 | 109.2359 | 189.9333 |
| ENSMUSG00000020639 | protein_coding | Pfn4 | 104.1861 | 44.50162 | 74.34384 |
| ENSMUSG00000044156 | protein_coding | Hepacam2 | 42.75753 | 10.48843 | 26.62298 |
| ENSMUSG00000043102 | protein_coding | Qrfp | 121.3866 | 54.7151 | 88.05083 |
| ENSMUSG00000030256 | protein_coding | Bhlhe41 | 347.3691 | 647.2686 | 497.3189 |
| ENSMUSG00000071547 | protein_coding | Nt5dc2 | 1613.375 | 1096.063 | 1354.719 |
| ENSMUSG00000042269 | protein_coding | Fam92b | 40.28159 | 7.204905 | 23.74325 |
| ENSMUSG00000040560 | protein_coding | Wdr7 | 1266.375 | 912.9933 | 1089.684 |
| ENSMUSG00000028024 | protein_coding | Enpep | 222.1221 | 423.2332 | 322.6777 |
| ENSMUSG00000009108 | protein_coding | Gnat2 | 220.9154 | 127.9646 | 174.44 |
| ENSMUSG00000060098 | protein_coding | Prmt7 | 2172.763 | 1632.692 | 1902.728 |
| ENSMUSG00000039621 | protein_coding | Prex1 | 1030.715 | 698.5477 | 864.6315 |
| ENSMUSG00000060733 | protein_coding | Ipmk | 1308.752 | 933.5691 | 1121.161 |
| ENSMUSG00000026980 | protein_coding | Ly75 | 356.9537 | 203.1822 | 280.068 |
| ENSMUSG00000087672 | processed_transcrip | Gm15122 | 52.01144 | 120.2077 | 86.10957 |
| ENSMUSG00000045467 | protein_coding | Ttll13 | 139.6387 | 62.67318 | 101.156 |
| ENSMUSG00000045004 | protein_coding | Spata21 | 80.91235 | 160.679 | 120.7957 |
| ENSMUSG00000039323 | protein_coding | Igfbp2 | 1373.041 | 857.9746 | 1115.508 |
| ENSMUSG00000028541 | protein_coding | B4galt2 | 879.4238 | 633.4697 | 756.4468 |
| ENSMUSG00000028132 | protein_coding | Tmem56 | 431.6999 | 245.4852 | 338.5926 |
| ENSMUSG00000025069 | protein_coding | Gsto2 | 171.2803 | 82.16881 | 126.7246 |
| ENSMUSG00000034303 | protein_coding | Ccdc15 | 342.0433 | 222.7356 | 282.3894 |
| ENSMUSG00000042426 | protein_coding | Dhx29 | 1625.685 | 1216.696 | 1421.19 |
| ENSMUSG00000054889 | protein_coding | Dsp | 67.19706 | 138.5934 | 102.8952 |
| ENSMUSG00000004035 | protein_coding | Gstm7 | 4731.227 | 6269.89 | 5500.558 |
| ENSMUSG00000092470 | protein_coding | Gm20518 | 29.76317 | 5.465308 | 17.61424 |
| ENSMUSG00000029446 | protein_coding | Psph | 852.6347 | 597.4657 | 725.0502 |
| ENSMUSG00000031748 | protein_coding | Gnao1 | 323.3647 | 206.5537 | 264.9592 |
| ENSMUSG00000035284 | protein_coding | Vps13c | 3663.793 | 2502.647 | 3083.22 |
| ENSMUSG00000032815 | protein_coding | Fanca | 1201.347 | 831.9899 | 1016.669 |
| ENSMUSG00000034560 | protein_coding | A230046K03Rik | 1871.177 | 2434.162 | 2152.669 |
| ENSMUSG00000042305 | protein_coding | Tmem183a | 2661.067 | 3401.153 | 3031.11 |
| ENSMUSG00000091444 | lincRNA | 2010300F17Rik | 3.558345 | 23.10603 | 13.33219 |
| ENSMUSG00000054727 | protein_coding | 1700013H16Rik | 1098.407 | 603.1971 | 850.8021 |
| ENSMUSG00000026009 | protein_coding | Icos | 5.193495 | 36.43891 | 20.8162 |
| ENSMUSG00000085196 | lincRNA | Gm14963 | 57.06427 | 19.94719 | 38.50573 |
| ENSMUSG00000059434 | protein_coding | Gckr | 16.85253 | 54.7324 | 35.79246 |
| ENSMUSG00000025983 | protein_coding | Ccdc150 | 120.713 | 39.15162 | 79.93229 |
| ENSMUSG00000023043 | protein_coding | Krt18 | 25.70672 | 72.64739 | 49.17705 |
| ENSMUSG00000003435 | protein_coding | Supt5h | 6678.518 | 4929.244 | 5803.881 |
| ENSMUSG00000034022 | protein_coding | Cpsf1 | 2901.283 | 2158.471 | 2529.877 |
| ENSMUSG00000028782 | protein_coding | Bai2 | 366.8915 | 235.8722 | 301.3819 |
| ENSMUSG00000026239 | protein_coding | Pde6d | 1209.873 | 858.1943 | 1034.034 |
| ENSMUSG00000000811 | protein_coding | Txnrd3 | 1707.98 | 1192.731 | 1450.355 |
| ENSMUSG00000034023 | protein_coding | Fancd2 | 1766.476 | 1261.51 | 1513.993 |

| | | | | | |
|---|---|---|---|---|---|
| ENSMUSG00000029176 | protein_coding | Anapc4 | 3731.517 | 2772.043 | 3251.78 |
| ENSMUSG00000031765 | protein_coding | Mt1 | 1396.129 | 1942.498 | 1669.313 |
| ENSMUSG00000001366 | protein_coding | Fbxo9 | 1578.269 | 1148.314 | 1363.291 |
| ENSMUSG00000024521 | protein_coding | Pmaip1 | 380.3851 | 639.5115 | 509.9483 |
| ENSMUSG00000022763 | protein_coding | Aifm3 | 729.4072 | 508.7646 | 619.0859 |
| ENSMUSG00000025287 | protein_coding | Acot9 | 971.6546 | 642.2806 | 806.9676 |
| ENSMUSG00000001829 | protein_coding | Clpb | 1955.99 | 1473.493 | 1714.742 |
| ENSMUSG00000021481 | protein_coding | Zfp346 | 1265.036 | 1686.106 | 1475.571 |
| ENSMUSG00000058006 | protein_coding | Mdn1 | 17024.73 | 12794.83 | 14909.78 |
| ENSMUSG00000025572 | protein_coding | Tmc6 | 557.6982 | 755.2722 | 656.4852 |
| ENSMUSG00000087433 | processed_transcrip | Gm14167 | 70.76069 | 22.75606 | 46.75838 |
| ENSMUSG00000043924 | protein_coding | A330049M08Rik | 224.7033 | 348.8348 | 286.7691 |
| ENSMUSG00000020669 | protein_coding | Sh3yl1 | 164.2368 | 88.36905 | 126.3029 |
| ENSMUSG00000074064 | protein_coding | Mlycd | 511.6356 | 346.1262 | 428.8809 |
| ENSMUSG00000040359 | protein_coding | 1810074P20Rik | 1516.929 | 1107.73 | 1312.329 |
| ENSMUSG00000053279 | protein_coding | Aldh1a1 | 13771.8 | 17496.75 | 15634.28 |
| ENSMUSG00000038000 | protein_coding | Acd | 668.1413 | 476.4374 | 572.2894 |
| ENSMUSG00000038932 | protein_coding | Tcfl5 | 1250.519 | 907.9151 | 1079.217 |
| ENSMUSG00000083618 | pseudogene | Gm11771 | 6.196204 | 33.72743 | 19.96182 |
| ENSMUSG00000050079 | protein_coding | Rspry1 | 2440.701 | 1702.376 | 2071.539 |
| ENSMUSG00000025060 | protein_coding | Slk | 5889.484 | 4560.872 | 5225.178 |
| ENSMUSG00000086635 | lincRNA | 4932415G12Rik | 761.7844 | 470.6941 | 616.2393 |
| ENSMUSG00000021032 | protein_coding | Ngb | 32.63757 | 79.31489 | 55.97623 |
| ENSMUSG00000022191 | protein_coding | Drosha | 4610.603 | 3444.456 | 4027.529 |
| ENSMUSG00000030207 | protein_coding | 8430419L09Rik | 316.4041 | 458.1141 | 387.2591 |
| ENSMUSG00000021022 | protein_coding | Ppp2r3c | 698.0499 | 491.6805 | 594.8652 |
| ENSMUSG00000028078 | protein_coding | Dclk2 | 477.0281 | 312.3521 | 394.6901 |
| ENSMUSG00000005493 | protein_coding | Msh4 | 243.5804 | 91.13739 | 167.3589 |
| ENSMUSG00000056596 | protein_coding | Trnp1 | 283.444 | 185.3394 | 234.3917 |
| ENSMUSG00000022652 | protein_coding | Morc1 | 826.0277 | 581.8386 | 703.9332 |
| ENSMUSG00000069053 | protein_coding | Ube1y1 | 3709.927 | 2607.623 | 3158.775 |
| ENSMUSG00000020250 | protein_coding | Txnrd1 | 1871.258 | 2679.316 | 2275.287 |
| ENSMUSG00000068101 | protein_coding | Cenpm | 488.8021 | 341.7182 | 415.2602 |
| ENSMUSG00000027346 | protein_coding | Gpcpd1 | 3197.008 | 4635.909 | 3916.458 |
| ENSMUSG00000025318 | protein_coding | Jph3 | 236.0063 | 131.068 | 183.5371 |
| ENSMUSG00000019790 | protein_coding | Stxbp5 | 1360.506 | 1025.197 | 1192.851 |
| ENSMUSG00000038368 | protein_coding | BC057079 | 999.6056 | 644.6955 | 822.1505 |
| ENSMUSG00000022456 | protein_coding | 3-Sep | 210.7351 | 113.247 | 161.9911 |
| ENSMUSG00000083977 | pseudogene | Mageb8-ps | 66.32577 | 129.4973 | 97.91154 |
| ENSMUSG00000024968 | protein_coding | Rcor2 | 1410.56 | 902.2496 | 1156.405 |
| ENSMUSG00000030271 | protein_coding | Ogg1 | 398.4331 | 273.9993 | 336.2162 |
| ENSMUSG00000038388 | protein_coding | Mpp6 | 1466.364 | 1074.831 | 1270.597 |
| ENSMUSG00000032705 | protein_coding | Exd2 | 1256.404 | 915.605 | 1086.005 |
| ENSMUSG00000026404 | protein_coding | Ddx59 | 359.5929 | 236.8578 | 298.2254 |
| ENSMUSG00000018189 | protein_coding | Uchl5 | 846.716 | 631.8728 | 739.2944 |
| ENSMUSG00000033730 | protein_coding | Egr3 | 14.7672 | 46.17675 | 30.47197 |
| ENSMUSG00000081965 | pseudogene | Gm11620 | 52.30664 | 108.0308 | 80.16872 |
| ENSMUSG00000026679 | protein_coding | Enkur | 134.2026 | 73.61067 | 103.9066 |
| ENSMUSG00000089774 | protein_coding | Slc5a3 | 2614.576 | 2006.015 | 2310.295 |
| ENSMUSG00000056938 | protein_coding | Acbd4 | 315.549 | 449.4965 | 382.5227 |
| ENSMUSG00000020773 | protein_coding | Trim47 | 1691.636 | 2398.935 | 2045.286 |
| ENSMUSG00000026496 | protein_coding | Parp1 | 9045.124 | 6798.658 | 7921.891 |
| ENSMUSG00000028698 | protein_coding | Pik3r3 | 4060.926 | 2882.561 | 3471.744 |
| ENSMUSG00000004880 | protein_coding | Lbr | 2191.325 | 1733.59 | 1962.457 |
| ENSMUSG00000030681 | protein_coding | Mvp | 393.9486 | 610.1902 | 502.0694 |
| ENSMUSG00000030528 | protein_coding | Blm | 1609.899 | 1215.936 | 1412.917 |
| ENSMUSG00000027490 | protein_coding | E2f1 | 1012.253 | 754.9629 | 883.6077 |
| ENSMUSG00000035964 | protein_coding | Tmem59l | 355.1556 | 538.1057 | 446.6307 |

| | | | | | |
|---|---|---|---|---|---|
| ENSMUSG00000040586 | protein_coding | Ofd1 | 1319.757 | 974.7534 | 1147.255 |
| ENSMUSG00000024054 | protein_coding | Smchd1 | 4735.64 | 3714.568 | 4225.104 |
| ENSMUSG00000039599 | protein_coding | Fam149b | 1225.652 | 908.6851 | 1067.168 |
| ENSMUSG00000023908 | protein_coding | Pkmyt1 | 204.7473 | 119.4671 | 162.1072 |
| ENSMUSG00000085470 | processed_transcrip | 1700012C14Rik | 2.911813 | 20.3537 | 11.63276 |
| ENSMUSG00000037773 | protein_coding | Fam113a | 560.2677 | 394.3536 | 477.3107 |
| ENSMUSG00000031458 | protein_coding | 2410022L05Rik | 769.6755 | 567.9252 | 668.8004 |
| ENSMUSG00000059834 | protein_coding | Sclt1 | 708.8946 | 492.795 | 600.8448 |
| ENSMUSG00000032936 | protein_coding | Camkv | 307.6145 | 193.6438 | 250.6291 |
| ENSMUSG00000047281 | protein_coding | Sfn | 202.9962 | 369.0895 | 286.0428 |
| ENSMUSG00000038564 | protein_coding | Ift172 | 1053.04 | 764.4831 | 908.7617 |
| ENSMUSG00000079508 | protein_coding | Apoo | 545.4709 | 398.9792 | 472.225 |
| ENSMUSG00000029034 | protein_coding | Cpsf3l | 1135 | 877.1616 | 1006.081 |
| ENSMUSG00000052373 | protein_coding | Mpp3 | 838.989 | 1231.063 | 1035.026 |
| ENSMUSG00000039431 | protein_coding | Mtmr7 | 768.6255 | 570.4595 | 669.5425 |
| ENSMUSG00000038242 | protein_coding | Aox4 | 67.49606 | 28.66936 | 48.08271 |
| ENSMUSG00000037161 | protein_coding | 4930583H14Rik | 2583.347 | 3645.167 | 3114.257 |
| ENSMUSG00000057215 | processed_transcrip | Gm15514 | 30.77557 | 85.195 | 57.98529 |
| ENSMUSG00000067702 | protein_coding | Tuba3a | 8952.388 | 6610.911 | 7781.649 |
| ENSMUSG00000075389 | lincRNA | 2810410L24Rik | 441.951 | 672.7879 | 557.3695 |
| ENSMUSG00000000378 | protein_coding | Ccm2 | 1573.736 | 1223.463 | 1398.599 |
| ENSMUSG00000025946 | protein_coding | Pth2r | 4.233054 | 22.46165 | 13.34735 |
| ENSMUSG00000028345 | protein_coding | Tex10 | 4195.223 | 3289.627 | 3742.425 |
| ENSMUSG00000040363 | protein_coding | Bcor | 619.9689 | 891.5525 | 755.7607 |
| ENSMUSG00000041238 | protein_coding | Rbbp8 | 2599.646 | 1964.718 | 2282.182 |
| ENSMUSG00000041921 | protein_coding | Metap1d | 1084.587 | 738.4156 | 911.5011 |
| ENSMUSG00000068587 | protein_coding | Mgam | 7.749131 | 28.77638 | 18.26276 |
| ENSMUSG00000014355 | protein_coding | Anapc1 | 7111.621 | 5717.497 | 6414.559 |
| ENSMUSG00000085259 | lincRNA | Gm4890 | 142.8438 | 82.95092 | 112.8974 |
| ENSMUSG00000058186 | protein_coding | Gm13242 | 176.511 | 308.2958 | 242.4034 |
| ENSMUSG00000010277 | protein_coding | 2610507B11Rik | 12095.26 | 9418.481 | 10756.87 |
| ENSMUSG00000029423 | protein_coding | Piwil1 | 184.7139 | 102.8743 | 143.7941 |
| ENSMUSG00000033488 | protein_coding | BC026585 | 360.8907 | 500.533 | 430.7119 |
| ENSMUSG00000015980 | protein_coding | Lrrc27 | 272.8913 | 176.0666 | 224.479 |
| ENSMUSG00000056342 | protein_coding | Usp34 | 12357.5 | 9721.658 | 11039.58 |
| ENSMUSG00000051043 | protein_coding | Gprc5c | 166.5845 | 277.5145 | 222.0495 |
| ENSMUSG00000056174 | protein_coding | Col8a2 | 249.4696 | 380.093 | 314.7813 |
| ENSMUSG00000006289 | protein_coding | Osgep | 1419.953 | 1096.293 | 1258.123 |
| ENSMUSG00000086788 | processed_transcrip | 1700029M20Rik | 83.97712 | 22.10811 | 53.04261 |
| ENSMUSG00000000253 | protein_coding | Gmpr | 281.1783 | 396.518 | 338.8482 |
| ENSMUSG00000025876 | protein_coding | Unc5a | 236.0653 | 148.8856 | 192.4754 |
| ENSMUSG00000087373 | processed_transcrip | Gm15892 | 29.31851 | 82.69715 | 56.00783 |
| ENSMUSG00000022822 | protein_coding | Abcc5 | 2407.69 | 3347.99 | 2877.84 |
| ENSMUSG00000026645 | protein_coding | Olah | 22.6526 | 4.370091 | 13.51134 |
| ENSMUSG00000029270 | protein_coding | Fam69a | 430.4386 | 306.5431 | 368.4909 |
| ENSMUSG00000019960 | protein_coding | Dusp6 | 1132.19 | 1673.163 | 1402.677 |
| ENSMUSG00000064369 | Mt_tRNA | mt-Te | 94.10984 | 44.0018 | 69.05582 |
| ENSMUSG00000027233 | protein_coding | Patl2 | 94.43575 | 185.6835 | 140.0596 |
| ENSMUSG00000060032 | protein_coding | H2afj | 903.2614 | 1222.551 | 1062.906 |
| ENSMUSG00000042462 | protein_coding | Dctpp1 | 1321.748 | 1012.221 | 1166.984 |
| ENSMUSG00000018809 | protein_coding | Smyd4 | 536.6294 | 391.246 | 463.9377 |
| ENSMUSG00000027650 | protein_coding | Tti1 | 1539.026 | 1165.471 | 1352.249 |
| ENSMUSG00000022672 | protein_coding | Prkdc | 2958.297 | 2356.174 | 2657.235 |
| ENSMUSG00000007570 | protein_coding | Fance | 842.3105 | 644.1969 | 743.2537 |
| ENSMUSG00000025571 | protein_coding | Tnrc6c | 3858.645 | 2798.384 | 3328.515 |
| ENSMUSG00000037270 | protein_coding | 4932438A13Rik | 8524.866 | 6174.168 | 7349.517 |
| ENSMUSG00000027167 | protein_coding | Elp4 | 485.4973 | 352.3876 | 418.9424 |
| ENSMUSG00000038838 | protein_coding | Vars2 | 981.7496 | 747.3706 | 864.5601 |

| | | | | | |
|---|---|---|---|---|---|
| ENSMUSG00000015889 | protein_coding | Lta4h | 1409.687 | 906.7954 | 1158.241 |
| ENSMUSG00000022762 | protein_coding | Ncam2 | 27.95433 | 6.789745 | 17.37204 |
| ENSMUSG00000043962 | protein_coding | Thrap3 | 7685.61 | 6310.913 | 6998.261 |
| ENSMUSG00000053337 | lincRNA | Gm9903 | 28.44782 | 7.549799 | 17.99881 |
| ENSMUSG00000027263 | protein_coding | Tubgcp4 | 819.7462 | 618.2626 | 719.0044 |
| ENSMUSG00000055923 | protein_coding | Aasdh | 594.1725 | 426.4892 | 510.3308 |
| ENSMUSG00000032449 | protein_coding | Slc25a36 | 4516.642 | 5855.049 | 5185.846 |
| ENSMUSG00000078773 | protein_coding | Rad54b | 725.3638 | 508.9177 | 617.1408 |
| ENSMUSG00000048067 | protein_coding | Olfr1349 | 5.862315 | 27.27714 | 16.56973 |
| ENSMUSG00000023443 | protein_coding | Esx1 | 1402.659 | 822.4119 | 1112.535 |
| ENSMUSG00000059895 | protein_coding | Ptp4a3 | 1297.779 | 1002.601 | 1150.19 |
| ENSMUSG00000021694 | protein_coding | Ercc8 | 763.7917 | 580.5301 | 672.1609 |
| ENSMUSG00000018983 | protein_coding | E2f2 | 465.8307 | 292.8176 | 379.3242 |
| ENSMUSG00000031453 | protein_coding | Rasa3 | 922.4972 | 699.8276 | 811.1624 |
| ENSMUSG00000021420 | protein_coding | Fars2 | 928.9083 | 723.6856 | 826.297 |
| ENSMUSG00000039187 | protein_coding | Fanci | 921.45 | 679.1608 | 800.3054 |
| ENSMUSG00000029815 | protein_coding | 2410003K15Rik | 598.5654 | 442.9664 | 520.7659 |
| ENSMUSG00000021311 | protein_coding | Mtr | 3576.192 | 2690.141 | 3133.166 |
| ENSMUSG00000008999 | protein_coding | Bmp7 | 526.9095 | 373.8911 | 450.4003 |
| ENSMUSG00000091900 | protein_coding | Gm4353 | 7.1108 | 41.9244 | 24.5176 |
| ENSMUSG00000027993 | protein_coding | Trim2 | 609.908 | 940.2044 | 775.0562 |
| ENSMUSG00000004885 | protein_coding | Crabp2 | 34.86774 | 81.54323 | 58.20548 |
| ENSMUSG00000052316 | protein_coding | Lrrc15 | 19.63627 | 56.16418 | 37.90022 |
| ENSMUSG00000050966 | protein_coding | Lin28a | 2199.289 | 1503.99 | 1851.639 |
| ENSMUSG00000038780 | protein_coding | Smurf1 | 2239.514 | 1770.161 | 2004.837 |
| ENSMUSG00000028927 | protein_coding | Padi2 | 1745.088 | 2493.266 | 2119.177 |
| ENSMUSG00000038764 | protein_coding | Ptpn3 | 479.2578 | 669.8657 | 574.5618 |
| ENSMUSG00000048550 | protein_coding | Thnsl1 | 580.1092 | 435.5597 | 507.8345 |
| ENSMUSG00000005370 | protein_coding | Msh6 | 4006.924 | 3234.616 | 3620.77 |
| ENSMUSG00000028707 | protein_coding | Dmbx1 | 128.0273 | 62.17585 | 95.1016 |
| ENSMUSG00000027071 | protein_coding | P2rx3 | 117.4626 | 60.55694 | 89.00976 |
| ENSMUSG00000059146 | protein_coding | Ntrk3 | 75.11459 | 35.86204 | 55.48831 |
| ENSMUSG00000040479 | protein_coding | Dgkz | 1762.546 | 1352.349 | 1557.448 |
| ENSMUSG00000027427 | protein_coding | Polr3f | 1619.223 | 2000.032 | 1809.628 |
| ENSMUSG00000024422 | protein_coding | Dhx16 | 1909.774 | 1454.81 | 1682.292 |
| ENSMUSG00000034453 | protein_coding | Polr3b | 2152.728 | 1561.008 | 1856.868 |
| ENSMUSG00000026683 | protein_coding | Nuf2 | 1114.926 | 837.6262 | 976.2761 |
| ENSMUSG00000056436 | protein_coding | Cyct | 287.2459 | 196.6665 | 241.9562 |
| ENSMUSG00000040675 | protein_coding | Mthfd1l | 360.2076 | 505.9617 | 433.0846 |
| ENSMUSG00000032952 | protein_coding | Ap4b1 | 891.0782 | 638.7229 | 764.9005 |
| ENSMUSG00000027330 | protein_coding | Cdc25b | 573.0134 | 757.38 | 665.1967 |
| ENSMUSG00000040548 | protein_coding | Tex2 | 1401.75 | 1049.754 | 1225.752 |
| ENSMUSG00000062480 | protein_coding | Acat3 | 177.8925 | 275.0837 | 226.4881 |
| ENSMUSG00000030877 | protein_coding | 4933427G17Rik | 103.0055 | 30.23627 | 66.62091 |
| ENSMUSG00000016386 | protein_coding | Mpped2 | 250.0129 | 157.2976 | 203.6552 |
| ENSMUSG00000042581 | protein_coding | Thsd7b | 41.80336 | 109.4909 | 75.64712 |
| ENSMUSG00000034773 | protein_coding | BC030867 | 473.1586 | 315.106 | 394.1323 |
| ENSMUSG00000048636 | protein_coding | A730049H05Rik | 61.82987 | 118.4356 | 90.13276 |
| ENSMUSG00000020871 | protein_coding | Dlx4 | 38.64346 | 78.49322 | 58.56834 |
| ENSMUSG00000024169 | protein_coding | Ift140 | 1605.292 | 1260.062 | 1432.677 |
| ENSMUSG00000028591 | protein_coding | Pramef12 | 1378.458 | 1085.979 | 1232.219 |
| ENSMUSG00000032264 | protein_coding | Zw10 | 1373.684 | 1091.525 | 1232.604 |
| ENSMUSG00000040489 | protein_coding | Sox30 | 146.7285 | 69.36454 | 108.0465 |
| ENSMUSG00000050812 | protein_coding | AI314180 | 6759.078 | 5557.121 | 6158.099 |
| ENSMUSG00000056155 | protein_coding | Nanos3 | 178.6077 | 105.9024 | 142.255 |
| ENSMUSG00000004892 | protein_coding | Bcan | 1324.96 | 1973.236 | 1649.098 |
| ENSMUSG00000042797 | protein_coding | Aqp11 | 159.0983 | 100.1505 | 129.6244 |
| ENSMUSG00000039458 | protein_coding | Mtmr12 | 1307.037 | 1013.796 | 1160.416 |

| | | | | | |
|---|---|---|---|---|---|
| ENSMUSG00000064284 | protein_coding | 2210021J22Rik | 271.7139 | 179.5891 | 225.6515 |
| ENSMUSG00000034731 | protein_coding | Dgkh | 284.7746 | 189.6079 | 237.1912 |
| ENSMUSG00000020461 | protein_coding | 1700034F02Rik | 46.07115 | 103.4198 | 74.74549 |
| ENSMUSG00000033152 | protein_coding | Podxl2 | 673.6118 | 478.3268 | 575.9693 |
| ENSMUSG00000086503 | lincRNA | Xist | 168.1604 | 59.17301 | 113.6667 |
| ENSMUSG00000023072 | protein_coding | Ccdc123 | 867.4912 | 660.8413 | 764.1662 |
| ENSMUSG00000035427 | protein_coding | Mageb4 | 983.2942 | 1441.132 | 1212.213 |
| ENSMUSG00000090671 | pseudogene | Gm5067 | 106.285 | 172.9441 | 139.6146 |
| ENSMUSG00000022471 | protein_coding | Xrcc6 | 2102.241 | 1665.774 | 1884.008 |
| ENSMUSG00000028575 | protein_coding | 4930579C15Rik | 17.22257 | 48.13289 | 32.67773 |
| ENSMUSG00000064288 | protein_coding | Hist1h4k | 19.29187 | 55.24697 | 37.26942 |
| ENSMUSG00000031075 | protein_coding | Ano1 | 447.0791 | 266.4501 | 356.7646 |
| ENSMUSG00000028496 | protein_coding | Mllt3 | 891.6686 | 670.4476 | 781.0581 |
| ENSMUSG00000006527 | protein_coding | Sfmbt1 | 2833.619 | 2104.647 | 2469.133 |
| ENSMUSG00000025792 | protein_coding | Slc25a10 | 613.8387 | 806.5696 | 710.2041 |
| ENSMUSG00000030851 | protein_coding | Ldhc | 39.70192 | 11.62869 | 25.66531 |
| ENSMUSG00000026399 | protein_coding | Cd55 | 209.9806 | 314.1589 | 262.0697 |
| ENSMUSG00000042826 | protein_coding | Fgf11 | 805.7164 | 1206.369 | 1006.043 |
| ENSMUSG00000026511 | protein_coding | Srp9 | 1633.449 | 1126.163 | 1379.806 |
| ENSMUSG00000056917 | protein_coding | Sipa1 | 1166.74 | 1476.846 | 1321.793 |
| ENSMUSG00000041415 | protein_coding | Dicer1 | 4479.026 | 3589.266 | 4034.146 |
| ENSMUSG00000030313 | protein_coding | Dennd5b | 634.1019 | 442.2528 | 538.1774 |
| ENSMUSG00000056413 | protein_coding | Adap1 | 177.4748 | 109.5318 | 143.5033 |
| ENSMUSG00000009739 | protein_coding | Pou6f1 | 442.3635 | 321.7415 | 382.0525 |
| ENSMUSG00000020513 | protein_coding | Tubd1 | 312.3098 | 216.9806 | 264.6452 |
| ENSMUSG00000032175 | protein_coding | Tyk2 | 1419.142 | 1089.313 | 1254.228 |
| ENSMUSG00000031928 | protein_coding | Mre11a | 1434.786 | 1104.942 | 1269.864 |
| ENSMUSG00000054003 | protein_coding | Tdrd9 | 1994.963 | 1390.537 | 1692.75 |
| ENSMUSG00000072244 | protein_coding | Trim6 | 687.2211 | 899.8699 | 793.5455 |
| ENSMUSG00000033400 | protein_coding | Agl | 1483.798 | 1196.13 | 1339.964 |
| ENSMUSG00000035582 | protein_coding | Gdpd4 | 76.44849 | 195.1521 | 135.8003 |
| ENSMUSG00000028430 | protein_coding | Nol6 | 2706.978 | 2057.912 | 2382.445 |
| ENSMUSG00000086493 | processed_transcrip | Gm12204 | 18.1725 | 3.399196 | 10.78585 |
| ENSMUSG00000039321 | protein_coding | Uts2r | 6.781758 | 27.66206 | 17.22191 |
| ENSMUSG00000038692 | protein_coding | Hoxb4 | 41.40454 | 83.06224 | 62.23339 |
| ENSMUSG00000038172 | protein_coding | Ttc39b | 550.9327 | 369.0493 | 459.991 |
| ENSMUSG00000020549 | protein_coding | Elac2 | 1817.003 | 1433.056 | 1625.029 |
| ENSMUSG00000031384 | protein_coding | Asb9 | 301.3536 | 189.6387 | 245.4961 |
| ENSMUSG00000026274 | protein_coding | Pask | 1897.384 | 1455.327 | 1676.355 |
| ENSMUSG00000065999 | protein_coding | Gm13154 | 54.10556 | 100.3328 | 77.21918 |
| ENSMUSG00000039318 | protein_coding | Rab3gap2 | 2241.765 | 1681.747 | 1961.756 |
| ENSMUSG00000041670 | protein_coding | Rims1 | 237.7818 | 145.8558 | 191.8188 |
| ENSMUSG00000026634 | protein_coding | Angel2 | 2661.599 | 2151.291 | 2406.445 |
| ENSMUSG00000078796 | protein_coding | Zfp541 | 650.2989 | 128.3287 | 389.3138 |
| ENSMUSG00000036218 | protein_coding | Pdzrn4 | 12.56312 | 36.74786 | 24.65549 |
| ENSMUSG00000017057 | protein_coding | Il13ra1 | 726.2192 | 929.6699 | 827.9445 |
| ENSMUSG00000055707 | protein_coding | Klhl26 | 581.5569 | 764.7754 | 673.1662 |
| ENSMUSG00000073664 | protein_coding | Nbeal1 | 2963.587 | 2342.194 | 2652.891 |
| ENSMUSG00000022913 | protein_coding | Psmg1 | 1047.491 | 836.3939 | 941.9425 |
| ENSMUSG00000015766 | protein_coding | Eps8 | 1670.893 | 1089.267 | 1380.08 |
| ENSMUSG00000061533 | protein_coding | 4930534B04Rik | 395.7637 | 273.849 | 334.8064 |
| ENSMUSG00000034557 | protein_coding | Zfyve9 | 2918.89 | 2274.657 | 2596.774 |
| ENSMUSG00000038241 | protein_coding | Cep250 | 1555.249 | 1196.917 | 1376.083 |
| ENSMUSG00000001604 | protein_coding | Tcea3 | 172.1839 | 98.6351 | 135.4095 |
| ENSMUSG00000018548 | protein_coding | Trim37 | 5098.57 | 3919.353 | 4508.962 |
| ENSMUSG00000002602 | protein_coding | Axl | 7614.038 | 9547.494 | 8580.766 |
| ENSMUSG00000037337 | protein_coding | Map4k1 | 206.0078 | 133.315 | 169.6614 |
| ENSMUSG00000032743 | protein_coding | D430042O09Rik | 1423.965 | 1131.375 | 1277.67 |

| | | | | | |
|---|---|---|---|---|---|
| ENSMUSG00000026547 | protein_coding | Tagln2 | 1027.6 | 1337.526 | 1182.563 |
| ENSMUSG00000042638 | protein_coding | Gucy2c | 40.18058 | 6.780736 | 23.48066 |
| ENSMUSG00000023067 | protein_coding | Cdkn1a | 149.7902 | 236.283 | 193.0366 |
| ENSMUSG00000029864 | protein_coding | Gstk1 | 226.7897 | 154.2936 | 190.5417 |
| ENSMUSG00000038545 | protein_coding | Cul7 | 2772.583 | 2186.5 | 2479.542 |
| ENSMUSG00000027014 | protein_coding | Cwc22 | 1343.754 | 6480.543 | 3912.149 |
| ENSMUSG00000024986 | protein_coding | Hhex | 252.7883 | 365.766 | 309.2772 |
| ENSMUSG00000022474 | protein_coding | Pmm1 | 1251.31 | 984.145 | 1117.728 |
| ENSMUSG00000024597 | protein_coding | Slc12a2 | 2978.704 | 2469.784 | 2724.244 |
| ENSMUSG00000030092 | protein_coding | Cntn6 | 75.97752 | 36.31681 | 56.14717 |
| ENSMUSG00000042156 | protein_coding | Dzip1 | 472.8723 | 350.2116 | 411.542 |
| ENSMUSG00000024533 | protein_coding | Spire1 | 1529.905 | 1149.874 | 1339.89 |
| ENSMUSG00000070934 | protein_coding | Rraga | 1654.718 | 2083.535 | 1869.126 |
| ENSMUSG00000092552 | pseudogene | Gm4287 | 10.97128 | 0.662038 | 5.816657 |
| ENSMUSG00000005267 | protein_coding | Zfp287 | 471.5578 | 346.048 | 408.8029 |
| ENSMUSG00000022092 | protein_coding | Ppp3cc | 1237.27 | 893.7439 | 1065.507 |
| ENSMUSG00000021647 | protein_coding | Cartpt | 6.806357 | 25.05575 | 15.93105 |
| ENSMUSG00000038122 | protein_coding | D630037F22Rik | 1025.284 | 792.4476 | 908.8659 |
| ENSMUSG00000038235 | protein_coding | F11r | 4482.5 | 5376.691 | 4929.596 |
| ENSMUSG00000019838 | protein_coding | Slc16a10 | 462.4634 | 314.3864 | 388.4249 |
| ENSMUSG00000017733 | protein_coding | Spinlw1 | 1338.449 | 1036.04 | 1187.244 |
| ENSMUSG00000037106 | protein_coding | Fer1l6 | 47.09339 | 86.40344 | 66.74842 |
| ENSMUSG00000036202 | protein_coding | Rif1 | 8073.936 | 9751.659 | 8912.797 |
| ENSMUSG00000027996 | protein_coding | Sfrp2 | 191.8149 | 272.0856 | 231.9503 |
| ENSMUSG00000033644 | protein_coding | Piwil2 | 4972.933 | 3664.341 | 4318.637 |
| ENSMUSG00000026670 | protein_coding | Uap1 | 1645.572 | 1280.495 | 1463.033 |
| ENSMUSG00000024369 | protein_coding | Rdbp | 1595.26 | 1160.657 | 1377.959 |
| ENSMUSG00000035754 | protein_coding | Wdr18 | 1861.915 | 1494.864 | 1678.389 |
| ENSMUSG00000042500 | protein_coding | Eif2c4 | 526.8445 | 334.1182 | 430.4814 |
| ENSMUSG00000018570 | protein_coding | 2810408A11Rik | 166.6793 | 96.03525 | 131.3573 |
| ENSMUSG00000028246 | protein_coding | 6230409E13Rik | 220.8456 | 140.3267 | 180.5861 |
| ENSMUSG00000031833 | protein_coding | Mast3 | 1364.447 | 1067.042 | 1215.744 |
| ENSMUSG00000029674 | protein_coding | Limk1 | 2232.364 | 1761.649 | 1997.007 |
| ENSMUSG00000038299 | protein_coding | Wdr36 | 3269.705 | 2612.929 | 2941.317 |
| ENSMUSG00000091475 | lincRNA | 2810468N07Rik | 161.5179 | 245.0308 | 203.2744 |
| ENSMUSG00000068373 | protein_coding | D430041D05Rik | 230.0514 | 158.0344 | 194.0429 |
| ENSMUSG00000043587 | protein_coding | Acpl2 | 351.5632 | 246.6687 | 299.116 |
| ENSMUSG00000034520 | protein_coding | Gjc1 | 1576.595 | 1234.733 | 1405.664 |
| ENSMUSG00000031661 | protein_coding | Nkd1 | 501.7018 | 377.6656 | 439.6837 |
| ENSMUSG00000082033 | protein_coding | Mageb10-ps | 291.0573 | 133.4522 | 212.2548 |
| ENSMUSG00000020546 | protein_coding | Stxbp4 | 790.3155 | 982.3534 | 886.3344 |
| ENSMUSG00000086826 | processed_transcrip | Gm11739 | 134.0638 | 203.9428 | 169.0033 |
| ENSMUSG00000092074 | protein_coding | Dynlt1a | 286.7191 | 448.2069 | 367.463 |
| ENSMUSG00000022422 | protein_coding | Dscc1 | 372.1604 | 265.0515 | 318.6059 |
| ENSMUSG00000041550 | protein_coding | Serpina5 | 3325.777 | 4119.429 | 3722.603 |
| ENSMUSG00000024740 | protein_coding | Ddb1 | 9546.824 | 7923.956 | 8735.39 |
| ENSMUSG00000029279 | protein_coding | Brdt | 928.5303 | 544.1664 | 736.3483 |
| ENSMUSG00000063063 | protein_coding | Ctnna2 | 1213.075 | 1627.78 | 1420.427 |
| ENSMUSG00000056055 | protein_coding | Sag | 21.75187 | 57.46044 | 39.60616 |
| ENSMUSG00000002068 | protein_coding | Ccne1 | 377.2448 | 266.1676 | 321.7062 |
| ENSMUSG00000052712 | protein_coding | BC004004 | 1523.055 | 1142.994 | 1333.024 |
| ENSMUSG00000044072 | protein_coding | Eml6 | 154.9189 | 98.02856 | 126.4737 |
| ENSMUSG00000006445 | protein_coding | Epha2 | 712.9392 | 1100.742 | 906.8405 |
| ENSMUSG00000022229 | protein_coding | Atp12a | 62.53656 | 115.0162 | 88.77639 |
| ENSMUSG00000056043 | protein_coding | Rgs9bp | 111.8918 | 186.1183 | 149.005 |
| ENSMUSG00000030064 | protein_coding | Frmd4b | 1247.548 | 1568.997 | 1408.273 |
| ENSMUSG00000019373 | protein_coding | Cops3 | 2367.849 | 1938.882 | 2153.366 |
| ENSMUSG00000052299 | protein_coding | Ltn1 | 2560.566 | 2069.71 | 2315.138 |

| | | | | | |
|---|---|---|---|---|---|
| ENSMUSG00000020451 | protein_coding | Limk2 | 1296.403 | 1648.135 | 1472.269 |
| ENSMUSG00000026427 | protein_coding | Eif2d | 908.0395 | 709.1249 | 808.5822 |
| ENSMUSG00000077034 | miRNA | AC167229.1 | 37.35442 | 86.43156 | 61.89299 |
| ENSMUSG00000048602 | protein_coding | Morc2b | 9.556704 | 0 | 4.778352 |
| ENSMUSG00000032908 | protein_coding | Sgpp2 | 160.1435 | 236.6154 | 198.3794 |
| ENSMUSG00000061633 | protein_coding | 1700029P11Rik | 72.11727 | 136.8367 | 104.477 |
| ENSMUSG00000038037 | protein_coding | Socs1 | 281.8437 | 197.2237 | 239.5337 |
| ENSMUSG00000044337 | protein_coding | Cxcr7 | 850.0045 | 633.2526 | 741.6285 |
| ENSMUSG00000074594 | protein_coding | Wfdc9 | 207.0823 | 140.4331 | 173.7577 |
| ENSMUSG00000073433 | protein_coding | Arhgdig | 371.1963 | 487.9064 | 429.5514 |
| ENSMUSG00000033763 | protein_coding | Mtss1l | 921.3931 | 720.173 | 820.783 |
| ENSMUSG00000047466 | protein_coding | 8030462N17Rik | 1431.411 | 1126.647 | 1279.029 |
| ENSMUSG00000045193 | protein_coding | Cirbp | 4021.477 | 3195.057 | 3608.267 |
| ENSMUSG00000025068 | protein_coding | Gsto1 | 442.4722 | 581.7604 | 512.1163 |
| ENSMUSG00000027635 | protein_coding | Dsn1 | 1573.875 | 1274.651 | 1424.263 |
| ENSMUSG00000052407 | protein_coding | 4930473A06Rik | 700.3182 | 495.8728 | 598.0955 |
| ENSMUSG00000041548 | protein_coding | Hspb8 | 244.1407 | 378.8787 | 311.5097 |
| ENSMUSG00000034239 | protein_coding | Gm884 | 415.8249 | 224.6346 | 320.2297 |
| ENSMUSG00000020333 | protein_coding | Acsl6 | 538.4171 | 311.713 | 425.0651 |
| ENSMUSG00000029361 | protein_coding | Nos1 | 252.7462 | 174.8106 | 213.7784 |
| ENSMUSG00000055660 | protein_coding | Mettl4 | 744.598 | 587.4904 | 666.0442 |
| ENSMUSG00000030359 | protein_coding | Pzp | 29.17658 | 62.99933 | 46.08795 |
| ENSMUSG00000010911 | protein_coding | Apip | 633.5568 | 476.2086 | 554.8827 |
| ENSMUSG00000020069 | protein_coding | Hnrnph3 | 909.7092 | 719.7148 | 814.712 |
| ENSMUSG00000035121 | protein_coding | Neil2 | 230.6691 | 314.9002 | 272.7847 |
| ENSMUSG00000074408 | lincRNA | Gm10687 | 23.88573 | 50.07183 | 36.97878 |
| ENSMUSG00000032740 | protein_coding | Ccdc88a | 3112.119 | 2605.645 | 2858.882 |
| ENSMUSG00000079108 | protein_coding | Srp54b | 97.52879 | 54.51291 | 76.02085 |
| ENSMUSG00000028610 | protein_coding | Dmrtb1 | 1705.876 | 1133.766 | 1419.821 |
| ENSMUSG00000045822 | protein_coding | Zswim3 | 626.0172 | 451.9907 | 539.0039 |
| ENSMUSG00000036046 | protein_coding | 5031439G07Rik | 1464.287 | 1180.744 | 1322.515 |
| ENSMUSG00000038910 | protein_coding | Plcl2 | 1338.802 | 1040.252 | 1189.527 |
| ENSMUSG00000086574 | lincRNA | Gm16960 | 35.87715 | 12.51343 | 24.19529 |
| ENSMUSG00000044224 | protein_coding | Dnajc21 | 1715.489 | 1379.652 | 1547.57 |
| ENSMUSG00000087166 | protein_coding | L1td1 | 24.25241 | 50.91695 | 37.58468 |
| ENSMUSG00000090918 | lincRNA | 1700007L15Rik | 94.65946 | 50.27615 | 72.4678 |
| ENSMUSG00000036196 | protein_coding | Slc26a8 | 107.2178 | 43.84502 | 75.53141 |
| ENSMUSG00000020775 | protein_coding | Mrpl38 | 1958.304 | 1570.985 | 1764.645 |
| ENSMUSG00000036875 | protein_coding | Dna2 | 532.2185 | 406.9788 | 469.5986 |
| ENSMUSG00000028334 | protein_coding | Nans | 1602.211 | 1281.441 | 1441.826 |
| ENSMUSG00000021725 | protein_coding | Parp8 | 738.0098 | 927.163 | 832.5864 |
| ENSMUSG00000027374 | protein_coding | Mrps5 | 2180.933 | 1807.383 | 1994.158 |
| ENSMUSG00000013629 | protein_coding | Cad | 3834.664 | 2874.043 | 3354.353 |
| ENSMUSG00000087222 | lincRNA | E030042O20Rik | 79.86342 | 42.31404 | 61.08873 |
| ENSMUSG00000020024 | protein_coding | Ccdc41 | 2707.168 | 1954.907 | 2331.038 |
| ENSMUSG00000062542 | protein_coding | Syt9 | 1175.829 | 898.2232 | 1037.026 |
| ENSMUSG00000020331 | protein_coding | Hcn2 | 177.492 | 105.7441 | 141.6181 |
| ENSMUSG00000029366 | protein_coding | Dck | 1440.432 | 1143.657 | 1292.045 |
| ENSMUSG00000027596 | protein_coding | a | 16.19399 | 3.027997 | 9.610995 |
| ENSMUSG00000092286 | processed_transcrip | Gm20448 | 5.512027 | 21.82048 | 13.66625 |
| ENSMUSG00000020156 | protein_coding | Mum1 | 2951.372 | 2446.993 | 2699.182 |
| ENSMUSG00000036918 | protein_coding | Ttc7 | 1056.565 | 738.7301 | 897.6476 |
| ENSMUSG00000077043 | miRNA | AC167229.2 | 503.259 | 741.9964 | 622.6277 |
| ENSMUSG00000029463 | protein_coding | 1500011H22Rik | 719.213 | 525.4977 | 622.3554 |
| ENSMUSG00000070427 | protein_coding | Il18bp | 104.5881 | 171.1144 | 137.8512 |
| ENSMUSG00000030001 | protein_coding | Figla | 125.0732 | 67.86173 | 96.46746 |
| ENSMUSG00000038011 | protein_coding | Dnahc10 | 280.3571 | 176.1509 | 228.254 |
| ENSMUSG00000024271 | protein_coding | Elp2 | 3020.227 | 2417.976 | 2719.101 |

| | | | | | |
|---|---|---|---|---|---|
| ENSMUSG00000072214 | protein_coding | 5-Sep | 424.2638 | 307.4532 | 365.8585 |
| ENSMUSG00000038312 | protein_coding | Edem2 | 1280.397 | 944.1434 | 1112.27 |
| ENSMUSG00000044220 | protein_coding | Nkx2-3 | 6.096313 | 25.4111 | 15.7537 |
| ENSMUSG00000042659 | protein_coding | Arrdc4 | 1054.506 | 834.3961 | 944.451 |
| ENSMUSG00000018470 | protein_coding | Kcnab3 | 324.0132 | 429.4592 | 376.7362 |
| ENSMUSG00000030319 | protein_coding | Cand2 | 803.3317 | 599.9846 | 701.6582 |
| ENSMUSG00000021707 | protein_coding | Dhfr | 1101.329 | 851.213 | 976.2712 |
| ENSMUSG00000018707 | protein_coding | Dync1h1 | 16841.13 | 13455.94 | 15148.53 |
| ENSMUSG00000002324 | protein_coding | Rec8 | 689.0623 | 482.5346 | 585.7984 |
| ENSMUSG00000018417 | protein_coding | Myo1b | 1625.627 | 1252.516 | 1439.072 |
| ENSMUSG00000028402 | protein_coding | Mpdz | 1544.118 | 1252.436 | 1398.277 |
| ENSMUSG00000043165 | protein_coding | Lor | 41.12067 | 82.03408 | 61.57737 |
| ENSMUSG00000069713 | protein_coding | 4933406P04Rik | 59.65926 | 28.32518 | 43.99222 |
| ENSMUSG00000021494 | protein_coding | Ddx41 | 1917.922 | 1560.407 | 1739.165 |
| ENSMUSG00000022504 | protein_coding | Ciita | 14.25336 | 35.43243 | 24.8429 |
| ENSMUSG00000003813 | protein_coding | Rad23a | 761.6515 | 954.9838 | 858.3176 |
| ENSMUSG00000025092 | protein_coding | Hspa12a | 767.7356 | 1021.392 | 894.5639 |
| ENSMUSG00000030546 | protein_coding | Plin1 | 241.104 | 352.0791 | 296.5916 |
| ENSMUSG00000028409 | protein_coding | Smu1 | 2714.876 | 2229.043 | 2471.96 |
| ENSMUSG00000032368 | protein_coding | Zic1 | 41.19491 | 15.11689 | 28.1559 |
| ENSMUSG00000058486 | protein_coding | Wdr91 | 711.9941 | 532.8864 | 622.4402 |
| ENSMUSG00000067455 | protein_coding | Hist1h4j | 9.386593 | 27.5554 | 18.471 |
| ENSMUSG00000031787 | protein_coding | Katnb1 | 771.7313 | 608.9209 | 690.3261 |
| ENSMUSG00000021147 | protein_coding | Wdr37 | 1972.703 | 1615.331 | 1794.017 |
| ENSMUSG00000036928 | protein_coding | Stag3 | 4830.34 | 3316.106 | 4073.223 |
| ENSMUSG00000047751 | protein_coding | Utf1 | 80.30465 | 156.7748 | 118.5397 |
| ENSMUSG00000092274 | processed_transcrip | Neat1 | 1034.819 | 1666.007 | 1350.413 |
| ENSMUSG00000023723 | protein_coding | Mrps23 | 1228.491 | 996.136 | 1112.313 |
| ENSMUSG00000051517 | protein_coding | E130306D19Rik | 105.453 | 178.5253 | 141.9892 |
| ENSMUSG00000079620 | protein_coding | Muc4 | 7.450578 | 24.53641 | 15.99349 |
| ENSMUSG00000024180 | protein_coding | Tmem8 | 653.193 | 509.9489 | 581.5709 |
| ENSMUSG00000056821 | protein_coding | 1700028I16Rik | 39.75299 | 16.87451 | 28.31375 |
| ENSMUSG00000062518 | protein_coding | Zfp534 | 117.0082 | 207.4011 | 162.2047 |
| ENSMUSG00000035325 | protein_coding | Sec31a | 3700.171 | 3038.199 | 3369.185 |
| ENSMUSG00000036211 | protein_coding | Hist1h1t | 2.275793 | 15.34824 | 8.812017 |
| ENSMUSG00000072694 | protein_coding | 2610524H06Rik | 349.7109 | 470.6133 | 410.1621 |
| ENSMUSG00000025982 | protein_coding | Sf3b1 | 9833.611 | 11697.4 | 10765.51 |
| ENSMUSG00000018861 | protein_coding | Fdxr | 2095.845 | 2845.794 | 2470.82 |
| ENSMUSG00000023467 | protein_coding | Tulp2 | 2436.515 | 3001.526 | 2719.02 |
| ENSMUSG00000064127 | protein_coding | Med14 | 940.8749 | 1166.92 | 1053.898 |
| ENSMUSG00000030016 | protein_coding | Zfml | 5929.694 | 4894.509 | 5412.102 |
| ENSMUSG00000039329 | protein_coding | Tex19.1 | 1633.095 | 1980.645 | 1806.87 |
| ENSMUSG00000074771 | protein_coding | Ankrd5 | 50.9854 | 24.78365 | 37.88452 |
| ENSMUSG00000036863 | protein_coding | Syde2 | 551.7038 | 401.6087 | 476.6563 |
| ENSMUSG00000024056 | protein_coding | Ndc80 | 1348.418 | 1065.365 | 1206.891 |
| ENSMUSG00000020176 | protein_coding | Grb10 | 2669.336 | 2117.59 | 2393.463 |
| ENSMUSG00000035270 | protein_coding | Impg2 | 15.49699 | 37.30572 | 26.40136 |
| ENSMUSG00000027513 | protein_coding | Pck1 | 32.99963 | 65.41541 | 49.20752 |
| ENSMUSG00000024120 | protein_coding | Lrpprc | 3123.481 | 2603.447 | 2863.464 |
| ENSMUSG00000025323 | protein_coding | Sp4 | 1679.687 | 1373.566 | 1526.627 |
| ENSMUSG00000031910 | protein_coding | Has3 | 308.8554 | 211.9343 | 260.3949 |
| ENSMUSG00000029173 | protein_coding | Sepsecs | 1055.565 | 828.4234 | 941.9943 |
| ENSMUSG00000026095 | protein_coding | Asnsd1 | 2406.688 | 1953.466 | 2180.077 |
| ENSMUSG00000076356 | miRNA | AL607145.1 | 4.826807 | 18.74531 | 11.78606 |
| ENSMUSG00000031174 | protein_coding | Rpgr | 677.2463 | 467.1308 | 572.1885 |
| ENSMUSG00000002100 | protein_coding | Mybpc3 | 62.22578 | 107.3966 | 84.81117 |
| ENSMUSG00000015053 | protein_coding | Gata2 | 272.2442 | 193.3906 | 232.8174 |
| ENSMUSG00000063804 | protein_coding | Lin28b | 1401.772 | 1088.089 | 1244.931 |

| | | | | | |
|---|---|---|---|---|---|
| ENSMUSG00000029120 | protein_coding | Ppp2r2c | 484.717 | 652.6407 | 568.6788 |
| ENSMUSG00000038070 | protein_coding | Cntln | 1072.665 | 791.5696 | 932.1173 |
| ENSMUSG00000059708 | protein_coding | Akap17b | 1067.408 | 861.0244 | 964.216 |
| ENSMUSG00000038077 | protein_coding | Kcna6 | 102.3549 | 198.6124 | 150.4837 |
| ENSMUSG00000020682 | protein_coding | Mmp28 | 56.83467 | 27.89237 | 42.36352 |
| ENSMUSG00000078495 | protein_coding | Gm13157 | 426.8941 | 662.6908 | 544.7925 |
| ENSMUSG00000002319 | protein_coding | Ipo4 | 3615.279 | 2826.288 | 3220.784 |
| ENSMUSG00000038827 | protein_coding | BC026590 | 1095.988 | 887.3887 | 991.6884 |
| ENSMUSG00000025728 | protein_coding | Pigq | 1483.631 | 1791.843 | 1637.737 |
| ENSMUSG00000090611 | lincRNA | C330013E15Rik | 50.14356 | 22.33946 | 36.24151 |
| ENSMUSG00000010311 | protein_coding | Optc | 54.33226 | 25.43668 | 39.88447 |
| ENSMUSG00000025579 | protein_coding | Gaa | 2784.686 | 2178.34 | 2481.513 |
| ENSMUSG00000048154 | protein_coding | Mll2 | 6493.943 | 7677.206 | 7085.574 |
| ENSMUSG00000007617 | protein_coding | Homer1 | 1917.324 | 1558.499 | 1737.912 |
| ENSMUSG00000028970 | protein_coding | Abcb1b | 43.65677 | 83.55844 | 63.6076 |
| ENSMUSG00000028431 | protein_coding | Ikbkap | 2899.861 | 2429.243 | 2664.552 |
| ENSMUSG00000046846 | protein_coding | Spesp1 | 60.9767 | 27.77918 | 44.37794 |
| ENSMUSG00000036964 | protein_coding | Trim17 | 51.25473 | 96.67948 | 73.96711 |
| ENSMUSG00000072921 | protein_coding | Gm15091 | 153.0781 | 99.39686 | 126.2375 |
| ENSMUSG00000037405 | protein_coding | Icam1 | 171.3654 | 284.9706 | 228.168 |
| ENSMUSG00000032641 | protein_coding | Gpr19 | 220.4738 | 112.7611 | 166.6174 |
| ENSMUSG00000017721 | protein_coding | Pigt | 2852.071 | 2194.969 | 2523.52 |
| ENSMUSG00000029544 | protein_coding | Cabp1 | 394.5485 | 513.397 | 453.9728 |
| ENSMUSG00000041193 | protein_coding | Pla2g5 | 815.2744 | 1181.412 | 998.3432 |
| ENSMUSG00000058740 | protein_coding | Kcnt1 | 760.0609 | 1110.832 | 935.4465 |
| ENSMUSG00000020828 | protein_coding | Pld2 | 169.8638 | 240.4704 | 205.1671 |
| ENSMUSG00000014786 | protein_coding | Slc9a5 | 285.3263 | 180.1321 | 232.7292 |
| ENSMUSG00000017307 | protein_coding | Acot8 | 315.2542 | 422.0627 | 368.6585 |
| ENSMUSG00000031090 | protein_coding | Nadsyn1 | 246.5465 | 167.7777 | 207.1621 |
| ENSMUSG00000025804 | protein_coding | Ccr1 | 29.61915 | 9.093725 | 19.35644 |
| ENSMUSG00000033222 | protein_coding | Ttf2 | 1118.507 | 907.7412 | 1013.124 |
| ENSMUSG00000091649 | protein_coding | Gm4902 | 9.698192 | 26.63856 | 18.16838 |
| ENSMUSG00000028465 | protein_coding | Tln1 | 4530.653 | 5385.347 | 4958 |
| ENSMUSG00000041329 | protein_coding | Atp1b2 | 7967.294 | 9893.997 | 8930.645 |
| ENSMUSG00000067825 | protein_coding | Pex26 | 580.0017 | 437.2831 | 508.6424 |
| ENSMUSG00000070407 | protein_coding | Hs3st3b1 | 154.9146 | 253.4261 | 204.1704 |
| ENSMUSG00000021385 | protein_coding | Ippk | 890.2152 | 1155.37 | 1022.793 |
| ENSMUSG00000070972 | protein_coding | Dnajc25 | 504.8904 | 643.35 | 574.1202 |
| ENSMUSG00000038738 | protein_coding | Shank1 | 129.8435 | 49.50341 | 89.67345 |
| ENSMUSG00000030208 | protein_coding | Emp1 | 916.2315 | 1239.408 | 1077.82 |
| ENSMUSG00000037466 | protein_coding | 4930427A07Rik | 581.203 | 445.2009 | 513.2019 |
| ENSMUSG00000001901 | protein_coding | Kcnh6 | 53.98741 | 26.57585 | 40.28163 |
| ENSMUSG00000020407 | protein_coding | Upp1 | 262.8709 | 169.3788 | 216.1249 |
| ENSMUSG00000059714 | protein_coding | Flot1 | 597.058 | 743.6402 | 670.3491 |
| ENSMUSG00000029415 | protein_coding | Sdad1 | 4502.228 | 3761.192 | 4131.71 |
| ENSMUSG00000041297 | protein_coding | Cdk13 | 3616.294 | 2966.359 | 3291.327 |
| ENSMUSG00000071533 | protein_coding | Pcnp | 2377.191 | 2835.901 | 2606.546 |
| ENSMUSG00000049709 | protein_coding | Nlrp10 | 30.20322 | 63.12483 | 46.66402 |
| ENSMUSG00000029701 | protein_coding | Rbm28 | 1916.361 | 2359.698 | 2138.03 |
| ENSMUSG00000087125 | processed_transcrip | A230108P19Rik | 17.84092 | 4.441442 | 11.14118 |
| ENSMUSG00000031379 | protein_coding | Pir | 104.1076 | 157.2394 | 130.6735 |
| ENSMUSG00000029848 | protein_coding | Stra8 | 3941.175 | 2684.865 | 3313.02 |
| ENSMUSG00000023032 | protein_coding | Slc4a8 | 365.449 | 272.3461 | 318.8976 |
| ENSMUSG00000031351 | protein_coding | Zfp185 | 447.2251 | 566.4526 | 506.8388 |
| ENSMUSG00000002799 | protein_coding | Jag2 | 1679.537 | 1211.376 | 1445.456 |
| ENSMUSG00000031651 | protein_coding | Triml1 | 10.97948 | 29.47482 | 20.22715 |
| ENSMUSG00000046169 | protein_coding | Adamts6 | 163.0943 | 267.6283 | 215.3613 |
| ENSMUSG00000024696 | protein_coding | Lpxn | 49.92215 | 22.84186 | 36.38201 |

| | | | | | |
|---|---|---|---|---|---|
| ENSMUSG00000087655 | lincRNA | Gm5091 | 40.84843 | 17.37583 | 29.11213 |
| ENSMUSG00000085494 | lincRNA | 3110045C21Rik | 58.12959 | 26.00427 | 42.06693 |
| ENSMUSG00000035172 | protein_coding | Plekhh3 | 701.5135 | 882.7804 | 792.147 |
| ENSMUSG00000002718 | protein_coding | Cse1l | 6708.708 | 5134.758 | 5921.733 |
| ENSMUSG00000057722 | protein_coding | Lepr | 76.81109 | 141.781 | 109.296 |
| ENSMUSG00000027086 | protein_coding | Fastkd1 | 1289.993 | 1018.349 | 1154.171 |
| ENSMUSG00000040852 | protein_coding | Plekhh2 | 547.5405 | 692.4912 | 620.0158 |
| ENSMUSG00000036885 | protein_coding | Arhgef26 | 331.7212 | 237.7325 | 284.7269 |
| ENSMUSG00000000530 | protein_coding | Acvrl1 | 589.0599 | 846.6707 | 717.8653 |
| ENSMUSG00000015023 | protein_coding | Ddx19a | 1140.688 | 1386.939 | 1263.814 |
| ENSMUSG00000073485 | processed_transcrip | Gm16067 | 113.8775 | 70.60898 | 92.24326 |
| ENSMUSG00000001542 | protein_coding | Ell2 | 726.7909 | 554.3152 | 640.5531 |
| ENSMUSG00000056749 | protein_coding | Nfil3 | 178.9735 | 263.4145 | 221.194 |
| ENSMUSG00000021876 | protein_coding | Rnase4 | 574.3971 | 758.0089 | 666.203 |
| ENSMUSG00000021831 | protein_coding | Ero1l | 1885.451 | 1554.579 | 1720.015 |
| ENSMUSG00000058290 | protein_coding | Espl1 | 2772.746 | 2105.534 | 2439.14 |
| ENSMUSG00000032609 | protein_coding | Klhdc8b | 710.9823 | 929.6376 | 820.3099 |
| ENSMUSG00000032839 | protein_coding | Trpc1 | 348.2989 | 202.2303 | 275.2646 |
| ENSMUSG00000021991 | protein_coding | Cacna2d3 | 72.66577 | 40.74888 | 56.70733 |
| ENSMUSG00000058441 | protein_coding | Panx2 | 43.41749 | 81.26828 | 62.34288 |
| ENSMUSG00000021066 | protein_coding | Atl1 | 393.6244 | 274.8782 | 334.2513 |
| ENSMUSG00000024843 | protein_coding | Chka | 3740.951 | 4794.073 | 4267.512 |
| ENSMUSG00000067377 | protein_coding | Tspan6 | 494.2115 | 692.5082 | 593.3598 |
| ENSMUSG00000038664 | protein_coding | Herc1 | 9728.104 | 7910.457 | 8819.28 |
| ENSMUSG00000037855 | protein_coding | Zfp365 | 85.33758 | 138.5795 | 111.9586 |
| ENSMUSG00000024251 | protein_coding | Thada | 1392.608 | 1073.837 | 1233.223 |
| ENSMUSG00000019990 | protein_coding | Pde7b | 219.3819 | 129.6343 | 174.5081 |
| ENSMUSG00000024502 | protein_coding | Jakmip2 | 87.8707 | 152.2604 | 120.0656 |
| ENSMUSG00000028838 | protein_coding | Extl1 | 273.3393 | 422.0244 | 347.6819 |
| ENSMUSG00000029859 | protein_coding | Epha1 | 197.1914 | 281.6997 | 239.4455 |
| ENSMUSG00000058328 | protein_coding | Xlr5a | 323.5784 | 423.9036 | 373.741 |
| ENSMUSG00000039715 | protein_coding | Wdr34 | 691.3285 | 540.5963 | 615.9624 |
| ENSMUSG00000042812 | protein_coding | Foxf1a | 276.7626 | 442.8516 | 359.8071 |
| ENSMUSG00000026490 | protein_coding | Cdc42bpa | 4637.104 | 3776.455 | 4206.78 |
| ENSMUSG00000048234 | protein_coding | Rnf149 | 1466.986 | 1159.786 | 1313.386 |
| ENSMUSG00000078500 | protein_coding | Gm13235 | 34.48867 | 78.9685 | 56.72858 |
| ENSMUSG00000027442 | protein_coding | Cst8 | 144.6128 | 287.2081 | 215.9105 |
| ENSMUSG00000021884 | protein_coding | Hacl1 | 370.4937 | 276.4657 | 323.4797 |
| ENSMUSG00000068259 | protein_coding | Olfr461 | 0.33031 | 6.941094 | 3.635702 |
| ENSMUSG00000018479 | protein_coding | 1700125H20Rik | 49.34398 | 19.41848 | 34.38123 |
| ENSMUSG00000003438 | protein_coding | Timm50 | 1666.413 | 1369.711 | 1518.062 |
| ENSMUSG00000035722 | protein_coding | Abca7 | 470.2381 | 353.0444 | 411.6413 |
| ENSMUSG00000023336 | protein_coding | Wfdc1 | 1990.369 | 1517.063 | 1753.716 |
| ENSMUSG00000034159 | protein_coding | 2310007B03Rik | 0.316221 | 7.017881 | 3.667051 |
| ENSMUSG00000020319 | protein_coding | Wdpcp | 205.0683 | 143.4225 | 174.2454 |
| ENSMUSG00000038555 | protein_coding | Reep2 | 3023.776 | 3595.337 | 3309.556 |
| ENSMUSG00000074476 | protein_coding | Spc24 | 667.9299 | 486.2828 | 577.1063 |
| ENSMUSG00000039414 | protein_coding | Heatr5b | 2360.113 | 1827.565 | 2093.839 |
| ENSMUSG00000018849 | protein_coding | Wwc1 | 534.3954 | 404.3354 | 469.3654 |
| ENSMUSG00000028653 | protein_coding | Trit1 | 668.4443 | 833.9228 | 751.1835 |
| ENSMUSG00000025429 | protein_coding | Pstpip2 | 69.20583 | 35.06336 | 52.13459 |
| ENSMUSG00000020027 | protein_coding | Socs2 | 1985.887 | 3008.241 | 2497.064 |
| ENSMUSG00000027111 | protein_coding | Itga6 | 4533.969 | 5812.111 | 5173.04 |
| ENSMUSG00000039686 | protein_coding | Zer1 | 1504.363 | 1211.46 | 1357.911 |
| ENSMUSG00000074923 | protein_coding | Pak6 | 847.2547 | 1121.953 | 984.6039 |
| ENSMUSG00000024993 | protein_coding | Fam45a | 1228.501 | 979.5484 | 1104.024 |
| ENSMUSG00000022177 | protein_coding | Haus4 | 1195.984 | 964.4879 | 1080.236 |
| ENSMUSG00000044201 | protein_coding | Cdc25c | 385.9032 | 274.7451 | 330.3241 |

| | | | | | |
|---|---|---|---|---|---|
| ENSMUSG00000061119 | protein_coding | Prcp | 525.8822 | 654.4342 | 590.1582 |
| ENSMUSG00000068740 | protein_coding | Celsr2 | 381.7595 | 267.9326 | 324.8461 |
| ENSMUSG00000022680 | protein_coding | Pdxdc1 | 3635.413 | 3009.817 | 3322.615 |
| ENSMUSG00000039047 | protein_coding | Pigk | 1857.473 | 1560.064 | 1708.768 |
| ENSMUSG00000029165 | protein_coding | Agbl5 | 1073.417 | 876.1353 | 974.7761 |
| ENSMUSG00000041362 | protein_coding | 4930506M07Rik | 2900.943 | 3582.087 | 3241.515 |
| ENSMUSG00000074577 | protein_coding | Fam65c | 72.51609 | 38.17575 | 55.34592 |
| ENSMUSG00000019863 | protein_coding | Qrsl1 | 667.1725 | 533.1693 | 600.1709 |
| ENSMUSG00000075555 | processed_transcrip | Gm10863 | 172.0147 | 250.3454 | 211.18 |
| ENSMUSG00000082860 | pseudogene | Gm13204 | 34.34547 | 63.64158 | 48.99353 |
| ENSMUSG00000079105 | protein_coding | C7 | 50.30375 | 87.2363 | 68.77003 |
| ENSMUSG00000021993 | protein_coding | Mipep | 874.0011 | 715.7079 | 794.8545 |
| ENSMUSG00000028654 | protein_coding | Mycl1 | 536.7181 | 408.8294 | 472.7738 |
| ENSMUSG00000033022 | protein_coding | Cdo1 | 1693.557 | 1311.118 | 1502.337 |
| ENSMUSG00000049404 | protein_coding | Rarres1 | 717.262 | 1024.875 | 871.0687 |
| ENSMUSG00000001507 | protein_coding | Itga3 | 293.8011 | 404.2035 | 349.0023 |
| ENSMUSG00000021177 | protein_coding | Tdp1 | 1094.961 | 894.0437 | 994.5024 |
| ENSMUSG00000035211 | protein_coding | Xrra1 | 91.28569 | 50.54281 | 70.91425 |
| ENSMUSG00000074240 | protein_coding | Cib3 | 0.32211 | 6.994426 | 3.658268 |
| ENSMUSG00000046196 | protein_coding | Ttc39d | 23.96899 | 7.831267 | 15.90013 |
| ENSMUSG00000028150 | protein_coding | Rorc | 514.2632 | 653.4655 | 583.8644 |
| ENSMUSG00000062031 | protein_coding | Athl1 | 400.2679 | 551.9171 | 476.0925 |
| ENSMUSG00000036030 | protein_coding | Prtg | 492.4523 | 357.894 | 425.1731 |
| ENSMUSG00000083360 | pseudogene | Gm13105 | 0 | 4.794622 | 2.397311 |
| ENSMUSG00000033847 | protein_coding | Pla2g4c | 53.11739 | 95.65743 | 74.38741 |
| ENSMUSG00000030512 | protein_coding | Snrpa1 | 1163.405 | 949.212 | 1056.308 |
| ENSMUSG00000023953 | protein_coding | Polh | 526.6474 | 668.1115 | 597.3795 |
| ENSMUSG00000025789 | protein_coding | St8sia2 | 607.2374 | 400.3449 | 503.7912 |
| ENSMUSG00000012609 | protein_coding | Ttll5 | 1434.099 | 1110.81 | 1272.454 |
| ENSMUSG00000027793 | protein_coding | Ccna1 | 33.4525 | 13.73949 | 23.59599 |
| ENSMUSG00000053205 | protein_coding | Styx | 439.5449 | 338.4909 | 389.0179 |
| ENSMUSG00000031344 | protein_coding | Gabrq | 49.43813 | 24.91734 | 37.17774 |
| ENSMUSG00000031146 | protein_coding | Plp2 | 558.4824 | 721.0006 | 639.7415 |
| ENSMUSG00000040022 | protein_coding | Rab11fip2 | 1164.52 | 947.6398 | 1056.08 |
| ENSMUSG00000024163 | protein_coding | Mapk8ip3 | 2922.107 | 3828.782 | 3375.445 |
| ENSMUSG00000022020 | protein_coding | Naa16 | 770.2829 | 948.486 | 859.3844 |
| ENSMUSG00000019823 | protein_coding | Mical1 | 258.2069 | 358.4005 | 308.3037 |
| ENSMUSG00000022574 | protein_coding | Naprt1 | 252.8921 | 363.0092 | 307.9507 |
| ENSMUSG00000030346 | protein_coding | Rad51ap1 | 779.292 | 621.5854 | 700.4387 |
| ENSMUSG00000020937 | protein_coding | Plcd3 | 539.9533 | 671.0503 | 605.5018 |
| ENSMUSG00000038291 | protein_coding | Snx25 | 1155.708 | 948.6381 | 1052.173 |
| ENSMUSG00000041642 | protein_coding | Kif21b | 1332.701 | 1068.477 | 1200.589 |
| ENSMUSG00000020766 | protein_coding | Galk1 | 1522.361 | 1131.782 | 1327.071 |
| ENSMUSG00000048376 | protein_coding | F2r | 373.0201 | 537.3522 | 455.1861 |
| ENSMUSG00000025473 | protein_coding | Adam8 | 62.0963 | 98.79219 | 80.44424 |
| ENSMUSG00000039512 | protein_coding | Uhrf1bp1 | 2164.281 | 1744.614 | 1954.447 |
| ENSMUSG00000061878 | protein_coding | Sphk1 | 458.1626 | 613.4476 | 535.8051 |
| ENSMUSG00000024999 | protein_coding | Noc3l | 1909.676 | 2348.267 | 2128.971 |
| ENSMUSG00000034108 | protein_coding | Ccs | 724.6245 | 590.6128 | 657.6186 |
| ENSMUSG00000039224 | protein_coding | D1Pas1 | 827.7524 | 1105.86 | 966.8064 |
| ENSMUSG00000020894 | protein_coding | Vamp2 | 1736.56 | 2112.356 | 1924.458 |
| ENSMUSG00000032418 | protein_coding | Me1 | 12301.15 | 15253.79 | 13777.47 |
| ENSMUSG00000085410 | lincRNA | 2010110K18Rik | 37.55122 | 16.58082 | 27.06602 |
| ENSMUSG00000085956 | lincRNA | 4930481B07Rik | 114.2837 | 67.90098 | 91.09235 |
| ENSMUSG00000020908 | protein_coding | Myh3 | 160.244 | 107.0227 | 133.6334 |
| ENSMUSG00000020961 | protein_coding | Ston2 | 259.4632 | 182.5666 | 221.0149 |
| ENSMUSG00000033031 | protein_coding | C330027C09Rik | 2492.726 | 2067.819 | 2280.272 |
| ENSMUSG00000020620 | protein_coding | Abca8b | 105.6948 | 161.7029 | 133.6988 |

| | | | | | |
|---|---|---|---|---|---|
| ENSMUSG00000025155 | protein_coding | Dus1l | 1203.659 | 994.1682 | 1098.914 |
| ENSMUSG00000026162 | protein_coding | Nhej1 | 180.476 | 125.4841 | 152.98 |
| ENSMUSG00000030510 | protein_coding | Lass3 | 81.34157 | 27.19963 | 54.2706 |
| ENSMUSG00000070737 | protein_coding | Gm12942 | 255.8988 | 354.7821 | 305.3404 |
| ENSMUSG00000072932 | protein_coding | Gm15128 | 280.3156 | 201.5187 | 240.9171 |
| ENSMUSG00000042489 | protein_coding | Clspn | 2346.249 | 1833.166 | 2089.707 |
| ENSMUSG00000092020 | protein_coding | Gm5176 | 8.433308 | 24.41209 | 16.4227 |
| ENSMUSG00000042213 | protein_coding | Anubl1 | 533.9653 | 387.0982 | 460.5317 |
| ENSMUSG00000034958 | protein_coding | Atcay | 39.51079 | 17.35781 | 28.4343 |
| ENSMUSG00000067023 | protein_coding | Gm626 | 8.73544 | 0.724379 | 4.72991 |
| ENSMUSG00000020374 | protein_coding | Rasgef1c | 155.25 | 292.4686 | 223.8593 |
| ENSMUSG00000031093 | protein_coding | Dock11 | 1567.3 | 1269.846 | 1418.573 |
| ENSMUSG00000016346 | protein_coding | Kcnq2 | 18.75448 | 39.36283 | 29.05865 |
| ENSMUSG00000037355 | protein_coding | 4933407H18Rik | 1195.464 | 906.8169 | 1051.14 |
| ENSMUSG00000079657 | protein_coding | Rab26 | 95.57152 | 54.01879 | 74.79516 |
| ENSMUSG00000028378 | protein_coding | Ptgr1 | 2051.387 | 2599.578 | 2325.482 |
| ENSMUSG00000041623 | protein_coding | D11Wsu47e | 1157.95 | 961.8411 | 1059.896 |
| ENSMUSG00000002475 | protein_coding | Abhd3 | 660.2055 | 850.075 | 755.1402 |
| ENSMUSG00000035699 | protein_coding | Osta | 25.30432 | 8.600331 | 16.95233 |
| ENSMUSG00000046753 | protein_coding | Ccdc66 | 721.2876 | 585.3816 | 653.3346 |
| ENSMUSG00000029061 | protein_coding | Mmp23 | 198.9997 | 360.5998 | 279.7998 |
| ENSMUSG00000087566 | lincRNA | C920006O11Rik | 81.11787 | 149.0323 | 115.0751 |
| ENSMUSG00000040434 | protein_coding | Gyltl1b | 632.08 | 824.4759 | 728.278 |
| ENSMUSG00000026608 | protein_coding | Kctd3 | 2272.98 | 1800.014 | 2036.497 |
| ENSMUSG00000007379 | protein_coding | Dennd2c | 982.3495 | 725.8684 | 854.109 |
| ENSMUSG00000045587 | protein_coding | BC049730 | 34.77776 | 12.16926 | 23.47351 |
| ENSMUSG00000031255 | protein_coding | Sytl4 | 2471.644 | 3022.752 | 2747.198 |
| ENSMUSG00000052293 | protein_coding | Taf9 | 3125.04 | 2482.705 | 2803.873 |
| ENSMUSG00000001027 | protein_coding | Scn4a | 4.196676 | 18.58128 | 11.38898 |
| ENSMUSG00000087239 | pseudogene | Gm7613 | 4.510586 | 18.52981 | 11.5202 |
| ENSMUSG00000050382 | protein_coding | Kif7 | 841.3926 | 654.5239 | 747.9583 |
| ENSMUSG00000087370 | protein_coding | Tmem170b | 1243.418 | 1032.944 | 1138.181 |
| ENSMUSG00000087676 | lincRNA | 9230114K14Rik | 85.60967 | 51.90836 | 68.75901 |
| ENSMUSG00000072843 | protein_coding | Gm10428 | 7.491577 | 23.32516 | 15.40837 |
| ENSMUSG00000047407 | protein_coding | Tgif1 | 1884.711 | 2354.599 | 2119.655 |
| ENSMUSG00000020913 | protein_coding | Krt24 | 28.11416 | 11.60202 | 19.85809 |
| ENSMUSG00000045835 | protein_coding | Hdgfl1 | 42.52793 | 17.8454 | 30.18667 |
| ENSMUSG00000044681 | protein_coding | Cnpy1 | 26.51665 | 55.14461 | 40.83063 |
| ENSMUSG00000028397 | protein_coding | Kdm4c | 1146.969 | 945.7014 | 1046.335 |
| ENSMUSG00000036743 | protein_coding | Psma8 | 507.2748 | 370.1966 | 438.7357 |
| ENSMUSG00000021982 | protein_coding | Cdadc1 | 877.8182 | 1076.927 | 977.3725 |
| ENSMUSG00000021012 | protein_coding | Zc3h14 | 5904.521 | 4995.818 | 5450.17 |
| ENSMUSG00000044005 | protein_coding | Gls2 | 69.64423 | 37.73963 | 53.69193 |
| ENSMUSG00000026211 | protein_coding | Obsl1 | 6502.864 | 8146.408 | 7324.636 |
| ENSMUSG00000026131 | protein_coding | Dst | 9244.692 | 13105.06 | 11174.88 |
| ENSMUSG00000036499 | protein_coding | Eea1 | 2182.823 | 1715.495 | 1949.159 |
| ENSMUSG00000046093 | protein_coding | Hpcal4 | 20.68587 | 7.1786 | 13.93223 |
| ENSMUSG00000000148 | protein_coding | Baat1 | 1257.295 | 1054.057 | 1155.676 |
| ENSMUSG00000001247 | protein_coding | Lsr | 2459.267 | 2932.092 | 2695.68 |
| ENSMUSG00000026158 | protein_coding | Ogfrl1 | 397.9957 | 288.2517 | 343.1237 |
| ENSMUSG00000024805 | protein_coding | Pcgf5 | 2074.406 | 1640.789 | 1857.597 |
| ENSMUSG00000048416 | protein_coding | Mlf1 | 992.0293 | 803.6217 | 897.8255 |
| ENSMUSG00000055633 | protein_coding | Zfp580 | 154.002 | 212.1704 | 183.0862 |
| ENSMUSG00000025558 | protein_coding | Dock9 | 2612.933 | 2060.91 | 2336.921 |
| ENSMUSG00000026646 | protein_coding | Suv39h2 | 1304.859 | 1053.088 | 1178.974 |
| ENSMUSG00000019791 | protein_coding | Hint3 | 478.4729 | 380.2763 | 429.3746 |
| ENSMUSG00000064016 | protein_coding | Gm648 | 70.99118 | 21.68964 | 46.34041 |
| ENSMUSG00000020736 | protein_coding | Nt5c | 664.6386 | 820.269 | 742.4538 |

| | | | | | |
|---|---|---|---|---|---|
| ENSMUSG00000039345 | protein_coding | Mettl22 | 262.2927 | 190.6192 | 226.4559 |
| ENSMUSG00000045273 | protein_coding | Cenph | 565.9286 | 709.1292 | 637.5289 |
| ENSMUSG00000039519 | protein_coding | Cyp7b1 | 65.92666 | 110.7103 | 88.31849 |
| ENSMUSG00000057933 | protein_coding | Gsta2 | 534.2863 | 836.4284 | 685.3574 |
| ENSMUSG00000029186 | protein_coding | Pi4k2b | 1243.376 | 1008.76 | 1126.068 |
| ENSMUSG00000037343 | protein_coding | Taf2 | 3327.437 | 2829.54 | 3078.488 |
| ENSMUSG00000032583 | protein_coding | Mon1a | 299.1975 | 225.4728 | 262.3351 |
| ENSMUSG00000023960 | protein_coding | Enpp5 | 1265.997 | 1532.682 | 1399.339 |
| ENSMUSG00000036699 | protein_coding | Zcchc12 | 136.4647 | 195.9946 | 166.2296 |
| ENSMUSG00000026188 | protein_coding | Tmem169 | 90.70454 | 56.72752 | 73.71603 |
| ENSMUSG00000030725 | protein_coding | Lipt2 | 367.2995 | 282.9067 | 325.1031 |
| ENSMUSG00000027287 | protein_coding | Snap23 | 1048.838 | 1312.65 | 1180.744 |
| ENSMUSG00000005413 | protein_coding | Hmox1 | 1348.036 | 1692.521 | 1520.279 |
| ENSMUSG00000049916 | protein_coding | 2610318N02Rik | 181.9753 | 251.1312 | 216.5532 |
| ENSMUSG00000027406 | protein_coding | Idh3b | 1717.755 | 2084.896 | 1901.326 |
| ENSMUSG00000066363 | protein_coding | Serpina3f | 12.69685 | 2.374969 | 7.53591 |
| ENSMUSG00000031214 | protein_coding | Ophn1 | 797.667 | 646.2588 | 721.9629 |
| ENSMUSG00000004668 | protein_coding | Abca13 | 22.97933 | 8.202466 | 15.5909 |
| ENSMUSG00000090554 | lincRNA | Gm17565 | 15.48544 | 40.77565 | 28.13054 |
| ENSMUSG00000021379 | protein_coding | Id4 | 314.6405 | 232.6863 | 273.6634 |
| ENSMUSG00000024084 | protein_coding | Qpct | 3855.854 | 4663.97 | 4259.912 |
| ENSMUSG00000035681 | protein_coding | Kcnc2 | 197.1546 | 289.2687 | 243.2116 |
| ENSMUSG00000022949 | protein_coding | Clic6 | 426.2247 | 315.8936 | 371.0592 |
| ENSMUSG00000035299 | protein_coding | Mid1 | 350.7838 | 263.1973 | 306.9905 |
| ENSMUSG00000053886 | protein_coding | Sh2d4a | 1215.941 | 1610.903 | 1413.422 |
| ENSMUSG00000054196 | protein_coding | Cthrc1 | 82.29352 | 49.65952 | 65.97652 |
| ENSMUSG00000040782 | protein_coding | Rfwd2 | 2247.562 | 1922.38 | 2084.971 |
| ENSMUSG00000026721 | protein_coding | Rabgap1l | 1629.751 | 1143.178 | 1386.464 |
| ENSMUSG00000029716 | protein_coding | Trfr2 | 142.9832 | 94.97095 | 118.9771 |
| ENSMUSG00000025500 | protein_coding | 1600016N20Rik | 232.0117 | 119.7257 | 175.8687 |
| ENSMUSG00000000690 | protein_coding | Hoxb6 | 19.39385 | 46.55519 | 32.97452 |
| ENSMUSG00000038822 | protein_coding | Hace1 | 1284.672 | 1042.971 | 1163.822 |
| ENSMUSG00000030788 | protein_coding | Rnf141 | 714.0357 | 574.5123 | 644.274 |
| ENSMUSG00000001517 | protein_coding | Foxm1 | 2057.98 | 1700.523 | 1879.251 |
| ENSMUSG00000044341 | pseudogene | Gm5601 | 371.3777 | 511.1852 | 441.2814 |
| ENSMUSG00000074170 | protein_coding | Plekhf1 | 237.569 | 308.4637 | 273.0163 |
| ENSMUSG00000044229 | protein_coding | Fam55d | 159.6067 | 226.8826 | 193.2447 |
| ENSMUSG00000026308 | protein_coding | Klhl30 | 45.86272 | 94.4179 | 70.14031 |
| ENSMUSG00000041358 | protein_coding | BC125332 | 618.527 | 465.8445 | 542.1858 |
| ENSMUSG00000021198 | protein_coding | Unc79 | 45.70379 | 77.27332 | 61.48855 |
| ENSMUSG00000050069 | protein_coding | Grem2 | 1134.411 | 1604.26 | 1369.336 |
| ENSMUSG00000031425 | protein_coding | Plp1 | 1056.459 | 1373.126 | 1214.792 |
| ENSMUSG00000058028 | protein_coding | Zscan5b | 23.25328 | 44.55246 | 33.90287 |
| ENSMUSG00000004383 | protein_coding | Large | 477.2211 | 348.9013 | 413.0612 |
| ENSMUSG00000006378 | protein_coding | Gcat | 957.1628 | 739.8939 | 848.5284 |
| ENSMUSG00000051176 | protein_coding | Zfp42 | 196.2601 | 142.7713 | 169.5157 |
| ENSMUSG00000029726 | protein_coding | Mepce | 2297.036 | 1770.253 | 2033.645 |
| ENSMUSG00000022769 | protein_coding | Sdf2l1 | 544.5925 | 381.4021 | 462.9973 |
| ENSMUSG00000053641 | protein_coding | Dennd4a | 1596.836 | 1296.589 | 1446.713 |
| ENSMUSG00000052056 | protein_coding | Zfp217 | 3020.693 | 2390.257 | 2705.475 |
| ENSMUSG00000022702 | protein_coding | Hira | 1675.119 | 1400.716 | 1537.918 |
| ENSMUSG00000031813 | protein_coding | Fam125a | 702.6705 | 553.0866 | 627.8785 |
| ENSMUSG00000053914 | protein_coding | Kdm4d | 78.33069 | 35.90378 | 57.11723 |
| ENSMUSG00000020090 | protein_coding | Npffr1 | 26.72352 | 50.74143 | 38.73247 |
| ENSMUSG00000038775 | protein_coding | Vill | 337.9287 | 243.4198 | 290.6743 |
| ENSMUSG00000026994 | protein_coding | Galnt3 | 152.0458 | 102.422 | 127.2339 |
| ENSMUSG00000032127 | protein_coding | Vps11 | 2212.227 | 1837.954 | 2025.09 |
| ENSMUSG00000070858 | protein_coding | Gm1673 | 354.1147 | 267.5699 | 310.8423 |

| | | | | | |
|---|---|---|---|---|---|
| ENSMUSG00000060257 | protein_coding | Scrt2 | 11.9353 | 28.37852 | 20.15691 |
| ENSMUSG00000038844 | protein_coding | Kif16b | 1298.954 | 1020.541 | 1159.748 |
| ENSMUSG00000070720 | protein_coding | Tmem200b | 864.407 | 1073.576 | 968.9914 |
| ENSMUSG00000032359 | protein_coding | Ctsh | 288.6215 | 404.1678 | 346.3946 |
| ENSMUSG00000029657 | protein_coding | Hsph1 | 1967.848 | 2380.858 | 2174.353 |
| ENSMUSG00000041429 | protein_coding | Nthl1 | 326.1586 | 251.774 | 288.9663 |
| ENSMUSG00000034771 | protein_coding | Tle2 | 412.0435 | 602.6716 | 507.3575 |
| ENSMUSG00000026739 | protein_coding | Bmi1 | 3061.303 | 2564.135 | 2812.719 |
| ENSMUSG00000026097 | protein_coding | Ormdl1 | 679.4824 | 846.3491 | 762.9157 |
| ENSMUSG00000033705 | protein_coding | Stard9 | 3639.141 | 4233.771 | 3936.456 |
| ENSMUSG00000023991 | protein_coding | Foxp4 | 2029.11 | 2473.715 | 2251.412 |
| ENSMUSG00000068794 | protein_coding | Col28a1 | 55.41801 | 27.25011 | 41.33406 |
| ENSMUSG00000025795 | protein_coding | Rassf3 | 1100.531 | 917.4386 | 1008.985 |
| ENSMUSG00000070738 | protein_coding | Dgkd | 6470.634 | 5340.577 | 5905.605 |
| ENSMUSG00000087700 | lincRNA | Gm15283 | 22.6936 | 46.74683 | 34.72021 |
| ENSMUSG00000030074 | protein_coding | Gxylt2 | 149.7118 | 204.0073 | 176.8595 |
| ENSMUSG00000034687 | protein_coding | Fras1 | 486.2379 | 357.5417 | 421.8898 |
| ENSMUSG00000040139 | protein_coding | 9430038I01Rik | 359.5415 | 277.6522 | 318.5969 |
| ENSMUSG00000044433 | protein_coding | Camsap3 | 915.465 | 1124.014 | 1019.74 |
| ENSMUSG00000025283 | protein_coding | Sat1 | 976.5863 | 1243.379 | 1109.983 |
| ENSMUSG00000018659 | protein_coding | Pnpo | 496.1428 | 397.2727 | 446.7078 |
| ENSMUSG00000034171 | protein_coding | Faah | 444.1042 | 328.2405 | 386.1724 |
| ENSMUSG00000002233 | protein_coding | Rhoc | 1219.502 | 1505.685 | 1362.594 |
| ENSMUSG00000031799 | protein_coding | Tpm4 | 2413.092 | 3111.421 | 2762.256 |
| ENSMUSG00000036923 | protein_coding | Stox1 | 174.9213 | 125.4924 | 150.2068 |
| ENSMUSG00000050737 | protein_coding | Ptges | 204.5629 | 311.4525 | 258.0077 |
| ENSMUSG00000040459 | protein_coding | Arglu1 | 2339.198 | 2871.237 | 2605.217 |
| ENSMUSG00000039375 | protein_coding | Wdr17 | 66.21015 | 33.0423 | 49.62622 |
| ENSMUSG00000001786 | protein_coding | Fbxo7 | 1119.676 | 888.8351 | 1004.256 |
| ENSMUSG00000044037 | protein_coding | Als2cl | 613.574 | 746.4514 | 680.0127 |
| ENSMUSG00000008690 | protein_coding | Ncaph2 | 2919.46 | 2510.087 | 2714.774 |
| ENSMUSG00000021811 | protein_coding | Dnajc9 | 1807.338 | 1500.926 | 1654.132 |
| ENSMUSG00000069135 | protein_coding | Fgfr1op | 2205.158 | 1860.052 | 2032.605 |
| ENSMUSG00000073469 | protein_coding | Rnaset2a | 1076.299 | 784.1421 | 930.2206 |
| ENSMUSG00000020079 | protein_coding | Supv3l1 | 1038.697 | 856.8856 | 947.7914 |
| ENSMUSG00000029056 | protein_coding | Pank4 | 1009.063 | 1229.868 | 1119.465 |
| ENSMUSG00000038425 | protein_coding | Poli | 636.9677 | 510.1738 | 573.5707 |
| ENSMUSG00000058806 | protein_coding | Col13a1 | 326.1311 | 440.2493 | 383.1902 |
| ENSMUSG00000052861 | protein_coding | Dnahc6 | 30.97364 | 9.749603 | 20.36162 |
| ENSMUSG00000021944 | protein_coding | Gata4 | 4186.664 | 5267.242 | 4726.953 |
| ENSMUSG00000022099 | protein_coding | Epb4.9 | 34.52207 | 60.74619 | 47.63413 |
| ENSMUSG00000041323 | protein_coding | Ak7 | 194.1027 | 268.6829 | 231.3928 |
| ENSMUSG00000081205 | pseudogene | Gm5940 | 9.372504 | 27.51579 | 18.44415 |
| ENSMUSG00000052369 | protein_coding | Tmem106c | 647.3544 | 515.9155 | 581.6349 |
| ENSMUSG00000054994 | protein_coding | AV320801 | 280.2241 | 214.9144 | 247.5693 |
| ENSMUSG00000004929 | protein_coding | Thop1 | 2535.146 | 2003.499 | 2269.323 |
| ENSMUSG00000034998 | protein_coding | Foxn2 | 1484.505 | 1164.077 | 1324.291 |
| ENSMUSG00000079008 | protein_coding | Gm14124 | 105.1578 | 150.7078 | 127.9328 |
| ENSMUSG00000036983 | protein_coding | Tfb1m | 432.0469 | 327.7363 | 379.8916 |
| ENSMUSG00000033632 | protein_coding | AW554918 | 707.1245 | 567.5784 | 637.3514 |
| ENSMUSG00000021090 | protein_coding | Lrrc9 | 176.3701 | 126.7126 | 151.5414 |
| ENSMUSG00000033508 | protein_coding | Asprv1 | 8.716505 | 23.9641 | 16.3403 |
| ENSMUSG00000034587 | lincRNA | 8430429K09Rik | 173.1004 | 120.3115 | 146.7059 |
| ENSMUSG00000005682 | protein_coding | Pan2 | 1844.236 | 1525.594 | 1684.915 |
| ENSMUSG00000018727 | protein_coding | Cpsf4l | 58.61131 | 93.17874 | 75.89502 |
| ENSMUSG00000022322 | protein_coding | Shcbp1 | 453.264 | 355.2715 | 404.2678 |
| ENSMUSG00000050675 | protein_coding | Gp1ba | 143.048 | 80.11963 | 111.5838 |
| ENSMUSG00000028766 | protein_coding | Alpl | 460.483 | 597.798 | 529.1405 |

| | | | | | |
|---|---|---|---|---|---|
| ENSMUSG00000015243 | protein_coding | Abca1 | 7015.353 | 8672.877 | 7844.115 |
| ENSMUSG00000064043 | protein_coding | Trerf1 | 814.1689 | 664.7053 | 739.4371 |
| ENSMUSG00000007827 | protein_coding | Ankrd26 | 1242.891 | 1023.225 | 1133.058 |
| ENSMUSG00000064036 | protein_coding | Mro | 5964.856 | 7508.805 | 6736.83 |
| ENSMUSG00000091497 | protein_coding | Gm44 | 247.6249 | 177.5992 | 212.6121 |
| ENSMUSG00000028718 | protein_coding | Stil | 724.9577 | 575.182 | 650.0698 |
| ENSMUSG00000045031 | protein_coding | Cetn4 | 100.0703 | 61.51634 | 80.79334 |
| ENSMUSG00000012126 | protein_coding | Ubxn11 | 185.6241 | 121.754 | 153.6891 |
| ENSMUSG00000025420 | protein_coding | Katnal2 | 183.417 | 126.162 | 154.7895 |
| ENSMUSG00000027088 | protein_coding | Phospho2 | 1011.601 | 804.0847 | 907.8428 |
| ENSMUSG00000026437 | protein_coding | Cdk18 | 53.35034 | 86.46398 | 69.90716 |
| ENSMUSG00000018362 | protein_coding | Kpna2 | 4640.414 | 5744.291 | 5192.352 |
| ENSMUSG00000019088 | protein_coding | Dnase1l1 | 154.2434 | 209.7814 | 182.0124 |
| ENSMUSG00000052133 | protein_coding | Sema5b | 3793.723 | 4510.538 | 4152.13 |
| ENSMUSG00000025190 | protein_coding | Got1 | 1068.415 | 1337.599 | 1203.007 |
| ENSMUSG00000057176 | protein_coding | Gm166 | 133.1877 | 88.39598 | 110.7918 |
| ENSMUSG00000032462 | protein_coding | Pik3cb | 969.4271 | 759.8013 | 864.6142 |
| ENSMUSG00000049686 | protein_coding | Orai1 | 479.3426 | 635.5093 | 557.4259 |
| ENSMUSG00000071650 | protein_coding | Ganab | 5675.607 | 6613.259 | 6144.433 |
| ENSMUSG00000027656 | protein_coding | Wisp2 | 4.569253 | 17.18301 | 10.87613 |
| ENSMUSG00000047129 | protein_coding | 1700113H08Rik | 6.387935 | 20.6507 | 13.51932 |
| ENSMUSG00000029547 | protein_coding | Ints1 | 4820.734 | 3909.92 | 4365.327 |
| ENSMUSG00000027869 | protein_coding | Hsd3b6 | 77.02183 | 37.60226 | 57.31204 |
| ENSMUSG00000021363 | protein_coding | Mak | 70.62465 | 39.38012 | 55.00238 |
| ENSMUSG00000043091 | protein_coding | Tuba1c | 2434.972 | 2838.951 | 2636.962 |
| ENSMUSG00000019848 | protein_coding | Popdc3 | 61.62278 | 36.43864 | 49.03071 |
| ENSMUSG00000053469 | protein_coding | Tg | 133.7705 | 83.43375 | 108.6021 |
| ENSMUSG00000055045 | processed_transcrip | Gm11190 | 18.12665 | 37.13672 | 27.63169 |
| ENSMUSG00000041977 | protein_coding | Arhgef11 | 2100.897 | 1739.367 | 1920.132 |
| ENSMUSG00000029625 | protein_coding | Cpsf4 | 1404.704 | 1186.039 | 1295.371 |
| ENSMUSG00000027433 | protein_coding | Xrn2 | 4918.339 | 5706.86 | 5312.599 |
| ENSMUSG00000037475 | protein_coding | Thoc2 | 5655.995 | 6670.57 | 6163.283 |
| ENSMUSG00000041062 | protein_coding | Mslnl | 48.41023 | 20.76316 | 34.5867 |
| ENSMUSG00000021263 | protein_coding | Degs2 | 252.9311 | 347.3998 | 300.1655 |
| ENSMUSG00000051235 | protein_coding | Gen1 | 534.8649 | 412.2573 | 473.5611 |
| ENSMUSG00000089365 | snoRNA | SNORA17 | 34.13354 | 8.532553 | 21.33305 |
| ENSMUSG00000036111 | protein_coding | Lmo1 | 258.6729 | 173.4701 | 216.0715 |
| ENSMUSG00000001510 | protein_coding | Dlx3 | 101.3392 | 165.5026 | 133.4209 |
| ENSMUSG00000074607 | protein_coding | Tox2 | 148.5295 | 220.4657 | 184.4976 |
| ENSMUSG00000027478 | protein_coding | Dnmt3b | 1837.766 | 1434.765 | 1636.266 |
| ENSMUSG00000037022 | protein_coding | Mmaa | 491.6241 | 393.1084 | 442.3663 |
| ENSMUSG00000025089 | protein_coding | Gfra1 | 792.4061 | 1083.421 | 937.9135 |
| ENSMUSG00000059248 | protein_coding | 9-Sep | 4573.505 | 5509.355 | 5041.43 |
| ENSMUSG00000022656 | protein_coding | Pvrl3 | 1055.896 | 867.0012 | 961.4488 |
| ENSMUSG00000028128 | protein_coding | F3 | 449.9708 | 569.548 | 509.7594 |
| ENSMUSG00000046287 | protein_coding | Pnma3 | 52.60728 | 103.3465 | 77.97687 |
| ENSMUSG00000029735 | protein_coding | Tpk1 | 373.1533 | 291.7845 | 332.4689 |
| ENSMUSG00000033486 | protein_coding | Catsper2 | 171.356 | 103.8113 | 137.5837 |
| ENSMUSG00000038141 | protein_coding | Tmem181a | 340.9304 | 469.3424 | 405.1364 |
| ENSMUSG00000026798 | protein_coding | Coq4 | 261.09 | 194.9201 | 228.005 |
| ENSMUSG00000030826 | protein_coding | Bcat2 | 3404.569 | 4011.091 | 3707.83 |
| ENSMUSG00000084364 | pseudogene | Gm15801 | 4.851407 | 26.24146 | 15.54643 |
| ENSMUSG00000027195 | protein_coding | Hsd17b12 | 2591.769 | 3139.224 | 2865.496 |
| ENSMUSG00000048230 | protein_coding | Fbxo43 | 56.88558 | 25.48385 | 41.18471 |
| ENSMUSG00000061536 | protein_coding | Sec22c | 693.6271 | 545.7076 | 619.6674 |
| ENSMUSG00000048029 | protein_coding | Eno4 | 142.7831 | 97.91718 | 120.3501 |
| ENSMUSG00000028457 | protein_coding | Atp8b5 | 225.6669 | 305.6148 | 265.6409 |
| ENSMUSG00000013698 | protein_coding | Pea15a | 3884.868 | 4649.457 | 4267.163 |

| | | | | | |
|---|---|---|---|---|---|
| ENSMUSG00000060152 | protein_coding | Pop5 | 615.1128 | 478.7797 | 546.9463 |
| ENSMUSG00000088494 | snRNA | U6 | 0 | 4.096909 | 2.048455 |
| ENSMUSG00000037568 | protein_coding | Vash2 | 97.6957 | 143.5173 | 120.6065 |
| ENSMUSG00000000627 | protein_coding | Sema4f | 377.9157 | 269.7441 | 323.8299 |
| ENSMUSG00000047045 | protein_coding | Tmem164 | 1661.615 | 2079.921 | 1870.768 |
| ENSMUSG00000043531 | protein_coding | Sorcs1 | 7.776266 | 26.33151 | 17.05389 |
| ENSMUSG00000074749 | protein_coding | Plk1s1 | 1215.715 | 1004.65 | 1110.183 |
| ENSMUSG00000031758 | protein_coding | Cdyl2 | 445.7756 | 343.3399 | 394.5578 |
| ENSMUSG00000067787 | protein_coding | Blcap | 1232.013 | 1478.16 | 1355.086 |
| ENSMUSG00000024381 | protein_coding | Bin1 | 709.2369 | 892.5734 | 800.9051 |
| ENSMUSG00000091430 | lincRNA | Gm17337 | 0 | 4.132585 | 2.066292 |
| ENSMUSG00000046179 | protein_coding | E2f8 | 263.8942 | 176.3049 | 220.0995 |
| ENSMUSG00000028784 | protein_coding | Spocd1 | 107.8048 | 58.55281 | 83.17879 |
| ENSMUSG00000039096 | protein_coding | Rsad1 | 398.0939 | 303.9696 | 351.0317 |
| ENSMUSG00000056300 | protein_coding | Gm13247 | 334.7613 | 551.7888 | 443.2751 |
| ENSMUSG00000042992 | protein_coding | Loh12cr1 | 347.226 | 244.7412 | 295.9836 |
| ENSMUSG00000044279 | protein_coding | Crb3 | 38.14266 | 71.26699 | 54.70482 |
| ENSMUSG00000034205 | protein_coding | Loxl2 | 526.1241 | 651.8466 | 588.9853 |
| ENSMUSG00000033669 | protein_coding | Zfp7 | 918.9107 | 1129.782 | 1024.346 |
| ENSMUSG00000070331 | protein_coding | Qrich2 | 4.857296 | 16.04274 | 10.45002 |
| ENSMUSG00000043439 | protein_coding | E130012A19Rik | 198.1341 | 135.5387 | 166.8364 |
| ENSMUSG00000049908 | protein_coding | Gja8 | 12.99771 | 3.063673 | 8.030694 |
| ENSMUSG00000037366 | protein_coding | Pafah2 | 526.5396 | 424.987 | 475.7633 |
| ENSMUSG00000091897 | protein_coding | Gm17019 | 18.03161 | 6.181039 | 12.10632 |
| ENSMUSG00000090548 | lincRNA | Gm17489 | 4.193097 | 15.15293 | 9.673014 |
| ENSMUSG00000086657 | processed_transcrip | 4921530L18Rik | 26.53998 | 10.78864 | 18.66431 |
| ENSMUSG00000025478 | protein_coding | Dpysl4 | 2874.707 | 3388.797 | 3131.752 |
| ENSMUSG00000067919 | protein_coding | Gm13138 | 49.76554 | 90.71037 | 70.23795 |
| ENSMUSG00000060950 | protein_coding | Trmt61a | 339.4519 | 264.4803 | 301.9661 |
| ENSMUSG00000045275 | protein_coding | Lca5l | 303.1035 | 203.8186 | 253.4611 |
| ENSMUSG00000034121 | protein_coding | Mks1 | 336.1247 | 257.937 | 297.0308 |
| ENSMUSG00000079356 | processed_transcrip | Mettl4-ps1 | 28.04058 | 8.255798 | 18.14819 |
| ENSMUSG00000034120 | protein_coding | Srsf2 | 8086.91 | 6798.461 | 7442.686 |
| ENSMUSG00000026723 | protein_coding | Trdmt1 | 549.9168 | 442.357 | 496.1369 |
| ENSMUSG00000024990 | protein_coding | Rbp4 | 524.4421 | 392.8814 | 458.6618 |
| ENSMUSG00000035064 | protein_coding | Eef2k | 1231.111 | 969.0997 | 1100.106 |
| ENSMUSG00000090236 | protein_coding | Car15 | 23.37524 | 8.475648 | 15.92544 |
| ENSMUSG00000035529 | protein_coding | Prdm4 | 2166.953 | 1776.72 | 1971.836 |
| ENSMUSG00000082095 | pseudogene | Gm11991 | 8.05842 | 1.024227 | 4.541324 |
| ENSMUSG00000028946 | protein_coding | Hes3 | 5.862315 | 0.36219 | 3.112252 |
| ENSMUSG00000022021 | protein_coding | Diap3 | 767.2698 | 600.2055 | 683.7377 |
| ENSMUSG00000092146 | pseudogene | Gm13694 | 0 | 5.783898 | 2.891949 |
| ENSMUSG00000062309 | protein_coding | Rpp25 | 198.5208 | 278.6657 | 238.5933 |
| ENSMUSG00000032064 | protein_coding | Dixdc1 | 949.0192 | 776.6673 | 862.8433 |
| ENSMUSG00000050088 | protein_coding | 1600012H06Rik | 1236.212 | 1532.151 | 1384.181 |
| ENSMUSG00000042360 | protein_coding | 4930433N12Rik | 16.27979 | 3.850393 | 10.06509 |
| ENSMUSG00000064936 | snoRNA | SNORA33 | 3.524277 | 13.34162 | 8.432949 |
| ENSMUSG00000065078 | snRNA | U6 | 4.188476 | 0 | 2.094238 |
| ENSMUSG00000063455 | protein_coding | D630045J12Rik | 1165.934 | 919.3749 | 1042.655 |
| ENSMUSG00000032534 | protein_coding | Cep63 | 1189.841 | 944.019 | 1066.93 |
| ENSMUSG00000073481 | protein_coding | Mosc2 | 1137.35 | 1405.787 | 1271.568 |
| ENSMUSG00000026525 | protein_coding | Opn3 | 289.2367 | 215.6078 | 252.4222 |
| ENSMUSG00000025787 | protein_coding | Tgm4 | 19.74309 | 39.32787 | 29.53548 |
| ENSMUSG00000024485 | protein_coding | Slc4a9 | 2.287571 | 11.82115 | 7.054361 |
| ENSMUSG00000029015 | protein_coding | Slc26a5 | 18.79443 | 6.471878 | 12.63315 |
| ENSMUSG00000024924 | protein_coding | Vldlr | 182.3567 | 288.8518 | 235.6042 |
| ENSMUSG00000001229 | protein_coding | Dpp9 | 639.6563 | 772.996 | 706.3262 |
| ENSMUSG00000074818 | protein_coding | Pdzd7 | 138.594 | 219.3617 | 178.9779 |

| | | | | | |
|---|---|---|---|---|---|
| ENSMUSG00000020876 | protein_coding | Snx11 | 1008.787 | 836.464 | 922.6254 |
| ENSMUSG00000022427 | protein_coding | Tomm22 | 1816.954 | 2155.159 | 1986.057 |
| ENSMUSG00000055816 | protein_coding | Magea3 | 126.882 | 75.5693 | 101.2256 |
| ENSMUSG00000059401 | protein_coding | Mamld1 | 1207.616 | 1478.806 | 1343.211 |
| ENSMUSG00000038116 | protein_coding | Phf20 | 4026.676 | 3357.793 | 3692.234 |
| ENSMUSG00000032218 | protein_coding | Ccnb2 | 952.596 | 1194.736 | 1073.666 |
| ENSMUSG00000068762 | protein_coding | Gstm6 | 6324.306 | 7550.05 | 6937.178 |
| ENSMUSG00000045915 | protein_coding | Ccdc42 | 16.23984 | 4.821288 | 10.53056 |
| ENSMUSG00000038683 | protein_coding | Pak1ip1 | 2746.504 | 3165.954 | 2956.229 |
| ENSMUSG00000060143 | protein_coding | Gm10076 | 52.70508 | 29.0056 | 40.85534 |
| ENSMUSG00000001948 | protein_coding | Spa17 | 218.1299 | 145.753 | 181.9414 |
| ENSMUSG00000020608 | protein_coding | Smc6 | 6998.757 | 6012.017 | 6505.387 |
| ENSMUSG00000054237 | protein_coding | 5730455O13Rik | 804.2036 | 645.7008 | 724.9522 |
| ENSMUSG00000084066 | pseudogene | Xlr4d-ps | 77.38307 | 43.76316 | 60.57311 |
| ENSMUSG00000000317 | protein_coding | Bcl6b | 295.7508 | 387.282 | 341.5164 |
| ENSMUSG00000031155 | protein_coding | Pim2 | 520.4223 | 408.4355 | 464.4289 |
| ENSMUSG00000078208 | protein_coding | Gm5634 | 94.72708 | 137.3654 | 116.0463 |
| ENSMUSG00000066009 | protein_coding | Gm13051 | 62.18232 | 106.3188 | 84.25059 |
| ENSMUSG00000026187 | protein_coding | Xrcc5 | 1311.653 | 1104.064 | 1207.858 |
| ENSMUSG00000027811 | protein_coding | 4930579G24Rik | 373.4309 | 292.3421 | 332.8865 |
| ENSMUSG00000003355 | protein_coding | Fkbp11 | 51.96097 | 84.53906 | 68.25002 |
| ENSMUSG00000022540 | protein_coding | Rogdi | 767.4572 | 611.071 | 689.2641 |
| ENSMUSG00000044288 | protein_coding | Cnr1 | 84.74972 | 48.56508 | 66.6574 |
| ENSMUSG00000084984 | processed_transcrip | 1700006P19Rik | 77.37107 | 118.0366 | 97.70383 |
| ENSMUSG00000015748 | protein_coding | Prpf3 | 1817.115 | 2262.594 | 2039.855 |
| ENSMUSG00000020471 | protein_coding | Pold2 | 1216.65 | 985.3947 | 1101.023 |
| ENSMUSG00000005947 | protein_coding | Itgae | 88.85537 | 57.3034 | 73.07938 |
| ENSMUSG00000029312 | protein_coding | Klhl8 | 830.2245 | 690.8952 | 760.5599 |
| ENSMUSG00000050751 | protein_coding | Pgbd5 | 362.12 | 488.7621 | 425.4411 |
| ENSMUSG00000022865 | protein_coding | Cxadr | 1661.376 | 2033.003 | 1847.189 |
| ENSMUSG00000039133 | protein_coding | 9330171B17Rik | 21.06791 | 6.833706 | 13.95081 |
| ENSMUSG00000001569 | protein_coding | Nom1 | 1860.641 | 1600.284 | 1730.462 |
| ENSMUSG00000071103 | protein_coding | 1700029J07Rik | 212.4833 | 128.2936 | 170.3884 |
| ENSMUSG00000028059 | protein_coding | Arhgef2 | 1195.418 | 1442.188 | 1318.803 |
| ENSMUSG00000033688 | protein_coding | 1300017J02Rik | 47.48974 | 92.45825 | 69.974 |
| ENSMUSG00000067338 | protein_coding | Tuba3b | 6019.628 | 5012.423 | 5516.025 |
| ENSMUSG00000031669 | protein_coding | Gins3 | 490.1097 | 392.443 | 441.2764 |
| ENSMUSG00000034349 | protein_coding | Smc4 | 6287.149 | 5244.038 | 5765.593 |
| ENSMUSG00000028309 | protein_coding | Rnf20 | 4196.502 | 4913.293 | 4554.898 |
| ENSMUSG00000021814 | protein_coding | Anxa7 | 1569.264 | 1818.784 | 1694.024 |
| ENSMUSG00000063760 | protein_coding | Rnf217 | 639.0675 | 506.045 | 572.5563 |
| ENSMUSG00000046223 | protein_coding | Plaur | 12.30198 | 27.66315 | 19.98257 |
| ENSMUSG00000073158 | protein_coding | 9030624G23Rik | 60.5017 | 28.04558 | 44.27364 |
| ENSMUSG00000000399 | protein_coding | Ndufa9 | 2425.641 | 2059.718 | 2242.679 |
| ENSMUSG00000037275 | protein_coding | Gemin5 | 3021.418 | 2455.24 | 2738.329 |
| ENSMUSG00000086646 | processed_transcrip | 5133400J02Rik | 56.25649 | 31.80402 | 44.03026 |
| ENSMUSG00000020932 | protein_coding | Gfap | 24.31041 | 63.00776 | 43.65908 |
| ENSMUSG00000040532 | protein_coding | Abhd11 | 868.1307 | 710.7643 | 789.4475 |
| ENSMUSG00000010608 | protein_coding | Rbm25 | 5692.578 | 6569.928 | 6131.253 |
| ENSMUSG00000075122 | protein_coding | Cd80 | 9.684103 | 23.2127 | 16.4484 |
| ENSMUSG00000027082 | protein_coding | Tfpi | 713.5245 | 992.2907 | 852.9076 |
| ENSMUSG00000067438 | protein_coding | Hmx1 | 182.9551 | 126.1224 | 154.5387 |
| ENSMUSG00000022507 | protein_coding | 1810013L24Rik | 2666.06 | 2270.753 | 2468.406 |
| ENSMUSG00000089775 | pseudogene | Gm16270 | 1066.885 | 1413.663 | 1240.274 |
| ENSMUSG00000025777 | protein_coding | Gdap1 | 64.66624 | 37.95518 | 51.31071 |
| ENSMUSG00000068600 | protein_coding | Hemt1 | 131.5562 | 58.80182 | 95.17903 |
| ENSMUSG00000074876 | processed_transcrip | Spata5l1 | 260.5449 | 181.8495 | 221.1972 |
| ENSMUSG00000026544 | protein_coding | Dusp23 | 58.24208 | 32.20877 | 45.22542 |

| | | | | | |
|---|---|---|---|---|---|
| ENSMUSG00000028923 | protein_coding | Necap2 | 1213.813 | 1420.839 | 1317.326 |
| ENSMUSG00000075415 | protein_coding | Fnbp1 | 1175.681 | 1407.286 | 1291.484 |
| ENSMUSG00000050822 | protein_coding | Slc29a4 | 113.154 | 70.50595 | 91.82997 |
| ENSMUSG00000038295 | protein_coding | Atg9b | 83.3303 | 48.90889 | 66.11959 |
| ENSMUSG00000039842 | protein_coding | Mcph1 | 775.6448 | 637.4907 | 706.5677 |
| ENSMUSG00000001054 | protein_coding | Rmnd5b | 1497.68 | 1740.394 | 1619.037 |
| ENSMUSG00000024208 | protein_coding | 2900010M23Rik | 1093.539 | 876.3351 | 984.937 |
| ENSMUSG00000042997 | protein_coding | Nhlrc3 | 761.6761 | 624.3901 | 693.0331 |
| ENSMUSG00000026349 | protein_coding | Ccnt2 | 3296.695 | 4048.778 | 3672.737 |
| ENSMUSG00000030424 | protein_coding | Zfp939 | 106.2402 | 72.25165 | 89.24594 |
| ENSMUSG00000031753 | protein_coding | Cog4 | 1741.703 | 1472.532 | 1607.117 |
| ENSMUSG00000092060 | protein_coding | Bend4 | 3475.804 | 2739.775 | 3107.79 |
| ENSMUSG00000062627 | protein_coding | Mysm1 | 2378.862 | 1963.298 | 2171.08 |
| ENSMUSG00000026209 | protein_coding | Dnpep | 1809.604 | 1418.793 | 1614.198 |
| ENSMUSG00000030772 | protein_coding | Dkk3 | 415.3625 | 310.0978 | 362.7302 |
| ENSMUSG00000026276 | protein_coding | 2-Sep | 7964.286 | 9340.982 | 8652.634 |
| ENSMUSG00000049350 | protein_coding | Zg16 | 0.64422 | 7.761002 | 4.202611 |
| ENSMUSG00000018395 | protein_coding | Kif3a | 2418.865 | 2077.082 | 2247.974 |
| ENSMUSG00000046230 | protein_coding | Vps13a | 4053.023 | 3274.333 | 3663.678 |
| ENSMUSG00000041594 | protein_coding | Tmtc4 | 870.289 | 711.0689 | 790.6789 |
| ENSMUSG00000034616 | protein_coding | Ssh3 | 326.7488 | 444.4259 | 385.5873 |
| ENSMUSG00000034936 | protein_coding | Arl4d | 902.8474 | 616.3388 | 759.5931 |
| ENSMUSG00000022623 | protein_coding | Shank3 | 461.5699 | 372.8404 | 417.2052 |
| ENSMUSG00000030303 | protein_coding | Far2 | 113.9459 | 184.9588 | 149.4523 |
| ENSMUSG00000043388 | protein_coding | Tmem130 | 58.09873 | 92.4464 | 75.27256 |
| ENSMUSG00000032482 | protein_coding | Cspg5 | 142.4704 | 101.0006 | 121.7355 |
| ENSMUSG00000031755 | protein_coding | Bbs2 | 717.1566 | 897.4944 | 807.3255 |
| ENSMUSG00000079637 | protein_coding | Rhox2c | 22.23649 | 8.74883 | 15.49266 |
| ENSMUSG00000024782 | protein_coding | Ak3 | 2450.547 | 2841.138 | 2645.843 |
| ENSMUSG00000032714 | protein_coding | Syde1 | 406.3771 | 545.7927 | 476.0849 |
| ENSMUSG00000027641 | protein_coding | Rbl1 | 1070.812 | 882.1719 | 976.4918 |
| ENSMUSG00000039781 | protein_coding | Azi1 | 656.22 | 532.6917 | 594.4558 |
| ENSMUSG00000022432 | protein_coding | Smc1b | 4228.744 | 1981.424 | 3105.084 |
| ENSMUSG00000074623 | protein_coding | Gm826 | 4.820918 | 16.228 | 10.52446 |
| ENSMUSG00000020656 | protein_coding | Grhl1 | 357.021 | 259.2864 | 308.1537 |
| ENSMUSG00000003644 | protein_coding | Rps6ka1 | 1312.513 | 1020.388 | 1166.451 |
| ENSMUSG00000046182 | protein_coding | Gsg1l | 216.7758 | 355.3597 | 286.0678 |
| ENSMUSG00000048834 | protein_coding | Vstm2a | 15.19255 | 33.11732 | 24.15494 |
| ENSMUSG00000027327 | protein_coding | 1700037H04Rik | 1076.198 | 906.071 | 991.1344 |
| ENSMUSG00000041406 | protein_coding | BC055324 | 2694.801 | 2183.681 | 2439.241 |
| ENSMUSG00000027973 | protein_coding | 1700006A11Rik | 6.267692 | 0 | 3.133846 |
| ENSMUSG00000031221 | protein_coding | Igbp1 | 935.4161 | 1154.043 | 1044.73 |
| ENSMUSG00000030498 | protein_coding | Gas2 | 444.7802 | 352.0698 | 398.425 |
| ENSMUSG00000025813 | protein_coding | Homer2 | 425.4024 | 329.6361 | 377.5193 |
| ENSMUSG00000034926 | protein_coding | Dhcr24 | 2771.812 | 2224.001 | 2497.907 |
| ENSMUSG00000061843 | pseudogene | Vmn1r-ps32 | 18.37497 | 6.454221 | 12.41459 |
| ENSMUSG00000020056 | protein_coding | Ccdc53 | 804.8519 | 660.2622 | 732.5571 |
| ENSMUSG00000002265 | protein_coding | Peg3 | 5119.474 | 4276.088 | 4697.781 |
| ENSMUSG00000055991 | protein_coding | Zkscan5 | 736.3658 | 593.9322 | 665.149 |
| ENSMUSG00000029149 | protein_coding | Krtcap3 | 276.063 | 206.7812 | 241.4221 |
| ENSMUSG00000025212 | protein_coding | Sfxn3 | 2262.636 | 2720.91 | 2491.773 |
| ENSMUSG00000037012 | protein_coding | Hk1 | 1117.34 | 944.3937 | 1030.867 |
| ENSMUSG00000009585 | protein_coding | Apobec3 | 918.953 | 1085.373 | 1002.163 |
| ENSMUSG00000040354 | protein_coding | Mars | 3208.75 | 2725.309 | 2967.029 |
| ENSMUSG00000091490 | protein_coding | Triml2 | 47.10934 | 81.1118 | 64.11057 |
| ENSMUSG00000039774 | protein_coding | Galnt12 | 672.1357 | 498.1025 | 585.1191 |
| ENSMUSG00000035078 | protein_coding | Mtmr9 | 1445.989 | 1200.637 | 1323.313 |
| ENSMUSG00000026894 | protein_coding | Morn5 | 26.33648 | 9.695909 | 18.01619 |

| | | | | | |
|---|---|---|---|---|---|
| ENSMUSG00000087207 | processed_transcrip | Gm13147 | 33.82112 | 70.36816 | 52.09464 |
| ENSMUSG00000021895 | protein_coding | Arhgef3 | 2073.705 | 2480.193 | 2276.949 |
| ENSMUSG00000059142 | protein_coding | Zfp945 | 1310.377 | 1710.454 | 1510.416 |
| ENSMUSG00000086003 | lincRNA | B230206L02Rik | 9.356104 | 22.5067 | 15.9314 |
| ENSMUSG00000047986 | protein_coding | Palm3 | 343.7518 | 240.7275 | 292.2397 |
| ENSMUSG00000038372 | protein_coding | Gmds | 559.3473 | 426.7347 | 493.041 |
| ENSMUSG00000054752 | protein_coding | Fsd1l | 618.8731 | 497.7173 | 558.2952 |
| ENSMUSG00000007029 | protein_coding | Vars | 2669.505 | 2154.786 | 2412.146 |
| ENSMUSG00000055401 | protein_coding | Fbxo6 | 508.3571 | 653.2942 | 580.8257 |
| ENSMUSG00000006127 | protein_coding | Inpp5k | 895.1502 | 740.73 | 817.9401 |
| ENSMUSG00000026080 | protein_coding | Chst10 | 429.8772 | 309.4379 | 369.6576 |
| ENSMUSG00000055874 | protein_coding | Foxi3 | 45.68485 | 23.03396 | 34.35941 |
| ENSMUSG00000030231 | protein_coding | Plekha5 | 1361.705 | 1099.542 | 1230.623 |
| ENSMUSG00000037188 | protein_coding | Grhl3 | 194.6263 | 133.3421 | 163.9842 |
| ENSMUSG00000035378 | protein_coding | Shq1 | 838.0673 | 686.3016 | 762.1844 |
| ENSMUSG00000038213 | protein_coding | Tapbpl | 166.3181 | 122.1739 | 144.246 |
| ENSMUSG00000018774 | protein_coding | Cd68 | 158.6934 | 213.5309 | 186.1122 |
| ENSMUSG00000020412 | protein_coding | Ascc2 | 2012.126 | 1689.695 | 1850.91 |
| ENSMUSG00000028385 | protein_coding | Snx30 | 878.9416 | 737.997 | 808.4693 |
| ENSMUSG00000085243 | lincRNA | 4930480K23Rik | 78.22827 | 50.22281 | 64.22554 |
| ENSMUSG00000062044 | protein_coding | Lmtk3 | 86.9927 | 54.14714 | 70.56992 |
| ENSMUSG00000056608 | protein_coding | Chd9 | 924.0124 | 1145.822 | 1034.917 |
| ENSMUSG00000029101 | protein_coding | Rgs12 | 1963.457 | 1671.921 | 1817.689 |
| ENSMUSG00000051790 | protein_coding | Nlgn2 | 1218.567 | 1007.314 | 1112.941 |
| ENSMUSG00000022178 | protein_coding | Jub | 2661.012 | 3228.266 | 2944.639 |
| ENSMUSG00000048142 | protein_coding | Nat8l | 314.9571 | 237.4162 | 276.1867 |
| ENSMUSG00000045503 | protein_coding | Sys1 | 469.9082 | 381.5182 | 425.7132 |
| ENSMUSG00000029491 | protein_coding | Pde6b | 12.62641 | 27.6181 | 20.12225 |
| ENSMUSG00000091412 | lincRNA | Gm2895 | 19.3741 | 7.240942 | 13.30752 |
| ENSMUSG00000024181 | protein_coding | Mrpl28 | 1263.56 | 1051.545 | 1157.552 |
| ENSMUSG00000051695 | protein_coding | Pcbp1 | 3900.466 | 4611.929 | 4256.198 |
| ENSMUSG00000079387 | protein_coding | Luzp4 | 124.1183 | 85.66969 | 104.894 |
| ENSMUSG00000025200 | protein_coding | Cwf19l1 | 694.9333 | 574.0124 | 634.4729 |
| ENSMUSG00000015653 | protein_coding | Steap2 | 304.3006 | 239.0678 | 271.6842 |
| ENSMUSG00000034848 | protein_coding | Ttc21b | 1705.662 | 1445.917 | 1575.789 |
| ENSMUSG00000057406 | protein_coding | Whsc1 | 5668.756 | 4713.96 | 5191.358 |
| ENSMUSG00000024493 | protein_coding | Lars | 3186.855 | 2702.258 | 2944.556 |
| ENSMUSG00000035183 | protein_coding | Slc24a5 | 238.6924 | 344.6035 | 291.6479 |
| ENSMUSG00000044148 | protein_coding | 1810030O07Rik | 788.0245 | 964.5451 | 876.2848 |
| ENSMUSG00000090672 | lincRNA | Gm17256 | 9.407839 | 27.35642 | 18.38213 |
| ENSMUSG00000031821 | protein_coding | Gins2 | 634.9984 | 516.5132 | 575.7558 |
| ENSMUSG00000052798 | protein_coding | Nup107 | 1968.983 | 1638.479 | 1803.731 |
| ENSMUSG00000040964 | protein_coding | Arhgef10l | 215.9967 | 305.6924 | 260.8445 |
| ENSMUSG00000038240 | protein_coding | Pdss2 | 316.1577 | 248.5903 | 282.374 |
| ENSMUSG00000044788 | protein_coding | Fads6 | 804.6302 | 1017.167 | 910.8986 |
| ENSMUSG00000054013 | protein_coding | Tmem179 | 30.00224 | 13.72147 | 21.86185 |
| ENSMUSG00000026618 | protein_coding | Iars2 | 2673.784 | 2315.639 | 2494.711 |
| ENSMUSG00000075590 | protein_coding | Nrbp2 | 2101.92 | 2764.427 | 2433.174 |
| ENSMUSG00000069170 | protein_coding | Gpr98 | 427.0185 | 329.7698 | 378.3942 |
| ENSMUSG00000027950 | protein_coding | Chrnb2 | 61.21196 | 102.0792 | 81.6456 |
| ENSMUSG00000031563 | protein_coding | Wwc2 | 2673.43 | 2274.988 | 2474.209 |
| ENSMUSG00000052831 | protein_coding | Rbmy1a1 | 276.9453 | 189.0641 | 233.0047 |
| ENSMUSG00000085273 | lincRNA | 4930467D21Rik | 27.96909 | 11.21317 | 19.59113 |
| ENSMUSG00000031626 | protein_coding | Sorbs2 | 1025.529 | 1221 | 1123.264 |
| ENSMUSG00000025082 | protein_coding | Vwa2 | 160.8841 | 110.4392 | 135.6616 |
| ENSMUSG00000053137 | protein_coding | Mapk11 | 68.9245 | 108.2864 | 88.60543 |
| ENSMUSG00000023927 | protein_coding | Satb1 | 617.4161 | 492.2891 | 554.8526 |
| ENSMUSG00000092981 | miRNA | AC122821.1 | 92.83131 | 158.9035 | 125.8674 |

| | | | | | |
|---|---|---|---|---|---|
| ENSMUSG00000030036 | protein_coding | Mogs | 1408.57 | 1150.341 | 1279.456 |
| ENSMUSG00000034744 | protein_coding | Nagk | 579.7123 | 473.7536 | 526.7329 |
| ENSMUSG00000013418 | protein_coding | B4galnt2 | 42.62634 | 71.46027 | 57.0433 |
| ENSMUSG00000030051 | protein_coding | Aplf | 917.9415 | 761.181 | 839.5613 |
| ENSMUSG00000044597 | protein_coding | Mycs | 54.88978 | 27.40369 | 41.14673 |
| ENSMUSG00000033581 | protein_coding | Igf2bp2 | 450.2894 | 571.8392 | 511.0643 |
| ENSMUSG00000089563 | snRNA | U6 | 12.95672 | 3.743729 | 8.350222 |
| ENSMUSG00000013523 | protein_coding | Bcas1 | 71.30018 | 106.8908 | 89.09551 |
| ENSMUSG00000060373 | protein_coding | Hnrnpc | 7537.693 | 8678.827 | 8108.26 |
| ENSMUSG00000074657 | protein_coding | Kif5a | 609.0877 | 480.7671 | 544.9274 |
| ENSMUSG00000025140 | protein_coding | Pycr1 | 141.7353 | 101.6054 | 121.6704 |
| ENSMUSG00000074221 | protein_coding | Zfp568 | 1375.903 | 1654.638 | 1515.27 |
| ENSMUSG00000045100 | protein_coding | Slc25a26 | 491.9237 | 602.5011 | 547.2124 |
| ENSMUSG00000037126 | protein_coding | Psd | 248.2415 | 165.6358 | 206.9387 |
| ENSMUSG00000026974 | protein_coding | Zmynd19 | 2387.857 | 2000.989 | 2194.423 |
| ENSMUSG00000046691 | protein_coding | Chtf8 | 3084.427 | 3641.533 | 3362.98 |
| ENSMUSG00000055912 | protein_coding | Tmem150a | 413.2316 | 540.1451 | 476.6883 |
| ENSMUSG00000010721 | protein_coding | Lmbr1 | 1036.504 | 876.3491 | 956.4266 |
| ENSMUSG00000048217 | protein_coding | Nags | 305.0807 | 232.4736 | 268.7771 |
| ENSMUSG00000045294 | protein_coding | Insig1 | 2543.799 | 2077.459 | 2310.629 |
| ENSMUSG00000071719 | protein_coding | Tmem28 | 129.6575 | 85.0087 | 107.3331 |
| ENSMUSG00000036768 | protein_coding | Kif15 | 1644.124 | 1374.389 | 1509.256 |
| ENSMUSG00000033965 | protein_coding | Slc16a2 | 1235.256 | 1453.597 | 1344.426 |
| ENSMUSG00000002147 | protein_coding | Stat6 | 724.7537 | 868.845 | 796.7994 |
| ENSMUSG00000049044 | protein_coding | Rapgef4 | 244.6008 | 168.1028 | 206.3518 |
| ENSMUSG00000090891 | protein_coding | D6Ertd527e | 4.585653 | 17.37039 | 10.97802 |
| ENSMUSG00000026826 | protein_coding | Nr4a2 | 143.519 | 75.7183 | 109.6186 |
| ENSMUSG00000085148 | processed_transcrip | 2210403K04Rik | 364.1876 | 476.7818 | 420.4847 |
| ENSMUSG00000045362 | protein_coding | Tnfrsf26 | 4.849096 | 16.99313 | 10.92111 |
| ENSMUSG00000030421 | protein_coding | C80913 | 2880.452 | 2461.133 | 2670.792 |
| ENSMUSG00000038486 | protein_coding | Sv2a | 82.19326 | 120.8173 | 101.5053 |
| ENSMUSG00000085081 | processed_transcrip | A430071A18Rik | 6.447869 | 18.80336 | 12.62561 |
| ENSMUSG00000081432 | pseudogene | Gm12593 | 30.44377 | 13.80183 | 22.1228 |
| ENSMUSG00000045928 | protein_coding | 4933440M02Rik | 10.41621 | 2.08413 | 6.250171 |
| ENSMUSG00000029348 | protein_coding | Asphd2 | 202.6969 | 150.9578 | 176.8274 |
| ENSMUSG00000059316 | protein_coding | Slc27a4 | 1310.207 | 1113.457 | 1211.832 |
| ENSMUSG00000028583 | protein_coding | Pdpn | 1281.822 | 1638.04 | 1459.931 |
| ENSMUSG00000034449 | protein_coding | Dhrs11 | 670.0182 | 833.0528 | 751.5355 |
| ENSMUSG00000030592 | protein_coding | Ryr1 | 268.8826 | 341.9007 | 305.3916 |
| ENSMUSG00000079019 | protein_coding | Insl3 | 690.4384 | 883.1488 | 786.7936 |
| ENSMUSG00000023806 | protein_coding | Rsph3b | 315.677 | 414.0664 | 364.8717 |
| ENSMUSG00000078859 | lincRNA | Gm17491 | 192.0417 | 275.4619 | 233.7518 |
| ENSMUSG00000015002 | protein_coding | Efr3a | 3655.575 | 3184.728 | 3420.151 |
| ENSMUSG00000024220 | protein_coding | Zfp523 | 1750.77 | 1504.166 | 1627.468 |
| ENSMUSG00000044566 | protein_coding | Cage1 | 198.467 | 136.3984 | 167.4327 |
| ENSMUSG00000040111 | protein_coding | Gramd1b | 2149.453 | 2654.538 | 2401.996 |
| ENSMUSG00000057614 | protein_coding | Gnai1 | 1588.241 | 1223.314 | 1405.777 |
| ENSMUSG00000084002 | pseudogene | Gm13146 | 30.82939 | 62.93368 | 46.88154 |
| ENSMUSG00000073380 | protein_coding | Arrdc5 | 5.209895 | 0.326514 | 2.768205 |
| ENSMUSG00000026204 | protein_coding | Ptprn | 6.407913 | 19.039 | 12.72346 |
| ENSMUSG00000091912 | lincRNA | Gm17468 | 21.40516 | 41.00513 | 31.20514 |
| ENSMUSG00000006154 | protein_coding | Eps8l1 | 22.33869 | 47.64343 | 34.99106 |
| ENSMUSG00000002297 | protein_coding | Dbf4 | 2043.585 | 1754.172 | 1898.879 |
| ENSMUSG00000024776 | protein_coding | Stambpl1 | 180.3781 | 264.5746 | 222.4763 |
| ENSMUSG00000058886 | protein_coding | Deaf1 | 1171.195 | 993.2168 | 1082.206 |
| ENSMUSG00000072249 | protein_coding | Gm5635 | 135.8297 | 97.0937 | 116.4617 |
| ENSMUSG00000028771 | protein_coding | Ptpn12 | 2532.381 | 2191.675 | 2362.028 |
| ENSMUSG00000038349 | protein_coding | Plcl1 | 143.9153 | 99.98325 | 121.9493 |

| | | | | | |
|---|---|---|---|---|---|
| ENSMUSG00000005950 | protein_coding | P2rx5 | 16.77724 | 33.03008 | 24.90366 |
| ENSMUSG00000056648 | protein_coding | Hoxb8 | 10.31066 | 24.81104 | 17.56085 |
| ENSMUSG00000020956 | protein_coding | 6530401N04Rik | 883.9889 | 727.8713 | 805.9301 |
| ENSMUSG00000062157 | protein_coding | Il28ra | 84.46689 | 121.1018 | 102.7843 |
| ENSMUSG00000050035 | protein_coding | Fhl4 | 123.3586 | 63.86998 | 93.61428 |
| ENSMUSG00000061815 | protein_coding | Rufy4 | 25.07428 | 53.80193 | 39.4381 |
| ENSMUSG00000008482 | protein_coding | Rnf151 | 2.561525 | 11.03764 | 6.799583 |
| ENSMUSG00000052912 | processed_transcrip | Smarca5-ps | 178.9157 | 250.053 | 214.4843 |
| ENSMUSG00000026972 | protein_coding | Arrdc1 | 420.7745 | 330.0393 | 375.4069 |
| ENSMUSG00000003778 | protein_coding | Brd8 | 2629.89 | 3043.176 | 2836.533 |
| ENSMUSG00000050545 | protein_coding | A830093I24Rik | 151.8331 | 96.93361 | 124.3833 |
| ENSMUSG00000074264 | protein_coding | Amy1 | 55.52021 | 100.1997 | 77.85997 |
| ENSMUSG00000038264 | protein_coding | Sema7a | 5795.463 | 7013.157 | 6404.31 |
| ENSMUSG00000031847 | protein_coding | 1700030J22Rik | 313.4987 | 237.8496 | 275.6741 |
| ENSMUSG00000018449 | protein_coding | Rpain | 706.2894 | 558.7112 | 632.5003 |
| ENSMUSG00000036814 | protein_coding | Slc6a20a | 7.124889 | 19.77203 | 13.44846 |
| ENSMUSG00000021555 | protein_coding | Naa35 | 4133.89 | 4711.769 | 4422.829 |
| ENSMUSG00000054423 | protein_coding | Cadps | 198.2129 | 304.5474 | 251.3801 |
| ENSMUSG00000078695 | protein_coding | Cisd3 | 194.8448 | 258.3587 | 226.6017 |
| ENSMUSG00000068299 | protein_coding | 1700019G17Rik | 383.3333 | 502.4057 | 442.8695 |
| ENSMUSG00000083773 | pseudogene | Gm13394 | 4332.796 | 5655.03 | 4993.913 |
| ENSMUSG00000069516 | protein_coding | Lyz2 | 1174.809 | 1507.825 | 1341.317 |
| ENSMUSG00000025328 | protein_coding | Padi3 | 312.73 | 222.6163 | 267.6732 |
| ENSMUSG00000041202 | protein_coding | Pla2g2d | 11.91763 | 25.84283 | 18.88023 |
| ENSMUSG00000033826 | protein_coding | Dnahc8 | 1497.358 | 785.1525 | 1141.255 |
| ENSMUSG00000051185 | protein_coding | Fam174a | 680.9262 | 558.2065 | 619.5663 |
| ENSMUSG00000037266 | protein_coding | D4Wsu53e | 6741.581 | 8682.955 | 7712.268 |
| ENSMUSG00000051339 | protein_coding | 2900026A02Rik | 667.6215 | 816.3285 | 741.975 |
| ENSMUSG00000086420 | pseudogene | Gm8865 | 16.53019 | 4.919305 | 10.72475 |
| ENSMUSG00000027387 | protein_coding | Zc3h8 | 487.3399 | 340.1794 | 413.7597 |
| ENSMUSG00000040562 | protein_coding | Gstm2 | 10924.29 | 13077.96 | 12001.13 |
| ENSMUSG00000023051 | protein_coding | Tarbp2 | 1294.621 | 1076.15 | 1185.385 |
| ENSMUSG00000066438 | protein_coding | 3830431G21Rik | 58.29113 | 90.24768 | 74.2694 |
| ENSMUSG00000036459 | protein_coding | Wtip | 621.9601 | 507.7672 | 564.8637 |
| ENSMUSG00000086179 | processed_transcrip | Gm14317 | 8.669617 | 22.09874 | 15.38418 |
| ENSMUSG00000042700 | protein_coding | Sipa1l1 | 4035.049 | 3384.53 | 3709.79 |
| ENSMUSG00000024413 | protein_coding | Npc1 | 1206.944 | 1489.039 | 1347.992 |
| ENSMUSG00000039395 | protein_coding | Mreg | 523.6595 | 394.3673 | 459.0134 |
| ENSMUSG00000016257 | protein_coding | Slmo2 | 1860.667 | 1590.381 | 1725.524 |
| ENSMUSG00000002769 | protein_coding | Gnmt | 20.99389 | 38.67127 | 29.83258 |
| ENSMUSG00000023266 | protein_coding | Frs3 | 306.4013 | 231.4967 | 268.949 |
| ENSMUSG00000035637 | protein_coding | Grhpr | 1373.549 | 1633.472 | 1503.51 |
| ENSMUSG00000004865 | protein_coding | Srpk1 | 3944.585 | 3417.234 | 3680.91 |
| ENSMUSG00000027403 | protein_coding | Tgm6 | 23.24091 | 6.860734 | 15.05082 |
| ENSMUSG00000017417 | protein_coding | Plxdc1 | 222.9084 | 287.0695 | 254.9889 |
| ENSMUSG00000029250 | protein_coding | Polr2b | 78.83231 | 50.20122 | 64.51676 |
| ENSMUSG00000041712 | protein_coding | Ubr7 | 2412.192 | 2099.478 | 2255.835 |
| ENSMUSG00000087141 | protein_coding | Plcxd2 | 90.31199 | 60.44386 | 75.37792 |
| ENSMUSG00000026694 | protein_coding | Mettl13 | 1055.075 | 895.6563 | 975.3659 |
| ENSMUSG00000038065 | protein_coding | 2410066E13Rik | 517.0887 | 626.8759 | 571.9823 |
| ENSMUSG00000028125 | protein_coding | Abca4 | 131.0471 | 88.60465 | 109.8259 |
| ENSMUSG00000054309 | protein_coding | Cpsf3 | 2597.865 | 2222.115 | 2409.99 |
| ENSMUSG00000037010 | protein_coding | Apln | 400.6146 | 303.2994 | 351.957 |
| ENSMUSG00000035875 | protein_coding | AI182371 | 38.70764 | 17.73874 | 28.22319 |
| ENSMUSG00000028381 | protein_coding | Ugcg | 1806.486 | 2160.468 | 1983.477 |
| ENSMUSG00000068851 | protein_coding | Hist4h4 | 1.953683 | 9.986748 | 5.970215 |
| ENSMUSG00000032135 | protein_coding | Mcam | 2997.343 | 3525.672 | 3261.508 |
| ENSMUSG00000061393 | protein_coding | Acvr2b | 398.3673 | 305.1035 | 351.7354 |

| | | | | | |
|---|---|---|---|---|---|
| ENSMUSG00000022199 | protein_coding | Slc22a17 | 1710.653 | 2065.275 | 1887.964 |
| ENSMUSG00000066149 | protein_coding | Cdc26 | 1074.174 | 1306.69 | 1190.432 |
| ENSMUSG00000022039 | protein_coding | Adam2 | 15.17786 | 1.422092 | 8.299978 |
| ENSMUSG00000015709 | protein_coding | Arnt2 | 1197.767 | 1448.569 | 1323.168 |
| ENSMUSG00000074497 | protein_coding | A430078G23Rik | 105.8054 | 144.5527 | 125.179 |
| ENSMUSG00000007594 | protein_coding | Hapln4 | 41.06394 | 71.86311 | 56.46353 |
| ENSMUSG00000047022 | protein_coding | Mipol1 | 518.192 | 425.121 | 471.6565 |
| ENSMUSG00000006019 | protein_coding | Dhx34 | 506.2077 | 417.6074 | 461.9075 |
| ENSMUSG00000052684 | protein_coding | Jun | 1938.623 | 2413.911 | 2176.267 |
| ENSMUSG00000027401 | protein_coding | Tgm3 | 10.28837 | 23.9872 | 17.13778 |
| ENSMUSG00000087365 | lincRNA | C430049B03Rik | 367.3764 | 502.3339 | 434.8552 |
| ENSMUSG00000027777 | protein_coding | Schip1 | 1371.284 | 1703.197 | 1537.241 |
| ENSMUSG00000046207 | protein_coding | Pik3r6 | 60.32741 | 36.67543 | 48.50142 |
| ENSMUSG00000017861 | protein_coding | Mybl2 | 1008.486 | 812.2446 | 910.3656 |
| ENSMUSG00000021549 | protein_coding | Rasa1 | 3798.854 | 3302.413 | 3550.634 |
| ENSMUSG00000021224 | protein_coding | Numb | 1573.342 | 1828.917 | 1701.13 |
| ENSMUSG00000029235 | protein_coding | Pdcl2 | 18.71392 | 4.150241 | 11.43208 |
| ENSMUSG00000007050 | protein_coding | Lsm2 | 1157.258 | 949.2304 | 1053.244 |
| ENSMUSG00000022437 | protein_coding | Samm50 | 2582.552 | 2222.192 | 2402.372 |
| ENSMUSG00000028182 | protein_coding | Lrriq3 | 118.1724 | 76.05405 | 97.11322 |
| ENSMUSG00000035852 | protein_coding | 9130017N09Rik | 36.81218 | 17.31312 | 27.06265 |
| ENSMUSG00000024791 | protein_coding | Cdca5 | 857.9082 | 696.0962 | 777.0022 |
| ENSMUSG00000060081 | protein_coding | Hist1h2aa | 6.178536 | 20.09782 | 13.13818 |
| ENSMUSG00000084765 | lincRNA | Gm16998 | 3.908633 | 0 | 1.954316 |
| ENSMUSG00000082532 | pseudogene | Rpl31-ps6 | 12.40985 | 2.763824 | 7.586837 |
| ENSMUSG00000087400 | lincRNA | Gm15270 | 41.34356 | 66.07998 | 53.71177 |
| ENSMUSG00000031441 | protein_coding | Atp11a | 1714.404 | 2019.131 | 1866.768 |
| ENSMUSG00000044628 | protein_coding | Rnf208 | 534.9547 | 686.8444 | 610.8996 |
| ENSMUSG00000020704 | protein_coding | Accn1 | 16.77366 | 35.58523 | 26.17944 |
| ENSMUSG00000018569 | protein_coding | Cldn7 | 84.35484 | 130.9439 | 107.6494 |
| ENSMUSG00000020214 | protein_coding | Glipr1l2 | 55.04863 | 19.31182 | 37.18022 |
| ENSMUSG00000029629 | protein_coding | Phf14 | 1904.496 | 1638.192 | 1771.344 |
| ENSMUSG00000032026 | protein_coding | Rexo2 | 1028.174 | 1228.176 | 1128.175 |
| ENSMUSG00000041757 | protein_coding | Plekha6 | 773.4429 | 598.3366 | 685.8898 |
| ENSMUSG00000024431 | protein_coding | Nr3c1 | 2214.759 | 1874.435 | 2044.597 |
| ENSMUSG00000000751 | protein_coding | Rpa1 | 2989.619 | 2544.797 | 2767.208 |
| ENSMUSG00000037339 | protein_coding | Fam53a | 2042.376 | 1777.706 | 1910.041 |
| ENSMUSG00000024151 | protein_coding | Msh2 | 3238.871 | 2774.327 | 3006.599 |
| ENSMUSG00000087612 | processed_transcrip | A230005M16Rik | 10.30119 | 24.80203 | 17.55161 |
| ENSMUSG00000079017 | protein_coding | Ifi27l2a | 73.46624 | 44.14772 | 58.80698 |
| ENSMUSG00000039828 | protein_coding | Wdr70 | 700.6744 | 885.8909 | 793.2826 |
| ENSMUSG00000021364 | protein_coding | Elovl2 | 9441.683 | 11396.39 | 10419.04 |
| ENSMUSG00000027750 | protein_coding | Postn | 453.1011 | 616.4407 | 534.7709 |
| ENSMUSG00000041702 | protein_coding | Btbd7 | 2459.005 | 2026.781 | 2242.893 |
| ENSMUSG00000030761 | protein_coding | Myo7a | 6565.037 | 7768.733 | 7166.885 |
| ENSMUSG00000026153 | protein_coding | Fam135a | 4480.127 | 3770.703 | 4125.415 |
| ENSMUSG00000000759 | protein_coding | Tubgcp3 | 2014.43 | 1754.228 | 1884.329 |
| ENSMUSG00000030979 | protein_coding | Uros | 566.3617 | 675.1076 | 620.7347 |
| ENSMUSG00000063659 | protein_coding | Zfp238 | 3055.249 | 2633.897 | 2844.573 |
| ENSMUSG00000012123 | protein_coding | Aim1l | 213.6787 | 149.3603 | 181.5195 |
| ENSMUSG00000020848 | protein_coding | Doc2b | 498.4564 | 685.361 | 591.9087 |
| ENSMUSG00000061923 | protein_coding | Odf1 | 4.867807 | 16.37283 | 10.62032 |
| ENSMUSG00000034993 | protein_coding | Vat1 | 3571.807 | 4231.767 | 3901.787 |
| ENSMUSG00000037217 | protein_coding | Syn1 | 63.96313 | 40.50889 | 52.23601 |
| ENSMUSG00000006678 | protein_coding | Pola1 | 1052.81 | 872.8506 | 962.8305 |
| ENSMUSG00000044595 | protein_coding | Dnd1 | 739.5711 | 901.9152 | 820.7431 |
| ENSMUSG00000051169 | protein_coding | Rpusd3 | 119.6685 | 84.48475 | 102.0766 |
| ENSMUSG00000054910 | lincRNA | 4931415C17Rik | 35.52753 | 14.18167 | 24.8546 |

| | | | | | |
|---|---|---|---|---|---|
| ENSMUSG00000091865 | protein_coding | Gm17244 | 12.63692 | 27.08111 | 19.85901 |
| ENSMUSG00000038233 | protein_coding | Fam198a | 190.3193 | 308.0045 | 249.1619 |
| ENSMUSG00000031431 | protein_coding | Tsc22d3 | 3814.314 | 3165.033 | 3489.674 |
| ENSMUSG00000000202 | protein_coding | Btbd17 | 10.00264 | 22.613 | 16.30782 |
| ENSMUSG00000039509 | protein_coding | Nup133 | 3191.253 | 2685.546 | 2938.399 |
| ENSMUSG00000031709 | protein_coding | Tbc1d9 | 364.4717 | 497.4375 | 430.9546 |
| ENSMUSG00000045441 | protein_coding | Gprin3 | 33.18905 | 55.37569 | 44.28237 |
| ENSMUSG00000001918 | protein_coding | Slc1a5 | 736.5081 | 572.9107 | 654.7094 |
| ENSMUSG00000022064 | protein_coding | Pibf1 | 713.7917 | 595.3994 | 654.5956 |
| ENSMUSG00000074987 | lincRNA | AI314831 | 262.9111 | 349.495 | 306.2031 |
| ENSMUSG00000018363 | protein_coding | Smurf2 | 3013.654 | 2646.884 | 2830.269 |
| ENSMUSG00000021614 | protein_coding | Vcan | 431.2466 | 641.0283 | 536.1375 |
| ENSMUSG00000027577 | protein_coding | Chrna4 | 190.9142 | 282.0209 | 236.4675 |
| ENSMUSG00000024331 | protein_coding | Dsc2 | 106.8873 | 191.5931 | 149.2402 |
| ENSMUSG00000040390 | protein_coding | Map3k10 | 1359.771 | 1171.119 | 1265.445 |
| ENSMUSG00000066705 | protein_coding | Fxyd6 | 5552.984 | 6355.297 | 5954.14 |
| ENSMUSG00000004789 | protein_coding | Dlst | 2881.601 | 3286.411 | 3084.006 |
| ENSMUSG00000070305 | protein_coding | Mpzl3 | 297.2238 | 367.3974 | 332.3106 |
| ENSMUSG00000041235 | protein_coding | Chd7 | 697.4587 | 899.0621 | 798.2604 |
| ENSMUSG00000041046 | protein_coding | Ramp3 | 95.67746 | 61.26232 | 78.46989 |
| ENSMUSG00000020900 | protein_coding | Myh10 | 2454.737 | 2086.598 | 2270.668 |
| ENSMUSG00000027455 | protein_coding | Nsfl1c | 3052.449 | 3466.291 | 3259.37 |
| ENSMUSG00000028797 | protein_coding | 2510006D16Rik | 1767.826 | 2047.301 | 1907.563 |
| ENSMUSG00000024301 | protein_coding | Kifc5b | 434.5234 | 353.8792 | 394.2013 |
| ENSMUSG00000028926 | protein_coding | Cdk14 | 603.8181 | 745.277 | 674.5476 |
| ENSMUSG00000020844 | protein_coding | Nxn | 598.3996 | 497.9612 | 548.1804 |
| ENSMUSG00000030539 | protein_coding | Sema4b | 1265.295 | 1461.179 | 1363.237 |
| ENSMUSG00000002763 | protein_coding | Pex6 | 1284.778 | 1084.087 | 1184.432 |
| ENSMUSG00000089788 | processed_transcrip | Gm16586 | 1128.729 | 1561.502 | 1345.116 |
| ENSMUSG00000060636 | protein_coding | Rpl35a | 2516.083 | 1793.445 | 2154.764 |
| ENSMUSG00000035569 | protein_coding | Ankrd11 | 6432.891 | 7523.667 | 6978.279 |
| ENSMUSG00000040936 | protein_coding | Ulk4 | 295.8559 | 233.0322 | 264.4441 |
| ENSMUSG00000079036 | protein_coding | Alkbh1 | 626.4406 | 771.0384 | 698.7395 |
| ENSMUSG00000016995 | protein_coding | Matn4 | 74.27342 | 120.0119 | 97.14268 |
| ENSMUSG00000003534 | protein_coding | Ddr1 | 4902.27 | 5923.495 | 5412.882 |
| ENSMUSG00000020883 | protein_coding | Fbxl20 | 2283.546 | 1970.155 | 2126.851 |
| ENSMUSG00000084844 | processed_transcrip | Hoxb3os | 26.16741 | 47.29107 | 36.72924 |
| ENSMUSG00000015837 | protein_coding | Sqstm1 | 7162.762 | 8164.538 | 7663.65 |
| ENSMUSG00000036941 | protein_coding | Elac1 | 659.9089 | 552.4399 | 606.1744 |
| ENSMUSG00000068680 | processed_transcrip | Gm10248 | 0 | 3.434871 | 1.717436 |
| ENSMUSG00000001128 | protein_coding | Cfp | 213.7637 | 286.2832 | 250.0234 |
| ENSMUSG00000087396 | lincRNA | 4933407K13Rik | 166.0618 | 235.4336 | 200.7477 |
| ENSMUSG00000015757 | protein_coding | Ppil4 | 2292.942 | 1970.629 | 2131.785 |
| ENSMUSG00000033122 | protein_coding | Hsd17b3 | 780.2346 | 1489.621 | 1134.928 |
| ENSMUSG00000027674 | protein_coding | Pex5l | 29.96922 | 52.42629 | 41.19775 |
| ENSMUSG00000032420 | protein_coding | Nt5e | 2633.402 | 3255.687 | 2944.545 |
| ENSMUSG00000036053 | protein_coding | Fmnl2 | 1375.209 | 1167.461 | 1271.335 |
| ENSMUSG00000079350 | protein_coding | Magea8 | 323.2989 | 460.7746 | 392.0368 |
| ENSMUSG00000024885 | protein_coding | Aldh3b1 | 421.0541 | 600.3681 | 510.7111 |
| ENSMUSG00000049300 | protein_coding | Prmt6 | 906.6424 | 1081.6 | 994.1213 |
| ENSMUSG00000024907 | protein_coding | Gal | 52.58417 | 82.64274 | 67.61345 |
| ENSMUSG00000026915 | protein_coding | Strbp | 4627.792 | 3642.703 | 4135.247 |
| ENSMUSG00000028195 | protein_coding | Cyr61 | 208.0588 | 301.6192 | 254.839 |
| ENSMUSG00000003282 | protein_coding | Plag1 | 452.9685 | 546.7044 | 499.8364 |
| ENSMUSG00000025359 | protein_coding | Pmel | 127.5781 | 72.75142 | 100.1648 |
| ENSMUSG00000034006 | protein_coding | Pqlc1 | 528.0637 | 708.8926 | 618.4782 |
| ENSMUSG00000074340 | protein_coding | Ovgp1 | 75.59406 | 38.65646 | 57.12526 |
| ENSMUSG00000028657 | protein_coding | Ppt1 | 11343.07 | 13162.5 | 12252.78 |

| | | | | | |
|---|---|---|---|---|---|
| ENSMUSG00000022887 | protein_coding | Masp1 | 470.8461 | 606.1493 | 538.4977 |
| ENSMUSG00000032855 | protein_coding | Pkd1 | 3548.595 | 3127.495 | 3338.045 |
| ENSMUSG00000029071 | protein_coding | Dvl1 | 1716.522 | 1415.229 | 1565.876 |
| ENSMUSG00000029860 | protein_coding | Zyx | 4193.196 | 4979.6 | 4586.398 |
| ENSMUSG00000002010 | protein_coding | Idh3g | 1940.188 | 2286.137 | 2113.162 |
| ENSMUSG00000040659 | protein_coding | Efhd2 | 5418.319 | 6259.392 | 5838.856 |
| ENSMUSG00000007817 | protein_coding | Zmiz1 | 838.8005 | 1067.118 | 952.9593 |
| ENSMUSG00000000416 | protein_coding | Cttnbp2 | 88.74959 | 122.6752 | 105.7124 |
| ENSMUSG00000032560 | protein_coding | Dnajc13 | 3585.117 | 3079.002 | 3332.059 |
| ENSMUSG00000073130 | protein_coding | Gm1141 | 564.0205 | 462.4594 | 513.2399 |
| ENSMUSG00000046337 | protein_coding | Fam178b | 362.3092 | 271.0338 | 316.6715 |
| ENSMUSG00000033450 | protein_coding | Tagap | 68.79098 | 34.96312 | 51.87705 |
| ENSMUSG00000041064 | protein_coding | Pif1 | 159.7314 | 231.4298 | 195.5806 |
| ENSMUSG00000032101 | protein_coding | Ddx25 | 1040.224 | 873.2036 | 956.7136 |
| ENSMUSG00000024505 | protein_coding | Dtwd2 | 336.6273 | 269.4846 | 303.056 |
| ENSMUSG00000002043 | protein_coding | Trappc6a | 400.1656 | 323.1542 | 361.6599 |
| ENSMUSG00000057440 | protein_coding | Mpp7 | 654.9939 | 521.6537 | 588.3238 |
| ENSMUSG00000021061 | protein_coding | Spnb1 | 2127.378 | 2737.841 | 2432.61 |
| ENSMUSG00000046694 | protein_coding | Fam46b | 50.00393 | 30.02911 | 40.01652 |
| ENSMUSG00000012429 | protein_coding | 2810021B07Rik | 882.5157 | 741.8224 | 812.1691 |
| ENSMUSG00000030086 | protein_coding | Chchd6 | 788.0823 | 554.9162 | 671.4992 |
| ENSMUSG00000034891 | protein_coding | Sncb | 225.1238 | 147.4172 | 186.2705 |
| ENSMUSG00000060600 | protein_coding | Eno3 | 323.5995 | 234.4766 | 279.0381 |
| ENSMUSG00000022964 | protein_coding | Tmem50b | 2469.229 | 2933.016 | 2701.122 |
| ENSMUSG00000074698 | protein_coding | Csnk2a1 | 3876.974 | 4404.94 | 4140.957 |
| ENSMUSG00000033233 | protein_coding | Trim45 | 385.6843 | 289.6803 | 337.6823 |
| ENSMUSG00000020264 | protein_coding | Slc36a2 | 3.876876 | 12.67995 | 8.278411 |
| ENSMUSG00000085704 | lincRNA | 4921531C22Rik | 317.0404 | 238.6768 | 277.8586 |
| ENSMUSG00000078706 | protein_coding | Gm53 | 838.422 | 1072.95 | 955.6858 |
| ENSMUSG00000057054 | protein_coding | Inca1 | 556.4582 | 210.7523 | 383.6052 |
| ENSMUSG00000037727 | protein_coding | Avp | 11.02174 | 3.063673 | 7.042708 |
| ENSMUSG00000091994 | lincRNA | E130317F20Rik | 120.6849 | 84.79718 | 102.7411 |
| ENSMUSG00000029752 | protein_coding | Asns | 1715.539 | 1986.543 | 1851.041 |
| ENSMUSG00000048251 | protein_coding | Bcl11b | 143.8246 | 98.89714 | 121.3609 |
| ENSMUSG00000021259 | protein_coding | Cyp46a1 | 85.95936 | 48.90951 | 67.43443 |
| ENSMUSG00000042225 | protein_coding | Ammecr1 | 2284.199 | 1901.527 | 2092.863 |
| ENSMUSG00000025977 | protein_coding | Boll | 775.6258 | 544.9577 | 660.2917 |
| ENSMUSG00000000365 | protein_coding | Rnf17 | 3008.402 | 2374.999 | 2691.701 |
| ENSMUSG00000085299 | lincRNA | Gm16627 | 25.87936 | 12.30875 | 19.09406 |
| ENSMUSG00000044030 | protein_coding | Irf2bp1 | 1075.552 | 1280.372 | 1177.962 |
| ENSMUSG00000060224 | protein_coding | Pyroxd2 | 343.5537 | 455.4504 | 399.502 |
| ENSMUSG00000059674 | protein_coding | Cdh24 | 221.5554 | 281.5999 | 251.5777 |
| ENSMUSG00000031378 | protein_coding | Abcd1 | 378.1161 | 269.0617 | 323.5889 |
| ENSMUSG00000034930 | protein_coding | Rtkn | 184.5506 | 251.0605 | 217.8056 |
| ENSMUSG00000074123 | protein_coding | 7420426K07Rik | 0 | 3.417215 | 1.708607 |
| ENSMUSG00000030695 | protein_coding | Aldoa | 9192.545 | 10548.2 | 9870.375 |
| ENSMUSG00000062169 | protein_coding | Cnih4 | 995.3229 | 1162.517 | 1078.92 |
| ENSMUSG00000079998 | pseudogene | Gm12947 | 3.532477 | 0 | 1.766239 |
| ENSMUSG00000091834 | pseudogene | Gm17177 | 10.99125 | 3.055025 | 7.02314 |
| ENSMUSG00000020492 | protein_coding | Fam33a | 698.7225 | 858.2324 | 778.4774 |
| ENSMUSG00000028341 | protein_coding | Nr4a3 | 130.8621 | 85.728 | 108.295 |
| ENSMUSG00000019734 | protein_coding | Tmc4 | 66.77341 | 103.811 | 85.29219 |
| ENSMUSG00000063160 | protein_coding | Numbl | 1045.535 | 891.3798 | 968.4574 |
| ENSMUSG00000022905 | protein_coding | Kpna1 | 2636.883 | 3001.289 | 2819.086 |
| ENSMUSG00000049086 | protein_coding | Bmyc | 202.0303 | 270.8939 | 236.4621 |
| ENSMUSG00000028466 | protein_coding | Creb3 | 2411.414 | 2808.331 | 2609.873 |
| ENSMUSG00000026664 | protein_coding | Phyh | 720.6494 | 856.5258 | 788.5876 |
| ENSMUSG00000044997 | protein_coding | E130304I02Rik | 4.515208 | 15.26788 | 9.891544 |

| | | | | | |
|---|---|---|---|---|---|
| ENSMUSG00000027528 | protein_coding | Fabp9 | 27.15185 | 9.271378 | 18.21161 |
| ENSMUSG00000022094 | protein_coding | Slc39a14 | 767.4415 | 902.7292 | 835.0854 |
| ENSMUSG00000091955 | pseudogene | Gm3788 | 61.65454 | 38.60893 | 50.13174 |
| ENSMUSG00000011589 | protein_coding | Fsd1 | 614.6341 | 493.2555 | 553.9448 |
| ENSMUSG00000070315 | lincRNA | 4930581F22Rik | 154.5994 | 214.5663 | 184.5829 |
| ENSMUSG00000074590 | pseudogene | F630111L10Rik | 0 | 3.390187 | 1.695093 |
| ENSMUSG00000069833 | protein_coding | Ahnak | 4096.692 | 4913.302 | 4504.997 |
| ENSMUSG00000025036 | protein_coding | Sfxn2 | 240.1528 | 327.9788 | 284.0658 |
| ENSMUSG00000020572 | protein_coding | Nampt | 3503.468 | 3066.684 | 3285.076 |
| ENSMUSG00000051896 | protein_coding | 1700011F03Rik | 0 | 3.381539 | 1.69077 |
| ENSMUSG00000043384 | protein_coding | Gprasp1 | 5868.459 | 7293.26 | 6580.86 |
| ENSMUSG00000081670 | pseudogene | Gm15697 | 41.07318 | 66.86774 | 53.97046 |
| ENSMUSG00000022454 | protein_coding | Nell2 | 349.7837 | 254.2011 | 301.9924 |
| ENSMUSG00000063447 | protein_coding | 1700013N18Rik | 7.524376 | 1.024227 | 4.274302 |
| ENSMUSG00000050953 | protein_coding | Gja1 | 9354.09 | 11894.83 | 10624.46 |
| ENSMUSG00000072922 | protein_coding | Gm15097 | 103.8822 | 72.43722 | 88.15972 |
| ENSMUSG00000028456 | protein_coding | Unc13b | 6395.415 | 7301.125 | 6848.27 |
| ENSMUSG00000042793 | protein_coding | Lgr6 | 24.4179 | 9.871074 | 17.14449 |
| ENSMUSG00000024170 | protein_coding | Telo2 | 862.1765 | 726.0909 | 794.1337 |
| ENSMUSG00000025038 | protein_coding | Efhc2 | 218.4746 | 278.1714 | 248.323 |
| ENSMUSG00000020253 | protein_coding | Ppm1m | 1361.216 | 1749.069 | 1555.142 |
| ENSMUSG00000048546 | protein_coding | Tob2 | 960.0759 | 785.7253 | 872.9006 |
| ENSMUSG00000027175 | protein_coding | Tcp11l1 | 131.8286 | 196.3172 | 164.0729 |
| ENSMUSG00000030126 | protein_coding | Tmcc1 | 1849.882 | 1606.158 | 1728.02 |
| ENSMUSG00000090262 | protein_coding | Mpv17 | 680.5115 | 816.0284 | 748.2699 |
| ENSMUSG00000032376 | protein_coding | Usp3 | 2565.65 | 3057.335 | 2811.493 |
| ENSMUSG00000021702 | protein_coding | Thbs4 | 74.6123 | 47.43088 | 61.02159 |
| ENSMUSG00000026650 | protein_coding | Meig1 | 248.3667 | 180.277 | 214.3218 |
| ENSMUSG00000010342 | protein_coding | Tex14 | 2983.665 | 2332.395 | 2658.03 |
| ENSMUSG00000017664 | protein_coding | Slc35c2 | 1320.377 | 1131.543 | 1225.96 |
| ENSMUSG00000062949 | protein_coding | Atp11c | 2338.262 | 1932.533 | 2135.398 |
| ENSMUSG00000050248 | protein_coding | Evc2 | 589.8194 | 713.2036 | 651.5115 |
| ENSMUSG00000030428 | protein_coding | Ttyh1 | 6499.026 | 7555.56 | 7027.293 |
| ENSMUSG00000021115 | protein_coding | Vrk1 | 1480.52 | 1260.036 | 1370.278 |
| ENSMUSG00000015083 | protein_coding | C8g | 92.47931 | 61.79025 | 77.13478 |
| ENSMUSG00000026456 | protein_coding | Cyb5r1 | 1523.19 | 1801.301 | 1662.245 |
| ENSMUSG00000025507 | protein_coding | Lrdd | 368.3493 | 289.4204 | 328.8848 |
| ENSMUSG00000035759 | protein_coding | Bbs10 | 254.9814 | 329.2561 | 292.1187 |
| ENSMUSG00000049647 | protein_coding | Purb | 1401.011 | 1680.997 | 1541.004 |
| ENSMUSG00000030042 | protein_coding | Pole4 | 1531.266 | 1320.826 | 1426.046 |
| ENSMUSG00000025232 | protein_coding | Hexa | 2266.282 | 2650.023 | 2458.152 |
| ENSMUSG00000045045 | protein_coding | Lrfn4 | 531.8626 | 644.0659 | 587.9642 |
| ENSMUSG00000042922 | lincRNA | D130020L05Rik | 96.80257 | 64.9116 | 80.85708 |
| ENSMUSG00000003824 | protein_coding | Syce2 | 2011.868 | 1569.526 | 1790.697 |
| ENSMUSG00000033102 | protein_coding | Cdc14b | 1723.44 | 1980.307 | 1851.874 |
| ENSMUSG00000022075 | protein_coding | Rhobtb2 | 502.4865 | 401.8786 | 452.1825 |
| ENSMUSG00000028670 | protein_coding | Lypla2 | 944.3725 | 1120.277 | 1032.325 |
| ENSMUSG00000032968 | protein_coding | Inha | 11102.33 | 12927.68 | 12015 |
| ENSMUSG00000031410 | protein_coding | Nxf7 | 3.876876 | 13.57297 | 8.724922 |
| ENSMUSG00000042492 | protein_coding | Tbc1d10b | 1798.022 | 1546.433 | 1672.228 |
| ENSMUSG00000034614 | protein_coding | Pik3ip1 | 1042.874 | 1245.86 | 1144.367 |
| ENSMUSG00000020658 | protein_coding | Efr3b | 424.4375 | 336.0008 | 380.2191 |
| ENSMUSG00000036572 | protein_coding | Upf3b | 1057.537 | 1334.541 | 1196.039 |
| ENSMUSG00000050592 | protein_coding | Fam78a | 732.352 | 575.1137 | 653.7329 |
| ENSMUSG00000021848 | protein_coding | Otx2 | 2.575614 | 10.25957 | 6.417591 |
| ENSMUSG00000054400 | protein_coding | Cklf | 182.633 | 244.0885 | 213.3607 |
| ENSMUSG00000022332 | protein_coding | Khdrbs3 | 597.3479 | 490.2954 | 543.8217 |
| ENSMUSG00000085016 | lincRNA | Gm11335 | 15.73099 | 4.212944 | 9.971969 |

| | | | | | |
|---|---|---|---|---|---|
| ENSMUSG00000024095 | protein_coding | Hnrpll | 2563.751 | 2255.146 | 2409.449 |
| ENSMUSG00000034247 | protein_coding | Plekhm1 | 1178.696 | 980.917 | 1079.807 |
| ENSMUSG00000027165 | protein_coding | B230118H07Rik | 430.8641 | 343.4106 | 387.1373 |
| ENSMUSG00000060962 | protein_coding | Dmkn | 31.7791 | 14.50891 | 23.14401 |
| ENSMUSG00000027298 | protein_coding | Tyro3 | 8421.439 | 10152.93 | 9287.183 |
| ENSMUSG00000025176 | protein_coding | Hoga1 | 102.2445 | 69.87124 | 86.05788 |
| ENSMUSG00000031303 | protein_coding | Map3k15 | 535.7768 | 429.3938 | 482.5853 |
| ENSMUSG00000007021 | protein_coding | Syngr3 | 25.20361 | 45.26675 | 35.23518 |
| ENSMUSG00000068854 | protein_coding | Hist2h2be | 284.0003 | 371.0916 | 327.5459 |
| ENSMUSG00000021109 | protein_coding | Hif1a | 4648.666 | 5348.234 | 4998.45 |
| ENSMUSG00000021108 | protein_coding | Prkch | 181.3357 | 137.3323 | 159.334 |
| ENSMUSG00000022206 | protein_coding | Npr3 | 715.2554 | 573.445 | 644.3502 |
| ENSMUSG00000027012 | protein_coding | Dync1i2 | 2792.856 | 2418.921 | 2605.888 |
| ENSMUSG00000007041 | protein_coding | Clic1 | 2859.015 | 3273.01 | 3066.013 |
| ENSMUSG00000025515 | protein_coding | Muc2 | 12.98594 | 28.47296 | 20.72945 |
| ENSMUSG00000049013 | protein_coding | Prss48 | 42.8654 | 21.02439 | 31.94489 |
| ENSMUSG00000026447 | protein_coding | Pik3c2b | 581.2173 | 465.055 | 523.1361 |
| ENSMUSG00000023262 | protein_coding | Acy1 | 576.2342 | 467.72 | 521.9771 |
| ENSMUSG00000021194 | protein_coding | Chga | 269.9617 | 201.0815 | 235.5216 |
| ENSMUSG00000000934 | protein_coding | Top1mt | 568.3452 | 473.6353 | 520.9902 |
| ENSMUSG00000081058 | pseudogene | Hist2h3c2 | 19.99349 | 36.18312 | 28.08831 |
| ENSMUSG00000030095 | protein_coding | Tmem43 | 1073.741 | 923.1783 | 998.4596 |
| ENSMUSG00000032517 | protein_coding | Mobp | 49.78865 | 27.24975 | 38.5192 |
| ENSMUSG00000006014 | protein_coding | Prg4 | 20.68356 | 37.64052 | 29.16204 |
| ENSMUSG00000017453 | protein_coding | Pipox | 87.46831 | 130.4903 | 108.9793 |
| ENSMUSG00000037016 | protein_coding | Frem2 | 175.4149 | 132.1419 | 153.7784 |
| ENSMUSG00000073146 | lincRNA | Gm10474 | 14.32842 | 4.194926 | 9.261674 |
| ENSMUSG00000020396 | protein_coding | Nefh | 541.238 | 416.604 | 478.921 |
| ENSMUSG00000067764 | protein_coding | Xlr5c | 355.8112 | 469.206 | 412.5086 |
| ENSMUSG00000054509 | protein_coding | Parp4 | 647.1656 | 786.5173 | 716.8414 |
| ENSMUSG00000037697 | protein_coding | Ddhd1 | 3129.958 | 2569.512 | 2849.735 |
| ENSMUSG00000022575 | protein_coding | Gsdmd | 336.7867 | 430.5386 | 383.6626 |
| ENSMUSG00000039244 | protein_coding | E130309D02Rik | 1052.271 | 880.1009 | 966.186 |
| ENSMUSG00000037601 | protein_coding | Nme1 | 478.2275 | 569.8709 | 524.0492 |
| ENSMUSG00000029033 | protein_coding | Acap3 | 810.7235 | 673.3904 | 742.0569 |
| ENSMUSG00000040978 | protein_coding | Gm11992 | 47.86903 | 27.75179 | 37.81041 |
| ENSMUSG00000003541 | protein_coding | Ier3 | 100.4557 | 160.0708 | 130.2633 |
| ENSMUSG00000025505 | protein_coding | Tmem80 | 377.7216 | 473.1012 | 425.4114 |
| ENSMUSG00000033885 | protein_coding | Pxk | 516.0344 | 627.2051 | 571.6198 |
| ENSMUSG00000032579 | protein_coding | Hemk1 | 480.0229 | 392.1685 | 436.0957 |
| ENSMUSG00000043190 | protein_coding | Rfesd | 545.461 | 443.6132 | 494.5371 |
| ENSMUSG00000038768 | protein_coding | 9130409I23Rik | 0.32211 | 4.557115 | 2.439613 |
| ENSMUSG00000052565 | protein_coding | Hist1h1d | 6.099892 | 16.04347 | 11.07168 |
| ENSMUSG00000021585 | protein_coding | Cast | 668.1219 | 790.9766 | 729.5493 |
| ENSMUSG00000072966 | protein_coding | Gprasp2 | 2532.799 | 2969.76 | 2751.28 |
| ENSMUSG00000026113 | protein_coding | Inpp4a | 524.1547 | 624.1935 | 574.1741 |
| ENSMUSG00000009376 | protein_coding | Met | 686.4365 | 564.3129 | 625.3747 |
| ENSMUSG00000027905 | protein_coding | Ddx20 | 2091.267 | 1822.106 | 1956.687 |
| ENSMUSG00000035069 | protein_coding | Oma1 | 330.3593 | 263.774 | 297.0666 |
| ENSMUSG00000086859 | processed_transcrip | 2810008D09Rik | 383.4629 | 499.292 | 441.3775 |
| ENSMUSG00000029306 | protein_coding | Ibsp | 29.25626 | 9.918608 | 19.58744 |
| ENSMUSG00000026821 | protein_coding | Ralgds | 1584.163 | 1344.758 | 1464.461 |
| ENSMUSG00000068117 | protein_coding | Mei1 | 768.1861 | 403.1313 | 585.6587 |
| ENSMUSG00000022270 | protein_coding | Fam134b | 840.3367 | 639.4808 | 739.9087 |
| ENSMUSG00000005357 | protein_coding | Slc1a6 | 450.6036 | 640.7943 | 545.699 |
| ENSMUSG00000021140 | protein_coding | Pcnx | 4357.683 | 3788.01 | 4072.846 |
| ENSMUSG00000041827 | protein_coding | Oasl1 | 44.31762 | 25.91418 | 35.1159 |
| ENSMUSG00000054702 | protein_coding | Ap1s3 | 196.2309 | 139.2586 | 167.7447 |

| | | | | | |
|---|---|---|---|---|---|
| ENSMUSG00000032186 | protein_coding | Tmod2 | 671.0137 | 888.242 | 779.6279 |
| ENSMUSG00000001661 | protein_coding | Hoxc6 | 97.67572 | 141.822 | 119.7489 |
| ENSMUSG00000024104 | protein_coding | D6Wsu116e | 2551.082 | 2227.501 | 2389.292 |
| ENSMUSG00000018995 | protein_coding | Nars2 | 589.2 | 485.1081 | 537.154 |
| ENSMUSG00000034371 | protein_coding | Dak | 577.0688 | 456.0462 | 516.5575 |
| ENSMUSG00000051375 | protein_coding | Pcdh1 | 113.786 | 153.9408 | 133.8634 |
| ENSMUSG00000031467 | protein_coding | Agpat5 | 1331.643 | 1122.458 | 1227.05 |
| ENSMUSG00000030824 | protein_coding | Nucb1 | 2499.318 | 2063.521 | 2281.42 |
| ENSMUSG00000054580 | protein_coding | Pla2r1 | 374.6279 | 469.1829 | 421.9054 |
| ENSMUSG00000039470 | protein_coding | Zdhhc2 | 2913.319 | 3478.987 | 3196.153 |
| ENSMUSG00000039801 | protein_coding | 2410089E03Rik | 2711.741 | 2153.725 | 2432.733 |
| ENSMUSG00000042851 | protein_coding | Zc3h6 | 715.0188 | 585.8307 | 650.4248 |
| ENSMUSG00000082776 | pseudogene | Gm7061 | 123.6844 | 171.5783 | 147.6314 |
| ENSMUSG00000067359 | protein_coding | Gm5128 | 85.23605 | 57.15997 | 71.19801 |
| ENSMUSG00000018377 | protein_coding | Vezf1 | 2220.463 | 1878.053 | 2049.258 |
| ENSMUSG00000046607 | protein_coding | Hrk | 337.8046 | 462.6103 | 400.2075 |
| ENSMUSG00000035086 | protein_coding | Becn1 | 2557.83 | 2238.605 | 2398.218 |
| ENSMUSG00000027340 | protein_coding | Slc23a2 | 1953.206 | 2332.675 | 2142.941 |
| ENSMUSG00000006301 | protein_coding | Tmbim1 | 603.0935 | 721.9392 | 662.5164 |
| ENSMUSG00000040666 | protein_coding | Sh3bgr | 603.4997 | 723.3245 | 663.4121 |
| ENSMUSG00000039629 | protein_coding | Fam40b | 3749.837 | 4463.366 | 4106.601 |
| ENSMUSG00000045216 | protein_coding | Hs6st1 | 1824.941 | 2167.008 | 1995.974 |
| ENSMUSG00000064247 | protein_coding | Plcxd1 | 19.03432 | 34.50586 | 26.77009 |
| ENSMUSG00000034640 | protein_coding | Tiparp | 2434.39 | 2031.682 | 2233.036 |
| ENSMUSG00000031934 | protein_coding | Panx1 | 556.7976 | 449.2454 | 503.0215 |
| ENSMUSG00000037172 | protein_coding | E330009J07Rik | 809.9348 | 659.2363 | 734.5855 |
| ENSMUSG00000008892 | protein_coding | Vdac3 | 7427.373 | 8589.049 | 8008.211 |
| ENSMUSG00000058620 | protein_coding | Adra2b | 32.20863 | 54.95908 | 43.58386 |
| ENSMUSG00000068245 | protein_coding | D14Ertd668e | 212.3354 | 270.231 | 241.2832 |
| ENSMUSG00000026430 | protein_coding | Rassf5 | 615.479 | 743.849 | 679.664 |
| ENSMUSG00000017737 | protein_coding | Mmp9 | 123.3219 | 162.7051 | 143.0135 |
| ENSMUSG00000064364 | Mt_tRNA | mt-Th | 63.72116 | 31.54627 | 47.63371 |
| ENSMUSG00000003812 | protein_coding | Dnase2a | 211.2452 | 268.2359 | 239.7406 |
| ENSMUSG00000028294 | protein_coding | 1700003M02Rik | 29.17553 | 14.37486 | 21.7752 |
| ENSMUSG00000042433 | protein_coding | E230019M04Rik | 39.14052 | 19.49884 | 29.31968 |
| ENSMUSG00000048355 | protein_coding | Arxes1 | 289.0586 | 360.362 | 324.7103 |
| ENSMUSG00000085085 | lincRNA | 1700086P04Rik | 54.52018 | 83.21033 | 68.86526 |
| ENSMUSG00000001288 | protein_coding | Rarg | 720.3711 | 600.9088 | 660.6399 |
| ENSMUSG00000031374 | protein_coding | Zfp92 | 41.22138 | 65.77252 | 53.49695 |
| ENSMUSG00000025408 | protein_coding | Ddit3 | 432.9513 | 535.8716 | 484.4114 |
| ENSMUSG00000033004 | protein_coding | Mycbp2 | 9867.047 | 8277.79 | 9072.418 |
| ENSMUSG00000030982 | protein_coding | 9030624J02Rik | 2870.961 | 2518.382 | 2694.671 |
| ENSMUSG00000014039 | protein_coding | Prdm15 | 584.3521 | 700.8705 | 642.6113 |
| ENSMUSG00000050700 | protein_coding | Emilin3 | 1640.763 | 2182.298 | 1911.53 |
| ENSMUSG00000022228 | protein_coding | Zfp187 | 1321.051 | 1617.369 | 1469.21 |
| ENSMUSG00000040564 | protein_coding | Apoc1 | 36.2346 | 20.06902 | 28.15181 |
| ENSMUSG00000041135 | protein_coding | Ripk2 | 674.1109 | 550.689 | 612.4 |
| ENSMUSG00000063931 | protein_coding | Pepd | 865.5272 | 1033.671 | 949.599 |
| ENSMUSG00000056228 | protein_coding | Cars2 | 611.137 | 504.0433 | 557.5901 |
| ENSMUSG00000035048 | protein_coding | Anapc13 | 813.1727 | 691.039 | 752.1058 |
| ENSMUSG00000049804 | protein_coding | Armcx4 | 647.1877 | 773.4709 | 710.3293 |
| ENSMUSG00000021913 | protein_coding | Ogdhl | 596.7952 | 491.842 | 544.3186 |
| ENSMUSG00000063146 | protein_coding | Clip2 | 644.6216 | 539.5452 | 592.0834 |
| ENSMUSG00000049092 | protein_coding | Gpr137c | 264.4357 | 204.0213 | 234.2285 |
| ENSMUSG00000006276 | protein_coding | Eps15l1 | 1312.25 | 1540.232 | 1426.241 |
| ENSMUSG00000034127 | protein_coding | Tspan8 | 156.7288 | 230.3785 | 193.5537 |
| ENSMUSG00000087114 | processed_transcrip | Gm16099 | 7.76322 | 1.712931 | 4.738076 |
| ENSMUSG00000089876 | protein_coding | Tmem102 | 44.56339 | 68.41498 | 56.48919 |

| | | | | | |
|---|---|---|---|---|---|
| ENSMUSG00000070691 | protein_coding | Runx3 | 485.9167 | 353.0285 | 419.4726 |
| ENSMUSG00000057246 | protein_coding | BC051142 | 308.1508 | 105.2037 | 206.6773 |
| ENSMUSG00000074637 | protein_coding | Sox2 | 12.87218 | 26.88435 | 19.87827 |
| ENSMUSG00000086606 | processed_transcrip | Gm13205 | 5.865894 | 17.42916 | 11.64753 |
| ENSMUSG00000070498 | protein_coding | Tmem132b | 0.98273 | 6.976407 | 3.979569 |
| ENSMUSG00000090677 | lincRNA | D930019O06Rik | 2.920013 | 10.3493 | 6.634656 |
| ENSMUSG00000027999 | protein_coding | Pla2g12a | 666.0807 | 566.7445 | 616.4126 |
| ENSMUSG00000036833 | protein_coding | Pnpla7 | 796.15 | 658.3598 | 727.2549 |
| ENSMUSG00000021294 | protein_coding | Kif26a | 1372.422 | 1128.524 | 1250.473 |
| ENSMUSG00000078289 | protein_coding | Gm10923 | 2.611992 | 9.880084 | 6.246038 |
| ENSMUSG00000031698 | protein_coding | Mylk3 | 62.12909 | 97.68728 | 79.90819 |
| ENSMUSG00000028825 | protein_coding | Rhd | 116.6174 | 172.9667 | 144.7921 |
| ENSMUSG00000040258 | protein_coding | Nxph4 | 20.20878 | 38.3444 | 29.27659 |
| ENSMUSG00000021892 | protein_coding | Sh3bp5 | 3709.38 | 4400.181 | 4054.78 |
| ENSMUSG00000087714 | snRNA | U6 | 3.927343 | 16.40778 | 10.16756 |
| ENSMUSG00000030795 | protein_coding | Fus | 11608.05 | 13401.07 | 12504.56 |
| ENSMUSG00000059743 | protein_coding | Fdps | 2070.371 | 1703.421 | 1886.896 |
| ENSMUSG00000004371 | protein_coding | Il11 | 6.470158 | 16.42295 | 11.44655 |
| ENSMUSG00000053046 | protein_coding | Brsk2 | 648.2969 | 507.0699 | 577.6834 |
| ENSMUSG00000037933 | protein_coding | Bicd2 | 1905.922 | 1656.637 | 1781.279 |
| ENSMUSG00000011960 | protein_coding | Ccnt1 | 2022.957 | 2391.222 | 2207.089 |
| ENSMUSG00000032271 | protein_coding | Nnmt | 32.77928 | 63.63765 | 48.20846 |
| ENSMUSG00000027488 | protein_coding | Snta1 | 211.0224 | 151.2457 | 181.1341 |
| ENSMUSG00000031578 | protein_coding | Mak16 | 1523.089 | 1754.429 | 1638.759 |
| ENSMUSG00000087391 | lincRNA | Gm2694 | 149.1439 | 111.3976 | 130.2707 |
| ENSMUSG00000023092 | protein_coding | Fhl1 | 3037.659 | 3482.396 | 3260.028 |
| ENSMUSG00000040740 | protein_coding | Slc25a34 | 121.0568 | 160.3194 | 140.6881 |
| ENSMUSG00000043251 | protein_coding | Exoc3l | 1187.779 | 1406.515 | 1297.147 |
| ENSMUSG00000041958 | protein_coding | Pigs | 2107.779 | 1750.982 | 1929.38 |
| ENSMUSG00000020288 | protein_coding | Ahsa2 | 640.5538 | 802.062 | 721.3079 |
| ENSMUSG00000036086 | protein_coding | Zranb3 | 696.175 | 586.4784 | 641.3267 |
| ENSMUSG00000025586 | protein_coding | Cpeb1 | 1330.495 | 980.0338 | 1155.265 |
| ENSMUSG00000020723 | protein_coding | Cacng4 | 260.7253 | 188.1734 | 224.4493 |
| ENSMUSG00000043913 | protein_coding | Ccdc60 | 14.26849 | 4.856964 | 9.562726 |
| ENSMUSG00000030878 | protein_coding | Cdr2 | 972.8865 | 774.1028 | 873.4947 |
| ENSMUSG00000021874 | processed_transcrip | 4933413J09Rik | 219.4724 | 293.2894 | 256.3809 |
| ENSMUSG00000037627 | protein_coding | Rgs22 | 791.921 | 957.0966 | 874.5088 |
| ENSMUSG00000050994 | protein_coding | 9130014G24Rik | 14.61289 | 4.812641 | 9.712764 |
| ENSMUSG00000032281 | protein_coding | Acsbg1 | 10424.42 | 11836.16 | 11130.29 |
| ENSMUSG00000031681 | protein_coding | Smad1 | 694.9917 | 574.9251 | 634.9584 |
| ENSMUSG00000062296 | protein_coding | Trank1 | 21695.08 | 24778.51 | 23236.79 |
| ENSMUSG00000091637 | lincRNA | Gm17281 | 29.0544 | 49.22132 | 39.13786 |
| ENSMUSG00000022790 | protein_coding | Igsf11 | 1289.295 | 1511.286 | 1400.291 |
| ENSMUSG00000024091 | protein_coding | Vapa | 6522.199 | 7470.942 | 6996.57 |
| ENSMUSG00000023150 | protein_coding | Ivns1abp | 10845.74 | 12415.64 | 11630.69 |
| ENSMUSG00000022686 | protein_coding | B3gnt5 | 128.0287 | 173.0155 | 150.5221 |
| ENSMUSG00000035173 | protein_coding | A630007B06Rik | 2722.16 | 2384.884 | 2553.522 |
| ENSMUSG00000032349 | protein_coding | Elovl5 | 4972.114 | 5783.25 | 5377.682 |
| ENSMUSG00000009670 | protein_coding | Tex11 | 3588.828 | 2896.044 | 3242.436 |
| ENSMUSG00000031166 | protein_coding | Wdr13 | 1856.339 | 2193.164 | 2024.751 |
| ENSMUSG00000053838 | protein_coding | Nudcd3 | 2132.814 | 2458.15 | 2295.482 |
| ENSMUSG00000028487 | protein_coding | Bnc2 | 1059.395 | 843.7129 | 951.5541 |
| ENSMUSG00000029570 | protein_coding | Lfng | 533.6949 | 676.9188 | 605.3068 |
| ENSMUSG00000021957 | protein_coding | Tkt | 2882.695 | 3281.636 | 3082.165 |
| ENSMUSG00000040841 | protein_coding | Six5 | 494.9497 | 649.148 | 572.0489 |
| ENSMUSG00000020262 | protein_coding | Adarb1 | 1386.859 | 1052.116 | 1219.488 |
| ENSMUSG00000090000 | protein_coding | Ier3ip1 | 866.3366 | 1017.112 | 941.7241 |
| ENSMUSG00000038777 | protein_coding | Sema6c | 5697.857 | 6741.734 | 6219.795 |

| | | | | | |
|---|---|---|---|---|---|
| ENSMUSG00000006784 | protein_coding | Ttc25 | 64.91873 | 42.23083 | 53.57478 |
| ENSMUSG00000021936 | protein_coding | Mapk8 | 2968.194 | 2593.748 | 2780.971 |
| ENSMUSG00000029810 | protein_coding | Tmem176b | 725.0467 | 897.222 | 811.1344 |
| ENSMUSG00000042814 | protein_coding | Mcts2 | 344.6922 | 435.7108 | 390.2015 |
| ENSMUSG00000030598 | protein_coding | Fbxo17 | 1246.196 | 1467.476 | 1356.836 |
| ENSMUSG00000020246 | protein_coding | Hcfc2 | 2074.381 | 1769.872 | 1922.127 |
| ENSMUSG00000000561 | protein_coding | Wdr77 | 2549.243 | 2892.767 | 2721.005 |
| ENSMUSG00000022176 | protein_coding | Rem2 | 6.78869 | 16.59812 | 11.6934 |
| ENSMUSG00000035521 | protein_coding | Gnptg | 545.586 | 445.6413 | 495.6137 |
| ENSMUSG00000043415 | protein_coding | Otud1 | 191.0034 | 137.3513 | 164.1774 |
| ENSMUSG00000073791 | protein_coding | Efcab7 | 223.5664 | 316.0982 | 269.8323 |
| ENSMUSG00000083693 | pseudogene | Gm11971 | 6.539335 | 30.87191 | 18.70562 |
| ENSMUSG00000003476 | protein_coding | Crhr2 | 7.1108 | 1.324076 | 4.217438 |
| ENSMUSG00000051238 | protein_coding | 2310047B19Rik | 424.6855 | 346.162 | 385.4237 |
| ENSMUSG00000061950 | protein_coding | Ppp4r1 | 4123.237 | 4730.843 | 4427.04 |
| ENSMUSG00000032115 | protein_coding | Hyou1 | 6135.886 | 5234.959 | 5685.423 |
| ENSMUSG00000039298 | protein_coding | Cdk5rap2 | 1962.171 | 1676.28 | 1819.225 |
| ENSMUSG00000046180 | protein_coding | 4930550L24Rik | 3268.749 | 3813.549 | 3541.149 |
| ENSMUSG00000038485 | protein_coding | Socs7 | 2738.621 | 2374.518 | 2556.569 |
| ENSMUSG00000020732 | protein_coding | Rab37 | 8.42153 | 2.012779 | 5.217155 |
| ENSMUSG00000044701 | protein_coding | Il27 | 0.962752 | 6.136355 | 3.549553 |
| ENSMUSG00000007656 | protein_coding | Arpp19 | 2992.901 | 3459.244 | 3226.072 |
| ENSMUSG00000047674 | protein_coding | Pdha2 | 286.6818 | 378.1962 | 332.439 |
| ENSMUSG00000053560 | protein_coding | Ier2 | 794.5387 | 958.0837 | 876.3112 |
| ENSMUSG00000079472 | protein_coding | Gm550 | 15.95284 | 5.049786 | 10.50131 |
| ENSMUSG00000033174 | protein_coding | Mgll | 518.6552 | 427.939 | 473.2971 |
| ENSMUSG00000054944 | processed_transcrip | 5330416C01Rik | 0.33031 | 4.070243 | 2.200277 |
| ENSMUSG00000055657 | protein_coding | E030030I06Rik | 25.80869 | 45.7457 | 35.7772 |
| ENSMUSG00000087580 | lincRNA | Gm16869 | 0.65242 | 8.043555 | 4.347988 |
| ENSMUSG00000032182 | protein_coding | Yipf2 | 653.6663 | 538.1796 | 595.9229 |
| ENSMUSG00000012819 | protein_coding | Cdh23 | 4855.362 | 5668.921 | 5262.142 |
| ENSMUSG00000038039 | protein_coding | Gcc2 | 2166.652 | 2454.941 | 2310.797 |
| ENSMUSG00000027849 | protein_coding | Syt6 | 7.049823 | 18.62855 | 12.83919 |
| ENSMUSG00000037509 | protein_coding | Arhgef4 | 1101.115 | 1522.481 | 1311.798 |
| ENSMUSG00000029409 | protein_coding | U90926 | 30.11033 | 13.7748 | 21.94257 |
| ENSMUSG00000071723 | protein_coding | Gspt2 | 1166.447 | 1374.699 | 1270.573 |
| ENSMUSG00000023906 | protein_coding | Cldn6 | 17.14161 | 34.31739 | 25.7295 |
| ENSMUSG00000085034 | lincRNA | Gm11240 | 7.478756 | 1.350742 | 4.414749 |
| ENSMUSG00000032500 | protein_coding | Dclk3 | 28.88622 | 54.62455 | 41.75538 |
| ENSMUSG00000061882 | protein_coding | Ccdc62 | 310.8663 | 198.3752 | 254.6208 |
| ENSMUSG00000057818 | protein_coding | 1600029O15Rik | 7.378865 | 19.5472 | 13.46303 |
| ENSMUSG00000074113 | processed_transcrip | Gm10629 | 4.559785 | 14.41017 | 9.484978 |
| ENSMUSG00000067276 | protein_coding | Capn6 | 4066.317 | 5005.821 | 4536.069 |
| ENSMUSG00000049422 | protein_coding | Chchd10 | 3072.309 | 3507.259 | 3289.784 |
| ENSMUSG00000074788 | lincRNA | 5830416P10Rik | 1.612862 | 7.549799 | 4.581331 |
| ENSMUSG00000042988 | protein_coding | Notum | 21.71191 | 39.41295 | 30.56243 |
| ENSMUSG00000028138 | protein_coding | Adh5 | 3392.105 | 3997.961 | 3695.033 |
| ENSMUSG00000085577 | pseudogene | Mageb6-ps | 82.82212 | 115.5853 | 99.20369 |
| ENSMUSG00000069094 | protein_coding | Pde7a | 1591.301 | 1337.484 | 1464.392 |
| ENSMUSG00000039787 | protein_coding | Cercam | 606.9162 | 721.3639 | 664.14 |
| ENSMUSG00000048498 | protein_coding | Cd300e | 2.583814 | 9.419877 | 6.001846 |
| ENSMUSG00000041852 | protein_coding | Tcf20 | 2608.283 | 2150.646 | 2379.465 |
| ENSMUSG00000020650 | protein_coding | Bcap29 | 1420.553 | 1219.95 | 1320.252 |
| ENSMUSG00000039748 | protein_coding | Exo1 | 1406.052 | 1209.276 | 1307.664 |
| ENSMUSG00000025510 | protein_coding | Cd151 | 3885.074 | 4534.462 | 4209.768 |
| ENSMUSG00000075243 | lincRNA | 4930547M16Rik | 27.84378 | 13.50778 | 20.67578 |
| ENSMUSG00000030543 | protein_coding | Mesp2 | 115.7178 | 80.30639 | 98.01212 |
| ENSMUSG00000086988 | lincRNA | 1700086O06Rik | 38.98219 | 68.65989 | 53.82104 |

| | | | | | |
|---|---|---|---|---|---|
| ENSMUSG00000021418 | protein_coding | Rpp40 | 760.6886 | 639.0532 | 699.8709 |
| ENSMUSG00000026688 | protein_coding | Mgst3 | 431.4791 | 537.2637 | 484.3714 |
| ENSMUSG00000022562 | protein_coding | Oplah | 3049.306 | 3557.599 | 3303.452 |
| ENSMUSG00000090374 | processed_transcrip | Gm17232 | 17.94543 | 5.599 | 11.77222 |
| ENSMUSG00000087982 | rRNA | 5S_rRNA | 0 | 3.099348 | 1.549674 |
| ENSMUSG00000030522 | protein_coding | Mtmr10 | 953.0969 | 824.316 | 888.7064 |
| ENSMUSG00000049538 | protein_coding | Adamts16 | 18.60412 | 48.49951 | 33.55182 |
| ENSMUSG00000034263 | protein_coding | 2010321M09Rik | 1148.206 | 1327.894 | 1238.05 |
| ENSMUSG00000046000 | protein_coding | Naa11 | 207.2348 | 157.3055 | 182.2702 |
| ENSMUSG00000003746 | protein_coding | Man1a | 228.6631 | 179.9822 | 204.3226 |
| ENSMUSG00000027984 | protein_coding | Hadh | 4865.407 | 5494.691 | 5180.049 |
| ENSMUSG00000028438 | protein_coding | Kif24 | 775.9386 | 614.5212 | 695.2299 |
| ENSMUSG00000047036 | protein_coding | Zfp445 | 1908.546 | 2214.743 | 2061.644 |
| ENSMUSG00000038055 | protein_coding | Dexi | 287.7321 | 233.3509 | 260.5415 |
| ENSMUSG00000034161 | protein_coding | Scx | 592.8855 | 742.9757 | 667.9306 |
| ENSMUSG00000036615 | protein_coding | Rfxap | 635.0362 | 526.0718 | 580.554 |
| ENSMUSG00000058997 | protein_coding | 1300010F03Rik | 1285.831 | 1115.314 | 1200.572 |
| ENSMUSG00000082345 | pseudogene | Gm13622 | 6.802779 | 19.68587 | 13.24432 |
| ENSMUSG00000029483 | protein_coding | Tmem132b | 6.752312 | 16.85328 | 11.8028 |
| ENSMUSG00000026546 | protein_coding | Ccdc19 | 36.55313 | 59.73382 | 48.14348 |
| ENSMUSG00000006143 | protein_coding | 2310043J07Rik | 10.00852 | 21.40139 | 15.70496 |
| ENSMUSG00000038384 | protein_coding | Setd1b | 913.0295 | 1102.146 | 1007.588 |
| ENSMUSG00000003559 | protein_coding | As3mt | 451.1875 | 556.56 | 503.8737 |
| ENSMUSG00000078671 | protein_coding | Chd2 | 1968.744 | 2237.136 | 2102.94 |
| ENSMUSG00000063108 | protein_coding | Zfp26 | 1608.938 | 1940.425 | 1774.681 |
| ENSMUSG00000044296 | protein_coding | Zfp879 | 72.74144 | 46.2218 | 59.48162 |
| ENSMUSG00000029007 | protein_coding | Agtrap | 2321.176 | 2683.648 | 2502.412 |
| ENSMUSG00000048616 | protein_coding | Nog | 16.36828 | 31.93558 | 24.15193 |
| ENSMUSG00000040009 | protein_coding | Gnaz | 547.577 | 429.5085 | 488.5427 |
| ENSMUSG00000049287 | protein_coding | A230051G13Rik | 412.448 | 330.6299 | 371.5389 |
| ENSMUSG00000034781 | protein_coding | Gna11 | 2772.468 | 2430.787 | 2601.627 |
| ENSMUSG00000078187 | pseudogene | Gm13820 | 0 | 3.135023 | 1.567512 |
| ENSMUSG00000071425 | protein_coding | Gm10332 | 276.6026 | 176.936 | 226.7693 |
| ENSMUSG00000022594 | protein_coding | Lynx1 | 326.129 | 250.744 | 288.4365 |
| ENSMUSG00000028943 | protein_coding | Espn | 2689.848 | 3252.064 | 2970.956 |
| ENSMUSG00000050702 | protein_coding | Rfpl3s | 15.87315 | 6.038338 | 10.95574 |
| ENSMUSG00000056486 | protein_coding | Chn1 | 997.8038 | 827.939 | 912.8714 |
| ENSMUSG00000022759 | protein_coding | 4930451C15Rik | 30.69566 | 16.32493 | 23.5103 |
| ENSMUSG00000026950 | protein_coding | Neb | 851.9909 | 677.2291 | 764.61 |
| ENSMUSG00000082082 | pseudogene | Gm13230 | 117.2527 | 169.4008 | 143.3268 |
| ENSMUSG00000085843 | processed_transcrip | A730085A09Rik | 115.0649 | 168.4875 | 141.7762 |
| ENSMUSG00000046793 | protein_coding | Gpr61 | 23.40931 | 9.6869 | 16.5481 |
| ENSMUSG00000042251 | protein_coding | Pm20d1 | 308.9999 | 389.6118 | 349.3058 |
| ENSMUSG00000053199 | protein_coding | Arhgap20 | 1838.696 | 1533.079 | 1685.888 |
| ENSMUSG00000073684 | protein_coding | 2610002J02Rik | 476.5796 | 566.0337 | 521.3066 |
| ENSMUSG00000025229 | protein_coding | Pitx3 | 2.569725 | 9.437896 | 6.00381 |
| ENSMUSG00000032262 | protein_coding | Elovl4 | 85.6418 | 120.7907 | 103.2162 |
| ENSMUSG00000088956 | snoRNA | SNORA25 | 0 | 3.037007 | 1.518503 |
| ENSMUSG00000026311 | protein_coding | Asb1 | 945.426 | 703.8958 | 824.6609 |
| ENSMUSG00000032180 | protein_coding | Tmed1 | 583.293 | 721.8359 | 652.5645 |
| ENSMUSG00000001280 | protein_coding | Sp1 | 4997.347 | 5723.303 | 5360.325 |
| ENSMUSG00000001095 | protein_coding | Slc13a2 | 29.43413 | 48.26989 | 38.85201 |
| ENSMUSG00000025907 | protein_coding | Rb1cc1 | 3690.013 | 3281.353 | 3485.683 |
| ENSMUSG00000044365 | protein_coding | Cxxc4 | 202.8202 | 299.4196 | 251.1199 |
| ENSMUSG00000091408 | protein_coding | Gm6728 | 13.57045 | 26.77841 | 20.17443 |
| ENSMUSG00000084958 | lincRNA | 4930558J18Rik | 94.34026 | 66.52688 | 80.43357 |
| ENSMUSG00000001774 | protein_coding | Chordc1 | 1619.021 | 1954.27 | 1786.645 |
| ENSMUSG00000017146 | protein_coding | Brca1 | 2205.821 | 1895.77 | 2050.796 |

| | | | | | |
|---|---|---|---|---|---|
| ENSMUSG00000038807 | protein_coding | Rap1gap2 | 137.3313 | 181.6728 | 159.5021 |
| ENSMUSG00000027547 | protein_coding | Sall4 | 1543.112 | 1242.965 | 1393.039 |
| ENSMUSG00000022685 | protein_coding | Parn | 1785.437 | 1558.511 | 1671.974 |
| ENSMUSG00000074207 | protein_coding | Adh1 | 757.2661 | 966.4375 | 861.8518 |
| ENSMUSG00000032937 | protein_coding | Fshr | 693.7022 | 875.5582 | 784.6302 |
| ENSMUSG00000002565 | protein_coding | Scin | 26.33118 | 47.57466 | 36.95292 |
| ENSMUSG00000020562 | protein_coding | Efcab10 | 23.97384 | 10.76482 | 17.36933 |
| ENSMUSG00000005161 | protein_coding | Prdx2 | 5192.013 | 6160.467 | 5676.24 |
| ENSMUSG00000055866 | protein_coding | Per2 | 315.997 | 391.0975 | 353.5472 |
| ENSMUSG00000029826 | protein_coding | Zc3hav1 | 1830.967 | 2230.959 | 2030.963 |
| ENSMUSG00000026614 | protein_coding | Slc30a10 | 21.30318 | 9.624558 | 15.46387 |
| ENSMUSG00000020712 | protein_coding | Tcam1 | 308.6824 | 220.3966 | 264.5395 |
| ENSMUSG00000091946 | lincRNA | Gm17518 | 46.58879 | 27.77918 | 37.18399 |
| ENSMUSG00000084965 | processed_transcrip | Gm13857 | 3.878144 | 11.97322 | 7.925683 |
| ENSMUSG00000035413 | protein_coding | Tmem98 | 185.961 | 261.3703 | 223.6656 |
| ENSMUSG00000070372 | protein_coding | Capza1 | 3395.708 | 3886.292 | 3641 |
| ENSMUSG00000023000 | protein_coding | Dhh | 4546.167 | 5486.75 | 5016.459 |
| ENSMUSG00000023070 | protein_coding | Rgn | 7.090822 | 17.34124 | 12.21603 |
| ENSMUSG00000031158 | protein_coding | Timm17b | 1025.865 | 882.575 | 954.2201 |
| ENSMUSG00000021457 | protein_coding | Sykb | 247.0795 | 325.8401 | 286.4598 |
| ENSMUSG00000045631 | protein_coding | Tmprss12 | 16.96144 | 5.845516 | 11.40348 |
| ENSMUSG00000025439 | protein_coding | Clns1a | 1733.574 | 1971.783 | 1852.679 |
| ENSMUSG00000078497 | protein_coding | Gm13145 | 99.40591 | 140.6823 | 120.0441 |
| ENSMUSG00000021678 | protein_coding | F2rl1 | 41.38054 | 70.22592 | 55.80323 |
| ENSMUSG00000037286 | protein_coding | Stag1 | 5605.518 | 4939.864 | 5272.691 |
| ENSMUSG00000071656 | protein_coding | Lrrn4cl | 47.3215 | 71.66961 | 59.49555 |
| ENSMUSG00000040482 | protein_coding | Dom3z | 1042.204 | 876.3127 | 959.2585 |
| ENSMUSG00000027933 | protein_coding | Ints3 | 3361.862 | 2894.602 | 3128.232 |
| ENSMUSG00000038180 | protein_coding | Spag4 | 111.2653 | 74.25066 | 92.75799 |
| ENSMUSG00000025270 | protein_coding | Alas2 | 154.2822 | 112.6513 | 133.4667 |
| ENSMUSG00000044749 | protein_coding | Abca6 | 262.1782 | 325.4416 | 293.8099 |
| ENSMUSG00000087310 | lincRNA | Gm16989 | 3.238545 | 0 | 1.619273 |
| ENSMUSG00000024530 | protein_coding | Slmo1 | 243.0313 | 305.9589 | 274.4951 |
| ENSMUSG00000092250 | processed_transcrip | Gm20467 | 0.98273 | 6.101041 | 3.541886 |
| ENSMUSG00000053931 | protein_coding | Cnn3 | 6586.284 | 8105.2 | 7345.742 |
| ENSMUSG00000031250 | protein_coding | Tnmd | 12.91803 | 26.31748 | 19.61775 |
| ENSMUSG00000001497 | protein_coding | Pax9 | 23.29555 | 8.819458 | 16.0575 |
| ENSMUSG00000033446 | protein_coding | Lpar6 | 173.4644 | 219.8896 | 196.677 |
| ENSMUSG00000078247 | lincRNA | Airn | 69.26151 | 101.9027 | 85.58212 |
| ENSMUSG00000058135 | protein_coding | Gstm1 | 12869.17 | 15105.43 | 13987.3 |
| ENSMUSG00000028949 | protein_coding | Smarcd3 | 461.9073 | 372.0881 | 416.9977 |
| ENSMUSG00000078486 | protein_coding | 2310042D19Rik | 35.77122 | 16.91956 | 26.34539 |
| ENSMUSG00000029790 | protein_coding | Tsga14 | 611.9974 | 510.1082 | 561.0528 |
| ENSMUSG00000035245 | protein_coding | A130022J15Rik | 289.478 | 374.312 | 331.895 |
| ENSMUSG00000028799 | protein_coding | Zfp362 | 1404.83 | 1646.874 | 1525.852 |
| ENSMUSG00000037801 | protein_coding | Iqch | 186.1043 | 132.5058 | 159.3051 |
| ENSMUSG00000022476 | protein_coding | Polr3h | 1525.587 | 1317.683 | 1421.635 |
| ENSMUSG00000000826 | protein_coding | Dnajc5 | 4164.724 | 4739.402 | 4452.063 |
| ENSMUSG00000024610 | protein_coding | Cd74 | 84.40158 | 52.97835 | 68.68997 |
| ENSMUSG00000038540 | protein_coding | Tmc3 | 107.6678 | 145.477 | 126.5724 |
| ENSMUSG00000055447 | protein_coding | Cd47 | 1107.519 | 961.1259 | 1034.322 |
| ENSMUSG00000078532 | protein_coding | Nkain1 | 1069.769 | 869.8695 | 969.8193 |
| ENSMUSG00000002910 | protein_coding | Arrdc2 | 412.1247 | 328.4948 | 370.3098 |
| ENSMUSG00000048495 | protein_coding | 1110034B05Rik | 362.1294 | 436.5314 | 399.3304 |
| ENSMUSG00000013878 | protein_coding | Rnf170 | 1031.443 | 867.8915 | 949.6671 |
| ENSMUSG00000032122 | protein_coding | Slc37a2 | 331.8443 | 398.9752 | 365.4097 |
| ENSMUSG00000025857 | protein_coding | Heatr2 | 1213.354 | 997.4309 | 1105.393 |
| ENSMUSG00000013033 | protein_coding | Lphn1 | 9375.199 | 10553.55 | 9964.375 |

| | | | | | |
|---|---|---|---|---|---|
| ENSMUSG00000046295 | protein_coding | Ankle1 | 208.8531 | 146.0221 | 177.4376 |
| ENSMUSG00000023104 | protein_coding | Rfc2 | 1549.225 | 1323.35 | 1436.287 |
| ENSMUSG00000020282 | protein_coding | Rhbdf1 | 471.7524 | 599.0063 | 535.3794 |
| ENSMUSG00000086604 | lincRNA | Gm15510 | 14.21802 | 28.08695 | 21.15249 |
| ENSMUSG00000018168 | protein_coding | Ikzf3 | 46.32304 | 27.09555 | 36.70929 |
| ENSMUSG00000070392 | protein_coding | Hist2h3c1 | 169.5303 | 226.1232 | 197.8267 |
| ENSMUSG00000058655 | protein_coding | Eif4b | 9605.175 | 10718.22 | 10161.7 |
| ENSMUSG00000029452 | protein_coding | Tmem116 | 133.9924 | 193.4009 | 163.6966 |
| ENSMUSG00000014245 | protein_coding | Pigl | 390.8446 | 464.7942 | 427.8194 |
| ENSMUSG00000022096 | protein_coding | Hr | 532.4989 | 412.1993 | 472.3491 |
| ENSMUSG00000032491 | protein_coding | Nradd | 146.5708 | 188.7264 | 167.6486 |
| ENSMUSG00000036009 | protein_coding | BC067068 | 388.5888 | 317.6914 | 353.1401 |
| ENSMUSG00000017767 | protein_coding | 1700020C07Rik | 11.2793 | 3.488204 | 7.383751 |
| ENSMUSG00000041936 | protein_coding | Agrn | 1922.402 | 1649.118 | 1785.76 |
| ENSMUSG00000020178 | protein_coding | Adora2a | 39.37348 | 60.33683 | 49.85515 |
| ENSMUSG00000033882 | protein_coding | Rbm46 | 1570.223 | 1324.635 | 1447.429 |
| ENSMUSG00000036529 | protein_coding | Sbf1 | 4332.762 | 3738.198 | 4035.48 |
| ENSMUSG00000033808 | protein_coding | Tmem87a | 2412.83 | 2776.058 | 2594.444 |
| ENSMUSG00000072437 | protein_coding | Nanos1 | 646.651 | 514.3566 | 580.5038 |
| ENSMUSG00000050192 | protein_coding | Eif5a2 | 2569.953 | 2157.687 | 2363.82 |
| ENSMUSG00000062632 | protein_coding | Gm12588 | 52.46057 | 30.30809 | 41.38433 |
| ENSMUSG00000045392 | protein_coding | Olfr1033 | 221.6881 | 170.6835 | 196.1858 |
| ENSMUSG00000056025 | protein_coding | Clca1 | 268.7206 | 368.4679 | 318.5942 |
| ENSMUSG00000051220 | protein_coding | Ercc6l | 1022.116 | 867.7566 | 944.9361 |
| ENSMUSG00000016626 | protein_coding | Nlrp14 | 144.8357 | 91.13247 | 117.9841 |
| ENSMUSG00000035949 | protein_coding | Fbxw2 | 2748.474 | 2427.374 | 2587.924 |
| ENSMUSG00000092572 | polymorphic_pseuc | Serpinb10-ps | 17.82348 | 7.213914 | 12.5187 |
| ENSMUSG00000020329 | protein_coding | Polrmt | 1227.505 | 1017.944 | 1122.725 |
| ENSMUSG00000062760 | protein_coding | 1810041L15Rik | 107.1584 | 141.0039 | 124.0812 |
| ENSMUSG00000078502 | protein_coding | Gm13212 | 473.5705 | 638.58 | 556.0753 |
| ENSMUSG00000037872 | protein_coding | Darc | 16.39392 | 33.83445 | 25.11419 |
| ENSMUSG00000027931 | protein_coding | Npr1 | 5560.158 | 6565.142 | 6062.65 |
| ENSMUSG00000017390 | protein_coding | Aldoc | 445.5426 | 365.8673 | 405.705 |
| ENSMUSG00000019856 | protein_coding | Fam184a | 117.5767 | 85.81276 | 101.6947 |
| ENSMUSG00000055421 | protein_coding | Pcdh9 | 24.44414 | 42.28996 | 33.36705 |
| ENSMUSG00000030683 | protein_coding | Sez6l2 | 178.8282 | 251.4102 | 215.1192 |
| ENSMUSG00000042985 | protein_coding | Upk3b | 20.7048 | 38.47053 | 29.58766 |
| ENSMUSG00000037221 | protein_coding | Mospd3 | 833.5095 | 685.739 | 759.6242 |
| ENSMUSG00000039263 | protein_coding | Npepl1 | 769.8406 | 948.106 | 858.9733 |
| ENSMUSG00000033436 | protein_coding | Armcx2 | 1706.863 | 2070.408 | 1888.635 |
| ENSMUSG00000019124 | protein_coding | Scrn1 | 2002.997 | 2380.17 | 2191.583 |
| ENSMUSG00000022010 | protein_coding | Tsc22d1 | 14020.28 | 16416.51 | 15218.4 |
| ENSMUSG00000026289 | protein_coding | Atg16l1 | 1894.387 | 1663.321 | 1778.854 |
| ENSMUSG00000050106 | protein_coding | Tmc8 | 96.13143 | 135.15 | 115.6407 |
| ENSMUSG00000046410 | protein_coding | Kcnk6 | 589.9981 | 484.0089 | 537.0035 |
| ENSMUSG00000075502 | protein_coding | Gm5465 | 225.8932 | 167.3675 | 196.6304 |
| ENSMUSG00000031383 | protein_coding | Dusp9 | 1093.205 | 910.1295 | 1001.667 |
| ENSMUSG00000080152 | pseudogene | Gm12260 | 44.84748 | 88.63484 | 66.74116 |
| ENSMUSG00000030792 | protein_coding | Dkkl1 | 40.55576 | 65.75487 | 53.15532 |
| ENSMUSG00000021712 | protein_coding | Trim23 | 1886.759 | 1630.584 | 1758.671 |
| ENSMUSG00000090041 | pseudogene | Gm16027 | 8.09122 | 2.021789 | 5.056504 |
| ENSMUSG00000022723 | protein_coding | Crybg3 | 760.9791 | 630.2736 | 695.6263 |
| ENSMUSG00000025940 | protein_coding | Tmem70 | 1000.22 | 861.7447 | 930.9821 |
| ENSMUSG00000027404 | protein_coding | Snrpb | 4402.187 | 3901.339 | 4151.763 |
| ENSMUSG00000053334 | protein_coding | Ficd | 204.7237 | 152.3707 | 178.5472 |
| ENSMUSG00000038782 | protein_coding | 1700028J19Rik | 50.39902 | 26.11057 | 38.2548 |
| ENSMUSG00000031986 | protein_coding | Gm505 | 906.7387 | 769.4859 | 838.1123 |
| ENSMUSG00000070632 | protein_coding | Btbd8 | 61.28681 | 40.20868 | 50.74774 |

| | | | | | |
|---|---|---|---|---|---|
| ENSMUSG00000046186 | protein_coding | Cd109 | 139.1712 | 196.5382 | 167.8547 |
| ENSMUSG00000021180 | protein_coding | Rps6ka5 | 844.2849 | 655.3546 | 749.8197 |
| ENSMUSG00000031864 | protein_coding | Ints10 | 1814.959 | 1590.256 | 1702.608 |
| ENSMUSG00000042354 | protein_coding | Gnl3 | 1915.921 | 1660.349 | 1788.135 |
| ENSMUSG00000034932 | protein_coding | Mrpl54 | 869.8325 | 741.2739 | 805.5532 |
| ENSMUSG00000002845 | protein_coding | Tmem39a | 1231.387 | 1408.995 | 1320.191 |
| ENSMUSG00000044716 | protein_coding | Dok7 | 80.38568 | 110.3906 | 95.38816 |
| ENSMUSG00000041477 | protein_coding | Dcp1b | 610.6718 | 506.1538 | 558.4128 |
| ENSMUSG00000020086 | protein_coding | H2afy2 | 761.8569 | 922.6931 | 842.275 |
| ENSMUSG00000046808 | lincRNA | Atp10d | 367.6949 | 491.3191 | 429.507 |
| ENSMUSG00000026604 | protein_coding | Ptpn14 | 627.3175 | 738.7108 | 683.0141 |
| ENSMUSG00000022225 | protein_coding | Cma1 | 14.3818 | 4.043577 | 9.212688 |
| ENSMUSG00000078851 | protein_coding | Hist3h2a | 102.0025 | 146.181 | 124.0918 |
| ENSMUSG00000054204 | protein_coding | Fam150b | 4.869074 | 13.76651 | 9.317794 |
| ENSMUSG00000032782 | protein_coding | Cntrob | 1061.025 | 927.1025 | 994.064 |
| ENSMUSG00000008373 | protein_coding | Prpf31 | 1489.565 | 1296.537 | 1393.051 |
| ENSMUSG00000031397 | protein_coding | Tktl1 | 2112.566 | 1715.45 | 1914.008 |
| ENSMUSG00000060147 | protein_coding | Serpinb6a | 4471.543 | 5542.466 | 5007.004 |
| ENSMUSG00000080316 | protein_coding | Ncrna00085 | 308.8559 | 418.0852 | 363.4706 |
| ENSMUSG00000026409 | protein_coding | Pfkfb2 | 1244.8 | 1436.001 | 1340.401 |
| ENSMUSG00000045282 | protein_coding | Tmem86b | 59.89072 | 37.33275 | 48.61174 |
| ENSMUSG00000027425 | protein_coding | Csrp2bp | 1394.397 | 1167.299 | 1280.848 |
| ENSMUSG00000027510 | protein_coding | Rbm38 | 1803.991 | 1495.543 | 1649.767 |
| ENSMUSG00000027983 | protein_coding | Cyp2u1 | 98.70996 | 68.29822 | 83.50409 |
| ENSMUSG00000034748 | protein_coding | Sirt6 | 529.8383 | 625.2258 | 577.532 |
| ENSMUSG00000007739 | protein_coding | Cct4 | 5745.801 | 5081.484 | 5413.642 |
| ENSMUSG00000031231 | protein_coding | Cox7b | 793.3885 | 962.8632 | 878.1258 |
| ENSMUSG00000030471 | protein_coding | Zdhhc13 | 1286.068 | 1088.939 | 1187.504 |
| ENSMUSG00000042129 | protein_coding | Rassf4 | 251.0541 | 324.2074 | 287.6308 |
| ENSMUSG00000062077 | protein_coding | Trim54 | 11.28392 | 23.34639 | 17.31515 |
| ENSMUSG00000024790 | protein_coding | Sac3d1 | 593.2225 | 479.21 | 536.2163 |
| ENSMUSG00000038208 | protein_coding | Pgap3 | 375.645 | 458.9462 | 417.2956 |
| ENSMUSG00000079502 | protein_coding | 1700101E01Rik | 43.24089 | 26.22267 | 34.73178 |
| ENSMUSG00000021432 | protein_coding | Slc35b3 | 492.8247 | 592.431 | 542.6278 |
| ENSMUSG00000062110 | protein_coding | Scfd2 | 561.6049 | 454.4008 | 508.0029 |
| ENSMUSG00000037731 | protein_coding | BC013712 | 174.5823 | 134.0689 | 154.3256 |
| ENSMUSG00000043631 | protein_coding | Ecm2 | 629.2309 | 769.3255 | 699.2782 |
| ENSMUSG00000024302 | protein_coding | Dtna | 2890.393 | 3521.805 | 3206.099 |
| ENSMUSG00000031311 | protein_coding | Nono | 7405.275 | 8692.798 | 8049.036 |
| ENSMUSG00000057322 | protein_coding | Rpl38 | 1438.092 | 1197.136 | 1317.614 |
| ENSMUSG00000030172 | protein_coding | Erc1 | 2020.857 | 1707.348 | 1864.102 |
| ENSMUSG00000087442 | processed_transcrip | Gm14453 | 17.40275 | 7.451783 | 12.42726 |
| ENSMUSG00000040746 | protein_coding | Rnf167 | 1577.372 | 1819.453 | 1698.413 |
| ENSMUSG00000082908 | pseudogene | Gm13736 | 704.7526 | 935.5031 | 820.1278 |
| ENSMUSG00000091540 | lincRNA | Gm17548 | 15.30757 | 5.402966 | 10.35527 |
| ENSMUSG00000033075 | protein_coding | Senp1 | 1518.13 | 1786.164 | 1652.147 |
| ENSMUSG00000018378 | protein_coding | Cuedc1 | 603.7645 | 750.2452 | 677.0048 |
| ENSMUSG00000038256 | protein_coding | Bcl9 | 2057.855 | 1816.858 | 1937.357 |
| ENSMUSG00000066000 | protein_coding | 2610305D13Rik | 302.1908 | 420.9908 | 361.5908 |
| ENSMUSG00000001436 | protein_coding | Slc19a1 | 386.3241 | 464.9163 | 425.6202 |
| ENSMUSG00000038199 | protein_coding | 4931409K22Rik | 14.93395 | 33.52337 | 24.22866 |
| ENSMUSG00000066839 | protein_coding | Ecsit | 935.33 | 751.943 | 843.6365 |
| ENSMUSG00000083307 | pseudogene | AA414768 | 626.9033 | 736.7729 | 681.8381 |
| ENSMUSG00000059891 | protein_coding | Tsks | 33.06732 | 16.3253 | 24.69631 |
| ENSMUSG00000038801 | protein_coding | Scgb1c1 | 1.601084 | 7.098241 | 4.349662 |
| ENSMUSG00000026245 | protein_coding | Farsb | 2290.709 | 2003.593 | 2147.151 |
| ENSMUSG00000022787 | protein_coding | Wdr53 | 332.6454 | 270.9079 | 301.7766 |
| ENSMUSG00000054942 | protein_coding | Fam73a | 1248.919 | 1452.185 | 1350.552 |

| | | | | | |
|---|---|---|---|---|---|
| ENSMUSG00000038552 | protein_coding | Fndc4 | 226.249 | 281.6698 | 253.9594 |
| ENSMUSG00000034902 | protein_coding | Pip5k1c | 2918.508 | 2562.422 | 2740.465 |
| ENSMUSG00000029470 | protein_coding | P2rx4 | 442.0446 | 530.773 | 486.4088 |
| ENSMUSG00000051550 | protein_coding | Zfp579 | 550.4292 | 732.8648 | 641.647 |
| ENSMUSG00000051243 | protein_coding | Islr2 | 425.3303 | 843.2136 | 634.2719 |
| ENSMUSG00000022607 | protein_coding | Ptk2 | 4319.865 | 3667.079 | 3993.472 |
| ENSMUSG00000090531 | lincRNA | Gm17696 | 2.225326 | 9.226693 | 5.72601 |
| ENSMUSG00000001418 | protein_coding | 0610031J06Rik | 766.4353 | 898.0647 | 832.25 |
| ENSMUSG00000068957 | protein_coding | 4930589L23Rik | 3.874566 | 0.36219 | 2.118378 |
| ENSMUSG00000051359 | protein_coding | Ncald | 206.7818 | 281.9135 | 244.3477 |
| ENSMUSG00000041343 | protein_coding | Ankrd42 | 431.7719 | 322.4917 | 377.1318 |
| ENSMUSG00000067148 | protein_coding | Polr1c | 2073.618 | 2381.634 | 2227.626 |
| ENSMUSG00000087042 | processed_transcrip | Gm11611 | 37.96353 | 61.39958 | 49.68155 |
| ENSMUSG00000042712 | protein_coding | Wbp5 | 4660.795 | 5807.947 | 5234.371 |
| ENSMUSG00000045629 | protein_coding | Sh3tc2 | 1651.868 | 2020.889 | 1836.378 |
| ENSMUSG00000084961 | lincRNA | 4933416M07Rik | 35.36287 | 19.1338 | 27.24834 |
| ENSMUSG00000073177 | protein_coding | Gm773 | 416.7742 | 310.0024 | 363.3883 |
| ENSMUSG00000050150 | protein_coding | Nhedc1 | 177.9355 | 229.6446 | 203.79 |
| ENSMUSG00000072923 | protein_coding | Gm10439 | 724.0707 | 592.3693 | 658.22 |
| ENSMUSG00000024030 | protein_coding | Abcg1 | 614.3947 | 737.7938 | 676.0942 |
| ENSMUSG00000057156 | protein_coding | Homez | 904.1199 | 746.7566 | 825.4382 |
| ENSMUSG00000066007 | protein_coding | Zfp600 | 85.3252 | 119.699 | 102.5121 |
| ENSMUSG00000003031 | protein_coding | Cdkn1b | 1511.282 | 1732.696 | 1621.989 |
| ENSMUSG00000017195 | protein_coding | Zpbp2 | 31.8321 | 16.28061 | 24.05636 |
| ENSMUSG00000028051 | protein_coding | Hcn3 | 228.0122 | 143.1168 | 185.5645 |
| ENSMUSG00000026018 | protein_coding | Ica1l | 126.4905 | 85.05804 | 105.7743 |
| ENSMUSG00000012519 | protein_coding | Mlkl | 212.3686 | 276.0027 | 244.1856 |
| ENSMUSG00000027792 | protein_coding | Bche | 222.9855 | 296.9548 | 259.9701 |
| ENSMUSG00000062124 | protein_coding | Defb45 | 45.79801 | 27.41699 | 36.6075 |
| ENSMUSG00000037400 | protein_coding | Atp11b | 4098.514 | 3569.523 | 3834.019 |
| ENSMUSG00000078768 | protein_coding | Zfp566 | 288.1974 | 232.4297 | 260.3136 |
| ENSMUSG00000090100 | protein_coding | Ttbk2 | 1248.512 | 1054.539 | 1151.526 |
| ENSMUSG00000027244 | protein_coding | Atg13 | 1740.681 | 1514.44 | 1627.56 |
| ENSMUSG00000020994 | protein_coding | Pnn | 4610.621 | 5186.194 | 4898.407 |
| ENSMUSG00000020886 | protein_coding | Dlg4 | 401.9618 | 475.8009 | 438.8814 |
| ENSMUSG00000085622 | lincRNA | 3110056K07Rik | 266.6159 | 334.8636 | 300.7398 |
| ENSMUSG00000021431 | protein_coding | Snrnp48 | 642.4276 | 764.3756 | 703.4016 |
| ENSMUSG00000022139 | protein_coding | Mbnl2 | 3021.308 | 3494.748 | 3258.028 |
| ENSMUSG00000037740 | protein_coding | Mrps26 | 921.6061 | 787.3504 | 854.4783 |
| ENSMUSG00000011752 | protein_coding | Pgam1 | 4667.47 | 5411.436 | 5039.453 |
| ENSMUSG00000081811 | pseudogene | Gm12713 | 0.638331 | 5.272485 | 2.955408 |
| ENSMUSG00000086457 | lincRNA | D930032P07Rik | 0.638331 | 5.272485 | 2.955408 |
| ENSMUSG00000066158 | lincRNA | AY512931 | 0.33031 | 3.73472 | 2.032515 |
| ENSMUSG00000007836 | protein_coding | Hnrnpa0 | 5823.814 | 6498.291 | 6161.052 |
| ENSMUSG00000037816 | protein_coding | Fbxw17 | 416.5901 | 508.1215 | 462.3558 |
| ENSMUSG00000029513 | protein_coding | Prkab1 | 650.4511 | 760.7767 | 705.6139 |
| ENSMUSG00000048949 | pseudogene | Gm6206 | 293.6372 | 239.5265 | 266.5818 |
| ENSMUSG00000050860 | protein_coding | Phospho1 | 56.87335 | 35.57084 | 46.2221 |
| ENSMUSG00000027514 | protein_coding | Zbp1 | 13.28934 | 4.767595 | 9.028466 |
| ENSMUSG00000041096 | protein_coding | Tspyl2 | 4146.65 | 4767.786 | 4457.218 |
| ENSMUSG00000030800 | protein_coding | Prss8 | 42.02185 | 64.99953 | 53.51069 |
| ENSMUSG00000035504 | protein_coding | Reep6 | 494.6637 | 395.2705 | 444.9671 |
| ENSMUSG00000034308 | protein_coding | Sdr42e1 | 77.9072 | 52.57433 | 65.24076 |
| ENSMUSG00000085421 | processed_transcrip | 4732490B19Rik | 7.743242 | 17.34088 | 12.54206 |
| ENSMUSG00000031543 | protein_coding | Ank1 | 44.2026 | 66.05472 | 55.12866 |
| ENSMUSG00000028688 | protein_coding | Toe1 | 903.112 | 779.137 | 841.1245 |
| ENSMUSG00000001827 | protein_coding | Folr1 | 933.6737 | 1093.429 | 1013.551 |
| ENSMUSG00000000142 | protein_coding | Axin2 | 1039.384 | 861.7233 | 950.5537 |

| | | | | | |
|---|---|---|---|---|---|
| ENSMUSG00000006362 | protein_coding | Cbfa2t3 | 461.9591 | 634.3138 | 548.1364 |
| ENSMUSG00000009566 | protein_coding | Fpgs | 950.7853 | 816.5148 | 883.6501 |
| ENSMUSG00000042073 | protein_coding | Abhd14b | 149.4921 | 205.2063 | 177.3492 |
| ENSMUSG00000014668 | protein_coding | Chfr | 1366.451 | 1188.479 | 1277.465 |
| ENSMUSG00000029014 | protein_coding | Dnajc2 | 3432.072 | 2964.876 | 3198.474 |
| ENSMUSG00000000730 | protein_coding | Dnmt3l | 26.45545 | 14.066 | 20.26073 |
| ENSMUSG00000074627 | protein_coding | 4922505G16Rik | 37.5788 | 16.39628 | 26.98754 |
| ENSMUSG00000048807 | protein_coding | Slc35e4 | 69.40472 | 46.33923 | 57.87197 |
| ENSMUSG00000082999 | pseudogene | Gm13238 | 57.57035 | 95.15643 | 76.36339 |
| ENSMUSG00000037103 | protein_coding | Dcaf15 | 1448.542 | 1223.985 | 1336.264 |
| ENSMUSG00000078752 | protein_coding | Gm12776 | 2.575614 | 9.083992 | 5.829803 |
| ENSMUSG00000040451 | protein_coding | Sgms1 | 1744.205 | 2028.711 | 1886.458 |
| ENSMUSG00000017167 | protein_coding | Cntnap1 | 189.5314 | 144.3591 | 166.9452 |
| ENSMUSG00000031536 | protein_coding | Polb | 752.2784 | 645.4082 | 698.8433 |
| ENSMUSG00000085615 | lincRNA | A330035P11Rik | 114.5039 | 82.35194 | 98.4279 |
| ENSMUSG00000023505 | protein_coding | Cdca3 | 1027.274 | 863.7582 | 945.5161 |
| ENSMUSG00000049680 | protein_coding | Urgcp | 1045.416 | 904.8167 | 975.1162 |
| ENSMUSG00000070348 | protein_coding | Ccnd1 | 1864.74 | 1490.614 | 1677.677 |
| ENSMUSG00000022119 | protein_coding | Rbm26 | 3060.313 | 3606.722 | 3333.517 |
| ENSMUSG00000000318 | protein_coding | Clec10a | 62.76996 | 41.50645 | 52.1382 |
| ENSMUSG00000033423 | protein_coding | Eri3 | 2303.614 | 2038.541 | 2171.078 |
| ENSMUSG00000017588 | protein_coding | Krt27 | 11.93045 | 3.859402 | 7.894926 |
| ENSMUSG00000019864 | protein_coding | Rtn4ip1 | 545.6738 | 425.4611 | 485.5674 |
| ENSMUSG00000020548 | protein_coding | 1700086D15Rik | 9.103171 | 2.410644 | 5.756908 |
| ENSMUSG00000055692 | protein_coding | Tmem191c | 112.8851 | 149.3776 | 131.1313 |
| ENSMUSG00000060149 | protein_coding | BC002059 | 241.2502 | 317.3593 | 279.3047 |
| ENSMUSG00000003680 | protein_coding | Taf6l | 753.5101 | 633.0282 | 693.2691 |
| ENSMUSG00000051361 | protein_coding | 6030498E09Rik | 23.20871 | 42.0366 | 32.62265 |
| ENSMUSG00000001525 | protein_coding | Tubb5 | 15180.78 | 17072.56 | 16126.67 |
| ENSMUSG00000028898 | protein_coding | Trnau1ap | 1069.276 | 908.6312 | 988.9537 |
| ENSMUSG00000086697 | lincRNA | 4933427G23Rik | 29.18709 | 50.12335 | 39.65522 |
| ENSMUSG00000057240 | protein_coding | Ms4a13 | 4.243564 | 0.326514 | 2.285039 |
| ENSMUSG00000079476 | protein_coding | 1700011M02Rik | 559.4914 | 378.1265 | 468.8089 |
| ENSMUSG00000055170 | protein_coding | Ifng | 6.115248 | 0.988552 | 3.5519 |
| ENSMUSG00000002983 | protein_coding | Relb | 362.5238 | 271.8602 | 317.192 |
| ENSMUSG00000042401 | protein_coding | Crtac1 | 100.937 | 139.2736 | 120.1053 |
| ENSMUSG00000074785 | protein_coding | Plxnc1 | 5721.763 | 7060.937 | 6391.35 |
| ENSMUSG00000020547 | protein_coding | Bzw2 | 626.1851 | 728.4962 | 677.3406 |
| ENSMUSG00000008489 | protein_coding | Elavl2 | 1843.73 | 1533.025 | 1688.378 |
| ENSMUSG00000004655 | protein_coding | Aqp1 | 49.54093 | 75.46454 | 62.50274 |
| ENSMUSG00000090401 | lincRNA | Snord123 | 458.2962 | 543.7496 | 501.0229 |
| ENSMUSG00000025007 | protein_coding | Aldh18a1 | 879.8027 | 1023.47 | 951.6363 |
| ENSMUSG00000025491 | protein_coding | Ifitm1 | 291.601 | 362.4463 | 327.0237 |
| ENSMUSG00000034274 | protein_coding | Thoc5 | 1138.474 | 1339.242 | 1238.858 |
| ENSMUSG00000075595 | protein_coding | Zfp652 | 2116.98 | 1837.654 | 1977.317 |
| ENSMUSG00000042686 | protein_coding | Jph1 | 335.6427 | 261.4414 | 298.542 |
| ENSMUSG00000034853 | protein_coding | Acot11 | 210.7995 | 163.2947 | 187.0471 |
| ENSMUSG00000025737 | protein_coding | Wdr24 | 885.7329 | 767.8643 | 826.7986 |
| ENSMUSG00000008028 | protein_coding | 1700008O03Rik | 29.34497 | 16.35196 | 22.84847 |
| ENSMUSG00000008682 | protein_coding | Rpl10 | 6402.754 | 7241.478 | 6822.116 |
| ENSMUSG00000022311 | protein_coding | Csmd3 | 2.914124 | 0 | 1.457062 |
| ENSMUSG00000038623 | protein_coding | Tm6sf1 | 349.6734 | 436.1164 | 392.8949 |
| ENSMUSG00000028982 | protein_coding | Slc25a33 | 726.3752 | 586.11 | 656.2426 |
| ENSMUSG00000087719 | misc_RNA | RNaseP_nuc | 1.298952 | 6.145364 | 3.722158 |
| ENSMUSG00000007207 | protein_coding | Stx1a | 794.3346 | 946.807 | 870.5708 |
| ENSMUSG00000038797 | protein_coding | Zscan2 | 285.6828 | 228.5484 | 257.1156 |
| ENSMUSG00000035606 | protein_coding | Ky | 17.73201 | 39.99467 | 28.86334 |
| ENSMUSG00000023232 | protein_coding | Serinc2 | 33.26643 | 51.77611 | 42.52127 |

| | | | | | |
|---|---|---|---|---|---|
| ENSMUSG00000004267 | protein_coding | Eno2 | 52.9141 | 82.76094 | 67.83752 |
| ENSMUSG00000074059 | protein_coding | Fbxw18 | 0.65242 | 4.758947 | 2.705684 |
| ENSMUSG00000022299 | protein_coding | Slc25a32 | 692.6198 | 591.5872 | 642.1035 |
| ENSMUSG00000078123 | pseudogene | Gm5071 | 46.93296 | 26.94126 | 36.93711 |
| ENSMUSG00000068480 | protein_coding | Gm7551 | 28.09567 | 52.75312 | 40.4244 |
| ENSMUSG00000031446 | protein_coding | Cul4a | 4022.174 | 3570.798 | 3796.486 |
| ENSMUSG00000033987 | protein_coding | Dnahc17 | 81.88337 | 129.9373 | 105.9103 |
| ENSMUSG00000022514 | protein_coding | Il1rap | 497.0211 | 421.914 | 459.4676 |
| ENSMUSG00000023473 | protein_coding | Celsr3 | 52.28479 | 26.55711 | 39.42095 |
| ENSMUSG00000038990 | protein_coding | Cables2 | 1174.769 | 986.4556 | 1080.612 |
| ENSMUSG00000010044 | protein_coding | Zmynd10 | 22.17953 | 8.944864 | 15.5622 |
| ENSMUSG00000036368 | protein_coding | Fam82a1 | 1500.393 | 1790.427 | 1645.41 |
| ENSMUSG00000028278 | protein_coding | Rragd | 1966.972 | 2250.581 | 2108.777 |
| ENSMUSG00000055850 | protein_coding | Rnf181 | 5489.171 | 6179.857 | 5834.514 |
| ENSMUSG00000000275 | protein_coding | Trim25 | 1998.526 | 2308.005 | 2153.265 |
| ENSMUSG00000043866 | protein_coding | Taf10 | 860.7967 | 660.2244 | 760.5105 |
| ENSMUSG00000033149 | protein_coding | Phldb2 | 1066.387 | 1471.489 | 1268.938 |
| ENSMUSG00000055817 | protein_coding | Mta3 | 1855.349 | 2124.262 | 1989.806 |
| ENSMUSG00000027840 | protein_coding | Wnt2b | 294.8665 | 241.3544 | 268.1104 |
| ENSMUSG00000026458 | protein_coding | Ppfia4 | 433.6222 | 343.7046 | 388.6634 |
| ENSMUSG00000041272 | protein_coding | Tox | 806.2973 | 965.0898 | 885.6935 |
| ENSMUSG00000092680 | snoRNA | AL671866.1 | 20.91882 | 9.871436 | 15.39513 |
| ENSMUSG00000073678 | protein_coding | Pgap1 | 292.6788 | 236.8031 | 264.7409 |
| ENSMUSG00000031558 | protein_coding | Slit2 | 617.4022 | 461.8393 | 539.6207 |
| ENSMUSG00000006310 | protein_coding | Zbtb32 | 598.5688 | 479.8897 | 539.2292 |
| ENSMUSG00000056257 | protein_coding | Gm5447 | 5.165317 | 0.653029 | 2.909173 |
| ENSMUSG00000022323 | protein_coding | Hrsp12 | 348.6648 | 276.8417 | 312.7533 |
| ENSMUSG00000059645 | protein_coding | Gm7361 | 42.74344 | 65.67347 | 54.20846 |
| ENSMUSG00000050619 | protein_coding | Zscan29 | 944.4081 | 822.5256 | 883.4668 |
| ENSMUSG00000031518 | protein_coding | Spata4 | 8.518885 | 1.748606 | 5.133746 |
| ENSMUSG00000018287 | protein_coding | Spag7 | 949.6789 | 1117.416 | 1033.548 |
| ENSMUSG00000043789 | protein_coding | Vwce | 23.3168 | 40.39606 | 31.85643 |
| ENSMUSG00000050945 | protein_coding | Zfp438 | 304.121 | 250.4723 | 277.2966 |
| ENSMUSG00000008393 | protein_coding | Carhsp1 | 5974.831 | 7032.651 | 6503.741 |
| ENSMUSG00000021221 | protein_coding | Dpf3 | 202.1058 | 252.3045 | 227.2051 |
| ENSMUSG00000033216 | protein_coding | Eefsec | 1031.731 | 888.3986 | 960.065 |
| ENSMUSG00000058717 | protein_coding | Gm4763 | 8.843306 | 1.739597 | 5.291452 |
| ENSMUSG00000043183 | protein_coding | 4732471D19Rik | 1909.452 | 1580.482 | 1744.967 |
| ENSMUSG00000025017 | protein_coding | Pik3ap1 | 267.6755 | 210.4366 | 239.056 |
| ENSMUSG00000085894 | processed_transcrip | Gm15832 | 58.61555 | 85.76015 | 72.18785 |
| ENSMUSG00000022436 | protein_coding | Sh3bp1 | 414.0461 | 497.271 | 455.6586 |
| ENSMUSG00000000308 | protein_coding | Ckmt1 | 11.57785 | 4.0879 | 7.832876 |
| ENSMUSG00000089759 | processed_transcrip | 3632454L22Rik | 85.74549 | 127.0084 | 106.3769 |
| ENSMUSG00000028332 | protein_coding | Hemgn | 1.959572 | 7.875952 | 4.917762 |
| ENSMUSG00000023017 | protein_coding | Accn2 | 185.9888 | 146.1827 | 166.0858 |
| ENSMUSG00000029762 | protein_coding | Akr1b8 | 3566.932 | 3979.378 | 3773.155 |
| ENSMUSG00000029916 | protein_coding | Agk | 885.5942 | 744.0247 | 814.8095 |
| ENSMUSG00000038126 | protein_coding | Mphosph9 | 2406.854 | 2058.261 | 2232.557 |
| ENSMUSG00000031617 | protein_coding | Tmem184c | 989.9398 | 1168.712 | 1079.326 |
| ENSMUSG00000023094 | protein_coding | Msrb2 | 324.6663 | 391.408 | 358.0371 |
| ENSMUSG00000046034 | protein_coding | Fam105b | 883.8707 | 765.7254 | 824.798 |
| ENSMUSG00000032417 | protein_coding | Rwdd2a | 124.8511 | 167.7072 | 146.2792 |
| ENSMUSG00000026834 | protein_coding | Acvr1c | 40.44656 | 73.30119 | 56.87387 |
| ENSMUSG00000046961 | protein_coding | Gpr156 | 68.93143 | 97.61304 | 83.27223 |
| ENSMUSG00000046637 | protein_coding | Ttc34 | 163.3014 | 119.143 | 141.2222 |
| ENSMUSG00000025915 | protein_coding | Sgk3 | 2628.713 | 3040.121 | 2834.417 |
| ENSMUSG00000025880 | protein_coding | Smad7 | 1189.065 | 1406.562 | 1297.813 |
| ENSMUSG00000031145 | protein_coding | Prickle3 | 177.2906 | 229.5531 | 203.4219 |

| | | | | | |
|---|---|---|---|---|---|
| ENSMUSG00000051331 | protein_coding | Cacna1c | 907.2363 | 723.5126 | 815.3745 |
| ENSMUSG00000021902 | protein_coding | Phf7 | 988.7103 | 771.7941 | 880.2522 |
| ENSMUSG00000045589 | protein_coding | 6430704M03Rik | 66.7963 | 92.15887 | 79.47758 |
| ENSMUSG00000026931 | protein_coding | 1700019N19Rik | 0.976841 | 5.943532 | 3.460187 |
| ENSMUSG00000074397 | protein_coding | Foxr1 | 312.2867 | 253.8218 | 283.0542 |
| ENSMUSG00000001467 | protein_coding | Cyp51 | 1638.411 | 1336.454 | 1487.433 |
| ENSMUSG00000075704 | protein_coding | Txnrd2 | 450.9155 | 380.1777 | 415.5466 |
| ENSMUSG00000029199 | protein_coding | Lias | 982.3837 | 857.0888 | 919.7362 |
| ENSMUSG00000022883 | protein_coding | Robo1 | 882.5665 | 760.1619 | 821.3642 |
| ENSMUSG00000030747 | protein_coding | Dgat2 | 1262.872 | 1022.051 | 1142.461 |
| ENSMUSG00000070644 | protein_coding | Etnk2 | 6763.047 | 7914.124 | 7338.585 |
| ENSMUSG00000046173 | protein_coding | Pabpc6 | 56.22033 | 32.20224 | 44.21129 |
| ENSMUSG00000031853 | protein_coding | BC021891 | 4438.902 | 5131.997 | 4785.449 |
| ENSMUSG00000030671 | protein_coding | Pde3b | 1032.664 | 839.2446 | 935.9541 |
| ENSMUSG00000034101 | protein_coding | Ctnnd1 | 6759.45 | 7673.502 | 7216.476 |
| ENSMUSG00000039701 | protein_coding | Usp53 | 1948.524 | 2262.32 | 2105.422 |
| ENSMUSG00000067713 | protein_coding | Prkag1 | 428.5712 | 505.0516 | 466.8114 |
| ENSMUSG00000040260 | protein_coding | Daam2 | 12285.38 | 13845.55 | 13065.47 |
| ENSMUSG00000015668 | protein_coding | Pdzd11 | 571.7118 | 677.7482 | 624.73 |
| ENSMUSG00000034118 | protein_coding | Tpst1 | 2246.471 | 2006.337 | 2126.404 |
| ENSMUSG00000052212 | protein_coding | Cd177 | 18.34783 | 34.3383 | 26.34307 |
| ENSMUSG00000014195 | protein_coding | Dnajc7 | 4111.249 | 4564.583 | 4337.916 |
| ENSMUSG00000057777 | protein_coding | Mab21l2 | 7.402422 | 17.00463 | 12.20353 |
| ENSMUSG00000048899 | protein_coding | Rimkla | 467.7021 | 368.1026 | 417.9024 |
| ENSMUSG00000034109 | protein_coding | Golim4 | 1706.579 | 1978.026 | 1842.303 |
| ENSMUSG00000021373 | protein_coding | Cap2 | 75.39547 | 105.565 | 90.48021 |
| ENSMUSG00000039427 | protein_coding | Alg1 | 988.9104 | 851.2386 | 920.0745 |
| ENSMUSG00000004771 | protein_coding | Rab11a | 4947.571 | 5548.843 | 5248.207 |
| ENSMUSG00000018973 | protein_coding | Hoxb1 | 4.549275 | 12.37109 | 8.460181 |
| ENSMUSG00000000901 | protein_coding | Mmp11 | 1717.51 | 2130.184 | 1923.847 |
| ENSMUSG00000034021 | protein_coding | Pds5b | 4780.492 | 4282.885 | 4531.689 |
| ENSMUSG00000046338 | protein_coding | Gpat2 | 1961.583 | 1523.605 | 1742.594 |
| ENSMUSG00000091596 | lincRNA | 3110083C13Rik | 90.00821 | 61.68105 | 75.84463 |
| ENSMUSG00000029502 | protein_coding | Golga3 | 2543.115 | 2195.835 | 2369.475 |
| ENSMUSG00000031360 | protein_coding | Ctps2 | 922.9857 | 1146.98 | 1034.983 |
| ENSMUSG00000030713 | protein_coding | Klk7 | 2.549747 | 10.16512 | 6.357436 |
| ENSMUSG00000016559 | protein_coding | H3f3b | 19702.46 | 22377.56 | 21040.01 |
| ENSMUSG00000051457 | protein_coding | Spn | 18.76163 | 33.5083 | 26.13497 |
| ENSMUSG00000045210 | protein_coding | Vcpip1 | 4178.098 | 4765.915 | 4472.006 |
| ENSMUSG00000027132 | protein_coding | 2410042D21Rik | 724.0493 | 856.719 | 790.3841 |
| ENSMUSG00000037035 | protein_coding | Inhbb | 5760.76 | 6929.726 | 6345.243 |
| ENSMUSG00000035000 | protein_coding | Dpp4 | 41.57845 | 69.15623 | 55.36734 |
| ENSMUSG00000042942 | protein_coding | Greb1l | 1233.439 | 1401.266 | 1317.353 |
| ENSMUSG00000071322 | protein_coding | Gm10326 | 3.199857 | 11.03515 | 7.117505 |
| ENSMUSG00000071379 | protein_coding | Hpcal1 | 1658.065 | 1936.619 | 1797.342 |
| ENSMUSG00000041669 | protein_coding | Prima1 | 25.79483 | 13.93231 | 19.86357 |
| ENSMUSG00000029718 | protein_coding | Pcolce | 1758.038 | 2081.657 | 1919.847 |
| ENSMUSG00000015542 | protein_coding | Nat9 | 303.4699 | 247.1462 | 275.3081 |
| ENSMUSG00000027203 | protein_coding | Dut | 1388.393 | 1599.859 | 1494.126 |
| ENSMUSG00000038331 | protein_coding | Satb2 | 75.82336 | 53.05763 | 64.4405 |
| ENSMUSG00000035298 | protein_coding | Klhl35 | 55.30761 | 35.9251 | 45.61636 |
| ENSMUSG00000047021 | protein_coding | Ccdc108 | 28.74935 | 11.58473 | 20.16704 |
| ENSMUSG00000007589 | protein_coding | Tinf2 | 1762.522 | 1509.664 | 1636.093 |
| ENSMUSG00000030322 | protein_coding | Mbd4 | 672.4186 | 574.9378 | 623.6782 |
| ENSMUSG00000089809 | protein_coding | A930011G23Rik | 32.43383 | 16.58331 | 24.50857 |
| ENSMUSG00000023456 | protein_coding | Tpi1 | 1882.133 | 2205.647 | 2043.89 |
| ENSMUSG00000009248 | protein_coding | Ascl2 | 197.5778 | 148.481 | 173.0294 |
| ENSMUSG00000079432 | protein_coding | Gm15023 | 260.0379 | 209.1009 | 234.5694 |

| | | | | | |
|---|---|---|---|---|---|
| ENSMUSG00000020098 | protein_coding | Pcbd1 | 62.28609 | 95.20741 | 78.74675 |
| ENSMUSG00000075015 | protein_coding | Gm10801 | 0.966331 | 7.381155 | 4.173743 |
| ENSMUSG00000079157 | protein_coding | Fam155a | 10.27786 | 22.23895 | 16.2584 |
| ENSMUSG00000063273 | protein_coding | Naa15 | 5397.757 | 4785.818 | 5091.788 |
| ENSMUSG00000015647 | protein_coding | Lama5 | 1426.891 | 1695.867 | 1561.379 |
| ENSMUSG00000082450 | pseudogene | Mageb7-ps | 1057.042 | 846.9917 | 952.0166 |
| ENSMUSG00000034160 | protein_coding | Ogt | 8956.799 | 10886.77 | 9921.784 |
| ENSMUSG00000033526 | protein_coding | Ppip5k1 | 779.4207 | 659.6797 | 719.5502 |
| ENSMUSG00000021710 | protein_coding | Nln | 1727.235 | 1497.483 | 1612.359 |
| ENSMUSG00000036766 | protein_coding | Dner | 195.8393 | 277.4065 | 236.6229 |
| ENSMUSG00000025410 | protein_coding | Dctn2 | 2445.352 | 2757.07 | 2601.211 |
| ENSMUSG00000022120 | protein_coding | Rnf219 | 632.9107 | 532.6604 | 582.7855 |
| ENSMUSG00000027454 | protein_coding | Gins1 | 516.6421 | 428.426 | 472.5341 |
| ENSMUSG00000029999 | protein_coding | Tgfa | 122.3147 | 164.8787 | 143.5967 |
| ENSMUSG00000035455 | protein_coding | Fignl1 | 1515.357 | 1308.904 | 1412.131 |
| ENSMUSG00000026615 | protein_coding | Eprs | 6145.178 | 5452.644 | 5798.911 |
| ENSMUSG00000033323 | protein_coding | Ctdp1 | 950.1961 | 814.751 | 882.4735 |
| ENSMUSG00000032827 | protein_coding | Ppp1r9a | 4831.202 | 5504.545 | 5167.873 |
| ENSMUSG00000030344 | protein_coding | Akap3 | 19.51349 | 7.311931 | 13.41271 |
| ENSMUSG00000031530 | protein_coding | Dusp4 | 97.87774 | 134.1731 | 116.0254 |
| ENSMUSG00000078810 | protein_coding | Gp6 | 22.31998 | 10.11179 | 16.21588 |
| ENSMUSG00000031434 | protein_coding | Morc4 | 329.6316 | 263.7649 | 296.6982 |
| ENSMUSG00000091028 | protein_coding | Gm10722 | 0 | 3.717425 | 1.858712 |
| ENSMUSG00000004113 | protein_coding | Cacna1b | 338.0637 | 261.9499 | 300.0068 |
| ENSMUSG00000037306 | protein_coding | Man1c1 | 379.9713 | 314.5561 | 347.2637 |
| ENSMUSG00000028560 | protein_coding | Usp1 | 3174.686 | 2747.209 | 2960.947 |
| ENSMUSG00000086164 | processed_transcrip | Gm13029 | 0.962752 | 5.519001 | 3.240877 |
| ENSMUSG00000038618 | protein_coding | Rassf7 | 339.8225 | 265.1175 | 302.47 |
| ENSMUSG00000021391 | protein_coding | Cenpp | 355.9803 | 296.062 | 326.0211 |
| ENSMUSG00000031134 | protein_coding | Rbmx | 1420.227 | 1825.994 | 1623.111 |
| ENSMUSG00000088205 | rRNA | 5S_rRNA | 4.873696 | 0.688704 | 2.7812 |
| ENSMUSG00000028773 | protein_coding | Fabp3 | 886.2184 | 1116.323 | 1001.271 |
| ENSMUSG00000020684 | protein_coding | Rasl10b | 107.7957 | 79.08106 | 93.43837 |
| ENSMUSG00000020332 | protein_coding | 4930404A10Rik | 21.24034 | 7.982978 | 14.61166 |
| ENSMUSG00000025234 | protein_coding | Arih1 | 4640.004 | 5173.066 | 4906.535 |
| ENSMUSG00000054079 | protein_coding | Utp18 | 2268.105 | 1946.573 | 2107.339 |
| ENSMUSG00000026305 | protein_coding | Lrrfip1 | 968.7611 | 1221.793 | 1095.277 |
| ENSMUSG00000023984 | protein_coding | Med20 | 32.75028 | 56.12343 | 44.43685 |
| ENSMUSG00000030272 | protein_coding | Camk1 | 455.9029 | 567.4727 | 511.6878 |
| ENSMUSG00000034152 | protein_coding | Exoc3 | 2787.103 | 2497.494 | 2642.298 |
| ENSMUSG00000057751 | protein_coding | Megf6 | 379.9667 | 308.3228 | 344.1447 |
| ENSMUSG00000032443 | protein_coding | Zcwpw2 | 128.1475 | 75.66043 | 101.904 |
| ENSMUSG00000092225 | protein_coding | Gm2381 | 30.98833 | 47.82118 | 39.40475 |
| ENSMUSG00000002771 | protein_coding | Grin2d | 192.8533 | 135.7051 | 164.2792 |
| ENSMUSG00000057469 | protein_coding | E2f6 | 1576.621 | 1862.661 | 1719.641 |
| ENSMUSG00000021646 | protein_coding | Mccc2 | 881.0612 | 1019.98 | 950.5208 |
| ENSMUSG00000023973 | protein_coding | Cnpy3 | 1277.728 | 1117.916 | 1197.822 |
| ENSMUSG00000060969 | protein_coding | Irx1 | 9.699235 | 21.83135 | 15.76529 |
| ENSMUSG00000085396 | lincRNA | 6720401G13Rik | 575.0289 | 717.794 | 646.4114 |
| ENSMUSG00000054999 | protein_coding | Naaladl1 | 80.41237 | 57.17799 | 68.79518 |
| ENSMUSG00000004364 | protein_coding | Cul3 | 6263.159 | 5610.052 | 5936.605 |
| ENSMUSG00000025401 | protein_coding | Myo1a | 118.7174 | 169.5658 | 144.1416 |
| ENSMUSG00000022774 | protein_coding | Ncbp2 | 2681.893 | 3081.342 | 2881.618 |
| ENSMUSG00000029206 | protein_coding | Nsun7 | 27.21052 | 9.951072 | 18.58079 |
| ENSMUSG00000058173 | protein_coding | BC017612 | 446.4704 | 555.9407 | 501.2055 |
| ENSMUSG00000051074 | protein_coding | 4930579K19Rik | 54.5427 | 77.59512 | 66.06891 |
| ENSMUSG00000031878 | protein_coding | Nae1 | 2518.648 | 2210.232 | 2364.44 |
| ENSMUSG00000034908 | protein_coding | Sidt2 | 4871.863 | 5483.536 | 5177.7 |

| | | | | | |
|---|---|---|---|---|---|
| ENSMUSG00000069114 | protein_coding | Zbtb10 | 1394.612 | 1190.746 | 1292.679 |
| ENSMUSG00000002748 | protein_coding | Baz1b | 5602.97 | 5028.987 | 5315.979 |
| ENSMUSG00000035351 | protein_coding | Nup37 | 709.8956 | 830.5348 | 770.2152 |
| ENSMUSG00000041660 | protein_coding | Bbox1 | 15.69655 | 4.283933 | 9.990242 |
| ENSMUSG00000079137 | protein_coding | Rpl27-ps1 | 5.729625 | 0.671047 | 3.200336 |
| ENSMUSG00000022753 | protein_coding | Tmem30c | 15.9354 | 30.55165 | 23.24352 |
| ENSMUSG00000020863 | protein_coding | Luc7l3 | 5065.043 | 5844.429 | 5454.736 |
| ENSMUSG00000048747 | protein_coding | E130114P18Rik | 160.831 | 227.1938 | 194.0124 |
| ENSMUSG00000029036 | protein_coding | Atad3a | 1498.666 | 1312.814 | 1405.74 |
| ENSMUSG00000021671 | protein_coding | Poc5 | 794.2582 | 680.7176 | 737.4879 |
| ENSMUSG00000035954 | protein_coding | Dock4 | 307.5676 | 247.3152 | 277.4414 |
| ENSMUSG00000063065 | protein_coding | Mapk3 | 3661.057 | 4094.964 | 3878.011 |
| ENSMUSG00000035594 | protein_coding | Chrna5 | 94.56144 | 67.20549 | 80.88346 |
| ENSMUSG00000034751 | protein_coding | Mast4 | 1012.144 | 846.4121 | 929.2781 |
| ENSMUSG00000078870 | protein_coding | Gm14410 | 162.4827 | 229.7157 | 196.0992 |
| ENSMUSG00000025316 | protein_coding | Banp | 779.7468 | 908.2003 | 843.9736 |
| ENSMUSG00000021477 | protein_coding | Ctsl | 20884.87 | 23742.62 | 22313.74 |
| ENSMUSG00000051827 | protein_coding | Rhox2a | 97.07585 | 70.55208 | 83.81396 |
| ENSMUSG00000046668 | protein_coding | Cxxc5 | 2476.195 | 3003.024 | 2739.61 |
| ENSMUSG00000058640 | pseudogene | Gm10045 | 3.866366 | 11.12344 | 7.494902 |
| ENSMUSG00000071711 | protein_coding | Mpst | 662.628 | 772.4578 | 717.5429 |
| ENSMUSG00000019590 | protein_coding | Cyb561 | 1276.101 | 1474.855 | 1375.478 |
| ENSMUSG00000086543 | lincRNA | 4933406C10Rik | 67.19608 | 35.73513 | 51.46561 |
| ENSMUSG00000033409 | protein_coding | Syce1l | 134.4778 | 101.4101 | 117.9439 |
| ENSMUSG00000069565 | protein_coding | Dazap1 | 2289.936 | 2032.009 | 2160.972 |
| ENSMUSG00000062168 | protein_coding | Ppef1 | 15.26889 | 6.198696 | 10.73379 |
| ENSMUSG00000017412 | protein_coding | Cacnb4 | 208.8065 | 275.7408 | 242.2737 |
| ENSMUSG00000015377 | protein_coding | Fam116b | 296.8819 | 394.3909 | 345.6364 |
| ENSMUSG00000001482 | protein_coding | Def8 | 614.5064 | 717.9976 | 666.252 |
| ENSMUSG00000072115 | protein_coding | Ang | 19.11527 | 34.59632 | 26.85579 |
| ENSMUSG00000019320 | protein_coding | Noxo1 | 70.67489 | 44.4342 | 57.55455 |
| ENSMUSG00000022519 | protein_coding | Srl | 874.956 | 707.3884 | 791.1722 |
| ENSMUSG00000073152 | processed_transcrip | Gm10475 | 9.392482 | 19.42428 | 14.40838 |
| ENSMUSG00000018446 | protein_coding | C1qbp | 1823.192 | 2062.32 | 1942.756 |
| ENSMUSG00000042750 | protein_coding | Bex2 | 2506.067 | 3117.209 | 2811.638 |
| ENSMUSG00000071815 | protein_coding | Fthl17 | 109.1764 | 149.9841 | 129.5803 |
| ENSMUSG00000039738 | protein_coding | Slx4 | 1091.075 | 901.3433 | 996.2091 |
| ENSMUSG00000047557 | protein_coding | Lxn | 340.929 | 430.1559 | 385.5425 |
| ENSMUSG00000033855 | protein_coding | Ston1 | 421.3995 | 568.7159 | 495.0577 |
| ENSMUSG00000043177 | pseudogene | Gm11993 | 84.51527 | 112.4502 | 98.48275 |
| ENSMUSG00000020592 | protein_coding | Sdc1 | 899.0071 | 769.5185 | 834.2628 |
| ENSMUSG00000084972 | lincRNA | 9030407P20Rik | 125.0239 | 94.35582 | 109.6899 |
| ENSMUSG00000031450 | protein_coding | Grk1 | 16.78186 | 30.1001 | 23.44098 |
| ENSMUSG00000039178 | protein_coding | Tbc1d19 | 707.2571 | 828.2031 | 767.7301 |
| ENSMUSG00000029436 | protein_coding | Mmp17 | 275.5496 | 217.1922 | 246.3709 |
| ENSMUSG00000024359 | protein_coding | Hspa9 | 7375.938 | 8206.291 | 7791.114 |
| ENSMUSG00000086857 | lincRNA | Gm16913 | 1.953683 | 7.920636 | 4.937159 |
| ENSMUSG00000025762 | protein_coding | Larp1b | 106.4089 | 60.65745 | 83.53318 |
| ENSMUSG00000048707 | protein_coding | Tprn | 579.0875 | 474.1987 | 526.6431 |
| ENSMUSG00000003585 | protein_coding | Sec14l2 | 246.4466 | 198.1423 | 222.2944 |
| ENSMUSG00000013593 | protein_coding | Ndufs2 | 3017.04 | 3364.145 | 3190.592 |
| ENSMUSG00000043943 | protein_coding | Naalad2 | 1635.622 | 1936.384 | 1786.003 |
| ENSMUSG00000037251 | protein_coding | 4930444A02Rik | 538.4459 | 656.018 | 597.2319 |
| ENSMUSG00000025474 | protein_coding | Tubgcp2 | 1530.288 | 1303.03 | 1416.659 |
| ENSMUSG00000031411 | protein_coding | Prame | 36.01804 | 18.42993 | 27.22399 |
| ENSMUSG00000038611 | protein_coding | Phrf1 | 2904.388 | 2538.355 | 2721.372 |
| ENSMUSG00000042167 | protein_coding | Papd4 | 1295.697 | 1484.952 | 1390.324 |
| ENSMUSG00000060675 | protein_coding | Pla2g16 | 758.7935 | 906.4951 | 832.6443 |

| | | | | | |
|---|---|---|---|---|---|
| ENSMUSG00000027428 | protein_coding | Rbbp9 | 1384.82 | 1756.111 | 1570.466 |
| ENSMUSG00000034105 | protein_coding | 4632415K11Rik | 329.5323 | 404.3487 | 366.9405 |
| ENSMUSG00000041840 | protein_coding | Haus1 | 824.7573 | 706.3804 | 765.5689 |
| ENSMUSG00000029581 | protein_coding | Fscn1 | 1191.377 | 1367.327 | 1279.352 |
| ENSMUSG00000020697 | protein_coding | Lig3 | 2114.627 | 1880.663 | 1997.645 |
| ENSMUSG00000048769 | pseudogene | H1f0 | 3943.746 | 4517.659 | 4230.702 |
| ENSMUSG00000054659 | protein_coding | Pm20d2 | 183.324 | 229.6039 | 206.464 |
| ENSMUSG00000027865 | protein_coding | Gdap2 | 771.3465 | 658.9141 | 715.1303 |
| ENSMUSG00000026807 | protein_coding | Ak8 | 62.55505 | 42.95484 | 52.75495 |
| ENSMUSG00000030131 | protein_coding | Mug2 | 10.65968 | 22.80727 | 16.73347 |
| ENSMUSG00000032802 | protein_coding | Srxn1 | 230.9414 | 298.7415 | 264.8415 |
| ENSMUSG00000031849 | protein_coding | Comp | 33.89656 | 53.08357 | 43.49007 |
| ENSMUSG00000023827 | protein_coding | Agpat4 | 2026.829 | 2334.001 | 2180.415 |
| ENSMUSG00000085146 | processed_transcrip | Eif2c5 | 83.68416 | 59.65098 | 71.66757 |
| ENSMUSG00000032436 | protein_coding | Cmtm7 | 233.6228 | 302.081 | 267.8519 |
| ENSMUSG00000038872 | protein_coding | Zfhx3 | 855.6036 | 1043.365 | 949.4842 |
| ENSMUSG00000046432 | protein_coding | Ngfrap1 | 2528.609 | 3040.811 | 2784.71 |
| ENSMUSG00000070476 | protein_coding | 9030418K01Rik | 643.4783 | 526.5029 | 584.9906 |
| ENSMUSG00000029505 | protein_coding | Ep400 | 6774.17 | 5678.921 | 6226.546 |
| ENSMUSG00000026185 | protein_coding | Igfbp5 | 451.3204 | 596.388 | 523.8542 |
| ENSMUSG00000038151 | protein_coding | Prdm1 | 61.72856 | 85.85599 | 73.79228 |
| ENSMUSG00000091387 | protein_coding | Gcnt4 | 797.9191 | 653.8616 | 725.8903 |
| ENSMUSG00000021589 | protein_coding | Rhobtb3 | 2669.441 | 3001.684 | 2835.562 |
| ENSMUSG00000050428 | protein_coding | Fbxo46 | 686.22 | 576.0935 | 631.1567 |
| ENSMUSG00000058183 | protein_coding | Mmel1 | 81.10475 | 116.9128 | 99.00877 |
| ENSMUSG00000062327 | protein_coding | T | 39.53241 | 63.99735 | 51.76488 |
| ENSMUSG00000043923 | protein_coding | Ccdc84 | 327.4318 | 401.4753 | 364.4535 |
| ENSMUSG00000056598 | protein_coding | Lrrc48 | 183.5109 | 124.5719 | 154.0414 |
| ENSMUSG00000027971 | protein_coding | Ndst4 | 22.87608 | 11.53031 | 17.2032 |
| ENSMUSG00000033707 | protein_coding | Lrrc24 | 38.44584 | 57.03565 | 47.74074 |
| ENSMUSG00000030685 | protein_coding | Kctd13 | 1561.375 | 1219.461 | 1390.418 |
| ENSMUSG00000032611 | protein_coding | 1700102P08Rik | 86.36898 | 41.25771 | 63.81334 |
| ENSMUSG00000036908 | protein_coding | Unc93b1 | 309.791 | 370.0643 | 339.9276 |
| ENSMUSG00000083012 | protein_coding | 2810453I06Rik | 54.82127 | 35.88078 | 45.35103 |
| ENSMUSG00000030138 | protein_coding | Bms1 | 3310.617 | 2926.361 | 3118.489 |
| ENSMUSG00000008763 | protein_coding | Man1a2 | 3810.433 | 3340.634 | 3575.533 |
| ENSMUSG00000048755 | protein_coding | Mcat | 725.7107 | 627.1676 | 676.4391 |
| ENSMUSG00000025329 | protein_coding | Padi1 | 10.96308 | 4.0879 | 7.525488 |
| ENSMUSG00000047514 | protein_coding | Tspyl1 | 2278.625 | 2597.126 | 2437.875 |
| ENSMUSG00000030680 | protein_coding | 2900092E17Rik | 645.4742 | 540.9663 | 593.2203 |
| ENSMUSG00000034586 | protein_coding | C630004H02Rik | 151.4692 | 193.1757 | 172.3225 |
| ENSMUSG00000054072 | protein_coding | Iigp1 | 127.2151 | 75.79236 | 101.5037 |
| ENSMUSG00000042097 | protein_coding | Zfp239 | 325.8517 | 404.4185 | 365.1351 |
| ENSMUSG00000000325 | protein_coding | Arvcf | 1144.813 | 970.9788 | 1057.896 |
| ENSMUSG00000066643 | protein_coding | Wdr35 | 1009.339 | 823.192 | 916.2652 |
| ENSMUSG00000070889 | protein_coding | Pgap1 | 505.1171 | 398.6909 | 451.904 |
| ENSMUSG00000061540 | protein_coding | Orm2 | 0.65242 | 4.459099 | 2.55576 |
| ENSMUSG00000085650 | lincRNA | 2310010J17Rik | 67.14853 | 43.46874 | 55.30864 |
| ENSMUSG00000031398 | protein_coding | Plxna3 | 412.2163 | 491.1971 | 451.7067 |
| ENSMUSG00000092893 | misc_RNA | AC124489.1 | 3.219835 | 10.05846 | 6.639147 |
| ENSMUSG00000020074 | protein_coding | Ccar1 | 3347.108 | 3707.797 | 3527.453 |
| ENSMUSG00000041268 | protein_coding | Dmxl2 | 3221.998 | 2753.805 | 2987.901 |
| ENSMUSG00000044350 | protein_coding | 9030625A04Rik | 558.3551 | 476.3108 | 517.333 |
| ENSMUSG00000021243 | protein_coding | Fcf1 | 1073.973 | 1223.785 | 1148.879 |
| ENSMUSG00000022351 | protein_coding | Sqle | 1799.052 | 1528.141 | 1663.597 |
| ENSMUSG00000039095 | protein_coding | En2 | 15.17869 | 5.782813 | 10.48075 |
| ENSMUSG00000015568 | protein_coding | Lpl | 273.3116 | 768.7906 | 521.0511 |
| ENSMUSG00000037979 | protein_coding | Ccdc92 | 681.3965 | 549.987 | 615.6918 |

| | | | | | |
|---|---|---|---|---|---|
| ENSMUSG00000030335 | protein_coding | Mrpl51 | 1296.695 | 1137.503 | 1217.099 |
| ENSMUSG00000053347 | protein_coding | Zfp943 | 549.4834 | 663.2547 | 606.369 |
| ENSMUSG00000035027 | protein_coding | Map2k2 | 3071.293 | 2734.816 | 2903.054 |
| ENSMUSG00000045039 | protein_coding | Megf8 | 3425.924 | 3060.882 | 3243.403 |
| ENSMUSG00000036206 | protein_coding | Sh3bp4 | 843.6819 | 725.9 | 784.791 |
| ENSMUSG00000037025 | protein_coding | Foxa2 | 10.41517 | 23.69884 | 17.05701 |
| ENSMUSG00000041491 | protein_coding | Cep78 | 1258.775 | 1100.663 | 1179.719 |
| ENSMUSG00000022429 | protein_coding | Dmc1 | 1496.291 | 623.351 | 1059.821 |
| ENSMUSG00000033685 | protein_coding | Ucp2 | 4916.48 | 5737.515 | 5326.997 |
| ENSMUSG00000070371 | protein_coding | Prss36 | 456.2401 | 369.5492 | 412.8947 |
| ENSMUSG00000042708 | protein_coding | 1700012A16Rik | 518.2694 | 367.1562 | 442.7128 |
| ENSMUSG00000042632 | protein_coding | Pla2g6 | 1136.548 | 988.1497 | 1062.349 |
| ENSMUSG00000021537 | protein_coding | Cetn3 | 2139.351 | 1827.578 | 1983.465 |
| ENSMUSG00000058013 | protein_coding | 11-Sep | 6132.33 | 7481.525 | 6806.927 |
| ENSMUSG00000030491 | protein_coding | Tdrd12 | 688.542 | 560.051 | 624.2965 |
| ENSMUSG00000090256 | pseudogene | Alms1-ps1 | 2.555636 | 9.342367 | 5.949001 |
| ENSMUSG00000041961 | protein_coding | Znrf3 | 1498.612 | 1315.513 | 1407.062 |
| ENSMUSG00000078360 | protein_coding | Gm10929 | 0.316221 | 4.723995 | 2.520108 |
| ENSMUSG00000021576 | protein_coding | Pdcd6 | 1689.251 | 1962.255 | 1825.753 |
| ENSMUSG00000032119 | protein_coding | Hinfp | 1006.383 | 883.8639 | 945.1235 |
| ENSMUSG00000036912 | protein_coding | Piwil4 | 292.4362 | 234.5837 | 263.5099 |
| ENSMUSG00000053062 | protein_coding | Jam2 | 4021.72 | 4788.691 | 4405.205 |
| ENSMUSG00000037608 | protein_coding | Bclaf1 | 9007.113 | 10119.76 | 9563.439 |
| ENSMUSG00000039601 | protein_coding | Rcan2 | 43.91791 | 64.3962 | 54.15705 |
| ENSMUSG00000025395 | protein_coding | Prim1 | 1304.323 | 1122.323 | 1213.323 |
| ENSMUSG00000084592 | miRNA | Mir1898 | 58.84031 | 82.75193 | 70.79612 |
| ENSMUSG00000029319 | protein_coding | Coq2 | 1285.045 | 1101.683 | 1193.364 |
| ENSMUSG00000041708 | protein_coding | Mpped1 | 119.6687 | 89.92945 | 104.7991 |
| ENSMUSG00000019689 | protein_coding | 1110001J03Rik | 166.7688 | 206.1483 | 186.4586 |
| ENSMUSG00000020838 | protein_coding | Slc6a4 | 38.23979 | 21.62409 | 29.93194 |
| ENSMUSG00000020092 | protein_coding | X99384 | 2651.247 | 3030.782 | 2841.014 |
| ENSMUSG00000059970 | protein_coding | Hspa2 | 1551.385 | 1201.072 | 1376.228 |
| ENSMUSG00000031994 | protein_coding | Adamts8 | 49.45409 | 78.95062 | 64.20235 |
| ENSMUSG00000032320 | protein_coding | Rcn2 | 2816.904 | 2497.694 | 2657.299 |
| ENSMUSG00000020070 | protein_coding | Rufy2 | 618.1738 | 720.9844 | 669.5791 |
| ENSMUSG00000025277 | protein_coding | Abhd6 | 1331.846 | 1548.952 | 1440.399 |
| ENSMUSG00000085633 | processed_transcrip | 4930580A09Rik | 20.10263 | 9.351738 | 14.72718 |
| ENSMUSG00000042828 | protein_coding | Trim72 | 19.90247 | 44.18153 | 32.042 |
| ENSMUSG00000028098 | protein_coding | Rnf115 | 1970.056 | 1751.269 | 1860.662 |
| ENSMUSG00000087624 | processed_transcrip | 9230111E07Rik | 6.452491 | 14.74606 | 10.59927 |
| ENSMUSG00000024974 | protein_coding | Smc3 | 6512.191 | 7481.684 | 6996.937 |
| ENSMUSG00000023919 | protein_coding | Cenpq | 738.106 | 631.6013 | 684.8536 |
| ENSMUSG00000081924 | pseudogene | Gm11191 | 1.929083 | 7.715232 | 4.822158 |
| ENSMUSG00000024338 | protein_coding | Psmb8 | 52.73222 | 34.21502 | 43.47362 |
| ENSMUSG00000014303 | protein_coding | Glis2 | 1181.469 | 1436.88 | 1309.174 |
| ENSMUSG00000076617 | IG_C_gene | Ighm | 153.1848 | 106.297 | 129.7409 |
| ENSMUSG00000038619 | protein_coding | Ensa | 4647.584 | 4187.221 | 4417.403 |
| ENSMUSG00000040688 | protein_coding | Tbl3 | 2102.501 | 1790.572 | 1946.536 |
| ENSMUSG00000020720 | protein_coding | Psmd12 | 3047.131 | 3393.695 | 3220.413 |
| ENSMUSG00000042289 | protein_coding | Hsd3b7 | 310.5849 | 387.2502 | 348.9176 |
| ENSMUSG00000021660 | protein_coding | Btf3 | 4369.009 | 4983.532 | 4676.27 |
| ENSMUSG00000063171 | protein_coding | Rps4y2 | 3129.279 | 3645.852 | 3387.566 |
| ENSMUSG00000087112 | processed_transcrip | Gm7237 | 3.941432 | 15.22713 | 9.584281 |
| ENSMUSG00000057058 | protein_coding | Skap1 | 30.69081 | 47.19482 | 38.94282 |
| ENSMUSG00000021055 | protein_coding | Esr2 | 93.07784 | 131.6035 | 112.3407 |
| ENSMUSG00000026728 | protein_coding | Vim | 13107.34 | 14734.95 | 13921.15 |
| ENSMUSG00000045411 | protein_coding | 2410002F23Rik | 6256.33 | 7045.45 | 6650.89 |
| ENSMUSG00000043760 | protein_coding | Pkhd1 | 9.353793 | 3.055025 | 6.204409 |

| | | | | | |
|---|---|---|---|---|---|
| ENSMUSG00000074674 | protein_coding | Gm10742 | 7.583043 | 0.724379 | 4.153711 |
| ENSMUSG00000087162 | processed_transcrip | Gm14244 | 4.624341 | 0 | 2.312171 |
| ENSMUSG00000040836 | protein_coding | Gpr161 | 147.1639 | 107.9591 | 127.5615 |
| ENSMUSG00000090516 | pseudogene | Gm6202 | 14.5906 | 6.127707 | 10.35915 |
| ENSMUSG00000026457 | protein_coding | Adipor1 | 5823.062 | 6577.975 | 6200.518 |
| ENSMUSG00000031591 | protein_coding | Asah1 | 3614.68 | 4173.356 | 3894.018 |
| ENSMUSG00000038482 | protein_coding | Tfdp1 | 3960.738 | 3476.839 | 3718.788 |
| ENSMUSG00000024659 | protein_coding | Anxa1 | 669.4331 | 824.1608 | 746.7969 |
| ENSMUSG00000044340 | protein_coding | Phlpp1 | 684.0462 | 561.6785 | 622.8624 |
| ENSMUSG00000074634 | protein_coding | Gm7120 | 195.1531 | 85.72339 | 140.4383 |
| ENSMUSG00000024566 | protein_coding | Atp9b | 3895.49 | 4390.327 | 4142.909 |
| ENSMUSG00000021081 | protein_coding | Serpina1f | 3.852277 | 10.93062 | 7.391446 |
| ENSMUSG00000019489 | protein_coding | Cd70 | 8.382841 | 2.43731 | 5.410076 |
| ENSMUSG00000040282 | protein_coding | BC052040 | 371.7672 | 309.9139 | 340.8405 |
| ENSMUSG00000045427 | protein_coding | Hnrnph2 | 2521.39 | 2807.592 | 2664.491 |
| ENSMUSG00000041995 | protein_coding | Zbed3 | 689.1266 | 819.36 | 754.2433 |
| ENSMUSG00000026398 | protein_coding | Nr5a2 | 176.1278 | 125.6255 | 150.8767 |
| ENSMUSG00000016256 | protein_coding | Ctsz | 1071.826 | 1215.575 | 1143.7 |
| ENSMUSG00000050272 | protein_coding | Dscam | 54.14761 | 34.08382 | 44.11571 |
| ENSMUSG00000056004 | protein_coding | 9330182L06Rik | 197.4903 | 154.3773 | 175.9338 |
| ENSMUSG00000028822 | protein_coding | Tmem50a | 2275.596 | 2634.395 | 2454.995 |
| ENSMUSG00000020536 | protein_coding | Llgl1 | 4015.713 | 3496.097 | 3755.905 |
| ENSMUSG00000030345 | protein_coding | Dyrk4 | 15.57818 | 32.95541 | 24.26679 |
| ENSMUSG00000022241 | protein_coding | Tars | 2496.051 | 2177.249 | 2336.65 |
| ENSMUSG00000065767 | snRNA | U2 | 11.39894 | 2.535327 | 6.967134 |
| ENSMUSG00000051316 | protein_coding | Taf7 | 2681.071 | 3043.488 | 2862.28 |
| ENSMUSG00000026126 | protein_coding | Ptpn18 | 38.1765 | 60.6781 | 49.4273 |
| ENSMUSG00000038766 | protein_coding | Gabpb2 | 3268.946 | 3677.374 | 3473.16 |
| ENSMUSG00000030641 | protein_coding | 4632434I11Rik | 1084.329 | 916.8137 | 1000.571 |
| ENSMUSG00000030870 | protein_coding | Ubfd1 | 3749.54 | 3232.987 | 3491.263 |
| ENSMUSG00000070871 | protein_coding | Ccnyl1 | 1509.814 | 1283.032 | 1396.423 |
| ENSMUSG00000079160 | lincRNA | Gm17608 | 6.472469 | 15.91335 | 11.19291 |
| ENSMUSG00000024944 | protein_coding | Arl2 | 569.7472 | 476.3121 | 523.0297 |
| ENSMUSG00000020007 | protein_coding | Il20ra | 9.692303 | 3.434871 | 6.563587 |
| ENSMUSG00000027580 | protein_coding | BC006779 | 281.2689 | 349.5142 | 315.3915 |
| ENSMUSG00000029134 | protein_coding | Plb1 | 96.71759 | 126.7184 | 111.718 |
| ENSMUSG00000021027 | protein_coding | Ralgapa1 | 3819.527 | 3255.125 | 3537.326 |
| ENSMUSG00000078202 | protein_coding | Nrarp | 815.3001 | 689.5537 | 752.4269 |
| ENSMUSG00000021880 | protein_coding | Rnase6 | 20.26409 | 9.942425 | 15.10326 |
| ENSMUSG00000081266 | pseudogene | Gm15820 | 0.33031 | 3.390187 | 1.860249 |
| ENSMUSG00000029627 | protein_coding | Zkscan14 | 290.907 | 238.2993 | 264.6032 |
| ENSMUSG00000038068 | protein_coding | Rnf144b | 1472.062 | 1709.794 | 1590.928 |
| ENSMUSG00000084373 | pseudogene | Rps2-ps4 | 15.55462 | 6.727042 | 11.14083 |
| ENSMUSG00000030854 | protein_coding | Ptpn5 | 20.92345 | 36.03969 | 28.48157 |
| ENSMUSG00000030431 | protein_coding | 2210411K11Rik | 66.98177 | 44.29838 | 55.64008 |
| ENSMUSG00000002083 | protein_coding | Bbc3 | 137.7842 | 190.3221 | 164.0532 |
| ENSMUSG00000006219 | protein_coding | Fblim1 | 1107.442 | 1327.666 | 1217.554 |
| ENSMUSG00000008734 | protein_coding | Gprc5b | 2833.585 | 3260.081 | 3046.833 |
| ENSMUSG00000022974 | protein_coding | Gcfc1 | 2255.66 | 2780.91 | 2518.285 |
| ENSMUSG00000033918 | protein_coding | Parl | 962.6155 | 1102.669 | 1032.642 |
| ENSMUSG00000054477 | protein_coding | Kcnn2 | 74.43272 | 51.38762 | 62.91017 |
| ENSMUSG00000002205 | protein_coding | Vrk3 | 978.0364 | 853.5176 | 915.777 |
| ENSMUSG00000079286 | protein_coding | Gm11084 | 13.91716 | 25.8349 | 19.87603 |
| ENSMUSG00000030762 | protein_coding | Aqp8 | 17.19335 | 7.585113 | 12.38923 |
| ENSMUSG00000060591 | protein_coding | Ifitm2 | 946.0237 | 1143.968 | 1044.996 |
| ENSMUSG00000040990 | protein_coding | Sh3kbp1 | 553.8633 | 475.6906 | 514.7769 |
| ENSMUSG00000042709 | protein_coding | Atpaf2 | 802.4276 | 667.0769 | 734.7523 |
| ENSMUSG00000051940 | protein_coding | 5031410I06Rik | 31.36679 | 50.42288 | 40.89484 |

| | | | | | |
|---|---|---|---|---|---|
| ENSMUSG00000071757 | protein_coding | Zhx2 | 634.0535 | 774.2453 | 704.1494 |
| ENSMUSG00000079437 | protein_coding | Tmem179b | 417.9021 | 495.0324 | 456.4672 |
| ENSMUSG00000031534 | protein_coding | AI316807 | 529.329 | 450.4807 | 489.9048 |
| ENSMUSG00000050410 | protein_coding | Tcf19 | 508.0301 | 417.1707 | 462.6004 |
| ENSMUSG00000002343 | protein_coding | Armc6 | 486.6272 | 412.3648 | 449.496 |
| ENSMUSG00000050677 | protein_coding | Ccdc96 | 65.77071 | 40.37152 | 53.07111 |
| ENSMUSG00000036752 | protein_coding | Tubb2c | 11440.14 | 9935.84 | 10687.99 |
| ENSMUSG00000036585 | protein_coding | Fgf1 | 13.27756 | 24.81932 | 19.04844 |
| ENSMUSG00000051037 | protein_coding | Zfp455 | 121.8979 | 85.08304 | 103.4905 |
| ENSMUSG00000043993 | lincRNA | 2900052L18Rik | 296.4233 | 390.8628 | 343.643 |
| ENSMUSG00000027569 | protein_coding | 1600027N09Rik | 1341.432 | 1150.455 | 1245.943 |
| ENSMUSG00000019971 | protein_coding | Cep290 | 1333.826 | 1035.881 | 1184.854 |
| ENSMUSG00000030002 | protein_coding | Dusp11 | 3656.446 | 4315.324 | 3985.885 |
| ENSMUSG00000031207 | protein_coding | Msn | 1774.824 | 2035.681 | 1905.252 |
| ENSMUSG00000033207 | protein_coding | Mamdc2 | 107.1666 | 139.8936 | 123.5301 |
| ENSMUSG00000023902 | protein_coding | Zscan10 | 17.42146 | 30.12355 | 23.7725 |
| ENSMUSG00000022710 | protein_coding | Usp7 | 8253.384 | 7449.117 | 7851.25 |
| ENSMUSG00000034522 | protein_coding | Zfp395 | 3102.586 | 3522.208 | 3312.397 |
| ENSMUSG00000055725 | protein_coding | Paqr3 | 221.3403 | 179.3307 | 200.3355 |
| ENSMUSG00000015944 | protein_coding | Gatsl2 | 988.2478 | 791.625 | 889.9364 |
| ENSMUSG00000029586 | protein_coding | 4933411G11Rik | 15.34626 | 5.0851 | 10.21568 |
| ENSMUSG00000062270 | protein_coding | Morf4l1 | 5085.029 | 5804.39 | 5444.709 |
| ENSMUSG00000031358 | protein_coding | Msl3 | 942.3837 | 1121.186 | 1031.785 |
| ENSMUSG00000073236 | lincRNA | 2500004C02Rik | 232.0346 | 289.6376 | 260.8361 |
| ENSMUSG00000004415 | protein_coding | Emid2 | 337.5106 | 270.4833 | 303.997 |
| ENSMUSG00000028836 | protein_coding | Slc30a2 | 2278.302 | 2611.167 | 2444.734 |
| ENSMUSG00000031785 | protein_coding | Gpr56 | 6169.542 | 7321.464 | 6745.503 |
| ENSMUSG00000023846 | protein_coding | Riok2 | 1516.522 | 1743.801 | 1630.161 |
| ENSMUSG00000044636 | protein_coding | Csrnp2 | 1191.536 | 1037.948 | 1114.742 |
| ENSMUSG00000027589 | protein_coding | Pcmtd2 | 2131.762 | 2508.874 | 2320.318 |
| ENSMUSG00000047658 | protein_coding | Gal3st3 | 192.0746 | 237.1163 | 214.5955 |
| ENSMUSG00000055900 | protein_coding | Tmem69 | 247.8408 | 296.9081 | 272.3745 |
| ENSMUSG00000022185 | protein_coding | Acin1 | 6183.925 | 6901.708 | 6542.817 |
| ENSMUSG00000048922 | protein_coding | Cdca2 | 1093.79 | 947.8325 | 1020.811 |
| ENSMUSG00000039661 | protein_coding | Dusp26 | 30.67926 | 17.51853 | 24.09889 |
| ENSMUSG00000030555 | protein_coding | Ttc23 | 714.5623 | 851.9438 | 783.253 |
| ENSMUSG00000090617 | lincRNA | Gm17395 | 4.749206 | 0.326514 | 2.53786 |
| ENSMUSG00000025732 | protein_coding | Fam195a | 467.6951 | 579.2426 | 523.4688 |
| ENSMUSG00000021451 | protein_coding | Sema4d | 1334.342 | 1085.597 | 1209.97 |
| ENSMUSG00000024269 | protein_coding | 5730494M16Rik | 1412.524 | 1159.997 | 1286.261 |
| ENSMUSG00000063229 | protein_coding | Ldha | 2027.561 | 2383.529 | 2205.545 |
| ENSMUSG00000042350 | protein_coding | 1110018G07Rik | 1999.623 | 1756.895 | 1878.259 |
| ENSMUSG00000013766 | protein_coding | Ly6g6e | 21.95329 | 10.7526 | 16.35294 |
| ENSMUSG00000032725 | protein_coding | Folr2 | 153.8336 | 193.4276 | 173.6306 |
| ENSMUSG00000028264 | protein_coding | Spaca1 | 60.63357 | 38.33968 | 49.48662 |
| ENSMUSG00000031897 | protein_coding | Psmb10 | 470.5918 | 395.4851 | 433.0385 |
| ENSMUSG00000085012 | processed_transcrip | Gm15492 | 25.53705 | 13.01511 | 19.27608 |
| ENSMUSG00000043633 | protein_coding | 4930412F15Rik | 34.00443 | 19.25813 | 26.63128 |
| ENSMUSG00000041225 | protein_coding | Arhgap12 | 2605.939 | 2916.474 | 2761.206 |
| ENSMUSG00000008129 | protein_coding | 4930432K21Rik | 492.07 | 353.4486 | 422.7593 |
| ENSMUSG00000066224 | protein_coding | Arid3c | 27.39845 | 46.86007 | 37.12926 |
| ENSMUSG00000005583 | protein_coding | Mef2c | 1279.862 | 1523.274 | 1401.568 |
| ENSMUSG00000031985 | protein_coding | Gnpat | 2226.375 | 1980.609 | 2103.492 |
| ENSMUSG00000081929 | pseudogene | Gm11741 | 15.91773 | 6.727042 | 11.32238 |
| ENSMUSG00000000706 | protein_coding | Btn1a1 | 9.352526 | 19.71476 | 14.53364 |
| ENSMUSG00000003573 | protein_coding | Homer3 | 363.0776 | 428.9643 | 396.0209 |
| ENSMUSG00000091829 | lincRNA | 9330159M07Rik | 81.58117 | 59.08447 | 70.33282 |
| ENSMUSG00000049100 | protein_coding | Pcdh10 | 20.33327 | 10.08192 | 15.20759 |

| | | | | | |
|---|---|---|---|---|---|
| ENSMUSG00000091687 | protein_coding | Gm4983 | 20.16949 | 8.713155 | 14.44132 |
| ENSMUSG00000015943 | protein_coding | Bola1 | 243.0819 | 302.5357 | 272.8088 |
| ENSMUSG00000027188 | protein_coding | Pamr1 | 19.40563 | 32.64194 | 26.02379 |
| ENSMUSG00000033382 | protein_coding | Trappc8 | 2947.859 | 2542.884 | 2745.371 |
| ENSMUSG00000063253 | protein_coding | Scoc | 552.1728 | 657.4626 | 604.8177 |
| ENSMUSG00000033819 | protein_coding | Ppp1r16a | 1368.7 | 1196.82 | 1282.76 |
| ENSMUSG00000090739 | lincRNA | D930016D06Rik | 845.9049 | 1013.7 | 929.8025 |
| ENSMUSG00000028134 | protein_coding | Ptbp2 | 2357.581 | 1964.844 | 2161.213 |
| ENSMUSG00000035776 | protein_coding | Cd99l2 | 2444.482 | 2915.968 | 2680.225 |
| ENSMUSG00000035891 | protein_coding | Cerk | 5950.325 | 6748.506 | 6349.416 |
| ENSMUSG00000087383 | processed_transcrip | Gm12446 | 7.150756 | 1.65059 | 4.400673 |
| ENSMUSG00000041696 | protein_coding | Rasl12 | 3857.296 | 4508.84 | 4183.068 |
| ENSMUSG00000041722 | protein_coding | Khdc1c | 5.476917 | 0.988552 | 3.232735 |
| ENSMUSG00000029403 | protein_coding | Cdkl2 | 694.4488 | 498.4635 | 596.4561 |
| ENSMUSG00000084140 | pseudogene | Gm14000 | 9.05755 | 2.808509 | 5.93303 |
| ENSMUSG00000048668 | protein_coding | 5930416I19Rik | 878.5007 | 755.8052 | 817.153 |
| ENSMUSG00000040751 | protein_coding | Lat2 | 21.93666 | 35.57477 | 28.75572 |
| ENSMUSG00000039496 | protein_coding | Cdnf | 146.8629 | 110.7257 | 128.7943 |
| ENSMUSG00000058669 | protein_coding | Nkx2-9 | 1.643351 | 9.318912 | 5.481131 |
| ENSMUSG00000027456 | protein_coding | Sdcbp2 | 50.1282 | 71.13841 | 60.63331 |
| ENSMUSG00000024960 | protein_coding | Plcb3 | 1649.026 | 1426.332 | 1537.679 |
| ENSMUSG00000033386 | protein_coding | Frrs1 | 56.36242 | 78.60749 | 67.48496 |
| ENSMUSG00000024477 | protein_coding | Pggt1b | 1054.358 | 1211.606 | 1132.982 |
| ENSMUSG00000019843 | protein_coding | Fyn | 4644.44 | 5158.983 | 4901.712 |
| ENSMUSG00000086013 | lincRNA | Gm15706 | 61.45736 | 36.47789 | 48.96763 |
| ENSMUSG00000024647 | protein_coding | Cbln2 | 108.1586 | 73.25143 | 90.70501 |
| ENSMUSG00000033722 | protein_coding | BC034090 | 1690.093 | 2249.65 | 1969.872 |
| ENSMUSG00000032254 | protein_coding | Kif23 | 2892.045 | 3418.853 | 3155.449 |
| ENSMUSG00000020447 | protein_coding | Npc1l1 | 25.26563 | 42.3043 | 33.78497 |
| ENSMUSG00000014837 | protein_coding | 4931428F04Rik | 410.5413 | 338.0017 | 374.2715 |
| ENSMUSG00000031790 | protein_coding | Mmp15 | 186.3123 | 243.2591 | 214.7857 |
| ENSMUSG00000024300 | protein_coding | Myo1f | 95.97877 | 124.3528 | 110.1658 |
| ENSMUSG00000086627 | lincRNA | Gm16702 | 20.4676 | 42.35918 | 31.41339 |
| ENSMUSG00000061603 | protein_coding | Akap6 | 110.1114 | 178.5793 | 144.3453 |
| ENSMUSG00000003378 | protein_coding | Grik5 | 660.7379 | 505.6936 | 583.2158 |
| ENSMUSG00000022969 | protein_coding | Il10rb | 608.4359 | 732.6046 | 670.5203 |
| ENSMUSG00000002835 | protein_coding | Chaf1a | 1358.515 | 1178.963 | 1268.739 |
| ENSMUSG00000040502 | protein_coding | 9-Mar | 198.7952 | 160.6869 | 179.7411 |
| ENSMUSG00000064140 | protein_coding | Trim38 | 13.24476 | 24.48272 | 18.86374 |
| ENSMUSG00000020739 | protein_coding | Nup85 | 2376.225 | 2075.778 | 2226.001 |
| ENSMUSG00000069855 | protein_coding | Slc47a2 | 2356.796 | 2729.573 | 2543.185 |
| ENSMUSG00000059555 | protein_coding | A830007P12Rik | 237.2012 | 330.2957 | 283.7484 |
| ENSMUSG00000048644 | protein_coding | Ctxn1 | 406.5688 | 489.8829 | 448.2258 |
| ENSMUSG00000050553 | protein_coding | Gk2 | 6.522936 | 1.333085 | 3.92801 |
| ENSMUSG00000003873 | protein_coding | Bax | 1532.835 | 1352.078 | 1442.457 |
| ENSMUSG00000028559 | protein_coding | Osbpl9 | 4198.996 | 4714.977 | 4456.987 |
| ENSMUSG00000033943 | protein_coding | Mga | 7429.987 | 8434.673 | 7932.33 |
| ENSMUSG00000087353 | processed_transcrip | Gm13727 | 0 | 18.31607 | 9.158033 |
| ENSMUSG00000031333 | protein_coding | Abcb7 | 929.8209 | 811.9524 | 870.8866 |
| ENSMUSG00000025464 | protein_coding | Paox | 224.7856 | 175.3455 | 200.0655 |
| ENSMUSG00000042155 | protein_coding | Klhl23 | 9156.664 | 11074.62 | 10115.64 |
| ENSMUSG00000063358 | protein_coding | Mapk1 | 5984.102 | 5435.864 | 5709.983 |
| ENSMUSG00000009487 | protein_coding | Otog | 2.549747 | 9.467411 | 6.008579 |
| ENSMUSG00000031976 | protein_coding | Urb2 | 1545.906 | 1230.866 | 1388.386 |
| ENSMUSG00000043411 | protein_coding | Usp48 | 4436.794 | 5031.164 | 4733.979 |
| ENSMUSG00000032194 | protein_coding | Kank2 | 2703.64 | 3153.369 | 2928.505 |
| ENSMUSG00000084970 | processed_transcrip | 1700060J05Rik | 8.071242 | 16.75847 | 12.41486 |
| ENSMUSG00000070939 | protein_coding | Tgfbrap1 | 1349.877 | 1171.048 | 1260.462 |

| | | | | | |
|---|---|---|---|---|---|
| ENSMUSG00000047534 | protein_coding | Mis18bp1 | 2003.049 | 2294.116 | 2148.583 |
| ENSMUSG00000006576 | protein_coding | Slc4a3 | 984.8939 | 1187.042 | 1085.968 |
| ENSMUSG00000034854 | protein_coding | F630110N24Rik | 381.7114 | 319.8404 | 350.7759 |
| ENSMUSG00000020810 | protein_coding | Cygb | 820.8083 | 967.9377 | 894.373 |
| ENSMUSG00000037754 | protein_coding | Ppp1r16b | 1530.733 | 1927.84 | 1729.286 |
| ENSMUSG00000073492 | protein_coding | Gm10521 | 3.582944 | 0.326514 | 1.954729 |
| ENSMUSG00000079186 | protein_coding | Gzmc | 55.0499 | 26.84717 | 40.94854 |
| ENSMUSG00000028995 | protein_coding | Fam126a | 4004.449 | 4729.535 | 4366.992 |
| ENSMUSG00000052848 | protein_coding | C130026L21Rik | 2.245304 | 7.522772 | 4.884038 |
| ENSMUSG00000037845 | protein_coding | Fdxacb1 | 490.9127 | 415.3874 | 453.15 |
| ENSMUSG00000000355 | protein_coding | Mcts1 | 382.811 | 464.9147 | 423.8628 |
| ENSMUSG00000089942 | protein_coding | Pira2 | 0.316221 | 3.532888 | 1.924555 |
| ENSMUSG00000028455 | protein_coding | Stoml2 | 1546.039 | 1738.204 | 1642.121 |
| ENSMUSG00000021508 | protein_coding | Cxcl14 | 39.24332 | 63.56345 | 51.40339 |
| ENSMUSG00000049556 | protein_coding | Lingo1 | 98.84287 | 71.26993 | 85.0564 |
| ENSMUSG00000084968 | lincRNA | Gm12743 | 54.79123 | 81.20692 | 67.99908 |
| ENSMUSG00000093304 | miRNA | AC132310.1 | 0 | 3.09971 | 1.549855 |
| ENSMUSG00000031521 | protein_coding | Aga | 675.1215 | 784.2777 | 729.6996 |
| ENSMUSG00000035473 | protein_coding | Galm | 144.8498 | 113.1552 | 129.0025 |
| ENSMUSG00000031634 | protein_coding | Ufsp2 | 884.0776 | 768.1856 | 826.1316 |
| ENSMUSG00000053398 | protein_coding | Phgdh | 5042.746 | 5603.515 | 5323.13 |
| ENSMUSG00000040351 | protein_coding | Ankib1 | 3903.09 | 3490.05 | 3696.57 |
| ENSMUSG00000000384 | protein_coding | Tbrg4 | 1400.995 | 1588.075 | 1494.535 |
| ENSMUSG00000028464 | protein_coding | Tpm2 | 1283.386 | 1137.568 | 1210.477 |
| ENSMUSG00000039041 | protein_coding | Adrm1 | 3754.121 | 4194.998 | 3974.56 |
| ENSMUSG00000020571 | protein_coding | Pdia6 | 7205.253 | 6415.357 | 6810.305 |
| ENSMUSG00000081219 | pseudogene | Bambi-ps1 | 13.61734 | 5.765518 | 9.691427 |
| ENSMUSG00000022871 | protein_coding | Fetub | 46.8907 | 68.04047 | 57.46558 |
| ENSMUSG00000034165 | protein_coding | Ccnd3 | 1732.82 | 2021.21 | 1877.015 |
| ENSMUSG00000026611 | protein_coding | Spata17 | 43.86222 | 25.09572 | 34.47897 |
| ENSMUSG00000023345 | protein_coding | Poc1a | 402.6943 | 327.5303 | 365.1123 |
| ENSMUSG00000040569 | protein_coding | Slc26a7 | 5772.461 | 7199.541 | 6486.001 |
| ENSMUSG00000028488 | protein_coding | Sh3gl2 | 2628.175 | 2995.853 | 2812.014 |
| ENSMUSG00000028519 | protein_coding | Dab1 | 223.2017 | 276.2858 | 249.7438 |
| ENSMUSG00000034595 | protein_coding | 2310014H01Rik | 1521.372 | 1738.214 | 1629.793 |
| ENSMUSG00000004270 | protein_coding | Lpcat3 | 1243.019 | 1428.23 | 1335.624 |
| ENSMUSG00000071266 | protein_coding | Zfp946 | 607.5526 | 729.3772 | 668.4649 |
| ENSMUSG00000090213 | protein_coding | Tmem189 | 378.0399 | 442.9574 | 410.4986 |
| ENSMUSG00000030493 | protein_coding | C230052I12Rik | 233.1497 | 186.1784 | 209.6641 |
| ENSMUSG00000022477 | protein_coding | Aco2 | 4454.452 | 4021.638 | 4238.045 |
| ENSMUSG00000028197 | protein_coding | Col24a1 | 69.41523 | 94.38135 | 81.89829 |
| ENSMUSG00000026848 | protein_coding | Tor1b | 1267.348 | 1460.983 | 1364.166 |
| ENSMUSG00000058594 | protein_coding | Fbxo18 | 2789.604 | 2473.64 | 2631.622 |
| ENSMUSG00000034075 | protein_coding | Zdhhc5 | 2766.121 | 3115.851 | 2940.986 |
| ENSMUSG00000021669 | protein_coding | Col4a3bp | 3684.45 | 3336.002 | 3510.226 |
| ENSMUSG00000027677 | protein_coding | Ttc14 | 3651.024 | 4397.518 | 4024.271 |
| ENSMUSG00000020444 | protein_coding | Guk1 | 1028.902 | 912.6826 | 970.7922 |
| ENSMUSG00000018008 | protein_coding | Cyth4 | 136.808 | 172.6825 | 154.7453 |
| ENSMUSG00000024410 | protein_coding | 3110002H16Rik | 910.7657 | 794.8923 | 852.829 |
| ENSMUSG00000025815 | protein_coding | Dhtkd1 | 464.7175 | 386.347 | 425.5322 |
| ENSMUSG00000021260 | protein_coding | Hhipl1 | 13.89256 | 5.8902 | 9.891379 |
| ENSMUSG00000024511 | protein_coding | Rab27b | 83.04136 | 115.9 | 99.47066 |
| ENSMUSG00000064068 | protein_coding | Mtx1 | 1264.046 | 1125.818 | 1194.932 |
| ENSMUSG00000056987 | protein_coding | Fam71d | 2.267593 | 7.576104 | 4.921848 |
| ENSMUSG00000039904 | protein_coding | Gpr37 | 2566.559 | 2938.58 | 2752.569 |
| ENSMUSG00000012211 | protein_coding | Tex22 | 6.46069 | 1.712931 | 4.086811 |
| ENSMUSG00000018238 | protein_coding | Gdf9 | 12.62156 | 25.08562 | 18.85359 |
| ENSMUSG00000087392 | lincRNA | Gm16994 | 7.088511 | 15.53278 | 11.31064 |

| | | | | | |
|---|---|---|---|---|---|
| ENSMUSG00000029059 | protein_coding | 2810405K02Rik | 201.2079 | 246.4362 | 223.822 |
| ENSMUSG00000068748 | protein_coding | Ptprz1 | 50.22071 | 78.96284 | 64.59178 |
| ENSMUSG00000081766 | pseudogene | 2210409E12Rik | 2.577925 | 0 | 1.288962 |
| ENSMUSG00000002329 | protein_coding | Mdp1 | 717.2825 | 854.3225 | 785.8025 |
| ENSMUSG00000037493 | protein_coding | Cib2 | 131.2489 | 98.77567 | 115.0123 |
| ENSMUSG00000083538 | pseudogene | Gm16016 | 0.976841 | 5.067804 | 3.022323 |
| ENSMUSG00000055602 | protein_coding | Tcp10b | 1.304841 | 5.872182 | 3.588511 |
| ENSMUSG00000040254 | protein_coding | Sema3d | 152.6254 | 305.2137 | 228.9195 |
| ENSMUSG00000035900 | protein_coding | Gramd4 | 560.6944 | 654.9745 | 607.8345 |
| ENSMUSG00000041625 | protein_coding | A2ld1 | 245.1319 | 293.662 | 269.397 |
| ENSMUSG00000020033 | processed_transcrip | 4930463O16Rik | 5.915093 | 1.024227 | 3.46966 |
| ENSMUSG00000021509 | protein_coding | Slc25a48 | 17.0043 | 31.63646 | 24.32038 |
| ENSMUSG00000024082 | protein_coding | 2410091C18Rik | 834.7087 | 729.1593 | 781.934 |
| ENSMUSG00000071332 | lincRNA | E230015B07Rik | 16.22702 | 7.258599 | 11.74281 |
| ENSMUSG00000046341 | pseudogene | Gm11223 | 97.02397 | 70.76541 | 83.89469 |
| ENSMUSG00000061244 | protein_coding | Exoc5 | 2684.692 | 2349.08 | 2516.886 |
| ENSMUSG00000001763 | protein_coding | Tspan33 | 461.3214 | 366.2578 | 413.7896 |
| ENSMUSG00000039452 | protein_coding | Snx22 | 74.11232 | 102.7532 | 88.43275 |
| ENSMUSG00000025104 | protein_coding | Hdgfrp3 | 3307.842 | 2971.819 | 3139.83 |
| ENSMUSG00000085180 | processed_transcrip | AI838599 | 93.83 | 64.23765 | 79.03383 |
| ENSMUSG00000085349 | lincRNA | Gm16956 | 9.370193 | 18.46312 | 13.91666 |
| ENSMUSG00000009863 | protein_coding | Sdhb | 2251.582 | 1998.584 | 2125.083 |
| ENSMUSG00000028661 | protein_coding | Epha8 | 688.9618 | 823.9044 | 756.4331 |
| ENSMUSG00000069835 | protein_coding | Sat2 | 590.1259 | 716.0937 | 653.1098 |
| ENSMUSG00000026074 | protein_coding | Map4k4 | 7722.868 | 8783.111 | 8252.989 |
| ENSMUSG00000022889 | protein_coding | Mrpl39 | 1249.789 | 1422.237 | 1336.013 |
| ENSMUSG00000073705 | protein_coding | Apitd1 | 519.9265 | 441.1319 | 480.5292 |
| ENSMUSG00000044080 | protein_coding | S100a1 | 111.2696 | 151.0985 | 131.184 |
| ENSMUSG00000091033 | lincRNA | 4930477E14Rik | 2.273482 | 7.549438 | 4.91146 |
| ENSMUSG00000013928 | protein_coding | Thap8 | 11.71285 | 4.459099 | 8.085976 |
| ENSMUSG00000033676 | protein_coding | Gabrb3 | 60.51534 | 39.78523 | 50.15028 |
| ENSMUSG00000021065 | protein_coding | Fut8 | 2511.575 | 2166.688 | 2339.132 |
| ENSMUSG00000084931 | lincRNA | 1110019D14Rik | 137.0877 | 175.3656 | 156.2266 |
| ENSMUSG00000030094 | protein_coding | Xpc | 1332.584 | 1168.727 | 1250.656 |
| ENSMUSG00000033831 | protein_coding | Fgb | 5.881026 | 0.997561 | 3.439294 |
| ENSMUSG00000018604 | protein_coding | Tbx3 | 218.8005 | 268.5924 | 243.6964 |
| ENSMUSG00000031465 | protein_coding | Angpt2 | 229.1369 | 181.5693 | 205.3531 |
| ENSMUSG00000033478 | protein_coding | Fam160b1 | 1879.15 | 2141.002 | 2010.076 |
| ENSMUSG00000078197 | protein_coding | Gm17374 | 3.239813 | 9.755039 | 6.497426 |
| ENSMUSG00000084279 | pseudogene | Gm13232 | 2.606103 | 0 | 1.303051 |
| ENSMUSG00000023795 | processed_transcrip | Pisd-ps2 | 365.2392 | 306.2743 | 335.7568 |
| ENSMUSG00000073147 | protein_coding | 5031425E22Rik | 405.1936 | 487.3583 | 446.276 |
| ENSMUSG00000054737 | protein_coding | Zfp182 | 399.7937 | 498.7388 | 449.2662 |
| ENSMUSG00000031451 | protein_coding | Gas6 | 5647.823 | 6246.832 | 5947.328 |
| ENSMUSG00000020733 | protein_coding | Slc9a3r1 | 562.3396 | 656.6955 | 609.5175 |
| ENSMUSG00000042035 | protein_coding | Igsf3 | 1111.901 | 954.4495 | 1033.175 |
| ENSMUSG00000001472 | protein_coding | Tcf25 | 5816.151 | 5295.765 | 5555.958 |
| ENSMUSG00000037353 | protein_coding | Letmd1 | 744.833 | 854.1443 | 799.4887 |
| ENSMUSG00000087177 | lincRNA | E130307A14Rik | 678.1286 | 564.4629 | 621.2958 |
| ENSMUSG00000033099 | protein_coding | Nol12 | 1346.011 | 1190.452 | 1268.232 |
| ENSMUSG00000030785 | protein_coding | Cox6a2 | 9.276192 | 20.35158 | 14.81388 |
| ENSMUSG00000083325 | pseudogene | Gm14121 | 109.405 | 83.45436 | 96.42966 |
| ENSMUSG00000021879 | protein_coding | Dnahc12 | 91.85238 | 50.84871 | 71.35055 |
| ENSMUSG00000029869 | protein_coding | Ephb6 | 727.7146 | 852.7425 | 790.2286 |
| ENSMUSG00000042372 | protein_coding | Dmrt3 | 138.5741 | 194.3655 | 166.4698 |
| ENSMUSG00000025582 | protein_coding | Nptx1 | 128.071 | 191.7473 | 159.9091 |
| ENSMUSG00000002064 | protein_coding | Sdf2 | 1246.198 | 1420.473 | 1333.336 |
| ENSMUSG00000034219 | protein_coding | 14-Sep | 795.8003 | 626.7714 | 711.2859 |

| | | | | | |
|---|---|---|---|---|---|
| ENSMUSG00000022358 | protein_coding | Fbxo32 | 829.2695 | 723.2087 | 776.2391 |
| ENSMUSG00000043501 | protein_coding | Lgals2 | 31.9393 | 48.62483 | 40.28207 |
| ENSMUSG00000022641 | protein_coding | Bbx | 5123.607 | 4553.339 | 4838.473 |
| ENSMUSG00000064194 | protein_coding | Zfp936 | 157.7046 | 198.9617 | 178.3331 |
| ENSMUSG00000029095 | protein_coding | Ablim2 | 112.0701 | 82.85394 | 97.46203 |
| ENSMUSG00000032988 | protein_coding | Slc16a8 | 40.49941 | 61.27739 | 50.8884 |
| ENSMUSG00000003161 | protein_coding | Sri | 1180.232 | 1387.975 | 1284.103 |
| ENSMUSG00000089235 | snoRNA | SCARNA17 | 0.65242 | 4.969788 | 2.811104 |
| ENSMUSG00000046722 | protein_coding | Cdc42se1 | 5836.948 | 6609.587 | 6223.267 |
| ENSMUSG00000007080 | protein_coding | Pole | 4093.134 | 3283.994 | 3688.564 |
| ENSMUSG00000005148 | protein_coding | Klf5 | 82.43814 | 59.49062 | 70.96438 |
| ENSMUSG00000038700 | protein_coding | Hoxb5 | 7.703286 | 17.48394 | 12.59361 |
| ENSMUSG00000033653 | protein_coding | Vps8 | 1567.94 | 1340.647 | 1454.294 |
| ENSMUSG00000020734 | protein_coding | Grin2c | 238.274 | 324.9534 | 281.6137 |
| ENSMUSG00000086252 | lincRNA | C030044B11Rik | 299.6166 | 364.8315 | 332.224 |
| ENSMUSG00000030166 | protein_coding | Rad52 | 295.0097 | 368.0532 | 331.5314 |
| ENSMUSG00000088171 | snRNA | U6 | 3.228034 | 0.326514 | 1.777274 |
| ENSMUSG00000042502 | protein_coding | Cd2bp2 | 2376.287 | 2633.315 | 2504.801 |
| ENSMUSG00000023845 | protein_coding | Lnpep | 1385.227 | 1196.195 | 1290.711 |
| ENSMUSG00000026019 | protein_coding | Wdr12 | 1127.395 | 996.9802 | 1062.188 |
| ENSMUSG00000020029 | protein_coding | Nudt4 | 7641.531 | 8542.875 | 8092.203 |
| ENSMUSG00000034953 | protein_coding | 1700047I17Rik2 | 27.77669 | 16.37863 | 22.07766 |
| ENSMUSG00000053286 | protein_coding | 1190005F20Rik | 1208.812 | 1062.731 | 1135.772 |
| ENSMUSG00000027445 | protein_coding | Cst9 | 5515.336 | 6277.414 | 5896.375 |
| ENSMUSG00000031299 | protein_coding | Pdha1 | 1126.156 | 1286.288 | 1206.222 |
| ENSMUSG00000061838 | protein_coding | Suclg2 | 2117.514 | 2366.859 | 2242.186 |
| ENSMUSG00000090112 | protein_coding | Shprh | 2201.81 | 1982.877 | 2092.343 |
| ENSMUSG00000019826 | protein_coding | Zbtb24 | 646.7974 | 738.9685 | 692.883 |
| ENSMUSG00000045813 | pseudogene | Gm9801 | 5.456939 | 13.59751 | 9.527223 |
| ENSMUSG00000032131 | protein_coding | Abcg4 | 70.08531 | 93.79288 | 81.9391 |
| ENSMUSG00000058979 | protein_coding | Cecr5 | 2688.11 | 3041.274 | 2864.692 |
| ENSMUSG00000020235 | protein_coding | Fzr1 | 1652.381 | 1853.471 | 1752.926 |
| ENSMUSG00000018697 | protein_coding | Aatf | 1044.423 | 898.3984 | 971.4106 |
| ENSMUSG00000026027 | protein_coding | Stradb | 1119.197 | 958.2792 | 1038.738 |
| ENSMUSG00000010021 | protein_coding | Kif19a | 59.35146 | 88.05507 | 73.70327 |
| ENSMUSG00000088439 | misc_RNA | 7SK | 5.171206 | 1.050893 | 3.11105 |
| ENSMUSG00000001036 | protein_coding | Epn2 | 2485.215 | 2138.866 | 2312.041 |
| ENSMUSG00000026339 | protein_coding | Ccdc93 | 1048.6 | 928.7783 | 988.689 |
| ENSMUSG00000009995 | protein_coding | Taz | 856.536 | 1008.46 | 932.4978 |
| ENSMUSG00000018841 | protein_coding | Rad51l3 | 533.8221 | 443.4695 | 488.6458 |
| ENSMUSG00000019122 | protein_coding | Ccl9 | 3.552455 | 9.491228 | 6.521842 |
| ENSMUSG00000020930 | protein_coding | Ccdc103 | 68.76661 | 49.37339 | 59.07 |
| ENSMUSG00000043279 | protein_coding | Trim56 | 499.7524 | 596.8269 | 548.2897 |
| ENSMUSG00000022536 | protein_coding | Glyr1 | 4745.588 | 5353.231 | 5049.41 |
| ENSMUSG00000038884 | protein_coding | A230050P20Rik | 213.7942 | 262.2451 | 238.0196 |
| ENSMUSG00000028194 | protein_coding | Ddah1 | 2732.418 | 3066.663 | 2899.541 |
| ENSMUSG00000035919 | protein_coding | Bbs9 | 1140.397 | 961.6292 | 1051.013 |
| ENSMUSG00000035208 | protein_coding | Slfn8 | 26.45545 | 15.38107 | 20.91826 |
| ENSMUSG00000023118 | protein_coding | Sympk | 2810.478 | 2438.132 | 2624.305 |
| ENSMUSG00000022247 | protein_coding | Brix1 | 1955.883 | 2185.39 | 2070.636 |
| ENSMUSG00000071202 | protein_coding | Ccdc78 | 24.40844 | 12.2374 | 18.32292 |
| ENSMUSG00000004667 | protein_coding | Polr2e | 2508.218 | 2210.94 | 2359.579 |
| ENSMUSG00000073591 | protein_coding | Pcdhb22 | 47.3871 | 66.67279 | 57.02994 |
| ENSMUSG00000030745 | protein_coding | Il21r | 10.96308 | 3.654722 | 7.308899 |
| ENSMUSG00000022755 | protein_coding | Gpr128 | 36.34604 | 20.47481 | 28.41043 |
| ENSMUSG00000027198 | protein_coding | Ext2 | 2435.223 | 2187.259 | 2311.241 |
| ENSMUSG00000011382 | protein_coding | Dhdh | 351.8558 | 291.8862 | 321.871 |
| ENSMUSG00000009097 | protein_coding | Tbx1 | 394.4076 | 279.0602 | 336.7339 |

| | | | | | |
|---|---|---|---|---|---|
| ENSMUSG00000040600 | protein_coding | Eps8l3 | 2.289882 | 7.965321 | 5.127601 |
| ENSMUSG00000032497 | protein_coding | Lrrfip2 | 891.7221 | 1087.754 | 989.738 |
| ENSMUSG00000035873 | protein_coding | Pawr | 528.9905 | 448.2134 | 488.602 |
| ENSMUSG00000066090 | protein_coding | Insl5 | 1.606973 | 6.3735 | 3.990236 |
| ENSMUSG00000055555 | protein_coding | 4930502E18Rik | 347.9967 | 273.7277 | 310.8622 |
| ENSMUSG00000035704 | protein_coding | Alg8 | 525.5797 | 644.2675 | 584.9236 |
| ENSMUSG00000087651 | protein_coding | 1500009L16Rik | 1078.554 | 1237.97 | 1158.262 |
| ENSMUSG00000082651 | pseudogene | Gm14001 | 9.050394 | 3.054663 | 6.052529 |
| ENSMUSG00000023764 | protein_coding | Sfi1 | 1040.268 | 810.7392 | 925.5037 |
| ENSMUSG00000042682 | protein_coding | Selk | 2054.377 | 2295.135 | 2174.756 |
| ENSMUSG00000033405 | protein_coding | Nudt15 | 238.11 | 194.5312 | 216.3206 |
| ENSMUSG00000049658 | protein_coding | Bdp1 | 2420.757 | 2776.331 | 2598.544 |
| ENSMUSG00000026455 | protein_coding | Klhl12 | 2639.695 | 3014.839 | 2827.267 |
| ENSMUSG00000028041 | protein_coding | Adam15 | 823.2344 | 696.0292 | 759.6318 |
| ENSMUSG00000034359 | protein_coding | Skint2 | 2.869546 | 9.722575 | 6.296061 |
| ENSMUSG00000034677 | protein_coding | Gpr142 | 0.65242 | 4.061234 | 2.356827 |
| ENSMUSG00000028337 | protein_coding | Coro2a | 1102.945 | 961.6597 | 1032.302 |
| ENSMUSG00000031438 | protein_coding | Rnf128 | 495.3357 | 402.4934 | 448.9146 |
| ENSMUSG00000021987 | protein_coding | Mtmr6 | 2209.169 | 1931.082 | 2070.126 |
| ENSMUSG00000086918 | lincRNA | 4930429F24Rik | 37.70493 | 21.62445 | 29.66469 |
| ENSMUSG00000021130 | protein_coding | Galntl1 | 287.5499 | 238.3029 | 262.9264 |
| ENSMUSG00000051860 | protein_coding | Samd7 | 13.20376 | 24.49172 | 18.84774 |
| ENSMUSG00000049553 | protein_coding | Polr1a | 2839.241 | 3263.253 | 3051.247 |
| ENSMUSG00000003657 | protein_coding | Calb2 | 43.809 | 27.17519 | 35.49209 |
| ENSMUSG00000027525 | protein_coding | Phactr3 | 8.686016 | 17.85405 | 13.27003 |
| ENSMUSG00000015750 | protein_coding | Aph1a | 1461.946 | 1634.572 | 1548.259 |
| ENSMUSG00000019039 | protein_coding | Dalrd3 | 432.8403 | 517.1092 | 474.9747 |
| ENSMUSG00000034110 | protein_coding | Kctd7 | 250.166 | 201.8457 | 226.0059 |
| ENSMUSG00000031562 | protein_coding | Dctd | 636.7945 | 739.6021 | 688.1983 |
| ENSMUSG00000044847 | protein_coding | Lsm11 | 451.2087 | 379.5701 | 415.3894 |
| ENSMUSG00000029338 | protein_coding | Antxr2 | 587.3459 | 693.543 | 640.4444 |
| ENSMUSG00000081535 | pseudogene | Gm15453 | 70.40429 | 49.24943 | 59.82686 |
| ENSMUSG00000017485 | protein_coding | Top2b | 5086.811 | 4474.538 | 4780.674 |
| ENSMUSG00000022884 | protein_coding | Eif4a2 | 9003.599 | 10628.36 | 9815.981 |
| ENSMUSG00000038886 | protein_coding | Man2a2 | 4167.739 | 4699.003 | 4433.371 |
| ENSMUSG00000063632 | protein_coding | Sox11 | 293.3454 | 218.8072 | 256.0763 |
| ENSMUSG00000022613 | protein_coding | Miox | 16.40362 | 31.40511 | 23.90436 |
| ENSMUSG00000093107 | snoRNA | AC167122.1 | 9.353793 | 18.92048 | 14.13713 |
| ENSMUSG00000016942 | protein_coding | Tmprss6 | 26.79061 | 41.71149 | 34.25105 |
| ENSMUSG00000033781 | protein_coding | Asb13 | 152.961 | 190.7709 | 171.866 |
| ENSMUSG00000091563 | protein_coding | Gm8108 | 3.219835 | 0.36219 | 1.791012 |
| ENSMUSG00000085684 | processed_transcrip | 4930469K13Rik | 65.91182 | 42.44203 | 54.17692 |
| ENSMUSG00000007815 | protein_coding | Rhoa | 7648.086 | 8536.081 | 8092.084 |
| ENSMUSG00000019326 | protein_coding | Aoc3 | 1142.395 | 953.1501 | 1047.772 |
| ENSMUSG00000060843 | protein_coding | Ctnna3 | 131.5996 | 171.4331 | 151.5163 |
| ENSMUSG00000024571 | protein_coding | Gm16286 | 1819.129 | 2062.066 | 1940.598 |
| ENSMUSG00000051527 | protein_coding | Usp29 | 31.08695 | 18.24327 | 24.66511 |
| ENSMUSG00000000392 | protein_coding | Fap | 31.5703 | 50.61964 | 41.09497 |
| ENSMUSG00000025482 | protein_coding | Odf3 | 2.908235 | 9.695547 | 6.301891 |
| ENSMUSG00000071636 | protein_coding | Rimbp3 | 48.84587 | 29.22908 | 39.03748 |
| ENSMUSG00000024186 | protein_coding | Rgs11 | 532.4895 | 906.6174 | 719.5534 |
| ENSMUSG00000038893 | protein_coding | Fam117a | 1060.156 | 901.0122 | 980.5843 |
| ENSMUSG00000051864 | protein_coding | Tbc1d22a | 1152.653 | 987.754 | 1070.204 |
| ENSMUSG00000033124 | protein_coding | Atg9a | 721.0076 | 612.7622 | 666.8849 |
| ENSMUSG00000070436 | protein_coding | Serpinh1 | 4858.545 | 5894.107 | 5376.326 |
| ENSMUSG00000020986 | protein_coding | Sec23a | 2931.972 | 2653.335 | 2792.653 |
| ENSMUSG00000059327 | protein_coding | Eda | 190.0455 | 148.6431 | 169.3443 |
| ENSMUSG00000059439 | protein_coding | Bcas3 | 1451.993 | 1224.596 | 1338.295 |

| | | | | | |
|---|---|---|---|---|---|
| ENSMUSG00000044359 | protein_coding | P2ry4 | 4.851407 | 12.05286 | 8.452133 |
| ENSMUSG00000025085 | protein_coding | Ablim1 | 627.6404 | 728.3993 | 678.0199 |
| ENSMUSG00000061397 | protein_coding | Krt79 | 89.41341 | 50.55751 | 69.98546 |
| ENSMUSG00000047712 | protein_coding | Ust | 173.67 | 214.4164 | 194.0432 |
| ENSMUSG00000087434 | processed_transcrip | Gm11202 | 0 | 2.419653 | 1.209827 |
| ENSMUSG00000080461 | snoRNA | SNORA11 | 0 | 2.419653 | 1.209827 |
| ENSMUSG00000086380 | processed_transcrip | Gm11195 | 1.601084 | 6.243381 | 3.922232 |
| ENSMUSG00000009563 | protein_coding | Tor2a | 951.9489 | 831.675 | 891.812 |
| ENSMUSG00000084791 | processed_transcrip | Gm13667 | 4.80914 | 11.86584 | 8.337488 |
| ENSMUSG00000022827 | protein_coding | Rabl3 | 684.8486 | 799.2797 | 742.0641 |
| ENSMUSG00000034612 | protein_coding | Chst11 | 454.2731 | 547.2234 | 500.7482 |
| ENSMUSG00000028107 | protein_coding | Tars2 | 1125.628 | 970.4472 | 1048.038 |
| ENSMUSG00000093352 | miRNA | AC159243.1 | 0 | 2.446319 | 1.22316 |
| ENSMUSG00000027087 | protein_coding | Itgav | 1891.266 | 2121.661 | 2006.464 |
| ENSMUSG00000019872 | protein_coding | Smpdl3a | 141.9935 | 192.1534 | 167.0734 |
| ENSMUSG00000055827 | protein_coding | Gsdmc3 | 0.316221 | 3.099348 | 1.707785 |
| ENSMUSG00000035621 | protein_coding | Midn | 2640.294 | 2994.907 | 2817.601 |
| ENSMUSG00000009630 | protein_coding | Ppp2cb | 2296.415 | 2572.424 | 2434.42 |
| ENSMUSG00000043154 | protein_coding | Ppp2r3a | 2224.399 | 1996.653 | 2110.526 |
| ENSMUSG00000061979 | protein_coding | Wbscr16 | 734.5229 | 637.7571 | 686.14 |
| ENSMUSG00000041359 | protein_coding | Tcl1 | 241.8741 | 323.8825 | 282.8783 |
| ENSMUSG00000026270 | protein_coding | Capn10 | 1468.911 | 1271.808 | 1370.36 |
| ENSMUSG00000007783 | protein_coding | Cpt1c | 345.2138 | 283.1374 | 314.1756 |
| ENSMUSG00000035798 | protein_coding | Zdhhc17 | 1040.162 | 1238.362 | 1139.262 |
| ENSMUSG00000022552 | protein_coding | Sharpin | 768.8312 | 657.368 | 713.0996 |
| ENSMUSG00000020402 | protein_coding | Vdac1 | 3603.973 | 4050.509 | 3827.241 |
| ENSMUSG00000006021 | protein_coding | Kptn | 358.7941 | 301.7925 | 330.2933 |
| ENSMUSG00000022818 | protein_coding | Cyp2ab1 | 4.176698 | 0.688704 | 2.432701 |
| ENSMUSG00000045211 | protein_coding | Nudt18 | 344.4354 | 290.6802 | 317.5578 |
| ENSMUSG00000071341 | protein_coding | Egr4 | 538.0262 | 676.0852 | 607.0557 |
| ENSMUSG00000085642 | processed_transcrip | 3110053B16Rik | 160.7978 | 116.926 | 138.8619 |
| ENSMUSG00000031963 | protein_coding | Bmper | 76.6421 | 106.195 | 91.41854 |
| ENSMUSG00000032383 | protein_coding | Ppib | 2020.446 | 2258.198 | 2139.322 |
| ENSMUSG00000041566 | protein_coding | Tssk1 | 31.0001 | 18.58744 | 24.79377 |
| ENSMUSG00000083282 | protein_coding | Ctsf | 2421.808 | 2100.795 | 2261.301 |
| ENSMUSG00000019979 | protein_coding | Apaf1 | 1015.534 | 890.4555 | 952.9948 |
| ENSMUSG00000091476 | protein_coding | Gm16432 | 2.259393 | 7.495744 | 4.877568 |
| ENSMUSG00000046844 | protein_coding | Vat1l | 71.96833 | 98.51756 | 85.24294 |
| ENSMUSG00000017679 | protein_coding | Ttpal | 1034.712 | 1209.84 | 1122.276 |
| ENSMUSG00000014074 | protein_coding | Rnf168 | 1193.24 | 1012.387 | 1102.813 |
| ENSMUSG00000024049 | protein_coding | Myom1 | 277.4023 | 229.8785 | 253.6404 |
| ENSMUSG00000050105 | protein_coding | Grrp1 | 63.00962 | 85.98969 | 74.49965 |
| ENSMUSG00000031403 | protein_coding | Dkc1 | 1776.558 | 2047.309 | 1911.933 |
| ENSMUSG00000031539 | protein_coding | Ap3m2 | 442.6262 | 376.4145 | 409.5204 |
| ENSMUSG00000068226 | protein_coding | Gm6723 | 28.39273 | 42.91016 | 35.65145 |
| ENSMUSG00000002279 | protein_coding | Lmf1 | 356.7787 | 417.1525 | 386.9656 |
| ENSMUSG00000004263 | protein_coding | Atn1 | 1461.117 | 1637.589 | 1549.353 |
| ENSMUSG00000043110 | protein_coding | Lrrn4 | 67.51596 | 44.20534 | 55.86065 |
| ENSMUSG00000024989 | protein_coding | Cep55 | 1499.861 | 1323.098 | 1411.48 |
| ENSMUSG00000026035 | protein_coding | Ppil3 | 392.7613 | 328.3789 | 360.5701 |
| ENSMUSG00000030329 | protein_coding | C530028O21Rik | 445.4206 | 563.0617 | 504.2412 |
| ENSMUSG00000086078 | processed_transcrip | Gm11657 | 2.620192 | 0 | 1.310096 |
| ENSMUSG00000028788 | protein_coding | Ptp4a2 | 8513.457 | 9363.055 | 8938.256 |
| ENSMUSG00000020388 | protein_coding | Pdlim4 | 1687.172 | 2191.426 | 1939.299 |
| ENSMUSG00000029591 | protein_coding | Ung | 1731.649 | 1516.332 | 1623.99 |
| ENSMUSG00000038352 | protein_coding | Arl5c | 29.30524 | 48.27998 | 38.79261 |
| ENSMUSG00000086109 | processed_transcrip | Gm13391 | 0.638331 | 4.034568 | 2.33645 |
| ENSMUSG00000022443 | protein_coding | Myh9 | 5342.366 | 6032.156 | 5687.261 |

| | | | | | |
|---|---|---|---|---|---|
| ENSMUSG00000026295 | protein_coding | Spp2 | 0.33031 | 3.135023 | 1.732667 |
| ENSMUSG00000041733 | protein_coding | Coq5 | 891.0011 | 770.092 | 830.5466 |
| ENSMUSG00000043549 | protein_coding | 4932442L08Rik | 1026.558 | 822.1437 | 924.3511 |
| ENSMUSG00000044703 | protein_coding | Phf11 | 0.316221 | 3.063673 | 1.689947 |
| ENSMUSG00000041390 | protein_coding | Mdfic | 355.5522 | 475.7521 | 415.6522 |
| ENSMUSG00000063015 | protein_coding | Ccni | 6381.685 | 5725.619 | 6053.652 |
| ENSMUSG00000045751 | protein_coding | Mms22l | 1447.673 | 1264.189 | 1355.931 |
| ENSMUSG00000048280 | protein_coding | Zfp738 | 606.2481 | 695.0317 | 650.6399 |
| ENSMUSG00000036777 | protein_coding | Anln | 2741.247 | 2456.978 | 2599.113 |
| ENSMUSG00000060621 | protein_coding | Nkpd1 | 14.56958 | 6.807402 | 10.68849 |
| ENSMUSG00000032352 | protein_coding | Lrrc1 | 724.5571 | 825.2413 | 774.8992 |
| ENSMUSG00000034796 | protein_coding | Cpne7 | 120.3549 | 156.4967 | 138.4258 |
| ENSMUSG00000091267 | lincRNA | Gm17322 | 67.41108 | 90.1493 | 78.78019 |
| ENSMUSG00000037408 | protein_coding | Cnnm4 | 802.9758 | 695.2728 | 749.1243 |
| ENSMUSG00000050891 | protein_coding | Tatdn1 | 376.1811 | 319.0952 | 347.6382 |
| ENSMUSG00000040524 | protein_coding | Zfp609 | 4327.922 | 3761.385 | 4044.654 |
| ENSMUSG00000003810 | protein_coding | Mast2 | 4958.474 | 4330.916 | 4644.695 |
| ENSMUSG00000035310 | protein_coding | Lin54 | 865.7079 | 757.6811 | 811.6945 |
| ENSMUSG00000078350 | lincRNA | 1190007F08Rik | 1418.505 | 1735.647 | 1577.076 |
| ENSMUSG00000079575 | protein_coding | Rbpsuh-rs3 | 49.03171 | 32.54284 | 40.78728 |
| ENSMUSG00000083449 | pseudogene | Gm13487 | 3.225724 | 0.36219 | 1.793957 |
| ENSMUSG00000043336 | protein_coding | Filip1l | 627.1836 | 791.8943 | 709.539 |
| ENSMUSG00000029420 | protein_coding | Rimbp2 | 36.40993 | 55.44957 | 45.92975 |
| ENSMUSG00000022602 | protein_coding | Arc | 406.3724 | 318.5972 | 362.4848 |
| ENSMUSG00000032719 | protein_coding | Gm106 | 62.48907 | 42.93719 | 52.71313 |
| ENSMUSG00000032400 | protein_coding | Zwilch | 1188.227 | 1384.125 | 1286.176 |
| ENSMUSG00000065775 | snRNA | U6 | 6.113981 | 13.53481 | 9.824393 |
| ENSMUSG00000074733 | processed_transcrip | 5830428H23Rik | 295.6083 | 372.6354 | 334.1219 |
| ENSMUSG00000030091 | protein_coding | Nup210 | 1798.302 | 1428.931 | 1613.616 |
| ENSMUSG00000034467 | protein_coding | Dynlrb2 | 6.845046 | 1.659599 | 4.252322 |
| ENSMUSG00000091696 | lincRNA | Gm17670 | 101.3394 | 135.9913 | 118.6653 |
| ENSMUSG00000036644 | protein_coding | Tbc1d9b | 2762.249 | 2469.26 | 2615.754 |
| ENSMUSG00000027347 | protein_coding | Rasgrp1 | 81.94241 | 54.83725 | 68.38983 |
| ENSMUSG00000038774 | protein_coding | Ascc3 | 2345.487 | 1995.159 | 2170.323 |
| ENSMUSG00000024085 | protein_coding | Man2a1 | 6218.619 | 7241.487 | 6730.053 |
| ENSMUSG00000027281 | protein_coding | 2210009G21Rik | 686.7846 | 779.097 | 732.9408 |
| ENSMUSG00000018672 | protein_coding | Copz2 | 363.7428 | 303.4936 | 333.6182 |
| ENSMUSG00000046727 | protein_coding | 0610010O12Rik | 359.941 | 431.9748 | 395.9579 |
| ENSMUSG00000087403 | processed_transcrip | 2900056M20Rik | 77.68812 | 103.5684 | 90.62827 |
| ENSMUSG00000030678 | protein_coding | Maz | 4366.24 | 4815.206 | 4590.723 |
| ENSMUSG00000030967 | protein_coding | Zranb1 | 3154.336 | 2781.968 | 2968.152 |
| ENSMUSG00000022180 | protein_coding | Slc7a8 | 1222.141 | 1053.79 | 1137.965 |
| ENSMUSG00000021086 | protein_coding | 4930403N07Rik | 10.31319 | 3.336855 | 6.825023 |
| ENSMUSG00000018593 | protein_coding | Sparc | 28743.84 | 31735.63 | 30239.74 |
| ENSMUSG00000056209 | protein_coding | Npm3 | 1368.106 | 1196.244 | 1282.175 |
| ENSMUSG00000046567 | protein_coding | 4930430F08Rik | 339.2982 | 277.9935 | 308.6458 |
| ENSMUSG00000024891 | protein_coding | Slc29a2 | 189.9403 | 236.8622 | 213.4012 |
| ENSMUSG00000032232 | protein_coding | Cgnl1 | 2808.446 | 3293.281 | 3050.864 |
| ENSMUSG00000050708 | protein_coding | Ftl1 | 8230.892 | 9172.988 | 8701.94 |
| ENSMUSG00000041845 | protein_coding | Rhod | 90.39398 | 115.7702 | 103.0821 |
| ENSMUSG00000019970 | protein_coding | Sgk1 | 554.9946 | 430.1791 | 492.5869 |
| ENSMUSG00000029512 | protein_coding | Ulk1 | 2156.725 | 1917.833 | 2037.279 |
| ENSMUSG00000030612 | protein_coding | Mrpl46 | 1020.36 | 909.0589 | 964.7093 |
| ENSMUSG00000042109 | protein_coding | Csdc2 | 1847.61 | 2129.713 | 1988.662 |
| ENSMUSG00000020949 | protein_coding | Fkbp3 | 2229.792 | 2002.591 | 2116.191 |
| ENSMUSG00000041939 | protein_coding | Mvk | 534.0515 | 448.125 | 491.0883 |
| ENSMUSG00000085448 | processed_transcrip | Gm13963 | 1.284863 | 5.412337 | 3.3486 |
| ENSMUSG00000027977 | protein_coding | Ndst3 | 15.51235 | 7.513762 | 11.51306 |

| | | | | | |
|---|---|---|---|---|---|
| ENSMUSG00000038194 | protein_coding | Lhb | 11.69645 | 23.63143 | 17.66394 |
| ENSMUSG00000079669 | lincRNA | Gm17396 | 15.79808 | 27.97816 | 21.88812 |
| ENSMUSG00000048626 | protein_coding | Klf17 | 8.693173 | 17.30484 | 12.99901 |
| ENSMUSG00000039103 | protein_coding | Nexn | 370.9064 | 445.4473 | 408.1768 |
| ENSMUSG00000031162 | protein_coding | Gata1 | 1407.951 | 1188.466 | 1298.208 |
| ENSMUSG00000050017 | protein_coding | Pitpnb | 2246.471 | 2487.624 | 2367.047 |
| ENSMUSG00000052812 | protein_coding | Atad2b | 997.7087 | 882.755 | 940.2319 |
| ENSMUSG00000075602 | protein_coding | Ly6a | 78.53234 | 115.0991 | 96.8157 |
| ENSMUSG00000033096 | protein_coding | 2310001A20Rik | 1022.38 | 906.4957 | 964.4379 |
| ENSMUSG00000021303 | protein_coding | Gng4 | 10.33421 | 19.73314 | 15.03368 |
| ENSMUSG00000040037 | protein_coding | Negr1 | 178.3185 | 139.6446 | 158.9815 |
| ENSMUSG00000062995 | protein_coding | Ica1 | 409.6123 | 473.8519 | 441.7321 |
| ENSMUSG00000047419 | protein_coding | Cmya5 | 47.18254 | 30.91892 | 39.05073 |
| ENSMUSG00000084897 | protein_coding | Gm14226 | 6160.062 | 9805.292 | 7982.677 |
| ENSMUSG00000051007 | protein_coding | Pddc1 | 677.7192 | 777.8872 | 727.8032 |
| ENSMUSG00000044068 | protein_coding | Zrsr1 | 630.5358 | 725.0249 | 677.7803 |
| ENSMUSG00000031004 | protein_coding | Mki67 | 9473.15 | 8604.967 | 9039.059 |
| ENSMUSG00000056215 | protein_coding | Lrguk | 75.03892 | 54.22098 | 64.62995 |
| ENSMUSG00000030407 | protein_coding | Qpctl | 321.3122 | 269.445 | 295.3786 |
| ENSMUSG00000038217 | protein_coding | Tlcd2 | 230.1961 | 185.0043 | 207.6002 |
| ENSMUSG00000092569 | processed_transcrip | RP24-263P17.1 | 32.96199 | 51.30441 | 42.1332 |
| ENSMUSG00000036879 | protein_coding | Phkb | 1178.004 | 1334.729 | 1256.366 |
| ENSMUSG00000091458 | lincRNA | Gm17444 | 8.087641 | 2.455329 | 5.271485 |
| ENSMUSG00000087274 | lincRNA | 1010001N08Rik | 109.5345 | 139.5681 | 124.5513 |
| ENSMUSG00000054252 | protein_coding | Fgfr3 | 465.8172 | 357.895 | 411.8561 |
| ENSMUSG00000062198 | protein_coding | 2700097O09Rik | 525.2648 | 606.6797 | 565.9722 |
| ENSMUSG00000045777 | protein_coding | 6330512M04Rik | 6.107048 | 14.54966 | 10.32835 |
| ENSMUSG00000033751 | protein_coding | Gadd45gip1 | 638.5034 | 753.1598 | 695.8316 |
| ENSMUSG00000063765 | protein_coding | Chadl | 80.80746 | 59.40197 | 70.10472 |
| ENSMUSG00000031703 | protein_coding | Itfg1 | 3110.599 | 2784.31 | 2947.455 |
| ENSMUSG00000020912 | protein_coding | Krt12 | 2.586125 | 8.485019 | 5.535572 |
| ENSMUSG00000092589 | processed_transcrip | Gm20466 | 0 | 2.472986 | 1.236493 |
| ENSMUSG00000066894 | protein_coding | Vsig10 | 2886.597 | 3175.503 | 3031.05 |
| ENSMUSG00000044518 | protein_coding | Foxe3 | 0.954552 | 4.776604 | 2.865578 |
| ENSMUSG00000031245 | protein_coding | Hmgn5 | 2390.432 | 2752.362 | 2571.397 |
| ENSMUSG00000069314 | protein_coding | Hist1h2bl | 1.290752 | 5.367291 | 3.329021 |
| ENSMUSG00000021495 | protein_coding | Fam193b | 1520.552 | 1259.114 | 1389.833 |
| ENSMUSG00000003623 | protein_coding | Crot | 1664.311 | 1445.547 | 1554.929 |
| ENSMUSG00000050973 | protein_coding | D330012F22Rik | 301.2121 | 247.3052 | 274.2587 |
| ENSMUSG00000038271 | protein_coding | Iffo1 | 1062.615 | 1217.59 | 1140.102 |
| ENSMUSG00000062580 | protein_coding | Timm17a | 1501.345 | 1686.789 | 1594.067 |
| ENSMUSG00000009350 | protein_coding | Mpo | 3.264412 | 0.335524 | 1.799968 |
| ENSMUSG00000086305 | lincRNA | Gm4262 | 22.3328 | 12.15704 | 17.24492 |
| ENSMUSG00000026047 | protein_coding | Kdelc1 | 1157.564 | 1342.854 | 1250.209 |
| ENSMUSG00000082342 | pseudogene | Gm5395 | 0.316221 | 3.117366 | 1.716794 |
| ENSMUSG00000032346 | protein_coding | Ooep | 10.0121 | 19.08011 | 14.54611 |
| ENSMUSG00000041609 | protein_coding | Ccdc64 | 832.8736 | 638.2014 | 735.5375 |
| ENSMUSG00000034438 | protein_coding | Gbp8 | 14.81893 | 5.996864 | 10.4079 |
| ENSMUSG00000041471 | protein_coding | Fam35a | 386.9103 | 327.7525 | 357.3314 |
| ENSMUSG00000086985 | lincRNA | Gm16973 | 279.9959 | 344.3539 | 312.1749 |
| ENSMUSG00000022455 | protein_coding | Wbp2nl | 133.3685 | 95.41418 | 114.3913 |
| ENSMUSG00000041078 | protein_coding | Grid1 | 51.63379 | 27.07966 | 39.35673 |
| ENSMUSG00000021749 | protein_coding | Oit1 | 13.24707 | 23.70609 | 18.47658 |
| ENSMUSG00000090207 | processed_transcrip | 4930524O07Rik | 27.19598 | 15.78794 | 21.49196 |
| ENSMUSG00000076392 | miRNA | AC116121.1 | 3.199857 | 0.36219 | 1.781023 |
| ENSMUSG00000030806 | protein_coding | Stx1b | 257.8153 | 312.7461 | 285.2807 |
| ENSMUSG00000003235 | protein_coding | Eif2b5 | 2252.952 | 1993.753 | 2123.353 |
| ENSMUSG00000085334 | processed_transcrip | Gm12940 | 1105.384 | 1391.324 | 1248.354 |

| | | | | | |
|---|---|---|---|---|---|
| ENSMUSG00000028354 | protein_coding | Fmn2 | 43.5668 | 28.18212 | 35.87446 |
| ENSMUSG00000016458 | protein_coding | Wt1 | 5471.214 | 6051.356 | 5761.285 |
| ENSMUSG00000025868 | protein_coding | Higd2a | 877.9965 | 1000.405 | 939.2008 |
| ENSMUSG00000044583 | protein_coding | Tlr7 | 40.63143 | 58.94606 | 49.78875 |
| ENSMUSG00000005883 | protein_coding | Spo11 | 243.9921 | 99.09111 | 171.5416 |
| ENSMUSG00000008590 | protein_coding | Htr3b | 21.76827 | 36.9875 | 29.37788 |
| ENSMUSG00000061576 | protein_coding | Dpp6 | 7.444689 | 15.7519 | 11.5983 |
| ENSMUSG00000051159 | protein_coding | Cited1 | 3348.857 | 3786.631 | 3567.744 |
| ENSMUSG00000015790 | protein_coding | Surf1 | 633.861 | 737.6091 | 685.735 |
| ENSMUSG00000027829 | protein_coding | Ccnl1 | 2325.06 | 2816.891 | 2570.976 |
| ENSMUSG00000030447 | protein_coding | Cyfip1 | 3636.446 | 4125.546 | 3880.996 |
| ENSMUSG00000055436 | protein_coding | Srsf11 | 5356.698 | 6213.386 | 5785.042 |
| ENSMUSG00000028640 | protein_coding | Tcfap2c | 78.87167 | 51.45892 | 65.1653 |
| ENSMUSG00000027584 | protein_coding | Oprl1 | 23.74677 | 39.87351 | 31.81014 |
| ENSMUSG00000002496 | protein_coding | Tsc2 | 2356.445 | 2116.672 | 2236.558 |
| ENSMUSG00000029713 | protein_coding | Gnb2 | 4492.67 | 4963.408 | 4728.039 |
| ENSMUSG00000030451 | protein_coding | Herc2 | 10223.46 | 8993.988 | 9608.726 |
| ENSMUSG00000029283 | protein_coding | Cdc7 | 1830.749 | 1618.358 | 1724.554 |
| ENSMUSG00000020085 | protein_coding | Aifm2 | 596.735 | 678.5351 | 637.6351 |
| ENSMUSG00000029471 | protein_coding | Camkk2 | 3146.574 | 3643.795 | 3395.185 |
| ENSMUSG00000027871 | protein_coding | Hsd3b1 | 4723.86 | 5568.897 | 5146.379 |
| ENSMUSG00000033080 | protein_coding | Vsx1 | 21.90029 | 34.71634 | 28.30831 |
| ENSMUSG00000027615 | protein_coding | Hps3 | 1861.158 | 2117.762 | 1989.46 |
| ENSMUSG00000040097 | protein_coding | Flywch1 | 839.5555 | 737.4482 | 788.5019 |
| ENSMUSG00000045464 | lincRNA | 2810002D19Rik | 192.5491 | 148.3412 | 170.4451 |
| ENSMUSG00000056515 | protein_coding | Rab31 | 9903.651 | 11154.56 | 10529.1 |
| ENSMUSG00000002033 | protein_coding | Cd3g | 14.27796 | 6.534219 | 10.40609 |
| ENSMUSG00000027015 | protein_coding | Cybrd1 | 126.1847 | 163.3696 | 144.7771 |
| ENSMUSG00000085562 | processed_transcrip | 2610028E06Rik | 2.897724 | 9.031383 | 5.964554 |
| ENSMUSG00000021947 | protein_coding | Cryl1 | 909.2003 | 1025.363 | 967.2819 |
| ENSMUSG00000018761 | protein_coding | Mpdu1 | 1117.766 | 1266.505 | 1192.135 |
| ENSMUSG00000093247 | snRNA | AC120136.2 | 1.945483 | 6.507553 | 4.226518 |
| ENSMUSG00000033703 | protein_coding | Fuk | 682.1836 | 598.6946 | 640.4391 |
| ENSMUSG00000024910 | protein_coding | Ctsw | 20.10978 | 10.55113 | 15.33046 |
| ENSMUSG00000031605 | protein_coding | Klhl2 | 964.9419 | 822.5954 | 893.7687 |
| ENSMUSG00000081858 | pseudogene | Gm14514 | 90.84096 | 123.5465 | 107.1937 |
| ENSMUSG00000028862 | protein_coding | Map3k6 | 131.9491 | 102.2213 | 117.0852 |
| ENSMUSG00000006587 | protein_coding | Snai3 | 149.343 | 107.3858 | 128.3644 |
| ENSMUSG00000005672 | protein_coding | Kit | 3236.968 | 2737.515 | 2987.242 |
| ENSMUSG00000032487 | protein_coding | Ptgs2 | 17.78039 | 4.450451 | 11.11542 |
| ENSMUSG00000035382 | protein_coding | Pcsk7 | 880.3911 | 745.7685 | 813.0798 |
| ENSMUSG00000051439 | protein_coding | Cd14 | 60.51057 | 41.26786 | 50.88921 |
| ENSMUSG00000026698 | protein_coding | Pigc | 469.2205 | 569.2649 | 519.2427 |
| ENSMUSG00000049265 | protein_coding | Kcnk3 | 937.6596 | 1089.017 | 1013.338 |
| ENSMUSG00000061615 | protein_coding | Hist1h2ab | 2.595592 | 7.956312 | 5.275952 |
| ENSMUSG00000036026 | protein_coding | Tmem63b | 607.0559 | 689.1198 | 648.0879 |
| ENSMUSG00000084007 | pseudogene | Gm9003 | 4.541075 | 11.24921 | 7.89514 |
| ENSMUSG00000073183 | protein_coding | Gm17680 | 18.82723 | 7.944091 | 13.38566 |
| ENSMUSG00000041846 | protein_coding | Smek1 | 3663.49 | 4019.772 | 3841.631 |
| ENSMUSG00000048332 | protein_coding | Lhfp | 839.6732 | 723.0578 | 781.3655 |
| ENSMUSG00000023004 | protein_coding | Tuba1b | 9720.71 | 10639.25 | 10179.98 |
| ENSMUSG00000026032 | protein_coding | Ndufb3 | 702.7501 | 603.615 | 653.1826 |
| ENSMUSG00000034462 | protein_coding | Pkd2 | 3422.766 | 3813.149 | 3617.958 |
| ENSMUSG00000075254 | protein_coding | Heg1 | 2220.282 | 2505.902 | 2363.092 |
| ENSMUSG00000032059 | protein_coding | Alg9 | 793.4244 | 695.2557 | 744.3401 |
| ENSMUSG00000050910 | protein_coding | Cdr2l | 961.605 | 1128.614 | 1045.11 |
| ENSMUSG00000057110 | protein_coding | Cep110 | 3318.788 | 2939.484 | 3129.136 |
| ENSMUSG00000042680 | protein_coding | Fam59a | 614.5753 | 515.947 | 565.2612 |

| | | | | | |
|---|---|---|---|---|---|
| ENSMUSG00000038502 | protein_coding | Ptov1 | 4072.186 | 4542.072 | 4307.129 |
| ENSMUSG00000027597 | protein_coding | Ahcy | 2428.528 | 3101.336 | 2764.932 |
| ENSMUSG00000025151 | protein_coding | Maged1 | 28920.88 | 31896.37 | 30408.62 |
| ENSMUSG00000062540 | protein_coding | Zfp781 | 1.606973 | 5.774165 | 3.690569 |
| ENSMUSG00000038010 | protein_coding | Ccdc138 | 472.7028 | 401.949 | 437.3259 |
| ENSMUSG00000051648 | protein_coding | Kctd19 | 139.2104 | 27.85872 | 83.53455 |
| ENSMUSG00000051736 | protein_coding | 1700025K23Rik | 108.3028 | 82.54725 | 95.42503 |
| ENSMUSG00000011263 | protein_coding | Exoc3l2 | 3437.645 | 4043.29 | 3740.467 |
| ENSMUSG00000029156 | protein_coding | Sgcb | 1537.242 | 1755.038 | 1646.14 |
| ENSMUSG00000025785 | protein_coding | Exosc7 | 841.4295 | 949.9794 | 895.7045 |
| ENSMUSG00000064952 | snoRNA | SNORD62 | 0.32211 | 3.054663 | 1.688387 |
| ENSMUSG00000029128 | protein_coding | Rab28 | 1181.721 | 1040.742 | 1111.232 |
| ENSMUSG00000014470 | protein_coding | Rnf166 | 1054.566 | 1185.915 | 1120.241 |
| ENSMUSG00000024425 | protein_coding | Ndfip1 | 6766.193 | 7701.467 | 7233.83 |
| ENSMUSG00000023931 | protein_coding | Efhb | 43.61808 | 29.07623 | 36.34716 |
| ENSMUSG00000086040 | protein_coding | Wipf3 | 6770.225 | 7553.021 | 7161.623 |
| ENSMUSG00000024957 | protein_coding | Kcnk4 | 6.172647 | 13.57913 | 9.875888 |
| ENSMUSG00000041261 | protein_coding | Car8 | 59.75423 | 38.93581 | 49.34502 |
| ENSMUSG00000088378 | snRNA | U1 | 4.190787 | 10.32263 | 7.25671 |
| ENSMUSG00000084457 | rRNA | n-R5s73 | 6.190315 | 1.422092 | 3.806203 |
| ENSMUSG00000078815 | protein_coding | Cacng6 | 32.72419 | 18.39969 | 25.56194 |
| ENSMUSG00000021871 | protein_coding | Pnp | 309.9622 | 378.0022 | 343.9822 |
| ENSMUSG00000034210 | protein_coding | 4732418C07Rik | 1910.842 | 1707.144 | 1808.993 |
| ENSMUSG00000020929 | protein_coding | Eftud2 | 4463.044 | 3925.566 | 4194.305 |
| ENSMUSG00000023066 | protein_coding | Rttn | 611.7135 | 538.2817 | 574.9976 |
| ENSMUSG00000034083 | protein_coding | C130022K22Rik | 906.6863 | 1023.983 | 965.3349 |
| ENSMUSG00000057762 | protein_coding | Gm6169 | 37.55979 | 58.05667 | 47.80823 |
| ENSMUSG00000092312 | pseudogene | Zfp419 | 5.447471 | 1.350742 | 3.399106 |
| ENSMUSG00000068566 | protein_coding | Myadm | 1540.835 | 1814.796 | 1677.816 |
| ENSMUSG00000027712 | protein_coding | Anxa5 | 2629.528 | 3227.273 | 2928.401 |
| ENSMUSG00000030897 | protein_coding | Cnga4 | 14.64927 | 6.189687 | 10.41948 |
| ENSMUSG00000029432 | protein_coding | Gbas | 2341.841 | 2617.474 | 2479.658 |
| ENSMUSG00000034714 | protein_coding | Ttyh2 | 2875.127 | 3201.873 | 3038.5 |
| ENSMUSG00000032267 | protein_coding | Usp28 | 4346.268 | 3931.745 | 4139.006 |
| ENSMUSG00000039656 | protein_coding | Rxrb | 1854.156 | 1640.186 | 1747.171 |
| ENSMUSG00000087183 | lincRNA | E030007J07Rik | 22.08575 | 11.60166 | 16.84371 |
| ENSMUSG00000021414 | protein_coding | 1700026J04Rik | 18.82365 | 9.526542 | 14.1751 |
| ENSMUSG00000061544 | protein_coding | Zfp229 | 739.4953 | 913.6858 | 826.5905 |
| ENSMUSG00000042279 | protein_coding | H1foo | 17.58106 | 7.692139 | 12.6366 |
| ENSMUSG00000061728 | protein_coding | Btnl7 | 107.0712 | 69.00241 | 88.0368 |
| ENSMUSG00000025591 | protein_coding | 1810029B16Rik | 718.2734 | 627.3925 | 672.833 |
| ENSMUSG00000028671 | protein_coding | Gale | 326.2417 | 381.3071 | 353.7744 |
| ENSMUSG00000049878 | protein_coding | Rlf | 3624.982 | 4031.432 | 3828.207 |
| ENSMUSG00000004099 | protein_coding | Dnmt1 | 6615.188 | 5746.305 | 6180.747 |
| ENSMUSG00000030881 | protein_coding | Arfip2 | 1411.998 | 1588.368 | 1500.183 |
| ENSMUSG00000026313 | protein_coding | Hdac4 | 676.245 | 567.995 | 622.12 |
| ENSMUSG00000041954 | protein_coding | Tnfrsf18 | 44.05649 | 64.41209 | 54.23429 |
| ENSMUSG00000046550 | protein_coding | Spin2 | 89.94657 | 116.5576 | 103.2521 |
| ENSMUSG00000031342 | protein_coding | Gpm6b | 2549.08 | 2893.647 | 2721.364 |
| ENSMUSG00000035829 | protein_coding | Gm347 | 658.6602 | 780.262 | 719.4611 |
| ENSMUSG00000025314 | protein_coding | Ptprj | 389.837 | 324.8766 | 357.3568 |
| ENSMUSG00000047656 | protein_coding | Trpt1 | 146.5552 | 117.1977 | 131.8764 |
| ENSMUSG00000001558 | protein_coding | Klhl10 | 2552.994 | 3002.924 | 2777.959 |
| ENSMUSG00000021901 | protein_coding | Bap1 | 3364.529 | 3044.457 | 3204.493 |
| ENSMUSG00000049692 | protein_coding | 4933425O20Rik | 8.401552 | 16.40566 | 12.4036 |
| ENSMUSG00000003423 | protein_coding | Pih1d1 | 839.0974 | 728.6001 | 783.8488 |
| ENSMUSG00000015120 | protein_coding | Ube2i | 6264.299 | 6836.965 | 6550.632 |
| ENSMUSG00000045466 | protein_coding | Zfp956 | 469.5862 | 403.7021 | 436.6441 |

| | | | | | |
|---|---|---|---|---|---|
| ENSMUSG00000068329 | protein_coding | Htra2 | 591.7502 | 500.7855 | 546.2679 |
| ENSMUSG00000019966 | protein_coding | Kitl | 7834.748 | 9701.553 | 8768.151 |
| ENSMUSG00000040896 | protein_coding | Kcnd3 | 2768.307 | 2354.505 | 2561.406 |
| ENSMUSG00000085362 | lincRNA | C030034L19Rik | 5.540205 | 1.350742 | 3.445473 |
| ENSMUSG00000090873 | processed_transcrip | Gm17112 | 28.72662 | 45.88736 | 37.30699 |
| ENSMUSG00000056091 | protein_coding | St3gal5 | 3800.553 | 4241.815 | 4021.184 |
| ENSMUSG00000064317 | protein_coding | Gm10146 | 281.2313 | 332.7911 | 307.0112 |
| ENSMUSG00000026193 | protein_coding | Fn1 | 4772.968 | 4258.829 | 4515.899 |
| ENSMUSG00000032532 | protein_coding | Cck | 183.3913 | 18.83364 | 101.1125 |
| ENSMUSG00000061186 | protein_coding | Sfmbt2 | 1652.174 | 1398.306 | 1525.24 |
| ENSMUSG00000038843 | protein_coding | Gcnt1 | 155.6154 | 222.9485 | 189.282 |
| ENSMUSG00000005225 | protein_coding | Plekha8 | 2348.176 | 2100.843 | 2224.51 |
| ENSMUSG00000059991 | protein_coding | Nptx2 | 906.332 | 717.1748 | 811.7534 |
| ENSMUSG00000003228 | protein_coding | Grk5 | 757.2142 | 650.7993 | 704.0068 |
| ENSMUSG00000037791 | protein_coding | Phf12 | 2489.677 | 2206.874 | 2348.275 |
| ENSMUSG00000038860 | protein_coding | Garnl3 | 3350.015 | 2867.712 | 3108.864 |
| ENSMUSG00000086130 | processed_transcrip | Gm16211 | 2.283993 | 7.858657 | 5.071325 |
| ENSMUSG00000040220 | protein_coding | Gas8 | 581.1844 | 508.1919 | 544.6882 |
| ENSMUSG00000024274 | protein_coding | Zscan30 | 177.2366 | 215.6117 | 196.4241 |
| ENSMUSG00000086468 | processed_transcrip | 2900053O20Rik | 35.57167 | 22.73483 | 29.15325 |
| ENSMUSG00000030894 | protein_coding | Tpp1 | 1452.865 | 1684.682 | 1568.773 |
| ENSMUSG00000030400 | protein_coding | Ercc2 | 677.2888 | 587.1696 | 632.2292 |
| ENSMUSG00000030802 | protein_coding | Bckdk | 1186.541 | 1048.724 | 1117.632 |
| ENSMUSG00000031682 | protein_coding | 1700011L22Rik | 0.33031 | 3.001331 | 1.665821 |
| ENSMUSG00000020585 | protein_coding | Laptm4a | 8503.558 | 9388.651 | 8946.104 |
| ENSMUSG00000020907 | protein_coding | Rcvrn | 3.902744 | 10.26858 | 7.085661 |
| ENSMUSG00000025371 | protein_coding | Chmp6 | 717.0998 | 610.5732 | 663.8365 |
| ENSMUSG00000022885 | protein_coding | St6gal1 | 1975.074 | 2432.844 | 2203.959 |
| ENSMUSG00000023048 | protein_coding | Prr13 | 487.6537 | 582.954 | 535.3038 |
| ENSMUSG00000028542 | protein_coding | Slc6a9 | 400.6987 | 336.6412 | 368.67 |
| ENSMUSG00000020263 | protein_coding | Appl2 | 4613.106 | 5286.784 | 4949.945 |
| ENSMUSG00000062510 | protein_coding | Nsl1 | 433.4861 | 365.2414 | 399.3638 |
| ENSMUSG00000046675 | protein_coding | D230037D09Rik | 361.9319 | 308.2572 | 335.0946 |
| ENSMUSG00000040738 | protein_coding | Ints8 | 3012.216 | 2663.135 | 2837.675 |
| ENSMUSG00000035126 | protein_coding | Wdr78 | 277.0874 | 232.9534 | 255.0204 |
| ENSMUSG00000030117 | protein_coding | Gdf3 | 0.976841 | 4.892639 | 2.93474 |
| ENSMUSG00000000326 | protein_coding | Comt | 918.5584 | 1033.099 | 975.8287 |
| ENSMUSG00000023249 | protein_coding | Parp3 | 130.4249 | 96.85144 | 113.6382 |
| ENSMUSG00000040720 | protein_coding | 1110037F02Rik | 3436.718 | 3097.43 | 3267.074 |
| ENSMUSG00000045662 | protein_coding | 4921515J06Rik | 358.354 | 298.3111 | 328.3325 |
| ENSMUSG00000026574 | protein_coding | Dpt | 717.7263 | 835.0583 | 776.3923 |
| ENSMUSG00000030796 | protein_coding | Tead2 | 1067.581 | 1226.191 | 1146.886 |
| ENSMUSG00000043243 | protein_coding | Fam129c | 133.7453 | 105.3041 | 119.5247 |
| ENSMUSG00000025266 | protein_coding | Gnl3l | 1109.322 | 1321.542 | 1215.432 |
| ENSMUSG00000087574 | lincRNA | C030037D09Rik | 103.7264 | 134.2271 | 118.9768 |
| ENSMUSG00000032374 | protein_coding | Plod2 | 4898.934 | 5540.869 | 5219.902 |
| ENSMUSG00000045103 | protein_coding | Dmd | 2064.609 | 2372.786 | 2218.698 |
| ENSMUSG00000032012 | protein_coding | Pvrl1 | 487.5543 | 606.395 | 546.9747 |
| ENSMUSG00000035429 | protein_coding | Ptprh | 1.284863 | 6.457071 | 3.870967 |
| ENSMUSG00000043602 | protein_coding | Zfp3 | 339.9184 | 281.2108 | 310.5646 |
| ENSMUSG00000020216 | protein_coding | Jsrp1 | 325.2428 | 396.0843 | 360.6635 |
| ENSMUSG00000024940 | protein_coding | Ltbp3 | 1750.316 | 2130.285 | 1940.301 |
| ENSMUSG00000043090 | protein_coding | Zfp866 | 1594.82 | 1359.107 | 1476.964 |
| ENSMUSG00000085804 | processed_transcrip | Gm12973 | 9.739192 | 3.743729 | 6.74146 |
| ENSMUSG00000023809 | protein_coding | Rps6ka2 | 4041.348 | 4821.765 | 4431.556 |
| ENSMUSG00000054452 | protein_coding | Aes | 6200.173 | 6773.73 | 6486.951 |
| ENSMUSG00000052934 | protein_coding | 1110003O08Rik | 277.129 | 230.8328 | 253.9809 |
| ENSMUSG00000031173 | protein_coding | Otc | 6.137537 | 1.677256 | 3.907396 |

| | | | | | |
|---|---|---|---|---|---|
| ENSMUSG00000019432 | protein_coding | Ddx39b | 5921.187 | 6629.668 | 6275.428 |
| ENSMUSG00000026102 | protein_coding | Inpp1 | 126.047 | 158.6919 | 142.3694 |
| ENSMUSG00000038026 | protein_coding | Kcnj9 | 3.594722 | 9.951072 | 6.772897 |
| ENSMUSG00000024542 | protein_coding | Cep192 | 2699.082 | 2357.56 | 2528.321 |
| ENSMUSG00000029389 | protein_coding | Ddx55 | 788.7728 | 679.9267 | 734.3498 |
| ENSMUSG00000031809 | protein_coding | 1700018B08Rik | 16.16835 | 8.184809 | 12.17658 |
| ENSMUSG00000042331 | protein_coding | Specc1 | 1404.69 | 1100.802 | 1252.746 |
| ENSMUSG00000029152 | protein_coding | Ociad1 | 3136.271 | 3439.983 | 3288.127 |
| ENSMUSG00000029179 | protein_coding | Zcchc4 | 635.0953 | 735.7218 | 685.4085 |
| ENSMUSG00000086294 | lincRNA | Gm16765 | 69.75038 | 51.43986 | 60.59512 |
| ENSMUSG00000042878 | protein_coding | Ccdc85b | 850.7876 | 749.9126 | 800.3501 |
| ENSMUSG00000019235 | protein_coding | Rps6kl1 | 14.61184 | 27.00002 | 20.80593 |
| ENSMUSG00000028931 | protein_coding | Kcnab2 | 385.451 | 321.1906 | 353.3208 |
| ENSMUSG00000079638 | protein_coding | Rhox2b | 26.16152 | 15.5829 | 20.87221 |
| ENSMUSG00000064366 | Mt_tRNA | mt-Tl2 | 9.097507 | 2.7995 | 5.948503 |
| ENSMUSG00000034485 | protein_coding | Uaca | 1740.483 | 1548.966 | 1644.724 |
| ENSMUSG00000051786 | protein_coding | Tubgcp6 | 1127.823 | 997.4603 | 1062.642 |
| ENSMUSG00000062761 | protein_coding | Zfp512 | 1536.257 | 1338.055 | 1437.156 |
| ENSMUSG00000030339 | protein_coding | Ltbr | 701.4413 | 803.1962 | 752.3187 |
| ENSMUSG00000002797 | protein_coding | Ggct | 682.5867 | 575.0405 | 628.8136 |
| ENSMUSG00000027508 | protein_coding | Pag1 | 866.5449 | 764.3008 | 815.4228 |
| ENSMUSG00000053216 | protein_coding | Btn2a2 | 27.36774 | 13.76724 | 20.56749 |
| ENSMUSG00000021365 | protein_coding | Nedd9 | 2259.193 | 2870.175 | 2564.684 |
| ENSMUSG00000067150 | protein_coding | Xpo5 | 1823.121 | 1619.342 | 1721.232 |
| ENSMUSG00000037710 | protein_coding | Cisd1 | 701.9489 | 616.3904 | 659.1696 |
| ENSMUSG00000039048 | protein_coding | Foxred1 | 856.6465 | 734.4846 | 795.5656 |
| ENSMUSG00000034175 | protein_coding | Rhbdd3 | 602.1816 | 507.2327 | 554.7071 |
| ENSMUSG00000086208 | lincRNA | Gm2716 | 6.488868 | 1.739597 | 4.114233 |
| ENSMUSG00000031880 | protein_coding | Rrad | 137.1762 | 176.3593 | 156.7677 |
| ENSMUSG00000022186 | protein_coding | Oxct1 | 3705.741 | 4245.489 | 3975.615 |
| ENSMUSG00000068037 | protein_coding | Mas1 | 4.227165 | 10.77098 | 7.499073 |
| ENSMUSG00000042477 | protein_coding | Tcfap2e | 33.5793 | 19.94326 | 26.76128 |
| ENSMUSG00000046782 | protein_coding | Ttc6 | 60.30095 | 38.93177 | 49.61636 |
| ENSMUSG00000001313 | protein_coding | Rnd2 | 4237.837 | 4921.95 | 4579.893 |
| ENSMUSG00000041565 | protein_coding | L3mbtl4 | 371.6425 | 296.7118 | 334.1772 |
| ENSMUSG00000029695 | protein_coding | Aass | 549.3554 | 639.7711 | 594.5632 |
| ENSMUSG00000018800 | protein_coding | Abca5 | 1200.235 | 921.4549 | 1060.845 |
| ENSMUSG00000022307 | protein_coding | Oxr1 | 2770.033 | 2450.79 | 2610.411 |
| ENSMUSG00000044276 | protein_coding | 4933427E11Rik | 14.51553 | 7.1786 | 10.84707 |
| ENSMUSG00000030269 | protein_coding | Mtmr14 | 1163.83 | 1316.628 | 1240.229 |
| ENSMUSG00000037992 | protein_coding | Rara | 2049.922 | 2339.884 | 2194.903 |
| ENSMUSG00000035246 | protein_coding | Pcyt1b | 794.1033 | 926.9852 | 860.5443 |
| ENSMUSG00000081443 | pseudogene | Gm3880 | 13.95354 | 6.462869 | 10.2082 |
| ENSMUSG00000029263 | protein_coding | Pigg | 694.561 | 601.2022 | 647.8816 |
| ENSMUSG00000032239 | protein_coding | Rp9 | 1549.134 | 1339.422 | 1444.278 |
| ENSMUSG00000024998 | protein_coding | Plce1 | 2729.043 | 3074.894 | 2901.968 |
| ENSMUSG00000091673 | lincRNA | Gm17525 | 28.80169 | 45.60801 | 37.20485 |
| ENSMUSG00000006998 | protein_coding | Psmd2 | 10468.73 | 9313.61 | 9891.171 |
| ENSMUSG00000064350 | Mt_tRNA | mt-Ty | 37.94899 | 22.87396 | 30.41148 |
| ENSMUSG00000054008 | protein_coding | Ndst1 | 5342.687 | 6094.584 | 5718.636 |
| ENSMUSG00000051554 | protein_coding | Gm9853 | 439.6666 | 371.4462 | 405.5564 |
| ENSMUSG00000004730 | protein_coding | Emr1 | 197.3638 | 245.142 | 221.2529 |
| ENSMUSG00000000184 | protein_coding | Ccnd2 | 5605.742 | 6695.512 | 6150.627 |
| ENSMUSG00000019082 | protein_coding | Slc25a22 | 312.5856 | 364.2708 | 338.4282 |
| ENSMUSG00000026315 | protein_coding | Serpinb8 | 13.79162 | 26.67211 | 20.23187 |
| ENSMUSG00000086542 | processed_transcrip | Gm13782 | 8.127598 | 2.683826 | 5.405712 |
| ENSMUSG00000008540 | protein_coding | Mgst1 | 3201.808 | 3636.109 | 3418.959 |
| ENSMUSG00000026960 | protein_coding | Arl6ip6 | 1359.611 | 1509.136 | 1434.373 |

| | | | | | |
|---|---|---|---|---|---|
| ENSMUSG00000091127 | lincRNA | Gm17238 | 52.93446 | 37.02897 | 44.98171 |
| ENSMUSG00000033728 | protein_coding | Lrrc14 | 1217.58 | 1079.27 | 1148.425 |
| ENSMUSG00000000876 | protein_coding | Pxmp4 | 522.6189 | 452.1676 | 487.3933 |
| ENSMUSG00000063052 | protein_coding | Lrrc40 | 2214.766 | 1997.714 | 2106.24 |
| ENSMUSG00000022200 | protein_coding | Golph3 | 4305.564 | 4786.977 | 4546.271 |
| ENSMUSG00000045062 | protein_coding | Pcdhb7 | 23.83295 | 36.96155 | 30.39725 |
| ENSMUSG00000039081 | protein_coding | Zfp503 | 189.2425 | 154.4818 | 171.8621 |
| ENSMUSG00000036986 | protein_coding | Pml | 740.9462 | 853.8801 | 797.4132 |
| ENSMUSG00000072978 | protein_coding | Gm5830 | 6.456069 | 2.048455 | 4.252262 |
| ENSMUSG00000053388 | protein_coding | Trim50 | 69.31645 | 49.18057 | 59.24851 |
| ENSMUSG00000021500 | protein_coding | Ddx46 | 4595.61 | 5021.683 | 4808.647 |
| ENSMUSG00000086917 | processed_transcrip | Gm11630 | 12.56185 | 5.81885 | 9.19035 |
| ENSMUSG00000025658 | protein_coding | Cnksr2 | 420.8756 | 340.6693 | 380.7724 |
| ENSMUSG00000024600 | protein_coding | Slc27a6 | 1473.018 | 1648.111 | 1560.565 |
| ENSMUSG00000028850 | protein_coding | Gpatch3 | 578.96 | 487.5964 | 533.2782 |
| ENSMUSG00000030246 | protein_coding | Ldhb | 11418.87 | 12534.24 | 11976.56 |
| ENSMUSG00000049295 | protein_coding | Zfp219 | 2369.62 | 2671.835 | 2520.727 |
| ENSMUSG00000084754 | processed_transcrip | Gm12532 | 1.945483 | 6.587913 | 4.266698 |
| ENSMUSG00000079845 | protein_coding | Xlr4a | 380.1145 | 285.555 | 332.8347 |
| ENSMUSG00000009079 | protein_coding | Ewsr1 | 8253.262 | 9245.471 | 8749.367 |
| ENSMUSG00000029484 | protein_coding | Anxa3 | 483.1075 | 628.9713 | 556.0394 |
| ENSMUSG00000005681 | protein_coding | Apoa2 | 18.18532 | 7.443497 | 12.81441 |
| ENSMUSG00000029777 | protein_coding | Gars | 3648.022 | 3259.698 | 3453.86 |
| ENSMUSG00000084868 | lincRNA | Gm16831 | 3.896855 | 0.662038 | 2.279446 |
| ENSMUSG00000036972 | protein_coding | Zic4 | 9.829615 | 2.7995 | 6.314557 |
| ENSMUSG00000013236 | protein_coding | Ptprs | 3609.712 | 3251.403 | 3430.557 |
| ENSMUSG00000004626 | protein_coding | Stxbp2 | 1236.55 | 1384.651 | 1310.6 |
| ENSMUSG00000025271 | protein_coding | Pfkfb1 | 129.2881 | 102.2098 | 115.7489 |
| ENSMUSG00000062248 | protein_coding | Cks2 | 993.7367 | 1122.383 | 1058.06 |
| ENSMUSG00000039307 | protein_coding | Hexdc | 401.9371 | 472.6821 | 437.3096 |
| ENSMUSG00000052102 | protein_coding | Gnpda1 | 504.4415 | 1065.007 | 784.7243 |
| ENSMUSG00000062234 | protein_coding | Gak | 2543.29 | 2283.596 | 2413.443 |
| ENSMUSG00000030607 | protein_coding | Acan | 158.0367 | 120.2671 | 139.1519 |
| ENSMUSG00000002791 | processed_transcrip | Pdxk-ps | 6.186736 | 1.677256 | 3.931996 |
| ENSMUSG00000038446 | protein_coding | Cdc40 | 1373.487 | 1571.579 | 1472.533 |
| ENSMUSG00000010825 | protein_coding | Grid2ip | 12.92496 | 22.56003 | 17.74249 |
| ENSMUSG00000035266 | protein_coding | Helq | 720.868 | 625.7363 | 673.3022 |
| ENSMUSG00000089682 | protein_coding | Bcl2l2 | 2061.912 | 2311.636 | 2186.774 |
| ENSMUSG00000000738 | protein_coding | Spg7 | 1430.476 | 1240.492 | 1335.484 |
| ENSMUSG00000026656 | protein_coding | Fcgr2b | 146.3641 | 114.3116 | 130.3378 |
| ENSMUSG00000036275 | protein_coding | 9530068E07Rik | 5520.946 | 6188.354 | 5854.65 |
| ENSMUSG00000033904 | protein_coding | 6330503K22Rik | 3700 | 3082.403 | 3391.202 |
| ENSMUSG00000028494 | protein_coding | Plin2 | 895.8583 | 1055.611 | 975.7348 |
| ENSMUSG00000061758 | protein_coding | Akr1b10 | 479.5407 | 573.3564 | 526.4486 |
| ENSMUSG00000031728 | protein_coding | Zfp821 | 718.7541 | 626.6709 | 672.7125 |
| ENSMUSG00000063905 | protein_coding | Gm10139 | 37.50724 | 21.12028 | 29.31376 |
| ENSMUSG00000025468 | protein_coding | Caly | 10.31066 | 19.50143 | 14.90604 |
| ENSMUSG00000059406 | protein_coding | Tmprss9 | 24.00768 | 38.44205 | 31.22487 |
| ENSMUSG00000018567 | protein_coding | Gabarap | 2724.003 | 3000.349 | 2862.176 |
| ENSMUSG00000077345 | snoRNA | Snord70 | 6.46069 | 13.6948 | 10.07775 |
| ENSMUSG00000059547 | protein_coding | Fbxw26 | 1.945483 | 6.516201 | 4.230842 |
| ENSMUSG00000045658 | protein_coding | Pid1 | 297.4272 | 372.0932 | 334.7602 |
| ENSMUSG00000032940 | protein_coding | Rbm11 | 95.97183 | 71.49263 | 83.73223 |
| ENSMUSG00000087159 | processed_transcrip | Gm15246 | 323.3727 | 242.5373 | 282.955 |
| ENSMUSG00000030727 | protein_coding | Rabep2 | 889.9685 | 1026.492 | 958.2301 |
| ENSMUSG00000052336 | protein_coding | Cx3cr1 | 266.8431 | 214.5725 | 240.7078 |
| ENSMUSG00000027883 | protein_coding | Gpsm2 | 1360.262 | 1210.116 | 1285.189 |
| ENSMUSG00000062151 | protein_coding | Unc13c | 24.56379 | 13.99465 | 19.27922 |

| | | | | | |
|---|---|---|---|---|---|
| ENSMUSG00000026958 | protein_coding | Dpp7 | 286.4376 | 231.6719 | 259.0548 |
| ENSMUSG00000030008 | protein_coding | 1700040I03Rik | 319.9433 | 267.3518 | 293.6476 |
| ENSMUSG00000063785 | protein_coding | Utp14a | 762.8291 | 867.8062 | 815.3176 |
| ENSMUSG00000084074 | pseudogene | Gm8310 | 11.04403 | 3.841384 | 7.442708 |
| ENSMUSG00000022155 | protein_coding | Heatr7b2 | 6.506536 | 1.73095 | 4.118743 |
| ENSMUSG00000030030 | protein_coding | 1700003E16Rik | 52.87392 | 35.12322 | 43.99857 |
| ENSMUSG00000064770 | rRNA | n-R5s29 | 3.860477 | 0.697713 | 2.279095 |
| ENSMUSG00000049521 | protein_coding | Cdc42ep1 | 340.5134 | 404.5195 | 372.5164 |
| ENSMUSG00000024883 | protein_coding | Rin1 | 31.7545 | 47.60169 | 39.6781 |
| ENSMUSG00000086544 | processed_transcrip | Gm13704 | 29.80902 | 16.96139 | 23.3852 |
| ENSMUSG00000022144 | protein_coding | Gdnf | 400.4046 | 547.5716 | 473.9881 |
| ENSMUSG00000086059 | processed_transcrip | Gm15797 | 0.65242 | 3.72571 | 2.189065 |
| ENSMUSG00000021758 | protein_coding | Ddx4 | 5895.656 | 5255.209 | 5575.433 |
| ENSMUSG00000042258 | protein_coding | Isl1 | 35.83511 | 22.73768 | 29.2864 |
| ENSMUSG00000078584 | protein_coding | AU022252 | 951.6898 | 833.8521 | 892.7709 |
| ENSMUSG00000041132 | protein_coding | N4bp2l1 | 934.066 | 1064.568 | 999.3169 |
| ENSMUSG00000028416 | protein_coding | Bag1 | 3291.06 | 2911.259 | 3101.16 |
| ENSMUSG00000028093 | protein_coding | Acp6 | 884.7232 | 782.689 | 833.7061 |
| ENSMUSG00000040594 | protein_coding | Ranbp17 | 614.3808 | 521.7069 | 568.0439 |
| ENSMUSG00000018776 | protein_coding | Amac1 | 12.36065 | 5.174469 | 8.76756 |
| ENSMUSG00000031357 | protein_coding | Syap1 | 6807.03 | 7674.211 | 7240.621 |
| ENSMUSG00000032353 | protein_coding | Tmed3 | 985.7501 | 869.2764 | 927.5133 |
| ENSMUSG00000061046 | protein_coding | Haghl | 303.8118 | 363.6729 | 333.7423 |
| ENSMUSG00000044328 | protein_coding | Trp53i13 | 407.1591 | 343.2655 | 375.2123 |
| ENSMUSG00000022938 | protein_coding | Fam3b | 80.13088 | 55.60704 | 67.86896 |
| ENSMUSG00000031955 | protein_coding | Bcar1 | 2882.767 | 3255.617 | 3069.192 |
| ENSMUSG00000036158 | protein_coding | Prickle1 | 1204.095 | 1346.853 | 1275.474 |
| ENSMUSG00000079326 | protein_coding | Gm15262 | 185.0572 | 145.9815 | 165.5193 |
| ENSMUSG00000020647 | protein_coding | Ncoa1 | 1774.283 | 1503.258 | 1638.771 |
| ENSMUSG00000053441 | protein_coding | Adamts19 | 182.9976 | 226.3694 | 204.6835 |
| ENSMUSG00000027932 | protein_coding | Slc27a3 | 1050.457 | 1279.919 | 1165.188 |
| ENSMUSG00000049112 | protein_coding | Oxtr | 715.0623 | 833.9622 | 774.5122 |
| ENSMUSG00000011305 | protein_coding | Plin5 | 31.3363 | 19.57635 | 25.45633 |
| ENSMUSG00000022824 | protein_coding | Muc13 | 3.242123 | 8.784144 | 6.013134 |
| ENSMUSG00000085236 | lincRNA | 2610206C17Rik | 62.87 | 86.91548 | 74.89274 |
| ENSMUSG00000032087 | protein_coding | Dscaml1 | 46.03097 | 68.77857 | 57.40477 |
| ENSMUSG00000038260 | protein_coding | Trpm4 | 537.4611 | 636.415 | 586.9381 |
| ENSMUSG00000001376 | protein_coding | Ccdc132 | 1895.322 | 2099.579 | 1997.45 |
| ENSMUSG00000048878 | protein_coding | Hexim1 | 2981.697 | 3464.881 | 3223.289 |
| ENSMUSG00000073374 | protein_coding | C030034I22Rik | 58.12922 | 78.82553 | 68.47738 |
| ENSMUSG00000032925 | protein_coding | Itgbl1 | 260.1293 | 317.4815 | 288.8054 |
| ENSMUSG00000027574 | protein_coding | Nkain4 | 107.0341 | 138.3115 | 122.6728 |
| ENSMUSG00000027010 | protein_coding | Slc25a12 | 499.1587 | 580.6193 | 539.889 |
| ENSMUSG00000058046 | protein_coding | 4933430I17Rik | 56.64651 | 39.80216 | 48.22434 |
| ENSMUSG00000026620 | protein_coding | Mark1 | 501.2915 | 424.9401 | 463.1158 |
| ENSMUSG00000028910 | protein_coding | Mecr | 617.1474 | 531.4471 | 574.2973 |
| ENSMUSG00000078630 | protein_coding | Tomt | 35.03554 | 21.28929 | 28.16241 |
| ENSMUSG00000046949 | protein_coding | Nqo2 | 807.5207 | 910.4287 | 858.9747 |
| ENSMUSG00000056328 | protein_coding | Myh1 | 120.5117 | 76.50048 | 98.50609 |
| ENSMUSG00000029265 | protein_coding | Dr1 | 2347.552 | 2094.796 | 2221.174 |
| ENSMUSG00000072663 | protein_coding | Spef2 | 32.41743 | 19.83152 | 26.12447 |
| ENSMUSG00000046618 | protein_coding | Olfml2a | 156.4057 | 211.6908 | 184.0483 |
| ENSMUSG00000024414 | protein_coding | Mrpl27 | 523.3351 | 596.9852 | 560.1602 |
| ENSMUSG00000051730 | protein_coding | Mettl5 | 360.7684 | 303.6384 | 332.2034 |
| ENSMUSG00000074580 | protein_coding | 4931440P22Rik | 61.6081 | 40.24684 | 50.92747 |
| ENSMUSG00000030780 | protein_coding | BC017158 | 386.4053 | 453.0738 | 419.7395 |
| ENSMUSG00000002885 | protein_coding | Cd97 | 202.0073 | 246.7326 | 224.37 |
| ENSMUSG00000023781 | protein_coding | Hes7 | 26.79858 | 41.54969 | 34.17413 |

| | | | | | |
|---|---|---|---|---|---|
| ENSMUSG00000035735 | protein_coding | Dagla | 347.8316 | 296.8213 | 322.3264 |
| ENSMUSG00000074277 | protein_coding | Phldb3 | 69.78907 | 49.53266 | 59.66087 |
| ENSMUSG00000004071 | protein_coding | 5730403B10Rik | 2286.222 | 2541.808 | 2414.015 |
| ENSMUSG00000032038 | protein_coding | St3gal4 | 2887.971 | 3338.73 | 3113.351 |
| ENSMUSG00000069706 | protein_coding | Taar5 | 0.968641 | 4.379101 | 2.673871 |
| ENSMUSG00000038319 | protein_coding | Kcnh2 | 2406.905 | 2693.417 | 2550.161 |
| ENSMUSG00000027667 | protein_coding | Zfp639 | 907.9803 | 789.2223 | 848.6013 |
| ENSMUSG00000027806 | protein_coding | Tsc22d2 | 2467.053 | 2172.062 | 2319.557 |
| ENSMUSG00000057234 | protein_coding | Mettl15 | 169.5091 | 210.452 | 189.9805 |
| ENSMUSG00000025372 | protein_coding | Baiap2 | 1244.056 | 1479.986 | 1362.021 |
| ENSMUSG00000043998 | protein_coding | Mgat2 | 1789.734 | 1617.541 | 1703.638 |
| ENSMUSG00000023036 | protein_coding | Pcdhgc5 | 2850.104 | 3212.444 | 3031.274 |
| ENSMUSG00000028950 | protein_coding | Tas1r1 | 90.02372 | 68.50647 | 79.26509 |
| ENSMUSG00000039239 | protein_coding | Tgfb2 | 274.0941 | 328.6188 | 301.3565 |
| ENSMUSG00000002908 | protein_coding | Kcnn1 | 140.7156 | 110.1603 | 125.438 |
| ENSMUSG00000040687 | protein_coding | Madd | 889.6911 | 691.3718 | 790.5314 |
| ENSMUSG00000030321 | processed_transcrip | BC060267 | 68.17517 | 49.89273 | 59.03395 |
| ENSMUSG00000085886 | lincRNA | D030047H15Rik | 92.79196 | 119.0281 | 105.91 |
| ENSMUSG00000025008 | protein_coding | Tctn3 | 450.2639 | 388.8014 | 419.5326 |
| ENSMUSG00000027568 | protein_coding | Ntsr1 | 15.43729 | 26.15169 | 20.79449 |
| ENSMUSG00000037583 | protein_coding | Nr0b2 | 26.44516 | 47.16825 | 36.80671 |
| ENSMUSG00000049764 | protein_coding | Zfp280b | 2152.96 | 1825.948 | 1989.454 |
| ENSMUSG00000045625 | protein_coding | Pigz | 6.182115 | 13.64147 | 9.911792 |
| ENSMUSG00000005882 | protein_coding | Uqcc | 968.0586 | 1111.864 | 1039.961 |
| ENSMUSG00000036599 | protein_coding | Chst12 | 406.2126 | 343.1066 | 374.6596 |
| ENSMUSG00000023367 | protein_coding | Tmem176a | 277.4032 | 333.2426 | 305.3229 |
| ENSMUSG00000073489 | protein_coding | Ifi204 | 29.63913 | 16.93436 | 23.28675 |
| ENSMUSG00000023235 | protein_coding | Ccl25 | 114.0218 | 149.5206 | 131.7712 |
| ENSMUSG00000036251 | protein_coding | Trpm8 | 13.24014 | 23.24008 | 18.24011 |
| ENSMUSG00000036855 | protein_coding | Gjd4 | 0.66062 | 4.025559 | 2.343089 |
| ENSMUSG00000071856 | protein_coding | Mcc | 130.1339 | 165.7637 | 147.9488 |
| ENSMUSG00000071350 | protein_coding | Setdb2 | 1307.376 | 1123.398 | 1215.387 |
| ENSMUSG00000024384 | protein_coding | Iws1 | 4097.955 | 3625.765 | 3861.86 |
| ENSMUSG00000025001 | protein_coding | Hells | 9176.656 | 8154.915 | 8665.785 |
| ENSMUSG00000086237 | processed_transcrip | Gm15591 | 9.129038 | 18.48942 | 13.80923 |
| ENSMUSG00000051900 | protein_coding | Abca16 | 129.0533 | 88.33576 | 108.6945 |
| ENSMUSG00000035171 | protein_coding | 1110059E24Rik | 872.854 | 1020.184 | 946.5188 |
| ENSMUSG00000034258 | protein_coding | Mfsd7c | 2392.432 | 2765.336 | 2578.884 |
| ENSMUSG00000039476 | protein_coding | Prrx2 | 115.6529 | 148.6576 | 132.1552 |
| ENSMUSG00000079343 | protein_coding | Gm5077 | 78.73272 | 110.0086 | 94.37067 |
| ENSMUSG00000028080 | protein_coding | Lrba | 4479.312 | 3791.048 | 4135.18 |
| ENSMUSG00000018821 | protein_coding | Avpi1 | 261.3125 | 215.6915 | 238.502 |
| ENSMUSG00000078786 | protein_coding | BC024978 | 261.1707 | 217.626 | 239.3984 |
| ENSMUSG00000029012 | protein_coding | Orc5 | 883.487 | 993.5659 | 938.5265 |
| ENSMUSG00000020832 | protein_coding | Eral1 | 735.8882 | 652.364 | 694.1261 |
| ENSMUSG00000090470 | protein_coding | 2410012M07Rik | 228.1416 | 187.3454 | 207.7435 |
| ENSMUSG00000022091 | protein_coding | Sorbs3 | 2675.935 | 2996.323 | 2836.129 |
| ENSMUSG00000018340 | protein_coding | Anxa6 | 2877.366 | 3393.422 | 3135.394 |
| ENSMUSG00000043036 | protein_coding | Ccdc63 | 11.61781 | 5.192487 | 8.405148 |
| ENSMUSG00000034329 | protein_coding | Brip1 | 932.5419 | 760.2084 | 846.3752 |
| ENSMUSG00000053950 | protein_coding | Adnp2 | 1831.85 | 1570.451 | 1701.151 |
| ENSMUSG00000001138 | protein_coding | Cnnm3 | 1171.006 | 1318.973 | 1244.99 |
| ENSMUSG00000082399 | pseudogene | Gm14036 | 7.144867 | 2.374969 | 4.759918 |
| ENSMUSG00000020564 | protein_coding | Atxn7l1 | 334.2251 | 409.3375 | 371.7813 |
| ENSMUSG00000020435 | protein_coding | Osbp2 | 177.2309 | 135.8973 | 156.5641 |
| ENSMUSG00000040875 | protein_coding | Osbpl10 | 340.187 | 403.4282 | 371.8076 |
| ENSMUSG00000015597 | protein_coding | Zfp318 | 3649.39 | 3109.78 | 3379.585 |
| ENSMUSG00000047215 | protein_coding | Rpl9 | 4673.415 | 5259.263 | 4966.339 |

| | | | | | |
|---|---|---|---|---|---|
| ENSMUSG00000020758 | protein_coding | Itgb4 | 77.47954 | 58.17012 | 67.82483 |
| ENSMUSG00000005873 | protein_coding | Reep5 | 1306.592 | 1462.991 | 1384.792 |
| ENSMUSG00000057729 | protein_coding | Prtn3 | 52.77448 | 36.17447 | 44.47448 |
| ENSMUSG00000041117 | protein_coding | Ccdc8 | 863.2755 | 1006.769 | 935.0225 |
| ENSMUSG00000061816 | protein_coding | Myl1 | 13.60094 | 6.489897 | 10.04542 |
| ENSMUSG00000049047 | protein_coding | Armcx3 | 684.4682 | 829.381 | 756.9246 |
| ENSMUSG00000021534 | protein_coding | 1700001L19Rik | 21.89924 | 11.6204 | 16.75982 |
| ENSMUSG00000041930 | protein_coding | BC057022 | 828.1837 | 1001.742 | 914.9626 |
| ENSMUSG00000070713 | protein_coding | Gm10282 | 569.5147 | 497.7966 | 533.6557 |
| ENSMUSG00000031242 | protein_coding | 2610002M06Rik | 1145.858 | 1283.97 | 1214.914 |
| ENSMUSG00000031139 | protein_coding | Mcf2 | 223.0662 | 156.8563 | 189.9613 |
| ENSMUSG00000028932 | protein_coding | Psmc2 | 2520.105 | 2775.913 | 2648.009 |
| ENSMUSG00000069792 | protein_coding | Gm11428 | 13.57738 | 23.26603 | 18.4217 |
| ENSMUSG00000015806 | protein_coding | Qdpr | 2453.401 | 2199.606 | 2326.504 |
| ENSMUSG00000071112 | protein_coding | B230216G23Rik | 61.27905 | 85.74825 | 73.51365 |
| ENSMUSG00000044627 | protein_coding | Swi5 | 3262.148 | 2958.956 | 3110.552 |
| ENSMUSG00000091145 | protein_coding | Gm4308 | 0.33031 | 3.30118 | 1.815745 |
| ENSMUSG00000077611 | snoRNA | SNORA74 | 10.67272 | 19.46897 | 15.07084 |
| ENSMUSG00000027365 | protein_coding | Trpm7 | 5358.26 | 5851.947 | 5605.103 |
| ENSMUSG00000030443 | protein_coding | Zfp583 | 364.2949 | 424.1466 | 394.2207 |
| ENSMUSG00000052131 | protein_coding | Akr1b7 | 86.00074 | 117.3868 | 101.6938 |
| ENSMUSG00000036155 | protein_coding | Mgat5 | 982.9264 | 844.3519 | 913.6391 |
| ENSMUSG00000034883 | protein_coding | Lrr1 | 418.3617 | 358.2263 | 388.294 |
| ENSMUSG00000042507 | protein_coding | C130039O16Rik | 673.9281 | 781.0894 | 727.5087 |
| ENSMUSG00000025235 | protein_coding | Bbs4 | 775.179 | 886.4871 | 830.8331 |
| ENSMUSG00000030309 | protein_coding | Caprin2 | 2993.253 | 2525.701 | 2759.477 |
| ENSMUSG00000024292 | protein_coding | Cyp4f14 | 46.94743 | 68.35765 | 57.65254 |
| ENSMUSG00000086968 | lincRNA | 4933431E20Rik | 511.5387 | 596.7189 | 554.1288 |
| ENSMUSG00000008855 | protein_coding | Hdac5 | 2426.148 | 2184.094 | 2305.121 |
| ENSMUSG00000068270 | protein_coding | Shroom4 | 721.7528 | 611.0038 | 666.3783 |
| ENSMUSG00000039568 | protein_coding | Fam100a | 678.3373 | 602.1121 | 640.2247 |
| ENSMUSG00000020423 | protein_coding | Btg2 | 1817.901 | 1537.389 | 1677.645 |
| ENSMUSG00000011958 | protein_coding | Bnip2 | 4642.284 | 5238.687 | 4940.485 |
| ENSMUSG00000086989 | pseudogene | Gm16441 | 39.11637 | 20.07115 | 29.59376 |
| ENSMUSG00000037239 | protein_coding | Spred3 | 72.07448 | 101.4818 | 86.77812 |
| ENSMUSG00000016495 | protein_coding | 5033414D02Rik | 406.8875 | 478.474 | 442.6807 |
| ENSMUSG00000066042 | protein_coding | Med18 | 309.4449 | 251.9209 | 280.6829 |
| ENSMUSG00000027371 | protein_coding | Fahd2a | 267.7931 | 319.6618 | 293.7274 |
| ENSMUSG00000074608 | protein_coding | Zfp850 | 70.85402 | 50.66971 | 60.76187 |
| ENSMUSG00000042184 | protein_coding | 1700069L16Rik | 3.910944 | 0.688704 | 2.299824 |
| ENSMUSG00000025738 | protein_coding | Fbxl16 | 119.3545 | 161.9909 | 140.6727 |
| ENSMUSG00000025189 | protein_coding | Cnnm1 | 31.06004 | 17.98738 | 24.52371 |
| ENSMUSG00000025337 | protein_coding | Sbds | 1112.168 | 1272.306 | 1192.237 |
| ENSMUSG00000009394 | protein_coding | Syn2 | 295.533 | 226.9841 | 261.2585 |
| ENSMUSG00000034394 | protein_coding | Lif | 47.39552 | 65.80214 | 56.59883 |
| ENSMUSG00000052374 | protein_coding | Actn2 | 231.6576 | 189.4778 | 210.5677 |
| ENSMUSG00000056579 | lincRNA | Tug1 | 4875.591 | 5426.329 | 5150.96 |
| ENSMUSG00000085667 | processed_transcrip | Gm12992 | 202.0651 | 255.704 | 228.8845 |
| ENSMUSG00000030616 | protein_coding | Sytl2 | 335.8817 | 414.9216 | 375.4017 |
| ENSMUSG00000032035 | protein_coding | Ets1 | 1190.442 | 1055.228 | 1122.835 |
| ENSMUSG00000086370 | lincRNA | B230206F22Rik | 215.4394 | 268.0234 | 241.7314 |
| ENSMUSG00000079578 | protein_coding | Gm1140 | 21.34649 | 41.19133 | 31.26891 |
| ENSMUSG00000024059 | protein_coding | Clip4 | 857.1051 | 986.1116 | 921.6083 |
| ENSMUSG00000042198 | protein_coding | Chchd7 | 680.877 | 588.8709 | 634.874 |
| ENSMUSG00000043885 | protein_coding | Slc36a4 | 1309.468 | 1139.401 | 1224.434 |
| ENSMUSG00000032399 | protein_coding | Rpl4 | 21965.71 | 24215.73 | 23090.72 |
| ENSMUSG00000004895 | protein_coding | Prcc | 1861.244 | 1668.49 | 1764.867 |
| ENSMUSG00000073672 | pseudogene | 9930005F22Rik | 37.97695 | 56.58088 | 47.27891 |

| | | | | | |
|---|---|---|---|---|---|
| ENSMUSG00000022843 | protein_coding | Clcn2 | 3287.087 | 3864.891 | 3575.989 |
| ENSMUSG00000039838 | protein_coding | Slc45a1 | 12.26665 | 21.9564 | 17.11152 |
| ENSMUSG00000030089 | protein_coding | Slc41a3 | 1160.977 | 1343.751 | 1252.364 |
| ENSMUSG00000054409 | protein_coding | Tmem74 | 9.004548 | 18.49558 | 13.75007 |
| ENSMUSG00000035105 | protein_coding | Egln3 | 167.7934 | 127.9965 | 147.8949 |
| ENSMUSG00000056032 | processed_transcrip | BC018473 | 1.929083 | 6.542867 | 4.235975 |
| ENSMUSG00000043782 | protein_coding | Ccdc64b | 9.994435 | 19.05272 | 14.52358 |
| ENSMUSG00000000282 | protein_coding | Mnt | 1189.922 | 1063.897 | 1126.91 |
| ENSMUSG00000030317 | protein_coding | Timp4 | 19.16447 | 9.817742 | 14.49111 |
| ENSMUSG00000030704 | protein_coding | Rab6 | 4334.165 | 4798.945 | 4566.555 |
| ENSMUSG00000040729 | protein_coding | AK129341 | 503.4709 | 378.5688 | 441.0198 |
| ENSMUSG00000015846 | protein_coding | Rxra | 2189.656 | 1930.949 | 2060.302 |
| ENSMUSG00000062937 | protein_coding | Mtap | 915.3386 | 1071.029 | 993.184 |
| ENSMUSG00000021638 | protein_coding | Ocln | 2142.026 | 2499.178 | 2320.602 |
| ENSMUSG00000021898 | protein_coding | Asb14 | 43.29664 | 25.65865 | 34.47765 |
| ENSMUSG00000016194 | protein_coding | Hsd11b1 | 22.84306 | 35.60688 | 29.22497 |
| ENSMUSG00000051111 | protein_coding | Sv2c | 41.61185 | 65.35297 | 53.48241 |
| ENSMUSG00000090828 | lincRNA | Sox2ot | 1.629262 | 5.943532 | 3.786397 |
| ENSMUSG00000087479 | lincRNA | Gm16835 | 21.24496 | 9.147419 | 15.19619 |
| ENSMUSG00000038132 | protein_coding | Rbm24 | 25.69725 | 40.40543 | 33.05134 |
| ENSMUSG00000051510 | protein_coding | Mafg | 1717.854 | 1899.912 | 1808.883 |
| ENSMUSG00000079732 | protein_coding | Dynlt1f | 86.96348 | 66.0252 | 76.49434 |
| ENSMUSG00000082217 | pseudogene | Gm1401 | 0.33031 | 2.737158 | 1.533734 |
| ENSMUSG00000092456 | protein_coding | V1rd19 | 0.33031 | 2.737158 | 1.533734 |
| ENSMUSG00000022053 | protein_coding | Ebf2 | 32.26626 | 18.25119 | 25.25872 |
| ENSMUSG00000028914 | protein_coding | Casp9 | 1413.175 | 1239.747 | 1326.461 |
| ENSMUSG00000030742 | protein_coding | Lat | 48.19472 | 68.87151 | 58.53311 |
| ENSMUSG00000079598 | protein_coding | Clec2l | 208.4275 | 160.1344 | 184.2809 |
| ENSMUSG00000025369 | protein_coding | Smarcc2 | 3539.444 | 4009.389 | 3774.417 |
| ENSMUSG00000068114 | protein_coding | Ccdc134 | 492.5264 | 427.6038 | 460.0651 |
| ENSMUSG00000083009 | pseudogene | Gm1335 | 0.316221 | 2.737158 | 1.52669 |
| ENSMUSG00000065374 | snRNA | U1 | 0.316221 | 2.737158 | 1.52669 |
| ENSMUSG00000023978 | protein_coding | Prph2 | 67.01457 | 45.93164 | 56.4731 |
| ENSMUSG00000023930 | protein_coding | Crisp2 | 7.546665 | 1.739597 | 4.643131 |
| ENSMUSG00000030188 | protein_coding | Magohb | 301.2012 | 256.1805 | 278.6909 |
| ENSMUSG00000034639 | protein_coding | Setmar | 224.901 | 179.1354 | 202.0182 |
| ENSMUSG00000006782 | protein_coding | Cnp | 910.2161 | 1079.434 | 994.8253 |
| ENSMUSG00000048349 | protein_coding | Pou4f1 | 460.8316 | 377.0287 | 418.9302 |
| ENSMUSG00000085416 | processed_transcrip | 1700019G24Rik | 0.316221 | 2.772834 | 1.544527 |
| ENSMUSG00000047515 | protein_coding | BC049715 | 10.6561 | 3.541897 | 7.098998 |
| ENSMUSG00000092660 | miRNA | AL845291.1 | 5.439271 | 12.25505 | 8.847162 |
| ENSMUSG00000027660 | protein_coding | Skil | 2913.185 | 3230.167 | 3071.676 |
| ENSMUSG00000063480 | protein_coding | Nhp2l1 | 4212.945 | 4618.625 | 4415.785 |
| ENSMUSG00000059482 | protein_coding | 2610301B20Rik | 991.4389 | 862.6721 | 927.0555 |
| ENSMUSG00000085538 | processed_transcrip | Gm11455 | 1.304841 | 5.156812 | 3.230826 |
| ENSMUSG00000072681 | protein_coding | Plac9 | 2.908235 | 0.36219 | 1.635212 |
| ENSMUSG00000090358 | lincRNA | Gm2822 | 12.64869 | 5.854525 | 9.25161 |
| ENSMUSG00000055341 | protein_coding | Zfp457 | 10.28964 | 22.37916 | 16.3344 |
| ENSMUSG00000019189 | protein_coding | Rnf145 | 3896.363 | 4325.928 | 4111.146 |
| ENSMUSG00000024012 | protein_coding | Mtch1 | 3515.793 | 3194.561 | 3355.177 |
| ENSMUSG00000052135 | protein_coding | Foxo6 | 761.1841 | 891.0543 | 826.1192 |
| ENSMUSG00000026335 | protein_coding | Pam | 2969.137 | 3374.76 | 3171.949 |
| ENSMUSG00000046240 | protein_coding | Hepacam | 140.0749 | 185.1604 | 162.6176 |
| ENSMUSG00000024914 | protein_coding | Drap1 | 2298.373 | 2073.14 | 2185.757 |
| ENSMUSG00000040850 | protein_coding | Psme4 | 3537.532 | 3204.124 | 3370.828 |
| ENSMUSG00000000739 | protein_coding | Sult5a1 | 69.09498 | 96.33909 | 82.71703 |
| ENSMUSG00000049191 | protein_coding | Rgag4 | 79.82704 | 102.5767 | 91.20185 |
| ENSMUSG00000023176 | protein_coding | Cpn2 | 1.929083 | 6.109689 | 4.019386 |

| | | | | | |
|---|---|---|---|---|---|
| ENSMUSG00000031770 | protein_coding | Herpud1 | 556.0361 | 632.8302 | 594.4331 |
| ENSMUSG00000082286 | processed_transcrip | Pisd-ps1 | 878.0525 | 711.6636 | 794.8581 |
| ENSMUSG00000021600 | protein_coding | 8030423J24Rik | 2.261704 | 9.712163 | 5.986933 |
| ENSMUSG00000081128 | pseudogene | Gm13328 | 5.803649 | 1.306057 | 3.554853 |
| ENSMUSG00000082898 | pseudogene | Gm12041 | 15.40322 | 29.74806 | 22.57564 |
| ENSMUSG00000042737 | protein_coding | Dpm3 | 279.8795 | 329.8431 | 304.8613 |
| ENSMUSG00000020163 | protein_coding | Uqcr11 | 1116.168 | 986.0354 | 1051.102 |
| ENSMUSG00000085726 | lincRNA | 9630001P10Rik | 64.70218 | 97.0051 | 80.85364 |
| ENSMUSG00000026806 | protein_coding | Ddx31 | 695.2025 | 604.4224 | 649.8124 |
| ENSMUSG00000035914 | protein_coding | Cd276 | 540.9121 | 638.4364 | 589.6743 |
| ENSMUSG00000044084 | protein_coding | 4933402P03Rik | 1.945483 | 6.136355 | 4.040919 |
| ENSMUSG00000082036 | pseudogene | Gm13724 | 23.32373 | 13.1488 | 18.23626 |
| ENSMUSG00000024614 | protein_coding | Tmx3 | 2026.594 | 2345.349 | 2185.972 |
| ENSMUSG00000085407 | lincRNA | 1700095J03Rik | 29.60297 | 15.84992 | 22.72645 |
| ENSMUSG00000081165 | pseudogene | Gm11783 | 5.209895 | 1.359751 | 3.284823 |
| ENSMUSG00000041112 | protein_coding | Elmo1 | 819.6628 | 959.3142 | 889.4885 |
| ENSMUSG00000083832 | pseudogene | Gm13771 | 8.081752 | 3.046016 | 5.563884 |
| ENSMUSG00000003809 | protein_coding | Gcdh | 647.9687 | 744.0122 | 695.9905 |
| ENSMUSG00000022751 | protein_coding | Nit2 | 910.779 | 1067.705 | 989.2422 |
| ENSMUSG00000020193 | protein_coding | Zpbp | 139.6667 | 105.046 | 122.3564 |
| ENSMUSG00000037784 | protein_coding | Dzip1l | 371.0792 | 305.2866 | 338.1829 |
| ENSMUSG00000062054 | protein_coding | Iah1 | 998.6208 | 865.883 | 932.2519 |
| ENSMUSG00000031865 | protein_coding | Dctn1 | 5641.576 | 6309.887 | 5975.731 |
| ENSMUSG00000079364 | protein_coding | Gm3558 | 2.865968 | 8.502314 | 5.684141 |
| ENSMUSG00000056673 | protein_coding | Kdm5d | 1062.753 | 1223.585 | 1143.169 |
| ENSMUSG00000029844 | protein_coding | Hoxa1 | 14.5332 | 25.93468 | 20.23394 |
| ENSMUSG00000036932 | protein_coding | Aifm1 | 1843.845 | 2068.73 | 1956.287 |
| ENSMUSG00000035373 | protein_coding | Ccl7 | 15.33806 | 6.60557 | 10.97182 |
| ENSMUSG00000033276 | protein_coding | Stk36 | 223.2216 | 174.7502 | 198.9859 |
| ENSMUSG00000023143 | protein_coding | Nagpa | 835.1858 | 735.7546 | 785.4702 |
| ENSMUSG00000091159 | protein_coding | Gm17545 | 7.384754 | 15.64845 | 11.5166 |
| ENSMUSG00000024421 | protein_coding | Lama3 | 738.5341 | 931.5016 | 835.0179 |
| ENSMUSG00000087084 | processed_transcrip | Gm13016 | 29.94335 | 17.50595 | 23.72465 |
| ENSMUSG00000055322 | protein_coding | Tns1 | 2098.503 | 2417.909 | 2258.206 |
| ENSMUSG00000027130 | protein_coding | Slc12a6 | 3418.99 | 3104.376 | 3261.683 |
| ENSMUSG00000046792 | protein_coding | Zfp787 | 1042.208 | 1187.47 | 1114.839 |
| ENSMUSG00000019817 | protein_coding | Plagl1 | 2170.303 | 2543.188 | 2356.745 |
| ENSMUSG00000084949 | processed_transcrip | Gm16249 | 0.646531 | 3.726072 | 2.186302 |
| ENSMUSG00000000632 | protein_coding | Sez6 | 73.01561 | 52.81799 | 62.9168 |
| ENSMUSG00000051435 | protein_coding | Fhad1 | 108.8123 | 83.69078 | 96.25152 |
| ENSMUSG00000085558 | lincRNA | 4930412C18Rik | 99.10587 | 75.47925 | 87.29256 |
| ENSMUSG00000002808 | protein_coding | Epdr1 | 499.5301 | 609.2886 | 554.4094 |
| ENSMUSG00000036528 | protein_coding | Ppfibp2 | 706.0733 | 845.0381 | 775.5557 |
| ENSMUSG00000066798 | protein_coding | Zbtb6 | 816.5262 | 951.8748 | 884.2005 |
| ENSMUSG00000020653 | protein_coding | Klf11 | 1657.76 | 1452.282 | 1555.021 |
| ENSMUSG00000031904 | protein_coding | Slc7a6 | 2350.173 | 2689.433 | 2519.803 |
| ENSMUSG00000022194 | protein_coding | Pabpn1 | 1685.993 | 2027.96 | 1856.977 |
| ENSMUSG00000041809 | protein_coding | Efhc1 | 134.5688 | 101.0148 | 117.7918 |
| ENSMUSG00000031367 | protein_coding | Ap1s2 | 1880.855 | 1621.87 | 1751.362 |
| ENSMUSG00000037681 | protein_coding | Esyt3 | 1633.529 | 1311.827 | 1472.678 |
| ENSMUSG00000056687 | protein_coding | Gm11696 | 48.99638 | 66.30739 | 57.65189 |
| ENSMUSG00000030663 | protein_coding | 1110004F10Rik | 2865.006 | 3143.474 | 3004.24 |
| ENSMUSG00000027419 | protein_coding | Pcsk2 | 6.123448 | 13.16361 | 9.643527 |
| ENSMUSG00000055494 | processed_transcrip | Gm14168 | 23.00497 | 36.33198 | 29.66848 |
| ENSMUSG00000029168 | protein_coding | Dpysl5 | 927.3106 | 1065.682 | 996.4963 |
| ENSMUSG00000010025 | protein_coding | Aldh3a2 | 2786.917 | 3133.581 | 2960.249 |
| ENSMUSG00000029126 | protein_coding | Nsg1 | 177.5305 | 218.5093 | 198.0199 |
| ENSMUSG00000002006 | protein_coding | Pdzd4 | 264.0369 | 220.1044 | 242.0707 |

| | | | | | |
|---|---|---|---|---|---|
| ENSMUSG00000087277 | processed_transcrip | 2010013B24Rik | 3.899165 | 0.662038 | 2.280601 |
| ENSMUSG00000039055 | protein_coding | Eme1 | 428.2843 | 364.8118 | 396.5481 |
| ENSMUSG00000026452 | protein_coding | Syt2 | 363.2892 | 485.6596 | 424.4744 |
| ENSMUSG00000091144 | protein_coding | Gm6907 | 103.3793 | 139.4332 | 121.4063 |
| ENSMUSG00000026413 | protein_coding | Pkp1 | 42.18644 | 28.18212 | 35.18428 |
| ENSMUSG00000030781 | protein_coding | Slc5a2 | 8.423841 | 3.018988 | 5.721414 |
| ENSMUSG00000079252 | protein_coding | Tor1aip2 | 2139.881 | 2525.484 | 2332.683 |
| ENSMUSG00000025504 | protein_coding | Eps8l2 | 23.73604 | 43.1703 | 33.45317 |
| ENSMUSG00000034463 | protein_coding | Scara3 | 1160.753 | 1314.257 | 1237.505 |
| ENSMUSG00000068545 | pseudogene | Gm10243 | 65.53164 | 91.08492 | 78.30828 |
| ENSMUSG00000016382 | protein_coding | Pls3 | 1981.815 | 2389.711 | 2185.763 |
| ENSMUSG00000054408 | processed_transcrip | Spcs3 | 2227.984 | 2007.364 | 2117.674 |
| ENSMUSG00000053310 | protein_coding | Nrgn | 21.35827 | 33.11116 | 27.23471 |
| ENSMUSG00000038648 | protein_coding | Creb3l2 | 1832.046 | 2220.264 | 2026.155 |
| ENSMUSG00000033460 | protein_coding | Armcx1 | 589.127 | 743.292 | 666.2095 |
| ENSMUSG00000029516 | protein_coding | Cit | 1059.061 | 913.8413 | 986.4511 |
| ENSMUSG00000001127 | protein_coding | Araf | 1551.744 | 1746.723 | 1649.234 |
| ENSMUSG00000031262 | protein_coding | Cenpi | 655.7463 | 569.8612 | 612.8038 |
| ENSMUSG00000078866 | protein_coding | Gm14420 | 213.5283 | 253.7851 | 233.6567 |
| ENSMUSG00000031512 | protein_coding | 1700018L24Rik | 0.33031 | 2.781843 | 1.556077 |
| ENSMUSG00000031023 | protein_coding | D930014E17Rik | 422.967 | 360.0193 | 391.4932 |
| ENSMUSG00000060681 | protein_coding | Slc9a6 | 1609.168 | 1441.567 | 1525.367 |
| ENSMUSG00000046999 | protein_coding | 1110032F04Rik | 32.44852 | 54.51803 | 43.48327 |
| ENSMUSG00000000532 | protein_coding | Acvr1b | 1120.742 | 1319.907 | 1220.324 |
| ENSMUSG00000045438 | protein_coding | Cox19 | 665.155 | 577.0136 | 621.0843 |
| ENSMUSG00000036167 | protein_coding | Pphln1 | 2526.52 | 2778.936 | 2652.728 |
| ENSMUSG00000010307 | protein_coding | Tmem86a | 2963.234 | 3518.032 | 3240.633 |
| ENSMUSG00000049600 | protein_coding | Zbtb45 | 303.1647 | 253.4877 | 278.3262 |
| ENSMUSG00000032939 | protein_coding | Nup93 | 3634.842 | 3164.47 | 3399.656 |
| ENSMUSG00000027506 | protein_coding | Tpd52 | 2591.213 | 2940.033 | 2765.623 |
| ENSMUSG00000042671 | protein_coding | Rgs8 | 9.370193 | 3.823727 | 6.59696 |
| ENSMUSG00000078177 | protein_coding | Gm4877 | 0.316221 | 2.781843 | 1.549032 |
| ENSMUSG00000000411 | protein_coding | Tssk3 | 16.73266 | 8.909188 | 12.82092 |
| ENSMUSG00000020941 | protein_coding | Map3k14 | 229.6889 | 191.2109 | 210.4499 |
| ENSMUSG00000035878 | protein_coding | Agphd1 | 269.8382 | 228.8939 | 249.3661 |
| ENSMUSG00000024793 | protein_coding | Tnfrsf25 | 47.12745 | 66.36253 | 56.74499 |
| ENSMUSG00000078635 | processed_transcrip | 1700028N14Rik | 2.942302 | 0.326514 | 1.634408 |
| ENSMUSG00000020003 | protein_coding | Pex7 | 795.8304 | 699.5329 | 747.6816 |
| ENSMUSG00000023737 | processed_transcrip | 1700082M22Rik | 9.126728 | 3.434871 | 6.2808 |
| ENSMUSG00000032818 | protein_coding | Loxhd1 | 12.94032 | 6.09167 | 9.515993 |
| ENSMUSG00000020882 | protein_coding | Cacnb1 | 127.1822 | 160.0714 | 143.6268 |
| ENSMUSG00000028876 | protein_coding | Epha10 | 10.68219 | 4.423424 | 7.552807 |
| ENSMUSG00000090095 | pseudogene | Rpl29-ps1 | 2.233526 | 0 | 1.116763 |
| ENSMUSG00000070306 | protein_coding | Ccdc153 | 0.632442 | 3.770395 | 2.201419 |
| ENSMUSG00000078498 | protein_coding | Gm13151 | 21.73778 | 37.15106 | 29.44442 |
| ENSMUSG00000091678 | protein_coding | Gm17527 | 5.140718 | 12.43379 | 8.787254 |
| ENSMUSG00000030423 | protein_coding | Pop4 | 1173.796 | 899.4272 | 1036.612 |
| ENSMUSG00000022587 | protein_coding | Ly6e | 1830.672 | 2047.662 | 1939.167 |
| ENSMUSG00000072494 | lincRNA | Ppp1r3e | 281.0555 | 340.069 | 310.5623 |
| ENSMUSG00000036381 | protein_coding | P2ry14 | 16.80206 | 29.4683 | 23.13518 |
| ENSMUSG00000024583 | protein_coding | Txnl1 | 2977.019 | 3295.464 | 3136.242 |
| ENSMUSG00000078901 | protein_coding | Gm14440 | 42.13724 | 26.68112 | 34.40918 |
| ENSMUSG00000057193 | protein_coding | Slc44a2 | 4940.499 | 5484.118 | 5212.308 |
| ENSMUSG00000079609 | lincRNA | Gm17371 | 3.578323 | 10.61632 | 7.097322 |
| ENSMUSG00000023020 | protein_coding | 2310016M24Rik | 1117.557 | 1266.177 | 1191.867 |
| ENSMUSG00000046731 | protein_coding | Kctd11 | 107.4069 | 140.6371 | 124.022 |
| ENSMUSG00000039813 | protein_coding | Tbc1d2 | 207.2849 | 170.1555 | 188.7202 |
| ENSMUSG00000049382 | protein_coding | Krt8 | 35.27684 | 59.33968 | 47.30826 |

| | | | | | |
|---|---|---|---|---|---|
| ENSMUSG00000073119 | protein_coding | Speer4a | 51.00873 | 68.45386 | 59.7313 |
| ENSMUSG00000040543 | protein_coding | Pitpnm3 | 46.59088 | 69.2457 | 57.91829 |
| ENSMUSG00000020719 | protein_coding | Ddx5 | 24283.38 | 26584.71 | 25434.04 |
| ENSMUSG00000036781 | protein_coding | Rps27l | 1444.137 | 1286.97 | 1365.553 |
| ENSMUSG00000031387 | protein_coding | Renbp | 227.2106 | 274.1337 | 250.6722 |
| ENSMUSG00000025010 | protein_coding | Ccnj | 796.0663 | 696.3409 | 746.2036 |
| ENSMUSG00000022992 | protein_coding | 2310037I24Rik | 3157.53 | 2807.452 | 2982.491 |
| ENSMUSG00000050240 | protein_coding | Hic2 | 655.2202 | 558.3957 | 606.808 |
| ENSMUSG00000023186 | protein_coding | Vwa5a | 1139.838 | 1337.682 | 1238.76 |
| ENSMUSG00000045149 | pseudogene | Gm8946 | 40.7065 | 27.54675 | 34.12662 |
| ENSMUSG00000036606 | protein_coding | Plxnb2 | 4783.751 | 5306.363 | 5045.057 |
| ENSMUSG00000027555 | protein_coding | Car13 | 55.79223 | 74.63097 | 65.2116 |
| ENSMUSG00000086058 | lincRNA | Gm12575 | 0.646531 | 3.681388 | 2.163959 |
| ENSMUSG00000035585 | protein_coding | Tsen34 | 2198.348 | 1984.536 | 2091.442 |
| ENSMUSG00000044496 | protein_coding | 2510039O18Rik | 2129.162 | 1917.244 | 2023.203 |
| ENSMUSG00000020330 | protein_coding | Hmmr | 1082.628 | 1236.329 | 1159.478 |
| ENSMUSG00000038421 | protein_coding | Fcrla | 1.290752 | 5.368014 | 3.329383 |
| ENSMUSG00000087616 | processed_transcrip | Gm14257 | 18.5907 | 8.327149 | 13.45892 |
| ENSMUSG00000027890 | protein_coding | Gstm4 | 327.0909 | 279.2108 | 303.1509 |
| ENSMUSG00000028607 | protein_coding | Cpt2 | 1391.292 | 1259.06 | 1325.176 |
| ENSMUSG00000047343 | protein_coding | A530098C11Rik | 8.345196 | 16.61686 | 12.48103 |
| ENSMUSG00000004054 | protein_coding | Map3k11 | 1571.074 | 1755.307 | 1663.191 |
| ENSMUSG00000073027 | protein_coding | Dmrtc1b | 40.86841 | 23.05804 | 31.96322 |
| ENSMUSG00000030077 | protein_coding | Chl1 | 4.504697 | 1.033237 | 2.768967 |
| ENSMUSG00000071671 | protein_coding | Gm10343 | 3.882766 | 9.182371 | 6.532568 |
| ENSMUSG00000019988 | protein_coding | Nedd1 | 1321.101 | 1172.445 | 1246.773 |
| ENSMUSG00000047384 | protein_coding | A730013G03Rik | 14.79075 | 26.12751 | 20.45913 |
| ENSMUSG00000032788 | protein_coding | Pdxk | 965.0377 | 854.0802 | 909.5589 |
| ENSMUSG00000031226 | protein_coding | 2610029G23Rik | 1422.614 | 1275.821 | 1349.218 |
| ENSMUSG00000074220 | protein_coding | Zfp382 | 140.7311 | 111.9316 | 126.3314 |
| ENSMUSG00000034910 | protein_coding | Pygo1 | 814.4914 | 688.997 | 751.7442 |
| ENSMUSG00000066613 | protein_coding | Zfp932 | 439.1754 | 550.7168 | 494.9461 |
| ENSMUSG00000069273 | protein_coding | Hist1h3e | 7.452888 | 2.737158 | 5.095023 |
| ENSMUSG00000046613 | protein_coding | Vwa5b2 | 50.98771 | 36.44729 | 43.7175 |
| ENSMUSG00000047496 | protein_coding | Rnf152 | 265.5976 | 214.7829 | 240.1903 |
| ENSMUSG00000022106 | protein_coding | Rcbtb2 | 978.7844 | 1104.334 | 1041.559 |
| ENSMUSG00000028136 | protein_coding | Snx27 | 3180.955 | 2818 | 2999.478 |
| ENSMUSG00000023243 | protein_coding | Kcnk5 | 320.9954 | 270.2137 | 295.6045 |
| ENSMUSG00000047773 | protein_coding | Ankfn1 | 0 | 2.075121 | 1.03756 |
| ENSMUSG00000055629 | protein_coding | B4galnt4 | 172.3134 | 139.2448 | 155.7791 |
| ENSMUSG00000024766 | protein_coding | Lipo1 | 481.318 | 553.3295 | 517.3238 |
| ENSMUSG00000079735 | protein_coding | Gm2792 | 23.22973 | 13.46309 | 18.34641 |
| ENSMUSG00000029843 | protein_coding | Slc13a4 | 98.07647 | 72.90929 | 85.49288 |
| ENSMUSG00000039438 | protein_coding | Ttc36 | 17.22257 | 8.873513 | 13.04804 |
| ENSMUSG00000039985 | protein_coding | Fam60a | 2199.914 | 2486.877 | 2343.395 |
| ENSMUSG00000058297 | protein_coding | Spock2 | 16185.39 | 18149.12 | 17167.25 |
| ENSMUSG00000004996 | protein_coding | Mri1 | 365.7523 | 442.8166 | 404.2844 |
| ENSMUSG00000045091 | protein_coding | Aqp12 | 23.11955 | 39.7741 | 31.44682 |
| ENSMUSG00000058388 | protein_coding | Phtf1 | 1740.963 | 1504.671 | 1622.817 |
| ENSMUSG00000044452 | protein_coding | Zfp507 | 984.3036 | 859.788 | 922.0458 |
| ENSMUSG00000074075 | protein_coding | Gm10621 | 15.70833 | 6.52521 | 11.11677 |
| ENSMUSG00000031732 | protein_coding | Phlpp2 | 1469.471 | 1298.236 | 1383.853 |
| ENSMUSG00000020826 | protein_coding | Nos2 | 7.12951 | 14.64768 | 10.88859 |
| ENSMUSG00000091698 | protein_coding | Gm6526 | 91.56554 | 68.11906 | 79.8423 |
| ENSMUSG00000028757 | protein_coding | Ddost | 6193.313 | 5605.933 | 5899.623 |
| ENSMUSG00000042185 | protein_coding | Nfrkb | 1548.612 | 1718.032 | 1633.322 |
| ENSMUSG00000037325 | protein_coding | Bbs7 | 489.1238 | 405.1576 | 447.1407 |
| ENSMUSG00000021645 | protein_coding | Smn1 | 652.3138 | 742.6427 | 697.4783 |

| | | | | | |
|---|---|---|---|---|---|
| ENSMUSG00000024517 | protein_coding | Grp | 2.581503 | 7.18761 | 4.884556 |
| ENSMUSG00000044734 | protein_coding | Serpinb1a | 584.9675 | 659.8061 | 622.3868 |
| ENSMUSG00000024154 | protein_coding | Gtf2a1l | 6.508847 | 2.075121 | 4.291984 |
| ENSMUSG00000066807 | pseudogene | Gm10179 | 30.94606 | 19.54896 | 25.24751 |
| ENSMUSG00000034617 | protein_coding | Mtrr | 795.8002 | 708.9021 | 752.3512 |
| ENSMUSG00000048249 | protein_coding | A930001N09Rik | 3491.661 | 3179.233 | 3335.447 |
| ENSMUSG00000036634 | protein_coding | Mag | 18.50027 | 9.298406 | 13.89934 |
| ENSMUSG00000051490 | protein_coding | Foxd4 | 0.976841 | 4.548106 | 2.762474 |
| ENSMUSG00000044252 | protein_coding | Osbpl1a | 2018.378 | 2284.31 | 2151.344 |
| ENSMUSG00000091811 | protein_coding | Prr24 | 327.7219 | 267.0131 | 297.3675 |
| ENSMUSG00000079510 | protein_coding | Mdga2 | 2.547436 | 0 | 1.273718 |
| ENSMUSG00000022044 | protein_coding | Stmn4 | 7.418821 | 14.35684 | 10.88783 |
| ENSMUSG00000029094 | protein_coding | Afap1 | 853.7615 | 1032.256 | 943.0087 |
| ENSMUSG00000057805 | protein_coding | 1700084M14Rik | 8.985838 | 3.381178 | 6.183508 |
| ENSMUSG00000086331 | processed_transcrip | Gm16310 | 66.30095 | 47.96932 | 57.13513 |
| ENSMUSG00000029009 | protein_coding | Mthfr | 2286.534 | 2676.021 | 2481.278 |
| ENSMUSG00000025964 | protein_coding | Adam23 | 403.7742 | 346.8184 | 375.2963 |
| ENSMUSG00000041859 | protein_coding | Mcm3 | 4068.976 | 4508.423 | 4288.7 |
| ENSMUSG00000085001 | processed_transcrip | BB114351 | 0 | 2.057464 | 1.028732 |
| ENSMUSG00000003072 | protein_coding | Atp5d | 2699.9 | 2428.358 | 2564.129 |
| ENSMUSG00000053841 | protein_coding | Txlna | 4134.676 | 3742.282 | 3938.479 |
| ENSMUSG00000045248 | protein_coding | Med26 | 1106.258 | 940.0025 | 1023.13 |
| ENSMUSG00000075279 | pseudogene | Mrpl23-ps1 | 10.06361 | 4.114566 | 7.089089 |
| ENSMUSG00000004948 | protein_coding | Zp3 | 46.31126 | 68.70971 | 57.51048 |
| ENSMUSG00000062345 | protein_coding | Serpinb2 | 62.4199 | 43.56034 | 52.99012 |
| ENSMUSG00000028062 | protein_coding | Lamtor2 | 905.2977 | 779.0378 | 842.1677 |
| ENSMUSG00000057707 | protein_coding | Rpl29-ps2 | 4.509319 | 0.988552 | 2.748935 |
| ENSMUSG00000026571 | protein_coding | Dcaf6 | 2619.864 | 2189.115 | 2404.49 |
| ENSMUSG00000044408 | protein_coding | 1110002B05Rik | 2084.334 | 2358.577 | 2221.456 |
| ENSMUSG00000063320 | protein_coding | 1190007I07Rik | 155.6069 | 196.941 | 176.2739 |
| ENSMUSG00000058258 | protein_coding | Idi1 | 1051.57 | 920.4347 | 986.0023 |
| ENSMUSG00000025665 | protein_coding | Rps6ka6 | 1662.554 | 1391.395 | 1526.975 |
| ENSMUSG00000028776 | protein_coding | Tinagl1 | 104.7937 | 134.6607 | 119.7272 |
| ENSMUSG00000037949 | protein_coding | Ano10 | 944.2869 | 840.0156 | 892.1513 |
| ENSMUSG00000089687 | protein_coding | Rab42-ps | 28.96986 | 43.95893 | 36.46439 |
| ENSMUSG00000064280 | protein_coding | Ccdc146 | 27.80845 | 17.37655 | 22.5925 |
| ENSMUSG00000042367 | protein_coding | Gjb3 | 14.77898 | 24.87266 | 19.82582 |
| ENSMUSG00000070509 | protein_coding | Rgma | 596.2225 | 504.1322 | 550.1774 |
| ENSMUSG00000059412 | protein_coding | Fxyd2 | 46.83919 | 64.07621 | 55.4577 |
| ENSMUSG00000021223 | protein_coding | Papln | 154.7398 | 120.1488 | 137.4443 |
| ENSMUSG00000040794 | protein_coding | C1qtnf4 | 282.5714 | 331.2962 | 306.9338 |
| ENSMUSG00000067231 | protein_coding | Cyp2c65 | 1.298952 | 4.856964 | 3.077958 |
| ENSMUSG00000056553 | protein_coding | Ptprn2 | 63.47911 | 85.63225 | 74.55568 |
| ENSMUSG00000000682 | protein_coding | Cd52 | 31.58566 | 19.61131 | 25.59848 |
| ENSMUSG00000035960 | protein_coding | Apex1 | 1054.112 | 1196.257 | 1125.185 |
| ENSMUSG00000034709 | protein_coding | Klraq1 | 960.0752 | 1063.213 | 1011.644 |
| ENSMUSG00000041355 | protein_coding | Ssr2 | 4037.467 | 3677.688 | 3857.578 |
| ENSMUSG00000077505 | snoRNA | SNORA26 | 6.446601 | 2.030798 | 4.2387 |
| ENSMUSG00000021728 | protein_coding | Emb | 529.5061 | 659.9433 | 594.7247 |
| ENSMUSG00000052629 | protein_coding | Gm9885 | 12.2633 | 5.756508 | 9.009902 |
| ENSMUSG00000067928 | protein_coding | Zfp760 | 627.2383 | 750.3562 | 688.7972 |
| ENSMUSG00000082176 | pseudogene | Gm13163 | 6.125759 | 13.46703 | 9.796393 |
| ENSMUSG00000089640 | pseudogene | Gm6397 | 31.09425 | 48.14448 | 39.61937 |
| ENSMUSG00000071847 | protein_coding | Apcdd1 | 390.8336 | 339.939 | 365.3863 |
| ENSMUSG00000083240 | pseudogene | Gm13453 | 22.84373 | 47.37686 | 35.1103 |
| ENSMUSG00000038866 | protein_coding | Zcchc2 | 775.3846 | 682.8291 | 729.1069 |
| ENSMUSG00000037143 | protein_coding | 4930529M08Rik | 83.7394 | 63.51975 | 73.62958 |
| ENSMUSG00000048960 | protein_coding | Prex2 | 67.06459 | 99.48632 | 83.27545 |

| | | | | | |
|---|---|---|---|---|---|
| ENSMUSG00000090628 | pseudogene | Gm17083 | 9.811947 | 20.76959 | 15.29077 |
| ENSMUSG00000034601 | protein_coding | 2700049A03Rik | 736.4492 | 645.4477 | 690.9484 |
| ENSMUSG00000089728 | pseudogene | Clec2f-ps | 0.32211 | 2.674817 | 1.498464 |
| ENSMUSG00000042599 | protein_coding | Jhdm1d | 10036.4 | 11277.38 | 10656.89 |
| ENSMUSG00000009406 | protein_coding | Elk1 | 772.3997 | 861.215 | 816.8073 |
| ENSMUSG00000046709 | protein_coding | Mapk10 | 355.9593 | 429.247 | 392.6032 |
| ENSMUSG00000025002 | protein_coding | Cyp2c55 | 44.1355 | 28.40089 | 36.2682 |
| ENSMUSG00000032511 | protein_coding | Scn5a | 639.3324 | 519.3094 | 579.3209 |
| ENSMUSG00000038542 | protein_coding | Pcid2 | 1160.736 | 1035.226 | 1097.981 |
| ENSMUSG00000060176 | protein_coding | Kif27 | 579.7226 | 452.1066 | 515.9146 |
| ENSMUSG00000024667 | protein_coding | Tmem216 | 250.7978 | 211.2118 | 231.0048 |
| ENSMUSG00000028164 | protein_coding | Manba | 448.8669 | 581.7887 | 515.3278 |
| ENSMUSG00000040843 | protein_coding | Tiprl | 1171.983 | 1322.045 | 1247.014 |
| ENSMUSG00000021997 | protein_coding | Lrrc63 | 8.079441 | 3.135023 | 5.607232 |
| ENSMUSG00000078908 | protein_coding | Mon1b | 421.3743 | 359.6624 | 390.5183 |
| ENSMUSG00000074680 | protein_coding | Defb26 | 0 | 2.110796 | 1.055398 |
| ENSMUSG00000003948 | protein_coding | Mmd | 1026.346 | 1180.834 | 1103.59 |
| ENSMUSG00000045917 | protein_coding | 6330416G13Rik | 360.237 | 305.1921 | 332.7146 |
| ENSMUSG00000021714 | protein_coding | Cenpk | 415.0834 | 357.5757 | 386.3296 |
| ENSMUSG00000034059 | protein_coding | Ypel4 | 42.68374 | 63.57856 | 53.13115 |
| ENSMUSG00000090867 | lincRNA | Snhg1 | 611.2306 | 723.5164 | 667.3735 |
| ENSMUSG00000027108 | protein_coding | Ola1 | 1769.729 | 1578.238 | 1673.984 |
| ENSMUSG00000024797 | protein_coding | 1110014N23Rik | 934.4223 | 1061.595 | 998.0086 |
| ENSMUSG00000020099 | protein_coding | Unc5b | 562.5113 | 486.8756 | 524.6934 |
| ENSMUSG00000027601 | protein_coding | Mtfr1 | 1243.571 | 1125.791 | 1184.681 |
| ENSMUSG00000044026 | protein_coding | Tmem20 | 1846.682 | 2081.402 | 1964.042 |
| ENSMUSG00000025782 | protein_coding | Taf3 | 711.2615 | 614.5162 | 662.8889 |
| ENSMUSG00000018651 | protein_coding | Tada2a | 1083.119 | 1212.197 | 1147.658 |
| ENSMUSG00000037885 | protein_coding | Stk35 | 1907.908 | 1733.078 | 1820.493 |
| ENSMUSG00000082381 | pseudogene | Gm12928 | 56.90869 | 41.32227 | 49.11548 |
| ENSMUSG00000079455 | pseudogene | Gm16026 | 3.208056 | 8.681052 | 5.944554 |
| ENSMUSG00000084892 | lincRNA | Gm14471 | 4.890095 | 1.050893 | 2.970494 |
| ENSMUSG00000032323 | protein_coding | Cyp11a1 | 1804.892 | 2192.023 | 1998.458 |
| ENSMUSG00000031816 | protein_coding | Mthfsd | 358.472 | 417.9861 | 388.2291 |
| ENSMUSG00000019578 | protein_coding | Ubxn6 | 2876.267 | 2594.718 | 2735.492 |
| ENSMUSG00000026473 | protein_coding | Glul | 7205.546 | 6518.107 | 6861.826 |
| ENSMUSG00000088309 | snoRNA | U3 | 11.99858 | 22.92434 | 17.46146 |
| ENSMUSG00000026649 | protein_coding | 1700009P17Rik | 41.04411 | 24.11369 | 32.5789 |
| ENSMUSG00000066315 | pseudogene | Gm12918 | 47.8009 | 32.73603 | 40.26846 |
| ENSMUSG00000031723 | protein_coding | Txnl4b | 333.7833 | 284.0488 | 308.9161 |
| ENSMUSG00000020895 | protein_coding | Tmem107 | 453.5786 | 390.3569 | 421.9678 |
| ENSMUSG00000025257 | protein_coding | Ribc1 | 189.3708 | 152.6628 | 171.0168 |
| ENSMUSG00000084459 | snoRNA | SNORD113 | 17.10963 | 33.43804 | 25.27383 |
| ENSMUSG00000040387 | protein_coding | Klhl32 | 62.44912 | 45.17267 | 53.81089 |
| ENSMUSG00000028356 | protein_coding | Ambp | 2.950502 | 0.36219 | 1.656346 |
| ENSMUSG00000087191 | lincRNA | 4933440J02Rik | 5.171206 | 1.395426 | 3.283316 |
| ENSMUSG00000030110 | protein_coding | Ret | 1419.687 | 1661.964 | 1540.825 |
| ENSMUSG00000034401 | protein_coding | Spata6 | 788.2359 | 680.9958 | 734.6159 |
| ENSMUSG00000032998 | protein_coding | Foxj3 | 2476.616 | 2834.632 | 2655.624 |
| ENSMUSG00000022809 | protein_coding | Nr1i2 | 0.638331 | 3.434871 | 2.036601 |
| ENSMUSG00000041624 | protein_coding | Gucy1a2 | 30.25264 | 44.28865 | 37.27065 |
| ENSMUSG00000032468 | protein_coding | Armc8 | 1711.662 | 1538.601 | 1625.132 |
| ENSMUSG00000019301 | protein_coding | Hsd17b1 | 438.1206 | 719.5565 | 578.8385 |
| ENSMUSG00000039116 | protein_coding | Gpr126 | 16.98075 | 29.08239 | 23.03157 |
| ENSMUSG00000032219 | protein_coding | Tex9 | 34.46676 | 49.82065 | 42.14371 |
| ENSMUSG00000018669 | protein_coding | Cdk5rap3 | 1692.477 | 1516.297 | 1604.387 |
| ENSMUSG00000036411 | protein_coding | 9530077C05Rik | 397.9155 | 341.1462 | 369.5309 |
| ENSMUSG00000052504 | protein_coding | Epha3 | 50.95618 | 71.48983 | 61.223 |

| | | | | | |
|---|---|---|---|---|---|
| ENSMUSG00000017009 | protein_coding | Sdc4 | 6528.581 | 7507.634 | 7018.107 |
| ENSMUSG00000032671 | protein_coding | A930018P22Rik | 16.24804 | 8.25616 | 12.2521 |
| ENSMUSG00000038712 | protein_coding | Fam63a | 964.6427 | 1075.19 | 1019.917 |
| ENSMUSG00000021665 | protein_coding | Hexb | 302.9163 | 354.9979 | 328.9571 |
| ENSMUSG00000043144 | protein_coding | Aqp6 | 1.623372 | 5.720833 | 3.672103 |
| ENSMUSG00000073295 | protein_coding | Nudt11 | 707.9012 | 831.8138 | 769.8575 |
| ENSMUSG00000023987 | protein_coding | Pgc | 5.845916 | 12.28172 | 9.063817 |
| ENSMUSG00000092358 | pseudogene | RP24-92H14.2 | 44.31635 | 30.30157 | 37.30896 |
| ENSMUSG00000079349 | protein_coding | Magea5 | 230.0479 | 283.3241 | 256.686 |
| ENSMUSG00000053194 | protein_coding | Cib4 | 0 | 2.428301 | 1.21415 |
| ENSMUSG00000029131 | protein_coding | Dnajb6 | 3416.279 | 3744.282 | 3580.281 |
| ENSMUSG00000007279 | protein_coding | Scube2 | 247.244 | 191.7252 | 219.4846 |
| ENSMUSG00000075265 | processed_transcrip | Gm10818 | 17.24486 | 29.29644 | 23.27065 |
| ENSMUSG00000086991 | processed_transcrip | Gm15334 | 0.316221 | 2.835175 | 1.575698 |
| ENSMUSG00000090145 | protein_coding | Ugt1a6b | 17.00303 | 8.359975 | 12.6815 |
| ENSMUSG00000046491 | protein_coding | C1qtnf2 | 131.5338 | 168.5097 | 150.0218 |
| ENSMUSG00000074781 | protein_coding | Ube2n | 3981.666 | 4336.659 | 4159.163 |
| ENSMUSG00000035095 | protein_coding | Fam167a | 77.91816 | 55.09241 | 66.50529 |
| ENSMUSG00000034292 | protein_coding | Traf3ip1 | 659.6791 | 582.8194 | 621.2492 |
| ENSMUSG00000084986 | processed_transcrip | Gm16224 | 2.259393 | 6.735689 | 4.497541 |
| ENSMUSG00000027692 | protein_coding | Tnik | 2716.767 | 3024.311 | 2870.539 |
| ENSMUSG00000064349 | Mt_tRNA | mt-Tc | 187.5372 | 148.9841 | 168.2606 |
| ENSMUSG00000028399 | protein_coding | Ptprd | 3089.523 | 3695.873 | 3392.698 |
| ENSMUSG00000024446 | protein_coding | Rpp21 | 226.3621 | 272.3912 | 249.3766 |
| ENSMUSG00000015968 | protein_coding | Cacna1d | 482.7602 | 391.3036 | 437.0319 |
| ENSMUSG00000028545 | protein_coding | Bend5 | 297.9896 | 249.8969 | 273.9432 |
| ENSMUSG00000035109 | protein_coding | Shc4 | 1431.537 | 968.0609 | 1199.799 |
| ENSMUSG00000074797 | protein_coding | Itpa | 2147.898 | 2417.135 | 2282.516 |
| ENSMUSG00000002820 | protein_coding | Atg4d | 679.1923 | 598.8968 | 639.0446 |
| ENSMUSG00000032192 | protein_coding | Gnb5 | 747.5815 | 876.0893 | 811.8354 |
| ENSMUSG00000067700 | protein_coding | Gm5862 | 61.36292 | 83.25578 | 72.30935 |
| ENSMUSG00000049028 | protein_coding | Olfr873 | 1.934972 | 7.036623 | 4.485797 |
| ENSMUSG00000026870 | protein_coding | D730039F16Rik | 5.532005 | 11.88385 | 8.707929 |
| ENSMUSG00000060044 | protein_coding | Tmem26 | 19.01434 | 10.19831 | 14.60633 |
| ENSMUSG00000066180 | protein_coding | Gm10155 | 18.46054 | 10.11759 | 14.28907 |
| ENSMUSG00000063767 | protein_coding | S100a7a | 1.284863 | 4.714262 | 2.999562 |
| ENSMUSG00000074346 | processed_transcrip | 1700095B22Rik | 57.36051 | 41.51473 | 49.43762 |
| ENSMUSG00000029524 | protein_coding | Sirt4 | 229.5621 | 280.0477 | 254.8049 |
| ENSMUSG00000024266 | protein_coding | Adad2 | 418.4772 | 358.4786 | 388.4779 |
| ENSMUSG00000079109 | protein_coding | Pms2 | 1189.853 | 1042.628 | 1116.241 |
| ENSMUSG00000028158 | protein_coding | Mttp | 362.0905 | 295.5143 | 328.8024 |
| ENSMUSG00000056763 | protein_coding | Cspp1 | 1183.886 | 1415.061 | 1299.474 |
| ENSMUSG00000004637 | protein_coding | Wwox | 483.1658 | 408.8843 | 446.0251 |
| ENSMUSG00000072844 | lincRNA | G530011O06Rik | 78.96209 | 53.35716 | 66.15963 |
| ENSMUSG00000025227 | protein_coding | Tmem180 | 745.5287 | 638.8277 | 692.1782 |
| ENSMUSG00000025722 | protein_coding | Wdr73 | 897.941 | 799.0832 | 848.5121 |
| ENSMUSG00000041346 | protein_coding | Wrap53 | 636.1003 | 559.2326 | 597.6664 |
| ENSMUSG00000058665 | protein_coding | En1 | 19.28703 | 10.57743 | 14.93223 |
| ENSMUSG00000002221 | protein_coding | Paxip1 | 1607.906 | 1427.992 | 1517.949 |
| ENSMUSG00000039191 | protein_coding | Rbpj | 3122.234 | 2834.628 | 2978.431 |
| ENSMUSG00000034652 | protein_coding | Cd300a | 52.68279 | 70.30877 | 61.49578 |
| ENSMUSG00000069631 | protein_coding | Strada | 904.804 | 808.1173 | 856.4607 |
| ENSMUSG00000022748 | protein_coding | 2610528E23Rik | 483.4224 | 551.0225 | 517.2224 |
| ENSMUSG00000033417 | protein_coding | 2700078E11Rik | 3349.178 | 3745.662 | 3547.42 |
| ENSMUSG00000056698 | protein_coding | Elmod3 | 410.0272 | 471.7263 | 440.8768 |
| ENSMUSG00000038954 | protein_coding | Supt3h | 445.167 | 382.4379 | 413.8025 |
| ENSMUSG00000046404 | protein_coding | Yod1 | 336.7208 | 390.04 | 363.3804 |
| ENSMUSG00000015932 | protein_coding | Dstn | 8351.825 | 9355.47 | 8853.648 |

| | | | | | | |
|---|---|---|---|---|---|---|
| ENSMUSG00000041837 | protein_coding | Pdcd7 | | 1286.937 | 1164.734 | 1225.835 |
| ENSMUSG00000026029 | protein_coding | Casp8 | | 705.2779 | 803.9964 | 754.6371 |
| ENSMUSG00000041075 | protein_coding | Fzd7 | | 893.3225 | 1049.624 | 971.4733 |
| ENSMUSG00000087547 | processed_transcrip | 2410019O14Rik | | 10.32832 | 18.40906 | 14.36869 |
| ENSMUSG00000033016 | protein_coding | Nfatc1 | | 89.51636 | 115.7327 | 102.6245 |
| ENSMUSG00000031737 | protein_coding | Irx5 | | 10.69501 | 4.821288 | 7.75815 |
| ENSMUSG00000018661 | protein_coding | Cog1 | | 756.8417 | 673.5842 | 715.213 |
| ENSMUSG00000074283 | protein_coding | Zfp109 | | 165.207 | 131.2176 | 148.2123 |
| ENSMUSG00000040943 | protein_coding | Tet2 | | 723.4186 | 836.6684 | 780.0435 |
| ENSMUSG00000026384 | protein_coding | Ptpn4 | | 1399.542 | 1223.046 | 1311.294 |
| ENSMUSG00000020385 | protein_coding | Clk4 | | 1709.621 | 2028.061 | 1868.841 |
| ENSMUSG00000039337 | protein_coding | Tex19.2 | | 1027.872 | 1160.636 | 1094.254 |
| ENSMUSG00000043661 | pseudogene | Gm9785 | | 163.3173 | 133.471 | 148.3942 |
| ENSMUSG00000021569 | protein_coding | Trip13 | | 1323.901 | 1174.762 | 1249.331 |
| ENSMUSG00000032263 | protein_coding | Bckdhb | | 649.0392 | 569.1526 | 609.0959 |
| ENSMUSG00000057421 | protein_coding | Las1l | | 2355.557 | 2601.029 | 2478.293 |
| ENSMUSG00000040268 | protein_coding | Plekha1 | | 2298.216 | 2556.677 | 2427.447 |
| ENSMUSG00000035215 | protein_coding | Lsm7 | | 1039.087 | 1183.491 | 1111.289 |
| ENSMUSG00000042408 | protein_coding | Zmym6 | | 1559.13 | 1307.544 | 1433.337 |
| ENSMUSG00000025757 | protein_coding | Hspa4l | | 3941.352 | 3304.893 | 3623.122 |
| ENSMUSG00000027166 | protein_coding | Dnajc24 | | 491.2976 | 427.0497 | 459.1736 |
| ENSMUSG00000080921 | pseudogene | Rpl38-ps2 | | 101.7135 | 79.46235 | 90.58791 |
| ENSMUSG00000024114 | protein_coding | Prss41 | | 151.9658 | 104.4239 | 128.1948 |
| ENSMUSG00000087351 | processed_transcrip | Gm11464 | | 15.9272 | 7.956312 | 11.94175 |
| ENSMUSG00000073867 | protein_coding | AA474408 | | 57.33404 | 40.11351 | 48.72378 |
| ENSMUSG00000051495 | protein_coding | Irf2bp2 | | 1714.359 | 1907.123 | 1810.741 |
| ENSMUSG00000045094 | protein_coding | Arhgef37 | | 4.518786 | 10.41164 | 7.465213 |
| ENSMUSG00000022558 | protein_coding | Heatr7a | | 1417.95 | 1212.299 | 1315.125 |
| ENSMUSG00000005705 | protein_coding | Agrp | | 34.95794 | 22.96618 | 28.96206 |
| ENSMUSG00000017670 | protein_coding | Elmo2 | | 1380.585 | 1240.923 | 1310.754 |
| ENSMUSG00000064871 | snoRNA | SNORD58 | | 11.69645 | 21.46517 | 16.58081 |
| ENSMUSG00000031400 | protein_coding | G6pdx | | 1468.931 | 1618.008 | 1543.47 |
| ENSMUSG00000087143 | lincRNA | A830082K12Rik | | 6.470158 | 12.88141 | 9.675786 |
| ENSMUSG00000016028 | protein_coding | Celsr1 | | 402.2484 | 488.0141 | 445.1313 |
| ENSMUSG00000065851 | snRNA | U6 | | 11.07579 | 20.8107 | 15.94324 |
| ENSMUSG00000082114 | pseudogene | Gm13489 | | 6.178536 | 2.048455 | 4.113496 |
| ENSMUSG00000071553 | protein_coding | Cpa2 | | 9.078572 | 16.76785 | 12.92321 |
| ENSMUSG00000039735 | protein_coding | Fnbp1l | | 4254.524 | 3900.064 | 4077.294 |
| ENSMUSG00000015994 | protein_coding | Fnta | | 1828.095 | 1996.336 | 1912.216 |
| ENSMUSG00000030243 | protein_coding | Recql | | 1110.097 | 940.4999 | 1025.299 |
| ENSMUSG00000059409 | protein_coding | Ppp2r5d | | 2260.499 | 2022.956 | 2141.728 |
| ENSMUSG00000014606 | protein_coding | Slc25a11 | | 989.9506 | 1117.656 | 1053.803 |
| ENSMUSG00000035597 | protein_coding | Prpf39 | | 1939.647 | 2285.821 | 2112.734 |
| ENSMUSG00000024160 | protein_coding | Spsb3 | | 201.1402 | 164.7193 | 182.9298 |
| ENSMUSG00000072528 | protein_coding | Rps19-ps4 | | 1.293062 | 5.014111 | 3.153587 |
| ENSMUSG00000070529 | protein_coding | Wfdc10 | | 2345.283 | 2103.449 | 2224.366 |
| ENSMUSG00000021339 | protein_coding | Mrs2 | | 807.0498 | 493.7122 | 650.381 |
| ENSMUSG00000086864 | processed_transcrip | A630077J23Rik | | 47.84674 | 64.89752 | 56.37213 |
| ENSMUSG00000020486 | protein_coding | | 4-Sep | 133.62 | 168.0125 | 150.8162 |
| ENSMUSG00000034460 | protein_coding | Six4 | | 142.9018 | 189.4012 | 166.1515 |
| ENSMUSG00000027519 | protein_coding | Rab22a | | 2597.433 | 2349.098 | 2473.265 |
| ENSMUSG00000057367 | protein_coding | Birc2 | | 4034.439 | 4625.497 | 4329.968 |
| ENSMUSG00000064215 | protein_coding | Ifi27l1 | | 1619.323 | 1861.334 | 1740.328 |
| ENSMUSG00000027881 | protein_coding | Prpf38b | | 3818.179 | 4261.203 | 4039.691 |
| ENSMUSG00000028011 | protein_coding | Tdo2 | | 12.61463 | 21.59493 | 17.10478 |
| ENSMUSG00000046922 | protein_coding | Gpr6 | | 5.487428 | 1.677256 | 3.582342 |
| ENSMUSG00000079139 | pseudogene | Gm4204 | | 1123.747 | 1012.082 | 1067.915 |
| ENSMUSG00000019471 | protein_coding | Cdc37 | | 3841.976 | 4238.999 | 4040.487 |

| | | | | | |
|---|---|---|---|---|---|
| ENSMUSG00000022957 | protein_coding | Itsn1 | 3853.444 | 3481.193 | 3667.318 |
| ENSMUSG00000024991 | protein_coding | Eif3a | 10959.05 | 12061.23 | 11510.14 |
| ENSMUSG00000038816 | protein_coding | Ctnnal1 | 4522.428 | 5061.647 | 4792.038 |
| ENSMUSG00000087658 | processed_transcrip | Gm15051 | 14.19931 | 24.99082 | 19.59506 |
| ENSMUSG00000028137 | protein_coding | Celf3 | 28.79349 | 41.63149 | 35.21249 |
| ENSMUSG00000019831 | protein_coding | Wasf1 | 1525.775 | 1723.458 | 1624.617 |
| ENSMUSG00000022994 | protein_coding | Adcy6 | 672.8551 | 794.7093 | 733.7822 |
| ENSMUSG00000090931 | lincRNA | 4632411P08Rik | 12.35118 | 5.747499 | 9.049341 |
| ENSMUSG00000020034 | protein_coding | Tcp11l2 | 430.1265 | 346.9639 | 388.5452 |
| ENSMUSG00000022961 | protein_coding | Son | 13231.96 | 14677.09 | 13954.53 |
| ENSMUSG00000000631 | protein_coding | Myo18a | 3782.341 | 4332.311 | 4057.326 |
| ENSMUSG00000053550 | protein_coding | Shisa7 | 45.88568 | 63.99657 | 54.94113 |
| ENSMUSG00000039128 | protein_coding | Cdc123 | 1493.517 | 1687.633 | 1590.575 |
| ENSMUSG00000091729 | pseudogene | Gm13697 | 0 | 2.401997 | 1.200998 |
| ENSMUSG00000089184 | snoRNA | SNORA70 | 0 | 2.499652 | 1.249826 |
| ENSMUSG00000029426 | protein_coding | Scarb2 | 1006.654 | 1128.66 | 1067.657 |
| ENSMUSG00000049303 | protein_coding | Syt12 | 125.3412 | 160.1323 | 142.7368 |
| ENSMUSG00000022263 | protein_coding | Trio | 2426.672 | 2765.579 | 2596.125 |
| ENSMUSG00000027978 | protein_coding | Prss12 | 462.7059 | 539.9476 | 501.3268 |
| ENSMUSG00000058189 | protein_coding | Hist1h2bm | 9.716903 | 4.16826 | 6.942581 |
| ENSMUSG00000026639 | protein_coding | Lamb3 | 4.524675 | 10.75332 | 7.639 |
| ENSMUSG00000066415 | protein_coding | Msl2 | 1426.076 | 1602.306 | 1514.191 |
| ENSMUSG00000073068 | protein_coding | Gm8914 | 0 | 2.030798 | 1.015399 |
| ENSMUSG00000022142 | protein_coding | Nup155 | 3116.946 | 2829.485 | 2973.216 |
| ENSMUSG00000069302 | protein_coding | Hist1h2ah | 4.208454 | 9.853056 | 7.030755 |
| ENSMUSG00000086281 | lincRNA | 9530082P21Rik | 103.7899 | 78.39298 | 91.09145 |
| ENSMUSG00000039007 | protein_coding | Pgcp | 1184.802 | 1344.405 | 1264.604 |
| ENSMUSG00000032058 | protein_coding | Ppp2r1b | 2336.786 | 2074.857 | 2205.822 |
| ENSMUSG00000046352 | protein_coding | Gjb2 | 124.1394 | 155.231 | 139.6852 |
| ENSMUSG00000000673 | protein_coding | Haao | 20.04523 | 31.64982 | 25.84752 |
| ENSMUSG00000043753 | protein_coding | Dmrta1 | 4191.509 | 3686.578 | 3939.044 |
| ENSMUSG00000045036 | protein_coding | Tmem232 | 44.23353 | 73.90675 | 59.07014 |
| ENSMUSG00000039577 | protein_coding | Nphp4 | 276.4826 | 229.8886 | 253.1856 |
| ENSMUSG00000025955 | protein_coding | Akr1cl | 4329.952 | 5048.156 | 4689.054 |
| ENSMUSG00000020160 | protein_coding | Meis1 | 611.801 | 741.7213 | 676.7611 |
| ENSMUSG00000030946 | protein_coding | Lhpp | 850.0361 | 757.5512 | 803.7937 |
| ENSMUSG00000019894 | protein_coding | Slc6a15 | 12.229 | 25.16173 | 18.69537 |
| ENSMUSG00000029417 | protein_coding | Cxcl9 | 13.42076 | 5.23681 | 9.328784 |
| ENSMUSG00000030513 | protein_coding | Pcsk6 | 1117.18 | 970.1237 | 1043.652 |
| ENSMUSG00000091786 | pseudogene | Gm8425 | 3.858166 | 0.724379 | 2.291273 |
| ENSMUSG00000022561 | protein_coding | Gpaa1 | 1198.117 | 1048.112 | 1123.115 |
| ENSMUSG00000015290 | protein_coding | Ubl4 | 1226.394 | 1391.603 | 1308.999 |
| ENSMUSG00000073375 | protein_coding | Lrrc30 | 1.598773 | 5.782813 | 3.690793 |
| ENSMUSG00000024816 | protein_coding | Frmd8 | 1109.057 | 1237.697 | 1173.377 |
| ENSMUSG00000013155 | protein_coding | E130303B06Rik | 266.1641 | 216.8679 | 241.516 |
| ENSMUSG00000005362 | protein_coding | Crbn | 1664.829 | 1828.69 | 1746.76 |
| ENSMUSG00000046434 | protein_coding | Hnrnpa1 | 12361 | 13773.95 | 13067.48 |
| ENSMUSG00000053646 | protein_coding | Plxnb1 | 1029.818 | 1275.494 | 1152.656 |
| ENSMUSG00000040813 | protein_coding | Tex264 | 1034.953 | 927.2052 | 981.0789 |
| ENSMUSG00000001588 | protein_coding | Acap1 | 68.39552 | 48.00608 | 58.2008 |
| ENSMUSG00000020017 | protein_coding | Hal | 5.143028 | 11.20452 | 8.173775 |
| ENSMUSG00000091570 | pseudogene | Gm7707 | 3.556034 | 0.688704 | 2.122369 |
| ENSMUSG00000052302 | protein_coding | Tbc1d30 | 1191.512 | 1485.632 | 1338.572 |
| ENSMUSG00000082274 | pseudogene | Gm14026 | 35.7798 | 23.55795 | 29.66887 |
| ENSMUSG00000089889 | processed_transcrip | 0610040B10Rik | 43.46966 | 28.52122 | 35.99544 |
| ENSMUSG00000015869 | protein_coding | Prpsap1 | 3250.989 | 2896.77 | 3073.879 |
| ENSMUSG00000062732 | protein_coding | Lypd4 | 24.40844 | 11.17097 | 17.7897 |
| ENSMUSG00000026874 | protein_coding | Hc | 27.03049 | 39.89153 | 33.46101 |

| | | | | | |
|---|---|---|---|---|---|
| ENSMUSG00000057113 | protein_coding | Npm1 | 14313.48 | 15657.98 | 14985.73 |
| ENSMUSG00000001082 | protein_coding | Mfsd10 | 587.3367 | 684.746 | 636.0413 |
| ENSMUSG00000026176 | protein_coding | Ctdsp1 | 2198.952 | 2455.528 | 2327.24 |
| ENSMUSG00000026181 | protein_coding | Ppm1f | 1059.355 | 1190.062 | 1124.708 |
| ENSMUSG00000059456 | protein_coding | Ptk2b | 574.3406 | 673.799 | 624.0698 |
| ENSMUSG00000049504 | protein_coding | 2810046L04Rik | 935.2606 | 1053.434 | 994.3474 |
| ENSMUSG00000042724 | protein_coding | Map3k9 | 923.4392 | 761.8701 | 842.6546 |
| ENSMUSG00000022679 | protein_coding | Mpv17l | 269.1442 | 313.8633 | 291.5037 |
| ENSMUSG00000031381 | protein_coding | Piga | 722.0672 | 823.9979 | 773.0326 |
| ENSMUSG00000061833 | pseudogene | Gm6311 | 10.65759 | 4.387748 | 7.52267 |
| ENSMUSG00000086651 | lincRNA | 1300002E11Rik | 204.4899 | 168.0678 | 186.2789 |
| ENSMUSG00000069804 | protein_coding | Gm10277 | 0 | 2.00377 | 1.001885 |
| ENSMUSG00000032402 | protein_coding | Smad3 | 2104.297 | 2465.148 | 2284.723 |
| ENSMUSG00000000037 | protein_coding | Scml2 | 4906.776 | 4119.347 | 4513.061 |
| ENSMUSG00000003949 | protein_coding | Hlf | 401.1692 | 325.7672 | 363.4682 |
| ENSMUSG00000043719 | protein_coding | Col6a6 | 173.1508 | 221.372 | 197.2614 |
| ENSMUSG00000028439 | protein_coding | 2310028H24Rik | 230.5162 | 176.4874 | 203.5018 |
| ENSMUSG00000082728 | pseudogene | Fam48b1 | 81.07574 | 119.1692 | 100.1225 |
| ENSMUSG00000022667 | protein_coding | Cd200r1 | 13.21427 | 22.39351 | 17.80389 |
| ENSMUSG00000054404 | protein_coding | Slfn5 | 199.9348 | 146.9919 | 173.4634 |
| ENSMUSG00000024048 | protein_coding | 2900073G15Rik | 5977.356 | 6555.15 | 6266.253 |
| ENSMUSG00000091726 | lincRNA | Gm17211 | 2.575614 | 0.36219 | 1.468902 |
| ENSMUSG00000040170 | protein_coding | Fmo2 | 62.99136 | 88.40965 | 75.7005 |
| ENSMUSG00000000295 | protein_coding | Hddc2 | 678.2264 | 600.8186 | 639.5225 |
| ENSMUSG00000074506 | pseudogene | Gm10705 | 4.832697 | 1.350742 | 3.091719 |
| ENSMUSG00000079501 | protein_coding | Gm5138 | 2.583814 | 0.335524 | 1.459669 |
| ENSMUSG00000073408 | protein_coding | Dpcr1 | 16.82181 | 9.280387 | 13.0511 |
| ENSMUSG00000086983 | lincRNA | Gm16972 | 12.34887 | 5.801193 | 9.075033 |
| ENSMUSG00000015714 | protein_coding | Lass2 | 1896.594 | 2109.233 | 2002.914 |
| ENSMUSG00000067158 | protein_coding | Col4a4 | 2547.011 | 2184.493 | 2365.752 |
| ENSMUSG00000035547 | protein_coding | Capn5 | 534.272 | 445.4944 | 489.8832 |
| ENSMUSG00000039646 | protein_coding | Vasn | 2109.718 | 2385.412 | 2247.565 |
| ENSMUSG00000028582 | protein_coding | Cc2d1b | 1405.339 | 1270.327 | 1337.833 |
| ENSMUSG00000043410 | protein_coding | Hfm1 | 1182.831 | 573.2657 | 878.0482 |
| ENSMUSG00000025090 | protein_coding | 1700011F14Rik | 445.8208 | 358.1529 | 401.9869 |
| ENSMUSG00000038002 | protein_coding | Cramp1l | 2101.554 | 2381.492 | 2241.523 |
| ENSMUSG00000072999 | protein_coding | Gm15401 | 29.038 | 44.83646 | 36.93723 |
| ENSMUSG00000036672 | protein_coding | Cenpt | 807.0412 | 709.6706 | 758.3559 |
| ENSMUSG00000090083 | protein_coding | Rnf8 | 1340.621 | 1211.818 | 1276.219 |
| ENSMUSG00000038268 | protein_coding | Ovca2 | 232.1933 | 272.8753 | 252.5343 |
| ENSMUSG00000024858 | protein_coding | Adrbk1 | 1727.391 | 1532.499 | 1629.945 |
| ENSMUSG00000081192 | pseudogene | Gm13243 | 9.039883 | 16.2716 | 12.65574 |
| ENSMUSG00000032458 | protein_coding | Copb2 | 8635.154 | 7896.402 | 8265.778 |
| ENSMUSG00000027245 | protein_coding | 2310003F16Rik | 100.0045 | 128.0803 | 114.0424 |
| ENSMUSG00000022752 | protein_coding | Tomm70a | 4502.419 | 4115.585 | 4309.002 |
| ENSMUSG00000027870 | protein_coding | Hao2 | 302.586 | 226.413 | 264.4995 |
| ENSMUSG00000025086 | protein_coding | Trub1 | 725.6179 | 830.0937 | 777.8558 |
| ENSMUSG00000022603 | protein_coding | 1700016M24Rik | 18.82767 | 0.662038 | 9.744856 |
| ENSMUSG00000036330 | protein_coding | Slc18a1 | 37.41793 | 22.44684 | 29.93239 |
| ENSMUSG00000090590 | protein_coding | Gm17570 | 18.74859 | 10.8953 | 14.82194 |
| ENSMUSG00000040731 | protein_coding | Eif4h | 6238.074 | 6774.045 | 6506.059 |
| ENSMUSG00000045709 | lincRNA | 1700023L04Rik | 55.89212 | 73.8777 | 64.88491 |
| ENSMUSG00000060419 | pseudogene | Rps16-ps2 | 842.955 | 943.1479 | 893.0514 |
| ENSMUSG00000041886 | protein_coding | Macc1 | 10.60586 | 4.38811 | 7.496983 |
| ENSMUSG00000028199 | protein_coding | Cryz | 802.7458 | 694.4923 | 748.619 |
| ENSMUSG00000036580 | protein_coding | Spg20 | 1267.061 | 1114.238 | 1190.649 |
| ENSMUSG00000079677 | protein_coding | Fdx1l | 376.4546 | 326.5066 | 351.4806 |
| ENSMUSG00000070425 | protein_coding | Trpc2 | 1105.115 | 983.9284 | 1044.522 |

| | | | | | | |
|---|---|---|---|---|---|---|
| ENSMUSG00000035930 | protein_coding | Chst4 | | 3.225724 | 8.582312 | 5.904018 |
| ENSMUSG00000070304 | protein_coding | Scn2b | | 53.88014 | 77.80203 | 65.84108 |
| ENSMUSG00000004127 | protein_coding | Rg9mtd2 | | 1131.072 | 885.0272 | 1008.049 |
| ENSMUSG00000031714 | protein_coding | Gab1 | | 2112.62 | 2373.973 | 2243.297 |
| ENSMUSG00000058546 | protein_coding | Rpl23a | | 3671.911 | 4044.221 | 3858.066 |
| ENSMUSG00000073430 | processed_transcrip | Gm10505 | | 8.571663 | 0 | 4.285831 |
| ENSMUSG00000026558 | protein_coding | Uck2 | | 875.8902 | 771.9434 | 823.9168 |
| ENSMUSG00000027381 | protein_coding | Bcl2l11 | | 1250.85 | 1109.931 | 1180.391 |
| ENSMUSG00000033854 | protein_coding | Kcnk10 | | 2.597903 | 0.326514 | 1.462209 |
| ENSMUSG00000083314 | pseudogene | Gm11742 | | 1.304841 | 4.785613 | 3.045227 |
| ENSMUSG00000057147 | protein_coding | Atpbd4 | | 724.3684 | 809.8706 | 767.1195 |
| ENSMUSG00000050876 | protein_coding | Fam75d3 | | 7.459821 | 15.02752 | 11.24367 |
| ENSMUSG00000021556 | protein_coding | Golm1 | | 978.2768 | 1138.132 | 1058.204 |
| ENSMUSG00000062417 | protein_coding | Hist1h3g | | 1.929083 | 6.302511 | 4.115797 |
| ENSMUSG00000080050 | pseudogene | Gm14864 | | 0.98273 | 4.132585 | 2.557657 |
| ENSMUSG00000038370 | protein_coding | Pcp4l1 | | 716.8105 | 842.9032 | 779.8569 |
| ENSMUSG00000063698 | protein_coding | Sfxn4 | | 198.729 | 165.709 | 182.219 |
| ENSMUSG00000020211 | protein_coding | Sf3a2 | | 1254.203 | 1388.53 | 1321.366 |
| ENSMUSG00000019917 | protein_coding | | 10-Sep | 1701.757 | 1881.88 | 1791.818 |
| ENSMUSG00000033948 | protein_coding | Zswim5 | | 1287.653 | 1057.003 | 1172.328 |
| ENSMUSG00000040717 | protein_coding | Il17rd | | 621.3349 | 730.8457 | 676.0903 |
| ENSMUSG00000093043 | misc_RNA | AC159379.1 | | 0.974531 | 4.105918 | 2.540225 |
| ENSMUSG00000091718 | protein_coding | Gm16506 | | 11.56861 | 5.165459 | 8.367034 |
| ENSMUSG00000084411 | pseudogene | Gm11510 | | 3.588833 | 0.688704 | 2.138769 |
| ENSMUSG00000025013 | protein_coding | Tll2 | | 53.41572 | 73.9141 | 63.66491 |
| ENSMUSG00000087294 | lincRNA | Gm13556 | | 2.303971 | 7.68313 | 4.99355 |
| ENSMUSG00000024682 | protein_coding | Gif | | 5.240384 | 1.086569 | 3.163476 |
| ENSMUSG00000031574 | protein_coding | Star | | 2077.75 | 2480.26 | 2279.005 |
| ENSMUSG00000059851 | protein_coding | Suv420h2 | | 1389.868 | 1202.796 | 1296.332 |
| ENSMUSG00000030059 | protein_coding | Tmf1 | | 1890.249 | 1712.292 | 1801.271 |
| ENSMUSG00000033712 | protein_coding | 2610301G19Rik | | 1701.56 | 1915.361 | 1808.46 |
| ENSMUSG00000047720 | protein_coding | 4922502D21Rik | | 5.284961 | 0.697713 | 2.991337 |
| ENSMUSG00000006642 | protein_coding | Tcf23 | | 503.9872 | 409.1967 | 456.5919 |
| ENSMUSG00000032179 | protein_coding | Bmp5 | | 25.48785 | 15.77028 | 20.62907 |
| ENSMUSG00000059939 | protein_coding | 9430015G10Rik | | 1067.343 | 932.2493 | 999.7962 |
| ENSMUSG00000030956 | protein_coding | Fam53b | | 1007.286 | 902.8783 | 955.0824 |
| ENSMUSG00000028937 | protein_coding | Acot7 | | 817.4224 | 697.7948 | 757.6086 |
| ENSMUSG00000030557 | protein_coding | Mef2a | | 6182.457 | 6789.966 | 6486.212 |
| ENSMUSG00000035984 | protein_coding | Nme5 | | 29.56071 | 17.45655 | 23.50863 |
| ENSMUSG00000021550 | protein_coding | 2210016F16Rik | | 1271.347 | 1130.003 | 1200.675 |
| ENSMUSG00000047921 | protein_coding | Trappc9 | | 915.2138 | 819.8872 | 867.5505 |
| ENSMUSG00000015468 | protein_coding | Notch4 | | 117.9684 | 94.83798 | 106.4032 |
| ENSMUSG00000059049 | protein_coding | Frem1 | | 231.9107 | 280.6204 | 256.2655 |
| ENSMUSG00000033227 | protein_coding | Wnt6 | | 2453.533 | 2763.101 | 2608.317 |
| ENSMUSG00000047446 | protein_coding | Arl4a | | 1643.16 | 1452.388 | 1547.774 |
| ENSMUSG00000030162 | protein_coding | Olr1 | | 7.417554 | 2.719502 | 5.068528 |
| ENSMUSG00000024565 | protein_coding | Sall3 | | 52.89346 | 69.8046 | 61.34903 |
| ENSMUSG00000040118 | protein_coding | Cacna2d1 | | 787.5055 | 940.2344 | 863.8699 |
| ENSMUSG00000091244 | lincRNA | Gm17690 | | 87.30811 | 65.90632 | 76.60721 |
| ENSMUSG00000046756 | protein_coding | Mrps7 | | 1496.751 | 1358.828 | 1427.79 |
| ENSMUSG00000083906 | pseudogene | Gm15186 | | 19.1199 | 30.58697 | 24.85343 |
| ENSMUSG00000057863 | protein_coding | Rpl36 | | 2007.148 | 1801.286 | 1904.217 |
| ENSMUSG00000003752 | protein_coding | Itpkc | | 524.0692 | 455.701 | 489.8851 |
| ENSMUSG00000037628 | protein_coding | Cdkn3 | | 288.9277 | 236.6844 | 262.8061 |
| ENSMUSG00000028358 | protein_coding | Zfp618 | | 935.9341 | 1131.498 | 1033.716 |
| ENSMUSG00000048138 | protein_coding | Dmrt2 | | 71.94075 | 98.43222 | 85.18649 |
| ENSMUSG00000035493 | protein_coding | Tgfbi | | 945.1345 | 1148.098 | 1046.616 |
| ENSMUSG00000048939 | protein_coding | Atp13a5 | | 5.817738 | 12.02692 | 8.922327 |

| | | | | | |
|---|---|---|---|---|---|
| ENSMUSG00000032103 | protein_coding | Pus3 | 3.866366 | 9.208675 | 6.53752 |
| ENSMUSG00000028633 | protein_coding | Ctps | 3647.071 | 3277.173 | 3462.122 |
| ENSMUSG00000029003 | protein_coding | Mad2l2 | 468.2686 | 388.181 | 428.2248 |
| ENSMUSG00000047866 | protein_coding | Lonp2 | 2273.683 | 2022.997 | 2148.34 |
| ENSMUSG00000030214 | protein_coding | Plbd1 | 733.1172 | 1604.12 | 1168.619 |
| ENSMUSG00000057596 | protein_coding | Trim30d | 39.57572 | 60.37612 | 49.97592 |
| ENSMUSG00000014846 | protein_coding | Tppp3 | 673.902 | 758.7987 | 716.3504 |
| ENSMUSG00000073753 | pseudogene | Gm10571 | 1.592884 | 5.411976 | 3.50243 |
| ENSMUSG00000006269 | protein_coding | Atp6v1b1 | 6.836846 | 2.004132 | 4.420489 |
| ENSMUSG00000089260 | miRNA | Mir1929 | 1.609283 | 5.420985 | 3.515134 |
| ENSMUSG00000037070 | protein_coding | Rbmxrt | 1969.732 | 2218.909 | 2094.321 |
| ENSMUSG00000026977 | protein_coding | 7-Mar | 4086.094 | 4488.626 | 4287.36 |
| ENSMUSG00000028359 | protein_coding | Orm3 | 1.937283 | 0 | 0.968641 |
| ENSMUSG00000090162 | pseudogene | Gm6808 | 1.937283 | 0 | 0.968641 |
| ENSMUSG00000066036 | protein_coding | Ubr4 | 12140.33 | 10871.4 | 11505.86 |
| ENSMUSG00000085335 | lincRNA | Gm13684 | 0.976841 | 4.610448 | 2.793644 |
| ENSMUSG00000036377 | protein_coding | C530008M17Rik | 716.1387 | 876.8692 | 796.5039 |
| ENSMUSG00000053040 | protein_coding | Aph1c | 116.3284 | 84.12178 | 100.2251 |
| ENSMUSG00000017405 | protein_coding | Nek8 | 510.4034 | 585.1984 | 547.8009 |
| ENSMUSG00000032307 | protein_coding | Ube2q2 | 1687.874 | 1852.445 | 1770.16 |
| ENSMUSG00000015656 | protein_coding | Hspa8 | 23696.53 | 26795.6 | 25246.06 |
| ENSMUSG00000052085 | protein_coding | Dock8 | 674.7254 | 567.3065 | 621.016 |
| ENSMUSG00000071722 | protein_coding | Spin4 | 174.2219 | 208.7957 | 191.5088 |
| ENSMUSG00000015981 | protein_coding | Stk32c | 162.7218 | 131.489 | 147.1054 |
| ENSMUSG00000084789 | processed_transcrip | Gm12974 | 9.77788 | 3.832736 | 6.805308 |
| ENSMUSG00000030142 | protein_coding | Clec4e | 4.877274 | 1.386417 | 3.131846 |
| ENSMUSG00000039633 | protein_coding | Lonrf1 | 690.2674 | 597.6126 | 643.94 |
| ENSMUSG00000092885 | miRNA | AL646020.1 | 135.7067 | 166.2762 | 150.9915 |
| ENSMUSG00000029468 | protein_coding | P2rx7 | 446.5464 | 537.1272 | 491.8368 |
| ENSMUSG00000024013 | protein_coding | Fgd2 | 52.87013 | 34.9337 | 43.90191 |
| ENSMUSG00000031805 | protein_coding | Jak3 | 75.69365 | 97.58736 | 86.64051 |
| ENSMUSG00000040018 | protein_coding | Cox15 | 1135.684 | 1265.638 | 1200.661 |
| ENSMUSG00000016921 | protein_coding | Srsf6 | 7345.872 | 8243.424 | 7794.648 |
| ENSMUSG00000032324 | protein_coding | Tspan3 | 3388.909 | 3701.595 | 3545.252 |
| ENSMUSG00000042404 | protein_coding | Dennd4b | 346.4227 | 300.2446 | 323.3337 |
| ENSMUSG00000021832 | protein_coding | Psmc6 | 3434.976 | 3793.364 | 3614.17 |
| ENSMUSG00000037846 | protein_coding | Rtkn2 | 377.2149 | 316.3325 | 346.7737 |
| ENSMUSG00000084881 | processed_transcrip | Gm15585 | 11.9927 | 20.4759 | 16.2343 |
| ENSMUSG00000074164 | processed_transcrip | Vmn2r-ps54 | 0.646531 | 3.354512 | 2.000521 |
| ENSMUSG00000052713 | protein_coding | Zfp608 | 482.9079 | 551.425 | 517.1664 |
| ENSMUSG00000086464 | processed_transcrip | Gm15835 | 19.82047 | 34.52093 | 27.1707 |
| ENSMUSG00000031774 | protein_coding | Fam192a | 1722.299 | 1926.966 | 1824.632 |
| ENSMUSG00000085480 | lincRNA | 4930432B10Rik | 27.84818 | 15.38392 | 21.61605 |
| ENSMUSG00000034673 | protein_coding | Pbx2 | 2572.958 | 2331.593 | 2452.276 |
| ENSMUSG00000032712 | protein_coding | 2810474O19Rik | 1983.058 | 2249.787 | 2116.422 |
| ENSMUSG00000087475 | lincRNA | 4933406I18Rik | 5.537894 | 12.55418 | 9.046036 |
| ENSMUSG00000039552 | protein_coding | Rsph4a | 6.754623 | 2.43731 | 4.595966 |
| ENSMUSG00000024736 | protein_coding | Tmem132a | 607.7392 | 695.4733 | 651.6062 |
| ENSMUSG00000041729 | protein_coding | Coro2b | 128.8049 | 157.6578 | 143.2314 |
| ENSMUSG00000059237 | pseudogene | Gm6435 | 15.18077 | 8.149134 | 11.66495 |
| ENSMUSG00000025480 | protein_coding | Syce1 | 1468.434 | 1197.171 | 1332.802 |
| ENSMUSG00000027893 | protein_coding | Ahcyl1 | 4590.905 | 5004.757 | 4797.831 |
| ENSMUSG00000034912 | protein_coding | Mdga2 | 40.25758 | 27.43972 | 33.84865 |
| ENSMUSG00000028229 | protein_coding | Fam82b | 234.2254 | 198.9496 | 216.5875 |
| ENSMUSG00000034570 | protein_coding | Inpp5j | 39.55455 | 63.84337 | 51.69896 |
| ENSMUSG00000018433 | protein_coding | Nol11 | 1256.116 | 1397.999 | 1327.057 |
| ENSMUSG00000078191 | protein_coding | Gm5559 | 1164.554 | 1293.307 | 1228.93 |
| ENSMUSG00000059447 | protein_coding | Hadhb | 3816.444 | 4180.542 | 3998.493 |

| | | | | | |
|---|---|---|---|---|---|
| ENSMUSG00000091344 | lincRNA | 1700037F24Rik | 2.581503 | 7.680642 | 5.131073 |
| ENSMUSG00000030590 | protein_coding | Fam98c | 82.47541 | 63.75726 | 73.11633 |
| ENSMUSG00000038523 | protein_coding | 1700003F12Rik | 28.50082 | 18.00291 | 23.25187 |
| ENSMUSG00000039994 | protein_coding | Timeless | 1354.336 | 1188.57 | 1271.453 |
| ENSMUSG00000067942 | protein_coding | Zfp160 | 727.2336 | 869.6659 | 798.4498 |
| ENSMUSG00000037926 | protein_coding | Ssh2 | 2823.062 | 2502.278 | 2662.67 |
| ENSMUSG00000085433 | processed_transcrip | Gm16001 | 86.8346 | 67.97564 | 77.40512 |
| ENSMUSG00000090086 | processed_transcrip | AI480526 | 596.0636 | 738.8239 | 667.4438 |
| ENSMUSG00000021218 | protein_coding | Gdi2 | 6498.552 | 7082.614 | 6790.583 |
| ENSMUSG00000036850 | protein_coding | Mrpl41 | 817.4328 | 711.5065 | 764.4696 |
| ENSMUSG00000044534 | protein_coding | Ccbp2 | 14.9432 | 25.18944 | 20.06632 |
| ENSMUSG00000078765 | protein_coding | U2af1l4 | 506.1495 | 567.4329 | 536.7912 |
| ENSMUSG00000084111 | pseudogene | Gm15710 | 252.5438 | 211.6028 | 232.0733 |
| ENSMUSG00000006736 | protein_coding | Tspan31 | 907.0079 | 1042.089 | 974.5487 |
| ENSMUSG00000033467 | protein_coding | Crlf2 | 362.7335 | 310.5009 | 336.6172 |
| ENSMUSG00000005481 | protein_coding | Ddx39 | 2162.573 | 2385.997 | 2274.285 |
| ENSMUSG00000079724 | protein_coding | Rnaset2b | 540.2253 | 645.041 | 592.6331 |
| ENSMUSG00000027703 | protein_coding | Lrriq4 | 18.11279 | 9.455914 | 13.78435 |
| ENSMUSG00000026816 | protein_coding | Gtf3c5 | 1226.538 | 1100.927 | 1163.733 |
| ENSMUSG00000025278 | protein_coding | Flnb | 1219.003 | 1079.253 | 1149.128 |
| ENSMUSG00000086827 | lincRNA | 4930461C15Rik | 11.08272 | 4.74129 | 7.912006 |
| ENSMUSG00000025894 | protein_coding | Aasdhppt | 1078.364 | 955.604 | 1016.984 |
| ENSMUSG00000009418 | protein_coding | Nav1 | 1284.954 | 1524.262 | 1404.608 |
| ENSMUSG00000021514 | protein_coding | Zfp369 | 336.0619 | 414.2382 | 375.1501 |
| ENSMUSG00000030351 | protein_coding | Tspan11 | 339.4155 | 394.0365 | 366.726 |
| ENSMUSG00000012443 | protein_coding | Kif11 | 2825.542 | 3132.014 | 2978.778 |
| ENSMUSG00000017144 | protein_coding | Rnd3 | 521.3507 | 589.1209 | 555.2358 |
| ENSMUSG00000088219 | snoRNA | snoU83B | 10.66914 | 18.47213 | 14.57064 |
| ENSMUSG00000053111 | protein_coding | Fank1 | 33.3699 | 21.01362 | 27.19176 |
| ENSMUSG00000027368 | protein_coding | Dusp2 | 117.7207 | 144.1083 | 130.9145 |
| ENSMUSG00000032766 | protein_coding | Gng11 | 142.9397 | 173.6664 | 158.303 |
| ENSMUSG00000066456 | protein_coding | Hmgn3 | 619.5847 | 732.5878 | 676.0863 |
| ENSMUSG00000025289 | protein_coding | Prdx4 | 2831.634 | 2548.266 | 2689.95 |
| ENSMUSG00000032009 | protein_coding | Sesn3 | 1074.749 | 916.0334 | 995.3913 |
| ENSMUSG00000037646 | protein_coding | Vps13b | 3416.843 | 2942.607 | 3179.725 |
| ENSMUSG00000040263 | protein_coding | Klhdc4 | 506.9489 | 450.4853 | 478.7171 |
| ENSMUSG00000023923 | protein_coding | Tbc1d5 | 1142.056 | 1018.075 | 1080.066 |
| ENSMUSG00000026237 | protein_coding | Nmur1 | 3.258523 | 8.73045 | 5.994487 |
| ENSMUSG00000031239 | protein_coding | Itm2a | 2172.901 | 2566.34 | 2369.62 |
| ENSMUSG00000073627 | lincRNA | C130036L24Rik | 78.90402 | 103.8768 | 91.39042 |
| ENSMUSG00000087443 | processed_transcrip | 5530401N12Rik | 14.83995 | 23.83077 | 19.33536 |
| ENSMUSG00000085553 | processed_transcrip | Gm14808 | 13.14025 | 22.69051 | 17.91538 |
| ENSMUSG00000087644 | lincRNA | Gm14703 | 6.143426 | 2.021789 | 4.082607 |
| ENSMUSG00000025290 | protein_coding | Rps24 | 9461.329 | 8617.475 | 9039.402 |
| ENSMUSG00000031877 | protein_coding | Ces2g | 234.7376 | 170.428 | 202.5828 |
| ENSMUSG00000042751 | protein_coding | Nmnat2 | 9.635947 | 17.38556 | 13.51075 |
| ENSMUSG00000028187 | protein_coding | Rpf1 | 978.4918 | 1095.996 | 1037.244 |
| ENSMUSG00000022483 | protein_coding | Col2a1 | 77.3707 | 113.4816 | 95.42613 |
| ENSMUSG00000085702 | lincRNA | Gm16722 | 2.577925 | 0.335524 | 1.456724 |
| ENSMUSG00000022855 | protein_coding | Senp2 | 2649.354 | 2264.263 | 2456.809 |
| ENSMUSG00000030306 | protein_coding | Tmtc1 | 818.0307 | 727.3517 | 772.6912 |
| ENSMUSG00000041012 | protein_coding | Cmtm8 | 52.55115 | 69.37179 | 60.96147 |
| ENSMUSG00000026121 | protein_coding | Sema4c | 487.3556 | 567.3408 | 527.3482 |
| ENSMUSG00000015126 | protein_coding | 0610007P22Rik | 701.5349 | 627.9171 | 664.726 |
| ENSMUSG00000091318 | protein_coding | Gm5415 | 1.951372 | 0 | 0.975686 |
| ENSMUSG00000003234 | protein_coding | Abcf3 | 1793.062 | 1636.176 | 1714.619 |
| ENSMUSG00000042078 | protein_coding | Svop | 7.378865 | 14.64732 | 11.01309 |
| ENSMUSG00000052087 | protein_coding | Rgs14 | 15.19486 | 7.947664 | 11.57126 |

| | | | | | |
|---|---|---|---|---|---|
| ENSMUSG00000029166 | protein_coding | Mapre3 | 389.7917 | 329.8657 | 359.8287 |
| ENSMUSG00000041164 | protein_coding | Zmiz2 | 1366.275 | 1212.004 | 1289.14 |
| ENSMUSG00000025466 | protein_coding | 1810014F10Rik | 52.39281 | 73.02759 | 62.7102 |
| ENSMUSG00000000381 | protein_coding | Wap | 16.23395 | 8.045319 | 12.13963 |
| ENSMUSG00000090522 | protein_coding | Gm5919 | 9.411192 | 17.56321 | 13.4872 |
| ENSMUSG00000048992 | protein_coding | Prss32 | 5.204006 | 1.422092 | 3.313049 |
| ENSMUSG00000046323 | protein_coding | Dppa3 | 55.67683 | 36.2029 | 45.93986 |
| ENSMUSG00000035367 | protein_coding | Rmi1 | 1431.259 | 1301.9 | 1366.579 |
| ENSMUSG00000025219 | protein_coding | Fgf8 | 12.25025 | 21.58805 | 16.91915 |
| ENSMUSG00000022948 | protein_coding | Setd4 | 187.5685 | 220.2158 | 203.8921 |
| ENSMUSG00000048856 | protein_coding | Slc25a47 | 72.27218 | 55.29963 | 63.7859 |
| ENSMUSG00000092954 | miRNA | AL808021.1 | 1.623372 | 5.536658 | 3.580015 |
| ENSMUSG00000041014 | protein_coding | Nrg3 | 1.945483 | 0 | 0.972741 |
| ENSMUSG00000026743 | protein_coding | Mllt10 | 2242.813 | 1952.853 | 2097.833 |
| ENSMUSG00000024528 | protein_coding | Srfbp1 | 934.5982 | 1044.211 | 989.4045 |
| ENSMUSG00000061411 | protein_coding | 8430427H17Rik | 1599.348 | 1789.206 | 1694.277 |
| ENSMUSG00000050855 | protein_coding | Zfp940 | 204.7025 | 169.2088 | 186.9556 |
| ENSMUSG00000021803 | protein_coding | Cdhr1 | 2.569725 | 6.762717 | 4.666221 |
| ENSMUSG00000090179 | pseudogene | Gm16307 | 1.943172 | 0 | 0.971586 |
| ENSMUSG00000081076 | pseudogene | Rpsa-ps4 | 1.943172 | 0 | 0.971586 |
| ENSMUSG00000088753 | snoRNA | SNORA61 | 1.943172 | 0 | 0.971586 |
| ENSMUSG00000026655 | protein_coding | Fam107b | 503.4739 | 444.2261 | 473.85 |
| ENSMUSG00000036687 | protein_coding | Tmem184a | 11761.26 | 13068.7 | 12414.98 |
| ENSMUSG00000041375 | protein_coding | Ccdc9 | 1463.546 | 1315.954 | 1389.75 |
| ENSMUSG00000072082 | protein_coding | Ccnf | 1319.803 | 1162.05 | 1240.927 |
| ENSMUSG00000074055 | lincRNA | Gm10614 | 24.21835 | 15.30107 | 19.75971 |
| ENSMUSG00000042379 | protein_coding | Esm1 | 25.49166 | 15.76164 | 20.62665 |
| ENSMUSG00000037577 | protein_coding | Ephx3 | 0.64422 | 3.65436 | 2.14929 |
| ENSMUSG00000054321 | protein_coding | Taf4b | 2726.242 | 2203.184 | 2464.713 |
| ENSMUSG00000002897 | protein_coding | Il17ra | 857.6692 | 745.9629 | 801.816 |
| ENSMUSG00000020576 | protein_coding | Nbas | 1571.257 | 1350.525 | 1460.891 |
| ENSMUSG00000003444 | protein_coding | Med29 | 822.5502 | 732.7882 | 777.6692 |
| ENSMUSG00000029482 | protein_coding | Aacs | 799.1036 | 706.4394 | 752.7715 |
| ENSMUSG00000052915 | protein_coding | Msl1 | 3385.384 | 3063.878 | 3224.631 |
| ENSMUSG00000020785 | protein_coding | Camkk1 | 216.8501 | 181.2198 | 199.035 |
| ENSMUSG00000038429 | protein_coding | Usp5 | 3051.265 | 2759.828 | 2905.547 |
| ENSMUSG00000036249 | protein_coding | Rbm43 | 263.8512 | 307.8445 | 285.8479 |
| ENSMUSG00000091397 | lincRNA | Gm17265 | 21.03489 | 12.36172 | 16.6983 |
| ENSMUSG00000043668 | protein_coding | Tox3 | 187.2367 | 155.1668 | 171.2017 |
| ENSMUSG00000060798 | protein_coding | Intu | 654.805 | 565.0346 | 609.9198 |
| ENSMUSG00000085628 | processed_transcrip | D630032N06Rik | 37.80519 | 25.19989 | 31.50254 |
| ENSMUSG00000028656 | protein_coding | Cap1 | 6231.549 | 6853.558 | 6542.554 |
| ENSMUSG00000042684 | protein_coding | Npl | 782.4529 | 878.2576 | 830.3553 |
| ENSMUSG00000085501 | processed_transcrip | Gm11772 | 11.25932 | 19.2996 | 15.27946 |
| ENSMUSG00000036622 | protein_coding | Atp13a2 | 2341.356 | 2015.144 | 2178.25 |
| ENSMUSG00000020334 | protein_coding | Slc22a4 | 182.3447 | 145.669 | 164.0069 |
| ENSMUSG00000060510 | protein_coding | Zfp266 | 3938.289 | 4602.526 | 4270.407 |
| ENSMUSG00000079355 | protein_coding | Ccrl1 | 50.95155 | 68.51372 | 59.73264 |
| ENSMUSG00000040415 | protein_coding | Dtx3 | 838.7825 | 1021.319 | 930.0506 |
| ENSMUSG00000026890 | protein_coding | Lhx6 | 169.7761 | 137.4487 | 153.6124 |
| ENSMUSG00000030123 | protein_coding | Plxnd1 | 1863.075 | 1678.883 | 1770.979 |
| ENSMUSG00000047592 | protein_coding | BC055004 | 12.21618 | 20.99808 | 16.60713 |
| ENSMUSG00000066755 | protein_coding | Tnfsf18 | 5.447471 | 11.53104 | 8.489253 |
| ENSMUSG00000082625 | pseudogene | Gm15204 | 5.47796 | 1.748606 | 3.613283 |
| ENSMUSG00000037916 | protein_coding | Ndufv1 | 2568.492 | 2333.005 | 2450.749 |
| ENSMUSG00000091562 | processed_transcrip | Gm9581 | 320.2003 | 263.6098 | 291.9051 |
| ENSMUSG00000057181 | protein_coding | 5730455P16Rik | 929.889 | 834.9389 | 882.414 |
| ENSMUSG00000018166 | protein_coding | Erbb3 | 528.882 | 647.5164 | 588.1992 |

| | | | | | |
|---|---|---|---|---|---|
| ENSMUSG00000022340 | protein_coding | Sybu | 125.928 | 153.3325 | 139.6303 |
| ENSMUSG00000040204 | protein_coding | 2810417H13Rik | 480.3463 | 583.579 | 531.9627 |
| ENSMUSG00000021478 | protein_coding | Drd1a | 1.929083 | 0 | 0.964541 |
| ENSMUSG00000073257 | protein_coding | Gm10488 | 1.929083 | 0 | 0.964541 |
| ENSMUSG00000025555 | protein_coding | Farp1 | 599.843 | 745.0701 | 672.4566 |
| ENSMUSG00000042757 | protein_coding | Tmem108 | 130.3163 | 162.7047 | 146.5105 |
| ENSMUSG00000075591 | protein_coding | Gm10874 | 6.206714 | 13.46667 | 9.83669 |
| ENSMUSG00000000078 | protein_coding | Klf6 | 2135.021 | 2354.782 | 2244.901 |
| ENSMUSG00000021918 | protein_coding | Nek4 | 884.3948 | 779.2412 | 831.818 |
| ENSMUSG00000024773 | protein_coding | Atg2a | 1798.623 | 1571.404 | 1685.014 |
| ENSMUSG00000092399 | processed_transcrip | RP23-243J8.2 | 1.939594 | 6.279056 | 4.109325 |
| ENSMUSG00000020495 | protein_coding | 1200011M11Rik | 1353.877 | 1219.181 | 1286.529 |
| ENSMUSG00000028793 | protein_coding | Rnf19b | 2042.207 | 1685.953 | 1864.08 |
| ENSMUSG00000049620 | protein_coding | Prss33 | 7.824198 | 2.808509 | 5.316353 |
| ENSMUSG00000039126 | protein_coding | Prune2 | 83.86329 | 112.6736 | 98.26842 |
| ENSMUSG00000024211 | protein_coding | Grm8 | 7.427021 | 14.16402 | 10.79552 |
| ENSMUSG00000042589 | protein_coding | Cux2 | 273.6902 | 318.3949 | 296.0426 |
| ENSMUSG00000081104 | pseudogene | Gm15989 | 1.923194 | 0 | 0.961597 |
| ENSMUSG00000058589 | protein_coding | Anks1b | 69.10489 | 49.41476 | 59.25983 |
| ENSMUSG00000024519 | protein_coding | Cplx4 | 3.550145 | 0.671047 | 2.110596 |
| ENSMUSG00000024388 | protein_coding | Myo7b | 2.592014 | 7.365624 | 4.978819 |
| ENSMUSG00000050373 | protein_coding | Snx21 | 1058.558 | 1168.822 | 1113.69 |
| ENSMUSG00000036955 | protein_coding | 2510003E04Rik | 1500.175 | 1363.025 | 1431.6 |
| ENSMUSG00000062075 | protein_coding | Lmnb2 | 954.5784 | 1070.206 | 1012.392 |
| ENSMUSG00000040651 | protein_coding | D14Abb1e | 3041.914 | 3376.161 | 3209.038 |
| ENSMUSG00000021395 | protein_coding | Spin1 | 6712.73 | 7416.151 | 7064.44 |
| ENSMUSG00000024873 | protein_coding | Cnih2 | 157.8562 | 127.7564 | 142.8063 |
| ENSMUSG00000071072 | protein_coding | Ptges3 | 4784.144 | 5221.315 | 5002.729 |
| ENSMUSG00000044348 | protein_coding | Mcart6 | 433.4366 | 375.0161 | 404.2264 |
| ENSMUSG00000032344 | protein_coding | E330016A19Rik | 135.0858 | 164.8376 | 149.9617 |
| ENSMUSG00000091561 | protein_coding | Gm6665 | 437.5957 | 494.5092 | 466.0524 |
| ENSMUSG00000042182 | protein_coding | Bend6 | 202.8227 | 169.4837 | 186.1532 |
| ENSMUSG00000002658 | protein_coding | Gtf2f1 | 3726.002 | 3401.987 | 3563.994 |
| ENSMUSG00000046679 | protein_coding | C87436 | 542.2582 | 610.2447 | 576.2515 |
| ENSMUSG00000000753 | protein_coding | Serpinf1 | 886.96 | 1035.49 | 961.2252 |
| ENSMUSG00000038417 | protein_coding | Fig4 | 1446.15 | 1249.097 | 1347.624 |
| ENSMUSG00000020527 | protein_coding | Myo19 | 891.693 | 798.9616 | 845.3273 |
| ENSMUSG00000038685 | protein_coding | Rtel1 | 1099.544 | 971.7595 | 1035.652 |
| ENSMUSG00000002228 | protein_coding | Ppm1j | 76.616 | 98.92339 | 87.7697 |
| ENSMUSG00000038855 | protein_coding | Itpkb | 5211.427 | 5807.158 | 5509.292 |
| ENSMUSG00000042447 | protein_coding | Mios | 2340.336 | 2100.687 | 2220.512 |
| ENSMUSG00000085152 | processed_transcrip | Gm11496 | 8.691906 | 3.690035 | 6.19097 |
| ENSMUSG00000039481 | protein_coding | Nrtn | 162.4492 | 127.2172 | 144.8332 |
| ENSMUSG00000029596 | protein_coding | Sdsl | 281.1086 | 335.0739 | 308.0913 |
| ENSMUSG00000086910 | processed_transcrip | Gm15755 | 5.811849 | 11.67301 | 8.742431 |
| ENSMUSG00000023988 | protein_coding | Bysl | 876.647 | 759.6182 | 818.1326 |
| ENSMUSG00000060260 | protein_coding | Pwwp2b | 448.9624 | 517.6712 | 483.3168 |
| ENSMUSG00000031089 | protein_coding | Slc6a14 | 606.6295 | 489.363 | 547.9963 |
| ENSMUSG00000020747 | protein_coding | 2310067B10Rik | 1551.027 | 1384.343 | 1467.685 |
| ENSMUSG00000020728 | protein_coding | Ccdc46 | 424.3504 | 359.6648 | 392.0076 |
| ENSMUSG00000004631 | protein_coding | Sgce | 752.4599 | 871.1677 | 811.8138 |
| ENSMUSG00000020474 | protein_coding | Polm | 297.9248 | 346.784 | 322.3544 |
| ENSMUSG00000074513 | protein_coding | Arfip1 | 2051.895 | 2248.654 | 2150.275 |
| ENSMUSG00000041301 | protein_coding | Cftr | 186.6579 | 226.5156 | 206.5868 |
| ENSMUSG00000021340 | protein_coding | Gpld1 | 732.3528 | 576.6077 | 654.4802 |
| ENSMUSG00000049285 | protein_coding | Mblac1 | 66.56037 | 89.39757 | 77.97897 |
| ENSMUSG00000041205 | protein_coding | Map6d1 | 126.4415 | 154.992 | 140.7168 |
| ENSMUSG00000091337 | protein_coding | Eid1 | 1359.098 | 1561.255 | 1460.176 |

| | | | | | |
|---|---|---|---|---|---|
| ENSMUSG00000013584 | protein_coding | Aldh1a2 | 1758.5 | 1084.851 | 1421.676 |
| ENSMUSG00000022577 | protein_coding | Ly6h | 19.04736 | 30.41905 | 24.73321 |
| ENSMUSG00000031781 | protein_coding | Ciapin1 | 2664.834 | 2349.207 | 2507.021 |
| ENSMUSG00000048001 | protein_coding | Hes5 | 18.46285 | 28.65998 | 23.56142 |
| ENSMUSG00000011008 | protein_coding | Mcoln2 | 12.93801 | 6.52521 | 9.731608 |
| ENSMUSG00000025736 | protein_coding | Jmjd8 | 334.362 | 391.7663 | 363.0641 |
| ENSMUSG00000024132 | protein_coding | Eci1 | 836.1087 | 976.4108 | 906.2597 |
| ENSMUSG00000037062 | protein_coding | Sh3glb1 | 2587.326 | 2379.143 | 2483.234 |
| ENSMUSG00000042763 | protein_coding | Maneal | 31.97695 | 21.56211 | 26.76953 |
| ENSMUSG00000085698 | processed_transcrip | Gm16022 | 3.242123 | 8.105173 | 5.673648 |
| ENSMUSG00000035595 | protein_coding | 1600002K03Rik | 125.6446 | 101.5892 | 113.6169 |
| ENSMUSG00000016308 | protein_coding | Ube2a | 549.0362 | 626.8375 | 587.9368 |
| ENSMUSG00000059263 | protein_coding | Usp47 | 6637.072 | 6068.979 | 6353.026 |
| ENSMUSG00000002963 | protein_coding | Pnkp | 566.1872 | 501.7061 | 533.9467 |
| ENSMUSG00000026735 | protein_coding | Ptf1a | 0.33031 | 2.383978 | 1.357144 |
| ENSMUSG00000091915 | protein_coding | Gm4312 | 2.259393 | 6.198696 | 4.229045 |
| ENSMUSG00000072553 | protein_coding | Gm525 | 12.92958 | 6.507553 | 9.718567 |
| ENSMUSG00000027752 | protein_coding | Exosc8 | 1634.402 | 1471.975 | 1553.188 |
| ENSMUSG00000087006 | processed_transcrip | Gm13889 | 19.42561 | 29.57755 | 24.50158 |
| ENSMUSG00000091986 | protein_coding | Rps2-ps5 | 0.32211 | 2.383978 | 1.353044 |
| ENSMUSG00000001334 | protein_coding | Fndc5 | 4317.852 | 5096.617 | 4707.234 |
| ENSMUSG00000042079 | protein_coding | Hnrnpf | 7688.171 | 8298.147 | 7993.159 |
| ENSMUSG00000026520 | protein_coding | Pycr2 | 705.4264 | 619.1972 | 662.3118 |
| ENSMUSG00000043671 | protein_coding | Dpy19l3 | 1436.178 | 1713.821 | 1575 |
| ENSMUSG00000049907 | protein_coding | Rasl11b | 601.1305 | 678.6103 | 639.8704 |
| ENSMUSG00000074284 | lincRNA | Gm10658 | 41.23674 | 27.98681 | 34.61177 |
| ENSMUSG00000030766 | protein_coding | Arhgap17 | 1407.302 | 1557.853 | 1482.577 |
| ENSMUSG00000035199 | protein_coding | Arl6ip5 | 647.6224 | 728.1459 | 687.8841 |
| ENSMUSG00000015143 | protein_coding | Actn1 | 742.3394 | 840.3712 | 791.3553 |
| ENSMUSG00000029434 | protein_coding | Vps33a | 1861.83 | 1675.977 | 1768.903 |
| ENSMUSG00000076516 | IG_V_gene | Igkv1-117 | 0 | 2.294609 | 1.147305 |
| ENSMUSG00000028729 | protein_coding | Ebna1bp2 | 2311.636 | 2550.422 | 2431.029 |
| ENSMUSG00000027227 | protein_coding | Sord | 480.3024 | 411.4194 | 445.8609 |
| ENSMUSG00000039221 | protein_coding | Rpl22l1 | 698.9367 | 597.1075 | 648.0221 |
| ENSMUSG00000086502 | processed_transcrip | B130055M24Rik | 120.339 | 151.5775 | 135.9582 |
| ENSMUSG00000028189 | protein_coding | Ctbs | 565.1807 | 644.5969 | 604.8888 |
| ENSMUSG00000015222 | protein_coding | Mtap2 | 978.0232 | 1207.52 | 1092.772 |
| ENSMUSG00000078771 | protein_coding | Evi2a | 35.61267 | 49.39105 | 42.50186 |
| ENSMUSG00000072941 | protein_coding | Sod3 | 1363.778 | 1175.021 | 1269.399 |
| ENSMUSG00000033752 | protein_coding | Mnd1 | 185.4248 | 150.0642 | 167.7445 |
| ENSMUSG00000020877 | protein_coding | Scrn2 | 1087.336 | 1213.864 | 1150.6 |
| ENSMUSG00000044211 | pseudogene | Gm7887 | 490.6536 | 619.5043 | 555.079 |
| ENSMUSG00000031980 | protein_coding | Agt | 475.6818 | 587.7157 | 531.6988 |
| ENSMUSG00000051335 | protein_coding | Gfod1 | 39.9155 | 55.68232 | 47.79891 |
| ENSMUSG00000036054 | protein_coding | Sugp2 | 1716.78 | 1495.157 | 1605.969 |
| ENSMUSG00000028116 | protein_coding | Myoz2 | 117.2479 | 150.0277 | 133.6378 |
| ENSMUSG00000079161 | protein_coding | Gm17607 | 0.316221 | 2.374969 | 1.345595 |
| ENSMUSG00000062961 | protein_coding | Gm1568 | 21.61709 | 12.89006 | 17.25358 |
| ENSMUSG00000020605 | protein_coding | Hs1bp3 | 351.8791 | 413.4878 | 382.6834 |
| ENSMUSG00000051000 | protein_coding | Fam160a1 | 164.8242 | 128.3755 | 146.5999 |
| ENSMUSG00000026751 | protein_coding | Nr5a1 | 4434.611 | 4971.918 | 4703.264 |
| ENSMUSG00000079479 | protein_coding | Gm9112 | 85.81891 | 53.61182 | 69.71536 |
| ENSMUSG00000087381 | processed_transcrip | Gm16008 | 50.71837 | 70.62989 | 60.67413 |
| ENSMUSG00000022150 | protein_coding | Dab2 | 1583.234 | 1771.199 | 1677.216 |
| ENSMUSG00000079629 | protein_coding | Rhox2g | 23.21922 | 14.59435 | 18.90678 |
| ENSMUSG00000078605 | protein_coding | E030025P04Rik | 5.187606 | 1.686265 | 3.436936 |
| ENSMUSG00000076576 | IG_V_gene | Igkv6-32 | 2.972791 | 0 | 1.486395 |
| ENSMUSG00000040818 | protein_coding | Fam116a | 1616.681 | 1400.117 | 1508.399 |

| | | | | | |
|---|---|---|---|---|---|
| ENSMUSG00000010080 | protein_coding | Epn3 | 781.7344 | 697.1086 | 739.4215 |
| ENSMUSG00000079666 | protein_coding | 1700015F17Rik | 7.493888 | 2.463976 | 4.978932 |
| ENSMUSG00000086158 | processed_transcrip | Gm5918 | 248.0944 | 288.4998 | 268.2971 |
| ENSMUSG00000028555 | protein_coding | Ttc39a | 93.39093 | 119.6289 | 106.5099 |
| ENSMUSG00000024764 | protein_coding | Naa40 | 2427.888 | 2219.767 | 2323.827 |
| ENSMUSG00000051853 | protein_coding | Arf3 | 7530.979 | 8421.583 | 7976.281 |
| ENSMUSG00000029472 | protein_coding | Anapc5 | 7786.007 | 7000.181 | 7393.094 |
| ENSMUSG00000042766 | protein_coding | Trim46 | 72.32033 | 93.14633 | 82.73333 |
| ENSMUSG00000045140 | protein_coding | Pigw | 425.2298 | 496.5004 | 460.8651 |
| ENSMUSG00000027006 | protein_coding | Dnajc10 | 3228.398 | 3590.663 | 3409.53 |
| ENSMUSG00000085578 | processed_transcrip | Gm11527 | 8.060731 | 3.408205 | 5.734468 |
| ENSMUSG00000033983 | protein_coding | Coil | 849.5562 | 744.606 | 797.0811 |
| ENSMUSG00000035354 | protein_coding | Uvrag | 900.6346 | 791.6299 | 846.1322 |
| ENSMUSG00000041263 | protein_coding | Rusc1 | 164.3326 | 195.4029 | 179.8678 |
| ENSMUSG00000030657 | protein_coding | Xylt1 | 347.5386 | 293.2661 | 320.4024 |
| ENSMUSG00000078938 | protein_coding | Syce3 | 259.2952 | 196.6836 | 227.9894 |
| ENSMUSG00000031831 | protein_coding | Lrrc50 | 306.6676 | 263.5227 | 285.0952 |
| ENSMUSG00000028779 | protein_coding | Pef1 | 957.874 | 1067.779 | 1012.827 |
| ENSMUSG00000071229 | protein_coding | Timm8a2 | 77.35147 | 98.07325 | 87.71236 |
| ENSMUSG00000074676 | protein_coding | Foxs1 | 218.5183 | 267.2562 | 242.8872 |
| ENSMUSG00000030222 | protein_coding | Rerg | 315.1877 | 384.1787 | 349.6832 |
| ENSMUSG00000070570 | protein_coding | Slc17a7 | 460.325 | 377.3139 | 418.8195 |
| ENSMUSG00000032796 | protein_coding | Lama1 | 3042.418 | 3410.076 | 3226.247 |
| ENSMUSG00000034377 | protein_coding | Tulp4 | 3215.135 | 2946.908 | 3081.022 |
| ENSMUSG00000039533 | protein_coding | Mmd2 | 6012.913 | 6609.243 | 6311.078 |
| ENSMUSG00000026003 | protein_coding | Acadl | 1780.938 | 2014.688 | 1897.813 |
| ENSMUSG00000033365 | protein_coding | Ipo13 | 1364.268 | 1189.16 | 1276.714 |
| ENSMUSG00000052921 | protein_coding | Arhgef15 | 371.1429 | 322.1107 | 346.6268 |
| ENSMUSG00000035916 | protein_coding | Ptprq | 23.89981 | 35.41727 | 29.65854 |
| ENSMUSG00000050424 | protein_coding | Pnma5 | 1088.669 | 985.4181 | 1037.044 |
| ENSMUSG00000039782 | protein_coding | Cpeb2 | 1095.449 | 923.8361 | 1009.643 |
| ENSMUSG00000061048 | protein_coding | Cdh3 | 364.5408 | 445.1151 | 404.828 |
| ENSMUSG00000083763 | pseudogene | Gm9050 | 0.968641 | 3.788052 | 2.378347 |
| ENSMUSG00000026734 | protein_coding | 4921504E06Rik | 0.33031 | 2.446319 | 1.388315 |
| ENSMUSG00000028809 | protein_coding | Srrm1 | 4866.487 | 4455.915 | 4661.201 |
| ENSMUSG00000030538 | protein_coding | Cib1 | 1151.673 | 1049.159 | 1100.416 |
| ENSMUSG00000028266 | protein_coding | Lmo4 | 1632.608 | 1861.628 | 1747.118 |
| ENSMUSG00000061410 | protein_coding | Zcchc14 | 3033.083 | 2728.368 | 2880.726 |
| ENSMUSG00000055493 | protein_coding | Epm2a | 322.5651 | 277.7294 | 300.1472 |
| ENSMUSG00000090678 | lincRNA | 6030442K20Rik | 20.9142 | 12.15704 | 16.53562 |
| ENSMUSG00000047126 | protein_coding | Cltc | 11676.14 | 12670.24 | 12173.19 |
| ENSMUSG00000093064 | misc_RNA | AC124613.1 | 1.937283 | 5.703176 | 3.820229 |
| ENSMUSG00000002028 | protein_coding | Mll1 | 5192.825 | 5806.991 | 5499.908 |
| ENSMUSG00000092464 | pseudogene | Gm8902 | 0.33031 | 2.43731 | 1.38381 |
| ENSMUSG00000037669 | protein_coding | 1110057K04Rik | 740.0605 | 836.8418 | 788.4512 |
| ENSMUSG00000060657 | protein_coding | 4921513D23Rik | 3460.054 | 3120.422 | 3290.238 |
| ENSMUSG00000052676 | protein_coding | Zmat1 | 1944.823 | 2229.587 | 2087.205 |
| ENSMUSG00000024587 | protein_coding | Nars | 2678.909 | 2904.851 | 2791.88 |
| ENSMUSG00000030316 | protein_coding | 1500001M20Rik | 646.1906 | 727.4374 | 686.814 |
| ENSMUSG00000072944 | protein_coding | Nup62cl | 1753.142 | 1470.962 | 1612.052 |
| ENSMUSG00000059159 | pseudogene | Gm8129 | 19.98298 | 11.9289 | 15.95594 |
| ENSMUSG00000057841 | protein_coding | Rpl32 | 6616.954 | 6000.004 | 6308.479 |
| ENSMUSG00000040840 | processed_transcrip | 4930579G18Rik | 172.1482 | 210.3155 | 191.2318 |
| ENSMUSG00000003309 | protein_coding | Ap1m2 | 288.7726 | 245.9269 | 267.3498 |
| ENSMUSG00000087571 | processed_transcrip | Gm15202 | 1.934972 | 0 | 0.967486 |
| ENSMUSG00000079463 | lincRNA | Gm17682 | 56.10718 | 41.80593 | 48.95656 |
| ENSMUSG00000038451 | protein_coding | Spsb2 | 79.51708 | 101.2357 | 90.37637 |
| ENSMUSG00000020482 | protein_coding | Ccdc117 | 1774.561 | 1563.063 | 1668.812 |

| | | | | | |
|---|---|---|---|---|---|
| ENSMUSG00000019528 | protein_coding | Gyg | 399.4172 | 477.7036 | 438.5604 |
| ENSMUSG00000049532 | protein_coding | Sall2 | 716.7666 | 826.8354 | 771.801 |
| ENSMUSG00000065500 | miRNA | Mir10b | 0.32211 | 2.401635 | 1.361873 |
| ENSMUSG00000056028 | protein_coding | Ovol3 | 7.811376 | 3.063673 | 5.437525 |
| ENSMUSG00000011158 | protein_coding | Brf1 | 1918.565 | 1721.866 | 1820.215 |
| ENSMUSG00000044737 | protein_coding | Klk14 | 0.316221 | 2.401635 | 1.358928 |
| ENSMUSG00000082816 | pseudogene | Gm11953 | 0.316221 | 2.401635 | 1.358928 |
| ENSMUSG00000037112 | protein_coding | Sik2 | 624.8433 | 524.4621 | 574.6527 |
| ENSMUSG00000087413 | processed_transcrip | Gm11266 | 21.33933 | 33.3584 | 27.34887 |
| ENSMUSG00000081544 | pseudogene | Gm13077 | 3.872255 | 8.918559 | 6.395407 |
| ENSMUSG00000037395 | protein_coding | Rcor3 | 553.0265 | 619.3144 | 586.1705 |
| ENSMUSG00000077704 | snoRNA | Snord89 | 40.81436 | 28.12299 | 34.46868 |
| ENSMUSG00000030499 | protein_coding | Kctd15 | 532.8938 | 466.9207 | 499.9073 |
| ENSMUSG00000046408 | protein_coding | 1700067K01Rik | 86.29466 | 67.13522 | 76.71494 |
| ENSMUSG00000045252 | protein_coding | Zfp574 | 861.3434 | 754.8707 | 808.1071 |
| ENSMUSG00000047767 | protein_coding | Atg16l2 | 146.546 | 180.8361 | 163.691 |
| ENSMUSG00000026872 | protein_coding | Zeb2 | 2086.077 | 2313.16 | 2199.619 |
| ENSMUSG00000071337 | protein_coding | Tia1 | 2800.545 | 3325.211 | 3062.878 |
| ENSMUSG00000028033 | protein_coding | Kcnq5 | 57.27031 | 79.35713 | 68.31372 |
| ENSMUSG00000024246 | protein_coding | Thumpd2 | 237.9748 | 275.6545 | 256.8146 |
| ENSMUSG00000037455 | protein_coding | 1110021L09Rik | 669.0241 | 829.274 | 749.149 |
| ENSMUSG00000049957 | protein_coding | Ccdc137 | 680.7567 | 779.3643 | 730.0605 |
| ENSMUSG00000025583 | protein_coding | Rptor | 1706.232 | 1515.748 | 1610.99 |
| ENSMUSG00000049476 | protein_coding | 1700104B16Rik | 2.944613 | 0.326514 | 1.635564 |
| ENSMUSG00000016520 | protein_coding | Lnx2 | 808.2347 | 698.8098 | 753.5222 |
| ENSMUSG00000024223 | protein_coding | 4930511I11Rik | 0.646531 | 3.318836 | 1.982684 |
| ENSMUSG00000030217 | protein_coding | Art4 | 129.1563 | 160.1443 | 144.6503 |
| ENSMUSG00000038793 | protein_coding | Lefty1 | 27.78616 | 13.20498 | 20.49557 |
| ENSMUSG00000032056 | protein_coding | Btg4 | 7.463399 | 3.072682 | 5.26804 |
| ENSMUSG00000051285 | protein_coding | Pcmtd1 | 2628.143 | 2977.257 | 2802.7 |
| ENSMUSG00000031568 | protein_coding | Rwdd4a | 1151.083 | 1308.506 | 1229.794 |
| ENSMUSG00000022054 | protein_coding | Nefm | 248.3264 | 297.2076 | 272.767 |
| ENSMUSG00000027217 | protein_coding | Tspan18 | 568.0298 | 636.8564 | 602.4431 |
| ENSMUSG00000028826 | protein_coding | Tmem57 | 3674.389 | 3226.596 | 3450.492 |
| ENSMUSG00000038201 | protein_coding | Kcna7 | 65.83124 | 83.35707 | 74.59415 |
| ENSMUSG00000016427 | protein_coding | Ndufa1 | 619.7775 | 541.9463 | 580.8619 |
| ENSMUSG00000082354 | pseudogene | Gm378 | 39.4437 | 27.39897 | 33.42134 |
| ENSMUSG00000075225 | protein_coding | Ccdc162 | 39.57408 | 55.21963 | 47.39685 |
| ENSMUSG00000069755 | protein_coding | Zfp125 | 120.385 | 80.83665 | 100.6108 |
| ENSMUSG00000087091 | lincRNA | 4930509E16Rik | 20.7704 | 11.99052 | 16.38046 |
| ENSMUSG00000071862 | protein_coding | Lrrtm2 | 2.606103 | 6.843077 | 4.72459 |
| ENSMUSG00000072925 | protein_coding | Ott | 14.18395 | 7.523133 | 10.85354 |
| ENSMUSG00000079225 | pseudogene | Gm9531 | 326.5774 | 373.1392 | 349.8583 |
| ENSMUSG00000025078 | protein_coding | Nhlrc2 | 973.4318 | 881.6234 | 927.5276 |
| ENSMUSG00000030417 | protein_coding | Pdcd5 | 1476.796 | 1325.1 | 1400.948 |
| ENSMUSG00000037007 | protein_coding | Zfp113 | 536.2273 | 625.8616 | 581.0444 |
| ENSMUSG00000085467 | processed_transcrip | Gm11842 | 0.960442 | 4.123937 | 2.542189 |
| ENSMUSG00000049760 | protein_coding | 2410015M20Rik | 1164.676 | 1045.366 | 1105.021 |
| ENSMUSG00000015217 | protein_coding | Hmgb3 | 7285.513 | 8289.522 | 7787.518 |
| ENSMUSG00000085436 | processed_transcrip | Gm11458 | 58.75495 | 82.62394 | 70.68944 |
| ENSMUSG00000070643 | protein_coding | Sox13 | 943.0702 | 1059.372 | 1001.221 |
| ENSMUSG00000090836 | lincRNA | Gm17475 | 2.614303 | 8.849696 | 5.732 |
| ENSMUSG00000006932 | protein_coding | Ctnnb1 | 18085.91 | 19906.21 | 18996.06 |
| ENSMUSG00000029851 | protein_coding | Fam115c | 205.4663 | 244.0333 | 224.7498 |
| ENSMUSG00000029227 | protein_coding | Fip1l1 | 2877.033 | 3169.502 | 3023.268 |
| ENSMUSG00000003346 | protein_coding | Fam108a | 2311.799 | 2113.387 | 2212.593 |
| ENSMUSG00000038047 | protein_coding | Haus6 | 1890.01 | 1709.978 | 1799.994 |
| ENSMUSG00000022095 | protein_coding | Fam160b2 | 1095.97 | 1219.527 | 1157.748 |

| | | | | | |
|---|---|---|---|---|---|
| ENSMUSG00000028617 | protein_coding | Lrrc42 | 3488.578 | 3944.948 | 3716.763 |
| ENSMUSG00000052281 | processed_transcrip | Gm15024 | 3.566544 | 8.582312 | 6.074428 |
| ENSMUSG00000082313 | pseudogene | Gm11701 | 7.390643 | 3.072682 | 5.231663 |
| ENSMUSG00000025203 | protein_coding | Scd2 | 24328.19 | 27095.21 | 25711.7 |
| ENSMUSG00000042607 | protein_coding | Asb4 | 13.47056 | 23.0354 | 18.25298 |
| ENSMUSG00000086421 | processed_transcrip | Gm14091 | 23.50495 | 36.97387 | 30.23941 |
| ENSMUSG00000030031 | protein_coding | Kbtbd8 | 770.7302 | 661.1855 | 715.9578 |
| ENSMUSG00000077524 | rRNA | n-R5s67 | 5.517916 | 1.73095 | 3.624433 |
| ENSMUSG00000074519 | protein_coding | Etohi1 | 200.0977 | 249.0211 | 224.5594 |
| ENSMUSG00000054715 | protein_coding | Zscan22 | 301.2461 | 360.3106 | 330.7784 |
| ENSMUSG00000091589 | lincRNA | Gm17278 | 4.822186 | 1.006571 | 2.914378 |
| ENSMUSG00000061517 | protein_coding | Sox21 | 1.264884 | 5.625666 | 3.445275 |
| ENSMUSG00000053830 | pseudogene | Gm9923 | 151.8578 | 126.2077 | 139.0328 |
| ENSMUSG00000056966 | protein_coding | Gjc3 | 43.55189 | 26.41192 | 34.98191 |
| ENSMUSG00000049799 | protein_coding | Lrrc19 | 10.63046 | 18.09156 | 14.36101 |
| ENSMUSG00000079555 | protein_coding | Haus3 | 1333.956 | 1492.638 | 1413.297 |
| ENSMUSG00000031201 | protein_coding | Brcc3 | 847.8075 | 955.3556 | 901.5815 |
| ENSMUSG00000018921 | protein_coding | Pelp1 | 1635.945 | 1452.264 | 1544.105 |
| ENSMUSG00000022546 | protein_coding | Gpt | 66.79399 | 85.37564 | 76.08482 |
| ENSMUSG00000024304 | protein_coding | Cdh2 | 2623.619 | 2391.831 | 2507.725 |
| ENSMUSG00000021567 | protein_coding | Nkd2 | 254.9142 | 216.2093 | 235.5617 |
| ENSMUSG00000093169 | miRNA | AC155074.1 | 0.66062 | 3.37253 | 2.016575 |
| ENSMUSG00000021967 | protein_coding | Mrp63 | 630.5408 | 703.964 | 667.2524 |
| ENSMUSG00000009772 | protein_coding | Nuak2 | 834.8576 | 1008.633 | 921.7451 |
| ENSMUSG00000027551 | protein_coding | Zfp64 | 580.3944 | 662.6865 | 621.5404 |
| ENSMUSG00000076431 | protein_coding | Sox4 | 1603.937 | 1818.293 | 1711.115 |
| ENSMUSG00000048371 | protein_coding | Pdp2 | 441.2138 | 371.9152 | 406.5645 |
| ENSMUSG00000026955 | protein_coding | 2010317E24Rik | 268.9197 | 228.5253 | 248.7225 |
| ENSMUSG00000001506 | protein_coding | Col1a1 | 5093.88 | 4475.319 | 4784.599 |
| ENSMUSG00000059278 | protein_coding | Lsmd1 | 797.5443 | 700.1423 | 748.8433 |
| ENSMUSG00000089984 | protein_coding | Fbxo24 | 40.06101 | 28.09311 | 34.07706 |
| ENSMUSG00000049551 | protein_coding | Fzd9 | 217.4719 | 176.7335 | 197.1027 |
| ENSMUSG00000073598 | protein_coding | 1700066B19Rik | 11.68005 | 5.845516 | 8.762784 |
| ENSMUSG00000019179 | protein_coding | Mdh2 | 4446.953 | 4890.417 | 4668.685 |
| ENSMUSG00000003198 | protein_coding | Zfp959 | 257.4025 | 309.341 | 283.3718 |
| ENSMUSG00000049550 | protein_coding | Clip1 | 2334.558 | 2081.523 | 2208.04 |
| ENSMUSG00000024347 | protein_coding | Psd2 | 57.22722 | 74.29866 | 65.76294 |
| ENSMUSG00000037234 | protein_coding | Hook3 | 2697.064 | 2921.171 | 2809.118 |
| ENSMUSG00000031594 | protein_coding | Fgl1 | 100.5564 | 80.03859 | 90.29752 |
| ENSMUSG00000069378 | protein_coding | Prdm6 | 4.210765 | 9.191018 | 6.700891 |
| ENSMUSG00000039234 | protein_coding | Sec24d | 817.9822 | 934.407 | 876.1946 |
| ENSMUSG00000026672 | protein_coding | Optn | 933.4179 | 1059.189 | 996.3036 |
| ENSMUSG00000021041 | protein_coding | 2700073G19Rik | 15.52517 | 25.16634 | 20.34576 |
| ENSMUSG00000086990 | lincRNA | 4833428L15Rik | 0.33031 | 2.348303 | 1.339306 |
| ENSMUSG00000025577 | protein_coding | Cbx2 | 1867.505 | 1620.569 | 1744.037 |
| ENSMUSG00000043241 | protein_coding | Upf2 | 3115.428 | 2857.608 | 2986.518 |
| ENSMUSG00000021643 | protein_coding | Serf1 | 716.1882 | 799.6546 | 757.9214 |
| ENSMUSG00000056305 | protein_coding | Usp39 | 1813.46 | 1655.986 | 1734.723 |
| ENSMUSG00000029672 | protein_coding | Fam3c | 2889.599 | 3177.706 | 3033.652 |
| ENSMUSG00000028012 | protein_coding | Rrh | 4.565675 | 10.80914 | 7.687409 |
| ENSMUSG00000037622 | protein_coding | Wdtc1 | 1808.149 | 1568.586 | 1688.368 |
| ENSMUSG00000022914 | protein_coding | Brwd1 | 6357.552 | 5888.405 | 6122.979 |
| ENSMUSG00000079261 | protein_coding | Gm15217 | 0.32211 | 2.348303 | 1.335207 |
| ENSMUSG00000055980 | protein_coding | Irs1 | 190.3152 | 240.0282 | 215.1717 |
| ENSMUSG00000052906 | protein_coding | Ubxn8 | 626.1647 | 695.8379 | 661.0013 |
| ENSMUSG00000027435 | protein_coding | Cd93 | 491.2095 | 575.3168 | 533.2632 |
| ENSMUSG00000040174 | protein_coding | Alkbh3 | 554.0546 | 463.1407 | 508.5977 |
| ENSMUSG00000035047 | protein_coding | Kri1 | 793.2406 | 703.5031 | 748.3718 |

| | | | | | |
|---|---|---|---|---|---|
| ENSMUSG00000021541 | protein_coding | Trpc7 | 9.390171 | 16.36962 | 12.87989 |
| ENSMUSG00000024725 | protein_coding | Ostf1 | 873.1999 | 782.91 | 828.055 |
| ENSMUSG00000091133 | processed_transcrip | Gm17108 | 22.60086 | 13.82886 | 18.21486 |
| ENSMUSG00000004609 | protein_coding | Cd33 | 38.71748 | 52.2183 | 45.46789 |
| ENSMUSG00000035773 | protein_coding | Kiss1r | 42.81576 | 29.0813 | 35.94853 |
| ENSMUSG00000028076 | protein_coding | Cd1d1 | 170.4192 | 216.8809 | 193.65 |
| ENSMUSG00000087927 | miRNA | AC124367.1 | 28.38811 | 39.88252 | 34.13532 |
| ENSMUSG00000021639 | protein_coding | Gtf2h2 | 1183.749 | 1066.046 | 1124.898 |
| ENSMUSG00000086539 | lincRNA | Gm16759 | 21.10765 | 12.29938 | 16.70351 |
| ENSMUSG00000052403 | processed_transcrip | Gm13520 | 22.3574 | 33.47226 | 27.91483 |
| ENSMUSG00000028553 | protein_coding | Angptl3 | 5.201695 | 1.712931 | 3.457313 |
| ENSMUSG00000034591 | protein_coding | Slc41a2 | 704.6587 | 590.8593 | 647.759 |
| ENSMUSG00000071226 | protein_coding | Cecr2 | 3509.972 | 2934.218 | 3222.095 |
| ENSMUSG00000074182 | protein_coding | Znhit6 | 1153.457 | 1047.886 | 1100.671 |
| ENSMUSG00000026837 | protein_coding | Col5a1 | 2133.826 | 1896.914 | 2015.37 |
| ENSMUSG00000078684 | pseudogene | 5830417I10Rik | 1410.97 | 1682.352 | 1546.661 |
| ENSMUSG00000084797 | processed_transcrip | Gm14321 | 9.96499 | 17.6137 | 13.78934 |
| ENSMUSG00000031783 | protein_coding | Polr2c | 1273.715 | 1401.295 | 1337.505 |
| ENSMUSG00000031424 | protein_coding | Kir3dl1 | 0.316221 | 2.36596 | 1.34109 |
| ENSMUSG00000074171 | protein_coding | Gm6658 | 5.562494 | 1.72194 | 3.642217 |
| ENSMUSG00000027955 | protein_coding | Fam198b | 2140.976 | 2488.764 | 2314.87 |
| ENSMUSG00000027642 | protein_coding | Rpn2 | 4914.744 | 4450.758 | 4682.751 |
| ENSMUSG00000029201 | protein_coding | Ugdh | 1015.608 | 1131.099 | 1073.354 |
| ENSMUSG00000002728 | protein_coding | Naa20 | 789.4738 | 898.4825 | 843.9781 |
| ENSMUSG00000033253 | protein_coding | Szt2 | 3008.533 | 3257.02 | 3132.776 |
| ENSMUSG00000056952 | protein_coding | Tatdn2 | 2048.88 | 1830.366 | 1939.623 |
| ENSMUSG00000037503 | protein_coding | Fam168b | 5647.164 | 6243.74 | 5945.452 |
| ENSMUSG00000037386 | protein_coding | Rims2 | 238.4842 | 126.2493 | 182.3667 |
| ENSMUSG00000030069 | protein_coding | Prok2 | 40.06228 | 58.86887 | 49.46557 |
| ENSMUSG00000037411 | protein_coding | Serpine1 | 164.2591 | 202.4907 | 183.3749 |
| ENSMUSG00000031792 | protein_coding | AA960436 | 1062.345 | 955.8503 | 1009.098 |
| ENSMUSG00000004187 | protein_coding | Kifc2 | 311.2134 | 259.6597 | 285.4365 |
| ENSMUSG00000041298 | protein_coding | Katnal1 | 621.1171 | 556.8529 | 588.985 |
| ENSMUSG00000005354 | protein_coding | Txn2 | 1577.909 | 1725.758 | 1651.833 |
| ENSMUSG00000031153 | protein_coding | Gripap1 | 596.5682 | 673.9075 | 635.2378 |
| ENSMUSG00000085212 | lincRNA | 4931440J10Rik | 5.201695 | 1.72194 | 3.461818 |
| ENSMUSG00000032496 | protein_coding | Ltf | 2.298082 | 0 | 1.149041 |
| ENSMUSG00000038650 | protein_coding | Rnh1 | 3083.704 | 3345.017 | 3214.36 |
| ENSMUSG00000031386 | protein_coding | Hcfc1 | 6172.777 | 5508.367 | 5840.572 |
| ENSMUSG00000086913 | processed_transcrip | Gm14019 | 7.128467 | 13.78381 | 10.45614 |
| ENSMUSG00000032415 | protein_coding | Ube2cbp | 157.2559 | 129.1453 | 143.2006 |
| ENSMUSG00000024101 | protein_coding | ORF19 | 1175.783 | 1073.98 | 1124.881 |
| ENSMUSG00000025204 | protein_coding | Ndufb8 | 1557.929 | 1401.771 | 1479.85 |
| ENSMUSG00000024122 | protein_coding | Pdpk1 | 3059.272 | 2707.221 | 2883.246 |
| ENSMUSG00000069308 | protein_coding | Hist1h2bp | 4.515208 | 9.642577 | 7.078892 |
| ENSMUSG00000085873 | processed_transcrip | Gm12750 | 14.84942 | 24.06864 | 19.45903 |
| ENSMUSG00000027652 | protein_coding | Ralgapb | 5149.055 | 4491.581 | 4820.318 |
| ENSMUSG00000068062 | processed_transcrip | Gm14164 | 4.504697 | 1.386417 | 2.945557 |
| ENSMUSG00000027160 | protein_coding | Ccdc34 | 760.319 | 670.1931 | 715.2561 |
| ENSMUSG00000055944 | protein_coding | E130018O15Rik | 12.29378 | 6.082661 | 9.188223 |
| ENSMUSG00000085207 | processed_transcrip | Gm11767 | 25.02806 | 14.85272 | 19.94039 |
| ENSMUSG00000085586 | lincRNA | Gm11613 | 76.53885 | 57.61946 | 67.07915 |
| ENSMUSG00000019854 | protein_coding | Reps1 | 2206.681 | 1949.131 | 2077.906 |
| ENSMUSG00000022894 | protein_coding | Adamts5 | 4577.772 | 4126.088 | 4351.93 |
| ENSMUSG00000028894 | protein_coding | Inpp5b | 3196.357 | 2881.457 | 3038.907 |
| ENSMUSG00000019837 | protein_coding | Gtf3c6 | 1012.772 | 899.9607 | 956.3664 |
| ENSMUSG00000052605 | protein_coding | Isoc2b | 142.4439 | 117.0355 | 129.7397 |
| ENSMUSG00000085654 | processed_transcrip | Gm12536 | 0.968641 | 3.690035 | 2.329338 |

| | | | | | |
|---|---|---|---|---|---|
| ENSMUSG00000082107 | pseudogene | Gm16216 | 4.515208 | 9.598254 | 7.056731 |
| ENSMUSG00000062383 | protein_coding | B130024G19Rik | 31.63024 | 47.40783 | 39.51903 |
| ENSMUSG00000028871 | protein_coding | Rspo1 | 86.38255 | 66.72612 | 76.55434 |
| ENSMUSG00000045257 | protein_coding | Morn2 | 148.3792 | 121.8946 | 135.1369 |
| ENSMUSG00000025986 | protein_coding | Slc39a10 | 2871.57 | 3204.221 | 3037.895 |
| ENSMUSG00000009292 | protein_coding | Trpm2 | 0.65242 | 3.072682 | 1.862551 |
| ENSMUSG00000021713 | protein_coding | Ppwd1 | 1051.114 | 1166.032 | 1108.573 |
| ENSMUSG00000027134 | protein_coding | Lpcat4 | 372.3725 | 322.1617 | 347.2671 |
| ENSMUSG00000031125 | protein_coding | 3830403N18Rik | 142.3258 | 109.007 | 125.6664 |
| ENSMUSG00000088990 | snoRNA | SCARNA1 | 2.928213 | 7.231933 | 5.080073 |
| ENSMUSG00000081392 | pseudogene | Gm11668 | 0.33031 | 2.339294 | 1.334802 |
| ENSMUSG00000015501 | protein_coding | Hivep2 | 672.8309 | 802.689 | 737.76 |
| ENSMUSG00000022105 | protein_coding | Rb1 | 2019.142 | 1824.368 | 1921.755 |
| ENSMUSG00000036775 | protein_coding | Decr2 | 408.2513 | 521.7778 | 465.0145 |
| ENSMUSG00000090925 | protein_coding | 1810064F22Rik | 22.30924 | 33.08485 | 27.69705 |
| ENSMUSG00000020978 | protein_coding | Klhdc2 | 2345.679 | 2602.9 | 2474.29 |
| ENSMUSG00000031982 | protein_coding | Arv1 | 312.0911 | 355.608 | 333.8496 |
| ENSMUSG00000072736 | protein_coding | Gm3591 | 6.816868 | 2.710492 | 4.76368 |
| ENSMUSG00000053907 | protein_coding | Mat2a | 13480.52 | 14844.77 | 14162.64 |
| ENSMUSG00000025809 | protein_coding | Itgb1 | 11206.58 | 12380.88 | 11793.73 |
| ENSMUSG00000019883 | protein_coding | Echdc1 | 328.1701 | 287.2215 | 307.6958 |
| ENSMUSG00000041560 | protein_coding | Gltscr2 | 3273.935 | 3530.072 | 3402.003 |
| ENSMUSG00000019923 | protein_coding | Zwint | 4280.248 | 4714.847 | 4497.547 |
| ENSMUSG00000034341 | protein_coding | Wbp2 | 2078.909 | 2338.84 | 2208.874 |
| ENSMUSG00000025221 | protein_coding | Kcnip2 | 43.57962 | 58.57663 | 51.07812 |
| ENSMUSG00000087516 | processed_transcrip | Gm2301 | 3.222145 | 7.576465 | 5.399305 |
| ENSMUSG00000066191 | protein_coding | Anks6 | 251.9266 | 215.8475 | 233.8871 |
| ENSMUSG00000021806 | protein_coding | Nid2 | 1665.634 | 1887.396 | 1776.515 |
| ENSMUSG00000034593 | protein_coding | Myo5a | 2758.344 | 2337.14 | 2547.742 |
| ENSMUSG00000071652 | protein_coding | Ints5 | 1351.287 | 1210.419 | 1280.853 |
| ENSMUSG00000044629 | protein_coding | Cnrip1 | 141.4914 | 170.1274 | 155.8094 |
| ENSMUSG00000053896 | protein_coding | 4933409G03Rik | 2.964591 | 0.36219 | 1.66339 |
| ENSMUSG00000020128 | protein_coding | Vps54 | 2250.24 | 2047.495 | 2148.868 |
| ENSMUSG00000052137 | protein_coding | C430048L16Rik | 561.9228 | 647.3761 | 604.6494 |
| ENSMUSG00000025014 | protein_coding | Dntt | 3.543213 | 0.36219 | 1.952701 |
| ENSMUSG00000036427 | protein_coding | Gpi1 | 3902.775 | 4248.294 | 4075.534 |
| ENSMUSG00000040147 | protein_coding | Maob | 339.9988 | 294.5822 | 317.2905 |
| ENSMUSG00000034889 | protein_coding | 2510012J08Rik | 1338.818 | 1210.114 | 1274.466 |
| ENSMUSG00000021266 | protein_coding | Wars | 1153.903 | 1287.222 | 1220.562 |
| ENSMUSG00000002058 | protein_coding | Unc119 | 3003.549 | 3269.233 | 3136.391 |
| ENSMUSG00000026429 | protein_coding | Ube2t | 1042.717 | 875.6636 | 959.1902 |
| ENSMUSG00000033373 | protein_coding | Fntb | 625.5043 | 555.3373 | 590.4208 |
| ENSMUSG00000059208 | protein_coding | Hnrnpm | 6381.286 | 6916.199 | 6648.742 |
| ENSMUSG00000028730 | protein_coding | Wdr65 | 39.29654 | 25.81036 | 32.55345 |
| ENSMUSG00000039463 | protein_coding | Slc9a8 | 961.4512 | 1095.85 | 1028.651 |
| ENSMUSG00000028015 | protein_coding | Ctso | 1617.807 | 1795.601 | 1706.704 |
| ENSMUSG00000026347 | protein_coding | Tmem163 | 192.8448 | 161.7534 | 177.2991 |
| ENSMUSG00000026579 | protein_coding | F5 | 59.9277 | 44.84688 | 52.38729 |
| ENSMUSG00000041774 | protein_coding | Ydjc | 161.1589 | 133.5125 | 147.3357 |
| ENSMUSG00000036306 | protein_coding | Lzts1 | 143.9733 | 115.0096 | 129.4915 |
| ENSMUSG00000033032 | protein_coding | Afap1l1 | 302.2665 | 258.9803 | 280.6234 |
| ENSMUSG00000021207 | protein_coding | Akr1c21 | 3.216256 | 0.697713 | 1.956985 |
| ENSMUSG00000086454 | lincRNA | 4930500J02Rik | 92.28296 | 118.833 | 105.558 |
| ENSMUSG00000027209 | protein_coding | 4930525F21Rik | 16.68621 | 7.012083 | 11.84915 |
| ENSMUSG00000028393 | protein_coding | Alad | 2984.201 | 3590.758 | 3287.479 |
| ENSMUSG00000021203 | protein_coding | Otub2 | 188.8678 | 157.503 | 173.1854 |
| ENSMUSG00000082234 | pseudogene | Gm16221 | 1.586995 | 5.617018 | 3.602006 |
| ENSMUSG00000031310 | protein_coding | Zmym3 | 6973.887 | 7643.375 | 7308.631 |

| | | | | | |
|---|---|---|---|---|---|
| ENSMUSG00000014329 | protein_coding | Bicc1 | 985.0537 | 886.1458 | 935.5997 |
| ENSMUSG00000059878 | protein_coding | Zfp422 | 1194.336 | 1405.487 | 1299.911 |
| ENSMUSG00000071074 | protein_coding | Yipf3 | 1200.229 | 1330.985 | 1265.607 |
| ENSMUSG00000078867 | protein_coding | Gm14418 | 15.48417 | 8.802524 | 12.14335 |
| ENSMUSG00000057335 | protein_coding | Cep170 | 2348.878 | 2116.972 | 2232.925 |
| ENSMUSG00000031111 | protein_coding | Igsf1 | 9.658236 | 17.60577 | 13.632 |
| ENSMUSG00000028957 | protein_coding | Per3 | 1064.778 | 892.0345 | 978.4062 |
| ENSMUSG00000039043 | protein_coding | 2610034B18Rik | 234.0352 | 282.1813 | 258.1082 |
| ENSMUSG00000016503 | protein_coding | Gtf3a | 433.0087 | 372.8828 | 402.9458 |
| ENSMUSG00000039910 | protein_coding | Cited2 | 3095.239 | 3449.961 | 3272.6 |
| ENSMUSG00000073676 | protein_coding | Hspe1 | 2437.122 | 2700.661 | 2568.892 |
| ENSMUSG00000030990 | protein_coding | Pgap2 | 1794.2 | 2020.364 | 1907.282 |
| ENSMUSG00000020171 | protein_coding | Yeats4 | 2171.407 | 1982.931 | 2077.169 |
| ENSMUSG00000001833 | protein_coding | 7-Sep | 5204.63 | 5819.546 | 5512.088 |
| ENSMUSG00000029136 | protein_coding | Rbks | 154.2997 | 181.6526 | 167.9762 |
| ENSMUSG00000028010 | protein_coding | Gar1 | 1111.218 | 1222.671 | 1166.945 |
| ENSMUSG00000024622 | protein_coding | Hmgxb3 | 2044.103 | 1819.563 | 1931.833 |
| ENSMUSG00000045664 | protein_coding | Cdc42ep2 | 236.388 | 276.856 | 256.622 |
| ENSMUSG00000024666 | protein_coding | Tmem138 | 320.602 | 367.4736 | 344.0378 |
| ENSMUSG00000022218 | protein_coding | Tgm1 | 75.7143 | 114.5198 | 95.11703 |
| ENSMUSG00000053528 | protein_coding | A530021J07Rik | 11.89176 | 20.28023 | 16.08599 |
| ENSMUSG00000091196 | protein_coding | Gm17330 | 5.125361 | 1.686265 | 3.405813 |
| ENSMUSG00000071567 | processed_transcrip | A830039N20Rik | 83.46463 | 108.2698 | 95.86721 |
| ENSMUSG00000021390 | protein_coding | Ogn | 2242.676 | 2002.535 | 2122.605 |
| ENSMUSG00000028423 | protein_coding | Nfx1 | 4026.142 | 4449.54 | 4237.841 |
| ENSMUSG00000049090 | protein_coding | Zadh2 | 1915.237 | 2149.209 | 2032.223 |
| ENSMUSG00000006818 | protein_coding | Sod2 | 2142.438 | 1947.93 | 2045.184 |
| ENSMUSG00000036402 | protein_coding | Gng12 | 5752.65 | 6405.559 | 6079.104 |
| ENSMUSG00000090693 | protein_coding | Trim43c | 22.27055 | 13.97735 | 18.12395 |
| ENSMUSG00000086147 | processed_transcrip | Gm13150 | 7.44111 | 13.80183 | 10.62147 |
| ENSMUSG00000024121 | protein_coding | Atp6v0c | 1212.928 | 1466.432 | 1339.68 |
| ENSMUSG00000051586 | protein_coding | Mical3 | 2203.935 | 2424.947 | 2314.441 |
| ENSMUSG00000036106 | protein_coding | Prr5 | 979.6814 | 871.1139 | 925.3977 |
| ENSMUSG00000020248 | protein_coding | Nfyb | 1271.046 | 1135.971 | 1203.509 |
| ENSMUSG00000036667 | protein_coding | Fam115a | 1103.559 | 1315.878 | 1209.719 |
| ENSMUSG00000025759 | protein_coding | Mfsd8 | 325.3893 | 277.3039 | 301.3466 |
| ENSMUSG00000032485 | protein_coding | Scap | 3746.317 | 3425.966 | 3586.142 |
| ENSMUSG00000006720 | protein_coding | Zfp184 | 410.6144 | 353.71 | 382.1622 |
| ENSMUSG00000036501 | protein_coding | Fam13b | 3343.887 | 3080.217 | 3212.052 |
| ENSMUSG00000023039 | protein_coding | Krt7 | 4.837318 | 9.853418 | 7.345368 |
| ENSMUSG00000045316 | protein_coding | Fahd1 | 368.4729 | 432.4224 | 400.4477 |
| ENSMUSG00000022370 | protein_coding | Mrpl13 | 1360.429 | 1228.163 | 1294.296 |
| ENSMUSG00000052310 | protein_coding | Slc39a1 | 1966.114 | 2135.556 | 2050.835 |
| ENSMUSG00000038323 | protein_coding | 1700066M21Rik | 677.8338 | 589.4647 | 633.6492 |
| ENSMUSG00000044966 | protein_coding | Fbxo48 | 81.77021 | 63.16149 | 72.46585 |
| ENSMUSG00000040747 | protein_coding | Cd53 | 101.8535 | 125.2137 | 113.5336 |
| ENSMUSG00000029647 | protein_coding | Pan3 | 2067.092 | 2271.847 | 2169.469 |
| ENSMUSG00000045994 | protein_coding | B3gat1 | 13.19429 | 7.10725 | 10.15077 |
| ENSMUSG00000002108 | protein_coding | Nr1h3 | 69.90871 | 102.7527 | 86.33069 |
| ENSMUSG00000091710 | lincRNA | 4930426I24Rik | 45.40464 | 30.42982 | 37.91723 |
| ENSMUSG00000064580 | snRNA | U1 | 4.496497 | 1.350742 | 2.923619 |
| ENSMUSG00000010936 | protein_coding | Vac14 | 1254.8 | 1119.808 | 1187.304 |
| ENSMUSG00000035540 | protein_coding | Gc | 5.276762 | 1.033237 | 3.154999 |
| ENSMUSG00000086691 | pseudogene | Gm15432 | 8.412062 | 14.94789 | 11.67998 |
| ENSMUSG00000085492 | lincRNA | Trmt61b | 156.2288 | 131.3469 | 143.7878 |
| ENSMUSG00000003929 | protein_coding | Zfp81 | 861.7845 | 767.7419 | 814.7632 |
| ENSMUSG00000058454 | protein_coding | Dhcr7 | 1806.802 | 1588.5 | 1697.651 |
| ENSMUSG00000028689 | protein_coding | Ccdc163 | 380.8204 | 320.6534 | 350.7369 |

| | | | | | |
|---|---|---|---|---|---|
| ENSMUSG00000034317 | protein_coding | Trim59 | 2016.27 | 2315.714 | 2165.992 |
| ENSMUSG00000031320 | protein_coding | Rps4x | 6824.786 | 7583.064 | 7203.925 |
| ENSMUSG00000029174 | protein_coding | Tbc1d1 | 1881.452 | 1633.811 | 1757.632 |
| ENSMUSG00000028621 | protein_coding | Cyb5rl | 720.4594 | 598.0985 | 659.279 |
| ENSMUSG00000057697 | protein_coding | Vmn1r53 | 11.33796 | 5.792184 | 8.565074 |
| ENSMUSG00000018160 | protein_coding | Med1 | 3396.845 | 3718.933 | 3557.889 |
| ENSMUSG00000040760 | protein_coding | Appl1 | 4134.266 | 4458.072 | 4296.169 |
| ENSMUSG00000005836 | protein_coding | Gata6 | 1405.312 | 1588.112 | 1496.712 |
| ENSMUSG00000039000 | protein_coding | Ube3c | 3192.991 | 2938.706 | 3065.848 |
| ENSMUSG00000043059 | protein_coding | Zfp513 | 731.9326 | 825.7665 | 778.8496 |
| ENSMUSG00000052752 | protein_coding | Traf7 | 2642.44 | 2428.707 | 2535.573 |
| ENSMUSG00000074682 | protein_coding | Zcchc3 | 1414.154 | 1579.232 | 1496.693 |
| ENSMUSG00000085617 | lincRNA | G730013B05Rik | 7.471599 | 3.081691 | 5.276645 |
| ENSMUSG00000034254 | protein_coding | Agpat1 | 1771.277 | 1616.986 | 1694.132 |
| ENSMUSG00000031775 | protein_coding | Pllp | 44.60566 | 59.02177 | 51.81371 |
| ENSMUSG00000059937 | lincRNA | Gdap10 | 229.298 | 194.7678 | 212.0329 |
| ENSMUSG00000043313 | protein_coding | Pcdhb19 | 55.9522 | 76.94685 | 66.44953 |
| ENSMUSG00000078182 | pseudogene | Gm6083 | 3.258523 | 0.697713 | 1.978118 |
| ENSMUSG00000046213 | protein_coding | Cym | 0.960442 | 3.761386 | 2.360914 |
| ENSMUSG00000044349 | protein_coding | Snhg11 | 511.6966 | 583.0811 | 547.3889 |
| ENSMUSG00000073716 | pseudogene | Gm13241 | 218.2985 | 288.1722 | 253.2353 |
| ENSMUSG00000089798 | protein_coding | 1700028K03Rik | 171.6147 | 118.8935 | 145.2541 |
| ENSMUSG00000006423 | protein_coding | C330007P06Rik | 1095.111 | 1228.561 | 1161.836 |
| ENSMUSG00000023050 | protein_coding | Map3k12 | 487.1949 | 562.564 | 524.8795 |
| ENSMUSG00000064267 | protein_coding | Hvcn1 | 388.3709 | 338.6103 | 363.4906 |
| ENSMUSG00000033208 | protein_coding | S100b | 443.844 | 393.7441 | 418.7941 |
| ENSMUSG00000041842 | protein_coding | Fhdc1 | 71.28736 | 95.80526 | 83.54631 |
| ENSMUSG00000033967 | protein_coding | 2310014L17Rik | 16.88995 | 26.56721 | 21.72858 |
| ENSMUSG00000020297 | protein_coding | Nsg2 | 13.87593 | 22.93014 | 18.40304 |
| ENSMUSG00000027257 | protein_coding | Pacsin3 | 337.008 | 294.6164 | 315.8122 |
| ENSMUSG00000028232 | protein_coding | Tmem68 | 665.497 | 753.7469 | 709.6219 |
| ENSMUSG00000037958 | protein_coding | Ccdc55 | 1611.136 | 1772.781 | 1691.959 |
| ENSMUSG00000045996 | protein_coding | Polr2k | 441.9407 | 387.2993 | 414.62 |
| ENSMUSG00000037580 | protein_coding | Gch1 | 160.8229 | 132.8605 | 146.8417 |
| ENSMUSG00000033809 | protein_coding | Alg3 | 424.5736 | 374.6982 | 399.6359 |
| ENSMUSG00000024259 | protein_coding | Slc25a46 | 1221.817 | 1110.57 | 1166.193 |
| ENSMUSG00000031756 | protein_coding | Cenpn | 756.2599 | 863.6394 | 809.9497 |
| ENSMUSG00000029142 | protein_coding | Mrpl33 | 848.4091 | 962.0779 | 905.2435 |
| ENSMUSG00000050394 | protein_coding | Armcx6 | 249.8077 | 303.974 | 276.8908 |
| ENSMUSG00000085137 | lincRNA | Gm16740 | 34.17871 | 23.77672 | 28.97772 |
| ENSMUSG00000020671 | protein_coding | Rab10 | 5130.478 | 5589.293 | 5359.885 |
| ENSMUSG00000054135 | protein_coding | A430110L20Rik | 37.21271 | 51.03407 | 44.12339 |
| ENSMUSG00000007036 | protein_coding | Abhd16a | 994.0327 | 896.297 | 945.1648 |
| ENSMUSG00000034353 | protein_coding | Ramp1 | 160.6088 | 190.6832 | 175.646 |
| ENSMUSG00000079227 | protein_coding | Ccr5 | 18.70528 | 11.01998 | 14.86263 |
| ENSMUSG00000052681 | protein_coding | Rap1b | 5246.933 | 5696.077 | 5471.505 |
| ENSMUSG00000020818 | protein_coding | Mfsd11 | 704.4189 | 627.3543 | 665.8866 |
| ENSMUSG00000005986 | protein_coding | Ankrd13d | 187.0525 | 158.1642 | 172.6084 |
| ENSMUSG00000062944 | protein_coding | 9130023H24Rik | 340.3757 | 290.0302 | 315.203 |
| ENSMUSG00000034435 | protein_coding | Tmem30b | 60.82821 | 41.92347 | 51.37584 |
| ENSMUSG00000055296 | protein_coding | D730040F13Rik | 2944.091 | 3212.89 | 3078.491 |
| ENSMUSG00000028173 | protein_coding | Wls | 1044.921 | 1236.92 | 1140.921 |
| ENSMUSG00000068522 | protein_coding | Aard | 13613.62 | 15318.77 | 14466.2 |
| ENSMUSG00000032565 | protein_coding | Nudt16 | 191.1952 | 162.7188 | 176.957 |
| ENSMUSG00000040034 | protein_coding | Nup43 | 850.1998 | 752.0041 | 801.1019 |
| ENSMUSG00000020490 | protein_coding | Butr1 | 2190.101 | 1800.888 | 1995.495 |
| ENSMUSG00000091550 | protein_coding | Gm6811 | 5.820048 | 11.21317 | 8.516608 |
| ENSMUSG00000045854 | protein_coding | Lyrm2 | 642.9329 | 733.1842 | 688.0585 |

| | | | | | |
|---|---|---|---|---|---|
| ENSMUSG00000015961 | protein_coding | Adss | 2323.714 | 2687.22 | 2505.467 |
| ENSMUSG00000028599 | protein_coding | Tnfrsf1b | 109.7481 | 132.8933 | 121.3207 |
| ENSMUSG00000005649 | protein_coding | Cabp5 | 6.162137 | 11.84818 | 9.005158 |
| ENSMUSG00000061306 | protein_coding | Slc38a10 | 3401.75 | 3078.727 | 3240.238 |
| ENSMUSG00000086943 | lincRNA | 4732414G09Rik | 6.162137 | 2.119805 | 4.140971 |
| ENSMUSG00000063895 | protein_coding | Nupl1 | 2791.839 | 2540.65 | 2666.245 |
| ENSMUSG00000028172 | protein_coding | Tacr3 | 81.09841 | 124.6709 | 102.8847 |
| ENSMUSG00000059552 | protein_coding | Trp53 | 1831.826 | 2049.423 | 1940.624 |
| ENSMUSG00000025917 | protein_coding | Cops5 | 2281.667 | 2493.323 | 2387.495 |
| ENSMUSG00000023341 | protein_coding | Mx2 | 42.87718 | 27.71104 | 35.29411 |
| ENSMUSG00000079235 | protein_coding | Ccdc13 | 64.45133 | 83.11521 | 73.78327 |
| ENSMUSG00000046962 | protein_coding | Zfp295 | 978.9169 | 885.3818 | 932.1493 |
| ENSMUSG00000032028 | protein_coding | Fam55b | 39.64892 | 55.75082 | 47.69987 |
| ENSMUSG00000044857 | protein_coding | Lemd2 | 1473.471 | 1322.534 | 1398.003 |
| ENSMUSG00000081004 | pseudogene | Gm9157 | 0.962752 | 3.980874 | 2.471813 |
| ENSMUSG00000090898 | protein_coding | Gm7732 | 1.945483 | 5.501345 | 3.723414 |
| ENSMUSG00000087639 | processed_transcrip | Gm15512 | 8.466107 | 3.318836 | 5.892472 |
| ENSMUSG00000044937 | protein_coding | BC030307 | 214.4725 | 165.258 | 189.8652 |
| ENSMUSG00000001986 | protein_coding | Gria3 | 240.3523 | 287.4714 | 263.9118 |
| ENSMUSG00000032531 | protein_coding | Amotl2 | 2503.768 | 2819.778 | 2661.773 |
| ENSMUSG00000059078 | pseudogene | Gm10060 | 81.33979 | 63.14783 | 72.24381 |
| ENSMUSG00000057894 | protein_coding | Zfp329 | 1187.391 | 1333.115 | 1260.253 |
| ENSMUSG00000037313 | protein_coding | Tacc3 | 2042.277 | 1855.102 | 1948.69 |
| ENSMUSG00000074166 | protein_coding | AW146154 | 243.1685 | 289.9578 | 266.5632 |
| ENSMUSG00000020310 | protein_coding | Madcam1 | 13.29291 | 7.10725 | 10.20008 |
| ENSMUSG00000035150 | protein_coding | Eif2s3x | 1577.834 | 1763.891 | 1670.862 |
| ENSMUSG00000031788 | protein_coding | Kifc3 | 472.3751 | 416.2049 | 444.29 |
| ENSMUSG00000021810 | protein_coding | Ecd | 1293.613 | 1179.514 | 1236.564 |
| ENSMUSG00000078564 | protein_coding | Gm9631 | 74.77041 | 49.54394 | 62.15718 |
| ENSMUSG00000029072 | protein_coding | Tas1r3 | 44.21415 | 31.23642 | 37.72529 |
| ENSMUSG00000040322 | protein_coding | Slc25a24 | 764.4078 | 859.8687 | 812.1383 |
| ENSMUSG00000026207 | protein_coding | Speg | 167.9777 | 203.0162 | 185.4969 |
| ENSMUSG00000025499 | protein_coding | Hras1 | 849.6989 | 767.7257 | 808.7123 |
| ENSMUSG00000018882 | protein_coding | Mrpl45 | 1153.008 | 1270.632 | 1211.82 |
| ENSMUSG00000025607 | protein_coding | Copg2 | 3532.392 | 3213.272 | 3372.832 |
| ENSMUSG00000058317 | protein_coding | Ube2e2 | 580.0346 | 511.4767 | 545.7556 |
| ENSMUSG00000020873 | protein_coding | Slc35b1 | 968.9194 | 1064.501 | 1016.71 |
| ENSMUSG00000040865 | protein_coding | Ino80d | 2412.86 | 2646.858 | 2529.859 |
| ENSMUSG00000041199 | protein_coding | Rpusd1 | 564.2556 | 499.7262 | 531.9909 |
| ENSMUSG00000015839 | protein_coding | Nfe2l2 | 2887.066 | 3285.171 | 3086.119 |
| ENSMUSG00000021699 | protein_coding | Pde4d | 265.6049 | 214.3723 | 239.9886 |
| ENSMUSG00000027583 | protein_coding | Zbtb46 | 1601.435 | 1850.11 | 1725.773 |
| ENSMUSG00000091348 | protein_coding | Gm13151 | 29.84435 | 42.74473 | 36.29454 |
| ENSMUSG00000032318 | protein_coding | Isl2 | 35.82102 | 52.11739 | 43.9692 |
| ENSMUSG00000015112 | protein_coding | Slc25a13 | 572.4422 | 640.876 | 606.6591 |
| ENSMUSG00000091438 | processed_transcrip | Gm17088 | 0.976841 | 3.73472 | 2.35578 |
| ENSMUSG00000090961 | protein_coding | Vmn2r8 | 2.885946 | 7.134278 | 5.010112 |
| ENSMUSG00000019782 | protein_coding | Rwdd1 | 1312.078 | 1190.486 | 1251.282 |
| ENSMUSG00000026114 | protein_coding | Cnga3 | 13.60787 | 21.85294 | 17.73041 |
| ENSMUSG00000002012 | protein_coding | Pnck | 107.9843 | 133.8073 | 120.8958 |
| ENSMUSG00000027680 | protein_coding | Fxr1 | 5322.521 | 5737.043 | 5529.782 |
| ENSMUSG00000070780 | protein_coding | Rbm47 | 105.5256 | 128.5052 | 117.0154 |
| ENSMUSG00000040151 | protein_coding | Hs2st1 | 1638.276 | 1483.846 | 1561.061 |
| ENSMUSG00000031729 | protein_coding | 2400003C14Rik | 3131.68 | 3408.512 | 3270.096 |
| ENSMUSG00000090800 | lincRNA | 4833407H14Rik | 1.957261 | 6.092393 | 4.024827 |
| ENSMUSG00000035283 | protein_coding | Adrb1 | 12.8604 | 20.95956 | 16.90998 |
| ENSMUSG00000066068 | pseudogene | Gm13611 | 3.264412 | 0.688704 | 1.976558 |
| ENSMUSG00000020087 | protein_coding | Tysnd1 | 536.5007 | 476.7491 | 506.6249 |

| | | | | | |
|---|---|---|---|---|---|
| ENSMUSG00000035840 | protein_coding | Lysmd3 | 1074.364 | 1217.497 | 1145.93 |
| ENSMUSG00000028018 | protein_coding | Gstcd | 666.8728 | 598.7255 | 632.7991 |
| ENSMUSG00000089937 | pseudogene | Gm7281 | 0 | 2.066473 | 1.033237 |
| ENSMUSG00000038150 | protein_coding | Ormdl3 | 4940.552 | 5400.023 | 5170.287 |
| ENSMUSG00000039137 | protein_coding | Whrn | 668.2786 | 755.4481 | 711.8634 |
| ENSMUSG00000013989 | lincRNA | D430018E03Rik | 61.0303 | 46.60019 | 53.81525 |
| ENSMUSG00000037946 | protein_coding | Fgd3 | 86.10398 | 65.34442 | 75.7242 |
| ENSMUSG00000022553 | protein_coding | Maf1 | 2496.769 | 2306.443 | 2401.606 |
| ENSMUSG00000052794 | protein_coding | 1700030K09Rik | 380.7566 | 433.5077 | 407.1321 |
| ENSMUSG00000036636 | protein_coding | Clcn7 | 1354.223 | 1226.134 | 1290.178 |
| ENSMUSG00000020493 | protein_coding | Prr11 | 564.8299 | 641.4737 | 603.1518 |
| ENSMUSG00000091753 | lincRNA | Gm17432 | 40.82152 | 28.45023 | 34.63587 |
| ENSMUSG00000086123 | pseudogene | Gm16060 | 0 | 1.72194 | 0.86097 |
| ENSMUSG00000051237 | protein_coding | 9230109A22Rik | 0 | 1.72194 | 0.86097 |
| ENSMUSG00000026317 | protein_coding | Cln8 | 645.3705 | 725.3597 | 685.3651 |
| ENSMUSG00000032386 | protein_coding | Trip4 | 1259.552 | 1382.938 | 1321.245 |
| ENSMUSG00000047844 | protein_coding | Bex4 | 1677.257 | 1826.844 | 1752.05 |
| ENSMUSG00000052271 | protein_coding | Bhlha15 | 8.371063 | 14.99185 | 11.68146 |
| ENSMUSG00000055319 | protein_coding | Sec23ip | 3866.444 | 3530.561 | 3698.503 |
| ENSMUSG00000039105 | protein_coding | Atp6v1g1 | 2378.654 | 2647.207 | 2512.931 |
| ENSMUSG00000087288 | lincRNA | Gm13061 | 19.29187 | 11.65536 | 15.47361 |
| ENSMUSG00000030096 | protein_coding | Slc6a6 | 1267.836 | 1137.286 | 1202.561 |
| ENSMUSG00000062753 | protein_coding | AI413582 | 137.1163 | 164.6743 | 150.8953 |
| ENSMUSG00000063019 | protein_coding | Manbal | 521.1131 | 601.7901 | 561.4516 |
| ENSMUSG00000042371 | protein_coding | Slc5a10 | 21.10429 | 12.31703 | 16.71066 |
| ENSMUSG00000033161 | protein_coding | Atp1a1 | 14965.72 | 16524.46 | 15745.09 |
| ENSMUSG00000059422 | pseudogene | Gm8116 | 413.0634 | 356.67 | 384.8667 |
| ENSMUSG00000037364 | protein_coding | Srrt | 3318.375 | 3622.366 | 3470.37 |
| ENSMUSG00000082348 | pseudogene | Gm12807 | 0.638331 | 3.161689 | 1.90001 |
| ENSMUSG00000049202 | pseudogene | 4930515G13Rik | 7.133089 | 13.50198 | 10.31753 |
| ENSMUSG00000079659 | protein_coding | 4930420K17Rik | 261.1315 | 215.7354 | 238.4334 |
| ENSMUSG00000023873 | protein_coding | 1700010I14Rik | 68.48781 | 52.80935 | 60.64858 |
| ENSMUSG00000037994 | protein_coding | Nhedc2 | 31.16977 | 19.56413 | 25.36695 |
| ENSMUSG00000057551 | protein_coding | Zfp317 | 719.7747 | 827.2353 | 773.505 |
| ENSMUSG00000090664 | lincRNA | Gm17568 | 47.3871 | 34.56784 | 40.97747 |
| ENSMUSG00000057229 | protein_coding | Atp5sl | 908.9946 | 821.7054 | 865.35 |
| ENSMUSG00000024997 | protein_coding | Prdx3 | 3615.393 | 4000.286 | 3807.839 |
| ENSMUSG00000010796 | protein_coding | Asz1 | 1128.993 | 998.5663 | 1063.78 |
| ENSMUSG00000022498 | protein_coding | Txndc11 | 1212.878 | 1094.202 | 1153.54 |
| ENSMUSG00000047284 | protein_coding | Neurl4 | 2582.567 | 2325.656 | 2454.112 |
| ENSMUSG00000053559 | protein_coding | Smagp | 454.6888 | 381.0424 | 417.8656 |
| ENSMUSG00000002477 | protein_coding | Snrpd1 | 1384.103 | 1522.842 | 1453.473 |
| ENSMUSG00000087604 | processed_transcrip | 4930452L02Rik | 0 | 1.712931 | 0.856466 |
| ENSMUSG00000085138 | lincRNA | Gm16738 | 0 | 1.712931 | 0.856466 |
| ENSMUSG00000034757 | protein_coding | Tmub2 | 863.7488 | 952.3665 | 908.0577 |
| ENSMUSG00000026116 | protein_coding | Tmem131 | 3142.188 | 2795.194 | 2968.691 |
| ENSMUSG00000022681 | protein_coding | Ntan1 | 748.2342 | 666.5867 | 707.4104 |
| ENSMUSG00000002015 | protein_coding | Bcap31 | 2271.609 | 2483.889 | 2377.749 |
| ENSMUSG00000056501 | protein_coding | Cebpb | 38.45448 | 23.34354 | 30.89901 |
| ENSMUSG00000020640 | protein_coding | Itsn2 | 2445.322 | 2732.334 | 2588.828 |
| ENSMUSG00000035933 | protein_coding | Cog5 | 1164.698 | 1045.662 | 1105.18 |
| ENSMUSG00000074158 | protein_coding | 9830147E19Rik | 794.3183 | 887.7187 | 841.0185 |
| ENSMUSG00000016637 | protein_coding | Ift27 | 247.1374 | 210.7303 | 228.9339 |
| ENSMUSG00000073882 | protein_coding | Gm10597 | 0 | 1.748606 | 0.874303 |
| ENSMUSG00000081979 | pseudogene | Gm8078 | 0 | 1.748606 | 0.874303 |
| ENSMUSG00000051323 | protein_coding | Pcdh19 | 77.75729 | 59.89428 | 68.82579 |
| ENSMUSG00000053604 | protein_coding | Rpia | 1600.271 | 1454.657 | 1527.464 |
| ENSMUSG00000032261 | protein_coding | Sh3bgrl2 | 2095.082 | 1864.82 | 1979.951 |

| | | | | | |
|---|---|---|---|---|---|
| ENSMUSG00000011114 | protein_coding | Tbrg1 | 2525.575 | 2726.355 | 2625.965 |
| ENSMUSG00000037514 | protein_coding | Pank2 | 1520.341 | 1373.487 | 1446.914 |
| ENSMUSG00000021610 | protein_coding | Clptm1l | 3341.753 | 3027.599 | 3184.676 |
| ENSMUSG00000042608 | protein_coding | Stk40 | 1473.614 | 1634.409 | 1554.012 |
| ENSMUSG00000035596 | protein_coding | Mboat7 | 1668.505 | 1809.194 | 1738.85 |
| ENSMUSG00000033350 | protein_coding | Chst2 | 532.7133 | 461.8681 | 497.2907 |
| ENSMUSG00000049515 | protein_coding | Espnl | 18.30854 | 32.37089 | 25.33972 |
| ENSMUSG00000003341 | protein_coding | Atp8b3 | 74.65837 | 58.14925 | 66.40381 |
| ENSMUSG00000015659 | protein_coding | Serac1 | 646.0164 | 581.8585 | 613.9375 |
| ENSMUSG00000058498 | protein_coding | Rnf207 | 10.98894 | 18.25772 | 14.62333 |
| ENSMUSG00000036995 | protein_coding | Asap3 | 327.0571 | 285.8565 | 306.4568 |
| ENSMUSG00000081497 | pseudogene | Gm15560 | 1.304841 | 4.503783 | 2.904312 |
| ENSMUSG00000047648 | protein_coding | Fbxo30 | 1475.598 | 1345.067 | 1410.332 |
| ENSMUSG00000002500 | protein_coding | Rpl3l | 109.9462 | 86.56914 | 98.25765 |
| ENSMUSG00000024201 | protein_coding | Kdm4b | 1803.456 | 1978.05 | 1890.753 |
| ENSMUSG00000020386 | protein_coding | Sar1b | 1460.888 | 1621.705 | 1541.296 |
| ENSMUSG00000040658 | protein_coding | BC048355 | 462.1193 | 394.7509 | 428.4351 |
| ENSMUSG00000005087 | protein_coding | Cd44 | 103.1444 | 126.7516 | 114.948 |
| ENSMUSG00000040219 | protein_coding | Ttc12 | 156.4462 | 193.647 | 175.0466 |
| ENSMUSG00000074136 | lincRNA | 4930513N10Rik | 143.7466 | 118.9927 | 131.3697 |
| ENSMUSG00000038508 | protein_coding | Gdf15 | 20.03218 | 29.72025 | 24.87621 |
| ENSMUSG00000034430 | protein_coding | Zxdc | 996.5807 | 898.5564 | 947.5685 |
| ENSMUSG00000017550 | protein_coding | Atad5 | 2039.001 | 1755.725 | 1897.363 |
| ENSMUSG00000037536 | protein_coding | Fbxo34 | 2572.078 | 2297.979 | 2435.028 |
| ENSMUSG00000029219 | protein_coding | Slc10a4 | 0.33031 | 2.710854 | 1.520582 |
| ENSMUSG00000073774 | protein_coding | Kncn | 55.91418 | 41.20303 | 48.5586 |
| ENSMUSG00000078817 | protein_coding | Nlrp12 | 15.23944 | 8.274178 | 11.75681 |
| ENSMUSG00000032269 | protein_coding | Htr3a | 3.211635 | 0.688704 | 1.950169 |
| ENSMUSG00000023286 | protein_coding | Ube2j2 | 2442.051 | 2185.337 | 2313.694 |
| ENSMUSG00000042106 | protein_coding | 6230427J02Rik | 72.64475 | 90.74107 | 81.69291 |
| ENSMUSG00000079144 | protein_coding | A130010J15Rik | 213.2918 | 177.0023 | 195.147 |
| ENSMUSG00000030801 | protein_coding | Myst1 | 782.0498 | 863.9956 | 823.0227 |
| ENSMUSG00000001349 | protein_coding | Cnn1 | 588.8893 | 519.2412 | 554.0652 |
| ENSMUSG00000045251 | protein_coding | Zfp688 | 394.7488 | 344.1088 | 369.4288 |
| ENSMUSG00000022771 | protein_coding | Ppil2 | 2215.526 | 2401.778 | 2308.652 |
| ENSMUSG00000025421 | protein_coding | Hdhd2 | 1686.269 | 1879.009 | 1782.639 |
| ENSMUSG00000052997 | protein_coding | Uba2 | 5959.736 | 5515.222 | 5737.479 |
| ENSMUSG00000043895 | protein_coding | S1pr2 | 321.8948 | 380.797 | 351.3459 |
| ENSMUSG00000074793 | protein_coding | Hspa12b | 276.8857 | 320.6171 | 298.7514 |
| ENSMUSG00000070999 | protein_coding | Ccin | 2.561525 | 6.52521 | 4.543368 |
| ENSMUSG00000020964 | protein_coding | Sel1l | 3648.212 | 3326.83 | 3487.521 |
| ENSMUSG00000056737 | protein_coding | Capg | 295.6755 | 341.2328 | 318.4542 |
| ENSMUSG00000028676 | protein_coding | Srsf10 | 11977.17 | 13338.4 | 12657.79 |
| ENSMUSG00000090885 | protein_coding | Gm3944 | 3.880455 | 1.059903 | 2.470179 |
| ENSMUSG00000038624 | protein_coding | Nepn | 3.876876 | 1.015218 | 2.446047 |
| ENSMUSG00000028184 | protein_coding | Lphn2 | 2817.319 | 3245.719 | 3031.519 |
| ENSMUSG00000024227 | protein_coding | 2610034M16Rik | 6.110402 | 2.066473 | 4.088438 |
| ENSMUSG00000028521 | protein_coding | Slc35d1 | 639.0261 | 716.4051 | 677.7156 |
| ENSMUSG00000090939 | pseudogene | Gm17147 | 2.267593 | 0.335524 | 1.301558 |
| ENSMUSG00000032883 | protein_coding | Acsl3 | 2562.168 | 2311.911 | 2437.04 |
| ENSMUSG00000056288 | processed_transcrip | Gm11961 | 5.198117 | 1.695274 | 3.446696 |
| ENSMUSG00000030731 | protein_coding | Syt3 | 19.40205 | 11.67337 | 15.53771 |
| ENSMUSG00000039632 | protein_coding | Ccdc151 | 41.68609 | 25.62619 | 33.65614 |
| ENSMUSG00000028221 | protein_coding | Tmem55a | 705.4301 | 840.5863 | 773.0082 |
| ENSMUSG00000028636 | protein_coding | Ppcs | 656.2836 | 733.9395 | 695.1115 |
| ENSMUSG00000028329 | protein_coding | Xpa | 237.6533 | 279.3301 | 258.4917 |
| ENSMUSG00000036825 | protein_coding | Ssx2ip | 2496.908 | 2275.3 | 2386.104 |
| ENSMUSG00000042523 | protein_coding | Dnalc1 | 862.2974 | 769.5714 | 815.9344 |

| | | | | | |
|---|---|---|---|---|---|
| ENSMUSG00000082255 | pseudogene | Gm11822 | 2.267593 | 0.326514 | 1.297054 |
| ENSMUSG00000086087 | processed_transcrip | Gm12916 | 0 | 2.030436 | 1.015218 |
| ENSMUSG00000077844 | miRNA | AC126023.1 | 0 | 2.030436 | 1.015218 |
| ENSMUSG00000091002 | protein_coding | Tcerg1l | 11.31337 | 19.4365 | 15.37493 |
| ENSMUSG00000073007 | protein_coding | Fam46d | 381.4606 | 304.3026 | 342.8816 |
| ENSMUSG00000086471 | lincRNA | A530020G20Rik | 133.5391 | 187.234 | 160.3865 |
| ENSMUSG00000030562 | protein_coding | Nox4 | 82.79223 | 64.83197 | 73.8121 |
| ENSMUSG00000045314 | protein_coding | Ankrd56 | 16.10715 | 24.85428 | 20.48071 |
| ENSMUSG00000081170 | pseudogene | Gm12922 | 0.948663 | 4.043577 | 2.49612 |
| ENSMUSG00000029641 | protein_coding | Rasl11a | 277.3532 | 325.9723 | 301.6627 |
| ENSMUSG00000067750 | protein_coding | Khdc1a | 8.040753 | 3.009979 | 5.525366 |
| ENSMUSG00000051236 | protein_coding | Msrb3 | 2282.974 | 2484.314 | 2383.644 |
| ENSMUSG00000031668 | protein_coding | Eif2ak3 | 912.6982 | 804.2332 | 858.4657 |
| ENSMUSG00000028532 | protein_coding | Cachd1 | 167.5768 | 216.5486 | 192.0627 |
| ENSMUSG00000040310 | protein_coding | Alx4 | 48.74852 | 69.44817 | 59.09834 |
| ENSMUSG00000078125 | protein_coding | Gm10916 | 11.94454 | 20.02506 | 15.9848 |
| ENSMUSG00000043648 | protein_coding | Pld6 | 451.9505 | 508.3121 | 480.1313 |
| ENSMUSG00000093321 | snoRNA | AC117663.1 | 2.259393 | 0.326514 | 1.292954 |
| ENSMUSG00000023055 | protein_coding | Calcoco1 | 2232.356 | 2473.186 | 2352.771 |
| ENSMUSG00000078868 | protein_coding | Gm14412 | 69.96127 | 88.79535 | 79.37831 |
| ENSMUSG00000019802 | protein_coding | Sec63 | 3820.049 | 4192.845 | 4006.447 |
| ENSMUSG00000090542 | lincRNA | Gm10814 | 32.81879 | 46.59336 | 39.70607 |
| ENSMUSG00000073739 | processed_transcrip | Gm16287 | 8.78695 | 3.523879 | 6.155414 |
| ENSMUSG00000062464 | protein_coding | Gm9705 | 0 | 1.686265 | 0.843133 |
| ENSMUSG00000066632 | pseudogene | Pgk1-rs7 | 11.98807 | 6.020681 | 9.004378 |
| ENSMUSG00000071181 | protein_coding | 3830408C21Rik | 26.58329 | 38.87704 | 32.73017 |
| ENSMUSG00000001403 | protein_coding | Ube2c | 935.4179 | 1107.835 | 1021.626 |
| ENSMUSG00000030980 | protein_coding | 2310008H09Rik | 2381.276 | 2676.411 | 2528.844 |
| ENSMUSG00000021497 | protein_coding | Txndc15 | 844.0268 | 947.5135 | 895.7701 |
| ENSMUSG00000069895 | protein_coding | Atxn1l | 1589.161 | 1757.811 | 1673.486 |
| ENSMUSG00000022261 | protein_coding | Sdc2 | 1094.004 | 1268.047 | 1181.025 |
| ENSMUSG00000033066 | protein_coding | Gas7 | 7008.566 | 7733.182 | 7370.874 |
| ENSMUSG00000033610 | protein_coding | Pank1 | 1596.658 | 1841.683 | 1719.17 |
| ENSMUSG00000075318 | protein_coding | Scn2a1 | 72.46726 | 95.30331 | 83.88528 |
| ENSMUSG00000025487 | protein_coding | Psmd13 | 2405.775 | 2202.553 | 2304.164 |
| ENSMUSG00000059852 | protein_coding | Kcng2 | 465.6596 | 557.5319 | 511.5957 |
| ENSMUSG00000022404 | protein_coding | Slc25a17 | 1217.035 | 1083.899 | 1150.467 |
| ENSMUSG00000025512 | protein_coding | Chid1 | 1166.315 | 1045.98 | 1106.147 |
| ENSMUSG00000026622 | protein_coding | Nek2 | 1727.489 | 1556.119 | 1641.804 |
| ENSMUSG00000038084 | protein_coding | Opa1 | 2835.997 | 3081.224 | 2958.611 |
| ENSMUSG00000068959 | protein_coding | Zfp619 | 210.3587 | 254.0003 | 232.1795 |
| ENSMUSG00000041828 | protein_coding | Abca8a | 78.14649 | 57.98418 | 68.06533 |
| ENSMUSG00000085923 | processed_transcrip | Gm12781 | 4.851407 | 9.791076 | 7.321242 |
| ENSMUSG00000078716 | protein_coding | Tmem8b | 942.3286 | 848.9985 | 895.6636 |
| ENSMUSG00000091514 | protein_coding | Gm17484 | 52.52573 | 38.12563 | 45.32568 |
| ENSMUSG00000058736 | protein_coding | Gm10047 | 30.94144 | 20.98586 | 25.96365 |
| ENSMUSG00000041794 | protein_coding | Myrip | 128.614 | 101.5419 | 115.0779 |
| ENSMUSG00000073184 | protein_coding | Gm10479 | 26.11336 | 17.03129 | 21.57233 |
| ENSMUSG00000025049 | protein_coding | Taf5 | 1080.537 | 969.1843 | 1024.861 |
| ENSMUSG00000027452 | protein_coding | Acss1 | 2301.38 | 2566.281 | 2433.831 |
| ENSMUSG00000028469 | protein_coding | Npr2 | 863.7245 | 1018.131 | 940.928 |
| ENSMUSG00000045532 | protein_coding | C1ql1 | 92.62982 | 115.5034 | 104.0666 |
| ENSMUSG00000005949 | protein_coding | Ctns | 598.6018 | 537.5541 | 568.078 |
| ENSMUSG00000005470 | protein_coding | Asf1b | 1788.431 | 1560.431 | 1674.431 |
| ENSMUSG00000057003 | protein_coding | Myh4 | 8.437929 | 16.24742 | 12.34268 |
| ENSMUSG00000047379 | protein_coding | B3gnt1 | 470.4347 | 540.2067 | 505.3207 |
| ENSMUSG00000005907 | protein_coding | Pex1 | 764.5879 | 678.0257 | 721.3068 |
| ENSMUSG00000087368 | processed_transcrip | BC065397 | 145.2003 | 109.2458 | 127.2231 |

| | | | | | |
|---|---|---|---|---|---|
| ENSMUSG00000019834 | protein_coding | Slc22a16 | 10.66557 | 5.483326 | 8.074446 |
| ENSMUSG00000078902 | protein_coding | Gm14443 | 87.28023 | 121.73 | 104.5051 |
| ENSMUSG00000074899 | protein_coding | Spnb5 | 350.5387 | 304.1019 | 327.3203 |
| ENSMUSG00000039232 | protein_coding | Stx11 | 84.11094 | 65.37688 | 74.74391 |
| ENSMUSG00000084063 | protein_coding | Gm5934 | 3.611122 | 21.15513 | 12.38313 |
| ENSMUSG00000029074 | protein_coding | Ttll10 | 25.53243 | 36.83687 | 31.18465 |
| ENSMUSG00000073761 | protein_coding | 4933427I04Rik | 8.09122 | 14.75543 | 11.42332 |
| ENSMUSG00000050989 | protein_coding | Sepn1 | 594.5315 | 519.9304 | 557.231 |
| ENSMUSG00000053656 | processed_transcrip | 2700023E23Rik | 129.4735 | 157.359 | 143.4163 |
| ENSMUSG00000041264 | protein_coding | Uspl1 | 2343.956 | 2125.624 | 2234.79 |
| ENSMUSG00000021752 | protein_coding | Kctd6 | 884.9692 | 793.5971 | 839.2832 |
| ENSMUSG00000055653 | protein_coding | Gpc3 | 1441.608 | 1691.406 | 1566.507 |
| ENSMUSG00000030838 | protein_coding | Ush1c | 17.77279 | 26.93877 | 22.35578 |
| ENSMUSG00000031860 | protein_coding | Pbx4 | 107.4622 | 86.07978 | 96.77101 |
| ENSMUSG00000020366 | protein_coding | Mapk9 | 6278.401 | 6791.51 | 6534.956 |
| ENSMUSG00000026820 | protein_coding | Ptges2 | 686.5373 | 612.279 | 649.4081 |
| ENSMUSG00000059173 | protein_coding | Pde1a | 468.2163 | 562.5971 | 515.4067 |
| ENSMUSG00000028691 | protein_coding | Prdx1 | 8616.364 | 9464.029 | 9040.197 |
| ENSMUSG00000028066 | protein_coding | Pmf1 | 937.0447 | 843.3187 | 890.1817 |
| ENSMUSG00000086513 | pseudogene | 9130208D14Rik | 15.23609 | 8.045319 | 11.6407 |
| ENSMUSG00000044117 | protein_coding | 2900011O08Rik | 14.89169 | 23.40624 | 19.14896 |
| ENSMUSG00000087203 | lincRNA | Gm13986 | 1.623372 | 5.076814 | 3.350093 |
| ENSMUSG00000028736 | protein_coding | Pax7 | 24.39957 | 36.08619 | 30.24288 |
| ENSMUSG00000024240 | protein_coding | Epc1 | 1512.493 | 1666.077 | 1589.285 |
| ENSMUSG00000073482 | protein_coding | Gm10517 | 5.489738 | 2.057464 | 3.773601 |
| ENSMUSG00000074508 | processed_transcrip | Gm10706 | 0.962752 | 3.716701 | 2.339727 |
| ENSMUSG00000087362 | processed_transcrip | Gm13710 | 14.47558 | 23.58498 | 19.03028 |
| ENSMUSG00000006241 | protein_coding | Ccdc159 | 46.90039 | 32.8445 | 39.87244 |
| ENSMUSG00000090071 | protein_coding | Cdk5r2 | 16.43515 | 9.481857 | 12.9585 |
| ENSMUSG00000085517 | processed_transcrip | Gm12963 | 44.50011 | 61.82416 | 53.16213 |
| ENSMUSG00000017004 | protein_coding | Svs5 | 35.16376 | 47.37827 | 41.27101 |
| ENSMUSG00000048175 | protein_coding | Asb8 | 838.7685 | 760.5444 | 799.6564 |
| ENSMUSG00000030084 | protein_coding | Plxna1 | 10009.02 | 11151.25 | 10580.13 |
| ENSMUSG00000006715 | protein_coding | Gmnn | 1192.375 | 1082.001 | 1137.188 |
| ENSMUSG00000085414 | processed_transcrip | Gm15705 | 0.65242 | 3.01935 | 1.835885 |
| ENSMUSG00000011658 | protein_coding | Fuz | 324.6899 | 383.4994 | 354.0946 |
| ENSMUSG00000026938 | protein_coding | Fcna | 18.53561 | 29.20956 | 23.87258 |
| ENSMUSG00000037706 | protein_coding | Cd81 | 5505.116 | 5976.359 | 5740.737 |
| ENSMUSG00000029211 | protein_coding | Gabra4 | 117.4915 | 87.69977 | 102.5956 |
| ENSMUSG00000018334 | protein_coding | Ksr1 | 750.0428 | 674.0809 | 712.0618 |
| ENSMUSG00000005410 | protein_coding | Mcm5 | 4317.88 | 4898.274 | 4608.077 |
| ENSMUSG00000006095 | protein_coding | Tbcb | 1898.356 | 1712.12 | 1805.238 |
| ENSMUSG00000031883 | protein_coding | Car7 | 565.8271 | 374.3291 | 470.0781 |
| ENSMUSG00000031803 | protein_coding | B3gnt3 | 23.88908 | 35.54919 | 29.71914 |
| ENSMUSG00000059375 | protein_coding | Vmn1r33 | 2.273482 | 0.36219 | 1.317836 |
| ENSMUSG00000053080 | protein_coding | 2700081O15Rik | 1267.548 | 1416.833 | 1342.19 |
| ENSMUSG00000083854 | pseudogene | Gm15118 | 41.24978 | 29.72962 | 35.4897 |
| ENSMUSG00000078954 | protein_coding | Arhgap8 | 29.95282 | 41.44374 | 35.69828 |
| ENSMUSG00000027882 | protein_coding | Stxbp3a | 1162.228 | 1289.903 | 1226.065 |
| ENSMUSG00000026926 | protein_coding | Pmpca | 2643.467 | 2438.856 | 2541.162 |
| ENSMUSG00000068699 | protein_coding | Flnc | 647.9704 | 719.2685 | 683.6194 |
| ENSMUSG00000046598 | protein_coding | Bdh1 | 653.5711 | 764.5343 | 709.0527 |
| ENSMUSG00000016763 | protein_coding | Scube1 | 516.7458 | 447.9826 | 482.3642 |
| ENSMUSG00000020901 | protein_coding | Pik3r5 | 34.26787 | 23.14963 | 28.70875 |
| ENSMUSG00000041966 | protein_coding | Dcaf17 | 1010.031 | 912.3845 | 961.2079 |
| ENSMUSG00000036285 | protein_coding | 2610024G14Rik | 802.7104 | 713.0531 | 757.8818 |
| ENSMUSG00000034780 | protein_coding | B3galt1 | 191.5261 | 150.4867 | 171.0064 |
| ENSMUSG00000040212 | protein_coding | Emp3 | 635.3358 | 706.1663 | 670.751 |

| | | | | | |
|---|---|---|---|---|---|
| ENSMUSG00000052188 | processed_transcrip | Gm14964 | 25.83248 | 36.59072 | 31.2116 |
| ENSMUSG00000085411 | processed_transcrip | Gm14319 | 0 | 1.695274 | 0.847637 |
| ENSMUSG00000039328 | protein_coding | Rnf122 | 333.4495 | 396.2721 | 364.8608 |
| ENSMUSG00000046441 | protein_coding | Ftsjd1 | 500.3559 | 429.9419 | 465.1489 |
| ENSMUSG00000043162 | protein_coding | Pigy | 655.8973 | 730.1551 | 693.0262 |
| ENSMUSG00000046997 | protein_coding | Spsb4 | 102.3818 | 138.0525 | 120.2172 |
| ENSMUSG00000072929 | pseudogene | Gm15109 | 7.082622 | 2.630133 | 4.856377 |
| ENSMUSG00000040420 | protein_coding | Cdh18 | 7.113111 | 3.063673 | 5.088392 |
| ENSMUSG00000085596 | processed_transcrip | Gm11476 | 8.077131 | 3.761386 | 5.919258 |
| ENSMUSG00000087605 | processed_transcrip | Gm15742 | 2.273482 | 0.326514 | 1.299998 |
| ENSMUSG00000074218 | protein_coding | Cox7a1 | 112.4715 | 92.12392 | 102.2977 |
| ENSMUSG00000029063 | protein_coding | Nadk | 3512.211 | 3807.569 | 3659.89 |
| ENSMUSG00000086680 | processed_transcrip | Gm11592 | 14.373 | 6.350406 | 10.3617 |
| ENSMUSG00000045671 | protein_coding | Spred2 | 913.9581 | 1056.611 | 985.2847 |
| ENSMUSG00000044006 | protein_coding | Cilp2 | 211.2389 | 249.9318 | 230.5853 |
| ENSMUSG00000086792 | processed_transcrip | Gm12364 | 2.609681 | 0.326514 | 1.468098 |
| ENSMUSG00000026878 | protein_coding | Rab14 | 6283.467 | 6823.588 | 6553.528 |
| ENSMUSG00000092003 | lincRNA | Gm3906 | 41.86918 | 56.70629 | 49.28773 |
| ENSMUSG00000021978 | protein_coding | Extl3 | 2059.077 | 2269.358 | 2164.218 |
| ENSMUSG00000071818 | processed_transcrip | 4930417O22Rik | 9.479327 | 4.052225 | 6.765776 |
| ENSMUSG00000026575 | protein_coding | Nme7 | 658.174 | 738.4952 | 698.3346 |
| ENSMUSG00000008193 | protein_coding | Spib | 8.996573 | 17.21655 | 13.10656 |
| ENSMUSG00000079304 | protein_coding | 4933413G19Rik | 8.810507 | 3.717063 | 6.263785 |
| ENSMUSG00000030263 | protein_coding | Lrmp | 49.88138 | 35.17183 | 42.52661 |
| ENSMUSG00000043067 | protein_coding | Dpy19l1 | 1739.985 | 1571.45 | 1655.718 |
| ENSMUSG00000086077 | protein_coding | Gm14396 | 20.02987 | 31.87754 | 25.9537 |
| ENSMUSG00000028590 | processed_transcrip | D530049N12Rik | 2.261704 | 0.335524 | 1.298614 |
| ENSMUSG00000036958 | protein_coding | Cst11 | 20.4386 | 36.25343 | 28.34601 |
| ENSMUSG00000057399 | pseudogene | Ska2l-ps | 2.275793 | 0.36219 | 1.318991 |
| ENSMUSG00000017376 | protein_coding | Nlk | 1649.002 | 1871.155 | 1760.078 |
| ENSMUSG00000029330 | protein_coding | Cds1 | 343.4258 | 298.6103 | 321.018 |
| ENSMUSG00000044676 | protein_coding | Zfp612 | 419.4158 | 479.7542 | 449.585 |
| ENSMUSG00000020300 | protein_coding | Cpeb4 | 1513.033 | 1802.955 | 1657.994 |
| ENSMUSG00000035478 | protein_coding | Mbd3 | 2352.594 | 2161.979 | 2257.286 |
| ENSMUSG00000022197 | protein_coding | Pdzd2 | 1703.285 | 1949.325 | 1826.305 |
| ENSMUSG00000021870 | protein_coding | Slmap | 10949.72 | 11979.27 | 11464.49 |
| ENSMUSG00000020279 | protein_coding | Il9r | 40.25065 | 54.51834 | 47.3845 |
| ENSMUSG00000032291 | protein_coding | Crabp1 | 1429.175 | 1263.785 | 1346.48 |
| ENSMUSG00000032446 | protein_coding | Eomes | 27.16781 | 40.73879 | 33.9533 |
| ENSMUSG00000085189 | processed_transcrip | Gm11963 | 7.1108 | 3.055025 | 5.082913 |
| ENSMUSG00000086779 | lincRNA | Gm13562 | 11.69056 | 19.53203 | 15.6113 |
| ENSMUSG00000030413 | protein_coding | Pglyrp1 | 10.38468 | 5.094471 | 7.739575 |
| ENSMUSG00000029054 | protein_coding | Gabrd | 8.763618 | 15.87695 | 12.32028 |
| ENSMUSG00000086212 | lincRNA | Gm13845 | 34.65223 | 22.8984 | 28.77531 |
| ENSMUSG00000024867 | protein_coding | Pip5k1b | 1195.474 | 1350.166 | 1272.82 |
| ENSMUSG00000047246 | protein_coding | Hist1h2be | 7.74451 | 15.01603 | 11.38027 |
| ENSMUSG00000016239 | protein_coding | Lonrf3 | 4831.385 | 4342.61 | 4586.997 |
| ENSMUSG00000021067 | protein_coding | Sav1 | 1452.496 | 1327.258 | 1389.877 |
| ENSMUSG00000050357 | protein_coding | Rltpr | 51.25055 | 68.65963 | 59.95509 |
| ENSMUSG00000020191 | protein_coding | 4930415F15Rik | 11.57785 | 6.118698 | 8.848275 |
| ENSMUSG00000084017 | pseudogene | Rpl21-ps9 | 2.281682 | 0.326514 | 1.304098 |
| ENSMUSG00000017707 | protein_coding | Serinc3 | 14551.57 | 15701.68 | 15126.63 |
| ENSMUSG00000051910 | protein_coding | Sox6 | 705.6628 | 632.7318 | 669.1973 |
| ENSMUSG00000045693 | protein_coding | Nlrp4e | 6.45018 | 11.90151 | 9.175845 |
| ENSMUSG00000033427 | protein_coding | Upb1 | 13.23298 | 7.017881 | 10.12543 |
| ENSMUSG00000027887 | protein_coding | Sypl2 | 40.2801 | 28.27113 | 34.27561 |
| ENSMUSG00000031633 | protein_coding | Slc25a4 | 3540.453 | 3879.278 | 3709.866 |
| ENSMUSG00000000605 | protein_coding | Clcn4-2 | 1298.389 | 1435.43 | 1366.909 |

| | | | | | |
|---|---|---|---|---|---|
| ENSMUSG00000033389 | protein_coding | Arhgap44 | 767.1328 | 688.3112 | 727.722 |
| ENSMUSG00000026966 | protein_coding | Ssna1 | 1220.449 | 1091.79 | 1156.119 |
| ENSMUSG00000029882 | protein_coding | 2210010C04Rik | 12.2551 | 6.489897 | 9.372496 |
| ENSMUSG00000019027 | protein_coding | Dnahc1 | 230.8014 | 191.1377 | 210.9695 |
| ENSMUSG00000090104 | processed_transcrip | Gm16241 | 25.90165 | 37.86218 | 31.88192 |
| ENSMUSG00000040356 | protein_coding | Skiv2l | 1498.611 | 1333.452 | 1416.032 |
| ENSMUSG00000038210 | protein_coding | Hoxa11 | 0 | 1.677256 | 0.838628 |
| ENSMUSG00000032268 | protein_coding | Tmprss5 | 74.23741 | 92.39487 | 83.31614 |
| ENSMUSG00000054342 | protein_coding | Kcnn4 | 3.860477 | 8.184809 | 6.022643 |
| ENSMUSG00000068860 | protein_coding | Gm128 | 36.61769 | 25.41286 | 31.01527 |
| ENSMUSG00000087424 | lincRNA | 5730405O15Rik | 33.87785 | 47.19305 | 40.53545 |
| ENSMUSG00000020393 | protein_coding | Kremen1 | 985.147 | 842.3855 | 913.7662 |
| ENSMUSG00000024215 | protein_coding | Spdef | 3.258523 | 7.576465 | 5.417494 |
| ENSMUSG00000087478 | lincRNA | 4930506C21Rik | 20.67871 | 31.08652 | 25.88262 |
| ENSMUSG00000031362 | protein_coding | Xlr4c | 290.2754 | 217.4843 | 253.8798 |
| ENSMUSG00000052584 | protein_coding | Serp2 | 101.3371 | 122.8392 | 112.0882 |
| ENSMUSG00000054766 | protein_coding | Set | 7403.629 | 7959.129 | 7681.379 |
| ENSMUSG00000030043 | protein_coding | Tacr1 | 3.558345 | 1.024227 | 2.291286 |
| ENSMUSG00000036805 | protein_coding | Noxa1 | 8.417951 | 3.779043 | 6.098497 |
| ENSMUSG00000028693 | protein_coding | Nasp | 9444.017 | 8693.42 | 9068.719 |
| ENSMUSG00000032803 | protein_coding | Cdv3 | 5845.334 | 6297.888 | 6071.611 |
| ENSMUSG00000046275 | protein_coding | Tusc5 | 16.54786 | 25.67382 | 21.11084 |
| ENSMUSG00000072575 | protein_coding | Eddm3b | 2.587392 | 6.462869 | 4.525131 |
| ENSMUSG00000041632 | protein_coding | Mrps27 | 777.9181 | 854.7599 | 816.339 |
| ENSMUSG00000001300 | protein_coding | Efnb2 | 418.6102 | 491.0464 | 454.8283 |
| ENSMUSG00000086492 | lincRNA | Gm16652 | 19.66907 | 11.94583 | 15.80745 |
| ENSMUSG00000016982 | protein_coding | Pom121l2 | 2.626081 | 0.36219 | 1.494135 |
| ENSMUSG00000045659 | protein_coding | Plekha7 | 1138.882 | 1003.912 | 1071.397 |
| ENSMUSG00000022360 | protein_coding | Atad2 | 2890.409 | 3216.781 | 3053.595 |
| ENSMUSG00000032314 | protein_coding | Etfa | 2925.079 | 3186.871 | 3055.975 |
| ENSMUSG00000027997 | protein_coding | Casp6 | 1322.204 | 1522.259 | 1422.232 |
| ENSMUSG00000030330 | protein_coding | Ing4 | 1345.929 | 1514.442 | 1430.185 |
| ENSMUSG00000054901 | protein_coding | Arhgef33 | 123.414 | 98.83972 | 111.1269 |
| ENSMUSG00000038462 | protein_coding | Uqcrfs1 | 1825.521 | 1979.355 | 1902.438 |
| ENSMUSG00000025610 | protein_coding | ORF63 | 24.81672 | 37.06966 | 30.94319 |
| ENSMUSG00000024426 | protein_coding | Atat1 | 466.175 | 536.4049 | 501.29 |
| ENSMUSG00000074273 | protein_coding | Gm10657 | 18.08081 | 10.97566 | 14.52824 |
| ENSMUSG00000086787 | lincRNA | Gm16706 | 23.86575 | 33.86764 | 28.86669 |
| ENSMUSG00000036292 | protein_coding | Gramd1c | 166.3277 | 136.92 | 151.6239 |
| ENSMUSG00000062352 | protein_coding | Itgb1bp1 | 669.1559 | 597.66 | 633.4079 |
| ENSMUSG00000076434 | protein_coding | Wfdc3 | 16.78186 | 25.75739 | 21.26963 |
| ENSMUSG00000024947 | protein_coding | Men1 | 1911.991 | 1744.926 | 1828.459 |
| ENSMUSG00000079632 | protein_coding | Rhox2f | 45.14872 | 32.56951 | 38.85912 |
| ENSMUSG00000030786 | protein_coding | Itgam | 39.35559 | 56.31982 | 47.8377 |
| ENSMUSG00000059669 | protein_coding | Taf1b | 1024.162 | 911.7099 | 967.936 |
| ENSMUSG00000070837 | protein_coding | Aurkc | 52.92499 | 40.07498 | 46.49999 |
| ENSMUSG00000020275 | protein_coding | Rel | 545.1169 | 649.9814 | 597.5492 |
| ENSMUSG00000044022 | protein_coding | Pcdhb21 | 18.30094 | 28.52774 | 23.41434 |
| ENSMUSG00000025794 | protein_coding | Rpl14 | 6029.955 | 6528.233 | 6279.094 |
| ENSMUSG00000058099 | protein_coding | Nfam1 | 41.34587 | 54.31108 | 47.82847 |
| ENSMUSG00000035578 | protein_coding | Iqcg | 227.2208 | 178.7092 | 202.965 |
| ENSMUSG00000090661 | lincRNA | Gm17724 | 184.365 | 153.8312 | 169.0981 |
| ENSMUSG00000035051 | protein_coding | Dhx57 | 985.9499 | 1117.887 | 1051.918 |
| ENSMUSG00000034173 | protein_coding | 2410018M08Rik | 876.6846 | 795.2086 | 835.9466 |
| ENSMUSG00000020305 | protein_coding | Asb3 | 626.0546 | 551.0833 | 588.5689 |
| ENSMUSG00000079536 | protein_coding | Gm6880 | 230.8337 | 269.7372 | 250.2855 |
| ENSMUSG00000081792 | pseudogene | Anp32b-ps1 | 24.5579 | 37.10281 | 30.83035 |
| ENSMUSG00000029408 | protein_coding | Abcb9 | 160.4247 | 131.6128 | 146.0187 |

| | | | | | |
|---|---|---|---|---|---|
| ENSMUSG00000020836 | protein_coding | Coro6 | 44.78166 | 61.27418 | 53.02792 |
| ENSMUSG00000067873 | protein_coding | Htatsf1 | 5851.74 | 6330.328 | 6091.034 |
| ENSMUSG00000006262 | protein_coding | Mob1b | 2721.04 | 2515.877 | 2618.459 |
| ENSMUSG00000060422 | protein_coding | Olfr1034 | 13.22478 | 7.433764 | 10.32927 |
| ENSMUSG00000075425 | protein_coding | Gm13547 | 19.77589 | 12.24604 | 16.01097 |
| ENSMUSG00000039218 | protein_coding | Srrm2 | 14864.03 | 16751.68 | 15807.85 |
| ENSMUSG00000067924 | protein_coding | Cxx1b | 166.3681 | 196.6662 | 181.5171 |
| ENSMUSG00000034951 | protein_coding | Cog7 | 920.7683 | 817.1338 | 868.951 |
| ENSMUSG00000034203 | protein_coding | Chchd4 | 744.9982 | 674.4239 | 709.7111 |
| ENSMUSG00000032380 | protein_coding | Dapk2 | 357.5682 | 305.79 | 331.6791 |
| ENSMUSG00000027860 | protein_coding | Vangl1 | 306.5384 | 356.5729 | 331.5557 |
| ENSMUSG00000032854 | protein_coding | Ugt8a | 1130.023 | 795.6718 | 962.8474 |
| ENSMUSG00000024222 | protein_coding | Fkbp5 | 3837.615 | 4228.132 | 4032.873 |
| ENSMUSG00000050908 | protein_coding | Fam18a | 79.03142 | 61.96148 | 70.49645 |
| ENSMUSG00000027612 | protein_coding | Mmp24 | 18.16765 | 29.49067 | 23.82916 |
| ENSMUSG00000034066 | protein_coding | Farp2 | 386.2605 | 322.4718 | 354.3661 |
| ENSMUSG00000090543 | lincRNA | Gm17694 | 5.195806 | 1.739597 | 3.467702 |
| ENSMUSG00000030403 | protein_coding | Vasp | 1397.987 | 1533.042 | 1465.515 |
| ENSMUSG00000035539 | protein_coding | E230008N13Rik | 80.6729 | 60.54757 | 70.61024 |
| ENSMUSG00000027133 | protein_coding | Nop10 | 1071.027 | 1175.829 | 1123.428 |
| ENSMUSG00000029394 | protein_coding | Cdk2ap1 | 1227.792 | 1343.162 | 1285.477 |
| ENSMUSG00000002409 | protein_coding | Dyrk1b | 789.9661 | 707.5696 | 748.7679 |
| ENSMUSG00000006403 | protein_coding | Adamts4 | 144.9114 | 117.9725 | 131.4419 |
| ENSMUSG00000086335 | processed_transcrip | Gm12107 | 0.65242 | 3.595229 | 2.123825 |
| ENSMUSG00000038721 | protein_coding | Hoxb7 | 63.01782 | 84.98103 | 73.99943 |
| ENSMUSG00000086277 | protein_coding | 4930558K02Rik | 7.807798 | 3.425862 | 5.61683 |
| ENSMUSG00000066861 | protein_coding | Oas1g | 9.416038 | 4.459099 | 6.937569 |
| ENSMUSG00000047963 | protein_coding | Stbd1 | 116.5791 | 139.495 | 128.037 |
| ENSMUSG00000007888 | protein_coding | Crlf1 | 245.7856 | 290.9149 | 268.3502 |
| ENSMUSG00000087231 | lincRNA | E230016M11Rik | 89.30741 | 114.1552 | 101.7313 |
| ENSMUSG00000032567 | protein_coding | Aste1 | 455.5878 | 400.672 | 428.1299 |
| ENSMUSG00000021071 | protein_coding | Trim9 | 121.3859 | 101.1311 | 111.2585 |
| ENSMUSG00000072653 | processed_transcrip | Zfp783 | 63.79578 | 47.78587 | 55.79082 |
| ENSMUSG00000086847 | lincRNA | Gm16065 | 2.275793 | 6.109327 | 4.19256 |
| ENSMUSG00000079184 | protein_coding | Mphosph8 | 4117.526 | 4720.197 | 4418.862 |
| ENSMUSG00000046822 | protein_coding | Slc39a3 | 876.06 | 789.2732 | 832.6666 |
| ENSMUSG00000026278 | protein_coding | Bok | 2079.058 | 2426.191 | 2252.625 |
| ENSMUSG00000068744 | protein_coding | Psrc1 | 173.6599 | 218.2507 | 195.9553 |
| ENSMUSG00000085039 | lincRNA | Gm15927 | 50.65299 | 34.188 | 42.4205 |
| ENSMUSG00000030282 | protein_coding | Cmas | 1888.155 | 2057.158 | 1972.657 |
| ENSMUSG00000040374 | protein_coding | Pxmp3 | 1679.104 | 1903.755 | 1791.429 |
| ENSMUSG00000034187 | protein_coding | Nsf | 1505.573 | 1334.08 | 1419.826 |
| ENSMUSG00000053664 | protein_coding | Uck2 | 67.04797 | 83.85367 | 75.45082 |
| ENSMUSG00000051048 | protein_coding | P4ha3 | 10.04952 | 18.02306 | 14.03629 |
| ENSMUSG00000030697 | protein_coding | Ppp4c | 2706.527 | 2925.993 | 2816.26 |
| ENSMUSG00000057411 | protein_coding | Fam173a | 709.3023 | 826.2013 | 767.7518 |
| ENSMUSG00000075308 | lincRNA | Gm10827 | 50.51844 | 36.62173 | 43.57009 |
| ENSMUSG00000067578 | protein_coding | Cbln4 | 23.58397 | 68.44209 | 46.01303 |
| ENSMUSG00000074852 | protein_coding | Hpse2 | 57.26844 | 75.74483 | 66.50664 |
| ENSMUSG00000022760 | protein_coding | Thap7 | 807.3342 | 721.65 | 764.4921 |
| ENSMUSG00000084093 | pseudogene | Gm16418 | 81.60934 | 65.32821 | 73.46878 |
| ENSMUSG00000031896 | protein_coding | Ctrl | 18.47925 | 28.09596 | 23.28761 |
| ENSMUSG00000043795 | protein_coding | Gm14492 | 65.38173 | 92.3276 | 78.85466 |
| ENSMUSG00000032235 | protein_coding | Narg2 | 1364.542 | 1487.239 | 1425.891 |
| ENSMUSG00000090839 | processed_transcrip | Gm17094 | 3.874566 | 8.247512 | 6.061039 |
| ENSMUSG00000078499 | protein_coding | Rex2 | 18.43363 | 28.28842 | 23.36103 |
| ENSMUSG00000037972 | protein_coding | Snn | 469.5637 | 542.9821 | 506.2729 |
| ENSMUSG00000021134 | protein_coding | Srsf5 | 10194.5 | 11602.92 | 10898.71 |

| | | | | | |
|---|---|---|---|---|---|
| ENSMUSG00000032114 | protein_coding | Slc37a4 | 298.6851 | 348.3574 | 323.5213 |
| ENSMUSG00000030871 | protein_coding | Ears2 | 703.4611 | 637.4628 | 670.462 |
| ENSMUSG00000024052 | protein_coding | Lpin2 | 3359.172 | 3087.345 | 3223.259 |
| ENSMUSG00000085780 | lincRNA | 4930509G22Rik | 36.51973 | 19.72118 | 28.12046 |
| ENSMUSG00000035934 | protein_coding | Pknox2 | 396.5858 | 482.0622 | 439.324 |
| ENSMUSG00000003464 | protein_coding | Pex19 | 3035.28 | 3314.365 | 3174.823 |
| ENSMUSG00000037808 | protein_coding | Fam76b | 1590.232 | 1450.07 | 1520.151 |
| ENSMUSG00000086589 | lincRNA | Gm12915 | 9.635947 | 4.714262 | 7.175105 |
| ENSMUSG00000039607 | protein_coding | Rbms3 | 415.0164 | 474.8399 | 444.9281 |
| ENSMUSG00000032698 | protein_coding | Lmo2 | 106.7366 | 129.874 | 118.3053 |
| ENSMUSG00000050201 | protein_coding | Otop2 | 162.2825 | 121.866 | 142.0742 |
| ENSMUSG00000078437 | protein_coding | Gm3055 | 9.224457 | 3.452528 | 6.338493 |
| ENSMUSG00000039648 | protein_coding | Ccbl1 | 267.4859 | 228.0881 | 247.787 |
| ENSMUSG00000035983 | protein_coding | Gm7008 | 26.17919 | 36.67936 | 31.42927 |
| ENSMUSG00000070527 | protein_coding | Mkrn3 | 201.9307 | 234.3276 | 218.1292 |
| ENSMUSG00000041765 | protein_coding | Ubac2 | 1987.323 | 1821.561 | 1904.442 |
| ENSMUSG00000090666 | protein_coding | Gm17567 | 2.239415 | 5.756508 | 3.997962 |
| ENSMUSG00000035726 | protein_coding | Supt16h | 5555.777 | 5104.75 | 5330.263 |
| ENSMUSG00000089901 | protein_coding | Gm8113 | 133.0571 | 234.3638 | 183.7105 |
| ENSMUSG00000002524 | protein_coding | Puf60 | 5642.114 | 6121.668 | 5881.891 |
| ENSMUSG00000086429 | lincRNA | Gt(ROSA)26Sor | 420.6661 | 483.8193 | 452.2427 |
| ENSMUSG00000048520 | protein_coding | Fbxl13 | 22.95808 | 32.72158 | 27.83983 |
| ENSMUSG00000042606 | protein_coding | Hirip3 | 1341.672 | 1173.258 | 1257.465 |
| ENSMUSG00000051579 | protein_coding | Tceal8 | 1814.736 | 2001.36 | 1908.048 |
| ENSMUSG00000085584 | protein_coding | Rgag1 | 6.424313 | 11.94656 | 9.185435 |
| ENSMUSG00000062905 | protein_coding | Vmn1r32 | 22.64902 | 32.43939 | 27.5442 |
| ENSMUSG00000051341 | protein_coding | Zfp52 | 187.7489 | 222.4531 | 205.101 |
| ENSMUSG00000023959 | protein_coding | Clic5 | 111.4747 | 136.3588 | 123.9168 |
| ENSMUSG00000066652 | protein_coding | Lefty2 | 25.18826 | 16.47628 | 20.83227 |
| ENSMUSG00000074892 | protein_coding | B3galt5 | 4.188476 | 8.74883 | 6.468653 |
| ENSMUSG00000064469 | snRNA | U6 | 2.576882 | 7.849286 | 5.213084 |
| ENSMUSG00000087412 | processed_transcrip | Gm15501 | 111.632 | 90.26828 | 100.9501 |
| ENSMUSG00000033491 | protein_coding | Prss35 | 8032.668 | 9010.776 | 8521.722 |
| ENSMUSG00000091488 | lincRNA | Gm6225 | 10.31655 | 17.68826 | 14.0024 |
| ENSMUSG00000070933 | protein_coding | Speer4d | 10.00621 | 5.147803 | 7.577008 |
| ENSMUSG00000008601 | protein_coding | Rab25 | 19.01561 | 28.26212 | 23.63886 |
| ENSMUSG00000069830 | protein_coding | Nlrp1a | 4.210765 | 1.386417 | 2.798591 |
| ENSMUSG00000035390 | protein_coding | Brsk1 | 275.8646 | 237.203 | 256.5338 |
| ENSMUSG00000068120 | pseudogene | Gm10231 | 0.66062 | 3.081691 | 1.871156 |
| ENSMUSG00000074256 | protein_coding | Gm10655 | 40.81496 | 28.17275 | 34.49386 |
| ENSMUSG00000025209 | protein_coding | Peo1 | 1014.446 | 922.5269 | 968.4865 |
| ENSMUSG00000087530 | processed_transcrip | Gm15533 | 1.32124 | 5.299151 | 3.310196 |
| ENSMUSG00000029191 | protein_coding | Rfc1 | 4372.229 | 4041.652 | 4206.94 |
| ENSMUSG00000060743 | protein_coding | H3f3a | 4687.171 | 5152.295 | 4919.733 |
| ENSMUSG00000045064 | protein_coding | Fam164c | 201.5848 | 170.9379 | 186.2613 |
| ENSMUSG00000015970 | protein_coding | Chdh | 261.2117 | 342.7568 | 301.9842 |
| ENSMUSG00000085663 | processed_transcrip | Gm15718 | 16.26802 | 9.137686 | 12.70285 |
| ENSMUSG00000035365 | protein_coding | 4930547N16Rik | 455.5919 | 401.7326 | 428.6623 |
| ENSMUSG00000052724 | processed_transcrip | Gm9888 | 10.62561 | 17.76504 | 14.19533 |
| ENSMUSG00000072295 | protein_coding | Als2cr11 | 150.4237 | 115.8717 | 133.1477 |
| ENSMUSG00000062115 | protein_coding | Rai1 | 2635.159 | 2872.729 | 2753.944 |
| ENSMUSG00000043398 | protein_coding | Gpr135 | 16.08128 | 26.55385 | 21.31756 |
| ENSMUSG00000074527 | protein_coding | Gm14296 | 11.59067 | 5.747499 | 8.669086 |
| ENSMUSG00000052544 | protein_coding | St6galnac3 | 69.4449 | 53.41376 | 61.42933 |
| ENSMUSG00000022089 | protein_coding | Bin3 | 521.5383 | 582.5342 | 552.0362 |
| ENSMUSG00000085576 | protein_coding | Dpy19l2 | 22.90195 | 14.47251 | 18.68723 |
| ENSMUSG00000044795 | protein_coding | Cyb5d1 | 644.8031 | 721.2607 | 683.0319 |
| ENSMUSG00000055531 | protein_coding | Cpsf6 | 3767.023 | 4123.839 | 3945.431 |

| | | | | | |
|---|---|---|---|---|---|
| ENSMUSG00000022556 | protein_coding | Hsf1 | 1189.774 | 1086.249 | 1138.012 |
| ENSMUSG00000037965 | protein_coding | Zc3h7a | 1599.107 | 1824.228 | 1711.667 |
| ENSMUSG00000085233 | lincRNA | B230208H11Rik | 25.48092 | 35.84868 | 30.6648 |
| ENSMUSG00000057236 | protein_coding | Rbbp4 | 9559.825 | 10327.8 | 9943.814 |
| ENSMUSG00000021236 | protein_coding | Entpd5 | 2179.735 | 2380.611 | 2280.173 |
| ENSMUSG00000032245 | protein_coding | Cln6 | 360.0793 | 405.6842 | 382.8817 |
| ENSMUSG00000051116 | pseudogene | Gm8121 | 159.4869 | 133.4278 | 146.4574 |
| ENSMUSG00000085535 | lincRNA | Gm16719 | 10.67146 | 5.545667 | 8.108561 |
| ENSMUSG00000013158 | protein_coding | 4933405L10Rik | 11.33208 | 5.907857 | 8.619966 |
| ENSMUSG00000027272 | protein_coding | Ubr1 | 1282.607 | 1432.086 | 1357.346 |
| ENSMUSG00000039349 | protein_coding | C130074G19Rik | 518.2018 | 595.6625 | 556.9322 |
| ENSMUSG00000037474 | protein_coding | Dtl | 1810.463 | 1593.15 | 1701.806 |
| ENSMUSG00000090137 | protein_coding | Uba52 | 329.2936 | 275.3821 | 302.3378 |
| ENSMUSG00000031353 | protein_coding | Rbbp7 | 8666.61 | 9337.313 | 9001.962 |
| ENSMUSG00000089871 | lincRNA | Speer7-ps1 | 7.777309 | 3.45289 | 5.6151 |
| ENSMUSG00000036091 | protein_coding | Hyal3 | 31.58185 | 21.75493 | 26.66839 |
| ENSMUSG00000017756 | protein_coding | Slc12a7 | 2039.859 | 2234.139 | 2136.999 |
| ENSMUSG00000026987 | protein_coding | Baz2b | 1873.051 | 2081.024 | 1977.038 |
| ENSMUSG00000032557 | protein_coding | Uba5 | 1443.883 | 1583.251 | 1513.567 |
| ENSMUSG00000062184 | protein_coding | Hs6st2 | 1745.814 | 1535.154 | 1640.484 |
| ENSMUSG00000031553 | protein_coding | Adam3 | 2.911813 | 0.662038 | 1.786926 |
| ENSMUSG00000073393 | lincRNA | B230354K17Rik | 1723.61 | 1578.123 | 1650.867 |
| ENSMUSG00000061013 | protein_coding | Mkx | 23.82139 | 36.63612 | 30.22876 |
| ENSMUSG00000026667 | protein_coding | Uhmk1 | 2750.379 | 3023.737 | 2887.058 |
| ENSMUSG00000005615 | protein_coding | Pcyt1a | 4099.356 | 4445.08 | 4272.218 |
| ENSMUSG00000087384 | processed_transcrip | Gm15558 | 1.592884 | 4.794622 | 3.193753 |
| ENSMUSG00000057880 | protein_coding | Abat | 1760.952 | 1999.601 | 1880.276 |
| ENSMUSG00000028248 | protein_coding | Sfrs18 | 4400.842 | 5072.337 | 4736.59 |
| ENSMUSG00000032300 | protein_coding | 1700017B05Rik | 2899.358 | 3309.856 | 3104.607 |
| ENSMUSG00000030223 | protein_coding | Ptpro | 84.35932 | 108.113 | 96.23614 |
| ENSMUSG00000026098 | protein_coding | Pms1 | 633.7637 | 556.1556 | 594.9597 |
| ENSMUSG00000062432 | protein_coding | Cyp26c1 | 13.95816 | 22.25946 | 18.10881 |
| ENSMUSG00000051279 | protein_coding | Gdf6 | 3.552455 | 7.983339 | 5.767897 |
| ENSMUSG00000040952 | protein_coding | Rps19 | 4924.944 | 5415.474 | 5170.209 |
| ENSMUSG00000036880 | protein_coding | Acaa2 | 3947.915 | 4290.794 | 4119.354 |
| ENSMUSG00000079111 | protein_coding | Kdelr2 | 2935.347 | 3175.169 | 3055.258 |
| ENSMUSG00000042978 | protein_coding | Sbk1 | 2606.126 | 2963.886 | 2785.006 |
| ENSMUSG00000084834 | processed_transcrip | 4930565N06Rik | 108.0547 | 86.32806 | 97.19139 |
| ENSMUSG00000046719 | protein_coding | Nxph3 | 116.4609 | 147.8753 | 132.1681 |
| ENSMUSG00000031028 | protein_coding | Tub | 639.3175 | 558.3869 | 598.8522 |
| ENSMUSG00000065591 | snRNA | U6 | 10.27659 | 17.3679 | 13.82225 |
| ENSMUSG00000078496 | protein_coding | Gm13152 | 127.5532 | 154.5064 | 141.0298 |
| ENSMUSG00000039682 | protein_coding | Lap3 | 1854.77 | 1697.254 | 1776.012 |
| ENSMUSG00000020717 | protein_coding | Pecam1 | 296.24 | 356.0272 | 326.1336 |
| ENSMUSG00000059810 | protein_coding | Rgs3 | 1241.984 | 1395.863 | 1318.923 |
| ENSMUSG00000025461 | protein_coding | Cd163l1 | 3.556034 | 1.024227 | 2.290131 |
| ENSMUSG00000042730 | protein_coding | Gm9775 | 2.239415 | 5.898848 | 4.069131 |
| ENSMUSG00000068856 | protein_coding | Sf3b4 | 1382.777 | 1534.931 | 1458.854 |
| ENSMUSG00000016179 | protein_coding | Camk1g | 40.3703 | 29.05536 | 34.71283 |
| ENSMUSG00000055061 | lincRNA | 4931431C16Rik | 2.905924 | 0.688704 | 1.797314 |
| ENSMUSG00000032340 | protein_coding | Neo1 | 7220.2 | 7943.767 | 7581.983 |
| ENSMUSG00000081441 | pseudogene | Gm6161 | 16.53377 | 9.889093 | 13.21143 |
| ENSMUSG00000060206 | protein_coding | Zfp462 | 2901.823 | 3267.025 | 3084.424 |
| ENSMUSG00000020251 | protein_coding | Glt8d2 | 168.2398 | 137.8224 | 153.0311 |
| ENSMUSG00000028140 | protein_coding | Mrpl9 | 1556.225 | 1684.478 | 1620.351 |
| ENSMUSG00000037827 | protein_coding | Gm5884 | 143.7904 | 120.9889 | 132.3897 |
| ENSMUSG00000024073 | protein_coding | Birc6 | 7077.642 | 6522.254 | 6799.948 |
| ENSMUSG00000048824 | processed_transcrip | Gm568 | 8.749529 | 4.114566 | 6.432048 |

| | | | | | |
|---|---|---|---|---|---|
| ENSMUSG00000067889 | protein_coding | Spnb3 | 1724.243 | 1938.745 | 1831.494 |
| ENSMUSG00000087548 | lincRNA | 1700028E10Rik | 74.09257 | 96.00015 | 85.04636 |
| ENSMUSG00000038594 | protein_coding | Gm9766 | 94.16299 | 75.79567 | 84.97933 |
| ENSMUSG00000028124 | protein_coding | Gclm | 1717.784 | 1565.202 | 1641.493 |
| ENSMUSG00000029684 | protein_coding | Wasl | 3567.073 | 3896.982 | 3732.028 |
| ENSMUSG00000025171 | protein_coding | Ubtd1 | 383.7887 | 339.5801 | 361.6844 |
| ENSMUSG00000031647 | protein_coding | Mfap3l | 953.3836 | 1172.26 | 1062.822 |
| ENSMUSG00000018143 | protein_coding | Mafk | 2069.812 | 2281.803 | 2175.807 |
| ENSMUSG00000023904 | protein_coding | Hcfc1r1 | 407.8492 | 463.7341 | 435.7917 |
| ENSMUSG00000035775 | protein_coding | Krt20 | 47.38687 | 62.8123 | 55.09959 |
| ENSMUSG00000079100 | pseudogene | Gm5456 | 0.98273 | 3.672378 | 2.327554 |
| ENSMUSG00000005656 | protein_coding | Snx6 | 3002.695 | 3307.493 | 3155.094 |
| ENSMUSG00000055782 | protein_coding | Abcd2 | 690.5884 | 623.2788 | 656.9336 |
| ENSMUSG00000026627 | protein_coding | Tmem206 | 3856.335 | 4349.176 | 4102.755 |
| ENSMUSG00000002847 | protein_coding | Pla1a | 63.73502 | 82.88743 | 73.31123 |
| ENSMUSG00000051502 | protein_coding | Ufsp1 | 86.74626 | 69.67127 | 78.20877 |
| ENSMUSG00000022048 | protein_coding | Dpysl2 | 1769.898 | 2026.35 | 1898.124 |
| ENSMUSG00000086597 | lincRNA | F420014N23Rik | 47.67708 | 64.12338 | 55.90023 |
| ENSMUSG00000046699 | protein_coding | Slitrk4 | 4.196676 | 8.511324 | 6.354 |
| ENSMUSG00000041653 | protein_coding | Pnpla3 | 46.13168 | 34.20565 | 40.16867 |
| ENSMUSG00000027855 | protein_coding | Sycp1 | 6444.669 | 4431.652 | 5438.16 |
| ENSMUSG00000014243 | protein_coding | Zswim7 | 535.1582 | 473.1058 | 504.132 |
| ENSMUSG00000044951 | protein_coding | Mylk4 | 12.05956 | 5.907857 | 8.98371 |
| ENSMUSG00000086488 | lincRNA | 1600020E01Rik | 437.3045 | 365.3176 | 401.3111 |
| ENSMUSG00000055067 | protein_coding | Smyd3 | 600.0249 | 530.9245 | 565.4747 |
| ENSMUSG00000037826 | protein_coding | Ppm1k | 1106.287 | 1264.316 | 1185.301 |
| ENSMUSG00000078377 | protein_coding | Gm4294 | 135.2333 | 111.7861 | 123.5097 |
| ENSMUSG00000030124 | protein_coding | Lag3 | 51.81382 | 38.67448 | 45.24415 |
| ENSMUSG00000060568 | protein_coding | Fam78b | 92.35773 | 114.7625 | 103.5601 |
| ENSMUSG00000031289 | protein_coding | Il13ra2 | 18.40545 | 11.16885 | 14.78715 |
| ENSMUSG00000032621 | protein_coding | Srek1 | 2224.149 | 2511.805 | 2367.977 |
| ENSMUSG00000063234 | protein_coding | Gpr84 | 14.18649 | 7.947664 | 11.06708 |
| ENSMUSG00000003436 | protein_coding | Dll3 | 124.6867 | 94.51686 | 109.6018 |
| ENSMUSG00000028741 | protein_coding | Mrto4 | 1145.124 | 1044.969 | 1095.047 |
| ENSMUSG00000027809 | protein_coding | Etfdh | 1583.441 | 1722.493 | 1652.967 |
| ENSMUSG00000043496 | protein_coding | Tril | 507.2385 | 606.0902 | 556.6643 |
| ENSMUSG00000069554 | protein_coding | I830134H01Rik | 9.072682 | 4.414414 | 6.743548 |
| ENSMUSG00000084761 | processed_transcrip | Gm12406 | 6.494758 | 2.719502 | 4.60713 |
| ENSMUSG00000027446 | protein_coding | 9230104L09Rik | 11.68825 | 6.065366 | 8.876809 |
| ENSMUSG00000071766 | protein_coding | Rhox12 | 1.965461 | 0 | 0.98273 |
| ENSMUSG00000031610 | protein_coding | Scrg1 | 1.601084 | 4.714624 | 3.157854 |
| ENSMUSG00000087242 | processed_transcrip | C78197 | 0.33031 | 2.392626 | 1.361468 |
| ENSMUSG00000043432 | protein_coding | Leng9 | 202.7639 | 174.5497 | 188.6568 |
| ENSMUSG00000022947 | protein_coding | Cbr3 | 61.3173 | 76.95582 | 69.13656 |
| ENSMUSG00000083695 | processed_transcrip | 1700045I19Rik | 598.2069 | 498.4345 | 548.3207 |
| ENSMUSG00000024833 | protein_coding | Pola2 | 940.7689 | 840.8445 | 890.8067 |
| ENSMUSG00000053930 | protein_coding | Shisa6 | 417.2201 | 624.8157 | 521.0179 |
| ENSMUSG00000039246 | protein_coding | Lyplal1 | 298.6946 | 341.3746 | 320.0346 |
| ENSMUSG00000038563 | protein_coding | Eftud1 | 659.7712 | 736.5774 | 698.1743 |
| ENSMUSG00000038497 | protein_coding | Tmco3 | 949.241 | 867.3063 | 908.2736 |
| ENSMUSG00000030374 | protein_coding | Strn4 | 2777.457 | 2571.758 | 2674.607 |
| ENSMUSG00000036555 | protein_coding | Iqce | 782.0764 | 712.7446 | 747.4105 |
| ENSMUSG00000008090 | protein_coding | Fgfrl1 | 848.0341 | 939.6996 | 893.8668 |
| ENSMUSG00000045838 | protein_coding | A430105I19Rik | 99.13912 | 120.8531 | 109.9961 |
| ENSMUSG00000031828 | protein_coding | Klhl36 | 812.8932 | 670.5068 | 741.7 |
| ENSMUSG00000002341 | protein_coding | Ncan | 61.48614 | 46.53169 | 54.00892 |
| ENSMUSG00000032571 | protein_coding | Pik3r4 | 1644.051 | 1489.87 | 1566.96 |
| ENSMUSG00000040785 | protein_coding | Ttc3 | 6743.388 | 7330.665 | 7037.027 |

| | | | | | |
|---|---|---|---|---|---|
| ENSMUSG00000058706 | protein_coding | 0610030E20Rik | 1184.729 | 1318.48 | 1251.604 |
| ENSMUSG00000027939 | protein_coding | Nup210l | 125.0152 | 90.04833 | 107.5318 |
| ENSMUSG00000026414 | protein_coding | Tnnt2 | 674.1977 | 568.7456 | 621.4716 |
| ENSMUSG00000074578 | protein_coding | 1500012F01Rik | 443.0363 | 518.082 | 480.5591 |
| ENSMUSG00000020097 | protein_coding | Sgpl1 | 4935.103 | 5557.851 | 5246.477 |
| ENSMUSG00000022365 | protein_coding | Derl1 | 2597.84 | 2818.462 | 2708.151 |
| ENSMUSG00000062515 | protein_coding | Fabp4 | 16.51044 | 27.47177 | 21.9911 |
| ENSMUSG00000070323 | protein_coding | Mmp27 | 1.609283 | 5.059157 | 3.33422 |
| ENSMUSG00000048503 | protein_coding | Tmem136 | 124.3147 | 157.7715 | 141.0431 |
| ENSMUSG00000029060 | protein_coding | Mib2 | 1055.162 | 1155.052 | 1105.107 |
| ENSMUSG00000032495 | protein_coding | Lrrc2 | 18.78854 | 28.45458 | 23.62156 |
| ENSMUSG00000048696 | protein_coding | Mex3d | 2207.772 | 1949.957 | 2078.865 |
| ENSMUSG00000050335 | protein_coding | Lgals3 | 28.34942 | 40.2573 | 34.30336 |
| ENSMUSG00000050503 | protein_coding | Fbxl22 | 256.3695 | 223.0715 | 239.7205 |
| ENSMUSG00000091586 | protein_coding | Cyp4f17 | 77.07982 | 101.9158 | 89.49779 |
| ENSMUSG00000027896 | protein_coding | Slc16a4 | 715.8227 | 797.2215 | 756.5221 |
| ENSMUSG00000039960 | protein_coding | Rhou | 2354.482 | 2581.607 | 2468.045 |
| ENSMUSG00000052616 | protein_coding | Ccdc79 | 278.5357 | 109.6834 | 194.1096 |
| ENSMUSG00000026509 | protein_coding | Capn2 | 916.9482 | 1035.828 | 976.3879 |
| ENSMUSG00000086012 | processed_transcrip | Gm15902 | 0.32211 | 2.048455 | 1.185282 |
| ENSMUSG00000082953 | pseudogene | Gm13217 | 31.29866 | 22.03712 | 26.66789 |
| ENSMUSG00000025264 | protein_coding | Tsr2 | 1015.863 | 928.5889 | 972.2258 |
| ENSMUSG00000038607 | protein_coding | Gng10 | 5.803649 | 10.90467 | 8.354161 |
| ENSMUSG00000052397 | protein_coding | Ezr | 1687.698 | 1533.187 | 1610.443 |
| ENSMUSG00000028920 | protein_coding | Fbxo42 | 1648.676 | 1503.811 | 1576.243 |
| ENSMUSG00000086261 | pseudogene | Gm3765 | 1.953683 | 0 | 0.976841 |
| ENSMUSG00000064080 | protein_coding | Fbln2 | 476.7079 | 606.0942 | 541.401 |
| ENSMUSG00000040249 | protein_coding | Lrp1 | 7071.064 | 7657.447 | 7364.256 |
| ENSMUSG00000085906 | lincRNA | Gm16882 | 55.71105 | 72.86641 | 64.28873 |
| ENSMUSG00000024948 | protein_coding | Map4k2 | 1088.543 | 987.1525 | 1037.848 |
| ENSMUSG00000039754 | protein_coding | Alkbh4 | 341.9939 | 295.6368 | 318.8153 |
| ENSMUSG00000065738 | snoRNA | SNORD45 | 16.43179 | 25.03301 | 20.7324 |
| ENSMUSG00000029598 | protein_coding | Plbd2 | 2440.68 | 2202.219 | 2321.449 |
| ENSMUSG00000040158 | protein_coding | Tax1bp3 | 642.5155 | 719.0508 | 680.7832 |
| ENSMUSG00000091914 | lincRNA | 2010015M23Rik | 49.09985 | 36.99365 | 43.04675 |
| ENSMUSG00000075376 | protein_coding | Rc3h2 | 3095.329 | 3404.972 | 3250.15 |
| ENSMUSG00000061740 | protein_coding | Cyp2d22 | 726.6766 | 850.517 | 788.5968 |
| ENSMUSG00000035021 | protein_coding | Baz1a | 1796.08 | 1505.125 | 1650.603 |
| ENSMUSG00000034793 | protein_coding | G6pc3 | 680.2061 | 750.0706 | 715.1384 |
| ENSMUSG00000057897 | protein_coding | Camk2b | 163.8938 | 195.1827 | 179.5382 |
| ENSMUSG00000029522 | protein_coding | Pla2g1b | 76.24663 | 56.02406 | 66.13535 |
| ENSMUSG00000028952 | protein_coding | Zbtb48 | 590.4887 | 528.6397 | 559.5642 |
| ENSMUSG00000067878 | protein_coding | Mtap7d3 | 700.8236 | 776.7853 | 738.8044 |
| ENSMUSG00000085936 | lincRNA | 2610307P16Rik | 131.6736 | 159.7576 | 145.7156 |
| ENSMUSG00000027863 | protein_coding | Cd2 | 9.009394 | 15.29242 | 12.15091 |
| ENSMUSG00000027199 | protein_coding | Gatm | 16136.87 | 17433.26 | 16785.07 |
| ENSMUSG00000018865 | protein_coding | Sult4a1 | 284.2357 | 324.5976 | 304.4167 |
| ENSMUSG00000054909 | lincRNA | Wbscr25 | 1.949061 | 0 | 0.974531 |
| ENSMUSG00000014418 | protein_coding | Hps5 | 1047.825 | 950.7436 | 999.2843 |
| ENSMUSG00000030108 | protein_coding | Slc6a13 | 4.207186 | 1.386417 | 2.796802 |
| ENSMUSG00000055540 | protein_coding | Epha6 | 9.659504 | 4.696606 | 7.178055 |
| ENSMUSG00000031365 | protein_coding | Zfp275 | 5378.316 | 6071.848 | 5725.082 |
| ENSMUSG00000052253 | protein_coding | Zfp622 | 2309.09 | 2529.411 | 2419.25 |
| ENSMUSG00000026648 | protein_coding | Dclre1c | 799.8972 | 898.8252 | 849.3612 |
| ENSMUSG00000041161 | protein_coding | Otud3 | 415.2374 | 367.1376 | 391.1875 |
| ENSMUSG00000021193 | protein_coding | Pitrm1 | 2372.748 | 2623.961 | 2498.354 |
| ENSMUSG00000021750 | protein_coding | Fam107a | 138.0877 | 109.5623 | 123.825 |
| ENSMUSG00000050022 | protein_coding | Amz1 | 25.78305 | 37.34896 | 31.56601 |

| | | | | | |
|---|---|---|---|---|---|
| ENSMUSG00000002379 | protein_coding | Ndufa11 | 1510.372 | 1342.902 | 1426.637 |
| ENSMUSG00000006024 | protein_coding | Napa | 2127.216 | 2287.243 | 2207.229 |
| ENSMUSG00000023800 | protein_coding | Tiam2 | 338.4008 | 292.1828 | 315.2918 |
| ENSMUSG00000066026 | protein_coding | Dhrs3 | 357.9623 | 417.3465 | 387.6544 |
| ENSMUSG00000024276 | protein_coding | Zfp397 | 1894.788 | 2059.838 | 1977.313 |
| ENSMUSG00000033557 | protein_coding | Fam20b | 2734.533 | 2489.237 | 2611.885 |
| ENSMUSG00000006675 | protein_coding | P4htm | 82.39654 | 65.20058 | 73.79856 |
| ENSMUSG00000034432 | protein_coding | Cops8 | 2821.829 | 2600.724 | 2711.276 |
| ENSMUSG00000031489 | protein_coding | Adrb3 | 7.776266 | 15.13999 | 11.45813 |
| ENSMUSG00000024277 | protein_coding | Mapre2 | 1106.92 | 1220.921 | 1163.921 |
| ENSMUSG00000028133 | protein_coding | Rwdd3 | 232.958 | 200.6765 | 216.8172 |
| ENSMUSG00000040950 | protein_coding | Mgl2 | 97.82839 | 78.62121 | 88.2248 |
| ENSMUSG00000040828 | protein_coding | Tmem146 | 59.75073 | 79.25943 | 69.50508 |
| ENSMUSG00000029911 | protein_coding | Ssbp1 | 1365.742 | 1515.751 | 1440.747 |
| ENSMUSG00000005534 | protein_coding | Insr | 973.3274 | 1090.859 | 1032.093 |
| ENSMUSG00000017943 | protein_coding | Gdap1l1 | 11.2957 | 18.21624 | 14.75597 |
| ENSMUSG00000042328 | protein_coding | Hps4 | 453.5914 | 505.3322 | 479.4618 |
| ENSMUSG00000026979 | protein_coding | Psd4 | 55.54876 | 42.78584 | 49.1673 |
| ENSMUSG00000022014 | protein_coding | Epsti1 | 25.87116 | 16.22076 | 21.04596 |
| ENSMUSG00000045569 | protein_coding | Mc2r | 2.897724 | 0.697713 | 1.797719 |
| ENSMUSG00000029032 | protein_coding | Arhgef16 | 93.1336 | 115.2638 | 104.1987 |
| ENSMUSG00000086106 | lincRNA | 1110036E04Rik | 4.193097 | 1.386417 | 2.789757 |
| ENSMUSG00000031485 | protein_coding | Prosc | 906.7573 | 1024.864 | 965.8108 |
| ENSMUSG00000020594 | protein_coding | Pum2 | 6873.116 | 7360.196 | 7116.656 |
| ENSMUSG00000087523 | processed_transcrip | Gm12319 | 94.89808 | 75.41225 | 85.15517 |
| ENSMUSG00000092247 | pseudogene | Gm20426 | 18.68761 | 11.49536 | 15.09148 |
| ENSMUSG00000039385 | protein_coding | Cdh6 | 4.257653 | 10.59866 | 7.428159 |
| ENSMUSG00000017667 | protein_coding | Zfp334 | 1107.826 | 962.569 | 1035.197 |
| ENSMUSG00000044624 | protein_coding | Gm4922 | 2.891835 | 0.697713 | 1.794774 |
| ENSMUSG00000023966 | protein_coding | Rsph9 | 136.2086 | 113.4243 | 124.8165 |
| ENSMUSG00000044244 | protein_coding | Il20rb | 40.13376 | 53.23388 | 46.68382 |
| ENSMUSG00000029171 | protein_coding | Pgm1 | 477.6279 | 581.3182 | 529.4731 |
| ENSMUSG00000074482 | pseudogene | Gm16589 | 854.0638 | 778.921 | 816.4924 |
| ENSMUSG00000052353 | protein_coding | 9930013L23Rik | 235.3487 | 177.2467 | 206.2977 |
| ENSMUSG00000087428 | processed_transcrip | Gm15017 | 87.83566 | 62.74986 | 75.29276 |
| ENSMUSG00000018574 | protein_coding | Acadvl | 1058.967 | 971.6185 | 1015.293 |
| ENSMUSG00000070504 | protein_coding | Fcrl6 | 1.621062 | 0 | 0.810531 |
| ENSMUSG00000043880 | protein_coding | Olfr323 | 1.621062 | 0 | 0.810531 |
| ENSMUSG00000040452 | protein_coding | Cdh12 | 1.621062 | 0 | 0.810531 |
| ENSMUSG00000025192 | protein_coding | Entpd7 | 234.1689 | 281.525 | 257.847 |
| ENSMUSG00000037535 | protein_coding | 1700021A07Rik | 10.62792 | 5.005101 | 7.816511 |
| ENSMUSG00000067973 | protein_coding | Gm6994 | 9.346637 | 15.65425 | 12.50044 |
| ENSMUSG00000022362 | protein_coding | 9130401M01Rik | 1211.418 | 1322.864 | 1267.141 |
| ENSMUSG00000023942 | protein_coding | Slc29a1 | 1989.501 | 2190.711 | 2090.106 |
| ENSMUSG00000024228 | protein_coding | Nudt12 | 195.6844 | 232.3069 | 213.9957 |
| ENSMUSG00000091500 | lincRNA | Gm17292 | 14.5496 | 8.54736 | 11.54848 |
| ENSMUSG00000018537 | protein_coding | Pcgf2 | 819.8803 | 907.9149 | 863.8976 |
| ENSMUSG00000031026 | protein_coding | Trim66 | 89.0351 | 72.12696 | 80.58103 |
| ENSMUSG00000026348 | protein_coding | Acmsd | 21.00798 | 13.5521 | 17.28004 |
| ENSMUSG00000073700 | protein_coding | Klhl21 | 1644.873 | 1442.938 | 1543.906 |
| ENSMUSG00000087331 | processed_transcrip | 1810021B22Rik | 16.86766 | 9.996119 | 13.43189 |
| ENSMUSG00000009640 | protein_coding | Dmap1 | 1083.428 | 1195.521 | 1139.475 |
| ENSMUSG00000076951 | miRNA | AC127431.1 | 0.32211 | 2.075121 | 1.198615 |
| ENSMUSG00000050121 | protein_coding | Opalin | 0.32211 | 2.075121 | 1.198615 |
| ENSMUSG00000026956 | protein_coding | Uap1l1 | 543.1852 | 488.669 | 515.9271 |
| ENSMUSG00000061175 | protein_coding | Fnip2 | 1243.999 | 1406.461 | 1325.23 |
| ENSMUSG00000072934 | processed_transcrip | Gm15070 | 23.31911 | 14.85272 | 19.08591 |
| ENSMUSG00000034533 | protein_coding | Scn10a | 32.8987 | 22.97234 | 27.93552 |

| | | | | | |
|---|---|---|---|---|---|
| ENSMUSG00000073090 | protein_coding | Gm7932 | 0.316221 | 2.075121 | 1.195671 |
| ENSMUSG00000056153 | protein_coding | Socs6 | 1604.361 | 1448.4 | 1526.381 |
| ENSMUSG00000021413 | protein_coding | Prpf4b | 3093.844 | 3454.719 | 3274.282 |
| ENSMUSG00000030083 | protein_coding | Abtb1 | 843.7502 | 757.4496 | 800.5999 |
| ENSMUSG00000050217 | protein_coding | Lgsn | 1.615173 | 0 | 0.807586 |
| ENSMUSG00000033044 | protein_coding | Dhrs7c | 1.615173 | 0 | 0.807586 |
| ENSMUSG00000024734 | protein_coding | Zp1 | 1.615173 | 0 | 0.807586 |
| ENSMUSG00000089177 | snoRNA | SNORA25 | 1.615173 | 0 | 0.807586 |
| ENSMUSG00000053593 | protein_coding | Fate1 | 1.615173 | 0 | 0.807586 |
| ENSMUSG00000032463 | protein_coding | Faim | 938.5556 | 854.2664 | 896.411 |
| ENSMUSG00000063873 | protein_coding | Slc24a3 | 749.8273 | 597.1938 | 673.5106 |
| ENSMUSG00000068742 | protein_coding | Cry2 | 2001.381 | 1845.169 | 1923.275 |
| ENSMUSG00000022234 | protein_coding | Cct5 | 7072.512 | 7637.526 | 7355.019 |
| ENSMUSG00000074575 | protein_coding | Kcng1 | 73.07555 | 94.73282 | 83.90419 |
| ENSMUSG00000046814 | protein_coding | Gchfr | 16.78081 | 26.78561 | 21.78321 |
| ENSMUSG00000034551 | protein_coding | Hdx | 86.78726 | 112.1847 | 99.48596 |
| ENSMUSG00000035189 | protein_coding | Ano4 | 259.2065 | 312.7503 | 285.9784 |
| ENSMUSG00000041147 | protein_coding | Brca2 | 3773.674 | 3118.112 | 3445.893 |
| ENSMUSG00000036432 | protein_coding | Siah2 | 333.6773 | 296.4576 | 315.0674 |
| ENSMUSG00000087161 | lincRNA | Gm16845 | 94.5381 | 121.1723 | 107.8552 |
| ENSMUSG00000004473 | protein_coding | Clec11a | 75.35156 | 58.45588 | 66.90372 |
| ENSMUSG00000033520 | protein_coding | Idi2 | 3.270301 | 7.795954 | 5.533128 |
| ENSMUSG00000017561 | protein_coding | Crlf3 | 2337.769 | 2176.096 | 2256.933 |
| ENSMUSG00000055799 | protein_coding | Tcf7l1 | 343.1003 | 303.5927 | 323.3465 |
| ENSMUSG00000039109 | protein_coding | F13a1 | 1007.675 | 905.0455 | 956.3605 |
| ENSMUSG00000022114 | protein_coding | Spry2 | 273.1184 | 315.5214 | 294.3199 |
| ENSMUSG00000020042 | protein_coding | Btbd11 | 90.12719 | 73.41785 | 81.77252 |
| ENSMUSG00000030866 | protein_coding | Ern2 | 17.78583 | 28.19719 | 22.99151 |
| ENSMUSG00000021048 | protein_coding | Mthfd1 | 1324.748 | 1470.73 | 1397.739 |
| ENSMUSG00000047025 | protein_coding | 4921510H08Rik | 5.828248 | 2.410644 | 4.119446 |
| ENSMUSG00000048429 | protein_coding | 1810026J23Rik | 1925.876 | 1724.724 | 1825.3 |
| ENSMUSG00000020701 | protein_coding | Tmem132e | 230.1457 | 278.9796 | 254.5627 |
| ENSMUSG00000041735 | protein_coding | Gm13178 | 0.954552 | 3.981236 | 2.467894 |
| ENSMUSG00000022037 | protein_coding | Clu | 45813.7 | 50360.79 | 48087.24 |
| ENSMUSG00000032558 | protein_coding | Nphp3 | 433.33 | 499.9274 | 466.6287 |
| ENSMUSG00000085945 | lincRNA | 2310014F06Rik | 3.250323 | 7.18761 | 5.218967 |
| ENSMUSG00000084370 | pseudogene | Gm15442 | 1.635151 | 4.767956 | 3.201553 |
| ENSMUSG00000064563 | snoRNA | Snora7a | 27.74978 | 19.03615 | 23.39297 |
| ENSMUSG00000028060 | protein_coding | 2810403A07Rik | 2566.505 | 2849.828 | 2708.166 |
| ENSMUSG00000025384 | protein_coding | 2310003H01Rik | 871.6142 | 973.8329 | 922.7236 |
| ENSMUSG00000059493 | protein_coding | Nhs | 492.5284 | 555.5314 | 524.0299 |
| ENSMUSG00000087020 | lincRNA | Gm16283 | 19.13294 | 11.9552 | 15.54407 |
| ENSMUSG00000088812 | snRNA | U6 | 1.606973 | 0 | 0.803486 |
| ENSMUSG00000090940 | lincRNA | AW121686 | 1.606973 | 0 | 0.803486 |
| ENSMUSG00000022043 | protein_coding | Trim35 | 7440.065 | 8130.064 | 7785.064 |
| ENSMUSG00000092373 | processed_transcrip | RP24-370C21.2 | 2.917703 | 6.861095 | 4.889399 |
| ENSMUSG00000042770 | protein_coding | Hebp1 | 121.8493 | 147.474 | 134.6617 |
| ENSMUSG00000074406 | protein_coding | Zfp628 | 548.8631 | 492.2342 | 520.5486 |
| ENSMUSG00000091272 | protein_coding | Gm17641 | 6.422002 | 12.69475 | 9.558377 |
| ENSMUSG00000005763 | protein_coding | Cd247 | 4.498808 | 9.280025 | 6.889417 |
| ENSMUSG00000030527 | protein_coding | Crtc3 | 2764.26 | 3026.732 | 2895.496 |
| ENSMUSG00000065855 | snRNA | U6 | 36.2838 | 49.41264 | 42.84822 |
| ENSMUSG00000081998 | pseudogene | Gm13245 | 62.23487 | 81.46602 | 71.85045 |
| ENSMUSG00000026568 | protein_coding | Brp44 | 1427.588 | 1300.494 | 1364.041 |
| ENSMUSG00000082455 | pseudogene | Gm13665 | 0.32211 | 2.057464 | 1.189787 |
| ENSMUSG00000087325 | pseudogene | Gm6038 | 108.5513 | 40.39524 | 74.47325 |
| ENSMUSG00000084942 | processed_transcrip | 7330404K18Rik | 3.264412 | 0.724379 | 1.994396 |
| ENSMUSG00000049231 | pseudogene | Gm12497 | 1.304841 | 4.123575 | 2.714208 |

| | | | | | |
|---|---|---|---|---|---|
| ENSMUSG00000026489 | protein_coding | Adck3 | 1422.007 | 1277.327 | 1349.667 |
| ENSMUSG00000087646 | processed_transcrip | Gm1667 | 0.316221 | 2.057464 | 1.186842 |
| ENSMUSG00000079067 | pseudogene | Hmgn2-ps1 | 127.9172 | 106.2227 | 117.07 |
| ENSMUSG00000061724 | pseudogene | Gm2423 | 89.43712 | 109.9257 | 99.6814 |
| ENSMUSG00000086553 | lincRNA | 9230116N13Rik | 60.99139 | 46.99951 | 53.99545 |
| ENSMUSG00000085482 | processed_transcrip | A230083N12Rik | 10.08128 | 5.112127 | 7.596704 |
| ENSMUSG00000021721 | protein_coding | Htr1a | 1.973661 | 0 | 0.98683 |
| ENSMUSG00000030811 | protein_coding | Fbxl19 | 4469.487 | 4156.324 | 4312.906 |
| ENSMUSG00000030560 | protein_coding | Ctsc | 386.3282 | 439.2292 | 412.7787 |
| ENSMUSG00000027254 | protein_coding | Mtap1a | 699.6923 | 622.313 | 661.0027 |
| ENSMUSG00000032328 | protein_coding | Tmem30a | 4095.612 | 3794.591 | 3945.101 |
| ENSMUSG00000004359 | protein_coding | Spic | 24.41388 | 37.90402 | 31.15895 |
| ENSMUSG00000032215 | protein_coding | Rsl24d1 | 1354.319 | 1471.39 | 1412.855 |
| ENSMUSG00000038181 | protein_coding | Chpf2 | 415.9534 | 466.5315 | 441.2424 |
| ENSMUSG00000073910 | protein_coding | Mob3b | 416.5326 | 489.1487 | 452.8406 |
| ENSMUSG00000037822 | protein_coding | 1110003E01Rik | 1717.468 | 1862.271 | 1789.87 |
| ENSMUSG00000026277 | protein_coding | Stk25 | 3842.858 | 3418.638 | 3630.748 |
| ENSMUSG00000021149 | protein_coding | Gtpbp4 | 3546.098 | 3877.191 | 3711.644 |
| ENSMUSG00000034755 | protein_coding | Pcdh11x | 13.69829 | 6.949741 | 10.32402 |
| ENSMUSG00000060038 | protein_coding | Dhps | 1128.902 | 1033.86 | 1081.381 |
| ENSMUSG00000084349 | pseudogene | Rpl3-ps1 | 5292.672 | 5697.359 | 5495.015 |
| ENSMUSG00000085211 | processed_transcrip | B430219N15Rik | 10.36932 | 5.014472 | 7.691898 |
| ENSMUSG00000073551 | protein_coding | Spink13 | 3.544256 | 7.585113 | 5.564684 |
| ENSMUSG00000039531 | protein_coding | Zufsp | 413.7257 | 358.0937 | 385.9097 |
| ENSMUSG00000085725 | processed_transcrip | Gm15873 | 1.909105 | 0 | 0.954552 |
| ENSMUSG00000050425 | protein_coding | Mrgprb2 | 1.909105 | 0 | 0.954552 |
| ENSMUSG00000048402 | protein_coding | Gli2 | 433.2614 | 377.2334 | 405.2474 |
| ENSMUSG00000030835 | protein_coding | Nomo1 | 3089.493 | 2732.705 | 2911.099 |
| ENSMUSG00000087495 | processed_transcrip | Gm14152 | 1.967772 | 0 | 0.983886 |
| ENSMUSG00000025702 | protein_coding | 8-Mar | 3765.618 | 3492.162 | 3628.89 |
| ENSMUSG00000068922 | protein_coding | Msto1 | 1185.124 | 1082.636 | 1133.88 |
| ENSMUSG00000026626 | protein_coding | Ppp2r5a | 1719.201 | 1865.779 | 1792.49 |
| ENSMUSG00000035640 | protein_coding | Dos | 780.9433 | 878.5518 | 829.7476 |
| ENSMUSG00000038412 | protein_coding | Higd1a | 1613.241 | 1483.188 | 1548.214 |
| ENSMUSG00000045515 | protein_coding | Pou3f3 | 6.123448 | 11.48635 | 8.804899 |
| ENSMUSG00000042628 | protein_coding | Zfyve1 | 1850.078 | 1686.295 | 1768.187 |
| ENSMUSG00000086477 | lincRNA | Gm15506 | 26.84443 | 18.27573 | 22.56008 |
| ENSMUSG00000053091 | protein_coding | Lins | 373.5154 | 416.9615 | 395.2384 |
| ENSMUSG00000087454 | processed_transcrip | Gm13174 | 0.33031 | 2.039445 | 1.184878 |
| ENSMUSG00000080775 | pseudogene | Gm6368 | 0.646531 | 2.772834 | 1.709682 |
| ENSMUSG00000079560 | protein_coding | Hoxa3 | 16.8441 | 25.94913 | 21.39662 |
| ENSMUSG00000058672 | protein_coding | Tubb2a | 342.9646 | 386.3745 | 364.6696 |
| ENSMUSG00000074146 | protein_coding | 4930579C12Rik | 1.629262 | 0 | 0.814631 |
| ENSMUSG00000080544 | misc_RNA | CPEB3_ribozyme | 1.629262 | 0 | 0.814631 |
| ENSMUSG00000019961 | protein_coding | Tmpo | 4479.328 | 4874.401 | 4676.865 |
| ENSMUSG00000026697 | protein_coding | Myoc | 21.66144 | 14.02132 | 17.84138 |
| ENSMUSG00000083811 | pseudogene | Gm13071 | 16.28673 | 8.381204 | 12.33397 |
| ENSMUSG00000046449 | protein_coding | C77370 | 2146.155 | 1918.642 | 2032.399 |
| ENSMUSG00000027349 | protein_coding | Fam98b | 1393.333 | 1517.675 | 1455.504 |
| ENSMUSG00000027714 | protein_coding | Exosc9 | 794.0401 | 700.1005 | 747.0703 |
| ENSMUSG00000080396 | snoRNA | Snord111 | 0.33031 | 2.110796 | 1.220553 |
| ENSMUSG00000090663 | protein_coding | Srcap | 1751.603 | 1925.394 | 1838.499 |
| ENSMUSG00000040312 | protein_coding | Cchcr1 | 478.1576 | 411.3487 | 444.7531 |
| ENSMUSG00000052779 | pseudogene | 4833418N17Rik | 9.062172 | 4.52144 | 6.791806 |
| ENSMUSG00000063275 | protein_coding | Ptpla | 290.2216 | 252.0945 | 271.158 |
| ENSMUSG00000092758 | miRNA | AL672100.1 | 0.32211 | 2.312628 | 1.317369 |
| ENSMUSG00000069743 | protein_coding | Zfp820 | 375.9847 | 430.3937 | 403.1892 |
| ENSMUSG00000031898 | protein_coding | Dpep3 | 1629.089 | 1018.123 | 1323.606 |

| | | | | | |
|---|---|---|---|---|---|
| ENSMUSG00000083907 | pseudogene | Plk-ps1 | 92.91958 | 73.56884 | 83.24421 |
| ENSMUSG00000027200 | protein_coding | Sema6d | 1442.937 | 1600.271 | 1521.604 |
| ENSMUSG00000022617 | protein_coding | Chkb | 417.8451 | 484.5004 | 451.1727 |
| ENSMUSG00000001739 | protein_coding | Cldn15 | 16.50559 | 25.20202 | 20.85381 |
| ENSMUSG00000029922 | protein_coding | Mkrn1 | 4511.687 | 4866.857 | 4689.272 |
| ENSMUSG00000032231 | protein_coding | Anxa2 | 7968.426 | 8650.827 | 8309.627 |
| ENSMUSG00000043909 | protein_coding | Trp53bp1 | 3686.661 | 3352.243 | 3519.452 |
| ENSMUSG00000026388 | protein_coding | 3110009E18Rik | 98.26232 | 77.44237 | 87.85234 |
| ENSMUSG00000047417 | protein_coding | Rexo1 | 3677.894 | 4003.681 | 3840.788 |
| ENSMUSG00000034216 | protein_coding | Vps18 | 1385.576 | 1231.129 | 1308.352 |
| ENSMUSG00000052656 | protein_coding | Rnf103 | 1089.267 | 980.4374 | 1034.852 |
| ENSMUSG00000086236 | lincRNA | 5830418P13Rik | 3.574744 | 7.796315 | 5.68553 |
| ENSMUSG00000042063 | protein_coding | Zfp386 | 950.4413 | 849.5374 | 899.9894 |
| ENSMUSG00000022369 | protein_coding | Mtbp | 988.8821 | 876.0583 | 932.4702 |
| ENSMUSG00000087446 | lincRNA | 4930578M01Rik | 13.55636 | 21.36965 | 17.463 |
| ENSMUSG00000022864 | protein_coding | D16Ertd472e | 436.543 | 375.2721 | 405.9076 |
| ENSMUSG00000046311 | protein_coding | Zfp62 | 1961.718 | 2180.803 | 2071.26 |
| ENSMUSG00000060002 | protein_coding | Chpt1 | 1791.222 | 1956.262 | 1873.742 |
| ENSMUSG00000074780 | protein_coding | Gm6843 | 19.67369 | 11.72386 | 15.69878 |
| ENSMUSG00000039233 | protein_coding | Tbce | 1039.088 | 1160.126 | 1099.607 |
| ENSMUSG00000020224 | protein_coding | Llph | 1124.251 | 1233.466 | 1178.858 |
| ENSMUSG00000047161 | protein_coding | Chst9 | 2.934102 | 0.688704 | 1.811403 |
| ENSMUSG00000037344 | protein_coding | Slc12a9 | 632.8853 | 569.9435 | 601.4144 |
| ENSMUSG00000070683 | lincRNA | Lactbl1 | 1.284863 | 4.069882 | 2.677372 |
| ENSMUSG00000032643 | protein_coding | Fhl3 | 397.2315 | 448.1302 | 422.6808 |
| ENSMUSG00000014782 | protein_coding | Plekhg4 | 878.4066 | 752.325 | 815.3658 |
| ENSMUSG00000026817 | protein_coding | Ak1 | 160.6866 | 186.4897 | 173.5882 |
| ENSMUSG00000028180 | protein_coding | Zranb2 | 3913.175 | 4265.582 | 4089.378 |
| ENSMUSG00000026249 | protein_coding | Serpine2 | 1573.947 | 2189.286 | 1881.616 |
| ENSMUSG00000087565 | processed_transcrip | Gm14662 | 38.99501 | 27.73921 | 33.36711 |
| ENSMUSG00000046372 | protein_coding | Gm5590 | 0.954552 | 3.399196 | 2.176874 |
| ENSMUSG00000054556 | processed_transcrip | Gm4876 | 18.78854 | 11.45968 | 15.12411 |
| ENSMUSG00000033837 | protein_coding | Foxh1 | 13.65834 | 7.558447 | 10.60839 |
| ENSMUSG00000055334 | protein_coding | Snupn | 581.1165 | 526.8073 | 553.9619 |
| ENSMUSG00000064365 | Mt_tRNA | mt-Ts2 | 8.765929 | 3.895078 | 6.330503 |
| ENSMUSG00000005237 | protein_coding | Dnahc2 | 437.7228 | 533.0688 | 485.3958 |
| ENSMUSG00000016487 | protein_coding | Ppfibp1 | 3810.228 | 4170.958 | 3990.593 |
| ENSMUSG00000063856 | protein_coding | Gpx1 | 1629.779 | 1781.568 | 1705.673 |
| ENSMUSG00000090641 | protein_coding | Zfp712 | 228.5432 | 187.8749 | 208.2091 |
| ENSMUSG00000043300 | protein_coding | B3galnt1 | 632.464 | 732.0368 | 682.2504 |
| ENSMUSG00000040681 | protein_coding | Hmgn1 | 8816.362 | 9542.738 | 9179.55 |
| ENSMUSG00000053460 | protein_coding | Ggcx | 523.6881 | 586.8201 | 555.2541 |
| ENSMUSG00000001018 | protein_coding | Snapin | 1554.388 | 1691.467 | 1622.927 |
| ENSMUSG00000065236 | rRNA | n-R5s197 | 4.237675 | 10.54533 | 7.391504 |
| ENSMUSG00000023755 | protein_coding | Rhebl1 | 156.9208 | 184.7195 | 170.8202 |
| ENSMUSG00000060424 | protein_coding | 2610017I09Rik | 0.33031 | 2.012779 | 1.171545 |
| ENSMUSG00000026504 | protein_coding | Sdccag8 | 438.8532 | 393.3974 | 416.1253 |
| ENSMUSG00000035042 | protein_coding | Ccl5 | 2.934102 | 0.697713 | 1.815908 |
| ENSMUSG00000073894 | protein_coding | Rbmxl2 | 590.0006 | 495.7695 | 542.885 |
| ENSMUSG00000032783 | protein_coding | Troap | 234.1004 | 268.8814 | 251.4909 |
| ENSMUSG00000031827 | protein_coding | Cotl1 | 622.499 | 691.6949 | 657.097 |
| ENSMUSG00000027901 | protein_coding | Dennd2d | 58.05266 | 44.82601 | 51.43933 |
| ENSMUSG00000007946 | protein_coding | Phox2a | 42.61098 | 56.00169 | 49.30634 |
| ENSMUSG00000070284 | protein_coding | Gmppb | 279.6245 | 243.6048 | 261.6146 |
| ENSMUSG00000021468 | protein_coding | Sptlc1 | 1252.171 | 1371.812 | 1311.991 |
| ENSMUSG00000086217 | lincRNA | Gm9530 | 24.2911 | 15.32194 | 19.80652 |
| ENSMUSG00000027620 | protein_coding | Rbm39 | 14879.38 | 16490.81 | 15685.09 |
| ENSMUSG00000091455 | protein_coding | Otogl | 2.908235 | 0.697713 | 1.802974 |

| | | | | | |
|---|---|---|---|---|---|
| ENSMUSG00000038526 | protein_coding | Car14 | 71.14922 | 51.72304 | 61.43613 |
| ENSMUSG00000031012 | protein_coding | Cask | 963.1352 | 884.2857 | 923.7105 |
| ENSMUSG00000032125 | protein_coding | Robo4 | 218.8706 | 260.7648 | 239.8177 |
| ENSMUSG00000034795 | protein_coding | Ccdc122 | 419.5024 | 357.7435 | 388.6229 |
| ENSMUSG00000085748 | processed_transcrip | Gm12280 | 0.316221 | 2.012779 | 1.1645 |
| ENSMUSG00000017176 | protein_coding | Nt5c3l | 2557.602 | 2831.307 | 2694.454 |
| ENSMUSG00000027611 | protein_coding | Procr | 57.85273 | 42.91233 | 50.38253 |
| ENSMUSG00000036112 | protein_coding | Metap2 | 5548.141 | 5969.281 | 5758.711 |
| ENSMUSG00000044024 | protein_coding | Rell2 | 85.50433 | 66.83646 | 76.1704 |
| ENSMUSG00000032966 | protein_coding | Fkbp1a | 8945.763 | 9783.943 | 9364.853 |
| ENSMUSG00000075023 | protein_coding | Accsl | 5.837716 | 10.958 | 8.39786 |
| ENSMUSG00000043541 | protein_coding | Casc1 | 94.93133 | 65.3569 | 80.14411 |
| ENSMUSG00000078789 | protein_coding | Dph1 | 382.8884 | 426.137 | 404.5127 |
| ENSMUSG00000021640 | protein_coding | Naip1 | 9.408882 | 16.52061 | 12.96474 |
| ENSMUSG00000074661 | protein_coding | Pde4d | 96.40785 | 74.16097 | 85.28441 |
| ENSMUSG00000001524 | protein_coding | Gtf2h4 | 570.6482 | 498.8931 | 534.7706 |
| ENSMUSG00000020744 | protein_coding | Slc25a19 | 541.366 | 491.0991 | 516.2325 |
| ENSMUSG00000028622 | protein_coding | Mrpl37 | 1860.118 | 2020.985 | 1940.552 |
| ENSMUSG00000081529 | pseudogene | Gm14884 | 2.575614 | 6.296713 | 4.436163 |
| ENSMUSG00000054618 | processed_transcrip | Gm9951 | 8.156819 | 3.08133 | 5.619074 |
| ENSMUSG00000008226 | protein_coding | Scrn3 | 699.3387 | 773.8887 | 736.6137 |
| ENSMUSG00000073675 | lincRNA | Gm10561 | 26.45903 | 37.07981 | 31.76942 |
| ENSMUSG00000015950 | protein_coding | Ncf1 | 88.48026 | 107.4303 | 97.95529 |
| ENSMUSG00000065952 | protein_coding | C330021F23Rik | 15.74277 | 24.61428 | 20.17853 |
| ENSMUSG00000087800 | snoRNA | SNORA48 | 1.601084 | 0 | 0.800542 |
| ENSMUSG00000025141 | protein_coding | Myadml2 | 2.617881 | 6.771365 | 4.694623 |
| ENSMUSG00000044042 | protein_coding | Fmn1 | 144.458 | 174.5865 | 159.5222 |
| ENSMUSG00000085859 | lincRNA | D030068K23Rik | 60.68948 | 46.06569 | 53.37758 |
| ENSMUSG00000032514 | protein_coding | Ttc21a | 113.9383 | 92.4291 | 103.1837 |
| ENSMUSG00000024664 | protein_coding | Fads3 | 338.6284 | 384.056 | 361.3422 |
| ENSMUSG00000032257 | protein_coding | Ankk1 | 0.976841 | 3.408205 | 2.192523 |
| ENSMUSG00000004988 | protein_coding | Fxyd4 | 6.77229 | 2.790852 | 4.781571 |
| ENSMUSG00000028933 | protein_coding | Xrcc2 | 510.7545 | 446.5015 | 478.628 |
| ENSMUSG00000034206 | protein_coding | Polq | 1075.306 | 958.9159 | 1017.111 |
| ENSMUSG00000031078 | protein_coding | Cttn | 4252.555 | 4586.429 | 4419.492 |
| ENSMUSG00000087430 | processed_transcrip | Gm13405 | 4.565675 | 1.422092 | 2.993883 |
| ENSMUSG00000022635 | protein_coding | Zcrb1 | 1876.215 | 2027.715 | 1951.965 |
| ENSMUSG00000032413 | protein_coding | Rasa2 | 2104.458 | 1913.995 | 2009.227 |
| ENSMUSG00000057060 | protein_coding | Slc35f3 | 57.09833 | 42.46109 | 49.77971 |
| ENSMUSG00000037971 | protein_coding | 1110032A03Rik | 447.5318 | 395.1817 | 421.3567 |
| ENSMUSG00000036537 | protein_coding | Rnf113a1 | 65.88044 | 81.77239 | 73.82641 |
| ENSMUSG00000047940 | protein_coding | B930007M17Rik | 26.15794 | 18.04724 | 22.10259 |
| ENSMUSG00000020392 | protein_coding | Cdkn2aipnl | 1168.265 | 1317.684 | 1242.975 |
| ENSMUSG00000054612 | protein_coding | Mgmt | 207.149 | 239.6015 | 223.3752 |
| ENSMUSG00000024646 | protein_coding | Cyb5 | 1761.74 | 1907.731 | 1834.735 |
| ENSMUSG00000056529 | protein_coding | Ptafr | 23.16055 | 33.37176 | 28.26615 |
| ENSMUSG00000012777 | protein_coding | Acpt | 24.96223 | 36.0166 | 30.48942 |
| ENSMUSG00000054823 | protein_coding | Whsc1l1 | 2316.448 | 2139.786 | 2228.117 |
| ENSMUSG00000000958 | protein_coding | Slc7a7 | 115.6583 | 136.7419 | 126.2001 |
| ENSMUSG00000086198 | lincRNA | Gm16598 | 14.30487 | 7.392291 | 10.84858 |
| ENSMUSG00000082465 | pseudogene | Rps6-ps3 | 4.196676 | 1.395426 | 2.796051 |
| ENSMUSG00000086774 | processed_transcrip | Gm11915 | 1.606973 | 4.405767 | 3.00637 |
| ENSMUSG00000022663 | protein_coding | Atg3 | 1010.14 | 907.034 | 958.5871 |
| ENSMUSG00000018931 | protein_coding | Gm16515 | 2424.473 | 2679.136 | 2551.804 |
| ENSMUSG00000061894 | protein_coding | Zscan20 | 565.316 | 503.2388 | 534.2774 |
| ENSMUSG00000066233 | protein_coding | Tmem42 | 228.0562 | 198.7744 | 213.4153 |
| ENSMUSG00000082154 | pseudogene | Gm16464 | 9.050394 | 4.343425 | 6.696909 |
| ENSMUSG00000047797 | protein_coding | Gjb1 | 9.378393 | 15.31909 | 12.34874 |

| | | | | | |
|---|---|---|---|---|---|
| ENSMUSG00000038806 | protein_coding | BC031781 | 1677.808 | 1831.628 | 1754.718 |
| ENSMUSG00000031608 | protein_coding | Galnt7 | 1323.313 | 1158.506 | 1240.909 |
| ENSMUSG00000030929 | protein_coding | Eri2 | 913.525 | 1023.381 | 968.453 |
| ENSMUSG00000032015 | protein_coding | Pou2f3 | 6.794579 | 12.00854 | 9.401558 |
| ENSMUSG00000092617 | pseudogene | RP24-92P15.2 | 1.31304 | 4.610448 | 2.961744 |
| ENSMUSG00000066568 | protein_coding | Lsm14a | 4571.588 | 4923.061 | 4747.324 |
| ENSMUSG00000035757 | protein_coding | 1300018J18Rik | 307.6609 | 353.4032 | 330.5321 |
| ENSMUSG00000036545 | protein_coding | Adamts2 | 1444.913 | 1587.566 | 1516.239 |
| ENSMUSG00000055748 | protein_coding | Gsdmc4 | 2.233526 | 5.934523 | 4.084024 |
| ENSMUSG00000028068 | protein_coding | Iqgap3 | 307.6637 | 366.647 | 337.1554 |
| ENSMUSG00000074796 | protein_coding | Slc4a11 | 186.6007 | 225.4926 | 206.0467 |
| ENSMUSG00000090863 | protein_coding | A530084C06Rik | 52.86401 | 67.08868 | 59.97634 |
| ENSMUSG00000001445 | protein_coding | Mrpl10 | 1293.552 | 1400.737 | 1347.145 |
| ENSMUSG00000026584 | protein_coding | Scyl3 | 1049.14 | 957.337 | 1003.239 |
| ENSMUSG00000037029 | protein_coding | Zfp146 | 2077.877 | 2363.833 | 2220.855 |
| ENSMUSG00000084993 | processed_transcrip | Gm12305 | 5.151228 | 2.039445 | 3.595337 |
| ENSMUSG00000057531 | protein_coding | Dtnbp1 | 604.6774 | 541.6585 | 573.168 |
| ENSMUSG00000052783 | protein_coding | Grk4 | 431.0576 | 336.7772 | 383.9174 |
| ENSMUSG00000029775 | protein_coding | Klhdc10 | 3077.401 | 2823.681 | 2950.541 |
| ENSMUSG00000068302 | protein_coding | Noto | 5.195806 | 2.012779 | 3.604293 |
| ENSMUSG00000029089 | protein_coding | Pacrgl | 605.8001 | 665.5104 | 635.6552 |
| ENSMUSG00000078117 | protein_coding | Gm16485 | 8.391041 | 14.76987 | 11.58046 |
| ENSMUSG00000082691 | pseudogene | Dynlt1-ps1 | 813.3222 | 893.8125 | 853.5673 |
| ENSMUSG00000004568 | protein_coding | Arhgef18 | 2992.06 | 3271.263 | 3131.662 |
| ENSMUSG00000026479 | protein_coding | Lamc2 | 63.75985 | 81.29738 | 72.52861 |
| ENSMUSG00000028111 | protein_coding | Ctsk | 126.001 | 99.57714 | 112.7891 |
| ENSMUSG00000015879 | protein_coding | Fam184b | 323.7096 | 386.2987 | 355.0042 |
| ENSMUSG00000040043 | protein_coding | Rbms2 | 2913.143 | 3166.055 | 3039.599 |
| ENSMUSG00000074881 | protein_coding | Mageb3 | 23.40931 | 14.78173 | 19.09552 |
| ENSMUSG00000009628 | protein_coding | Tex15 | 14761.2 | 10715.24 | 12738.22 |
| ENSMUSG00000004897 | protein_coding | Hdgf | 4794.629 | 4439.124 | 4616.877 |
| ENSMUSG00000020609 | protein_coding | Apob | 23.05902 | 14.0833 | 18.57116 |
| ENSMUSG00000037720 | protein_coding | Tmem33 | 2089.195 | 2261.604 | 2175.4 |
| ENSMUSG00000078578 | protein_coding | Ube2d3 | 7344.76 | 8021.885 | 7683.323 |
| ENSMUSG00000004044 | protein_coding | Ptrf | 5181.948 | 5657.961 | 5419.954 |
| ENSMUSG00000070524 | protein_coding | Fcrlb | 7.320198 | 2.321637 | 4.820918 |
| ENSMUSG00000051056 | protein_coding | Gja10 | 8.507107 | 3.506222 | 6.006664 |
| ENSMUSG00000045440 | protein_coding | Insm2 | 3.597033 | 1.024227 | 2.31063 |
| ENSMUSG00000020926 | protein_coding | Adam11 | 171.6737 | 144.7589 | 158.2163 |
| ENSMUSG00000024563 | protein_coding | Smad2 | 2238.918 | 2062.983 | 2150.95 |
| ENSMUSG00000037049 | protein_coding | Smpd1 | 560.6331 | 504.0878 | 532.3604 |
| ENSMUSG00000047501 | protein_coding | Cldn4 | 19.25423 | 29.2413 | 24.24776 |
| ENSMUSG00000032578 | protein_coding | Cish | 534.8664 | 604.9441 | 569.9052 |
| ENSMUSG00000048583 | protein_coding | Igf2 | 3691.252 | 3400.797 | 3546.024 |
| ENSMUSG00000036281 | protein_coding | Snapc4 | 969.214 | 879.9048 | 924.5594 |
| ENSMUSG00000051234 | protein_coding | Rnf7 | 2669.307 | 2479.635 | 2574.471 |
| ENSMUSG00000017466 | protein_coding | Timp2 | 5377.598 | 4582.554 | 4980.076 |
| ENSMUSG00000060715 | protein_coding | 1700019A02Rik | 19.88734 | 11.67337 | 15.78036 |
| ENSMUSG00000033813 | protein_coding | Tcea1 | 2195.167 | 2489.805 | 2342.486 |
| ENSMUSG00000002052 | protein_coding | Supt6h | 4657.388 | 5169.389 | 4913.389 |
| ENSMUSG00000027706 | protein_coding | Sec62 | 4337.853 | 4640.586 | 4489.219 |
| ENSMUSG00000001039 | protein_coding | B9d1 | 228.058 | 260.5596 | 244.3088 |
| ENSMUSG00000049881 | protein_coding | 2810025M15Rik | 672.665 | 606.4291 | 639.5471 |
| ENSMUSG00000014747 | protein_coding | Ankrd53 | 13.81496 | 21.32175 | 17.56835 |
| ENSMUSG00000006717 | protein_coding | Acot13 | 402.6388 | 337.5312 | 370.085 |
| ENSMUSG00000024869 | protein_coding | Nudt8 | 146.7304 | 122.2853 | 134.5079 |
| ENSMUSG00000068205 | protein_coding | Macrod2 | 384.3868 | 312.0204 | 348.2036 |
| ENSMUSG00000092390 | pseudogene | Gm20541 | 58.46057 | 72.72014 | 65.59036 |

| | | | | | |
|---|---|---|---|---|---|
| ENSMUSG00000028411 | protein_coding | Aptx | 900.9147 | 982.0703 | 941.4925 |
| ENSMUSG00000031838 | protein_coding | Ifi30 | 502.1343 | 566.1871 | 534.1607 |
| ENSMUSG00000072451 | protein_coding | Gm10359 | 0.33031 | 2.030798 | 1.180554 |
| ENSMUSG00000086082 | lincRNA | 1700113A16Rik | 52.0291 | 39.59748 | 45.81329 |
| ENSMUSG00000053175 | protein_coding | Bcl3 | 334.8382 | 281.2026 | 308.0204 |
| ENSMUSG00000080800 | pseudogene | Gm11582 | 0.65242 | 2.7995 | 1.72596 |
| ENSMUSG00000014503 | protein_coding | Pkd2l2 | 775.9863 | 851.2016 | 813.594 |
| ENSMUSG00000017723 | protein_coding | Wfdc2 | 46.23723 | 60.09674 | 53.16699 |
| ENSMUSG00000021973 | protein_coding | Efha1 | 2177.426 | 2000.68 | 2089.053 |
| ENSMUSG00000074472 | protein_coding | Zfp872 | 83.50309 | 67.51792 | 75.51051 |
| ENSMUSG00000024454 | protein_coding | Hdac3 | 2613.753 | 2418.684 | 2516.219 |
| ENSMUSG00000024217 | protein_coding | Snrpc | 1827.445 | 1977.874 | 1902.66 |
| ENSMUSG00000030158 | protein_coding | Clec12b | 3647.403 | 3939.223 | 3793.313 |
| ENSMUSG00000027886 | protein_coding | 1700013F07Rik | 6.498336 | 2.755177 | 4.626756 |
| ENSMUSG00000033578 | protein_coding | Tmem35 | 2442.732 | 2668.543 | 2555.637 |
| ENSMUSG00000060791 | protein_coding | Gmfg | 60.77923 | 46.34575 | 53.56249 |
| ENSMUSG00000040838 | protein_coding | Gm11639 | 61.63039 | 44.83098 | 53.23068 |
| ENSMUSG00000035671 | protein_coding | Zswim4 | 2129.591 | 1822.126 | 1975.859 |
| ENSMUSG00000086046 | processed_transcrip | 1700095A21Rik | 3.550145 | 7.611779 | 5.580962 |
| ENSMUSG00000005320 | protein_coding | Fgfr4 | 35.28616 | 48.47172 | 41.87894 |
| ENSMUSG00000078566 | protein_coding | Bnip3 | 2039.368 | 2275.69 | 2157.529 |
| ENSMUSG00000028551 | protein_coding | Cdkn2c | 721.7002 | 617.9455 | 669.8228 |
| ENSMUSG00000006519 | protein_coding | Cyba | 383.6676 | 342.6595 | 363.1635 |
| ENSMUSG00000031818 | protein_coding | Cox4i1 | 4810.655 | 5190.09 | 5000.373 |
| ENSMUSG00000000959 | protein_coding | Oxa1l | 1736.749 | 1930.423 | 1833.586 |
| ENSMUSG00000032959 | protein_coding | Pebp1 | 8564.776 | 9172.866 | 8868.821 |
| ENSMUSG00000037110 | protein_coding | Ralgapa2 | 2771.431 | 3092.709 | 2932.07 |
| ENSMUSG00000037446 | protein_coding | Tulp1 | 35.60573 | 25.42694 | 30.51634 |
| ENSMUSG00000048126 | protein_coding | Col6a3 | 1231.675 | 1100.267 | 1165.971 |
| ENSMUSG00000074521 | protein_coding | Gm14327 | 30.53941 | 20.23912 | 25.38926 |
| ENSMUSG00000021572 | protein_coding | Cep72 | 735.0986 | 656.1645 | 695.6315 |
| ENSMUSG00000041341 | protein_coding | Atg2b | 2467.118 | 2172.844 | 2319.981 |
| ENSMUSG00000091666 | lincRNA | Gm17717 | 37.12944 | 27.0721 | 32.10077 |
| ENSMUSG00000041567 | protein_coding | Serpina12 | 12.43482 | 27.38136 | 19.90809 |
| ENSMUSG00000048497 | protein_coding | Mmgt2 | 711.0655 | 790.298 | 750.6818 |
| ENSMUSG00000085287 | lincRNA | 4833418N02Rik | 85.55815 | 64.71728 | 75.13772 |
| ENSMUSG00000016619 | protein_coding | Nup50 | 2433.889 | 2228.83 | 2331.36 |
| ENSMUSG00000025402 | protein_coding | Nab2 | 1335.511 | 1468.958 | 1402.234 |
| ENSMUSG00000053801 | protein_coding | Grwd1 | 1170.012 | 1069.155 | 1119.583 |
| ENSMUSG00000026628 | protein_coding | Atf3 | 224.7336 | 278.2303 | 251.482 |
| ENSMUSG00000020593 | protein_coding | Lpin1 | 866.6598 | 988.3257 | 927.4928 |
| ENSMUSG00000048040 | protein_coding | Arxes2 | 1066.286 | 1175.406 | 1120.846 |
| ENSMUSG00000026880 | protein_coding | Stom | 1201.577 | 1437.268 | 1319.423 |
| ENSMUSG00000034634 | protein_coding | Ly6d | 4.192054 | 9.342367 | 6.767211 |
| ENSMUSG00000028441 | protein_coding | 1110017D15Rik | 5.173517 | 2.00377 | 3.588644 |
| ENSMUSG00000021597 | protein_coding | Ankrd32 | 901.7455 | 806.5829 | 854.1642 |
| ENSMUSG00000028864 | protein_coding | Hgf | 47.95729 | 63.16838 | 55.56284 |
| ENSMUSG00000025034 | protein_coding | Trim8 | 558.9956 | 628.0149 | 593.5053 |
| ENSMUSG00000031314 | protein_coding | Taf1 | 4760.719 | 4337.655 | 4549.187 |
| ENSMUSG00000045215 | protein_coding | Asxl3 | 245.6716 | 209.8561 | 227.7638 |
| ENSMUSG00000020988 | protein_coding | L2hgdh | 671.5458 | 613.3289 | 642.4373 |
| ENSMUSG00000025574 | protein_coding | Tk1 | 682.1047 | 603.5013 | 642.803 |
| ENSMUSG00000032478 | protein_coding | Nme6 | 392.28 | 347.6576 | 369.9688 |
| ENSMUSG00000026142 | protein_coding | Rhbdd1 | 382.6374 | 338.9098 | 360.7736 |
| ENSMUSG00000048440 | protein_coding | Cyp4f16 | 217.6786 | 251.3456 | 234.5121 |
| ENSMUSG00000000686 | protein_coding | Abhd15 | 10.02261 | 4.616246 | 7.31943 |
| ENSMUSG00000057103 | protein_coding | Cml1 | 123.2998 | 145.081 | 134.1904 |
| ENSMUSG00000028745 | protein_coding | Capzb | 4029.113 | 4318.056 | 4173.585 |

| | | | | | |
|---|---|---|---|---|---|
| ENSMUSG00000028121 | protein_coding | Bcar3 | 1001.655 | 1116.688 | 1059.172 |
| ENSMUSG00000019945 | protein_coding | 1700040L02Rik | 154.4048 | 179.6579 | 167.0314 |
| ENSMUSG00000019820 | protein_coding | Utrn | 3431.304 | 3114.935 | 3273.12 |
| ENSMUSG00000046032 | protein_coding | Snx12 | 1375.141 | 1266.61 | 1320.875 |
| ENSMUSG00000041577 | protein_coding | Prelp | 435.7049 | 368.9364 | 402.3207 |
| ENSMUSG00000028539 | protein_coding | Artn | 24.81337 | 35.1373 | 29.97533 |
| ENSMUSG00000029823 | protein_coding | Luc7l2 | 5465.33 | 6056.146 | 5760.738 |
| ENSMUSG00000031637 | protein_coding | Lrp2bp | 27.52607 | 18.76261 | 23.14434 |
| ENSMUSG00000074896 | protein_coding | Ifit3 | 42.38727 | 31.26702 | 36.82715 |
| ENSMUSG00000041417 | protein_coding | Pik3r1 | 887.0709 | 798.3762 | 842.7235 |
| ENSMUSG00000091509 | processed_transcrip | Gm17066 | 205.6191 | 243.4544 | 224.5368 |
| ENSMUSG00000013083 | protein_coding | 2200002J24Rik | 73.25826 | 90.64456 | 81.95141 |
| ENSMUSG00000027820 | protein_coding | Mme | 2766.579 | 3726.245 | 3246.412 |
| ENSMUSG00000072489 | protein_coding | Gm10362 | 1.307151 | 4.150241 | 2.728696 |
| ENSMUSG00000033671 | protein_coding | Cep350 | 3034.67 | 2759.21 | 2896.94 |
| ENSMUSG00000025731 | protein_coding | 0610011F06Rik | 369.1388 | 425.3804 | 397.2596 |
| ENSMUSG00000015488 | protein_coding | 5930434B04Rik | 1369.961 | 1530.419 | 1450.19 |
| ENSMUSG00000058440 | protein_coding | Nrf1 | 1628.496 | 1508.1 | 1568.298 |
| ENSMUSG00000020570 | protein_coding | Sypl | 2465.117 | 2279.751 | 2372.434 |
| ENSMUSG00000050288 | protein_coding | Fzd2 | 565.0343 | 635.8075 | 600.4209 |
| ENSMUSG00000066637 | protein_coding | Ttc32 | 124.2382 | 102.2159 | 113.2271 |
| ENSMUSG00000065118 | snoRNA | SNORA54 | 4.193097 | 1.315066 | 2.754082 |
| ENSMUSG00000018965 | protein_coding | Ywhah | 6356.284 | 6847.975 | 6602.13 |
| ENSMUSG00000043004 | protein_coding | Gng2 | 292.4079 | 329.2985 | 310.8532 |
| ENSMUSG00000037876 | protein_coding | Jmjd1c | 4610.161 | 4268.341 | 4439.251 |
| ENSMUSG00000025825 | protein_coding | Iscu | 1004.289 | 1090.648 | 1047.469 |
| ENSMUSG00000055409 | protein_coding | Nell1 | 11.30032 | 18.35537 | 14.82784 |
| ENSMUSG00000023982 | protein_coding | Guca1a | 48.71087 | 37.08339 | 42.89713 |
| ENSMUSG00000090599 | protein_coding | A730068I03Rik | 11.07221 | 18.6678 | 14.87001 |
| ENSMUSG00000034164 | protein_coding | Emid1 | 98.10696 | 79.57616 | 88.84156 |
| ENSMUSG00000090969 | lincRNA | Gm17656 | 7.421132 | 13.00574 | 10.21343 |
| ENSMUSG00000068876 | protein_coding | Cgn | 7759.58 | 8468.701 | 8114.141 |
| ENSMUSG00000015165 | protein_coding | Hnrnpl | 8995.626 | 9654.74 | 9325.183 |
| ENSMUSG00000042312 | protein_coding | S100a13 | 59.24606 | 75.85019 | 67.54812 |
| ENSMUSG00000038622 | protein_coding | Med30 | 584.4377 | 525.3333 | 554.8855 |
| ENSMUSG00000036564 | protein_coding | Ndrg4 | 1801.001 | 2075.516 | 1938.258 |
| ENSMUSG00000087044 | processed_transcrip | 1700042G15Rik | 2.312171 | 0 | 1.156085 |
| ENSMUSG00000005503 | protein_coding | Evx1 | 2.312171 | 0 | 1.156085 |
| ENSMUSG00000074652 | protein_coding | Myh7b | 141.3771 | 105.9926 | 123.6849 |
| ENSMUSG00000030534 | protein_coding | Vps33b | 619.2628 | 547.784 | 583.5234 |
| ENSMUSG00000068009 | protein_coding | Bpifb6 | 1.296641 | 4.052225 | 2.674433 |
| ENSMUSG00000083500 | pseudogene | Gm15470 | 45.05621 | 58.88843 | 51.97232 |
| ENSMUSG00000059486 | protein_coding | Kbtbd2 | 1954.576 | 2196.644 | 2075.61 |
| ENSMUSG00000020363 | protein_coding | Gfpt2 | 515.8786 | 466.6261 | 491.2523 |
| ENSMUSG00000040167 | protein_coding | Ikzf5 | 1004.576 | 913.8947 | 959.2356 |
| ENSMUSG00000042429 | protein_coding | Adora1 | 5459.303 | 6084.111 | 5771.707 |
| ENSMUSG00000021730 | protein_coding | Hcn1 | 6.776912 | 2.683465 | 4.730188 |
| ENSMUSG00000063727 | protein_coding | Tnfrsf11b | 134.307 | 164.3172 | 149.3121 |
| ENSMUSG00000022024 | protein_coding | Sugt1 | 2262.756 | 2455.225 | 2358.991 |
| ENSMUSG00000031586 | protein_coding | Rbpms | 1467.711 | 1616.206 | 1541.959 |
| ENSMUSG00000000579 | protein_coding | Dynlt1c | 24.23706 | 15.91298 | 20.07502 |
| ENSMUSG00000075000 | protein_coding | Nrbf2 | 651.8125 | 586.2821 | 619.0473 |
| ENSMUSG00000033933 | protein_coding | Vhl | 2614.045 | 2420.337 | 2517.191 |
| ENSMUSG00000035007 | protein_coding | Rundc1 | 955.771 | 1046.069 | 1000.92 |
| ENSMUSG00000053211 | protein_coding | Zfy1 | 201.6228 | 147.446 | 174.5344 |
| ENSMUSG00000062300 | protein_coding | Pvrl2 | 3923.586 | 4240.593 | 4082.089 |
| ENSMUSG00000039050 | protein_coding | Osbpl2 | 1602.927 | 1476.719 | 1539.823 |
| ENSMUSG00000079577 | protein_coding | Gm14692 | 56.2606 | 31.48728 | 43.87394 |

| | | | | | |
|---|---|---|---|---|---|
| ENSMUSG00000092320 | processed_transcrip | RP24-276N7.2 | 39.06777 | 28.09239 | 33.58008 |
| ENSMUSG00000080783 | pseudogene | Gm8250 | 1.951372 | 5.121498 | 3.536435 |
| ENSMUSG00000022338 | protein_coding | Eny2 | 1424.284 | 1303.879 | 1364.081 |
| ENSMUSG00000020541 | protein_coding | Tom1l1 | 1034.412 | 1138.625 | 1086.518 |
| ENSMUSG00000063935 | protein_coding | Zar1 | 5.201695 | 2.057464 | 3.62958 |
| ENSMUSG00000032253 | protein_coding | Phip | 4584.541 | 4968.241 | 4776.391 |
| ENSMUSG00000093042 | miRNA | CT009708.1 | 0.968641 | 3.318836 | 2.143739 |
| ENSMUSG00000086700 | processed_transcrip | Gm15747 | 1.604662 | 4.80327 | 3.203966 |
| ENSMUSG00000031953 | protein_coding | Tmem170 | 469.5329 | 400.0386 | 434.7858 |
| ENSMUSG00000030787 | protein_coding | Lyve1 | 40.01539 | 52.18734 | 46.10136 |
| ENSMUSG00000025465 | protein_coding | Echs1 | 2174.752 | 2346.654 | 2260.703 |
| ENSMUSG00000025040 | protein_coding | Fundc1 | 810.3635 | 907.3324 | 858.848 |
| ENSMUSG00000004455 | protein_coding | Ppp1cc | 7281.489 | 6757.778 | 7019.633 |
| ENSMUSG00000038379 | protein_coding | Ttk | 1535.965 | 1420.209 | 1478.087 |
| ENSMUSG00000079608 | protein_coding | Stard6 | 70.63329 | 50.62032 | 60.6268 |
| ENSMUSG00000027523 | protein_coding | Gnas | 555.0417 | 631.2703 | 593.156 |
| ENSMUSG00000028677 | protein_coding | Rnf220 | 2840.942 | 2631.993 | 2736.468 |
| ENSMUSG00000029660 | protein_coding | 4930588N13Rik | 18.65712 | 11.62833 | 15.14272 |
| ENSMUSG00000085449 | processed_transcrip | Gm15520 | 7.1108 | 3.434871 | 5.272836 |
| ENSMUSG00000049357 | protein_coding | 4933408B17Rik | 33.87114 | 20.53208 | 27.20161 |
| ENSMUSG00000060279 | protein_coding | Ap2a1 | 2384.825 | 2171.083 | 2277.954 |
| ENSMUSG00000023994 | protein_coding | Nfya | 1866 | 2088.249 | 1977.124 |
| ENSMUSG00000033157 | protein_coding | Abhd10 | 710.9004 | 777.9437 | 744.422 |
| ENSMUSG00000075267 | protein_coding | Dfnb59 | 8.776439 | 4.423424 | 6.599931 |
| ENSMUSG00000027162 | protein_coding | Lin7c | 2920.454 | 3143.387 | 3031.921 |
| ENSMUSG00000091094 | protein_coding | Gm9222 | 89.35236 | 108.3231 | 98.83772 |
| ENSMUSG00000055839 | protein_coding | Tceb2 | 2243.172 | 2078.002 | 2160.587 |
| ENSMUSG00000090035 | protein_coding | Galnt4 | 232.717 | 271.0001 | 251.8586 |
| ENSMUSG00000029838 | protein_coding | Ptn | 794.4807 | 691.8119 | 743.1463 |
| ENSMUSG00000083223 | pseudogene | Gm15016 | 4.190787 | 8.25616 | 6.223473 |
| ENSMUSG00000028565 | protein_coding | Nfia | 2019.187 | 2313.307 | 2166.247 |
| ENSMUSG00000040726 | protein_coding | Hesx1 | 4.170809 | 1.333085 | 2.751947 |
| ENSMUSG00000015749 | protein_coding | Anp32e | 3929.199 | 4256.984 | 4093.092 |
| ENSMUSG00000024430 | protein_coding | Cabyr | 82.68756 | 64.33029 | 73.50893 |
| ENSMUSG00000051107 | protein_coding | Gm15440 | 10.97359 | 6.17203 | 8.572809 |
| ENSMUSG00000035967 | protein_coding | Ddx26b | 1838.905 | 2123.111 | 1981.008 |
| ENSMUSG00000030850 | protein_coding | Ate1 | 1643.723 | 1775.677 | 1709.7 |
| ENSMUSG00000085191 | lincRNA | Gm16847 | 53.00468 | 71.90322 | 62.45395 |
| ENSMUSG00000054579 | protein_coding | 9130219A07Rik | 14.12887 | 21.93946 | 18.03417 |
| ENSMUSG00000090924 | pseudogene | Gm17390 | 75.78534 | 59.79948 | 67.79241 |
| ENSMUSG00000091138 | lincRNA | E530011L22Rik | 71.49527 | 89.17379 | 80.33453 |
| ENSMUSG00000074782 | processed_transcrip | 4833422C13Rik | 18.4311 | 26.71027 | 22.57068 |
| ENSMUSG00000030397 | protein_coding | Mark4 | 3179.13 | 3543.969 | 3361.55 |
| ENSMUSG00000038765 | protein_coding | Lmx1b | 24.85772 | 16.21211 | 20.53492 |
| ENSMUSG00000026609 | protein_coding | Ush2a | 37.26191 | 52.61684 | 44.93937 |
| ENSMUSG00000054044 | protein_coding | Gm9933 | 3.541945 | 1.059903 | 2.300924 |
| ENSMUSG00000027794 | protein_coding | Sohlh2 | 1334.048 | 1487.693 | 1410.87 |
| ENSMUSG00000044816 | protein_coding | D630023F18Rik | 25.47503 | 34.69004 | 30.08253 |
| ENSMUSG00000027784 | protein_coding | Ppm1l | 2573.65 | 2924.611 | 2749.13 |
| ENSMUSG00000034379 | protein_coding | Wdr5b | 118.8536 | 144.8616 | 131.8576 |
| ENSMUSG00000089170 | misc_RNA | Y_RNA | 8.138108 | 3.832736 | 5.985422 |
| ENSMUSG00000039031 | protein_coding | Arhgap18 | 1579.544 | 1733.869 | 1656.707 |
| ENSMUSG00000066885 | protein_coding | Ptprv | 594.0902 | 712.7188 | 653.4045 |
| ENSMUSG00000041617 | protein_coding | Ccdc74a | 88.76137 | 106.2256 | 97.49348 |
| ENSMUSG00000027324 | protein_coding | Rpusd2 | 359.5871 | 319.151 | 339.3691 |
| ENSMUSG00000074812 | protein_coding | Gm355 | 0.974531 | 3.45289 | 2.21371 |
| ENSMUSG00000034282 | protein_coding | Evpl | 409.3483 | 348.6198 | 378.984 |
| ENSMUSG00000049124 | pseudogene | Gm8186 | 115.3131 | 96.39091 | 105.852 |

| | | | | | |
|---|---|---|---|---|---|
| ENSMUSG00000026637 | protein_coding | Traf5 | 139.7352 | 116.6881 | 128.2116 |
| ENSMUSG00000028295 | protein_coding | 1810030N24Rik | 340.177 | 300.2486 | 320.2128 |
| ENSMUSG00000026255 | protein_coding | Efhd1 | 3009.387 | 3314.037 | 3161.712 |
| ENSMUSG00000041671 | protein_coding | Pyroxd1 | 252.4561 | 221.0022 | 236.7291 |
| ENSMUSG00000058966 | protein_coding | Fam57b | 243.1461 | 279.2126 | 261.1793 |
| ENSMUSG00000059182 | protein_coding | Skap2 | 911.5863 | 811.5104 | 861.5483 |
| ENSMUSG00000085170 | processed_transcrip | 4930557C09Rik | 11.01817 | 19.26134 | 15.13975 |
| ENSMUSG00000035262 | protein_coding | Amh | 4048.294 | 4610.254 | 4329.274 |
| ENSMUSG00000082686 | pseudogene | Gm12961 | 2.287571 | 0.335524 | 1.311547 |
| ENSMUSG00000001988 | protein_coding | Npas1 | 6.424313 | 11.56671 | 8.995511 |
| ENSMUSG00000091214 | lincRNA | 4732491K20Rik | 132.5956 | 110.7453 | 121.6704 |
| ENSMUSG00000081296 | pseudogene | Gm12203 | 14.86686 | 8.918198 | 11.89253 |
| ENSMUSG00000047371 | protein_coding | Zfp768 | 708.2808 | 645.6534 | 676.9671 |
| ENSMUSG00000036832 | protein_coding | Lpar3 | 1884.313 | 1591.402 | 1737.857 |
| ENSMUSG00000086272 | processed_transcrip | BC039966 | 3.574744 | 7.629797 | 5.602271 |
| ENSMUSG00000029599 | protein_coding | Ddx54 | 2577.191 | 2345.636 | 2461.414 |
| ENSMUSG00000057572 | protein_coding | Zbtb8os | 1046.031 | 958.4179 | 1002.224 |
| ENSMUSG00000054850 | protein_coding | Ncrna00086 | 940.0434 | 815.9368 | 877.9901 |
| ENSMUSG00000026833 | protein_coding | Olfm1 | 201.9439 | 170.7161 | 186.33 |
| ENSMUSG00000086494 | processed_transcrip | 2210417A02Rik | 3.574744 | 7.523133 | 5.548939 |
| ENSMUSG00000081684 | pseudogene | Rps2-ps13 | 6552.572 | 5611.059 | 6081.815 |
| ENSMUSG00000038495 | protein_coding | Otud7b | 2200.523 | 2368.686 | 2284.604 |
| ENSMUSG00000056962 | protein_coding | Jmjd6 | 1377.735 | 1506.047 | 1441.891 |
| ENSMUSG00000035198 | protein_coding | Tubg1 | 1128.05 | 1009.976 | 1069.013 |
| ENSMUSG00000024273 | protein_coding | 2700062C07Rik | 388.3174 | 437.5063 | 412.9119 |
| ENSMUSG00000008958 | protein_coding | Vps72 | 1601.282 | 1723.544 | 1662.413 |
| ENSMUSG00000037910 | protein_coding | 1700018B24Rik | 317.8791 | 357.6068 | 337.7429 |
| ENSMUSG00000021286 | protein_coding | Zfyve21 | 1865.781 | 2055.974 | 1960.877 |
| ENSMUSG00000066202 | protein_coding | Ccl27b | 0.65242 | 2.692836 | 1.672628 |
| ENSMUSG00000079351 | protein_coding | 9930021J03Rik | 466.8758 | 400.3218 | 433.5988 |
| ENSMUSG00000033411 | protein_coding | Ctdspl2 | 2855.07 | 2611.825 | 2733.448 |
| ENSMUSG00000027570 | protein_coding | Col9a3 | 191.9549 | 226.7499 | 209.3524 |
| ENSMUSG00000062397 | protein_coding | Zfp706 | 2633.231 | 2847.988 | 2740.61 |
| ENSMUSG00000051890 | protein_coding | Klhdc1 | 369.7855 | 327.8628 | 348.8241 |
| ENSMUSG00000003279 | protein_coding | Dlgap1 | 329.0935 | 371.3271 | 350.2103 |
| ENSMUSG00000075534 | processed_transcrip | Gm13262 | 0 | 1.739597 | 0.869799 |
| ENSMUSG00000085555 | lincRNA | 2610035F20Rik | 0 | 1.739597 | 0.869799 |
| ENSMUSG00000035845 | protein_coding | Alg12 | 360.0249 | 402.4241 | 381.2245 |
| ENSMUSG00000026469 | protein_coding | Xpr1 | 2793.319 | 2577.199 | 2685.259 |
| ENSMUSG00000074355 | protein_coding | Gm10676 | 22.98626 | 32.05954 | 27.5229 |
| ENSMUSG00000036924 | protein_coding | Cst13 | 2.287571 | 5.756508 | 4.02204 |
| ENSMUSG00000044060 | protein_coding | A830010M20Rik | 430.3038 | 378.9868 | 404.6453 |
| ENSMUSG00000075070 | lincRNA | 4932412D23Rik | 7.153067 | 13.1751 | 10.16409 |
| ENSMUSG00000003863 | protein_coding | Ppfia3 | 222.2143 | 256.0544 | 239.1344 |
| ENSMUSG00000086519 | processed_transcrip | Gm13393 | 2.279371 | 0.326514 | 1.302943 |
| ENSMUSG00000040274 | protein_coding | Cdk6 | 546.8251 | 476.2981 | 511.5616 |
| ENSMUSG00000082487 | pseudogene | Gm11628 | 5.151228 | 9.508885 | 7.330057 |
| ENSMUSG00000050856 | protein_coding | Atp5k | 338.1941 | 290.6069 | 314.4005 |
| ENSMUSG00000025035 | protein_coding | Arl3 | 769.6841 | 850.8824 | 810.2832 |
| ENSMUSG00000064692 | snoRNA | SNORA67 | 1.290752 | 3.743729 | 2.51724 |
| ENSMUSG00000045095 | protein_coding | Magi1 | 913.0918 | 807.6504 | 860.3711 |
| ENSMUSG00000060198 | protein_coding | Gm11353 | 76.30843 | 61.16896 | 68.73869 |
| ENSMUSG00000038354 | protein_coding | Ankrd35 | 191.508 | 163.8022 | 177.6551 |
| ENSMUSG00000073730 | protein_coding | 4933415F23Rik | 11.44352 | 5.545667 | 8.494594 |
| ENSMUSG00000026585 | protein_coding | Kifap3 | 1199.511 | 1103.456 | 1151.484 |
| ENSMUSG00000002076 | protein_coding | Hsf2bp | 513.5978 | 347.6221 | 430.6099 |
| ENSMUSG00000001674 | protein_coding | Ddx18 | 2261.913 | 2091.057 | 2176.485 |
| ENSMUSG00000091056 | lincRNA | Gm17536 | 12.8686 | 19.71584 | 16.29222 |

| | | | | | |
|---|---|---|---|---|---|
| ENSMUSG00000048652 | protein_coding | Samd13 | 9.706392 | 5.192487 | 7.44944 |
| ENSMUSG00000033114 | protein_coding | Slc35d2 | 151.4243 | 179.0766 | 165.2504 |
| ENSMUSG00000037664 | protein_coding | Cdkn1c | 585.7089 | 532.3442 | 559.0266 |
| ENSMUSG00000031176 | protein_coding | Dynlt3 | 1794.053 | 1971.214 | 1882.634 |
| ENSMUSG00000037852 | protein_coding | Cpe | 14626.88 | 15948.48 | 15287.68 |
| ENSMUSG00000040331 | protein_coding | Nsmce4a | 1615.483 | 1747.755 | 1681.619 |
| ENSMUSG00000030523 | protein_coding | Trpm1 | 20.86187 | 11.86004 | 16.36095 |
| ENSMUSG00000090077 | protein_coding | Lime1 | 4.179008 | 8.672043 | 6.425526 |
| ENSMUSG00000086712 | processed_transcrip | AI427809 | 31.82554 | 22.38165 | 27.1036 |
| ENSMUSG00000038578 | protein_coding | Susd1 | 236.0672 | 207.2983 | 221.6827 |
| ENSMUSG00000039100 | protein_coding | 6-Mar | 4211.653 | 4636.552 | 4424.103 |
| ENSMUSG00000068264 | protein_coding | 2310035K24Rik | 499.2314 | 562.2715 | 530.7515 |
| ENSMUSG00000077557 | snoRNA | SNORD86 | 1.937283 | 4.758947 | 3.348115 |
| ENSMUSG00000070469 | protein_coding | Adamtsl3 | 28.00584 | 39.83276 | 33.9193 |
| ENSMUSG00000026078 | protein_coding | Pdcl3 | 1380.553 | 1503.452 | 1442.003 |
| ENSMUSG00000070471 | protein_coding | Fam194a | 13.92536 | 8.291835 | 11.1086 |
| ENSMUSG00000060036 | protein_coding | Rpl3 | 8306.409 | 8940.69 | 8623.55 |
| ENSMUSG00000039887 | protein_coding | Alg14 | 377.8868 | 339.9044 | 358.8956 |
| ENSMUSG00000086391 | processed_transcrip | 1700042O10Rik | 2.275793 | 5.545667 | 3.91073 |
| ENSMUSG00000070568 | protein_coding | 1700039E15Rik | 41.70809 | 58.07541 | 49.89175 |
| ENSMUSG00000030629 | protein_coding | Zfand6 | 2896.757 | 3135.503 | 3016.13 |
| ENSMUSG00000066896 | protein_coding | Olfr18 | 0 | 1.73095 | 0.865475 |
| ENSMUSG00000086674 | processed_transcrip | Gm12283 | 0 | 1.73095 | 0.865475 |
| ENSMUSG00000028343 | protein_coding | Erp44 | 2610.224 | 2807.189 | 2708.706 |
| ENSMUSG00000071141 | pseudogene | Rpl36a-ps3 | 22.24364 | 14.93888 | 18.59126 |
| ENSMUSG00000045345 | pseudogene | 9530056K15Rik | 23.27788 | 32.2621 | 27.76999 |
| ENSMUSG00000041115 | protein_coding | Iqsec2 | 387.4235 | 431.904 | 409.6638 |
| ENSMUSG00000021950 | protein_coding | Anxa8 | 17.41452 | 28.34461 | 22.87956 |
| ENSMUSG00000041718 | protein_coding | Alg13 | 1807.01 | 2051.891 | 1929.451 |
| ENSMUSG00000041592 | protein_coding | Sdk2 | 498.7687 | 404.6155 | 451.6921 |
| ENSMUSG00000085800 | lincRNA | 1700066O22Rik | 3.236234 | 1.024227 | 2.130231 |
| ENSMUSG00000009073 | protein_coding | Nf2 | 4177.358 | 3727.381 | 3952.37 |
| ENSMUSG00000086915 | processed_transcrip | Gm16365 | 71.81447 | 90.23468 | 81.02457 |
| ENSMUSG00000043987 | protein_coding | Cep164 | 956.8349 | 869.7009 | 913.2679 |
| ENSMUSG00000081210 | pseudogene | Gm12933 | 2.905924 | 0.724379 | 1.815152 |
| ENSMUSG00000085860 | lincRNA | 2410003L11Rik | 12.60181 | 19.5223 | 16.06205 |
| ENSMUSG00000028478 | protein_coding | Clta | 3122.123 | 3357.936 | 3240.03 |
| ENSMUSG00000021609 | protein_coding | Slc6a3 | 1.284863 | 3.752377 | 2.51862 |
| ENSMUSG00000032615 | protein_coding | Nt5m | 458.4151 | 408.4421 | 433.4286 |
| ENSMUSG00000090115 | protein_coding | Usp49 | 288.4014 | 328.5872 | 308.4943 |
| ENSMUSG00000037104 | protein_coding | Socs5 | 911.6285 | 804.7087 | 858.1686 |
| ENSMUSG00000022516 | protein_coding | Nudt16l1 | 648.4202 | 568.1809 | 608.3006 |
| ENSMUSG00000087060 | lincRNA | 2810442I21Rik | 87.15671 | 65.60972 | 76.38321 |
| ENSMUSG00000090697 | protein_coding | Nsun4 | 6.178536 | 2.701483 | 4.44001 |
| ENSMUSG00000078960 | protein_coding | Tob2 | 11.93761 | 6.861095 | 9.399351 |
| ENSMUSG00000085455 | processed_transcrip | 1810059H22Rik | 14.23211 | 21.64283 | 17.93747 |
| ENSMUSG00000070035 | protein_coding | Gm4885 | 35.44129 | 26.00319 | 30.72224 |
| ENSMUSG00000054932 | protein_coding | Afp | 234.8425 | 270.045 | 252.4437 |
| ENSMUSG00000044250 | protein_coding | Fam113b | 811.2381 | 931.5913 | 871.4147 |
| ENSMUSG00000021850 | protein_coding | 1700011H14Rik | 2.583814 | 5.872182 | 4.227998 |
| ENSMUSG00000021136 | protein_coding | Smoc1 | 1191.409 | 1072.667 | 1132.038 |
| ENSMUSG00000034501 | protein_coding | 1810048J11Rik | 591.0791 | 506.051 | 548.565 |
| ENSMUSG00000086315 | processed_transcrip | 4930555J06Rik | 7.485688 | 3.153042 | 5.319365 |
| ENSMUSG00000063808 | protein_coding | Gpatch1 | 647.7435 | 581.6755 | 614.7095 |
| ENSMUSG00000022560 | protein_coding | Gpr172b | 389.1938 | 347.4464 | 368.3201 |
| ENSMUSG00000090034 | processed_transcrip | Gm15917 | 9.983925 | 5.420985 | 7.702455 |
| ENSMUSG00000044505 | protein_coding | Lingo4 | 7.119 | 12.29037 | 9.704683 |
| ENSMUSG00000027122 | protein_coding | 2700007P21Rik | 1384.209 | 1537.177 | 1460.693 |

| | | | | | |
|---|---|---|---|---|---|
| ENSMUSG00000019539 | protein_coding | Rcn3 | 1627.023 | 1450.609 | 1538.816 |
| ENSMUSG00000087938 | snRNA | U6 | 3.242123 | 6.825058 | 5.033591 |
| ENSMUSG00000045886 | pseudogene | Gm9803 | 2.259393 | 5.393957 | 3.826675 |
| ENSMUSG00000031021 | protein_coding | Tmem9b | 1045.317 | 957.2126 | 1001.265 |
| ENSMUSG00000034035 | protein_coding | Ccdc17 | 55.06772 | 70.67313 | 62.87042 |
| ENSMUSG00000029642 | protein_coding | Polr1d | 1933.32 | 2156.677 | 2044.999 |
| ENSMUSG00000017386 | protein_coding | Traf4 | 1355.422 | 1474.962 | 1415.192 |
| ENSMUSG00000029474 | protein_coding | Rnf34 | 3610.29 | 3888.076 | 3749.183 |
| ENSMUSG00000026708 | protein_coding | Cenpl | 483.2867 | 430.3407 | 456.8137 |
| ENSMUSG00000022414 | protein_coding | Tab1 | 838.2743 | 950.04 | 894.1571 |
| ENSMUSG00000091299 | lincRNA | Snhg7 | 214.3423 | 248.8671 | 231.6047 |
| ENSMUSG00000089714 | processed_transcrip | Gm16092 | 1.937283 | 0.335524 | 1.136403 |
| ENSMUSG00000084624 | rRNA | n-R5s189 | 1.937283 | 0.335524 | 1.136403 |
| ENSMUSG00000091558 | protein_coding | Gm17227 | 1.937283 | 0.335524 | 1.136403 |
| ENSMUSG00000036109 | protein_coding | Mbnl3 | 870.7721 | 777.5309 | 824.1515 |
| ENSMUSG00000014778 | protein_coding | Fhod1 | 404.541 | 359.5457 | 382.0433 |
| ENSMUSG00000084766 | processed_transcrip | Gm12714 | 1.637461 | 4.767595 | 3.202528 |
| ENSMUSG00000007837 | protein_coding | Prrg2 | 319.1542 | 356.6157 | 337.885 |
| ENSMUSG00000034269 | protein_coding | Setd5 | 7390.166 | 8003.59 | 7696.878 |
| ENSMUSG00000004268 | protein_coding | Emg1 | 2671.61 | 2463.393 | 2567.502 |
| ENSMUSG00000084790 | processed_transcrip | Gm15879 | 25.86401 | 18.02922 | 21.94661 |
| ENSMUSG00000091878 | lincRNA | C430002E04Rik | 20.87887 | 13.68615 | 17.28251 |
| ENSMUSG00000034265 | protein_coding | Zdhhc14 | 403.7203 | 448.8076 | 426.264 |
| ENSMUSG00000026961 | protein_coding | Lrrc26 | 1.937283 | 0.326514 | 1.131899 |
| ENSMUSG00000081732 | pseudogene | Gm15495 | 2.920013 | 0.724379 | 1.822196 |
| ENSMUSG00000030249 | protein_coding | Abcc9 | 153.5007 | 129.7233 | 141.612 |
| ENSMUSG00000061298 | protein_coding | Agbl4 | 26.91949 | 38.65895 | 32.78922 |
| ENSMUSG00000080370 | snoRNA | SNORA42 | 4.881896 | 10.05561 | 7.468753 |
| ENSMUSG00000079497 | processed_transcrip | Gm13420 | 1.937283 | 4.776604 | 3.356943 |
| ENSMUSG00000014402 | protein_coding | Tsg101 | 2225.589 | 2383.941 | 2304.765 |
| ENSMUSG00000051977 | protein_coding | Prdm9 | 1033.306 | 634.4707 | 833.8884 |
| ENSMUSG00000020456 | protein_coding | Ogdh | 3971.922 | 3633.713 | 3802.818 |
| ENSMUSG00000004788 | protein_coding | Eif2b2 | 992.6596 | 903.425 | 948.0423 |
| ENSMUSG00000023328 | protein_coding | Ache | 17.4919 | 25.35052 | 21.42121 |
| ENSMUSG00000064802 | snRNA | U1 | 3.902744 | 1.350742 | 2.626743 |
| ENSMUSG00000086398 | processed_transcrip | Gm15639 | 11.90585 | 6.763079 | 9.334465 |
| ENSMUSG00000052563 | protein_coding | D930048N14Rik | 272.9811 | 317.7258 | 295.3535 |
| ENSMUSG00000030122 | protein_coding | Ptms | 3121.29 | 3426.254 | 3273.772 |
| ENSMUSG00000086873 | processed_transcrip | Gm15672 | 51.01439 | 38.54944 | 44.78192 |
| ENSMUSG00000085135 | processed_transcrip | Gm13713 | 4.218965 | 8.422678 | 6.320821 |
| ENSMUSG00000030029 | protein_coding | Lrig1 | 2245.15 | 2440.583 | 2342.866 |
| ENSMUSG00000029253 | protein_coding | Cenpc1 | 1270.643 | 1150.334 | 1210.489 |
| ENSMUSG00000085437 | processed_transcrip | 1700047A11Rik | 0 | 1.703922 | 0.851961 |
| ENSMUSG00000063410 | protein_coding | Stk24 | 3602.709 | 3937.644 | 3770.177 |
| ENSMUSG00000050914 | protein_coding | Ankrd37 | 60.4273 | 47.10296 | 53.76513 |
| ENSMUSG00000032306 | protein_coding | Mpi | 955.5487 | 1044.539 | 1000.044 |
| ENSMUSG00000032563 | protein_coding | Mrpl3 | 2048.241 | 2212.903 | 2130.572 |
| ENSMUSG00000016940 | protein_coding | Kctd2 | 1102.747 | 986.2875 | 1044.517 |
| ENSMUSG00000022610 | protein_coding | Mapk12 | 135.7366 | 115.462 | 125.5993 |
| ENSMUSG00000037617 | protein_coding | Spag1 | 394.2535 | 437.9422 | 416.0978 |
| ENSMUSG00000039485 | protein_coding | Tspyl4 | 117.7889 | 138.1738 | 127.9813 |
| ENSMUSG00000021496 | protein_coding | Pcbd2 | 157.4619 | 181.1834 | 169.3227 |
| ENSMUSG00000019989 | protein_coding | Enpp3 | 16.7091 | 24.55479 | 20.63195 |
| ENSMUSG00000022715 | protein_coding | Cldn26 | 3762.299 | 4125.988 | 3944.143 |
| ENSMUSG00000014498 | protein_coding | Ankrd52 | 1467.814 | 1601.106 | 1534.46 |
| ENSMUSG00000030104 | protein_coding | Edem1 | 1429.556 | 1323.981 | 1376.769 |
| ENSMUSG00000025380 | protein_coding | Fscn2 | 44.23204 | 33.34758 | 38.78981 |
| ENSMUSG00000064337 | Mt_rRNA | mt-Rnr1 | 5344.474 | 5971.349 | 5657.911 |

| | | | | | |
|---|---|---|---|---|---|
| ENSMUSG00000075053 | pseudogene | Vdac3-ps1 | 1198.311 | 1314.649 | 1256.48 |
| ENSMUSG00000019132 | protein_coding | BC005537 | 8832.893 | 8287.693 | 8560.293 |
| ENSMUSG00000042662 | protein_coding | Dusp15 | 1086.121 | 1213.104 | 1149.613 |
| ENSMUSG00000022673 | protein_coding | Mcm4 | 2956.906 | 3235.821 | 3096.363 |
| ENSMUSG00000053522 | protein_coding | Lgals7 | 219.0428 | 335.6339 | 277.3383 |
| ENSMUSG00000037533 | protein_coding | Rapgef6 | 3446.683 | 3101.824 | 3274.253 |
| ENSMUSG00000057370 | protein_coding | Rpl21-ps12 | 2.900035 | 0.724379 | 1.812207 |
| ENSMUSG00000031329 | protein_coding | Tsx | 221.146 | 165.9365 | 193.5413 |
| ENSMUSG00000033963 | protein_coding | 4931417G12Rik | 3.919143 | 8.202466 | 6.060805 |
| ENSMUSG00000080791 | pseudogene | Gm11442 | 1.629262 | 4.370091 | 2.999676 |
| ENSMUSG00000069020 | protein_coding | Urm1 | 1053.743 | 1144.178 | 1098.96 |
| ENSMUSG00000042675 | protein_coding | Ypel3 | 811.4212 | 887.3121 | 849.3667 |
| ENSMUSG00000041779 | protein_coding | Tram2 | 506.8516 | 440.3961 | 473.6238 |
| ENSMUSG00000033658 | protein_coding | Ddx19b | 1781.567 | 1650.53 | 1716.048 |
| ENSMUSG00000063802 | protein_coding | Hspbp1 | 841.574 | 768.4868 | 805.0304 |
| ENSMUSG00000057899 | protein_coding | Gpr111 | 6.113981 | 2.665808 | 4.389894 |
| ENSMUSG00000011257 | protein_coding | Pabpc4 | 2339.471 | 2549.892 | 2444.682 |
| ENSMUSG00000027386 | protein_coding | Fbln7 | 151.4961 | 175.6363 | 163.5662 |
| ENSMUSG00000081944 | pseudogene | Gm12276 | 36.2756 | 49.20252 | 42.73906 |
| ENSMUSG00000035370 | protein_coding | Adat3 | 103.0934 | 123.1117 | 113.1025 |
| ENSMUSG00000027225 | protein_coding | Duoxa2 | 2.245304 | 5.563324 | 3.904314 |
| ENSMUSG00000022637 | protein_coding | Cblb | 1771.803 | 1636.757 | 1704.28 |
| ENSMUSG00000086321 | processed_transcrip | Gm11413 | 10.98999 | 5.75687 | 8.373429 |
| ENSMUSG00000015937 | protein_coding | H2afy | 3150.143 | 3413.672 | 3281.907 |
| ENSMUSG00000024942 | protein_coding | Capn1 | 761.7362 | 863.3649 | 812.5506 |
| ENSMUSG00000073468 | protein_coding | Sft2d1 | 1082.918 | 1202.983 | 1142.951 |
| ENSMUSG00000048351 | protein_coding | 2010305A19Rik | 803.6029 | 720.2527 | 761.9278 |
| ENSMUSG00000060212 | protein_coding | Pcnxl2 | 17.77174 | 26.26156 | 22.01665 |
| ENSMUSG00000006342 | protein_coding | Susd2 | 216.3965 | 185.17 | 200.7832 |
| ENSMUSG00000033294 | protein_coding | Noc4l | 1214.096 | 1341.705 | 1277.901 |
| ENSMUSG00000074817 | protein_coding | Papolb | 119.4487 | 94.5475 | 106.9981 |
| ENSMUSG00000032872 | protein_coding | Cyb5r4 | 1023.78 | 1122.855 | 1073.317 |
| ENSMUSG00000085977 | pseudogene | Gm5970 | 1.951372 | 0.36219 | 1.156781 |
| ENSMUSG00000082002 | pseudogene | Gm6568 | 1.951372 | 0.36219 | 1.156781 |
| ENSMUSG00000061759 | protein_coding | 1700052N19Rik | 749.7206 | 683.5418 | 716.6312 |
| ENSMUSG00000002458 | protein_coding | Rgs19 | 349.193 | 402.3584 | 375.7757 |
| ENSMUSG00000039952 | protein_coding | Dag1 | 6730.329 | 7516.381 | 7123.355 |
| ENSMUSG00000084772 | lincRNA | Gm15473 | 1.290752 | 3.699044 | 2.494898 |
| ENSMUSG00000092050 | lincRNA | 5330439K02Rik | 29.06282 | 38.94445 | 34.00364 |
| ENSMUSG00000056310 | protein_coding | Tyw1 | 913.0917 | 992.2815 | 952.6866 |
| ENSMUSG00000036959 | protein_coding | Bcorl1 | 1488.577 | 1337.361 | 1412.969 |
| ENSMUSG00000030401 | protein_coding | Rtn2 | 157.3865 | 135.3645 | 146.3755 |
| ENSMUSG00000076437 | protein_coding | 2700094K13Rik | 1527.669 | 1673.74 | 1600.705 |
| ENSMUSG00000055826 | protein_coding | 1700008P20Rik | 21.76745 | 33.92491 | 27.84618 |
| ENSMUSG00000020651 | protein_coding | Slc26a4 | 4.207186 | 1.413083 | 2.810135 |
| ENSMUSG00000005980 | protein_coding | Dnase1 | 113.6775 | 95.33857 | 104.5081 |
| ENSMUSG00000009575 | protein_coding | Cbx5 | 5597.818 | 6136.754 | 5867.286 |
| ENSMUSG00000013611 | protein_coding | Snx31 | 38.00281 | 28.36086 | 33.18184 |
| ENSMUSG00000020610 | protein_coding | Amz2 | 1291.172 | 1187.134 | 1239.153 |
| ENSMUSG00000016933 | protein_coding | Plcg1 | 3350.968 | 3110.109 | 3230.538 |
| ENSMUSG00000027194 | protein_coding | Ttc17 | 1057.774 | 960.3964 | 1009.085 |
| ENSMUSG00000023018 | protein_coding | Smarcd1 | 1985.95 | 2186.558 | 2086.254 |
| ENSMUSG00000069255 | protein_coding | Dusp22 | 221.218 | 253.2365 | 237.2272 |
| ENSMUSG00000046314 | protein_coding | Stxbp6 | 1265.555 | 1403.81 | 1334.683 |
| ENSMUSG00000090445 | lincRNA | Gm17653 | 1.937283 | 4.88363 | 3.410456 |
| ENSMUSG00000072931 | protein_coding | Gm15080 | 35.82587 | 25.88715 | 30.85651 |
| ENSMUSG00000006457 | protein_coding | Actn3 | 353.1115 | 232.975 | 293.0432 |
| ENSMUSG00000085009 | processed_transcrip | Gm12977 | 1.943172 | 0.36219 | 1.152681 |

| | | | | | |
|---|---|---|---|---|---|
| ENSMUSG00000032079 | protein_coding | Apoa5 | 1.943172 | 0.36219 | 1.152681 |
| ENSMUSG00000044018 | protein_coding | Mrpl50 | 1220.665 | 1347.762 | 1284.213 |
| ENSMUSG00000087484 | lincRNA | 2900089D17Rik | 55.12236 | 70.29328 | 62.70782 |
| ENSMUSG00000043003 | protein_coding | Rasef | 88.80886 | 71.09228 | 79.95057 |
| ENSMUSG00000043867 | pseudogene | Foxl1 | 14.77898 | 22.3103 | 18.54464 |
| ENSMUSG00000020649 | protein_coding | Rrm2 | 1501.149 | 1358.923 | 1430.036 |
| ENSMUSG00000026343 | protein_coding | Gpr39 | 4.843207 | 9.119667 | 6.981437 |
| ENSMUSG00000020783 | protein_coding | 1200014J11Rik | 1945.665 | 2099.781 | 2022.723 |
| ENSMUSG00000091794 | protein_coding | Vmn2r18 | 2.597903 | 5.836506 | 4.217205 |
| ENSMUSG00000082605 | pseudogene | Gm16148 | 11.61781 | 17.76504 | 14.69143 |
| ENSMUSG00000037993 | protein_coding | Dhx38 | 1915.34 | 2165.621 | 2040.48 |
| ENSMUSG00000027007 | protein_coding | Ssfa2 | 1962.52 | 1760.887 | 1861.704 |
| ENSMUSG00000087876 | misc_RNA | 7SK | 1.951372 | 0.326514 | 1.138943 |
| ENSMUSG00000034675 | protein_coding | Dbn1 | 1334.526 | 1447.216 | 1390.871 |
| ENSMUSG00000024379 | protein_coding | Tslp | 3.236234 | 6.736051 | 4.986143 |
| ENSMUSG00000000168 | protein_coding | Dlat | 1518.284 | 1407.225 | 1462.754 |
| ENSMUSG00000029603 | protein_coding | Dtx1 | 3585.611 | 4018.904 | 3802.257 |
| ENSMUSG00000021123 | protein_coding | Rdh12 | 24.3195 | 15.78758 | 20.05354 |
| ENSMUSG00000072704 | protein_coding | 2700089E24Rik | 1274.665 | 1447.697 | 1361.181 |
| ENSMUSG00000083505 | pseudogene | Gm7541 | 2.925902 | 6.427194 | 4.676548 |
| ENSMUSG00000092182 | processed_transcrip | RP24-92P15.1 | 0.66062 | 2.746168 | 1.703394 |
| ENSMUSG00000027210 | protein_coding | Meis2 | 52.00093 | 64.68347 | 58.3422 |
| ENSMUSG00000048277 | protein_coding | Syngr2 | 973.5035 | 1078.366 | 1025.935 |
| ENSMUSG00000028293 | protein_coding | Slc35a1 | 855.1957 | 955.7434 | 905.4696 |
| ENSMUSG00000046750 | protein_coding | BC089491 | 10.33883 | 16.52749 | 13.43316 |
| ENSMUSG00000038205 | protein_coding | Prkab2 | 1010.578 | 911.7153 | 961.1468 |
| ENSMUSG00000080971 | pseudogene | Gm16128 | 0.976841 | 3.734358 | 2.3556 |
| ENSMUSG00000040380 | protein_coding | Cbln3 | 143.235 | 118.2889 | 130.762 |
| ENSMUSG00000036617 | protein_coding | Etl4 | 4431.822 | 4892.548 | 4662.185 |
| ENSMUSG00000037437 | protein_coding | Adam32 | 12.01036 | 18.94062 | 15.47549 |
| ENSMUSG00000040229 | protein_coding | Gpr34 | 69.91058 | 85.38786 | 77.64922 |
| ENSMUSG00000081330 | pseudogene | Gm13013 | 1.943172 | 0.335524 | 1.139348 |
| ENSMUSG00000020364 | protein_coding | Zfp354a | 108.4746 | 134.6096 | 121.5421 |
| ENSMUSG00000087690 | processed_transcrip | Gm16031 | 6.125759 | 11.1065 | 8.616131 |
| ENSMUSG00000084020 | pseudogene | Gm12282 | 1.609283 | 4.468108 | 3.038696 |
| ENSMUSG00000053141 | protein_coding | Ptprt | 147.2316 | 124.3013 | 135.7664 |
| ENSMUSG00000085254 | pseudogene | Gm15881 | 2.303971 | 0.36219 | 1.33308 |
| ENSMUSG00000078154 | protein_coding | Gm12184 | 198.1192 | 234.9471 | 216.5331 |
| ENSMUSG00000031644 | protein_coding | Nek1 | 3013.62 | 2640.849 | 2827.234 |
| ENSMUSG00000047603 | protein_coding | Zfp235 | 244.6148 | 283.8395 | 264.2272 |
| ENSMUSG00000042464 | protein_coding | Hoxd4 | 27.14067 | 38.67308 | 32.90688 |
| ENSMUSG00000030047 | protein_coding | Arhgap25 | 25.5539 | 17.27853 | 21.41622 |
| ENSMUSG00000084914 | lincRNA | Gm16786 | 1.943172 | 0.326514 | 1.134843 |
| ENSMUSG00000084951 | processed_transcrip | Gm15919 | 1.943172 | 0.326514 | 1.134843 |
| ENSMUSG00000051098 | protein_coding | Mblac2 | 694.8893 | 802.5424 | 748.7158 |
| ENSMUSG00000031323 | protein_coding | Dmrtc1a | 1270.453 | 935.5272 | 1102.99 |
| ENSMUSG00000069223 | processed_transcrip | 4930451E10Rik | 4.188476 | 8.185171 | 6.186823 |
| ENSMUSG00000034429 | protein_coding | Zfp707 | 605.9679 | 546.9275 | 576.4477 |
| ENSMUSG00000029192 | protein_coding | Tbc1d14 | 2375 | 2158.503 | 2266.751 |
| ENSMUSG00000085602 | lincRNA | 1500002F19Rik | 10.34472 | 5.765518 | 8.055121 |
| ENSMUSG00000028669 | protein_coding | 1110049F12Rik | 809.0259 | 907.6918 | 858.3589 |
| ENSMUSG00000030882 | protein_coding | Dnhd1 | 261.5347 | 316.8349 | 289.1848 |
| ENSMUSG00000014198 | protein_coding | Zfp385c | 19.41972 | 27.84116 | 23.63044 |
| ENSMUSG00000025225 | protein_coding | Nfkb2 | 273.9156 | 310.0812 | 291.9984 |
| ENSMUSG00000093003 | miRNA | AC182412.1 | 1.929083 | 0.36219 | 1.145636 |
| ENSMUSG00000006651 | protein_coding | Aplp1 | 259.9132 | 297.2639 | 278.5886 |
| ENSMUSG00000004317 | protein_coding | Clcn5 | 3301.93 | 3060.597 | 3181.264 |
| ENSMUSG00000079096 | protein_coding | Gm6309 | 7.173045 | 13.13979 | 10.15642 |

| | | | | | |
|---|---|---|---|---|---|
| ENSMUSG00000020257 | protein_coding | Wdr82 | 5885.523 | 6303.496 | 6094.51 |
| ENSMUSG00000050132 | protein_coding | Sarm1 | 61.43105 | 47.96823 | 54.69964 |
| ENSMUSG00000055013 | protein_coding | Agap1 | 1715.494 | 1576.193 | 1645.844 |
| ENSMUSG00000027868 | protein_coding | Tbx15 | 6.794579 | 11.64671 | 9.220644 |
| ENSMUSG00000055235 | protein_coding | Wdr86 | 68.52038 | 53.16833 | 60.84435 |
| ENSMUSG00000038457 | protein_coding | Fam70b | 8.088909 | 4.0879 | 6.088404 |
| ENSMUSG00000004508 | protein_coding | Gab2 | 2004.748 | 2271.459 | 2138.103 |
| ENSMUSG00000064264 | protein_coding | Zfp428 | 489.6933 | 440.1606 | 464.9269 |
| ENSMUSG00000038879 | protein_coding | Nipal2 | 112.4469 | 134.859 | 123.6529 |
| ENSMUSG00000026779 | protein_coding | Mastl | 972.2511 | 887.9503 | 930.1007 |
| ENSMUSG00000050087 | protein_coding | Cby3 | 1.290752 | 3.841746 | 2.566249 |
| ENSMUSG00000055424 | protein_coding | Gm9970 | 11.73514 | 6.234371 | 8.984756 |
| ENSMUSG00000042506 | protein_coding | Usp22 | 5766.014 | 6254.978 | 6010.496 |
| ENSMUSG00000092257 | pseudogene | Gm2308 | 1.931394 | 0.335524 | 1.133459 |
| ENSMUSG00000060961 | protein_coding | Slc4a4 | 119.6395 | 141.9648 | 130.8021 |
| ENSMUSG00000020299 | protein_coding | 4930524B15Rik | 182.2286 | 105.025 | 143.6268 |
| ENSMUSG00000009894 | protein_coding | Snap47 | 2119.592 | 1972.138 | 2045.865 |
| ENSMUSG00000052609 | protein_coding | Plekhg3 | 524.3694 | 590.1128 | 557.2411 |
| ENSMUSG00000060924 | protein_coding | Csmd1 | 5.475649 | 9.888731 | 7.68219 |
| ENSMUSG00000084983 | processed_transcrip | Gm11789 | 4.502386 | 1.712931 | 3.107659 |
| ENSMUSG00000030199 | protein_coding | Etv6 | 1443.162 | 1584.958 | 1514.06 |
| ENSMUSG00000044702 | protein_coding | Palb2 | 740.5472 | 673.3362 | 706.9417 |
| ENSMUSG00000007030 | protein_coding | D17H6S56E-3 | 30.80711 | 21.82628 | 26.31669 |
| ENSMUSG00000028772 | protein_coding | Zcchc17 | 963.1353 | 888.0334 | 925.5844 |
| ENSMUSG00000032258 | protein_coding | Lca5 | 400.5555 | 349.5648 | 375.0602 |
| ENSMUSG00000028879 | protein_coding | Stx12 | 3491.646 | 3766.697 | 3629.171 |
| ENSMUSG00000059468 | protein_coding | Gm4891 | 1.929083 | 0.326514 | 1.127799 |
| ENSMUSG00000090987 | protein_coding | Gm4781 | 22.1896 | 31.03532 | 26.61246 |
| ENSMUSG00000003974 | protein_coding | Grm3 | 18.05286 | 11.41785 | 14.73535 |
| ENSMUSG00000002055 | protein_coding | Spag5 | 1101.32 | 994.2369 | 1047.779 |
| ENSMUSG00000089817 | pseudogene | Gm7162 | 9.358415 | 15.29278 | 12.3256 |
| ENSMUSG00000080901 | pseudogene | Gm6215 | 248.7127 | 195.9624 | 222.3375 |
| ENSMUSG00000038987 | protein_coding | 1700019L03Rik | 45.14962 | 59.95983 | 52.55473 |
| ENSMUSG00000040795 | protein_coding | Iqcc | 221.3122 | 257.2819 | 239.297 |
| ENSMUSG00000022965 | protein_coding | Ifngr2 | 3941.923 | 4264.704 | 4103.313 |
| ENSMUSG00000065817 | snoRNA | SNORA3 | 5.495627 | 2.401635 | 3.948631 |
| ENSMUSG00000021823 | protein_coding | Vcl | 4538.409 | 5008.219 | 4773.314 |
| ENSMUSG00000058756 | protein_coding | Thra | 5036.159 | 5491.507 | 5263.833 |
| ENSMUSG00000031444 | protein_coding | F10 | 3.258523 | 1.024227 | 2.141375 |
| ENSMUSG00000079705 | protein_coding | Ssxb1 | 3.258523 | 1.024227 | 2.141375 |
| ENSMUSG00000083524 | pseudogene | Mup-ps21 | 1.923194 | 0.335524 | 1.129359 |
| ENSMUSG00000008167 | protein_coding | Fbxw9 | 747.5842 | 683.7486 | 715.6664 |
| ENSMUSG00000027472 | protein_coding | Pdrg1 | 1488.22 | 1631.269 | 1559.745 |
| ENSMUSG00000023932 | protein_coding | Cdc5l | 4022.144 | 4307.237 | 4164.691 |
| ENSMUSG00000028347 | protein_coding | Tmeff1 | 3628.079 | 4025.541 | 3826.81 |
| ENSMUSG00000028692 | protein_coding | Akr1a4 | 6569.981 | 7032.279 | 6801.13 |
| ENSMUSG00000034685 | protein_coding | Fam171a2 | 111.8038 | 137.9389 | 124.8713 |
| ENSMUSG00000009549 | protein_coding | Srp14 | 2520.505 | 2322.987 | 2421.746 |
| ENSMUSG00000088752 | miRNA | Mir1934 | 4.877274 | 9.227055 | 7.052165 |
| ENSMUSG00000025016 | protein_coding | Tm9sf3 | 7711.101 | 8234.047 | 7972.574 |
| ENSMUSG00000050783 | protein_coding | Htr1f | 0 | 1.704284 | 0.852142 |
| ENSMUSG00000030770 | protein_coding | Parva | 1662.613 | 1804.159 | 1733.386 |
| ENSMUSG00000020899 | protein_coding | Pfas | 1860.032 | 2012.62 | 1936.326 |
| ENSMUSG00000056445 | processed_transcrip | 5730446D14Rik | 1.923194 | 0.326514 | 1.124854 |
| ENSMUSG00000082988 | pseudogene | Gm5903 | 337.8083 | 299.0741 | 318.4412 |
| ENSMUSG00000052914 | protein_coding | Cyp2j6 | 285.9192 | 335.0035 | 310.4613 |
| ENSMUSG00000087490 | lincRNA | A330076H08Rik | 37.26422 | 51.21354 | 44.23888 |
| ENSMUSG00000001444 | protein_coding | Tbx21 | 13.5246 | 8.104449 | 10.81453 |

| | | | | | |
|---|---|---|---|---|---|
| ENSMUSG00000042485 | protein_coding | Mustn1 | 23.94312 | 33.11116 | 28.52714 |
| ENSMUSG00000083626 | pseudogene | Gm4459 | 58.78962 | 46.62795 | 52.70878 |
| ENSMUSG00000086202 | processed_transcrip | 1700019E08Rik | 11.66492 | 18.76297 | 15.21394 |
| ENSMUSG00000029517 | protein_coding | Ankrd7 | 7.532576 | 3.099348 | 5.315962 |
| ENSMUSG00000092475 | processed_transcrip | RP23-329P9.11 | 0 | 1.659599 | 0.8298 |
| ENSMUSG00000020413 | protein_coding | Hus1 | 939.6791 | 1028.713 | 984.1959 |
| ENSMUSG00000052675 | protein_coding | Zfp112 | 123.32 | 147.1003 | 135.2102 |
| ENSMUSG00000030652 | protein_coding | Coq7 | 1195.075 | 1093.702 | 1144.388 |
| ENSMUSG00000039391 | protein_coding | Ccdc81 | 15.26531 | 22.89519 | 19.08025 |
| ENSMUSG00000027502 | protein_coding | 2410001C21Rik | 1804.839 | 1951.139 | 1877.989 |
| ENSMUSG00000007603 | protein_coding | Dus3l | 1283.955 | 1169.838 | 1226.897 |
| ENSMUSG00000026179 | protein_coding | Pnkd | 4347.345 | 4703.027 | 4525.186 |
| ENSMUSG00000089317 | snoRNA | Snord123 | 3.902744 | 7.991987 | 5.947365 |
| ENSMUSG00000039706 | protein_coding | Ldb2 | 310.3845 | 348.9733 | 329.6789 |
| ENSMUSG00000033220 | protein_coding | Rac2 | 75.93839 | 92.25289 | 84.09564 |
| ENSMUSG00000020183 | protein_coding | Cpm | 87.69119 | 114.097 | 100.8941 |
| ENSMUSG00000031147 | protein_coding | Magix | 9.358415 | 14.96591 | 12.16216 |
| ENSMUSG00000059229 | protein_coding | Gm6802 | 6.10347 | 2.674817 | 4.389144 |
| ENSMUSG00000075077 | protein_coding | Olfr1251 | 8.74364 | 14.92443 | 11.83404 |
| ENSMUSG00000086773 | processed_transcrip | Gm16192 | 1.598773 | 4.414414 | 3.006594 |
| ENSMUSG00000022130 | protein_coding | Tgds | 346.555 | 389.8628 | 368.2089 |
| ENSMUSG00000032773 | protein_coding | Chrm1 | 10.34472 | 5.801193 | 8.072958 |
| ENSMUSG00000031708 | protein_coding | Tecr | 2923.259 | 3156.081 | 3039.67 |
| ENSMUSG00000034145 | protein_coding | Tmem63c | 73.38006 | 55.53605 | 64.45806 |
| ENSMUSG00000017724 | protein_coding | Etv4 | 178.147 | 207.7561 | 192.9516 |
| ENSMUSG00000001642 | protein_coding | Akr1b3 | 11893.03 | 12856.45 | 12374.74 |
| ENSMUSG00000034295 | protein_coding | Fhod3 | 111.7769 | 133.758 | 122.7674 |
| ENSMUSG00000027694 | protein_coding | Gm8325 | 148.6314 | 126.3995 | 137.5155 |
| ENSMUSG00000058444 | protein_coding | Map2k5 | 623.5474 | 570.8558 | 597.2016 |
| ENSMUSG00000081208 | pseudogene | Gm11400 | 34.30216 | 24.60884 | 29.4555 |
| ENSMUSG00000039578 | protein_coding | Fam190a | 10.65506 | 6.136355 | 8.395705 |
| ENSMUSG00000049321 | protein_coding | Zfp2 | 196.8352 | 229.9629 | 213.399 |
| ENSMUSG00000027077 | protein_coding | Smtnl1 | 4.532875 | 8.565017 | 6.548946 |
| ENSMUSG00000013921 | protein_coding | Clip3 | 993.2127 | 1090.567 | 1041.89 |
| ENSMUSG00000034729 | protein_coding | Mrps10 | 1157.955 | 1061.332 | 1109.643 |
| ENSMUSG00000037260 | protein_coding | Hgsnat | 1554.101 | 1738.676 | 1646.389 |
| ENSMUSG00000022711 | protein_coding | Pmm2 | 765.7139 | 830.1681 | 797.941 |
| ENSMUSG00000051727 | protein_coding | Kctd14 | 3768.875 | 5205.184 | 4487.029 |
| ENSMUSG00000024525 | protein_coding | Impa2 | 331.7791 | 371.8231 | 351.8011 |
| ENSMUSG00000032864 | protein_coding | Rag2 | 2.262971 | 0.36219 | 1.312581 |
| ENSMUSG00000070424 | protein_coding | Art5 | 60.36379 | 47.94193 | 54.15286 |
| ENSMUSG00000082935 | pseudogene | Gm7658 | 58.82622 | 77.90982 | 68.36802 |
| ENSMUSG00000064602 | snoRNA | Snora41 | 5.520227 | 10.56951 | 8.044869 |
| ENSMUSG00000037426 | protein_coding | Depdc5 | 2291.33 | 1993.328 | 2142.329 |
| ENSMUSG00000085354 | processed_transcrip | Gm2044 | 1795.867 | 2498.965 | 2147.416 |
| ENSMUSG00000029454 | protein_coding | Mapkapk5 | 1321.359 | 1184.326 | 1252.842 |
| ENSMUSG00000025486 | protein_coding | Sirt3 | 625.8168 | 569.7508 | 597.7838 |
| ENSMUSG00000051136 | protein_coding | Ghsr | 0 | 1.65059 | 0.825295 |
| ENSMUSG00000072188 | protein_coding | Gm10354 | 9.336126 | 5.130146 | 7.233136 |
| ENSMUSG00000083111 | pseudogene | Gm14421 | 15.79451 | 23.42711 | 19.61081 |
| ENSMUSG00000018830 | protein_coding | Myh11 | 4614.308 | 4195.772 | 4405.04 |
| ENSMUSG00000035498 | protein_coding | Cdcp1 | 13.50105 | 20.62689 | 17.06397 |
| ENSMUSG00000086330 | lincRNA | 1700007J10Rik | 30.97886 | 43.41945 | 37.19915 |
| ENSMUSG00000028614 | protein_coding | Tmem48 | 1937.218 | 2094.587 | 2015.902 |
| ENSMUSG00000083678 | pseudogene | Gm12989 | 138.644 | 162.2789 | 150.4614 |
| ENSMUSG00000000555 | protein_coding | Itga5 | 250.3245 | 282.9917 | 266.6581 |
| ENSMUSG00000028896 | protein_coding | Rcc1 | 1175.08 | 1302.798 | 1238.939 |
| ENSMUSG00000034723 | protein_coding | Tmx4 | 3183.537 | 2922.551 | 3053.044 |

| | | | | | |
|---|---|---|---|---|---|
| ENSMUSG00000027379 | protein_coding | Bub1 | 3820.22 | 2864.095 | 3342.157 |
| ENSMUSG00000069083 | pseudogene | Gm10259 | 16.82412 | 10.61311 | 13.71862 |
| ENSMUSG00000039021 | protein_coding | Ttc16 | 53.76459 | 35.77329 | 44.76894 |
| ENSMUSG00000025860 | protein_coding | Xiap | 2807.365 | 3091.813 | 2949.589 |
| ENSMUSG00000085921 | processed_transcrip | Gm13752 | 1.923194 | 4.839307 | 3.38125 |
| ENSMUSG00000029138 | protein_coding | 4930548H24Rik | 3.597033 | 1.050893 | 2.323963 |
| ENSMUSG00000003283 | protein_coding | Hck | 196.6516 | 163.9576 | 180.3046 |
| ENSMUSG00000050189 | protein_coding | Etos1 | 0.960442 | 3.345502 | 2.152972 |
| ENSMUSG00000039033 | protein_coding | Tasp1 | 540.1708 | 489.0876 | 514.6292 |
| ENSMUSG00000016534 | protein_coding | Lamp2 | 6091.553 | 6612.656 | 6352.104 |
| ENSMUSG00000037922 | protein_coding | Bank1 | 23.90339 | 33.09386 | 28.49863 |
| ENSMUSG00000042807 | protein_coding | Hecw2 | 437.553 | 393.516 | 415.5345 |
| ENSMUSG00000050243 | pseudogene | Gm5446 | 20.96802 | 29.09425 | 25.03114 |
| ENSMUSG00000027201 | protein_coding | Myef2 | 4235.885 | 4707.293 | 4471.589 |
| ENSMUSG00000087014 | processed_transcrip | Gm16364 | 58.53922 | 73.33097 | 65.93509 |
| ENSMUSG00000081719 | pseudogene | Gm13726 | 0.33031 | 2.093139 | 1.211725 |
| ENSMUSG00000038965 | protein_coding | Ube2l3 | 1708.933 | 1867.167 | 1788.05 |
| ENSMUSG00000075256 | protein_coding | Cerkl | 23.9139 | 16.73217 | 20.32304 |
| ENSMUSG00000024366 | protein_coding | Gfra3 | 80.06208 | 64.35266 | 72.20737 |
| ENSMUSG00000064998 | snRNA | U6 | 1.959572 | 0.335524 | 1.147548 |
| ENSMUSG00000051732 | protein_coding | Pabpc2 | 1.959572 | 0.335524 | 1.147548 |
| ENSMUSG00000057649 | protein_coding | Brd9 | 1466.385 | 1347.081 | 1406.733 |
| ENSMUSG00000006740 | protein_coding | Kif5b | 9777.366 | 10823.03 | 10300.2 |
| ENSMUSG00000042790 | protein_coding | Rnf214 | 1215.731 | 1076.306 | 1146.018 |
| ENSMUSG00000039079 | protein_coding | Trhr2 | 0.66062 | 2.781843 | 1.721232 |
| ENSMUSG00000026069 | protein_coding | Il1rl1 | 13.42113 | 7.071936 | 10.24653 |
| ENSMUSG00000086877 | lincRNA | A230072C01Rik | 121.5485 | 148.5126 | 135.0306 |
| ENSMUSG00000058835 | protein_coding | Abi1 | 4090.373 | 4459.744 | 4275.059 |
| ENSMUSG00000068663 | protein_coding | Clec16a | 1130.676 | 1039.606 | 1085.141 |
| ENSMUSG00000073129 | protein_coding | Gm10472 | 1.959572 | 0.326514 | 1.143043 |
| ENSMUSG00000024042 | protein_coding | Sik1 | 1256.151 | 1109.23 | 1182.691 |
| ENSMUSG00000015090 | protein_coding | Ptgds | 49.86178 | 70.16471 | 60.01324 |
| ENSMUSG00000027314 | protein_coding | Dll4 | 88.15688 | 105.2143 | 96.68559 |
| ENSMUSG00000074596 | protein_coding | Spint3 | 96.32503 | 116.3482 | 106.3366 |
| ENSMUSG00000032519 | protein_coding | Slc25a38 | 430.1251 | 473.6192 | 451.8721 |
| ENSMUSG00000050050 | protein_coding | Ccdc158 | 486.9558 | 392.4884 | 439.7221 |
| ENSMUSG00000081852 | pseudogene | Gm12281 | 0.316221 | 2.093139 | 1.20468 |
| ENSMUSG00000021636 | protein_coding | Marveld2 | 435.5212 | 501.6136 | 468.5674 |
| ENSMUSG00000006288 | protein_coding | Ttc5 | 1367.343 | 1490.732 | 1429.038 |
| ENSMUSG00000087263 | processed_transcrip | Gm15726 | 23.98793 | 15.31981 | 19.65387 |
| ENSMUSG00000019810 | protein_coding | Fuca2 | 1015.747 | 1115.097 | 1065.422 |
| ENSMUSG00000081738 | pseudogene | Hmgb1-ps2 | 108.6313 | 90.52915 | 99.5802 |
| ENSMUSG00000040123 | protein_coding | Zmym5 | 3186.753 | 2983.765 | 3085.259 |
| ENSMUSG00000086924 | processed_transcrip | Gm11766 | 54.23639 | 38.75697 | 46.49668 |
| ENSMUSG00000073455 | protein_coding | Gm3435 | 24.89917 | 34.84755 | 29.87336 |
| ENSMUSG00000031990 | protein_coding | Jam3 | 3354.966 | 3638.348 | 3496.657 |
| ENSMUSG00000033126 | protein_coding | Ybey | 176.1857 | 152.478 | 164.3319 |
| ENSMUSG00000025959 | protein_coding | Klf7 | 601.3262 | 537.7195 | 569.5229 |
| ENSMUSG00000043557 | protein_coding | Mdga1 | 95.76706 | 78.35052 | 87.05879 |
| ENSMUSG00000028992 | protein_coding | Nmnat1 | 178.4951 | 206.3711 | 192.4331 |
| ENSMUSG00000025626 | protein_coding | Phf6 | 1024.287 | 1151.383 | 1087.835 |
| ENSMUSG00000004040 | protein_coding | Stat3 | 2144.764 | 2413.039 | 2278.901 |
| ENSMUSG00000036800 | protein_coding | Fam135b | 15.89313 | 23.24837 | 19.57075 |
| ENSMUSG00000027709 | protein_coding | Mccc1 | 648.4434 | 720.8368 | 684.6401 |
| ENSMUSG00000050627 | protein_coding | Gpd1l | 692.0553 | 760.0525 | 726.0539 |
| ENSMUSG00000035397 | protein_coding | Klf16 | 924.9329 | 845.7374 | 885.3352 |
| ENSMUSG00000043029 | protein_coding | Trpv3 | 7.153067 | 12.39775 | 9.77541 |
| ENSMUSG00000038534 | protein_coding | Osbpl7 | 622.5324 | 683.3405 | 652.9364 |

| | | | | | |
|---|---|---|---|---|---|
| ENSMUSG00000071506 | protein_coding | Tmem139 | 19.67854 | 13.25261 | 16.46558 |
| ENSMUSG00000024462 | protein_coding | Gabbr1 | 430.1827 | 507.3981 | 468.7904 |
| ENSMUSG00000068218 | protein_coding | Ssxb9 | 3.860477 | 1.324076 | 2.592276 |
| ENSMUSG00000050197 | protein_coding | Rhox13 | 588.7127 | 516.9634 | 552.838 |
| ENSMUSG00000081309 | pseudogene | Gm12397 | 58.05393 | 45.9147 | 51.98432 |
| ENSMUSG00000027009 | protein_coding | Itga4 | 230.3418 | 285.1748 | 257.7583 |
| ENSMUSG00000075573 | protein_coding | Defb47 | 8.792839 | 4.45009 | 6.621464 |
| ENSMUSG00000032640 | protein_coding | Chsy1 | 669.8315 | 601.2518 | 635.5416 |
| ENSMUSG00000069274 | protein_coding | Hist1h4f | 0.66062 | 2.763824 | 1.712222 |
| ENSMUSG00000065408 | miRNA | Mir31 | 8.057153 | 13.27892 | 10.66804 |
| ENSMUSG00000035392 | protein_coding | Dennd1a | 1020.444 | 874.2435 | 947.3436 |
| ENSMUSG00000029364 | protein_coding | Wsb2 | 4040.726 | 4341.39 | 4191.058 |
| ENSMUSG00000082127 | pseudogene | Gm13577 | 217.5646 | 191.297 | 204.4308 |
| ENSMUSG00000062762 | protein_coding | Ei24 | 2592.607 | 2404.413 | 2498.51 |
| ENSMUSG00000038587 | protein_coding | Akap12 | 9497.788 | 8268.28 | 8883.034 |
| ENSMUSG00000062646 | protein_coding | Ganc | 447.0542 | 393.1055 | 420.0799 |
| ENSMUSG00000090210 | protein_coding | Itga10 | 35.3528 | 48.82055 | 42.08667 |
| ENSMUSG00000075525 | pseudogene | Gm10849 | 2.939991 | 0.688704 | 1.814348 |
| ENSMUSG00000068806 | protein_coding | Olfr1259 | 6.125759 | 11.0917 | 8.608728 |
| ENSMUSG00000010517 | protein_coding | Faf1 | 2398.158 | 2163.18 | 2280.669 |
| ENSMUSG00000090438 | lincRNA | Gm17454 | 1.923194 | 4.812641 | 3.367917 |
| ENSMUSG00000072738 | protein_coding | Gm16440 | 0 | 1.668247 | 0.834123 |
| ENSMUSG00000029546 | protein_coding | Uncx | 4.198987 | 8.680329 | 6.439658 |
| ENSMUSG00000018844 | protein_coding | Fndc8 | 2.275793 | 5.456298 | 3.866046 |
| ENSMUSG00000006390 | protein_coding | Elovl1 | 507.7268 | 556.5265 | 532.1266 |
| ENSMUSG00000092001 | protein_coding | Tmem200a | 2.227637 | 0.36219 | 1.294913 |
| ENSMUSG00000032422 | protein_coding | Snx14 | 1356.441 | 1475.355 | 1415.898 |
| ENSMUSG00000033852 | protein_coding | Pla2g4b | 936.9058 | 1036.345 | 986.6255 |
| ENSMUSG00000019699 | protein_coding | Akt3 | 2050.324 | 1870.184 | 1960.254 |
| ENSMUSG00000087081 | lincRNA | 6430590A07Rik | 31.7873 | 21.98627 | 26.88679 |
| ENSMUSG00000029918 | protein_coding | Mrps33 | 579.461 | 644.173 | 611.817 |
| ENSMUSG00000020463 | protein_coding | Smek2 | 4591.019 | 4982.954 | 4786.987 |
| ENSMUSG00000087264 | processed_transcrip | 1500002O10Rik | 2.891835 | 6.216714 | 4.554275 |
| ENSMUSG00000044034 | protein_coding | Npb | 5.46976 | 2.357312 | 3.913536 |
| ENSMUSG00000064090 | protein_coding | Vrk2 | 219.025 | 189.8775 | 204.4513 |
| ENSMUSG00000014426 | protein_coding | Map3k4 | 1335.063 | 1204.46 | 1269.762 |
| ENSMUSG00000032377 | protein_coding | Plscr4 | 197.426 | 237.4352 | 217.4306 |
| ENSMUSG00000054514 | processed_transcrip | 2610204G22Rik | 25.25385 | 17.42124 | 21.33754 |
| ENSMUSG00000085368 | processed_transcrip | Gm16188 | 2.597903 | 0.688704 | 1.643303 |
| ENSMUSG00000031292 | protein_coding | Cdkl5 | 313.275 | 267.2614 | 290.2682 |
| ENSMUSG00000023393 | protein_coding | Slc17a9 | 18.45823 | 26.84324 | 22.65074 |
| ENSMUSG00000031875 | protein_coding | Cmtm3 | 1943.791 | 2165.423 | 2054.607 |
| ENSMUSG00000043510 | protein_coding | Hscb | 573.2948 | 521.4407 | 547.3678 |
| ENSMUSG00000082374 | pseudogene | Gm12741 | 0.33031 | 2.066111 | 1.198211 |
| ENSMUSG00000033268 | protein_coding | Duox1 | 60.70424 | 44.19168 | 52.44796 |
| ENSMUSG00000021279 | protein_coding | Cdc42bpb | 5967.009 | 5266.827 | 5616.918 |
| ENSMUSG00000043207 | protein_coding | Zmpste24 | 1957.72 | 2111.083 | 2034.401 |
| ENSMUSG00000032118 | protein_coding | Fez1 | 166.0117 | 193.1509 | 179.5813 |
| ENSMUSG00000090291 | protein_coding | Lrrc10b | 11.70592 | 6.45386 | 9.07989 |
| ENSMUSG00000084757 | processed_transcrip | 1700057H15Rik | 1.934972 | 0.326514 | 1.130743 |
| ENSMUSG00000019866 | protein_coding | Aim1 | 382.7921 | 452.641 | 417.7166 |
| ENSMUSG00000025727 | protein_coding | A930017K11Rik | 24.1927 | 33.6204 | 28.90655 |
| ENSMUSG00000032528 | protein_coding | Vipr1 | 43.3126 | 32.14783 | 37.73021 |
| ENSMUSG00000091657 | protein_coding | Gm3072 | 0.32211 | 2.066111 | 1.194111 |
| ENSMUSG00000028996 | protein_coding | Rbp7 | 7.108489 | 11.91088 | 9.509685 |
| ENSMUSG00000025650 | protein_coding | Col7a1 | 83.98718 | 112.8757 | 98.43146 |
| ENSMUSG00000019461 | protein_coding | Plscr3 | 751.9934 | 824.2943 | 788.1438 |
| ENSMUSG00000053693 | protein_coding | Mast1 | 66.89746 | 81.78363 | 74.34054 |

| | | | | | |
|---|---|---|---|---|---|
| ENSMUSG00000000439 | protein_coding | Mkrn2 | 1305.998 | 1207.826 | 1256.912 |
| ENSMUSG00000035765 | protein_coding | Dym | 1000.481 | 919.7289 | 960.1052 |
| ENSMUSG00000061981 | protein_coding | Flot2 | 2057.215 | 1910.33 | 1983.773 |
| ENSMUSG00000004031 | protein_coding | Fam5b | 56.01907 | 42.88893 | 49.454 |
| ENSMUSG00000037996 | protein_coding | Slc24a2 | 269.9357 | 237.371 | 253.6534 |
| ENSMUSG00000024353 | protein_coding | 2010001M09Rik | 10.36008 | 5.448012 | 7.904046 |
| ENSMUSG00000056544 | protein_coding | Defb21 | 1.917305 | 0.36219 | 1.139747 |
| ENSMUSG00000030994 | protein_coding | D7Ertd443e | 2.259393 | 5.121137 | 3.690265 |
| ENSMUSG00000054863 | protein_coding | Fam19a5 | 1264.817 | 1420.427 | 1342.622 |
| ENSMUSG00000024697 | protein_coding | Gna14 | 1439.386 | 1574.27 | 1506.828 |
| ENSMUSG00000022952 | protein_coding | Runx1 | 76.10745 | 60.89827 | 68.50286 |
| ENSMUSG00000067925 | protein_coding | Cxx1a | 142.798 | 165.4124 | 154.1052 |
| ENSMUSG00000045160 | protein_coding | Bola3 | 461.5523 | 409.8677 | 435.71 |
| ENSMUSG00000031935 | protein_coding | Med17 | 2789.547 | 2611.12 | 2700.333 |
| ENSMUSG00000054779 | lincRNA | Gm12541 | 12.23385 | 18.41807 | 15.32596 |
| ENSMUSG00000038604 | protein_coding | Fam65a | 1339.338 | 1476.75 | 1408.044 |
| ENSMUSG00000093350 | miRNA | AC118603.1 | 0.33031 | 2.146471 | 1.238391 |
| ENSMUSG00000029510 | protein_coding | Gpc2 | 635.3648 | 563.9511 | 599.658 |
| ENSMUSG00000025058 | protein_coding | 5430427O19Rik | 2.567414 | 5.73849 | 4.152952 |
| ENSMUSG00000035649 | protein_coding | Zcchc7 | 2150.135 | 2391.289 | 2270.712 |
| ENSMUSG00000006611 | protein_coding | Hfe | 760.907 | 860.8172 | 810.8621 |
| ENSMUSG00000064137 | protein_coding | Rhox8 | 2123.38 | 2340.984 | 2232.182 |
| ENSMUSG00000063286 | pseudogene | Gm8995 | 3.874566 | 7.549438 | 5.712002 |
| ENSMUSG00000072683 | processed_transcrip | Gm7457 | 1.284863 | 4.388472 | 2.836667 |
| ENSMUSG00000026933 | protein_coding | Camsap1 | 3570.694 | 3279.484 | 3425.089 |
| ENSMUSG00000039879 | protein_coding | Heca | 1739.236 | 1613.742 | 1676.489 |
| ENSMUSG00000025348 | protein_coding | Itga7 | 313.6464 | 273.2302 | 293.4383 |
| ENSMUSG00000038335 | protein_coding | Tsr1 | 1580.828 | 1726.717 | 1653.772 |
| ENSMUSG00000036110 | protein_coding | Slc17a2 | 1.293062 | 3.80607 | 2.549566 |
| ENSMUSG00000066595 | protein_coding | Mfsd7b | 128.0735 | 107.1294 | 117.6015 |
| ENSMUSG00000035285 | protein_coding | Nat14 | 111.9583 | 94.3248 | 103.1415 |
| ENSMUSG00000071054 | protein_coding | Safb | 4262.759 | 3964.616 | 4113.687 |
| ENSMUSG00000023921 | protein_coding | Mut | 1283.465 | 1412.727 | 1348.096 |
| ENSMUSG00000027966 | protein_coding | Col11a1 | 147.074 | 177.4268 | 162.2504 |
| ENSMUSG00000078967 | protein_coding | Gm3272 | 1.917305 | 0.335524 | 1.126414 |
| ENSMUSG00000014444 | protein_coding | Fam38a | 1727.947 | 1907.784 | 1817.866 |
| ENSMUSG00000026248 | protein_coding | Mrpl44 | 835.7967 | 915.1654 | 875.481 |
| ENSMUSG00000024939 | protein_coding | Fam89b | 1.606973 | 4.123575 | 2.865274 |
| ENSMUSG00000022012 | protein_coding | Enox1 | 260.0766 | 228.3162 | 244.1964 |
| ENSMUSG00000002980 | protein_coding | Bcam | 434.327 | 494.9809 | 464.6539 |
| ENSMUSG00000047037 | protein_coding | Nipa1 | 383.8129 | 346.5669 | 365.1899 |
| ENSMUSG00000091240 | protein_coding | Gm9182 | 16.12019 | 23.15973 | 19.63996 |
| ENSMUSG00000027399 | protein_coding | Il1a | 11.75743 | 6.216714 | 8.987072 |
| ENSMUSG00000042854 | protein_coding | Trp53rk | 194.362 | 226.9334 | 210.6477 |
| ENSMUSG00000018442 | protein_coding | Derl2 | 1382.645 | 1494.606 | 1438.626 |
| ENSMUSG00000086461 | lincRNA | Gm16899 | 59.5335 | 73.77968 | 66.65659 |
| ENSMUSG00000037977 | protein_coding | 6430571L13Rik | 53.64047 | 40.87035 | 47.25541 |
| ENSMUSG00000037904 | protein_coding | Ankrd9 | 139.2294 | 108.5603 | 123.8948 |
| ENSMUSG00000002833 | protein_coding | Hdgfrp2 | 2171.908 | 1897.84 | 2034.874 |
| ENSMUSG00000012296 | protein_coding | Tjap1 | 1196.099 | 1106.897 | 1151.498 |
| ENSMUSG00000050824 | protein_coding | Sstr5 | 15.51235 | 22.39895 | 18.95565 |
| ENSMUSG00000081355 | pseudogene | Gm15264 | 17.43577 | 10.68446 | 14.06012 |
| ENSMUSG00000030058 | protein_coding | Copg | 3252.375 | 3026.887 | 3139.631 |
| ENSMUSG00000065127 | snRNA | U6 | 0 | 1.386417 | 0.693208 |
| ENSMUSG00000087742 | snoRNA | SNORA17 | 0 | 1.386417 | 0.693208 |
| ENSMUSG00000029788 | protein_coding | Cpa5 | 0 | 1.386417 | 0.693208 |
| ENSMUSG00000081343 | pseudogene | Gm16071 | 0 | 1.386417 | 0.693208 |
| ENSMUSG00000062873 | protein_coding | Olfr1355 | 0 | 1.386417 | 0.693208 |

| | | | | | |
|---|---|---|---|---|---|
| ENSMUSG00000082626 | pseudogene | Gm12270 | 0 | 1.386417 | 0.693208 |
| ENSMUSG00000084072 | pseudogene | Gm11285 | 0 | 1.386417 | 0.693208 |
| ENSMUSG00000085568 | lincRNA | Gm14858 | 42.43416 | 55.70899 | 49.07157 |
| ENSMUSG00000032894 | protein_coding | 1700031F05Rik | 32.99374 | 44.46165 | 38.7277 |
| ENSMUSG00000086929 | processed_transcrip | Gm11788 | 4.585653 | 9.307053 | 6.946353 |
| ENSMUSG00000048967 | protein_coding | Yjefn3 | 34.61354 | 45.77381 | 40.19367 |
| ENSMUSG00000024856 | protein_coding | Cdk2ap2 | 969.988 | 889.221 | 929.6045 |
| ENSMUSG00000036980 | protein_coding | Taf6 | 2451.7 | 2256.472 | 2354.086 |
| ENSMUSG00000040165 | protein_coding | Cd209c | 0.316221 | 2.137462 | 1.226842 |
| ENSMUSG00000045672 | protein_coding | Col27a1 | 127.6826 | 152.9422 | 140.3124 |
| ENSMUSG00000020021 | protein_coding | Fgd6 | 1938.394 | 1676.976 | 1807.685 |
| ENSMUSG00000024912 | protein_coding | Fosl1 | 16.78081 | 24.25422 | 20.51752 |
| ENSMUSG00000057764 | pseudogene | Rpl35a-ps7 | 4.843207 | 2.057464 | 3.450335 |
| ENSMUSG00000070000 | protein_coding | Fcho1 | 317.4244 | 284.8322 | 301.1283 |
| ENSMUSG00000047228 | protein_coding | BC048546 | 32.28728 | 23.89239 | 28.08983 |
| ENSMUSG00000019873 | protein_coding | Reep3 | 658.1616 | 756.4139 | 707.2878 |
| ENSMUSG00000064310 | protein_coding | Zpld1 | 0 | 1.641581 | 0.82079 |
| ENSMUSG00000047150 | protein_coding | 1700001C19Rik | 40.39027 | 30.49475 | 35.44251 |
| ENSMUSG00000020741 | protein_coding | 1300001I01Rik | 2924.967 | 2696.499 | 2810.733 |
| ENSMUSG00000021917 | protein_coding | Spcs1 | 1583.684 | 1736.316 | 1660 |
| ENSMUSG00000027177 | protein_coding | Hipk3 | 3816.597 | 3543.282 | 3679.94 |
| ENSMUSG00000024982 | protein_coding | Zdhhc6 | 1191.461 | 1282.995 | 1237.228 |
| ENSMUSG00000084128 | protein_coding | Esrp2 | 53.2553 | 66.33986 | 59.79758 |
| ENSMUSG00000074811 | protein_coding | Hps6 | 254.5742 | 218.0272 | 236.3007 |
| ENSMUSG00000063687 | protein_coding | Pcdhb5 | 12.60054 | 18.70927 | 15.65491 |
| ENSMUSG00000021820 | protein_coding | Camk2g | 1576.485 | 1459.323 | 1517.904 |
| ENSMUSG00000021280 | protein_coding | 1200009I06Rik | 34.55718 | 44.57048 | 39.56383 |
| ENSMUSG00000057329 | protein_coding | Bcl2 | 219.7695 | 255.5305 | 237.65 |
| ENSMUSG00000022945 | protein_coding | Chaf1b | 508.9622 | 450.6397 | 479.8009 |
| ENSMUSG00000086552 | lincRNA | A730090H04Rik | 7.442378 | 12.70625 | 10.07431 |
| ENSMUSG00000087600 | processed_transcrip | Gm14454 | 3.527856 | 7.433402 | 5.480629 |
| ENSMUSG00000031167 | protein_coding | Rbm3 | 4626.719 | 5057.802 | 4842.26 |
| ENSMUSG00000071646 | protein_coding | Mta2 | 4250.379 | 4547.257 | 4398.818 |
| ENSMUSG00000030638 | protein_coding | Sh3gl3 | 1290.969 | 1396.954 | 1343.961 |
| ENSMUSG00000086121 | processed_transcrip | Gm16068 | 2.603792 | 5.863172 | 4.233482 |
| ENSMUSG00000051596 | protein_coding | Otop1 | 23.21355 | 34.11556 | 28.66456 |
| ENSMUSG00000038398 | protein_coding | Upf3a | 1198.159 | 1291.085 | 1244.622 |
| ENSMUSG00000017404 | protein_coding | Rpl19 | 9189.08 | 9839.104 | 9514.092 |
| ENSMUSG00000050697 | protein_coding | Prkaa1 | 936.6888 | 1061.412 | 999.0505 |
| ENSMUSG00000030068 | protein_coding | Eif4e3 | 275.8565 | 243.7178 | 259.7872 |
| ENSMUSG00000036561 | protein_coding | Ppp6r2 | 1200.261 | 1094.838 | 1147.549 |
| ENSMUSG00000053063 | protein_coding | Clec12a | 17.09472 | 11.23983 | 14.16728 |
| ENSMUSG00000031024 | protein_coding | St5 | 3626.048 | 3906.481 | 3766.264 |
| ENSMUSG00000042558 | protein_coding | Adprhl2 | 882.0229 | 958.3958 | 920.2093 |
| ENSMUSG00000066979 | protein_coding | Bub3 | 2029.081 | 2223.425 | 2126.253 |
| ENSMUSG00000089664 | pseudogene | Gm12494 | 13.28114 | 19.85782 | 16.56948 |
| ENSMUSG00000033444 | protein_coding | Specc1l | 4287.168 | 3976.781 | 4131.974 |
| ENSMUSG00000079242 | protein_coding | C730034F03Rik | 25.61488 | 35.87571 | 30.74529 |
| ENSMUSG00000091655 | pseudogene | Gm8741 | 17.18388 | 11.02899 | 14.10644 |
| ENSMUSG00000029605 | protein_coding | Oas1b | 42.59145 | 30.46228 | 36.52687 |
| ENSMUSG00000038754 | protein_coding | Elovl3 | 11.31232 | 18.2224 | 14.76736 |
| ENSMUSG00000001700 | protein_coding | Gramd3 | 572.1468 | 639.6863 | 605.9166 |
| ENSMUSG00000033306 | protein_coding | Lpp | 1669.193 | 1807.114 | 1738.153 |
| ENSMUSG00000091345 | protein_coding | Col6a5 | 31.97821 | 42.84062 | 37.40942 |
| ENSMUSG00000038463 | protein_coding | Olfml2b | 2711.375 | 2981.32 | 2846.348 |
| ENSMUSG00000090170 | protein_coding | Tnfsf12 | 120.0823 | 142.1975 | 131.1399 |
| ENSMUSG00000022749 | protein_coding | Tbc1d23 | 1302.19 | 1177.599 | 1239.894 |
| ENSMUSG00000059562 | protein_coding | Ccdc154 | 3.272612 | 1.024227 | 2.14842 |

| | | | | | |
|---|---|---|---|---|---|
| ENSMUSG00000078129 | protein_coding | 1700007I08Rik | 14.25798 | 8.592045 | 11.42501 |
| ENSMUSG00000032590 | protein_coding | Apeh | 1135.774 | 1051.961 | 1093.867 |
| ENSMUSG00000075324 | protein_coding | Fign | 130.2066 | 156.2268 | 143.2167 |
| ENSMUSG00000024218 | protein_coding | Taf11 | 1211.077 | 1124.433 | 1167.755 |
| ENSMUSG00000087522 | pseudogene | Gm371 | 329.7113 | 280.0969 | 304.9041 |
| ENSMUSG00000027900 | protein_coding | Dram2 | 895.5091 | 995.7902 | 945.6496 |
| ENSMUSG00000027848 | protein_coding | Olfml3 | 1131.256 | 963.7274 | 1047.492 |
| ENSMUSG00000071497 | protein_coding | Nutf2-ps1 | 142.1547 | 168.8397 | 155.4972 |
| ENSMUSG00000037974 | protein_coding | Muc5ac | 0.99093 | 3.461538 | 2.226234 |
| ENSMUSG00000001020 | protein_coding | S100a4 | 9.361993 | 14.73705 | 12.04952 |
| ENSMUSG00000025351 | protein_coding | Cd63 | 2588.338 | 2819.919 | 2704.129 |
| ENSMUSG00000044819 | protein_coding | Oxgr1 | 2.956391 | 0.688704 | 1.822547 |
| ENSMUSG00000089146 | misc_RNA | 7SK | 18.41954 | 26.83283 | 22.62619 |
| ENSMUSG00000025781 | protein_coding | Atp5c1 | 6073.26 | 6572.554 | 6322.907 |
| ENSMUSG00000031659 | protein_coding | Adcy7 | 2273.821 | 2586.879 | 2430.35 |
| ENSMUSG00000043822 | protein_coding | Adamtsl5 | 68.40394 | 85.50603 | 76.95498 |
| ENSMUSG00000020721 | protein_coding | Helz | 5523.631 | 5077.845 | 5300.738 |
| ENSMUSG00000021701 | protein_coding | Plk2 | 537.2528 | 462.5392 | 499.896 |
| ENSMUSG00000001281 | protein_coding | Itgb7 | 42.44258 | 57.55059 | 49.99659 |
| ENSMUSG00000026499 | protein_coding | Acbd3 | 2371.611 | 2576.428 | 2474.02 |
| ENSMUSG00000029376 | protein_coding | Mthfd2l | 136.6722 | 159.861 | 148.2666 |
| ENSMUSG00000065204 | snoRNA | SNORA32 | 6.132691 | 2.463976 | 4.298334 |
| ENSMUSG00000005621 | protein_coding | Zfp592 | 887.1526 | 966.3062 | 926.7294 |
| ENSMUSG00000040855 | protein_coding | Reps2 | 1102.087 | 1014.835 | 1058.461 |
| ENSMUSG00000085260 | processed_transcrip | 1700013G23Rik | 22.42889 | 14.72804 | 18.57846 |
| ENSMUSG00000040848 | protein_coding | Sft2d2 | 3085.66 | 3403.182 | 3244.421 |
| ENSMUSG00000029307 | protein_coding | Dmp1 | 4.541075 | 8.448982 | 6.495029 |
| ENSMUSG00000062064 | protein_coding | Slc2a7 | 6.476047 | 3.010341 | 4.743194 |
| ENSMUSG00000086660 | processed_transcrip | Gm12594 | 7.76322 | 12.67958 | 10.2214 |
| ENSMUSG00000040127 | protein_coding | Sdr9c7 | 5.78956 | 10.14426 | 7.966908 |
| ENSMUSG00000004110 | protein_coding | Cacna1e | 19.0379 | 12.72427 | 15.88108 |
| ENSMUSG00000016831 | protein_coding | Tox4 | 2474.902 | 2765.416 | 2620.159 |
| ENSMUSG00000039145 | protein_coding | Camk1d | 778.363 | 707.2933 | 742.8282 |
| ENSMUSG00000026577 | protein_coding | Blzf1 | 1094.657 | 1197.702 | 1146.179 |
| ENSMUSG00000071179 | protein_coding | Gm46 | 6.766401 | 11.65499 | 9.210698 |
| ENSMUSG00000030922 | protein_coding | Lyrm1 | 207.8988 | 177.9827 | 192.9407 |
| ENSMUSG00000040044 | protein_coding | Orc3 | 2113.932 | 2284.056 | 2198.994 |
| ENSMUSG00000026805 | protein_coding | Barhl1 | 20.32738 | 13.93231 | 17.12984 |
| ENSMUSG00000050931 | protein_coding | Sgms2 | 295.6471 | 258.0671 | 276.8571 |
| ENSMUSG00000071294 | pseudogene | Gm5196 | 1.629262 | 4.610448 | 3.119855 |
| ENSMUSG00000029560 | protein_coding | Snx8 | 1263.069 | 1163.201 | 1213.135 |
| ENSMUSG00000087184 | lincRNA | Gm11650 | 5.840026 | 2.710492 | 4.275259 |
| ENSMUSG00000053839 | protein_coding | Gm9924 | 2.245304 | 5.482964 | 3.864134 |
| ENSMUSG00000027422 | protein_coding | Rrbp1 | 3832.483 | 3538.744 | 3685.613 |
| ENSMUSG00000028854 | protein_coding | Slc9a1 | 1202.711 | 1302.883 | 1252.797 |
| ENSMUSG00000081348 | pseudogene | Gm13878 | 7.471599 | 3.770395 | 5.620997 |
| ENSMUSG00000060923 | protein_coding | Acyp2 | 118.1617 | 139.2492 | 128.7054 |
| ENSMUSG00000019359 | protein_coding | Gdpd2 | 48.11712 | 59.96957 | 54.04334 |
| ENSMUSG00000024486 | protein_coding | Hbegf | 146.542 | 174.9496 | 160.7458 |
| ENSMUSG00000020950 | protein_coding | Foxg1 | 4.879585 | 2.012779 | 3.446182 |
| ENSMUSG00000084980 | processed_transcrip | 4921508A21Rik | 0.32211 | 2.030436 | 1.176273 |
| ENSMUSG00000074121 | protein_coding | Ntf5 | 450.9036 | 516.796 | 483.8498 |
| ENSMUSG00000039155 | protein_coding | Cdh26 | 1.621062 | 4.0879 | 2.854481 |
| ENSMUSG00000069515 | protein_coding | Lyz1 | 1.276663 | 3.752738 | 2.5147 |
| ENSMUSG00000000290 | protein_coding | Itgb2 | 48.85072 | 63.84191 | 56.34632 |
| ENSMUSG00000074215 | protein_coding | Gm10643 | 56.90973 | 43.77144 | 50.34059 |
| ENSMUSG00000020694 | protein_coding | Tlk2 | 3317.374 | 3563.056 | 3440.215 |
| ENSMUSG00000031640 | protein_coding | Klkb1 | 3.216256 | 0.997561 | 2.106909 |

| | | | | | |
|---|---|---|---|---|---|
| ENSMUSG00000025489 | protein_coding | Ifitm5 | 2.934102 | 6.445574 | 4.689838 |
| ENSMUSG00000079633 | protein_coding | Rhox4d | 2.603792 | 0.697713 | 1.650753 |
| ENSMUSG00000025816 | protein_coding | Sec61a2 | 1357.652 | 1498.53 | 1428.091 |
| ENSMUSG00000031380 | protein_coding | Figf | 106.555 | 87.32929 | 96.94216 |
| ENSMUSG00000072772 | protein_coding | Grcc10 | 211.4385 | 245.1221 | 228.2803 |
| ENSMUSG00000079147 | protein_coding | Gm11053 | 3.224456 | 6.834429 | 5.029443 |
| ENSMUSG00000002900 | protein_coding | Lamb1 | 5291.66 | 5871.211 | 5581.436 |
| ENSMUSG00000005802 | protein_coding | Slc30a4 | 2790.802 | 2495.182 | 2642.992 |
| ENSMUSG00000022503 | protein_coding | Nubp1 | 546.1691 | 494.9514 | 520.5602 |
| ENSMUSG00000021754 | protein_coding | Map3k1 | 2984.218 | 3306.204 | 3145.211 |
| ENSMUSG00000044456 | protein_coding | Rin3 | 797.8741 | 884.8606 | 841.3673 |
| ENSMUSG00000086171 | lincRNA | C630020P19Rik | 3.264412 | 6.852086 | 5.058249 |
| ENSMUSG00000021113 | protein_coding | Snapc1 | 968.575 | 885.4972 | 927.0361 |
| ENSMUSG00000060794 | protein_coding | Tssk5 | 0.64422 | 2.983675 | 1.813947 |
| ENSMUSG00000055681 | protein_coding | Cope | 1585.496 | 1465.639 | 1525.567 |
| ENSMUSG00000028145 | protein_coding | Them4 | 240.1493 | 209.3392 | 224.7442 |
| ENSMUSG00000009621 | protein_coding | Vav2 | 1529.787 | 1401.159 | 1465.473 |
| ENSMUSG00000087395 | processed_transcrip | Gm12705 | 1.270774 | 3.779404 | 2.525089 |
| ENSMUSG00000085527 | lincRNA | Gm15535 | 39.63043 | 27.88657 | 33.7585 |
| ENSMUSG00000039335 | protein_coding | Spata16 | 7.114378 | 3.45289 | 5.283634 |
| ENSMUSG00000031246 | protein_coding | Sh3bgrl | 3252.874 | 3581.053 | 3416.964 |
| ENSMUSG00000005364 | protein_coding | Il5ra | 0 | 1.359751 | 0.679875 |
| ENSMUSG00000074228 | protein_coding | Gm10645 | 0 | 1.359751 | 0.679875 |
| ENSMUSG00000082736 | pseudogene | Gm5381 | 0 | 1.359751 | 0.679875 |
| ENSMUSG00000068267 | protein_coding | Cenpb | 3710.642 | 4014.52 | 3862.581 |
| ENSMUSG00000086677 | processed_transcrip | Gm12284 | 16.52199 | 10.61347 | 13.56773 |
| ENSMUSG00000068221 | protein_coding | Pdxp | 180.5437 | 150.4476 | 165.4956 |
| ENSMUSG00000006575 | protein_coding | Rundc3a | 71.46418 | 58.23826 | 64.85122 |
| ENSMUSG00000087102 | processed_transcrip | Gm13661 | 2.592014 | 0.697713 | 1.644863 |
| ENSMUSG00000032869 | protein_coding | Psmf1 | 961.9716 | 1046.56 | 1004.266 |
| ENSMUSG00000065797 | snRNA | U6 | 4.863185 | 2.057464 | 3.460325 |
| ENSMUSG00000057173 | protein_coding | Rfx8 | 30.5611 | 45.75575 | 38.15843 |
| ENSMUSG00000037336 | protein_coding | Mfsd2b | 37.08404 | 51.86295 | 44.47349 |
| ENSMUSG00000060180 | protein_coding | Myh13 | 49.95622 | 36.71395 | 43.33509 |
| ENSMUSG00000074006 | protein_coding | Omp | 49.78425 | 35.59574 | 42.69 |
| ENSMUSG00000035329 | protein_coding | Fbxo33 | 1068.405 | 982.0191 | 1025.212 |
| ENSMUSG00000031740 | protein_coding | Mmp2 | 1226.986 | 1815.715 | 1521.35 |
| ENSMUSG00000092429 | pseudogene | Gm18958 | 2.261704 | 5.121137 | 3.69142 |
| ENSMUSG00000086076 | pseudogene | Ncr3-ps | 2.597903 | 6.403739 | 4.500821 |
| ENSMUSG00000058799 | protein_coding | Nap1l1 | 6232.678 | 5833.226 | 6032.952 |
| ENSMUSG00000035824 | protein_coding | Tk2 | 753.3716 | 692.5032 | 722.9374 |
| ENSMUSG00000075701 | protein_coding | H47 | 1262.08 | 1359.418 | 1310.749 |
| ENSMUSG00000039217 | protein_coding | Il18 | 71.57562 | 89.37837 | 80.477 |
| ENSMUSG00000005373 | protein_coding | Mlxipl | 1115.981 | 1297.546 | 1206.763 |
| ENSMUSG00000022515 | protein_coding | Anks3 | 1001.975 | 1093.216 | 1047.596 |
| ENSMUSG00000027185 | protein_coding | Nat10 | 1805.098 | 1664.169 | 1734.633 |
| ENSMUSG00000050633 | pseudogene | 1110035H17Rik | 3.896855 | 7.505115 | 5.700985 |
| ENSMUSG00000026785 | protein_coding | Pkn3 | 303.6919 | 269.7289 | 286.7104 |
| ENSMUSG00000093305 | miRNA | AC124576.2 | 195.5981 | 165.9694 | 180.7838 |
| ENSMUSG00000027469 | protein_coding | Tpx2 | 3839.74 | 4094.8 | 3967.27 |
| ENSMUSG00000068328 | protein_coding | Aup1 | 1033.477 | 1119.457 | 1076.467 |
| ENSMUSG00000045414 | protein_coding | 1190002N15Rik | 1431.157 | 1316.895 | 1374.026 |
| ENSMUSG00000022571 | protein_coding | Pycrl | 776.7932 | 870.3959 | 823.5945 |
| ENSMUSG00000078919 | protein_coding | Dpm1 | 1579.44 | 1430.953 | 1505.197 |
| ENSMUSG00000027763 | protein_coding | Mbnl1 | 3218.835 | 3473.495 | 3346.165 |
| ENSMUSG00000058914 | protein_coding | C1qtnf3 | 876.7045 | 1258.064 | 1067.384 |
| ENSMUSG00000073442 | lincRNA | Gm16386 | 62.83489 | 77.85422 | 70.34456 |
| ENSMUSG00000047636 | protein_coding | Cdcp2 | 3.210367 | 6.96776 | 5.089063 |

| | | | | | |
|---|---|---|---|---|---|
| ENSMUSG00000025027 | protein_coding | Xpnpep1 | 3865.364 | 3544.953 | 3705.158 |
| ENSMUSG00000064302 | protein_coding | Clasp1 | 2704.846 | 2487.235 | 2596.04 |
| ENSMUSG00000021037 | protein_coding | Ahsa1 | 2252.452 | 2085.766 | 2169.109 |
| ENSMUSG00000002104 | protein_coding | Rapsn | 45.61299 | 57.28502 | 51.449 |
| ENSMUSG00000033880 | protein_coding | Lgals3bp | 167.492 | 131.0761 | 149.284 |
| ENSMUSG00000026464 | protein_coding | Zc3h11a | 5674.72 | 6133.75 | 5904.235 |
| ENSMUSG00000029048 | protein_coding | Rer1 | 2194.43 | 2012.78 | 2103.605 |
| ENSMUSG00000087328 | processed_transcrip | Gm12120 | 2.606103 | 0.688704 | 1.647403 |
| ENSMUSG00000045521 | protein_coding | Tssk2 | 24.54046 | 17.47421 | 21.00733 |
| ENSMUSG00000058385 | protein_coding | Hist1h2bg | 6.12218 | 10.86035 | 8.491265 |
| ENSMUSG00000032348 | protein_coding | Gsta4 | 5803.331 | 6256.136 | 6029.734 |
| ENSMUSG00000088359 | rRNA | 5S_rRNA | 0 | 1.350742 | 0.675371 |
| ENSMUSG00000092252 | protein_coding | Gm20499 | 0 | 1.350742 | 0.675371 |
| ENSMUSG00000044903 | protein_coding | Psg22 | 0 | 1.350742 | 0.675371 |
| ENSMUSG00000090948 | lincRNA | Gm17512 | 0 | 1.350742 | 0.675371 |
| ENSMUSG00000086838 | processed_transcrip | Gm16212 | 0 | 1.350742 | 0.675371 |
| ENSMUSG00000020383 | protein_coding | Il13 | 0 | 1.350742 | 0.675371 |
| ENSMUSG00000089679 | processed_transcrip | Gm16299 | 12.92265 | 20.0726 | 16.49762 |
| ENSMUSG00000025403 | protein_coding | Shmt2 | 1424.048 | 1546.929 | 1485.488 |
| ENSMUSG00000057691 | protein_coding | Zfp746 | 1260.109 | 1167.264 | 1213.687 |
| ENSMUSG00000090919 | protein_coding | Pabpc4l | 193.7413 | 234.8524 | 214.2969 |
| ENSMUSG00000087101 | lincRNA | Gm16953 | 9.692303 | 5.519001 | 7.605652 |
| ENSMUSG00000030102 | protein_coding | Itpr1 | 3505.42 | 3769.397 | 3637.409 |
| ENSMUSG00000056586 | protein_coding | Zar1l | 3.258523 | 0.997561 | 2.128042 |
| ENSMUSG00000012282 | protein_coding | Wnt8a | 4.863185 | 10.31647 | 7.589829 |
| ENSMUSG00000020925 | protein_coding | Ccdc43 | 572.6234 | 640.9328 | 606.7781 |
| ENSMUSG00000081619 | pseudogene | Gm13351 | 4.881896 | 9.262369 | 7.072132 |
| ENSMUSG00000087283 | processed_transcrip | Gm13580 | 1.635151 | 0 | 0.817575 |
| ENSMUSG00000092915 | miRNA | CT010508.1 | 1.635151 | 0 | 0.817575 |
| ENSMUSG00000045008 | protein_coding | 9030612E09Rik | 62.90891 | 78.17245 | 70.54068 |
| ENSMUSG00000033737 | protein_coding | Fndc3c1 | 63.76529 | 48.62411 | 56.1947 |
| ENSMUSG00000032470 | protein_coding | Mras | 1250.586 | 1403.8 | 1327.193 |
| ENSMUSG00000086316 | processed_transcrip | 2210013O21Rik | 418.1555 | 469.1237 | 443.6396 |
| ENSMUSG00000038984 | protein_coding | Tspyl5 | 405.6312 | 360.7059 | 383.1685 |
| ENSMUSG00000026492 | protein_coding | Tfb2m | 1073.77 | 991.0153 | 1032.392 |
| ENSMUSG00000047669 | protein_coding | Msl3l2 | 395.2586 | 473.7863 | 434.5224 |
| ENSMUSG00000034445 | protein_coding | Cybasc3 | 520.8719 | 470.6128 | 495.7423 |
| ENSMUSG00000084241 | pseudogene | Gm13416 | 7.387065 | 3.770395 | 5.57873 |
| ENSMUSG00000079000 | processed_transcrip | 7530422B04Rik | 5.462828 | 10.33993 | 7.901378 |
| ENSMUSG00000005718 | protein_coding | Tcfap4 | 554.1878 | 608.0668 | 581.1273 |
| ENSMUSG00000026970 | protein_coding | Rbms1 | 1364.944 | 1499.812 | 1432.378 |
| ENSMUSG00000037129 | protein_coding | Tmprss13 | 195.44 | 296.4007 | 245.9203 |
| ENSMUSG00000029814 | protein_coding | Igf2bp3 | 402.0891 | 351.5733 | 376.8312 |
| ENSMUSG00000074203 | protein_coding | G430095P16Rik | 10.34241 | 15.97212 | 13.15726 |
| ENSMUSG00000091047 | lincRNA | 4930532G15Rik | 14.935 | 9.025224 | 11.98011 |
| ENSMUSG00000056536 | protein_coding | Pign | 1441.061 | 1568.791 | 1504.926 |
| ENSMUSG00000030340 | protein_coding | Scnn1a | 38.66366 | 50.45488 | 44.55927 |
| ENSMUSG00000049488 | protein_coding | Tmem67 | 484.1689 | 535.8032 | 509.9861 |
| ENSMUSG00000030324 | protein_coding | Rho | 39.44601 | 29.23695 | 34.34148 |
| ENSMUSG00000047205 | protein_coding | Dusp18 | 315.0981 | 272.8383 | 293.9682 |
| ENSMUSG00000020612 | protein_coding | Prkar1a | 9636.552 | 10303.83 | 9970.189 |
| ENSMUSG00000021428 | protein_coding | Riok1 | 1086.556 | 1177.473 | 1132.015 |
| ENSMUSG00000044312 | protein_coding | Neurog3 | 116.4222 | 96.34986 | 106.386 |
| ENSMUSG00000019952 | protein_coding | Poc1b | 372.4875 | 335.5542 | 354.0208 |
| ENSMUSG00000046952 | pseudogene | Gm5815 | 10.28837 | 5.872182 | 8.080275 |
| ENSMUSG00000049036 | protein_coding | Tmem121 | 35.84458 | 26.87606 | 31.36032 |
| ENSMUSG00000058147 | protein_coding | Xlr3c | 687.069 | 616.6468 | 651.8579 |
| ENSMUSG00000036534 | protein_coding | Slc38a7 | 545.2581 | 603.9565 | 574.6073 |

| | | | | | |
|---|---|---|---|---|---|
| ENSMUSG00000038279 | protein_coding | Nop2 | 1659.035 | 1528.566 | 1593.801 |
| ENSMUSG00000003934 | protein_coding | Efnb3 | 484.6536 | 438.0018 | 461.3277 |
| ENSMUSG00000024248 | protein_coding | Cox7a2l | 2947.458 | 3159.318 | 3053.388 |
| ENSMUSG00000092273 | processed_transcrip | RP23-428N8.1 | 9.672325 | 14.9832 | 12.32776 |
| ENSMUSG00000085042 | processed_transcrip | 2010001M06Rik | 23.48139 | 31.96189 | 27.72164 |
| ENSMUSG00000086545 | processed_transcrip | Gm12 | 1.626951 | 0 | 0.813475 |
| ENSMUSG00000082260 | pseudogene | Gm7429 | 1.626951 | 0 | 0.813475 |
| ENSMUSG00000055884 | protein_coding | Fancm | 1324.85 | 1228.547 | 1276.698 |
| ENSMUSG00000046774 | protein_coding | 8030474K03Rik | 1426.676 | 1279.545 | 1353.111 |
| ENSMUSG00000026824 | protein_coding | Kcnj3 | 28.22135 | 42.83021 | 35.52578 |
| ENSMUSG00000002881 | protein_coding | Nab1 | 2091.661 | 2304.818 | 2198.239 |
| ENSMUSG00000066880 | protein_coding | Zfp617 | 833.2828 | 902.925 | 868.1039 |
| ENSMUSG00000024247 | protein_coding | Pkdcc | 1857.99 | 2009.389 | 1933.69 |
| ENSMUSG00000030472 | protein_coding | Ceacam18 | 6.78049 | 11.32884 | 9.054666 |
| ENSMUSG00000017747 | protein_coding | Ghdc | 171.3932 | 147.1544 | 159.2738 |
| ENSMUSG00000050875 | protein_coding | A730017C20Rik | 429.3071 | 363.3074 | 396.3073 |
| ENSMUSG00000027284 | protein_coding | Cdan1 | 1425.508 | 1572.189 | 1498.848 |
| ENSMUSG00000089475 | misc_RNA | 7SK | 3.256212 | 6.75407 | 5.005141 |
| ENSMUSG00000021709 | protein_coding | Erbb2ip | 3786.45 | 4067.008 | 3926.729 |
| ENSMUSG00000026705 | protein_coding | Klhl20 | 1298.94 | 1198.883 | 1248.912 |
| ENSMUSG00000024811 | protein_coding | Tnks2 | 9169.534 | 9831.551 | 9500.543 |
| ENSMUSG00000068349 | protein_coding | Gml | 138.3399 | 63.02911 | 100.6845 |
| ENSMUSG00000021492 | protein_coding | F12 | 1.615173 | 4.114928 | 2.86505 |
| ENSMUSG00000068969 | protein_coding | Rpl32-ps | 1.623372 | 0 | 0.811686 |
| ENSMUSG00000031216 | protein_coding | Stard8 | 929.1221 | 854.2174 | 891.6697 |
| ENSMUSG00000015224 | protein_coding | Cyp2j9 | 56.11769 | 71.24973 | 63.68371 |
| ENSMUSG00000029082 | protein_coding | Bst1 | 7.147178 | 12.77832 | 9.962751 |
| ENSMUSG00000052406 | protein_coding | Rexo4 | 1527.035 | 1649.241 | 1588.138 |
| ENSMUSG00000052726 | protein_coding | Kcnt2 | 28.18102 | 19.90323 | 24.04213 |
| ENSMUSG00000087471 | lincRNA | Gm16836 | 210.3481 | 251.4298 | 230.8889 |
| ENSMUSG00000085370 | lincRNA | 2310002F09Rik | 0.98273 | 3.108357 | 2.045544 |
| ENSMUSG00000032536 | protein_coding | Trak1 | 3461.072 | 3731.235 | 3596.153 |
| ENSMUSG00000000568 | protein_coding | Hnrnpd | 4180.777 | 4492.958 | 4336.868 |
| ENSMUSG00000020101 | protein_coding | 4632428N05Rik | 245.4996 | 216.5279 | 231.0138 |
| ENSMUSG00000085636 | lincRNA | Gm11769 | 19.65394 | 12.73871 | 16.19633 |
| ENSMUSG00000047613 | protein_coding | A430005L14Rik | 665.5444 | 599.888 | 632.7162 |
| ENSMUSG00000065715 | snoRNA | Snord7 | 7.777309 | 13.6743 | 10.7258 |
| ENSMUSG00000033774 | protein_coding | Npbwr1 | 1.31304 | 3.80607 | 2.559555 |
| ENSMUSG00000031179 | protein_coding | 3830417A13Rik | 29.4928 | 39.41652 | 34.45466 |
| ENSMUSG00000085807 | processed_transcrip | Gm13069 | 2.611992 | 5.916866 | 4.264429 |
| ENSMUSG00000023013 | protein_coding | Aqp2 | 197.7797 | 242.6894 | 220.2345 |
| ENSMUSG00000074825 | protein_coding | Itpripl1 | 388.7054 | 441.4782 | 415.0918 |
| ENSMUSG00000065475 | miRNA | Mir27b | 2.592014 | 5.738851 | 4.165433 |
| ENSMUSG00000013973 | protein_coding | Dedd | 196.0241 | 223.9138 | 209.9689 |
| ENSMUSG00000039257 | protein_coding | Vstm2b | 14.29204 | 21.39346 | 17.84275 |
| ENSMUSG00000019173 | protein_coding | Rab5c | 3978.558 | 4261.269 | 4119.913 |
| ENSMUSG00000039988 | protein_coding | Ankrd13c | 1758.837 | 1639.795 | 1699.316 |
| ENSMUSG00000056476 | protein_coding | Med12l | 762.7742 | 682.1974 | 722.4858 |
| ENSMUSG00000035228 | protein_coding | Ccdc106 | 233.5991 | 206.2312 | 219.9151 |
| ENSMUSG00000030402 | protein_coding | Ppm1n | 19.10372 | 12.32604 | 15.71488 |
| ENSMUSG00000068732 | protein_coding | Tmem167b | 1572.294 | 1458.056 | 1515.175 |
| ENSMUSG00000044217 | protein_coding | Aqp5 | 185.8742 | 213.4948 | 199.6845 |
| ENSMUSG00000022098 | protein_coding | Bmp1 | 1292.182 | 1489.497 | 1390.84 |
| ENSMUSG00000020850 | protein_coding | Prpf8 | 9643.441 | 8773.773 | 9208.607 |
| ENSMUSG00000025076 | protein_coding | Casp7 | 977.7583 | 903.4708 | 940.6145 |
| ENSMUSG00000061356 | protein_coding | Gm600 | 36.78966 | 27.94534 | 32.3675 |
| ENSMUSG00000024286 | protein_coding | Ccny | 3643.732 | 3415.336 | 3529.534 |
| ENSMUSG00000049502 | protein_coding | Dtx3l | 200.2992 | 227.3464 | 213.8228 |

| | | | | | |
|---|---|---|---|---|---|
| ENSMUSG00000027834 | protein_coding | Serpini1 | 726.8848 | 601.4868 | 664.1858 |
| ENSMUSG00000038762 | protein_coding | Abcf1 | 4006.423 | 3651.321 | 3828.872 |
| ENSMUSG00000067879 | protein_coding | 3110035E14Rik | 901.2361 | 1011.427 | 956.3314 |
| ENSMUSG00000029847 | protein_coding | 2010107G12Rik | 17.79172 | 24.92599 | 21.35886 |
| ENSMUSG00000064339 | Mt_rRNA | mt-Rnr2 | 9103.307 | 8211.686 | 8657.497 |
| ENSMUSG00000090015 | protein_coding | Gm15446 | 340.38 | 399.3263 | 369.8531 |
| ENSMUSG00000062078 | protein_coding | Qk | 10239 | 10908.25 | 10573.63 |
| ENSMUSG00000070368 | protein_coding | Prok1 | 0.974531 | 3.135023 | 2.054777 |
| ENSMUSG00000063524 | protein_coding | Eno1 | 6981.678 | 7745.396 | 7363.537 |
| ENSMUSG00000047259 | protein_coding | Mc4r | 22.53273 | 15.53858 | 19.03565 |
| ENSMUSG00000029521 | protein_coding | Chek2 | 374.1701 | 337.0758 | 355.623 |
| ENSMUSG00000066800 | protein_coding | Rnasel | 716.3657 | 813.2012 | 764.7834 |
| ENSMUSG00000020695 | protein_coding | Mrc2 | 450.5181 | 402.607 | 426.5626 |
| ENSMUSG00000025823 | protein_coding | Pdia4 | 5567.706 | 5132.158 | 5349.932 |
| ENSMUSG00000070690 | protein_coding | 5830473C10Rik | 2.620192 | 0.662038 | 1.641115 |
| ENSMUSG00000026811 | protein_coding | St6galnac6 | 817.9639 | 902.6515 | 860.3077 |
| ENSMUSG00000033849 | protein_coding | B3galt2 | 5.143028 | 9.26273 | 7.202879 |
| ENSMUSG00000059741 | protein_coding | Myl3 | 7.751442 | 12.89871 | 10.32508 |
| ENSMUSG00000082941 | pseudogene | Gm12850 | 4.204876 | 7.956312 | 6.080594 |
| ENSMUSG00000085848 | lincRNA | Gm16790 | 0.638331 | 2.374969 | 1.50665 |
| ENSMUSG00000027285 | protein_coding | Haus2 | 1168.878 | 1066.589 | 1117.734 |
| ENSMUSG00000033416 | protein_coding | 1110038D17Rik | 970.9451 | 861.9157 | 916.4304 |
| ENSMUSG00000057719 | protein_coding | Sh3rf2 | 16.46101 | 10.83296 | 13.64699 |
| ENSMUSG00000031442 | protein_coding | Mcf2l | 406.1161 | 461.521 | 433.8186 |
| ENSMUSG00000086723 | lincRNA | Gm16601 | 59.29824 | 75.884 | 67.59112 |
| ENSMUSG00000091939 | lincRNA | Gm17595 | 0.638331 | 2.648151 | 1.643241 |
| ENSMUSG00000020399 | protein_coding | Havcr2 | 15.80039 | 22.6757 | 19.23805 |
| ENSMUSG00000057219 | protein_coding | Armc7 | 346.31 | 304.9039 | 325.6069 |
| ENSMUSG00000052221 | protein_coding | Gm70 | 56.13096 | 70.73517 | 63.43306 |
| ENSMUSG00000001750 | protein_coding | Tcirg1 | 334.2605 | 387.4748 | 360.8676 |
| ENSMUSG00000029703 | protein_coding | Lrwd1 | 1651.461 | 1505.002 | 1578.231 |
| ENSMUSG00000030605 | protein_coding | Mfge8 | 2367.6 | 2186.737 | 2277.169 |
| ENSMUSG00000067106 | pseudogene | Gm5529 | 44.20848 | 55.31692 | 49.7627 |
| ENSMUSG00000027634 | protein_coding | Ndrg3 | 2302.68 | 2475.399 | 2389.039 |
| ENSMUSG00000083847 | pseudogene | Xlr3d-ps | 16.17528 | 23.17774 | 19.67651 |
| ENSMUSG00000026307 | protein_coding | Scly | 102.1096 | 80.89335 | 91.50147 |
| ENSMUSG00000021969 | protein_coding | Zdhhc20 | 2867.216 | 3089.706 | 2978.461 |
| ENSMUSG00000024423 | protein_coding | Impact | 810.5746 | 896.7848 | 853.6797 |
| ENSMUSG00000091188 | lincRNA | 2700016F22Rik | 11.32283 | 17.36718 | 14.34501 |
| ENSMUSG00000085463 | processed_transcrip | 9430024E24Rik | 1.643351 | 0 | 0.821675 |
| ENSMUSG00000072707 | protein_coding | Olfr31 | 1.643351 | 0 | 0.821675 |
| ENSMUSG00000038015 | protein_coding | Prm2 | 1.643351 | 0 | 0.821675 |
| ENSMUSG00000067722 | pseudogene | BC003965 | 917.3508 | 1007.765 | 962.5578 |
| ENSMUSG00000039545 | processed_transcrip | 4930524L23Rik | 35.40558 | 25.561 | 30.48329 |
| ENSMUSG00000060678 | protein_coding | Hist1h4c | 6.802779 | 12.70946 | 9.75612 |
| ENSMUSG00000034190 | protein_coding | Chmp7 | 1951.105 | 1809.306 | 1880.206 |
| ENSMUSG00000074824 | protein_coding | Zfp708 | 51.73115 | 67.63835 | 59.68475 |
| ENSMUSG00000041000 | protein_coding | Trim62 | 1293.361 | 1467.094 | 1380.227 |
| ENSMUSG00000006930 | protein_coding | Hap1 | 441.4754 | 490.039 | 465.7572 |
| ENSMUSG00000005202 | protein_coding | Shbg | 2232.897 | 2451.208 | 2342.053 |
| ENSMUSG00000081534 | protein_coding | Slc48a1 | 754.4191 | 825.0991 | 789.7591 |
| ENSMUSG00000031749 | protein_coding | St3gal2 | 4271.314 | 4631.986 | 4451.65 |
| ENSMUSG00000086443 | lincRNA | 4933421A08Rik | 24.16661 | 17.03166 | 20.59913 |
| ENSMUSG00000041734 | protein_coding | Kirrel | 722.1045 | 826.418 | 774.2612 |
| ENSMUSG00000080859 | pseudogene | Rpl10-ps1 | 15.56871 | 9.951434 | 12.76007 |
| ENSMUSG00000004056 | protein_coding | Akt2 | 4677.154 | 5016.652 | 4846.903 |
| ENSMUSG00000085289 | processed_transcrip | Gm15337 | 18.81545 | 29.35475 | 24.0851 |
| ENSMUSG00000081833 | pseudogene | Gm13669 | 5.473339 | 9.606901 | 7.54012 |

| | | | | | |
|---|---|---|---|---|---|
| ENSMUSG00000059658 | pseudogene | Gm16379 | 0.646531 | 2.374969 | 1.51075 |
| ENSMUSG00000078347 | protein_coding | 1700012L04Rik | 0.646531 | 2.374969 | 1.51075 |
| ENSMUSG00000073400 | protein_coding | Trim10 | 16.18706 | 23.8956 | 20.04133 |
| ENSMUSG00000090354 | lincRNA | Gm17556 | 375.7025 | 330.8656 | 353.284 |
| ENSMUSG00000031889 | protein_coding | D230025D16Rik | 1145.939 | 1054.643 | 1100.291 |
| ENSMUSG00000006728 | protein_coding | Cdk4 | 4020.084 | 4371.567 | 4195.825 |
| ENSMUSG00000022868 | protein_coding | Ahsg | 373.4277 | 331.2373 | 352.3325 |
| ENSMUSG00000068040 | protein_coding | Tm9sf4 | 2020.401 | 2189.15 | 2104.776 |
| ENSMUSG00000086789 | processed_transcrip | Gm12228 | 19.41152 | 26.84976 | 23.13064 |
| ENSMUSG00000051413 | protein_coding | Plagl2 | 1455.148 | 1328.29 | 1391.719 |
| ENSMUSG00000068601 | protein_coding | Gm10244 | 21.6604 | 29.5516 | 25.606 |
| ENSMUSG00000087609 | processed_transcrip | 4930442J19Rik | 1.637461 | 0 | 0.818731 |
| ENSMUSG00000075408 | protein_coding | 6030408B16Rik | 791.9894 | 914.0036 | 852.9965 |
| ENSMUSG00000085793 | protein_coding | Lin52 | 397.7348 | 439.9135 | 418.8242 |
| ENSMUSG00000027581 | protein_coding | Stmn3 | 13.57738 | 8.439973 | 11.00868 |
| ENSMUSG00000028081 | protein_coding | Rps3a | 12502.37 | 13446.87 | 12974.62 |
| ENSMUSG00000077349 | snRNA | U6 | 1.598773 | 0 | 0.799386 |
| ENSMUSG00000036862 | protein_coding | Dchs1 | 427.5789 | 383.1174 | 405.3481 |
| ENSMUSG00000038044 | protein_coding | Cct8l1 | 0.954552 | 3.144033 | 2.049292 |
| ENSMUSG00000038520 | protein_coding | Tbc1d17 | 806.3097 | 871.3723 | 838.841 |
| ENSMUSG00000085272 | protein_coding | Gm1078 | 33.64132 | 23.61848 | 28.6299 |
| ENSMUSG00000061273 | protein_coding | Mmgt1 | 1138.893 | 1230.037 | 1184.465 |
| ENSMUSG00000078252 | protein_coding | Krtap17-1 | 0.638331 | 2.43731 | 1.537821 |
| ENSMUSG00000041645 | protein_coding | Ddx24 | 3835.382 | 3470.639 | 3653.01 |
| ENSMUSG00000021242 | protein_coding | Npc2 | 3520.547 | 3285.74 | 3403.144 |
| ENSMUSG00000027665 | protein_coding | Pik3ca | 2360.68 | 2152.137 | 2256.409 |
| ENSMUSG00000024812 | protein_coding | Tjp2 | 3414.366 | 3738.438 | 3576.402 |
| ENSMUSG00000055963 | lincRNA | Gm11818 | 125.3288 | 105.9452 | 115.637 |
| ENSMUSG00000024786 | protein_coding | 1700123I01Rik | 405.8839 | 276.2442 | 341.0641 |
| ENSMUSG00000073647 | pseudogene | Gm10557 | 437.5557 | 398.5856 | 418.0707 |
| ENSMUSG00000044033 | protein_coding | Ccdc141 | 680.8891 | 619.2926 | 650.0908 |
| ENSMUSG00000028647 | protein_coding | Mycbp | 1440.759 | 1338.192 | 1389.475 |
| ENSMUSG00000021087 | protein_coding | Rtn1 | 95.69617 | 117.8118 | 106.754 |
| ENSMUSG00000022701 | protein_coding | 2610015P09Rik | 769.6701 | 682.0329 | 725.8515 |
| ENSMUSG00000077416 | snoRNA | SNORA17 | 2.911813 | 6.056356 | 4.484085 |
| ENSMUSG00000045404 | protein_coding | Kcnk13 | 582.3804 | 657.4846 | 619.9325 |
| ENSMUSG00000085658 | lincRNA | Gm15704 | 13.98633 | 7.454632 | 10.72048 |
| ENSMUSG00000048442 | processed_transcrip | 2210020M01Rik | 10.69501 | 16.35196 | 13.52349 |
| ENSMUSG00000092174 | pseudogene | Gm7029 | 0.646531 | 2.419653 | 1.533092 |
| ENSMUSG00000032226 | protein_coding | Gcnt3 | 11.31441 | 17.56036 | 14.43739 |
| ENSMUSG00000031391 | protein_coding | L1cam | 20.426 | 28.32482 | 24.37541 |
| ENSMUSG00000047977 | protein_coding | D930020E02Rik | 2.620192 | 6.887038 | 4.753615 |
| ENSMUSG00000032504 | protein_coding | Pdcd6ip | 3519.169 | 3775.163 | 3647.166 |
| ENSMUSG00000052419 | protein_coding | 2610001J05Rik | 597.6868 | 661.4154 | 629.5511 |
| ENSMUSG00000024778 | protein_coding | Fas | 25.21054 | 34.02256 | 29.61655 |
| ENSMUSG00000029076 | protein_coding | Sdf4 | 5238.948 | 4931.056 | 5085.002 |
| ENSMUSG00000024155 | protein_coding | 4930528F23Rik | 405.9572 | 162.2792 | 284.1182 |
| ENSMUSG00000040055 | protein_coding | Gjb6 | 9.336126 | 14.55867 | 11.9474 |
| ENSMUSG00000036052 | protein_coding | Dnajb5 | 211.3866 | 241.5033 | 226.445 |
| ENSMUSG00000020226 | protein_coding | Slc5a4b | 1.626951 | 4.36973 | 2.99834 |
| ENSMUSG00000025963 | protein_coding | Mdh1b | 9.062172 | 5.058795 | 7.060484 |
| ENSMUSG00000030268 | protein_coding | Bcat1 | 26.5423 | 35.84003 | 31.19116 |
| ENSMUSG00000051228 | protein_coding | Nyx | 30.01737 | 39.40715 | 34.71226 |
| ENSMUSG00000025937 | protein_coding | Lactb2 | 511.987 | 561.4235 | 536.7052 |
| ENSMUSG00000048782 | protein_coding | Insc | 26.78449 | 36.67222 | 31.72835 |
| ENSMUSG00000026450 | protein_coding | Chit1 | 33.49416 | 22.86236 | 28.17826 |
| ENSMUSG00000067189 | pseudogene | Gm7335 | 12.28663 | 7.407098 | 9.846863 |
| ENSMUSG00000092178 | processed_transcrip | Gm3912 | 11.28645 | 17.32286 | 14.30466 |

| | | | | | |
|---|---|---|---|---|---|
| ENSMUSG00000073460 | protein_coding | Pnldc1 | 802.2458 | 739.0133 | 770.6296 |
| ENSMUSG00000080727 | protein_coding | C920021L13Rik | 48.2296 | 37.37422 | 42.80191 |
| ENSMUSG00000022543 | protein_coding | 4930451G09Rik | 116.3709 | 91.22826 | 103.7996 |
| ENSMUSG00000029017 | protein_coding | Pmpcb | 2386.276 | 2238.88 | 2312.578 |
| ENSMUSG00000028268 | protein_coding | Gbp3 | 234.2745 | 181.429 | 207.8517 |
| ENSMUSG00000086255 | processed_transcrip | Gm11534 | 3.510188 | 7.142925 | 5.326557 |
| ENSMUSG00000083679 | pseudogene | Gm12892 | 1.939594 | 4.758947 | 3.34927 |
| ENSMUSG00000087411 | processed_transcrip | Gm16048 | 6.160869 | 10.958 | 8.559437 |
| ENSMUSG00000022816 | protein_coding | Fstl1 | 4456.262 | 4921.424 | 4688.843 |
| ENSMUSG00000026784 | protein_coding | Pdss1 | 395.0128 | 350.1131 | 372.5629 |
| ENSMUSG00000051022 | protein_coding | Hs3st1 | 86.34327 | 105.7193 | 96.03128 |
| ENSMUSG00000062901 | protein_coding | Klhl24 | 2490.322 | 2329.049 | 2409.685 |
| ENSMUSG00000054855 | protein_coding | Rnd1 | 78.08067 | 96.91885 | 87.49976 |
| ENSMUSG00000050930 | protein_coding | 4933403G14Rik | 234.6896 | 202.9462 | 218.8179 |
| ENSMUSG00000029169 | protein_coding | Dhx15 | 6002.049 | 6421.844 | 6211.946 |
| ENSMUSG00000079277 | protein_coding | Hoxd3 | 90.07001 | 120.2896 | 105.1798 |
| ENSMUSG00000018547 | protein_coding | Pip4k2b | 1938.533 | 1793.544 | 1866.038 |
| ENSMUSG00000026527 | protein_coding | Rgs7 | 10.65863 | 16.81724 | 13.73794 |
| ENSMUSG00000089975 | pseudogene | Gm7420 | 12.3207 | 7.303283 | 9.811989 |
| ENSMUSG00000028053 | protein_coding | Ash1l | 4990.432 | 5406.926 | 5198.679 |
| ENSMUSG00000048915 | protein_coding | Efna5 | 44.48579 | 56.27668 | 50.38124 |
| ENSMUSG00000043999 | protein_coding | Gpr75 | 49.37149 | 62.77202 | 56.07175 |
| ENSMUSG00000086033 | lincRNA | 2900076A07Rik | 127.9534 | 153.2969 | 140.6251 |
| ENSMUSG00000060166 | protein_coding | Zdhhc8 | 963.5129 | 1073.877 | 1018.695 |
| ENSMUSG00000058587 | protein_coding | Tmod3 | 3050.973 | 3282.52 | 3166.747 |
| ENSMUSG00000034738 | protein_coding | Nostrin | 44.83608 | 55.89932 | 50.3677 |
| ENSMUSG00000032370 | protein_coding | Lactb | 763.1684 | 654.0004 | 708.5844 |
| ENSMUSG00000029275 | processed_transcrip | Gfi1 | 29.64375 | 21.27991 | 25.46183 |
| ENSMUSG00000055994 | protein_coding | Nod2 | 18.01625 | 11.99088 | 15.00357 |
| ENSMUSG00000019815 | protein_coding | Fam164b | 0.976841 | 3.51487 | 2.245855 |
| ENSMUSG00000051675 | protein_coding | Trim32 | 2336.458 | 2617.417 | 2476.937 |
| ENSMUSG00000092991 | misc_RNA | AC158379.1 | 0.33031 | 1.72194 | 1.026125 |
| ENSMUSG00000081911 | pseudogene | Mrps36-ps2 | 0.33031 | 1.72194 | 1.026125 |
| ENSMUSG00000031812 | protein_coding | Map1lc3b | 4650.159 | 4965.396 | 4807.778 |
| ENSMUSG00000064741 | snoRNA | Snord14a | 7.416511 | 12.22875 | 9.822629 |
| ENSMUSG00000024063 | protein_coding | Lbh | 1539.536 | 1798.879 | 1669.208 |
| ENSMUSG00000042082 | protein_coding | Arsb | 290.6082 | 259.6748 | 275.1415 |
| ENSMUSG00000000787 | protein_coding | Ddx3x | 8778.298 | 9505.462 | 9141.88 |
| ENSMUSG00000028963 | protein_coding | Uts2 | 3.882766 | 1.422092 | 2.652429 |
| ENSMUSG00000032599 | protein_coding | Ip6k2 | 1980.517 | 2127.927 | 2054.222 |
| ENSMUSG00000039372 | protein_coding | 4-Mar | 0.316221 | 1.72194 | 1.019081 |
| ENSMUSG00000031364 | protein_coding | Grpr | 0.316221 | 1.72194 | 1.019081 |
| ENSMUSG00000053411 | protein_coding | Cbx7 | 459.8045 | 505.5528 | 482.6787 |
| ENSMUSG00000038305 | protein_coding | Spats2l | 437.867 | 492.1188 | 464.9929 |
| ENSMUSG00000024483 | protein_coding | Ankhd1 | 5125.99 | 4472.989 | 4799.489 |
| ENSMUSG00000025262 | protein_coding | Fam120c | 495.2243 | 440.1012 | 467.6627 |
| ENSMUSG00000030689 | protein_coding | Ino80e | 848.9367 | 921.0156 | 884.9762 |
| ENSMUSG00000016253 | protein_coding | Th1l | 1658.05 | 1535.884 | 1596.967 |
| ENSMUSG00000034452 | protein_coding | Slc24a1 | 5.489738 | 9.482219 | 7.485978 |
| ENSMUSG00000028433 | protein_coding | Ubap2 | 3377.424 | 3127.283 | 3252.354 |
| ENSMUSG00000043986 | protein_coding | 4932411G14Rik | 1.629262 | 4.114566 | 2.871914 |
| ENSMUSG00000048600 | pseudogene | Gm5763 | 9.624169 | 15.26575 | 12.44496 |
| ENSMUSG00000072601 | protein_coding | Ear1 | 13.49203 | 23.29922 | 18.39562 |
| ENSMUSG00000036327 | protein_coding | Qsox2 | 1933.172 | 1768.766 | 1850.969 |
| ENSMUSG00000038415 | protein_coding | Foxq1 | 332.507 | 377.2479 | 354.8774 |
| ENSMUSG00000032745 | protein_coding | Gpbp1 | 9317.478 | 8664.083 | 8990.781 |
| ENSMUSG00000086782 | processed_transcrip | E130102H24Rik | 121.0304 | 144.713 | 132.8717 |
| ENSMUSG00000043456 | protein_coding | Zfp536 | 135.2586 | 102.5433 | 118.9009 |

| | | | | | |
|---|---|---|---|---|---|
| ENSMUSG00000054422 | protein_coding | Fabp1 | 1.981861 | 0 | 0.99093 |
| ENSMUSG00000083896 | pseudogene | Scgb2b1-ps | 1.981861 | 0 | 0.99093 |
| ENSMUSG00000030556 | protein_coding | Lrrc28 | 616.608 | 560.8543 | 588.7311 |
| ENSMUSG00000082093 | pseudogene | Gm15928 | 0.33031 | 1.712931 | 1.021621 |
| ENSMUSG00000089678 | protein_coding | Agxt2 | 0.33031 | 1.712931 | 1.021621 |
| ENSMUSG00000090121 | protein_coding | Abhd12b | 1.293062 | 4.266276 | 2.779669 |
| ENSMUSG00000090110 | protein_coding | Mtcp1 | 34.816 | 45.30459 | 40.0603 |
| ENSMUSG00000088544 | miRNA | Mir1945 | 0.65242 | 2.401635 | 1.527028 |
| ENSMUSG00000087142 | processed_transcrip | Gm12454 | 12.57109 | 19.47005 | 16.02057 |
| ENSMUSG00000078863 | protein_coding | Gm14325 | 337.6702 | 387.2031 | 362.4367 |
| ENSMUSG00000090272 | protein_coding | Mndal | 49.00122 | 38.41647 | 43.70885 |
| ENSMUSG00000024905 | protein_coding | Mtl5 | 553.7998 | 261.5873 | 407.6936 |
| ENSMUSG00000028394 | protein_coding | Pole3 | 1826.665 | 1685.616 | 1756.14 |
| ENSMUSG00000059772 | protein_coding | Slx1b | 306.2823 | 339.4283 | 322.8553 |
| ENSMUSG00000031409 | protein_coding | Tceal6 | 255.1698 | 287.2785 | 271.2242 |
| ENSMUSG00000039523 | protein_coding | BC046331 | 1027.6 | 952.5695 | 990.0846 |
| ENSMUSG00000090902 | lincRNA | Gm17246 | 17.25306 | 10.39077 | 13.82192 |
| ENSMUSG00000083183 | pseudogene | Gm15575 | 2.589703 | 0.671047 | 1.630375 |
| ENSMUSG00000047898 | protein_coding | Ccr4 | 0.99093 | 3.523879 | 2.257405 |
| ENSMUSG00000032342 | protein_coding | Mto1 | 479.1984 | 436.4553 | 457.8269 |
| ENSMUSG00000083878 | pseudogene | Gm12034 | 4.208454 | 1.686265 | 2.94736 |
| ENSMUSG00000031673 | protein_coding | Cdh11 | 619.5553 | 735.1779 | 677.3666 |
| ENSMUSG00000038991 | protein_coding | Txndc5 | 3420.671 | 3719.954 | 3570.313 |
| ENSMUSG00000034158 | protein_coding | Lrrc58 | 3978.157 | 4343.02 | 4160.589 |
| ENSMUSG00000055184 | protein_coding | Fam72a | 172.3797 | 149.3692 | 160.8745 |
| ENSMUSG00000053094 | protein_coding | 0610007L01Rik | 2085.25 | 1934.378 | 2009.814 |
| ENSMUSG00000029001 | protein_coding | Fbxo44 | 166.6287 | 199.6976 | 183.1631 |
| ENSMUSG00000043020 | protein_coding | Wdr63 | 5.562494 | 2.383978 | 3.973236 |
| ENSMUSG00000032044 | protein_coding | Rpusd4 | 632.7814 | 687.3752 | 660.0783 |
| ENSMUSG00000038422 | protein_coding | Hdhd3 | 131.4615 | 168.0208 | 149.7411 |
| ENSMUSG00000086083 | processed_transcrip | Gm15829 | 4.523408 | 8.739459 | 6.631434 |
| ENSMUSG00000035456 | protein_coding | Prdm8 | 4.820918 | 8.954235 | 6.887576 |
| ENSMUSG00000073243 | protein_coding | Gm9 | 52.84992 | 64.93863 | 58.89428 |
| ENSMUSG00000087258 | processed_transcrip | Gm15461 | 0.316221 | 1.748606 | 1.032414 |
| ENSMUSG00000083875 | pseudogene | Gm13831 | 0.316221 | 1.748606 | 1.032414 |
| ENSMUSG00000052850 | protein_coding | Tas2r137 | 0.316221 | 1.748606 | 1.032414 |
| ENSMUSG00000077338 | snRNA | U1 | 0.316221 | 1.748606 | 1.032414 |
| ENSMUSG00000087057 | processed_transcrip | Gm11730 | 8.725973 | 4.714624 | 6.720298 |
| ENSMUSG00000086711 | processed_transcrip | Gm15482 | 4.535186 | 8.229494 | 6.38234 |
| ENSMUSG00000090498 | protein_coding | Kcnb2 | 11.25932 | 6.798392 | 9.028856 |
| ENSMUSG00000049327 | protein_coding | Setd8 | 3667.621 | 3991.507 | 3829.564 |
| ENSMUSG00000038255 | protein_coding | Neurod2 | 4.806829 | 9.007205 | 6.907017 |
| ENSMUSG00000034758 | protein_coding | Tle6 | 447.9177 | 404.1047 | 426.0112 |
| ENSMUSG00000030056 | protein_coding | Isy1 | 1024.039 | 1114.378 | 1069.209 |
| ENSMUSG00000065126 | snoRNA | Snord104 | 42.93667 | 55.81176 | 49.37422 |
| ENSMUSG00000020083 | protein_coding | 2010107G23Rik | 484.0447 | 430.659 | 457.3519 |
| ENSMUSG00000046380 | protein_coding | Jrk | 613.969 | 560.618 | 587.2935 |
| ENSMUSG00000087629 | processed_transcrip | Gm12246 | 9.050394 | 5.130146 | 7.09027 |
| ENSMUSG00000027668 | protein_coding | Mfn1 | 2299.573 | 2058.687 | 2179.13 |
| ENSMUSG00000020483 | protein_coding | Dynll2 | 6921.754 | 6406.017 | 6663.886 |
| ENSMUSG00000044378 | protein_coding | Slc15a5 | 18.89663 | 29.28635 | 24.09149 |
| ENSMUSG00000091347 | protein_coding | Gm17353 | 13.81749 | 21.2504 | 17.53395 |
| ENSMUSG00000043498 | pseudogene | 9330132A10Rik | 11.57323 | 17.46556 | 14.51939 |
| ENSMUSG00000041929 | pseudogene | Gm6363 | 21.04562 | 14.26747 | 17.65655 |
| ENSMUSG00000036856 | protein_coding | Wnt4 | 50.24255 | 62.52006 | 56.38131 |
| ENSMUSG00000084930 | processed_transcrip | Gm11706 | 9.084461 | 5.094471 | 7.089466 |
| ENSMUSG00000044424 | protein_coding | Gm9493 | 160.9053 | 134.3444 | 147.6249 |
| ENSMUSG00000018924 | protein_coding | Alox15 | 7.158956 | 12.45973 | 9.809345 |

| | | | | | |
|---|---|---|---|---|---|
| ENSMUSG00000032396 | protein_coding | Dis3l | 470.5248 | 406.5932 | 438.559 |
| ENSMUSG00000018581 | protein_coding | Dnahc11 | 52.06317 | 40.35459 | 46.20888 |
| ENSMUSG00000030789 | protein_coding | Itgax | 15.54873 | 22.28648 | 18.91761 |
| ENSMUSG00000046109 | protein_coding | AB041806 | 2.569725 | 0.653029 | 1.611377 |
| ENSMUSG00000020256 | protein_coding | Aldh1l2 | 115.4627 | 140.5541 | 128.0084 |
| ENSMUSG00000087241 | processed_transcrip | Gm11844 | 3.197546 | 7.353043 | 5.275294 |
| ENSMUSG00000070544 | protein_coding | Top1 | 5885.071 | 6317.415 | 6101.243 |
| ENSMUSG00000028427 | protein_coding | Aqp7 | 4.218965 | 7.911627 | 6.065296 |
| ENSMUSG00000060519 | protein_coding | Tor3a | 179.3461 | 152.2981 | 165.8221 |
| ENSMUSG00000085391 | processed_transcrip | Gm16150 | 8.782329 | 4.785613 | 6.783971 |
| ENSMUSG00000075224 | protein_coding | Lrrc55 | 38.06275 | 48.20967 | 43.13621 |
| ENSMUSG00000027835 | protein_coding | Pdcd10 | 1579.007 | 1713.706 | 1646.357 |
| ENSMUSG00000034245 | protein_coding | Hdac11 | 453.7336 | 412.9753 | 433.3544 |
| ENSMUSG00000082516 | pseudogene | Gm13342 | 1.937283 | 4.432433 | 3.184858 |
| ENSMUSG00000088290 | snoRNA | SCARNA4 | 1.296641 | 3.81508 | 2.55586 |
| ENSMUSG00000029458 | protein_coding | Brap | 2318.096 | 2082.401 | 2200.249 |
| ENSMUSG00000041747 | protein_coding | Utp15 | 1293.035 | 1204.097 | 1248.566 |
| ENSMUSG00000086950 | lincRNA | Gm16907 | 53.89408 | 70.89759 | 62.39584 |
| ENSMUSG00000048076 | protein_coding | Arf1 | 9466.555 | 10051.72 | 9759.137 |
| ENSMUSG00000073875 | protein_coding | Gm10595 | 2.269904 | 6.481611 | 4.375757 |
| ENSMUSG00000027168 | protein_coding | Pax6 | 8.10173 | 13.06844 | 10.58508 |
| ENSMUSG00000005981 | protein_coding | Trap1 | 2878.587 | 3082.081 | 2980.334 |
| ENSMUSG00000065919 | snRNA | U6 | 0.646531 | 2.392987 | 1.519759 |
| ENSMUSG00000021595 | protein_coding | Nsun2 | 3337.488 | 3120.524 | 3229.006 |
| ENSMUSG00000029178 | protein_coding | Klf3 | 451.7295 | 409.9563 | 430.8429 |
| ENSMUSG00000039166 | protein_coding | Akap7 | 987.0874 | 1068.184 | 1027.636 |
| ENSMUSG00000031974 | protein_coding | Abcb10 | 1626.339 | 1779.193 | 1702.766 |
| ENSMUSG00000031548 | protein_coding | Sfrp1 | 1154.961 | 1277.862 | 1216.412 |
| ENSMUSG00000056459 | protein_coding | Zbtb25 | 498.9664 | 456.6281 | 477.7973 |
| ENSMUSG00000035161 | protein_coding | Ints6 | 4221.657 | 3797.54 | 4009.598 |
| ENSMUSG00000046330 | protein_coding | Rpl37a | 2778.669 | 2555.798 | 2667.234 |
| ENSMUSG00000043157 | protein_coding | Arl11 | 10.34472 | 15.68128 | 13.013 |
| ENSMUSG00000085399 | lincRNA | 9130206I24Rik | 20.69049 | 28.41383 | 24.55216 |
| ENSMUSG00000045092 | protein_coding | S1pr1 | 264.0795 | 386.9721 | 325.5258 |
| ENSMUSG00000029602 | protein_coding | Rasal1 | 152.1881 | 174.2246 | 163.2064 |
| ENSMUSG00000031601 | protein_coding | Cnot7 | 5196.921 | 5564.08 | 5380.501 |
| ENSMUSG00000017734 | protein_coding | Dbndd2 | 121.0644 | 142.7817 | 131.9231 |
| ENSMUSG00000031104 | protein_coding | Rab33a | 31.70657 | 23.38786 | 27.54722 |
| ENSMUSG00000086609 | lincRNA | 5830432E09Rik | 7.731464 | 4.043577 | 5.887521 |
| ENSMUSG00000025213 | protein_coding | Kazald1 | 2054.218 | 2291.855 | 2173.036 |
| ENSMUSG00000084960 | lincRNA | B430010I23Rik | 2.888257 | 7.381155 | 5.134706 |
| ENSMUSG00000067586 | protein_coding | S1pr3 | 498.0639 | 435.4384 | 466.7511 |
| ENSMUSG00000002396 | protein_coding | Ocel1 | 573.4091 | 516.0536 | 544.7314 |
| ENSMUSG00000086219 | processed_transcrip | 2410137F16Rik | 13.82084 | 20.32455 | 17.0727 |
| ENSMUSG00000084133 | pseudogene | Gm12856 | 3.272612 | 6.771726 | 5.022169 |
| ENSMUSG00000048327 | protein_coding | Ckap2l | 1660.041 | 1520.837 | 1590.439 |
| ENSMUSG00000065662 | rRNA | n-R5s161 | 0.646531 | 2.472986 | 1.559758 |
| ENSMUSG00000031156 | protein_coding | Slc35a2 | 373.1067 | 410.3689 | 391.7378 |
| ENSMUSG00000087596 | processed_transcrip | Gm11715 | 0.638331 | 2.36596 | 1.502145 |
| ENSMUSG00000029757 | protein_coding | Dync1i1 | 1031.008 | 1124.581 | 1077.794 |
| ENSMUSG00000034064 | protein_coding | Poglut1 | 1392.196 | 1284.223 | 1338.209 |
| ENSMUSG00000026718 | protein_coding | Stam | 1217.482 | 1310.757 | 1264.119 |
| ENSMUSG00000020303 | protein_coding | Stc2 | 395.0041 | 468.7522 | 431.8781 |
| ENSMUSG00000089944 | pseudogene | Gm8292 | 100.0442 | 80.63725 | 90.34075 |
| ENSMUSG00000052955 | protein_coding | Cpvl | 35.61036 | 26.93877 | 31.27456 |
| ENSMUSG00000044367 | protein_coding | Slc16a13 | 142.95 | 163.8804 | 153.4152 |
| ENSMUSG00000062691 | protein_coding | 1110001A16Rik | 116.5501 | 97.57809 | 107.0641 |
| ENSMUSG00000051951 | protein_coding | Xkr4 | 50.50622 | 65.23635 | 57.87129 |

| | | | | | |
|---|---|---|---|---|---|
| ENSMUSG00000085737 | processed_transcrip | Gm13442 | 0.32211 | 1.686265 | 1.004188 |
| ENSMUSG00000087054 | processed_transcrip | Gm12405 | 0.32211 | 1.686265 | 1.004188 |
| ENSMUSG00000084419 | misc_RNA | 7SK | 0.32211 | 1.686265 | 1.004188 |
| ENSMUSG00000023938 | protein_coding | Aars2 | 460.7224 | 502.8932 | 481.8078 |
| ENSMUSG00000090389 | pseudogene | Gm7236 | 8.700105 | 4.830298 | 6.765201 |
| ENSMUSG00000069270 | protein_coding | Hist1h2ac | 6.760512 | 11.33749 | 9.049001 |
| ENSMUSG00000018102 | protein_coding | Hist1h2bc | 220.5803 | 273.3129 | 246.9466 |
| ENSMUSG00000024507 | protein_coding | Hsd17b4 | 2626.887 | 2451.499 | 2539.193 |
| ENSMUSG00000020186 | protein_coding | Csrp2 | 620.9771 | 710.1852 | 665.5812 |
| ENSMUSG00000004612 | protein_coding | Nkg7 | 6.196204 | 2.481995 | 4.339099 |
| ENSMUSG00000020196 | protein_coding | Cabin1 | 4065.573 | 3735.798 | 3900.685 |
| ENSMUSG00000063382 | protein_coding | Bcl9l | 1763.048 | 1609.501 | 1686.275 |
| ENSMUSG00000086556 | processed_transcrip | Gm15444 | 1.290752 | 3.408205 | 2.349479 |
| ENSMUSG00000043289 | protein_coding | Mei4 | 209.6639 | 181.0687 | 195.3663 |
| ENSMUSG00000085747 | processed_transcrip | Gm11194 | 7.737353 | 12.51058 | 10.12397 |
| ENSMUSG00000070530 | protein_coding | Wfdc16 | 2.579193 | 0.697713 | 1.638453 |
| ENSMUSG00000033430 | protein_coding | Terf2ip | 986.4687 | 907.3589 | 946.9138 |
| ENSMUSG00000058174 | protein_coding | Gm5148 | 6.786379 | 11.415 | 9.100689 |
| ENSMUSG00000031196 | protein_coding | F8 | 219.6478 | 191.7745 | 205.7112 |
| ENSMUSG00000040297 | protein_coding | AI848100 | 5642.408 | 6112.798 | 5877.603 |
| ENSMUSG00000029784 | protein_coding | 1700025E21Rik | 27.61649 | 18.72113 | 23.16881 |
| ENSMUSG00000027259 | protein_coding | Adal | 748.8772 | 679.4238 | 714.1505 |
| ENSMUSG00000024781 | protein_coding | Lipa | 714.2914 | 786.3135 | 750.3024 |
| ENSMUSG00000049154 | protein_coding | Fam183b | 14.51784 | 8.686851 | 11.60235 |
| ENSMUSG00000019718 | protein_coding | 2810055F11Rik | 156.1228 | 187.3499 | 171.7364 |
| ENSMUSG00000083548 | pseudogene | Gm11514 | 0.646531 | 2.36596 | 1.506245 |
| ENSMUSG00000001964 | protein_coding | Emd | 1110.241 | 1204.066 | 1157.154 |
| ENSMUSG00000067541 | protein_coding | A630073D07Rik | 2.295771 | 5.572334 | 3.934052 |
| ENSMUSG00000079644 | protein_coding | Gm1110 | 1.939594 | 4.839307 | 3.38945 |
| ENSMUSG00000036620 | protein_coding | Mgat4b | 3703.774 | 4084.012 | 3893.893 |
| ENSMUSG00000024424 | protein_coding | Ttc39c | 1273.495 | 1377.119 | 1325.307 |
| ENSMUSG00000037606 | protein_coding | Osbpl5 | 936.6343 | 1010.572 | 973.6029 |
| ENSMUSG00000089281 | snoRNA | Scarna6 | 3.888655 | 7.701148 | 5.794901 |
| ENSMUSG00000037860 | protein_coding | Aim2 | 42.01596 | 52.71346 | 47.36471 |
| ENSMUSG00000083093 | pseudogene | Gm15385 | 0.65242 | 2.348303 | 1.500362 |
| ENSMUSG00000064916 | snRNA | U6 | 7.794977 | 13.12213 | 10.45855 |
| ENSMUSG00000023944 | protein_coding | Hsp90ab1 | 32951.13 | 35423.5 | 34187.31 |
| ENSMUSG00000023045 | protein_coding | Soat2 | 7.461088 | 12.15704 | 9.809062 |
| ENSMUSG00000047841 | protein_coding | BC051628 | 3.558345 | 1.386417 | 2.472381 |
| ENSMUSG00000024862 | protein_coding | Klc2 | 2675.823 | 2392.663 | 2534.243 |
| ENSMUSG00000009076 | protein_coding | Zmat5 | 280.8107 | 251.6223 | 266.2165 |
| ENSMUSG00000032298 | protein_coding | Neil1 | 112.2461 | 133.5407 | 122.8934 |
| ENSMUSG00000024927 | protein_coding | Rela | 1737.459 | 1910.49 | 1823.974 |
| ENSMUSG00000024645 | protein_coding | 1700034H14Rik | 697.6562 | 771.2925 | 734.4744 |
| ENSMUSG00000031066 | protein_coding | Usp11 | 1139.816 | 1227.138 | 1183.477 |
| ENSMUSG00000089917 | protein_coding | Uckl1 | 438.1664 | 489.4714 | 463.8189 |
| ENSMUSG00000053166 | protein_coding | Cdh22 | 9.039883 | 5.139155 | 7.089519 |
| ENSMUSG00000063239 | protein_coding | Grm4 | 154.0956 | 190.8359 | 172.4658 |
| ENSMUSG00000028223 | protein_coding | Decr1 | 957.3231 | 1043.826 | 1000.575 |
| ENSMUSG00000084764 | processed_transcrip | 4930564G21Rik | 0.646531 | 2.357312 | 1.501922 |
| ENSMUSG00000091831 | pseudogene | Gm4707 | 1.298952 | 3.850755 | 2.574853 |
| ENSMUSG00000042389 | protein_coding | Tsen2 | 539.269 | 492.8747 | 516.0719 |
| ENSMUSG00000083961 | pseudogene | Gm13725 | 3.886344 | 1.422092 | 2.654218 |
| ENSMUSG00000028524 | protein_coding | Sgip1 | 86.52464 | 69.35843 | 77.94153 |
| ENSMUSG00000020737 | protein_coding | Hn1 | 5029.551 | 5319.957 | 5174.754 |
| ENSMUSG00000022420 | protein_coding | Dnalc4 | 592.7341 | 644.3263 | 618.5302 |
| ENSMUSG00000067399 | protein_coding | Trim43a | 21.03154 | 14.45522 | 17.74338 |
| ENSMUSG00000085502 | processed_transcrip | Gm12320 | 0.638331 | 2.339294 | 1.488812 |

| | | | | | |
|---|---|---|---|---|---|
| ENSMUSG00000024851 | protein_coding | Pitpnm1 | 422.677 | 378.8856 | 400.7813 |
| ENSMUSG00000025647 | protein_coding | Shisa5 | 577.2671 | 633.2255 | 605.2463 |
| ENSMUSG00000084648 | snRNA | U6 | 3.205746 | 1.050893 | 2.12832 |
| ENSMUSG00000082070 | pseudogene | Gm1866 | 8.687284 | 14.25302 | 11.47015 |
| ENSMUSG00000020059 | protein_coding | Sycp3 | 1915.39 | 1287.923 | 1601.657 |
| ENSMUSG00000072582 | protein_coding | Ptrh2 | 680.9705 | 744.6561 | 712.8133 |
| ENSMUSG00000014791 | protein_coding | Elmo3 | 182.7909 | 152.3179 | 167.5544 |
| ENSMUSG00000026553 | protein_coding | Copa | 7872.665 | 7364.073 | 7618.369 |
| ENSMUSG00000021732 | protein_coding | Fgf10 | 21.87464 | 30.8879 | 26.38127 |
| ENSMUSG00000036295 | protein_coding | Lrrn3 | 29.32395 | 40.75364 | 35.0388 |
| ENSMUSG00000074115 | protein_coding | Saa1 | 5.811849 | 9.817742 | 7.814795 |
| ENSMUSG00000087198 | processed_transcrip | Gm13097 | 19.89092 | 12.18691 | 16.03891 |
| ENSMUSG00000058396 | protein_coding | Gpr182 | 42.24996 | 32.00336 | 37.12666 |
| ENSMUSG00000018819 | protein_coding | Lsp1 | 1197.961 | 1360.241 | 1279.101 |
| ENSMUSG00000050623 | protein_coding | 1700019N12Rik | 15.49364 | 10.17092 | 12.83228 |
| ENSMUSG00000045179 | protein_coding | Sox3 | 808.5295 | 707.6037 | 758.0666 |
| ENSMUSG00000063661 | protein_coding | Krt73 | 1.923194 | 4.468108 | 3.195651 |
| ENSMUSG00000079444 | protein_coding | Prickle4 | 21.3488 | 14.9832 | 18.166 |
| ENSMUSG00000006931 | protein_coding | Leprel4 | 1440.686 | 1316.297 | 1378.492 |
| ENSMUSG00000010048 | protein_coding | Ifrd2 | 875.2548 | 783.9467 | 829.6007 |
| ENSMUSG00000020611 | protein_coding | Gna13 | 2983.132 | 3195.713 | 3089.422 |
| ENSMUSG00000029102 | protein_coding | Hgfac | 4.551586 | 8.55637 | 6.553978 |
| ENSMUSG00000026736 | protein_coding | 4930426L09Rik | 7.044977 | 12.22875 | 9.636862 |
| ENSMUSG00000068428 | protein_coding | Gm606 | 4.202565 | 1.748606 | 2.975586 |
| ENSMUSG00000021176 | protein_coding | 2610021K21Rik | 193.2771 | 171.1376 | 182.2073 |
| ENSMUSG00000074127 | protein_coding | Cmtm2a | 0.33031 | 1.695274 | 1.012792 |
| ENSMUSG00000091828 | lincRNA | 5033417F24Rik | 40.38647 | 30.97297 | 35.67972 |
| ENSMUSG00000031629 | protein_coding | Mlf1ip | 304.4513 | 273.8349 | 289.1431 |
| ENSMUSG00000071041 | pseudogene | Gm15210 | 720.7529 | 661.9828 | 691.3678 |
| ENSMUSG00000040883 | protein_coding | Tmem205 | 462.5931 | 420.311 | 441.452 |
| ENSMUSG00000078881 | protein_coding | Gm14434 | 0.32211 | 1.695274 | 1.008692 |
| ENSMUSG00000039462 | protein_coding | Col10a1 | 0.32211 | 1.695274 | 1.008692 |
| ENSMUSG00000089223 | snRNA | U6 | 0.32211 | 1.695274 | 1.008692 |
| ENSMUSG00000055407 | protein_coding | Mtap6 | 258.1807 | 286.3087 | 272.2447 |
| ENSMUSG00000091395 | lincRNA | Gm6297 | 115.6147 | 95.79774 | 105.7062 |
| ENSMUSG00000075297 | protein_coding | H60b | 14.70368 | 23.28192 | 18.9928 |
| ENSMUSG00000032899 | protein_coding | Styk1 | 1.284863 | 3.417215 | 2.351039 |
| ENSMUSG00000028986 | protein_coding | Klhl7 | 1785.411 | 1633.215 | 1709.313 |
| ENSMUSG00000070357 | protein_coding | E030019B06Rik | 6.174958 | 2.7995 | 4.487229 |
| ENSMUSG00000004462 | protein_coding | Tbccd1 | 786.715 | 727.0574 | 756.8862 |
| ENSMUSG00000074303 | processed_transcrip | Gm10664 | 2.569725 | 5.465308 | 4.017516 |
| ENSMUSG00000061859 | protein_coding | Inadl | 648.9833 | 709.5357 | 679.2595 |
| ENSMUSG00000091633 | lincRNA | Gm17280 | 5.185295 | 2.348303 | 3.766799 |
| ENSMUSG00000034040 | protein_coding | Wbscr17 | 28.78439 | 41.34314 | 35.06377 |
| ENSMUSG00000037936 | protein_coding | Scarb1 | 4930.613 | 5415.349 | 5172.981 |
| ENSMUSG00000065453 | miRNA | Mirlet7d | 41.36503 | 30.94703 | 36.15603 |
| ENSMUSG00000028988 | protein_coding | Ctnnbip1 | 582.5389 | 536.8368 | 559.6879 |
| ENSMUSG00000015355 | protein_coding | Cd48 | 27.80256 | 20.32383 | 24.06319 |
| ENSMUSG00000006345 | protein_coding | Ggt1 | 29.79851 | 38.81361 | 34.30606 |
| ENSMUSG00000022101 | protein_coding | Fgf17 | 10.41032 | 5.783174 | 8.096748 |
| ENSMUSG00000037020 | protein_coding | Wdr62 | 961.1174 | 827.263 | 894.1902 |
| ENSMUSG00000078651 | protein_coding | Aoc2 | 134.3172 | 157.3018 | 145.8095 |
| ENSMUSG00000078491 | protein_coding | Gm13090 | 15.34037 | 7.682768 | 11.51157 |
| ENSMUSG00000039720 | protein_coding | Got1l1 | 68.97936 | 56.64064 | 62.81 |
| ENSMUSG00000028989 | protein_coding | Angptl7 | 16.43179 | 24.32417 | 20.37798 |
| ENSMUSG00000081676 | pseudogene | Gm11643 | 6.814557 | 3.470547 | 5.142552 |
| ENSMUSG00000037761 | protein_coding | Actr5 | 599.9805 | 545.3541 | 572.6673 |
| ENSMUSG00000018326 | protein_coding | Ywhab | 7677.702 | 8229.247 | 7953.475 |

| | | | | | |
|---|---|---|---|---|---|
| ENSMUSG00000045594 | protein_coding | Glb1 | 626.1824 | 679.2175 | 652.6999 |
| ENSMUSG00000081604 | pseudogene | Gm11518 | 88.67326 | 74.58986 | 81.63156 |
| ENSMUSG00000085071 | lincRNA | Gm14066 | 13.82084 | 20.03335 | 16.9271 |
| ENSMUSG00000033352 | protein_coding | Map2k4 | 3019.6 | 3207.686 | 3113.643 |
| ENSMUSG00000029314 | protein_coding | Agpat9 | 11.5991 | 7.142925 | 9.371011 |
| ENSMUSG00000037148 | protein_coding | Arhgap10 | 976.6622 | 897.2587 | 936.9604 |
| ENSMUSG00000020390 | protein_coding | Ube2b | 3551.75 | 3848.165 | 3699.957 |
| ENSMUSG00000053977 | protein_coding | Cd8a | 5.864626 | 2.719502 | 4.292064 |
| ENSMUSG00000089812 | processed_transcrip | Gm15867 | 23.18284 | 31.41267 | 27.29776 |
| ENSMUSG00000034620 | protein_coding | Tmem5 | 1175.883 | 1083.964 | 1129.924 |
| ENSMUSG00000035104 | protein_coding | Fam176a | 62.87254 | 50.98038 | 56.92646 |
| ENSMUSG00000004530 | protein_coding | Coro1c | 3088.135 | 2857.94 | 2973.038 |
| ENSMUSG00000037432 | processed_transcrip | Fer1l5 | 275.509 | 241.3835 | 258.4463 |
| ENSMUSG00000036553 | protein_coding | Sh3tc1 | 176.3791 | 150.4458 | 163.4125 |
| ENSMUSG00000035561 | protein_coding | Aldh1b1 | 1165.308 | 1265.231 | 1215.269 |
| ENSMUSG00000068706 | protein_coding | Gm10250 | 200.8462 | 175.0944 | 187.9703 |
| ENSMUSG00000090378 | protein_coding | Gm10925 | 1437.729 | 1215.374 | 1326.551 |
| ENSMUSG00000027207 | protein_coding | Galk2 | 569.6755 | 630.7438 | 600.2096 |
| ENSMUSG00000042594 | protein_coding | Sh2b3 | 1012.648 | 940.5079 | 976.5779 |
| ENSMUSG00000067629 | protein_coding | Syngap1 | 455.655 | 384.3954 | 420.0252 |
| ENSMUSG00000055313 | protein_coding | Pgbd1 | 56.72389 | 70.61442 | 63.66915 |
| ENSMUSG00000061702 | protein_coding | Tmem91 | 67.05684 | 53.66276 | 60.3598 |
| ENSMUSG00000030518 | protein_coding | Fam189a1 | 21.01387 | 28.32518 | 24.66953 |
| ENSMUSG00000034573 | protein_coding | Ptpn13 | 3099.154 | 3332.103 | 3215.628 |
| ENSMUSG00000037519 | protein_coding | Ppfia1 | 3885.706 | 3599.641 | 3742.674 |
| ENSMUSG00000027004 | protein_coding | Frzb | 706.2314 | 1010.756 | 858.4936 |
| ENSMUSG00000092212 | polymorphic_pseuc | Slc22a13b | 19.89912 | 12.53688 | 16.218 |
| ENSMUSG00000019814 | protein_coding | Ltv1 | 1799.458 | 2013.274 | 1906.366 |
| ENSMUSG00000034574 | protein_coding | Daam1 | 956.4671 | 887.2149 | 921.841 |
| ENSMUSG00000038704 | protein_coding | Aspdh | 5.157117 | 9.280025 | 7.218571 |
| ENSMUSG00000024099 | protein_coding | Ndufv2 | 2585.986 | 2418.686 | 2502.336 |
| ENSMUSG00000035629 | protein_coding | 1700021K19Rik | 1855.896 | 1708.231 | 1782.064 |
| ENSMUSG00000001105 | protein_coding | Ift20 | 1160.05 | 1256.048 | 1208.049 |
| ENSMUSG00000061079 | protein_coding | Zfp143 | 623.8523 | 682.2026 | 653.0274 |
| ENSMUSG00000031109 | protein_coding | Enox2 | 402.0209 | 365.0428 | 383.5318 |
| ENSMUSG00000047238 | protein_coding | Mageh1 | 326.1745 | 368.9044 | 347.5395 |
| ENSMUSG00000090452 | lincRNA | 1700022N22Rik | 45.75261 | 33.87406 | 39.81334 |
| ENSMUSG00000050493 | protein_coding | Fam167b | 7.785509 | 3.734358 | 5.759934 |
| ENSMUSG00000025237 | protein_coding | Parp6 | 912.6053 | 817.5224 | 865.0639 |
| ENSMUSG00000053134 | protein_coding | Supt7l | 710.0952 | 771.166 | 740.6306 |
| ENSMUSG00000026771 | protein_coding | Spopl | 991.2525 | 899.1235 | 945.188 |
| ENSMUSG00000069727 | protein_coding | Gm5595 | 525.6701 | 599.8069 | 562.7385 |
| ENSMUSG00000084888 | lincRNA | Gm16908 | 26.42563 | 41.77373 | 34.09968 |
| ENSMUSG00000041912 | protein_coding | Tdrkh | 2051.939 | 1846.792 | 1949.365 |
| ENSMUSG00000092099 | protein_coding | Gm17530 | 10.29657 | 15.66362 | 12.98009 |
| ENSMUSG00000086654 | processed_transcrip | Gm13165 | 5.405204 | 2.08413 | 3.744667 |
| ENSMUSG00000022425 | protein_coding | Enpp2 | 3318.247 | 3597.27 | 3457.758 |
| ENSMUSG00000032285 | protein_coding | Dnaja4 | 238.3653 | 210.473 | 224.4191 |
| ENSMUSG00000033590 | protein_coding | Myo5c | 45.52741 | 57.91894 | 51.72318 |
| ENSMUSG00000091427 | lincRNA | Gm17508 | 0.33031 | 1.677256 | 1.003783 |
| ENSMUSG00000000049 | protein_coding | Apoh | 0.33031 | 1.677256 | 1.003783 |
| ENSMUSG00000060673 | protein_coding | 4930595M18Rik | 148.0934 | 182.8494 | 165.4714 |
| ENSMUSG00000000794 | protein_coding | Kcnn3 | 5.825937 | 10.05489 | 7.940412 |
| ENSMUSG00000048185 | protein_coding | Gm7075 | 5.201695 | 2.410644 | 3.80617 |
| ENSMUSG00000020164 | processed_transcrip | 1700058G18Rik | 1.264884 | 4.016911 | 2.640898 |
| ENSMUSG00000019927 | protein_coding | Ube2d1 | 900.6744 | 989.5223 | 945.0984 |
| ENSMUSG00000005054 | protein_coding | Cstb | 692.7156 | 748.2761 | 720.4958 |
| ENSMUSG00000039156 | protein_coding | Stim2 | 1521.896 | 1377.702 | 1449.799 |

| | | | | | |
|---|---|---|---|---|---|
| ENSMUSG00000040650 | protein_coding | Cyp2b23 | 2.577925 | 0.671047 | 1.624486 |
| ENSMUSG00000030235 | protein_coding | Slco1c1 | 6.825068 | 3.461538 | 5.143303 |
| ENSMUSG00000032702 | protein_coding | Kank1 | 2302.04 | 2133.484 | 2217.762 |
| ENSMUSG00000083382 | pseudogene | Gm6433 | 2.583814 | 0.724379 | 1.654097 |
| ENSMUSG00000045083 | protein_coding | Lingo2 | 186.8918 | 163.3967 | 175.1443 |
| ENSMUSG00000038324 | protein_coding | Trpc4ap | 3321.802 | 3093.245 | 3207.524 |
| ENSMUSG00000020290 | protein_coding | Xpo1 | 12262.9 | 13057.02 | 12659.96 |
| ENSMUSG00000052688 | protein_coding | 5430435G22Rik | 1758.787 | 1920.959 | 1839.873 |
| ENSMUSG00000046879 | protein_coding | Irgm1 | 187.4514 | 217.1999 | 202.3257 |
| ENSMUSG00000026814 | protein_coding | Eng | 654.4184 | 576.0321 | 615.2252 |
| ENSMUSG00000082186 | pseudogene | Gm12403 | 1.945483 | 4.423424 | 3.184453 |
| ENSMUSG00000047735 | protein_coding | Samd9l | 545.196 | 637.6202 | 591.4081 |
| ENSMUSG00000033629 | protein_coding | Ptplad1 | 3702.426 | 3946.508 | 3824.467 |
| ENSMUSG00000066116 | protein_coding | Gm10154 | 39.31839 | 29.72641 | 34.5224 |
| ENSMUSG00000044647 | protein_coding | Csrnp3 | 115.148 | 95.26939 | 105.2087 |
| ENSMUSG00000028467 | protein_coding | Gba2 | 865.957 | 930.6494 | 898.3032 |
| ENSMUSG00000030630 | protein_coding | Fah | 266.3276 | 298.4863 | 282.4069 |
| ENSMUSG00000073069 | protein_coding | Mageb2 | 43.89584 | 55.7137 | 49.80477 |
| ENSMUSG00000047842 | protein_coding | Diras2 | 76.81176 | 91.1523 | 83.98203 |
| ENSMUSG00000086264 | lincRNA | Gm15850 | 9.984968 | 5.854525 | 7.919746 |
| ENSMUSG00000027654 | protein_coding | Fam83d | 737.8075 | 652.9332 | 695.3704 |
| ENSMUSG00000085988 | lincRNA | Gm16896 | 62.07401 | 76.53024 | 69.30212 |
| ENSMUSG00000025920 | protein_coding | Stau2 | 1077.047 | 1153.582 | 1115.315 |
| ENSMUSG00000082332 | pseudogene | Gm13384 | 2.561525 | 5.438642 | 4.000083 |
| ENSMUSG00000043843 | protein_coding | Tmem145 | 22.42195 | 33.15911 | 27.79053 |
| ENSMUSG00000078920 | protein_coding | Ifi47 | 44.47133 | 33.08734 | 38.77934 |
| ENSMUSG00000027861 | protein_coding | Casq2 | 1399.013 | 1568.245 | 1483.629 |
| ENSMUSG00000064220 | protein_coding | Hist2h2aa2 | 75.44154 | 91.13169 | 83.28662 |
| ENSMUSG00000078439 | protein_coding | 2210404O07Rik | 75.92072 | 62.73588 | 69.3283 |
| ENSMUSG00000082208 | pseudogene | Gm15379 | 2.911813 | 6.011672 | 4.461743 |
| ENSMUSG00000034799 | protein_coding | Unc13a | 98.17621 | 72.68292 | 85.42956 |
| ENSMUSG00000032666 | protein_coding | 1700025G04Rik | 1715.827 | 1896.596 | 1806.211 |
| ENSMUSG00000026304 | protein_coding | Rab17 | 6.376156 | 11.67374 | 9.024946 |
| ENSMUSG00000075528 | protein_coding | Aarsd1 | 878.1929 | 814.2228 | 846.2079 |
| ENSMUSG00000090107 | protein_coding | Gm11492 | 1.609283 | 4.36072 | 2.985002 |
| ENSMUSG00000032292 | protein_coding | Nr2e3 | 10.00495 | 5.546029 | 7.775488 |
| ENSMUSG00000092149 | lincRNA | Gm17453 | 16.63701 | 28.20004 | 22.41853 |
| ENSMUSG00000050350 | protein_coding | Gpr18 | 2.611992 | 5.685158 | 4.148575 |
| ENSMUSG00000049001 | protein_coding | A930038C07Rik | 14.14169 | 8.351327 | 11.24651 |
| ENSMUSG00000064347 | Mt_tRNA | mt-Ta | 2.597903 | 0.724379 | 1.661141 |
| ENSMUSG00000044876 | protein_coding | Zfp444 | 704.1264 | 767.7054 | 735.9159 |
| ENSMUSG00000050312 | protein_coding | Nsun3 | 535.6392 | 479.6284 | 507.6338 |
| ENSMUSG00000024668 | protein_coding | Sdhaf2 | 1125.143 | 1034.99 | 1080.067 |
| ENSMUSG00000090634 | protein_coding | Gm8126 | 30.41224 | 42.03941 | 36.22582 |
| ENSMUSG00000020911 | protein_coding | Krt19 | 9.694614 | 14.73705 | 12.21583 |
| ENSMUSG00000026576 | protein_coding | Atp1b1 | 4994.446 | 4565.076 | 4779.761 |
| ENSMUSG00000020859 | protein_coding | Spag9 | 7145.582 | 7678.607 | 7412.094 |
| ENSMUSG00000019992 | protein_coding | Fam54a | 428.2274 | 390.6185 | 409.4229 |
| ENSMUSG00000006800 | protein_coding | Sulf2 | 3557.09 | 3239.871 | 3398.48 |
| ENSMUSG00000032076 | protein_coding | Cadm1 | 6778.334 | 6237.231 | 6507.782 |
| ENSMUSG00000035642 | protein_coding | 1810020D17Rik | 528.8447 | 486.8104 | 507.8276 |
| ENSMUSG00000071001 | protein_coding | Hrct1 | 29.26864 | 38.74335 | 34.00599 |
| ENSMUSG00000019188 | protein_coding | H13 | 2058.825 | 2221.998 | 2140.411 |
| ENSMUSG00000063851 | protein_coding | Rnf183 | 7.496198 | 13.61765 | 10.55693 |
| ENSMUSG00000036596 | protein_coding | Cpz | 138.7165 | 117.8086 | 128.2626 |
| ENSMUSG00000029135 | protein_coding | Fosl2 | 1022.518 | 891.2192 | 956.8689 |
| ENSMUSG00000014301 | protein_coding | Pam16 | 403.4535 | 442.7655 | 423.1095 |
| ENSMUSG00000065202 | snoRNA | SNORA20 | 1.298952 | 0 | 0.649476 |

| | | | | | |
|---|---|---|---|---|---|
| ENSMUSG00000080354 | miRNA | CT571271.1 | 1.298952 | 0 | 0.649476 |
| ENSMUSG00000084849 | processed_transcrip | Gm16105 | 1.298952 | 0 | 0.649476 |
| ENSMUSG00000077649 | snoRNA | SNORD86 | 1.298952 | 0 | 0.649476 |
| ENSMUSG00000063911 | pseudogene | Tdgf1-ps2 | 1.298952 | 0 | 0.649476 |
| ENSMUSG00000049123 | protein_coding | Catsperg2 | 31.7457 | 19.71833 | 25.73202 |
| ENSMUSG00000022244 | protein_coding | Amacr | 312.6316 | 273.5665 | 293.099 |
| ENSMUSG00000014773 | protein_coding | Dll1 | 30.48664 | 21.79024 | 26.13844 |
| ENSMUSG00000027344 | protein_coding | Fsip1 | 140.2479 | 169.1076 | 154.6777 |
| ENSMUSG00000065343 | misc_RNA | 7SK | 10.64455 | 6.445212 | 8.544879 |
| ENSMUSG00000058318 | protein_coding | Phf21a | 2097.396 | 1936.057 | 2016.727 |
| ENSMUSG00000092103 | lincRNA | Gm17039 | 3.536056 | 6.861095 | 5.198576 |
| ENSMUSG00000004698 | protein_coding | Hdac9 | 323.2153 | 290.3001 | 306.7577 |
| ENSMUSG00000066452 | protein_coding | Gm8226 | 4.521097 | 2.048455 | 3.284776 |
| ENSMUSG00000030751 | protein_coding | Psma1 | 2944.228 | 3156.083 | 3050.156 |
| ENSMUSG00000034336 | protein_coding | Ina | 171.2828 | 148.8684 | 160.0756 |
| ENSMUSG00000087023 | lincRNA | 1110020A21Rik | 9.678214 | 14.68335 | 12.18078 |
| ENSMUSG00000030145 | protein_coding | Zfp248 | 255.5001 | 289.6457 | 272.5729 |
| ENSMUSG00000028703 | protein_coding | Lrrc41 | 2488.707 | 2317.111 | 2402.909 |
| ENSMUSG00000047221 | protein_coding | Fam185a | 375.7969 | 341.9284 | 358.8626 |
| ENSMUSG00000075511 | protein_coding | 1700001L05Rik | 1189.277 | 828.345 | 1008.811 |
| ENSMUSG00000039699 | protein_coding | Batf2 | 5.506138 | 9.660595 | 7.583367 |
| ENSMUSG00000032040 | protein_coding | Dcps | 1113.222 | 1037.679 | 1075.451 |
| ENSMUSG00000085419 | processed_transcrip | Gm11734 | 7.729153 | 12.39775 | 10.06345 |
| ENSMUSG00000055775 | protein_coding | Myh8 | 101.6954 | 75.05245 | 88.37393 |
| ENSMUSG00000024083 | protein_coding | Pja2 | 3729.383 | 3513.874 | 3621.629 |
| ENSMUSG00000086417 | processed_transcrip | Gm12996 | 16.22344 | 9.747115 | 12.98528 |
| ENSMUSG00000032484 | protein_coding | Ngp | 5.195806 | 2.401635 | 3.79872 |
| ENSMUSG00000062591 | protein_coding | Tubb4 | 36.33486 | 48.38706 | 42.36096 |
| ENSMUSG00000029026 | protein_coding | Trp73 | 13.64425 | 21.04205 | 17.34315 |
| ENSMUSG00000031229 | protein_coding | Atrx | 5652.259 | 6225.862 | 5939.061 |
| ENSMUSG00000078490 | protein_coding | C030017K20Rik | 24.75872 | 35.52036 | 30.13954 |
| ENSMUSG00000085164 | lincRNA | Gm16704 | 3.196278 | 6.976407 | 5.086343 |
| ENSMUSG00000085982 | lincRNA | 9530051G07Rik | 9.342015 | 14.25017 | 11.7961 |
| ENSMUSG00000049225 | protein_coding | Pdp1 | 3364.832 | 3601.909 | 3483.371 |
| ENSMUSG00000090377 | protein_coding | Gm8281 | 1.914994 | 0.335524 | 1.125259 |
| ENSMUSG00000027867 | protein_coding | Spag17 | 6.79689 | 3.452528 | 5.124709 |
| ENSMUSG00000030255 | protein_coding | Sspn | 972.4363 | 702.8775 | 837.6569 |
| ENSMUSG00000029098 | protein_coding | Acox3 | 384.3825 | 433.7014 | 409.042 |
| ENSMUSG00000025758 | protein_coding | Plk4 | 2031.795 | 1884.404 | 1958.099 |
| ENSMUSG00000021506 | protein_coding | Pitx1 | 14.77309 | 9.32471 | 12.0489 |
| ENSMUSG00000021072 | protein_coding | Tmx1 | 2112.403 | 1952.475 | 2032.439 |
| ENSMUSG00000056271 | protein_coding | Lman1l | 5.157117 | 2.419653 | 3.788385 |
| ENSMUSG00000036961 | protein_coding | Wnt8b | 10.68912 | 6.073652 | 8.381387 |
| ENSMUSG00000020788 | protein_coding | Atp2a3 | 549.7129 | 476.7881 | 513.2505 |
| ENSMUSG00000047591 | protein_coding | Mafa | 171.2143 | 209.0078 | 190.1111 |
| ENSMUSG00000024604 | protein_coding | Rbm22 | 1530.39 | 1646.599 | 1588.495 |
| ENSMUSG00000091997 | pseudogene | Gm6611 | 1.931394 | 4.530449 | 3.230922 |
| ENSMUSG00000037152 | protein_coding | Ndufc1 | 605.3973 | 550.0634 | 577.7304 |
| ENSMUSG00000085857 | processed_transcrip | Gm15392 | 1.290752 | 0 | 0.645376 |
| ENSMUSG00000087079 | processed_transcrip | Gm13389 | 1.290752 | 0 | 0.645376 |
| ENSMUSG00000056494 | protein_coding | Cngb3 | 1.290752 | 0 | 0.645376 |
| ENSMUSG00000067156 | protein_coding | Gm7337 | 1.290752 | 0 | 0.645376 |
| ENSMUSG00000048015 | protein_coding | Neurod4 | 1.290752 | 0 | 0.645376 |
| ENSMUSG00000063885 | protein_coding | Gm6498 | 1.290752 | 0 | 0.645376 |
| ENSMUSG00000064623 | snRNA | U6 | 1.290752 | 0 | 0.645376 |
| ENSMUSG00000066392 | protein_coding | Nrxn3 | 27.76849 | 20.28887 | 24.02868 |
| ENSMUSG00000035212 | protein_coding | Leprot | 2627.02 | 2801.486 | 2714.253 |
| ENSMUSG00000021708 | protein_coding | Rasgrf2 | 59.01541 | 71.76514 | 65.39028 |

| | | | | | |
|---|---|---|---|---|---|
| ENSMUSG00000025817 | protein_coding | Nudt5 | 1001.103 | 927.0633 | 964.0834 |
| ENSMUSG00000060981 | protein_coding | Hist1h4h | 6.125759 | 10.85383 | 8.489793 |
| ENSMUSG00000044400 | protein_coding | Ankrd58 | 23.79366 | 35.35886 | 29.57626 |
| ENSMUSG00000092164 | protein_coding | Rergl | 12.93212 | 18.73594 | 15.83403 |
| ENSMUSG00000079018 | protein_coding | Ly6c1 | 56.12678 | 71.40005 | 63.76342 |
| ENSMUSG00000020973 | protein_coding | 1110034A24Rik | 764.9873 | 685.2608 | 725.1241 |
| ENSMUSG00000022146 | protein_coding | Osmr | 115.3407 | 139.2322 | 127.2864 |
| ENSMUSG00000043298 | protein_coding | C030030A07Rik | 20.39656 | 13.96762 | 17.18209 |
| ENSMUSG00000021096 | protein_coding | Ppm1a | 3234.805 | 3479.303 | 3357.054 |
| ENSMUSG00000030201 | protein_coding | Lrp6 | 3642.311 | 3417.155 | 3529.733 |
| ENSMUSG00000047875 | protein_coding | Gpr157 | 211.7109 | 183.9943 | 197.8526 |
| ENSMUSG00000055301 | protein_coding | Adh7 | 6.097581 | 10.58717 | 8.342374 |
| ENSMUSG00000032252 | protein_coding | Glce | 2872.427 | 2663.011 | 2767.719 |
| ENSMUSG00000034259 | protein_coding | Exosc4 | 715.7156 | 773.5089 | 744.6122 |
| ENSMUSG00000092353 | processed_transcrip | RP23-396N6.7 | 3.888655 | 7.603493 | 5.746074 |
| ENSMUSG00000057342 | protein_coding | Sphk2 | 1648.469 | 1770.045 | 1709.257 |
| ENSMUSG00000028639 | protein_coding | Ybx1 | 7515.723 | 7033.891 | 7274.807 |
| ENSMUSG00000056978 | protein_coding | Hamp2 | 5.509716 | 9.553931 | 7.531824 |
| ENSMUSG00000015354 | protein_coding | Pcolce2 | 22.32922 | 30.08208 | 26.20565 |
| ENSMUSG00000023110 | protein_coding | Prmt5 | 2188.321 | 2340.626 | 2264.473 |
| ENSMUSG00000031382 | protein_coding | Asb11 | 2.928213 | 1.024227 | 1.97622 |
| ENSMUSG00000051225 | protein_coding | Fam83a | 2.597903 | 5.402966 | 4.000435 |
| ENSMUSG00000034855 | protein_coding | Cxcl10 | 14.3566 | 8.33652 | 11.34656 |
| ENSMUSG00000091989 | pseudogene | Gm17257 | 40.02001 | 31.35318 | 35.6866 |
| ENSMUSG00000021423 | protein_coding | Ly86 | 56.77287 | 45.3208 | 51.04684 |
| ENSMUSG00000065829 | snoRNA | Snord16a | 13.61376 | 20.3101 | 16.96193 |
| ENSMUSG00000075706 | protein_coding | Gpx4 | 2721.251 | 2530.708 | 2625.98 |
| ENSMUSG00000028917 | protein_coding | Plekhm2 | 893.9458 | 959.8881 | 926.9169 |
| ENSMUSG00000050533 | pseudogene | Gm9845 | 1.920883 | 0.335524 | 1.128203 |
| ENSMUSG00000029778 | protein_coding | Adcyap1r1 | 394.9552 | 545.7887 | 470.3719 |
| ENSMUSG00000041378 | protein_coding | Cldn5 | 168.3792 | 198.9444 | 183.6618 |
| ENSMUSG00000037529 | protein_coding | Prss40 | 1.284863 | 0 | 0.642431 |
| ENSMUSG00000083115 | pseudogene | Gm13386 | 1.284863 | 0 | 0.642431 |
| ENSMUSG00000051788 | protein_coding | 4930564D02Rik | 1.284863 | 0 | 0.642431 |
| ENSMUSG00000083720 | pseudogene | Gm12901 | 1.284863 | 0 | 0.642431 |
| ENSMUSG00000083203 | pseudogene | Gm11421 | 1.284863 | 0 | 0.642431 |
| ENSMUSG00000028085 | protein_coding | Pet112l | 576.4981 | 527.0757 | 551.7869 |
| ENSMUSG00000046958 | protein_coding | 4930432E11Rik | 0.64422 | 2.826166 | 1.735193 |
| ENSMUSG00000002020 | protein_coding | Ltbp2 | 301.5331 | 363.8787 | 332.7059 |
| ENSMUSG00000072774 | protein_coding | Zfp951 | 340.3227 | 288.5501 | 314.4364 |
| ENSMUSG00000020401 | protein_coding | Fam71b | 4.215386 | 1.712931 | 2.964159 |
| ENSMUSG00000028073 | protein_coding | Pear1 | 404.1601 | 456.9208 | 430.5404 |
| ENSMUSG00000063281 | protein_coding | Zfp35 | 835.7734 | 919.7426 | 877.758 |
| ENSMUSG00000067773 | protein_coding | Defb41 | 1.920883 | 0.326514 | 1.123699 |
| ENSMUSG00000024261 | protein_coding | Syt4 | 24.60665 | 15.98112 | 20.29389 |
| ENSMUSG00000024976 | protein_coding | Shoc2 | 2715.022 | 2504.824 | 2609.923 |
| ENSMUSG00000058638 | protein_coding | Zfp110 | 1217.782 | 1334.845 | 1276.314 |
| ENSMUSG00000059923 | protein_coding | Grb2 | 2510.27 | 2746.726 | 2628.498 |
| ENSMUSG00000057789 | protein_coding | Bak1 | 426.7255 | 473.5987 | 450.1621 |
| ENSMUSG00000079966 | pseudogene | Gm14058 | 9.068061 | 14.04798 | 11.55802 |
| ENSMUSG00000085721 | processed_transcrip | Gm12796 | 2.905924 | 5.801193 | 4.353559 |
| ENSMUSG00000041775 | protein_coding | Mapk1ip1 | 477.4 | 425.9929 | 451.6964 |
| ENSMUSG00000015804 | protein_coding | Med28 | 1486.405 | 1380.741 | 1433.573 |
| ENSMUSG00000087968 | snoRNA | SCARNA6 | 1.967772 | 0.36219 | 1.164981 |
| ENSMUSG00000022875 | protein_coding | Kng1 | 1.967772 | 0.36219 | 1.164981 |
| ENSMUSG00000028675 | protein_coding | Pnrc2 | 2311.994 | 2544.379 | 2428.186 |
| ENSMUSG00000009569 | protein_coding | Mkl2 | 2206.436 | 2380.984 | 2293.71 |
| ENSMUSG00000001305 | protein_coding | Rrp15 | 1004.721 | 1076.252 | 1040.486 |

| | | | | | |
|---|---|---|---|---|---|
| ENSMUSG00000007564 | protein_coding | Ppp2r1a | 4736.2 | 5073.988 | 4905.094 |
| ENSMUSG00000048007 | protein_coding | Timm8a1 | 553.0726 | 504.9997 | 529.0361 |
| ENSMUSG00000026761 | protein_coding | Orc4 | 1610.005 | 1741.165 | 1675.585 |
| ENSMUSG00000032238 | protein_coding | Rora | 583.3271 | 650.399 | 616.863 |
| ENSMUSG00000063296 | protein_coding | Tmem117 | 134.0167 | 110.0861 | 122.0514 |
| ENSMUSG00000067562 | protein_coding | Dmrtc1c1 | 5.864626 | 2.728149 | 4.296388 |
| ENSMUSG00000020515 | protein_coding | Cnot8 | 1614.869 | 1724.652 | 1669.761 |
| ENSMUSG00000038917 | protein_coding | 3930402G23Rik | 1.917305 | 4.494774 | 3.206039 |
| ENSMUSG00000039765 | protein_coding | Cc2d2a | 546.3701 | 628.4724 | 587.4213 |
| ENSMUSG00000020175 | protein_coding | Rab36 | 614.0238 | 677.2452 | 645.6345 |
| ENSMUSG00000029047 | protein_coding | Pex10 | 319.5408 | 282.621 | 301.0809 |
| ENSMUSG00000087021 | pseudogene | Gm5753 | 1.973661 | 0.335524 | 1.154592 |
| ENSMUSG00000029153 | protein_coding | Ociad2 | 133.7773 | 159.6545 | 146.7159 |
| ENSMUSG00000039231 | protein_coding | Suv39h1 | 1952.754 | 1774.132 | 1863.443 |
| ENSMUSG00000046994 | protein_coding | Mars2 | 273.9264 | 245.8279 | 259.8772 |
| ENSMUSG00000086839 | processed_transcrip | Gm11973 | 2.914124 | 5.836506 | 4.375315 |
| ENSMUSG00000032294 | protein_coding | Pkm2 | 13844.6 | 14648.62 | 14246.61 |
| ENSMUSG00000090923 | protein_coding | Gm17391 | 1.909105 | 0.335524 | 1.122314 |
| ENSMUSG00000048410 | protein_coding | Zfp407 | 1095.782 | 1223.094 | 1159.438 |
| ENSMUSG00000089847 | protein_coding | Fxc1 | 78.46562 | 93.52944 | 85.99753 |
| ENSMUSG00000050089 | protein_coding | Akap4 | 8.073552 | 4.476756 | 6.275154 |
| ENSMUSG00000035165 | protein_coding | Kcne3 | 5.165317 | 8.962882 | 7.0641 |
| ENSMUSG00000018341 | protein_coding | Il12rb2 | 49.11766 | 63.34862 | 56.23314 |
| ENSMUSG00000042121 | protein_coding | Ssh1 | 2440.27 | 2730.838 | 2585.554 |
| ENSMUSG00000021322 | protein_coding | Aoah | 30.28417 | 21.45653 | 25.87035 |
| ENSMUSG00000027164 | protein_coding | Traf6 | 1060.477 | 1137.746 | 1099.111 |
| ENSMUSG00000024901 | protein_coding | Peli3 | 101.6868 | 82.19377 | 91.94027 |
| ENSMUSG00000085828 | lincRNA | Gm15612 | 52.06086 | 41.29276 | 46.67681 |
| ENSMUSG00000047604 | protein_coding | Frat2 | 171.2231 | 151.0008 | 161.1119 |
| ENSMUSG00000079523 | protein_coding | Tmsb10 | 3996.273 | 3722.023 | 3859.148 |
| ENSMUSG00000031389 | protein_coding | Arhgap4 | 80.76855 | 95.76776 | 88.26816 |
| ENSMUSG00000043419 | lincRNA | A030009H04Rik | 1042.985 | 948.6966 | 995.8408 |
| ENSMUSG00000078956 | protein_coding | Gm14221 | 12.93316 | 8.238503 | 10.58583 |
| ENSMUSG00000030619 | protein_coding | Eed | 1896.931 | 2037.749 | 1967.34 |
| ENSMUSG00000080388 | miRNA | AC160543.1 | 2.581503 | 5.510354 | 4.045929 |
| ENSMUSG00000037798 | protein_coding | Mat1a | 28.53004 | 20.1411 | 24.33557 |
| ENSMUSG00000029561 | protein_coding | Oasl2 | 530.6041 | 449.0026 | 489.8033 |
| ENSMUSG00000022978 | protein_coding | 2610039C10Rik | 846.8399 | 916.6523 | 881.7461 |
| ENSMUSG00000029863 | protein_coding | Casp2 | 2235.188 | 2425.676 | 2330.432 |
| ENSMUSG00000082855 | pseudogene | Gm14537 | 3.538366 | 6.870105 | 5.204236 |
| ENSMUSG00000074639 | protein_coding | BC089597 | 2.561525 | 5.394319 | 3.977922 |
| ENSMUSG00000030055 | protein_coding | Rab43 | 1497.093 | 1338.452 | 1417.772 |
| ENSMUSG00000032309 | protein_coding | Fbxo22 | 1355.313 | 1458.148 | 1406.73 |
| ENSMUSG00000067206 | protein_coding | Lrrc66 | 1.951372 | 4.476756 | 3.214064 |
| ENSMUSG00000027805 | protein_coding | Pfn2 | 2684.383 | 2525.479 | 2604.931 |
| ENSMUSG00000056537 | protein_coding | Rlim | 7229.321 | 7828.123 | 7528.722 |
| ENSMUSG00000037625 | protein_coding | Cldn11 | 10516.06 | 11272.78 | 10894.42 |
| ENSMUSG00000080465 | snoRNA | SNORD94 | 30.11831 | 21.98415 | 26.05123 |
| ENSMUSG00000082088 | pseudogene | Gm15753 | 65.42966 | 51.56382 | 58.49674 |
| ENSMUSG00000038214 | protein_coding | Bend3 | 754.2082 | 681.613 | 717.9106 |
| ENSMUSG00000043391 | protein_coding | 2510009E07Rik | 746.5257 | 813.2939 | 779.9098 |
| ENSMUSG00000001741 | protein_coding | Il16 | 54.0217 | 43.26442 | 48.64306 |
| ENSMUSG00000078160 | protein_coding | Gm16503 | 46.36866 | 59.97599 | 53.17232 |
| ENSMUSG00000072723 | processed_transcrip | Gm10044 | 55.81683 | 67.96228 | 61.88955 |
| ENSMUSG00000021840 | protein_coding | Mapk1ip1l | 3610.244 | 3870.826 | 3740.535 |
| ENSMUSG00000025544 | protein_coding | Tm9sf2 | 4414.885 | 4669.611 | 4542.248 |
| ENSMUSG00000050526 | protein_coding | 4933406M09Rik | 13.23887 | 19.25813 | 16.2485 |
| ENSMUSG00000067219 | protein_coding | Nipal1 | 199.7981 | 161.6844 | 180.7412 |

| | | | | | |
|---|---|---|---|---|---|
| ENSMUSG00000028702 | protein_coding | Rad54l | 922.6895 | 849.229 | 885.9593 |
| ENSMUSG00000010476 | protein_coding | Ebf3 | 527.9348 | 432.4421 | 480.1884 |
| ENSMUSG00000067360 | protein_coding | Pramel3 | 415.5678 | 378.1954 | 396.8816 |
| ENSMUSG00000024671 | protein_coding | Ms4a14 | 9.10675 | 14.40189 | 11.75432 |
| ENSMUSG00000089987 | pseudogene | Gpx2-ps1 | 2.231215 | 5.447289 | 3.839252 |
| ENSMUSG00000082910 | pseudogene | Gm14758 | 21.972 | 15.71659 | 18.84429 |
| ENSMUSG00000086780 | lincRNA | Gm16707 | 115.032 | 97.56908 | 106.3005 |
| ENSMUSG00000056023 | protein_coding | Gm9989 | 9.344326 | 14.26819 | 11.80626 |
| ENSMUSG00000026466 | protein_coding | Tor1aip1 | 2124.492 | 2295.717 | 2210.104 |
| ENSMUSG00000036902 | protein_coding | Neto2 | 904.0181 | 775.6882 | 839.8531 |
| ENSMUSG00000060450 | protein_coding | Rnf14 | 2260.824 | 2412.646 | 2336.735 |
| ENSMUSG00000091966 | lincRNA | Gm2670 | 6.162137 | 10.22462 | 8.193377 |
| ENSMUSG00000071180 | protein_coding | 2810008M24Rik | 1299.895 | 1437.388 | 1368.642 |
| ENSMUSG00000031791 | protein_coding | Tmem38a | 684.0893 | 739.3653 | 711.7273 |
| ENSMUSG00000046470 | protein_coding | Sox18 | 485.2197 | 559.1452 | 522.1825 |
| ENSMUSG00000046818 | protein_coding | Ddit4l | 916.9495 | 1045.436 | 981.1926 |
| ENSMUSG00000039652 | protein_coding | Cpeb3 | 290.0983 | 333.233 | 311.6657 |
| ENSMUSG00000040537 | protein_coding | Adam22 | 361.6248 | 324.1233 | 342.874 |
| ENSMUSG00000065503 | miRNA | Mir351 | 8.416908 | 13.87318 | 11.14504 |
| ENSMUSG00000090514 | lincRNA | Gm7782 | 7.446999 | 4.034568 | 5.740784 |
| ENSMUSG00000005958 | protein_coding | Ephb3 | 171.267 | 142.0406 | 156.6538 |
| ENSMUSG00000022565 | protein_coding | Plec | 6931.196 | 7581.995 | 7256.595 |
| ENSMUSG00000029651 | protein_coding | Mtus2 | 144.5168 | 124.3719 | 134.4443 |
| ENSMUSG00000026418 | protein_coding | Tnni1 | 13.67011 | 22.29663 | 17.98337 |
| ENSMUSG00000060530 | pseudogene | A930017M01Rik | 1.298952 | 3.532888 | 2.41592 |
| ENSMUSG00000022557 | protein_coding | Bop1 | 2158.742 | 1979.392 | 2069.067 |
| ENSMUSG00000039835 | protein_coding | Nhsl1 | 3196.573 | 3468.329 | 3332.451 |
| ENSMUSG00000034761 | protein_coding | Map4k5 | 1394.59 | 1501.223 | 1447.907 |
| ENSMUSG00000005779 | protein_coding | Psmb4 | 4612.312 | 4900.354 | 4756.333 |
| ENSMUSG00000006522 | protein_coding | Itih3 | 25.90165 | 34.81829 | 30.35997 |
| ENSMUSG00000092044 | lincRNA | 4930556M19Rik | 19.4315 | 13.40432 | 16.41791 |
| ENSMUSG00000033111 | protein_coding | 3830406C13Rik | 895.6509 | 972.042 | 933.8464 |
| ENSMUSG00000026058 | protein_coding | Khdrbs2 | 51.7682 | 41.25056 | 46.50938 |
| ENSMUSG00000053965 | protein_coding | Pde5a | 919.1046 | 1033.399 | 976.2519 |
| ENSMUSG00000027361 | protein_coding | Gabpb1 | 1444.232 | 1352.908 | 1398.57 |
| ENSMUSG00000020068 | protein_coding | Pbld2 | 79.82286 | 62.7308 | 71.27683 |
| ENSMUSG00000020774 | protein_coding | Aspa | 75.37601 | 53.00171 | 64.18886 |
| ENSMUSG00000023010 | protein_coding | Tmbim6 | 6986.764 | 7423.115 | 7204.939 |
| ENSMUSG00000024409 | protein_coding | Psors1c2 | 7.151799 | 3.479556 | 5.315678 |
| ENSMUSG00000074414 | protein_coding | Gm10691 | 7.128467 | 3.717063 | 5.422765 |
| ENSMUSG00000055368 | protein_coding | Slc6a2 | 43.22113 | 33.14932 | 38.18523 |
| ENSMUSG00000026857 | protein_coding | Mettl11a | 460.4415 | 501.2855 | 480.8635 |
| ENSMUSG00000003131 | protein_coding | Pafah1b2 | 5291.058 | 5661.7 | 5476.379 |
| ENSMUSG00000089651 | pseudogene | Gm16353 | 3.888655 | 7.276617 | 5.582636 |
| ENSMUSG00000027845 | protein_coding | Dclre1b | 390.8167 | 353.6892 | 372.253 |
| ENSMUSG00000036990 | protein_coding | Otud4 | 8401.397 | 8866.926 | 8634.161 |
| ENSMUSG00000025933 | protein_coding | Tmem14a | 117.8654 | 137.2839 | 127.5747 |
| ENSMUSG00000001441 | protein_coding | Npepps | 2175.947 | 2317.725 | 2246.836 |
| ENSMUSG00000028955 | protein_coding | Vamp3 | 3524.439 | 3765.769 | 3645.104 |
| ENSMUSG00000036816 | protein_coding | Atoh7 | 1.626951 | 4.052225 | 2.839588 |
| ENSMUSG00000050721 | protein_coding | Plekho2 | 2491.192 | 2693.466 | 2592.329 |
| ENSMUSG00000025169 | protein_coding | 1110031I02Rik | 238.2048 | 210.1252 | 224.165 |
| ENSMUSG00000023191 | protein_coding | Leprel2 | 463.0491 | 414.6511 | 438.8501 |
| ENSMUSG00000022055 | protein_coding | Nefl | 8.052531 | 4.361082 | 6.206807 |
| ENSMUSG00000024134 | protein_coding | Six2 | 19.07763 | 13.01475 | 16.04619 |
| ENSMUSG00000025142 | protein_coding | Aspscr1 | 1503.26 | 1403.395 | 1453.327 |
| ENSMUSG00000073738 | protein_coding | Gm10567 | 250.2369 | 286.7352 | 268.4861 |
| ENSMUSG00000028750 | protein_coding | Pla2g2c | 86.33082 | 71.52075 | 78.92578 |

| | | | | | |
|---|---|---|---|---|---|
| ENSMUSG00000026275 | protein_coding | Ppp1r7 | 1897.544 | 1768.881 | 1833.212 |
| ENSMUSG00000073877 | protein_coding | Gm13306 | 4.154409 | 1.72194 | 2.938175 |
| ENSMUSG00000053747 | protein_coding | Sox14 | 1.276663 | 3.408205 | 2.342434 |
| ENSMUSG00000020434 | protein_coding | 4921536K21Rik | 36.01931 | 47.84997 | 41.93464 |
| ENSMUSG00000038521 | protein_coding | C1s | 1428.813 | 1562.538 | 1495.675 |
| ENSMUSG00000025538 | protein_coding | Sumf2 | 731.4479 | 801.6593 | 766.5536 |
| ENSMUSG00000061802 | protein_coding | Armc4 | 2.303971 | 5.572334 | 3.938152 |
| ENSMUSG00000024780 | protein_coding | Cdc37l1 | 2148.7 | 2366.103 | 2257.402 |
| ENSMUSG00000027803 | protein_coding | Wwtr1 | 3295.883 | 3605.778 | 3450.831 |
| ENSMUSG00000031271 | protein_coding | Serpina7 | 4.512897 | 2.057464 | 3.28518 |
| ENSMUSG00000071104 | protein_coding | Ccdc110 | 3.586523 | 1.386417 | 2.48647 |
| ENSMUSG00000022306 | protein_coding | Zfpm2 | 298.5101 | 343.1463 | 320.8282 |
| ENSMUSG00000086645 | processed_transcrip | Gm15743 | 7.056755 | 3.699044 | 5.3779 |
| ENSMUSG00000049493 | protein_coding | Pls1 | 739.9892 | 673.132 | 706.5606 |
| ENSMUSG00000068154 | protein_coding | Insm1 | 4.835007 | 8.422316 | 6.628662 |
| ENSMUSG00000090593 | lincRNA | 4930539J05Rik | 54.06605 | 43.10655 | 48.5863 |
| ENSMUSG00000038658 | protein_coding | C030046E11Rik | 1586.676 | 1715.607 | 1651.142 |
| ENSMUSG00000029070 | protein_coding | Mxra8 | 1003.82 | 1119.591 | 1061.706 |
| ENSMUSG00000078492 | processed_transcrip | 1700045H11Rik | 3.913254 | 1.413083 | 2.663169 |
| ENSMUSG00000053783 | protein_coding | 1700016K19Rik | 93.42589 | 78.01536 | 85.72062 |
| ENSMUSG00000084917 | lincRNA | Gm17477 | 30.89201 | 41.59405 | 36.24303 |
| ENSMUSG00000090812 | protein_coding | Samd15 | 43.80482 | 33.20809 | 38.50646 |
| ENSMUSG00000022555 | protein_coding | Dgat1 | 985.7706 | 868.6852 | 927.2279 |
| ENSMUSG00000024750 | protein_coding | Zfand5 | 4720.979 | 5078.379 | 4899.679 |
| ENSMUSG00000012117 | protein_coding | Dhdds | 1575.554 | 1461.767 | 1518.66 |
| ENSMUSG00000024253 | protein_coding | Dync2li1 | 380.4582 | 340.366 | 360.4121 |
| ENSMUSG00000019731 | protein_coding | Slc35e1 | 1049.722 | 1140.548 | 1095.135 |
| ENSMUSG00000090862 | protein_coding | Rps13 | 3066.663 | 3279.806 | 3173.234 |
| ENSMUSG00000085727 | lincRNA | 4930471M09Rik | 7.820619 | 3.788052 | 5.804336 |
| ENSMUSG00000032875 | protein_coding | Arhgef17 | 3550.145 | 4061.913 | 3806.029 |
| ENSMUSG00000034248 | protein_coding | Slc25a37 | 554.0302 | 500.2896 | 527.1599 |
| ENSMUSG00000019852 | protein_coding | D10Bwg1379e | 205.713 | 179.0841 | 192.3986 |
| ENSMUSG00000042348 | protein_coding | Arl15 | 691.1435 | 752.0944 | 721.619 |
| ENSMUSG00000020114 | protein_coding | Cand1 | 4781.294 | 4470.897 | 4626.095 |
| ENSMUSG00000028251 | protein_coding | 2610029I01Rik | 106.6328 | 90.20703 | 98.4199 |
| ENSMUSG00000020722 | protein_coding | Cacng1 | 4.218965 | 1.73095 | 2.974957 |
| ENSMUSG00000030551 | protein_coding | Nr2f2 | 1614.438 | 1752.859 | 1683.649 |
| ENSMUSG00000052751 | protein_coding | Repin1 | 467.2332 | 519.9275 | 493.5803 |
| ENSMUSG00000042195 | protein_coding | Slc35f2 | 1174.339 | 1303.206 | 1238.773 |
| ENSMUSG00000074358 | protein_coding | Ccdc61 | 916.1008 | 850.9046 | 883.5027 |
| ENSMUSG00000027559 | protein_coding | Car3 | 11.23807 | 20.65323 | 15.94565 |
| ENSMUSG00000061313 | protein_coding | Ddhd2 | 2469.763 | 2328.278 | 2399.021 |
| ENSMUSG00000003068 | protein_coding | Stk11 | 3924.858 | 3705.231 | 3815.045 |
| ENSMUSG00000079267 | protein_coding | Gm5930 | 37.49486 | 28.292 | 32.89343 |
| ENSMUSG00000021886 | protein_coding | Gpr65 | 31.97821 | 24.18395 | 28.08108 |
| ENSMUSG00000086895 | lincRNA | 9330136K24Rik | 25.44223 | 34.10587 | 29.77405 |
| ENSMUSG00000064360 | protein_coding | mt-Nd3 | 40.57552 | 30.03232 | 35.30392 |
| ENSMUSG00000022211 | protein_coding | Lrrc16b | 469.3179 | 412.9902 | 441.1541 |
| ENSMUSG00000058248 | protein_coding | Kcnh1 | 18.36296 | 25.77112 | 22.06704 |
| ENSMUSG00000059195 | pseudogene | Gm12715 | 288.9304 | 257.1913 | 273.0609 |
| ENSMUSG00000054920 | protein_coding | Klhl5 | 2254.345 | 2449.344 | 2351.844 |
| ENSMUSG00000029189 | protein_coding | Sel1l3 | 2306.704 | 2948.755 | 2627.729 |
| ENSMUSG00000021611 | protein_coding | Tert | 245.9508 | 216.9194 | 231.4351 |
| ENSMUSG00000050965 | protein_coding | Prkca | 246.3265 | 275.8083 | 261.0674 |
| ENSMUSG00000047213 | protein_coding | Ythdf3 | 3538.323 | 3856.937 | 3697.63 |
| ENSMUSG00000066510 | protein_coding | Ankdd1a | 3.925032 | 7.496105 | 5.710569 |
| ENSMUSG00000047085 | protein_coding | Lrrc4b | 107.9503 | 138.4124 | 123.1814 |
| ENSMUSG00000032763 | protein_coding | Ilvbl | 616.7785 | 686.5557 | 651.6671 |

| | | | | | |
|---|---|---|---|---|---|
| ENSMUSG00000043259 | protein_coding | Fam13c | 131.603 | 152.8358 | 142.2194 |
| ENSMUSG00000020432 | protein_coding | Tcn2 | 880.4826 | 808.9881 | 844.7354 |
| ENSMUSG00000085186 | processed_transcrip | Gm14377 | 1.290752 | 3.345864 | 2.318308 |
| ENSMUSG00000013539 | protein_coding | D16H22S680E | 568.2549 | 521.7744 | 545.0147 |
| ENSMUSG00000021222 | protein_coding | Dcaf4 | 1969.649 | 1850.588 | 1910.118 |
| ENSMUSG00000048281 | protein_coding | Dleu7 | 137.8238 | 116.1234 | 126.9736 |
| ENSMUSG00000041523 | protein_coding | Upk2 | 15.20075 | 21.56174 | 18.38125 |
| ENSMUSG00000086644 | processed_transcrip | Gm13470 | 12.5749 | 7.505476 | 10.04019 |
| ENSMUSG00000065934 | snRNA | U1 | 2.267593 | 4.767956 | 3.517775 |
| ENSMUSG00000030844 | protein_coding | Rgs10 | 166.8649 | 187.9364 | 177.4006 |
| ENSMUSG00000074873 | protein_coding | AI606181 | 227.4457 | 262.4501 | 244.9479 |
| ENSMUSG00000090456 | protein_coding | Vmn2r-ps100 | 2.942302 | 6.288065 | 4.615184 |
| ENSMUSG00000063245 | protein_coding | 2610036A22Rik | 124.8194 | 148.5738 | 136.6966 |
| ENSMUSG00000066150 | protein_coding | Slc31a1 | 4532.188 | 4846.291 | 4689.24 |
| ENSMUSG00000041498 | protein_coding | Kif14 | 851.4275 | 777.5023 | 814.4649 |
| ENSMUSG00000086582 | processed_transcrip | Gm16272 | 5.517916 | 2.746168 | 4.132042 |
| ENSMUSG00000052392 | protein_coding | Acot4 | 10.31528 | 6.216714 | 8.265996 |
| ENSMUSG00000028071 | protein_coding | Sh2d2a | 8.731862 | 13.83714 | 11.2845 |
| ENSMUSG00000003379 | protein_coding | Cd79a | 16.42255 | 24.24629 | 20.33442 |
| ENSMUSG00000038500 | protein_coding | Prr3 | 851.0148 | 916.4978 | 883.7563 |
| ENSMUSG00000081146 | pseudogene | Gm6207 | 88.05683 | 63.85802 | 75.95743 |
| ENSMUSG00000042845 | protein_coding | Wfdc12 | 3.558345 | 1.341732 | 2.450038 |
| ENSMUSG00000025064 | protein_coding | Col17a1 | 180.7666 | 212.4578 | 196.6122 |
| ENSMUSG00000024795 | protein_coding | Kif20b | 2292.309 | 2466.703 | 2379.506 |
| ENSMUSG00000054675 | protein_coding | Tmem119 | 247.621 | 282.0212 | 264.8211 |
| ENSMUSG00000087590 | lincRNA | 2410004N09Rik | 175.317 | 200.7173 | 188.0171 |
| ENSMUSG00000025413 | protein_coding | Ttc4 | 1480.22 | 1350.035 | 1415.127 |
| ENSMUSG00000029020 | protein_coding | Mfn2 | 2076.783 | 1885.251 | 1981.017 |
| ENSMUSG00000032525 | protein_coding | Nktr | 5974.16 | 6536.164 | 6255.162 |
| ENSMUSG00000021559 | protein_coding | Dapk1 | 356.8823 | 405.5121 | 381.1972 |
| ENSMUSG00000050600 | protein_coding | Zfp831 | 20.6682 | 14.60372 | 17.63596 |
| ENSMUSG00000030983 | protein_coding | Bccip | 1662.177 | 1801.202 | 1731.69 |
| ENSMUSG00000026601 | protein_coding | 9430070O13Rik | 23.29197 | 16.59268 | 19.94233 |
| ENSMUSG00000083396 | pseudogene | Gm15542 | 309.5298 | 340.9006 | 325.2152 |
| ENSMUSG00000010538 | protein_coding | 1700021C14Rik | 52.3996 | 41.46249 | 46.93104 |
| ENSMUSG00000091537 | protein_coding | Ccdc72 | 1093.489 | 1173.043 | 1133.266 |
| ENSMUSG00000085129 | processed_transcrip | 5031425F14Rik | 3.544256 | 6.905057 | 5.224656 |
| ENSMUSG00000066475 | protein_coding | Gm10268 | 2.239415 | 5.652332 | 3.945873 |
| ENSMUSG00000089665 | processed_transcrip | 2400009B08Rik | 5.860005 | 10.13561 | 7.997807 |
| ENSMUSG00000086154 | processed_transcrip | Gm16196 | 7.107222 | 3.80607 | 5.456646 |
| ENSMUSG00000004947 | protein_coding | Dtx2 | 1504.404 | 1388.55 | 1446.477 |
| ENSMUSG00000087439 | processed_transcrip | Gm15788 | 61.42852 | 49.5193 | 55.47391 |
| ENSMUSG00000011832 | protein_coding | Evi5l | 1667.617 | 1477.613 | 1572.615 |
| ENSMUSG00000059857 | protein_coding | Ntng1 | 5.117161 | 9.093363 | 7.105262 |
| ENSMUSG00000050796 | protein_coding | B3galt6 | 403.5385 | 361.4273 | 382.4829 |
| ENSMUSG00000073859 | protein_coding | Itpripl2 | 228.9034 | 262.3077 | 245.6055 |
| ENSMUSG00000017615 | protein_coding | Tnfaip1 | 2309.131 | 2471.739 | 2390.435 |
| ENSMUSG00000038112 | protein_coding | AW551984 | 77.79911 | 95.89435 | 86.84673 |
| ENSMUSG00000052305 | protein_coding | Hbb-b1 | 1108.345 | 978.4324 | 1043.389 |
| ENSMUSG00000034212 | protein_coding | Ankmy1 | 23.21922 | 16.50295 | 19.86108 |
| ENSMUSG00000062593 | protein_coding | Lilrb4 | 46.78119 | 60.42361 | 53.6024 |
| ENSMUSG00000039763 | protein_coding | Dnajc28 | 584.5127 | 671.7026 | 628.1077 |
| ENSMUSG00000028243 | protein_coding | Ubxn2b | 831.8916 | 746.2095 | 789.0506 |
| ENSMUSG00000000916 | protein_coding | Nsun5 | 948.8107 | 874.6466 | 911.7287 |
| ENSMUSG00000058398 | protein_coding | Prss43 | 1.934972 | 4.476756 | 3.205864 |
| ENSMUSG00000092129 | lincRNA | Gm17578 | 2.593282 | 0.688704 | 1.640993 |
| ENSMUSG00000029658 | protein_coding | Wdr95 | 10.32243 | 6.046986 | 8.18471 |
| ENSMUSG00000068263 | protein_coding | Ccdc48 | 41.41632 | 52.60824 | 47.01228 |

| | | | | | |
|---|---|---|---|---|---|
| ENSMUSG00000086993 | lincRNA | Gm15623 | 24.49461 | 17.61691 | 21.05576 |
| ENSMUSG00000028861 | protein_coding | Mrps15 | 1013.834 | 933.7617 | 973.7977 |
| ENSMUSG00000040824 | protein_coding | Snrpd2 | 2338.169 | 2177.272 | 2257.72 |
| ENSMUSG00000086748 | lincRNA | Gm13261 | 83.42199 | 69.13936 | 76.28067 |
| ENSMUSG00000066443 | pseudogene | Gm10163 | 53.25448 | 68.1811 | 60.71779 |
| ENSMUSG00000074918 | processed_transcrip | 5430417L22Rik | 555.7737 | 509.4211 | 532.5974 |
| ENSMUSG00000075334 | protein_coding | Rprm | 7.997218 | 13.00646 | 10.50184 |
| ENSMUSG00000086671 | processed_transcrip | Gm13618 | 6.75589 | 11.11515 | 8.935521 |
| ENSMUSG00000020077 | protein_coding | Srgn | 55.98463 | 68.10141 | 62.04302 |
| ENSMUSG00000030554 | protein_coding | Synm | 1726.04 | 1952.409 | 1839.225 |
| ENSMUSG00000021959 | protein_coding | Lats2 | 3102.246 | 2838.6 | 2970.423 |
| ENSMUSG00000049939 | protein_coding | Lrrc4 | 390.2802 | 343.52 | 366.9001 |
| ENSMUSG00000052525 | protein_coding | Spdya | 239.9001 | 132.2465 | 186.0733 |
| ENSMUSG00000001998 | protein_coding | Ap4e1 | 1707.032 | 1838.055 | 1772.544 |
| ENSMUSG00000030327 | protein_coding | Necap1 | 1388.668 | 1501.013 | 1444.84 |
| ENSMUSG00000053825 | protein_coding | Ppfia2 | 6.721823 | 11.72386 | 9.22284 |
| ENSMUSG00000020890 | protein_coding | Gucy2e | 23.28608 | 30.92037 | 27.10322 |
| ENSMUSG00000025226 | protein_coding | Fbxl15 | 189.9913 | 167.7902 | 178.8907 |
| ENSMUSG00000020440 | protein_coding | Arf5 | 2347.227 | 2489.693 | 2418.46 |
| ENSMUSG00000061689 | protein_coding | Dlgap4 | 3103.37 | 3312.017 | 3207.693 |
| ENSMUSG00000074910 | protein_coding | Tes3-ps | 20.71278 | 28.16768 | 24.44023 |
| ENSMUSG00000022971 | protein_coding | Ifnar2 | 652.6661 | 728.2617 | 690.4639 |
| ENSMUSG00000055172 | protein_coding | C1ra | 735.8991 | 803.4787 | 769.6889 |
| ENSMUSG00000086572 | lincRNA | Gm16962 | 13.5328 | 19.57563 | 16.55422 |
| ENSMUSG00000023170 | protein_coding | Gps2 | 926.1595 | 851.5522 | 888.8559 |
| ENSMUSG00000047143 | protein_coding | Dmrta2 | 37.20682 | 47.67703 | 42.44192 |
| ENSMUSG00000046245 | protein_coding | Pilra | 9.75917 | 16.55985 | 13.15951 |
| ENSMUSG00000054134 | protein_coding | Umodl1 | 2.905924 | 1.015218 | 1.960571 |
| ENSMUSG00000001248 | protein_coding | Gramd1a | 1648.036 | 1782.916 | 1715.476 |
| ENSMUSG00000066553 | pseudogene | Gm6969 | 32.5177 | 23.456 | 27.98685 |
| ENSMUSG00000028165 | protein_coding | Cisd2 | 1151.627 | 1252.17 | 1201.898 |
| ENSMUSG00000036278 | protein_coding | Macrod1 | 71.67067 | 57.37045 | 64.52056 |
| ENSMUSG00000053613 | processed_transcrip | 4732444A12Rik | 5.473339 | 2.674817 | 4.074078 |
| ENSMUSG00000038203 | protein_coding | Hoxa13 | 0.632442 | 2.36596 | 1.499201 |
| ENSMUSG00000090614 | lincRNA | Gm17629 | 5.532005 | 2.710492 | 4.121249 |
| ENSMUSG00000042115 | protein_coding | Klhdc8a | 238.3106 | 267.2914 | 252.801 |
| ENSMUSG00000047631 | protein_coding | Apof | 6.478358 | 3.443881 | 4.961119 |
| ENSMUSG00000059546 | protein_coding | Vmn1r40 | 0 | 1.422092 | 0.711046 |
| ENSMUSG00000091045 | protein_coding | Vmn2r55 | 0 | 1.422092 | 0.711046 |
| ENSMUSG00000064624 | snRNA | U6 | 0 | 1.422092 | 0.711046 |
| ENSMUSG00000009210 | protein_coding | 2310007L24Rik | 0 | 1.422092 | 0.711046 |
| ENSMUSG00000005625 | protein_coding | Psmd4 | 3554.217 | 3767.395 | 3660.806 |
| ENSMUSG00000027204 | protein_coding | Fbn1 | 3151.669 | 3459.971 | 3305.82 |
| ENSMUSG00000001847 | protein_coding | Rac1 | 4395.822 | 4704.19 | 4550.006 |
| ENSMUSG00000050103 | protein_coding | Agmo | 45.0451 | 33.34499 | 39.19505 |
| ENSMUSG00000082938 | pseudogene | Gm2810 | 50.7432 | 40.94206 | 45.84263 |
| ENSMUSG00000062933 | protein_coding | Gm10123 | 243.9461 | 217.4077 | 230.6769 |
| ENSMUSG00000063646 | protein_coding | Jakmip1 | 1282.832 | 1379.721 | 1331.276 |
| ENSMUSG00000032593 | protein_coding | Amigo3 | 75.85825 | 91.34506 | 83.60166 |
| ENSMUSG00000064595 | snoRNA | SNORA44 | 1.264884 | 3.708054 | 2.486469 |
| ENSMUSG00000073240 | protein_coding | 2410017P09Rik | 89.05045 | 75.57085 | 82.31065 |
| ENSMUSG00000030717 | protein_coding | Nupr1 | 119.1429 | 135.9721 | 127.5575 |
| ENSMUSG00000086953 | processed_transcrip | 4933437N03Rik | 10.36112 | 15.44341 | 12.90227 |
| ENSMUSG00000092260 | protein_coding | Zfp963 | 93.38698 | 78.85437 | 86.12067 |
| ENSMUSG00000022738 | protein_coding | Gsc2 | 14.83533 | 9.6869 | 12.26112 |
| ENSMUSG00000025352 | protein_coding | Gdf11 | 242.0371 | 209.42 | 225.7285 |
| ENSMUSG00000038437 | protein_coding | Mllt6 | 5195.933 | 5509.436 | 5352.685 |
| ENSMUSG00000027261 | protein_coding | Hao1 | 1.621062 | 0.36219 | 0.991626 |

| | | | | | |
|---|---|---|---|---|---|
| ENSMUSG00000075570 | protein_coding | Krt26 | 1.621062 | 0.36219 | 0.991626 |
| ENSMUSG00000044062 | processed_transcrip | 0610009B14Rik | 1.621062 | 0.36219 | 0.991626 |
| ENSMUSG00000090307 | processed_transcrip | 1700071M16Rik | 21.76238 | 15.0369 | 18.39964 |
| ENSMUSG00000022987 | protein_coding | Zfp641 | 330.644 | 367.3805 | 349.0123 |
| ENSMUSG00000087094 | processed_transcrip | Gm13093 | 1.621062 | 3.761386 | 2.691224 |
| ENSMUSG00000073987 | protein_coding | Ggh | 655.1583 | 714.4493 | 684.8038 |
| ENSMUSG00000022388 | protein_coding | Ttll8 | 13.61734 | 8.837838 | 11.22759 |
| ENSMUSG00000026468 | protein_coding | Lhx4 | 8.695484 | 5.130146 | 6.912815 |
| ENSMUSG00000067833 | protein_coding | 2900097C17Rik | 469.6077 | 424.3859 | 446.9968 |
| ENSMUSG00000061665 | protein_coding | Cd2ap | 6185.649 | 6524.615 | 6355.132 |
| ENSMUSG00000037159 | protein_coding | Wee2 | 13.50462 | 8.846847 | 11.17574 |
| ENSMUSG00000031957 | protein_coding | Ctrb1 | 6.492447 | 10.55978 | 8.526112 |
| ENSMUSG00000022533 | protein_coding | Atp13a3 | 3256.927 | 2993.087 | 3125.007 |
| ENSMUSG00000085519 | processed_transcrip | Gm13703 | 0 | 1.395426 | 0.697713 |
| ENSMUSG00000089783 | protein_coding | Gm454 | 0 | 1.395426 | 0.697713 |
| ENSMUSG00000000183 | protein_coding | Fgf6 | 0 | 1.395426 | 0.697713 |
| ENSMUSG00000086032 | lincRNA | Gm15929 | 0 | 1.395426 | 0.697713 |
| ENSMUSG00000089172 | snoRNA | snoU13 | 0 | 1.395426 | 0.697713 |
| ENSMUSG00000052534 | protein_coding | Pbx1 | 612.0953 | 691.97 | 652.0327 |
| ENSMUSG00000049076 | protein_coding | Acap2 | 2102.394 | 2275.648 | 2189.021 |
| ENSMUSG00000091343 | lincRNA | Gm17328 | 31.94877 | 40.91291 | 36.43084 |
| ENSMUSG00000048826 | protein_coding | Dact2 | 149.9273 | 174.0404 | 161.9839 |
| ENSMUSG00000022285 | protein_coding | Ywhaz | 11632.44 | 12419.47 | 12025.96 |
| ENSMUSG00000025241 | protein_coding | Fyco1 | 1909.765 | 2117.669 | 2013.717 |
| ENSMUSG00000020817 | protein_coding | Rabep1 | 2595.456 | 2412.902 | 2504.179 |
| ENSMUSG00000030872 | protein_coding | Gga2 | 1746.817 | 1604.347 | 1675.582 |
| ENSMUSG00000089129 | misc_RNA | 7SK | 8.696752 | 4.812641 | 6.754696 |
| ENSMUSG00000009145 | protein_coding | Dqx1 | 71.82415 | 54.87261 | 63.34838 |
| ENSMUSG00000031927 | protein_coding | 1700012B09Rik | 2.972791 | 0.662038 | 1.817414 |
| ENSMUSG00000021190 | protein_coding | Lgmn | 3667.037 | 4062.034 | 3864.536 |
| ENSMUSG00000032540 | protein_coding | Abhd5 | 1019.685 | 953.6131 | 986.649 |
| ENSMUSG00000022629 | protein_coding | Kif21a | 4170.997 | 4678.705 | 4424.851 |
| ENSMUSG00000059901 | protein_coding | Adamts14 | 289.2506 | 255.7141 | 272.4824 |
| ENSMUSG00000039457 | protein_coding | Ppl | 17.82348 | 25.64421 | 21.73384 |
| ENSMUSG00000083332 | pseudogene | Gm7599 | 4.509319 | 2.012779 | 3.261049 |
| ENSMUSG00000027438 | protein_coding | Napb | 215.9417 | 191.3032 | 203.6224 |
| ENSMUSG00000001909 | protein_coding | Trmt1 | 1606.96 | 1472.646 | 1539.803 |
| ENSMUSG00000032366 | protein_coding | Tpm1 | 5370.829 | 5832.804 | 5601.816 |
| ENSMUSG00000083569 | pseudogene | Gm13666 | 1.615173 | 0.36219 | 0.988681 |
| ENSMUSG00000042189 | protein_coding | Tekt3 | 1.615173 | 0.36219 | 0.988681 |
| ENSMUSG00000029528 | protein_coding | Pxn | 5039.662 | 5446.263 | 5242.962 |
| ENSMUSG00000065023 | snRNA | U6 | 0 | 1.413083 | 0.706541 |
| ENSMUSG00000073290 | protein_coding | Gm10490 | 0 | 1.413083 | 0.706541 |
| ENSMUSG00000022346 | protein_coding | Myc | 233.2349 | 199.2387 | 216.2368 |
| ENSMUSG00000044807 | protein_coding | Zfp354c | 780.2091 | 688.0478 | 734.1284 |
| ENSMUSG00000056493 | protein_coding | Foxk1 | 1330.758 | 1442.325 | 1386.541 |
| ENSMUSG00000078087 | protein_coding | Gm15887 | 1.934972 | 4.414776 | 3.174874 |
| ENSMUSG00000054106 | protein_coding | Try4 | 1.621062 | 0.335524 | 0.978293 |
| ENSMUSG00000007867 | protein_coding | 1700019E19Rik | 772.4222 | 832.5054 | 802.4638 |
| ENSMUSG00000022159 | protein_coding | Rab2b | 833.0021 | 894.2545 | 863.6283 |
| ENSMUSG00000035311 | protein_coding | Gnptab | 2537.286 | 2334.119 | 2435.702 |
| ENSMUSG00000055125 | protein_coding | M5C1000I18Rik | 5.268562 | 1.775272 | 3.521917 |
| ENSMUSG00000020525 | protein_coding | Ppm1d | 1360.654 | 1267.849 | 1314.251 |
| ENSMUSG00000032493 | protein_coding | Prss44 | 19.82636 | 27.99759 | 23.91197 |
| ENSMUSG00000027312 | protein_coding | Atrn | 2674.487 | 2484.411 | 2579.449 |
| ENSMUSG00000002017 | protein_coding | Fam98a | 1762.352 | 1634.691 | 1698.521 |
| ENSMUSG00000071661 | protein_coding | Zbtb3 | 144.2941 | 121.0921 | 132.6931 |
| ENSMUSG00000035835 | protein_coding | BC005764 | 373.0262 | 335.2105 | 354.1183 |

| | | | | | |
|---|---|---|---|---|---|
| ENSMUSG00000042148 | protein_coding | Cox10 | 724.9933 | 650.9068 | 687.95 |
| ENSMUSG00000040997 | protein_coding | Abhd4 | 10444.25 | 11251.85 | 10848.05 |
| ENSMUSG00000072324 | protein_coding | Gm8420 | 1.621062 | 0.326514 | 0.973788 |
| ENSMUSG00000070605 | protein_coding | Gm13251 | 792.7306 | 895.2491 | 843.9899 |
| ENSMUSG00000051950 | protein_coding | B3galtl | 359.2785 | 326.5574 | 342.918 |
| ENSMUSG00000020793 | protein_coding | Galr2 | 36.85168 | 28.04227 | 32.44698 |
| ENSMUSG00000033237 | protein_coding | Arid2 | 3375.931 | 3151.586 | 3263.759 |
| ENSMUSG00000051428 | pseudogene | Gm5644 | 6.78049 | 3.73472 | 5.257605 |
| ENSMUSG00000033902 | protein_coding | Mapkbp1 | 2682.188 | 2454.358 | 2568.273 |
| ENSMUSG00000079042 | protein_coding | Trim61 | 14.54729 | 8.986337 | 11.76681 |
| ENSMUSG00000036594 | protein_coding | H2-Aa | 33.69305 | 25.34731 | 29.52018 |
| ENSMUSG00000019080 | protein_coding | Mfsd3 | 245.7552 | 273.7322 | 259.7437 |
| ENSMUSG00000028420 | protein_coding | Tmem38b | 801.778 | 741.6523 | 771.7151 |
| ENSMUSG00000090783 | protein_coding | Gm17485 | 1.606973 | 0.36219 | 0.984581 |
| ENSMUSG00000073868 | protein_coding | Gm10590 | 1.606973 | 0.36219 | 0.984581 |
| ENSMUSG00000024205 | pseudogene | Rpl36-ps2 | 1.606973 | 0.36219 | 0.984581 |
| ENSMUSG00000084086 | pseudogene | Gm5761 | 1.606973 | 0.36219 | 0.984581 |
| ENSMUSG00000067285 | protein_coding | Gm16223 | 1.615173 | 0.335524 | 0.975348 |
| ENSMUSG00000084065 | pseudogene | Gm15159 | 1.615173 | 0.335524 | 0.975348 |
| ENSMUSG00000091748 | lincRNA | Gm17733 | 26.95356 | 18.33155 | 22.64256 |
| ENSMUSG00000037725 | protein_coding | Ckap2 | 1486.572 | 1383.697 | 1435.134 |
| ENSMUSG00000027962 | protein_coding | Vcam1 | 2120.768 | 1814.801 | 1967.785 |
| ENSMUSG00000013974 | protein_coding | 1810033B17Rik | 1.304841 | 3.497213 | 2.401027 |
| ENSMUSG00000019256 | protein_coding | Ahr | 406.4128 | 361.1332 | 383.773 |
| ENSMUSG00000049721 | protein_coding | Gal3st1 | 235.1243 | 147.5199 | 191.3221 |
| ENSMUSG00000024712 | protein_coding | Rfk | 1635.687 | 1492.437 | 1564.062 |
| ENSMUSG00000028587 | protein_coding | Orc1 | 970.9198 | 1062.871 | 1016.895 |
| ENSMUSG00000043991 | protein_coding | Pura | 2943.415 | 3168.135 | 3055.775 |
| ENSMUSG00000054051 | protein_coding | Ercc6 | 2667.313 | 2831.486 | 2749.4 |
| ENSMUSG00000083075 | pseudogene | Gm11980 | 1.615173 | 0.326514 | 0.970843 |
| ENSMUSG00000024055 | protein_coding | Cyp4f13 | 559.5838 | 626.2248 | 592.9043 |
| ENSMUSG00000046317 | protein_coding | BC107364 | 17.88908 | 25.1635 | 21.52629 |
| ENSMUSG00000023999 | protein_coding | Kif6 | 165.9758 | 190.6536 | 178.3147 |
| ENSMUSG00000037531 | protein_coding | Mrpl47 | 696.9675 | 755.8736 | 726.4206 |
| ENSMUSG00000041570 | protein_coding | Camsap2 | 2458.059 | 2299.358 | 2378.708 |
| ENSMUSG00000018648 | protein_coding | Dusp14 | 253.5269 | 224.3372 | 238.9321 |
| ENSMUSG00000068523 | protein_coding | Gng5 | 2232.047 | 2096.561 | 2164.304 |
| ENSMUSG00000042046 | protein_coding | Dstyk | 2573.287 | 2396.243 | 2484.765 |
| ENSMUSG00000031834 | protein_coding | Pik3r2 | 2731.89 | 2555.071 | 2643.481 |
| ENSMUSG00000079363 | protein_coding | Gbp4 | 36.83469 | 25.58875 | 31.21172 |
| ENSMUSG00000032596 | protein_coding | Uba7 | 87.26062 | 106.0371 | 96.64884 |
| ENSMUSG00000010358 | protein_coding | Ifi35 | 237.0399 | 267.4928 | 252.2664 |
| ENSMUSG00000081940 | pseudogene | Xlr3e-ps | 0.66062 | 2.472986 | 1.566803 |
| ENSMUSG00000040693 | protein_coding | Slco4c1 | 1.943172 | 4.38811 | 3.165641 |
| ENSMUSG00000030775 | protein_coding | Trat1 | 16.5079 | 10.8005 | 13.6542 |
| ENSMUSG00000071286 | pseudogene | Ankrd57 | 2317.73 | 2489.814 | 2403.772 |
| ENSMUSG00000061068 | protein_coding | Mcpt4 | 16.69628 | 11.15083 | 13.92355 |
| ENSMUSG00000030804 | protein_coding | Vkorc1 | 911.5145 | 847.8475 | 879.681 |
| ENSMUSG00000027531 | protein_coding | Impa1 | 2873.367 | 3193.045 | 3033.206 |
| ENSMUSG00000018398 | protein_coding | 8-Sep | 1256.891 | 1362.783 | 1309.837 |
| ENSMUSG00000005142 | protein_coding | Man2b1 | 1497.494 | 1349.441 | 1423.468 |
| ENSMUSG00000084104 | pseudogene | Gm13578 | 1.606973 | 0.335524 | 0.971248 |
| ENSMUSG00000070289 | protein_coding | Rpl7a-ps10 | 1.606973 | 0.335524 | 0.971248 |
| ENSMUSG00000083994 | pseudogene | Gm16465 | 1.606973 | 0.335524 | 0.971248 |
| ENSMUSG00000028868 | protein_coding | Wasf2 | 4022.149 | 4308.97 | 4165.56 |
| ENSMUSG00000024992 | protein_coding | Pde6c | 1.965461 | 4.794261 | 3.379861 |
| ENSMUSG00000015134 | protein_coding | Aldh1a3 | 411.1133 | 448.8302 | 429.9717 |
| ENSMUSG00000075569 | protein_coding | Rsph10b2 | 167.3873 | 267.8754 | 217.6313 |

| | | | | | |
|---|---|---|---|---|---|
| ENSMUSG00000082052 | pseudogene | Gm13819 | 2.891835 | 1.015218 | 1.953527 |
| ENSMUSG00000031701 | protein_coding | Dnaja2 | 7118.563 | 7607.118 | 7362.84 |
| ENSMUSG00000022297 | protein_coding | Fzd6 | 786.9718 | 858.1553 | 822.5636 |
| ENSMUSG00000026930 | protein_coding | Gpsm1 | 1954.274 | 2088.081 | 2021.178 |
| ENSMUSG00000044231 | protein_coding | Nhlrc1 | 211.9066 | 240.7667 | 226.3366 |
| ENSMUSG00000086830 | processed_transcrip | Gm13467 | 0.33031 | 1.757616 | 1.043963 |
| ENSMUSG00000091431 | lincRNA | Gm17336 | 2.928213 | 5.898848 | 4.41353 |
| ENSMUSG00000018920 | protein_coding | Cxcl16 | 188.2722 | 213.1536 | 200.7129 |
| ENSMUSG00000088262 | misc_RNA | 7SK | 4.516475 | 2.039445 | 3.27796 |
| ENSMUSG00000081590 | pseudogene | Gm14413 | 1.606973 | 0.326514 | 0.966744 |
| ENSMUSG00000041347 | protein_coding | Bdkrb1 | 0 | 1.377408 | 0.688704 |
| ENSMUSG00000063406 | protein_coding | Tmed5 | 917.2619 | 842.1344 | 879.6982 |
| ENSMUSG00000052459 | protein_coding | Atp6v1a | 2331.945 | 2197.976 | 2264.96 |
| ENSMUSG00000071562 | protein_coding | Stfa1 | 1.629262 | 0.36219 | 0.995726 |
| ENSMUSG00000032171 | protein_coding | Pin1 | 1747.042 | 1640.667 | 1693.854 |
| ENSMUSG00000031422 | protein_coding | Morf4l2 | 10470.99 | 11271.35 | 10871.17 |
| ENSMUSG00000072618 | protein_coding | Gm10384 | 3.852277 | 7.134278 | 5.493277 |
| ENSMUSG00000086587 | processed_transcrip | Gm11837 | 106.0674 | 86.72452 | 96.39597 |
| ENSMUSG00000091764 | protein_coding | Zfp964 | 92.23622 | 77.18291 | 84.70957 |
| ENSMUSG00000023007 | protein_coding | Prpf40b | 2018.327 | 1855.446 | 1936.887 |
| ENSMUSG00000088442 | misc_RNA | 7SK | 0.32211 | 1.757616 | 1.039863 |
| ENSMUSG00000034664 | protein_coding | Itga2b | 55.30634 | 66.82093 | 61.06363 |
| ENSMUSG00000003134 | protein_coding | Tbc1d8 | 1467.144 | 1318.676 | 1392.91 |
| ENSMUSG00000036975 | protein_coding | Tmem177 | 299.6331 | 269.6862 | 284.6597 |
| ENSMUSG00000020228 | protein_coding | Helb | 399.8444 | 440.3343 | 420.0894 |
| ENSMUSG00000015291 | protein_coding | Gdi1 | 1878.822 | 2047.349 | 1963.085 |
| ENSMUSG00000041130 | protein_coding | Zfp598 | 1864.715 | 1732.341 | 1798.528 |
| ENSMUSG00000047250 | protein_coding | Ptgs1 | 9.400682 | 14.85059 | 12.12564 |
| ENSMUSG00000025521 | protein_coding | Tmem192 | 486.2981 | 448.1669 | 467.2325 |
| ENSMUSG00000046008 | protein_coding | Pnlip | 32.52023 | 24.74761 | 28.63392 |
| ENSMUSG00000055760 | protein_coding | Gemin6 | 299.671 | 266.8381 | 283.2545 |
| ENSMUSG00000022658 | protein_coding | Tagln3 | 4.775073 | 8.900541 | 6.837807 |
| ENSMUSG00000025899 | protein_coding | Alkbh8 | 718.7468 | 777.0217 | 747.8842 |
| ENSMUSG00000040940 | protein_coding | Arhgef1 | 1647.53 | 1526.293 | 1586.911 |
| ENSMUSG00000091088 | lincRNA | 4921514A10Rik | 17.47654 | 11.82187 | 14.64921 |
| ENSMUSG00000051456 | protein_coding | Hspb3 | 4.820918 | 2.039807 | 3.430363 |
| ENSMUSG00000073568 | protein_coding | Gm949 | 490.5654 | 537.7901 | 514.1778 |
| ENSMUSG00000085644 | lincRNA | 2310015A10Rik | 392.8717 | 432.2926 | 412.5821 |
| ENSMUSG00000090559 | processed_transcrip | Gm17137 | 1.945483 | 4.592791 | 3.269137 |
| ENSMUSG00000081476 | pseudogene | Itpa-ps1 | 55.75123 | 67.27792 | 61.51458 |
| ENSMUSG00000021254 | protein_coding | 1700020O03Rik | 1155.117 | 1079.703 | 1117.41 |
| ENSMUSG00000042747 | protein_coding | Krtcap2 | 816.634 | 886.9358 | 851.7849 |
| ENSMUSG00000028082 | protein_coding | Sh3d19 | 3761.428 | 4062.146 | 3911.787 |
| ENSMUSG00000057346 | protein_coding | Apol9b | 2.928213 | 1.015218 | 1.971716 |
| ENSMUSG00000090222 | pseudogene | Gm16340 | 4.526986 | 7.911989 | 6.219487 |
| ENSMUSG00000024900 | protein_coding | Cpt1a | 4414.61 | 4734.492 | 4574.551 |
| ENSMUSG00000035383 | protein_coding | Pmch | 5.111272 | 9.084354 | 7.097813 |
| ENSMUSG00000051949 | protein_coding | 2010005H15Rik | 2.905924 | 1.050893 | 1.978409 |
| ENSMUSG00000086689 | processed_transcrip | Gm16876 | 1.629262 | 0.335524 | 0.982393 |
| ENSMUSG00000091519 | protein_coding | Skor2 | 1.629262 | 0.335524 | 0.982393 |
| ENSMUSG00000057069 | protein_coding | Ero1lb | 978.4778 | 829.6111 | 904.0444 |
| ENSMUSG00000056185 | protein_coding | Snx32 | 141.3961 | 173.7186 | 157.5574 |
| ENSMUSG00000053877 | protein_coding | Srcap | 1516.535 | 1711.38 | 1613.958 |
| ENSMUSG00000039063 | protein_coding | Echdc3 | 210.0339 | 244.1943 | 227.1141 |
| ENSMUSG00000036693 | protein_coding | Nop14 | 2339.314 | 2491.604 | 2415.459 |
| ENSMUSG00000042202 | protein_coding | Slc35e2 | 1124.027 | 1201.596 | 1162.812 |
| ENSMUSG00000001768 | protein_coding | Rin2 | 1190.708 | 1298.296 | 1244.502 |
| ENSMUSG00000086205 | processed_transcrip | Gm12679 | 1.629262 | 0.326514 | 0.977888 |

| | | | | | |
|---|---|---|---|---|---|
| ENSMUSG00000070408 | protein_coding | Gm10290 | 1.629262 | 0.326514 | 0.977888 |
| ENSMUSG00000082045 | pseudogene | Gm11585 | 16.75853 | 23.1154 | 19.93696 |
| ENSMUSG00000048916 | pseudogene | Gm5540 | 13.61376 | 19.5223 | 16.56803 |
| ENSMUSG00000028383 | protein_coding | Hsdl2 | 1254.987 | 1178.183 | 1216.585 |
| ENSMUSG00000034471 | protein_coding | Caskin2 | 556.7868 | 621.5452 | 589.166 |
| ENSMUSG00000028567 | protein_coding | Txndc12 | 1106.996 | 1216.173 | 1161.585 |
| ENSMUSG00000040867 | protein_coding | Begain | 301.385 | 270.1012 | 285.7431 |
| ENSMUSG00000026888 | protein_coding | Grb14 | 1947.74 | 2143.435 | 2045.587 |
| ENSMUSG00000042834 | protein_coding | D0H4S114 | 4632.156 | 5114.105 | 4873.13 |
| ENSMUSG00000039089 | protein_coding | L3mbtl3 | 726.302 | 794.157 | 760.2295 |
| ENSMUSG00000032523 | protein_coding | Hhatl | 23.32477 | 31.30057 | 27.31267 |
| ENSMUSG00000039153 | protein_coding | Runx2 | 18.29863 | 25.57866 | 21.93864 |
| ENSMUSG00000040690 | protein_coding | Col16a1 | 639.6132 | 583.2234 | 611.4183 |
| ENSMUSG00000088602 | snRNA | U6atac | 2.281682 | 4.785613 | 3.533647 |
| ENSMUSG00000058818 | protein_coding | Lilrb3 | 19.07301 | 25.72136 | 22.39718 |
| ENSMUSG00000051262 | protein_coding | Cml3 | 1.909105 | 4.468108 | 3.188606 |
| ENSMUSG00000072928 | protein_coding | Gm15127 | 98.75014 | 81.47001 | 90.11008 |
| ENSMUSG00000020953 | protein_coding | Coch | 86.99561 | 104.0808 | 95.53822 |
| ENSMUSG00000066440 | protein_coding | Zfyve26 | 1759.842 | 1620.46 | 1690.151 |
| ENSMUSG00000057666 | protein_coding | Gapdh | 706.2849 | 520.0401 | 613.1625 |
| ENSMUSG00000091171 | lincRNA | Gm10060 | 14.86813 | 10.00477 | 12.43645 |
| ENSMUSG00000028962 | protein_coding | Slc4a2 | 3613.749 | 3860.262 | 3737.006 |
| ENSMUSG00000047257 | protein_coding | Prss45 | 18.51101 | 12.59021 | 15.55061 |
| ENSMUSG00000073377 | protein_coding | AU016765 | 1.612862 | 0.36219 | 0.987526 |
| ENSMUSG00000029406 | protein_coding | Pitpnm2 | 2424.862 | 2255.832 | 2340.347 |
| ENSMUSG00000059647 | protein_coding | Gm10068 | 6.804046 | 11.15047 | 8.977256 |
| ENSMUSG00000073802 | protein_coding | Cdkn2b | 171.1102 | 147.0105 | 159.0603 |
| ENSMUSG00000037315 | protein_coding | Phf16 | 7096.696 | 6647.922 | 6872.309 |
| ENSMUSG00000053332 | lincRNA | Gas5 | 1634.584 | 1840.408 | 1737.496 |
| ENSMUSG00000061126 | protein_coding | Cyp4f39 | 20.96467 | 14.48225 | 17.72346 |
| ENSMUSG00000042138 | protein_coding | BC024479 | 804.1171 | 899.5815 | 851.8493 |
| ENSMUSG00000036438 | protein_coding | Calm2 | 9496.856 | 8975.946 | 9236.401 |
| ENSMUSG00000073775 | protein_coding | Kti12 | 360.0865 | 397.79 | 378.9382 |
| ENSMUSG00000038515 | protein_coding | Grtp1 | 254.8155 | 285.7736 | 270.2946 |
| ENSMUSG00000025199 | protein_coding | Chuk | 3849.58 | 3531.414 | 3690.497 |
| ENSMUSG00000089956 | processed_transcrip | Gm16553 | 0.32211 | 1.739597 | 1.030854 |
| ENSMUSG00000027443 | protein_coding | Cst12 | 3961.199 | 4273.342 | 4117.271 |
| ENSMUSG00000022439 | protein_coding | Parvg | 22.91246 | 16.71451 | 19.81349 |
| ENSMUSG00000022570 | protein_coding | Tsta3 | 949.6461 | 878.8184 | 914.2322 |
| ENSMUSG00000055305 | protein_coding | Zfp93 | 301.2775 | 263.915 | 282.5962 |
| ENSMUSG00000039725 | protein_coding | 2810408M09Rik | 684.0464 | 629.825 | 656.9357 |
| ENSMUSG00000068735 | protein_coding | Trp53i11 | 863.6947 | 949.5059 | 906.6003 |
| ENSMUSG00000078206 | protein_coding | Gm14458 | 87.34635 | 102.9666 | 95.15648 |
| ENSMUSG00000001794 | protein_coding | Capns1 | 7205.288 | 7655.069 | 7430.179 |
| ENSMUSG00000029093 | protein_coding | Sorcs2 | 1110.655 | 1245.835 | 1178.245 |
| ENSMUSG00000029804 | protein_coding | Herc3 | 1052.124 | 962.7231 | 1007.423 |
| ENSMUSG00000034285 | protein_coding | Nipsnap1 | 670.321 | 724.6577 | 697.4894 |
| ENSMUSG00000024479 | protein_coding | Mal2 | 28.62442 | 36.976 | 32.80021 |
| ENSMUSG00000035401 | protein_coding | 2210018M11Rik | 1619.366 | 1481.859 | 1550.613 |
| ENSMUSG00000078510 | protein_coding | Gm13101 | 0.32211 | 1.784282 | 1.053196 |
| ENSMUSG00000046056 | protein_coding | Sbsn | 140.9217 | 161.7173 | 151.3195 |
| ENSMUSG00000088018 | snRNA | U6 | 2.281682 | 0.688704 | 1.485193 |
| ENSMUSG00000091253 | protein_coding | Gm17505 | 2.281682 | 0.688704 | 1.485193 |
| ENSMUSG00000021099 | protein_coding | Six6 | 1.635151 | 4.530088 | 3.082619 |
| ENSMUSG00000031016 | protein_coding | Wee1 | 1472.287 | 1589.288 | 1530.788 |
| ENSMUSG00000029439 | protein_coding | Sfswap | 2049.567 | 2201.968 | 2125.767 |
| ENSMUSG00000083258 | pseudogene | Gm5939 | 2.273482 | 0.697713 | 1.485598 |
| ENSMUSG00000081868 | pseudogene | Gm12039 | 0.316221 | 1.784282 | 1.050251 |

| | | | | | |
|---|---|---|---|---|---|
| ENSMUSG00000022521 | protein_coding | Crebbp | 2969.045 | 3248.26 | 3108.652 |
| ENSMUSG00000029196 | protein_coding | Tada2b | 1084.659 | 1168.278 | 1126.468 |
| ENSMUSG00000037843 | protein_coding | Vstm2l | 3.864055 | 7.098241 | 5.481148 |
| ENSMUSG00000047123 | protein_coding | Ticam1 | 260.4975 | 295.2523 | 277.8749 |
| ENSMUSG00000065624 | snRNA | U6 | 0.33031 | 1.73095 | 1.03063 |
| ENSMUSG00000089781 | processed_transcrip | Gm15756 | 0.33031 | 1.73095 | 1.03063 |
| ENSMUSG00000024459 | protein_coding | H2-M5 | 9.666436 | 14.31252 | 11.98948 |
| ENSMUSG00000083528 | pseudogene | Gm13432 | 1.612862 | 0.335524 | 0.974193 |
| ENSMUSG00000085564 | lincRNA | Gm12198 | 5.463871 | 9.227055 | 7.345463 |
| ENSMUSG00000019944 | protein_coding | Rhobtb1 | 1242.565 | 1138.15 | 1190.358 |
| ENSMUSG00000003032 | protein_coding | Klf4 | 845.6622 | 774.1279 | 809.895 |
| ENSMUSG00000062609 | protein_coding | Kcnj15 | 33.35872 | 46.99308 | 40.1759 |
| ENSMUSG00000028086 | protein_coding | Fbxw7 | 1461.222 | 1339.664 | 1400.443 |
| ENSMUSG00000048827 | protein_coding | Pkd1l3 | 54.41634 | 43.29865 | 48.8575 |
| ENSMUSG00000028832 | protein_coding | Stmn1 | 5702.896 | 5355.756 | 5529.326 |
| ENSMUSG00000027524 | protein_coding | Edn3 | 55.55696 | 45.70137 | 50.62917 |
| ENSMUSG00000058900 | protein_coding | Rsl1 | 147.1455 | 169.4117 | 158.2786 |
| ENSMUSG00000038156 | protein_coding | Spon1 | 115.6829 | 100.1602 | 107.9216 |
| ENSMUSG00000029384 | protein_coding | 2010109A12Rik | 10.71499 | 6.092393 | 8.403692 |
| ENSMUSG00000084280 | pseudogene | Gm11972 | 0.33031 | 1.775272 | 1.052791 |
| ENSMUSG00000041870 | protein_coding | Ankrd13a | 8147.746 | 8656.28 | 8402.013 |
| ENSMUSG00000071292 | protein_coding | Gm5451 | 10.97717 | 15.95446 | 13.46581 |
| ENSMUSG00000020307 | protein_coding | Cdc34 | 1726.66 | 1606.875 | 1666.767 |
| ENSMUSG00000091067 | protein_coding | Gm7889 | 1.612862 | 0.326514 | 0.969688 |
| ENSMUSG00000015405 | protein_coding | Ace2 | 105.5344 | 126.1465 | 115.8405 |
| ENSMUSG00000089988 | pseudogene | Gm16238 | 9.064483 | 5.447651 | 7.256067 |
| ENSMUSG00000067931 | protein_coding | Zfp948 | 446.0008 | 494.1381 | 470.0694 |
| ENSMUSG00000039001 | protein_coding | Rps21 | 1577.964 | 1460.424 | 1519.194 |
| ENSMUSG00000092097 | protein_coding | Gm5819 | 21.28298 | 14.8884 | 18.08569 |
| ENSMUSG00000064105 | protein_coding | Cnnm2 | 633.9192 | 580.5935 | 607.2563 |
| ENSMUSG00000071151 | protein_coding | Gm4799 | 3.890965 | 1.686265 | 2.788615 |
| ENSMUSG00000000394 | protein_coding | Gcg | 0.32211 | 1.775272 | 1.048691 |
| ENSMUSG00000077387 | snRNA | U1 | 0.32211 | 1.775272 | 1.048691 |
| ENSMUSG00000047324 | protein_coding | 4931429P17Rik | 0.32211 | 1.775272 | 1.048691 |
| ENSMUSG00000049436 | protein_coding | Upk1b | 9.384282 | 14.05699 | 11.72064 |
| ENSMUSG00000048911 | protein_coding | Rnf24 | 896.8325 | 972.3035 | 934.568 |
| ENSMUSG00000015087 | protein_coding | B230208H17Rik | 2687.602 | 2535.765 | 2611.684 |
| ENSMUSG00000001131 | protein_coding | Timp1 | 133.2449 | 150.8854 | 142.0651 |
| ENSMUSG00000045679 | protein_coding | Pqlc3 | 190.1086 | 167.1433 | 178.6259 |
| ENSMUSG00000053483 | protein_coding | Usp21 | 927.4312 | 1004.261 | 965.8463 |
| ENSMUSG00000062797 | protein_coding | I7Rn6 | 736.478 | 682.8009 | 709.6394 |
| ENSMUSG00000084221 | pseudogene | Hmgb1-ps4 | 2.261704 | 0.697713 | 1.479708 |
| ENSMUSG00000001166 | protein_coding | Oas1c | 77.61707 | 64.94728 | 71.28218 |
| ENSMUSG00000020311 | protein_coding | Erlec1 | 1065.921 | 1179.994 | 1122.958 |
| ENSMUSG00000067916 | protein_coding | Gm13139 | 493.8919 | 576.106 | 534.9989 |
| ENSMUSG00000023279 | protein_coding | Bmp15 | 1.282552 | 3.381539 | 2.332046 |
| ENSMUSG00000029530 | protein_coding | Ccr9 | 2.889525 | 5.791822 | 4.340673 |
| ENSMUSG00000092242 | processed_transcrip | Gm20515 | 2.281682 | 0.697713 | 1.489697 |
| ENSMUSG00000069733 | protein_coding | Ube2u | 11.44017 | 6.207705 | 8.823936 |
| ENSMUSG00000086035 | lincRNA | Gm12610 | 12.90036 | 18.57486 | 15.73761 |
| ENSMUSG00000002413 | protein_coding | Braf | 1946.069 | 2122.682 | 2034.376 |
| ENSMUSG00000084478 | snRNA | U4 | 2.265282 | 0.688704 | 1.476993 |
| ENSMUSG00000080741 | processed_transcrip | Gm11398 | 6.741801 | 10.93098 | 8.836389 |
| ENSMUSG00000009207 | protein_coding | Lnp | 986.0234 | 1139.257 | 1062.64 |
| ENSMUSG00000030771 | protein_coding | Micalcl | 46.55703 | 36.81564 | 41.68634 |
| ENSMUSG00000022982 | protein_coding | Sod1 | 4313.957 | 4033.557 | 4173.757 |
| ENSMUSG00000002345 | protein_coding | 2310045N01Rik | 502.925 | 545.1283 | 524.0267 |
| ENSMUSG00000042444 | protein_coding | Fam63b | 608.2626 | 555.7853 | 582.024 |

| | | | | | |
|---|---|---|---|---|---|
| ENSMUSG00000071735 | processed_transcrip | Gm16462 | 16.28211 | 10.63113 | 13.45662 |
| ENSMUSG00000030654 | protein_coding | Arl6ip1 | 7626.563 | 7190.95 | 7408.756 |
| ENSMUSG00000019920 | protein_coding | Lims1 | 3159.828 | 3377.482 | 3268.655 |
| ENSMUSG00000084301 | pseudogene | Gm7856 | 12.96849 | 8.193457 | 10.58098 |
| ENSMUSG00000057137 | protein_coding | Tmem140 | 66.21075 | 78.2889 | 72.24982 |
| ENSMUSG00000058101 | protein_coding | 1700084J12Rik | 1.598773 | 3.998893 | 2.798833 |
| ENSMUSG00000028597 | protein_coding | Gpx7 | 441.9185 | 492.7152 | 467.3168 |
| ENSMUSG00000063177 | protein_coding | Klk1b27 | 0 | 1.36876 | 0.68438 |
| ENSMUSG00000049008 | protein_coding | BB014433 | 0 | 1.36876 | 0.68438 |
| ENSMUSG00000031621 | protein_coding | Isx | 0 | 1.36876 | 0.68438 |
| ENSMUSG00000051703 | protein_coding | Tmem198 | 104.5832 | 123.6716 | 114.1274 |
| ENSMUSG00000073650 | protein_coding | Gm216 | 25.65923 | 34.63955 | 30.14939 |
| ENSMUSG00000032456 | protein_coding | Nmnat3 | 274.9711 | 306.4715 | 290.7213 |
| ENSMUSG00000008976 | protein_coding | Gabpa | 2829.526 | 2672.949 | 2751.238 |
| ENSMUSG00000028556 | protein_coding | Dock7 | 5861.427 | 6305.596 | 6083.511 |
| ENSMUSG00000044757 | pseudogene | Gm6430 | 106.198 | 121.8856 | 114.0418 |
| ENSMUSG00000057244 | protein_coding | Gm6139 | 7.77962 | 4.45009 | 6.114855 |
| ENSMUSG00000081593 | pseudogene | Gm11841 | 7.789088 | 12.43343 | 10.11126 |
| ENSMUSG00000086541 | processed_transcrip | Has2as | 2.261704 | 4.740929 | 3.501316 |
| ENSMUSG00000079460 | protein_coding | 4933403O08Rik | 105.464 | 131.4004 | 118.4322 |
| ENSMUSG00000073940 | protein_coding | Hbb-b2 | 217.0754 | 186.4458 | 201.7606 |
| ENSMUSG00000035678 | protein_coding | Tnfsf9 | 11.30159 | 7.044908 | 9.173248 |
| ENSMUSG00000082491 | pseudogene | Gm5909 | 14.5496 | 9.871074 | 12.21034 |
| ENSMUSG00000006398 | protein_coding | Cdc20 | 2521.097 | 2342.42 | 2431.759 |
| ENSMUSG00000058145 | protein_coding | Adamts17 | 153.2976 | 131.6237 | 142.4607 |
| ENSMUSG00000053862 | protein_coding | Ostb | 24.87561 | 18.32042 | 21.59802 |
| ENSMUSG00000056872 | pseudogene | Gm10011 | 5.125361 | 8.802524 | 6.963943 |
| ENSMUSG00000040938 | protein_coding | Slc16a11 | 138.5158 | 163.6044 | 151.0601 |
| ENSMUSG00000051065 | protein_coding | 1600021P15Rik | 1050.811 | 1154.634 | 1102.723 |
| ENSMUSG00000079254 | protein_coding | Itprip | 209.9482 | 245.6385 | 227.7934 |
| ENSMUSG00000090756 | protein_coding | Gm17635 | 89.30361 | 72.77321 | 81.03841 |
| ENSMUSG00000020212 | protein_coding | Mdm1 | 773.7132 | 837.867 | 805.7901 |
| ENSMUSG00000053898 | protein_coding | Ech1 | 2467.804 | 2640.89 | 2554.347 |
| ENSMUSG00000030365 | protein_coding | Clec2i | 6.142159 | 3.161689 | 4.651924 |
| ENSMUSG00000029722 | protein_coding | Agfg2 | 2111.124 | 1954.581 | 2032.852 |
| ENSMUSG00000038894 | protein_coding | Irs2 | 453.0182 | 514.7759 | 483.897 |
| ENSMUSG00000080950 | pseudogene | Gm7278 | 2.900035 | 1.059903 | 1.979969 |
| ENSMUSG00000080205 | pseudogene | Gm15526 | 2.245304 | 0.697713 | 1.471509 |
| ENSMUSG00000083603 | pseudogene | Gm14810 | 2.245304 | 0.697713 | 1.471509 |
| ENSMUSG00000060093 | protein_coding | Hist1h4a | 3.231613 | 6.418184 | 4.824899 |
| ENSMUSG00000068614 | protein_coding | Actc1 | 4.843207 | 8.282826 | 6.563017 |
| ENSMUSG00000085336 | lincRNA | Gm11732 | 2.569725 | 5.130146 | 3.849935 |
| ENSMUSG00000043483 | pseudogene | Gm6863 | 543.8738 | 591.6554 | 567.7646 |
| ENSMUSG00000028634 | protein_coding | Hivep3 | 363.5962 | 315.7071 | 339.6517 |
| ENSMUSG00000035314 | protein_coding | Gdpd5 | 123.4945 | 106.9731 | 115.2338 |
| ENSMUSG00000024575 | protein_coding | Pde6a | 18.15252 | 25.77397 | 21.96324 |
| ENSMUSG00000079450 | protein_coding | 3110007F17Rik | 41.05656 | 50.67116 | 45.86386 |
| ENSMUSG00000085286 | lincRNA | 1700121F15Rik | 15.20433 | 21.16496 | 18.18465 |
| ENSMUSG00000044664 | protein_coding | Prss42 | 62.65412 | 80.87353 | 71.76382 |
| ENSMUSG00000032452 | protein_coding | Clstn2 | 267.6972 | 306.2294 | 286.9633 |
| ENSMUSG00000032083 | protein_coding | Apoa1 | 15.77162 | 8.555646 | 12.16363 |
| ENSMUSG00000019951 | protein_coding | Uhrf1bp1l | 3354.294 | 3146.687 | 3250.491 |
| ENSMUSG00000082364 | pseudogene | Gm11955 | 2.247615 | 0.688704 | 1.468159 |
| ENSMUSG00000031443 | protein_coding | F7 | 6.508847 | 3.417215 | 4.963031 |
| ENSMUSG00000028643 | protein_coding | Ccdc23 | 848.4584 | 777.2791 | 812.8688 |
| ENSMUSG00000048365 | pseudogene | Gm9825 | 1672.269 | 1281.367 | 1476.818 |
| ENSMUSG00000031284 | protein_coding | Pak3 | 1878.966 | 2113.402 | 1996.184 |
| ENSMUSG00000067440 | protein_coding | Dnahc7a | 0.962752 | 2.737158 | 1.849955 |

| | | | | | |
|---|---|---|---|---|---|
| ENSMUSG00000022707 | protein_coding | Gbe1 | 461.7736 | 503.3254 | 482.5495 |
| ENSMUSG00000036748 | protein_coding | Cuedc2 | 839.6291 | 901.4202 | 870.5247 |
| ENSMUSG00000053024 | protein_coding | Cntn2 | 16.81257 | 11.53932 | 14.17595 |
| ENSMUSG00000085604 | processed_transcrip | Gm11547 | 3.902744 | 1.712931 | 2.807837 |
| ENSMUSG00000059479 | protein_coding | B3gnt8 | 213.6055 | 188.571 | 201.0883 |
| ENSMUSG00000024011 | protein_coding | Pi16 | 122.3512 | 143.2124 | 132.7818 |
| ENSMUSG00000039693 | protein_coding | 5730528L13Rik | 1084.424 | 1006.018 | 1045.221 |
| ENSMUSG00000078049 | miRNA | AL626775.1 | 12.58056 | 17.90453 | 15.24255 |
| ENSMUSG00000039159 | protein_coding | Ube2h | 5152.895 | 5509.929 | 5331.412 |
| ENSMUSG00000020919 | protein_coding | Stat5b | 1705.671 | 1869.071 | 1787.371 |
| ENSMUSG00000044864 | protein_coding | Ankrd50 | 2398.851 | 2247.177 | 2323.014 |
| ENSMUSG00000022305 | protein_coding | Lrp12 | 544.4542 | 501.1335 | 522.7939 |
| ENSMUSG00000086905 | processed_transcrip | Gm13716 | 11.60603 | 7.24058 | 9.423305 |
| ENSMUSG00000047220 | protein_coding | Ccdc36 | 1018.749 | 784.6355 | 901.6922 |
| ENSMUSG00000006705 | protein_coding | Pknox1 | 992.9273 | 928.0351 | 960.4812 |
| ENSMUSG00000078429 | protein_coding | Ctdsp2 | 4519.161 | 4793.622 | 4656.392 |
| ENSMUSG00000031059 | protein_coding | Ndufb11 | 1191.123 | 1283.548 | 1237.336 |
| ENSMUSG00000027935 | protein_coding | Rab13 | 313.1632 | 348.3675 | 330.7654 |
| ENSMUSG00000045545 | protein_coding | Krt14 | 1.601084 | 4.159612 | 2.880348 |
| ENSMUSG00000029712 | protein_coding | Actl6b | 6.792268 | 10.86827 | 8.830271 |
| ENSMUSG00000025532 | protein_coding | Crcp | 792.6136 | 739.7272 | 766.1704 |
| ENSMUSG00000026775 | protein_coding | Yme1l1 | 4921.211 | 5264.895 | 5093.053 |
| ENSMUSG00000037788 | protein_coding | Vopp1 | 724.9817 | 792.837 | 758.9094 |
| ENSMUSG00000037210 | protein_coding | Fam193a | 2599.553 | 2392.931 | 2496.242 |
| ENSMUSG00000017716 | protein_coding | Birc5 | 861.9776 | 777.0428 | 819.5102 |
| ENSMUSG00000031519 | protein_coding | Asb5 | 32.49563 | 24.29605 | 28.39584 |
| ENSMUSG00000037935 | protein_coding | Smarce1 | 3948.132 | 4178.337 | 4063.234 |
| ENSMUSG00000027605 | protein_coding | Acss2 | 1712.485 | 1485.937 | 1599.211 |
| ENSMUSG00000085762 | processed_transcrip | 4930443B20Rik | 5.526116 | 2.692836 | 4.109476 |
| ENSMUSG00000049775 | protein_coding | Tmsb4x | 6316.01 | 5897.17 | 6106.59 |
| ENSMUSG00000084822 | processed_transcrip | Gm11768 | 0.316221 | 1.703922 | 1.010071 |
| ENSMUSG00000046088 | protein_coding | Gm9805 | 10.64686 | 15.80632 | 13.22659 |
| ENSMUSG00000021171 | protein_coding | Esyt2 | 3157.22 | 2962.015 | 3059.617 |
| ENSMUSG00000020219 | protein_coding | Timm13 | 1138.306 | 1063.44 | 1100.873 |
| ENSMUSG00000065037 | misc_RNA | 7SK | 69.83134 | 88.11296 | 78.97215 |
| ENSMUSG00000028096 | protein_coding | Gpr89 | 671.5973 | 730.1637 | 700.8805 |
| ENSMUSG00000081325 | pseudogene | Gm12599 | 0.968641 | 2.665808 | 1.817225 |
| ENSMUSG00000073532 | protein_coding | Gm7276 | 2.589703 | 5.174469 | 3.882086 |
| ENSMUSG00000021182 | protein_coding | Ccdc88c | 2784.887 | 2530.237 | 2657.562 |
| ENSMUSG00000042548 | protein_coding | Asxl1 | 4203.113 | 3951.193 | 4077.153 |
| ENSMUSG00000064590 | snRNA | U6 | 2.547436 | 5.420985 | 3.98421 |
| ENSMUSG00000027699 | protein_coding | Ect2 | 1857.767 | 1732.857 | 1795.312 |
| ENSMUSG00000027828 | protein_coding | Ssr3 | 6518.936 | 6939.328 | 6729.132 |
| ENSMUSG00000082599 | pseudogene | Gm13141 | 3.939121 | 7.982978 | 5.96105 |
| ENSMUSG00000044626 | protein_coding | Liph | 7.1354 | 11.5213 | 9.328351 |
| ENSMUSG00000068964 | pseudogene | Gm6361 | 2.247615 | 0.697713 | 1.472664 |
| ENSMUSG00000005540 | protein_coding | Fcer2a | 4.865496 | 2.348303 | 3.606899 |
| ENSMUSG00000028100 | protein_coding | Nudt17 | 128.6221 | 106.4097 | 117.5159 |
| ENSMUSG00000005251 | protein_coding | Ripk4 | 614.8892 | 551.1998 | 583.0445 |
| ENSMUSG00000031823 | protein_coding | Zdhhc7 | 834.2065 | 774.3248 | 804.2656 |
| ENSMUSG00000036123 | protein_coding | Slc9a3 | 3.849966 | 7.151934 | 5.50095 |
| ENSMUSG00000090954 | protein_coding | Gm17345 | 14.22391 | 19.90287 | 17.06339 |
| ENSMUSG00000042340 | protein_coding | Ctf1 | 71.05395 | 59.6254 | 65.33968 |
| ENSMUSG00000083616 | pseudogene | H2afb3-ps | 1.293062 | 3.426224 | 2.359643 |
| ENSMUSG00000039768 | protein_coding | Dnajc11 | 2241.645 | 2383.153 | 2312.399 |
| ENSMUSG00000048833 | protein_coding | Slc39a9 | 2104.883 | 2252.754 | 2178.818 |
| ENSMUSG00000093245 | snoRNA | AC131120.1 | 1.31304 | 3.390187 | 2.351614 |
| ENSMUSG00000058779 | protein_coding | Tomm20 | 2994.266 | 3194.786 | 3094.526 |

| | | | | | |
|---|---|---|---|---|---|
| ENSMUSG00000039936 | protein_coding | Pik3cd | 596.2609 | 644.1833 | 620.2221 |
| ENSMUSG00000040888 | protein_coding | Gfer | 700.1106 | 639.6674 | 669.889 |
| ENSMUSG00000090473 | lincRNA | Gm17262 | 54.21954 | 43.27887 | 48.74921 |
| ENSMUSG00000034361 | protein_coding | Cpne2 | 395.7381 | 353.0832 | 374.4106 |
| ENSMUSG00000042272 | protein_coding | Sestd1 | 2660.537 | 2813.963 | 2737.25 |
| ENSMUSG00000024072 | protein_coding | Yipf4 | 1505.354 | 1404.08 | 1454.717 |
| ENSMUSG00000052889 | protein_coding | Prkcb | 230.8895 | 254.8871 | 242.8883 |
| ENSMUSG00000021983 | protein_coding | Atp8a2 | 225.697 | 250.9959 | 238.3464 |
| ENSMUSG00000068617 | protein_coding | Efcab1 | 38.96221 | 30.13686 | 34.54953 |
| ENSMUSG00000025875 | protein_coding | Tspan17 | 1666.338 | 1468.996 | 1567.667 |
| ENSMUSG00000045128 | protein_coding | Rpl18a | 8502.428 | 8952.399 | 8727.413 |
| ENSMUSG00000013076 | protein_coding | Amotl1 | 859.8089 | 943.0852 | 901.447 |
| ENSMUSG00000070282 | processed_transcrip | 3000002C10Rik | 194.8239 | 221.2757 | 208.0498 |
| ENSMUSG00000045165 | protein_coding | AI467606 | 19.12221 | 26.46412 | 22.79316 |
| ENSMUSG00000087227 | lincRNA | Gm16958 | 0.98273 | 2.746168 | 1.864449 |
| ENSMUSG00000047996 | protein_coding | Prrg1 | 857.0456 | 946.2174 | 901.6315 |
| ENSMUSG00000046826 | protein_coding | Fam187b | 83.73881 | 69.63016 | 76.68448 |
| ENSMUSG00000074646 | protein_coding | 6430550D23Rik | 58.93848 | 47.44962 | 53.19405 |
| ENSMUSG00000026885 | protein_coding | Ttll11 | 87.75113 | 73.54217 | 80.64665 |
| ENSMUSG00000026860 | protein_coding | Sh3glb2 | 1398.994 | 1492.659 | 1445.827 |
| ENSMUSG00000083230 | pseudogene | Gm14158 | 13.23887 | 8.891532 | 11.0652 |
| ENSMUSG00000020808 | protein_coding | Fam64a | 283.7507 | 335.6212 | 309.6859 |
| ENSMUSG00000065211 | snoRNA | SNORA76 | 0.65242 | 2.321637 | 1.487029 |
| ENSMUSG00000078676 | protein_coding | Casc3 | 2755.188 | 2991.952 | 2873.57 |
| ENSMUSG00000026156 | protein_coding | B3gat2 | 46.71663 | 58.87792 | 52.79728 |
| ENSMUSG00000006494 | protein_coding | Pdk1 | 3149.973 | 3373.609 | 3261.791 |
| ENSMUSG00000021745 | protein_coding | Ptprg | 2540.967 | 2393.976 | 2467.472 |
| ENSMUSG00000073000 | protein_coding | Gm10451 | 113.8346 | 96.65463 | 105.2446 |
| ENSMUSG00000092052 | lincRNA | Gm5101 | 1.931394 | 4.352435 | 3.141914 |
| ENSMUSG00000087019 | lincRNA | 4930593A02Rik | 6.200825 | 3.090339 | 4.645582 |
| ENSMUSG00000074939 | protein_coding | Chrm5 | 0 | 1.324076 | 0.662038 |
| ENSMUSG00000085964 | lincRNA | Gm16983 | 0 | 1.324076 | 0.662038 |
| ENSMUSG00000063314 | pseudogene | Gm12657 | 23.39648 | 33.39833 | 28.3974 |
| ENSMUSG00000060288 | protein_coding | Ppih | 1022.392 | 950.8408 | 986.6164 |
| ENSMUSG00000051855 | protein_coding | Mest | 673.3011 | 738.6923 | 705.9967 |
| ENSMUSG00000054362 | protein_coding | BC055111 | 25.52214 | 18.91395 | 22.21805 |
| ENSMUSG00000010097 | protein_coding | Nxf1 | 2986.288 | 3180.016 | 3083.152 |
| ENSMUSG00000052415 | protein_coding | Tchh | 324.977 | 384.1238 | 354.5504 |
| ENSMUSG00000086881 | processed_transcrip | Gm13594 | 17.84785 | 11.90187 | 14.87486 |
| ENSMUSG00000039269 | protein_coding | 2300002M23Rik | 0 | 1.341732 | 0.670866 |
| ENSMUSG00000042102 | protein_coding | Dmgdh | 4.571564 | 2.00377 | 3.287667 |
| ENSMUSG00000033634 | protein_coding | Cml2 | 17.76123 | 24.98217 | 21.3717 |
| ENSMUSG00000024260 | protein_coding | Sap130 | 2329.263 | 2153.072 | 2241.168 |
| ENSMUSG00000028843 | protein_coding | Sh3bgrl3 | 814.2982 | 755.8398 | 785.069 |
| ENSMUSG00000036745 | protein_coding | Ttll7 | 255.8021 | 282.7214 | 269.2617 |
| ENSMUSG00000090656 | lincRNA | Gm17559 | 29.04158 | 37.18176 | 33.11167 |
| ENSMUSG00000021573 | protein_coding | Tppp | 280.7896 | 312.9497 | 296.8697 |
| ENSMUSG00000035506 | protein_coding | Slc12a8 | 56.35616 | 68.08266 | 62.21941 |
| ENSMUSG00000046959 | protein_coding | Slc26a1 | 34.43985 | 44.14202 | 39.29093 |
| ENSMUSG00000072660 | lincRNA | Gm6288 | 32.5045 | 23.69056 | 28.09753 |
| ENSMUSG00000084217 | pseudogene | Gm15488 | 6.825068 | 3.72571 | 5.275389 |
| ENSMUSG00000025648 | protein_coding | Pfkfb4 | 233.2443 | 258.19 | 245.7172 |
| ENSMUSG00000060862 | protein_coding | Zbtb40 | 858.1274 | 923.34 | 890.7337 |
| ENSMUSG00000089952 | processed_transcrip | 4933413C19Rik | 2.289882 | 0.688704 | 1.489293 |
| ENSMUSG00000019737 | protein_coding | AI428936 | 38.74819 | 48.15634 | 43.45227 |
| ENSMUSG00000028889 | protein_coding | Yrdc | 1130.474 | 1022.265 | 1076.369 |
| ENSMUSG00000039342 | protein_coding | Ankar | 2.942302 | 1.015218 | 1.97876 |
| ENSMUSG00000031620 | protein_coding | 1700007B14Rik | 2.583814 | 5.076452 | 3.830133 |

| | | | | | |
|---|---|---|---|---|---|
| ENSMUSG00000018572 | protein_coding | Phf23 | 2589.442 | 2745.625 | 2667.533 |
| ENSMUSG00000024036 | protein_coding | Slc37a1 | 159.3759 | 184.5115 | 171.9437 |
| ENSMUSG00000062132 | processed_transcrip | Gm1082 | 12.63334 | 18.12723 | 15.38029 |
| ENSMUSG00000059089 | protein_coding | Fcgr4 | 3.905054 | 1.712931 | 2.808993 |
| ENSMUSG00000081137 | pseudogene | BC022960 | 793.7755 | 676.7665 | 735.271 |
| ENSMUSG00000045827 | protein_coding | Serpinb9 | 255.6812 | 287.3925 | 271.5368 |
| ENSMUSG00000036062 | protein_coding | N28178 | 258.8098 | 224.7093 | 241.7596 |
| ENSMUSG00000022074 | protein_coding | Tnfrsf10b | 244.0171 | 274.1734 | 259.0952 |
| ENSMUSG00000017858 | protein_coding | Ift52 | 1237.983 | 1161.43 | 1199.706 |
| ENSMUSG00000087597 | processed_transcrip | Gm15345 | 4.535186 | 7.902618 | 6.218902 |
| ENSMUSG00000060126 | protein_coding | Tpt1 | 14634.81 | 15563.18 | 15098.99 |
| ENSMUSG00000038402 | protein_coding | Foxf2 | 2.259393 | 5.218792 | 3.739092 |
| ENSMUSG00000027720 | protein_coding | Il2 | 7.522066 | 3.814718 | 5.668392 |
| ENSMUSG00000020184 | protein_coding | Mdm2 | 2404.726 | 2270.334 | 2337.53 |
| ENSMUSG00000007122 | protein_coding | Casq1 | 20.32738 | 26.78706 | 23.55722 |
| ENSMUSG00000085303 | lincRNA | Gm16751 | 31.69121 | 24.0704 | 27.88081 |
| ENSMUSG00000022829 | protein_coding | Stxbp5l | 209.5009 | 333.8916 | 271.6962 |
| ENSMUSG00000027782 | protein_coding | Kpna4 | 3378.377 | 3591.354 | 3484.865 |
| ENSMUSG00000049659 | protein_coding | Aftph | 2538.592 | 2383.855 | 2461.223 |
| ENSMUSG00000044393 | protein_coding | Dsg2 | 41.72702 | 53.98751 | 47.85727 |
| ENSMUSG00000031494 | protein_coding | Cd209a | 0 | 1.333085 | 0.666542 |
| ENSMUSG00000040328 | protein_coding | Olfr56 | 0 | 1.333085 | 0.666542 |
| ENSMUSG00000074194 | protein_coding | Zfp791 | 54.77312 | 66.36725 | 60.57018 |
| ENSMUSG00000078185 | protein_coding | Chml | 423.7748 | 381.8241 | 402.7995 |
| ENSMUSG00000047261 | protein_coding | Gap43 | 6.410224 | 10.50608 | 8.458154 |
| ENSMUSG00000003119 | protein_coding | Cdk12 | 3152.68 | 3434.759 | 3293.72 |
| ENSMUSG00000087259 | lincRNA | 2610035D17Rik | 315.5253 | 360.4615 | 337.9934 |
| ENSMUSG00000028573 | protein_coding | Fggy | 323.6483 | 289.7824 | 306.7153 |
| ENSMUSG00000084067 | pseudogene | Gm14269 | 79.14331 | 64.98984 | 72.06658 |
| ENSMUSG00000024500 | protein_coding | Ppp2r2b | 2.917703 | 5.729481 | 4.323592 |
| ENSMUSG00000074635 | protein_coding | 3110070M22Rik | 19.07659 | 13.06559 | 16.07109 |
| ENSMUSG00000005699 | protein_coding | Pard6a | 178.8013 | 155.9504 | 167.3758 |
| ENSMUSG00000024827 | protein_coding | Gldc | 128.5027 | 151.1596 | 139.8311 |
| ENSMUSG00000027276 | protein_coding | Jag1 | 657.0324 | 603.9264 | 630.4794 |
| ENSMUSG00000049299 | protein_coding | Trappc1 | 782.7045 | 839.3337 | 811.0191 |
| ENSMUSG00000074034 | protein_coding | Gm5921 | 5.177096 | 2.392626 | 3.784861 |
| ENSMUSG00000070462 | protein_coding | Mesdc1 | 1509.165 | 1611.233 | 1560.199 |
| ENSMUSG00000061111 | protein_coding | Fam195b | 1873.991 | 1767.483 | 1820.737 |
| ENSMUSG00000031712 | protein_coding | Il15 | 65.75386 | 77.92164 | 71.83775 |
| ENSMUSG00000031738 | protein_coding | Irx6 | 4.798629 | 2.066111 | 3.43237 |
| ENSMUSG00000022634 | protein_coding | Yaf2 | 1052.215 | 986.906 | 1019.561 |
| ENSMUSG00000021025 | protein_coding | Nfkbia | 1398.876 | 1531.273 | 1465.074 |
| ENSMUSG00000054320 | protein_coding | Lrrc36 | 73.17253 | 55.15683 | 64.16468 |
| ENSMUSG00000049539 | protein_coding | Hist1h1a | 35.35742 | 47.1213 | 41.23936 |
| ENSMUSG00000039953 | protein_coding | Clstn1 | 8075.026 | 7421.844 | 7748.435 |
| ENSMUSG00000022833 | protein_coding | Ccdc14 | 499.4693 | 457.35 | 478.4097 |
| ENSMUSG00000083657 | pseudogene | Gm12245 | 60.13464 | 72.03107 | 66.08286 |
| ENSMUSG00000025178 | protein_coding | Pi4k2a | 1827.673 | 1714.106 | 1770.89 |
| ENSMUSG00000015027 | protein_coding | Galns | 175.6461 | 198.4053 | 187.0257 |
| ENSMUSG00000038633 | protein_coding | Degs1 | 1115.871 | 1191.245 | 1153.558 |
| ENSMUSG00000001666 | protein_coding | Ddt | 147.6873 | 167.2439 | 157.4656 |
| ENSMUSG00000029661 | protein_coding | Col1a2 | 10323.84 | 9436.218 | 9880.027 |
| ENSMUSG00000060679 | protein_coding | Mrps9 | 1538.128 | 1635.61 | 1586.869 |
| ENSMUSG00000026235 | protein_coding | Epha4 | 53.41527 | 65.32893 | 59.3721 |
| ENSMUSG00000029276 | protein_coding | Glmn | 732.2547 | 676.1121 | 704.1834 |
| ENSMUSG00000016255 | protein_coding | Tubb1 | 0.962752 | 2.701483 | 1.832118 |
| ENSMUSG00000039194 | protein_coding | Rlbp1 | 8.107619 | 4.767956 | 6.437788 |
| ENSMUSG00000041596 | protein_coding | Vmn1r90 | 51.65257 | 65.18944 | 58.42101 |

| | | | | | |
|---|---|---|---|---|---|
| ENSMUSG00000084799 | lincRNA | Gm11602 | 57.5424 | 69.24203 | 63.39221 |
| ENSMUSG00000022554 | protein_coding | Fam203a | 581.0431 | 536.1494 | 558.5962 |
| ENSMUSG00000041528 | protein_coding | Rnf123 | 706.8698 | 771.1087 | 738.9893 |
| ENSMUSG00000053768 | protein_coding | Chchd3 | 1986.496 | 1858.82 | 1922.658 |
| ENSMUSG00000028635 | protein_coding | Edn2 | 4.484719 | 8.060488 | 6.272604 |
| ENSMUSG00000026087 | protein_coding | Mrpl30 | 1186.587 | 1104.326 | 1145.456 |
| ENSMUSG00000079942 | pseudogene | Rpl28-ps3 | 31.65946 | 24.60812 | 28.13379 |
| ENSMUSG00000037868 | protein_coding | Egr2 | 304.9658 | 343.5825 | 324.2741 |
| ENSMUSG00000027940 | protein_coding | Tpm3 | 4204.621 | 4844.836 | 4524.729 |
| ENSMUSG00000067653 | protein_coding | Ankrd23 | 172.6501 | 199.9334 | 186.2918 |
| ENSMUSG00000020668 | protein_coding | Kif3c | 578.4373 | 646.4298 | 612.4336 |
| ENSMUSG00000084159 | pseudogene | Gm12696 | 80.91339 | 94.58318 | 87.74828 |
| ENSMUSG00000036551 | protein_coding | Akap14 | 0.32211 | 1.704284 | 1.013197 |
| ENSMUSG00000038608 | protein_coding | Dock10 | 73.87788 | 87.02188 | 80.44988 |
| ENSMUSG00000032582 | protein_coding | Rbm6 | 3621.61 | 3390.736 | 3506.173 |
| ENSMUSG00000047731 | protein_coding | D19Wsu162e | 2681.321 | 2909.88 | 2795.601 |
| ENSMUSG00000059900 | protein_coding | Tmem40 | 4.229475 | 1.73095 | 2.980212 |
| ENSMUSG00000049670 | protein_coding | Morn4 | 484.0124 | 440.9575 | 462.4849 |
| ENSMUSG00000087075 | protein_coding | A230065H16Rik | 65.59665 | 51.35593 | 58.47629 |
| ENSMUSG00000046667 | protein_coding | Rbm12b | 497.7078 | 551.6494 | 524.6786 |
| ENSMUSG00000033794 | protein_coding | Lpcat2b | 11.49399 | 5.325817 | 8.409902 |
| ENSMUSG00000064373 | protein_coding | Sepp1 | 3820.232 | 4076.272 | 3948.252 |
| ENSMUSG00000038416 | protein_coding | Cdc16 | 2698.333 | 2547.801 | 2623.067 |
| ENSMUSG00000050310 | protein_coding | Rictor | 3968.132 | 4202.773 | 4085.452 |
| ENSMUSG00000047098 | protein_coding | Rnf31 | 1156.277 | 1076.381 | 1116.329 |
| ENSMUSG00000029221 | protein_coding | Slc30a9 | 3479.996 | 3675.528 | 3577.762 |
| ENSMUSG00000053821 | protein_coding | Gm9922 | 6.444291 | 10.19795 | 8.32112 |
| ENSMUSG00000078572 | protein_coding | 1810043H04Rik | 535.194 | 486.3503 | 510.7721 |
| ENSMUSG00000025358 | protein_coding | Cdk2 | 3266.602 | 3068 | 3167.301 |
| ENSMUSG00000029556 | protein_coding | Hnf1a | 9.688725 | 5.916866 | 7.802796 |
| ENSMUSG00000062526 | protein_coding | Mppe1 | 170.7923 | 197.1598 | 183.9761 |
| ENSMUSG00000025154 | protein_coding | Arhgap19 | 2359.627 | 2529.111 | 2444.369 |
| ENSMUSG00000026864 | protein_coding | Hspa5 | 16693.58 | 15205.94 | 15949.76 |
| ENSMUSG00000028999 | protein_coding | Rint1 | 944.8614 | 1011.371 | 978.1164 |
| ENSMUSG00000060685 | protein_coding | Gm14511 | 13.81853 | 19.37996 | 16.59925 |
| ENSMUSG00000051067 | protein_coding | Lingo3 | 11.57681 | 17.20755 | 14.39218 |
| ENSMUSG00000040447 | protein_coding | Spns2 | 251.1265 | 221.8648 | 236.4957 |
| ENSMUSG00000059325 | protein_coding | Hopx | 205.7161 | 164.2704 | 184.9932 |
| ENSMUSG00000019948 | protein_coding | Actr6 | 1049.008 | 965.929 | 1007.469 |
| ENSMUSG00000055926 | protein_coding | Gm14137 | 44.1273 | 34.88358 | 39.50544 |
| ENSMUSG00000005580 | protein_coding | Adcy9 | 792.1277 | 735.768 | 763.9479 |
| ENSMUSG00000018634 | protein_coding | Crhr1 | 11.61676 | 17.5701 | 14.59343 |
| ENSMUSG00000033845 | protein_coding | Mrpl15 | 1210.699 | 1126.94 | 1168.82 |
| ENSMUSG00000044502 | protein_coding | Bod1 | 1570.861 | 1463.148 | 1517.005 |
| ENSMUSG00000086392 | lincRNA | A330050B17Rik | 10.34115 | 14.95654 | 12.64884 |
| ENSMUSG00000078317 | protein_coding | F8a | 216.0932 | 241.3435 | 228.7184 |
| ENSMUSG00000029016 | protein_coding | Clcn6 | 1274.704 | 1182.945 | 1228.825 |
| ENSMUSG00000031430 | protein_coding | Vsig1 | 1805.27 | 1663.553 | 1734.411 |
| ENSMUSG00000022722 | protein_coding | Arl6 | 524.7966 | 485.2031 | 504.9998 |
| ENSMUSG00000025812 | protein_coding | Pard3 | 492.2779 | 534.1191 | 513.1985 |
| ENSMUSG00000031072 | protein_coding | Oraov1 | 685.716 | 624.1638 | 654.9399 |
| ENSMUSG00000020309 | protein_coding | Chac2 | 130.0961 | 149.5409 | 139.8185 |
| ENSMUSG00000050390 | protein_coding | C77080 | 627.4048 | 576.4101 | 601.9075 |
| ENSMUSG00000036078 | protein_coding | Sigmar1 | 585.6473 | 647.8226 | 616.735 |
| ENSMUSG00000081143 | pseudogene | Gm15823 | 0 | 1.315066 | 0.657533 |
| ENSMUSG00000015787 | protein_coding | Abo | 14.2462 | 9.455191 | 11.8507 |
| ENSMUSG00000057895 | protein_coding | Zfp105 | 626.1538 | 575.9291 | 601.0415 |
| ENSMUSG00000055945 | protein_coding | Prr18 | 27.59189 | 20.211 | 23.90145 |

| | | | | | |
|---|---|---|---|---|---|
| ENSMUSG00000074579 | protein_coding | Lekr1 | 103.7502 | 119.6962 | 111.7232 |
| ENSMUSG00000046442 | protein_coding | Ppm1e | 696.0758 | 754.4541 | 725.265 |
| ENSMUSG00000020260 | protein_coding | Pofut2 | 2146.442 | 2289.637 | 2218.04 |
| ENSMUSG00000040554 | protein_coding | Aipl1 | 33.32278 | 41.61311 | 37.46795 |
| ENSMUSG00000047654 | protein_coding | Tssk6 | 32.92688 | 41.15327 | 37.04007 |
| ENSMUSG00000032109 | protein_coding | Nlrx1 | 334.1251 | 303.6922 | 318.9086 |
| ENSMUSG00000023913 | protein_coding | Pla2g7 | 945.3585 | 856.4584 | 900.9084 |
| ENSMUSG00000046318 | protein_coding | Ccbe1 | 1270.993 | 1372.489 | 1321.741 |
| ENSMUSG00000038227 | protein_coding | Hoxa9 | 10.01903 | 6.199058 | 8.109046 |
| ENSMUSG00000052974 | protein_coding | Cyp2f2 | 4.221275 | 1.775272 | 2.998274 |
| ENSMUSG00000062997 | protein_coding | Rpl35 | 3306.121 | 3081.692 | 3193.907 |
| ENSMUSG00000087667 | processed_transcrip | Gm13381 | 10.36112 | 6.498906 | 8.430015 |
| ENSMUSG00000031631 | protein_coding | 4933411K20Rik | 1538.02 | 1629.271 | 1583.646 |
| ENSMUSG00000057522 | protein_coding | Spop | 3068.679 | 3275.315 | 3171.997 |
| ENSMUSG00000028522 | protein_coding | Mier1 | 4247.539 | 3923.539 | 4085.539 |
| ENSMUSG00000053746 | protein_coding | Ptrh1 | 160.2548 | 140.3491 | 150.3019 |
| ENSMUSG00000020738 | protein_coding | Sumo2 | 4145.304 | 4423.921 | 4284.613 |
| ENSMUSG00000037463 | protein_coding | Fbxo27 | 424.8425 | 372.8141 | 398.8283 |
| ENSMUSG00000050936 | protein_coding | Hist2h2bb | 77.90795 | 150.1006 | 114.0043 |
| ENSMUSG00000073427 | protein_coding | Gm4924 | 178.1316 | 201.007 | 189.5693 |
| ENSMUSG00000037166 | protein_coding | Ppp1r14a | 370.1615 | 410.1653 | 390.1634 |
| ENSMUSG00000032338 | protein_coding | Hcn4 | 23.53417 | 17.21868 | 20.37642 |
| ENSMUSG00000084781 | lincRNA | D930015M05Rik | 38.86784 | 30.24932 | 34.55858 |
| ENSMUSG00000038580 | protein_coding | Sct | 247.3209 | 219.1702 | 233.2456 |
| ENSMUSG00000001120 | protein_coding | Pcbp3 | 528.8166 | 460.7445 | 494.7806 |
| ENSMUSG00000028176 | protein_coding | Lrrc7 | 4.518786 | 2.066111 | 3.292449 |
| ENSMUSG00000032078 | protein_coding | Zfp259 | 1443.962 | 1353.215 | 1398.589 |
| ENSMUSG00000059273 | protein_coding | Zc3h4 | 1976.22 | 1855.911 | 1916.066 |
| ENSMUSG00000001767 | protein_coding | Crnkl1 | 1822.948 | 1946.343 | 1884.646 |
| ENSMUSG00000035399 | protein_coding | 3230401D17Rik | 1138.797 | 1063.946 | 1101.371 |
| ENSMUSG00000055897 | lincRNA | Ppp4r1l-ps | 941.0379 | 852.3932 | 896.7155 |
| ENSMUSG00000023074 | protein_coding | Mospd1 | 407.8543 | 449.9488 | 428.9016 |
| ENSMUSG00000029229 | protein_coding | Chic2 | 1909.644 | 1712.813 | 1811.229 |
| ENSMUSG00000071662 | protein_coding | Polr2g | 1646.169 | 1770.008 | 1708.089 |
| ENSMUSG00000086719 | processed_transcrip | Gm16275 | 11.62496 | 7.531781 | 9.578373 |
| ENSMUSG00000024539 | protein_coding | Ptpn2 | 1843.661 | 1971.896 | 1907.778 |
| ENSMUSG00000036913 | protein_coding | Trim67 | 17.44963 | 24.12016 | 20.7849 |
| ENSMUSG00000026617 | protein_coding | Bpnt1 | 729.4916 | 681.2977 | 705.3947 |
| ENSMUSG00000032549 | protein_coding | Rab6b | 518.9643 | 467.569 | 493.2667 |
| ENSMUSG00000053909 | protein_coding | Rhox10 | 99.70007 | 115.7824 | 107.7412 |
| ENSMUSG00000032243 | protein_coding | Itga11 | 184.7707 | 220.2077 | 202.4892 |
| ENSMUSG00000075391 | pseudogene | Gm13443 | 731.3696 | 532.0951 | 631.7324 |
| ENSMUSG00000014763 | protein_coding | Fam120b | 1184.02 | 1101.277 | 1142.648 |
| ENSMUSG00000090231 | protein_coding | Cfb | 8.797461 | 14.89741 | 11.84743 |
| ENSMUSG00000034845 | protein_coding | Plvap | 194.4723 | 232.2422 | 213.3573 |
| ENSMUSG00000056158 | protein_coding | Car10 | 1.929083 | 4.096909 | 3.012996 |
| ENSMUSG00000089941 | processed_transcrip | Gm16168 | 2.883635 | 1.059903 | 1.971769 |
| ENSMUSG00000071235 | protein_coding | Vrtn | 2.233526 | 0.688704 | 1.461115 |
| ENSMUSG00000025867 | protein_coding | Cplx2 | 605.7399 | 682.067 | 643.9035 |
| ENSMUSG00000091164 | lincRNA | Gm17716 | 24.19248 | 31.55574 | 27.87411 |
| ENSMUSG00000092558 | protein_coding | Med20 | 914.868 | 993.9744 | 954.4212 |
| ENSMUSG00000022856 | protein_coding | Tmem41a | 567.5011 | 526.0912 | 546.7961 |
| ENSMUSG00000030307 | protein_coding | Slc6a11 | 16.74906 | 23.97166 | 20.36036 |
| ENSMUSG00000002307 | protein_coding | Daxx | 2221.027 | 2377.109 | 2299.068 |
| ENSMUSG00000045767 | protein_coding | B230219D22Rik | 3247.071 | 3490.385 | 3368.728 |
| ENSMUSG00000021719 | protein_coding | Rgs7bp | 86.31904 | 72.6405 | 79.47977 |
| ENSMUSG00000018566 | protein_coding | Slc2a4 | 108.2948 | 91.56989 | 99.93236 |
| ENSMUSG00000021799 | protein_coding | Opn4 | 6.427891 | 3.452528 | 4.94021 |

| | | | | | |
|---|---|---|---|---|---|
| ENSMUSG00000074269 | protein_coding | 2410076I21Rik | 342.7792 | 310.3041 | 326.5417 |
| ENSMUSG00000085239 | processed_transcrip | Gm12150 | 1.307151 | 3.426224 | 2.366688 |
| ENSMUSG00000046435 | protein_coding | Gm13078 | 0.960442 | 2.965656 | 1.963049 |
| ENSMUSG00000082969 | pseudogene | Gm12630 | 4.846786 | 8.220485 | 6.533635 |
| ENSMUSG00000051177 | protein_coding | Plcb1 | 85.13824 | 99.73419 | 92.43622 |
| ENSMUSG00000023175 | protein_coding | Bsg | 6996.915 | 6603.9 | 6800.407 |
| ENSMUSG00000049871 | protein_coding | Nlrc3 | 49.96442 | 38.98805 | 44.47624 |
| ENSMUSG00000049891 | pseudogene | Gm7984 | 14.71777 | 9.455553 | 12.08666 |
| ENSMUSG00000032553 | protein_coding | Srprb | 747.8586 | 800.3863 | 774.1224 |
| ENSMUSG00000057541 | protein_coding | Pus7 | 2200.288 | 2055.188 | 2127.738 |
| ENSMUSG00000005897 | protein_coding | Nr2c1 | 383.9917 | 419.6338 | 401.8128 |
| ENSMUSG00000082414 | pseudogene | Gm13303 | 9.367658 | 4.159612 | 6.763635 |
| ENSMUSG00000039084 | protein_coding | Chad | 35.1686 | 45.42285 | 40.29573 |
| ENSMUSG00000043463 | protein_coding | Rab9b | 40.7065 | 49.72657 | 45.21653 |
| ENSMUSG00000018486 | protein_coding | Wnt9b | 12.89805 | 8.52898 | 10.71351 |
| ENSMUSG00000010142 | protein_coding | Tnfrsf13b | 22.52706 | 29.99379 | 26.26043 |
| ENSMUSG00000091058 | pseudogene | Gm17538 | 9.471127 | 5.411976 | 7.441551 |
| ENSMUSG00000063268 | protein_coding | Parp10 | 328.8175 | 361.3505 | 345.084 |
| ENSMUSG00000035476 | protein_coding | Tab3 | 3044.692 | 3304.956 | 3174.824 |
| ENSMUSG00000000440 | protein_coding | Pparg | 180.2996 | 143.9528 | 162.1262 |
| ENSMUSG00000019761 | protein_coding | Krt10 | 164.9655 | 184.6828 | 174.8241 |
| ENSMUSG00000054385 | protein_coding | Ceacam2 | 12.08774 | 19.49848 | 15.79311 |
| ENSMUSG00000031181 | protein_coding | Ctag2 | 6.721823 | 10.949 | 8.835409 |
| ENSMUSG00000021621 | protein_coding | Zcchc9 | 516.8283 | 564.1196 | 540.4739 |
| ENSMUSG00000027384 | protein_coding | 2310003L22Rik | 315.3642 | 285.6082 | 300.4862 |
| ENSMUSG00000079554 | protein_coding | Aox3l1 | 18.33016 | 24.72995 | 21.53006 |
| ENSMUSG00000053263 | lincRNA | Gm12592 | 60.7515 | 50.1702 | 55.46085 |
| ENSMUSG00000084855 | pseudogene | Gm9850 | 3.230345 | 1.359751 | 2.295048 |
| ENSMUSG00000033192 | protein_coding | Lpcat2 | 713.7332 | 636.1304 | 674.9318 |
| ENSMUSG00000024299 | protein_coding | Adamts10 | 1026.039 | 1158.498 | 1092.268 |
| ENSMUSG00000087481 | processed_transcrip | Gm15608 | 24.18219 | 31.98783 | 28.08501 |
| ENSMUSG00000086126 | lincRNA | 5730457N03Rik | 4.261232 | 1.677256 | 2.969244 |
| ENSMUSG00000087171 | processed_transcrip | Gm12962 | 1.929083 | 4.159251 | 3.044167 |
| ENSMUSG00000090325 | lincRNA | 4930505N22Rik | 3.250323 | 1.359751 | 2.305037 |
| ENSMUSG00000000805 | protein_coding | Car4 | 9.479327 | 14.92987 | 12.2046 |
| ENSMUSG00000083674 | lincRNA | Zfp133-ps | 51.23013 | 41.48227 | 46.3562 |
| ENSMUSG00000057388 | protein_coding | Mrpl18 | 868.0673 | 805.215 | 836.6411 |
| ENSMUSG00000090841 | protein_coding | Myl6 | 4663.214 | 5008.655 | 4835.935 |
| ENSMUSG00000003271 | protein_coding | Sult2b1 | 17.7013 | 24.23407 | 20.96769 |
| ENSMUSG00000031133 | protein_coding | Arhgef6 | 240.1979 | 272.3489 | 256.2734 |
| ENSMUSG00000057363 | protein_coding | Uxs1 | 4397.293 | 4668.693 | 4532.993 |
| ENSMUSG00000084497 | snRNA | U6 | 1.937283 | 4.194926 | 3.066104 |
| ENSMUSG00000081214 | pseudogene | Rpl35a-ps2 | 32.01921 | 40.70279 | 36.361 |
| ENSMUSG00000090767 | lincRNA | Gm17685 | 3.236234 | 1.386417 | 2.311326 |
| ENSMUSG00000071645 | protein_coding | Tut1 | 1140.92 | 1070.725 | 1105.823 |
| ENSMUSG00000068130 | protein_coding | Zfp442 | 66.9401 | 80.38928 | 73.66469 |
| ENSMUSG00000028484 | protein_coding | Psip1 | 4673.995 | 4983.718 | 4828.856 |
| ENSMUSG00000035237 | protein_coding | Lcat | 32.36234 | 41.49495 | 36.92865 |
| ENSMUSG00000045377 | protein_coding | Tmem88 | 67.83434 | 79.81589 | 73.82512 |
| ENSMUSG00000037034 | protein_coding | Pax1 | 0.33031 | 1.668247 | 0.999278 |
| ENSMUSG00000032332 | protein_coding | Col12a1 | 57.82895 | 70.60758 | 64.21826 |
| ENSMUSG00000072915 | protein_coding | Gm12258 | 302.5041 | 339.0439 | 320.774 |
| ENSMUSG00000025453 | protein_coding | Nnt | 908.2765 | 1001.302 | 954.7892 |
| ENSMUSG00000078957 | protein_coding | 1700060C20Rik | 0.638331 | 2.075121 | 1.356726 |
| ENSMUSG00000047606 | protein_coding | Ankrd34c | 0.638331 | 2.075121 | 1.356726 |
| ENSMUSG00000090009 | processed_transcrip | Gm16282 | 0.32211 | 1.668247 | 0.995178 |
| ENSMUSG00000085048 | processed_transcrip | Gm15537 | 0.32211 | 1.668247 | 0.995178 |
| ENSMUSG00000042846 | protein_coding | Lrrtm3 | 10.32012 | 6.463231 | 8.391677 |

| | | | | | |
|---|---|---|---|---|---|
| ENSMUSG00000028445 | processed_transcrip | Enho | 171.2214 | 148.039 | 159.6302 |
| ENSMUSG00000074345 | protein_coding | Tnfaip8l3 | 6.12807 | 3.37253 | 4.7503 |
| ENSMUSG00000036446 | protein_coding | Lum | 591.4749 | 538.8252 | 565.1501 |
| ENSMUSG00000028863 | protein_coding | Meaf6 | 1034.601 | 1113.124 | 1073.862 |
| ENSMUSG00000084568 | misc_RNA | 7SK | 0.316221 | 1.668608 | 0.992415 |
| ENSMUSG00000086547 | processed_transcrip | Gm11755 | 7.088511 | 10.92197 | 9.005239 |
| ENSMUSG00000010609 | protein_coding | Psen2 | 386.293 | 422.3768 | 404.3349 |
| ENSMUSG00000050383 | protein_coding | Svs3b | 0.316221 | 1.668247 | 0.992234 |
| ENSMUSG00000088377 | snoRNA | SNORA40 | 0.316221 | 1.668247 | 0.992234 |
| ENSMUSG00000080021 | pseudogene | Gm5915 | 4.871385 | 2.419653 | 3.645519 |
| ENSMUSG00000074206 | protein_coding | Adh6b | 6.518314 | 11.038 | 8.778158 |
| ENSMUSG00000077964 | miRNA | AC122270.1 | 1.953683 | 4.450451 | 3.202067 |
| ENSMUSG00000040610 | protein_coding | Tlx3 | 6.461958 | 10.25092 | 8.356439 |
| ENSMUSG00000089735 | pseudogene | Gm8428 | 1.284863 | 3.099348 | 2.192105 |
| ENSMUSG00000065167 | snoRNA | SNORA19 | 0.646531 | 2.08413 | 1.365331 |
| ENSMUSG00000049950 | protein_coding | Rpp38 | 252.2188 | 224.298 | 238.2584 |
| ENSMUSG00000074456 | protein_coding | 4930467E23Rik | 4.535186 | 7.74226 | 6.138723 |
| ENSMUSG00000033985 | protein_coding | Tesk2 | 488.3852 | 440.098 | 464.2416 |
| ENSMUSG00000045699 | pseudogene | Gm8935 | 6.504225 | 10.58681 | 8.545515 |
| ENSMUSG00000090870 | protein_coding | Gm17722 | 4.816297 | 9.304204 | 7.06025 |
| ENSMUSG00000013353 | protein_coding | 4931406B18Rik | 3.838188 | 1.686265 | 2.762226 |
| ENSMUSG00000000600 | protein_coding | Krit1 | 1977.276 | 1863.312 | 1920.294 |
| ENSMUSG00000039206 | protein_coding | Daglb | 455.7042 | 419.1214 | 437.4128 |
| ENSMUSG00000054484 | protein_coding | Tmem62 | 184.5385 | 207.7805 | 196.1595 |
| ENSMUSG00000032744 | protein_coding | Heyl | 743.1469 | 866.6298 | 804.8884 |
| ENSMUSG00000083397 | pseudogene | Gm5466 | 7.819576 | 4.423424 | 6.1215 |
| ENSMUSG00000090761 | processed_transcrip | Gm17201 | 87.77126 | 102.2818 | 95.02655 |
| ENSMUSG00000090906 | protein_coding | Mcart1 | 20.03576 | 26.92183 | 23.4788 |
| ENSMUSG00000013663 | protein_coding | Pten | 3387.969 | 3203.192 | 3295.581 |
| ENSMUSG00000064202 | protein_coding | 4430402I18Rik | 30.70721 | 23.4444 | 27.07581 |
| ENSMUSG00000084022 | pseudogene | Gm16459 | 2.905924 | 5.438642 | 4.172283 |
| ENSMUSG00000014956 | protein_coding | Ppp1cb | 6987.703 | 7405.999 | 7196.851 |
| ENSMUSG00000073116 | protein_coding | Gm10471 | 22.99573 | 29.97614 | 26.48593 |
| ENSMUSG00000025075 | protein_coding | Habp2 | 8.446129 | 4.87462 | 6.660375 |
| ENSMUSG00000049115 | protein_coding | Agtr1a | 37.43388 | 29.3768 | 33.40534 |
| ENSMUSG00000079470 | protein_coding | Utp14b | 538.3441 | 607.6699 | 573.007 |
| ENSMUSG00000091158 | lincRNA | Mirg | 57.6446 | 70.24539 | 63.94499 |
| ENSMUSG00000038840 | protein_coding | Birc7 | 0.98273 | 2.728149 | 1.85544 |
| ENSMUSG00000020255 | protein_coding | D10Wsu102e | 3057.358 | 2860.4 | 2958.879 |
| ENSMUSG00000036114 | protein_coding | 2810432D09Rik | 481.4673 | 528.0021 | 504.7347 |
| ENSMUSG00000021670 | protein_coding | Hmgcr | 2972.914 | 2780.656 | 2876.785 |
| ENSMUSG00000028959 | protein_coding | Fastk | 1718.141 | 1604.866 | 1661.503 |
| ENSMUSG00000032288 | protein_coding | Imp3 | 1075.725 | 1160.41 | 1118.068 |
| ENSMUSG00000055745 | protein_coding | Ldoc1l | 871.916 | 950.1465 | 911.0313 |
| ENSMUSG00000038973 | protein_coding | Cldnd2 | 1.290752 | 3.099348 | 2.19505 |
| ENSMUSG00000020185 | protein_coding | E2f7 | 506.9273 | 460.3976 | 483.6625 |
| ENSMUSG00000086578 | processed_transcrip | Gm13583 | 0.646531 | 2.075121 | 1.360826 |
| ENSMUSG00000084756 | processed_transcrip | Gm13881 | 0.646531 | 2.075121 | 1.360826 |
| ENSMUSG00000090290 | protein_coding | Gm17296 | 169.4916 | 190.2879 | 179.8898 |
| ENSMUSG00000085343 | processed_transcrip | Gm15463 | 19.43612 | 26.33079 | 22.88345 |
| ENSMUSG00000029112 | protein_coding | Nkx1-1 | 93.58504 | 77.25199 | 85.41852 |
| ENSMUSG00000088239 | misc_RNA | 7SK | 0.65242 | 2.08413 | 1.368275 |
| ENSMUSG00000020604 | protein_coding | Arsg | 172.7055 | 196.345 | 184.5252 |
| ENSMUSG00000053647 | protein_coding | Gpr30 | 99.65646 | 117.251 | 108.4537 |
| ENSMUSG00000074912 | lincRNA | Gm14207 | 11.00892 | 15.91842 | 13.46367 |
| ENSMUSG00000040596 | protein_coding | Pogk | 2173.181 | 2038.508 | 2105.844 |
| ENSMUSG00000059022 | protein_coding | Kcp | 432.589 | 491.0343 | 461.8116 |
| ENSMUSG00000030342 | protein_coding | Cd9 | 1277.742 | 1393.567 | 1335.654 |

| | | | | | |
|---|---|---|---|---|---|
| ENSMUSG00000035762 | protein_coding | Tmem161b | 681.8802 | 741.2332 | 711.5567 |
| ENSMUSG00000034192 | processed_transcrip | Lsm3 | 14.894 | 20.42184 | 17.65792 |
| ENSMUSG00000051499 | protein_coding | Zfp786 | 198.4487 | 176.254 | 187.3514 |
| ENSMUSG00000064018 | pseudogene | Mageb16-ps2 | 17.43197 | 23.36984 | 20.4009 |
| ENSMUSG00000020538 | protein_coding | Srebf1 | 2095.173 | 2246.985 | 2171.079 |
| ENSMUSG00000026082 | protein_coding | Rev1 | 1734.074 | 1618.268 | 1676.171 |
| ENSMUSG00000026407 | protein_coding | Cacna1s | 135.8882 | 165.1321 | 150.5101 |
| ENSMUSG00000029669 | protein_coding | Tspan12 | 300.3539 | 330.9106 | 315.6323 |
| ENSMUSG00000020954 | protein_coding | Strn3 | 3003.45 | 3202.297 | 3102.873 |
| ENSMUSG00000001656 | protein_coding | Hoxc11 | 0.638331 | 2.057464 | 1.347898 |
| ENSMUSG00000031951 | protein_coding | Tmem231 | 426.6244 | 390.9281 | 408.7762 |
| ENSMUSG00000079487 | protein_coding | Med12 | 2197.49 | 1994.682 | 2096.086 |
| ENSMUSG00000085184 | lincRNA | 4933439K11Rik | 1.626951 | 3.770395 | 2.698673 |
| ENSMUSG00000045546 | pseudogene | Rnf113a2 | 382.9039 | 416.4127 | 399.6583 |
| ENSMUSG00000051223 | protein_coding | Bzw1 | 12332.71 | 13161.83 | 12747.27 |
| ENSMUSG00000070473 | protein_coding | Cldn3 | 51.85668 | 41.99948 | 46.92808 |
| ENSMUSG00000038644 | protein_coding | Pold1 | 2714.13 | 2470.962 | 2592.546 |
| ENSMUSG00000072621 | protein_coding | Slfn10-ps | 1.931394 | 4.070243 | 3.000818 |
| ENSMUSG00000006215 | protein_coding | Zbtb17 | 1039.093 | 968.726 | 1003.909 |
| ENSMUSG00000041717 | protein_coding | Golt1a | 4.204876 | 7.1786 | 5.691738 |
| ENSMUSG00000032691 | protein_coding | Nlrp3 | 5.137139 | 8.53799 | 6.837564 |
| ENSMUSG00000041935 | protein_coding | AW549877 | 3557.207 | 3811.803 | 3684.505 |
| ENSMUSG00000086181 | lincRNA | C230034O21Rik | 3.858166 | 1.748606 | 2.803386 |
| ENSMUSG00000006333 | protein_coding | Rps9 | 8167.442 | 8684.434 | 8425.938 |
| ENSMUSG00000037692 | protein_coding | Ahdc1 | 1836.471 | 1964.213 | 1900.342 |
| ENSMUSG00000070461 | protein_coding | 9230112E08Rik | 61.09716 | 72.17129 | 66.63423 |
| ENSMUSG00000028292 | protein_coding | Rars2 | 932.4856 | 864.4048 | 898.4452 |
| ENSMUSG00000041515 | protein_coding | Irf8 | 305.4263 | 266.107 | 285.7666 |
| ENSMUSG00000037003 | protein_coding | Tenc1 | 581.9872 | 624.3899 | 603.1885 |
| ENSMUSG00000019846 | protein_coding | Lama4 | 578.5725 | 659.9507 | 619.2616 |
| ENSMUSG00000057554 | protein_coding | Lgals8 | 738.2551 | 797.3825 | 767.8188 |
| ENSMUSG00000041431 | protein_coding | Ccnb1 | 2880.923 | 3087.13 | 2984.027 |
| ENSMUSG00000076430 | protein_coding | Hus1b | 69.03176 | 83.39093 | 76.21135 |
| ENSMUSG00000068394 | protein_coding | Cep152 | 435.6927 | 473.7183 | 454.7055 |
| ENSMUSG00000032062 | protein_coding | 2310030G06Rik | 83.89125 | 98.81316 | 91.3522 |
| ENSMUSG00000004894 | protein_coding | Hapln2 | 16.49508 | 11.64635 | 14.07071 |
| ENSMUSG00000079588 | protein_coding | Tmem182 | 3.208056 | 1.359751 | 2.283904 |
| ENSMUSG00000049149 | protein_coding | Olfr1258 | 3.208056 | 1.359751 | 2.283904 |
| ENSMUSG00000040370 | protein_coding | Lyrm5 | 969.3764 | 1048.399 | 1008.888 |
| ENSMUSG00000035958 | protein_coding | Tdp2 | 1872.731 | 1718.53 | 1795.631 |
| ENSMUSG00000059288 | protein_coding | Cdyl | 1878.369 | 1757.362 | 1817.865 |
| ENSMUSG00000092539 | processed_transcrip | Gm20468 | 1.945483 | 4.0879 | 3.016691 |
| ENSMUSG00000042719 | protein_coding | Naa25 | 1010.72 | 948.2036 | 979.4616 |
| ENSMUSG00000031786 | protein_coding | Ccdc135 | 9.041151 | 4.856602 | 6.948876 |
| ENSMUSG00000027070 | protein_coding | Lrp2 | 115.27 | 96.29213 | 105.7811 |
| ENSMUSG00000051147 | protein_coding | Nat2 | 119.8664 | 102.8917 | 111.379 |
| ENSMUSG00000078933 | protein_coding | Ipo11 | 38.42019 | 48.18658 | 43.30339 |
| ENSMUSG00000053178 | protein_coding | Gm9897 | 48.78489 | 59.12951 | 53.9572 |
| ENSMUSG00000008932 | protein_coding | Slc1a7 | 1.609283 | 3.832736 | 2.72101 |
| ENSMUSG00000092232 | protein_coding | Gm20521 | 3.856898 | 7.106888 | 5.481893 |
| ENSMUSG00000030067 | protein_coding | Foxp1 | 943.7887 | 1018.026 | 980.9074 |
| ENSMUSG00000087338 | processed_transcrip | 4930405P13Rik | 6.773333 | 3.381178 | 5.077255 |
| ENSMUSG00000027273 | protein_coding | Snap25 | 8.399241 | 5.058795 | 6.729018 |
| ENSMUSG00000078877 | protein_coding | Gm14295 | 133.9879 | 105.7301 | 119.859 |
| ENSMUSG00000039542 | protein_coding | Ncam1 | 127.5404 | 148.7027 | 138.1215 |
| ENSMUSG00000044522 | protein_coding | A730020M07Rik | 0.646531 | 2.057464 | 1.351998 |
| ENSMUSG00000070526 | protein_coding | Peg12 | 72.14329 | 86.77143 | 79.45736 |
| ENSMUSG00000064612 | snoRNA | SNORD51 | 1.609283 | 3.743729 | 2.676506 |

| | | | | | |
|---|---|---|---|---|---|
| ENSMUSG00000079429 | protein_coding | Heatr7b1 | 363.6254 | 318.4031 | 341.0143 |
| ENSMUSG00000030283 | protein_coding | St8sia1 | 663.054 | 767.0615 | 715.0578 |
| ENSMUSG00000036943 | protein_coding | Rab8b | 905.1595 | 845.3292 | 875.2444 |
| ENSMUSG00000036598 | protein_coding | Ccdc113 | 58.54406 | 47.50223 | 53.02315 |
| ENSMUSG00000021091 | protein_coding | Serpina3n | 5.546094 | 10.26822 | 7.907155 |
| ENSMUSG00000024501 | protein_coding | Dpysl3 | 1614.579 | 1718.95 | 1666.765 |
| ENSMUSG00000070495 | protein_coding | Ctcfl | 390.9166 | 342.098 | 366.5073 |
| ENSMUSG00000025324 | protein_coding | Atp10a | 632.7652 | 577.9954 | 605.3803 |
| ENSMUSG00000085235 | processed_transcrip | Gm12576 | 4.873696 | 8.211475 | 6.542586 |
| ENSMUSG00000082446 | pseudogene | Gm13863 | 5.254473 | 2.137462 | 3.695967 |
| ENSMUSG00000056144 | protein_coding | Trim34a | 183.0459 | 208.1895 | 195.6177 |
| ENSMUSG00000025246 | protein_coding | Tbl1x | 4107.208 | 4341.249 | 4224.229 |
| ENSMUSG00000026031 | protein_coding | Cflar | 1712.049 | 1922.101 | 1817.075 |
| ENSMUSG00000052581 | protein_coding | Lrrtm4 | 0.974531 | 2.683826 | 1.829178 |
| ENSMUSG00000002111 | protein_coding | Sfpi1 | 74.03182 | 87.12995 | 80.58088 |
| ENSMUSG00000081376 | pseudogene | Gm11694 | 2.589703 | 5.094109 | 3.841906 |
| ENSMUSG00000062960 | protein_coding | Kdr | 665.4638 | 593.4001 | 629.432 |
| ENSMUSG00000087168 | lincRNA | Gm15983 | 19.66549 | 25.95886 | 22.81218 |
| ENSMUSG00000027544 | protein_coding | Nfatc2 | 842.9684 | 916.2316 | 879.6 |
| ENSMUSG00000061315 | protein_coding | Naca | 5867.773 | 6253.737 | 6060.755 |
| ENSMUSG00000039917 | protein_coding | Rhbdd2 | 1091.488 | 1010.336 | 1050.912 |
| ENSMUSG00000087063 | processed_transcrip | Gm15857 | 0.638331 | 2.039445 | 1.338888 |
| ENSMUSG00000022025 | protein_coding | Lect1 | 54.50967 | 68.10937 | 61.30952 |
| ENSMUSG00000048905 | protein_coding | 4930539E08Rik | 95.20252 | 116.4151 | 105.8088 |
| ENSMUSG00000004945 | protein_coding | 5730437N04Rik | 940.151 | 1011.734 | 975.9426 |
| ENSMUSG00000051669 | protein_coding | AU021092 | 260.9435 | 221.5958 | 241.2697 |
| ENSMUSG00000052371 | lincRNA | 1700109F18Rik | 34.49851 | 42.55734 | 38.52793 |
| ENSMUSG00000034226 | protein_coding | Rhov | 175.6833 | 202.8837 | 189.2835 |
| ENSMUSG00000039108 | protein_coding | Lsm14b | 2708.706 | 2544.324 | 2626.515 |
| ENSMUSG00000080818 | pseudogene | Gm9434 | 4.492919 | 2.066111 | 3.279515 |
| ENSMUSG00000042647 | protein_coding | Acad12 | 277.5699 | 303.1497 | 290.3598 |
| ENSMUSG00000030204 | protein_coding | Ddx47 | 1829.596 | 1934.539 | 1882.068 |
| ENSMUSG00000032089 | protein_coding | Il10ra | 956.7286 | 864.2331 | 910.4808 |
| ENSMUSG00000038538 | protein_coding | Ubn2 | 4365.883 | 4626.633 | 4496.258 |
| ENSMUSG00000030621 | protein_coding | Me3 | 6.461958 | 10.27759 | 8.369772 |
| ENSMUSG00000019877 | protein_coding | Serinc1 | 5983.166 | 6292.928 | 6138.047 |
| ENSMUSG00000067649 | protein_coding | Mageb18 | 195.6063 | 219.7897 | 207.698 |
| ENSMUSG00000072884 | lincRNA | Gm10433 | 39.05241 | 31.35318 | 35.2028 |
| ENSMUSG00000087677 | processed_transcrip | Gm15226 | 9.728681 | 6.109689 | 7.919185 |
| ENSMUSG00000021815 | protein_coding | Zmynd17 | 48.48969 | 58.9399 | 53.7148 |
| ENSMUSG00000026253 | protein_coding | Chrng | 1.623372 | 3.998531 | 2.810952 |
| ENSMUSG00000000567 | protein_coding | Sox9 | 3920.114 | 4303.195 | 4111.655 |
| ENSMUSG00000083283 | pseudogene | Gm15361 | 27.96343 | 20.0362 | 23.99981 |
| ENSMUSG00000045903 | protein_coding | Npas4 | 4.893674 | 2.410644 | 3.652159 |
| ENSMUSG00000059027 | lincRNA | 9630013D21Rik | 28.58446 | 38.84825 | 33.71636 |
| ENSMUSG00000021824 | protein_coding | Ap3m1 | 3166.256 | 2988.784 | 3077.52 |
| ENSMUSG00000087179 | processed_transcrip | Gm14230 | 405.9168 | 370.5372 | 388.227 |
| ENSMUSG00000031486 | protein_coding | Gpr124 | 408.3702 | 446.5516 | 427.4609 |
| ENSMUSG00000028003 | protein_coding | Lrat | 37.73244 | 46.96704 | 42.34974 |
| ENSMUSG00000050144 | protein_coding | Slc25a44 | 1115.608 | 1037.801 | 1076.704 |
| ENSMUSG00000024173 | protein_coding | Tpsab1 | 7.014488 | 3.743729 | 5.379108 |
| ENSMUSG00000023967 | protein_coding | Mrps18a | 772.0745 | 827.4438 | 799.7592 |
| ENSMUSG00000047495 | protein_coding | Dlgap2 | 5.173517 | 9.006843 | 7.09018 |
| ENSMUSG00000031711 | protein_coding | Zfp330 | 1404.204 | 1501.758 | 1452.981 |
| ENSMUSG00000058331 | protein_coding | Zfp85-rs1 | 176.565 | 200.1025 | 188.3337 |
| ENSMUSG00000021047 | protein_coding | Nova1 | 223.9858 | 199.1028 | 211.5443 |
| ENSMUSG00000037341 | protein_coding | Slc9a7 | 200.2501 | 227.2507 | 213.7504 |
| ENSMUSG00000056820 | protein_coding | Tsnax | 3108.349 | 3278.222 | 3193.286 |

| | | | | | |
|---|---|---|---|---|---|
| ENSMUSG00000092015 | lincRNA | Gm17551 | 0.65242 | 2.110796 | 1.381608 |
| ENSMUSG00000078632 | protein_coding | Lrrc37a | 0.65242 | 2.110796 | 1.381608 |
| ENSMUSG00000075259 | protein_coding | Nkain2 | 6.76409 | 10.57743 | 8.670763 |
| ENSMUSG00000037513 | protein_coding | Samd4b | 3360.26 | 3042.059 | 3201.16 |
| ENSMUSG00000049832 | pseudogene | Gm9840 | 8.802307 | 13.57913 | 11.19072 |
| ENSMUSG00000090266 | protein_coding | 1110005A03Rik | 413.5456 | 362.4136 | 387.9796 |
| ENSMUSG00000025470 | protein_coding | Zfp511 | 290.268 | 316.4719 | 303.3699 |
| ENSMUSG00000057254 | protein_coding | Zfp870 | 375.2775 | 331.7772 | 353.5273 |
| ENSMUSG00000073542 | protein_coding | Cep76 | 566.3415 | 523.9397 | 545.1406 |
| ENSMUSG00000029486 | protein_coding | Mrpl1 | 748.5765 | 799.7855 | 774.181 |
| ENSMUSG00000051251 | protein_coding | Nhlh1 | 1.304841 | 0 | 0.65242 |
| ENSMUSG00000082789 | pseudogene | Gm13284 | 1.304841 | 0 | 0.65242 |
| ENSMUSG00000044609 | pseudogene | Gm9294 | 1.304841 | 0 | 0.65242 |
| ENSMUSG00000089655 | processed_transcrip | Gm16004 | 1.304841 | 0 | 0.65242 |
| ENSMUSG00000021538 | protein_coding | Il9 | 1.304841 | 0 | 0.65242 |
| ENSMUSG00000082662 | pseudogene | Gm15516 | 1.304841 | 0 | 0.65242 |
| ENSMUSG00000045636 | protein_coding | Mtus1 | 1722.137 | 1904.198 | 1813.168 |
| ENSMUSG00000022993 | protein_coding | 4930415O20Rik | 3.530167 | 6.623589 | 5.076878 |
| ENSMUSG00000028972 | protein_coding | Car6 | 32.22064 | 41.35474 | 36.78769 |
| ENSMUSG00000005686 | protein_coding | Ampd3 | 116.0759 | 100.2023 | 108.1391 |
| ENSMUSG00000089429 | rRNA | 5S_rRNA | 7.731464 | 4.503783 | 6.117624 |
| ENSMUSG00000087282 | processed_transcrip | Gm15317 | 5.79187 | 3.135023 | 4.463447 |
| ENSMUSG00000032564 | protein_coding | Cpne4 | 2.273482 | 4.936962 | 3.605222 |
| ENSMUSG00000043019 | protein_coding | Edem3 | 2312.723 | 2468.065 | 2390.394 |
| ENSMUSG00000071726 | protein_coding | Gm5941 | 1.284863 | 3.037007 | 2.160935 |
| ENSMUSG00000051242 | protein_coding | Pcdhb9 | 33.69343 | 44.01904 | 38.85623 |
| ENSMUSG00000034163 | protein_coding | Zfc3h1 | 2924.495 | 3190.901 | 3057.698 |
| ENSMUSG00000030849 | protein_coding | Fgfr2 | 392.5617 | 436.1608 | 414.3612 |
| ENSMUSG00000086127 | lincRNA | Gm11934 | 2.606103 | 5.192487 | 3.899295 |
| ENSMUSG00000086444 | processed_transcrip | Gm15961 | 3.570123 | 6.507553 | 5.038838 |
| ENSMUSG00000021539 | protein_coding | Lect2 | 0.638331 | 2.021789 | 1.33006 |
| ENSMUSG00000022136 | protein_coding | Dnajc3 | 2472.416 | 2228.321 | 2350.369 |
| ENSMUSG00000003190 | protein_coding | Bcl2l12 | 458.873 | 501.8411 | 480.357 |
| ENSMUSG00000028364 | protein_coding | Tnc | 14.52731 | 20.00704 | 17.26718 |
| ENSMUSG00000037705 | protein_coding | Tecta | 28.08471 | 21.45472 | 24.76972 |
| ENSMUSG00000030553 | protein_coding | Pgpep1l | 7.058022 | 11.15083 | 9.104425 |
| ENSMUSG00000084314 | pseudogene | Gm13048 | 15.89902 | 10.99332 | 13.44617 |
| ENSMUSG00000026829 | protein_coding | Gbgt1 | 61.0795 | 71.94243 | 66.51096 |
| ENSMUSG00000053110 | protein_coding | Yap1 | 4415.759 | 4794.171 | 4604.965 |
| ENSMUSG00000048978 | protein_coding | Nrsn1 | 15.95768 | 10.50608 | 13.23188 |
| ENSMUSG00000031493 | protein_coding | Ggn | 23.84704 | 30.61078 | 27.22891 |
| ENSMUSG00000069972 | protein_coding | Gm10159 | 11.95401 | 16.80316 | 14.37858 |
| ENSMUSG00000092469 | processed_transcrip | Gm20395 | 2.901303 | 1.015218 | 1.95826 |
| ENSMUSG00000040728 | protein_coding | Esrp1 | 1440.851 | 1313.292 | 1377.071 |
| ENSMUSG00000055485 | protein_coding | 9830001H06Rik | 763.5732 | 834.2398 | 798.9065 |
| ENSMUSG00000036502 | protein_coding | Fam70a | 1927.587 | 1705.58 | 1816.583 |
| ENSMUSG00000090444 | lincRNA | D930048G16Rik | 14.1314 | 20.94226 | 17.53683 |
| ENSMUSG00000038152 | protein_coding | 5033430I15Rik | 21.54053 | 15.51512 | 18.52783 |
| ENSMUSG00000084715 | miRNA | AC153790.1 | 2.956391 | 7.29034 | 5.123365 |
| ENSMUSG00000072501 | protein_coding | Phf20l1 | 1801.192 | 1921.002 | 1861.097 |
| ENSMUSG00000024182 | protein_coding | Axin1 | 3392.43 | 3158.08 | 3275.255 |
| ENSMUSG00000048758 | protein_coding | Rpl29 | 3055.371 | 3813.554 | 3434.463 |
| ENSMUSG00000056899 | protein_coding | Immp2l | 57.75664 | 47.83562 | 52.79613 |
| ENSMUSG00000055746 | protein_coding | Magea2 | 61.93438 | 85.11412 | 73.52425 |
| ENSMUSG00000055312 | protein_coding | 0610012H03Rik | 1.606973 | 3.735081 | 2.671027 |
| ENSMUSG00000051952 | protein_coding | Olfr371 | 1.981861 | 0.36219 | 1.172025 |
| ENSMUSG00000090638 | processed_transcrip | Gm17028 | 1.981861 | 0.36219 | 1.172025 |
| ENSMUSG00000035951 | protein_coding | Ascl3 | 1.945483 | 4.185917 | 3.0657 |

| | | | | | |
|---|---|---|---|---|---|
| ENSMUSG00000047420 | protein_coding | Fam180a | 11.58143 | 16.40566 | 13.99354 |
| ENSMUSG00000042745 | protein_coding | Id1 | 932.6032 | 847.1925 | 889.8979 |
| ENSMUSG00000031672 | protein_coding | Got2 | 1852.206 | 1972.512 | 1912.359 |
| ENSMUSG00000091697 | pseudogene | Eif3s6-ps2 | 63.36461 | 81.19212 | 72.27836 |
| ENSMUSG00000025150 | protein_coding | Cbr2 | 307.2326 | 346.8672 | 327.0499 |
| ENSMUSG00000025979 | protein_coding | Mob4 | 1409.746 | 1509.299 | 1459.523 |
| ENSMUSG00000019842 | protein_coding | Traf3ip2 | 389.8497 | 351.6889 | 370.7693 |
| ENSMUSG00000085133 | processed_transcrip | B930095G15Rik | 54.92929 | 69.7538 | 62.34155 |
| ENSMUSG00000091203 | protein_coding | Gm8011 | 0.638331 | 2.012779 | 1.325555 |
| ENSMUSG00000060716 | protein_coding | Plekhh1 | 1237.807 | 1341.912 | 1289.86 |
| ENSMUSG00000039347 | protein_coding | Atp6v0e2 | 891.6503 | 971.3577 | 931.504 |
| ENSMUSG00000026110 | protein_coding | Mgat4a | 602.542 | 670.5775 | 636.5598 |
| ENSMUSG00000081578 | pseudogene | Gm12611 | 3.238545 | 1.350742 | 2.294643 |
| ENSMUSG00000025193 | protein_coding | Cutc | 448.2962 | 401.6722 | 424.9842 |
| ENSMUSG00000021018 | protein_coding | Polr2h | 931.7765 | 997.6151 | 964.6958 |
| ENSMUSG00000069720 | protein_coding | 4930572O03Rik | 4.14032 | 1.695274 | 2.917797 |
| ENSMUSG00000041073 | protein_coding | Nacad | 59.40528 | 46.98692 | 53.1961 |
| ENSMUSG00000020286 | protein_coding | 1700093K21Rik | 21.47202 | 15.09059 | 18.28131 |
| ENSMUSG00000084903 | lincRNA | Gm16624 | 32.16241 | 41.63185 | 36.89713 |
| ENSMUSG00000071042 | protein_coding | Rasgrp3 | 132.8059 | 150.2868 | 141.5463 |
| ENSMUSG00000015733 | protein_coding | Capza2 | 4676.444 | 4959.637 | 4818.041 |
| ENSMUSG00000044471 | protein_coding | AB041803 | 3.540677 | 6.52521 | 5.032944 |
| ENSMUSG00000034789 | protein_coding | Rab24 | 1206.619 | 1083.427 | 1145.023 |
| ENSMUSG00000082195 | pseudogene | Gm13034 | 79.11871 | 98.58809 | 88.8534 |
| ENSMUSG00000046847 | pseudogene | Gm7094 | 1.31304 | 0 | 0.65652 |
| ENSMUSG00000037033 | protein_coding | Clca4 | 1.31304 | 0 | 0.65652 |
| ENSMUSG00000081127 | pseudogene | Gm12677 | 1.31304 | 0 | 0.65652 |
| ENSMUSG00000089062 | snRNA | U6 | 1.31304 | 0 | 0.65652 |
| ENSMUSG00000001496 | protein_coding | Nkx2-1 | 1.31304 | 0 | 0.65652 |
| ENSMUSG00000046070 | protein_coding | Igfals | 2.911813 | 5.581343 | 4.246578 |
| ENSMUSG00000028459 | protein_coding | Cd72 | 34.51663 | 25.09536 | 29.80599 |
| ENSMUSG00000029798 | protein_coding | Herc6 | 267.3368 | 301.0665 | 284.2017 |
| ENSMUSG00000048799 | protein_coding | Cep120 | 1693.772 | 1602.249 | 1648.01 |
| ENSMUSG00000000782 | protein_coding | Tcf7 | 1978.344 | 2143.745 | 2061.045 |
| ENSMUSG00000049792 | protein_coding | Bag5 | 736.3993 | 685.6519 | 711.0256 |
| ENSMUSG00000024742 | protein_coding | Fen1 | 221.3188 | 196.5533 | 208.9361 |
| ENSMUSG00000065547 | miRNA | Mir199a-1 | 4.877274 | 8.291835 | 6.584555 |
| ENSMUSG00000055471 | protein_coding | Alk | 4.218965 | 7.169591 | 5.694278 |
| ENSMUSG00000034275 | protein_coding | Igsf9b | 323.9079 | 286.0914 | 304.9997 |
| ENSMUSG00000085786 | processed_transcrip | Gm15987 | 1.293062 | 0 | 0.646531 |
| ENSMUSG00000071207 | pseudogene | Gm8587 | 1.293062 | 0 | 0.646531 |
| ENSMUSG00000009075 | protein_coding | Cabp7 | 12.18696 | 7.656463 | 9.921713 |
| ENSMUSG00000041991 | protein_coding | Hrnr | 2.950502 | 1.050893 | 2.000698 |
| ENSMUSG00000018042 | protein_coding | Cyb5r3 | 2797.926 | 2622.84 | 2710.383 |
| ENSMUSG00000033319 | protein_coding | Fem1c | 1550.238 | 1431.117 | 1490.678 |
| ENSMUSG00000081603 | pseudogene | Gm14681 | 292.9122 | 325.8408 | 309.3765 |
| ENSMUSG00000039508 | protein_coding | Fam26d | 6.11167 | 9.818104 | 7.964887 |
| ENSMUSG00000008450 | protein_coding | Nutf2 | 1955.266 | 2081.665 | 2018.465 |
| ENSMUSG00000037742 | protein_coding | Eef1a1 | 59619.19 | 62536.48 | 61077.84 |
| ENSMUSG00000026831 | protein_coding | 1700007K13Rik | 41.35869 | 50.9429 | 46.15079 |
| ENSMUSG00000033219 | protein_coding | Gm9758 | 5.815427 | 3.090339 | 4.452883 |
| ENSMUSG00000041020 | protein_coding | Mtap7d2 | 1474.16 | 1315.893 | 1395.026 |
| ENSMUSG00000053684 | protein_coding | BC048403 | 350.1724 | 318.7592 | 334.4658 |
| ENSMUSG00000020993 | protein_coding | Trappc6b | 1173.535 | 1098.097 | 1135.816 |
| ENSMUSG00000048039 | protein_coding | Isg20l2 | 1696.554 | 1834.964 | 1765.759 |
| ENSMUSG00000040283 | protein_coding | Btnl9 | 10.35523 | 6.507915 | 8.431575 |
| ENSMUSG00000030986 | protein_coding | Dhx32 | 3271.242 | 3077.344 | 3174.293 |
| ENSMUSG00000030061 | protein_coding | Uba3 | 2769.614 | 2965.94 | 2867.777 |

| | | | | | |
|---|---|---|---|---|---|
| ENSMUSG00000063011 | protein_coding | Msln | 15.96126 | 22.94925 | 19.45525 |
| ENSMUSG00000039183 | protein_coding | Nubp2 | 1136.447 | 1060.147 | 1098.297 |
| ENSMUSG00000000827 | protein_coding | Tpd52l2 | 2442.269 | 2583.996 | 2513.133 |
| ENSMUSG00000091975 | lincRNA | Gm7822 | 6.102427 | 2.719502 | 4.410964 |
| ENSMUSG00000026943 | protein_coding | Lcn12 | 1.307151 | 0 | 0.653576 |
| ENSMUSG00000086129 | processed_transcrip | Gm13558 | 1.307151 | 0 | 0.653576 |
| ENSMUSG00000092494 | processed_transcrip | Gm20420 | 1.307151 | 0 | 0.653576 |
| ENSMUSG00000056829 | protein_coding | Foxb2 | 1.307151 | 0 | 0.653576 |
| ENSMUSG00000060416 | protein_coding | Gm839 | 0.646531 | 2.012779 | 1.329655 |
| ENSMUSG00000079635 | protein_coding | Rhox4c | 0.646531 | 2.012779 | 1.329655 |
| ENSMUSG00000035545 | protein_coding | Leng8 | 2951.575 | 3363.109 | 3157.342 |
| ENSMUSG00000064637 | snoRNA | Snora20 | 9.43937 | 14.52621 | 11.98279 |
| ENSMUSG00000059108 | protein_coding | Ifitm6 | 0.65242 | 2.021789 | 1.337104 |
| ENSMUSG00000044794 | pseudogene | 9330133O14Rik | 124.9758 | 109.6479 | 117.3118 |
| ENSMUSG00000030976 | protein_coding | 4930404H21Rik | 2.269904 | 0.662038 | 1.465971 |
| ENSMUSG00000022434 | protein_coding | Fam118a | 1060.077 | 990.8877 | 1025.482 |
| ENSMUSG00000053153 | protein_coding | Spag16 | 31.07517 | 23.54242 | 27.3088 |
| ENSMUSG00000032216 | protein_coding | Nedd4 | 21293.52 | 22904.34 | 22098.93 |
| ENSMUSG00000027011 | protein_coding | Ube2e3 | 3502.235 | 3313.954 | 3408.094 |
| ENSMUSG00000035342 | protein_coding | Lzts2 | 2604.283 | 2791.886 | 2698.084 |
| ENSMUSG00000041534 | protein_coding | Rbp3 | 6.200825 | 10.17957 | 8.190198 |
| ENSMUSG00000036570 | protein_coding | Fxyd1 | 472.1534 | 426.0122 | 449.0828 |
| ENSMUSG00000002778 | protein_coding | Kdelr1 | 1192.766 | 1273.834 | 1233.3 |
| ENSMUSG00000040703 | protein_coding | Cyp2s1 | 600.8302 | 551.8811 | 576.3557 |
| ENSMUSG00000063709 | protein_coding | Rhox2e | 20.31918 | 15.00987 | 17.66452 |
| ENSMUSG00000021716 | protein_coding | Srek1ip1 | 881.3225 | 972.0935 | 926.708 |
| ENSMUSG00000025260 | protein_coding | Hsd17b10 | 1148.071 | 1237.36 | 1192.716 |
| ENSMUSG00000025481 | protein_coding | 1190003J15Rik | 9.364304 | 13.60616 | 11.48523 |
| ENSMUSG00000002968 | protein_coding | Med25 | 2148.895 | 2027.681 | 2088.288 |
| ENSMUSG00000091932 | lincRNA | Gm17596 | 35.27647 | 27.15033 | 31.2134 |
| ENSMUSG00000032633 | protein_coding | Flcn | 979.0562 | 1057.818 | 1018.437 |
| ENSMUSG00000034663 | protein_coding | Bmp2k | 3115.339 | 3338.503 | 3226.921 |
| ENSMUSG00000005892 | protein_coding | Trh | 5.195806 | 8.511685 | 6.853746 |
| ENSMUSG00000022983 | protein_coding | Scaf4 | 1684.489 | 1557.24 | 1620.864 |
| ENSMUSG00000085441 | pseudogene | Gm8289 | 5.864626 | 3.099348 | 4.481987 |
| ENSMUSG00000030682 | protein_coding | Cdipt | 1234.868 | 1317.818 | 1276.343 |
| ENSMUSG00000050830 | protein_coding | Vwc2 | 11.63779 | 7.611779 | 9.624782 |
| ENSMUSG00000017002 | protein_coding | Slpi | 4.194365 | 7.558085 | 5.876225 |
| ENSMUSG00000019303 | protein_coding | Psmc3ip | 494.9281 | 455.2594 | 475.0938 |
| ENSMUSG00000047766 | protein_coding | Lrrc49 | 825.8851 | 770.802 | 798.3436 |
| ENSMUSG00000051705 | protein_coding | Senp8 | 633.2058 | 572.1179 | 602.6618 |
| ENSMUSG00000031903 | protein_coding | Pla2g15 | 427.7957 | 394.2043 | 411 |
| ENSMUSG00000068466 | pseudogene | Gm5518 | 409.692 | 371.7969 | 390.7445 |
| ENSMUSG00000083974 | pseudogene | Gm16053 | 1.923194 | 4.043577 | 2.983385 |
| ENSMUSG00000037820 | protein_coding | Tgm2 | 1248.868 | 1339.501 | 1294.184 |
| ENSMUSG00000006395 | protein_coding | Hyi | 245.909 | 219.2649 | 232.587 |
| ENSMUSG00000063511 | protein_coding | Snrnp70 | 5123.572 | 5444.673 | 5284.122 |
| ENSMUSG00000085603 | processed_transcrip | Gm11346 | 13.24476 | 8.025175 | 10.63497 |
| ENSMUSG00000035559 | protein_coding | Mpv17l2 | 430.3936 | 393.1681 | 411.7809 |
| ENSMUSG00000014873 | protein_coding | Surf2 | 541.3152 | 585.6326 | 563.4739 |
| ENSMUSG00000038605 | protein_coding | Samd10 | 410.1892 | 449.6317 | 429.9104 |
| ENSMUSG00000031371 | protein_coding | Haus7 | 2176.883 | 2325.992 | 2251.438 |
| ENSMUSG00000013160 | protein_coding | Atp6v0d1 | 1707.332 | 1588.716 | 1648.024 |
| ENSMUSG00000026199 | processed_transcrip | Ankzf1 | 701.0774 | 626.3362 | 663.7068 |
| ENSMUSG00000031376 | protein_coding | Atp2b3 | 54.5919 | 64.85791 | 59.7249 |
| ENSMUSG00000019813 | protein_coding | Cep57l1 | 392.8039 | 426.7255 | 409.7647 |
| ENSMUSG00000064514 | snoRNA | SNORD18 | 5.137139 | 2.710492 | 3.923816 |
| ENSMUSG00000039013 | protein_coding | Siglec5 | 5.822359 | 9.428887 | 7.625623 |

| | | | | | |
|---|---|---|---|---|---|
| ENSMUSG00000027804 | protein_coding | Ppid | 2559.993 | 2747.952 | 2653.973 |
| ENSMUSG00000089631 | pseudogene | Gm16326 | 9.366615 | 5.881191 | 7.623903 |
| ENSMUSG00000061082 | protein_coding | Plac1 | 414.3854 | 460.5335 | 437.4594 |
| ENSMUSG00000050567 | protein_coding | Maml1 | 1513.546 | 1405.91 | 1459.728 |
| ENSMUSG00000044197 | protein_coding | Gpr146 | 336.1778 | 307.5745 | 321.8762 |
| ENSMUSG00000019558 | protein_coding | Slc6a8 | 873.4886 | 954.6275 | 914.058 |
| ENSMUSG00000085440 | processed_transcrip | D330022K07Rik | 11.32514 | 16.08779 | 13.70647 |
| ENSMUSG00000054808 | protein_coding | Actn4 | 3353.125 | 3562.171 | 3457.648 |
| ENSMUSG00000086390 | lincRNA | 1810019D21Rik | 18.35499 | 24.46578 | 21.41038 |
| ENSMUSG00000055717 | protein_coding | Slain1 | 1814.006 | 1949.188 | 1881.597 |
| ENSMUSG00000083303 | pseudogene | Gm12110 | 1.296641 | 0 | 0.64832 |
| ENSMUSG00000009185 | protein_coding | Ccl8 | 6.176226 | 3.408205 | 4.792216 |
| ENSMUSG00000019726 | protein_coding | Lyst | 1961.983 | 1733.59 | 1847.786 |
| ENSMUSG00000038143 | protein_coding | Stox2 | 570.1435 | 529.8691 | 550.0063 |
| ENSMUSG00000086658 | processed_transcrip | Gm14260 | 8.403862 | 5.139155 | 6.771509 |
| ENSMUSG00000057400 | protein_coding | Ces1c | 2.972791 | 0.671047 | 1.821919 |
| ENSMUSG00000058207 | protein_coding | Serpina3k | 2.972791 | 0.671047 | 1.821919 |
| ENSMUSG00000024427 | protein_coding | Spry4 | 255.6759 | 285.3718 | 270.5239 |
| ENSMUSG00000049672 | protein_coding | Zfp161 | 859.4086 | 773.6267 | 816.5176 |
| ENSMUSG00000074997 | pseudogene | Pin1-ps1 | 1.276663 | 0 | 0.638331 |
| ENSMUSG00000053044 | protein_coding | Cd8b1 | 1.276663 | 0 | 0.638331 |
| ENSMUSG00000083196 | pseudogene | Gm15272 | 1.276663 | 0 | 0.638331 |
| ENSMUSG00000032000 | protein_coding | Birc3 | 388.1737 | 428.333 | 408.2534 |
| ENSMUSG00000084785 | lincRNA | Gm13972 | 1.635151 | 0.36219 | 0.99867 |
| ENSMUSG00000026767 | protein_coding | Fam188a | 884.8289 | 824.2211 | 854.525 |
| ENSMUSG00000045775 | protein_coding | Slc16a5 | 50.28147 | 60.83416 | 55.55781 |
| ENSMUSG00000087163 | processed_transcrip | Gm16230 | 10.41159 | 6.534219 | 8.472905 |
| ENSMUSG00000033585 | protein_coding | Ndn | 1964.509 | 2125.281 | 2044.895 |
| ENSMUSG00000033498 | protein_coding | Strc | 4.209497 | 7.920998 | 6.065248 |
| ENSMUSG00000088901 | miRNA | Mir1943 | 3.866366 | 1.668247 | 2.767306 |
| ENSMUSG00000020357 | protein_coding | Flt4 | 44.26715 | 53.53373 | 48.90044 |
| ENSMUSG00000017493 | protein_coding | Igfbp4 | 2976.348 | 2761.485 | 2868.917 |
| ENSMUSG00000074063 | protein_coding | Osgin1 | 141.8556 | 122.4067 | 132.1312 |
| ENSMUSG00000047454 | protein_coding | Gphn | 930.3315 | 863.8961 | 897.1138 |
| ENSMUSG00000020230 | protein_coding | Prmt2 | 1348.91 | 1458.43 | 1403.67 |
| ENSMUSG00000003037 | protein_coding | Rab8a | 1559.54 | 1472.635 | 1516.088 |
| ENSMUSG00000083817 | pseudogene | Gm14400 | 1.298952 | 3.090339 | 2.194645 |
| ENSMUSG00000073187 | protein_coding | Gm5784 | 42.17235 | 33.49893 | 37.83564 |
| ENSMUSG00000034880 | protein_coding | Mrpl34 | 492.2878 | 542.6664 | 517.4771 |
| ENSMUSG00000087615 | processed_transcrip | Gm16190 | 1.288441 | 3.470547 | 2.379494 |
| ENSMUSG00000038195 | protein_coding | Rilp | 75.64885 | 88.93085 | 82.28985 |
| ENSMUSG00000026005 | protein_coding | Rpe | 1172.613 | 1083.02 | 1127.817 |
| ENSMUSG00000032057 | protein_coding | 4833427G06Rik | 3.963721 | 1.006571 | 2.485146 |
| ENSMUSG00000039878 | protein_coding | Slc39a5 | 4.247143 | 7.478449 | 5.862796 |
| ENSMUSG00000004896 | protein_coding | Rrnad1 | 619.8779 | 672.1601 | 646.019 |
| ENSMUSG00000037652 | protein_coding | Phc3 | 2203.636 | 2349.422 | 2276.529 |
| ENSMUSG00000080753 | pseudogene | Gm15613 | 1.635151 | 3.859402 | 2.747277 |
| ENSMUSG00000091162 | lincRNA | Gm7677 | 11.62243 | 7.594122 | 9.608276 |
| ENSMUSG00000063434 | protein_coding | Sorcs3 | 37.77113 | 30.22514 | 33.99813 |
| ENSMUSG00000020778 | protein_coding | 2310004N24Rik | 385.6094 | 418.429 | 402.0192 |
| ENSMUSG00000030722 | protein_coding | Nfatc2ip | 3084.699 | 2834.911 | 2959.805 |
| ENSMUSG00000040247 | protein_coding | Tbc1d10c | 112.2311 | 94.25879 | 103.2449 |
| ENSMUSG00000026319 | protein_coding | 2310035C23Rik | 2863.763 | 2680.656 | 2772.209 |
| ENSMUSG00000086823 | processed_transcrip | 1700028P15Rik | 7.450578 | 3.998531 | 5.724554 |
| ENSMUSG00000071454 | protein_coding | Dtnb | 1880.57 | 1754.744 | 1817.657 |
| ENSMUSG00000090565 | processed_transcrip | 4930405O22Rik | 1.635151 | 0.335524 | 0.985337 |
| ENSMUSG00000071494 | protein_coding | Olfr455 | 8.689595 | 12.89079 | 10.79019 |
| ENSMUSG00000086714 | lincRNA | 0610009E02Rik | 24.62499 | 18.47141 | 21.5482 |

| | | | | | |
|---|---|---|---|---|---|
| ENSMUSG00000038535 | protein_coding | Zfp280d | 1650.192 | 1747.048 | 1698.62 |
| ENSMUSG00000083027 | pseudogene | Gm13140 | 60.58101 | 74.31336 | 67.44719 |
| ENSMUSG00000075703 | protein_coding | Ept1 | 1116.597 | 1202.027 | 1159.312 |
| ENSMUSG00000033863 | protein_coding | Klf9 | 1806.581 | 1626.124 | 1716.353 |
| ENSMUSG00000072980 | protein_coding | Oip5 | 449.3936 | 406.599 | 427.9963 |
| ENSMUSG00000005150 | protein_coding | Wdr83 | 535.4739 | 491.8523 | 513.6631 |
| ENSMUSG00000076441 | protein_coding | Ass1 | 959.0508 | 1060.726 | 1009.888 |
| ENSMUSG00000092389 | processed_transcrip | Gm20483 | 1.932661 | 0.36219 | 1.147425 |
| ENSMUSG00000024576 | protein_coding | Csnk1a1 | 9286.586 | 9777.462 | 9532.024 |
| ENSMUSG00000010054 | protein_coding | Tusc2 | 1082.657 | 1155.994 | 1119.326 |
| ENSMUSG00000086978 | processed_transcrip | 4933435F18Rik | 19.14472 | 13.47531 | 16.31002 |
| ENSMUSG00000062284 | pseudogene | Gm6030 | 6.522936 | 3.188355 | 4.855645 |
| ENSMUSG00000092957 | miRNA | AC084822.1 | 1.623372 | 0.36219 | 0.992781 |
| ENSMUSG00000022371 | protein_coding | Col14a1 | 277.9519 | 314.3528 | 296.1524 |
| ENSMUSG00000048938 | protein_coding | Nr1h5 | 50.50413 | 38.32664 | 44.41538 |
| ENSMUSG00000025645 | protein_coding | Ccdc51 | 162.4136 | 183.1501 | 172.7819 |
| ENSMUSG00000089581 | snRNA | U6 | 9.725103 | 6.17203 | 7.948566 |
| ENSMUSG00000028602 | protein_coding | Tnfrsf8 | 26.12872 | 20.10434 | 23.11653 |
| ENSMUSG00000061559 | protein_coding | Wdr61 | 1973.761 | 1851.528 | 1912.644 |
| ENSMUSG00000022510 | protein_coding | Trp63 | 65.11299 | 54.70609 | 59.90954 |
| ENSMUSG00000091820 | pseudogene | Gm1815 | 145.5771 | 129.7028 | 137.64 |
| ENSMUSG00000021592 | protein_coding | Arsk | 483.9047 | 526.2141 | 505.0594 |
| ENSMUSG00000049643 | protein_coding | 2310022A10Rik | 491.8658 | 530.5047 | 511.1853 |
| ENSMUSG00000031939 | protein_coding | Taf1d | 1842.831 | 1715.986 | 1779.408 |
| ENSMUSG00000039967 | protein_coding | Zfp292 | 5246.86 | 5604.35 | 5425.605 |
| ENSMUSG00000040605 | protein_coding | Bace2 | 263.916 | 294.0579 | 278.987 |
| ENSMUSG00000076335 | miRNA | Mir670 | 1.282552 | 0 | 0.641276 |
| ENSMUSG00000084969 | processed_transcrip | Gm11205 | 10.02955 | 15.25996 | 12.64475 |
| ENSMUSG00000031843 | protein_coding | Mphosph6 | 1203.967 | 1290.723 | 1247.345 |
| ENSMUSG00000074199 | protein_coding | Krtdap | 1.298952 | 3.0907 | 2.194826 |
| ENSMUSG00000041141 | protein_coding | Pnmal1 | 94.34444 | 110.0205 | 102.1825 |
| ENSMUSG00000027907 | protein_coding | S100a11 | 1244.799 | 1356.882 | 1300.84 |
| ENSMUSG00000038812 | protein_coding | Trmt112 | 781.13 | 845.2608 | 813.1954 |
| ENSMUSG00000031163 | protein_coding | Glod5 | 1.637461 | 3.797061 | 2.717261 |
| ENSMUSG00000027533 | protein_coding | Fabp5 | 785.606 | 855.6246 | 820.6153 |
| ENSMUSG00000052560 | protein_coding | Cpne8 | 702.3864 | 756.3379 | 729.3621 |
| ENSMUSG00000048330 | protein_coding | Ric3 | 896.9712 | 772.9168 | 834.944 |
| ENSMUSG00000065434 | miRNA | Mir7-1 | 53.41744 | 65.87484 | 59.64614 |
| ENSMUSG00000021577 | protein_coding | Sdha | 6091.123 | 6408.136 | 6249.63 |
| ENSMUSG00000033964 | protein_coding | Zbtb41 | 2226.275 | 2078.48 | 2152.377 |
| ENSMUSG00000043263 | protein_coding | Pyhin1 | 5.795449 | 3.064034 | 4.429742 |
| ENSMUSG00000052951 | lincRNA | C130021I20Rik | 2.259393 | 4.52144 | 3.390417 |
| ENSMUSG00000092624 | pseudogene | Gm3654 | 6.419691 | 3.399558 | 4.909625 |
| ENSMUSG00000028899 | protein_coding | Taf12 | 907.4391 | 850.3553 | 878.8972 |
| ENSMUSG00000038831 | protein_coding | Ralgps1 | 791.0698 | 733.5922 | 762.331 |
| ENSMUSG00000059898 | protein_coding | Dsc3 | 7.703286 | 12.09506 | 9.899171 |
| ENSMUSG00000029925 | protein_coding | Tbxas1 | 22.862 | 16.90734 | 19.88467 |
| ENSMUSG00000065623 | snRNA | U6 | 5.842337 | 3.064034 | 4.453186 |
| ENSMUSG00000053870 | protein_coding | Fpgt | 625.0693 | 577.9178 | 601.4935 |
| ENSMUSG00000018554 | protein_coding | Ybx2 | 704.293 | 442.1879 | 573.2405 |
| ENSMUSG00000007440 | protein_coding | Pcdha11 | 45.68508 | 57.63395 | 51.65951 |
| ENSMUSG00000027742 | protein_coding | Cog6 | 1711.851 | 1594.398 | 1653.124 |
| ENSMUSG00000086594 | processed_transcrip | 4930505H01Rik | 1.278973 | 0 | 0.639487 |
| ENSMUSG00000027479 | protein_coding | Mapre1 | 9277.035 | 9747.878 | 9512.456 |
| ENSMUSG00000039315 | protein_coding | Clnk | 8.391041 | 5.067443 | 6.729242 |
| ENSMUSG00000003518 | protein_coding | Dusp3 | 1018.39 | 1111.8 | 1065.095 |
| ENSMUSG00000026395 | protein_coding | Ptprc | 60.40963 | 72.4336 | 66.42162 |
| ENSMUSG00000071477 | protein_coding | Zfp777 | 1023.044 | 1091.615 | 1057.33 |

| | | | | | |
|---|---|---|---|---|---|
| ENSMUSG00000045576 | protein_coding | St7l | 612.8182 | 572.1727 | 592.4955 |
| ENSMUSG00000028291 | protein_coding | Akirin2 | 1124.139 | 1192.544 | 1158.341 |
| ENSMUSG00000073982 | protein_coding | Rhog | 731.8017 | 783.9191 | 757.8604 |
| ENSMUSG00000073758 | protein_coding | 1700029G01Rik | 248.2396 | 224.6504 | 236.445 |
| ENSMUSG00000026355 | protein_coding | Mcm6 | 5080.036 | 5353.182 | 5216.609 |
| ENSMUSG00000053863 | protein_coding | Mepe | 2.295771 | 0.688704 | 1.492237 |
| ENSMUSG00000033623 | protein_coding | Pcgf3 | 3520.789 | 3256.267 | 3388.528 |
| ENSMUSG00000028175 | protein_coding | Depdc1a | 388.3791 | 333.7784 | 361.0788 |
| ENSMUSG00000039197 | protein_coding | Adk | 2411.345 | 2608.954 | 2510.15 |
| ENSMUSG00000085645 | processed_transcrip | 0610040B09Rik | 1.270774 | 3.717425 | 2.494099 |
| ENSMUSG00000032840 | protein_coding | 2410131K14Rik | 935.1026 | 1041.341 | 988.2219 |
| ENSMUSG00000078966 | protein_coding | Fam81b | 5.481538 | 8.989187 | 7.235362 |
| ENSMUSG00000031688 | protein_coding | Pou4f2 | 0.66062 | 2.499652 | 1.580136 |
| ENSMUSG00000093182 | misc_RNA | AC136021.1 | 2.259393 | 4.441442 | 3.350418 |
| ENSMUSG00000032735 | protein_coding | Ablim3 | 50.61998 | 41.02564 | 45.82281 |
| ENSMUSG00000033392 | protein_coding | Clasp2 | 2850.607 | 3017.472 | 2934.039 |
| ENSMUSG00000007035 | protein_coding | Msh5 | 530.2991 | 343.7315 | 437.0153 |
| ENSMUSG00000038550 | protein_coding | Gm129 | 332.313 | 368.9189 | 350.6159 |
| ENSMUSG00000022449 | protein_coding | Adamts20 | 58.72171 | 46.94369 | 52.8327 |
| ENSMUSG00000015652 | protein_coding | Steap1 | 184.2484 | 163.9481 | 174.0982 |
| ENSMUSG00000074629 | protein_coding | 4930518I15Rik | 81.01999 | 94.36555 | 87.69277 |
| ENSMUSG00000018339 | protein_coding | Gpx3 | 264.0297 | 293.8107 | 278.9202 |
| ENSMUSG00000034382 | protein_coding | AI661453 | 219.7645 | 195.5104 | 207.6374 |
| ENSMUSG00000043079 | protein_coding | Synpo | 1493.995 | 1664.579 | 1579.287 |
| ENSMUSG00000080364 | snoRNA | SNORA77 | 11.9599 | 16.68785 | 14.32387 |
| ENSMUSG00000041762 | protein_coding | Gpr155 | 129.34 | 112.5068 | 120.9234 |
| ENSMUSG00000025776 | protein_coding | Crispld1 | 134.9848 | 112.5995 | 123.7922 |
| ENSMUSG00000052496 | protein_coding | Pkdrej | 30.34053 | 23.80374 | 27.07214 |
| ENSMUSG00000053889 | processed_transcrip | A130066N16Rik | 20.69765 | 27.12615 | 23.9119 |
| ENSMUSG00000031428 | protein_coding | Zcchc18 | 265.3126 | 179.9681 | 222.6403 |
| ENSMUSG00000025437 | protein_coding | Usp33 | 2534.127 | 2690.307 | 2612.217 |
| ENSMUSG00000056824 | protein_coding | Zfp663 | 1.604662 | 3.752377 | 2.678519 |
| ENSMUSG00000025103 | protein_coding | Btbd1 | 4374.131 | 4125.471 | 4249.801 |
| ENSMUSG00000085010 | processed_transcrip | 0610038P03Rik | 2.603792 | 1.024227 | 1.81401 |
| ENSMUSG00000020952 | protein_coding | Scfd1 | 1744.87 | 1869.919 | 1807.395 |
| ENSMUSG00000055385 | protein_coding | Rnf212 | 132.7624 | 109.6953 | 121.2289 |
| ENSMUSG00000038679 | protein_coding | Trps1 | 664.2982 | 731.0986 | 697.6984 |
| ENSMUSG00000042570 | protein_coding | Mier2 | 729.0535 | 776.3331 | 752.6933 |
| ENSMUSG00000022181 | protein_coding | C6 | 1.290752 | 3.054663 | 2.172708 |
| ENSMUSG00000020781 | protein_coding | Tsen54 | 582.7708 | 541.4482 | 562.1095 |
| ENSMUSG00000051346 | protein_coding | Spryd4 | 287.5135 | 316.5022 | 302.0079 |
| ENSMUSG00000085546 | processed_transcrip | Gm14252 | 1.270774 | 0 | 0.635387 |
| ENSMUSG00000082196 | pseudogene | Gm14231 | 1.270774 | 0 | 0.635387 |
| ENSMUSG00000029286 | protein_coding | Enam | 1.270774 | 0 | 0.635387 |
| ENSMUSG00000090157 | processed_transcrip | Gm16534 | 0.632442 | 2.321637 | 1.477039 |
| ENSMUSG00000053856 | protein_coding | Dnajc5g | 97.2681 | 115.4486 | 106.3584 |
| ENSMUSG00000018770 | protein_coding | Atp5g3 | 2474.403 | 2640.069 | 2557.236 |
| ENSMUSG00000081752 | pseudogene | Gm14680 | 147.1227 | 129.7252 | 138.424 |
| ENSMUSG00000021981 | protein_coding | Cab39l | 1816.516 | 1713.459 | 1764.987 |
| ENSMUSG00000032424 | lincRNA | Snhg5 | 740.2201 | 807.8929 | 774.0565 |
| ENSMUSG00000031070 | protein_coding | Mrgprf | 83.63832 | 71.89195 | 77.76513 |
| ENSMUSG00000049517 | protein_coding | Rps23 | 5571.28 | 5872.202 | 5721.741 |
| ENSMUSG00000024642 | protein_coding | Tle4 | 1895.054 | 2009.768 | 1952.411 |
| ENSMUSG00000078952 | lincRNA | Gm11019 | 43.35084 | 33.13461 | 38.24273 |
| ENSMUSG00000041959 | protein_coding | S100a10 | 2366.843 | 2538.506 | 2452.674 |
| ENSMUSG00000006273 | protein_coding | Atp6v1b2 | 3024.381 | 2873.657 | 2949.019 |
| ENSMUSG00000044071 | protein_coding | Fam19a2 | 16.37775 | 11.33749 | 13.85762 |
| ENSMUSG00000022283 | protein_coding | Pabpc1 | 15888.74 | 15014.36 | 15451.55 |

| | | | | | |
|---|---|---|---|---|---|
| ENSMUSG00000038930 | protein_coding | Rccd1 | 302.0137 | 332.2084 | 317.111 |
| ENSMUSG00000048661 | protein_coding | Lemd3 | 1728.643 | 1622.453 | 1675.548 |
| ENSMUSG00000064030 | protein_coding | Wibg | 758.3923 | 704.5602 | 731.4762 |
| ENSMUSG00000026063 | protein_coding | 4930521A18Rik | 1.643351 | 0.36219 | 1.00277 |
| ENSMUSG00000036357 | protein_coding | Gpr101 | 1.643351 | 0.36219 | 1.00277 |
| ENSMUSG00000005124 | protein_coding | Wisp1 | 1073.062 | 1189.45 | 1131.256 |
| ENSMUSG00000087367 | processed_transcrip | Gm15491 | 1.276663 | 3.108357 | 2.19251 |
| ENSMUSG00000015850 | protein_coding | Adamtsl4 | 164.44 | 139.6801 | 152.06 |
| ENSMUSG00000009588 | protein_coding | St6galnac1 | 3.898122 | 7.709796 | 5.803959 |
| ENSMUSG00000091052 | protein_coding | Gm5551 | 3.228034 | 1.333085 | 2.28056 |
| ENSMUSG00000032387 | protein_coding | Rbpms2 | 656.3476 | 765.5026 | 710.9251 |
| ENSMUSG00000059816 | protein_coding | Fam159a | 9.41246 | 13.95861 | 11.68554 |
| ENSMUSG00000024064 | protein_coding | Galnt14 | 38.67648 | 30.71709 | 34.69678 |
| ENSMUSG00000086533 | processed_transcrip | Gm16266 | 21.58638 | 27.92768 | 24.75703 |
| ENSMUSG00000020868 | protein_coding | Xylt2 | 738.9268 | 688.9603 | 713.9436 |
| ENSMUSG00000026600 | protein_coding | Soat1 | 3918.272 | 4349.089 | 4133.681 |
| ENSMUSG00000028938 | protein_coding | Galntl5 | 2.295771 | 0.697713 | 1.496742 |
| ENSMUSG00000028549 | protein_coding | Itgb3bp | 292.0178 | 245.7992 | 268.9085 |
| ENSMUSG00000021070 | protein_coding | Bdkrb2 | 22.862 | 29.74655 | 26.30427 |
| ENSMUSG00000041120 | protein_coding | Nbl1 | 1446.522 | 1561.177 | 1503.85 |
| ENSMUSG00000026792 | protein_coding | Lrsam1 | 1003.891 | 1065.968 | 1034.93 |
| ENSMUSG00000074555 | processed_transcrip | Gm10714 | 2.592014 | 5.02312 | 3.807567 |
| ENSMUSG00000056752 | protein_coding | Dnahc9 | 23.9467 | 17.93441 | 20.94056 |
| ENSMUSG00000037124 | protein_coding | Trim58 | 2.592014 | 1.024227 | 1.808121 |
| ENSMUSG00000026988 | protein_coding | Wdsub1 | 762.5582 | 705.5038 | 734.031 |
| ENSMUSG00000029687 | protein_coding | Ezh2 | 1620.296 | 1741.143 | 1680.72 |
| ENSMUSG00000024033 | protein_coding | Rsph1 | 590.0561 | 435.8724 | 512.9642 |
| ENSMUSG00000030446 | protein_coding | Zfp273 | 207.2047 | 228.6517 | 217.9282 |
| ENSMUSG00000071679 | protein_coding | Zcchc16 | 187.0104 | 207.0976 | 197.054 |
| ENSMUSG00000021759 | protein_coding | Ppap2a | 325.3944 | 296.5643 | 310.9793 |
| ENSMUSG00000030232 | protein_coding | Aebp2 | 2636.705 | 2486.058 | 2561.382 |
| ENSMUSG00000030519 | protein_coding | Apba2 | 170.4095 | 144.8791 | 157.6443 |
| ENSMUSG00000032739 | protein_coding | Pram1 | 1.626951 | 3.814718 | 2.720834 |
| ENSMUSG00000090363 | protein_coding | Gm3512 | 3.219835 | 5.774165 | 4.497 |
| ENSMUSG00000083953 | pseudogene | Gm14049 | 1.637461 | 0.36219 | 0.999825 |
| ENSMUSG00000024958 | protein_coding | Gpr137 | 440.2592 | 408.2423 | 424.2507 |
| ENSMUSG00000020834 | protein_coding | Dhrs13 | 396.0378 | 361.9193 | 378.9786 |
| ENSMUSG00000040616 | protein_coding | Tmem51 | 46.13399 | 37.93245 | 42.03322 |
| ENSMUSG00000085277 | processed_transcrip | Gm13943 | 1.284863 | 3.010341 | 2.147602 |
| ENSMUSG00000045867 | protein_coding | Cradd | 269.8618 | 294.0957 | 281.9787 |
| ENSMUSG00000027947 | protein_coding | Il6ra | 311.672 | 275.4425 | 293.5573 |
| ENSMUSG00000021287 | protein_coding | Xrcc3 | 394.1004 | 428.2162 | 411.1583 |
| ENSMUSG00000025958 | protein_coding | Creb1 | 2864.772 | 3134.974 | 2999.873 |
| ENSMUSG00000002825 | protein_coding | Qtrt1 | 297.584 | 325.1479 | 311.3659 |
| ENSMUSG00000030450 | protein_coding | Oca2 | 119.2028 | 103.2711 | 111.237 |
| ENSMUSG00000083833 | pseudogene | Gm13841 | 457.906 | 417.2541 | 437.5801 |
| ENSMUSG00000064354 | protein_coding | mt-Co2 | 1917.751 | 2122.647 | 2020.199 |
| ENSMUSG00000052295 | protein_coding | 8030423F21Rik | 22.22031 | 15.87374 | 19.04702 |
| ENSMUSG00000002384 | protein_coding | Bmp8b | 59.13655 | 49.16799 | 54.15227 |
| ENSMUSG00000026701 | protein_coding | Prdx6 | 4354.321 | 4614.575 | 4484.448 |
| ENSMUSG00000054499 | protein_coding | Dedd2 | 303.2312 | 330.931 | 317.0811 |
| ENSMUSG00000039069 | protein_coding | Gtpbp5 | 342.0074 | 372.4199 | 357.2137 |
| ENSMUSG00000084243 | pseudogene | Gm13784 | 0.954552 | 2.656799 | 1.805676 |
| ENSMUSG00000035745 | protein_coding | Grin3b | 23.35653 | 17.39385 | 20.37519 |
| ENSMUSG00000024319 | protein_coding | Vps52 | 1358.222 | 1232.731 | 1295.477 |
| ENSMUSG00000063611 | protein_coding | Gm10134 | 21.32986 | 28.41419 | 24.87203 |
| ENSMUSG00000086965 | protein_coding | Gm16314 | 146.5346 | 128.0364 | 137.2855 |
| ENSMUSG00000015095 | protein_coding | Fbxw5 | 1418.713 | 1329.345 | 1374.029 |

| | | | | | |
|---|---|---|---|---|---|
| ENSMUSG00000030830 | protein_coding | Itgal | 71.5792 | 85.20545 | 78.39233 |
| ENSMUSG00000023033 | protein_coding | Scn8a | 160.0185 | 137.1319 | 148.5752 |
| ENSMUSG00000046876 | protein_coding | Atxn1 | 179.5624 | 157.9991 | 168.7807 |
| ENSMUSG00000073884 | protein_coding | Ccl21b | 2.283993 | 0.697713 | 1.490853 |
| ENSMUSG00000029437 | protein_coding | Il31 | 1.643351 | 0.326514 | 0.984932 |
| ENSMUSG00000028333 | protein_coding | Anp32b | 6721.494 | 6312.869 | 6517.182 |
| ENSMUSG00000069089 | protein_coding | Cdk7 | 1594.508 | 1724.672 | 1659.59 |
| ENSMUSG00000037157 | protein_coding | Il22ra1 | 9.692303 | 14.39867 | 12.04549 |
| ENSMUSG00000026322 | protein_coding | Htr4 | 7.088511 | 10.8326 | 8.960555 |
| ENSMUSG00000035131 | protein_coding | Fam5c | 3.896855 | 6.727042 | 5.311948 |
| ENSMUSG00000042525 | protein_coding | 4933428M09Rik | 10.71014 | 6.852086 | 8.781115 |
| ENSMUSG00000074595 | protein_coding | Wfdc6a | 613.2304 | 667.8989 | 640.5647 |
| ENSMUSG00000077711 | snoRNA | SNORA40 | 18.36065 | 24.51083 | 21.43574 |
| ENSMUSG00000041707 | protein_coding | 1810011H11Rik | 1.598773 | 0.335524 | 0.967148 |
| ENSMUSG00000075470 | protein_coding | Alg10b | 1767.217 | 1887.34 | 1827.279 |
| ENSMUSG00000058355 | protein_coding | Abce1 | 5152.406 | 4844.11 | 4998.258 |
| ENSMUSG00000033285 | protein_coding | Wdr3 | 2188.861 | 2320.681 | 2254.771 |
| ENSMUSG00000092106 | lincRNA | Gm17715 | 7.418821 | 4.494774 | 5.956798 |
| ENSMUSG00000000223 | protein_coding | Drp2 | 1767.466 | 1591.824 | 1679.645 |
| ENSMUSG00000064208 | pseudogene | Gm10145 | 25.20697 | 19.39798 | 22.30247 |
| ENSMUSG00000033210 | protein_coding | Slc9a10 | 1.637461 | 0.335524 | 0.986492 |
| ENSMUSG00000089125 | snRNA | U6 | 9.048083 | 13.70997 | 11.37903 |
| ENSMUSG00000057230 | protein_coding | Aak1 | 648.5196 | 585.3208 | 616.9202 |
| ENSMUSG00000015776 | protein_coding | Med22 | 2134.27 | 2262.834 | 2198.552 |
| ENSMUSG00000087588 | pseudogene | Gm16355 | 1.598773 | 0.326514 | 0.962644 |
| ENSMUSG00000020708 | protein_coding | Psmc5 | 2635.648 | 2797.813 | 2716.731 |
| ENSMUSG00000073144 | processed_transcrip | 4930599N23Rik | 166.7899 | 144.8741 | 155.832 |
| ENSMUSG00000090248 | processed_transcrip | Gm14027 | 16.17886 | 11.54869 | 13.86378 |
| ENSMUSG00000026288 | protein_coding | Inpp5d | 135.141 | 117.2351 | 126.188 |
| ENSMUSG00000086710 | processed_transcrip | Gm13465 | 1.586995 | 3.717063 | 2.652029 |
| ENSMUSG00000029438 | protein_coding | Bcl7a | 627.8375 | 567.0775 | 597.4575 |
| ENSMUSG00000055452 | protein_coding | Gm7353 | 278.4355 | 250.3407 | 264.3881 |
| ENSMUSG00000064648 | snRNA | U1 | 5.185295 | 2.772834 | 3.979065 |
| ENSMUSG00000038074 | protein_coding | Fkbp14 | 1317.345 | 1408.114 | 1362.729 |
| ENSMUSG00000087496 | processed_transcrip | Gm13523 | 1.951372 | 4.043215 | 2.997294 |
| ENSMUSG00000025144 | protein_coding | Stra13 | 497.4749 | 458.2994 | 477.8871 |
| ENSMUSG00000026147 | protein_coding | Col9a1 | 31.61592 | 40.1979 | 35.90691 |
| ENSMUSG00000032437 | protein_coding | Stt3b | 4815.134 | 4587.416 | 4701.275 |
| ENSMUSG00000021368 | protein_coding | Tbc1d7 | 443.8654 | 410.8346 | 427.35 |
| ENSMUSG00000084915 | lincRNA | C230037L18Rik | 6.184426 | 10.00513 | 8.094777 |
| ENSMUSG00000026893 | protein_coding | Gca | 3541.815 | 3821.426 | 3681.62 |
| ENSMUSG00000009216 | protein_coding | Fam163b | 44.8243 | 54.16729 | 49.4958 |
| ENSMUSG00000037390 | protein_coding | Muc3 | 45.52048 | 37.03798 | 41.27923 |
| ENSMUSG00000081111 | pseudogene | Gm5913 | 19.05534 | 13.93267 | 16.494 |
| ENSMUSG00000024150 | protein_coding | Mcfd2 | 3003.42 | 3193.25 | 3098.335 |
| ENSMUSG00000040314 | protein_coding | Ctsg | 1.592884 | 0.335524 | 0.964204 |
| ENSMUSG00000024913 | protein_coding | Lrp5 | 1106.475 | 998.1465 | 1052.311 |
| ENSMUSG00000029110 | protein_coding | Rnf4 | 5164.043 | 5449.736 | 5306.889 |
| ENSMUSG00000044991 | protein_coding | 1110034G24Rik | 157.979 | 175.9513 | 166.9651 |
| ENSMUSG00000083988 | pseudogene | Gm15633 | 2.267593 | 4.512431 | 3.390012 |
| ENSMUSG00000063714 | processed_transcrip | 1700011J10Rik | 117.0831 | 133.8105 | 125.4468 |
| ENSMUSG00000047264 | protein_coding | Zfp358 | 339.8644 | 374.4899 | 357.1772 |
| ENSMUSG00000045942 | protein_coding | BC049762 | 104.5837 | 83.48957 | 94.03662 |
| ENSMUSG00000005803 | protein_coding | Sqrdl | 148.9184 | 167.0198 | 157.9691 |
| ENSMUSG00000019792 | protein_coding | Trmt11 | 530.572 | 487.9618 | 509.2669 |
| ENSMUSG00000024118 | protein_coding | 1600002H07Rik | 510.3765 | 470.7228 | 490.5496 |
| ENSMUSG00000049537 | protein_coding | Tecrl | 27.84945 | 21.51778 | 24.68361 |
| ENSMUSG00000000531 | protein_coding | Grasp | 265.1916 | 294.4062 | 279.7989 |

| | | | | | |
|---|---|---|---|---|---|
| ENSMUSG00000090846 | protein_coding | Lsm3 | 912.4938 | 852.077 | 882.2854 |
| ENSMUSG00000081829 | pseudogene | Gm15460 | 0.33031 | 1.386417 | 0.858363 |
| ENSMUSG00000082917 | pseudogene | Gm11440 | 0.33031 | 1.386417 | 0.858363 |
| ENSMUSG00000090785 | processed_transcrip | Gm17116 | 0.33031 | 1.386417 | 0.858363 |
| ENSMUSG00000015357 | protein_coding | Clpx | 1815.476 | 1709.041 | 1762.258 |
| ENSMUSG00000040046 | protein_coding | Tph1 | 21.94024 | 16.22184 | 19.08104 |
| ENSMUSG00000054263 | protein_coding | Lifr | 2427.861 | 2591.878 | 2509.87 |
| ENSMUSG00000090433 | lincRNA | 9430037G07Rik | 75.67808 | 89.29811 | 82.48809 |
| ENSMUSG00000032388 | protein_coding | Spg21 | 1780.832 | 1878.741 | 1829.787 |
| ENSMUSG00000031298 | protein_coding | Gpr64 | 32.98785 | 40.64258 | 36.81522 |
| ENSMUSG00000080574 | miRNA | Mir466i | 16.2388 | 22.59607 | 19.41743 |
| ENSMUSG00000080662 | miRNA | Mir1b | 2.239415 | 0.724379 | 1.481897 |
| ENSMUSG00000071893 | protein_coding | Vmn1r4 | 3.228034 | 1.395426 | 2.31173 |
| ENSMUSG00000005609 | protein_coding | Ctr9 | 3611.147 | 3370.93 | 3491.039 |
| ENSMUSG00000047040 | protein_coding | Prr15l | 10.0285 | 6.489535 | 8.259019 |
| ENSMUSG00000085671 | lincRNA | C330046G13Rik | 0.32211 | 1.386417 | 0.854264 |
| ENSMUSG00000079563 | protein_coding | Pglyrp2 | 0.32211 | 1.386417 | 0.854264 |
| ENSMUSG00000006464 | protein_coding | Bbs1 | 398.1016 | 358.6937 | 378.3976 |
| ENSMUSG00000042903 | protein_coding | Foxo4 | 917.0279 | 979.7588 | 948.3933 |
| ENSMUSG00000034032 | protein_coding | Rpap1 | 1683.026 | 1572.789 | 1627.907 |
| ENSMUSG00000038192 | protein_coding | Cer1 | 3.208056 | 1.315066 | 2.261561 |
| ENSMUSG00000040829 | protein_coding | Zmynd15 | 71.94738 | 59.47332 | 65.71035 |
| ENSMUSG00000090002 | processed_transcrip | Gm16006 | 3.222145 | 1.395426 | 2.308786 |
| ENSMUSG00000060336 | protein_coding | Zfp937 | 121.9872 | 141.4927 | 131.7399 |
| ENSMUSG00000014453 | protein_coding | Blk | 7.376554 | 11.52239 | 9.449471 |
| ENSMUSG00000028869 | protein_coding | Gnl2 | 3171.814 | 2996.369 | 3084.092 |
| ENSMUSG00000054074 | protein_coding | 2810030E01Rik | 110.9261 | 97.15641 | 104.0413 |
| ENSMUSG00000074088 | protein_coding | Snrnp40 | 2526.969 | 2383.152 | 2455.061 |
| ENSMUSG00000014294 | protein_coding | Ndufa2 | 1046.667 | 967.2514 | 1006.959 |
| ENSMUSG00000054753 | protein_coding | AU018091 | 14.94678 | 9.740232 | 12.3435 |
| ENSMUSG00000055240 | protein_coding | Zfp101 | 336.1194 | 301.6762 | 318.8978 |
| ENSMUSG00000062931 | protein_coding | Zfp938 | 291.1248 | 325.0464 | 308.0856 |
| ENSMUSG00000078517 | protein_coding | C230096C10Rik | 1467.372 | 1373.376 | 1420.374 |
| ENSMUSG00000087576 | lincRNA | Gm16596 | 10.65737 | 15.04554 | 12.85145 |
| ENSMUSG00000091556 | protein_coding | Gm14569 | 15330.09 | 13539.68 | 14434.88 |
| ENSMUSG00000027651 | protein_coding | Rprd1b | 966.8871 | 894.9892 | 930.9381 |
| ENSMUSG00000052368 | protein_coding | Gm9873 | 7.437532 | 11.28416 | 9.360844 |
| ENSMUSG00000048701 | protein_coding | Ccdc6 | 3594.762 | 3332.785 | 3463.774 |
| ENSMUSG00000044139 | protein_coding | Prss53 | 4.220232 | 7.902618 | 6.061425 |
| ENSMUSG00000062444 | protein_coding | Ap3b2 | 33.30892 | 25.83739 | 29.57316 |
| ENSMUSG00000054027 | protein_coding | Nt5dc3 | 1038.984 | 1173.831 | 1106.407 |
| ENSMUSG00000087380 | lincRNA | 2210408F21Rik | 18.37497 | 24.42146 | 21.39821 |
| ENSMUSG00000028141 | protein_coding | Oaz3 | 101.424 | 79.07407 | 90.24904 |
| ENSMUSG00000083462 | pseudogene | Gm12501 | 4.216654 | 2.08413 | 3.150392 |
| ENSMUSG00000085639 | processed_transcrip | Gm15410 | 1.618751 | 0.335524 | 0.977137 |
| ENSMUSG00000008318 | protein_coding | Relt | 189.8432 | 163.1097 | 176.4765 |
| ENSMUSG00000028778 | protein_coding | Hcrtr1 | 10.03543 | 6.436203 | 8.235819 |
| ENSMUSG00000065524 | miRNA | Mir135a-2 | 2.553325 | 5.094471 | 3.823898 |
| ENSMUSG00000029161 | protein_coding | Cgref1 | 28.08725 | 36.03969 | 32.06347 |
| ENSMUSG00000020962 | protein_coding | Gtf2a1 | 2679.047 | 2817.646 | 2748.346 |
| ENSMUSG00000022538 | protein_coding | Lsg1 | 1655.714 | 1759.344 | 1707.529 |
| ENSMUSG00000036934 | protein_coding | 4921524J17Rik | 777.9485 | 829.6356 | 803.7921 |
| ENSMUSG00000047733 | protein_coding | BC048609 | 1.304841 | 3.072682 | 2.188761 |
| ENSMUSG00000021765 | protein_coding | Fst | 192.3143 | 212.0277 | 202.171 |
| ENSMUSG00000040265 | protein_coding | Dnm3 | 466.964 | 423.1755 | 445.0698 |
| ENSMUSG00000082665 | pseudogene | Gm11470 | 4.213076 | 7.080584 | 5.64683 |
| ENSMUSG00000031879 | protein_coding | Fam96b | 498.5457 | 448.4629 | 473.5043 |
| ENSMUSG00000020745 | protein_coding | Pafah1b1 | 8170.685 | 8563.091 | 8366.888 |

| | | | | | |
|---|---|---|---|---|---|
| ENSMUSG00000015314 | protein_coding | Slamf6 | 2.262971 | 0.662038 | 1.462505 |
| ENSMUSG00000070420 | protein_coding | Zfp498 | 449.1514 | 413.6705 | 431.4109 |
| ENSMUSG00000032303 | protein_coding | Chrna3 | 6.133959 | 3.452528 | 4.793244 |
| ENSMUSG00000085151 | lincRNA | 1110018N20Rik | 10.98768 | 15.78509 | 13.38638 |
| ENSMUSG00000085413 | processed_transcrip | 1700010K10Rik | 5.165317 | 2.508661 | 3.836989 |
| ENSMUSG00000028453 | protein_coding | Fancg | 667.5156 | 621.8327 | 644.6741 |
| ENSMUSG00000021933 | protein_coding | Gucy1b2 | 5.495627 | 3.063673 | 4.27965 |
| ENSMUSG00000040152 | protein_coding | Thbs1 | 416.1265 | 458.7273 | 437.4269 |
| ENSMUSG00000024620 | protein_coding | Pdgfrb | 287.6082 | 248.4834 | 268.0458 |
| ENSMUSG00000079634 | protein_coding | Rhox2d | 51.09095 | 41.06069 | 46.07582 |
| ENSMUSG00000022325 | protein_coding | Pop1 | 850.227 | 781.0543 | 815.6407 |
| ENSMUSG00000091136 | lincRNA | Gm17437 | 0.99093 | 2.746168 | 1.868549 |
| ENSMUSG00000042308 | protein_coding | Setd1a | 1624.868 | 1472.643 | 1548.755 |
| ENSMUSG00000071748 | protein_coding | Gm14698 | 69.21425 | 58.31753 | 63.76589 |
| ENSMUSG00000077019 | miRNA | AC154322.1 | 1.604662 | 0.36219 | 0.983426 |
| ENSMUSG00000024298 | protein_coding | Zfp871 | 901.6186 | 987.3308 | 944.4747 |
| ENSMUSG00000006010 | protein_coding | BC003331 | 1097.886 | 1023.768 | 1060.827 |
| ENSMUSG00000050917 | protein_coding | Fgf4 | 5.804916 | 3.055025 | 4.429971 |
| ENSMUSG00000086279 | processed_transcrip | Gm15634 | 2.265282 | 4.485765 | 3.375524 |
| ENSMUSG00000003545 | protein_coding | Fosb | 26.05514 | 37.16908 | 31.61211 |
| ENSMUSG00000041420 | protein_coding | Meis3 | 63.2062 | 73.9826 | 68.5944 |
| ENSMUSG00000036412 | protein_coding | Arsi | 265.2654 | 242.2313 | 253.7483 |
| ENSMUSG00000078444 | protein_coding | Gm10941 | 44.68744 | 34.46262 | 39.57503 |
| ENSMUSG00000030862 | protein_coding | Cpxm2 | 15.50311 | 21.64174 | 18.57243 |
| ENSMUSG00000004285 | protein_coding | Atp6v1f | 1816.95 | 1919.847 | 1868.399 |
| ENSMUSG00000033569 | protein_coding | Bai3 | 36.54955 | 29.0856 | 32.81758 |
| ENSMUSG00000086401 | lincRNA | Gm15559 | 116.6161 | 100.073 | 108.3445 |
| ENSMUSG00000013465 | protein_coding | Cobra1 | 2346.235 | 2196.816 | 2271.526 |
| ENSMUSG00000028840 | protein_coding | Zfp593 | 30.26546 | 23.79474 | 27.0301 |
| ENSMUSG00000046215 | protein_coding | Rprml | 120.0373 | 139.2186 | 129.6279 |
| ENSMUSG00000040591 | protein_coding | 1110051M20Rik | 650.8016 | 714.1995 | 682.5006 |
| ENSMUSG00000026873 | protein_coding | Phf19 | 166.2457 | 147.0307 | 156.6382 |
| ENSMUSG00000034311 | protein_coding | Kif4 | 1315.676 | 1233.611 | 1274.643 |
| ENSMUSG00000015474 | protein_coding | Ppt2 | 283.2508 | 309.4158 | 296.3333 |
| ENSMUSG00000078746 | protein_coding | Gm13298 | 2.241726 | 0.697713 | 1.469719 |
| ENSMUSG00000047227 | protein_coding | Gm527 | 409.5844 | 371.5169 | 390.5506 |
| ENSMUSG00000037204 | protein_coding | 9430023L20Rik | 1142.975 | 1208.572 | 1175.774 |
| ENSMUSG00000090026 | processed_transcrip | Gm15996 | 14.59291 | 20.19407 | 17.39349 |
| ENSMUSG00000009733 | protein_coding | Tcfcp2 | 838.932 | 784.5826 | 811.7573 |
| ENSMUSG00000091896 | protein_coding | Ube2d2 | 3732.636 | 3914.904 | 3823.77 |
| ENSMUSG00000020903 | protein_coding | Stx8 | 514.6414 | 560.663 | 537.6522 |
| ENSMUSG00000087573 | lincRNA | 9530026P05Rik | 46.99708 | 56.61433 | 51.8057 |
| ENSMUSG00000087028 | processed_transcrip | Gm13387 | 8.736483 | 12.89006 | 10.81327 |
| ENSMUSG00000027313 | protein_coding | Chac1 | 17.37561 | 12.62625 | 15.00093 |
| ENSMUSG00000008686 | protein_coding | Zfp955a | 394.8565 | 356.2309 | 375.5437 |
| ENSMUSG00000082550 | pseudogene | Gm12344 | 0.99093 | 3.188355 | 2.089643 |
| ENSMUSG00000081671 | pseudogene | Gm13167 | 23.96817 | 17.19201 | 20.58009 |
| ENSMUSG00000060445 | protein_coding | Sycp2 | 5614.73 | 3936.373 | 4775.551 |
| ENSMUSG00000079104 | protein_coding | Prps1l3 | 1611.829 | 1717.727 | 1664.778 |
| ENSMUSG00000075611 | pseudogene | Gm11545 | 25.42814 | 19.25564 | 22.34189 |
| ENSMUSG00000032680 | protein_coding | 6820408C15Rik | 71.11747 | 82.46581 | 76.79164 |
| ENSMUSG00000085692 | lincRNA | Gm2061 | 10.04132 | 6.252028 | 8.146676 |
| ENSMUSG00000045930 | protein_coding | Clec14a | 143.1379 | 162.3524 | 152.7451 |
| ENSMUSG00000025887 | protein_coding | Casp12 | 125.5098 | 141.8707 | 133.6902 |
| ENSMUSG00000007033 | protein_coding | Hspa1l | 26.42623 | 33.60274 | 30.01449 |
| ENSMUSG00000026853 | protein_coding | Crat | 941.1857 | 859.2599 | 900.2228 |
| ENSMUSG00000051920 | protein_coding | Rspo2 | 175.4902 | 152.9119 | 164.2011 |
| ENSMUSG00000042118 | protein_coding | Bhmt2 | 8.105309 | 4.494412 | 6.299861 |

| | | | | | |
|---|---|---|---|---|---|
| ENSMUSG00000024191 | protein_coding | Bnip1 | 477.9595 | 515.2006 | 496.58 |
| ENSMUSG00000025784 | protein_coding | Clec3b | 780.1057 | 685.7405 | 732.9231 |
| ENSMUSG00000087652 | processed_transcrip | Gm15918 | 4.901874 | 2.43731 | 3.669592 |
| ENSMUSG00000086588 | processed_transcrip | Gm11729 | 1.290752 | 3.028359 | 2.159555 |
| ENSMUSG00000031286 | protein_coding | Glt28d2 | 12.58876 | 8.564656 | 10.57671 |
| ENSMUSG00000037134 | protein_coding | Prmt10 | 1097.138 | 1032.857 | 1064.997 |
| ENSMUSG00000071317 | protein_coding | Bves | 123.2704 | 109.2485 | 116.2595 |
| ENSMUSG00000019143 | protein_coding | Hars2 | 874.7792 | 950.5102 | 912.6447 |
| ENSMUSG00000001655 | protein_coding | Hoxc13 | 5.79187 | 3.161689 | 4.47678 |
| ENSMUSG00000026748 | protein_coding | Plxdc2 | 1018.418 | 1097.252 | 1057.835 |
| ENSMUSG00000046490 | protein_coding | Rnf222 | 175.7193 | 196.1673 | 185.9433 |
| ENSMUSG00000079357 | processed_transcrip | Gm11100 | 5.185295 | 2.763824 | 3.97456 |
| ENSMUSG00000080542 | snRNA | U4atac | 0.33031 | 1.359751 | 0.84503 |
| ENSMUSG00000074679 | protein_coding | Defb28 | 0.33031 | 1.359751 | 0.84503 |
| ENSMUSG00000035020 | protein_coding | Epgn | 0.33031 | 1.359751 | 0.84503 |
| ENSMUSG00000030147 | protein_coding | Clec4b1 | 0.33031 | 1.359751 | 0.84503 |
| ENSMUSG00000077520 | snoRNA | SNORA17 | 0.33031 | 1.359751 | 0.84503 |
| ENSMUSG00000083209 | pseudogene | Gm11969 | 0.33031 | 1.359751 | 0.84503 |
| ENSMUSG00000047547 | protein_coding | Cltb | 2556.393 | 2414.337 | 2485.365 |
| ENSMUSG00000062382 | protein_coding | Gm10116 | 76.64589 | 90.48223 | 83.56406 |
| ENSMUSG00000086085 | lincRNA | 5730422E09Rik | 23.59537 | 18.04724 | 20.8213 |
| ENSMUSG00000017801 | protein_coding | Mlx | 535.0093 | 580.4755 | 557.7424 |
| ENSMUSG00000035024 | protein_coding | Ncapd3 | 2581.641 | 2449.401 | 2515.521 |
| ENSMUSG00000078631 | protein_coding | Gm10967 | 1.939594 | 4.159251 | 3.049422 |
| ENSMUSG00000070336 | protein_coding | Fbxo47 | 544.4029 | 331.772 | 438.0874 |
| ENSMUSG00000037278 | protein_coding | Tmem97 | 1168.213 | 1103.638 | 1135.925 |
| ENSMUSG00000085557 | lincRNA | Gm16600 | 67.61652 | 79.2731 | 73.44481 |
| ENSMUSG00000041789 | protein_coding | 2700046A07Rik | 136.0108 | 120.3677 | 128.1892 |
| ENSMUSG00000089739 | protein_coding | Tmem189 | 0.32211 | 1.359751 | 0.840931 |
| ENSMUSG00000089392 | rRNA | 5S_rRNA | 0.32211 | 1.359751 | 0.840931 |
| ENSMUSG00000058057 | protein_coding | Mettl7a3 | 0.32211 | 1.359751 | 0.840931 |
| ENSMUSG00000079427 | protein_coding | Gm2382 | 141.028 | 157.5152 | 149.2716 |
| ENSMUSG00000091877 | lincRNA | Gm17699 | 0 | 1.024227 | 0.512114 |
| ENSMUSG00000037307 | protein_coding | Banf2 | 0 | 1.024227 | 0.512114 |
| ENSMUSG00000081470 | pseudogene | Gm14131 | 0 | 1.024227 | 0.512114 |
| ENSMUSG00000092127 | lincRNA | Gm17580 | 0 | 1.024227 | 0.512114 |
| ENSMUSG00000090642 | lincRNA | Gm17182 | 0 | 1.024227 | 0.512114 |
| ENSMUSG00000080759 | pseudogene | Gm15573 | 0 | 1.024227 | 0.512114 |
| ENSMUSG00000077773 | snoRNA | SNORA17 | 0 | 1.024227 | 0.512114 |
| ENSMUSG00000089556 | snoRNA | SNORA70 | 0 | 1.024227 | 0.512114 |
| ENSMUSG00000035191 | protein_coding | Rfpl4 | 0 | 1.024227 | 0.512114 |
| ENSMUSG00000093055 | miRNA | AC117644.2 | 0 | 1.024227 | 0.512114 |
| ENSMUSG00000043948 | protein_coding | Olfr691 | 0 | 1.024227 | 0.512114 |
| ENSMUSG00000000216 | protein_coding | Scnn1g | 0 | 1.024227 | 0.512114 |
| ENSMUSG00000091564 | lincRNA | Gm17683 | 0 | 1.024227 | 0.512114 |
| ENSMUSG00000084231 | pseudogene | Gm11975 | 0 | 1.024227 | 0.512114 |
| ENSMUSG00000059397 | protein_coding | Olfr54 | 0 | 1.024227 | 0.512114 |
| ENSMUSG00000081686 | pseudogene | Gm11774 | 0 | 1.024227 | 0.512114 |
| ENSMUSG00000087092 | lincRNA | 4930511A02Rik | 0 | 1.024227 | 0.512114 |
| ENSMUSG00000021907 | protein_coding | Msmb | 0 | 1.024227 | 0.512114 |
| ENSMUSG00000072596 | protein_coding | Ear2 | 0 | 1.024227 | 0.512114 |
| ENSMUSG00000089405 | rRNA | 5S_rRNA | 0 | 1.024227 | 0.512114 |
| ENSMUSG00000052363 | protein_coding | Zdhhc19 | 0 | 1.024227 | 0.512114 |
| ENSMUSG00000077708 | snoRNA | SNORA17 | 0 | 1.024227 | 0.512114 |
| ENSMUSG00000067103 | protein_coding | AY702103 | 0 | 1.024227 | 0.512114 |
| ENSMUSG00000082974 | pseudogene | Gm15129 | 0 | 1.024227 | 0.512114 |
| ENSMUSG00000052539 | protein_coding | Magi3 | 2104.577 | 1923.885 | 2014.231 |
| ENSMUSG00000086620 | processed_transcrip | Gm14157 | 3.546566 | 6.207705 | 4.877136 |

| | | | | | |
|---|---|---|---|---|---|
| ENSMUSG00000027315 | protein_coding | Spint1 | 820.5163 | 742.1804 | 781.3484 |
| ENSMUSG00000014361 | protein_coding | Mertk | 811.4878 | 886.9319 | 849.2099 |
| ENSMUSG00000083921 | pseudogene | Gm15750 | 0.64422 | 2.075121 | 1.359671 |
| ENSMUSG00000088969 | snRNA | U6 | 0.64422 | 2.075121 | 1.359671 |
| ENSMUSG00000089524 | snoRNA | SNORA70 | 0 | 1.448758 | 0.724379 |
| ENSMUSG00000023993 | protein_coding | Treml1 | 0 | 1.448758 | 0.724379 |
| ENSMUSG00000028795 | protein_coding | Ccdc28b | 998.1645 | 1075.49 | 1036.827 |
| ENSMUSG00000091354 | protein_coding | 6030419C18Rik | 42.95538 | 34.71955 | 38.83747 |
| ENSMUSG00000026072 | protein_coding | Il1r1 | 630.3939 | 568.2634 | 599.3287 |
| ENSMUSG00000076376 | miRNA | Mir674 | 0.316221 | 1.359751 | 0.837986 |
| ENSMUSG00000090303 | protein_coding | Gm9479 | 0.316221 | 1.359751 | 0.837986 |
| ENSMUSG00000082415 | pseudogene | Gm12486 | 0.316221 | 1.359751 | 0.837986 |
| ENSMUSG00000089957 | processed_transcrip | A830011K09Rik | 0.316221 | 1.359751 | 0.837986 |
| ENSMUSG00000074336 | protein_coding | Apoc4 | 0.316221 | 1.359751 | 0.837986 |
| ENSMUSG00000080999 | pseudogene | Gm11336 | 0.316221 | 1.359751 | 0.837986 |
| ENSMUSG00000090669 | lincRNA | Gm2990 | 0.316221 | 1.359751 | 0.837986 |
| ENSMUSG00000086004 | pseudogene | Gm7805 | 0.316221 | 1.359751 | 0.837986 |
| ENSMUSG00000050732 | protein_coding | Vamp8 | 772.6239 | 828.0384 | 800.3311 |
| ENSMUSG00000073079 | protein_coding | Srp54c | 785.0807 | 836.3967 | 810.7387 |
| ENSMUSG00000040652 | processed_transcrip | Oaz2 | 3330.693 | 3150.584 | 3240.638 |
| ENSMUSG00000037944 | protein_coding | Ccr7 | 2.875436 | 1.015218 | 1.945327 |
| ENSMUSG00000029772 | protein_coding | Ahcyl2 | 2022.331 | 1842.318 | 1932.324 |
| ENSMUSG00000029309 | protein_coding | Sparcl1 | 3895.859 | 3502.988 | 3699.423 |
| ENSMUSG00000087293 | processed_transcrip | Gm14341 | 1.943172 | 4.069882 | 3.006527 |
| ENSMUSG00000040653 | protein_coding | Ppp1r14c | 14.81766 | 10.47978 | 12.64872 |
| ENSMUSG00000021852 | protein_coding | Slc35f4 | 14.62109 | 9.9781 | 12.29959 |
| ENSMUSG00000055676 | protein_coding | Gm5069 | 25.8207 | 32.53813 | 29.17941 |
| ENSMUSG00000030161 | protein_coding | Gabarapl1 | 2346.591 | 2484.467 | 2415.529 |
| ENSMUSG00000001034 | protein_coding | Mapk7 | 1618.457 | 1513.128 | 1565.793 |
| ENSMUSG00000040963 | protein_coding | Asgr2 | 8.402819 | 5.067443 | 6.735131 |
| ENSMUSG00000020660 | protein_coding | Pomc | 41.80171 | 51.28924 | 46.54548 |
| ENSMUSG00000029208 | protein_coding | Guf1 | 1280.043 | 1415.121 | 1347.582 |
| ENSMUSG00000085597 | lincRNA | 4932441J04Rik | 3.572434 | 1.686265 | 2.629349 |
| ENSMUSG00000090400 | lincRNA | A330094K24Rik | 73.9317 | 85.47366 | 79.70268 |
| ENSMUSG00000032410 | protein_coding | Xrn1 | 1515.464 | 1419.471 | 1467.468 |
| ENSMUSG00000029480 | protein_coding | Dhx37 | 1016.924 | 930.5618 | 973.7427 |
| ENSMUSG00000031107 | protein_coding | Rbmx2 | 792.7384 | 726.8017 | 759.7701 |
| ENSMUSG00000074933 | lincRNA | Gm17719 | 4.507008 | 2.374969 | 3.440988 |
| ENSMUSG00000090933 | lincRNA | Gm17703 | 98.8624 | 85.88126 | 92.37183 |
| ENSMUSG00000082379 | pseudogene | Gm13884 | 0.33031 | 1.350742 | 0.840526 |
| ENSMUSG00000006462 | processed_transcrip | A530013C23Rik | 0.33031 | 1.350742 | 0.840526 |
| ENSMUSG00000022335 | protein_coding | Zfat | 453.704 | 411.954 | 432.829 |
| ENSMUSG00000046806 | protein_coding | 3110062M04Rik | 105.2232 | 119.2735 | 112.2484 |
| ENSMUSG00000004562 | protein_coding | Arhgef40 | 1176.363 | 1268.754 | 1222.559 |
| ENSMUSG00000063889 | protein_coding | Crem | 388.4985 | 354.0801 | 371.2893 |
| ENSMUSG00000090653 | lincRNA | Gm17645 | 22.99342 | 29.91701 | 26.45521 |
| ENSMUSG00000087693 | processed_transcrip | Gm16191 | 2.253504 | 4.361082 | 3.307293 |
| ENSMUSG00000091109 | protein_coding | Gm17622 | 7.088511 | 10.61347 | 8.850992 |
| ENSMUSG00000068924 | protein_coding | Gm10253 | 2.923592 | 1.033237 | 1.978414 |
| ENSMUSG00000078375 | protein_coding | Gm9257 | 1.615173 | 3.399196 | 2.507184 |
| ENSMUSG00000087052 | processed_transcrip | Gm8093 | 6.813289 | 10.64879 | 8.731037 |
| ENSMUSG00000073822 | lincRNA | Gm2529 | 11.29108 | 15.70758 | 13.49933 |
| ENSMUSG00000085261 | lincRNA | Gm13814 | 11.93172 | 8.202828 | 10.06727 |
| ENSMUSG00000038729 | protein_coding | Akap2 | 1.586995 | 0.335524 | 0.961259 |
| ENSMUSG00000067581 | protein_coding | AY702102 | 0.32211 | 1.350742 | 0.836426 |
| ENSMUSG00000087743 | snRNA | U1 | 0.32211 | 1.350742 | 0.836426 |
| ENSMUSG00000065820 | snRNA | U2 | 0.32211 | 1.350742 | 0.836426 |
| ENSMUSG00000005813 | protein_coding | Metap1 | 1776.998 | 1870.816 | 1823.907 |

| | | | | | |
|---|---|---|---|---|---|
| ENSMUSG00000028820 | protein_coding | Sfpq | 9826.439 | 9348.169 | 9587.304 |
| ENSMUSG00000091449 | protein_coding | Gm10269 | 14.83533 | 10.02858 | 12.43196 |
| ENSMUSG00000070717 | protein_coding | Gm10300 | 0.316221 | 1.350742 | 0.833481 |
| ENSMUSG00000029618 | protein_coding | Ocm | 0.316221 | 1.350742 | 0.833481 |
| ENSMUSG00000085371 | pseudogene | Gm3160 | 0.316221 | 1.350742 | 0.833481 |
| ENSMUSG00000086028 | processed_transcrip | Gm12243 | 0.316221 | 1.350742 | 0.833481 |
| ENSMUSG00000040904 | protein_coding | Rnasek | 786.4135 | 843.4447 | 814.9291 |
| ENSMUSG00000028149 | protein_coding | Rap1gds1 | 1665.199 | 1562.548 | 1613.874 |
| ENSMUSG00000021760 | protein_coding | Gpx8 | 1214.061 | 1387.384 | 1300.723 |
| ENSMUSG00000041126 | protein_coding | H2afv | 2491.497 | 2652.338 | 2571.917 |
| ENSMUSG00000031826 | protein_coding | Usp10 | 1429.877 | 1516.464 | 1473.171 |
| ENSMUSG00000039478 | protein_coding | Efha2 | 662.5078 | 608.5557 | 635.5317 |
| ENSMUSG00000081743 | pseudogene | Gm4911 | 40.03685 | 26.29429 | 33.16557 |
| ENSMUSG00000025025 | protein_coding | Mxi1 | 4190.936 | 4434.703 | 4312.82 |
| ENSMUSG00000046861 | protein_coding | Hectd3 | 760.0099 | 681.6839 | 720.8469 |
| ENSMUSG00000027610 | protein_coding | Gss | 613.8435 | 661.9293 | 637.8864 |
| ENSMUSG00000089672 | protein_coding | Gp49a | 17.94774 | 25.5379 | 21.74282 |
| ENSMUSG00000002204 | protein_coding | Napsa | 9.033994 | 5.801193 | 7.417593 |
| ENSMUSG00000022677 | protein_coding | 0610037P05Rik | 1088.695 | 1162.661 | 1125.678 |
| ENSMUSG00000054428 | protein_coding | Atpif1 | 1679.24 | 1788.383 | 1733.811 |
| ENSMUSG00000011751 | protein_coding | Spnb4 | 38.8653 | 30.92249 | 34.8939 |
| ENSMUSG00000074183 | protein_coding | Gsta1 | 0.64422 | 2.057464 | 1.350842 |
| ENSMUSG00000016918 | protein_coding | Sulf1 | 1584.753 | 1425.741 | 1505.247 |
| ENSMUSG00000033249 | protein_coding | Hsf4 | 24.25957 | 31.06162 | 27.66059 |
| ENSMUSG00000023460 | protein_coding | Rab12 | 2120.611 | 2235.63 | 2178.12 |
| ENSMUSG00000041571 | protein_coding | Sepw1 | 1164.342 | 1241.156 | 1202.749 |
| ENSMUSG00000086347 | processed_transcrip | Gm11626 | 0.99093 | 2.781843 | 1.886387 |
| ENSMUSG00000045514 | protein_coding | Olfr460 | 0.66062 | 2.08413 | 1.372375 |
| ENSMUSG00000075276 | pseudogene | Gm13653 | 3.874566 | 6.534219 | 5.204393 |
| ENSMUSG00000081121 | pseudogene | Gm12791 | 10.87496 | 16.04274 | 13.45885 |
| ENSMUSG00000027322 | protein_coding | Siglec1 | 33.31585 | 41.57128 | 37.44356 |
| ENSMUSG00000018476 | protein_coding | Kdm6b | 3116.95 | 3374.16 | 3245.555 |
| ENSMUSG00000064615 | snRNA | U6 | 11.95296 | 17.13941 | 14.54618 |
| ENSMUSG00000082103 | pseudogene | Gm15320 | 3.916833 | 1.668247 | 2.79254 |
| ENSMUSG00000006764 | protein_coding | Tph2 | 0.638331 | 2.101787 | 1.370059 |
| ENSMUSG00000046985 | protein_coding | Tapt1 | 1424.164 | 1510.826 | 1467.495 |
| ENSMUSG00000040669 | protein_coding | Phc1 | 2088.841 | 1881.26 | 1985.051 |
| ENSMUSG00000049624 | protein_coding | Slc17a5 | 363.0268 | 331.3081 | 347.1675 |
| ENSMUSG00000029108 | protein_coding | Pcdh7 | 169.6317 | 189.2017 | 179.4167 |
| ENSMUSG00000018677 | protein_coding | Slc25a39 | 2798.928 | 2663.5 | 2731.214 |
| ENSMUSG00000028261 | protein_coding | Ndufaf4 | 658.1724 | 705.1186 | 681.6455 |
| ENSMUSG00000036449 | protein_coding | Lcn8 | 0.976841 | 2.790491 | 1.883666 |
| ENSMUSG00000032369 | protein_coding | Plscr1 | 228.9929 | 260.6095 | 244.8012 |
| ENSMUSG00000004264 | protein_coding | Phb2 | 3411.878 | 3598.172 | 3505.025 |
| ENSMUSG00000038914 | protein_coding | Dido1 | 3272.862 | 3494.913 | 3383.888 |
| ENSMUSG00000028681 | protein_coding | Ptch2 | 897.2831 | 810.4814 | 853.8823 |
| ENSMUSG00000036225 | protein_coding | Kctd1 | 259.519 | 288.5846 | 274.0518 |
| ENSMUSG00000032021 | protein_coding | Crtam | 50.10278 | 40.22053 | 45.16166 |
| ENSMUSG00000026875 | protein_coding | Traf1 | 50.19529 | 40.8833 | 45.53929 |
| ENSMUSG00000041710 | protein_coding | Trpc5 | 14.80358 | 10.56879 | 12.68618 |
| ENSMUSG00000085961 | processed_transcrip | Gm15904 | 4.549275 | 2.374969 | 3.462122 |
| ENSMUSG00000080715 | pseudogene | Gm5406 | 0.66062 | 2.303618 | 1.482119 |
| ENSMUSG00000022800 | protein_coding | Fyttd1 | 4834.634 | 4601.233 | 4717.934 |
| ENSMUSG00000069171 | protein_coding | Nr2f1 | 15.10802 | 20.49392 | 17.80097 |
| ENSMUSG00000043644 | protein_coding | 0610009L18Rik | 46.5053 | 55.23729 | 50.87129 |
| ENSMUSG00000039198 | protein_coding | Ptchd3 | 26.04948 | 18.08907 | 22.06927 |
| ENSMUSG00000021377 | protein_coding | Dek | 6761.143 | 7148.699 | 6954.921 |
| ENSMUSG00000062101 | protein_coding | Zfp119b | 171.3543 | 192.0204 | 181.6873 |

| | | | | | |
|---|---|---|---|---|---|
| ENSMUSG00000028780 | protein_coding | Sema3c | 5222.641 | 5576.199 | 5399.42 |
| ENSMUSG00000091870 | lincRNA | 1700101I11Rik | 15.55231 | 20.9271 | 18.2397 |
| ENSMUSG00000037257 | protein_coding | Aagab | 2436.055 | 2571.739 | 2503.897 |
| ENSMUSG00000080469 | snoRNA | Snord98 | 2.601481 | 5.130146 | 3.865814 |
| ENSMUSG00000074794 | protein_coding | Arrdc3 | 3697.98 | 4508.298 | 4103.139 |
| ENSMUSG00000027360 | protein_coding | Hdc | 611.0859 | 662.4209 | 636.7534 |
| ENSMUSG00000086832 | lincRNA | 2810425M01Rik | 3.580633 | 1.686265 | 2.633449 |
| ENSMUSG00000032290 | protein_coding | Ptpn9 | 1305.439 | 1378.339 | 1341.889 |
| ENSMUSG00000043311 | protein_coding | D17H6S53E | 315.5888 | 359.8551 | 337.722 |
| ENSMUSG00000056602 | protein_coding | Fry | 643.0788 | 583.6217 | 613.3503 |
| ENSMUSG00000042116 | protein_coding | Vwa1 | 681.5671 | 746.7491 | 714.1581 |
| ENSMUSG00000028007 | protein_coding | Snx7 | 1089.212 | 1164.557 | 1126.884 |
| ENSMUSG00000020453 | protein_coding | Patz1 | 2080.288 | 1949.042 | 2014.665 |
| ENSMUSG00000004328 | protein_coding | Hif3a | 226.1348 | 254.5328 | 240.3338 |
| ENSMUSG00000022773 | protein_coding | Ypel1 | 561.5632 | 608.4753 | 585.0192 |
| ENSMUSG00000078942 | protein_coding | Naip6 | 13.64909 | 19.04444 | 16.34676 |
| ENSMUSG00000022706 | protein_coding | Mrpl40 | 905.7653 | 845.0283 | 875.3968 |
| ENSMUSG00000054206 | protein_coding | Gzmm | 50.84055 | 41.77891 | 46.30973 |
| ENSMUSG00000016481 | protein_coding | Cr1l | 814.3173 | 876.689 | 845.5031 |
| ENSMUSG00000036887 | protein_coding | C1qa | 388.6601 | 422.4216 | 405.5409 |
| ENSMUSG00000078127 | protein_coding | Fam170b | 9.022216 | 13.41012 | 11.21617 |
| ENSMUSG00000092852 | miRNA | AC106841.1 | 1.612862 | 3.434871 | 2.523867 |
| ENSMUSG00000041986 | protein_coding | Elmod1 | 1.612862 | 3.434871 | 2.523867 |
| ENSMUSG00000085918 | lincRNA | Gm13032 | 2.603792 | 0.988552 | 1.796172 |
| ENSMUSG00000021703 | protein_coding | Serinc5 | 3986.979 | 4340.705 | 4163.842 |
| ENSMUSG00000029370 | protein_coding | Rassf6 | 6.430202 | 9.915397 | 8.172799 |
| ENSMUSG00000038375 | protein_coding | Trp53inp2 | 2605.469 | 2782.529 | 2693.999 |
| ENSMUSG00000033039 | protein_coding | Micall1 | 1565.277 | 1477.57 | 1521.424 |
| ENSMUSG00000084941 | processed_transcrip | Gm11944 | 52.1363 | 62.34997 | 57.24314 |
| ENSMUSG00000070493 | protein_coding | Chchd2 | 6720.306 | 7089.443 | 6904.874 |
| ENSMUSG00000031538 | protein_coding | Plat | 2281.164 | 2130.558 | 2205.861 |
| ENSMUSG00000027954 | protein_coding | Efna1 | 170.094 | 188.574 | 179.334 |
| ENSMUSG00000032489 | protein_coding | Kif9 | 148.8692 | 122.2985 | 135.5838 |
| ENSMUSG00000092634 | miRNA | AC164093.1 | 4.532875 | 2.348303 | 3.440589 |
| ENSMUSG00000018907 | protein_coding | Alox12e | 4.200254 | 2.075121 | 3.137688 |
| ENSMUSG00000042508 | protein_coding | Dmtf1 | 2141.751 | 2265.004 | 2203.377 |
| ENSMUSG00000017713 | protein_coding | Tha1 | 57.12271 | 68.50615 | 62.81443 |
| ENSMUSG00000067872 | protein_coding | Ccdc87 | 30.96708 | 24.37999 | 27.67353 |
| ENSMUSG00000060601 | protein_coding | Nr1h2 | 2127.244 | 2021.274 | 2074.259 |
| ENSMUSG00000036845 | protein_coding | Lin37 | 391.5878 | 362.6014 | 377.0946 |
| ENSMUSG00000057093 | protein_coding | C030039L03Rik | 165.2773 | 147.5003 | 156.3888 |
| ENSMUSG00000030254 | protein_coding | Rad18 | 1787.741 | 1684.681 | 1736.211 |
| ENSMUSG00000030774 | protein_coding | Pak1 | 1335.825 | 1168.669 | 1252.247 |
| ENSMUSG00000032806 | protein_coding | Slc10a3 | 415.5823 | 448.9601 | 432.2712 |
| ENSMUSG00000026889 | protein_coding | Rbm18 | 1447.138 | 1542.491 | 1494.814 |
| ENSMUSG00000046168 | protein_coding | Kcnrg | 17.15339 | 22.51534 | 19.83437 |
| ENSMUSG00000026341 | protein_coding | Actr3 | 6634.161 | 7039.232 | 6836.697 |
| ENSMUSG00000073424 | protein_coding | Cyp4f15 | 10.0449 | 6.44485 | 8.244876 |
| ENSMUSG00000005233 | protein_coding | Spc25 | 1300.287 | 1220.064 | 1260.175 |
| ENSMUSG00000027253 | protein_coding | Lrp4 | 196.4254 | 174.859 | 185.6422 |
| ENSMUSG00000044442 | protein_coding | N6amt1 | 599.5966 | 556.4764 | 578.0365 |
| ENSMUSG00000085348 | processed_transcrip | Gm12300 | 5.497938 | 3.099348 | 4.298643 |
| ENSMUSG00000037415 | protein_coding | Ranbp10 | 1784.111 | 1897.485 | 1840.798 |
| ENSMUSG00000035948 | protein_coding | Acss3 | 138.7674 | 157.0632 | 147.9153 |
| ENSMUSG00000056671 | protein_coding | Prelid2 | 50.67887 | 62.40547 | 56.54217 |
| ENSMUSG00000020601 | protein_coding | Trib2 | 1844.566 | 1993.215 | 1918.89 |
| ENSMUSG00000030549 | protein_coding | Rhcg | 50.68371 | 38.98511 | 44.83441 |
| ENSMUSG00000029490 | protein_coding | Mfsd7a | 24.28648 | 30.95604 | 27.62126 |

| | | | | | |
|---|---|---|---|---|---|
| ENSMUSG00000078921 | protein_coding | Tgtp2 | 8.723662 | 5.536658 | 7.13016 |
| ENSMUSG00000047905 | pseudogene | Gm8566 | 82.77918 | 69.88683 | 76.33301 |
| ENSMUSG00000024787 | protein_coding | Snx15 | 408.0484 | 377.7991 | 392.9238 |
| ENSMUSG00000087013 | lincRNA | 2610027K06Rik | 19.51014 | 14.04834 | 16.77924 |
| ENSMUSG00000069712 | protein_coding | 4930444G20Rik | 2.589703 | 1.033237 | 1.81147 |
| ENSMUSG00000083732 | pseudogene | Gm14197 | 3.244434 | 5.810202 | 4.527318 |
| ENSMUSG00000074024 | lincRNA | 4632427E13Rik | 99.63365 | 116.8858 | 108.2597 |
| ENSMUSG00000025422 | protein_coding | Agap2 | 163.1376 | 181.418 | 172.2778 |
| ENSMUSG00000046020 | protein_coding | Pofut1 | 1339.201 | 1420.443 | 1379.822 |
| ENSMUSG00000073725 | protein_coding | Lmbrd1 | 1216.096 | 1294.048 | 1255.072 |
| ENSMUSG00000061669 | protein_coding | Gm6404 | 3.560655 | 1.448758 | 2.504707 |
| ENSMUSG00000029822 | protein_coding | Osbpl3 | 386.906 | 430.4429 | 408.6745 |
| ENSMUSG00000031881 | protein_coding | Cdh16 | 14.53782 | 20.88966 | 17.71374 |
| ENSMUSG00000044155 | protein_coding | Naa38 | 1610.633 | 1725.244 | 1667.939 |
| ENSMUSG00000019818 | protein_coding | Cd164 | 10784.06 | 11334.31 | 11059.18 |
| ENSMUSG00000027309 | protein_coding | 4930402H24Rik | 2370.613 | 2498.517 | 2434.565 |
| ENSMUSG00000051486 | protein_coding | Pcdhb11 | 48.26554 | 61.786 | 55.02577 |
| ENSMUSG00000032350 | protein_coding | Gclc | 2125.03 | 2280.209 | 2202.62 |
| ENSMUSG00000091581 | lincRNA | Gm17277 | 3.564234 | 6.109689 | 4.836961 |
| ENSMUSG00000078485 | protein_coding | Plekhn1 | 84.88702 | 71.52727 | 78.20715 |
| ENSMUSG00000028249 | protein_coding | Sdcbp | 5237.582 | 5590.761 | 5414.171 |
| ENSMUSG00000024234 | protein_coding | Mtpap | 659.8676 | 703.8587 | 681.8631 |
| ENSMUSG00000034243 | protein_coding | Golgb1 | 5724.176 | 5393.368 | 5558.772 |
| ENSMUSG00000039783 | protein_coding | Kmo | 14.8776 | 10.53347 | 12.70554 |
| ENSMUSG00000020312 | protein_coding | Shc2 | 1650.849 | 1784.442 | 1717.646 |
| ENSMUSG00000049676 | protein_coding | Catsperg1 | 209.7519 | 177.1188 | 193.4353 |
| ENSMUSG00000025531 | protein_coding | Chm | 944.7689 | 1009.147 | 976.958 |
| ENSMUSG00000025375 | protein_coding | Aatk | 39.5711 | 48.13905 | 43.85507 |
| ENSMUSG00000044707 | protein_coding | Ccnjl | 415.4576 | 378.7649 | 397.1113 |
| ENSMUSG00000028964 | protein_coding | Park7 | 2729.902 | 2584.321 | 2657.112 |
| ENSMUSG00000032776 | protein_coding | Mctp2 | 18.69119 | 24.55515 | 21.62317 |
| ENSMUSG00000074653 | protein_coding | Lrrc31 | 0.638331 | 2.137462 | 1.387897 |
| ENSMUSG00000005732 | protein_coding | Ranbp1 | 3137.613 | 3341.715 | 3239.664 |
| ENSMUSG00000039704 | protein_coding | Lmbrd2 | 782.8152 | 721.5148 | 752.165 |
| ENSMUSG00000081305 | pseudogene | Gm12879 | 2.922324 | 1.086569 | 2.004446 |
| ENSMUSG00000053121 | protein_coding | Gm5129 | 1.629262 | 3.434871 | 2.532067 |
| ENSMUSG00000087202 | processed_transcrip | Gm15813 | 3.242123 | 1.413083 | 2.327603 |
| ENSMUSG00000038459 | protein_coding | Fam108c | 1015.103 | 936.7445 | 975.9236 |
| ENSMUSG00000042677 | protein_coding | Zc3h12a | 90.06241 | 78.69614 | 84.37927 |
| ENSMUSG00000028883 | protein_coding | Sema3a | 24.60203 | 36.14712 | 30.37458 |
| ENSMUSG00000037913 | protein_coding | Tmem156 | 2.575614 | 1.033237 | 1.804425 |
| ENSMUSG00000061897 | pseudogene | Gm14292 | 273.9618 | 324.8587 | 299.4103 |
| ENSMUSG00000081904 | pseudogene | Gm13419 | 0.66062 | 2.110796 | 1.385708 |
| ENSMUSG00000048285 | protein_coding | Frmd6 | 1519.885 | 1616.965 | 1568.425 |
| ENSMUSG00000030224 | protein_coding | Strap | 4254.304 | 4061.616 | 4157.96 |
| ENSMUSG00000057262 | protein_coding | Gm10020 | 9.361993 | 6.092032 | 7.727012 |
| ENSMUSG00000021239 | protein_coding | Vsx2 | 3.844077 | 1.668247 | 2.756162 |
| ENSMUSG00000078739 | protein_coding | Gm1987 | 0.646531 | 2.146471 | 1.396501 |
| ENSMUSG00000082220 | pseudogene | Gm12943 | 2.597903 | 1.033237 | 1.81557 |
| ENSMUSG00000065490 | miRNA | Mir30c-1 | 0.64422 | 2.021789 | 1.333005 |
| ENSMUSG00000054663 | protein_coding | Gm9953 | 0.64422 | 2.021789 | 1.333005 |
| ENSMUSG00000028626 | protein_coding | Col9a2 | 80.72434 | 95.49862 | 88.11148 |
| ENSMUSG00000025245 | protein_coding | Lztfl1 | 1185.571 | 1277.891 | 1231.731 |
| ENSMUSG00000036552 | protein_coding | 2210404J11Rik | 233.8181 | 268.7456 | 251.2819 |
| ENSMUSG00000022797 | protein_coding | Tfrc | 4851.666 | 4571.47 | 4711.568 |
| ENSMUSG00000020458 | protein_coding | Rtn4 | 4694.369 | 4433.513 | 4563.941 |
| ENSMUSG00000057808 | pseudogene | Gm10031 | 246.9875 | 270.5067 | 258.7471 |
| ENSMUSG00000078907 | protein_coding | Fam186b | 25.28792 | 33.58214 | 29.43503 |

| | | | | | |
|---|---|---|---|---|---|
| ENSMUSG00000029204 | protein_coding | Rhoh | 36.55335 | 29.18869 | 32.87102 |
| ENSMUSG00000046971 | protein_coding | Pla2g4f | 6.506536 | 10.11723 | 8.311882 |
| ENSMUSG00000001419 | protein_coding | Mef2d | 2138.16 | 2275.595 | 2206.877 |
| ENSMUSG00000091778 | lincRNA | Gm17306 | 14.17344 | 9.737744 | 11.95559 |
| ENSMUSG00000018411 | protein_coding | Mapt | 114.7789 | 100.9916 | 107.8852 |
| ENSMUSG00000086368 | lincRNA | Gm13830 | 41.13289 | 32.90824 | 37.02057 |
| ENSMUSG00000010051 | protein_coding | Hyal1 | 66.38861 | 79.55933 | 72.97397 |
| ENSMUSG00000037348 | protein_coding | Paqr7 | 1683.983 | 1784.957 | 1734.47 |
| ENSMUSG00000015337 | protein_coding | Endog | 126.6526 | 107.9148 | 117.2837 |
| ENSMUSG00000021313 | protein_coding | Ryr2 | 238.8906 | 335.5136 | 287.2021 |
| ENSMUSG00000062545 | protein_coding | Tlr12 | 14.86224 | 10.50608 | 12.68416 |
| ENSMUSG00000053472 | pseudogene | Gm13534 | 39.25085 | 14.43616 | 26.84351 |
| ENSMUSG00000086522 | processed_transcrip | Gm4473 | 10.28144 | 6.534219 | 8.407828 |
| ENSMUSG00000023868 | protein_coding | Pde10a | 212.484 | 122.9077 | 167.6958 |
| ENSMUSG00000090630 | lincRNA | Gm17403 | 21.04205 | 26.93877 | 23.99041 |
| ENSMUSG00000041629 | protein_coding | D11Wsu99e | 1289.79 | 1212.881 | 1251.336 |
| ENSMUSG00000047182 | protein_coding | Irs3 | 2.942302 | 5.465308 | 4.203805 |
| ENSMUSG00000051716 | protein_coding | Apon | 4.512897 | 2.36596 | 3.439428 |
| ENSMUSG00000039330 | protein_coding | Tsga10ip | 4.549275 | 7.424755 | 5.987015 |
| ENSMUSG00000031278 | protein_coding | Acsl4 | 2201.092 | 2342.805 | 2271.948 |
| ENSMUSG00000046721 | pseudogene | Rpl14-ps1 | 1763.344 | 1856.924 | 1810.134 |
| ENSMUSG00000040003 | protein_coding | Magi2 | 223.8634 | 247.3856 | 235.6245 |
| ENSMUSG00000043155 | protein_coding | Hpdl | 109.5578 | 124.9449 | 117.2514 |
| ENSMUSG00000005374 | protein_coding | Tbl2 | 2047.667 | 1905.097 | 1976.382 |
| ENSMUSG00000030588 | protein_coding | Yif1b | 808.0563 | 760.2436 | 784.15 |
| ENSMUSG00000088070 | misc_RNA | 7SK | 1.601084 | 3.734358 | 2.667721 |
| ENSMUSG00000087589 | lincRNA | D430040D24Rik | 14.2544 | 9.960082 | 12.10724 |
| ENSMUSG00000082016 | pseudogene | Pgam1-ps2 | 38.96452 | 47.2089 | 43.08671 |
| ENSMUSG00000074830 | protein_coding | Zfp640 | 106.8245 | 88.04398 | 97.43425 |
| ENSMUSG00000086770 | processed_transcrip | Gm15351 | 14.80842 | 20.33356 | 17.57099 |
| ENSMUSG00000015452 | protein_coding | Ager | 27.60702 | 36.13988 | 31.87345 |
| ENSMUSG00000034334 | protein_coding | Fam151b | 68.80068 | 79.50952 | 74.1551 |
| ENSMUSG00000057132 | protein_coding | Rpgrip1 | 35.40893 | 27.08111 | 31.24502 |
| ENSMUSG00000041138 | protein_coding | Txndc3 | 7.797287 | 4.714262 | 6.255775 |
| ENSMUSG00000091507 | pseudogene | Gm17194 | 22.99215 | 29.46472 | 26.22844 |
| ENSMUSG00000064585 | snoRNA | SNORA71 | 0.66062 | 2.039445 | 1.350033 |
| ENSMUSG00000020265 | protein_coding | Sumo3 | 4136.274 | 3830.937 | 3983.605 |
| ENSMUSG00000027076 | protein_coding | Timm10 | 404.2067 | 437.1267 | 420.6667 |
| ENSMUSG00000024672 | protein_coding | Ms4a7 | 100.6062 | 88.16106 | 94.38365 |
| ENSMUSG00000081747 | pseudogene | Rpl7a-ps9 | 6.829689 | 3.770395 | 5.300042 |
| ENSMUSG00000068979 | protein_coding | Gm5848 | 1.606973 | 3.363521 | 2.485247 |
| ENSMUSG00000063887 | protein_coding | Nlgn1 | 70.55562 | 59.72377 | 65.1397 |
| ENSMUSG00000032902 | protein_coding | Slc16a1 | 3932.837 | 3697.051 | 3814.944 |
| ENSMUSG00000060376 | protein_coding | Bckdha | 2039.856 | 1933.402 | 1986.629 |
| ENSMUSG00000070666 | protein_coding | Ldlrad2 | 3.580633 | 1.72194 | 2.651287 |
| ENSMUSG00000058925 | protein_coding | 1700011I03Rik | 3.580633 | 1.72194 | 2.651287 |
| ENSMUSG00000091866 | pseudogene | Gm6257 | 21.62633 | 15.95337 | 18.78985 |
| ENSMUSG00000050765 | protein_coding | Gm5084 | 13.17454 | 21.7092 | 17.44187 |
| ENSMUSG00000017291 | protein_coding | Taok1 | 7265.498 | 7624.861 | 7445.179 |
| ENSMUSG00000086956 | lincRNA | 1700125G22Rik | 4.832697 | 7.875952 | 6.354324 |
| ENSMUSG00000063049 | protein_coding | Ing2 | 953.5699 | 1015.891 | 984.7305 |
| ENSMUSG00000034701 | protein_coding | Neurod1 | 4.512897 | 2.401635 | 3.457266 |
| ENSMUSG00000071604 | protein_coding | Fam189a2 | 1011.566 | 1107.474 | 1059.52 |
| ENSMUSG00000021460 | protein_coding | Auh | 497.347 | 535.9601 | 516.6535 |
| ENSMUSG00000071550 | protein_coding | Wdr52 | 44.33506 | 53.19784 | 48.76645 |
| ENSMUSG00000070576 | protein_coding | Mn1 | 197.5183 | 220.6709 | 209.0946 |
| ENSMUSG00000087292 | processed_transcrip | Gm13832 | 18.99205 | 24.59046 | 21.79126 |
| ENSMUSG00000024245 | protein_coding | Tmem178 | 77.9734 | 66.84252 | 72.40796 |

| | | | | | |
|---|---|---|---|---|---|
| ENSMUSG00000030159 | protein_coding | Clec1b | 2.589703 | 4.776965 | 3.683334 |
| ENSMUSG00000082920 | pseudogene | Gm13864 | 8.09122 | 5.112127 | 6.601674 |
| ENSMUSG00000002325 | protein_coding | Irf9 | 782.4179 | 857.0063 | 819.7121 |
| ENSMUSG00000025407 | protein_coding | Gli1 | 860.3631 | 959.1393 | 909.7512 |
| ENSMUSG00000033952 | protein_coding | Aspm | 2310.799 | 2160.227 | 2235.513 |
| ENSMUSG00000052160 | protein_coding | Pld4 | 80.96751 | 95.87665 | 88.42208 |
| ENSMUSG00000029141 | protein_coding | Slc4a1ap | 1503.83 | 1422.072 | 1462.951 |
| ENSMUSG00000043230 | protein_coding | Fam124b | 8.415641 | 12.3001 | 10.35787 |
| ENSMUSG00000058301 | protein_coding | Upf1 | 3455.114 | 3715.362 | 3585.238 |
| ENSMUSG00000039384 | protein_coding | Dusp10 | 164.7687 | 147.9122 | 156.3404 |
| ENSMUSG00000086813 | lincRNA | Gm13657 | 2.595592 | 1.015218 | 1.805405 |
| ENSMUSG00000017740 | protein_coding | Slc12a5 | 176.0703 | 157.3357 | 166.703 |
| ENSMUSG00000020346 | protein_coding | Mgat1 | 1351.304 | 1271.317 | 1311.311 |
| ENSMUSG00000042388 | protein_coding | Dlgap3 | 180.1973 | 199.1899 | 189.6936 |
| ENSMUSG00000086665 | processed_transcrip | Gm13067 | 17.46499 | 12.54553 | 15.00526 |
| ENSMUSG00000038456 | protein_coding | Dennd2a | 778.0747 | 727.7992 | 752.9369 |
| ENSMUSG00000048617 | protein_coding | Rtbdn | 6.194936 | 10.53596 | 8.365449 |
| ENSMUSG00000082104 | pseudogene | Gm15933 | 2.279371 | 0.653029 | 1.4662 |
| ENSMUSG00000031343 | protein_coding | Gabra3 | 59.02048 | 69.56389 | 64.29219 |
| ENSMUSG00000090093 | protein_coding | Gm14399 | 108.1408 | 131.7939 | 119.9674 |
| ENSMUSG00000042096 | protein_coding | Dao | 3.248013 | 1.36876 | 2.308386 |
| ENSMUSG00000032108 | protein_coding | D730048I06Rik | 83.25165 | 71.92332 | 77.58749 |
| ENSMUSG00000032469 | protein_coding | Dbr1 | 709.6966 | 662.7643 | 686.2305 |
| ENSMUSG00000022508 | protein_coding | Bcl6 | 158.3307 | 137.1565 | 147.7436 |
| ENSMUSG00000030609 | protein_coding | Aen | 1451.876 | 1355.689 | 1403.782 |
| ENSMUSG00000048164 | protein_coding | Gm7204 | 6.461958 | 3.65436 | 5.058159 |
| ENSMUSG00000039670 | protein_coding | 1810049H13Rik | 141.3426 | 124.986 | 133.1643 |
| ENSMUSG00000026471 | protein_coding | Mr1 | 583.562 | 639.9258 | 611.7439 |
| ENSMUSG00000024654 | protein_coding | Asrgl1 | 681.3599 | 638.1637 | 659.7618 |
| ENSMUSG00000051255 | pseudogene | Gm6563 | 858.1432 | 915.3052 | 886.7242 |
| ENSMUSG00000055159 | lincRNA | 4930583K01Rik | 6.806357 | 4.096909 | 5.451633 |
| ENSMUSG00000022184 | protein_coding | Fbxo4 | 448.3345 | 495.3339 | 471.8342 |
| ENSMUSG00000035049 | protein_coding | Rrp12 | 2323.444 | 2110.084 | 2216.764 |
| ENSMUSG00000043323 | protein_coding | Fbrsl1 | 4601.796 | 4884.937 | 4743.366 |
| ENSMUSG00000036992 | protein_coding | Nxt1 | 677.3882 | 629.0711 | 653.2296 |
| ENSMUSG00000030125 | protein_coding | Lrrc23 | 9.053972 | 5.881191 | 7.467581 |
| ENSMUSG00000036019 | protein_coding | Tmtc2 | 1670.465 | 1818.861 | 1744.663 |
| ENSMUSG00000037060 | protein_coding | Prkcdbp | 661.0356 | 710.0389 | 685.5372 |
| ENSMUSG00000038496 | protein_coding | Slc19a3 | 18.10205 | 23.75978 | 20.93092 |
| ENSMUSG00000026573 | protein_coding | Xcl1 | 10.06257 | 6.489535 | 8.276052 |
| ENSMUSG00000081643 | pseudogene | Gm11605 | 3.250323 | 5.738851 | 4.494587 |
| ENSMUSG00000062319 | protein_coding | Gm10115 | 0.65242 | 2.066473 | 1.359447 |
| ENSMUSG00000042417 | protein_coding | Ccno | 106.1254 | 92.75204 | 99.43873 |
| ENSMUSG00000074647 | protein_coding | Fam83c | 17.77637 | 13.08646 | 15.43141 |
| ENSMUSG00000035364 | protein_coding | 4930524J08Rik | 21.27142 | 16.11482 | 18.69312 |
| ENSMUSG00000006304 | protein_coding | Arpc2 | 5491.812 | 5749.743 | 5620.777 |
| ENSMUSG00000028398 | protein_coding | 3110001D03Rik | 562.0105 | 608.6868 | 585.3486 |
| ENSMUSG00000038903 | protein_coding | Ccdc68 | 141.471 | 164.5121 | 152.9916 |
| ENSMUSG00000073197 | protein_coding | 5730507C01Rik | 45.64661 | 30.71646 | 38.18154 |
| ENSMUSG00000020381 | protein_coding | 3010026O09Rik | 341.8838 | 306.708 | 324.2959 |
| ENSMUSG00000044081 | protein_coding | 4930441O14Rik | 16.17193 | 11.53932 | 13.85562 |
| ENSMUSG00000062252 | protein_coding | Lhfpl5 | 2.269904 | 0.653029 | 1.461466 |
| ENSMUSG00000020173 | protein_coding | Cobl | 275.2232 | 303.3862 | 289.3047 |
| ENSMUSG00000005089 | protein_coding | Slc1a2 | 32.05201 | 25.3952 | 28.72361 |
| ENSMUSG00000024357 | protein_coding | Sil1 | 713.6724 | 656.2895 | 684.9809 |
| ENSMUSG00000021056 | protein_coding | Tex21 | 17.10881 | 12.53688 | 14.82285 |
| ENSMUSG00000087501 | lincRNA | Gm12116 | 45.59577 | 33.91802 | 39.7569 |
| ENSMUSG00000020875 | protein_coding | Hoxb9 | 609.3174 | 548.8005 | 579.059 |

| | | | | | |
|---|---|---|---|---|---|
| ENSMUSG00000091264 | protein_coding | BC024659 | 951.6728 | 887.2415 | 919.4571 |
| ENSMUSG00000038491 | processed_transcrip | 4930520K10Rik | 5.457982 | 9.033871 | 7.245926 |
| ENSMUSG00000045005 | protein_coding | Fzd5 | 1714.541 | 1876.839 | 1795.69 |
| ENSMUSG00000087174 | lincRNA | 5530601H04Rik | 425.4077 | 389.7666 | 407.5871 |
| ENSMUSG00000025138 | protein_coding | Sirt7 | 907.7571 | 818.6959 | 863.2265 |
| ENSMUSG00000036822 | protein_coding | Topors | 2660.513 | 2502.097 | 2581.305 |
| ENSMUSG00000026436 | protein_coding | Elk4 | 1175.373 | 1241.27 | 1208.322 |
| ENSMUSG00000031402 | protein_coding | Mpp1 | 539.1112 | 591.4149 | 565.263 |
| ENSMUSG00000041771 | protein_coding | Slc24a4 | 73.46304 | 85.32294 | 79.39299 |
| ENSMUSG00000066607 | protein_coding | 6030419C18Rik | 63.01171 | 75.35964 | 69.18568 |
| ENSMUSG00000088185 | snoRNA | Scarna2 | 3.538366 | 6.288065 | 4.913216 |
| ENSMUSG00000047490 | protein_coding | 4932411E22Rik | 2.587392 | 0.997561 | 1.792477 |
| ENSMUSG00000045896 | protein_coding | Paip2b | 3287.119 | 3061.184 | 3174.151 |
| ENSMUSG00000023087 | protein_coding | Ccrn4l | 788.0049 | 738.8462 | 763.4256 |
| ENSMUSG00000091635 | protein_coding | Vmn2r13 | 43.54742 | 56.94731 | 50.24736 |
| ENSMUSG00000031652 | protein_coding | N4bp1 | 2656.057 | 2505.986 | 2581.022 |
| ENSMUSG00000002372 | protein_coding | Ranbp3 | 2527.155 | 2407.221 | 2467.188 |
| ENSMUSG00000031210 | protein_coding | Gpr165 | 1.931394 | 0.662038 | 1.296716 |
| ENSMUSG00000050666 | protein_coding | E130203B14Rik | 1447.558 | 1559.715 | 1503.637 |
| ENSMUSG00000078489 | processed_transcrip | Gm17106 | 1.949061 | 4.114928 | 3.031994 |
| ENSMUSG00000034868 | protein_coding | Myl12b | 2859.567 | 3076.841 | 2968.204 |
| ENSMUSG00000039253 | protein_coding | Fn3krp | 295.9174 | 330.9911 | 313.4543 |
| ENSMUSG00000079362 | protein_coding | Gbp6 | 71.09816 | 57.61366 | 64.35591 |
| ENSMUSG00000000544 | protein_coding | Gpa33 | 5.766003 | 9.307415 | 7.536709 |
| ENSMUSG00000028245 | protein_coding | Nsmaf | 2606.562 | 2765.927 | 2686.245 |
| ENSMUSG00000088010 | rRNA | 5S_rRNA | 2.597903 | 1.059903 | 1.828903 |
| ENSMUSG00000022935 | protein_coding | Grik1 | 2.597903 | 1.059903 | 1.828903 |
| ENSMUSG00000035235 | protein_coding | Trim13 | 652.4726 | 712.3764 | 682.4245 |
| ENSMUSG00000029624 | protein_coding | Ptcd1 | 715.4815 | 770.7221 | 743.1018 |
| ENSMUSG00000036769 | protein_coding | Wdr44 | 633.8609 | 579.6804 | 606.7706 |
| ENSMUSG00000058833 | protein_coding | 2810428I15Rik | 1006.237 | 941.7294 | 973.9834 |
| ENSMUSG00000059690 | protein_coding | 1700020N15Rik | 1.929083 | 0.662038 | 1.29556 |
| ENSMUSG00000092200 | pseudogene | Tnxa | 0.638331 | 2.030436 | 1.334384 |
| ENSMUSG00000090962 | lincRNA | Dio3os | 9.138506 | 5.429632 | 7.284069 |
| ENSMUSG00000046219 | lincRNA | C230012O17Rik | 5.895115 | 3.037007 | 4.466061 |
| ENSMUSG00000029500 | protein_coding | Pgam5 | 849.7773 | 792.1239 | 820.9506 |
| ENSMUSG00000021097 | protein_coding | Clmn | 224.4172 | 203.8112 | 214.1142 |
| ENSMUSG00000030670 | protein_coding | Cyp2r1 | 87.26815 | 72.63932 | 79.95373 |
| ENSMUSG00000070337 | protein_coding | Gpr179 | 122.5392 | 103.6408 | 113.09 |
| ENSMUSG00000054519 | protein_coding | Zfp867 | 700.0435 | 657.4366 | 678.7401 |
| ENSMUSG00000023079 | protein_coding | Gtf2ird1 | 760.379 | 824.2077 | 792.2934 |
| ENSMUSG00000003527 | protein_coding | Dgcr14 | 969.3416 | 905.0501 | 937.1958 |
| ENSMUSG00000079388 | protein_coding | 2610042L04Rik | 10.86803 | 6.780736 | 8.824384 |
| ENSMUSG00000083714 | pseudogene | Gm14499 | 63.60591 | 73.9141 | 68.76 |
| ENSMUSG00000018217 | protein_coding | Pmp22 | 1594.291 | 1716.163 | 1655.227 |
| ENSMUSG00000063383 | protein_coding | Zfp947 | 97.92254 | 115.4455 | 106.684 |
| ENSMUSG00000075184 | protein_coding | F930017D23Rik | 5.14765 | 8.386279 | 6.766965 |
| ENSMUSG00000070823 | protein_coding | Gm1043 | 0.968641 | 2.348303 | 1.658472 |
| ENSMUSG00000035435 | protein_coding | Abca17 | 39.24884 | 49.98826 | 44.61855 |
| ENSMUSG00000054387 | protein_coding | Mdm4 | 2664.48 | 2832.851 | 2748.666 |
| ENSMUSG00000034656 | protein_coding | Cacna1a | 297.0709 | 328.4847 | 312.7778 |
| ENSMUSG00000052334 | protein_coding | 1700024B05Rik | 8.989416 | 12.96177 | 10.9756 |
| ENSMUSG00000004567 | protein_coding | Mcoln1 | 920.1795 | 831.9856 | 876.0826 |
| ENSMUSG00000084146 | pseudogene | Gm12104 | 3.278501 | 1.350742 | 2.314621 |
| ENSMUSG00000064472 | snRNA | U6 | 4.518786 | 2.419653 | 3.46922 |
| ENSMUSG00000052926 | protein_coding | Rnaseh2a | 1459.754 | 1338.637 | 1399.195 |
| ENSMUSG00000078636 | protein_coding | Gm3200 | 0.968641 | 2.36596 | 1.667301 |
| ENSMUSG00000089706 | processed_transcrip | B230216N24Rik | 5.497938 | 8.574027 | 7.035982 |

| | | | | | |
|---|---|---|---|---|---|
| ENSMUSG00000025436 | protein_coding | Xrcc6bp1 | 307.216 | 337.555 | 322.3855 |
| ENSMUSG00000021606 | protein_coding | Ndufs6 | 616.8528 | 660.4478 | 638.6503 |
| ENSMUSG00000046139 | protein_coding | Patl1 | 2095.052 | 2245.317 | 2170.184 |
| ENSMUSG00000020841 | protein_coding | Cpd | 3338.436 | 3537.736 | 3438.086 |
| ENSMUSG00000039115 | protein_coding | Itga9 | 799.0355 | 890.2529 | 844.6442 |
| ENSMUSG00000085862 | lincRNA | Gm13483 | 9.019905 | 5.703176 | 7.361541 |
| ENSMUSG00000046671 | protein_coding | Fam54b | 3924.652 | 4105.508 | 4015.08 |
| ENSMUSG00000020812 | protein_coding | 1810032O08Rik | 255.4929 | 280.399 | 267.9459 |
| ENSMUSG00000052854 | protein_coding | Nrk | 5602.844 | 5269.114 | 5435.979 |
| ENSMUSG00000046585 | protein_coding | Ccdc147 | 5.425182 | 2.639142 | 4.032162 |
| ENSMUSG00000019312 | protein_coding | Grb7 | 259.7203 | 289.7844 | 274.7523 |
| ENSMUSG00000082200 | pseudogene | Gm12871 | 2.561525 | 1.015218 | 1.788372 |
| ENSMUSG00000040506 | protein_coding | Ambra1 | 1985.272 | 1879.992 | 1932.632 |
| ENSMUSG00000043468 | protein_coding | Adam30 | 6.182115 | 3.416853 | 4.799484 |
| ENSMUSG00000024242 | protein_coding | Map4k3 | 2449.439 | 2320.877 | 2385.158 |
| ENSMUSG00000089854 | processed_transcrip | Gm16133 | 2.911813 | 5.39468 | 4.153247 |
| ENSMUSG00000070025 | pseudogene | Gm10284 | 1.293062 | 3.010341 | 2.151701 |
| ENSMUSG00000021461 | protein_coding | Fancc | 529.6936 | 473.3744 | 501.534 |
| ENSMUSG00000068284 | protein_coding | Gm608 | 1276.609 | 1206.551 | 1241.58 |
| ENSMUSG00000023992 | protein_coding | Trem2 | 64.50896 | 54.21057 | 59.35976 |
| ENSMUSG00000025723 | protein_coding | Nmb | 127.9469 | 111.8513 | 119.8991 |
| ENSMUSG00000071654 | protein_coding | AI462493 | 576.7287 | 619.1607 | 597.9447 |
| ENSMUSG00000020325 | protein_coding | Fstl3 | 518.6538 | 564.0979 | 541.3759 |
| ENSMUSG00000028001 | protein_coding | Fga | 3.303101 | 1.033237 | 2.168169 |
| ENSMUSG00000035910 | protein_coding | Dcdc2a | 29.77622 | 21.51602 | 25.64612 |
| ENSMUSG00000033857 | protein_coding | Engase | 137.1624 | 119.8846 | 128.5235 |
| ENSMUSG00000022269 | protein_coding | 11-Mar | 4.867807 | 2.428301 | 3.648054 |
| ENSMUSG00000054161 | protein_coding | Fam83e | 3.524277 | 1.72194 | 2.623109 |
| ENSMUSG00000068877 | protein_coding | Selenbp2 | 34.76367 | 26.22552 | 30.4946 |
| ENSMUSG00000029185 | protein_coding | Fam114a1 | 848.0295 | 909.4168 | 878.7232 |
| ENSMUSG00000067242 | protein_coding | Lgi1 | 10.65968 | 15.16194 | 12.91081 |
| ENSMUSG00000023192 | protein_coding | Grm2 | 3.588833 | 1.677256 | 2.633045 |
| ENSMUSG00000039058 | protein_coding | Ak5 | 25.20592 | 31.95573 | 28.58083 |
| ENSMUSG00000085749 | processed_transcrip | Gm12843 | 18.21581 | 13.0061 | 15.61095 |
| ENSMUSG00000041740 | protein_coding | Rnf10 | 7557.419 | 7201.814 | 7379.617 |
| ENSMUSG00000053702 | protein_coding | Nebl | 20.04754 | 25.71271 | 22.88012 |
| ENSMUSG00000083964 | pseudogene | Gm14122 | 11.34154 | 7.353766 | 9.347654 |
| ENSMUSG00000037262 | protein_coding | Kin | 615.718 | 570.5529 | 593.1355 |
| ENSMUSG00000080790 | pseudogene | Gm7638 | 8.521196 | 4.292943 | 6.407069 |
| ENSMUSG00000059751 | protein_coding | Gm9000 | 71.65531 | 60.35842 | 66.00687 |
| ENSMUSG00000040714 | protein_coding | Klc3 | 205.3098 | 178.6974 | 192.0036 |
| ENSMUSG00000085174 | lincRNA | Gm16206 | 20.69176 | 15.09639 | 17.89407 |
| ENSMUSG00000021809 | protein_coding | Nudt13 | 261.672 | 288.6286 | 275.1503 |
| ENSMUSG00000069310 | protein_coding | Hist1h3c | 10.6069 | 6.659264 | 8.633082 |
| ENSMUSG00000072671 | lincRNA | Gm10390 | 13.90076 | 18.65594 | 16.27835 |
| ENSMUSG00000020088 | protein_coding | Sar1a | 4752.659 | 5022.875 | 4887.767 |
| ENSMUSG00000023912 | protein_coding | Slc25a27 | 247.4559 | 274.9539 | 261.2049 |
| ENSMUSG00000089827 | lincRNA | 1700023H06Rik | 10.08359 | 16.3137 | 13.19864 |
| ENSMUSG00000054662 | protein_coding | Ano9 | 6.814557 | 10.19759 | 8.506073 |
| ENSMUSG00000045394 | protein_coding | Epcam | 1674.578 | 1804.126 | 1739.352 |
| ENSMUSG00000063535 | protein_coding | Zfp773 | 73.40206 | 61.87211 | 67.63708 |
| ENSMUSG00000060771 | protein_coding | Tsga10 | 900.1052 | 664.3284 | 782.2168 |
| ENSMUSG00000029217 | protein_coding | Tec | 248.2802 | 272.6293 | 260.4547 |
| ENSMUSG00000015478 | protein_coding | Rnf5 | 551.401 | 592.386 | 571.8935 |
| ENSMUSG00000029038 | protein_coding | Ssu72 | 1946.683 | 2056.772 | 2001.727 |
| ENSMUSG00000081256 | pseudogene | Gm15791 | 3.921454 | 1.695274 | 2.808364 |
| ENSMUSG00000041949 | protein_coding | Tmco7 | 499.02 | 458.2091 | 478.6146 |
| ENSMUSG00000078670 | protein_coding | Fam174b | 119.5609 | 104.366 | 111.9634 |

| | | | | | |
|---|---|---|---|---|---|
| ENSMUSG00000031861 | protein_coding | Lpar2 | 1876.306 | 2037.357 | 1956.832 |
| ENSMUSG00000092540 | pseudogene | RP23-326K2.4 | 1.937283 | 3.770395 | 2.853839 |
| ENSMUSG00000029507 | protein_coding | Pus1 | 470.82 | 436.5624 | 453.6912 |
| ENSMUSG00000025608 | protein_coding | Podxl | 697.8775 | 752.1408 | 725.0092 |
| ENSMUSG00000086498 | processed_transcrip | Gm12681 | 3.868677 | 6.409537 | 5.139107 |
| ENSMUSG00000055302 | protein_coding | Mrfap1 | 12121.33 | 12678.58 | 12399.96 |
| ENSMUSG00000091155 | protein_coding | Serpine3 | 49.97471 | 59.52086 | 54.74779 |
| ENSMUSG00000019803 | protein_coding | Nr2e1 | 59.09913 | 45.98326 | 52.54119 |
| ENSMUSG00000074060 | protein_coding | Fbxw15 | 1.629262 | 3.37253 | 2.500896 |
| ENSMUSG00000004535 | protein_coding | Tax1bp1 | 8241.701 | 8620.719 | 8431.21 |
| ENSMUSG00000092102 | lincRNA | Gm17712 | 16.77955 | 12.36208 | 14.57081 |
| ENSMUSG00000050323 | protein_coding | 2310030N02Rik | 161.9801 | 144.9977 | 153.4889 |
| ENSMUSG00000048486 | protein_coding | Fitm2 | 626.2387 | 576.3984 | 601.3185 |
| ENSMUSG00000086291 | processed_transcrip | Gm15513 | 74.07423 | 89.54788 | 81.81106 |
| ENSMUSG00000064348 | Mt_tRNA | mt-Tn | 104.5063 | 91.0229 | 97.7646 |
| ENSMUSG00000075335 | processed_transcrip | 4930588K23Rik | 4.241254 | 7.089231 | 5.665242 |
| ENSMUSG00000020349 | protein_coding | Ppp2ca | 5636.537 | 5947.687 | 5792.112 |
| ENSMUSG00000040061 | protein_coding | Plcb2 | 40.01516 | 49.09337 | 44.55427 |
| ENSMUSG00000046991 | protein_coding | Wdr27 | 25.39348 | 18.28723 | 21.84035 |
| ENSMUSG00000073681 | protein_coding | 1500002C15Rik | 11.30748 | 7.831629 | 9.569552 |
| ENSMUSG00000020523 | protein_coding | Fam114a2 | 1672.454 | 1770.069 | 1721.262 |
| ENSMUSG00000025872 | protein_coding | Thoc3 | 2179.931 | 2314.412 | 2247.171 |
| ENSMUSG00000085551 | processed_transcrip | 1810063I02Rik | 4.516475 | 2.339294 | 3.427885 |
| ENSMUSG00000086373 | processed_transcrip | Gm11978 | 2.620192 | 0.988552 | 1.804372 |
| ENSMUSG00000085962 | lincRNA | Gm16984 | 20.9634 | 27.00183 | 23.98262 |
| ENSMUSG00000022131 | protein_coding | Gpr180 | 926.2586 | 866.2219 | 896.2403 |
| ENSMUSG00000047793 | protein_coding | Sned1 | 883.4913 | 993.825 | 938.6582 |
| ENSMUSG00000036957 | protein_coding | Lrfn3 | 178.6413 | 197.1821 | 187.9117 |
| ENSMUSG00000067575 | pseudogene | Rpl35a-ps3 | 7.730421 | 4.812279 | 6.27135 |
| ENSMUSG00000081627 | pseudogene | Gm13158 | 0.954552 | 2.410644 | 1.682598 |
| ENSMUSG00000022249 | protein_coding | Ttc23l | 0.954552 | 2.410644 | 1.682598 |
| ENSMUSG00000082297 | pseudogene | Rhox3-ps | 1.937283 | 3.761386 | 2.849334 |
| ENSMUSG00000054672 | protein_coding | 5830411N06Rik | 1.276663 | 3.028359 | 2.152511 |
| ENSMUSG00000042604 | protein_coding | Kcna4 | 3.193967 | 1.395426 | 2.294697 |
| ENSMUSG00000066543 | pseudogene | Gm10166 | 3.538366 | 1.748606 | 2.643486 |
| ENSMUSG00000045365 | protein_coding | Rbm15b | 1372.298 | 1287.991 | 1330.144 |
| ENSMUSG00000000385 | protein_coding | Tmprss2 | 4.198987 | 6.852086 | 5.525536 |
| ENSMUSG00000090191 | processed_transcrip | 9230105E05Rik | 3.896855 | 6.52521 | 5.211032 |
| ENSMUSG00000007458 | protein_coding | M6pr | 4106.163 | 4308.209 | 4207.186 |
| ENSMUSG00000078616 | protein_coding | Trim30c | 1.304841 | 3.01935 | 2.162095 |
| ENSMUSG00000018427 | protein_coding | Ypel2 | 863.5418 | 956.327 | 909.9344 |
| ENSMUSG00000029920 | protein_coding | Smarcad1 | 2138.712 | 2033.906 | 2086.309 |
| ENSMUSG00000085049 | processed_transcrip | A230071N21Rik | 2.905924 | 5.210144 | 4.058034 |
| ENSMUSG00000092977 | misc_RNA | AC108434.1 | 2.592014 | 1.050893 | 1.821454 |
| ENSMUSG00000080268 | protein_coding | Brms1 | 810.2876 | 760.6279 | 785.4578 |
| ENSMUSG00000075027 | lincRNA | 4631405J19Rik | 9.005816 | 5.881191 | 7.443503 |
| ENSMUSG00000033128 | protein_coding | Gga1 | 1336.191 | 1248.079 | 1292.135 |
| ENSMUSG00000049988 | protein_coding | Lrrc25 | 23.74319 | 30.89334 | 27.31827 |
| ENSMUSG00000074151 | lincRNA | Nlrc5 | 31.90948 | 24.0441 | 27.97679 |
| ENSMUSG00000070285 | protein_coding | BY080835 | 27.18063 | 33.85247 | 30.51655 |
| ENSMUSG00000080530 | miRNA | mmu-mir-1194 | 1.931394 | 0.688704 | 1.310049 |
| ENSMUSG00000026668 | protein_coding | Ucma | 1.945483 | 0.697713 | 1.321598 |
| ENSMUSG00000087308 | processed_transcrip | Gm16195 | 1.945483 | 0.697713 | 1.321598 |
| ENSMUSG00000053025 | protein_coding | Sv2b | 38.92397 | 30.72325 | 34.82361 |
| ENSMUSG00000002486 | protein_coding | Tchp | 614.5466 | 568.3719 | 591.4592 |
| ENSMUSG00000028737 | protein_coding | Aldh4a1 | 701.321 | 654.076 | 677.6985 |
| ENSMUSG00000064340 | Mt_tRNA | mt-Tl1 | 57.94315 | 47.30003 | 52.62159 |
| ENSMUSG00000090413 | protein_coding | Gm17583 | 1.31304 | 3.027997 | 2.170519 |

| | | | | | |
|---|---|---|---|---|---|
| ENSMUSG00000000088 | protein_coding | Cox5a | 1581.554 | 1496.521 | 1539.037 |
| ENSMUSG00000038718 | protein_coding | Pbx3 | 718.5299 | 524.7881 | 621.659 |
| ENSMUSG00000025321 | protein_coding | Itgb8 | 1516.422 | 1691.129 | 1603.775 |
| ENSMUSG00000038393 | protein_coding | Txnip | 6684.707 | 8154.554 | 7419.631 |
| ENSMUSG00000021384 | protein_coding | Susd3 | 2319.621 | 2501.84 | 2410.73 |
| ENSMUSG00000036655 | protein_coding | Colec11 | 52.01166 | 61.10082 | 56.55624 |
| ENSMUSG00000064768 | snoRNA | SNORD60 | 8.060731 | 5.067804 | 6.564268 |
| ENSMUSG00000091160 | processed_transcrip | Gm17133 | 15.49826 | 20.34221 | 17.92023 |
| ENSMUSG00000085040 | lincRNA | A230028O05Rik | 3.568855 | 6.145364 | 4.85711 |
| ENSMUSG00000086797 | processed_transcrip | Gm12965 | 1.937283 | 3.80607 | 2.871677 |
| ENSMUSG00000082118 | pseudogene | Gm13862 | 2.626081 | 5.210144 | 3.918112 |
| ENSMUSG00000030376 | protein_coding | Slc8a2 | 143.273 | 123.1325 | 133.2027 |
| ENSMUSG00000089707 | processed_transcrip | 4921525B02Rik | 29.74319 | 38.85643 | 34.29981 |
| ENSMUSG00000026259 | protein_coding | Ngef | 143.2216 | 127.1905 | 135.2061 |
| ENSMUSG00000006526 | protein_coding | Tmem110 | 825.0804 | 777.4918 | 801.2861 |
| ENSMUSG00000091802 | lincRNA | Gm17638 | 43.03864 | 35.15998 | 39.09931 |
| ENSMUSG00000075707 | protein_coding | Dio3 | 27.73465 | 20.57827 | 24.15646 |
| ENSMUSG00000086863 | processed_transcrip | Gm15543 | 32.63697 | 23.01371 | 27.82534 |
| ENSMUSG00000036197 | protein_coding | Gxylt1 | 2204.624 | 2336.764 | 2270.694 |
| ENSMUSG00000001134 | protein_coding | Uxt | 278.4587 | 254.9058 | 266.6823 |
| ENSMUSG00000020261 | protein_coding | Slc36a1 | 688.9701 | 733.0932 | 711.0316 |
| ENSMUSG00000056812 | protein_coding | St8sia3 | 8.399241 | 5.447651 | 6.923446 |
| ENSMUSG00000033740 | protein_coding | St18 | 13.41972 | 7.392291 | 10.406 |
| ENSMUSG00000053414 | protein_coding | Hunk | 131.6087 | 147.4959 | 139.5523 |
| ENSMUSG00000000085 | protein_coding | Scmh1 | 1632.449 | 1771.578 | 1702.014 |
| ENSMUSG00000032425 | protein_coding | Zfp949 | 375.0086 | 345.7021 | 360.3554 |
| ENSMUSG00000085033 | processed_transcrip | Gm11646 | 1.934972 | 0.662038 | 1.298505 |
| ENSMUSG00000000276 | protein_coding | Dgke | 1191.23 | 1113.538 | 1152.384 |
| ENSMUSG00000040913 | protein_coding | Fbxw4 | 239.176 | 265.6316 | 252.4038 |
| ENSMUSG00000046487 | protein_coding | Mospd4 | 1.621062 | 3.354512 | 2.487787 |
| ENSMUSG00000091421 | pseudogene | Gm17510 | 276.5575 | 301.404 | 288.9808 |
| ENSMUSG00000017286 | protein_coding | Glod4 | 1533.224 | 1419.188 | 1476.206 |
| ENSMUSG00000020321 | protein_coding | Mdh1 | 3542.374 | 3758.824 | 3650.599 |
| ENSMUSG00000054555 | protein_coding | Adam12 | 1074.293 | 990.3836 | 1032.338 |
| ENSMUSG00000091712 | protein_coding | Sec14l5 | 23.30606 | 29.31337 | 26.30972 |
| ENSMUSG00000030382 | protein_coding | Slc27a5 | 5.80007 | 3.363521 | 4.581796 |
| ENSMUSG00000008496 | protein_coding | Pou2f2 | 47.17643 | 39.27346 | 43.22494 |
| ENSMUSG00000031377 | protein_coding | Bmx | 18.05748 | 23.62029 | 20.83889 |
| ENSMUSG00000001123 | protein_coding | Lgals9 | 267.3043 | 291.5743 | 279.4393 |
| ENSMUSG00000082279 | pseudogene | Gm12870 | 8.043064 | 5.067443 | 6.555253 |
| ENSMUSG00000073519 | lincRNA | Gm10524 | 54.31012 | 66.81766 | 60.56389 |
| ENSMUSG00000084032 | pseudogene | Gm12991 | 1.629262 | 3.488204 | 2.558733 |
| ENSMUSG00000055069 | protein_coding | Rab39 | 76.73736 | 61.76639 | 69.25187 |
| ENSMUSG00000061451 | protein_coding | Tmem151a | 15.51928 | 20.7671 | 18.14319 |
| ENSMUSG00000090386 | protein_coding | 2810055G20Rik | 20.65769 | 26.15169 | 23.40469 |
| ENSMUSG00000040498 | protein_coding | 2210010C17Rik | 20.7458 | 26.74273 | 23.74427 |
| ENSMUSG00000073514 | protein_coding | Dok6 | 2.575614 | 4.705615 | 3.640615 |
| ENSMUSG00000001983 | protein_coding | Taco1 | 311.3256 | 287.3114 | 299.3185 |
| ENSMUSG00000085402 | processed_transcrip | Gm12111 | 0.638331 | 1.977104 | 1.307718 |
| ENSMUSG00000051769 | protein_coding | Wfdc15a | 75.48425 | 64.40164 | 69.94295 |
| ENSMUSG00000082956 | pseudogene | Naip3 | 1.923194 | 3.797061 | 2.860127 |
| ENSMUSG00000085938 | pseudogene | Gm14371 | 2.237104 | 0.671047 | 1.454076 |
| ENSMUSG00000036309 | protein_coding | Skp1a | 6737.482 | 7081.52 | 6909.501 |
| ENSMUSG00000038570 | protein_coding | Fam154b | 114.056 | 130.1283 | 122.0922 |
| ENSMUSG00000020267 | protein_coding | Hint1 | 2287.947 | 2424.427 | 2356.187 |
| ENSMUSG00000074671 | protein_coding | Tspyl3 | 157.2285 | 175.223 | 166.2257 |
| ENSMUSG00000061898 | protein_coding | Rbak | 617.722 | 674.0793 | 645.9006 |
| ENSMUSG00000032890 | protein_coding | Rims3 | 711.4547 | 653.776 | 682.6153 |

| | | | | | |
|---|---|---|---|---|---|
| ENSMUSG00000044667 | protein_coding | D3Bwg0562e | 1045.432 | 1109.996 | 1077.714 |
| ENSMUSG00000026596 | protein_coding | Abl2 | 3057.006 | 3293.153 | 3175.079 |
| ENSMUSG00000090322 | processed_transcrip | Gm17090 | 1.929083 | 0.697713 | 1.313398 |
| ENSMUSG00000025902 | protein_coding | Sox17 | 86.82304 | 103.1482 | 94.98564 |
| ENSMUSG00000024069 | protein_coding | Slc30a6 | 481.8407 | 516.5297 | 499.1852 |
| ENSMUSG00000079070 | processed_transcrip | Gm3985 | 0.954552 | 2.383978 | 1.669265 |
| ENSMUSG00000028558 | protein_coding | Calr4 | 284.6405 | 308.4893 | 296.5649 |
| ENSMUSG00000087557 | processed_transcrip | Gm15869 | 15.88389 | 11.29317 | 13.58853 |
| ENSMUSG00000037001 | protein_coding | Zfp39 | 718.4292 | 661.8599 | 690.1446 |
| ENSMUSG00000031309 | protein_coding | Rps6ka3 | 3863.583 | 3619.612 | 3741.598 |
| ENSMUSG00000079733 | protein_coding | Tmem181b-ps | 724.0241 | 787.6774 | 755.8507 |
| ENSMUSG00000038127 | protein_coding | Ccdc50 | 2209.611 | 2359.819 | 2284.715 |
| ENSMUSG00000042613 | protein_coding | Pbxip1 | 1291.771 | 1217.257 | 1254.514 |
| ENSMUSG00000029993 | protein_coding | Nfu1 | 1124.206 | 1064.143 | 1094.174 |
| ENSMUSG00000020457 | protein_coding | Drg1 | 2897.391 | 3034.22 | 2965.805 |
| ENSMUSG00000088250 | snRNA | U4 | 6.512425 | 3.752377 | 5.132401 |
| ENSMUSG00000027746 | protein_coding | Ufm1 | 1233.598 | 1166.713 | 1200.155 |
| ENSMUSG00000057036 | protein_coding | Gm7536 | 287.5142 | 261.3684 | 274.4413 |
| ENSMUSG00000089567 | snoRNA | snoU13 | 0.954552 | 2.446319 | 1.700436 |
| ENSMUSG00000024728 | protein_coding | 1700025F22Rik | 0.954552 | 2.446319 | 1.700436 |
| ENSMUSG00000024403 | protein_coding | Atp6v1g2 | 145.0574 | 166.543 | 155.8002 |
| ENSMUSG00000045382 | protein_coding | Cxcr4 | 255.6561 | 226.3407 | 240.9984 |
| ENSMUSG00000092926 | miRNA | AC090489.2 | 2.905924 | 1.350742 | 2.128333 |
| ENSMUSG00000043366 | protein_coding | Olfr78 | 3.560655 | 6.065366 | 4.813011 |
| ENSMUSG00000064402 | snRNA | U6 | 2.920013 | 5.165821 | 4.042917 |
| ENSMUSG00000085315 | lincRNA | A430018G15Rik | 2.601481 | 1.024227 | 1.812854 |
| ENSMUSG00000034863 | protein_coding | Ano8 | 236.5276 | 261.8099 | 249.1688 |
| ENSMUSG00000038351 | protein_coding | Sgsm2 | 893.9668 | 956.6261 | 925.2965 |
| ENSMUSG00000061531 | protein_coding | Fam23a | 2.614303 | 5.228162 | 3.921233 |
| ENSMUSG00000024966 | protein_coding | Stip1 | 4276.125 | 4531.023 | 4403.574 |
| ENSMUSG00000087401 | processed_transcrip | Gm14664 | 1.606973 | 3.336855 | 2.471914 |
| ENSMUSG00000027787 | protein_coding | Nmd3 | 2413.206 | 2604.086 | 2508.646 |
| ENSMUSG00000038042 | protein_coding | Ptpdc1 | 793.8527 | 698.6938 | 746.2732 |
| ENSMUSG00000027463 | protein_coding | 2310046K01Rik | 4033.851 | 4321.969 | 4177.91 |
| ENSMUSG00000030515 | protein_coding | Tarsl2 | 821.03 | 878.0267 | 849.5283 |
| ENSMUSG00000029427 | protein_coding | Zcchc8 | 1860.378 | 1770.532 | 1815.455 |
| ENSMUSG00000028967 | protein_coding | Errfi1 | 1964.543 | 1827.658 | 1896.101 |
| ENSMUSG00000032409 | protein_coding | Atr | 6714.445 | 5410.437 | 6062.441 |
| ENSMUSG00000092486 | protein_coding | RP23-3M10.7 | 683.6027 | 626.4926 | 655.0477 |
| ENSMUSG00000025956 | protein_coding | Mettl21a | 286.5622 | 312.5513 | 299.5567 |
| ENSMUSG00000002274 | protein_coding | Metrn | 46.20548 | 38.23737 | 42.22142 |
| ENSMUSG00000083097 | pseudogene | Gm14494 | 268.7449 | 293.3093 | 281.0271 |
| ENSMUSG00000027229 | protein_coding | 4933406J08Rik | 256.35 | 188.8253 | 222.5876 |
| ENSMUSG00000015289 | protein_coding | Lage3 | 501.3937 | 548.6467 | 525.0202 |
| ENSMUSG00000078861 | protein_coding | Zfp931 | 148.919 | 171.7341 | 160.3266 |
| ENSMUSG00000063932 | protein_coding | Poteg | 0.962752 | 2.383978 | 1.673365 |
| ENSMUSG00000005846 | protein_coding | Rsl1d1 | 3693.906 | 3893.675 | 3793.791 |
| ENSMUSG00000030022 | protein_coding | Adamts9 | 2619.695 | 2793.226 | 2706.461 |
| ENSMUSG00000027678 | protein_coding | Ncoa3 | 2262.56 | 2483.987 | 2373.273 |
| ENSMUSG00000084271 | pseudogene | Gm14846 | 2.620192 | 1.015218 | 1.817705 |
| ENSMUSG00000040640 | protein_coding | Erc2 | 74.58181 | 64.74545 | 69.66363 |
| ENSMUSG00000020740 | protein_coding | Gga3 | 1145.803 | 1079.596 | 1112.699 |
| ENSMUSG00000034872 | protein_coding | Gipc3 | 247.5844 | 273.3969 | 260.4906 |
| ENSMUSG00000075588 | protein_coding | Hoxb2 | 7.121311 | 11.40092 | 9.261113 |
| ENSMUSG00000092443 | protein_coding | CT009512.1 | 2.281682 | 4.38811 | 3.334896 |
| ENSMUSG00000037679 | protein_coding | Inf2 | 492.7355 | 545.7756 | 519.2556 |
| ENSMUSG00000064919 | snRNA | U6 | 11.24061 | 7.469801 | 9.355205 |
| ENSMUSG00000032401 | protein_coding | Lctl | 14.85993 | 19.61783 | 17.23888 |

| | | | | | |
|---|---|---|---|---|---|
| ENSMUSG00000039110 | protein_coding | Mycbpap | 106.162 | 121.0722 | 113.6171 |
| ENSMUSG00000002393 | protein_coding | Nr2f6 | 648.0932 | 606.4278 | 627.2605 |
| ENSMUSG00000091530 | protein_coding | Cldn20 | 12.60285 | 8.873875 | 10.73836 |
| ENSMUSG00000016319 | protein_coding | Slc25a5 | 4591.257 | 4824.332 | 4707.795 |
| ENSMUSG00000031129 | protein_coding | Slc9a9 | 105.891 | 93.71859 | 99.80478 |
| ENSMUSG00000003269 | protein_coding | Cyth2 | 3276.464 | 3095.444 | 3185.954 |
| ENSMUSG00000043702 | protein_coding | Pde12 | 900.7471 | 847.985 | 874.3661 |
| ENSMUSG00000083083 | pseudogene | Gm15382 | 2.905924 | 1.359751 | 2.132838 |
| ENSMUSG00000062456 | protein_coding | Rpl9-ps6 | 366.2194 | 394.0649 | 380.1422 |
| ENSMUSG00000032193 | protein_coding | Ldlr | 897.4979 | 823.6034 | 860.5506 |
| ENSMUSG00000023122 | protein_coding | Sult1c2 | 3.560655 | 1.659599 | 2.610127 |
| ENSMUSG00000030737 | protein_coding | Slco2b1 | 91.23687 | 80.02021 | 85.62854 |
| ENSMUSG00000045007 | protein_coding | Tubg2 | 96.03617 | 82.07555 | 89.05586 |
| ENSMUSG00000068391 | protein_coding | Chrac1 | 1117.645 | 1043.516 | 1080.581 |
| ENSMUSG00000057072 | protein_coding | 1700022P22Rik | 5.876404 | 9.244712 | 7.560558 |
| ENSMUSG00000075410 | protein_coding | Gm11744 | 731.1643 | 775.4238 | 753.2941 |
| ENSMUSG00000022100 | protein_coding | Xpo7 | 4313.3 | 4095.616 | 4204.458 |
| ENSMUSG00000045752 | protein_coding | Tssc4 | 821.4275 | 769.2455 | 795.3365 |
| ENSMUSG00000069307 | protein_coding | Hist1h2bq | 0.962752 | 2.374969 | 1.668861 |
| ENSMUSG00000088958 | snoRNA | Scarna8 | 1.943172 | 3.797061 | 2.870117 |
| ENSMUSG00000034641 | protein_coding | Cd300ld | 2.606103 | 4.758947 | 3.682525 |
| ENSMUSG00000078713 | protein_coding | Tomm5 | 498.3598 | 541.5249 | 519.9423 |
| ENSMUSG00000049576 | protein_coding | Zfa | 7.147178 | 4.423424 | 5.785301 |
| ENSMUSG00000035248 | protein_coding | Zcchc6 | 1564.431 | 1646.204 | 1605.317 |
| ENSMUSG00000033020 | protein_coding | Polr2f | 1430.79 | 1348.076 | 1389.433 |
| ENSMUSG00000025730 | protein_coding | Rab40c | 1611.045 | 1695.606 | 1653.325 |
| ENSMUSG00000022390 | protein_coding | Zc3h7b | 13057.15 | 13942.28 | 13499.72 |
| ENSMUSG00000084950 | protein_coding | Gm5577 | 55.99663 | 66.32934 | 61.16299 |
| ENSMUSG00000034957 | protein_coding | Cebpa | 609.8205 | 549.0612 | 579.4409 |
| ENSMUSG00000085428 | processed_transcrip | Gm14344 | 10.96077 | 15.09023 | 13.0255 |
| ENSMUSG00000027173 | protein_coding | Depdc7 | 297.9959 | 326.899 | 312.4475 |
| ENSMUSG00000056145 | protein_coding | AI504432 | 167.6277 | 188.6046 | 178.1161 |
| ENSMUSG00000022223 | protein_coding | Sdr39u1 | 692.5052 | 740.9098 | 716.7075 |
| ENSMUSG00000073176 | protein_coding | Zfp449 | 454.6701 | 499.7753 | 477.2227 |
| ENSMUSG00000070369 | protein_coding | Itgad | 17.0289 | 22.78925 | 19.90908 |
| ENSMUSG00000019822 | protein_coding | Smpd2 | 649.3877 | 605.5648 | 627.4763 |
| ENSMUSG00000000552 | protein_coding | Zfp385a | 815.9166 | 755.6802 | 785.7984 |
| ENSMUSG00000038274 | protein_coding | Fau | 4261.175 | 4493.845 | 4377.51 |
| ENSMUSG00000022792 | protein_coding | Yars2 | 685.6841 | 731.7016 | 708.6929 |
| ENSMUSG00000031189 | protein_coding | Aff2 | 289.2949 | 319.926 | 304.6105 |
| ENSMUSG00000081650 | protein_coding | Gm16181 | 8.071242 | 11.74473 | 9.907983 |
| ENSMUSG00000006498 | protein_coding | Ptbp1 | 7532.436 | 7959.658 | 7746.047 |
| ENSMUSG00000025094 | protein_coding | Slc18a2 | 78.70469 | 68.56125 | 73.63297 |
| ENSMUSG00000025745 | protein_coding | Hadha | 5372.673 | 5633.855 | 5503.264 |
| ENSMUSG00000048924 | protein_coding | Ccdc125 | 74.31144 | 86.17318 | 80.24231 |
| ENSMUSG00000038708 | protein_coding | Golga4 | 4144.877 | 3914.747 | 4029.812 |
| ENSMUSG00000035041 | protein_coding | Creb3l3 | 24.67904 | 18.56041 | 21.61972 |
| ENSMUSG00000025743 | protein_coding | Sdc3 | 695.7862 | 751.423 | 723.6046 |
| ENSMUSG00000049439 | protein_coding | Cyp20a1 | 422.832 | 455.6048 | 439.2184 |
| ENSMUSG00000086687 | lincRNA | 4930547E08Rik | 13.59147 | 9.472848 | 11.53216 |
| ENSMUSG00000015759 | protein_coding | Cnih | 1825.591 | 1921.857 | 1873.724 |
| ENSMUSG00000031885 | protein_coding | Cbfb | 4039.21 | 4338.468 | 4188.839 |
| ENSMUSG00000029111 | protein_coding | Whsc2 | 1243.047 | 1331.478 | 1287.263 |
| ENSMUSG00000005501 | protein_coding | Usp40 | 1559.329 | 1680.371 | 1619.85 |
| ENSMUSG00000084163 | pseudogene | Gm11901 | 2.298082 | 0.671047 | 1.484564 |
| ENSMUSG00000056682 | pseudogene | 4930465K10Rik | 8.375684 | 5.402966 | 6.889325 |
| ENSMUSG00000020821 | protein_coding | Kif1c | 3362.07 | 3164.604 | 3263.337 |
| ENSMUSG00000054793 | protein_coding | Cadm4 | 535.0539 | 588.7895 | 561.9217 |

| | | | | | |
|---|---|---|---|---|---|
| ENSMUSG00000024570 | protein_coding | Rbfa | 1464.68 | 1388.105 | 1426.393 |
| ENSMUSG00000008301 | protein_coding | Phax | 1378.207 | 1467.848 | 1423.027 |
| ENSMUSG00000022208 | protein_coding | Jph4 | 116.463 | 103.1493 | 109.8062 |
| ENSMUSG00000001415 | protein_coding | Smg5 | 3084.169 | 2928.5 | 3006.334 |
| ENSMUSG00000022912 | protein_coding | Pros1 | 1164.234 | 1243.668 | 1203.951 |
| ENSMUSG00000090946 | protein_coding | 2010109K11Rik | 538.9181 | 487.8148 | 513.3664 |
| ENSMUSG00000085162 | processed_transcrip | Gm12295 | 9.070372 | 12.89079 | 10.98058 |
| ENSMUSG00000045980 | protein_coding | Tmem104 | 355.8176 | 384.9117 | 370.3647 |
| ENSMUSG00000078650 | protein_coding | G6pc | 3.561923 | 6.216714 | 4.889319 |
| ENSMUSG00000074457 | protein_coding | S100a16 | 192.3088 | 212.6487 | 202.4788 |
| ENSMUSG00000032786 | protein_coding | Alas1 | 1633.809 | 1757.593 | 1695.701 |
| ENSMUSG00000087502 | lincRNA | Gm16091 | 38.45404 | 46.74719 | 42.60061 |
| ENSMUSG00000060342 | pseudogene | Gm15454 | 9.970879 | 13.99501 | 11.98295 |
| ENSMUSG00000054083 | protein_coding | Capn12 | 21.65451 | 27.44081 | 24.54766 |
| ENSMUSG00000092741 | miRNA | AC130827.1 | 19.32929 | 25.68392 | 22.50661 |
| ENSMUSG00000090488 | lincRNA | A430105J06Rik | 32.28266 | 39.22986 | 35.75626 |
| ENSMUSG00000003531 | protein_coding | Dgcr6 | 598.8004 | 557.6307 | 578.2155 |
| ENSMUSG00000029050 | protein_coding | Ski | 3968.555 | 3755.819 | 3862.187 |
| ENSMUSG00000067847 | protein_coding | Romo1 | 465.6718 | 502.0568 | 483.8643 |
| ENSMUSG00000087126 | lincRNA | 1700109K24Rik | 18.72272 | 24.14864 | 21.43568 |
| ENSMUSG00000031995 | protein_coding | St14 | 48.58511 | 57.89051 | 53.23781 |
| ENSMUSG00000078762 | protein_coding | Haus5 | 660.7173 | 604.3962 | 632.5567 |
| ENSMUSG00000091321 | lincRNA | Gm17480 | 29.36831 | 36.04114 | 32.70472 |
| ENSMUSG00000022683 | protein_coding | Pla2g10 | 17.79172 | 13.35063 | 15.57118 |
| ENSMUSG00000026070 | protein_coding | Il18r1 | 1.598773 | 3.425862 | 2.512318 |
| ENSMUSG00000035769 | protein_coding | Xylb | 394.8081 | 365.4376 | 380.1229 |
| ENSMUSG00000029090 | protein_coding | Gpr125 | 6128.715 | 6542.21 | 6335.463 |
| ENSMUSG00000019777 | protein_coding | Hdac2 | 5443.885 | 5765.307 | 5604.596 |
| ENSMUSG00000021620 | protein_coding | Acot12 | 5.179406 | 8.416518 | 6.797962 |
| ENSMUSG00000021039 | protein_coding | Snw1 | 1842.229 | 1954.87 | 1898.549 |
| ENSMUSG00000061371 | protein_coding | Zfp873 | 304.9584 | 340.7039 | 322.8312 |
| ENSMUSG00000028497 | protein_coding | Ptplad2 | 119.5234 | 134.6207 | 127.0721 |
| ENSMUSG00000049421 | protein_coding | Zfp260 | 1759.356 | 1922.942 | 1841.149 |
| ENSMUSG00000025888 | protein_coding | Casp1 | 26.38732 | 20.7934 | 23.59036 |
| ENSMUSG00000078595 | protein_coding | Dnahc7c | 5.229873 | 2.499652 | 3.864762 |
| ENSMUSG00000048188 | protein_coding | Gm8181 | 4.213076 | 6.780736 | 5.496906 |
| ENSMUSG00000054364 | protein_coding | Rhob | 3523.921 | 3730.058 | 3626.99 |
| ENSMUSG00000070822 | protein_coding | Zscan18 | 700.4482 | 654.3516 | 677.3999 |
| ENSMUSG00000086564 | protein_coding | Cd101 | 2.905924 | 5.049786 | 3.977855 |
| ENSMUSG00000087948 | miRNA | AC124515.1 | 0.32211 | 1.632571 | 0.977341 |
| ENSMUSG00000071036 | protein_coding | Gm10309 | 4.831429 | 2.710492 | 3.770961 |
| ENSMUSG00000020258 | protein_coding | Glyctk | 61.53534 | 51.26827 | 56.40181 |
| ENSMUSG00000082129 | pseudogene | Gm14502 | 3.228034 | 5.492335 | 4.360185 |
| ENSMUSG00000085605 | lincRNA | 5033403F01Rik | 32.53141 | 24.97968 | 28.75555 |
| ENSMUSG00000039676 | protein_coding | Capsl | 36.48395 | 29.31953 | 32.90174 |
| ENSMUSG00000063787 | protein_coding | Chchd1 | 637.6035 | 678.5263 | 658.0649 |
| ENSMUSG00000035770 | protein_coding | Dync1li2 | 4677.784 | 4937.034 | 4807.409 |
| ENSMUSG00000008822 | protein_coding | Acyp1 | 222.3552 | 202.1751 | 212.2652 |
| ENSMUSG00000010086 | protein_coding | Rnf112 | 19.38461 | 14.53522 | 16.95991 |
| ENSMUSG00000030861 | protein_coding | Acadsb | 2485.526 | 2616.792 | 2551.159 |
| ENSMUSG00000051483 | protein_coding | Cbr1 | 527.2475 | 571.5076 | 549.3776 |
| ENSMUSG00000024855 | protein_coding | Pacs1 | 2190.887 | 2018.258 | 2104.573 |
| ENSMUSG00000039850 | protein_coding | A730011L01Rik | 822.0575 | 879.1133 | 850.5854 |
| ENSMUSG00000087620 | lincRNA | 5330434G04Rik | 0.33031 | 1.422092 | 0.876201 |
| ENSMUSG00000021408 | protein_coding | Ripk1 | 694.2396 | 746.4295 | 720.3346 |
| ENSMUSG00000025332 | protein_coding | Kdm5c | 2846.175 | 3048.509 | 2947.342 |
| ENSMUSG00000028475 | processed_transcrip | 5430416O09Rik | 159.8847 | 179.212 | 169.5483 |
| ENSMUSG00000037108 | protein_coding | Zcwpw1 | 3600.086 | 3186.472 | 3393.279 |

| | | | | | |
|---|---|---|---|---|---|
| ENSMUSG00000078862 | protein_coding | Gm14326 | 198.9184 | 227.2518 | 213.0851 |
| ENSMUSG00000031822 | protein_coding | Gse1 | 1209.586 | 1102.186 | 1155.886 |
| ENSMUSG00000033931 | protein_coding | Rbm34 | 1076.431 | 1136.65 | 1106.54 |
| ENSMUSG00000030364 | protein_coding | Clec2h | 8.158086 | 12.17469 | 10.16639 |
| ENSMUSG00000019899 | protein_coding | Lama2 | 4167.548 | 4499.709 | 4333.628 |
| ENSMUSG00000038602 | protein_coding | Slc35f1 | 9.407839 | 13.83501 | 11.62143 |
| ENSMUSG00000042726 | protein_coding | Trafd1 | 908.9004 | 966.8897 | 937.8951 |
| ENSMUSG00000029656 | protein_coding | C8b | 60.38146 | 51.78403 | 56.08275 |
| ENSMUSG00000077592 | snoRNA | SNORA17 | 0.32211 | 1.422092 | 0.872101 |
| ENSMUSG00000081252 | pseudogene | Gm16032 | 0.32211 | 1.422092 | 0.872101 |
| ENSMUSG00000065421 | miRNA | Mirlet7a-1 | 0.32211 | 1.422092 | 0.872101 |
| ENSMUSG00000072770 | protein_coding | Acrbp | 61.10179 | 70.96905 | 66.03542 |
| ENSMUSG00000087254 | processed_transcrip | 2310005A03Rik | 0.646531 | 2.004132 | 1.325331 |
| ENSMUSG00000043153 | protein_coding | Ispd | 114.1872 | 100.5477 | 107.3674 |
| ENSMUSG00000070280 | protein_coding | Slc22a14 | 4.202565 | 2.119805 | 3.161185 |
| ENSMUSG00000069678 | protein_coding | Pcgf1 | 360.7209 | 333.6157 | 347.1683 |
| ENSMUSG00000022956 | protein_coding | Atp5o | 3206.76 | 3037.808 | 3122.284 |
| ENSMUSG00000056124 | protein_coding | B4galt6 | 622.9436 | 441.0827 | 532.0132 |
| ENSMUSG00000034278 | protein_coding | Dnajc17 | 329.357 | 303.0106 | 316.1838 |
| ENSMUSG00000092482 | processed_transcrip | RP24-297C1.11 | 0.33031 | 1.395426 | 0.862868 |
| ENSMUSG00000083193 | protein_coding | 4930595D18Rik | 0.33031 | 1.395426 | 0.862868 |
| ENSMUSG00000063548 | protein_coding | Gm4825 | 0.33031 | 1.395426 | 0.862868 |
| ENSMUSG00000064911 | snRNA | U6 | 0.33031 | 1.395426 | 0.862868 |
| ENSMUSG00000038406 | protein_coding | Scaf1 | 2881.532 | 2739.551 | 2810.542 |
| ENSMUSG00000031996 | protein_coding | Aplp2 | 9561.706 | 10102.9 | 9832.304 |
| ENSMUSG00000046111 | protein_coding | 5830418K08Rik | 2444.638 | 2285.194 | 2364.916 |
| ENSMUSG00000083669 | pseudogene | Gm10169 | 31.38319 | 25.07449 | 28.22884 |
| ENSMUSG00000064367 | protein_coding | mt-Nd5 | 58781.98 | 61773.85 | 60277.91 |
| ENSMUSG00000071793 | processed_transcrip | 2610005L07Rik | 1251.554 | 1327.664 | 1289.609 |
| ENSMUSG00000026196 | protein_coding | Bard1 | 898.1822 | 839.4275 | 868.8049 |
| ENSMUSG00000074978 | pseudogene | Actg-ps1 | 0.316221 | 1.422092 | 0.869157 |
| ENSMUSG00000084322 | pseudogene | Gm14778 | 0.316221 | 1.422092 | 0.869157 |
| ENSMUSG00000050974 | protein_coding | Gm9847 | 2.269904 | 4.441442 | 3.355673 |
| ENSMUSG00000031232 | protein_coding | Magt1 | 1860.214 | 1968.048 | 1914.131 |
| ENSMUSG00000091105 | pseudogene | Gm5633 | 5.161739 | 2.66617 | 3.913954 |
| ENSMUSG00000090510 | lincRNA | Gm17554 | 0.98273 | 2.446319 | 1.714525 |
| ENSMUSG00000091636 | protein_coding | A330050F15Rik | 0.98273 | 2.446319 | 1.714525 |
| ENSMUSG00000048930 | protein_coding | Tada3 | 766.0906 | 817.1311 | 791.6108 |
| ENSMUSG00000079884 | pseudogene | Gm10698 | 584.2758 | 545.2811 | 564.7784 |
| ENSMUSG00000021098 | protein_coding | 4930447C04Rik | 2200.637 | 1531.734 | 1866.185 |
| ENSMUSG00000088978 | rRNA | 5S_rRNA | 0.32211 | 1.395426 | 0.858768 |
| ENSMUSG00000077170 | snoRNA | SNORA43 | 2.908235 | 5.147803 | 4.028019 |
| ENSMUSG00000020946 | protein_coding | Gosr2 | 4784.639 | 5041.958 | 4913.298 |
| ENSMUSG00000017421 | protein_coding | Zfp207 | 6848.1 | 7226.95 | 7037.525 |
| ENSMUSG00000030107 | protein_coding | Usp18 | 63.80502 | 52.30078 | 58.0529 |
| ENSMUSG00000020846 | protein_coding | Fam101b | 304.225 | 278.0544 | 291.1397 |
| ENSMUSG00000022598 | protein_coding | Psca | 10.01672 | 6.753708 | 8.385216 |
| ENSMUSG00000074465 | processed_transcrip | Gm10701 | 2.905924 | 1.386417 | 2.146171 |
| ENSMUSG00000091559 | lincRNA | Gm17726 | 0.98273 | 2.374969 | 1.67885 |
| ENSMUSG00000084320 | processed_transcrip | Gm14125 | 2.891835 | 1.359751 | 2.125793 |
| ENSMUSG00000087881 | snoRNA | SCARNA21 | 2.891835 | 1.359751 | 2.125793 |
| ENSMUSG00000070564 | protein_coding | Ntn5 | 35.9394 | 28.50248 | 32.22094 |
| ENSMUSG00000089997 | processed_transcrip | 1810020O05Rik | 6.46658 | 10.04981 | 8.258196 |
| ENSMUSG00000080406 | miRNA | Mir1188 | 0.316221 | 1.395426 | 0.855824 |
| ENSMUSG00000027420 | protein_coding | Bfsp1 | 118.7968 | 104.2409 | 111.5189 |
| ENSMUSG00000020803 | protein_coding | Txndc17 | 896.6783 | 958.7093 | 927.6938 |
| ENSMUSG00000062619 | protein_coding | 2310039H08Rik | 299.6496 | 325.4924 | 312.571 |
| ENSMUSG00000085670 | lincRNA | A830052D11Rik | 3.540677 | 6.09167 | 4.816174 |

| | | | | | |
|---|---|---|---|---|---|
| ENSMUSG00000083016 | pseudogene | Gm11703 | 18.81187 | 13.74886 | 16.28037 |
| ENSMUSG00000049823 | protein_coding | Zbtb12 | 660.9646 | 716.4071 | 688.6859 |
| ENSMUSG00000036921 | protein_coding | 4930549C01Rik | 0.33031 | 1.413083 | 0.871697 |
| ENSMUSG00000093016 | misc_RNA | AC122266.1 | 0.33031 | 1.413083 | 0.871697 |
| ENSMUSG00000080933 | pseudogene | Rhox3g-ps | 0.33031 | 1.413083 | 0.871697 |
| ENSMUSG00000050249 | lincRNA | Gm17291 | 36.18205 | 29.02326 | 32.60265 |
| ENSMUSG00000090124 | protein_coding | Ugt1a7c | 47.9769 | 38.66905 | 43.32297 |
| ENSMUSG00000069227 | protein_coding | Gprin1 | 51.58735 | 43.49184 | 47.53959 |
| ENSMUSG00000023979 | protein_coding | Guca1b | 77.87671 | 67.89885 | 72.88778 |
| ENSMUSG00000068921 | protein_coding | Dap3 | 2925.055 | 2774.039 | 2849.547 |
| ENSMUSG00000031480 | protein_coding | Thsd1 | 67.19117 | 57.60904 | 62.40011 |
| ENSMUSG00000084998 | lincRNA | Gm16279 | 27.85071 | 34.35487 | 31.10279 |
| ENSMUSG00000004768 | protein_coding | Rab23 | 1027.377 | 1111.162 | 1069.27 |
| ENSMUSG00000028049 | protein_coding | Scamp3 | 778.6387 | 828.8963 | 803.7675 |
| ENSMUSG00000029776 | protein_coding | Hibadh | 2021.66 | 2120.25 | 2070.955 |
| ENSMUSG00000063488 | protein_coding | Zfp167 | 195.8483 | 214.7523 | 205.3003 |
| ENSMUSG00000059634 | pseudogene | Gm11942 | 31.3489 | 24.07765 | 27.71327 |
| ENSMUSG00000038296 | protein_coding | Galntl4 | 624.858 | 691.2933 | 658.0757 |
| ENSMUSG00000093249 | misc_RNA | AC148990.1 | 1.298952 | 0.36219 | 0.830571 |
| ENSMUSG00000063260 | protein_coding | Syt10 | 1.298952 | 0.36219 | 0.830571 |
| ENSMUSG00000064565 | snRNA | U1 | 1.298952 | 0.36219 | 0.830571 |
| ENSMUSG00000087190 | lincRNA | D430001F17Rik | 1.298952 | 0.36219 | 0.830571 |
| ENSMUSG00000090757 | protein_coding | Gm13277 | 0 | 1.306057 | 0.653029 |
| ENSMUSG00000029819 | protein_coding | Npy | 0 | 1.306057 | 0.653029 |
| ENSMUSG00000065026 | snRNA | U6 | 0 | 1.306057 | 0.653029 |
| ENSMUSG00000018999 | protein_coding | Slc35b4 | 1424.662 | 1329.094 | 1376.878 |
| ENSMUSG00000042532 | protein_coding | Golga7b | 7.45751 | 11.07368 | 9.265594 |
| ENSMUSG00000042369 | protein_coding | Rbm45 | 1079.593 | 1021.207 | 1050.4 |
| ENSMUSG00000082512 | pseudogene | Gm13937 | 0.32211 | 1.413083 | 0.867597 |
| ENSMUSG00000059657 | protein_coding | Stfa2l1 | 0.32211 | 1.413083 | 0.867597 |
| ENSMUSG00000051391 | protein_coding | Ywhag | 5755.953 | 6165.268 | 5960.611 |
| ENSMUSG00000079408 | protein_coding | D830030K20Rik | 1.943172 | 3.743729 | 2.84345 |
| ENSMUSG00000038517 | protein_coding | Tbkbp1 | 448.2665 | 416.8714 | 432.569 |
| ENSMUSG00000041797 | protein_coding | Abca9 | 190.5395 | 213.8717 | 202.2056 |
| ENSMUSG00000085304 | processed_transcrip | Gm12802 | 1.937283 | 0.653029 | 1.295156 |
| ENSMUSG00000038383 | protein_coding | Pigu | 1177.298 | 1252.148 | 1214.723 |
| ENSMUSG00000020116 | protein_coding | Pno1 | 1263.821 | 1195.011 | 1229.416 |
| ENSMUSG00000002428 | protein_coding | Hltf | 1465.547 | 1385.564 | 1425.556 |
| ENSMUSG00000050944 | protein_coding | Efcab5 | 213.2401 | 174.0398 | 193.64 |
| ENSMUSG00000020801 | protein_coding | Med31 | 746.7357 | 804.639 | 775.6873 |
| ENSMUSG00000040549 | protein_coding | Ckap5 | 7785.001 | 7318.897 | 7551.949 |
| ENSMUSG00000014633 | protein_coding | 2310061C15Rik | 462.7568 | 422.0702 | 442.4135 |
| ENSMUSG00000090453 | protein_coding | Gm17343 | 0.968641 | 0 | 0.484321 |
| ENSMUSG00000091703 | protein_coding | Gm4222 | 0.968641 | 0 | 0.484321 |
| ENSMUSG00000027965 | protein_coding | Olfm3 | 0.968641 | 0 | 0.484321 |
| ENSMUSG00000089743 | processed_transcrip | Gm16226 | 0.968641 | 0 | 0.484321 |
| ENSMUSG00000076462 | IG_V_gene | Trbv2 | 0.968641 | 0 | 0.484321 |
| ENSMUSG00000058700 | pseudogene | Gm8724 | 0.968641 | 0 | 0.484321 |
| ENSMUSG00000072460 | protein_coding | Atp5l-ps1 | 0.968641 | 0 | 0.484321 |
| ENSMUSG00000088037 | snoRNA | SNORA70 | 0.968641 | 0 | 0.484321 |
| ENSMUSG00000090569 | lincRNA | Gm17466 | 0.968641 | 0 | 0.484321 |
| ENSMUSG00000089404 | misc_RNA | Y_RNA | 0.968641 | 0 | 0.484321 |
| ENSMUSG00000091851 | protein_coding | Gm8024 | 0.968641 | 0 | 0.484321 |
| ENSMUSG00000038591 | protein_coding | Colec10 | 0.968641 | 0 | 0.484321 |
| ENSMUSG00000054905 | protein_coding | Stfa3 | 0.968641 | 0 | 0.484321 |
| ENSMUSG00000029683 | protein_coding | Lmod2 | 0.976841 | 2.401635 | 1.689238 |
| ENSMUSG00000066516 | protein_coding | Klk1b21 | 3.272612 | 5.943532 | 4.608072 |
| ENSMUSG00000087272 | protein_coding | Gm15409 | 1.951372 | 3.788052 | 2.869712 |

| | | | | | |
|---|---|---|---|---|---|
| ENSMUSG00000017760 | protein_coding | Ctsa | 1766.497 | 1871.204 | 1818.851 |
| ENSMUSG00000056666 | protein_coding | Retsat | 494.304 | 460.1577 | 477.2308 |
| ENSMUSG00000039997 | protein_coding | Ifi203 | 95.9166 | 112.2779 | 104.0973 |
| ENSMUSG00000075702 | protein_coding | Selm | 498.5215 | 465.5363 | 482.0289 |
| ENSMUSG00000038530 | protein_coding | Rgs4 | 84.32167 | 98.79648 | 91.55908 |
| ENSMUSG00000021650 | protein_coding | Ptcd2 | 515.8935 | 553.7464 | 534.82 |
| ENSMUSG00000061104 | protein_coding | Gm10094 | 704.8622 | 662.2903 | 683.5762 |
| ENSMUSG00000084812 | lincRNA | Gm17445 | 8.069974 | 11.87557 | 9.972771 |
| ENSMUSG00000090418 | lincRNA | Gm17585 | 31.96643 | 25.84283 | 28.90463 |
| ENSMUSG00000090159 | pseudogene | Gm8580 | 1.298952 | 0.335524 | 0.817238 |
| ENSMUSG00000085123 | processed_transcript | Gm15219 | 1.298952 | 0.335524 | 0.817238 |
| ENSMUSG00000038805 | protein_coding | Six3 | 1.298952 | 0.335524 | 0.817238 |
| ENSMUSG00000053861 | protein_coding | Gm9925 | 1.298952 | 0.335524 | 0.817238 |
| ENSMUSG00000042808 | protein_coding | Gpx2 | 3.211635 | 5.693805 | 4.45272 |
| ENSMUSG00000080659 | miRNA | AC132253.2 | 1.917305 | 0.697713 | 1.307509 |
| ENSMUSG00000090624 | pseudogene | Gm17060 | 9.43937 | 13.71246 | 11.57591 |
| ENSMUSG00000027380 | protein_coding | Acoxl | 48.93838 | 40.22597 | 44.58218 |
| ENSMUSG00000020100 | protein_coding | Slc29a3 | 589.8676 | 642.529 | 616.1983 |
| ENSMUSG00000089955 | processed_transcript | C230030N03Rik | 1.290752 | 0.36219 | 0.826471 |
| ENSMUSG00000078528 | pseudogene | Gm17068 | 1.290752 | 0.36219 | 0.826471 |
| ENSMUSG00000039789 | protein_coding | Zfp597 | 1220.839 | 1138.477 | 1179.658 |
| ENSMUSG00000051811 | protein_coding | Cox6b2 | 232.6937 | 207.3622 | 220.0279 |
| ENSMUSG00000055202 | protein_coding | Zfp811 | 49.56531 | 59.62648 | 54.59589 |
| ENSMUSG00000019302 | protein_coding | Atp6v0a1 | 1343.628 | 1430.941 | 1387.284 |
| ENSMUSG00000046834 | protein_coding | Krt1 | 4.521097 | 7.362775 | 5.941936 |
| ENSMUSG00000028893 | protein_coding | Sesn2 | 2617.313 | 2333.593 | 2475.453 |
| ENSMUSG00000048920 | protein_coding | Fkrp | 619.5561 | 676.5151 | 648.0356 |
| ENSMUSG00000015451 | processed_transcript | C4a | 83.81581 | 100.3317 | 92.07376 |
| ENSMUSG00000091017 | protein_coding | Fam71a | 1.298952 | 0.326514 | 0.812733 |
| ENSMUSG00000083170 | pseudogene | Gm14405 | 1.298952 | 0.326514 | 0.812733 |
| ENSMUSG00000087787 | snRNA | U7 | 1.298952 | 0.326514 | 0.812733 |
| ENSMUSG00000087423 | lincRNA | Gm16875 | 1.298952 | 0.326514 | 0.812733 |
| ENSMUSG00000083090 | pseudogene | Gm11736 | 1.298952 | 0.326514 | 0.812733 |
| ENSMUSG00000074991 | protein_coding | Gabrr3 | 1.298952 | 0.326514 | 0.812733 |
| ENSMUSG00000073529 | lincRNA | F830208F22Rik | 1.298952 | 0.326514 | 0.812733 |
| ENSMUSG00000049723 | protein_coding | Mmp12 | 0.33031 | 1.377408 | 0.853859 |
| ENSMUSG00000084013 | pseudogene | Gm14270 | 8.731862 | 5.765156 | 7.248509 |
| ENSMUSG00000059203 | protein_coding | Il1rapl2 | 7.827776 | 4.803632 | 6.315704 |
| ENSMUSG00000092104 | lincRNA | Gm17713 | 11.35436 | 7.804963 | 9.579664 |
| ENSMUSG00000066362 | protein_coding | Rps13-ps1 | 60.26121 | 49.85886 | 55.06004 |
| ENSMUSG00000092223 | pseudogene | Gm19807 | 4.869074 | 2.710492 | 3.789783 |
| ENSMUSG00000034544 | protein_coding | Rsrc1 | 1252.723 | 1187.196 | 1219.959 |
| ENSMUSG00000040592 | protein_coding | Cd79b | 7.360155 | 11.57608 | 9.468118 |
| ENSMUSG00000034706 | protein_coding | Dnaic2 | 36.49804 | 43.57292 | 40.03548 |
| ENSMUSG00000020630 | protein_coding | Rnaseh1 | 560.0308 | 523.2336 | 541.6322 |
| ENSMUSG00000022262 | protein_coding | Dnahc5 | 72.87166 | 56.76594 | 64.8188 |
| ENSMUSG00000047507 | protein_coding | Baiap3 | 147.8607 | 165.5749 | 156.7178 |
| ENSMUSG00000041144 | protein_coding | Dnahc7b | 693.9851 | 781.4248 | 737.7049 |
| ENSMUSG00000085521 | processed_transcript | 4930526A20Rik | 16.88175 | 12.25469 | 14.56822 |
| ENSMUSG00000027397 | protein_coding | Slc20a1 | 2524.097 | 2405.75 | 2464.923 |
| ENSMUSG00000029767 | protein_coding | Calu | 9611.231 | 10191.78 | 9901.506 |
| ENSMUSG00000038069 | protein_coding | Cdkn2aip | 1397.252 | 1315.331 | 1356.291 |
| ENSMUSG00000017000 | protein_coding | Svs6 | 7.060333 | 4.432433 | 5.746383 |
| ENSMUSG00000006134 | protein_coding | Crkl | 2062.912 | 2231.572 | 2147.242 |
| ENSMUSG00000020225 | protein_coding | Tmbim4 | 1035.575 | 955.167 | 995.3712 |
| ENSMUSG00000060814 | protein_coding | Gm4980 | 161.8795 | 93.74837 | 127.814 |
| ENSMUSG00000090317 | protein_coding | Gm17324 | 0.954552 | 2.348303 | 1.651428 |
| ENSMUSG00000077091 | miRNA | AC122806.2 | 10.66221 | 7.214276 | 8.938244 |

| | | | | | |
|---|---|---|---|---|---|
| ENSMUSG00000037487 | protein_coding | Ubr5 | 11504.15 | 10978.57 | 11241.36 |
| ENSMUSG00000051593 | protein_coding | Olfr272 | 1.284863 | 0.36219 | 0.823526 |
| ENSMUSG00000030483 | protein_coding | Cyp2b10 | 1.284863 | 0.36219 | 0.823526 |
| ENSMUSG00000065440 | miRNA | Mirlet7g | 1.284863 | 0.36219 | 0.823526 |
| ENSMUSG00000085279 | processed_transcrip | Gm15965 | 1.284863 | 0.36219 | 0.823526 |
| ENSMUSG00000019261 | protein_coding | Mtap1s | 1642.658 | 1553.573 | 1598.115 |
| ENSMUSG00000029247 | protein_coding | Paics | 4138.913 | 4358.918 | 4248.916 |
| ENSMUSG00000091918 | protein_coding | Gm4301 | 0.316221 | 1.377408 | 0.846814 |
| ENSMUSG00000080862 | pseudogene | Gm14523 | 0.316221 | 1.377408 | 0.846814 |
| ENSMUSG00000044548 | protein_coding | Dact1 | 233.2757 | 210.807 | 222.0413 |
| ENSMUSG00000058793 | protein_coding | Cds2 | 2805.681 | 2967.553 | 2886.617 |
| ENSMUSG00000091709 | processed_transcrip | Gm17189 | 3.546566 | 1.641581 | 2.594073 |
| ENSMUSG00000085733 | processed_transcrip | Gm11641 | 9.088039 | 13.05114 | 11.06959 |
| ENSMUSG00000025742 | protein_coding | Prps2 | 546.1862 | 587.5845 | 566.8854 |
| ENSMUSG00000082727 | pseudogene | Gm13260 | 1.290752 | 0.335524 | 0.813138 |
| ENSMUSG00000087033 | processed_transcrip | Gm14155 | 1.290752 | 0.335524 | 0.813138 |
| ENSMUSG00000086193 | processed_transcrip | Gm11508 | 1.290752 | 0.335524 | 0.813138 |
| ENSMUSG00000075042 | processed_transcrip | 4930431P03Rik | 1.290752 | 0.335524 | 0.813138 |
| ENSMUSG00000000708 | protein_coding | Kat2b | 1846.308 | 1949.172 | 1897.74 |
| ENSMUSG00000087238 | processed_transcrip | Gm12865 | 5.842337 | 3.390549 | 4.616443 |
| ENSMUSG00000018930 | protein_coding | Ccl4 | 0.974531 | 2.472986 | 1.723758 |
| ENSMUSG00000070423 | protein_coding | Olfr558 | 21.99787 | 27.93669 | 24.96728 |
| ENSMUSG00000084867 | lincRNA | Enox | 37.76062 | 45.90465 | 41.83263 |
| ENSMUSG00000026643 | protein_coding | Nmt2 | 9201.819 | 9762.232 | 9482.025 |
| ENSMUSG00000020376 | protein_coding | Rnf130 | 2055.848 | 2159.464 | 2107.656 |
| ENSMUSG00000027075 | protein_coding | Slc43a1 | 131.5141 | 117.561 | 124.5375 |
| ENSMUSG00000024491 | protein_coding | Rbm27 | 2835.104 | 3127.515 | 2981.31 |
| ENSMUSG00000012705 | protein_coding | Retn | 4.213076 | 6.976769 | 5.594922 |
| ENSMUSG00000030287 | protein_coding | Itpr2 | 248.4573 | 210.1851 | 229.3212 |
| ENSMUSG00000039182 | protein_coding | AW209491 | 634.01 | 677.0808 | 655.5454 |
| ENSMUSG00000031842 | protein_coding | Pde4c | 219.347 | 244.9897 | 232.1684 |
| ENSMUSG00000038374 | protein_coding | Rbm8a | 2784.297 | 2932.926 | 2858.612 |
| ENSMUSG00000027332 | protein_coding | Ivd | 1791.575 | 1927.741 | 1859.658 |
| ENSMUSG00000063739 | protein_coding | Gm4963 | 1.290752 | 0.326514 | 0.808633 |
| ENSMUSG00000064491 | snRNA | U6 | 1.290752 | 0.326514 | 0.808633 |
| ENSMUSG00000064857 | rRNA | n-R5s25 | 1.290752 | 0.326514 | 0.808633 |
| ENSMUSG00000041328 | protein_coding | Pcf11 | 2640.038 | 2791.168 | 2715.603 |
| ENSMUSG00000037254 | protein_coding | Itih2 | 134.1191 | 152.4744 | 143.2967 |
| ENSMUSG00000018286 | protein_coding | Psmb6 | 2968.123 | 3113.43 | 3040.777 |
| ENSMUSG00000074227 | protein_coding | Spint2 | 3638.652 | 3808.719 | 3723.685 |
| ENSMUSG00000003153 | protein_coding | Slc2a3 | 685.769 | 635.7379 | 660.7535 |
| ENSMUSG00000074822 | protein_coding | Gm10766 | 12.5995 | 17.03166 | 14.81558 |
| ENSMUSG00000025912 | protein_coding | Mybl1 | 1119.758 | 1266.37 | 1193.064 |
| ENSMUSG00000033420 | protein_coding | Antxr1 | 1790.336 | 1951.744 | 1871.04 |
| ENSMUSG00000044576 | protein_coding | Fam59b | 320.8856 | 295.2558 | 308.0707 |
| ENSMUSG00000044573 | protein_coding | Acp1 | 1315.562 | 1188.637 | 1252.099 |
| ENSMUSG00000069301 | protein_coding | Hist1h2ag | 9.675904 | 6.525572 | 8.100738 |
| ENSMUSG00000051031 | protein_coding | Gm9726 | 3.896855 | 2.039445 | 2.96815 |
| ENSMUSG00000085900 | processed_transcrip | A930041C12Rik | 18.17377 | 23.87509 | 21.02443 |
| ENSMUSG00000008575 | protein_coding | Nfib | 2396.388 | 2583.142 | 2489.765 |
| ENSMUSG00000067321 | pseudogene | Gm7931 | 36.169 | 29.68494 | 32.92697 |
| ENSMUSG00000062391 | processed_transcrip | 4932435O22Rik | 6.156248 | 3.690035 | 4.923141 |
| ENSMUSG00000089093 | snoRNA | Snord11 | 1.957261 | 4.007902 | 2.982581 |
| ENSMUSG00000007682 | protein_coding | Dio2 | 24.20888 | 18.35682 | 21.28285 |
| ENSMUSG00000032834 | protein_coding | Pwp2 | 1277.551 | 1186.731 | 1232.141 |
| ENSMUSG00000024193 | protein_coding | Phf1 | 569.9188 | 608.3022 | 589.1105 |
| ENSMUSG00000079007 | protein_coding | Tcl1b4 | 1.284863 | 0.335524 | 0.810193 |
| ENSMUSG00000080546 | miRNA | AC129309.1 | 1.284863 | 0.335524 | 0.810193 |

| | | | | | |
|---|---|---|---|---|---|
| ENSMUSG00000024729 | protein_coding | 1700017D01Rik | 1.284863 | 0.335524 | 0.810193 |
| ENSMUSG00000077063 | miRNA | AC143329.1 | 64.97129 | 26.88192 | 45.92661 |
| ENSMUSG00000032985 | protein_coding | 5730522E02Rik | 10.95361 | 14.95654 | 12.95507 |
| ENSMUSG00000046718 | protein_coding | Bst2 | 609.501 | 657.6185 | 633.5598 |
| ENSMUSG00000085996 | lincRNA | A830012C17Rik | 8.060731 | 11.74115 | 9.900942 |
| ENSMUSG00000041423 | protein_coding | Paqr6 | 79.18326 | 93.4 | 86.29163 |
| ENSMUSG00000029681 | protein_coding | Bcl7b | 810.4243 | 858.2854 | 834.3549 |
| ENSMUSG00000033799 | protein_coding | BC016423 | 7652.64 | 7192.424 | 7422.532 |
| ENSMUSG00000038902 | protein_coding | Pogz | 3545.718 | 3751.035 | 3648.376 |
| ENSMUSG00000022523 | protein_coding | Fgf12 | 33.60159 | 40.73811 | 37.16985 |
| ENSMUSG00000033565 | protein_coding | Rbfox2 | 2476.211 | 2607.851 | 2542.031 |
| ENSMUSG00000086359 | processed_transcrip | 9630013K17Rik | 22.41942 | 29.37572 | 25.89757 |
| ENSMUSG00000032977 | protein_coding | 1810008A18Rik | 891.7426 | 837.0236 | 864.3831 |
| ENSMUSG00000083716 | pseudogene | Gm13436 | 49.10216 | 41.44411 | 45.27313 |
| ENSMUSG00000029328 | protein_coding | Hnrpdl | 7038.85 | 7391.703 | 7215.277 |
| ENSMUSG00000036323 | protein_coding | Srp72 | 3759.469 | 3934.459 | 3846.964 |
| ENSMUSG00000060780 | protein_coding | Lrrtm1 | 4.849096 | 2.692836 | 3.770966 |
| ENSMUSG00000089875 | lincRNA | Etohd2 | 63.89291 | 73.62548 | 68.7592 |
| ENSMUSG00000075405 | protein_coding | 9430097D07Rik | 1.284863 | 0.326514 | 0.805688 |
| ENSMUSG00000081030 | pseudogene | Gm12704 | 1.284863 | 0.326514 | 0.805688 |
| ENSMUSG00000070194 | snRNA | U1 | 1.284863 | 0.326514 | 0.805688 |
| ENSMUSG00000085360 | processed_transcrip | Gm11647 | 1.284863 | 0.326514 | 0.805688 |
| ENSMUSG00000091565 | protein_coding | Gm3317 | 1.284863 | 0.326514 | 0.805688 |
| ENSMUSG00000089686 | pseudogene | Gm3833 | 1.284863 | 0.326514 | 0.805688 |
| ENSMUSG00000039515 | protein_coding | Ppp2r4 | 4403.332 | 4215.722 | 4309.527 |
| ENSMUSG00000005474 | protein_coding | Myl10 | 1.943172 | 0.653029 | 1.2981 |
| ENSMUSG00000082927 | pseudogene | Gm5863 | 1.943172 | 0.653029 | 1.2981 |
| ENSMUSG00000074128 | processed_transcrip | Gm10631 | 2.575614 | 1.006571 | 1.791092 |
| ENSMUSG00000031618 | protein_coding | Nr3c2 | 115.0661 | 101.6829 | 108.3745 |
| ENSMUSG00000027350 | protein_coding | Chgb | 19.40332 | 24.59911 | 22.00121 |
| ENSMUSG00000033792 | protein_coding | Atp7a | 1667.197 | 1555.047 | 1611.122 |
| ENSMUSG00000059839 | protein_coding | Zfp874b | 271.4956 | 295.6703 | 283.583 |
| ENSMUSG00000079469 | protein_coding | Pigb | 141.2667 | 127.0248 | 134.1457 |
| ENSMUSG00000001173 | protein_coding | Ocrl | 1953.936 | 2114.74 | 2034.338 |
| ENSMUSG00000062519 | protein_coding | Zfp398 | 1028.146 | 1096.2 | 1062.173 |
| ENSMUSG00000079639 | protein_coding | Rhox4a | 9.686414 | 6.409898 | 8.048156 |
| ENSMUSG00000052430 | protein_coding | Bmpr1b | 65.14706 | 74.74157 | 69.94431 |
| ENSMUSG00000053581 | protein_coding | Zfand2a | 1115.856 | 1048.318 | 1082.087 |
| ENSMUSG00000028990 | protein_coding | Lzic | 926.1382 | 870.8275 | 898.4828 |
| ENSMUSG00000037735 | protein_coding | 2810032G03Rik | 32.70235 | 25.89652 | 29.29943 |
| ENSMUSG00000030433 | protein_coding | Sbk2 | 9.019905 | 12.57256 | 10.79623 |
| ENSMUSG00000031965 | protein_coding | Tbx20 | 15.13493 | 10.63077 | 12.88285 |
| ENSMUSG00000026764 | protein_coding | Kif5c | 1368.082 | 1295.021 | 1331.552 |
| ENSMUSG00000045731 | protein_coding | Pnoc | 8.071242 | 4.713901 | 6.392571 |
| ENSMUSG00000020553 | protein_coding | Pctp | 329.4734 | 358.9013 | 344.1874 |
| ENSMUSG00000078126 | protein_coding | Rpl23a-ps3 | 184.0897 | 205.3876 | 194.7386 |
| ENSMUSG00000090771 | lincRNA | Gm17501 | 4.535186 | 7.160944 | 5.848065 |
| ENSMUSG00000081991 | pseudogene | Gm13859 | 3.550145 | 1.739597 | 2.644871 |
| ENSMUSG00000088789 | snoRNA | Scarna13 | 13.82084 | 9.773419 | 11.79713 |
| ENSMUSG00000049526 | protein_coding | Tmem202 | 12.90163 | 17.30484 | 15.10323 |
| ENSMUSG00000047242 | protein_coding | Taf9b | 2696.476 | 2542.269 | 2619.372 |
| ENSMUSG00000044881 | protein_coding | Chchd8 | 153.7966 | 178.739 | 166.2678 |
| ENSMUSG00000037098 | protein_coding | Rab11fip3 | 3354.611 | 3168.389 | 3261.5 |
| ENSMUSG00000078906 | protein_coding | Gm14444 | 5.173517 | 8.086431 | 6.629974 |
| ENSMUSG00000033972 | protein_coding | Zfp944 | 632.1955 | 678.6874 | 655.4415 |
| ENSMUSG00000027300 | protein_coding | Ubox5 | 736.3855 | 692.1998 | 714.2926 |
| ENSMUSG00000091405 | protein_coding | Hist2h4 | 3.893276 | 6.851725 | 5.3725 |
| ENSMUSG00000048677 | protein_coding | Tpcn2 | 302.3068 | 276.0438 | 289.1753 |

| | | | | | |
|---|---|---|---|---|---|
| ENSMUSG00000040774 | protein_coding | Cept1 | 900.7712 | 964.4686 | 932.6199 |
| ENSMUSG00000021676 | protein_coding | Iqgap2 | 625.5406 | 575.26 | 600.4003 |
| ENSMUSG00000070695 | protein_coding | Cntnap5a | 4.186165 | 2.093139 | 3.139652 |
| ENSMUSG00000086576 | processed_transcrip | Gm13660 | 1.284863 | 2.737158 | 2.01101 |
| ENSMUSG00000015575 | protein_coding | Atp6v0e | 2021.738 | 2146.48 | 2084.109 |
| ENSMUSG00000082262 | pseudogene | Gm14730 | 1.290752 | 2.746168 | 2.01846 |
| ENSMUSG00000033009 | protein_coding | Ogfod1 | 932.4282 | 875.4676 | 903.9479 |
| ENSMUSG00000038510 | protein_coding | Rpf2 | 863.0902 | 925.8907 | 894.4904 |
| ENSMUSG00000024480 | protein_coding | Ap3s1 | 2239.056 | 2359.229 | 2299.142 |
| ENSMUSG00000064128 | protein_coding | Cenpj | 1221.463 | 1156.463 | 1188.963 |
| ENSMUSG00000050148 | protein_coding | Ubqln2 | 3046.884 | 3247.921 | 3147.403 |
| ENSMUSG00000042579 | protein_coding | 4632404H12Rik | 208.8098 | 190.2147 | 199.5123 |
| ENSMUSG00000073117 | protein_coding | Gm7347 | 18.78243 | 24.88167 | 21.83205 |
| ENSMUSG00000078606 | protein_coding | Gm4070 | 5.811849 | 3.452528 | 4.632188 |
| ENSMUSG00000037096 | protein_coding | Gm9762 | 83.09883 | 71.79036 | 77.4446 |
| ENSMUSG00000020134 | protein_coding | Peli1 | 1253.122 | 1176.95 | 1215.036 |
| ENSMUSG00000002249 | protein_coding | Tead3 | 351.2592 | 376.8257 | 364.0424 |
| ENSMUSG00000029648 | protein_coding | Flt1 | 179.1975 | 198.6104 | 188.904 |
| ENSMUSG00000061835 | protein_coding | Olfr316 | 17.76228 | 12.39527 | 15.07877 |
| ENSMUSG00000081778 | pseudogene | Gm6325 | 1.65155 | 0.36219 | 1.00687 |
| ENSMUSG00000078731 | protein_coding | Gm7819 | 20.23547 | 12.30983 | 16.27265 |
| ENSMUSG00000054728 | protein_coding | Phactr1 | 663.3653 | 586.8254 | 625.0953 |
| ENSMUSG00000033544 | protein_coding | Angptl1 | 60.19517 | 71.90427 | 66.04972 |
| ENSMUSG00000058755 | protein_coding | Osm | 3.246745 | 6.377072 | 4.811909 |
| ENSMUSG00000091366 | processed_transcrip | Gm17040 | 1.595194 | 3.497213 | 2.546204 |
| ENSMUSG00000028601 | protein_coding | Echdc2 | 951.3629 | 1021.019 | 986.1909 |
| ENSMUSG00000031627 | protein_coding | Irf2 | 354.4466 | 384.5537 | 369.5002 |
| ENSMUSG00000074342 | protein_coding | I830077J02Rik | 4.490608 | 7.18761 | 5.839109 |
| ENSMUSG00000004038 | protein_coding | Gstm3 | 20.52858 | 26.56793 | 23.54825 |
| ENSMUSG00000084769 | processed_transcrip | Gm12061 | 12.92623 | 9.024862 | 10.97554 |
| ENSMUSG00000058447 | protein_coding | Zfp82 | 214.2956 | 233.1167 | 223.7061 |
| ENSMUSG00000086284 | processed_transcrip | 2900093L17Rik | 7.511555 | 4.343425 | 5.92749 |
| ENSMUSG00000016528 | protein_coding | Mapkapk2 | 2968.226 | 3122.961 | 3045.593 |
| ENSMUSG00000075031 | protein_coding | Hist1h2bb | 4.544653 | 2.401635 | 3.473144 |
| ENSMUSG00000025537 | protein_coding | Phkg1 | 44.354 | 52.50914 | 48.43157 |
| ENSMUSG00000070366 | protein_coding | Ppapdc1a | 5.492049 | 2.974665 | 4.233357 |
| ENSMUSG00000036840 | protein_coding | Siah1a | 829.8752 | 888.5182 | 859.1967 |
| ENSMUSG00000072761 | protein_coding | Gm6712 | 89.65956 | 101.4804 | 95.56997 |
| ENSMUSG00000023495 | protein_coding | Pcbp4 | 916.2714 | 988.44 | 952.3557 |
| ENSMUSG00000033177 | protein_coding | Tmprss7 | 1.298952 | 2.746168 | 2.02256 |
| ENSMUSG00000083892 | pseudogene | Gm1848 | 1.298952 | 2.746168 | 2.02256 |
| ENSMUSG00000085293 | lincRNA | Gm13817 | 1.32124 | 0 | 0.66062 |
| ENSMUSG00000027157 | protein_coding | 4930430A15Rik | 1.32124 | 0 | 0.66062 |
| ENSMUSG00000058400 | protein_coding | Qrfpr | 1.32124 | 0 | 0.66062 |
| ENSMUSG00000093230 | miRNA | AC139241.1 | 1.32124 | 0 | 0.66062 |
| ENSMUSG00000023140 | protein_coding | Reg2 | 1.32124 | 0 | 0.66062 |
| ENSMUSG00000066092 | protein_coding | Gm10152 | 1.32124 | 0 | 0.66062 |
| ENSMUSG00000079012 | protein_coding | Serpina3m | 1.32124 | 0 | 0.66062 |
| ENSMUSG00000085746 | lincRNA | 4930455B14Rik | 1.32124 | 0 | 0.66062 |
| ENSMUSG00000078097 | pseudogene | Gm10913 | 1.32124 | 0 | 0.66062 |
| ENSMUSG00000057817 | protein_coding | Olfr1446 | 1.32124 | 0 | 0.66062 |
| ENSMUSG00000073405 | pseudogene | C920025E04Rik | 6.480669 | 10.18314 | 8.331906 |
| ENSMUSG00000028974 | protein_coding | Dffa | 666.1569 | 621.9056 | 644.0312 |
| ENSMUSG00000056031 | protein_coding | 9330154J02Rik | 7.797287 | 11.35551 | 9.576398 |
| ENSMUSG00000083567 | pseudogene | Gm11451 | 22.08702 | 16.59848 | 19.34275 |
| ENSMUSG00000090685 | protein_coding | Gm9047 | 1.951372 | 0.671047 | 1.311209 |
| ENSMUSG00000040972 | protein_coding | Igsf21 | 43.02537 | 51.90944 | 47.46741 |
| ENSMUSG00000083374 | pseudogene | Gm11449 | 0.33031 | 1.36876 | 0.849535 |

| | | | | | |
|---|---|---|---|---|---|
| ENSMUSG00000090620 | protein_coding | Gm6724 | 6.116291 | 3.443519 | 4.779905 |
| ENSMUSG00000031760 | protein_coding | Mt3 | 151.2129 | 184.1204 | 167.6667 |
| ENSMUSG00000064158 | protein_coding | Izumo1 | 45.8411 | 38.58548 | 42.21329 |
| ENSMUSG00000073795 | protein_coding | 6430531B16Rik | 7.739664 | 4.865973 | 6.302818 |
| ENSMUSG00000002308 | protein_coding | Cd320 | 1421.728 | 1322.946 | 1372.337 |
| ENSMUSG00000039987 | protein_coding | Phtf2 | 1926.185 | 1832.532 | 1879.359 |
| ENSMUSG00000069300 | protein_coding | Hist1h2bj | 10.58461 | 6.940732 | 8.762671 |
| ENSMUSG00000056214 | protein_coding | Pard6g | 848.5228 | 801.6141 | 825.0684 |
| ENSMUSG00000037523 | protein_coding | Mavs | 1307.346 | 1419.341 | 1363.343 |
| ENSMUSG00000026159 | protein_coding | Agfg1 | 2208.476 | 2317.57 | 2263.023 |
| ENSMUSG00000015133 | protein_coding | Lrrk1 | 367.204 | 338.6839 | 352.944 |
| ENSMUSG00000060992 | protein_coding | Copz1 | 2459.219 | 2586.794 | 2523.007 |
| ENSMUSG00000084851 | processed_transcrip | Gm11267 | 2.303971 | 0.724379 | 1.514175 |
| ENSMUSG00000085372 | processed_transcrip | Gm16145 | 2.303971 | 0.724379 | 1.514175 |
| ENSMUSG00000072872 | protein_coding | Rybp | 2197.47 | 2341.863 | 2269.667 |
| ENSMUSG00000090422 | lincRNA | Gm17272 | 4.184898 | 6.772088 | 5.478493 |
| ENSMUSG00000069581 | protein_coding | C330046G03Rik | 12.92854 | 9.182009 | 11.05527 |
| ENSMUSG00000068923 | protein_coding | Syt11 | 492.6612 | 530.933 | 511.7971 |
| ENSMUSG00000014867 | protein_coding | Surf4 | 4621.987 | 4426.138 | 4524.063 |
| ENSMUSG00000051295 | processed_transcrip | 9630028B13Rik | 7.100289 | 10.69963 | 8.89996 |
| ENSMUSG00000020462 | protein_coding | Ccdc104 | 1050.934 | 992.7291 | 1021.832 |
| ENSMUSG00000072531 | pseudogene | Gm6755 | 1.934972 | 3.779404 | 2.857188 |
| ENSMUSG00000055148 | protein_coding | Klf2 | 259.8358 | 285.2379 | 272.5368 |
| ENSMUSG00000031294 | protein_coding | D630029K05Rik | 3.574744 | 1.73095 | 2.652847 |
| ENSMUSG00000054256 | protein_coding | Msi1 | 3949.491 | 4192.556 | 4071.023 |
| ENSMUSG00000073599 | protein_coding | Ecscr | 144.1653 | 128.0724 | 136.1189 |
| ENSMUSG00000022811 | protein_coding | Zfp148 | 3894.466 | 4110.683 | 4002.575 |
| ENSMUSG00000046841 | protein_coding | Ckap4 | 1318.948 | 1212.428 | 1265.688 |
| ENSMUSG00000016327 | protein_coding | Atp1b4 | 5.134829 | 8.398861 | 6.766845 |
| ENSMUSG00000037470 | protein_coding | Uggt1 | 3620.324 | 3417.653 | 3518.989 |
| ENSMUSG00000037236 | protein_coding | Matr3 | 13094.56 | 12477.15 | 12785.86 |
| ENSMUSG00000053490 | protein_coding | Trim60 | 7.116689 | 4.503783 | 5.810236 |
| ENSMUSG00000040918 | protein_coding | Slc19a2 | 1678.253 | 1770.59 | 1724.421 |
| ENSMUSG00000071262 | protein_coding | Zfp957 | 2.925902 | 1.386417 | 2.15616 |
| ENSMUSG00000052760 | protein_coding | A630001G21Rik | 9.770948 | 14.18167 | 11.97631 |
| ENSMUSG00000050852 | pseudogene | Gm12115 | 8.10993 | 11.54833 | 9.82913 |
| ENSMUSG00000081564 | pseudogene | Gm13717 | 11.9681 | 16.08815 | 14.02812 |
| ENSMUSG00000032041 | protein_coding | Tirap | 171.8143 | 190.8578 | 181.336 |
| ENSMUSG00000071176 | protein_coding | Arhgef10 | 1268.672 | 1349.954 | 1309.313 |
| ENSMUSG00000022842 | protein_coding | Ece2 | 332.8178 | 358.6902 | 345.754 |
| ENSMUSG00000090993 | lincRNA | Gm17587 | 0 | 1.059903 | 0.529951 |
| ENSMUSG00000066063 | protein_coding | Olfr1338 | 0 | 1.059903 | 0.529951 |
| ENSMUSG00000087487 | processed_transcrip | 4933401P06Rik | 0 | 1.059903 | 0.529951 |
| ENSMUSG00000087914 | misc_RNA | 7SK | 0 | 1.059903 | 0.529951 |
| ENSMUSG00000040017 | protein_coding | Saa4 | 0 | 1.059903 | 0.529951 |
| ENSMUSG00000021214 | protein_coding | Akr1c18 | 0 | 1.059903 | 0.529951 |
| ENSMUSG00000091894 | pseudogene | Gm3030 | 0 | 1.059903 | 0.529951 |
| ENSMUSG00000089796 | lincRNA | Gm16575 | 0 | 1.059903 | 0.529951 |
| ENSMUSG00000090790 | pseudogene | Gm17377 | 0 | 1.059903 | 0.529951 |
| ENSMUSG00000071230 | protein_coding | Npw | 12.25383 | 17.49471 | 14.87427 |
| ENSMUSG00000074593 | protein_coding | Gm14316 | 29.64144 | 23.34318 | 26.49231 |
| ENSMUSG00000046447 | protein_coding | Camk2n1 | 394.8271 | 424.0446 | 409.4358 |
| ENSMUSG00000031938 | protein_coding | 4931406C07Rik | 1473.442 | 1403.524 | 1438.483 |
| ENSMUSG00000071113 | protein_coding | Mboat4 | 2.253504 | 4.0879 | 3.170702 |
| ENSMUSG00000018381 | protein_coding | Abi3 | 16.78081 | 21.49112 | 19.13597 |
| ENSMUSG00000049960 | protein_coding | Mrps16 | 958.5774 | 895.7125 | 927.145 |
| ENSMUSG00000051557 | protein_coding | Pusl1 | 432.5107 | 401.3416 | 416.9262 |
| ENSMUSG00000026357 | protein_coding | Rgs18 | 10.0449 | 6.691366 | 8.368134 |

| | | | | | |
|---|---|---|---|---|---|
| ENSMUSG00000088478 | miRNA | Mir1946b | 2.934102 | 1.386417 | 2.16026 |
| ENSMUSG00000086287 | processed_transcrip | Gm15972 | 2.934102 | 1.386417 | 2.16026 |
| ENSMUSG00000054957 | pseudogene | Gm9959 | 2.549747 | 1.050893 | 1.80032 |
| ENSMUSG00000036437 | protein_coding | Npy1r | 18.0905 | 13.34198 | 15.71624 |
| ENSMUSG00000038056 | protein_coding | Mll3 | 5551.052 | 6094.909 | 5822.981 |
| ENSMUSG00000078784 | protein_coding | 1810022K09Rik | 733.6943 | 785.907 | 759.8007 |
| ENSMUSG00000058586 | protein_coding | Serhl | 192.3331 | 214.8963 | 203.6147 |
| ENSMUSG00000036721 | protein_coding | Zscan12 | 394.3511 | 362.0451 | 378.1981 |
| ENSMUSG00000024841 | protein_coding | Eif1ad | 1659.206 | 1548.156 | 1603.681 |
| ENSMUSG00000039117 | protein_coding | Taf4a | 1174.188 | 1104.165 | 1139.176 |
| ENSMUSG00000025223 | protein_coding | Ldb1 | 3166.1 | 2997.524 | 3081.812 |
| ENSMUSG00000045538 | protein_coding | Ddx28 | 352.0005 | 327.5467 | 339.7736 |
| ENSMUSG00000086536 | processed_transcrip | Gm12264 | 40.39132 | 48.44542 | 44.41837 |
| ENSMUSG00000090340 | protein_coding | C030048H21Rik | 9.684103 | 13.33261 | 11.50836 |
| ENSMUSG00000054099 | protein_coding | Slc25a40 | 521.365 | 561.9939 | 541.6795 |
| ENSMUSG00000022415 | protein_coding | Syngr1 | 556.0754 | 597.6592 | 576.8673 |
| ENSMUSG00000032249 | protein_coding | Anp32a | 3431.043 | 3701.189 | 3566.116 |
| ENSMUSG00000005973 | protein_coding | Rcn1 | 1409.96 | 1526.262 | 1468.111 |
| ENSMUSG00000023348 | protein_coding | Trip6 | 1263.22 | 1347.962 | 1305.591 |
| ENSMUSG00000016477 | protein_coding | E2f3 | 1003.132 | 928.7916 | 965.962 |
| ENSMUSG00000065904 | snoRNA | SNORD63 | 6.150358 | 9.147057 | 7.648708 |
| ENSMUSG00000074365 | protein_coding | Crxos1 | 12.28432 | 8.680691 | 10.4825 |
| ENSMUSG00000072964 | protein_coding | Bhlhb9 | 1922.013 | 2058.193 | 1990.103 |
| ENSMUSG00000086602 | lincRNA | Gm15609 | 13.25296 | 9.62492 | 11.43894 |
| ENSMUSG00000068227 | protein_coding | Il2rb | 23.31806 | 30.11775 | 26.71791 |
| ENSMUSG00000055761 | protein_coding | Nkain3 | 4.867807 | 2.683826 | 3.775816 |
| ENSMUSG00000020659 | protein_coding | Cbll1 | 922.3235 | 980.2065 | 951.265 |
| ENSMUSG00000003184 | protein_coding | Irf3 | 1083.939 | 1165.793 | 1124.866 |
| ENSMUSG00000067941 | protein_coding | Gm7168 | 0.66062 | 2.101787 | 1.381203 |
| ENSMUSG00000075594 | protein_coding | K230010J24Rik | 48.44005 | 57.26845 | 52.85425 |
| ENSMUSG00000032750 | protein_coding | Gab3 | 263.5493 | 241.8913 | 252.7203 |
| ENSMUSG00000021613 | protein_coding | Hapln1 | 389.3715 | 335.7556 | 362.5636 |
| ENSMUSG00000084416 | pseudogene | Rpl10a-ps1 | 266.5735 | 240.5959 | 253.5847 |
| ENSMUSG00000030917 | protein_coding | Tmem159 | 665.7699 | 709.9207 | 687.8453 |
| ENSMUSG00000067358 | protein_coding | Gm7903 | 1.931394 | 0.671047 | 1.30122 |
| ENSMUSG00000004936 | protein_coding | Map2k1 | 1590.302 | 1502.984 | 1546.643 |
| ENSMUSG00000093080 | miRNA | AL807825.1 | 8.74364 | 12.22839 | 10.48601 |
| ENSMUSG00000090602 | protein_coding | Gm5611 | 10.38699 | 14.38351 | 12.38525 |
| ENSMUSG00000081809 | pseudogene | Gm15539 | 1.951372 | 3.717063 | 2.834217 |
| ENSMUSG00000019368 | protein_coding | Sec14l4 | 16.25982 | 11.58364 | 13.92173 |
| ENSMUSG00000051154 | protein_coding | Commd3 | 1269.145 | 1355.036 | 1312.09 |
| ENSMUSG00000073535 | processed_transcrip | Gm5532 | 33.11525 | 25.99998 | 29.55761 |
| ENSMUSG00000052981 | protein_coding | Ube2ql1 | 12.88627 | 17.03202 | 14.95914 |
| ENSMUSG00000049353 | protein_coding | Rd3 | 1.929083 | 0.671047 | 1.300065 |
| ENSMUSG00000033777 | protein_coding | Tlr13 | 45.14306 | 53.32004 | 49.23155 |
| ENSMUSG00000087476 | processed_transcrip | 1810058N05Rik | 19.69852 | 24.90761 | 22.30306 |
| ENSMUSG00000052593 | protein_coding | Adam17 | 2920.211 | 3066.053 | 2993.132 |
| ENSMUSG00000062729 | protein_coding | Ppox | 487.7516 | 548.816 | 518.2838 |
| ENSMUSG00000031448 | protein_coding | Adprhl1 | 4.851407 | 7.670909 | 6.261158 |
| ENSMUSG00000022475 | protein_coding | Hdac7 | 588.3622 | 632.7487 | 610.5555 |
| ENSMUSG00000061032 | protein_coding | Rrp1 | 3659.003 | 3834.36 | 3746.681 |
| ENSMUSG00000020081 | protein_coding | Tacr2 | 9.638258 | 6.355843 | 7.997051 |
| ENSMUSG00000091527 | lincRNA | Gm8330 | 4.873696 | 2.692836 | 3.783266 |
| ENSMUSG00000046785 | protein_coding | Epm2aip1 | 2574.021 | 2739.103 | 2656.562 |
| ENSMUSG00000072109 | processed_transcrip | A530040E14Rik | 0 | 1.050893 | 0.525447 |
| ENSMUSG00000092397 | lincRNA | C130080G10Rik | 0 | 1.050893 | 0.525447 |
| ENSMUSG00000086999 | processed_transcrip | 1700040N02Rik | 0 | 1.050893 | 0.525447 |
| ENSMUSG00000079988 | pseudogene | Gm11970 | 0 | 1.050893 | 0.525447 |

| | | | | | |
|---|---|---|---|---|---|
| ENSMUSG00000084278 | pseudogene | Gm12189 | 0 | 1.050893 | 0.525447 |
| ENSMUSG00000072675 | protein_coding | Gm10394 | 0 | 1.050893 | 0.525447 |
| ENSMUSG00000075572 | protein_coding | Defb43 | 0 | 1.050893 | 0.525447 |
| ENSMUSG00000034473 | protein_coding | Sec22a | 790.857 | 739.6946 | 765.2758 |
| ENSMUSG00000034829 | protein_coding | Nxnl1 | 27.20187 | 19.99338 | 23.59763 |
| ENSMUSG00000037376 | protein_coding | Trmt6 | 1901.712 | 1991.576 | 1946.644 |
| ENSMUSG00000045319 | protein_coding | 5430407P10Rik | 631.3774 | 585.6745 | 608.5259 |
| ENSMUSG00000047714 | protein_coding | Ppp1r2 | 3018.028 | 2863.838 | 2940.933 |
| ENSMUSG00000021619 | protein_coding | Atg10 | 299.8713 | 323.4815 | 311.6764 |
| ENSMUSG00000091825 | pseudogene | Gm5778 | 1.298952 | 2.710492 | 2.004722 |
| ENSMUSG00000078600 | protein_coding | 1700092K14Rik | 1.298952 | 2.710492 | 2.004722 |
| ENSMUSG00000006335 | protein_coding | Tfpt | 643.0387 | 606.7244 | 624.8815 |
| ENSMUSG00000020153 | protein_coding | Ndufs7 | 1009.051 | 1068.337 | 1038.694 |
| ENSMUSG00000048756 | protein_coding | Foxo3 | 6385.073 | 6773.776 | 6579.425 |
| ENSMUSG00000019487 | protein_coding | Trip10 | 715.7516 | 760.5996 | 738.1756 |
| ENSMUSG00000044434 | pseudogene | Gm9791 | 122.2454 | 135.6615 | 128.9535 |
| ENSMUSG00000020395 | protein_coding | Itk | 6.152669 | 9.561855 | 7.857262 |
| ENSMUSG00000024209 | protein_coding | 1700061G19Rik | 38.11784 | 31.69771 | 34.90778 |
| ENSMUSG00000006313 | protein_coding | Upk1a | 11.61319 | 15.5829 | 13.59804 |
| ENSMUSG00000056851 | protein_coding | Pcbp2 | 4947.385 | 5222.375 | 5084.88 |
| ENSMUSG00000056569 | protein_coding | Mpz | 19.26541 | 13.62381 | 16.44461 |
| ENSMUSG00000025137 | protein_coding | Pcyt2 | 670.1883 | 620.299 | 645.2436 |
| ENSMUSG00000085051 | lincRNA | Gm11542 | 6.814557 | 4.141232 | 5.477895 |
| ENSMUSG00000037548 | protein_coding | H2-DMb2 | 5.834137 | 3.497213 | 4.665675 |
| ENSMUSG00000012535 | protein_coding | Tnpo3 | 3613.762 | 3781.146 | 3697.454 |
| ENSMUSG00000067998 | protein_coding | Bpifb9a | 0.66062 | 2.146471 | 1.403546 |
| ENSMUSG00000039296 | protein_coding | Spdyb | 1.953683 | 0.662038 | 1.30786 |
| ENSMUSG00000051682 | protein_coding | Treml4 | 1.953683 | 0.662038 | 1.30786 |
| ENSMUSG00000041079 | protein_coding | Rwdd2b | 254.5759 | 228.5427 | 241.5593 |
| ENSMUSG00000032502 | protein_coding | Stac | 7.157688 | 4.387748 | 5.772718 |
| ENSMUSG00000090920 | lincRNA | 9130213A22Rik | 11.05812 | 15.35404 | 13.20608 |
| ENSMUSG00000005043 | protein_coding | Sgsh | 630.4275 | 545.3212 | 587.8743 |
| ENSMUSG00000048307 | protein_coding | Ankrd46 | 1563.832 | 1656.878 | 1610.355 |
| ENSMUSG00000034487 | protein_coding | Kdelc2 | 1427.816 | 1511.986 | 1469.901 |
| ENSMUSG00000084835 | processed_transcrip | Gm12352 | 17.1393 | 12.81006 | 14.97468 |
| ENSMUSG00000030530 | protein_coding | Furin | 3183.668 | 3388.334 | 3286.001 |
| ENSMUSG00000051747 | protein_coding | Ttn | 360.5149 | 321.9462 | 341.2305 |
| ENSMUSG00000070719 | protein_coding | Pla2g4d | 14.84584 | 10.73779 | 12.79182 |
| ENSMUSG00000058571 | protein_coding | Gpc6 | 160.4427 | 179.1335 | 169.7881 |
| ENSMUSG00000047383 | protein_coding | Als2cr11 | 14.63518 | 10.27831 | 12.45674 |
| ENSMUSG00000084779 | lincRNA | 4921504A21Rik | 29.31113 | 36.48876 | 32.89995 |
| ENSMUSG00000091745 | processed_transcrip | Gm17098 | 20.39887 | 15.23257 | 17.81572 |
| ENSMUSG00000087794 | rRNA | 5S_rRNA | 4.555164 | 7.1786 | 5.866882 |
| ENSMUSG00000075553 | protein_coding | Gm5464 | 26.15332 | 32.29813 | 29.22573 |
| ENSMUSG00000086949 | processed_transcrip | Gm13066 | 0.316221 | 1.324076 | 0.820148 |
| ENSMUSG00000053113 | protein_coding | Socs3 | 198.8554 | 217.6865 | 208.271 |
| ENSMUSG00000083287 | pseudogene | Gm13502 | 49.10909 | 57.28217 | 53.19563 |
| ENSMUSG00000052485 | protein_coding | Tmem171 | 4.15672 | 6.798754 | 5.477737 |
| ENSMUSG00000032333 | protein_coding | Stoml1 | 501.4725 | 537.3019 | 519.3872 |
| ENSMUSG00000028163 | protein_coding | Nfkb1 | 972.3179 | 1054.562 | 1013.44 |
| ENSMUSG00000053289 | protein_coding | Ddx10 | 4398.114 | 4085.837 | 4241.976 |
| ENSMUSG00000030276 | protein_coding | Ttll3 | 160.0731 | 179.5479 | 169.8105 |
| ENSMUSG00000031373 | protein_coding | Car5b | 874.1793 | 788.9322 | 831.5558 |
| ENSMUSG00000025935 | protein_coding | Tram1 | 2671.17 | 2832.818 | 2751.994 |
| ENSMUSG00000041879 | protein_coding | Ipo9 | 3356.1 | 3529.769 | 3442.934 |
| ENSMUSG00000020642 | protein_coding | Rnf144a | 3266.789 | 3450.595 | 3358.692 |
| ENSMUSG00000039062 | protein_coding | Anpep | 264.0082 | 238.9942 | 251.5012 |
| ENSMUSG00000086124 | processed_transcrip | A530076I17Rik | 10.25557 | 6.771726 | 8.513647 |

| | | | | | |
|---|---|---|---|---|---|
| ENSMUSG00000083619 | pseudogene | Gm14414 | 5.167628 | 3.063673 | 4.11565 |
| ENSMUSG00000041674 | lincRNA | BC006965 | 49.80124 | 58.61018 | 54.20571 |
| ENSMUSG00000088712 | snoRNA | SCARNA23 | 4.247143 | 7.4013 | 5.824221 |
| ENSMUSG00000037321 | protein_coding | Tap1 | 138.5591 | 119.3981 | 128.9786 |
| ENSMUSG00000067313 | protein_coding | Gm7205 | 0.32211 | 1.341732 | 0.831921 |
| ENSMUSG00000060807 | protein_coding | Serpina6 | 0.32211 | 1.341732 | 0.831921 |
| ENSMUSG00000086485 | processed_transcrip | Gm15458 | 0.32211 | 1.341732 | 0.831921 |
| ENSMUSG00000056770 | protein_coding | Setd3 | 3571.673 | 3389.309 | 3480.491 |
| ENSMUSG00000028538 | protein_coding | St3gal3 | 531.2012 | 497.7804 | 514.4908 |
| ENSMUSG00000039903 | protein_coding | 4931408A02Rik | 14.87275 | 19.54933 | 17.21104 |
| ENSMUSG00000091773 | lincRNA | Gm17308 | 3.546566 | 5.80984 | 4.678203 |
| ENSMUSG00000024327 | protein_coding | Slc39a7 | 77.30816 | 67.57197 | 72.44007 |
| ENSMUSG00000091577 | pseudogene | Gm6211 | 0.316221 | 1.341732 | 0.828977 |
| ENSMUSG00000088008 | snoRNA | SCARNA20 | 6.500647 | 9.553569 | 8.027108 |
| ENSMUSG00000025511 | protein_coding | Tspan4 | 946.8278 | 896.9928 | 921.9103 |
| ENSMUSG00000091587 | processed_transcrip | Gm17191 | 0.962752 | 2.897516 | 1.930134 |
| ENSMUSG00000091110 | protein_coding | Gm2832 | 29.45075 | 23.02816 | 26.23946 |
| ENSMUSG00000060572 | protein_coding | Mfap2 | 467.5069 | 503.2698 | 485.3884 |
| ENSMUSG00000000538 | protein_coding | Tom1l2 | 2320.343 | 2187.716 | 2254.03 |
| ENSMUSG00000089292 | snRNA | U6 | 1.288441 | 0 | 0.64422 |
| ENSMUSG00000026927 | protein_coding | Sdccag3 | 1439.953 | 1523.336 | 1481.645 |
| ENSMUSG00000036450 | protein_coding | Hif1an | 4057.855 | 4255.513 | 4156.684 |
| ENSMUSG00000074238 | protein_coding | Ap1ar | 2101.88 | 1997.416 | 2049.648 |
| ENSMUSG00000052428 | protein_coding | Tmco1 | 1446.43 | 1520.677 | 1483.554 |
| ENSMUSG00000050645 | protein_coding | Defb19 | 2091.94 | 1972.912 | 2032.426 |
| ENSMUSG00000077450 | protein_coding | Rab11b | 2282.163 | 2462.229 | 2372.196 |
| ENSMUSG00000039628 | protein_coding | Hs3st6 | 39.31861 | 30.91643 | 35.11752 |
| ENSMUSG00000001417 | protein_coding | Rhbg | 9.043461 | 13.43891 | 11.24119 |
| ENSMUSG00000021953 | protein_coding | Tdh | 65.53544 | 54.85092 | 60.19318 |
| ENSMUSG00000030284 | protein_coding | Creld1 | 429.7336 | 459.1015 | 444.4176 |
| ENSMUSG00000046145 | pseudogene | 1700018M17Rik | 6.847356 | 4.016911 | 5.432134 |
| ENSMUSG00000063077 | protein_coding | Kif1b | 4242.895 | 4042.59 | 4142.743 |
| ENSMUSG00000087339 | processed_transcrip | Gm11586 | 4.859607 | 7.620788 | 6.240197 |
| ENSMUSG00000082545 | pseudogene | Gm12196 | 0.33031 | 1.333085 | 0.831697 |
| ENSMUSG00000065678 | snRNA | U6 | 0.33031 | 1.333085 | 0.831697 |
| ENSMUSG00000079234 | protein_coding | Gm11067 | 0.33031 | 1.333085 | 0.831697 |
| ENSMUSG00000028436 | protein_coding | Dcaf12 | 2835.007 | 2995.691 | 2915.349 |
| ENSMUSG00000042705 | protein_coding | Commd10 | 1243.374 | 1311.086 | 1277.23 |
| ENSMUSG00000034826 | protein_coding | Nup54 | 1464.056 | 1534.223 | 1499.139 |
| ENSMUSG00000081490 | pseudogene | Gm11830 | 2.936413 | 1.386417 | 2.161415 |
| ENSMUSG00000036686 | protein_coding | Cc2d1a | 1404.485 | 1336.397 | 1370.441 |
| ENSMUSG00000021079 | protein_coding | Timm9 | 401.2651 | 434.3705 | 417.8178 |
| ENSMUSG00000051246 | protein_coding | A930005I04Rik | 35.40685 | 44.09832 | 39.75258 |
| ENSMUSG00000037331 | protein_coding | Larp1 | 5137.776 | 4853.978 | 4995.877 |
| ENSMUSG00000031349 | protein_coding | Nsdhl | 753.8152 | 810.5851 | 782.2002 |
| ENSMUSG00000051851 | protein_coding | Cxx1c | 207.6861 | 228.8677 | 218.2769 |
| ENSMUSG00000020419 | protein_coding | Hormad2 | 491.5451 | 373.0207 | 432.2829 |
| ENSMUSG00000039361 | protein_coding | Picalm | 5414.61 | 5191.571 | 5303.091 |
| ENSMUSG00000054758 | protein_coding | Gm9956 | 0.32211 | 1.333085 | 0.827597 |
| ENSMUSG00000091814 | protein_coding | Gm8050 | 0.32211 | 1.333085 | 0.827597 |
| ENSMUSG00000028212 | protein_coding | Ccne2 | 1979.331 | 1804.33 | 1891.83 |
| ENSMUSG00000090489 | protein_coding | Gm17415 | 4.81734 | 7.513762 | 6.165551 |
| ENSMUSG00000019518 | protein_coding | Ap4m1 | 534.4599 | 495.7085 | 515.0842 |
| ENSMUSG00000038975 | protein_coding | Rabggtb | 833.3345 | 900.4287 | 866.8816 |
| ENSMUSG00000026356 | protein_coding | Dars | 3498.214 | 3327.077 | 3412.646 |
| ENSMUSG00000072423 | protein_coding | Psmb11 | 1.917305 | 0.653029 | 1.285167 |
| ENSMUSG00000005982 | protein_coding | Nat15 | 919.0949 | 869.5423 | 894.3186 |
| ENSMUSG00000031807 | protein_coding | Pgls | 1050.543 | 985.466 | 1018.004 |

| | | | | | |
|---|---|---|---|---|---|
| ENSMUSG00000026765 | protein_coding | Lypd6b | 3.550145 | 5.863534 | 4.706839 |
| ENSMUSG00000077440 | snoRNA | SNORD101 | 45.21015 | 56.1825 | 50.69633 |
| ENSMUSG00000024661 | protein_coding | Fth1 | 8422.029 | 8773.596 | 8597.813 |
| ENSMUSG00000045876 | protein_coding | Pcdhb8 | 14.83533 | 19.73635 | 17.28584 |
| ENSMUSG00000002699 | protein_coding | Lcp2 | 106.4557 | 94.60083 | 100.5283 |
| ENSMUSG00000029910 | protein_coding | Mad2l1 | 1776.188 | 1667.639 | 1721.914 |
| ENSMUSG00000074909 | protein_coding | Ranbp6 | 1089.126 | 1148.84 | 1118.983 |
| ENSMUSG00000026222 | protein_coding | Sp100 | 52.76583 | 44.21114 | 48.48849 |
| ENSMUSG00000020131 | protein_coding | Pcsk4 | 96.41128 | 108.4964 | 102.4538 |
| ENSMUSG00000026125 | protein_coding | Prss39 | 0 | 1.033237 | 0.516618 |
| ENSMUSG00000089518 | snRNA | U7 | 0 | 1.033237 | 0.516618 |
| ENSMUSG00000065826 | snRNA | U6 | 0 | 1.033237 | 0.516618 |
| ENSMUSG00000090604 | pseudogene | Gm17378 | 0 | 1.033237 | 0.516618 |
| ENSMUSG00000081656 | pseudogene | Gm11246 | 0 | 1.033237 | 0.516618 |
| ENSMUSG00000092199 | pseudogene | Gm18358 | 0 | 1.033237 | 0.516618 |
| ENSMUSG00000074108 | pseudogene | Rpl10-ps2 | 0 | 1.033237 | 0.516618 |
| ENSMUSG00000065100 | snoRNA | SNORA57 | 0 | 1.033237 | 0.516618 |
| ENSMUSG00000088600 | rRNA | 5S_rRNA | 0 | 1.033237 | 0.516618 |
| ENSMUSG00000087622 | processed_transcrip | Gm12290 | 0 | 1.033237 | 0.516618 |
| ENSMUSG00000051748 | protein_coding | 1100001G20Rik | 0 | 1.033237 | 0.516618 |
| ENSMUSG00000091913 | protein_coding | Gm17558 | 0 | 1.033237 | 0.516618 |
| ENSMUSG00000077227 | snoRNA | SNORA17 | 0 | 1.033237 | 0.516618 |
| ENSMUSG00000068457 | protein_coding | Uty | 1092.826 | 1009.53 | 1051.178 |
| ENSMUSG00000006711 | protein_coding | D130043K22Rik | 20.94573 | 16.36962 | 18.65768 |
| ENSMUSG00000084330 | pseudogene | Gm15019 | 19.08121 | 14.68335 | 16.88228 |
| ENSMUSG00000027286 | protein_coding | Lrrc57 | 402.8747 | 430.101 | 416.4879 |
| ENSMUSG00000033760 | protein_coding | Rbm4b | 880.5958 | 831.8585 | 856.2271 |
| ENSMUSG00000092772 | miRNA | AL596130.1 | 0.64422 | 2.030436 | 1.337328 |
| ENSMUSG00000090643 | protein_coding | Gm3453 | 0.64422 | 2.030436 | 1.337328 |
| ENSMUSG00000020627 | protein_coding | Klhl29 | 381.8909 | 352.0344 | 366.9626 |
| ENSMUSG00000086063 | processed_transcrip | Gm12257 | 2.905924 | 1.377408 | 2.141666 |
| ENSMUSG00000036087 | protein_coding | Slain2 | 2883.299 | 3023.971 | 2953.635 |
| ENSMUSG00000037708 | protein_coding | BC061194 | 10.06488 | 14.22636 | 12.14562 |
| ENSMUSG00000018387 | protein_coding | Shroom1 | 25.85223 | 20.23518 | 23.04371 |
| ENSMUSG00000012076 | protein_coding | Brms1l | 1537.941 | 1615.798 | 1576.87 |
| ENSMUSG00000045991 | protein_coding | Onecut2 | 75.33368 | 88.00138 | 81.66753 |
| ENSMUSG00000053552 | protein_coding | Ebf4 | 158.835 | 179.0066 | 168.9208 |
| ENSMUSG00000068407 | protein_coding | Rnase12 | 1.290752 | 2.728149 | 2.00945 |
| ENSMUSG00000039450 | protein_coding | Dcxr | 71.64517 | 81.71512 | 76.68015 |
| ENSMUSG00000060371 | protein_coding | Caln1 | 4.206143 | 7.044547 | 5.625345 |
| ENSMUSG00000058070 | protein_coding | Eml1 | 618.0475 | 577.9315 | 597.9895 |
| ENSMUSG00000079426 | protein_coding | Arpc4 | 1619.05 | 1709.716 | 1664.383 |
| ENSMUSG00000060128 | pseudogene | Gm10075 | 6.46658 | 4.052225 | 5.259402 |
| ENSMUSG00000086383 | lincRNA | Gm16630 | 16.56784 | 12.24604 | 14.40694 |
| ENSMUSG00000089926 | lincRNA | Gm15734 | 1.937283 | 0.724379 | 1.330831 |
| ENSMUSG00000091728 | lincRNA | Gm8705 | 2.914124 | 1.333085 | 2.123604 |
| ENSMUSG00000086567 | pseudogene | Gm16247 | 22.94526 | 18.07426 | 20.50976 |
| ENSMUSG00000056370 | protein_coding | Sftpb | 1.945483 | 3.743367 | 2.844425 |
| ENSMUSG00000084845 | protein_coding | Gm5151 | 13.8608 | 18.09192 | 15.97636 |
| ENSMUSG00000050188 | protein_coding | Lsm10 | 385.4769 | 356.801 | 371.139 |
| ENSMUSG00000037686 | protein_coding | Aspg | 42.3406 | 34.02655 | 38.18358 |
| ENSMUSG00000071644 | protein_coding | Eef1g | 8903.692 | 8379.386 | 8641.539 |
| ENSMUSG00000085968 | processed_transcrip | Gm13027 | 0.638331 | 1.72194 | 1.180136 |
| ENSMUSG00000043319 | protein_coding | Cox8c | 57.07418 | 47.94586 | 52.51002 |
| ENSMUSG00000078380 | protein_coding | Gm6096 | 9.692303 | 13.51099 | 11.60165 |
| ENSMUSG00000042032 | protein_coding | Mat2b | 2751.298 | 2924.189 | 2837.743 |
| ENSMUSG00000034959 | protein_coding | 5031414D18Rik | 233.4881 | 168.1436 | 200.8159 |
| ENSMUSG00000032134 | protein_coding | Muc16 | 0.968641 | 2.321637 | 1.645139 |

| | | | | | |
|---|---|---|---|---|---|
| ENSMUSG00000022321 | protein_coding | Cdh10 | 56.41311 | 65.89295 | 61.15303 |
| ENSMUSG00000020679 | protein_coding | Hnf1b | 30.02557 | 24.43875 | 27.23216 |
| ENSMUSG00000057969 | protein_coding | Sema3b | 57.31891 | 68.637 | 62.97795 |
| ENSMUSG00000086681 | processed_transcrip | Gm16178 | 15.00082 | 20.45788 | 17.72935 |
| ENSMUSG00000088273 | snoRNA | SNORA48 | 3.908633 | 6.47224 | 5.190436 |
| ENSMUSG00000045639 | protein_coding | Zfp629 | 990.1656 | 1069.497 | 1029.831 |
| ENSMUSG00000089375 | snoRNA | SNORD107 | 2.920013 | 1.341732 | 2.130873 |
| ENSMUSG00000054455 | protein_coding | Vapb | 4720.888 | 4944.406 | 4832.647 |
| ENSMUSG00000000056 | protein_coding | Narf | 1100.261 | 1186.807 | 1143.534 |
| ENSMUSG00000090528 | pseudogene | Gm17381 | 0.638331 | 1.748606 | 1.193469 |
| ENSMUSG00000032584 | protein_coding | Mst1r | 95.29966 | 109.2212 | 102.2604 |
| ENSMUSG00000047508 | protein_coding | Mbd3l2 | 7.446999 | 10.54212 | 8.99456 |
| ENSMUSG00000048970 | protein_coding | C1galt1c1 | 171.6049 | 195.5656 | 183.5852 |
| ENSMUSG00000042066 | protein_coding | Tmcc2 | 299.8293 | 268.0825 | 283.9559 |
| ENSMUSG00000047084 | protein_coding | Ngrn | 742.0683 | 790.3462 | 766.2072 |
| ENSMUSG00000024535 | protein_coding | Snx24 | 175.9578 | 192.3598 | 184.1588 |
| ENSMUSG00000030726 | protein_coding | Pold3 | 1878.254 | 1778.956 | 1828.605 |
| ENSMUSG00000039164 | protein_coding | Naif1 | 255.3841 | 233.9237 | 244.6539 |
| ENSMUSG00000061751 | protein_coding | Kalrn | 383.5102 | 340.9965 | 362.2533 |
| ENSMUSG00000036977 | protein_coding | Anapc10 | 715.7975 | 675.0905 | 695.444 |
| ENSMUSG00000065439 | miRNA | Mir140 | 1.967772 | 0.662038 | 1.314905 |
| ENSMUSG00000085837 | processed_transcrip | 4933400F03Rik | 29.72113 | 23.59906 | 26.6601 |
| ENSMUSG00000026383 | protein_coding | Epb4.1l5 | 2457.774 | 2295.387 | 2376.58 |
| ENSMUSG00000026134 | protein_coding | Prim2 | 1365.646 | 1289.363 | 1327.504 |
| ENSMUSG00000082073 | pseudogene | Gm13114 | 0.33031 | 1.315066 | 0.822688 |
| ENSMUSG00000086424 | processed_transcrip | Gm15569 | 1.959572 | 3.752738 | 2.856155 |
| ENSMUSG00000003555 | protein_coding | Cyp17a1 | 2624.95 | 3458.652 | 3041.801 |
| ENSMUSG00000029402 | protein_coding | Snrnp35 | 378.7782 | 408.7689 | 393.7735 |
| ENSMUSG00000091931 | protein_coding | Moap1 | 95.40581 | 107.6971 | 101.5514 |
| ENSMUSG00000036931 | protein_coding | Nfkbid | 132.7467 | 148.397 | 140.5719 |
| ENSMUSG00000026435 | protein_coding | Slc45a3 | 277.9031 | 307.157 | 292.5301 |
| ENSMUSG00000051735 | protein_coding | Rinl | 101.3902 | 89.17483 | 95.28253 |
| ENSMUSG00000037490 | protein_coding | Slc2a12 | 465.1875 | 431.8058 | 448.4967 |
| ENSMUSG00000031750 | protein_coding | Il34 | 11.9763 | 16.27481 | 14.12555 |
| ENSMUSG00000041926 | protein_coding | Rnpep | 308.3215 | 272.2029 | 290.2622 |
| ENSMUSG00000060988 | protein_coding | Galnt13 | 22.44819 | 29.05609 | 25.75214 |
| ENSMUSG00000091329 | processed_transcrip | 1700112D23Rik | 29.7186 | 36.08474 | 32.90167 |
| ENSMUSG00000063514 | protein_coding | Gm6756 | 2.559214 | 4.838945 | 3.69908 |
| ENSMUSG00000044303 | protein_coding | Cdkn2a | 830.5145 | 775.0363 | 802.7754 |
| ENSMUSG00000080888 | pseudogene | Gm14387 | 13.13205 | 18.00327 | 15.56766 |
| ENSMUSG00000082536 | pseudogene | Gm13456 | 151.6535 | 135.9977 | 143.8256 |
| ENSMUSG00000088054 | miRNA | Mir1968 | 2.267593 | 1.024227 | 1.64591 |
| ENSMUSG00000029862 | protein_coding | Clcn1 | 67.1975 | 55.69169 | 61.4446 |
| ENSMUSG00000091243 | protein_coding | Vgll3 | 411.9727 | 442.4018 | 427.1872 |
| ENSMUSG00000043542 | protein_coding | Fam164a | 668.5984 | 624.2499 | 646.4242 |
| ENSMUSG00000090561 | processed_transcrip | Gm17207 | 0.32211 | 1.315066 | 0.818588 |
| ENSMUSG00000039911 | protein_coding | Spsb1 | 448.0075 | 499.6417 | 473.8246 |
| ENSMUSG00000047759 | protein_coding | Hs3st3a1 | 132.7009 | 148.9927 | 140.8468 |
| ENSMUSG00000080877 | pseudogene | Rpl22-ps1 | 10.69143 | 7.514124 | 9.102779 |
| ENSMUSG00000040732 | protein_coding | Erg | 561.9397 | 622.1024 | 592.0211 |
| ENSMUSG00000087290 | processed_transcrip | Gm15866 | 3.584212 | 1.712931 | 2.648571 |
| ENSMUSG00000059101 | protein_coding | Olfr157 | 0.316221 | 1.315066 | 0.815644 |
| ENSMUSG00000074092 | pseudogene | Gm6181 | 0.316221 | 1.315066 | 0.815644 |
| ENSMUSG00000091156 | protein_coding | Gm3500 | 0.316221 | 1.315066 | 0.815644 |
| ENSMUSG00000041872 | protein_coding | Il17f | 8.541174 | 4.892639 | 6.716906 |
| ENSMUSG00000021986 | protein_coding | Fam123a | 3.250323 | 5.402966 | 4.326645 |
| ENSMUSG00000068798 | protein_coding | Rap1a | 1872.491 | 1995.797 | 1934.144 |
| ENSMUSG00000034621 | protein_coding | Gpatch8 | 3843.902 | 4028.04 | 3935.971 |

| | | | | | |
|---|---|---|---|---|---|
| ENSMUSG00000092889 | miRNA | AL669944.1 | 19.76054 | 14.75543 | 17.25798 |
| ENSMUSG00000089874 | processed_transcrip | 9230117E06Rik | 2.589703 | 1.086569 | 1.838136 |
| ENSMUSG00000078619 | protein_coding | Smarcd2 | 2129.077 | 2020.205 | 2074.641 |
| ENSMUSG00000026578 | protein_coding | 4930455F23Rik | 688.0778 | 622.7756 | 655.4267 |
| ENSMUSG00000050714 | protein_coding | Zbtb26 | 727.2497 | 782.247 | 754.7483 |
| ENSMUSG00000091277 | protein_coding | Gm1818 | 124.8059 | 112.2668 | 118.5363 |
| ENSMUSG00000020865 | protein_coding | Abcc3 | 67.46281 | 76.69273 | 72.07777 |
| ENSMUSG00000040661 | protein_coding | Rad54l2 | 2825.202 | 3013.687 | 2919.444 |
| ENSMUSG00000078853 | protein_coding | Igtp | 172.5793 | 150.9038 | 161.7416 |
| ENSMUSG00000091941 | pseudogene | Gm7399 | 2.895414 | 1.386417 | 2.140915 |
| ENSMUSG00000045328 | protein_coding | Cenpe | 3230.279 | 3070.555 | 3150.417 |
| ENSMUSG00000014980 | protein_coding | Tsen15 | 496.7593 | 531.817 | 514.2882 |
| ENSMUSG00000028530 | protein_coding | Jak1 | 8415.521 | 8833.712 | 8624.617 |
| ENSMUSG00000033282 | protein_coding | Rpgrip1l | 1137.79 | 1050.304 | 1094.047 |
| ENSMUSG00000022768 | protein_coding | Ccdc116 | 28.12005 | 22.40759 | 25.26382 |
| ENSMUSG00000072744 | protein_coding | Gm8165 | 2.259393 | 1.024227 | 1.64181 |
| ENSMUSG00000045555 | protein_coding | 9030224M15Rik | 27.02565 | 33.28016 | 30.15291 |
| ENSMUSG00000078151 | pseudogene | Gm12226 | 3.572434 | 5.792184 | 4.682309 |
| ENSMUSG00000023027 | protein_coding | Atf1 | 2377.373 | 2251.794 | 2314.584 |
| ENSMUSG00000014504 | protein_coding | Srp19 | 1161.678 | 1099.487 | 1130.582 |
| ENSMUSG00000071773 | protein_coding | Rhox1 | 121.676 | 134.7194 | 128.1977 |
| ENSMUSG00000064669 | snRNA | U6 | 23.99151 | 31.63315 | 27.81233 |
| ENSMUSG00000053161 | protein_coding | Wdr69 | 5.204006 | 2.535327 | 3.869666 |
| ENSMUSG00000022032 | protein_coding | Scara5 | 6044.93 | 6505.67 | 6275.3 |
| ENSMUSG00000029249 | protein_coding | Rest | 4749.701 | 5072.599 | 4911.15 |
| ENSMUSG00000072620 | protein_coding | Slfn2 | 50.13871 | 42.45316 | 46.29594 |
| ENSMUSG00000089820 | processed_transcrip | Gm15775 | 7.444689 | 10.6668 | 9.055746 |
| ENSMUSG00000030660 | protein_coding | Pik3c2a | 5531.042 | 5245.009 | 5388.026 |
| ENSMUSG00000087385 | processed_transcrip | AA415398 | 31.4101 | 25.49866 | 28.45438 |
| ENSMUSG00000029447 | protein_coding | Cct6a | 4939.183 | 5183.047 | 5061.115 |
| ENSMUSG00000088056 | snoRNA | SNORA48 | 0.646531 | 1.712931 | 1.179731 |
| ENSMUSG00000082617 | pseudogene | Smt3h2-ps | 0.646531 | 1.712931 | 1.179731 |
| ENSMUSG00000071753 | lincRNA | C230004F18Rik | 0.646531 | 1.712931 | 1.179731 |
| ENSMUSG00000067704 | protein_coding | Wfdc13 | 3.204478 | 1.341732 | 2.273105 |
| ENSMUSG00000007805 | protein_coding | Twist2 | 78.76686 | 91.40746 | 85.08716 |
| ENSMUSG00000084190 | pseudogene | Gm11869 | 0 | 1.015218 | 0.507609 |
| ENSMUSG00000086071 | processed_transcrip | Gm12354 | 0 | 1.015218 | 0.507609 |
| ENSMUSG00000089929 | protein_coding | Bcl2a1b | 0 | 1.015218 | 0.507609 |
| ENSMUSG00000055872 | pseudogene | Gm9985 | 0 | 1.015218 | 0.507609 |
| ENSMUSG00000072742 | protein_coding | Gm3483 | 0 | 1.015218 | 0.507609 |
| ENSMUSG00000076121 | miRNA | Mir687 | 0 | 1.015218 | 0.507609 |
| ENSMUSG00000088130 | snRNA | U7 | 0 | 1.015218 | 0.507609 |
| ENSMUSG00000039963 | protein_coding | Ccdc40 | 179.8966 | 159.9896 | 169.9431 |
| ENSMUSG00000000157 | protein_coding | Itgb2l | 1.945483 | 0.724379 | 1.334931 |
| ENSMUSG00000030057 | protein_coding | Cnbp | 9910.319 | 10381.14 | 10145.73 |
| ENSMUSG00000089657 | pseudogene | Gm16585 | 2.956391 | 1.086569 | 2.02148 |
| ENSMUSG00000073838 | protein_coding | Tufm | 1890.917 | 1999.207 | 1945.062 |
| ENSMUSG00000036291 | protein_coding | Mudeng | 894.2791 | 846.7892 | 870.5342 |
| ENSMUSG00000065306 | snRNA | U6 | 17.74357 | 13.63282 | 15.68819 |
| ENSMUSG00000031513 | protein_coding | Leprotl1 | 1103.895 | 1169.082 | 1136.488 |
| ENSMUSG00000065406 | miRNA | Mirlet7i | 9.424238 | 13.53765 | 11.48095 |
| ENSMUSG00000021301 | protein_coding | Hecw1 | 6.136269 | 3.743367 | 4.939818 |
| ENSMUSG00000031375 | protein_coding | Bgn | 3162.632 | 3391.011 | 3276.822 |
| ENSMUSG00000078917 | pseudogene | Gm11281 | 2.885946 | 1.315066 | 2.100506 |
| ENSMUSG00000068220 | protein_coding | Lgals1 | 2790.338 | 3019.763 | 2905.051 |
| ENSMUSG00000064493 | snoRNA | Snora28 | 48.04965 | 58.06702 | 53.05834 |
| ENSMUSG00000054302 | protein_coding | Eapp | 1001.294 | 1062.399 | 1031.846 |
| ENSMUSG00000029352 | protein_coding | Crybb3 | 15.87673 | 20.62689 | 18.25181 |

| | | | | | |
|---|---|---|---|---|---|
| ENSMUSG00000027823 | protein_coding | Gmps | 3750.286 | 3601.703 | 3675.994 |
| ENSMUSG00000071392 | protein_coding | Ect2l | 6.791 | 4.176907 | 5.483954 |
| ENSMUSG00000091478 | pseudogene | Gm10039 | 478.1648 | 510.7461 | 494.4554 |
| ENSMUSG00000036893 | protein_coding | Ehmt1 | 3115.363 | 2964.437 | 3039.9 |
| ENSMUSG00000047115 | protein_coding | D330028D13Rik | 330.2685 | 305.0349 | 317.6517 |
| ENSMUSG00000078872 | protein_coding | Gm14401 | 10.31423 | 14.02132 | 12.16778 |
| ENSMUSG00000024034 | protein_coding | Tmprss3 | 0.99093 | 2.71914 | 1.855035 |
| ENSMUSG00000039680 | protein_coding | Mrps6 | 897.1161 | 952.6182 | 924.8672 |
| ENSMUSG00000040723 | protein_coding | Rcsd1 | 410.737 | 458.5722 | 434.6546 |
| ENSMUSG00000038168 | protein_coding | Leprel1 | 56.77831 | 47.81041 | 52.29436 |
| ENSMUSG00000025184 | protein_coding | D19Ertd386e | 824.6391 | 874.5175 | 849.5783 |
| ENSMUSG00000020577 | protein_coding | Tspan13 | 241.8631 | 261.3784 | 251.6208 |
| ENSMUSG00000091012 | protein_coding | Vmn1r34 | 3.550145 | 1.775272 | 2.662709 |
| ENSMUSG00000022193 | protein_coding | Psmb5 | 1907.135 | 2011.289 | 1959.212 |
| ENSMUSG00000024168 | protein_coding | Tmem204 | 489.5407 | 533.0868 | 511.3137 |
| ENSMUSG00000035200 | protein_coding | Chrnb4 | 7.739888 | 11.86941 | 9.804648 |
| ENSMUSG00000028415 | protein_coding | Spink4 | 19.37999 | 24.75118 | 22.06558 |
| ENSMUSG00000030048 | protein_coding | Gkn3 | 5.515606 | 8.574388 | 7.044997 |
| ENSMUSG00000092918 | miRNA | AL928590.1 | 1.290752 | 2.683826 | 1.987289 |
| ENSMUSG00000085723 | processed_transcrip | Gm15915 | 10.67377 | 14.62174 | 12.64775 |
| ENSMUSG00000044147 | protein_coding | Arf6 | 2894.178 | 3036.646 | 2965.412 |
| ENSMUSG00000023826 | protein_coding | Park2 | 47.92852 | 58.00251 | 52.96551 |
| ENSMUSG00000027937 | protein_coding | Jtb | 952.1061 | 1022.793 | 987.4496 |
| ENSMUSG00000024498 | protein_coding | Tcerg1 | 2844.955 | 2713.351 | 2779.153 |
| ENSMUSG00000048222 | protein_coding | Mfap1b | 390.0698 | 356.4647 | 373.2672 |
| ENSMUSG00000087511 | lincRNA | A330009N23Rik | 37.36344 | 30.85766 | 34.11055 |
| ENSMUSG00000035274 | protein_coding | Tpbg | 214.744 | 196.7667 | 205.7554 |
| ENSMUSG00000038518 | protein_coding | Jarid2 | 4312.468 | 4028.234 | 4170.351 |
| ENSMUSG00000085941 | processed_transcrip | Gm11201 | 2.247615 | 4.114928 | 3.181271 |
| ENSMUSG00000092454 | pseudogene | Gm2991 | 28.9888 | 23.43291 | 26.21085 |
| ENSMUSG00000019789 | protein_coding | Hey2 | 57.74106 | 49.75324 | 53.74715 |
| ENSMUSG00000030509 | protein_coding | Asb7 | 1085.017 | 1146.448 | 1115.732 |
| ENSMUSG00000038593 | protein_coding | Tctn1 | 327.5196 | 300.8644 | 314.192 |
| ENSMUSG00000026109 | protein_coding | Tmeff2 | 101.1304 | 89.19787 | 95.16416 |
| ENSMUSG00000002546 | protein_coding | Golga2 | 2277.363 | 2416.371 | 2346.867 |
| ENSMUSG00000056366 | pseudogene | Fabp3-ps1 | 15.29937 | 10.89494 | 13.09716 |
| ENSMUSG00000001910 | protein_coding | Nacc1 | 4419.077 | 4645.76 | 4532.418 |
| ENSMUSG00000030970 | protein_coding | Ctbp2 | 699.1479 | 759.9336 | 729.5407 |
| ENSMUSG00000084184 | pseudogene | Gm13474 | 0.638331 | 1.686265 | 1.162298 |
| ENSMUSG00000064925 | snoRNA | Snora62 | 1.307151 | 3.08133 | 2.19424 |
| ENSMUSG00000020709 | protein_coding | Adap2 | 79.39349 | 69.95559 | 74.67454 |
| ENSMUSG00000037197 | protein_coding | Rbm17 | 2731.41 | 2853.879 | 2792.644 |
| ENSMUSG00000004356 | protein_coding | Utp20 | 4967.125 | 4677.666 | 4822.396 |
| ENSMUSG00000082593 | pseudogene | Gm11331 | 12.87934 | 18.21044 | 15.54489 |
| ENSMUSG00000082429 | pseudogene | Gm13171 | 0.962752 | 2.455329 | 1.709041 |
| ENSMUSG00000063558 | protein_coding | Aox1 | 331.0252 | 363.608 | 347.3166 |
| ENSMUSG00000021490 | protein_coding | Slc34a1 | 1.931394 | 0.724379 | 1.327886 |
| ENSMUSG00000083465 | pseudogene | Gm11652 | 14.51091 | 10.66717 | 12.58904 |
| ENSMUSG00000081896 | pseudogene | Gm5389 | 3.848698 | 2.08413 | 2.966414 |
| ENSMUSG00000020115 | protein_coding | Tbk1 | 1349.439 | 1270.403 | 1309.921 |
| ENSMUSG00000057530 | protein_coding | Ece1 | 2820.885 | 2979.743 | 2900.314 |
| ENSMUSG00000083284 | pseudogene | Gm13688 | 10.64917 | 7.549799 | 9.099483 |
| ENSMUSG00000033439 | protein_coding | Ccdc76 | 261.915 | 284.0379 | 272.9764 |
| ENSMUSG00000040472 | protein_coding | Rabggta | 798.7441 | 742.4606 | 770.6023 |
| ENSMUSG00000021282 | protein_coding | Eif5 | 8577.251 | 8113.883 | 8345.567 |
| ENSMUSG00000046811 | protein_coding | Gltpd2 | 10.29784 | 7.10725 | 8.702543 |
| ENSMUSG00000090878 | lincRNA | Gm17640 | 8.381574 | 5.349996 | 6.865785 |
| ENSMUSG00000040298 | protein_coding | Btbd16 | 14.90913 | 10.77062 | 12.83987 |

| | | | | | |
|---|---|---|---|---|---|
| ENSMUSG00000064061 | protein_coding | Dzip3 | 2580.276 | 2440.261 | 2510.269 |
| ENSMUSG00000039158 | protein_coding | Akna | 247.0328 | 270.1276 | 258.5802 |
| ENSMUSG00000066002 | pseudogene | Gm13237 | 488.2568 | 207.375 | 347.8159 |
| ENSMUSG00000002032 | protein_coding | Tmem25 | 142.3579 | 126.7891 | 134.5735 |
| ENSMUSG00000075700 | protein_coding | 2810407C02Rik | 5984.601 | 6323.922 | 6154.262 |
| ENSMUSG00000058656 | protein_coding | Samd12 | 24.55119 | 18.70819 | 21.62969 |
| ENSMUSG00000054763 | protein_coding | Defb42 | 85.46095 | 104.6245 | 95.04272 |
| ENSMUSG00000038569 | protein_coding | Rad9b | 575.9758 | 521.1745 | 548.5751 |
| ENSMUSG00000020891 | protein_coding | Alox8 | 10.62919 | 7.478087 | 9.053638 |
| ENSMUSG00000038612 | protein_coding | Mcl1 | 4408.037 | 4598.137 | 4503.087 |
| ENSMUSG00000043623 | pseudogene | 4933412A08Rik | 7.527955 | 4.212944 | 5.87045 |
| ENSMUSG00000028270 | protein_coding | Gbp2 | 148.674 | 132.6627 | 140.6683 |
| ENSMUSG00000084306 | pseudogene | Gm11743 | 3.564234 | 1.775272 | 2.669753 |
| ENSMUSG00000024068 | protein_coding | Spast | 2279.461 | 2168.099 | 2223.78 |
| ENSMUSG00000022179 | protein_coding | 4931414P19Rik | 304.9554 | 274.5458 | 289.7506 |
| ENSMUSG00000067049 | protein_coding | Unc93a | 3.846388 | 6.383232 | 5.11481 |
| ENSMUSG00000034412 | protein_coding | Tbc1d10a | 1262.828 | 1338.202 | 1300.515 |
| ENSMUSG00000022216 | protein_coding | Psme1 | 1660.662 | 1740.42 | 1700.541 |
| ENSMUSG00000080731 | pseudogene | Rps2-ps9 | 2.253504 | 4.212583 | 3.233043 |
| ENSMUSG00000022512 | protein_coding | Cldn1 | 538.7274 | 597.7319 | 568.2296 |
| ENSMUSG00000020752 | protein_coding | Recql5 | 811.4341 | 754.6861 | 783.0601 |
| ENSMUSG00000055150 | protein_coding | Zfp78 | 81.60934 | 72.03723 | 76.82329 |
| ENSMUSG00000004561 | protein_coding | Mettl17 | 409.9839 | 448.6831 | 429.3335 |
| ENSMUSG00000050967 | protein_coding | Creg2 | 21.71571 | 28.33275 | 25.02423 |
| ENSMUSG00000090622 | protein_coding | A930033H14Rik | 207.9812 | 227.9839 | 217.9825 |
| ENSMUSG00000028255 | protein_coding | Clca3 | 33.60032 | 26.21144 | 29.90588 |
| ENSMUSG00000036858 | IG_C_gene | Ptcra | 4.194365 | 7.065776 | 5.630071 |
| ENSMUSG00000006386 | protein_coding | Tek | 171.8429 | 191.3745 | 181.6087 |
| ENSMUSG00000040488 | protein_coding | Ltbp4 | 5554.771 | 5193.744 | 5374.258 |
| ENSMUSG00000041444 | protein_coding | Arhgap32 | 3707.526 | 3498.577 | 3603.051 |
| ENSMUSG00000028426 | protein_coding | Rad23b | 5816.298 | 6093.587 | 5954.942 |
| ENSMUSG00000000167 | protein_coding | Pih1d2 | 103.7403 | 89.67822 | 96.70928 |
| ENSMUSG00000041881 | protein_coding | Ndufa7 | 1384.589 | 1474.709 | 1429.649 |
| ENSMUSG00000035451 | protein_coding | Foxa1 | 19.37283 | 24.84454 | 22.10869 |
| ENSMUSG00000040649 | protein_coding | Rimklb | 1683.025 | 1499.388 | 1591.207 |
| ENSMUSG00000091747 | protein_coding | D17H6S56E-5 | 712.8977 | 792.8058 | 752.8518 |
| ENSMUSG00000021024 | protein_coding | Psma6 | 3045.686 | 3192.692 | 3119.189 |
| ENSMUSG00000032777 | protein_coding | Gtf3c1 | 5078.244 | 4829.455 | 4953.85 |
| ENSMUSG00000040007 | protein_coding | Bahd1 | 2045.287 | 1895.376 | 1970.331 |
| ENSMUSG00000008036 | protein_coding | Ap2s1 | 1304.589 | 1228.483 | 1266.536 |
| ENSMUSG00000036894 | protein_coding | Rap2b | 770.8152 | 818.1541 | 794.4846 |
| ENSMUSG00000034450 | protein_coding | Gulo | 40.98969 | 48.33472 | 44.66221 |
| ENSMUSG00000037295 | protein_coding | Ldlrap1 | 690.272 | 644.2799 | 667.276 |
| ENSMUSG00000048388 | protein_coding | Fam171b | 328.025 | 369.1901 | 348.6075 |
| ENSMUSG00000057130 | protein_coding | Txnl4a | 582.4594 | 547.1372 | 564.7983 |
| ENSMUSG00000028382 | protein_coding | Rod1 | 4959.073 | 5186.346 | 5072.71 |
| ENSMUSG00000034892 | protein_coding | Rps29 | 1411.793 | 1313.215 | 1362.504 |
| ENSMUSG00000048521 | protein_coding | Cxcr6 | 15.53464 | 20.42505 | 17.97985 |
| ENSMUSG00000080904 | pseudogene | Gm11966 | 0.646531 | 1.686265 | 1.166398 |
| ENSMUSG00000023828 | protein_coding | Slc22a3 | 3.246745 | 5.738851 | 4.492798 |
| ENSMUSG00000078362 | protein_coding | Gm10930 | 3.919143 | 2.030798 | 2.974971 |
| ENSMUSG00000067017 | pseudogene | Gm3608 | 205.0486 | 224.4327 | 214.7406 |
| ENSMUSG00000045038 | protein_coding | Prkce | 773.7729 | 823.3164 | 798.5447 |
| ENSMUSG00000071456 | protein_coding | 1110002L01Rik | 130.7157 | 145.9031 | 138.3094 |
| ENSMUSG00000027530 | protein_coding | Fabp12 | 5.199384 | 8.212199 | 6.705792 |
| ENSMUSG00000066170 | protein_coding | E230001N04Rik | 46.36232 | 54.52119 | 50.44176 |
| ENSMUSG00000032733 | protein_coding | Snx33 | 2347.654 | 2519.654 | 2433.654 |
| ENSMUSG00000029866 | protein_coding | Kel | 2.275793 | 1.024227 | 1.65001 |

| | | | | | |
|---|---|---|---|---|---|
| ENSMUSG00000023151 | protein_coding | Lrrc69 | 0.966331 | 2.446319 | 1.706325 |
| ENSMUSG00000044792 | protein_coding | Isca1 | 1729.87 | 1827.693 | 1778.782 |
| ENSMUSG00000036864 | protein_coding | BC053749 | 268.6509 | 289.3504 | 279.0007 |
| ENSMUSG00000024640 | protein_coding | Psat1 | 3979.367 | 4205.596 | 4092.481 |
| ENSMUSG00000052727 | protein_coding | Mtap1b | 5804.205 | 6161.768 | 5982.986 |
| ENSMUSG00000044617 | protein_coding | Zbtb39 | 1241.297 | 1312.269 | 1276.783 |
| ENSMUSG00000060438 | pseudogene | Rps10-ps1 | 133.7386 | 120.4675 | 127.103 |
| ENSMUSG00000042487 | protein_coding | Leo1 | 1253.409 | 1332.153 | 1292.781 |
| ENSMUSG00000088869 | snoRNA | snoU13 | 1.909105 | 0.688704 | 1.298904 |
| ENSMUSG00000079889 | pseudogene | Gm11951 | 5.159428 | 7.964959 | 6.562194 |
| ENSMUSG00000079627 | protein_coding | Rhox2h | 17.51188 | 13.19027 | 15.35108 |
| ENSMUSG00000037636 | protein_coding | Slc25a43 | 24.53919 | 30.24932 | 27.39425 |
| ENSMUSG00000019297 | protein_coding | 2610027L16Rik | 1606.408 | 1503.866 | 1555.137 |
| ENSMUSG00000070870 | protein_coding | Cryge | 28.22433 | 22.36684 | 25.29559 |
| ENSMUSG00000010064 | protein_coding | Slc38a3 | 24.6355 | 31.22162 | 27.92856 |
| ENSMUSG00000035851 | protein_coding | Ythdc1 | 3586.878 | 3738.976 | 3662.927 |
| ENSMUSG00000022002 | protein_coding | 4930564B18Rik | 5.212206 | 2.675179 | 3.943692 |
| ENSMUSG00000031490 | protein_coding | Eif4ebp1 | 1073.877 | 1147.262 | 1110.569 |
| ENSMUSG00000056307 | protein_coding | Gm9994 | 0 | 0.997561 | 0.498781 |
| ENSMUSG00000087062 | processed_transcrip | Gm13587 | 0 | 0.997561 | 0.498781 |
| ENSMUSG00000093306 | miRNA | AL731857.1 | 0 | 0.997561 | 0.498781 |
| ENSMUSG00000082310 | pseudogene | Gm12904 | 0 | 0.997561 | 0.498781 |
| ENSMUSG00000084947 | processed_transcrip | Gm15594 | 0 | 0.997561 | 0.498781 |
| ENSMUSG00000087705 | snoRNA | SNORA7 | 0 | 0.997561 | 0.498781 |
| ENSMUSG00000066771 | protein_coding | Gm5614 | 0 | 0.997561 | 0.498781 |
| ENSMUSG00000091661 | processed_transcrip | Gm17021 | 0 | 0.997561 | 0.498781 |
| ENSMUSG00000087398 | processed_transcrip | Gm12343 | 0 | 0.997561 | 0.498781 |
| ENSMUSG00000068392 | protein_coding | Rnase13 | 0 | 0.997561 | 0.498781 |
| ENSMUSG00000086146 | processed_transcrip | Gm15729 | 0 | 0.997561 | 0.498781 |
| ENSMUSG00000000204 | protein_coding | Slfn4 | 26.84927 | 21.5358 | 24.19254 |
| ENSMUSG00000060284 | protein_coding | Sp7 | 19.43738 | 15.05455 | 17.24597 |
| ENSMUSG00000006442 | protein_coding | Srm | 690.1208 | 646.6117 | 668.3662 |
| ENSMUSG00000022639 | protein_coding | 5330426P16Rik | 175.487 | 194.426 | 184.9565 |
| ENSMUSG00000045657 | protein_coding | Pcdhb10 | 12.21514 | 16.53935 | 14.37724 |
| ENSMUSG00000027189 | protein_coding | Trim44 | 5307.705 | 5544.33 | 5426.018 |
| ENSMUSG00000039795 | protein_coding | Zfand1 | 646.2728 | 698.5609 | 672.4169 |
| ENSMUSG00000000120 | protein_coding | Ngfr | 114.3598 | 100.6605 | 107.5101 |
| ENSMUSG00000084033 | pseudogene | Gm14447 | 41.01221 | 34.38082 | 37.69651 |
| ENSMUSG00000074619 | protein_coding | 1700034I23Rik | 5.206317 | 8.238141 | 6.722229 |
| ENSMUSG00000038697 | protein_coding | Taf5l | 1532.983 | 1459.16 | 1496.071 |
| ENSMUSG00000086023 | lincRNA | 2810011L19Rik | 42.01596 | 49.27895 | 45.64745 |
| ENSMUSG00000068048 | protein_coding | Rhox9 | 6.427891 | 9.784916 | 8.106404 |
| ENSMUSG00000040711 | protein_coding | Sh3pxd2b | 544.615 | 582.8864 | 563.7507 |
| ENSMUSG00000078591 | protein_coding | Hs3st4 | 9.692303 | 6.798392 | 8.245348 |
| ENSMUSG00000002320 | protein_coding | Tm9sf1 | 1284.21 | 1349.685 | 1316.948 |
| ENSMUSG00000024137 | protein_coding | E4f1 | 813.3788 | 762.6297 | 788.0042 |
| ENSMUSG00000006724 | protein_coding | Cyp27b1 | 13.96636 | 10.02242 | 11.99439 |
| ENSMUSG00000078519 | processed_transcrip | 2310026L22Rik | 31.10104 | 25.27052 | 28.18578 |
| ENSMUSG00000004661 | protein_coding | Arid3b | 1040.817 | 1107.437 | 1074.127 |
| ENSMUSG00000090963 | protein_coding | Gm17655 | 16.43895 | 11.74151 | 14.09023 |
| ENSMUSG00000046807 | protein_coding | AI646023 | 1465.15 | 1604.658 | 1534.904 |
| ENSMUSG00000050555 | protein_coding | Hyls1 | 588.1935 | 544.6611 | 566.4273 |
| ENSMUSG00000030615 | protein_coding | Tmem126a | 440.89 | 469.8552 | 455.3726 |
| ENSMUSG00000044149 | protein_coding | Nkrf | 593.257 | 560.2852 | 576.7711 |
| ENSMUSG00000088249 | rRNA | 5S_rRNA | 6.798157 | 9.835761 | 8.316959 |
| ENSMUSG00000086981 | processed_transcrip | Gm12171 | 1.959572 | 0.724379 | 1.341975 |
| ENSMUSG00000092002 | lincRNA | Gm17375 | 14.90578 | 10.89494 | 12.90036 |
| ENSMUSG00000038072 | protein_coding | Galnt11 | 1581.473 | 1475.977 | 1528.725 |

| | | | | | |
|---|---|---|---|---|---|
| ENSMUSG00000064495 | snoRNA | SNORA5 | 0.65242 | 1.686265 | 1.169343 |
| ENSMUSG00000091230 | protein_coding | Gm6970 | 0.65242 | 1.686265 | 1.169343 |
| ENSMUSG00000078923 | protein_coding | Ube2v1 | 35.24367 | 41.84125 | 38.54246 |
| ENSMUSG00000065341 | snRNA | U6 | 1.276663 | 2.781843 | 2.029253 |
| ENSMUSG00000004748 | protein_coding | Mtfp1 | 217.6049 | 235.8747 | 226.7398 |
| ENSMUSG00000031068 | protein_coding | Glrx3 | 3880.047 | 3691.49 | 3785.769 |
| ENSMUSG00000053541 | protein_coding | Gm4759 | 2.265282 | 1.024227 | 1.644755 |
| ENSMUSG00000035566 | protein_coding | Pcdh17 | 91.03865 | 71.61499 | 81.32682 |
| ENSMUSG00000063952 | protein_coding | Brpf3 | 3507.495 | 3737.213 | 3622.354 |
| ENSMUSG00000019055 | protein_coding | Plod1 | 2251.743 | 2123.774 | 2187.759 |
| ENSMUSG00000076432 | protein_coding | Ywhaq | 9164.251 | 9567.937 | 9366.094 |
| ENSMUSG00000037458 | protein_coding | Azin1 | 3850.98 | 3658.941 | 3754.96 |
| ENSMUSG00000031198 | protein_coding | Fundc2 | 983.2583 | 1042.404 | 1012.831 |
| ENSMUSG00000034923 | protein_coding | Ly6g6f | 1.967772 | 0.697713 | 1.332742 |
| ENSMUSG00000020151 | protein_coding | Ptprr | 135.8901 | 154.1205 | 145.0053 |
| ENSMUSG00000030007 | protein_coding | Cct7 | 6098.828 | 5843.125 | 5970.976 |
| ENSMUSG00000080850 | pseudogene | Gm12439 | 13.98171 | 18.88801 | 16.43486 |
| ENSMUSG00000085219 | lincRNA | Gm16617 | 10.67503 | 7.504753 | 9.089893 |
| ENSMUSG00000011148 | protein_coding | Adssl1 | 60.0545 | 51.48926 | 55.77188 |
| ENSMUSG00000026220 | protein_coding | Slc16a14 | 57.69715 | 48.88973 | 53.29344 |
| ENSMUSG00000026172 | protein_coding | Bcs1l | 506.5002 | 473.4399 | 489.97 |
| ENSMUSG00000036214 | protein_coding | Znrd1as | 178.0793 | 159.1208 | 168.6 |
| ENSMUSG00000032295 | protein_coding | Man2c1 | 751.4698 | 805.4972 | 778.4835 |
| ENSMUSG00000031584 | protein_coding | Gsr | 4257.574 | 4009.781 | 4133.678 |
| ENSMUSG00000054091 | protein_coding | 1810037I17Rik | 1683 | 1778.717 | 1730.858 |
| ENSMUSG00000065315 | snRNA | U6 | 2.253504 | 4.123937 | 3.18872 |
| ENSMUSG00000037169 | protein_coding | Mycn | 1768.001 | 1914.203 | 1841.102 |
| ENSMUSG00000025019 | protein_coding | Lcor | 980.5529 | 912.7501 | 946.6515 |
| ENSMUSG00000081487 | pseudogene | Gm13689 | 5.153539 | 3.054663 | 4.104101 |
| ENSMUSG00000079103 | protein_coding | Tgm7 | 1.304841 | 2.772834 | 2.038837 |
| ENSMUSG00000070047 | protein_coding | Fat1 | 9477.298 | 10002.15 | 9739.723 |
| ENSMUSG00000041040 | protein_coding | Fam117b | 1888.826 | 1780.352 | 1834.589 |
| ENSMUSG00000021843 | protein_coding | Ktn1 | 2297.612 | 2480.33 | 2388.971 |
| ENSMUSG00000022812 | protein_coding | Gsk3b | 3427.923 | 3267.952 | 3347.937 |
| ENSMUSG00000031858 | protein_coding | Mau2 | 3000.747 | 3162.075 | 3081.411 |
| ENSMUSG00000053070 | protein_coding | 9230110C19Rik | 708.2828 | 668.3221 | 688.3024 |
| ENSMUSG00000091730 | pseudogene | Gm17230 | 2.595592 | 1.086569 | 1.84108 |
| ENSMUSG00000020799 | protein_coding | Tekt1 | 23.93619 | 18.90567 | 21.42093 |
| ENSMUSG00000023259 | protein_coding | Slc26a6 | 181.5148 | 197.9794 | 189.7471 |
| ENSMUSG00000085819 | lincRNA | A930028C08Rik | 6.14678 | 9.307415 | 7.727097 |
| ENSMUSG00000082064 | pseudogene | Gm14217 | 3.927343 | 2.021789 | 2.974566 |
| ENSMUSG00000042622 | protein_coding | Maff | 89.75655 | 77.88488 | 83.82071 |
| ENSMUSG00000069136 | pseudogene | A130006I12Rik | 19.76076 | 25.10152 | 22.43114 |
| ENSMUSG00000024679 | protein_coding | Ms4a6d | 92.9406 | 105.9693 | 99.45494 |
| ENSMUSG00000034647 | protein_coding | Ankrd12 | 2528.36 | 2366.018 | 2447.189 |
| ENSMUSG00000024972 | protein_coding | Lgals12 | 6.724134 | 9.826751 | 8.275443 |
| ENSMUSG00000041817 | protein_coding | Fam169a | 2672.359 | 2510.065 | 2591.212 |
| ENSMUSG00000068551 | protein_coding | Zfp467 | 586.1063 | 655.0264 | 620.5664 |
| ENSMUSG00000083245 | pseudogene | Gm11185 | 0 | 0.988552 | 0.494276 |
| ENSMUSG00000078512 | protein_coding | Pramef6 | 0 | 0.988552 | 0.494276 |
| ENSMUSG00000058567 | pseudogene | Gm10043 | 0 | 0.988552 | 0.494276 |
| ENSMUSG00000039364 | protein_coding | Sectm1b | 0 | 0.988552 | 0.494276 |
| ENSMUSG00000091216 | pseudogene | Gm17208 | 0 | 0.988552 | 0.494276 |
| ENSMUSG00000024497 | protein_coding | Pou4f3 | 0 | 0.988552 | 0.494276 |
| ENSMUSG00000090930 | lincRNA | Gm17704 | 0 | 0.988552 | 0.494276 |
| ENSMUSG00000081147 | pseudogene | Gm6024 | 0 | 0.988552 | 0.494276 |
| ENSMUSG00000059539 | protein_coding | Magea1 | 0 | 0.988552 | 0.494276 |
| ENSMUSG00000082629 | pseudogene | Gm5639 | 2.875436 | 5.094471 | 3.984953 |

| | | | | | |
|---|---|---|---|---|---|
| ENSMUSG00000057497 | protein_coding | Fam136a | 2432.138 | 2250.484 | 2341.311 |
| ENSMUSG00000020481 | protein_coding | Ankrd36 | 26.51583 | 18.89594 | 22.70588 |
| ENSMUSG00000025239 | protein_coding | Limd1 | 1186.627 | 1285.841 | 1236.234 |
| ENSMUSG00000079871 | pseudogene | Gm4984 | 18.43594 | 23.23035 | 20.83315 |
| ENSMUSG00000050786 | protein_coding | Ccdc126 | 288.0836 | 266.0836 | 277.0836 |
| ENSMUSG00000076928 | IG_C_gene | Trac | 99.01709 | 83.36328 | 91.19018 |
| ENSMUSG00000029103 | protein_coding | Lrpap1 | 4325.733 | 4095.369 | 4210.551 |
| ENSMUSG00000037280 | protein_coding | Galnt6 | 700.3532 | 643.6505 | 672.0018 |
| ENSMUSG00000083909 | pseudogene | Gm15842 | 4.901874 | 2.755177 | 3.828525 |
| ENSMUSG00000085062 | lincRNA | 4933404O12Rik | 223.9793 | 197.0949 | 210.5371 |
| ENSMUSG00000054619 | protein_coding | Mettl7a1 | 151.3385 | 167.2007 | 159.2696 |
| ENSMUSG00000020315 | protein_coding | Spnb2 | 10901.01 | 11480.75 | 11190.88 |
| ENSMUSG00000084511 | misc_RNA | 7SK | 1.940861 | 0.688704 | 1.314783 |
| ENSMUSG00000050854 | protein_coding | Tmem125 | 1.278973 | 2.746168 | 2.012571 |
| ENSMUSG00000031273 | protein_coding | Col4a6 | 103.2657 | 204.7934 | 154.0296 |
| ENSMUSG00000017837 | protein_coding | Nkiras2 | 490.1762 | 523.417 | 506.7966 |
| ENSMUSG00000034194 | protein_coding | R3hcc1 | 1434.743 | 1369.541 | 1402.142 |
| ENSMUSG00000009647 | protein_coding | Ccdc109a | 294.5281 | 317.8518 | 306.19 |
| ENSMUSG00000046865 | protein_coding | Fbl | 3781.036 | 3613.227 | 3697.132 |
| ENSMUSG00000004032 | protein_coding | Gstm5 | 1648.475 | 1548.102 | 1598.289 |
| ENSMUSG00000083902 | pseudogene | Gm15975 | 31.33332 | 20.12168 | 25.7275 |
| ENSMUSG00000085772 | processed_transcrip | D630024D03Rik | 1.276663 | 2.7995 | 2.038081 |
| ENSMUSG00000037349 | protein_coding | Nudt22 | 264.287 | 242.6588 | 253.4729 |
| ENSMUSG00000090625 | protein_coding | AF085738 | 3.894544 | 6.216714 | 5.055629 |
| ENSMUSG00000031970 | protein_coding | Dbndd1 | 218.6784 | 197.9725 | 208.3254 |
| ENSMUSG00000059734 | protein_coding | Ndufs8 | 2008.235 | 1885.597 | 1946.916 |
| ENSMUSG00000053574 | lincRNA | 4930563E22Rik | 114.0234 | 101.8 | 107.9117 |
| ENSMUSG00000019861 | protein_coding | Gopc | 1169.968 | 1110.53 | 1140.249 |
| ENSMUSG00000027878 | protein_coding | Notch2 | 4156.115 | 4407.22 | 4281.667 |
| ENSMUSG00000022915 | processed_transcrip | 1700093J21Rik | 7.1108 | 4.432794 | 5.771797 |
| ENSMUSG00000089643 | processed_transcrip | Gm16301 | 1.615173 | 3.108357 | 2.361765 |
| ENSMUSG00000029119 | protein_coding | Man2b2 | 569.1623 | 507.4839 | 538.3231 |
| ENSMUSG00000092080 | protein_coding | Vmn2r16 | 0.638331 | 1.677256 | 1.157794 |
| ENSMUSG00000048982 | protein_coding | Gphb5 | 0.962752 | 2.508661 | 1.735707 |
| ENSMUSG00000081608 | pseudogene | Gm16210 | 0.974531 | 2.428301 | 1.701416 |
| ENSMUSG00000086320 | processed_transcrip | Gm12840 | 3.242123 | 1.712931 | 2.477527 |
| ENSMUSG00000042254 | protein_coding | Cilp | 52.95294 | 44.97772 | 48.96533 |
| ENSMUSG00000003345 | protein_coding | Csnk1g2 | 3449.762 | 3309.081 | 3379.421 |
| ENSMUSG00000055373 | protein_coding | Fut9 | 1.278973 | 2.737158 | 2.008066 |
| ENSMUSG00000023089 | protein_coding | Ndufa5 | 780.933 | 730.4085 | 755.6708 |
| ENSMUSG00000079573 | processed_transcrip | Zfp783 | 18.44414 | 14.27684 | 16.36049 |
| ENSMUSG00000021994 | protein_coding | Wnt5a | 1640.802 | 1788.818 | 1714.81 |
| ENSMUSG00000024853 | protein_coding | Sf3b2 | 5963.271 | 6218.65 | 6090.961 |
| ENSMUSG00000038925 | protein_coding | E330034G19Rik | 0.65242 | 1.695274 | 1.173847 |
| ENSMUSG00000063972 | protein_coding | Nr6a1 | 1629.334 | 1488.394 | 1558.864 |
| ENSMUSG00000059994 | protein_coding | Fcrl1 | 2.925902 | 1.395426 | 2.160664 |
| ENSMUSG00000056394 | protein_coding | Lig1 | 3643.462 | 3937.494 | 3790.478 |
| ENSMUSG00000033200 | protein_coding | Tpsg1 | 3.566544 | 1.632571 | 2.599558 |
| ENSMUSG00000050821 | protein_coding | Fam131a | 196.9533 | 214.8759 | 205.9146 |
| ENSMUSG00000064147 | protein_coding | Rab44 | 3.538366 | 5.702814 | 4.62059 |
| ENSMUSG00000082895 | pseudogene | Rpsa-ps9 | 2.569725 | 4.476756 | 3.52324 |
| ENSMUSG00000030942 | protein_coding | Thumpd1 | 2874.949 | 2747.384 | 2811.166 |
| ENSMUSG00000066418 | lincRNA | Gm17627 | 5.195806 | 3.090339 | 4.143072 |
| ENSMUSG00000068206 | protein_coding | Pick1 | 1074.327 | 1010.96 | 1042.644 |
| ENSMUSG00000000740 | protein_coding | Rpl13 | 11017.58 | 10494.47 | 10756.03 |
| ENSMUSG00000022312 | protein_coding | Eif3h | 5470.435 | 5760.11 | 5615.272 |
| ENSMUSG00000062210 | protein_coding | Tnfaip8 | 311.2094 | 286.0637 | 298.6366 |
| ENSMUSG00000087532 | processed_transcrip | Gm15938 | 1.615173 | 3.072682 | 2.343927 |

| | | | | | |
|---|---|---|---|---|---|
| ENSMUSG00000034659 | protein_coding | Tmem109 | 919.7868 | 969.8459 | 944.8164 |
| ENSMUSG00000048387 | protein_coding | Osr1 | 517.2855 | 484.0967 | 500.6911 |
| ENSMUSG00000078767 | pseudogene | Gm10988 | 1.621062 | 3.108357 | 2.364709 |
| ENSMUSG00000079043 | protein_coding | Fastkd5 | 237.0304 | 217.9472 | 227.4888 |
| ENSMUSG00000028644 | protein_coding | Ermap | 16.47153 | 21.26878 | 18.87015 |
| ENSMUSG00000032548 | protein_coding | Slco2a1 | 57.39018 | 68.19575 | 62.79297 |
| ENSMUSG00000023034 | protein_coding | Nr4a1 | 1621.793 | 1086.05 | 1353.921 |
| ENSMUSG00000021235 | protein_coding | Coq6 | 497.7434 | 466.1108 | 481.9271 |
| ENSMUSG00000086395 | processed_transcrip | A630014C17Rik | 25.9109 | 19.802 | 22.85645 |
| ENSMUSG00000024670 | protein_coding | Cd6 | 7.709175 | 5.147803 | 6.428489 |
| ENSMUSG00000045180 | protein_coding | Shroom2 | 549.4921 | 511.6597 | 530.5759 |
| ENSMUSG00000081733 | pseudogene | Gm12852 | 2.934102 | 1.395426 | 2.164764 |
| ENSMUSG00000024283 | protein_coding | Wac | 4385.968 | 4574.328 | 4480.148 |
| ENSMUSG00000086966 | lincRNA | Gm16866 | 3.244434 | 1.677256 | 2.460845 |
| ENSMUSG00000044447 | protein_coding | Dock5 | 1651.086 | 1737.329 | 1694.208 |
| ENSMUSG00000075229 | protein_coding | Ccdc58 | 617.0733 | 580.3474 | 598.7103 |
| ENSMUSG00000021209 | protein_coding | Ppp4r4 | 519.284 | 401.1801 | 460.2321 |
| ENSMUSG00000020657 | protein_coding | Dnajc27 | 750.5646 | 705.6324 | 728.0985 |
| ENSMUSG00000070330 | protein_coding | Cldn27 | 4.543386 | 7.241303 | 5.892345 |
| ENSMUSG00000045932 | protein_coding | Ifit2 | 124.6156 | 140.3365 | 132.4761 |
| ENSMUSG00000029545 | protein_coding | Acads | 869.651 | 928.3934 | 899.0222 |
| ENSMUSG00000055978 | protein_coding | Fut2 | 16.27391 | 11.99088 | 14.13239 |
| ENSMUSG00000020892 | protein_coding | Aloxe3 | 92.05731 | 107.8987 | 99.97801 |
| ENSMUSG00000034818 | protein_coding | Celf5 | 66.7017 | 77.91082 | 72.30626 |
| ENSMUSG00000009905 | protein_coding | Kdsr | 918.0226 | 967.9819 | 943.0023 |
| ENSMUSG00000055116 | protein_coding | Arntl | 466.0474 | 496.1994 | 481.1234 |
| ENSMUSG00000019943 | protein_coding | Atp2b1 | 5548.701 | 5773.491 | 5661.096 |
| ENSMUSG00000061322 | protein_coding | Dnaic1 | 50.38053 | 39.56098 | 44.97076 |
| ENSMUSG00000005640 | protein_coding | Insrr | 34.27711 | 41.96774 | 38.12243 |
| ENSMUSG00000015829 | protein_coding | Tnr | 1.304841 | 0.36219 | 0.833515 |
| ENSMUSG00000064719 | snRNA | U6 | 1.304841 | 0.36219 | 0.833515 |
| ENSMUSG00000083087 | pseudogene | Gm11249 | 15.14022 | 23.32231 | 19.23127 |
| ENSMUSG00000020743 | protein_coding | Mif4gd | 1244.591 | 1317.76 | 1281.175 |
| ENSMUSG00000026991 | protein_coding | Pkp4 | 1068.511 | 1131.393 | 1099.952 |
| ENSMUSG00000022766 | protein_coding | Serpind1 | 1.621062 | 3.072682 | 2.346872 |
| ENSMUSG00000081615 | pseudogene | Gm12539 | 0.646531 | 1.677256 | 1.161894 |
| ENSMUSG00000084989 | protein_coding | E030010N08Rik | 17.40714 | 12.71526 | 15.0612 |
| ENSMUSG00000002679 | protein_coding | Med6 | 1126.371 | 1189.645 | 1158.008 |
| ENSMUSG00000030533 | protein_coding | Unc45a | 1210.529 | 1129.062 | 1169.796 |
| ENSMUSG00000017652 | protein_coding | Cd40 | 14.56265 | 19.39725 | 16.97995 |
| ENSMUSG00000032508 | protein_coding | Myd88 | 328.727 | 306.3032 | 317.5151 |
| ENSMUSG00000086796 | processed_transcrip | 4932702P03Rik | 19.68778 | 14.74678 | 17.21728 |
| ENSMUSG00000073652 | protein_coding | Apol7d | 25.3079 | 31.74996 | 28.52893 |
| ENSMUSG00000083715 | pseudogene | Gm14202 | 0.99093 | 2.374969 | 1.68295 |
| ENSMUSG00000022125 | protein_coding | Cln5 | 829.7681 | 899.9038 | 864.8359 |
| ENSMUSG00000029616 | protein_coding | Erp29 | 2143.358 | 2243.688 | 2193.523 |
| ENSMUSG00000001435 | protein_coding | Col18a1 | 9998.87 | 9435.935 | 9717.403 |
| ENSMUSG00000078238 | pseudogene | Gm12854 | 37.76755 | 44.61553 | 41.19154 |
| ENSMUSG00000071419 | pseudogene | Rps15-ps2 | 1.304841 | 2.710492 | 2.007667 |
| ENSMUSG00000020904 | protein_coding | Wdr16 | 20.40371 | 15.9541 | 18.17891 |
| ENSMUSG00000040969 | protein_coding | D630013G24Rik | 6.12807 | 3.550545 | 4.839307 |
| ENSMUSG00000030505 | protein_coding | Prmt3 | 1019.376 | 965.6942 | 992.5351 |
| ENSMUSG00000022013 | protein_coding | Dnajc15 | 920.3556 | 868.7022 | 894.5289 |
| ENSMUSG00000011350 | processed_transcrip | Gm5893 | 1.621062 | 3.135023 | 2.378043 |
| ENSMUSG00000032356 | protein_coding | Rasgrf1 | 30.48626 | 24.40344 | 27.44485 |
| ENSMUSG00000014748 | protein_coding | Tex261 | 2266.203 | 2404.71 | 2335.456 |
| ENSMUSG00000082668 | pseudogene | Gm13258 | 7.404732 | 10.4351 | 8.919914 |
| ENSMUSG00000091973 | pseudogene | Gm4109 | 6.78869 | 4.441442 | 5.615066 |

| | | | | | |
|---|---|---|---|---|---|
| ENSMUSG00000001289 | protein_coding | Pfdn5 | 2595.484 | 2708.943 | 2652.213 |
| ENSMUSG00000078734 | protein_coding | Gm2506 | 1.304841 | 2.781843 | 2.043342 |
| ENSMUSG00000063694 | protein_coding | Cycs | 5046.599 | 5308.518 | 5177.558 |
| ENSMUSG00000028284 | protein_coding | Map3k7 | 3655.706 | 3507.666 | 3581.686 |
| ENSMUSG00000090460 | protein_coding | Gm17511 | 3.230345 | 1.72194 | 2.476143 |
| ENSMUSG00000083109 | pseudogene | Gm12876 | 3.852277 | 6.136716 | 4.994497 |
| ENSMUSG00000044770 | protein_coding | Scml4 | 34.27249 | 28.03362 | 31.15306 |
| ENSMUSG00000061731 | protein_coding | Ext1 | 1091.637 | 1182.465 | 1137.051 |
| ENSMUSG00000069367 | pseudogene | Rps2-ps10 | 177.9277 | 160.0666 | 168.9972 |
| ENSMUSG00000032014 | protein_coding | Oaf | 556.7211 | 596.1521 | 576.4366 |
| ENSMUSG00000029587 | protein_coding | Zfp12 | 926.1057 | 859.1779 | 892.6418 |
| ENSMUSG00000070960 | pseudogene | Gm10307 | 29.67402 | 35.58958 | 32.6318 |
| ENSMUSG00000046079 | protein_coding | Lrrc8d | 2043.442 | 2157.891 | 2100.667 |
| ENSMUSG00000037896 | protein_coding | Rcor1 | 2166.561 | 2286.18 | 2226.37 |
| ENSMUSG00000038301 | protein_coding | Snx10 | 742.8004 | 787.7797 | 765.29 |
| ENSMUSG00000071078 | protein_coding | Nr2c2ap | 21.89209 | 27.39649 | 24.64429 |
| ENSMUSG00000049940 | protein_coding | Pgrmc2 | 1440.117 | 1519.961 | 1480.039 |
| ENSMUSG00000085158 | processed_transcrip | Gm14264 | 5.46156 | 3.399196 | 4.430378 |
| ENSMUSG00000030406 | protein_coding | Gipr | 21.36185 | 26.87027 | 24.11606 |
| ENSMUSG00000071252 | protein_coding | 2210408I21Rik | 210.7961 | 191.1176 | 200.9568 |
| ENSMUSG00000019715 | protein_coding | Gle1 | 2233.09 | 2120.623 | 2176.857 |
| ENSMUSG00000089968 | processed_transcrip | 4921518J05Rik | 30.37944 | 38.00524 | 34.19234 |
| ENSMUSG00000021054 | protein_coding | Sgpp1 | 2362.66 | 2259.262 | 2310.961 |
| ENSMUSG00000002617 | protein_coding | Zfp40 | 624.8353 | 674.0865 | 649.4609 |
| ENSMUSG00000081739 | pseudogene | Mdm4-ps | 1.304841 | 0.335524 | 0.820182 |
| ENSMUSG00000052446 | protein_coding | Zfp961 | 663.5577 | 618.5449 | 641.0513 |
| ENSMUSG00000036478 | protein_coding | Btg1 | 1689.556 | 1590.567 | 1640.061 |
| ENSMUSG00000031596 | protein_coding | Slc7a2 | 2116.532 | 2261.774 | 2189.153 |
| ENSMUSG00000004043 | protein_coding | Stat5a | 443.3074 | 472.6947 | 458.001 |
| ENSMUSG00000022219 | protein_coding | Cideb | 69.74323 | 60.62731 | 65.18527 |
| ENSMUSG00000088555 | snRNA | U4 | 0.65242 | 1.677256 | 1.164838 |
| ENSMUSG00000050397 | protein_coding | Foxl2 | 4.221275 | 2.410644 | 3.31596 |
| ENSMUSG00000024713 | protein_coding | Pcsk5 | 546.5206 | 502.2766 | 524.3986 |
| ENSMUSG00000075327 | protein_coding | Zbtb2 | 602.8978 | 643.4345 | 623.1661 |
| ENSMUSG00000060131 | protein_coding | Atp8b4 | 27.78154 | 33.59482 | 30.68818 |
| ENSMUSG00000002981 | protein_coding | Clptm1 | 3255.371 | 3407.135 | 3331.253 |
| ENSMUSG00000032327 | protein_coding | Stra6 | 1231.679 | 1348.951 | 1290.315 |
| ENSMUSG00000085505 | processed_transcrip | Gm12868 | 1.304841 | 0.326514 | 0.815677 |
| ENSMUSG00000091680 | protein_coding | Klhdc7b | 1.304841 | 0.326514 | 0.815677 |
| ENSMUSG00000051297 | protein_coding | 2410124H12Rik | 1.304841 | 0.326514 | 0.815677 |
| ENSMUSG00000077733 | snoRNA | SNORA17 | 2.905924 | 4.830298 | 3.868111 |
| ENSMUSG00000061477 | protein_coding | Rps7 | 7383.1 | 6977.079 | 7180.09 |
| ENSMUSG00000024593 | protein_coding | Megf10 | 16.43664 | 21.78159 | 19.10912 |
| ENSMUSG00000037712 | protein_coding | Fermt2 | 3294.892 | 3149.276 | 3222.084 |
| ENSMUSG00000022187 | protein_coding | Gm5546 | 1.621062 | 3.081691 | 2.351376 |
| ENSMUSG00000029084 | protein_coding | Cd38 | 89.87828 | 106.4197 | 98.149 |
| ENSMUSG00000092545 | processed_transcrip | RP24-345H5.2 | 2.259393 | 0.997561 | 1.628477 |
| ENSMUSG00000027367 | protein_coding | Stard7 | 3853.4 | 3655.313 | 3754.356 |
| ENSMUSG00000061510 | protein_coding | Gm10101 | 21.63222 | 26.97159 | 24.30191 |
| ENSMUSG00000030782 | protein_coding | Tgfb1i1 | 643.395 | 687.9617 | 665.6784 |
| ENSMUSG00000015966 | protein_coding | Il17rb | 44.84368 | 56.18069 | 50.51219 |
| ENSMUSG00000020122 | protein_coding | Egfr | 645.393 | 594.6343 | 620.0137 |
| ENSMUSG00000041730 | protein_coding | Prrxl1 | 3.199857 | 5.666778 | 4.433317 |
| ENSMUSG00000081499 | pseudogene | Gm12517 | 1.31304 | 0.36219 | 0.837615 |
| ENSMUSG00000085773 | lincRNA | Gm16233 | 1.31304 | 0.36219 | 0.837615 |
| ENSMUSG00000040601 | protein_coding | Nlrp4a | 1.304841 | 2.719502 | 2.012171 |
| ENSMUSG00000020963 | protein_coding | Tshr | 2.583814 | 4.450451 | 3.517133 |
| ENSMUSG00000069271 | protein_coding | Hist1h2bn | 4.190787 | 6.632236 | 5.411511 |

| | | | | | |
|---|---|---|---|---|---|
| ENSMUSG00000087130 | lincRNA | D230004N17Rik | 15.2302 | 11.38217 | 13.30619 |
| ENSMUSG00000061436 | protein_coding | Hipk2 | 761.803 | 702.661 | 732.232 |
| ENSMUSG00000021546 | protein_coding | Hnrnpk | 17587.32 | 18274.48 | 17930.9 |
| ENSMUSG00000082463 | pseudogene | Gm15765 | 0.98273 | 2.508661 | 1.745696 |
| ENSMUSG00000027540 | protein_coding | Ptpn1 | 2691.719 | 2822.986 | 2757.352 |
| ENSMUSG00000050075 | protein_coding | Gpr171 | 5.177096 | 3.018988 | 4.098042 |
| ENSMUSG00000018500 | protein_coding | Adora2b | 94.27862 | 82.339 | 88.30881 |
| ENSMUSG00000086579 | lincRNA | A930012L18Rik | 3.239813 | 5.599 | 4.419406 |
| ENSMUSG00000019806 | protein_coding | Aig1 | 220.8587 | 239.0047 | 229.9317 |
| ENSMUSG00000028492 | protein_coding | Fam154a | 4.202565 | 2.419653 | 3.311109 |
| ENSMUSG00000051401 | protein_coding | Kctd16 | 5.473339 | 3.390187 | 4.431763 |
| ENSMUSG00000086768 | lincRNA | Gm16827 | 18.03981 | 13.61553 | 15.82767 |
| ENSMUSG00000032086 | protein_coding | Bace1 | 1071.433 | 1015.559 | 1043.496 |
| ENSMUSG00000082809 | pseudogene | Gm14150 | 42.91058 | 49.9021 | 46.40634 |
| ENSMUSG00000008604 | protein_coding | Ubqln4 | 1306.554 | 1241.983 | 1274.269 |
| ENSMUSG00000018428 | protein_coding | Akap1 | 1073.503 | 1012.832 | 1043.167 |
| ENSMUSG00000026303 | protein_coding | Mlph | 11.58374 | 8.309492 | 9.946616 |
| ENSMUSG00000062209 | protein_coding | Erbb4 | 625.7377 | 780.5853 | 703.1615 |
| ENSMUSG00000042831 | protein_coding | Alkbh6 | 512.6304 | 482.8523 | 497.7414 |
| ENSMUSG00000073778 | processed_transcrip | Gm10574 | 12.90856 | 16.68749 | 14.79802 |
| ENSMUSG00000024925 | protein_coding | Rnaseh2c | 617.9723 | 570.5341 | 594.2532 |
| ENSMUSG00000073843 | processed_transcrip | Gm12542 | 2.902346 | 1.350742 | 2.126544 |
| ENSMUSG00000021302 | protein_coding | Ggps1 | 1288.102 | 1210.865 | 1249.483 |
| ENSMUSG00000081274 | pseudogene | Gm15727 | 34.9718 | 42.58509 | 38.77845 |
| ENSMUSG00000078190 | processed_transcrip | Dnm3os | 326.1003 | 375.7862 | 350.9432 |
| ENSMUSG00000092789 | miRNA | AL732624.1 | 1.307151 | 0.36219 | 0.83467 |
| ENSMUSG00000085488 | processed_transcrip | 4930557F10Rik | 1.307151 | 0.36219 | 0.83467 |
| ENSMUSG00000024029 | protein_coding | Tff3 | 1.307151 | 0.36219 | 0.83467 |
| ENSMUSG00000053624 | protein_coding | Gykl1 | 1.307151 | 0.36219 | 0.83467 |
| ENSMUSG00000082272 | pseudogene | Gm11675 | 3.208056 | 1.677256 | 2.442656 |
| ENSMUSG00000048058 | protein_coding | Ldlrad3 | 1128.982 | 1061.709 | 1095.345 |
| ENSMUSG00000024065 | protein_coding | Ehd3 | 194.0033 | 177.1467 | 185.575 |
| ENSMUSG00000006599 | protein_coding | Gtf2h1 | 2911.453 | 2760.119 | 2835.786 |
| ENSMUSG00000026417 | protein_coding | Pigr | 1.31304 | 2.772834 | 2.042937 |
| ENSMUSG00000086131 | processed_transcrip | BC048594 | 1.31304 | 2.772834 | 2.042937 |
| ENSMUSG00000026582 | protein_coding | Sele | 1.606973 | 3.046016 | 2.326494 |
| ENSMUSG00000092233 | protein_coding | Gm20403 | 2.273482 | 0.988552 | 1.631017 |
| ENSMUSG00000031131 | protein_coding | Vgll1 | 3.600612 | 6.199058 | 4.899835 |
| ENSMUSG00000020009 | protein_coding | Ifngr1 | 827.4579 | 784.2181 | 805.838 |
| ENSMUSG00000028713 | protein_coding | Cyp4b1 | 2.561525 | 4.396757 | 3.479141 |
| ENSMUSG00000091210 | lincRNA | 1110054M08Rik | 33.89194 | 40.50997 | 37.20096 |
| ENSMUSG00000029466 | protein_coding | Anapc7 | 1797.912 | 1879.935 | 1838.923 |
| ENSMUSG00000085154 | lincRNA | C130046K22Rik | 34.41927 | 14.45191 | 24.43559 |
| ENSMUSG00000089151 | miRNA | Mir1950 | 1.31304 | 0.335524 | 0.824282 |
| ENSMUSG00000054057 | protein_coding | A930004D18Rik | 66.08544 | 57.28646 | 61.68595 |
| ENSMUSG00000064210 | protein_coding | Ano6 | 1177.19 | 1242.093 | 1209.642 |
| ENSMUSG00000086360 | processed_transcrip | Gm16214 | 1.293062 | 0.335524 | 0.814293 |
| ENSMUSG00000021196 | protein_coding | Pfkp | 232.0461 | 254.2512 | 243.1486 |
| ENSMUSG00000086603 | processed_transcrip | Gm13620 | 1.270774 | 2.701483 | 1.986128 |
| ENSMUSG00000086785 | pseudogene | Gm6081 | 1.307151 | 2.737158 | 2.022155 |
| ENSMUSG00000066357 | protein_coding | Wdr6 | 2585.042 | 2448.784 | 2516.913 |
| ENSMUSG00000057123 | protein_coding | Gja5 | 56.45389 | 64.77356 | 60.61372 |
| ENSMUSG00000020271 | protein_coding | Fbxw11 | 3508.205 | 3678.202 | 3593.203 |
| ENSMUSG00000028017 | protein_coding | Egf | 57.88993 | 48.23525 | 53.06259 |
| ENSMUSG00000087701 | pseudogene | Gm13493 | 55.67936 | 47.16779 | 51.42358 |
| ENSMUSG00000037851 | protein_coding | Iars | 2915.632 | 2757.853 | 2836.742 |
| ENSMUSG00000026659 | protein_coding | Dusp12 | 399.2408 | 366.7882 | 383.0145 |
| ENSMUSG00000092744 | miRNA | AC107641.1 | 3.525545 | 6.101041 | 4.813293 |

| | | | | | |
|---|---|---|---|---|---|
| ENSMUSG00000078144 | protein_coding | Capns2 | 7.719686 | 11.17785 | 9.44877 |
| ENSMUSG00000091418 | protein_coding | Gm17143 | 1.282552 | 2.7995 | 2.041026 |
| ENSMUSG00000077677 | snRNA | U6 | 1.31304 | 0.326514 | 0.819777 |
| ENSMUSG00000084861 | lincRNA | 1700095J07Rik | 1.31304 | 0.326514 | 0.819777 |
| ENSMUSG00000082171 | pseudogene | Rpl27-ps2 | 1.31304 | 0.326514 | 0.819777 |
| ENSMUSG00000072769 | protein_coding | Gm10419 | 25.81712 | 31.80909 | 28.81311 |
| ENSMUSG00000089710 | processed_transcrip | Gm15810 | 1.293062 | 0.326514 | 0.809788 |
| ENSMUSG00000090564 | processed_transcrip | A430057M04Rik | 21.27731 | 26.2234 | 23.75035 |
| ENSMUSG00000086563 | processed_transcrip | Gm14136 | 0.33031 | 1.448758 | 0.889534 |
| ENSMUSG00000063600 | protein_coding | Egfem1 | 0.33031 | 1.448758 | 0.889534 |
| ENSMUSG00000081108 | pseudogene | Gm4234 | 0.33031 | 1.448758 | 0.889534 |
| ENSMUSG00000091580 | pseudogene | Gm6467 | 60.96805 | 52.90379 | 56.93592 |
| ENSMUSG00000047824 | protein_coding | Pygo2 | 1967.809 | 2084.064 | 2025.937 |
| ENSMUSG00000033228 | protein_coding | Scaf11 | 6618.856 | 6287.648 | 6453.252 |
| ENSMUSG00000089491 | snoRNA | SNORA19 | 5.512027 | 3.425862 | 4.468945 |
| ENSMUSG00000032841 | protein_coding | Prr5l | 38.6893 | 32.66825 | 35.67877 |
| ENSMUSG00000022972 | protein_coding | 1110004E09Rik | 741.2336 | 781.3882 | 761.3109 |
| ENSMUSG00000015312 | protein_coding | Gadd45b | 678.5036 | 738.8524 | 708.678 |
| ENSMUSG00000065939 | snoRNA | Snora2b | 4.229475 | 6.736051 | 5.482763 |
| ENSMUSG00000021965 | protein_coding | F630043A04Rik | 720.229 | 672.3588 | 696.2939 |
| ENSMUSG00000086670 | pseudogene | Gm13194 | 2.603792 | 4.432433 | 3.518112 |
| ENSMUSG00000085669 | lincRNA | Gm14817 | 2.942302 | 1.36876 | 2.155531 |
| ENSMUSG00000048763 | protein_coding | Hoxb3 | 16.44208 | 21.72935 | 19.08571 |
| ENSMUSG00000029049 | protein_coding | Morn1 | 159.7197 | 145.4486 | 152.5841 |
| ENSMUSG00000089296 | snRNA | U6 | 7.472866 | 4.66994 | 6.071403 |
| ENSMUSG00000005198 | protein_coding | Polr2a | 9126.203 | 8388.178 | 8757.191 |
| ENSMUSG00000034211 | protein_coding | Mrps17 | 1665.985 | 1748.191 | 1707.088 |
| ENSMUSG00000009545 | protein_coding | Kcnq1 | 12.90625 | 16.73181 | 14.81903 |
| ENSMUSG00000067147 | pseudogene | Rpl7a-ps11 | 37.23269 | 30.84865 | 34.04067 |
| ENSMUSG00000024758 | protein_coding | Rtn3 | 4114.795 | 3920.119 | 4017.457 |
| ENSMUSG00000085201 | processed_transcrip | Gm13507 | 2.895414 | 5.094832 | 3.995123 |
| ENSMUSG00000033998 | protein_coding | Kcnk1 | 106.7893 | 92.61332 | 99.70132 |
| ENSMUSG00000011632 | protein_coding | 2310033E01Rik | 1.307151 | 0.335524 | 0.821337 |
| ENSMUSG00000059132 | pseudogene | Gm9242 | 54.78616 | 45.29636 | 50.04126 |
| ENSMUSG00000027030 | protein_coding | Stk39 | 2359.296 | 2486.049 | 2422.673 |
| ENSMUSG00000060262 | protein_coding | Hist1h2ao | 1.957261 | 0.653029 | 1.305145 |
| ENSMUSG00000021264 | protein_coding | Yy1 | 2615.683 | 2737.982 | 2676.832 |
| ENSMUSG00000077426 | snoRNA | SNORA66 | 10.37521 | 7.116621 | 8.745917 |
| ENSMUSG00000006941 | protein_coding | Eif1b | 2085.802 | 1983.411 | 2034.606 |
| ENSMUSG00000073883 | protein_coding | Gm10598 | 3.822831 | 1.704284 | 2.763557 |
| ENSMUSG00000003882 | protein_coding | Il7r | 3.225724 | 5.367653 | 4.296688 |
| ENSMUSG00000056220 | protein_coding | Pla2g4a | 572.3675 | 614.8989 | 593.6332 |
| ENSMUSG00000025535 | protein_coding | 4930579G22Rik | 124.3669 | 112.3929 | 118.3799 |
| ENSMUSG00000031149 | protein_coding | Praf2 | 284.86 | 310.3259 | 297.593 |
| ENSMUSG00000047260 | protein_coding | Tmem93 | 1584.111 | 1505.876 | 1544.993 |
| ENSMUSG00000001025 | protein_coding | S100a6 | 284.9856 | 309.0635 | 297.0245 |
| ENSMUSG00000079507 | protein_coding | H2-Q2 | 19.54167 | 29.00586 | 24.27377 |
| ENSMUSG00000081721 | pseudogene | Gm13882 | 1.307151 | 0.326514 | 0.816833 |
| ENSMUSG00000006948 | protein_coding | Klk4 | 1.307151 | 0.326514 | 0.816833 |
| ENSMUSG00000038691 | protein_coding | Mbd3l1 | 1.307151 | 0.326514 | 0.816833 |
| ENSMUSG00000081570 | pseudogene | Gm12192 | 1.307151 | 0.326514 | 0.816833 |
| ENSMUSG00000087505 | processed_transcrip | Gm15241 | 1.307151 | 0.326514 | 0.816833 |
| ENSMUSG00000090217 | processed_transcrip | Gm16116 | 4.521097 | 2.710492 | 3.615795 |
| ENSMUSG00000029027 | protein_coding | Dffb | 201.6839 | 218.4751 | 210.0795 |
| ENSMUSG00000092543 | pseudogene | Skiv2l-ps1 | 1.296641 | 0.36219 | 0.829415 |
| ENSMUSG00000036461 | protein_coding | Elf1 | 1169.013 | 1107.167 | 1138.09 |
| ENSMUSG00000086228 | protein_coding | Gm514 | 10.04721 | 13.66777 | 11.85749 |
| ENSMUSG00000037772 | protein_coding | Mrpl23 | 764.1352 | 809.7238 | 786.9295 |

| | | | | | |
|---|---|---|---|---|---|
| ENSMUSG00000079580 | protein_coding | Tmem217 | 11.35668 | 15.58611 | 13.47139 |
| ENSMUSG00000071359 | protein_coding | Tbpl1 | 1441.284 | 1373.112 | 1407.198 |
| ENSMUSG00000063849 | protein_coding | Ppcdc | 446.1445 | 482.7247 | 464.4346 |
| ENSMUSG00000087539 | processed_transcrip | Gm13584 | 3.205746 | 5.349634 | 4.27769 |
| ENSMUSG00000019370 | protein_coding | Calm3 | 9255.232 | 9661.411 | 9458.321 |
| ENSMUSG00000021187 | protein_coding | Tc2n | 604.6785 | 474.7875 | 539.733 |
| ENSMUSG00000031949 | protein_coding | Adat1 | 385.2203 | 359.7526 | 372.4864 |
| ENSMUSG00000005469 | protein_coding | Prkaca | 2783.748 | 2922.97 | 2853.359 |
| ENSMUSG00000046573 | protein_coding | Lyrm4 | 412.8755 | 388.5192 | 400.6973 |
| ENSMUSG00000049691 | protein_coding | Nkx3-2 | 6.407913 | 9.500599 | 7.954256 |
| ENSMUSG00000064179 | protein_coding | Tnnt1 | 146.3565 | 160.0076 | 153.1821 |
| ENSMUSG00000041319 | protein_coding | Thoc6 | 374.5211 | 348.8988 | 361.7099 |
| ENSMUSG00000073733 | protein_coding | 6330545A04Rik | 19.49143 | 26.25576 | 22.8736 |
| ENSMUSG00000042178 | protein_coding | Armc5 | 564.453 | 532.848 | 548.6505 |
| ENSMUSG00000051606 | pseudogene | 2010001K21Rik | 9.009394 | 12.39775 | 10.70357 |
| ENSMUSG00000028975 | protein_coding | Pex14 | 907.5718 | 854.6571 | 881.1145 |
| ENSMUSG00000032652 | protein_coding | Crebl2 | 983.6138 | 911.2601 | 947.4369 |
| ENSMUSG00000040618 | protein_coding | Pck2 | 771.7883 | 824.0037 | 797.896 |
| ENSMUSG00000045176 | protein_coding | 2310047M10Rik | 268.7979 | 288.8365 | 278.8172 |
| ENSMUSG00000026034 | protein_coding | Clk1 | 4085.834 | 5010.888 | 4548.361 |
| ENSMUSG00000027808 | protein_coding | Serp1 | 4838.331 | 5085.073 | 4961.702 |
| ENSMUSG00000024365 | protein_coding | Cyp21a1 | 34.14301 | 25.81725 | 29.98013 |
| ENSMUSG00000037418 | protein_coding | Best1 | 60.36841 | 68.82221 | 64.59531 |
| ENSMUSG00000026439 | protein_coding | Rbbp5 | 1784.918 | 1877.137 | 1831.027 |
| ENSMUSG00000079071 | protein_coding | Gm14085 | 1.293062 | 2.835175 | 2.064119 |
| ENSMUSG00000028709 | protein_coding | Mob3c | 324.8859 | 298.7931 | 311.8395 |
| ENSMUSG00000063254 | protein_coding | B230325K18Rik | 2.592014 | 4.927953 | 3.759983 |
| ENSMUSG00000036646 | protein_coding | Man1b1 | 2624.309 | 2513.048 | 2568.679 |
| ENSMUSG00000007812 | protein_coding | Zfp655 | 2896.441 | 3050.456 | 2973.448 |
| ENSMUSG00000047369 | protein_coding | Dnahc14 | 49.80505 | 42.37969 | 46.09237 |
| ENSMUSG00000052479 | protein_coding | A330008L17Rik | 1.296641 | 0.335524 | 0.816082 |
| ENSMUSG00000045534 | protein_coding | Kcna5 | 21.63684 | 27.26279 | 24.44982 |
| ENSMUSG00000020105 | protein_coding | Lrig3 | 369.7682 | 394.9749 | 382.3716 |
| ENSMUSG00000029919 | protein_coding | Hpgds | 95.72204 | 84.82063 | 90.27133 |
| ENSMUSG00000036718 | protein_coding | Micall2 | 385.6265 | 420.5745 | 403.1005 |
| ENSMUSG00000086856 | processed_transcrip | Gm16080 | 1.276663 | 0.335524 | 0.806093 |
| ENSMUSG00000027959 | protein_coding | Sass6 | 478.4828 | 450.9748 | 464.7288 |
| ENSMUSG00000005371 | protein_coding | Fbxo11 | 3074.588 | 3214.338 | 3144.463 |
| ENSMUSG00000020411 | protein_coding | Nipal4 | 5.493317 | 3.417215 | 4.455266 |
| ENSMUSG00000044949 | protein_coding | Ubtd2 | 1027.602 | 966.196 | 996.8991 |
| ENSMUSG00000035203 | protein_coding | Epn1 | 3237.102 | 3087.742 | 3162.422 |
| ENSMUSG00000080180 | pseudogene | Gm17235 | 8.504796 | 12.42442 | 10.46461 |
| ENSMUSG00000021905 | protein_coding | Dph3 | 744.6615 | 703.1003 | 723.8809 |
| ENSMUSG00000022941 | protein_coding | Ripply3 | 4.82554 | 2.65716 | 3.74135 |
| ENSMUSG00000044364 | protein_coding | 5430405G05Rik | 49.62882 | 42.4777 | 46.05326 |
| ENSMUSG00000085064 | processed_transcrip | Gm14495 | 7.411664 | 4.767956 | 6.08981 |
| ENSMUSG00000028672 | protein_coding | Hmgcl | 716.8992 | 759.4086 | 738.1539 |
| ENSMUSG00000022899 | protein_coding | Slc15a2 | 134.0527 | 154.4076 | 144.2301 |
| ENSMUSG00000073418 | protein_coding | C4b | 26.93507 | 34.07589 | 30.50548 |
| ENSMUSG00000067006 | protein_coding | Serpinb5 | 1.296641 | 0.326514 | 0.811578 |
| ENSMUSG00000073145 | processed_transcrip | Gm12000 | 1.296641 | 0.326514 | 0.811578 |
| ENSMUSG00000031105 | protein_coding | Slc25a14 | 425.559 | 400.1825 | 412.8708 |
| ENSMUSG00000090817 | protein_coding | Gm4450 | 1.276663 | 0.326514 | 0.801588 |
| ENSMUSG00000057612 | protein_coding | Vmn1r37 | 1.276663 | 0.326514 | 0.801588 |
| ENSMUSG00000086490 | processed_transcrip | Gm12292 | 1.276663 | 0.326514 | 0.801588 |
| ENSMUSG00000036565 | protein_coding | Ttyh3 | 2992.414 | 3169.968 | 3081.191 |
| ENSMUSG00000020241 | protein_coding | Col6a2 | 2718.872 | 2532.267 | 2625.57 |
| ENSMUSG00000043051 | protein_coding | Disc1 | 20.02398 | 15.63695 | 17.83047 |

| | | | | | |
|---|---|---|---|---|---|
| ENSMUSG00000074305 | protein_coding | C230081A13Rik | 2217.22 | 2343.224 | 2280.222 |
| ENSMUSG00000084274 | processed_transcrip | Gm12504 | 9.718171 | 6.843077 | 8.280624 |
| ENSMUSG00000030889 | protein_coding | Vwa3a | 13.92074 | 18.19895 | 16.05984 |
| ENSMUSG00000031819 | protein_coding | Cox4nb | 2360.464 | 2478.045 | 2419.254 |
| ENSMUSG00000075078 | protein_coding | Olfr1250 | 4.213076 | 6.587913 | 5.400494 |
| ENSMUSG00000030662 | protein_coding | Ipo5 | 4778.367 | 4467.647 | 4623.007 |
| ENSMUSG00000041119 | protein_coding | Pde9a | 158.3483 | 172.7536 | 165.551 |
| ENSMUSG00000021392 | protein_coding | Nol8 | 3140.956 | 2904.583 | 3022.769 |
| ENSMUSG00000078955 | processed_transcrip | Gm14222 | 83.47641 | 73.71449 | 78.59545 |
| ENSMUSG00000028789 | protein_coding | Adc | 458.0488 | 491.9941 | 475.0214 |
| ENSMUSG00000067203 | pseudogene | H2-K2 | 10.73012 | 14.75434 | 12.74223 |
| ENSMUSG00000001999 | protein_coding | Blvra | 690.9063 | 650.3086 | 670.6074 |
| ENSMUSG00000026224 | protein_coding | 4933407L21Rik | 22.90299 | 28.49528 | 25.69914 |
| ENSMUSG00000072720 | protein_coding | Myo18b | 142.8968 | 159.7831 | 151.34 |
| ENSMUSG00000058831 | protein_coding | Opn1sw | 2.289882 | 4.114566 | 3.202224 |
| ENSMUSG00000021718 | protein_coding | 4933425L06Rik | 82.70225 | 73.35479 | 78.02852 |
| ENSMUSG00000025091 | protein_coding | Pnliprp2 | 6.77229 | 4.494774 | 5.633532 |
| ENSMUSG00000081075 | pseudogene | Gm13160 | 8.742597 | 5.527649 | 7.135123 |
| ENSMUSG00000041731 | protein_coding | Pgm5 | 108.0204 | 96.12307 | 102.0718 |
| ENSMUSG00000033470 | protein_coding | Cysltr2 | 0.632442 | 1.72194 | 1.177191 |
| ENSMUSG00000038170 | protein_coding | Pde4dip | 902.9996 | 853.8767 | 878.4381 |
| ENSMUSG00000025726 | protein_coding | Slc28a1 | 21.05845 | 16.44061 | 18.74953 |
| ENSMUSG00000081692 | pseudogene | Gm15971 | 1.282552 | 0.36219 | 0.822371 |
| ENSMUSG00000031862 | protein_coding | Atp13a1 | 1870.58 | 1978.914 | 1924.747 |
| ENSMUSG00000038250 | protein_coding | Usp38 | 1212.941 | 1155.658 | 1184.3 |
| ENSMUSG00000079477 | protein_coding | Rab7 | 4440.895 | 4648.616 | 4544.756 |
| ENSMUSG00000036513 | protein_coding | Commd2 | 704.8329 | 746.7961 | 725.8145 |
| ENSMUSG00000041035 | protein_coding | Dpcd | 456.3178 | 484.4247 | 470.3712 |
| ENSMUSG00000057098 | protein_coding | Ebf1 | 775.2759 | 720.9192 | 748.0975 |
| ENSMUSG00000087613 | processed_transcrip | Gm13855 | 0.632442 | 1.748606 | 1.190524 |
| ENSMUSG00000085141 | lincRNA | Gm13429 | 4.198987 | 2.366321 | 3.282654 |
| ENSMUSG00000044835 | protein_coding | Ankrd45 | 143.4849 | 161.4101 | 152.4475 |
| ENSMUSG00000079372 | protein_coding | Gm3468 | 41.76958 | 32.2448 | 37.00719 |
| ENSMUSG00000074467 | pseudogene | Gm10702 | 6.825068 | 4.45009 | 5.637579 |
| ENSMUSG00000087748 | rRNA | 5S_rRNA | 0.638331 | 1.757616 | 1.197973 |
| ENSMUSG00000043284 | protein_coding | Tmem11 | 1272.163 | 1339.366 | 1305.764 |
| ENSMUSG00000029326 | protein_coding | Enoph1 | 1224.758 | 1287.719 | 1256.238 |
| ENSMUSG00000025950 | protein_coding | Idh1 | 4514.719 | 4763.192 | 4638.956 |
| ENSMUSG00000088357 | misc_RNA | 7SK | 10.01084 | 13.35964 | 11.68524 |
| ENSMUSG00000001383 | protein_coding | Zmat2 | 2167.518 | 2280.737 | 2224.127 |
| ENSMUSG00000028104 | protein_coding | Polr3gl | 468.5247 | 497.5862 | 483.0555 |
| ENSMUSG00000067577 | protein_coding | A430093F15Rik | 2.273482 | 1.015218 | 1.64435 |
| ENSMUSG00000041449 | processed_transcrip | Serpina3h | 2.281682 | 4.105557 | 3.193619 |
| ENSMUSG00000085069 | lincRNA | Gm13111 | 14.43562 | 19.0084 | 16.72201 |
| ENSMUSG00000056498 | protein_coding | Tmem154 | 12.58772 | 16.44133 | 14.51452 |
| ENSMUSG00000085589 | lincRNA | A430078I02Rik | 17.11575 | 21.70481 | 19.41028 |
| ENSMUSG00000081436 | pseudogene | Gm5912 | 7.692775 | 5.076814 | 6.384795 |
| ENSMUSG00000028998 | protein_coding | Tomm7 | 662.696 | 703.8002 | 683.2481 |
| ENSMUSG00000035371 | protein_coding | Ssx9 | 1.278973 | 0.36219 | 0.820581 |
| ENSMUSG00000081860 | pseudogene | Gm11533 | 1.32124 | 2.992322 | 2.156781 |
| ENSMUSG00000083412 | pseudogene | Gm12268 | 1.304841 | 2.835175 | 2.070008 |
| ENSMUSG00000085014 | processed_transcrip | Gm13490 | 4.567985 | 7.638807 | 6.103396 |
| ENSMUSG00000084058 | pseudogene | Gm15263 | 1.629262 | 3.081691 | 2.355476 |
| ENSMUSG00000031445 | protein_coding | Proz | 24.49461 | 19.68918 | 22.09189 |
| ENSMUSG00000038115 | protein_coding | Ano2 | 3.225724 | 1.72194 | 2.473832 |
| ENSMUSG00000085389 | processed_transcrip | 1700003M07Rik | 11.67059 | 8.300483 | 9.985534 |
| ENSMUSG00000056076 | protein_coding | Eif3b | 6979.673 | 6643.264 | 6811.468 |
| ENSMUSG00000086442 | processed_transcrip | Gm11722 | 2.259393 | 1.033237 | 1.646315 |

| | | | | | |
|---|---|---|---|---|---|
| ENSMUSG00000092812 | miRNA | AL669982.1 | 2.245304 | 0.997561 | 1.621433 |
| ENSMUSG00000040877 | protein_coding | Wdr25 | 237.2928 | 217.8141 | 227.5534 |
| ENSMUSG00000021013 | protein_coding | Ttc8 | 432.6356 | 400.0305 | 416.333 |
| ENSMUSG00000028803 | protein_coding | Nipal3 | 362.1732 | 388.2169 | 375.1951 |
| ENSMUSG00000087562 | lincRNA | Gm16754 | 20.73276 | 16.35196 | 18.54236 |
| ENSMUSG00000033589 | protein_coding | Reep4 | 522.582 | 490.9442 | 506.7631 |
| ENSMUSG00000048170 | protein_coding | Mcmbp | 3446.969 | 3294.922 | 3370.946 |
| ENSMUSG00000034800 | protein_coding | Zfp661 | 218.4174 | 200.7854 | 209.6014 |
| ENSMUSG00000031488 | protein_coding | Rab11fip1 | 936.4585 | 843.4555 | 889.957 |
| ENSMUSG00000061360 | protein_coding | Phf5a | 1319.541 | 1394.858 | 1357.199 |
| ENSMUSG00000019808 | protein_coding | Adat2 | 159.1273 | 175.9643 | 167.5458 |
| ENSMUSG00000030655 | protein_coding | Smg1 | 11140.76 | 11831.21 | 11485.98 |
| ENSMUSG00000028413 | protein_coding | B4galt1 | 2812.797 | 2968.699 | 2890.748 |
| ENSMUSG00000032913 | protein_coding | Lrig2 | 1587.207 | 1669.513 | 1628.36 |
| ENSMUSG00000048766 | protein_coding | Skint10 | 1.282552 | 0.335524 | 0.809038 |
| ENSMUSG00000090702 | lincRNA | Gm17552 | 42.97222 | 33.6989 | 38.33556 |
| ENSMUSG00000025393 | protein_coding | Atp5b | 15140.13 | 15746.3 | 15443.21 |
| ENSMUSG00000024165 | protein_coding | Hn1l | 2040.097 | 2172.927 | 2106.512 |
| ENSMUSG00000005355 | protein_coding | Casp14 | 1.953683 | 3.779043 | 2.866363 |
| ENSMUSG00000026796 | protein_coding | Fam129b | 643.7238 | 682.5655 | 663.1447 |
| ENSMUSG00000068240 | protein_coding | Gm11808 | 1747.885 | 1823.16 | 1785.523 |
| ENSMUSG00000002831 | protein_coding | Plin4 | 21.71802 | 27.68401 | 24.70102 |
| ENSMUSG00000093333 | snRNA | AC120136.3 | 4.501119 | 7.036261 | 5.76869 |
| ENSMUSG00000045087 | protein_coding | S1pr5 | 5.479228 | 3.327846 | 4.403537 |
| ENSMUSG00000043099 | protein_coding | Hic1 | 619.7036 | 664.2809 | 641.9923 |
| ENSMUSG00000032085 | protein_coding | Tagln | 1582.875 | 1677.461 | 1630.168 |
| ENSMUSG00000084557 | snRNA | U4 | 0.98273 | 0 | 0.491365 |
| ENSMUSG00000089902 | processed_transcrip | Gm13625 | 0.98273 | 0 | 0.491365 |
| ENSMUSG00000087789 | snRNA | U6 | 0.98273 | 0 | 0.491365 |
| ENSMUSG00000088115 | misc_RNA | 7SK | 0.98273 | 0 | 0.491365 |
| ENSMUSG00000085153 | processed_transcrip | B230314M03Rik | 0.98273 | 0 | 0.491365 |
| ENSMUSG00000079264 | protein_coding | Gm11077 | 0.98273 | 0 | 0.491365 |
| ENSMUSG00000092505 | pseudogene | Gm19246 | 0.98273 | 0 | 0.491365 |
| ENSMUSG00000070563 | protein_coding | Spaca4 | 0.98273 | 0 | 0.491365 |
| ENSMUSG00000087542 | pseudogene | Gm15878 | 0.98273 | 0 | 0.491365 |
| ENSMUSG00000084498 | rRNA | n-R5s98 | 0.98273 | 0 | 0.491365 |
| ENSMUSG00000084703 | snRNA | U4 | 0.98273 | 0 | 0.491365 |
| ENSMUSG00000064691 | snRNA | U6 | 0.98273 | 0 | 0.491365 |
| ENSMUSG00000069189 | protein_coding | Zdhhc11 | 0.98273 | 0 | 0.491365 |
| ENSMUSG00000064822 | snRNA | U6 | 0.98273 | 0 | 0.491365 |
| ENSMUSG00000005696 | protein_coding | Sh2d1a | 0.98273 | 0 | 0.491365 |
| ENSMUSG00000088445 | snoRNA | SCARNA24 | 0.98273 | 0 | 0.491365 |
| ENSMUSG00000081055 | pseudogene | Gm6987 | 0.98273 | 0 | 0.491365 |
| ENSMUSG00000093157 | miRNA | AC166162.1 | 4.849096 | 7.196257 | 6.022677 |
| ENSMUSG00000046683 | processed_transcrip | 0610025J13Rik | 11.31105 | 8.12283 | 9.716942 |
| ENSMUSG00000063427 | pseudogene | Gm8017 | 1797.692 | 1883.009 | 1840.35 |
| ENSMUSG00000058745 | pseudogene | Gm9174 | 1.282552 | 0.326514 | 0.804533 |
| ENSMUSG00000092692 | miRNA | CT030723.1 | 1.282552 | 0.326514 | 0.804533 |
| ENSMUSG00000037130 | protein_coding | H2-M10.6 | 1.282552 | 0.326514 | 0.804533 |
| ENSMUSG00000005506 | protein_coding | Celf1 | 6677.759 | 6409.387 | 6543.573 |
| ENSMUSG00000026012 | protein_coding | Cd28 | 4.780962 | 7.751992 | 6.266477 |
| ENSMUSG00000078997 | protein_coding | Vmn2r-ps105 | 1.967772 | 4.167898 | 3.067835 |
| ENSMUSG00000025616 | protein_coding | Usp16 | 1617.804 | 1520.684 | 1569.244 |
| ENSMUSG00000091875 | lincRNA | Mir155 | 4.535186 | 2.719502 | 3.627344 |
| ENSMUSG00000060204 | protein_coding | Gm5868 | 5.436961 | 8.476372 | 6.956666 |
| ENSMUSG00000032060 | protein_coding | Cryab | 172.1915 | 187.4769 | 179.8342 |
| ENSMUSG00000088179 | snRNA | U6 | 1.278973 | 0.335524 | 0.807248 |
| ENSMUSG00000086112 | protein_coding | 4930563I02Rik | 1.278973 | 0.335524 | 0.807248 |

| | | | | | |
|---|---|---|---|---|---|
| ENSMUSG00000065196 | snoRNA | Snord85 | 0.638331 | 1.784282 | 1.211307 |
| ENSMUSG00000087541 | processed_transcrip | Gm15830 | 2.231215 | 4.114566 | 3.172891 |
| ENSMUSG00000045690 | protein_coding | Wdr89 | 216.1724 | 238.4659 | 227.3191 |
| ENSMUSG00000090551 | protein_coding | A730015C16Rik | 8.697795 | 11.93791 | 10.31785 |
| ENSMUSG00000081552 | pseudogene | Gm12328 | 3.202167 | 5.492697 | 4.347432 |
| ENSMUSG00000071291 | protein_coding | Zfp58 | 324.8665 | 298.4334 | 311.6499 |
| ENSMUSG00000085932 | processed_transcrip | Gm15556 | 0.646531 | 1.757616 | 1.202073 |
| ENSMUSG00000073632 | pseudogene | Gm15433 | 1.270774 | 0.36219 | 0.816482 |
| ENSMUSG00000074547 | pseudogene | Gm10712 | 1.270774 | 0.36219 | 0.816482 |
| ENSMUSG00000023333 | protein_coding | Gcm1 | 1.270774 | 0.36219 | 0.816482 |
| ENSMUSG00000024487 | protein_coding | Yipf5 | 1463.902 | 1401.848 | 1432.875 |
| ENSMUSG00000020664 | protein_coding | Dld | 3673.07 | 3834.023 | 3753.546 |
| ENSMUSG00000072419 | protein_coding | Dppa2 | 14.44867 | 18.87543 | 16.66205 |
| ENSMUSG00000083038 | pseudogene | Gm13233 | 2.883635 | 4.794622 | 3.839129 |
| ENSMUSG00000002768 | protein_coding | Mea1 | 1561.507 | 1486.252 | 1523.88 |
| ENSMUSG00000049641 | protein_coding | Vgll2 | 1.612862 | 3.336493 | 2.474678 |
| ENSMUSG00000084876 | processed_transcrip | Gm14965 | 7.805487 | 5.058434 | 6.43196 |
| ENSMUSG00000047002 | protein_coding | Msgn1 | 1.278973 | 0.326514 | 0.802744 |
| ENSMUSG00000089766 | processed_transcrip | Gm16538 | 1.278973 | 0.326514 | 0.802744 |
| ENSMUSG00000029720 | protein_coding | Lrch4 | 1129.427 | 1059.39 | 1094.408 |
| ENSMUSG00000038079 | protein_coding | Als2cr4 | 855.589 | 902.4534 | 879.0212 |
| ENSMUSG00000022450 | protein_coding | Ndufa6 | 1205.214 | 1265.348 | 1235.281 |
| ENSMUSG00000084786 | protein_coding | Ubl5 | 1160.285 | 1223.924 | 1192.105 |
| ENSMUSG00000019769 | protein_coding | Syne1 | 1193.33 | 1264.001 | 1228.666 |
| ENSMUSG00000050818 | protein_coding | Olfr330 | 8.752883 | 12.74845 | 10.75066 |
| ENSMUSG00000022831 | protein_coding | Hcls1 | 76.97076 | 67.00003 | 71.9854 |
| ENSMUSG00000040093 | protein_coding | Bmf | 966.401 | 905.6286 | 936.0148 |
| ENSMUSG00000089261 | miRNA | Mir669n | 0.64422 | 1.72194 | 1.18308 |
| ENSMUSG00000022102 | protein_coding | Dok2 | 111.156 | 95.15476 | 103.1554 |
| ENSMUSG00000001630 | protein_coding | Stk38l | 2080.788 | 2193.831 | 2137.309 |
| ENSMUSG00000054493 | protein_coding | Gm9947 | 3.250323 | 1.65059 | 2.450457 |
| ENSMUSG00000030649 | protein_coding | 3200002M19Rik | 820.2118 | 773.4872 | 796.8495 |
| ENSMUSG00000089698 | pseudogene | Gm2541 | 18.32427 | 23.35146 | 20.83787 |
| ENSMUSG00000026234 | protein_coding | Ncl | 21452.76 | 20522.9 | 20987.83 |
| ENSMUSG00000041220 | protein_coding | Elovl6 | 670.2795 | 732.6883 | 701.4839 |
| ENSMUSG00000093345 | misc_RNA | AC124584.1 | 0.64422 | 1.748606 | 1.196413 |
| ENSMUSG00000064484 | snoRNA | SNORD103 | 8.09942 | 11.13281 | 9.616114 |
| ENSMUSG00000092072 | protein_coding | Gm4540 | 5.863583 | 9.200027 | 7.531805 |
| ENSMUSG00000084923 | processed_transcrip | Gm15611 | 3.239813 | 1.712931 | 2.476372 |
| ENSMUSG00000024866 | protein_coding | Acy3 | 127.7518 | 140.4327 | 134.0922 |
| ENSMUSG00000086453 | processed_transcrip | Gm11457 | 19.01076 | 23.58389 | 21.29733 |
| ENSMUSG00000026254 | protein_coding | Eif4e2 | 2601.478 | 2486.13 | 2543.804 |
| ENSMUSG00000029913 | protein_coding | Prdm5 | 171.1074 | 191.2751 | 181.1913 |
| ENSMUSG00000085471 | processed_transcrip | 4933423P22Rik | 0.976841 | 0 | 0.488421 |
| ENSMUSG00000074435 | protein_coding | Smcp | 0.976841 | 0 | 0.488421 |
| ENSMUSG00000086845 | processed_transcrip | Gm13010 | 0.976841 | 0 | 0.488421 |
| ENSMUSG00000073500 | protein_coding | Gm3106 | 0.976841 | 0 | 0.488421 |
| ENSMUSG00000075930 | snRNA | U5 | 0.976841 | 0 | 0.488421 |
| ENSMUSG00000015890 | protein_coding | Amdhd1 | 0.976841 | 0 | 0.488421 |
| ENSMUSG00000085561 | processed_transcrip | 1700046C09Rik | 0.976841 | 0 | 0.488421 |
| ENSMUSG00000071489 | protein_coding | Ptgdr | 0.976841 | 0 | 0.488421 |
| ENSMUSG00000092604 | protein_coding | Gm20508 | 0.976841 | 0 | 0.488421 |
| ENSMUSG00000032900 | protein_coding | 1700065I17Rik | 0.976841 | 0 | 0.488421 |
| ENSMUSG00000028979 | protein_coding | Masp2 | 4.213076 | 6.516201 | 5.364638 |
| ENSMUSG00000033106 | protein_coding | Slc7a6os | 1143.7 | 1089.979 | 1116.84 |
| ENSMUSG00000054622 | protein_coding | D730045B01Rik | 2.920013 | 4.776604 | 3.848309 |
| ENSMUSG00000051319 | processed_transcrip | 1500011K16Rik | 285.1514 | 312.1167 | 298.6341 |
| ENSMUSG00000045102 | protein_coding | Poln | 335.4506 | 252.1677 | 293.8091 |

| | | | | | |
|---|---|---|---|---|---|
| ENSMUSG00000062846 | pseudogene | Gm14176 | 45.36468 | 38.0532 | 41.70894 |
| ENSMUSG00000027001 | protein_coding | Dusp19 | 187.2827 | 168.5698 | 177.9263 |
| ENSMUSG00000032807 | protein_coding | Alox12b | 63.46018 | 54.68947 | 59.07483 |
| ENSMUSG00000085077 | processed_transcrip | 1700049E15Rik | 1.270774 | 0.335524 | 0.803149 |
| ENSMUSG00000071540 | protein_coding | 3425401B19Rik | 1.270774 | 0.335524 | 0.803149 |
| ENSMUSG00000040399 | protein_coding | C630050I24Rik | 0.65242 | 1.757616 | 1.205018 |
| ENSMUSG00000038848 | protein_coding | Ythdf1 | 3922.944 | 4086.792 | 4004.868 |
| ENSMUSG00000074968 | protein_coding | Ano3 | 38.27699 | 31.42961 | 34.8533 |
| ENSMUSG00000023009 | protein_coding | Nckap5l | 1165.234 | 1222.767 | 1194.001 |
| ENSMUSG00000084211 | pseudogene | Gm11949 | 2.265282 | 1.015218 | 1.64025 |
| ENSMUSG00000078860 | protein_coding | Gm11006 | 2.265282 | 1.015218 | 1.64025 |
| ENSMUSG00000089761 | processed_transcrip | Vmn2r-ps111 | 2.265282 | 1.015218 | 1.64025 |
| ENSMUSG00000048312 | protein_coding | Gm4884 | 2.597903 | 4.512793 | 3.555348 |
| ENSMUSG00000089158 | misc_RNA | 7SK | 0.974531 | 0 | 0.487265 |
| ENSMUSG00000026830 | protein_coding | Ermn | 0.974531 | 0 | 0.487265 |
| ENSMUSG00000028238 | protein_coding | Atp6v0d2 | 0.974531 | 0 | 0.487265 |
| ENSMUSG00000050810 | protein_coding | Oog3 | 0.974531 | 0 | 0.487265 |
| ENSMUSG00000086259 | processed_transcrip | 5830403M04Rik | 0.974531 | 0 | 0.487265 |
| ENSMUSG00000072778 | protein_coding | Vmn2r27 | 0.974531 | 0 | 0.487265 |
| ENSMUSG00000093239 | miRNA | AC160118.1 | 0.974531 | 0 | 0.487265 |
| ENSMUSG00000089336 | snoRNA | SNORA17 | 0.974531 | 0 | 0.487265 |
| ENSMUSG00000085718 | pseudogene | Rpl9-ps3 | 0.974531 | 0 | 0.487265 |
| ENSMUSG00000052909 | protein_coding | Gm9894 | 0.974531 | 0 | 0.487265 |
| ENSMUSG00000069208 | protein_coding | Zfp825 | 197.1355 | 216.0847 | 206.6101 |
| ENSMUSG00000092953 | miRNA | AC162893.1 | 1.612862 | 3.161689 | 2.387276 |
| ENSMUSG00000086430 | processed_transcrip | 4930551O13Rik | 7.477488 | 4.892639 | 6.185063 |
| ENSMUSG00000024008 | protein_coding | Cpne5 | 542.7664 | 493.9168 | 518.3416 |
| ENSMUSG00000030816 | protein_coding | Rnf40 | 2972.891 | 2832.187 | 2902.539 |
| ENSMUSG00000045648 | protein_coding | Vwc2l | 3.205746 | 1.712931 | 2.459338 |
| ENSMUSG00000046413 | lincRNA | D230002A01Rik | 2.255815 | 4.194926 | 3.22537 |
| ENSMUSG00000021771 | protein_coding | Vdac2 | 4256.354 | 4428.738 | 4342.546 |
| ENSMUSG00000051582 | protein_coding | Otud6a | 211.0234 | 236.3662 | 223.6948 |
| ENSMUSG00000005871 | protein_coding | Apc | 6723.439 | 6399.26 | 6561.35 |
| ENSMUSG00000082182 | pseudogene | Gm6660 | 1.270774 | 0.326514 | 0.798644 |
| ENSMUSG00000071669 | protein_coding | Snx29 | 200.1763 | 181.3317 | 190.754 |
| ENSMUSG00000031832 | protein_coding | Taf1c | 586.1943 | 619.1758 | 602.685 |
| ENSMUSG00000085318 | processed_transcrip | Gm12524 | 0.638331 | 1.73095 | 1.18464 |
| ENSMUSG00000019876 | protein_coding | Pkib | 9.677171 | 13.35964 | 11.51841 |
| ENSMUSG00000083894 | pseudogene | Gm11489 | 9.477016 | 13.90849 | 11.69275 |
| ENSMUSG00000034333 | protein_coding | Zbed4 | 977.7978 | 921.0801 | 949.4389 |
| ENSMUSG00000032172 | protein_coding | Olfm2 | 37.46415 | 44.92185 | 41.193 |
| ENSMUSG00000039419 | protein_coding | Cntnap2 | 11.98345 | 8.654025 | 10.31874 |
| ENSMUSG00000024176 | protein_coding | Sox8 | 2449.237 | 2935.421 | 2692.329 |
| ENSMUSG00000049882 | protein_coding | Mettl21d | 515.088 | 554.7398 | 534.9139 |
| ENSMUSG00000046191 | pseudogene | Pcdhb20 | 78.8388 | 89.79508 | 84.31694 |
| ENSMUSG00000028971 | protein_coding | Cort | 7.463399 | 4.848316 | 6.155858 |
| ENSMUSG00000038252 | protein_coding | Ncapd2 | 3257.136 | 3024.595 | 3140.866 |
| ENSMUSG00000028684 | protein_coding | Urod | 1033.554 | 1083.795 | 1058.675 |
| ENSMUSG00000024096 | protein_coding | Ralbp1 | 2882.055 | 2727.531 | 2804.793 |
| ENSMUSG00000090936 | lincRNA | Gm17705 | 63.73965 | 55.18753 | 59.46359 |
| ENSMUSG00000039086 | protein_coding | Ss18l1 | 724.4064 | 685.3948 | 704.9006 |
| ENSMUSG00000020692 | protein_coding | Nle1 | 405.017 | 381.3752 | 393.1961 |
| ENSMUSG00000038544 | protein_coding | 1110054O05Rik | 926.9766 | 973.3719 | 950.1742 |
| ENSMUSG00000037410 | protein_coding | Tbc1d2b | 2051.222 | 2194.376 | 2122.799 |
| ENSMUSG00000025733 | protein_coding | Rhot2 | 1311.087 | 1370.668 | 1340.878 |
| ENSMUSG00000075538 | protein_coding | Gm10855 | 2.267593 | 1.059903 | 1.663748 |
| ENSMUSG00000048279 | protein_coding | Sacs | 2058.386 | 2295.063 | 2176.725 |
| ENSMUSG00000051832 | lincRNA | E230016K23Rik | 0.646531 | 1.739597 | 1.193064 |

| | | | | | |
|---|---|---|---|---|---|
| ENSMUSG00000040373 | protein_coding | Cacng5 | 576.7951 | 514.5847 | 545.6899 |
| ENSMUSG00000028747 | protein_coding | Htr6 | 22.87355 | 18.40042 | 20.63698 |
| ENSMUSG00000072731 | protein_coding | Gm3715 | 4.255343 | 2.383978 | 3.31966 |
| ENSMUSG00000015599 | protein_coding | Ttbk1 | 110.3475 | 126.518 | 118.4328 |
| ENSMUSG00000023707 | protein_coding | Ogfod2 | 340.6746 | 316.9857 | 328.8302 |
| ENSMUSG00000005447 | protein_coding | Pafah1b3 | 1202.233 | 1280.772 | 1241.502 |
| ENSMUSG00000030137 | protein_coding | Tuba8 | 11.91174 | 8.820181 | 10.36596 |
| ENSMUSG00000078348 | protein_coding | Sf3b5 | 1318.543 | 1393.396 | 1355.969 |
| ENSMUSG00000028280 | protein_coding | Gabrr1 | 4.507008 | 7.151211 | 5.829109 |
| ENSMUSG00000028327 | protein_coding | 1300002K09Rik | 10.37521 | 7.195896 | 8.785554 |
| ENSMUSG00000071745 | protein_coding | DXBay18 | 12.88627 | 16.74046 | 14.81336 |
| ENSMUSG00000044322 | protein_coding | Dsc1 | 0.632442 | 1.686265 | 1.159354 |
| ENSMUSG00000056606 | protein_coding | Gm10000 | 0.646531 | 1.775272 | 1.210902 |
| ENSMUSG00000028882 | protein_coding | Ppp1r8 | 2823.217 | 2948.678 | 2885.948 |
| ENSMUSG00000021587 | protein_coding | Pcsk1 | 5.465139 | 8.060127 | 6.762633 |
| ENSMUSG00000045875 | protein_coding | Adra1a | 3.211635 | 1.677256 | 2.444445 |
| ENSMUSG00000034116 | protein_coding | Vav1 | 46.42688 | 39.68934 | 43.05811 |
| ENSMUSG00000079259 | protein_coding | Trim71 | 1083.531 | 1162.986 | 1123.259 |
| ENSMUSG00000083263 | pseudogene | Gm14874 | 2.251193 | 1.015218 | 1.633206 |
| ENSMUSG00000027857 | protein_coding | Tshb | 10.47488 | 15.17923 | 12.82706 |
| ENSMUSG00000022376 | protein_coding | Adcy8 | 2.261704 | 1.033237 | 1.64747 |
| ENSMUSG00000080920 | pseudogene | Gm6228 | 36.25942 | 42.90151 | 39.58047 |
| ENSMUSG00000040701 | protein_coding | Ap1g2 | 137.9367 | 151.2747 | 144.6057 |
| ENSMUSG00000024817 | protein_coding | Uhrf2 | 1675.793 | 1759.765 | 1717.779 |
| ENSMUSG00000026380 | protein_coding | Tcfcp2l1 | 649.9913 | 737.5247 | 693.758 |
| ENSMUSG00000034007 | protein_coding | Scaper | 2394.512 | 2263.94 | 2329.226 |
| ENSMUSG00000049323 | protein_coding | Smcr8 | 1432.014 | 1522.71 | 1477.362 |
| ENSMUSG00000085274 | lincRNA | BC046401 | 9.972147 | 13.43063 | 11.70139 |
| ENSMUSG00000036782 | protein_coding | Klhl13 | 583.4719 | 548.6372 | 566.0545 |
| ENSMUSG00000041324 | protein_coding | Inhba | 185.9162 | 164.904 | 175.4101 |
| ENSMUSG00000057637 | protein_coding | Prdm2 | 3192.36 | 3038.484 | 3115.422 |
| ENSMUSG00000032221 | protein_coding | Mns1 | 1533.414 | 1415.279 | 1474.347 |
| ENSMUSG00000055228 | protein_coding | Zfp935 | 235.4613 | 260.9723 | 248.2168 |
| ENSMUSG00000091639 | pseudogene | Gm3756 | 56.40827 | 64.97896 | 60.69362 |
| ENSMUSG00000025083 | protein_coding | Afap1l2 | 527.7877 | 562.8827 | 545.3352 |
| ENSMUSG00000041408 | protein_coding | Wapal | 7200.005 | 6851.446 | 7025.726 |
| ENSMUSG00000087721 | rRNA | 5S_rRNA | 14.22749 | 10.72014 | 12.47381 |
| ENSMUSG00000052533 | protein_coding | Nup188 | 3430.524 | 3214.023 | 3322.273 |
| ENSMUSG00000030228 | protein_coding | Pik3c2g | 15.18204 | 19.81671 | 17.49938 |
| ENSMUSG00000030610 | protein_coding | Det1 | 596.0017 | 550.8184 | 573.41 |
| ENSMUSG00000044017 | protein_coding | Gpr133 | 35.49891 | 29.9401 | 32.71951 |
| ENSMUSG00000001076 | protein_coding | C1ql4 | 3.919143 | 1.994761 | 2.956952 |
| ENSMUSG00000031390 | protein_coding | Avpr2 | 0.66062 | 1.72194 | 1.19128 |
| ENSMUSG00000086955 | processed_transcrip | 1700021L23Rik | 11.43301 | 7.67412 | 9.553565 |
| ENSMUSG00000023577 | protein_coding | Iqcf3 | 0.65242 | 1.775272 | 1.213846 |
| ENSMUSG00000085591 | processed_transcrip | Gm13479 | 0.962752 | 0 | 0.481376 |
| ENSMUSG00000074620 | protein_coding | Gm10731 | 0.962752 | 0 | 0.481376 |
| ENSMUSG00000089431 | snRNA | U6 | 0.962752 | 0 | 0.481376 |
| ENSMUSG00000087349 | lincRNA | Gm12680 | 0.962752 | 0 | 0.481376 |
| ENSMUSG00000080732 | pseudogene | Gm13222 | 0.962752 | 0 | 0.481376 |
| ENSMUSG00000082150 | pseudogene | Gm13144 | 0.962752 | 0 | 0.481376 |
| ENSMUSG00000080972 | pseudogene | Gm16061 | 0.962752 | 0 | 0.481376 |
| ENSMUSG00000086919 | processed_transcrip | Gm11497 | 0.962752 | 0 | 0.481376 |
| ENSMUSG00000091756 | protein_coding | Gm3095 | 0.962752 | 0 | 0.481376 |
| ENSMUSG00000091911 | pseudogene | Gm9587 | 0.962752 | 0 | 0.481376 |
| ENSMUSG00000089645 | processed_transcrip | Gm5766 | 0.962752 | 0 | 0.481376 |
| ENSMUSG00000087793 | snoRNA | SNORA38 | 0.962752 | 0 | 0.481376 |
| ENSMUSG00000085520 | lincRNA | Gm16871 | 0.962752 | 0 | 0.481376 |

| | | | | | |
|---|---|---|---|---|---|
| ENSMUSG00000065031 | snRNA | U6 | 0.962752 | 0 | 0.481376 |
| ENSMUSG00000083821 | pseudogene | Gm14631 | 0.962752 | 0 | 0.481376 |
| ENSMUSG00000081414 | pseudogene | Gm3943 | 0.962752 | 0 | 0.481376 |
| ENSMUSG00000084110 | pseudogene | Gm4992 | 0.962752 | 0 | 0.481376 |
| ENSMUSG00000034858 | protein_coding | BC031353 | 3616.66 | 3793.351 | 3705.005 |
| ENSMUSG00000021792 | protein_coding | 5730469M10Rik | 3341.633 | 3474.067 | 3407.85 |
| ENSMUSG00000028139 | protein_coding | Riiad1 | 11.4225 | 15.61894 | 13.52072 |
| ENSMUSG00000024219 | protein_coding | Anks1 | 1265.365 | 1354.427 | 1309.896 |
| ENSMUSG00000001240 | protein_coding | Ramp2 | 257.4816 | 277.1894 | 267.3355 |
| ENSMUSG00000012889 | protein_coding | Podnl1 | 6.795847 | 4.423424 | 5.609635 |
| ENSMUSG00000054034 | protein_coding | Tceal5 | 41.52247 | 49.35754 | 45.44001 |
| ENSMUSG00000005566 | protein_coding | Trim28 | 7166.11 | 6853.672 | 7009.891 |
| ENSMUSG00000087647 | processed_transcrip | Gm15407 | 0.98273 | 2.330646 | 1.656688 |
| ENSMUSG00000083380 | pseudogene | Gm3244 | 47.81141 | 41.0902 | 44.4508 |
| ENSMUSG00000087632 | pseudogene | Gm6058 | 0 | 1.086569 | 0.543284 |
| ENSMUSG00000086517 | processed_transcrip | Gm12534 | 0 | 1.086569 | 0.543284 |
| ENSMUSG00000082928 | pseudogene | Gm12785 | 0 | 1.086569 | 0.543284 |
| ENSMUSG00000075546 | protein_coding | Gm10858 | 0 | 1.086569 | 0.543284 |
| ENSMUSG00000076941 | miRNA | AC134447.1 | 0 | 1.086569 | 0.543284 |
| ENSMUSG00000088298 | misc_RNA | 7SK | 0 | 1.086569 | 0.543284 |
| ENSMUSG00000025527 | protein_coding | Satl1 | 0 | 1.086569 | 0.543284 |
| ENSMUSG00000031022 | protein_coding | BC051019 | 14.16524 | 18.13661 | 16.15092 |
| ENSMUSG00000038034 | protein_coding | Igsf8 | 1042.384 | 1116.198 | 1079.291 |
| ENSMUSG00000070990 | protein_coding | Foxe1 | 5.526116 | 3.470547 | 4.498331 |
| ENSMUSG00000077635 | snoRNA | SNORA17 | 0.960442 | 0 | 0.480221 |
| ENSMUSG00000064547 | misc_RNA | 7SK | 0.960442 | 0 | 0.480221 |
| ENSMUSG00000087525 | processed_transcrip | Gm13062 | 0.960442 | 0 | 0.480221 |
| ENSMUSG00000084458 | miRNA | mmu-mir-1907 | 0.960442 | 0 | 0.480221 |
| ENSMUSG00000089386 | snoRNA | SNORA84 | 0.960442 | 0 | 0.480221 |
| ENSMUSG00000085285 | processed_transcrip | Gm14968 | 0.960442 | 0 | 0.480221 |
| ENSMUSG00000080626 | miRNA | Mir1192 | 0.960442 | 0 | 0.480221 |
| ENSMUSG00000048573 | protein_coding | Cypt3 | 0.960442 | 0 | 0.480221 |
| ENSMUSG00000064361 | Mt_tRNA | mt-Tr | 0.960442 | 0 | 0.480221 |
| ENSMUSG00000066964 | protein_coding | Tmem211 | 7.438799 | 10.25128 | 8.845041 |
| ENSMUSG00000024442 | protein_coding | 0610009O20Rik | 860.1905 | 909.4277 | 884.8091 |
| ENSMUSG00000017119 | protein_coding | Nbr1 | 5315.338 | 5546.919 | 5431.128 |
| ENSMUSG00000067194 | protein_coding | Eif1ax | 3248.981 | 3375.163 | 3312.072 |
| ENSMUSG00000086977 | lincRNA | 9430065F17Rik | 17.49883 | 13.21114 | 15.35499 |
| ENSMUSG00000069670 | protein_coding | Nkain2 | 8.397973 | 5.774527 | 7.08625 |
| ENSMUSG00000041313 | protein_coding | Slc7a1 | 5049.207 | 5403.872 | 5226.54 |
| ENSMUSG00000053333 | protein_coding | Dis3l2 | 597.8239 | 631.7325 | 614.7782 |
| ENSMUSG00000022550 | protein_coding | Adck5 | 774.3764 | 721.4254 | 747.9009 |
| ENSMUSG00000032220 | protein_coding | Myo1e | 237.4479 | 261.8941 | 249.671 |
| ENSMUSG00000086357 | lincRNA | 1700112J16Rik | 12.27023 | 9.173361 | 10.72179 |
| ENSMUSG00000025821 | protein_coding | Zfp282 | 884.8886 | 966.1711 | 925.5298 |
| ENSMUSG00000086549 | processed_transcrip | Gm13648 | 8.028975 | 11.17785 | 9.603415 |
| ENSMUSG00000044948 | protein_coding | Wdr96 | 104.493 | 92.85327 | 98.67315 |
| ENSMUSG00000018239 | protein_coding | Zcchc10 | 415.8717 | 445.144 | 430.5078 |
| ENSMUSG00000092262 | pseudogene | Gm20434 | 5.48616 | 3.434871 | 4.460516 |
| ENSMUSG00000051062 | protein_coding | Fbll1 | 1.592884 | 3.108357 | 2.35062 |
| ENSMUSG00000077457 | snoRNA | Snord65 | 1.923194 | 3.434871 | 2.679033 |
| ENSMUSG00000015396 | protein_coding | Cd83 | 45.03564 | 52.86517 | 48.9504 |
| ENSMUSG00000085185 | lincRNA | BC028777 | 21.3406 | 16.24742 | 18.79401 |
| ENSMUSG00000090912 | lincRNA | 4931403G20Rik | 0.64422 | 1.686265 | 1.165243 |
| ENSMUSG00000049969 | protein_coding | Plekhf2 | 1316.509 | 1247.461 | 1281.985 |
| ENSMUSG00000019969 | protein_coding | Psen1 | 1651.267 | 1737.177 | 1694.222 |
| ENSMUSG00000014602 | protein_coding | Kif1a | 1997.459 | 2127.332 | 2062.395 |
| ENSMUSG00000074398 | lincRNA | Gm15441 | 20.35325 | 24.89896 | 22.6261 |

| | | | | | |
|---|---|---|---|---|---|
| ENSMUSG00000086935 | processed_transcrip | Gm15956 | 3.216256 | 5.121137 | 4.168696 |
| ENSMUSG00000048439 | protein_coding | Nupl2 | 308.5879 | 287.4616 | 298.0247 |
| ENSMUSG00000004207 | protein_coding | Psap | 16082.06 | 16819.33 | 16450.7 |
| ENSMUSG00000056735 | lincRNA | A930024E05Rik | 90.77476 | 81.39726 | 86.08601 |
| ENSMUSG00000022999 | protein_coding | Lmbr1l | 1351.285 | 1428.175 | 1389.73 |
| ENSMUSG00000028847 | protein_coding | Trappc3 | 1880.512 | 1800.448 | 1840.48 |
| ENSMUSG00000028936 | protein_coding | Rpl22 | 4635.744 | 4866.634 | 4751.189 |
| ENSMUSG00000043629 | protein_coding | 1700019D03Rik | 16.12377 | 20.33284 | 18.2283 |
| ENSMUSG00000093155 | miRNA | AC117610.1 | 0.954552 | 0 | 0.477276 |
| ENSMUSG00000012187 | protein_coding | Mogat1 | 0.954552 | 0 | 0.477276 |
| ENSMUSG00000080994 | pseudogene | Gm13464 | 0.954552 | 0 | 0.477276 |
| ENSMUSG00000057290 | pseudogene | Gm15732 | 0.954552 | 0 | 0.477276 |
| ENSMUSG00000093238 | miRNA | AC110909.1 | 0.954552 | 0 | 0.477276 |
| ENSMUSG00000037145 | protein_coding | 2210407C18Rik | 0.954552 | 0 | 0.477276 |
| ENSMUSG00000088985 | miRNA | Mir1946a | 0.954552 | 0 | 0.477276 |
| ENSMUSG00000034472 | protein_coding | Rasd2 | 1002.122 | 938.2603 | 970.1913 |
| ENSMUSG00000084127 | pseudogene | Gm13169 | 7.75502 | 10.59545 | 9.175237 |
| ENSMUSG00000030088 | protein_coding | Aldh1l1 | 360.6616 | 335.1926 | 347.9271 |
| ENSMUSG00000040429 | protein_coding | Mterf | 147.5468 | 164.9573 | 156.252 |
| ENSMUSG00000047394 | protein_coding | Odf3b | 5.522538 | 8.404659 | 6.963598 |
| ENSMUSG00000028865 | protein_coding | Cd164l2 | 26.2919 | 20.89971 | 23.5958 |
| ENSMUSG00000046519 | protein_coding | Golph3l | 1558.471 | 1659.913 | 1609.192 |
| ENSMUSG00000017720 | protein_coding | Trp53tg5 | 7.697397 | 5.041138 | 6.369268 |
| ENSMUSG00000091083 | protein_coding | Gm4535 | 8.713151 | 11.86584 | 10.28949 |
| ENSMUSG00000020600 | protein_coding | Slc7a15 | 2.555636 | 4.361082 | 3.458359 |
| ENSMUSG00000051113 | protein_coding | Fam71e1 | 126.0736 | 144.7649 | 135.4193 |
| ENSMUSG00000056531 | protein_coding | Ccdc18 | 695.5893 | 617.0461 | 656.3177 |
| ENSMUSG00000024959 | protein_coding | Bad | 535.4381 | 502.9275 | 519.1828 |
| ENSMUSG00000085925 | protein_coding | Rtl1 | 17.47781 | 13.54381 | 15.51081 |
| ENSMUSG00000022684 | protein_coding | Bfar | 1268.172 | 1341.948 | 1305.06 |
| ENSMUSG00000020422 | protein_coding | Tns3 | 4009.491 | 4223.224 | 4116.357 |
| ENSMUSG00000069475 | protein_coding | Gm6020 | 15.77706 | 11.7862 | 13.78163 |
| ENSMUSG00000059854 | protein_coding | Hydin | 103.0618 | 88.42617 | 95.74401 |
| ENSMUSG00000038224 | protein_coding | Serpinf2 | 4.543386 | 6.780736 | 5.662061 |
| ENSMUSG00000030144 | protein_coding | Clec4d | 9.434749 | 6.409898 | 7.922324 |
| ENSMUSG00000030148 | protein_coding | Clec4a2 | 47.99747 | 55.90548 | 51.95148 |
| ENSMUSG00000036473 | protein_coding | Tbc1d24 | 897.2174 | 949.8133 | 923.5153 |
| ENSMUSG00000047923 | protein_coding | Gm9821 | 2.269904 | 1.024227 | 1.647065 |
| ENSMUSG00000026385 | protein_coding | Dbi | 3254.989 | 3450.22 | 3352.605 |
| ENSMUSG00000007646 | protein_coding | Rad51c | 781.5253 | 829.3322 | 805.4288 |
| ENSMUSG00000022160 | protein_coding | Mettl3 | 905.4157 | 953.1757 | 929.2957 |
| ENSMUSG00000016756 | protein_coding | Cmah | 49.35151 | 58.16841 | 53.75996 |
| ENSMUSG00000040225 | protein_coding | Prrc2c | 8387.437 | 8915.152 | 8651.295 |
| ENSMUSG00000037921 | protein_coding | Ddx60 | 250.9505 | 279.6773 | 265.3139 |
| ENSMUSG00000030041 | protein_coding | D6Mm5e | 1953.559 | 1777.489 | 1865.524 |
| ENSMUSG00000025609 | protein_coding | Mkln1 | 2878.249 | 2769.343 | 2823.796 |
| ENSMUSG00000027679 | protein_coding | Dnajc19 | 691.7831 | 650.345 | 671.0641 |
| ENSMUSG00000061086 | protein_coding | Myl4 | 98.67149 | 87.94628 | 93.30889 |
| ENSMUSG00000031561 | protein_coding | Odz3 | 1296.232 | 1391.691 | 1343.961 |
| ENSMUSG00000091363 | pseudogene | Gm7701 | 9.056283 | 6.445212 | 7.750747 |
| ENSMUSG00000020459 | protein_coding | Mtif2 | 1056.375 | 1128.291 | 1092.333 |
| ENSMUSG00000037224 | protein_coding | Zfyve28 | 431.2882 | 400.3798 | 415.834 |
| ENSMUSG00000025494 | protein_coding | Sigirr | 17.12059 | 21.48247 | 19.30153 |
| ENSMUSG00000042406 | protein_coding | Atf4 | 2451.704 | 2597.405 | 2524.555 |
| ENSMUSG00000027859 | protein_coding | Ngf | 4.235364 | 6.534219 | 5.384792 |
| ENSMUSG00000012405 | protein_coding | Rpl15 | 8295.263 | 8693.944 | 8494.604 |
| ENSMUSG00000021120 | protein_coding | Pigh | 522.9768 | 555.4083 | 539.1925 |
| ENSMUSG00000037525 | protein_coding | Bcdin3d | 202.8956 | 221.0801 | 211.9878 |

| | | | | | |
|---|---|---|---|---|---|
| ENSMUSG00000041736 | protein_coding | Tspo | 595.6175 | 630.5614 | 613.0895 |
| ENSMUSG00000020143 | protein_coding | Dock2 | 56.3578 | 48.84183 | 52.59982 |
| ENSMUSG00000073768 | protein_coding | Olfr1330 | 2.611992 | 4.44108 | 3.526536 |
| ENSMUSG00000087549 | processed_transcrip | Gm13427 | 15.51235 | 19.51401 | 17.51318 |
| ENSMUSG00000032280 | protein_coding | Tle3 | 3990.982 | 4260.057 | 4125.52 |
| ENSMUSG00000085030 | processed_transcrip | 2810455O05Rik | 39.79988 | 33.68347 | 36.74167 |
| ENSMUSG00000079164 | protein_coding | Tlr5 | 13.28576 | 9.561855 | 11.42381 |
| ENSMUSG00000075296 | protein_coding | Aldh3b2 | 2.579193 | 4.45009 | 3.514641 |
| ENSMUSG00000032481 | protein_coding | Smarcc1 | 6017.161 | 5667.559 | 5842.36 |
| ENSMUSG00000078958 | protein_coding | Atp6ap1l | 2.239415 | 1.033237 | 1.636326 |
| ENSMUSG00000032601 | protein_coding | Prkar2a | 1528.178 | 1623.184 | 1575.681 |
| ENSMUSG00000044768 | protein_coding | D1Ertd622e | 662.2032 | 702.544 | 682.3736 |
| ENSMUSG00000029501 | protein_coding | Ankle2 | 2219.553 | 2134.578 | 2177.065 |
| ENSMUSG00000039615 | protein_coding | Stub1 | 1274.004 | 1330.485 | 1302.244 |
| ENSMUSG00000065557 | miRNA | Mirlet7c-1 | 1.307151 | 2.674817 | 1.990984 |
| ENSMUSG00000007415 | protein_coding | Gatad1 | 1630.662 | 1553.409 | 1592.035 |
| ENSMUSG00000049897 | protein_coding | Gm711 | 27.27567 | 21.69544 | 24.48555 |
| ENSMUSG00000084277 | pseudogene | Gm11702 | 25.97314 | 11.76999 | 18.87156 |
| ENSMUSG00000077737 | snoRNA | Snord72 | 18.31503 | 13.94204 | 16.12854 |
| ENSMUSG00000047988 | protein_coding | 4933428G20Rik | 15.46188 | 11.98187 | 13.72188 |
| ENSMUSG00000021124 | protein_coding | Vti1b | 1865.25 | 1778.004 | 1821.627 |
| ENSMUSG00000082329 | pseudogene | Gm14287 | 46.55703 | 53.57411 | 50.06557 |
| ENSMUSG00000026301 | protein_coding | Iqca | 0.632442 | 1.677256 | 1.154849 |
| ENSMUSG00000031523 | protein_coding | Dlc1 | 1086.133 | 1031.94 | 1059.036 |
| ENSMUSG00000072829 | protein_coding | Gm7292 | 110.7949 | 62.34139 | 86.56817 |
| ENSMUSG00000082878 | pseudogene | Gm12583 | 8.490707 | 5.581343 | 7.036025 |
| ENSMUSG00000073366 | lincRNA | Gm10492 | 21.13746 | 16.35232 | 18.74489 |
| ENSMUSG00000085232 | pseudogene | Gm11962 | 5.512027 | 3.426224 | 4.469126 |
| ENSMUSG00000025218 | protein_coding | Poll | 329.5907 | 352.6078 | 341.0993 |
| ENSMUSG00000030291 | protein_coding | Med21 | 1198.985 | 1129.949 | 1164.467 |
| ENSMUSG00000027765 | protein_coding | P2ry1 | 111.5163 | 95.90151 | 103.7089 |
| ENSMUSG00000021474 | protein_coding | Sfxn1 | 773.3108 | 705.181 | 739.2459 |
| ENSMUSG00000002227 | protein_coding | Mov10 | 3372.651 | 3195.734 | 3284.193 |
| ENSMUSG00000008140 | protein_coding | 2310044H10Rik | 2140.951 | 2022.138 | 2081.545 |
| ENSMUSG00000012848 | protein_coding | Rps5 | 9581.624 | 9953.484 | 9767.554 |
| ENSMUSG00000024053 | protein_coding | Emilin2 | 130.2828 | 117.2294 | 123.7561 |
| ENSMUSG00000046160 | protein_coding | Olig1 | 26.39261 | 18.65227 | 22.52244 |
| ENSMUSG00000078899 | protein_coding | Gm4631 | 1.965461 | 4.079614 | 3.022537 |
| ENSMUSG00000025557 | protein_coding | Slc15a1 | 28.42911 | 33.69248 | 31.06079 |
| ENSMUSG00000065676 | snoRNA | Snord42b | 8.717773 | 6.136716 | 7.427245 |
| ENSMUSG00000024295 | pseudogene | Cyp4f41-ps | 10.93848 | 15.01603 | 12.97725 |
| ENSMUSG00000073406 | polymorphic_pseuc | H2-Bl | 4.610252 | 2.137462 | 3.373857 |
| ENSMUSG00000069267 | protein_coding | Hist1h3b | 3.228034 | 1.668247 | 2.448141 |
| ENSMUSG00000003135 | protein_coding | D1Bwg0212e | 1225.082 | 1166.205 | 1195.643 |
| ENSMUSG00000037058 | protein_coding | Paip2 | 5397.036 | 5648.615 | 5522.825 |
| ENSMUSG00000087208 | pseudogene | Gm5125 | 0.968641 | 2.039445 | 1.504043 |
| ENSMUSG00000087035 | processed_transcrip | BC052486 | 6.094002 | 8.882161 | 7.488082 |
| ENSMUSG00000083620 | pseudogene | Gm15529 | 42.92533 | 34.55303 | 38.73918 |
| ENSMUSG00000044933 | protein_coding | Sstr3 | 5.799027 | 8.502314 | 7.150671 |
| ENSMUSG00000068749 | protein_coding | Psma5 | 1926.347 | 2019.037 | 1972.692 |
| ENSMUSG00000027496 | protein_coding | Aurka | 820.6704 | 871.1555 | 845.9129 |
| ENSMUSG00000069806 | protein_coding | Cacng7 | 368.4073 | 343.9729 | 356.1901 |
| ENSMUSG00000081051 | pseudogene | Gm15427 | 1058.218 | 1109.338 | 1083.778 |
| ENSMUSG00000034390 | protein_coding | 4933407C03Rik | 930.8146 | 1023.116 | 976.9652 |
| ENSMUSG00000021417 | protein_coding | Eci2 | 1377.625 | 1445.211 | 1411.418 |
| ENSMUSG00000022622 | protein_coding | Acr | 5.220406 | 3.099348 | 4.159877 |
| ENSMUSG00000052642 | protein_coding | 4930504O13Rik | 25.58081 | 20.67773 | 23.12927 |
| ENSMUSG00000000563 | protein_coding | Atp5f1 | 5240.281 | 5445.668 | 5342.975 |

| | | | | | |
|---|---|---|---|---|---|
| ENSMUSG00000047810 | protein_coding | Ccdc88b | 63.68791 | 55.28917 | 59.48854 |
| ENSMUSG00000034777 | protein_coding | Vax2 | 25.96725 | 20.60602 | 23.28663 |
| ENSMUSG00000091461 | lincRNA | 2210008F06Rik | 7.723264 | 10.56879 | 9.146026 |
| ENSMUSG00000071660 | protein_coding | Ttc9c | 1204.998 | 1308.121 | 1256.56 |
| ENSMUSG00000037351 | protein_coding | Actr1b | 1505.783 | 1582.766 | 1544.275 |
| ENSMUSG00000037999 | protein_coding | Arap2 | 1966.884 | 1813.106 | 1889.995 |
| ENSMUSG00000086893 | processed_transcrip | Gm11365 | 5.163007 | 7.567456 | 6.365231 |
| ENSMUSG00000046031 | protein_coding | Fam26f | 7.068533 | 4.705615 | 5.887074 |
| ENSMUSG00000083344 | pseudogene | Gm7363 | 7.382443 | 10.28696 | 8.834701 |
| ENSMUSG00000088763 | snRNA | U6 | 8.683706 | 6.154373 | 7.419039 |
| ENSMUSG00000082383 | pseudogene | Gm9670 | 1.951372 | 3.434871 | 2.693122 |
| ENSMUSG00000020716 | protein_coding | Nf1 | 4309.759 | 4609.93 | 4459.845 |
| ENSMUSG00000046688 | protein_coding | Tifa | 514.5201 | 545.0256 | 529.7729 |
| ENSMUSG00000047388 | protein_coding | Atmin | 1575.903 | 1487.329 | 1531.616 |
| ENSMUSG00000069188 | pseudogene | Gm13192 | 18.73912 | 14.78173 | 16.76043 |
| ENSMUSG00000018412 | protein_coding | 1700081L11Rik | 4180.158 | 3953.07 | 4066.614 |
| ENSMUSG00000024824 | protein_coding | Rad9 | 344.4978 | 370.3546 | 357.4262 |
| ENSMUSG00000035890 | protein_coding | Rnf126 | 1465.43 | 1400.063 | 1432.746 |
| ENSMUSG00000009535 | protein_coding | Rnmt | 1818.904 | 1737.908 | 1778.406 |
| ENSMUSG00000050010 | protein_coding | Shisa3 | 41.93605 | 48.60754 | 45.27179 |
| ENSMUSG00000048865 | protein_coding | Arhgap30 | 106.6036 | 118.1252 | 112.3644 |
| ENSMUSG00000047067 | protein_coding | Dusp28 | 216.9175 | 234.556 | 225.7367 |
| ENSMUSG00000041287 | protein_coding | Sox15 | 13.6432 | 17.77369 | 15.70845 |
| ENSMUSG00000085776 | lincRNA | 5430402O13Rik | 54.89761 | 62.50204 | 58.69983 |
| ENSMUSG00000055065 | protein_coding | Ddx17 | 11954.69 | 12526.59 | 12240.64 |
| ENSMUSG00000033054 | protein_coding | Npat | 3062.37 | 3249.88 | 3156.125 |
| ENSMUSG00000040891 | protein_coding | Foxa3 | 15.743 | 11.90151 | 13.82225 |
| ENSMUSG00000041695 | protein_coding | Kcnj2 | 39.01752 | 31.95003 | 35.48378 |
| ENSMUSG00000001942 | protein_coding | Siae | 1257.287 | 1319.741 | 1288.514 |
| ENSMUSG00000052572 | protein_coding | Dlg2 | 220.4114 | 240.1179 | 230.2647 |
| ENSMUSG00000059626 | protein_coding | Rhox3a | 1.937283 | 3.506222 | 2.721752 |
| ENSMUSG00000039286 | protein_coding | Fndc3b | 3953.895 | 4166.067 | 4059.981 |
| ENSMUSG00000072381 | protein_coding | Gm10355 | 29.68475 | 24.64416 | 27.16446 |
| ENSMUSG00000018196 | protein_coding | Glrx2 | 510.7142 | 552.9287 | 531.8215 |
| ENSMUSG00000033701 | protein_coding | Acbd6 | 1149.419 | 1088.159 | 1118.789 |
| ENSMUSG00000035458 | protein_coding | Tnni3 | 3607.844 | 3407.883 | 3507.863 |
| ENSMUSG00000046711 | protein_coding | Hmga1 | 3460.145 | 3256.311 | 3358.228 |
| ENSMUSG00000021213 | protein_coding | Akr1c13 | 30.54239 | 24.64344 | 27.59291 |
| ENSMUSG00000020053 | protein_coding | Igf1 | 3576.476 | 3374.489 | 3475.482 |
| ENSMUSG00000028217 | protein_coding | Cdh17 | 64.92357 | 74.05182 | 69.4877 |
| ENSMUSG00000030492 | protein_coding | Slc7a9 | 26.85263 | 32.21125 | 29.53194 |
| ENSMUSG00000040189 | protein_coding | Ccdc114 | 222.3109 | 240.4578 | 231.3844 |
| ENSMUSG00000029761 | protein_coding | Cald1 | 2682.592 | 2827.308 | 2754.95 |
| ENSMUSG00000034566 | protein_coding | Atp5h | 2102.128 | 1997.712 | 2049.92 |
| ENSMUSG00000034575 | protein_coding | Papd7 | 1361.491 | 1294.2 | 1327.845 |
| ENSMUSG00000044712 | protein_coding | Slc38a6 | 313.7811 | 284.6643 | 299.2227 |
| ENSMUSG00000069744 | protein_coding | Psmb3 | 2466.929 | 2341.918 | 2404.424 |
| ENSMUSG00000038646 | protein_coding | Fam103a1 | 1579.722 | 1649.296 | 1614.509 |
| ENSMUSG00000083114 | pseudogene | Gm14790 | 223.2702 | 202.6911 | 212.9806 |
| ENSMUSG00000031931 | protein_coding | Ankrd49 | 975.3902 | 929.7204 | 952.5553 |
| ENSMUSG00000035437 | protein_coding | Rabgap1 | 3621.82 | 3490.048 | 3555.934 |
| ENSMUSG00000020089 | protein_coding | Ppa1 | 2497.438 | 2388.904 | 2443.171 |
| ENSMUSG00000066571 | protein_coding | 4931406P16Rik | 3228.486 | 3022.548 | 3125.517 |
| ENSMUSG00000020802 | protein_coding | Ube2o | 3072.542 | 2911.623 | 2992.083 |
| ENSMUSG00000055835 | protein_coding | Zfp1 | 866.6193 | 820.9435 | 843.7814 |
| ENSMUSG00000070699 | protein_coding | Sars2 | 599.7199 | 565.1842 | 582.4521 |
| ENSMUSG00000070056 | protein_coding | Mfhas1 | 490.3898 | 534.8284 | 512.6091 |
| ENSMUSG00000037196 | protein_coding | Pacrg | 61.49016 | 47.76123 | 54.62569 |

| | | | | | |
|---|---|---|---|---|---|
| ENSMUSG00000074826 | protein_coding | Gm10767 | 44.63615 | 38.09897 | 41.36756 |
| ENSMUSG00000083166 | pseudogene | Gm8648 | 3.236234 | 1.739597 | 2.487916 |
| ENSMUSG00000032224 | protein_coding | Fam81a | 139.5205 | 124.9983 | 132.2594 |
| ENSMUSG00000091306 | protein_coding | Gm17606 | 2.295771 | 4.0879 | 3.191835 |
| ENSMUSG00000025127 | protein_coding | Gcgr | 31.45595 | 25.78913 | 28.62254 |
| ENSMUSG00000079566 | protein_coding | 4930408F14Rik | 4.496497 | 2.674817 | 3.585657 |
| ENSMUSG00000068882 | protein_coding | Ssb | 7319.97 | 7626.043 | 7473.006 |
| ENSMUSG00000005610 | protein_coding | Eif4g2 | 24980.91 | 25907.97 | 25444.44 |
| ENSMUSG00000019929 | protein_coding | Dcn | 7275.097 | 7624.24 | 7449.668 |
| ENSMUSG00000070111 | pseudogene | Gm10286 | 6.170337 | 8.793153 | 7.481745 |
| ENSMUSG00000027489 | protein_coding | Necab3 | 163.9664 | 143.1172 | 153.5418 |
| ENSMUSG00000007107 | protein_coding | Atp1a4 | 11.75408 | 17.07054 | 14.41231 |
| ENSMUSG00000083139 | pseudogene | Gm12418 | 30.36662 | 24.96995 | 27.66828 |
| ENSMUSG00000005951 | protein_coding | Shpk | 112.6996 | 102.2036 | 107.4516 |
| ENSMUSG00000002266 | protein_coding | Zim1 | 609.8363 | 572.1996 | 591.018 |
| ENSMUSG00000054069 | protein_coding | 6030452D12Rik | 1.615173 | 0.662038 | 1.138605 |
| ENSMUSG00000031101 | protein_coding | Sash3 | 28.07293 | 33.42002 | 30.74648 |
| ENSMUSG00000010607 | protein_coding | Pigyl | 813.6311 | 771.3621 | 792.4966 |
| ENSMUSG00000019505 | protein_coding | Ubb | 16670.56 | 17261.72 | 16966.14 |
| ENSMUSG00000022416 | protein_coding | Cacna1i | 50.60566 | 43.90772 | 47.25669 |
| ENSMUSG00000029255 | protein_coding | Gnrhr | 19.9384 | 24.53677 | 22.23759 |
| ENSMUSG00000087236 | protein_coding | Gm1305 | 1.592884 | 3.046016 | 2.31945 |
| ENSMUSG00000084846 | lincRNA | A730011C13Rik | 79.15471 | 65.07983 | 72.11727 |
| ENSMUSG00000041782 | protein_coding | Lad1 | 86.212 | 98.73239 | 92.47219 |
| ENSMUSG00000074802 | protein_coding | Gas2l3 | 280.9062 | 309.2419 | 295.074 |
| ENSMUSG00000064701 | snRNA | U6 | 12.35476 | 16.51884 | 14.4368 |
| ENSMUSG00000085728 | processed_transcrip | Gm14339 | 10.37394 | 13.87898 | 12.12646 |
| ENSMUSG00000025272 | protein_coding | Tro | 1096.151 | 1174.907 | 1135.529 |
| ENSMUSG00000064389 | snRNA | U6 | 1.937283 | 3.381539 | 2.659411 |
| ENSMUSG00000015522 | protein_coding | Arnt | 2566.832 | 2690.489 | 2628.66 |
| ENSMUSG00000079297 | pseudogene | Gm2223 | 59.19582 | 68.1814 | 63.68861 |
| ENSMUSG00000060332 | protein_coding | Tmc2 | 10.03901 | 7.258599 | 8.648806 |
| ENSMUSG00000025724 | protein_coding | Sec11a | 2007.748 | 2092.258 | 2050.003 |
| ENSMUSG00000066154 | protein_coding | Mup3 | 2.634281 | 1.006571 | 1.820426 |
| ENSMUSG00000026675 | protein_coding | Hsd17b7 | 912.467 | 856.7177 | 884.5924 |
| ENSMUSG00000086560 | lincRNA | Gm13372 | 2.575614 | 4.352435 | 3.464024 |
| ENSMUSG00000042459 | protein_coding | Bpifa2e | 0.32211 | 1.342094 | 0.832102 |
| ENSMUSG00000049612 | protein_coding | Omg | 0.32211 | 1.342094 | 0.832102 |
| ENSMUSG00000064254 | protein_coding | Ethe1 | 121.3312 | 108.3526 | 114.8419 |
| ENSMUSG00000021958 | protein_coding | Pinx1 | 547.9789 | 581.5718 | 564.7753 |
| ENSMUSG00000062808 | protein_coding | Hist1h3d | 6.391513 | 3.663731 | 5.027622 |
| ENSMUSG00000089840 | protein_coding | Gm4491 | 3.848698 | 2.119805 | 2.984252 |
| ENSMUSG00000061780 | protein_coding | Cfd | 0.316221 | 1.342094 | 0.829158 |
| ENSMUSG00000092011 | lincRNA | Gm9793 | 50.60991 | 62.78875 | 56.69933 |
| ENSMUSG00000020830 | protein_coding | Vmo1 | 4.196676 | 2.294609 | 3.245642 |
| ENSMUSG00000049728 | protein_coding | Zfp668 | 665.6117 | 702.7669 | 684.1893 |
| ENSMUSG00000031917 | protein_coding | Nip7 | 1992.507 | 1901.634 | 1947.07 |
| ENSMUSG00000027684 | protein_coding | Mecom | 50.88908 | 58.51252 | 54.7008 |
| ENSMUSG00000078604 | protein_coding | Gm10961 | 3.222145 | 1.739597 | 2.480871 |
| ENSMUSG00000043068 | protein_coding | Fam89a | 108.0701 | 120.2267 | 114.1484 |
| ENSMUSG00000023961 | protein_coding | Enpp4 | 721.2101 | 683.6172 | 702.4137 |
| ENSMUSG00000065489 | miRNA | Mir365-2 | 0.968641 | 2.012779 | 1.49071 |
| ENSMUSG00000068479 | protein_coding | Mfap1a | 611.9113 | 576.8618 | 594.3866 |
| ENSMUSG00000017310 | protein_coding | Spint4 | 1.953683 | 0.724379 | 1.339031 |
| ENSMUSG00000051503 | protein_coding | Gm6583 | 3.572434 | 2.039445 | 2.805939 |
| ENSMUSG00000086843 | lincRNA | E030013I19Rik | 2.881325 | 1.324076 | 2.1027 |
| ENSMUSG00000073073 | lincRNA | Gm8098 | 22.98984 | 28.36738 | 25.67861 |
| ENSMUSG00000026478 | protein_coding | Lamc1 | 3683.971 | 3881.119 | 3782.545 |

| | | | | | |
|---|---|---|---|---|---|
| ENSMUSG00000034906 | protein_coding | Ncaph | 1004.872 | 940.0002 | 972.4361 |
| ENSMUSG00000022382 | protein_coding | Wnt7b | 36.77789 | 30.70264 | 33.74026 |
| ENSMUSG00000027995 | protein_coding | Tlr2 | 62.79456 | 72.71366 | 67.75411 |
| ENSMUSG00000043881 | protein_coding | Kbtbd7 | 1140.461 | 1089.097 | 1114.779 |
| ENSMUSG00000015962 | protein_coding | 1700016C15Rik | 1.606973 | 0.662038 | 1.134505 |
| ENSMUSG00000062546 | protein_coding | V1ra8 | 1.606973 | 0.662038 | 1.134505 |
| ENSMUSG00000005800 | protein_coding | Mmp8 | 1.606973 | 0.662038 | 1.134505 |
| ENSMUSG00000087192 | lincRNA | 4930412L05Rik | 17.42608 | 13.26447 | 15.34528 |
| ENSMUSG00000044308 | protein_coding | Ubr3 | 4769.411 | 4581.873 | 4675.642 |
| ENSMUSG00000054934 | protein_coding | Kcnmb4 | 87.64572 | 98.05409 | 92.8499 |
| ENSMUSG00000040570 | protein_coding | Rundc3b | 1488.785 | 1384.434 | 1436.609 |
| ENSMUSG00000027459 | protein_coding | Fam110a | 270.212 | 292.7886 | 281.5003 |
| ENSMUSG00000026914 | protein_coding | Psmd14 | 1801.52 | 1879.794 | 1840.657 |
| ENSMUSG00000081785 | pseudogene | Gm12424 | 7.104911 | 4.856964 | 5.980937 |
| ENSMUSG00000007039 | protein_coding | Ddah2 | 2052.234 | 2175.476 | 2113.855 |
| ENSMUSG00000030323 | protein_coding | Ift122 | 1347.525 | 1243.567 | 1295.546 |
| ENSMUSG00000086426 | processed_transcrip | Gm15295 | 2.259393 | 0.979543 | 1.619468 |
| ENSMUSG00000092778 | miRNA | AC104325.1 | 2.597903 | 1.350742 | 1.974322 |
| ENSMUSG00000055197 | protein_coding | Fev | 1.951372 | 3.425862 | 2.688617 |
| ENSMUSG00000042087 | protein_coding | 4933440N22Rik | 31.38423 | 38.00629 | 34.69526 |
| ENSMUSG00000058581 | pseudogene | Gm5801 | 10.37879 | 7.415746 | 8.897268 |
| ENSMUSG00000040478 | protein_coding | Prdm13 | 1.629262 | 0.662038 | 1.14565 |
| ENSMUSG00000021244 | protein_coding | Ylpm1 | 3027.645 | 2884.443 | 2956.044 |
| ENSMUSG00000034947 | protein_coding | Tmem106a | 169.055 | 153.9977 | 161.5263 |
| ENSMUSG00000021250 | protein_coding | Fos | 224.3506 | 165.4314 | 194.891 |
| ENSMUSG00000044387 | processed_transcrip | 2410080I02Rik | 4.507008 | 6.727403 | 5.617206 |
| ENSMUSG00000032575 | protein_coding | Manf | 4081.119 | 3902.213 | 3991.666 |
| ENSMUSG00000025573 | protein_coding | 6030468B19Rik | 4.216654 | 6.614218 | 5.415436 |
| ENSMUSG00000031780 | protein_coding | Ccl17 | 6.447869 | 9.075345 | 7.761607 |
| ENSMUSG00000032480 | protein_coding | Dhx30 | 3162.728 | 3022.783 | 3092.756 |
| ENSMUSG00000027366 | protein_coding | 2010106G01Rik | 2580.997 | 2723.914 | 2652.455 |
| ENSMUSG00000036860 | protein_coding | Mrpl55 | 680.0473 | 640.0498 | 660.0486 |
| ENSMUSG00000079020 | protein_coding | Slc45a4 | 5726.113 | 5969.349 | 5847.731 |
| ENSMUSG00000020839 | protein_coding | Tmigd1 | 3.205746 | 5.130146 | 4.167946 |
| ENSMUSG00000043833 | lincRNA | 2900005J15Rik | 38.59135 | 45.57053 | 42.08094 |
| ENSMUSG00000020150 | protein_coding | Gamt | 2491.847 | 2588.101 | 2539.974 |
| ENSMUSG00000064293 | protein_coding | Cntn4 | 29.73455 | 49.96308 | 39.84882 |
| ENSMUSG00000085627 | pseudogene | Gm11222 | 2.241726 | 1.024227 | 1.632976 |
| ENSMUSG00000040345 | protein_coding | Arhgap9 | 52.31103 | 61.01322 | 56.66212 |
| ENSMUSG00000028435 | protein_coding | Aqp3 | 1.635151 | 3.135023 | 2.385087 |
| ENSMUSG00000073209 | protein_coding | Klf14 | 7.478756 | 10.55978 | 9.019267 |
| ENSMUSG00000092005 | protein_coding | C1rb | 47.72061 | 56.44641 | 52.08351 |
| ENSMUSG00000021696 | protein_coding | Elovl7 | 33.68947 | 40.53411 | 37.11179 |
| ENSMUSG00000089932 | pseudogene | Gm16114 | 2.253504 | 0.979543 | 1.616523 |
| ENSMUSG00000042133 | protein_coding | Ppig | 5005.527 | 4804.279 | 4904.903 |
| ENSMUSG00000041685 | protein_coding | Fcho2 | 3221.761 | 3375.293 | 3298.527 |
| ENSMUSG00000028706 | protein_coding | Nsun4 | 221.9455 | 238.2767 | 230.1111 |
| ENSMUSG00000072847 | processed_transcrip | A530017D24Rik | 240.2067 | 221.1365 | 230.6716 |
| ENSMUSG00000031264 | protein_coding | Btk | 44.00371 | 37.68272 | 40.84321 |
| ENSMUSG00000033256 | protein_coding | Shf | 574.9762 | 544.2878 | 559.632 |
| ENSMUSG00000064435 | snoRNA | SNORA4 | 12.98363 | 9.423088 | 11.20336 |
| ENSMUSG00000048921 | protein_coding | Zfp689 | 136.0768 | 123.9885 | 130.0326 |
| ENSMUSG00000059981 | protein_coding | Taok2 | 2896.83 | 2775.963 | 2836.396 |
| ENSMUSG00000037486 | protein_coding | Asxl2 | 2572.086 | 2705.014 | 2638.55 |
| ENSMUSG00000036834 | protein_coding | Plch1 | 10.29657 | 13.5258 | 11.91118 |
| ENSMUSG00000035031 | protein_coding | C8a | 3.900433 | 2.119805 | 3.010119 |
| ENSMUSG00000039620 | protein_coding | 6430573F11Rik | 103.3564 | 92.87061 | 98.11349 |
| ENSMUSG00000091383 | protein_coding | Hist1h2al | 314.8574 | 603.4208 | 459.1391 |

| | | | | | |
|---|---|---|---|---|---|
| ENSMUSG00000023008 | protein_coding | Fmnl3 | 1564.916 | 1735.891 | 1650.403 |
| ENSMUSG00000029390 | protein_coding | Tmed2 | 3537.335 | 3397.899 | 3467.617 |
| ENSMUSG00000039473 | protein_coding | Ubn1 | 3479.472 | 3288.65 | 3384.061 |
| ENSMUSG00000028357 | protein_coding | Kif12 | 0.65242 | 1.704284 | 1.178352 |
| ENSMUSG00000027942 | protein_coding | 4933434E20Rik | 1588.456 | 1676.692 | 1632.574 |
| ENSMUSG00000025217 | protein_coding | Btrc | 1551.698 | 1487.479 | 1519.589 |
| ENSMUSG00000090206 | protein_coding | Tepp | 95.34655 | 85.30859 | 90.32757 |
| ENSMUSG00000025129 | protein_coding | Dysfip1 | 5.845916 | 8.582312 | 7.214114 |
| ENSMUSG00000036376 | protein_coding | Abt1 | 879.8963 | 924.4873 | 902.1918 |
| ENSMUSG00000086306 | processed_transcrip | Gm11754 | 3.546566 | 5.509992 | 4.528279 |
| ENSMUSG00000053774 | protein_coding | Ubxn7 | 3982.853 | 3808.919 | 3895.886 |
| ENSMUSG00000086753 | processed_transcrip | Gm15751 | 205.2835 | 226.6599 | 215.9717 |
| ENSMUSG00000034266 | protein_coding | Batf | 22.47302 | 28.03077 | 25.2519 |
| ENSMUSG00000080845 | pseudogene | Gm9115 | 2.928213 | 4.767595 | 3.847904 |
| ENSMUSG00000091534 | pseudogene | Gm17195 | 109.1248 | 95.55345 | 102.3391 |
| ENSMUSG00000022205 | protein_coding | Sub1 | 3696.631 | 3550.68 | 3623.656 |
| ENSMUSG00000042439 | protein_coding | Zfp532 | 1686.348 | 1775.139 | 1730.743 |
| ENSMUSG00000021868 | protein_coding | Ppif | 708.2975 | 747.994 | 728.1458 |
| ENSMUSG00000062328 | protein_coding | Rpl17 | 6539.353 | 6840.962 | 6690.158 |
| ENSMUSG00000042104 | protein_coding | Uggt2 | 410.1505 | 373.4802 | 391.8153 |
| ENSMUSG00000053358 | protein_coding | Gm9905 | 21.43102 | 16.95166 | 19.19134 |
| ENSMUSG00000005698 | protein_coding | Ctcf | 2945.757 | 3084.269 | 3015.013 |
| ENSMUSG00000091102 | protein_coding | 5830462I19Rik | 9.42886 | 12.67094 | 11.0499 |
| ENSMUSG00000025602 | protein_coding | Zfp202 | 344.6456 | 318.1176 | 331.3816 |
| ENSMUSG00000073437 | protein_coding | D330041H03Rik | 57.13911 | 49.78348 | 53.46129 |
| ENSMUSG00000091740 | protein_coding | Gm7929 | 1.609283 | 3.037007 | 2.323145 |
| ENSMUSG00000026107 | protein_coding | Obfc2a | 2924.872 | 2748.044 | 2836.458 |
| ENSMUSG00000001755 | protein_coding | Coasy | 971.9776 | 1020.638 | 996.3077 |
| ENSMUSG00000024346 | protein_coding | Pfdn1 | 1412.776 | 1472.28 | 1442.528 |
| ENSMUSG00000092216 | protein_coding | RP23-191I16.2 | 15.21484 | 11.70905 | 13.46195 |
| ENSMUSG00000035601 | protein_coding | Rg9mtd3 | 440.0278 | 412.8874 | 426.4576 |
| ENSMUSG00000037017 | protein_coding | Zscan21 | 1377.504 | 1454.401 | 1415.953 |
| ENSMUSG00000091063 | pseudogene | Gm13698 | 0 | 1.006571 | 0.503285 |
| ENSMUSG00000028738 | protein_coding | Tas1r2 | 0 | 1.006571 | 0.503285 |
| ENSMUSG00000081690 | pseudogene | Gm12317 | 0 | 1.006571 | 0.503285 |
| ENSMUSG00000064807 | snoRNA | U3 | 0 | 1.006571 | 0.503285 |
| ENSMUSG00000082696 | pseudogene | Gm15040 | 0 | 1.006571 | 0.503285 |
| ENSMUSG00000025173 | protein_coding | Wdr45l | 2872.246 | 2994.226 | 2933.236 |
| ENSMUSG00000036270 | protein_coding | Edc4 | 2337.013 | 2200.318 | 2268.665 |
| ENSMUSG00000087036 | lincRNA | Gm11748 | 3.193967 | 1.748606 | 2.471287 |
| ENSMUSG00000084890 | processed_transcrip | A830036E02Rik | 7.761953 | 11.18034 | 9.471147 |
| ENSMUSG00000027713 | protein_coding | 1810062G17Rik | 14.58829 | 18.41843 | 16.50336 |
| ENSMUSG00000033313 | protein_coding | Fbxl8 | 58.70404 | 67.26462 | 62.98433 |
| ENSMUSG00000092527 | processed_transcrip | Gm20423 | 1.612862 | 0.662038 | 1.13745 |
| ENSMUSG00000086837 | lincRNA | Gm16618 | 8.764661 | 6.056356 | 7.410509 |
| ENSMUSG00000062127 | protein_coding | Cttnbp2nl | 1108.543 | 1172.953 | 1140.748 |
| ENSMUSG00000040321 | protein_coding | Zfp770 | 952.6021 | 898.74 | 925.6711 |
| ENSMUSG00000086043 | processed_transcrip | Gm12473 | 38.67082 | 45.7342 | 42.20251 |
| ENSMUSG00000049164 | protein_coding | Zfp518a | 6976.784 | 6557.783 | 6767.284 |
| ENSMUSG00000048731 | protein_coding | Ggnbp1 | 140.5792 | 157.7288 | 149.154 |
| ENSMUSG00000029028 | protein_coding | Lrrc47 | 1834.463 | 1937.902 | 1886.182 |
| ENSMUSG00000078885 | protein_coding | Gm14432 | 5.45336 | 3.426224 | 4.439792 |
| ENSMUSG00000015533 | protein_coding | Itga2 | 9.993168 | 13.27964 | 11.6364 |
| ENSMUSG00000089959 | processed_transcrip | Gm16268 | 0.968641 | 2.030798 | 1.49972 |
| ENSMUSG00000081516 | pseudogene | Gm12470 | 1.929083 | 3.45289 | 2.690986 |
| ENSMUSG00000039254 | protein_coding | Pomt1 | 584.4073 | 542.9832 | 563.6953 |
| ENSMUSG00000087529 | lincRNA | C130060C02Rik | 22.96524 | 28.26393 | 25.61458 |
| ENSMUSG00000049387 | protein_coding | Cox7b2 | 306.1101 | 330.0719 | 318.091 |

| | | | | | |
|---|---|---|---|---|---|
| ENSMUSG00000074529 | protein_coding | C330013J21Rik | 10.60101 | 13.90564 | 12.25333 |
| ENSMUSG00000022324 | protein_coding | Matn2 | 816.5573 | 762.7095 | 789.6334 |
| ENSMUSG00000031118 | protein_coding | 1700080O16Rik | 98.72516 | 80.10011 | 89.41263 |
| ENSMUSG00000029433 | protein_coding | Diablo | 2491.936 | 2621.729 | 2556.833 |
| ENSMUSG00000038943 | protein_coding | Prc1 | 2486.583 | 2614.579 | 2550.581 |
| ENSMUSG00000090486 | protein_coding | BC035947 | 0.962752 | 2.535327 | 1.74904 |
| ENSMUSG00000082588 | pseudogene | Gm15443 | 12.86167 | 9.049402 | 10.95554 |
| ENSMUSG00000018167 | protein_coding | Stard3 | 1036.035 | 1096.704 | 1066.369 |
| ENSMUSG00000069919 | protein_coding | Hba-a1 | 956.8186 | 799.6337 | 878.2261 |
| ENSMUSG00000023064 | protein_coding | Sncg | 4.508276 | 6.923075 | 5.715675 |
| ENSMUSG00000025362 | protein_coding | Rps26 | 5456.501 | 5690.792 | 5573.647 |
| ENSMUSG00000035472 | protein_coding | Slc25a21 | 235.1706 | 215.6091 | 225.3899 |
| ENSMUSG00000081999 | pseudogene | Gm13461 | 31.33146 | 25.92246 | 28.62696 |
| ENSMUSG00000053687 | protein_coding | Dpep2 | 16.25751 | 21.10547 | 18.68149 |
| ENSMUSG00000026687 | protein_coding | Aldh9a1 | 2639.687 | 2520.61 | 2580.148 |
| ENSMUSG00000050628 | protein_coding | Fam100b | 663.8545 | 702.5741 | 683.2143 |
| ENSMUSG00000064357 | protein_coding | mt-Atp6 | 1726.279 | 1916.438 | 1821.358 |
| ENSMUSG00000049103 | protein_coding | Ccr2 | 52.26206 | 45.35068 | 48.80637 |
| ENSMUSG00000063060 | protein_coding | Sox7 | 41.28236 | 48.16322 | 44.72279 |
| ENSMUSG00000048612 | protein_coding | Myof | 1370.483 | 1239.381 | 1304.932 |
| ENSMUSG00000065562 | miRNA | Mir215 | 3.190389 | 1.342094 | 2.266241 |
| ENSMUSG00000088308 | snoRNA | snoU109 | 3.199857 | 5.10348 | 4.151668 |
| ENSMUSG00000050296 | protein_coding | Abca12 | 71.50771 | 62.53369 | 67.0207 |
| ENSMUSG00000021684 | protein_coding | Pde8b | 923.3128 | 865.5841 | 894.4484 |
| ENSMUSG00000039270 | protein_coding | Megf9 | 1396.963 | 1327.147 | 1362.055 |
| ENSMUSG00000036480 | processed_transcrip | 1700027L20Rik | 1.626951 | 3.046016 | 2.336483 |
| ENSMUSG00000035107 | protein_coding | Dcbld2 | 2908.575 | 3099.596 | 3004.086 |
| ENSMUSG00000053226 | protein_coding | Dand5 | 105.7411 | 117.078 | 111.4095 |
| ENSMUSG00000081094 | pseudogene | Rpl19-ps11 | 102.9792 | 113.1404 | 108.0598 |
| ENSMUSG00000069045 | protein_coding | Ddx3y | 2660.726 | 2548.002 | 2604.364 |
| ENSMUSG00000037610 | protein_coding | Kcnmb2 | 3.544256 | 2.030798 | 2.787527 |
| ENSMUSG00000031840 | protein_coding | Rab3a | 312.815 | 332.2916 | 322.5533 |
| ENSMUSG00000037211 | protein_coding | Spry1 | 485.6785 | 518.0599 | 501.8692 |
| ENSMUSG00000081520 | pseudogene | Gm16200 | 3.578323 | 2.048455 | 2.813389 |
| ENSMUSG00000024845 | processed_transcrip | Tmem134 | 1124.14 | 1070.152 | 1097.146 |
| ENSMUSG00000026171 | protein_coding | Rnf25 | 709.1605 | 754.5983 | 731.8794 |
| ENSMUSG00000009687 | protein_coding | Fxyd5 | 137.9985 | 126.2261 | 132.1123 |
| ENSMUSG00000002102 | protein_coding | Psmc3 | 4979.246 | 4780.775 | 4880.01 |
| ENSMUSG00000088648 | snRNA | U7 | 0.960442 | 2.08413 | 1.522286 |
| ENSMUSG00000087346 | protein_coding | Gm5699 | 0.33031 | 1.306057 | 0.818184 |
| ENSMUSG00000035032 | protein_coding | Nek11 | 35.9476 | 29.67841 | 32.81301 |
| ENSMUSG00000046523 | protein_coding | Kctd4 | 2.559214 | 4.396757 | 3.477986 |
| ENSMUSG00000045350 | protein_coding | 1700030F18Rik | 10.34703 | 14.24365 | 12.29534 |
| ENSMUSG00000022337 | protein_coding | Ttc35 | 1436.493 | 1354.17 | 1395.332 |
| ENSMUSG00000061848 | protein_coding | Gm5805 | 94.7036 | 85.10888 | 89.90624 |
| ENSMUSG00000051343 | protein_coding | Rab11fip5 | 663.3683 | 704.9269 | 684.1476 |
| ENSMUSG00000022838 | protein_coding | Eaf2 | 238.8611 | 271.3033 | 255.0822 |
| ENSMUSG00000032826 | protein_coding | Ank2 | 354.0091 | 330.7724 | 342.3907 |
| ENSMUSG00000026219 | protein_coding | Trip12 | 18340.09 | 17540.66 | 17940.37 |
| ENSMUSG00000043445 | protein_coding | Pgp | 2502.944 | 2387.078 | 2445.011 |
| ENSMUSG00000027487 | protein_coding | Cdk5rap1 | 422.1075 | 399.0271 | 410.5673 |
| ENSMUSG00000052192 | pseudogene | Gm5963 | 4.521097 | 2.639142 | 3.580119 |
| ENSMUSG00000093232 | miRNA | AC147162.1 | 3.872255 | 6.135993 | 5.004124 |
| ENSMUSG00000065778 | snoRNA | SNORA55 | 0.960442 | 2.048455 | 1.504448 |
| ENSMUSG00000069814 | protein_coding | E130309D14Rik | 82.09925 | 73.5123 | 77.80578 |
| ENSMUSG00000048100 | protein_coding | Taf13 | 1141.151 | 1078.152 | 1109.651 |
| ENSMUSG00000066129 | protein_coding | Kndc1 | 37.60444 | 28.24188 | 32.92316 |
| ENSMUSG00000070426 | protein_coding | Rnf121 | 944.8861 | 989.7355 | 967.3108 |

| | | | | | |
|---|---|---|---|---|---|
| ENSMUSG00000079711 | protein_coding | Smok4a | 0.32211 | 1.306057 | 0.814084 |
| ENSMUSG00000064930 | snoRNA | SNORA41 | 1.621062 | 0.697713 | 1.159387 |
| ENSMUSG00000054226 | protein_coding | Tprkb | 1165.598 | 1227.618 | 1196.608 |
| ENSMUSG00000019809 | protein_coding | Pex3 | 857.9642 | 808.8091 | 833.3866 |
| ENSMUSG00000032717 | protein_coding | Mdfi | 209.0924 | 228.2269 | 218.6597 |
| ENSMUSG00000073947 | protein_coding | Olfr615 | 2.583814 | 1.386417 | 1.985115 |
| ENSMUSG00000078622 | protein_coding | Ccdc47 | 2444.393 | 2578.897 | 2511.645 |
| ENSMUSG00000079471 | protein_coding | Gm7325 | 11.98219 | 15.32773 | 13.65496 |
| ENSMUSG00000035062 | protein_coding | Zc4h2 | 350.3617 | 378.0972 | 364.2294 |
| ENSMUSG00000051278 | protein_coding | 4930422G04Rik | 1009.689 | 936.9966 | 973.3427 |
| ENSMUSG00000025791 | protein_coding | Pgm2 | 1816.35 | 1733 | 1774.675 |
| ENSMUSG00000090799 | protein_coding | Klhl33 | 4.170809 | 2.392626 | 3.281717 |
| ENSMUSG00000071178 | protein_coding | Serpina1b | 7.588932 | 4.185917 | 5.887424 |
| ENSMUSG00000000058 | protein_coding | Cav2 | 392.3517 | 419.0343 | 405.693 |
| ENSMUSG00000045934 | protein_coding | Mtmr11 | 76.61749 | 67.65021 | 72.13385 |
| ENSMUSG00000084886 | lincRNA | 4732487G21Rik | 95.66001 | 85.88659 | 90.7733 |
| ENSMUSG00000062683 | protein_coding | Atp5g2 | 1862.197 | 1950.553 | 1906.375 |
| ENSMUSG00000058873 | pseudogene | Gm5582 | 1.629262 | 0.688704 | 1.158983 |
| ENSMUSG00000077367 | snRNA | U6 | 1.629262 | 0.688704 | 1.158983 |
| ENSMUSG00000031497 | protein_coding | Tnfsf13b | 12.96618 | 16.68749 | 14.82683 |
| ENSMUSG00000048334 | protein_coding | Gm8258 | 3.560655 | 2.075121 | 2.817888 |
| ENSMUSG00000065979 | protein_coding | Cpped1 | 1140.676 | 1193.231 | 1166.953 |
| ENSMUSG00000073293 | protein_coding | Nudt10 | 190.9996 | 208.4342 | 199.7169 |
| ENSMUSG00000071767 | protein_coding | Rhox7 | 58.9349 | 67.8012 | 63.36805 |
| ENSMUSG00000079558 | protein_coding | Gm6981 | 76.8494 | 68.50103 | 72.67522 |
| ENSMUSG00000081804 | pseudogene | Xlr5d-ps | 5.471028 | 7.929646 | 6.700337 |
| ENSMUSG00000064417 | snoRNA | SNORD113 | 3.231613 | 1.659599 | 2.445606 |
| ENSMUSG00000025425 | protein_coding | St8sia5 | 43.5794 | 37.54251 | 40.56095 |
| ENSMUSG00000084863 | processed_transcrip | Gm12523 | 1.615173 | 0.697713 | 1.156443 |
| ENSMUSG00000087140 | processed_transcrip | Gm11376 | 1.615173 | 0.697713 | 1.156443 |
| ENSMUSG00000024293 | protein_coding | Esco1 | 3165.851 | 3001.215 | 3083.533 |
| ENSMUSG00000073409 | protein_coding | H2-Q6 | 49.95771 | 42.28344 | 46.12057 |
| ENSMUSG00000065608 | miRNA | Mirlet7c-2 | 2.253504 | 4.319609 | 3.286556 |
| ENSMUSG00000028801 | protein_coding | 4930555I21Rik | 28.33302 | 23.28368 | 25.80835 |
| ENSMUSG00000046733 | protein_coding | Gprc5a | 12.20567 | 15.7256 | 13.96564 |
| ENSMUSG00000000126 | protein_coding | Wnt9a | 155.4704 | 140.1776 | 147.824 |
| ENSMUSG00000092659 | miRNA | AC170864.1 | 0.962752 | 2.075121 | 1.518936 |
| ENSMUSG00000012422 | protein_coding | Tmem167 | 1712.773 | 1819.089 | 1765.931 |
| ENSMUSG00000091716 | protein_coding | Gm17676 | 0 | 0.979543 | 0.489771 |
| ENSMUSG00000058523 | protein_coding | Mup5 | 0 | 0.979543 | 0.489771 |
| ENSMUSG00000092418 | processed_transcrip | RP24-286J21.3 | 0 | 0.979543 | 0.489771 |
| ENSMUSG00000083683 | pseudogene | Gm6539 | 0 | 0.979543 | 0.489771 |
| ENSMUSG00000049902 | protein_coding | 4921517D22Rik | 4.15903 | 2.330284 | 3.244657 |
| ENSMUSG00000048581 | protein_coding | E130311K13Rik | 284.6801 | 265.0753 | 274.8777 |
| ENSMUSG00000042323 | protein_coding | Pbrm1 | 4415.685 | 4234.606 | 4325.146 |
| ENSMUSG00000062818 | protein_coding | Vmn1r51 | 8.96817 | 12.2374 | 10.60278 |
| ENSMUSG00000040856 | protein_coding | Dlk1 | 4223.275 | 4003.503 | 4113.389 |
| ENSMUSG00000071984 | protein_coding | Fndc1 | 51.20828 | 43.43592 | 47.3221 |
| ENSMUSG00000047554 | protein_coding | Tmem41b | 1372.941 | 1438.239 | 1405.59 |
| ENSMUSG00000083534 | pseudogene | H2-M6-ps | 1.604662 | 3.063673 | 2.334167 |
| ENSMUSG00000024800 | protein_coding | Rpp30 | 681.4518 | 645.4036 | 663.4277 |
| ENSMUSG00000022210 | protein_coding | Dhrs4 | 365.9172 | 399.8079 | 382.8625 |
| ENSMUSG00000065883 | snoRNA | U3 | 2.930524 | 1.395426 | 2.162975 |
| ENSMUSG00000031506 | protein_coding | Ptpn7 | 16.48688 | 12.79205 | 14.63946 |
| ENSMUSG00000085364 | lincRNA | Gm16641 | 8.772861 | 6.118698 | 7.445779 |
| ENSMUSG00000053228 | protein_coding | Ceacam3 | 0.65242 | 1.668247 | 1.160333 |
| ENSMUSG00000047248 | protein_coding | C2cd3 | 1311.49 | 1247.31 | 1279.4 |
| ENSMUSG00000079376 | protein_coding | Gm3383 | 3.547834 | 2.012779 | 2.780307 |

| | | | | | |
|---|---|---|---|---|---|
| ENSMUSG00000075752 | snRNA | U2 | 2.267593 | 3.761386 | 3.014489 |
| ENSMUSG00000030032 | protein_coding | Wdr54 | 115.5904 | 104.747 | 110.1687 |
| ENSMUSG00000090784 | lincRNA | Gm17531 | 4.563364 | 2.763824 | 3.663594 |
| ENSMUSG00000020806 | protein_coding | Rhbdf2 | 2134.102 | 2014.188 | 2074.145 |
| ENSMUSG00000029992 | protein_coding | Gfpt1 | 2575.835 | 2478.808 | 2527.321 |
| ENSMUSG00000039834 | protein_coding | Zfp335 | 847.4528 | 908.6223 | 878.0375 |
| ENSMUSG00000028637 | protein_coding | Ccdc30 | 105.2554 | 115.9208 | 110.5881 |
| ENSMUSG00000015702 | protein_coding | Anxa9 | 123.3077 | 107.3924 | 115.35 |
| ENSMUSG00000027411 | protein_coding | Vps16 | 2084.073 | 2208.168 | 2146.121 |
| ENSMUSG00000028618 | protein_coding | Tmem59 | 4386.611 | 4544.907 | 4465.759 |
| ENSMUSG00000049922 | protein_coding | Slc35c1 | 390.6204 | 361.8739 | 376.2471 |
| ENSMUSG00000079311 | protein_coding | Gm3222 | 10.25661 | 13.74813 | 12.00237 |
| ENSMUSG00000041293 | protein_coding | Gpr110 | 29.97742 | 25.02437 | 27.50089 |
| ENSMUSG00000056679 | protein_coding | Gpr173 | 148.2263 | 164.2952 | 156.2608 |
| ENSMUSG00000051548 | pseudogene | Gm6365 | 14.55653 | 11.19515 | 12.87584 |
| ENSMUSG00000048988 | protein_coding | Elfn1 | 112.0664 | 100.6892 | 106.3778 |
| ENSMUSG00000042215 | protein_coding | Bag2 | 809.1331 | 856.9777 | 833.0554 |
| ENSMUSG00000079509 | protein_coding | Zfx | 3014.124 | 2888.274 | 2951.199 |
| ENSMUSG00000049235 | pseudogene | Gm7324 | 21.95202 | 17.86306 | 19.90754 |
| ENSMUSG00000008307 | protein_coding | 1700109H08Rik | 35.57398 | 29.91701 | 32.74549 |
| ENSMUSG00000003721 | protein_coding | Insig2 | 1588.37 | 1661.685 | 1625.027 |
| ENSMUSG00000020847 | protein_coding | Rph3al | 50.4553 | 59.12667 | 54.79098 |
| ENSMUSG00000084938 | lincRNA | BB557941 | 1.923194 | 3.426224 | 2.674709 |
| ENSMUSG00000093110 | miRNA | AC115767.1 | 43.50269 | 36.222 | 39.86235 |
| ENSMUSG00000031388 | protein_coding | Naa10 | 200.1318 | 216.2076 | 208.1697 |
| ENSMUSG00000026832 | protein_coding | Cytip | 1008.782 | 944.0938 | 976.438 |
| ENSMUSG00000085072 | processed_transcrip | C920011F04Rik | 17.12983 | 21.28644 | 19.20814 |
| ENSMUSG00000029878 | protein_coding | Dbpht2 | 3.270301 | 1.712931 | 2.491616 |
| ENSMUSG00000086754 | processed_transcrip | Gm16098 | 4.535186 | 2.65716 | 3.596173 |
| ENSMUSG00000052142 | protein_coding | Rasal3 | 38.88319 | 45.89777 | 42.39048 |
| ENSMUSG00000087445 | processed_transcrip | Gm14286 | 35.68184 | 29.42149 | 32.55166 |
| ENSMUSG00000033871 | protein_coding | Ppargc1b | 19.9466 | 15.81424 | 17.88042 |
| ENSMUSG00000064181 | protein_coding | Rab3ip | 1143.613 | 1197.268 | 1170.44 |
| ENSMUSG00000039543 | protein_coding | Ttc18 | 53.82617 | 46.16086 | 49.99351 |
| ENSMUSG00000032512 | protein_coding | Wdr48 | 3098.587 | 2981.991 | 3040.289 |
| ENSMUSG00000006369 | protein_coding | Fbln1 | 1310.89 | 1221.076 | 1265.983 |
| ENSMUSG00000036526 | protein_coding | Card11 | 24.82492 | 29.69394 | 27.25943 |
| ENSMUSG00000086969 | lincRNA | 4930443O20Rik | 2.920013 | 4.723633 | 3.821823 |
| ENSMUSG00000063354 | protein_coding | Slc39a4 | 22.56911 | 27.23789 | 24.9035 |
| ENSMUSG00000089862 | protein_coding | Gm16039 | 438.0372 | 414.0438 | 426.0405 |
| ENSMUSG00000081453 | pseudogene | Gm6767 | 1.629262 | 0.697713 | 1.163487 |
| ENSMUSG00000086860 | processed_transcrip | Gm1720 | 1.629262 | 0.697713 | 1.163487 |
| ENSMUSG00000076982 | miRNA | AC117684.1 | 99.09059 | 111.7424 | 105.4165 |
| ENSMUSG00000017868 | protein_coding | Sgk2 | 10.40339 | 7.558447 | 8.980919 |
| ENSMUSG00000092528 | polymorphic_pseuc | Nlrp1c-ps | 2.611992 | 1.359751 | 1.985871 |
| ENSMUSG00000073769 | protein_coding | Olfr1331 | 0.974531 | 2.048455 | 1.511493 |
| ENSMUSG00000049742 | protein_coding | C330006D17Rik | 142.4708 | 155.8206 | 149.1457 |
| ENSMUSG00000010047 | protein_coding | Hyal2 | 998.601 | 1068.909 | 1033.755 |
| ENSMUSG00000039057 | protein_coding | Myo16 | 10.3319 | 7.531781 | 8.931842 |
| ENSMUSG00000055435 | protein_coding | Maf | 908.6258 | 859.653 | 884.1394 |
| ENSMUSG00000071724 | protein_coding | Gm10345 | 39.26606 | 46.59807 | 42.93206 |
| ENSMUSG00000062380 | protein_coding | Tubb3 | 4298.786 | 4091.59 | 4195.188 |
| ENSMUSG00000028804 | protein_coding | Csmd2 | 33.24645 | 27.81056 | 30.52851 |
| ENSMUSG00000053580 | protein_coding | Tanc2 | 2951.335 | 3234.611 | 3092.973 |
| ENSMUSG00000024081 | protein_coding | Cebpz | 3229.133 | 3083.942 | 3156.537 |
| ENSMUSG00000036403 | protein_coding | Cep135 | 743.1234 | 784.0903 | 763.6068 |
| ENSMUSG00000047428 | protein_coding | Dlk2 | 32.2957 | 26.96507 | 29.63039 |
| ENSMUSG00000038861 | protein_coding | Pi4kb | 1414.401 | 1353.579 | 1383.99 |

| | | | | | |
|---|---|---|---|---|---|
| ENSMUSG00000005417 | protein_coding | Mprip | 7289.677 | 7029.434 | 7159.555 |
| ENSMUSG00000083563 | pseudogene | Gm13340 | 161.4186 | 147.4578 | 154.4382 |
| ENSMUSG00000019986 | protein_coding | Ahi1 | 1170.664 | 1107.106 | 1138.885 |
| ENSMUSG00000087204 | processed_transcrip | 1700073E17Rik | 1.637461 | 3.099348 | 2.368405 |
| ENSMUSG00000003062 | protein_coding | Stard3nl | 922.8014 | 868.3169 | 895.5591 |
| ENSMUSG00000028127 | protein_coding | Abcd3 | 4397.358 | 4580.286 | 4488.822 |
| ENSMUSG00000074169 | protein_coding | Gm16387 | 2.614303 | 1.086569 | 1.850436 |
| ENSMUSG00000021893 | protein_coding | Capn7 | 2636.621 | 2522.452 | 2579.536 |
| ENSMUSG00000026922 | protein_coding | Agpat2 | 175.4 | 189.7708 | 182.5854 |
| ENSMUSG00000031665 | protein_coding | Sall1 | 492.5971 | 459.5366 | 476.0669 |
| ENSMUSG00000031972 | protein_coding | Acta1 | 2.577925 | 1.359751 | 1.968838 |
| ENSMUSG00000090893 | protein_coding | Gm4840 | 2.577925 | 1.359751 | 1.968838 |
| ENSMUSG00000082401 | pseudogene | Gm14328 | 2.577925 | 1.359751 | 1.968838 |
| ENSMUSG00000028444 | protein_coding | Cntfr | 244.8998 | 219.2415 | 232.0706 |
| ENSMUSG00000057580 | pseudogene | Gm10012 | 6.147005 | 3.80607 | 4.976538 |
| ENSMUSG00000036915 | protein_coding | Kirrel2 | 33.45503 | 27.46675 | 30.46089 |
| ENSMUSG00000040029 | protein_coding | Ipo8 | 1695.462 | 1794.171 | 1744.817 |
| ENSMUSG00000024395 | protein_coding | Lims2 | 99.32056 | 111.5207 | 105.4206 |
| ENSMUSG00000011179 | protein_coding | Odc1 | 3719.93 | 3977.641 | 3848.786 |
| ENSMUSG00000083061 | pseudogene | Gm12191 | 35.95013 | 30.02118 | 32.98566 |
| ENSMUSG00000087414 | lincRNA | Gm16720 | 6.474779 | 4.212583 | 5.343681 |
| ENSMUSG00000032673 | protein_coding | Prorsd1 | 294.3282 | 313.0871 | 303.7077 |
| ENSMUSG00000026393 | protein_coding | Nek7 | 1555.158 | 1484.985 | 1520.071 |
| ENSMUSG00000085637 | processed_transcrip | Gm15980 | 12.8768 | 16.56316 | 14.71998 |
| ENSMUSG00000026309 | protein_coding | Ilkap | 1621.367 | 1547.672 | 1584.52 |
| ENSMUSG00000024290 | protein_coding | Rock1 | 4148.785 | 3948.323 | 4048.554 |
| ENSMUSG00000044814 | protein_coding | Olfr543 | 9.373771 | 12.68031 | 11.02704 |
| ENSMUSG00000015721 | protein_coding | Nlrp5 | 9.667704 | 6.638034 | 8.152869 |
| ENSMUSG00000039067 | protein_coding | Psmd7 | 4110.508 | 4282.148 | 4196.328 |
| ENSMUSG00000090721 | lincRNA | Gm17632 | 53.37263 | 46.57425 | 49.97344 |
| ENSMUSG00000087118 | lincRNA | Gm16793 | 11.28392 | 14.76335 | 13.02364 |
| ENSMUSG00000051008 | processed_transcrip | 4930412M03Rik | 15.55589 | 19.86647 | 17.71118 |
| ENSMUSG00000076436 | protein_coding | Oxct2a | 4.532875 | 2.7995 | 3.666187 |
| ENSMUSG00000042744 | protein_coding | Gm15800 | 1978.555 | 2132.849 | 2055.702 |
| ENSMUSG00000069763 | protein_coding | Tmem100 | 261.1051 | 232.7316 | 246.9183 |
| ENSMUSG00000093104 | miRNA | AL845455.1 | 3.205746 | 5.112489 | 4.159117 |
| ENSMUSG00000092448 | processed_transcrip | RP24-346E16.1 | 2.569725 | 1.386417 | 1.978071 |
| ENSMUSG00000081819 | pseudogene | Gm12722 | 8.40513 | 11.24884 | 9.826987 |
| ENSMUSG00000089803 | pseudogene | Gm10171 | 10.98074 | 8.140125 | 9.560434 |
| ENSMUSG00000040473 | protein_coding | A330021E22Rik | 157.1847 | 135.0195 | 146.1021 |
| ENSMUSG00000020102 | protein_coding | Slc16a7 | 49.68518 | 56.97476 | 53.32997 |
| ENSMUSG00000005899 | protein_coding | Smpd4 | 2181.444 | 2289.542 | 2235.493 |
| ENSMUSG00000067579 | pseudogene | Gm8210 | 405.7455 | 248.2345 | 326.99 |
| ENSMUSG00000075592 | protein_coding | Nynrin | 436.9801 | 473.5879 | 455.284 |
| ENSMUSG00000042284 | protein_coding | Itga1 | 210.8335 | 226.7643 | 218.7989 |
| ENSMUSG00000074732 | protein_coding | Zfp950 | 95.6384 | 109.2508 | 102.4446 |
| ENSMUSG00000089667 | pseudogene | Gm15914 | 2.603792 | 1.386417 | 1.995105 |
| ENSMUSG00000027115 | protein_coding | Kif18a | 1156.489 | 1231.268 | 1193.879 |
| ENSMUSG00000037643 | protein_coding | Prkci | 3983.046 | 3814.594 | 3898.82 |
| ENSMUSG00000013415 | protein_coding | Igf2bp1 | 1737.121 | 1846.949 | 1792.035 |
| ENSMUSG00000045326 | protein_coding | Fndc7 | 10.41748 | 7.531781 | 8.97463 |
| ENSMUSG00000054312 | protein_coding | Mrps21 | 642.2825 | 601.7199 | 622.0012 |
| ENSMUSG00000073486 | protein_coding | Gm10518 | 3.199857 | 1.668247 | 2.434052 |
| ENSMUSG00000027827 | protein_coding | Kcnab1 | 22.83844 | 27.90386 | 25.37115 |
| ENSMUSG00000001924 | protein_coding | Uba1 | 8019.489 | 8406.842 | 8213.166 |
| ENSMUSG00000028370 | protein_coding | Pappa | 1214.599 | 1430.509 | 1322.554 |
| ENSMUSG00000086186 | processed_transcrip | 1700024P03Rik | 1.612862 | 0.697713 | 1.155287 |
| ENSMUSG00000026895 | protein_coding | Ndufa8 | 1423.603 | 1366.282 | 1394.943 |

| | | | | | |
|---|---|---|---|---|---|
| ENSMUSG00000035033 | protein_coding | Tbr1 | 8.798728 | 5.970198 | 7.384463 |
| ENSMUSG00000085850 | lincRNA | 4833412C05Rik | 4.48703 | 6.833706 | 5.660368 |
| ENSMUSG00000025952 | protein_coding | Crygc | 6.519357 | 3.904087 | 5.211722 |
| ENSMUSG00000074863 | processed_transcrip | Gm5665 | 74.44979 | 85.48153 | 79.96566 |
| ENSMUSG00000028443 | protein_coding | Nudt2 | 312.3661 | 290.7099 | 301.538 |
| ENSMUSG00000032594 | protein_coding | Ip6k1 | 3272.944 | 3410.93 | 3341.937 |
| ENSMUSG00000038781 | protein_coding | Stap2 | 36.25353 | 30.28536 | 33.26944 |
| ENSMUSG00000084800 | processed_transcrip | Gm12205 | 15.29937 | 11.61103 | 13.4552 |
| ENSMUSG00000086067 | processed_transcrip | Gm16183 | 3.564234 | 5.465308 | 4.514771 |
| ENSMUSG00000080829 | pseudogene | Gm8545 | 3.858166 | 5.854525 | 4.856345 |
| ENSMUSG00000038014 | protein_coding | Fam120a | 4932.592 | 5139.445 | 5036.018 |
| ENSMUSG00000035407 | protein_coding | Kank4 | 90.60652 | 103.0102 | 96.80836 |
| ENSMUSG00000056130 | protein_coding | Ticam2 | 21.28193 | 17.35817 | 19.32005 |
| ENSMUSG00000002957 | protein_coding | Ap2a2 | 2824.801 | 2695.789 | 2760.295 |
| ENSMUSG00000069609 | protein_coding | Gm11710 | 1.618751 | 3.046016 | 2.332383 |
| ENSMUSG00000026200 | protein_coding | Glb1l | 360.3383 | 394.6258 | 377.4821 |
| ENSMUSG00000086986 | pseudogene | Gm12103 | 0.960442 | 2.039445 | 1.499943 |
| ENSMUSG00000079299 | protein_coding | Klrb1 | 6.142159 | 8.600331 | 7.371245 |
| ENSMUSG00000057667 | protein_coding | Bloc1s3 | 388.958 | 411.8273 | 400.3926 |
| ENSMUSG00000090098 | processed_transcrip | Alms1-ps2 | 2.928213 | 4.794261 | 3.861237 |
| ENSMUSG00000025509 | protein_coding | Pnpla2 | 787.8012 | 830.5969 | 809.1991 |
| ENSMUSG00000021631 | protein_coding | Mrps36-ps1 | 9.017594 | 11.91989 | 10.46874 |
| ENSMUSG00000050900 | pseudogene | Gm7327 | 20.98107 | 17.04967 | 19.01537 |
| ENSMUSG00000024767 | protein_coding | Otub1 | 2131.347 | 2039.828 | 2085.587 |
| ENSMUSG00000014932 | protein_coding | Yes1 | 2276.752 | 2186.882 | 2231.817 |
| ENSMUSG00000036853 | protein_coding | Mcoln3 | 5.232184 | 3.170699 | 4.201441 |
| ENSMUSG00000086447 | processed_transcrip | Gm13522 | 0.974531 | 2.057464 | 1.515997 |
| ENSMUSG00000091391 | protein_coding | Gm5373 | 0.974531 | 2.057464 | 1.515997 |
| ENSMUSG00000035227 | protein_coding | Spcs2 | 3089.135 | 3237.527 | 3163.331 |
| ENSMUSG00000081282 | pseudogene | Gm13836 | 5.814159 | 8.140486 | 6.977323 |
| ENSMUSG00000092814 | miRNA | AL928557.1 | 0.33031 | 1.024227 | 0.677269 |
| ENSMUSG00000084717 | snRNA | U6 | 0.33031 | 1.024227 | 0.677269 |
| ENSMUSG00000029680 | protein_coding | Hyal4 | 0.33031 | 1.024227 | 0.677269 |
| ENSMUSG00000087003 | processed_transcrip | 1700067G03Rik | 0.33031 | 1.024227 | 0.677269 |
| ENSMUSG00000065590 | miRNA | Mir212 | 0.33031 | 1.024227 | 0.677269 |
| ENSMUSG00000074901 | protein_coding | Gm8765 | 0.33031 | 1.024227 | 0.677269 |
| ENSMUSG00000073394 | processed_transcrip | Gm10497 | 0.33031 | 1.024227 | 0.677269 |
| ENSMUSG00000022394 | protein_coding | L3mbtl2 | 1249.412 | 1178.965 | 1214.188 |
| ENSMUSG00000079494 | protein_coding | Cml5 | 0.962752 | 2.039445 | 1.501099 |
| ENSMUSG00000047938 | lincRNA | 4930483J18Rik | 3.256212 | 1.757616 | 2.506914 |
| ENSMUSG00000021262 | protein_coding | Evl | 2751.662 | 2905.077 | 2828.369 |
| ENSMUSG00000073646 | lincRNA | Gm10556 | 2.592014 | 1.386417 | 1.989215 |
| ENSMUSG00000004891 | protein_coding | Nes | 988.4225 | 1045.026 | 1016.724 |
| ENSMUSG00000066035 | lincRNA | Gm17372 | 4.210765 | 6.329177 | 5.269971 |
| ENSMUSG00000030532 | protein_coding | Hddc3 | 340.6207 | 263.5065 | 302.0636 |
| ENSMUSG00000039716 | protein_coding | Dock3 | 2199.497 | 1873.515 | 2036.506 |
| ENSMUSG00000050295 | protein_coding | Foxc1 | 30.34411 | 35.46775 | 32.90593 |
| ENSMUSG00000044145 | protein_coding | 1810024B03Rik | 43.88891 | 50.79263 | 47.34077 |
| ENSMUSG00000073198 | pseudogene | Gm10480 | 348.5366 | 370.2457 | 359.3911 |
| ENSMUSG00000087795 | snoRNA | SNORA17 | 8.748261 | 11.56635 | 10.15731 |
| ENSMUSG00000027716 | protein_coding | Trpc3 | 342.1263 | 375.9669 | 359.0466 |
| ENSMUSG00000071343 | protein_coding | Gm10327 | 3.492521 | 1.659599 | 2.57606 |
| ENSMUSG00000041506 | protein_coding | Rrp9 | 1202.465 | 1141.587 | 1172.026 |
| ENSMUSG00000036574 | pseudogene | 1700019O17Rik | 0.32211 | 1.024227 | 0.673169 |
| ENSMUSG00000044294 | protein_coding | Krt84 | 0.32211 | 1.024227 | 0.673169 |
| ENSMUSG00000088224 | snoRNA | SNORA17 | 0.32211 | 1.024227 | 0.673169 |
| ENSMUSG00000071771 | protein_coding | Rhox4b | 0.32211 | 1.024227 | 0.673169 |
| ENSMUSG00000020940 | protein_coding | 1700023F06Rik | 11.89534 | 8.829552 | 10.36245 |

| | | | | | |
|---|---|---|---|---|---|
| ENSMUSG00000002031 | protein_coding | Ift46 | 1315.058 | 1231.564 | 1273.311 |
| ENSMUSG00000042197 | protein_coding | Zfp451 | 4236.293 | 4407.974 | 4322.134 |
| ENSMUSG00000085056 | lincRNA | 4930455D15Rik | 2.295771 | 1.015218 | 1.655494 |
| ENSMUSG00000026023 | protein_coding | Cdk15 | 7.44931 | 4.978435 | 6.213873 |
| ENSMUSG00000013495 | protein_coding | Tmem175 | 593.9635 | 558.3771 | 576.1703 |
| ENSMUSG00000076931 | protein_coding | Iglc3 | 0.632442 | 1.73095 | 1.181696 |
| ENSMUSG00000055805 | protein_coding | Fmnl1 | 318.2185 | 293.5991 | 305.9088 |
| ENSMUSG00000028613 | protein_coding | Lrp8 | 2408.727 | 1860.12 | 2134.423 |
| ENSMUSG00000028057 | protein_coding | Rit1 | 673.4112 | 708.7406 | 691.0759 |
| ENSMUSG00000089896 | pseudogene | Wnk1 | 41.17158 | 50.47752 | 45.82455 |
| ENSMUSG00000032473 | protein_coding | Cldn18 | 3.574744 | 5.536658 | 4.555701 |
| ENSMUSG00000081740 | pseudogene | Gm14279 | 0.316221 | 1.024227 | 0.670224 |
| ENSMUSG00000079127 | protein_coding | Gm1977 | 0.316221 | 1.024227 | 0.670224 |
| ENSMUSG00000088643 | snRNA | U6 | 0.316221 | 1.024227 | 0.670224 |
| ENSMUSG00000092323 | processed_transcrip | Fam13a | 0.316221 | 1.024227 | 0.670224 |
| ENSMUSG00000074412 | pseudogene | Gm10689 | 0.316221 | 1.024227 | 0.670224 |
| ENSMUSG00000049946 | protein_coding | BC030500 | 0.316221 | 1.024227 | 0.670224 |
| ENSMUSG00000020125 | protein_coding | Elane | 0.316221 | 1.024227 | 0.670224 |
| ENSMUSG00000092147 | protein_coding | Gm7052 | 0.316221 | 1.024227 | 0.670224 |
| ENSMUSG00000045999 | pseudogene | AY036118 | 0.316221 | 1.024227 | 0.670224 |
| ENSMUSG00000035580 | protein_coding | Kcnh8 | 0.316221 | 1.024227 | 0.670224 |
| ENSMUSG00000059455 | protein_coding | Plac8l1 | 0.316221 | 1.024227 | 0.670224 |
| ENSMUSG00000081220 | pseudogene | Gm14542 | 0.316221 | 1.024227 | 0.670224 |
| ENSMUSG00000019230 | protein_coding | Lhx9 | 311.8581 | 336 | 323.9291 |
| ENSMUSG00000030790 | protein_coding | Adm | 25.87348 | 30.61979 | 28.24663 |
| ENSMUSG00000027831 | protein_coding | Veph1 | 10.65506 | 7.733974 | 9.194515 |
| ENSMUSG00000032595 | protein_coding | Cdhr4 | 17.90548 | 13.49949 | 15.70248 |
| ENSMUSG00000067626 | lincRNA | Gm17245 | 55.20101 | 47.69401 | 51.44751 |
| ENSMUSG00000031736 | protein_coding | 4933436C20Rik | 32.50681 | 25.71628 | 29.11155 |
| ENSMUSG00000061787 | protein_coding | Rps17 | 4215.805 | 4004.528 | 4110.167 |
| ENSMUSG00000025364 | protein_coding | Pa2g4 | 3597.374 | 3748.555 | 3672.965 |
| ENSMUSG00000031508 | protein_coding | Ankrd10 | 3407.373 | 3231.248 | 3319.311 |
| ENSMUSG00000040435 | protein_coding | Ppp1r15a | 924.3163 | 868.9159 | 896.6161 |
| ENSMUSG00000040350 | protein_coding | Trim7 | 1454.691 | 1545.071 | 1499.881 |
| ENSMUSG00000078134 | protein_coding | Gm12355 | 28.86267 | 23.74425 | 26.30346 |
| ENSMUSG00000086996 | processed_transcrip | 4933416E14Rik | 204.7845 | 186.6922 | 195.7383 |
| ENSMUSG00000039157 | protein_coding | Fam102a | 6691.025 | 6126.023 | 6408.524 |
| ENSMUSG00000029066 | protein_coding | Mrpl20 | 1091.695 | 1045.22 | 1068.457 |
| ENSMUSG00000085912 | lincRNA | Gm16197 | 6.097581 | 8.556008 | 7.326794 |
| ENSMUSG00000089711 | processed_transcrip | Gm16240 | 1.601084 | 0.697713 | 1.149398 |
| ENSMUSG00000073880 | protein_coding | Gm10596 | 1.264884 | 3.355235 | 2.31006 |
| ENSMUSG00000035356 | protein_coding | Nfkbiz | 172.0647 | 187.7803 | 179.9225 |
| ENSMUSG00000066151 | protein_coding | Fkbp15 | 1438.139 | 1552.393 | 1495.266 |
| ENSMUSG00000058816 | pseudogene | Gm5972 | 0.638331 | 1.810948 | 1.22464 |
| ENSMUSG00000054733 | protein_coding | Msra | 213.6545 | 197.629 | 205.6418 |
| ENSMUSG00000021270 | protein_coding | Hsp90aa1 | 22948.93 | 23909.9 | 23429.41 |
| ENSMUSG00000034653 | protein_coding | Ythdc2 | 1864.319 | 1710.081 | 1787.2 |
| ENSMUSG00000093126 | miRNA | AC161763.1 | 16.78313 | 20.65034 | 18.71673 |
| ENSMUSG00000014776 | protein_coding | Nol3 | 33.52883 | 39.45691 | 36.49287 |
| ENSMUSG00000013622 | protein_coding | 0610007C21Rik | 1315.537 | 1249.704 | 1282.621 |
| ENSMUSG00000090264 | protein_coding | Eif4ebp3 | 18.54843 | 23.76263 | 21.15553 |
| ENSMUSG00000014852 | protein_coding | Adamts13 | 6.46069 | 4.396757 | 5.428724 |
| ENSMUSG00000032580 | protein_coding | Rbm5 | 3525.395 | 3734.651 | 3630.023 |
| ENSMUSG00000026580 | protein_coding | Selp | 12.31481 | 15.8056 | 14.0602 |
| ENSMUSG00000026036 | protein_coding | Nif3l1 | 833.0089 | 793.6502 | 813.3296 |
| ENSMUSG00000087314 | processed_transcrip | Gm15552 | 14.57778 | 11.07948 | 12.82863 |
| ENSMUSG00000090541 | lincRNA | Gm17487 | 8.107619 | 5.783174 | 6.945397 |
| ENSMUSG00000084616 | snRNA | U6 | 408.9946 | 380.6862 | 394.8404 |

| | | | | | |
|---|---|---|---|---|---|
| ENSMUSG00000020283 | protein_coding | Pex13 | 1671.523 | 1744.372 | 1707.947 |
| ENSMUSG00000026640 | protein_coding | Plxna2 | 154.625 | 169.0772 | 161.8511 |
| ENSMUSG00000035278 | protein_coding | Plekhj1 | 1541.276 | 1611.782 | 1576.529 |
| ENSMUSG00000085213 | processed_transcrip | Gm13091 | 60.41321 | 51.53027 | 55.97174 |
| ENSMUSG00000041133 | protein_coding | Smc1a | 2619.76 | 2500.89 | 2560.325 |
| ENSMUSG00000002871 | protein_coding | Tpra1 | 593.5572 | 626.2778 | 609.9175 |
| ENSMUSG00000013338 | protein_coding | Fer1l4 | 27.56856 | 22.2591 | 24.91383 |
| ENSMUSG00000019906 | protein_coding | Lin7a | 34.40012 | 28.4917 | 31.44591 |
| ENSMUSG00000071414 | protein_coding | Gm6736 | 22.96039 | 18.69162 | 20.82601 |
| ENSMUSG00000038212 | protein_coding | Hiatl1 | 1950.452 | 2041.4 | 1995.926 |
| ENSMUSG00000084803 | lincRNA | 5830444B04Rik | 16.63113 | 22.87422 | 19.75267 |
| ENSMUSG00000020273 | protein_coding | Papolg | 1777.7 | 1895.814 | 1836.757 |
| ENSMUSG00000071655 | protein_coding | Ubxn1 | 3474.095 | 3348.929 | 3411.512 |
| ENSMUSG00000084304 | pseudogene | Gm6142 | 5.822359 | 3.779043 | 4.800701 |
| ENSMUSG00000032915 | protein_coding | Emr4 | 6.13038 | 8.663034 | 7.396707 |
| ENSMUSG00000030341 | protein_coding | Tnfrsf1a | 1857.358 | 1966.655 | 1912.006 |
| ENSMUSG00000046062 | protein_coding | Ppp1r15b | 4294.84 | 4504.722 | 4399.781 |
| ENSMUSG00000020519 | protein_coding | Sap30l | 291.1557 | 309.1452 | 300.1504 |
| ENSMUSG00000037750 | protein_coding | BC017647 | 1511.287 | 1426.676 | 1468.982 |
| ENSMUSG00000079260 | protein_coding | Glb1 | 231.1774 | 210.6204 | 220.8989 |
| ENSMUSG00000018347 | protein_coding | Zkscan6 | 450.8259 | 426.4358 | 438.6309 |
| ENSMUSG00000081789 | pseudogene | Gm11893 | 5.474606 | 3.390549 | 4.432577 |
| ENSMUSG00000073747 | protein_coding | Gm7534 | 1.32124 | 0.36219 | 0.841715 |
| ENSMUSG00000000248 | protein_coding | Clec2g | 1.32124 | 0.36219 | 0.841715 |
| ENSMUSG00000089949 | processed_transcrip | Gm16137 | 1.32124 | 0.36219 | 0.841715 |
| ENSMUSG00000083885 | pseudogene | Gm13213 | 2.261704 | 3.73472 | 2.998212 |
| ENSMUSG00000051390 | protein_coding | Zbtb22 | 887.4082 | 927.0325 | 907.2203 |
| ENSMUSG00000054391 | processed_transcrip | 4930517O19Rik | 0.64422 | 1.739597 | 1.191909 |
| ENSMUSG00000054065 | protein_coding | Pkp3 | 11.02532 | 14.40116 | 12.71324 |
| ENSMUSG00000019892 | protein_coding | Lrriq1 | 28.77247 | 23.77957 | 26.27602 |
| ENSMUSG00000020415 | protein_coding | Pttg1 | 705.5847 | 657.728 | 681.6564 |
| ENSMUSG00000035513 | protein_coding | Ntng2 | 165.6819 | 146.6573 | 156.1696 |
| ENSMUSG00000052151 | protein_coding | Ppap2c | 116.9352 | 127.7383 | 122.3368 |
| ENSMUSG00000038489 | protein_coding | Polr2l | 938.3172 | 894.0928 | 916.205 |
| ENSMUSG00000019738 | protein_coding | Polr2i | 733.2515 | 693.8606 | 713.5561 |
| ENSMUSG00000033543 | protein_coding | Gtf2a2 | 1462.723 | 1544.262 | 1503.493 |
| ENSMUSG00000086730 | processed_transcrip | Gm11607 | 0.99093 | 0 | 0.495465 |
| ENSMUSG00000085205 | processed_transcrip | Gm13185 | 0.99093 | 0 | 0.495465 |
| ENSMUSG00000086006 | processed_transcrip | Gm13293 | 0.99093 | 0 | 0.495465 |
| ENSMUSG00000056329 | lincRNA | 1810010K12Rik | 0.99093 | 0 | 0.495465 |
| ENSMUSG00000093001 | misc_RNA | AL954388.2 | 0.99093 | 0 | 0.495465 |
| ENSMUSG00000082850 | pseudogene | Gm14299 | 0.99093 | 0 | 0.495465 |
| ENSMUSG00000027902 | protein_coding | BC051070 | 0.99093 | 0 | 0.495465 |
| ENSMUSG00000091273 | pseudogene | Gm6673 | 0.99093 | 0 | 0.495465 |
| ENSMUSG00000089719 | processed_transcrip | Gm15758 | 0.99093 | 0 | 0.495465 |
| ENSMUSG00000080560 | miRNA | AC131777.1 | 0.99093 | 0 | 0.495465 |
| ENSMUSG00000072963 | protein_coding | Gm10447 | 0.99093 | 0 | 0.495465 |
| ENSMUSG00000062128 | protein_coding | Olfr20 | 0.99093 | 0 | 0.495465 |
| ENSMUSG00000092054 | protein_coding | Kif4-ps | 0.99093 | 0 | 0.495465 |
| ENSMUSG00000076057 | miRNA | AC122313.1 | 0.99093 | 0 | 0.495465 |
| ENSMUSG00000034623 | protein_coding | Prss55 | 0.99093 | 0 | 0.495465 |
| ENSMUSG00000083192 | pseudogene | Gm7689 | 0.99093 | 0 | 0.495465 |
| ENSMUSG00000054827 | protein_coding | Cyp2c50 | 0.99093 | 0 | 0.495465 |
| ENSMUSG00000083048 | pseudogene | Gm14595 | 0.99093 | 0 | 0.495465 |
| ENSMUSG00000034732 | protein_coding | Pabpc5 | 5.441582 | 7.804963 | 6.623273 |
| ENSMUSG00000031872 | protein_coding | Bean1 | 41.00587 | 34.04244 | 37.52416 |
| ENSMUSG00000031730 | protein_coding | Dhodh | 426.0641 | 449.9769 | 438.0205 |
| ENSMUSG00000092642 | miRNA | AC130216.1 | 3.880455 | 2.348303 | 3.114379 |

| | | | | | |
|---|---|---|---|---|---|
| ENSMUSG00000027799 | protein_coding | Nbea | 1843.592 | 1712.578 | 1778.085 |
| ENSMUSG00000081052 | pseudogene | Gm6305 | 11.37076 | 8.300844 | 9.835804 |
| ENSMUSG00000083123 | pseudogene | Gm6826 | 12.25741 | 15.60956 | 13.93349 |
| ENSMUSG00000089048 | misc_RNA | 7SK | 4.198987 | 6.216714 | 5.207851 |
| ENSMUSG00000029121 | protein_coding | Crmp1 | 15.11264 | 19.11579 | 17.11421 |
| ENSMUSG00000018736 | protein_coding | Ndel1 | 2170.053 | 2254.83 | 2212.442 |
| ENSMUSG00000034437 | pseudogene | Gm9761 | 222.4833 | 207.4803 | 214.9818 |
| ENSMUSG00000063838 | protein_coding | Cdc42ep5 | 1222.395 | 1139.835 | 1181.115 |
| ENSMUSG00000087078 | lincRNA | 6720483E21Rik | 17.80812 | 22.04934 | 19.92873 |
| ENSMUSG00000062647 | protein_coding | Rpl7a | 7569.375 | 7862.279 | 7715.827 |
| ENSMUSG00000062906 | protein_coding | Hdac10 | 206.3638 | 188.171 | 197.2674 |
| ENSMUSG00000073856 | protein_coding | Iqck | 112.3798 | 102.6156 | 107.4977 |
| ENSMUSG00000015672 | protein_coding | Mrpl32 | 634.0836 | 669.9393 | 652.0114 |
| ENSMUSG00000031779 | protein_coding | Ccl22 | 2.273482 | 3.823727 | 3.048605 |
| ENSMUSG00000032612 | protein_coding | Usp4 | 3233.696 | 3373.476 | 3303.586 |
| ENSMUSG00000086802 | lincRNA | 2700038G22Rik | 148.0402 | 161.0139 | 154.5271 |
| ENSMUSG00000091641 | lincRNA | Gm17593 | 23.26886 | 29.01353 | 26.1412 |
| ENSMUSG00000021273 | protein_coding | Fdft1 | 1848.792 | 1771.611 | 1810.201 |
| ENSMUSG00000069041 | protein_coding | Slc25a31 | 3547.063 | 3756.302 | 3651.683 |
| ENSMUSG00000083220 | pseudogene | Gm12919 | 7.475177 | 5.174469 | 6.324823 |
| ENSMUSG00000078570 | protein_coding | 1110065P20Rik | 846.7952 | 803.0319 | 824.9135 |
| ENSMUSG00000075370 | protein_coding | Igll1 | 8.696976 | 2.321637 | 5.509307 |
| ENSMUSG00000074178 | protein_coding | Gm10638 | 33.63081 | 28.63368 | 31.13224 |
| ENSMUSG00000029754 | protein_coding | Dlx6 | 2.557947 | 1.086569 | 1.822258 |
| ENSMUSG00000029836 | protein_coding | Cbx3 | 3301.344 | 3473.477 | 3387.411 |
| ENSMUSG00000030930 | protein_coding | Chst15 | 183.5552 | 200.4942 | 192.0247 |
| ENSMUSG00000056184 | protein_coding | Olfr283 | 0.64422 | 1.73095 | 1.187585 |
| ENSMUSG00000047409 | protein_coding | Ctdspl | 2036.356 | 1946.679 | 1991.518 |
| ENSMUSG00000025900 | protein_coding | Rp1 | 1.32124 | 0.335524 | 0.828382 |
| ENSMUSG00000032081 | protein_coding | Apoc3 | 1.32124 | 0.335524 | 0.828382 |
| ENSMUSG00000021872 | protein_coding | Rnase10 | 1.32124 | 0.335524 | 0.828382 |
| ENSMUSG00000072612 | protein_coding | Gm10382 | 7.167156 | 9.880084 | 8.52362 |
| ENSMUSG00000014353 | protein_coding | Tmem87b | 2452.467 | 2351.681 | 2402.074 |
| ENSMUSG00000043487 | protein_coding | Acot6 | 151.3354 | 136.7654 | 144.0504 |
| ENSMUSG00000091118 | lincRNA | Mir1900 | 3.197546 | 1.712931 | 2.455238 |
| ENSMUSG00000071285 | protein_coding | Zfp87 | 513.4232 | 473.2371 | 493.3301 |
| ENSMUSG00000045302 | protein_coding | Preb | 1556.491 | 1632.242 | 1594.367 |
| ENSMUSG00000079462 | protein_coding | Gm15737 | 1.284863 | 2.410644 | 1.847753 |
| ENSMUSG00000058357 | pseudogene | Gm6612 | 2.275793 | 3.770395 | 3.023094 |
| ENSMUSG00000031710 | protein_coding | Ucp1 | 1.32124 | 2.763824 | 2.042532 |
| ENSMUSG00000022485 | protein_coding | Hoxc5 | 16.78313 | 12.98916 | 14.88614 |
| ENSMUSG00000033053 | protein_coding | 1700028P14Rik | 2.242993 | 0.997561 | 1.620277 |
| ENSMUSG00000033872 | pseudogene | Best4-ps | 1.621062 | 0.653029 | 1.137045 |
| ENSMUSG00000052749 | protein_coding | Trim30b | 1.621062 | 0.653029 | 1.137045 |
| ENSMUSG00000046456 | protein_coding | Tmem150b | 3.866366 | 2.348303 | 3.107334 |
| ENSMUSG00000030469 | protein_coding | Zfp719 | 558.9993 | 609.9099 | 584.4546 |
| ENSMUSG00000018733 | protein_coding | Pex12 | 525.4875 | 496.2808 | 510.8841 |
| ENSMUSG00000086942 | lincRNA | Gm15489 | 23.98539 | 19.32948 | 21.65743 |
| ENSMUSG00000050730 | protein_coding | Arhgap42 | 528.4559 | 561.1133 | 544.7846 |
| ENSMUSG00000056204 | protein_coding | Pgpep1 | 819.7717 | 883.5678 | 851.6698 |
| ENSMUSG00000084782 | processed_transcrip | Gm11213 | 1.32124 | 0.326514 | 0.823877 |
| ENSMUSG00000083406 | pseudogene | Gm8410 | 1.32124 | 0.326514 | 0.823877 |
| ENSMUSG00000032407 | protein_coding | 2610101N10Rik | 4237.249 | 4386.41 | 4311.83 |
| ENSMUSG00000081136 | pseudogene | Gm12990 | 5.171206 | 3.408205 | 4.289706 |
| ENSMUSG00000041482 | protein_coding | Fam38b | 170.3562 | 157.073 | 163.7146 |
| ENSMUSG00000043192 | protein_coding | Gm1840 | 5.837716 | 8.211475 | 7.024596 |
| ENSMUSG00000030521 | protein_coding | Mphosph10 | 1680.842 | 1598.493 | 1639.668 |
| ENSMUSG00000020140 | protein_coding | Lgr5 | 45.25726 | 53.06058 | 49.15892 |

| | | | | | |
|---|---|---|---|---|---|
| ENSMUSG00000071647 | protein_coding | Eml3 | 791.8548 | 833.2475 | 812.5512 |
| ENSMUSG00000091450 | protein_coding | Vmn2r11 | 2.575614 | 4.159251 | 3.367432 |
| ENSMUSG00000029461 | protein_coding | Fam168a | 2165.639 | 2256.936 | 2211.287 |
| ENSMUSG00000072387 | pseudogene | Gm10356 | 6.478358 | 4.379101 | 5.428729 |
| ENSMUSG00000025597 | protein_coding | Klhl4 | 120.5326 | 109.8514 | 115.192 |
| ENSMUSG00000079265 | protein_coding | Gm8257 | 2.595592 | 1.324076 | 1.959834 |
| ENSMUSG00000021756 | protein_coding | Il6st | 4599.031 | 4352.287 | 4475.659 |
| ENSMUSG00000070923 | protein_coding | Klhl9 | 2904.651 | 3089.347 | 2996.999 |
| ENSMUSG00000000001 | protein_coding | Gnai3 | 6539.192 | 6801.137 | 6670.164 |
| ENSMUSG00000049152 | protein_coding | Ugt3a2 | 5.207584 | 3.073044 | 4.140314 |
| ENSMUSG00000028528 | protein_coding | Dnajc6 | 26.28743 | 32.08693 | 29.18718 |
| ENSMUSG00000040463 | protein_coding | Mybbp1a | 4177.742 | 4400.641 | 4289.191 |
| ENSMUSG00000026149 | protein_coding | Tm4sf20 | 0.632442 | 1.703922 | 1.168182 |
| ENSMUSG00000081378 | pseudogene | Gm6834 | 10.34472 | 13.36829 | 11.85651 |
| ENSMUSG00000062604 | protein_coding | Srpk2 | 5955.615 | 6280.402 | 6118.009 |
| ENSMUSG00000027223 | protein_coding | Mapk8ip1 | 669.5722 | 629.8244 | 649.6983 |
| ENSMUSG00000056131 | protein_coding | Pgm3 | 1203.523 | 1143.097 | 1173.31 |
| ENSMUSG00000086251 | processed_transcrip | Gm12694 | 1.615173 | 0.653029 | 1.134101 |
| ENSMUSG00000035725 | protein_coding | Prkx | 774.8013 | 732.2684 | 753.5348 |
| ENSMUSG00000070891 | protein_coding | Gm12689 | 2.592014 | 1.324076 | 1.958045 |
| ENSMUSG00000086628 | processed_transcrip | Gm16157 | 2.592014 | 1.324076 | 1.958045 |
| ENSMUSG00000069913 | pseudogene | Anp32-ps | 1.917305 | 3.354512 | 2.635908 |
| ENSMUSG00000074489 | protein_coding | Bglap-rs1 | 2.273482 | 3.743729 | 3.008605 |
| ENSMUSG00000039046 | protein_coding | Usp6nl | 1454.803 | 1391.748 | 1423.276 |
| ENSMUSG00000052075 | protein_coding | 1700029F12Rik | 0.66062 | 1.784282 | 1.222451 |
| ENSMUSG00000037570 | protein_coding | Mcrs1 | 2281.167 | 2181.735 | 2231.451 |
| ENSMUSG00000084828 | lincRNA | Gm12367 | 7.485688 | 5.165821 | 6.325754 |
| ENSMUSG00000045348 | protein_coding | 6430598A04Rik | 352.1273 | 329.9317 | 341.0295 |
| ENSMUSG00000083483 | pseudogene | Gm14044 | 1.939594 | 3.443881 | 2.691737 |
| ENSMUSG00000085105 | lincRNA | Gm12758 | 36.20053 | 42.12774 | 39.16413 |
| ENSMUSG00000025330 | protein_coding | Padi4 | 43.19653 | 37.07329 | 40.13491 |
| ENSMUSG00000046982 | protein_coding | Tshz1 | 2208.1 | 2303.48 | 2255.79 |
| ENSMUSG00000086034 | lincRNA | Gm15201 | 16.87228 | 21.14731 | 19.00979 |
| ENSMUSG00000040557 | protein_coding | Wbscr27 | 593.0556 | 627.6914 | 610.3735 |
| ENSMUSG00000040612 | protein_coding | Ildr2 | 2008.227 | 2161.68 | 2084.954 |
| ENSMUSG00000083328 | pseudogene | Gm11826 | 2.583814 | 1.333085 | 1.958449 |
| ENSMUSG00000003992 | protein_coding | Ssbp2 | 711.9322 | 746.1735 | 729.0528 |
| ENSMUSG00000056411 | protein_coding | Gm12500 | 8.08302 | 5.792184 | 6.937602 |
| ENSMUSG00000060314 | protein_coding | Zfp941 | 123.8749 | 135.1173 | 129.4961 |
| ENSMUSG00000091474 | protein_coding | 2610021A01Rik | 776.3771 | 833.2963 | 804.8367 |
| ENSMUSG00000086400 | lincRNA | Gm16789 | 7.178934 | 10.34894 | 8.763936 |
| ENSMUSG00000007877 | protein_coding | Tcap | 6.44198 | 9.058049 | 7.750015 |
| ENSMUSG00000002731 | protein_coding | Prkra | 2994.454 | 3144.964 | 3069.709 |
| ENSMUSG00000038524 | protein_coding | Fchsd1 | 559.5465 | 595.2168 | 577.3817 |
| ENSMUSG00000085687 | lincRNA | Gm16153 | 101.2696 | 113.0516 | 107.1606 |
| ENSMUSG00000079941 | protein_coding | Gm11273 | 142.8675 | 131.0857 | 136.9766 |
| ENSMUSG00000021326 | protein_coding | Trim27 | 1496.962 | 1567.706 | 1532.334 |
| ENSMUSG00000051344 | protein_coding | Plekhm3 | 980.4191 | 937.0099 | 958.7145 |
| ENSMUSG00000047904 | protein_coding | Sstr2 | 40.21532 | 46.48488 | 43.3501 |
| ENSMUSG00000029127 | protein_coding | Zbtb49 | 549.5272 | 517.5763 | 533.5518 |
| ENSMUSG00000085563 | lincRNA | 2210411M09Rik | 5.159428 | 3.434871 | 4.29715 |
| ENSMUSG00000015340 | protein_coding | Cybb | 78.25794 | 88.0821 | 83.17002 |
| ENSMUSG00000072791 | protein_coding | Abcb5 | 85.31239 | 104.8403 | 95.07637 |
| ENSMUSG00000022203 | protein_coding | Efs | 616.4868 | 664.313 | 640.3999 |
| ENSMUSG00000087135 | lincRNA | Gm16096 | 6.168026 | 8.704146 | 7.436086 |
| ENSMUSG00000079339 | protein_coding | Gm14446 | 3.266723 | 5.183478 | 4.2251 |
| ENSMUSG00000038175 | protein_coding | Mylip | 292.1524 | 315.821 | 303.9867 |
| ENSMUSG00000078306 | protein_coding | Gm16493 | 21.91438 | 17.96688 | 19.94063 |

| | | | | | |
|---|---|---|---|---|---|
| ENSMUSG00000086922 | pseudogene | Gm13835 | 1005.235 | 956.5601 | 980.8977 |
| ENSMUSG00000021974 | protein_coding | Fgf9 | 31.56821 | 37.60236 | 34.58529 |
| ENSMUSG00000036792 | protein_coding | Mbd5 | 918.91 | 874.0604 | 896.4852 |
| ENSMUSG00000028063 | protein_coding | Lmna | 1073.888 | 1121.585 | 1097.736 |
| ENSMUSG00000028495 | protein_coding | Rps6 | 10523.93 | 10933.61 | 10728.77 |
| ENSMUSG00000074140 | protein_coding | Zfp319 | 222.2726 | 202.11 | 212.1913 |
| ENSMUSG00000083557 | pseudogene | Gm6877 | 122.8541 | 111.9669 | 117.4105 |
| ENSMUSG00000004085 | protein_coding | B230120H23Rik | 515.6584 | 544.6462 | 530.1523 |
| ENSMUSG00000034607 | protein_coding | Pof1b | 7.394222 | 5.165821 | 6.280021 |
| ENSMUSG00000000134 | protein_coding | Tcfe3 | 2463.298 | 2575.437 | 2519.367 |
| ENSMUSG00000020676 | protein_coding | Ccl11 | 1.945483 | 3.336855 | 2.641169 |
| ENSMUSG00000065359 | snoRNA | SNORD16 | 5.507406 | 7.769288 | 6.638347 |
| ENSMUSG00000064345 | protein_coding | mt-Nd2 | 30483.48 | 29337.07 | 29910.27 |
| ENSMUSG00000019796 | protein_coding | Lrp11 | 127.2858 | 138.372 | 132.8289 |
| ENSMUSG00000073749 | lincRNA | A330069E16Rik | 76.34168 | 85.28333 | 80.8125 |
| ENSMUSG00000022148 | protein_coding | Fyb | 45.17124 | 51.38689 | 48.27906 |
| ENSMUSG00000034442 | protein_coding | Trmt5 | 824.3559 | 787.0573 | 805.7066 |
| ENSMUSG00000028360 | protein_coding | Slc44a5 | 8.401552 | 6.109689 | 7.25562 |
| ENSMUSG00000063129 | protein_coding | Aldoart2 | 5.884604 | 3.752738 | 4.818671 |
| ENSMUSG00000042351 | protein_coding | Grap2 | 35.1484 | 29.83628 | 32.49234 |
| ENSMUSG00000055320 | protein_coding | Tead1 | 923.411 | 976.7354 | 950.0732 |
| ENSMUSG00000025905 | protein_coding | Oprk1 | 0.966331 | 0 | 0.483165 |
| ENSMUSG00000083001 | pseudogene | Gm13250 | 0.966331 | 0 | 0.483165 |
| ENSMUSG00000066478 | protein_coding | Gm5745 | 0.966331 | 0 | 0.483165 |
| ENSMUSG00000089155 | snoRNA | SNORA17 | 0.966331 | 0 | 0.483165 |
| ENSMUSG00000085803 | processed_transcrip | Gm12056 | 0.966331 | 0 | 0.483165 |
| ENSMUSG00000039005 | protein_coding | Tlr4 | 51.06531 | 44.64075 | 47.85303 |
| ENSMUSG00000086526 | processed_transcrip | Gm12762 | 9.708703 | 12.81328 | 11.26099 |
| ENSMUSG00000052551 | protein_coding | Adarb2 | 1.298952 | 2.410644 | 1.854798 |
| ENSMUSG00000029559 | protein_coding | 2210016L21Rik | 736.8702 | 777.1794 | 757.0248 |
| ENSMUSG00000086626 | lincRNA | A630072L19Rik | 0.66062 | 1.739597 | 1.200109 |
| ENSMUSG00000049489 | protein_coding | Fam58b | 432.0364 | 457.7675 | 444.9019 |
| ENSMUSG00000032217 | protein_coding | Rnf111 | 1484.19 | 1560.381 | 1522.285 |
| ENSMUSG00000086926 | pseudogene | Gm6226 | 1.629262 | 0.653029 | 1.141145 |
| ENSMUSG00000000320 | protein_coding | Alox12 | 71.17464 | 62.16181 | 66.66822 |
| ENSMUSG00000071192 | protein_coding | Wfikkn1 | 77.66418 | 90.23287 | 83.94852 |
| ENSMUSG00000027206 | protein_coding | Cops2 | 4758.77 | 4945.699 | 4852.234 |
| ENSMUSG00000032555 | protein_coding | Topbp1 | 4786.939 | 4546.404 | 4666.672 |
| ENSMUSG00000046862 | protein_coding | Pramef8 | 1047.654 | 1099.379 | 1073.517 |
| ENSMUSG00000060803 | protein_coding | Gstp1 | 1368.283 | 1491.394 | 1429.839 |
| ENSMUSG00000024002 | protein_coding | Brd4 | 4146.399 | 3946.745 | 4046.572 |
| ENSMUSG00000001225 | protein_coding | Slc26a3 | 3.586523 | 5.483326 | 4.534924 |
| ENSMUSG00000027219 | protein_coding | Slc28a2 | 5.198117 | 7.585475 | 6.391796 |
| ENSMUSG00000021127 | protein_coding | Zfp36l1 | 1032.723 | 1085.393 | 1059.058 |
| ENSMUSG00000033308 | protein_coding | Dpyd | 247.7302 | 230.432 | 239.0811 |
| ENSMUSG00000030595 | protein_coding | Nfkbib | 558.57 | 522.1191 | 540.3445 |
| ENSMUSG00000017400 | protein_coding | Stac2 | 854.1229 | 897.697 | 875.9099 |
| ENSMUSG00000020023 | protein_coding | Tmcc3 | 2787.532 | 2979.672 | 2883.602 |
| ENSMUSG00000022408 | protein_coding | Fam83f | 177.8997 | 165.3407 | 171.6202 |
| ENSMUSG00000086288 | processed_transcrip | Gm15265 | 86.5716 | 76.00796 | 81.28978 |
| ENSMUSG00000058325 | protein_coding | Dock1 | 1396.285 | 1470.52 | 1433.403 |
| ENSMUSG00000086101 | lincRNA | 2610316D01Rik | 4.182587 | 6.127345 | 5.154966 |
| ENSMUSG00000083364 | pseudogene | Gm14535 | 22.3574 | 18.22525 | 20.29132 |
| ENSMUSG00000075307 | protein_coding | Kbtbd10 | 10.66914 | 13.78997 | 12.22956 |
| ENSMUSG00000024621 | protein_coding | Csf1r | 442.0268 | 470.6789 | 456.3529 |
| ENSMUSG00000022090 | protein_coding | Pdlim2 | 280.6163 | 299.8335 | 290.2249 |
| ENSMUSG00000059890 | protein_coding | Ube4a | 2805.906 | 2931.127 | 2868.516 |
| ENSMUSG00000056692 | protein_coding | D17Wsu92e | 4874.751 | 4666.392 | 4770.572 |

| | | | | | |
|---|---|---|---|---|---|
| ENSMUSG00000030541 | protein_coding | Idh2 | 1476.544 | 1575.007 | 1525.775 |
| ENSMUSG00000086615 | lincRNA | Gm16862 | 2.303971 | 1.015218 | 1.659594 |
| ENSMUSG00000035576 | protein_coding | L3mbtl1 | 23.96206 | 19.78683 | 21.87445 |
| ENSMUSG00000027593 | protein_coding | Raly | 5921.346 | 6150.839 | 6036.092 |
| ENSMUSG00000027002 | protein_coding | Nckap1 | 7894.667 | 8170.725 | 8032.696 |
| ENSMUSG00000027305 | protein_coding | Ndufaf1 | 743.4169 | 706.2621 | 724.8395 |
| ENSMUSG00000041849 | protein_coding | Card6 | 181.3594 | 167.787 | 174.5732 |
| ENSMUSG00000010122 | protein_coding | Slc47a1 | 82.49539 | 92.24461 | 87.37 |
| ENSMUSG00000006699 | protein_coding | Cdc42 | 10975.68 | 11354.79 | 11165.24 |
| ENSMUSG00000029621 | protein_coding | Arpc1a | 4726.035 | 4563.701 | 4644.868 |
| ENSMUSG00000078453 | protein_coding | 3110003A17Rik | 512.7824 | 542.3887 | 527.5855 |
| ENSMUSG00000038094 | protein_coding | Atp13a4 | 427.7714 | 458.7348 | 443.2531 |
| ENSMUSG00000062866 | protein_coding | Phactr2 | 1113.29 | 1168.355 | 1140.823 |
| ENSMUSG00000056643 | protein_coding | Chst13 | 14.49682 | 18.03823 | 16.26752 |
| ENSMUSG00000063450 | protein_coding | Syne2 | 12902.15 | 13526.22 | 13214.18 |
| ENSMUSG00000078440 | protein_coding | Dohh | 1394.36 | 1334.564 | 1364.462 |
| ENSMUSG00000064390 | snoRNA | SNORD73 | 21.03258 | 16.854 | 18.94329 |
| ENSMUSG00000086596 | protein_coding | Susd5 | 24.24652 | 20.11371 | 22.18012 |
| ENSMUSG00000078896 | protein_coding | Gm8923 | 5.173517 | 3.417215 | 4.295366 |
| ENSMUSG00000022898 | protein_coding | Dscr3 | 1146.202 | 1208.06 | 1177.131 |
| ENSMUSG00000053375 | protein_coding | Atp6v1e2 | 0.66062 | 1.73095 | 1.195785 |
| ENSMUSG00000076490 | IG_C_gene | Trbc1 | 1.635151 | 0.662038 | 1.148594 |
| ENSMUSG00000084399 | pseudogene | Gm15666 | 1.635151 | 0.662038 | 1.148594 |
| ENSMUSG00000092500 | processed_transcrip | RP24-323I5.2 | 4.222543 | 2.428301 | 3.325422 |
| ENSMUSG00000027306 | protein_coding | Nusap1 | 1322.446 | 1406.768 | 1364.607 |
| ENSMUSG00000032036 | protein_coding | Kirrel3 | 52.09135 | 58.99903 | 55.54519 |
| ENSMUSG00000037578 | protein_coding | Pkd2l1 | 255.3939 | 273.7254 | 264.5597 |
| ENSMUSG00000087472 | lincRNA | A630075F10Rik | 5.507406 | 7.787306 | 6.647356 |
| ENSMUSG00000086138 | processed_transcrip | 4930471C06Rik | 2.298082 | 1.015218 | 1.65665 |
| ENSMUSG00000035258 | protein_coding | Abi3bp | 456.3726 | 495.6873 | 476.0299 |
| ENSMUSG00000003273 | protein_coding | Car11 | 82.95377 | 93.08974 | 88.02175 |
| ENSMUSG00000091760 | lincRNA | Gm17619 | 2.589703 | 4.105918 | 3.347811 |
| ENSMUSG00000028307 | protein_coding | Aldob | 5.187606 | 3.425862 | 4.306734 |
| ENSMUSG00000085778 | lincRNA | Gm16892 | 40.74392 | 47.73942 | 44.24167 |
| ENSMUSG00000082031 | pseudogene | Gm8097 | 9.664125 | 7.187248 | 8.425687 |
| ENSMUSG00000067261 | protein_coding | Foxd3 | 3.568855 | 5.536658 | 4.552757 |
| ENSMUSG00000007097 | protein_coding | Atp1a2 | 1092.984 | 1016.322 | 1054.653 |
| ENSMUSG00000023892 | protein_coding | Zfp51 | 411.579 | 443.1427 | 427.3609 |
| ENSMUSG00000092067 | lincRNA | A230107N01Rik | 35.12969 | 41.0203 | 38.07499 |
| ENSMUSG00000041608 | protein_coding | Entpd3 | 51.12375 | 58.6296 | 54.87667 |
| ENSMUSG00000015127 | protein_coding | Unkl | 502.4985 | 550.2672 | 526.3829 |
| ENSMUSG00000035632 | protein_coding | Cnot3 | 2035.513 | 2125.624 | 2080.569 |
| ENSMUSG00000022113 | protein_coding | Trim52 | 509.3556 | 384.3718 | 446.8637 |
| ENSMUSG00000085181 | processed_transcrip | Gm12709 | 1.621062 | 0.671047 | 1.146054 |
| ENSMUSG00000085920 | processed_transcrip | Gm12951 | 1.621062 | 0.671047 | 1.146054 |
| ENSMUSG00000025389 | protein_coding | Mip | 1.621062 | 0.671047 | 1.146054 |
| ENSMUSG00000059279 | protein_coding | Olfr224 | 1.621062 | 0.671047 | 1.146054 |
| ENSMUSG00000052125 | lincRNA | F730043M19Rik | 51.17526 | 43.92931 | 47.55229 |
| ENSMUSG00000038572 | protein_coding | Bpifb5 | 15.90468 | 20.72278 | 18.31373 |
| ENSMUSG00000089185 | snRNA | U1 | 1.290752 | 2.383978 | 1.837365 |
| ENSMUSG00000083207 | pseudogene | Gm14780 | 1.290752 | 2.383978 | 1.837365 |
| ENSMUSG00000036591 | protein_coding | Arhgap21 | 2910.014 | 2798.732 | 2854.373 |
| ENSMUSG00000082292 | pseudogene | Gm12250 | 17.45426 | 21.82949 | 19.64187 |
| ENSMUSG00000083829 | pseudogene | Gm2199 | 1.284863 | 2.374969 | 1.829916 |
| ENSMUSG00000028902 | protein_coding | Sf3a3 | 2933.695 | 3055.185 | 2994.44 |
| ENSMUSG00000033579 | protein_coding | Fa2h | 35.29518 | 29.78223 | 32.5387 |
| ENSMUSG00000000278 | protein_coding | Scpep1 | 1766.483 | 1838.907 | 1802.695 |
| ENSMUSG00000014313 | protein_coding | Cox6c | 2098.629 | 2003.695 | 2051.162 |

| | | | | | |
|---|---|---|---|---|---|
| ENSMUSG00000063975 | protein_coding | Slco1a5 | 90.07217 | 76.4238 | 83.24798 |
| ENSMUSG00000042333 | protein_coding | Tnfrsf14 | 9.39606 | 12.37073 | 10.88339 |
| ENSMUSG00000083392 | pseudogene | Gm6335 | 5.829516 | 3.814718 | 4.822117 |
| ENSMUSG00000033295 | protein_coding | Ptprf | 8322.984 | 8679.552 | 8501.268 |
| ENSMUSG00000024936 | protein_coding | Kcnk7 | 2.597903 | 1.377408 | 1.987655 |
| ENSMUSG00000024675 | protein_coding | Ms4a4c | 2.620192 | 1.386417 | 2.003304 |
| ENSMUSG00000032092 | protein_coding | Mpzl2 | 248.5293 | 322.2787 | 285.404 |
| ENSMUSG00000010914 | protein_coding | Pdhx | 1340.847 | 1280.844 | 1310.846 |
| ENSMUSG00000021023 | protein_coding | 1110008L16Rik | 526.6644 | 554.9404 | 540.8024 |
| ENSMUSG00000085423 | processed_transcrip | Gm15735 | 3.578323 | 5.500983 | 4.539653 |
| ENSMUSG00000041215 | protein_coding | Yeats2 | 1892.753 | 1997.355 | 1945.054 |
| ENSMUSG00000078995 | protein_coding | Zfp456 | 119.2776 | 133.0325 | 126.1551 |
| ENSMUSG00000025980 | protein_coding | Hspd1 | 6944.203 | 7232.104 | 7088.154 |
| ENSMUSG00000055809 | protein_coding | 6030429G01Rik | 121.2482 | 110.5398 | 115.894 |
| ENSMUSG00000030880 | protein_coding | Polr3e | 1954.903 | 2046.903 | 2000.903 |
| ENSMUSG00000055691 | protein_coding | Gja6 | 412.0223 | 315.0206 | 363.5215 |
| ENSMUSG00000056429 | protein_coding | Tgoln1 | 5437.562 | 5641.007 | 5539.285 |
| ENSMUSG00000087344 | processed_transcrip | Gm15627 | 1.929083 | 3.363883 | 2.646483 |
| ENSMUSG00000047686 | protein_coding | Zcchc5 | 5.783671 | 8.99607 | 7.38987 |
| ENSMUSG00000037605 | protein_coding | Lphn3 | 113.5366 | 99.88606 | 106.7114 |
| ENSMUSG00000029287 | protein_coding | Tgfbr3 | 1200.918 | 1129.193 | 1165.055 |
| ENSMUSG00000083208 | pseudogene | Gm11522 | 0.948663 | 0 | 0.474332 |
| ENSMUSG00000089343 | misc_RNA | 7SK | 0.948663 | 0 | 0.474332 |
| ENSMUSG00000057829 | pseudogene | Gm5278 | 2.579193 | 1.359751 | 1.969472 |
| ENSMUSG00000067144 | protein_coding | Slc22a7 | 11.01585 | 8.193457 | 9.604656 |
| ENSMUSG00000090509 | protein_coding | Sfta2 | 1.612862 | 0.653029 | 1.132945 |
| ENSMUSG00000000154 | protein_coding | Slc22a18 | 9.08215 | 6.551876 | 7.817013 |
| ENSMUSG00000020677 | protein_coding | Ddx52 | 1969.445 | 2056.22 | 2012.832 |
| ENSMUSG00000016541 | protein_coding | Atxn10 | 3511.321 | 3655.051 | 3583.186 |
| ENSMUSG00000088332 | snRNA | U1 | 1.615173 | 0.671047 | 1.14311 |
| ENSMUSG00000089723 | processed_transcrip | Gm16117 | 1.615173 | 0.671047 | 1.14311 |
| ENSMUSG00000028961 | protein_coding | Pgd | 3782.909 | 3601.284 | 3692.097 |
| ENSMUSG00000019913 | protein_coding | Sim1 | 1.284863 | 2.401635 | 1.843249 |
| ENSMUSG00000019194 | protein_coding | Scn1b | 53.9687 | 47.56602 | 50.76736 |
| ENSMUSG00000028919 | protein_coding | Arhgef19 | 109.7336 | 122.1374 | 115.9355 |
| ENSMUSG00000041247 | protein_coding | Lamp3 | 6.78869 | 4.785613 | 5.787151 |
| ENSMUSG00000046909 | protein_coding | 1110002N22Rik | 425.6445 | 402.2612 | 413.9528 |
| ENSMUSG00000047694 | protein_coding | Yipf6 | 1648.179 | 1579.693 | 1613.936 |
| ENSMUSG00000085922 | processed_transcrip | Gm13643 | 1.298952 | 2.43731 | 1.868131 |
| ENSMUSG00000059796 | protein_coding | Eif4a1 | 17452.48 | 18080.26 | 17766.37 |
| ENSMUSG00000087825 | snRNA | U6 | 1.623372 | 0.662038 | 1.142705 |
| ENSMUSG00000032042 | protein_coding | Srpr | 3791.377 | 3938.995 | 3865.186 |
| ENSMUSG00000027221 | protein_coding | Chst1 | 74.21743 | 66.6555 | 70.43647 |
| ENSMUSG00000028821 | protein_coding | Syf2 | 1889.701 | 1970.952 | 1930.326 |
| ENSMUSG00000086718 | processed_transcrip | Gm16069 | 1.298952 | 2.419653 | 1.859302 |
| ENSMUSG00000026442 | protein_coding | Nfasc | 181.292 | 196.6029 | 188.9474 |
| ENSMUSG00000082145 | pseudogene | Gm12312 | 5.856426 | 8.318501 | 7.087464 |
| ENSMUSG00000020427 | protein_coding | Igfbp3 | 910.4776 | 843.4742 | 876.9759 |
| ENSMUSG00000029917 | protein_coding | C130060K24Rik | 3.202167 | 5.157174 | 4.17967 |
| ENSMUSG00000026630 | protein_coding | Batf3 | 37.58297 | 43.83057 | 40.70677 |
| ENSMUSG00000051498 | protein_coding | Tlr6 | 11.53685 | 15.06392 | 13.30039 |
| ENSMUSG00000026754 | protein_coding | Golga1 | 1179.721 | 1237.959 | 1208.84 |
| ENSMUSG00000024826 | protein_coding | Dpf2 | 3535.557 | 3687.149 | 3611.353 |
| ENSMUSG00000083808 | pseudogene | Gm3828 | 0.974531 | 2.030798 | 1.502664 |
| ENSMUSG00000036989 | protein_coding | Trim3 | 652.0815 | 618.8977 | 635.4896 |
| ENSMUSG00000074800 | pseudogene | Gm4149 | 39.75299 | 34.29181 | 37.0224 |
| ENSMUSG00000006418 | protein_coding | Rnf114 | 3697.978 | 3541.787 | 3619.883 |
| ENSMUSG00000007038 | protein_coding | Neu1 | 410.586 | 433.6955 | 422.1407 |

| | | | | | |
|---|---|---|---|---|---|
| ENSMUSG00000029188 | protein_coding | Slc34a2 | 31.34219 | 26.5946 | 28.96839 |
| ENSMUSG00000033597 | protein_coding | Caskin1 | 2403.239 | 2296.576 | 2349.907 |
| ENSMUSG00000067356 | protein_coding | B430203G13Rik | 16.06615 | 20.15876 | 18.11245 |
| ENSMUSG00000053519 | protein_coding | Kcnip1 | 137.7834 | 155.9635 | 146.8735 |
| ENSMUSG00000092483 | processed_transcrip | RP24-89N4.5 | 8.444862 | 12.02262 | 10.23374 |
| ENSMUSG00000041737 | protein_coding | Tmem45b | 3.633411 | 1.748606 | 2.691009 |
| ENSMUSG00000029538 | protein_coding | Srsf9 | 3506.042 | 3630.899 | 3568.471 |
| ENSMUSG00000052423 | protein_coding | B4galt3 | 744.1283 | 785.7885 | 764.9584 |
| ENSMUSG00000024526 | protein_coding | Cidea | 916.857 | 963.2727 | 940.0649 |
| ENSMUSG00000086024 | lincRNA | Gm16639 | 1.606973 | 0.671047 | 1.13901 |
| ENSMUSG00000021112 | protein_coding | Mpp5 | 2887.075 | 2734.638 | 2810.856 |
| ENSMUSG00000039717 | protein_coding | Ralyl | 2.253504 | 1.086569 | 1.670036 |
| ENSMUSG00000040495 | protein_coding | Chrm4 | 9.386593 | 6.605208 | 7.995901 |
| ENSMUSG00000052837 | protein_coding | Junb | 712.7708 | 513.3673 | 613.069 |
| ENSMUSG00000034209 | protein_coding | Rasl10a | 793.3215 | 851.4509 | 822.3862 |
| ENSMUSG00000026883 | protein_coding | Dab2ip | 4310.383 | 4533.387 | 4421.885 |
| ENSMUSG00000006538 | protein_coding | Ihh | 13.53511 | 10.56014 | 12.04763 |
| ENSMUSG00000029291 | protein_coding | Rufy3 | 1764.014 | 1838.837 | 1801.425 |
| ENSMUSG00000046096 | protein_coding | BC030336 | 970.1585 | 1025.624 | 997.8915 |
| ENSMUSG00000030302 | protein_coding | Atp2b2 | 20.97876 | 17.04931 | 19.01404 |
| ENSMUSG00000079179 | protein_coding | 1700012B15Rik | 293.7278 | 273.3716 | 283.5497 |
| ENSMUSG00000021519 | protein_coding | Mterfd1 | 938.2091 | 898.0346 | 918.1218 |
| ENSMUSG00000022034 | protein_coding | Esco2 | 3118.905 | 2914.372 | 3016.638 |
| ENSMUSG00000024975 | protein_coding | Pdcd4 | 2734.515 | 2609.933 | 2672.224 |
| ENSMUSG00000071324 | protein_coding | Armc2 | 110.5416 | 121.745 | 116.1433 |
| ENSMUSG00000043614 | protein_coding | Vps37d | 269.3999 | 288.0245 | 278.7122 |
| ENSMUSG00000085614 | lincRNA | 1700123M08Rik | 70.12885 | 62.69156 | 66.4102 |
| ENSMUSG00000047237 | protein_coding | Fbxw21 | 2.589703 | 1.36876 | 1.979232 |
| ENSMUSG00000085464 | processed_transcrip | Gm16208 | 1.32124 | 2.728511 | 2.024876 |
| ENSMUSG00000031727 | protein_coding | Pmfbp1 | 3.844077 | 5.792184 | 4.81813 |
| ENSMUSG00000089660 | processed_transcrip | Vmn2r-ps110 | 1.601084 | 0.653029 | 1.127056 |
| ENSMUSG00000010057 | protein_coding | Nprl2 | 387.9747 | 364.0381 | 376.0064 |
| ENSMUSG00000044982 | protein_coding | Sft2d3 | 346.4658 | 370.852 | 358.6589 |
| ENSMUSG00000041923 | protein_coding | Nol4 | 25.09425 | 30.29219 | 27.69322 |
| ENSMUSG00000005505 | protein_coding | Kbtbd4 | 782.7238 | 819.5158 | 801.1198 |
| ENSMUSG00000042249 | protein_coding | Adrbk2 | 200.0868 | 185.1477 | 192.6172 |
| ENSMUSG00000042670 | protein_coding | Immp1l | 705.958 | 671.8345 | 688.8962 |
| ENSMUSG00000033335 | protein_coding | Dnm2 | 2755.531 | 2615.964 | 2685.747 |
| ENSMUSG00000070343 | protein_coding | Gm10288 | 32.1071 | 26.92111 | 29.51411 |
| ENSMUSG00000091769 | lincRNA | 4921507G05Rik | 9.036305 | 6.587913 | 7.812109 |
| ENSMUSG00000004846 | protein_coding | Plod3 | 1096.4 | 1039.24 | 1067.82 |
| ENSMUSG00000065097 | snoRNA | Snora16a | 11.94812 | 15.39981 | 13.67396 |
| ENSMUSG00000087440 | processed_transcrip | 4930577N17Rik | 34.4514 | 29.06401 | 31.75771 |
| ENSMUSG00000026768 | protein_coding | Itga8 | 175.5252 | 195.9905 | 185.7578 |
| ENSMUSG00000022441 | protein_coding | Efcab6 | 51.71669 | 44.08796 | 47.90232 |
| ENSMUSG00000021265 | protein_coding | Slc25a29 | 95.29161 | 105.5264 | 100.409 |
| ENSMUSG00000081187 | pseudogene | 3110067C02Rik | 12.22923 | 15.61821 | 13.92372 |
| ENSMUSG00000081633 | pseudogene | Gm8522 | 4.526986 | 2.835175 | 3.681081 |
| ENSMUSG00000062074 | protein_coding | Wisp3 | 30.36639 | 25.69433 | 28.03036 |
| ENSMUSG00000005125 | protein_coding | Ndrg1 | 1191.287 | 1128.246 | 1159.766 |
| ENSMUSG00000042650 | protein_coding | Alkbh5 | 4686.288 | 4436.42 | 4561.354 |
| ENSMUSG00000021764 | protein_coding | Ndufs4 | 1134.897 | 1189.78 | 1162.338 |
| ENSMUSG00000043929 | protein_coding | Klhl15 | 1926.17 | 1837.962 | 1882.066 |
| ENSMUSG00000010362 | protein_coding | Rdm1 | 284.6163 | 265.2334 | 274.9249 |
| ENSMUSG00000074868 | pseudogene | Gm6478 | 14.2298 | 10.80013 | 12.51497 |
| ENSMUSG00000044150 | protein_coding | A830080D01Rik | 1750.662 | 1856.434 | 1803.548 |
| ENSMUSG00000036510 | protein_coding | Cdh8 | 1.609283 | 0.662038 | 1.135661 |
| ENSMUSG00000024077 | protein_coding | Strn | 3257.641 | 3117.011 | 3187.326 |

| | | | | | |
|---|---|---|---|---|---|
| ENSMUSG00000046572 | protein_coding | Zfp518b | 1591.629 | 1498.624 | 1545.127 |
| ENSMUSG00000028848 | protein_coding | Gpn2 | 617.1581 | 588.5265 | 602.8423 |
| ENSMUSG00000065950 | protein_coding | Gm5908 | 5.137139 | 3.381539 | 4.259339 |
| ENSMUSG00000031533 | protein_coding | Mrps31 | 723.4183 | 689.5999 | 706.5091 |
| ENSMUSG00000010154 | protein_coding | Spire2 | 1375.515 | 1436.393 | 1405.954 |
| ENSMUSG00000027291 | protein_coding | Vps39 | 2025.407 | 1936.561 | 1980.984 |
| ENSMUSG00000055670 | protein_coding | Zzef1 | 1934.536 | 1824.151 | 1879.343 |
| ENSMUSG00000026663 | protein_coding | Atf6 | 2093.41 | 2012.812 | 2053.111 |
| ENSMUSG00000038756 | protein_coding | Ttll6 | 15.23251 | 11.81571 | 13.52411 |
| ENSMUSG00000018171 | protein_coding | Vmp1 | 2160.835 | 2247.363 | 2204.099 |
| ENSMUSG00000080893 | pseudogene | Gm15920 | 275.2905 | 309.6376 | 292.464 |
| ENSMUSG00000063870 | protein_coding | Chd4 | 12683.99 | 13252.31 | 12968.15 |
| ENSMUSG00000021051 | protein_coding | Ppp2r5e | 2470.028 | 2369.962 | 2419.995 |
| ENSMUSG00000047033 | protein_coding | Pcdhb15 | 18.15483 | 22.30486 | 20.22985 |
| ENSMUSG00000085078 | processed_transcrip | C030013C21Rik | 6.180847 | 4.088262 | 5.134554 |
| ENSMUSG00000021179 | protein_coding | BC002230 | 1104.802 | 1051.855 | 1078.329 |
| ENSMUSG00000030869 | protein_coding | Ndufab1 | 1336.682 | 1276.011 | 1306.346 |
| ENSMUSG00000032892 | protein_coding | Rangrf | 5.836448 | 8.149496 | 6.992972 |
| ENSMUSG00000052105 | protein_coding | 1110012J17Rik | 4067.834 | 4257.61 | 4162.722 |
| ENSMUSG00000050234 | protein_coding | Gja4 | 113.0093 | 124.1849 | 118.5971 |
| ENSMUSG00000059005 | protein_coding | Hnrnpa3 | 2734.276 | 2607.15 | 2670.713 |
| ENSMUSG00000065449 | miRNA | Mir365-1 | 1.296641 | 2.648151 | 1.972396 |
| ENSMUSG00000015484 | protein_coding | Fam163a | 26.77652 | 21.92357 | 24.35004 |
| ENSMUSG00000025133 | protein_coding | Ints4 | 2574.277 | 2685.089 | 2629.683 |
| ENSMUSG00000033499 | protein_coding | Larp4b | 1859.859 | 1773.175 | 1816.517 |
| ENSMUSG00000031808 | protein_coding | Slc27a1 | 3221.171 | 3428.987 | 3325.079 |
| ENSMUSG00000086419 | lincRNA | 4933408J17Rik | 3.851009 | 2.030436 | 2.940723 |
| ENSMUSG00000024339 | protein_coding | Tap2 | 74.87895 | 65.43193 | 70.15544 |
| ENSMUSG00000025871 | protein_coding | 4833439L19Rik | 3541.167 | 3381.805 | 3461.486 |
| ENSMUSG00000032358 | protein_coding | Fam83b | 37.33168 | 43.11271 | 40.2222 |
| ENSMUSG00000064326 | protein_coding | Siva1 | 1743.658 | 1676.575 | 1710.117 |
| ENSMUSG00000086276 | lincRNA | 2500002B13Rik | 15.13977 | 11.79521 | 13.46749 |
| ENSMUSG00000029144 | protein_coding | Gtf3c2 | 3434.953 | 3574.267 | 3504.61 |
| ENSMUSG00000056999 | protein_coding | Ide | 2486.54 | 2395.818 | 2441.179 |
| ENSMUSG00000038119 | protein_coding | Cdon | 1848.258 | 1691.227 | 1769.743 |
| ENSMUSG00000045795 | protein_coding | Whamm | 297.64 | 319.1219 | 308.3809 |
| ENSMUSG00000058402 | protein_coding | Zfp420 | 553.8717 | 586.4944 | 570.1831 |
| ENSMUSG00000061039 | protein_coding | Olfr920 | 12.01498 | 9.007205 | 10.51109 |
| ENSMUSG00000050213 | protein_coding | Snip1 | 1306.076 | 1241.377 | 1273.727 |
| ENSMUSG00000090459 | processed_transcrip | Gm17210 | 45.4059 | 52.35163 | 48.87877 |
| ENSMUSG00000029840 | protein_coding | Mtpn | 8133.302 | 8443.405 | 8288.354 |
| ENSMUSG00000086435 | lincRNA | D930007P13Rik | 3.239813 | 5.210506 | 4.225159 |
| ENSMUSG00000030494 | protein_coding | Rhpn2 | 1099.204 | 1046.169 | 1072.687 |
| ENSMUSG00000049565 | protein_coding | Aknad1 | 2.889525 | 4.651921 | 3.770723 |
| ENSMUSG00000051452 | protein_coding | Gm11437 | 3.248013 | 1.784282 | 2.516147 |
| ENSMUSG00000014158 | protein_coding | Trpv4 | 196.0638 | 180.933 | 188.4984 |
| ENSMUSG00000075272 | protein_coding | Ttc30a2 | 6.140891 | 8.820543 | 7.480717 |
| ENSMUSG00000024818 | protein_coding | Slc25a45 | 1211.189 | 1279.419 | 1245.304 |
| ENSMUSG00000032030 | protein_coding | Cul5 | 2941.524 | 2825.214 | 2883.369 |
| ENSMUSG00000090904 | lincRNA | Gm17248 | 10.96539 | 8.256522 | 9.610955 |
| ENSMUSG00000035238 | protein_coding | Kcnk15 | 8.139376 | 5.572334 | 6.855855 |
| ENSMUSG00000039016 | protein_coding | Timm8b | 1036.785 | 1086.677 | 1061.731 |
| ENSMUSG00000081861 | pseudogene | Gm14441 | 0.954552 | 2.119805 | 1.537179 |
| ENSMUSG00000053510 | protein_coding | Nrd1 | 4453.369 | 4632.702 | 4543.036 |
| ENSMUSG00000033594 | protein_coding | Spata2L | 77.42907 | 69.63953 | 73.5343 |
| ENSMUSG00000026887 | protein_coding | Mrrf | 802.4026 | 837.3702 | 819.8864 |
| ENSMUSG00000040112 | protein_coding | Mrps35 | 850.3523 | 887.0186 | 868.6854 |
| ENSMUSG00000019102 | protein_coding | Aldh3a1 | 208.9727 | 192.3524 | 200.6626 |

| | | | | | |
|---|---|---|---|---|---|
| ENSMUSG00000032077 | protein_coding | Bud13 | 639.7191 | 676.5773 | 658.1482 |
| ENSMUSG00000085760 | processed_transcrip | Gm14302 | 3.905054 | 2.357312 | 3.131183 |
| ENSMUSG00000025981 | protein_coding | Coq10b | 1118.385 | 1168.022 | 1143.203 |
| ENSMUSG00000005667 | protein_coding | Mthfd2 | 1954.981 | 1876.222 | 1915.602 |
| ENSMUSG00000074678 | protein_coding | Defb25 | 2.569725 | 4.079252 | 3.324489 |
| ENSMUSG00000085631 | processed_transcrip | 9630028H03Rik | 7.729153 | 10.25993 | 8.994542 |
| ENSMUSG00000058922 | pseudogene | Gm10052 | 1080.398 | 1131.214 | 1105.806 |
| ENSMUSG00000048261 | pseudogene | Gm4879 | 33.22185 | 28.40482 | 30.81334 |
| ENSMUSG00000030650 | protein_coding | Tmc5 | 19.0133 | 23.13234 | 21.07282 |
| ENSMUSG00000084381 | pseudogene | AA413626 | 12.72734 | 9.227417 | 10.97738 |
| ENSMUSG00000020460 | protein_coding | Rps27a | 5502.211 | 5747.988 | 5625.1 |
| ENSMUSG00000025417 | protein_coding | Pip4k2c | 2029.399 | 2134.878 | 2082.138 |
| ENSMUSG00000039703 | protein_coding | Nploc4 | 3917.291 | 3728.659 | 3822.975 |
| ENSMUSG00000085282 | lincRNA | Gm15663 | 64.19378 | 71.44582 | 67.8198 |
| ENSMUSG00000032120 | protein_coding | C2cd2l | 362.0547 | 339.0015 | 350.5281 |
| ENSMUSG00000052557 | protein_coding | Gan | 176.0496 | 162.509 | 169.2793 |
| ENSMUSG00000052296 | protein_coding | Ppp6r1 | 4520.01 | 4323.449 | 4421.729 |
| ENSMUSG00000090188 | pseudogene | Gm7418 | 3.862787 | 2.410644 | 3.136716 |
| ENSMUSG00000038366 | protein_coding | Lasp1 | 3447.366 | 3586.563 | 3516.964 |
| ENSMUSG00000029868 | protein_coding | Trpv6 | 42.90595 | 37.32882 | 40.11739 |
| ENSMUSG00000059995 | protein_coding | Atxn7l3 | 2402.03 | 2294.231 | 2348.131 |
| ENSMUSG00000021501 | protein_coding | Caml | 1128.095 | 1179.852 | 1153.973 |
| ENSMUSG00000007476 | protein_coding | Lrrc8a | 1284.892 | 1213.924 | 1249.408 |
| ENSMUSG00000021768 | protein_coding | Dusp13 | 4.541075 | 6.471878 | 5.506477 |
| ENSMUSG00000086328 | processed_transcrip | 2700033N17Rik | 3.896855 | 2.419653 | 3.158254 |
| ENSMUSG00000007891 | protein_coding | Ctsd | 6640.774 | 6872.055 | 6756.415 |
| ENSMUSG00000091217 | lincRNA | 5430400D12Rik | 26.57047 | 21.55667 | 24.06357 |
| ENSMUSG00000021720 | protein_coding | Rnf180 | 600.1852 | 631.8101 | 615.9976 |
| ENSMUSG00000084141 | pseudogene | Olfr1372-ps1 | 50.5868 | 58.70675 | 54.64678 |
| ENSMUSG00000053436 | protein_coding | Mapk14 | 3044.354 | 2931.313 | 2987.834 |
| ENSMUSG00000090888 | lincRNA | Gm17429 | 6.186736 | 8.972253 | 7.579495 |
| ENSMUSG00000024287 | protein_coding | Thoc1 | 2493.444 | 2611.009 | 2552.227 |
| ENSMUSG00000027957 | protein_coding | Slc35a3 | 1297.424 | 1350.104 | 1323.764 |
| ENSMUSG00000048562 | protein_coding | Sp8 | 2.592014 | 1.377408 | 1.984711 |
| ENSMUSG00000081984 | protein_coding | Dnajb3 | 163.5594 | 151.0783 | 157.3188 |
| ENSMUSG00000091841 | lincRNA | 4930519A11Rik | 6.492447 | 8.837838 | 7.665142 |
| ENSMUSG00000017007 | protein_coding | Rbpjl | 22.28337 | 27.0357 | 24.65954 |
| ENSMUSG00000086820 | processed_transcrip | Gm11465 | 1.307151 | 2.65716 | 1.982156 |
| ENSMUSG00000067561 | protein_coding | Dmrtc1c2 | 28.61144 | 15.59947 | 22.10546 |
| ENSMUSG00000065513 | miRNA | Mir26a-1 | 4.221275 | 6.216714 | 5.218995 |
| ENSMUSG00000025405 | protein_coding | Inhbc | 3.546566 | 2.066111 | 2.806339 |
| ENSMUSG00000073077 | protein_coding | Gm7173 | 26.92665 | 21.27375 | 24.1002 |
| ENSMUSG00000032246 | protein_coding | Calml4 | 67.78492 | 60.57863 | 64.18178 |
| ENSMUSG00000029346 | protein_coding | Srrd | 242.4684 | 224.508 | 233.4882 |
| ENSMUSG00000079915 | pseudogene | Gm16073 | 3.852277 | 5.783536 | 4.817906 |
| ENSMUSG00000065774 | snRNA | U6 | 11.63548 | 8.944864 | 10.29017 |
| ENSMUSG00000032589 | protein_coding | Bsn | 153.1994 | 139.6695 | 146.4345 |
| ENSMUSG00000026004 | protein_coding | 1110028C15Rik | 1126.701 | 1188.734 | 1157.717 |
| ENSMUSG00000067547 | pseudogene | Gm7666 | 206.775 | 193.0827 | 199.9288 |
| ENSMUSG00000041805 | protein_coding | Pramel1 | 1318.126 | 1225.587 | 1271.857 |
| ENSMUSG00000063820 | protein_coding | Arl9 | 17.53648 | 13.04462 | 15.29055 |
| ENSMUSG00000034973 | protein_coding | Dopey1 | 1843.234 | 1691.813 | 1767.523 |
| ENSMUSG00000046367 | protein_coding | 4931440L10Rik | 4.585653 | 6.923075 | 5.754364 |
| ENSMUSG00000047090 | protein_coding | 1110012D08Rik | 243.7406 | 262.4315 | 253.086 |
| ENSMUSG00000025701 | protein_coding | Alox5 | 28.8368 | 24.01707 | 26.42694 |
| ENSMUSG00000025525 | protein_coding | Apool | 632.3351 | 668.633 | 650.484 |
| ENSMUSG00000009112 | protein_coding | Bcl2l13 | 1911.075 | 1830.207 | 1870.641 |
| ENSMUSG00000045509 | protein_coding | Gpr150 | 4.518786 | 6.587552 | 5.553169 |

| | | | | | |
|---|---|---|---|---|---|
| ENSMUSG00000066324 | protein_coding | Impad1 | 3996.102 | 4194.459 | 4095.28 |
| ENSMUSG00000070034 | protein_coding | Sp110 | 27.0328 | 32.38999 | 29.7114 |
| ENSMUSG00000036188 | protein_coding | Ankmy2 | 662.628 | 711.3537 | 686.9908 |
| ENSMUSG00000032726 | protein_coding | Bmp8a | 13.78216 | 10.55077 | 12.16646 |
| ENSMUSG00000035179 | protein_coding | 4930579J09Rik | 62.90496 | 55.02572 | 58.96534 |
| ENSMUSG00000024603 | protein_coding | Dctn4 | 3366.341 | 3515.199 | 3440.77 |
| ENSMUSG00000020205 | protein_coding | Phlda1 | 123.0509 | 135.5744 | 129.3126 |
| ENSMUSG00000006299 | protein_coding | Aamp | 4307.664 | 4169.067 | 4238.365 |
| ENSMUSG00000086161 | processed_transcrip | Gm15389 | 1.626951 | 0.688704 | 1.157827 |
| ENSMUSG00000079258 | protein_coding | Calhm1 | 6.422002 | 8.820543 | 7.621272 |
| ENSMUSG00000058975 | protein_coding | Kcnc1 | 4.221275 | 6.243381 | 5.232328 |
| ENSMUSG00000031595 | protein_coding | Pdgfrl | 123.157 | 136.2471 | 129.7021 |
| ENSMUSG00000043556 | protein_coding | Fbxl7 | 84.01425 | 75.03955 | 79.5269 |
| ENSMUSG00000081227 | pseudogene | Gm13082 | 0.632442 | 1.668247 | 1.150344 |
| ENSMUSG00000020477 | protein_coding | Mrps24 | 912.0341 | 858.2841 | 885.1591 |
| ENSMUSG00000022620 | protein_coding | Arsa | 152.3612 | 164.5338 | 158.4475 |
| ENSMUSG00000022265 | protein_coding | Ank | 564.0027 | 595.4424 | 579.7225 |
| ENSMUSG00000029234 | protein_coding | Tmem165 | 1845.66 | 1942.272 | 1893.966 |
| ENSMUSG00000040021 | protein_coding | Lats1 | 2763.603 | 2894.337 | 2828.97 |
| ENSMUSG00000084830 | pseudogene | Gm14539 | 42.29349 | 48.36827 | 45.33088 |
| ENSMUSG00000079671 | protein_coding | 2610203C22Rik | 24.24295 | 28.69638 | 26.46966 |
| ENSMUSG00000084555 | snRNA | U4 | 1.618751 | 0.662038 | 1.140394 |
| ENSMUSG00000026208 | protein_coding | Des | 417.3818 | 387.2259 | 402.3039 |
| ENSMUSG00000009378 | protein_coding | Slc16a12 | 16.7867 | 13.33334 | 15.06002 |
| ENSMUSG00000062794 | protein_coding | Zfp599 | 55.68876 | 63.3306 | 59.50968 |
| ENSMUSG00000038651 | protein_coding | Sycp2l | 9.336126 | 6.932084 | 8.134105 |
| ENSMUSG00000045288 | protein_coding | Ush1g | 37.77575 | 32.71293 | 35.24434 |
| ENSMUSG00000051224 | protein_coding | Tceanc | 528.8801 | 503.5826 | 516.2314 |
| ENSMUSG00000022657 | protein_coding | Cd96 | 3.564234 | 2.066473 | 2.815353 |
| ENSMUSG00000020078 | protein_coding | Vps26a | 3184.905 | 3302.006 | 3243.455 |
| ENSMUSG00000031148 | protein_coding | Gpkow | 2270.436 | 2186.088 | 2228.262 |
| ENSMUSG00000020889 | protein_coding | Nr1d1 | 589.4432 | 645.2237 | 617.3334 |
| ENSMUSG00000081249 | pseudogene | Gm11517 | 8.77055 | 11.664 | 10.21728 |
| ENSMUSG00000092335 | protein_coding | Gm7221 | 174.1449 | 190.1273 | 182.1361 |
| ENSMUSG00000080969 | pseudogene | Gm12372 | 3.878144 | 2.357312 | 3.117728 |
| ENSMUSG00000038583 | protein_coding | Pln | 3.840499 | 2.383978 | 3.112238 |
| ENSMUSG00000024116 | protein_coding | Prss21 | 0.64422 | 1.65059 | 1.147405 |
| ENSMUSG00000086878 | lincRNA | Miat | 3.580633 | 5.599361 | 4.589997 |
| ENSMUSG00000005917 | protein_coding | Otx1 | 55.76763 | 47.42658 | 51.5971 |
| ENSMUSG00000053465 | protein_coding | Hs6st3 | 2.609681 | 1.359751 | 1.984716 |
| ENSMUSG00000016349 | protein_coding | Eef1a2 | 10.0531 | 7.513762 | 8.783432 |
| ENSMUSG00000062260 | protein_coding | AA415398 | 51.514 | 44.54558 | 48.02979 |
| ENSMUSG00000091968 | lincRNA | Gm17115 | 2.905924 | 4.432433 | 3.669179 |
| ENSMUSG00000026676 | protein_coding | Ccdc3 | 21.98586 | 26.47784 | 24.23185 |
| ENSMUSG00000037957 | protein_coding | Wdr20a | 855.2602 | 901.5472 | 878.4037 |
| ENSMUSG00000038344 | protein_coding | Txlng | 1221.205 | 1325.003 | 1273.104 |
| ENSMUSG00000015605 | protein_coding | Srf | 2187.828 | 2087.925 | 2137.877 |
| ENSMUSG00000085795 | protein_coding | Zfp703 | 1024.439 | 953.2021 | 988.8204 |
| ENSMUSG00000073407 | protein_coding | Gm6034 | 2.891835 | 4.52144 | 3.706638 |
| ENSMUSG00000020400 | protein_coding | Tnip1 | 1317.598 | 1251.426 | 1284.512 |
| ENSMUSG00000022051 | protein_coding | Bnip3l | 5339.358 | 5534.202 | 5436.78 |
| ENSMUSG00000040812 | protein_coding | Agbl2 | 107.2392 | 122.4802 | 114.8597 |
| ENSMUSG00000031481 | protein_coding | Tpte | 3.910944 | 2.401635 | 3.156289 |
| ENSMUSG00000036371 | protein_coding | Serbp1 | 10212.13 | 10633.04 | 10422.58 |
| ENSMUSG00000024524 | protein_coding | Gnal | 57.77707 | 65.98989 | 61.88348 |
| ENSMUSG00000086465 | processed_transcrip | Gm16049 | 8.740062 | 6.471878 | 7.60597 |
| ENSMUSG00000040035 | protein_coding | Disp2 | 33.84087 | 26.6818 | 30.26133 |
| ENSMUSG00000035152 | protein_coding | Ap2b1 | 4017.427 | 3854.068 | 3935.747 |

| | | | | | |
|---|---|---|---|---|---|
| ENSMUSG00000029649 | protein_coding | Pomp | 1910.243 | 2006.834 | 1958.539 |
| ENSMUSG00000025224 | protein_coding | Gbf1 | 2722.847 | 2520.753 | 2621.8 |
| ENSMUSG00000022421 | protein_coding | Nptxr | 8.11813 | 5.845516 | 6.981823 |
| ENSMUSG00000022124 | protein_coding | Fbxl3 | 3199.784 | 3075.028 | 3137.406 |
| ENSMUSG00000064938 | snoRNA | SNORA58 | 4.512897 | 6.427555 | 5.470226 |
| ENSMUSG00000090345 | protein_coding | Gm5798 | 3.874566 | 2.43731 | 3.155938 |
| ENSMUSG00000089715 | protein_coding | Cbx6 | 1948.271 | 2050.555 | 1999.413 |
| ENSMUSG00000018583 | protein_coding | G3bp1 | 4473.909 | 4656.349 | 4565.129 |
| ENSMUSG00000081400 | pseudogene | Gm13680 | 61.62047 | 68.69209 | 65.15628 |
| ENSMUSG00000040054 | protein_coding | Baz2a | 5400.888 | 5790.178 | 5595.533 |
| ENSMUSG00000031097 | protein_coding | Tnni2 | 19.96785 | 24.26431 | 22.11608 |
| ENSMUSG00000037440 | protein_coding | Vnn1 | 477.7112 | 685.9564 | 581.8338 |
| ENSMUSG00000055371 | protein_coding | Stam2 | 2308.388 | 2219.824 | 2264.106 |
| ENSMUSG00000030890 | protein_coding | Ilk | 87.14008 | 79.22769 | 83.18389 |
| ENSMUSG00000029343 | protein_coding | Crybb1 | 9.696925 | 7.008872 | 8.352898 |
| ENSMUSG00000032053 | protein_coding | Pou2af1 | 16.51633 | 12.96214 | 14.73923 |
| ENSMUSG00000036333 | protein_coding | Kidins220 | 3536.873 | 3411.558 | 3474.216 |
| ENSMUSG00000029064 | protein_coding | Gnb1 | 11365.47 | 11763.1 | 11564.28 |
| ENSMUSG00000021360 | protein_coding | Gcnt2 | 90.70767 | 82.23482 | 86.47125 |
| ENSMUSG00000081683 | protein_coding | Fzd10 | 10.38341 | 13.39495 | 11.88918 |
| ENSMUSG00000092016 | protein_coding | Gm3252 | 2.922324 | 4.687958 | 3.805141 |
| ENSMUSG00000042015 | protein_coding | Wdr41 | 1103.068 | 1150.585 | 1126.826 |
| ENSMUSG00000072952 | protein_coding | Gm5878 | 5.173517 | 7.196257 | 6.184887 |
| ENSMUSG00000002944 | protein_coding | Cd36 | 114.3074 | 128.869 | 121.5882 |
| ENSMUSG00000086387 | processed_transcrip | Gm15563 | 4.193097 | 6.092032 | 5.142565 |
| ENSMUSG00000091711 | lincRNA | Gm2885 | 35.41967 | 29.63088 | 32.52527 |
| ENSMUSG00000070489 | pseudogene | 4930527J03Rik | 8.10173 | 5.863534 | 6.982632 |
| ENSMUSG00000001053 | protein_coding | N4bp3 | 650.1255 | 612.0503 | 631.0879 |
| ENSMUSG00000033063 | protein_coding | Cntnap3 | 1.276663 | 2.410644 | 1.843653 |
| ENSMUSG00000011486 | protein_coding | Slc25a41 | 11.62012 | 8.855495 | 10.23781 |
| ENSMUSG00000045231 | protein_coding | BC106179 | 67.10828 | 57.59719 | 62.35273 |
| ENSMUSG00000085504 | processed_transcrip | Gm14055 | 3.874566 | 5.694167 | 4.784366 |
| ENSMUSG00000085818 | processed_transcrip | Gm13267 | 4.559785 | 2.7995 | 3.679643 |
| ENSMUSG00000030337 | protein_coding | Vamp1 | 731.0218 | 774.2308 | 752.6263 |
| ENSMUSG00000024107 | protein_coding | Lhcgr | 403.6921 | 436.3123 | 420.0022 |
| ENSMUSG00000092349 | processed_transcrip | BC033916 | 20.70227 | 17.05868 | 18.88048 |
| ENSMUSG00000006585 | protein_coding | Cdt1 | 1122.476 | 1181.203 | 1151.839 |
| ENSMUSG00000064901 | snoRNA | Snora21 | 1.604662 | 0.662038 | 1.13335 |
| ENSMUSG00000049604 | protein_coding | Hoxb13 | 20.34147 | 16.66118 | 18.50133 |
| ENSMUSG00000031511 | protein_coding | Arhgef7 | 2704.917 | 2596.326 | 2650.622 |
| ENSMUSG00000086794 | lincRNA | Gm11642 | 25.72968 | 10.5035 | 18.11659 |
| ENSMUSG00000020137 | protein_coding | Thap2 | 308.6478 | 344.588 | 326.6179 |
| ENSMUSG00000090440 | protein_coding | Gm9732 | 221.6886 | 203.2868 | 212.4877 |
| ENSMUSG00000031987 | protein_coding | Egln1 | 2278.432 | 2363.015 | 2320.723 |
| ENSMUSG00000047880 | protein_coding | Cxcr5 | 21.31011 | 25.50694 | 23.40853 |
| ENSMUSG00000028156 | protein_coding | Eif4e | 3893.888 | 4032.941 | 3963.415 |
| ENSMUSG00000025128 | protein_coding | Bhlhe22 | 83.25903 | 94.92275 | 89.09089 |
| ENSMUSG00000048728 | protein_coding | Zfp454 | 98.12694 | 107.6401 | 102.8835 |
| ENSMUSG00000029304 | protein_coding | Spp1 | 1279.484 | 1409.814 | 1344.649 |
| ENSMUSG00000086810 | lincRNA | Gm13110 | 31.35986 | 26.66559 | 29.01272 |
| ENSMUSG00000026727 | protein_coding | Rsu1 | 1328.664 | 1391.752 | 1360.208 |
| ENSMUSG00000030823 | protein_coding | 9130019O22Rik | 137.4488 | 124.4499 | 130.9493 |
| ENSMUSG00000033773 | protein_coding | Rpap2 | 829.0256 | 790.9253 | 809.9754 |
| ENSMUSG00000037287 | protein_coding | Tbcel | 2016.37 | 1930.976 | 1973.673 |
| ENSMUSG00000085275 | processed_transcrip | Gm14487 | 2.920013 | 4.494774 | 3.707394 |
| ENSMUSG00000068323 | protein_coding | Slc4a5 | 357.7739 | 331.1049 | 344.4394 |
| ENSMUSG00000024308 | protein_coding | Tapbp | 4346.02 | 4526.395 | 4436.208 |
| ENSMUSG00000048087 | protein_coding | Gm4737 | 1016.435 | 868.2518 | 942.3436 |

| | | | | | |
|---|---|---|---|---|---|
| ENSMUSG00000024143 | protein_coding | Rhoq | 1129.722 | 1219.84 | 1174.781 |
| ENSMUSG00000020792 | protein_coding | Exoc7 | 1392.223 | 1464.113 | 1428.168 |
| ENSMUSG00000016946 | protein_coding | Kctd5 | 1873.166 | 1954.15 | 1913.658 |
| ENSMUSG00000037797 | protein_coding | Adh4 | 174.5596 | 190.0453 | 182.3025 |
| ENSMUSG00000068452 | protein_coding | Duox2 | 20.19536 | 25.53682 | 22.86609 |
| ENSMUSG00000025059 | protein_coding | Gyk | 199.5421 | 183.5087 | 191.5254 |
| ENSMUSG00000086346 | processed_transcrip | Gm12510 | 1.298952 | 2.36596 | 1.832456 |
| ENSMUSG00000022841 | protein_coding | Ap2m1 | 6116.075 | 6319.658 | 6217.867 |
| ENSMUSG00000027832 | protein_coding | Ptx3 | 5.841294 | 3.80607 | 4.823682 |
| ENSMUSG00000085262 | lincRNA | Gm11574 | 2.900035 | 1.677256 | 2.288645 |
| ENSMUSG00000028407 | processed_transcrip | 2010003O02Rik | 24.21604 | 20.2474 | 22.23172 |
| ENSMUSG00000026669 | protein_coding | Mcm10 | 979.5673 | 936.3345 | 957.9509 |
| ENSMUSG00000084839 | processed_transcrip | Gm14097 | 0.33031 | 1.059903 | 0.695106 |
| ENSMUSG00000074764 | protein_coding | Sel1l2 | 0.33031 | 1.059903 | 0.695106 |
| ENSMUSG00000059252 | pseudogene | Gm6790 | 0.33031 | 1.059903 | 0.695106 |
| ENSMUSG00000068083 | protein_coding | Cyp2d40 | 0.33031 | 1.059903 | 0.695106 |
| ENSMUSG00000084475 | misc_RNA | 7SK | 0.33031 | 1.059903 | 0.695106 |
| ENSMUSG00000064524 | snRNA | U6 | 0.33031 | 1.059903 | 0.695106 |
| ENSMUSG00000058715 | protein_coding | Fcer1g | 156.3922 | 171.1861 | 163.7892 |
| ENSMUSG00000062472 | protein_coding | Gm4945 | 8.146308 | 10.92197 | 9.534138 |
| ENSMUSG00000017677 | protein_coding | Wsb1 | 3734.471 | 3934.562 | 3834.517 |
| ENSMUSG00000072825 | protein_coding | AW555464 | 4132.581 | 4339.46 | 4236.02 |
| ENSMUSG00000073376 | protein_coding | Gm10495 | 10.0203 | 13.14916 | 11.58473 |
| ENSMUSG00000046532 | protein_coding | Ar | 2141.031 | 2052.485 | 2096.758 |
| ENSMUSG00000055958 | pseudogene | Gm9987 | 10.66326 | 8.149134 | 9.406195 |
| ENSMUSG00000024399 | protein_coding | Ltb | 74.55005 | 104.8047 | 89.67737 |
| ENSMUSG00000024074 | protein_coding | Crim1 | 1429.326 | 1513.59 | 1471.458 |
| ENSMUSG00000044254 | protein_coding | Pcsk9 | 23.80775 | 18.57807 | 21.19291 |
| ENSMUSG00000074884 | protein_coding | Serf2 | 2519.38 | 2614.35 | 2566.865 |
| ENSMUSG00000051314 | protein_coding | Ffar2 | 2.620192 | 1.333085 | 1.976638 |
| ENSMUSG00000021116 | protein_coding | Eif2s1 | 3288.494 | 3171.236 | 3229.865 |
| ENSMUSG00000092268 | processed_transcrip | RP23-127F20.2 | 0.32211 | 1.059903 | 0.691006 |
| ENSMUSG00000090737 | protein_coding | Gm6788 | 0.32211 | 1.059903 | 0.691006 |
| ENSMUSG00000085935 | lincRNA | A830009L08Rik | 0.966331 | 2.048455 | 1.507393 |
| ENSMUSG00000038276 | protein_coding | Accn3 | 19.14576 | 23.24873 | 21.19725 |
| ENSMUSG00000081960 | pseudogene | Mageb17-ps | 88.57008 | 100.7681 | 94.66909 |
| ENSMUSG00000054939 | protein_coding | Zfp174 | 133.2915 | 121.1014 | 127.1965 |
| ENSMUSG00000032093 | protein_coding | Cd3e | 79.3902 | 96.36452 | 87.87736 |
| ENSMUSG00000082676 | pseudogene | Gm11843 | 36.09311 | 29.56854 | 32.83083 |
| ENSMUSG00000028557 | protein_coding | Rnf11 | 2511.961 | 2419.433 | 2465.697 |
| ENSMUSG00000032999 | protein_coding | Nlrp4f | 353.1745 | 317.2504 | 335.2124 |
| ENSMUSG00000001228 | protein_coding | Uhrf1 | 3557.387 | 3402.233 | 3479.81 |
| ENSMUSG00000089800 | pseudogene | Rps19-ps10 | 5.204006 | 3.354873 | 4.27944 |
| ENSMUSG00000034683 | protein_coding | Ppp1r1c | 0.316221 | 1.059903 | 0.688062 |
| ENSMUSG00000077547 | snRNA | U6 | 0.316221 | 1.059903 | 0.688062 |
| ENSMUSG00000087925 | snRNA | U6 | 0.316221 | 1.059903 | 0.688062 |
| ENSMUSG00000091504 | protein_coding | Vmn2r118 | 0.316221 | 1.059903 | 0.688062 |
| ENSMUSG00000067545 | protein_coding | Olfr1419 | 0.316221 | 1.059903 | 0.688062 |
| ENSMUSG00000090416 | IG_V_gene | Gm4242 | 0.316221 | 1.059903 | 0.688062 |
| ENSMUSG00000060214 | pseudogene | Gm8203 | 238.268 | 218.7815 | 228.5247 |
| ENSMUSG00000032405 | protein_coding | Pias1 | 1771.022 | 1702.386 | 1736.704 |
| ENSMUSG00000039853 | protein_coding | Trim14 | 242.3499 | 259.4075 | 250.8787 |
| ENSMUSG00000089900 | processed_transcrip | 0610038L08Rik | 2.911813 | 4.432433 | 3.672123 |
| ENSMUSG00000040102 | protein_coding | Klhdc5 | 891.9083 | 962.9207 | 927.4145 |
| ENSMUSG00000067843 | protein_coding | Dcpp2 | 15.2548 | 20.01238 | 17.63359 |
| ENSMUSG00000087698 | processed_transcrip | Gm13031 | 7.397575 | 5.147803 | 6.272689 |
| ENSMUSG00000024006 | protein_coding | Stk38 | 2493.26 | 2604.213 | 2548.737 |
| ENSMUSG00000067567 | protein_coding | Hdac8 | 165.7289 | 179.0193 | 172.3741 |

| | | | | | |
|---|---|---|---|---|---|
| ENSMUSG00000030357 | protein_coding | Fkbp4 | 9417.326 | 9741.852 | 9579.589 |
| ENSMUSG00000049926 | protein_coding | Olfr921 | 1.643351 | 0.697713 | 1.170532 |
| ENSMUSG00000078139 | protein_coding | AK157302 | 4.812718 | 6.869743 | 5.841231 |
| ENSMUSG00000020295 | protein_coding | Hbq1a | 0.66062 | 1.65059 | 1.155605 |
| ENSMUSG00000024397 | protein_coding | Aif1 | 74.73888 | 66.61874 | 70.67881 |
| ENSMUSG00000021319 | protein_coding | Sfrp4 | 175.3682 | 195.5302 | 185.4492 |
| ENSMUSG00000032024 | protein_coding | 9030425E11Rik | 412.3607 | 436.6732 | 424.517 |
| ENSMUSG00000086352 | processed_transcrip | 4930401G09Rik | 18.09743 | 21.88898 | 19.99321 |
| ENSMUSG00000046687 | pseudogene | Gm5424 | 159.4739 | 174.0804 | 166.7771 |
| ENSMUSG00000036613 | protein_coding | Tssc1 | 739.5722 | 705.2509 | 722.4116 |
| ENSMUSG00000031639 | protein_coding | Tlr3 | 299.3518 | 280.55 | 289.9509 |
| ENSMUSG00000000171 | protein_coding | Sdhd | 2330.973 | 2419.419 | 2375.196 |
| ENSMUSG00000049562 | protein_coding | Gm962 | 229.9517 | 245.9952 | 237.9735 |
| ENSMUSG00000025962 | protein_coding | Fastkd2 | 914.9985 | 960.728 | 937.8633 |
| ENSMUSG00000060183 | processed_transcrip | Cxcl11 | 1.609283 | 0.697713 | 1.153498 |
| ENSMUSG00000086685 | processed_transcrip | Gm15015 | 0.976841 | 2.101787 | 1.539314 |
| ENSMUSG00000085341 | processed_transcrip | Gm15863 | 29.33089 | 24.98797 | 27.15943 |
| ENSMUSG00000018541 | protein_coding | Cwc25 | 989.6009 | 1032.347 | 1010.974 |
| ENSMUSG00000036223 | protein_coding | Ska1 | 311.2737 | 290.7831 | 301.0284 |
| ENSMUSG00000079557 | protein_coding | 2-Mar | 1715.273 | 1646.802 | 1681.037 |
| ENSMUSG00000047911 | protein_coding | Npm2 | 29.61371 | 24.30827 | 26.96099 |
| ENSMUSG00000058407 | protein_coding | Txndc9 | 1748.978 | 1829.718 | 1789.348 |
| ENSMUSG00000072852 | processed_transcrip | 2310040G07Rik | 13.85722 | 10.958 | 12.40761 |
| ENSMUSG00000092564 | processed_transcrip | BC051226 | 285.0077 | 318.9795 | 301.9936 |
| ENSMUSG00000022799 | protein_coding | Arhgap31 | 1673.15 | 1789.595 | 1731.373 |
| ENSMUSG00000075524 | protein_coding | 4930407I10Rik | 2.620192 | 1.341732 | 1.980962 |
| ENSMUSG00000061132 | protein_coding | Blnk | 22.84686 | 27.95906 | 25.40296 |
| ENSMUSG00000091302 | lincRNA | 2310043M15Rik | 11.97041 | 14.96554 | 13.46798 |
| ENSMUSG00000020327 | protein_coding | Fgf22 | 39.56163 | 33.52202 | 36.54183 |
| ENSMUSG00000036835 | protein_coding | Psenen | 875.8967 | 916.2121 | 896.0544 |
| ENSMUSG00000021927 | pseudogene | Gm5803 | 1.592884 | 0.688704 | 1.140794 |
| ENSMUSG00000026815 | protein_coding | Gfi1b | 4.221275 | 6.127345 | 5.17431 |
| ENSMUSG00000034783 | protein_coding | Cd207 | 1.637461 | 0.697713 | 1.167587 |
| ENSMUSG00000073490 | protein_coding | AI607873 | 40.3366 | 33.14756 | 36.74208 |
| ENSMUSG00000034055 | protein_coding | Phka1 | 1095.192 | 1033.052 | 1064.122 |
| ENSMUSG00000071952 | protein_coding | Gm10351 | 8.76004 | 6.480887 | 7.620464 |
| ENSMUSG00000021118 | protein_coding | Plek2 | 45.79548 | 38.05578 | 41.92563 |
| ENSMUSG00000022440 | protein_coding | C1qtnf6 | 1641.429 | 1714.447 | 1677.938 |
| ENSMUSG00000085810 | processed_transcrip | Gm16325 | 0.32211 | 1.050893 | 0.686502 |
| ENSMUSG00000083826 | pseudogene | Gm13039 | 0.32211 | 1.050893 | 0.686502 |
| ENSMUSG00000086190 | processed_transcrip | 4930579M01Rik | 0.32211 | 1.050893 | 0.686502 |
| ENSMUSG00000088622 | snRNA | U6 | 0.32211 | 1.050893 | 0.686502 |
| ENSMUSG00000034482 | protein_coding | Ly6g5c | 0.32211 | 1.050893 | 0.686502 |
| ENSMUSG00000062785 | protein_coding | Kcnc3 | 47.84778 | 42.118 | 44.98289 |
| ENSMUSG00000085797 | processed_transcrip | A530010F05Rik | 4.786851 | 3.010341 | 3.898596 |
| ENSMUSG00000091255 | protein_coding | Speer4e | 4.206143 | 2.375331 | 3.290737 |
| ENSMUSG00000056758 | protein_coding | Hmga2 | 7.428289 | 9.90675 | 8.667519 |
| ENSMUSG00000086369 | processed_transcrip | E330017L17Rik | 125.2007 | 137.8148 | 131.5078 |
| ENSMUSG00000057354 | protein_coding | 4930430O22Rik | 5.077205 | 3.046016 | 4.06161 |
| ENSMUSG00000028749 | protein_coding | Pla2g2f | 4.873696 | 7.018242 | 5.945969 |
| ENSMUSG00000038236 | protein_coding | Hoxa7 | 16.73728 | 20.47518 | 18.60623 |
| ENSMUSG00000066270 | pseudogene | Gm10157 | 7.090822 | 9.437534 | 8.264178 |
| ENSMUSG00000071083 | protein_coding | Gm10311 | 45.75552 | 40.2094 | 42.98246 |
| ENSMUSG00000043017 | protein_coding | Ptgir | 4.194365 | 6.216353 | 5.205359 |
| ENSMUSG00000025766 | protein_coding | D3Ertd751e | 546.01 | 505.8855 | 525.9477 |
| ENSMUSG00000092425 | pseudogene | Gm8018 | 0.960442 | 2.066473 | 1.513457 |
| ENSMUSG00000082570 | pseudogene | Gm15711 | 1.615173 | 0.724379 | 1.169776 |
| ENSMUSG00000091804 | protein_coding | Gm17636 | 0.316221 | 1.050893 | 0.683557 |

| | | | | | |
|---|---|---|---|---|---|
| ENSMUSG00000084783 | processed_transcrip | Gm15419 | 0.316221 | 1.050893 | 0.683557 |
| ENSMUSG00000032556 | protein_coding | Bfsp2 | 0.316221 | 1.050893 | 0.683557 |
| ENSMUSG00000064567 | snoRNA | SNORA38 | 0.316221 | 1.050893 | 0.683557 |
| ENSMUSG00000056487 | protein_coding | Mettl7a2 | 0.316221 | 1.050893 | 0.683557 |
| ENSMUSG00000047003 | protein_coding | Zfp41 | 654.6282 | 618.4016 | 636.5149 |
| ENSMUSG00000050043 | protein_coding | Tmx2 | 2881.886 | 2990.682 | 2936.284 |
| ENSMUSG00000061167 | pseudogene | Rpl15-ps3 | 16.8113 | 13.63246 | 15.22188 |
| ENSMUSG00000078931 | processed_transcrip | Pdf | 13.20607 | 16.45899 | 14.83253 |
| ENSMUSG00000032334 | protein_coding | Loxl1 | 641.1181 | 679.1725 | 660.1453 |
| ENSMUSG00000025995 | protein_coding | Wdr75 | 2769.416 | 2673.185 | 2721.301 |
| ENSMUSG00000042817 | protein_coding | Flt3 | 53.03748 | 46.1374 | 49.58744 |
| ENSMUSG00000018012 | protein_coding | Rac3 | 119.9062 | 131.201 | 125.5536 |
| ENSMUSG00000026657 | protein_coding | Frmd4a | 1609.751 | 1747.454 | 1678.603 |
| ENSMUSG00000001021 | protein_coding | S100a3 | 1.937283 | 1.024227 | 1.480755 |
| ENSMUSG00000081974 | pseudogene | Gm11960 | 1.937283 | 1.024227 | 1.480755 |
| ENSMUSG00000069236 | protein_coding | Gm7251 | 1.937283 | 1.024227 | 1.480755 |
| ENSMUSG00000088909 | snoRNA | SNORD86 | 1.937283 | 1.024227 | 1.480755 |
| ENSMUSG00000085363 | processed_transcrip | Gm15478 | 4.538764 | 6.471878 | 5.505321 |
| ENSMUSG00000033379 | protein_coding | Atp6v0b | 1698.188 | 1769.325 | 1733.757 |
| ENSMUSG00000082985 | pseudogene | Gm14042 | 8.44024 | 11.17785 | 9.809047 |
| ENSMUSG00000041203 | protein_coding | 2310036O22Rik | 2258.398 | 2351.326 | 2304.862 |
| ENSMUSG00000058579 | protein_coding | Cela2a | 163.2788 | 149.1429 | 156.2108 |
| ENSMUSG00000015619 | protein_coding | Gata3 | 8.441508 | 6.038338 | 7.239923 |
| ENSMUSG00000048478 | protein_coding | 4732415M23Rik | 193.1178 | 177.5795 | 185.3486 |
| ENSMUSG00000032363 | protein_coding | Adamts7 | 575.2147 | 616.7873 | 596.001 |
| ENSMUSG00000086904 | processed_transcrip | Gm13404 | 10.65401 | 7.778659 | 9.216336 |
| ENSMUSG00000090500 | processed_transcrip | Gm17057 | 1.298952 | 2.339294 | 1.819123 |
| ENSMUSG00000030120 | protein_coding | Mlf2 | 4104.429 | 3948.57 | 4026.5 |
| ENSMUSG00000024538 | protein_coding | Ppic | 1276.155 | 1356.512 | 1316.334 |
| ENSMUSG00000003923 | protein_coding | Tfam | 1285.833 | 1342.775 | 1314.304 |
| ENSMUSG00000003352 | protein_coding | Cacnb3 | 716.7268 | 680.0659 | 698.3963 |
| ENSMUSG00000021943 | protein_coding | Gdf10 | 39.0419 | 45.1446 | 42.09325 |
| ENSMUSG00000029310 | protein_coding | Nudt9 | 1685.169 | 1609.849 | 1647.509 |
| ENSMUSG00000085994 | lincRNA | Gm16733 | 1.278973 | 2.410644 | 1.844809 |
| ENSMUSG00000063412 | pseudogene | Gm10131 | 452.9459 | 482.2921 | 467.619 |
| ENSMUSG00000043458 | protein_coding | Pcdhb12 | 19.98425 | 24.26467 | 22.12446 |
| ENSMUSG00000082087 | pseudogene | Gm12138 | 78.72698 | 86.46542 | 82.5962 |
| ENSMUSG00000037661 | protein_coding | Gpr160 | 143.7025 | 159.8872 | 151.7949 |
| ENSMUSG00000022878 | protein_coding | Adipoq | 2.883635 | 4.52144 | 3.702538 |
| ENSMUSG00000037347 | protein_coding | Chst7 | 16.80772 | 13.58814 | 15.19793 |
| ENSMUSG00000046658 | protein_coding | Zfp316 | 664.086 | 622.0797 | 643.0829 |
| ENSMUSG00000018372 | protein_coding | Ccdc45 | 817.6648 | 860.8288 | 839.2468 |
| ENSMUSG00000052763 | protein_coding | Zfp212 | 1204.67 | 1153.559 | 1179.115 |
| ENSMUSG00000034583 | protein_coding | Olfr1347 | 2.611992 | 4.096909 | 3.354451 |
| ENSMUSG00000072889 | protein_coding | Nfxl1 | 2364.28 | 2218.907 | 2291.593 |
| ENSMUSG00000048215 | processed_transcrip | A630023P12Rik | 3.902744 | 2.446319 | 3.174532 |
| ENSMUSG00000027968 | protein_coding | Larp7 | 2205.333 | 2296.674 | 2251.003 |
| ENSMUSG00000018565 | protein_coding | Rai12 | 2150.711 | 2068.525 | 2109.618 |
| ENSMUSG00000002814 | protein_coding | Top3a | 1401.718 | 1339.14 | 1370.429 |
| ENSMUSG00000055818 | protein_coding | A230083G16Rik | 17.72948 | 21.33941 | 19.53444 |
| ENSMUSG00000024135 | protein_coding | Srbd1 | 1392.615 | 1302.028 | 1347.322 |
| ENSMUSG00000084628 | misc_RNA | 7SK | 2.891835 | 1.695274 | 2.293555 |
| ENSMUSG00000070730 | protein_coding | Fam82a2 | 817.7393 | 775.449 | 796.5942 |
| ENSMUSG00000049606 | protein_coding | Zfp644 | 4711.537 | 4920.619 | 4816.078 |
| ENSMUSG00000091909 | lincRNA | Gm17282 | 371.8898 | 397.0667 | 384.4782 |
| ENSMUSG00000084866 | lincRNA | A930006K02Rik | 44.06237 | 50.27366 | 47.16802 |
| ENSMUSG00000015094 | protein_coding | Npdc1 | 448.8498 | 423.2286 | 436.0392 |
| ENSMUSG00000092773 | misc_RNA | AC157822.1 | 1.606973 | 0.724379 | 1.165676 |

| | | | | | |
|---|---|---|---|---|---|
| ENSMUSG00000044375 | protein_coding | BC027072 | 3.894544 | 2.321637 | 3.10809 |
| ENSMUSG00000031824 | protein_coding | 6430548M08Rik | 522.9561 | 477.1334 | 500.0447 |
| ENSMUSG00000058881 | protein_coding | Zfp516 | 2333.106 | 2224.471 | 2278.788 |
| ENSMUSG00000019853 | protein_coding | Hebp2 | 350.6745 | 373.296 | 361.9852 |
| ENSMUSG00000089684 | pseudogene | Gm14163 | 2.905924 | 4.44108 | 3.673502 |
| ENSMUSG00000021963 | protein_coding | Sap18 | 2869.379 | 2770.434 | 2819.906 |
| ENSMUSG00000048485 | protein_coding | Zbtb8b | 185.8427 | 204.6057 | 195.2242 |
| ENSMUSG00000089744 | processed_transcrip | Gm16146 | 1.973661 | 3.497213 | 2.735437 |
| ENSMUSG00000075419 | protein_coding | Dolk | 465.85 | 443.5032 | 454.6766 |
| ENSMUSG00000032434 | protein_coding | Cmtm6 | 3200.335 | 3332.043 | 3266.189 |
| ENSMUSG00000086762 | processed_transcrip | Gm14546 | 14.91629 | 18.56041 | 16.73835 |
| ENSMUSG00000018899 | protein_coding | Irf1 | 788.1141 | 751.9461 | 770.0301 |
| ENSMUSG00000039982 | protein_coding | Dtx4 | 4487.739 | 4670.223 | 4578.981 |
| ENSMUSG00000077637 | snoRNA | SNORD91 | 5.483849 | 3.752377 | 4.618113 |
| ENSMUSG00000066829 | protein_coding | Zfp810 | 247.6124 | 232.6222 | 240.1173 |
| ENSMUSG00000081871 | pseudogene | Gm11488 | 12.55596 | 9.844408 | 11.20018 |
| ENSMUSG00000060216 | protein_coding | Arrb2 | 1243.498 | 1189.304 | 1216.401 |
| ENSMUSG00000039278 | protein_coding | Pcsk1n | 1228.245 | 907.2076 | 1067.726 |
| ENSMUSG00000089832 | protein_coding | Shkbp1 | 235.4398 | 250.8845 | 243.1621 |
| ENSMUSG00000091018 | protein_coding | Rplp2-ps1 | 217.0936 | 231.5872 | 224.3404 |
| ENSMUSG00000089716 | pseudogene | Gm6264 | 1.610551 | 3.144033 | 2.377292 |
| ENSMUSG00000022231 | protein_coding | Sema5a | 1889.803 | 1977.144 | 1933.474 |
| ENSMUSG00000039521 | protein_coding | Foxp3 | 13.67138 | 10.06675 | 11.86906 |
| ENSMUSG00000085306 | lincRNA | Gm16750 | 4.202565 | 6.082661 | 5.142613 |
| ENSMUSG00000035372 | protein_coding | 1810055G02Rik | 781.3024 | 821.1187 | 801.2106 |
| ENSMUSG00000092592 | protein_coding | Gm20449 | 12.89216 | 15.98112 | 14.43664 |
| ENSMUSG00000086771 | processed_transcrip | 1700080G11Rik | 1.951372 | 1.024227 | 1.4878 |
| ENSMUSG00000030270 | protein_coding | Cpne9 | 27.50505 | 22.85408 | 25.17956 |
| ENSMUSG00000024079 | protein_coding | Eif2ak2 | 983.4096 | 1025.075 | 1004.242 |
| ENSMUSG00000045973 | protein_coding | Mcart1 | 2437.137 | 2544.093 | 2490.615 |
| ENSMUSG00000028101 | protein_coding | Pias3 | 866.628 | 828.2701 | 847.449 |
| ENSMUSG00000023088 | protein_coding | Abcc1 | 4812.61 | 4623.936 | 4718.273 |
| ENSMUSG00000074479 | pseudogene | Gm10704 | 216.3158 | 187.3341 | 201.825 |
| ENSMUSG00000053318 | protein_coding | Slamf8 | 5.085405 | 2.983675 | 4.03454 |
| ENSMUSG00000083764 | pseudogene | Gm11516 | 1.293062 | 2.419653 | 1.856358 |
| ENSMUSG00000084832 | lincRNA | 4930563E18Rik | 1.615173 | 2.772834 | 2.194003 |
| ENSMUSG00000010554 | protein_coding | Mettl16 | 2678.239 | 2798.545 | 2738.392 |
| ENSMUSG00000022788 | protein_coding | Fgd4 | 1037.732 | 1104.046 | 1070.889 |
| ENSMUSG00000040033 | protein_coding | Stat2 | 604.6654 | 575.1461 | 589.9057 |
| ENSMUSG00000085498 | lincRNA | Gm14023 | 17.81401 | 14.44621 | 16.13011 |
| ENSMUSG00000011267 | protein_coding | Zfp296 | 81.9609 | 72.94326 | 77.45208 |
| ENSMUSG00000024349 | protein_coding | Tmem173 | 58.69166 | 67.22096 | 62.95631 |
| ENSMUSG00000091166 | protein_coding | Tstd1 | 39.29319 | 46.48669 | 42.88994 |
| ENSMUSG00000017607 | protein_coding | Tns4 | 7.77962 | 10.22425 | 9.001937 |
| ENSMUSG00000079729 | protein_coding | Tmem181d-ps | 2.934102 | 1.712931 | 2.323517 |
| ENSMUSG00000026073 | protein_coding | Il1r2 | 22.47048 | 27.71793 | 25.0942 |
| ENSMUSG00000086897 | lincRNA | 4632428C04Rik | 9.44757 | 12.64355 | 11.04556 |
| ENSMUSG00000029380 | protein_coding | Cxcl1 | 11.73052 | 8.434536 | 10.08253 |
| ENSMUSG00000036916 | protein_coding | Zfp280c | 1037.818 | 1091.722 | 1064.77 |
| ENSMUSG00000020537 | protein_coding | Drg2 | 844.4718 | 887.2034 | 865.8376 |
| ENSMUSG00000091154 | protein_coding | 1700024N20Rik | 4.230743 | 6.17203 | 5.201386 |
| ENSMUSG00000040389 | protein_coding | Wdr47 | 1379.245 | 1283.233 | 1331.239 |
| ENSMUSG00000091071 | protein_coding | 1700030C10Rik | 76.09672 | 85.46569 | 80.78121 |
| ENSMUSG00000086192 | processed_transcrip | Gm13609 | 1.945483 | 1.024227 | 1.484855 |
| ENSMUSG00000066629 | protein_coding | Rpl36-ps3 | 1.945483 | 1.024227 | 1.484855 |
| ENSMUSG00000086480 | processed_transcrip | Gm15287 | 6.830957 | 9.173361 | 8.002159 |
| ENSMUSG00000038745 | protein_coding | Nlrp6 | 3.236234 | 4.794622 | 4.015428 |
| ENSMUSG00000085218 | lincRNA | BB218582 | 13.23298 | 16.42367 | 14.82833 |

| | | | | | |
|---|---|---|---|---|---|
| ENSMUSG00000080773 | pseudogene | Gm12955 | 3.896855 | 2.173137 | 3.034996 |
| ENSMUSG00000089789 | protein_coding | Rdh1 | 5.811849 | 7.90298 | 6.857414 |
| ENSMUSG00000064177 | protein_coding | Ghrl | 19.73594 | 16.16815 | 17.95204 |
| ENSMUSG00000085093 | processed_transcrip | Gm12737 | 4.895985 | 3.126014 | 4.010999 |
| ENSMUSG00000021028 | protein_coding | Mbip | 754.9261 | 796.9137 | 775.9199 |
| ENSMUSG00000087267 | processed_transcrip | 4933427J07Rik | 0.33031 | 1.033237 | 0.681773 |
| ENSMUSG00000046178 | protein_coding | Nxph1 | 0.33031 | 1.033237 | 0.681773 |
| ENSMUSG00000057293 | protein_coding | 1700054O19Rik | 0.33031 | 1.033237 | 0.681773 |
| ENSMUSG00000083844 | processed_transcrip | 2210015D19Rik | 399.1499 | 376.8892 | 388.0195 |
| ENSMUSG00000074569 | protein_coding | Gcnt7 | 14.77182 | 11.69103 | 13.23143 |
| ENSMUSG00000002105 | protein_coding | Slc39a13 | 435.9017 | 461.2465 | 448.5741 |
| ENSMUSG00000093087 | miRNA | AC093451.2 | 2.903614 | 1.72194 | 2.312777 |
| ENSMUSG00000035234 | protein_coding | Fam175a | 554.613 | 581.523 | 568.068 |
| ENSMUSG00000074388 | processed_transcrip | Gm5544 | 1.943172 | 1.024227 | 1.4837 |
| ENSMUSG00000064452 | snoRNA | SNORD113 | 1.943172 | 1.024227 | 1.4837 |
| ENSMUSG00000047462 | protein_coding | A530099J19Rik | 1.943172 | 1.024227 | 1.4837 |
| ENSMUSG00000050244 | protein_coding | Heatr1 | 4941.37 | 4706.319 | 4823.845 |
| ENSMUSG00000048544 | protein_coding | 5930422O12Rik | 3.888655 | 2.330646 | 3.10965 |
| ENSMUSG00000048538 | pseudogene | Gm9826 | 48.79175 | 57.5961 | 53.19393 |
| ENSMUSG00000035522 | protein_coding | Tsga8 | 7.4142 | 9.862427 | 8.638313 |
| ENSMUSG00000022718 | protein_coding | Dgcr8 | 1774.287 | 1845.413 | 1809.85 |
| ENSMUSG00000034610 | protein_coding | Zcchc11 | 3609.729 | 3861.641 | 3735.685 |
| ENSMUSG00000087343 | processed_transcrip | 1700021N21Rik | 6.169069 | 4.043215 | 5.106142 |
| ENSMUSG00000084641 | rRNA | n-R5s21 | 0.976841 | 2.066473 | 1.521657 |
| ENSMUSG00000082436 | pseudogene | Gm11688 | 0.32211 | 1.033237 | 0.677673 |
| ENSMUSG00000081522 | pseudogene | Gm13170 | 0.32211 | 1.033237 | 0.677673 |
| ENSMUSG00000065389 | snRNA | U1 | 0.32211 | 1.033237 | 0.677673 |
| ENSMUSG00000090990 | processed_transcrip | Gm17197 | 0.32211 | 1.033237 | 0.677673 |
| ENSMUSG00000084678 | snRNA | U2 | 0.32211 | 1.033237 | 0.677673 |
| ENSMUSG00000015189 | protein_coding | Casd1 | 955.9564 | 1018.132 | 987.0443 |
| ENSMUSG00000031504 | protein_coding | Rab20 | 624.071 | 666.6041 | 645.3375 |
| ENSMUSG00000021094 | protein_coding | Dhrs7 | 1356.643 | 1416.876 | 1386.76 |
| ENSMUSG00000028369 | protein_coding | Svep1 | 122.2318 | 112.2055 | 117.2186 |
| ENSMUSG00000024906 | protein_coding | Mus81 | 546.2432 | 575.1554 | 560.6993 |
| ENSMUSG00000083152 | pseudogene | Apc-ps1 | 8.677817 | 11.27551 | 9.976663 |
| ENSMUSG00000067698 | protein_coding | Gm10220 | 8.417951 | 11.02035 | 9.719149 |
| ENSMUSG00000039883 | protein_coding | Lrrc17 | 780.885 | 834.4766 | 807.6808 |
| ENSMUSG00000026049 | protein_coding | 1700029F09Rik | 2201.697 | 2083.54 | 2142.619 |
| ENSMUSG00000089844 | protein_coding | A530032D15Rik | 0.316221 | 1.033237 | 0.674729 |
| ENSMUSG00000065518 | miRNA | Mir423 | 0.316221 | 1.033237 | 0.674729 |
| ENSMUSG00000089753 | protein_coding | Gm11709 | 0.316221 | 1.033237 | 0.674729 |
| ENSMUSG00000052562 | protein_coding | Slc22a30 | 0.316221 | 1.033237 | 0.674729 |
| ENSMUSG00000083416 | pseudogene | Gm6036 | 0.316221 | 1.033237 | 0.674729 |
| ENSMUSG00000057439 | protein_coding | Kir3dl2 | 0.316221 | 1.033237 | 0.674729 |
| ENSMUSG00000036139 | protein_coding | Hoxc9 | 31.67586 | 26.84868 | 29.26227 |
| ENSMUSG00000024677 | protein_coding | Ms4a6b | 74.24017 | 83.01067 | 78.62542 |
| ENSMUSG00000091645 | lincRNA | Gm17591 | 7.503355 | 5.148164 | 6.32576 |
| ENSMUSG00000051515 | pseudogene | Fam181b | 9.963947 | 12.85511 | 11.40953 |
| ENSMUSG00000045034 | protein_coding | Ankrd34b | 137.1223 | 125.3875 | 131.2549 |
| ENSMUSG00000006476 | protein_coding | Nelf | 1820.296 | 1756.042 | 1788.169 |
| ENSMUSG00000034525 | protein_coding | BC018507 | 2341.355 | 2431.646 | 2386.501 |
| ENSMUSG00000034255 | protein_coding | Arhgap27 | 127.1178 | 138.4636 | 132.7907 |
| ENSMUSG00000055194 | protein_coding | Actbl2 | 2.603792 | 1.422092 | 2.012942 |
| ENSMUSG00000036568 | protein_coding | BC032203 | 1178.356 | 1226.26 | 1202.308 |
| ENSMUSG00000008398 | protein_coding | Elk3 | 227.6808 | 243.8523 | 235.7665 |
| ENSMUSG00000083904 | pseudogene | Gm12805 | 3.278501 | 1.739597 | 2.509049 |
| ENSMUSG00000047153 | protein_coding | Khnyn | 755.2368 | 803.0364 | 779.1366 |
| ENSMUSG00000028036 | protein_coding | Ptgfr | 1.621062 | 2.746168 | 2.183615 |

| | | | | | |
|---|---|---|---|---|---|
| ENSMUSG00000018451 | processed_transcrip | 6330403K07Rik | 182.204 | 199.1475 | 190.6757 |
| ENSMUSG00000064065 | protein_coding | Ipcef1 | 45.91803 | 53.72406 | 49.82105 |
| ENSMUSG00000080789 | pseudogene | Gm14007 | 1.615173 | 2.737158 | 2.176166 |
| ENSMUSG00000020623 | protein_coding | Map2k6 | 206.4541 | 223.7109 | 215.0825 |
| ENSMUSG00000028790 | protein_coding | Khdrbs1 | 4611.869 | 4761.86 | 4686.865 |
| ENSMUSG00000025147 | protein_coding | Mob2 | 1068.763 | 1110.325 | 1089.544 |
| ENSMUSG00000024588 | protein_coding | Fech | 1993.095 | 2067.388 | 2030.242 |
| ENSMUSG00000020638 | protein_coding | Cmpk2 | 135.1962 | 124.2177 | 129.707 |
| ENSMUSG00000087361 | processed_transcrip | 0610043K17Rik | 9.664125 | 12.36208 | 11.0131 |
| ENSMUSG00000007872 | protein_coding | Id3 | 1633.166 | 1536.069 | 1584.618 |
| ENSMUSG00000032656 | protein_coding | 3-Mar | 339.3722 | 359.5619 | 349.467 |
| ENSMUSG00000045048 | pseudogene | Gm9795 | 16.3067 | 12.52787 | 14.41729 |
| ENSMUSG00000024127 | protein_coding | Prepl | 1404.642 | 1355.475 | 1380.058 |
| ENSMUSG00000028159 | protein_coding | Dapp1 | 515.669 | 564.9261 | 540.2975 |
| ENSMUSG00000024087 | protein_coding | Cyp1b1 | 268.0192 | 244.0922 | 256.0557 |
| ENSMUSG00000025353 | protein_coding | Ormdl2 | 621.182 | 590.0448 | 605.6134 |
| ENSMUSG00000027824 | protein_coding | Vmn2r1 | 13.30678 | 17.39457 | 15.35067 |
| ENSMUSG00000059518 | protein_coding | Znhit1 | 670.6551 | 640.6887 | 655.6719 |
| ENSMUSG00000025479 | protein_coding | Cyp2e1 | 27.10011 | 34.77573 | 30.93792 |
| ENSMUSG00000046947 | protein_coding | Adck2 | 351.8247 | 371.6667 | 361.7457 |
| ENSMUSG00000050164 | protein_coding | Mchr1 | 11.94939 | 9.217684 | 10.58353 |
| ENSMUSG00000022665 | protein_coding | Ccdc80 | 681.0775 | 732.5118 | 706.7947 |
| ENSMUSG00000042626 | protein_coding | Shc1 | 2261.805 | 2181.564 | 2221.684 |
| ENSMUSG00000074250 | processed_transcrip | Gm10653 | 4.815029 | 6.74506 | 5.780045 |
| ENSMUSG00000065046 | snoRNA | SNORD62 | 1.931394 | 1.024227 | 1.477811 |
| ENSMUSG00000081151 | pseudogene | Gm11448 | 1.931394 | 1.024227 | 1.477811 |
| ENSMUSG00000030098 | protein_coding | Grip2 | 68.71152 | 61.21836 | 64.96494 |
| ENSMUSG00000028468 | protein_coding | Rgp1 | 1223.935 | 1173.846 | 1198.89 |
| ENSMUSG00000032423 | protein_coding | Syncrip | 6432.759 | 6655.733 | 6544.246 |
| ENSMUSG00000079467 | processed_transcrip | Gm14966 | 79.2274 | 87.71484 | 83.47112 |
| ENSMUSG00000081370 | pseudogene | Gm9105 | 6.76178 | 9.191741 | 7.97676 |
| ENSMUSG00000027376 | protein_coding | Prom2 | 17.09472 | 13.90564 | 15.50018 |
| ENSMUSG00000063334 | protein_coding | Krr1 | 1605.443 | 1667.569 | 1636.506 |
| ENSMUSG00000037653 | protein_coding | Kctd8 | 6.11167 | 8.368622 | 7.240146 |
| ENSMUSG00000028517 | protein_coding | Ppap2b | 3133.946 | 2973.929 | 3053.937 |
| ENSMUSG00000022821 | protein_coding | Hgd | 4.546964 | 6.463231 | 5.505097 |
| ENSMUSG00000035357 | protein_coding | Pdzrn3 | 496.2387 | 471.8494 | 484.0441 |
| ENSMUSG00000079168 | protein_coding | Cd209g | 9.680525 | 12.29974 | 10.99013 |
| ENSMUSG00000001930 | protein_coding | Vwf | 95.94171 | 107.3659 | 101.6538 |
| ENSMUSG00000020359 | protein_coding | Agxt2l2 | 522.0786 | 497.4524 | 509.7655 |
| ENSMUSG00000085355 | processed_transcrip | 3010003L21Rik | 127.2959 | 115.1336 | 121.2148 |
| ENSMUSG00000028651 | protein_coding | Ppie | 1029.872 | 1075.134 | 1052.503 |
| ENSMUSG00000084711 | misc_RNA | Y_RNA | 2.592014 | 1.422092 | 2.007053 |
| ENSMUSG00000086962 | pseudogene | Gm12248 | 14.93731 | 11.18899 | 13.06315 |
| ENSMUSG00000057715 | protein_coding | A830018L16Rik | 10.83926 | 15.9425 | 13.39088 |
| ENSMUSG00000054457 | lincRNA | 9430021M05Rik | 12.93316 | 10.17092 | 11.55204 |
| ENSMUSG00000030237 | protein_coding | Slco1a4 | 113.9818 | 98.77403 | 106.3779 |
| ENSMUSG00000037419 | protein_coding | Endod1 | 402.6548 | 422.9739 | 412.8143 |
| ENSMUSG00000027222 | protein_coding | Pex16 | 543.5288 | 512.0948 | 527.8118 |
| ENSMUSG00000021298 | protein_coding | Gpr132 | 1.290752 | 2.463976 | 1.877364 |
| ENSMUSG00000070528 | pseudogene | Gm10297 | 337.0462 | 318.2785 | 327.6624 |
| ENSMUSG00000020635 | protein_coding | Fkbp1b | 12.57594 | 15.84992 | 14.21293 |
| ENSMUSG00000041559 | protein_coding | Fmod | 154.5629 | 135.3243 | 144.9436 |
| ENSMUSG00000017478 | protein_coding | Zc3h18 | 2403.971 | 2513.29 | 2458.63 |
| ENSMUSG00000062203 | protein_coding | Gspt1 | 4465.679 | 4311.778 | 4388.728 |
| ENSMUSG00000042476 | protein_coding | Abcb4 | 35.26596 | 40.37483 | 37.82039 |
| ENSMUSG00000024402 | protein_coding | Lta | 14.63391 | 20.57998 | 17.60694 |
| ENSMUSG00000032355 | protein_coding | 2310046A06Rik | 2.920013 | 1.659599 | 2.289806 |

| | | | | | |
|---|---|---|---|---|---|
| ENSMUSG00000080717 | protein_coding | B230307C23Rik | 96.79288 | 88.06304 | 92.42796 |
| ENSMUSG00000090536 | protein_coding | Scn8a | 25.57365 | 21.38694 | 23.4803 |
| ENSMUSG00000061887 | protein_coding | Ssbp3 | 2921.29 | 3038.296 | 2979.793 |
| ENSMUSG00000015843 | protein_coding | Rxrg | 5.148918 | 7.285265 | 6.217091 |
| ENSMUSG00000042729 | protein_coding | Wdr74 | 1140.583 | 1092.771 | 1116.677 |
| ENSMUSG00000044976 | protein_coding | Wdr72 | 12.00805 | 9.271378 | 10.63972 |
| ENSMUSG00000043488 | processed_transcrip | Gm9783 | 1.923194 | 1.024227 | 1.473711 |
| ENSMUSG00000073113 | protein_coding | Heatr5b | 101.0524 | 110.1005 | 105.5765 |
| ENSMUSG00000072568 | protein_coding | Fam84b | 227.5096 | 244.1026 | 235.8061 |
| ENSMUSG00000074403 | protein_coding | Hist2h3b | 1.601084 | 2.737158 | 2.169121 |
| ENSMUSG00000086187 | processed_transcrip | Gm12860 | 1.284863 | 2.508661 | 1.896762 |
| ENSMUSG00000086586 | processed_transcrip | Gm11192 | 1.959572 | 1.024227 | 1.491899 |
| ENSMUSG00000091856 | lincRNA | Gm17701 | 7.380133 | 9.969453 | 8.674793 |
| ENSMUSG00000091742 | protein_coding | Gm5093 | 2.611992 | 1.422092 | 2.017042 |
| ENSMUSG00000026602 | protein_coding | Nphs2 | 12.91929 | 9.960082 | 11.43969 |
| ENSMUSG00000067336 | protein_coding | Bmpr2 | 1469.307 | 1389.665 | 1429.486 |
| ENSMUSG00000081712 | pseudogene | Gm12096 | 1.284863 | 2.330284 | 1.807573 |
| ENSMUSG00000028743 | protein_coding | Akr7a5 | 1366.079 | 1311.826 | 1338.952 |
| ENSMUSG00000053740 | protein_coding | Gm6457 | 97.64269 | 89.34528 | 93.49399 |
| ENSMUSG00000049288 | protein_coding | Lix1l | 1262.819 | 1313.651 | 1288.235 |
| ENSMUSG00000082901 | pseudogene | Gm14323 | 0.968641 | 0.36219 | 0.665415 |
| ENSMUSG00000046203 | protein_coding | Sprr2g | 0.968641 | 0.36219 | 0.665415 |
| ENSMUSG00000089381 | snRNA | U6 | 0.968641 | 0.36219 | 0.665415 |
| ENSMUSG00000089731 | protein_coding | Gm10013 | 0.968641 | 0.36219 | 0.665415 |
| ENSMUSG00000082744 | pseudogene | Gm15625 | 0.968641 | 0.36219 | 0.665415 |
| ENSMUSG00000084913 | processed_transcrip | Gm7616 | 0.968641 | 0.36219 | 0.665415 |
| ENSMUSG00000081304 | pseudogene | Gm11509 | 0.968641 | 0.36219 | 0.665415 |
| ENSMUSG00000081191 | pseudogene | Gm11271 | 0.968641 | 0.36219 | 0.665415 |
| ENSMUSG00000090469 | protein_coding | Gm3401 | 0.968641 | 0.36219 | 0.665415 |
| ENSMUSG00000089785 | pseudogene | Gm8429 | 0.968641 | 0.36219 | 0.665415 |
| ENSMUSG00000080779 | pseudogene | Gm8731 | 0.968641 | 0.36219 | 0.665415 |
| ENSMUSG00000081881 | pseudogene | Gm14752 | 0.968641 | 0.36219 | 0.665415 |
| ENSMUSG00000024769 | protein_coding | Cdc42bpg | 843.7494 | 884.9765 | 864.363 |
| ENSMUSG00000069867 | protein_coding | Pabpn1l | 2.567414 | 1.413083 | 1.990249 |
| ENSMUSG00000056699 | protein_coding | Gm5533 | 3.862787 | 2.303618 | 3.083203 |
| ENSMUSG00000022814 | protein_coding | Umps | 1656.193 | 1596.053 | 1626.123 |
| ENSMUSG00000050844 | protein_coding | 1700020N01Rik | 70.51976 | 56.87804 | 63.6989 |
| ENSMUSG00000032313 | protein_coding | AI118078 | 96.16125 | 86.74829 | 91.45477 |
| ENSMUSG00000027643 | protein_coding | Ghrh | 1.635151 | 0.653029 | 1.14409 |
| ENSMUSG00000091207 | protein_coding | Purb | 394.566 | 370.0568 | 382.3114 |
| ENSMUSG00000074384 | protein_coding | AI429214 | 68.91816 | 77.99516 | 73.45666 |
| ENSMUSG00000038473 | protein_coding | Nos1ap | 56.39947 | 48.44387 | 52.42167 |
| ENSMUSG00000084558 | snRNA | U6 | 0.33031 | 1.015218 | 0.672764 |
| ENSMUSG00000087722 | snoRNA | SNORA70 | 0.33031 | 1.015218 | 0.672764 |
| ENSMUSG00000072679 | processed_transcrip | D6Ertd474e | 0.33031 | 1.015218 | 0.672764 |
| ENSMUSG00000093328 | misc_RNA | AL646096.2 | 0.33031 | 1.015218 | 0.672764 |
| ENSMUSG00000089654 | pseudogene | Ear-ps12 | 0.33031 | 1.015218 | 0.672764 |
| ENSMUSG00000080155 | pseudogene | Gm14777 | 0.33031 | 1.015218 | 0.672764 |
| ENSMUSG00000051212 | protein_coding | Gpr183 | 25.8289 | 30.05577 | 27.94233 |
| ENSMUSG00000045794 | protein_coding | 4930526D03Rik | 1.296641 | 2.43731 | 1.866976 |
| ENSMUSG00000085223 | processed_transcrip | Gm11210 | 2.922324 | 4.485765 | 3.704044 |
| ENSMUSG00000067771 | protein_coding | Gm14685 | 6.137537 | 8.421954 | 7.279746 |
| ENSMUSG00000040432 | protein_coding | Ltb4r2 | 2.603792 | 4.034568 | 3.31918 |
| ENSMUSG00000056552 | protein_coding | Zfp953 | 206.8872 | 191.3659 | 199.1266 |
| ENSMUSG00000023945 | protein_coding | Slc5a7 | 5.134829 | 7.169591 | 6.15221 |
| ENSMUSG00000065282 | snoRNA | SNORA18 | 43.55018 | 49.35682 | 46.4535 |
| ENSMUSG00000051124 | protein_coding | Gimap9 | 79.17649 | 68.39514 | 73.78581 |
| ENSMUSG00000087211 | lincRNA | 1500016L03Rik | 13.24245 | 16.63488 | 14.93866 |

| | | | | | |
|---|---|---|---|---|---|
| ENSMUSG00000023206 | protein_coding | Il15ra | 260.3343 | 279.4241 | 269.8792 |
| ENSMUSG00000075558 | processed_transcrip | Gm17151 | 0.32211 | 1.015218 | 0.668664 |
| ENSMUSG00000085732 | processed_transcrip | Gm4279 | 0.32211 | 1.015218 | 0.668664 |
| ENSMUSG00000015627 | protein_coding | Gata5 | 1.290752 | 2.330284 | 1.810518 |
| ENSMUSG00000031452 | protein_coding | 1700029H14Rik | 1.290752 | 2.330284 | 1.810518 |
| ENSMUSG00000024847 | protein_coding | Aip | 1195.371 | 1142.052 | 1168.711 |
| ENSMUSG00000030611 | protein_coding | Mrps11 | 1050.314 | 1003.889 | 1027.102 |
| ENSMUSG00000046636 | pseudogene | Gm7729 | 109.5849 | 119.2807 | 114.4328 |
| ENSMUSG00000055553 | protein_coding | 2810422J05Rik | 924.437 | 962.594 | 943.5155 |
| ENSMUSG00000007670 | protein_coding | Khsrp | 3354.795 | 3507.86 | 3431.327 |
| ENSMUSG00000043635 | protein_coding | Adamts3 | 64.42628 | 72.22969 | 68.32799 |
| ENSMUSG00000091751 | lincRNA | C030010L15Rik | 5.512027 | 7.576465 | 6.544246 |
| ENSMUSG00000086022 | protein_coding | Rad51ap2 | 398.3203 | 271.0734 | 334.6969 |
| ENSMUSG00000050747 | protein_coding | Trim15 | 34.11334 | 40.33159 | 37.22247 |
| ENSMUSG00000001751 | protein_coding | Naglu | 582.457 | 610.5985 | 596.5277 |
| ENSMUSG00000039759 | protein_coding | Thap3 | 277.0115 | 295.853 | 286.4323 |
| ENSMUSG00000086172 | processed_transcrip | 2700068H02Rik | 12.03265 | 9.057326 | 10.54499 |
| ENSMUSG00000029416 | protein_coding | Slc15a4 | 700.9828 | 734.1312 | 717.557 |
| ENSMUSG00000035201 | protein_coding | B020004J07Rik | 0.316221 | 1.015218 | 0.66572 |
| ENSMUSG00000041460 | protein_coding | Cacna2d4 | 1.937283 | 0.988552 | 1.462917 |
| ENSMUSG00000073658 | protein_coding | Crygb | 4.198987 | 6.02969 | 5.114339 |
| ENSMUSG00000073543 | pseudogene | Chmp1b | 1255.257 | 1308.057 | 1281.657 |
| ENSMUSG00000085006 | processed_transcrip | BC021767 | 59.89035 | 50.21049 | 55.05042 |
| ENSMUSG00000026726 | protein_coding | Cubn | 70.30187 | 77.57674 | 73.9393 |
| ENSMUSG00000053395 | protein_coding | Cacng8 | 23.54364 | 27.69561 | 25.61962 |
| ENSMUSG00000027864 | protein_coding | Ptgfrn | 1084.394 | 1027.456 | 1055.925 |
| ENSMUSG00000038369 | protein_coding | Ncoa6 | 2842.205 | 2979.263 | 2910.734 |
| ENSMUSG00000036246 | protein_coding | Gmip | 287.4659 | 303.6798 | 295.5729 |
| ENSMUSG00000082159 | pseudogene | Gm7910 | 0.968641 | 0.335524 | 0.652082 |
| ENSMUSG00000092012 | protein_coding | Gm4782 | 0.968641 | 0.335524 | 0.652082 |
| ENSMUSG00000053338 | protein_coding | Tarm1 | 0.968641 | 0.335524 | 0.652082 |
| ENSMUSG00000074311 | protein_coding | Vmn1r139 | 0.968641 | 0.335524 | 0.652082 |
| ENSMUSG00000081059 | pseudogene | Gm11945 | 0.968641 | 0.335524 | 0.652082 |
| ENSMUSG00000085091 | processed_transcrip | E230015J15Rik | 0.968641 | 0.335524 | 0.652082 |
| ENSMUSG00000087912 | snoRNA | SNORD19B | 0.968641 | 0.335524 | 0.652082 |
| ENSMUSG00000022041 | protein_coding | Chrna2 | 0.968641 | 0.335524 | 0.652082 |
| ENSMUSG00000084229 | pseudogene | Gm7958 | 0.968641 | 0.335524 | 0.652082 |
| ENSMUSG00000086695 | processed_transcrip | Gm15247 | 12.71325 | 9.633567 | 11.17341 |
| ENSMUSG00000037138 | protein_coding | Aff3 | 1151.282 | 1198.882 | 1175.082 |
| ENSMUSG00000035694 | protein_coding | Caps2 | 15.40031 | 11.14467 | 13.27249 |
| ENSMUSG00000038543 | protein_coding | BC028528 | 70.4766 | 63.39579 | 66.9362 |
| ENSMUSG00000033961 | protein_coding | Zfp446 | 427.6067 | 405.6202 | 416.6134 |
| ENSMUSG00000028282 | protein_coding | Casp8ap2 | 3675.239 | 3860.514 | 3767.876 |
| ENSMUSG00000083218 | pseudogene | Gm16425 | 7.4306 | 5.394319 | 6.412459 |
| ENSMUSG00000070572 | pseudogene | Gm13072 | 171.0544 | 159.2858 | 165.1701 |
| ENSMUSG00000078878 | protein_coding | Gm14305 | 4.806829 | 6.798754 | 5.802792 |
| ENSMUSG00000038064 | protein_coding | Cldn22 | 2.600214 | 1.324076 | 1.962145 |
| ENSMUSG00000051906 | protein_coding | Cd209f | 86.93956 | 77.73746 | 82.33851 |
| ENSMUSG00000002608 | protein_coding | Ccdc97 | 1172.839 | 1125.357 | 1149.098 |
| ENSMUSG00000027303 | protein_coding | Ptpra | 1957.196 | 2032.72 | 1994.958 |
| ENSMUSG00000007908 | protein_coding | Hmgcll1 | 16.86177 | 13.51642 | 15.1891 |
| ENSMUSG00000003849 | protein_coding | Nqo1 | 144.5298 | 156.1083 | 150.319 |
| ENSMUSG00000034825 | protein_coding | Nrip3 | 672.5024 | 608.5333 | 640.5178 |
| ENSMUSG00000061099 | protein_coding | Gapdhs | 284.0086 | 266.2645 | 275.1365 |
| ENSMUSG00000091279 | lincRNA | Gm17643 | 0.968641 | 0.326514 | 0.647578 |
| ENSMUSG00000083599 | pseudogene | Gm12821 | 0.968641 | 0.326514 | 0.647578 |
| ENSMUSG00000025746 | protein_coding | Il6 | 0.968641 | 0.326514 | 0.647578 |
| ENSMUSG00000087611 | lincRNA | 4930458D05Rik | 0.968641 | 0.326514 | 0.647578 |

| | | | | | |
|---|---|---|---|---|---|
| ENSMUSG00000092238 | pseudogene | Gm6004 | 0.968641 | 0.326514 | 0.647578 |
| ENSMUSG00000090938 | protein_coding | Gm6905 | 0.968641 | 0.326514 | 0.647578 |
| ENSMUSG00000008874 | protein_coding | Clec3a | 0.968641 | 0.326514 | 0.647578 |
| ENSMUSG00000085594 | processed_transcrip | Gm11551 | 0.968641 | 0.326514 | 0.647578 |
| ENSMUSG00000085651 | processed_transcrip | Gm11695 | 0.968641 | 0.326514 | 0.647578 |
| ENSMUSG00000092512 | pseudogene | Gm4152 | 0.968641 | 0.326514 | 0.647578 |
| ENSMUSG00000076229 | miRNA | AC155261.1 | 0.968641 | 0.326514 | 0.647578 |
| ENSMUSG00000062814 | protein_coding | Ssxa1 | 0.968641 | 0.326514 | 0.647578 |
| ENSMUSG00000081942 | pseudogene | Gm15149 | 0.968641 | 0.326514 | 0.647578 |
| ENSMUSG00000055497 | pseudogene | Gm9974 | 7.124889 | 5.050148 | 6.087518 |
| ENSMUSG00000087269 | lincRNA | D330023K18Rik | 78.12987 | 70.70379 | 74.41683 |
| ENSMUSG00000025484 | protein_coding | Bet1l | 552.033 | 588.006 | 570.0195 |
| ENSMUSG00000080942 | pseudogene | Gm14556 | 23.81886 | 28.02534 | 25.9221 |
| ENSMUSG00000029413 | protein_coding | Naaa | 595.6554 | 630.9233 | 613.2894 |
| ENSMUSG00000078448 | protein_coding | Gm10943 | 1.623372 | 0.653029 | 1.1382 |
| ENSMUSG00000040181 | protein_coding | Fmo1 | 504.6229 | 532.72 | 518.6715 |
| ENSMUSG00000028310 | protein_coding | Ppp3r2 | 9.733303 | 7.276617 | 8.50496 |
| ENSMUSG00000021773 | protein_coding | Comtd1 | 28.12594 | 33.0423 | 30.58412 |
| ENSMUSG00000029797 | protein_coding | Sspo | 47.36652 | 41.00441 | 44.18546 |
| ENSMUSG00000059355 | protein_coding | BC056474 | 372.524 | 393.3631 | 382.9436 |
| ENSMUSG00000083268 | pseudogene | Gm11511 | 2.897724 | 1.748606 | 2.323165 |
| ENSMUSG00000063145 | protein_coding | Bbs5 | 206.4514 | 193.547 | 199.9992 |
| ENSMUSG00000084929 | processed_transcrip | 4732418A04Rik | 8.720083 | 6.552238 | 7.636161 |
| ENSMUSG00000059058 | pseudogene | Gm15431 | 135.4635 | 146.7211 | 141.0923 |
| ENSMUSG00000090221 | protein_coding | Gm4245 | 7.369397 | 5.139155 | 6.254276 |
| ENSMUSG00000017428 | protein_coding | Psmd11 | 2989.115 | 2880.574 | 2934.845 |
| ENSMUSG00000070880 | protein_coding | Gad1 | 3.230345 | 4.776604 | 4.003475 |
| ENSMUSG00000072592 | protein_coding | Gm10373 | 10.31297 | 7.454632 | 8.8838 |
| ENSMUSG00000074673 | protein_coding | Ttll9 | 65.70511 | 58.78462 | 62.24486 |
| ENSMUSG00000020189 | protein_coding | Osbpl8 | 2124.992 | 2005.983 | 2065.488 |
| ENSMUSG00000019467 | protein_coding | Arhgef25 | 761.8155 | 716.1924 | 739.0039 |
| ENSMUSG00000070263 | snRNA | U5 | 1.937283 | 0.997561 | 1.467422 |
| ENSMUSG00000055254 | protein_coding | Ntrk2 | 413.1907 | 386.5388 | 399.8647 |
| ENSMUSG00000071769 | protein_coding | Rhox3h | 3.568855 | 5.706025 | 4.63744 |
| ENSMUSG00000053536 | protein_coding | Cstf2t | 2013.429 | 2102.432 | 2057.93 |
| ENSMUSG00000043969 | protein_coding | Emx2 | 12.23512 | 15.2744 | 13.75476 |
| ENSMUSG00000066955 | protein_coding | Gm6396 | 5.117161 | 7.303283 | 6.210222 |
| ENSMUSG00000041058 | protein_coding | Wwp1 | 2018.926 | 1944.765 | 1981.846 |
| ENSMUSG00000003363 | protein_coding | Pld3 | 1790.816 | 1870.101 | 1830.458 |
| ENSMUSG00000001521 | protein_coding | Tulp3 | 1687.409 | 1790.542 | 1738.976 |
| ENSMUSG00000063409 | protein_coding | Lrrc43 | 7.737353 | 10.43794 | 9.087649 |
| ENSMUSG00000024743 | protein_coding | Syt7 | 1928.932 | 2006.933 | 1967.933 |
| ENSMUSG00000019996 | protein_coding | Mtap7 | 2566.601 | 2661.197 | 2613.899 |
| ENSMUSG00000033953 | protein_coding | Ppp3r1 | 3566.158 | 3696.896 | 3631.527 |
| ENSMUSG00000036199 | protein_coding | Ndufa13 | 1727.268 | 1646.11 | 1686.689 |
| ENSMUSG00000085767 | processed_transcrip | Gm13563 | 84.85967 | 95.85397 | 90.35682 |
| ENSMUSG00000028351 | protein_coding | Dbc1 | 6.139848 | 8.220485 | 7.180166 |
| ENSMUSG00000025165 | protein_coding | Sectm1a | 13.95816 | 10.9753 | 12.46673 |
| ENSMUSG00000044783 | protein_coding | Hjurp | 4814.237 | 4462.228 | 4638.232 |
| ENSMUSG00000031258 | protein_coding | Xkrx | 51.70595 | 45.70717 | 48.70656 |
| ENSMUSG00000066667 | protein_coding | Gm16432 | 48.96596 | 60.40156 | 54.68376 |
| ENSMUSG00000081485 | pseudogene | Gm12338 | 151.7556 | 137.1406 | 144.4481 |
| ENSMUSG00000038859 | protein_coding | Baiap2l1 | 374.4998 | 339.498 | 356.9989 |
| ENSMUSG00000062588 | protein_coding | Gm6104 | 210.3854 | 225.7327 | 218.0591 |
| ENSMUSG00000080440 | snoRNA | SNORA79 | 4.233054 | 2.692474 | 3.462764 |
| ENSMUSG00000032921 | protein_coding | Odf4 | 2.283993 | 3.73472 | 3.009356 |
| ENSMUSG00000002416 | protein_coding | Ndufb2 | 121.6786 | 134.5177 | 128.0981 |
| ENSMUSG00000062991 | protein_coding | Nrg1 | 49.11267 | 43.12245 | 46.11756 |

| | | | | | |
|---|---|---|---|---|---|
| ENSMUSG00000028572 | protein_coding | Hook1 | 3330.101 | 3210.71 | 3270.405 |
| ENSMUSG00000043670 | protein_coding | Diras1 | 40.15143 | 45.95545 | 43.05344 |
| ENSMUSG00000005312 | protein_coding | Ubqln1 | 3432.166 | 3559.827 | 3495.996 |
| ENSMUSG00000036356 | protein_coding | Csgalnact1 | 48.6812 | 55.23837 | 51.95979 |
| ENSMUSG00000085696 | lincRNA | 2700086A05Rik | 9.434749 | 6.95875 | 8.19675 |
| ENSMUSG00000027080 | protein_coding | Med19 | 2392.398 | 2479.19 | 2435.794 |
| ENSMUSG00000034088 | protein_coding | Hdlbp | 8487.345 | 8773.821 | 8630.583 |
| ENSMUSG00000083819 | pseudogene | Gm11977 | 1.284863 | 2.392626 | 1.838744 |
| ENSMUSG00000048003 | protein_coding | Catsper4 | 1.31304 | 2.383978 | 1.848509 |
| ENSMUSG00000021816 | protein_coding | Ppp3cb | 3115.644 | 3007.287 | 3061.466 |
| ENSMUSG00000092371 | processed_transcrip | RP23-341C5.10 | 1.945483 | 0.988552 | 1.467017 |
| ENSMUSG00000060240 | protein_coding | Cend1 | 66.64811 | 74.25941 | 70.45376 |
| ENSMUSG00000089396 | snRNA | U1 | 10.31886 | 12.94376 | 11.63131 |
| ENSMUSG00000088634 | snoRNA | SNORD125 | 3.552455 | 5.130146 | 4.341301 |
| ENSMUSG00000028649 | protein_coding | Macf1 | 10968.46 | 10610.1 | 10789.28 |
| ENSMUSG00000030310 | protein_coding | Slc6a1 | 54.17139 | 47.91526 | 51.04332 |
| ENSMUSG00000034762 | protein_coding | Glis1 | 291.3386 | 271.1258 | 281.2322 |
| ENSMUSG00000004540 | protein_coding | Psg17 | 0.99093 | 2.021789 | 1.506359 |
| ENSMUSG00000047189 | pseudogene | Gm9818 | 0.99093 | 2.021789 | 1.506359 |
| ENSMUSG00000042190 | protein_coding | Cmklr1 | 64.60966 | 57.77696 | 61.19331 |
| ENSMUSG00000083043 | pseudogene | Gm13433 | 7.841865 | 5.500983 | 6.671424 |
| ENSMUSG00000000197 | protein_coding | Nalcn | 176.7448 | 195.5469 | 186.1458 |
| ENSMUSG00000044134 | protein_coding | Fam109a | 457.8911 | 487.0406 | 472.4658 |
| ENSMUSG00000090150 | protein_coding | Acad11 | 925.8866 | 885.8161 | 905.8513 |
| ENSMUSG00000076974 | miRNA | AL691478.1 | 0.33031 | 0.997561 | 0.663936 |
| ENSMUSG00000027913 | protein_coding | Crct1 | 0.33031 | 0.997561 | 0.663936 |
| ENSMUSG00000083485 | pseudogene | Gm12308 | 0.33031 | 0.997561 | 0.663936 |
| ENSMUSG00000024771 | protein_coding | Lipk | 0.33031 | 0.997561 | 0.663936 |
| ENSMUSG00000070565 | protein_coding | Rasal2 | 1944.064 | 1819.11 | 1881.587 |
| ENSMUSG00000066640 | protein_coding | Fbxl18 | 1618.706 | 1677.132 | 1647.919 |
| ENSMUSG00000021767 | protein_coding | Myst4 | 2428.079 | 2340.553 | 2384.316 |
| ENSMUSG00000036040 | protein_coding | Adamtsl2 | 121.5 | 139.9727 | 130.7364 |
| ENSMUSG00000040767 | protein_coding | Snrnp25 | 606.0066 | 572.0368 | 589.0217 |
| ENSMUSG00000052852 | protein_coding | Reep1 | 7953.522 | 8270.517 | 8112.019 |
| ENSMUSG00000086663 | lincRNA | 9530036O11Rik | 5.78136 | 7.876313 | 6.828837 |
| ENSMUSG00000083022 | pseudogene | Gm11968 | 16.16373 | 13.01511 | 14.58942 |
| ENSMUSG00000074922 | protein_coding | Fam122a | 385.227 | 363.9937 | 374.6104 |
| ENSMUSG00000032946 | protein_coding | Rasgrp2 | 534.8941 | 563.1008 | 548.9974 |
| ENSMUSG00000058794 | protein_coding | Nfe2 | 9.650036 | 12.28208 | 10.96606 |
| ENSMUSG00000044330 | pseudogene | Gm9790 | 4.224854 | 2.772834 | 3.498844 |
| ENSMUSG00000032403 | protein_coding | 2300009A05Rik | 141.5537 | 153.939 | 147.7464 |
| ENSMUSG00000057359 | lincRNA | Gm17494 | 41.57569 | 34.85324 | 38.21447 |
| ENSMUSG00000065578 | miRNA | Mir181b-2 | 0.32211 | 0.997561 | 0.659836 |
| ENSMUSG00000078949 | protein_coding | R3hdml | 0.32211 | 0.997561 | 0.659836 |
| ENSMUSG00000048373 | protein_coding | Fgfbp1 | 0.32211 | 0.997561 | 0.659836 |
| ENSMUSG00000086911 | processed_transcrip | Gm12027 | 0.32211 | 0.997561 | 0.659836 |
| ENSMUSG00000065968 | protein_coding | Ifitm7 | 0.32211 | 0.997561 | 0.659836 |
| ENSMUSG00000026976 | protein_coding | Pax8 | 75.28492 | 85.45952 | 80.37222 |
| ENSMUSG00000001260 | protein_coding | Gabrg1 | 2.586125 | 1.315066 | 1.950596 |
| ENSMUSG00000017204 | protein_coding | Gsdma | 5.848226 | 4.016549 | 4.932388 |
| ENSMUSG00000074994 | protein_coding | Qser1 | 2915.718 | 2796.761 | 2856.239 |
| ENSMUSG00000021794 | protein_coding | Glud1 | 3076.033 | 3179.77 | 3127.901 |
| ENSMUSG00000042179 | protein_coding | Pnliprp1 | 7.062644 | 5.147803 | 6.105223 |
| ENSMUSG00000064145 | protein_coding | Arih2 | 2171.371 | 2074.698 | 2123.035 |
| ENSMUSG00000000125 | protein_coding | Wnt3 | 55.09396 | 61.75136 | 58.42266 |
| ENSMUSG00000022221 | protein_coding | Ripk3 | 459.1323 | 428.8167 | 443.9745 |
| ENSMUSG00000073737 | protein_coding | Gm10566 | 0.316221 | 0.997561 | 0.656891 |
| ENSMUSG00000086800 | processed_transcrip | 4930556L07Rik | 0.316221 | 0.997561 | 0.656891 |

| | | | | | |
|---|---|---|---|---|---|
| ENSMUSG00000000562 | protein_coding | Adora3 | 0.316221 | 0.997561 | 0.656891 |
| ENSMUSG00000081873 | pseudogene | Gm15197 | 0.316221 | 0.997561 | 0.656891 |
| ENSMUSG00000090185 | processed_transcrip | Gm15523 | 0.316221 | 0.997561 | 0.656891 |
| ENSMUSG00000085995 | lincRNA | Gm2788 | 4.162609 | 2.719502 | 3.441055 |
| ENSMUSG00000091078 | processed_transcrip | Gm17218 | 3.866366 | 2.472986 | 3.169676 |
| ENSMUSG00000086504 | processed_transcrip | 4930511F01Rik | 4.202565 | 2.656799 | 3.429682 |
| ENSMUSG00000030452 | protein_coding | Nipa2 | 2066.149 | 2146.669 | 2106.409 |
| ENSMUSG00000029580 | protein_coding | Actb | 19639.26 | 20288.21 | 19963.74 |
| ENSMUSG00000028753 | protein_coding | Vwa5b1 | 3.193967 | 4.856964 | 4.025466 |
| ENSMUSG00000077493 | snoRNA | Snord91a | 15.43706 | 12.24604 | 13.84155 |
| ENSMUSG00000048721 | protein_coding | C030019I05Rik | 0.638331 | 1.386417 | 1.012374 |
| ENSMUSG00000065935 | snRNA | U6 | 0.638331 | 1.386417 | 1.012374 |
| ENSMUSG00000078970 | protein_coding | Wdr92 | 625.2175 | 656.9945 | 641.106 |
| ENSMUSG00000029571 | protein_coding | Tmem106b | 2533.452 | 2651.42 | 2592.436 |
| ENSMUSG00000026565 | protein_coding | Pou2f1 | 1860.126 | 1773.976 | 1817.051 |
| ENSMUSG00000078618 | processed_transcrip | 4930456L15Rik | 5.803649 | 4.132585 | 4.968117 |
| ENSMUSG00000021306 | protein_coding | Gpr137b | 225.041 | 240.908 | 232.9745 |
| ENSMUSG00000028664 | protein_coding | Ephb2 | 329.6351 | 311.9485 | 320.7918 |
| ENSMUSG00000035505 | protein_coding | Cox18 | 423.168 | 399.9705 | 411.5693 |
| ENSMUSG00000085613 | lincRNA | 5730420D15Rik | 9.010437 | 11.69103 | 10.35073 |
| ENSMUSG00000020044 | protein_coding | Timp3 | 2563.933 | 2687.525 | 2625.729 |
| ENSMUSG00000059659 | lincRNA | Gm10069 | 142.5245 | 131.7711 | 137.1478 |
| ENSMUSG00000027951 | protein_coding | Adar | 1827.216 | 1746.757 | 1786.987 |
| ENSMUSG00000089838 | pseudogene | Gm2962 | 22.3164 | 18.70026 | 20.50833 |
| ENSMUSG00000084252 | pseudogene | Gm12036 | 1.31304 | 2.401635 | 1.857338 |
| ENSMUSG00000077652 | snRNA | U1 | 3.530167 | 5.165821 | 4.347994 |
| ENSMUSG00000026849 | protein_coding | Tor1a | 893.9781 | 939.0402 | 916.5092 |
| ENSMUSG00000073574 | protein_coding | Grxcr2 | 1.943172 | 0.997561 | 1.470367 |
| ENSMUSG00000082791 | pseudogene | Gm4875 | 6.105781 | 8.273817 | 7.189799 |
| ENSMUSG00000035439 | protein_coding | Haus8 | 1108.753 | 1048.006 | 1078.379 |
| ENSMUSG00000034739 | protein_coding | Mfrp | 6.464269 | 8.654025 | 7.559147 |
| ENSMUSG00000086416 | processed_transcrip | Gm14002 | 15.43184 | 10.26822 | 12.85003 |
| ENSMUSG00000029175 | protein_coding | 4930471M23Rik | 667.1139 | 694.9748 | 681.0443 |
| ENSMUSG00000007034 | protein_coding | Slc44a4 | 37.14919 | 32.25521 | 34.7022 |
| ENSMUSG00000042303 | protein_coding | Sgsm3 | 2405.521 | 2247.028 | 2326.275 |
| ENSMUSG00000015755 | protein_coding | Tab2 | 3344.928 | 3237.069 | 3290.998 |
| ENSMUSG00000081862 | pseudogene | Gm12413 | 0.33031 | 0.988552 | 0.659431 |
| ENSMUSG00000054169 | protein_coding | Ceacam10 | 0.33031 | 0.988552 | 0.659431 |
| ENSMUSG00000090038 | processed_transcrip | Gm16573 | 0.33031 | 0.988552 | 0.659431 |
| ENSMUSG00000070106 | miRNA | Mir363 | 0.33031 | 0.988552 | 0.659431 |
| ENSMUSG00000074037 | protein_coding | Mc1r | 16.14479 | 19.3623 | 17.75355 |
| ENSMUSG00000032006 | protein_coding | Pdgfd | 277.2764 | 294.9506 | 286.1135 |
| ENSMUSG00000077360 | snoRNA | SNORA24 | 1.937283 | 1.015218 | 1.47625 |
| ENSMUSG00000022280 | protein_coding | Rnf19a | 2724.634 | 2815.18 | 2769.907 |
| ENSMUSG00000088066 | snRNA | U6 | 0.976841 | 1.995123 | 1.485982 |
| ENSMUSG00000036578 | protein_coding | Fxyd7 | 53.87954 | 47.78302 | 50.83128 |
| ENSMUSG00000024210 | protein_coding | Ip6k3 | 5.783671 | 7.786944 | 6.785307 |
| ENSMUSG00000032459 | protein_coding | Mrps22 | 1384.056 | 1321.252 | 1352.654 |
| ENSMUSG00000035337 | protein_coding | Uchl4 | 21.69551 | 18.10057 | 19.89804 |
| ENSMUSG00000090427 | processed_transcrip | Gm17225 | 0.32211 | 0.988552 | 0.655331 |
| ENSMUSG00000074181 | protein_coding | Gm3776 | 0.32211 | 0.988552 | 0.655331 |
| ENSMUSG00000091468 | protein_coding | Vmn2r82 | 0.32211 | 0.988552 | 0.655331 |
| ENSMUSG00000084996 | processed_transcrip | Gm11419 | 0.32211 | 0.988552 | 0.655331 |
| ENSMUSG00000092116 | protein_coding | Gm10320 | 0.32211 | 0.988552 | 0.655331 |
| ENSMUSG00000022315 | protein_coding | Slc30a8 | 0.32211 | 0.988552 | 0.655331 |
| ENSMUSG00000038357 | protein_coding | Camp | 23.01631 | 16.82842 | 19.92236 |
| ENSMUSG00000048191 | protein_coding | Muc6 | 7.141289 | 9.56294 | 8.352114 |
| ENSMUSG00000077394 | snoRNA | snoU2_19 | 1.592884 | 0.653029 | 1.122956 |

| | | | | | |
|---|---|---|---|---|---|
| ENSMUSG00000091100 | lincRNA | Gm17626 | 7.401154 | 5.281495 | 6.341324 |
| ENSMUSG00000056258 | protein_coding | Kcnq3 | 28.53295 | 34.77764 | 31.6553 |
| ENSMUSG00000025938 | protein_coding | Slco5a1 | 11.02532 | 8.546999 | 9.78616 |
| ENSMUSG00000037709 | protein_coding | Fam13a | 140.6527 | 128.3092 | 134.481 |
| ENSMUSG00000061589 | protein_coding | Dot1l | 4562.314 | 4792.454 | 4677.384 |
| ENSMUSG00000032498 | protein_coding | Mlh1 | 794.0333 | 757.2293 | 775.6313 |
| ENSMUSG00000087685 | processed_transcrip | 1700122E12Rik | 27.79078 | 23.84807 | 25.81942 |
| ENSMUSG00000087151 | lincRNA | Gm14022 | 13.91485 | 17.08499 | 15.49992 |
| ENSMUSG00000062232 | protein_coding | Rapgef2 | 1227.743 | 1275.55 | 1251.646 |
| ENSMUSG00000093187 | miRNA | AC107641.3 | 0.316221 | 0.988552 | 0.652387 |
| ENSMUSG00000038059 | protein_coding | 2010002N04Rik | 140.2085 | 129.3413 | 134.7749 |
| ENSMUSG00000006763 | protein_coding | Saal1 | 716.0583 | 746.6389 | 731.3486 |
| ENSMUSG00000024038 | protein_coding | Ndufv3 | 892.2531 | 856.6311 | 874.4421 |
| ENSMUSG00000083081 | pseudogene | Gm13223 | 5.735514 | 8.11382 | 6.924667 |
| ENSMUSG00000052270 | protein_coding | Fpr2 | 1.923194 | 0.988552 | 1.455873 |
| ENSMUSG00000083642 | pseudogene | Gm13148 | 2.897724 | 4.38811 | 3.642917 |
| ENSMUSG00000085311 | processed_transcrip | Gm15445 | 12.98489 | 10.16227 | 11.57358 |
| ENSMUSG00000090136 | processed_transcrip | Gm10177 | 94.82742 | 86.14507 | 90.48624 |
| ENSMUSG00000087120 | processed_transcrip | Gm12279 | 45.97968 | 40.00105 | 42.99036 |
| ENSMUSG00000039219 | protein_coding | Arid4b | 4341.836 | 4525.14 | 4433.488 |
| ENSMUSG00000063522 | protein_coding | 2010109I03Rik | 1.959572 | 0.988552 | 1.474062 |
| ENSMUSG00000053253 | protein_coding | Ndfip2 | 1151.803 | 1199.64 | 1175.722 |
| ENSMUSG00000022708 | protein_coding | Zbtb20 | 3364.805 | 3207.198 | 3286.002 |
| ENSMUSG00000030876 | protein_coding | Mettl9 | 1371.85 | 1430.75 | 1401.3 |
| ENSMUSG00000038576 | protein_coding | Susd4 | 420.9511 | 442.3327 | 431.6419 |
| ENSMUSG00000080490 | miRNA | AL845174.1 | 2.922324 | 1.748606 | 2.335465 |
| ENSMUSG00000085739 | processed_transcrip | Gm15862 | 0.646531 | 1.386417 | 1.016474 |
| ENSMUSG00000019797 | protein_coding | 1700021F05Rik | 477.1977 | 499.9142 | 488.556 |
| ENSMUSG00000091115 | protein_coding | BC027582 | 2.573303 | 1.306057 | 1.93968 |
| ENSMUSG00000082305 | pseudogene | Gm2420 | 20.46596 | 16.22076 | 18.34336 |
| ENSMUSG00000087175 | processed_transcrip | Gm15133 | 19.62092 | 16.0795 | 17.85021 |
| ENSMUSG00000064341 | protein_coding | mt-Nd1 | 48408.54 | 49989.64 | 49199.09 |
| ENSMUSG00000027457 | protein_coding | Snph | 31.86423 | 37.46111 | 34.66267 |
| ENSMUSG00000027163 | protein_coding | Commd9 | 682.1474 | 712.9667 | 697.5571 |
| ENSMUSG00000047044 | protein_coding | D030056L22Rik | 2020.769 | 2087.689 | 2054.229 |
| ENSMUSG00000014959 | protein_coding | Gorasp2 | 1767.457 | 1827.952 | 1797.705 |
| ENSMUSG00000078652 | protein_coding | Psme3 | 4718.566 | 4918.908 | 4818.737 |
| ENSMUSG00000028410 | protein_coding | Dnaja1 | 8228.953 | 8530.012 | 8379.482 |
| ENSMUSG00000014030 | protein_coding | Pax5 | 4.230743 | 2.665808 | 3.448275 |
| ENSMUSG00000084607 | snRNA | U6 | 3.82641 | 5.792545 | 4.809477 |
| ENSMUSG00000003872 | protein_coding | Lin7b | 79.75726 | 89.09225 | 84.42475 |
| ENSMUSG00000037902 | protein_coding | Sirpa | 767.0464 | 734.4797 | 750.7631 |
| ENSMUSG00000027184 | protein_coding | Caprin1 | 11722.85 | 12107.27 | 11915.06 |
| ENSMUSG00000027534 | protein_coding | Snx16 | 551.0235 | 525.2666 | 538.145 |
| ENSMUSG00000039179 | protein_coding | Tekt5 | 11.97987 | 9.280025 | 10.62995 |
| ENSMUSG00000029710 | protein_coding | Ephb4 | 1573.979 | 1505.186 | 1539.582 |
| ENSMUSG00000020755 | protein_coding | Sap30bp | 1518.848 | 1581.45 | 1550.149 |
| ENSMUSG00000068859 | protein_coding | Sp9 | 42.63178 | 36.78173 | 39.70675 |
| ENSMUSG00000087638 | processed_transcrip | Gm11773 | 3.242123 | 4.732281 | 3.987202 |
| ENSMUSG00000036138 | protein_coding | Acaa1a | 800.0692 | 831.3902 | 815.7297 |
| ENSMUSG00000044177 | protein_coding | Wfikkn2 | 155.4008 | 167.6911 | 161.546 |
| ENSMUSG00000073633 | protein_coding | Fbxo36 | 176.7806 | 189.8013 | 183.2909 |
| ENSMUSG00000054414 | protein_coding | Slc30a7 | 1483.821 | 1420.365 | 1452.093 |
| ENSMUSG00000090024 | processed_transcrip | Gm16350 | 4.202565 | 2.781843 | 3.492204 |
| ENSMUSG00000041703 | protein_coding | Zic5 | 12.27612 | 9.570864 | 10.92349 |
| ENSMUSG00000049791 | protein_coding | Fzd4 | 244.5545 | 229.0518 | 236.8031 |
| ENSMUSG00000028013 | protein_coding | Ppa2 | 720.0618 | 690.5788 | 705.3203 |
| ENSMUSG00000050534 | protein_coding | Htr5b | 3.866366 | 5.519001 | 4.692684 |

| | | | | | |
|---|---|---|---|---|---|
| ENSMUSG00000074210 | protein_coding | E130208F15Rik | 13.19071 | 10.49744 | 11.84408 |
| ENSMUSG00000024815 | protein_coding | Trpd52l3 | 34.78469 | 29.12091 | 31.9528 |
| ENSMUSG00000030301 | protein_coding | Ccdc91 | 1335.526 | 1390.271 | 1362.898 |
| ENSMUSG00000084809 | processed_transcrip | Gm12631 | 5.473339 | 3.832736 | 4.653037 |
| ENSMUSG00000078747 | protein_coding | Gm13308 | 2.592014 | 4.097271 | 3.344642 |
| ENSMUSG00000045842 | pseudogene | Gm7148 | 10.96897 | 13.65913 | 12.31405 |
| ENSMUSG00000045078 | protein_coding | Rnf216 | 4413.031 | 4162.508 | 4287.77 |
| ENSMUSG00000074748 | protein_coding | Atxn7l3b | 4079.403 | 4283.036 | 4181.219 |
| ENSMUSG00000089648 | pseudogene | Gm15790 | 19.36105 | 22.97234 | 21.16669 |
| ENSMUSG00000044641 | protein_coding | Pard6b | 2386.018 | 2519.783 | 2452.901 |
| ENSMUSG00000052435 | protein_coding | Cebpe | 5.865894 | 8.149496 | 7.007695 |
| ENSMUSG00000035085 | protein_coding | 1700020L24Rik | 9.400682 | 7.125268 | 8.262975 |
| ENSMUSG00000086233 | processed_transcrip | Gm11816 | 15.84855 | 19.34749 | 17.59802 |
| ENSMUSG00000021000 | protein_coding | Ctage5 | 2983.229 | 2883.51 | 2933.369 |
| ENSMUSG00000077167 | snoRNA | SNORA53 | 13.02589 | 9.898464 | 11.46218 |
| ENSMUSG00000027481 | protein_coding | Bpifb2 | 0.65242 | 1.386417 | 1.019419 |
| ENSMUSG00000086234 | processed_transcrip | 1700112M01Rik | 0.65242 | 1.386417 | 1.019419 |
| ENSMUSG00000047565 | protein_coding | Acot10 | 0.65242 | 1.386417 | 1.019419 |
| ENSMUSG00000088591 | rRNA | 5S_rRNA | 7.781931 | 10.44732 | 9.114623 |
| ENSMUSG00000039809 | protein_coding | Gabbr2 | 24.25137 | 20.49356 | 22.37246 |
| ENSMUSG00000032715 | protein_coding | Trib3 | 124.0536 | 134.6435 | 129.3485 |
| ENSMUSG00000047643 | protein_coding | Gm5454 | 16.8031 | 20.122 | 18.46255 |
| ENSMUSG00000028042 | protein_coding | Zbtb7b | 1077.626 | 1128.984 | 1103.305 |
| ENSMUSG00000025145 | protein_coding | Lrrc45 | 539.0435 | 502.1584 | 520.601 |
| ENSMUSG00000089726 | processed_transcrip | Mir17hg | 431.6586 | 394.1696 | 412.9141 |
| ENSMUSG00000082130 | pseudogene | Gm15799 | 1.945483 | 1.015218 | 1.48035 |
| ENSMUSG00000029578 | protein_coding | Wipi2 | 3053.754 | 2949.614 | 3001.684 |
| ENSMUSG00000081600 | pseudogene | Gm12286 | 3.919143 | 5.702814 | 4.810979 |
| ENSMUSG00000036305 | processed_transcrip | Gm15430 | 15.45011 | 12.56391 | 14.00701 |
| ENSMUSG00000049414 | pseudogene | Gm5417 | 18.07746 | 21.49976 | 19.78861 |
| ENSMUSG00000063445 | protein_coding | Nmral1 | 273.4186 | 251.2736 | 262.3461 |
| ENSMUSG00000031960 | protein_coding | Aars | 3338.771 | 3463.555 | 3401.163 |
| ENSMUSG00000078940 | protein_coding | Wfdc11 | 37.16686 | 42.07948 | 39.62317 |
| ENSMUSG00000082778 | pseudogene | Gm15191 | 0.960442 | 1.994761 | 1.477601 |
| ENSMUSG00000045608 | protein_coding | Dbx2 | 23.85986 | 20.22902 | 22.04444 |
| ENSMUSG00000034403 | protein_coding | Pja1 | 7476.13 | 7717.817 | 7596.973 |
| ENSMUSG00000028755 | protein_coding | Cda | 36.09371 | 41.68669 | 38.8902 |
| ENSMUSG00000035711 | protein_coding | Dok3 | 31.28457 | 35.92868 | 33.60662 |
| ENSMUSG00000085560 | lincRNA | C230038L03Rik | 13.30931 | 10.5241 | 11.91671 |
| ENSMUSG00000074139 | protein_coding | 1700057G04Rik | 4.543386 | 3.063673 | 3.803529 |
| ENSMUSG00000052962 | protein_coding | Mrpl35 | 987.9184 | 1032.831 | 1010.374 |
| ENSMUSG00000002948 | protein_coding | Map2k7 | 2289.129 | 2204.224 | 2246.677 |
| ENSMUSG00000092869 | snoRNA | AL808027.3 | 5.517916 | 3.761747 | 4.639832 |
| ENSMUSG00000028678 | protein_coding | Kif2c | 1641.716 | 1704.584 | 1673.15 |
| ENSMUSG00000024371 | protein_coding | C2 | 226.629 | 203.105 | 214.867 |
| ENSMUSG00000087828 | snRNA | U7 | 6.142159 | 8.149496 | 7.145827 |
| ENSMUSG00000085385 | lincRNA | 9430008C03Rik | 569.4836 | 534.9673 | 552.2255 |
| ENSMUSG00000087326 | lincRNA | Gm12503 | 1.937283 | 1.033237 | 1.48526 |
| ENSMUSG00000043733 | protein_coding | Ptpn11 | 4230.665 | 4064.534 | 4147.6 |
| ENSMUSG00000029725 | protein_coding | 2010007H12Rik | 470.0342 | 443.9693 | 457.0017 |
| ENSMUSG00000073608 | protein_coding | Gm6086 | 2.883635 | 1.659599 | 2.271617 |
| ENSMUSG00000029675 | protein_coding | Eln | 3888.394 | 4077.425 | 3982.91 |
| ENSMUSG00000080981 | pseudogene | Gm12161 | 0.638331 | 1.359751 | 0.999041 |
| ENSMUSG00000062312 | protein_coding | Erbb2 | 1082.775 | 1163.81 | 1123.292 |
| ENSMUSG00000023236 | protein_coding | Scg5 | 47.39425 | 39.99861 | 43.69643 |
| ENSMUSG00000085948 | lincRNA | Gm16684 | 10.98768 | 13.65949 | 12.32358 |
| ENSMUSG00000028648 | protein_coding | Ndufs5 | 1016.684 | 975.5625 | 996.1231 |
| ENSMUSG00000074734 | protein_coding | 4933416C03Rik | 295.1395 | 325.5077 | 310.3236 |

| | | | | | |
|---|---|---|---|---|---|
| ENSMUSG00000080540 | snoRNA | SNORA76 | 6.133959 | 8.166791 | 7.150375 |
| ENSMUSG00000020887 | protein_coding | A230052G05Rik | 5.165317 | 7.195896 | 6.180606 |
| ENSMUSG00000027394 | protein_coding | Ttl | 468.987 | 445.7034 | 457.3452 |
| ENSMUSG00000046934 | protein_coding | Csl | 34.16 | 39.4465 | 36.80325 |
| ENSMUSG00000029029 | protein_coding | Wdr8 | 1021.896 | 980.3157 | 1001.106 |
| ENSMUSG00000030677 | protein_coding | Kif22 | 1108.781 | 1060.322 | 1084.551 |
| ENSMUSG00000039913 | protein_coding | Pak7 | 2.559214 | 4.079252 | 3.319233 |
| ENSMUSG00000090186 | processed_transcrip | Gm7592 | 2.865968 | 4.503783 | 3.684876 |
| ENSMUSG00000022621 | protein_coding | Rabl2 | 513.2483 | 535.5906 | 524.4195 |
| ENSMUSG00000038095 | protein_coding | Sbno1 | 12676.9 | 12170.7 | 12423.8 |
| ENSMUSG00000080956 | pseudogene | Gm16179 | 3.536056 | 5.254829 | 4.395442 |
| ENSMUSG00000040464 | protein_coding | Gtpbp10 | 1293.823 | 1242.66 | 1268.242 |
| ENSMUSG00000034893 | protein_coding | Cog3 | 1534.963 | 1610.646 | 1572.805 |
| ENSMUSG00000019278 | protein_coding | Dpep1 | 237.1024 | 219.7465 | 228.4245 |
| ENSMUSG00000034342 | protein_coding | Cbl | 980.4219 | 1043.79 | 1012.106 |
| ENSMUSG00000065304 | snoRNA | SNORA22 | 3.250323 | 4.732281 | 3.991302 |
| ENSMUSG00000030103 | protein_coding | Bhlhe40 | 468.5387 | 500.7225 | 484.6306 |
| ENSMUSG00000027193 | protein_coding | Api5 | 4745.434 | 4595.494 | 4670.464 |
| ENSMUSG00000049580 | protein_coding | Tsku | 247.7437 | 232.9563 | 240.35 |
| ENSMUSG00000076433 | protein_coding | BC100451 | 5.817738 | 4.159251 | 4.988494 |
| ENSMUSG00000067860 | protein_coding | Zic3 | 190.4662 | 203.2154 | 196.8408 |
| ENSMUSG00000090779 | processed_transcrip | Gm17110 | 7.785509 | 5.765156 | 6.775333 |
| ENSMUSG00000087483 | lincRNA | D830044I16Rik | 58.05206 | 51.17569 | 54.61388 |
| ENSMUSG00000023805 | protein_coding | Synj2 | 1373.593 | 1435.342 | 1404.467 |
| ENSMUSG00000045075 | protein_coding | Gm9796 | 10.62099 | 8.282826 | 9.451907 |
| ENSMUSG00000028945 | protein_coding | Rheb | 2419.623 | 2341.548 | 2380.586 |
| ENSMUSG00000024197 | protein_coding | Plin3 | 1711.518 | 1803.131 | 1757.325 |
| ENSMUSG00000031557 | protein_coding | Plekha2 | 242.6111 | 263.5189 | 253.065 |
| ENSMUSG00000071551 | protein_coding | Akr1c19 | 2.905924 | 1.739597 | 2.322761 |
| ENSMUSG00000019139 | protein_coding | Isyna1 | 6987.813 | 6776.459 | 6882.136 |
| ENSMUSG00000003955 | protein_coding | Fam162a | 949.4973 | 993.4011 | 971.4492 |
| ENSMUSG00000025754 | protein_coding | Agbl1 | 2.626081 | 1.377408 | 2.001744 |
| ENSMUSG00000052429 | protein_coding | Prmt1 | 4557.771 | 4383.24 | 4470.506 |
| ENSMUSG00000028273 | protein_coding | Pdlim5 | 1201.52 | 1111.177 | 1156.348 |
| ENSMUSG00000042302 | protein_coding | Ehbp1 | 896.9673 | 861.5375 | 879.2524 |
| ENSMUSG00000035295 | protein_coding | Wdr38 | 19.76538 | 23.40552 | 21.58545 |
| ENSMUSG00000091113 | lincRNA | Gm17311 | 0.948663 | 2.101787 | 1.525225 |
| ENSMUSG00000021215 | protein_coding | Net1 | 1328.719 | 1389.239 | 1358.979 |
| ENSMUSG00000047696 | protein_coding | Ccdc144b | 1.592884 | 2.737158 | 2.165021 |
| ENSMUSG00000079559 | protein_coding | Gm684 | 69.40576 | 76.47252 | 72.93914 |
| ENSMUSG00000081817 | pseudogene | Gm13590 | 18.77214 | 15.27725 | 17.0247 |
| ENSMUSG00000039205 | protein_coding | Ciz1 | 755.5428 | 788.7658 | 772.1543 |
| ENSMUSG00000047787 | protein_coding | Flrt1 | 1245.203 | 1313.19 | 1279.196 |
| ENSMUSG00000071047 | protein_coding | Ces1a | 6.695956 | 9.298044 | 7.997 |
| ENSMUSG00000045107 | protein_coding | 1810063B07Rik | 275.5835 | 259.1681 | 267.3758 |
| ENSMUSG00000024420 | protein_coding | Zfp521 | 180.9804 | 169.4905 | 175.2355 |
| ENSMUSG00000091862 | lincRNA | C230036H16Rik | 20.02167 | 16.67811 | 18.34989 |
| ENSMUSG00000009030 | protein_coding | Pdcl | 1603.904 | 1686.091 | 1644.998 |
| ENSMUSG00000025006 | protein_coding | Sorbs1 | 377.758 | 356.7125 | 367.2353 |
| ENSMUSG00000024581 | protein_coding | Napg | 1277.955 | 1330.681 | 1304.318 |
| ENSMUSG00000041308 | protein_coding | Sntb2 | 1653.301 | 1582.252 | 1617.776 |
| ENSMUSG00000049625 | protein_coding | Tifab | 35.63264 | 40.62492 | 38.12878 |
| ENSMUSG00000051378 | protein_coding | Kif18b | 713.1161 | 682.9882 | 698.0522 |
| ENSMUSG00000078137 | protein_coding | Gm1337 | 131.2967 | 143.9714 | 137.634 |
| ENSMUSG00000035352 | protein_coding | Ccl12 | 9.085728 | 6.834068 | 7.959898 |
| ENSMUSG00000048897 | protein_coding | Zfp710 | 1367.229 | 1316.606 | 1341.918 |
| ENSMUSG00000085495 | lincRNA | Gm16796 | 4.873696 | 3.15268 | 4.013188 |
| ENSMUSG00000070519 | pseudogene | Rpl35a-ps6 | 0.646531 | 1.359751 | 1.003141 |

| | | | | | |
|---|---|---|---|---|---|
| ENSMUSG00000048108 | protein_coding | Tmem72 | 0.646531 | 1.359751 | 1.003141 |
| ENSMUSG00000074561 | protein_coding | Gm10717 | 0.646531 | 1.359751 | 1.003141 |
| ENSMUSG00000044938 | protein_coding | Klhl31 | 27.50274 | 31.92005 | 29.71139 |
| ENSMUSG00000022235 | protein_coding | Cmbl | 161.9332 | 176.3437 | 169.1384 |
| ENSMUSG00000073787 | lincRNA | Gm10575 | 35.56578 | 30.76865 | 33.16722 |
| ENSMUSG00000074026 | pseudogene | Gm6091 | 2.925902 | 4.378739 | 3.652321 |
| ENSMUSG00000022893 | protein_coding | Adamts1 | 623.7256 | 674.1136 | 648.9196 |
| ENSMUSG00000029610 | protein_coding | Aimp2 | 782.0352 | 744.8344 | 763.4348 |
| ENSMUSG00000030707 | protein_coding | Coro1a | 177.2036 | 191.2977 | 184.2507 |
| ENSMUSG00000030386 | protein_coding | Zfp606 | 1006.448 | 1079.847 | 1043.148 |
| ENSMUSG00000031487 | protein_coding | Brf2 | 397.6387 | 377.1436 | 387.3911 |
| ENSMUSG00000024378 | protein_coding | Stard4 | 1447.057 | 1382.562 | 1414.81 |
| ENSMUSG00000074841 | pseudogene | Gm6917 | 3.199857 | 4.723272 | 3.961564 |
| ENSMUSG00000040771 | protein_coding | AI314976 | 921.4472 | 885.3973 | 903.4222 |
| ENSMUSG00000086605 | processed_transcrip | Gm14290 | 75.74599 | 68.6193 | 72.18264 |
| ENSMUSG00000027249 | protein_coding | F2 | 49.41294 | 43.01755 | 46.21524 |
| ENSMUSG00000062661 | protein_coding | Ncs1 | 390.8445 | 419.2141 | 405.0293 |
| ENSMUSG00000021911 | protein_coding | Parg | 3301.457 | 3192.857 | 3247.157 |
| ENSMUSG00000084981 | processed_transcrip | Gm15962 | 0.974531 | 1.994761 | 1.484646 |
| ENSMUSG00000033453 | protein_coding | Adamts15 | 168.0738 | 155.2804 | 161.6771 |
| ENSMUSG00000029146 | protein_coding | Snx17 | 1884.154 | 1822.734 | 1853.444 |
| ENSMUSG00000075043 | protein_coding | Tmem223 | 544.2555 | 574.1489 | 559.2022 |
| ENSMUSG00000085992 | processed_transcrip | Gm11515 | 4.518786 | 3.099348 | 3.809067 |
| ENSMUSG00000081254 | pseudogene | Gm12112 | 39.84006 | 34.44673 | 37.14339 |
| ENSMUSG00000020277 | protein_coding | Pfkl | 1682.991 | 1762.446 | 1722.718 |
| ENSMUSG00000084686 | snoRNA | SNORA19 | 5.501517 | 7.469801 | 6.485659 |
| ENSMUSG00000028479 | protein_coding | Gne | 651.9457 | 610.672 | 631.3088 |
| ENSMUSG00000083774 | pseudogene | Gm7180 | 14.50018 | 11.68202 | 13.0911 |
| ENSMUSG00000029345 | protein_coding | Tfip11 | 1638.818 | 1571.926 | 1605.372 |
| ENSMUSG00000063692 | protein_coding | Gm5576 | 1.937283 | 3.063673 | 2.500478 |
| ENSMUSG00000021687 | protein_coding | Scamp1 | 2888.633 | 2994.114 | 2941.373 |
| ENSMUSG00000057654 | protein_coding | Olfr328 | 2.908235 | 4.661292 | 3.784764 |
| ENSMUSG00000050325 | protein_coding | 1700034J04Rik | 1.943172 | 1.033237 | 1.488204 |
| ENSMUSG00000047298 | protein_coding | Kcnv2 | 19.60914 | 24.63593 | 22.12253 |
| ENSMUSG00000022097 | protein_coding | Sftpc | 6.170337 | 8.211475 | 7.190906 |
| ENSMUSG00000078681 | protein_coding | Tm2d3 | 763.2326 | 798.7586 | 780.9956 |
| ENSMUSG00000056071 | protein_coding | S100a9 | 4.579764 | 6.569895 | 5.574829 |
| ENSMUSG00000013150 | protein_coding | Gfod2 | 660.6369 | 693.179 | 676.908 |
| ENSMUSG00000037553 | protein_coding | Zdhhc18 | 924.4761 | 967.07 | 945.7731 |
| ENSMUSG00000015852 | protein_coding | Fcrls | 269.6632 | 284.6159 | 277.1396 |
| ENSMUSG00000080451 | miRNA | AC159290.2 | 0.962752 | 2.173137 | 1.567945 |
| ENSMUSG00000044700 | protein_coding | Tmem201 | 1603.728 | 1546.174 | 1574.951 |
| ENSMUSG00000082029 | pseudogene | H3f3c | 18.97438 | 15.77028 | 17.37233 |
| ENSMUSG00000028040 | protein_coding | Efna4 | 172.1233 | 158.9615 | 165.5424 |
| ENSMUSG00000045374 | protein_coding | Wdr81 | 1352.989 | 1293.692 | 1323.341 |
| ENSMUSG00000032852 | protein_coding | Rspo4 | 292.4937 | 271.0977 | 281.7957 |
| ENSMUSG00000038888 | protein_coding | Ctu1 | 261.8918 | 244.5822 | 253.237 |
| ENSMUSG00000078656 | protein_coding | Vps25 | 29.0992 | 33.47879 | 31.28899 |
| ENSMUSG00000020354 | protein_coding | Sgcd | 18.98743 | 22.85879 | 20.92311 |
| ENSMUSG00000091617 | protein_coding | Gm3752 | 2.295771 | 3.716701 | 3.006236 |
| ENSMUSG00000029442 | protein_coding | Wdr66 | 45.31906 | 51.00052 | 48.15979 |
| ENSMUSG00000086729 | processed_transcrip | Gm15589 | 2.972791 | 1.422092 | 2.197441 |
| ENSMUSG00000025132 | protein_coding | Arhgdia | 5629.317 | 5824.906 | 5727.112 |
| ENSMUSG00000060586 | protein_coding | H2-Eb1 | 36.71124 | 43.01941 | 39.86533 |
| ENSMUSG00000085716 | processed_transcrip | Gm13388 | 0.65242 | 1.359751 | 1.006086 |
| ENSMUSG00000086410 | processed_transcrip | Gm16158 | 0.65242 | 1.359751 | 1.006086 |
| ENSMUSG00000026605 | protein_coding | Cenpf | 7957.728 | 8377.119 | 8167.424 |
| ENSMUSG00000015247 | protein_coding | Nipsnap3b | 853.5528 | 817.915 | 835.7339 |

| | | | | | |
|---|---|---|---|---|---|
| ENSMUSG00000031483 | protein_coding | Erlin2 | 3127.754 | 3018.021 | 3072.888 |
| ENSMUSG00000087692 | processed_transcrip | Gm11940 | 5.814159 | 7.831267 | 6.822713 |
| ENSMUSG00000002949 | protein_coding | Timm44 | 1491.055 | 1548.845 | 1519.95 |
| ENSMUSG00000087498 | processed_transcrip | 2210416O15Rik | 20.34147 | 17.10337 | 18.72242 |
| ENSMUSG00000054582 | protein_coding | Pabpc1l | 23.31553 | 27.21195 | 25.26374 |
| ENSMUSG00000090151 | processed_transcrip | Gm16043 | 0.646531 | 1.350742 | 0.998636 |
| ENSMUSG00000000182 | protein_coding | Fgf23 | 0.646531 | 1.350742 | 0.998636 |
| ENSMUSG00000034427 | protein_coding | Myo15b | 34.53385 | 29.61902 | 32.07644 |
| ENSMUSG00000052143 | protein_coding | Gm9869 | 2.889525 | 4.352073 | 3.620799 |
| ENSMUSG00000066900 | protein_coding | Suds3 | 2936.561 | 3034.756 | 2985.658 |
| ENSMUSG00000086938 | protein_coding | 4930481A15Rik | 36.94971 | 29.03797 | 32.99384 |
| ENSMUSG00000040138 | protein_coding | Ndp | 31.83837 | 37.63591 | 34.73714 |
| ENSMUSG00000064330 | protein_coding | Pde6h | 1.298952 | 2.35695 | 1.827951 |
| ENSMUSG00000029833 | protein_coding | Trim24 | 2502.138 | 2393.738 | 2447.938 |
| ENSMUSG00000042271 | protein_coding | Nxt2 | 786.4432 | 824.2565 | 805.3498 |
| ENSMUSG00000039770 | protein_coding | Ypel5 | 2540.616 | 2626.136 | 2583.376 |
| ENSMUSG00000037703 | protein_coding | Prosapip1 | 565.9452 | 526.9041 | 546.4246 |
| ENSMUSG00000045284 | protein_coding | Dcaf12l1 | 892.2818 | 937.0224 | 914.6521 |
| ENSMUSG00000034903 | protein_coding | Cobll1 | 1241.291 | 1170.503 | 1205.897 |
| ENSMUSG00000067736 | protein_coding | Gm10222 | 312.0773 | 285.5613 | 298.8193 |
| ENSMUSG00000027546 | protein_coding | Atp9a | 1984.861 | 1896.441 | 1940.651 |
| ENSMUSG00000087739 | snoRNA | SCARNA21 | 10.35523 | 12.9524 | 11.65382 |
| ENSMUSG00000030693 | protein_coding | Klk10 | 9.028105 | 6.646682 | 7.837393 |
| ENSMUSG00000023011 | protein_coding | Faim2 | 113.2137 | 123.6552 | 118.4344 |
| ENSMUSG00000083708 | pseudogene | Gm13123 | 30.34515 | 34.81472 | 32.57993 |
| ENSMUSG00000066860 | protein_coding | Cdkn2d | 257.0234 | 242.943 | 249.9832 |
| ENSMUSG00000028348 | protein_coding | Murc | 69.82023 | 78.53899 | 74.17961 |
| ENSMUSG00000058253 | protein_coding | BC016201 | 6.172647 | 4.361082 | 5.266865 |
| ENSMUSG00000087556 | processed_transcrip | Gm15764 | 36.79302 | 41.69311 | 39.24306 |
| ENSMUSG00000026203 | protein_coding | Dnajb2 | 447.6824 | 422.9207 | 435.3015 |
| ENSMUSG00000058267 | protein_coding | Mrps14 | 1051.18 | 1097.727 | 1074.454 |
| ENSMUSG00000027722 | protein_coding | Spata5 | 5511.265 | 5156.167 | 5333.716 |
| ENSMUSG00000005823 | protein_coding | Gpr108 | 652.7659 | 681.9995 | 667.3827 |
| ENSMUSG00000039697 | protein_coding | Ncoa7 | 1367.453 | 1435.138 | 1401.295 |
| ENSMUSG00000081100 | pseudogene | Gm14630 | 3.838188 | 2.330646 | 3.084417 |
| ENSMUSG00000026843 | protein_coding | Fubp3 | 2166.945 | 2242.137 | 2204.541 |
| ENSMUSG00000031029 | protein_coding | Eif3f | 3161.18 | 3304.828 | 3233.004 |
| ENSMUSG00000090998 | lincRNA | Gm17586 | 92.17465 | 84.38166 | 88.27815 |
| ENSMUSG00000057454 | protein_coding | Lypd3 | 3.248013 | 2.012779 | 2.630396 |
| ENSMUSG00000028830 | protein_coding | AU040320 | 1348.307 | 1280.376 | 1314.342 |
| ENSMUSG00000086638 | lincRNA | 4930405A21Rik | 43.5548 | 48.85157 | 46.20318 |
| ENSMUSG00000066687 | protein_coding | Zbtb16 | 2237.923 | 2122.194 | 2180.059 |
| ENSMUSG00000081824 | pseudogene | BC002163 | 6.131648 | 4.378739 | 5.255193 |
| ENSMUSG00000086256 | processed_transcrip | Gm12052 | 3.250323 | 2.057464 | 2.653894 |
| ENSMUSG00000078307 | protein_coding | AI593442 | 6.478358 | 8.707357 | 7.592857 |
| ENSMUSG00000038668 | protein_coding | Lpar1 | 699.0337 | 739.0376 | 719.0357 |
| ENSMUSG00000025268 | protein_coding | Maged2 | 6995.078 | 6778.119 | 6886.598 |
| ENSMUSG00000025469 | protein_coding | Msx3 | 3.256212 | 2.048455 | 2.652334 |
| ENSMUSG00000026065 | protein_coding | Slc9a4 | 9.655925 | 7.496105 | 8.576015 |
| ENSMUSG00000024644 | protein_coding | Cndp2 | 988.5276 | 952.622 | 970.5748 |
| ENSMUSG00000021566 | processed_transcrip | 5430425J12Rik | 2.942302 | 4.396757 | 3.66953 |
| ENSMUSG00000082276 | pseudogene | Gm13885 | 1.290752 | 2.312628 | 1.80169 |
| ENSMUSG00000021388 | protein_coding | Aspn | 119.0332 | 130.0311 | 124.5322 |
| ENSMUSG00000049583 | protein_coding | Grm5 | 17.68028 | 14.08909 | 15.88469 |
| ENSMUSG00000025889 | protein_coding | Snca | 47.08288 | 52.85295 | 49.96791 |
| ENSMUSG00000029246 | protein_coding | Ppat | 2717.818 | 2620.703 | 2669.261 |
| ENSMUSG00000069971 | protein_coding | 4933402J07Rik | 25.90523 | 29.99343 | 27.94933 |
| ENSMUSG00000026020 | protein_coding | Nop58 | 3730.86 | 3587.956 | 3659.408 |

| | | | | | |
|---|---|---|---|---|---|
| ENSMUSG00000063672 | protein_coding | Nkx6-3 | 0.65242 | 1.350742 | 1.001581 |
| ENSMUSG00000064778 | snoRNA | SNORD59 | 0.65242 | 1.350742 | 1.001581 |
| ENSMUSG00000069376 | protein_coding | Gm5507 | 0.65242 | 1.350742 | 1.001581 |
| ENSMUSG00000081891 | pseudogene | Gm14660 | 0.65242 | 1.350742 | 1.001581 |
| ENSMUSG00000090063 | processed_transcrip | Dlx6os1 | 2.891835 | 1.739597 | 2.315716 |
| ENSMUSG00000051256 | protein_coding | Jagn1 | 816.3497 | 782.3017 | 799.3257 |
| ENSMUSG00000083935 | pseudogene | Gm12294 | 1.951372 | 1.050893 | 1.501133 |
| ENSMUSG00000035268 | protein_coding | Pkig | 1207.07 | 1159.901 | 1183.486 |
| ENSMUSG00000036131 | protein_coding | Frmd7 | 125.2563 | 137.853 | 131.5546 |
| ENSMUSG00000027187 | protein_coding | Cat | 1485.036 | 1552.867 | 1518.951 |
| ENSMUSG00000078183 | pseudogene | Gm15610 | 13.58916 | 16.53614 | 15.06265 |
| ENSMUSG00000033343 | protein_coding | Magea4 | 143.7603 | 125.8857 | 134.823 |
| ENSMUSG00000034911 | protein_coding | Ushbp1 | 71.46105 | 79.87968 | 75.67036 |
| ENSMUSG00000035551 | protein_coding | Igfbpl1 | 69.45876 | 77.76196 | 73.61036 |
| ENSMUSG00000082806 | pseudogene | Rpl13-ps1 | 7.1108 | 5.228162 | 6.169481 |
| ENSMUSG00000068739 | protein_coding | Sars | 2294.206 | 2372.927 | 2333.566 |
| ENSMUSG00000023861 | protein_coding | Brp44l | 324.4235 | 346.6577 | 335.5406 |
| ENSMUSG00000021733 | protein_coding | Slc4a7 | 2187.967 | 2288.756 | 2238.361 |
| ENSMUSG00000042642 | protein_coding | Flad1 | 508.3021 | 531.2176 | 519.7599 |
| ENSMUSG00000030325 | protein_coding | Klrb1c | 3.874566 | 5.411976 | 4.643271 |
| ENSMUSG00000059183 | protein_coding | Mtfmt | 703.8421 | 675.1313 | 689.4867 |
| ENSMUSG00000081989 | pseudogene | Gm13300 | 7.854686 | 4.708464 | 6.281575 |
| ENSMUSG00000039193 | protein_coding | Nlrc4 | 13.55867 | 10.90395 | 12.23131 |
| ENSMUSG00000082510 | pseudogene | Gm11793 | 5.829516 | 7.795954 | 6.812735 |
| ENSMUSG00000037072 | protein_coding | 15-Sep | 4771.072 | 4973.83 | 4872.451 |
| ENSMUSG00000063564 | protein_coding | Col23a1 | 490.6933 | 465.1493 | 477.9213 |
| ENSMUSG00000006392 | protein_coding | Med8 | 1101.499 | 1141.951 | 1121.725 |
| ENSMUSG00000077148 | snRNA | U6 | 6.464269 | 4.723272 | 5.59377 |
| ENSMUSG00000089736 | protein_coding | Gm14378 | 49.32863 | 56.8508 | 53.08971 |
| ENSMUSG00000071295 | protein_coding | Gm10323 | 48.56477 | 39.56398 | 44.06437 |
| ENSMUSG00000089635 | processed_transcrip | Gm16559 | 6.226693 | 8.96252 | 7.594607 |
| ENSMUSG00000072855 | protein_coding | Gm10430 | 3.213945 | 4.759309 | 3.986627 |
| ENSMUSG00000073155 | lincRNA | 1810058I24Rik | 2009.95 | 2076.583 | 2043.266 |
| ENSMUSG00000036185 | protein_coding | Ng23 | 58.30731 | 65.54547 | 61.92639 |
| ENSMUSG00000071690 | protein_coding | Gm10344 | 16.16119 | 19.37167 | 17.76643 |
| ENSMUSG00000022248 | protein_coding | Rad1 | 449.0099 | 425.121 | 437.0654 |
| ENSMUSG00000037101 | protein_coding | Ttc29 | 59.34953 | 53.42634 | 56.38793 |
| ENSMUSG00000022111 | protein_coding | Uchl3 | 1342.006 | 1288.93 | 1315.468 |
| ENSMUSG00000012042 | protein_coding | 4930579F01Rik | 1.945483 | 1.050893 | 1.498188 |
| ENSMUSG00000058050 | protein_coding | Gm9234 | 11.64956 | 14.3572 | 13.00338 |
| ENSMUSG00000026241 | protein_coding | Nppc | 37.38319 | 32.03976 | 34.71148 |
| ENSMUSG00000030703 | protein_coding | Gdpd3 | 30.13829 | 35.05839 | 32.59834 |
| ENSMUSG00000030079 | protein_coding | Ruvbl1 | 2002.71 | 2093.972 | 2048.341 |
| ENSMUSG00000041769 | protein_coding | Ppp2r2d | 2204.797 | 2273.334 | 2239.065 |
| ENSMUSG00000029267 | protein_coding | Mtf2 | 2808.068 | 2930.185 | 2869.126 |
| ENSMUSG00000022750 | protein_coding | Klhl22 | 915.8581 | 874.5712 | 895.2146 |
| ENSMUSG00000024889 | protein_coding | Rce1 | 805.3099 | 839.0544 | 822.1821 |
| ENSMUSG00000029673 | protein_coding | Auts2 | 1357.568 | 1448.656 | 1403.112 |
| ENSMUSG00000033088 | protein_coding | Triobp | 1857.77 | 1781.148 | 1819.459 |
| ENSMUSG00000022463 | protein_coding | Srebf2 | 5641.456 | 5427.349 | 5534.402 |
| ENSMUSG00000001052 | protein_coding | Sec24b | 1648.316 | 1709.583 | 1678.949 |
| ENSMUSG00000085294 | lincRNA | Gm16629 | 36.34895 | 41.39077 | 38.86986 |
| ENSMUSG00000030105 | protein_coding | Arl8b | 2828.536 | 2936.706 | 2882.621 |
| ENSMUSG00000061613 | protein_coding | U2af1 | 1574.447 | 1631.989 | 1603.218 |
| ENSMUSG00000074576 | protein_coding | Mocs3 | 84.01194 | 76.98357 | 80.49775 |
| ENSMUSG00000025265 | protein_coding | Fgd1 | 614.0369 | 582.6003 | 598.3186 |
| ENSMUSG00000034024 | protein_coding | Cct2 | 7332.396 | 7119.431 | 7225.914 |
| ENSMUSG00000081541 | pseudogene | Gm13940 | 0.32211 | 1.086569 | 0.704339 |

| | | | | | |
|---|---|---|---|---|---|
| ENSMUSG00000055801 | protein_coding | Gm1045 | 0.32211 | 1.086569 | 0.704339 |
| ENSMUSG00000050641 | protein_coding | BC048562 | 0.32211 | 1.086569 | 0.704339 |
| ENSMUSG00000025971 | protein_coding | 9430016H08Rik | 310.9259 | 327.4545 | 319.1902 |
| ENSMUSG00000090905 | lincRNA | Gm17249 | 114.6712 | 123.3376 | 119.0044 |
| ENSMUSG00000020467 | protein_coding | Efemp1 | 700.6784 | 734.4503 | 717.5643 |
| ENSMUSG00000070031 | protein_coding | Sp140 | 8.075863 | 10.88086 | 9.478359 |
| ENSMUSG00000006056 | protein_coding | Calcoco2 | 1.635151 | 2.772834 | 2.203992 |
| ENSMUSG00000039860 | protein_coding | Srrm3 | 31.3609 | 26.98237 | 29.17163 |
| ENSMUSG00000079002 | protein_coding | C030006K11Rik | 315.054 | 332.4377 | 323.7459 |
| ENSMUSG00000050107 | protein_coding | Gsg2 | 340.2854 | 323.5975 | 331.9414 |
| ENSMUSG00000036242 | protein_coding | 3632451O06Rik | 102.0416 | 111.4134 | 106.7275 |
| ENSMUSG00000054640 | protein_coding | Slc8a1 | 197.9207 | 184.0049 | 190.9628 |
| ENSMUSG00000001333 | protein_coding | Sync | 119.9355 | 111.1507 | 115.5431 |
| ENSMUSG00000023886 | protein_coding | Smoc2 | 3385.367 | 3556.915 | 3471.141 |
| ENSMUSG00000026117 | protein_coding | Zap70 | 29.56593 | 25.27052 | 27.41822 |
| ENSMUSG00000034139 | protein_coding | Serpini2 | 0.316221 | 1.086569 | 0.701395 |
| ENSMUSG00000080412 | miRNA | AC111115.1 | 0.316221 | 1.086569 | 0.701395 |
| ENSMUSG00000073242 | processed_transcrip | Gm10485 | 0.316221 | 1.086569 | 0.701395 |
| ENSMUSG00000084562 | rRNA | n-R5s39 | 4.563364 | 3.081691 | 3.822528 |
| ENSMUSG00000090005 | processed_transcrip | Gm16540 | 3.196278 | 4.749938 | 3.973108 |
| ENSMUSG00000090612 | lincRNA | D930030I03Rik | 18.8695 | 22.75285 | 20.81117 |
| ENSMUSG00000028070 | protein_coding | Apoa1bp | 1379.145 | 1331.148 | 1355.146 |
| ENSMUSG00000023918 | protein_coding | Gpr115 | 9.694614 | 7.523133 | 8.608874 |
| ENSMUSG00000040340 | protein_coding | 1700019B03Rik | 11.76459 | 8.582312 | 10.17345 |
| ENSMUSG00000090282 | protein_coding | Gm17688 | 1.951372 | 1.059903 | 1.505637 |
| ENSMUSG00000081196 | pseudogene | Gm13858 | 1.951372 | 1.059903 | 1.505637 |
| ENSMUSG00000042535 | protein_coding | Gtpbp1 | 1612.121 | 1550.814 | 1581.468 |
| ENSMUSG00000031907 | protein_coding | Zfp90 | 145.7556 | 134.0517 | 139.9036 |
| ENSMUSG00000026198 | protein_coding | Abcb6 | 891.2653 | 856.3801 | 873.8227 |
| ENSMUSG00000074129 | protein_coding | Rpl13a | 13608.93 | 14112.36 | 13860.65 |
| ENSMUSG00000057706 | protein_coding | Mex3b | 1733.13 | 1848.931 | 1791.03 |
| ENSMUSG00000005514 | protein_coding | Por | 1578.483 | 1671.561 | 1625.022 |
| ENSMUSG00000028451 | protein_coding | 1700022I11Rik | 6.875534 | 4.821288 | 5.848411 |
| ENSMUSG00000041351 | protein_coding | Rap1gap | 524.4 | 499.79 | 512.095 |
| ENSMUSG00000064811 | snoRNA | SNORA32 | 5.844648 | 8.069497 | 6.957073 |
| ENSMUSG00000050447 | protein_coding | Lypd6 | 123.0965 | 132.9556 | 128.0261 |
| ENSMUSG00000085331 | processed_transcrip | Gm11274 | 8.355706 | 6.181401 | 7.268554 |
| ENSMUSG00000002455 | protein_coding | Prpf6 | 2195.929 | 2285.052 | 2240.49 |
| ENSMUSG00000078251 | pseudogene | Gm16487 | 2.897724 | 1.757616 | 2.32767 |
| ENSMUSG00000038009 | protein_coding | Dnajc22 | 9.695882 | 12.25469 | 10.97529 |
| ENSMUSG00000042694 | protein_coding | Obfc1 | 435.7987 | 410.3536 | 423.0762 |
| ENSMUSG00000040213 | protein_coding | Ccbl2 | 463.1038 | 489.7654 | 476.4346 |
| ENSMUSG00000085990 | lincRNA | Gm16731 | 5.501517 | 7.47881 | 6.490163 |
| ENSMUSG00000059820 | protein_coding | AU019823 | 1527.065 | 1578.552 | 1552.808 |
| ENSMUSG00000031662 | protein_coding | Snx20 | 16.4915 | 13.56111 | 15.02631 |
| ENSMUSG00000045763 | protein_coding | Basp1 | 6157.4 | 6390.802 | 6274.101 |
| ENSMUSG00000027243 | protein_coding | Harbi1 | 343.7886 | 325.4164 | 334.6025 |
| ENSMUSG00000024027 | protein_coding | Glp1r | 4.496497 | 3.063673 | 3.780085 |
| ENSMUSG00000047635 | protein_coding | 2810006K23Rik | 540.2115 | 564.482 | 552.3467 |
| ENSMUSG00000005983 | protein_coding | 1700037C18Rik | 72.55373 | 65.00739 | 68.78056 |
| ENSMUSG00000064841 | snoRNA | SNORA42 | 24.86972 | 29.76353 | 27.31663 |
| ENSMUSG00000064315 | protein_coding | Rpl21-ps10 | 11.66827 | 14.36585 | 13.01706 |
| ENSMUSG00000023947 | protein_coding | Nfkbie | 80.1988 | 72.21168 | 76.20524 |
| ENSMUSG00000062704 | protein_coding | 9430002A10Rik | 1.934972 | 3.108357 | 2.521665 |
| ENSMUSG00000027377 | protein_coding | Mall | 4.15672 | 6.101403 | 5.129061 |
| ENSMUSG00000024437 | pseudogene | Gm8615 | 282.822 | 198.0592 | 240.4406 |
| ENSMUSG00000081223 | pseudogene | Gm12247 | 23.27326 | 29.75189 | 26.51258 |
| ENSMUSG00000043716 | protein_coding | Rpl7 | 10951.33 | 11389.57 | 11170.45 |

| | | | | | |
|---|---|---|---|---|---|
| ENSMUSG00000083235 | pseudogene | Gm13246 | 14.64107 | 18.15354 | 16.3973 |
| ENSMUSG00000034538 | protein_coding | Zfp418 | 63.92735 | 72.64417 | 68.28576 |
| ENSMUSG00000073729 | protein_coding | Gm6420 | 1.615173 | 2.826166 | 2.220669 |
| ENSMUSG00000085689 | lincRNA | Gm16838 | 1.943172 | 1.059903 | 1.501537 |
| ENSMUSG00000078554 | protein_coding | 1700125D06Rik | 13.54458 | 10.99332 | 12.26895 |
| ENSMUSG00000075394 | protein_coding | Hoxc4 | 86.8422 | 78.12776 | 82.48498 |
| ENSMUSG00000091032 | protein_coding | Gm17299 | 6.77229 | 8.856218 | 7.814254 |
| ENSMUSG00000093284 | misc_RNA | AC131316.1 | 0 | 0.724379 | 0.36219 |
| ENSMUSG00000016529 | protein_coding | Il10 | 0 | 0.724379 | 0.36219 |
| ENSMUSG00000077967 | miRNA | AL845457.1 | 0 | 0.724379 | 0.36219 |
| ENSMUSG00000082987 | pseudogene | Gm11654 | 0 | 0.724379 | 0.36219 |
| ENSMUSG00000087194 | protein_coding | Skint6 | 0 | 0.724379 | 0.36219 |
| ENSMUSG00000084526 | rRNA | n-R5s192 | 0 | 0.724379 | 0.36219 |
| ENSMUSG00000087394 | lincRNA | Gm12945 | 0 | 0.724379 | 0.36219 |
| ENSMUSG00000083054 | pseudogene | Gm12986 | 0 | 0.724379 | 0.36219 |
| ENSMUSG00000091674 | protein_coding | C630004L07Rik | 0 | 0.724379 | 0.36219 |
| ENSMUSG00000085424 | processed_transcrip | Gm13037 | 0 | 0.724379 | 0.36219 |
| ENSMUSG00000085593 | processed_transcrip | 4930589P08Rik | 0 | 0.724379 | 0.36219 |
| ENSMUSG00000090692 | processed_transcrip | Gm17172 | 0 | 0.724379 | 0.36219 |
| ENSMUSG00000049093 | protein_coding | Il23r | 0 | 0.724379 | 0.36219 |
| ENSMUSG00000065750 | snoRNA | SNORA5 | 0 | 0.724379 | 0.36219 |
| ENSMUSG00000051046 | protein_coding | Olfr214 | 0 | 0.724379 | 0.36219 |
| ENSMUSG00000092451 | pseudogene | Vmn2r-ps56 | 0 | 0.724379 | 0.36219 |
| ENSMUSG00000070551 | protein_coding | Mrgprb5 | 0 | 0.724379 | 0.36219 |
| ENSMUSG00000065822 | snoRNA | Snord15a | 0 | 0.724379 | 0.36219 |
| ENSMUSG00000060777 | pseudogene | Gm10087 | 0 | 0.724379 | 0.36219 |
| ENSMUSG00000088778 | snoRNA | snoU13 | 0 | 0.724379 | 0.36219 |
| ENSMUSG00000080334 | miRNA | AC159266.1 | 0 | 0.724379 | 0.36219 |
| ENSMUSG00000092970 | miRNA | AC091763.1 | 0 | 0.724379 | 0.36219 |
| ENSMUSG00000066080 | miRNA | Mir297-2 | 0 | 0.724379 | 0.36219 |
| ENSMUSG00000086375 | processed_transcrip | Gm12127 | 0 | 0.724379 | 0.36219 |
| ENSMUSG00000020436 | protein_coding | Gabrg2 | 0 | 0.724379 | 0.36219 |
| ENSMUSG00000080823 | pseudogene | Gm11477 | 0 | 0.724379 | 0.36219 |
| ENSMUSG00000079381 | protein_coding | 4930555G01Rik | 0 | 0.724379 | 0.36219 |
| ENSMUSG00000084382 | pseudogene | Gm3267 | 0 | 0.724379 | 0.36219 |
| ENSMUSG00000093330 | miRNA | AC166903.1 | 0 | 0.724379 | 0.36219 |
| ENSMUSG00000004366 | protein_coding | Sst | 0 | 0.724379 | 0.36219 |
| ENSMUSG00000082387 | pseudogene | Gm15724 | 0 | 0.724379 | 0.36219 |
| ENSMUSG00000068167 | protein_coding | Csnka2ip | 0 | 0.724379 | 0.36219 |
| ENSMUSG00000086899 | processed_transcrip | 1600002D24Rik | 0 | 0.724379 | 0.36219 |
| ENSMUSG00000092600 | processed_transcrip | Gm20442 | 0 | 0.724379 | 0.36219 |
| ENSMUSG00000088617 | misc_RNA | 7SK | 0 | 0.724379 | 0.36219 |
| ENSMUSG00000077976 | miRNA | AC142100.1 | 0 | 0.724379 | 0.36219 |
| ENSMUSG00000081020 | pseudogene | Gm14509 | 0 | 0.724379 | 0.36219 |
| ENSMUSG00000084394 | pseudogene | Gm8111 | 0 | 0.724379 | 0.36219 |
| ENSMUSG00000083830 | pseudogene | Gm8839 | 0 | 0.724379 | 0.36219 |
| ENSMUSG00000080813 | pseudogene | Gm14828 | 0 | 0.724379 | 0.36219 |
| ENSMUSG00000088114 | snoRNA | SNORA72 | 0 | 0.724379 | 0.36219 |
| ENSMUSG00000066878 | pseudogene | Gm10184 | 46.57634 | 53.07171 | 49.82403 |
| ENSMUSG00000086486 | processed_transcrip | 2310043D23Rik | 11.9845 | 14.79038 | 13.38744 |
| ENSMUSG00000092255 | processed_transcrip | RP23-11C16.11 | 1.945483 | 3.063673 | 2.504578 |
| ENSMUSG00000078141 | protein_coding | Gm2399 | 9.745081 | 12.43307 | 11.08907 |
| ENSMUSG00000031529 | protein_coding | Tnks | 2635.362 | 2769.297 | 2702.33 |
| ENSMUSG00000089662 | pseudogene | Gm14057 | 8.004375 | 10.74431 | 9.374345 |
| ENSMUSG00000079001 | protein_coding | 4930404H24Rik | 1.929083 | 1.050893 | 1.489988 |
| ENSMUSG00000040667 | protein_coding | Nup88 | 3636.04 | 3530.946 | 3583.493 |
| ENSMUSG00000066258 | protein_coding | Trim12a | 557.116 | 668.4396 | 612.7778 |
| ENSMUSG00000083055 | pseudogene | Gm14140 | 6.12218 | 4.405405 | 5.263793 |

| | | | | | |
|---|---|---|---|---|---|
| ENSMUSG00000067369 | protein_coding | Trmt2b | 949.8059 | 987.6775 | 968.7417 |
| ENSMUSG00000049588 | protein_coding | Ccdc69 | 239.7618 | 260.9151 | 250.3384 |
| ENSMUSG00000005881 | protein_coding | Ergic3 | 2617.184 | 2517.089 | 2567.137 |
| ENSMUSG00000000215 | protein_coding | Ins2 | 107.6622 | 117.2773 | 112.4697 |
| ENSMUSG00000065388 | snRNA | U1 | 1.598773 | 2.701483 | 2.150128 |
| ENSMUSG00000089224 | misc_RNA | 7SK | 3.258523 | 4.945971 | 4.102247 |
| ENSMUSG00000085342 | pseudogene | Gm12254 | 11.01354 | 13.60616 | 12.30985 |
| ENSMUSG00000001504 | protein_coding | Irx2 | 19.71261 | 16.4943 | 18.10345 |
| ENSMUSG00000081185 | pseudogene | Gm4852 | 1.629262 | 2.692474 | 2.160868 |
| ENSMUSG00000085974 | lincRNA | 4930444M15Rik | 1.290752 | 2.303618 | 1.797185 |
| ENSMUSG00000044801 | protein_coding | Olfr159 | 1.914994 | 1.024227 | 1.469611 |
| ENSMUSG00000073765 | processed_transcrip | Gm12863 | 1.914994 | 1.024227 | 1.469611 |
| ENSMUSG00000069303 | protein_coding | Hist1h2br | 1.31304 | 2.366321 | 1.839681 |
| ENSMUSG00000071691 | protein_coding | Gm960 | 388.2434 | 318.8986 | 353.571 |
| ENSMUSG00000054720 | protein_coding | Lrrc8c | 1037.777 | 985.6064 | 1011.692 |
| ENSMUSG00000029373 | protein_coding | Pf4 | 225.176 | 209.6358 | 217.4059 |
| ENSMUSG00000079737 | protein_coding | 3110001I22Rik | 146.4465 | 157.7314 | 152.089 |
| ENSMUSG00000028417 | protein_coding | Tal2 | 14.4802 | 17.91427 | 16.19723 |
| ENSMUSG00000043621 | protein_coding | Ubxn10 | 221.3046 | 235.0335 | 228.169 |
| ENSMUSG00000034156 | protein_coding | Bzrap1 | 100.7835 | 92.96718 | 96.87534 |
| ENSMUSG00000077550 | miRNA | CT009748.1 | 4.49523 | 6.374223 | 5.434726 |
| ENSMUSG00000028167 | protein_coding | Bdh2 | 137.2079 | 126.7923 | 132.0001 |
| ENSMUSG00000037563 | protein_coding | Rps16 | 6151.736 | 6364.509 | 6258.123 |
| ENSMUSG00000037370 | protein_coding | Enpp1 | 85.08129 | 93.47999 | 89.28064 |
| ENSMUSG00000022757 | protein_coding | Tfg | 3137.85 | 3239.828 | 3188.839 |
| ENSMUSG00000031007 | protein_coding | Atp6ap2 | 1270.285 | 1313.194 | 1291.74 |
| ENSMUSG00000056222 | protein_coding | Spock1 | 41.4386 | 36.58134 | 39.00997 |
| ENSMUSG00000085816 | processed_transcrip | Gm13052 | 3.568855 | 5.192487 | 4.380671 |
| ENSMUSG00000063047 | protein_coding | Zfp780b | 203.694 | 182.3019 | 192.998 |
| ENSMUSG00000067786 | protein_coding | Nnat | 310.6154 | 345.1823 | 327.8989 |
| ENSMUSG00000061207 | protein_coding | Stk19 | 511.5833 | 537.919 | 524.7512 |
| ENSMUSG00000020646 | protein_coding | Mboat2 | 2428.341 | 2510.782 | 2469.561 |
| ENSMUSG00000006586 | protein_coding | Runx1t1 | 153.4911 | 143.6618 | 148.5764 |
| ENSMUSG00000067818 | protein_coding | Myl9 | 5439.191 | 5605.956 | 5522.573 |
| ENSMUSG00000047441 | protein_coding | Antxrl | 3.210367 | 4.785975 | 3.998171 |
| ENSMUSG00000035818 | protein_coding | 9930023K05Rik | 24.55581 | 20.75157 | 22.65369 |
| ENSMUSG00000028419 | protein_coding | Chmp5 | 1768.536 | 1846.033 | 1807.284 |
| ENSMUSG00000020375 | protein_coding | Rufy1 | 644.6265 | 670.9542 | 657.7903 |
| ENSMUSG00000086914 | processed_transcrip | Gm16124 | 25.85119 | 22.25982 | 24.0555 |
| ENSMUSG00000080957 | pseudogene | Gm15739 | 2.242993 | 1.086569 | 1.664781 |
| ENSMUSG00000086804 | processed_transcrip | Gm13847 | 0.638331 | 1.422092 | 1.030212 |
| ENSMUSG00000066897 | protein_coding | Olfr872 | 5.128939 | 7.044908 | 6.086924 |
| ENSMUSG00000029410 | protein_coding | Ppef2 | 15.10698 | 12.21974 | 13.66336 |
| ENSMUSG00000049761 | protein_coding | 4930479M11Rik | 71.71144 | 63.12696 | 67.4192 |
| ENSMUSG00000047497 | protein_coding | Adamts12 | 266.3234 | 251.6474 | 258.9854 |
| ENSMUSG00000093233 | misc_RNA | AL591070.1 | 10.66094 | 8.33652 | 9.498732 |
| ENSMUSG00000057738 | protein_coding | Spna2 | 20094.4 | 20815.28 | 20454.84 |
| ENSMUSG00000032518 | protein_coding | Rpsa | 16202.8 | 16765.53 | 16484.17 |
| ENSMUSG00000078864 | protein_coding | Gm14322 | 16.71753 | 20.27987 | 18.4987 |
| ENSMUSG00000026750 | protein_coding | Psmb7 | 4146.398 | 3982.858 | 4064.628 |
| ENSMUSG00000030653 | protein_coding | Pde2a | 301.5989 | 324.1072 | 312.8531 |
| ENSMUSG00000015176 | protein_coding | Nolc1 | 2479.859 | 2570.229 | 2525.044 |
| ENSMUSG00000032187 | protein_coding | Smarca4 | 6338.437 | 6681.817 | 6510.127 |
| ENSMUSG00000092190 | processed_transcrip | RP24-258P4.8 | 6.176226 | 8.238141 | 7.207184 |
| ENSMUSG00000030315 | protein_coding | Vgll4 | 1168.367 | 1220.055 | 1194.211 |
| ENSMUSG00000019865 | protein_coding | Nmbr | 31.03059 | 27.04543 | 29.03801 |
| ENSMUSG00000041303 | protein_coding | Gtf3c3 | 1263.06 | 1311.536 | 1287.298 |
| ENSMUSG00000079162 | protein_coding | Trim43b | 4.806829 | 3.15268 | 3.979755 |

| | | | | | |
|---|---|---|---|---|---|
| ENSMUSG00000073821 | lincRNA | 8030451A03Rik | 15.44064 | 11.86728 | 13.65396 |
| ENSMUSG00000037660 | protein_coding | Gdf7 | 4.856028 | 7.062927 | 5.959478 |
| ENSMUSG00000048153 | protein_coding | Olfr49 | 5.218095 | 7.365624 | 6.29186 |
| ENSMUSG00000036890 | protein_coding | Gtdc1 | 995.0869 | 955.3301 | 975.2085 |
| ENSMUSG00000026197 | protein_coding | Zfand2b | 301.2112 | 284.869 | 293.0401 |
| ENSMUSG00000047026 | protein_coding | Acsm4 | 1.929083 | 1.059903 | 1.494493 |
| ENSMUSG00000025188 | protein_coding | Hps1 | 533.7167 | 510.2487 | 521.9827 |
| ENSMUSG00000022890 | protein_coding | Atp5j | 1845.298 | 1919.172 | 1882.235 |
| ENSMUSG00000042625 | protein_coding | Safb2 | 3597.331 | 3733.674 | 3665.503 |
| ENSMUSG00000092667 | misc_RNA | AC159897.1 | 2.259393 | 3.470547 | 2.86497 |
| ENSMUSG00000029190 | protein_coding | D5Ertd579e | 3503.25 | 3651.016 | 3577.133 |
| ENSMUSG00000001143 | protein_coding | Lman2l | 900.9778 | 856.5569 | 878.7674 |
| ENSMUSG00000084350 | pseudogene | 1700029I01Rik | 386.1389 | 419.1242 | 402.6315 |
| ENSMUSG00000050608 | protein_coding | 2310028O11Rik | 2181.674 | 2256.41 | 2219.042 |
| ENSMUSG00000089085 | snRNA | U1 | 1.929083 | 3.197365 | 2.563224 |
| ENSMUSG00000042473 | protein_coding | Tbc1d8b | 739.3043 | 704.3627 | 721.8335 |
| ENSMUSG00000049971 | protein_coding | Glt1d1 | 32.22988 | 27.9266 | 30.07824 |
| ENSMUSG00000065946 | snRNA | U6 | 0.98273 | 0.36219 | 0.67246 |
| ENSMUSG00000086939 | processed_transcrip | Gm13530 | 0.98273 | 0.36219 | 0.67246 |
| ENSMUSG00000087187 | processed_transcrip | Gm13431 | 0.98273 | 0.36219 | 0.67246 |
| ENSMUSG00000057216 | protein_coding | 4933403O03Rik | 0.98273 | 0.36219 | 0.67246 |
| ENSMUSG00000033731 | protein_coding | 3300002A11Rik | 0.98273 | 0.36219 | 0.67246 |
| ENSMUSG00000089704 | protein_coding | Galnt2 | 11.20759 | 8.654025 | 9.930805 |
| ENSMUSG00000070251 | snoRNA | U3 | 1.609283 | 0.724379 | 1.166831 |
| ENSMUSG00000086767 | processed_transcrip | Gm13070 | 3.199857 | 2.048455 | 2.624156 |
| ENSMUSG00000024212 | protein_coding | Mllt1 | 1490.454 | 1436.421 | 1463.437 |
| ENSMUSG00000073902 | protein_coding | Gm1966 | 4.48703 | 3.081691 | 3.78436 |
| ENSMUSG00000003316 | protein_coding | Glg1 | 7459.966 | 7173.764 | 7316.865 |
| ENSMUSG00000074466 | processed_transcrip | Gm15417 | 59.85226 | 66.03493 | 62.9436 |
| ENSMUSG00000037594 | protein_coding | BC022687 | 611.343 | 574.5279 | 592.9354 |
| ENSMUSG00000086356 | processed_transcrip | Gm13441 | 0.638331 | 1.395426 | 1.016879 |
| ENSMUSG00000036466 | protein_coding | Megf11 | 88.3009 | 79.63669 | 83.9688 |
| ENSMUSG00000010663 | protein_coding | Fads1 | 4025.052 | 4179.993 | 4102.522 |
| ENSMUSG00000062202 | protein_coding | Btbd9 | 1416.384 | 1305.284 | 1360.834 |
| ENSMUSG00000004096 | protein_coding | Cwc15 | 1784.914 | 1842.873 | 1813.893 |
| ENSMUSG00000022901 | protein_coding | Cd86 | 57.78817 | 63.70936 | 60.74877 |
| ENSMUSG00000069072 | protein_coding | Slc7a14 | 51.90842 | 57.65658 | 54.7825 |
| ENSMUSG00000032322 | protein_coding | Pstpip1 | 32.58352 | 28.5032 | 30.54336 |
| ENSMUSG00000055831 | protein_coding | Gm9982 | 17.76921 | 21.40139 | 19.5853 |
| ENSMUSG00000080928 | pseudogene | Hmgb1-ps6 | 6.150358 | 8.077783 | 7.114071 |
| ENSMUSG00000026312 | protein_coding | Cdh7 | 3.202167 | 2.039445 | 2.620806 |
| ENSMUSG00000078812 | protein_coding | Eif5a | 9969.715 | 9671.832 | 9820.774 |
| ENSMUSG00000035572 | protein_coding | Dcaf10 | 1062.714 | 1024.334 | 1043.524 |
| ENSMUSG00000081156 | pseudogene | Gm14425 | 2.267593 | 3.470547 | 2.86907 |
| ENSMUSG00000022389 | protein_coding | Tef | 4343.616 | 4174.454 | 4259.035 |
| ENSMUSG00000009013 | protein_coding | Dynll1 | 4299.048 | 4098.622 | 4198.835 |
| ENSMUSG00000001155 | protein_coding | Ftcd | 4.535186 | 6.181039 | 5.358113 |
| ENSMUSG00000081164 | pseudogene | Gm8722 | 2.267593 | 3.434871 | 2.851232 |
| ENSMUSG00000066383 | protein_coding | Iqcf1 | 1.612862 | 2.826166 | 2.219514 |
| ENSMUSG00000027409 | protein_coding | 1700020A23Rik | 9.10086 | 6.914428 | 8.007644 |
| ENSMUSG00000079707 | protein_coding | Tcte3 | 3.264412 | 2.057464 | 2.660938 |
| ENSMUSG00000070873 | protein_coding | Lilra5 | 1.951372 | 0.979543 | 1.465457 |
| ENSMUSG00000092086 | protein_coding | Gm6793 | 205.4166 | 220.0463 | 212.7315 |
| ENSMUSG00000079319 | protein_coding | Gm11092 | 0.646531 | 1.422092 | 1.034312 |
| ENSMUSG00000090088 | processed_transcrip | Gm15934 | 0.646531 | 1.422092 | 1.034312 |
| ENSMUSG00000002280 | protein_coding | Narfl | 625.6953 | 652.2773 | 638.9863 |
| ENSMUSG00000087421 | lincRNA | Gm5432 | 4.849096 | 3.434871 | 4.141984 |
| ENSMUSG00000054383 | protein_coding | Pnma1 | 26.63272 | 31.18666 | 28.90969 |

| | | | | | |
|---|---|---|---|---|---|
| ENSMUSG00000050232 | protein_coding | Cxcr3 | 6.091692 | 4.432433 | 5.262062 |
| ENSMUSG00000084979 | processed_transcript | Gm16267 | 4.873696 | 3.399196 | 4.136446 |
| ENSMUSG00000028099 | protein_coding | Polr3c | 1081.096 | 1124.335 | 1102.715 |
| ENSMUSG00000090907 | processed_transcript | C030017D09Rik | 1.609283 | 2.728149 | 2.168716 |
| ENSMUSG00000055541 | protein_coding | Lair1 | 110.4666 | 101.4861 | 105.9763 |
| ENSMUSG00000029130 | protein_coding | Rnf32 | 202.8296 | 217.227 | 210.0283 |
| ENSMUSG00000042842 | protein_coding | Serpinb6b | 158.1545 | 173.4449 | 165.7997 |
| ENSMUSG00000074731 | protein_coding | Zfp345 | 88.94848 | 81.6081 | 85.27829 |
| ENSMUSG00000085317 | processed_transcript | Gm14256 | 2.936413 | 1.739597 | 2.338005 |
| ENSMUSG00000028008 | protein_coding | Accn5 | 0.98273 | 0.335524 | 0.659127 |
| ENSMUSG00000085121 | processed_transcript | 5730437C11Rik | 0.98273 | 0.335524 | 0.659127 |
| ENSMUSG00000050440 | protein_coding | Hamp | 0.98273 | 0.335524 | 0.659127 |
| ENSMUSG00000090129 | protein_coding | Olfr287 | 0.98273 | 0.335524 | 0.659127 |
| ENSMUSG00000080740 | pseudogene | Gm15013 | 0.98273 | 0.335524 | 0.659127 |
| ENSMUSG00000087340 | processed_transcript | Gm15228 | 0.98273 | 0.335524 | 0.659127 |
| ENSMUSG00000083915 | pseudogene | Gm13932 | 0.976841 | 0.36219 | 0.669515 |
| ENSMUSG00000082140 | pseudogene | Gm13094 | 0.976841 | 0.36219 | 0.669515 |
| ENSMUSG00000054165 | protein_coding | 4922502B01Rik | 0.976841 | 0.36219 | 0.669515 |
| ENSMUSG00000051354 | protein_coding | Samd3 | 0.976841 | 0.36219 | 0.669515 |
| ENSMUSG00000048399 | protein_coding | Tprg | 0.976841 | 0.36219 | 0.669515 |
| ENSMUSG00000086673 | lincRNA | Gm16736 | 0.976841 | 0.36219 | 0.669515 |
| ENSMUSG00000084205 | pseudogene | Gm8061 | 0.976841 | 0.36219 | 0.669515 |
| ENSMUSG00000005575 | protein_coding | Ube2m | 1367.744 | 1416.31 | 1392.027 |
| ENSMUSG00000066620 | protein_coding | Gm4883 | 1.917305 | 3.046016 | 2.48166 |
| ENSMUSG00000000881 | protein_coding | Dlg3 | 495.618 | 517.5939 | 506.606 |
| ENSMUSG00000086119 | lincRNA | Gm2415 | 19.39512 | 16.34295 | 17.86903 |
| ENSMUSG00000027669 | protein_coding | Gnb4 | 868.4552 | 899.5366 | 883.9959 |
| ENSMUSG00000076609 | IG_C_gene | Igkc | 10.01441 | 12.69439 | 11.3544 |
| ENSMUSG00000087387 | processed_transcript | Gm15420 | 3.570123 | 5.112127 | 4.341125 |
| ENSMUSG00000002290 | pseudogene | Rab11b-ps2 | 297.791 | 327.035 | 312.413 |
| ENSMUSG00000018820 | protein_coding | Zfyve27 | 1419.32 | 1476.991 | 1448.155 |
| ENSMUSG00000086397 | lincRNA | 4933405D12Rik | 18.39472 | 21.81691 | 20.10581 |
| ENSMUSG00000018293 | protein_coding | Pfn1 | 4720.874 | 4889.595 | 4805.234 |
| ENSMUSG00000060377 | pseudogene | Rpl36a-ps1 | 13.00949 | 15.96311 | 14.4863 |
| ENSMUSG00000085556 | processed_transcript | Gm15020 | 10.37983 | 7.973968 | 9.176901 |
| ENSMUSG00000030701 | protein_coding | Plekhb1 | 195.0764 | 210.3459 | 202.7111 |
| ENSMUSG00000048489 | protein_coding | 8430408G22Rik | 21.29498 | 25.11301 | 23.204 |
| ENSMUSG00000036027 | protein_coding | 1810046K07Rik | 3.902744 | 5.501345 | 4.702044 |
| ENSMUSG00000045730 | protein_coding | Adrb2 | 19.91485 | 16.5628 | 18.23882 |
| ENSMUSG00000045232 | protein_coding | Rln3 | 16.87355 | 20.25248 | 18.56301 |
| ENSMUSG00000052469 | protein_coding | Gm9880 | 1.945483 | 0.979543 | 1.462513 |
| ENSMUSG00000030463 | protein_coding | 4933421I07Rik | 0.98273 | 0.326514 | 0.654622 |
| ENSMUSG00000039691 | protein_coding | Tspan10 | 0.98273 | 0.326514 | 0.654622 |
| ENSMUSG00000091725 | protein_coding | Gm8220 | 0.98273 | 0.326514 | 0.654622 |
| ENSMUSG00000083468 | pseudogene | Gm9431 | 0.98273 | 0.326514 | 0.654622 |
| ENSMUSG00000038759 | protein_coding | Nup205 | 4691.687 | 4496.899 | 4594.293 |
| ENSMUSG00000055833 | protein_coding | 1700034H15Rik | 146.3071 | 156.797 | 151.552 |
| ENSMUSG00000020469 | protein_coding | Myl7 | 18.1315 | 22.26339 | 20.19745 |
| ENSMUSG00000064844 | snoRNA | SNORD58 | 7.737353 | 5.836868 | 6.787111 |
| ENSMUSG00000067015 | pseudogene | Gm10193 | 0.974531 | 0.36219 | 0.66836 |
| ENSMUSG00000027556 | protein_coding | Car1 | 0.974531 | 0.36219 | 0.66836 |
| ENSMUSG00000091070 | lincRNA | Gm3764 | 0.974531 | 0.36219 | 0.66836 |
| ENSMUSG00000078487 | protein_coding | E230028L10Rik | 0.974531 | 0.36219 | 0.66836 |
| ENSMUSG00000076754 | IG_V_gene | Tcrg-V2 | 0.974531 | 0.36219 | 0.66836 |
| ENSMUSG00000033383 | protein_coding | Rtp1 | 0.974531 | 0.36219 | 0.66836 |
| ENSMUSG00000084019 | pseudogene | Gm8754 | 0.974531 | 0.36219 | 0.66836 |
| ENSMUSG00000081666 | pseudogene | Gm11677 | 1.957261 | 0.988552 | 1.472906 |
| ENSMUSG00000038408 | processed_transcript | 1700018A04Rik | 1.598773 | 0.724379 | 1.161576 |

| | | | | | |
|---|---|---|---|---|---|
| ENSMUSG00000050395 | protein_coding | Tnfsf15 | 2.885946 | 4.361444 | 3.623695 |
| ENSMUSG00000069769 | protein_coding | Msi2 | 4473.64 | 4688.6 | 4581.12 |
| ENSMUSG00000037815 | protein_coding | Ctnna1 | 6580.197 | 6797.053 | 6688.625 |
| ENSMUSG00000027359 | protein_coding | Slc27a2 | 285.1348 | 303.8024 | 294.4686 |
| ENSMUSG00000006777 | protein_coding | Krt23 | 13.26705 | 16.05248 | 14.65976 |
| ENSMUSG00000040276 | protein_coding | Pacsin1 | 123.6261 | 112.3651 | 117.9956 |
| ENSMUSG00000092509 | processed_transcrip | RP23-392I3.14 | 4.526986 | 2.983675 | 3.75533 |
| ENSMUSG00000045291 | protein_coding | E330021D16Rik | 1.934972 | 3.063673 | 2.499322 |
| ENSMUSG00000028029 | protein_coding | Aimp1 | 1871.907 | 1937.103 | 1904.505 |
| ENSMUSG00000060121 | protein_coding | Sip1 | 576.963 | 549.6516 | 563.3073 |
| ENSMUSG00000075040 | protein_coding | Zfp408 | 755.0401 | 783.8174 | 769.4288 |
| ENSMUSG00000000792 | protein_coding | Slc5a5 | 66.58377 | 77.3525 | 71.96813 |
| ENSMUSG00000039358 | protein_coding | Drd5 | 1.586995 | 2.701483 | 2.144239 |
| ENSMUSG00000086451 | lincRNA | 4933431K23Rik | 24.22655 | 20.64205 | 22.4343 |
| ENSMUSG00000036810 | protein_coding | Tmem188 | 1966.357 | 2056.639 | 2011.498 |
| ENSMUSG00000048665 | pseudogene | A430010J10Rik | 0.65242 | 1.422092 | 1.037256 |
| ENSMUSG00000047216 | protein_coding | Cdh19 | 24.70848 | 30.14913 | 27.42881 |
| ENSMUSG00000090511 | lincRNA | Gm3510 | 2.253504 | 3.399196 | 2.82635 |
| ENSMUSG00000011256 | protein_coding | Adam19 | 366.2899 | 346.5176 | 356.4037 |
| ENSMUSG00000087578 | processed_transcrip | Gm15605 | 14.62236 | 17.95207 | 16.28721 |
| ENSMUSG00000081662 | pseudogene | Gm14098 | 0.646531 | 1.413083 | 1.029807 |
| ENSMUSG00000037824 | protein_coding | Tspan14 | 1703.723 | 1787.626 | 1745.675 |
| ENSMUSG00000038732 | protein_coding | Mboat1 | 55.4312 | 49.71285 | 52.57202 |
| ENSMUSG00000087103 | processed_transcrip | Gm13344 | 0.976841 | 0.335524 | 0.656182 |
| ENSMUSG00000088764 | snoRNA | SNORA17 | 0.976841 | 0.335524 | 0.656182 |
| ENSMUSG00000077428 | snoRNA | SNORA42 | 0.976841 | 0.335524 | 0.656182 |
| ENSMUSG00000038253 | protein_coding | Hoxa5 | 5.821316 | 4.150241 | 4.985779 |
| ENSMUSG00000028031 | protein_coding | Dkk2 | 9.619548 | 12.3001 | 10.95982 |
| ENSMUSG00000025156 | protein_coding | Gps1 | 2511.441 | 2592.914 | 2552.178 |
| ENSMUSG00000020566 | protein_coding | Atp6v1c2 | 55.36858 | 62.26951 | 58.81905 |
| ENSMUSG00000033542 | protein_coding | Arhgef5 | 356.8229 | 377.0326 | 366.9277 |
| ENSMUSG00000018698 | protein_coding | Lhx1 | 113.0913 | 127.0065 | 120.0489 |
| ENSMUSG00000083562 | pseudogene | Gm13234 | 1.931394 | 3.027997 | 2.479696 |
| ENSMUSG00000023277 | protein_coding | Twf2 | 1020.735 | 1062.745 | 1041.74 |
| ENSMUSG00000038642 | protein_coding | Ctss | 235.2355 | 250.1738 | 242.7046 |
| ENSMUSG00000005553 | protein_coding | Atp4a | 118.9925 | 127.9236 | 123.4581 |
| ENSMUSG00000024184 | protein_coding | Pdia2 | 11.39894 | 14.47003 | 12.93448 |
| ENSMUSG00000066043 | protein_coding | Phactr4 | 1863.334 | 1775.744 | 1819.539 |
| ENSMUSG00000091283 | processed_transcrip | Gm17234 | 0 | 0.697713 | 0.348857 |
| ENSMUSG00000069085 | protein_coding | Dytn | 0 | 0.697713 | 0.348857 |
| ENSMUSG00000081488 | pseudogene | Gm15368 | 0 | 0.697713 | 0.348857 |
| ENSMUSG00000040113 | protein_coding | Mettl11b | 0 | 0.697713 | 0.348857 |
| ENSMUSG00000092085 | processed_transcrip | Gm17224 | 0 | 0.697713 | 0.348857 |
| ENSMUSG00000086980 | processed_transcrip | Gm13791 | 0 | 0.697713 | 0.348857 |
| ENSMUSG00000083495 | pseudogene | Gm13877 | 0 | 0.697713 | 0.348857 |
| ENSMUSG00000086764 | processed_transcrip | 4930527A07Rik | 0 | 0.697713 | 0.348857 |
| ENSMUSG00000075978 | snRNA | U1 | 0 | 0.697713 | 0.348857 |
| ENSMUSG00000071015 | protein_coding | Gm136 | 0 | 0.697713 | 0.348857 |
| ENSMUSG00000087378 | processed_transcrip | Gm12414 | 0 | 0.697713 | 0.348857 |
| ENSMUSG00000086940 | processed_transcrip | Gm12746 | 0 | 0.697713 | 0.348857 |
| ENSMUSG00000075390 | pseudogene | Rpl36-ps8 | 0 | 0.697713 | 0.348857 |
| ENSMUSG00000088902 | misc_RNA | 7SK | 0 | 0.697713 | 0.348857 |
| ENSMUSG00000087010 | processed_transcrip | 1700041M05Rik | 0 | 0.697713 | 0.348857 |
| ENSMUSG00000063324 | protein_coding | Gm5299 | 0 | 0.697713 | 0.348857 |
| ENSMUSG00000085771 | processed_transcrip | C230066G23Rik | 0 | 0.697713 | 0.348857 |
| ENSMUSG00000085026 | processed_transcrip | 4930521C21Rik | 0 | 0.697713 | 0.348857 |
| ENSMUSG00000085540 | processed_transcrip | Gm16036 | 0 | 0.697713 | 0.348857 |
| ENSMUSG00000082524 | pseudogene | Gm6272 | 0 | 0.697713 | 0.348857 |

| | | | | | |
|---|---|---|---|---|---|
| ENSMUSG00000085740 | processed_transcrip | 4930505K14Rik | 0 | 0.697713 | 0.348857 |
| ENSMUSG00000076508 | IG_V_gene | Igkv17-127 | 0 | 0.697713 | 0.348857 |
| ENSMUSG00000077311 | snoRNA | SNORA17 | 0 | 0.697713 | 0.348857 |
| ENSMUSG00000090967 | protein_coding | Vmn2r61 | 0 | 0.697713 | 0.348857 |
| ENSMUSG00000093108 | miRNA | AC117644.4 | 0 | 0.697713 | 0.348857 |
| ENSMUSG00000085194 | processed_transcrip | Gm16012 | 0 | 0.697713 | 0.348857 |
| ENSMUSG00000053360 | protein_coding | F730035P03Rik | 0 | 0.697713 | 0.348857 |
| ENSMUSG00000072481 | pseudogene | Gm8556 | 0 | 0.697713 | 0.348857 |
| ENSMUSG00000047286 | protein_coding | Olfr370 | 0 | 0.697713 | 0.348857 |
| ENSMUSG00000084848 | processed_transcrip | 4933408N05Rik | 0 | 0.697713 | 0.348857 |
| ENSMUSG00000031998 | protein_coding | 1700128F08Rik | 0 | 0.697713 | 0.348857 |
| ENSMUSG00000061486 | pseudogene | Gm5161 | 0 | 0.697713 | 0.348857 |
| ENSMUSG00000077973 | miRNA | AC162467.1 | 0 | 0.697713 | 0.348857 |
| ENSMUSG00000065686 | snoRNA | Snora5c | 0 | 0.697713 | 0.348857 |
| ENSMUSG00000020429 | protein_coding | Igfbp1 | 0 | 0.697713 | 0.348857 |
| ENSMUSG00000080978 | pseudogene | Gm12297 | 0 | 0.697713 | 0.348857 |
| ENSMUSG00000082983 | pseudogene | Gm12318 | 0 | 0.697713 | 0.348857 |
| ENSMUSG00000085646 | processed_transcrip | Gm11493 | 0 | 0.697713 | 0.348857 |
| ENSMUSG00000048981 | protein_coding | Krt31 | 0 | 0.697713 | 0.348857 |
| ENSMUSG00000086222 | processed_transcrip | Gm11665 | 0 | 0.697713 | 0.348857 |
| ENSMUSG00000077264 | snoRNA | SNORA17 | 0 | 0.697713 | 0.348857 |
| ENSMUSG00000083348 | pseudogene | Gm11289 | 0 | 0.697713 | 0.348857 |
| ENSMUSG00000091185 | protein_coding | Gm3278 | 0 | 0.697713 | 0.348857 |
| ENSMUSG00000092036 | protein_coding | Gm2244 | 0 | 0.697713 | 0.348857 |
| ENSMUSG00000021789 | protein_coding | Sftpa1 | 0 | 0.697713 | 0.348857 |
| ENSMUSG00000076873 | IG_V_gene | Trdv5 | 0 | 0.697713 | 0.348857 |
| ENSMUSG00000092109 | protein_coding | Gm9376 | 0 | 0.697713 | 0.348857 |
| ENSMUSG00000093012 | misc_RNA | AC122247.1 | 0 | 0.697713 | 0.348857 |
| ENSMUSG00000091373 | pseudogene | Gm8810 | 0 | 0.697713 | 0.348857 |
| ENSMUSG00000037477 | protein_coding | Tbx10 | 0 | 0.697713 | 0.348857 |
| ENSMUSG00000085114 | processed_transcrip | 1600025M17Rik | 0 | 0.697713 | 0.348857 |
| ENSMUSG00000065542 | miRNA | Mir224 | 0 | 0.697713 | 0.348857 |
| ENSMUSG00000082244 | pseudogene | Gm8503 | 0 | 0.697713 | 0.348857 |
| ENSMUSG00000084040 | pseudogene | Gm14763 | 0 | 0.697713 | 0.348857 |
| ENSMUSG00000085314 | lincRNA | Gm14866 | 0 | 0.697713 | 0.348857 |
| ENSMUSG00000084837 | processed_transcrip | 1700108N11Rik | 9.003505 | 6.656053 | 7.829779 |
| ENSMUSG00000087156 | lincRNA | 1110050K14Rik | 89.25821 | 98.02738 | 93.64279 |
| ENSMUSG00000092169 | lincRNA | B930082K07Rik | 11.64368 | 9.298044 | 10.47086 |
| ENSMUSG00000031600 | protein_coding | Vps37a | 3324.141 | 3439.805 | 3381.973 |
| ENSMUSG00000086496 | lincRNA | Gm14204 | 4.532875 | 6.17203 | 5.352453 |
| ENSMUSG00000020840 | protein_coding | Blmh | 2849.836 | 2758.306 | 2804.071 |
| ENSMUSG00000022837 | protein_coding | Iqcb1 | 2323.678 | 2050.966 | 2187.322 |
| ENSMUSG00000074753 | processed_transcrip | Gm14092 | 0.976841 | 0.326514 | 0.651678 |
| ENSMUSG00000072673 | protein_coding | Gm10392 | 0.976841 | 0.326514 | 0.651678 |
| ENSMUSG00000091762 | pseudogene | Gm3191 | 0.976841 | 0.326514 | 0.651678 |
| ENSMUSG00000046957 | protein_coding | Spz1 | 0.976841 | 0.326514 | 0.651678 |
| ENSMUSG00000090385 | pseudogene | Gm7316 | 0.976841 | 0.326514 | 0.651678 |
| ENSMUSG00000059648 | protein_coding | Rnase11 | 0.976841 | 0.326514 | 0.651678 |
| ENSMUSG00000055789 | protein_coding | E330017A01Rik | 0.976841 | 0.326514 | 0.651678 |
| ENSMUSG00000020096 | protein_coding | Tbata | 278.3774 | 262.6045 | 270.4909 |
| ENSMUSG00000080242 | pseudogene | Gm15487 | 916.4276 | 793.0618 | 854.7447 |
| ENSMUSG00000078370 | protein_coding | Gm6316 | 0.974531 | 0.335524 | 0.655027 |
| ENSMUSG00000055795 | protein_coding | Gm5160 | 0.974531 | 0.335524 | 0.655027 |
| ENSMUSG00000017831 | protein_coding | Rab5a | 2626.981 | 2713.748 | 2670.365 |
| ENSMUSG00000091223 | pseudogene | Gm8775 | 14.44636 | 17.6137 | 16.03003 |
| ENSMUSG00000082544 | pseudogene | Gm11667 | 2.259393 | 1.350742 | 1.805067 |
| ENSMUSG00000093075 | miRNA | AL807824.1 | 2.259393 | 1.350742 | 1.805067 |
| ENSMUSG00000028039 | protein_coding | Efna3 | 134.4895 | 124.0446 | 129.2671 |

| | | | | | |
|---|---|---|---|---|---|
| ENSMUSG00000038028 | protein_coding | 9630033F20Rik | 657.3256 | 626.5187 | 641.9221 |
| ENSMUSG00000084827 | lincRNA | B230344G16Rik | 1.623372 | 2.728149 | 2.175761 |
| ENSMUSG00000044708 | protein_coding | Kcnj10 | 55.13981 | 61.06049 | 58.10015 |
| ENSMUSG00000002109 | protein_coding | Ddb2 | 997.7316 | 916.5923 | 957.162 |
| ENSMUSG00000028673 | protein_coding | Fuca1 | 1890.683 | 1823.6 | 1857.142 |
| ENSMUSG00000084904 | processed_transcrip | Gm14827 | 21.88433 | 27.37267 | 24.6285 |
| ENSMUSG00000027800 | protein_coding | Tm4sf1 | 204.2063 | 219.8009 | 212.0036 |
| ENSMUSG00000025997 | protein_coding | Ikzf2 | 159.0319 | 147.6665 | 153.3492 |
| ENSMUSG00000086821 | pseudogene | Gm13377 | 0.974531 | 0.326514 | 0.650522 |
| ENSMUSG00000081504 | pseudogene | Gm12508 | 0.974531 | 0.326514 | 0.650522 |
| ENSMUSG00000090346 | protein_coding | Vmn1r5 | 0.974531 | 0.326514 | 0.650522 |
| ENSMUSG00000064710 | snRNA | U6 | 0.974531 | 0.326514 | 0.650522 |
| ENSMUSG00000062083 | protein_coding | Rpl13a-ps1 | 0.974531 | 0.326514 | 0.650522 |
| ENSMUSG00000083912 | pseudogene | Gm5391 | 0.974531 | 0.326514 | 0.650522 |
| ENSMUSG00000078998 | protein_coding | Bpifa6 | 2.267593 | 3.408205 | 2.837899 |
| ENSMUSG00000001891 | protein_coding | Ugp2 | 2349.838 | 2435.509 | 2392.674 |
| ENSMUSG00000031592 | protein_coding | Pcm1 | 6042.116 | 5846.207 | 5944.161 |
| ENSMUSG00000066621 | protein_coding | Tecpr1 | 760.1446 | 790.888 | 775.5163 |
| ENSMUSG00000024941 | protein_coding | Scyl1 | 1874.898 | 1812.031 | 1843.465 |
| ENSMUSG00000021228 | protein_coding | Acot3 | 12.24794 | 9.817381 | 11.03266 |
| ENSMUSG00000033615 | protein_coding | Cplx1 | 20.44702 | 24.55764 | 22.50233 |
| ENSMUSG00000043747 | protein_coding | 1520401A03Rik | 4.194365 | 2.683465 | 3.438915 |
| ENSMUSG00000024254 | protein_coding | Abcg8 | 4.840896 | 3.434871 | 4.137884 |
| ENSMUSG00000085719 | processed_transcrip | 4933424L21Rik | 0.638331 | 1.377408 | 1.007869 |
| ENSMUSG00000085270 | lincRNA | 9530052E02Rik | 1.931394 | 0.979543 | 1.455468 |
| ENSMUSG00000028109 | protein_coding | Hormad1 | 1677.834 | 1381.342 | 1529.588 |
| ENSMUSG00000055865 | protein_coding | Fam19a3 | 7.720953 | 9.817742 | 8.769348 |
| ENSMUSG00000029763 | protein_coding | Exoc4 | 2582.577 | 2686.423 | 2634.5 |
| ENSMUSG00000027247 | protein_coding | Arhgap1 | 2557.633 | 2649.419 | 2603.526 |
| ENSMUSG00000044320 | protein_coding | 1700001O22Rik | 19.52423 | 16.05211 | 17.78817 |
| ENSMUSG00000047370 | protein_coding | Gm7367 | 167.8977 | 179.8875 | 173.8926 |
| ENSMUSG00000043673 | protein_coding | Kcns3 | 63.97678 | 72.55517 | 68.26597 |
| ENSMUSG00000087324 | processed_transcrip | Gm14818 | 4.15672 | 5.80984 | 4.98328 |
| ENSMUSG00000036422 | protein_coding | Pcdh8 | 9.739192 | 7.549438 | 8.644315 |
| ENSMUSG00000068129 | protein_coding | Cst7 | 0.65242 | 1.395426 | 1.023923 |
| ENSMUSG00000045027 | protein_coding | Prss22 | 0.65242 | 1.395426 | 1.023923 |
| ENSMUSG00000039891 | protein_coding | Txlnb | 119.3056 | 131.0269 | 125.1662 |
| ENSMUSG00000020917 | protein_coding | Acly | 9706.1 | 9371.714 | 9538.907 |
| ENSMUSG00000040712 | protein_coding | Camta2 | 873.3309 | 922.4789 | 897.9049 |
| ENSMUSG00000032265 | protein_coding | Fam46a | 321.8879 | 297.3574 | 309.6227 |
| ENSMUSG00000027470 | protein_coding | Mylk2 | 3.238545 | 2.075121 | 2.656833 |
| ENSMUSG00000021518 | protein_coding | Ptdss1 | 1735.308 | 1790.296 | 1762.802 |
| ENSMUSG00000024870 | protein_coding | Rab1b | 2970.89 | 3078.409 | 3024.65 |
| ENSMUSG00000006542 | protein_coding | Prkag3 | 32.02383 | 27.83867 | 29.93125 |
| ENSMUSG00000086841 | lincRNA | 2410006H16Rik | 474.8535 | 496.0978 | 485.4757 |
| ENSMUSG00000022614 | protein_coding | Lmf2 | 1020.285 | 979.3422 | 999.8136 |
| ENSMUSG00000056508 | protein_coding | 1700001K19Rik | 29.70935 | 25.47992 | 27.59463 |
| ENSMUSG00000039990 | protein_coding | 2700050L05Rik | 2242.904 | 2170.074 | 2206.489 |
| ENSMUSG00000021359 | protein_coding | Tcfap2a | 54.62447 | 48.82666 | 51.72557 |
| ENSMUSG00000050640 | protein_coding | Tmem150c | 40.07152 | 35.12865 | 37.60009 |
| ENSMUSG00000086799 | lincRNA | 4930502E09Rik | 4.851407 | 6.507553 | 5.67948 |
| ENSMUSG00000022805 | protein_coding | 4932425I24Rik | 15.53002 | 12.52207 | 14.02605 |
| ENSMUSG00000046768 | protein_coding | Rhoj | 546.4131 | 571.796 | 559.1046 |
| ENSMUSG00000078696 | pseudogene | Gm12537 | 4.517518 | 3.063673 | 3.790596 |
| ENSMUSG00000060550 | protein_coding | H2-Q7 | 65.70071 | 59.17311 | 62.43691 |
| ENSMUSG00000087281 | processed_transcrip | Gm16015 | 3.272612 | 2.057464 | 2.665038 |
| ENSMUSG00000083116 | pseudogene | Gm13410 | 8.045374 | 6.163021 | 7.104198 |
| ENSMUSG00000028655 | protein_coding | Mfsd2a | 788.4192 | 756.9486 | 772.6839 |

| | | | | | |
|---|---|---|---|---|---|
| ENSMUSG00000054717 | protein_coding | Hmgb2 | 7059.952 | 7323.077 | 7191.515 |
| ENSMUSG00000042567 | protein_coding | Nek10 | 15.18793 | 12.4601 | 13.82401 |
| ENSMUSG00000089829 | processed_transcrip | Gm16565 | 3.882766 | 5.599361 | 4.741063 |
| ENSMUSG00000087322 | processed_transcrip | Gm16075 | 1.643351 | 2.746168 | 2.194759 |
| ENSMUSG00000043421 | protein_coding | 2310016C08Rik | 278.4925 | 262.3479 | 270.4202 |
| ENSMUSG00000038216 | protein_coding | Pnmt | 27.58727 | 23.47651 | 25.53189 |
| ENSMUSG00000092124 | protein_coding | B930094E09Rik | 6.794579 | 5.094471 | 5.944525 |
| ENSMUSG00000024232 | protein_coding | Bambi | 755.504 | 815.4981 | 785.501 |
| ENSMUSG00000082212 | pseudogene | Gm15826 | 0 | 0.688704 | 0.344352 |
| ENSMUSG00000077274 | snoRNA | SNORA26 | 0 | 0.688704 | 0.344352 |
| ENSMUSG00000085319 | processed_transcrip | Gm13330 | 0 | 0.688704 | 0.344352 |
| ENSMUSG00000085025 | processed_transcrip | Gm13715 | 0 | 0.688704 | 0.344352 |
| ENSMUSG00000083688 | pseudogene | Gm13743 | 0 | 0.688704 | 0.344352 |
| ENSMUSG00000087473 | processed_transcrip | Gm13883 | 0 | 0.688704 | 0.344352 |
| ENSMUSG00000083487 | pseudogene | Gm13888 | 0 | 0.688704 | 0.344352 |
| ENSMUSG00000033156 | protein_coding | Cst10 | 0 | 0.688704 | 0.344352 |
| ENSMUSG00000081758 | pseudogene | Gm11460 | 0 | 0.688704 | 0.344352 |
| ENSMUSG00000083436 | pseudogene | Gm11471 | 0 | 0.688704 | 0.344352 |
| ENSMUSG00000068890 | protein_coding | Lce1a2 | 0 | 0.688704 | 0.344352 |
| ENSMUSG00000077561 | snoRNA | SNORA17 | 0 | 0.688704 | 0.344352 |
| ENSMUSG00000083377 | pseudogene | Gm12653 | 0 | 0.688704 | 0.344352 |
| ENSMUSG00000081553 | pseudogene | Gm12699 | 0 | 0.688704 | 0.344352 |
| ENSMUSG00000087807 | miRNA | AL671520.1 | 0 | 0.688704 | 0.344352 |
| ENSMUSG00000082009 | pseudogene | Gm12954 | 0 | 0.688704 | 0.344352 |
| ENSMUSG00000064655 | snoRNA | SNORA55 | 0 | 0.688704 | 0.344352 |
| ENSMUSG00000048686 | protein_coding | Hmgb4 | 0 | 0.688704 | 0.344352 |
| ENSMUSG00000086884 | processed_transcrip | Gm16225 | 0 | 0.688704 | 0.344352 |
| ENSMUSG00000085542 | processed_transcrip | 1700037C06Rik | 0 | 0.688704 | 0.344352 |
| ENSMUSG00000088774 | snoRNA | SNORA48 | 0 | 0.688704 | 0.344352 |
| ENSMUSG00000088137 | snoRNA | snoU13 | 0 | 0.688704 | 0.344352 |
| ENSMUSG00000067101 | protein_coding | 1700010H22Rik | 0 | 0.688704 | 0.344352 |
| ENSMUSG00000029829 | protein_coding | Tmem213 | 0 | 0.688704 | 0.344352 |
| ENSMUSG00000076587 | IG_V_gene | Igkv6-20 | 0 | 0.688704 | 0.344352 |
| ENSMUSG00000088031 | misc_RNA | 7SK | 0 | 0.688704 | 0.344352 |
| ENSMUSG00000087317 | processed_transcrip | 4930466I24Rik | 0 | 0.688704 | 0.344352 |
| ENSMUSG00000081532 | pseudogene | Gm15781 | 0 | 0.688704 | 0.344352 |
| ENSMUSG00000091797 | protein_coding | Vmn2r53 | 0 | 0.688704 | 0.344352 |
| ENSMUSG00000087127 | processed_transcrip | Gm12756 | 0 | 0.688704 | 0.344352 |
| ENSMUSG00000066189 | protein_coding | Cacng3 | 0 | 0.688704 | 0.344352 |
| ENSMUSG00000080726 | pseudogene | Gm15366 | 0 | 0.688704 | 0.344352 |
| ENSMUSG00000051095 | protein_coding | Olfr986 | 0 | 0.688704 | 0.344352 |
| ENSMUSG00000074385 | protein_coding | Gm10684 | 0 | 0.688704 | 0.344352 |
| ENSMUSG00000065107 | rRNA | n-R5s88 | 0 | 0.688704 | 0.344352 |
| ENSMUSG00000075241 | protein_coding | Gm8055 | 0 | 0.688704 | 0.344352 |
| ENSMUSG00000042774 | protein_coding | Olfr1353 | 0 | 0.688704 | 0.344352 |
| ENSMUSG00000068314 | protein_coding | Gm6899 | 0 | 0.688704 | 0.344352 |
| ENSMUSG00000086666 | processed_transcrip | Gm12249 | 0 | 0.688704 | 0.344352 |
| ENSMUSG00000082032 | pseudogene | Gm12271 | 0 | 0.688704 | 0.344352 |
| ENSMUSG00000054146 | protein_coding | Krt15 | 0 | 0.688704 | 0.344352 |
| ENSMUSG00000087265 | processed_transcrip | Gm12349 | 0 | 0.688704 | 0.344352 |
| ENSMUSG00000020624 | processed_transcrip | 4933434M16Rik | 0 | 0.688704 | 0.344352 |
| ENSMUSG00000047828 | protein_coding | A730018C14Rik | 0 | 0.688704 | 0.344352 |
| ENSMUSG00000091222 | protein_coding | Gm17723 | 0 | 0.688704 | 0.344352 |
| ENSMUSG00000004344 | protein_coding | Gpx5 | 0 | 0.688704 | 0.344352 |
| ENSMUSG00000043145 | lincRNA | Gm11292 | 0 | 0.688704 | 0.344352 |
| ENSMUSG00000089973 | pseudogene | Vmn1r190-ps | 0 | 0.688704 | 0.344352 |
| ENSMUSG00000091022 | protein_coding | Gm3115 | 0 | 0.688704 | 0.344352 |
| ENSMUSG00000072728 | protein_coding | Gm16434 | 0 | 0.688704 | 0.344352 |

| | | | | | |
|---|---|---|---|---|---|
| ENSMUSG00000077158 | snoRNA | SNORA17 | 0 | 0.688704 | 0.344352 |
| ENSMUSG00000090616 | lincRNA | Gm17628 | 0 | 0.688704 | 0.344352 |
| ENSMUSG00000091293 | pseudogene | Gm6047 | 0 | 0.688704 | 0.344352 |
| ENSMUSG00000076921 | IG_J_gene | Gm16919 | 0 | 0.688704 | 0.344352 |
| ENSMUSG00000076927 | IG_J_gene | Traj1 | 0 | 0.688704 | 0.344352 |
| ENSMUSG00000091686 | processed_transcrip | 4930471C04Rik | 0 | 0.688704 | 0.344352 |
| ENSMUSG00000022056 | protein_coding | Adam7 | 0 | 0.688704 | 0.344352 |
| ENSMUSG00000044021 | protein_coding | Muc19 | 0 | 0.688704 | 0.344352 |
| ENSMUSG00000048699 | protein_coding | 4732456N10Rik | 0 | 0.688704 | 0.344352 |
| ENSMUSG00000090294 | lincRNA | Gm17298 | 0 | 0.688704 | 0.344352 |
| ENSMUSG00000075829 | snRNA | U1 | 0 | 0.688704 | 0.344352 |
| ENSMUSG00000050102 | protein_coding | Vmn1r235 | 0 | 0.688704 | 0.344352 |
| ENSMUSG00000088342 | rRNA | 5S_rRNA | 0 | 0.688704 | 0.344352 |
| ENSMUSG00000064421 | snRNA | U6 | 0 | 0.688704 | 0.344352 |
| ENSMUSG00000078209 | protein_coding | Gm14374 | 0 | 0.688704 | 0.344352 |
| ENSMUSG00000071772 | protein_coding | Rhox3e | 0 | 0.688704 | 0.344352 |
| ENSMUSG00000082467 | pseudogene | Gm6743 | 0 | 0.688704 | 0.344352 |
| ENSMUSG00000083438 | pseudogene | Gm5402 | 0 | 0.688704 | 0.344352 |
| ENSMUSG00000081793 | pseudogene | 5730416F02Rik | 0 | 0.688704 | 0.344352 |
| ENSMUSG00000025333 | protein_coding | Gpr143 | 0 | 0.688704 | 0.344352 |
| ENSMUSG00000026433 | protein_coding | Rab7l1 | 228.2075 | 214.8856 | 221.5466 |
| ENSMUSG00000075006 | protein_coding | Gm10799 | 0.960442 | 0.36219 | 0.661316 |
| ENSMUSG00000082709 | pseudogene | Gm12362 | 0.960442 | 0.36219 | 0.661316 |
| ENSMUSG00000083100 | pseudogene | Gm6044 | 0.960442 | 0.36219 | 0.661316 |
| ENSMUSG00000021919 | protein_coding | Chat | 0.960442 | 0.36219 | 0.661316 |
| ENSMUSG00000045498 | protein_coding | Pcdhb3 | 29.38113 | 24.57032 | 26.97572 |
| ENSMUSG00000022860 | protein_coding | Chodl | 9.598527 | 13.39247 | 11.4955 |
| ENSMUSG00000040724 | protein_coding | Kcna2 | 6.447869 | 4.776604 | 5.612236 |
| ENSMUSG00000027858 | protein_coding | Tspan2 | 314.1507 | 297.9036 | 306.0272 |
| ENSMUSG00000019977 | protein_coding | Hbs1l | 2074.859 | 2003.788 | 2039.324 |
| ENSMUSG00000088373 | miRNA | Mir1956 | 3.550145 | 2.374969 | 2.962557 |
| ENSMUSG00000026062 | protein_coding | Slc9a2 | 93.66436 | 81.79466 | 87.72951 |
| ENSMUSG00000006356 | protein_coding | Crip2 | 577.7219 | 548.4504 | 563.0862 |
| ENSMUSG00000001855 | protein_coding | Nup214 | 1921.884 | 1828.836 | 1875.36 |
| ENSMUSG00000092994 | rRNA | AC113124.1 | 2.603792 | 1.448758 | 2.026275 |
| ENSMUSG00000085408 | lincRNA | C530005A16Rik | 28.32356 | 24.37677 | 26.35017 |
| ENSMUSG00000084666 | misc_RNA | 7SK | 1.581105 | 0.688704 | 1.134905 |
| ENSMUSG00000082265 | pseudogene | Gm15547 | 8.812817 | 6.578904 | 7.695861 |
| ENSMUSG00000063388 | protein_coding | BC023105 | 6.472469 | 4.750299 | 5.611384 |
| ENSMUSG00000054150 | protein_coding | 4831426I19Rik | 819.0041 | 859.9313 | 839.4677 |
| ENSMUSG00000067719 | pseudogene | Gm10221 | 243.5602 | 228.9538 | 236.257 |
| ENSMUSG00000092344 | pseudogene | RP23-171F16.3 | 1.635151 | 2.728149 | 2.18165 |
| ENSMUSG00000021276 | protein_coding | Cinp | 1278.857 | 1233.033 | 1255.945 |
| ENSMUSG00000032604 | protein_coding | Qars | 3494.107 | 3596.681 | 3545.394 |
| ENSMUSG00000039456 | protein_coding | Morc3 | 2676.343 | 2580.941 | 2628.642 |
| ENSMUSG00000030336 | protein_coding | Cd27 | 26.17203 | 29.95776 | 28.06489 |
| ENSMUSG00000048148 | protein_coding | Nwd1 | 29.92926 | 35.01396 | 32.47161 |
| ENSMUSG00000052471 | protein_coding | Gm9881 | 3.544256 | 2.383978 | 2.964117 |
| ENSMUSG00000030672 | protein_coding | Mylpf | 59.02592 | 65.01827 | 62.0221 |
| ENSMUSG00000085876 | lincRNA | Gm12409 | 54.37005 | 60.64641 | 57.50823 |
| ENSMUSG00000039376 | protein_coding | Synpo2l | 22.13033 | 26.27637 | 24.20335 |
| ENSMUSG00000093154 | snRNA | AC126942.1 | 4.820918 | 6.60557 | 5.713244 |
| ENSMUSG00000026662 | protein_coding | Sephs1 | 1753.124 | 1847.659 | 1800.391 |
| ENSMUSG00000021285 | protein_coding | Ppp1r13b | 1711.146 | 1613.063 | 1662.105 |
| ENSMUSG00000093119 | miRNA | AC122463.1 | 1.953683 | 0.997561 | 1.475622 |
| ENSMUSG00000051674 | protein_coding | Dcun1d4 | 815.7113 | 876.462 | 846.0867 |
| ENSMUSG00000090030 | lincRNA | A430072P03Rik | 1.276663 | 2.35695 | 1.816807 |
| ENSMUSG00000029387 | protein_coding | Gtf2h3 | 1815.451 | 1878.133 | 1846.792 |

| | | | | | |
|---|---|---|---|---|---|
| ENSMUSG00000025580 | protein_coding | Eif4a3 | 3294.778 | 3419.441 | 3357.109 |
| ENSMUSG00000020368 | protein_coding | Canx | 9096.793 | 9363.75 | 9230.272 |
| ENSMUSG00000088113 | miRNA | Mir1960 | 0.962752 | 0.335524 | 0.649138 |
| ENSMUSG00000082630 | pseudogene | Gm14834 | 0.962752 | 0.335524 | 0.649138 |
| ENSMUSG00000021998 | protein_coding | Lcp1 | 383.4113 | 365.062 | 374.2367 |
| ENSMUSG00000024387 | protein_coding | Csnk2b | 2024.154 | 2100.645 | 2062.4 |
| ENSMUSG00000050368 | protein_coding | Hoxd10 | 203.581 | 191.3813 | 197.4812 |
| ENSMUSG00000030873 | protein_coding | Scnn1b | 34.89234 | 39.69943 | 37.29589 |
| ENSMUSG00000082283 | pseudogene | Gm14472 | 10.34472 | 8.12283 | 9.233777 |
| ENSMUSG00000077714 | snoRNA | Snord17 | 8.047685 | 10.23326 | 9.140474 |
| ENSMUSG00000050471 | protein_coding | Fam118b | 1138.585 | 1099.535 | 1119.06 |
| ENSMUSG00000028668 | protein_coding | Tceb3 | 2078.924 | 2005.981 | 2042.453 |
| ENSMUSG00000033186 | protein_coding | Mzt1 | 1629.933 | 1687.539 | 1658.736 |
| ENSMUSG00000042043 | protein_coding | Tbca | 1254.054 | 1304.633 | 1279.343 |
| ENSMUSG00000058446 | protein_coding | Znrf2 | 1893.742 | 1961.198 | 1927.47 |
| ENSMUSG00000086583 | processed_transcrip | Gm15500 | 948.2462 | 906.2798 | 927.263 |
| ENSMUSG00000073889 | protein_coding | Il11ra1 | 603.1015 | 631.629 | 617.3652 |
| ENSMUSG00000074247 | protein_coding | Dda1 | 1237.772 | 1191.818 | 1214.795 |
| ENSMUSG00000080916 | pseudogene | Gm12463 | 6.452491 | 4.821288 | 5.636889 |
| ENSMUSG00000069911 | protein_coding | Fam196b | 2.279371 | 3.877059 | 3.078215 |
| ENSMUSG00000032812 | protein_coding | Arap1 | 2044.699 | 2139.134 | 2091.917 |
| ENSMUSG00000039092 | protein_coding | Sptlc3 | 0.962752 | 0.326514 | 0.644633 |
| ENSMUSG00000083121 | pseudogene | Gm6341 | 0.962752 | 0.326514 | 0.644633 |
| ENSMUSG00000085301 | processed_transcrip | Gm12132 | 0.962752 | 0.326514 | 0.644633 |
| ENSMUSG00000046345 | protein_coding | 2310010M20Rik | 0.962752 | 0.326514 | 0.644633 |
| ENSMUSG00000081892 | pseudogene | Gm8864 | 0.962752 | 0.326514 | 0.644633 |
| ENSMUSG00000067650 | protein_coding | Gm5072 | 0.962752 | 0.326514 | 0.644633 |
| ENSMUSG00000048490 | protein_coding | Nrip1 | 2033.742 | 2118.377 | 2076.06 |
| ENSMUSG00000078639 | protein_coding | Gm12695 | 0.960442 | 0.335524 | 0.647983 |
| ENSMUSG00000086655 | lincRNA | C330018A13Rik | 0.960442 | 0.335524 | 0.647983 |
| ENSMUSG00000089248 | misc_RNA | 7SK | 0.960442 | 0.335524 | 0.647983 |
| ENSMUSG00000077798 | snoRNA | SNORA17 | 0.960442 | 0.335524 | 0.647983 |
| ENSMUSG00000087966 | snRNA | U6 | 0.960442 | 0.335524 | 0.647983 |
| ENSMUSG00000020159 | protein_coding | Gabrp | 0.960442 | 0.335524 | 0.647983 |
| ENSMUSG00000081238 | pseudogene | Gm15836 | 0.960442 | 0.335524 | 0.647983 |
| ENSMUSG00000004842 | protein_coding | Pou1f1 | 0.960442 | 0.335524 | 0.647983 |
| ENSMUSG00000077795 | snoRNA | SNORA17 | 0.954552 | 0.36219 | 0.658371 |
| ENSMUSG00000064689 | snRNA | U6 | 0.954552 | 0.36219 | 0.658371 |
| ENSMUSG00000045620 | protein_coding | Odf3l1 | 0.954552 | 0.36219 | 0.658371 |
| ENSMUSG00000068506 | protein_coding | Gm5800 | 0.954552 | 0.36219 | 0.658371 |
| ENSMUSG00000092458 | pseudogene | Gm18737 | 0.954552 | 0.36219 | 0.658371 |
| ENSMUSG00000039671 | protein_coding | Zmynd8 | 1790.899 | 1854.991 | 1822.945 |
| ENSMUSG00000081887 | pseudogene | Gm12097 | 1.934972 | 3.055025 | 2.494999 |
| ENSMUSG00000026725 | protein_coding | Tnn | 8.427419 | 6.454221 | 7.44082 |
| ENSMUSG00000016510 | protein_coding | Mtif3 | 411.2817 | 392.3902 | 401.836 |
| ENSMUSG00000078787 | protein_coding | Cyp2t4 | 21.95202 | 18.53991 | 20.24596 |
| ENSMUSG00000079478 | protein_coding | Sssca1 | 421.6332 | 440.3826 | 431.0079 |
| ENSMUSG00000087674 | processed_transcrip | 4930447M23Rik | 0.968641 | 1.748606 | 1.358624 |
| ENSMUSG00000031217 | protein_coding | Efnb1 | 2167.652 | 2248.634 | 2208.143 |
| ENSMUSG00000022313 | protein_coding | Utp23 | 381.5614 | 407.6334 | 394.5974 |
| ENSMUSG00000087360 | processed_transcrip | Gm15104 | 3.544256 | 5.005101 | 4.274679 |
| ENSMUSG00000029553 | protein_coding | Tcfec | 16.42695 | 13.51715 | 14.97205 |
| ENSMUSG00000076677 | IG_V_gene | Gm16930 | 0.960442 | 0.326514 | 0.643478 |
| ENSMUSG00000024306 | protein_coding | 4921528I01Rik | 0.960442 | 0.326514 | 0.643478 |
| ENSMUSG00000084824 | processed_transcrip | Gm16344 | 0.960442 | 0.326514 | 0.643478 |
| ENSMUSG00000025432 | protein_coding | Avil | 21.36647 | 24.8986 | 23.13253 |
| ENSMUSG00000040234 | protein_coding | Tm7sf3 | 2109.361 | 2181.741 | 2145.551 |
| ENSMUSG00000084446 | rRNA | n-R5s207 | 0.632442 | 1.350742 | 0.991592 |

| | | | | | |
|---|---|---|---|---|---|
| ENSMUSG00000066487 | pseudogene | Gm5786 | 3.239813 | 4.705253 | 3.972533 |
| ENSMUSG00000035275 | protein_coding | Raver2 | 797.1797 | 837.5791 | 817.3794 |
| ENSMUSG00000022678 | protein_coding | Nde1 | 1249.593 | 1324.905 | 1287.249 |
| ENSMUSG00000073403 | processed_transcrip | Gm10499 | 4.887785 | 3.390187 | 4.138986 |
| ENSMUSG00000043122 | protein_coding | A530016L24Rik | 5.475874 | 9.003271 | 7.239572 |
| ENSMUSG00000034457 | protein_coding | Eda2r | 128.6974 | 140.001 | 134.3492 |
| ENSMUSG00000025024 | protein_coding | Smndc1 | 1796.517 | 1858.483 | 1827.5 |
| ENSMUSG00000087695 | processed_transcrip | Gm16291 | 3.272612 | 2.075121 | 2.673866 |
| ENSMUSG00000013483 | protein_coding | Card14 | 61.31626 | 55.44777 | 58.38201 |
| ENSMUSG00000026064 | protein_coding | Ptp4a1 | 1379.473 | 1430.785 | 1405.129 |
| ENSMUSG00000037149 | protein_coding | Ddx1 | 4504.28 | 4632.215 | 4568.248 |
| ENSMUSG00000003452 | protein_coding | Bicd1 | 350.3115 | 329.9051 | 340.1083 |
| ENSMUSG00000084763 | lincRNA | Gm5106 | 0.65242 | 1.377408 | 1.014914 |
| ENSMUSG00000038637 | protein_coding | Lrrc56 | 249.5614 | 233.895 | 241.7282 |
| ENSMUSG00000041700 | protein_coding | Lhfpl1 | 4.823229 | 6.614579 | 5.718904 |
| ENSMUSG00000071335 | protein_coding | G630090E17Rik | 3.874566 | 5.457022 | 4.665794 |
| ENSMUSG00000026463 | protein_coding | Atp2b4 | 3862.424 | 3718.779 | 3790.602 |
| ENSMUSG00000032202 | protein_coding | Rab27a | 390.2734 | 416.6387 | 403.4561 |
| ENSMUSG00000075289 | protein_coding | Carns1 | 185.6922 | 170.8956 | 178.2939 |
| ENSMUSG00000024457 | protein_coding | Trim26 | 4813.005 | 4984.915 | 4898.96 |
| ENSMUSG00000006850 | protein_coding | Tmco6 | 355.4027 | 338.4058 | 346.9043 |
| ENSMUSG00000054160 | protein_coding | Nkx2-4 | 8.426151 | 10.97494 | 9.700545 |
| ENSMUSG00000091262 | protein_coding | D430020J02Rik | 15.81448 | 19.21308 | 17.51378 |
| ENSMUSG00000085887 | processed_transcrip | Gm11648 | 3.239813 | 2.08413 | 2.661971 |
| ENSMUSG00000005220 | protein_coding | Corin | 228.1555 | 184.5518 | 206.3537 |
| ENSMUSG00000026344 | protein_coding | Lypd1 | 2.253504 | 3.37253 | 2.813017 |
| ENSMUSG00000092536 | processed_transcrip | RP23-118G3.2 | 8.393352 | 6.489535 | 7.441443 |
| ENSMUSG00000015341 | protein_coding | Golga7 | 2883.138 | 2967.79 | 2925.464 |
| ENSMUSG00000084957 | lincRNA | Bbip1 | 800.1694 | 768.602 | 784.3857 |
| ENSMUSG00000081850 | pseudogene | Gm15667 | 8.129908 | 10.86248 | 9.496192 |
| ENSMUSG00000021057 | protein_coding | Akap5 | 281.0206 | 264 | 272.5103 |
| ENSMUSG00000045983 | protein_coding | Eif4g1 | 10330.93 | 9991.629 | 10161.28 |
| ENSMUSG00000069372 | protein_coding | Ctxn3 | 10.37879 | 8.140486 | 9.259639 |
| ENSMUSG00000075609 | protein_coding | Gm7935 | 5.173517 | 6.896409 | 6.034963 |
| ENSMUSG00000067058 | pseudogene | Gm10196 | 3.538366 | 2.383978 | 2.961172 |
| ENSMUSG00000065362 | snoRNA | SNORA71 | 17.55519 | 14.36621 | 15.9607 |
| ENSMUSG00000082461 | pseudogene | Gm8844 | 0.66062 | 1.386417 | 1.023519 |
| ENSMUSG00000028777 | protein_coding | Gnat3 | 0.968641 | 1.72194 | 1.345291 |
| ENSMUSG00000043282 | protein_coding | Gm5531 | 0.954552 | 0.335524 | 0.645038 |
| ENSMUSG00000064906 | rRNA | n-R5s204 | 0.954552 | 0.335524 | 0.645038 |
| ENSMUSG00000083076 | pseudogene | Gm11408 | 0.954552 | 0.335524 | 0.645038 |
| ENSMUSG00000089504 | snRNA | U7 | 0.954552 | 0.335524 | 0.645038 |
| ENSMUSG00000035242 | protein_coding | Oaz1 | 7424.286 | 7650.987 | 7537.636 |
| ENSMUSG00000087164 | lincRNA | 4933433P16Rik | 27.35134 | 23.62894 | 25.49014 |
| ENSMUSG00000072946 | protein_coding | Ptgr2 | 1385.919 | 1336.155 | 1361.037 |
| ENSMUSG00000069385 | protein_coding | Gm10267 | 1.920883 | 3.135023 | 2.527953 |
| ENSMUSG00000078887 | protein_coding | Gm6710 | 10.04259 | 12.46553 | 11.25406 |
| ENSMUSG00000033697 | protein_coding | Arhgap39 | 1518.625 | 1578.696 | 1548.661 |
| ENSMUSG00000031393 | protein_coding | Mecp2 | 1779.967 | 1715.404 | 1747.686 |
| ENSMUSG00000040734 | protein_coding | Ppp1r13l | 331.0756 | 349.25 | 340.1628 |
| ENSMUSG00000011034 | protein_coding | Slc5a1 | 11.29339 | 8.873151 | 10.08327 |
| ENSMUSG00000086189 | processed_transcrip | Gm15462 | 2.245304 | 3.488204 | 2.866754 |
| ENSMUSG00000004558 | protein_coding | Ndrg2 | 1039.474 | 1000.545 | 1020.01 |
| ENSMUSG00000035202 | protein_coding | Lars2 | 526.8961 | 506.2661 | 516.5811 |
| ENSMUSG00000026495 | protein_coding | Efcab2 | 370.659 | 340.7727 | 355.7159 |
| ENSMUSG00000031604 | protein_coding | Sc4mol | 1923.306 | 1817.466 | 1870.386 |
| ENSMUSG00000042962 | protein_coding | Gm5436 | 37.70426 | 33.39227 | 35.54826 |
| ENSMUSG00000071267 | protein_coding | Zfp942 | 1126.084 | 1171.358 | 1148.721 |

| | | | | | |
|---|---|---|---|---|---|
| ENSMUSG00000025044 | protein_coding | Msr1 | 55.05848 | 61.97111 | 58.51479 |
| ENSMUSG00000001555 | protein_coding | Fkbp10 | 1251.662 | 1204.172 | 1227.917 |
| ENSMUSG00000043140 | protein_coding | Tmem186 | 813.5622 | 780.0327 | 796.7974 |
| ENSMUSG00000059039 | protein_coding | Gm5900 | 26.40126 | 18.99137 | 22.69631 |
| ENSMUSG00000029145 | protein_coding | Eif2b4 | 1164.854 | 1214.522 | 1189.688 |
| ENSMUSG00000046993 | pseudogene | Gm5637 | 85.59916 | 78.23448 | 81.91682 |
| ENSMUSG00000056629 | protein_coding | Fkbp2 | 762.535 | 793.2762 | 777.9056 |
| ENSMUSG00000081093 | pseudogene | Gm12565 | 0.954552 | 0.326514 | 0.640533 |
| ENSMUSG00000089890 | processed_transcrip | Gm16055 | 0.954552 | 0.326514 | 0.640533 |
| ENSMUSG00000024803 | protein_coding | Ankrd1 | 0.954552 | 0.326514 | 0.640533 |
| ENSMUSG00000084331 | pseudogene | Gm8163 | 0.954552 | 0.326514 | 0.640533 |
| ENSMUSG00000040048 | protein_coding | Ndufb10 | 1087.95 | 1051.555 | 1069.752 |
| ENSMUSG00000091588 | lincRNA | Gm17279 | 4.521097 | 6.234733 | 5.377915 |
| ENSMUSG00000073439 | protein_coding | Gm10506 | 0.966331 | 1.986113 | 1.476222 |
| ENSMUSG00000028751 | protein_coding | Pla2g2e | 1.609283 | 2.692474 | 2.150879 |
| ENSMUSG00000065273 | snoRNA | SNORD83 | 2.261704 | 1.359751 | 1.810727 |
| ENSMUSG00000027301 | protein_coding | Oxt | 2.239415 | 3.417215 | 2.828315 |
| ENSMUSG00000083410 | pseudogene | Gm14300 | 0.64422 | 1.359751 | 1.001986 |
| ENSMUSG00000032343 | protein_coding | Impg1 | 0.64422 | 1.359751 | 1.001986 |
| ENSMUSG00000091793 | lincRNA | Rian | 2039.917 | 2158.882 | 2099.399 |
| ENSMUSG00000024735 | protein_coding | Prpf19 | 4917.899 | 5060.76 | 4989.33 |
| ENSMUSG00000015697 | protein_coding | Setdb1 | 4430.132 | 4663.128 | 4546.63 |
| ENSMUSG00000045598 | protein_coding | Zfp553 | 1286.589 | 1344.126 | 1315.357 |
| ENSMUSG00000004933 | protein_coding | Matk | 99.174 | 91.11305 | 95.14353 |
| ENSMUSG00000026180 | protein_coding | Cxcr2 | 0 | 0.671047 | 0.335524 |
| ENSMUSG00000026272 | protein_coding | Agxt | 0 | 0.671047 | 0.335524 |
| ENSMUSG00000088779 | snRNA | U6 | 0 | 0.671047 | 0.335524 |
| ENSMUSG00000075316 | protein_coding | Scn9a | 0 | 0.671047 | 0.335524 |
| ENSMUSG00000017003 | protein_coding | Svs3a | 0 | 0.671047 | 0.335524 |
| ENSMUSG00000027761 | protein_coding | Aadac | 0 | 0.671047 | 0.335524 |
| ENSMUSG00000091315 | pseudogene | Gm17214 | 0 | 0.671047 | 0.335524 |
| ENSMUSG00000078655 | protein_coding | Gm10972 | 0 | 0.671047 | 0.335524 |
| ENSMUSG00000074344 | protein_coding | Adora3 | 0 | 0.671047 | 0.335524 |
| ENSMUSG00000084787 | processed_transcrip | Gm12436 | 0 | 0.671047 | 0.335524 |
| ENSMUSG00000083359 | pseudogene | Gm12656 | 0 | 0.671047 | 0.335524 |
| ENSMUSG00000078504 | protein_coding | Gm438 | 0 | 0.671047 | 0.335524 |
| ENSMUSG00000086766 | processed_transcrip | Gm15969 | 0 | 0.671047 | 0.335524 |
| ENSMUSG00000080335 | snoRNA | SNORD93 | 0 | 0.671047 | 0.335524 |
| ENSMUSG00000065329 | snRNA | U1 | 0 | 0.671047 | 0.335524 |
| ENSMUSG00000053873 | protein_coding | Aym1 | 0 | 0.671047 | 0.335524 |
| ENSMUSG00000083728 | pseudogene | Gm13854 | 0 | 0.671047 | 0.335524 |
| ENSMUSG00000076608 | IG_J_gene | Igkj5 | 0 | 0.671047 | 0.335524 |
| ENSMUSG00000065187 | snoRNA | SNORA36 | 0 | 0.671047 | 0.335524 |
| ENSMUSG00000060407 | protein_coding | Cyp2a12 | 0 | 0.671047 | 0.335524 |
| ENSMUSG00000088629 | misc_RNA | 7SK | 0 | 0.671047 | 0.335524 |
| ENSMUSG00000057074 | protein_coding | Ces1g | 0 | 0.671047 | 0.335524 |
| ENSMUSG00000077125 | miRNA | AC164603.1 | 0 | 0.671047 | 0.335524 |
| ENSMUSG00000078107 | pseudogene | Gm4478 | 0 | 0.671047 | 0.335524 |
| ENSMUSG00000046974 | protein_coding | BC053393 | 0 | 0.671047 | 0.335524 |
| ENSMUSG00000092378 | pseudogene | RP23-71I22.3 | 0 | 0.671047 | 0.335524 |
| ENSMUSG00000071342 | protein_coding | Gm889 | 0 | 0.671047 | 0.335524 |
| ENSMUSG00000076698 | IG_V_gene | Gm16860 | 0 | 0.671047 | 0.335524 |
| ENSMUSG00000086531 | processed_transcrip | Gm11351 | 0 | 0.671047 | 0.335524 |
| ENSMUSG00000087460 | processed_transcrip | 4930401O12Rik | 0 | 0.671047 | 0.335524 |
| ENSMUSG00000076913 | IG_J_gene | Gm16656 | 0 | 0.671047 | 0.335524 |
| ENSMUSG00000045655 | protein_coding | AU021034 | 0 | 0.671047 | 0.335524 |
| ENSMUSG00000058869 | pseudogene | Gm4832 | 0 | 0.671047 | 0.335524 |
| ENSMUSG00000056632 | protein_coding | Dsg3 | 0 | 0.671047 | 0.335524 |

| | | | | | |
|---|---|---|---|---|---|
| ENSMUSG00000065922 | rRNA | n-R5-8s1 | 0 | 0.671047 | 0.335524 |
| ENSMUSG00000077199 | snoRNA | SNORA17 | 0 | 0.671047 | 0.335524 |
| ENSMUSG00000086907 | processed_transcrip | Gm15298 | 0 | 0.671047 | 0.335524 |
| ENSMUSG00000087307 | processed_transcrip | Gm12925 | 22.28464 | 19.10678 | 20.69571 |
| ENSMUSG00000027783 | pseudogene | Arl14 | 7.034466 | 9.384564 | 8.209515 |
| ENSMUSG00000090066 | protein_coding | 1110002E22Rik | 4.82554 | 3.363521 | 4.09453 |
| ENSMUSG00000020607 | protein_coding | Fam84a | 2526.969 | 2393.085 | 2460.027 |
| ENSMUSG00000026094 | protein_coding | Stk17b | 1217.814 | 1139.067 | 1178.441 |
| ENSMUSG00000039954 | protein_coding | Stk32a | 37.2423 | 43.14984 | 40.19607 |
| ENSMUSG00000049305 | protein_coding | Ccdc71 | 1662.278 | 1600.281 | 1631.28 |
| ENSMUSG00000074046 | protein_coding | Gm16378 | 24.14164 | 19.67116 | 21.9064 |
| ENSMUSG00000007721 | protein_coding | Ccdc124 | 903.6066 | 936.4262 | 920.0164 |
| ENSMUSG00000038365 | protein_coding | Fbxo25 | 795.9944 | 764.7763 | 780.3853 |
| ENSMUSG00000014551 | protein_coding | Mrps25 | 781.4347 | 751.5923 | 766.5135 |
| ENSMUSG00000089199 | snRNA | U6 | 13.93587 | 16.90118 | 15.41852 |
| ENSMUSG00000047414 | protein_coding | Flrt2 | 871.1264 | 924.9074 | 898.0169 |
| ENSMUSG00000024754 | protein_coding | Tmem2 | 1044.137 | 991.4975 | 1017.817 |
| ENSMUSG00000064543 | snRNA | U1 | 2.628392 | 1.422092 | 2.025242 |
| ENSMUSG00000029816 | protein_coding | Gpnmb | 11.3121 | 8.811895 | 10.062 |
| ENSMUSG00000033706 | protein_coding | Smyd5 | 1149.384 | 1197.983 | 1173.684 |
| ENSMUSG00000061461 | protein_coding | 1810013D10Rik | 848.9128 | 889.7137 | 869.3133 |
| ENSMUSG00000087030 | processed_transcrip | Gm16143 | 1.925505 | 3.099348 | 2.512426 |
| ENSMUSG00000079628 | protein_coding | Rhox4g | 3.233924 | 4.66994 | 3.951932 |
| ENSMUSG00000047832 | protein_coding | Cdca4 | 1507.899 | 1572.47 | 1540.185 |
| ENSMUSG00000027109 | protein_coding | Sp3 | 3485.342 | 3357.558 | 3421.45 |
| ENSMUSG00000002588 | protein_coding | Pon1 | 1.967772 | 0.997561 | 1.482666 |
| ENSMUSG00000002846 | protein_coding | 4930455C21Rik | 962.0501 | 921.7762 | 941.9132 |
| ENSMUSG00000078779 | protein_coding | Zfp59 | 395.75 | 369.9799 | 382.8649 |
| ENSMUSG00000047139 | protein_coding | Cd24a | 792.0293 | 827.0342 | 809.5317 |
| ENSMUSG00000081262 | pseudogene | Gm12261 | 3.544256 | 2.410644 | 2.97745 |
| ENSMUSG00000031921 | protein_coding | Terf2 | 1243.968 | 1196.015 | 1219.991 |
| ENSMUSG00000014599 | protein_coding | Csf1 | 419.5798 | 398.9312 | 409.2555 |
| ENSMUSG00000083337 | pseudogene | Gm11539 | 16.74906 | 19.9984 | 18.37373 |
| ENSMUSG00000040528 | protein_coding | Gm885 | 12.61463 | 15.49461 | 14.05462 |
| ENSMUSG00000089389 | snoRNA | SCARNA20 | 4.815029 | 6.561247 | 5.688138 |
| ENSMUSG00000022668 | protein_coding | Gtpbp8 | 725.3749 | 759.282 | 742.3285 |
| ENSMUSG00000029575 | protein_coding | Mmab | 695.414 | 662.0701 | 678.742 |
| ENSMUSG00000045948 | protein_coding | Mrps12 | 744.2982 | 773.9979 | 759.148 |
| ENSMUSG00000047180 | protein_coding | Neurl3 | 29.42131 | 25.68568 | 27.5535 |
| ENSMUSG00000044317 | protein_coding | Gpr4 | 41.1777 | 46.63944 | 43.90857 |
| ENSMUSG00000070443 | protein_coding | Gm10291 | 2.275793 | 3.443881 | 2.859837 |
| ENSMUSG00000021775 | protein_coding | Nr1d2 | 3198.377 | 3089.646 | 3144.012 |
| ENSMUSG00000027107 | protein_coding | Chrna1 | 2.583814 | 3.832736 | 3.208275 |
| ENSMUSG00000055216 | pseudogene | 9430025C20Rik | 2.583814 | 3.832736 | 3.208275 |
| ENSMUSG00000063543 | protein_coding | Gm5616 | 7.780888 | 9.933416 | 8.857152 |
| ENSMUSG00000090200 | processed_transcrip | 1700025N21Rik | 1.65155 | 0.653029 | 1.152289 |
| ENSMUSG00000024660 | protein_coding | Incenp | 2312.518 | 2211.005 | 2261.761 |
| ENSMUSG00000073805 | protein_coding | Fam196a | 102.0582 | 93.35101 | 97.70463 |
| ENSMUSG00000029455 | protein_coding | Aldh2 | 5898.869 | 6097.328 | 5998.099 |
| ENSMUSG00000072432 | protein_coding | Gm10358 | 0.64422 | 1.350742 | 0.997481 |
| ENSMUSG00000082564 | pseudogene | Gm15586 | 0.64422 | 1.350742 | 0.997481 |
| ENSMUSG00000082464 | pseudogene | Rab9b-ps1 | 0.64422 | 1.350742 | 0.997481 |
| ENSMUSG00000078905 | protein_coding | Gm14393 | 7.074422 | 9.236426 | 8.155424 |
| ENSMUSG00000030664 | protein_coding | 1110006G14Rik | 5.820048 | 7.679919 | 6.749983 |
| ENSMUSG00000058240 | protein_coding | Cryzl1 | 715.6336 | 752.4668 | 734.0502 |
| ENSMUSG00000081777 | pseudogene | Gm14159 | 2.267593 | 1.324076 | 1.795834 |
| ENSMUSG00000029088 | protein_coding | Kcnip4 | 65.89706 | 72.20769 | 69.05237 |
| ENSMUSG00000074236 | pseudogene | Gm9387 | 13.86184 | 11.23046 | 12.54615 |

| | | | | | |
|---|---|---|---|---|---|
| ENSMUSG00000087660 | processed_transcrip | B230398E01Rik | 3.572434 | 2.410644 | 2.991539 |
| ENSMUSG00000032113 | protein_coding | Chek1 | 1201.115 | 1158.336 | 1179.725 |
| ENSMUSG00000029781 | protein_coding | Fkbp9 | 6350.708 | 6149.449 | 6250.078 |
| ENSMUSG00000082336 | pseudogene | Gm8401 | 6.113981 | 4.512431 | 5.313206 |
| ENSMUSG00000085156 | lincRNA | Gm11974 | 100.4555 | 92.25584 | 96.35568 |
| ENSMUSG00000031478 | protein_coding | Nek3 | 307.0234 | 284.0884 | 295.5559 |
| ENSMUSG00000087790 | snoRNA | SNORD127 | 4.518786 | 6.101041 | 5.309914 |
| ENSMUSG00000021469 | protein_coding | Msx2 | 24.49946 | 28.31296 | 26.40621 |
| ENSMUSG00000031312 | protein_coding | Itgb1bp2 | 13.01307 | 10.31326 | 11.66317 |
| ENSMUSG00000027447 | protein_coding | Cst3 | 5452.279 | 5285.306 | 5368.793 |
| ENSMUSG00000041544 | protein_coding | Ptchd2 | 210.8707 | 192.3263 | 201.5985 |
| ENSMUSG00000048483 | protein_coding | Zdhhc22 | 0.646531 | 1.36876 | 1.007646 |
| ENSMUSG00000002997 | protein_coding | Prkar2b | 4355.511 | 4663.785 | 4509.648 |
| ENSMUSG00000029705 | protein_coding | Cux1 | 1755.994 | 1840.494 | 1798.244 |
| ENSMUSG00000032360 | protein_coding | Hcrtr2 | 3.556034 | 2.383978 | 2.970006 |
| ENSMUSG00000065429 | miRNA | Mir345 | 3.521967 | 5.041138 | 4.281553 |
| ENSMUSG00000024014 | protein_coding | Pim1 | 1632.746 | 1544.706 | 1588.726 |
| ENSMUSG00000031706 | protein_coding | Rfx1 | 710.6467 | 740.5567 | 725.6017 |
| ENSMUSG00000000420 | protein_coding | Galnt1 | 7929.657 | 8227.876 | 8078.767 |
| ENSMUSG00000087374 | processed_transcrip | Gm15457 | 0 | 0.662038 | 0.331019 |
| ENSMUSG00000082745 | pseudogene | Gm15181 | 0 | 0.662038 | 0.331019 |
| ENSMUSG00000067001 | protein_coding | Serpinb7 | 0 | 0.662038 | 0.331019 |
| ENSMUSG00000025716 | protein_coding | Myo3a | 0 | 0.662038 | 0.331019 |
| ENSMUSG00000087196 | processed_transcrip | Gm13373 | 0 | 0.662038 | 0.331019 |
| ENSMUSG00000064652 | snoRNA | SNORA31 | 0 | 0.662038 | 0.331019 |
| ENSMUSG00000082132 | pseudogene | Gm13541 | 0 | 0.662038 | 0.331019 |
| ENSMUSG00000082330 | pseudogene | Ldha-ps | 0 | 0.662038 | 0.331019 |
| ENSMUSG00000081801 | pseudogene | Dnmt3l-ps1 | 0 | 0.662038 | 0.331019 |
| ENSMUSG00000086957 | processed_transcrip | Gm13869 | 0 | 0.662038 | 0.331019 |
| ENSMUSG00000065083 | rRNA | n-R5s205 | 0 | 0.662038 | 0.331019 |
| ENSMUSG00000083630 | pseudogene | Gm15602 | 0 | 0.662038 | 0.331019 |
| ENSMUSG00000028009 | protein_coding | 1700061I17Rik | 0 | 0.662038 | 0.331019 |
| ENSMUSG00000082978 | pseudogene | Rpsa-ps11 | 0 | 0.662038 | 0.331019 |
| ENSMUSG00000092698 | miRNA | AC022236.1 | 0 | 0.662038 | 0.331019 |
| ENSMUSG00000053129 | protein_coding | Gsx1 | 0 | 0.662038 | 0.331019 |
| ENSMUSG00000083812 | pseudogene | Gm5054 | 0 | 0.662038 | 0.331019 |
| ENSMUSG00000085745 | processed_transcrip | Gm14372 | 0 | 0.662038 | 0.331019 |
| ENSMUSG00000091371 | lincRNA | Gm17397 | 0 | 0.662038 | 0.331019 |
| ENSMUSG00000032357 | protein_coding | Tinag | 0 | 0.662038 | 0.331019 |
| ENSMUSG00000064299 | protein_coding | 4921528I07Rik | 0 | 0.662038 | 0.331019 |
| ENSMUSG00000082247 | pseudogene | Gm16074 | 0 | 0.662038 | 0.331019 |
| ENSMUSG00000085902 | processed_transcrip | Gm12200 | 0 | 0.662038 | 0.331019 |
| ENSMUSG00000088091 | rRNA | 5S_rRNA | 0 | 0.662038 | 0.331019 |
| ENSMUSG00000088485 | snRNA | U6 | 0 | 0.662038 | 0.331019 |
| ENSMUSG00000082767 | pseudogene | Gm11622 | 0 | 0.662038 | 0.331019 |
| ENSMUSG00000085511 | processed_transcrip | Gm11738 | 0 | 0.662038 | 0.331019 |
| ENSMUSG00000082947 | pseudogene | Gm12587 | 0 | 0.662038 | 0.331019 |
| ENSMUSG00000093173 | miRNA | AC139568.1 | 0 | 0.662038 | 0.331019 |
| ENSMUSG00000086786 | lincRNA | Gm15908 | 0 | 0.662038 | 0.331019 |
| ENSMUSG00000090027 | pseudogene | Gm15740 | 0 | 0.662038 | 0.331019 |
| ENSMUSG00000072726 | protein_coding | Gm5797 | 0 | 0.662038 | 0.331019 |
| ENSMUSG00000045776 | protein_coding | Lrtm1 | 0 | 0.662038 | 0.331019 |
| ENSMUSG00000021795 | protein_coding | Sftpd | 0 | 0.662038 | 0.331019 |
| ENSMUSG00000077685 | snoRNA | SNORD86 | 0 | 0.662038 | 0.331019 |
| ENSMUSG00000065612 | miRNA | Mir151 | 0 | 0.662038 | 0.331019 |
| ENSMUSG00000022991 | protein_coding | Lalba | 0 | 0.662038 | 0.331019 |
| ENSMUSG00000022650 | protein_coding | Retnlb | 0 | 0.662038 | 0.331019 |
| ENSMUSG00000045504 | protein_coding | Olfr209 | 0 | 0.662038 | 0.331019 |

| | | | | | |
|---|---|---|---|---|---|
| ENSMUSG00000051728 | protein_coding | 4930563D23Rik | 0 | 0.662038 | 0.331019 |
| ENSMUSG00000093320 | miRNA | AC163629.1 | 0 | 0.662038 | 0.331019 |
| ENSMUSG00000073594 | protein_coding | Gm10545 | 0 | 0.662038 | 0.331019 |
| ENSMUSG00000065155 | snRNA | U1 | 0 | 0.662038 | 0.331019 |
| ENSMUSG00000086562 | pseudogene | Gm14651 | 0 | 0.662038 | 0.331019 |
| ENSMUSG00000081923 | pseudogene | Gm9062 | 0 | 0.662038 | 0.331019 |
| ENSMUSG00000083302 | pseudogene | Gm7905 | 0 | 0.662038 | 0.331019 |
| ENSMUSG00000079318 | protein_coding | Gm11091 | 0 | 0.662038 | 0.331019 |
| ENSMUSG00000086258 | processed_transcrip | Gm15239 | 0 | 0.662038 | 0.331019 |
| ENSMUSG00000021156 | protein_coding | Zmynd11 | 3465.037 | 3610.662 | 3537.85 |
| ENSMUSG00000001672 | protein_coding | Marveld3 | 10.62457 | 8.422316 | 9.523442 |
| ENSMUSG00000026333 | protein_coding | Gin1 | 530.9747 | 555.5667 | 543.2707 |
| ENSMUSG00000021785 | protein_coding | Ngly1 | 878.9588 | 845.788 | 862.3734 |
| ENSMUSG00000052819 | protein_coding | Best2 | 6.151626 | 8.220123 | 7.185875 |
| ENSMUSG00000086865 | lincRNA | Gm16978 | 1.909105 | 3.161689 | 2.535397 |
| ENSMUSG00000028322 | protein_coding | Exosc3 | 644.5245 | 674.8556 | 659.69 |
| ENSMUSG00000031340 | protein_coding | Gabre | 60.16199 | 52.38187 | 56.27193 |
| ENSMUSG00000034413 | protein_coding | Neurl1b | 443.2489 | 420.31 | 431.7795 |
| ENSMUSG00000078452 | protein_coding | Raet1d | 51.94144 | 45.63183 | 48.78664 |
| ENSMUSG00000032305 | protein_coding | 2310046O06Rik | 753.1034 | 725.8227 | 739.4631 |
| ENSMUSG00000004994 | protein_coding | Ccdc130 | 322.8116 | 338.5213 | 330.6665 |
| ENSMUSG00000042549 | processed_transcrip | Gm16516 | 39.6323 | 44.63826 | 42.13528 |
| ENSMUSG00000085256 | processed_transcrip | 3010003L10Rik | 5.157117 | 6.807402 | 5.98226 |
| ENSMUSG00000058761 | protein_coding | Rnf169 | 1279.659 | 1225.139 | 1252.399 |
| ENSMUSG00000026354 | protein_coding | Lct | 45.47739 | 38.96709 | 42.22224 |
| ENSMUSG00000035151 | protein_coding | Elmod2 | 1280.484 | 1323.445 | 1301.965 |
| ENSMUSG00000064647 | snoRNA | SNORD58 | 9.460616 | 12.17505 | 10.81784 |
| ENSMUSG00000091007 | protein_coding | D630036H23Rik | 8.748261 | 6.789383 | 7.768822 |
| ENSMUSG00000025158 | protein_coding | Rfng | 385.7185 | 367.2025 | 376.4605 |
| ENSMUSG00000062421 | protein_coding | Arf2 | 2613.025 | 2711.828 | 2662.427 |
| ENSMUSG00000034842 | protein_coding | Art3 | 1798.404 | 1864.297 | 1831.35 |
| ENSMUSG00000041037 | protein_coding | Irgq | 620.7439 | 660.0251 | 640.3845 |
| ENSMUSG00000085445 | lincRNA | Gm16348 | 18.83208 | 22.54165 | 20.68686 |
| ENSMUSG00000037542 | protein_coding | Aldh8a1 | 1.965461 | 1.033237 | 1.499349 |
| ENSMUSG00000003458 | protein_coding | Ncstn | 3092.792 | 3217.386 | 3155.089 |
| ENSMUSG00000025381 | protein_coding | Cnpy2 | 1253.757 | 1312.865 | 1283.311 |
| ENSMUSG00000028763 | protein_coding | Hspg2 | 5304.001 | 5131.933 | 5217.967 |
| ENSMUSG00000062181 | protein_coding | Ces3b | 2.273482 | 1.386417 | 1.829949 |
| ENSMUSG00000059309 | protein_coding | Hist1h3f | 2.273482 | 1.386417 | 1.829949 |
| ENSMUSG00000049354 | protein_coding | Dcaf7 | 4303.392 | 4459.083 | 4381.237 |
| ENSMUSG00000082431 | pseudogene | Gm7110 | 11.98092 | 9.41123 | 10.69607 |
| ENSMUSG00000019880 | protein_coding | Rspo3 | 10.31655 | 8.096164 | 9.206355 |
| ENSMUSG00000028744 | protein_coding | Pqlc2 | 252.9501 | 269.9861 | 261.4681 |
| ENSMUSG00000027500 | protein_coding | Stmn2 | 14.20878 | 17.09472 | 15.65175 |
| ENSMUSG00000082926 | pseudogene | Gm8172 | 10.69732 | 8.511685 | 9.604504 |
| ENSMUSG00000000731 | protein_coding | Aire | 6.490136 | 4.714624 | 5.60238 |
| ENSMUSG00000024663 | protein_coding | Rab3il1 | 553.0315 | 580.3648 | 566.6981 |
| ENSMUSG00000077655 | snoRNA | SNORA17 | 0.65242 | 1.36876 | 1.01059 |
| ENSMUSG00000056481 | protein_coding | Cd248 | 706.9832 | 669.831 | 688.4071 |
| ENSMUSG00000026155 | protein_coding | Smap1 | 807.56 | 774.9015 | 791.2308 |
| ENSMUSG00000046556 | protein_coding | Zfp319 | 762.5438 | 793.2463 | 777.895 |
| ENSMUSG00000073823 | pseudogene | Gm10586 | 1.973661 | 1.015218 | 1.494439 |
| ENSMUSG00000038696 | protein_coding | Mapkap1 | 2108.405 | 2042.197 | 2075.301 |
| ENSMUSG00000026516 | protein_coding | Nvl | 1890.013 | 1951.409 | 1920.711 |
| ENSMUSG00000040935 | protein_coding | Padi6 | 2.253504 | 1.324076 | 1.78879 |
| ENSMUSG00000042943 | protein_coding | 4922501L14Rik | 14.25798 | 17.9196 | 16.08879 |
| ENSMUSG00000022412 | protein_coding | Smcr7l | 1597.795 | 1541.592 | 1569.693 |
| ENSMUSG00000082284 | pseudogene | H3f3a-ps1 | 91.67698 | 84.20649 | 87.94174 |

| | | | | | |
|---|---|---|---|---|---|
| ENSMUSG00000021681 | protein_coding | Aggf1 | 3062.947 | 3156.81 | 3109.878 |
| ENSMUSG00000007338 | protein_coding | Mrpl49 | 1421.28 | 1483.405 | 1452.342 |
| ENSMUSG00000047945 | protein_coding | Marcksl1 | 12531.07 | 12148.91 | 12339.99 |
| ENSMUSG00000051257 | protein_coding | Trap1a | 668.9043 | 636.5669 | 652.7356 |
| ENSMUSG00000087129 | processed_transcrip | Gm16316 | 1.957261 | 3.099348 | 2.528304 |
| ENSMUSG00000057716 | protein_coding | Gm5567 | 47.80358 | 54.36332 | 51.08345 |
| ENSMUSG00000078932 | protein_coding | CN725425 | 443.517 | 479.8328 | 461.6749 |
| ENSMUSG00000044499 | protein_coding | Hs3st5 | 4.832697 | 6.471878 | 5.652287 |
| ENSMUSG00000032551 | protein_coding | 1110059G10Rik | 467.837 | 488.2635 | 478.0503 |
| ENSMUSG00000022847 | protein_coding | Thpo | 2.265282 | 3.417215 | 2.841248 |
| ENSMUSG00000040722 | protein_coding | Scamp5 | 1001.092 | 1045.834 | 1023.463 |
| ENSMUSG00000087275 | lincRNA | 1110002J07Rik | 11.6237 | 14.1882 | 12.90595 |
| ENSMUSG00000082112 | pseudogene | Gm14445 | 0.32211 | 1.006571 | 0.66434 |
| ENSMUSG00000086887 | processed_transcrip | 1700060C16Rik | 0.32211 | 1.006571 | 0.66434 |
| ENSMUSG00000065086 | snoRNA | SNORA4 | 0.32211 | 1.006571 | 0.66434 |
| ENSMUSG00000089109 | misc_RNA | RNaseP_nuc | 0.32211 | 1.006571 | 0.66434 |
| ENSMUSG00000063684 | pseudogene | Gm13910 | 988.16 | 1028.242 | 1008.201 |
| ENSMUSG00000087095 | lincRNA | Emx2os | 5.163007 | 6.843077 | 6.003042 |
| ENSMUSG00000064970 | snRNA | U6 | 2.289882 | 1.359751 | 1.824816 |
| ENSMUSG00000022022 | protein_coding | Mtrf1 | 214.5247 | 201.4406 | 207.9826 |
| ENSMUSG00000044258 | protein_coding | Ctla2a | 88.52506 | 96.85754 | 92.6913 |
| ENSMUSG00000085019 | processed_transcrip | Gm12747 | 0 | 0.653029 | 0.326514 |
| ENSMUSG00000044103 | protein_coding | Il1f9 | 0 | 0.653029 | 0.326514 |
| ENSMUSG00000000889 | protein_coding | Dbh | 0 | 0.653029 | 0.326514 |
| ENSMUSG00000077999 | miRNA | AL589870.1 | 0 | 0.653029 | 0.326514 |
| ENSMUSG00000092849 | miRNA | AL831766.1 | 0 | 0.653029 | 0.326514 |
| ENSMUSG00000078876 | protein_coding | Gm14408 | 0 | 0.653029 | 0.326514 |
| ENSMUSG00000068999 | protein_coding | Vmn2r5 | 0 | 0.653029 | 0.326514 |
| ENSMUSG00000064867 | snRNA | U6 | 0 | 0.653029 | 0.326514 |
| ENSMUSG00000090278 | protein_coding | D130062J21Rik | 0 | 0.653029 | 0.326514 |
| ENSMUSG00000059343 | protein_coding | Aldoart1 | 0 | 0.653029 | 0.326514 |
| ENSMUSG00000091038 | protein_coding | Gm17662 | 0 | 0.653029 | 0.326514 |
| ENSMUSG00000092785 | misc_RNA | AL672076.1 | 0 | 0.653029 | 0.326514 |
| ENSMUSG00000087813 | snoRNA | SNORA40 | 0 | 0.653029 | 0.326514 |
| ENSMUSG00000084469 | snRNA | U4 | 0 | 0.653029 | 0.326514 |
| ENSMUSG00000084945 | protein_coding | C030037F17Rik | 0 | 0.653029 | 0.326514 |
| ENSMUSG00000090802 | protein_coding | Gm8074 | 0 | 0.653029 | 0.326514 |
| ENSMUSG00000082587 | pseudogene | Gm12129 | 0 | 0.653029 | 0.326514 |
| ENSMUSG00000075832 | snRNA | U6 | 0 | 0.653029 | 0.326514 |
| ENSMUSG00000021101 | protein_coding | 4930408O17Rik | 0 | 0.653029 | 0.326514 |
| ENSMUSG00000088149 | snRNA | U6 | 0 | 0.653029 | 0.326514 |
| ENSMUSG00000074840 | protein_coding | Gm17449 | 0 | 0.653029 | 0.326514 |
| ENSMUSG00000093334 | miRNA | AC165250.1 | 0 | 0.653029 | 0.326514 |
| ENSMUSG00000021741 | pseudogene | Gm5457 | 0 | 0.653029 | 0.326514 |
| ENSMUSG00000037246 | protein_coding | H2-M10.5 | 0 | 0.653029 | 0.326514 |
| ENSMUSG00000025431 | protein_coding | Crisp1 | 0 | 0.653029 | 0.326514 |
| ENSMUSG00000024770 | protein_coding | Lipn | 0 | 0.653029 | 0.326514 |
| ENSMUSG00000080186 | pseudogene | Gm14448 | 0 | 0.653029 | 0.326514 |
| ENSMUSG00000081180 | pseudogene | Gm7212 | 0 | 0.653029 | 0.326514 |
| ENSMUSG00000072640 | protein_coding | 1810012P15Rik | 721.41 | 754.6988 | 738.0544 |
| ENSMUSG00000023802 | protein_coding | Nox3 | 0.316221 | 1.006571 | 0.661396 |
| ENSMUSG00000031988 | protein_coding | Vps26b | 3238.537 | 3338.366 | 3288.451 |
| ENSMUSG00000036257 | protein_coding | Pnpla8 | 3235.578 | 3129.237 | 3182.407 |
| ENSMUSG00000020076 | protein_coding | Ddx50 | 3702.872 | 3833.718 | 3768.295 |
| ENSMUSG00000023411 | protein_coding | Nfatc4 | 244.8964 | 258.85 | 251.8732 |
| ENSMUSG00000084323 | pseudogene | Gm14438 | 6.123448 | 4.503783 | 5.313616 |
| ENSMUSG00000067279 | protein_coding | Ppp1r3c | 240.314 | 225.6714 | 232.9927 |
| ENSMUSG00000075270 | protein_coding | Pde11a | 20.63898 | 17.50051 | 19.06975 |

| | | | | | |
|---|---|---|---|---|---|
| ENSMUSG00000037847 | protein_coding | BC016495 | 166.1211 | 154.1072 | 160.1142 |
| ENSMUSG00000048232 | protein_coding | Fbxo10 | 1115.038 | 1068.416 | 1091.727 |
| ENSMUSG00000048347 | protein_coding | Pcdhb18 | 26.67432 | 30.78952 | 28.73192 |
| ENSMUSG00000064252 | pseudogene | Olfr329-ps | 6.746423 | 9.058411 | 7.902417 |
| ENSMUSG00000040287 | protein_coding | Stac3 | 34.46743 | 40.11532 | 37.29137 |
| ENSMUSG00000022548 | protein_coding | Apod | 15.17973 | 18.15354 | 16.66663 |
| ENSMUSG00000085755 | processed_transcrip | Gm13496 | 8.031285 | 6.198696 | 7.114991 |
| ENSMUSG00000022385 | protein_coding | Gtse1 | 924.6297 | 875.1689 | 899.8993 |
| ENSMUSG00000082357 | pseudogene | Gm13542 | 2.273482 | 1.324076 | 1.798779 |
| ENSMUSG00000021686 | protein_coding | Ap3b1 | 3026.497 | 3134.722 | 3080.609 |
| ENSMUSG00000023852 | protein_coding | Chd1 | 2570.547 | 2495.505 | 2533.026 |
| ENSMUSG00000086308 | lincRNA | G630016G05Rik | 2.239415 | 1.359751 | 1.799583 |
| ENSMUSG00000028603 | protein_coding | Scp2 | 2337.65 | 2431.498 | 2384.574 |
| ENSMUSG00000036712 | protein_coding | Cyld | 1944.572 | 1810.077 | 1877.325 |
| ENSMUSG00000080002 | pseudogene | Gm5566 | 1.914994 | 1.015218 | 1.465106 |
| ENSMUSG00000030659 | protein_coding | Nucb2 | 532.0901 | 508.1422 | 520.1162 |
| ENSMUSG00000086566 | lincRNA | 2810429I04Rik | 157.6116 | 146.4392 | 152.0254 |
| ENSMUSG00000003437 | protein_coding | Paf1 | 2493.807 | 2386.27 | 2440.039 |
| ENSMUSG00000091754 | protein_coding | Gm3636 | 15.67426 | 19.77927 | 17.72677 |
| ENSMUSG00000090576 | processed_transcrip | Gm17055 | 19.92432 | 16.81253 | 18.36842 |
| ENSMUSG00000061374 | protein_coding | Fiz1 | 1270.042 | 1213.895 | 1241.969 |
| ENSMUSG00000029238 | protein_coding | Clock | 4399.933 | 4556.201 | 4478.067 |
| ENSMUSG00000021962 | protein_coding | Dcp1a | 1528.28 | 1581.967 | 1555.124 |
| ENSMUSG00000028860 | protein_coding | Sytl1 | 19.4069 | 16.45034 | 17.92862 |
| ENSMUSG00000065579 | miRNA | Mir425 | 4.873696 | 3.470547 | 4.172121 |
| ENSMUSG00000053617 | protein_coding | Sh3pxd2a | 2062.908 | 1952.875 | 2007.891 |
| ENSMUSG00000032648 | protein_coding | Pygm | 578.9343 | 612.1404 | 595.5374 |
| ENSMUSG00000021906 | protein_coding | Oxnad1 | 532.3405 | 504.8547 | 518.5976 |
| ENSMUSG00000006235 | protein_coding | Epor | 32.65523 | 28.65998 | 30.65761 |
| ENSMUSG00000032412 | protein_coding | Atp1b3 | 4322.082 | 4210.954 | 4266.518 |
| ENSMUSG00000027582 | protein_coding | Zgpat | 1069.474 | 1106.075 | 1087.775 |
| ENSMUSG00000092192 | protein_coding | Dyx1c1 | 33.25003 | 29.41139 | 31.33071 |
| ENSMUSG00000085826 | processed_transcrip | Gm15638 | 1.920883 | 1.015218 | 1.468051 |
| ENSMUSG00000086245 | lincRNA | Gm16170 | 210.5365 | 227.9592 | 219.2478 |
| ENSMUSG00000026202 | protein_coding | Tuba4a | 360.7073 | 328.7394 | 344.7233 |
| ENSMUSG00000003227 | protein_coding | Edar | 57.30669 | 65.51171 | 61.4092 |
| ENSMUSG00000037716 | protein_coding | Ccdc33 | 3.250323 | 4.651921 | 3.951122 |
| ENSMUSG00000070044 | protein_coding | Fam149a | 159.6055 | 170.4831 | 165.0443 |
| ENSMUSG00000055430 | protein_coding | Nap1l5 | 53.95692 | 59.37567 | 56.66629 |
| ENSMUSG00000025159 | protein_coding | Mms19 | 1616.063 | 1676.728 | 1646.395 |
| ENSMUSG00000065990 | protein_coding | Aurkaip1 | 1379.391 | 1327.313 | 1353.352 |
| ENSMUSG00000026104 | protein_coding | Stat1 | 3076.445 | 2908.818 | 2992.632 |
| ENSMUSG00000062743 | protein_coding | Zfp677 | 270.6542 | 255.4457 | 263.05 |
| ENSMUSG00000041781 | protein_coding | Cpsf2 | 3042.524 | 2940.731 | 2991.627 |
| ENSMUSG00000090042 | pseudogene | Gm8550 | 8.435619 | 10.75581 | 9.595715 |
| ENSMUSG00000080772 | pseudogene | Gm12543 | 0.638331 | 1.333085 | 0.985708 |
| ENSMUSG00000031901 | protein_coding | Dus2l | 278.7784 | 295.1651 | 286.9718 |
| ENSMUSG00000074364 | protein_coding | Ehd2 | 1430.91 | 1382.572 | 1406.741 |
| ENSMUSG00000030209 | protein_coding | Grin2b | 1.965461 | 1.050893 | 1.508177 |
| ENSMUSG00000030435 | protein_coding | U2af2 | 5383.768 | 5227.051 | 5305.409 |
| ENSMUSG00000034185 | lincRNA | 6430628N08Rik | 5.165317 | 3.65436 | 4.409839 |
| ENSMUSG00000075515 | protein_coding | Rps2-ps6 | 25.75704 | 51.34738 | 38.55221 |
| ENSMUSG00000031200 | protein_coding | Mtcp1 | 301.7107 | 321.5401 | 311.6254 |
| ENSMUSG00000034881 | protein_coding | Tbxa2r | 85.78269 | 93.46388 | 89.62329 |
| ENSMUSG00000014850 | protein_coding | Msh3 | 1363.488 | 1420.731 | 1392.11 |
| ENSMUSG00000020654 | protein_coding | Adcy3 | 462.3203 | 487.6662 | 474.9933 |
| ENSMUSG00000059395 | protein_coding | Nkapl | 62.80313 | 56.95602 | 59.87958 |
| ENSMUSG00000068105 | protein_coding | Tnfrsf13c | 63.21678 | 53.68933 | 58.45305 |

| | | | | | |
|---|---|---|---|---|---|
| ENSMUSG00000021068 | protein_coding | Nin | 456.3788 | 479.8705 | 468.1247 |
| ENSMUSG00000029564 | protein_coding | 4930519G04Rik | 2.273482 | 1.315066 | 1.794274 |
| ENSMUSG00000024039 | protein_coding | Cbs | 23.08846 | 19.46032 | 21.27439 |
| ENSMUSG00000020641 | protein_coding | Rsad2 | 22.91351 | 26.41907 | 24.66629 |
| ENSMUSG00000024213 | protein_coding | Nudt3 | 4367.287 | 4496.492 | 4431.89 |
| ENSMUSG00000028514 | protein_coding | Usp24 | 5122.234 | 4939.305 | 5030.769 |
| ENSMUSG00000060393 | pseudogene | Gm5580 | 2.257082 | 1.350742 | 1.803912 |
| ENSMUSG00000025135 | protein_coding | Anapc11 | 1377.41 | 1422.464 | 1399.937 |
| ENSMUSG00000030245 | protein_coding | Golt1b | 1508.53 | 1562.535 | 1535.532 |
| ENSMUSG00000085147 | lincRNA | Gm12609 | 10.98305 | 8.891532 | 9.937293 |
| ENSMUSG00000082616 | pseudogene | Gm6025 | 2.958702 | 1.65059 | 2.304646 |
| ENSMUSG00000028851 | protein_coding | Nudc | 6517.766 | 6300.388 | 6409.077 |
| ENSMUSG00000072950 | pseudogene | Gm10444 | 0.646531 | 1.341732 | 0.994132 |
| ENSMUSG00000070980 | protein_coding | Actl7b | 3.260834 | 2.012779 | 2.636807 |
| ENSMUSG00000016984 | protein_coding | Etaa1 | 1432.323 | 1371.058 | 1401.691 |
| ENSMUSG00000025534 | protein_coding | Gusb | 870.4142 | 911.8485 | 891.1313 |
| ENSMUSG00000052234 | protein_coding | Epx | 6.830957 | 8.820543 | 7.82575 |
| ENSMUSG00000087382 | processed_transcrip | 1300015D01Rik | 99.53212 | 89.64037 | 94.58624 |
| ENSMUSG00000044430 | protein_coding | Klk12 | 0.646531 | 1.324076 | 0.985303 |
| ENSMUSG00000036492 | protein_coding | Rnf39 | 48.28723 | 53.98528 | 51.13626 |
| ENSMUSG00000039357 | protein_coding | Fut11 | 779.3635 | 739.9967 | 759.6801 |
| ENSMUSG00000035277 | protein_coding | Arx | 595.3271 | 620.5658 | 607.9465 |
| ENSMUSG00000050668 | protein_coding | Ccdc75 | 1094.567 | 1134.354 | 1114.46 |
| ENSMUSG00000021139 | protein_coding | Synj2bp | 13.81853 | 16.58911 | 15.20382 |
| ENSMUSG00000038347 | protein_coding | Tcte2 | 44.36831 | 38.81977 | 41.59404 |
| ENSMUSG00000057378 | protein_coding | Ryr3 | 53.24628 | 47.48887 | 50.36757 |
| ENSMUSG00000049717 | protein_coding | Lig4 | 578.7117 | 542.6496 | 560.6806 |
| ENSMUSG00000026532 | protein_coding | Spna1 | 22.69859 | 27.85778 | 25.27818 |
| ENSMUSG00000027718 | protein_coding | Il21 | 8.043064 | 6.243381 | 7.143222 |
| ENSMUSG00000031157 | protein_coding | Pqbp1 | 791.8388 | 829.6934 | 810.7661 |
| ENSMUSG00000078305 | protein_coding | Gm3436 | 12.32889 | 15.11653 | 13.72271 |
| ENSMUSG00000086393 | lincRNA | Gm16794 | 50.255 | 57.06294 | 53.65897 |
| ENSMUSG00000086002 | lincRNA | Gm16744 | 1.957261 | 3.063673 | 2.510467 |
| ENSMUSG00000026502 | protein_coding | Pppde1 | 1689.165 | 1631.979 | 1660.572 |
| ENSMUSG00000027690 | protein_coding | Slc2a2 | 24.69819 | 32.58245 | 28.64032 |
| ENSMUSG00000092187 | processed_transcrip | Gm20457 | 74.57026 | 81.01492 | 77.79259 |
| ENSMUSG00000075054 | protein_coding | 1600012F09Rik | 794.3273 | 829.0043 | 811.6658 |
| ENSMUSG00000091604 | protein_coding | Gm17349 | 10.34935 | 12.62625 | 11.4878 |
| ENSMUSG00000022391 | protein_coding | Rangap1 | 2686.937 | 2780.598 | 2733.768 |
| ENSMUSG00000040183 | protein_coding | Ankrd6 | 384.2578 | 403.7718 | 394.0148 |
| ENSMUSG00000033770 | protein_coding | Clcnka | 3.217524 | 1.977104 | 2.597314 |
| ENSMUSG00000086184 | processed_transcrip | Gm12764 | 16.6511 | 13.21114 | 14.93112 |
| ENSMUSG00000091124 | protein_coding | Gm17239 | 43.44633 | 49.36216 | 46.40424 |
| ENSMUSG00000090837 | protein_coding | Gm7233 | 9.664125 | 12.20457 | 10.93435 |
| ENSMUSG00000093124 | miRNA | AC114917.1 | 4.869074 | 3.470547 | 4.169811 |
| ENSMUSG00000029272 | protein_coding | Sult1e1 | 0.646531 | 1.333085 | 0.989808 |
| ENSMUSG00000068252 | protein_coding | Apol7b | 0.646531 | 1.333085 | 0.989808 |
| ENSMUSG00000028759 | protein_coding | Hp1bp3 | 5881.166 | 6058.076 | 5969.621 |
| ENSMUSG00000082576 | pseudogene | Gm12058 | 0.65242 | 1.341732 | 0.997076 |
| ENSMUSG00000025497 | protein_coding | Cdhr5 | 43.09142 | 38.34797 | 40.7197 |
| ENSMUSG00000076271 | miRNA | Mir697 | 6.412534 | 4.749938 | 5.581236 |
| ENSMUSG00000087535 | lincRNA | D930049A15Rik | 10.99125 | 13.28829 | 12.13977 |
| ENSMUSG00000020829 | protein_coding | Slc46a1 | 460.7164 | 481.081 | 470.8987 |
| ENSMUSG00000067297 | protein_coding | 2010002M12Rik | 3.538366 | 2.419653 | 2.97901 |
| ENSMUSG00000022808 | protein_coding | Snx4 | 3333.416 | 3428.823 | 3381.12 |
| ENSMUSG00000030385 | protein_coding | 2900092C05Rik | 0.65242 | 1.324076 | 0.988248 |
| ENSMUSG00000039637 | protein_coding | Coro7 | 510.3735 | 481.7722 | 496.0728 |
| ENSMUSG00000026564 | protein_coding | Dusp27 | 13.71991 | 10.37312 | 12.04651 |

| | | | | | |
|---|---|---|---|---|---|
| ENSMUSG00000001380 | protein_coding | Hars | 1474.551 | 1521.276 | 1497.914 |
| ENSMUSG00000019933 | protein_coding | 2310015B20Rik | 3.540677 | 2.348303 | 2.94449 |
| ENSMUSG00000021738 | protein_coding | Atxn7 | 1122.328 | 1087.741 | 1105.035 |
| ENSMUSG00000059921 | protein_coding | Unc5c | 10.71603 | 13.54703 | 12.13153 |
| ENSMUSG00000066175 | protein_coding | 2510046G10Rik | 22.7018 | 26.49006 | 24.59593 |
| ENSMUSG00000016409 | protein_coding | Nkap | 513.2982 | 492.4741 | 502.8861 |
| ENSMUSG00000028392 | protein_coding | Bspry | 140.342 | 150.7356 | 145.5388 |
| ENSMUSG00000087594 | lincRNA | BC039771 | 46.81161 | 40.48766 | 43.64963 |
| ENSMUSG00000085007 | lincRNA | Gm11549 | 50.03777 | 40.5006 | 45.26919 |
| ENSMUSG00000078493 | protein_coding | A930039A15Rik | 15.55231 | 12.83709 | 14.1947 |
| ENSMUSG00000063728 | protein_coding | Magea6 | 145.755 | 134.4809 | 140.118 |
| ENSMUSG00000086741 | processed_transcrip | Gm15816 | 5.167628 | 3.797061 | 4.482345 |
| ENSMUSG00000060301 | protein_coding | 2610008E11Rik | 1359.901 | 1459.381 | 1409.641 |
| ENSMUSG00000092937 | miRNA | AC158384.1 | 0.99093 | 2.004132 | 1.497531 |
| ENSMUSG00000003039 | protein_coding | Fam32a | 2709.165 | 2815.203 | 2762.184 |
| ENSMUSG00000027331 | protein_coding | D2Ertd750e | 863.4279 | 814.8161 | 839.122 |
| ENSMUSG00000028405 | protein_coding | Aco1 | 1458.448 | 1507.584 | 1483.016 |
| ENSMUSG00000074838 | protein_coding | Gm16512 | 9.041151 | 11.39698 | 10.21907 |
| ENSMUSG00000080709 | pseudogene | Gm14324 | 0.33031 | 0.979543 | 0.654926 |
| ENSMUSG00000087799 | rRNA | 5S_rRNA | 0.33031 | 0.979543 | 0.654926 |
| ENSMUSG00000085409 | processed_transcrip | Gm16155 | 0.33031 | 0.979543 | 0.654926 |
| ENSMUSG00000084397 | pseudogene | Gm9133 | 0.33031 | 0.979543 | 0.654926 |
| ENSMUSG00000031636 | protein_coding | Pdlim3 | 1310.693 | 1372.657 | 1341.675 |
| ENSMUSG00000079399 | protein_coding | Gm3002 | 3.199857 | 1.968095 | 2.583976 |
| ENSMUSG00000071253 | protein_coding | Slc25a16 | 429.84 | 408.6636 | 419.2518 |
| ENSMUSG00000039831 | protein_coding | Arhgap29 | 5766.028 | 5582.639 | 5674.333 |
| ENSMUSG00000062758 | protein_coding | Gm16477 | 38.55162 | 43.29902 | 40.92532 |
| ENSMUSG00000016664 | protein_coding | Pacsin2 | 2636.481 | 2732.29 | 2684.385 |
| ENSMUSG00000030226 | protein_coding | Lmo3 | 6.41738 | 8.901264 | 7.659322 |
| ENSMUSG00000047909 | protein_coding | Ankrd16 | 906.868 | 957.9702 | 932.4191 |
| ENSMUSG00000082814 | pseudogene | Gm12956 | 0.954552 | 1.72194 | 1.338246 |
| ENSMUSG00000032392 | protein_coding | Parp16 | 263.8889 | 278.6205 | 271.2547 |
| ENSMUSG00000052384 | protein_coding | Lrrc33 | 92.60776 | 100.3941 | 96.50093 |
| ENSMUSG00000085710 | lincRNA | Gm12676 | 9.305637 | 11.575 | 10.44032 |
| ENSMUSG00000038188 | protein_coding | Scarf1 | 42.06851 | 47.40638 | 44.73745 |
| ENSMUSG00000088997 | snRNA | U6 | 0.638331 | 1.315066 | 0.976699 |
| ENSMUSG00000033065 | protein_coding | Pfkm | 1256.203 | 1298.033 | 1277.118 |
| ENSMUSG00000036362 | protein_coding | P2ry13 | 66.90268 | 60.43485 | 63.66876 |
| ENSMUSG00000048047 | protein_coding | Zbtb33 | 1799.645 | 1879.803 | 1839.724 |
| ENSMUSG00000054199 | protein_coding | Gon4l | 2127.526 | 2206.919 | 2167.223 |
| ENSMUSG00000022214 | protein_coding | Dcaf11 | 1923.403 | 1864.595 | 1893.999 |
| ENSMUSG00000005864 | protein_coding | Cnga2 | 4.865496 | 3.479556 | 4.172526 |
| ENSMUSG00000086506 | processed_transcrip | Gm15911 | 12.58645 | 15.07257 | 13.82951 |
| ENSMUSG00000022844 | protein_coding | Pdia5 | 1863.197 | 1784.34 | 1823.768 |
| ENSMUSG00000060560 | protein_coding | Ces4a | 0.32211 | 0.979543 | 0.650827 |
| ENSMUSG00000085832 | lincRNA | D430036J16Rik | 0.968641 | 1.677256 | 1.322949 |
| ENSMUSG00000089791 | pseudogene | Gm3555 | 14.48735 | 12.04421 | 13.26578 |
| ENSMUSG00000020782 | protein_coding | Llgl2 | 539.1485 | 510.3068 | 524.7277 |
| ENSMUSG00000053925 | lincRNA | Gm9929 | 26.62236 | 22.37337 | 24.49786 |
| ENSMUSG00000048787 | protein_coding | Dcun1d3 | 1353.658 | 1306.759 | 1330.209 |
| ENSMUSG00000074860 | protein_coding | Gm10778 | 12.19874 | 15.03726 | 13.618 |
| ENSMUSG00000020019 | protein_coding | Ntn4 | 1333.852 | 1281.36 | 1307.606 |
| ENSMUSG00000046593 | protein_coding | Tmem215 | 0.65242 | 1.333085 | 0.992753 |
| ENSMUSG00000036442 | protein_coding | Thap11 | 1667.797 | 1605.269 | 1636.533 |
| ENSMUSG00000082062 | pseudogene | Ftl2 | 2.563836 | 3.859402 | 3.211619 |
| ENSMUSG00000086634 | lincRNA | 4930448N21Rik | 4.893674 | 3.416853 | 4.155263 |
| ENSMUSG00000087508 | processed_transcrip | Gm14174 | 0.316221 | 0.979543 | 0.647882 |
| ENSMUSG00000056815 | protein_coding | Gm6812 | 0.316221 | 0.979543 | 0.647882 |

| | | | | | |
|---|---|---|---|---|---|
| ENSMUSG00000060466 | protein_coding | Thap6 | 534.1673 | 558.4915 | 546.3294 |
| ENSMUSG00000030748 | protein_coding | Il4ra | 439.7016 | 469.2553 | 454.4785 |
| ENSMUSG00000085035 | processed_transcrip | Gm12031 | 3.278501 | 4.812279 | 4.04539 |
| ENSMUSG00000028600 | protein_coding | Podn | 26.88438 | 23.19576 | 25.04007 |
| ENSMUSG00000092560 | pseudogene | Gm8750 | 1.298952 | 0.662038 | 0.980495 |
| ENSMUSG00000027628 | protein_coding | 0610011L14Rik | 1036.874 | 997.7827 | 1017.329 |
| ENSMUSG00000026774 | protein_coding | 4931423N10Rik | 3.552455 | 2.43731 | 2.994883 |
| ENSMUSG00000031171 | protein_coding | Ftsj1 | 799.9592 | 772.0572 | 786.0082 |
| ENSMUSG00000087558 | lincRNA | 1700030G06Rik | 83.58889 | 90.30825 | 86.94857 |
| ENSMUSG00000036264 | protein_coding | Fstl4 | 81.63201 | 92.35258 | 86.99229 |
| ENSMUSG00000045817 | protein_coding | Zfp36l2 | 3322.756 | 3214.014 | 3268.385 |
| ENSMUSG00000032527 | protein_coding | Pccb | 1207.849 | 1249.187 | 1228.518 |
| ENSMUSG00000024194 | protein_coding | Cuta | 1052.672 | 1013.232 | 1032.952 |
| ENSMUSG00000087149 | processed_transcrip | Itih5l-ps | 10.6315 | 8.546999 | 9.589249 |
| ENSMUSG00000047678 | protein_coding | Gpr82 | 5.489738 | 4.052225 | 4.770981 |
| ENSMUSG00000020358 | protein_coding | Hnrnpab | 8989.289 | 8673.823 | 8831.556 |
| ENSMUSG00000064307 | protein_coding | Lrrc51 | 284.1367 | 265.2704 | 274.7035 |
| ENSMUSG00000019773 | protein_coding | Fbxo5 | 660.9866 | 631.8232 | 646.4049 |
| ENSMUSG00000030834 | protein_coding | Abcc6 | 12.36065 | 9.942425 | 11.15154 |
| ENSMUSG00000091454 | protein_coding | Dynlt1b | 326.224 | 347.4605 | 336.8423 |
| ENSMUSG00000024594 | protein_coding | Prrc1 | 1399.274 | 1342.88 | 1371.077 |
| ENSMUSG00000024140 | protein_coding | Epas1 | 4426.812 | 4216.493 | 4321.653 |
| ENSMUSG00000031983 | protein_coding | 2310022B05Rik | 2309.313 | 2394.979 | 2352.146 |
| ENSMUSG00000029321 | protein_coding | Slc10a6 | 17.61617 | 14.15537 | 15.88577 |
| ENSMUSG00000032198 | protein_coding | Dock6 | 1336.813 | 1285.085 | 1310.949 |
| ENSMUSG00000087519 | lincRNA | AV039307 | 15.83319 | 19.08368 | 17.45844 |
| ENSMUSG00000085991 | processed_transcrip | Gm11479 | 1.909105 | 1.033237 | 1.471171 |
| ENSMUSG00000054293 | protein_coding | A630033H20Rik | 19.91127 | 23.84951 | 21.88039 |
| ENSMUSG00000017897 | protein_coding | Eya2 | 30.60524 | 26.85261 | 28.72892 |
| ENSMUSG00000071640 | protein_coding | Stxbp3b | 29.62944 | 33.59518 | 31.61231 |
| ENSMUSG00000044562 | protein_coding | Rasip1 | 365.4028 | 386.5744 | 375.9886 |
| ENSMUSG00000063142 | protein_coding | Kcnma1 | 438.4305 | 472.9427 | 455.6866 |
| ENSMUSG00000029736 | protein_coding | Nobox | 77.20431 | 83.70916 | 80.45674 |
| ENSMUSG00000027236 | protein_coding | Eif3j | 2218.83 | 2127.945 | 2173.388 |
| ENSMUSG00000079228 | protein_coding | Gm4694 | 2.267593 | 1.377408 | 1.8225 |
| ENSMUSG00000063953 | protein_coding | Amd2 | 223.0433 | 178.6868 | 200.8651 |
| ENSMUSG00000037593 | protein_coding | BC030499 | 33.2835 | 28.00732 | 30.64541 |
| ENSMUSG00000010066 | protein_coding | Cacna2d2 | 158.808 | 168.2739 | 163.5409 |
| ENSMUSG00000033458 | protein_coding | Fan1 | 379.4828 | 361.3722 | 370.4275 |
| ENSMUSG00000083692 | pseudogene | Gm9575 | 23.63406 | 20.44887 | 22.04147 |
| ENSMUSG00000033966 | protein_coding | Cdkl4 | 17.83988 | 14.8347 | 16.33729 |
| ENSMUSG00000054641 | protein_coding | Mmrn1 | 8.094798 | 10.30498 | 9.199887 |
| ENSMUSG00000056590 | pseudogene | H2afy3 | 1.635151 | 2.790852 | 2.213001 |
| ENSMUSG00000021125 | protein_coding | Arg2 | 28.53004 | 24.69677 | 26.61341 |
| ENSMUSG00000064982 | snRNA | U2 | 3.564234 | 2.321637 | 2.942935 |
| ENSMUSG00000011306 | protein_coding | Sugp1 | 2175.38 | 2061.241 | 2118.311 |
| ENSMUSG00000041476 | protein_coding | Smpx | 2137.244 | 2210.655 | 2173.949 |
| ENSMUSG00000024268 | protein_coding | Celf4 | 113.8435 | 105.9701 | 109.9068 |
| ENSMUSG00000028207 | protein_coding | Asph | 729.9255 | 756.7954 | 743.3604 |
| ENSMUSG00000033684 | protein_coding | Qsox1 | 3172.428 | 3372.287 | 3272.357 |
| ENSMUSG00000001962 | protein_coding | Fam50a | 722.563 | 750.3063 | 736.4346 |
| ENSMUSG00000084433 | snoRNA | SNORA42 | 18.72168 | 21.77331 | 20.24749 |
| ENSMUSG00000062014 | protein_coding | Gmfb | 3853.686 | 3974.136 | 3913.911 |
| ENSMUSG00000079610 | protein_coding | Ankrd39 | 276.0243 | 290.1083 | 283.0663 |
| ENSMUSG00000007613 | protein_coding | Tgfbr1 | 3236.678 | 3088.914 | 3162.796 |
| ENSMUSG00000083007 | pseudogene | Gm13623 | 0.646531 | 1.448758 | 1.047645 |
| ENSMUSG00000066178 | protein_coding | 6030445D17Rik | 5.165317 | 6.736051 | 5.950684 |
| ENSMUSG00000030114 | protein_coding | Klrg1 | 0.646531 | 1.315066 | 0.980799 |

| | | | | | |
|---|---|---|---|---|---|
| ENSMUSG00000059912 | protein_coding | Rpl21-ps6 | 0.646531 | 1.315066 | 0.980799 |
| ENSMUSG00000090826 | protein_coding | Gm17285 | 6.816868 | 5.174469 | 5.995668 |
| ENSMUSG00000032278 | protein_coding | Paqr5 | 40.49121 | 46.56986 | 43.53053 |
| ENSMUSG00000019295 | protein_coding | Tmem129 | 1045.421 | 998.5335 | 1021.977 |
| ENSMUSG00000037942 | protein_coding | Crp | 1.290752 | 0.662038 | 0.976395 |
| ENSMUSG00000066058 | protein_coding | Cldn19 | 1.290752 | 0.662038 | 0.976395 |
| ENSMUSG00000073208 | protein_coding | Speer4c | 1.290752 | 0.662038 | 0.976395 |
| ENSMUSG00000088563 | snRNA | U7 | 1.290752 | 0.662038 | 0.976395 |
| ENSMUSG00000019947 | protein_coding | Arid5b | 992.9134 | 945.1276 | 969.0205 |
| ENSMUSG00000034509 | protein_coding | Mad2l1bp | 222.5844 | 235.9825 | 229.2835 |
| ENSMUSG00000090586 | lincRNA | Gm17260 | 16.6845 | 13.83429 | 15.2594 |
| ENSMUSG00000071064 | protein_coding | Zfp827 | 1108.397 | 1165.695 | 1137.046 |
| ENSMUSG00000077066 | miRNA | AC124080.1 | 9.979303 | 12.53761 | 11.25845 |
| ENSMUSG00000020524 | protein_coding | Gria1 | 3.554766 | 5.005101 | 4.279934 |
| ENSMUSG00000028480 | protein_coding | Glipr2 | 652.7463 | 683.2862 | 668.0163 |
| ENSMUSG00000036298 | protein_coding | Slc2a13 | 124.997 | 116.5619 | 120.7795 |
| ENSMUSG00000034768 | protein_coding | Asb16 | 21.69678 | 18.36759 | 20.03218 |
| ENSMUSG00000079697 | protein_coding | Gm5751 | 68.78137 | 61.99063 | 65.386 |
| ENSMUSG00000032050 | protein_coding | Rdx | 7352.738 | 7592.307 | 7472.522 |
| ENSMUSG00000079374 | protein_coding | Gm8334 | 15.70475 | 20.31187 | 18.00831 |
| ENSMUSG00000054976 | protein_coding | 9430031J16Rik | 0.66062 | 0 | 0.33031 |
| ENSMUSG00000067125 | protein_coding | Rpl30-ps6 | 0.66062 | 0 | 0.33031 |
| ENSMUSG00000082413 | pseudogene | Gm13401 | 0.66062 | 0 | 0.33031 |
| ENSMUSG00000085668 | processed_transcrip | Gm13447 | 0.66062 | 0 | 0.33031 |
| ENSMUSG00000086196 | processed_transcrip | Gm13571 | 0.66062 | 0 | 0.33031 |
| ENSMUSG00000001823 | protein_coding | Hoxd12 | 0.66062 | 0 | 0.33031 |
| ENSMUSG00000093174 | misc_RNA | BX323066.1 | 0.66062 | 0 | 0.33031 |
| ENSMUSG00000045493 | protein_coding | Bhlhe23 | 0.66062 | 0 | 0.33031 |
| ENSMUSG00000074728 | protein_coding | Gm10748 | 0.66062 | 0 | 0.33031 |
| ENSMUSG00000042092 | protein_coding | Lce1c | 0.66062 | 0 | 0.33031 |
| ENSMUSG00000083842 | pseudogene | Gm12485 | 0.66062 | 0 | 0.33031 |
| ENSMUSG00000083122 | pseudogene | Gm15578 | 0.66062 | 0 | 0.33031 |
| ENSMUSG00000081190 | pseudogene | Gm11807 | 0.66062 | 0 | 0.33031 |
| ENSMUSG00000046450 | protein_coding | Olfr71 | 0.66062 | 0 | 0.33031 |
| ENSMUSG00000078680 | protein_coding | Mup10 | 0.66062 | 0 | 0.33031 |
| ENSMUSG00000052520 | protein_coding | Cyp2j5 | 0.66062 | 0 | 0.33031 |
| ENSMUSG00000081678 | pseudogene | Gm12959 | 0.66062 | 0 | 0.33031 |
| ENSMUSG00000086262 | processed_transcrip | A930031H19Rik | 0.66062 | 0 | 0.33031 |
| ENSMUSG00000084108 | pseudogene | Gm15794 | 0.66062 | 0 | 0.33031 |
| ENSMUSG00000029269 | protein_coding | Sult1b1 | 0.66062 | 0 | 0.33031 |
| ENSMUSG00000076475 | IG_V_gene | Trbv19 | 0.66062 | 0 | 0.33031 |
| ENSMUSG00000056197 | protein_coding | 4931417E11Rik | 0.66062 | 0 | 0.33031 |
| ENSMUSG00000005547 | protein_coding | Cyp2a5 | 0.66062 | 0 | 0.33031 |
| ENSMUSG00000077059 | miRNA | AC110903.1 | 0.66062 | 0 | 0.33031 |
| ENSMUSG00000033533 | protein_coding | Acsm1 | 0.66062 | 0 | 0.33031 |
| ENSMUSG00000031603 | protein_coding | Fgf20 | 0.66062 | 0 | 0.33031 |
| ENSMUSG00000065492 | miRNA | Mir34b | 0.66062 | 0 | 0.33031 |
| ENSMUSG00000064735 | snRNA | U6 | 0.66062 | 0 | 0.33031 |
| ENSMUSG00000087311 | lincRNA | Gm16988 | 0.66062 | 0 | 0.33031 |
| ENSMUSG00000074695 | protein_coding | Il22 | 0.66062 | 0 | 0.33031 |
| ENSMUSG00000064948 | snRNA | U6 | 0.66062 | 0 | 0.33031 |
| ENSMUSG00000084905 | processed_transcrip | Gm12081 | 0.66062 | 0 | 0.33031 |
| ENSMUSG00000087306 | lincRNA | A230004M16Rik | 0.66062 | 0 | 0.33031 |
| ENSMUSG00000084224 | pseudogene | Gm12337 | 0.66062 | 0 | 0.33031 |
| ENSMUSG00000001103 | protein_coding | Sebox | 0.66062 | 0 | 0.33031 |
| ENSMUSG00000084079 | pseudogene | Gm11631 | 0.66062 | 0 | 0.33031 |
| ENSMUSG00000075996 | snRNA | U1 | 0.66062 | 0 | 0.33031 |
| ENSMUSG00000021210 | protein_coding | Akr1c6 | 0.66062 | 0 | 0.33031 |

| | | | | | |
|---|---|---|---|---|---|
| ENSMUSG00000092884 | miRNA | AC125484.1 | 0.66062 | 0 | 0.33031 |
| ENSMUSG00000081433 | pseudogene | Serpinb1-ps1 | 0.66062 | 0 | 0.33031 |
| ENSMUSG00000076333 | miRNA | Mir599 | 0.66062 | 0 | 0.33031 |
| ENSMUSG00000081771 | pseudogene | Gm15381 | 0.66062 | 0 | 0.33031 |
| ENSMUSG00000082653 | pseudogene | Gm15897 | 0.66062 | 0 | 0.33031 |
| ENSMUSG00000089690 | pseudogene | Gm16569 | 0.66062 | 0 | 0.33031 |
| ENSMUSG00000091426 | pseudogene | Gm17228 | 0.66062 | 0 | 0.33031 |
| ENSMUSG00000035420 | protein_coding | Fam170a | 0.66062 | 0 | 0.33031 |
| ENSMUSG00000050865 | protein_coding | Olfr1494 | 0.66062 | 0 | 0.33031 |
| ENSMUSG00000024774 | protein_coding | Ankrd22 | 0.66062 | 0 | 0.33031 |
| ENSMUSG00000067225 | protein_coding | Cyp2c54 | 0.66062 | 0 | 0.33031 |
| ENSMUSG00000084559 | miRNA | Mir1906-2 | 0.66062 | 0 | 0.33031 |
| ENSMUSG00000081807 | pseudogene | Gm14849 | 0.66062 | 0 | 0.33031 |
| ENSMUSG00000080788 | pseudogene | Gm7841 | 0.66062 | 0 | 0.33031 |
| ENSMUSG00000087595 | processed_transcrip | 1810012K08Rik | 56.93225 | 62.6375 | 59.78487 |
| ENSMUSG00000019889 | protein_coding | Ptprk | 745.3991 | 774.6927 | 760.0459 |
| ENSMUSG00000076315 | miRNA | Mir343 | 3.882766 | 2.683826 | 3.283296 |
| ENSMUSG00000074735 | protein_coding | Gm10749 | 0.960442 | 1.712931 | 1.336686 |
| ENSMUSG00000031546 | protein_coding | Gins4 | 1177.794 | 1136.364 | 1157.079 |
| ENSMUSG00000032997 | protein_coding | Chpf | 1569.679 | 1483.86 | 1526.77 |
| ENSMUSG00000049281 | protein_coding | Scn3b | 43.27495 | 47.99707 | 45.63601 |
| ENSMUSG00000021464 | protein_coding | Ror2 | 173.6345 | 185.2345 | 179.4345 |
| ENSMUSG00000021575 | protein_coding | Ahrr | 42.65891 | 38.08131 | 40.37011 |
| ENSMUSG00000090610 | pseudogene | Gm3571 | 138.5186 | 129.2924 | 133.9055 |
| ENSMUSG00000028412 | protein_coding | Slc44a1 | 2064.857 | 2154.502 | 2109.68 |
| ENSMUSG00000087606 | processed_transcrip | Gm16121 | 1.937283 | 1.086569 | 1.511926 |
| ENSMUSG00000080746 | pseudogene | Rpsa-ps12 | 3.584212 | 5.174469 | 4.37934 |
| ENSMUSG00000004931 | protein_coding | Apba3 | 878.9315 | 910.9461 | 894.9388 |
| ENSMUSG00000039396 | protein_coding | Neil3 | 217.352 | 196.5658 | 206.9589 |
| ENSMUSG00000033107 | protein_coding | Rnf125 | 42.6238 | 47.20346 | 44.91363 |
| ENSMUSG00000036985 | protein_coding | Zdhhc9 | 997.9219 | 957.3764 | 977.6492 |
| ENSMUSG00000057778 | protein_coding | Cyb5d2 | 228.3603 | 240.5597 | 234.46 |
| ENSMUSG00000026866 | protein_coding | Kynu | 0.962752 | 1.712931 | 1.337842 |
| ENSMUSG00000030739 | protein_coding | Myh14 | 519.384 | 548.5855 | 533.9847 |
| ENSMUSG00000030427 | protein_coding | Lilra6 | 0.974531 | 1.748606 | 1.361568 |
| ENSMUSG00000082861 | pseudogene | Gm11362 | 0.974531 | 1.748606 | 1.361568 |
| ENSMUSG00000090403 | protein_coding | Lca5 | 1.284863 | 0.662038 | 0.97345 |
| ENSMUSG00000052337 | protein_coding | Immt | 4112.205 | 4220.917 | 4166.561 |
| ENSMUSG00000028034 | protein_coding | Fubp1 | 6806.875 | 7041.369 | 6924.122 |
| ENSMUSG00000081206 | pseudogene | Gm11935 | 2.239415 | 1.315066 | 1.777241 |
| ENSMUSG00000085058 | processed_transcrip | 8030453O22Rik | 6.168026 | 4.548106 | 5.358066 |
| ENSMUSG00000075437 | processed_transcrip | Gm11681 | 5.204006 | 6.878752 | 6.041379 |
| ENSMUSG00000022339 | protein_coding | Ebag9 | 678.1323 | 654.619 | 666.3756 |
| ENSMUSG00000022501 | protein_coding | Prm1 | 6.464269 | 8.202828 | 7.333548 |
| ENSMUSG00000047117 | protein_coding | Ankdd1b | 72.75135 | 78.98154 | 75.86644 |
| ENSMUSG00000037960 | protein_coding | 1110007C09Rik | 356.7605 | 373.0139 | 364.8872 |
| ENSMUSG00000078590 | protein_coding | Gm10959 | 2.569725 | 3.717063 | 3.143394 |
| ENSMUSG00000065643 | snRNA | U6 | 3.594722 | 2.401635 | 2.998179 |
| ENSMUSG00000024829 | protein_coding | Mrpl21 | 898.2254 | 934.2803 | 916.2529 |
| ENSMUSG00000021796 | protein_coding | Bmpr1a | 5332.892 | 5537.754 | 5435.323 |
| ENSMUSG00000030718 | protein_coding | Ppme1 | 1677.316 | 1736.944 | 1707.13 |
| ENSMUSG00000085321 | lincRNA | 4930412F09Rik | 0.65242 | 1.448758 | 1.050589 |
| ENSMUSG00000034708 | protein_coding | Grn | 2138.302 | 2065.25 | 2101.776 |
| ENSMUSG00000045624 | protein_coding | Esf1 | 2791.183 | 2679.338 | 2735.261 |
| ENSMUSG00000038298 | protein_coding | Pdzk1 | 16.53839 | 13.73084 | 15.13462 |
| ENSMUSG00000082264 | pseudogene | Gm12799 | 2.555636 | 3.72571 | 3.140673 |
| ENSMUSG00000082054 | pseudogene | Psmb6-ps | 2.555636 | 3.72571 | 3.140673 |
| ENSMUSG00000059179 | protein_coding | Gm8991 | 30.70744 | 34.63634 | 32.67189 |

| | | | | | |
|---|---|---|---|---|---|
| ENSMUSG00000042873 | protein_coding | Lhfpl4 | 17.77868 | 15.05455 | 16.41661 |
| ENSMUSG00000065013 | snoRNA | SNORD113 | 0.976841 | 1.748606 | 1.362724 |
| ENSMUSG00000081272 | pseudogene | Gm13509 | 712.9553 | 688.1428 | 700.5491 |
| ENSMUSG00000026260 | protein_coding | Ndufa10 | 1853.128 | 1906.69 | 1879.909 |
| ENSMUSG00000049858 | protein_coding | Suox | 314.9318 | 332.1651 | 323.5485 |
| ENSMUSG00000085271 | processed_transcript | E130006D01Rik | 3.902744 | 2.674817 | 3.28878 |
| ENSMUSG00000031015 | protein_coding | Swap70 | 1254.327 | 1298.03 | 1276.178 |
| ENSMUSG00000092029 | pseudogene | Gm9027 | 51.27344 | 56.29325 | 53.78335 |
| ENSMUSG00000047583 | protein_coding | Tyw3 | 188.4494 | 176.3744 | 182.4119 |
| ENSMUSG00000080932 | pseudogene | Gm10224 | 0.64422 | 1.413083 | 1.028652 |
| ENSMUSG00000064658 | snoRNA | SNORA2 | 0.64422 | 1.413083 | 1.028652 |
| ENSMUSG00000085024 | processed_transcript | C230035I16Rik | 49.886 | 56.87809 | 53.38204 |
| ENSMUSG00000042363 | protein_coding | 1110067D22Rik | 969.4617 | 1008.477 | 988.9693 |
| ENSMUSG00000023153 | protein_coding | Tmem52 | 256.8829 | 280.3597 | 268.6213 |
| ENSMUSG00000085971 | processed_transcript | Gm15411 | 5.186338 | 7.142925 | 6.164632 |
| ENSMUSG00000054178 | protein_coding | Gm9938 | 148.5869 | 157.7541 | 153.1705 |
| ENSMUSG00000083736 | pseudogene | Gm6039 | 140.5041 | 131.4648 | 135.9844 |
| ENSMUSG00000004610 | protein_coding | Etfb | 1069.394 | 1034.181 | 1051.788 |
| ENSMUSG00000060090 | protein_coding | Rp2h | 477.0511 | 504.3875 | 490.7193 |
| ENSMUSG00000070639 | protein_coding | Lrrc8b | 689.8244 | 720.1741 | 704.9992 |
| ENSMUSG00000076135 | miRNA | AC163389.1 | 0.65242 | 0 | 0.32621 |
| ENSMUSG00000050711 | protein_coding | Scg2 | 0.65242 | 0 | 0.32621 |
| ENSMUSG00000093146 | snoRNA | AC131793.1 | 0.65242 | 0 | 0.32621 |
| ENSMUSG00000026691 | protein_coding | Fmo3 | 0.65242 | 0 | 0.32621 |
| ENSMUSG00000084143 | pseudogene | Gm13400 | 0.65242 | 0 | 0.32621 |
| ENSMUSG00000085950 | processed_transcript | Gm13589 | 0.65242 | 0 | 0.32621 |
| ENSMUSG00000088365 | misc_RNA | 7SK | 0.65242 | 0 | 0.32621 |
| ENSMUSG00000090953 | lincRNA | Gm17250 | 0.65242 | 0 | 0.32621 |
| ENSMUSG00000084213 | pseudogene | Hspe1-ps5 | 0.65242 | 0 | 0.32621 |
| ENSMUSG00000093242 | miRNA | AL772404.1 | 0.65242 | 0 | 0.32621 |
| ENSMUSG00000088639 | miRNA | Mir1955 | 0.65242 | 0 | 0.32621 |
| ENSMUSG00000080802 | pseudogene | Gm13804 | 0.65242 | 0 | 0.32621 |
| ENSMUSG00000041255 | protein_coding | Tmco5b | 0.65242 | 0 | 0.32621 |
| ENSMUSG00000087678 | processed_transcript | Gm14120 | 0.65242 | 0 | 0.32621 |
| ENSMUSG00000078972 | protein_coding | Gm15557 | 0.65242 | 0 | 0.32621 |
| ENSMUSG00000093120 | miRNA | AL663091.1 | 0.65242 | 0 | 0.32621 |
| ENSMUSG00000086090 | processed_transcript | Gm12531 | 0.65242 | 0 | 0.32621 |
| ENSMUSG00000089689 | pseudogene | Gm4982 | 0.65242 | 0 | 0.32621 |
| ENSMUSG00000082122 | pseudogene | Gm11809 | 0.65242 | 0 | 0.32621 |
| ENSMUSG00000086080 | processed_transcript | 1700123O12Rik | 0.65242 | 0 | 0.32621 |
| ENSMUSG00000093374 | miRNA | AL669940.1 | 0.65242 | 0 | 0.32621 |
| ENSMUSG00000082888 | pseudogene | Gm12934 | 0.65242 | 0 | 0.32621 |
| ENSMUSG00000093031 | misc_RNA | BX537327.1 | 0.65242 | 0 | 0.32621 |
| ENSMUSG00000083326 | pseudogene | Rpl38-ps1 | 0.65242 | 0 | 0.32621 |
| ENSMUSG00000054284 | protein_coding | Gm6543 | 0.65242 | 0 | 0.32621 |
| ENSMUSG00000085839 | processed_transcript | Gm15949 | 0.65242 | 0 | 0.32621 |
| ENSMUSG00000086167 | pseudogene | Gm13827 | 0.65242 | 0 | 0.32621 |
| ENSMUSG00000066867 | protein_coding | Oas1e | 0.65242 | 0 | 0.32621 |
| ENSMUSG00000086885 | processed_transcript | Gm13846 | 0.65242 | 0 | 0.32621 |
| ENSMUSG00000093105 | miRNA | AC153918.1 | 0.65242 | 0 | 0.32621 |
| ENSMUSG00000086527 | processed_transcript | Gm15856 | 0.65242 | 0 | 0.32621 |
| ENSMUSG00000081550 | pseudogene | Gm15877 | 0.65242 | 0 | 0.32621 |
| ENSMUSG00000074377 | protein_coding | Sult2a4 | 0.65242 | 0 | 0.32621 |
| ENSMUSG00000044748 | protein_coding | Defb1 | 0.65242 | 0 | 0.32621 |
| ENSMUSG00000088414 | miRNA | Mir1967 | 0.65242 | 0 | 0.32621 |
| ENSMUSG00000090402 | lincRNA | Gm17400 | 0.65242 | 0 | 0.32621 |
| ENSMUSG00000077258 | snoRNA | SNORA17 | 0.65242 | 0 | 0.32621 |
| ENSMUSG00000057626 | pseudogene | Gm5621 | 0.65242 | 0 | 0.32621 |

| | | | | | |
|---|---|---|---|---|---|
| ENSMUSG00000066368 | protein_coding | 4921517D21Rik | 0.65242 | 0 | 0.32621 |
| ENSMUSG00000088086 | misc_RNA | 7SK | 0.65242 | 0 | 0.32621 |
| ENSMUSG00000077090 | miRNA | AC155640.1 | 0.65242 | 0 | 0.32621 |
| ENSMUSG00000075854 | snRNA | U6 | 0.65242 | 0 | 0.32621 |
| ENSMUSG00000084819 | processed_transcrip | Gm11967 | 0.65242 | 0 | 0.32621 |
| ENSMUSG00000085452 | processed_transcrip | 4930554G24Rik | 0.65242 | 0 | 0.32621 |
| ENSMUSG00000078924 | protein_coding | Gm12169 | 0.65242 | 0 | 0.32621 |
| ENSMUSG00000080487 | miRNA | AL606479.1 | 0.65242 | 0 | 0.32621 |
| ENSMUSG00000018916 | protein_coding | Csf2 | 0.65242 | 0 | 0.32621 |
| ENSMUSG00000081472 | pseudogene | Gm12620 | 0.65242 | 0 | 0.32621 |
| ENSMUSG00000086636 | processed_transcrip | Gm12949 | 0.65242 | 0 | 0.32621 |
| ENSMUSG00000082252 | pseudogene | Gm11204 | 0.65242 | 0 | 0.32621 |
| ENSMUSG00000087669 | processed_transcrip | Gm11724 | 0.65242 | 0 | 0.32621 |
| ENSMUSG00000020581 | protein_coding | Agr2 | 0.65242 | 0 | 0.32621 |
| ENSMUSG00000086372 | processed_transcrip | Gm16246 | 0.65242 | 0 | 0.32621 |
| ENSMUSG00000079034 | protein_coding | Gm8300 | 0.65242 | 0 | 0.32621 |
| ENSMUSG00000078312 | pseudogene | Gm6344 | 0.65242 | 0 | 0.32621 |
| ENSMUSG00000087319 | processed_transcrip | Gm15907 | 0.65242 | 0 | 0.32621 |
| ENSMUSG00000063162 | protein_coding | Gm16416 | 0.65242 | 0 | 0.32621 |
| ENSMUSG00000078128 | protein_coding | Gm2178 | 0.65242 | 0 | 0.32621 |
| ENSMUSG00000093191 | misc_RNA | AC113598.1 | 0.65242 | 0 | 0.32621 |
| ENSMUSG00000091788 | pseudogene | Gm8032 | 0.65242 | 0 | 0.32621 |
| ENSMUSG00000072607 | protein_coding | Gm10378 | 0.65242 | 0 | 0.32621 |
| ENSMUSG00000087212 | protein_coding | 9330188P03Rik | 0.65242 | 0 | 0.32621 |
| ENSMUSG00000082233 | pseudogene | Gm6506 | 0.65242 | 0 | 0.32621 |
| ENSMUSG00000014372 | protein_coding | Cyp2d10 | 0.65242 | 0 | 0.32621 |
| ENSMUSG00000022534 | protein_coding | Mefv | 0.65242 | 0 | 0.32621 |
| ENSMUSG00000070225 | rRNA | n-R5s33 | 0.65242 | 0 | 0.32621 |
| ENSMUSG00000073420 | processed_transcrip | Btnl5 | 0.65242 | 0 | 0.32621 |
| ENSMUSG00000048231 | protein_coding | H2-M10.4 | 0.65242 | 0 | 0.32621 |
| ENSMUSG00000081724 | protein_coding | Olfr129 | 0.65242 | 0 | 0.32621 |
| ENSMUSG00000043972 | protein_coding | Opn5 | 0.65242 | 0 | 0.32621 |
| ENSMUSG00000033993 | protein_coding | 1700018A14Rik | 0.65242 | 0 | 0.32621 |
| ENSMUSG00000049247 | protein_coding | Rpl37-ps1 | 0.65242 | 0 | 0.32621 |
| ENSMUSG00000078017 | miRNA | Mir883a | 0.65242 | 0 | 0.32621 |
| ENSMUSG00000082759 | pseudogene | Gm14826 | 0.65242 | 0 | 0.32621 |
| ENSMUSG00000083971 | pseudogene | Gm5943 | 0.65242 | 0 | 0.32621 |
| ENSMUSG00000089255 | snoRNA | Snora78 | 7.407043 | 5.377024 | 6.392033 |
| ENSMUSG00000028414 | protein_coding | Fktn | 1159.931 | 1116.653 | 1138.292 |
| ENSMUSG00000052477 | protein_coding | C130026I21Rik | 2.920013 | 4.105918 | 3.512966 |
| ENSMUSG00000066037 | protein_coding | Hnrnpr | 4476.151 | 4601.968 | 4539.06 |
| ENSMUSG00000036764 | protein_coding | Dnajc12 | 209.1197 | 198.6266 | 203.8732 |
| ENSMUSG00000022401 | protein_coding | Xpnpep3 | 789.5636 | 822.9157 | 806.2396 |
| ENSMUSG00000000194 | protein_coding | Gpr107 | 1627.67 | 1676.869 | 1652.269 |
| ENSMUSG00000084843 | processed_transcrip | B230312C02Rik | 11.35205 | 9.173723 | 10.26289 |
| ENSMUSG00000093205 | misc_RNA | AC122417.1 | 1.951372 | 1.086569 | 1.51897 |
| ENSMUSG00000028544 | protein_coding | Slc5a9 | 5.175828 | 3.690397 | 4.433112 |
| ENSMUSG00000070531 | protein_coding | Wfdc6b | 115.4437 | 124.7787 | 120.1112 |
| ENSMUSG00000020064 | protein_coding | Herc4 | 3142.58 | 3234.48 | 3188.53 |
| ENSMUSG00000055093 | pseudogene | Gm8430 | 38.47782 | 34.00169 | 36.23976 |
| ENSMUSG00000050108 | protein_coding | Bpifc | 23.36242 | 19.95584 | 21.65913 |
| ENSMUSG00000052488 | protein_coding | Cherp | 3017.654 | 2885.72 | 2951.687 |
| ENSMUSG00000025931 | protein_coding | Paqr8 | 1563.435 | 1620.497 | 1591.966 |
| ENSMUSG00000033715 | protein_coding | Akr1c14 | 291.4286 | 312.6746 | 302.0516 |
| ENSMUSG00000073067 | lincRNA | 9130019P16Rik | 37.72193 | 33.63371 | 35.67782 |
| ENSMUSG00000021486 | protein_coding | Prelid1 | 2948.173 | 3035.63 | 2991.902 |
| ENSMUSG00000034765 | protein_coding | Dusp5 | 443.5057 | 419.737 | 431.6214 |
| ENSMUSG00000006673 | protein_coding | Qrich1 | 4701.618 | 4554.075 | 4627.847 |

| | | | | | |
|---|---|---|---|---|---|
| ENSMUSG00000078503 | protein_coding | Gm13225 | 179.9681 | 260.8878 | 220.4279 |
| ENSMUSG00000073207 | protein_coding | Ccdc160 | 1352.025 | 1408.849 | 1380.437 |
| ENSMUSG00000090146 | processed_transcrip | 4930405H06Rik | 4.529297 | 3.126376 | 3.827836 |
| ENSMUSG00000040414 | protein_coding | Slc25a28 | 719.2916 | 683.5643 | 701.4279 |
| ENSMUSG00000081788 | pseudogene | Gm5898 | 87.60257 | 130.3154 | 108.959 |
| ENSMUSG00000031628 | protein_coding | Casp3 | 429.1951 | 448.3538 | 438.7745 |
| ENSMUSG00000030244 | protein_coding | Gys2 | 0.98273 | 1.748606 | 1.365668 |
| ENSMUSG00000021337 | protein_coding | Scgn | 0.98273 | 1.748606 | 1.365668 |
| ENSMUSG00000021819 | protein_coding | 2310021P13Rik | 4548.685 | 4343.836 | 4446.26 |
| ENSMUSG00000067224 | pseudogene | Gm3695 | 3.213945 | 4.494774 | 3.85436 |
| ENSMUSG00000027444 | protein_coding | 8030411F24Rik | 499.1915 | 531.7087 | 515.4501 |
| ENSMUSG00000021278 | protein_coding | Amn | 28.84187 | 34.35084 | 31.59635 |
| ENSMUSG00000030200 | protein_coding | Bcl2l14 | 2.883635 | 4.096909 | 3.490272 |
| ENSMUSG00000031119 | protein_coding | Gpc4 | 1753.647 | 1817.679 | 1785.663 |
| ENSMUSG00000092098 | lincRNA | Gm17529 | 9.052704 | 11.18614 | 10.11942 |
| ENSMUSG00000035125 | protein_coding | AW146020 | 339.3166 | 320.751 | 330.0338 |
| ENSMUSG00000026942 | protein_coding | Traf2 | 711.0556 | 677.9726 | 694.5141 |
| ENSMUSG00000022500 | protein_coding | Litaf | 2332.903 | 2404.494 | 2368.698 |
| ENSMUSG00000050612 | protein_coding | Txndc2 | 28.3448 | 32.616 | 30.4804 |
| ENSMUSG00000032637 | protein_coding | Atxn2l | 3478.228 | 3633.732 | 3555.98 |
| ENSMUSG00000050963 | protein_coding | Kcns2 | 138.8731 | 153.0584 | 145.9657 |
| ENSMUSG00000086575 | processed_transcrip | Gm14104 | 3.854588 | 2.710492 | 3.28254 |
| ENSMUSG00000079496 | protein_coding | Gm11129 | 1.945483 | 1.086569 | 1.516026 |
| ENSMUSG00000003418 | protein_coding | St8sia6 | 37.44335 | 33.38362 | 35.41348 |
| ENSMUSG00000013997 | protein_coding | Nit1 | 553.9308 | 577.9735 | 565.9522 |
| ENSMUSG00000090409 | lincRNA | Gm17401 | 70.32647 | 64.49313 | 67.4098 |
| ENSMUSG00000022009 | protein_coding | Nufip1 | 786.0026 | 814.6644 | 800.3335 |
| ENSMUSG00000032330 | protein_coding | Cox7a2 | 1300.187 | 1253.342 | 1276.765 |
| ENSMUSG00000035469 | protein_coding | Rcbtb1 | 2065.523 | 2006.91 | 2036.216 |
| ENSMUSG00000085217 | processed_transcrip | 6030471H07Rik | 4.180276 | 5.694167 | 4.937222 |
| ENSMUSG00000083191 | pseudogene | Gm15571 | 2.269904 | 1.350742 | 1.810323 |
| ENSMUSG00000060808 | processed_transcrip | 3110043A19Rik | 3.910944 | 5.358643 | 4.634793 |
| ENSMUSG00000085177 | processed_transcrip | Gm11209 | 11.32619 | 13.65048 | 12.48833 |
| ENSMUSG00000084347 | pseudogene | Akt2-ps | 119.155 | 127.756 | 123.4555 |
| ENSMUSG00000085280 | processed_transcrip | Gm16151 | 0.646531 | 0 | 0.323266 |
| ENSMUSG00000065520 | miRNA | Mir128-1 | 0.646531 | 0 | 0.323266 |
| ENSMUSG00000091743 | protein_coding | Gm5705 | 0.646531 | 0 | 0.323266 |
| ENSMUSG00000069875 | processed_transcrip | Frmd4a | 0.646531 | 0 | 0.323266 |
| ENSMUSG00000069080 | protein_coding | Lcn11 | 0.646531 | 0 | 0.323266 |
| ENSMUSG00000084226 | pseudogene | Gm13426 | 0.646531 | 0 | 0.323266 |
| ENSMUSG00000082931 | pseudogene | Gm13586 | 0.646531 | 0 | 0.323266 |
| ENSMUSG00000082066 | pseudogene | Gm13904 | 0.646531 | 0 | 0.323266 |
| ENSMUSG00000084089 | pseudogene | Gm14099 | 0.646531 | 0 | 0.323266 |
| ENSMUSG00000081243 | pseudogene | Gm14037 | 0.646531 | 0 | 0.323266 |
| ENSMUSG00000078882 | protein_coding | Gm14431 | 0.646531 | 0 | 0.323266 |
| ENSMUSG00000077293 | snoRNA | SNORA17 | 0.646531 | 0 | 0.323266 |
| ENSMUSG00000084518 | misc_RNA | 7SK | 0.646531 | 0 | 0.323266 |
| ENSMUSG00000084148 | pseudogene | Gm12380 | 0.646531 | 0 | 0.323266 |
| ENSMUSG00000082141 | pseudogene | Gm11212 | 0.646531 | 0 | 0.323266 |
| ENSMUSG00000086592 | processed_transcrip | Gm12682 | 0.646531 | 0 | 0.323266 |
| ENSMUSG00000088014 | misc_RNA | 7SK | 0.646531 | 0 | 0.323266 |
| ENSMUSG00000092863 | misc_RNA | BX000694.1 | 0.646531 | 0 | 0.323266 |
| ENSMUSG00000073226 | protein_coding | Gm10482 | 0.646531 | 0 | 0.323266 |
| ENSMUSG00000084745 | snoRNA | SNORA57 | 0.646531 | 0 | 0.323266 |
| ENSMUSG00000088085 | snRNA | U6 | 0.646531 | 0 | 0.323266 |
| ENSMUSG00000055408 | lincRNA | Gm15053 | 0.646531 | 0 | 0.323266 |
| ENSMUSG00000084454 | snRNA | U6 | 0.646531 | 0 | 0.323266 |
| ENSMUSG00000082992 | pseudogene | Sult2a7 | 0.646531 | 0 | 0.323266 |

| | | | | | |
|---|---|---|---|---|---|
| ENSMUSG00000089230 | snRNA | U7 | 0.646531 | 0 | 0.323266 |
| ENSMUSG00000050063 | protein_coding | Klk6 | 0.646531 | 0 | 0.323266 |
| ENSMUSG00000065483 | miRNA | Mir181c | 0.646531 | 0 | 0.323266 |
| ENSMUSG00000091098 | protein_coding | Cldn21 | 0.646531 | 0 | 0.323266 |
| ENSMUSG00000041748 | protein_coding | Ccrl1 | 0.646531 | 0 | 0.323266 |
| ENSMUSG00000074686 | pseudogene | Gm9046 | 0.646531 | 0 | 0.323266 |
| ENSMUSG00000082737 | pseudogene | Gm12026 | 0.646531 | 0 | 0.323266 |
| ENSMUSG00000084362 | pseudogene | 2610024D14Rik | 0.646531 | 0 | 0.323266 |
| ENSMUSG00000086311 | processed_transcrip | Gm12122 | 0.646531 | 0 | 0.323266 |
| ENSMUSG00000084152 | pseudogene | Gm11617 | 0.646531 | 0 | 0.323266 |
| ENSMUSG00000077958 | miRNA | AC157352.1 | 0.646531 | 0 | 0.323266 |
| ENSMUSG00000085004 | processed_transcrip | 5430427M07Rik | 0.646531 | 0 | 0.323266 |
| ENSMUSG00000086265 | pseudogene | Marcksl1-ps4 | 0.646531 | 0 | 0.323266 |
| ENSMUSG00000054258 | protein_coding | Gm5082 | 0.646531 | 0 | 0.323266 |
| ENSMUSG00000076246 | miRNA | AC122357.1 | 0.646531 | 0 | 0.323266 |
| ENSMUSG00000086743 | pseudogene | Hnrnpa1l2-ps | 0.646531 | 0 | 0.323266 |
| ENSMUSG00000084452 | misc_RNA | 7SK | 0.646531 | 0 | 0.323266 |
| ENSMUSG00000091508 | protein_coding | Gm7951 | 0.646531 | 0 | 0.323266 |
| ENSMUSG00000077890 | miRNA | Mir875 | 0.646531 | 0 | 0.323266 |
| ENSMUSG00000022366 | protein_coding | Slc22a22 | 0.646531 | 0 | 0.323266 |
| ENSMUSG00000062543 | protein_coding | Gsdmcl1 | 0.646531 | 0 | 0.323266 |
| ENSMUSG00000067594 | protein_coding | Krt77 | 0.646531 | 0 | 0.323266 |
| ENSMUSG00000077476 | snoRNA | SNORA17 | 0.646531 | 0 | 0.323266 |
| ENSMUSG00000090525 | protein_coding | Gm7742 | 0.646531 | 0 | 0.323266 |
| ENSMUSG00000092975 | miRNA | AC122252.1 | 0.646531 | 0 | 0.323266 |
| ENSMUSG00000090012 | processed_transcrip | Gm16555 | 0.646531 | 0 | 0.323266 |
| ENSMUSG00000091842 | protein_coding | Gm16490 | 0.646531 | 0 | 0.323266 |
| ENSMUSG00000024471 | protein_coding | Myot | 0.646531 | 0 | 0.323266 |
| ENSMUSG00000092154 | lincRNA | Gm17270 | 0.646531 | 0 | 0.323266 |
| ENSMUSG00000067085 | protein_coding | Gm10197 | 0.646531 | 0 | 0.323266 |
| ENSMUSG00000089127 | snoRNA | SNORA40 | 0.646531 | 0 | 0.323266 |
| ENSMUSG00000082324 | pseudogene | Gm14615 | 0.646531 | 0 | 0.323266 |
| ENSMUSG00000070081 | miRNA | Mir505 | 0.646531 | 0 | 0.323266 |
| ENSMUSG00000081703 | pseudogene | Gm6285 | 0.646531 | 0 | 0.323266 |
| ENSMUSG00000082197 | pseudogene | Gm8577 | 0.646531 | 0 | 0.323266 |
| ENSMUSG00000086008 | processed_transcrip | Gm8817 | 0.646531 | 0 | 0.323266 |
| ENSMUSG00000085418 | processed_transcrip | 4930483C13Rik | 0.974531 | 1.712931 | 1.343731 |
| ENSMUSG00000091845 | pseudogene | Gm4604 | 32.38001 | 28.59837 | 30.48919 |
| ENSMUSG00000032537 | protein_coding | Ephb1 | 485.3369 | 385.7925 | 435.5647 |
| ENSMUSG00000010529 | protein_coding | Gm266 | 8.720308 | 11.36094 | 10.04063 |
| ENSMUSG00000039653 | protein_coding | Baat | 2.255815 | 3.408205 | 2.83201 |
| ENSMUSG00000092595 | processed_transcrip | Gm20427 | 7.084933 | 5.367653 | 6.226293 |
| ENSMUSG00000065259 | snoRNA | Snora30 | 4.82554 | 6.409537 | 5.617538 |
| ENSMUSG00000073555 | protein_coding | Gm4951 | 15.86264 | 19.14602 | 17.50433 |
| ENSMUSG00000082454 | pseudogene | Gm12183 | 244.1433 | 256.5653 | 250.3543 |
| ENSMUSG00000038665 | protein_coding | Dgki | 110.7071 | 120.4468 | 115.5769 |
| ENSMUSG00000022452 | protein_coding | 1500032L24Rik | 1166.164 | 1127.531 | 1146.848 |
| ENSMUSG00000017548 | protein_coding | Suz12 | 4319.768 | 4466.387 | 4393.078 |
| ENSMUSG00000078365 | protein_coding | Mos | 3.536056 | 4.969426 | 4.252741 |
| ENSMUSG00000050705 | protein_coding | 2310061I04Rik | 675.0901 | 645.9832 | 660.5367 |
| ENSMUSG00000053916 | protein_coding | Nanp | 116.6356 | 108.8402 | 112.7379 |
| ENSMUSG00000029363 | protein_coding | Rfc5 | 1090.469 | 1056.324 | 1073.396 |
| ENSMUSG00000021217 | protein_coding | Tshz3 | 403.0314 | 385.5993 | 394.3153 |
| ENSMUSG00000091403 | protein_coding | 9530085L02Rik | 0.976841 | 1.712931 | 1.344886 |
| ENSMUSG00000087429 | processed_transcrip | Gm16235 | 4.853718 | 6.427194 | 5.640456 |
| ENSMUSG00000087453 | pseudogene | Gm16327 | 0.64422 | 0 | 0.32211 |
| ENSMUSG00000045174 | protein_coding | Fam123c | 0.64422 | 0 | 0.32211 |
| ENSMUSG00000027790 | protein_coding | Sis | 0.64422 | 0 | 0.32211 |

| | | | | | |
|---|---|---|---|---|---|
| ENSMUSG00000092701 | miRNA | AC098732.1 | 0.64422 | 0 | 0.32211 |
| ENSMUSG00000042240 | protein_coding | Crybb2 | 0.64422 | 0 | 0.32211 |
| ENSMUSG00000084392 | pseudogene | Gm15757 | 0.64422 | 0 | 0.32211 |
| ENSMUSG00000092760 | miRNA | AC153910.1 | 0.64422 | 0 | 0.32211 |
| ENSMUSG00000055048 | protein_coding | Gm9962 | 0.64422 | 0 | 0.32211 |
| ENSMUSG00000092921 | miRNA | AC091333.3 | 0.64422 | 0 | 0.32211 |
| ENSMUSG00000089179 | snoRNA | SNORA17 | 0.64422 | 0 | 0.32211 |
| ENSMUSG00000065252 | snRNA | U6 | 0.64422 | 0 | 0.32211 |
| ENSMUSG00000093282 | miRNA | AC153974.1 | 0.64422 | 0 | 0.32211 |
| ENSMUSG00000043916 | pseudogene | D630028G08Rik | 0.64422 | 0 | 0.32211 |
| ENSMUSG00000092434 | processed_transcrip | RP23-25D18.6 | 0.64422 | 0 | 0.32211 |
| ENSMUSG00000087393 | processed_transcrip | 1700023C21Rik | 0.64422 | 0 | 0.32211 |
| ENSMUSG00000051804 | protein_coding | Adam6b | 0.64422 | 0 | 0.32211 |
| ENSMUSG00000076712 | IG_V_gene | Gm16829 | 0.64422 | 0 | 0.32211 |
| ENSMUSG00000076714 | IG_V_gene | AB069917 | 0.64422 | 0 | 0.32211 |
| ENSMUSG00000087276 | processed_transcrip | Gm11378 | 0.64422 | 0 | 0.32211 |
| ENSMUSG00000068399 | protein_coding | Gm7247 | 0.64422 | 0 | 0.32211 |
| ENSMUSG00000076857 | IG_V_gene | Gm13933 | 0.64422 | 0 | 0.32211 |
| ENSMUSG00000021966 | protein_coding | Prss52 | 0.64422 | 0 | 0.32211 |
| ENSMUSG00000060417 | protein_coding | Gm8603 | 0.64422 | 0 | 0.32211 |
| ENSMUSG00000079693 | protein_coding | Gm11165 | 0.64422 | 0 | 0.32211 |
| ENSMUSG00000091992 | lincRNA | Gm17458 | 0.64422 | 0 | 0.32211 |
| ENSMUSG00000067909 | protein_coding | Slxl1 | 0.64422 | 0 | 0.32211 |
| ENSMUSG00000081167 | pseudogene | Gm8880 | 0.64422 | 0 | 0.32211 |
| ENSMUSG00000008845 | protein_coding | Cd163 | 60.05219 | 54.81488 | 57.43354 |
| ENSMUSG00000057133 | protein_coding | Chd6 | 5184.06 | 5047.46 | 5115.76 |
| ENSMUSG00000038717 | protein_coding | Atp5l | 1103.655 | 1145.206 | 1124.431 |
| ENSMUSG00000056947 | protein_coding | Mab21l1 | 5.180674 | 7.329588 | 6.255131 |
| ENSMUSG00000078627 | protein_coding | 10-Mar | 8.414373 | 10.80914 | 9.611758 |
| ENSMUSG00000081612 | pseudogene | Xlr5e-ps | 1.926772 | 3.0907 | 2.508736 |
| ENSMUSG00000052273 | protein_coding | Dnahc3 | 11.9681 | 14.29486 | 13.13148 |
| ENSMUSG00000026839 | protein_coding | Upp2 | 29.32626 | 33.28669 | 31.30648 |
| ENSMUSG00000030353 | protein_coding | Tead4 | 21.56767 | 18.3766 | 19.97213 |
| ENSMUSG00000029534 | protein_coding | St7 | 239.3554 | 223.6091 | 231.4822 |
| ENSMUSG00000032501 | protein_coding | Trib1 | 618.6311 | 646.4066 | 632.5188 |
| ENSMUSG00000049130 | protein_coding | C5ar1 | 10.70425 | 13.16361 | 11.93393 |
| ENSMUSG00000083823 | pseudogene | Amd-ps1 | 0.66062 | 1.422092 | 1.041356 |
| ENSMUSG00000068099 | protein_coding | 1500009C09Rik | 33.99809 | 39.3928 | 36.69545 |
| ENSMUSG00000026374 | protein_coding | Tsn | 3722.865 | 3622.89 | 3672.877 |
| ENSMUSG00000039826 | protein_coding | Trub2 | 631.6052 | 607.3436 | 619.4744 |
| ENSMUSG00000085799 | pseudogene | Gm6963 | 2.312171 | 1.086569 | 1.69937 |
| ENSMUSG00000050663 | protein_coding | Trhde | 1.911416 | 1.033237 | 1.472326 |
| ENSMUSG00000073639 | protein_coding | Rab18 | 4362.429 | 4234.664 | 4298.546 |
| ENSMUSG00000085763 | processed_transcrip | 2700081L22Rik | 5.532005 | 4.096909 | 4.814457 |
| ENSMUSG00000085879 | lincRNA | C430014B12Rik | 7.833665 | 10.08192 | 8.95779 |
| ENSMUSG00000031224 | protein_coding | Magee2 | 27.00798 | 23.23651 | 25.12225 |
| ENSMUSG00000027242 | protein_coding | Wdr76 | 1103.188 | 1068.134 | 1085.661 |
| ENSMUSG00000047676 | protein_coding | Rpsa-ps10 | 215.4901 | 204.097 | 209.7935 |
| ENSMUSG00000082110 | pseudogene | Gm11196 | 0.960442 | 1.686265 | 1.323353 |
| ENSMUSG00000031886 | protein_coding | Ces2e | 13.88771 | 11.54869 | 12.7182 |
| ENSMUSG00000030724 | protein_coding | Cd19 | 17.11597 | 13.93339 | 15.52468 |
| ENSMUSG00000074744 | lincRNA | Gm807 | 10.60817 | 13.05042 | 11.82929 |
| ENSMUSG00000037683 | protein_coding | Armc3 | 4.893674 | 6.560886 | 5.72728 |
| ENSMUSG00000071868 | protein_coding | Gm10349 | 5.503827 | 4.132585 | 4.818206 |
| ENSMUSG00000069920 | protein_coding | B3gnt9-ps | 239.1612 | 253.6683 | 246.4148 |
| ENSMUSG00000037544 | protein_coding | Dlgap5 | 854.1833 | 886.4669 | 870.3251 |
| ENSMUSG00000085933 | processed_transcrip | Tmem61 | 2.289882 | 3.452528 | 2.871205 |
| ENSMUSG00000031887 | protein_coding | Tradd | 278.9523 | 294.3134 | 286.6328 |

| | | | | | |
|---|---|---|---|---|---|
| ENSMUSG00000039634 | protein_coding | Zfp189 | 526.4999 | 495.8539 | 511.1769 |
| ENSMUSG00000050912 | protein_coding | Tmem123 | 6352.903 | 6585.676 | 6469.29 |
| ENSMUSG00000041915 | protein_coding | Ammecr1l | 953.7772 | 920.0833 | 936.9303 |
| ENSMUSG00000085509 | processed_transcrip | Gm11521 | 2.908235 | 4.123575 | 3.515905 |
| ENSMUSG00000037185 | protein_coding | Krt80 | 2.889525 | 4.096909 | 3.493217 |
| ENSMUSG00000080613 | miRNA | AC121604.1 | 0.954552 | 1.695274 | 1.324913 |
| ENSMUSG00000082170 | pseudogene | Gm8617 | 0.962752 | 1.686265 | 1.324509 |
| ENSMUSG00000020475 | protein_coding | Pgam2 | 37.59348 | 33.35731 | 35.4754 |
| ENSMUSG00000038209 | protein_coding | Itln1 | 0.98273 | 1.712931 | 1.347831 |
| ENSMUSG00000091229 | pseudogene | Gm7177 | 0.98273 | 1.712931 | 1.347831 |
| ENSMUSG00000087147 | processed_transcrip | Gm14228 | 5.497938 | 4.007902 | 4.75292 |
| ENSMUSG00000030753 | protein_coding | Prkrir | 2753.326 | 2670.851 | 2712.089 |
| ENSMUSG00000049560 | protein_coding | Defb20 | 2.928213 | 4.123575 | 3.525894 |
| ENSMUSG00000077314 | snoRNA | SNORA17 | 0.638331 | 0 | 0.319166 |
| ENSMUSG00000082160 | pseudogene | Gm11578 | 0.638331 | 0 | 0.319166 |
| ENSMUSG00000089730 | processed_transcrip | 1700007P06Rik | 0.638331 | 0 | 0.319166 |
| ENSMUSG00000083533 | pseudogene | Gm13450 | 0.638331 | 0 | 0.319166 |
| ENSMUSG00000087694 | lincRNA | A530058N18Rik | 0.638331 | 0 | 0.319166 |
| ENSMUSG00000084620 | misc_RNA | 7SK | 0.638331 | 0 | 0.319166 |
| ENSMUSG00000091691 | lincRNA | Gm17667 | 0.638331 | 0 | 0.319166 |
| ENSMUSG00000028174 | protein_coding | Rpe65 | 0.638331 | 0 | 0.319166 |
| ENSMUSG00000087402 | processed_transcrip | Gm11867 | 0.638331 | 0 | 0.319166 |
| ENSMUSG00000028362 | protein_coding | Tnfsf8 | 0.638331 | 0 | 0.319166 |
| ENSMUSG00000082416 | pseudogene | Gm11414 | 0.638331 | 0 | 0.319166 |
| ENSMUSG00000083272 | pseudogene | Gm12550 | 0.638331 | 0 | 0.319166 |
| ENSMUSG00000086074 | processed_transcrip | Gm13274 | 0.638331 | 0 | 0.319166 |
| ENSMUSG00000065299 | snRNA | U6 | 0.638331 | 0 | 0.319166 |
| ENSMUSG00000082877 | pseudogene | Gm13009 | 0.638331 | 0 | 0.319166 |
| ENSMUSG00000086159 | processed_transcrip | Gm13025 | 0.638331 | 0 | 0.319166 |
| ENSMUSG00000043430 | protein_coding | Psapl1 | 0.638331 | 0 | 0.319166 |
| ENSMUSG00000080700 | pseudogene | Gm15981 | 0.638331 | 0 | 0.319166 |
| ENSMUSG00000079223 | pseudogene | Gm8778 | 0.638331 | 0 | 0.319166 |
| ENSMUSG00000078041 | miRNA | Mir292 | 0.638331 | 0 | 0.319166 |
| ENSMUSG00000082782 | pseudogene | Gm5319 | 0.638331 | 0 | 0.319166 |
| ENSMUSG00000055193 | protein_coding | Klk15 | 0.638331 | 0 | 0.319166 |
| ENSMUSG00000030500 | protein_coding | Slc17a6 | 0.638331 | 0 | 0.319166 |
| ENSMUSG00000059867 | protein_coding | Olfr960 | 0.638331 | 0 | 0.319166 |
| ENSMUSG00000093316 | misc_RNA | AC110235.1 | 0.638331 | 0 | 0.319166 |
| ENSMUSG00000092565 | pseudogene | RP23-469I15.4 | 0.638331 | 0 | 0.319166 |
| ENSMUSG00000084995 | processed_transcrip | 4930596M17Rik | 0.638331 | 0 | 0.319166 |
| ENSMUSG00000025396 | protein_coding | Hsd17b6 | 0.638331 | 0 | 0.319166 |
| ENSMUSG00000087080 | processed_transcrip | Gm12199 | 0.638331 | 0 | 0.319166 |
| ENSMUSG00000087668 | processed_transcrip | Gm11186 | 0.638331 | 0 | 0.319166 |
| ENSMUSG00000083052 | pseudogene | Gm12285 | 0.638331 | 0 | 0.319166 |
| ENSMUSG00000082945 | pseudogene | Gm11540 | 0.638331 | 0 | 0.319166 |
| ENSMUSG00000091963 | protein_coding | Gm17658 | 0.638331 | 0 | 0.319166 |
| ENSMUSG00000091328 | protein_coding | Gm3542 | 0.638331 | 0 | 0.319166 |
| ENSMUSG00000021867 | protein_coding | Gm9746 | 0.638331 | 0 | 0.319166 |
| ENSMUSG00000093034 | miRNA | AC154327.1 | 0.638331 | 0 | 0.319166 |
| ENSMUSG00000090305 | protein_coding | Gm5459 | 0.638331 | 0 | 0.319166 |
| ENSMUSG00000085500 | lincRNA | Gm16976 | 0.638331 | 0 | 0.319166 |
| ENSMUSG00000090228 | pseudogene | Gm16289 | 0.638331 | 0 | 0.319166 |
| ENSMUSG00000076039 | miRNA | AC115005.1 | 0.638331 | 0 | 0.319166 |
| ENSMUSG00000065572 | miRNA | Mir130b | 0.638331 | 0 | 0.319166 |
| ENSMUSG00000075330 | protein_coding | A930003A15Rik | 0.638331 | 0 | 0.319166 |
| ENSMUSG00000083226 | pseudogene | Gm7831 | 0.638331 | 0 | 0.319166 |
| ENSMUSG00000075780 | snRNA | U6 | 0.638331 | 0 | 0.319166 |
| ENSMUSG00000073445 | protein_coding | Vmn2r111 | 0.638331 | 0 | 0.319166 |

| | | | | | |
|---|---|---|---|---|---|
| ENSMUSG00000092277 | protein_coding | RP23-108F2.4 | 0.638331 | 0 | 0.319166 |
| ENSMUSG00000080468 | miRNA | Mir1195 | 0.638331 | 0 | 0.319166 |
| ENSMUSG00000037346 | protein_coding | Hrh4 | 0.638331 | 0 | 0.319166 |
| ENSMUSG00000085300 | pseudogene | Gm16345 | 0.638331 | 0 | 0.319166 |
| ENSMUSG00000088493 | snoRNA | SNORA40 | 0.638331 | 0 | 0.319166 |
| ENSMUSG00000048292 | protein_coding | Olfr1417 | 0.638331 | 0 | 0.319166 |
| ENSMUSG00000082458 | pseudogene | Gm9430 | 0.638331 | 0 | 0.319166 |
| ENSMUSG00000088394 | snRNA | U1 | 0.638331 | 0 | 0.319166 |
| ENSMUSG00000084831 | pseudogene | Gm14710 | 0.638331 | 0 | 0.319166 |
| ENSMUSG00000031326 | protein_coding | Cdx4 | 0.638331 | 0 | 0.319166 |
| ENSMUSG00000081415 | pseudogene | Gm15176 | 0.638331 | 0 | 0.319166 |
| ENSMUSG00000084806 | processed_transcrip | Gm15232 | 0.638331 | 0 | 0.319166 |
| ENSMUSG00000017288 | protein_coding | Vps53 | 1192.85 | 1142.068 | 1167.459 |
| ENSMUSG00000090397 | processed_transcrip | Gm17096 | 45.82537 | 51.6986 | 48.76198 |
| ENSMUSG00000030116 | protein_coding | Mfap5 | 299.154 | 314.4332 | 306.7936 |
| ENSMUSG00000057182 | protein_coding | Scn3a | 43.81086 | 49.65382 | 46.73234 |
| ENSMUSG00000008333 | protein_coding | Snrpb2 | 1771.278 | 1830.122 | 1800.7 |
| ENSMUSG00000026088 | protein_coding | Mitd1 | 285.6475 | 302.2348 | 293.9411 |
| ENSMUSG00000021371 | protein_coding | Ccdc90a | 236.715 | 250.6494 | 243.6822 |
| ENSMUSG00000045757 | protein_coding | Zfp764 | 80.08355 | 87.00966 | 83.54661 |
| ENSMUSG00000041309 | protein_coding | Nkx6-2 | 28.71022 | 32.65567 | 30.68294 |
| ENSMUSG00000024527 | protein_coding | Afg3l2 | 3060.681 | 2959.806 | 3010.244 |
| ENSMUSG00000053716 | protein_coding | Dusp7 | 618.5143 | 648.7734 | 633.6439 |
| ENSMUSG00000037541 | protein_coding | Shank2 | 443.735 | 471.1341 | 457.4345 |
| ENSMUSG00000034949 | protein_coding | Zfr2 | 269.1173 | 286.6052 | 277.8613 |
| ENSMUSG00000050439 | protein_coding | Enthd1 | 61.51536 | 54.54926 | 58.03231 |
| ENSMUSG00000033726 | protein_coding | Emx1 | 3.858166 | 2.701483 | 3.279825 |
| ENSMUSG00000046242 | protein_coding | 1600029I14Rik | 5.501517 | 4.052586 | 4.777051 |
| ENSMUSG00000086784 | protein_coding | Isoc2a | 917.5062 | 951.4534 | 934.4798 |
| ENSMUSG00000021990 | protein_coding | Spata13 | 1088.002 | 1141.221 | 1114.611 |
| ENSMUSG00000034660 | protein_coding | Galp | 0.66062 | 1.413083 | 1.036852 |
| ENSMUSG00000032724 | protein_coding | Abtb2 | 2055.475 | 2120.779 | 2088.127 |
| ENSMUSG00000003123 | protein_coding | Lipe | 394.6496 | 376.3772 | 385.5134 |
| ENSMUSG00000026163 | protein_coding | Sphkap | 26.68885 | 22.22166 | 24.45525 |
| ENSMUSG00000003848 | protein_coding | Nob1 | 986.3731 | 1024.457 | 1005.415 |
| ENSMUSG00000029623 | protein_coding | Pdap1 | 3548.047 | 3659.165 | 3603.606 |
| ENSMUSG00000085312 | processed_transcrip | Gm12763 | 0.99093 | 0.36219 | 0.67656 |
| ENSMUSG00000038994 | protein_coding | Hils1 | 0.99093 | 0.36219 | 0.67656 |
| ENSMUSG00000085472 | processed_transcrip | Gm15691 | 0.99093 | 0.36219 | 0.67656 |
| ENSMUSG00000079712 | protein_coding | Gm8597 | 0.99093 | 0.36219 | 0.67656 |
| ENSMUSG00000055943 | protein_coding | 2900064A13Rik | 1556.468 | 1495.909 | 1526.188 |
| ENSMUSG00000008859 | protein_coding | Rala | 2185.859 | 2119.432 | 2152.645 |
| ENSMUSG00000089993 | pseudogene | Gm5822 | 0.66062 | 1.395426 | 1.028023 |
| ENSMUSG00000005886 | protein_coding | Ncoa2 | 7198.575 | 6975.832 | 7087.203 |
| ENSMUSG00000074665 | protein_coding | Bpifb4 | 71.82834 | 64.52492 | 68.17663 |
| ENSMUSG00000056753 | protein_coding | C330011M18Rik | 60.55515 | 54.41987 | 57.48751 |
| ENSMUSG00000046351 | protein_coding | Zfp322a | 1330.561 | 1408.486 | 1369.524 |
| ENSMUSG00000030054 | protein_coding | Gp9 | 2.281682 | 1.395426 | 1.838554 |
| ENSMUSG00000063903 | protein_coding | Klk1 | 10.62561 | 13.20213 | 11.91387 |
| ENSMUSG00000061272 | pseudogene | Gm14173 | 0.960442 | 1.695274 | 1.327858 |
| ENSMUSG00000073881 | processed_transcrip | Gm3892 | 0.960442 | 1.695274 | 1.327858 |
| ENSMUSG00000089612 | snRNA | U6 | 2.555636 | 3.717063 | 3.136349 |
| ENSMUSG00000033278 | protein_coding | Ptprm | 796.7998 | 839.3534 | 818.0766 |
| ENSMUSG00000044957 | protein_coding | 4921523A10Rik | 3.216256 | 4.530449 | 3.873353 |
| ENSMUSG00000028960 | protein_coding | Ube4b | 3643.145 | 3779.483 | 3711.314 |
| ENSMUSG00000085888 | processed_transcrip | Gm12224 | 2.920013 | 4.061234 | 3.490624 |
| ENSMUSG00000020496 | protein_coding | Rnf187 | 4916.506 | 4784.452 | 4850.479 |
| ENSMUSG00000006344 | protein_coding | Ggt5 | 339.8605 | 360.7637 | 350.3121 |

| | | | | | |
|---|---|---|---|---|---|
| ENSMUSG00000042631 | protein_coding | Xkr7 | 3.866366 | 2.692474 | 3.27942 |
| ENSMUSG00000060739 | protein_coding | Nsa2 | 4172.161 | 4309.641 | 4240.901 |
| ENSMUSG00000022015 | protein_coding | Tnfsf11 | 6.091692 | 4.512793 | 5.302242 |
| ENSMUSG00000083670 | pseudogene | Gm6829 | 7.066222 | 9.128315 | 8.097269 |
| ENSMUSG00000031433 | protein_coding | Rbm41 | 190.7009 | 202.9636 | 196.8322 |
| ENSMUSG00000085079 | protein_coding | Khdc1b | 0.632442 | 0 | 0.316221 |
| ENSMUSG00000083797 | pseudogene | Gm13800 | 0.632442 | 0 | 0.316221 |
| ENSMUSG00000078736 | protein_coding | Gm2023 | 0.632442 | 0 | 0.316221 |
| ENSMUSG00000073869 | protein_coding | Gm10591 | 0.632442 | 0 | 0.316221 |
| ENSMUSG00000084090 | pseudogene | Gm11245 | 0.632442 | 0 | 0.316221 |
| ENSMUSG00000086326 | processed_transcrip | Gm13200 | 0.632442 | 0 | 0.316221 |
| ENSMUSG00000085497 | processed_transcrip | Gm15985 | 0.632442 | 0 | 0.316221 |
| ENSMUSG00000044867 | processed_transcrip | Gm3345 | 0.632442 | 0 | 0.316221 |
| ENSMUSG00000076566 | IG_V_gene | Igkv12-44 | 0.632442 | 0 | 0.316221 |
| ENSMUSG00000077526 | snoRNA | SNORA65 | 0.632442 | 0 | 0.316221 |
| ENSMUSG00000064496 | snoRNA | SNORD113 | 0.632442 | 0 | 0.316221 |
| ENSMUSG00000021700 | protein_coding | Rab3c | 0.632442 | 0 | 0.316221 |
| ENSMUSG00000091700 | protein_coding | Gm7876 | 0.632442 | 0 | 0.316221 |
| ENSMUSG00000090772 | pseudogene | Gm2930 | 0.632442 | 0 | 0.316221 |
| ENSMUSG00000090821 | pseudogene | Gm8161 | 0.632442 | 0 | 0.316221 |
| ENSMUSG00000010601 | protein_coding | Apol7a | 0.632442 | 0 | 0.316221 |
| ENSMUSG00000089207 | rRNA | 5S_rRNA | 0.632442 | 0 | 0.316221 |
| ENSMUSG00000080355 | miRNA | AC148019.1 | 0.632442 | 0 | 0.316221 |
| ENSMUSG00000045030 | protein_coding | Olfr1443 | 0.632442 | 0 | 0.316221 |
| ENSMUSG00000082621 | pseudogene | Rpl7a-ps13 | 0.632442 | 0 | 0.316221 |
| ENSMUSG00000081814 | pseudogene | Gm14695 | 0.632442 | 0 | 0.316221 |
| ENSMUSG00000086081 | pseudogene | Gm14759 | 0.632442 | 0 | 0.316221 |
| ENSMUSG00000081349 | pseudogene | Gm15041 | 0.632442 | 0 | 0.316221 |
| ENSMUSG00000040146 | protein_coding | Rgl3 | 76.89316 | 84.26987 | 80.58152 |
| ENSMUSG00000081669 | pseudogene | Npm3-ps1 | 5.485117 | 4.096909 | 4.791013 |
| ENSMUSG00000091750 | lincRNA | 2810029C07Rik | 82.84545 | 76.25375 | 79.5496 |
| ENSMUSG00000041124 | protein_coding | 8430410K20Rik | 1577.662 | 1654.198 | 1615.93 |
| ENSMUSG00000077285 | snoRNA | SNORA42 | 4.851407 | 6.454583 | 5.652995 |
| ENSMUSG00000068303 | protein_coding | Spr-ps1 | 4.583342 | 3.108357 | 3.84585 |
| ENSMUSG00000069309 | protein_coding | Hist1h2an | 1.917305 | 2.983313 | 2.450309 |
| ENSMUSG00000089591 | snoRNA | SNORA17 | 1.298952 | 0.697713 | 0.998332 |
| ENSMUSG00000056696 | protein_coding | Olfr1350 | 1.298952 | 0.697713 | 0.998332 |
| ENSMUSG00000077283 | rRNA | n-R5s158 | 1.298952 | 0.697713 | 0.998332 |
| ENSMUSG00000081930 | pseudogene | Gm12345 | 18.1725 | 15.30143 | 16.73696 |
| ENSMUSG00000021948 | protein_coding | Prkcd | 758.0913 | 730.0708 | 744.081 |
| ENSMUSG00000058159 | processed_transcrip | T2 | 7.446999 | 9.366184 | 8.406591 |
| ENSMUSG00000090553 | protein_coding | Snrpe | 1449.982 | 1497.677 | 1473.83 |
| ENSMUSG00000002395 | protein_coding | Use1 | 1617.605 | 1675.601 | 1646.603 |
| ENSMUSG00000041895 | protein_coding | Wipi1 | 934.7077 | 967.2579 | 950.9828 |
| ENSMUSG00000037171 | protein_coding | Nodal | 2.880057 | 4.16826 | 3.524158 |
| ENSMUSG00000089656 | processed_transcrip | Gm16271 | 4.561053 | 6.127707 | 5.34438 |
| ENSMUSG00000056973 | protein_coding | Ces1d | 5.440539 | 3.752738 | 4.596639 |
| ENSMUSG00000090517 | lincRNA | 6330403N20Rik | 4.839629 | 3.497213 | 4.168421 |
| ENSMUSG00000034312 | protein_coding | Iqsec1 | 2140.816 | 2232.134 | 2186.475 |
| ENSMUSG00000078898 | protein_coding | Gm4723 | 14.41795 | 11.64707 | 13.03251 |
| ENSMUSG00000014550 | protein_coding | Zfyve20 | 1869.312 | 1931.813 | 1900.562 |
| ENSMUSG00000044646 | protein_coding | Zbtb7c | 179.9335 | 168.5392 | 174.2363 |
| ENSMUSG00000083646 | pseudogene | Gm6088 | 39.21917 | 34.60316 | 36.91116 |
| ENSMUSG00000079484 | protein_coding | Phyhd1 | 12.9872 | 10.65816 | 11.82268 |
| ENSMUSG00000039983 | protein_coding | Ccdc32 | 486.9372 | 463.9481 | 475.4427 |
| ENSMUSG00000035936 | protein_coding | Aldh5a1 | 812.2312 | 867.7042 | 839.9677 |
| ENSMUSG00000020177 | protein_coding | 9530003J23Rik | 2.271171 | 1.359751 | 1.815461 |
| ENSMUSG00000004872 | protein_coding | Pax3 | 2.303971 | 1.350742 | 1.827356 |

| | | | | | |
|---|---|---|---|---|---|
| ENSMUSG00000071035 | pseudogene | Gm5499 | 7.124889 | 5.421346 | 6.273118 |
| ENSMUSG00000086555 | lincRNA | Gm13446 | 9.339704 | 11.67917 | 10.50944 |
| ENSMUSG00000024440 | protein_coding | Pcdh12 | 71.576 | 64.78402 | 68.18001 |
| ENSMUSG00000066477 | processed_transcrip | Gm16551 | 4.869074 | 3.488204 | 4.178639 |
| ENSMUSG00000026285 | protein_coding | Pdcd1 | 0.99093 | 0.335524 | 0.663227 |
| ENSMUSG00000084625 | snoRNA | SNORA42 | 0.99093 | 0.335524 | 0.663227 |
| ENSMUSG00000078964 | protein_coding | Ces1b | 0.99093 | 0.335524 | 0.663227 |
| ENSMUSG00000029686 | protein_coding | Cul1 | 5175.982 | 5023.996 | 5099.989 |
| ENSMUSG00000031697 | protein_coding | Orc6 | 1306.85 | 1356.051 | 1331.451 |
| ENSMUSG00000040105 | protein_coding | Ppapdc2 | 228.9146 | 218.0898 | 223.5022 |
| ENSMUSG00000014077 | protein_coding | 1500003O03Rik | 2059.329 | 2126.946 | 2093.137 |
| ENSMUSG00000058897 | protein_coding | Col25a1 | 204.4422 | 183.9982 | 194.2202 |
| ENSMUSG00000085609 | lincRNA | 1700016P03Rik | 2.922324 | 4.132585 | 3.527454 |
| ENSMUSG00000071076 | protein_coding | Jund | 3402.057 | 3507.863 | 3454.96 |
| ENSMUSG00000009281 | protein_coding | Rarres2 | 369.2995 | 349.0152 | 359.1573 |
| ENSMUSG00000087004 | processed_transcrip | Gm14154 | 2.247615 | 1.377408 | 1.812511 |
| ENSMUSG00000041801 | protein_coding | Phlda3 | 472.9382 | 449.8382 | 461.3882 |
| ENSMUSG00000032419 | protein_coding | Tbx18 | 11.68825 | 9.190656 | 10.43945 |
| ENSMUSG00000037296 | protein_coding | Lsm1 | 1158.943 | 1116.456 | 1137.7 |
| ENSMUSG00000020898 | protein_coding | 1500010J02Rik | 1404.833 | 1453.564 | 1429.198 |
| ENSMUSG00000028527 | protein_coding | Ak4 | 981.4528 | 1022.392 | 1001.922 |
| ENSMUSG00000046791 | protein_coding | 2410016O06Rik | 663.7958 | 635.6343 | 649.715 |
| ENSMUSG00000004460 | protein_coding | Dnajb11 | 3057.873 | 2928.255 | 2993.064 |
| ENSMUSG00000045364 | protein_coding | Ifne | 0.99093 | 0.326514 | 0.658722 |
| ENSMUSG00000090223 | protein_coding | Pcp4 | 0.99093 | 0.326514 | 0.658722 |
| ENSMUSG00000088212 | snRNA | U1 | 0.99093 | 0.326514 | 0.658722 |
| ENSMUSG00000027282 | protein_coding | Mtch2 | 2685.749 | 2613.035 | 2649.392 |
| ENSMUSG00000030536 | protein_coding | Iqgap1 | 2435.528 | 2525.591 | 2480.56 |
| ENSMUSG00000039956 | protein_coding | Mrap | 65.05694 | 57.53759 | 61.29727 |
| ENSMUSG00000081957 | pseudogene | Ak3l2-ps | 16.09664 | 18.80729 | 17.45196 |
| ENSMUSG00000037857 | protein_coding | Nufip2 | 3145.459 | 3244.184 | 3194.822 |
| ENSMUSG00000024726 | protein_coding | 2410127L17Rik | 956.7118 | 905.3347 | 931.0233 |
| ENSMUSG00000023940 | protein_coding | Sgol1 | 731.9455 | 696.8315 | 714.3885 |
| ENSMUSG00000020010 | protein_coding | Vnn3 | 7.737353 | 5.970198 | 6.853776 |
| ENSMUSG00000088412 | snoRNA | SNORA79 | 1.284863 | 2.075121 | 1.679992 |
| ENSMUSG00000067835 | processed_transcrip | Gm14661 | 86.95193 | 93.40294 | 90.17744 |
| ENSMUSG00000079662 | protein_coding | Ntn3 | 206.4061 | 191.7732 | 199.0897 |
| ENSMUSG00000057457 | protein_coding | Phex | 40.00107 | 44.45517 | 42.22812 |
| ENSMUSG00000081787 | pseudogene | Gm13991 | 48.95284 | 44.29658 | 46.62471 |
| ENSMUSG00000088059 | snoRNA | SNORA74 | 5.79776 | 4.396757 | 5.097258 |
| ENSMUSG00000001249 | protein_coding | Hpn | 11.29697 | 13.57949 | 12.43823 |
| ENSMUSG00000090330 | protein_coding | 9130221H12Rik | 138.128 | 129.5032 | 133.8156 |
| ENSMUSG00000084593 | snRNA | U6 | 3.552455 | 4.821288 | 4.186872 |
| ENSMUSG00000053603 | protein_coding | 4930442H23Rik | 1.290752 | 0.697713 | 0.994232 |
| ENSMUSG00000078763 | protein_coding | Slfn1 | 1.290752 | 0.697713 | 0.994232 |
| ENSMUSG00000022517 | protein_coding | Mgrn1 | 2137.188 | 2065.674 | 2101.431 |
| ENSMUSG00000052769 | pseudogene | Gm9889 | 1.626951 | 2.73752 | 2.182235 |
| ENSMUSG00000020680 | protein_coding | Taf15 | 2349.679 | 2226.914 | 2288.297 |
| ENSMUSG00000049184 | protein_coding | Purg | 335.9986 | 319.887 | 327.9428 |
| ENSMUSG00000020705 | protein_coding | Ddx42 | 4521.218 | 4402.75 | 4461.984 |
| ENSMUSG00000047330 | protein_coding | Kcne4 | 131.3785 | 142.1744 | 136.7764 |
| ENSMUSG00000058773 | protein_coding | Hist1h1b | 7.053176 | 9.538762 | 8.295969 |
| ENSMUSG00000041180 | protein_coding | Hectd2 | 689.3705 | 737.4118 | 713.3911 |
| ENSMUSG00000013822 | protein_coding | Elof1 | 1036.996 | 995.3957 | 1016.196 |
| ENSMUSG00000047990 | protein_coding | C2cd4a | 12.62514 | 15.02789 | 13.82651 |
| ENSMUSG00000004221 | protein_coding | Ikbkg | 1310.372 | 1351.57 | 1330.971 |
| ENSMUSG00000040124 | protein_coding | Gorab | 608.0185 | 630.326 | 619.1723 |
| ENSMUSG00000079652 | protein_coding | Fam71f2 | 4.543386 | 3.15268 | 3.848033 |

| | | | | | |
|---|---|---|---|---|---|
| ENSMUSG00000061723 | protein_coding | Tnnt3 | 13.94407 | 11.45068 | 12.69737 |
| ENSMUSG00000062980 | protein_coding | A430107O13Rik | 1346.46 | 1398.844 | 1372.652 |
| ENSMUSG00000091575 | protein_coding | 2010016I18Rik | 1.284863 | 2.048455 | 1.666659 |
| ENSMUSG00000030149 | protein_coding | Klrk1 | 1.284863 | 2.048455 | 1.666659 |
| ENSMUSG00000052144 | protein_coding | Ppp4r2 | 3918.986 | 4038.5 | 3978.743 |
| ENSMUSG00000026632 | protein_coding | Tatdn3 | 241.8268 | 227.168 | 234.4974 |
| ENSMUSG00000030647 | protein_coding | Ndufc2 | 1155.298 | 1085.26 | 1120.279 |
| ENSMUSG00000064168 | protein_coding | Hist1h2bh | 1.949061 | 3.01935 | 2.484205 |
| ENSMUSG00000084632 | snRNA | U2 | 2.595592 | 1.686265 | 2.140929 |
| ENSMUSG00000075028 | protein_coding | Prdm11 | 689.7841 | 721.778 | 705.781 |
| ENSMUSG00000019470 | protein_coding | Xab2 | 2276.647 | 2203.745 | 2240.196 |
| ENSMUSG00000065521 | miRNA | Mir296 | 5.840026 | 4.423424 | 5.131725 |
| ENSMUSG00000080373 | snoRNA | Snord88a | 3.611122 | 2.36596 | 2.988541 |
| ENSMUSG00000028048 | protein_coding | Gba | 971.7904 | 939.2236 | 955.507 |
| ENSMUSG00000020124 | protein_coding | Usp15 | 2179.141 | 2252.881 | 2216.011 |
| ENSMUSG00000029477 | protein_coding | Morn3 | 18.06799 | 20.9271 | 19.49754 |
| ENSMUSG00000002365 | protein_coding | Snx9 | 1587.45 | 1638.495 | 1612.972 |
| ENSMUSG00000063681 | protein_coding | Crb1 | 15.43371 | 12.92646 | 14.18008 |
| ENSMUSG00000017950 | protein_coding | Hnf4a | 2.251193 | 1.36876 | 1.809977 |
| ENSMUSG00000026401 | protein_coding | Daf2 | 1.290752 | 2.075121 | 1.682936 |
| ENSMUSG00000036057 | protein_coding | Ptpn23 | 2232.944 | 2324.485 | 2278.715 |
| ENSMUSG00000024050 | protein_coding | Wiz | 2413.105 | 2325.81 | 2369.457 |
| ENSMUSG00000061532 | protein_coding | Zfp955b | 279.7565 | 259.6444 | 269.7005 |
| ENSMUSG00000024456 | protein_coding | Diap1 | 1117.933 | 1175.081 | 1146.507 |
| ENSMUSG00000081270 | pseudogene | Gm11653 | 0.66062 | 1.377408 | 1.019014 |
| ENSMUSG00000030735 | protein_coding | Gm9755 | 13.25989 | 10.79765 | 12.02877 |
| ENSMUSG00000062028 | protein_coding | Irgc1 | 37.08591 | 41.28411 | 39.18501 |
| ENSMUSG00000090263 | processed_transcrip | D730045A05Rik | 2.583814 | 1.712931 | 2.148373 |
| ENSMUSG00000028088 | protein_coding | Fmo5 | 205.9692 | 195.1362 | 200.5527 |
| ENSMUSG00000067558 | pseudogene | Rpl35a-ps5 | 0.98273 | 1.686265 | 1.334498 |
| ENSMUSG00000087213 | processed_transcrip | 2810408I11Rik | 106.3256 | 98.04228 | 102.1839 |
| ENSMUSG00000066537 | protein_coding | Vmn2r57 | 1.284863 | 0.697713 | 0.991288 |
| ENSMUSG00000090506 | lincRNA | Gm17734 | 1.284863 | 0.697713 | 0.991288 |
| ENSMUSG00000069540 | processed_transcrip | Gm4925 | 9.359683 | 7.54079 | 8.450236 |
| ENSMUSG00000087348 | processed_transcrip | Gm15582 | 1.298952 | 2.08413 | 1.691541 |
| ENSMUSG00000040713 | protein_coding | Creg1 | 1287.267 | 1357.851 | 1322.559 |
| ENSMUSG00000021245 | protein_coding | Mlh3 | 989.9016 | 1025.077 | 1007.49 |
| ENSMUSG00000028367 | protein_coding | Txn1 | 1428.924 | 1478.246 | 1453.585 |
| ENSMUSG00000031520 | protein_coding | Vegfc | 27.02818 | 30.81619 | 28.92219 |
| ENSMUSG00000039497 | protein_coding | Dse | 404.7787 | 387.2474 | 396.0131 |
| ENSMUSG00000072893 | lincRNA | 4933439C10Rik | 140.9331 | 127.1678 | 134.0505 |
| ENSMUSG00000031776 | protein_coding | Arl2bp | 2063.337 | 2118.97 | 2091.153 |
| ENSMUSG00000041354 | protein_coding | Rgl2 | 979.9802 | 1018.756 | 999.3682 |
| ENSMUSG00000086916 | processed_transcrip | Gm15903 | 3.228034 | 4.414414 | 3.821224 |
| ENSMUSG00000013662 | protein_coding | Atad1 | 3467.204 | 3581.898 | 3524.551 |
| ENSMUSG00000025991 | protein_coding | Cps1 | 22.10678 | 18.44795 | 20.27736 |
| ENSMUSG00000020015 | protein_coding | Cdk17 | 933.0003 | 893.4744 | 913.2373 |
| ENSMUSG00000053835 | protein_coding | H2-T24 | 94.81877 | 84.39724 | 89.60801 |
| ENSMUSG00000020700 | protein_coding | Map3k3 | 1683.517 | 1631.359 | 1657.438 |
| ENSMUSG00000034218 | protein_coding | Atm | 4724.495 | 4490.876 | 4607.685 |
| ENSMUSG00000086148 | processed_transcrip | Gm15271 | 0.962752 | 1.677256 | 1.320004 |
| ENSMUSG00000026173 | protein_coding | Plcd4 | 55.75003 | 73.82308 | 64.78655 |
| ENSMUSG00000040133 | protein_coding | Gpr176 | 20.75631 | 17.65222 | 19.20427 |
| ENSMUSG00000051638 | protein_coding | Gm9857 | 3.835877 | 5.402966 | 4.619422 |
| ENSMUSG00000054247 | pseudogene | Gm9939 | 6.488868 | 8.282826 | 7.385847 |
| ENSMUSG00000022245 | protein_coding | Skor1 | 17.78099 | 21.10475 | 19.44287 |
| ENSMUSG00000083782 | pseudogene | Gm11660 | 3.580633 | 2.392626 | 2.98663 |
| ENSMUSG00000028641 | protein_coding | Lepre1 | 1944.599 | 1870.837 | 1907.718 |

| | | | | | |
|---|---|---|---|---|---|
| ENSMUSG00000020530 | protein_coding | Ggnbp2 | 3437.75 | 3525.916 | 3481.833 |
| ENSMUSG00000084801 | lincRNA | Gm16929 | 5.814159 | 4.361082 | 5.087621 |
| ENSMUSG00000081044 | protein_coding | AU015836 | 7.072111 | 8.936216 | 8.004164 |
| ENSMUSG00000035831 | protein_coding | Krt25 | 2.589703 | 1.72194 | 2.155822 |
| ENSMUSG00000032034 | protein_coding | Kcnj5 | 12.9872 | 15.52092 | 14.25406 |
| ENSMUSG00000009035 | protein_coding | Tmem184b | 1270.325 | 1320.332 | 1295.328 |
| ENSMUSG00000026825 | protein_coding | Dnm1 | 270.33 | 284.0281 | 277.1791 |
| ENSMUSG00000066442 | protein_coding | Mthfs | 101.1018 | 108.4719 | 104.7869 |
| ENSMUSG00000031924 | protein_coding | Cyb5b | 3808.657 | 3910.541 | 3859.599 |
| ENSMUSG00000047443 | protein_coding | Fam132b | 86.85793 | 80.38711 | 83.62252 |
| ENSMUSG00000064597 | snoRNA | SNORA2 | 5.753182 | 4.194926 | 4.974054 |
| ENSMUSG00000042641 | processed_transcrip | Rgsl1 | 11.31694 | 9.26273 | 10.28984 |
| ENSMUSG00000028458 | protein_coding | Tesk1 | 565.8387 | 543.6783 | 554.7585 |
| ENSMUSG00000036304 | protein_coding | Zdhhc23 | 155.4918 | 146.0194 | 150.7556 |
| ENSMUSG00000027339 | protein_coding | Rassf2 | 3630.304 | 3504.084 | 3567.194 |
| ENSMUSG00000077734 | snoRNA | Snord83b | 3.231613 | 2.039807 | 2.63571 |
| ENSMUSG00000048603 | processed_transcrip | Gm9828 | 28.05549 | 31.88546 | 29.97048 |
| ENSMUSG00000062822 | protein_coding | 4833420G17Rik | 1357.526 | 1402.96 | 1380.243 |
| ENSMUSG00000045333 | protein_coding | Zfp423 | 230.4698 | 241.3346 | 235.9022 |
| ENSMUSG00000091594 | protein_coding | Gm17067 | 23.28504 | 26.52252 | 24.90378 |
| ENSMUSG00000091358 | pseudogene | Speer5-ps1 | 2.575614 | 1.712931 | 2.144273 |
| ENSMUSG00000059003 | protein_coding | Grin2a | 3.179878 | 2.057464 | 2.618671 |
| ENSMUSG00000082535 | pseudogene | Gm7860 | 4.565675 | 3.126376 | 3.846025 |
| ENSMUSG00000035898 | protein_coding | Uba6 | 4567.298 | 4354.381 | 4460.839 |
| ENSMUSG00000038023 | protein_coding | Atp6v0a2 | 1562.388 | 1626.205 | 1594.296 |
| ENSMUSG00000080582 | snoRNA | SNORA11 | 2.914124 | 4.088262 | 3.501193 |
| ENSMUSG00000021248 | protein_coding | Tmed10 | 4458.816 | 4576.252 | 4517.534 |
| ENSMUSG00000057788 | protein_coding | Ddx49 | 1081.323 | 1046.095 | 1063.709 |
| ENSMUSG00000021635 | protein_coding | Rad17 | 1701.985 | 1645.153 | 1673.569 |
| ENSMUSG00000021375 | protein_coding | Kif13a | 824.2446 | 792.4699 | 808.3572 |
| ENSMUSG00000017309 | protein_coding | Cd300lg | 17.13446 | 14.43756 | 15.78601 |
| ENSMUSG00000086389 | lincRNA | Gm15998 | 8.794107 | 11.02899 | 9.91155 |
| ENSMUSG00000038770 | protein_coding | Kpna7 | 11.36972 | 9.083992 | 10.22686 |
| ENSMUSG00000086499 | processed_transcrip | Gm16217 | 2.583814 | 1.72194 | 2.152877 |
| ENSMUSG00000083595 | pseudogene | Gm9200 | 2.247615 | 1.395426 | 1.82152 |
| ENSMUSG00000085316 | processed_transcrip | D330050G23Rik | 7.407043 | 9.182371 | 8.294707 |
| ENSMUSG00000015942 | protein_coding | Gtf2ird2 | 450.241 | 467.5344 | 458.8877 |
| ENSMUSG00000079051 | protein_coding | Gm14025 | 4.861918 | 3.45289 | 4.157404 |
| ENSMUSG00000082373 | pseudogene | Gm13754 | 0.632442 | 1.341732 | 0.987087 |
| ENSMUSG00000003402 | protein_coding | Prkcsh | 2697.246 | 2779.16 | 2738.203 |
| ENSMUSG00000071866 | protein_coding | Ppia | 10501.35 | 10819.6 | 10660.47 |
| ENSMUSG00000041143 | protein_coding | Tmco4 | 516.2887 | 492.4218 | 504.3552 |
| ENSMUSG00000000301 | protein_coding | Pemt | 235.7636 | 223.505 | 229.6343 |
| ENSMUSG00000020362 | protein_coding | Cnot6 | 5547.382 | 5733.39 | 5640.386 |
| ENSMUSG00000085465 | processed_transcrip | Gm15347 | 23.23688 | 26.58201 | 24.90945 |
| ENSMUSG00000079584 | protein_coding | Gm364 | 2256.715 | 1952.513 | 2104.614 |
| ENSMUSG00000027316 | protein_coding | Gfra4 | 127.7642 | 118.9002 | 123.3322 |
| ENSMUSG00000047992 | protein_coding | Fam69c | 1.973661 | 1.006571 | 1.490116 |
| ENSMUSG00000029811 | protein_coding | Abp1 | 0.966331 | 0.36219 | 0.66426 |
| ENSMUSG00000092813 | miRNA | AC102604.1 | 0.966331 | 0.36219 | 0.66426 |
| ENSMUSG00000064853 | snoRNA | SNORA38 | 1.298952 | 2.048455 | 1.673703 |
| ENSMUSG00000028680 | protein_coding | Plk3 | 99.33651 | 92.4962 | 95.91636 |
| ENSMUSG00000089621 | misc_RNA | 7SK | 1.298952 | 0.653029 | 0.97599 |
| ENSMUSG00000085068 | processed_transcrip | Gm15895 | 1.298952 | 0.653029 | 0.97599 |
| ENSMUSG00000004151 | protein_coding | Etv1 | 446.4709 | 468.4495 | 457.4602 |
| ENSMUSG00000085768 | processed_transcrip | Gm13707 | 4.577453 | 6.225362 | 5.401407 |
| ENSMUSG00000029676 | protein_coding | Pot1a | 1465.294 | 1515.578 | 1490.436 |
| ENSMUSG00000089026 | snRNA | U6 | 3.870987 | 2.701483 | 3.286235 |

| | | | | | |
|---|---|---|---|---|---|
| ENSMUSG00000068962 | protein_coding | Zfp114 | 37.74086 | 33.70148 | 35.72117 |
| ENSMUSG00000040467 | protein_coding | 4921522P10Rik | 4.196676 | 3.046016 | 3.621346 |
| ENSMUSG00000032591 | protein_coding | Mst1 | 5.576583 | 7.478087 | 6.527335 |
| ENSMUSG00000080885 | pseudogene | Gm4167 | 7.427021 | 9.173361 | 8.300191 |
| ENSMUSG00000028546 | protein_coding | Elavl4 | 48.57989 | 43.25682 | 45.91836 |
| ENSMUSG00000029650 | protein_coding | Slc46a3 | 102.1602 | 94.35406 | 98.25713 |
| ENSMUSG00000075431 | processed_transcrip | Gm11691 | 2.255815 | 3.363521 | 2.809668 |
| ENSMUSG00000090420 | lincRNA | 1700040D17Rik | 35.5376 | 39.7333 | 37.63545 |
| ENSMUSG00000037526 | protein_coding | Atg14 | 1473.393 | 1518.003 | 1495.698 |
| ENSMUSG00000020787 | protein_coding | P2rx1 | 10.66557 | 12.8998 | 11.78268 |
| ENSMUSG00000040329 | protein_coding | Il7 | 1.290752 | 2.057464 | 1.674108 |
| ENSMUSG00000034235 | protein_coding | Usp54 | 1222.154 | 1273.728 | 1247.941 |
| ENSMUSG00000030591 | protein_coding | Psmd8 | 2991.761 | 3074.918 | 3033.339 |
| ENSMUSG00000022336 | protein_coding | Eif3e | 5107.356 | 5271.663 | 5189.51 |
| ENSMUSG00000022472 | protein_coding | Pppde2 | 1187.557 | 1141.74 | 1164.649 |
| ENSMUSG00000091089 | protein_coding | A930018M24Rik | 2.279371 | 1.324076 | 1.801723 |
| ENSMUSG00000020936 | protein_coding | Nmt1 | 2047.773 | 2110.874 | 2079.324 |
| ENSMUSG00000066176 | processed_transcrip | Gm12511 | 3.250323 | 4.45009 | 3.850206 |
| ENSMUSG00000072487 | protein_coding | Gm628 | 17.72717 | 15.13455 | 16.43086 |
| ENSMUSG00000004934 | protein_coding | Pias4 | 1373.444 | 1330.17 | 1351.807 |
| ENSMUSG00000006638 | lincRNA | Abhd1 | 9.716903 | 7.822981 | 8.769942 |
| ENSMUSG00000074604 | protein_coding | Mgst2 | 14.82124 | 17.42124 | 16.12124 |
| ENSMUSG00000060073 | protein_coding | Psma3 | 2239.988 | 2167.87 | 2203.929 |
| ENSMUSG00000075600 | protein_coding | Zc3h3 | 500.4102 | 520.8712 | 510.6407 |
| ENSMUSG00000023963 | protein_coding | Cyp39a1 | 336.5647 | 310.8952 | 323.7299 |
| ENSMUSG00000009941 | protein_coding | Nxf2 | 3708.948 | 3870.359 | 3789.653 |
| ENSMUSG00000043850 | protein_coding | Clrn1 | 5.12305 | 3.788052 | 4.455551 |
| ENSMUSG00000043248 | protein_coding | Gm9780 | 4.255343 | 5.756508 | 5.005925 |
| ENSMUSG00000032067 | protein_coding | Pts | 700.8411 | 674.1468 | 687.4939 |
| ENSMUSG00000025196 | protein_coding | Cpn1 | 15.5746 | 13.07745 | 14.32602 |
| ENSMUSG00000038776 | protein_coding | Ephx1 | 635.2941 | 678.8215 | 657.0578 |
| ENSMUSG00000086649 | processed_transcrip | Gm15286 | 65.34684 | 59.81713 | 62.58199 |
| ENSMUSG00000064634 | snoRNA | SNORA1 | 5.187606 | 6.736413 | 5.962009 |
| ENSMUSG00000037243 | protein_coding | Zfp692 | 388.3132 | 368.3155 | 378.3143 |
| ENSMUSG00000071657 | protein_coding | Bscl2 | 510.2573 | 537.2032 | 523.7303 |
| ENSMUSG00000079480 | protein_coding | Pin4 | 309.8505 | 326.7016 | 318.276 |
| ENSMUSG00000021466 | protein_coding | Ptch1 | 4095.736 | 4256.952 | 4176.344 |
| ENSMUSG00000030822 | protein_coding | Prr14 | 923.172 | 893.7963 | 908.4841 |
| ENSMUSG00000091059 | protein_coding | Vmn2r14 | 9.363036 | 11.73608 | 10.54956 |
| ENSMUSG00000049511 | protein_coding | Htr1b | 0.632442 | 1.333085 | 0.982763 |
| ENSMUSG00000029162 | protein_coding | Khk | 508.9667 | 490.1113 | 499.539 |
| ENSMUSG00000049577 | protein_coding | Zfpm1 | 1403.218 | 1461.265 | 1432.242 |
| ENSMUSG00000031098 | protein_coding | Syt8 | 0.966331 | 0.335524 | 0.650927 |
| ENSMUSG00000086052 | processed_transcrip | Gm11802 | 2.603792 | 1.695274 | 2.149533 |
| ENSMUSG00000023935 | protein_coding | Spats1 | 73.84486 | 68.31152 | 71.07819 |
| ENSMUSG00000021922 | protein_coding | Itih4 | 27.54143 | 24.01386 | 25.77764 |
| ENSMUSG00000073565 | protein_coding | Prr16 | 27.90096 | 23.49017 | 25.69556 |
| ENSMUSG00000045969 | protein_coding | Ing1 | 1421.622 | 1374.188 | 1397.905 |
| ENSMUSG00000053442 | protein_coding | 4930597O21Rik | 11.22883 | 9.182371 | 10.2056 |
| ENSMUSG00000003421 | protein_coding | Nosip | 1534.693 | 1488.573 | 1511.633 |
| ENSMUSG00000087178 | lincRNA | A230056P14Rik | 89.61812 | 83.07632 | 86.34722 |
| ENSMUSG00000066107 | protein_coding | Gm12666 | 0.976841 | 1.677256 | 1.327049 |
| ENSMUSG00000086825 | processed_transcrip | Gm15675 | 17.19208 | 14.22423 | 15.70816 |
| ENSMUSG00000062867 | protein_coding | Impdh2 | 2597.471 | 2527.721 | 2562.596 |
| ENSMUSG00000045968 | protein_coding | 5830403L16Rik | 5.822359 | 4.079614 | 4.950987 |
| ENSMUSG00000086224 | lincRNA | 2700069I18Rik | 1.290752 | 0.653029 | 0.97189 |
| ENSMUSG00000029372 | protein_coding | Ppbp | 1.290752 | 0.653029 | 0.97189 |
| ENSMUSG00000056854 | protein_coding | Pou3f4 | 1.290752 | 0.653029 | 0.97189 |

| | | | | | |
|---|---|---|---|---|---|
| ENSMUSG00000091555 | lincRNA | Gm17725 | 17.13572 | 19.92917 | 18.53245 |
| ENSMUSG00000084885 | lincRNA | 3010001F23Rik | 23.18411 | 26.63607 | 24.91009 |
| ENSMUSG00000030409 | protein_coding | Dmpk | 762.9857 | 805.6065 | 784.2961 |
| ENSMUSG00000073002 | protein_coding | Vamp5 | 136.5126 | 128.5903 | 132.5515 |
| ENSMUSG00000063594 | protein_coding | Gng8 | 22.96397 | 26.15241 | 24.55819 |
| ENSMUSG00000083701 | pseudogene | Gm4912 | 118.3283 | 126.221 | 122.2747 |
| ENSMUSG00000026841 | protein_coding | Fibcd1 | 86.8712 | 94.45167 | 90.66143 |
| ENSMUSG00000047013 | protein_coding | Fbxo41 | 470.9797 | 491.2134 | 481.0966 |
| ENSMUSG00000006676 | protein_coding | Usp19 | 7234.425 | 7473.303 | 7353.864 |
| ENSMUSG00000042414 | protein_coding | Prdm14 | 4.829118 | 3.443519 | 4.136319 |
| ENSMUSG00000091186 | lincRNA | Gm2078 | 4.832697 | 6.418908 | 5.625802 |
| ENSMUSG00000000127 | protein_coding | Fer | 852.3342 | 826.0055 | 839.1698 |
| ENSMUSG00000019710 | protein_coding | Mrpl24 | 1654.717 | 1709.349 | 1682.033 |
| ENSMUSG00000058743 | protein_coding | Kcnj14 | 15.43833 | 18.10922 | 16.77377 |
| ENSMUSG00000090446 | protein_coding | A930017M01Rik | 0.960442 | 1.775272 | 1.367857 |
| ENSMUSG00000055675 | protein_coding | Kbtbd11 | 318.3028 | 334.3368 | 326.3198 |
| ENSMUSG00000057967 | protein_coding | Fgf18 | 78.10355 | 85.38885 | 81.7462 |
| ENSMUSG00000091380 | lincRNA | Gm17575 | 2.242993 | 3.416853 | 2.829923 |
| ENSMUSG00000086414 | processed_transcrip | Gm15645 | 1.284863 | 2.039445 | 1.662154 |
| ENSMUSG00000090585 | processed_transcrip | 4933406F09Rik | 1.298952 | 2.057464 | 1.678208 |
| ENSMUSG00000017446 | protein_coding | C1qtnf1 | 142.8566 | 151.4502 | 147.1534 |
| ENSMUSG00000047187 | protein_coding | Rab2a | 3333.613 | 3421.751 | 3377.682 |
| ENSMUSG00000028447 | protein_coding | Dctn3 | 825.9449 | 797.9779 | 811.9614 |
| ENSMUSG00000071632 | protein_coding | 2510002D24Rik | 161.3767 | 172.5799 | 166.9783 |
| ENSMUSG00000010205 | protein_coding | Raver1 | 2395.892 | 2316.122 | 2356.007 |
| ENSMUSG00000031527 | protein_coding | Eri1 | 1492.665 | 1452.243 | 1472.454 |
| ENSMUSG00000090648 | lincRNA | Gm17446 | 1.925505 | 1.006571 | 1.466038 |
| ENSMUSG00000071369 | protein_coding | Map3k5 | 830.9626 | 802.4764 | 816.7195 |
| ENSMUSG00000045689 | protein_coding | Pcdhb4 | 7.715064 | 9.607263 | 8.661164 |
| ENSMUSG00000022108 | protein_coding | Itm2b | 7953.709 | 8149.561 | 8051.635 |
| ENSMUSG00000082575 | pseudogene | Gm13050 | 171.0826 | 162.3296 | 166.7061 |
| ENSMUSG00000066838 | protein_coding | Zfp772 | 213.628 | 225.9471 | 219.7876 |
| ENSMUSG00000026240 | protein_coding | Cops7b | 899.5766 | 927.61 | 913.5933 |
| ENSMUSG00000078963 | protein_coding | Hsbp1l1 | 51.45951 | 56.99241 | 54.22596 |
| ENSMUSG00000021583 | protein_coding | Erap1 | 1645.348 | 1696.629 | 1670.988 |
| ENSMUSG00000032911 | protein_coding | Cspg4 | 306.2984 | 325.7957 | 316.047 |
| ENSMUSG00000036641 | protein_coding | Ccdc148 | 22.67235 | 26.11094 | 24.39164 |
| ENSMUSG00000091055 | protein_coding | Siglec15 | 1.31304 | 0.662038 | 0.987539 |
| ENSMUSG00000050558 | protein_coding | Prokr2 | 16.68219 | 19.90939 | 18.29579 |
| ENSMUSG00000082884 | pseudogene | Gm13339 | 2.894146 | 4.052225 | 3.473185 |
| ENSMUSG00000044574 | protein_coding | 5031434C07Rik | 11.29928 | 9.325072 | 10.31217 |
| ENSMUSG00000091759 | lincRNA | Gm17433 | 2.569725 | 1.695274 | 2.1325 |
| ENSMUSG00000040775 | protein_coding | Gm9772 | 7.116689 | 8.811172 | 7.96393 |
| ENSMUSG00000081723 | pseudogene | Gm15931 | 3.866366 | 5.219153 | 4.54276 |
| ENSMUSG00000082456 | pseudogene | Gm11598 | 2.597903 | 1.65059 | 2.124246 |
| ENSMUSG00000090567 | lincRNA | Gm17465 | 14.84353 | 12.53724 | 13.69039 |
| ENSMUSG00000042436 | protein_coding | Mfap4 | 4221.992 | 4393.65 | 4307.821 |
| ENSMUSG00000003847 | protein_coding | Nfat5 | 10100.36 | 10511.77 | 10306.06 |
| ENSMUSG00000089401 | rRNA | 5S_rRNA | 0.968641 | 1.659599 | 1.31412 |
| ENSMUSG00000031844 | protein_coding | Hsd17b2 | 0.968641 | 1.659599 | 1.31412 |
| ENSMUSG00000082896 | pseudogene | Gm5844 | 14.87887 | 17.57802 | 16.22844 |
| ENSMUSG00000046707 | protein_coding | Csnk2a2 | 1334.546 | 1387.291 | 1360.918 |
| ENSMUSG00000022783 | protein_coding | Spag6 | 13.32802 | 10.93962 | 12.13382 |
| ENSMUSG00000068745 | protein_coding | Mybphl | 23.81782 | 27.39612 | 25.60697 |
| ENSMUSG00000062773 | protein_coding | Tex101 | 3521.282 | 3082.768 | 3302.025 |
| ENSMUSG00000063317 | protein_coding | Usp31 | 2988.763 | 2896.614 | 2942.688 |
| ENSMUSG00000048155 | protein_coding | 1700014N06Rik | 117.3892 | 128.7755 | 123.0823 |
| ENSMUSG00000087261 | lincRNA | Gm15477 | 6.808668 | 5.245819 | 6.027244 |

| | | | | | |
|---|---|---|---|---|---|
| ENSMUSG00000031073 | protein_coding | Fgf15 | 3.239813 | 1.959086 | 2.599449 |
| ENSMUSG00000084884 | processed_transcrip | Gm12289 | 1.284863 | 0.653029 | 0.968946 |
| ENSMUSG00000053164 | protein_coding | Gpr21 | 14.84353 | 12.45973 | 13.65163 |
| ENSMUSG00000029004 | protein_coding | Mll5 | 3707.221 | 3845.652 | 3776.437 |
| ENSMUSG00000040536 | protein_coding | Necab1 | 191.122 | 202.8838 | 197.0029 |
| ENSMUSG00000041777 | protein_coding | Cir1 | 940.5887 | 904.6707 | 922.6297 |
| ENSMUSG00000045114 | protein_coding | Prrt2 | 58.75286 | 64.85283 | 61.80285 |
| ENSMUSG00000049649 | protein_coding | Gpr3 | 13.55509 | 15.89176 | 14.72342 |
| ENSMUSG00000026856 | protein_coding | Dolpp1 | 496.2928 | 473.9069 | 485.0998 |
| ENSMUSG00000086652 | processed_transcrip | Gm14029 | 2.592014 | 1.695274 | 2.143644 |
| ENSMUSG00000009471 | protein_coding | Myod1 | 2.567414 | 1.677256 | 2.122335 |
| ENSMUSG00000072594 | pseudogene | Gm16439 | 3.225724 | 4.423424 | 3.824574 |
| ENSMUSG00000040761 | protein_coding | Spen | 3354.554 | 3464.164 | 3409.359 |
| ENSMUSG00000087076 | pseudogene | Gm13229 | 5.169939 | 3.823727 | 4.496833 |
| ENSMUSG00000085566 | processed_transcrip | A730017L22Rik | 190.988 | 181.085 | 186.0365 |
| ENSMUSG00000068262 | protein_coding | Gm5879 | 2.920013 | 4.025559 | 3.472786 |
| ENSMUSG00000047459 | protein_coding | Dynlrb1 | 2128.977 | 2210.332 | 2169.654 |
| ENSMUSG00000044055 | protein_coding | Otos | 6.853246 | 5.165459 | 6.009353 |
| ENSMUSG00000033790 | protein_coding | Tubgcp5 | 2153.233 | 2228.751 | 2190.992 |
| ENSMUSG00000077471 | snRNA | U1 | 1.307151 | 0.662038 | 0.984595 |
| ENSMUSG00000085431 | processed_transcrip | 4930440I19Rik | 1.307151 | 0.662038 | 0.984595 |
| ENSMUSG00000072878 | protein_coding | 1700123L14Rik | 1.307151 | 0.662038 | 0.984595 |
| ENSMUSG00000082189 | pseudogene | Gm4784 | 1.307151 | 0.662038 | 0.984595 |
| ENSMUSG00000026494 | protein_coding | Kif26b | 24.79466 | 21.0597 | 22.92718 |
| ENSMUSG00000023052 | protein_coding | Npff | 29.62526 | 34.74766 | 32.18646 |
| ENSMUSG00000074029 | protein_coding | Gm10608 | 1.293062 | 0.662038 | 0.97755 |
| ENSMUSG00000019867 | protein_coding | Gje1 | 1.293062 | 0.662038 | 0.97755 |
| ENSMUSG00000087978 | snoRNA | SNORA32 | 1.293062 | 0.662038 | 0.97755 |
| ENSMUSG00000091050 | processed_transcrip | 9330020H09Rik | 36.731 | 41.33387 | 39.03243 |
| ENSMUSG00000085842 | processed_transcrip | 4930448K12Rik | 0.974531 | 1.757616 | 1.366073 |
| ENSMUSG00000085769 | lincRNA | D230017M19Rik | 158.8008 | 168.1834 | 163.4921 |
| ENSMUSG00000025427 | protein_coding | Rnf165 | 75.40285 | 82.34682 | 78.87484 |
| ENSMUSG00000067608 | pseudogene | Pcna-ps2 | 492.4574 | 351.7849 | 422.1211 |
| ENSMUSG00000051146 | protein_coding | Camk2n2 | 47.21661 | 51.77539 | 49.496 |
| ENSMUSG00000038067 | protein_coding | Csf3 | 3.233924 | 2.146471 | 2.690197 |
| ENSMUSG00000039377 | protein_coding | Hlx | 30.44526 | 26.69189 | 28.56858 |
| ENSMUSG00000059602 | protein_coding | Syn3 | 62.8984 | 68.23909 | 65.56875 |
| ENSMUSG00000038997 | protein_coding | Asb17 | 21.16527 | 17.62628 | 19.39577 |
| ENSMUSG00000028044 | protein_coding | Cks1b | 1288.956 | 1231.94 | 1260.448 |
| ENSMUSG00000034297 | protein_coding | Med13 | 5249.218 | 5409.113 | 5329.165 |
| ENSMUSG00000059554 | protein_coding | Ccdc28a | 616.4325 | 642.3802 | 629.4063 |
| ENSMUSG00000029407 | protein_coding | Uso1 | 3419.764 | 3330.512 | 3375.138 |
| ENSMUSG00000085125 | lincRNA | Gm16070 | 1.965461 | 3.018988 | 2.492224 |
| ENSMUSG00000031559 | protein_coding | 4930555F03Rik | 2.592014 | 1.72194 | 2.156977 |
| ENSMUSG00000073590 | protein_coding | 3222401L13Rik | 68.41401 | 73.78584 | 71.09992 |
| ENSMUSG00000007653 | protein_coding | Gabrb2 | 24.56968 | 28.64993 | 26.60981 |
| ENSMUSG00000013091 | protein_coding | Tmem190 | 3.866366 | 5.121137 | 4.493751 |
| ENSMUSG00000052920 | protein_coding | Prkg1 | 57.27769 | 62.86092 | 60.06931 |
| ENSMUSG00000082180 | pseudogene | Gm12230 | 2.295771 | 3.417215 | 2.856493 |
| ENSMUSG00000083356 | pseudogene | Gm11708 | 2.271171 | 1.324076 | 1.797623 |
| ENSMUSG00000038058 | protein_coding | Nod1 | 185.1259 | 196.2297 | 190.6778 |
| ENSMUSG00000081575 | pseudogene | Gm12090 | 2.611992 | 1.72194 | 2.166966 |
| ENSMUSG00000092734 | miRNA | AL596215.1 | 2.611992 | 1.72194 | 2.166966 |
| ENSMUSG00000081651 | pseudogene | Gm15530 | 2.611992 | 1.72194 | 2.166966 |
| ENSMUSG00000032867 | protein_coding | Fbxw8 | 2226.007 | 2154.632 | 2190.32 |
| ENSMUSG00000021111 | protein_coding | Papola | 8763.281 | 9014.738 | 8889.01 |
| ENSMUSG00000027884 | protein_coding | Clcc1 | 1169.438 | 1206.315 | 1187.876 |
| ENSMUSG00000085814 | lincRNA | 1810014B01Rik | 97.51783 | 104.3357 | 100.9268 |

| | | | | | |
|---|---|---|---|---|---|
| ENSMUSG00000035799 | protein_coding | Twist1 | 349.5187 | 335.5001 | 342.5094 |
| ENSMUSG00000093288 | miRNA | AC153909.1 | 0.968641 | 1.65059 | 1.309616 |
| ENSMUSG00000083816 | pseudogene | Gm13033 | 58.36426 | 63.41024 | 60.88725 |
| ENSMUSG00000059908 | protein_coding | Mug1 | 3.552455 | 4.776965 | 4.16471 |
| ENSMUSG00000067869 | pseudogene | Tcea1-ps1 | 458.1006 | 373.0861 | 415.5933 |
| ENSMUSG00000003206 | protein_coding | Ebi3 | 9.658236 | 7.884961 | 8.771599 |
| ENSMUSG00000029158 | protein_coding | Yipf7 | 2.577925 | 1.712931 | 2.145428 |
| ENSMUSG00000047368 | protein_coding | Fam108b | 792.2287 | 819.5712 | 805.9 |
| ENSMUSG00000024145 | protein_coding | Pigf | 289.3373 | 272.7777 | 281.0575 |
| ENSMUSG00000042292 | protein_coding | Mkl1 | 1001.616 | 964.7407 | 983.1784 |
| ENSMUSG00000038346 | protein_coding | Zfp384 | 1796.363 | 1742.826 | 1769.594 |
| ENSMUSG00000022246 | protein_coding | Rai14 | 3231.928 | 3323.28 | 3277.604 |
| ENSMUSG00000020888 | protein_coding | Dvl2 | 1829.278 | 1774.96 | 1802.119 |
| ENSMUSG00000081178 | pseudogene | Gm5490 | 2.577925 | 3.707692 | 3.142808 |
| ENSMUSG00000026205 | protein_coding | Slc23a3 | 10.77239 | 8.54736 | 9.659875 |
| ENSMUSG00000016494 | protein_coding | Cd34 | 2581.357 | 2655.952 | 2618.655 |
| ENSMUSG00000022494 | protein_coding | Shisa9 | 2.581503 | 1.65059 | 2.116047 |
| ENSMUSG00000092673 | miRNA | AC124398.1 | 0.632442 | 1.315066 | 0.973754 |
| ENSMUSG00000020914 | protein_coding | Top2a | 10878.68 | 10463.08 | 10670.88 |
| ENSMUSG00000043664 | protein_coding | Tmem221 | 55.07987 | 49.89775 | 52.48881 |
| ENSMUSG00000051219 | protein_coding | 3100002H09Rik | 1.298952 | 0.671047 | 0.984999 |
| ENSMUSG00000032185 | protein_coding | Carm1 | 1222.434 | 1180.112 | 1201.273 |
| ENSMUSG00000000983 | protein_coding | Expi | 22.48711 | 25.97724 | 24.23218 |
| ENSMUSG00000043889 | pseudogene | Gm8399 | 1580.736 | 2241.575 | 1911.156 |
| ENSMUSG00000037579 | protein_coding | Kcnh3 | 81.00612 | 74.19339 | 77.59976 |
| ENSMUSG00000001632 | protein_coding | Brpf1 | 2351.919 | 2265.833 | 2308.876 |
| ENSMUSG00000040624 | protein_coding | Plekhg1 | 2861.732 | 2961.638 | 2911.685 |
| ENSMUSG00000079709 | protein_coding | 9030025P20Rik | 65.39813 | 71.79574 | 68.59693 |
| ENSMUSG00000081118 | pseudogene | Gm6758 | 4.196676 | 3.081691 | 3.639184 |
| ENSMUSG00000051768 | protein_coding | Xrcc1 | 1223.311 | 1261.887 | 1242.599 |
| ENSMUSG00000087504 | processed_transcrip | Gm13658 | 0.948663 | 1.712931 | 1.330797 |
| ENSMUSG00000089373 | snoRNA | SNORA17 | 0.948663 | 1.712931 | 1.330797 |
| ENSMUSG00000029638 | protein_coding | Glcci1 | 116.4944 | 106.0957 | 111.2951 |
| ENSMUSG00000030825 | protein_coding | Hsd17b14 | 3.250323 | 4.414414 | 3.832369 |
| ENSMUSG00000032121 | protein_coding | Tmem218 | 258.9159 | 244.438 | 251.677 |
| ENSMUSG00000086537 | lincRNA | Nespas | 55.49412 | 49.95828 | 52.7262 |
| ENSMUSG00000036036 | protein_coding | Zfp57 | 163.5915 | 177.8175 | 170.7045 |
| ENSMUSG00000089764 | pseudogene | Gm16580 | 25.87936 | 22.73804 | 24.3087 |
| ENSMUSG00000090490 | protein_coding | Gm17553 | 3.532477 | 4.910296 | 4.221387 |
| ENSMUSG00000045573 | protein_coding | Penk | 836.4704 | 872.5189 | 854.4946 |
| ENSMUSG00000053007 | protein_coding | Creb5 | 182.1823 | 197.4426 | 189.8124 |
| ENSMUSG00000063430 | protein_coding | Wscd2 | 42.54389 | 38.08602 | 40.31496 |
| ENSMUSG00000082253 | pseudogene | Gm14639 | 7.740931 | 6.136716 | 6.938824 |
| ENSMUSG00000082480 | pseudogene | Gm11687 | 4.233054 | 3.010341 | 3.621697 |
| ENSMUSG00000049690 | protein_coding | Nckap5 | 762.8229 | 797.3018 | 780.0623 |
| ENSMUSG00000091384 | lincRNA | Gm17574 | 24.34977 | 21.09325 | 22.72151 |
| ENSMUSG00000042569 | protein_coding | Dhrs7b | 527.6604 | 505.2845 | 516.4725 |
| ENSMUSG00000011349 | protein_coding | Dmrtc2 | 317.5084 | 254.0072 | 285.7578 |
| ENSMUSG00000003549 | protein_coding | Ercc1 | 489.2207 | 467.6203 | 478.4205 |
| ENSMUSG00000084333 | pseudogene | Gm11599 | 0.948663 | 0.335524 | 0.642093 |
| ENSMUSG00000078626 | protein_coding | Gm12790 | 0.948663 | 0.335524 | 0.642093 |
| ENSMUSG00000053754 | protein_coding | Chd8 | 4820.847 | 4973.342 | 4897.094 |
| ENSMUSG00000025139 | protein_coding | Tollip | 3678.439 | 3559.352 | 3618.895 |
| ENSMUSG00000027378 | protein_coding | Nphp1 | 1537.278 | 1470.466 | 1503.872 |
| ENSMUSG00000052776 | protein_coding | Oas1a | 36.058 | 30.75017 | 33.40409 |
| ENSMUSG00000076248 | miRNA | CT573034.1 | 1.296641 | 0.662038 | 0.979339 |
| ENSMUSG00000081958 | pseudogene | Gm11594 | 2.555636 | 1.686265 | 2.120951 |
| ENSMUSG00000085338 | processed_transcrip | 2410004I01Rik | 6.451448 | 8.211475 | 7.331461 |

| | | | | | |
|---|---|---|---|---|---|
| ENSMUSG00000086495 | lincRNA | Gm13778 | 16.21166 | 19.24155 | 17.72661 |
| ENSMUSG00000079244 | protein_coding | Gm5622 | 8.107619 | 10.01378 | 9.060697 |
| ENSMUSG00000065734 | snoRNA | Snord49a | 17.07475 | 14.53237 | 15.80356 |
| ENSMUSG00000036815 | protein_coding | Dpp10 | 2.231215 | 3.354873 | 2.793044 |
| ENSMUSG00000061414 | protein_coding | Efcab4b | 7.412932 | 9.138048 | 8.27549 |
| ENSMUSG00000024340 | protein_coding | Btnl2 | 30.23899 | 25.84712 | 28.04306 |
| ENSMUSG00000046463 | protein_coding | 5930403N24Rik | 61.22919 | 56.35881 | 58.794 |
| ENSMUSG00000042298 | protein_coding | Ttc19 | 1409.442 | 1453.273 | 1431.357 |
| ENSMUSG00000052031 | protein_coding | Tagap1 | 939.234 | 982.1526 | 960.6933 |
| ENSMUSG00000020052 | protein_coding | Ascl1 | 2.889525 | 4.105557 | 3.497541 |
| ENSMUSG00000029659 | protein_coding | 6330406I15Rik | 20.03934 | 22.9669 | 21.50312 |
| ENSMUSG00000083914 | pseudogene | Rps18-ps1 | 0.948663 | 0.326514 | 0.637589 |
| ENSMUSG00000027566 | protein_coding | Psma7 | 4643.096 | 4519.909 | 4581.502 |
| ENSMUSG00000084094 | pseudogene | Gm13961 | 9.053972 | 11.01998 | 10.03698 |
| ENSMUSG00000012483 | protein_coding | Rpa3 | 720.4694 | 691.2842 | 705.8768 |
| ENSMUSG00000040405 | protein_coding | Havcr1 | 12.93443 | 15.61241 | 14.27342 |
| ENSMUSG00000018906 | protein_coding | P4ha2 | 297.8335 | 282.0137 | 289.9236 |
| ENSMUSG00000042938 | pseudogene | Gm14117 | 155.126 | 146.1388 | 150.6324 |
| ENSMUSG00000034917 | protein_coding | Tjp3 | 126.5865 | 140.4206 | 133.5036 |
| ENSMUSG00000083454 | pseudogene | Gm13226 | 1.284863 | 2.012779 | 1.648821 |
| ENSMUSG00000047719 | protein_coding | Ubiad1 | 749.7363 | 791.2659 | 770.5011 |
| ENSMUSG00000073388 | protein_coding | A330017A19Rik | 18.74396 | 15.99806 | 17.37101 |
| ENSMUSG00000014554 | protein_coding | Dguok | 698.8002 | 672.8719 | 685.8361 |
| ENSMUSG00000085446 | lincRNA | A430046D13Rik | 96.18652 | 103.501 | 99.84377 |
| ENSMUSG00000065847 | snoRNA | SNORA32 | 3.230345 | 4.610448 | 3.920396 |
| ENSMUSG00000086049 | processed_transcrip | Gm12735 | 3.572434 | 4.803632 | 4.188033 |
| ENSMUSG00000078240 | protein_coding | Gm3550 | 4.198987 | 3.055025 | 3.627006 |
| ENSMUSG00000002257 | protein_coding | Def6 | 36.46166 | 40.26273 | 38.3622 |
| ENSMUSG00000048709 | pseudogene | Gm8666 | 40.00361 | 44.14015 | 42.07188 |
| ENSMUSG00000020706 | protein_coding | Ftsj3 | 3370.966 | 3290.012 | 3330.489 |
| ENSMUSG00000051969 | protein_coding | Tlr11 | 1.290752 | 0.671047 | 0.980899 |
| ENSMUSG00000085182 | processed_transcrip | Gm13596 | 10.34472 | 8.520333 | 9.432528 |
| ENSMUSG00000019338 | protein_coding | Zfp687 | 3123.256 | 3023.501 | 3073.378 |
| ENSMUSG00000079188 | protein_coding | Gm7694 | 32.96892 | 29.25497 | 31.11194 |
| ENSMUSG00000020831 | protein_coding | 0610010K14Rik | 1591.636 | 1543.265 | 1567.451 |
| ENSMUSG00000029313 | protein_coding | Aff1 | 1337.983 | 1382.985 | 1360.484 |
| ENSMUSG00000093356 | rRNA | AC142112.1 | 9.360726 | 7.362775 | 8.36175 |
| ENSMUSG00000024400 | protein_coding | Wdr33 | 2808.518 | 2884.222 | 2846.37 |
| ENSMUSG00000027944 | protein_coding | Hax1 | 1439.859 | 1492.692 | 1466.276 |
| ENSMUSG00000044162 | protein_coding | Tnip3 | 1.276663 | 0.662038 | 0.96935 |
| ENSMUSG00000082518 | pseudogene | Gm6221 | 0.98273 | 1.775272 | 1.379001 |
| ENSMUSG00000024513 | protein_coding | Mbd2 | 1334.868 | 1376.211 | 1355.539 |
| ENSMUSG00000031502 | protein_coding | Col4a1 | 6929.506 | 7224.35 | 7076.928 |
| ENSMUSG00000031700 | protein_coding | Gpt2 | 1418.506 | 1482.446 | 1450.476 |
| ENSMUSG00000027395 | protein_coding | Polr1b | 1376.652 | 1333.191 | 1354.921 |
| ENSMUSG00000076949 | miRNA | AC121540.1 | 1.276663 | 2.075121 | 1.675892 |
| ENSMUSG00000086610 | processed_transcrip | Gm15408 | 10.99588 | 8.84757 | 9.921723 |
| ENSMUSG00000086683 | processed_transcrip | Gm12867 | 1.290752 | 2.012779 | 1.651765 |
| ENSMUSG00000082481 | pseudogene | Gm12334 | 1.290752 | 2.012779 | 1.651765 |
| ENSMUSG00000067870 | protein_coding | Gm8759 | 452.5002 | 435.8478 | 444.174 |
| ENSMUSG00000091149 | pseudogene | Gm17020 | 2.567414 | 1.72194 | 2.144677 |
| ENSMUSG00000081748 | pseudogene | Gm14870 | 0.66062 | 1.341732 | 1.001176 |
| ENSMUSG00000054958 | protein_coding | Nt5c1a | 7.052133 | 5.509992 | 6.281063 |
| ENSMUSG00000086867 | processed_transcrip | Gm4577 | 4.196676 | 3.099348 | 3.648012 |
| ENSMUSG00000018415 | protein_coding | 4933439F18Rik | 2425.321 | 2336.284 | 2380.803 |
| ENSMUSG00000057609 | protein_coding | Lce1a1 | 1.967772 | 3.010341 | 2.489056 |
| ENSMUSG00000051590 | protein_coding | Ysk4 | 5.204006 | 3.841746 | 4.522876 |
| ENSMUSG00000086891 | processed_transcrip | Gm15963 | 1.298952 | 2.021789 | 1.66037 |

| | | | | | |
|---|---|---|---|---|---|
| ENSMUSG00000090329 | processed_transcrip | Gm17160 | 1.284863 | 0.671047 | 0.977955 |
| ENSMUSG00000089925 | pseudogene | Ang-ps2 | 1.284863 | 0.671047 | 0.977955 |
| ENSMUSG00000073233 | pseudogene | Gm9144 | 9.048083 | 10.8953 | 9.971692 |
| ENSMUSG00000034321 | protein_coding | Exosc1 | 1289.703 | 1253.478 | 1271.59 |
| ENSMUSG00000049420 | protein_coding | Tmem200a | 24.42797 | 28.02534 | 26.22665 |
| ENSMUSG00000063904 | protein_coding | Dpp3 | 2283.626 | 2216.537 | 2250.081 |
| ENSMUSG00000031967 | protein_coding | Afg3l1 | 1722.93 | 1785.014 | 1753.972 |
| ENSMUSG00000073988 | protein_coding | Ttpa | 53.02257 | 48.36102 | 50.6918 |
| ENSMUSG00000020814 | protein_coding | Mxra7 | 1055.742 | 1122.998 | 1089.37 |
| ENSMUSG00000019762 | protein_coding | Iyd | 2.583814 | 1.748606 | 2.16621 |
| ENSMUSG00000063362 | protein_coding | Alg11 | 1151.818 | 1103.999 | 1127.909 |
| ENSMUSG00000024978 | protein_coding | Gpam | 3538.736 | 3442.508 | 3490.622 |
| ENSMUSG00000040125 | protein_coding | Gpr26 | 0.64422 | 1.315066 | 0.979643 |
| ENSMUSG00000082089 | pseudogene | Gm12431 | 1.304841 | 0.688704 | 0.996772 |
| ENSMUSG00000091029 | protein_coding | Gm8138 | 1.304841 | 0.688704 | 0.996772 |
| ENSMUSG00000084390 | pseudogene | Gm15425 | 1.304841 | 0.688704 | 0.996772 |
| ENSMUSG00000026269 | protein_coding | Rnpepl1 | 1517.891 | 1569.531 | 1543.711 |
| ENSMUSG00000063704 | protein_coding | Mapk15 | 13.32214 | 15.86473 | 14.59343 |
| ENSMUSG00000059333 | protein_coding | Gm10063 | 3.864055 | 2.755177 | 3.309616 |
| ENSMUSG00000020220 | protein_coding | Vps13d | 4024.854 | 3891.677 | 3958.266 |
| ENSMUSG00000034107 | protein_coding | Ano7 | 5.834137 | 4.239249 | 5.036693 |
| ENSMUSG00000053799 | protein_coding | Exoc6 | 1753.471 | 1825.409 | 1789.44 |
| ENSMUSG00000022892 | protein_coding | App | 10458.34 | 10846.51 | 10652.42 |
| ENSMUSG00000028035 | protein_coding | Dnajb4 | 1264.17 | 1226.671 | 1245.42 |
| ENSMUSG00000023484 | protein_coding | Prph | 9.679257 | 11.91124 | 10.79525 |
| ENSMUSG00000047181 | protein_coding | Samd14 | 384.2451 | 369.2784 | 376.7617 |
| ENSMUSG00000032577 | protein_coding | Mapkapk3 | 631.711 | 545.0642 | 588.3876 |
| ENSMUSG00000087691 | processed_transcrip | Gm15674 | 3.902744 | 5.368014 | 4.635379 |
| ENSMUSG00000032310 | protein_coding | Cyp1a2 | 17.18492 | 20.22866 | 18.70679 |
| ENSMUSG00000046192 | protein_coding | Iqub | 23.95617 | 20.79625 | 22.37621 |
| ENSMUSG00000072766 | protein_coding | Gm10417 | 9.652347 | 11.78905 | 10.7207 |
| ENSMUSG00000004069 | protein_coding | Dnaja3 | 1827.384 | 1885.109 | 1856.247 |
| ENSMUSG00000024078 | protein_coding | Ttc27 | 1753.194 | 1694.516 | 1723.855 |
| ENSMUSG00000031954 | protein_coding | Cfdp1 | 3113.539 | 3200.177 | 3156.858 |
| ENSMUSG00000047112 | pseudogene | 0610012G03Rik | 645.416 | 667.1953 | 656.3057 |
| ENSMUSG00000022946 | protein_coding | Dopey2 | 758.5109 | 792.6359 | 775.5734 |
| ENSMUSG00000056941 | protein_coding | Commd7 | 1004.466 | 1043.956 | 1024.211 |
| ENSMUSG00000066721 | protein_coding | Zfp575 | 33.85325 | 30.31166 | 32.08245 |
| ENSMUSG00000067215 | protein_coding | Usp51 | 20.33685 | 17.72072 | 19.02879 |
| ENSMUSG00000087271 | processed_transcrip | 2900008C10Rik | 9.339704 | 11.25749 | 10.2986 |
| ENSMUSG00000028792 | protein_coding | Ak2 | 1819.786 | 1887.661 | 1853.723 |
| ENSMUSG00000068855 | protein_coding | Hist2h2ac | 2.581503 | 3.797423 | 3.189463 |
| ENSMUSG00000038239 | protein_coding | Hrc | 43.16433 | 48.35097 | 45.75765 |
| ENSMUSG00000071398 | protein_coding | 2410004P03Rik | 17.79634 | 15.31909 | 16.55772 |
| ENSMUSG00000072573 | protein_coding | Gm10369 | 9.692303 | 11.60202 | 10.64716 |
| ENSMUSG00000019210 | protein_coding | Atp6v1e1 | 2119.491 | 2177.154 | 2148.323 |
| ENSMUSG00000026459 | protein_coding | Myog | 3.908633 | 2.763824 | 3.336229 |
| ENSMUSG00000030769 | protein_coding | Slc5a11 | 3.224456 | 4.44108 | 3.832768 |
| ENSMUSG00000049494 | pseudogene | Gm12669 | 22.86073 | 19.93497 | 21.39785 |
| ENSMUSG00000081865 | pseudogene | Gm15484 | 7.704554 | 6.065004 | 6.884779 |
| ENSMUSG00000042529 | protein_coding | Kcnj12 | 14.19342 | 11.87448 | 13.03395 |
| ENSMUSG00000085787 | pseudogene | Gm13092 | 72.44185 | 94.36275 | 83.4023 |
| ENSMUSG00000028948 | protein_coding | Nol9 | 1690.889 | 1747.365 | 1719.127 |
| ENSMUSG00000035158 | protein_coding | Mitf | 269.5512 | 256.7153 | 263.1333 |
| ENSMUSG00000004552 | protein_coding | Ctse | 60.22499 | 65.88716 | 63.05607 |
| ENSMUSG00000042759 | protein_coding | Apobr | 33.89887 | 30.48931 | 32.19409 |
| ENSMUSG00000036136 | protein_coding | Fam110c | 254.4081 | 238.2343 | 246.3212 |
| ENSMUSG00000028271 | protein_coding | Gtf2b | 1628.747 | 1580.489 | 1604.618 |

| | | | | | |
|---|---|---|---|---|---|
| ENSMUSG00000028954 | protein_coding | Nub1 | 1692.439 | 1645.77 | 1669.105 |
| ENSMUSG00000085750 | processed_transcrip | Gm14009 | 1.31304 | 0.688704 | 1.000872 |
| ENSMUSG00000088623 | snRNA | U6atac | 1.31304 | 0.688704 | 1.000872 |
| ENSMUSG00000075480 | protein_coding | Gm10840 | 1.31304 | 0.688704 | 1.000872 |
| ENSMUSG00000048528 | protein_coding | Nkx1-2 | 176.9932 | 165.6513 | 171.3223 |
| ENSMUSG00000039929 | protein_coding | Urb1 | 2365.148 | 2252.24 | 2308.694 |
| ENSMUSG00000064115 | protein_coding | Cadm2 | 9.982657 | 8.09544 | 9.039049 |
| ENSMUSG00000020464 | protein_coding | Pnpt1 | 1543.984 | 1481.719 | 1512.852 |
| ENSMUSG00000081726 | pseudogene | Gm13202 | 21.62402 | 24.46578 | 23.0449 |
| ENSMUSG00000071763 | pseudogene | 4933416I08Rik | 25.28539 | 21.78196 | 23.53367 |
| ENSMUSG00000026854 | protein_coding | Usp20 | 908.5658 | 879.5242 | 894.045 |
| ENSMUSG00000021458 | protein_coding | 2010111I01Rik | 402.4668 | 427.2069 | 414.8369 |
| ENSMUSG00000023949 | protein_coding | Tcte1 | 34.16716 | 29.87346 | 32.02031 |
| ENSMUSG00000022419 | protein_coding | Deptor | 632.6657 | 666.8562 | 649.7609 |
| ENSMUSG00000069633 | protein_coding | Pex11c | 120.1738 | 113.2474 | 116.7106 |
| ENSMUSG00000054931 | protein_coding | Zkscan4 | 112.9637 | 104.5877 | 108.7757 |
| ENSMUSG00000034764 | lincRNA | 1700006J14Rik | 20.50338 | 17.2994 | 18.90139 |
| ENSMUSG00000000804 | protein_coding | Usp32 | 6227.384 | 6026.289 | 6126.837 |
| ENSMUSG00000087785 | snoRNA | SNORA25 | 1.284863 | 2.093139 | 1.689001 |
| ENSMUSG00000092241 | protein_coding | Gm20522 | 29.75035 | 33.19623 | 31.47329 |
| ENSMUSG00000034126 | protein_coding | Pomt2 | 1416.18 | 1347.409 | 1381.794 |
| ENSMUSG00000033769 | protein_coding | Exoc6b | 2017.705 | 2098.065 | 2057.885 |
| ENSMUSG00000034850 | protein_coding | Tmem127 | 2562.121 | 2630.248 | 2596.184 |
| ENSMUSG00000021275 | protein_coding | Tecpr2 | 1012.522 | 1076.737 | 1044.63 |
| ENSMUSG00000087355 | lincRNA | Gm13187 | 0.948663 | 1.686265 | 1.317464 |
| ENSMUSG00000081718 | pseudogene | Gm8261 | 1.290752 | 2.146471 | 1.718611 |
| ENSMUSG00000050550 | pseudogene | Gm11868 | 98.33865 | 70.05719 | 84.19792 |
| ENSMUSG00000032690 | protein_coding | Oas2 | 24.38972 | 28.03471 | 26.21222 |
| ENSMUSG00000026638 | protein_coding | Irf6 | 147.2445 | 136.6695 | 141.957 |
| ENSMUSG00000029563 | protein_coding | Foxp2 | 17.12983 | 14.70101 | 15.91542 |
| ENSMUSG00000042041 | protein_coding | 2010003K11Rik | 11.68005 | 9.499514 | 10.58978 |
| ENSMUSG00000040128 | protein_coding | Pnrc1 | 2315.146 | 2235.9 | 2275.523 |
| ENSMUSG00000083767 | pseudogene | Gm11405 | 18.05748 | 15.59191 | 16.82469 |
| ENSMUSG00000018893 | protein_coding | Mb | 1.276663 | 2.057464 | 1.667063 |
| ENSMUSG00000028115 | protein_coding | Bnipl | 8.694216 | 10.63113 | 9.662672 |
| ENSMUSG00000089940 | lincRNA | Gm4117 | 33.26539 | 36.89921 | 35.0823 |
| ENSMUSG00000039662 | protein_coding | Icmt | 2164.384 | 2101.838 | 2133.111 |
| ENSMUSG00000031932 | protein_coding | Gpr83 | 5.795449 | 4.44108 | 5.118265 |
| ENSMUSG00000067199 | pseudogene | Frat1 | 36.99384 | 32.91761 | 34.95573 |
| ENSMUSG00000033501 | protein_coding | Crygs | 12.55954 | 14.97383 | 13.76668 |
| ENSMUSG00000024937 | protein_coding | Ehbp1l1 | 721.8707 | 749.953 | 735.9118 |
| ENSMUSG00000060063 | protein_coding | Alox5ap | 76.95392 | 83.34946 | 80.15169 |
| ENSMUSG00000085572 | processed_transcrip | Gm16234 | 10.30708 | 8.484657 | 9.395868 |
| ENSMUSG00000081087 | pseudogene | Gm14166 | 28.78298 | 25.64136 | 27.21217 |
| ENSMUSG00000049470 | protein_coding | Aff4 | 6189.78 | 6429.022 | 6309.401 |
| ENSMUSG00000081161 | pseudogene | Gm12783 | 2.587392 | 1.65059 | 2.118991 |
| ENSMUSG00000001663 | protein_coding | Gstt1 | 232.4491 | 246.1425 | 239.2958 |
| ENSMUSG00000029634 | protein_coding | Rnf6 | 1944.519 | 1993.435 | 1968.977 |
| ENSMUSG00000065870 | snoRNA | U3 | 4.176698 | 5.447651 | 4.812174 |
| ENSMUSG00000028256 | protein_coding | Odf2l | 694.5236 | 665.6465 | 680.085 |
| ENSMUSG00000055024 | protein_coding | Ep300 | 3865.527 | 4019.802 | 3942.665 |
| ENSMUSG00000082891 | pseudogene | Gm12881 | 1.307151 | 0.688704 | 0.997928 |
| ENSMUSG00000091956 | protein_coding | C2cd4b | 1.307151 | 0.688704 | 0.997928 |
| ENSMUSG00000044916 | protein_coding | 1700029I15Rik | 19.40794 | 22.26883 | 20.83838 |
| ENSMUSG00000081113 | pseudogene | Gm7308 | 22.89248 | 20.131 | 21.51174 |
| ENSMUSG00000031657 | protein_coding | Heatr3 | 2089.911 | 2163.935 | 2126.923 |
| ENSMUSG00000053714 | protein_coding | 4732471J01Rik | 1.293062 | 0.688704 | 0.990883 |
| ENSMUSG00000086868 | processed_transcrip | Gm15883 | 1.293062 | 0.688704 | 0.990883 |

| | | | | | |
|---|---|---|---|---|---|
| ENSMUSG00000030859 | protein_coding | Fam24a | 1.293062 | 0.688704 | 0.990883 |
| ENSMUSG00000086870 | processed_transcrip | Gm15288 | 1.293062 | 0.688704 | 0.990883 |
| ENSMUSG00000080594 | miRNA | Mir669h | 1.304841 | 0.697713 | 1.001277 |
| ENSMUSG00000068604 | protein_coding | Gm8225 | 1.304841 | 0.697713 | 1.001277 |
| ENSMUSG00000061742 | protein_coding | Slc22a12 | 1.304841 | 0.697713 | 1.001277 |
| ENSMUSG00000022661 | protein_coding | Cd200 | 1428.931 | 1501.13 | 1465.03 |
| ENSMUSG00000039059 | protein_coding | Hrh3 | 2.577925 | 1.659599 | 2.118762 |
| ENSMUSG00000032398 | protein_coding | Snapc5 | 470.3785 | 490.7712 | 480.5748 |
| ENSMUSG00000045725 | protein_coding | Prr15 | 819.1307 | 853.6742 | 836.4024 |
| ENSMUSG00000091020 | pseudogene | Gm5828 | 3.834609 | 2.692474 | 3.263542 |
| ENSMUSG00000034602 | protein_coding | Mon2 | 2200.748 | 2279.611 | 2240.18 |
| ENSMUSG00000048473 | protein_coding | Gm766 | 9.678214 | 11.7804 | 10.72931 |
| ENSMUSG00000018845 | protein_coding | Unc45b | 25.21643 | 28.59516 | 26.90579 |
| ENSMUSG00000032475 | protein_coding | Nck1 | 1268.484 | 1310.688 | 1289.586 |
| ENSMUSG00000091511 | protein_coding | Vmn2r87 | 3.568855 | 2.401997 | 2.985426 |
| ENSMUSG00000001552 | protein_coding | Jup | 1020.703 | 1057.059 | 1038.881 |
| ENSMUSG00000074582 | protein_coding | Arfgef2 | 2169.152 | 1950.33 | 2059.741 |
| ENSMUSG00000034898 | protein_coding | Filip1 | 202.3731 | 192.8946 | 197.6338 |
| ENSMUSG00000006469 | protein_coding | Slc34a3 | 3.902744 | 2.7995 | 3.351122 |
| ENSMUSG00000025485 | protein_coding | Ric8 | 1063.965 | 1098.678 | 1081.322 |
| ENSMUSG00000048078 | protein_coding | Odz4 | 2004.633 | 1923.549 | 1964.091 |
| ENSMUSG00000061171 | protein_coding | Slc38a11 | 11.56965 | 9.62492 | 10.59729 |
| ENSMUSG00000038332 | protein_coding | Sesn1 | 2348.745 | 2421.589 | 2385.167 |
| ENSMUSG00000046500 | protein_coding | Fam19a4 | 4.194365 | 3.055025 | 3.624695 |
| ENSMUSG00000043939 | protein_coding | A530064D06Rik | 5.870515 | 7.67412 | 6.772318 |
| ENSMUSG00000077400 | snoRNA | SNORA17 | 1.298952 | 2.030798 | 1.664875 |
| ENSMUSG00000024842 | protein_coding | Cabp4 | 11.32156 | 9.369395 | 10.34548 |
| ENSMUSG00000029212 | protein_coding | Gabrb1 | 4.204876 | 5.501345 | 4.85311 |
| ENSMUSG00000004815 | protein_coding | Dgkq | 586.9164 | 565.1899 | 576.0532 |
| ENSMUSG00000025578 | protein_coding | Cbx8 | 633.6458 | 668.1376 | 650.8917 |
| ENSMUSG00000090652 | protein_coding | Gm4064 | 213.2042 | 225.1369 | 219.1705 |
| ENSMUSG00000033721 | protein_coding | Vav3 | 142.7658 | 134.9933 | 138.8795 |
| ENSMUSG00000004980 | protein_coding | Hnrnpa2b1 | 20877.74 | 21593.72 | 21235.73 |
| ENSMUSG00000021396 | protein_coding | Nxnl2 | 566.4415 | 588.8216 | 577.6315 |
| ENSMUSG00000037685 | protein_coding | Atp8a1 | 1416.007 | 1465.702 | 1440.854 |
| ENSMUSG00000087364 | processed_transcrip | Gm13398 | 12.57594 | 10.40807 | 11.492 |
| ENSMUSG00000026273 | protein_coding | Mterfd2 | 511.1768 | 491.2368 | 501.2068 |
| ENSMUSG00000086472 | processed_transcrip | Gm16172 | 3.242123 | 4.379101 | 3.810612 |
| ENSMUSG00000062175 | protein_coding | Tgif2 | 2160.918 | 2242.509 | 2201.713 |
| ENSMUSG00000017832 | protein_coding | Hspb9 | 10.33063 | 8.565017 | 9.447826 |
| ENSMUSG00000078091 | protein_coding | Gm10912 | 1.31304 | 0.697713 | 1.005377 |
| ENSMUSG00000079307 | protein_coding | Gm4461 | 1.31304 | 0.697713 | 1.005377 |
| ENSMUSG00000030275 | protein_coding | Etnk1 | 5876.003 | 5637.641 | 5756.822 |
| ENSMUSG00000076651 | IG_V_gene | Gm16700 | 0.638331 | 1.306057 | 0.972194 |
| ENSMUSG00000077497 | snoRNA | SNORA26 | 0.638331 | 1.306057 | 0.972194 |
| ENSMUSG00000085128 | lincRNA | Gm2115 | 2.569725 | 1.748606 | 2.159166 |
| ENSMUSG00000062470 | processed_transcrip | 5730577I03Rik | 59.61506 | 65.88575 | 62.75041 |
| ENSMUSG00000083168 | pseudogene | Gm14201 | 1.581105 | 2.754815 | 2.16796 |
| ENSMUSG00000035184 | protein_coding | Fam124a | 550.4539 | 574.0909 | 562.2724 |
| ENSMUSG00000030965 | protein_coding | Fam175b | 1395.342 | 1346.634 | 1370.988 |
| ENSMUSG00000065944 | snRNA | U2 | 1.606973 | 2.472986 | 2.039979 |
| ENSMUSG00000091066 | lincRNA | Gm17598 | 16.14942 | 13.73912 | 14.94427 |
| ENSMUSG00000025780 | protein_coding | Itih5 | 3215.311 | 3326.85 | 3271.08 |
| ENSMUSG00000073995 | protein_coding | Gm10604 | 8.720083 | 10.52446 | 9.622274 |
| ENSMUSG00000037759 | protein_coding | Ptger2 | 10.34241 | 8.449344 | 9.395878 |
| ENSMUSG00000004347 | protein_coding | Pde1c | 117.8938 | 109.464 | 113.6789 |
| ENSMUSG00000050064 | protein_coding | Zfp697 | 193.4417 | 178.2226 | 185.8321 |
| ENSMUSG00000024943 | protein_coding | Smc5 | 2813.719 | 2890.045 | 2851.882 |

| | | | | | |
|---|---|---|---|---|---|
| ENSMUSG00000069893 | protein_coding | 9930111J21Rik1 | 3.890965 | 2.7995 | 3.345233 |
| ENSMUSG00000092444 | processed_transcrip | Gm18733 | 0.99093 | 1.748606 | 1.369768 |
| ENSMUSG00000018846 | protein_coding | Pank3 | 6921.335 | 7153.4 | 7037.368 |
| ENSMUSG00000021033 | protein_coding | Gstz1 | 915.3921 | 888.0276 | 901.7099 |
| ENSMUSG00000026037 | protein_coding | Orc2 | 2270.792 | 2202.917 | 2236.854 |
| ENSMUSG00000040552 | protein_coding | C3ar1 | 85.52222 | 92.29079 | 88.90651 |
| ENSMUSG00000000131 | protein_coding | Xpo6 | 3297.143 | 3160.5 | 3228.822 |
| ENSMUSG00000021448 | protein_coding | Shc3 | 10.68092 | 8.82919 | 9.755057 |
| ENSMUSG00000020328 | protein_coding | Nudcd2 | 880.8522 | 845.412 | 863.1321 |
| ENSMUSG00000042605 | protein_coding | Atxn2 | 2481.066 | 2571.316 | 2526.191 |
| ENSMUSG00000014601 | protein_coding | Fam40a | 1609.938 | 1562.709 | 1586.324 |
| ENSMUSG00000089965 | pseudogene | Gm16236 | 4.210765 | 5.474317 | 4.842541 |
| ENSMUSG00000026415 | protein_coding | Fcamr | 14.46149 | 12.27307 | 13.36728 |
| ENSMUSG00000054200 | protein_coding | O3far1 | 21.94151 | 18.84913 | 20.39532 |
| ENSMUSG00000068917 | protein_coding | Clk2 | 1454.74 | 1408.343 | 1431.541 |
| ENSMUSG00000039684 | pseudogene | Gm5422 | 73.8049 | 68.36698 | 71.08594 |
| ENSMUSG00000050709 | protein_coding | 4932442E05Rik | 30.68724 | 34.36637 | 32.5268 |
| ENSMUSG00000073179 | pseudogene | Gm10478 | 20.37092 | 23.78324 | 22.07708 |
| ENSMUSG00000078161 | protein_coding | BC007180 | 4.887785 | 6.533858 | 5.710821 |
| ENSMUSG00000061755 | protein_coding | Bod1l | 3677.873 | 3823.493 | 3750.683 |
| ENSMUSG00000091624 | protein_coding | Vmn2r9 | 19.70754 | 15.39401 | 17.55077 |
| ENSMUSG00000042111 | protein_coding | Ccdc115 | 1113.156 | 1065.909 | 1089.532 |
| ENSMUSG00000070705 | protein_coding | Eid2b | 417.1027 | 397.2097 | 407.1562 |
| ENSMUSG00000044176 | protein_coding | Spink10 | 4.504697 | 5.81885 | 5.161773 |
| ENSMUSG00000060019 | protein_coding | Gm10073 | 28.67951 | 25.36533 | 27.02242 |
| ENSMUSG00000081402 | pseudogene | Gm15455 | 5.843605 | 4.45009 | 5.146847 |
| ENSMUSG00000021891 | protein_coding | Mettl6 | 1014.967 | 972.9704 | 993.9689 |
| ENSMUSG00000060890 | protein_coding | Arr3 | 6.522936 | 8.238503 | 7.380719 |
| ENSMUSG00000074902 | protein_coding | Gm904 | 4.532875 | 3.363521 | 3.948198 |
| ENSMUSG00000027639 | protein_coding | Samhd1 | 1519.329 | 1475.782 | 1497.555 |
| ENSMUSG00000006311 | protein_coding | Etv2 | 2.561525 | 1.659599 | 2.110562 |
| ENSMUSG00000034674 | protein_coding | Tdg | 1103.267 | 1311.497 | 1207.382 |
| ENSMUSG00000022756 | protein_coding | Slc7a4 | 558.1294 | 582.4655 | 570.2975 |
| ENSMUSG00000074874 | protein_coding | Ctla2b | 31.9513 | 28.13888 | 30.04509 |
| ENSMUSG00000027079 | protein_coding | Clp1 | 717.332 | 748.5078 | 732.9199 |
| ENSMUSG00000081996 | pseudogene | Gm7153 | 15.90826 | 18.65558 | 17.28192 |
| ENSMUSG00000039308 | protein_coding | Ndst2 | 1055.87 | 1015.78 | 1035.825 |
| ENSMUSG00000088583 | snRNA | U6 | 1.304841 | 2.075121 | 1.689981 |
| ENSMUSG00000029994 | protein_coding | Anxa4 | 1347.208 | 1398.02 | 1372.614 |
| ENSMUSG00000053475 | protein_coding | Tnfaip6 | 138.7842 | 126.2013 | 132.4928 |
| ENSMUSG00000026425 | protein_coding | Srgap2 | 2038.094 | 1945.729 | 1991.911 |
| ENSMUSG00000072875 | protein_coding | Gpr27 | 40.77187 | 36.12326 | 38.44757 |
| ENSMUSG00000020121 | protein_coding | Srgap1 | 113.0956 | 122.3424 | 117.719 |
| ENSMUSG00000035781 | protein_coding | C030046I01Rik | 1216.071 | 1258.448 | 1237.26 |
| ENSMUSG00000020372 | protein_coding | Gnb2l1 | 11558.11 | 11242.87 | 11400.49 |
| ENSMUSG00000022721 | protein_coding | Trmt2a | 1000.762 | 972.2759 | 986.5189 |
| ENSMUSG00000017830 | protein_coding | Dhx58 | 49.62651 | 45.4008 | 47.51366 |
| ENSMUSG00000026246 | protein_coding | Alppl2 | 4.176698 | 3.108357 | 3.642528 |
| ENSMUSG00000078235 | protein_coding | Fam43b | 6.142159 | 4.812279 | 5.477219 |
| ENSMUSG00000028874 | protein_coding | Fgr | 25.57954 | 22.61336 | 24.09645 |
| ENSMUSG00000048481 | protein_coding | Mypop | 156.84 | 165.3436 | 161.0918 |
| ENSMUSG00000091839 | lincRNA | Gm17315 | 8.43204 | 6.753708 | 7.592874 |
| ENSMUSG00000051984 | protein_coding | Sec31b | 89.93203 | 97.82677 | 93.8794 |
| ENSMUSG00000082319 | pseudogene | Gm8822 | 174.3537 | 183.9548 | 179.1543 |
| ENSMUSG00000022109 | protein_coding | Med4 | 2402.973 | 2343.02 | 2372.997 |
| ENSMUSG00000084342 | pseudogene | Gm11224 | 4.521097 | 5.863534 | 5.192316 |
| ENSMUSG00000001627 | protein_coding | Ifrd1 | 1449.725 | 1402.791 | 1426.258 |
| ENSMUSG00000038193 | protein_coding | Hand2 | 1.606973 | 2.383978 | 1.995475 |

| | | | | | |
|---|---|---|---|---|---|
| ENSMUSG00000068184 | protein_coding | Ndufaf2 | 426.3131 | 443.4941 | 434.9036 |
| ENSMUSG00000037015 | pseudogene | Tmem185b | 1134.078 | 1183.079 | 1158.579 |
| ENSMUSG00000082057 | pseudogene | Gm15789 | 21.26658 | 18.3139 | 19.79024 |
| ENSMUSG00000056290 | protein_coding | Ms4a4b | 4.518786 | 3.399196 | 3.958991 |
| ENSMUSG00000037469 | protein_coding | C330005M16Rik | 725.2943 | 776.9847 | 751.1395 |
| ENSMUSG00000062070 | protein_coding | Pgk1 | 2057.829 | 2111.549 | 2084.689 |
| ENSMUSG00000010290 | protein_coding | AI597479 | 1231.203 | 1265.073 | 1248.138 |
| ENSMUSG00000024188 | protein_coding | Luc7l | 2871.447 | 2985.788 | 2928.617 |
| ENSMUSG00000024386 | protein_coding | Proc | 3.886344 | 2.621123 | 3.253734 |
| ENSMUSG00000031447 | protein_coding | Lamp1 | 8612.452 | 8808.143 | 8710.298 |
| ENSMUSG00000027649 | protein_coding | Ctnnbl1 | 1544.513 | 1494.819 | 1519.666 |
| ENSMUSG00000026809 | protein_coding | 1700026L06Rik | 17.12059 | 19.68017 | 18.40038 |
| ENSMUSG00000040325 | protein_coding | Vprbp | 3156.597 | 3030.94 | 3093.769 |
| ENSMUSG00000087032 | processed_transcrip | Gm13874 | 1.601084 | 2.419653 | 2.010369 |
| ENSMUSG00000058052 | protein_coding | Defb35 | 0.66062 | 1.448758 | 1.054689 |
| ENSMUSG00000021044 | protein_coding | Adck1 | 752.2721 | 729.6439 | 740.958 |
| ENSMUSG00000023083 | protein_coding | H2-M10.2 | 9.051661 | 10.93962 | 9.995643 |
| ENSMUSG00000040424 | protein_coding | Hipk4 | 65.7274 | 60.26434 | 62.99587 |
| ENSMUSG00000029622 | protein_coding | Arpc1b | 5190.03 | 5345.683 | 5267.857 |
| ENSMUSG00000049719 | protein_coding | Prss46 | 2.950502 | 4.185917 | 3.568209 |
| ENSMUSG00000045349 | protein_coding | Sh2d5 | 269.1177 | 257.5839 | 263.3508 |
| ENSMUSG00000082872 | pseudogene | Gm15773 | 3.860477 | 5.049786 | 4.455131 |
| ENSMUSG00000020340 | protein_coding | Cyfip2 | 320.473 | 303.7598 | 312.1164 |
| ENSMUSG00000081002 | pseudogene | Gm14240 | 1.621062 | 2.472986 | 2.047024 |
| ENSMUSG00000029233 | protein_coding | Srd5a3 | 415.8257 | 434.7293 | 425.2775 |
| ENSMUSG00000035168 | protein_coding | Tanc1 | 1628.782 | 1678.692 | 1653.737 |
| ENSMUSG00000065045 | snRNA | U1 | 0.98273 | 1.703922 | 1.343326 |
| ENSMUSG00000083608 | pseudogene | Gm14863 | 0.98273 | 1.703922 | 1.343326 |
| ENSMUSG00000079636 | protein_coding | Rhox3c | 0.98273 | 1.703922 | 1.343326 |
| ENSMUSG00000066797 | protein_coding | Zfp648 | 20.22436 | 16.89796 | 18.56116 |
| ENSMUSG00000091761 | lincRNA | Gm4349 | 61.42479 | 68.34285 | 64.88382 |
| ENSMUSG00000027104 | protein_coding | Atf2 | 2586.464 | 2656.609 | 2621.536 |
| ENSMUSG00000030827 | protein_coding | Fgf21 | 7.805487 | 6.207705 | 7.006596 |
| ENSMUSG00000039798 | protein_coding | 2600006K01Rik | 7.108489 | 8.739821 | 7.924155 |
| ENSMUSG00000034707 | protein_coding | Gns | 2109.37 | 2036.153 | 2072.762 |
| ENSMUSG00000083353 | pseudogene | Gm12540 | 4.521097 | 5.783174 | 5.152136 |
| ENSMUSG00000041187 | protein_coding | Prkd2 | 162.1744 | 171.8122 | 166.9933 |
| ENSMUSG00000015957 | protein_coding | Wnt11 | 28.34689 | 31.91612 | 30.1315 |
| ENSMUSG00000086561 | processed_transcrip | Gm15540 | 2.239415 | 1.342094 | 1.790754 |
| ENSMUSG00000085864 | processed_transcrip | C330019F10Rik | 6.486558 | 5.040777 | 5.763667 |
| ENSMUSG00000028940 | protein_coding | Hes2 | 4.507008 | 5.800831 | 5.15392 |
| ENSMUSG00000022354 | protein_coding | Ndufb9 | 2046.042 | 1981.961 | 2014.002 |
| ENSMUSG00000040699 | protein_coding | Limd2 | 785.3962 | 759.6325 | 772.5144 |
| ENSMUSG00000057000 | protein_coding | Nxf3 | 4505.338 | 4017.925 | 4261.632 |
| ENSMUSG00000026786 | protein_coding | Apbb1ip | 68.31897 | 63.12768 | 65.72332 |
| ENSMUSG00000067604 | protein_coding | Nms | 0.99093 | 1.712931 | 1.351931 |
| ENSMUSG00000089708 | processed_transcrip | 5330439A09Rik | 0.99093 | 1.712931 | 1.351931 |
| ENSMUSG00000030879 | protein_coding | Mrpl17 | 1317.679 | 1277.941 | 1297.81 |
| ENSMUSG00000074843 | protein_coding | Gm6901 | 3.521967 | 4.987806 | 4.254887 |
| ENSMUSG00000058952 | protein_coding | Cfi | 102.8949 | 134.427 | 118.661 |
| ENSMUSG00000085895 | processed_transcrip | Gm12861 | 1.296641 | 0.697713 | 0.997177 |
| ENSMUSG00000074684 | protein_coding | Gm9049 | 1.296641 | 0.697713 | 0.997177 |
| ENSMUSG00000082389 | pseudogene | Mkrn1-ps1 | 1.606973 | 2.374969 | 1.990971 |
| ENSMUSG00000035273 | protein_coding | Hpse | 217.3524 | 205.1758 | 211.2641 |
| ENSMUSG00000048794 | protein_coding | Ccdc37 | 18.0539 | 15.70794 | 16.88092 |
| ENSMUSG00000025932 | protein_coding | Eya1 | 76.83971 | 84.96151 | 80.90061 |
| ENSMUSG00000041633 | protein_coding | Kctd12b | 310.9022 | 330.3175 | 320.6099 |
| ENSMUSG00000028969 | protein_coding | Cdk5 | 2159.239 | 2227.065 | 2193.152 |

| | | | | | |
|---|---|---|---|---|---|
| ENSMUSG00000056856 | protein_coding | Jakmip3 | 187.4416 | 201.6267 | 194.5341 |
| ENSMUSG00000020878 | protein_coding | Lrrc46 | 64.5422 | 58.30236 | 61.42228 |
| ENSMUSG00000046364 | protein_coding | Rpl27a | 6126.796 | 6297.499 | 6212.147 |
| ENSMUSG00000056054 | protein_coding | S100a8 | 2.914124 | 2.048455 | 2.481289 |
| ENSMUSG00000016498 | protein_coding | Pdcd1lg2 | 3.554766 | 4.945971 | 4.250369 |
| ENSMUSG00000048109 | protein_coding | Rbm15 | 1143.855 | 1189.704 | 1166.78 |
| ENSMUSG00000059430 | protein_coding | Actg2 | 296.7323 | 281.3306 | 289.0314 |
| ENSMUSG00000013858 | protein_coding | ORF61 | 2130.478 | 2191.055 | 2160.766 |
| ENSMUSG00000074354 | processed_transcrip | 9230115E21Rik | 19.47838 | 16.62835 | 18.05337 |
| ENSMUSG00000027341 | protein_coding | 5730494N06Rik | 1249.006 | 1300.145 | 1274.576 |
| ENSMUSG00000051726 | protein_coding | Kcnf1 | 1.282552 | 0.688704 | 0.985628 |
| ENSMUSG00000033033 | protein_coding | Calhm2 | 600.5092 | 628.0971 | 614.3032 |
| ENSMUSG00000051965 | protein_coding | Nanos2 | 27.66756 | 31.10418 | 29.38587 |
| ENSMUSG00000026781 | protein_coding | Acbd5 | 2451.609 | 2521.62 | 2486.614 |
| ENSMUSG00000028944 | protein_coding | Prkag2 | 1225.401 | 1179.752 | 1202.577 |
| ENSMUSG00000074887 | protein_coding | EU599041 | 55.89062 | 60.84788 | 58.36925 |
| ENSMUSG00000040985 | protein_coding | Sun3 | 5.840026 | 4.52144 | 5.180733 |
| ENSMUSG00000060244 | protein_coding | Refbp2 | 54.9363 | 59.43729 | 57.18679 |
| ENSMUSG00000031266 | protein_coding | Gla | 334.7206 | 321.3296 | 328.0251 |
| ENSMUSG00000030738 | protein_coding | Eif3c | 8280.604 | 8491.148 | 8385.876 |
| ENSMUSG00000091399 | protein_coding | Gm3164 | 2.573303 | 1.641581 | 2.107442 |
| ENSMUSG00000075510 | protein_coding | Fam187a | 4.16492 | 3.010341 | 3.58763 |
| ENSMUSG00000039611 | protein_coding | 2810432L12Rik | 506.7612 | 529.8589 | 518.31 |
| ENSMUSG00000085145 | processed_transcrip | Gm11614 | 3.558345 | 4.696606 | 4.127475 |
| ENSMUSG00000021830 | protein_coding | Txndc16 | 3168.644 | 3049.607 | 3109.126 |
| ENSMUSG00000042423 | protein_coding | Fbrs | 1257.224 | 1203.379 | 1230.301 |
| ENSMUSG00000055560 | protein_coding | Zfp459 | 104.1923 | 93.2315 | 98.71191 |
| ENSMUSG00000069601 | protein_coding | Ank3 | 705.2767 | 668.6333 | 686.955 |
| ENSMUSG00000091269 | pseudogene | Gm6682 | 4.521097 | 5.925876 | 5.223486 |
| ENSMUSG00000040505 | protein_coding | Abcg5 | 4.543386 | 3.408205 | 3.975796 |
| ENSMUSG00000022070 | protein_coding | 6720463M24Rik | 824.56 | 852.1806 | 838.3703 |
| ENSMUSG00000090108 | processed_transcrip | Gm15717 | 14.81766 | 12.59995 | 13.70881 |
| ENSMUSG00000044860 | protein_coding | Gm1123 | 2.869546 | 4.052225 | 3.460886 |
| ENSMUSG00000091890 | protein_coding | A830073O21Rik | 8.044331 | 9.880084 | 8.962207 |
| ENSMUSG00000056900 | protein_coding | Usp13 | 84.57013 | 90.61209 | 87.59111 |
| ENSMUSG00000020521 | protein_coding | Rnft1 | 1404.059 | 1365.151 | 1384.605 |
| ENSMUSG00000037326 | protein_coding | Solh | 1054.118 | 1016.053 | 1035.086 |
| ENSMUSG00000085229 | processed_transcrip | Gm11672 | 1.601084 | 2.383978 | 1.992531 |
| ENSMUSG00000081710 | pseudogene | Gm13240 | 5.159428 | 6.543229 | 5.851328 |
| ENSMUSG00000087056 | lincRNA | Gm14004 | 4.168498 | 5.438642 | 4.80357 |
| ENSMUSG00000053706 | protein_coding | B430305J03Rik | 9.365572 | 11.23983 | 10.3027 |
| ENSMUSG00000021327 | protein_coding | Zkscan3 | 2203.591 | 2334.318 | 2268.954 |
| ENSMUSG00000020827 | protein_coding | Mink1 | 2395.953 | 2306.228 | 2351.091 |
| ENSMUSG00000035509 | protein_coding | Fbxl21 | 50.46567 | 55.32878 | 52.89722 |
| ENSMUSG00000090176 | protein_coding | Cd200r2 | 3.588833 | 2.463976 | 3.026405 |
| ENSMUSG00000086336 | processed_transcrip | Gm12979 | 1.307151 | 2.110796 | 1.708974 |
| ENSMUSG00000030361 | protein_coding | Klrb1a | 10.03081 | 11.96385 | 10.99733 |
| ENSMUSG00000037814 | protein_coding | Larp1b | 1114.067 | 982.4625 | 1048.265 |
| ENSMUSG00000083788 | pseudogene | Gm11499 | 1.276663 | 0.697713 | 0.987188 |
| ENSMUSG00000020982 | protein_coding | Nemf | 1855.058 | 1917.279 | 1886.169 |
| ENSMUSG00000003410 | protein_coding | Elavl3 | 241.1727 | 263.8938 | 252.5332 |
| ENSMUSG00000034327 | protein_coding | Kctd9 | 1530.543 | 1484.652 | 1507.597 |
| ENSMUSG00000056412 | pseudogene | Gm12396 | 6.45018 | 5.112127 | 5.781154 |
| ENSMUSG00000090019 | protein_coding | Gimap1 | 23.91621 | 21.19127 | 22.55374 |
| ENSMUSG00000034345 | protein_coding | Gtf2h5 | 1336.577 | 1296.82 | 1316.698 |
| ENSMUSG00000051149 | protein_coding | Adnp | 110.328 | 101.9311 | 106.1295 |
| ENSMUSG00000005952 | protein_coding | Trpv1 | 3.260834 | 4.856602 | 4.058718 |
| ENSMUSG00000040264 | protein_coding | Gbp5 | 27.28872 | 23.89275 | 25.59073 |

| | | | | | |
|---|---|---|---|---|---|
| ENSMUSG00000079614 | protein_coding | Seh1l | 2218.286 | 2287.451 | 2252.869 |
| ENSMUSG00000082706 | pseudogene | Gm11663 | 3.205746 | 4.38811 | 3.796928 |
| ENSMUSG00000023015 | protein_coding | Racgap1 | 3655.017 | 3759.46 | 3707.239 |
| ENSMUSG00000052911 | protein_coding | Lamb2 | 4296.178 | 4114.118 | 4205.148 |
| ENSMUSG00000092064 | protein_coding | Gm9312 | 6.170337 | 4.678949 | 5.424643 |
| ENSMUSG00000003070 | protein_coding | Efna2 | 515.2224 | 542.0343 | 528.6284 |
| ENSMUSG00000088200 | snRNA | U1 | 2.597903 | 1.73095 | 2.164426 |
| ENSMUSG00000032842 | protein_coding | Abcc10 | 437.5271 | 417.5355 | 427.5313 |
| ENSMUSG00000079395 | protein_coding | Gm15114 | 5.79187 | 4.503783 | 5.147827 |
| ENSMUSG00000071537 | protein_coding | Klrg2 | 115.3119 | 123.2132 | 119.2626 |
| ENSMUSG00000019732 | protein_coding | Calr3 | 140.9494 | 132.6363 | 136.7928 |
| ENSMUSG00000029707 | protein_coding | Fscn3 | 1.276663 | 2.012779 | 1.644721 |
| ENSMUSG00000092417 | protein_coding | RP23-115O3.9 | 75.24854 | 81.17311 | 78.21083 |
| ENSMUSG00000081854 | pseudogene | Gm11447 | 1.307151 | 2.075121 | 1.691136 |
| ENSMUSG00000092805 | misc_RNA | CT025673.1 | 1.307151 | 2.075121 | 1.691136 |
| ENSMUSG00000085131 | lincRNA | Gm16739 | 3.860477 | 5.121498 | 4.490987 |
| ENSMUSG00000035790 | protein_coding | 1500031L02Rik | 451.4949 | 432.0552 | 441.775 |
| ENSMUSG00000071659 | protein_coding | Hnrnpul2 | 7755.296 | 7968.326 | 7861.811 |
| ENSMUSG00000063568 | protein_coding | Jazf1 | 1190.725 | 1233.397 | 1212.061 |
| ENSMUSG00000022377 | protein_coding | Asap1 | 2288.176 | 2207.893 | 2248.034 |
| ENSMUSG00000062410 | protein_coding | Hsd3b3 | 1.621062 | 2.401635 | 2.011348 |
| ENSMUSG00000023791 | protein_coding | Pigx | 838.6869 | 867.1488 | 852.9178 |
| ENSMUSG00000082185 | pseudogene | Gm12428 | 2.897724 | 2.048455 | 2.47309 |
| ENSMUSG00000093319 | misc_RNA | AC155810.1 | 8.437929 | 6.682357 | 7.560143 |
| ENSMUSG00000020590 | protein_coding | Snx13 | 2633.841 | 2707.788 | 2670.815 |
| ENSMUSG00000026581 | protein_coding | Sell | 2.931792 | 4.221592 | 3.576692 |
| ENSMUSG00000059301 | protein_coding | Gm5434 | 2.891835 | 2.021789 | 2.456812 |
| ENSMUSG00000001566 | protein_coding | Mnx1 | 4.507008 | 3.327846 | 3.917427 |
| ENSMUSG00000024007 | protein_coding | Ppil1 | 1644.43 | 1689.83 | 1667.13 |
| ENSMUSG00000040423 | protein_coding | Rc3h1 | 3694.738 | 3811.827 | 3753.282 |
| ENSMUSG00000026986 | protein_coding | Hnmt | 118.5436 | 126.3109 | 122.4272 |
| ENSMUSG00000085089 | processed_transcrip | A430092G05Rik | 1.296641 | 2.057464 | 1.677052 |
| ENSMUSG00000022426 | protein_coding | Josd1 | 2641.456 | 2716.699 | 2679.077 |
| ENSMUSG00000039844 | protein_coding | Rapgef1 | 1657.874 | 1709.798 | 1683.836 |
| ENSMUSG00000064378 | snRNA | U6 | 1.304841 | 0.653029 | 0.978935 |
| ENSMUSG00000049999 | protein_coding | Ppp1r3d | 121.1994 | 129.2434 | 125.2214 |
| ENSMUSG00000025105 | protein_coding | Bnc1 | 885.1524 | 853.3851 | 869.2687 |
| ENSMUSG00000073771 | protein_coding | Btbd19 | 80.6176 | 86.99522 | 83.80641 |
| ENSMUSG00000081194 | pseudogene | Gm8424 | 1.307151 | 2.048455 | 1.677803 |
| ENSMUSG00000032046 | protein_coding | Abhd12 | 1833.991 | 1773.223 | 1803.607 |
| ENSMUSG00000081730 | pseudogene | Gm13785 | 2.573303 | 1.748606 | 2.160955 |
| ENSMUSG00000091498 | protein_coding | Gm3452 | 461.2304 | 482.7299 | 471.9802 |
| ENSMUSG00000072491 | lincRNA | Gm17515 | 2.875436 | 4.088262 | 3.481849 |
| ENSMUSG00000074911 | lincRNA | Gm17362 | 6.403291 | 8.176162 | 7.289727 |
| ENSMUSG00000037991 | protein_coding | A630055G03Rik | 132.7089 | 142.2127 | 137.4608 |
| ENSMUSG00000072610 | protein_coding | Gm7995 | 1.282552 | 0.697713 | 0.990132 |
| ENSMUSG00000086824 | processed_transcrip | 4930448D08Rik | 1.282552 | 0.697713 | 0.990132 |
| ENSMUSG00000008200 | protein_coding | Fnbp4 | 1858.387 | 1793.012 | 1825.699 |
| ENSMUSG00000058073 | pseudogene | Gm11189 | 27.09378 | 24.18395 | 25.63887 |
| ENSMUSG00000092325 | pseudogene | RP24-216B4.2 | 33.89783 | 30.60214 | 32.24998 |
| ENSMUSG00000001870 | protein_coding | Ltbp1 | 980.9807 | 933.3008 | 957.1408 |
| ENSMUSG00000048874 | protein_coding | Phf3 | 2548.717 | 2465.051 | 2506.884 |
| ENSMUSG00000084384 | pseudogene | Gm12251 | 52.94266 | 57.33622 | 55.13944 |
| ENSMUSG00000085200 | lincRNA | 4930552P12Rik | 22.75226 | 19.74215 | 21.24721 |
| ENSMUSG00000045929 | protein_coding | Gm16532 | 16.7785 | 14.48188 | 15.63019 |
| ENSMUSG00000038736 | protein_coding | Nudcd1 | 989.2458 | 947.2764 | 968.2611 |
| ENSMUSG00000044724 | protein_coding | Gpr152 | 3.578323 | 2.463976 | 3.021149 |
| ENSMUSG00000029601 | protein_coding | Iqcd | 13.23656 | 15.36341 | 14.29998 |

| | | | | | |
|---|---|---|---|---|---|
| ENSMUSG00000049097 | protein_coding | Ankrd34a | 26.86567 | 23.74068 | 25.30318 |
| ENSMUSG00000022303 | protein_coding | Tm7sf4 | 12.90267 | 10.90467 | 11.90367 |
| ENSMUSG00000056755 | protein_coding | Grm7 | 11.60372 | 13.8912 | 12.74746 |
| ENSMUSG00000029830 | protein_coding | Svopl | 4.524675 | 5.898848 | 5.211762 |
| ENSMUSG00000087357 | processed_transcrip | Gm12498 | 1.612862 | 2.383978 | 1.99842 |
| ENSMUSG00000086118 | lincRNA | Gm14169 | 50.53022 | 46.06284 | 48.29653 |
| ENSMUSG00000087673 | lincRNA | Gm16884 | 10.32348 | 12.31739 | 11.32044 |
| ENSMUSG00000051578 | protein_coding | Cmtm4 | 326.4239 | 312.4157 | 319.4198 |
| ENSMUSG00000030545 | protein_coding | Pex11a | 921.6619 | 883.7359 | 902.6989 |
| ENSMUSG00000071302 | protein_coding | Gm10324 | 17.32999 | 20.34542 | 18.8377 |
| ENSMUSG00000023965 | protein_coding | Fbxl17 | 864.873 | 839.5201 | 852.1966 |
| ENSMUSG00000065301 | snRNA | U6 | 0.966331 | 1.677256 | 1.321793 |
| ENSMUSG00000086621 | processed_transcrip | Gm13348 | 1.31304 | 0.653029 | 0.983035 |
| ENSMUSG00000070533 | protein_coding | Wfdc8 | 94.97979 | 102.1716 | 98.57569 |
| ENSMUSG00000026042 | protein_coding | Col5a2 | 1084.63 | 1136.157 | 1110.394 |
| ENSMUSG00000020622 | protein_coding | Nt5c1b | 6.116291 | 4.776965 | 5.446628 |
| ENSMUSG00000066060 | processed_transcrip | Gm12866 | 1.298952 | 0.724379 | 1.011665 |
| ENSMUSG00000022770 | protein_coding | Dlg1 | 3317.306 | 3231.962 | 3274.634 |
| ENSMUSG00000085523 | processed_transcrip | Gm15945 | 2.942302 | 4.052586 | 3.497444 |
| ENSMUSG00000074141 | protein_coding | Il4i1 | 101.6174 | 109.3299 | 105.4736 |
| ENSMUSG00000031715 | protein_coding | Smarca5 | 10974.07 | 11349.82 | 11161.95 |
| ENSMUSG00000041697 | protein_coding | Cox6a1 | 3003.581 | 2924.275 | 2963.928 |
| ENSMUSG00000031509 | protein_coding | 1700016D06Rik | 4.529297 | 3.434871 | 3.982084 |
| ENSMUSG00000054746 | protein_coding | Abca15 | 8.510685 | 6.596561 | 7.553623 |
| ENSMUSG00000061353 | protein_coding | Cxcl12 | 2257.218 | 2326.188 | 2291.703 |
| ENSMUSG00000086740 | processed_transcrip | Gm17029 | 22.03171 | 24.8813 | 23.45651 |
| ENSMUSG00000027274 | protein_coding | Mkks | 815.9684 | 779.1149 | 797.5417 |
| ENSMUSG00000087621 | lincRNA | 1700003G18Rik | 11.02763 | 9.069546 | 10.04859 |
| ENSMUSG00000006200 | protein_coding | Rhox6 | 9.639526 | 11.84238 | 10.74095 |
| ENSMUSG00000053675 | protein_coding | Tgm5 | 1.270774 | 2.012779 | 1.641776 |
| ENSMUSG00000033021 | protein_coding | Gmppa | 626.1415 | 605.3682 | 615.7549 |
| ENSMUSG00000081179 | pseudogene | Gm13136 | 118.1995 | 127.5847 | 122.8921 |
| ENSMUSG00000072623 | protein_coding | Zfp9 | 178.1625 | 167.6977 | 172.9301 |
| ENSMUSG00000071347 | protein_coding | C1qtnf9 | 25.79945 | 22.59643 | 24.19794 |
| ENSMUSG00000052942 | protein_coding | Glis3 | 783.364 | 816.9686 | 800.1663 |
| ENSMUSG00000085266 | lincRNA | 9330111N05Rik | 31.38908 | 28.15903 | 29.77405 |
| ENSMUSG00000079645 | processed_transcrip | Gm17193 | 33.5286 | 36.99402 | 35.26131 |
| ENSMUSG00000048482 | protein_coding | Bdnf | 4.803251 | 6.338548 | 5.570899 |
| ENSMUSG00000030218 | protein_coding | Mgp | 1015.727 | 1063.261 | 1039.494 |
| ENSMUSG00000086631 | lincRNA | Gm12784 | 4.213076 | 3.126014 | 3.669545 |
| ENSMUSG00000079736 | protein_coding | Gm11169 | 9.031683 | 7.338958 | 8.185321 |
| ENSMUSG00000023452 | protein_coding | Pisd | 539.2679 | 556.8446 | 548.0562 |
| ENSMUSG00000029759 | protein_coding | Pon3 | 282.2304 | 270.733 | 276.4817 |
| ENSMUSG00000001864 | protein_coding | Aif1l | 292.593 | 309.0456 | 300.8193 |
| ENSMUSG00000027676 | protein_coding | Ccdc39 | 369.3639 | 391.8143 | 380.5891 |
| ENSMUSG00000054123 | protein_coding | Gm9936 | 1.307151 | 2.057464 | 1.682308 |
| ENSMUSG00000053615 | protein_coding | Gm9913 | 2.875436 | 4.043577 | 3.459506 |
| ENSMUSG00000018459 | protein_coding | Slc13a3 | 17.17926 | 19.87548 | 18.52737 |
| ENSMUSG00000033487 | protein_coding | Fndc3a | 3075.807 | 2997.32 | 3036.563 |
| ENSMUSG00000022545 | protein_coding | Ercc4 | 499.0225 | 517.8336 | 508.428 |
| ENSMUSG00000022350 | protein_coding | E430025E21Rik | 2336.734 | 2402.008 | 2369.371 |
| ENSMUSG00000040441 | protein_coding | Slc26a10 | 43.35718 | 47.70763 | 45.5324 |
| ENSMUSG00000090008 | pseudogene | Gm16111 | 6.502957 | 5.076452 | 5.789705 |
| ENSMUSG00000027518 | protein_coding | 1700021F07Rik | 6.109359 | 4.705615 | 5.407487 |
| ENSMUSG00000029469 | protein_coding | Ift81 | 1105.254 | 1061.749 | 1083.502 |
| ENSMUSG00000063757 | protein_coding | 4632433K11Rik | 6.200825 | 7.822981 | 7.011903 |
| ENSMUSG00000022033 | protein_coding | Pbk | 1327.078 | 1275.895 | 1301.487 |
| ENSMUSG00000042410 | protein_coding | Agps | 3130.175 | 3209.857 | 3170.016 |

| | | | | | |
|---|---|---|---|---|---|
| ENSMUSG00000085415 | processed_transcrip | Gm12060 | 2.936413 | 2.012779 | 2.474596 |
| ENSMUSG00000039747 | protein_coding | Orai2 | 216.3281 | 229.3972 | 222.8627 |
| ENSMUSG00000084407 | pseudogene | Gm14018 | 41.71972 | 37.669 | 39.69436 |
| ENSMUSG00000081250 | pseudogene | Gm15554 | 0.960442 | 1.668247 | 1.314344 |
| ENSMUSG00000033545 | protein_coding | Znrf1 | 1447.758 | 1493.584 | 1470.671 |
| ENSMUSG00000077051 | miRNA | AC122806.1 | 10.09433 | 7.745471 | 8.919898 |
| ENSMUSG00000047675 | protein_coding | Rps8 | 9277.964 | 9041.512 | 9159.738 |
| ENSMUSG00000042210 | protein_coding | Abhd14a | 288.3734 | 304.4937 | 296.4336 |
| ENSMUSG00000087218 | lincRNA | Gm16997 | 7.451621 | 9.147057 | 8.299339 |
| ENSMUSG00000036944 | protein_coding | Tmem71 | 77.1156 | 83.8458 | 80.4807 |
| ENSMUSG00000072800 | pseudogene | Cecr6 | 38.24143 | 42.45104 | 40.34624 |
| ENSMUSG00000025220 | protein_coding | Mgea5 | 6459.98 | 6625.139 | 6542.56 |
| ENSMUSG00000092831 | misc_RNA | AC114668.1 | 1.293062 | 0.653029 | 0.973045 |
| ENSMUSG00000085347 | processed_transcrip | 2210417K05Rik | 19.61629 | 16.68857 | 18.15243 |
| ENSMUSG00000060477 | protein_coding | Irak2 | 250.5155 | 238.2587 | 244.3871 |
| ENSMUSG00000078945 | protein_coding | Naip2 | 15.85802 | 13.6148 | 14.73641 |
| ENSMUSG00000073567 | protein_coding | Tnfaip8 | 6.794579 | 5.411976 | 6.103277 |
| ENSMUSG00000068335 | protein_coding | Dok1 | 318.362 | 331.3408 | 324.8514 |
| ENSMUSG00000022357 | protein_coding | Klhl38 | 9.010662 | 11.09749 | 10.05408 |
| ENSMUSG00000033862 | protein_coding | Cdk10 | 901.9292 | 931.5953 | 916.7623 |
| ENSMUSG00000035226 | protein_coding | Rims4 | 1837.76 | 1762.055 | 1799.908 |
| ENSMUSG00000053600 | protein_coding | Zfp472 | 380.2273 | 360.0028 | 370.115 |
| ENSMUSG00000042282 | protein_coding | Gucy2f | 1.270774 | 0.697713 | 0.984243 |
| ENSMUSG00000032561 | protein_coding | Acpp | 67.99059 | 73.51193 | 70.75126 |
| ENSMUSG00000022861 | protein_coding | Dgkg | 23.20997 | 20.46653 | 21.83825 |
| ENSMUSG00000051627 | protein_coding | Hist1h1e | 8.469686 | 10.62176 | 9.545722 |
| ENSMUSG00000073643 | protein_coding | Wdfy1 | 1260.82 | 1297.266 | 1279.043 |
| ENSMUSG00000086775 | processed_transcrip | 5730588L14Rik | 4.515208 | 3.434871 | 3.97504 |
| ENSMUSG00000089910 | pseudogene | Gm16113 | 1.290752 | 0.724379 | 1.007565 |
| ENSMUSG00000056306 | protein_coding | 6030405A18Rik | 12.53009 | 14.87975 | 13.70492 |
| ENSMUSG00000061130 | protein_coding | Ppm1b | 2386.637 | 2450.927 | 2418.782 |
| ENSMUSG00000074768 | protein_coding | Bhmt | 76.95772 | 71.30814 | 74.13293 |
| ENSMUSG00000015437 | protein_coding | Gzmb | 6.73718 | 5.112489 | 5.924834 |
| ENSMUSG00000022789 | protein_coding | Dnm1l | 4456.853 | 4574.806 | 4515.83 |
| ENSMUSG00000023019 | protein_coding | Gpd1 | 1141.992 | 1201.81 | 1171.901 |
| ENSMUSG00000042246 | protein_coding | Tmc7 | 1560.763 | 1515.144 | 1537.954 |
| ENSMUSG00000026623 | protein_coding | Lpgat1 | 1562.535 | 1506.406 | 1534.471 |
| ENSMUSG00000043219 | protein_coding | Hoxa6 | 4.204876 | 3.144033 | 3.674454 |
| ENSMUSG00000078821 | lincRNA | Gm17414 | 75.15284 | 51.31782 | 63.23533 |
| ENSMUSG00000089617 | snoRNA | Scarna10 | 2.911813 | 2.057464 | 2.484639 |
| ENSMUSG00000092341 | processed_transcrip | Malat1 | 8045.009 | 8543.807 | 8294.408 |
| ENSMUSG00000029915 | protein_coding | Clec5a | 31.59744 | 28.38716 | 29.9923 |
| ENSMUSG00000062478 | protein_coding | Ctrc | 1.621062 | 1.024227 | 1.322645 |
| ENSMUSG00000030911 | protein_coding | Zp2 | 1.621062 | 1.024227 | 1.322645 |
| ENSMUSG00000088549 | snRNA | U1 | 1.621062 | 1.024227 | 1.322645 |
| ENSMUSG00000035941 | protein_coding | Ibtk | 2687.231 | 2617.474 | 2652.353 |
| ENSMUSG00000038481 | protein_coding | Cdk19 | 2486.535 | 2552.255 | 2519.395 |
| ENSMUSG00000046417 | protein_coding | BC046404 | 725.9914 | 751.1626 | 738.577 |
| ENSMUSG00000029203 | protein_coding | Ube2k | 2901.087 | 2967.218 | 2934.153 |
| ENSMUSG00000027239 | protein_coding | Mdk | 1172.866 | 1130.814 | 1151.84 |
| ENSMUSG00000074861 | lincRNA | 1810034E14Rik | 77.45702 | 83.23881 | 80.34791 |
| ENSMUSG00000084440 | snRNA | U4 | 14.62467 | 17.27201 | 15.94834 |
| ENSMUSG00000032507 | protein_coding | Fbxl2 | 97.66588 | 91.30686 | 94.48637 |
| ENSMUSG00000046717 | protein_coding | Igbp1b | 37.37522 | 41.22969 | 39.30246 |
| ENSMUSG00000060275 | protein_coding | Nrg2 | 99.06912 | 92.35091 | 95.71001 |
| ENSMUSG00000018919 | protein_coding | Tm4sf5 | 23.01697 | 20.122 | 21.56948 |
| ENSMUSG00000038178 | protein_coding | Slc43a2 | 1013.62 | 963.8651 | 988.7425 |
| ENSMUSG00000032942 | protein_coding | Ucp3 | 7.142556 | 5.590352 | 6.366454 |

| | | | | | |
|---|---|---|---|---|---|
| ENSMUSG00000091683 | lincRNA | Gm17357 | 17.70361 | 15.40773 | 16.55567 |
| ENSMUSG00000065593 | miRNA | Mir339 | 11.95401 | 14.25339 | 13.1037 |
| ENSMUSG00000024922 | protein_coding | Ovol1 | 116.9588 | 108.945 | 112.9519 |
| ENSMUSG00000034833 | protein_coding | 5830405N20Rik | 2.603792 | 1.739597 | 2.171695 |
| ENSMUSG00000084139 | pseudogene | Gm12322 | 2.603792 | 1.739597 | 2.171695 |
| ENSMUSG00000000159 | protein_coding | Igsf5 | 12.55596 | 14.70101 | 13.62849 |
| ENSMUSG00000073062 | protein_coding | Zxdb | 502.0971 | 521.1779 | 511.6375 |
| ENSMUSG00000087291 | lincRNA | Gm11946 | 4.854985 | 6.190048 | 5.522517 |
| ENSMUSG00000054648 | protein_coding | Zfp869 | 782.9113 | 814.3617 | 798.6365 |
| ENSMUSG00000019897 | protein_coding | Ccdc59 | 1374.48 | 1411.898 | 1393.189 |
| ENSMUSG00000026836 | protein_coding | Acvr1 | 497.0524 | 515.7905 | 506.4214 |
| ENSMUSG00000052928 | protein_coding | Ctif | 904.9704 | 947.4525 | 926.2114 |
| ENSMUSG00000092131 | processed_transcrip | BC050972 | 4.537497 | 5.854525 | 5.196011 |
| ENSMUSG00000034484 | protein_coding | Snx2 | 2138.351 | 2190.363 | 2164.357 |
| ENSMUSG00000072571 | protein_coding | G630016D24Rik | 61.8875 | 57.05476 | 59.47113 |
| ENSMUSG00000087784 | misc_RNA | 7SK | 1.629262 | 2.383978 | 2.00662 |
| ENSMUSG00000075486 | protein_coding | Commd6 | 777.0812 | 800.9352 | 789.0082 |
| ENSMUSG00000078794 | protein_coding | Dact3 | 365.6488 | 379.1433 | 372.396 |
| ENSMUSG00000039154 | protein_coding | Shd | 31.29046 | 28.20014 | 29.7453 |
| ENSMUSG00000027439 | protein_coding | Gzf1 | 1339.096 | 1383.52 | 1361.308 |
| ENSMUSG00000081700 | pseudogene | Gm14337 | 4.840896 | 6.199058 | 5.519977 |
| ENSMUSG00000052621 | protein_coding | Pou3f2 | 11.95401 | 14.10131 | 13.02766 |
| ENSMUSG00000000386 | protein_coding | Mx1 | 17.09346 | 19.52266 | 18.30806 |
| ENSMUSG00000064132 | pseudogene | Gm6421 | 71.20364 | 76.50891 | 73.85628 |
| ENSMUSG00000082438 | pseudogene | Gm15682 | 8.43204 | 6.878752 | 7.655396 |
| ENSMUSG00000084883 | protein_coding | Ccdc85c | 284.6156 | 301.5069 | 293.0612 |
| ENSMUSG00000077633 | snRNA | U6 | 1.284863 | 0.724379 | 1.004621 |
| ENSMUSG00000082049 | pseudogene | Gm12105 | 1.284863 | 0.724379 | 1.004621 |
| ENSMUSG00000032905 | protein_coding | Atg12 | 2521.7 | 2584.326 | 2553.013 |
| ENSMUSG00000057835 | protein_coding | Zfp119a | 179.4873 | 189.0533 | 184.2703 |
| ENSMUSG00000086231 | processed_transcrip | 4732447D17Rik | 6.150358 | 4.759309 | 5.454834 |
| ENSMUSG00000089837 | protein_coding | Npcd | 1.296641 | 0.653029 | 0.974835 |
| ENSMUSG00000062813 | protein_coding | Gm10121 | 1.270774 | 2.119805 | 1.695289 |
| ENSMUSG00000042761 | protein_coding | Mrap2 | 3.856898 | 5.085461 | 4.47118 |
| ENSMUSG00000034681 | protein_coding | Rnps1 | 2380.982 | 2638.844 | 2509.913 |
| ENSMUSG00000028191 | protein_coding | Bcl10 | 1070.261 | 1107.017 | 1088.639 |
| ENSMUSG00000030399 | protein_coding | Ckm | 2.563836 | 1.668608 | 2.116222 |
| ENSMUSG00000086616 | lincRNA | Gm16863 | 1.615173 | 1.024227 | 1.3197 |
| ENSMUSG00000050271 | protein_coding | D8Ertd82e | 96.2979 | 102.6668 | 99.48233 |
| ENSMUSG00000091546 | lincRNA | Gm2366 | 4.198987 | 5.403328 | 4.801157 |
| ENSMUSG00000040533 | protein_coding | Matn1 | 1.290752 | 2.030436 | 1.660594 |
| ENSMUSG00000086108 | lincRNA | Gm5602 | 27.46047 | 30.49475 | 28.97761 |
| ENSMUSG00000085581 | processed_transcrip | Gm12786 | 11.97987 | 14.08366 | 13.03177 |
| ENSMUSG00000035057 | protein_coding | 4933434I20Rik | 3.289012 | 2.093139 | 2.691076 |
| ENSMUSG00000041488 | protein_coding | Stx3 | 293.0096 | 281.2715 | 287.1405 |
| ENSMUSG00000066235 | protein_coding | C85492 | 323.72 | 338.0978 | 330.9089 |
| ENSMUSG00000018474 | protein_coding | Chd3 | 6794.522 | 6962.061 | 6878.291 |
| ENSMUSG00000018858 | protein_coding | Ict1 | 726.3307 | 748.9397 | 737.6352 |
| ENSMUSG00000078694 | lincRNA | AC152164.1 | 1.929083 | 2.772834 | 2.350958 |
| ENSMUSG00000090788 | pseudogene | Gm9597 | 1.929083 | 2.772834 | 2.350958 |
| ENSMUSG00000045391 | protein_coding | 1700120B22Rik | 1.629262 | 1.024227 | 1.326744 |
| ENSMUSG00000084413 | pseudogene | Gm12017 | 1.629262 | 1.024227 | 1.326744 |
| ENSMUSG00000088402 | snRNA | U2 | 1.629262 | 1.024227 | 1.326744 |
| ENSMUSG00000027875 | protein_coding | Hmgcs2 | 4259.648 | 4135.972 | 4197.81 |
| ENSMUSG00000044501 | protein_coding | Zfp758 | 335.7119 | 349.8105 | 342.7612 |
| ENSMUSG00000085043 | processed_transcrip | Gm13184 | 16.47868 | 14.17846 | 15.32857 |
| ENSMUSG00000043252 | protein_coding | Tmem64 | 4666.841 | 4802.927 | 4734.884 |
| ENSMUSG00000088832 | snRNA | U6 | 2.928213 | 2.030798 | 2.479505 |

| | | | | | |
|---|---|---|---|---|---|
| ENSMUSG00000088217 | snRNA | U6 | 2.905924 | 2.075121 | 2.490522 |
| ENSMUSG00000063021 | protein_coding | Hist1h2ak | 4.818608 | 6.270047 | 5.544327 |
| ENSMUSG00000007591 | protein_coding | Tssk4 | 20.93291 | 18.24042 | 19.58667 |
| ENSMUSG00000029554 | protein_coding | Mad1l1 | 715.7979 | 690.1782 | 702.988 |
| ENSMUSG00000056364 | lincRNA | Six3os1 | 1.304841 | 0.671047 | 0.987944 |
| ENSMUSG00000026712 | protein_coding | Mrc1 | 1144.032 | 1107.717 | 1125.874 |
| ENSMUSG00000029557 | protein_coding | Ftsj2 | 198.009 | 209.9502 | 203.9796 |
| ENSMUSG00000087228 | processed_transcrip | Gm12827 | 2.889525 | 2.012779 | 2.451152 |
| ENSMUSG00000070583 | protein_coding | Fv1 | 178.0999 | 187.9172 | 183.0085 |
| ENSMUSG00000056293 | protein_coding | Gsdmc2 | 2.269904 | 1.413083 | 1.841493 |
| ENSMUSG00000026484 | protein_coding | Rnf2 | 3465.662 | 3551.483 | 3508.573 |
| ENSMUSG00000091667 | lincRNA | Gm17718 | 1.629262 | 2.374969 | 2.002115 |
| ENSMUSG00000043618 | pseudogene | Eif5al3-ps | 1.629262 | 2.374969 | 2.002115 |
| ENSMUSG00000070687 | protein_coding | Htr1d | 26.68378 | 30.27019 | 28.47698 |
| ENSMUSG00000062257 | protein_coding | Opcml | 22.89248 | 20.25569 | 21.57409 |
| ENSMUSG00000022490 | protein_coding | Ppp1r1a | 6.802779 | 5.429994 | 6.116386 |
| ENSMUSG00000015202 | protein_coding | Cnksr3 | 1217.085 | 1159.197 | 1188.141 |
| ENSMUSG00000087497 | lincRNA | 2810001G20Rik | 240.8414 | 252.5013 | 246.6714 |
| ENSMUSG00000039356 | protein_coding | Exosc2 | 792.3816 | 816.6684 | 804.525 |
| ENSMUSG00000082101 | pseudogene | Slfn14-ps | 4.512897 | 3.381178 | 3.947037 |
| ENSMUSG00000004098 | protein_coding | Col5a3 | 331.7087 | 344.3909 | 338.0498 |
| ENSMUSG00000074766 | protein_coding | Ism1 | 62.26157 | 57.20574 | 59.73365 |
| ENSMUSG00000053897 | protein_coding | Slc39a8 | 345.9651 | 258.8911 | 302.4281 |
| ENSMUSG00000090255 | processed_transcrip | 4921534H16Rik | 1.615173 | 2.36596 | 1.990566 |
| ENSMUSG00000020918 | protein_coding | Kat2a | 1971.115 | 1922.904 | 1947.009 |
| ENSMUSG00000014232 | protein_coding | Cluap1 | 549.1076 | 568.1925 | 558.65 |
| ENSMUSG00000021314 | protein_coding | Amph | 12.58183 | 14.62101 | 13.60142 |
| ENSMUSG00000033272 | protein_coding | Slc35a4 | 1513.637 | 1560.848 | 1537.243 |
| ENSMUSG00000008658 | protein_coding | Rbfox1 | 27.3772 | 31.8664 | 29.6218 |
| ENSMUSG00000086568 | processed_transcrip | 9130020K20Rik | 35.97347 | 32.17309 | 34.07328 |
| ENSMUSG00000077323 | snRNA | U11 | 1.296641 | 2.021789 | 1.659215 |
| ENSMUSG00000080478 | snoRNA | Snord23 | 1.296641 | 2.021789 | 1.659215 |
| ENSMUSG00000082457 | pseudogene | Gm13776 | 5.144071 | 3.672378 | 4.408225 |
| ENSMUSG00000004296 | protein_coding | Il12b | 2.900035 | 2.030798 | 2.465416 |
| ENSMUSG00000036391 | protein_coding | Sec24a | 1475.594 | 1513.531 | 1494.563 |
| ENSMUSG00000092577 | pseudogene | RP24-221F4.3 | 18.1643 | 20.71304 | 19.43867 |
| ENSMUSG00000024172 | protein_coding | St6gal2 | 40.30343 | 36.70318 | 38.5033 |
| ENSMUSG00000024370 | protein_coding | Cdc23 | 2471.484 | 2549.671 | 2510.578 |
| ENSMUSG00000084796 | processed_transcrip | A430104N18Rik | 4.224854 | 5.465308 | 4.845081 |
| ENSMUSG00000085766 | lincRNA | 2810430I11Rik | 15.2056 | 17.58739 | 16.39649 |
| ENSMUSG00000081782 | pseudogene | Gm15152 | 1.606973 | 1.024227 | 1.3156 |
| ENSMUSG00000031754 | protein_coding | Nudt21 | 3032.871 | 2944.066 | 2988.468 |
| ENSMUSG00000086322 | lincRNA | E130218I03Rik | 2.579193 | 1.748606 | 2.1639 |
| ENSMUSG00000038773 | protein_coding | Kdm3b | 3871.629 | 3780.71 | 3826.17 |
| ENSMUSG00000029714 | protein_coding | Gigyf1 | 2268.005 | 2213.985 | 2240.995 |
| ENSMUSG00000087696 | lincRNA | Gm16957 | 103.3353 | 95.46429 | 99.39981 |
| ENSMUSG00000088604 | snoRNA | SCARNA15 | 6.774601 | 5.429994 | 6.102297 |
| ENSMUSG00000035683 | protein_coding | Melk | 1328.384 | 1287.474 | 1307.929 |
| ENSMUSG00000065057 | snRNA | U12 | 0.98273 | 1.810948 | 1.396839 |
| ENSMUSG00000092357 | pseudogene | Vmn2r-ps83 | 0.98273 | 1.810948 | 1.396839 |
| ENSMUSG00000083745 | pseudogene | Gm13543 | 0.98273 | 1.65059 | 1.31666 |
| ENSMUSG00000042228 | protein_coding | Lyn | 154.3258 | 162.7052 | 158.5155 |
| ENSMUSG00000002346 | protein_coding | Slc25a42 | 544.7304 | 509.3293 | 527.0298 |
| ENSMUSG00000053218 | protein_coding | Gm9900 | 106.9431 | 100.5026 | 103.7229 |
| ENSMUSG00000044581 | protein_coding | 4932415D10Rik | 4.210765 | 5.58999 | 4.900378 |
| ENSMUSG00000049608 | protein_coding | Gpr55 | 566.4622 | 535.6034 | 551.0328 |
| ENSMUSG00000027599 | protein_coding | Armc1 | 1595.186 | 1643.486 | 1619.336 |
| ENSMUSG00000083922 | pseudogene | Gm3835 | 4.484719 | 5.765156 | 5.124937 |

| | | | | | |
|---|---|---|---|---|---|
| ENSMUSG00000046589 | protein_coding | Lrrc8e | 73.24208 | 68.09416 | 70.66812 |
| ENSMUSG00000060034 | protein_coding | Ctf2 | 5.834137 | 4.325769 | 5.079953 |
| ENSMUSG00000050304 | protein_coding | Slc25a2 | 11.72105 | 14.1824 | 12.95172 |
| ENSMUSG00000049872 | protein_coding | Fam26e | 20.05574 | 22.65483 | 21.35529 |
| ENSMUSG00000071531 | protein_coding | Gprin2 | 29.54155 | 26.12466 | 27.8331 |
| ENSMUSG00000023909 | protein_coding | Paqr4 | 197.2517 | 206.246 | 201.7489 |
| ENSMUSG00000024926 | protein_coding | Kat5 | 1023.488 | 1050.816 | 1037.152 |
| ENSMUSG00000087244 | pseudogene | Gm7284 | 0.99093 | 1.677256 | 1.334093 |
| ENSMUSG00000014813 | protein_coding | Stc1 | 307.0974 | 285.0843 | 296.0908 |
| ENSMUSG00000019984 | protein_coding | Med23 | 2241.445 | 2182.597 | 2212.021 |
| ENSMUSG00000039099 | protein_coding | Wdr93 | 86.9078 | 94.72148 | 90.81464 |
| ENSMUSG00000027843 | protein_coding | Ptpn22 | 6.766401 | 5.429994 | 6.098197 |
| ENSMUSG00000085171 | processed_transcrip | D830026I12Rik | 14.27669 | 16.81289 | 15.54479 |
| ENSMUSG00000042275 | protein_coding | Pelo | 411.0383 | 429.9533 | 420.4958 |
| ENSMUSG00000028980 | protein_coding | H6pd | 764.4343 | 737.346 | 750.8901 |
| ENSMUSG00000025496 | protein_coding | Drd4 | 1671.214 | 1488.84 | 1580.027 |
| ENSMUSG00000087449 | processed_transcrip | Gm12971 | 4.216654 | 5.411976 | 4.814315 |
| ENSMUSG00000071068 | protein_coding | Treml2 | 5.795449 | 7.205266 | 6.500358 |
| ENSMUSG00000019982 | protein_coding | Myb | 21.31854 | 24.50467 | 22.9116 |
| ENSMUSG00000031060 | protein_coding | Rbm10 | 1455.914 | 1501.422 | 1478.668 |
| ENSMUSG00000071169 | protein_coding | Defb46 | 1.282552 | 0.653029 | 0.96779 |
| ENSMUSG00000063364 | protein_coding | 3300002I08Rik | 67.36568 | 62.17372 | 64.7697 |
| ENSMUSG00000090708 | processed_transcrip | Gm17196 | 1.307151 | 0.671047 | 0.989099 |
| ENSMUSG00000072066 | protein_coding | 6720489N17Rik | 30.64982 | 27.65378 | 29.1518 |
| ENSMUSG00000053654 | protein_coding | Krt42 | 1.296641 | 2.012779 | 1.65471 |
| ENSMUSG00000068036 | protein_coding | Mllt4 | 3145.373 | 3222.026 | 3183.699 |
| ENSMUSG00000001815 | protein_coding | Evx2 | 2.255815 | 1.413083 | 1.834449 |
| ENSMUSG00000071303 | protein_coding | Rps8-ps1 | 11.30748 | 9.562579 | 10.43503 |
| ENSMUSG00000016150 | protein_coding | Odz1 | 2.903614 | 2.057464 | 2.480539 |
| ENSMUSG00000092091 | lincRNA | 1700066J03Rik | 10.93848 | 9.155704 | 10.04709 |
| ENSMUSG00000029655 | protein_coding | N4bp2l2 | 3577.659 | 3685.438 | 3631.548 |
| ENSMUSG00000040177 | protein_coding | 2310057M21Rik | 518.0548 | 500.3655 | 509.2101 |
| ENSMUSG00000070661 | protein_coding | Rnf186 | 2.900035 | 1.977104 | 2.43857 |
| ENSMUSG00000073016 | protein_coding | Uprt | 81.96232 | 88.35709 | 85.15971 |
| ENSMUSG00000038560 | protein_coding | Sp6 | 5.750871 | 7.505115 | 6.627993 |
| ENSMUSG00000091705 | protein_coding | H2-Q2 | 98.77809 | 92.85399 | 95.81604 |
| ENSMUSG00000041445 | protein_coding | Mmrn2 | 95.84712 | 102.5694 | 99.20824 |
| ENSMUSG00000086862 | processed_transcrip | Gm13546 | 17.50114 | 15.07257 | 16.28686 |
| ENSMUSG00000092155 | protein_coding | Gm17269 | 1.293062 | 0.671047 | 0.982055 |
| ENSMUSG00000037174 | protein_coding | Elf2 | 3821.712 | 3733.387 | 3777.55 |
| ENSMUSG00000086607 | lincRNA | 4930511M06Rik | 14.19931 | 16.70835 | 15.45383 |
| ENSMUSG00000086199 | processed_transcrip | 8430403D17Rik | 3.902744 | 5.049786 | 4.476265 |
| ENSMUSG00000032068 | protein_coding | 1600029D21Rik | 13.26116 | 11.11551 | 12.18834 |
| ENSMUSG00000054723 | protein_coding | Vmac | 714.2211 | 691.4815 | 702.8513 |
| ENSMUSG00000083340 | pseudogene | Gm13156 | 6.176226 | 8.160993 | 7.168609 |
| ENSMUSG00000089634 | protein_coding | Nat8b | 0.98273 | 1.668608 | 1.325669 |
| ENSMUSG00000047657 | pseudogene | Crry-ps | 0.98273 | 1.668608 | 1.325669 |
| ENSMUSG00000044052 | protein_coding | Ccr10 | 14.48735 | 16.80932 | 15.64834 |
| ENSMUSG00000026566 | protein_coding | Mpzl1 | 2693.475 | 2609.63 | 2651.553 |
| ENSMUSG00000036093 | protein_coding | Arl5a | 1742.671 | 1648.39 | 1695.53 |
| ENSMUSG00000000244 | protein_coding | Tspan32 | 3.580633 | 4.696606 | 4.13862 |
| ENSMUSG00000084149 | pseudogene | Gm12944 | 14.894 | 17.14769 | 16.02084 |
| ENSMUSG00000062859 | protein_coding | Tcp11 | 84.3465 | 76.83575 | 80.59112 |
| ENSMUSG00000021952 | protein_coding | Xpo4 | 1288.845 | 1249.921 | 1269.383 |
| ENSMUSG00000090621 | lincRNA | Gm4419 | 126.9631 | 134.5821 | 130.7726 |
| ENSMUSG00000052291 | pseudogene | 5330438D12Rik | 191.4838 | 182.7817 | 187.1327 |
| ENSMUSG00000029097 | protein_coding | 2310079F23Rik | 371.1732 | 358.3622 | 364.7677 |
| ENSMUSG00000078580 | protein_coding | E430018J23Rik | 171.6408 | 182.2798 | 176.9603 |

| | | | | | |
|---|---|---|---|---|---|
| ENSMUSG00000028699 | protein_coding | Tspan1 | 6.439669 | 5.10348 | 5.771575 |
| ENSMUSG00000031841 | protein_coding | Cdh13 | 44.20699 | 40.26309 | 42.23504 |
| ENSMUSG00000024830 | protein_coding | Rps6kb2 | 458.5868 | 480.9922 | 469.7895 |
| ENSMUSG00000083803 | pseudogene | Gm12455 | 1.278973 | 0.653029 | 0.966001 |
| ENSMUSG00000010803 | protein_coding | Gabra1 | 18.70863 | 16.27233 | 17.49048 |
| ENSMUSG00000025936 | protein_coding | Gm4956 | 1.621062 | 2.357312 | 1.989187 |
| ENSMUSG00000032769 | protein_coding | Trpa1 | 1.270774 | 2.00377 | 1.637272 |
| ENSMUSG00000073164 | processed_transcrip | 2410018L13Rik | 101.2279 | 75.31806 | 88.27301 |
| ENSMUSG00000022435 | protein_coding | Upk3a | 4.241254 | 5.572334 | 4.906794 |
| ENSMUSG00000059070 | protein_coding | Rpl18 | 6668.589 | 6485.263 | 6576.926 |
| ENSMUSG00000079418 | protein_coding | Atg4a | 363.2062 | 349.3654 | 356.2858 |
| ENSMUSG00000033209 | lincRNA | Ttc28 | 721.5998 | 700.456 | 711.0279 |
| ENSMUSG00000015016 | protein_coding | Acsf3 | 283.735 | 272.1392 | 277.9371 |
| ENSMUSG00000092484 | pseudogene | RP24-165O16.1 | 12.61001 | 14.67434 | 13.64218 |
| ENSMUSG00000024044 | protein_coding | Epb4.1l3 | 3730.551 | 3852.515 | 3791.533 |
| ENSMUSG00000032228 | protein_coding | Tcf12 | 4539.298 | 4442.219 | 4490.758 |
| ENSMUSG00000052414 | protein_coding | Atf7 | 1004.851 | 1054.753 | 1029.802 |
| ENSMUSG00000020902 | protein_coding | Ntn1 | 242.739 | 255.4967 | 249.1178 |
| ENSMUSG00000023990 | protein_coding | Tcfeb | 325.5438 | 309.825 | 317.6844 |
| ENSMUSG00000000957 | protein_coding | Mmp14 | 4349.613 | 4498.204 | 4423.908 |
| ENSMUSG00000025163 | protein_coding | Cd7 | 16.77724 | 14.49918 | 15.63821 |
| ENSMUSG00000022438 | protein_coding | Parvb | 570.5433 | 505.4129 | 537.9781 |
| ENSMUSG00000025047 | protein_coding | Pdcd11 | 3530.269 | 3648.466 | 3589.367 |
| ENSMUSG00000092087 | protein_coding | Gm10772 | 10.88339 | 8.007518 | 9.445453 |
| ENSMUSG00000081800 | pseudogene | Gm14200 | 1.931394 | 2.737158 | 2.334276 |
| ENSMUSG00000043592 | protein_coding | Unc5cl | 2.877746 | 2.021789 | 2.449767 |
| ENSMUSG00000078897 | protein_coding | Gm4724 | 3.540677 | 4.696606 | 4.118641 |
| ENSMUSG00000026281 | protein_coding | Dtymk | 1547.263 | 1595.546 | 1571.405 |
| ENSMUSG00000092035 | protein_coding | Peg10 | 4638.276 | 4395.198 | 4516.737 |
| ENSMUSG00000032297 | protein_coding | Celf6 | 29.60066 | 24.79727 | 27.19897 |
| ENSMUSG00000079174 | protein_coding | Gm3054 | 4.840896 | 3.73472 | 4.287808 |
| ENSMUSG00000040842 | protein_coding | D4Ertd22e | 2638.254 | 2701.859 | 2670.056 |
| ENSMUSG00000003344 | protein_coding | Btbd2 | 1078.894 | 1110.404 | 1094.649 |
| ENSMUSG00000043832 | protein_coding | Clec4a3 | 38.06737 | 41.56843 | 39.8179 |
| ENSMUSG00000027485 | protein_coding | Bpifb1 | 8.44024 | 6.861095 | 7.650668 |
| ENSMUSG00000090582 | processed_transcrip | Gm17024 | 46.84172 | 51.11987 | 48.9808 |
| ENSMUSG00000029019 | protein_coding | Nppb | 1.951372 | 2.772834 | 2.362103 |
| ENSMUSG00000082711 | pseudogene | Gm12987 | 3.250323 | 4.637114 | 3.943718 |
| ENSMUSG00000087645 | lincRNA | F630040L22Rik | 4.845518 | 3.708054 | 4.276786 |
| ENSMUSG00000033577 | protein_coding | Myo6 | 2045.105 | 2109.125 | 2077.115 |
| ENSMUSG00000029073 | protein_coding | Gltpd1 | 353.1333 | 337.6071 | 345.3702 |
| ENSMUSG00000087623 | lincRNA | Gm12404 | 12.34761 | 10.29561 | 11.32161 |
| ENSMUSG00000054770 | protein_coding | Kctd18 | 1368.01 | 1405.643 | 1386.827 |
| ENSMUSG00000040385 | protein_coding | Ppp1ca | 6115.85 | 6270.761 | 6193.305 |
| ENSMUSG00000024844 | protein_coding | Banf1 | 2082.767 | 2133.003 | 2107.885 |
| ENSMUSG00000021704 | protein_coding | Mtx3 | 1581.385 | 1635.977 | 1608.681 |
| ENSMUSG00000031304 | protein_coding | Il2rg | 25.24796 | 22.3845 | 23.81623 |
| ENSMUSG00000087257 | processed_transcrip | Gm15548 | 4.849096 | 6.208067 | 5.528582 |
| ENSMUSG00000083146 | pseudogene | Gm15921 | 4.552853 | 3.408205 | 3.980529 |
| ENSMUSG00000091238 | processed_transcrip | Gm17103 | 8.454329 | 6.878752 | 7.666541 |
| ENSMUSG00000042390 | protein_coding | Gatad2b | 1275.244 | 1329.672 | 1302.458 |
| ENSMUSG00000082396 | pseudogene | Zfp389 | 32.47148 | 28.96019 | 30.71584 |
| ENSMUSG00000084874 | pseudogene | Gm12906 | 4.849096 | 3.761386 | 4.305241 |
| ENSMUSG00000025885 | protein_coding | Myo5b | 2256.354 | 2341.82 | 2299.087 |
| ENSMUSG00000053467 | protein_coding | A530088E08Rik | 5.167628 | 6.463231 | 5.815429 |
| ENSMUSG00000000149 | protein_coding | Gna12 | 4666.035 | 4782.519 | 4724.277 |
| ENSMUSG00000078915 | pseudogene | Hsp25-ps1 | 2.895414 | 2.021789 | 2.458601 |
| ENSMUSG00000086459 | lincRNA | 1700030C12Rik | 1.307151 | 2.012779 | 1.659965 |

| | | | | | |
|---|---|---|---|---|---|
| ENSMUSG00000043346 | protein_coding | Gm6741 | 1.304841 | 2.146471 | 1.725656 |
| ENSMUSG00000024070 | protein_coding | Prkd3 | 5480.698 | 5620.279 | 5550.488 |
| ENSMUSG00000092117 | lincRNA | Gm17520 | 45.37601 | 41.4351 | 43.40556 |
| ENSMUSG00000083748 | pseudogene | Gm11662 | 1.609283 | 2.472986 | 2.041135 |
| ENSMUSG00000091370 | processed_transcrip | 5730435O14Rik | 1.945483 | 2.746168 | 2.345825 |
| ENSMUSG00000030814 | protein_coding | Bcl7c | 1548.545 | 1493.571 | 1521.058 |
| ENSMUSG00000049477 | pseudogene | Gm8508 | 14.50964 | 16.63379 | 15.57172 |
| ENSMUSG00000021548 | protein_coding | Ccnh | 1395.655 | 1432.165 | 1413.91 |
| ENSMUSG00000033909 | protein_coding | Usp36 | 1329.437 | 1285.12 | 1307.279 |
| ENSMUSG00000026443 | protein_coding | Lrrn2 | 128.4847 | 137.1863 | 132.8355 |
| ENSMUSG00000087182 | protein_coding | Gm13334 | 4.563364 | 5.854525 | 5.208944 |
| ENSMUSG00000001348 | protein_coding | Acp5 | 181.7507 | 173.3446 | 177.5476 |
| ENSMUSG00000020280 | protein_coding | Pus10 | 1049.173 | 1082.383 | 1065.778 |
| ENSMUSG00000036073 | protein_coding | Galt | 215.5065 | 225.6155 | 220.561 |
| ENSMUSG00000033788 | protein_coding | Dysf | 275.1555 | 264.2929 | 269.7242 |
| ENSMUSG00000044092 | protein_coding | C130050O18Rik | 11.28981 | 13.20829 | 12.24905 |
| ENSMUSG00000061286 | protein_coding | Exosc5 | 1211.911 | 1172.177 | 1192.044 |
| ENSMUSG00000024902 | protein_coding | Mrpl11 | 1009.9 | 1036.256 | 1023.078 |
| ENSMUSG00000090687 | lincRNA | 9330151L19Rik | 275.6199 | 290.7054 | 283.1626 |
| ENSMUSG00000084808 | lincRNA | 9430091E24Rik | 358.8696 | 372.5574 | 365.7135 |
| ENSMUSG00000092684 | misc_RNA | AC158995.1 | 13.57738 | 15.61857 | 14.59798 |
| ENSMUSG00000033860 | protein_coding | Fgg | 13.29523 | 15.44377 | 14.3695 |
| ENSMUSG00000000247 | protein_coding | Lhx2 | 225.2231 | 213.8107 | 219.5169 |
| ENSMUSG00000041538 | protein_coding | H2-Ob | 1.606973 | 0.988552 | 1.297762 |
| ENSMUSG00000032096 | protein_coding | Arcn1 | 8023.806 | 8215.389 | 8119.598 |
| ENSMUSG00000026548 | protein_coding | Slamf9 | 8.725973 | 7.222923 | 7.974448 |
| ENSMUSG00000025317 | protein_coding | Car5a | 5.787249 | 4.467746 | 5.127498 |
| ENSMUSG00000033900 | protein_coding | Mtap9 | 452.9959 | 473.6337 | 463.3148 |
| ENSMUSG00000087551 | lincRNA | Gm16912 | 4.530564 | 3.417215 | 3.97389 |
| ENSMUSG00000005397 | protein_coding | Nid1 | 4739.153 | 4583.589 | 4661.371 |
| ENSMUSG00000083857 | pseudogene | Gm12584 | 4.579764 | 3.408205 | 3.993984 |
| ENSMUSG00000039678 | protein_coding | Tbc1d13 | 1632.281 | 1591.379 | 1611.83 |
| ENSMUSG00000020884 | protein_coding | Asgr1 | 11.60961 | 13.54309 | 12.57635 |
| ENSMUSG00000073229 | protein_coding | Rpl15-ps2 | 2.909503 | 2.039445 | 2.474474 |
| ENSMUSG00000019779 | protein_coding | Frk | 91.01294 | 99.43812 | 95.22553 |
| ENSMUSG00000088984 | snoRNA | SNORA17 | 3.919143 | 5.139155 | 4.529149 |
| ENSMUSG00000018974 | protein_coding | Sart3 | 2236.237 | 2296.945 | 2266.591 |
| ENSMUSG00000005069 | protein_coding | Pex5 | 1335.389 | 1369.9 | 1352.644 |
| ENSMUSG00000021095 | protein_coding | Gsc | 8.39693 | 10.55434 | 9.475636 |
| ENSMUSG00000086041 | processed_transcrip | Gm12869 | 1.615173 | 0.997561 | 1.306367 |
| ENSMUSG00000037393 | protein_coding | Nmur2 | 1.615173 | 0.997561 | 1.306367 |
| ENSMUSG00000034987 | protein_coding | Hrh2 | 1.951372 | 2.746168 | 2.34877 |
| ENSMUSG00000004070 | protein_coding | Hmox2 | 2078.316 | 2021.613 | 2049.964 |
| ENSMUSG00000022061 | protein_coding | Nkx3-1 | 1784.327 | 1855.538 | 1819.932 |
| ENSMUSG00000053852 | protein_coding | Gpr112 | 4.865496 | 6.136716 | 5.501106 |
| ENSMUSG00000024759 | protein_coding | Atl3 | 4972.006 | 5102.471 | 5037.239 |
| ENSMUSG00000039683 | protein_coding | Sdk1 | 776.118 | 631.8261 | 703.972 |
| ENSMUSG00000053588 | protein_coding | A730061H03Rik | 4.798629 | 3.488565 | 4.143597 |
| ENSMUSG00000063439 | protein_coding | B9d2 | 250.7383 | 240.7735 | 245.7559 |
| ENSMUSG00000070604 | protein_coding | Vsig10l | 95.23301 | 88.84039 | 92.0367 |
| ENSMUSG00000069206 | protein_coding | Zfp874a | 285.1553 | 270.7005 | 277.9279 |
| ENSMUSG00000006007 | protein_coding | Pdc | 1.629262 | 0.997561 | 1.313411 |
| ENSMUSG00000076269 | miRNA | Mir374 | 1.629262 | 0.997561 | 1.313411 |
| ENSMUSG00000046897 | protein_coding | Zfp740 | 3832.885 | 3955.899 | 3894.392 |
| ENSMUSG00000035448 | protein_coding | Ccr3 | 2.289882 | 3.310189 | 2.800035 |
| ENSMUSG00000084826 | processed_transcrip | AI847159 | 1.264884 | 2.048455 | 1.65667 |
| ENSMUSG00000091980 | pseudogene | Gm3719 | 3.197546 | 1.959086 | 2.578316 |
| ENSMUSG00000018752 | protein_coding | BC096441 | 26.63607 | 30.1455 | 28.39079 |

| | | | | | |
|---|---|---|---|---|---|
| ENSMUSG00000040540 | pseudogene | Gm9770 | 156.4436 | 148.4041 | 152.4239 |
| ENSMUSG00000024130 | protein_coding | Abca3 | 1431.447 | 1481.722 | 1456.584 |
| ENSMUSG00000039275 | protein_coding | Foxk2 | 2409.752 | 2476.183 | 2442.967 |
| ENSMUSG00000038024 | protein_coding | Dennd4c | 2510.892 | 2573.733 | 2542.312 |
| ENSMUSG00000019845 | protein_coding | Tube1 | 397.8202 | 412.9678 | 405.394 |
| ENSMUSG00000027876 | protein_coding | Reg4 | 0.99093 | 1.757616 | 1.374273 |
| ENSMUSG00000005057 | protein_coding | Sh2b2 | 183.6191 | 193.3766 | 188.4978 |
| ENSMUSG00000028108 | protein_coding | Ecm1 | 214.0074 | 229.3213 | 221.6644 |
| ENSMUSG00000044813 | protein_coding | Shb | 620.2408 | 652.4292 | 636.335 |
| ENSMUSG00000039639 | protein_coding | Kcne1 | 28.26466 | 25.05683 | 26.66075 |
| ENSMUSG00000078816 | protein_coding | Prkcc | 20.59291 | 17.52862 | 19.06077 |
| ENSMUSG00000062908 | protein_coding | Acadm | 2297.981 | 2367.624 | 2332.802 |
| ENSMUSG00000036813 | protein_coding | Entpd8 | 3.925032 | 5.165821 | 4.545427 |
| ENSMUSG00000039199 | protein_coding | Zdhhc1 | 511.3639 | 488.8009 | 500.0824 |
| ENSMUSG00000088929 | snoRNA | SNORD5 | 4.860874 | 6.225724 | 5.543299 |
| ENSMUSG00000081975 | pseudogene | Gm12482 | 5.501517 | 6.82542 | 6.163468 |
| ENSMUSG00000031654 | protein_coding | Cbln1 | 81.6247 | 87.25427 | 84.43949 |
| ENSMUSG00000015536 | protein_coding | Mocs2 | 888.2529 | 988.4076 | 938.3302 |
| ENSMUSG00000034486 | protein_coding | Gbx2 | 10.34935 | 12.37036 | 11.35985 |
| ENSMUSG00000040725 | protein_coding | Hnrnpul1 | 8614.062 | 8386.204 | 8500.133 |
| ENSMUSG00000089999 | pseudogene | Gm6485 | 144.5309 | 152.0909 | 148.3109 |
| ENSMUSG00000026283 | protein_coding | Ing5 | 1048.579 | 1094.475 | 1071.527 |
| ENSMUSG00000075411 | protein_coding | Bin2 | 448.4612 | 475.3634 | 461.9123 |
| ENSMUSG00000061718 | protein_coding | Ppp1r1b | 368.9919 | 350.9089 | 359.9504 |
| ENSMUSG00000084345 | pseudogene | Rpl17-ps4 | 1.282552 | 0.671047 | 0.976799 |
| ENSMUSG00000033364 | protein_coding | Usp37 | 4687.244 | 4589.326 | 4638.285 |
| ENSMUSG00000085914 | processed_transcrip | Gm14320 | 3.228034 | 2.348303 | 2.788169 |
| ENSMUSG00000087819 | snoRNA | SNORA48 | 3.852277 | 5.059157 | 4.455717 |
| ENSMUSG00000057228 | protein_coding | Aadat | 2.583814 | 3.618685 | 3.101249 |
| ENSMUSG00000086446 | processed_transcrip | 4933426D04Rik | 6.525246 | 5.112127 | 5.818687 |
| ENSMUSG00000087037 | lincRNA | 4930432L08Rik | 1.32124 | 0.688704 | 1.004972 |
| ENSMUSG00000081884 | pseudogene | Gm11821 | 16.16119 | 18.3824 | 17.2718 |
| ENSMUSG00000003660 | protein_coding | Snrnp200 | 6756.934 | 6544.049 | 6650.492 |
| ENSMUSG00000035067 | protein_coding | Xkr6 | 25.20108 | 28.07831 | 26.63969 |
| ENSMUSG00000041245 | processed_transcrip | Wnk3-ps | 6153.883 | 5992.845 | 6073.364 |
| ENSMUSG00000086340 | lincRNA | 1810059C17Rik | 9.378393 | 7.81361 | 8.596002 |
| ENSMUSG00000000374 | protein_coding | Trappc10 | 1774.903 | 1843.206 | 1809.055 |
| ENSMUSG00000085881 | processed_transcrip | Gm15912 | 16.80079 | 19.09777 | 17.94928 |
| ENSMUSG00000027810 | protein_coding | Eif2a | 1952.175 | 1997.443 | 1974.809 |
| ENSMUSG00000021062 | protein_coding | Rab15 | 63.6818 | 69.57797 | 66.62989 |
| ENSMUSG00000029397 | protein_coding | Rchy1 | 1333.785 | 1300.83 | 1317.307 |
| ENSMUSG00000067929 | protein_coding | Gm10226 | 66.95441 | 72.33708 | 69.64575 |
| ENSMUSG00000063236 | protein_coding | 1110038F14Rik | 759.5413 | 782.7972 | 771.1692 |
| ENSMUSG00000047959 | protein_coding | Kcna3 | 99.46369 | 107.3604 | 103.412 |
| ENSMUSG00000053985 | protein_coding | Zfp14 | 165.2702 | 156.8056 | 161.0379 |
| ENSMUSG00000072589 | processed_transcrip | Gm10371 | 1.934972 | 2.746168 | 2.34057 |
| ENSMUSG00000081967 | pseudogene | Gm14017 | 3.228034 | 2.374969 | 2.801502 |
| ENSMUSG00000030839 | protein_coding | Sergef | 182.8104 | 174.402 | 178.6062 |
| ENSMUSG00000003779 | protein_coding | Kif20a | 2084.212 | 2025.77 | 2054.991 |
| ENSMUSG00000019936 | protein_coding | Epyc | 92.98205 | 85.06301 | 89.02253 |
| ENSMUSG00000079293 | protein_coding | Clec7a | 4.867807 | 3.73472 | 4.301263 |
| ENSMUSG00000029576 | protein_coding | Radil | 893.8565 | 940.9949 | 917.4257 |
| ENSMUSG00000077358 | snoRNA | SNORD70 | 5.473339 | 6.932446 | 6.202892 |
| ENSMUSG00000081404 | pseudogene | Gm15694 | 17.73537 | 15.53278 | 16.63407 |
| ENSMUSG00000021374 | protein_coding | Nup153 | 4700.854 | 4868.112 | 4784.483 |
| ENSMUSG00000023914 | protein_coding | Mep1a | 1.586995 | 2.401635 | 1.994315 |
| ENSMUSG00000081885 | pseudogene | Gm13231 | 64.83613 | 70.75381 | 67.79497 |
| ENSMUSG00000024360 | protein_coding | Etf1 | 5202.842 | 5331.539 | 5267.19 |

| | | | | | |
|---|---|---|---|---|---|
| ENSMUSG00000064525 | snoRNA | SNORA65 | 12.25256 | 14.26747 | 13.26002 |
| ENSMUSG00000026127 | protein_coding | Imp4 | 1166.664 | 1199.19 | 1182.927 |
| ENSMUSG00000026791 | protein_coding | Slc2a8 | 272.1708 | 260.9012 | 266.536 |
| ENSMUSG00000086944 | processed_transcrip | Gm15859 | 1.621062 | 1.015218 | 1.31814 |
| ENSMUSG00000038677 | protein_coding | Scube3 | 704.3357 | 741.6338 | 722.9847 |
| ENSMUSG00000029147 | protein_coding | Ppm1g | 3295.58 | 3202.833 | 3249.206 |
| ENSMUSG00000038007 | protein_coding | Acer2 | 208.7374 | 222.6338 | 215.6856 |
| ENSMUSG00000050199 | protein_coding | Lgr4 | 1924.076 | 1972.123 | 1948.099 |
| ENSMUSG00000055780 | protein_coding | Usp26 | 3095.645 | 3198.719 | 3147.182 |
| ENSMUSG00000049339 | protein_coding | Fam134a | 2245.572 | 2189.747 | 2217.66 |
| ENSMUSG00000041798 | protein_coding | Gck | 8.745951 | 10.42645 | 9.586199 |
| ENSMUSG00000067480 | protein_coding | Gm14403 | 90.85274 | 96.5113 | 93.68202 |
| ENSMUSG00000047423 | protein_coding | AI837181 | 1055.754 | 1084.961 | 1070.357 |
| ENSMUSG00000041439 | protein_coding | Mfsd6 | 136.336 | 143.6686 | 140.0023 |
| ENSMUSG00000046805 | protein_coding | Mpeg1 | 1149.909 | 1276.034 | 1212.972 |
| ENSMUSG00000092951 | misc_RNA | AL928889.1 | 2.942302 | 2.08413 | 2.513216 |
| ENSMUSG00000018501 | protein_coding | Ncor1 | 5703.375 | 5835.187 | 5769.281 |
| ENSMUSG00000071960 | protein_coding | Gm10352 | 1.282552 | 2.066111 | 1.674332 |
| ENSMUSG00000072688 | protein_coding | Gm10399 | 50.78293 | 46.67299 | 48.72796 |
| ENSMUSG00000091549 | pseudogene | Gm6548 | 417.4615 | 431.615 | 424.5382 |
| ENSMUSG00000026803 | protein_coding | Ttf1 | 791.7265 | 815.5253 | 803.6259 |
| ENSMUSG00000044694 | protein_coding | 2010007H06Rik | 27.45816 | 24.52885 | 25.9935 |
| ENSMUSG00000082144 | pseudogene | Gm12788 | 3.588833 | 4.705615 | 4.147224 |
| ENSMUSG00000049291 | protein_coding | Prss38 | 35.41251 | 39.94699 | 37.67975 |
| ENSMUSG00000075571 | protein_coding | Defb30 | 2.245304 | 3.108357 | 2.676831 |
| ENSMUSG00000020867 | protein_coding | Spata20 | 6.45018 | 7.920998 | 7.185589 |
| ENSMUSG00000039630 | protein_coding | Hnrnpu | 21904.61 | 21447.02 | 21675.82 |
| ENSMUSG00000076009 | miRNA | Mir688 | 1.293062 | 2.066473 | 1.679768 |
| ENSMUSG00000062372 | protein_coding | Otof | 190.4567 | 200.1911 | 195.3239 |
| ENSMUSG00000022253 | protein_coding | 1110020G09Rik | 1372.116 | 1406.427 | 1389.272 |
| ENSMUSG00000057816 | protein_coding | 1700007G11Rik | 6.522936 | 5.022758 | 5.772847 |
| ENSMUSG00000030468 | protein_coding | Siglecg | 2.901303 | 4.070605 | 3.485954 |
| ENSMUSG00000085875 | processed_transcrip | Gm12905 | 34.52773 | 38.11082 | 36.31928 |
| ENSMUSG00000031429 | protein_coding | Psmd10 | 750.2815 | 727.2413 | 738.7614 |
| ENSMUSG00000090194 | processed_transcrip | Gm16161 | 16.51864 | 18.95615 | 17.7374 |
| ENSMUSG00000077127 | miRNA | Mir743 | 5.814159 | 7.347606 | 6.580883 |
| ENSMUSG00000072812 | protein_coding | Ahnak2 | 96.16089 | 104.2907 | 100.2258 |
| ENSMUSG00000089782 | pseudogene | Gm3531 | 116.7933 | 124.4477 | 120.6205 |
| ENSMUSG00000025898 | protein_coding | Cwf19l2 | 1054.702 | 1022.06 | 1038.381 |
| ENSMUSG00000048997 | protein_coding | Atxn7l2 | 470.7877 | 455.7298 | 463.2587 |
| ENSMUSG00000040511 | protein_coding | Pvr | 756.5886 | 726.9315 | 741.76 |
| ENSMUSG00000025208 | protein_coding | Mrpl43 | 873.0459 | 896.6359 | 884.8409 |
| ENSMUSG00000091803 | protein_coding | Cox16 | 169.8971 | 178.1684 | 174.0327 |
| ENSMUSG00000027102 | protein_coding | Hoxd8 | 538.2082 | 561.1096 | 549.6589 |
| ENSMUSG00000045009 | protein_coding | Prrt3 | 42.05107 | 37.69857 | 39.87482 |
| ENSMUSG00000056724 | protein_coding | Nbeal2 | 1180.469 | 1136.479 | 1158.474 |
| ENSMUSG00000042816 | protein_coding | Gpr151 | 1.615173 | 1.015218 | 1.315195 |
| ENSMUSG00000085458 | lincRNA | G630030J09Rik | 4.857296 | 3.779404 | 4.31835 |
| ENSMUSG00000047757 | protein_coding | Fancb | 93.88121 | 85.84304 | 89.86213 |
| ENSMUSG00000001323 | protein_coding | Srr | 844.2503 | 819.2422 | 831.7463 |
| ENSMUSG00000083378 | pseudogene | Gm13196 | 1.270774 | 0.671047 | 0.97091 |
| ENSMUSG00000074109 | protein_coding | Mrgprx2 | 1.270774 | 0.671047 | 0.97091 |
| ENSMUSG00000025400 | protein_coding | Tac2 | 1.270774 | 0.671047 | 0.97091 |
| ENSMUSG00000091275 | protein_coding | Gm3248 | 4.269432 | 3.046016 | 3.657724 |
| ENSMUSG00000082394 | pseudogene | Gm4596 | 13.92178 | 11.85139 | 12.88658 |
| ENSMUSG00000092261 | pseudogene | RP23-411N10.3 | 4.537497 | 3.30118 | 3.919338 |
| ENSMUSG00000024985 | protein_coding | Tcf7l2 | 658.1546 | 679.3241 | 668.7394 |
| ENSMUSG00000044934 | protein_coding | Zfp367 | 1049.244 | 1091.991 | 1070.618 |

| | | | | | |
|---|---|---|---|---|---|
| ENSMUSG00000002985 | protein_coding | Apoe | 3336.481 | 3224.388 | 3280.434 |
| ENSMUSG00000030060 | protein_coding | 8430410A17Rik | 1010.485 | 1044.261 | 1027.373 |
| ENSMUSG00000086704 | processed_transcrip | Gm14582 | 2.883635 | 1.986113 | 2.434874 |
| ENSMUSG00000050896 | protein_coding | Rtn4rl2 | 33.44139 | 29.91628 | 31.67883 |
| ENSMUSG00000049687 | protein_coding | Fam109b | 135.4977 | 144.7052 | 140.1015 |
| ENSMUSG00000080977 | pseudogene | Gm13772 | 1.917305 | 2.719502 | 2.318403 |
| ENSMUSG00000053030 | protein_coding | Spink2 | 9.384282 | 7.679919 | 8.5321 |
| ENSMUSG00000025056 | protein_coding | Nr0b1 | 1759.073 | 1833.235 | 1796.154 |
| ENSMUSG00000036770 | protein_coding | 4933433C11Rik | 14.12529 | 16.30728 | 15.21628 |
| ENSMUSG00000032530 | protein_coding | Lyzl4 | 1.623372 | 2.401635 | 2.012504 |
| ENSMUSG00000030093 | protein_coding | Wnt7a | 10.58819 | 8.882522 | 9.735356 |
| ENSMUSG00000031328 | protein_coding | Flna | 7085.975 | 6859.882 | 6972.929 |
| ENSMUSG00000031491 | protein_coding | Chrna6 | 1.967772 | 3.259706 | 2.613739 |
| ENSMUSG00000031372 | protein_coding | Trex2 | 1.264884 | 2.039445 | 1.652165 |
| ENSMUSG00000040694 | protein_coding | Apobec2 | 13.16611 | 11.29317 | 12.22964 |
| ENSMUSG00000021208 | protein_coding | Ifi27l2b | 3.242123 | 2.357312 | 2.799718 |
| ENSMUSG00000075273 | protein_coding | Ttc30b | 200.9229 | 210.6396 | 205.7813 |
| ENSMUSG00000026514 | protein_coding | Cnih3 | 7.080311 | 5.711824 | 6.396068 |
| ENSMUSG00000041945 | protein_coding | Mfsd9 | 130.4308 | 123.4421 | 126.9365 |
| ENSMUSG00000031204 | protein_coding | Asb12 | 16.45639 | 14.39288 | 15.42463 |
| ENSMUSG00000035045 | protein_coding | Zc3h12b | 303.8266 | 292.5528 | 298.1897 |
| ENSMUSG00000089906 | pseudogene | Gm6428 | 1.635151 | 1.024227 | 1.329689 |
| ENSMUSG00000028186 | protein_coding | Uox | 1.635151 | 1.024227 | 1.329689 |
| ENSMUSG00000091942 | pseudogene | Gm17138 | 1.635151 | 1.024227 | 1.329689 |
| ENSMUSG00000058704 | protein_coding | Memo1 | 925.2364 | 950.7636 | 938 |
| ENSMUSG00000049230 | protein_coding | Gm9833 | 319.5282 | 306.1901 | 312.8591 |
| ENSMUSG00000020561 | protein_coding | Twistnb | 1033.255 | 1004.867 | 1019.061 |
| ENSMUSG00000020702 | protein_coding | Ccl1 | 1.288441 | 2.048455 | 1.668448 |
| ENSMUSG00000030763 | protein_coding | Lcmt1 | 1140.143 | 1108.325 | 1124.234 |
| ENSMUSG00000032047 | protein_coding | Acat1 | 3371.024 | 3261.346 | 3316.185 |
| ENSMUSG00000018199 | protein_coding | Trove2 | 1105.428 | 1158.33 | 1131.879 |
| ENSMUSG00000031997 | protein_coding | Trpc6 | 15.08931 | 13.05078 | 14.07004 |
| ENSMUSG00000087545 | processed_transcrip | Gm13966 | 1.606973 | 1.015218 | 1.311095 |
| ENSMUSG00000044141 | pseudogene | E130201H02Rik | 1.606973 | 1.015218 | 1.311095 |
| ENSMUSG00000030730 | protein_coding | Atp2a1 | 33.44243 | 37.04699 | 35.24471 |
| ENSMUSG00000090004 | pseudogene | Gm16125 | 2.213548 | 3.345864 | 2.779706 |
| ENSMUSG00000085101 | processed_transcrip | Gm13227 | 11.31694 | 13.19027 | 12.25361 |
| ENSMUSG00000022018 | protein_coding | 1190002H23Rik | 166.0197 | 176.6534 | 171.3365 |
| ENSMUSG00000059187 | protein_coding | Fam19a1 | 4.535186 | 3.497213 | 4.016199 |
| ENSMUSG00000023391 | protein_coding | Dlx2 | 10.5823 | 12.73255 | 11.65743 |
| ENSMUSG00000086166 | processed_transcrip | Gm14342 | 6.088113 | 7.647454 | 6.867784 |
| ENSMUSG00000054885 | protein_coding | 4930578G10Rik | 2.289882 | 1.448758 | 1.86932 |
| ENSMUSG00000085438 | lincRNA | 1700020I14Rik | 6369.836 | 6611.653 | 6490.745 |
| ENSMUSG00000000693 | protein_coding | Loxl3 | 279.5766 | 267.7735 | 273.675 |
| ENSMUSG00000068134 | protein_coding | Zfp120 | 470.0915 | 444.8892 | 457.4904 |
| ENSMUSG00000037451 | protein_coding | Slc22a20 | 2.259393 | 3.081691 | 2.670542 |
| ENSMUSG00000068697 | protein_coding | Myoz1 | 57.54598 | 61.94988 | 59.74793 |
| ENSMUSG00000035279 | protein_coding | A430110N23Rik | 60.66994 | 65.95778 | 63.31386 |
| ENSMUSG00000066141 | protein_coding | Gm11232 | 4.172076 | 2.983675 | 3.577875 |
| ENSMUSG00000086216 | lincRNA | Gm13610 | 3.889922 | 2.755177 | 3.32255 |
| ENSMUSG00000022453 | protein_coding | Naga | 395.0734 | 412.8734 | 403.9734 |
| ENSMUSG00000050141 | protein_coding | BC049635 | 46.78872 | 50.92809 | 48.8584 |
| ENSMUSG00000090659 | protein_coding | Zfp493 | 64.11305 | 69.8263 | 66.96967 |
| ENSMUSG00000082028 | pseudogene | Gm1826 | 2.920013 | 2.110796 | 2.515405 |
| ENSMUSG00000041605 | protein_coding | 5730559C18Rik | 35.72471 | 39.43604 | 37.58037 |
| ENSMUSG00000078153 | protein_coding | Psme2b-ps | 467.8256 | 452.6164 | 460.221 |
| ENSMUSG00000081139 | pseudogene | Gm13333 | 3.213945 | 2.374969 | 2.794457 |
| ENSMUSG00000021036 | protein_coding | Sptlc2 | 2637.727 | 2562.654 | 2600.191 |

| | | | | | |
|---|---|---|---|---|---|
| ENSMUSG00000035764 | protein_coding | Fbxo45 | 868.9917 | 841.2915 | 855.1416 |
| ENSMUSG00000038690 | protein_coding | Atp5j2 | 1983.395 | 1931.591 | 1957.493 |
| ENSMUSG00000081042 | pseudogene | Psmb7-ps2 | 2.905924 | 2.110796 | 2.50836 |
| ENSMUSG00000028911 | protein_coding | Srsf4 | 1048.665 | 1020.252 | 1034.459 |
| ENSMUSG00000074807 | protein_coding | Gm10762 | 186.5683 | 178.4384 | 182.5033 |
| ENSMUSG00000015461 | protein_coding | Atf6b | 1984.72 | 2040.695 | 2012.708 |
| ENSMUSG00000029245 | protein_coding | Epha5 | 7.986708 | 10.01994 | 9.003322 |
| ENSMUSG00000030525 | protein_coding | Chrna7 | 105.2358 | 98.04405 | 101.6399 |
| ENSMUSG00000028274 | protein_coding | Rngtt | 1027.777 | 1055.521 | 1041.649 |
| ENSMUSG00000020648 | protein_coding | Dus4l | 263.7344 | 253.0513 | 258.3928 |
| ENSMUSG00000085392 | lincRNA | Gm16606 | 13.98067 | 11.6377 | 12.80918 |
| ENSMUSG00000031782 | protein_coding | Coq9 | 1216.962 | 1183.792 | 1200.377 |
| ENSMUSG00000061877 | protein_coding | BC048679 | 3.586523 | 4.794984 | 4.190753 |
| ENSMUSG00000002984 | protein_coding | Tomm40 | 1333.23 | 1295.801 | 1314.516 |
| ENSMUSG00000053730 | protein_coding | Tmem39b | 537.3767 | 554.6564 | 546.0165 |
| ENSMUSG00000033059 | protein_coding | Pygb | 5043.62 | 5158.968 | 5101.294 |
| ENSMUSG00000035638 | protein_coding | Muc20 | 3.913254 | 2.790491 | 3.351872 |
| ENSMUSG00000087437 | processed_transcrip | Gm13112 | 7.729153 | 9.244712 | 8.486933 |
| ENSMUSG00000057101 | protein_coding | Zfp180 | 1010.058 | 980.8257 | 995.4417 |
| ENSMUSG00000021975 | protein_coding | Ints9 | 833.387 | 807.0205 | 820.2038 |
| ENSMUSG00000025050 | protein_coding | Pcgf6 | 894.6433 | 859.4951 | 877.0692 |
| ENSMUSG00000002733 | protein_coding | Plekha3 | 1383.618 | 1351.049 | 1367.334 |
| ENSMUSG00000086155 | processed_transcrip | 9430041J12Rik | 110.0948 | 116.4566 | 113.2757 |
| ENSMUSG00000047502 | protein_coding | Heatr8 | 38.85248 | 42.48055 | 40.66652 |
| ENSMUSG00000000094 | protein_coding | Tbx4 | 16.86177 | 14.48116 | 15.67147 |
| ENSMUSG00000027078 | protein_coding | Ube2l6 | 554.7448 | 572.4362 | 563.5905 |
| ENSMUSG00000019428 | protein_coding | Fkbp8 | 3390.175 | 3470.599 | 3430.387 |
| ENSMUSG00000023026 | protein_coding | Dip2b | 2722.707 | 2637.105 | 2679.906 |
| ENSMUSG00000091423 | protein_coding | Gm17509 | 3.252634 | 4.352435 | 3.802534 |
| ENSMUSG00000034189 | protein_coding | Hsdl1 | 1735.61 | 1680.222 | 1707.916 |
| ENSMUSG00000081451 | pseudogene | Gm12734 | 4.530564 | 3.470547 | 4.000556 |
| ENSMUSG00000082981 | pseudogene | Gm11526 | 1.626951 | 1.024227 | 1.325589 |
| ENSMUSG00000087268 | processed_transcrip | Gm14486 | 19.91843 | 22.47066 | 21.19454 |
| ENSMUSG00000090632 | protein_coding | Gm17404 | 15.40091 | 13.14559 | 14.27325 |
| ENSMUSG00000031327 | protein_coding | Chic1 | 655.3629 | 676.2951 | 665.829 |
| ENSMUSG00000085621 | lincRNA | 4930467K11Rik | 2.956391 | 2.012779 | 2.484585 |
| ENSMUSG00000017715 | protein_coding | Pgs1 | 1223.948 | 1261.753 | 1242.851 |
| ENSMUSG00000001156 | protein_coding | Mxd1 | 2023.176 | 2075.327 | 2049.252 |
| ENSMUSG00000041957 | protein_coding | Pkp2 | 389.3631 | 373.9315 | 381.6473 |
| ENSMUSG00000087026 | lincRNA | A230103J11Rik | 15.84959 | 18.18057 | 17.01508 |
| ENSMUSG00000083004 | pseudogene | Gm13413 | 1.635151 | 2.401635 | 2.018393 |
| ENSMUSG00000090118 | processed_transcrip | Gm16163 | 1.615173 | 2.401997 | 2.008585 |
| ENSMUSG00000079101 | pseudogene | Gm2904 | 1.288441 | 2.057464 | 1.672952 |
| ENSMUSG00000031933 | protein_coding | Folr4 | 2.267593 | 3.081691 | 2.674642 |
| ENSMUSG00000085891 | lincRNA | Gm14634 | 30.61053 | 34.69397 | 32.65225 |
| ENSMUSG00000079163 | processed_transcrip | Gm15498 | 10.96308 | 8.9503 | 9.956689 |
| ENSMUSG00000086894 | processed_transcrip | Gm15708 | 20.92218 | 23.65276 | 22.28747 |
| ENSMUSG00000002222 | protein_coding | Rmnd5a | 3719.384 | 3796.452 | 3757.918 |
| ENSMUSG00000039068 | protein_coding | Zzz3 | 3590.402 | 3713.299 | 3651.851 |
| ENSMUSG00000087016 | processed_transcrip | Gm15979 | 1.612862 | 1.015218 | 1.31404 |
| ENSMUSG00000087671 | lincRNA | A330048O09Rik | 1.612862 | 1.015218 | 1.31404 |
| ENSMUSG00000031253 | protein_coding | Srpx2 | 133.8467 | 126.1717 | 130.0092 |
| ENSMUSG00000087153 | protein_coding | Gm6483 | 921.9842 | 953.2142 | 937.5992 |
| ENSMUSG00000066760 | protein_coding | Psg16 | 4.854985 | 3.779404 | 4.317195 |
| ENSMUSG00000035247 | protein_coding | Hectd1 | 6447.083 | 6293.54 | 6370.311 |
| ENSMUSG00000058905 | pseudogene | Gm10051 | 6.782801 | 8.238141 | 7.510471 |
| ENSMUSG00000090484 | lincRNA | Gm17417 | 2.600214 | 1.703922 | 2.152068 |
| ENSMUSG00000024256 | protein_coding | Adcyap1 | 3.213945 | 2.383978 | 2.798962 |

| | | | | | |
|---|---|---|---|---|---|
| ENSMUSG00000031577 | protein_coding | BC019943 | 843.957 | 817.9399 | 830.9484 |
| ENSMUSG00000076998 | miRNA | AC122386.1 | 3.222145 | 2.330284 | 2.776215 |
| ENSMUSG00000034636 | protein_coding | Zyg11b | 3490.391 | 3404.687 | 3447.539 |
| ENSMUSG00000055114 | protein_coding | Anxa13 | 13.35031 | 11.14467 | 12.24749 |
| ENSMUSG00000073139 | protein_coding | BC023829 | 979.6699 | 953.1933 | 966.4316 |
| ENSMUSG00000078667 | protein_coding | 1700094D03Rik | 100.3654 | 91.17144 | 95.76841 |
| ENSMUSG00000093176 | miRNA | AL591205.1 | 16.27264 | 18.85197 | 17.56231 |
| ENSMUSG00000063130 | protein_coding | Calml3 | 1.598773 | 2.348303 | 1.973538 |
| ENSMUSG00000054951 | protein_coding | 9130008F23Rik | 23.20975 | 20.7307 | 21.97022 |
| ENSMUSG00000046580 | pseudogene | Gm7862 | 20.72814 | 23.21306 | 21.9706 |
| ENSMUSG00000043461 | protein_coding | 1110032A04Rik | 1.937283 | 2.683826 | 2.310555 |
| ENSMUSG00000086690 | processed_transcrip | 4933406J10Rik | 1.937283 | 2.683826 | 2.310555 |
| ENSMUSG00000069682 | pseudogene | Gm10275 | 1167.274 | 1134.042 | 1150.658 |
| ENSMUSG00000071658 | protein_coding | Gng3 | 30.49088 | 27.10705 | 28.79897 |
| ENSMUSG00000037990 | protein_coding | Sh3rf3 | 110.2544 | 103.6376 | 106.946 |
| ENSMUSG00000032849 | protein_coding | Abcc4 | 548.4512 | 575.4094 | 561.9303 |
| ENSMUSG00000041773 | protein_coding | Enc1 | 2931.784 | 2841.812 | 2886.798 |
| ENSMUSG00000052298 | protein_coding | Cdc42se2 | 4898.082 | 4797.818 | 4847.95 |
| ENSMUSG00000054520 | protein_coding | Sh3bp2 | 582.0833 | 563.3297 | 572.7065 |
| ENSMUSG00000072968 | protein_coding | Gm17728 | 1.951372 | 2.701483 | 2.326427 |
| ENSMUSG00000021939 | protein_coding | Ctsb | 6088.841 | 6241.386 | 6165.113 |
| ENSMUSG00000022428 | protein_coding | Cby1 | 815.363 | 787.5098 | 801.4364 |
| ENSMUSG00000052658 | protein_coding | 5830454E08Rik | 26.10054 | 23.45885 | 24.7797 |
| ENSMUSG00000088119 | snRNA | U6 | 6.427891 | 7.840277 | 7.134084 |
| ENSMUSG00000036707 | protein_coding | Cab39 | 3683.674 | 3761.019 | 3722.347 |
| ENSMUSG00000079334 | protein_coding | Nat6 | 129.209 | 138.4837 | 133.8464 |
| ENSMUSG00000037300 | protein_coding | Ttc13 | 1746.883 | 1705.455 | 1726.169 |
| ENSMUSG00000063919 | protein_coding | Srrm4 | 203.4549 | 217.7685 | 210.6117 |
| ENSMUSG00000045055 | pseudogene | Rpsa-ps2 | 20.06163 | 17.79207 | 18.92685 |
| ENSMUSG00000055078 | protein_coding | Gabra5 | 6.436091 | 5.049424 | 5.742758 |
| ENSMUSG00000020455 | protein_coding | Trim11 | 771.5484 | 795.0563 | 783.3024 |
| ENSMUSG00000087332 | processed_transcrip | Gm12690 | 7.44111 | 6.118698 | 6.779904 |
| ENSMUSG00000024325 | protein_coding | Ring1 | 730.0981 | 754.9587 | 742.5284 |
| ENSMUSG00000027994 | protein_coding | Ccdc109b | 13.828 | 11.84818 | 12.83809 |
| ENSMUSG00000073166 | pseudogene | Gm6950 | 9.976768 | 8.336158 | 9.156463 |
| ENSMUSG00000044645 | protein_coding | Gm7334 | 158.5539 | 199.1911 | 178.8725 |
| ENSMUSG00000074500 | protein_coding | Zfp558 | 231.0381 | 243.4696 | 237.2538 |
| ENSMUSG00000050370 | protein_coding | Ch25h | 6.45018 | 7.929284 | 7.189732 |
| ENSMUSG00000045071 | protein_coding | E130308A19Rik | 1583.975 | 1531.476 | 1557.725 |
| ENSMUSG00000078974 | protein_coding | Sec61g | 509.3811 | 487.299 | 498.3401 |
| ENSMUSG00000021774 | protein_coding | Ube2e1 | 1123.206 | 1095.434 | 1109.32 |
| ENSMUSG00000038135 | protein_coding | Crygn | 11.38023 | 13.41261 | 12.39642 |
| ENSMUSG00000024878 | protein_coding | Cbwd1 | 1002.59 | 1056.081 | 1029.336 |
| ENSMUSG00000087503 | processed_transcrip | Gm11459 | 5.153539 | 4.052225 | 4.602882 |
| ENSMUSG00000083579 | pseudogene | Gm15538 | 1.923194 | 2.683826 | 2.30351 |
| ENSMUSG00000085192 | processed_transcrip | Gm12195 | 4.545696 | 5.899209 | 5.222453 |
| ENSMUSG00000006221 | protein_coding | Hspb7 | 63.73227 | 69.00566 | 66.36897 |
| ENSMUSG00000086433 | processed_transcrip | 1700006D18Rik | 1.304841 | 0.724379 | 1.01461 |
| ENSMUSG00000052707 | protein_coding | Tnrc6a | 4477.644 | 4578.159 | 4527.902 |
| ENSMUSG00000026918 | protein_coding | Brd3 | 4088.018 | 3962.109 | 4025.064 |
| ENSMUSG00000062825 | protein_coding | Actg1 | 13934.06 | 14311.17 | 14122.61 |
| ENSMUSG00000061118 | protein_coding | Dnajc30 | 256.1353 | 265.8332 | 260.9842 |
| ENSMUSG00000049739 | protein_coding | Zfp646 | 2093.386 | 2035.067 | 2064.226 |
| ENSMUSG00000029923 | protein_coding | Rab19 | 26.81983 | 29.66764 | 28.24373 |
| ENSMUSG00000021557 | protein_coding | Agtpbp1 | 1178.371 | 1146.248 | 1162.31 |
| ENSMUSG00000025968 | protein_coding | Ndufs1 | 2836.55 | 2899.972 | 2868.261 |
| ENSMUSG00000091685 | protein_coding | Gm17359 | 5.173517 | 4.043215 | 4.608366 |
| ENSMUSG00000083306 | pseudogene | Gm13868 | 2.583814 | 3.470547 | 3.02718 |

| | | | | | |
|---|---|---|---|---|---|
| ENSMUSG00000028563 | protein_coding | Tm2d1 | 672.0375 | 700.8808 | 686.4591 |
| ENSMUSG00000086241 | lincRNA | 4930483K19Rik | 1.959572 | 2.763824 | 2.361698 |
| ENSMUSG00000002688 | protein_coding | Prkd1 | 1148.871 | 1178.626 | 1163.748 |
| ENSMUSG00000020000 | protein_coding | Moxd1 | 18.01879 | 15.82362 | 16.9212 |
| ENSMUSG00000081597 | pseudogene | Gm14506 | 2.247615 | 3.117366 | 2.682491 |
| ENSMUSG00000010751 | protein_coding | Tnfrsf22 | 40.04461 | 36.78354 | 38.41407 |
| ENSMUSG00000087128 | lincRNA | Gm12655 | 54.21008 | 58.43501 | 56.32254 |
| ENSMUSG00000028629 | protein_coding | Dem1 | 540.7193 | 514.4264 | 527.5728 |
| ENSMUSG00000033082 | protein_coding | Clec1a | 20.07698 | 22.56868 | 21.32283 |
| ENSMUSG00000061288 | protein_coding | Taok3 | 996.5332 | 963.9182 | 980.2257 |
| ENSMUSG00000043583 | protein_coding | 4930470P17Rik | 15.21969 | 17.35853 | 16.28911 |
| ENSMUSG00000034786 | protein_coding | Gpsm3 | 202.6937 | 193.5243 | 198.109 |
| ENSMUSG00000030101 | protein_coding | Sumf1 | 1365.119 | 1399.215 | 1382.167 |
| ENSMUSG00000002580 | protein_coding | 1810046J19Rik | 229.8163 | 220.8392 | 225.3278 |
| ENSMUSG00000025366 | protein_coding | Esyt1 | 415.4142 | 430.7216 | 423.0679 |
| ENSMUSG00000081934 | pseudogene | Gm12193 | 1.637461 | 2.419653 | 2.028557 |
| ENSMUSG00000074999 | protein_coding | Gm10797 | 1.606973 | 1.033237 | 1.320105 |
| ENSMUSG00000068196 | protein_coding | Col8a1 | 26.69892 | 29.55124 | 28.12508 |
| ENSMUSG00000025077 | protein_coding | Dclre1a | 492.5003 | 475.9469 | 484.2236 |
| ENSMUSG00000016206 | protein_coding | H2-M3 | 88.21257 | 94.02636 | 91.11946 |
| ENSMUSG00000085798 | processed_transcrip | Gm2018 | 1.31304 | 0.724379 | 1.01871 |
| ENSMUSG00000024669 | protein_coding | Cd5 | 1.31304 | 0.724379 | 1.01871 |
| ENSMUSG00000021578 | protein_coding | Ccdc127 | 1387.444 | 1425.016 | 1406.23 |
| ENSMUSG00000027894 | protein_coding | Slc6a17 | 93.73704 | 99.74253 | 96.73979 |
| ENSMUSG00000060467 | pseudogene | Gm10080 | 5.187606 | 4.105918 | 4.646762 |
| ENSMUSG00000020472 | protein_coding | Zkscan17 | 859.4991 | 884.6763 | 872.0877 |
| ENSMUSG00000018862 | protein_coding | Otop3 | 2.261704 | 3.126014 | 2.693859 |
| ENSMUSG00000049037 | protein_coding | Clec4a1 | 32.3285 | 29.34941 | 30.83896 |
| ENSMUSG00000087406 | lincRNA | E130215H24Rik | 7.745553 | 9.307053 | 8.526303 |
| ENSMUSG00000064494 | snRNA | U6 | 2.900035 | 2.066111 | 2.483073 |
| ENSMUSG00000061808 | protein_coding | Ttr | 2.312171 | 1.36876 | 1.840465 |
| ENSMUSG00000021288 | protein_coding | Klc1 | 1610.838 | 1575.23 | 1593.034 |
| ENSMUSG00000022674 | protein_coding | Ube2v2 | 1131.389 | 1166.632 | 1149.011 |
| ENSMUSG00000091393 | protein_coding | 5330438I03Rik | 16.1307 | 13.85303 | 14.99187 |
| ENSMUSG00000082377 | pseudogene | Gm14415 | 2.549747 | 1.739597 | 2.144672 |
| ENSMUSG00000086102 | lincRNA | Gm16885 | 1.931394 | 2.683826 | 2.30761 |
| ENSMUSG00000085412 | processed_transcrip | Gm15055 | 7.122578 | 8.573665 | 7.848122 |
| ENSMUSG00000069539 | protein_coding | Scyl2 | 1409.042 | 1448.608 | 1428.825 |
| ENSMUSG00000091919 | lincRNA | Gm17470 | 3.213945 | 2.330284 | 2.772115 |
| ENSMUSG00000028785 | protein_coding | Hpca | 40.70269 | 37.09561 | 38.89915 |
| ENSMUSG00000035885 | protein_coding | Cox8a | 1747.116 | 1797.847 | 1772.482 |
| ENSMUSG00000090879 | lincRNA | Gm17721 | 2.259393 | 3.046016 | 2.652704 |
| ENSMUSG00000032375 | protein_coding | Aph1b | 377.1181 | 395.3487 | 386.2334 |
| ENSMUSG00000063235 | protein_coding | Ptpmt1 | 236.5935 | 246.5794 | 241.5864 |
| ENSMUSG00000071749 | protein_coding | 4933412E24Rik | 12.61232 | 10.78828 | 11.7003 |
| ENSMUSG00000027317 | protein_coding | Ppp1r14d | 17.47297 | 20.29965 | 18.88631 |
| ENSMUSG00000001520 | protein_coding | Nrip2 | 215.5386 | 203.4136 | 209.4761 |
| ENSMUSG00000065387 | rRNA | n-R5s85 | 3.272612 | 2.374969 | 2.823791 |
| ENSMUSG00000071855 | protein_coding | Ccdc112 | 683.9367 | 663.0818 | 673.5092 |
| ENSMUSG00000079009 | protein_coding | Gm14139 | 102.8715 | 108.8803 | 105.8759 |
| ENSMUSG00000031906 | protein_coding | Smpd3 | 176.2209 | 148.7308 | 162.4758 |
| ENSMUSG00000079224 | pseudogene | Gm6565 | 27.84378 | 24.98797 | 26.41587 |
| ENSMUSG00000055882 | protein_coding | Abhd16b | 9.381971 | 11.20488 | 10.29343 |
| ENSMUSG00000017299 | protein_coding | Dnttip1 | 979.5351 | 948.4504 | 963.9927 |
| ENSMUSG00000020614 | protein_coding | Fam20a | 15.54157 | 13.38667 | 14.46412 |
| ENSMUSG00000082762 | pseudogene | Gm12366 | 16.83255 | 14.71038 | 15.77147 |
| ENSMUSG00000086815 | protein_coding | 3110082J24Rik | 8.766972 | 7.1786 | 7.972786 |
| ENSMUSG00000036905 | protein_coding | C1qb | 409.8568 | 426.3164 | 418.0866 |

| | | | | | |
|---|---|---|---|---|---|
| ENSMUSG00000085269 | processed_transcrip | Gm15777 | 163.0772 | 156.0008 | 159.539 |
| ENSMUSG00000041907 | protein_coding | Gpr45 | 1.307151 | 0.724379 | 1.015765 |
| ENSMUSG00000038980 | protein_coding | BC066135 | 1.307151 | 0.724379 | 1.015765 |
| ENSMUSG00000086188 | processed_transcrip | Gm15169 | 1.307151 | 0.724379 | 1.015765 |
| ENSMUSG00000008384 | protein_coding | Sertad1 | 494.8475 | 479.2654 | 487.0564 |
| ENSMUSG00000084768 | processed_transcrip | Gm13605 | 1.621062 | 1.050893 | 1.335978 |
| ENSMUSG00000080573 | miRNA | Mir467f | 1.621062 | 1.050893 | 1.335978 |
| ENSMUSG00000064356 | protein_coding | mt-Atp8 | 58.11662 | 53.16937 | 55.64299 |
| ENSMUSG00000002732 | protein_coding | Fkbp7 | 368.4603 | 353.9138 | 361.1871 |
| ENSMUSG00000026229 | protein_coding | Psmd1 | 4560.038 | 4445.542 | 4502.79 |
| ENSMUSG00000032312 | protein_coding | Csk | 1531.921 | 1569.975 | 1550.948 |
| ENSMUSG00000031962 | protein_coding | Cdh15 | 125.7727 | 118.9924 | 122.3825 |
| ENSMUSG00000050541 | protein_coding | Adra1b | 6.144469 | 4.865611 | 5.50504 |
| ENSMUSG00000065476 | miRNA | Mir30b | 1.293062 | 0.724379 | 1.008721 |
| ENSMUSG00000015093 | protein_coding | Clic3 | 32.9069 | 35.99682 | 34.45186 |
| ENSMUSG00000071584 | protein_coding | Atf7 | 246.9796 | 259.8103 | 253.3949 |
| ENSMUSG00000027936 | protein_coding | Crtc2 | 1234.833 | 1272.823 | 1253.828 |
| ENSMUSG00000050029 | protein_coding | Rap2c | 2116.421 | 2067.965 | 2092.193 |
| ENSMUSG00000030847 | protein_coding | Bag3 | 408.9735 | 423.6867 | 416.3301 |
| ENSMUSG00000020123 | protein_coding | Avpr1a | 127.7583 | 120.6789 | 124.2186 |
| ENSMUSG00000090984 | lincRNA | Gm17276 | 10.27659 | 12.13866 | 11.20762 |
| ENSMUSG00000060708 | protein_coding | Cno | 582.4929 | 549.8514 | 566.1722 |
| ENSMUSG00000086992 | processed_transcrip | Gm15941 | 1.612862 | 1.033237 | 1.323049 |
| ENSMUSG00000059974 | protein_coding | Ntm | 26.77652 | 24.02323 | 25.39988 |
| ENSMUSG00000007216 | protein_coding | Zfp775 | 154.4015 | 146.4999 | 150.4507 |
| ENSMUSG00000081895 | pseudogene | Gm10294 | 312.8456 | 300.4267 | 306.6361 |
| ENSMUSG00000060594 | protein_coding | Layn | 93.55724 | 100.3152 | 96.93621 |
| ENSMUSG00000036390 | protein_coding | Gadd45a | 476.1482 | 460.4377 | 468.293 |
| ENSMUSG00000046387 | protein_coding | Pcdhb17 | 82.81787 | 87.91491 | 85.36639 |
| ENSMUSG00000032382 | protein_coding | Snx1 | 2183.196 | 2137.321 | 2160.258 |
| ENSMUSG00000085738 | pseudogene | Gm12335 | 2.267593 | 3.046016 | 2.656804 |
| ENSMUSG00000055639 | protein_coding | Dach1 | 20.60238 | 23.15035 | 21.87637 |
| ENSMUSG00000038722 | protein_coding | Bud31 | 1765.648 | 1814.857 | 1790.253 |
| ENSMUSG00000081024 | pseudogene | Gm14627 | 15.15029 | 13.21694 | 14.18361 |
| ENSMUSG00000043964 | protein_coding | Orai3 | 425.0751 | 439.4867 | 432.2809 |
| ENSMUSG00000001518 | protein_coding | Itfg2 | 560.3373 | 578.4114 | 569.3744 |
| ENSMUSG00000085655 | processed_transcrip | Gm15952 | 15.54284 | 17.60469 | 16.57376 |
| ENSMUSG00000007850 | protein_coding | Hnrnph1 | 15769.63 | 15241.9 | 15505.77 |
| ENSMUSG00000056380 | protein_coding | Gpr50 | 36.79533 | 33.36488 | 35.0801 |
| ENSMUSG00000052389 | protein_coding | Gm9875 | 2.905924 | 2.093139 | 2.499532 |
| ENSMUSG00000027091 | protein_coding | Zc3h15 | 4518.552 | 4618.154 | 4568.353 |
| ENSMUSG00000050074 | protein_coding | Spink8 | 7.746821 | 6.270408 | 7.008614 |
| ENSMUSG00000077549 | snoRNA | Snord71 | 8.415641 | 10.04945 | 9.232546 |
| ENSMUSG00000044854 | protein_coding | 1700056E22Rik | 6.792268 | 5.545667 | 6.168968 |
| ENSMUSG00000050490 | protein_coding | Gm8394 | 116.7861 | 111.0916 | 113.9389 |
| ENSMUSG00000076435 | protein_coding | Acsf2 | 933.2148 | 968.3048 | 950.7598 |
| ENSMUSG00000083424 | pseudogene | Rpl35a-ps4 | 1.618751 | 1.024227 | 1.321489 |
| ENSMUSG00000070577 | protein_coding | Gm572 | 1.618751 | 1.024227 | 1.321489 |
| ENSMUSG00000092384 | processed_transcrip | RP24-341K21.6 | 6.841467 | 5.331977 | 6.086722 |
| ENSMUSG00000023284 | protein_coding | Zfp605 | 385.8729 | 401.3582 | 393.6155 |
| ENSMUSG00000058163 | protein_coding | Gm5431 | 8.359285 | 9.89774 | 9.128513 |
| ENSMUSG00000028020 | protein_coding | Glrb | 60.62723 | 55.51342 | 58.07032 |
| ENSMUSG00000001270 | protein_coding | Ckb | 492.8971 | 511.7113 | 502.3042 |
| ENSMUSG00000066382 | protein_coding | Iqcf5 | 1.629262 | 1.050893 | 1.340077 |
| ENSMUSG00000085917 | pseudogene | Gm15899 | 5.13945 | 4.070243 | 4.604847 |
| ENSMUSG00000022220 | protein_coding | Adcy4 | 53.31732 | 49.40907 | 51.36319 |
| ENSMUSG00000028833 | protein_coding | Ncdn | 1274.306 | 1234.943 | 1254.625 |
| ENSMUSG00000000402 | protein_coding | Egfl6 | 67.51261 | 72.62828 | 70.07045 |

| | | | | | |
|---|---|---|---|---|---|
| ENSMUSG00000042515 | protein_coding | Mum1l1 | 9865.109 | 9428.179 | 9646.644 |
| ENSMUSG00000081340 | pseudogene | Gm12275 | 3.256212 | 2.410644 | 2.833428 |
| ENSMUSG00000022849 | protein_coding | Hspbap1 | 718.5568 | 694.0525 | 706.3046 |
| ENSMUSG00000053317 | protein_coding | Sec61b | 828.6837 | 857.9479 | 843.3158 |
| ENSMUSG00000027180 | protein_coding | Fbxo3 | 2805.301 | 2875.711 | 2840.506 |
| ENSMUSG00000083049 | pseudogene | Gm14822 | 1.604662 | 2.36596 | 1.985311 |
| ENSMUSG00000085852 | processed_transcrip | Gm13807 | 27.92682 | 31.16036 | 29.54359 |
| ENSMUSG00000028211 | protein_coding | Trp53inp1 | 3416.862 | 3305.554 | 3361.208 |
| ENSMUSG00000026360 | protein_coding | Rgs2 | 3256.47 | 3409.989 | 3333.23 |
| ENSMUSG00000058093 | protein_coding | AA987161 | 1065.911 | 1022.816 | 1044.364 |
| ENSMUSG00000073886 | protein_coding | Gm10600 | 2.283993 | 3.621896 | 2.952944 |
| ENSMUSG00000037406 | protein_coding | Htra4 | 26.80701 | 29.71232 | 28.25967 |
| ENSMUSG00000026819 | protein_coding | Slc25a25 | 965.9045 | 1007.158 | 986.5315 |
| ENSMUSG00000038204 | protein_coding | Asb10 | 35.04538 | 31.58276 | 33.31407 |
| ENSMUSG00000039529 | protein_coding | Atp8b1 | 72.9805 | 78.00531 | 75.4929 |
| ENSMUSG00000079562 | protein_coding | Maea | 3340.547 | 3424.037 | 3382.292 |
| ENSMUSG00000030219 | protein_coding | Erp27 | 1.621062 | 1.059903 | 1.340482 |
| ENSMUSG00000065360 | snoRNA | SNORA32 | 2.620192 | 1.784282 | 2.202237 |
| ENSMUSG00000049173 | protein_coding | Myoz3 | 4.860874 | 3.707692 | 4.284283 |
| ENSMUSG00000027208 | protein_coding | Fgf7 | 48.59793 | 52.66085 | 50.62939 |
| ENSMUSG00000018543 | protein_coding | 1700001P01Rik | 3.191657 | 4.352435 | 3.772046 |
| ENSMUSG00000056880 | protein_coding | Gadl1 | 9.378393 | 7.929646 | 8.654019 |
| ENSMUSG00000061911 | protein_coding | Myt1l | 260.0684 | 242.0565 | 251.0625 |
| ENSMUSG00000026917 | protein_coding | Wdr5 | 3857.073 | 3944.136 | 3900.604 |
| ENSMUSG00000087666 | protein_coding | B230359F08Rik | 3.199857 | 2.348303 | 2.77408 |
| ENSMUSG00000047197 | protein_coding | Gjd3 | 19.78767 | 22.45585 | 21.12176 |
| ENSMUSG00000056468 | protein_coding | 5730596B20Rik | 1.949061 | 2.772834 | 2.360947 |
| ENSMUSG00000040121 | protein_coding | Rep15 | 17.46372 | 19.83224 | 18.64798 |
| ENSMUSG00000091139 | protein_coding | Gm6190 | 6.752312 | 5.528011 | 6.140161 |
| ENSMUSG00000083506 | pseudogene | Gm13993 | 0.99093 | 1.704284 | 1.347607 |
| ENSMUSG00000014226 | protein_coding | Cacybp | 4826.552 | 4954.952 | 4890.752 |
| ENSMUSG00000089994 | processed_transcrip | Gm16239 | 2.575614 | 3.399196 | 2.987405 |
| ENSMUSG00000086068 | processed_transcrip | 1700120G11Rik | 2.573303 | 1.784282 | 2.178793 |
| ENSMUSG00000031554 | protein_coding | Adam5 | 28.41733 | 25.75739 | 27.08736 |
| ENSMUSG00000064141 | protein_coding | Zfp69 | 157.1062 | 148.2518 | 152.679 |
| ENSMUSG00000044719 | protein_coding | E230025N22Rik | 14.24516 | 16.81502 | 15.53009 |
| ENSMUSG00000048310 | protein_coding | Pskh1 | 1958.849 | 2010.983 | 1984.916 |
| ENSMUSG00000091824 | protein_coding | Gm6553 | 1.606973 | 1.050893 | 1.328933 |
| ENSMUSG00000084975 | lincRNA | Gm16890 | 2.275793 | 3.081691 | 2.678742 |
| ENSMUSG00000053769 | protein_coding | Lysmd1 | 177.7275 | 169.9511 | 173.8393 |
| ENSMUSG00000004018 | protein_coding | Fancl | 402.6278 | 387.1211 | 394.8745 |
| ENSMUSG00000029335 | protein_coding | Bmp3 | 1.601084 | 1.033237 | 1.31716 |
| ENSMUSG00000042029 | protein_coding | Ncapg2 | 3270.111 | 3395.413 | 3332.762 |
| ENSMUSG00000020544 | protein_coding | Cox11 | 538.7176 | 558.0018 | 548.3597 |
| ENSMUSG00000085607 | lincRNA | Gm16854 | 1380.336 | 1421.354 | 1400.845 |
| ENSMUSG00000051592 | protein_coding | Ccnb3 | 915.2162 | 1120.177 | 1017.697 |
| ENSMUSG00000083849 | pseudogene | Gm13477 | 5.193495 | 4.123575 | 4.658535 |
| ENSMUSG00000048574 | pseudogene | Gm5593 | 32.54968 | 29.52422 | 31.03695 |
| ENSMUSG00000071415 | protein_coding | Rpl23 | 4881.635 | 5005.905 | 4943.77 |
| ENSMUSG00000021520 | protein_coding | Uqcrb | 1223.012 | 1260.871 | 1241.941 |
| ENSMUSG00000079396 | protein_coding | Gm5796 | 5.842337 | 7.125268 | 6.483803 |
| ENSMUSG00000085037 | lincRNA | 4933421O10Rik | 254.836 | 269.4257 | 262.1309 |
| ENSMUSG00000015747 | protein_coding | Vps45 | 540.5372 | 523.49 | 532.0136 |
| ENSMUSG00000081406 | pseudogene | Gm13654 | 520.1495 | 535.5619 | 527.8557 |
| ENSMUSG00000021338 | protein_coding | Lrrc16a | 2977.418 | 3067.095 | 3022.257 |
| ENSMUSG00000055396 | pseudogene | D830050J10Rik | 7.087243 | 8.582674 | 7.834959 |
| ENSMUSG00000018585 | protein_coding | Atox1 | 620.9966 | 602.054 | 611.5253 |
| ENSMUSG00000020805 | protein_coding | Slc13a5 | 1.945483 | 2.692474 | 2.318978 |

| | | | | | |
|---|---|---|---|---|---|
| ENSMUSG00000062554 | pseudogene | Gm12751 | 273.1824 | 347.5777 | 310.38 |
| ENSMUSG00000066113 | protein_coding | Adamtsl1 | 45.93674 | 42.13602 | 44.03638 |
| ENSMUSG00000031993 | protein_coding | Snx19 | 1272.48 | 1305.802 | 1289.141 |
| ENSMUSG00000026797 | protein_coding | Stxbp1 | 1316.359 | 1253.149 | 1284.754 |
| ENSMUSG00000070167 | snoRNA | SNORA57 | 35.15936 | 38.8832 | 37.02128 |
| ENSMUSG00000085168 | processed_transcrip | Gm16252 | 3.230345 | 2.419653 | 2.824999 |
| ENSMUSG00000042472 | protein_coding | Zfp410 | 1925.946 | 1884.949 | 1905.448 |
| ENSMUSG00000084105 | pseudogene | Gm13252 | 1.629262 | 1.059903 | 1.344582 |
| ENSMUSG00000082419 | pseudogene | Gm11425 | 1.629262 | 1.059903 | 1.344582 |
| ENSMUSG00000020823 | protein_coding | Sec14l1 | 2416.695 | 2508.147 | 2462.421 |
| ENSMUSG00000030898 | protein_coding | Cckbr | 4.847829 | 3.716701 | 4.282265 |
| ENSMUSG00000006641 | protein_coding | Slc5a6 | 553.6998 | 578.7871 | 566.2434 |
| ENSMUSG00000092130 | protein_coding | D030025P21Rik | 4.854985 | 6.029329 | 5.442157 |
| ENSMUSG00000000617 | protein_coding | Grm6 | 1.293062 | 1.995123 | 1.644092 |
| ENSMUSG00000024515 | protein_coding | Smad4 | 2495.321 | 2555.126 | 2525.223 |
| ENSMUSG00000078601 | processed_transcrip | Gm12525 | 1.293062 | 1.986113 | 1.639588 |
| ENSMUSG00000037989 | protein_coding | Wnk2 | 89.64361 | 83.40093 | 86.52227 |
| ENSMUSG00000009907 | protein_coding | Vps4b | 3108.17 | 3181.731 | 3144.95 |
| ENSMUSG00000079076 | protein_coding | Gm3086 | 10.00032 | 11.68238 | 10.84135 |
| ENSMUSG00000053214 | pseudogene | Gm9899 | 6.778179 | 5.510354 | 6.144267 |
| ENSMUSG00000024650 | protein_coding | Slc22a6 | 8.224953 | 5.768367 | 6.99666 |
| ENSMUSG00000086160 | processed_transcrip | 4933438A12Rik | 2.609681 | 1.757616 | 2.183648 |
| ENSMUSG00000021253 | protein_coding | Tgfb3 | 1630.796 | 1693.047 | 1661.921 |
| ENSMUSG00000030225 | protein_coding | Dera | 812.604 | 841.4913 | 827.0476 |
| ENSMUSG00000084792 | processed_transcrip | 1700056N10Rik | 6.856824 | 5.420985 | 6.138904 |
| ENSMUSG00000010136 | protein_coding | 1700027A23Rik | 3.566544 | 2.710492 | 3.138518 |
| ENSMUSG00000089767 | processed_transcrip | 4930556I23Rik | 2.569725 | 3.417215 | 2.99347 |
| ENSMUSG00000063576 | protein_coding | Klhdc3 | 4067.332 | 4151.86 | 4109.596 |
| ENSMUSG00000091198 | protein_coding | Gm5218 | 3.233924 | 2.419653 | 2.826789 |
| ENSMUSG00000030170 | protein_coding | Wnt5b | 271.6492 | 283.0806 | 277.3649 |
| ENSMUSG00000028106 | protein_coding | Rprd2 | 2252.823 | 2312.188 | 2282.505 |
| ENSMUSG00000042293 | protein_coding | Gm5617 | 213.169 | 221.8342 | 217.5016 |
| ENSMUSG00000000942 | protein_coding | Hoxa4 | 12.63334 | 14.62065 | 13.62699 |
| ENSMUSG00000032625 | protein_coding | Thsd7a | 24.77572 | 27.43108 | 26.1034 |
| ENSMUSG00000034271 | protein_coding | Jdp2 | 206.8887 | 217.9843 | 212.4365 |
| ENSMUSG00000008035 | protein_coding | Mid1ip1 | 2625.963 | 2705.12 | 2665.541 |
| ENSMUSG00000086031 | lincRNA | Gm12348 | 1.612862 | 0.979543 | 1.296202 |
| ENSMUSG00000033538 | protein_coding | Casp4 | 18.45003 | 16.31629 | 17.38316 |
| ENSMUSG00000026975 | protein_coding | Wdr85 | 471.9498 | 486.1747 | 479.0622 |
| ENSMUSG00000035296 | protein_coding | Sgcg | 6.502957 | 5.15645 | 5.829704 |
| ENSMUSG00000029125 | protein_coding | Stx18 | 439.1592 | 426.0884 | 432.6238 |
| ENSMUSG00000027956 | protein_coding | Tmem144 | 455.2917 | 473.9825 | 464.6371 |
| ENSMUSG00000036896 | protein_coding | C1qc | 414.8545 | 398.9465 | 406.9005 |
| ENSMUSG00000078894 | protein_coding | 2210418O10Rik | 1.606973 | 1.059903 | 1.333438 |
| ENSMUSG00000054280 | protein_coding | C330019G07Rik | 2531.025 | 2602.777 | 2566.901 |
| ENSMUSG00000039285 | protein_coding | Azi2 | 2219.82 | 2173.992 | 2196.906 |
| ENSMUSG00000083718 | processed_transcrip | Ccnb2-ps | 102.3486 | 109.5942 | 105.9714 |
| ENSMUSG00000088474 | misc_RNA | 7SK | 2.891835 | 2.093139 | 2.492487 |
| ENSMUSG00000078875 | protein_coding | Gm14419 | 4.851407 | 6.33891 | 5.595158 |
| ENSMUSG00000049314 | protein_coding | Fbxw13 | 10.0203 | 8.466639 | 9.243471 |
| ENSMUSG00000063652 | protein_coding | Slc22a21 | 746.4438 | 709.89 | 728.1669 |
| ENSMUSG00000066148 | protein_coding | Prpf4 | 1355.683 | 1387.794 | 1371.739 |
| ENSMUSG00000035232 | protein_coding | Pdk3 | 2764.484 | 2702.151 | 2733.317 |
| ENSMUSG00000089945 | protein_coding | AF064781 | 2.237104 | 3.135023 | 2.686064 |
| ENSMUSG00000022052 | protein_coding | Ppp2r2a | 2956.404 | 3032.817 | 2994.611 |
| ENSMUSG00000034875 | protein_coding | Nudt19 | 1607.877 | 1549.988 | 1578.932 |
| ENSMUSG00000038304 | protein_coding | Cd160 | 30.74613 | 27.7039 | 29.22501 |
| ENSMUSG00000083757 | pseudogene | Gm13253 | 5.509716 | 4.343064 | 4.92639 |

| | | | | | |
|---|---|---|---|---|---|
| ENSMUSG00000058935 | protein_coding | 1700018C11Rik | 1.925505 | 2.719502 | 2.322503 |
| ENSMUSG00000090031 | processed_transcrip | 4732440D04Rik | 50.76064 | 55.00191 | 52.88127 |
| ENSMUSG00000014075 | protein_coding | Tctex1d2 | 463.8071 | 482.3111 | 473.0591 |
| ENSMUSG00000049928 | protein_coding | Glp2r | 10.32117 | 12.10407 | 11.21262 |
| ENSMUSG00000079410 | protein_coding | Gm2897 | 16.09917 | 13.71789 | 14.90853 |
| ENSMUSG00000042514 | protein_coding | Klhl14 | 65.10777 | 70.74815 | 67.92796 |
| ENSMUSG00000033991 | protein_coding | Ttc37 | 1273.542 | 1232.851 | 1253.197 |
| ENSMUSG00000038751 | protein_coding | Ptk6 | 6.768712 | 5.554677 | 6.161694 |
| ENSMUSG00000081745 | pseudogene | Gm15549 | 1.595194 | 1.024227 | 1.309711 |
| ENSMUSG00000087327 | processed_transcrip | Gm15884 | 4.557475 | 5.792545 | 5.17501 |
| ENSMUSG00000021679 | protein_coding | S100z | 40.33898 | 35.99858 | 38.16878 |
| ENSMUSG00000084050 | pseudogene | Gm14439 | 3.832299 | 5.023482 | 4.42789 |
| ENSMUSG00000071037 | protein_coding | 1700106N22Rik | 249.3278 | 264.0185 | 256.6731 |
| ENSMUSG00000025498 | protein_coding | Irf7 | 131.2797 | 141.3761 | 136.3279 |
| ENSMUSG00000072754 | protein_coding | Gm1679 | 3.238545 | 2.401635 | 2.82009 |
| ENSMUSG00000026523 | protein_coding | Wdr64 | 4.559785 | 5.854163 | 5.206974 |
| ENSMUSG00000050122 | protein_coding | Vwa3b | 9.700503 | 11.28452 | 10.49251 |
| ENSMUSG00000079056 | protein_coding | Kcnip3 | 586.2932 | 570.1192 | 578.2062 |
| ENSMUSG00000068747 | protein_coding | Sort1 | 3006.242 | 3079.953 | 3043.097 |
| ENSMUSG00000005677 | protein_coding | Nr1i3 | 6.436091 | 7.751631 | 7.093861 |
| ENSMUSG00000049699 | protein_coding | Ucn2 | 1.635151 | 1.015218 | 1.325184 |
| ENSMUSG00000022651 | protein_coding | Retnlg | 1.635151 | 1.015218 | 1.325184 |
| ENSMUSG00000083836 | pseudogene | Ldha-ps2 | 11.32976 | 9.704918 | 10.51734 |
| ENSMUSG00000027323 | protein_coding | Rad51 | 1856.002 | 1806.416 | 1831.209 |
| ENSMUSG00000016526 | protein_coding | Dyrk3 | 262.5878 | 251.9991 | 257.2934 |
| ENSMUSG00000087397 | processed_transcrip | Gm16256 | 2.281682 | 3.063673 | 2.672677 |
| ENSMUSG00000020323 | protein_coding | Prss57 | 2.891835 | 3.761386 | 3.326611 |
| ENSMUSG00000033955 | protein_coding | Tnks1bp1 | 1888.862 | 1935.786 | 1912.324 |
| ENSMUSG00000056612 | protein_coding | Ppp1r14b | 775.5152 | 753.7735 | 764.6444 |
| ENSMUSG00000085554 | lincRNA | AI854517 | 178.1169 | 169.234 | 173.6755 |
| ENSMUSG00000038507 | protein_coding | Parp12 | 2433.587 | 2319.866 | 2376.726 |
| ENSMUSG00000028756 | protein_coding | Pink1 | 2306.89 | 2232.43 | 2269.66 |
| ENSMUSG00000021185 | protein_coding | 9030617O03Rik | 97.07123 | 91.81071 | 94.44097 |
| ENSMUSG00000086162 | pseudogene | Gm16342 | 2.905924 | 3.761386 | 3.333655 |
| ENSMUSG00000043061 | protein_coding | Tmem18 | 1429.697 | 1385.386 | 1407.541 |
| ENSMUSG00000091810 | lincRNA | Gm17442 | 4.236632 | 5.608009 | 4.92232 |
| ENSMUSG00000090558 | processed_transcrip | Gm17202 | 4.616141 | 6.394729 | 5.505435 |
| ENSMUSG00000034919 | protein_coding | Ttc22 | 6.456069 | 7.805325 | 7.130697 |
| ENSMUSG00000092215 | processed_transcrip | Gm20419 | 34.25356 | 46.96792 | 40.61074 |
| ENSMUSG00000004843 | protein_coding | Chmp2b | 1430.226 | 1393.731 | 1411.979 |
| ENSMUSG00000052482 | pseudogene | Gm8174 | 0.98273 | 1.677618 | 1.330174 |
| ENSMUSG00000025551 | protein_coding | Fgf14 | 27.4264 | 30.71962 | 29.07301 |
| ENSMUSG00000061980 | protein_coding | Dcpp1 | 4.573874 | 5.961189 | 5.267532 |
| ENSMUSG00000060887 | pseudogene | Gm12960 | 220.1686 | 211.6966 | 215.9326 |
| ENSMUSG00000001440 | protein_coding | Kpnb1 | 8577.087 | 8759.27 | 8668.178 |
| ENSMUSG00000060205 | protein_coding | Olfr57 | 1.609283 | 0.997561 | 1.303422 |
| ENSMUSG00000042200 | protein_coding | Cdrt4 | 1.635151 | 2.339294 | 1.987222 |
| ENSMUSG00000054690 | protein_coding | Emcn | 100.3117 | 94.19826 | 97.25498 |
| ENSMUSG00000022747 | protein_coding | St3gal6 | 981.0928 | 1014.015 | 997.5537 |
| ENSMUSG00000092923 | miRNA | BX005039.1 | 7.138978 | 8.484657 | 7.811818 |
| ENSMUSG00000020522 | protein_coding | Mfap3 | 3537.969 | 3632.856 | 3585.413 |
| ENSMUSG00000063316 | protein_coding | Rpl27 | 3582.611 | 3484.028 | 3533.32 |
| ENSMUSG00000079738 | protein_coding | Snx29 | 108.2904 | 102.6361 | 105.4632 |
| ENSMUSG00000029334 | protein_coding | Prkg2 | 19.74078 | 17.3794 | 18.56009 |
| ENSMUSG00000024098 | protein_coding | Twsg1 | 4778.997 | 4921.052 | 4850.025 |
| ENSMUSG00000036949 | protein_coding | Slc39a12 | 3.239813 | 2.401635 | 2.820724 |
| ENSMUSG00000068674 | protein_coding | Gm10247 | 1.270774 | 0.724379 | 0.997576 |
| ENSMUSG00000028247 | protein_coding | Coq3 | 633.7798 | 652.2678 | 643.0238 |

| | | | | | |
|---|---|---|---|---|---|
| ENSMUSG00000046541 | protein_coding | Zfp526 | 358.8299 | 344.808 | 351.819 |
| ENSMUSG00000080797 | pseudogene | Gm15760 | 17.45448 | 15.25602 | 16.35525 |
| ENSMUSG00000021552 | protein_coding | Gkap1 | 2888.1 | 2824.089 | 2856.094 |
| ENSMUSG00000044345 | protein_coding | Marveld1 | 538.8146 | 555.5934 | 547.204 |
| ENSMUSG00000078729 | processed_transcrip | Gm7819 | 46.38282 | 67.09342 | 56.73812 |
| ENSMUSG00000048120 | protein_coding | Entpd1 | 618.8638 | 643.9381 | 631.401 |
| ENSMUSG00000019916 | protein_coding | P4ha1 | 2232.199 | 2290.903 | 2261.551 |
| ENSMUSG00000011254 | protein_coding | Thg1l | 529.3171 | 511.6872 | 520.5021 |
| ENSMUSG00000049744 | protein_coding | Arhgap15 | 37.50433 | 34.47019 | 35.98726 |
| ENSMUSG00000078890 | protein_coding | Gm11007 | 6.10347 | 7.416107 | 6.759789 |
| ENSMUSG00000021219 | protein_coding | Rgs6 | 9.024526 | 10.63113 | 9.827828 |
| ENSMUSG00000029832 | protein_coding | Nfe2l3 | 55.74742 | 51.98007 | 53.86375 |
| ENSMUSG00000024335 | protein_coding | Brd2 | 10085.81 | 9878.972 | 9982.393 |
| ENSMUSG00000038145 | protein_coding | Snrk | 1860.359 | 1907.111 | 1883.735 |
| ENSMUSG00000033257 | protein_coding | Ttll4 | 3662.362 | 3573.127 | 3617.744 |
| ENSMUSG00000063586 | pseudogene | Gm5513 | 1.288441 | 0.653029 | 0.970735 |
| ENSMUSG00000089628 | pseudogene | Gm9727 | 8.053574 | 9.482219 | 8.767896 |
| ENSMUSG00000026773 | protein_coding | Pfkfb3 | 1022.992 | 984.9047 | 1003.948 |
| ENSMUSG00000004961 | protein_coding | Syt5 | 55.45498 | 51.46544 | 53.46021 |
| ENSMUSG00000030034 | protein_coding | Ino80b | 631.1949 | 613.6401 | 622.4175 |
| ENSMUSG00000044887 | pseudogene | Gm4766 | 1.615173 | 1.006571 | 1.310872 |
| ENSMUSG00000032308 | protein_coding | Ulk3 | 488.82 | 503.5834 | 496.2017 |
| ENSMUSG00000088464 | miRNA | Mir1970 | 5.159428 | 4.141594 | 4.650511 |
| ENSMUSG00000038400 | protein_coding | Pmepa1 | 650.0561 | 677.4104 | 663.7332 |
| ENSMUSG00000046798 | protein_coding | Cldn12 | 1344.289 | 1382.871 | 1363.58 |
| ENSMUSG00000004266 | protein_coding | Ptpn6 | 95.02608 | 100.5949 | 97.81047 |
| ENSMUSG00000021078 | protein_coding | Tomm20l | 3.558345 | 2.737158 | 3.147752 |
| ENSMUSG00000046402 | protein_coding | Rbp1 | 1304.058 | 1257.349 | 1280.703 |
| ENSMUSG00000044927 | protein_coding | H1fx | 420.379 | 443.141 | 431.76 |
| ENSMUSG00000022132 | protein_coding | Cldn10 | 2.583814 | 3.390187 | 2.987 |
| ENSMUSG00000027985 | protein_coding | Lef1 | 130.6018 | 140.5381 | 135.5699 |
| ENSMUSG00000026869 | protein_coding | Psmd5 | 2641.928 | 2721.976 | 2681.952 |
| ENSMUSG00000087193 | processed_transcrip | Gm14820 | 3.566544 | 2.737158 | 3.151851 |
| ENSMUSG00000054679 | protein_coding | Srsf12 | 54.79541 | 58.70819 | 56.7518 |
| ENSMUSG00000029205 | protein_coding | Chrna9 | 1.623372 | 1.015218 | 1.319295 |
| ENSMUSG00000091881 | processed_transcrip | Gm17146 | 7.097979 | 5.845516 | 6.471747 |
| ENSMUSG00000033970 | protein_coding | Rfc3 | 821.8053 | 847.4632 | 834.6343 |
| ENSMUSG00000054459 | protein_coding | Vsnl1 | 28.83255 | 32.66648 | 30.74952 |
| ENSMUSG00000044067 | protein_coding | Gpr22 | 1.643351 | 1.015218 | 1.329284 |
| ENSMUSG00000084249 | pseudogene | Gm12267 | 2.261704 | 3.054663 | 2.658184 |
| ENSMUSG00000079298 | protein_coding | Klrb1b | 1.951372 | 1.350742 | 1.651057 |
| ENSMUSG00000086436 | pseudogene | Gm13690 | 11.99858 | 10.22498 | 11.11178 |
| ENSMUSG00000035783 | protein_coding | Acta2 | 9188.606 | 9006.159 | 9097.383 |
| ENSMUSG00000020169 | protein_coding | Best3 | 10.63277 | 12.38838 | 11.51057 |
| ENSMUSG00000026368 | protein_coding | F13b | 0.99093 | 1.810948 | 1.400939 |
| ENSMUSG00000072974 | protein_coding | Gm4787 | 9.025794 | 10.61383 | 9.819814 |
| ENSMUSG00000088334 | misc_RNA | HAR1F | 2.569725 | 3.37253 | 2.971128 |
| ENSMUSG00000060882 | protein_coding | Kcnd2 | 55.17641 | 51.09248 | 53.13444 |
| ENSMUSG00000058503 | protein_coding | Fam133b | 1025.436 | 1053.106 | 1039.271 |
| ENSMUSG00000023224 | protein_coding | Serping1 | 1439.963 | 1489.689 | 1464.826 |
| ENSMUSG00000062929 | protein_coding | Cfl2 | 2786.402 | 2732.936 | 2759.669 |
| ENSMUSG00000028228 | protein_coding | Cpne3 | 1809.484 | 1857.003 | 1833.244 |
| ENSMUSG00000012640 | protein_coding | Zfp715 | 1171.239 | 1206.779 | 1189.009 |
| ENSMUSG00000023882 | protein_coding | Zfp54 | 120.3735 | 129.1458 | 124.7596 |
| ENSMUSG00000085451 | processed_transcrip | Gm15137 | 55.04372 | 59.34936 | 57.19654 |
| ENSMUSG00000040520 | protein_coding | Manea | 1050.088 | 1019.457 | 1034.772 |
| ENSMUSG00000059586 | protein_coding | Nsmce2 | 762.2023 | 742.3307 | 752.2665 |
| ENSMUSG00000029828 | protein_coding | 4921507P07Rik | 212.3381 | 202.5869 | 207.4625 |

| | | | | | |
|---|---|---|---|---|---|
| ENSMUSG00000067424 | protein_coding | Zfp563 | 264.9433 | 253.6993 | 259.3213 |
| ENSMUSG00000030486 | protein_coding | Zfp108 | 146.2612 | 136.6495 | 141.4553 |
| ENSMUSG00000037465 | protein_coding | Klf10 | 1867.809 | 1800.663 | 1834.236 |
| ENSMUSG00000084814 | lincRNA | 9430076G02Rik | 3.199857 | 2.410644 | 2.80525 |
| ENSMUSG00000040372 | protein_coding | Gpr63 | 28.98902 | 26.16106 | 27.57504 |
| ENSMUSG00000021252 | protein_coding | 0610007P14Rik | 1365.195 | 1325.179 | 1345.187 |
| ENSMUSG00000031979 | protein_coding | Cog2 | 1636.675 | 1597.87 | 1617.273 |
| ENSMUSG00000078893 | protein_coding | Gm2004 | 8.329839 | 6.896409 | 7.613124 |
| ENSMUSG00000040078 | pseudogene | Gm9769 | 852.6694 | 877.046 | 864.8577 |
| ENSMUSG00000037242 | protein_coding | Clic4 | 4547.875 | 4410.16 | 4479.018 |
| ENSMUSG00000029422 | protein_coding | Rsrc2 | 3751.698 | 3854.267 | 3802.983 |
| ENSMUSG00000022595 | protein_coding | Lypd2 | 2.575614 | 3.426224 | 3.000919 |
| ENSMUSG00000086268 | processed_transcrip | Gm11670 | 3.278501 | 4.352073 | 3.815287 |
| ENSMUSG00000022568 | protein_coding | Scrib | 6298.199 | 6418.685 | 6358.442 |
| ENSMUSG00000065036 | snoRNA | SNORA70 | 4.579764 | 3.488204 | 4.033984 |
| ENSMUSG00000070617 | protein_coding | Gm13089 | 1.604662 | 0.988552 | 1.296607 |
| ENSMUSG00000081494 | pseudogene | Gm14130 | 1.284863 | 2.013141 | 1.649002 |
| ENSMUSG00000026696 | protein_coding | Vamp4 | 1169.676 | 1138.086 | 1153.881 |
| ENSMUSG00000046605 | protein_coding | B3gntl1 | 675.5211 | 655.0711 | 665.2961 |
| ENSMUSG00000030082 | protein_coding | Sec61a1 | 8617.473 | 8805.612 | 8711.542 |
| ENSMUSG00000044164 | protein_coding | Rnf182 | 46.78552 | 42.33894 | 44.56223 |
| ENSMUSG00000026790 | protein_coding | Odf2 | 2177.355 | 2125.667 | 2151.511 |
| ENSMUSG00000046167 | protein_coding | Gldn | 2.891835 | 3.743729 | 3.317782 |
| ENSMUSG00000071713 | protein_coding | Csf2rb | 56.33133 | 51.7862 | 54.05877 |
| ENSMUSG00000060187 | protein_coding | Lrrc10 | 1.637461 | 1.015218 | 1.32634 |
| ENSMUSG00000055723 | protein_coding | Rras2 | 3491.585 | 3416.138 | 3453.862 |
| ENSMUSG00000084900 | lincRNA | Gm16625 | 1.606973 | 1.006571 | 1.306772 |
| ENSMUSG00000064796 | misc_RNA | Telomerase-vert | 36.54597 | 33.47263 | 35.0093 |
| ENSMUSG00000000902 | protein_coding | Smarcb1 | 2078.913 | 2129.23 | 2104.072 |
| ENSMUSG00000031590 | protein_coding | Frg1 | 1180.437 | 1207.958 | 1194.197 |
| ENSMUSG00000079197 | protein_coding | Psme2 | 1540.83 | 1578.706 | 1559.768 |
| ENSMUSG00000078586 | protein_coding | Gm11735 | 2.581503 | 3.390187 | 2.985845 |
| ENSMUSG00000024691 | protein_coding | Fam111a | 280.4249 | 296.1911 | 288.308 |
| ENSMUSG00000030350 | protein_coding | Prmt8 | 5.128939 | 4.096909 | 4.612924 |
| ENSMUSG00000030259 | protein_coding | Rassf8 | 796.5921 | 825.5306 | 811.0613 |
| ENSMUSG00000023307 | protein_coding | 5-Mar | 2029.407 | 1973.804 | 2001.605 |
| ENSMUSG00000085665 | lincRNA | Gm12059 | 33.94241 | 36.85416 | 35.39829 |
| ENSMUSG00000037202 | protein_coding | Prf1 | 1.943172 | 1.350742 | 1.646957 |
| ENSMUSG00000039518 | protein_coding | Cdsn | 25.38565 | 28.30753 | 26.84659 |
| ENSMUSG00000084693 | snoRNA | SNORA33 | 2.259393 | 3.126376 | 2.692884 |
| ENSMUSG00000045746 | protein_coding | B230317F23Rik | 35.46716 | 32.46321 | 33.96518 |
| ENSMUSG00000072548 | protein_coding | Rps19-ps2 | 2.245304 | 3.01935 | 2.632327 |
| ENSMUSG00000020109 | protein_coding | Dnajb12 | 1130.471 | 1159.077 | 1144.774 |
| ENSMUSG00000030603 | protein_coding | Psmc4 | 2164.568 | 2211.588 | 2188.078 |
| ENSMUSG00000024019 | protein_coding | Ftsjd2 | 331.9354 | 320.1276 | 326.0315 |
| ENSMUSG00000079177 | protein_coding | 4930417G10Rik | 31.93766 | 36.33011 | 34.13389 |
| ENSMUSG00000028161 | protein_coding | Ppp3ca | 2296.027 | 2233.201 | 2264.614 |
| ENSMUSG00000026563 | protein_coding | Tada1 | 762.257 | 786.261 | 774.259 |
| ENSMUSG00000008461 | protein_coding | Fut1 | 2.877746 | 3.761386 | 3.319566 |
| ENSMUSG00000027460 | protein_coding | Angpt4 | 9.051661 | 10.53311 | 9.792387 |
| ENSMUSG00000079391 | protein_coding | Gm2974 | 19.28494 | 16.91634 | 18.10064 |
| ENSMUSG00000084096 | pseudogene | Gm11332 | 1.635151 | 2.428301 | 2.031726 |
| ENSMUSG00000040148 | protein_coding | Hmx3 | 9.995703 | 8.362824 | 9.179264 |
| ENSMUSG00000019794 | protein_coding | Katna1 | 1018.76 | 1043.394 | 1031.077 |
| ENSMUSG00000041025 | protein_coding | Iffo2 | 1213.488 | 1178.906 | 1196.197 |
| ENSMUSG00000004937 | protein_coding | Sgta | 2254.54 | 2308.264 | 2281.402 |
| ENSMUSG00000027215 | protein_coding | Cd82 | 753.4632 | 777.5082 | 765.4857 |
| ENSMUSG00000088608 | rRNA | 5S_rRNA | 2.900035 | 3.73472 | 3.317377 |

| | | | | | |
|---|---|---|---|---|---|
| ENSMUSG00000022906 | protein_coding | Parp9 | 294.9497 | 305.6475 | 300.2986 |
| ENSMUSG00000007659 | protein_coding | Bcl2l1 | 2092.95 | 2045.922 | 2069.436 |
| ENSMUSG00000020182 | protein_coding | Ddc | 29.64249 | 32.61849 | 31.13049 |
| ENSMUSG00000026115 | protein_coding | Vwa3b | 2.267593 | 3.01935 | 2.643471 |
| ENSMUSG00000028461 | protein_coding | Ccdc107 | 395.1006 | 409.9497 | 402.5252 |
| ENSMUSG00000077222 | snoRNA | SNORA66 | 18.37139 | 16.14728 | 17.25933 |
| ENSMUSG00000032293 | protein_coding | Ireb2 | 6330.959 | 6202.316 | 6266.637 |
| ENSMUSG00000021945 | protein_coding | Zmym2 | 4437.767 | 4341.017 | 4389.392 |
| ENSMUSG00000070243 | snoRNA | U3 | 6.200825 | 7.692139 | 6.946482 |
| ENSMUSG00000040721 | protein_coding | Zfhx2 | 675.6426 | 649.2521 | 662.4474 |
| ENSMUSG00000087250 | processed_transcrip | 1700094C10Rik | 2.261704 | 3.188355 | 2.72503 |
| ENSMUSG00000089265 | snoRNA | SNORD121A | 3.560655 | 2.737158 | 3.148907 |
| ENSMUSG00000090944 | lincRNA | Gm17513 | 19.9794 | 17.71492 | 18.84716 |
| ENSMUSG00000031991 | protein_coding | Spata19 | 7.455199 | 6.145726 | 6.800462 |
| ENSMUSG00000062093 | protein_coding | Gm10110 | 35.00147 | 38.16995 | 36.58571 |
| ENSMUSG00000033213 | protein_coding | AA467197 | 104.8647 | 137.075 | 120.9699 |
| ENSMUSG00000033633 | protein_coding | Clec18a | 1.959572 | 1.350742 | 1.655157 |
| ENSMUSG00000014547 | protein_coding | Wdfy2 | 472.57 | 490.846 | 481.708 |
| ENSMUSG00000048379 | protein_coding | Socs4 | 1688.333 | 1729.284 | 1708.809 |
| ENSMUSG00000060860 | protein_coding | Ube2s | 3379.543 | 3457.013 | 3418.278 |
| ENSMUSG00000013275 | protein_coding | Slc41a1 | 2160.127 | 2220.223 | 2190.175 |
| ENSMUSG00000030206 | protein_coding | Gsg1 | 1.939594 | 2.728511 | 2.334052 |
| ENSMUSG00000044172 | protein_coding | Ptx4 | 3.256212 | 2.43731 | 2.846761 |
| ENSMUSG00000090780 | protein_coding | Gm17532 | 1.595194 | 0.988552 | 1.291873 |
| ENSMUSG00000071867 | pseudogene | Gm6304 | 16.43873 | 14.40225 | 15.42049 |
| ENSMUSG00000048752 | protein_coding | Prss50 | 1637.438 | 1410.087 | 1523.763 |
| ENSMUSG00000032554 | protein_coding | Trf | 15.89186 | 17.98489 | 16.93838 |
| ENSMUSG00000039209 | protein_coding | Rpl39l | 865.6772 | 841.064 | 853.3706 |
| ENSMUSG00000086412 | lincRNA | Gm16626 | 23.55079 | 26.01256 | 24.78168 |
| ENSMUSG00000030292 | protein_coding | 1700023A16Rik | 22.26466 | 24.81104 | 23.53785 |
| ENSMUSG00000036061 | protein_coding | Smug1 | 480.0487 | 498.3699 | 489.2093 |
| ENSMUSG00000055632 | protein_coding | Hmcn2 | 25.96032 | 28.86503 | 27.41267 |
| ENSMUSG00000039210 | protein_coding | Gpatch2 | 852.288 | 874.7642 | 863.5261 |
| ENSMUSG00000070166 | snoRNA | SNORA21 | 3.222145 | 4.105918 | 3.664032 |
| ENSMUSG00000056718 | protein_coding | Gm13199 | 34.54667 | 31.56475 | 33.05571 |
| ENSMUSG00000037736 | protein_coding | Limch1 | 400.5077 | 418.0374 | 409.2725 |
| ENSMUSG00000048706 | protein_coding | D4Bwg0951e | 35.11351 | 38.20527 | 36.65939 |
| ENSMUSG00000009828 | protein_coding | Ick | 1025.988 | 1058.093 | 1042.041 |
| ENSMUSG00000044098 | protein_coding | Rsbn1 | 944.7727 | 920.4795 | 932.6261 |
| ENSMUSG00000054626 | protein_coding | Xlr | 266.2434 | 251.7511 | 258.9972 |
| ENSMUSG00000022761 | protein_coding | Lztr1 | 1594.402 | 1556.371 | 1575.387 |
| ENSMUSG00000022330 | protein_coding | Osr2 | 1472.43 | 1511.614 | 1492.022 |
| ENSMUSG00000027879 | protein_coding | Sec22b | 2662.867 | 2608.897 | 2635.882 |
| ENSMUSG00000017929 | protein_coding | B4galt5 | 1107.612 | 1071.271 | 1089.442 |
| ENSMUSG00000046747 | pseudogene | Gm9812 | 5.526116 | 4.45009 | 4.988103 |
| ENSMUSG00000047843 | protein_coding | Bri3 | 654.8813 | 674.396 | 664.6386 |
| ENSMUSG00000020014 | protein_coding | 4930485B16Rik | 116.0264 | 108.4754 | 112.2509 |
| ENSMUSG00000092887 | snoRNA | CT010429.2 | 14.51195 | 16.60169 | 15.55682 |
| ENSMUSG00000028716 | protein_coding | Pdzk1ip1 | 5.805959 | 4.723272 | 5.264616 |
| ENSMUSG00000046312 | protein_coding | AI464131 | 63.69775 | 59.29459 | 61.49617 |
| ENSMUSG00000050919 | protein_coding | Zfp366 | 20.58725 | 18.38312 | 19.48518 |
| ENSMUSG00000025666 | protein_coding | Tmem47 | 1127.322 | 1166.039 | 1146.68 |
| ENSMUSG00000072789 | pseudogene | Gm10420 | 13.20838 | 11.53968 | 12.37403 |
| ENSMUSG00000066809 | protein_coding | Gm10180 | 2.609681 | 3.645712 | 3.127697 |
| ENSMUSG00000044550 | protein_coding | Tceal3 | 379.6483 | 365.8201 | 372.7342 |
| ENSMUSG00000064481 | snRNA | U1 | 1.643351 | 2.508661 | 2.076006 |
| ENSMUSG00000025261 | protein_coding | Huwe1 | 18958.12 | 18551.22 | 18754.67 |
| ENSMUSG00000087150 | processed_transcrip | BC064078 | 13.63836 | 11.74473 | 12.69154 |

| | | | | | |
|---|---|---|---|---|---|
| ENSMUSG00000085143 | processed_transcrip | Gm11520 | 19.82636 | 22.42018 | 21.12327 |
| ENSMUSG00000025503 | protein_coding | Taldo1 | 3062.304 | 2995.88 | 3029.092 |
| ENSMUSG00000067768 | protein_coding | Xlr4b | 424.9805 | 441.0274 | 433.004 |
| ENSMUSG00000020389 | protein_coding | Cdkl3 | 452.3195 | 432.4731 | 442.3963 |
| ENSMUSG00000028470 | protein_coding | Hint2 | 460.4954 | 476.0653 | 468.2803 |
| ENSMUSG00000020534 | protein_coding | Shmt1 | 1803.04 | 1757.423 | 1780.232 |
| ENSMUSG00000055137 | protein_coding | 5033411D12Rik | 83.44487 | 78.1011 | 80.77298 |
| ENSMUSG00000024146 | protein_coding | Cript | 1092.963 | 1066.619 | 1079.791 |
| ENSMUSG00000047746 | protein_coding | Fbxo40 | 117.1913 | 111.5272 | 114.3593 |
| ENSMUSG00000036299 | protein_coding | BC031181 | 1601.166 | 1642.503 | 1621.834 |
| ENSMUSG00000049598 | protein_coding | Vsig8 | 2.279371 | 3.135023 | 2.707197 |
| ENSMUSG00000038418 | protein_coding | Egr1 | 1418.455 | 1261.186 | 1339.82 |
| ENSMUSG00000092226 | pseudogene | RP23-71I22.5 | 25.84172 | 28.62824 | 27.23498 |
| ENSMUSG00000085582 | processed_transcrip | 3110099E03Rik | 3.574744 | 2.746168 | 3.160456 |
| ENSMUSG00000025162 | protein_coding | Csnk1d | 5267.222 | 5394.859 | 5331.041 |
| ENSMUSG00000030888 | protein_coding | Rrp8 | 887.3218 | 866.3104 | 876.8161 |
| ENSMUSG00000086725 | lincRNA | A630052C17Rik | 202.0504 | 210.3548 | 206.2026 |
| ENSMUSG00000085327 | processed_transcrip | Gm16104 | 4.559785 | 5.729481 | 5.144633 |
| ENSMUSG00000026167 | protein_coding | Wnt10a | 8.433308 | 10.02278 | 9.228046 |
| ENSMUSG00000021624 | protein_coding | Cd180 | 34.56538 | 37.51335 | 36.03937 |
| ENSMUSG00000026715 | protein_coding | Serpinc1 | 8.09122 | 9.490866 | 8.791043 |
| ENSMUSG00000078776 | protein_coding | 9530053A07Rik | 7.847754 | 6.279056 | 7.063405 |
| ENSMUSG00000082515 | pseudogene | Gm12902 | 7.150756 | 8.662672 | 7.906714 |
| ENSMUSG00000031010 | protein_coding | Usp9x | 11425.08 | 11674.24 | 11549.66 |
| ENSMUSG00000023832 | protein_coding | Acat2 | 1376.943 | 1430.571 | 1403.757 |
| ENSMUSG00000027737 | protein_coding | Slc7a11 | 50.62736 | 46.34431 | 48.48583 |
| ENSMUSG00000041654 | protein_coding | Slc39a11 | 877.0425 | 901.1028 | 889.0726 |
| ENSMUSG00000005107 | protein_coding | Slc2a9 | 35.0828 | 38.23266 | 36.65773 |
| ENSMUSG00000087579 | lincRNA | 1500017E21Rik | 7.813687 | 6.32374 | 7.068714 |
| ENSMUSG00000091844 | protein_coding | Gm8251 | 9.451149 | 7.920636 | 8.685892 |
| ENSMUSG00000037907 | protein_coding | Ankrd13b | 276.7142 | 286.4893 | 281.6017 |
| ENSMUSG00000040945 | protein_coding | Rcc2 | 6621.864 | 6467.467 | 6544.665 |
| ENSMUSG00000090605 | lincRNA | E230013L22Rik | 76.18752 | 71.95144 | 74.06948 |
| ENSMUSG00000036282 | protein_coding | Naa30 | 1962.569 | 2003.851 | 1983.21 |
| ENSMUSG00000015745 | protein_coding | Plekho1 | 796.3741 | 767.8421 | 782.1081 |
| ENSMUSG00000017999 | protein_coding | Ddx27 | 2459.608 | 2526.744 | 2493.176 |
| ENSMUSG00000032177 | protein_coding | Pde4a | 249.0009 | 238.902 | 243.9515 |
| ENSMUSG00000030220 | protein_coding | Arhgdib | 199.1681 | 209.1381 | 204.1531 |
| ENSMUSG00000088475 | rRNA | 5S_rRNA | 1.595194 | 0.997561 | 1.296378 |
| ENSMUSG00000074361 | protein_coding | Gpr77 | 1.923194 | 1.350742 | 1.636968 |
| ENSMUSG00000030793 | protein_coding | Pycard | 236.6398 | 246.171 | 241.4054 |
| ENSMUSG00000058603 | pseudogene | Rpl28-ps1 | 290.8464 | 281.5584 | 286.2024 |
| ENSMUSG00000003308 | protein_coding | Keap1 | 1039.36 | 1069.162 | 1054.261 |
| ENSMUSG00000047539 | protein_coding | Fbxo28 | 1894.25 | 1946.189 | 1920.219 |
| ENSMUSG00000089542 | snoRNA | snoU6-77 | 11.72001 | 9.951434 | 10.83572 |
| ENSMUSG00000004446 | protein_coding | Bid | 821.5501 | 843.7256 | 832.6379 |
| ENSMUSG00000040901 | protein_coding | Kcnk18 | 2.289882 | 3.063673 | 2.676777 |
| ENSMUSG00000043484 | pseudogene | Gm5867 | 3.558345 | 2.648151 | 3.103248 |
| ENSMUSG00000036469 | protein_coding | 1-Mar | 24.67546 | 22.02195 | 23.3487 |
| ENSMUSG00000031492 | protein_coding | Chrnb3 | 3.244434 | 4.159251 | 3.701842 |
| ENSMUSG00000056600 | protein_coding | Olfr90 | 1.637461 | 1.033237 | 1.335349 |
| ENSMUSG00000052155 | protein_coding | Acvr2a | 1248.943 | 1218.302 | 1233.623 |
| ENSMUSG00000024601 | protein_coding | Isoc1 | 2342.729 | 2397.899 | 2370.314 |
| ENSMUSG00000052698 | protein_coding | Tln2 | 1220.392 | 1259.022 | 1239.707 |
| ENSMUSG00000052241 | pseudogene | A930035D04Rik | 19.68547 | 21.83529 | 20.76038 |
| ENSMUSG00000022212 | protein_coding | Cpne6 | 3.580633 | 2.728149 | 3.154391 |
| ENSMUSG00000038179 | protein_coding | Slamf7 | 6.127027 | 4.741652 | 5.434339 |
| ENSMUSG00000053644 | protein_coding | Aldh7a1 | 2349.75 | 2263.352 | 2306.551 |

| | | | | | |
|---|---|---|---|---|---|
| ENSMUSG00000031112 | protein_coding | 2610018G03Rik | 498.6099 | 518.2192 | 508.4145 |
| ENSMUSG00000020598 | protein_coding | Nrcam | 675.6979 | 712.3971 | 694.0475 |
| ENSMUSG00000064443 | snRNA | U6 | 1.592884 | 1.015218 | 1.304051 |
| ENSMUSG00000029062 | protein_coding | Cdk11b | 3182.889 | 3067.077 | 3124.983 |
| ENSMUSG00000024592 | protein_coding | C330018D20Rik | 589.3797 | 609.2116 | 599.2957 |
| ENSMUSG00000002289 | protein_coding | Angptl4 | 742.7404 | 766.7344 | 754.7374 |
| ENSMUSG00000023075 | protein_coding | Akirin1 | 4204.245 | 4291.783 | 4248.014 |
| ENSMUSG00000032017 | protein_coding | Grik4 | 85.03351 | 90.33171 | 87.68261 |
| ENSMUSG00000033540 | protein_coding | Idua | 376.9243 | 363.9419 | 370.4331 |
| ENSMUSG00000057286 | protein_coding | St6galnac2 | 125.9903 | 132.9705 | 129.4804 |
| ENSMUSG00000000982 | protein_coding | Ccl3 | 1.929083 | 1.359751 | 1.644417 |
| ENSMUSG00000037833 | protein_coding | Sh2d4b | 22.24595 | 19.89029 | 21.06812 |
| ENSMUSG00000022367 | protein_coding | Has2 | 7.696354 | 9.182732 | 8.439543 |
| ENSMUSG00000047230 | protein_coding | Cldn2 | 5.79187 | 7.089955 | 6.440913 |
| ENSMUSG00000055138 | protein_coding | Gm4861 | 0.33031 | 0.697713 | 0.514012 |
| ENSMUSG00000087477 | processed_transcrip | Gm13822 | 0.33031 | 0.697713 | 0.514012 |
| ENSMUSG00000092004 | protein_coding | Gm17482 | 0.33031 | 0.697713 | 0.514012 |
| ENSMUSG00000090007 | pseudogene | Rpl30-ps2 | 0.33031 | 0.697713 | 0.514012 |
| ENSMUSG00000082484 | pseudogene | Gm16177 | 0.33031 | 0.697713 | 0.514012 |
| ENSMUSG00000059571 | pseudogene | Gm16218 | 0.33031 | 0.697713 | 0.514012 |
| ENSMUSG00000070324 | protein_coding | Fbxw22 | 0.33031 | 0.697713 | 0.514012 |
| ENSMUSG00000085993 | pseudogene | Gm15905 | 0.33031 | 0.697713 | 0.514012 |
| ENSMUSG00000069873 | protein_coding | 4930438A08Rik | 0.33031 | 0.697713 | 0.514012 |
| ENSMUSG00000075960 | snRNA | U1 | 0.33031 | 0.697713 | 0.514012 |
| ENSMUSG00000084629 | snRNA | U6 | 0.33031 | 0.697713 | 0.514012 |
| ENSMUSG00000021335 | protein_coding | Slc17a1 | 0.33031 | 0.697713 | 0.514012 |
| ENSMUSG00000084739 | snRNA | U6 | 0.33031 | 0.697713 | 0.514012 |
| ENSMUSG00000079546 | protein_coding | Gm11146 | 0.33031 | 0.697713 | 0.514012 |
| ENSMUSG00000048875 | protein_coding | Hdhd1a | 0.33031 | 0.697713 | 0.514012 |
| ENSMUSG00000083130 | pseudogene | Gm14560 | 0.33031 | 0.697713 | 0.514012 |
| ENSMUSG00000064530 | snoRNA | SNORA36 | 0.33031 | 0.697713 | 0.514012 |
| ENSMUSG00000076140 | miRNA | Mir542 | 0.33031 | 0.697713 | 0.514012 |
| ENSMUSG00000081217 | pseudogene | Gm15268 | 0.33031 | 0.697713 | 0.514012 |
| ENSMUSG00000083157 | pseudogene | Gm13511 | 1.635151 | 1.050893 | 1.343022 |
| ENSMUSG00000059303 | protein_coding | Olfr836 | 1.635151 | 1.050893 | 1.343022 |
| ENSMUSG00000035491 | protein_coding | 1600014K23Rik | 1.635151 | 1.050893 | 1.343022 |
| ENSMUSG00000063875 | protein_coding | Rps6-ps1 | 10.00032 | 11.51265 | 10.75649 |
| ENSMUSG00000074828 | protein_coding | Gm10768 | 8.09353 | 6.843077 | 7.468304 |
| ENSMUSG00000032607 | protein_coding | Amt | 131.1831 | 123.4689 | 127.326 |
| ENSMUSG00000039530 | protein_coding | Tusc3 | 1715.608 | 1675.803 | 1695.705 |
| ENSMUSG00000067851 | protein_coding | Arfgef1 | 4462.095 | 4563.398 | 4512.746 |
| ENSMUSG00000021103 | protein_coding | Mnat1 | 631.9421 | 647.9918 | 639.9669 |
| ENSMUSG00000039200 | protein_coding | Atf7ip2 | 891.2291 | 841.9793 | 866.6042 |
| ENSMUSG00000086844 | lincRNA | B230206H07Rik | 2.908235 | 3.779404 | 3.34382 |
| ENSMUSG00000038900 | protein_coding | Rpl12 | 6774.21 | 6925.404 | 6849.807 |
| ENSMUSG00000064448 | snRNA | U6 | 0.32211 | 0.697713 | 0.509912 |
| ENSMUSG00000085967 | processed_transcrip | Gm12530 | 0.32211 | 0.697713 | 0.509912 |
| ENSMUSG00000080793 | pseudogene | Cbx3-ps3 | 0.32211 | 0.697713 | 0.509912 |
| ENSMUSG00000064900 | snoRNA | SNORA71 | 0.32211 | 0.697713 | 0.509912 |
| ENSMUSG00000086678 | lincRNA | Gm16737 | 0.32211 | 0.697713 | 0.509912 |
| ENSMUSG00000056777 | protein_coding | Rpl21-ps13 | 0.32211 | 0.697713 | 0.509912 |
| ENSMUSG00000031645 | protein_coding | F11 | 0.32211 | 0.697713 | 0.509912 |
| ENSMUSG00000000766 | protein_coding | Oprm1 | 0.32211 | 0.697713 | 0.509912 |
| ENSMUSG00000091355 | pseudogene | Gm17142 | 0.32211 | 0.697713 | 0.509912 |
| ENSMUSG00000021403 | protein_coding | Serpinb9b | 0.32211 | 0.697713 | 0.509912 |
| ENSMUSG00000067614 | protein_coding | Krt86 | 0.32211 | 0.697713 | 0.509912 |
| ENSMUSG00000082019 | pseudogene | Gm15231 | 0.32211 | 0.697713 | 0.509912 |
| ENSMUSG00000037434 | protein_coding | Slc30a1 | 2393.349 | 2308.255 | 2350.802 |

| | | | | | |
|---|---|---|---|---|---|
| ENSMUSG00000025269 | protein_coding | Apex2 | 260.7207 | 251.0612 | 255.891 |
| ENSMUSG00000048337 | protein_coding | Ppyr1 | 12.62178 | 10.90395 | 11.76287 |
| ENSMUSG00000086286 | processed_transcrip | Gm15138 | 37.69837 | 34.70153 | 36.19995 |
| ENSMUSG00000077057 | miRNA | AC124426.1 | 130.4762 | 110.1161 | 120.2961 |
| ENSMUSG00000044820 | protein_coding | AY074887 | 5.831827 | 4.740929 | 5.286378 |
| ENSMUSG00000054000 | protein_coding | Tusc1 | 337.2328 | 350.5035 | 343.8682 |
| ENSMUSG00000077420 | miRNA | AC161183.1 | 0.316221 | 0.697713 | 0.506967 |
| ENSMUSG00000074591 | protein_coding | Gm410 | 0.316221 | 0.697713 | 0.506967 |
| ENSMUSG00000083735 | pseudogene | Gm12495 | 0.316221 | 0.697713 | 0.506967 |
| ENSMUSG00000087270 | pseudogene | Gm12983 | 0.316221 | 0.697713 | 0.506967 |
| ENSMUSG00000068689 | pseudogene | Rps19-ps9 | 0.316221 | 0.697713 | 0.506967 |
| ENSMUSG00000004542 | protein_coding | Psg19 | 0.316221 | 0.697713 | 0.506967 |
| ENSMUSG00000046411 | protein_coding | Gm6816 | 0.316221 | 0.697713 | 0.506967 |
| ENSMUSG00000047619 | protein_coding | Ddi1 | 0.316221 | 0.697713 | 0.506967 |
| ENSMUSG00000081259 | pseudogene | Gm6893 | 0.316221 | 0.697713 | 0.506967 |
| ENSMUSG00000090914 | pseudogene | Gm17563 | 0.316221 | 0.697713 | 0.506967 |
| ENSMUSG00000079143 | processed_transcrip | Gm11052 | 0.316221 | 0.697713 | 0.506967 |
| ENSMUSG00000089593 | snRNA | U6 | 0.316221 | 0.697713 | 0.506967 |
| ENSMUSG00000022705 | protein_coding | Drd3 | 0.316221 | 0.697713 | 0.506967 |
| ENSMUSG00000073980 | pseudogene | Gm9574 | 0.316221 | 0.697713 | 0.506967 |
| ENSMUSG00000092556 | pseudogene | Olfr755-ps1 | 0.316221 | 0.697713 | 0.506967 |
| ENSMUSG00000024411 | protein_coding | Aqp4 | 0.316221 | 0.697713 | 0.506967 |
| ENSMUSG00000054822 | protein_coding | 1700041G16Rik | 2.909503 | 3.832736 | 3.37112 |
| ENSMUSG00000086266 | lincRNA | Igf2as | 59.59806 | 54.66022 | 57.12914 |
| ENSMUSG00000046080 | protein_coding | Clec9a | 3.202167 | 2.330646 | 2.766407 |
| ENSMUSG00000019874 | protein_coding | Fabp7 | 9.353793 | 7.876675 | 8.615234 |
| ENSMUSG00000026470 | protein_coding | Stx6 | 2152.236 | 2102.969 | 2127.602 |
| ENSMUSG00000039480 | protein_coding | Nt5dc1 | 479.0176 | 492.6474 | 485.8325 |
| ENSMUSG00000040565 | protein_coding | Btaf1 | 3905.045 | 4034.047 | 3969.546 |
| ENSMUSG00000065226 | snoRNA | SNORA8 | 67.22881 | 71.64403 | 69.43642 |
| ENSMUSG00000035394 | protein_coding | Ccdc11 | 56.31412 | 52.01466 | 54.16439 |
| ENSMUSG00000088153 | misc_RNA | 7SK | 4.901874 | 3.814718 | 4.358296 |
| ENSMUSG00000065818 | snoRNA | Snord35a | 1.923194 | 1.359751 | 1.641472 |
| ENSMUSG00000092859 | miRNA | AC139317.1 | 1.609283 | 1.033237 | 1.32126 |
| ENSMUSG00000060534 | protein_coding | Dcc | 1.609283 | 1.033237 | 1.32126 |
| ENSMUSG00000079540 | lincRNA | Gm4485 | 20.31791 | 22.52435 | 21.42113 |
| ENSMUSG00000027797 | protein_coding | Dclk1 | 35.82207 | 38.67992 | 37.25099 |
| ENSMUSG00000042743 | protein_coding | Sgtb | 418.0578 | 406.2089 | 412.1333 |
| ENSMUSG00000076258 | miRNA | mmu-mir-689-2 | 7.372976 | 6.136355 | 6.754665 |
| ENSMUSG00000023068 | protein_coding | Nus1 | 2727.072 | 2785.483 | 2756.278 |
| ENSMUSG00000025020 | protein_coding | Slit1 | 30.82813 | 34.1822 | 32.50516 |
| ENSMUSG00000001424 | protein_coding | Snd1 | 3205.555 | 3292.174 | 3248.865 |
| ENSMUSG00000038506 | protein_coding | Dcun1d2 | 922.6731 | 948.6681 | 935.6706 |
| ENSMUSG00000022484 | protein_coding | Hoxc10 | 27.39845 | 24.81932 | 26.10889 |
| ENSMUSG00000088545 | snRNA | U6 | 3.250323 | 4.132585 | 3.691454 |
| ENSMUSG00000064437 | snoRNA | Snord49b | 5.848226 | 7.116621 | 6.482424 |
| ENSMUSG00000077309 | rRNA | n-R5s8 | 3.550145 | 4.468108 | 4.009126 |
| ENSMUSG00000060727 | pseudogene | Gm12571 | 9.318459 | 10.90395 | 10.1112 |
| ENSMUSG00000033318 | protein_coding | Gstt2 | 152.534 | 161.2896 | 156.9118 |
| ENSMUSG00000038670 | protein_coding | Mybpc2 | 49.2382 | 53.00036 | 51.11928 |
| ENSMUSG00000050761 | protein_coding | Gp1bb | 9.09855 | 10.59545 | 9.847002 |
| ENSMUSG00000089960 | protein_coding | Ugt1a1 | 1.959572 | 1.324076 | 1.641824 |
| ENSMUSG00000036022 | protein_coding | Fam122b | 582.0456 | 564.6187 | 573.3322 |
| ENSMUSG00000032679 | protein_coding | Cd59a | 654.4242 | 620.7114 | 637.5678 |
| ENSMUSG00000042165 | protein_coding | Gm9774 | 43.30336 | 39.82231 | 41.56283 |
| ENSMUSG00000025134 | protein_coding | Thoc4 | 2245.297 | 2292.993 | 2269.145 |
| ENSMUSG00000040471 | protein_coding | Ggt6 | 1.626951 | 1.050893 | 1.338922 |
| ENSMUSG00000092644 | miRNA | AL645623.1 | 3.530167 | 2.746168 | 3.138167 |

| | | | | | |
|---|---|---|---|---|---|
| ENSMUSG00000020484 | protein_coding | Xbp1 | 1827.579 | 1775.342 | 1801.46 |
| ENSMUSG00000029022 | protein_coding | Miip | 1249.001 | 1313.614 | 1281.307 |
| ENSMUSG00000005533 | protein_coding | Igf1r | 1773.201 | 1829.933 | 1801.567 |
| ENSMUSG00000026941 | protein_coding | Mamdc4 | 123.1594 | 117.2168 | 120.1881 |
| ENSMUSG00000028645 | protein_coding | Slc2a1 | 832.3355 | 812.2243 | 822.2799 |
| ENSMUSG00000024909 | protein_coding | Efemp2 | 879.4532 | 859.3521 | 869.4026 |
| ENSMUSG00000085984 | lincRNA | 1700001G11Rik | 16.09433 | 18.01156 | 17.05294 |
| ENSMUSG00000022676 | protein_coding | Snai2 | 225.4041 | 235.8363 | 230.6202 |
| ENSMUSG00000011171 | protein_coding | Vipr2 | 22.58215 | 24.86365 | 23.7229 |
| ENSMUSG00000053101 | protein_coding | Gpr141 | 19.24245 | 21.75601 | 20.49923 |
| ENSMUSG00000040364 | protein_coding | Sec1 | 188.3542 | 179.855 | 184.1046 |
| ENSMUSG00000003166 | protein_coding | Dgcr2 | 2106.062 | 2052.791 | 2079.427 |
| ENSMUSG00000055041 | protein_coding | Commd5 | 491.1549 | 505.8145 | 498.4847 |
| ENSMUSG00000019832 | protein_coding | Rab32 | 80.71115 | 76.23324 | 78.4722 |
| ENSMUSG00000088873 | snRNA | U6 | 1.643351 | 1.050893 | 1.347122 |
| ENSMUSG00000042002 | protein_coding | Foxn4 | 57.08618 | 62.23311 | 59.65965 |
| ENSMUSG00000042976 | processed_transcrip | 9930038B18Rik | 3.541945 | 2.656799 | 3.099372 |
| ENSMUSG00000025477 | protein_coding | Inpp5a | 1249.303 | 1276.681 | 1262.992 |
| ENSMUSG00000027465 | protein_coding | Tbc1d20 | 3747.896 | 3676.226 | 3712.061 |
| ENSMUSG00000044165 | protein_coding | Bcl2l15 | 1.945483 | 1.386417 | 1.66595 |
| ENSMUSG00000037738 | protein_coding | Nek5 | 12.62872 | 14.33053 | 13.47963 |
| ENSMUSG00000020672 | protein_coding | Sntg2 | 173.2871 | 162.5932 | 167.9401 |
| ENSMUSG00000028289 | protein_coding | Epha7 | 243.9539 | 259.8571 | 251.9055 |
| ENSMUSG00000092944 | miRNA | AC119249.2 | 6.517046 | 7.795954 | 7.1565 |
| ENSMUSG00000085028 | processed_transcrip | Slc2a4rg-ps | 91.38305 | 84.87142 | 88.12723 |
| ENSMUSG00000022881 | protein_coding | Rfc4 | 1278.849 | 1247.161 | 1263.005 |
| ENSMUSG00000077448 | snoRNA | SNORA17 | 0.33031 | 0.688704 | 0.509507 |
| ENSMUSG00000067744 | pseudogene | Gm5829 | 0.33031 | 0.688704 | 0.509507 |
| ENSMUSG00000091876 | pseudogene | Gm3851 | 0.33031 | 0.688704 | 0.509507 |
| ENSMUSG00000026432 | protein_coding | Avpr1b | 0.33031 | 0.688704 | 0.509507 |
| ENSMUSG00000075063 | protein_coding | Olfr142 | 0.33031 | 0.688704 | 0.509507 |
| ENSMUSG00000080870 | pseudogene | Gm14111 | 0.33031 | 0.688704 | 0.509507 |
| ENSMUSG00000081751 | pseudogene | Gm14251 | 0.33031 | 0.688704 | 0.509507 |
| ENSMUSG00000083702 | pseudogene | Gm14428 | 0.33031 | 0.688704 | 0.509507 |
| ENSMUSG00000089394 | snRNA | U6 | 0.33031 | 0.688704 | 0.509507 |
| ENSMUSG00000041984 | protein_coding | Rptn | 0.33031 | 0.688704 | 0.509507 |
| ENSMUSG00000073766 | protein_coding | Olfr1328 | 0.33031 | 0.688704 | 0.509507 |
| ENSMUSG00000083182 | pseudogene | Gm13828 | 0.33031 | 0.688704 | 0.509507 |
| ENSMUSG00000088849 | misc_RNA | 7SK | 0.33031 | 0.688704 | 0.509507 |
| ENSMUSG00000047104 | protein_coding | Pbp2 | 0.33031 | 0.688704 | 0.509507 |
| ENSMUSG00000081338 | pseudogene | Gm7618 | 0.33031 | 0.688704 | 0.509507 |
| ENSMUSG00000066723 | protein_coding | Vmn1r183 | 0.33031 | 0.688704 | 0.509507 |
| ENSMUSG00000089904 | pseudogene | Gm15664 | 0.33031 | 0.688704 | 0.509507 |
| ENSMUSG00000017316 | protein_coding | Ppy | 0.33031 | 0.688704 | 0.509507 |
| ENSMUSG00000086982 | lincRNA | Gm8327 | 0.33031 | 0.688704 | 0.509507 |
| ENSMUSG00000091365 | pseudogene | Gm17045 | 0.33031 | 0.688704 | 0.509507 |
| ENSMUSG00000091783 | protein_coding | Gm8212 | 0.33031 | 0.688704 | 0.509507 |
| ENSMUSG00000021961 | protein_coding | 4930578I06Rik | 0.33031 | 0.688704 | 0.509507 |
| ENSMUSG00000078648 | protein_coding | Gm17546 | 0.33031 | 0.688704 | 0.509507 |
| ENSMUSG00000085333 | processed_transcrip | 1700030A11Rik | 0.33031 | 0.688704 | 0.509507 |
| ENSMUSG00000084467 | rRNA | n-R5s24 | 0.33031 | 0.688704 | 0.509507 |
| ENSMUSG00000079630 | protein_coding | Rhox4f | 0.33031 | 0.688704 | 0.509507 |
| ENSMUSG00000081289 | pseudogene | Gm14857 | 0.33031 | 0.688704 | 0.509507 |
| ENSMUSG00000029875 | protein_coding | AI836003 | 15.80628 | 14.05699 | 14.93164 |
| ENSMUSG00000048022 | protein_coding | Tmem229a | 1.934972 | 1.359751 | 1.647361 |
| ENSMUSG00000061390 | protein_coding | Gm7866 | 2.589703 | 3.354512 | 2.972107 |
| ENSMUSG00000087088 | lincRNA | Gm16638 | 29.89057 | 32.73996 | 31.31527 |
| ENSMUSG00000059830 | protein_coding | Gm16380 | 13.54816 | 11.83052 | 12.68934 |

| | | | | | |
|---|---|---|---|---|---|
| ENSMUSG00000030019 | protein_coding | Fbxl14 | 1047.249 | 1076.994 | 1062.122 |
| ENSMUSG00000080708 | pseudogene | Ccnd3-ps | 1.618751 | 2.401997 | 2.010374 |
| ENSMUSG00000047822 | protein_coding | Gm6484 | 2.287571 | 3.072682 | 2.680126 |
| ENSMUSG00000050405 | protein_coding | Tmem151b | 62.59396 | 58.63504 | 60.6145 |
| ENSMUSG00000008435 | protein_coding | Rdh13 | 770.0931 | 795.8283 | 782.9607 |
| ENSMUSG00000061863 | pseudogene | Gm6822 | 0.32211 | 0.688704 | 0.505407 |
| ENSMUSG00000067299 | protein_coding | Crygd | 0.32211 | 0.688704 | 0.505407 |
| ENSMUSG00000079434 | protein_coding | Neu2 | 0.32211 | 0.688704 | 0.505407 |
| ENSMUSG00000085867 | pseudogene | Gm5834 | 0.32211 | 0.688704 | 0.505407 |
| ENSMUSG00000062061 | protein_coding | Lcn13 | 0.32211 | 0.688704 | 0.505407 |
| ENSMUSG00000078587 | lincRNA | Gm2464 | 0.32211 | 0.688704 | 0.505407 |
| ENSMUSG00000081810 | pseudogene | Gm11878 | 0.32211 | 0.688704 | 0.505407 |
| ENSMUSG00000073846 | lincRNA | Gm12526 | 0.32211 | 0.688704 | 0.505407 |
| ENSMUSG00000086952 | processed_transcrip | Gm12596 | 0.32211 | 0.688704 | 0.505407 |
| ENSMUSG00000078582 | protein_coding | Hivep3 | 0.32211 | 0.688704 | 0.505407 |
| ENSMUSG00000086317 | lincRNA | Gm16766 | 0.32211 | 0.688704 | 0.505407 |
| ENSMUSG00000064963 | snRNA | U6 | 0.32211 | 0.688704 | 0.505407 |
| ENSMUSG00000080518 | snoRNA | SNORD111 | 0.32211 | 0.688704 | 0.505407 |
| ENSMUSG00000019890 | protein_coding | Nts | 0.32211 | 0.688704 | 0.505407 |
| ENSMUSG00000017211 | protein_coding | Gsdma2 | 0.32211 | 0.688704 | 0.505407 |
| ENSMUSG00000090216 | processed_transcrip | Gm16549 | 0.32211 | 0.688704 | 0.505407 |
| ENSMUSG00000076288 | miRNA | Mir301b | 0.32211 | 0.688704 | 0.505407 |
| ENSMUSG00000092557 | pseudogene | Gm8696 | 0.32211 | 0.688704 | 0.505407 |
| ENSMUSG00000073383 | protein_coding | Kcnh8 | 0.32211 | 0.688704 | 0.505407 |
| ENSMUSG00000065131 | snRNA | U6 | 0.32211 | 0.688704 | 0.505407 |
| ENSMUSG00000085880 | pseudogene | Gm15229 | 0.32211 | 0.688704 | 0.505407 |
| ENSMUSG00000033014 | protein_coding | Trim33 | 3745.079 | 3817.113 | 3781.096 |
| ENSMUSG00000075392 | protein_coding | Gm5561 | 3.880455 | 3.010341 | 3.445398 |
| ENSMUSG00000085825 | lincRNA | A930038B10Rik | 18.10437 | 20.211 | 19.15768 |
| ENSMUSG00000027246 | protein_coding | Ell3 | 13.55047 | 11.91088 | 12.73068 |
| ENSMUSG00000024658 | lincRNA | Gm9750 | 3.244434 | 4.105918 | 3.675176 |
| ENSMUSG00000005078 | protein_coding | Jkamp | 1151.28 | 1113.489 | 1132.384 |
| ENSMUSG00000079658 | protein_coding | Tceb1 | 1485.396 | 1528.151 | 1506.774 |
| ENSMUSG00000013846 | protein_coding | St3gal1 | 556.937 | 541.9579 | 549.4474 |
| ENSMUSG00000039037 | protein_coding | St6galnac5 | 10.9759 | 12.84931 | 11.91261 |
| ENSMUSG00000048271 | protein_coding | Rbm33 | 2667.546 | 2590.419 | 2628.983 |
| ENSMUSG00000070191 | snRNA | U6 | 0.316221 | 0.688704 | 0.502462 |
| ENSMUSG00000083671 | pseudogene | Gm14304 | 0.316221 | 0.688704 | 0.502462 |
| ENSMUSG00000074677 | protein_coding | Sirpb1c | 0.316221 | 0.688704 | 0.502462 |
| ENSMUSG00000063730 | protein_coding | Hsd3b2 | 0.316221 | 0.688704 | 0.502462 |
| ENSMUSG00000085020 | processed_transcrip | 2310081O03Rik | 0.316221 | 0.688704 | 0.502462 |
| ENSMUSG00000082143 | pseudogene | Gm12864 | 0.316221 | 0.688704 | 0.502462 |
| ENSMUSG00000091297 | protein_coding | Gm8439 | 0.316221 | 0.688704 | 0.502462 |
| ENSMUSG00000090331 | pseudogene | Gm17199 | 0.316221 | 0.688704 | 0.502462 |
| ENSMUSG00000092235 | processed_transcrip | Gm20393 | 0.316221 | 0.688704 | 0.502462 |
| ENSMUSG00000039760 | protein_coding | Il22ra2 | 0.316221 | 0.688704 | 0.502462 |
| ENSMUSG00000081016 | pseudogene | Olfr1397-ps1 | 0.316221 | 0.688704 | 0.502462 |
| ENSMUSG00000011052 | processed_transcrip | 1700049J03Rik | 0.316221 | 0.688704 | 0.502462 |
| ENSMUSG00000020152 | protein_coding | Actr2 | 3156.695 | 3222.771 | 3189.733 |
| ENSMUSG00000085736 | lincRNA | Gm16682 | 16.26675 | 14.11933 | 15.19304 |
| ENSMUSG00000074281 | lincRNA | Gm12522 | 15.18435 | 17.00535 | 16.09485 |
| ENSMUSG00000020108 | protein_coding | Ddit4 | 2913.199 | 3019.796 | 2966.498 |
| ENSMUSG00000052566 | protein_coding | Hook2 | 224.672 | 216.8108 | 220.7414 |
| ENSMUSG00000012350 | protein_coding | Ehf | 7.108489 | 8.431687 | 7.770088 |
| ENSMUSG00000033960 | protein_coding | 9430020K01Rik | 4284.613 | 4376.66 | 4330.637 |
| ENSMUSG00000017491 | protein_coding | Rarb | 25.16239 | 22.85123 | 24.00681 |
| ENSMUSG00000073043 | protein_coding | Atoh1 | 1.923194 | 1.324076 | 1.623635 |
| ENSMUSG00000020776 | protein_coding | Fbf1 | 991.0648 | 1015.808 | 1003.436 |

| | | | | | |
|---|---|---|---|---|---|
| ENSMUSG00000087614 | processed_transcrip | Gm12514 | 2.579193 | 3.399196 | 2.989194 |
| ENSMUSG00000025967 | protein_coding | Eef1b2 | 4471.049 | 4576.736 | 4523.892 |
| ENSMUSG00000035778 | protein_coding | Ggta1 | 221.473 | 231.9548 | 226.7139 |
| ENSMUSG00000050505 | protein_coding | Pcdh20 | 1.626951 | 1.059903 | 1.343427 |
| ENSMUSG00000073008 | protein_coding | Gpr174 | 7.384754 | 8.731174 | 8.057964 |
| ENSMUSG00000085376 | processed_transcrip | Gm14508 | 16.19884 | 14.22387 | 15.21135 |
| ENSMUSG00000077114 | miRNA | AC117684.2 | 14.59544 | 12.74229 | 13.66887 |
| ENSMUSG00000021693 | protein_coding | Kif2a | 2507.374 | 2452.617 | 2479.996 |
| ENSMUSG00000064125 | protein_coding | BC068157 | 116.3387 | 122.2906 | 119.3147 |
| ENSMUSG00000030109 | protein_coding | Slc6a12 | 1.959572 | 1.386417 | 1.672994 |
| ENSMUSG00000081818 | pseudogene | Gm12652 | 3.829988 | 2.710854 | 3.270421 |
| ENSMUSG00000021211 | protein_coding | Akr1c12 | 1.923194 | 2.648151 | 2.285672 |
| ENSMUSG00000081605 | pseudogene | Gm15953 | 8.681395 | 10.09957 | 9.390483 |
| ENSMUSG00000024737 | protein_coding | Slc15a3 | 16.59602 | 18.77125 | 17.68363 |
| ENSMUSG00000086880 | lincRNA | C430042M11Rik | 32.59299 | 29.99307 | 31.29303 |
| ENSMUSG00000022433 | protein_coding | Csnk1e | 1161.308 | 1189.293 | 1175.301 |
| ENSMUSG00000073752 | protein_coding | Gm10570 | 6.730023 | 8.105173 | 7.417598 |
| ENSMUSG00000005442 | protein_coding | Cic | 5436.636 | 5319.048 | 5377.842 |
| ENSMUSG00000032002 | protein_coding | Dcun1d5 | 2032.007 | 1974.436 | 2003.222 |
| ENSMUSG00000008153 | protein_coding | Clstn3 | 109.6637 | 103.5046 | 106.5842 |
| ENSMUSG00000055733 | protein_coding | Nap1l3 | 80.25404 | 85.3061 | 82.78007 |
| ENSMUSG00000083507 | pseudogene | Gm12842 | 1.643351 | 1.059903 | 1.351627 |
| ENSMUSG00000092626 | processed_transcrip | RP23-242C19.5 | 1.923194 | 1.315066 | 1.61913 |
| ENSMUSG00000087741 | snRNA | U1 | 2.592014 | 3.568563 | 3.080289 |
| ENSMUSG00000059361 | protein_coding | Nrsn2 | 3.894544 | 3.046016 | 3.47028 |
| ENSMUSG00000058748 | protein_coding | Zfp958 | 478.0002 | 496.7297 | 487.3649 |
| ENSMUSG00000031948 | protein_coding | Kars | 3461.53 | 3370.809 | 3416.169 |
| ENSMUSG00000036819 | protein_coding | Jmjd4 | 789.81 | 770.8303 | 780.3202 |
| ENSMUSG00000030583 | protein_coding | Sipa1l3 | 2903.058 | 2994.554 | 2948.806 |
| ENSMUSG00000083473 | pseudogene | Gm13207 | 1.931394 | 1.386417 | 1.658905 |
| ENSMUSG00000086805 | protein_coding | 4932443L11Rik | 1.609283 | 1.050893 | 1.330088 |
| ENSMUSG00000039617 | pseudogene | Gm7488 | 24.89328 | 22.52399 | 23.70864 |
| ENSMUSG00000079065 | protein_coding | BC005561 | 1038.112 | 1071.604 | 1054.858 |
| ENSMUSG00000018678 | protein_coding | Sp2 | 843.8432 | 815.3388 | 829.591 |
| ENSMUSG00000022551 | protein_coding | Cyc1 | 3287.983 | 3224.147 | 3256.065 |
| ENSMUSG00000031154 | protein_coding | Otud5 | 2631.285 | 2560.983 | 2596.134 |
| ENSMUSG00000064917 | snoRNA | snoZ39 | 3.866366 | 4.803632 | 4.334999 |
| ENSMUSG00000062098 | protein_coding | Btbd3 | 1287.097 | 1333.498 | 1310.298 |
| ENSMUSG00000021175 | protein_coding | Cdca7l | 1297.687 | 1265.511 | 1281.599 |
| ENSMUSG00000057135 | protein_coding | A430084P05Rik | 1.951372 | 1.341732 | 1.646552 |
| ENSMUSG00000081265 | pseudogene | Gm11282 | 9.745081 | 11.23983 | 10.49246 |
| ENSMUSG00000029536 | protein_coding | Gatc | 683.5139 | 701.1325 | 692.3232 |
| ENSMUSG00000030852 | protein_coding | Tacc2 | 1642.655 | 1680.845 | 1661.75 |
| ENSMUSG00000087939 | snRNA | U6 | 3.874566 | 3.01935 | 3.446958 |
| ENSMUSG00000015671 | protein_coding | Psma2 | 2671.743 | 2738.246 | 2704.994 |
| ENSMUSG00000051034 | protein_coding | Zfp11 | 305.144 | 293.5155 | 299.3298 |
| ENSMUSG00000027499 | protein_coding | Pkia | 1243.07 | 1292.603 | 1267.837 |
| ENSMUSG00000017817 | protein_coding | Jph2 | 222.9965 | 214.7069 | 218.8517 |
| ENSMUSG00000066407 | pseudogene | Gm10263 | 25.24081 | 22.59607 | 23.91844 |
| ENSMUSG00000040367 | protein_coding | 4932412H11Rik | 3.846388 | 4.812279 | 4.329333 |
| ENSMUSG00000036372 | protein_coding | 1810006K21Rik | 939.2311 | 913.4926 | 926.3618 |
| ENSMUSG00000046058 | protein_coding | Eid2 | 588.6581 | 573.1797 | 580.9189 |
| ENSMUSG00000051036 | protein_coding | Ttc24 | 2.583814 | 3.336855 | 2.960334 |
| ENSMUSG00000084154 | pseudogene | Gm15644 | 1.939594 | 2.665808 | 2.302701 |
| ENSMUSG00000059689 | protein_coding | Zfp637 | 456.4161 | 472.5691 | 464.4926 |
| ENSMUSG00000083864 | pseudogene | Gm13719 | 1.937283 | 2.639142 | 2.288212 |
| ENSMUSG00000041750 | protein_coding | Cd1d2 | 1.934972 | 1.324076 | 1.629524 |
| ENSMUSG00000060671 | protein_coding | Atp8b2 | 6101.702 | 6246.294 | 6173.998 |

| | | | | | |
|---|---|---|---|---|---|
| ENSMUSG00000031570 | protein_coding | Ppapdc1b | 374.4088 | 388.5778 | 381.4933 |
| ENSMUSG00000087405 | processed_transcrip | Gm14232 | 2.936413 | 3.823727 | 3.38007 |
| ENSMUSG00000044361 | protein_coding | BC024139 | 3.902744 | 2.992322 | 3.447533 |
| ENSMUSG00000084422 | snoRNA | SNORD88 | 8.745951 | 7.433764 | 8.089857 |
| ENSMUSG00000075312 | pseudogene | Gm13597 | 82.62637 | 87.35275 | 84.98956 |
| ENSMUSG00000001508 | protein_coding | Sgca | 5.548405 | 4.468108 | 5.008256 |
| ENSMUSG00000069196 | protein_coding | Gm3511 | 1.959572 | 1.333085 | 1.646328 |
| ENSMUSG00000088626 | snoRNA | SNORA28 | 1.959572 | 1.333085 | 1.646328 |
| ENSMUSG00000025916 | protein_coding | Lrrc67 | 5.232184 | 4.150241 | 4.691213 |
| ENSMUSG00000071052 | protein_coding | Rpl7a-ps5 | 20.28154 | 22.41733 | 21.34943 |
| ENSMUSG00000044791 | protein_coding | Setd2 | 5468.263 | 5588.741 | 5528.502 |
| ENSMUSG00000079597 | protein_coding | Gm5483 | 1.957261 | 1.350742 | 1.654001 |
| ENSMUSG00000046101 | protein_coding | 6030422M02Rik | 140.0659 | 118.2717 | 129.1688 |
| ENSMUSG00000020687 | protein_coding | Cdc27 | 3059.846 | 3151.011 | 3105.429 |
| ENSMUSG00000015363 | protein_coding | Trabd | 933.2088 | 954.8122 | 944.0105 |
| ENSMUSG00000027796 | protein_coding | Smad9 | 68.93352 | 73.40134 | 71.16743 |
| ENSMUSG00000054892 | protein_coding | Txk | 89.56959 | 83.27474 | 86.42216 |
| ENSMUSG00000042918 | protein_coding | Mamstr | 94.56941 | 99.84675 | 97.20808 |
| ENSMUSG00000087313 | lincRNA | Dleu2 | 164.1422 | 170.6189 | 167.3805 |
| ENSMUSG00000037379 | protein_coding | Spon2 | 98.25031 | 92.89479 | 95.57255 |
| ENSMUSG00000009731 | protein_coding | Kcnd1 | 51.58839 | 48.29583 | 49.94211 |
| ENSMUSG00000024354 | protein_coding | Slc23a1 | 30.6639 | 33.30434 | 31.98412 |
| ENSMUSG00000093085 | miRNA | AC153815.1 | 1.926772 | 2.692474 | 2.309623 |
| ENSMUSG00000085055 | lincRNA | Gm15958 | 34.25147 | 31.48366 | 32.86757 |
| ENSMUSG00000065530 | miRNA | Mir99a | 20.19305 | 17.78342 | 18.98824 |
| ENSMUSG00000089936 | pseudogene | Gm16199 | 2.589703 | 3.336855 | 2.963279 |
| ENSMUSG00000025779 | protein_coding | Ly96 | 55.04834 | 58.89708 | 56.97271 |
| ENSMUSG00000082678 | pseudogene | Gm12818 | 1.943172 | 1.341732 | 1.642452 |
| ENSMUSG00000064281 | pseudogene | Rpl19-ps1 | 3.905054 | 3.027997 | 3.466526 |
| ENSMUSG00000078640 | protein_coding | Gm11627 | 47.75378 | 44.61517 | 46.18447 |
| ENSMUSG00000015013 | protein_coding | Trappc2l | 551.123 | 569.2411 | 560.182 |
| ENSMUSG00000021200 | protein_coding | Asb2 | 884.472 | 854.1303 | 869.3011 |
| ENSMUSG00000021504 | protein_coding | B4galt7 | 449.8111 | 462.4154 | 456.1132 |
| ENSMUSG00000085307 | processed_transcrip | Gm11525 | 3.574744 | 4.557115 | 4.06593 |
| ENSMUSG00000035653 | protein_coding | Lrfn5 | 17.42735 | 15.55623 | 16.49179 |
| ENSMUSG00000074805 | processed_transcrip | Gm14047 | 8.202664 | 10.15612 | 9.179389 |
| ENSMUSG00000025644 | lincRNA | Gm7628 | 15.85086 | 17.75676 | 16.80381 |
| ENSMUSG00000069808 | protein_coding | Fam57a | 167.2961 | 175.6083 | 171.4522 |
| ENSMUSG00000026170 | protein_coding | Cyp27a1 | 653.3951 | 688.7193 | 671.0572 |
| ENSMUSG00000022840 | protein_coding | Adcy5 | 696.4855 | 677.4961 | 686.9908 |
| ENSMUSG00000048027 | protein_coding | Rgmb | 1159.928 | 1188.479 | 1174.203 |
| ENSMUSG00000018750 | protein_coding | Zbtb4 | 990.5923 | 1019.178 | 1004.885 |
| ENSMUSG00000085820 | processed_transcrip | 4933406K04Rik | 3.233924 | 2.375331 | 2.804627 |
| ENSMUSG00000067365 | protein_coding | Tmem128 | 1202.148 | 1235.609 | 1218.878 |
| ENSMUSG00000024875 | protein_coding | Yif1a | 493.8298 | 477.7356 | 485.7827 |
| ENSMUSG00000065258 | snoRNA | SNORD24 | 6.139848 | 5.067804 | 5.603826 |
| ENSMUSG00000091523 | processed_transcrip | AU040972 | 1.609283 | 1.059903 | 1.334593 |
| ENSMUSG00000042380 | protein_coding | BC003266 | 480.7065 | 467.708 | 474.2072 |
| ENSMUSG00000034168 | protein_coding | 6430527G18Rik | 2265.014 | 2325.962 | 2295.488 |
| ENSMUSG00000000628 | protein_coding | Hk2 | 4019.473 | 4116.111 | 4067.792 |
| ENSMUSG00000056919 | protein_coding | 4922501C03Rik | 1242.022 | 1208.931 | 1225.476 |
| ENSMUSG00000039474 | protein_coding | Wfs1 | 359.5637 | 371.0429 | 365.3033 |
| ENSMUSG00000086925 | pseudogene | Gm6286 | 3.238545 | 4.096909 | 3.667727 |
| ENSMUSG00000049349 | protein_coding | Gm5105 | 0.638331 | 1.024227 | 0.831279 |
| ENSMUSG00000090903 | protein_coding | Gm17247 | 0.638331 | 1.024227 | 0.831279 |
| ENSMUSG00000073051 | processed_transcrip | Gm14812 | 14.25567 | 15.98977 | 15.12272 |
| ENSMUSG00000035395 | protein_coding | Pet2 | 968.3518 | 850.8428 | 909.5973 |
| ENSMUSG00000074834 | protein_coding | Vmn2r-ps104 | 2.592014 | 3.51487 | 3.053442 |

| | | | | | |
|---|---|---|---|---|---|
| ENSMUSG00000074232 | protein_coding | Gm10647 | 15.32397 | 13.20213 | 14.26305 |
| ENSMUSG00000020872 | protein_coding | Tac4 | 2.289882 | 3.188355 | 2.739119 |
| ENSMUSG00000050147 | protein_coding | F2rl3 | 16.13659 | 17.95823 | 17.04741 |
| ENSMUSG00000059791 | protein_coding | Nrm | 381.714 | 393.3741 | 387.5441 |
| ENSMUSG00000090381 | protein_coding | Gm6158 | 11.02532 | 9.526542 | 10.27593 |
| ENSMUSG00000047804 | protein_coding | Akap10 | 1038.425 | 1014.009 | 1026.217 |
| ENSMUSG00000080723 | pseudogene | Gm12564 | 2.597903 | 3.479194 | 3.038549 |
| ENSMUSG00000091168 | lincRNA | 1700026J14Rik | 3.866366 | 3.063673 | 3.465019 |
| ENSMUSG00000089661 | protein_coding | Mia1 | 22.55629 | 24.88203 | 23.71916 |
| ENSMUSG00000002014 | protein_coding | Ssr4 | 1099.964 | 1071.197 | 1085.58 |
| ENSMUSG00000030589 | protein_coding | Rasgrp4 | 36.98564 | 40.47751 | 38.73157 |
| ENSMUSG00000028330 | protein_coding | Ncbp1 | 3026.274 | 3085.546 | 3055.91 |
| ENSMUSG00000049985 | protein_coding | Ankrd55 | 19.0543 | 21.09361 | 20.07396 |
| ENSMUSG00000018169 | protein_coding | Mfng | 80.80157 | 76.50462 | 78.65309 |
| ENSMUSG00000024732 | protein_coding | Ccdc86 | 810.7907 | 785.118 | 797.9543 |
| ENSMUSG00000089525 | misc_RNA | 7SK | 5.546094 | 4.485765 | 5.01593 |
| ENSMUSG00000089780 | processed_transcrip | Gm16306 | 6.11987 | 5.076452 | 5.598161 |
| ENSMUSG00000063193 | protein_coding | Cd300lb | 3.564234 | 2.781843 | 3.173038 |
| ENSMUSG00000028344 | protein_coding | Invs | 742.137 | 767.7658 | 754.9514 |
| ENSMUSG00000085789 | lincRNA | 4732419C18Rik | 49.43224 | 53.4951 | 51.46367 |
| ENSMUSG00000057606 | protein_coding | Colq | 3.550145 | 4.414414 | 3.982279 |
| ENSMUSG00000047141 | protein_coding | Zfp654 | 1352.502 | 1289.459 | 1320.98 |
| ENSMUSG00000034037 | protein_coding | Fgd5 | 416.9345 | 434.0057 | 425.4701 |
| ENSMUSG00000039065 | protein_coding | Fam173b | 626.8575 | 646.63 | 636.7438 |
| ENSMUSG00000069237 | protein_coding | C78339 | 1679.146 | 1645.073 | 1662.11 |
| ENSMUSG00000021999 | protein_coding | Cpb2 | 3.910944 | 3.072682 | 3.491813 |
| ENSMUSG00000060487 | protein_coding | Samd5 | 82.99738 | 76.37273 | 79.68506 |
| ENSMUSG00000082674 | pseudogene | Gm11914 | 79.60624 | 84.648 | 82.12712 |
| ENSMUSG00000031984 | protein_coding | 2810004N23Rik | 1437.387 | 1403.632 | 1420.509 |
| ENSMUSG00000049536 | protein_coding | Tceal1 | 430.0342 | 444.9422 | 437.4882 |
| ENSMUSG00000034690 | protein_coding | Nlrp4c | 1433.704 | 1469.454 | 1451.579 |
| ENSMUSG00000034177 | protein_coding | Rnf43 | 117.8957 | 123.3895 | 120.6426 |
| ENSMUSG00000086995 | processed_transcrip | Gm13544 | 6.11987 | 5.032129 | 5.575999 |
| ENSMUSG00000028152 | protein_coding | Tspan5 | 2333.994 | 2391.138 | 2362.566 |
| ENSMUSG00000073792 | protein_coding | Alg6 | 466.1608 | 481.9035 | 474.0322 |
| ENSMUSG00000020111 | protein_coding | Cbara1 | 3120.368 | 3187.259 | 3153.813 |
| ENSMUSG00000078922 | protein_coding | Tgtp1 | 10.72319 | 9.200027 | 9.961609 |
| ENSMUSG00000087220 | processed_transcrip | Gm11377 | 3.872255 | 3.072682 | 3.472468 |
| ENSMUSG00000086210 | lincRNA | A230087F16Rik | 8.994262 | 7.406736 | 8.200499 |
| ENSMUSG00000090053 | protein_coding | Palm2 | 11.97399 | 10.42029 | 11.19714 |
| ENSMUSG00000021188 | protein_coding | Trip11 | 1583.53 | 1547.15 | 1565.34 |
| ENSMUSG00000084056 | pseudogene | Rhox3b | 2.620192 | 3.425862 | 3.023027 |
| ENSMUSG00000040521 | protein_coding | Tsfm | 542.0677 | 558.9147 | 550.4912 |
| ENSMUSG00000061474 | protein_coding | Mrps36 | 430.4142 | 416.9159 | 423.665 |
| ENSMUSG00000055972 | protein_coding | 2810407A14Rik | 3.876876 | 4.812279 | 4.344578 |
| ENSMUSG00000066554 | pseudogene | Gm10167 | 90.93332 | 96.32382 | 93.62857 |
| ENSMUSG00000009070 | protein_coding | Rtdr1 | 8.368752 | 9.781705 | 9.075229 |
| ENSMUSG00000037190 | protein_coding | Cyb561d2 | 388.8806 | 376.5306 | 382.7056 |
| ENSMUSG00000038342 | protein_coding | Mlxip | 2443.618 | 2372.055 | 2407.837 |
| ENSMUSG00000051041 | protein_coding | Olfml1 | 281.9558 | 291.108 | 286.5319 |
| ENSMUSG00000036097 | protein_coding | Fam178a | 5978.774 | 6116.781 | 6047.777 |
| ENSMUSG00000049755 | protein_coding | Zfp672 | 811.2727 | 779.8668 | 795.5697 |
| ENSMUSG00000041536 | protein_coding | Serpina3a | 876.5018 | 1124.487 | 1000.495 |
| ENSMUSG00000025922 | protein_coding | Gm5045 | 3.538366 | 4.414776 | 3.976571 |
| ENSMUSG00000072662 | protein_coding | Gm5887 | 72.13046 | 76.49126 | 74.31086 |
| ENSMUSG00000033316 | protein_coding | Galnt9 | 15.17153 | 17.03129 | 16.10141 |
| ENSMUSG00000090467 | protein_coding | Gm4613 | 2.577925 | 3.354873 | 2.966399 |
| ENSMUSG00000090523 | protein_coding | Gypc | 170.6945 | 163.9175 | 167.306 |

| | | | | | |
|---|---|---|---|---|---|
| ENSMUSG00000024393 | protein_coding | Prrc2a | 15808.12 | 15433.79 | 15620.95 |
| ENSMUSG00000020051 | protein_coding | Pah | 5.204006 | 4.177269 | 4.690637 |
| ENSMUSG00000027603 | protein_coding | Ggt7 | 143.488 | 137.5352 | 140.5116 |
| ENSMUSG00000029708 | protein_coding | Gcc1 | 1109.524 | 1076.241 | 1092.882 |
| ENSMUSG00000008305 | protein_coding | Tle1 | 1943.385 | 1895.323 | 1919.354 |
| ENSMUSG00000026904 | protein_coding | Slc4a10 | 25.89323 | 23.11717 | 24.5052 |
| ENSMUSG00000040599 | protein_coding | Mis12 | 1464.112 | 1418.566 | 1441.339 |
| ENSMUSG00000000305 | protein_coding | Cdh4 | 45.64743 | 49.333 | 47.49022 |
| ENSMUSG00000009681 | protein_coding | Bcr | 1925.257 | 1979.45 | 1952.354 |
| ENSMUSG00000058809 | pseudogene | Gm12141 | 108.3638 | 114.433 | 111.3984 |
| ENSMUSG00000039672 | protein_coding | Kcne2 | 5.850537 | 4.750299 | 5.300418 |
| ENSMUSG00000075266 | protein_coding | Cenpw | 305.7072 | 317.5305 | 311.6189 |
| ENSMUSG00000039824 | protein_coding | Myl6b | 437.1682 | 425.7982 | 431.4832 |
| ENSMUSG00000025969 | protein_coding | Nrp2 | 416.3297 | 398.466 | 407.3978 |
| ENSMUSG00000069312 | protein_coding | Hist1h3h | 12.55134 | 10.9753 | 11.76332 |
| ENSMUSG00000082610 | pseudogene | Gm13829 | 0.646531 | 1.024227 | 0.835379 |
| ENSMUSG00000030205 | protein_coding | Gprc5d | 0.646531 | 1.024227 | 0.835379 |
| ENSMUSG00000086761 | processed_transcrip | Gm11666 | 0.646531 | 1.024227 | 0.835379 |
| ENSMUSG00000071273 | protein_coding | Gm5145 | 0.646531 | 1.024227 | 0.835379 |
| ENSMUSG00000065314 | snRNA | U6 | 0.646531 | 1.024227 | 0.835379 |
| ENSMUSG00000022615 | protein_coding | Tymp | 127.3239 | 132.9629 | 130.1434 |
| ENSMUSG00000026353 | protein_coding | Ubxn4 | 3469.961 | 3534.95 | 3502.455 |
| ENSMUSG00000026039 | protein_coding | Sgol2 | 536.0433 | 521.2865 | 528.6649 |
| ENSMUSG00000069011 | pseudogene | Gm10254 | 99.13129 | 93.57019 | 96.35074 |
| ENSMUSG00000020698 | protein_coding | Cct6b | 36.20053 | 38.98298 | 37.59176 |
| ENSMUSG00000043453 | protein_coding | Magea10 | 294.1056 | 282.8197 | 288.4626 |
| ENSMUSG00000040026 | protein_coding | Saa3 | 2.942302 | 3.850393 | 3.396348 |
| ENSMUSG00000040250 | protein_coding | 4933424B01Rik | 1929.912 | 1886.522 | 1908.217 |
| ENSMUSG00000087221 | lincRNA | BC037032 | 150.8414 | 144.4705 | 147.656 |
| ENSMUSG00000029386 | protein_coding | Tctn2 | 960.2009 | 988.0766 | 974.1388 |
| ENSMUSG00000025930 | protein_coding | Msc | 11.6401 | 10.0639 | 10.852 |
| ENSMUSG00000077614 | snoRNA | SNORA40 | 12.52063 | 10.91296 | 11.71679 |
| ENSMUSG00000074660 | protein_coding | Gm10736 | 23.93746 | 21.71925 | 22.82836 |
| ENSMUSG00000038060 | protein_coding | Dlec1 | 95.05932 | 99.78038 | 97.41985 |
| ENSMUSG00000090327 | processed_transcrip | Gm17111 | 9.417081 | 10.90467 | 10.16088 |
| ENSMUSG00000087657 | processed_transcrip | Gm16764 | 3.566544 | 4.414414 | 3.990479 |
| ENSMUSG00000044390 | protein_coding | Tigd3 | 145.8475 | 139.1115 | 142.4795 |
| ENSMUSG00000031432 | protein_coding | Prps1 | 1536.92 | 1503.357 | 1520.138 |
| ENSMUSG00000005483 | protein_coding | Dnajb1 | 1901.461 | 1864.314 | 1882.887 |
| ENSMUSG00000055489 | protein_coding | Ano5 | 25.04908 | 22.20105 | 23.62506 |
| ENSMUSG00000092713 | snoRNA | CT010429.1 | 14.22876 | 16.05175 | 15.14025 |
| ENSMUSG00000028476 | protein_coding | Reck | 1346.996 | 1375.224 | 1361.11 |
| ENSMUSG00000074704 | protein_coding | Rad21l | 568.4124 | 499.0295 | 533.721 |
| ENSMUSG00000025873 | protein_coding | Faf2 | 3533.928 | 3602.274 | 3568.101 |
| ENSMUSG00000033576 | protein_coding | Apol6 | 6.11987 | 5.121137 | 5.620503 |
| ENSMUSG00000082111 | pseudogene | Gm12479 | 9.112639 | 7.629436 | 8.371037 |
| ENSMUSG00000042505 | protein_coding | Acn9 | 446.353 | 434.1704 | 440.2617 |
| ENSMUSG00000090637 | protein_coding | Gm6189 | 3.225724 | 2.428301 | 2.827012 |
| ENSMUSG00000029478 | protein_coding | Ncor2 | 8334.369 | 8514.396 | 8424.382 |
| ENSMUSG00000022619 | protein_coding | Mapk8ip2 | 59.19268 | 62.94279 | 61.06773 |
| ENSMUSG00000045237 | protein_coding | 1110012L19Rik | 415.1558 | 431.5523 | 423.3541 |
| ENSMUSG00000055762 | protein_coding | Eef1d | 3795.717 | 3715.343 | 3755.53 |
| ENSMUSG00000036790 | protein_coding | Slitrk2 | 3.566544 | 4.450451 | 4.008498 |
| ENSMUSG00000000884 | protein_coding | Gnb1l | 220.7556 | 213.0419 | 216.8987 |
| ENSMUSG00000021782 | protein_coding | Dlg5 | 1254.425 | 1299.664 | 1277.045 |
| ENSMUSG00000070997 | protein_coding | 1700055D18Rik | 35.34691 | 38.29428 | 36.82059 |
| ENSMUSG00000074603 | protein_coding | Gm10729 | 2.902346 | 3.80607 | 3.354208 |
| ENSMUSG00000036430 | protein_coding | Tbcc | 612.588 | 592.2096 | 602.3988 |

| | | | | | |
|---|---|---|---|---|---|
| ENSMUSG00000037995 | protein_coding | Igsf9 | 292.9301 | 306.2825 | 299.6063 |
| ENSMUSG00000032845 | protein_coding | Alpk2 | 9.605459 | 8.176162 | 8.89081 |
| ENSMUSG00000021411 | protein_coding | 1300014I06Rik | 42.19151 | 38.93581 | 40.56366 |
| ENSMUSG00000080254 | pseudogene | Gm6036 | 1.939594 | 1.359751 | 1.649672 |
| ENSMUSG00000076216 | miRNA | Mir695 | 0.33031 | 0.662038 | 0.496174 |
| ENSMUSG00000064738 | snRNA | U1 | 0.33031 | 0.662038 | 0.496174 |
| ENSMUSG00000028148 | protein_coding | Them5 | 0.33031 | 0.662038 | 0.496174 |
| ENSMUSG00000052767 | processed_transcrip | Gm12703 | 0.33031 | 0.662038 | 0.496174 |
| ENSMUSG00000028571 | protein_coding | Cyp2j13 | 0.33031 | 0.662038 | 0.496174 |
| ENSMUSG00000082699 | pseudogene | Gm12736 | 0.33031 | 0.662038 | 0.496174 |
| ENSMUSG00000090220 | processed_transcrip | 4930568K20Rik | 0.33031 | 0.662038 | 0.496174 |
| ENSMUSG00000079346 | protein_coding | 1700013D24Rik | 0.33031 | 0.662038 | 0.496174 |
| ENSMUSG00000054986 | protein_coding | Sec14l3 | 0.33031 | 0.662038 | 0.496174 |
| ENSMUSG00000083792 | pseudogene | Rpl12-ps2 | 0.33031 | 0.662038 | 0.496174 |
| ENSMUSG00000082857 | pseudogene | Gm12293 | 0.33031 | 0.662038 | 0.496174 |
| ENSMUSG00000081675 | pseudogene | Gm12712 | 0.33031 | 0.662038 | 0.496174 |
| ENSMUSG00000093028 | misc_RNA | AL596246.1 | 0.33031 | 0.662038 | 0.496174 |
| ENSMUSG00000090478 | pseudogene | Gm5656 | 0.33031 | 0.662038 | 0.496174 |
| ENSMUSG00000091682 | protein_coding | Gm17356 | 0.33031 | 0.662038 | 0.496174 |
| ENSMUSG00000084038 | pseudogene | Gm11295 | 0.33031 | 0.662038 | 0.496174 |
| ENSMUSG00000084024 | pseudogene | Gm15937 | 0.33031 | 0.662038 | 0.496174 |
| ENSMUSG00000084493 | snRNA | U4 | 0.33031 | 0.662038 | 0.496174 |
| ENSMUSG00000022586 | protein_coding | Ly6i | 0.33031 | 0.662038 | 0.496174 |
| ENSMUSG00000078314 | processed_transcrip | Gm14762 | 0.33031 | 0.662038 | 0.496174 |
| ENSMUSG00000083019 | pseudogene | Gm6222 | 0.33031 | 0.662038 | 0.496174 |
| ENSMUSG00000085536 | processed_transcrip | Gm14950 | 0.33031 | 0.662038 | 0.496174 |
| ENSMUSG00000064426 | snRNA | U6 | 0.638331 | 1.059903 | 0.849117 |
| ENSMUSG00000081413 | pseudogene | Gm14088 | 0.638331 | 1.059903 | 0.849117 |
| ENSMUSG00000088294 | snoRNA | SNORD113 | 0.638331 | 1.059903 | 0.849117 |
| ENSMUSG00000079386 | protein_coding | Gm3164 | 0.638331 | 1.059903 | 0.849117 |
| ENSMUSG00000041552 | protein_coding | Ptchd1 | 117.9543 | 124.7459 | 121.3501 |
| ENSMUSG00000052372 | protein_coding | Il1rapl1 | 1.288441 | 1.995123 | 1.641782 |
| ENSMUSG00000026770 | protein_coding | Il2ra | 34.86647 | 32.17485 | 33.52066 |
| ENSMUSG00000068615 | protein_coding | Gjd2 | 2.267593 | 2.983313 | 2.625453 |
| ENSMUSG00000025892 | protein_coding | Gria4 | 24.1069 | 26.5146 | 25.31075 |
| ENSMUSG00000039148 | protein_coding | Sart1 | 3162.72 | 3231.09 | 3196.905 |
| ENSMUSG00000062190 | protein_coding | Lancl2 | 959.9103 | 936.231 | 948.0707 |
| ENSMUSG00000052957 | protein_coding | Gas1 | 614.2765 | 591.3701 | 602.8233 |
| ENSMUSG00000080816 | pseudogene | Gm12447 | 0.65242 | 1.024227 | 0.838324 |
| ENSMUSG00000046652 | protein_coding | Tas2r143 | 0.65242 | 1.024227 | 0.838324 |
| ENSMUSG00000049396 | protein_coding | Gemin4 | 13.25549 | 15.14035 | 14.19792 |
| ENSMUSG00000074746 | protein_coding | Pdzd8 | 2344.27 | 2300.031 | 2322.15 |
| ENSMUSG00000081480 | pseudogene | Gm13608 | 0.32211 | 0.662038 | 0.492074 |
| ENSMUSG00000083530 | pseudogene | Gm13867 | 0.32211 | 0.662038 | 0.492074 |
| ENSMUSG00000082932 | protein_coding | Cyp2j8 | 0.32211 | 0.662038 | 0.492074 |
| ENSMUSG00000070877 | protein_coding | Ldlrad1 | 0.32211 | 0.662038 | 0.492074 |
| ENSMUSG00000081585 | pseudogene | Gm12926 | 0.32211 | 0.662038 | 0.492074 |
| ENSMUSG00000080523 | miRNA | AC117578.1 | 0.32211 | 0.662038 | 0.492074 |
| ENSMUSG00000085919 | processed_transcrip | Gm16019 | 0.32211 | 0.662038 | 0.492074 |
| ENSMUSG00000071204 | processed_transcrip | Gm10319 | 0.32211 | 0.662038 | 0.492074 |
| ENSMUSG00000091392 | lincRNA | Gm17263 | 0.32211 | 0.662038 | 0.492074 |
| ENSMUSG00000073997 | protein_coding | Olfr521 | 0.32211 | 0.662038 | 0.492074 |
| ENSMUSG00000084738 | snoRNA | SNORD88 | 0.32211 | 0.662038 | 0.492074 |
| ENSMUSG00000083760 | pseudogene | Gm14524 | 0.32211 | 0.662038 | 0.492074 |
| ENSMUSG00000081505 | pseudogene | Gm8644 | 0.32211 | 0.662038 | 0.492074 |
| ENSMUSG00000029106 | protein_coding | Add1 | 5944.216 | 6055.57 | 5999.893 |
| ENSMUSG00000022817 | protein_coding | Itgb5 | 2190.313 | 2247.548 | 2218.93 |
| ENSMUSG00000002812 | protein_coding | Flii | 2231.617 | 2277.538 | 2254.577 |

| | | | | | |
|---|---|---|---|---|---|
| ENSMUSG00000070847 | protein_coding | Vmn2r30 | 2.279371 | 3.054663 | 2.667017 |
| ENSMUSG00000090989 | pseudogene | Gm17042 | 5.830559 | 7.499316 | 6.664938 |
| ENSMUSG00000022314 | protein_coding | Rad21 | 9333.044 | 9522.236 | 9427.64 |
| ENSMUSG00000016024 | protein_coding | Lbp | 149.3166 | 139.7846 | 144.5506 |
| ENSMUSG00000071653 | protein_coding | 1810009A15Rik | 704.376 | 686.211 | 695.2935 |
| ENSMUSG00000025175 | protein_coding | Fn3k | 10.00264 | 11.56707 | 10.78485 |
| ENSMUSG00000085322 | lincRNA | Gm14261 | 12.55723 | 10.84482 | 11.70102 |
| ENSMUSG00000078888 | protein_coding | Gm2007 | 0.316221 | 0.662038 | 0.489129 |
| ENSMUSG00000065773 | snRNA | U1 | 0.316221 | 0.662038 | 0.489129 |
| ENSMUSG00000093163 | miRNA | AL607123.1 | 0.316221 | 0.662038 | 0.489129 |
| ENSMUSG00000089453 | misc_RNA | 7SK | 0.316221 | 0.662038 | 0.489129 |
| ENSMUSG00000084780 | processed_transcrip | Gm15350 | 0.316221 | 0.662038 | 0.489129 |
| ENSMUSG00000056943 | pseudogene | Gm10015 | 0.316221 | 0.662038 | 0.489129 |
| ENSMUSG00000065080 | snRNA | U6 | 0.316221 | 0.662038 | 0.489129 |
| ENSMUSG00000022076 | protein_coding | Klhl1 | 0.316221 | 0.662038 | 0.489129 |
| ENSMUSG00000023926 | protein_coding | Rhag | 0.316221 | 0.662038 | 0.489129 |
| ENSMUSG00000081298 | pseudogene | Gm14622 | 0.316221 | 0.662038 | 0.489129 |
| ENSMUSG00000061143 | protein_coding | Maml3 | 550.4656 | 532.2942 | 541.3799 |
| ENSMUSG00000031027 | protein_coding | Stk33 | 182.5883 | 157.8356 | 170.2119 |
| ENSMUSG00000078552 | protein_coding | Dcdc2b | 50.26193 | 46.50896 | 48.38545 |
| ENSMUSG00000043811 | protein_coding | Rtn4r | 5.79776 | 6.973196 | 6.385478 |
| ENSMUSG00000006412 | protein_coding | Pfdn2 | 1250.36 | 1281.517 | 1265.938 |
| ENSMUSG00000046748 | protein_coding | 2310005G13Rik | 17.73179 | 15.87374 | 16.80276 |
| ENSMUSG00000011877 | protein_coding | Git1 | 2340.003 | 2281.266 | 2310.634 |
| ENSMUSG00000081992 | pseudogene | Gm13408 | 31.97821 | 29.32347 | 30.65084 |
| ENSMUSG00000049148 | protein_coding | Plcxd3 | 11.94812 | 13.7121 | 12.83011 |
| ENSMUSG00000032661 | protein_coding | Oas3 | 3.878144 | 3.063673 | 3.470908 |
| ENSMUSG00000048240 | protein_coding | Gng7 | 312.1319 | 301.0064 | 306.5691 |
| ENSMUSG00000084021 | pseudogene | Gm5832 | 0.33031 | 0.724379 | 0.527345 |
| ENSMUSG00000084749 | misc_RNA | 7SK | 0.33031 | 0.724379 | 0.527345 |
| ENSMUSG00000069132 | protein_coding | Nxph2 | 0.33031 | 0.724379 | 0.527345 |
| ENSMUSG00000026919 | protein_coding | Lcn4 | 0.33031 | 0.724379 | 0.527345 |
| ENSMUSG00000086879 | processed_transcrip | Gm14012 | 0.33031 | 0.724379 | 0.527345 |
| ENSMUSG00000083750 | pseudogene | Gm14108 | 0.33031 | 0.724379 | 0.527345 |
| ENSMUSG00000083546 | pseudogene | Gm12487 | 0.33031 | 0.724379 | 0.527345 |
| ENSMUSG00000041616 | protein_coding | Nppa | 0.33031 | 0.724379 | 0.527345 |
| ENSMUSG00000088482 | snRNA | U6 | 0.33031 | 0.724379 | 0.527345 |
| ENSMUSG00000039728 | protein_coding | Slc6a5 | 0.33031 | 0.724379 | 0.527345 |
| ENSMUSG00000065611 | miRNA | Mir23a | 0.33031 | 0.724379 | 0.527345 |
| ENSMUSG00000092841 | miRNA | AC124992.1 | 0.33031 | 0.724379 | 0.527345 |
| ENSMUSG00000078131 | protein_coding | Krtap1-3 | 0.33031 | 0.724379 | 0.527345 |
| ENSMUSG00000081360 | pseudogene | Gm11718 | 0.33031 | 0.724379 | 0.527345 |
| ENSMUSG00000087486 | processed_transcrip | Gm11723 | 0.33031 | 0.724379 | 0.527345 |
| ENSMUSG00000083149 | pseudogene | Gm11380 | 0.33031 | 0.724379 | 0.527345 |
| ENSMUSG00000082433 | pseudogene | Gm9025 | 0.33031 | 0.724379 | 0.527345 |
| ENSMUSG00000085883 | processed_transcrip | Gm3848 | 0.33031 | 0.724379 | 0.527345 |
| ENSMUSG00000064411 | snRNA | U6 | 0.33031 | 0.724379 | 0.527345 |
| ENSMUSG00000088128 | snRNA | U6 | 0.33031 | 0.724379 | 0.527345 |
| ENSMUSG00000077460 | snoRNA | SNORA17 | 0.33031 | 0.724379 | 0.527345 |
| ENSMUSG00000091259 | protein_coding | Vmn2r110 | 0.33031 | 0.724379 | 0.527345 |
| ENSMUSG00000092198 | processed_transcrip | Gm20527 | 0.33031 | 0.724379 | 0.527345 |
| ENSMUSG00000044526 | protein_coding | Znrf4 | 0.33031 | 0.724379 | 0.527345 |
| ENSMUSG00000092333 | protein_coding | AL808133.1 | 0.33031 | 0.724379 | 0.527345 |
| ENSMUSG00000031130 | protein_coding | Brs3 | 0.33031 | 0.724379 | 0.527345 |
| ENSMUSG00000092081 | lincRNA | Gm17360 | 27.11711 | 24.70293 | 25.91002 |
| ENSMUSG00000020149 | protein_coding | Rab1 | 4317.028 | 4411.137 | 4364.083 |
| ENSMUSG00000028233 | protein_coding | Tgs1 | 1719.737 | 1674.201 | 1696.969 |
| ENSMUSG00000040272 | protein_coding | Accs | 291.6522 | 303.6792 | 297.6657 |

| | | | | | |
|---|---|---|---|---|---|
| ENSMUSG00000027770 | protein_coding | Dhx36 | 2378.834 | 2422.458 | 2400.646 |
| ENSMUSG00000031074 | protein_coding | Fgf3 | 27.656 | 30.58919 | 29.1226 |
| ENSMUSG00000074643 | protein_coding | Cpne1 | 2093.033 | 2021.662 | 2057.348 |
| ENSMUSG00000037013 | protein_coding | Ss18 | 3540.221 | 3606.418 | 3573.319 |
| ENSMUSG00000082507 | pseudogene | Gm9378 | 9.330237 | 10.77098 | 10.05061 |
| ENSMUSG00000020130 | protein_coding | Tbc1d15 | 3014.326 | 2955.705 | 2985.016 |
| ENSMUSG00000026700 | protein_coding | Tnfsf4 | 5.132518 | 4.114566 | 4.623542 |
| ENSMUSG00000067212 | protein_coding | H2-T23 | 86.54342 | 91.87062 | 89.20702 |
| ENSMUSG00000075020 | processed_transcrip | E530001K10Rik | 41.39403 | 38.28418 | 39.8391 |
| ENSMUSG00000086064 | processed_transcrip | 4930439A04Rik | 0.32211 | 0.724379 | 0.523245 |
| ENSMUSG00000081429 | pseudogene | Gm11603 | 0.32211 | 0.724379 | 0.523245 |
| ENSMUSG00000090847 | protein_coding | Gm17672 | 0.32211 | 0.724379 | 0.523245 |
| ENSMUSG00000082328 | pseudogene | Gm13921 | 0.32211 | 0.724379 | 0.523245 |
| ENSMUSG00000081423 | pseudogene | Gm12882 | 0.32211 | 0.724379 | 0.523245 |
| ENSMUSG00000082289 | pseudogene | Gm15596 | 0.32211 | 0.724379 | 0.523245 |
| ENSMUSG00000066512 | protein_coding | Klk1b5 | 0.32211 | 0.724379 | 0.523245 |
| ENSMUSG00000073866 | protein_coding | B430319F04Rik | 0.32211 | 0.724379 | 0.523245 |
| ENSMUSG00000086909 | processed_transcrip | Gm4926 | 0.32211 | 0.724379 | 0.523245 |
| ENSMUSG00000063166 | protein_coding | Gm5449 | 0.32211 | 0.724379 | 0.523245 |
| ENSMUSG00000091106 | lincRNA | Gm17625 | 0.32211 | 0.724379 | 0.523245 |
| ENSMUSG00000084575 | snRNA | U6 | 0.32211 | 0.724379 | 0.523245 |
| ENSMUSG00000070385 | protein_coding | Ampd1 | 6.113981 | 4.978435 | 5.546208 |
| ENSMUSG00000047789 | protein_coding | Slc38a9 | 740.4406 | 720.5561 | 730.4984 |
| ENSMUSG00000029223 | protein_coding | Uchl1 | 5864.049 | 5741.815 | 5802.932 |
| ENSMUSG00000062861 | protein_coding | Zfp28 | 259.5795 | 269.7334 | 264.6564 |
| ENSMUSG00000078869 | protein_coding | Gm14409 | 19.10245 | 17.09364 | 18.09804 |
| ENSMUSG00000006463 | protein_coding | Zdhhc24 | 535.9664 | 521.4006 | 528.6835 |
| ENSMUSG00000082851 | pseudogene | Gm11931 | 0.316221 | 0.724379 | 0.5203 |
| ENSMUSG00000081503 | pseudogene | Gm11214 | 0.316221 | 0.724379 | 0.5203 |
| ENSMUSG00000093077 | miRNA | AC113316.1 | 0.316221 | 0.724379 | 0.5203 |
| ENSMUSG00000091271 | processed_transcrip | Gm17104 | 0.316221 | 0.724379 | 0.5203 |
| ENSMUSG00000025488 | protein_coding | Cox8b | 0.316221 | 0.724379 | 0.5203 |
| ENSMUSG00000076144 | miRNA | Mir709 | 0.316221 | 0.724379 | 0.5203 |
| ENSMUSG00000087754 | snRNA | U1 | 0.316221 | 0.724379 | 0.5203 |
| ENSMUSG00000084944 | processed_transcrip | Gm14684 | 0.316221 | 0.724379 | 0.5203 |
| ENSMUSG00000075118 | lincRNA | Gpr137b-ps | 319.3653 | 328.7028 | 324.034 |
| ENSMUSG00000091886 | lincRNA | AW047730 | 8.774129 | 7.425117 | 8.099623 |
| ENSMUSG00000041426 | protein_coding | Hibch | 416.3708 | 402.5611 | 409.4659 |
| ENSMUSG00000016262 | protein_coding | Sertad4 | 1078.436 | 1106.564 | 1092.5 |
| ENSMUSG00000025575 | protein_coding | Cant1 | 1119.864 | 1094.119 | 1106.992 |
| ENSMUSG00000047819 | protein_coding | Tigd4 | 1.937283 | 1.395426 | 1.666354 |
| ENSMUSG00000090451 | protein_coding | Gm6133 | 1.937283 | 1.395426 | 1.666354 |
| ENSMUSG00000021240 | protein_coding | Abcd4 | 157.6941 | 164.4574 | 161.0757 |
| ENSMUSG00000073083 | protein_coding | Fam177a | 3.846388 | 3.072682 | 3.459535 |
| ENSMUSG00000051306 | protein_coding | Usp42 | 2010.966 | 1970.563 | 1990.765 |
| ENSMUSG00000020870 | protein_coding | Cdc34-ps | 12.64743 | 11.04665 | 11.84704 |
| ENSMUSG00000084773 | processed_transcrip | Gm12159 | 3.862787 | 3.055025 | 3.458906 |
| ENSMUSG00000062713 | protein_coding | Sim2 | 3.564234 | 2.7995 | 3.181867 |
| ENSMUSG00000071701 | protein_coding | Gm6604 | 1.914994 | 1.350742 | 1.632868 |
| ENSMUSG00000027220 | protein_coding | Syt13 | 450.0424 | 466.5416 | 458.292 |
| ENSMUSG00000036940 | protein_coding | Kdm1a | 3239.741 | 3316.08 | 3277.911 |
| ENSMUSG00000044763 | protein_coding | Rg9mtd1 | 1000.29 | 1031.933 | 1016.111 |
| ENSMUSG00000021846 | protein_coding | Peli2 | 414.6325 | 403.4818 | 409.0571 |
| ENSMUSG00000056133 | protein_coding | Gm9992 | 3.211635 | 4.052225 | 3.63193 |
| ENSMUSG00000041236 | protein_coding | Vps41 | 3305.884 | 3247.49 | 3276.687 |
| ENSMUSG00000081720 | pseudogene | Gm12391 | 1.957261 | 1.386417 | 1.671839 |
| ENSMUSG00000085952 | processed_transcrip | Gm12315 | 0.638331 | 1.050893 | 0.844612 |
| ENSMUSG00000073534 | lincRNA | Gm10532 | 0.638331 | 1.050893 | 0.844612 |

| | | | | | |
|---|---|---|---|---|---|
| ENSMUSG00000084289 | pseudogene | Gm6977 | 0.638331 | 1.050893 | 0.844612 |
| ENSMUSG00000035142 | protein_coding | Nubpl | 283.4862 | 293.597 | 288.5416 |
| ENSMUSG00000082192 | pseudogene | Gm14719 | 18.06799 | 16.1916 | 17.1298 |
| ENSMUSG00000085167 | processed_transcrip | Gm12063 | 0.646531 | 1.059903 | 0.853217 |
| ENSMUSG00000083890 | pseudogene | Gm15703 | 0.646531 | 1.059903 | 0.853217 |
| ENSMUSG00000089437 | snRNA | U6 | 0.646531 | 1.059903 | 0.853217 |
| ENSMUSG00000032397 | protein_coding | Tipin | 2150.931 | 2097.268 | 2124.1 |
| ENSMUSG00000030165 | protein_coding | Klrd1 | 3.266723 | 4.221592 | 3.744157 |
| ENSMUSG00000031731 | protein_coding | Ap1g1 | 3248.444 | 3166.435 | 3207.439 |
| ENSMUSG00000079110 | protein_coding | Capn3 | 15.52644 | 13.81047 | 14.66846 |
| ENSMUSG00000069939 | pseudogene | Gm12070 | 23.60461 | 21.361 | 22.48281 |
| ENSMUSG00000091183 | protein_coding | Gm3604 | 214.0414 | 227.2454 | 220.6434 |
| ENSMUSG00000035666 | protein_coding | Gtf3c4 | 1997.24 | 1951.875 | 1974.558 |
| ENSMUSG00000047747 | protein_coding | Rnf150 | 73.02993 | 69.15443 | 71.09218 |
| ENSMUSG00000040446 | protein_coding | Rprd1a | 1288.269 | 1316.816 | 1302.543 |
| ENSMUSG00000031918 | protein_coding | Mtmr2 | 2708.641 | 2755.899 | 2732.27 |
| ENSMUSG00000021662 | protein_coding | Rgnef | 195.1262 | 188.2841 | 191.7052 |
| ENSMUSG00000025326 | protein_coding | Ube3a | 3032.148 | 2943.562 | 2987.855 |
| ENSMUSG00000035673 | protein_coding | Sbno2 | 1403.717 | 1445.694 | 1424.706 |
| ENSMUSG00000032606 | protein_coding | Nicn1 | 483.8069 | 497.953 | 490.88 |
| ENSMUSG00000079481 | protein_coding | Nhsl2 | 822.2318 | 800.8637 | 811.5478 |
| ENSMUSG00000024270 | protein_coding | Slc39a6 | 4140.383 | 4228.282 | 4184.332 |
| ENSMUSG00000038836 | protein_coding | Agbl3 | 489.7093 | 463.3999 | 476.5546 |
| ENSMUSG00000064984 | snoRNA | SNORD73 | 4.218965 | 3.345864 | 3.782414 |
| ENSMUSG00000029381 | protein_coding | Shroom3 | 613.5158 | 595.0246 | 604.2702 |
| ENSMUSG00000026603 | protein_coding | Smyd2 | 1629.858 | 1584.686 | 1607.272 |
| ENSMUSG00000053957 | lincRNA | Gm12474 | 14.81766 | 16.55415 | 15.68591 |
| ENSMUSG00000040013 | protein_coding | Fkbp6 | 4029.499 | 3934.809 | 3982.154 |
| ENSMUSG00000020634 | protein_coding | Ubxn2a | 800.7086 | 823.3169 | 812.0127 |
| ENSMUSG00000027238 | protein_coding | Frmd5 | 409.1326 | 390.8895 | 400.011 |
| ENSMUSG00000040280 | protein_coding | Ndufa4l2 | 48.22856 | 51.37752 | 49.80304 |
| ENSMUSG00000032649 | protein_coding | Glt25d2 | 31.23388 | 34.43311 | 32.83349 |
| ENSMUSG00000056940 | protein_coding | Gm3629 | 1.610551 | 0.997561 | 1.304056 |
| ENSMUSG00000042590 | protein_coding | Ipo11 | 1974.047 | 2014.785 | 1994.416 |
| ENSMUSG00000051368 | pseudogene | 1700065D16Rik | 10.37752 | 8.953511 | 9.665517 |
| ENSMUSG00000060863 | protein_coding | Tcl1b2 | 1.949061 | 1.324076 | 1.636568 |
| ENSMUSG00000045954 | protein_coding | Sdpr | 839.8121 | 867.3914 | 853.6017 |
| ENSMUSG00000038949 | protein_coding | Cnst | 1454.465 | 1418.203 | 1436.334 |
| ENSMUSG00000049751 | protein_coding | Rpl36al | 1778.981 | 1822.589 | 1800.785 |
| ENSMUSG00000030411 | protein_coding | Nova2 | 80.69348 | 76.35218 | 78.52283 |
| ENSMUSG00000059824 | protein_coding | Dbp | 845.4482 | 872.3545 | 858.9014 |
| ENSMUSG00000084319 | pseudogene | Gm14456 | 718.9484 | 737.965 | 728.4567 |
| ENSMUSG00000034220 | protein_coding | Gpc1 | 483.6884 | 468.785 | 476.2367 |
| ENSMUSG00000031479 | protein_coding | Vps36 | 1000.241 | 978.0974 | 989.1693 |
| ENSMUSG00000001687 | protein_coding | Ubl3 | 2806.571 | 2862.755 | 2834.663 |
| ENSMUSG00000089015 | snRNA | U6 | 2.259393 | 1.677256 | 1.968324 |
| ENSMUSG00000091864 | lincRNA | Gm17102 | 8.363906 | 7.151934 | 7.75792 |
| ENSMUSG00000021186 | protein_coding | Fbln5 | 994.988 | 1024.552 | 1009.77 |
| ENSMUSG00000018559 | protein_coding | Ctdnep1 | 1281.327 | 1253.847 | 1267.587 |
| ENSMUSG00000006920 | protein_coding | Ezh1 | 1922.277 | 1876.878 | 1899.577 |
| ENSMUSG00000073142 | protein_coding | 6030469F06Rik | 16.10022 | 17.9049 | 17.00256 |
| ENSMUSG00000087323 | processed_transcrip | Gm14170 | 0.65242 | 1.059903 | 0.856161 |
| ENSMUSG00000082589 | pseudogene | Gm12433 | 0.65242 | 1.059903 | 0.856161 |
| ENSMUSG00000089250 | snRNA | U6 | 0.65242 | 1.059903 | 0.856161 |
| ENSMUSG00000064308 | protein_coding | 2410137M14Rik | 0.65242 | 1.059903 | 0.856161 |
| ENSMUSG00000090227 | processed_transcrip | Gm16554 | 0.65242 | 1.059903 | 0.856161 |
| ENSMUSG00000082245 | pseudogene | Gm6292 | 0.65242 | 1.059903 | 0.856161 |
| ENSMUSG00000046138 | protein_coding | 9930021J03Rik | 1130.314 | 1101.09 | 1115.702 |

| | | | | | |
|---|---|---|---|---|---|
| ENSMUSG00000044405 | protein_coding | Adig | 2.281682 | 1.677256 | 1.979469 |
| ENSMUSG00000022445 | protein_coding | Cyp2d26 | 2.281682 | 1.677256 | 1.979469 |
| ENSMUSG00000022008 | protein_coding | 1200011I18Rik | 1345.337 | 1308.989 | 1327.163 |
| ENSMUSG00000041258 | protein_coding | Zfp236 | 1738.04 | 1705.852 | 1721.946 |
| ENSMUSG00000019878 | protein_coding | Hsf2 | 1340.297 | 1371.447 | 1355.872 |
| ENSMUSG00000028314 | protein_coding | 4930547C10Rik | 6.447869 | 5.420985 | 5.934427 |
| ENSMUSG00000038695 | protein_coding | Josd2 | 667.2566 | 646.4614 | 656.859 |
| ENSMUSG00000026567 | protein_coding | Adcy10 | 90.73078 | 96.45729 | 93.59404 |
| ENSMUSG00000025877 | protein_coding | Hk3 | 38.47655 | 35.59352 | 37.03503 |
| ENSMUSG00000005968 | protein_coding | Tuft1 | 614.3971 | 640.1331 | 627.2651 |
| ENSMUSG00000054611 | protein_coding | Kdm2a | 5366.873 | 5243.743 | 5305.308 |
| ENSMUSG00000074224 | protein_coding | 4932431P20Rik | 73.73162 | 60.50528 | 67.11845 |
| ENSMUSG00000022967 | protein_coding | Ifnar1 | 3235.522 | 3293.993 | 3264.758 |
| ENSMUSG00000040820 | protein_coding | Hlcs | 742.1902 | 768.9402 | 755.5652 |
| ENSMUSG00000020361 | protein_coding | Hspa4 | 9117.222 | 9281.215 | 9199.219 |
| ENSMUSG00000067344 | pseudogene | Rps25-ps1 | 4.210765 | 5.085461 | 4.648113 |
| ENSMUSG00000037214 | protein_coding | Thap1 | 384.367 | 372.7908 | 378.5789 |
| ENSMUSG00000079679 | protein_coding | Vwde | 1.923194 | 1.36876 | 1.645977 |
| ENSMUSG00000030314 | protein_coding | Atg7 | 644.3632 | 659.8851 | 652.1242 |
| ENSMUSG00000005131 | protein_coding | 4930550C14Rik | 51.62753 | 48.26016 | 49.94384 |
| ENSMUSG00000027480 | protein_coding | Sun5 | 0.646531 | 1.050893 | 0.848712 |
| ENSMUSG00000077449 | snoRNA | SNORA42 | 0.646531 | 1.050893 | 0.848712 |
| ENSMUSG00000024503 | protein_coding | Spink3 | 0.646531 | 1.050893 | 0.848712 |
| ENSMUSG00000005299 | protein_coding | Letm1 | 1522.005 | 1556.813 | 1539.409 |
| ENSMUSG00000035901 | protein_coding | Dennd5a | 7029.93 | 6906.086 | 6968.008 |
| ENSMUSG00000078607 | processed_transcrip | 1810010H24Rik | 37.18095 | 40.01694 | 38.59895 |
| ENSMUSG00000069520 | protein_coding | Tmem19 | 1208.099 | 1232.34 | 1220.219 |
| ENSMUSG00000028794 | protein_coding | A3galt2 | 12.39576 | 14.84656 | 13.62116 |
| ENSMUSG00000017686 | protein_coding | Rhot1 | 2623.92 | 2574.983 | 2599.451 |
| ENSMUSG00000020437 | protein_coding | Myo1g | 25.44812 | 27.84152 | 26.64482 |
| ENSMUSG00000086054 | processed_transcrip | Gm13824 | 3.854588 | 3.099348 | 3.476968 |
| ENSMUSG00000087354 | lincRNA | 4930404I05Rik | 34.72036 | 37.93933 | 36.32985 |
| ENSMUSG00000086515 | processed_transcrip | Gm16292 | 3.594722 | 4.557115 | 4.075919 |
| ENSMUSG00000075725 | snoRNA | SNORD50 | 3.252634 | 4.079252 | 3.665943 |
| ENSMUSG00000035443 | protein_coding | Thyn1 | 725.5932 | 745.494 | 735.5436 |
| ENSMUSG00000038880 | protein_coding | Mrps34 | 519.9262 | 535.3153 | 527.6207 |
| ENSMUSG00000031169 | protein_coding | Porcn | 247.7778 | 259.2829 | 253.5304 |
| ENSMUSG00000077002 | miRNA | AC137969.2 | 95.6662 | 88.79162 | 92.22891 |
| ENSMUSG00000048965 | protein_coding | Mrgpre | 38.41766 | 41.08155 | 39.74961 |
| ENSMUSG00000024598 | protein_coding | Fbn2 | 2373.18 | 2300.169 | 2336.675 |
| ENSMUSG00000086769 | processed_transcrip | Gm15587 | 3.888655 | 4.723272 | 4.305963 |
| ENSMUSG00000075972 | snoRNA | SNORD50 | 5.474606 | 4.352435 | 4.91352 |
| ENSMUSG00000052833 | protein_coding | Sae1 | 4018.295 | 3937.349 | 3977.822 |
| ENSMUSG00000035139 | protein_coding | Secisbp2 | 1087.071 | 1113.994 | 1100.532 |
| ENSMUSG00000053846 | protein_coding | Lipg | 703.3228 | 681.6703 | 692.4965 |
| ENSMUSG00000021940 | protein_coding | Ptpn20 | 157.1933 | 147.9554 | 152.5743 |
| ENSMUSG00000084775 | lincRNA | Gm16741 | 4.839629 | 3.824089 | 4.331859 |
| ENSMUSG00000064451 | snoRNA | Snora23 | 4.179008 | 5.10348 | 4.641244 |
| ENSMUSG00000022525 | protein_coding | Hrasls | 85.45491 | 90.10321 | 87.77906 |
| ENSMUSG00000044186 | protein_coding | Nkx2-6 | 4.190787 | 5.058795 | 4.624791 |
| ENSMUSG00000042787 | protein_coding | Exog | 424.9108 | 412.1094 | 418.5101 |
| ENSMUSG00000029005 | protein_coding | 2610109H07Rik | 90.47763 | 83.01947 | 86.74855 |
| ENSMUSG00000006456 | protein_coding | Rbm14 | 3004.572 | 3064.945 | 3034.758 |
| ENSMUSG00000084864 | processed_transcrip | 1700027A07Rik | 1.957261 | 1.333085 | 1.645173 |
| ENSMUSG00000042320 | protein_coding | Prox2 | 115.9952 | 110.0011 | 112.9981 |
| ENSMUSG00000092021 | protein_coding | Gbp11 | 3.536056 | 2.7995 | 3.167778 |
| ENSMUSG00000059475 | protein_coding | Zfp426 | 1346.733 | 1310.891 | 1328.812 |
| ENSMUSG00000059291 | protein_coding | Rpl11 | 5277.363 | 5385.817 | 5331.59 |

| | | | | | |
|---|---|---|---|---|---|
| ENSMUSG00000022487 | protein_coding | Gtsf1 | 580.8099 | 564.7072 | 572.7585 |
| ENSMUSG00000079681 | protein_coding | Zglp1 | 250.2361 | 240.2963 | 245.2662 |
| ENSMUSG00000055413 | processed_transcrip | H2-Q8 | 3.215213 | 2.383978 | 2.799596 |
| ENSMUSG00000029320 | protein_coding | 1700016H13Rik | 5.213473 | 4.177269 | 4.695371 |
| ENSMUSG00000006270 | protein_coding | Vax1 | 4.538764 | 5.509992 | 5.024378 |
| ENSMUSG00000037813 | protein_coding | D630003M21Rik | 326.5465 | 338.6623 | 332.6044 |
| ENSMUSG00000072647 | protein_coding | Adam1a | 39.34403 | 42.33573 | 40.83988 |
| ENSMUSG00000042419 | protein_coding | Nfkbil1 | 471.9261 | 485.4704 | 478.6982 |
| ENSMUSG00000068115 | protein_coding | Ninl | 3652.953 | 3585.509 | 3619.231 |
| ENSMUSG00000031845 | protein_coding | Bcmo1 | 8.745951 | 7.567456 | 8.156703 |
| ENSMUSG00000054446 | protein_coding | Cpa1 | 9.036305 | 7.787306 | 8.411805 |
| ENSMUSG00000079442 | protein_coding | St6galnac4 | 219.0298 | 211.7373 | 215.3836 |
| ENSMUSG00000061584 | protein_coding | Lyg2 | 1.934972 | 1.36876 | 1.651866 |
| ENSMUSG00000046204 | protein_coding | Pnma2 | 10.68681 | 9.325072 | 10.00594 |
| ENSMUSG00000073538 | protein_coding | E330020D12Rik | 3.233924 | 2.481995 | 2.857959 |
| ENSMUSG00000006057 | protein_coding | Atp5g1 | 1731.801 | 1764.791 | 1748.296 |
| ENSMUSG00000064272 | protein_coding | Gpbar1 | 0.638331 | 1.033237 | 0.835784 |
| ENSMUSG00000089916 | processed_transcrip | Gm16160 | 0.638331 | 1.033237 | 0.835784 |
| ENSMUSG00000083813 | pseudogene | Gm15502 | 0.638331 | 1.033237 | 0.835784 |
| ENSMUSG00000086481 | processed_transcrip | Gm11707 | 0.638331 | 1.033237 | 0.835784 |
| ENSMUSG00000066677 | protein_coding | Pydc3 | 0.65242 | 1.050893 | 0.851657 |
| ENSMUSG00000091593 | lincRNA | 4631405K08Rik | 0.65242 | 1.050893 | 0.851657 |
| ENSMUSG00000023159 | protein_coding | Psg29 | 0.65242 | 1.050893 | 0.851657 |
| ENSMUSG00000075046 | protein_coding | Gm10807 | 0.65242 | 1.050893 | 0.851657 |
| ENSMUSG00000084853 | processed_transcrip | Gm11791 | 0.65242 | 1.050893 | 0.851657 |
| ENSMUSG00000027636 | protein_coding | Sla2 | 8.723662 | 7.567456 | 8.145559 |
| ENSMUSG00000023132 | protein_coding | Gzma | 4.224854 | 3.408205 | 3.81653 |
| ENSMUSG00000074625 | protein_coding | Arhgap40 | 11.97987 | 10.55978 | 11.26983 |
| ENSMUSG00000091234 | lincRNA | Gm17393 | 49.17596 | 45.31112 | 47.24354 |
| ENSMUSG00000052117 | protein_coding | D630039A03Rik | 2.917703 | 4.026282 | 3.471992 |
| ENSMUSG00000047361 | protein_coding | Gm973 | 177.1424 | 169.0054 | 173.0739 |
| ENSMUSG00000074030 | protein_coding | Exoc8 | 990.0585 | 963.4953 | 976.7769 |
| ENSMUSG00000090691 | protein_coding | Gm3667 | 16.23291 | 14.42855 | 15.33073 |
| ENSMUSG00000091543 | lincRNA | Gm17549 | 72.52742 | 76.5666 | 74.54701 |
| ENSMUSG00000020707 | protein_coding | Rnf135 | 543.8161 | 529.6941 | 536.7551 |
| ENSMUSG00000018986 | protein_coding | Slfn3 | 7.164845 | 8.431325 | 7.798085 |
| ENSMUSG00000070645 | protein_coding | Ren1 | 658.5849 | 732.6705 | 695.6277 |
| ENSMUSG00000031337 | protein_coding | Mtm1 | 785.4699 | 804.8145 | 795.1422 |
| ENSMUSG00000059920 | protein_coding | 4930453N24Rik | 1098.947 | 1120.85 | 1109.898 |
| ENSMUSG00000033326 | protein_coding | Kdm4a | 2136.09 | 2183.049 | 2159.57 |
| ENSMUSG00000082962 | pseudogene | Gm10601 | 7.657441 | 9.81018 | 8.73381 |
| ENSMUSG00000029787 | protein_coding | Avl9 | 2871.341 | 2939.228 | 2905.285 |
| ENSMUSG00000086682 | lincRNA | Gm16023 | 144.1645 | 137.555 | 140.8597 |
| ENSMUSG00000025584 | protein_coding | Pde8a | 1299.791 | 1262.294 | 1281.043 |
| ENSMUSG00000026828 | protein_coding | Galnt5 | 0.632442 | 1.024227 | 0.828335 |
| ENSMUSG00000034456 | protein_coding | Uroc1 | 0.632442 | 1.024227 | 0.828335 |
| ENSMUSG00000064508 | rRNA | n-R5s96 | 0.632442 | 1.024227 | 0.828335 |
| ENSMUSG00000076137 | miRNA | CT009594.1 | 0.632442 | 1.024227 | 0.828335 |
| ENSMUSG00000091084 | lincRNA | BC065403 | 4.849096 | 5.774165 | 5.311631 |
| ENSMUSG00000019850 | protein_coding | Tnfaip3 | 124.6549 | 130.0084 | 127.3316 |
| ENSMUSG00000030298 | protein_coding | Sec13 | 1845.906 | 1886.492 | 1866.199 |
| ENSMUSG00000004451 | protein_coding | Ralb | 3522.225 | 3598.742 | 3560.483 |
| ENSMUSG00000022848 | protein_coding | Dirc2 | 832.0443 | 813.8712 | 822.9578 |
| ENSMUSG00000053119 | protein_coding | Chmp3 | 2521.966 | 2474.911 | 2498.439 |
| ENSMUSG00000043940 | protein_coding | Wdfy3 | 3007.233 | 3085.761 | 3046.497 |
| ENSMUSG00000026644 | protein_coding | Acbd7 | 11.94581 | 10.50608 | 11.22595 |
| ENSMUSG00000021675 | protein_coding | F2rl2 | 6.198515 | 7.38007 | 6.789292 |
| ENSMUSG00000080768 | pseudogene | Gm12219 | 35.01429 | 38.40343 | 36.70886 |

| | | | | | |
|---|---|---|---|---|---|
| ENSMUSG00000042557 | protein_coding | Sin3a | 4683.76 | 4576.306 | 4630.033 |
| ENSMUSG00000083246 | pseudogene | Gm11839 | 9.264638 | 11.15984 | 10.21224 |
| ENSMUSG00000024109 | protein_coding | Nrxn1 | 23.55899 | 25.78406 | 24.67153 |
| ENSMUSG00000032299 | protein_coding | Commd4 | 666.8767 | 688.3225 | 677.5996 |
| ENSMUSG00000002332 | protein_coding | Dhrs1 | 630.2568 | 646.216 | 638.2364 |
| ENSMUSG00000086075 | lincRNA | Gm15728 | 54.57005 | 58.78675 | 56.6784 |
| ENSMUSG00000066877 | protein_coding | Nck2 | 1384.582 | 1417.014 | 1400.798 |
| ENSMUSG00000023977 | protein_coding | Ubr2 | 3324.522 | 3257.638 | 3291.08 |
| ENSMUSG00000091024 | lincRNA | Gm17351 | 6.46889 | 5.394319 | 5.931604 |
| ENSMUSG00000024837 | protein_coding | Dmrt1 | 6749.151 | 6602.683 | 6675.917 |
| ENSMUSG00000079517 | protein_coding | Gm8787 | 27.59189 | 30.22478 | 28.90833 |
| ENSMUSG00000024451 | protein_coding | Arap3 | 188.1681 | 197.5035 | 192.8358 |
| ENSMUSG00000000838 | protein_coding | Fmr1 | 5451.832 | 5240.123 | 5345.977 |
| ENSMUSG00000016252 | protein_coding | Atp5e | 1238.046 | 1206.564 | 1222.305 |
| ENSMUSG00000034071 | protein_coding | Zfp551 | 176 | 167.5017 | 171.7508 |
| ENSMUSG00000039004 | protein_coding | Bmp6 | 163.8055 | 170.8405 | 167.323 |
| ENSMUSG00000033916 | protein_coding | Chmp2a | 1670.34 | 1702.992 | 1686.666 |
| ENSMUSG00000043131 | protein_coding | Mob1a | 6934.984 | 7076.606 | 7005.795 |
| ENSMUSG00000056458 | protein_coding | Stk30 | 188.3012 | 180.7887 | 184.545 |
| ENSMUSG00000018507 | protein_coding | Trpv2 | 87.23408 | 92.0493 | 89.64169 |
| ENSMUSG00000067724 | protein_coding | Gbx1 | 9.080882 | 7.706585 | 8.393733 |
| ENSMUSG00000033713 | protein_coding | Foxn3 | 1428.664 | 1385.648 | 1407.156 |
| ENSMUSG00000059706 | processed_transcrip | A830035A12Rik | 9.028105 | 7.858295 | 8.4432 |
| ENSMUSG00000074122 | lincRNA | Foxl2os | 5.177096 | 6.181039 | 5.679067 |
| ENSMUSG00000040327 | protein_coding | Cul9 | 771.2301 | 751.204 | 761.2171 |
| ENSMUSG00000087356 | processed_transcrip | Gm13856 | 2.259393 | 1.65059 | 1.954991 |
| ENSMUSG00000038976 | protein_coding | Ppp1r9b | 2682.72 | 2630.182 | 2656.451 |
| ENSMUSG00000048004 | protein_coding | Tmem196 | 10.2543 | 11.76818 | 11.01124 |
| ENSMUSG00000084340 | pseudogene | Gm14766 | 6.150358 | 7.276617 | 6.713488 |
| ENSMUSG00000003865 | protein_coding | Gys1 | 402.2117 | 415.2859 | 408.7488 |
| ENSMUSG00000039814 | protein_coding | Xkr5 | 64.62376 | 70.48041 | 67.55208 |
| ENSMUSG00000026526 | protein_coding | Fh1 | 2331.872 | 2382.397 | 2357.135 |
| ENSMUSG00000090447 | lincRNA | Gm17652 | 34.70165 | 37.57062 | 36.13613 |
| ENSMUSG00000084230 | pseudogene | Gm14388 | 28.01427 | 25.64716 | 26.83071 |
| ENSMUSG00000092837 | misc_RNA | AC027184.1 | 24.42379 | 21.81147 | 23.11763 |
| ENSMUSG00000000594 | protein_coding | Gm2a | 11499.16 | 11126.68 | 11312.92 |
| ENSMUSG00000001995 | protein_coding | Sipa1l2 | 1193.384 | 1151.071 | 1172.228 |
| ENSMUSG00000038025 | protein_coding | Phf2 | 1054.603 | 1086.791 | 1070.697 |
| ENSMUSG00000018599 | protein_coding | Smcr7 | 582.8027 | 566.2611 | 574.5319 |
| ENSMUSG00000030846 | protein_coding | Tial1 | 2217.42 | 2270.374 | 2243.897 |
| ENSMUSG00000003154 | protein_coding | Foxj2 | 1095.421 | 1068.939 | 1082.18 |
| ENSMUSG00000003762 | protein_coding | Adck4 | 343.2624 | 352.6438 | 347.9531 |
| ENSMUSG00000090069 | lincRNA | D030054H15Rik | 7.0944 | 8.25616 | 7.67528 |
| ENSMUSG00000091513 | lincRNA | 4731419I09Rik | 12.26792 | 10.82431 | 11.54612 |
| ENSMUSG00000046334 | pseudogene | Gm6195 | 2.273482 | 1.65059 | 1.962036 |
| ENSMUSG00000082106 | pseudogene | Mageb16-ps1 | 2.259393 | 1.712931 | 1.986162 |
| ENSMUSG00000037656 | protein_coding | Slc20a2 | 2055.422 | 2112.185 | 2083.803 |
| ENSMUSG00000065191 | snRNA | U1 | 9.088039 | 7.840638 | 8.464339 |
| ENSMUSG00000025612 | protein_coding | Bach1 | 1035.876 | 1062.885 | 1049.381 |
| ENSMUSG00000079215 | protein_coding | Zfp664 | 3676.031 | 3604.764 | 3640.397 |
| ENSMUSG00000043925 | protein_coding | Olfr544 | 3.896855 | 4.723272 | 4.310063 |
| ENSMUSG00000067647 | protein_coding | 1700003E24Rik | 1.920883 | 1.324076 | 1.622479 |
| ENSMUSG00000091049 | protein_coding | Gm1979 | 34.92871 | 37.48954 | 36.20913 |
| ENSMUSG00000056875 | protein_coding | Gm3386 | 2.936413 | 3.743367 | 3.33989 |
| ENSMUSG00000032274 | protein_coding | Cyp19a1 | 2.281682 | 1.712931 | 1.997307 |
| ENSMUSG00000003033 | protein_coding | Ap1m1 | 1745.421 | 1697.924 | 1721.673 |
| ENSMUSG00000010830 | protein_coding | Kdelr3 | 388.1038 | 377.7132 | 382.9085 |
| ENSMUSG00000091722 | protein_coding | Siah3 | 2.253504 | 1.695274 | 1.974389 |

| | | | | | |
|---|---|---|---|---|---|
| ENSMUSG00000083014 | pseudogene | Gm11764 | 0.968641 | 1.386417 | 1.177529 |
| ENSMUSG00000087286 | pseudogene | Gm9013 | 0.968641 | 1.386417 | 1.177529 |
| ENSMUSG00000081176 | pseudogene | H3f3a-ps2 | 4.518786 | 5.483688 | 5.001237 |
| ENSMUSG00000022123 | protein_coding | Scel | 11.28519 | 9.916121 | 10.60065 |
| ENSMUSG00000062590 | protein_coding | Armc9 | 621.2707 | 639.9479 | 630.6093 |
| ENSMUSG00000043024 | pseudogene | Gm16433 | 27.31265 | 25.03301 | 26.17283 |
| ENSMUSG00000088632 | misc_RNA | 7SK | 4.213076 | 3.434871 | 3.823974 |
| ENSMUSG00000011884 | protein_coding | Gltp | 915.9208 | 891.3671 | 903.644 |
| ENSMUSG00000070547 | protein_coding | Mrgprb1 | 1.581105 | 0.997561 | 1.289333 |
| ENSMUSG00000085782 | processed_transcrip | Gm15624 | 4.865496 | 5.792184 | 5.32884 |
| ENSMUSG00000032393 | protein_coding | Dpp8 | 1953.035 | 1909.029 | 1931.032 |
| ENSMUSG00000022451 | protein_coding | Twf1 | 2387.91 | 2431.188 | 2409.549 |
| ENSMUSG00000068250 | protein_coding | Amn1 | 408.4709 | 420.0892 | 414.28 |
| ENSMUSG00000090646 | protein_coding | Gm17448 | 6.446601 | 7.567456 | 7.007029 |
| ENSMUSG00000081857 | pseudogene | Gm13624 | 0.646531 | 1.033237 | 0.839884 |
| ENSMUSG00000087115 | processed_transcrip | Gm12360 | 0.646531 | 1.033237 | 0.839884 |
| ENSMUSG00000091460 | pseudogene | Gm7808 | 0.646531 | 1.033237 | 0.839884 |
| ENSMUSG00000082600 | pseudogene | Gm9673 | 0.646531 | 1.033237 | 0.839884 |
| ENSMUSG00000022589 | protein_coding | Cyp11b2 | 1.953683 | 1.341732 | 1.647707 |
| ENSMUSG00000021133 | protein_coding | 4933426M11Rik | 2395.922 | 2462.069 | 2428.995 |
| ENSMUSG00000054117 | pseudogene | Zdhhc25 | 5.815427 | 6.878752 | 6.34709 |
| ENSMUSG00000086062 | lincRNA | Gm16853 | 4.517518 | 5.519001 | 5.01826 |
| ENSMUSG00000081344 | pseudogene | Gm14303 | 48.10534 | 44.9291 | 46.51722 |
| ENSMUSG00000027536 | protein_coding | Chmp4c | 131.076 | 140.1809 | 135.6285 |
| ENSMUSG00000087067 | lincRNA | Gm11532 | 3.844077 | 4.723272 | 4.283674 |
| ENSMUSG00000004952 | protein_coding | Rasa4 | 198.9848 | 192.3061 | 195.6455 |
| ENSMUSG00000078592 | protein_coding | Gm4609 | 3.866366 | 3.117366 | 3.491866 |
| ENSMUSG00000025130 | protein_coding | P4hb | 9540.702 | 9383.151 | 9461.926 |
| ENSMUSG00000024481 | protein_coding | 4833403I15Rik | 207.3686 | 194.8193 | 201.0939 |
| ENSMUSG00000039316 | protein_coding | Rftn1 | 802.5945 | 781.259 | 791.9268 |
| ENSMUSG00000026554 | protein_coding | Dcaf8 | 3490.991 | 3565.952 | 3528.472 |
| ENSMUSG00000040410 | protein_coding | Fbxl4 | 546.5572 | 560.9794 | 553.7683 |
| ENSMUSG00000041353 | protein_coding | Tmem29 | 197.9744 | 190.9499 | 194.4621 |
| ENSMUSG00000069622 | pseudogene | Gm10273 | 9.09855 | 7.796315 | 8.447432 |
| ENSMUSG00000054046 | protein_coding | Klk13 | 10.20741 | 8.386641 | 9.297026 |
| ENSMUSG00000090754 | protein_coding | C230091D08Rik | 2.275793 | 1.712931 | 1.994362 |
| ENSMUSG00000091732 | protein_coding | Gm17541 | 1.937283 | 1.342094 | 1.639688 |
| ENSMUSG00000039899 | protein_coding | Fgl2 | 2086.635 | 2126.946 | 2106.79 |
| ENSMUSG00000034028 | protein_coding | Cd226 | 1.940861 | 1.315066 | 1.627964 |
| ENSMUSG00000027981 | protein_coding | Rnpc3 | 1375.493 | 1413.709 | 1394.601 |
| ENSMUSG00000028841 | protein_coding | Cnksr1 | 61.57925 | 65.06947 | 63.32436 |
| ENSMUSG00000036002 | protein_coding | B230312A22Rik | 650.2429 | 666.537 | 658.39 |
| ENSMUSG00000040929 | protein_coding | Rfx3 | 659.3921 | 677.7054 | 668.5488 |
| ENSMUSG00000090062 | processed_transcrip | 1810009N23Rik | 4.200254 | 5.165459 | 4.682857 |
| ENSMUSG00000086273 | processed_transcrip | 4930564K09Rik | 29.81722 | 27.27678 | 28.547 |
| ENSMUSG00000018595 | protein_coding | Glra4 | 6.096313 | 7.267608 | 6.681961 |
| ENSMUSG00000053470 | protein_coding | Kdm3a | 2815.257 | 2749.581 | 2782.419 |
| ENSMUSG00000044600 | protein_coding | 9130011J15Rik | 1993.222 | 2032.112 | 2012.667 |
| ENSMUSG00000027423 | protein_coding | Snx5 | 4381.14 | 4301.718 | 4341.429 |
| ENSMUSG00000081157 | pseudogene | Gm13552 | 26.40014 | 28.8207 | 27.61042 |
| ENSMUSG00000023030 | protein_coding | Slc11a2 | 4198.762 | 4103.923 | 4151.342 |
| ENSMUSG00000090778 | processed_transcrip | Gm3235 | 0.638331 | 1.086569 | 0.86245 |
| ENSMUSG00000008730 | protein_coding | Hipk1 | 2723.157 | 2774.184 | 2748.67 |
| ENSMUSG00000037492 | protein_coding | Zmat4 | 93.601 | 97.97156 | 95.78628 |
| ENSMUSG00000079553 | protein_coding | Kifc1 | 745.4609 | 717.837 | 731.649 |
| ENSMUSG00000028837 | protein_coding | Psmb2 | 2484.794 | 2530.401 | 2507.597 |
| ENSMUSG00000001802 | protein_coding | Lrp3 | 209.9398 | 201.3258 | 205.6328 |
| ENSMUSG00000024871 | protein_coding | Doc2g | 20.69049 | 22.82492 | 21.75771 |

| | | | | | |
|---|---|---|---|---|---|
| ENSMUSG00000032513 | protein_coding | Gorasp1 | 629.3046 | 644.9813 | 637.1429 |
| ENSMUSG00000064566 | snRNA | U6 | 3.905054 | 3.117366 | 3.51121 |
| ENSMUSG00000022204 | protein_coding | Ngdn | 1214.861 | 1184.098 | 1199.479 |
| ENSMUSG00000050195 | protein_coding | Scd4 | 114.1358 | 121.6174 | 117.8766 |
| ENSMUSG00000047544 | protein_coding | Olfr616 | 3.554766 | 2.808509 | 3.181638 |
| ENSMUSG00000092046 | pseudogene | Gm6450 | 0.64422 | 1.024227 | 0.834224 |
| ENSMUSG00000063179 | protein_coding | Pstk | 460.8347 | 472.3192 | 466.5769 |
| ENSMUSG00000022542 | protein_coding | 12-Sep | 27.68463 | 24.89352 | 26.28907 |
| ENSMUSG00000024516 | protein_coding | Sec11c | 402.8647 | 416.0755 | 409.4701 |
| ENSMUSG00000054510 | processed_transcrip | Gm14461 | 0.65242 | 1.033237 | 0.842828 |
| ENSMUSG00000084956 | processed_transcrip | Gm16194 | 0.65242 | 1.033237 | 0.842828 |
| ENSMUSG00000081512 | protein_coding | Gm15821 | 0.65242 | 1.033237 | 0.842828 |
| ENSMUSG00000086312 | processed_transcrip | Gm15336 | 0.65242 | 1.033237 | 0.842828 |
| ENSMUSG00000080923 | pseudogene | Gm5400 | 0.65242 | 1.033237 | 0.842828 |
| ENSMUSG00000040706 | protein_coding | Agmat | 4.204876 | 5.085823 | 4.645349 |
| ENSMUSG00000025858 | protein_coding | Get4 | 3242.094 | 3175.637 | 3208.866 |
| ENSMUSG00000050926 | protein_coding | Dcaf12l2 | 2.575614 | 3.319198 | 2.947406 |
| ENSMUSG00000087538 | processed_transcrip | Gm16341 | 2.253504 | 1.72194 | 1.987722 |
| ENSMUSG00000029723 | protein_coding | Tsc22d4 | 1963.074 | 1922.581 | 1942.827 |
| ENSMUSG00000006546 | protein_coding | Cryba2 | 0.638331 | 1.015218 | 0.826775 |
| ENSMUSG00000045797 | protein_coding | 4930402K13Rik | 0.638331 | 1.015218 | 0.826775 |
| ENSMUSG00000087359 | lincRNA | Gm17478 | 12.29021 | 10.93098 | 11.61059 |
| ENSMUSG00000026024 | protein_coding | Als2 | 2254.227 | 2203.693 | 2228.96 |
| ENSMUSG00000056201 | protein_coding | Cfl1 | 10478.02 | 10637.7 | 10557.86 |
| ENSMUSG00000071281 | protein_coding | Zfp71-rs1 | 525.3416 | 551.6808 | 538.5112 |
| ENSMUSG00000027424 | protein_coding | 8430406I07Rik | 777.9328 | 795.9928 | 786.9628 |
| ENSMUSG00000030729 | protein_coding | Pgm2l1 | 2636.867 | 2728.925 | 2682.896 |
| ENSMUSG00000078691 | protein_coding | Gm6821 | 1.959572 | 1.413083 | 1.686327 |
| ENSMUSG00000024231 | protein_coding | Cul2 | 1732.643 | 1699.08 | 1715.861 |
| ENSMUSG00000019158 | protein_coding | Tmem160 | 122.9982 | 129.4791 | 126.2386 |
| ENSMUSG00000020599 | protein_coding | Rgs9 | 28.03909 | 25.83418 | 26.93664 |
| ENSMUSG00000034684 | protein_coding | Sema3f | 259.6556 | 267.4437 | 263.5497 |
| ENSMUSG00000003665 | protein_coding | Has1 | 11.29339 | 10.00477 | 10.64908 |
| ENSMUSG00000078700 | lincRNA | D030028A08Rik | 180.7749 | 188.0316 | 184.4033 |
| ENSMUSG00000031392 | protein_coding | Irak1 | 1250.096 | 1274.909 | 1262.503 |
| ENSMUSG00000071816 | protein_coding | Ssxb5 | 3.846388 | 4.687596 | 4.266992 |
| ENSMUSG00000038871 | protein_coding | Bpgm | 999.963 | 978.5579 | 989.2605 |
| ENSMUSG00000021904 | protein_coding | Sema3g | 97.99694 | 103.9887 | 100.9928 |
| ENSMUSG00000055403 | protein_coding | 4933427D06Rik | 1172.247 | 1212.758 | 1192.502 |
| ENSMUSG00000023021 | protein_coding | Lass5 | 3379.015 | 3287.092 | 3333.054 |
| ENSMUSG00000066530 | protein_coding | Hmg1l1 | 41.38016 | 38.57683 | 39.9785 |
| ENSMUSG00000084932 | processed_transcrip | Gm15156 | 3.570123 | 4.467746 | 4.018935 |
| ENSMUSG00000035620 | protein_coding | Ric8b | 1123.081 | 1098.758 | 1110.919 |
| ENSMUSG00000082693 | pseudogene | Gm15190 | 71.10465 | 67.53702 | 69.32083 |
| ENSMUSG00000045680 | protein_coding | Tcf21 | 1682.628 | 1618.948 | 1650.788 |
| ENSMUSG00000085897 | pseudogene | Gm11604 | 1.965461 | 1.377408 | 1.671434 |
| ENSMUSG00000090293 | lincRNA | Gm17034 | 10.92774 | 12.60031 | 11.76403 |
| ENSMUSG00000090928 | lincRNA | C530050E15Rik | 7.389376 | 8.740544 | 8.06496 |
| ENSMUSG00000031691 | protein_coding | Tnpo2 | 2336.862 | 2286.985 | 2311.924 |
| ENSMUSG00000066108 | protein_coding | Muc5b | 10.43619 | 12.29358 | 11.36488 |
| ENSMUSG00000033883 | protein_coding | D3Ertd254e | 442.5258 | 424.9347 | 433.7302 |
| ENSMUSG00000052632 | protein_coding | Asap2 | 417.9144 | 404.875 | 411.3947 |
| ENSMUSG00000045281 | protein_coding | Gpr20 | 38.95297 | 41.91332 | 40.43315 |
| ENSMUSG00000026174 | protein_coding | Rqcd1 | 3074.277 | 3127.639 | 3100.958 |
| ENSMUSG00000020018 | protein_coding | Snrpf | 840.2537 | 819.3771 | 829.8154 |
| ENSMUSG00000061684 | pseudogene | Rpl21-ps8 | 47.50972 | 51.92467 | 49.71719 |
| ENSMUSG00000091415 | protein_coding | Akd1 | 138.4712 | 146.7111 | 142.5911 |
| ENSMUSG00000066278 | protein_coding | Vps37b | 1587.053 | 1622.458 | 1604.755 |

| | | | | | |
|---|---|---|---|---|---|
| ENSMUSG00000090535 | pseudogene | Gm8540 | 7.454156 | 6.039061 | 6.746609 |
| ENSMUSG00000039914 | protein_coding | Coq10a | 279.2593 | 270.3646 | 274.812 |
| ENSMUSG00000024552 | protein_coding | Slc14a2 | 1.957261 | 1.377408 | 1.667334 |
| ENSMUSG00000065089 | snoRNA | SNORA46 | 9.042194 | 10.30461 | 9.673404 |
| ENSMUSG00000021996 | protein_coding | Esd | 2601.254 | 2546.832 | 2574.043 |
| ENSMUSG00000086438 | processed_transcrip | 2510003D18Rik | 5.238073 | 4.096548 | 4.66731 |
| ENSMUSG00000029369 | protein_coding | Afm | 7.597132 | 12.04706 | 9.822096 |
| ENSMUSG00000019935 | protein_coding | Slc17a8 | 1.943172 | 1.422092 | 1.682632 |
| ENSMUSG00000030905 | protein_coding | Crym | 34.62382 | 31.52504 | 33.07443 |
| ENSMUSG00000050052 | protein_coding | 2610019F03Rik | 2367.013 | 2420.173 | 2393.593 |
| ENSMUSG00000020928 | protein_coding | Higd1b | 28.00018 | 30.26625 | 29.13321 |
| ENSMUSG00000062461 | protein_coding | Gm5453 | 4.227165 | 5.129784 | 4.678474 |
| ENSMUSG00000072955 | protein_coding | Tmsb15l | 19.97351 | 21.94195 | 20.95773 |
| ENSMUSG00000034354 | protein_coding | Mtmr3 | 5428.471 | 5304.108 | 5366.289 |
| ENSMUSG00000029385 | protein_coding | Ccng2 | 1085.97 | 1057.605 | 1071.787 |
| ENSMUSG00000086095 | processed_transcrip | Gm15328 | 18.78265 | 17.03202 | 17.90734 |
| ENSMUSG00000091905 | pseudogene | Gm6395 | 80.08228 | 84.11567 | 82.09898 |
| ENSMUSG00000087488 | lincRNA | Gm16990 | 1.917305 | 1.395426 | 1.656365 |
| ENSMUSG00000086483 | processed_transcrip | 8030443G20Rik | 0.646531 | 1.086569 | 0.86655 |
| ENSMUSG00000001865 | protein_coding | Cpa3 | 7.986708 | 6.771726 | 7.379217 |
| ENSMUSG00000033029 | protein_coding | 1700088E04Rik | 43.59475 | 40.9781 | 42.28643 |
| ENSMUSG00000087330 | processed_transcrip | Gm11616 | 2.265282 | 1.659599 | 1.962441 |
| ENSMUSG00000022744 | protein_coding | Cldn25 | 2148.856 | 2190.381 | 2169.618 |
| ENSMUSG00000090955 | pseudogene | Gm17097 | 0.968641 | 1.422092 | 1.195367 |
| ENSMUSG00000041459 | protein_coding | Tardbp | 6887.297 | 7025.241 | 6956.269 |
| ENSMUSG00000040859 | protein_coding | Bsdc1 | 1697.095 | 1732.292 | 1714.694 |
| ENSMUSG00000080968 | pseudogene | Gm1862 | 3.919143 | 3.117366 | 3.518255 |
| ENSMUSG00000085462 | lincRNA | 5031434O11Rik | 34.20145 | 30.81583 | 32.50864 |
| ENSMUSG00000093030 | miRNA | AC140381.1 | 0.632442 | 1.059903 | 0.846172 |
| ENSMUSG00000024041 | protein_coding | Cryaa | 0.632442 | 1.059903 | 0.846172 |
| ENSMUSG00000086960 | processed_transcrip | Gm13017 | 2.245304 | 1.65059 | 1.947947 |
| ENSMUSG00000044286 | protein_coding | Olfr221 | 3.280812 | 2.463976 | 2.872394 |
| ENSMUSG00000051844 | lincRNA | B230319C09Rik | 16.8031 | 18.69447 | 17.74878 |
| ENSMUSG00000000346 | protein_coding | Dazap2 | 5373.611 | 5251.923 | 5312.767 |
| ENSMUSG00000086807 | lincRNA | C330013F16Rik | 19.67496 | 17.85369 | 18.76433 |
| ENSMUSG00000046610 | protein_coding | 5330437I02Rik | 27.8446 | 25.45433 | 26.64947 |
| ENSMUSG00000068579 | protein_coding | Rpl7a-ps3 | 3.217524 | 4.069882 | 3.643703 |
| ENSMUSG00000091625 | protein_coding | Lsm5 | 366.9642 | 355.367 | 361.1656 |
| ENSMUSG00000054412 | protein_coding | Gm4793 | 6.083492 | 7.336109 | 6.709801 |
| ENSMUSG00000049119 | protein_coding | Fam110b | 458.293 | 446.0656 | 452.1793 |
| ENSMUSG00000090958 | protein_coding | Lrrc32 | 98.79949 | 103.745 | 101.2723 |
| ENSMUSG00000077787 | snoRNA | SNORA17 | 5.820048 | 6.870105 | 6.345077 |
| ENSMUSG00000042256 | protein_coding | 3110082D06Rik | 4.176698 | 3.390549 | 3.783623 |
| ENSMUSG00000061455 | protein_coding | Stx17 | 1671.938 | 1637.766 | 1654.852 |
| ENSMUSG00000074001 | protein_coding | Kbtbd5 | 9.042194 | 10.27795 | 9.660071 |
| ENSMUSG00000070733 | protein_coding | Fryl | 3277.256 | 3201.049 | 3239.153 |
| ENSMUSG00000087266 | processed_transcrip | Gm15991 | 3.539634 | 4.467746 | 4.00369 |
| ENSMUSG00000033327 | protein_coding | Tnxb | 1113.132 | 1161.383 | 1137.257 |
| ENSMUSG00000034724 | protein_coding | Cnot6l | 3141.667 | 3089.566 | 3115.617 |
| ENSMUSG00000029430 | protein_coding | Ran | 6637.92 | 6755.831 | 6696.876 |
| ENSMUSG00000061311 | protein_coding | Rag1 | 7.404957 | 5.685881 | 6.545419 |
| ENSMUSG00000015467 | protein_coding | Egfl8 | 23.98666 | 21.82084 | 22.90375 |
| ENSMUSG00000026021 | protein_coding | Sumo1 | 2614.587 | 2667.396 | 2640.991 |
| ENSMUSG00000040907 | protein_coding | Atp1a3 | 32.73134 | 30.23379 | 31.48256 |
| ENSMUSG00000030045 | protein_coding | Mrpl19 | 793.4815 | 776.5036 | 784.9926 |
| ENSMUSG00000026482 | protein_coding | Rgl1 | 2800.866 | 2862.926 | 2831.896 |
| ENSMUSG00000046262 | protein_coding | C87977 | 0.646531 | 1.015218 | 0.830875 |
| ENSMUSG00000062742 | protein_coding | Gm5239 | 0.646531 | 1.015218 | 0.830875 |

| | | | | | |
|---|---|---|---|---|---|
| ENSMUSG00000035305 | protein_coding | Ror1 | 150.8772 | 144.8273 | 147.8523 |
| ENSMUSG00000086716 | processed_transcrip | Gm11629 | 7.749131 | 8.900179 | 8.324655 |
| ENSMUSG00000071649 | protein_coding | B3gat3 | 1041.908 | 1068.969 | 1055.439 |
| ENSMUSG00000086388 | lincRNA | Gm16631 | 6.816868 | 7.965321 | 7.391094 |
| ENSMUSG00000059811 | protein_coding | Atl2 | 2980.704 | 3032.613 | 3006.658 |
| ENSMUSG00000035805 | protein_coding | Mlc1 | 231.6894 | 216.9082 | 224.2988 |
| ENSMUSG00000024908 | protein_coding | Ppp6r3 | 5908.834 | 5815.74 | 5862.287 |
| ENSMUSG00000028953 | protein_coding | Abcf2 | 3828.177 | 3749.729 | 3788.953 |
| ENSMUSG00000082008 | pseudogene | Gm12390 | 1.923194 | 1.413083 | 1.668138 |
| ENSMUSG00000063458 | protein_coding | 1700112E06Rik | 10.03081 | 8.74883 | 9.389822 |
| ENSMUSG00000006169 | protein_coding | Clint1 | 2489.536 | 2536.449 | 2512.992 |
| ENSMUSG00000070003 | protein_coding | Ssbp4 | 1104.768 | 1129.209 | 1116.988 |
| ENSMUSG00000058624 | protein_coding | Gda | 36.43326 | 39.67381 | 38.05354 |
| ENSMUSG00000065420 | miRNA | Mir142 | 1.953683 | 1.377408 | 1.665545 |
| ENSMUSG00000080473 | miRNA | AC024607.1 | 6.192625 | 5.148164 | 5.670395 |
| ENSMUSG00000078996 | protein_coding | Vmn2r-ps106 | 9.084461 | 7.866943 | 8.475702 |
| ENSMUSG00000043441 | protein_coding | Gpr149 | 3.548877 | 2.648151 | 3.098514 |
| ENSMUSG00000014609 | protein_coding | Chrne | 3.246745 | 4.114566 | 3.680655 |
| ENSMUSG00000048424 | protein_coding | Ranbp3l | 6.492447 | 5.501345 | 5.996896 |
| ENSMUSG00000081068 | pseudogene | Gm12804 | 2.597903 | 3.309827 | 2.953865 |
| ENSMUSG00000074149 | protein_coding | Gm10634 | 2.247615 | 1.712931 | 1.980273 |
| ENSMUSG00000050473 | protein_coding | Slc35d3 | 9.706392 | 11.038 | 10.3722 |
| ENSMUSG00000035355 | protein_coding | Kcnh4 | 18.5497 | 16.54763 | 17.54867 |
| ENSMUSG00000069265 | protein_coding | Hist1h3a | 11.31568 | 12.74192 | 12.0288 |
| ENSMUSG00000001017 | protein_coding | 2500003M10Rik | 5088.754 | 4990.789 | 5039.772 |
| ENSMUSG00000006471 | protein_coding | Ndor1 | 1401.358 | 1367.653 | 1384.506 |
| ENSMUSG00000069324 | pseudogene | Gm5096 | 8.071242 | 9.209037 | 8.640139 |
| ENSMUSG00000086386 | processed_transcrip | Gm12108 | 6.427891 | 5.340987 | 5.884439 |
| ENSMUSG00000059033 | pseudogene | Rpl18a-ps1 | 0.968641 | 1.359751 | 1.164196 |
| ENSMUSG00000088448 | rRNA | 5S_rRNA | 0.968641 | 1.359751 | 1.164196 |
| ENSMUSG00000024738 | protein_coding | Pga5 | 0.968641 | 1.359751 | 1.164196 |
| ENSMUSG00000083929 | pseudogene | Gm13301 | 74.91935 | 111.8071 | 93.36324 |
| ENSMUSG00000045455 | protein_coding | Gm9797 | 5.167628 | 6.09167 | 5.629649 |
| ENSMUSG00000031613 | protein_coding | Hpgd | 447.4119 | 432.8734 | 440.1426 |
| ENSMUSG00000021361 | protein_coding | Tmem14c | 878.6483 | 897.8153 | 888.2318 |
| ENSMUSG00000083879 | pseudogene | Gm14038 | 54.17221 | 57.24069 | 55.70645 |
| ENSMUSG00000087458 | processed_transcrip | Gm13999 | 4.238943 | 3.345502 | 3.792223 |
| ENSMUSG00000043873 | protein_coding | Chi3l7 | 0.968641 | 1.413083 | 1.190862 |
| ENSMUSG00000026491 | protein_coding | Ahctf1 | 6794.533 | 6677.736 | 6736.134 |
| ENSMUSG00000027663 | protein_coding | Zmat3 | 379.0376 | 392.2623 | 385.6499 |
| ENSMUSG00000043518 | protein_coding | Rai2 | 18.10563 | 19.92053 | 19.01308 |
| ENSMUSG00000039309 | protein_coding | Edaradd | 21.64758 | 19.8135 | 20.73054 |
| ENSMUSG00000036160 | protein_coding | Surf6 | 1533.507 | 1568.734 | 1551.12 |
| ENSMUSG00000004849 | protein_coding | Ap1s1 | 1456.154 | 1490.51 | 1473.332 |
| ENSMUSG00000048648 | protein_coding | Gm9830 | 7.134357 | 8.37727 | 7.755813 |
| ENSMUSG00000042064 | protein_coding | Myo3b | 2.253504 | 2.974304 | 2.613904 |
| ENSMUSG00000016181 | protein_coding | Diexf | 901.0012 | 882.1937 | 891.5974 |
| ENSMUSG00000089998 | processed_transcrip | A130049A11Rik | 63.36118 | 66.67895 | 65.02007 |
| ENSMUSG00000025255 | protein_coding | Zfhx4 | 1034.163 | 1062.714 | 1048.439 |
| ENSMUSG00000025854 | protein_coding | Fam20c | 254.0681 | 245.4157 | 249.7419 |
| ENSMUSG00000086280 | lincRNA | 4930540M05Rik | 4.490608 | 5.457022 | 4.973815 |
| ENSMUSG00000075010 | protein_coding | AW112010 | 25.53854 | 23.47651 | 24.50753 |
| ENSMUSG00000057982 | protein_coding | Zfp809 | 597.2265 | 615.8659 | 606.5462 |
| ENSMUSG00000021493 | protein_coding | Pdlim7 | 1074.372 | 1101.543 | 1087.957 |
| ENSMUSG00000030699 | protein_coding | Tbx6 | 25.57365 | 23.54858 | 24.56112 |
| ENSMUSG00000028518 | protein_coding | Prkaa2 | 377.9833 | 388.8088 | 383.3961 |
| ENSMUSG00000027335 | protein_coding | Adra1d | 2.259393 | 1.668247 | 1.96382 |
| ENSMUSG00000082738 | pseudogene | Gm15422 | 3.252634 | 4.061596 | 3.657115 |

| | | | | | |
|---|---|---|---|---|---|
| ENSMUSG00000030711 | protein_coding | Sult1a1 | 568.7933 | 555.7918 | 562.2926 |
| ENSMUSG00000028878 | protein_coding | Fam76a | 1758.615 | 1727.155 | 1742.885 |
| ENSMUSG00000056234 | protein_coding | Ncoa4 | 2030.383 | 2081.037 | 2055.71 |
| ENSMUSG00000032898 | protein_coding | Fbxo21 | 8013.963 | 7812.435 | 7913.199 |
| ENSMUSG00000090502 | lincRNA | Gm17735 | 10.27808 | 12.6861 | 11.48209 |
| ENSMUSG00000063415 | protein_coding | Cyp26b1 | 5152.252 | 5046.823 | 5099.537 |
| ENSMUSG00000079414 | protein_coding | Gm11110 | 10.9513 | 12.40283 | 11.67706 |
| ENSMUSG00000084992 | processed_transcrip | Gm13842 | 0.65242 | 1.015218 | 0.833819 |
| ENSMUSG00000092570 | processed_transcrip | 2210019I11Rik | 0.65242 | 1.015218 | 0.833819 |
| ENSMUSG00000056203 | protein_coding | Tas2r135 | 0.65242 | 1.015218 | 0.833819 |
| ENSMUSG00000085672 | processed_transcrip | Gm16193 | 0.65242 | 1.015218 | 0.833819 |
| ENSMUSG00000092544 | protein_coding | Gm20422 | 0.65242 | 1.015218 | 0.833819 |
| ENSMUSG00000084307 | pseudogene | Gm12151 | 0.65242 | 1.015218 | 0.833819 |
| ENSMUSG00000021711 | protein_coding | 2410002O22Rik | 1266.199 | 1302.051 | 1284.125 |
| ENSMUSG00000019893 | protein_coding | Ros1 | 0.638331 | 0.997561 | 0.817946 |
| ENSMUSG00000065232 | snRNA | U2 | 0.638331 | 0.997561 | 0.817946 |
| ENSMUSG00000076749 | IG_C_gene | Gm17004 | 0.638331 | 0.997561 | 0.817946 |
| ENSMUSG00000081843 | pseudogene | Gm7851 | 0.638331 | 0.997561 | 0.817946 |
| ENSMUSG00000057672 | protein_coding | Pkn1 | 1492.556 | 1464.259 | 1478.407 |
| ENSMUSG00000044083 | processed_transcrip | Efcab8 | 6.178536 | 7.258599 | 6.718568 |
| ENSMUSG00000071089 | protein_coding | Trim75 | 2.239415 | 1.712931 | 1.976173 |
| ENSMUSG00000082769 | pseudogene | Gm15793 | 6.476047 | 5.501345 | 5.988696 |
| ENSMUSG00000091091 | protein_coding | Kcnmb3 | 0.66062 | 1.024227 | 0.842424 |
| ENSMUSG00000081640 | pseudogene | Rplp0-ps1 | 0.66062 | 1.024227 | 0.842424 |
| ENSMUSG00000093074 | snRNA | CAAA01037531.1.3 | 0.66062 | 1.024227 | 0.842424 |
| ENSMUSG00000021216 | protein_coding | Tubal3 | 0.66062 | 1.024227 | 0.842424 |
| ENSMUSG00000019564 | protein_coding | Arid3a | 648.6712 | 628.4048 | 638.538 |
| ENSMUSG00000020062 | protein_coding | Slc5a8 | 10.64581 | 12.14766 | 11.39674 |
| ENSMUSG00000078611 | protein_coding | Gm5901 | 43.70784 | 40.88909 | 42.29847 |
| ENSMUSG00000037600 | protein_coding | 1810019J16Rik | 92.248 | 96.59601 | 94.42201 |
| ENSMUSG00000050157 | protein_coding | Gm867 | 12.61463 | 14.07501 | 13.34482 |
| ENSMUSG00000041241 | protein_coding | Mul1 | 942.0739 | 961.8615 | 951.9677 |
| ENSMUSG00000030741 | protein_coding | Spns1 | 937.7235 | 958.9918 | 948.3577 |
| ENSMUSG00000035969 | protein_coding | Rusc2 | 752.8048 | 734.8016 | 743.8032 |
| ENSMUSG00000025790 | protein_coding | Slco3a1 | 1512.089 | 1554.655 | 1533.372 |
| ENSMUSG00000001783 | protein_coding | D10Wsu52e | 3983.571 | 4048.609 | 4016.09 |
| ENSMUSG00000054453 | protein_coding | Sytl5 | 9.700503 | 10.93999 | 10.32024 |
| ENSMUSG00000037119 | protein_coding | D15Ertd621e | 4694.205 | 4772.817 | 4733.511 |
| ENSMUSG00000087567 | lincRNA | Gm4755 | 2.923592 | 3.708054 | 3.315823 |
| ENSMUSG00000025409 | protein_coding | Mbd6 | 1356.42 | 1386.061 | 1371.241 |
| ENSMUSG00000036587 | protein_coding | Fut7 | 0.968641 | 1.395426 | 1.182034 |
| ENSMUSG00000020430 | protein_coding | Pes1 | 3176.648 | 3113.7 | 3145.174 |
| ENSMUSG00000085149 | lincRNA | Gm16825 | 28.79707 | 31.04505 | 29.92106 |
| ENSMUSG00000036339 | protein_coding | 6720456H20Rik | 928.7712 | 954.3794 | 941.5753 |
| ENSMUSG00000036353 | protein_coding | P2ry12 | 66.19204 | 62.89955 | 64.54579 |
| ENSMUSG00000028710 | protein_coding | Atpaf1 | 854.9453 | 838.1266 | 846.5359 |
| ENSMUSG00000041361 | protein_coding | Gcom1 | 436.2671 | 447.8524 | 442.0598 |
| ENSMUSG00000086559 | processed_transcrip | Gm14426 | 0.64422 | 1.059903 | 0.852062 |
| ENSMUSG00000085677 | processed_transcrip | 4930594O21Rik | 0.64422 | 1.059903 | 0.852062 |
| ENSMUSG00000070601 | protein_coding | Vmn2r84 | 0.64422 | 1.059903 | 0.852062 |
| ENSMUSG00000085765 | processed_transcrip | Gm12333 | 0.64422 | 1.059903 | 0.852062 |
| ENSMUSG00000024534 | protein_coding | Sncaip | 98.88529 | 103.4182 | 101.1517 |
| ENSMUSG00000024206 | protein_coding | Rfx2 | 285.2533 | 272.4008 | 278.8271 |
| ENSMUSG00000091213 | protein_coding | Gm17253 | 110.9178 | 116.9324 | 113.9251 |
| ENSMUSG00000083355 | pseudogene | Gm11581 | 0.968641 | 1.350742 | 1.159691 |
| ENSMUSG00000059873 | protein_coding | Olfr1029 | 0.968641 | 1.350742 | 1.159691 |
| ENSMUSG00000090257 | processed_transcrip | Gm4524 | 11.2957 | 9.818466 | 10.55708 |
| ENSMUSG00000049122 | protein_coding | Frmd3 | 5.755493 | 6.798392 | 6.276942 |

| | | | | | |
|---|---|---|---|---|---|
| ENSMUSG00000083065 | pseudogene | Gm13195 | 20.66485 | 22.56003 | 21.61244 |
| ENSMUSG00000027333 | protein_coding | Smox | 1503.517 | 1544.607 | 1524.062 |
| ENSMUSG00000061458 | protein_coding | Nol10 | 1311.98 | 1280.632 | 1296.306 |
| ENSMUSG00000028262 | protein_coding | Clca2 | 72.56424 | 68.1374 | 70.35082 |
| ENSMUSG00000043857 | protein_coding | Mgat5b | 201.4376 | 188.7107 | 195.0741 |
| ENSMUSG00000023885 | protein_coding | Thbs2 | 1052.933 | 1021.107 | 1037.02 |
| ENSMUSG00000087041 | processed_transcrip | Gm16295 | 86.07177 | 90.46649 | 88.26913 |
| ENSMUSG00000048065 | protein_coding | Cyb5r2 | 4.170809 | 3.425862 | 3.798335 |
| ENSMUSG00000029248 | protein_coding | 1700023E05Rik | 0.962752 | 1.386417 | 1.174585 |
| ENSMUSG00000032754 | protein_coding | Slc24a6 | 226.0843 | 233.1913 | 229.6378 |
| ENSMUSG00000092754 | snRNA | AC121599.1 | 9.700503 | 10.93098 | 10.31574 |
| ENSMUSG00000032479 | protein_coding | Mtap4 | 8599.133 | 8398.049 | 8498.591 |
| ENSMUSG00000061904 | protein_coding | Slc25a3 | 6922.571 | 6818.292 | 6870.432 |
| ENSMUSG00000073563 | protein_coding | Csnk1g3 | 1883.353 | 1916.521 | 1899.937 |
| ENSMUSG00000000103 | protein_coding | Zfy2 | 148.3541 | 128.9612 | 138.6576 |
| ENSMUSG00000022019 | protein_coding | Tdrd3 | 1145.967 | 1176.887 | 1161.427 |
| ENSMUSG00000052139 | protein_coding | Bre | 508.0834 | 496.0057 | 502.0446 |
| ENSMUSG00000035239 | protein_coding | Neu3 | 140.8703 | 135.1844 | 138.0273 |
| ENSMUSG00000039202 | protein_coding | Abhd2 | 1701.493 | 1744.197 | 1722.845 |
| ENSMUSG00000073096 | protein_coding | Lrrc61 | 214.6948 | 207.8242 | 211.2595 |
| ENSMUSG00000090952 | lincRNA | Gm17251 | 61.01852 | 64.33386 | 62.67619 |
| ENSMUSG00000040084 | protein_coding | Bub1b | 2463.868 | 2417.421 | 2440.645 |
| ENSMUSG00000074651 | protein_coding | Gm6320 | 4.869074 | 5.854163 | 5.361619 |
| ENSMUSG00000073422 | protein_coding | H2-Ke6 | 4.238943 | 5.112127 | 4.675535 |
| ENSMUSG00000007892 | protein_coding | Rplp1 | 6626.406 | 6512.343 | 6569.374 |
| ENSMUSG00000004500 | protein_coding | Zfp324 | 328.0668 | 339.8254 | 333.9461 |
| ENSMUSG00000061482 | protein_coding | Hist1h4d | 7.113111 | 6.055995 | 6.584553 |
| ENSMUSG00000067028 | protein_coding | Cntnap5b | 0.33031 | 0.671047 | 0.500679 |
| ENSMUSG00000082350 | pseudogene | Gm14008 | 0.33031 | 0.671047 | 0.500679 |
| ENSMUSG00000087409 | processed_transcrip | Gm14271 | 0.33031 | 0.671047 | 0.500679 |
| ENSMUSG00000080638 | miRNA | AC122490.1 | 0.33031 | 0.671047 | 0.500679 |
| ENSMUSG00000029273 | protein_coding | Sult1d1 | 0.33031 | 0.671047 | 0.500679 |
| ENSMUSG00000089830 | processed_transcrip | Gm15749 | 0.33031 | 0.671047 | 0.500679 |
| ENSMUSG00000065227 | snRNA | U6 | 0.33031 | 0.671047 | 0.500679 |
| ENSMUSG00000069922 | protein_coding | Ces3a | 0.33031 | 0.671047 | 0.500679 |
| ENSMUSG00000043522 | pseudogene | A430060F13Rik | 0.33031 | 0.671047 | 0.500679 |
| ENSMUSG00000090660 | pseudogene | Gm17030 | 0.33031 | 0.671047 | 0.500679 |
| ENSMUSG00000089779 | processed_transcrip | Gm15641 | 0.33031 | 0.671047 | 0.500679 |
| ENSMUSG00000089701 | processed_transcrip | Gm15978 | 0.33031 | 0.671047 | 0.500679 |
| ENSMUSG00000003053 | protein_coding | Cyp2c29 | 0.33031 | 0.671047 | 0.500679 |
| ENSMUSG00000081674 | pseudogene | Gm6184 | 0.33031 | 0.671047 | 0.500679 |
| ENSMUSG00000030960 | protein_coding | Mettl10 | 572.5791 | 585.5644 | 579.0717 |
| ENSMUSG00000060268 | protein_coding | Gm1661 | 18.14074 | 16.45863 | 17.29968 |
| ENSMUSG00000039977 | protein_coding | Ccdc67 | 12.05725 | 10.56879 | 11.31302 |
| ENSMUSG00000085052 | lincRNA | Gm16675 | 14.87864 | 13.35099 | 14.11482 |
| ENSMUSG00000082675 | pseudogene | Gm6382 | 88.75063 | 84.92978 | 86.84021 |
| ENSMUSG00000030474 | protein_coding | Siglece | 17.21899 | 15.3807 | 16.29985 |
| ENSMUSG00000061232 | protein_coding | H2-K1 | 1447.418 | 1600.015 | 1523.717 |
| ENSMUSG00000083726 | pseudogene | Gm13309 | 1.623372 | 1.086569 | 1.354971 |
| ENSMUSG00000027171 | protein_coding | Prrg4 | 137.4075 | 144.2867 | 140.8471 |
| ENSMUSG00000078349 | protein_coding | AW011738 | 8.694216 | 9.960443 | 9.32733 |
| ENSMUSG00000081232 | pseudogene | Gm14373 | 4.551586 | 3.681026 | 4.116306 |
| ENSMUSG00000024406 | protein_coding | Pou5f1 | 250.8993 | 240.8923 | 245.8958 |
| ENSMUSG00000028909 | protein_coding | Ptpru | 209.6103 | 218.9825 | 214.2964 |
| ENSMUSG00000087111 | processed_transcrip | Gm11399 | 3.902744 | 4.705253 | 4.303998 |
| ENSMUSG00000022129 | protein_coding | Dct | 74.31837 | 79.33544 | 76.82691 |
| ENSMUSG00000029780 | protein_coding | Nt5c3 | 2735.63 | 2791.425 | 2763.528 |
| ENSMUSG00000036632 | protein_coding | Alg5 | 738.0022 | 753.1997 | 745.6009 |

| | | | | | |
|---|---|---|---|---|---|
| ENSMUSG00000026971 | protein_coding | Itgb6 | 0.32211 | 0.671047 | 0.496579 |
| ENSMUSG00000084918 | processed_transcrip | Gm12708 | 0.32211 | 0.671047 | 0.496579 |
| ENSMUSG00000028536 | protein_coding | 2610528J11Rik | 0.32211 | 0.671047 | 0.496579 |
| ENSMUSG00000083494 | pseudogene | Gm12873 | 0.32211 | 0.671047 | 0.496579 |
| ENSMUSG00000087248 | processed_transcrip | Gm16120 | 0.32211 | 0.671047 | 0.496579 |
| ENSMUSG00000091981 | protein_coding | Gm8038 | 0.32211 | 0.671047 | 0.496579 |
| ENSMUSG00000048400 | protein_coding | Prss54 | 0.32211 | 0.671047 | 0.496579 |
| ENSMUSG00000090039 | processed_transcrip | Gm15894 | 0.32211 | 0.671047 | 0.496579 |
| ENSMUSG00000092210 | protein_coding | RP23-43M12.1 | 0.32211 | 0.671047 | 0.496579 |
| ENSMUSG00000092159 | protein_coding | Gm2135 | 0.32211 | 0.671047 | 0.496579 |
| ENSMUSG00000088384 | rRNA | 5S_rRNA | 0.32211 | 0.671047 | 0.496579 |
| ENSMUSG00000029711 | protein_coding | Epo | 4.210765 | 5.02312 | 4.616942 |
| ENSMUSG00000048012 | protein_coding | Zfp473 | 202.5534 | 188.9415 | 195.7474 |
| ENSMUSG00000021590 | protein_coding | Spata9 | 57.77535 | 54.71831 | 56.24683 |
| ENSMUSG00000036435 | protein_coding | Exoc1 | 2600.852 | 2648.419 | 2624.636 |
| ENSMUSG00000022559 | protein_coding | Fbxl6 | 1245.541 | 1268.98 | 1257.261 |
| ENSMUSG00000030760 | protein_coding | Acer3 | 1170.073 | 1125.208 | 1147.64 |
| ENSMUSG00000053565 | protein_coding | Eif3k | 2611.689 | 2665.858 | 2638.773 |
| ENSMUSG00000083404 | pseudogene | Gm13019 | 0.638331 | 0.988552 | 0.813442 |
| ENSMUSG00000032623 | protein_coding | Oas1d | 0.638331 | 0.988552 | 0.813442 |
| ENSMUSG00000069785 | protein_coding | Gm11444 | 0.638331 | 0.988552 | 0.813442 |
| ENSMUSG00000051729 | protein_coding | Gm5087 | 0.638331 | 0.988552 | 0.813442 |
| ENSMUSG00000021010 | protein_coding | Npas3 | 35.07356 | 32.44483 | 33.75919 |
| ENSMUSG00000051444 | protein_coding | Bbs12 | 314.5835 | 304.2485 | 309.416 |
| ENSMUSG00000085238 | processed_transcrip | 4930479D17Rik | 9.367882 | 8.238503 | 8.803193 |
| ENSMUSG00000039262 | protein_coding | Prrc2b | 5674.26 | 5801.826 | 5738.043 |
| ENSMUSG00000079492 | protein_coding | Gm11127 | 1.637461 | 1.086569 | 1.362015 |
| ENSMUSG00000081070 | pseudogene | Gm13181 | 0.316221 | 0.671047 | 0.493634 |
| ENSMUSG00000065824 | misc_RNA | 7SK | 0.316221 | 0.671047 | 0.493634 |
| ENSMUSG00000090545 | protein_coding | Gm17488 | 0.316221 | 0.671047 | 0.493634 |
| ENSMUSG00000082509 | pseudogene | Gm15673 | 0.316221 | 0.671047 | 0.493634 |
| ENSMUSG00000073979 | protein_coding | Olfr547 | 0.316221 | 0.671047 | 0.493634 |
| ENSMUSG00000074562 | protein_coding | Gm10718 | 0.316221 | 0.671047 | 0.493634 |
| ENSMUSG00000073253 | protein_coding | Gm10487 | 0.316221 | 0.671047 | 0.493634 |
| ENSMUSG00000014177 | protein_coding | Fam18b | 953.5716 | 977.5353 | 965.5534 |
| ENSMUSG00000083391 | pseudogene | Gm14148 | 0.646531 | 0.997561 | 0.822046 |
| ENSMUSG00000090166 | protein_coding | Ear10 | 0.646531 | 0.997561 | 0.822046 |
| ENSMUSG00000092495 | processed_transcrip | RP23-167B4.2 | 16.94861 | 15.09023 | 16.01942 |
| ENSMUSG00000049807 | protein_coding | Arhgap23 | 1780.635 | 1740.575 | 1760.605 |
| ENSMUSG00000080657 | miRNA | Mir669i | 0.65242 | 0.36219 | 0.507305 |
| ENSMUSG00000075036 | pseudogene | Gm10805 | 0.65242 | 0.36219 | 0.507305 |
| ENSMUSG00000067996 | protein_coding | Bpifb9b | 0.65242 | 0.36219 | 0.507305 |
| ENSMUSG00000078781 | protein_coding | Sirpb1b | 0.65242 | 0.36219 | 0.507305 |
| ENSMUSG00000081647 | pseudogene | Gm13022 | 0.65242 | 0.36219 | 0.507305 |
| ENSMUSG00000063297 | protein_coding | Luzp2 | 0.65242 | 0.36219 | 0.507305 |
| ENSMUSG00000092969 | miRNA | AC160534.1 | 0.65242 | 0.36219 | 0.507305 |
| ENSMUSG00000091663 | protein_coding | Gm2783 | 0.65242 | 0.36219 | 0.507305 |
| ENSMUSG00000064664 | snRNA | U4 | 0.65242 | 0.36219 | 0.507305 |
| ENSMUSG00000088324 | rRNA | 5S_rRNA | 0.65242 | 0.36219 | 0.507305 |
| ENSMUSG00000086025 | processed_transcrip | 4933406G16Rik | 0.65242 | 0.36219 | 0.507305 |
| ENSMUSG00000047861 | protein_coding | Foxi1 | 0.65242 | 0.36219 | 0.507305 |
| ENSMUSG00000087172 | processed_transcrip | Gm12153 | 0.65242 | 0.36219 | 0.507305 |
| ENSMUSG00000084034 | pseudogene | Gm12617 | 0.65242 | 0.36219 | 0.507305 |
| ENSMUSG00000089978 | pseudogene | Gm16245 | 0.65242 | 0.36219 | 0.507305 |
| ENSMUSG00000076912 | IG_J_gene | Gm17010 | 0.65242 | 0.36219 | 0.507305 |
| ENSMUSG00000050982 | protein_coding | Apol10a | 0.65242 | 0.36219 | 0.507305 |
| ENSMUSG00000093139 | snoRNA | AC161198.1 | 0.65242 | 0.36219 | 0.507305 |
| ENSMUSG00000090078 | pseudogene | Gm16147 | 0.65242 | 0.36219 | 0.507305 |

| | | | | | |
|---|---|---|---|---|---|
| ENSMUSG00000082090 | pseudogene | Gm16481 | 0.65242 | 0.36219 | 0.507305 |
| ENSMUSG00000030858 | protein_coding | 1700007K09Rik | 2.281682 | 1.748606 | 2.015144 |
| ENSMUSG00000069910 | protein_coding | Ccdc99 | 981.2412 | 1002.851 | 992.0461 |
| ENSMUSG00000086853 | processed_transcrip | Gm14720 | 2.273482 | 1.748606 | 2.011044 |
| ENSMUSG00000021608 | protein_coding | Lpcat1 | 595.2188 | 608.3634 | 601.7911 |
| ENSMUSG00000043342 | protein_coding | Hoxd9 | 661.0784 | 680.115 | 670.5967 |
| ENSMUSG00000075604 | protein_coding | Cyp11b1 | 14.84584 | 16.53862 | 15.69223 |
| ENSMUSG00000092079 | lincRNA | Gm17440 | 9.992125 | 8.784506 | 9.388315 |
| ENSMUSG00000045667 | protein_coding | Smtnl2 | 729.1319 | 750.9094 | 740.0206 |
| ENSMUSG00000010797 | protein_coding | Wnt2 | 23.28146 | 25.18079 | 24.23113 |
| ENSMUSG00000049866 | protein_coding | Arl4c | 352.8076 | 373.3375 | 363.0725 |
| ENSMUSG00000055204 | protein_coding | Ankrd17 | 8570.05 | 8794.951 | 8682.501 |
| ENSMUSG00000024949 | protein_coding | Sf1 | 6277.004 | 6160.01 | 6218.507 |
| ENSMUSG00000045868 | protein_coding | Gvin1 | 4.543386 | 3.770395 | 4.15689 |
| ENSMUSG00000028552 | protein_coding | Eps15 | 9236.155 | 9387.812 | 9311.984 |
| ENSMUSG00000087047 | lincRNA | 1700110K17Rik | 0.64422 | 1.050893 | 0.847557 |
| ENSMUSG00000027131 | protein_coding | Tmem85 | 1133.493 | 1155.462 | 1144.478 |
| ENSMUSG00000027509 | protein_coding | Rae1 | 2734.222 | 2668.656 | 2701.439 |
| ENSMUSG00000087601 | lincRNA | Gm16832 | 47.98383 | 44.84724 | 46.41553 |
| ENSMUSG00000023031 | protein_coding | Cela1 | 19.99961 | 21.94811 | 20.97386 |
| ENSMUSG00000037737 | protein_coding | Arpm1 | 2.583814 | 2.012779 | 2.298297 |
| ENSMUSG00000061469 | processed_transcrip | Gm12569 | 37.57746 | 34.75129 | 36.16438 |
| ENSMUSG00000022858 | protein_coding | Tra2b | 5972.901 | 6087.936 | 6030.418 |
| ENSMUSG00000022215 | protein_coding | Fitm1 | 11.31568 | 12.61724 | 11.96646 |
| ENSMUSG00000019795 | protein_coding | Pcmt1 | 1757.961 | 1724.743 | 1741.352 |
| ENSMUSG00000020990 | protein_coding | Cdkl1 | 13.83851 | 15.35476 | 14.59664 |
| ENSMUSG00000074505 | protein_coding | Fat3 | 112.8817 | 105.7672 | 109.3245 |
| ENSMUSG00000022066 | protein_coding | Entpd4 | 806.6375 | 770.9246 | 788.781 |
| ENSMUSG00000022580 | protein_coding | Rhpn1 | 288.1769 | 277.7121 | 282.9445 |
| ENSMUSG00000027708 | protein_coding | Dcun1d1 | 1592.892 | 1629.319 | 1611.106 |
| ENSMUSG00000033068 | protein_coding | Entpd6 | 1351.309 | 1326.262 | 1338.786 |
| ENSMUSG00000085119 | lincRNA | Gm13644 | 15.76171 | 14.17882 | 14.97027 |
| ENSMUSG00000028023 | protein_coding | Pitx2 | 4.518786 | 3.752377 | 4.135581 |
| ENSMUSG00000008475 | protein_coding | Arpc5 | 4527.998 | 4601.372 | 4564.685 |
| ENSMUSG00000082596 | pseudogene | Gm14227 | 3.594722 | 4.405405 | 4.000064 |
| ENSMUSG00000005936 | protein_coding | Kctd20 | 3095.725 | 3150.512 | 3123.118 |
| ENSMUSG00000035004 | protein_coding | Igsf6 | 67.84612 | 64.53461 | 66.19036 |
| ENSMUSG00000067010 | protein_coding | Vmn2r10 | 4.530564 | 3.72571 | 4.128137 |
| ENSMUSG00000035148 | protein_coding | Gpr33 | 0.632442 | 1.033237 | 0.832839 |
| ENSMUSG00000084999 | processed_transcrip | Gm15839 | 0.632442 | 1.033237 | 0.832839 |
| ENSMUSG00000091695 | protein_coding | Gm17669 | 0.632442 | 1.033237 | 0.832839 |
| ENSMUSG00000075908 | snRNA | U6 | 0.632442 | 1.033237 | 0.832839 |
| ENSMUSG00000081562 | pseudogene | Gm11575 | 0.65242 | 0.335524 | 0.493972 |
| ENSMUSG00000052759 | protein_coding | Gpr25 | 0.65242 | 0.335524 | 0.493972 |
| ENSMUSG00000087720 | snRNA | U6 | 0.65242 | 0.335524 | 0.493972 |
| ENSMUSG00000089493 | snRNA | U1 | 0.65242 | 0.335524 | 0.493972 |
| ENSMUSG00000087045 | processed_transcrip | 4930515B02Rik | 0.65242 | 0.335524 | 0.493972 |
| ENSMUSG00000089734 | processed_transcrip | Gm16108 | 0.65242 | 0.335524 | 0.493972 |
| ENSMUSG00000029697 | protein_coding | Fezf1 | 0.65242 | 0.335524 | 0.493972 |
| ENSMUSG00000080609 | miRNA | AC158296.1 | 0.65242 | 0.335524 | 0.493972 |
| ENSMUSG00000090439 | protein_coding | Gm17455 | 0.65242 | 0.335524 | 0.493972 |
| ENSMUSG00000064441 | snoRNA | Snord37 | 0.65242 | 0.335524 | 0.493972 |
| ENSMUSG00000088889 | snRNA | U6 | 0.65242 | 0.335524 | 0.493972 |
| ENSMUSG00000069607 | protein_coding | Cd300lh | 0.65242 | 0.335524 | 0.493972 |
| ENSMUSG00000078982 | pseudogene | Gm11025 | 0.65242 | 0.335524 | 0.493972 |
| ENSMUSG00000064063 | protein_coding | BC048507 | 0.65242 | 0.335524 | 0.493972 |
| ENSMUSG00000071616 | protein_coding | Gm3488 | 0.65242 | 0.335524 | 0.493972 |
| ENSMUSG00000022243 | protein_coding | Slc45a2 | 0.65242 | 0.335524 | 0.493972 |

| | | | | | |
|---|---|---|---|---|---|
| ENSMUSG00000082393 | pseudogene | Gm15028 | 0.65242 | 0.335524 | 0.493972 |
| ENSMUSG00000084197 | pseudogene | Gm9089 | 0.65242 | 0.335524 | 0.493972 |
| ENSMUSG00000082602 | pseudogene | Gm15021 | 0.65242 | 0.335524 | 0.493972 |
| ENSMUSG00000082598 | pseudogene | Gm15212 | 0.65242 | 0.335524 | 0.493972 |
| ENSMUSG00000036611 | protein_coding | Eepd1 | 476.8809 | 459.8802 | 468.3806 |
| ENSMUSG00000063894 | protein_coding | Zfp192 | 2144.611 | 2203.047 | 2173.829 |
| ENSMUSG00000058492 | pseudogene | Scp2-ps2 | 84.95404 | 80.84012 | 82.89708 |
| ENSMUSG00000038876 | protein_coding | Rnf146 | 1178.764 | 1209.745 | 1194.254 |
| ENSMUSG00000016493 | protein_coding | Cd46 | 46.19855 | 49.2952 | 47.74687 |
| ENSMUSG00000076498 | IG_C_gene | Trbc2 | 2.603792 | 2.012779 | 2.308286 |
| ENSMUSG00000026788 | protein_coding | Zbtb43 | 1830.758 | 1793.985 | 1812.372 |
| ENSMUSG00000026778 | protein_coding | Prkcq | 114.6586 | 109.471 | 112.0648 |
| ENSMUSG00000067995 | protein_coding | Gtf2f2 | 1314.132 | 1287.771 | 1300.951 |
| ENSMUSG00000090850 | protein_coding | Orai2-ps | 2.597903 | 2.021789 | 2.309846 |
| ENSMUSG00000034613 | protein_coding | Ppm1h | 53.57048 | 56.6032 | 55.08684 |
| ENSMUSG00000022016 | protein_coding | Akap11 | 6640.331 | 6517.25 | 6578.791 |
| ENSMUSG00000085454 | processed_transcrip | Gm16320 | 6.808668 | 5.782813 | 6.29574 |
| ENSMUSG00000083939 | pseudogene | Gm14451 | 21.29371 | 23.20912 | 22.25142 |
| ENSMUSG00000087024 | lincRNA | B230119M05Rik | 6.740534 | 5.65813 | 6.199332 |
| ENSMUSG00000026011 | protein_coding | Ctla4 | 0.65242 | 0.326514 | 0.489467 |
| ENSMUSG00000089808 | pseudogene | Gm7241 | 0.65242 | 0.326514 | 0.489467 |
| ENSMUSG00000069008 | pseudogene | Gm5537 | 0.65242 | 0.326514 | 0.489467 |
| ENSMUSG00000055891 | protein_coding | Ubl4b | 0.65242 | 0.326514 | 0.489467 |
| ENSMUSG00000078593 | protein_coding | 1700042G07Rik | 0.65242 | 0.326514 | 0.489467 |
| ENSMUSG00000076123 | miRNA | Mir700 | 0.65242 | 0.326514 | 0.489467 |
| ENSMUSG00000082308 | pseudogene | Gm15770 | 0.65242 | 0.326514 | 0.489467 |
| ENSMUSG00000066704 | protein_coding | Cyp2b19 | 0.65242 | 0.326514 | 0.489467 |
| ENSMUSG00000082210 | pseudogene | Gm14382 | 0.65242 | 0.326514 | 0.489467 |
| ENSMUSG00000074485 | protein_coding | Gm7381 | 0.65242 | 0.326514 | 0.489467 |
| ENSMUSG00000045591 | protein_coding | Olig3 | 0.65242 | 0.326514 | 0.489467 |
| ENSMUSG00000075986 | snRNA | U6 | 0.65242 | 0.326514 | 0.489467 |
| ENSMUSG00000079010 | protein_coding | Gm11032 | 0.65242 | 0.326514 | 0.489467 |
| ENSMUSG00000082529 | pseudogene | Gm11199 | 0.65242 | 0.326514 | 0.489467 |
| ENSMUSG00000085851 | processed_transcrip | 4921518K17Rik | 0.65242 | 0.326514 | 0.489467 |
| ENSMUSG00000035431 | protein_coding | Sstr1 | 0.65242 | 0.326514 | 0.489467 |
| ENSMUSG00000076621 | IG_J_gene | Gm16878 | 0.65242 | 0.326514 | 0.489467 |
| ENSMUSG00000090475 | pseudogene | Gm6245 | 0.65242 | 0.326514 | 0.489467 |
| ENSMUSG00000091613 | pseudogene | Gm17046 | 0.65242 | 0.326514 | 0.489467 |
| ENSMUSG00000076871 | IG_J_gene | Trdj2 | 0.65242 | 0.326514 | 0.489467 |
| ENSMUSG00000089044 | snRNA | U1 | 0.65242 | 0.326514 | 0.489467 |
| ENSMUSG00000090319 | pseudogene | Gm4462 | 0.65242 | 0.326514 | 0.489467 |
| ENSMUSG00000085846 | processed_transcrip | Gm15597 | 0.65242 | 0.326514 | 0.489467 |
| ENSMUSG00000092171 | processed_transcrip | 4833427F10Rik | 0.65242 | 0.326514 | 0.489467 |
| ENSMUSG00000077588 | misc_RNA | Y_RNA | 0.65242 | 0.326514 | 0.489467 |
| ENSMUSG00000091744 | protein_coding | Gm17732 | 0.65242 | 0.326514 | 0.489467 |
| ENSMUSG00000087251 | lincRNA | Gm16795 | 0.65242 | 0.326514 | 0.489467 |
| ENSMUSG00000087280 | lincRNA | Gm17557 | 5.540205 | 6.507553 | 6.023879 |
| ENSMUSG00000026535 | protein_coding | Ifi202b | 20.63965 | 24.01635 | 22.328 |
| ENSMUSG00000022564 | protein_coding | Grina | 1304.278 | 1333.596 | 1318.937 |
| ENSMUSG00000036907 | protein_coding | C1ql2 | 0.974531 | 1.386417 | 1.180474 |
| ENSMUSG00000086970 | processed_transcrip | Gm14259 | 0.974531 | 1.386417 | 1.180474 |
| ENSMUSG00000082072 | pseudogene | Gm15785 | 0.974531 | 1.386417 | 1.180474 |
| ENSMUSG00000021900 | protein_coding | Btd | 556.4128 | 569.9372 | 563.175 |
| ENSMUSG00000026223 | protein_coding | Itm2c | 7300.925 | 7414.83 | 7357.877 |
| ENSMUSG00000082766 | protein_coding | 1700064H15Rik | 4.221275 | 3.470547 | 3.845911 |
| ENSMUSG00000089991 | pseudogene | Gm16332 | 1.284863 | 1.748606 | 1.516734 |
| ENSMUSG00000036099 | protein_coding | Vezt | 1040.333 | 1064.128 | 1052.23 |
| ENSMUSG00000085021 | processed_transcrip | Gm12051 | 0.65242 | 0.997561 | 0.824991 |

| | | | | | |
|---|---|---|---|---|---|
| ENSMUSG00000080485 | miRNA | AC129021.1 | 0.65242 | 0.997561 | 0.824991 |
| ENSMUSG00000025216 | protein_coding | Lbx1 | 0.65242 | 0.997561 | 0.824991 |
| ENSMUSG00000085929 | lincRNA | Gm13421 | 62.51427 | 65.99926 | 64.25677 |
| ENSMUSG00000092246 | pseudogene | Gm20493 | 2.597903 | 2.00377 | 2.300837 |
| ENSMUSG00000056313 | protein_coding | 1810011O10Rik | 47.75759 | 50.96345 | 49.36052 |
| ENSMUSG00000009114 | protein_coding | 2610028H24Rik | 5.49436 | 6.543229 | 6.018794 |
| ENSMUSG00000088005 | snoRNA | SNORA70 | 0.954552 | 1.422092 | 1.188322 |
| ENSMUSG00000005774 | protein_coding | Rfx5 | 609.9806 | 595.6776 | 602.8291 |
| ENSMUSG00000058056 | protein_coding | Palld | 296.4779 | 286.7176 | 291.5978 |
| ENSMUSG00000050299 | protein_coding | Gm9843 | 68.25023 | 64.91554 | 66.58288 |
| ENSMUSG00000048772 | protein_coding | Tmem53 | 166.0201 | 172.2512 | 169.1357 |
| ENSMUSG00000038845 | protein_coding | Phb | 3075.514 | 3010.787 | 3043.15 |
| ENSMUSG00000029551 | protein_coding | Psmg3 | 334.4174 | 343.5506 | 338.984 |
| ENSMUSG00000060461 | protein_coding | Dppa5a | 66.95635 | 63.19976 | 65.07805 |
| ENSMUSG00000089967 | pseudogene | Gm16550 | 0.646531 | 0.36219 | 0.50436 |
| ENSMUSG00000080223 | pseudogene | Gm13768 | 0.646531 | 0.36219 | 0.50436 |
| ENSMUSG00000080873 | pseudogene | Rpl30-ps3 | 0.646531 | 0.36219 | 0.50436 |
| ENSMUSG00000088695 | snoRNA | SNORA70 | 0.646531 | 0.36219 | 0.50436 |
| ENSMUSG00000080807 | pseudogene | Gm13117 | 0.646531 | 0.36219 | 0.50436 |
| ENSMUSG00000084921 | processed_transcrip | Gm13838 | 0.646531 | 0.36219 | 0.50436 |
| ENSMUSG00000086715 | processed_transcrip | B230112J18Rik | 0.646531 | 0.36219 | 0.50436 |
| ENSMUSG00000083351 | pseudogene | Gm15783 | 0.646531 | 0.36219 | 0.50436 |
| ENSMUSG00000066515 | protein_coding | Klk1b3 | 0.646531 | 0.36219 | 0.50436 |
| ENSMUSG00000086403 | lincRNA | 1500012K07Rik | 0.646531 | 0.36219 | 0.50436 |
| ENSMUSG00000025475 | protein_coding | Gpr123 | 0.646531 | 0.36219 | 0.50436 |
| ENSMUSG00000090067 | pseudogene | Gm2976 | 0.646531 | 0.36219 | 0.50436 |
| ENSMUSG00000084048 | pseudogene | Gm12042 | 0.646531 | 0.36219 | 0.50436 |
| ENSMUSG00000022584 | protein_coding | Ly6c2 | 0.646531 | 0.36219 | 0.50436 |
| ENSMUSG00000024124 | protein_coding | Prss30 | 0.646531 | 0.36219 | 0.50436 |
| ENSMUSG00000077956 | miRNA | Mir743b | 0.646531 | 0.36219 | 0.50436 |
| ENSMUSG00000027575 | protein_coding | Arfgap1 | 1575.727 | 1544.408 | 1560.067 |
| ENSMUSG00000040627 | protein_coding | Aicda | 0.968641 | 1.377408 | 1.173025 |
| ENSMUSG00000032932 | protein_coding | Hspa13 | 1841.374 | 1797.169 | 1819.271 |
| ENSMUSG00000083199 | pseudogene | Gm9432 | 2.233526 | 1.695274 | 1.9644 |
| ENSMUSG00000080134 | lincRNA | Gm17516 | 0.954552 | 1.359751 | 1.157152 |
| ENSMUSG00000014351 | protein_coding | Gip | 0.976841 | 1.386417 | 1.181629 |
| ENSMUSG00000020591 | protein_coding | Ntsr2 | 0.646531 | 0.988552 | 0.817542 |
| ENSMUSG00000091815 | pseudogene | Gm17153 | 0.646531 | 0.988552 | 0.817542 |
| ENSMUSG00000085635 | processed_transcrip | Gm14565 | 0.646531 | 0.988552 | 0.817542 |
| ENSMUSG00000017639 | protein_coding | Rab11fip4 | 4799.606 | 4917.73 | 4858.668 |
| ENSMUSG00000025993 | protein_coding | Slc40a1 | 695.7736 | 714.5827 | 705.1781 |
| ENSMUSG00000021277 | protein_coding | Traf3 | 954.2766 | 932.2023 | 943.2395 |
| ENSMUSG00000022296 | protein_coding | Baalc | 14.48631 | 16.13827 | 15.31229 |
| ENSMUSG00000020439 | protein_coding | Smtn | 697.4643 | 680.9573 | 689.2108 |
| ENSMUSG00000003617 | protein_coding | Cp | 1134.927 | 1168.614 | 1151.77 |
| ENSMUSG00000026135 | protein_coding | Zfp142 | 1812.329 | 1761.412 | 1786.87 |
| ENSMUSG00000032212 | protein_coding | Sltm | 2159.811 | 2198.538 | 2179.175 |
| ENSMUSG00000066232 | protein_coding | Ipo7 | 3924.083 | 3847.162 | 3885.622 |
| ENSMUSG00000030157 | protein_coding | Clec2d | 99.11302 | 103.3883 | 101.2507 |
| ENSMUSG00000034940 | protein_coding | Synrg | 2146.357 | 2105.781 | 2126.069 |
| ENSMUSG00000059866 | protein_coding | Tnip2 | 701.5739 | 723.926 | 712.7499 |
| ENSMUSG00000039740 | protein_coding | Alg2 | 1361.217 | 1324.565 | 1342.891 |
| ENSMUSG00000058192 | protein_coding | Zfp846 | 502.6903 | 487.7148 | 495.2026 |
| ENSMUSG00000016200 | protein_coding | Syt14 | 372.6945 | 359.5073 | 366.1009 |
| ENSMUSG00000053626 | protein_coding | Tll1 | 22.24469 | 24.08594 | 23.16531 |
| ENSMUSG00000050604 | protein_coding | 3110021A11Rik | 29.94693 | 27.81128 | 28.87911 |
| ENSMUSG00000017188 | protein_coding | Ccdc56 | 584.1742 | 600.6214 | 592.3978 |
| ENSMUSG00000037894 | protein_coding | H2afz | 4210.488 | 4302.76 | 4256.624 |

| | | | | | |
|---|---|---|---|---|---|
| ENSMUSG00000026896 | protein_coding | Ifih1 | 294.4004 | 307.3836 | 300.892 |
| ENSMUSG00000059150 | protein_coding | Rpl26-ps4 | 0.968641 | 1.448758 | 1.2087 |
| ENSMUSG00000091247 | lincRNA | Gm16529 | 34.82651 | 37.16266 | 35.99459 |
| ENSMUSG00000037316 | protein_coding | Bag4 | 1168.145 | 1145.681 | 1156.913 |
| ENSMUSG00000012428 | protein_coding | Steap4 | 341.0431 | 326.0559 | 333.5495 |
| ENSMUSG00000017264 | protein_coding | Exosc10 | 2377.941 | 2421.25 | 2399.595 |
| ENSMUSG00000083104 | pseudogene | Gm12917 | 7.394222 | 8.484657 | 7.93944 |
| ENSMUSG00000085393 | processed_transcrip | Gm11280 | 2.247615 | 1.668608 | 1.958112 |
| ENSMUSG00000078374 | pseudogene | Gm10476 | 2.606103 | 2.012779 | 2.309441 |
| ENSMUSG00000089536 | snoRNA | Scarna3a | 7.466977 | 8.565017 | 8.015997 |
| ENSMUSG00000073471 | protein_coding | Rsph3a | 312.244 | 303.8799 | 308.0619 |
| ENSMUSG00000054381 | protein_coding | Zfp747 | 190.279 | 198.5569 | 194.4179 |
| ENSMUSG00000035441 | protein_coding | Myo1d | 1052.151 | 1078.099 | 1065.125 |
| ENSMUSG00000032846 | protein_coding | Zswim6 | 1672.793 | 1630.602 | 1651.697 |
| ENSMUSG00000037573 | protein_coding | Tob1 | 1285.465 | 1308.98 | 1297.222 |
| ENSMUSG00000027895 | protein_coding | Kcnc4 | 11.66365 | 13.04177 | 12.35271 |
| ENSMUSG00000031482 | protein_coding | Slc25a15 | 356.0293 | 366.1584 | 361.0939 |
| ENSMUSG00000052395 | protein_coding | Rft1 | 620.9647 | 633.7046 | 627.3347 |
| ENSMUSG00000024747 | protein_coding | Aldh1a7 | 129.5857 | 154.4524 | 142.0191 |
| ENSMUSG00000027550 | protein_coding | Lrrcc1 | 1186.17 | 1155.695 | 1170.933 |
| ENSMUSG00000089943 | protein_coding | Ugt1a5 | 0.646531 | 0.335524 | 0.491027 |
| ENSMUSG00000092700 | miRNA | AC161805.1 | 0.646531 | 0.335524 | 0.491027 |
| ENSMUSG00000081275 | pseudogene | Gm13875 | 0.646531 | 0.335524 | 0.491027 |
| ENSMUSG00000084696 | snRNA | U6 | 0.646531 | 0.335524 | 0.491027 |
| ENSMUSG00000090017 | processed_transcrip | Gm16213 | 0.646531 | 0.335524 | 0.491027 |
| ENSMUSG00000085378 | processed_transcrip | Gm11415 | 0.646531 | 0.335524 | 0.491027 |
| ENSMUSG00000092674 | misc_RNA | AC122546.1 | 0.646531 | 0.335524 | 0.491027 |
| ENSMUSG00000061762 | protein_coding | Tac1 | 0.646531 | 0.335524 | 0.491027 |
| ENSMUSG00000083962 | pseudogene | Gm15707 | 0.646531 | 0.335524 | 0.491027 |
| ENSMUSG00000085197 | processed_transcrip | 1700125H03Rik | 0.646531 | 0.335524 | 0.491027 |
| ENSMUSG00000050097 | protein_coding | Ces2b | 0.646531 | 0.335524 | 0.491027 |
| ENSMUSG00000087817 | misc_RNA | 7SK | 0.646531 | 0.335524 | 0.491027 |
| ENSMUSG00000089124 | snRNA | U6 | 0.646531 | 0.335524 | 0.491027 |
| ENSMUSG00000055010 | protein_coding | A830031A19Rik | 0.646531 | 0.335524 | 0.491027 |
| ENSMUSG00000085834 | processed_transcrip | Gm15622 | 0.646531 | 0.335524 | 0.491027 |
| ENSMUSG00000091883 | lincRNA | 1700039M10Rik | 0.646531 | 0.335524 | 0.491027 |
| ENSMUSG00000075605 | protein_coding | 2300005B03Rik | 0.646531 | 0.335524 | 0.491027 |
| ENSMUSG00000082241 | pseudogene | Gm15776 | 0.646531 | 0.335524 | 0.491027 |
| ENSMUSG00000092973 | miRNA | AC117949.1 | 0.646531 | 0.335524 | 0.491027 |
| ENSMUSG00000090117 | processed_transcrip | Gm16541 | 0.646531 | 0.335524 | 0.491027 |
| ENSMUSG00000080914 | pseudogene | Gm7129 | 0.646531 | 0.335524 | 0.491027 |
| ENSMUSG00000079534 | protein_coding | Gm5640 | 0.646531 | 0.335524 | 0.491027 |
| ENSMUSG00000081034 | pseudogene | Gm5944 | 0.646531 | 0.335524 | 0.491027 |
| ENSMUSG00000081559 | pseudogene | Gm12411 | 47.11508 | 51.62554 | 49.37031 |
| ENSMUSG00000039492 | protein_coding | Ccdc27 | 32.19454 | 34.6046 | 33.39957 |
| ENSMUSG00000028064 | protein_coding | Sema4a | 89.21572 | 93.40475 | 91.31024 |
| ENSMUSG00000018401 | protein_coding | Mtmr4 | 1807.809 | 1841.238 | 1824.523 |
| ENSMUSG00000036098 | protein_coding | Gm98 | 814.3805 | 786.1434 | 800.2619 |
| ENSMUSG00000087181 | pseudogene | Gm13570 | 0.66062 | 1.059903 | 0.860261 |
| ENSMUSG00000092162 | protein_coding | Vmn2r86 | 0.66062 | 1.059903 | 0.860261 |
| ENSMUSG00000040025 | protein_coding | Ythdf2 | 1849.804 | 1882.027 | 1865.915 |
| ENSMUSG00000085214 | lincRNA | 0610005C13Rik | 23.01929 | 21.03992 | 22.0296 |
| ENSMUSG00000067274 | protein_coding | Rplp0 | 21888.53 | 22271.25 | 22079.89 |
| ENSMUSG00000086798 | processed_transcrip | Gm16222 | 4.555164 | 5.394319 | 4.974741 |
| ENSMUSG00000083650 | pseudogene | Gm13357 | 7.153067 | 8.336158 | 7.744612 |
| ENSMUSG00000084009 | pseudogene | Gm7590 | 68.76601 | 64.56376 | 66.66488 |
| ENSMUSG00000027630 | protein_coding | Tbl1xr1 | 1451.312 | 1478.501 | 1464.907 |
| ENSMUSG00000086735 | processed_transcrip | Gm13977 | 6.465537 | 7.531781 | 6.998659 |

| | | | | | |
|---|---|---|---|---|---|
| ENSMUSG00000089787 | processed_transcrip | Gm15825 | 0 | 0.36219 | 0.181095 |
| ENSMUSG00000065405 | miRNA | Mir30a | 0 | 0.326514 | 0.163257 |
| ENSMUSG00000086726 | pseudogene | Gm9458 | 0 | 0.335524 | 0.167762 |
| ENSMUSG00000064688 | snRNA | U6 | 0 | 0.36219 | 0.181095 |
| ENSMUSG00000077939 | miRNA | AC084389.1 | 0 | 0.36219 | 0.181095 |
| ENSMUSG00000026085 | protein_coding | Lyg1 | 0 | 0.36219 | 0.181095 |
| ENSMUSG00000077132 | miRNA | AC125134.1 | 0 | 0.326514 | 0.163257 |
| ENSMUSG00000089877 | pseudogene | Gm16102 | 0 | 0.326514 | 0.163257 |
| ENSMUSG00000044457 | protein_coding | Gm4776 | 0 | 0.326514 | 0.163257 |
| ENSMUSG00000088353 | snRNA | U1 | 0 | 0.326514 | 0.163257 |
| ENSMUSG00000085447 | processed_transcrip | 9530026F06Rik | 0 | 0.335524 | 0.167762 |
| ENSMUSG00000086836 | processed_transcrip | Gm13748 | 0 | 0.326514 | 0.163257 |
| ENSMUSG00000086721 | pseudogene | Gm13750 | 0 | 0.335524 | 0.167762 |
| ENSMUSG00000073656 | lincRNA | Gm10558 | 0 | 0.326514 | 0.163257 |
| ENSMUSG00000084332 | pseudogene | Gm8840 | 0 | 0.335524 | 0.167762 |
| ENSMUSG00000089345 | misc_RNA | 7SK | 0 | 0.335524 | 0.167762 |
| ENSMUSG00000060125 | protein_coding | Gm5528 | 0 | 0.326514 | 0.163257 |
| ENSMUSG00000086053 | processed_transcrip | Gm15178 | 0 | 0.335524 | 0.167762 |
| ENSMUSG00000065055 | rRNA | n-R5s213 | 0 | 0.36219 | 0.181095 |
| ENSMUSG00000090246 | processed_transcrip | Gm17017 | 0 | 0.36219 | 0.181095 |
| ENSMUSG00000026251 | protein_coding | Chrnd | 0 | 0.335524 | 0.167762 |
| ENSMUSG00000026258 | protein_coding | 3110079O15Rik | 0 | 0.36219 | 0.181095 |
| ENSMUSG00000067081 | protein_coding | Asb18 | 0 | 0.335524 | 0.167762 |
| ENSMUSG00000061616 | protein_coding | Olfr12 | 0 | 0.36219 | 0.181095 |
| ENSMUSG00000059956 | protein_coding | Serpinb12 | 0 | 0.326514 | 0.163257 |
| ENSMUSG00000073596 | protein_coding | 2900060B14Rik | 0 | 0.326514 | 0.163257 |
| ENSMUSG00000092245 | pseudogene | RP23-95F15.1 | 0 | 0.335524 | 0.167762 |
| ENSMUSG00000073586 | pseudogene | Gm10543 | 0 | 0.326514 | 0.163257 |
| ENSMUSG00000089256 | misc_RNA | 7SK | 0 | 0.326514 | 0.163257 |
| ENSMUSG00000066936 | protein_coding | Gm10188 | 0 | 0.335524 | 0.167762 |
| ENSMUSG00000073559 | protein_coding | Gm8618 | 0 | 0.36219 | 0.181095 |
| ENSMUSG00000088564 | rRNA | 5S_rRNA | 0 | 0.335524 | 0.167762 |
| ENSMUSG00000026394 | protein_coding | Atp6v1g3 | 0 | 0.326514 | 0.163257 |
| ENSMUSG00000079255 | protein_coding | Gm11074 | 0 | 0.36219 | 0.181095 |
| ENSMUSG00000066752 | protein_coding | Gm10176 | 0 | 0.335524 | 0.167762 |
| ENSMUSG00000092188 | processed_transcrip | Gm20471 | 0 | 0.326514 | 0.163257 |
| ENSMUSG00000073494 | protein_coding | Sh2d1b2 | 0 | 0.36219 | 0.181095 |
| ENSMUSG00000045381 | protein_coding | Olfr433 | 0 | 0.36219 | 0.181095 |
| ENSMUSG00000089713 | processed_transcrip | Gm16564 | 0 | 0.36219 | 0.181095 |
| ENSMUSG00000085764 | lincRNA | Gm16621 | 0 | 0.335524 | 0.167762 |
| ENSMUSG00000064881 | snRNA | U5 | 0 | 0.326514 | 0.163257 |
| ENSMUSG00000090080 | processed_transcrip | Gm15872 | 0 | 0.36219 | 0.181095 |
| ENSMUSG00000089688 | processed_transcrip | 1600010F14Rik | 0 | 0.335524 | 0.167762 |
| ENSMUSG00000082580 | pseudogene | Gm13182 | 0 | 0.326514 | 0.163257 |
| ENSMUSG00000086445 | processed_transcrip | Gm13191 | 0 | 0.335524 | 0.167762 |
| ENSMUSG00000084859 | processed_transcrip | 1700080N15Rik | 0 | 0.335524 | 0.167762 |
| ENSMUSG00000092757 | miRNA | AL807778.1 | 0 | 0.335524 | 0.167762 |
| ENSMUSG00000085070 | processed_transcrip | Gm13211 | 0 | 0.335524 | 0.167762 |
| ENSMUSG00000076222 | miRNA | Mir297b | 0 | 0.36219 | 0.181095 |
| ENSMUSG00000080686 | miRNA | AL772216.40 | 0 | 0.335524 | 0.167762 |
| ENSMUSG00000083900 | pseudogene | Gm13294 | 0 | 0.326514 | 0.163257 |
| ENSMUSG00000080905 | pseudogene | Gm13295 | 0 | 0.335524 | 0.167762 |
| ENSMUSG00000086491 | processed_transcrip | Gm13291 | 0 | 0.36219 | 0.181095 |
| ENSMUSG00000083545 | pseudogene | Gm13320 | 0 | 0.326514 | 0.163257 |
| ENSMUSG00000084901 | processed_transcrip | Gm13266 | 0 | 0.36219 | 0.181095 |
| ENSMUSG00000083305 | pseudogene | Gm13315 | 0 | 0.326514 | 0.163257 |
| ENSMUSG00000087219 | processed_transcrip | Gm13376 | 0 | 0.335524 | 0.167762 |
| ENSMUSG00000080846 | pseudogene | Gm13379 | 0 | 0.36219 | 0.181095 |

| | | | | | |
|---|---|---|---|---|---|
| ENSMUSG00000085999 | processed_transcrip | Gm13411 | 0 | 0.335524 | 0.167762 |
| ENSMUSG00000085330 | processed_transcrip | Gm13417 | 0 | 0.36219 | 0.181095 |
| ENSMUSG00000088571 | misc_RNA | 7SK | 0 | 0.36219 | 0.181095 |
| ENSMUSG00000092845 | miRNA | AL732585.1 | 0 | 0.36219 | 0.181095 |
| ENSMUSG00000079539 | protein_coding | Lcn14 | 0 | 0.326514 | 0.163257 |
| ENSMUSG00000078010 | miRNA | AL954388.1 | 0 | 0.335524 | 0.167762 |
| ENSMUSG00000092765 | miRNA | AL808027.1 | 0 | 0.36219 | 0.181095 |
| ENSMUSG00000086353 | processed_transcrip | Gm13481 | 0 | 0.335524 | 0.167762 |
| ENSMUSG00000082879 | pseudogene | Gm13525 | 0 | 0.335524 | 0.167762 |
| ENSMUSG00000086396 | lincRNA | 4930555B11Rik | 0 | 0.326514 | 0.163257 |
| ENSMUSG00000084115 | pseudogene | Gm13566 | 0 | 0.335524 | 0.167762 |
| ENSMUSG00000080547 | miRNA | AL928581.1 | 0 | 0.36219 | 0.181095 |
| ENSMUSG00000087702 | processed_transcrip | Gm13575 | 0 | 0.326514 | 0.163257 |
| ENSMUSG00000092851 | miRNA | AL844840.1 | 0 | 0.335524 | 0.167762 |
| ENSMUSG00000092984 | miRNA | AL844840.2 | 0 | 0.36219 | 0.181095 |
| ENSMUSG00000083072 | pseudogene | Gm13668 | 0 | 0.36219 | 0.181095 |
| ENSMUSG00000082973 | pseudogene | Gm13672 | 0 | 0.36219 | 0.181095 |
| ENSMUSG00000082152 | pseudogene | Gm13655 | 0 | 0.326514 | 0.163257 |
| ENSMUSG00000087527 | processed_transcrip | Gm13656 | 0 | 0.326514 | 0.163257 |
| ENSMUSG00000082701 | pseudogene | Gm14460 | 0 | 0.326514 | 0.163257 |
| ENSMUSG00000082046 | pseudogene | Gm14464 | 0 | 0.335524 | 0.167762 |
| ENSMUSG00000083691 | pseudogene | Gm14465 | 0 | 0.326514 | 0.163257 |
| ENSMUSG00000065484 | miRNA | Mir130a | 0 | 0.335524 | 0.167762 |
| ENSMUSG00000082534 | pseudogene | Gm13712 | 0 | 0.335524 | 0.167762 |
| ENSMUSG00000075217 | protein_coding | 4833423E24Rik | 0 | 0.326514 | 0.163257 |
| ENSMUSG00000042796 | protein_coding | Olfr1032 | 0 | 0.36219 | 0.181095 |
| ENSMUSG00000075177 | protein_coding | Olfr1084 | 0 | 0.36219 | 0.181095 |
| ENSMUSG00000081836 | pseudogene | Olfr1192-ps1 | 0 | 0.36219 | 0.181095 |
| ENSMUSG00000075073 | protein_coding | Olfr1256 | 0 | 0.36219 | 0.181095 |
| ENSMUSG00000075072 | protein_coding | Olfr48 | 0 | 0.326514 | 0.163257 |
| ENSMUSG00000082574 | pseudogene | Olfr1268-ps1 | 0 | 0.36219 | 0.181095 |
| ENSMUSG00000075065 | protein_coding | Olfr1270 | 0 | 0.36219 | 0.181095 |
| ENSMUSG00000085547 | pseudogene | Gm13767 | 0 | 0.326514 | 0.163257 |
| ENSMUSG00000082700 | pseudogene | Gm13792 | 0 | 0.335524 | 0.167762 |
| ENSMUSG00000081774 | pseudogene | Gm13866 | 0 | 0.36219 | 0.181095 |
| ENSMUSG00000088078 | snoRNA | SCARNA4 | 0 | 0.36219 | 0.181095 |
| ENSMUSG00000041644 | protein_coding | Slc5a12 | 0 | 0.326514 | 0.163257 |
| ENSMUSG00000087309 | processed_transcrip | 4930528P14Rik | 0 | 0.326514 | 0.163257 |
| ENSMUSG00000086197 | processed_transcrip | Gm13976 | 0 | 0.326514 | 0.163257 |
| ENSMUSG00000065794 | snRNA | U1 | 0 | 0.335524 | 0.167762 |
| ENSMUSG00000086221 | processed_transcrip | 4930412B13Rik | 0 | 0.335524 | 0.167762 |
| ENSMUSG00000064722 | snoRNA | SNORA44 | 0 | 0.326514 | 0.163257 |
| ENSMUSG00000081056 | pseudogene | Gm13998 | 0 | 0.36219 | 0.181095 |
| ENSMUSG00000081264 | pseudogene | Gm14021 | 0 | 0.335524 | 0.167762 |
| ENSMUSG00000065084 | snRNA | U6 | 0 | 0.326514 | 0.163257 |
| ENSMUSG00000093203 | miRNA | AL844481.1 | 0 | 0.36219 | 0.181095 |
| ENSMUSG00000092719 | miRNA | AL833780.1 | 0 | 0.335524 | 0.167762 |
| ENSMUSG00000043727 | protein_coding | F830045P16Rik | 0 | 0.36219 | 0.181095 |
| ENSMUSG00000084328 | pseudogene | Gm14046 | 0 | 0.36219 | 0.181095 |
| ENSMUSG00000083589 | pseudogene | Gm14043 | 0 | 0.36219 | 0.181095 |
| ENSMUSG00000087524 | processed_transcrip | Gm14285 | 0 | 0.326514 | 0.163257 |
| ENSMUSG00000083469 | pseudogene | Gm14281 | 0 | 0.326514 | 0.163257 |
| ENSMUSG00000064413 | snRNA | U6 | 0 | 0.36219 | 0.181095 |
| ENSMUSG00000086928 | processed_transcrip | Gm14210 | 0 | 0.36219 | 0.181095 |
| ENSMUSG00000086214 | processed_transcrip | Gm14062 | 0 | 0.36219 | 0.181095 |
| ENSMUSG00000083110 | pseudogene | Gm14073 | 0 | 0.326514 | 0.163257 |
| ENSMUSG00000080711 | pseudogene | Gm11686 | 0 | 0.335524 | 0.167762 |
| ENSMUSG00000085796 | processed_transcrip | 4933406D12Rik | 0 | 0.335524 | 0.167762 |

| | | | | | |
|---|---|---|---|---|---|
| ENSMUSG00000053545 | processed_transcrip | 6430503K07Rik | 0 | 0.36219 | 0.181095 |
| ENSMUSG00000051986 | processed_transcrip | A530006G24Rik | 0 | 0.326514 | 0.163257 |
| ENSMUSG00000086141 | processed_transcrip | 9030622O22Rik | 0 | 0.36219 | 0.181095 |
| ENSMUSG00000027468 | protein_coding | Defb22 | 0 | 0.326514 | 0.163257 |
| ENSMUSG00000027483 | protein_coding | Bpifa1 | 0 | 0.36219 | 0.181095 |
| ENSMUSG00000088637 | misc_RNA | 7SK | 0 | 0.326514 | 0.163257 |
| ENSMUSG00000085524 | processed_transcrip | Gm14224 | 0 | 0.326514 | 0.163257 |
| ENSMUSG00000081616 | pseudogene | Gm14247 | 0 | 0.36219 | 0.181095 |
| ENSMUSG00000086653 | processed_transcrip | 9130015L21Rik | 0 | 0.36219 | 0.181095 |
| ENSMUSG00000092788 | miRNA | AL591905.1 | 0 | 0.36219 | 0.181095 |
| ENSMUSG00000081099 | pseudogene | Gm5270 | 0 | 0.36219 | 0.181095 |
| ENSMUSG00000082514 | pseudogene | Gm11452 | 0 | 0.36219 | 0.181095 |
| ENSMUSG00000092791 | miRNA | AL591584.1 | 0 | 0.326514 | 0.163257 |
| ENSMUSG00000040154 | protein_coding | Wfdc5 | 0 | 0.335524 | 0.167762 |
| ENSMUSG00000018211 | protein_coding | Wfdc15b | 0 | 0.326514 | 0.163257 |
| ENSMUSG00000089145 | misc_RNA | 7SK | 0 | 0.335524 | 0.167762 |
| ENSMUSG00000087684 | processed_transcrip | 1200007C13Rik | 0 | 0.36219 | 0.181095 |
| ENSMUSG00000080415 | miRNA | AL772258.1 | 0 | 0.326514 | 0.163257 |
| ENSMUSG00000085369 | processed_transcrip | 9430093N23Rik | 0 | 0.326514 | 0.163257 |
| ENSMUSG00000086840 | processed_transcrip | Gm14250 | 0 | 0.326514 | 0.163257 |
| ENSMUSG00000085905 | processed_transcrip | Gm14273 | 0 | 0.326514 | 0.163257 |
| ENSMUSG00000086177 | processed_transcrip | Gm14616 | 0 | 0.36219 | 0.181095 |
| ENSMUSG00000082343 | pseudogene | Gm14404 | 0 | 0.335524 | 0.167762 |
| ENSMUSG00000074507 | processed_transcrip | Gm14340 | 0 | 0.335524 | 0.167762 |
| ENSMUSG00000065454 | miRNA | Mir124a-3 | 0 | 0.36219 | 0.181095 |
| ENSMUSG00000081594 | pseudogene | Gm15467 | 0 | 0.326514 | 0.163257 |
| ENSMUSG00000089794 | processed_transcrip | Gm16337 | 0 | 0.326514 | 0.163257 |
| ENSMUSG00000039710 | protein_coding | Slc7a12 | 0 | 0.36219 | 0.181095 |
| ENSMUSG00000076975 | miRNA | AC098884.1 | 0 | 0.36219 | 0.181095 |
| ENSMUSG00000088644 | snRNA | U6 | 0 | 0.36219 | 0.181095 |
| ENSMUSG00000083388 | pseudogene | Gm11550 | 0 | 0.335524 | 0.167762 |
| ENSMUSG00000083575 | pseudogene | Gm12533 | 0 | 0.326514 | 0.163257 |
| ENSMUSG00000087457 | pseudogene | Gm7799 | 0 | 0.326514 | 0.163257 |
| ENSMUSG00000078233 | protein_coding | Gm16508 | 0 | 0.36219 | 0.181095 |
| ENSMUSG00000084517 | snoRNA | SNORA66 | 0 | 0.326514 | 0.163257 |
| ENSMUSG00000077276 | snoRNA | SNORA69 | 0 | 0.326514 | 0.163257 |
| ENSMUSG00000089039 | snRNA | U6 | 0 | 0.326514 | 0.163257 |
| ENSMUSG00000090408 | protein_coding | Gm17402 | 0 | 0.335524 | 0.167762 |
| ENSMUSG00000077459 | snoRNA | SNORA17 | 0 | 0.326514 | 0.163257 |
| ENSMUSG00000087902 | misc_RNA | 7SK | 0 | 0.326514 | 0.163257 |
| ENSMUSG00000084596 | misc_RNA | 7SK | 0 | 0.326514 | 0.163257 |
| ENSMUSG00000088597 | snRNA | U6 | 0 | 0.36219 | 0.181095 |
| ENSMUSG00000076255 | miRNA | Mir92b | 0 | 0.326514 | 0.163257 |
| ENSMUSG00000042306 | protein_coding | S100a14 | 0 | 0.36219 | 0.181095 |
| ENSMUSG00000046259 | protein_coding | Sprr2h | 0 | 0.335524 | 0.167762 |
| ENSMUSG00000042157 | protein_coding | Sprr2i | 0 | 0.335524 | 0.167762 |
| ENSMUSG00000050359 | protein_coding | Sprr1a | 0 | 0.335524 | 0.167762 |
| ENSMUSG00000027912 | protein_coding | Lce1m | 0 | 0.326514 | 0.163257 |
| ENSMUSG00000092163 | protein_coding | Flg | 0 | 0.326514 | 0.163257 |
| ENSMUSG00000083577 | pseudogene | Gm12432 | 0 | 0.335524 | 0.167762 |
| ENSMUSG00000064573 | snoRNA | SNORD81 | 0 | 0.326514 | 0.163257 |
| ENSMUSG00000088016 | snRNA | U6 | 0 | 0.36219 | 0.181095 |
| ENSMUSG00000080351 | miRNA | AC111029.1 | 0 | 0.36219 | 0.181095 |
| ENSMUSG00000074248 | protein_coding | 4930432M17Rik | 0 | 0.36219 | 0.181095 |
| ENSMUSG00000091611 | pseudogene | Gm9364 | 0 | 0.335524 | 0.167762 |
| ENSMUSG00000078164 | protein_coding | Gm4392 | 0 | 0.36219 | 0.181095 |
| ENSMUSG00000065482 | miRNA | Mir302c | 0 | 0.36219 | 0.181095 |
| ENSMUSG00000088537 | rRNA | 5S_rRNA | 0 | 0.36219 | 0.181095 |

| | | | | | |
|---|---|---|---|---|---|
| ENSMUSG00000092797 | misc_RNA | AC165333.1 | 0 | 0.36219 | 0.181095 |
| ENSMUSG00000093048 | miRNA | AC135118.1 | 0 | 0.326514 | 0.163257 |
| ENSMUSG00000080506 | miRNA | AC125040.1 | 0 | 0.335524 | 0.167762 |
| ENSMUSG00000045520 | protein_coding | Gm9799 | 0 | 0.326514 | 0.163257 |
| ENSMUSG00000078574 | protein_coding | Gm10955 | 0 | 0.36219 | 0.181095 |
| ENSMUSG00000087752 | snRNA | U1 | 0 | 0.326514 | 0.163257 |
| ENSMUSG00000090306 | protein_coding | Adh6-ps1 | 0 | 0.36219 | 0.181095 |
| ENSMUSG00000074195 | protein_coding | AI747448 | 0 | 0.36219 | 0.181095 |
| ENSMUSG00000040086 | protein_coding | Tnni3k | 0 | 0.36219 | 0.181095 |
| ENSMUSG00000092930 | miRNA | AL732617.1 | 0 | 0.36219 | 0.181095 |
| ENSMUSG00000077271 | snoRNA | SNORA17 | 0 | 0.326514 | 0.163257 |
| ENSMUSG00000084360 | pseudogene | Gm11797 | 0 | 0.335524 | 0.167762 |
| ENSMUSG00000081913 | pseudogene | Gm11803 | 0 | 0.326514 | 0.163257 |
| ENSMUSG00000083023 | pseudogene | Gm11804 | 0 | 0.36219 | 0.181095 |
| ENSMUSG00000082151 | pseudogene | Gm11810 | 0 | 0.335524 | 0.167762 |
| ENSMUSG00000084215 | pseudogene | Gm11814 | 0 | 0.335524 | 0.167762 |
| ENSMUSG00000067549 | protein_coding | Gm17283 | 0 | 0.36219 | 0.181095 |
| ENSMUSG00000080869 | pseudogene | Gm11856 | 0 | 0.335524 | 0.167762 |
| ENSMUSG00000041052 | protein_coding | Slc7a13 | 0 | 0.326514 | 0.163257 |
| ENSMUSG00000086017 | processed_transcrip | Gm11872 | 0 | 0.326514 | 0.163257 |
| ENSMUSG00000083727 | pseudogene | Gm11887 | 0 | 0.36219 | 0.181095 |
| ENSMUSG00000082451 | pseudogene | Gm11894 | 0 | 0.326514 | 0.163257 |
| ENSMUSG00000066252 | protein_coding | Bhmt-ps1 | 0 | 0.326514 | 0.163257 |
| ENSMUSG00000082398 | pseudogene | Gm11927 | 0 | 0.36219 | 0.181095 |
| ENSMUSG00000084611 | misc_RNA | 7SK | 0 | 0.36219 | 0.181095 |
| ENSMUSG00000073878 | protein_coding | Gm13304 | 0 | 0.36219 | 0.181095 |
| ENSMUSG00000071005 | protein_coding | Ccl19 | 0 | 0.36219 | 0.181095 |
| ENSMUSG00000089028 | snRNA | U7 | 0 | 0.326514 | 0.163257 |
| ENSMUSG00000071000 | protein_coding | Olfr155 | 0 | 0.335524 | 0.167762 |
| ENSMUSG00000092677 | miRNA | AL805952.1 | 0 | 0.335524 | 0.167762 |
| ENSMUSG00000083541 | pseudogene | Gm12585 | 0 | 0.36219 | 0.181095 |
| ENSMUSG00000085980 | processed_transcrip | Gm12408 | 0 | 0.36219 | 0.181095 |
| ENSMUSG00000073860 | processed_transcrip | Gm829 | 0 | 0.326514 | 0.163257 |
| ENSMUSG00000092924 | miRNA | AL806523.1 | 0 | 0.36219 | 0.181095 |
| ENSMUSG00000081483 | pseudogene | Gm12445 | 0 | 0.326514 | 0.163257 |
| ENSMUSG00000087844 | miRNA | Mir1958 | 0 | 0.36219 | 0.181095 |
| ENSMUSG00000093240 | rRNA | AL807771.1 | 0 | 0.335524 | 0.167762 |
| ENSMUSG00000083338 | pseudogene | Gm12475 | 0 | 0.36219 | 0.181095 |
| ENSMUSG00000087077 | processed_transcrip | Gm12480 | 0 | 0.36219 | 0.181095 |
| ENSMUSG00000081290 | pseudogene | Gm12520 | 0 | 0.326514 | 0.163257 |
| ENSMUSG00000085404 | processed_transcrip | Gm12909 | 0 | 0.335524 | 0.167762 |
| ENSMUSG00000065913 | snRNA | U6 | 0 | 0.335524 | 0.167762 |
| ENSMUSG00000083219 | pseudogene | Gm11410 | 0 | 0.36219 | 0.181095 |
| ENSMUSG00000087540 | processed_transcrip | Gm11412 | 0 | 0.335524 | 0.167762 |
| ENSMUSG00000085022 | processed_transcrip | Gm5860 | 0 | 0.335524 | 0.167762 |
| ENSMUSG00000082637 | pseudogene | Gm11248 | 0 | 0.326514 | 0.163257 |
| ENSMUSG00000080303 | pseudogene | Gm12419 | 0 | 0.335524 | 0.167762 |
| ENSMUSG00000085108 | processed_transcrip | 4930457A20Rik | 0 | 0.326514 | 0.163257 |
| ENSMUSG00000086512 | processed_transcrip | Gm12600 | 0 | 0.335524 | 0.167762 |
| ENSMUSG00000081501 | pseudogene | Gm13286 | 0 | 0.36219 | 0.181095 |
| ENSMUSG00000092000 | protein_coding | Gm13272 | 0 | 0.335524 | 0.167762 |
| ENSMUSG00000081876 | pseudogene | Gm12644 | 0 | 0.326514 | 0.163257 |
| ENSMUSG00000085209 | processed_transcrip | Gm12637 | 0 | 0.36219 | 0.181095 |
| ENSMUSG00000082325 | pseudogene | Gm12642 | 0 | 0.36219 | 0.181095 |
| ENSMUSG00000077921 | miRNA | Mir872 | 0 | 0.326514 | 0.163257 |
| ENSMUSG00000085835 | processed_transcrip | Gm12707 | 0 | 0.36219 | 0.181095 |
| ENSMUSG00000065745 | snRNA | U1 | 0 | 0.326514 | 0.163257 |
| ENSMUSG00000088548 | snRNA | U6 | 0 | 0.335524 | 0.167762 |

| | | | | | |
|---|---|---|---|---|---|
| ENSMUSG00000076294 | miRNA | AL627124.1 | 0 | 0.326514 | 0.163257 |
| ENSMUSG00000080967 | pseudogene | Gm12706 | 0 | 0.36219 | 0.181095 |
| ENSMUSG00000073788 | protein_coding | Gm10576 | 0 | 0.335524 | 0.167762 |
| ENSMUSG00000085863 | processed_transcrip | Gm12801 | 0 | 0.36219 | 0.181095 |
| ENSMUSG00000085790 | processed_transcrip | Gm12729 | 0 | 0.36219 | 0.181095 |
| ENSMUSG00000082958 | pseudogene | Gm12731 | 0 | 0.335524 | 0.167762 |
| ENSMUSG00000087051 | processed_transcrip | Gm12730 | 0 | 0.36219 | 0.181095 |
| ENSMUSG00000081110 | pseudogene | Gm12745 | 0 | 0.326514 | 0.163257 |
| ENSMUSG00000080460 | miRNA | mmu-mir-1194 | 0 | 0.36219 | 0.181095 |
| ENSMUSG00000087195 | processed_transcrip | Gm12907 | 0 | 0.326514 | 0.163257 |
| ENSMUSG00000081734 | pseudogene | Gm12740 | 0 | 0.326514 | 0.163257 |
| ENSMUSG00000083255 | pseudogene | Gm12738 | 0 | 0.326514 | 0.163257 |
| ENSMUSG00000083783 | pseudogene | Gm12739 | 0 | 0.36219 | 0.181095 |
| ENSMUSG00000081813 | pseudogene | Gm12806 | 0 | 0.36219 | 0.181095 |
| ENSMUSG00000049214 | protein_coding | Skint7 | 0 | 0.326514 | 0.163257 |
| ENSMUSG00000049972 | protein_coding | Skint9 | 0 | 0.36219 | 0.181095 |
| ENSMUSG00000083384 | pseudogene | Gm12813 | 0 | 0.326514 | 0.163257 |
| ENSMUSG00000081138 | pseudogene | Gm12834 | 0 | 0.335524 | 0.167762 |
| ENSMUSG00000028715 | protein_coding | Cyp4a14 | 0 | 0.335524 | 0.167762 |
| ENSMUSG00000063929 | protein_coding | Cyp4a32 | 0 | 0.335524 | 0.167762 |
| ENSMUSG00000082207 | pseudogene | Gm12998 | 0 | 0.36219 | 0.181095 |
| ENSMUSG00000081528 | pseudogene | Gm12924 | 0 | 0.36219 | 0.181095 |
| ENSMUSG00000085263 | processed_transcrip | 1700125G02Rik | 0 | 0.326514 | 0.163257 |
| ENSMUSG00000081609 | pseudogene | Gm12939 | 0 | 0.326514 | 0.163257 |
| ENSMUSG00000046623 | protein_coding | Gjb4 | 0 | 0.335524 | 0.167762 |
| ENSMUSG00000080944 | pseudogene | Gm12967 | 0 | 0.326514 | 0.163257 |
| ENSMUSG00000086548 | processed_transcrip | Gm12972 | 0 | 0.335524 | 0.167762 |
| ENSMUSG00000078531 | processed_transcrip | Gm831 | 0 | 0.36219 | 0.181095 |
| ENSMUSG00000089298 | misc_RNA | 7SK | 0 | 0.36219 | 0.181095 |
| ENSMUSG00000088614 | snRNA | U6 | 0 | 0.36219 | 0.181095 |
| ENSMUSG00000085574 | processed_transcrip | Gm13053 | 0 | 0.36219 | 0.181095 |
| ENSMUSG00000066688 | protein_coding | Gm13083 | 0 | 0.326514 | 0.163257 |
| ENSMUSG00000080943 | pseudogene | Gm13130 | 0 | 0.335524 | 0.167762 |
| ENSMUSG00000073721 | protein_coding | Gm13125 | 0 | 0.335524 | 0.167762 |
| ENSMUSG00000028589 | protein_coding | 1700012P22Rik | 0 | 0.335524 | 0.167762 |
| ENSMUSG00000054345 | protein_coding | Gm9944 | 0 | 0.326514 | 0.163257 |
| ENSMUSG00000081140 | pseudogene | Gm8178 | 0 | 0.36219 | 0.181095 |
| ENSMUSG00000087217 | processed_transcrip | Gm13209 | 0 | 0.36219 | 0.181095 |
| ENSMUSG00000085390 | processed_transcrip | Gm13115 | 0 | 0.36219 | 0.181095 |
| ENSMUSG00000051276 | protein_coding | Actrt2 | 0 | 0.36219 | 0.181095 |
| ENSMUSG00000092798 | misc_RNA | AL627204.1 | 0 | 0.335524 | 0.167762 |
| ENSMUSG00000093279 | miRNA | AC090443.1 | 0 | 0.335524 | 0.167762 |
| ENSMUSG00000085352 | processed_transcrip | Gm4128 | 0 | 0.36219 | 0.181095 |
| ENSMUSG00000088668 | snRNA | U6 | 0 | 0.36219 | 0.181095 |
| ENSMUSG00000079624 | protein_coding | Gm11160 | 0 | 0.36219 | 0.181095 |
| ENSMUSG00000090689 | protein_coding | Gm17695 | 0 | 0.335524 | 0.167762 |
| ENSMUSG00000092275 | processed_transcrip | Gm20465 | 0 | 0.335524 | 0.167762 |
| ENSMUSG00000086479 | processed_transcrip | Gm16014 | 0 | 0.326514 | 0.163257 |
| ENSMUSG00000029193 | protein_coding | Cckar | 0 | 0.326514 | 0.163257 |
| ENSMUSG00000093258 | snRNA | AC132284.1 | 0 | 0.326514 | 0.163257 |
| ENSMUSG00000084544 | misc_RNA | 7SK | 0 | 0.36219 | 0.181095 |
| ENSMUSG00000093151 | miRNA | AC123662.1 | 0 | 0.326514 | 0.163257 |
| ENSMUSG00000081849 | pseudogene | Gm15847 | 0 | 0.36219 | 0.181095 |
| ENSMUSG00000047652 | pseudogene | Pea15b | 0 | 0.36219 | 0.181095 |
| ENSMUSG00000064859 | snRNA | U1 | 0 | 0.36219 | 0.181095 |
| ENSMUSG00000035861 | protein_coding | Tmprss11bnl | 0 | 0.326514 | 0.163257 |
| ENSMUSG00000054537 | protein_coding | Tmprss11e | 0 | 0.335524 | 0.167762 |
| ENSMUSG00000088949 | rRNA | 5S_rRNA | 0 | 0.36219 | 0.181095 |

| | | | | | |
|---|---|---|---|---|---|
| ENSMUSG00000072826 | protein_coding | Gm16427 | 0 | 0.335524 | 0.167762 |
| ENSMUSG00000091252 | protein_coding | Gm6346 | 0 | 0.326514 | 0.163257 |
| ENSMUSG00000089627 | pseudogene | Gm16228 | 0 | 0.36219 | 0.181095 |
| ENSMUSG00000084126 | pseudogene | Gm15844 | 0 | 0.36219 | 0.181095 |
| ENSMUSG00000064523 | snRNA | U6 | 0 | 0.36219 | 0.181095 |
| ENSMUSG00000086871 | lincRNA | Gm16834 | 0 | 0.36219 | 0.181095 |
| ENSMUSG00000092911 | miRNA | AC137107.1 | 0 | 0.36219 | 0.181095 |
| ENSMUSG00000088429 | snRNA | U6 | 0 | 0.36219 | 0.181095 |
| ENSMUSG00000004821 | protein_coding | Tmed11 | 0 | 0.335524 | 0.167762 |
| ENSMUSG00000083771 | pseudogene | Gm15988 | 0 | 0.335524 | 0.167762 |
| ENSMUSG00000087124 | processed_transcrip | Gm13839 | 0 | 0.326514 | 0.163257 |
| ENSMUSG00000084587 | rRNA | n-R5s174 | 0 | 0.326514 | 0.163257 |
| ENSMUSG00000059352 | protein_coding | Gm10064 | 0 | 0.335524 | 0.167762 |
| ENSMUSG00000040576 | protein_coding | Wbscr28 | 0 | 0.335524 | 0.167762 |
| ENSMUSG00000008843 | protein_coding | Cldn13 | 0 | 0.335524 | 0.167762 |
| ENSMUSG00000088188 | snRNA | U12 | 0 | 0.326514 | 0.163257 |
| ENSMUSG00000065300 | snRNA | U6 | 0 | 0.36219 | 0.181095 |
| ENSMUSG00000079094 | protein_coding | Gm6370 | 0 | 0.36219 | 0.181095 |
| ENSMUSG00000041505 | protein_coding | Gm3402 | 0 | 0.36219 | 0.181095 |
| ENSMUSG00000079091 | protein_coding | Gm3404 | 0 | 0.36219 | 0.181095 |
| ENSMUSG00000085237 | processed_transcrip | Gm15406 | 0 | 0.326514 | 0.163257 |
| ENSMUSG00000029700 | protein_coding | Slc13a1 | 0 | 0.326514 | 0.163257 |
| ENSMUSG00000029678 | protein_coding | Hyal5 | 0 | 0.36219 | 0.181095 |
| ENSMUSG00000065501 | miRNA | Mir335 | 0 | 0.326514 | 0.163257 |
| ENSMUSG00000065610 | miRNA | Mir29a | 0 | 0.36219 | 0.181095 |
| ENSMUSG00000087312 | processed_transcrip | Gm13833 | 0 | 0.36219 | 0.181095 |
| ENSMUSG00000086732 | processed_transcrip | Gm6117 | 0 | 0.326514 | 0.163257 |
| ENSMUSG00000092877 | miRNA | AC166251.1 | 0 | 0.36219 | 0.181095 |
| ENSMUSG00000029837 | protein_coding | 1700111E14Rik | 0 | 0.36219 | 0.181095 |
| ENSMUSG00000029909 | protein_coding | Prss37 | 0 | 0.326514 | 0.163257 |
| ENSMUSG00000058250 | protein_coding | Tas2r138 | 0 | 0.36219 | 0.181095 |
| ENSMUSG00000090480 | protein_coding | Gm2663 | 0 | 0.36219 | 0.181095 |
| ENSMUSG00000076467 | IG_V_gene | Trbv13-1 | 0 | 0.326514 | 0.163257 |
| ENSMUSG00000076468 | IG_V_gene | Trbv12-2 | 0 | 0.326514 | 0.163257 |
| ENSMUSG00000057163 | protein_coding | Prss2 | 0 | 0.335524 | 0.167762 |
| ENSMUSG00000065206 | snRNA | U6 | 0 | 0.36219 | 0.181095 |
| ENSMUSG00000059411 | protein_coding | Olfr434 | 0 | 0.335524 | 0.167762 |
| ENSMUSG00000087670 | processed_transcrip | 9530036M11Rik | 0 | 0.326514 | 0.163257 |
| ENSMUSG00000002930 | protein_coding | Gsbs | 0 | 0.335524 | 0.167762 |
| ENSMUSG00000068476 | protein_coding | Vmn1r10 | 0 | 0.335524 | 0.167762 |
| ENSMUSG00000076502 | IG_V_gene | Igkv1-135 | 0 | 0.326514 | 0.163257 |
| ENSMUSG00000089157 | snoRNA | SNORA17 | 0 | 0.335524 | 0.167762 |
| ENSMUSG00000076509 | IG_V_gene | Igkv14-126 | 0 | 0.335524 | 0.167762 |
| ENSMUSG00000076518 | IG_V_gene | Igkv2-112 | 0 | 0.326514 | 0.163257 |
| ENSMUSG00000076522 | IG_V_gene | Igkv16-104 | 0 | 0.335524 | 0.167762 |
| ENSMUSG00000076529 | IG_V_gene | Igkv10-94 | 0 | 0.326514 | 0.163257 |
| ENSMUSG00000091274 | IG_V_gene | Igkv4-53 | 0 | 0.36219 | 0.181095 |
| ENSMUSG00000076569 | IG_V_gene | Igkv5-39 | 0 | 0.335524 | 0.167762 |
| ENSMUSG00000076572 | IG_V_gene | Igkv18-36 | 0 | 0.326514 | 0.163257 |
| ENSMUSG00000076577 | IG_V_gene | Igkv8-30 | 0 | 0.335524 | 0.167762 |
| ENSMUSG00000076580 | IG_V_gene | Igkv8-27 | 0 | 0.335524 | 0.167762 |
| ENSMUSG00000076590 | IG_V_gene | Igkv6-17 | 0 | 0.335524 | 0.167762 |
| ENSMUSG00000076602 | IG_V_gene | Igkv3-2 | 0 | 0.335524 | 0.167762 |
| ENSMUSG00000076605 | IG_J_gene | Igkj2 | 0 | 0.335524 | 0.167762 |
| ENSMUSG00000076606 | IG_J_gene | Igkj3 | 0 | 0.335524 | 0.167762 |
| ENSMUSG00000076607 | IG_J_gene | Igkj4 | 0 | 0.335524 | 0.167762 |
| ENSMUSG00000071356 | protein_coding | Reg3b | 0 | 0.36219 | 0.181095 |
| ENSMUSG00000059654 | protein_coding | Reg1 | 0 | 0.335524 | 0.167762 |

| | | | | | |
|---|---|---|---|---|---|
| ENSMUSG00000087001 | processed_transcrip | Gm15475 | 0 | 0.326514 | 0.163257 |
| ENSMUSG00000088560 | snoRNA | snoU13 | 0 | 0.36219 | 0.181095 |
| ENSMUSG00000030046 | protein_coding | Bmp10 | 0 | 0.36219 | 0.181095 |
| ENSMUSG00000077852 | miRNA | AC158626.1 | 0 | 0.36219 | 0.181095 |
| ENSMUSG00000068230 | protein_coding | Vmn1r41 | 0 | 0.36219 | 0.181095 |
| ENSMUSG00000058595 | protein_coding | Vmn1r50 | 0 | 0.335524 | 0.167762 |
| ENSMUSG00000090667 | protein_coding | Gm765 | 0 | 0.36219 | 0.181095 |
| ENSMUSG00000089084 | misc_RNA | 7SK | 0 | 0.326514 | 0.163257 |
| ENSMUSG00000087631 | lincRNA | 1700015O11Rik | 0 | 0.326514 | 0.163257 |
| ENSMUSG00000090813 | lincRNA | Gm4651 | 0 | 0.36219 | 0.181095 |
| ENSMUSG00000088177 | misc_RNA | 7SK | 0 | 0.335524 | 0.167762 |
| ENSMUSG00000014543 | protein_coding | Klra17 | 0 | 0.36219 | 0.181095 |
| ENSMUSG00000030173 | protein_coding | Klra5 | 0 | 0.335524 | 0.167762 |
| ENSMUSG00000079852 | protein_coding | Klra4 | 0 | 0.326514 | 0.163257 |
| ENSMUSG00000082777 | pseudogene | Gm15861 | 0 | 0.36219 | 0.181095 |
| ENSMUSG00000061969 | protein_coding | Gm5581 | 0 | 0.36219 | 0.181095 |
| ENSMUSG00000077015 | miRNA | AC124506.1 | 0 | 0.335524 | 0.167762 |
| ENSMUSG00000088372 | snoRNA | SNORA17 | 0 | 0.335524 | 0.167762 |
| ENSMUSG00000086239 | processed_transcrip | Gm14329 | 0 | 0.326514 | 0.163257 |
| ENSMUSG00000060782 | protein_coding | Gm8994 | 0 | 0.326514 | 0.163257 |
| ENSMUSG00000093364 | miRNA | AC131766.1 | 0 | 0.326514 | 0.163257 |
| ENSMUSG00000030230 | protein_coding | Plcz1 | 0 | 0.36219 | 0.181095 |
| ENSMUSG00000065882 | rRNA | n-R5s168 | 0 | 0.335524 | 0.167762 |
| ENSMUSG00000030236 | protein_coding | Slco1b2 | 0 | 0.335524 | 0.167762 |
| ENSMUSG00000079263 | protein_coding | Gm6614 | 0 | 0.36219 | 0.181095 |
| ENSMUSG00000072677 | processed_transcrip | Gm10396 | 0 | 0.335524 | 0.167762 |
| ENSMUSG00000085785 | processed_transcrip | 4933425H06Rik | 0 | 0.36219 | 0.181095 |
| ENSMUSG00000086282 | processed_transcrip | Gm15686 | 0 | 0.335524 | 0.167762 |
| ENSMUSG00000088867 | rRNA | 5S_rRNA | 0 | 0.335524 | 0.167762 |
| ENSMUSG00000086643 | processed_transcrip | Gm15687 | 0 | 0.326514 | 0.163257 |
| ENSMUSG00000080760 | pseudogene | Gm7571 | 0 | 0.326514 | 0.163257 |
| ENSMUSG00000065664 | snRNA | U6 | 0 | 0.326514 | 0.163257 |
| ENSMUSG00000074402 | protein_coding | Vmn1r55 | 0 | 0.326514 | 0.163257 |
| ENSMUSG00000066843 | protein_coding | Olfr1348 | 0 | 0.335524 | 0.167762 |
| ENSMUSG00000084654 | rRNA | n-R5s150 | 0 | 0.326514 | 0.163257 |
| ENSMUSG00000070816 | protein_coding | Vmn1r86 | 0 | 0.335524 | 0.167762 |
| ENSMUSG00000078799 | protein_coding | Sult2a5 | 0 | 0.36219 | 0.181095 |
| ENSMUSG00000078795 | protein_coding | Ceacam15 | 0 | 0.36219 | 0.181095 |
| ENSMUSG00000030373 | protein_coding | Psg28 | 0 | 0.335524 | 0.167762 |
| ENSMUSG00000030484 | protein_coding | Lypd5 | 0 | 0.36219 | 0.181095 |
| ENSMUSG00000092999 | misc_RNA | AC160471.1 | 0 | 0.326514 | 0.163257 |
| ENSMUSG00000051976 | protein_coding | 6330444E15Rik | 0 | 0.326514 | 0.163257 |
| ENSMUSG00000078758 | protein_coding | Gm4684 | 0 | 0.335524 | 0.167762 |
| ENSMUSG00000078759 | protein_coding | Gm6662 | 0 | 0.335524 | 0.167762 |
| ENSMUSG00000083434 | pseudogene | Gm12760 | 0 | 0.36219 | 0.181095 |
| ENSMUSG00000077469 | snoRNA | SNORA17 | 0 | 0.326514 | 0.163257 |
| ENSMUSG00000089273 | misc_RNA | 7SK | 0 | 0.36219 | 0.181095 |
| ENSMUSG00000076180 | miRNA | AC102628.1 | 0 | 0.335524 | 0.167762 |
| ENSMUSG00000067616 | protein_coding | Klk11 | 0 | 0.326514 | 0.163257 |
| ENSMUSG00000087492 | processed_transcrip | Gm15700 | 0 | 0.36219 | 0.181095 |
| ENSMUSG00000052303 | protein_coding | Mrgpra6 | 0 | 0.36219 | 0.181095 |
| ENSMUSG00000093101 | miRNA | AC119249.5 | 0 | 0.326514 | 0.163257 |
| ENSMUSG00000092646 | miRNA | AC117644.1 | 0 | 0.335524 | 0.167762 |
| ENSMUSG00000087926 | snoRNA | SNORA70 | 0 | 0.326514 | 0.163257 |
| ENSMUSG00000070511 | protein_coding | Gm10295 | 0 | 0.335524 | 0.167762 |
| ENSMUSG00000091647 | pseudogene | Gm4971 | 0 | 0.335524 | 0.167762 |
| ENSMUSG00000065609 | miRNA | Mir7-2 | 0 | 0.326514 | 0.163257 |
| ENSMUSG00000066372 | protein_coding | Vmn2r65 | 0 | 0.36219 | 0.181095 |

| | | | | | |
|---|---|---|---|---|---|
| ENSMUSG00000092623 | pseudogene | Gm20413 | 0 | 0.36219 | 0.181095 |
| ENSMUSG00000092259 | pseudogene | Vmn2r-ps68 | 0 | 0.36219 | 0.181095 |
| ENSMUSG00000062235 | pseudogene | Gm5341 | 0 | 0.36219 | 0.181095 |
| ENSMUSG00000081359 | pseudogene | Gm15683 | 0 | 0.36219 | 0.181095 |
| ENSMUSG00000073975 | protein_coding | Olfr550 | 0 | 0.326514 | 0.163257 |
| ENSMUSG00000066272 | protein_coding | Olfr559 | 0 | 0.335524 | 0.167762 |
| ENSMUSG00000073966 | protein_coding | Olfr561 | 0 | 0.326514 | 0.163257 |
| ENSMUSG00000060888 | protein_coding | Olfr566 | 0 | 0.335524 | 0.167762 |
| ENSMUSG00000080891 | pseudogene | Olfr587-ps1 | 0 | 0.36219 | 0.181095 |
| ENSMUSG00000057321 | protein_coding | Dub2a | 0 | 0.335524 | 0.167762 |
| ENSMUSG00000045540 | protein_coding | Olfr600 | 0 | 0.335524 | 0.167762 |
| ENSMUSG00000058976 | protein_coding | Usp17l5 | 0 | 0.36219 | 0.181095 |
| ENSMUSG00000080367 | miRNA | AC162877.1 | 0 | 0.335524 | 0.167762 |
| ENSMUSG00000051437 | protein_coding | Ubqlnl | 0 | 0.36219 | 0.181095 |
| ENSMUSG00000073925 | protein_coding | Olfr654 | 0 | 0.335524 | 0.167762 |
| ENSMUSG00000062369 | protein_coding | Dub1a | 0 | 0.335524 | 0.167762 |
| ENSMUSG00000073909 | protein_coding | Olfr688 | 0 | 0.36219 | 0.181095 |
| ENSMUSG00000073907 | protein_coding | Olfr689 | 0 | 0.326514 | 0.163257 |
| ENSMUSG00000050266 | protein_coding | Olfr690 | 0 | 0.36219 | 0.181095 |
| ENSMUSG00000088116 | miRNA | AC141566.1 | 0 | 0.36219 | 0.181095 |
| ENSMUSG00000065874 | snoRNA | SNORA71 | 0 | 0.335524 | 0.167762 |
| ENSMUSG00000088172 | snRNA | U6 | 0 | 0.36219 | 0.181095 |
| ENSMUSG00000064429 | snRNA | U6 | 0 | 0.335524 | 0.167762 |
| ENSMUSG00000074184 | pseudogene | Smt3h2-ps2 | 0 | 0.326514 | 0.163257 |
| ENSMUSG00000093020 | misc_RNA | AC149222.1 | 0 | 0.36219 | 0.181095 |
| ENSMUSG00000025467 | protein_coding | Prap1 | 0 | 0.326514 | 0.163257 |
| ENSMUSG00000060112 | protein_coding | Olfr60 | 0 | 0.36219 | 0.181095 |
| ENSMUSG00000060974 | protein_coding | Olfr530 | 0 | 0.36219 | 0.181095 |
| ENSMUSG00000070140 | miRNA | Mir483 | 0 | 0.326514 | 0.163257 |
| ENSMUSG00000023073 | protein_coding | Slc10a2 | 0 | 0.326514 | 0.163257 |
| ENSMUSG00000070157 | snRNA | U6 | 0 | 0.36219 | 0.181095 |
| ENSMUSG00000086500 | processed_transcrip | Gm15875 | 0 | 0.326514 | 0.163257 |
| ENSMUSG00000079146 | processed_transcrip | Gm15353 | 0 | 0.335524 | 0.167762 |
| ENSMUSG00000082682 | pseudogene | Gm15349 | 0 | 0.326514 | 0.163257 |
| ENSMUSG00000046354 | protein_coding | Defb14 | 0 | 0.326514 | 0.163257 |
| ENSMUSG00000085173 | pseudogene | Gm2869 | 0 | 0.36219 | 0.181095 |
| ENSMUSG00000082957 | pseudogene | Gm15303 | 0 | 0.335524 | 0.167762 |
| ENSMUSG00000082698 | pseudogene | Gm15316 | 0 | 0.326514 | 0.163257 |
| ENSMUSG00000031535 | protein_coding | Dkk4 | 0 | 0.335524 | 0.167762 |
| ENSMUSG00000084543 | snoRNA | SNORA33 | 0 | 0.326514 | 0.163257 |
| ENSMUSG00000092649 | misc_RNA | AC123616.1 | 0 | 0.326514 | 0.163257 |
| ENSMUSG00000054764 | protein_coding | Mtnr1a | 0 | 0.335524 | 0.167762 |
| ENSMUSG00000044014 | protein_coding | Npy5r | 0 | 0.335524 | 0.167762 |
| ENSMUSG00000089114 | snoRNA | SNORA17 | 0 | 0.326514 | 0.163257 |
| ENSMUSG00000092037 | pseudogene | Gm7850 | 0 | 0.36219 | 0.181095 |
| ENSMUSG00000092750 | misc_RNA | AC158898.1 | 0 | 0.36219 | 0.181095 |
| ENSMUSG00000074235 | lincRNA | Gm10649 | 0 | 0.326514 | 0.163257 |
| ENSMUSG00000091998 | pseudogene | Gm17072 | 0 | 0.36219 | 0.181095 |
| ENSMUSG00000062687 | protein_coding | Gm6531 | 0 | 0.335524 | 0.167762 |
| ENSMUSG00000084895 | processed_transcrip | AA672651 | 0 | 0.326514 | 0.163257 |
| ENSMUSG00000055968 | pseudogene | Gm9988 | 0 | 0.36219 | 0.181095 |
| ENSMUSG00000061959 | protein_coding | Ces1e | 0 | 0.335524 | 0.167762 |
| ENSMUSG00000093005 | miRNA | AC138118.1 | 0 | 0.326514 | 0.163257 |
| ENSMUSG00000065512 | miRNA | Mir138-2 | 0 | 0.36219 | 0.181095 |
| ENSMUSG00000031884 | protein_coding | Ces2d-ps | 0 | 0.335524 | 0.167762 |
| ENSMUSG00000092755 | misc_RNA | AC133195.1 | 0 | 0.326514 | 0.163257 |
| ENSMUSG00000074072 | pseudogene | Gm10620 | 0 | 0.36219 | 0.181095 |
| ENSMUSG00000064642 | snoRNA | SNORA32 | 0 | 0.326514 | 0.163257 |

| | | | | | |
|---|---|---|---|---|---|
| ENSMUSG00000090174 | processed_transcrip | Gm10612 | 0 | 0.335524 | 0.167762 |
| ENSMUSG00000065915 | snRNA | U6 | 0 | 0.326514 | 0.163257 |
| ENSMUSG00000083911 | pseudogene | Gm4342 | 0 | 0.335524 | 0.167762 |
| ENSMUSG00000092968 | miRNA | AC118255.1 | 0 | 0.36219 | 0.181095 |
| ENSMUSG00000079719 | protein_coding | Gm11167 | 0 | 0.335524 | 0.167762 |
| ENSMUSG00000064956 | snoRNA | SNORA24 | 0 | 0.36219 | 0.181095 |
| ENSMUSG00000092959 | miRNA | CT030685.2 | 0 | 0.36219 | 0.181095 |
| ENSMUSG00000051118 | protein_coding | Olfr77 | 0 | 0.335524 | 0.167762 |
| ENSMUSG00000093267 | misc_RNA | AC122525.1 | 0 | 0.36219 | 0.181095 |
| ENSMUSG00000085969 | processed_transcrip | Gm15600 | 0 | 0.335524 | 0.167762 |
| ENSMUSG00000088876 | snoRNA | SNORA70 | 0 | 0.326514 | 0.163257 |
| ENSMUSG00000080606 | miRNA | AC141438.1 | 0 | 0.36219 | 0.181095 |
| ENSMUSG00000093088 | miRNA | AC160635.1 | 0 | 0.326514 | 0.163257 |
| ENSMUSG00000032099 | protein_coding | Pate4 | 0 | 0.36219 | 0.181095 |
| ENSMUSG00000078934 | protein_coding | 9230113P08Rik | 0 | 0.326514 | 0.163257 |
| ENSMUSG00000077401 | rRNA | n-R5s82 | 0 | 0.326514 | 0.163257 |
| ENSMUSG00000058628 | protein_coding | Olfr875 | 0 | 0.335524 | 0.167762 |
| ENSMUSG00000066748 | protein_coding | Olfr145 | 0 | 0.36219 | 0.181095 |
| ENSMUSG00000059106 | protein_coding | Olfr961 | 0 | 0.326514 | 0.163257 |
| ENSMUSG00000049073 | protein_coding | Olfr985 | 0 | 0.335524 | 0.167762 |
| ENSMUSG00000040541 | protein_coding | Tmem225 | 0 | 0.36219 | 0.181095 |
| ENSMUSG00000088044 | snRNA | U6 | 0 | 0.326514 | 0.163257 |
| ENSMUSG00000088398 | snoRNA | SNORA40 | 0 | 0.36219 | 0.181095 |
| ENSMUSG00000080358 | snRNA | U6atac | 0 | 0.36219 | 0.181095 |
| ENSMUSG00000032080 | protein_coding | Apoa4 | 0 | 0.326514 | 0.163257 |
| ENSMUSG00000088090 | rRNA | 5S_rRNA | 0 | 0.36219 | 0.181095 |
| ENSMUSG00000064855 | snRNA | U1 | 0 | 0.36219 | 0.181095 |
| ENSMUSG00000076379 | miRNA | Mir190 | 0 | 0.36219 | 0.181095 |
| ENSMUSG00000088908 | misc_RNA | 7SK | 0 | 0.326514 | 0.163257 |
| ENSMUSG00000081273 | pseudogene | Gm15896 | 0 | 0.326514 | 0.163257 |
| ENSMUSG00000083731 | pseudogene | Gm16202 | 0 | 0.326514 | 0.163257 |
| ENSMUSG00000075724 | snoRNA | SNORD50 | 0 | 0.36219 | 0.181095 |
| ENSMUSG00000086585 | processed_transcrip | Gm16126 | 0 | 0.326514 | 0.163257 |
| ENSMUSG00000090915 | protein_coding | Gm17562 | 0 | 0.335524 | 0.167762 |
| ENSMUSG00000032451 | protein_coding | Trim42 | 0 | 0.36219 | 0.181095 |
| ENSMUSG00000032454 | protein_coding | Rbp2 | 0 | 0.335524 | 0.167762 |
| ENSMUSG00000089935 | processed_transcrip | Gm16343 | 0 | 0.36219 | 0.181095 |
| ENSMUSG00000064877 | snoRNA | SNORA17 | 0 | 0.36219 | 0.181095 |
| ENSMUSG00000077156 | rRNA | n-R5s90 | 0 | 0.326514 | 0.163257 |
| ENSMUSG00000087992 | snoRNA | SNORA17 | 0 | 0.36219 | 0.181095 |
| ENSMUSG00000088713 | snoRNA | SNORA19 | 0 | 0.326514 | 0.163257 |
| ENSMUSG00000091336 | pseudogene | Gm17157 | 0 | 0.335524 | 0.167762 |
| ENSMUSG00000065792 | snRNA | U1 | 0 | 0.335524 | 0.167762 |
| ENSMUSG00000064609 | snoRNA | SNORA36 | 0 | 0.335524 | 0.167762 |
| ENSMUSG00000087802 | snoRNA | SNORA70 | 0 | 0.36219 | 0.181095 |
| ENSMUSG00000088467 | snoRNA | snoU13 | 0 | 0.36219 | 0.181095 |
| ENSMUSG00000056379 | protein_coding | Taar1 | 0 | 0.36219 | 0.181095 |
| ENSMUSG00000077923 | miRNA | AC125010.1 | 0 | 0.36219 | 0.181095 |
| ENSMUSG00000080384 | snRNA | U6atac | 0 | 0.335524 | 0.167762 |
| ENSMUSG00000089673 | pseudogene | Gm16546 | 0 | 0.326514 | 0.163257 |
| ENSMUSG00000071320 | protein_coding | Gm9034 | 0 | 0.335524 | 0.167762 |
| ENSMUSG00000019909 | protein_coding | Fam162b | 0 | 0.36219 | 0.181095 |
| ENSMUSG00000019900 | protein_coding | Rfx6 | 0 | 0.335524 | 0.167762 |
| ENSMUSG00000078447 | protein_coding | Gm10942 | 0 | 0.326514 | 0.163257 |
| ENSMUSG00000071280 | protein_coding | Gm10322 | 0 | 0.335524 | 0.167762 |
| ENSMUSG00000091184 | processed_transcrip | Gm17059 | 0 | 0.335524 | 0.167762 |
| ENSMUSG00000088875 | snoRNA | SNORA70 | 0 | 0.36219 | 0.181095 |
| ENSMUSG00000089003 | snRNA | U6 | 0 | 0.36219 | 0.181095 |

| | | | | | |
|---|---|---|---|---|---|
| ENSMUSG00000052426 | pseudogene | Gm9877 | 0 | 0.36219 | 0.181095 |
| ENSMUSG00000084622 | misc_RNA | 7SK | 0 | 0.326514 | 0.163257 |
| ENSMUSG00000055515 | protein_coding | Vmn2r81 | 0 | 0.326514 | 0.163257 |
| ENSMUSG00000089228 | snRNA | U6 | 0 | 0.326514 | 0.163257 |
| ENSMUSG00000090816 | protein_coding | Gm6729 | 0 | 0.36219 | 0.181095 |
| ENSMUSG00000090308 | protein_coding | Gm5174 | 0 | 0.326514 | 0.163257 |
| ENSMUSG00000065005 | rRNA | n-R5s79 | 0 | 0.326514 | 0.163257 |
| ENSMUSG00000091739 | protein_coding | Gm8701 | 0 | 0.36219 | 0.181095 |
| ENSMUSG00000091101 | protein_coding | Gm4302 | 0 | 0.335524 | 0.167762 |
| ENSMUSG00000091552 | pseudogene | Gm8711 | 0 | 0.36219 | 0.181095 |
| ENSMUSG00000036602 | protein_coding | Alx1 | 0 | 0.36219 | 0.181095 |
| ENSMUSG00000079044 | lincRNA | C230021G24Rik | 0 | 0.335524 | 0.167762 |
| ENSMUSG00000065653 | snoRNA | SNORA38 | 0 | 0.36219 | 0.181095 |
| ENSMUSG00000043800 | pseudogene | Kifc5c-ps | 0 | 0.36219 | 0.181095 |
| ENSMUSG00000090461 | protein_coding | Iltifb | 0 | 0.36219 | 0.181095 |
| ENSMUSG00000074687 | pseudogene | Gm9045 | 0 | 0.36219 | 0.181095 |
| ENSMUSG00000054031 | processed_transcrip | Rdh18-ps | 0 | 0.326514 | 0.163257 |
| ENSMUSG00000090036 | pseudogene | Gm15900 | 0 | 0.326514 | 0.163257 |
| ENSMUSG00000045559 | protein_coding | Olfr827 | 0 | 0.326514 | 0.163257 |
| ENSMUSG00000088688 | snoRNA | SNORA70 | 0 | 0.36219 | 0.181095 |
| ENSMUSG00000083553 | pseudogene | Gm11989 | 0 | 0.326514 | 0.163257 |
| ENSMUSG00000082875 | pseudogene | Gm12598 | 0 | 0.36219 | 0.181095 |
| ENSMUSG00000084183 | pseudogene | Gm12009 | 0 | 0.335524 | 0.167762 |
| ENSMUSG00000091489 | protein_coding | Gm17664 | 0 | 0.335524 | 0.167762 |
| ENSMUSG00000083752 | pseudogene | Gm12014 | 0 | 0.335524 | 0.167762 |
| ENSMUSG00000082099 | pseudogene | Gm12013 | 0 | 0.36219 | 0.181095 |
| ENSMUSG00000087833 | misc_RNA | 7SK | 0 | 0.335524 | 0.167762 |
| ENSMUSG00000084085 | protein_coding | Gm16140 | 0 | 0.335524 | 0.167762 |
| ENSMUSG00000089210 | snoRNA | SCARNA21 | 0 | 0.335524 | 0.167762 |
| ENSMUSG00000081346 | pseudogene | Gm12030 | 0 | 0.335524 | 0.167762 |
| ENSMUSG00000084259 | pseudogene | Gm12040 | 0 | 0.326514 | 0.163257 |
| ENSMUSG00000084004 | pseudogene | Gm12047 | 0 | 0.36219 | 0.181095 |
| ENSMUSG00000085979 | processed_transcrip | Gm12055 | 0 | 0.335524 | 0.167762 |
| ENSMUSG00000077621 | snoRNA | SNORA63 | 0 | 0.36219 | 0.181095 |
| ENSMUSG00000086366 | processed_transcrip | 1700061J23Rik | 0 | 0.36219 | 0.181095 |
| ENSMUSG00000086117 | processed_transcrip | Gm12065 | 0 | 0.36219 | 0.181095 |
| ENSMUSG00000086892 | processed_transcrip | 4933430M04Rik | 0 | 0.335524 | 0.167762 |
| ENSMUSG00000084966 | processed_transcrip | 2810471M01Rik | 0 | 0.326514 | 0.163257 |
| ENSMUSG00000085510 | processed_transcrip | Gm12082 | 0 | 0.36219 | 0.181095 |
| ENSMUSG00000081695 | pseudogene | Rpsa-ps5 | 0 | 0.335524 | 0.167762 |
| ENSMUSG00000086707 | processed_transcrip | Gm12088 | 0 | 0.36219 | 0.181095 |
| ENSMUSG00000079890 | pseudogene | Gm12106 | 0 | 0.36219 | 0.181095 |
| ENSMUSG00000073057 | protein_coding | Gm10462 | 0 | 0.335524 | 0.167762 |
| ENSMUSG00000087318 | processed_transcrip | Gm12114 | 0 | 0.326514 | 0.163257 |
| ENSMUSG00000083471 | pseudogene | Gm12118 | 0 | 0.36219 | 0.181095 |
| ENSMUSG00000082688 | pseudogene | Gm12124 | 0 | 0.326514 | 0.163257 |
| ENSMUSG00000087388 | processed_transcrip | Gm12128 | 0 | 0.326514 | 0.163257 |
| ENSMUSG00000082614 | pseudogene | Gm12149 | 0 | 0.335524 | 0.167762 |
| ENSMUSG00000083662 | pseudogene | Gm12152 | 0 | 0.326514 | 0.163257 |
| ENSMUSG00000087089 | processed_transcrip | Gm12160 | 0 | 0.36219 | 0.181095 |
| ENSMUSG00000081251 | pseudogene | Gm12164 | 0 | 0.335524 | 0.167762 |
| ENSMUSG00000069899 | protein_coding | Gm12166 | 0 | 0.326514 | 0.163257 |
| ENSMUSG00000082250 | pseudogene | Gm12181 | 0 | 0.36219 | 0.181095 |
| ENSMUSG00000050763 | protein_coding | Olfr1395 | 0 | 0.36219 | 0.181095 |
| ENSMUSG00000092894 | miRNA | AL606479.2 | 0 | 0.36219 | 0.181095 |
| ENSMUSG00000076450 | miRNA | Mir804 | 0 | 0.36219 | 0.181095 |
| ENSMUSG00000044542 | protein_coding | Prop1 | 0 | 0.335524 | 0.167762 |
| ENSMUSG00000062204 | protein_coding | Olfr1373 | 0 | 0.335524 | 0.167762 |

| | | | | | |
|---|---|---|---|---|---|
| ENSMUSG00000082380 | pseudogene | Gm12218 | 0 | 0.326514 | 0.163257 |
| ENSMUSG00000088437 | snRNA | U6 | 0 | 0.36219 | 0.181095 |
| ENSMUSG00000065061 | snoRNA | SNORA29 | 0 | 0.36219 | 0.181095 |
| ENSMUSG00000086460 | processed_transcrip | Gm12236 | 0 | 0.36219 | 0.181095 |
| ENSMUSG00000084169 | pseudogene | Gm12240 | 0 | 0.36219 | 0.181095 |
| ENSMUSG00000063549 | protein_coding | Olfr179 | 0 | 0.335524 | 0.167762 |
| ENSMUSG00000087654 | processed_transcrip | Gm12256 | 0 | 0.326514 | 0.163257 |
| ENSMUSG00000088950 | rRNA | 5S_rRNA | 0 | 0.36219 | 0.181095 |
| ENSMUSG00000085132 | processed_transcrip | Gm12265 | 0 | 0.326514 | 0.163257 |
| ENSMUSG00000081753 | pseudogene | Gm12626 | 0 | 0.36219 | 0.181095 |
| ENSMUSG00000082748 | pseudogene | Gm12616 | 0 | 0.326514 | 0.163257 |
| ENSMUSG00000086310 | processed_transcrip | Gm12278 | 0 | 0.326514 | 0.163257 |
| ENSMUSG00000083142 | pseudogene | Gm12288 | 0 | 0.335524 | 0.167762 |
| ENSMUSG00000081756 | pseudogene | Gm12291 | 0 | 0.36219 | 0.181095 |
| ENSMUSG00000048070 | protein_coding | Pirt | 0 | 0.36219 | 0.181095 |
| ENSMUSG00000087410 | processed_transcrip | 2310065F04Rik | 0 | 0.36219 | 0.181095 |
| ENSMUSG00000093210 | misc_RNA | AL662894.1 | 0 | 0.36219 | 0.181095 |
| ENSMUSG00000083300 | pseudogene | Gm12309 | 0 | 0.36219 | 0.181095 |
| ENSMUSG00000081333 | pseudogene | Gm12307 | 0 | 0.36219 | 0.181095 |
| ENSMUSG00000084251 | pseudogene | Gm12310 | 0 | 0.36219 | 0.181095 |
| ENSMUSG00000087116 | processed_transcrip | Gm12324 | 0 | 0.335524 | 0.167762 |
| ENSMUSG00000065298 | snRNA | U1 | 0 | 0.36219 | 0.181095 |
| ENSMUSG00000055971 | protein_coding | Olfr378 | 0 | 0.36219 | 0.181095 |
| ENSMUSG00000093100 | miRNA | AL591496.1 | 0 | 0.335524 | 0.167762 |
| ENSMUSG00000080763 | pseudogene | Gm12341 | 0 | 0.335524 | 0.167762 |
| ENSMUSG00000085115 | pseudogene | Gm11197 | 0 | 0.36219 | 0.181095 |
| ENSMUSG00000081663 | pseudogene | Gm11207 | 0 | 0.36219 | 0.181095 |
| ENSMUSG00000086703 | processed_transcrip | 4930507D10Rik | 0 | 0.326514 | 0.163257 |
| ENSMUSG00000053184 | protein_coding | Spaca3 | 0 | 0.326514 | 0.163257 |
| ENSMUSG00000069796 | processed_transcrip | Gm11426 | 0 | 0.36219 | 0.181095 |
| ENSMUSG00000086327 | processed_transcrip | 1700003F17Rik | 0 | 0.326514 | 0.163257 |
| ENSMUSG00000087407 | processed_transcrip | Gm11431 | 0 | 0.326514 | 0.163257 |
| ENSMUSG00000087779 | snRNA | U1 | 0 | 0.36219 | 0.181095 |
| ENSMUSG00000093278 | miRNA | AL596324.1 | 0 | 0.326514 | 0.163257 |
| ENSMUSG00000088230 | misc_RNA | Y_RNA | 0 | 0.335524 | 0.167762 |
| ENSMUSG00000080976 | pseudogene | Gm11490 | 0 | 0.36219 | 0.181095 |
| ENSMUSG00000085972 | processed_transcrip | 1110028F11Rik | 0 | 0.326514 | 0.163257 |
| ENSMUSG00000091228 | protein_coding | Nme2 | 0 | 0.326514 | 0.163257 |
| ENSMUSG00000081007 | pseudogene | Gm11543 | 0 | 0.36219 | 0.181095 |
| ENSMUSG00000084856 | processed_transcrip | Gm11528 | 0 | 0.36219 | 0.181095 |
| ENSMUSG00000065515 | miRNA | Mir152 | 0 | 0.326514 | 0.163257 |
| ENSMUSG00000084368 | pseudogene | Gm11572 | 0 | 0.326514 | 0.163257 |
| ENSMUSG00000047253 | protein_coding | Krtap1-5 | 0 | 0.326514 | 0.163257 |
| ENSMUSG00000069717 | protein_coding | Gm11568 | 0 | 0.335524 | 0.167762 |
| ENSMUSG00000086163 | processed_transcrip | Gm14206 | 0 | 0.36219 | 0.181095 |
| ENSMUSG00000064759 | snRNA | U2 | 0 | 0.326514 | 0.163257 |
| ENSMUSG00000065251 | snRNA | U2 | 0 | 0.36219 | 0.181095 |
| ENSMUSG00000017311 | protein_coding | Pyy | 0 | 0.326514 | 0.163257 |
| ENSMUSG00000049506 | protein_coding | 4933407P14Rik | 0 | 0.335524 | 0.167762 |
| ENSMUSG00000075485 | protein_coding | Gm10842 | 0 | 0.36219 | 0.181095 |
| ENSMUSG00000088400 | snRNA | U6 | 0 | 0.36219 | 0.181095 |
| ENSMUSG00000084978 | processed_transcrip | Gm11655 | 0 | 0.36219 | 0.181095 |
| ENSMUSG00000081548 | pseudogene | Gm11692 | 0 | 0.36219 | 0.181095 |
| ENSMUSG00000083237 | pseudogene | Gm11698 | 0 | 0.326514 | 0.163257 |
| ENSMUSG00000092823 | miRNA | AL672228.1 | 0 | 0.36219 | 0.181095 |
| ENSMUSG00000080810 | pseudogene | Gm11737 | 0 | 0.36219 | 0.181095 |
| ENSMUSG00000086514 | processed_transcrip | Gm11747 | 0 | 0.36219 | 0.181095 |
| ENSMUSG00000092780 | miRNA | AL662835.1 | 0 | 0.326514 | 0.163257 |

| | | | | | |
|---|---|---|---|---|---|
| ENSMUSG00000083870 | pseudogene | Gm11761 | 0 | 0.335524 | 0.167762 |
| ENSMUSG00000092950 | miRNA | AL662901.1 | 0 | 0.36219 | 0.181095 |
| ENSMUSG00000085970 | processed_transcrip | Gm15643 | 0 | 0.36219 | 0.181095 |
| ENSMUSG00000092288 | pseudogene | RP23-71I22.2 | 0 | 0.335524 | 0.167762 |
| ENSMUSG00000020636 | protein_coding | Allc | 0 | 0.335524 | 0.167762 |
| ENSMUSG00000089583 | rRNA | 5S_rRNA | 0 | 0.36219 | 0.181095 |
| ENSMUSG00000091040 | pseudogene | Gm9359 | 0 | 0.36219 | 0.181095 |
| ENSMUSG00000088242 | snoRNA | SCARNA17 | 0 | 0.326514 | 0.163257 |
| ENSMUSG00000077750 | snoRNA | SNORA17 | 0 | 0.326514 | 0.163257 |
| ENSMUSG00000020545 | protein_coding | 1700108M19Rik | 0 | 0.36219 | 0.181095 |
| ENSMUSG00000046620 | protein_coding | Gm9808 | 0 | 0.326514 | 0.163257 |
| ENSMUSG00000071329 | pseudogene | Gm7555 | 0 | 0.326514 | 0.163257 |
| ENSMUSG00000092515 | processed_transcrip | C87198 | 0 | 0.335524 | 0.167762 |
| ENSMUSG00000084793 | pseudogene | Gm2568 | 0 | 0.36219 | 0.181095 |
| ENSMUSG00000091396 | protein_coding | Spanxn4 | 0 | 0.36219 | 0.181095 |
| ENSMUSG00000066457 | protein_coding | Gm5068 | 0 | 0.36219 | 0.181095 |
| ENSMUSG00000088039 | snoRNA | SNORA17 | 0 | 0.326514 | 0.163257 |
| ENSMUSG00000088822 | snRNA | U6 | 0 | 0.36219 | 0.181095 |
| ENSMUSG00000090883 | protein_coding | Gm17301 | 0 | 0.36219 | 0.181095 |
| ENSMUSG00000087426 | lincRNA | Gm805 | 0 | 0.335524 | 0.167762 |
| ENSMUSG00000059114 | protein_coding | Gm6772 | 0 | 0.36219 | 0.181095 |
| ENSMUSG00000071217 | protein_coding | BB287469 | 0 | 0.36219 | 0.181095 |
| ENSMUSG00000090722 | lincRNA | Gm8378 | 0 | 0.326514 | 0.163257 |
| ENSMUSG00000090050 | processed_transcrip | Gm16339 | 0 | 0.36219 | 0.181095 |
| ENSMUSG00000089324 | miRNA | Mir1936 | 0 | 0.36219 | 0.181095 |
| ENSMUSG00000090555 | pseudogene | Gm8893 | 0 | 0.335524 | 0.167762 |
| ENSMUSG00000091307 | pseudogene | Gm8895 | 0 | 0.36219 | 0.181095 |
| ENSMUSG00000066359 | protein_coding | Tcl1b1 | 0 | 0.335524 | 0.167762 |
| ENSMUSG00000068940 | protein_coding | Tcl1b3 | 0 | 0.326514 | 0.163257 |
| ENSMUSG00000087854 | rRNA | 5S_rRNA | 0 | 0.36219 | 0.181095 |
| ENSMUSG00000091280 | processed_transcrip | Gm17032 | 0 | 0.36219 | 0.181095 |
| ENSMUSG00000091583 | processed_transcrip | Gm17033 | 0 | 0.36219 | 0.181095 |
| ENSMUSG00000065526 | miRNA | Mir337 | 0 | 0.335524 | 0.167762 |
| ENSMUSG00000070101 | miRNA | Mir341 | 0 | 0.36219 | 0.181095 |
| ENSMUSG00000065757 | snoRNA | SNORD113 | 0 | 0.326514 | 0.163257 |
| ENSMUSG00000064726 | snoRNA | SNORD113 | 0 | 0.335524 | 0.167762 |
| ENSMUSG00000065566 | miRNA | Mir381 | 0 | 0.335524 | 0.167762 |
| ENSMUSG00000076063 | miRNA | Mir539 | 0 | 0.36219 | 0.181095 |
| ENSMUSG00000076613 | IG_C_gene | Ighg | 0 | 0.326514 | 0.163257 |
| ENSMUSG00000076614 | IG_C_gene | Ighg1 | 0 | 0.36219 | 0.181095 |
| ENSMUSG00000076643 | IG_V_gene | Gm16886 | 0 | 0.36219 | 0.181095 |
| ENSMUSG00000076645 | IG_V_gene | Gm16595 | 0 | 0.335524 | 0.167762 |
| ENSMUSG00000076648 | IG_V_gene | Gm16888 | 0 | 0.326514 | 0.163257 |
| ENSMUSG00000076650 | IG_V_gene | Gm16609 | 0 | 0.36219 | 0.181095 |
| ENSMUSG00000076652 | IG_V_gene | Gm16698 | 0 | 0.335524 | 0.167762 |
| ENSMUSG00000076665 | IG_V_gene | Gm16842 | 0 | 0.326514 | 0.163257 |
| ENSMUSG00000076701 | IG_V_gene | Gm16717 | 0 | 0.326514 | 0.163257 |
| ENSMUSG00000076718 | IG_V_gene | Gm16791 | 0 | 0.326514 | 0.163257 |
| ENSMUSG00000076726 | IG_V_gene | Gm16865 | 0 | 0.36219 | 0.181095 |
| ENSMUSG00000076735 | IG_V_gene | Gm16811 | 0 | 0.36219 | 0.181095 |
| ENSMUSG00000077164 | snoRNA | SNORA17 | 0 | 0.326514 | 0.163257 |
| ENSMUSG00000088366 | misc_RNA | 7SK | 0 | 0.36219 | 0.181095 |
| ENSMUSG00000086178 | lincRNA | Gm16950 | 0 | 0.36219 | 0.181095 |
| ENSMUSG00000051258 | protein_coding | Olfr1362 | 0 | 0.326514 | 0.163257 |
| ENSMUSG00000069296 | protein_coding | Vmn1r195 | 0 | 0.36219 | 0.181095 |
| ENSMUSG00000081717 | pseudogene | Gm11303 | 0 | 0.326514 | 0.163257 |
| ENSMUSG00000085221 | processed_transcrip | Gm11309 | 0 | 0.36219 | 0.181095 |
| ENSMUSG00000069266 | protein_coding | Hist1h4b | 0 | 0.36219 | 0.181095 |

| | | | | | |
|---|---|---|---|---|---|
| ENSMUSG00000087603 | processed_transcrip | Gm11339 | 0 | 0.36219 | 0.181095 |
| ENSMUSG00000092699 | miRNA | AL512647.1 | 0 | 0.326514 | 0.163257 |
| ENSMUSG00000083676 | pseudogene | Gm11367 | 0 | 0.335524 | 0.167762 |
| ENSMUSG00000086263 | processed_transcrip | 4930519D14Rik | 0 | 0.36219 | 0.181095 |
| ENSMUSG00000021404 | protein_coding | Serpinb9c | 0 | 0.335524 | 0.167762 |
| ENSMUSG00000055732 | protein_coding | Gm9979 | 0 | 0.326514 | 0.163257 |
| ENSMUSG00000085896 | processed_transcrip | 5330429C05Rik | 0 | 0.326514 | 0.163257 |
| ENSMUSG00000077279 | snoRNA | SNORA17 | 0 | 0.335524 | 0.167762 |
| ENSMUSG00000076249 | miRNA | AC159290.1 | 0 | 0.36219 | 0.181095 |
| ENSMUSG00000090232 | processed_transcrip | Gm16132 | 0 | 0.36219 | 0.181095 |
| ENSMUSG00000049806 | protein_coding | Olfr466 | 0 | 0.335524 | 0.167762 |
| ENSMUSG00000021598 | protein_coding | Med10 | 0 | 0.36219 | 0.181095 |
| ENSMUSG00000063180 | protein_coding | Gm10126 | 0 | 0.36219 | 0.181095 |
| ENSMUSG00000091547 | lincRNA | Gm17547 | 0 | 0.335524 | 0.167762 |
| ENSMUSG00000091518 | protein_coding | Gm4066 | 0 | 0.335524 | 0.167762 |
| ENSMUSG00000089972 | pseudogene | Gm16318 | 0 | 0.326514 | 0.163257 |
| ENSMUSG00000093106 | miRNA | AC136976.1 | 0 | 0.326514 | 0.163257 |
| ENSMUSG00000093109 | misc_RNA | AC165159.1 | 0 | 0.36219 | 0.181095 |
| ENSMUSG00000074662 | pseudogene | Gm10737 | 0 | 0.335524 | 0.167762 |
| ENSMUSG00000086057 | processed_transcrip | Gm15324 | 0 | 0.36219 | 0.181095 |
| ENSMUSG00000051758 | protein_coding | 4930544M13Rik | 0 | 0.326514 | 0.163257 |
| ENSMUSG00000092101 | protein_coding | Gm2956 | 0 | 0.326514 | 0.163257 |
| ENSMUSG00000079377 | protein_coding | Gm3029 | 0 | 0.335524 | 0.167762 |
| ENSMUSG00000091114 | protein_coding | Gm3138 | 0 | 0.36219 | 0.181095 |
| ENSMUSG00000091379 | protein_coding | Gm3159 | 0 | 0.326514 | 0.163257 |
| ENSMUSG00000090827 | protein_coding | Gm8297 | 0 | 0.326514 | 0.163257 |
| ENSMUSG00000090547 | protein_coding | Gm8265 | 0 | 0.326514 | 0.163257 |
| ENSMUSG00000091126 | protein_coding | Gm8246 | 0 | 0.36219 | 0.181095 |
| ENSMUSG00000090596 | pseudogene | Gm17145 | 0 | 0.36219 | 0.181095 |
| ENSMUSG00000090512 | protein_coding | Gm3424 | 0 | 0.326514 | 0.163257 |
| ENSMUSG00000090552 | pseudogene | Gm17158 | 0 | 0.36219 | 0.181095 |
| ENSMUSG00000090703 | pseudogene | Gm17048 | 0 | 0.335524 | 0.167762 |
| ENSMUSG00000079371 | protein_coding | Gm3476 | 0 | 0.326514 | 0.163257 |
| ENSMUSG00000091202 | protein_coding | Gm3685 | 0 | 0.335524 | 0.167762 |
| ENSMUSG00000091042 | protein_coding | Gm3727 | 0 | 0.335524 | 0.167762 |
| ENSMUSG00000090597 | pseudogene | Gm17121 | 0 | 0.36219 | 0.181095 |
| ENSMUSG00000063277 | protein_coding | Gm10128 | 0 | 0.36219 | 0.181095 |
| ENSMUSG00000049460 | pseudogene | Gm8396 | 0 | 0.335524 | 0.167762 |
| ENSMUSG00000083606 | pseudogene | Gm15916 | 0 | 0.335524 | 0.167762 |
| ENSMUSG00000091557 | pseudogene | Gm8584 | 0 | 0.326514 | 0.163257 |
| ENSMUSG00000057584 | pseudogene | Gm6128 | 0 | 0.335524 | 0.167762 |
| ENSMUSG00000072616 | protein_coding | Gm7853 | 0 | 0.36219 | 0.181095 |
| ENSMUSG00000041044 | protein_coding | Lrit1 | 0 | 0.36219 | 0.181095 |
| ENSMUSG00000092055 | pseudogene | Gm7925 | 0 | 0.36219 | 0.181095 |
| ENSMUSG00000052790 | pseudogene | Gm9890 | 0 | 0.36219 | 0.181095 |
| ENSMUSG00000091116 | pseudogene | Gm7991 | 0 | 0.36219 | 0.181095 |
| ENSMUSG00000090690 | protein_coding | Gm8020 | 0 | 0.335524 | 0.167762 |
| ENSMUSG00000091140 | protein_coding | Gm17124 | 0 | 0.36219 | 0.181095 |
| ENSMUSG00000091470 | pseudogene | Gm17159 | 0 | 0.326514 | 0.163257 |
| ENSMUSG00000079269 | protein_coding | Gm3676 | 0 | 0.326514 | 0.163257 |
| ENSMUSG00000090796 | pseudogene | Gm3141 | 0 | 0.36219 | 0.181095 |
| ENSMUSG00000090332 | pseudogene | Gm3216 | 0 | 0.36219 | 0.181095 |
| ENSMUSG00000090180 | pseudogene | Ang-ps1 | 0 | 0.326514 | 0.163257 |
| ENSMUSG00000091923 | protein_coding | Gm8267 | 0 | 0.36219 | 0.181095 |
| ENSMUSG00000085866 | processed_transcrip | Gm15218 | 0 | 0.36219 | 0.181095 |
| ENSMUSG00000093204 | snRNA | AC166574.2 | 0 | 0.36219 | 0.181095 |
| ENSMUSG00000053815 | protein_coding | Olfr744 | 0 | 0.335524 | 0.167762 |
| ENSMUSG00000070448 | protein_coding | Vmn2r89 | 0 | 0.335524 | 0.167762 |

| | | | | | |
|---|---|---|---|---|---|
| ENSMUSG00000091189 | pseudogene | Ear-ps3 | 0 | 0.36219 | 0.181095 |
| ENSMUSG00000050961 | protein_coding | AY358078 | 0 | 0.335524 | 0.167762 |
| ENSMUSG00000008813 | protein_coding | Tppp2 | 0 | 0.335524 | 0.167762 |
| ENSMUSG00000063867 | protein_coding | Olfr1511 | 0 | 0.335524 | 0.167762 |
| ENSMUSG00000076798 | IG_V_gene | Tcra-V2.6 | 0 | 0.326514 | 0.163257 |
| ENSMUSG00000076823 | IG_V_gene | Gm10907 | 0 | 0.326514 | 0.163257 |
| ENSMUSG00000076841 | IG_V_gene | Gm16591 | 0 | 0.326514 | 0.163257 |
| ENSMUSG00000076846 | IG_V_gene | Trav13-2 | 0 | 0.326514 | 0.163257 |
| ENSMUSG00000076865 | IG_V_gene | Trdv2-1 | 0 | 0.326514 | 0.163257 |
| ENSMUSG00000089469 | misc_RNA | 7SK | 0 | 0.326514 | 0.163257 |
| ENSMUSG00000076882 | IG_J_gene | Gm17012 | 0 | 0.335524 | 0.167762 |
| ENSMUSG00000076885 | IG_J_gene | Gm16927 | 0 | 0.326514 | 0.163257 |
| ENSMUSG00000076886 | IG_J_gene | Gm16926 | 0 | 0.326514 | 0.163257 |
| ENSMUSG00000076905 | IG_J_gene | Gm16824 | 0 | 0.326514 | 0.163257 |
| ENSMUSG00000076909 | IG_J_gene | Gm16816 | 0 | 0.326514 | 0.163257 |
| ENSMUSG00000076923 | IG_J_gene | Traj5 | 0 | 0.335524 | 0.167762 |
| ENSMUSG00000022226 | protein_coding | Mcpt2 | 0 | 0.326514 | 0.163257 |
| ENSMUSG00000091132 | pseudogene | Gm17109 | 0 | 0.36219 | 0.181095 |
| ENSMUSG00000091456 | lincRNA | A930011O12Rik | 0 | 0.335524 | 0.167762 |
| ENSMUSG00000014725 | protein_coding | Adam28 | 0 | 0.326514 | 0.163257 |
| ENSMUSG00000034913 | protein_coding | Spert | 0 | 0.335524 | 0.167762 |
| ENSMUSG00000088971 | rRNA | 5S_rRNA | 0 | 0.36219 | 0.181095 |
| ENSMUSG00000022149 | protein_coding | C9 | 0 | 0.335524 | 0.167762 |
| ENSMUSG00000072664 | protein_coding | Ugt3a1 | 0 | 0.335524 | 0.167762 |
| ENSMUSG00000093248 | miRNA | AC135292.1 | 0 | 0.36219 | 0.181095 |
| ENSMUSG00000088062 | rRNA | 5S_rRNA | 0 | 0.36219 | 0.181095 |
| ENSMUSG00000093021 | miRNA | AC138604.1 | 0 | 0.36219 | 0.181095 |
| ENSMUSG00000089811 | pseudogene | Gm16138 | 0 | 0.36219 | 0.181095 |
| ENSMUSG00000076032 | miRNA | AC122459.1 | 0 | 0.326514 | 0.163257 |
| ENSMUSG00000077470 | snoRNA | SNORA17 | 0 | 0.326514 | 0.163257 |
| ENSMUSG00000042895 | protein_coding | Abra | 0 | 0.326514 | 0.163257 |
| ENSMUSG00000053168 | protein_coding | 9030619P08Rik | 0 | 0.335524 | 0.167762 |
| ENSMUSG00000089242 | snRNA | U1 | 0 | 0.326514 | 0.163257 |
| ENSMUSG00000068246 | protein_coding | Apol9a | 0 | 0.326514 | 0.163257 |
| ENSMUSG00000091694 | protein_coding | Apol11b | 0 | 0.335524 | 0.167762 |
| ENSMUSG00000092974 | misc_RNA | AL589670.1 | 0 | 0.335524 | 0.167762 |
| ENSMUSG00000088740 | snoRNA | SNORA17 | 0 | 0.335524 | 0.167762 |
| ENSMUSG00000075517 | protein_coding | Cyp2d37-ps | 0 | 0.335524 | 0.167762 |
| ENSMUSG00000064764 | snRNA | U2 | 0 | 0.335524 | 0.167762 |
| ENSMUSG00000088071 | snRNA | U6 | 0 | 0.335524 | 0.167762 |
| ENSMUSG00000078847 | pseudogene | Gm6746 | 0 | 0.36219 | 0.181095 |
| ENSMUSG00000088454 | snRNA | U6 | 0 | 0.335524 | 0.167762 |
| ENSMUSG00000088325 | snoRNA | SNORA17 | 0 | 0.326514 | 0.163257 |
| ENSMUSG00000048077 | protein_coding | H1fnt | 0 | 0.36219 | 0.181095 |
| ENSMUSG00000064723 | snRNA | U6 | 0 | 0.36219 | 0.181095 |
| ENSMUSG00000064201 | protein_coding | Krt2 | 0 | 0.36219 | 0.181095 |
| ENSMUSG00000090274 | protein_coding | Gm17671 | 0 | 0.326514 | 0.163257 |
| ENSMUSG00000091182 | protein_coding | Gm17649 | 0 | 0.335524 | 0.167762 |
| ENSMUSG00000086903 | processed_transcrip | Gm16258 | 0 | 0.335524 | 0.167762 |
| ENSMUSG00000065560 | miRNA | Mir148b | 0 | 0.335524 | 0.167762 |
| ENSMUSG00000053508 | protein_coding | BC048502 | 0 | 0.326514 | 0.163257 |
| ENSMUSG00000076972 | miRNA | Mir193b | 0 | 0.335524 | 0.167762 |
| ENSMUSG00000082277 | pseudogene | Gm15807 | 0 | 0.36219 | 0.181095 |
| ENSMUSG00000048101 | protein_coding | Olfr19 | 0 | 0.335524 | 0.167762 |
| ENSMUSG00000081426 | pseudogene | Gm15714 | 0 | 0.36219 | 0.181095 |
| ENSMUSG00000076939 | IG_V_gene | Iglv3 | 0 | 0.326514 | 0.163257 |
| ENSMUSG00000080447 | miRNA | AC087898.1 | 0 | 0.335524 | 0.167762 |
| ENSMUSG00000092078 | lincRNA | Gm17439 | 0 | 0.326514 | 0.163257 |

| | | | | | |
|---|---|---|---|---|---|
| ENSMUSG00000090380 | lincRNA | 2610020F03Rik | 0 | 0.326514 | 0.163257 |
| ENSMUSG00000047531 | protein_coding | Rtp2 | 0 | 0.36219 | 0.181095 |
| ENSMUSG00000091972 | protein_coding | Tmem207 | 0 | 0.36219 | 0.181095 |
| ENSMUSG00000090214 | processed_transcrip | Gm15657 | 0 | 0.36219 | 0.181095 |
| ENSMUSG00000022832 | protein_coding | Ropn1 | 0 | 0.335524 | 0.167762 |
| ENSMUSG00000064769 | snRNA | U1 | 0 | 0.36219 | 0.181095 |
| ENSMUSG00000087689 | processed_transcrip | Gm15845 | 0 | 0.335524 | 0.167762 |
| ENSMUSG00000065188 | snRNA | U1 | 0 | 0.326514 | 0.163257 |
| ENSMUSG00000077530 | snoRNA | SNORA17 | 0 | 0.335524 | 0.167762 |
| ENSMUSG00000092022 | protein_coding | Gm17497 | 0 | 0.36219 | 0.181095 |
| ENSMUSG00000073976 | processed_transcrip | 1700026J12Rik | 0 | 0.36219 | 0.181095 |
| ENSMUSG00000063137 | protein_coding | Olfr177 | 0 | 0.36219 | 0.181095 |
| ENSMUSG00000090951 | protein_coding | Olfr181 | 0 | 0.36219 | 0.181095 |
| ENSMUSG00000093149 | miRNA | AC163694.1 | 0 | 0.335524 | 0.167762 |
| ENSMUSG00000087970 | snRNA | U7 | 0 | 0.335524 | 0.167762 |
| ENSMUSG00000092779 | miRNA | AC122218.1 | 0 | 0.36219 | 0.181095 |
| ENSMUSG00000047109 | protein_coding | Cldn14 | 0 | 0.335524 | 0.167762 |
| ENSMUSG00000076304 | miRNA | CT573086.1 | 0 | 0.326514 | 0.163257 |
| ENSMUSG00000088269 | rRNA | 5S_rRNA | 0 | 0.326514 | 0.163257 |
| ENSMUSG00000081783 | pseudogene | Gm15599 | 0 | 0.36219 | 0.181095 |
| ENSMUSG00000079730 | protein_coding | Gm2833 | 0 | 0.36219 | 0.181095 |
| ENSMUSG00000089907 | processed_transcrip | Gm16169 | 0 | 0.36219 | 0.181095 |
| ENSMUSG00000091640 | protein_coding | Gm17592 | 0 | 0.335524 | 0.167762 |
| ENSMUSG00000075815 | snRNA | U6 | 0 | 0.335524 | 0.167762 |
| ENSMUSG00000090355 | protein_coding | Vmn2r-ps128 | 0 | 0.335524 | 0.167762 |
| ENSMUSG00000057417 | protein_coding | Dcpp3 | 0 | 0.335524 | 0.167762 |
| ENSMUSG00000077184 | snoRNA | SNORA17 | 0 | 0.326514 | 0.163257 |
| ENSMUSG00000082741 | pseudogene | Gm9703 | 0 | 0.326514 | 0.163257 |
| ENSMUSG00000086149 | processed_transcrip | Gm15598 | 0 | 0.335524 | 0.167762 |
| ENSMUSG00000024224 | protein_coding | Gm749 | 0 | 0.326514 | 0.163257 |
| ENSMUSG00000089222 | snRNA | U6 | 0 | 0.326514 | 0.163257 |
| ENSMUSG00000089559 | snRNA | U6 | 0 | 0.326514 | 0.163257 |
| ENSMUSG00000086010 | processed_transcrip | Gm15318 | 0 | 0.326514 | 0.163257 |
| ENSMUSG00000092412 | processed_transcrip | Gm20507 | 0 | 0.335524 | 0.167762 |
| ENSMUSG00000092338 | processed_transcrip | Mir219-1 | 0 | 0.36219 | 0.181095 |
| ENSMUSG00000092550 | protein_coding | Gm20496 | 0 | 0.335524 | 0.167762 |
| ENSMUSG00000083522 | pseudogene | Gm15321 | 0 | 0.335524 | 0.167762 |
| ENSMUSG00000092406 | pseudogene | Gm20462 | 0 | 0.335524 | 0.167762 |
| ENSMUSG00000092586 | protein_coding | Ly6g6c | 0 | 0.36219 | 0.181095 |
| ENSMUSG00000092224 | pseudogene | Gm8815 | 0 | 0.335524 | 0.167762 |
| ENSMUSG00000092366 | pseudogene | Gm17782 | 0 | 0.36219 | 0.181095 |
| ENSMUSG00000092457 | pseudogene | Gm8835 | 0 | 0.335524 | 0.167762 |
| ENSMUSG00000092392 | processed_transcrip | Gm20546 | 0 | 0.335524 | 0.167762 |
| ENSMUSG00000058124 | protein_coding | H2-M10.3 | 0 | 0.36219 | 0.181095 |
| ENSMUSG00000058063 | protein_coding | Trim31 | 0 | 0.335524 | 0.167762 |
| ENSMUSG00000067192 | protein_coding | Olfr91 | 0 | 0.36219 | 0.181095 |
| ENSMUSG00000092524 | pseudogene | Olfr756-ps1 | 0 | 0.36219 | 0.181095 |
| ENSMUSG00000090894 | protein_coding | Olfr110 | 0 | 0.36219 | 0.181095 |
| ENSMUSG00000043312 | protein_coding | Olfr131 | 0 | 0.36219 | 0.181095 |
| ENSMUSG00000025433 | protein_coding | Crisp3 | 0 | 0.36219 | 0.181095 |
| ENSMUSG00000089849 | processed_transcrip | 9530072K05Rik | 0 | 0.36219 | 0.181095 |
| ENSMUSG00000065027 | snRNA | U1 | 0 | 0.335524 | 0.167762 |
| ENSMUSG00000064748 | rRNA | n-R5s28 | 0 | 0.335524 | 0.167762 |
| ENSMUSG00000021545 | protein_coding | 1700067P10Rik | 0 | 0.36219 | 0.181095 |
| ENSMUSG00000042265 | protein_coding | Trem1 | 0 | 0.335524 | 0.167762 |
| ENSMUSG00000041754 | protein_coding | Trem3 | 0 | 0.335524 | 0.167762 |
| ENSMUSG00000084583 | snRNA | U4 | 0 | 0.36219 | 0.181095 |
| ENSMUSG00000090426 | lincRNA | Gm17392 | 0 | 0.326514 | 0.163257 |

| | | | | | |
|---|---|---|---|---|---|
| ENSMUSG00000090224 | pseudogene | Gm15974 | 0 | 0.326514 | 0.163257 |
| ENSMUSG00000062196 | pseudogene | Fabp5l2 | 0 | 0.36219 | 0.181095 |
| ENSMUSG00000065941 | snRNA | U1 | 0 | 0.326514 | 0.163257 |
| ENSMUSG00000088077 | snRNA | U6 | 0 | 0.335524 | 0.167762 |
| ENSMUSG00000091428 | protein_coding | Gm17507 | 0 | 0.326514 | 0.163257 |
| ENSMUSG00000084959 | processed_transcrip | 4933407I08Rik | 0 | 0.326514 | 0.163257 |
| ENSMUSG00000061144 | protein_coding | Spink12 | 0 | 0.335524 | 0.167762 |
| ENSMUSG00000088804 | snRNA | U6 | 0 | 0.335524 | 0.167762 |
| ENSMUSG00000065402 | miRNA | Mir122a | 0 | 0.335524 | 0.167762 |
| ENSMUSG00000093283 | misc_RNA | AC102240.1 | 0 | 0.335524 | 0.167762 |
| ENSMUSG00000077593 | snoRNA | SNORA17 | 0 | 0.335524 | 0.167762 |
| ENSMUSG00000073525 | protein_coding | Gm10527 | 0 | 0.335524 | 0.167762 |
| ENSMUSG00000073521 | protein_coding | Gm10525 | 0 | 0.335524 | 0.167762 |
| ENSMUSG00000093259 | misc_RNA | AC133523.1 | 0 | 0.335524 | 0.167762 |
| ENSMUSG00000065523 | miRNA | Mir192 | 0 | 0.36219 | 0.181095 |
| ENSMUSG00000088680 | rRNA | 5S_rRNA | 0 | 0.335524 | 0.167762 |
| ENSMUSG00000075044 | protein_coding | Slc22a29 | 0 | 0.36219 | 0.181095 |
| ENSMUSG00000076370 | miRNA | AC132253.1 | 0 | 0.335524 | 0.167762 |
| ENSMUSG00000024730 | protein_coding | Ms4a8a | 0 | 0.326514 | 0.163257 |
| ENSMUSG00000058470 | protein_coding | Gm8369 | 0 | 0.326514 | 0.163257 |
| ENSMUSG00000067519 | protein_coding | Olfr262 | 0 | 0.326514 | 0.163257 |
| ENSMUSG00000046650 | protein_coding | Olfr1440 | 0 | 0.335524 | 0.167762 |
| ENSMUSG00000057503 | protein_coding | Olfr1459 | 0 | 0.326514 | 0.163257 |
| ENSMUSG00000090147 | pseudogene | Gm3329 | 0 | 0.36219 | 0.181095 |
| ENSMUSG00000086875 | protein_coding | Gm8975 | 0 | 0.36219 | 0.181095 |
| ENSMUSG00000086812 | processed_transcrip | Gm8978 | 0 | 0.36219 | 0.181095 |
| ENSMUSG00000065594 | miRNA | Mir107 | 0 | 0.335524 | 0.167762 |
| ENSMUSG00000076323 | miRNA | AL603804.1 | 0 | 0.36219 | 0.181095 |
| ENSMUSG00000089377 | rRNA | 5S_rRNA | 0 | 0.36219 | 0.181095 |
| ENSMUSG00000086005 | lincRNA | Gm16745 | 0 | 0.326514 | 0.163257 |
| ENSMUSG00000065143 | snRNA | U6 | 0 | 0.36219 | 0.181095 |
| ENSMUSG00000081661 | pseudogene | Gm14450 | 0 | 0.36219 | 0.181095 |
| ENSMUSG00000083402 | pseudogene | Gm14394 | 0 | 0.326514 | 0.163257 |
| ENSMUSG00000031143 | protein_coding | Ccdc22 | 0 | 0.335524 | 0.167762 |
| ENSMUSG00000088211 | snRNA | U6 | 0 | 0.36219 | 0.181095 |
| ENSMUSG00000086518 | pseudogene | Gm14503 | 0 | 0.326514 | 0.163257 |
| ENSMUSG00000081898 | pseudogene | Gm5754 | 0 | 0.335524 | 0.167762 |
| ENSMUSG00000081461 | pseudogene | Rpl30-ps11 | 0 | 0.335524 | 0.167762 |
| ENSMUSG00000083551 | pseudogene | Gm14516 | 0 | 0.36219 | 0.181095 |
| ENSMUSG00000023257 | protein_coding | Cypt1 | 0 | 0.335524 | 0.167762 |
| ENSMUSG00000081641 | pseudogene | Gm9439 | 0 | 0.36219 | 0.181095 |
| ENSMUSG00000081475 | pseudogene | Gm14558 | 0 | 0.326514 | 0.163257 |
| ENSMUSG00000088701 | rRNA | 5S_rRNA | 0 | 0.335524 | 0.167762 |
| ENSMUSG00000082175 | pseudogene | Gm14563 | 0 | 0.335524 | 0.167762 |
| ENSMUSG00000092421 | protein_coding | Gm20464 | 0 | 0.335524 | 0.167762 |
| ENSMUSG00000065739 | snoRNA | SNORA67 | 0 | 0.326514 | 0.163257 |
| ENSMUSG00000084039 | pseudogene | Gm14551 | 0 | 0.326514 | 0.163257 |
| ENSMUSG00000086223 | processed_transcrip | Gm362 | 0 | 0.36219 | 0.181095 |
| ENSMUSG00000083529 | pseudogene | Gm15035 | 0 | 0.326514 | 0.163257 |
| ENSMUSG00000087369 | processed_transcrip | Gm14696 | 0 | 0.36219 | 0.181095 |
| ENSMUSG00000093018 | miRNA | AL672099.1 | 0 | 0.335524 | 0.167762 |
| ENSMUSG00000065613 | miRNA | Mir92-2 | 0 | 0.36219 | 0.181095 |
| ENSMUSG00000081509 | pseudogene | Gm14598 | 0 | 0.335524 | 0.167762 |
| ENSMUSG00000081795 | pseudogene | Gm14590 | 0 | 0.326514 | 0.163257 |
| ENSMUSG00000083051 | pseudogene | Gm14625 | 0 | 0.36219 | 0.181095 |
| ENSMUSG00000081482 | pseudogene | Gm16404 | 0 | 0.36219 | 0.181095 |
| ENSMUSG00000083249 | pseudogene | Gm14623 | 0 | 0.36219 | 0.181095 |
| ENSMUSG00000081373 | pseudogene | Gm16436 | 0 | 0.335524 | 0.167762 |

| | | | | | |
|---|---|---|---|---|---|
| ENSMUSG00000070104 | miRNA | Mir471 | 0 | 0.36219 | 0.181095 |
| ENSMUSG00000070078 | miRNA | Mir463 | 0 | 0.335524 | 0.167762 |
| ENSMUSG00000082568 | pseudogene | Gm14683 | 0 | 0.36219 | 0.181095 |
| ENSMUSG00000084409 | pseudogene | Gm14700 | 0 | 0.36219 | 0.181095 |
| ENSMUSG00000082538 | pseudogene | Gm14704 | 0 | 0.335524 | 0.167762 |
| ENSMUSG00000084193 | pseudogene | Gm6823 | 0 | 0.335524 | 0.167762 |
| ENSMUSG00000077275 | snoRNA | SNORA17 | 0 | 0.36219 | 0.181095 |
| ENSMUSG00000080938 | pseudogene | Gm6062 | 0 | 0.36219 | 0.181095 |
| ENSMUSG00000085284 | processed_transcrip | Gm16189 | 0 | 0.326514 | 0.163257 |
| ENSMUSG00000070138 | miRNA | Mir452 | 0 | 0.335524 | 0.167762 |
| ENSMUSG00000088823 | snoRNA | SNORA70 | 0 | 0.36219 | 0.181095 |
| ENSMUSG00000080777 | pseudogene | Gm15365 | 0 | 0.335524 | 0.167762 |
| ENSMUSG00000085877 | pseudogene | Gm16388 | 0 | 0.36219 | 0.181095 |
| ENSMUSG00000086937 | processed_transcrip | Gm15063 | 0 | 0.36219 | 0.181095 |
| ENSMUSG00000082755 | pseudogene | Gm8692 | 0 | 0.326514 | 0.163257 |
| ENSMUSG00000086104 | processed_transcrip | Gm14707 | 0 | 0.326514 | 0.163257 |
| ENSMUSG00000044121 | protein_coding | 5430402E10Rik | 0 | 0.335524 | 0.167762 |
| ENSMUSG00000080890 | pseudogene | Gm16458 | 0 | 0.36219 | 0.181095 |
| ENSMUSG00000081212 | pseudogene | Gm6964 | 0 | 0.36219 | 0.181095 |
| ENSMUSG00000065758 | rRNA | n-R5s10 | 0 | 0.36219 | 0.181095 |
| ENSMUSG00000083421 | pseudogene | Gm14776 | 0 | 0.335524 | 0.167762 |
| ENSMUSG00000088663 | snRNA | U6 | 0 | 0.326514 | 0.163257 |
| ENSMUSG00000065266 | snoRNA | SNORA16 | 0 | 0.335524 | 0.167762 |
| ENSMUSG00000080298 | pseudogene | Gm14807 | 0 | 0.326514 | 0.163257 |
| ENSMUSG00000015665 | protein_coding | Awat1 | 0 | 0.36219 | 0.181095 |
| ENSMUSG00000077303 | snRNA | U11 | 0 | 0.326514 | 0.163257 |
| ENSMUSG00000082541 | pseudogene | Gm9095 | 0 | 0.335524 | 0.167762 |
| ENSMUSG00000081969 | pseudogene | Gm16471 | 0 | 0.326514 | 0.163257 |
| ENSMUSG00000083775 | pseudogene | Gm14837 | 0 | 0.36219 | 0.181095 |
| ENSMUSG00000081779 | pseudogene | Gm5126 | 0 | 0.326514 | 0.163257 |
| ENSMUSG00000082812 | pseudogene | Gm14860 | 0 | 0.335524 | 0.167762 |
| ENSMUSG00000083605 | pseudogene | Gm14893 | 0 | 0.335524 | 0.167762 |
| ENSMUSG00000081978 | pseudogene | Gm14895 | 0 | 0.36219 | 0.181095 |
| ENSMUSG00000081160 | pseudogene | Gm14897 | 0 | 0.326514 | 0.163257 |
| ENSMUSG00000083537 | pseudogene | Gm14942 | 0 | 0.335524 | 0.167762 |
| ENSMUSG00000080419 | miRNA | AL807401.1 | 0 | 0.36219 | 0.181095 |
| ENSMUSG00000082117 | pseudogene | Gm4994 | 0 | 0.335524 | 0.167762 |
| ENSMUSG00000081972 | pseudogene | Gm16397 | 0 | 0.335524 | 0.167762 |
| ENSMUSG00000081709 | pseudogene | Gm15022 | 0 | 0.335524 | 0.167762 |
| ENSMUSG00000084216 | pseudogene | Gm7973 | 0 | 0.326514 | 0.163257 |
| ENSMUSG00000083493 | pseudogene | Gm15044 | 0 | 0.36219 | 0.181095 |
| ENSMUSG00000082955 | pseudogene | Gm8036 | 0 | 0.326514 | 0.163257 |
| ENSMUSG00000083180 | pseudogene | Gm15007 | 0 | 0.36219 | 0.181095 |
| ENSMUSG00000081869 | pseudogene | Gm15002 | 0 | 0.36219 | 0.181095 |
| ENSMUSG00000084645 | misc_RNA | 7SK | 0 | 0.36219 | 0.181095 |
| ENSMUSG00000081408 | pseudogene | Gm15047 | 0 | 0.326514 | 0.163257 |
| ENSMUSG00000083710 | pseudogene | Gm15078 | 0 | 0.335524 | 0.167762 |
| ENSMUSG00000071680 | pseudogene | Gm7123 | 0 | 0.335524 | 0.167762 |
| ENSMUSG00000082060 | pseudogene | Gm15048 | 0 | 0.335524 | 0.167762 |
| ENSMUSG00000089282 | misc_RNA | 7SK | 0 | 0.36219 | 0.181095 |
| ENSMUSG00000082423 | pseudogene | Gm15148 | 0 | 0.36219 | 0.181095 |
| ENSMUSG00000064665 | snoRNA | SNORA4 | 0 | 0.335524 | 0.167762 |
| ENSMUSG00000081306 | pseudogene | Gm15266 | 0 | 0.326514 | 0.163257 |
| ENSMUSG00000091863 | protein_coding | Gm7157 | 0 | 0.326514 | 0.163257 |
| ENSMUSG00000080743 | pseudogene | Gm7150 | 0 | 0.36219 | 0.181095 |
| ENSMUSG00000080940 | pseudogene | Gm15145 | 0 | 0.326514 | 0.163257 |
| ENSMUSG00000088232 | snoRNA | SNORA17 | 0 | 0.335524 | 0.167762 |
| ENSMUSG00000082560 | pseudogene | Gm15157 | 0 | 0.326514 | 0.163257 |

| | | | | | |
|---|---|---|---|---|---|
| ENSMUSG00000083636 | pseudogene | Gm15175 | 0 | 0.335524 | 0.167762 |
| ENSMUSG00000082026 | pseudogene | Gm5764 | 0 | 0.335524 | 0.167762 |
| ENSMUSG00000083437 | pseudogene | Gm15187 | 0 | 0.36219 | 0.181095 |
| ENSMUSG00000092898 | rRNA | AL672284.1 | 0 | 0.36219 | 0.181095 |
| ENSMUSG00000082571 | pseudogene | Gm8737 | 0 | 0.36219 | 0.181095 |
| ENSMUSG00000082539 | pseudogene | Gm15207 | 0 | 0.326514 | 0.163257 |
| ENSMUSG00000084689 | rRNA | n-R5s18 | 0 | 0.36219 | 0.181095 |
| ENSMUSG00000084920 | processed_transcrip | Gm15230 | 0 | 0.335524 | 0.167762 |
| ENSMUSG00000084014 | pseudogene | Gm7209 | 0 | 0.36219 | 0.181095 |
| ENSMUSG00000087201 | processed_transcrip | Gm15261 | 0 | 0.326514 | 0.163257 |
| ENSMUSG00000090600 | protein_coding | Gm3395 | 0 | 0.326514 | 0.163257 |
| ENSMUSG00000064371 | Mt_tRNA | mt-Tt | 0 | 0.36219 | 0.181095 |
| ENSMUSG00000003299 | protein_coding | Mrpl4 | 1220.694 | 1197.597 | 1209.145 |
| ENSMUSG00000085400 | processed_transcrip | Gm11610 | 0.646531 | 0.326514 | 0.486523 |
| ENSMUSG00000084177 | pseudogene | Gm13508 | 0.646531 | 0.326514 | 0.486523 |
| ENSMUSG00000058360 | pseudogene | Gm10040 | 0.646531 | 0.326514 | 0.486523 |
| ENSMUSG00000033027 | protein_coding | Klrc3 | 0.646531 | 0.326514 | 0.486523 |
| ENSMUSG00000091953 | lincRNA | Gm17710 | 0.646531 | 0.326514 | 0.486523 |
| ENSMUSG00000085126 | processed_transcrip | Gm12589 | 0.646531 | 0.326514 | 0.486523 |
| ENSMUSG00000092066 | processed_transcrip | 1700121N20Rik | 0.646531 | 0.326514 | 0.486523 |
| ENSMUSG00000083409 | pseudogene | Gm11340 | 0.646531 | 0.326514 | 0.486523 |
| ENSMUSG00000072613 | protein_coding | 1700049E17Rik2 | 0.646531 | 0.326514 | 0.486523 |
| ENSMUSG00000037438 | pseudogene | Gm9763 | 0.646531 | 0.326514 | 0.486523 |
| ENSMUSG00000059043 | protein_coding | Olfr15 | 0.646531 | 0.326514 | 0.486523 |
| ENSMUSG00000092383 | pseudogene | Gm9566 | 0.646531 | 0.326514 | 0.486523 |
| ENSMUSG00000065816 | snRNA | U6 | 0.646531 | 0.326514 | 0.486523 |
| ENSMUSG00000024868 | protein_coding | Dkk1 | 0.646531 | 0.326514 | 0.486523 |
| ENSMUSG00000092838 | miRNA | AL671885.1 | 0.646531 | 0.326514 | 0.486523 |
| ENSMUSG00000080718 | pseudogene | Gm8019 | 0.646531 | 0.326514 | 0.486523 |
| ENSMUSG00000088625 | snRNA | U6 | 8.708305 | 9.898102 | 9.303204 |
| ENSMUSG00000047721 | protein_coding | Bola2 | 514.1218 | 502.2626 | 508.1922 |
| ENSMUSG00000041741 | protein_coding | Pde3a | 93.46957 | 98.01909 | 95.74433 |
| ENSMUSG00000043088 | protein_coding | Il17re | 175.1316 | 183.6112 | 179.3714 |
| ENSMUSG00000083649 | protein_coding | Rasl2-9-ps | 18.97438 | 20.70403 | 19.83921 |
| ENSMUSG00000081440 | pseudogene | Gm12464 | 0.960442 | 1.359751 | 1.160096 |
| ENSMUSG00000030127 | protein_coding | Cops7a | 2747.445 | 2799.292 | 2773.368 |
| ENSMUSG00000024953 | protein_coding | Prdx5 | 2312.111 | 2267.034 | 2289.572 |
| ENSMUSG00000047415 | protein_coding | Gpr68 | 18.39137 | 16.7592 | 17.57528 |
| ENSMUSG00000092904 | miRNA | AC124426.2 | 0.962752 | 1.422092 | 1.192422 |
| ENSMUSG00000091818 | pseudogene | Rpl19-ps7 | 0.962752 | 1.422092 | 1.192422 |
| ENSMUSG00000084131 | pseudogene | Rpl3-ps2 | 50.79135 | 47.8713 | 49.33133 |
| ENSMUSG00000022364 | protein_coding | Wdr67 | 747.2824 | 728.8325 | 738.0574 |
| ENSMUSG00000091852 | lincRNA | 4930534D22Rik | 0.65242 | 0.988552 | 0.820486 |
| ENSMUSG00000087659 | processed_transcrip | Gm12606 | 0.65242 | 0.988552 | 0.820486 |
| ENSMUSG00000092593 | processed_transcrip | RP24-341K21.5 | 8.073552 | 9.227417 | 8.650485 |
| ENSMUSG00000062116 | protein_coding | Zfp954 | 174.8937 | 168.2955 | 171.5946 |
| ENSMUSG00000047638 | protein_coding | Nr1h4 | 15.50773 | 14.066 | 14.78687 |
| ENSMUSG00000086961 | processed_transcrip | Gm12946 | 0.954552 | 1.350742 | 1.152647 |
| ENSMUSG00000010392 | protein_coding | Gosr1 | 1679.265 | 1712.231 | 1695.748 |
| ENSMUSG00000039238 | protein_coding | Zfp750 | 19.42687 | 17.5773 | 18.50209 |
| ENSMUSG00000028958 | protein_coding | Tmub1 | 294.1169 | 286.4669 | 290.2919 |
| ENSMUSG00000019857 | protein_coding | Asf1a | 1108.224 | 1130.278 | 1119.251 |
| ENSMUSG00000023951 | protein_coding | Vegfa | 858.8117 | 885.1002 | 871.9559 |
| ENSMUSG00000056656 | protein_coding | Apol8 | 0.962752 | 1.359751 | 1.161252 |
| ENSMUSG00000045053 | protein_coding | Kcng3 | 227.433 | 236.3038 | 231.8684 |
| ENSMUSG00000051455 | protein_coding | Gm1564 | 4266.476 | 3976.892 | 4121.684 |
| ENSMUSG00000083820 | pseudogene | Gm6415 | 22.25184 | 20.35158 | 21.30171 |
| ENSMUSG00000019767 | protein_coding | Gm221 | 351.0942 | 364.0978 | 357.596 |

| | | | | | |
|---|---|---|---|---|---|
| ENSMUSG00000088947 | rRNA | 5S_rRNA | 4.859607 | 4.052225 | 4.455916 |
| ENSMUSG00000035133 | protein_coding | Arhgap5 | 5635.806 | 5528.466 | 5582.136 |
| ENSMUSG00000026141 | protein_coding | Col19a1 | 4.569253 | 3.743729 | 4.156491 |
| ENSMUSG00000089742 | processed_transcrip | Gm15898 | 2.597903 | 2.039445 | 2.318674 |
| ENSMUSG00000037375 | protein_coding | Hhat | 114.2995 | 118.9718 | 116.6356 |
| ENSMUSG00000049892 | protein_coding | Rasd1 | 1133.597 | 1049.756 | 1091.676 |
| ENSMUSG00000079317 | protein_coding | Trappc2 | 621.785 | 636.384 | 629.0845 |
| ENSMUSG00000003354 | protein_coding | Ccdc65 | 31.05392 | 28.84452 | 29.94922 |
| ENSMUSG00000031347 | protein_coding | Cetn2 | 948.5016 | 926.9676 | 937.7346 |
| ENSMUSG00000080115 | protein_coding | Fam119b | 10.97001 | 12.31739 | 11.6437 |
| ENSMUSG00000081236 | pseudogene | Gm13574 | 0.638331 | 0.36219 | 0.50026 |
| ENSMUSG00000043385 | protein_coding | Olfr267 | 0.638331 | 0.36219 | 0.50026 |
| ENSMUSG00000065240 | snRNA | U6 | 0.638331 | 0.36219 | 0.50026 |
| ENSMUSG00000083461 | pseudogene | Gm12935 | 0.638331 | 0.36219 | 0.50026 |
| ENSMUSG00000058661 | pseudogene | Gm7452 | 0.638331 | 0.36219 | 0.50026 |
| ENSMUSG00000088722 | snRNA | U1 | 0.638331 | 0.36219 | 0.50026 |
| ENSMUSG00000056959 | protein_coding | Olfr315 | 0.638331 | 0.36219 | 0.50026 |
| ENSMUSG00000090607 | pseudogene | Gm17052 | 0.638331 | 0.36219 | 0.50026 |
| ENSMUSG00000036760 | protein_coding | Kcnk9 | 0.638331 | 0.36219 | 0.50026 |
| ENSMUSG00000077152 | snoRNA | SNORA17 | 0.638331 | 0.36219 | 0.50026 |
| ENSMUSG00000089755 | processed_transcrip | 0610012D04Rik | 0.638331 | 0.36219 | 0.50026 |
| ENSMUSG00000045506 | protein_coding | A930002H24Rik | 39.16281 | 41.77311 | 40.46796 |
| ENSMUSG00000025856 | protein_coding | Pdgfa | 443.465 | 427.4862 | 435.4756 |
| ENSMUSG00000087586 | processed_transcrip | Gm6938 | 4.523408 | 3.770395 | 4.146901 |
| ENSMUSG00000084101 | pseudogene | Gm17014 | 0.33031 | 0.653029 | 0.491669 |
| ENSMUSG00000081013 | pseudogene | Gm14497 | 0.33031 | 0.653029 | 0.491669 |
| ENSMUSG00000085643 | processed_transcrip | Gm12519 | 0.33031 | 0.653029 | 0.491669 |
| ENSMUSG00000029371 | protein_coding | Cxcl5 | 0.33031 | 0.653029 | 0.491669 |
| ENSMUSG00000054272 | protein_coding | Zscan4c | 0.33031 | 0.653029 | 0.491669 |
| ENSMUSG00000085443 | processed_transcrip | Gm15416 | 0.33031 | 0.653029 | 0.491669 |
| ENSMUSG00000092125 | pseudogene | AA543401 | 0.33031 | 0.653029 | 0.491669 |
| ENSMUSG00000035349 | protein_coding | Mia2 | 0.33031 | 0.653029 | 0.491669 |
| ENSMUSG00000089814 | pseudogene | Gm15310 | 0.33031 | 0.653029 | 0.491669 |
| ENSMUSG00000093346 | miRNA | AC109138.1 | 0.33031 | 0.653029 | 0.491669 |
| ENSMUSG00000088584 | snRNA | U7 | 0.33031 | 0.653029 | 0.491669 |
| ENSMUSG00000089076 | snoRNA | U3 | 0.33031 | 0.653029 | 0.491669 |
| ENSMUSG00000047798 | protein_coding | Cd300lf | 7.438799 | 6.471878 | 6.955339 |
| ENSMUSG00000088254 | snoRNA | SCARNA14 | 4.184898 | 3.372892 | 3.778895 |
| ENSMUSG00000029462 | protein_coding | Vps29 | 1781.757 | 1749.315 | 1765.536 |
| ENSMUSG00000030887 | protein_coding | Pdzd9 | 13.27167 | 14.75507 | 14.01337 |
| ENSMUSG00000051038 | protein_coding | Rhox11 | 12.04085 | 10.5093 | 11.27507 |
| ENSMUSG00000027405 | protein_coding | Nop56 | 3317.708 | 3258.06 | 3287.884 |
| ENSMUSG00000010755 | protein_coding | Cars | 935.8971 | 955.8218 | 945.8595 |
| ENSMUSG00000084383 | pseudogene | Gm13370 | 53.92815 | 58.10498 | 56.01656 |
| ENSMUSG00000033159 | protein_coding | 1810031K17Rik | 943.4387 | 923.5801 | 933.5094 |
| ENSMUSG00000087820 | miRNA | Mir1928 | 0.32211 | 0.653029 | 0.487569 |
| ENSMUSG00000092955 | misc_RNA | AL732525.1 | 0.32211 | 0.653029 | 0.487569 |
| ENSMUSG00000039146 | protein_coding | H28 | 0.32211 | 0.653029 | 0.487569 |
| ENSMUSG00000092189 | pseudogene | Gm3644 | 0.32211 | 0.653029 | 0.487569 |
| ENSMUSG00000056509 | protein_coding | Gm9999 | 0.32211 | 0.653029 | 0.487569 |
| ENSMUSG00000070460 | protein_coding | Olfr291 | 0.32211 | 0.653029 | 0.487569 |
| ENSMUSG00000076755 | IG_V_gene | Gm16602 | 0.32211 | 0.653029 | 0.487569 |
| ENSMUSG00000083420 | pseudogene | Gm15665 | 0.32211 | 0.653029 | 0.487569 |
| ENSMUSG00000073554 | pseudogene | Gm6960 | 0.32211 | 0.653029 | 0.487569 |
| ENSMUSG00000069862 | pseudogene | Gm10279 | 23.01213 | 25.1213 | 24.06671 |
| ENSMUSG00000073058 | protein_coding | Gm10463 | 11.33208 | 12.68823 | 12.01015 |
| ENSMUSG00000058351 | protein_coding | 2010107H07Rik | 125.5595 | 120.7662 | 123.1628 |
| ENSMUSG00000031576 | protein_coding | Kcnu1 | 43.56889 | 46.48665 | 45.02777 |

| | | | | | |
|---|---|---|---|---|---|
| ENSMUSG00000032586 | protein_coding | Traip | 719.5087 | 734.4775 | 726.9931 |
| ENSMUSG00000029068 | protein_coding | Ccnl2 | 3614.211 | 3707.399 | 3660.805 |
| ENSMUSG00000040433 | protein_coding | Zbtb38 | 1311.478 | 1287.302 | 1299.39 |
| ENSMUSG00000030674 | protein_coding | Qprt | 27.10556 | 25.09251 | 26.09903 |
| ENSMUSG00000056708 | protein_coding | Ier5 | 469.4866 | 456.1938 | 462.8402 |
| ENSMUSG00000091432 | lincRNA | Gm17338 | 6.174958 | 5.210144 | 5.692551 |
| ENSMUSG00000020154 | protein_coding | Ptprb | 316.0979 | 326.8052 | 321.4516 |
| ENSMUSG00000083979 | pseudogene | Gm12527 | 0.66062 | 1.050893 | 0.855757 |
| ENSMUSG00000084545 | rRNA | n-R5s80 | 0.66062 | 1.050893 | 0.855757 |
| ENSMUSG00000075708 | snRNA | U6 | 0.66062 | 1.050893 | 0.855757 |
| ENSMUSG00000022209 | protein_coding | Dhrs2 | 0.66062 | 1.050893 | 0.855757 |
| ENSMUSG00000081872 | pseudogene | Gm15572 | 0.316221 | 0.653029 | 0.484625 |
| ENSMUSG00000071459 | protein_coding | Vmn2r31 | 0.316221 | 0.653029 | 0.484625 |
| ENSMUSG00000065987 | protein_coding | Cd209b | 0.316221 | 0.653029 | 0.484625 |
| ENSMUSG00000069518 | protein_coding | Gm10271 | 0.316221 | 0.653029 | 0.484625 |
| ENSMUSG00000050762 | protein_coding | Prss27 | 0.316221 | 0.653029 | 0.484625 |
| ENSMUSG00000082497 | pseudogene | Gm40 | 0.316221 | 0.653029 | 0.484625 |
| ENSMUSG00000002664 | protein_coding | Pspn | 5.79776 | 6.718033 | 6.257896 |
| ENSMUSG00000051615 | protein_coding | Rap2a | 1534.179 | 1495.421 | 1514.8 |
| ENSMUSG00000050222 | protein_coding | Il17d | 197.2244 | 191.4999 | 194.3621 |
| ENSMUSG00000052126 | protein_coding | Gm1604A | 0.632442 | 1.015218 | 0.82383 |
| ENSMUSG00000061080 | protein_coding | Lsamp | 81.18526 | 77.48017 | 79.33271 |
| ENSMUSG00000069056 | protein_coding | Slc38a9 | 157.1377 | 150.661 | 153.8994 |
| ENSMUSG00000070271 | pseudogene | Gm13268 | 2.583814 | 1.977104 | 2.280459 |
| ENSMUSG00000045877 | protein_coding | 4933415A04Rik | 42.02751 | 39.34268 | 40.6851 |
| ENSMUSG00000044061 | protein_coding | Olfr225 | 0.960442 | 1.350742 | 1.155592 |
| ENSMUSG00000070074 | miRNA | Mir484 | 0.960442 | 1.350742 | 1.155592 |
| ENSMUSG00000085337 | lincRNA | Gm15964 | 78.62589 | 73.80127 | 76.21358 |
| ENSMUSG00000077265 | snRNA | U6 | 8.412062 | 9.579874 | 8.995968 |
| ENSMUSG00000081572 | pseudogene | Gm14138 | 0.638331 | 0.335524 | 0.486927 |
| ENSMUSG00000082493 | pseudogene | Gm14214 | 0.638331 | 0.335524 | 0.486927 |
| ENSMUSG00000083740 | pseudogene | Gm11456 | 0.638331 | 0.335524 | 0.486927 |
| ENSMUSG00000084777 | lincRNA | Gm16742 | 0.638331 | 0.335524 | 0.486927 |
| ENSMUSG00000088418 | snoRNA | SNORA17 | 0.638331 | 0.335524 | 0.486927 |
| ENSMUSG00000091169 | lincRNA | Gm17493 | 0.638331 | 0.335524 | 0.486927 |
| ENSMUSG00000087984 | snoRNA | snoU13 | 0.638331 | 0.335524 | 0.486927 |
| ENSMUSG00000092394 | pseudogene | RP24-82B18.2 | 0.638331 | 0.335524 | 0.486927 |
| ENSMUSG00000054568 | protein_coding | Dub1 | 0.638331 | 0.335524 | 0.486927 |
| ENSMUSG00000066100 | pseudogene | Krtap5-1 | 0.638331 | 0.335524 | 0.486927 |
| ENSMUSG00000054360 | protein_coding | Bsx | 0.638331 | 0.335524 | 0.486927 |
| ENSMUSG00000090298 | protein_coding | Gm4794 | 0.638331 | 0.335524 | 0.486927 |
| ENSMUSG00000089313 | snoRNA | U8 | 0.638331 | 0.335524 | 0.486927 |
| ENSMUSG00000091947 | lincRNA | 6530401F13Rik | 0.638331 | 0.335524 | 0.486927 |
| ENSMUSG00000091079 | processed_transcrip | Gm17105 | 0.638331 | 0.335524 | 0.486927 |
| ENSMUSG00000091976 | protein_coding | Gm2104 | 0.638331 | 0.335524 | 0.486927 |
| ENSMUSG00000090337 | protein_coding | Gm8122 | 0.638331 | 0.335524 | 0.486927 |
| ENSMUSG00000093046 | miRNA | AC124180.1 | 0.638331 | 0.335524 | 0.486927 |
| ENSMUSG00000086174 | pseudogene | Gm6425 | 0.638331 | 0.335524 | 0.486927 |
| ENSMUSG00000081255 | pseudogene | Gm5380 | 0.638331 | 0.335524 | 0.486927 |
| ENSMUSG00000082543 | pseudogene | Gm14336 | 0.968641 | 1.36876 | 1.168701 |
| ENSMUSG00000064622 | snoRNA | SNORA61 | 0.968641 | 1.36876 | 1.168701 |
| ENSMUSG00000024317 | protein_coding | Rnf138 | 2864.883 | 2932.681 | 2898.782 |
| ENSMUSG00000059040 | protein_coding | Gm5506 | 587.5498 | 677.8849 | 632.7174 |
| ENSMUSG00000019054 | protein_coding | Fis1 | 1874.785 | 1841.452 | 1858.118 |
| ENSMUSG00000002625 | protein_coding | Akap8l | 1823.321 | 1769.001 | 1796.161 |
| ENSMUSG00000069106 | protein_coding | Gm8910 | 12.87218 | 14.23216 | 13.55217 |
| ENSMUSG00000040919 | protein_coding | 4930505A04Rik | 6.464269 | 7.407098 | 6.935683 |
| ENSMUSG00000091191 | protein_coding | Gm17334 | 4.221275 | 3.337217 | 3.779246 |

| | | | | | |
|---|---|---|---|---|---|
| ENSMUSG00000044221 | protein_coding | Grsf1 | 3534.649 | 3478.24 | 3506.444 |
| ENSMUSG00000029228 | protein_coding | Lnx1 | 238.7904 | 231.6951 | 235.2428 |
| ENSMUSG00000052180 | protein_coding | Serpinb6c | 2.255815 | 1.712931 | 1.984373 |
| ENSMUSG00000081416 | pseudogene | Gm14383 | 0.638331 | 0.326514 | 0.482423 |
| ENSMUSG00000090441 | protein_coding | Gm17651 | 0.638331 | 0.326514 | 0.482423 |
| ENSMUSG00000076361 | miRNA | Mir182 | 0.638331 | 0.326514 | 0.482423 |
| ENSMUSG00000079524 | processed_transcrip | Gm15402 | 0.638331 | 0.326514 | 0.482423 |
| ENSMUSG00000087560 | lincRNA | Gm15396 | 0.638331 | 0.326514 | 0.482423 |
| ENSMUSG00000030954 | protein_coding | Gp2 | 0.638331 | 0.326514 | 0.482423 |
| ENSMUSG00000085199 | processed_transcrip | Gm15346 | 0.638331 | 0.326514 | 0.482423 |
| ENSMUSG00000091381 | protein_coding | Vmn2r83 | 0.638331 | 0.326514 | 0.482423 |
| ENSMUSG00000091738 | pseudogene | Gm8723 | 0.638331 | 0.326514 | 0.482423 |
| ENSMUSG00000082116 | pseudogene | Gm12188 | 0.638331 | 0.326514 | 0.482423 |
| ENSMUSG00000088148 | miRNA | Mir1983 | 0.638331 | 0.326514 | 0.482423 |
| ENSMUSG00000090742 | pseudogene | Gm3008 | 0.638331 | 0.326514 | 0.482423 |
| ENSMUSG00000090237 | processed_transcrip | Gm16308 | 0.638331 | 0.326514 | 0.482423 |
| ENSMUSG00000071311 | protein_coding | Gpr31c | 0.638331 | 0.326514 | 0.482423 |
| ENSMUSG00000092202 | pseudogene | Stk19-ps1 | 0.638331 | 0.326514 | 0.482423 |
| ENSMUSG00000086570 | lincRNA | Gm16961 | 3.274923 | 4.114928 | 3.694925 |
| ENSMUSG00000080198 | pseudogene | Gm13573 | 1.284863 | 1.712931 | 1.498897 |
| ENSMUSG00000044690 | pseudogene | Gm4798 | 1.284863 | 1.712931 | 1.498897 |
| ENSMUSG00000051537 | processed_transcrip | Gm5124 | 3.858166 | 4.651921 | 4.255044 |
| ENSMUSG00000049038 | protein_coding | Mterfd3 | 198.4805 | 191.8975 | 195.189 |
| ENSMUSG00000041836 | protein_coding | Ptpre | 1028.65 | 1052.279 | 1040.465 |
| ENSMUSG00000044468 | protein_coding | Fam46c | 1383.897 | 1484.075 | 1433.986 |
| ENSMUSG00000040407 | protein_coding | Akap9 | 4868.238 | 4771.633 | 4819.936 |
| ENSMUSG00000029405 | protein_coding | G3bp2 | 4179.597 | 4248.584 | 4214.09 |
| ENSMUSG00000044894 | protein_coding | Uqcrq | 1440.763 | 1414.075 | 1427.419 |
| ENSMUSG00000064351 | protein_coding | mt-Co1 | 121083.8 | 122906 | 121994.9 |
| ENSMUSG00000020916 | protein_coding | Krt36 | 4.484719 | 3.716701 | 4.10071 |
| ENSMUSG00000043085 | protein_coding | Tmem82 | 20.03472 | 21.72862 | 20.88167 |
| ENSMUSG00000005465 | protein_coding | Il27ra | 31.62792 | 33.81716 | 32.72254 |
| ENSMUSG00000043015 | protein_coding | Tmem194b | 528.153 | 542.8701 | 535.5116 |
| ENSMUSG00000060012 | protein_coding | Kif13b | 1569.515 | 1618.027 | 1593.771 |
| ENSMUSG00000037640 | protein_coding | Zfp60 | 1207.208 | 1253.606 | 1230.407 |
| ENSMUSG00000039714 | protein_coding | Cplx3 | 3.930922 | 4.847954 | 4.389438 |
| ENSMUSG00000040563 | protein_coding | BC018242 | 135.0428 | 128.7175 | 131.8802 |
| ENSMUSG00000050212 | protein_coding | Fam176b | 644.5221 | 662.7302 | 653.6261 |
| ENSMUSG00000006411 | protein_coding | Pvrl4 | 19.09172 | 17.4652 | 18.27846 |
| ENSMUSG00000021131 | protein_coding | Erh | 2605.819 | 2654.912 | 2630.366 |
| ENSMUSG00000021255 | protein_coding | Esrrb | 47.63458 | 44.36684 | 46.00071 |
| ENSMUSG00000052549 | protein_coding | Arl13a | 2.620192 | 2.00377 | 2.311981 |
| ENSMUSG00000056089 | protein_coding | Gm5468 | 7.124889 | 6.056718 | 6.590804 |
| ENSMUSG00000030815 | protein_coding | Phkg2 | 808.3637 | 826.0437 | 817.2037 |
| ENSMUSG00000031952 | protein_coding | Chst5 | 13.3234 | 15.01815 | 14.17078 |
| ENSMUSG00000054162 | protein_coding | Spock3 | 6.744112 | 5.721195 | 6.232653 |
| ENSMUSG00000070394 | protein_coding | 1810027O10Rik | 468.9648 | 456.6842 | 462.8245 |
| ENSMUSG00000035459 | protein_coding | Stab2 | 185.0309 | 177.5026 | 181.2668 |
| ENSMUSG00000032411 | protein_coding | Tfdp2 | 3904.442 | 3968.913 | 3936.678 |
| ENSMUSG00000009090 | protein_coding | Ap1b1 | 2551.647 | 2494.493 | 2523.07 |
| ENSMUSG00000031684 | protein_coding | Slc10a7 | 930.8802 | 910.6303 | 920.7553 |
| ENSMUSG00000040430 | protein_coding | Pitpnc1 | 788.8468 | 806.6512 | 797.749 |
| ENSMUSG00000021972 | protein_coding | Hmbox1 | 1676.583 | 1711.141 | 1693.862 |
| ENSMUSG00000018126 | protein_coding | Baiap2l2 | 27.97685 | 25.76211 | 26.86948 |
| ENSMUSG00000069117 | protein_coding | Gm17352 | 1.939594 | 1.413083 | 1.676338 |
| ENSMUSG00000081302 | pseudogene | Gm12020 | 2.575614 | 1.986113 | 2.280864 |
| ENSMUSG00000034042 | protein_coding | Gpbp1l1 | 3488.441 | 3429.93 | 3459.185 |
| ENSMUSG00000028581 | protein_coding | Laptm5 | 260.0923 | 267.3284 | 263.7104 |

| | | | | | |
|---|---|---|---|---|---|
| ENSMUSG00000082315 | pseudogene | Gm16523 | 0.974531 | 1.359751 | 1.167141 |
| ENSMUSG00000048776 | protein_coding | Pthlh | 0.974531 | 1.359751 | 1.167141 |
| ENSMUSG00000075286 | protein_coding | Gm1968 | 0.974531 | 1.359751 | 1.167141 |
| ENSMUSG00000069305 | protein_coding | Hist1h4n | 11.35332 | 12.80427 | 12.07879 |
| ENSMUSG00000030265 | protein_coding | Kras | 2982.75 | 2919.307 | 2951.028 |
| ENSMUSG00000090084 | protein_coding | Srpx | 110.8521 | 116.07 | 113.4611 |
| ENSMUSG00000088170 | misc_RNA | 7SK | 13.53511 | 12.21938 | 12.87724 |
| ENSMUSG00000091879 | protein_coding | Vmn2r17 | 0.962752 | 1.395426 | 1.179089 |
| ENSMUSG00000076122 | miRNA | Mir503 | 0.976841 | 1.422092 | 1.199467 |
| ENSMUSG00000085742 | processed_transcrip | Gm13536 | 1.290752 | 1.784282 | 1.537517 |
| ENSMUSG00000030203 | protein_coding | Dusp16 | 1570.11 | 1540.73 | 1555.42 |
| ENSMUSG00000024382 | protein_coding | Ercc3 | 2238.325 | 2290.779 | 2264.552 |
| ENSMUSG00000034117 | protein_coding | Gpr44 | 4.901874 | 5.800831 | 5.351352 |
| ENSMUSG00000022528 | protein_coding | Hes1 | 502.5194 | 521.9208 | 512.2201 |
| ENSMUSG00000047509 | pseudogene | Gm6776 | 0.948663 | 1.386417 | 1.16754 |
| ENSMUSG00000060899 | protein_coding | Rpl21-ps11 | 1.298952 | 1.72194 | 1.510446 |
| ENSMUSG00000029817 | protein_coding | Tra2a | 2642.064 | 2696.378 | 2669.221 |
| ENSMUSG00000027498 | protein_coding | Cstf1 | 1550.034 | 1523.616 | 1536.825 |
| ENSMUSG00000029299 | protein_coding | Abcg3 | 19.94429 | 21.67493 | 20.80961 |
| ENSMUSG00000091208 | protein_coding | Gm3494 | 0.976841 | 1.359751 | 1.168296 |
| ENSMUSG00000021791 | protein_coding | Dydc2 | 25.14599 | 27.26637 | 26.20618 |
| ENSMUSG00000023968 | protein_coding | Crip3 | 82.37723 | 86.91843 | 84.64783 |
| ENSMUSG00000021910 | protein_coding | Nisch | 8567.469 | 8733.281 | 8650.375 |
| ENSMUSG00000000093 | protein_coding | Tbx2 | 509.0872 | 526.6647 | 517.8759 |
| ENSMUSG00000022003 | protein_coding | Slc25a30 | 963.874 | 987.842 | 975.858 |
| ENSMUSG00000031778 | protein_coding | Cx3cl1 | 408.7152 | 397.9239 | 403.3196 |
| ENSMUSG00000075467 | protein_coding | Dnlz | 602.2286 | 618.2766 | 610.2526 |
| ENSMUSG00000085861 | processed_transcrip | 1700016G14Rik | 1.284863 | 1.757616 | 1.521239 |
| ENSMUSG00000028578 | protein_coding | 5830433M19Rik | 513.9519 | 525.9011 | 519.9265 |
| ENSMUSG00000020227 | protein_coding | Irak3 | 105.7453 | 101.0251 | 103.3852 |
| ENSMUSG00000084880 | processed_transcrip | Gm14872 | 23.64353 | 25.56064 | 24.60208 |
| ENSMUSG00000042451 | protein_coding | Mybph | 10.30246 | 9.031022 | 9.666739 |
| ENSMUSG00000065551 | miRNA | Mir210 | 4.496497 | 3.752377 | 4.124437 |
| ENSMUSG00000085398 | lincRNA | 4931406G06Rik | 4.541075 | 3.779043 | 4.160059 |
| ENSMUSG00000020155 | protein_coding | Kcnmb1 | 21.02669 | 19.32984 | 20.17826 |
| ENSMUSG00000021916 | protein_coding | Glt8d1 | 1305.33 | 1273.686 | 1289.508 |
| ENSMUSG00000070298 | protein_coding | Trcg1 | 2.567414 | 2.021789 | 2.294601 |
| ENSMUSG00000038045 | protein_coding | Sult6b1 | 32.28243 | 34.47055 | 33.37649 |
| ENSMUSG00000084862 | lincRNA | Gm16278 | 9.706392 | 11.01962 | 10.36301 |
| ENSMUSG00000047030 | protein_coding | Spata2 | 1537.446 | 1570.336 | 1553.891 |
| ENSMUSG00000081798 | pseudogene | Gm8152 | 4.559785 | 3.761386 | 4.160586 |
| ENSMUSG00000080712 | pseudogene | Hist3h2bb-ps | 3.578323 | 4.334416 | 3.956369 |
| ENSMUSG00000024619 | protein_coding | Cdx1 | 7.078001 | 6.145364 | 6.611682 |
| ENSMUSG00000092465 | pseudogene | RP23-138L1.1 | 4.879585 | 4.078891 | 4.479238 |
| ENSMUSG00000020284 | protein_coding | 1810043G02Rik | 285.4122 | 276.4773 | 280.9448 |
| ENSMUSG00000072898 | pseudogene | Gm10434 | 4.476519 | 5.349634 | 4.913077 |
| ENSMUSG00000083477 | pseudogene | Gm5555 | 56.2304 | 59.19434 | 57.71237 |
| ENSMUSG00000031517 | protein_coding | Gpm6a | 29.35213 | 31.62093 | 30.48653 |
| ENSMUSG00000087059 | processed_transcrip | Gm12339 | 8.400284 | 9.517894 | 8.959089 |
| ENSMUSG00000052631 | protein_coding | Sh2d6 | 8.111198 | 9.316424 | 8.713811 |
| ENSMUSG00000041638 | protein_coding | Gcn1l1 | 4618.437 | 4708.554 | 4663.495 |
| ENSMUSG00000041841 | protein_coding | Rpl37 | 2179.275 | 2135.687 | 2157.481 |
| ENSMUSG00000028973 | protein_coding | Abcb8 | 1029.816 | 1009.612 | 1019.714 |
| ENSMUSG00000036503 | protein_coding | Rnf13 | 1891.608 | 1856.66 | 1874.134 |
| ENSMUSG00000064960 | snoRNA | SNORA2 | 2.245304 | 1.704284 | 1.974794 |
| ENSMUSG00000042377 | protein_coding | Fam83g | 476.7823 | 457.745 | 467.2636 |
| ENSMUSG00000049741 | protein_coding | Rxfp4 | 0.98273 | 1.422092 | 1.202411 |
| ENSMUSG00000031916 | protein_coding | Cog8 | 880.1815 | 864.1943 | 872.1879 |

| | | | | | |
|---|---|---|---|---|---|
| ENSMUSG00000022103 | protein_coding | Gfra2 | 56.57644 | 59.94906 | 58.26275 |
| ENSMUSG00000021772 | protein_coding | Nkiras1 | 656.7912 | 643.6361 | 650.2137 |
| ENSMUSG00000071561 | protein_coding | BC100530 | 2.261704 | 1.739597 | 2.00065 |
| ENSMUSG00000078062 | miRNA | AC167132.1 | 0.64422 | 1.015218 | 0.829719 |
| ENSMUSG00000091654 | lincRNA | Gm17406 | 0.64422 | 1.015218 | 0.829719 |
| ENSMUSG00000084597 | misc_RNA | 7SK | 0.64422 | 1.015218 | 0.829719 |
| ENSMUSG00000090769 | lincRNA | Gm2824 | 6.739491 | 5.729481 | 6.234486 |
| ENSMUSG00000077202 | snoRNA | SNORA61 | 4.840896 | 4.052586 | 4.446741 |
| ENSMUSG00000066361 | protein_coding | Serpina3c | 125.4526 | 118.0327 | 121.7427 |
| ENSMUSG00000090243 | pseudogene | Gm16103 | 1.290752 | 1.775272 | 1.533012 |
| ENSMUSG00000005804 | protein_coding | Pldn | 972.7525 | 990.5009 | 981.6267 |
| ENSMUSG00000031370 | protein_coding | Zrsr2 | 2562.338 | 2610.673 | 2586.506 |
| ENSMUSG00000091614 | protein_coding | Gm17657 | 7.505666 | 6.436203 | 6.970934 |
| ENSMUSG00000081446 | pseudogene | Gm14068 | 0.976841 | 1.413083 | 1.194962 |
| ENSMUSG00000078930 | lincRNA | Gm17594 | 0.976841 | 1.413083 | 1.194962 |
| ENSMUSG00000050114 | protein_coding | Prdx6b | 0.98273 | 1.359751 | 1.171241 |
| ENSMUSG00000024653 | protein_coding | Scgb1a1 | 0.98273 | 1.359751 | 1.171241 |
| ENSMUSG00000085913 | processed_transcrip | Gm15601 | 223.2226 | 212.0593 | 217.641 |
| ENSMUSG00000068463 | processed_transcrip | B630019A10Rik | 13.54331 | 12.1574 | 12.85035 |
| ENSMUSG00000085098 | lincRNA | Gm4221 | 11.01712 | 12.28208 | 11.6496 |
| ENSMUSG00000030606 | protein_coding | Hapln3 | 37.01419 | 34.76175 | 35.88797 |
| ENSMUSG00000030617 | protein_coding | Ccdc83 | 1.298952 | 1.712931 | 1.505941 |
| ENSMUSG00000075833 | snRNA | U6 | 1.298952 | 1.712931 | 1.505941 |
| ENSMUSG00000028318 | protein_coding | Polr1e | 550.7977 | 562.4531 | 556.6254 |
| ENSMUSG00000040606 | protein_coding | Kazn | 1244.231 | 1269.215 | 1256.723 |
| ENSMUSG00000075066 | protein_coding | Olfr32 | 1.290752 | 1.757616 | 1.524184 |
| ENSMUSG00000005360 | protein_coding | Slc1a3 | 271.3155 | 280.3316 | 275.8236 |
| ENSMUSG00000039616 | protein_coding | Mocos | 26.08287 | 28.04299 | 27.06293 |
| ENSMUSG00000037982 | lincRNA | Gm9725 | 0.66062 | 1.033237 | 0.846928 |
| ENSMUSG00000019478 | protein_coding | Rab4a | 560.8288 | 547.7827 | 554.3058 |
| ENSMUSG00000026111 | protein_coding | Unc50 | 1268.629 | 1291.817 | 1280.223 |
| ENSMUSG00000037363 | protein_coding | Letm2 | 238.181 | 229.0567 | 233.6188 |
| ENSMUSG00000074628 | protein_coding | Gm1332 | 0.976841 | 1.350742 | 1.163791 |
| ENSMUSG00000090105 | processed_transcrip | Gm15890 | 0.976841 | 1.350742 | 1.163791 |
| ENSMUSG00000092033 | lincRNA | A730056A06Rik | 38.91048 | 36.50171 | 37.70609 |
| ENSMUSG00000033147 | protein_coding | Slc22a15 | 66.87495 | 70.48683 | 68.68089 |
| ENSMUSG00000021178 | protein_coding | Psmc1 | 4498.737 | 4428.396 | 4463.567 |
| ENSMUSG00000034839 | protein_coding | Larp6 | 51.33591 | 54.20985 | 52.77288 |
| ENSMUSG00000020135 | protein_coding | Apc2 | 114.9221 | 109.9373 | 112.4297 |
| ENSMUSG00000085295 | processed_transcrip | 4930430E12Rik | 14.84942 | 13.43928 | 14.14435 |
| ENSMUSG00000046562 | protein_coding | Unc119b | 2368.627 | 2330.956 | 2349.791 |
| ENSMUSG00000050605 | protein_coding | Zfp61 | 635.2855 | 655.9684 | 645.6269 |
| ENSMUSG00000007944 | protein_coding | Ttc9b | 3.930922 | 3.126014 | 3.528468 |
| ENSMUSG00000032301 | protein_coding | Psma4 | 4120.693 | 4042.269 | 4081.481 |
| ENSMUSG00000066956 | pseudogene | Gm10191 | 2.581503 | 2.057464 | 2.319484 |
| ENSMUSG00000086809 | lincRNA | 2610037D02Rik | 50.78293 | 53.5067 | 52.14481 |
| ENSMUSG00000032199 | protein_coding | Grinl1a | 3553.625 | 3624.991 | 3589.308 |
| ENSMUSG00000035772 | protein_coding | Mrps2 | 1226.128 | 1250.352 | 1238.24 |
| ENSMUSG00000064358 | protein_coding | mt-Co3 | 1745.951 | 1889.179 | 1817.565 |
| ENSMUSG00000091477 | protein_coding | Gm5799 | 11.98807 | 10.70212 | 11.3451 |
| ENSMUSG00000081952 | pseudogene | Gm12178 | 9.689993 | 10.98395 | 10.33697 |
| ENSMUSG00000040899 | protein_coding | Ccr6 | 0.974531 | 1.395426 | 1.184978 |
| ENSMUSG00000029123 | protein_coding | Stk32b | 4.15672 | 3.461538 | 3.809129 |
| ENSMUSG00000077563 | snoRNA | Snora68 | 9.72637 | 8.600331 | 9.163351 |
| ENSMUSG00000086139 | processed_transcrip | Gm11211 | 4.535186 | 3.823727 | 4.179456 |
| ENSMUSG00000046005 | processed_transcrip | D830044D21Rik | 2.242993 | 1.65059 | 1.946792 |
| ENSMUSG00000022035 | protein_coding | Ccdc25 | 2186.887 | 2145.395 | 2166.141 |
| ENSMUSG00000020057 | protein_coding | Dram1 | 133.9225 | 139.2286 | 136.5756 |

| | | | | | |
|---|---|---|---|---|---|
| ENSMUSG00000085611 | pseudogene | Ap3s1-ps1 | 98.00223 | 94.25273 | 96.12748 |
| ENSMUSG00000048832 | protein_coding | Vps37c | 767.1334 | 751.2645 | 759.199 |
| ENSMUSG00000092522 | processed_transcrip | Gm20389 | 0.960442 | 1.377408 | 1.168925 |
| ENSMUSG00000056219 | protein_coding | Gm5423 | 0.960442 | 1.377408 | 1.168925 |
| ENSMUSG00000026182 | protein_coding | Tnp1 | 7.751442 | 8.766487 | 8.258965 |
| ENSMUSG00000004231 | protein_coding | Pax2 | 122.1528 | 115.1811 | 118.6669 |
| ENSMUSG00000026279 | protein_coding | Thap4 | 1397.004 | 1373.42 | 1385.212 |
| ENSMUSG00000086448 | lincRNA | 9330162012Rik | 40.64298 | 38.22401 | 39.4335 |
| ENSMUSG00000027474 | protein_coding | BC020535 | 32.00848 | 29.8601 | 30.93429 |
| ENSMUSG00000076867 | IG_V_gene | Trdv4 | 3.856898 | 4.66093 | 4.258914 |
| ENSMUSG00000090466 | pseudogene | Gm17043 | 0.98273 | 1.413083 | 1.197907 |
| ENSMUSG00000059305 | protein_coding | Vpreb1 | 10.29448 | 8.811533 | 9.553007 |
| ENSMUSG00000047910 | protein_coding | Pcdhb16 | 72.23557 | 75.45481 | 73.84519 |
| ENSMUSG00000079654 | protein_coding | Prrt4 | 7.1108 | 8.069136 | 7.589968 |
| ENSMUSG00000030604 | protein_coding | Zfp626 | 137.3198 | 131.3582 | 134.339 |
| ENSMUSG00000027270 | protein_coding | 6330527O06Rik | 0.638331 | 1.006571 | 0.822451 |
| ENSMUSG00000086679 | processed_transcrip | Gm15551 | 0.638331 | 1.006571 | 0.822451 |
| ENSMUSG00000039208 | protein_coding | Metrnl | 84.29536 | 87.7095 | 86.00243 |
| ENSMUSG00000032598 | protein_coding | Nckipsd | 539.2801 | 527.8488 | 533.5645 |
| ENSMUSG00000044697 | pseudogene | Gm5479 | 0.976841 | 1.395426 | 1.186134 |
| ENSMUSG00000084976 | lincRNA | Gm16889 | 29.49995 | 27.41699 | 28.45847 |
| ENSMUSG00000066647 | protein_coding | Gm5113 | 164.6736 | 158.4039 | 161.5388 |
| ENSMUSG00000051989 | protein_coding | Fam165b | 303.6649 | 313.7366 | 308.7008 |
| ENSMUSG00000025198 | protein_coding | Erlin1 | 1315.022 | 1345.672 | 1330.347 |
| ENSMUSG00000028102 | protein_coding | Pex11b | 724.0867 | 738.3932 | 731.2399 |
| ENSMUSG00000083863 | pseudogene | Gm13341 | 26.85054 | 24.76491 | 25.80772 |
| ENSMUSG00000051232 | protein_coding | Tmem199 | 1189.925 | 1214.031 | 1201.978 |
| ENSMUSG00000016624 | protein_coding | Phf21b | 326.0958 | 312.3919 | 319.2439 |
| ENSMUSG00000009633 | protein_coding | G0s2 | 198.935 | 192.8433 | 195.8892 |
| ENSMUSG00000077571 | snoRNA | Snora36b | 0.962752 | 1.377408 | 1.17008 |
| ENSMUSG00000035963 | protein_coding | Odf3l2 | 0.962752 | 1.377408 | 1.17008 |
| ENSMUSG00000051397 | protein_coding | Tacstd2 | 0.98273 | 1.350742 | 1.166736 |
| ENSMUSG00000043301 | protein_coding | Kcnj6 | 0.98273 | 1.350742 | 1.166736 |
| ENSMUSG00000034555 | protein_coding | Tex16 | 1540.577 | 1404.428 | 1472.502 |
| ENSMUSG00000028609 | protein_coding | Magoh | 1581.617 | 1613.763 | 1597.69 |
| ENSMUSG00000080410 | miRNA | AC084287.1 | 1.298952 | 1.757616 | 1.528284 |
| ENSMUSG00000084973 | processed_transcrip | Gm13848 | 1.284863 | 1.686265 | 1.485564 |
| ENSMUSG00000030286 | protein_coding | Tmem111 | 2529.269 | 2490.044 | 2509.657 |
| ENSMUSG00000044863 | protein_coding | Defb36 | 1562.84 | 1592.569 | 1577.705 |
| ENSMUSG00000084767 | processed_transcrip | Gm13098 | 89.35236 | 85.66897 | 87.51067 |
| ENSMUSG00000021928 | protein_coding | Ebpl | 467.3202 | 456.0877 | 461.7039 |
| ENSMUSG00000062638 | protein_coding | Btnl3 | 2.279371 | 1.748606 | 2.013989 |
| ENSMUSG00000029924 | protein_coding | Slc37a3 | 1041.54 | 1064.098 | 1052.819 |
| ENSMUSG00000086015 | lincRNA | 4833417C18Rik | 30.68388 | 32.73023 | 31.70706 |
| ENSMUSG00000022807 | protein_coding | Osbpl11 | 3633.767 | 3698.018 | 3665.892 |
| ENSMUSG00000025925 | protein_coding | Terf1 | 893.0208 | 910.2094 | 901.6151 |
| ENSMUSG00000062939 | protein_coding | Stat4 | 7.466977 | 8.529704 | 7.998341 |
| ENSMUSG00000066366 | protein_coding | Serpina1a | 1.981861 | 1.36876 | 1.67531 |
| ENSMUSG00000067949 | pseudogene | Vmn1r-ps144 | 0.632442 | 0.988552 | 0.810497 |
| ENSMUSG00000057990 | pseudogene | E030024N20Rik | 0.632442 | 0.988552 | 0.810497 |
| ENSMUSG00000093111 | snRNA | AC120136.1 | 12.62641 | 11.38254 | 12.00447 |
| ENSMUSG00000068874 | protein_coding | Selenbp1 | 726.9502 | 741.4425 | 734.1963 |
| ENSMUSG00000057506 | protein_coding | Bloc1s2 | 953.3007 | 971.577 | 962.4389 |
| ENSMUSG00000038371 | protein_coding | Sbf2 | 3666.841 | 3730.103 | 3698.472 |
| ENSMUSG00000042510 | protein_coding | AA986860 | 49.35233 | 46.68956 | 48.02095 |
| ENSMUSG00000022636 | protein_coding | Alcam | 1691.679 | 1637.655 | 1664.667 |
| ENSMUSG00000044968 | protein_coding | Napepld | 1421.752 | 1392.528 | 1407.14 |
| ENSMUSG00000028300 | protein_coding | 3110043O21Rik | 1707.034 | 1681.275 | 1694.155 |

| | | | | | |
|---|---|---|---|---|---|
| ENSMUSG00000066152 | protein_coding | Slc31a2 | 517.3124 | 527.75 | 522.5312 |
| ENSMUSG00000090546 | protein_coding | Cdr1 | 2.233526 | 1.748606 | 1.991066 |
| ENSMUSG00000007610 | protein_coding | Gtpbp3 | 532.5156 | 547.2397 | 539.8776 |
| ENSMUSG00000086047 | lincRNA | 9530046B11Rik | 14.59649 | 16.00743 | 15.30196 |
| ENSMUSG00000086808 | processed_transcrip | Gm11981 | 2.242993 | 1.659599 | 1.951296 |
| ENSMUSG00000024695 | protein_coding | Zfp91 | 5190.376 | 5273.7 | 5232.038 |
| ENSMUSG00000035129 | pseudogene | Gm6781 | 1.606973 | 2.075121 | 1.841047 |
| ENSMUSG00000086934 | lincRNA | Gm16801 | 1.606973 | 2.075121 | 1.841047 |
| ENSMUSG00000031540 | protein_coding | Myst3 | 4604.917 | 4503.379 | 4554.148 |
| ENSMUSG00000065147 | snoRNA | Snora31 | 34.11483 | 36.7039 | 35.40936 |
| ENSMUSG00000076438 | protein_coding | Oxct2b | 9.40426 | 10.50645 | 9.955353 |
| ENSMUSG00000078937 | protein_coding | Cpt1b | 1.615173 | 2.08413 | 1.849651 |
| ENSMUSG00000004360 | protein_coding | 9330159F19Rik | 19.635 | 21.27706 | 20.45603 |
| ENSMUSG00000013562 | processed_transcrip | 4833413E03Rik | 71.73597 | 75.48619 | 73.61108 |
| ENSMUSG00000022529 | protein_coding | Zfp263 | 1368.563 | 1344.309 | 1356.436 |
| ENSMUSG00000090260 | processed_transcrip | 2810442N19Rik | 13.56098 | 12.09718 | 12.82908 |
| ENSMUSG00000020037 | protein_coding | Rfx4 | 7.801909 | 6.798392 | 7.300151 |
| ENSMUSG00000024810 | protein_coding | Il33 | 9.653615 | 10.80629 | 10.22995 |
| ENSMUSG00000057068 | protein_coding | Fam47e | 1.925505 | 1.422092 | 1.673798 |
| ENSMUSG00000031820 | protein_coding | 5430437P03Rik | 1437.695 | 1463.847 | 1450.771 |
| ENSMUSG00000051166 | protein_coding | Eml5 | 1617.955 | 1516.028 | 1566.992 |
| ENSMUSG00000085206 | processed_transcrip | Gm13380 | 0.968641 | 0.662038 | 0.81534 |
| ENSMUSG00000070834 | pseudogene | Gm13015 | 0.968641 | 0.662038 | 0.81534 |
| ENSMUSG00000035785 | protein_coding | Cmtm2b | 0.968641 | 0.662038 | 0.81534 |
| ENSMUSG00000074919 | protein_coding | Gm10787 | 0.968641 | 0.662038 | 0.81534 |
| ENSMUSG00000088843 | snRNA | U6atac | 0.968641 | 0.662038 | 0.81534 |
| ENSMUSG00000024553 | protein_coding | Galr1 | 0.968641 | 0.662038 | 0.81534 |
| ENSMUSG00000081308 | pseudogene | Gm14480 | 0.968641 | 0.662038 | 0.81534 |
| ENSMUSG00000066279 | protein_coding | Chrna10 | 10.39057 | 9.083992 | 9.737281 |
| ENSMUSG00000031505 | protein_coding | Carkd | 860.4975 | 877.5529 | 869.0252 |
| ENSMUSG00000031095 | protein_coding | Cul4b | 6039.322 | 5947.196 | 5993.259 |
| ENSMUSG00000081664 | pseudogene | Gm15544 | 0.954552 | 1.36876 | 1.161656 |
| ENSMUSG00000006205 | protein_coding | Htra1 | 955.2531 | 983.7788 | 969.5159 |
| ENSMUSG00000031609 | protein_coding | Sap30 | 148.9712 | 153.9062 | 151.4387 |
| ENSMUSG00000026674 | protein_coding | Ddr2 | 945.6699 | 968.2835 | 956.9767 |
| ENSMUSG00000042428 | protein_coding | Mgat3 | 441.4725 | 453.321 | 447.3967 |
| ENSMUSG00000086407 | lincRNA | Gm14123 | 1.290752 | 1.695274 | 1.493013 |
| ENSMUSG00000032279 | protein_coding | Idh3a | 2104.555 | 2146.549 | 2125.552 |
| ENSMUSG00000032602 | protein_coding | Slc25a20 | 653.3342 | 640.5418 | 646.938 |
| ENSMUSG00000059483 | pseudogene | Rpl21-ps1 | 0.962752 | 1.448758 | 1.205755 |
| ENSMUSG00000025153 | protein_coding | Fasn | 13966.9 | 13693.01 | 13829.95 |
| ENSMUSG00000083856 | pseudogene | Gm13886 | 2.583814 | 2.08413 | 2.333972 |
| ENSMUSG00000054988 | protein_coding | Agtr1b | 2.583814 | 2.08413 | 2.333972 |
| ENSMUSG00000031893 | protein_coding | Tsnaxip1 | 17.8691 | 19.54933 | 18.70921 |
| ENSMUSG00000006731 | protein_coding | B4galnt1 | 971.1797 | 932.3324 | 951.7561 |
| ENSMUSG00000029182 | protein_coding | 1700001C02Rik | 0.64422 | 0.997561 | 0.820891 |
| ENSMUSG00000026782 | protein_coding | Abi2 | 2219.505 | 2270.685 | 2245.095 |
| ENSMUSG00000055323 | protein_coding | Gm9967 | 32.91152 | 30.78631 | 31.84892 |
| ENSMUSG00000086292 | processed_transcrip | Gm16052 | 5.479228 | 6.347195 | 5.913212 |
| ENSMUSG00000088856 | snoRNA | SNORA48 | 10.39057 | 11.70004 | 11.0453 |
| ENSMUSG00000055022 | protein_coding | Cntn1 | 13.14383 | 14.60372 | 13.87377 |
| ENSMUSG00000078770 | protein_coding | Gm10989 | 2.905924 | 2.339294 | 2.622609 |
| ENSMUSG00000085998 | processed_transcrip | AW822252 | 31.0493 | 29.03227 | 30.04079 |
| ENSMUSG00000084505 | snRNA | U6 | 1.278973 | 1.72194 | 1.500457 |
| ENSMUSG00000031772 | protein_coding | Cntnap4 | 4.860874 | 4.105918 | 4.483396 |
| ENSMUSG00000053012 | protein_coding | Krcc1 | 1438.677 | 1470.932 | 1454.805 |
| ENSMUSG00000040188 | protein_coding | Scamp2 | 1643.369 | 1669.19 | 1656.28 |
| ENSMUSG00000088689 | snoRNA | Scarna17 | 2.567414 | 2.030798 | 2.299106 |

| | | | | | |
|---|---|---|---|---|---|
| ENSMUSG00000062981 | protein_coding | Mrpl42 | 1465.343 | 1435.539 | 1450.441 |
| ENSMUSG00000020048 | protein_coding | Hsp90b1 | 19083.83 | 19383 | 19233.42 |
| ENSMUSG00000022518 | protein_coding | 4930562C15Rik | 17.1557 | 18.62928 | 17.89249 |
| ENSMUSG00000081342 | pseudogene | Gm11434 | 1.615173 | 2.075121 | 1.845147 |
| ENSMUSG00000038582 | protein_coding | Pptc7 | 1632.824 | 1597.253 | 1615.039 |
| ENSMUSG00000083342 | pseudogene | Gm14237 | 0.66062 | 1.015218 | 0.837919 |
| ENSMUSG00000037973 | protein_coding | Ccdc129 | 0.66062 | 1.015218 | 0.837919 |
| ENSMUSG00000089498 | snRNA | U6 | 0.66062 | 1.015218 | 0.837919 |
| ENSMUSG00000024190 | protein_coding | Dusp1 | 301.4828 | 312.6952 | 307.089 |
| ENSMUSG00000031959 | protein_coding | Wdr59 | 1316.271 | 1294.637 | 1305.454 |
| ENSMUSG00000091737 | lincRNA | Gm17543 | 6.454801 | 5.572334 | 6.013567 |
| ENSMUSG00000059625 | protein_coding | Sohlh1 | 1104.37 | 1083.097 | 1093.733 |
| ENSMUSG00000006958 | protein_coding | Chrd | 25.8617 | 27.6866 | 26.77415 |
| ENSMUSG00000031734 | protein_coding | Irx3 | 37.08024 | 34.72499 | 35.90262 |
| ENSMUSG00000053038 | protein_coding | Gm6180 | 23.52031 | 25.48385 | 24.50208 |
| ENSMUSG00000085491 | processed_transcrip | 4930527E20Rik | 2.972791 | 2.146471 | 2.559631 |
| ENSMUSG00000060441 | protein_coding | Trim5 | 103.0039 | 97.2584 | 100.1312 |
| ENSMUSG00000036198 | protein_coding | Arhgap36 | 6.46658 | 7.39845 | 6.932515 |
| ENSMUSG00000080076 | pseudogene | Hist1h2aj | 5.823627 | 6.709023 | 6.266325 |
| ENSMUSG00000028631 | protein_coding | Kcnq4 | 103.9396 | 107.6982 | 105.8189 |
| ENSMUSG00000021189 | protein_coding | Atxn3 | 1292.673 | 1266.852 | 1279.762 |
| ENSMUSG00000059323 | protein_coding | Tonsl | 1264.374 | 1233.295 | 1248.834 |
| ENSMUSG00000079165 | protein_coding | Sap25 | 211.219 | 218.5335 | 214.8762 |
| ENSMUSG00000073844 | pseudogene | Gm6252 | 57.70349 | 53.77201 | 55.73775 |
| ENSMUSG00000086018 | processed_transcrip | Gm13179 | 1.293062 | 1.72194 | 1.507501 |
| ENSMUSG00000030733 | protein_coding | Sh2b1 | 2245.924 | 2279.389 | 2262.656 |
| ENSMUSG00000046942 | protein_coding | Mageb16 | 497.9873 | 480.1769 | 489.0821 |
| ENSMUSG00000073530 | protein_coding | Pappa2 | 6.166758 | 7.071575 | 6.619166 |
| ENSMUSG00000019836 | pseudogene | Amd-ps4 | 8.055067 | 9.881169 | 8.968118 |
| ENSMUSG00000029608 | protein_coding | Rph3a | 1.606973 | 2.048455 | 1.827714 |
| ENSMUSG00000093286 | miRNA | AC116712.1 | 0.66062 | 0.36219 | 0.511405 |
| ENSMUSG00000086593 | lincRNA | Gm16548 | 0.66062 | 0.36219 | 0.511405 |
| ENSMUSG00000081353 | pseudogene | Gm13818 | 0.66062 | 0.36219 | 0.511405 |
| ENSMUSG00000027923 | protein_coding | Lce1b | 0.66062 | 0.36219 | 0.511405 |
| ENSMUSG00000080744 | pseudogene | Gm12710 | 0.66062 | 0.36219 | 0.511405 |
| ENSMUSG00000086850 | processed_transcrip | Gm13257 | 0.66062 | 0.36219 | 0.511405 |
| ENSMUSG00000087552 | processed_transcrip | Gm15819 | 0.66062 | 0.36219 | 0.511405 |
| ENSMUSG00000086931 | processed_transcrip | Gm14532 | 0.66062 | 0.36219 | 0.511405 |
| ENSMUSG00000051614 | protein_coding | 4930533L02Rik | 0.66062 | 0.36219 | 0.511405 |
| ENSMUSG00000062726 | protein_coding | Olfr857 | 0.66062 | 0.36219 | 0.511405 |
| ENSMUSG00000037953 | protein_coding | A4gnt | 0.66062 | 0.36219 | 0.511405 |
| ENSMUSG00000089103 | rRNA | 5S_rRNA | 0.66062 | 0.36219 | 0.511405 |
| ENSMUSG00000072585 | protein_coding | Gm6616 | 0.66062 | 0.36219 | 0.511405 |
| ENSMUSG00000065156 | snRNA | U6 | 0.66062 | 0.36219 | 0.511405 |
| ENSMUSG00000063886 | pseudogene | Gm10138 | 13.20249 | 11.93791 | 12.5702 |
| ENSMUSG00000090344 | protein_coding | Gm17237 | 2.567414 | 2.057464 | 2.312439 |
| ENSMUSG00000086405 | processed_transcrip | 9330198N18Rik | 0.976841 | 1.377408 | 1.177124 |
| ENSMUSG00000002633 | protein_coding | Shh | 11.89072 | 13.26991 | 12.58031 |
| ENSMUSG00000078234 | protein_coding | Klhdc7a | 178.6112 | 172.3362 | 175.4737 |
| ENSMUSG00000087687 | lincRNA | 2900053A13Rik | 137.3387 | 132.8998 | 135.1193 |
| ENSMUSG00000048450 | protein_coding | Msx1 | 9.961636 | 8.864866 | 9.413251 |
| ENSMUSG00000090369 | protein_coding | 4933411K16Rik | 7.781931 | 8.811533 | 8.296732 |
| ENSMUSG00000028630 | protein_coding | Dyrk2 | 538.8712 | 525.6991 | 532.2851 |
| ENSMUSG00000032203 | protein_coding | Pigb | 161.4101 | 156.177 | 158.7935 |
| ENSMUSG00000028807 | protein_coding | Zbtb8a | 398.154 | 408.2474 | 403.2007 |
| ENSMUSG00000051373 | protein_coding | Ppapdc3 | 128.2267 | 132.9392 | 130.5829 |
| ENSMUSG00000029207 | protein_coding | Apbb2 | 757.0677 | 771.1013 | 764.0845 |
| ENSMUSG00000053291 | protein_coding | Rab4b | 88.14928 | 92.90167 | 90.52547 |

| | | | | | |
|---|---|---|---|---|---|
| ENSMUSG00000064558 | snoRNA | SNORA55 | 0.65242 | 1.006571 | 0.829495 |
| ENSMUSG00000001665 | protein_coding | Gstt3 | 125.6992 | 130.4142 | 128.0567 |
| ENSMUSG00000057465 | protein_coding | Saa2 | 1.606973 | 2.057464 | 1.832218 |
| ENSMUSG00000055538 | protein_coding | Zcchc24 | 988.8503 | 968.9832 | 978.9168 |
| ENSMUSG00000025495 | protein_coding | Ptdss2 | 1149.508 | 1129.745 | 1139.626 |
| ENSMUSG00000009646 | protein_coding | Pla2g12b | 5.560183 | 6.52521 | 6.042697 |
| ENSMUSG00000087305 | lincRNA | 2810488G03Rik | 53.81566 | 50.68659 | 52.25112 |
| ENSMUSG00000083621 | pseudogene | Gm14586 | 120.9605 | 125.4168 | 123.1886 |
| ENSMUSG00000078442 | protein_coding | Ccdc105 | 25.86528 | 24.00558 | 24.93543 |
| ENSMUSG00000064380 | snoRNA | SNORA73 | 13.34107 | 11.86656 | 12.60381 |
| ENSMUSG00000024952 | protein_coding | Rps6ka4 | 448.4333 | 458.4619 | 453.4476 |
| ENSMUSG00000004100 | protein_coding | Ppan | 1963.079 | 1994.787 | 1978.933 |
| ENSMUSG00000079731 | protein_coding | Tmem181c-ps | 1.959572 | 1.448758 | 1.704165 |
| ENSMUSG00000005045 | protein_coding | Chd5 | 559.0025 | 580.539 | 569.7707 |
| ENSMUSG00000035696 | protein_coding | Rnf38 | 2886.547 | 2841.65 | 2864.099 |
| ENSMUSG00000067389 | processed_transcrip | Gm17080 | 18.18763 | 16.35305 | 17.27034 |
| ENSMUSG00000055026 | protein_coding | Gabrg3 | 14.54371 | 13.21658 | 13.88014 |
| ENSMUSG00000059000 | protein_coding | Zfp799 | 287.9965 | 296.9248 | 292.4607 |
| ENSMUSG00000069986 | protein_coding | Gm8054 | 0.64422 | 0.988552 | 0.816386 |
| ENSMUSG00000037318 | protein_coding | Traf3ip3 | 15.51593 | 16.91634 | 16.21614 |
| ENSMUSG00000061468 | pseudogene | Gm3724 | 5.151228 | 4.414414 | 4.782821 |
| ENSMUSG00000034310 | protein_coding | Tmem132d | 12.87449 | 11.68238 | 12.27844 |
| ENSMUSG00000072940 | pseudogene | Gm10443 | 163.2063 | 168.8491 | 166.0277 |
| ENSMUSG00000028834 | protein_coding | Trim63 | 45.4513 | 48.16499 | 46.80815 |
| ENSMUSG00000036169 | protein_coding | Sostdc1 | 21.77647 | 23.82249 | 22.79948 |
| ENSMUSG00000022742 | protein_coding | Cpox | 789.3257 | 772.019 | 780.6723 |
| ENSMUSG00000022496 | protein_coding | Tnfrsf17 | 2.592014 | 2.08413 | 2.338072 |
| ENSMUSG00000047878 | protein_coding | A4galt | 148.4332 | 153.4096 | 150.9214 |
| ENSMUSG00000025157 | protein_coding | Zdhhc16 | 1215.884 | 1195.891 | 1205.888 |
| ENSMUSG00000009927 | protein_coding | Rps25 | 5216.173 | 5295.409 | 5255.791 |
| ENSMUSG00000055480 | protein_coding | Zfp458 | 95.74977 | 101.5792 | 98.66446 |
| ENSMUSG00000024235 | protein_coding | Map3k8 | 110.492 | 105.813 | 108.1525 |
| ENSMUSG00000042357 | protein_coding | Gjb5 | 5.13945 | 4.387748 | 4.763599 |
| ENSMUSG00000085204 | processed_transcrip | Gm15327 | 17.46372 | 16.07013 | 16.76693 |
| ENSMUSG00000047053 | protein_coding | Teddm1 | 0.66062 | 0.335524 | 0.498072 |
| ENSMUSG00000075416 | lincRNA | Gm14488 | 0.66062 | 0.335524 | 0.498072 |
| ENSMUSG00000028287 | protein_coding | 1700009N14Rik | 0.66062 | 0.335524 | 0.498072 |
| ENSMUSG00000086000 | processed_transcrip | Gm12493 | 0.66062 | 0.335524 | 0.498072 |
| ENSMUSG00000073212 | protein_coding | Gm10481 | 0.66062 | 0.335524 | 0.498072 |
| ENSMUSG00000039264 | protein_coding | Gimap3 | 0.66062 | 0.335524 | 0.498072 |
| ENSMUSG00000065212 | snRNA | U1 | 0.66062 | 0.335524 | 0.498072 |
| ENSMUSG00000060816 | protein_coding | Vmn1r52 | 0.66062 | 0.335524 | 0.498072 |
| ENSMUSG00000038968 | protein_coding | Klk1b16 | 0.66062 | 0.335524 | 0.498072 |
| ENSMUSG00000066197 | protein_coding | Gpr139 | 0.66062 | 0.335524 | 0.498072 |
| ENSMUSG00000085652 | lincRNA | Gm11999 | 0.66062 | 0.335524 | 0.498072 |
| ENSMUSG00000087630 | processed_transcrip | Gm11513 | 0.66062 | 0.335524 | 0.498072 |
| ENSMUSG00000076615 | IG_C_gene | AI324046 | 0.66062 | 0.335524 | 0.498072 |
| ENSMUSG00000074553 | pseudogene | Gm10713 | 0.66062 | 0.335524 | 0.498072 |
| ENSMUSG00000092375 | processed_transcrip | RP23-385C21.2 | 0.66062 | 0.335524 | 0.498072 |
| ENSMUSG00000022877 | protein_coding | Hrg | 0.66062 | 0.335524 | 0.498072 |
| ENSMUSG00000087950 | miRNA | Mir1948 | 0.66062 | 0.335524 | 0.498072 |
| ENSMUSG00000092009 | protein_coding | Myh15 | 1.615173 | 2.146471 | 1.880822 |
| ENSMUSG00000004105 | protein_coding | Angptl2 | 843.8045 | 871.7771 | 857.7908 |
| ENSMUSG00000026090 | protein_coding | 2010300C02Rik | 268.458 | 277.848 | 273.153 |
| ENSMUSG00000028906 | protein_coding | Epb4.1 | 2649.667 | 2688.197 | 2668.932 |
| ENSMUSG00000082183 | pseudogene | Gm15994 | 13.27756 | 12.01791 | 12.64773 |
| ENSMUSG00000087399 | processed_transcrip | Gm11899 | 0.948663 | 1.413083 | 1.180873 |
| ENSMUSG00000060096 | protein_coding | Amd-ps3 | 75.75419 | 72.37125 | 74.06272 |

| | | | | | |
|---|---|---|---|---|---|
| ENSMUSG00000045975 | protein_coding | C2cd2 | 300.8042 | 310.2106 | 305.5074 |
| ENSMUSG00000072919 | protein_coding | 4933437F05Rik | 36.0006 | 38.58796 | 37.29428 |
| ENSMUSG00000033061 | protein_coding | Resp18 | 0.66062 | 0.326514 | 0.493567 |
| ENSMUSG00000092689 | miRNA | AL844842.1 | 0.66062 | 0.326514 | 0.493567 |
| ENSMUSG00000086007 | processed_transcrip | 1700057H21Rik | 0.66062 | 0.326514 | 0.493567 |
| ENSMUSG00000084674 | misc_RNA | 7SK | 0.66062 | 0.326514 | 0.493567 |
| ENSMUSG00000085824 | lincRNA | 2410114N07Rik | 0.66062 | 0.326514 | 0.493567 |
| ENSMUSG00000086817 | processed_transcrip | Gm11482 | 0.66062 | 0.326514 | 0.493567 |
| ENSMUSG00000088102 | misc_RNA | 7SK | 0.66062 | 0.326514 | 0.493567 |
| ENSMUSG00000048377 | protein_coding | Foxi2 | 0.66062 | 0.326514 | 0.493567 |
| ENSMUSG00000047562 | protein_coding | Mmp10 | 0.66062 | 0.326514 | 0.493567 |
| ENSMUSG00000085468 | processed_transcrip | Gm15343 | 0.66062 | 0.326514 | 0.493567 |
| ENSMUSG00000075586 | lincRNA | Gm11529 | 0.66062 | 0.326514 | 0.493567 |
| ENSMUSG00000078297 | protein_coding | Gm3150 | 0.66062 | 0.326514 | 0.493567 |
| ENSMUSG00000090247 | protein_coding | Bloc1s1 | 23.24866 | 25.04818 | 24.14842 |
| ENSMUSG00000054836 | protein_coding | 2610002I17Rik | 327.5629 | 316.9528 | 322.2579 |
| ENSMUSG00000035187 | protein_coding | Nkx6-1 | 1.278973 | 1.784282 | 1.531628 |
| ENSMUSG00000086180 | lincRNA | Rmst | 15.91773 | 14.45522 | 15.18647 |
| ENSMUSG00000039713 | protein_coding | Plekhg5 | 1422.329 | 1448.986 | 1435.658 |
| ENSMUSG00000044362 | protein_coding | Ccdc89 | 25.44201 | 23.37994 | 24.41097 |
| ENSMUSG00000000732 | protein_coding | Icosl | 260.8442 | 267.8964 | 264.3703 |
| ENSMUSG00000028454 | protein_coding | Pigo | 669.0826 | 683.7228 | 676.4027 |
| ENSMUSG00000035385 | protein_coding | Ccl2 | 8.781061 | 7.647454 | 8.214258 |
| ENSMUSG00000025646 | protein_coding | Atrip | 510.0733 | 497.4739 | 503.7736 |
| ENSMUSG00000050621 | protein_coding | Gm9846 | 195.7173 | 201.0816 | 198.3994 |
| ENSMUSG00000041377 | protein_coding | Ninj2 | 2.914124 | 2.357312 | 2.635718 |
| ENSMUSG00000026981 | protein_coding | Il1rn | 10.93848 | 12.30481 | 11.62164 |
| ENSMUSG00000084847 | processed_transcrip | Gm15507 | 1.917305 | 2.472986 | 2.195145 |
| ENSMUSG00000039304 | protein_coding | Tnfsf10 | 39.44265 | 41.66681 | 40.55473 |
| ENSMUSG00000088183 | snRNA | U6 | 2.634281 | 2.021789 | 2.328035 |
| ENSMUSG00000051367 | protein_coding | Six1 | 68.38017 | 64.74079 | 66.56048 |
| ENSMUSG00000050890 | protein_coding | Pdik1l | 1285.41 | 1314.807 | 1300.109 |
| ENSMUSG00000024187 | protein_coding | Itfg3 | 3276.658 | 3345.721 | 3311.189 |
| ENSMUSG00000030643 | protein_coding | Rab30 | 296.2499 | 288.8329 | 292.5414 |
| ENSMUSG00000038615 | protein_coding | Nfe2l1 | 8330.229 | 8198.428 | 8264.328 |
| ENSMUSG00000004233 | protein_coding | Wars2 | 289.3357 | 297.3057 | 293.3207 |
| ENSMUSG00000084755 | pseudogene | Gm16276 | 2.911813 | 2.357312 | 2.634563 |
| ENSMUSG00000039958 | protein_coding | 4833442J19Rik | 372.4386 | 363.244 | 367.8413 |
| ENSMUSG00000087303 | protein_coding | Lipo2 | 34.38878 | 31.91462 | 33.1517 |
| ENSMUSG00000044122 | protein_coding | Proca1 | 160.8668 | 166.5512 | 163.709 |
| ENSMUSG00000079626 | protein_coding | Gm14543 | 54.5837 | 44.84611 | 49.7149 |
| ENSMUSG00000086625 | processed_transcrip | Gm11831 | 3.588833 | 2.87085 | 3.229842 |
| ENSMUSG00000026707 | protein_coding | Nsun6 | 427.0236 | 415.327 | 421.1753 |
| ENSMUSG00000042800 | protein_coding | 1700015E13Rik | 2.917703 | 2.339294 | 2.628498 |
| ENSMUSG00000024687 | protein_coding | Osbp | 2374.475 | 2337.768 | 2356.122 |
| ENSMUSG00000042229 | protein_coding | Rabif | 890.8417 | 911.3007 | 901.0712 |
| ENSMUSG00000045019 | protein_coding | Acer1 | 8.367485 | 9.41123 | 8.889357 |
| ENSMUSG00000058239 | protein_coding | Usf2 | 1013.03 | 988.1815 | 1000.606 |
| ENSMUSG00000049411 | protein_coding | 6030446N20Rik | 282.3145 | 273.8292 | 278.0718 |
| ENSMUSG00000028608 | protein_coding | 0610037L13Rik | 1152.85 | 1124.116 | 1138.483 |
| ENSMUSG00000023572 | protein_coding | Ccndbp1 | 1008.506 | 1025.098 | 1016.802 |
| ENSMUSG00000091985 | lincRNA | Gm17354 | 95.68819 | 99.42792 | 97.55805 |
| ENSMUSG00000006360 | protein_coding | Crip1 | 122.8898 | 118.441 | 120.6654 |
| ENSMUSG00000054136 | protein_coding | Adm2 | 2.239415 | 1.757616 | 1.998515 |
| ENSMUSG00000092819 | snoRNA | AC150277.1 | 2.917703 | 2.348303 | 2.633003 |
| ENSMUSG00000038702 | protein_coding | Dsel | 994.0579 | 974.7531 | 984.4055 |
| ENSMUSG00000035164 | protein_coding | Zc3h12c | 835.2873 | 852.1055 | 843.6964 |
| ENSMUSG00000092625 | processed_transcrip | RP23-223C23.3 | 10.68912 | 9.588883 | 10.139 |

| | | | | | |
|---|---|---|---|---|---|
| ENSMUSG00000025279 | protein_coding | Dnase1l3 | 8.115819 | 7.169591 | 7.642705 |
| ENSMUSG00000084778 | lincRNA | Gm16743 | 2.628392 | 2.021789 | 2.32509 |
| ENSMUSG00000080997 | pseudogene | Gm11885 | 0.638331 | 0.979543 | 0.808937 |
| ENSMUSG00000061028 | protein_coding | Clasrp | 871.4004 | 890.5539 | 880.9772 |
| ENSMUSG00000040296 | protein_coding | Ddx58 | 241.176 | 250.4559 | 245.8159 |
| ENSMUSG00000078525 | pseudogene | Gm5776 | 92.93911 | 89.39649 | 91.1678 |
| ENSMUSG00000038453 | protein_coding | Srcin1 | 341.7918 | 330.5619 | 336.1769 |
| ENSMUSG00000013701 | protein_coding | Timm23 | 2529.943 | 2566.43 | 2548.186 |
| ENSMUSG00000025588 | protein_coding | Nat1 | 4.504697 | 3.814718 | 4.159708 |
| ENSMUSG00000028859 | protein_coding | Csf3r | 10.35292 | 9.24435 | 9.798637 |
| ENSMUSG00000091868 | protein_coding | Gm6657 | 12.05725 | 10.61275 | 11.335 |
| ENSMUSG00000024558 | protein_coding | Mapk4 | 263.3004 | 273.0699 | 268.1852 |
| ENSMUSG00000030584 | protein_coding | Dpf1 | 782.3837 | 765.5112 | 773.9474 |
| ENSMUSG00000027329 | protein_coding | Spef1 | 453.9582 | 465.287 | 459.6226 |
| ENSMUSG00000063550 | protein_coding | Nup98 | 2538.943 | 2493.238 | 2516.091 |
| ENSMUSG00000006732 | protein_coding | Mettl1 | 467.867 | 458.7033 | 463.2851 |
| ENSMUSG00000034151 | protein_coding | Zbbx | 1.621062 | 2.057464 | 1.839263 |
| ENSMUSG00000073493 | lincRNA | Gm17286 | 4.241254 | 3.523879 | 3.882566 |
| ENSMUSG00000086997 | processed_transcrip | Gm12981 | 8.782329 | 9.92513 | 9.353729 |
| ENSMUSG00000055027 | protein_coding | Smyd1 | 136.9762 | 141.4825 | 139.2294 |
| ENSMUSG00000029401 | protein_coding | Rilpl2 | 789.6273 | 808.2509 | 798.9391 |
| ENSMUSG00000091785 | protein_coding | Rpl29-ps5 | 0.66062 | 0.997561 | 0.829091 |
| ENSMUSG00000027671 | protein_coding | Actl6a | 1924.366 | 1956.648 | 1940.507 |
| ENSMUSG00000020661 | protein_coding | Dnmt3a | 5691.075 | 5579.605 | 5635.34 |
| ENSMUSG00000048096 | protein_coding | Lmod1 | 572.7316 | 560.805 | 566.7683 |
| ENSMUSG00000063087 | protein_coding | Gm10125 | 76.5788 | 79.80108 | 78.18994 |
| ENSMUSG00000022803 | protein_coding | Popdc2 | 48.16505 | 45.76408 | 46.96456 |
| ENSMUSG00000065396 | miRNA | Mir99b | 1.284863 | 1.703922 | 1.494392 |
| ENSMUSG00000065399 | miRNA | Mir133a-1 | 1.284863 | 1.703922 | 1.494392 |
| ENSMUSG00000062393 | protein_coding | Dgkk | 203.862 | 211.0392 | 207.4506 |
| ENSMUSG00000019942 | protein_coding | Cdk1 | 1321.762 | 1357.63 | 1339.696 |
| ENSMUSG00000052748 | protein_coding | 1200016B10Rik | 2090.896 | 2137.808 | 2114.352 |
| ENSMUSG00000022383 | protein_coding | Ppara | 67.97606 | 64.48707 | 66.23157 |
| ENSMUSG00000020715 | protein_coding | Ern1 | 1290.073 | 1318.534 | 1304.304 |
| ENSMUSG00000005510 | protein_coding | Ndufs3 | 1680.245 | 1705.651 | 1692.948 |
| ENSMUSG00000024112 | protein_coding | Cacna1h | 1560.186 | 1585.917 | 1573.051 |
| ENSMUSG00000026766 | protein_coding | Mmadhc | 1668.165 | 1642.671 | 1655.418 |
| ENSMUSG00000032251 | protein_coding | Irak1bp1 | 356.9658 | 348.9008 | 352.9333 |
| ENSMUSG00000051985 | protein_coding | Igfn1 | 17.45679 | 19.03651 | 18.24665 |
| ENSMUSG00000047976 | protein_coding | Kcna1 | 25.4719 | 27.64477 | 26.55833 |
| ENSMUSG00000070327 | protein_coding | Rnf213 | 5549.558 | 5872.334 | 5710.946 |
| ENSMUSG00000057946 | protein_coding | Vmn1r181 | 4.261232 | 3.452528 | 3.85688 |
| ENSMUSG00000082193 | pseudogene | Rpl5-ps1 | 5.199384 | 4.405405 | 4.802395 |
| ENSMUSG00000039840 | protein_coding | 5430411K18Rik | 1658.408 | 1622.477 | 1640.442 |
| ENSMUSG00000011831 | protein_coding | Evi5 | 4075.863 | 4021.411 | 4048.637 |
| ENSMUSG00000040339 | protein_coding | Fam102b | 1473.911 | 1502.736 | 1488.324 |
| ENSMUSG00000068606 | protein_coding | Gm4841 | 21.28298 | 19.59044 | 20.43671 |
| ENSMUSG00000062381 | protein_coding | Vps28 | 702.2555 | 715.4378 | 708.8466 |
| ENSMUSG00000024018 | protein_coding | 1110021J02Rik | 176.6195 | 181.6505 | 179.135 |
| ENSMUSG00000020526 | protein_coding | Znhit3 | 447.3112 | 436.1791 | 441.7451 |
| ENSMUSG00000021822 | protein_coding | Plau | 84.1816 | 79.84106 | 82.01133 |
| ENSMUSG00000085649 | processed_transcrip | A730032A03Rik | 0.98273 | 0.662038 | 0.822384 |
| ENSMUSG00000068102 | pseudogene | Gm6749 | 0.98273 | 0.662038 | 0.822384 |
| ENSMUSG00000085678 | processed_transcrip | Gm16349 | 0.98273 | 0.662038 | 0.822384 |
| ENSMUSG00000026939 | protein_coding | Tmem141 | 322.2614 | 331.8629 | 327.0621 |
| ENSMUSG00000033530 | protein_coding | Ttc7b | 820.5584 | 834.788 | 827.6732 |
| ENSMUSG00000022659 | protein_coding | Gcet2 | 1.929083 | 2.410644 | 2.169864 |
| ENSMUSG00000023433 | protein_coding | Cela3b | 2.922324 | 2.374969 | 2.648646 |

| | | | | | |
|---|---|---|---|---|---|
| ENSMUSG00000050511 | protein_coding | Oprd1 | 18.38421 | 19.91152 | 19.14786 |
| ENSMUSG00000059883 | protein_coding | Irak4 | 128.0255 | 123.9823 | 126.0039 |
| ENSMUSG00000020948 | protein_coding | Klhl28 | 482.5177 | 471.6807 | 477.0992 |
| ENSMUSG00000083623 | pseudogene | Gm7224 | 17.26901 | 12.80541 | 15.03721 |
| ENSMUSG00000060802 | protein_coding | B2m | 2033.238 | 2100.271 | 2066.754 |
| ENSMUSG00000031103 | protein_coding | Elf4 | 1338.206 | 1366.272 | 1352.239 |
| ENSMUSG00000029503 | protein_coding | P2rx2 | 471.185 | 437.2874 | 454.2362 |
| ENSMUSG00000025243 | protein_coding | Slc6a20b | 2.283993 | 1.73095 | 2.007471 |
| ENSMUSG00000085169 | lincRNA | Gm10785 | 73.40608 | 76.54387 | 74.97497 |
| ENSMUSG00000041763 | protein_coding | Tpp2 | 4563.799 | 4500.891 | 4532.345 |
| ENSMUSG00000091891 | lincRNA | Gm17422 | 3.913254 | 4.678949 | 4.296102 |
| ENSMUSG00000090113 | protein_coding | Nhlrc4 | 4.526986 | 3.859402 | 4.193194 |
| ENSMUSG00000087635 | pseudogene | Gm13414 | 34.21509 | 36.2509 | 35.23299 |
| ENSMUSG00000081568 | pseudogene | Gm15032 | 2.573303 | 1.994761 | 2.284032 |
| ENSMUSG00000032727 | protein_coding | Mier3 | 5919.726 | 5792.244 | 5855.985 |
| ENSMUSG00000049653 | protein_coding | Spatc1 | 8.043064 | 7.115897 | 7.579481 |
| ENSMUSG00000026175 | protein_coding | Vil1 | 24.32413 | 22.5067 | 23.41541 |
| ENSMUSG00000085053 | processed_transcrip | Gm14809 | 2.922324 | 2.357312 | 2.639818 |
| ENSMUSG00000087528 | lincRNA | 9830144P21Rik | 105.9762 | 110.5578 | 108.267 |
| ENSMUSG00000059897 | protein_coding | Zfp930 | 378.4812 | 367.7079 | 373.0945 |
| ENSMUSG00000026621 | protein_coding | Mosc1 | 1.929083 | 2.419653 | 2.174368 |
| ENSMUSG00000085957 | protein_coding | Gm52 | 7.391911 | 6.471878 | 6.931895 |
| ENSMUSG00000044715 | protein_coding | 4933433P14Rik | 717.7878 | 735.6313 | 726.7096 |
| ENSMUSG00000048796 | protein_coding | Cyb561d1 | 348.8648 | 356.6067 | 352.7358 |
| ENSMUSG00000049482 | protein_coding | Ctu2 | 945.8426 | 924.7597 | 935.3011 |
| ENSMUSG00000024131 | protein_coding | Slc3a1 | 4.887785 | 4.150241 | 4.519013 |
| ENSMUSG00000019464 | protein_coding | Ptger1 | 1137.734 | 1119.658 | 1128.696 |
| ENSMUSG00000054115 | protein_coding | Skp2 | 989.943 | 1020.53 | 1005.236 |
| ENSMUSG00000074056 | protein_coding | Gm10615 | 170.1823 | 177.1551 | 173.6687 |
| ENSMUSG00000040957 | protein_coding | Cables1 | 377.542 | 394.8156 | 386.1788 |
| ENSMUSG00000070168 | snoRNA | U3 | 0.64422 | 0.36219 | 0.503205 |
| ENSMUSG00000085985 | processed_transcrip | Gm11682 | 0.64422 | 0.36219 | 0.503205 |
| ENSMUSG00000059408 | protein_coding | Mrgprh | 0.64422 | 0.36219 | 0.503205 |
| ENSMUSG00000090507 | pseudogene | Gm6476 | 0.64422 | 0.36219 | 0.503205 |
| ENSMUSG00000092983 | miRNA | AC123853.1 | 0.64422 | 0.36219 | 0.503205 |
| ENSMUSG00000059244 | protein_coding | Gm10062 | 5.540205 | 4.732281 | 5.136243 |
| ENSMUSG00000086001 | lincRNA | Gm13448 | 0.968641 | 0.653029 | 0.810835 |
| ENSMUSG00000032094 | protein_coding | Cd3d | 0.968641 | 0.653029 | 0.810835 |
| ENSMUSG00000081253 | pseudogene | Gm8501 | 0.968641 | 0.653029 | 0.810835 |
| ENSMUSG00000031227 | protein_coding | Magee1 | 233.3255 | 239.267 | 236.2963 |
| ENSMUSG00000022292 | protein_coding | Rrm2b | 727.3975 | 742.648 | 735.0228 |
| ENSMUSG00000087350 | pseudogene | Gm16254 | 11.29928 | 10.24227 | 10.77077 |
| ENSMUSG00000025450 | processed_transcrip | Gm9752 | 76.96636 | 72.94936 | 74.95786 |
| ENSMUSG00000053244 | pseudogene | Gm9901 | 1.951372 | 2.472986 | 2.212179 |
| ENSMUSG00000028708 | protein_coding | Mknk1 | 877.7092 | 861.2751 | 869.4921 |
| ENSMUSG00000085003 | processed_transcrip | 4930418C01Rik | 0.646531 | 0.979543 | 0.813037 |
| ENSMUSG00000033475 | protein_coding | Tomm6 | 1.615173 | 2.039445 | 1.827309 |
| ENSMUSG00000075032 | protein_coding | Hist1h3i | 4.812718 | 4.123575 | 4.468147 |
| ENSMUSG00000020923 | protein_coding | Ubtf | 8016.964 | 8160.957 | 8088.961 |
| ENSMUSG00000036854 | protein_coding | Hspb6 | 115.8303 | 119.6267 | 117.7285 |
| ENSMUSG00000041684 | protein_coding | Bivm | 761.2563 | 775.7612 | 768.5088 |
| ENSMUSG00000027852 | protein_coding | Nras | 2659.141 | 2712.993 | 2686.067 |
| ENSMUSG00000070802 | protein_coding | Pnmal2 | 152.8002 | 147.2448 | 150.0225 |
| ENSMUSG00000081239 | pseudogene | Gm11836 | 5.503827 | 4.767956 | 5.135892 |
| ENSMUSG00000044469 | protein_coding | Tnfaip8l1 | 59.53306 | 62.56982 | 61.05144 |
| ENSMUSG00000039756 | protein_coding | Dnttip2 | 2901.32 | 2857.404 | 2879.362 |
| ENSMUSG00000050959 | protein_coding | Olfr1392 | 0.954552 | 1.324076 | 1.139314 |
| ENSMUSG00000073623 | protein_coding | 5830472F04Rik | 0.968641 | 0.688704 | 0.828673 |

| | | | | | |
|---|---|---|---|---|---|
| ENSMUSG00000027670 | protein_coding | 4833422F24Rik | 0.968641 | 0.688704 | 0.828673 |
| ENSMUSG00000081838 | pseudogene | Gm13038 | 0.968641 | 0.688704 | 0.828673 |
| ENSMUSG00000045502 | protein_coding | Niacr1 | 0.968641 | 0.688704 | 0.828673 |
| ENSMUSG00000072576 | pseudogene | Gm16020 | 0.968641 | 0.688704 | 0.828673 |
| ENSMUSG00000090631 | protein_coding | Olfr456 | 0.968641 | 0.688704 | 0.828673 |
| ENSMUSG00000031919 | protein_coding | Tmed6 | 0.968641 | 0.688704 | 0.828673 |
| ENSMUSG00000082287 | pseudogene | Gm15333 | 0.968641 | 0.688704 | 0.828673 |
| ENSMUSG00000077133 | miRNA | AC122775.1 | 0.966331 | 1.413083 | 1.189707 |
| ENSMUSG00000065694 | snRNA | U1 | 0.976841 | 0.662038 | 0.81944 |
| ENSMUSG00000065246 | snRNA | U6 | 0.976841 | 0.662038 | 0.81944 |
| ENSMUSG00000050468 | protein_coding | Astl | 0.66062 | 0.988552 | 0.824586 |
| ENSMUSG00000083917 | pseudogene | Gm12093 | 0.66062 | 0.988552 | 0.824586 |
| ENSMUSG00000034330 | protein_coding | Plcg2 | 568.9949 | 583.8387 | 576.4168 |
| ENSMUSG00000087165 | lincRNA | 2010001A14Rik | 36.82224 | 34.46547 | 35.64386 |
| ENSMUSG00000036244 | protein_coding | Tbc1d21 | 1.951372 | 2.446319 | 2.198846 |
| ENSMUSG00000022604 | protein_coding | Cep97 | 710.6485 | 696.2626 | 703.4555 |
| ENSMUSG00000014349 | protein_coding | Ube2z | 4095.048 | 4169.13 | 4132.089 |
| ENSMUSG00000053128 | protein_coding | Rnf26 | 1260.885 | 1235.944 | 1248.415 |
| ENSMUSG00000020905 | protein_coding | Usp43 | 93.70231 | 97.43679 | 95.56955 |
| ENSMUSG00000089768 | protein_coding | Tmsb15b1 | 10.66661 | 8.956722 | 9.811666 |
| ENSMUSG00000041440 | protein_coding | Gk5 | 885.0446 | 868.4246 | 876.7346 |
| ENSMUSG00000076752 | IG_C_gene | Gm16603 | 1.937283 | 2.481995 | 2.209639 |
| ENSMUSG00000058298 | protein_coding | Mcm9 | 695.1272 | 682.0714 | 688.5993 |
| ENSMUSG00000026123 | protein_coding | Plekhb2 | 1327.731 | 1355.019 | 1341.375 |
| ENSMUSG00000020022 | protein_coding | Ndufa12 | 915.029 | 929.9341 | 922.4816 |
| ENSMUSG00000073491 | protein_coding | Pydc4 | 0.64422 | 0.335524 | 0.489872 |
| ENSMUSG00000074934 | protein_coding | Grem1 | 0.64422 | 0.335524 | 0.489872 |
| ENSMUSG00000074804 | pseudogene | AU015228 | 0.64422 | 0.335524 | 0.489872 |
| ENSMUSG00000093040 | miRNA | AL663077.1 | 0.64422 | 0.335524 | 0.489872 |
| ENSMUSG00000082684 | pseudogene | Gm12774 | 0.64422 | 0.335524 | 0.489872 |
| ENSMUSG00000057375 | protein_coding | Yipf1 | 1010.804 | 1030.126 | 1020.465 |
| ENSMUSG00000028701 | protein_coding | 1520402A15Rik | 340.8953 | 350.0471 | 345.4712 |
| ENSMUSG00000082670 | pseudogene | Gm14050 | 0.974531 | 0.662038 | 0.818284 |
| ENSMUSG00000093289 | snoRNA | CT010429.3 | 0.974531 | 0.662038 | 0.818284 |
| ENSMUSG00000082998 | pseudogene | Gm11221 | 63.71565 | 66.8725 | 65.29407 |
| ENSMUSG00000020491 | protein_coding | 2810021J22Rik | 744.026 | 760.4431 | 752.2345 |
| ENSMUSG00000028437 | protein_coding | Ubap1 | 1775.076 | 1802.559 | 1788.817 |
| ENSMUSG00000016524 | protein_coding | Il19 | 1.282552 | 1.686265 | 1.484408 |
| ENSMUSG00000083725 | pseudogene | Gm11865 | 1.282552 | 1.686265 | 1.484408 |
| ENSMUSG00000033906 | protein_coding | Zdhhc15 | 277.8636 | 270.8189 | 274.3412 |
| ENSMUSG00000037818 | protein_coding | 3110057O12Rik | 291.7317 | 305.5852 | 298.6584 |
| ENSMUSG00000026729 | protein_coding | 4930562F07Rik | 2.611992 | 2.066111 | 2.339052 |
| ENSMUSG00000026692 | protein_coding | Fmo4 | 88.5633 | 91.96677 | 90.26503 |
| ENSMUSG00000013367 | protein_coding | Iglon5 | 35.4518 | 37.53458 | 36.49319 |
| ENSMUSG00000091987 | protein_coding | Gm3376 | 331.1401 | 340.2517 | 335.6959 |
| ENSMUSG00000038537 | protein_coding | Mc3r | 0.64422 | 0.326514 | 0.485367 |
| ENSMUSG00000074664 | protein_coding | A830092H15Rik | 0.64422 | 0.326514 | 0.485367 |
| ENSMUSG00000085855 | processed_transcrip | Gm16520 | 0.64422 | 0.326514 | 0.485367 |
| ENSMUSG00000023247 | protein_coding | Guca2a | 0.64422 | 0.326514 | 0.485367 |
| ENSMUSG00000020686 | protein_coding | Gas2l2 | 0.64422 | 0.326514 | 0.485367 |
| ENSMUSG00000089815 | processed_transcrip | Gm5083 | 0.64422 | 0.326514 | 0.485367 |
| ENSMUSG00000045609 | protein_coding | Gm5455 | 0.64422 | 0.326514 | 0.485367 |
| ENSMUSG00000065864 | snoRNA | SNORA71 | 0.64422 | 0.326514 | 0.485367 |
| ENSMUSG00000058613 | protein_coding | Gm5062 | 0.64422 | 0.326514 | 0.485367 |
| ENSMUSG00000087447 | lincRNA | 2310026I22Rik | 0.64422 | 0.326514 | 0.485367 |
| ENSMUSG00000039098 | protein_coding | Gm9767 | 1.629262 | 2.048455 | 1.838858 |
| ENSMUSG00000070803 | protein_coding | Cited4 | 170.4778 | 151.3939 | 160.9359 |
| ENSMUSG00000055102 | protein_coding | Zfp819 | 70.27727 | 67.41126 | 68.84426 |

| | | | | | |
|---|---|---|---|---|---|
| ENSMUSG00000001943 | protein_coding | Vsig2 | 5.234495 | 6.127707 | 5.681101 |
| ENSMUSG00000089883 | pseudogene | Gm3355 | 1.606973 | 2.021789 | 1.814381 |
| ENSMUSG00000038916 | protein_coding | 6330407J23Rik | 6.781758 | 5.889839 | 6.335798 |
| ENSMUSG00000081103 | pseudogene | Gm11717 | 6.794579 | 5.908219 | 6.351399 |
| ENSMUSG00000072572 | protein_coding | Slc39a2 | 9.055015 | 7.832352 | 8.443684 |
| ENSMUSG00000046201 | protein_coding | Scaf8 | 3574.982 | 3641.123 | 3608.053 |
| ENSMUSG00000054418 | lincRNA | 2900041M22Rik | 35.28758 | 32.46036 | 33.87397 |
| ENSMUSG00000031209 | protein_coding | Heph | 544.8266 | 567.5911 | 556.2088 |
| ENSMUSG00000058246 | protein_coding | Gm10037 | 4.867807 | 4.150241 | 4.509024 |
| ENSMUSG00000074217 | protein_coding | 2210011C24Rik | 27.70982 | 29.49719 | 28.60351 |
| ENSMUSG00000040657 | protein_coding | 1700063H04Rik | 1.298952 | 1.703922 | 1.501437 |
| ENSMUSG00000089530 | snRNA | U6 | 1.298952 | 1.703922 | 1.501437 |
| ENSMUSG00000001062 | protein_coding | 1300018I17Rik | 593.6037 | 581.9419 | 587.7728 |
| ENSMUSG00000087686 | lincRNA | 1500026H17Rik | 2.303971 | 1.739597 | 2.021784 |
| ENSMUSG00000009115 | protein_coding | 1700027D21Rik | 3.617011 | 2.826166 | 3.221589 |
| ENSMUSG00000020020 | protein_coding | Usp44 | 123.365 | 130.4132 | 126.8891 |
| ENSMUSG00000038174 | protein_coding | Fam126b | 1360.414 | 1340.199 | 1350.307 |
| ENSMUSG00000001227 | protein_coding | Sema6b | 197.6093 | 191.0275 | 194.3184 |
| ENSMUSG00000017774 | protein_coding | Myo1c | 2251.627 | 2291.936 | 2271.782 |
| ENSMUSG00000026730 | protein_coding | Pter | 181.0493 | 186.5882 | 183.8187 |
| ENSMUSG00000002996 | protein_coding | Hbp1 | 5811.883 | 5895.875 | 5853.879 |
| ENSMUSG00000040697 | protein_coding | Dnajc16 | 1570.96 | 1545.132 | 1558.046 |
| ENSMUSG00000020673 | protein_coding | Tpo | 9.692303 | 10.789 | 10.24065 |
| ENSMUSG00000085882 | lincRNA | 2610507I01Rik | 184.844 | 210.3375 | 197.5908 |
| ENSMUSG00000058537 | protein_coding | Dux | 1.304841 | 1.775272 | 1.540057 |
| ENSMUSG00000091119 | protein_coding | Ccdc152 | 184.9694 | 175.9832 | 180.4763 |
| ENSMUSG00000031835 | protein_coding | Mbtps1 | 7619.273 | 7734.054 | 7676.664 |
| ENSMUSG00000001119 | protein_coding | Col6a1 | 4569.827 | 4459.27 | 4514.549 |
| ENSMUSG00000073617 | protein_coding | Hdac4 | 390.8151 | 379.0848 | 384.95 |
| ENSMUSG00000037499 | protein_coding | Nenf | 1169.402 | 1189.454 | 1179.428 |
| ENSMUSG00000085911 | processed_transcrip | Gm14061 | 0.65242 | 0.979543 | 0.815982 |
| ENSMUSG00000089694 | protein_coding | Gm4477 | 0.65242 | 0.979543 | 0.815982 |
| ENSMUSG00000026096 | protein_coding | Osgepl1 | 601.9172 | 615.1451 | 608.5312 |
| ENSMUSG00000085592 | processed_transcrip | Gm16321 | 0.960442 | 1.324076 | 1.142259 |
| ENSMUSG00000064679 | snoRNA | SNORD113 | 0.960442 | 1.324076 | 1.142259 |
| ENSMUSG00000034867 | protein_coding | Ankrd27 | 1135.011 | 1108.995 | 1122.003 |
| ENSMUSG00000058934 | processed_transcrip | Gm16176 | 1.937283 | 2.401635 | 2.169459 |
| ENSMUSG00000034290 | protein_coding | Nek9 | 3583.842 | 3531.406 | 3557.624 |
| ENSMUSG00000083101 | pseudogene | Gm13430 | 1.612862 | 2.093139 | 1.853001 |
| ENSMUSG00000086765 | processed_transcrip | Gm11827 | 8.026664 | 7.053556 | 7.54011 |
| ENSMUSG00000047710 | protein_coding | Zfp828 | 1650.043 | 1625.14 | 1637.591 |
| ENSMUSG00000038290 | protein_coding | Smg6 | 3091.561 | 3140.74 | 3116.151 |
| ENSMUSG00000081277 | pseudogene | Gm15285 | 2.911813 | 2.410644 | 2.661229 |
| ENSMUSG00000067034 | protein_coding | Zfp960 | 161.9607 | 155.66 | 158.8104 |
| ENSMUSG00000006050 | protein_coding | Sra1 | 960.7981 | 976.9766 | 968.8874 |
| ENSMUSG00000051934 | protein_coding | Spats2 | 2285.134 | 2244.747 | 2264.94 |
| ENSMUSG00000071909 | protein_coding | Gm806 | 1.270774 | 1.677256 | 1.474015 |
| ENSMUSG00000024579 | protein_coding | Pcyox1l | 175.5421 | 170.1289 | 172.8355 |
| ENSMUSG00000006191 | protein_coding | Cdkal1 | 491.6794 | 481.8742 | 486.7768 |
| ENSMUSG00000091082 | processed_transcrip | Gm17130 | 2.295771 | 1.757616 | 2.026693 |
| ENSMUSG00000074867 | protein_coding | Zfp808 | 490.832 | 505.8273 | 498.3296 |
| ENSMUSG00000027358 | protein_coding | Bmp2 | 141.1311 | 125.3841 | 133.2576 |
| ENSMUSG00000056492 | protein_coding | Gpr116 | 155.4334 | 161.1695 | 158.3014 |
| ENSMUSG00000030587 | protein_coding | 2200002D01Rik | 24.13739 | 26.08613 | 25.11176 |
| ENSMUSG00000035623 | protein_coding | Rsf1 | 2941.67 | 2996.585 | 2969.127 |
| ENSMUSG00000032207 | protein_coding | Lipc | 2.928213 | 2.401635 | 2.664924 |
| ENSMUSG00000050549 | protein_coding | 5730508B09Rik | 402.0298 | 388.9603 | 395.4951 |
| ENSMUSG00000052613 | protein_coding | Pcdh15 | 8.39693 | 7.416107 | 7.906519 |

| | | | | | |
|---|---|---|---|---|---|
| ENSMUSG00000020397 | protein_coding | Med7 | 640.3124 | 653.0569 | 646.6847 |
| ENSMUSG00000045871 | protein_coding | Slitrk6 | 0.962752 | 1.324076 | 1.143414 |
| ENSMUSG00000052922 | protein_coding | Bpi | 2.609681 | 2.075121 | 2.342401 |
| ENSMUSG00000090373 | lincRNA | Gm17435 | 96.71401 | 92.56978 | 94.64189 |
| ENSMUSG00000079537 | protein_coding | C030048H21Rik | 16.4587 | 17.85441 | 17.15656 |
| ENSMUSG00000047641 | protein_coding | Krt83 | 1.929083 | 2.401635 | 2.165359 |
| ENSMUSG00000069892 | protein_coding | 9930111J21Rik2 | 17.72486 | 16.38764 | 17.05625 |
| ENSMUSG00000026000 | protein_coding | Lancl1 | 1852.85 | 1822.627 | 1837.739 |
| ENSMUSG00000020713 | protein_coding | Gh | 1.282552 | 1.739597 | 1.511074 |
| ENSMUSG00000070806 | protein_coding | Zmynd12 | 27.2006 | 25.31769 | 26.25915 |
| ENSMUSG00000030465 | protein_coding | Psd3 | 1370.824 | 1398.215 | 1384.519 |
| ENSMUSG00000085112 | lincRNA | A530072M11Rik | 55.75838 | 52.8065 | 54.28244 |
| ENSMUSG00000079175 | protein_coding | Gm11060 | 2.267593 | 2.772834 | 2.520213 |
| ENSMUSG00000045330 | protein_coding | 4933402E13Rik | 4330.68 | 4253.283 | 4291.981 |
| ENSMUSG00000026189 | protein_coding | Pecr | 238.7574 | 244.6462 | 241.7018 |
| ENSMUSG00000013089 | protein_coding | Etv5 | 1094.894 | 1113.268 | 1104.081 |
| ENSMUSG00000057914 | protein_coding | Cacnb2 | 234.7033 | 241.7899 | 238.2466 |
| ENSMUSG00000087390 | processed_transcrip | Gm7598 | 23.94081 | 25.58766 | 24.76424 |
| ENSMUSG00000086727 | protein_coding | 4931428L18Rik | 5.509716 | 4.794622 | 5.152169 |
| ENSMUSG00000027661 | protein_coding | Slc2a10 | 41.66589 | 39.26232 | 40.46411 |
| ENSMUSG00000091332 | lincRNA | 4930500F10Rik | 5.159428 | 4.485765 | 4.822597 |
| ENSMUSG00000002992 | protein_coding | Apoc2 | 67.53296 | 79.03958 | 73.28627 |
| ENSMUSG00000070342 | protein_coding | Gm10287 | 1.606973 | 2.012779 | 1.809876 |
| ENSMUSG00000090759 | lincRNA | Gm17634 | 1.598773 | 2.057464 | 1.828118 |
| ENSMUSG00000080896 | pseudogene | Gm14567 | 5.499206 | 4.749938 | 5.124572 |
| ENSMUSG00000073414 | protein_coding | AU023871 | 15.16437 | 16.58367 | 15.87402 |
| ENSMUSG00000087022 | lincRNA | 9130024F11Rik | 0.968641 | 0.697713 | 0.833177 |
| ENSMUSG00000084758 | processed_transcrip | Gm12798 | 0.968641 | 0.697713 | 0.833177 |
| ENSMUSG00000028813 | protein_coding | CK137956 | 0.968641 | 0.697713 | 0.833177 |
| ENSMUSG00000079295 | protein_coding | Klrb1-ps1 | 0.968641 | 0.697713 | 0.833177 |
| ENSMUSG00000092039 | protein_coding | Gm5501 | 0.968641 | 0.697713 | 0.833177 |
| ENSMUSG00000029475 | protein_coding | Kdm2b | 1869.796 | 1830.636 | 1850.216 |
| ENSMUSG00000075585 | protein_coding | 6330403L08Rik | 158.0115 | 151.8035 | 154.9075 |
| ENSMUSG00000058838 | protein_coding | Rps27a-ps2 | 264.6282 | 257.8495 | 261.2388 |
| ENSMUSG00000073874 | protein_coding | 4933409K07Rik | 1.278973 | 1.73095 | 1.504961 |
| ENSMUSG00000065130 | snRNA | U6 | 1.937283 | 2.455329 | 2.196306 |
| ENSMUSG00000050014 | protein_coding | Apol10b | 14.89273 | 16.34839 | 15.62056 |
| ENSMUSG00000052673 | protein_coding | Gm9887 | 12.84989 | 11.65499 | 12.25244 |
| ENSMUSG00000073478 | lincRNA | D730003I15Rik | 43.43813 | 46.10173 | 44.76993 |
| ENSMUSG00000038167 | protein_coding | Plekhg6 | 25.10014 | 23.49489 | 24.29751 |
| ENSMUSG00000046873 | protein_coding | Mbtps2 | 852.9384 | 867.3892 | 860.1638 |
| ENSMUSG00000091199 | processed_transcrip | Gm2619 | 0.632442 | 0.36219 | 0.497316 |
| ENSMUSG00000065666 | snRNA | U6 | 0.632442 | 0.36219 | 0.497316 |
| ENSMUSG00000087467 | processed_transcrip | Gm13601 | 0.632442 | 0.36219 | 0.497316 |
| ENSMUSG00000081041 | pseudogene | Nlk-ps1 | 0.632442 | 0.36219 | 0.497316 |
| ENSMUSG00000083341 | pseudogene | Gm14238 | 0.632442 | 0.36219 | 0.497316 |
| ENSMUSG00000070979 | protein_coding | Actl7a | 0.632442 | 0.36219 | 0.497316 |
| ENSMUSG00000084267 | pseudogene | Gm12417 | 0.632442 | 0.36219 | 0.497316 |
| ENSMUSG00000082592 | pseudogene | Gm12950 | 0.632442 | 0.36219 | 0.497316 |
| ENSMUSG00000043066 | protein_coding | Vmn1r66 | 0.632442 | 0.36219 | 0.497316 |
| ENSMUSG00000086828 | processed_transcrip | Gm15579 | 0.632442 | 0.36219 | 0.497316 |
| ENSMUSG00000086428 | processed_transcrip | Gm15199 | 0.632442 | 0.36219 | 0.497316 |
| ENSMUSG00000081968 | pseudogene | Rpl23a-ps2 | 0.632442 | 0.36219 | 0.497316 |
| ENSMUSG00000083876 | pseudogene | Gm11439 | 0.632442 | 0.36219 | 0.497316 |
| ENSMUSG00000079271 | protein_coding | 1700049E17Rik1 | 0.632442 | 0.36219 | 0.497316 |
| ENSMUSG00000076919 | IG_J_gene | Gm16653 | 0.632442 | 0.36219 | 0.497316 |
| ENSMUSG00000033187 | protein_coding | BC016579 | 0.632442 | 0.36219 | 0.497316 |
| ENSMUSG00000023943 | protein_coding | Sult1c1 | 0.632442 | 0.36219 | 0.497316 |

| | | | | | |
|---|---|---|---|---|---|
| ENSMUSG00000004837 | protein_coding | Grap | 44.80686 | 41.92492 | 43.36589 |
| ENSMUSG00000073879 | pseudogene | Gm5859 | 5.875137 | 9.779118 | 7.827127 |
| ENSMUSG00000041992 | protein_coding | Rapgef5 | 204.0583 | 196.0702 | 200.0643 |
| ENSMUSG00000079465 | protein_coding | Col4a3 | 2540.234 | 2482.998 | 2511.616 |
| ENSMUSG00000035403 | protein_coding | Crb2 | 65.1086 | 62.28111 | 63.69485 |
| ENSMUSG00000023274 | protein_coding | Cd4 | 35.93836 | 38.1728 | 37.05558 |
| ENSMUSG00000065528 | miRNA | Mir320 | 4.835007 | 4.025197 | 4.430102 |
| ENSMUSG00000073702 | protein_coding | Rpl31 | 4087.213 | 4022.503 | 4054.858 |
| ENSMUSG00000027695 | protein_coding | Pld1 | 177.9653 | 172.737 | 175.3511 |
| ENSMUSG00000059013 | protein_coding | Sh2d3c | 311.5763 | 303.3774 | 307.4768 |
| ENSMUSG00000026201 | protein_coding | Stk16 | 1220.728 | 1240.281 | 1230.504 |
| ENSMUSG00000084194 | pseudogene | Gm12407 | 2.241726 | 1.704284 | 1.973005 |
| ENSMUSG00000048578 | protein_coding | Mlec | 12606.51 | 12437.98 | 12522.25 |
| ENSMUSG00000079532 | protein_coding | Gm6890 | 35.58114 | 37.67692 | 36.62903 |
| ENSMUSG00000049313 | protein_coding | Sorl1 | 1055.942 | 1024.026 | 1039.984 |
| ENSMUSG00000078735 | protein_coding | Gm2002 | 0.632442 | 1.006571 | 0.819506 |
| ENSMUSG00000000776 | protein_coding | Polr3d | 1597.033 | 1569.282 | 1583.157 |
| ENSMUSG00000031150 | protein_coding | Ccdc120 | 445.8977 | 437.0835 | 441.4906 |
| ENSMUSG00000034974 | protein_coding | Dapk3 | 704.2116 | 716.5802 | 710.3959 |
| ENSMUSG00000084744 | snoRNA | SNORA48 | 2.575614 | 3.108357 | 2.841986 |
| ENSMUSG00000064606 | snRNA | U4 | 1.307151 | 1.775272 | 1.541212 |
| ENSMUSG00000081459 | pseudogene | Gm12952 | 0.962752 | 0.662038 | 0.812395 |
| ENSMUSG00000047692 | protein_coding | 4930533K18Rik | 0.962752 | 0.662038 | 0.812395 |
| ENSMUSG00000032570 | protein_coding | Atp2c1 | 2698.949 | 2739.041 | 2718.995 |
| ENSMUSG00000031848 | protein_coding | Lsm4 | 1937.005 | 1970.217 | 1953.611 |
| ENSMUSG00000030004 | protein_coding | Nat8 | 7.737353 | 6.887761 | 7.312557 |
| ENSMUSG00000045802 | protein_coding | Hsf3 | 25.22128 | 27.35786 | 26.28957 |
| ENSMUSG00000047347 | protein_coding | Gm5806 | 5.175828 | 4.485765 | 4.830796 |
| ENSMUSG00000038503 | protein_coding | Mesdc2 | 3048.22 | 3005.762 | 3026.991 |
| ENSMUSG00000052076 | protein_coding | Gm9866 | 0.954552 | 1.315066 | 1.134809 |
| ENSMUSG00000073741 | protein_coding | 4732440D04Rik | 0.33031 | 0 | 0.165155 |
| ENSMUSG00000093057 | miRNA | AC156988.1 | 0.33031 | 0 | 0.165155 |
| ENSMUSG00000090142 | pseudogene | Gm15795 | 0.33031 | 0 | 0.165155 |
| ENSMUSG00000073722 | protein_coding | 4931408C20Rik | 0.316221 | 0 | 0.158111 |
| ENSMUSG00000049830 | protein_coding | Gm9839 | 0.32211 | 0 | 0.161055 |
| ENSMUSG00000080054 | protein_coding | Rpl12-ps1 | 0.32211 | 0 | 0.161055 |
| ENSMUSG00000092553 | pseudogene | RP24-180L10.1 | 0.33031 | 0 | 0.165155 |
| ENSMUSG00000092563 | pseudogene | RP24-539E18.2 | 0.33031 | 0 | 0.165155 |
| ENSMUSG00000088848 | snoRNA | SNORA17 | 0.33031 | 0 | 0.165155 |
| ENSMUSG00000064498 | snRNA | U6 | 0.32211 | 0 | 0.161055 |
| ENSMUSG00000025945 | protein_coding | Crygf | 0.316221 | 0 | 0.158111 |
| ENSMUSG00000088850 | snoRNA | SNORA26 | 0.33031 | 0 | 0.165155 |
| ENSMUSG00000077328 | snoRNA | SNORA17 | 0.33031 | 0 | 0.165155 |
| ENSMUSG00000059999 | pseudogene | Gm10072 | 0.33031 | 0 | 0.165155 |
| ENSMUSG00000082528 | pseudogene | Gm15182 | 0.32211 | 0 | 0.161055 |
| ENSMUSG00000087008 | pseudogene | Gm5530 | 0.316221 | 0 | 0.158111 |
| ENSMUSG00000026166 | protein_coding | Ccl20 | 0.316221 | 0 | 0.158111 |
| ENSMUSG00000089976 | pseudogene | Gm2389 | 0.32211 | 0 | 0.161055 |
| ENSMUSG00000090128 | pseudogene | Gm16038 | 0.316221 | 0 | 0.158111 |
| ENSMUSG00000079457 | processed_transcrip | Gm7609 | 0.32211 | 0 | 0.161055 |
| ENSMUSG00000089821 | processed_transcrip | Gm16535 | 0.33031 | 0 | 0.165155 |
| ENSMUSG00000077075 | miRNA | AC087801.1 | 0.316221 | 0 | 0.158111 |
| ENSMUSG00000084195 | pseudogene | Gm15372 | 0.32211 | 0 | 0.161055 |
| ENSMUSG00000082677 | pseudogene | Gm15371 | 0.316221 | 0 | 0.158111 |
| ENSMUSG00000090171 | protein_coding | Ugt1a2 | 0.316221 | 0 | 0.158111 |
| ENSMUSG00000089169 | misc_RNA | 7SK | 0.316221 | 0 | 0.158111 |
| ENSMUSG00000048775 | protein_coding | Serpinb13 | 0.32211 | 0 | 0.161055 |
| ENSMUSG00000026327 | protein_coding | Serpinb11 | 0.32211 | 0 | 0.161055 |

| | | | | | |
|---|---|---|---|---|---|
| ENSMUSG00000090277 | pseudogene | Gm8338 | 0.33031 | 0 | 0.165155 |
| ENSMUSG00000026387 | protein_coding | Sctr | 0.32211 | 0 | 0.161055 |
| ENSMUSG00000042554 | protein_coding | Zp3r | 0.316221 | 0 | 0.158111 |
| ENSMUSG00000076287 | miRNA | AC109299.1 | 0.32211 | 0 | 0.161055 |
| ENSMUSG00000065435 | miRNA | Mir135b | 0.316221 | 0 | 0.158111 |
| ENSMUSG00000026424 | protein_coding | Gpr37l1 | 0.32211 | 0 | 0.161055 |
| ENSMUSG00000051480 | protein_coding | A130050O07Rik | 0.32211 | 0 | 0.161055 |
| ENSMUSG00000065458 | miRNA | Mir181b-1 | 0.33031 | 0 | 0.165155 |
| ENSMUSG00000085361 | pseudogene | Gm16305 | 0.33031 | 0 | 0.165155 |
| ENSMUSG00000089221 | misc_RNA | Y_RNA | 0.316221 | 0 | 0.158111 |
| ENSMUSG00000046057 | pseudogene | Gm15428 | 0.32211 | 0 | 0.161055 |
| ENSMUSG00000090335 | lincRNA | Gm17698 | 0.33031 | 0 | 0.165155 |
| ENSMUSG00000086116 | lincRNA | Gm16608 | 0.32211 | 0 | 0.161055 |
| ENSMUSG00000087886 | misc_RNA | 7SK | 0.33031 | 0 | 0.165155 |
| ENSMUSG00000026560 | protein_coding | Fmo9 | 0.32211 | 0 | 0.161055 |
| ENSMUSG00000040485 | protein_coding | Lrrc52 | 0.316221 | 0 | 0.158111 |
| ENSMUSG00000055033 | protein_coding | Olfr420 | 0.316221 | 0 | 0.158111 |
| ENSMUSG00000065821 | snRNA | U6 | 0.33031 | 0 | 0.165155 |
| ENSMUSG00000092793 | miRNA | AL772216.43 | 0.33031 | 0 | 0.165155 |
| ENSMUSG00000076126 | miRNA | Mir669b | 0.32211 | 0 | 0.161055 |
| ENSMUSG00000077049 | miRNA | Mir467c | 0.33031 | 0 | 0.165155 |
| ENSMUSG00000080362 | miRNA | AL772216.7 | 0.316221 | 0 | 0.158111 |
| ENSMUSG00000076948 | miRNA | Mir467e | 0.316221 | 0 | 0.158111 |
| ENSMUSG00000070099 | miRNA | Mir466 | 0.32211 | 0 | 0.161055 |
| ENSMUSG00000077086 | miRNA | Mir669o | 0.32211 | 0 | 0.161055 |
| ENSMUSG00000080801 | pseudogene | Gm13310 | 0.33031 | 0 | 0.165155 |
| ENSMUSG00000085281 | pseudogene | Gm13316 | 0.316221 | 0 | 0.158111 |
| ENSMUSG00000064964 | snRNA | U6 | 0.32211 | 0 | 0.161055 |
| ENSMUSG00000084071 | pseudogene | Gm13378 | 0.33031 | 0 | 0.165155 |
| ENSMUSG00000065798 | snRNA | U6 | 0.32211 | 0 | 0.161055 |
| ENSMUSG00000089513 | snRNA | U6 | 0.316221 | 0 | 0.158111 |
| ENSMUSG00000076364 | miRNA | AL731552.1 | 0.33031 | 0 | 0.165155 |
| ENSMUSG00000088836 | snoRNA | SNORA48 | 0.316221 | 0 | 0.158111 |
| ENSMUSG00000082844 | pseudogene | Gm13537 | 0.316221 | 0 | 0.158111 |
| ENSMUSG00000079421 | protein_coding | 4930402F06Rik | 0.33031 | 0 | 0.165155 |
| ENSMUSG00000051052 | pseudogene | Olfr335-ps | 0.33031 | 0 | 0.165155 |
| ENSMUSG00000049018 | protein_coding | Olfr368 | 0.316221 | 0 | 0.158111 |
| ENSMUSG00000077756 | snoRNA | Snord90 | 0.32211 | 0 | 0.161055 |
| ENSMUSG00000092958 | miRNA | BX545905.1 | 0.33031 | 0 | 0.165155 |
| ENSMUSG00000064468 | snRNA | U6 | 0.316221 | 0 | 0.158111 |
| ENSMUSG00000083270 | pseudogene | Gm13498 | 0.32211 | 0 | 0.161055 |
| ENSMUSG00000084334 | pseudogene | Gm13494 | 0.32211 | 0 | 0.161055 |
| ENSMUSG00000080782 | pseudogene | Gm13495 | 0.33031 | 0 | 0.165155 |
| ENSMUSG00000085815 | pseudogene | Gm13548 | 0.32211 | 0 | 0.161055 |
| ENSMUSG00000089272 | snoRNA | SNORA17 | 0.316221 | 0 | 0.158111 |
| ENSMUSG00000082578 | pseudogene | Gm13527 | 0.33031 | 0 | 0.165155 |
| ENSMUSG00000080982 | pseudogene | Gm13549 | 0.32211 | 0 | 0.161055 |
| ENSMUSG00000026989 | protein_coding | Dapl1 | 0.316221 | 0 | 0.158111 |
| ENSMUSG00000086701 | processed_transcrip | Gm13595 | 0.33031 | 0 | 0.165155 |
| ENSMUSG00000081299 | pseudogene | Gm13603 | 0.33031 | 0 | 0.165155 |
| ENSMUSG00000005232 | protein_coding | G6pc2 | 0.33031 | 0 | 0.165155 |
| ENSMUSG00000082190 | pseudogene | Gm13641 | 0.316221 | 0 | 0.158111 |
| ENSMUSG00000086641 | lincRNA | Gm13663 | 0.32211 | 0 | 0.161055 |
| ENSMUSG00000084794 | lincRNA | Gm13662 | 0.32211 | 0 | 0.161055 |
| ENSMUSG00000083976 | pseudogene | Gm13664 | 0.33031 | 0 | 0.165155 |
| ENSMUSG00000086546 | processed_transcrip | Gm13709 | 0.33031 | 0 | 0.165155 |
| ENSMUSG00000086069 | processed_transcrip | Gm13708 | 0.33031 | 0 | 0.165155 |
| ENSMUSG00000092315 | processed_transcrip | Gm20440 | 0.33031 | 0 | 0.165155 |

| | | | | | |
|---|---|---|---|---|---|
| ENSMUSG00000082006 | pseudogene | Gm13649 | 0.33031 | 0 | 0.165155 |
| ENSMUSG00000092843 | miRNA | AL935159.1 | 0.316221 | 0 | 0.158111 |
| ENSMUSG00000082712 | pseudogene | Olfr990-ps1 | 0.32211 | 0 | 0.161055 |
| ENSMUSG00000046076 | protein_coding | Olfr998 | 0.32211 | 0 | 0.161055 |
| ENSMUSG00000082892 | pseudogene | Olfr1027-ps1 | 0.316221 | 0 | 0.158111 |
| ENSMUSG00000075156 | protein_coding | Olfr1128 | 0.32211 | 0 | 0.161055 |
| ENSMUSG00000075062 | protein_coding | Olfr1271 | 0.33031 | 0 | 0.165155 |
| ENSMUSG00000077883 | miRNA | AL683882.1 | 0.316221 | 0 | 0.158111 |
| ENSMUSG00000083643 | pseudogene | Gm13876 | 0.316221 | 0 | 0.158111 |
| ENSMUSG00000077236 | snoRNA | SNORA17 | 0.32211 | 0 | 0.161055 |
| ENSMUSG00000082934 | pseudogene | Gm13887 | 0.32211 | 0 | 0.161055 |
| ENSMUSG00000087226 | processed_transcrip | Gm14015 | 0.33031 | 0 | 0.165155 |
| ENSMUSG00000087225 | processed_transcrip | Gm14014 | 0.33031 | 0 | 0.165155 |
| ENSMUSG00000052005 | pseudogene | Gm9864 | 0.33031 | 0 | 0.165155 |
| ENSMUSG00000081746 | pseudogene | Gm13925 | 0.32211 | 0 | 0.161055 |
| ENSMUSG00000083320 | pseudogene | Gm13935 | 0.316221 | 0 | 0.158111 |
| ENSMUSG00000050808 | protein_coding | Muc15 | 0.32211 | 0 | 0.161055 |
| ENSMUSG00000079171 | processed_transcrip | Gm13942 | 0.33031 | 0 | 0.165155 |
| ENSMUSG00000093299 | miRNA | AL844901.1 | 0.32211 | 0 | 0.161055 |
| ENSMUSG00000027355 | protein_coding | Tmco5 | 0.32211 | 0 | 0.161055 |
| ENSMUSG00000074930 | pseudogene | Gm13981 | 0.33031 | 0 | 0.165155 |
| ENSMUSG00000084015 | pseudogene | Gm14175 | 0.33031 | 0 | 0.165155 |
| ENSMUSG00000087682 | processed_transcrip | Gm14003 | 0.316221 | 0 | 0.158111 |
| ENSMUSG00000079053 | processed_transcrip | Gm14010 | 0.33031 | 0 | 0.165155 |
| ENSMUSG00000082097 | pseudogene | Gm14039 | 0.316221 | 0 | 0.158111 |
| ENSMUSG00000027345 | processed_transcrip | 4921508D12Rik | 0.32211 | 0 | 0.161055 |
| ENSMUSG00000085709 | processed_transcrip | Gm14211 | 0.33031 | 0 | 0.165155 |
| ENSMUSG00000074763 | processed_transcrip | Gm14105 | 0.33031 | 0 | 0.165155 |
| ENSMUSG00000087158 | processed_transcrip | Gm14093 | 0.316221 | 0 | 0.158111 |
| ENSMUSG00000088213 | misc_RNA | 7SK | 0.33031 | 0 | 0.165155 |
| ENSMUSG00000082158 | pseudogene | Gm14116 | 0.316221 | 0 | 0.158111 |
| ENSMUSG00000074837 | protein_coding | Gm10770 | 0.316221 | 0 | 0.158111 |
| ENSMUSG00000082341 | pseudogene | Gm14143 | 0.33031 | 0 | 0.165155 |
| ENSMUSG00000088491 | miRNA | Mir1953 | 0.33031 | 0 | 0.165155 |
| ENSMUSG00000082689 | pseudogene | Gm14156 | 0.32211 | 0 | 0.161055 |
| ENSMUSG00000089499 | snRNA | U6 | 0.33031 | 0 | 0.165155 |
| ENSMUSG00000086872 | processed_transcrip | Gm14225 | 0.33031 | 0 | 0.165155 |
| ENSMUSG00000067745 | processed_transcrip | Gm14243 | 0.316221 | 0 | 0.158111 |
| ENSMUSG00000077143 | snoRNA | SNORA17 | 0.32211 | 0 | 0.161055 |
| ENSMUSG00000070708 | protein_coding | Gtsf1l | 0.33031 | 0 | 0.165155 |
| ENSMUSG00000053353 | protein_coding | 2310001K24Rik | 0.316221 | 0 | 0.158111 |
| ENSMUSG00000085618 | processed_transcrip | Gm11466 | 0.33031 | 0 | 0.165155 |
| ENSMUSG00000086238 | processed_transcrip | Gm14258 | 0.33031 | 0 | 0.165155 |
| ENSMUSG00000085426 | processed_transcrip | Gm14272 | 0.33031 | 0 | 0.165155 |
| ENSMUSG00000087100 | processed_transcrip | Gm14641 | 0.316221 | 0 | 0.158111 |
| ENSMUSG00000093160 | miRNA | AL845460.1 | 0.33031 | 0 | 0.165155 |
| ENSMUSG00000065410 | miRNA | Mir298 | 0.33031 | 0 | 0.165155 |
| ENSMUSG00000082376 | pseudogene | Gm14442 | 0.316221 | 0 | 0.158111 |
| ENSMUSG00000091984 | pseudogene | Gm17258 | 0.33031 | 0 | 0.165155 |
| ENSMUSG00000078886 | protein_coding | Gm2026 | 0.316221 | 0 | 0.158111 |
| ENSMUSG00000089951 | protein_coding | Gm14435 | 0.32211 | 0 | 0.161055 |
| ENSMUSG00000081326 | pseudogene | Gm14417 | 0.32211 | 0 | 0.161055 |
| ENSMUSG00000083317 | pseudogene | Gm14402 | 0.33031 | 0 | 0.165155 |
| ENSMUSG00000074518 | protein_coding | Gm10711 | 0.316221 | 0 | 0.158111 |
| ENSMUSG00000064422 | snRNA | U6 | 0.33031 | 0 | 0.165155 |
| ENSMUSG00000017688 | protein_coding | Hnf4g | 0.316221 | 0 | 0.158111 |
| ENSMUSG00000069118 | protein_coding | 1700008P02Rik | 0.33031 | 0 | 0.165155 |
| ENSMUSG00000044138 | protein_coding | Sirpb1a | 0.32211 | 0 | 0.161055 |

| | | | | | |
|---|---|---|---|---|---|
| ENSMUSG00000093073 | miRNA | AC124807.1 | 0.33031 | 0 | 0.165155 |
| ENSMUSG00000049796 | protein_coding | Crh | 0.33031 | 0 | 0.165155 |
| ENSMUSG00000092196 | processed_transcrip | Gm20446 | 0.33031 | 0 | 0.165155 |
| ENSMUSG00000065945 | snRNA | U1 | 0.33031 | 0 | 0.165155 |
| ENSMUSG00000093166 | misc_RNA | AC160757.1 | 0.33031 | 0 | 0.165155 |
| ENSMUSG00000092769 | miRNA | AC116729.1 | 0.316221 | 0 | 0.158111 |
| ENSMUSG00000091376 | protein_coding | Aadacl2 | 0.33031 | 0 | 0.165155 |
| ENSMUSG00000027762 | protein_coding | Sucnr1 | 0.33031 | 0 | 0.165155 |
| ENSMUSG00000043897 | protein_coding | Vmn2r2 | 0.33031 | 0 | 0.165155 |
| ENSMUSG00000090941 | pseudogene | Gm17212 | 0.316221 | 0 | 0.158111 |
| ENSMUSG00000086538 | lincRNA | Gm6634 | 0.32211 | 0 | 0.161055 |
| ENSMUSG00000064693 | snRNA | U6 | 0.316221 | 0 | 0.158111 |
| ENSMUSG00000087123 | pseudogene | Gm6706 | 0.32211 | 0 | 0.161055 |
| ENSMUSG00000064840 | snRNA | U6 | 0.316221 | 0 | 0.158111 |
| ENSMUSG00000048031 | protein_coding | Fcrl5 | 0.316221 | 0 | 0.158111 |
| ENSMUSG00000077376 | snoRNA | SNORA17 | 0.316221 | 0 | 0.158111 |
| ENSMUSG00000074486 | protein_coding | Bglap2 | 0.32211 | 0 | 0.161055 |
| ENSMUSG00000001023 | protein_coding | S100a5 | 0.33031 | 0 | 0.165155 |
| ENSMUSG00000042250 | protein_coding | Pglyrp4 | 0.33031 | 0 | 0.165155 |
| ENSMUSG00000042244 | protein_coding | Pglyrp3 | 0.33031 | 0 | 0.165155 |
| ENSMUSG00000074445 | protein_coding | Sprr2a3 | 0.316221 | 0 | 0.158111 |
| ENSMUSG00000042212 | protein_coding | Sprr2d | 0.33031 | 0 | 0.165155 |
| ENSMUSG00000050635 | protein_coding | Sprr2f | 0.316221 | 0 | 0.158111 |
| ENSMUSG00000054215 | protein_coding | Sprr2k | 0.32211 | 0 | 0.161055 |
| ENSMUSG00000086848 | protein_coding | Lce6a | 0.316221 | 0 | 0.158111 |
| ENSMUSG00000088511 | snRNA | U6 | 0.33031 | 0 | 0.165155 |
| ENSMUSG00000082000 | pseudogene | Gm12488 | 0.32211 | 0 | 0.161055 |
| ENSMUSG00000086745 | processed_transcrip | 2410057H14Rik | 0.32211 | 0 | 0.161055 |
| ENSMUSG00000065708 | snRNA | U1 | 0.316221 | 0 | 0.158111 |
| ENSMUSG00000088479 | snoRNA | SNORA17 | 0.32211 | 0 | 0.161055 |
| ENSMUSG00000042861 | protein_coding | Kcna10 | 0.33031 | 0 | 0.165155 |
| ENSMUSG00000081688 | pseudogene | Gm12499 | 0.33031 | 0 | 0.165155 |
| ENSMUSG00000092718 | miRNA | AL683823.1 | 0.33031 | 0 | 0.165155 |
| ENSMUSG00000080483 | miRNA | AC111029.2 | 0.316221 | 0 | 0.158111 |
| ENSMUSG00000093177 | miRNA | AC110195.1 | 0.33031 | 0 | 0.165155 |
| ENSMUSG00000092934 | miRNA | AC134918.1 | 0.32211 | 0 | 0.161055 |
| ENSMUSG00000076252 | miRNA | Mir302b | 0.33031 | 0 | 0.165155 |
| ENSMUSG00000065788 | snRNA | U6 | 0.316221 | 0 | 0.158111 |
| ENSMUSG00000091721 | protein_coding | Gm5549 | 0.33031 | 0 | 0.165155 |
| ENSMUSG00000092810 | miRNA | AC164408.1 | 0.32211 | 0 | 0.161055 |
| ENSMUSG00000083045 | pseudogene | Gm16100 | 0.316221 | 0 | 0.158111 |
| ENSMUSG00000091791 | pseudogene | Gm16500 | 0.33031 | 0 | 0.165155 |
| ENSMUSG00000078366 | protein_coding | Vmn1r3 | 0.33031 | 0 | 0.165155 |
| ENSMUSG00000081660 | pseudogene | Gm11787 | 0.33031 | 0 | 0.165155 |
| ENSMUSG00000086066 | processed_transcrip | 4930423M02Rik | 0.33031 | 0 | 0.165155 |
| ENSMUSG00000086070 | processed_transcrip | 8430436N08Rik | 0.316221 | 0 | 0.158111 |
| ENSMUSG00000083025 | pseudogene | Gm11829 | 0.33031 | 0 | 0.165155 |
| ENSMUSG00000087919 | snoRNA | SNORA17 | 0.32211 | 0 | 0.161055 |
| ENSMUSG00000092822 | miRNA | AL807379.1 | 0.316221 | 0 | 0.158111 |
| ENSMUSG00000073991 | protein_coding | Cnbd1 | 0.33031 | 0 | 0.165155 |
| ENSMUSG00000084201 | pseudogene | Gm11926 | 0.33031 | 0 | 0.165155 |
| ENSMUSG00000083565 | pseudogene | Gm11941 | 0.316221 | 0 | 0.158111 |
| ENSMUSG00000077397 | snoRNA | SNORA17 | 0.33031 | 0 | 0.165155 |
| ENSMUSG00000082649 | pseudogene | Gm12754 | 0.32211 | 0 | 0.161055 |
| ENSMUSG00000084898 | processed_transcrip | Gm12371 | 0.33031 | 0 | 0.165155 |
| ENSMUSG00000092971 | miRNA | AL833775.3 | 0.32211 | 0 | 0.161055 |
| ENSMUSG00000092862 | miRNA | AL837521.1 | 0.32211 | 0 | 0.161055 |
| ENSMUSG00000088528 | snRNA | U6 | 0.33031 | 0 | 0.165155 |

| | | | | | |
|---|---|---|---|---|---|
| ENSMUSG00000036162 | processed_transcrip | 1700066J24Rik | 0.316221 | 0 | 0.158111 |
| ENSMUSG00000078744 | protein_coding | Gm3893 | 0.33031 | 0 | 0.165155 |
| ENSMUSG00000078727 | protein_coding | Gm2542 | 0.33031 | 0 | 0.165155 |
| ENSMUSG00000077552 | snoRNA | SNORA17 | 0.33031 | 0 | 0.165155 |
| ENSMUSG00000092675 | miRNA | AL732626.1 | 0.33031 | 0 | 0.165155 |
| ENSMUSG00000083491 | pseudogene | Gm12423 | 0.33031 | 0 | 0.165155 |
| ENSMUSG00000083153 | pseudogene | Gm12459 | 0.32211 | 0 | 0.161055 |
| ENSMUSG00000082216 | pseudogene | Olfr271-ps1 | 0.32211 | 0 | 0.161055 |
| ENSMUSG00000077403 | snoRNA | U3 | 0.32211 | 0 | 0.161055 |
| ENSMUSG00000084144 | pseudogene | Gm12516 | 0.32211 | 0 | 0.161055 |
| ENSMUSG00000092753 | rRNA | AL805972.1 | 0.33031 | 0 | 0.165155 |
| ENSMUSG00000092712 | miRNA | AL824704.1 | 0.33031 | 0 | 0.165155 |
| ENSMUSG00000077161 | snoRNA | SNORA17 | 0.32211 | 0 | 0.161055 |
| ENSMUSG00000086364 | processed_transcrip | Gm11751 | 0.33031 | 0 | 0.165155 |
| ENSMUSG00000092492 | processed_transcrip | RP23-405M24.1 | 0.33031 | 0 | 0.165155 |
| ENSMUSG00000085840 | processed_transcrip | Gm11261 | 0.316221 | 0 | 0.158111 |
| ENSMUSG00000084192 | pseudogene | Gm13282 | 0.32211 | 0 | 0.161055 |
| ENSMUSG00000085473 | protein_coding | Gm16686 | 0.32211 | 0 | 0.161055 |
| ENSMUSG00000091535 | lincRNA | Gm17612 | 0.33031 | 0 | 0.165155 |
| ENSMUSG00000083881 | pseudogene | Gm12605 | 0.32211 | 0 | 0.161055 |
| ENSMUSG00000080808 | pseudogene | Gm12608 | 0.32211 | 0 | 0.161055 |
| ENSMUSG00000077589 | snoRNA | SNORA17 | 0.33031 | 0 | 0.165155 |
| ENSMUSG00000083724 | pseudogene | Gm12651 | 0.316221 | 0 | 0.158111 |
| ENSMUSG00000070900 | protein_coding | Gm10306 | 0.33031 | 0 | 0.165155 |
| ENSMUSG00000037028 | protein_coding | Gm12800 | 0.316221 | 0 | 0.158111 |
| ENSMUSG00000081189 | pseudogene | Gm12725 | 0.33031 | 0 | 0.165155 |
| ENSMUSG00000087289 | processed_transcrip | 4933424M12Rik | 0.316221 | 0 | 0.158111 |
| ENSMUSG00000028612 | processed_transcrip | 4930430J20Rik | 0.32211 | 0 | 0.161055 |
| ENSMUSG00000060491 | protein_coding | 4930522H14Rik | 0.33031 | 0 | 0.165155 |
| ENSMUSG00000082771 | pseudogene | Gm12803 | 0.32211 | 0 | 0.161055 |
| ENSMUSG00000066071 | protein_coding | Cyp4a12a | 0.33031 | 0 | 0.165155 |
| ENSMUSG00000061075 | protein_coding | Olfr1337 | 0.316221 | 0 | 0.158111 |
| ENSMUSG00000084082 | pseudogene | Gm12872 | 0.316221 | 0 | 0.158111 |
| ENSMUSG00000086153 | processed_transcrip | Gm12923 | 0.33031 | 0 | 0.165155 |
| ENSMUSG00000082634 | pseudogene | Gm12900 | 0.33031 | 0 | 0.165155 |
| ENSMUSG00000076035 | miRNA | Mir692-2a | 0.316221 | 0 | 0.158111 |
| ENSMUSG00000082468 | pseudogene | Gm12980 | 0.316221 | 0 | 0.158111 |
| ENSMUSG00000087451 | processed_transcrip | Gm12970 | 0.33031 | 0 | 0.165155 |
| ENSMUSG00000064739 | snRNA | U6 | 0.33031 | 0 | 0.165155 |
| ENSMUSG00000089321 | snRNA | U6 | 0.32211 | 0 | 0.161055 |
| ENSMUSG00000085002 | processed_transcrip | Gm12984 | 0.33031 | 0 | 0.165155 |
| ENSMUSG00000087417 | processed_transcrip | Gm13001 | 0.316221 | 0 | 0.158111 |
| ENSMUSG00000085833 | lincRNA | Gm13003 | 0.316221 | 0 | 0.158111 |
| ENSMUSG00000080806 | pseudogene | Gm13005 | 0.33031 | 0 | 0.165155 |
| ENSMUSG00000084804 | processed_transcrip | 1700095J12Rik | 0.33031 | 0 | 0.165155 |
| ENSMUSG00000078518 | protein_coding | Gm13030 | 0.32211 | 0 | 0.161055 |
| ENSMUSG00000093285 | miRNA | AL807811.2 | 0.316221 | 0 | 0.158111 |
| ENSMUSG00000092637 | miRNA | BX000454.1 | 0.33031 | 0 | 0.165155 |
| ENSMUSG00000087628 | processed_transcrip | Gm13028 | 0.33031 | 0 | 0.165155 |
| ENSMUSG00000085928 | processed_transcrip | 4933427I22Rik | 0.316221 | 0 | 0.158111 |
| ENSMUSG00000086818 | processed_transcrip | 1810035I16Rik | 0.316221 | 0 | 0.158111 |
| ENSMUSG00000082201 | pseudogene | Gm13018 | 0.32211 | 0 | 0.161055 |
| ENSMUSG00000087903 | snoRNA | SNORA17 | 0.33031 | 0 | 0.165155 |
| ENSMUSG00000081448 | pseudogene | Gm13086 | 0.32211 | 0 | 0.161055 |
| ENSMUSG00000070619 | protein_coding | Gm13119 | 0.32211 | 0 | 0.161055 |
| ENSMUSG00000082418 | pseudogene | Gm13126 | 0.33031 | 0 | 0.165155 |
| ENSMUSG00000081983 | pseudogene | Gm13249 | 0.32211 | 0 | 0.161055 |
| ENSMUSG00000082615 | pseudogene | Gm13239 | 0.33031 | 0 | 0.165155 |

| | | | | | |
|---|---|---|---|---|---|
| ENSMUSG00000087513 | processed_transcrip | Gm13236 | 0.316221 | 0 | 0.158111 |
| ENSMUSG00000082944 | pseudogene | Gm13137 | 0.316221 | 0 | 0.158111 |
| ENSMUSG00000087027 | processed_transcrip | Gm13206 | 0.316221 | 0 | 0.158111 |
| ENSMUSG00000087273 | processed_transcrip | Gm13203 | 0.316221 | 0 | 0.158111 |
| ENSMUSG00000085097 | processed_transcrip | Gm13049 | 0.33031 | 0 | 0.165155 |
| ENSMUSG00000085179 | lincRNA | Gm13645 | 0.32211 | 0 | 0.161055 |
| ENSMUSG00000080471 | miRNA | AL670236.1 | 0.32211 | 0 | 0.161055 |
| ENSMUSG00000065400 | miRNA | Mir200a | 0.33031 | 0 | 0.165155 |
| ENSMUSG00000088131 | snoRNA | SCARNA15 | 0.316221 | 0 | 0.158111 |
| ENSMUSG00000040514 | protein_coding | 4921511H03Rik | 0.33031 | 0 | 0.165155 |
| ENSMUSG00000081226 | pseudogene | Gm8802 | 0.32211 | 0 | 0.161055 |
| ENSMUSG00000082867 | pseudogene | Gm15730 | 0.32211 | 0 | 0.161055 |
| ENSMUSG00000093337 | miRNA | AC154124.1 | 0.32211 | 0 | 0.161055 |
| ENSMUSG00000090226 | processed_transcrip | Gm16110 | 0.33031 | 0 | 0.165155 |
| ENSMUSG00000092704 | snRNA | AC112688.1 | 0.33031 | 0 | 0.165155 |
| ENSMUSG00000090571 | protein_coding | Gm17434 | 0.33031 | 0 | 0.165155 |
| ENSMUSG00000092181 | pseudogene | RP23-171F16.1 | 0.316221 | 0 | 0.158111 |
| ENSMUSG00000029044 | protein_coding | 4930584F24Rik | 0.33031 | 0 | 0.165155 |
| ENSMUSG00000039106 | protein_coding | Htr5a | 0.316221 | 0 | 0.158111 |
| ENSMUSG00000088150 | misc_RNA | 7SK | 0.33031 | 0 | 0.165155 |
| ENSMUSG00000088327 | snRNA | U6 | 0.32211 | 0 | 0.161055 |
| ENSMUSG00000081618 | pseudogene | 5033423K11Rik | 0.32211 | 0 | 0.161055 |
| ENSMUSG00000080556 | miRNA | Mir467g | 0.33031 | 0 | 0.165155 |
| ENSMUSG00000072962 | protein_coding | Gm16401 | 0.32211 | 0 | 0.161055 |
| ENSMUSG00000092559 | processed_transcrip | RP23-195A18.2 | 0.316221 | 0 | 0.158111 |
| ENSMUSG00000065603 | miRNA | Mir218-1 | 0.33031 | 0 | 0.165155 |
| ENSMUSG00000077042 | miRNA | Mir574 | 0.32211 | 0 | 0.161055 |
| ENSMUSG00000080601 | miRNA | AC119834.1 | 0.33031 | 0 | 0.165155 |
| ENSMUSG00000061259 | protein_coding | Tmprss11d | 0.32211 | 0 | 0.161055 |
| ENSMUSG00000048764 | protein_coding | Tmprss11f | 0.33031 | 0 | 0.165155 |
| ENSMUSG00000089851 | processed_transcrip | Gm16579 | 0.33031 | 0 | 0.165155 |
| ENSMUSG00000035811 | protein_coding | Ugt2b35 | 0.33031 | 0 | 0.165155 |
| ENSMUSG00000054630 | protein_coding | Ugt2b5 | 0.33031 | 0 | 0.165155 |
| ENSMUSG00000007457 | protein_coding | 2310003L06Rik | 0.32211 | 0 | 0.161055 |
| ENSMUSG00000029280 | protein_coding | Smr3a | 0.32211 | 0 | 0.161055 |
| ENSMUSG00000029379 | protein_coding | Cxcl3 | 0.316221 | 0 | 0.158111 |
| ENSMUSG00000072813 | protein_coding | E330014E10Rik | 0.33031 | 0 | 0.165155 |
| ENSMUSG00000064550 | snRNA | U2 | 0.316221 | 0 | 0.158111 |
| ENSMUSG00000090006 | processed_transcrip | Gm16227 | 0.32211 | 0 | 0.161055 |
| ENSMUSG00000088742 | miRNA | AC109196.1 | 0.33031 | 0 | 0.165155 |
| ENSMUSG00000082527 | pseudogene | Gm8200 | 0.33031 | 0 | 0.165155 |
| ENSMUSG00000065942 | snRNA | U6 | 0.316221 | 0 | 0.158111 |
| ENSMUSG00000083609 | pseudogene | Gm16007 | 0.33031 | 0 | 0.165155 |
| ENSMUSG00000076163 | miRNA | Mir701 | 0.32211 | 0 | 0.161055 |
| ENSMUSG00000085713 | processed_transcrip | Gm15736 | 0.32211 | 0 | 0.161055 |
| ENSMUSG00000086302 | processed_transcrip | Gm13790 | 0.32211 | 0 | 0.161055 |
| ENSMUSG00000078186 | protein_coding | Gm13821 | 0.33031 | 0 | 0.165155 |
| ENSMUSG00000075779 | snRNA | U4 | 0.32211 | 0 | 0.161055 |
| ENSMUSG00000082365 | pseudogene | Gm15754 | 0.316221 | 0 | 0.158111 |
| ENSMUSG00000018263 | protein_coding | Tbx5 | 0.316221 | 0 | 0.158111 |
| ENSMUSG00000001168 | protein_coding | Oas1h | 0.316221 | 0 | 0.158111 |
| ENSMUSG00000029541 | protein_coding | Cyp2w1 | 0.32211 | 0 | 0.161055 |
| ENSMUSG00000085673 | processed_transcrip | Gm16122 | 0.33031 | 0 | 0.165155 |
| ENSMUSG00000084934 | processed_transcrip | Gm16035 | 0.33031 | 0 | 0.165155 |
| ENSMUSG00000061707 | protein_coding | Gm4871 | 0.32211 | 0 | 0.161055 |
| ENSMUSG00000029620 | protein_coding | 1700018F24Rik | 0.33031 | 0 | 0.165155 |
| ENSMUSG00000075552 | protein_coding | Cyp3a41b | 0.33031 | 0 | 0.165155 |
| ENSMUSG00000054417 | protein_coding | Cyp3a44 | 0.33031 | 0 | 0.165155 |

| | | | | | |
|---|---|---|---|---|---|
| ENSMUSG00000029630 | protein_coding | Cyp3a25 | 0.33031 | 0 | 0.165155 |
| ENSMUSG00000041468 | protein_coding | Gpr12 | 0.33031 | 0 | 0.165155 |
| ENSMUSG00000079069 | protein_coding | 8430423G03Rik | 0.32211 | 0 | 0.161055 |
| ENSMUSG00000092764 | snoRNA | AC122546.2 | 0.316221 | 0 | 0.158111 |
| ENSMUSG00000086744 | processed_transcrip | Gm15997 | 0.316221 | 0 | 0.158111 |
| ENSMUSG00000075784 | snRNA | U4 | 0.33031 | 0 | 0.165155 |
| ENSMUSG00000029755 | protein_coding | Dlx5 | 0.33031 | 0 | 0.165155 |
| ENSMUSG00000080767 | pseudogene | Gm15595 | 0.33031 | 0 | 0.165155 |
| ENSMUSG00000029706 | protein_coding | Pax4 | 0.33031 | 0 | 0.165155 |
| ENSMUSG00000065586 | miRNA | Mir96 | 0.32211 | 0 | 0.161055 |
| ENSMUSG00000087846 | rRNA | 5S_rRNA | 0.33031 | 0 | 0.165155 |
| ENSMUSG00000086854 | processed_transcrip | Gm13781 | 0.316221 | 0 | 0.158111 |
| ENSMUSG00000084894 | processed_transcrip | Gm13834 | 0.33031 | 0 | 0.165155 |
| ENSMUSG00000029766 | protein_coding | 1700012A03Rik | 0.33031 | 0 | 0.165155 |
| ENSMUSG00000073154 | lincRNA | 9330158H04Rik | 0.316221 | 0 | 0.158111 |
| ENSMUSG00000076461 | IG_V_gene | Trbv1 | 0.33031 | 0 | 0.165155 |
| ENSMUSG00000076465 | IG_V_gene | Trbv5 | 0.316221 | 0 | 0.158111 |
| ENSMUSG00000076470 | IG_V_gene | Trbv13-3 | 0.316221 | 0 | 0.158111 |
| ENSMUSG00000076471 | IG_V_gene | Trbv14 | 0.33031 | 0 | 0.165155 |
| ENSMUSG00000076473 | IG_V_gene | Trbv16 | 0.33031 | 0 | 0.165155 |
| ENSMUSG00000076479 | IG_V_gene | Trbv26 | 0.32211 | 0 | 0.161055 |
| ENSMUSG00000076494 | IG_J_gene | Trbj2-3 | 0.316221 | 0 | 0.158111 |
| ENSMUSG00000076497 | IG_J_gene | Trbj2-7 | 0.33031 | 0 | 0.165155 |
| ENSMUSG00000065701 | misc_RNA | Y_RNA | 0.32211 | 0 | 0.161055 |
| ENSMUSG00000029813 | protein_coding | 1600015I10Rik | 0.316221 | 0 | 0.158111 |
| ENSMUSG00000092936 | miRNA | AC153877.1 | 0.33031 | 0 | 0.165155 |
| ENSMUSG00000077501 | snoRNA | SNORA17 | 0.32211 | 0 | 0.161055 |
| ENSMUSG00000058923 | protein_coding | Vmn1r6 | 0.316221 | 0 | 0.158111 |
| ENSMUSG00000065754 | snoRNA | SNORA44 | 0.33031 | 0 | 0.165155 |
| ENSMUSG00000063974 | protein_coding | Vmn1r36 | 0.32211 | 0 | 0.161055 |
| ENSMUSG00000088245 | snoRNA | SNORA17 | 0.32211 | 0 | 0.161055 |
| ENSMUSG00000076511 | IG_V_gene | Igkv9-124 | 0.32211 | 0 | 0.161055 |
| ENSMUSG00000076542 | IG_V_gene | Igkv4-78 | 0.32211 | 0 | 0.161055 |
| ENSMUSG00000076562 | IG_V_gene | Igkv4-50 | 0.316221 | 0 | 0.158111 |
| ENSMUSG00000076573 | IG_V_gene | Igkv1-35 | 0.33031 | 0 | 0.165155 |
| ENSMUSG00000076586 | IG_V_gene | Igkv8-21 | 0.33031 | 0 | 0.165155 |
| ENSMUSG00000076592 | IG_V_gene | Igkv6-15 | 0.316221 | 0 | 0.158111 |
| ENSMUSG00000076601 | IG_V_gene | Igkv3-3 | 0.32211 | 0 | 0.161055 |
| ENSMUSG00000076603 | IG_V_gene | Igkv3-1 | 0.32211 | 0 | 0.161055 |
| ENSMUSG00000079516 | protein_coding | Reg3a | 0.316221 | 0 | 0.158111 |
| ENSMUSG00000014542 | protein_coding | Clec4f | 0.316221 | 0 | 0.158111 |
| ENSMUSG00000030050 | protein_coding | Gkn1 | 0.32211 | 0 | 0.161055 |
| ENSMUSG00000048206 | protein_coding | Dnajb8 | 0.316221 | 0 | 0.158111 |
| ENSMUSG00000060724 | protein_coding | Vmn1r47 | 0.33031 | 0 | 0.165155 |
| ENSMUSG00000049694 | protein_coding | BC048671 | 0.33031 | 0 | 0.165155 |
| ENSMUSG00000085624 | processed_transcrip | Gm14573 | 0.33031 | 0 | 0.165155 |
| ENSMUSG00000089069 | snoRNA | SNORA44 | 0.316221 | 0 | 0.158111 |
| ENSMUSG00000056743 | protein_coding | Gm10009 | 0.33031 | 0 | 0.165155 |
| ENSMUSG00000085067 | lincRNA | Gm15631 | 0.33031 | 0 | 0.165155 |
| ENSMUSG00000077262 | rRNA | n-R5s165 | 0.316221 | 0 | 0.158111 |
| ENSMUSG00000087083 | processed_transcrip | Gm15083 | 0.33031 | 0 | 0.165155 |
| ENSMUSG00000054488 | processed_transcrip | Gm9946 | 0.32211 | 0 | 0.161055 |
| ENSMUSG00000071237 | pseudogene | Gm6253 | 0.316221 | 0 | 0.158111 |
| ENSMUSG00000080719 | pseudogene | Gm15532 | 0.33031 | 0 | 0.165155 |
| ENSMUSG00000089378 | miRNA | AC113092.1 | 0.32211 | 0 | 0.161055 |
| ENSMUSG00000089931 | pseudogene | Gm8459 | 0.33031 | 0 | 0.165155 |
| ENSMUSG00000059639 | protein_coding | Clec4a4 | 0.33031 | 0 | 0.165155 |
| ENSMUSG00000090280 | protein_coding | Vmn2r20 | 0.33031 | 0 | 0.165155 |

| | | | | | |
|---|---|---|---|---|---|
| ENSMUSG00000030141 | protein_coding | Vmn2r26 | 0.32211 | 0 | 0.161055 |
| ENSMUSG00000030155 | protein_coding | Clec2e | 0.316221 | 0 | 0.158111 |
| ENSMUSG00000083120 | pseudogene | Gm15854 | 0.32211 | 0 | 0.161055 |
| ENSMUSG00000079853 | protein_coding | Klra1 | 0.32211 | 0 | 0.161055 |
| ENSMUSG00000030143 | protein_coding | Gm8882 | 0.316221 | 0 | 0.158111 |
| ENSMUSG00000088409 | snRNA | U4 | 0.33031 | 0 | 0.165155 |
| ENSMUSG00000079274 | protein_coding | Gm11078 | 0.33031 | 0 | 0.165155 |
| ENSMUSG00000088865 | misc_RNA | 7SK | 0.33031 | 0 | 0.165155 |
| ENSMUSG00000092488 | pseudogene | RP24-323I5.3 | 0.32211 | 0 | 0.161055 |
| ENSMUSG00000088362 | snoRNA | SNORA17 | 0.33031 | 0 | 0.165155 |
| ENSMUSG00000089948 | processed_transcrip | Gm16583 | 0.32211 | 0 | 0.161055 |
| ENSMUSG00000082633 | pseudogene | Gm15779 | 0.33031 | 0 | 0.165155 |
| ENSMUSG00000081335 | pseudogene | Gm15786 | 0.316221 | 0 | 0.158111 |
| ENSMUSG00000077815 | miRNA | Mir290 | 0.33031 | 0 | 0.165155 |
| ENSMUSG00000078032 | miRNA | Mir291b | 0.33031 | 0 | 0.165155 |
| ENSMUSG00000077886 | miRNA | Mir295 | 0.316221 | 0 | 0.158111 |
| ENSMUSG00000081665 | pseudogene | Gm15922 | 0.33031 | 0 | 0.165155 |
| ENSMUSG00000087297 | processed_transcrip | Gm15494 | 0.316221 | 0 | 0.158111 |
| ENSMUSG00000093045 | miRNA | AC162893.2 | 0.33031 | 0 | 0.165155 |
| ENSMUSG00000048398 | protein_coding | Gm5065 | 0.32211 | 0 | 0.161055 |
| ENSMUSG00000092815 | miRNA | AC149277.1 | 0.316221 | 0 | 0.158111 |
| ENSMUSG00000092221 | pseudogene | Gm7230 | 0.32211 | 0 | 0.161055 |
| ENSMUSG00000092620 | pseudogene | Gm18201 | 0.32211 | 0 | 0.161055 |
| ENSMUSG00000091544 | protein_coding | Vmn2r48 | 0.316221 | 0 | 0.158111 |
| ENSMUSG00000058685 | protein_coding | Vmn2r51 | 0.33031 | 0 | 0.165155 |
| ENSMUSG00000090714 | protein_coding | Zscan4d | 0.33031 | 0 | 0.165155 |
| ENSMUSG00000070826 | protein_coding | Zscan4-ps2 | 0.32211 | 0 | 0.161055 |
| ENSMUSG00000043308 | protein_coding | Vmn1r75 | 0.33031 | 0 | 0.165155 |
| ENSMUSG00000092285 | pseudogene | Vmn1r-ps56 | 0.33031 | 0 | 0.165155 |
| ENSMUSG00000092398 | pseudogene | Vmn1r-ps59 | 0.32211 | 0 | 0.161055 |
| ENSMUSG00000087915 | misc_RNA | 7SK | 0.316221 | 0 | 0.158111 |
| ENSMUSG00000074378 | protein_coding | Bsph1 | 0.33031 | 0 | 0.165155 |
| ENSMUSG00000070811 | protein_coding | Sult2a2 | 0.33031 | 0 | 0.165155 |
| ENSMUSG00000041578 | protein_coding | Crx | 0.33031 | 0 | 0.165155 |
| ENSMUSG00000065893 | rRNA | n-R5s151 | 0.33031 | 0 | 0.165155 |
| ENSMUSG00000090442 | protein_coding | Psg-ps1 | 0.33031 | 0 | 0.165155 |
| ENSMUSG00000063830 | pseudogene | Gm6065 | 0.316221 | 0 | 0.158111 |
| ENSMUSG00000063305 | protein_coding | Psg20 | 0.33031 | 0 | 0.165155 |
| ENSMUSG00000082802 | pseudogene | Gm15465 | 0.33031 | 0 | 0.165155 |
| ENSMUSG00000049685 | protein_coding | Cyp2g1 | 0.33031 | 0 | 0.165155 |
| ENSMUSG00000060747 | protein_coding | Il28b | 0.316221 | 0 | 0.158111 |
| ENSMUSG00000019429 | protein_coding | Ffar3 | 0.316221 | 0 | 0.158111 |
| ENSMUSG00000083381 | pseudogene | Gm12767 | 0.33031 | 0 | 0.165155 |
| ENSMUSG00000081491 | pseudogene | Gm12778 | 0.32211 | 0 | 0.161055 |
| ENSMUSG00000070621 | pseudogene | Vmn2r-ps60 | 0.32211 | 0 | 0.161055 |
| ENSMUSG00000092301 | pseudogene | Gm6887 | 0.32211 | 0 | 0.161055 |
| ENSMUSG00000088281 | snRNA | U6 | 0.32211 | 0 | 0.161055 |
| ENSMUSG00000091510 | pseudogene | Mtag2 | 0.32211 | 0 | 0.161055 |
| ENSMUSG00000074111 | protein_coding | Mrgpra9 | 0.32211 | 0 | 0.161055 |
| ENSMUSG00000061869 | pseudogene | Gm9354 | 0.33031 | 0 | 0.165155 |
| ENSMUSG00000087315 | lincRNA | 4930554H23Rik | 0.316221 | 0 | 0.158111 |
| ENSMUSG00000074067 | protein_coding | Gm10619 | 0.33031 | 0 | 0.165155 |
| ENSMUSG00000082400 | pseudogene | Gm16017 | 0.32211 | 0 | 0.161055 |
| ENSMUSG00000070459 | protein_coding | Olfr290 | 0.32211 | 0 | 0.161055 |
| ENSMUSG00000092621 | pseudogene | Vmn2r-ps66 | 0.32211 | 0 | 0.161055 |
| ENSMUSG00000092071 | protein_coding | A230065N10Rik | 0.32211 | 0 | 0.161055 |
| ENSMUSG00000054061 | protein_coding | Gm9934 | 0.316221 | 0 | 0.158111 |
| ENSMUSG00000085792 | processed_transcrip | Gm15414 | 0.33031 | 0 | 0.165155 |

| | | | | | |
|---|---|---|---|---|---|
| ENSMUSG00000087304 | processed_transcrip | Gm15415 | 0.33031 | 0 | 0.165155 |
| ENSMUSG00000089501 | snoRNA | SNORA17 | 0.33031 | 0 | 0.165155 |
| ENSMUSG00000030651 | protein_coding | Art2b | 0.33031 | 0 | 0.165155 |
| ENSMUSG00000073969 | protein_coding | Olfr556 | 0.316221 | 0 | 0.158111 |
| ENSMUSG00000073964 | protein_coding | Olfr570 | 0.32211 | 0 | 0.161055 |
| ENSMUSG00000078080 | protein_coding | Olfr585 | 0.33031 | 0 | 0.165155 |
| ENSMUSG00000080576 | miRNA | AC108410.1 | 0.32211 | 0 | 0.161055 |
| ENSMUSG00000082739 | pseudogene | Olfr602-ps1 | 0.32211 | 0 | 0.161055 |
| ENSMUSG00000058079 | protein_coding | Olfr605 | 0.316221 | 0 | 0.158111 |
| ENSMUSG00000073929 | processed_transcrip | Trim30e-ps1 | 0.316221 | 0 | 0.158111 |
| ENSMUSG00000073896 | protein_coding | Olfr716 | 0.33031 | 0 | 0.165155 |
| ENSMUSG00000089306 | snRNA | U6 | 0.33031 | 0 | 0.165155 |
| ENSMUSG00000091117 | pseudogene | Gm9064 | 0.33031 | 0 | 0.165155 |
| ENSMUSG00000093002 | snoRNA | AC127694.1 | 0.33031 | 0 | 0.165155 |
| ENSMUSG00000089467 | miRNA | AC103664.1 | 0.33031 | 0 | 0.165155 |
| ENSMUSG00000057945 | pseudogene | Gm17136 | 0.32211 | 0 | 0.161055 |
| ENSMUSG00000084453 | snRNA | U6 | 0.33031 | 0 | 0.165155 |
| ENSMUSG00000064734 | snRNA | U6 | 0.33031 | 0 | 0.165155 |
| ENSMUSG00000064677 | snRNA | U1 | 0.33031 | 0 | 0.165155 |
| ENSMUSG00000047517 | protein_coding | Dmbt1 | 0.32211 | 0 | 0.161055 |
| ENSMUSG00000084630 | rRNA | n-R5s159 | 0.316221 | 0 | 0.158111 |
| ENSMUSG00000088542 | snRNA | U6 | 0.33031 | 0 | 0.165155 |
| ENSMUSG00000090471 | protein_coding | Gm4553 | 0.33031 | 0 | 0.165155 |
| ENSMUSG00000054759 | protein_coding | Krtap5-2 | 0.32211 | 0 | 0.161055 |
| ENSMUSG00000066101 | protein_coding | Gm10153 | 0.32211 | 0 | 0.161055 |
| ENSMUSG00000073785 | protein_coding | Krtap5-5 | 0.33031 | 0 | 0.165155 |
| ENSMUSG00000000214 | protein_coding | Th | 0.316221 | 0 | 0.158111 |
| ENSMUSG00000086065 | processed_transcrip | Gm16180 | 0.316221 | 0 | 0.158111 |
| ENSMUSG00000057116 | protein_coding | AF366264 | 0.32211 | 0 | 0.161055 |
| ENSMUSG00000088320 | snoRNA | SNORA17 | 0.33031 | 0 | 0.165155 |
| ENSMUSG00000053695 | protein_coding | Defb37 | 0.316221 | 0 | 0.158111 |
| ENSMUSG00000061847 | protein_coding | Defb39 | 0.32211 | 0 | 0.161055 |
| ENSMUSG00000058618 | protein_coding | AY761184 | 0.32211 | 0 | 0.161055 |
| ENSMUSG00000058568 | protein_coding | Defb50 | 0.32211 | 0 | 0.161055 |
| ENSMUSG00000079112 | protein_coding | C86695 | 0.32211 | 0 | 0.161055 |
| ENSMUSG00000089484 | snRNA | U6 | 0.32211 | 0 | 0.161055 |
| ENSMUSG00000031552 | protein_coding | Adam18 | 0.33031 | 0 | 0.165155 |
| ENSMUSG00000083588 | pseudogene | Gm8051 | 0.32211 | 0 | 0.161055 |
| ENSMUSG00000039539 | protein_coding | Sgcz | 0.33031 | 0 | 0.165155 |
| ENSMUSG00000064682 | snRNA | U2 | 0.316221 | 0 | 0.158111 |
| ENSMUSG00000077585 | snoRNA | SNORA17 | 0.316221 | 0 | 0.158111 |
| ENSMUSG00000091848 | protein_coding | Gm7697 | 0.33031 | 0 | 0.165155 |
| ENSMUSG00000069998 | protein_coding | Olfr372 | 0.33031 | 0 | 0.165155 |
| ENSMUSG00000064758 | snRNA | U6 | 0.316221 | 0 | 0.158111 |
| ENSMUSG00000031725 | protein_coding | Ces1f | 0.33031 | 0 | 0.165155 |
| ENSMUSG00000055730 | protein_coding | Ces2a | 0.33031 | 0 | 0.165155 |
| ENSMUSG00000092749 | misc_RNA | AC152826.1 | 0.316221 | 0 | 0.158111 |
| ENSMUSG00000044287 | protein_coding | Nrn1l | 0.316221 | 0 | 0.158111 |
| ENSMUSG00000076382 | miRNA | AC163625.1 | 0.33031 | 0 | 0.165155 |
| ENSMUSG00000089745 | processed_transcrip | 1810008B01Rik | 0.316221 | 0 | 0.158111 |
| ENSMUSG00000088580 | rRNA | 5S_rRNA | 0.316221 | 0 | 0.158111 |
| ENSMUSG00000085253 | protein_coding | Gm16833 | 0.316221 | 0 | 0.158111 |
| ENSMUSG00000089702 | lincRNA | Gm16568 | 0.32211 | 0 | 0.161055 |
| ENSMUSG00000066903 | protein_coding | Olfr859 | 0.32211 | 0 | 0.161055 |
| ENSMUSG00000043659 | protein_coding | Npsr1 | 0.316221 | 0 | 0.158111 |
| ENSMUSG00000041248 | protein_coding | Kcnj1 | 0.33031 | 0 | 0.165155 |
| ENSMUSG00000070313 | protein_coding | A630095E13Rik | 0.316221 | 0 | 0.158111 |
| ENSMUSG00000052058 | protein_coding | Olfr901 | 0.33031 | 0 | 0.165155 |

| | | | | | |
|---|---|---|---|---|---|
| ENSMUSG00000044798 | protein_coding | Olfr923 | 0.33031 | 0 | 0.165155 |
| ENSMUSG00000059473 | protein_coding | Olfr979 | 0.33031 | 0 | 0.165155 |
| ENSMUSG00000093011 | miRNA | CT009696.2 | 0.33031 | 0 | 0.165155 |
| ENSMUSG00000093354 | miRNA | CT025619.1 | 0.33031 | 0 | 0.165155 |
| ENSMUSG00000032048 | protein_coding | 4930510E17Rik | 0.32211 | 0 | 0.161055 |
| ENSMUSG00000076281 | miRNA | mmu-mir-2134-4 | 0.32211 | 0 | 0.161055 |
| ENSMUSG00000086624 | processed_transcrip | Gm16130 | 0.316221 | 0 | 0.158111 |
| ENSMUSG00000093137 | snRNA | AC122057.1 | 0.33031 | 0 | 0.165155 |
| ENSMUSG00000088988 | rRNA | 5S_rRNA | 0.33031 | 0 | 0.165155 |
| ENSMUSG00000075763 | snRNA | U5 | 0.33031 | 0 | 0.165155 |
| ENSMUSG00000084437 | snRNA | U6 | 0.316221 | 0 | 0.158111 |
| ENSMUSG00000066690 | pseudogene | F830001A07Rik | 0.33031 | 0 | 0.165155 |
| ENSMUSG00000092670 | miRNA | AC111135.1 | 0.33031 | 0 | 0.165155 |
| ENSMUSG00000090001 | pseudogene | Gm9719 | 0.33031 | 0 | 0.165155 |
| ENSMUSG00000085159 | lincRNA | Gm16662 | 0.33031 | 0 | 0.165155 |
| ENSMUSG00000084502 | misc_RNA | 7SK | 0.33031 | 0 | 0.165155 |
| ENSMUSG00000086608 | processed_transcrip | 4930562D21Rik | 0.316221 | 0 | 0.158111 |
| ENSMUSG00000077208 | snoRNA | SNORA17 | 0.32211 | 0 | 0.161055 |
| ENSMUSG00000086225 | pseudogene | Gm8661 | 0.33031 | 0 | 0.165155 |
| ENSMUSG00000077438 | snoRNA | SNORA17 | 0.33031 | 0 | 0.165155 |
| ENSMUSG00000090639 | protein_coding | Trf | 0.33031 | 0 | 0.165155 |
| ENSMUSG00000088204 | miRNA | AC117688.1 | 0.32211 | 0 | 0.161055 |
| ENSMUSG00000032566 | protein_coding | 1700080E11Rik | 0.32211 | 0 | 0.161055 |
| ENSMUSG00000091129 | protein_coding | Iqcf6 | 0.32211 | 0 | 0.161055 |
| ENSMUSG00000088955 | snoRNA | SNORA61 | 0.33031 | 0 | 0.165155 |
| ENSMUSG00000086365 | processed_transcrip | 4930593C16Rik | 0.33031 | 0 | 0.165155 |
| ENSMUSG00000084706 | misc_RNA | 7SK | 0.316221 | 0 | 0.158111 |
| ENSMUSG00000081926 | pseudogene | Gm15536 | 0.32211 | 0 | 0.161055 |
| ENSMUSG00000019772 | protein_coding | Vip | 0.316221 | 0 | 0.158111 |
| ENSMUSG00000083257 | pseudogene | Gm15568 | 0.316221 | 0 | 0.158111 |
| ENSMUSG00000091893 | protein_coding | Taar8c | 0.32211 | 0 | 0.161055 |
| ENSMUSG00000093298 | miRNA | AC161426.1 | 0.316221 | 0 | 0.158111 |
| ENSMUSG00000065317 | snRNA | U6 | 0.33031 | 0 | 0.165155 |
| ENSMUSG00000062224 | protein_coding | 4933411G06Rik | 0.33031 | 0 | 0.165155 |
| ENSMUSG00000070214 | snRNA | U6 | 0.32211 | 0 | 0.161055 |
| ENSMUSG00000091526 | protein_coding | Gm6494 | 0.316221 | 0 | 0.158111 |
| ENSMUSG00000082619 | pseudogene | Gm16219 | 0.33031 | 0 | 0.165155 |
| ENSMUSG00000065691 | rRNA | n-R5s77 | 0.316221 | 0 | 0.158111 |
| ENSMUSG00000089533 | rRNA | 5S_rRNA | 0.32211 | 0 | 0.161055 |
| ENSMUSG00000071186 | protein_coding | Olfr1358 | 0.33031 | 0 | 0.165155 |
| ENSMUSG00000090414 | protein_coding | Gm6713 | 0.33031 | 0 | 0.165155 |
| ENSMUSG00000074810 | protein_coding | Gm10764 | 0.32211 | 0 | 0.161055 |
| ENSMUSG00000090412 | protein_coding | Gm16484 | 0.33031 | 0 | 0.165155 |
| ENSMUSG00000065296 | snRNA | U6 | 0.33031 | 0 | 0.165155 |
| ENSMUSG00000053420 | protein_coding | Gm4792 | 0.33031 | 0 | 0.165155 |
| ENSMUSG00000019932 | protein_coding | Kera | 0.33031 | 0 | 0.165155 |
| ENSMUSG00000093313 | miRNA | AC122210.1 | 0.316221 | 0 | 0.158111 |
| ENSMUSG00000088672 | snRNA | U6atac | 0.32211 | 0 | 0.161055 |
| ENSMUSG00000085731 | processed_transcrip | Gm16229 | 0.316221 | 0 | 0.158111 |
| ENSMUSG00000054052 | protein_coding | Rdh19 | 0.33031 | 0 | 0.165155 |
| ENSMUSG00000088519 | snoRNA | U8 | 0.32211 | 0 | 0.161055 |
| ENSMUSG00000084170 | pseudogene | Gm11954 | 0.33031 | 0 | 0.165155 |
| ENSMUSG00000084012 | pseudogene | Gm11956 | 0.33031 | 0 | 0.165155 |
| ENSMUSG00000085512 | processed_transcrip | Gm11958 | 0.32211 | 0 | 0.161055 |
| ENSMUSG00000081213 | pseudogene | Gm11983 | 0.33031 | 0 | 0.165155 |
| ENSMUSG00000084045 | pseudogene | Gm11997 | 0.33031 | 0 | 0.165155 |
| ENSMUSG00000087452 | processed_transcrip | Gm11998 | 0.33031 | 0 | 0.165155 |
| ENSMUSG00000081631 | pseudogene | Gm12005 | 0.32211 | 0 | 0.161055 |

| | | | | | |
|---|---|---|---|---|---|
| ENSMUSG00000084135 | pseudogene | Pom121l12 | 0.33031 | 0 | 0.165155 |
| ENSMUSG00000084442 | snRNA | U6 | 0.33031 | 0 | 0.165155 |
| ENSMUSG00000083555 | pseudogene | Gm12035 | 0.33031 | 0 | 0.165155 |
| ENSMUSG00000082874 | pseudogene | Rpl15-ps1 | 0.33031 | 0 | 0.165155 |
| ENSMUSG00000083008 | pseudogene | Gm12053 | 0.33031 | 0 | 0.165155 |
| ENSMUSG00000084258 | pseudogene | Gm12054 | 0.33031 | 0 | 0.165155 |
| ENSMUSG00000084206 | pseudogene | Gm12062 | 0.33031 | 0 | 0.165155 |
| ENSMUSG00000084064 | pseudogene | Gm12079 | 0.316221 | 0 | 0.158111 |
| ENSMUSG00000084850 | processed_transcrip | Gm12092 | 0.316221 | 0 | 0.158111 |
| ENSMUSG00000080851 | pseudogene | Gm12094 | 0.32211 | 0 | 0.161055 |
| ENSMUSG00000080906 | pseudogene | Gm12095 | 0.32211 | 0 | 0.161055 |
| ENSMUSG00000090842 | protein_coding | Gm17674 | 0.33031 | 0 | 0.165155 |
| ENSMUSG00000092110 | protein_coding | Gm17438 | 0.316221 | 0 | 0.158111 |
| ENSMUSG00000065583 | miRNA | Mir218-2 | 0.316221 | 0 | 0.158111 |
| ENSMUSG00000088256 | snoRNA | snoMe28S-Cm322 | 0.32211 | 0 | 0.161055 |
| ENSMUSG00000084627 | misc_RNA | 7SK | 0.316221 | 0 | 0.158111 |
| ENSMUSG00000085533 | processed_transcrip | Gm12146 | 0.33031 | 0 | 0.165155 |
| ENSMUSG00000082750 | pseudogene | Gm12156 | 0.33031 | 0 | 0.165155 |
| ENSMUSG00000082792 | pseudogene | Gm12176 | 0.32211 | 0 | 0.161055 |
| ENSMUSG00000084153 | pseudogene | Gm12186 | 0.32211 | 0 | 0.161055 |
| ENSMUSG00000083178 | pseudogene | Gm12187 | 0.316221 | 0 | 0.158111 |
| ENSMUSG00000047511 | protein_coding | Olfr1396 | 0.33031 | 0 | 0.165155 |
| ENSMUSG00000072983 | protein_coding | 4933414I15Rik | 0.33031 | 0 | 0.165155 |
| ENSMUSG00000083442 | pseudogene | Gm11187 | 0.32211 | 0 | 0.161055 |
| ENSMUSG00000083986 | pseudogene | Gm12213 | 0.33031 | 0 | 0.165155 |
| ENSMUSG00000081098 | pseudogene | Gm12220 | 0.33031 | 0 | 0.165155 |
| ENSMUSG00000088231 | snRNA | U1 | 0.33031 | 0 | 0.165155 |
| ENSMUSG00000081107 | pseudogene | Gm12235 | 0.32211 | 0 | 0.161055 |
| ENSMUSG00000050813 | protein_coding | Olfr332 | 0.33031 | 0 | 0.165155 |
| ENSMUSG00000061344 | protein_coding | Olfr320 | 0.32211 | 0 | 0.161055 |
| ENSMUSG00000058222 | protein_coding | Olfr318 | 0.32211 | 0 | 0.161055 |
| ENSMUSG00000060030 | protein_coding | Olfr317 | 0.316221 | 0 | 0.158111 |
| ENSMUSG00000059610 | protein_coding | Olfr222 | 0.33031 | 0 | 0.165155 |
| ENSMUSG00000081808 | pseudogene | Gm12622 | 0.33031 | 0 | 0.165155 |
| ENSMUSG00000081754 | pseudogene | Gm12623 | 0.33031 | 0 | 0.165155 |
| ENSMUSG00000050667 | pseudogene | Gm12612 | 0.316221 | 0 | 0.158111 |
| ENSMUSG00000083212 | pseudogene | Gm12274 | 0.32211 | 0 | 0.161055 |
| ENSMUSG00000085532 | processed_transcrip | B430319H21Rik | 0.32211 | 0 | 0.161055 |
| ENSMUSG00000065104 | snRNA | U6 | 0.32211 | 0 | 0.161055 |
| ENSMUSG00000085479 | processed_transcrip | 9430073C21Rik | 0.33031 | 0 | 0.165155 |
| ENSMUSG00000085937 | processed_transcrip | 4930535C22Rik | 0.33031 | 0 | 0.165155 |
| ENSMUSG00000088910 | snRNA | U6 | 0.32211 | 0 | 0.161055 |
| ENSMUSG00000088787 | snRNA | U6 | 0.33031 | 0 | 0.165155 |
| ENSMUSG00000082370 | pseudogene | Gm12313 | 0.316221 | 0 | 0.158111 |
| ENSMUSG00000070388 | protein_coding | Fbxo39 | 0.33031 | 0 | 0.165155 |
| ENSMUSG00000063881 | protein_coding | Olfr376 | 0.33031 | 0 | 0.165155 |
| ENSMUSG00000065401 | miRNA | Mir144 | 0.33031 | 0 | 0.165155 |
| ENSMUSG00000070065 | miRNA | Mir451 | 0.33031 | 0 | 0.165155 |
| ENSMUSG00000088748 | snRNA | U6 | 0.33031 | 0 | 0.165155 |
| ENSMUSG00000082304 | pseudogene | Gm11198 | 0.33031 | 0 | 0.165155 |
| ENSMUSG00000083395 | pseudogene | Gm11203 | 0.33031 | 0 | 0.165155 |
| ENSMUSG00000086507 | processed_transcrip | Gm11206 | 0.33031 | 0 | 0.165155 |
| ENSMUSG00000086971 | processed_transcrip | 4930527B05Rik | 0.316221 | 0 | 0.158111 |
| ENSMUSG00000080980 | pseudogene | 1700071K01Rik | 0.316221 | 0 | 0.158111 |
| ENSMUSG00000077217 | snoRNA | SNORA17 | 0.33031 | 0 | 0.165155 |
| ENSMUSG00000065307 | snRNA | U6 | 0.33031 | 0 | 0.165155 |
| ENSMUSG00000081632 | pseudogene | Gm11443 | 0.32211 | 0 | 0.161055 |
| ENSMUSG00000088816 | snoRNA | SCARNA3 | 0.33031 | 0 | 0.165155 |

| | | | | | |
|---|---|---|---|---|---|
| ENSMUSG00000082453 | pseudogene | Gm11502 | 0.32211 | 0 | 0.161055 |
| ENSMUSG00000087117 | processed_transcrip | Gm11523 | 0.32211 | 0 | 0.161055 |
| ENSMUSG00000086394 | processed_transcrip | Gm11612 | 0.32211 | 0 | 0.161055 |
| ENSMUSG00000064165 | protein_coding | Krt39 | 0.33031 | 0 | 0.165155 |
| ENSMUSG00000058725 | protein_coding | Gm11937 | 0.33031 | 0 | 0.165155 |
| ENSMUSG00000060756 | protein_coding | Krtap2-4 | 0.33031 | 0 | 0.165155 |
| ENSMUSG00000082900 | pseudogene | Gm11557 | 0.316221 | 0 | 0.158111 |
| ENSMUSG00000048294 | protein_coding | Krtap4-13 | 0.32211 | 0 | 0.161055 |
| ENSMUSG00000082531 | pseudogene | Gm11556 | 0.316221 | 0 | 0.158111 |
| ENSMUSG00000078256 | protein_coding | Gm11565 | 0.316221 | 0 | 0.158111 |
| ENSMUSG00000044041 | protein_coding | Krt13 | 0.33031 | 0 | 0.165155 |
| ENSMUSG00000065657 | snRNA | U6 | 0.33031 | 0 | 0.165155 |
| ENSMUSG00000081588 | pseudogene | Gm11661 | 0.33031 | 0 | 0.165155 |
| ENSMUSG00000084120 | pseudogene | Gm11624 | 0.32211 | 0 | 0.161055 |
| ENSMUSG00000089479 | snRNA | U6 | 0.33031 | 0 | 0.165155 |
| ENSMUSG00000088259 | misc_RNA | 7SK | 0.32211 | 0 | 0.161055 |
| ENSMUSG00000081995 | pseudogene | Gm11719 | 0.316221 | 0 | 0.158111 |
| ENSMUSG00000085245 | processed_transcrip | Gm11713 | 0.33031 | 0 | 0.165155 |
| ENSMUSG00000086733 | processed_transcrip | Gm11685 | 0.316221 | 0 | 0.158111 |
| ENSMUSG00000087139 | processed_transcrip | Gm11683 | 0.32211 | 0 | 0.161055 |
| ENSMUSG00000081318 | pseudogene | Rpl36-ps1 | 0.32211 | 0 | 0.161055 |
| ENSMUSG00000084887 | processed_transcrip | Gm11749 | 0.316221 | 0 | 0.158111 |
| ENSMUSG00000065600 | miRNA | Mir338 | 0.33031 | 0 | 0.165155 |
| ENSMUSG00000093310 | miRNA | AC159324.1 | 0.32211 | 0 | 0.161055 |
| ENSMUSG00000093145 | miRNA | AC140457.1 | 0.32211 | 0 | 0.161055 |
| ENSMUSG00000020633 | protein_coding | Dcdc2c | 0.32211 | 0 | 0.161055 |
| ENSMUSG00000092653 | miRNA | AC159204.1 | 0.33031 | 0 | 0.165155 |
| ENSMUSG00000093150 | miRNA | AC160932.1 | 0.316221 | 0 | 0.158111 |
| ENSMUSG00000088906 | snoRNA | SNORA17 | 0.32211 | 0 | 0.161055 |
| ENSMUSG00000077613 | snoRNA | SNORA17 | 0.32211 | 0 | 0.161055 |
| ENSMUSG00000081636 | pseudogene | Gm15901 | 0.33031 | 0 | 0.165155 |
| ENSMUSG00000089922 | protein_coding | 3110039M20Rik | 0.32211 | 0 | 0.161055 |
| ENSMUSG00000045946 | protein_coding | Gm9804 | 0.32211 | 0 | 0.161055 |
| ENSMUSG00000075923 | snRNA | U1 | 0.33031 | 0 | 0.165155 |
| ENSMUSG00000088389 | misc_RNA | 7SK | 0.316221 | 0 | 0.158111 |
| ENSMUSG00000060566 | protein_coding | Rpl21-ps3 | 0.316221 | 0 | 0.158111 |
| ENSMUSG00000089071 | snRNA | U6 | 0.316221 | 0 | 0.158111 |
| ENSMUSG00000079062 | protein_coding | Gm11043 | 0.33031 | 0 | 0.165155 |
| ENSMUSG00000034402 | protein_coding | Kcnh5 | 0.32211 | 0 | 0.161055 |
| ENSMUSG00000088002 | snRNA | U6 | 0.316221 | 0 | 0.158111 |
| ENSMUSG00000080443 | miRNA | AC153147.1 | 0.32211 | 0 | 0.161055 |
| ENSMUSG00000092100 | lincRNA | 4930544I03Rik | 0.32211 | 0 | 0.161055 |
| ENSMUSG00000066389 | pseudogene | Rpl31-ps1 | 0.33031 | 0 | 0.165155 |
| ENSMUSG00000062582 | protein_coding | Rpl30-ps8 | 0.316221 | 0 | 0.158111 |
| ENSMUSG00000072880 | protein_coding | Gm10431 | 0.32211 | 0 | 0.161055 |
| ENSMUSG00000064424 | snRNA | U6 | 0.33031 | 0 | 0.165155 |
| ENSMUSG00000077058 | miRNA | AC123824.1 | 0.33031 | 0 | 0.165155 |
| ENSMUSG00000065436 | miRNA | Mir342 | 0.33031 | 0 | 0.165155 |
| ENSMUSG00000076316 | miRNA | Mir673 | 0.33031 | 0 | 0.165155 |
| ENSMUSG00000077025 | miRNA | Mir493 | 0.32211 | 0 | 0.161055 |
| ENSMUSG00000089449 | snoRNA | SNORD112 | 0.33031 | 0 | 0.165155 |
| ENSMUSG00000084487 | snoRNA | SNORD113 | 0.33031 | 0 | 0.165155 |
| ENSMUSG00000065498 | miRNA | Mir379 | 0.316221 | 0 | 0.158111 |
| ENSMUSG00000093291 | miRNA | AC121784.4 | 0.32211 | 0 | 0.161055 |
| ENSMUSG00000076052 | miRNA | Mir541 | 0.316221 | 0 | 0.158111 |
| ENSMUSG00000090193 | processed_transcrip | Gm15995 | 0.33031 | 0 | 0.165155 |
| ENSMUSG00000065574 | miRNA | Mir203 | 0.316221 | 0 | 0.158111 |
| ENSMUSG00000078993 | protein_coding | Gm11029 | 0.32211 | 0 | 0.161055 |

| | | | | | |
|---|---|---|---|---|---|
| ENSMUSG00000076618 | IG_J_gene | Gm16734 | 0.33031 | 0 | 0.165155 |
| ENSMUSG00000076642 | IG_V_gene | Gm16594 | 0.32211 | 0 | 0.161055 |
| ENSMUSG00000076646 | IG_V_gene | Gm16887 | 0.32211 | 0 | 0.161055 |
| ENSMUSG00000076660 | IG_V_gene | Ighv14-3 | 0.33031 | 0 | 0.165155 |
| ENSMUSG00000076666 | IG_V_gene | Gm16841 | 0.32211 | 0 | 0.161055 |
| ENSMUSG00000076688 | IG_V_gene | Ighv15-2 | 0.32211 | 0 | 0.161055 |
| ENSMUSG00000076693 | IG_V_gene | Gm16858 | 0.316221 | 0 | 0.158111 |
| ENSMUSG00000076715 | IG_V_gene | Ighv8-6 | 0.33031 | 0 | 0.165155 |
| ENSMUSG00000076719 | IG_V_gene | Gm9740 | 0.316221 | 0 | 0.158111 |
| ENSMUSG00000090959 | IG_V_gene | Gm9232 | 0.32211 | 0 | 0.161055 |
| ENSMUSG00000076734 | IG_V_gene | Gm16710 | 0.32211 | 0 | 0.161055 |
| ENSMUSG00000076742 | IG_V_gene | Ighv1-84 | 0.33031 | 0 | 0.165155 |
| ENSMUSG00000065348 | snRNA | U6 | 0.32211 | 0 | 0.161055 |
| ENSMUSG00000054757 | protein_coding | Akr1c20 | 0.33031 | 0 | 0.165155 |
| ENSMUSG00000091053 | protein_coding | Gm9742 | 0.32211 | 0 | 0.161055 |
| ENSMUSG00000055360 | protein_coding | Prl2c5 | 0.316221 | 0 | 0.158111 |
| ENSMUSG00000064653 | snRNA | U6 | 0.32211 | 0 | 0.161055 |
| ENSMUSG00000076745 | IG_V_gene | Tcrg-V4 | 0.316221 | 0 | 0.158111 |
| ENSMUSG00000054890 | protein_coding | Olfr1535 | 0.33031 | 0 | 0.165155 |
| ENSMUSG00000069304 | protein_coding | Hist1h2ap | 0.32211 | 0 | 0.161055 |
| ENSMUSG00000085827 | processed_transcrip | Gm11290 | 0.33031 | 0 | 0.165155 |
| ENSMUSG00000081334 | pseudogene | Gm11286 | 0.33031 | 0 | 0.165155 |
| ENSMUSG00000061222 | protein_coding | Vmn1r189 | 0.33031 | 0 | 0.165155 |
| ENSMUSG00000083319 | pseudogene | Gm11284 | 0.33031 | 0 | 0.165155 |
| ENSMUSG00000083297 | pseudogene | Gm11316 | 0.316221 | 0 | 0.158111 |
| ENSMUSG00000021342 | protein_coding | Prl | 0.33031 | 0 | 0.165155 |
| ENSMUSG00000086423 | processed_transcrip | Gm11364 | 0.33031 | 0 | 0.165155 |
| ENSMUSG00000087662 | processed_transcrip | Gm11373 | 0.316221 | 0 | 0.158111 |
| ENSMUSG00000085620 | processed_transcrip | G630018N14Rik | 0.32211 | 0 | 0.161055 |
| ENSMUSG00000082808 | pseudogene | Gm11391 | 0.33031 | 0 | 0.165155 |
| ENSMUSG00000087909 | misc_RNA | 7SK | 0.33031 | 0 | 0.165155 |
| ENSMUSG00000076054 | miRNA | AC122194.1 | 0.33031 | 0 | 0.165155 |
| ENSMUSG00000087335 | processed_transcrip | 4930526F13Rik | 0.33031 | 0 | 0.165155 |
| ENSMUSG00000044444 | protein_coding | Pfn3 | 0.32211 | 0 | 0.161055 |
| ENSMUSG00000048904 | protein_coding | Neurog1 | 0.33031 | 0 | 0.165155 |
| ENSMUSG00000074885 | protein_coding | Gm10782 | 0.33031 | 0 | 0.165155 |
| ENSMUSG00000055298 | protein_coding | Ctsj | 0.33031 | 0 | 0.165155 |
| ENSMUSG00000076244 | miRNA | CT030240.1 | 0.32211 | 0 | 0.161055 |
| ENSMUSG00000069200 | protein_coding | Gm6132 | 0.32211 | 0 | 0.161055 |
| ENSMUSG00000077337 | snoRNA | SNORA24 | 0.33031 | 0 | 0.165155 |
| ENSMUSG00000082488 | protein_coding | 1700119I11Rik | 0.32211 | 0 | 0.161055 |
| ENSMUSG00000084423 | miRNA | AC123043.1 | 0.33031 | 0 | 0.165155 |
| ENSMUSG00000091451 | lincRNA | C430039J16Rik | 0.316221 | 0 | 0.158111 |
| ENSMUSG00000079172 | lincRNA | Gm17290 | 0.32211 | 0 | 0.161055 |
| ENSMUSG00000084651 | miRNA | Mir1904 | 0.33031 | 0 | 0.165155 |
| ENSMUSG00000085634 | processed_transcrip | Gm15290 | 0.33031 | 0 | 0.165155 |
| ENSMUSG00000065575 | miRNA | Mir449a | 0.33031 | 0 | 0.165155 |
| ENSMUSG00000051002 | protein_coding | Gm9848 | 0.32211 | 0 | 0.161055 |
| ENSMUSG00000079407 | protein_coding | 1700110I01Rik | 0.32211 | 0 | 0.161055 |
| ENSMUSG00000079394 | protein_coding | Gm3099 | 0.316221 | 0 | 0.158111 |
| ENSMUSG00000091221 | pseudogene | Gm3084 | 0.316221 | 0 | 0.158111 |
| ENSMUSG00000090279 | protein_coding | Gm9603 | 0.316221 | 0 | 0.158111 |
| ENSMUSG00000079369 | protein_coding | Gm3358 | 0.32211 | 0 | 0.161055 |
| ENSMUSG00000091850 | protein_coding | Gm3460 | 0.32211 | 0 | 0.161055 |
| ENSMUSG00000092133 | protein_coding | Gm3642 | 0.33031 | 0 | 0.165155 |
| ENSMUSG00000091227 | protein_coding | Gm3755 | 0.33031 | 0 | 0.165155 |
| ENSMUSG00000092756 | misc_RNA | AC190403.1 | 0.33031 | 0 | 0.165155 |
| ENSMUSG00000072672 | protein_coding | Gm10391 | 0.33031 | 0 | 0.165155 |

| | | | | | |
|---|---|---|---|---|---|
| ENSMUSG00000072624 | protein_coding | Gm5460 | 0.316221 | 0 | 0.158111 |
| ENSMUSG00000037780 | protein_coding | Mbl1 | 0.316221 | 0 | 0.158111 |
| ENSMUSG00000091131 | protein_coding | Gm7945 | 0.316221 | 0 | 0.158111 |
| ENSMUSG00000079266 | pseudogene | Gm6490 | 0.316221 | 0 | 0.158111 |
| ENSMUSG00000090731 | pseudogene | Gm2959 | 0.32211 | 0 | 0.161055 |
| ENSMUSG00000072608 | protein_coding | Gm3633 | 0.33031 | 0 | 0.165155 |
| ENSMUSG00000091961 | pseudogene | Gm3123 | 0.32211 | 0 | 0.161055 |
| ENSMUSG00000090657 | pseudogene | Gm8114 | 0.316221 | 0 | 0.158111 |
| ENSMUSG00000091707 | pseudogene | Gm8224 | 0.33031 | 0 | 0.165155 |
| ENSMUSG00000090736 | pseudogene | Gm9593 | 0.33031 | 0 | 0.165155 |
| ENSMUSG00000090296 | pseudogene | Gm6541 | 0.33031 | 0 | 0.165155 |
| ENSMUSG00000000606 | protein_coding | Vmn2r88 | 0.32211 | 0 | 0.161055 |
| ENSMUSG00000065853 | snoRNA | SNORD58 | 0.33031 | 0 | 0.165155 |
| ENSMUSG00000076771 | IG_V_gene | Trav9d-1 | 0.316221 | 0 | 0.158111 |
| ENSMUSG00000076780 | IG_V_gene | Trav15d-1-dv6d-1 | 0.32211 | 0 | 0.161055 |
| ENSMUSG00000076792 | IG_V_gene | Trav6d-7 | 0.316221 | 0 | 0.158111 |
| ENSMUSG00000076833 | IG_V_gene | Trav6-5 | 0.32211 | 0 | 0.161055 |
| ENSMUSG00000076851 | IG_V_gene | Gm13908 | 0.32211 | 0 | 0.161055 |
| ENSMUSG00000076855 | IG_V_gene | Gm13896 | 0.33031 | 0 | 0.165155 |
| ENSMUSG00000076866 | IG_V_gene | Gm17015 | 0.32211 | 0 | 0.161055 |
| ENSMUSG00000076915 | IG_J_gene | Gm16657 | 0.316221 | 0 | 0.158111 |
| ENSMUSG00000076918 | IG_J_gene | Gm16654 | 0.32211 | 0 | 0.161055 |
| ENSMUSG00000076924 | IG_J_gene | Gm16915 | 0.32211 | 0 | 0.161055 |
| ENSMUSG00000076926 | IG_J_gene | Traj2 | 0.32211 | 0 | 0.161055 |
| ENSMUSG00000065432 | miRNA | Mir208a | 0.32211 | 0 | 0.161055 |
| ENSMUSG00000022157 | protein_coding | Mcpt8 | 0.33031 | 0 | 0.165155 |
| ENSMUSG00000053868 | protein_coding | Gm5142 | 0.316221 | 0 | 0.158111 |
| ENSMUSG00000093007 | miRNA | AC154660.2 | 0.316221 | 0 | 0.158111 |
| ENSMUSG00000089531 | snoRNA | SNORA73 | 0.33031 | 0 | 0.165155 |
| ENSMUSG00000052099 | protein_coding | Prss51 | 0.316221 | 0 | 0.158111 |
| ENSMUSG00000075512 | protein_coding | Gm1587 | 0.316221 | 0 | 0.158111 |
| ENSMUSG00000084447 | snoRNA | SNORA40 | 0.316221 | 0 | 0.158111 |
| ENSMUSG00000083933 | pseudogene | Gm15515 | 0.33031 | 0 | 0.165155 |
| ENSMUSG00000092901 | miRNA | AC102815.1 | 0.33031 | 0 | 0.165155 |
| ENSMUSG00000075478 | protein_coding | Slitrk1 | 0.316221 | 0 | 0.158111 |
| ENSMUSG00000090284 | protein_coding | Gm17613 | 0.33031 | 0 | 0.165155 |
| ENSMUSG00000084476 | snRNA | U6 | 0.33031 | 0 | 0.165155 |
| ENSMUSG00000086297 | processed_transcrip | Gm15632 | 0.316221 | 0 | 0.158111 |
| ENSMUSG00000084873 | lincRNA | C030047K22Rik | 0.316221 | 0 | 0.158111 |
| ENSMUSG00000055416 | protein_coding | Fam136b-ps | 0.33031 | 0 | 0.165155 |
| ENSMUSG00000045267 | protein_coding | Tas2r119 | 0.316221 | 0 | 0.158111 |
| ENSMUSG00000092230 | pseudogene | RP23-244N5.1 | 0.32211 | 0 | 0.161055 |
| ENSMUSG00000088201 | snRNA | U6 | 0.33031 | 0 | 0.165155 |
| ENSMUSG00000072511 | protein_coding | Hhla1 | 0.316221 | 0 | 0.158111 |
| ENSMUSG00000090464 | processed_transcrip | Gm17140 | 0.316221 | 0 | 0.158111 |
| ENSMUSG00000065437 | miRNA | Mir30d | 0.316221 | 0 | 0.158111 |
| ENSMUSG00000069996 | pseudogene | Gm7125 | 0.32211 | 0 | 0.161055 |
| ENSMUSG00000047728 | protein_coding | BC025446 | 0.33031 | 0 | 0.165155 |
| ENSMUSG00000071716 | protein_coding | Apol7e | 0.33031 | 0 | 0.165155 |
| ENSMUSG00000068085 | protein_coding | Cyp2d11 | 0.33031 | 0 | 0.165155 |
| ENSMUSG00000093261 | miRNA | AC142474.1 | 0.32211 | 0 | 0.161055 |
| ENSMUSG00000087965 | misc_RNA | 7SK | 0.32211 | 0 | 0.161055 |
| ENSMUSG00000089560 | snRNA | U6 | 0.316221 | 0 | 0.158111 |
| ENSMUSG00000092709 | miRNA | AC109201.1 | 0.32211 | 0 | 0.161055 |
| ENSMUSG00000063971 | protein_coding | 1700011A15Rik | 0.32211 | 0 | 0.161055 |
| ENSMUSG00000059668 | protein_coding | Krt4 | 0.316221 | 0 | 0.158111 |
| ENSMUSG00000065111 | snRNA | U6 | 0.33031 | 0 | 0.165155 |
| ENSMUSG00000084737 | snRNA | U6 | 0.33031 | 0 | 0.165155 |

| | | | | | |
|---|---|---|---|---|---|
| ENSMUSG00000089861 | pseudogene | Gm16139 | 0.32211 | 0 | 0.161055 |
| ENSMUSG00000087951 | snRNA | U6 | 0.33031 | 0 | 0.165155 |
| ENSMUSG00000065710 | snRNA | U6 | 0.32211 | 0 | 0.161055 |
| ENSMUSG00000078100 | pseudogene | Gm6551 | 0.33031 | 0 | 0.165155 |
| ENSMUSG00000060459 | protein_coding | Kng2 | 0.33031 | 0 | 0.165155 |
| ENSMUSG00000084299 | pseudogene | Gm6630 | 0.316221 | 0 | 0.158111 |
| ENSMUSG00000087861 | snRNA | U7 | 0.33031 | 0 | 0.165155 |
| ENSMUSG00000089011 | snoRNA | SNORA48 | 0.316221 | 0 | 0.158111 |
| ENSMUSG00000092662 | misc_RNA | AC087556.1 | 0.316221 | 0 | 0.158111 |
| ENSMUSG00000092940 | miRNA | AC117662.1 | 0.33031 | 0 | 0.165155 |
| ENSMUSG00000083827 | pseudogene | Gm15712 | 0.316221 | 0 | 0.158111 |
| ENSMUSG00000085499 | processed_transcrip | Gm15713 | 0.33031 | 0 | 0.165155 |
| ENSMUSG00000088806 | snoRNA | SNORA84 | 0.316221 | 0 | 0.158111 |
| ENSMUSG00000065667 | snRNA | U6 | 0.33031 | 0 | 0.165155 |
| ENSMUSG00000076360 | miRNA | AC171202.1 | 0.33031 | 0 | 0.165155 |
| ENSMUSG00000077595 | snoRNA | SNORA17 | 0.32211 | 0 | 0.161055 |
| ENSMUSG00000049362 | protein_coding | Olfr173 | 0.316221 | 0 | 0.158111 |
| ENSMUSG00000066257 | protein_coding | Olfr206 | 0.33031 | 0 | 0.165155 |
| ENSMUSG00000077839 | miRNA | AC154473.1 | 0.33031 | 0 | 0.165155 |
| ENSMUSG00000089685 | processed_transcrip | Gm9017 | 0.33031 | 0 | 0.165155 |
| ENSMUSG00000088288 | misc_RNA | 7SK | 0.33031 | 0 | 0.165155 |
| ENSMUSG00000087214 | processed_transcrip | Gm15555 | 0.32211 | 0 | 0.161055 |
| ENSMUSG00000071475 | pseudogene | Rpl21-ps5 | 0.316221 | 0 | 0.158111 |
| ENSMUSG00000088684 | snoRNA | SNORA17 | 0.32211 | 0 | 0.161055 |
| ENSMUSG00000050520 | protein_coding | Cldn8 | 0.33031 | 0 | 0.165155 |
| ENSMUSG00000050224 | protein_coding | Krtap13 | 0.33031 | 0 | 0.165155 |
| ENSMUSG00000087240 | processed_transcrip | Gm15976 | 0.32211 | 0 | 0.161055 |
| ENSMUSG00000073457 | protein_coding | Smok2b | 0.33031 | 0 | 0.165155 |
| ENSMUSG00000092120 | protein_coding | Vmn2r90 | 0.316221 | 0 | 0.158111 |
| ENSMUSG00000090851 | protein_coding | Vmn2r98 | 0.32211 | 0 | 0.161055 |
| ENSMUSG00000090572 | protein_coding | Vmn2r109 | 0.32211 | 0 | 0.161055 |
| ENSMUSG00000062165 | protein_coding | Vmn1r232 | 0.32211 | 0 | 0.161055 |
| ENSMUSG00000045575 | protein_coding | Vmn1r233 | 0.33031 | 0 | 0.165155 |
| ENSMUSG00000057203 | protein_coding | Vmn1r234 | 0.33031 | 0 | 0.165155 |
| ENSMUSG00000054142 | protein_coding | Vmn1r236 | 0.33031 | 0 | 0.165155 |
| ENSMUSG00000073444 | protein_coding | Vmn2r112 | 0.33031 | 0 | 0.165155 |
| ENSMUSG00000090725 | lincRNA | Gm17633 | 0.33031 | 0 | 0.165155 |
| ENSMUSG00000090101 | processed_transcrip | Snhg9 | 0.33031 | 0 | 0.165155 |
| ENSMUSG00000091548 | pseudogene | Gm17572 | 0.32211 | 0 | 0.161055 |
| ENSMUSG00000079308 | protein_coding | Olfr239 | 0.33031 | 0 | 0.165155 |
| ENSMUSG00000084601 | snRNA | U6 | 0.32211 | 0 | 0.161055 |
| ENSMUSG00000036322 | pseudogene | H2-Ea-ps | 0.316221 | 0 | 0.158111 |
| ENSMUSG00000058435 | protein_coding | Btn3a3 | 0.33031 | 0 | 0.165155 |
| ENSMUSG00000092525 | processed_transcrip | Gm20461 | 0.32211 | 0 | 0.161055 |
| ENSMUSG00000092609 | protein_coding | Gm20481 | 0.316221 | 0 | 0.158111 |
| ENSMUSG00000065139 | snRNA | U6 | 0.32211 | 0 | 0.161055 |
| ENSMUSG00000077807 | miRNA | CR974466.1 | 0.33031 | 0 | 0.165155 |
| ENSMUSG00000092278 | pseudogene | Gm8752 | 0.32211 | 0 | 0.161055 |
| ENSMUSG00000092219 | processed_transcrip | Gm20443 | 0.32211 | 0 | 0.161055 |
| ENSMUSG00000092368 | processed_transcrip | A930015D03Rik | 0.33031 | 0 | 0.165155 |
| ENSMUSG00000092126 | protein_coding | Gm17579 | 0.32211 | 0 | 0.161055 |
| ENSMUSG00000038311 | protein_coding | 2410017I17Rik | 0.32211 | 0 | 0.161055 |
| ENSMUSG00000092374 | pseudogene | Gm6623 | 0.32211 | 0 | 0.161055 |
| ENSMUSG00000092240 | pseudogene | Gm4246 | 0.32211 | 0 | 0.161055 |
| ENSMUSG00000092306 | pseudogene | Gm20447 | 0.33031 | 0 | 0.165155 |
| ENSMUSG00000092362 | pseudogene | Gm20392 | 0.33031 | 0 | 0.165155 |
| ENSMUSG00000037334 | protein_coding | H2-M1 | 0.32211 | 0 | 0.161055 |
| ENSMUSG00000092460 | pseudogene | Gm8878 | 0.316221 | 0 | 0.158111 |

| | | | | | |
|---|---|---|---|---|---|
| ENSMUSG00000073399 | protein_coding | Trim40 | 0.33031 | 0 | 0.165155 |
| ENSMUSG00000092860 | misc_RNA | CR956641.1 | 0.33031 | 0 | 0.165155 |
| ENSMUSG00000092340 | pseudogene | Olfr754-ps1 | 0.33031 | 0 | 0.165155 |
| ENSMUSG00000016283 | protein_coding | H2-M2 | 0.32211 | 0 | 0.161055 |
| ENSMUSG00000064582 | snRNA | U4 | 0.316221 | 0 | 0.158111 |
| ENSMUSG00000076141 | miRNA | Mir693 | 0.33031 | 0 | 0.165155 |
| ENSMUSG00000082492 | pseudogene | Gm14871 | 0.316221 | 0 | 0.158111 |
| ENSMUSG00000088499 | snRNA | U6 | 0.32211 | 0 | 0.161055 |
| ENSMUSG00000093051 | miRNA | CT030727.1 | 0.316221 | 0 | 0.158111 |
| ENSMUSG00000087829 | misc_RNA | 7SK | 0.33031 | 0 | 0.165155 |
| ENSMUSG00000092828 | miRNA | AC154187.1 | 0.316221 | 0 | 0.158111 |
| ENSMUSG00000080632 | miRNA | AC098726.1 | 0.33031 | 0 | 0.165155 |
| ENSMUSG00000084025 | pseudogene | Gm6276 | 0.316221 | 0 | 0.158111 |
| ENSMUSG00000079333 | protein_coding | Gm11096 | 0.32211 | 0 | 0.161055 |
| ENSMUSG00000065732 | snRNA | U6 | 0.32211 | 0 | 0.161055 |
| ENSMUSG00000037689 | protein_coding | 1700090G07Rik | 0.33031 | 0 | 0.165155 |
| ENSMUSG00000088791 | snRNA | U6 | 0.33031 | 0 | 0.165155 |
| ENSMUSG00000092883 | miRNA | AC121802.1 | 0.33031 | 0 | 0.165155 |
| ENSMUSG00000091165 | processed_transcrip | Gm17036 | 0.316221 | 0 | 0.158111 |
| ENSMUSG00000039540 | protein_coding | 4921524L21Rik | 0.32211 | 0 | 0.161055 |
| ENSMUSG00000091019 | pseudogene | Gm7502 | 0.316221 | 0 | 0.158111 |
| ENSMUSG00000069441 | protein_coding | Dsg1a | 0.32211 | 0 | 0.161055 |
| ENSMUSG00000061928 | protein_coding | Dsg1b | 0.33031 | 0 | 0.165155 |
| ENSMUSG00000084537 | misc_RNA | 7SK | 0.33031 | 0 | 0.165155 |
| ENSMUSG00000057455 | protein_coding | Rit2 | 0.32211 | 0 | 0.161055 |
| ENSMUSG00000076996 | miRNA | AC161167.1 | 0.316221 | 0 | 0.158111 |
| ENSMUSG00000077263 | snoRNA | SNORA17 | 0.316221 | 0 | 0.158111 |
| ENSMUSG00000089983 | lincRNA | 2010320O07Rik | 0.32211 | 0 | 0.161055 |
| ENSMUSG00000087857 | snoRNA | SNORA17 | 0.316221 | 0 | 0.158111 |
| ENSMUSG00000077243 | snoRNA | SNORA17 | 0.316221 | 0 | 0.158111 |
| ENSMUSG00000064706 | misc_RNA | Y_RNA | 0.33031 | 0 | 0.165155 |
| ENSMUSG00000064527 | misc_RNA | Y_RNA | 0.33031 | 0 | 0.165155 |
| ENSMUSG00000073524 | protein_coding | Gm10526 | 0.32211 | 0 | 0.161055 |
| ENSMUSG00000024857 | protein_coding | Cabp2 | 0.33031 | 0 | 0.165155 |
| ENSMUSG00000065582 | miRNA | Mir194-2 | 0.33031 | 0 | 0.165155 |
| ENSMUSG00000080414 | miRNA | AC167245.1 | 0.33031 | 0 | 0.165155 |
| ENSMUSG00000093314 | miRNA | AC129217.1 | 0.316221 | 0 | 0.158111 |
| ENSMUSG00000024681 | protein_coding | Ms4a3 | 0.33031 | 0 | 0.165155 |
| ENSMUSG00000055895 | protein_coding | Plac1l | 0.316221 | 0 | 0.158111 |
| ENSMUSG00000040065 | protein_coding | Pfpl | 0.32211 | 0 | 0.161055 |
| ENSMUSG00000091014 | protein_coding | Gm5244 | 0.33031 | 0 | 0.165155 |
| ENSMUSG00000046272 | protein_coding | Olfr1444 | 0.33031 | 0 | 0.165155 |
| ENSMUSG00000060303 | protein_coding | Olfr1447 | 0.316221 | 0 | 0.158111 |
| ENSMUSG00000048456 | protein_coding | Olfr1448 | 0.32211 | 0 | 0.161055 |
| ENSMUSG00000046388 | pseudogene | Gm9806 | 0.316221 | 0 | 0.158111 |
| ENSMUSG00000079382 | protein_coding | Gm3443 | 0.316221 | 0 | 0.158111 |
| ENSMUSG00000074913 | protein_coding | Gm815 | 0.32211 | 0 | 0.161055 |
| ENSMUSG00000065556 | miRNA | Mir101b | 0.316221 | 0 | 0.158111 |
| ENSMUSG00000087937 | snRNA | U1 | 0.33031 | 0 | 0.165155 |
| ENSMUSG00000025003 | protein_coding | Cyp2c39 | 0.33031 | 0 | 0.165155 |
| ENSMUSG00000062624 | protein_coding | Cyp2c67 | 0.33031 | 0 | 0.165155 |
| ENSMUSG00000058350 | pseudogene | Gm5246 | 0.32211 | 0 | 0.161055 |
| ENSMUSG00000089807 | pseudogene | Gm16298 | 0.316221 | 0 | 0.158111 |
| ENSMUSG00000088894 | snoRNA | SNORA44 | 0.33031 | 0 | 0.165155 |
| ENSMUSG00000076251 | miRNA | Mir501 | 0.33031 | 0 | 0.165155 |
| ENSMUSG00000088477 | snoRNA | SNORA70 | 0.33031 | 0 | 0.165155 |
| ENSMUSG00000079702 | protein_coding | Ssxb6 | 0.32211 | 0 | 0.161055 |
| ENSMUSG00000081680 | pseudogene | Gm14473 | 0.32211 | 0 | 0.161055 |

| | | | | | |
|---|---|---|---|---|---|
| ENSMUSG00000083288 | pseudogene | Csnk2a1-ps | 0.33031 | 0 | 0.165155 |
| ENSMUSG00000068113 | protein_coding | Gm4907 | 0.316221 | 0 | 0.158111 |
| ENSMUSG00000064334 | protein_coding | Gm10147 | 0.33031 | 0 | 0.165155 |
| ENSMUSG00000073255 | protein_coding | Gm14632 | 0.32211 | 0 | 0.161055 |
| ENSMUSG00000085989 | processed_transcrip | Gm7437 | 0.316221 | 0 | 0.158111 |
| ENSMUSG00000078331 | protein_coding | Gm5926 | 0.33031 | 0 | 0.165155 |
| ENSMUSG00000087007 | pseudogene | Gm2231 | 0.33031 | 0 | 0.165155 |
| ENSMUSG00000083762 | pseudogene | Gm2318 | 0.32211 | 0 | 0.161055 |
| ENSMUSG00000085107 | pseudogene | Gm4764 | 0.32211 | 0 | 0.161055 |
| ENSMUSG00000079631 | protein_coding | Rhox3f | 0.33031 | 0 | 0.165155 |
| ENSMUSG00000081880 | pseudogene | Gm5385 | 0.32211 | 0 | 0.161055 |
| ENSMUSG00000081551 | pseudogene | Gm14654 | 0.33031 | 0 | 0.165155 |
| ENSMUSG00000064806 | snRNA | U1 | 0.33031 | 0 | 0.165155 |
| ENSMUSG00000089443 | snRNA | U6 | 0.32211 | 0 | 0.161055 |
| ENSMUSG00000083112 | pseudogene | Gm14620 | 0.32211 | 0 | 0.161055 |
| ENSMUSG00000081350 | pseudogene | Gm5388 | 0.32211 | 0 | 0.161055 |
| ENSMUSG00000076214 | miRNA | Mir717 | 0.33031 | 0 | 0.165155 |
| ENSMUSG00000076050 | miRNA | Mir450b | 0.33031 | 0 | 0.165155 |
| ENSMUSG00000084076 | pseudogene | Gm14600 | 0.32211 | 0 | 0.161055 |
| ENSMUSG00000084308 | pseudogene | Gm14593 | 0.33031 | 0 | 0.165155 |
| ENSMUSG00000081790 | pseudogene | Gm14596 | 0.32211 | 0 | 0.161055 |
| ENSMUSG00000082441 | pseudogene | Gm6664 | 0.32211 | 0 | 0.161055 |
| ENSMUSG00000031132 | protein_coding | Cd40lg | 0.316221 | 0 | 0.158111 |
| ENSMUSG00000081922 | pseudogene | Gm14646 | 0.316221 | 0 | 0.158111 |
| ENSMUSG00000031138 | protein_coding | F9 | 0.32211 | 0 | 0.161055 |
| ENSMUSG00000070071 | miRNA | Mir465 | 0.32211 | 0 | 0.161055 |
| ENSMUSG00000080467 | miRNA | AL808131.1 | 0.316221 | 0 | 0.158111 |
| ENSMUSG00000079531 | protein_coding | Gm5936 | 0.32211 | 0 | 0.161055 |
| ENSMUSG00000080193 | pseudogene | Gm14756 | 0.33031 | 0 | 0.165155 |
| ENSMUSG00000089014 | snoRNA | SNORA36 | 0.33031 | 0 | 0.165155 |
| ENSMUSG00000083333 | pseudogene | Magea7-ps | 0.33031 | 0 | 0.165155 |
| ENSMUSG00000081358 | pseudogene | Gm14746 | 0.32211 | 0 | 0.161055 |
| ENSMUSG00000088335 | snoRNA | SNORA25 | 0.33031 | 0 | 0.165155 |
| ENSMUSG00000083637 | pseudogene | Gm14754 | 0.32211 | 0 | 0.161055 |
| ENSMUSG00000081241 | pseudogene | Gm14737 | 0.316221 | 0 | 0.158111 |
| ENSMUSG00000065661 | snRNA | U6 | 0.33031 | 0 | 0.165155 |
| ENSMUSG00000081933 | pseudogene | Gm14767 | 0.32211 | 0 | 0.161055 |
| ENSMUSG00000080266 | pseudogene | Gm14772 | 0.316221 | 0 | 0.158111 |
| ENSMUSG00000082832 | pseudogene | Gm14786 | 0.316221 | 0 | 0.158111 |
| ENSMUSG00000080737 | pseudogene | Gm6982 | 0.33031 | 0 | 0.165155 |
| ENSMUSG00000067646 | protein_coding | BC061195 | 0.32211 | 0 | 0.161055 |
| ENSMUSG00000080995 | pseudogene | Gm15165 | 0.33031 | 0 | 0.165155 |
| ENSMUSG00000081796 | pseudogene | Gm9005 | 0.32211 | 0 | 0.161055 |
| ENSMUSG00000073060 | pseudogene | Zxda | 0.33031 | 0 | 0.165155 |
| ENSMUSG00000081606 | pseudogene | Gm9059 | 0.33031 | 0 | 0.165155 |
| ENSMUSG00000092463 | protein_coding | Gm20489 | 0.316221 | 0 | 0.158111 |
| ENSMUSG00000083213 | pseudogene | Gm14875 | 0.316221 | 0 | 0.158111 |
| ENSMUSG00000082996 | pseudogene | Gm6238 | 0.32211 | 0 | 0.161055 |
| ENSMUSG00000085791 | pseudogene | Rpl30-ps9 | 0.33031 | 0 | 0.165155 |
| ENSMUSG00000063442 | pseudogene | Gm7117 | 0.316221 | 0 | 0.158111 |
| ENSMUSG00000073010 | protein_coding | Gm5127 | 0.33031 | 0 | 0.165155 |
| ENSMUSG00000083560 | pseudogene | Gm14939 | 0.33031 | 0 | 0.165155 |
| ENSMUSG00000082718 | pseudogene | Gm14928 | 0.32211 | 0 | 0.161055 |
| ENSMUSG00000067402 | protein_coding | Gm2411 | 0.316221 | 0 | 0.158111 |
| ENSMUSG00000049815 | protein_coding | 4921511C20Rik | 0.316221 | 0 | 0.158111 |
| ENSMUSG00000082382 | pseudogene | Gm14970 | 0.32211 | 0 | 0.161055 |
| ENSMUSG00000083250 | pseudogene | Gm5403 | 0.33031 | 0 | 0.165155 |
| ENSMUSG00000083236 | pseudogene | Gm7222 | 0.32211 | 0 | 0.161055 |

| | | | | | |
|---|---|---|---|---|---|
| ENSMUSG00000081292 | pseudogene | Gm15018 | 0.33031 | 0 | 0.165155 |
| ENSMUSG00000080874 | pseudogene | Gm15010 | 0.32211 | 0 | 0.161055 |
| ENSMUSG00000031270 | protein_coding | 4930513O06Rik | 0.316221 | 0 | 0.158111 |
| ENSMUSG00000082223 | pseudogene | Gm15079 | 0.33031 | 0 | 0.165155 |
| ENSMUSG00000083200 | pseudogene | Gm15066 | 0.32211 | 0 | 0.161055 |
| ENSMUSG00000065536 | miRNA | Mir98 | 0.32211 | 0 | 0.161055 |
| ENSMUSG00000087235 | processed_transcrip | Gm4750 | 0.33031 | 0 | 0.165155 |
| ENSMUSG00000084275 | pseudogene | Gm5396 | 0.33031 | 0 | 0.165155 |
| ENSMUSG00000082733 | pseudogene | Gm15153 | 0.33031 | 0 | 0.165155 |
| ENSMUSG00000083488 | pseudogene | Gm8534 | 0.33031 | 0 | 0.165155 |
| ENSMUSG00000090349 | protein_coding | Gm17261 | 0.316221 | 0 | 0.158111 |
| ENSMUSG00000081222 | pseudogene | Gm15173 | 0.316221 | 0 | 0.158111 |
| ENSMUSG00000065943 | snRNA | U6 | 0.316221 | 0 | 0.158111 |
| ENSMUSG00000084598 | misc_RNA | 7SK | 0.32211 | 0 | 0.161055 |
| ENSMUSG00000082007 | pseudogene | Gm15206 | 0.32211 | 0 | 0.161055 |
| ENSMUSG00000083963 | pseudogene | Gm15203 | 0.32211 | 0 | 0.161055 |
| ENSMUSG00000080394 | snoRNA | SNORA76 | 0.316221 | 0 | 0.158111 |
| ENSMUSG00000092752 | miRNA | AL807822.1 | 0.33031 | 0 | 0.165155 |
| ENSMUSG00000040808 | protein_coding | S100g | 0.33031 | 0 | 0.165155 |
| ENSMUSG00000082134 | pseudogene | Gm15236 | 0.33031 | 0 | 0.165155 |
| ENSMUSG00000082360 | pseudogene | Gm8814 | 0.316221 | 0 | 0.158111 |
| ENSMUSG00000031354 | protein_coding | Amelx | 0.316221 | 0 | 0.158111 |
| ENSMUSG00000087587 | processed_transcrip | Gm15245 | 0.316221 | 0 | 0.158111 |
| ENSMUSG00000064355 | Mt_tRNA | mt-Tk | 0.33031 | 0 | 0.165155 |
| ENSMUSG00000071318 | protein_coding | Gm4795 | 1.621062 | 2.021789 | 1.821425 |
| ENSMUSG00000081673 | pseudogene | Gm14794 | 1.621062 | 2.021789 | 1.821425 |
| ENSMUSG00000045287 | protein_coding | Rtn4rl1 | 913.7896 | 887.8422 | 900.8159 |
| ENSMUSG00000036533 | protein_coding | Cdc42ep3 | 269.1494 | 261.3854 | 265.2674 |
| ENSMUSG00000027224 | protein_coding | Duoxa1 | 2.239415 | 1.775272 | 2.007344 |
| ENSMUSG00000039579 | protein_coding | Grin3a | 42.16944 | 39.63601 | 40.90273 |
| ENSMUSG00000058812 | protein_coding | 0610039K10Rik | 24.53583 | 26.35781 | 25.44682 |
| ENSMUSG00000050100 | protein_coding | Hmx2 | 2.253504 | 2.737158 | 2.495331 |
| ENSMUSG00000025855 | protein_coding | Prkar1b | 177.8988 | 183.1584 | 180.5286 |
| ENSMUSG00000065637 | snoRNA | SNORA14 | 42.57796 | 45.46993 | 44.02394 |
| ENSMUSG00000056268 | protein_coding | Dennd1b | 943.0921 | 959.7698 | 951.431 |
| ENSMUSG00000075284 | protein_coding | Wipf1 | 320.597 | 328.4995 | 324.5482 |
| ENSMUSG00000054116 | protein_coding | E130116L18Rik | 2.589703 | 3.135023 | 2.862363 |
| ENSMUSG00000047168 | pseudogene | Gm6684 | 2.891835 | 3.506222 | 3.199029 |
| ENSMUSG00000034459 | protein_coding | Ifit1 | 15.92131 | 14.37771 | 15.14951 |
| ENSMUSG00000089806 | pseudogene | Gm16570 | 1.307151 | 1.757616 | 1.532384 |
| ENSMUSG00000059436 | protein_coding | Max | 1487.745 | 1515.085 | 1501.415 |
| ENSMUSG00000017781 | protein_coding | Pitpna | 2826.619 | 2781.754 | 2804.186 |
| ENSMUSG00000001985 | protein_coding | Grik3 | 471.8222 | 455.1787 | 463.5005 |
| ENSMUSG00000036698 | protein_coding | Eif2c2 | 1049.764 | 1073.911 | 1061.837 |
| ENSMUSG00000039114 | protein_coding | Nrn1 | 205.8984 | 199.1601 | 202.5292 |
| ENSMUSG00000078157 | protein_coding | 4931440F15Rik | 2.593282 | 2.057464 | 2.325373 |
| ENSMUSG00000083585 | pseudogene | Gm15885 | 0.632442 | 0.335524 | 0.483983 |
| ENSMUSG00000023972 | protein_coding | Ptk7 | 2187.454 | 2147.521 | 2167.488 |
| ENSMUSG00000050866 | protein_coding | Clrn3 | 0.960442 | 0.662038 | 0.81124 |
| ENSMUSG00000079373 | protein_coding | Gm10021 | 0.960442 | 0.662038 | 0.81124 |
| ENSMUSG00000089816 | pseudogene | Ear-ps8 | 0.960442 | 0.662038 | 0.81124 |
| ENSMUSG00000090777 | protein_coding | Gm7873 | 0.960442 | 0.662038 | 0.81124 |
| ENSMUSG00000089417 | snoRNA | snoU90 | 23.48982 | 25.52781 | 24.50881 |
| ENSMUSG00000090406 | processed_transcrip | Gm17058 | 2.259393 | 2.763824 | 2.511609 |
| ENSMUSG00000028619 | protein_coding | 2210012G02Rik | 669.9789 | 657.7581 | 663.8685 |
| ENSMUSG00000020674 | protein_coding | Pxdn | 2487.978 | 2544.283 | 2516.13 |
| ENSMUSG00000026928 | protein_coding | Card9 | 59.1332 | 61.84793 | 60.49056 |
| ENSMUSG00000086084 | processed_transcrip | 2610528B01Rik | 99.52175 | 103.1572 | 101.3395 |

| | | | | | |
|---|---|---|---|---|---|
| ENSMUSG00000047617 | protein_coding | BC029214 | 252.1392 | 259.1755 | 255.6573 |
| ENSMUSG00000013736 | protein_coding | Trnt1 | 1425.963 | 1403.408 | 1414.685 |
| ENSMUSG00000001576 | protein_coding | Ergic1 | 2402.647 | 2439.479 | 2421.063 |
| ENSMUSG00000085252 | processed_transcrip | 8430437L04Rik | 1.931394 | 2.374969 | 2.153181 |
| ENSMUSG00000073173 | protein_coding | Gm10477 | 2.900035 | 3.497213 | 3.198624 |
| ENSMUSG00000047155 | protein_coding | Cyp4x1 | 0.632442 | 0.326514 | 0.479478 |
| ENSMUSG00000092452 | pseudogene | Vmn2r-ps45 | 0.632442 | 0.326514 | 0.479478 |
| ENSMUSG00000085116 | pseudogene | Gm2735 | 0.632442 | 0.326514 | 0.479478 |
| ENSMUSG00000077925 | miRNA | Mir453 | 0.632442 | 0.326514 | 0.479478 |
| ENSMUSG00000058254 | protein_coding | Tspan7 | 912.7301 | 931.7272 | 922.2286 |
| ENSMUSG00000077212 | snoRNA | Snord69 | 22.3656 | 24.15149 | 23.25854 |
| ENSMUSG00000093023 | misc_RNA | AC142099.1 | 1.959572 | 2.446319 | 2.202946 |
| ENSMUSG00000004633 | protein_coding | Chn2 | 130.4451 | 125.1808 | 127.8129 |
| ENSMUSG00000026420 | protein_coding | Il24 | 2.903614 | 2.410644 | 2.657129 |
| ENSMUSG00000055177 | protein_coding | Cstl1 | 0.968641 | 0.671047 | 0.819844 |
| ENSMUSG00000090665 | protein_coding | Gad1-ps | 0.968641 | 0.671047 | 0.819844 |
| ENSMUSG00000055415 | protein_coding | Atp10b | 0.968641 | 0.671047 | 0.819844 |
| ENSMUSG00000046006 | protein_coding | Gapt | 0.968641 | 0.671047 | 0.819844 |
| ENSMUSG00000023829 | protein_coding | Slc22a1 | 0.968641 | 0.671047 | 0.819844 |
| ENSMUSG00000090700 | protein_coding | Cyp4f40 | 0.968641 | 0.671047 | 0.819844 |
| ENSMUSG00000022141 | protein_coding | Nipbl | 4854.483 | 4738.581 | 4796.532 |
| ENSMUSG00000022758 | protein_coding | P2rx6 | 47.22585 | 49.5251 | 48.37548 |
| ENSMUSG00000054792 | protein_coding | Klhl18 | 681.4691 | 667.6646 | 674.5668 |
| ENSMUSG00000046561 | protein_coding | Arsj | 53.22459 | 50.39585 | 51.81022 |
| ENSMUSG00000002342 | protein_coding | Tmem161a | 859.9994 | 875.1118 | 867.5556 |
| ENSMUSG00000048537 | protein_coding | Phldb1 | 1536.096 | 1513.415 | 1524.755 |
| ENSMUSG00000069014 | pseudogene | Gm5641 | 810.0944 | 825.7976 | 817.946 |
| ENSMUSG00000047989 | protein_coding | Ino80c | 747.308 | 734.7337 | 741.0208 |
| ENSMUSG00000050921 | protein_coding | P2ry10 | 2.583814 | 3.108357 | 2.846086 |
| ENSMUSG00000068696 | protein_coding | Gpr88 | 143.2499 | 138.3611 | 140.8055 |
| ENSMUSG00000029550 | protein_coding | Sppl3 | 2144.555 | 2111.474 | 2128.014 |
| ENSMUSG00000087419 | protein_coding | Gm16721 | 0.974531 | 1.324076 | 1.149303 |
| ENSMUSG00000076934 | IG_V_gene | Iglv1 | 0.974531 | 1.324076 | 1.149303 |
| ENSMUSG00000044751 | pseudogene | Gm12231 | 33.58623 | 31.79573 | 32.69098 |
| ENSMUSG00000061242 | protein_coding | Rpl21-ps7 | 3.222145 | 2.674817 | 2.948481 |
| ENSMUSG00000040483 | protein_coding | Xaf1 | 155.3088 | 147.0716 | 151.1902 |
| ENSMUSG00000084659 | snRNA | U4 | 1.917305 | 2.392987 | 2.155146 |
| ENSMUSG00000054021 | protein_coding | Sirt5 | 105.1519 | 101.6681 | 103.41 |
| ENSMUSG00000029552 | protein_coding | Tes | 289.8239 | 298.4483 | 294.1361 |
| ENSMUSG00000034543 | protein_coding | Morc2a | 3683.213 | 3769.344 | 3726.278 |
| ENSMUSG00000085095 | processed_transcrip | Gm15635 | 1.621062 | 2.030798 | 1.82593 |
| ENSMUSG00000004939 | protein_coding | Itgb1bp3 | 0.98273 | 1.341732 | 1.162231 |
| ENSMUSG00000039087 | protein_coding | Rreb1 | 1954.653 | 1993.212 | 1973.933 |
| ENSMUSG00000048852 | protein_coding | Gm12185 | 2.577925 | 3.135023 | 2.856474 |
| ENSMUSG00000072473 | protein_coding | 1700024G13Rik | 1.626951 | 2.075121 | 1.851036 |
| ENSMUSG00000064363 | protein_coding | mt-Nd4 | 28495.15 | 28870.18 | 28682.67 |
| ENSMUSG00000061972 | protein_coding | Olfr99 | 1.635151 | 2.08413 | 1.85964 |
| ENSMUSG00000088786 | misc_RNA | 7SK | 0.99093 | 1.350742 | 1.170836 |
| ENSMUSG00000037167 | protein_coding | Spaca5 | 0.99093 | 1.350742 | 1.170836 |
| ENSMUSG00000030168 | protein_coding | Adipor2 | 3604.192 | 3668.99 | 3636.591 |
| ENSMUSG00000082035 | pseudogene | Gm14934 | 11.64725 | 10.56014 | 11.1037 |
| ENSMUSG00000039563 | lincRNA | 2210406O10Rik | 55.44723 | 52.34081 | 53.89402 |
| ENSMUSG00000057177 | protein_coding | Gsk3a | 1404.152 | 1382.619 | 1393.386 |
| ENSMUSG00000030347 | protein_coding | D6Wsu163e | 311.4492 | 318.3949 | 314.922 |
| ENSMUSG00000022696 | protein_coding | Sidt1 | 15.48775 | 14.25882 | 14.87329 |
| ENSMUSG00000092279 | protein_coding | RP23-3M10.11 | 87.30893 | 83.08668 | 85.1978 |
| ENSMUSG00000042216 | protein_coding | Sgsm1 | 112.5949 | 108.4589 | 110.5269 |
| ENSMUSG00000071340 | protein_coding | Bet3l | 0.954552 | 0.662038 | 0.808295 |

| | | | | | |
|---|---|---|---|---|---|
| ENSMUSG00000085954 | lincRNA | 4930478K11Rik | 0.954552 | 0.662038 | 0.808295 |
| ENSMUSG00000071244 | protein_coding | Gm6219 | 0.954552 | 0.662038 | 0.808295 |
| ENSMUSG00000072924 | protein_coding | Gm15085 | 0.954552 | 0.662038 | 0.808295 |
| ENSMUSG00000057604 | protein_coding | Lmcd1 | 99.82329 | 95.70475 | 97.76402 |
| ENSMUSG00000074681 | protein_coding | Defb23 | 14.21444 | 12.99745 | 13.60595 |
| ENSMUSG00000065369 | snRNA | U1 | 0.962752 | 1.315066 | 1.138909 |
| ENSMUSG00000042821 | protein_coding | Snai1 | 151.2164 | 156.6003 | 153.9083 |
| ENSMUSG00000061577 | protein_coding | Gpr114 | 7.814955 | 6.798392 | 7.306674 |
| ENSMUSG00000030062 | protein_coding | Rpn1 | 6256.465 | 6175.129 | 6215.797 |
| ENSMUSG00000035297 | protein_coding | Cops4 | 2561.595 | 2593.927 | 2577.761 |
| ENSMUSG00000066319 | protein_coding | Rtp3 | 2.581503 | 2.093139 | 2.337321 |
| ENSMUSG00000044043 | protein_coding | Pcdhb14 | 46.40668 | 44.22595 | 45.31631 |
| ENSMUSG00000020696 | protein_coding | Rffl | 1117.939 | 1095.48 | 1106.71 |
| ENSMUSG00000087626 | lincRNA | Gm15050 | 1.270774 | 1.703922 | 1.487348 |
| ENSMUSG00000038187 | protein_coding | Btbd10 | 2649.119 | 2600.741 | 2624.93 |
| ENSMUSG00000040663 | protein_coding | Clcf1 | 94.37686 | 98.8026 | 96.58973 |
| ENSMUSG00000032013 | protein_coding | Trim29 | 5.473339 | 4.794622 | 5.13398 |
| ENSMUSG00000029359 | protein_coding | Tesc | 297.2536 | 306.5058 | 301.8797 |
| ENSMUSG00000087704 | processed_transcrip | Gm14978 | 0.98273 | 0.688704 | 0.835717 |
| ENSMUSG00000037482 | protein_coding | Erv3 | 0.98273 | 0.688704 | 0.835717 |
| ENSMUSG00000044249 | protein_coding | Defb29 | 0.98273 | 0.688704 | 0.835717 |
| ENSMUSG00000080831 | pseudogene | Gm12529 | 0.98273 | 0.688704 | 0.835717 |
| ENSMUSG00000083989 | pseudogene | Gm12607 | 0.98273 | 0.688704 | 0.835717 |
| ENSMUSG00000086656 | processed_transcrip | Gm15701 | 0.98273 | 0.688704 | 0.835717 |
| ENSMUSG00000085565 | lincRNA | Gm15721 | 0.98273 | 0.688704 | 0.835717 |
| ENSMUSG00000065444 | miRNA | Mir27a | 0.98273 | 0.688704 | 0.835717 |
| ENSMUSG00000068165 | pseudogene | Gm10233 | 0.98273 | 0.688704 | 0.835717 |
| ENSMUSG00000024578 | protein_coding | Il17b | 0.98273 | 0.688704 | 0.835717 |
| ENSMUSG00000083428 | pseudogene | Gm14591 | 0.98273 | 0.688704 | 0.835717 |
| ENSMUSG00000049232 | protein_coding | Tigd2 | 477.6815 | 469.1729 | 473.4272 |
| ENSMUSG00000047730 | protein_coding | Fcgbp | 2.628392 | 2.075121 | 2.351756 |
| ENSMUSG00000011427 | protein_coding | Zfp790 | 730.5467 | 706.2008 | 718.3738 |
| ENSMUSG00000019987 | protein_coding | Arg1 | 18.71094 | 17.37619 | 18.04356 |
| ENSMUSG00000072849 | protein_coding | Serpina1e | 1.307151 | 1.695274 | 1.501213 |
| ENSMUSG00000073779 | protein_coding | B230314M03Rik | 2.259393 | 2.817518 | 2.538456 |
| ENSMUSG00000058281 | pseudogene | Gm10038 | 3.530167 | 4.194926 | 3.862546 |
| ENSMUSG00000085583 | processed_transcrip | Gm15676 | 19.09067 | 20.48455 | 19.78761 |
| ENSMUSG00000084821 | processed_transcrip | Gm15880 | 3.219835 | 3.859402 | 3.539619 |
| ENSMUSG00000024238 | protein_coding | Zeb1 | 2185.103 | 2222.949 | 2204.026 |
| ENSMUSG00000022772 | protein_coding | Senp5 | 1259.027 | 1284.179 | 1271.603 |
| ENSMUSG00000035992 | protein_coding | Fnip1 | 2274.234 | 2200.628 | 2237.431 |
| ENSMUSG00000024429 | protein_coding | Gnl1 | 2222.455 | 2179.045 | 2200.75 |
| ENSMUSG00000025519 | protein_coding | Tktl2 | 159.7916 | 133.9324 | 146.862 |
| ENSMUSG00000027775 | protein_coding | Mfsd1 | 2038.372 | 2075.473 | 2056.923 |
| ENSMUSG00000081883 | pseudogene | Gm15574 | 0.98273 | 0.653029 | 0.817879 |
| ENSMUSG00000029867 | protein_coding | 1700034O15Rik | 0.98273 | 0.653029 | 0.817879 |
| ENSMUSG00000074028 | protein_coding | Slc22a13 | 0.98273 | 0.653029 | 0.817879 |
| ENSMUSG00000049800 | protein_coding | Sertad2 | 1579.753 | 1547.401 | 1563.577 |
| ENSMUSG00000088793 | snRNA | U6 | 0.64422 | 1.006571 | 0.825395 |
| ENSMUSG00000021697 | protein_coding | Depdc1b | 175.4139 | 170.3462 | 172.88 |
| ENSMUSG00000018848 | protein_coding | Rars | 3209.824 | 3161.052 | 3185.438 |
| ENSMUSG00000084088 | pseudogene | Gm12941 | 22.86073 | 21.32175 | 22.09124 |
| ENSMUSG00000001729 | protein_coding | Akt1 | 4443.13 | 4504.062 | 4473.596 |
| ENSMUSG00000030720 | protein_coding | Cln3 | 936.1918 | 952.054 | 944.1229 |
| ENSMUSG00000034111 | protein_coding | Tmed8 | 1369.17 | 1389.221 | 1379.195 |
| ENSMUSG00000086662 | processed_transcrip | BC046251 | 12.34761 | 11.118 | 11.7328 |
| ENSMUSG00000021007 | protein_coding | Spata7 | 360.6473 | 353.5938 | 357.1205 |
| ENSMUSG00000041219 | protein_coding | Arhgap11a | 1170.849 | 1148.325 | 1159.587 |

| | | | | | |
|---|---|---|---|---|---|
| ENSMUSG00000034009 | protein_coding | Rxfp1 | 45.30721 | 36.36761 | 40.83741 |
| ENSMUSG00000031789 | protein_coding | Cngb1 | 284.8232 | 277.9945 | 281.4088 |
| ENSMUSG00000002844 | protein_coding | Adprh | 1779.771 | 1749.988 | 1764.879 |
| ENSMUSG00000074483 | protein_coding | Bglap | 7.777309 | 6.941094 | 7.359201 |
| ENSMUSG00000035614 | protein_coding | Fam179b | 2302.139 | 2265.705 | 2283.922 |
| ENSMUSG00000039294 | protein_coding | BC017643 | 753.6715 | 766.437 | 760.0543 |
| ENSMUSG00000024694 | protein_coding | Keg1 | 1.31304 | 1.686265 | 1.499653 |
| ENSMUSG00000028259 | protein_coding | Fhl5 | 13.84671 | 12.73292 | 13.28981 |
| ENSMUSG00000086534 | lincRNA | Gm16758 | 1.592884 | 2.021789 | 1.807336 |
| ENSMUSG00000035713 | processed_transcrip | Usp35 | 349.0282 | 341.0818 | 345.055 |
| ENSMUSG00000080840 | pseudogene | Gm11349 | 1.284863 | 1.668247 | 1.476555 |
| ENSMUSG00000047935 | protein_coding | Gm5607 | 2.914124 | 3.506222 | 3.210173 |
| ENSMUSG00000091209 | protein_coding | Gm3264 | 5.114851 | 4.387748 | 4.751299 |
| ENSMUSG00000035834 | protein_coding | Polr3g | 2287.741 | 2249.066 | 2268.403 |
| ENSMUSG00000061024 | protein_coding | Rrs1 | 1199.178 | 1224.589 | 1211.884 |
| ENSMUSG00000020617 | protein_coding | 1700012B07Rik | 35.86791 | 33.98616 | 34.92704 |
| ENSMUSG00000091512 | protein_coding | Lamtor3 | 981.5106 | 1001.57 | 991.5403 |
| ENSMUSG00000092193 | pseudogene | Cd9-ps | 0.976841 | 0.688704 | 0.832773 |
| ENSMUSG00000088252 | snoRNA | Snord13 | 0.976841 | 0.688704 | 0.832773 |
| ENSMUSG00000088329 | misc_RNA | RNaseP_nuc | 0.976841 | 0.688704 | 0.832773 |
| ENSMUSG00000000263 | protein_coding | Glra1 | 0.976841 | 0.688704 | 0.832773 |
| ENSMUSG00000060765 | protein_coding | Olfr325 | 0.976841 | 0.688704 | 0.832773 |
| ENSMUSG00000093322 | rRNA | AC155256.1 | 0.976841 | 0.688704 | 0.832773 |
| ENSMUSG00000091798 | lincRNA | Gm17691 | 0.976841 | 0.688704 | 0.832773 |
| ENSMUSG00000085761 | processed_transcrip | 4930455G09Rik | 23.63071 | 25.3325 | 24.4816 |
| ENSMUSG00000005378 | protein_coding | Wbscr22 | 960.1432 | 974.9679 | 967.5556 |
| ENSMUSG00000039741 | protein_coding | Bahcc1 | 3304.984 | 3243.816 | 3274.4 |
| ENSMUSG00000084823 | processed_transcrip | Gm17216 | 28.76173 | 30.66769 | 29.71471 |
| ENSMUSG00000062154 | protein_coding | 1700061J05Rik | 3.264412 | 2.710492 | 2.987452 |
| ENSMUSG00000016128 | protein_coding | Stard13 | 181.8165 | 176.1737 | 178.9951 |
| ENSMUSG00000092284 | pseudogene | Gm8801 | 14.32231 | 20.08482 | 17.20356 |
| ENSMUSG00000023971 | protein_coding | Rrp36 | 758.32 | 770.6367 | 764.4784 |
| ENSMUSG00000001707 | protein_coding | Eef1e1 | 1180.205 | 1157.679 | 1168.942 |
| ENSMUSG00000073018 | protein_coding | Gm1070 | 23.89646 | 22.30486 | 23.10066 |
| ENSMUSG00000022601 | protein_coding | Rpl24 | 6269.077 | 6183.936 | 6226.507 |
| ENSMUSG00000056019 | protein_coding | Zfp709 | 230.6848 | 238.647 | 234.6659 |
| ENSMUSG00000037373 | protein_coding | Ctbp1 | 3231.766 | 3183.715 | 3207.74 |
| ENSMUSG00000041216 | protein_coding | Clvs1 | 3.947321 | 4.714262 | 4.330792 |
| ENSMUSG00000000823 | protein_coding | Znf512b | 2110.761 | 2145.321 | 2128.041 |
| ENSMUSG00000055737 | protein_coding | Ghr | 405.408 | 417.0755 | 411.2418 |
| ENSMUSG00000086269 | processed_transcrip | Gm16154 | 5.193495 | 4.45946 | 4.826478 |
| ENSMUSG00000030669 | protein_coding | Calca | 2.573303 | 3.108357 | 2.84083 |
| ENSMUSG00000085210 | lincRNA | Gm16615 | 2.936413 | 2.419653 | 2.678033 |
| ENSMUSG00000048174 | protein_coding | Tmem81 | 144.0801 | 148.9307 | 146.5054 |
| ENSMUSG00000085110 | processed_transcrip | Gm13920 | 0.974531 | 0.688704 | 0.831617 |
| ENSMUSG00000084919 | processed_transcrip | C330008G21Rik | 0.974531 | 0.688704 | 0.831617 |
| ENSMUSG00000087334 | processed_transcrip | AW495222 | 0.974531 | 0.688704 | 0.831617 |
| ENSMUSG00000045052 | protein_coding | Prlhr | 0.974531 | 0.688704 | 0.831617 |
| ENSMUSG00000086473 | pseudogene | Gm16265 | 0.974531 | 0.688704 | 0.831617 |
| ENSMUSG00000082729 | pseudogene | Gm14845 | 0.974531 | 0.688704 | 0.831617 |
| ENSMUSG00000038628 | protein_coding | Polr3k | 1185.222 | 1214.705 | 1199.963 |
| ENSMUSG00000064731 | snoRNA | Snord45b | 2.900035 | 3.443881 | 3.171958 |
| ENSMUSG00000083793 | pseudogene | Gm14274 | 0.976841 | 0.653029 | 0.814935 |
| ENSMUSG00000092597 | pseudogene | RP23-71I22.1 | 0.976841 | 0.653029 | 0.814935 |
| ENSMUSG00000033287 | protein_coding | Kctd17 | 623.9891 | 634.9641 | 629.4766 |
| ENSMUSG00000088891 | snoRNA | SNORA76 | 2.245304 | 2.710492 | 2.477898 |
| ENSMUSG00000019868 | protein_coding | Vta1 | 796.5992 | 809.3822 | 802.9907 |
| ENSMUSG00000084284 | pseudogene | Gm16469 | 2.239415 | 2.710492 | 2.474954 |

| | | | | | |
|---|---|---|---|---|---|
| ENSMUSG00000084943 | processed_transcrip | Gm13014 | 2.583814 | 3.081691 | 2.832753 |
| ENSMUSG00000090197 | pseudogene | Dnaja1-ps | 1.290752 | 1.668608 | 1.47968 |
| ENSMUSG00000092867 | miRNA | AC159335.1 | 7.412932 | 6.578542 | 6.995737 |
| ENSMUSG00000037805 | protein_coding | Rpl10a | 7938.583 | 7823.726 | 7881.154 |
| ENSMUSG00000043801 | pseudogene | Gm9786 | 85.56807 | 81.87155 | 83.71981 |
| ENSMUSG00000084771 | lincRNA | Gm15139 | 1.929083 | 2.36596 | 2.147521 |
| ENSMUSG00000056457 | protein_coding | Prl2c3 | 2.225326 | 2.781843 | 2.503584 |
| ENSMUSG00000091303 | lincRNA | 4831440E17Rik | 41.48445 | 39.32715 | 40.4058 |
| ENSMUSG00000078925 | processed_transcrip | D030018L15Rik | 0.974531 | 1.315066 | 1.144798 |
| ENSMUSG00000085845 | processed_transcrip | Gm13944 | 1.623372 | 2.137462 | 1.880417 |
| ENSMUSG00000046010 | protein_coding | Zfp830 | 797.627 | 814.7701 | 806.1986 |
| ENSMUSG00000081434 | pseudogene | Gm14165 | 0.99093 | 0.662038 | 0.826484 |
| ENSMUSG00000068046 | protein_coding | 4930430D24Rik | 10.97359 | 12.09791 | 11.53575 |
| ENSMUSG00000081775 | pseudogene | Gm12420 | 0.98273 | 0.697713 | 0.840222 |
| ENSMUSG00000081153 | pseudogene | Gm11401 | 0.98273 | 0.697713 | 0.840222 |
| ENSMUSG00000025528 | protein_coding | 2010106E10Rik | 0.98273 | 0.697713 | 0.840222 |
| ENSMUSG00000080922 | pseudogene | Gm2214 | 0.98273 | 0.697713 | 0.840222 |
| ENSMUSG00000068580 | protein_coding | Zfyve19 | 897.8063 | 879.0219 | 888.4141 |
| ENSMUSG00000088184 | snRNA | U6 | 0.974531 | 0.653029 | 0.81378 |
| ENSMUSG00000020945 | protein_coding | Lyzl6 | 0.974531 | 0.653029 | 0.81378 |
| ENSMUSG00000085442 | pseudogene | Gm3362 | 32.90332 | 34.734 | 33.81866 |
| ENSMUSG00000060397 | protein_coding | Zfp128 | 223.7292 | 215.7381 | 219.7337 |
| ENSMUSG00000033196 | protein_coding | Myh2 | 8.401552 | 7.415384 | 7.908468 |
| ENSMUSG00000051396 | protein_coding | Hspa14 | 2006.581 | 2034.839 | 2020.71 |
| ENSMUSG00000040620 | protein_coding | Dhx33 | 2350.105 | 2318.774 | 2334.439 |
| ENSMUSG00000021540 | protein_coding | Smad5 | 2898.825 | 2954.021 | 2926.423 |
| ENSMUSG00000043290 | protein_coding | Zfp784 | 115.3234 | 119.1593 | 117.2414 |
| ENSMUSG00000050799 | protein_coding | Hist1h2ba | 27.68463 | 30.90981 | 29.29722 |
| ENSMUSG00000087237 | lincRNA | Gm16703 | 13.21069 | 14.3572 | 13.78395 |
| ENSMUSG00000049929 | protein_coding | Lpar4 | 126.5634 | 131.2615 | 128.9124 |
| ENSMUSG00000020272 | protein_coding | Stk10 | 1606.879 | 1640.67 | 1623.775 |
| ENSMUSG00000046886 | protein_coding | Zfp474 | 1.586995 | 2.021789 | 1.804392 |
| ENSMUSG00000071256 | protein_coding | Zfp213 | 328.9472 | 321.8697 | 325.4085 |
| ENSMUSG00000038671 | protein_coding | Arfrp1 | 1093.433 | 1075.594 | 1084.514 |
| ENSMUSG00000040428 | protein_coding | Plekha4 | 164.3093 | 169.2228 | 166.7661 |
| ENSMUSG00000078879 | protein_coding | Gm14306 | 7.014488 | 6.154373 | 6.58443 |
| ENSMUSG00000034667 | protein_coding | Xpot | 2656.213 | 2691.275 | 2673.744 |
| ENSMUSG00000031293 | protein_coding | Rs1 | 3.236234 | 2.737158 | 2.986696 |
| ENSMUSG00000002190 | protein_coding | Clgn | 322.5401 | 335.6566 | 329.0983 |
| ENSMUSG00000087553 | lincRNA | 5730412P04Rik | 2.261704 | 2.826166 | 2.543935 |
| ENSMUSG00000029202 | protein_coding | Pds5a | 3685.672 | 3632.626 | 3659.149 |
| ENSMUSG00000045435 | protein_coding | Tmem60 | 425.2132 | 433.1236 | 429.1684 |
| ENSMUSG00000037253 | protein_coding | Mex3c | 3113.256 | 3184.998 | 3149.127 |
| ENSMUSG00000048118 | protein_coding | Arid4a | 1387.697 | 1410.597 | 1399.147 |
| ENSMUSG00000042396 | protein_coding | Rbm7 | 2277.23 | 2319.86 | 2298.545 |
| ENSMUSG00000024983 | protein_coding | Vti1a | 955.4788 | 937.2663 | 946.3726 |
| ENSMUSG00000089308 | snoRNA | SNORD121A | 2.597903 | 3.126014 | 2.861959 |
| ENSMUSG00000090275 | protein_coding | Cbln1 | 2.547436 | 1.995123 | 2.271279 |
| ENSMUSG00000066952 | protein_coding | Myo1h | 89.04546 | 92.84576 | 90.94561 |
| ENSMUSG00000073294 | protein_coding | AU022751 | 62.66776 | 65.54874 | 64.10825 |
| ENSMUSG00000059277 | lincRNA | R74862 | 118.4472 | 114.7514 | 116.5993 |
| ENSMUSG00000054676 | protein_coding | 1600014C10Rik | 365.8629 | 374.6648 | 370.2639 |
| ENSMUSG00000051998 | protein_coding | Lax1 | 24.80956 | 23.19468 | 24.00212 |
| ENSMUSG00000000031 | processed_transcrip | H19 | 6150.383 | 6506.746 | 6328.565 |
| ENSMUSG00000047953 | protein_coding | Gp5 | 11.96579 | 10.95764 | 11.46171 |
| ENSMUSG00000085573 | processed_transcrip | Gm15418 | 1.943172 | 2.428663 | 2.185917 |
| ENSMUSG00000070610 | pseudogene | Gm13127 | 33.81076 | 32.01522 | 32.91299 |
| ENSMUSG00000085944 | lincRNA | 1700003D09Rik | 2.267593 | 2.710492 | 2.489043 |

| | | | | | |
|---|---|---|---|---|---|
| ENSMUSG00000081201 | pseudogene | Smt3h2-ps4 | 4.166187 | 3.479194 | 3.822691 |
| ENSMUSG00000024831 | protein_coding | Ighmbp2 | 463.7054 | 453.9725 | 458.839 |
| ENSMUSG00000092665 | misc_RNA | AC099934.1 | 2.583814 | 2.119805 | 2.35181 |
| ENSMUSG00000034918 | protein_coding | Cdhr2 | 2.583814 | 2.119805 | 2.35181 |
| ENSMUSG00000084701 | rRNA | n-R5s87 | 1.637461 | 2.075121 | 1.856291 |
| ENSMUSG00000029213 | protein_coding | Commd8 | 1250.37 | 1270.258 | 1260.314 |
| ENSMUSG00000061520 | protein_coding | Olfr153 | 0.976841 | 0.697713 | 0.837277 |
| ENSMUSG00000064747 | snRNA | U6 | 0.976841 | 0.697713 | 0.837277 |
| ENSMUSG00000092602 | processed_transcrip | 4931413I07Rik | 0.976841 | 0.697713 | 0.837277 |
| ENSMUSG00000052013 | protein_coding | Btla | 6.172647 | 5.411976 | 5.792311 |
| ENSMUSG00000073467 | lincRNA | 6530411M01Rik | 2.895414 | 2.410644 | 2.653029 |
| ENSMUSG00000027552 | protein_coding | E2f5 | 1121.28 | 1097.068 | 1109.174 |
| ENSMUSG00000016758 | protein_coding | Bik | 159.3747 | 154.1916 | 156.7831 |
| ENSMUSG00000086622 | lincRNA | Gm16701 | 37.84746 | 39.74919 | 38.79833 |
| ENSMUSG00000053481 | pseudogene | F930015N05Rik | 2.563836 | 3.072682 | 2.818259 |
| ENSMUSG00000055917 | protein_coding | Zfp277 | 577.8469 | 537.4224 | 557.6347 |
| ENSMUSG00000091026 | protein_coding | Gm6900 | 1.592884 | 2.066111 | 1.829498 |
| ENSMUSG00000088151 | snoRNA | SCARNA11 | 3.242123 | 2.65716 | 2.949642 |
| ENSMUSG00000048540 | protein_coding | Nhlh2 | 2.905924 | 3.425862 | 3.165893 |
| ENSMUSG00000069874 | protein_coding | Irgm2 | 230.7823 | 224.4273 | 227.6048 |
| ENSMUSG00000027048 | protein_coding | Abcb11 | 2.905924 | 2.392626 | 2.649275 |
| ENSMUSG00000039449 | protein_coding | Prpf18 | 1605.691 | 1582.634 | 1594.163 |
| ENSMUSG00000022309 | protein_coding | Angpt1 | 27.42767 | 29.14794 | 28.28781 |
| ENSMUSG00000024334 | protein_coding | H2-Oa | 3.560655 | 4.194926 | 3.877791 |
| ENSMUSG00000079283 | protein_coding | 2310009B15Rik | 147.9307 | 152.5224 | 150.2266 |
| ENSMUSG00000041483 | protein_coding | Zfp281 | 2745.378 | 2789.232 | 2767.305 |
| ENSMUSG00000026226 | protein_coding | Spata3 | 2.897724 | 3.45289 | 3.175307 |
| ENSMUSG00000078300 | protein_coding | Gm2606 | 10.46079 | 11.81571 | 11.13825 |
| ENSMUSG00000050530 | protein_coding | Fam171a1 | 936.1033 | 920.9218 | 928.5126 |
| ENSMUSG00000021456 | protein_coding | Fbp2 | 17.15235 | 15.82362 | 16.48798 |
| ENSMUSG00000024436 | protein_coding | Mrps18b | 459.6068 | 450.7272 | 455.167 |
| ENSMUSG00000024617 | protein_coding | Camk2a | 28.04729 | 29.83665 | 28.94197 |
| ENSMUSG00000028842 | protein_coding | Eif2c3 | 2224.133 | 2156.776 | 2190.454 |
| ENSMUSG00000065599 | miRNA | Mir23b | 3.236234 | 2.746168 | 2.991201 |
| ENSMUSG00000043223 | pseudogene | Gm4835 | 2.903614 | 3.434871 | 3.169243 |
| ENSMUSG00000057278 | protein_coding | Snrpg | 1401.204 | 1376.854 | 1389.029 |
| ENSMUSG00000036960 | protein_coding | Clca5 | 2.233526 | 2.719502 | 2.476514 |
| ENSMUSG00000024979 | protein_coding | Tectb | 0.974531 | 0.697713 | 0.836122 |
| ENSMUSG00000014686 | protein_coding | Ceacam16 | 22.49702 | 20.54145 | 21.51924 |
| ENSMUSG00000027963 | protein_coding | Extl2 | 677.0245 | 689.689 | 683.3568 |
| ENSMUSG00000020232 | protein_coding | Hmg20b | 1451.308 | 1431.015 | 1441.161 |
| ENSMUSG00000052396 | protein_coding | Mogat2 | 7.735042 | 8.627359 | 8.181201 |
| ENSMUSG00000022031 | protein_coding | Elp3 | 2302.857 | 2338.472 | 2320.665 |
| ENSMUSG00000020032 | protein_coding | Nuak1 | 497.8719 | 511.6542 | 504.763 |
| ENSMUSG00000087420 | lincRNA | Gm6277 | 31.16135 | 28.91375 | 30.03755 |
| ENSMUSG00000022895 | protein_coding | Ets2 | 812.6096 | 826.5651 | 819.5874 |
| ENSMUSG00000085890 | processed_transcrip | 2010012P19Rik | 28.18796 | 26.49943 | 27.34369 |
| ENSMUSG00000062081 | protein_coding | Gm6055 | 425.4997 | 433.9934 | 429.7466 |
| ENSMUSG00000001773 | protein_coding | Folh1 | 13.24476 | 14.33918 | 13.79197 |
| ENSMUSG00000073872 | processed_transcrip | Gm10593 | 5.711958 | 4.741652 | 5.226805 |
| ENSMUSG00000038828 | protein_coding | Tmem214 | 1597.803 | 1619.88 | 1608.841 |
| ENSMUSG00000085531 | processed_transcrip | Gm12233 | 3.242123 | 2.746168 | 2.994146 |
| ENSMUSG00000011096 | protein_coding | Akt1s1 | 1938.844 | 1969.104 | 1953.974 |
| ENSMUSG00000036968 | protein_coding | Cnpy4 | 998.1305 | 1019.593 | 1008.862 |
| ENSMUSG00000072259 | protein_coding | Gm5592 | 2.261704 | 2.710492 | 2.486098 |
| ENSMUSG00000028786 | protein_coding | Tmem54 | 1.943172 | 2.36596 | 2.154566 |
| ENSMUSG00000048480 | protein_coding | Cxcr1 | 0.962752 | 0.688704 | 0.825728 |
| ENSMUSG00000027022 | protein_coding | Xirp2 | 0.962752 | 0.688704 | 0.825728 |

| | | | | | |
|---|---|---|---|---|---|
| ENSMUSG00000089659 | processed_transcrip | Gm3470 | 0.962752 | 0.688704 | 0.825728 |
| ENSMUSG00000051956 | protein_coding | Rnf133 | 0.962752 | 0.688704 | 0.825728 |
| ENSMUSG00000064833 | snRNA | U1 | 0.962752 | 0.688704 | 0.825728 |
| ENSMUSG00000081583 | pseudogene | Gm14769 | 0.962752 | 0.688704 | 0.825728 |
| ENSMUSG00000085382 | processed_transcrip | Gm13861 | 3.213945 | 3.761386 | 3.487666 |
| ENSMUSG00000028690 | protein_coding | Mmachc | 312.0846 | 319.0487 | 315.5667 |
| ENSMUSG00000034201 | protein_coding | Gas2l1 | 5658.561 | 5540.522 | 5599.541 |
| ENSMUSG00000044461 | protein_coding | Shisa2 | 554.2967 | 539.8528 | 547.0747 |
| ENSMUSG00000023952 | protein_coding | Gtpbp2 | 1238.902 | 1218.469 | 1228.685 |
| ENSMUSG00000074516 | protein_coding | Gm10709 | 155.5507 | 163.0138 | 159.2822 |
| ENSMUSG00000081319 | pseudogene | Gm12936 | 1.32124 | 1.748606 | 1.534923 |
| ENSMUSG00000041598 | protein_coding | Cdc42ep4 | 1684.296 | 1715.246 | 1699.771 |
| ENSMUSG00000022110 | protein_coding | Sucla2 | 3334.643 | 3286.063 | 3310.353 |
| ENSMUSG00000039138 | protein_coding | 4930591A17Rik | 12.61821 | 13.73949 | 13.17885 |
| ENSMUSG00000070883 | protein_coding | 4930578N16Rik | 127.1269 | 131.0456 | 129.0863 |
| ENSMUSG00000060559 | protein_coding | Gm10083 | 6.436091 | 7.258599 | 6.847345 |
| ENSMUSG00000056716 | protein_coding | Gm5420 | 2.555636 | 3.063673 | 2.809654 |
| ENSMUSG00000061983 | protein_coding | Rps12 | 3724.086 | 3667.385 | 3695.735 |
| ENSMUSG00000018405 | protein_coding | Mrm1 | 616.6214 | 628.3378 | 622.4796 |
| ENSMUSG00000079383 | protein_coding | Gm3298 | 2.589703 | 3.090339 | 2.840021 |
| ENSMUSG00000036867 | protein_coding | Smad6 | 412.7739 | 397.275 | 405.0245 |
| ENSMUSG00000046794 | protein_coding | Ppp1r3b | 106.6022 | 101.1568 | 103.8795 |
| ENSMUSG00000091784 | pseudogene | Gm17022 | 23.00035 | 21.52643 | 22.26339 |
| ENSMUSG00000049401 | protein_coding | Ogfr | 1479.654 | 1501.725 | 1490.69 |
| ENSMUSG00000080951 | pseudogene | Gm12208 | 10.98768 | 12.03556 | 11.51162 |
| ENSMUSG00000030779 | protein_coding | Rbbp6 | 5559.784 | 5624.589 | 5592.186 |
| ENSMUSG00000054191 | protein_coding | Klf1 | 14.54371 | 15.68091 | 15.11231 |
| ENSMUSG00000088615 | snRNA | U6 | 3.219835 | 3.770395 | 3.495115 |
| ENSMUSG00000005262 | protein_coding | Ufd1l | 1792.465 | 1816.853 | 1804.659 |
| ENSMUSG00000074148 | protein_coding | Bcl2a1d | 5.495627 | 4.847954 | 5.171791 |
| ENSMUSG00000020166 | protein_coding | Cnot2 | 2792.658 | 2832.522 | 2812.59 |
| ENSMUSG00000080525 | miRNA | AC132471.1 | 0.960442 | 0.688704 | 0.824573 |
| ENSMUSG00000069306 | protein_coding | Hist1h4m | 4.213076 | 3.550545 | 3.88181 |
| ENSMUSG00000032435 | protein_coding | Dync1li1 | 1568.47 | 1590.882 | 1579.676 |
| ENSMUSG00000005628 | protein_coding | Tmod4 | 12.87345 | 14.10204 | 13.48774 |
| ENSMUSG00000026867 | protein_coding | Gapvd1 | 2195.693 | 2149.166 | 2172.429 |
| ENSMUSG00000033047 | protein_coding | Eif3l | 4801.858 | 4742.297 | 4772.078 |
| ENSMUSG00000034488 | protein_coding | Edil3 | 1.923194 | 2.401997 | 2.162595 |
| ENSMUSG00000029729 | protein_coding | Zkscan1 | 2301.852 | 2351.296 | 2326.574 |
| ENSMUSG00000054978 | pseudogene | Kbtbd13 | 59.01668 | 56.02804 | 57.52236 |
| ENSMUSG00000043505 | protein_coding | Gimap5 | 15.77453 | 17.14841 | 16.46147 |
| ENSMUSG00000042660 | protein_coding | Wdr55 | 847.0688 | 861.675 | 854.3719 |
| ENSMUSG00000050423 | protein_coding | Ppp1r3g | 6.11987 | 5.322968 | 5.721419 |
| ENSMUSG00000069793 | protein_coding | Slfn9 | 179.6187 | 172.8109 | 176.2148 |
| ENSMUSG00000090173 | protein_coding | Fbxw10 | 0.98273 | 0.671047 | 0.826889 |
| ENSMUSG00000002103 | protein_coding | Acp2 | 1128.14 | 1146.021 | 1137.081 |
| ENSMUSG00000029290 | protein_coding | Zfp326 | 1568.142 | 1538.819 | 1553.48 |
| ENSMUSG00000086218 | lincRNA | Gm16755 | 16.071 | 17.70922 | 16.89011 |
| ENSMUSG00000004677 | protein_coding | Myo9b | 3571.03 | 3502.078 | 3536.554 |
| ENSMUSG00000048107 | pseudogene | Kctd12 | 2.609681 | 2.066473 | 2.338077 |
| ENSMUSG00000090244 | processed_transcrip | Gm16572 | 2.255815 | 2.781843 | 2.518829 |
| ENSMUSG00000040302 | protein_coding | C030048B08Rik | 518.8973 | 509.025 | 513.9611 |
| ENSMUSG00000085031 | lincRNA | Gm16982 | 13.01538 | 11.74115 | 12.37827 |
| ENSMUSG00000036315 | protein_coding | Znrd1 | 454.6316 | 463.7525 | 459.192 |
| ENSMUSG00000083496 | pseudogene | Gm11263 | 16.4669 | 17.65802 | 17.06246 |
| ENSMUSG00000044513 | lincRNA | 9930014A18Rik | 39.38503 | 41.39399 | 40.38951 |
| ENSMUSG00000091041 | protein_coding | Gm17720 | 3.558345 | 3.001331 | 3.279838 |
| ENSMUSG00000046924 | protein_coding | Vmn1r179 | 1.949061 | 2.419653 | 2.184357 |

| | | | | | |
|---|---|---|---|---|---|
| ENSMUSG00000032562 | protein_coding | Gnai2 | 7231.975 | 7341.533 | 7286.754 |
| ENSMUSG00000072479 | protein_coding | Xlr5b | 173.1695 | 180.4251 | 176.7973 |
| ENSMUSG00000047632 | protein_coding | Fgfbp3 | 48.70245 | 50.9299 | 49.81617 |
| ENSMUSG00000032110 | protein_coding | Acrv1 | 1.939594 | 2.401635 | 2.170614 |
| ENSMUSG00000078184 | protein_coding | B020018G12Rik | 11.27468 | 10.18822 | 10.73145 |
| ENSMUSG00000059119 | protein_coding | Nap1l4 | 5725.802 | 5800.367 | 5763.085 |
| ENSMUSG00000092438 | pseudogene | Gm18734 | 2.298082 | 1.784282 | 2.041182 |
| ENSMUSG00000081953 | pseudogene | Gm12435 | 25.66639 | 27.3691 | 26.51774 |
| ENSMUSG00000079243 | protein_coding | Xirp1 | 2.275793 | 2.826166 | 2.550979 |
| ENSMUSG00000026673 | protein_coding | Sh2d1b1 | 2.569725 | 3.055025 | 2.812375 |
| ENSMUSG00000084166 | pseudogene | Gm6451 | 12.54776 | 11.4413 | 11.99453 |
| ENSMUSG00000034416 | protein_coding | Pkd1l2 | 21.43333 | 22.9842 | 22.20877 |
| ENSMUSG00000032477 | protein_coding | Cdc25a | 2141.333 | 2110.34 | 2125.837 |
| ENSMUSG00000050552 | protein_coding | 0910001L09Rik | 612.0304 | 624.2813 | 618.1559 |
| ENSMUSG00000026048 | protein_coding | Ercc5 | 794.5164 | 782.7016 | 788.609 |
| ENSMUSG00000047485 | protein_coding | Klhl34 | 6.13038 | 5.358282 | 5.744331 |
| ENSMUSG00000083411 | pseudogene | Rpl30-ps10 | 5.805959 | 5.138793 | 5.472376 |
| ENSMUSG00000086427 | lincRNA | Hoxa11as | 0.64422 | 0.979543 | 0.811882 |
| ENSMUSG00000044465 | protein_coding | Fam160a2 | 1408.939 | 1376.015 | 1392.477 |
| ENSMUSG00000086191 | processed_transcrip | 1110035M17Rik | 8.031285 | 7.223285 | 7.627285 |
| ENSMUSG00000049307 | protein_coding | Fut4 | 69.19361 | 66.51171 | 67.85266 |
| ENSMUSG00000039410 | protein_coding | Prdm16 | 432.0232 | 441.5396 | 436.7814 |
| ENSMUSG00000019888 | protein_coding | Mgat4c | 12.59003 | 11.50437 | 12.0472 |
| ENSMUSG00000046324 | protein_coding | Ermp1 | 1358.386 | 1340.152 | 1349.269 |
| ENSMUSG00000043168 | lincRNA | 4930426D05Rik | 51.93831 | 48.50092 | 50.21961 |
| ENSMUSG00000081025 | pseudogene | Gm11943 | 0.99093 | 1.448758 | 1.219844 |
| ENSMUSG00000066626 | protein_coding | Gm5121 | 0.954552 | 0.688704 | 0.821628 |
| ENSMUSG00000017764 | protein_coding | Zswim1 | 488.8798 | 479.0617 | 483.9707 |
| ENSMUSG00000084882 | processed_transcrip | Gm16047 | 7.382443 | 8.25616 | 7.819302 |
| ENSMUSG00000078994 | protein_coding | Zfp429 | 140.7816 | 145.4422 | 143.1119 |
| ENSMUSG00000027598 | protein_coding | Itch | 3370.097 | 3421.467 | 3395.782 |
| ENSMUSG00000084858 | pseudogene | Gm1980 | 5.763692 | 5.040777 | 5.402235 |
| ENSMUSG00000066724 | pseudogene | Gm10175 | 20.68714 | 22.03712 | 21.36213 |
| ENSMUSG00000025377 | protein_coding | 2410002I01Rik | 503.2504 | 511.9376 | 507.594 |
| ENSMUSG00000093162 | miRNA | AC158662.1 | 0.976841 | 0.671047 | 0.823944 |
| ENSMUSG00000052481 | protein_coding | 1700036A12Rik | 3.230345 | 3.761386 | 3.495865 |
| ENSMUSG00000055053 | protein_coding | Nfic | 4486.47 | 4559.716 | 4523.093 |
| ENSMUSG00000014164 | protein_coding | Klhl3 | 68.79039 | 71.80661 | 70.2985 |
| ENSMUSG00000012017 | protein_coding | Scarf2 | 423.5193 | 435.0474 | 429.2833 |
| ENSMUSG00000085144 | lincRNA | A930029G22Rik | 33.38168 | 31.44291 | 32.41229 |
| ENSMUSG00000022911 | protein_coding | Arl13b | 662.5096 | 649.9492 | 656.2294 |
| ENSMUSG00000033720 | protein_coding | Sfxn5 | 148.4999 | 153.1497 | 150.8248 |
| ENSMUSG00000060579 | protein_coding | Fhit | 30.53941 | 32.35903 | 31.44922 |
| ENSMUSG00000086510 | lincRNA | Gm16661 | 5.187606 | 5.943532 | 5.565569 |
| ENSMUSG00000027967 | protein_coding | Neurog2 | 1.917305 | 2.392626 | 2.154965 |
| ENSMUSG00000074165 | protein_coding | Zfp788 | 672.3915 | 684.4484 | 678.4199 |
| ENSMUSG00000040270 | protein_coding | Bach2 | 271.8946 | 280.6605 | 276.2776 |
| ENSMUSG00000069495 | protein_coding | Epc2 | 1675.994 | 1703.738 | 1689.866 |
| ENSMUSG00000006315 | protein_coding | Tmem147 | 1410.013 | 1431.081 | 1420.547 |
| ENSMUSG00000088208 | snoRNA | SCARNA11 | 0.962752 | 0.697713 | 0.830233 |
| ENSMUSG00000093086 | miRNA | AC127419.1 | 0.962752 | 0.697713 | 0.830233 |
| ENSMUSG00000086816 | lincRNA | Gm16726 | 0.962752 | 0.697713 | 0.830233 |
| ENSMUSG00000082906 | pseudogene | Smt3h1-ps | 0.962752 | 0.697713 | 0.830233 |
| ENSMUSG00000020573 | protein_coding | Pik3cg | 71.88297 | 68.90729 | 70.39513 |
| ENSMUSG00000030987 | protein_coding | Stim1 | 4214.358 | 4128.38 | 4171.369 |
| ENSMUSG00000024105 | protein_coding | 9130404H23Rik | 0.966331 | 1.341732 | 1.154032 |
| ENSMUSG00000081142 | pseudogene | Gm15497 | 7.396532 | 8.238503 | 7.817518 |
| ENSMUSG00000042055 | protein_coding | Wdr11 | 1901.222 | 1871.974 | 1886.598 |

| | | | | | |
|---|---|---|---|---|---|
| ENSMUSG00000060647 | pseudogene | Gm7099 | 31.28688 | 29.64962 | 30.46825 |
| ENSMUSG00000060703 | protein_coding | Cd302 | 576.7007 | 590.011 | 583.3558 |
| ENSMUSG00000085377 | processed_transcrip | 4930551L18Rik | 0.974531 | 0.671047 | 0.822789 |
| ENSMUSG00000040966 | protein_coding | Slc22a2 | 0.974531 | 0.671047 | 0.822789 |
| ENSMUSG00000022544 | protein_coding | Fam86 | 623.7218 | 633.4476 | 628.5847 |
| ENSMUSG00000088000 | snRNA | U6 | 8.400284 | 7.549438 | 7.974861 |
| ENSMUSG00000031385 | protein_coding | Plxnb3 | 39.08186 | 41.36877 | 40.22531 |
| ENSMUSG00000086016 | processed_transcrip | 1700084C06Rik | 5.794181 | 6.596561 | 6.195371 |
| ENSMUSG00000068290 | protein_coding | Ddrgk1 | 1513.641 | 1536.138 | 1524.889 |
| ENSMUSG00000085242 | processed_transcrip | Gm11537 | 4.202565 | 4.821288 | 4.511927 |
| ENSMUSG00000034997 | protein_coding | Htr2a | 3.208056 | 3.73472 | 3.471388 |
| ENSMUSG00000001285 | protein_coding | Myg1 | 666.3803 | 679.3395 | 672.8599 |
| ENSMUSG00000065651 | snoRNA | SNORA51 | 7.370665 | 6.596561 | 6.983613 |
| ENSMUSG00000024537 | protein_coding | Psmg2 | 834.0287 | 847.5037 | 840.7662 |
| ENSMUSG00000093256 | miRNA | AC134560.1 | 1.951372 | 2.348303 | 2.149837 |
| ENSMUSG00000086976 | processed_transcrip | Gm12316 | 2.275793 | 2.719502 | 2.497647 |
| ENSMUSG00000029198 | protein_coding | Grpel1 | 1659.762 | 1683.015 | 1671.389 |
| ENSMUSG00000032572 | protein_coding | Col6a4 | 541.2904 | 525.3663 | 533.3284 |
| ENSMUSG00000064032 | lincRNA | Gm10143 | 33.59212 | 31.72117 | 32.65664 |
| ENSMUSG00000091311 | protein_coding | Gm4934 | 0.99093 | 1.36876 | 1.179845 |
| ENSMUSG00000088705 | snRNA | U6 | 0.960442 | 0.697713 | 0.829077 |
| ENSMUSG00000085701 | processed_transcrip | Gm12968 | 0.960442 | 0.697713 | 0.829077 |
| ENSMUSG00000093263 | miRNA | AL929371.2 | 0.960442 | 0.697713 | 0.829077 |
| ENSMUSG00000081677 | pseudogene | Mdk-ps1 | 0.960442 | 0.697713 | 0.829077 |
| ENSMUSG00000021607 | protein_coding | Mrpl36 | 992.4446 | 1007.382 | 999.9134 |
| ENSMUSG00000027248 | protein_coding | Pdia3 | 12093.99 | 11959.62 | 12026.81 |
| ENSMUSG00000026822 | protein_coding | Lcn2 | 12.02318 | 10.93134 | 11.47726 |
| ENSMUSG00000021290 | protein_coding | 2010107E04Rik | 1124.801 | 1106.538 | 1115.67 |
| ENSMUSG00000080866 | pseudogene | Gm5687 | 2.275793 | 2.710492 | 2.493143 |
| ENSMUSG00000078865 | protein_coding | Gm14406 | 4.854985 | 5.572334 | 5.213659 |
| ENSMUSG00000074854 | protein_coding | Gm8290 | 7.431643 | 8.476733 | 7.954188 |
| ENSMUSG00000028991 | protein_coding | Mtor | 4299.498 | 4200.006 | 4249.752 |
| ENSMUSG00000085435 | processed_transcrip | 4930486F22Rik | 0.966331 | 0.662038 | 0.814184 |
| ENSMUSG00000042364 | protein_coding | Snx18 | 2937.919 | 2981.41 | 2959.665 |
| ENSMUSG00000091400 | protein_coding | Gm6356 | 53.76706 | 37.78256 | 45.77481 |
| ENSMUSG00000019494 | protein_coding | Cops6 | 3137.552 | 3085.904 | 3111.728 |
| ENSMUSG00000068122 | protein_coding | Agtr2 | 416.2227 | 428.9138 | 422.5683 |
| ENSMUSG00000021779 | protein_coding | Thrb | 178.222 | 172.6435 | 175.4328 |
| ENSMUSG00000042644 | protein_coding | Itpr3 | 1807.768 | 1841.674 | 1824.721 |
| ENSMUSG00000082925 | pseudogene | Gm13135 | 266.5197 | 275.8672 | 271.1934 |
| ENSMUSG00000031958 | protein_coding | Ldhd | 92.02265 | 95.84989 | 93.93627 |
| ENSMUSG00000021238 | protein_coding | Aldh6a1 | 2204.972 | 2165.703 | 2185.337 |
| ENSMUSG00000092381 | processed_transcrip | Gm20512 | 11.39432 | 10.28732 | 10.84082 |
| ENSMUSG00000025370 | protein_coding | Cdh9 | 10.97128 | 12.0716 | 11.52144 |
| ENSMUSG00000075033 | protein_coding | Fam55c | 208.816 | 201.5559 | 205.186 |
| ENSMUSG00000068243 | pseudogene | Gm7079 | 3.238545 | 3.80607 | 3.522308 |
| ENSMUSG00000022237 | protein_coding | Ankrd33b | 9.368925 | 8.448982 | 8.908954 |
| ENSMUSG00000082530 | pseudogene | Gm12168 | 7.75502 | 8.582674 | 8.168847 |
| ENSMUSG00000070697 | protein_coding | Utp3 | 1489.033 | 1507.877 | 1498.455 |
| ENSMUSG00000069379 | protein_coding | Gm4950 | 40.76285 | 38.84028 | 39.80157 |
| ENSMUSG00000092134 | processed_transcrip | Gm17089 | 2.603792 | 3.072682 | 2.838237 |
| ENSMUSG00000003662 | protein_coding | Ciao1 | 1332.733 | 1313.157 | 1322.945 |
| ENSMUSG00000089818 | pseudogene | Gm15950 | 33.0747 | 31.25838 | 32.16654 |
| ENSMUSG00000026959 | protein_coding | Grin1 | 30.30773 | 28.42755 | 29.36764 |
| ENSMUSG00000086296 | lincRNA | D030055H07Rik | 19.21979 | 17.65222 | 18.436 |
| ENSMUSG00000006574 | protein_coding | Slc4a1 | 5.439271 | 6.32374 | 5.881506 |
| ENSMUSG00000029404 | protein_coding | Arl6ip4 | 976.3007 | 993.3396 | 984.8201 |
| ENSMUSG00000024921 | protein_coding | Smarca2 | 5492.237 | 5407.973 | 5450.105 |

| | | | | | |
|---|---|---|---|---|---|
| ENSMUSG00000027562 | protein_coding | Car2 | 40.01181 | 41.86828 | 40.94004 |
| ENSMUSG00000085013 | lincRNA | 4930556M19Rik | 103.4604 | 100.0197 | 101.7401 |
| ENSMUSG00000088036 | miRNA | AL671963.1 | 9.666436 | 10.56879 | 10.11761 |
| ENSMUSG00000091246 | lincRNA | Gm3834 | 1.949061 | 2.401635 | 2.175348 |
| ENSMUSG00000049516 | protein_coding | Spty2d1 | 1316.798 | 1292.716 | 1304.757 |
| ENSMUSG00000029569 | protein_coding | Tmem168 | 1694.246 | 1662.229 | 1678.238 |
| ENSMUSG00000046085 | protein_coding | 4931422A03Rik | 24.32286 | 25.85256 | 25.08771 |
| ENSMUSG00000045826 | protein_coding | Ptprcap | 12.61821 | 13.65949 | 13.13885 |
| ENSMUSG00000030410 | protein_coding | Dmwd | 1891.367 | 1920.941 | 1906.154 |
| ENSMUSG00000085351 | processed_transcrip | Gm12472 | 2.942302 | 2.401997 | 2.672149 |
| ENSMUSG00000027475 | protein_coding | Kif3b | 2022.715 | 2049.953 | 2036.334 |
| ENSMUSG00000004709 | protein_coding | Cd244 | 3.538366 | 3.001331 | 3.269849 |
| ENSMUSG00000024548 | protein_coding | Setbp1 | 145.2192 | 140.521 | 142.8701 |
| ENSMUSG00000061136 | protein_coding | Prpf40a | 4677.335 | 4619.559 | 4648.447 |
| ENSMUSG00000068014 | pseudogene | Gm14648 | 13.31058 | 14.49597 | 13.90327 |
| ENSMUSG00000074971 | protein_coding | Fibin | 65.08504 | 68.07769 | 66.58137 |
| ENSMUSG00000004565 | protein_coding | Pnpla6 | 804.1905 | 817.0753 | 810.6329 |
| ENSMUSG00000064994 | snoRNA | SNORA70 | 2.267593 | 1.810948 | 2.03927 |
| ENSMUSG00000027854 | protein_coding | Sike1 | 1336.641 | 1359.781 | 1348.211 |
| ENSMUSG00000038013 | protein_coding | Wipf2 | 1414.562 | 1387.1 | 1400.831 |
| ENSMUSG00000028965 | protein_coding | Tnfrsf9 | 13.89718 | 12.83994 | 13.36856 |
| ENSMUSG00000083518 | pseudogene | Gm9429 | 6.142159 | 5.483326 | 5.812742 |
| ENSMUSG00000026280 | protein_coding | Atg4b | 2058.571 | 2086.422 | 2072.496 |
| ENSMUSG00000001946 | protein_coding | Esam | 144.3467 | 149.7437 | 147.0452 |
| ENSMUSG00000081126 | pseudogene | Gm15784 | 11.91994 | 10.93062 | 11.42528 |
| ENSMUSG00000026342 | protein_coding | Slc35f5 | 1924.207 | 1950.456 | 1937.331 |
| ENSMUSG00000027606 | protein_coding | Dnajc5b | 0.954552 | 0.697713 | 0.826133 |
| ENSMUSG00000089712 | processed_transcrip | Gm15889 | 0.954552 | 0.697713 | 0.826133 |
| ENSMUSG00000090676 | protein_coding | Gm6721 | 0.954552 | 0.697713 | 0.826133 |
| ENSMUSG00000085297 | processed_transcrip | Gm11651 | 0.954552 | 0.697713 | 0.826133 |
| ENSMUSG00000025862 | protein_coding | Stag2 | 4281.111 | 4344.099 | 4312.605 |
| ENSMUSG00000029595 | protein_coding | Lhx5 | 9.037572 | 8.053967 | 8.545769 |
| ENSMUSG00000083610 | pseudogene | Gm9329 | 45.26144 | 43.09542 | 44.17843 |
| ENSMUSG00000048962 | pseudogene | Rassf10 | 81.59383 | 77.49959 | 79.54671 |
| ENSMUSG00000022407 | protein_coding | Adsl | 1544.734 | 1566.758 | 1555.746 |
| ENSMUSG00000030752 | protein_coding | Jmjd5 | 294.6808 | 301.3032 | 297.992 |
| ENSMUSG00000044678 | protein_coding | Ly6k | 405.4714 | 448.2916 | 426.8815 |
| ENSMUSG00000031667 | protein_coding | Aktip | 1169.846 | 1149.347 | 1159.596 |
| ENSMUSG00000015092 | protein_coding | Edf1 | 1346.041 | 1368.308 | 1357.175 |
| ENSMUSG00000086706 | processed_transcrip | Gm15848 | 7.729153 | 6.932446 | 7.3308 |
| ENSMUSG00000040987 | protein_coding | Mill2 | 107.3155 | 110.9207 | 109.1181 |
| ENSMUSG00000029337 | protein_coding | Fgf5 | 1.606973 | 1.986113 | 1.796543 |
| ENSMUSG00000026405 | protein_coding | C4bp | 0.99093 | 0.688704 | 0.839817 |
| ENSMUSG00000046082 | protein_coding | Tmem174 | 0.99093 | 0.688704 | 0.839817 |
| ENSMUSG00000090823 | lincRNA | 2700012I20Rik | 16.11431 | 14.93272 | 15.52351 |
| ENSMUSG00000047807 | processed_transcrip | 9330154K18Rik | 6.798157 | 7.68313 | 7.240643 |
| ENSMUSG00000086363 | processed_transcrip | A330102I10Rik | 8.687284 | 9.553569 | 9.120427 |
| ENSMUSG00000085829 | processed_transcrip | Gm4285 | 92.73306 | 96.15875 | 94.4459 |
| ENSMUSG00000081295 | pseudogene | Gm6275 | 0.974531 | 1.342094 | 1.158312 |
| ENSMUSG00000072918 | processed_transcrip | 1700022K14Rik | 44.93933 | 46.92743 | 45.93338 |
| ENSMUSG00000091043 | protein_coding | Glyatl3 | 3.199857 | 3.779043 | 3.48945 |
| ENSMUSG00000018481 | protein_coding | Appbp2 | 8139.39 | 8248.699 | 8194.044 |
| ENSMUSG00000040164 | protein_coding | Kcns1 | 271.0207 | 277.3988 | 274.2097 |
| ENSMUSG00000020798 | protein_coding | Spns3 | 5.193495 | 5.934523 | 5.564009 |
| ENSMUSG00000026321 | protein_coding | Tnfrsf11a | 38.95863 | 40.88946 | 39.92404 |
| ENSMUSG00000086648 | processed_transcrip | Gm15511 | 3.244434 | 2.772834 | 3.008634 |
| ENSMUSG00000090942 | protein_coding | F830016B08Rik | 5.501517 | 4.865973 | 5.183745 |
| ENSMUSG00000041650 | protein_coding | Pcca | 667.2951 | 679.2767 | 673.2859 |

| | | | | | |
|---|---|---|---|---|---|
| ENSMUSG00000031825 | protein_coding | Crispld2 | 671.8673 | 684.7104 | 678.2889 |
| ENSMUSG00000008348 | protein_coding | Ubc | 2892.606 | 2960.161 | 2926.384 |
| ENSMUSG00000047166 | pseudogene | Tigd5 | 92.02407 | 95.58152 | 93.80279 |
| ENSMUSG00000081828 | pseudogene | Gm11420 | 1.953683 | 2.383978 | 2.16883 |
| ENSMUSG00000085096 | lincRNA | B230217O12Rik | 43.84157 | 46.34317 | 45.09237 |
| ENSMUSG00000018001 | protein_coding | Cyth3 | 4456.116 | 4389.271 | 4422.693 |
| ENSMUSG00000028978 | protein_coding | Nos3 | 51.75977 | 53.90063 | 52.8302 |
| ENSMUSG00000044086 | protein_coding | Lmod3 | 6.113981 | 5.429632 | 5.771806 |
| ENSMUSG00000010492 | processed_transcrip | Gm16119 | 98.50489 | 102.8898 | 100.6974 |
| ENSMUSG00000059463 | protein_coding | Spag11b | 0.962752 | 0.671047 | 0.8169 |
| ENSMUSG00000075946 | snoRNA | SNORA25 | 0.962752 | 0.671047 | 0.8169 |
| ENSMUSG00000022304 | protein_coding | Dpys | 0.962752 | 0.671047 | 0.8169 |
| ENSMUSG00000064343 | Mt_tRNA | mt-Tq | 0.962752 | 0.671047 | 0.8169 |
| ENSMUSG00000026177 | protein_coding | Slc11a1 | 45.59197 | 47.54479 | 46.56838 |
| ENSMUSG00000032336 | protein_coding | Nptn | 3169.039 | 3206.45 | 3187.745 |
| ENSMUSG00000088015 | miRNA | Mir1932 | 9.356104 | 10.21561 | 9.785856 |
| ENSMUSG00000029594 | protein_coding | Rbm19 | 897.2095 | 884.0683 | 890.6389 |
| ENSMUSG00000022876 | protein_coding | Samsn1 | 3.258523 | 3.797061 | 3.527792 |
| ENSMUSG00000085250 | lincRNA | Gm15234 | 5.495627 | 6.199058 | 5.847343 |
| ENSMUSG00000052229 | protein_coding | Gpr17 | 1.951372 | 2.392626 | 2.171999 |
| ENSMUSG00000036639 | protein_coding | Nudt1 | 268.4755 | 262.4409 | 265.4582 |
| ENSMUSG00000074039 | lincRNA | 4930520O04Rik | 58.36322 | 56.0035 | 57.18336 |
| ENSMUSG00000048264 | protein_coding | Dip2c | 1664.623 | 1711.764 | 1688.193 |
| ENSMUSG00000022375 | protein_coding | Lrrc6 | 33.99101 | 31.41516 | 32.70308 |
| ENSMUSG00000064542 | snoRNA | SNORD38 | 1.284863 | 1.677618 | 1.48124 |
| ENSMUSG00000045761 | protein_coding | Fam179a | 27.16296 | 25.44641 | 26.30468 |
| ENSMUSG00000027646 | protein_coding | Src | 2056.566 | 2087.425 | 2071.996 |
| ENSMUSG00000091833 | lincRNA | Gm17317 | 5.159428 | 4.557115 | 4.858272 |
| ENSMUSG00000086411 | processed_transcrip | Gm14236 | 1.298952 | 0.988552 | 1.143752 |
| ENSMUSG00000049409 | protein_coding | Prokr1 | 97.81825 | 94.24916 | 96.0337 |
| ENSMUSG00000030978 | protein_coding | Rrm1 | 5144.915 | 5067.607 | 5106.261 |
| ENSMUSG00000092135 | lincRNA | Gm17399 | 24.19964 | 25.6774 | 24.93852 |
| ENSMUSG00000031796 | protein_coding | Gtl3 | 1316.04 | 1334.313 | 1325.176 |
| ENSMUSG00000079360 | protein_coding | Gm3839 | 5.489738 | 4.82165 | 5.155694 |
| ENSMUSG00000030614 | protein_coding | Tmem126b | 580.8099 | 564.6132 | 572.7116 |
| ENSMUSG00000079415 | protein_coding | Cntf | 7.421132 | 8.193819 | 7.807475 |
| ENSMUSG00000086455 | processed_transcrip | Gm11815 | 0.960442 | 0.671047 | 0.815744 |
| ENSMUSG00000093197 | misc_RNA | AC156032.1 | 5.177096 | 4.548106 | 4.862601 |
| ENSMUSG00000033717 | protein_coding | Adra2a | 14.5332 | 15.60092 | 15.06706 |
| ENSMUSG00000023078 | protein_coding | Cxcl13 | 2.281682 | 2.728511 | 2.505096 |
| ENSMUSG00000030363 | protein_coding | Clec2j | 0.66062 | 0.979543 | 0.820081 |
| ENSMUSG00000092931 | miRNA | AC132354.1 | 1.626951 | 2.066473 | 1.846712 |
| ENSMUSG00000056296 | protein_coding | Synpr | 7.075465 | 6.048071 | 6.561768 |
| ENSMUSG00000065493 | miRNA | Mir34a | 7.438799 | 8.247512 | 7.843156 |
| ENSMUSG00000091015 | lincRNA | Gm17284 | 17.41557 | 16.15593 | 16.78575 |
| ENSMUSG00000025545 | protein_coding | Clybl | 260.1103 | 265.9535 | 263.0319 |
| ENSMUSG00000071478 | protein_coding | Hist1h2ad | 1.914994 | 2.366321 | 2.140658 |
| ENSMUSG00000002007 | protein_coding | Srpk3 | 20.30867 | 19.02714 | 19.66791 |
| ENSMUSG00000075232 | protein_coding | Amd1 | 4309.293 | 4371.006 | 4340.15 |
| ENSMUSG00000087461 | processed_transcrip | C230014O12Rik | 2.928213 | 3.408205 | 3.168209 |
| ENSMUSG00000029440 | protein_coding | Psmd9 | 806.3683 | 794.1213 | 800.2448 |
| ENSMUSG00000039908 | protein_coding | Slc26a11 | 231.2872 | 250.9534 | 241.1203 |
| ENSMUSG00000091619 | protein_coding | Gm3625 | 9.380704 | 10.31326 | 9.846983 |
| ENSMUSG00000042788 | protein_coding | Fam166b | 5.803649 | 5.183478 | 5.493563 |
| ENSMUSG00000025793 | protein_coding | Hgs | 1624.889 | 1651.749 | 1638.319 |
| ENSMUSG00000037624 | protein_coding | Kcnk2 | 51.44944 | 49.33228 | 50.39086 |
| ENSMUSG00000079420 | lincRNA | Gm17418 | 0.99093 | 0.653029 | 0.821979 |
| ENSMUSG00000027517 | protein_coding | Ankrd60 | 0.99093 | 0.653029 | 0.821979 |

| | | | | | |
|---|---|---|---|---|---|
| ENSMUSG00000047492 | protein_coding | Inhbe | 0.99093 | 0.653029 | 0.821979 |
| ENSMUSG00000085057 | processed_transcrip | Gm13415 | 1.618751 | 2.066111 | 1.842431 |
| ENSMUSG00000039873 | protein_coding | Neurl2 | 40.76158 | 38.921 | 39.84129 |
| ENSMUSG00000087561 | processed_transcrip | Gm11608 | 6.508847 | 5.765518 | 6.137182 |
| ENSMUSG00000087389 | processed_transcrip | Gm15592 | 3.890965 | 4.539097 | 4.215031 |
| ENSMUSG00000003484 | protein_coding | Cyp4f18 | 4.233054 | 4.95498 | 4.594017 |
| ENSMUSG00000055198 | protein_coding | Gm12830 | 1.282552 | 1.65059 | 1.466571 |
| ENSMUSG00000086629 | lincRNA | 2810403D21Rik | 68.83959 | 71.41988 | 70.12973 |
| ENSMUSG00000028884 | protein_coding | Rpa2 | 2729.325 | 2692.251 | 2710.788 |
| ENSMUSG00000057223 | protein_coding | Gm6578 | 0.98273 | 1.342094 | 1.162412 |
| ENSMUSG00000070867 | protein_coding | Gm12824 | 101.9027 | 98.68988 | 100.2963 |
| ENSMUSG00000079037 | protein_coding | Prnp | 684.4861 | 697.2319 | 690.859 |
| ENSMUSG00000046378 | protein_coding | Asphd1 | 27.94911 | 26.16391 | 27.05651 |
| ENSMUSG00000084408 | pseudogene | Gm11870 | 24.28163 | 22.79138 | 23.5365 |
| ENSMUSG00000043467 | protein_coding | Zbtb37 | 563.0601 | 552.8823 | 557.9712 |
| ENSMUSG00000023353 | protein_coding | Agap3 | 2495.229 | 2528.792 | 2512.01 |
| ENSMUSG00000082229 | protein_coding | Nap1l2 | 7.724532 | 8.529342 | 8.126937 |
| ENSMUSG00000075549 | protein_coding | Gm6878 | 3.222145 | 2.763824 | 2.992985 |
| ENSMUSG00000080880 | pseudogene | Gm14278 | 1.32124 | 1.775272 | 1.548256 |
| ENSMUSG00000081357 | pseudogene | Xlr4e-ps | 64.52289 | 60.74433 | 62.63361 |
| ENSMUSG00000029148 | protein_coding | Nrbp1 | 5386.629 | 5309.002 | 5347.815 |
| ENSMUSG00000020229 | protein_coding | Slc5a4a | 5.171206 | 5.80984 | 5.490523 |
| ENSMUSG00000086746 | processed_transcrip | Gm15222 | 4.823229 | 5.491974 | 5.157601 |
| ENSMUSG00000025428 | protein_coding | Atp5a1 | 13363.81 | 13507.44 | 13435.63 |
| ENSMUSG00000048621 | protein_coding | Gm6377 | 2.914124 | 2.472986 | 2.693555 |
| ENSMUSG00000087298 | pseudogene | Gm9392 | 203.2778 | 207.9979 | 205.6379 |
| ENSMUSG00000047642 | protein_coding | D930020B18Rik | 4.852675 | 5.572334 | 5.212504 |
| ENSMUSG00000075025 | processed_transcrip | Gm10804 | 5.517916 | 4.848316 | 5.183116 |
| ENSMUSG00000082503 | pseudogene | Gm12229 | 0.99093 | 0.697713 | 0.844322 |
| ENSMUSG00000028796 | protein_coding | Phc2 | 2517.056 | 2480.424 | 2498.74 |
| ENSMUSG00000024556 | protein_coding | Me2 | 3856.95 | 3908.179 | 3882.565 |
| ENSMUSG00000017344 | protein_coding | Vtn | 55.11669 | 52.49361 | 53.80515 |
| ENSMUSG00000018068 | protein_coding | Ints2 | 1959.872 | 1933.555 | 1946.713 |
| ENSMUSG00000004591 | protein_coding | Pkn2 | 4790.034 | 4843.448 | 4816.741 |
| ENSMUSG00000084010 | pseudogene | Gm13302 | 7.828819 | 6.588275 | 7.208547 |
| ENSMUSG00000023147 | protein_coding | Wrb | 962.1628 | 978.1173 | 970.1401 |
| ENSMUSG00000086404 | processed_transcrip | Gm6787 | 10.71961 | 11.73572 | 11.22766 |
| ENSMUSG00000031168 | protein_coding | Ebp | 717.0305 | 705.5305 | 711.2805 |
| ENSMUSG00000022996 | protein_coding | Wnt10b | 6.481936 | 5.756508 | 6.119222 |
| ENSMUSG00000071860 | protein_coding | 2900055J20Rik | 1.290752 | 0.988552 | 1.139652 |
| ENSMUSG00000038600 | protein_coding | Atp6v0a4 | 5.823627 | 6.507553 | 6.16559 |
| ENSMUSG00000080006 | pseudogene | Rps19-ps7 | 2.253504 | 2.665808 | 2.459656 |
| ENSMUSG00000087514 | processed_transcrip | Gm16076 | 0.948663 | 0.662038 | 0.805351 |
| ENSMUSG00000077962 | miRNA | Mir874 | 0.948663 | 0.662038 | 0.805351 |
| ENSMUSG00000054469 | protein_coding | Lclat1 | 1023.703 | 1039.57 | 1031.637 |
| ENSMUSG00000039737 | protein_coding | Prkrip1 | 881.706 | 867.5803 | 874.6431 |
| ENSMUSG00000054426 | protein_coding | A930005H10Rik | 44.8489 | 46.9426 | 45.89575 |
| ENSMUSG00000085811 | processed_transcrip | Gm11669 | 2.891835 | 2.428301 | 2.660068 |
| ENSMUSG00000031966 | protein_coding | Glb1l3 | 1925.803 | 1964.178 | 1944.99 |
| ENSMUSG00000064797 | snoRNA | snoZ40 | 75.8915 | 78.45935 | 77.17542 |
| ENSMUSG00000074280 | pseudogene | Gm6166 | 4.194365 | 4.812641 | 4.503503 |
| ENSMUSG00000026150 | protein_coding | Mff | 2537.514 | 2571.876 | 2554.695 |
| ENSMUSG00000038909 | protein_coding | Myst2 | 3734.052 | 3681.543 | 3707.797 |
| ENSMUSG00000000409 | protein_coding | Lck | 66.37415 | 63.78677 | 65.08046 |
| ENSMUSG00000054203 | protein_coding | Ifi205 | 1.298952 | 0.997561 | 1.148256 |
| ENSMUSG00000087223 | processed_transcrip | 4930442L01Rik | 1.298952 | 0.997561 | 1.148256 |
| ENSMUSG00000086632 | processed_transcrip | 1700112J05Rik | 1.298952 | 0.997561 | 1.148256 |
| ENSMUSG00000040413 | protein_coding | Timd2 | 1.298952 | 0.997561 | 1.148256 |

| | | | | | |
|---|---|---|---|---|---|
| ENSMUSG00000086152 | processed_transcrip | Gm11454 | 1.298952 | 1.024227 | 1.161589 |
| ENSMUSG00000028004 | protein_coding | Npy2r | 1.298952 | 1.024227 | 1.161589 |
| ENSMUSG00000087534 | processed_transcrip | Gm11418 | 1.296641 | 1.659599 | 1.47812 |
| ENSMUSG00000082820 | pseudogene | Gm13803 | 30.00686 | 31.546 | 30.77643 |
| ENSMUSG00000044268 | pseudogene | Gm4895 | 5.526116 | 6.25239 | 5.889253 |
| ENSMUSG00000074093 | protein_coding | Svip | 418.8399 | 427.3301 | 423.085 |
| ENSMUSG00000066944 | protein_coding | 4921513D11Rik | 18.01648 | 16.82118 | 17.41883 |
| ENSMUSG00000038206 | protein_coding | Fbxo8 | 673.9152 | 662.8073 | 668.3613 |
| ENSMUSG00000087610 | protein_coding | Gm16253 | 9.392482 | 8.573665 | 8.983073 |
| ENSMUSG00000040209 | protein_coding | Zfp704 | 3043.206 | 3106.438 | 3074.822 |
| ENSMUSG00000039242 | protein_coding | B3galnt2 | 1577.096 | 1555.924 | 1566.51 |
| ENSMUSG00000020198 | protein_coding | Ap3d1 | 4332.793 | 4414.715 | 4373.754 |
| ENSMUSG00000015711 | protein_coding | Prune | 1515.954 | 1491.946 | 1503.95 |
| ENSMUSG00000028389 | protein_coding | Zfp37 | 296.0623 | 288.1332 | 292.0978 |
| ENSMUSG00000000489 | protein_coding | Pdgfb | 380.5743 | 373.0647 | 376.8195 |
| ENSMUSG00000010461 | protein_coding | Eya4 | 9.652347 | 8.802162 | 9.227255 |
| ENSMUSG00000085485 | processed_transcrip | Gm14024 | 2.559214 | 2.066473 | 2.312844 |
| ENSMUSG00000067430 | protein_coding | Zfp763 | 461.0035 | 476.6786 | 468.841 |
| ENSMUSG00000076999 | miRNA | AC132082.1 | 0.968641 | 0.724379 | 0.84651 |
| ENSMUSG00000058287 | protein_coding | Gm12253 | 0.968641 | 0.724379 | 0.84651 |
| ENSMUSG00000081835 | pseudogene | Gm11790 | 0.968641 | 0.724379 | 0.84651 |
| ENSMUSG00000077577 | snRNA | U11 | 0.968641 | 0.724379 | 0.84651 |
| ENSMUSG00000036962 | protein_coding | Gm101 | 3.568855 | 4.141594 | 3.855224 |
| ENSMUSG00000035849 | protein_coding | Krt222 | 55.31014 | 53.02552 | 54.16783 |
| ENSMUSG00000024164 | protein_coding | C3 | 88.12788 | 84.77336 | 86.45062 |
| ENSMUSG00000036095 | protein_coding | Dgkb | 15.84497 | 14.76407 | 15.30452 |
| ENSMUSG00000086851 | lincRNA | A430108G06Rik | 5.820048 | 6.638396 | 6.229222 |
| ENSMUSG00000031930 | protein_coding | Wwp2 | 1895.839 | 1871.086 | 1883.462 |
| ENSMUSG00000020133 | protein_coding | 2310011J03Rik | 830.2462 | 843.801 | 837.0236 |
| ENSMUSG00000030352 | protein_coding | Tspan9 | 1313.54 | 1289.208 | 1301.374 |
| ENSMUSG00000026043 | protein_coding | Col3a1 | 10924.1 | 11126.14 | 11025.12 |
| ENSMUSG00000021807 | protein_coding | 2700060E02Rik | 2717.731 | 2676.488 | 2697.109 |
| ENSMUSG00000038733 | protein_coding | Wdr26 | 5405.775 | 5343.034 | 5374.405 |
| ENSMUSG00000021416 | protein_coding | Eci3 | 3.586523 | 3.072682 | 3.329602 |
| ENSMUSG00000015401 | protein_coding | Tmem27 | 6.760512 | 7.478449 | 7.11948 |
| ENSMUSG00000035860 | protein_coding | Cdhr3 | 9.307948 | 10.19795 | 9.752949 |
| ENSMUSG00000040648 | protein_coding | Ppip5k2 | 2282.305 | 2309.32 | 2295.813 |
| ENSMUSG00000023830 | protein_coding | Igf2r | 2928.138 | 2886.769 | 2907.454 |
| ENSMUSG00000069044 | protein_coding | Usp9y | 978.9586 | 1005.282 | 992.1205 |
| ENSMUSG00000086309 | lincRNA | A930007I19Rik | 53.65628 | 55.88203 | 54.76915 |
| ENSMUSG00000028177 | processed_transcrip | 1810013D15Rik | 1.637461 | 2.030798 | 1.83413 |
| ENSMUSG00000083658 | pseudogene | Gm15798 | 3.266723 | 2.772834 | 3.019778 |
| ENSMUSG00000034931 | protein_coding | Dhx8 | 1735.51 | 1768.189 | 1751.85 |
| ENSMUSG00000067161 | pseudogene | Gm5560 | 3.530167 | 3.01935 | 3.274758 |
| ENSMUSG00000063457 | protein_coding | Rps15 | 5806.329 | 5883.459 | 5844.894 |
| ENSMUSG00000020897 | protein_coding | Aurkb | 875.9356 | 892.221 | 884.0783 |
| ENSMUSG00000040010 | protein_coding | Slc7a5 | 8186.936 | 8060.047 | 8123.491 |
| ENSMUSG00000086295 | lincRNA | 4933433G15Rik | 59.65717 | 56.72778 | 58.19248 |
| ENSMUSG00000041658 | protein_coding | Rragb | 1380.978 | 1358.597 | 1369.788 |
| ENSMUSG00000043372 | protein_coding | Hexim2 | 241.4951 | 247.2854 | 244.3903 |
| ENSMUSG00000019087 | protein_coding | Atp6ap1 | 2311.559 | 2283.417 | 2297.488 |
| ENSMUSG00000023057 | protein_coding | Fabp2 | 1.637461 | 2.012779 | 1.82512 |
| ENSMUSG00000083339 | pseudogene | Gm11693 | 3.572434 | 4.0879 | 3.830167 |
| ENSMUSG00000035093 | protein_coding | Secisbp2l | 4970.673 | 5048.515 | 5009.594 |
| ENSMUSG00000002504 | protein_coding | Slc9a3r2 | 564.7464 | 574.1756 | 569.461 |
| ENSMUSG00000047412 | protein_coding | Zbtb44 | 2269.391 | 2302.069 | 2285.73 |
| ENSMUSG00000071172 | protein_coding | Srsf3 | 8924.59 | 9079.129 | 9001.859 |
| ENSMUSG00000042784 | protein_coding | Muc1 | 34.08099 | 36.1593 | 35.12014 |

| | | | | | |
|---|---|---|---|---|---|
| ENSMUSG00000073803 | protein_coding | 4930544L04Rik | 3.888655 | 3.363521 | 3.626088 |
| ENSMUSG00000089092 | snRNA | U6 | 6.170337 | 5.519001 | 5.844669 |
| ENSMUSG00000022682 | protein_coding | Rrn3 | 2573.194 | 2609.771 | 2591.483 |
| ENSMUSG00000025978 | protein_coding | Rftn2 | 274.6619 | 280.4196 | 277.5407 |
| ENSMUSG00000078435 | protein_coding | AU041133 | 137.7126 | 133.1151 | 135.4138 |
| ENSMUSG00000031936 | protein_coding | Hephl1 | 2.289882 | 2.728149 | 2.509015 |
| ENSMUSG00000084113 | pseudogene | Gm14277 | 8.703684 | 9.499876 | 9.10178 |
| ENSMUSG00000037111 | protein_coding | Setd7 | 983.6048 | 999.6327 | 991.6187 |
| ENSMUSG00000092203 | processed_transcrip | 1110038B12Rik | 408.6906 | 421.0083 | 414.8494 |
| ENSMUSG00000020218 | protein_coding | Wif1 | 242.4506 | 248.6187 | 245.5346 |
| ENSMUSG00000065592 | miRNA | Mir145 | 36.4598 | 39.37033 | 37.91507 |
| ENSMUSG00000020528 | protein_coding | Prpsap2 | 2351.258 | 2382.388 | 2366.823 |
| ENSMUSG00000091822 | lincRNA | Gm17502 | 25.12832 | 23.7414 | 24.43486 |
| ENSMUSG00000042688 | protein_coding | Mapk6 | 4773.882 | 4844.03 | 4808.956 |
| ENSMUSG00000045246 | protein_coding | Kcng4 | 151.3953 | 156.1485 | 153.7719 |
| ENSMUSG00000046282 | protein_coding | 4930529F22Rik | 1.290752 | 1.024227 | 1.157489 |
| ENSMUSG00000057387 | protein_coding | 4922502B01Rik | 1.290752 | 1.024227 | 1.157489 |
| ENSMUSG00000001098 | protein_coding | Kctd10 | 1521.025 | 1543.552 | 1532.288 |
| ENSMUSG00000071265 | protein_coding | 1700086L19Rik | 3.560655 | 3.054663 | 3.307659 |
| ENSMUSG00000027398 | protein_coding | Il1b | 7.810109 | 6.985416 | 7.397763 |
| ENSMUSG00000028800 | protein_coding | Hdac1 | 2968.058 | 2924.541 | 2946.299 |
| ENSMUSG00000061607 | protein_coding | Mdc1 | 3331.266 | 3162.808 | 3247.037 |
| ENSMUSG00000044726 | protein_coding | BC030476 | 7.12951 | 8.03631 | 7.58291 |
| ENSMUSG00000022387 | protein_coding | Brd1 | 4100.682 | 4154.983 | 4127.832 |
| ENSMUSG00000083614 | pseudogene | Gm12795 | 1.621062 | 1.977104 | 1.799083 |
| ENSMUSG00000034990 | protein_coding | Otoa | 4.857296 | 5.465308 | 5.161302 |
| ENSMUSG00000068200 | pseudogene | Gm5931 | 14.26387 | 13.16324 | 13.71356 |
| ENSMUSG00000025231 | protein_coding | Sufu | 785.0916 | 773.3973 | 779.2444 |
| ENSMUSG00000027035 | protein_coding | Lass6 | 2635.576 | 2675.131 | 2655.353 |
| ENSMUSG00000037361 | protein_coding | 0610009D07Rik | 1730.241 | 1755.893 | 1743.067 |
| ENSMUSG00000090379 | protein_coding | Gm8229 | 4.218965 | 4.80327 | 4.511117 |
| ENSMUSG00000025911 | protein_coding | Adhfe1 | 117.4647 | 120.8055 | 119.1351 |
| ENSMUSG00000085664 | lincRNA | 4930556N08Rik | 3.228034 | 3.699044 | 3.463539 |
| ENSMUSG00000042616 | protein_coding | Oscp1 | 222.7075 | 216.9924 | 219.8499 |
| ENSMUSG00000031839 | protein_coding | Hsbp1 | 2598.727 | 2562.609 | 2580.668 |
| ENSMUSG00000034031 | protein_coding | 1700106J16Rik | 2.617881 | 3.144033 | 2.880957 |
| ENSMUSG00000089065 | misc_RNA | 7SK | 4.938252 | 4.060872 | 4.499562 |
| ENSMUSG00000030577 | protein_coding | Cd22 | 5.163007 | 5.801193 | 5.4821 |
| ENSMUSG00000047884 | protein_coding | Klk9 | 3.193967 | 3.716701 | 3.455334 |
| ENSMUSG00000052033 | protein_coding | Pfdn4 | 596.7225 | 586.4529 | 591.5877 |
| ENSMUSG00000087132 | lincRNA | A930001C03Rik | 5.46156 | 6.100679 | 5.78112 |
| ENSMUSG00000025592 | protein_coding | Dach2 | 14.20878 | 15.32846 | 14.76862 |
| ENSMUSG00000055134 | lincRNA | 9130017K11Rik | 11.54736 | 12.55526 | 12.05131 |
| ENSMUSG00000023915 | protein_coding | Tnfrsf21 | 338.4868 | 346.8926 | 342.6897 |
| ENSMUSG00000085661 | lincRNA | 1700034P13Rik | 27.52271 | 26.07454 | 26.79863 |
| ENSMUSG00000030636 | protein_coding | 1700010L04Rik | 4.218965 | 4.776604 | 4.497784 |
| ENSMUSG00000040822 | protein_coding | 1700123O20Rik | 845.096 | 831.1868 | 838.1414 |
| ENSMUSG00000019907 | protein_coding | Ppp1r12a | 3380.088 | 3434.4 | 3407.244 |
| ENSMUSG00000024897 | protein_coding | Apba1 | 620.9603 | 610.262 | 615.6111 |
| ENSMUSG00000021144 | protein_coding | Mta1 | 2191.905 | 2221.594 | 2206.75 |
| ENSMUSG00000044095 | pseudogene | 2410075B13Rik | 234.9789 | 228.978 | 231.9785 |
| ENSMUSG00000045790 | protein_coding | Gm447 | 37.25371 | 35.47096 | 36.36233 |
| ENSMUSG00000066057 | protein_coding | 2610044O15Rik | 158.2027 | 162.2313 | 160.217 |
| ENSMUSG00000085246 | processed_transcrip | Gm15893 | 74.42497 | 77.59906 | 76.01201 |
| ENSMUSG00000080533 | miRNA | Mir1191 | 17.73537 | 18.91147 | 18.32342 |
| ENSMUSG00000029765 | protein_coding | Plxna4 | 210.218 | 216.7272 | 213.4726 |
| ENSMUSG00000047193 | protein_coding | Dync2h1 | 2280.354 | 2243.33 | 2261.842 |
| ENSMUSG00000029053 | protein_coding | Prkcz | 352.338 | 359.2324 | 355.7852 |

| | | | | | |
|---|---|---|---|---|---|
| ENSMUSG00000026365 | protein_coding | Cfh | 275.4254 | 283.1169 | 279.2711 |
| ENSMUSG00000007950 | protein_coding | Abhd8 | 862.7033 | 848.6093 | 855.6563 |
| ENSMUSG00000020621 | protein_coding | Rdh14 | 538.7462 | 528.7966 | 533.7714 |
| ENSMUSG00000082280 | pseudogene | Gm7901 | 226.8269 | 257.0912 | 241.9591 |
| ENSMUSG00000081816 | pseudogene | Gm15528 | 5.489738 | 4.865611 | 5.177675 |
| ENSMUSG00000022400 | protein_coding | Rbx1 | 1890.722 | 1919.101 | 1904.912 |
| ENSMUSG00000017221 | protein_coding | Psmd3 | 3205.547 | 3167.294 | 3186.42 |
| ENSMUSG00000001819 | protein_coding | Hoxd13 | 1.32124 | 1.739597 | 1.530419 |
| ENSMUSG00000031144 | protein_coding | Syp | 72.95844 | 75.46165 | 74.21004 |
| ENSMUSG00000073682 | protein_coding | Gm10563 | 7.43291 | 8.149134 | 7.791022 |
| ENSMUSG00000046714 | protein_coding | Foxc2 | 280.9495 | 273.7529 | 277.3512 |
| ENSMUSG00000058542 | pseudogene | Gm15590 | 6.78049 | 6.118698 | 6.449594 |
| ENSMUSG00000090797 | lincRNA | Gm17335 | 1.284863 | 0.997561 | 1.141212 |
| ENSMUSG00000082788 | pseudogene | Gm13396 | 1.284863 | 1.024227 | 1.154545 |
| ENSMUSG00000089077 | snRNA | U7 | 1.284863 | 1.024227 | 1.154545 |
| ENSMUSG00000064409 | rRNA | n-R5s40 | 1.284863 | 1.024227 | 1.154545 |
| ENSMUSG00000024350 | protein_coding | Dnajc18 | 1063.497 | 1048.844 | 1056.171 |
| ENSMUSG00000057315 | protein_coding | Arhgap24 | 224.4392 | 229.7351 | 227.0871 |
| ENSMUSG00000046415 | lincRNA | B430212C06Rik | 42.62611 | 40.80045 | 41.71328 |
| ENSMUSG00000021706 | protein_coding | Zfyve16 | 1619.102 | 1639.391 | 1629.246 |
| ENSMUSG00000037443 | protein_coding | Ccdc21 | 4134.736 | 4200.002 | 4167.369 |
| ENSMUSG00000071460 | pseudogene | Gm7102 | 4.557475 | 5.165821 | 4.861648 |
| ENSMUSG00000029998 | protein_coding | Pcyox1 | 3701.55 | 3651.583 | 3676.566 |
| ENSMUSG00000053749 | protein_coding | Gm9920 | 7.409354 | 8.167152 | 7.788253 |
| ENSMUSG00000038957 | protein_coding | Edc3 | 1707.672 | 1730.471 | 1719.072 |
| ENSMUSG00000069204 | protein_coding | Zfp748 | 1025.197 | 1050.285 | 1037.741 |
| ENSMUSG00000028717 | protein_coding | Tal1 | 29.01698 | 27.60981 | 28.3134 |
| ENSMUSG00000084948 | processed_transcript | 1700061H18Rik | 0.948663 | 1.342094 | 1.145379 |
| ENSMUSG00000036817 | protein_coding | Sun1 | 3251.239 | 3196.175 | 3223.707 |
| ENSMUSG00000029260 | protein_coding | Ugt2b34 | 0.99093 | 0.671047 | 0.830989 |
| ENSMUSG00000067047 | pseudogene | Olfr719-ps | 0.99093 | 0.671047 | 0.830989 |
| ENSMUSG00000040134 | protein_coding | Rdh7 | 0.99093 | 0.671047 | 0.830989 |
| ENSMUSG00000074154 | protein_coding | Ap3s1-ps2 | 12.93443 | 12.00854 | 12.47148 |
| ENSMUSG00000090608 | processed_transcript | Gm17200 | 5.165317 | 5.765156 | 5.465237 |
| ENSMUSG00000073474 | lincRNA | A330023F24Rik | 33.95523 | 32.30678 | 33.131 |
| ENSMUSG00000050587 | protein_coding | Lrrc4c | 3.204478 | 2.665808 | 2.935143 |
| ENSMUSG00000020794 | protein_coding | Ube2g1 | 2608.132 | 2579.059 | 2593.595 |
| ENSMUSG00000026737 | protein_coding | Pip4k2a | 609.6877 | 599.2598 | 604.4737 |
| ENSMUSG00000004630 | protein_coding | Pcp2 | 8.507107 | 7.585113 | 8.04611 |
| ENSMUSG00000066684 | protein_coding | Pilrb1 | 1.304841 | 0.988552 | 1.146696 |
| ENSMUSG00000045409 | protein_coding | Trim39 | 1332.339 | 1300.432 | 1316.386 |
| ENSMUSG00000028277 | protein_coding | Ube2j1 | 2068.796 | 2091.98 | 2080.388 |
| ENSMUSG00000027698 | protein_coding | Nceh1 | 489.5297 | 498.2654 | 493.8976 |
| ENSMUSG00000059588 | protein_coding | Calcrl | 238.3263 | 232.9138 | 235.6201 |
| ENSMUSG00000075271 | protein_coding | Ttc30a1 | 234.3893 | 239.6081 | 236.9987 |
| ENSMUSG00000042993 | protein_coding | Ifnk | 1.307151 | 1.65059 | 1.478871 |
| ENSMUSG00000001670 | protein_coding | Tat | 5.497938 | 6.136716 | 5.817327 |
| ENSMUSG00000075574 | protein_coding | Defb48 | 4.869074 | 5.501345 | 5.185209 |
| ENSMUSG00000085227 | lincRNA | 6330418K02Rik | 18.08208 | 16.95166 | 17.51687 |
| ENSMUSG00000062006 | protein_coding | Rpl34 | 2669.256 | 2549.397 | 2609.327 |
| ENSMUSG00000082420 | pseudogene | Gm6517 | 5.825937 | 6.454221 | 6.140079 |
| ENSMUSG00000037887 | protein_coding | Dusp8 | 166.0861 | 170.5901 | 168.3381 |
| ENSMUSG00000070332 | protein_coding | 4933422H20Rik | 4.512897 | 5.067804 | 4.790351 |
| ENSMUSG00000091378 | lincRNA | Gm4219 | 3.896855 | 3.381539 | 3.639197 |
| ENSMUSG00000065041 | snoRNA | SNORA62 | 21.9228 | 23.37093 | 22.64686 |
| ENSMUSG00000049336 | protein_coding | Odz2 | 51.36782 | 54.32484 | 52.84633 |
| ENSMUSG00000001158 | protein_coding | Snrnp27 | 1019.405 | 1002.769 | 1011.087 |
| ENSMUSG00000042520 | protein_coding | Ubap2l | 6161.114 | 6275.914 | 6218.514 |

| | | | | | |
|---|---|---|---|---|---|
| ENSMUSG00000031950 | protein_coding | Gabarapl2 | 1378.613 | 1396.674 | 1387.644 |
| ENSMUSG00000082199 | pseudogene | Gm15763 | 1.288441 | 1.704284 | 1.496362 |
| ENSMUSG00000038292 | protein_coding | Ccdc155 | 394.2466 | 364.7478 | 379.4972 |
| ENSMUSG00000087648 | lincRNA | E130018N17Rik | 4.546964 | 5.121137 | 4.83405 |
| ENSMUSG00000043831 | protein_coding | Lysmd4 | 534.9488 | 543.6595 | 539.3042 |
| ENSMUSG00000085884 | lincRNA | Gm15342 | 5.212206 | 5.952542 | 5.582374 |
| ENSMUSG00000022466 | protein_coding | Rpap3 | 944.9392 | 957.7677 | 951.3534 |
| ENSMUSG00000037892 | protein_coding | Pcdh18 | 378.9372 | 366.9142 | 372.9257 |
| ENSMUSG00000037089 | protein_coding | Slc35b2 | 1437.173 | 1455.032 | 1446.102 |
| ENSMUSG00000003038 | protein_coding | Hmgn2 | 7166.838 | 7078.889 | 7122.863 |
| ENSMUSG00000082474 | pseudogene | Gm8213 | 9.086771 | 9.915759 | 9.501265 |
| ENSMUSG00000092038 | lincRNA | Gm17369 | 5.509716 | 6.127707 | 5.818712 |
| ENSMUSG00000024258 | protein_coding | Polr2d | 1422.449 | 1398.667 | 1410.558 |
| ENSMUSG00000064263 | protein_coding | Gm1631 | 4.804519 | 5.447651 | 5.126085 |
| ENSMUSG00000028696 | protein_coding | Ipp | 1223.236 | 1203.878 | 1213.557 |
| ENSMUSG00000065669 | snoRNA | SNORA5 | 3.592412 | 3.027997 | 3.310205 |
| ENSMUSG00000009596 | protein_coding | Taf7l | 6585.321 | 6441.265 | 6513.293 |
| ENSMUSG00000085241 | processed_transcrip | Snhg3 | 9.658236 | 8.873875 | 9.266055 |
| ENSMUSG00000085711 | pseudogene | Gm15163 | 102.775 | 99.92236 | 101.3487 |
| ENSMUSG00000090537 | pseudogene | Gm17222 | 2.265282 | 2.665808 | 2.465545 |
| ENSMUSG00000087408 | protein_coding | Lass1 | 57.44482 | 55.28741 | 56.36611 |
| ENSMUSG00000019828 | protein_coding | Grm1 | 1.307151 | 0.988552 | 1.147852 |
| ENSMUSG00000038522 | protein_coding | AI317395 | 1.31304 | 1.024227 | 1.168634 |
| ENSMUSG00000055214 | protein_coding | Pld5 | 4.846786 | 4.248258 | 4.547522 |
| ENSMUSG00000038641 | protein_coding | Akr1d1 | 1.967772 | 2.374969 | 2.17137 |
| ENSMUSG00000019659 | protein_coding | Ccdc12 | 921.4786 | 909.001 | 915.2398 |
| ENSMUSG00000031099 | protein_coding | Smarca1 | 3231.074 | 3281.934 | 3256.504 |
| ENSMUSG00000045733 | protein_coding | Sprn | 83.45344 | 80.59252 | 82.02298 |
| ENSMUSG00000070520 | protein_coding | Ndnl2 | 602.9081 | 594.3493 | 598.6287 |
| ENSMUSG00000084644 | misc_RNA | Y_RNA | 8.443819 | 9.262369 | 8.853094 |
| ENSMUSG00000035557 | protein_coding | Krt17 | 10.37879 | 11.32848 | 10.85364 |
| ENSMUSG00000070390 | protein_coding | Nlrp1b | 3.532477 | 4.052586 | 3.792532 |
| ENSMUSG00000029445 | protein_coding | Hpd | 1.965461 | 2.428663 | 2.197062 |
| ENSMUSG00000073063 | protein_coding | Hbq1b | 2.897724 | 3.345864 | 3.121794 |
| ENSMUSG00000053093 | protein_coding | Myh7 | 149.3033 | 143.4909 | 146.3971 |
| ENSMUSG00000040140 | protein_coding | Tdrd6 | 19.09276 | 17.73838 | 18.41557 |
| ENSMUSG00000085228 | processed_transcrip | Gm14376 | 1.618751 | 2.030436 | 1.824594 |
| ENSMUSG00000089459 | rRNA | 5S_rRNA | 3.258523 | 2.7995 | 3.029011 |
| ENSMUSG00000020583 | protein_coding | Matn3 | 13.21196 | 12.28172 | 12.74684 |
| ENSMUSG00000057191 | protein_coding | AB124611 | 39.39808 | 41.21669 | 40.30739 |
| ENSMUSG00000093351 | miRNA | AC121784.5 | 3.874566 | 3.354873 | 3.61472 |
| ENSMUSG00000024158 | protein_coding | Hagh | 758.4761 | 771.2063 | 764.8412 |
| ENSMUSG00000034063 | protein_coding | 4930590J08Rik | 5.206317 | 6.005874 | 5.606095 |
| ENSMUSG00000059280 | protein_coding | Vpreb2 | 0.966331 | 0.697713 | 0.832022 |
| ENSMUSG00000077575 | snoRNA | SNORA3 | 13.87258 | 12.89943 | 13.38601 |
| ENSMUSG00000040752 | protein_coding | Myh6 | 452.0327 | 437.8467 | 444.9397 |
| ENSMUSG00000021499 | protein_coding | Catsper3 | 3.902744 | 3.390187 | 3.646465 |
| ENSMUSG00000048732 | protein_coding | Klhl11 | 2181.179 | 2226.114 | 2203.646 |
| ENSMUSG00000075705 | protein_coding | Sepx1 | 471.0083 | 479.1698 | 475.0891 |
| ENSMUSG00000028339 | protein_coding | Col15a1 | 1229.542 | 1202.361 | 1215.951 |
| ENSMUSG00000086529 | processed_transcrip | 4930471I20Rik | 3.899165 | 4.476756 | 4.18796 |
| ENSMUSG00000038811 | protein_coding | Gngt2 | 31.09157 | 32.57924 | 31.83541 |
| ENSMUSG00000013936 | protein_coding | Myl2 | 3.536056 | 3.081691 | 3.308873 |
| ENSMUSG00000028224 | protein_coding | Nbn | 1227.244 | 1243.264 | 1235.254 |
| ENSMUSG00000091176 | protein_coding | Gm17678 | 3.564234 | 3.108357 | 3.336295 |
| ENSMUSG00000022836 | protein_coding | Mylk | 6969.721 | 7048.696 | 7009.208 |
| ENSMUSG00000074261 | protein_coding | Gm7092 | 3.236234 | 3.690035 | 3.463135 |
| ENSMUSG00000056972 | protein_coding | Magel2 | 13.21658 | 12.3264 | 12.77149 |

| | | | | | |
|---|---|---|---|---|---|
| ENSMUSG00000086044 | processed_transcrip | A330043C09Rik | 6.407913 | 5.729842 | 6.068878 |
| ENSMUSG00000036560 | protein_coding | Lgi4 | 110.8494 | 114.509 | 112.6792 |
| ENSMUSG00000003575 | protein_coding | Crtc1 | 2025.58 | 2051.52 | 2038.55 |
| ENSMUSG00000044313 | protein_coding | Mab21l3 | 25.22799 | 26.59423 | 25.91111 |
| ENSMUSG00000027176 | protein_coding | Cstf3 | 1593.609 | 1613.368 | 1603.488 |
| ENSMUSG00000073858 | protein_coding | Itpripl2 | 149.4083 | 154.3247 | 151.8665 |
| ENSMUSG00000024174 | protein_coding | Pot1b | 771.7458 | 760.4215 | 766.0837 |
| ENSMUSG00000032503 | protein_coding | Arpp21 | 2.575614 | 2.992322 | 2.783968 |
| ENSMUSG00000022986 | protein_coding | Krt75 | 2.575614 | 2.992322 | 2.783968 |
| ENSMUSG00000085208 | processed_transcrip | 4632419I22Rik | 932.2645 | 946.5612 | 939.4129 |
| ENSMUSG00000072476 | protein_coding | Gm9008 | 1.290752 | 1.015218 | 1.152985 |
| ENSMUSG00000092711 | miRNA | AC160138.1 | 1.290752 | 1.015218 | 1.152985 |
| ENSMUSG00000073686 | protein_coding | Gm10564 | 1.307151 | 1.024227 | 1.165689 |
| ENSMUSG00000031355 | protein_coding | Arhgap6 | 115.7307 | 112.5029 | 114.1168 |
| ENSMUSG00000003868 | protein_coding | Ruvbl2 | 1186.01 | 1202.616 | 1194.313 |
| ENSMUSG00000073125 | protein_coding | Xlr3b | 440.1824 | 428.006 | 434.0942 |
| ENSMUSG00000048703 | protein_coding | Speer4b | 2.902346 | 3.399196 | 3.150771 |
| ENSMUSG00000056148 | protein_coding | Rdh9 | 10.0449 | 9.226693 | 9.635798 |
| ENSMUSG00000020843 | protein_coding | Timm22 | 914.9717 | 901.2731 | 908.1224 |
| ENSMUSG00000085102 | processed_transcrip | 1700010K24Rik | 10.05668 | 10.8953 | 10.47599 |
| ENSMUSG00000048534 | protein_coding | Amica1 | 6.464269 | 7.115897 | 6.790083 |
| ENSMUSG00000020167 | protein_coding | Tcf3 | 4354.199 | 4301.531 | 4327.865 |
| ENSMUSG00000050957 | protein_coding | Insl6 | 22.96986 | 24.23692 | 23.60339 |
| ENSMUSG00000007880 | protein_coding | Arid1a | 4966.001 | 4898.704 | 4932.353 |
| ENSMUSG00000087046 | lincRNA | AU022754 | 1.293062 | 0.988552 | 1.140807 |
| ENSMUSG00000060029 | lincRNA | 4930473A02Rik | 11.99523 | 13.15817 | 12.5767 |
| ENSMUSG00000074243 | pseudogene | Gm10651 | 62.37152 | 60.215 | 61.29326 |
| ENSMUSG00000029821 | protein_coding | Dfna5 | 220.9178 | 215.9321 | 218.4249 |
| ENSMUSG00000089476 | snRNA | U6 | 1.609283 | 1.995123 | 1.802203 |
| ENSMUSG00000085735 | processed_transcrip | 4930550G17Rik | 14.60238 | 15.60956 | 15.10597 |
| ENSMUSG00000065361 | misc_RNA | Telomerase-vert | 2.626081 | 3.108357 | 2.867219 |
| ENSMUSG00000028226 | protein_coding | Mmp16 | 109.7474 | 113.3996 | 111.5735 |
| ENSMUSG00000093257 | miRNA | CT010471.1 | 2.601481 | 3.0907 | 2.846091 |
| ENSMUSG00000003360 | protein_coding | Ddx23 | 2857.192 | 2895.207 | 2876.199 |
| ENSMUSG00000078965 | protein_coding | Gm12033 | 2.231215 | 2.665808 | 2.448511 |
| ENSMUSG00000021241 | protein_coding | Isca2 | 579.3336 | 569.6562 | 574.4949 |
| ENSMUSG00000082226 | pseudogene | Gm715 | 221.9915 | 216.7229 | 219.3572 |
| ENSMUSG00000034300 | protein_coding | Fam53c | 2798.739 | 2835.549 | 2817.144 |
| ENSMUSG00000025939 | protein_coding | Ube2w | 1601.496 | 1628.021 | 1614.759 |
| ENSMUSG00000022537 | protein_coding | Tmem44 | 330.7476 | 322.7926 | 326.7701 |
| ENSMUSG00000036882 | protein_coding | Arhgap33 | 643.5648 | 665.2958 | 654.4303 |
| ENSMUSG00000040269 | protein_coding | Mrps28 | 432.2311 | 425.0639 | 428.6475 |
| ENSMUSG00000044285 | pseudogene | Gm1821 | 1563.675 | 1589.136 | 1576.405 |
| ENSMUSG00000038280 | protein_coding | Ostm1 | 809.0574 | 795.943 | 802.5002 |
| ENSMUSG00000092827 | miRNA | AL663027.1 | 6.419691 | 7.098241 | 6.758966 |
| ENSMUSG00000018932 | protein_coding | Map2k3 | 909.4852 | 897.6729 | 903.5791 |
| ENSMUSG00000064129 | protein_coding | Gm14781 | 0.948663 | 0.688704 | 0.818684 |
| ENSMUSG00000020063 | protein_coding | Sirt1 | 2765.374 | 2809.599 | 2787.487 |
| ENSMUSG00000081496 | pseudogene | Gm13428 | 1.914994 | 2.35695 | 2.135972 |
| ENSMUSG00000024785 | protein_coding | Rcl1 | 1103.724 | 1090.139 | 1096.931 |
| ENSMUSG00000034810 | protein_coding | Scn7a | 47.84212 | 50.01057 | 48.92635 |
| ENSMUSG00000090336 | protein_coding | 4932443I19Rik | 1.284863 | 1.015218 | 1.15004 |
| ENSMUSG00000059540 | protein_coding | Tcea2 | 395.6906 | 406.0442 | 400.8674 |
| ENSMUSG00000063605 | protein_coding | Ccdc102a | 310.6917 | 304.6879 | 307.6898 |
| ENSMUSG00000046440 | pseudogene | Gm5564 | 9.068061 | 9.826751 | 9.447406 |
| ENSMUSG00000074839 | pseudogene | Gm5792 | 2.620192 | 3.259706 | 2.939949 |
| ENSMUSG00000025037 | protein_coding | Maoa | 764.0882 | 746.5694 | 755.3288 |
| ENSMUSG00000038102 | protein_coding | D030016E14Rik | 2242.299 | 2210.53 | 2226.415 |

| | | | | | |
|---|---|---|---|---|---|
| ENSMUSG00000033007 | protein_coding | Accn4 | 185.8877 | 181.1007 | 183.4942 |
| ENSMUSG00000042810 | protein_coding | Krba1 | 1551.596 | 1571.656 | 1561.626 |
| ENSMUSG00000083425 | pseudogene | Gm13992 | 0.98273 | 0.724379 | 0.853555 |
| ENSMUSG00000066592 | protein_coding | Gm7589 | 0.98273 | 0.724379 | 0.853555 |
| ENSMUSG00000027466 | protein_coding | Rbck1 | 2525.788 | 2497.588 | 2511.688 |
| ENSMUSG00000061518 | protein_coding | Cox5b | 2024.773 | 2052.549 | 2038.661 |
| ENSMUSG00000083261 | pseudogene | Gm7816 | 40.78052 | 39.04532 | 39.91292 |
| ENSMUSG00000082395 | pseudogene | Gm15480 | 1.293062 | 1.024227 | 1.158645 |
| ENSMUSG00000030167 | protein_coding | Klrc1 | 1.293062 | 1.024227 | 1.158645 |
| ENSMUSG00000087731 | rRNA | 5S_rRNA | 1.293062 | 1.024227 | 1.158645 |
| ENSMUSG00000073470 | protein_coding | Gm1604b | 1.293062 | 1.024227 | 1.158645 |
| ENSMUSG00000064918 | snoRNA | SNORD18 | 7.084933 | 6.382871 | 6.733902 |
| ENSMUSG00000081431 | pseudogene | Gm15483 | 4.238943 | 4.812279 | 4.525611 |
| ENSMUSG00000028037 | protein_coding | Ifi44 | 11.35921 | 10.42029 | 10.88975 |
| ENSMUSG00000087098 | processed_transcrip | Gm11884 | 2.298082 | 2.861841 | 2.579961 |
| ENSMUSG00000037428 | protein_coding | Vgf | 6.810979 | 7.469439 | 7.140209 |
| ENSMUSG00000020770 | protein_coding | Unk | 1439.525 | 1456.472 | 1447.998 |
| ENSMUSG00000022442 | protein_coding | Ttll1 | 269.1725 | 263.6499 | 266.4112 |
| ENSMUSG00000075045 | protein_coding | Gm4981 | 2.626081 | 3.099348 | 2.862714 |
| ENSMUSG00000058883 | protein_coding | Zfp708 | 205.3429 | 209.8305 | 207.5867 |
| ENSMUSG00000073600 | protein_coding | Gm1614 | 75.14739 | 77.58539 | 76.36639 |
| ENSMUSG00000001056 | protein_coding | Nhp2 | 2254.958 | 2283.558 | 2269.258 |
| ENSMUSG00000032112 | protein_coding | Trappc4 | 1446.616 | 1427.966 | 1437.291 |
| ENSMUSG00000008683 | protein_coding | Rps15a | 4421.522 | 4474.677 | 4448.099 |
| ENSMUSG00000024761 | pseudogene | Gm16437 | 20.68125 | 21.90628 | 21.29376 |
| ENSMUSG00000029377 | protein_coding | Ereg | 4.563364 | 5.121137 | 4.84225 |
| ENSMUSG00000045912 | protein_coding | C2cd4c | 226.2029 | 232.3268 | 229.2649 |
| ENSMUSG00000079550 | protein_coding | Mpp4 | 49.30507 | 51.64776 | 50.47641 |
| ENSMUSG00000079587 | protein_coding | Gpr45 | 23.89646 | 25.34223 | 24.61935 |
| ENSMUSG00000002660 | protein_coding | Clpp | 914.6792 | 927.2479 | 920.9636 |
| ENSMUSG00000067813 | protein_coding | Xkr9 | 0.948663 | 0.653029 | 0.800846 |
| ENSMUSG00000062524 | protein_coding | Ncr1 | 0.948663 | 0.653029 | 0.800846 |
| ENSMUSG00000021281 | protein_coding | Tnfaip2 | 47.31247 | 49.71534 | 48.5139 |
| ENSMUSG00000038705 | protein_coding | Gmeb2 | 1247.57 | 1227.767 | 1237.668 |
| ENSMUSG00000029600 | protein_coding | 1110008J03Rik | 367.0373 | 373.5837 | 370.3105 |
| ENSMUSG00000059326 | protein_coding | Csf2ra | 420.6539 | 430.8437 | 425.7488 |
| ENSMUSG00000007987 | protein_coding | Rabl5 | 418.4455 | 411.3464 | 414.8959 |
| ENSMUSG00000091648 | protein_coding | C2cd4d | 65.45277 | 67.99697 | 66.72487 |
| ENSMUSG00000063506 | protein_coding | Arhgap22 | 73.37075 | 70.87031 | 72.12053 |
| ENSMUSG00000025764 | protein_coding | Phf17 | 3485.753 | 3440.75 | 3463.252 |
| ENSMUSG00000073610 | protein_coding | Gm10549 | 3.238545 | 2.710854 | 2.9747 |
| ENSMUSG00000078725 | protein_coding | Gm2163 | 1.293062 | 0.997561 | 1.145312 |
| ENSMUSG00000044243 | protein_coding | Bhlha9 | 1.293062 | 0.997561 | 1.145312 |
| ENSMUSG00000019146 | protein_coding | Cacng2 | 2.295771 | 2.728149 | 2.51196 |
| ENSMUSG00000055720 | protein_coding | Ubl7 | 955.2822 | 968.6301 | 961.9562 |
| ENSMUSG00000022272 | protein_coding | Myo10 | 4899.157 | 4820.075 | 4859.616 |
| ENSMUSG00000091795 | lincRNA | Gm17692 | 33.90811 | 32.21234 | 33.06023 |
| ENSMUSG00000006307 | protein_coding | Wbp7 | 4063.839 | 3999.727 | 4031.783 |
| ENSMUSG00000021453 | protein_coding | Gadd45g | 303.9058 | 297.625 | 300.7654 |
| ENSMUSG00000005716 | protein_coding | Pvalb | 4.881896 | 5.474317 | 5.178106 |
| ENSMUSG00000077382 | rRNA | n-R5s200 | 5.191185 | 5.783174 | 5.487179 |
| ENSMUSG00000020935 | protein_coding | Dcakd | 2178.174 | 2146.053 | 2162.114 |
| ENSMUSG00000002767 | protein_coding | Mrpl2 | 1415.997 | 1433.565 | 1424.781 |
| ENSMUSG00000087029 | processed_transcrip | Gm14133 | 4.907763 | 5.634675 | 5.271219 |
| ENSMUSG00000027495 | protein_coding | 2010011I20Rik | 626.7303 | 638.2727 | 632.5015 |
| ENSMUSG00000031585 | protein_coding | Gtf2e2 | 2261.908 | 2232.94 | 2247.424 |
| ENSMUSG00000072407 | pseudogene | Gm6419 | 31.31044 | 29.8774 | 30.59392 |
| ENSMUSG00000087119 | pseudogene | Atg4a-ps | 0.976841 | 0.724379 | 0.85061 |

| | | | | | |
|---|---|---|---|---|---|
| ENSMUSG00000084964 | processed_transcrip | Gm15503 | 0.976841 | 0.724379 | 0.85061 |
| ENSMUSG00000056742 | protein_coding | Gm10008 | 0.976841 | 0.724379 | 0.85061 |
| ENSMUSG00000050625 | protein_coding | Ccdc121 | 3.211635 | 2.781843 | 2.996739 |
| ENSMUSG00000037086 | protein_coding | 1110059M19Rik | 0.966331 | 0.671047 | 0.818689 |
| ENSMUSG00000065509 | miRNA | Mir421 | 3.560655 | 4.061596 | 3.811125 |
| ENSMUSG00000028642 | protein_coding | 4930538K18Rik | 12.30534 | 11.31947 | 11.8124 |
| ENSMUSG00000024963 | protein_coding | Dnajc4 | 247.9211 | 242.8641 | 245.3926 |
| ENSMUSG00000086591 | processed_transcrip | 6030455H03Rik | 11.83772 | 10.87801 | 11.35786 |
| ENSMUSG00000081648 | pseudogene | Gm13423 | 3.913254 | 3.399196 | 3.656225 |
| ENSMUSG00000058443 | protein_coding | Rpl10-ps3 | 5.475649 | 6.074375 | 5.775012 |
| ENSMUSG00000053390 | protein_coding | Zfp952 | 460.8481 | 448.9291 | 454.8886 |
| ENSMUSG00000037638 | protein_coding | Zbtb42 | 144.5733 | 140.7047 | 142.639 |
| ENSMUSG00000092076 | pseudogene | Gm4482 | 20.94216 | 22.11748 | 21.52982 |
| ENSMUSG00000074570 | protein_coding | Cass4 | 26.91487 | 28.55581 | 27.73534 |
| ENSMUSG00000040205 | protein_coding | Cuzd1 | 15.25122 | 16.26295 | 15.75709 |
| ENSMUSG00000032020 | protein_coding | Ubash3b | 152.4213 | 157.0283 | 154.7248 |
| ENSMUSG00000026377 | protein_coding | Mki67ip | 1695.408 | 1718.583 | 1706.996 |
| ENSMUSG00000075420 | protein_coding | 1110017F19Rik | 7.469288 | 6.771726 | 7.120507 |
| ENSMUSG00000091781 | protein_coding | Gm9590 | 11.60499 | 10.73779 | 11.17139 |
| ENSMUSG00000028907 | protein_coding | Utp11l | 1380.109 | 1401.271 | 1390.69 |
| ENSMUSG00000089954 | pseudogene | Gm8783 | 10.40108 | 11.35515 | 10.87811 |
| ENSMUSG00000022801 | protein_coding | Lrch3 | 1832.823 | 1807.031 | 1819.927 |
| ENSMUSG00000063754 | protein_coding | Gm10136 | 33.97521 | 35.4854 | 34.73031 |
| ENSMUSG00000041556 | protein_coding | Fbxo2 | 55.08956 | 53.03712 | 54.06334 |
| ENSMUSG00000026426 | protein_coding | Arl8a | 1451.835 | 1435.163 | 1443.499 |
| ENSMUSG00000055567 | protein_coding | Unc80 | 26.2837 | 24.83983 | 25.56176 |
| ENSMUSG00000079547 | protein_coding | H2-DMb1 | 9.327926 | 8.360698 | 8.844312 |
| ENSMUSG00000066735 | protein_coding | Vkorc1l1 | 1388.368 | 1370.656 | 1379.512 |
| ENSMUSG00000039168 | protein_coding | Dap | 742.7893 | 730.1801 | 736.4847 |
| ENSMUSG00000091749 | pseudogene | Tspy-ps | 1.31304 | 1.015218 | 1.164129 |
| ENSMUSG00000087543 | lincRNA | Gm16576 | 127.0205 | 123.9516 | 125.4861 |
| ENSMUSG00000021903 | protein_coding | Galntl2 | 38.50323 | 36.75723 | 37.63023 |
| ENSMUSG00000073728 | lincRNA | Gm10565 | 3.864055 | 4.370091 | 4.117073 |
| ENSMUSG00000042099 | protein_coding | Kank3 | 849.5652 | 863.6365 | 856.6008 |
| ENSMUSG00000020268 | protein_coding | Lyrm7 | 59.55602 | 61.79247 | 60.67424 |
| ENSMUSG00000020091 | protein_coding | Eif4ebp2 | 1617.697 | 1645.235 | 1631.466 |
| ENSMUSG00000057886 | protein_coding | Gm6984 | 109.0305 | 105.8011 | 107.4158 |
| ENSMUSG00000055200 | protein_coding | Sertad3 | 404.7356 | 413.077 | 408.9063 |
| ENSMUSG00000032191 | protein_coding | Bcl2l10 | 3.230345 | 3.681388 | 3.455866 |
| ENSMUSG00000086134 | lincRNA | Gm16159 | 3.242123 | 3.707692 | 3.474908 |
| ENSMUSG00000082765 | pseudogene | Gm14411 | 6.057625 | 6.789745 | 6.423685 |
| ENSMUSG00000056888 | protein_coding | Glipr1 | 6.170337 | 6.780736 | 6.475536 |
| ENSMUSG00000092167 | protein_coding | Gm3696 | 33.92012 | 35.36108 | 34.6406 |
| ENSMUSG00000083709 | pseudogene | Gm14688 | 1.304841 | 1.015218 | 1.160029 |
| ENSMUSG00000035828 | protein_coding | Pim3 | 999.404 | 1019.238 | 1009.321 |
| ENSMUSG00000008429 | protein_coding | Herpud2 | 1225.021 | 1209.062 | 1217.041 |
| ENSMUSG00000079354 | protein_coding | Gm2237 | 47.61148 | 49.85958 | 48.73553 |
| ENSMUSG00000001911 | protein_coding | Nfix | 2738.357 | 2773.678 | 2756.017 |
| ENSMUSG00000020454 | protein_coding | Eif4enif1 | 3125.783 | 3072.934 | 3099.359 |
| ENSMUSG00000044125 | lincRNA | 9530080O11Rik | 17.14877 | 16.09644 | 16.6226 |
| ENSMUSG00000086111 | processed_transcrip | Gm15326 | 18.01394 | 19.14281 | 18.57838 |
| ENSMUSG00000022469 | protein_coding | Rapgef3 | 197.5789 | 202.1108 | 199.8449 |
| ENSMUSG00000033361 | protein_coding | Prrg3 | 612.24 | 601.8783 | 607.0591 |
| ENSMUSG00000081123 | pseudogene | Gm11469 | 62.56526 | 72.80921 | 67.68723 |
| ENSMUSG00000032431 | protein_coding | Crtap | 938.1144 | 923.5714 | 930.8429 |
| ENSMUSG00000019978 | protein_coding | Epb4.1l2 | 10962.97 | 10817.12 | 10890.05 |
| ENSMUSG00000084264 | pseudogene | Gm14142 | 13.91716 | 13.00646 | 13.46181 |
| ENSMUSG00000022951 | protein_coding | Rcan1 | 1418.002 | 1436.433 | 1427.217 |

| | | | | | |
|---|---|---|---|---|---|
| ENSMUSG00000086274 | lincRNA | Gm16677 | 7.185866 | 8.045319 | 7.615593 |
| ENSMUSG00000017776 | protein_coding | Crk | 4382.199 | 4457.14 | 4419.67 |
| ENSMUSG00000039886 | protein_coding | Tmem120a | 510.385 | 500.9633 | 505.6742 |
| ENSMUSG00000020190 | protein_coding | Mknk2 | 4808.766 | 4731.549 | 4770.158 |
| ENSMUSG00000043535 | protein_coding | Setx | 8123.938 | 7681.646 | 7902.792 |
| ENSMUSG00000083011 | pseudogene | Gm12816 | 10.32832 | 9.518256 | 9.92329 |
| ENSMUSG00000005103 | protein_coding | Wdr1 | 3011.104 | 3048.599 | 3029.852 |
| ENSMUSG00000071714 | protein_coding | Csf2rb2 | 16.55144 | 17.68577 | 17.1186 |
| ENSMUSG00000071613 | protein_coding | Gm10340 | 1.604662 | 1.994761 | 1.799711 |
| ENSMUSG00000079014 | protein_coding | Serpina3i | 2.279371 | 2.692836 | 2.486103 |
| ENSMUSG00000088769 | misc_RNA | 7SK | 7.104911 | 7.814334 | 7.459622 |
| ENSMUSG00000031399 | protein_coding | Fam3a | 484.2811 | 492.8899 | 488.5855 |
| ENSMUSG00000034648 | protein_coding | Lrrn1 | 146.2104 | 150.2761 | 148.2432 |
| ENSMUSG00000027776 | protein_coding | Il12a | 2.903614 | 3.327846 | 3.11573 |
| ENSMUSG00000041975 | protein_coding | Mettl8 | 565.5711 | 557.5182 | 561.5446 |
| ENSMUSG00000083481 | pseudogene | Rps8-ps2 | 1.307151 | 1.015218 | 1.161185 |
| ENSMUSG00000084985 | processed_transcrip | Gm16135 | 1.307151 | 1.015218 | 1.161185 |
| ENSMUSG00000052301 | protein_coding | Doc2a | 66.87159 | 64.49598 | 65.68379 |
| ENSMUSG00000071568 | pseudogene | Gm5874 | 36.83946 | 38.98049 | 37.90998 |
| ENSMUSG00000063663 | protein_coding | Brwd3 | 2517.931 | 2484.123 | 2501.027 |
| ENSMUSG00000038147 | protein_coding | Cd84 | 60.26643 | 62.60151 | 61.43397 |
| ENSMUSG00000089105 | rRNA | 5S_rRNA | 5.167628 | 5.711824 | 5.439726 |
| ENSMUSG00000024392 | protein_coding | Bag6 | 6494.973 | 6419.878 | 6457.425 |
| ENSMUSG00000021930 | protein_coding | 6330409N04Rik | 937.6555 | 913.0944 | 925.375 |
| ENSMUSG00000033060 | protein_coding | Lmo7 | 120.7025 | 117.0207 | 118.8616 |
| ENSMUSG00000028760 | protein_coding | Eif4g3 | 3695.879 | 3736.717 | 3716.298 |
| ENSMUSG00000087703 | processed_transcrip | Gm15650 | 3.228034 | 2.790852 | 3.009443 |
| ENSMUSG00000084142 | pseudogene | Gm9103 | 1.304841 | 0.979543 | 1.142192 |
| ENSMUSG00000000948 | protein_coding | Snrpn | 377.433 | 385.6878 | 381.5604 |
| ENSMUSG00000051029 | protein_coding | Serpinb1b | 11.63421 | 12.4749 | 12.05456 |
| ENSMUSG00000034584 | protein_coding | Exph5 | 25.80765 | 24.48416 | 25.14591 |
| ENSMUSG00000084052 | pseudogene | Gm11745 | 1.276663 | 1.024227 | 1.150445 |
| ENSMUSG00000084372 | pseudogene | Gm13988 | 5.598872 | 4.672789 | 5.13583 |
| ENSMUSG00000046516 | protein_coding | Cox17 | 614.9196 | 625.6277 | 620.2737 |
| ENSMUSG00000052450 | protein_coding | 2810055G20Rik | 48.67449 | 46.82077 | 47.74763 |
| ENSMUSG00000063531 | protein_coding | Sema3e | 10.00032 | 9.262369 | 9.631347 |
| ENSMUSG00000003208 | protein_coding | Ccdc94 | 398.7552 | 392.2608 | 395.508 |
| ENSMUSG00000040525 | protein_coding | Cblc | 15.85906 | 14.74891 | 15.30398 |
| ENSMUSG00000047518 | protein_coding | Slfnl1 | 3.239813 | 2.764186 | 3.001999 |
| ENSMUSG00000026271 | protein_coding | Gpr35 | 6.847356 | 7.576104 | 7.21173 |
| ENSMUSG00000084235 | pseudogene | Gm15421 | 1.284863 | 1.033237 | 1.15905 |
| ENSMUSG00000085657 | processed_transcrip | Gm15984 | 2.569725 | 3.046378 | 2.808051 |
| ENSMUSG00000053553 | protein_coding | 3110082I17Rik | 700.5903 | 710.4254 | 705.5078 |
| ENSMUSG00000058126 | protein_coding | Tpm3-rs7 | 2327.166 | 2560.23 | 2443.698 |
| ENSMUSG00000056216 | protein_coding | Cebpg | 996.0941 | 1014.535 | 1005.315 |
| ENSMUSG00000025201 | protein_coding | Gm13540 | 9.394793 | 10.17056 | 9.782677 |
| ENSMUSG00000058392 | protein_coding | Rrp1b | 1261.608 | 1280.761 | 1271.185 |
| ENSMUSG00000036395 | protein_coding | Glb1l2 | 856.8295 | 872.2485 | 864.539 |
| ENSMUSG00000025374 | protein_coding | Obfc2b | 335.3405 | 329.2916 | 332.3161 |
| ENSMUSG00000059334 | protein_coding | Zfp36l3 | 7.147178 | 6.507553 | 6.827366 |
| ENSMUSG00000090723 | pseudogene | Gm9625 | 10.95719 | 10.17092 | 10.56406 |
| ENSMUSG00000073412 | protein_coding | Lst1 | 22.90888 | 21.76394 | 22.33641 |
| ENSMUSG00000065589 | miRNA | Mir301 | 3.546566 | 3.117366 | 3.331966 |
| ENSMUSG00000077569 | snoRNA | SNORA58 | 2.298082 | 2.710492 | 2.504287 |
| ENSMUSG00000041396 | protein_coding | 2810422O20Rik | 189.0999 | 193.5538 | 191.3269 |
| ENSMUSG00000050671 | protein_coding | Ism2 | 56.50898 | 54.62858 | 55.56878 |
| ENSMUSG00000003581 | protein_coding | Rnf215 | 427.4951 | 436.2143 | 431.8547 |
| ENSMUSG00000031079 | protein_coding | Zfp300 | 132.5153 | 128.1659 | 130.3406 |

| | | | | | |
|---|---|---|---|---|---|
| ENSMUSG00000086456 | lincRNA | Gm16861 | 46.76285 | 48.44397 | 47.60341 |
| ENSMUSG00000085958 | processed_transcrip | A930019D19Rik | 14.53193 | 13.61444 | 14.07319 |
| ENSMUSG00000041028 | protein_coding | Ghitm | 5314.732 | 5257.308 | 5286.02 |
| ENSMUSG00000029614 | protein_coding | Rpl6 | 9868.642 | 9983.771 | 9926.207 |
| ENSMUSG00000006649 | protein_coding | Nphs1 | 21.6194 | 20.52022 | 21.06981 |
| ENSMUSG00000058945 | pseudogene | Gm10056 | 3.872255 | 3.434871 | 3.653563 |
| ENSMUSG00000092143 | lincRNA | Gm17452 | 1.293062 | 1.015218 | 1.15414 |
| ENSMUSG00000073413 | protein_coding | Ly6g6d | 8.989416 | 9.755039 | 9.372228 |
| ENSMUSG00000083733 | pseudogene | Gm13035 | 1.298952 | 1.050893 | 1.174922 |
| ENSMUSG00000032897 | protein_coding | Nfyc | 1734.587 | 1754.443 | 1744.515 |
| ENSMUSG00000072902 | processed_transcrip | Gm10435 | 2.303971 | 2.719502 | 2.511736 |
| ENSMUSG00000021629 | protein_coding | Slc30a5 | 1936.416 | 1966.181 | 1951.298 |
| ENSMUSG00000021366 | protein_coding | Hivep1 | 1548.784 | 1525.43 | 1537.107 |
| ENSMUSG00000020520 | protein_coding | Galnt10 | 1184.243 | 1200.964 | 1192.604 |
| ENSMUSG00000081133 | pseudogene | Gm14544 | 9.43117 | 10.26858 | 9.849874 |
| ENSMUSG00000035692 | protein_coding | Isg15 | 19.46556 | 20.75157 | 20.10857 |
| ENSMUSG00000033624 | protein_coding | Pdpr | 2706.352 | 2671.106 | 2688.729 |
| ENSMUSG00000084087 | pseudogene | Gm13650 | 4.188476 | 3.708054 | 3.948265 |
| ENSMUSG00000022962 | protein_coding | Gart | 3416.328 | 3456.226 | 3436.277 |
| ENSMUSG00000061535 | protein_coding | C1qtnf7 | 554.5502 | 546.0695 | 550.3098 |
| ENSMUSG00000046856 | protein_coding | Gpr1 | 0.99093 | 1.342094 | 1.166512 |
| ENSMUSG00000027505 | protein_coding | 1700029J11Rik | 11.82199 | 13.03313 | 12.42756 |
| ENSMUSG00000034832 | protein_coding | Tet3 | 5535.766 | 5465.647 | 5500.707 |
| ENSMUSG00000043314 | protein_coding | Olfr30 | 0.960442 | 0.724379 | 0.84241 |
| ENSMUSG00000088864 | misc_RNA | 7SK | 0.960442 | 0.724379 | 0.84241 |
| ENSMUSG00000056260 | protein_coding | 4933421E11Rik | 805.7604 | 817.2851 | 811.5227 |
| ENSMUSG00000043858 | protein_coding | Nup62 | 3067.983 | 3032.242 | 3050.112 |
| ENSMUSG00000039252 | protein_coding | Lgi2 | 365.6701 | 373.7138 | 369.6919 |
| ENSMUSG00000040383 | protein_coding | Aqr | 2793.273 | 2757.36 | 2775.316 |
| ENSMUSG00000033981 | protein_coding | Gria2 | 6.497068 | 5.881191 | 6.18913 |
| ENSMUSG00000082023 | pseudogene | Gm14407 | 1.296641 | 1.015218 | 1.155929 |
| ENSMUSG00000090843 | protein_coding | Gm17673 | 1.296641 | 1.015218 | 1.155929 |
| ENSMUSG00000009551 | protein_coding | 6330409D20Rik | 4.198987 | 3.73472 | 3.966853 |
| ENSMUSG00000055491 | protein_coding | Pprc1 | 3164.29 | 3118.041 | 3141.166 |
| ENSMUSG00000087347 | lincRNA | 1700047K16Rik | 13.60787 | 12.67922 | 13.14354 |
| ENSMUSG00000062012 | protein_coding | Zfp13 | 401.0905 | 393.8989 | 397.4947 |
| ENSMUSG00000088326 | misc_RNA | 7SK | 0.948663 | 0.671047 | 0.809855 |
| ENSMUSG00000025195 | protein_coding | Dnmbp | 1262.977 | 1291.88 | 1277.429 |
| ENSMUSG00000092622 | protein_coding | 2410004A20Rik | 5.820048 | 6.409898 | 6.114973 |
| ENSMUSG00000003814 | protein_coding | Calr | 13725.51 | 13509.42 | 13617.46 |
| ENSMUSG00000073616 | protein_coding | Myeov2 | 687.0535 | 677.5994 | 682.3265 |
| ENSMUSG00000069049 | protein_coding | Eif2s3y | 2507.375 | 2474.986 | 2491.18 |
| ENSMUSG00000074118 | protein_coding | Gm5897 | 7.189445 | 6.471878 | 6.830661 |
| ENSMUSG00000039851 | protein_coding | 4932438H23Rik | 9.433481 | 10.25128 | 9.842382 |
| ENSMUSG00000039428 | protein_coding | Tmem135 | 1636.106 | 1618.566 | 1627.336 |
| ENSMUSG00000058690 | protein_coding | Gcap14 | 2713.931 | 2685.22 | 2699.576 |
| ENSMUSG00000069917 | protein_coding | Hba-a2 | 32.45881 | 29.84503 | 31.15192 |
| ENSMUSG00000035376 | protein_coding | Ptplb | 1931.713 | 1957.947 | 1944.83 |
| ENSMUSG00000032657 | protein_coding | Fam189b | 352.7943 | 358.3506 | 355.5724 |
| ENSMUSG00000017631 | protein_coding | Abr | 1839.804 | 1870.69 | 1855.247 |
| ENSMUSG00000042742 | protein_coding | B630005N14Rik | 1158.266 | 1140.943 | 1149.604 |
| ENSMUSG00000090738 | protein_coding | Gm17727 | 15.19382 | 16.24815 | 15.72098 |
| ENSMUSG00000050947 | protein_coding | Amigo1 | 470.5004 | 461.0875 | 465.794 |
| ENSMUSG00000078291 | protein_coding | Gm3940 | 1.284863 | 1.006571 | 1.145717 |
| ENSMUSG00000025630 | protein_coding | Hprt | 2000.02 | 1976.274 | 1988.147 |
| ENSMUSG00000088707 | rRNA | 5S_rRNA | 1.270774 | 1.024227 | 1.1475 |
| ENSMUSG00000005824 | protein_coding | Tnfsf14 | 1.270774 | 1.024227 | 1.1475 |
| ENSMUSG00000045518 | protein_coding | Onecut3 | 5.528427 | 6.127345 | 5.827886 |

| | | | | | |
|---|---|---|---|---|---|
| ENSMUSG00000020690 | protein_coding | Efcab3 | 58.77597 | 56.02898 | 57.40248 |
| ENSMUSG00000085427 | processed_transcrip | 6430710C18Rik | 22.63724 | 21.52679 | 22.08202 |
| ENSMUSG00000040455 | protein_coding | Usp45 | 836.9404 | 826 | 831.4702 |
| ENSMUSG00000000296 | protein_coding | Tpd52l1 | 1588.617 | 1568.941 | 1578.779 |
| ENSMUSG00000081705 | pseudogene | Gm12582 | 1.959572 | 2.312628 | 2.1361 |
| ENSMUSG00000086742 | lincRNA | Gm16201 | 50.89587 | 52.93403 | 51.91495 |
| ENSMUSG00000034493 | protein_coding | 4930556J24Rik | 3.278501 | 3.814718 | 3.54661 |
| ENSMUSG00000038286 | protein_coding | Bphl | 315.1259 | 321.2247 | 318.1753 |
| ENSMUSG00000045482 | protein_coding | Trrap | 14318.8 | 14476.86 | 14397.83 |
| ENSMUSG00000009246 | protein_coding | Trpm5 | 167.0064 | 163.1304 | 165.0684 |
| ENSMUSG00000024777 | protein_coding | Ppp2r5b | 693.5985 | 684.9873 | 689.2929 |
| ENSMUSG00000068141 | pseudogene | Gm10232 | 168.7107 | 172.0731 | 170.3919 |
| ENSMUSG00000000441 | protein_coding | Raf1 | 4023.762 | 3983.696 | 4003.729 |
| ENSMUSG00000025170 | protein_coding | Rab40b | 42.18771 | 40.64294 | 41.41533 |
| ENSMUSG00000037730 | protein_coding | Mynn | 1655.425 | 1633.319 | 1644.372 |
| ENSMUSG00000040770 | protein_coding | Il25 | 6.782801 | 6.207705 | 6.495253 |
| ENSMUSG00000025240 | protein_coding | Sacm1l | 2147.191 | 2120.875 | 2134.033 |
| ENSMUSG00000029333 | protein_coding | Rasgef1b | 1087.76 | 1071.41 | 1079.585 |
| ENSMUSG00000020790 | protein_coding | Ankfy1 | 3296.273 | 3261.769 | 3279.021 |
| ENSMUSG00000003477 | protein_coding | Inmt | 1.629262 | 1.315066 | 1.472164 |
| ENSMUSG00000073234 | protein_coding | Gm8773 | 4.546964 | 3.989883 | 4.268424 |
| ENSMUSG00000077506 | snoRNA | Scarna9 | 1.290752 | 1.050893 | 1.170823 |
| ENSMUSG00000057842 | protein_coding | Zfp595 | 200.1576 | 194.732 | 197.4448 |
| ENSMUSG00000012114 | protein_coding | Med15 | 1349.363 | 1368.077 | 1358.72 |
| ENSMUSG00000036820 | protein_coding | Amdhd2 | 316.6402 | 310.4524 | 313.5463 |
| ENSMUSG00000003528 | protein_coding | Slc25a1 | 2934.391 | 2890.244 | 2912.317 |
| ENSMUSG00000083853 | pseudogene | Gm15696 | 13.0036 | 13.89699 | 13.4503 |
| ENSMUSG00000074461 | protein_coding | Gm10699 | 18.06799 | 17.12103 | 17.59451 |
| ENSMUSG00000054874 | protein_coding | Pcnxl3 | 2646.129 | 2617.361 | 2631.745 |
| ENSMUSG00000036168 | protein_coding | Ccdc38 | 25.28792 | 26.51387 | 25.9009 |
| ENSMUSG00000026799 | protein_coding | Med27 | 486.0015 | 478.9986 | 482.5 |
| ENSMUSG00000091568 | protein_coding | Gm8206 | 6.081181 | 6.860372 | 6.470777 |
| ENSMUSG00000051379 | protein_coding | Flrt3 | 88.80043 | 85.42866 | 87.11454 |
| ENSMUSG00000092042 | processed_transcrip | Gm17163 | 1.621062 | 1.315066 | 1.468064 |
| ENSMUSG00000088367 | misc_RNA | 7SK | 4.509319 | 5.031767 | 4.770543 |
| ENSMUSG00000091080 | protein_coding | Prr23a | 41.60574 | 39.91426 | 40.76 |
| ENSMUSG00000063954 | protein_coding | Hist2h2aa1 | 1.298952 | 1.059903 | 1.179427 |
| ENSMUSG00000061947 | protein_coding | Serpina10 | 1.298952 | 1.059903 | 1.179427 |
| ENSMUSG00000071234 | protein_coding | Tmem90a | 3.532477 | 3.117366 | 3.324922 |
| ENSMUSG00000048592 | pseudogene | Gm5946 | 1.637461 | 1.986113 | 1.811787 |
| ENSMUSG00000038267 | protein_coding | Slc22a23 | 57.11935 | 58.92375 | 58.02155 |
| ENSMUSG00000031290 | protein_coding | Lrch2 | 686.3782 | 695.807 | 691.0926 |
| ENSMUSG00000024892 | protein_coding | Pcx | 1966.614 | 2018.52 | 1992.567 |
| ENSMUSG00000030921 | protein_coding | Trim30a | 95.31919 | 92.86088 | 94.09003 |
| ENSMUSG00000034098 | protein_coding | Fstl5 | 3.884033 | 3.381178 | 3.632605 |
| ENSMUSG00000082108 | pseudogene | Gm13777 | 3.272612 | 3.743367 | 3.50799 |
| ENSMUSG00000027573 | protein_coding | 2310003C23Rik | 1766.219 | 1789.968 | 1778.093 |
| ENSMUSG00000024784 | protein_coding | Gpha2 | 24.3195 | 22.95826 | 23.63888 |
| ENSMUSG00000013646 | protein_coding | Sh3bp5l | 1378.768 | 1360.146 | 1369.457 |
| ENSMUSG00000073448 | protein_coding | Gm10509 | 54.79183 | 56.62986 | 55.71085 |
| ENSMUSG00000034785 | protein_coding | Dio1 | 4.210765 | 3.761386 | 3.986075 |
| ENSMUSG00000024835 | protein_coding | Coro1b | 3439.552 | 3474.333 | 3456.942 |
| ENSMUSG00000051054 | protein_coding | 1700014D04Rik | 10.00032 | 10.76125 | 10.38079 |
| ENSMUSG00000064289 | protein_coding | Tank | 1230.853 | 1248.301 | 1239.577 |
| ENSMUSG00000029727 | protein_coding | Cyp3a13 | 2.303971 | 2.701483 | 2.502727 |
| ENSMUSG00000085575 | processed_transcrip | Gm15485 | 1.307151 | 1.033237 | 1.170194 |
| ENSMUSG00000025386 | protein_coding | Pde6g | 23.5531 | 22.34634 | 22.94972 |
| ENSMUSG00000024731 | protein_coding | Ms4a10 | 34.63351 | 36.07718 | 35.35535 |

| | | | | | |
|---|---|---|---|---|---|
| ENSMUSG00000086351 | lincRNA | 6230400D17Rik | 53.72165 | 55.58182 | 54.65174 |
| ENSMUSG00000025383 | protein_coding | Il23a | 7.757331 | 7.116259 | 7.436795 |
| ENSMUSG00000048406 | pseudogene | B330016D10Rik | 55.12594 | 57.13688 | 56.13141 |
| ENSMUSG00000066720 | protein_coding | Cldn9 | 6.09527 | 5.483688 | 5.789479 |
| ENSMUSG00000066196 | protein_coding | Spag8 | 23.01213 | 21.66701 | 22.33957 |
| ENSMUSG00000058488 | protein_coding | Kl | 39.26412 | 40.88946 | 40.07679 |
| ENSMUSG00000053742 | protein_coding | Gm5114 | 2.900035 | 2.499652 | 2.699843 |
| ENSMUSG00000041607 | protein_coding | Mbp | 365.6762 | 378.3288 | 372.0025 |
| ENSMUSG00000030732 | protein_coding | Chrdl2 | 58.47489 | 60.42548 | 59.45018 |
| ENSMUSG00000062561 | protein_coding | Gm10118 | 4.243564 | 4.776965 | 4.510265 |
| ENSMUSG00000089072 | snoRNA | SNORA4 | 14.49786 | 13.56183 | 14.02985 |
| ENSMUSG00000029311 | protein_coding | Hsd17b11 | 1223.774 | 1238.942 | 1231.358 |
| ENSMUSG00000035847 | protein_coding | Ids | 1317.419 | 1301.835 | 1309.627 |
| ENSMUSG00000024955 | protein_coding | Esrra | 460.4559 | 453.8232 | 457.1395 |
| ENSMUSG00000002107 | protein_coding | Celf2 | 4600.594 | 4651.282 | 4625.938 |
| ENSMUSG00000074003 | protein_coding | Gucy2d | 1.629262 | 1.350742 | 1.490002 |
| ENSMUSG00000085715 | lincRNA | Tsix | 1.629262 | 1.350742 | 1.490002 |
| ENSMUSG00000085283 | lincRNA | 9530027J09Rik | 15.5336 | 14.39867 | 14.96614 |
| ENSMUSG00000015947 | protein_coding | Fcgr1 | 40.46176 | 42.03764 | 41.2497 |
| ENSMUSG00000085730 | processed_transcrip | Gm13307 | 1.284863 | 1.050893 | 1.167878 |
| ENSMUSG00000084445 | snoRNA | SNORD33 | 1.284863 | 1.050893 | 1.167878 |
| ENSMUSG00000046159 | protein_coding | Chrm3 | 1.284863 | 1.050893 | 1.167878 |
| ENSMUSG00000076757 | IG_C_gene | Tcrg-C | 1.284863 | 1.050893 | 1.167878 |
| ENSMUSG00000028890 | protein_coding | Mtf1 | 958.5911 | 971.4985 | 965.0448 |
| ENSMUSG00000091927 | lincRNA | Gm17408 | 87.20233 | 89.68841 | 88.44537 |
| ENSMUSG00000037752 | protein_coding | Xkr8 | 373.2174 | 365.3575 | 369.2875 |
| ENSMUSG00000028901 | protein_coding | Gmeb1 | 912.8754 | 923.8474 | 918.3614 |
| ENSMUSG00000041237 | protein_coding | Pklr | 4.218965 | 3.770395 | 3.99468 |
| ENSMUSG00000054435 | protein_coding | Gimap4 | 60.58266 | 58.75252 | 59.66759 |
| ENSMUSG00000065445 | miRNA | Mir143 | 10.45975 | 11.66758 | 11.06366 |
| ENSMUSG00000028550 | protein_coding | Atg4c | 335.5886 | 341.1204 | 338.3545 |
| ENSMUSG00000078308 | protein_coding | Gm7293 | 1.621062 | 1.350742 | 1.485902 |
| ENSMUSG00000086749 | processed_transcrip | Gm12037 | 1.621062 | 1.350742 | 1.485902 |
| ENSMUSG00000021790 | protein_coding | Dydc1 | 7.446999 | 8.105173 | 7.776086 |
| ENSMUSG00000031256 | protein_coding | Cstf2 | 3253.921 | 3219.746 | 3236.834 |
| ENSMUSG00000018909 | protein_coding | Arrb1 | 824.9486 | 838.5391 | 831.7438 |
| ENSMUSG00000067571 | protein_coding | Ms4a15 | 7.099022 | 7.715594 | 7.407308 |
| ENSMUSG00000053181 | protein_coding | A830005F24Rik | 18.6935 | 17.7027 | 18.1981 |
| ENSMUSG00000020955 | protein_coding | Ap4s1 | 276.5826 | 282.8463 | 279.7144 |
| ENSMUSG00000025185 | protein_coding | Loxl4 | 47.86418 | 46.11938 | 46.99178 |
| ENSMUSG00000025041 | protein_coding | Nt5c2 | 2556.64 | 2529.142 | 2542.891 |
| ENSMUSG00000082705 | pseudogene | Gm15616 | 1.282552 | 1.015218 | 1.148885 |
| ENSMUSG00000076460 | miRNA | Mir744 | 1.282552 | 1.015218 | 1.148885 |
| ENSMUSG00000047749 | protein_coding | Zc3hav1l | 750.6492 | 764.6057 | 757.6274 |
| ENSMUSG00000025529 | protein_coding | Zfp711 | 3036.106 | 3006.779 | 3021.443 |
| ENSMUSG00000084817 | pseudogene | Gm5526 | 3.880455 | 4.361444 | 4.120949 |
| ENSMUSG00000067780 | protein_coding | Pi15 | 336.9998 | 344.3428 | 340.6713 |
| ENSMUSG00000074925 | protein_coding | Ptar1 | 142.8219 | 146.3906 | 144.6062 |
| ENSMUSG00000085339 | lincRNA | Gm16714 | 18.46516 | 17.39706 | 17.93111 |
| ENSMUSG00000079428 | protein_coding | Tceal7 | 7.421132 | 6.771365 | 7.096248 |
| ENSMUSG00000046229 | protein_coding | Scand1 | 339.6134 | 345.7304 | 342.6719 |
| ENSMUSG00000087011 | processed_transcrip | Gm12930 | 4.208454 | 3.743729 | 3.976092 |
| ENSMUSG00000088323 | snoRNA | snoU109 | 12.23847 | 13.24682 | 12.74264 |
| ENSMUSG00000044352 | protein_coding | Ankrd43 | 4.538764 | 5.058434 | 4.798599 |
| ENSMUSG00000085757 | processed_transcrip | 1700019B21Rik | 110.4695 | 114.0611 | 112.2653 |
| ENSMUSG00000024142 | protein_coding | Mlst8 | 624.4181 | 634.6226 | 629.5203 |
| ENSMUSG00000004394 | protein_coding | Tmed4 | 1731.026 | 1710.155 | 1720.591 |
| ENSMUSG00000026361 | protein_coding | Cdc73 | 3196.592 | 3237.548 | 3217.07 |

| | | | | | |
|---|---|---|---|---|---|
| ENSMUSG00000030935 | protein_coding | Acsm3 | 63.93831 | 65.98196 | 64.96014 |
| ENSMUSG00000001761 | protein_coding | Smo | 2581.152 | 2619.647 | 2600.4 |
| ENSMUSG00000044519 | protein_coding | Zfp488 | 19.33183 | 20.35986 | 19.84585 |
| ENSMUSG00000047821 | protein_coding | Trim16 | 56.11515 | 58.21552 | 57.16534 |
| ENSMUSG00000010376 | protein_coding | Nedd8 | 2252.224 | 2274.408 | 2263.316 |
| ENSMUSG00000044338 | protein_coding | Aplnr | 129.2992 | 126.0139 | 127.6565 |
| ENSMUSG00000065468 | miRNA | Mir26b | 1.293062 | 1.033237 | 1.163149 |
| ENSMUSG00000083407 | pseudogene | Gm13176 | 1.293062 | 1.033237 | 1.163149 |
| ENSMUSG00000042208 | protein_coding | 0610010F05Rik | 1133.131 | 1118.494 | 1125.812 |
| ENSMUSG00000027304 | protein_coding | Rtf1 | 2656 | 2686.237 | 2671.118 |
| ENSMUSG00000021743 | protein_coding | Fezf2 | 1.32124 | 1.668247 | 1.494743 |
| ENSMUSG00000044407 | protein_coding | Qk | 54.84945 | 56.65974 | 55.7546 |
| ENSMUSG00000018740 | protein_coding | Slc25a35 | 231.7462 | 227.8073 | 229.7768 |
| ENSMUSG00000080935 | pseudogene | Got2-ps1 | 1.615173 | 1.350742 | 1.482957 |
| ENSMUSG00000084310 | pseudogene | Gm11950 | 1.615173 | 1.350742 | 1.482957 |
| ENSMUSG00000083394 | pseudogene | Gm12618 | 1.615173 | 1.350742 | 1.482957 |
| ENSMUSG00000065649 | snoRNA | Snora74a | 1.615173 | 1.350742 | 1.482957 |
| ENSMUSG00000062563 | protein_coding | Cys1 | 64.39855 | 62.14995 | 63.27425 |
| ENSMUSG00000034135 | protein_coding | Sik3 | 1876.276 | 1854.455 | 1865.366 |
| ENSMUSG00000018822 | protein_coding | Sfrp5 | 22.39966 | 21.19955 | 21.79961 |
| ENSMUSG00000030895 | protein_coding | Hpx | 23.03084 | 21.8443 | 22.43757 |
| ENSMUSG00000037369 | protein_coding | Kdm6a | 1443.032 | 1469.115 | 1456.073 |
| ENSMUSG00000033805 | protein_coding | Ephx4 | 32.18865 | 33.72235 | 32.9555 |
| ENSMUSG00000090398 | lincRNA | Gm17609 | 6.704156 | 6.02969 | 6.366923 |
| ENSMUSG00000085457 | lincRNA | 1110046J04Rik | 11.64241 | 10.82431 | 11.23336 |
| ENSMUSG00000020067 | protein_coding | Mypn | 42.60844 | 44.35312 | 43.48078 |
| ENSMUSG00000064554 | rRNA | n-R5s173 | 1.296641 | 1.033237 | 1.164939 |
| ENSMUSG00000026594 | protein_coding | Ralgps2 | 3896.599 | 3852.118 | 3874.358 |
| ENSMUSG00000052388 | protein_coding | Gm9874 | 1.612862 | 1.315066 | 1.463964 |
| ENSMUSG00000030963 | protein_coding | Umod | 1.278973 | 0.979543 | 1.129258 |
| ENSMUSG00000082687 | pseudogene | Gm11474 | 1.32124 | 1.015218 | 1.168229 |
| ENSMUSG00000019787 | protein_coding | Trdn | 1.32124 | 1.015218 | 1.168229 |
| ENSMUSG00000066682 | protein_coding | Pilrb2 | 1.629262 | 1.359751 | 1.494506 |
| ENSMUSG00000090122 | protein_coding | Kcne1l | 46.69368 | 44.49773 | 45.5957 |
| ENSMUSG00000024580 | protein_coding | Grpel2 | 1047.193 | 1061.031 | 1054.112 |
| ENSMUSG00000037014 | protein_coding | Sstr4 | 4.207186 | 3.761386 | 3.984286 |
| ENSMUSG00000085403 | processed_transcrip | Gm13068 | 17.22026 | 18.45696 | 17.83861 |
| ENSMUSG00000002550 | protein_coding | Uck1 | 1550.424 | 1534.727 | 1542.575 |
| ENSMUSG00000021040 | protein_coding | 1810035L17Rik | 333.9307 | 339.5416 | 336.7361 |
| ENSMUSG00000085744 | processed_transcrip | Gm12259 | 3.236234 | 2.826166 | 3.0312 |
| ENSMUSG00000040613 | protein_coding | Apobec1 | 148.6252 | 151.6001 | 150.1126 |
| ENSMUSG00000084099 | pseudogene | Gm12929 | 5.81647 | 5.227801 | 5.522135 |
| ENSMUSG00000071269 | protein_coding | Rpl9-ps4 | 247.9551 | 252.1553 | 250.0552 |
| ENSMUSG00000028047 | protein_coding | Thbs3 | 626.647 | 612.695 | 619.671 |
| ENSMUSG00000058729 | protein_coding | Lin9 | 763.6193 | 751.2854 | 757.4524 |
| ENSMUSG00000027618 | protein_coding | Nfs1 | 1235.435 | 1253.063 | 1244.249 |
| ENSMUSG00000075012 | protein_coding | Fjx1 | 145.1385 | 140.8274 | 142.983 |
| ENSMUSG00000047854 | protein_coding | Stx19 | 6.405602 | 5.836506 | 6.121054 |
| ENSMUSG00000030759 | protein_coding | Far1 | 3558.267 | 3605.871 | 3582.069 |
| ENSMUSG00000026940 | protein_coding | 4921530D09Rik | 36.6967 | 38.20527 | 37.45099 |
| ENSMUSG00000022820 | protein_coding | Ndufb4 | 801.9785 | 815.9748 | 808.9766 |
| ENSMUSG00000086213 | lincRNA | A330040F15Rik | 23.21586 | 25.01717 | 24.11651 |
| ENSMUSG00000074417 | protein_coding | Pira5 | 1.31304 | 1.050893 | 1.181967 |
| ENSMUSG00000086469 | lincRNA | Gm16897 | 25.34912 | 23.85816 | 24.60364 |
| ENSMUSG00000081822 | pseudogene | Gm15626 | 1.606973 | 1.324076 | 1.465524 |
| ENSMUSG00000080069 | pseudogene | Gm41 | 630.2404 | 620.8 | 625.5202 |
| ENSMUSG00000026500 | protein_coding | Fam36a | 36.15387 | 34.64571 | 35.39979 |
| ENSMUSG00000022151 | protein_coding | Ttc33 | 649.532 | 640.7611 | 645.1466 |

| | | | | | |
|---|---|---|---|---|---|
| ENSMUSG00000036966 | protein_coding | Spryd3 | 1667.869 | 1637.337 | 1652.603 |
| ENSMUSG00000020849 | protein_coding | Ywhae | 11507.51 | 11634.19 | 11570.85 |
| ENSMUSG00000041153 | protein_coding | Osgin2 | 895.097 | 884.0722 | 889.5846 |
| ENSMUSG00000050138 | protein_coding | Kcnk12 | 1.629262 | 1.333085 | 1.481173 |
| ENSMUSG00000022217 | protein_coding | Fam158a | 290.5939 | 286.0189 | 288.3064 |
| ENSMUSG00000092354 | processed_transcrip | WI1-1377A22.2 | 18.35014 | 19.32663 | 18.83838 |
| ENSMUSG00000024309 | protein_coding | H2-Ke2 | 1314.192 | 1296.967 | 1305.58 |
| ENSMUSG00000051355 | protein_coding | Commd1 | 959.4011 | 946.7877 | 953.0944 |
| ENSMUSG00000091952 | pseudogene | Gm17709 | 52.30664 | 54.15429 | 53.23046 |
| ENSMUSG00000075281 | lincRNA | Gm10822 | 14.82713 | 15.81424 | 15.32069 |
| ENSMUSG00000009741 | protein_coding | Ubp1 | 2438.308 | 2467.473 | 2452.89 |
| ENSMUSG00000030996 | protein_coding | Art1 | 19.65036 | 18.62891 | 19.13964 |
| ENSMUSG00000073593 | lincRNA | Gm10544 | 51.62962 | 49.8848 | 50.75721 |
| ENSMUSG00000001844 | protein_coding | Zdhhc4 | 836.5788 | 826.0622 | 831.3205 |
| ENSMUSG00000064262 | protein_coding | Gimap8 | 20.39783 | 19.31798 | 19.8579 |
| ENSMUSG00000031274 | protein_coding | Col4a5 | 1090.101 | 1075.267 | 1082.684 |
| ENSMUSG00000027673 | protein_coding | Ndufb5 | 1262.977 | 1245.551 | 1254.264 |
| ENSMUSG00000004707 | protein_coding | Ly9 | 13.86542 | 14.74606 | 14.30574 |
| ENSMUSG00000085220 | processed_transcrip | Gm12521 | 34.54898 | 33.01822 | 33.7836 |
| ENSMUSG00000045962 | protein_coding | Wnk1 | 15953.18 | 15707.22 | 15830.2 |
| ENSMUSG00000061578 | protein_coding | Ksr2 | 18.02214 | 17.09436 | 17.55825 |
| ENSMUSG00000070532 | protein_coding | 1700084C01Rik | 1.606973 | 1.350742 | 1.478857 |
| ENSMUSG00000015214 | protein_coding | Mtmr1 | 1588.843 | 1563.778 | 1576.31 |
| ENSMUSG00000084899 | processed_transcrip | Gm15344 | 46.72841 | 44.43896 | 45.58369 |
| ENSMUSG00000092253 | pseudogene | Gm6571 | 1.612862 | 1.350742 | 1.481802 |
| ENSMUSG00000033793 | protein_coding | Atp6v1h | 2308.597 | 2278.978 | 2293.787 |
| ENSMUSG00000020377 | protein_coding | Ltc4s | 7.043933 | 6.39188 | 6.717907 |
| ENSMUSG00000034324 | protein_coding | Tmem132c | 173.925 | 178.4867 | 176.2059 |
| ENSMUSG00000085849 | lincRNA | A730020E08Rik | 1.635151 | 1.315066 | 1.475108 |
| ENSMUSG00000056069 | protein_coding | Fam105a | 428.5598 | 434.9173 | 431.7385 |
| ENSMUSG00000036151 | protein_coding | Tm6sf2 | 27.92347 | 29.25067 | 28.58707 |
| ENSMUSG00000026999 | protein_coding | Nup35 | 618.4522 | 627.9872 | 623.2197 |
| ENSMUSG00000091434 | lincRNA | 1010001B22Rik | 11.29108 | 10.56014 | 10.92561 |
| ENSMUSG00000085084 | lincRNA | 4930570G19Rik | 23.12126 | 24.59331 | 23.85729 |
| ENSMUSG00000090673 | protein_coding | Gm340 | 1637.446 | 1611.236 | 1624.341 |
| ENSMUSG00000071516 | protein_coding | Hist1h2ai | 7.097979 | 6.516563 | 6.807271 |
| ENSMUSG00000086342 | processed_transcrip | Gm12932 | 54.17474 | 55.88094 | 55.02784 |
| ENSMUSG00000028032 | protein_coding | Papss1 | 2382.18 | 2347.703 | 2364.941 |
| ENSMUSG00000027434 | protein_coding | Nkx2-2 | 1.270774 | 0.979543 | 1.125158 |
| ENSMUSG00000033184 | protein_coding | Tmed7 | 3094.38 | 3125.698 | 3110.039 |
| ENSMUSG00000046546 | protein_coding | Fam43a | 169.1382 | 172.8981 | 171.0182 |
| ENSMUSG00000028849 | protein_coding | Mtap7d1 | 1534.434 | 1517.212 | 1525.823 |
| ENSMUSG00000051805 | protein_coding | A530095I07Rik | 1.307151 | 1.050893 | 1.179022 |
| ENSMUSG00000083474 | pseudogene | Gm15267 | 1.635151 | 1.350742 | 1.492946 |
| ENSMUSG00000022897 | protein_coding | Dyrk1a | 2934.071 | 2970.921 | 2952.496 |
| ENSMUSG00000042857 | protein_coding | Gm9776 | 94.06229 | 96.81876 | 95.44052 |
| ENSMUSG00000089769 | pseudogene | Gm16574 | 11.93403 | 11.09749 | 11.51576 |
| ENSMUSG00000046280 | protein_coding | She | 180.0518 | 184.0125 | 182.0322 |
| ENSMUSG00000041911 | protein_coding | Dlx1 | 169.8522 | 175.4192 | 172.6357 |
| ENSMUSG00000002059 | protein_coding | Rab34 | 1721.611 | 1740.594 | 1731.103 |
| ENSMUSG00000063556 | protein_coding | Gm10132 | 137.8251 | 134.6351 | 136.2301 |
| ENSMUSG00000085080 | processed_transcrip | 2310044K18Rik | 3.222145 | 2.826166 | 3.024156 |
| ENSMUSG00000086889 | processed_transcrip | Gm8619 | 32.01206 | 33.28632 | 32.64919 |
| ENSMUSG00000027624 | protein_coding | Epb4.1l1 | 1711.136 | 1687.177 | 1699.157 |
| ENSMUSG00000077565 | snoRNA | SNORD12 | 36.43244 | 38.09648 | 37.26446 |
| ENSMUSG00000089729 | processed_transcrip | Gm16552 | 1.635151 | 1.324076 | 1.479613 |
| ENSMUSG00000083367 | pseudogene | Gm8806 | 28.71611 | 27.49378 | 28.10495 |
| ENSMUSG00000080824 | pseudogene | Gm9001 | 26.84212 | 25.70334 | 26.27273 |

| | | | | | |
|---|---|---|---|---|---|
| ENSMUSG00000001065 | protein_coding | Zfp276 | 532.3777 | 539.9425 | 536.1601 |
| ENSMUSG00000083335 | pseudogene | Gm13397 | 1.293062 | 1.006571 | 1.149816 |
| ENSMUSG00000071470 | protein_coding | Ccnb1ip1 | 287.3209 | 292.7838 | 290.0524 |
| ENSMUSG00000044528 | protein_coding | Tram1l1 | 116.1691 | 113.2597 | 114.7144 |
| ENSMUSG00000043740 | protein_coding | B430306N03Rik | 44.8006 | 46.77277 | 45.78668 |
| ENSMUSG00000066041 | lincRNA | Gm10151 | 13.54689 | 14.41017 | 13.97853 |
| ENSMUSG00000036873 | protein_coding | 2410004B18Rik | 835.6685 | 823.9609 | 829.8147 |
| ENSMUSG00000006179 | protein_coding | Prss16 | 19.49732 | 18.39141 | 18.94436 |
| ENSMUSG00000042460 | protein_coding | C1galt1 | 903.4212 | 885.4481 | 894.4346 |
| ENSMUSG00000025228 | protein_coding | Actr1a | 2637.247 | 2611.958 | 2624.602 |
| ENSMUSG00000053819 | protein_coding | Camk2d | 918.3926 | 929.2252 | 923.8089 |
| ENSMUSG00000085267 | lincRNA | Gm14235 | 1.31304 | 1.059903 | 1.186472 |
| ENSMUSG00000081657 | pseudogene | Gm15466 | 1.31304 | 1.059903 | 1.186472 |
| ENSMUSG00000047014 | protein_coding | Catsperb | 2.269904 | 2.683465 | 2.476684 |
| ENSMUSG00000075576 | processed_transcrip | Gm12359 | 45.04637 | 46.64524 | 45.84581 |
| ENSMUSG00000038155 | protein_coding | Gstp2 | 74.37875 | 77.94648 | 76.16261 |
| ENSMUSG00000057924 | protein_coding | Gm10033 | 108.74 | 105.9856 | 107.3628 |
| ENSMUSG00000030304 | protein_coding | Ergic2 | 2422.019 | 2451.513 | 2436.766 |
| ENSMUSG00000053173 | pseudogene | Rpl18-ps2 | 4.213076 | 3.788052 | 4.000564 |
| ENSMUSG00000030470 | protein_coding | Csrp3 | 4.213076 | 3.788052 | 4.000564 |
| ENSMUSG00000034422 | protein_coding | Parp14 | 257.0365 | 261.5643 | 259.3004 |
| ENSMUSG00000013419 | protein_coding | Zfp651 | 1076.986 | 1059.694 | 1068.34 |
| ENSMUSG00000080836 | pseudogene | Gm6444 | 1.282552 | 1.033237 | 1.157894 |
| ENSMUSG00000042655 | protein_coding | Fam159b | 1.601084 | 1.324076 | 1.46258 |
| ENSMUSG00000064344 | Mt_tRNA | mt-Tm | 1.601084 | 1.324076 | 1.46258 |
| ENSMUSG00000000486 | protein_coding | 1-Sep | 42.34627 | 40.90675 | 41.62651 |
| ENSMUSG00000024177 | protein_coding | Nme4 | 309.2424 | 303.6659 | 306.4541 |
| ENSMUSG00000077540 | snoRNA | SNORA17 | 4.851407 | 4.361082 | 4.606245 |
| ENSMUSG00000002129 | protein_coding | Sf3a1 | 3594.458 | 3639.435 | 3616.947 |
| ENSMUSG00000029360 | protein_coding | Gm9754 | 15.27709 | 16.46184 | 15.86946 |
| ENSMUSG00000084036 | pseudogene | Ccl19-ps3 | 1.304841 | 1.059903 | 1.182372 |
| ENSMUSG00000007777 | protein_coding | 0610009B22Rik | 526.1362 | 515.5486 | 520.8424 |
| ENSMUSG00000037005 | protein_coding | Xpnpep2 | 20.08034 | 18.90852 | 19.49443 |
| ENSMUSG00000026247 | protein_coding | Ecel1 | 18.72756 | 19.67981 | 19.20369 |
| ENSMUSG00000053054 | protein_coding | Adh6a | 1.612862 | 1.359751 | 1.486306 |
| ENSMUSG00000028523 | protein_coding | Tctex1d1 | 1.612862 | 1.359751 | 1.486306 |
| ENSMUSG00000037075 | protein_coding | Rnf139 | 1476.697 | 1461.217 | 1468.957 |
| ENSMUSG00000053219 | protein_coding | Raet1c | 63.73436 | 65.95281 | 64.84358 |
| ENSMUSG00000038936 | protein_coding | Sccpdh | 1551.091 | 1567.131 | 1559.111 |
| ENSMUSG00000024561 | protein_coding | Mbd1 | 1204.031 | 1217.151 | 1210.591 |
| ENSMUSG00000082936 | pseudogene | Gm16166 | 5.828248 | 6.374223 | 6.101236 |
| ENSMUSG00000082873 | pseudogene | Gm15367 | 3.890965 | 4.325407 | 4.108186 |
| ENSMUSG00000086324 | processed_transcrip | Gm15564 | 14.5742 | 13.7575 | 14.16585 |
| ENSMUSG00000001016 | protein_coding | Ilf2 | 3250.841 | 3284.061 | 3267.451 |
| ENSMUSG00000049160 | processed_transcrip | 4930469G21Rik | 4.526986 | 4.096909 | 4.311948 |
| ENSMUSG00000055704 | protein_coding | Gm9978 | 1.293062 | 1.050893 | 1.171978 |
| ENSMUSG00000020075 | protein_coding | Ddx21 | 5277.315 | 5206.595 | 5241.955 |
| ENSMUSG00000085255 | processed_transcrip | 5330430P22Rik | 4.543386 | 4.078891 | 4.311138 |
| ENSMUSG00000067288 | protein_coding | Rps28 | 1574.434 | 1553.478 | 1563.956 |
| ENSMUSG00000080610 | snoRNA | Snord110 | 1.601084 | 1.350742 | 1.475913 |
| ENSMUSG00000069962 | protein_coding | Gm5356 | 1.626951 | 1.324076 | 1.475513 |
| ENSMUSG00000026969 | protein_coding | Fam166a | 4.585653 | 5.103118 | 4.844385 |
| ENSMUSG00000039536 | protein_coding | Stau1 | 4035.279 | 3997.489 | 4016.384 |
| ENSMUSG00000035929 | protein_coding | H2-Q4 | 99.74301 | 103.4512 | 101.5971 |
| ENSMUSG00000040629 | protein_coding | Mael | 4202.205 | 4142.824 | 4172.515 |
| ENSMUSG00000062564 | protein_coding | Tex28 | 4.285831 | 3.699044 | 3.992438 |
| ENSMUSG00000074000 | lincRNA | Gm10606 | 22.63008 | 21.46517 | 22.04763 |
| ENSMUSG00000006529 | protein_coding | Itih1 | 1.307151 | 1.059903 | 1.183527 |

| | | | | | |
|---|---|---|---|---|---|
| ENSMUSG00000075427 | protein_coding | Olfr288 | 1.307151 | 1.059903 | 1.183527 |
| ENSMUSG00000025903 | protein_coding | Lypla1 | 2128.228 | 2151.401 | 2139.815 |
| ENSMUSG00000018209 | protein_coding | Stk4 | 4101.464 | 4142.907 | 4122.185 |
| ENSMUSG00000021615 | protein_coding | Xrcc4 | 373.316 | 367.6166 | 370.4663 |
| ENSMUSG00000086890 | processed_transcrip | 1700021J08Rik | 1.65155 | 2.030436 | 1.840993 |
| ENSMUSG00000037447 | protein_coding | Arid5a | 865.7449 | 851.3877 | 858.5663 |
| ENSMUSG00000004665 | protein_coding | Cnn2 | 1856.552 | 1875.342 | 1865.947 |
| ENSMUSG00000031285 | protein_coding | Dcx | 20.06498 | 18.95543 | 19.5102 |
| ENSMUSG00000056014 | protein_coding | A430033K04Rik | 328.2359 | 321.4815 | 324.8587 |
| ENSMUSG00000038803 | protein_coding | Ost4 | 1040.017 | 1026.224 | 1033.121 |
| ENSMUSG00000030189 | protein_coding | Csda | 1613.914 | 1591.151 | 1602.533 |
| ENSMUSG00000046108 | protein_coding | Il17c | 4.857296 | 4.387748 | 4.622522 |
| ENSMUSG00000075581 | pseudogene | Gm16409 | 4.837318 | 4.352073 | 4.594695 |
| ENSMUSG00000080321 | pseudogene | Gm14644 | 10.41621 | 9.60654 | 10.01138 |
| ENSMUSG00000082165 | pseudogene | Gm7091 | 7.158956 | 6.569895 | 6.864425 |
| ENSMUSG00000056035 | protein_coding | Cyp3a11 | 1.32124 | 1.033237 | 1.177238 |
| ENSMUSG00000063393 | protein_coding | Gm10130 | 150.8577 | 147.9324 | 149.395 |
| ENSMUSG00000034563 | protein_coding | Ccpg1 | 17.48832 | 16.58367 | 17.036 |
| ENSMUSG00000004393 | protein_coding | Ddx56 | 1111.649 | 1100.042 | 1105.845 |
| ENSMUSG00000026519 | protein_coding | Tmem63a | 440.176 | 447.5421 | 443.859 |
| ENSMUSG00000038598 | protein_coding | AI481877 | 1018.32 | 1067.862 | 1043.091 |
| ENSMUSG00000084145 | pseudogene | Gm12263 | 32.02845 | 30.78916 | 31.40881 |
| ENSMUSG00000026068 | protein_coding | Il18rap | 4.907763 | 4.352073 | 4.629918 |
| ENSMUSG00000002635 | protein_coding | Pdcd2l | 776.9041 | 786.1198 | 781.512 |
| ENSMUSG00000089886 | pseudogene | Gm16184 | 28.42911 | 27.28258 | 27.85584 |
| ENSMUSG00000028646 | protein_coding | Rragc | 2090.621 | 2068.749 | 2079.685 |
| ENSMUSG00000057594 | protein_coding | Arl16 | 299.3836 | 304.2317 | 301.8077 |
| ENSMUSG00000087683 | processed_transcrip | Gm11712 | 8.085331 | 8.695498 | 8.390414 |
| ENSMUSG00000027357 | protein_coding | Crls1 | 1342.941 | 1325.651 | 1334.296 |
| ENSMUSG00000053158 | protein_coding | Fes | 94.53393 | 96.94878 | 95.74135 |
| ENSMUSG00000029613 | protein_coding | Eif2ak1 | 1612.048 | 1592.088 | 1602.068 |
| ENSMUSG00000016344 | protein_coding | Ppdpf | 855.9804 | 843.2975 | 849.6389 |
| ENSMUSG00000049630 | protein_coding | C1ql3 | 7.068533 | 6.516201 | 6.792367 |
| ENSMUSG00000039220 | protein_coding | Ppp1r10 | 2158.006 | 2187.296 | 2172.651 |
| ENSMUSG00000074415 | protein_coding | 2610203C20Rik | 419.2771 | 439.6476 | 429.4623 |
| ENSMUSG00000092985 | miRNA | AL732539.1 | 9.647726 | 10.49164 | 10.06968 |
| ENSMUSG00000029388 | protein_coding | Eif2b1 | 1234.119 | 1221.662 | 1227.89 |
| ENSMUSG00000085456 | processed_transcrip | Gm15398 | 20.93858 | 21.96577 | 21.45217 |
| ENSMUSG00000072100 | protein_coding | Gm16390 | 45.77259 | 47.52962 | 46.6511 |
| ENSMUSG00000086920 | processed_transcrip | Gm12207 | 18.43825 | 19.32663 | 18.88244 |
| ENSMUSG00000021661 | protein_coding | Ankra2 | 1368.245 | 1349.111 | 1358.678 |
| ENSMUSG00000084870 | processed_transcrip | Gm13954 | 3.894544 | 3.488204 | 3.691374 |
| ENSMUSG00000029428 | protein_coding | Stx2 | 654.7321 | 662.5314 | 658.6317 |
| ENSMUSG00000043687 | protein_coding | 1190005I06Rik | 19.76665 | 20.78403 | 20.27534 |
| ENSMUSG00000036103 | protein_coding | Colec12 | 494.8465 | 488.3929 | 491.6197 |
| ENSMUSG00000051652 | protein_coding | Lrrc3 | 54.17914 | 55.96239 | 55.07076 |
| ENSMUSG00000090311 | lincRNA | Gm17327 | 8.065352 | 7.496105 | 7.780729 |
| ENSMUSG00000066551 | protein_coding | Hmgb1 | 8408.672 | 8330.941 | 8369.807 |
| ENSMUSG00000064193 | pseudogene | Gm13699 | 541.4286 | 611.1821 | 576.3053 |
| ENSMUSG00000081281 | pseudogene | Gm6274 | 20.3238 | 19.35365 | 19.83873 |
| ENSMUSG00000036676 | protein_coding | Tmtc3 | 1734.82 | 1715.129 | 1724.975 |
| ENSMUSG00000031551 | protein_coding | Ido1 | 1.601084 | 1.359751 | 1.480417 |
| ENSMUSG00000043286 | protein_coding | Pnpla1 | 17.16517 | 18.23354 | 17.69935 |
| ENSMUSG00000033055 | protein_coding | Ankrd54 | 1036.152 | 1046.876 | 1041.514 |
| ENSMUSG00000026924 | protein_coding | Sec16a | 3347.521 | 3381.932 | 3364.726 |
| ENSMUSG00000037905 | protein_coding | Bri3bp | 950.588 | 939.8647 | 945.2263 |
| ENSMUSG00000024846 | protein_coding | Cst6 | 12.31018 | 11.55734 | 11.93376 |
| ENSMUSG00000071573 | protein_coding | Rnls | 231.6122 | 226.0957 | 228.8539 |

| | | | | | |
|---|---|---|---|---|---|
| ENSMUSG00000039163 | protein_coding | Cmc1 | 275.1997 | 270.3943 | 272.797 |
| ENSMUSG00000050215 | protein_coding | Olfr70 | 1.293062 | 1.059903 | 1.176482 |
| ENSMUSG00000092369 | processed_transcrip | 1700039E22Rik | 1.276663 | 1.006571 | 1.141617 |
| ENSMUSG00000090858 | protein_coding | Gm17365 | 0.966331 | 0.724379 | 0.845355 |
| ENSMUSG00000053724 | lincRNA | Gm9918 | 27.51451 | 28.64813 | 28.08132 |
| ENSMUSG00000084805 | processed_transcrip | Gm12273 | 1.621062 | 1.386417 | 1.503739 |
| ENSMUSG00000092526 | pseudogene | RP23-26J4.2 | 15.19128 | 16.1595 | 15.67539 |
| ENSMUSG00000074743 | protein_coding | Thbd | 2250.578 | 2212.781 | 2231.679 |
| ENSMUSG00000085983 | lincRNA | Gm11538 | 15.14797 | 15.9541 | 15.55104 |
| ENSMUSG00000035842 | protein_coding | Ddx11 | 755.742 | 747.3153 | 751.5287 |
| ENSMUSG00000067220 | protein_coding | Cnga1 | 4.482408 | 5.0415 | 4.761954 |
| ENSMUSG00000040412 | protein_coding | 5330417C22Rik | 102.3478 | 99.9425 | 101.1451 |
| ENSMUSG00000084421 | snoRNA | SNORA35 | 1.623372 | 1.359751 | 1.491562 |
| ENSMUSG00000086888 | lincRNA | A330033J07Rik | 7.773731 | 7.205266 | 7.489499 |
| ENSMUSG00000022329 | protein_coding | Stk3 | 1722.025 | 1738.784 | 1730.404 |
| ENSMUSG00000079316 | protein_coding | Rab9 | 1099.257 | 1111.673 | 1105.465 |
| ENSMUSG00000087697 | lincRNA | Gm2093 | 20.04418 | 18.79973 | 19.42196 |
| ENSMUSG00000029456 | protein_coding | Acad10 | 276.0231 | 280.0279 | 278.0255 |
| ENSMUSG00000020532 | protein_coding | Acaca | 5352.963 | 5422.656 | 5387.809 |
| ENSMUSG00000020807 | protein_coding | 4933427D14Rik | 510.153 | 516.1292 | 513.1411 |
| ENSMUSG00000029155 | protein_coding | Spata18 | 7.418821 | 6.887761 | 7.153291 |
| ENSMUSG00000028540 | protein_coding | Dph2 | 412.9206 | 418.868 | 415.8943 |
| ENSMUSG00000090861 | protein_coding | 4930570N19Rik | 4.221275 | 3.80607 | 4.013673 |
| ENSMUSG00000024772 | protein_coding | Ehd1 | 3433.796 | 3393.464 | 3413.63 |
| ENSMUSG00000088993 | snRNA | U6 | 8.734172 | 8.095802 | 8.414987 |
| ENSMUSG00000039236 | protein_coding | Isg20 | 26.26707 | 24.98724 | 25.62716 |
| ENSMUSG00000074394 | protein_coding | Vmn2r29 | 23.66328 | 24.775 | 24.21914 |
| ENSMUSG00000023022 | protein_coding | Lima1 | 1841.884 | 1862.922 | 1852.403 |
| ENSMUSG00000038390 | protein_coding | Gpr162 | 136.1475 | 132.2613 | 134.2044 |
| ENSMUSG00000022995 | protein_coding | Enah | 4372.889 | 4324.118 | 4348.503 |
| ENSMUSG00000026238 | protein_coding | Ptma | 14915.39 | 14796.93 | 14856.16 |
| ENSMUSG00000062353 | pseudogene | Gm15772 | 3892.367 | 3527.54 | 3709.953 |
| ENSMUSG00000080848 | pseudogene | Gm9385 | 14.22391 | 15.01924 | 14.62157 |
| ENSMUSG00000064767 | snoRNA | Snord35b | 4.873696 | 4.405767 | 4.639731 |
| ENSMUSG00000071665 | protein_coding | Foxr2 | 4.890095 | 4.361082 | 4.625589 |
| ENSMUSG00000028577 | protein_coding | Plaa | 5462.776 | 5512.921 | 5487.848 |
| ENSMUSG00000022026 | protein_coding | Olfm4 | 1.618751 | 1.350742 | 1.484746 |
| ENSMUSG00000037722 | protein_coding | Gnpnat1 | 1149.06 | 1135.524 | 1142.292 |
| ENSMUSG00000044167 | protein_coding | Foxo1 | 2214.261 | 2186.347 | 2200.304 |
| ENSMUSG00000042572 | protein_coding | Ube2q1 | 2381.434 | 2359.071 | 2370.252 |
| ENSMUSG00000085379 | lincRNA | 2310058D17Rik | 96.63999 | 98.99687 | 97.81843 |
| ENSMUSG00000090270 | processed_transcrip | Gm17226 | 3.594722 | 3.15268 | 3.373701 |
| ENSMUSG00000085011 | lincRNA | Gm16880 | 11.94454 | 11.22182 | 11.58318 |
| ENSMUSG00000021376 | protein_coding | Tpmt | 749.9442 | 740.9296 | 745.4369 |
| ENSMUSG00000017969 | protein_coding | Ptgis | 489.776 | 496.8574 | 493.3167 |
| ENSMUSG00000086165 | processed_transcrip | Gm15690 | 1.643351 | 1.341732 | 1.492541 |
| ENSMUSG00000062073 | protein_coding | Gm10109 | 7.46571 | 6.896409 | 7.181059 |
| ENSMUSG00000052248 | lincRNA | Gm13476 | 11.57912 | 10.84233 | 11.21073 |
| ENSMUSG00000045691 | protein_coding | Thtpa | 508.9805 | 502.2049 | 505.5927 |
| ENSMUSG00000037140 | protein_coding | Tas2r108 | 1.615173 | 1.386417 | 1.500795 |
| ENSMUSG00000088575 | snRNA | U6 | 1.615173 | 1.386417 | 1.500795 |
| ENSMUSG00000088761 | snRNA | U6 | 1.615173 | 1.386417 | 1.500795 |
| ENSMUSG00000076005 | miRNA | Mir547 | 1.282552 | 1.050893 | 1.166723 |
| ENSMUSG00000082163 | pseudogene | Gm14276 | 156.8419 | 153.6356 | 155.2388 |
| ENSMUSG00000062458 | pseudogene | Gm8623 | 1.276663 | 1.050893 | 1.163778 |
| ENSMUSG00000036523 | protein_coding | Greb1 | 778.0068 | 789.941 | 783.9739 |
| ENSMUSG00000092571 | pseudogene | Gm4134 | 14.9104 | 15.75263 | 15.33151 |
| ENSMUSG00000001379 | protein_coding | Apbb3 | 464.9324 | 473.7349 | 469.3336 |

| | | | | | |
|---|---|---|---|---|---|
| ENSMUSG00000059363 | protein_coding | Fxn | 229.5758 | 233.7283 | 231.652 |
| ENSMUSG00000082050 | pseudogene | Gm13989 | 4.602052 | 5.432843 | 5.017448 |
| ENSMUSG00000053477 | protein_coding | Tcf4 | 1976.758 | 2001.619 | 1989.189 |
| ENSMUSG00000025207 | protein_coding | Sema4g | 299.6733 | 292.7484 | 296.2108 |
| ENSMUSG00000031937 | protein_coding | 2200002K05Rik | 44.48833 | 46.04378 | 45.26605 |
| ENSMUSG00000042496 | protein_coding | Prdm10 | 357.2272 | 352.0611 | 354.6441 |
| ENSMUSG00000054814 | protein_coding | Usp46 | 2421.43 | 2394.685 | 2408.057 |
| ENSMUSG00000057836 | protein_coding | Xlr3a | 484.6321 | 475.9841 | 480.3081 |
| ENSMUSG00000021234 | protein_coding | Fam161b | 184.6071 | 179.6959 | 182.1515 |
| ENSMUSG00000028452 | protein_coding | Vcp | 10671.44 | 10827.04 | 10749.24 |
| ENSMUSG00000024076 | protein_coding | Vit | 226.0243 | 230.7661 | 228.3952 |
| ENSMUSG00000048720 | protein_coding | Tbc1d12 | 705.2826 | 713.1012 | 709.1919 |
| ENSMUSG00000040621 | protein_coding | Gemin8 | 366.5794 | 361.5916 | 364.0855 |
| ENSMUSG00000026879 | protein_coding | Gsn | 2779.57 | 2818.828 | 2799.199 |
| ENSMUSG00000046185 | protein_coding | Zfp84 | 601.0585 | 613.6515 | 607.355 |
| ENSMUSG00000010592 | protein_coding | Dazl | 14565.22 | 14354.51 | 14459.86 |
| ENSMUSG00000025066 | protein_coding | Sfr1 | 4629.522 | 4671.956 | 4650.739 |
| ENSMUSG00000085939 | processed_transcrip | Cd63-ps | 190.3913 | 193.7109 | 192.0511 |
| ENSMUSG00000029679 | protein_coding | Hyal6 | 4.873696 | 4.414414 | 4.644055 |
| ENSMUSG00000079499 | processed_transcrip | 6530402F18Rik | 65.18009 | 63.49381 | 64.33695 |
| ENSMUSG00000029861 | protein_coding | Fam131b | 90.36916 | 87.99677 | 89.18296 |
| ENSMUSG00000063628 | protein_coding | Gm7665 | 84.96351 | 87.29113 | 86.12732 |
| ENSMUSG00000021877 | protein_coding | Arf4 | 4132.089 | 4170.067 | 4151.078 |
| ENSMUSG00000026421 | protein_coding | Csrp1 | 3123.395 | 3155.035 | 3139.215 |
| ENSMUSG00000076456 | miRNA | Mir760 | 1.278973 | 1.050893 | 1.164933 |
| ENSMUSG00000068758 | protein_coding | Il3ra | 212.4599 | 207.5621 | 210.011 |
| ENSMUSG00000055546 | protein_coding | Timd4 | 28.21382 | 26.90522 | 27.55952 |
| ENSMUSG00000085027 | processed_transcrip | Gm11840 | 7.793709 | 8.387002 | 8.090356 |
| ENSMUSG00000090744 | protein_coding | Gm6871 | 270.8534 | 266.0285 | 268.4409 |
| ENSMUSG00000084168 | pseudogene | Atp5l2 | 4.546964 | 4.123575 | 4.33527 |
| ENSMUSG00000059975 | protein_coding | Zfp74 | 391.9208 | 383.5144 | 387.7176 |
| ENSMUSG00000000903 | protein_coding | Vpreb3 | 51.94353 | 50.32771 | 51.13562 |
| ENSMUSG00000039976 | protein_coding | Tbc1d16 | 1002.287 | 1013.715 | 1008.001 |
| ENSMUSG00000051184 | protein_coding | Zfp524 | 148.3777 | 151.0956 | 149.7366 |
| ENSMUSG00000006589 | protein_coding | Aprt | 813.2677 | 822.1582 | 817.7129 |
| ENSMUSG00000085422 | lincRNA | Gm6846 | 3.865098 | 3.434871 | 3.649985 |
| ENSMUSG00000060087 | pseudogene | Gm10074 | 64.23604 | 62.4397 | 63.33787 |
| ENSMUSG00000042268 | protein_coding | Slc26a9 | 13.55509 | 12.83129 | 13.19319 |
| ENSMUSG00000076993 | miRNA | AC137969.1 | 4.182587 | 3.80607 | 3.994329 |
| ENSMUSG00000028218 | protein_coding | Fam92a | 1019.57 | 1005.518 | 1012.544 |
| ENSMUSG00000027351 | protein_coding | Spred1 | 1961.682 | 1941.139 | 1951.411 |
| ENSMUSG00000036661 | protein_coding | Dennd3 | 165.2887 | 162.4352 | 163.8619 |
| ENSMUSG00000027739 | protein_coding | Rab33b | 843.0114 | 831.1251 | 837.0682 |
| ENSMUSG00000029518 | protein_coding | Rab35 | 1744.546 | 1760.095 | 1752.321 |
| ENSMUSG00000069844 | protein_coding | Sco1 | 500.9244 | 493.2915 | 497.108 |
| ENSMUSG00000074404 | processed_transcrip | Gm1698 | 7.167156 | 6.570256 | 6.868706 |
| ENSMUSG00000052364 | protein_coding | B630019K06Rik | 611.9184 | 620.2787 | 616.0985 |
| ENSMUSG00000088338 | misc_RNA | 7SK | 1.612862 | 1.386417 | 1.499639 |
| ENSMUSG00000083668 | pseudogene | Gm5648 | 12.56185 | 13.29694 | 12.92939 |
| ENSMUSG00000003500 | protein_coding | Impdh1 | 553.4585 | 544.2906 | 548.8745 |
| ENSMUSG00000026587 | protein_coding | Astn1 | 11.54863 | 12.38838 | 11.96851 |
| ENSMUSG00000041997 | protein_coding | Tlk1 | 3685.348 | 3718.364 | 3701.856 |
| ENSMUSG00000034981 | protein_coding | Parm1 | 146.0771 | 148.7927 | 147.4349 |
| ENSMUSG00000052821 | protein_coding | Cysltr1 | 10.93848 | 10.29561 | 10.61704 |
| ENSMUSG00000085601 | protein_coding | Gm4969 | 1523.907 | 1424.752 | 1474.33 |
| ENSMUSG00000033429 | protein_coding | Mcee | 251.1182 | 255.1657 | 253.142 |
| ENSMUSG00000030966 | protein_coding | Trim21 | 394.6121 | 389.1125 | 391.8623 |
| ENSMUSG00000046460 | protein_coding | Sh2d7 | 11.06528 | 10.29561 | 10.68044 |

| | | | | | |
|---|---|---|---|---|---|
| ENSMUSG00000019579 | protein_coding | D17Wsu104e | 1235.632 | 1247.571 | 1241.601 |
| ENSMUSG00000053329 | protein_coding | D10Jhu81e | 921.7745 | 931.9672 | 926.8708 |
| ENSMUSG00000080966 | pseudogene | Gm6263 | 8.745951 | 8.149134 | 8.447542 |
| ENSMUSG00000025735 | protein_coding | Rhbdl1 | 133.7198 | 136.8976 | 135.3087 |
| ENSMUSG00000030134 | protein_coding | Rasgef1a | 11.98219 | 12.71526 | 12.34872 |
| ENSMUSG00000039230 | protein_coding | Tbcd | 1697.142 | 1679.107 | 1688.125 |
| ENSMUSG00000089595 | snRNA | U7 | 1.276663 | 1.059903 | 1.168283 |
| ENSMUSG00000030924 | protein_coding | 2610020H08Rik | 1411.983 | 1383.654 | 1397.818 |
| ENSMUSG00000088951 | snRNA | U6atac | 1.635151 | 1.386417 | 1.510784 |
| ENSMUSG00000091578 | protein_coding | Gstm2-ps1 | 98.84176 | 96.7019 | 97.77183 |
| ENSMUSG00000090338 | pseudogene | Gm17081 | 4.596163 | 5.299513 | 4.947838 |
| ENSMUSG00000006435 | protein_coding | Neurl1a | 131.5718 | 134.131 | 132.8514 |
| ENSMUSG00000091506 | lincRNA | Gm17294 | 1.643351 | 1.386417 | 1.514884 |
| ENSMUSG00000082071 | pseudogene | Pramel | 434.8275 | 427.0459 | 430.9367 |
| ENSMUSG00000082044 | pseudogene | Gm14284 | 105.1816 | 107.6189 | 106.4002 |
| ENSMUSG00000086325 | lincRNA | Gm16938 | 11.36152 | 10.65816 | 11.00984 |
| ENSMUSG00000029592 | protein_coding | Usp30 | 925.3859 | 915.8228 | 920.6044 |
| ENSMUSG00000059423 | protein_coding | Zfp933 | 561.6634 | 553.5118 | 557.5876 |
| ENSMUSG00000027375 | protein_coding | Mal | 25.18341 | 26.20502 | 25.69421 |
| ENSMUSG00000054304 | pseudogene | D130007C19Rik | 29.6776 | 28.60701 | 29.1423 |
| ENSMUSG00000086557 | lincRNA | Gm15999 | 1.945483 | 1.65059 | 1.798036 |
| ENSMUSG00000089639 | pseudogene | Gm2862 | 1.615173 | 1.36876 | 1.491966 |
| ENSMUSG00000033940 | protein_coding | 6720456B07Rik | 3355.948 | 3386.315 | 3371.132 |
| ENSMUSG00000064294 | protein_coding | Aox3 | 13.91358 | 14.65705 | 14.28531 |
| ENSMUSG00000089762 | protein_coding | Ier5l | 315.1206 | 309.8605 | 312.4906 |
| ENSMUSG00000081486 | pseudogene | Gm14452 | 29.02875 | 27.81341 | 28.42108 |
| ENSMUSG00000041278 | protein_coding | Ttc1 | 1315.099 | 1328.205 | 1321.652 |
| ENSMUSG00000083722 | pseudogene | Gpr144 | 9.09035 | 9.710355 | 9.400352 |
| ENSMUSG00000087055 | processed_transcrip | Gm11948 | 3.838188 | 3.470547 | 3.654367 |
| ENSMUSG00000039623 | protein_coding | C330006K01Rik | 511.5734 | 505.1644 | 508.3689 |
| ENSMUSG00000081888 | pseudogene | Gm14045 | 10.33652 | 11.02899 | 10.68276 |
| ENSMUSG00000081152 | pseudogene | Gm12430 | 22.56217 | 21.65111 | 22.10664 |
| ENSMUSG00000085401 | pseudogene | Slc39a1-ps | 3.81821 | 3.399196 | 3.608703 |
| ENSMUSG00000050379 | protein_coding | 6-Sep | 4265.002 | 4319.725 | 4292.364 |
| ENSMUSG00000024207 | protein_coding | Acsbg2 | 1.943172 | 1.65059 | 1.796881 |
| ENSMUSG00000025949 | protein_coding | Pikfyve | 2230.936 | 2195.844 | 2213.39 |
| ENSMUSG00000003380 | protein_coding | Rabac1 | 1646.908 | 1631.195 | 1639.052 |
| ENSMUSG00000000743 | protein_coding | Chmp1a | 3467.838 | 3498.914 | 3483.376 |
| ENSMUSG00000053963 | protein_coding | 6330403A02Rik | 19.35158 | 18.47141 | 18.91149 |
| ENSMUSG00000045519 | protein_coding | Zfp560 | 347.1583 | 355.1078 | 351.133 |
| ENSMUSG00000042105 | protein_coding | Inpp5f | 1968.272 | 1989.092 | 1978.682 |
| ENSMUSG00000032889 | protein_coding | Gm6685 | 20.60365 | 19.64377 | 20.12371 |
| ENSMUSG00000020629 | protein_coding | Adi1 | 773.1786 | 783.6627 | 778.4206 |
| ENSMUSG00000064225 | protein_coding | Paqr9 | 118.9825 | 121.5732 | 120.2778 |
| ENSMUSG00000024665 | protein_coding | Fads2 | 6352.989 | 6272.238 | 6312.614 |
| ENSMUSG00000030247 | protein_coding | Kcnj8 | 66.47717 | 64.47656 | 65.47687 |
| ENSMUSG00000078880 | protein_coding | Gm14308 | 1.595194 | 1.315066 | 1.45513 |
| ENSMUSG00000046743 | protein_coding | Fat4 | 505.195 | 496.3709 | 500.7829 |
| ENSMUSG00000083754 | pseudogene | Gm7803 | 5.171206 | 4.723272 | 4.947239 |
| ENSMUSG00000085074 | processed_transcrip | Gm11494 | 1.637461 | 1.386417 | 1.511939 |
| ENSMUSG00000071203 | protein_coding | Naip5 | 30.18748 | 31.38922 | 30.78835 |
| ENSMUSG00000047965 | pseudogene | Rpl9-ps7 | 36.84371 | 38.04455 | 37.44413 |
| ENSMUSG00000027952 | protein_coding | Pmvk | 252.7596 | 248.465 | 250.6123 |
| ENSMUSG00000060512 | protein_coding | 0610040J01Rik | 9.08215 | 8.422678 | 8.752414 |
| ENSMUSG00000038725 | protein_coding | Pkhd1l1 | 31.38088 | 32.58245 | 31.98167 |
| ENSMUSG00000038259 | protein_coding | Gdf5 | 1.626951 | 1.386417 | 1.506684 |
| ENSMUSG00000072255 | protein_coding | Gm16382 | 1.626951 | 1.386417 | 1.506684 |
| ENSMUSG00000022791 | protein_coding | Tnk2 | 4272.589 | 4318.98 | 4295.784 |

| | | | | | |
|---|---|---|---|---|---|
| ENSMUSG00000066491 | pseudogene | Gm6265 | 2.295771 | 2.648151 | 2.471961 |
| ENSMUSG00000041372 | protein_coding | B4galnt3 | 154.916 | 158.0536 | 156.4848 |
| ENSMUSG00000090070 | processed_transcrip | Gm16577 | 10.0203 | 10.70248 | 10.36139 |
| ENSMUSG00000074491 | protein_coding | Clec4g | 1.943172 | 1.677256 | 1.810214 |
| ENSMUSG00000038630 | protein_coding | Zkscan16 | 165.038 | 168.568 | 166.803 |
| ENSMUSG00000003233 | protein_coding | Dvl3 | 1208.212 | 1194.753 | 1201.483 |
| ENSMUSG00000084967 | lincRNA | Gm12296 | 1.601084 | 1.386417 | 1.49375 |
| ENSMUSG00000017718 | protein_coding | Afmid | 331.4446 | 324.6419 | 328.0432 |
| ENSMUSG00000031137 | protein_coding | Fgf13 | 634.861 | 644.8154 | 639.8382 |
| ENSMUSG00000085514 | processed_transcrip | 9530048J24Rik | 1.606973 | 1.36876 | 1.487866 |
| ENSMUSG00000090695 | protein_coding | Gm17379 | 1.945483 | 1.686265 | 1.815874 |
| ENSMUSG00000020869 | protein_coding | Lrrc59 | 3483.821 | 3448.391 | 3466.106 |
| ENSMUSG00000030035 | protein_coding | Wbp1 | 1076.921 | 1089.264 | 1083.092 |
| ENSMUSG00000085870 | processed_transcrip | Gm12829 | 1.612862 | 1.36876 | 1.490811 |
| ENSMUSG00000045106 | protein_coding | Ccdc73 | 407.0202 | 395.6656 | 401.3429 |
| ENSMUSG00000089253 | snRNA | U6 | 1.615173 | 1.342094 | 1.478633 |
| ENSMUSG00000093131 | miRNA | AC124576.1 | 10.9841 | 11.7177 | 11.3509 |
| ENSMUSG00000087633 | lincRNA | Gm14455 | 5.195806 | 4.749938 | 4.972872 |
| ENSMUSG00000040323 | pseudogene | Gm15429 | 13.03178 | 12.21037 | 12.62107 |
| ENSMUSG00000036504 | protein_coding | Phpt1 | 24.8733 | 23.81312 | 24.34321 |
| ENSMUSG00000041168 | protein_coding | Lonp1 | 2799.64 | 2777.118 | 2788.379 |
| ENSMUSG00000030706 | protein_coding | Mrpl48 | 1199.837 | 1214.373 | 1207.105 |
| ENSMUSG00000021268 | lincRNA | Meg3 | 1883.36 | 1958.244 | 1920.802 |
| ENSMUSG00000058152 | protein_coding | Chsy3 | 8.711884 | 8.158143 | 8.435013 |
| ENSMUSG00000084303 | pseudogene | Gm8216 | 1.623372 | 1.386417 | 1.504895 |
| ENSMUSG00000023025 | protein_coding | Larp4 | 4477.593 | 4438.006 | 4457.8 |
| ENSMUSG00000038525 | protein_coding | Armc10 | 1721.291 | 1741.983 | 1731.637 |
| ENSMUSG00000023106 | protein_coding | Denr | 3320.239 | 3290.114 | 3305.176 |
| ENSMUSG00000057534 | protein_coding | Gm15698 | 1.943172 | 1.659599 | 1.801385 |
| ENSMUSG00000032515 | protein_coding | Csrnp1 | 335.5347 | 342.2931 | 338.9139 |
| ENSMUSG00000074886 | protein_coding | Grk6 | 1241.276 | 1253.356 | 1247.316 |
| ENSMUSG00000026228 | protein_coding | Htr2b | 14.60238 | 15.36341 | 14.98289 |
| ENSMUSG00000018425 | protein_coding | Dhx40 | 4088.864 | 4035.725 | 4062.294 |
| ENSMUSG00000015085 | protein_coding | Entpd2 | 497.0978 | 506.037 | 501.5674 |
| ENSMUSG00000064476 | snRNA | U1 | 1.298952 | 1.086569 | 1.19276 |
| ENSMUSG00000034157 | protein_coding | 2310044G17Rik | 1412.258 | 1426.882 | 1419.57 |
| ENSMUSG00000092416 | protein_coding | Zfp141 | 1077.271 | 1088.537 | 1082.904 |
| ENSMUSG00000073452 | protein_coding | Zfp97 | 114.3483 | 111.489 | 112.9186 |
| ENSMUSG00000039461 | protein_coding | Tcta | 563.2634 | 569.9538 | 566.6086 |
| ENSMUSG00000039483 | protein_coding | Asb6 | 738.0967 | 746.2476 | 742.1722 |
| ENSMUSG00000074813 | lincRNA | Gm14005 | 9.025794 | 9.660595 | 9.343195 |
| ENSMUSG00000060509 | protein_coding | Xcr1 | 42.65429 | 41.41164 | 42.03297 |
| ENSMUSG00000089908 | pseudogene | Gm16558 | 1.592884 | 1.359751 | 1.476317 |
| ENSMUSG00000042540 | protein_coding | Acot5 | 3.566544 | 3.188355 | 3.37745 |
| ENSMUSG00000040710 | protein_coding | St8sia4 | 45.47106 | 44.03893 | 44.75499 |
| ENSMUSG00000059149 | protein_coding | Mfsd4 | 673.5275 | 681.5962 | 677.5619 |
| ENSMUSG00000082645 | pseudogene | Gm15481 | 10.37752 | 11.09133 | 10.73443 |
| ENSMUSG00000035877 | protein_coding | Zhx3 | 2722.148 | 2689.062 | 2705.605 |
| ENSMUSG00000020780 | protein_coding | Srp68 | 2476.661 | 2499.556 | 2488.108 |
| ENSMUSG00000078193 | protein_coding | Gm2000 | 173.4787 | 169.8049 | 171.6418 |
| ENSMUSG00000040253 | protein_coding | Gbp7 | 336.0558 | 329.7747 | 332.9152 |
| ENSMUSG00000031811 | protein_coding | Fbxo31 | 1269.759 | 1252.711 | 1261.235 |
| ENSMUSG00000036144 | protein_coding | Meox2 | 20.23927 | 19.19366 | 19.71646 |
| ENSMUSG00000078772 | protein_coding | Gm12353 | 7.75502 | 7.249589 | 7.502305 |
| ENSMUSG00000002603 | protein_coding | Tgfb1 | 658.3958 | 666.626 | 662.5109 |
| ENSMUSG00000038539 | protein_coding | Atf5 | 544.3693 | 550.7011 | 547.5352 |
| ENSMUSG00000029236 | protein_coding | Nmu | 5.195806 | 4.731919 | 4.963863 |
| ENSMUSG00000090544 | protein_coding | Gm6576 | 4.204876 | 4.634264 | 4.41957 |

| | | | | | |
|---|---|---|---|---|---|
| ENSMUSG00000035944 | protein_coding | Ttc38 | 386.5129 | 391.1583 | 388.8356 |
| ENSMUSG00000088952 | snoRNA | snoU13 | 1.635151 | 1.36876 | 1.501955 |
| ENSMUSG00000021381 | protein_coding | Barx1 | 40.31498 | 41.56843 | 40.94171 |
| ENSMUSG00000086470 | processed_transcrip | Gm12580 | 9.039883 | 9.70528 | 9.372581 |
| ENSMUSG00000086341 | processed_transcrip | Gm15932 | 6.883734 | 7.505476 | 7.194605 |
| ENSMUSG00000090370 | pseudogene | Gm11055 | 1.621062 | 1.395426 | 1.508244 |
| ENSMUSG00000033356 | protein_coding | Pus7l | 341.3964 | 337.0343 | 339.2153 |
| ENSMUSG00000002210 | protein_coding | 1500002O20Rik | 486.6167 | 480.1015 | 483.3591 |
| ENSMUSG00000020420 | protein_coding | Zfp607 | 100.9332 | 98.45889 | 99.69604 |
| ENSMUSG00000007480 | protein_coding | Mc5r | 4.484719 | 4.061596 | 4.273157 |
| ENSMUSG00000090935 | protein_coding | Synj2bp | 1203.515 | 1189.453 | 1196.484 |
| ENSMUSG00000000359 | protein_coding | Rem1 | 266.9546 | 263.1389 | 265.0467 |
| ENSMUSG00000037890 | protein_coding | Wdr19 | 677.7814 | 686.4953 | 682.1383 |
| ENSMUSG00000089911 | protein_coding | Hiat1 | 2497.022 | 2472.993 | 2485.008 |
| ENSMUSG00000026946 | protein_coding | Nmi | 236.4359 | 232.9556 | 234.6958 |
| ENSMUSG00000031565 | protein_coding | Fgfr1 | 1131.329 | 1121.012 | 1126.171 |
| ENSMUSG00000064138 | protein_coding | Fam172a | 1246.606 | 1232.385 | 1239.496 |
| ENSMUSG00000035530 | protein_coding | Eif1 | 3364.356 | 3338.845 | 3351.6 |
| ENSMUSG00000027655 | protein_coding | Dhx35 | 1283.389 | 1271.248 | 1277.319 |
| ENSMUSG00000081916 | pseudogene | Gm12899 | 1.643351 | 1.377408 | 1.510379 |
| ENSMUSG00000092473 | protein_coding | Vmn1r180 | 1.643351 | 1.377408 | 1.510379 |
| ENSMUSG00000062082 | protein_coding | Cd200r4 | 1.643351 | 1.377408 | 1.510379 |
| ENSMUSG00000075998 | snRNA | U4 | 1.931394 | 1.677256 | 1.804325 |
| ENSMUSG00000078737 | protein_coding | Gm2128 | 1.931394 | 1.677256 | 1.804325 |
| ENSMUSG00000040136 | protein_coding | Abcc8 | 227.3228 | 231.4847 | 229.4037 |
| ENSMUSG00000022174 | protein_coding | Dad1 | 1563.716 | 1577.628 | 1570.672 |
| ENSMUSG00000083512 | pseudogene | Gm12749 | 46.78872 | 45.53522 | 46.16197 |
| ENSMUSG00000022286 | protein_coding | Grhl2 | 194.0708 | 191.008 | 192.5394 |
| ENSMUSG00000066826 | pseudogene | Gm10182 | 5.191185 | 4.643274 | 4.917229 |
| ENSMUSG00000033083 | protein_coding | Tbc1d4 | 1273.584 | 1258.559 | 1266.072 |
| ENSMUSG00000090230 | processed_transcrip | Gm16315 | 4.168498 | 3.734358 | 3.951428 |
| ENSMUSG00000000938 | protein_coding | Hoxa10 | 8.095841 | 7.576465 | 7.836153 |
| ENSMUSG00000078624 | protein_coding | Olfr613 | 1.595194 | 1.359751 | 1.477473 |
| ENSMUSG00000027293 | protein_coding | Ehd4 | 1732.439 | 1709.144 | 1720.792 |
| ENSMUSG00000019998 | protein_coding | Stx7 | 1815.311 | 1832.731 | 1824.021 |
| ENSMUSG00000081021 | pseudogene | Gm11964 | 12.30198 | 13.01475 | 12.65836 |
| ENSMUSG00000090215 | protein_coding | Trim34b | 21.54373 | 22.87137 | 22.20755 |
| ENSMUSG00000039131 | protein_coding | Gipc2 | 5.187606 | 4.767956 | 4.977781 |
| ENSMUSG00000054967 | protein_coding | Zfp647 | 182.7015 | 178.9332 | 180.8174 |
| ENSMUSG00000053935 | protein_coding | Atf7ip | 1275.072 | 1259.935 | 1267.504 |
| ENSMUSG00000037151 | protein_coding | Lrrc20 | 230.9714 | 234.6249 | 232.7982 |
| ENSMUSG00000084729 | misc_RNA | 7SK | 4.521097 | 4.159251 | 4.340174 |
| ENSMUSG00000025202 | protein_coding | Scd3 | 81.55411 | 79.44682 | 80.50046 |
| ENSMUSG00000018923 | protein_coding | Med11 | 340.4225 | 345.775 | 343.0987 |
| ENSMUSG00000081049 | pseudogene | Rps24-ps3 | 9.043461 | 8.421954 | 8.732708 |
| ENSMUSG00000085015 | processed_transcrip | Gm14424 | 1.290752 | 1.086569 | 1.18866 |
| ENSMUSG00000040462 | protein_coding | Os9 | 1955.638 | 1976.329 | 1965.983 |
| ENSMUSG00000059775 | protein_coding | Rps26-ps1 | 138.4381 | 135.4814 | 136.9598 |
| ENSMUSG00000085788 | lincRNA | 4930470G03Rik | 4.873696 | 4.468108 | 4.670902 |
| ENSMUSG00000041481 | protein_coding | Serpina3g | 215.8429 | 200.086 | 207.9645 |
| ENSMUSG00000034801 | protein_coding | Sos2 | 1640.622 | 1658.241 | 1649.432 |
| ENSMUSG00000049241 | protein_coding | Gpr81 | 11.91532 | 12.57292 | 12.24412 |
| ENSMUSG00000036898 | protein_coding | Zfp157 | 969.5313 | 982.8949 | 976.2131 |
| ENSMUSG00000030750 | protein_coding | Nsmce1 | 852.4541 | 842.9665 | 847.7103 |
| ENSMUSG00000022000 | protein_coding | Zc3h13 | 2827.105 | 2865.949 | 2846.527 |
| ENSMUSG00000020181 | protein_coding | Nav3 | 88.11901 | 85.71153 | 86.91527 |
| ENSMUSG00000082063 | pseudogene | Gm12993 | 21.71676 | 20.79304 | 21.2549 |
| ENSMUSG00000074758 | processed_transcrip | Gm5535 | 1.965461 | 1.659599 | 1.81253 |

| | | | | | |
|---|---|---|---|---|---|
| ENSMUSG00000047342 | protein_coding | Zfp286 | 289.2382 | 283.9696 | 286.6039 |
| ENSMUSG00000051451 | protein_coding | Crebzf | 2497.585 | 2527.68 | 2512.632 |
| ENSMUSG00000063882 | protein_coding | Uqcrh | 2089.111 | 2110.457 | 2099.784 |
| ENSMUSG00000032065 | protein_coding | Tex12 | 732.4142 | 718.1153 | 725.2647 |
| ENSMUSG00000091266 | lincRNA | Gm17321 | 5.223984 | 4.767956 | 4.99597 |
| ENSMUSG00000050954 | protein_coding | Zfp169 | 188.2907 | 185.2792 | 186.7849 |
| ENSMUSG00000087823 | snRNA | U6 | 1.937283 | 1.695274 | 1.816279 |
| ENSMUSG00000059249 | pseudogene | Gm12350 | 19.33414 | 20.26542 | 19.79978 |
| ENSMUSG00000014905 | protein_coding | Dnajb9 | 1530.87 | 1517.07 | 1523.97 |
| ENSMUSG00000085569 | lincRNA | Gm12602 | 1.626951 | 1.377408 | 1.502179 |
| ENSMUSG00000038740 | protein_coding | Fam125b | 692.726 | 701.1243 | 696.9251 |
| ENSMUSG00000025026 | protein_coding | Add3 | 5910.864 | 5854.595 | 5882.73 |
| ENSMUSG00000067235 | protein_coding | H2-Q10 | 466.5898 | 540.9235 | 503.7567 |
| ENSMUSG00000027342 | protein_coding | Pcna | 3574.988 | 3613.9 | 3594.444 |
| ENSMUSG00000026556 | protein_coding | Vangl2 | 409.764 | 403.1018 | 406.4329 |
| ENSMUSG00000091448 | lincRNA | Gm17388 | 113.1427 | 115.9551 | 114.5489 |
| ENSMUSG00000091982 | lincRNA | Gm17259 | 97.50501 | 95.52161 | 96.51331 |
| ENSMUSG00000085612 | processed_transcrip | Gm15868 | 11.90712 | 11.28452 | 11.59582 |
| ENSMUSG00000064621 | snoRNA | AF357355 | 1.288441 | 1.050893 | 1.169667 |
| ENSMUSG00000038057 | protein_coding | Dbil5 | 63.5708 | 61.98991 | 62.78036 |
| ENSMUSG00000068397 | protein_coding | Gm10240 | 74.96788 | 72.47083 | 73.71936 |
| ENSMUSG00000046049 | protein_coding | Rp1l1 | 1.592884 | 1.341732 | 1.467308 |
| ENSMUSG00000086573 | processed_transcrip | Gm15384 | 1.592884 | 1.341732 | 1.467308 |
| ENSMUSG00000020231 | protein_coding | Dip2a | 2009.285 | 1991.3 | 2000.293 |
| ENSMUSG00000060227 | protein_coding | Casc4 | 3927.702 | 3958.407 | 3943.055 |
| ENSMUSG00000024613 | protein_coding | Tcof1 | 1544.373 | 1525.786 | 1535.079 |
| ENSMUSG00000083327 | pseudogene | Vcp-rs | 2763.288 | 2610.262 | 2686.775 |
| ENSMUSG00000084977 | lincRNA | 4933417D19Rik | 28.91671 | 30.04712 | 29.48192 |
| ENSMUSG00000064370 | protein_coding | mt-Cytb | 55427.34 | 55849.65 | 55638.49 |
| ENSMUSG00000047879 | protein_coding | Usp14 | 2986.81 | 3009.532 | 2998.171 |
| ENSMUSG00000015854 | protein_coding | Cd5l | 10.25661 | 10.90431 | 10.58046 |
| ENSMUSG00000026589 | protein_coding | Sec16b | 67.08919 | 68.92747 | 68.00833 |
| ENSMUSG00000016833 | protein_coding | Mrps18c | 679.1895 | 687.2096 | 683.1995 |
| ENSMUSG00000028483 | protein_coding | Snapc3 | 1057.807 | 1046.553 | 1052.18 |
| ENSMUSG00000021258 | protein_coding | Ccnk | 1526.084 | 1543.74 | 1534.912 |
| ENSMUSG00000050315 | protein_coding | Synpo2 | 126.9723 | 129.4674 | 128.2199 |
| ENSMUSG00000089695 | pseudogene | Gm15946 | 9.390171 | 8.793153 | 9.091662 |
| ENSMUSG00000022853 | protein_coding | Ehhadh | 58.18431 | 59.67008 | 58.92719 |
| ENSMUSG00000081539 | pseudogene | Gm11990 | 1.65155 | 1.386417 | 1.518984 |
| ENSMUSG00000038086 | protein_coding | Hspb2 | 36.38279 | 37.58434 | 36.98357 |
| ENSMUSG00000092455 | processed_transcrip | RP23-242C19.6 | 1.923194 | 1.686265 | 1.804729 |
| ENSMUSG00000064023 | protein_coding | Klk8 | 72.86787 | 74.90084 | 73.88435 |
| ENSMUSG00000087049 | processed_transcrip | Gm11932 | 1.623372 | 1.377408 | 1.50039 |
| ENSMUSG00000003546 | protein_coding | Klc4 | 1109.821 | 1099.372 | 1104.597 |
| ENSMUSG00000029163 | protein_coding | Emilin1 | 668.3212 | 678.1037 | 673.2124 |
| ENSMUSG00000004791 | protein_coding | Pgf | 41.17807 | 39.98955 | 40.58381 |
| ENSMUSG00000041438 | protein_coding | Cirh1a | 2916.752 | 2943.494 | 2930.123 |
| ENSMUSG00000027715 | protein_coding | Ccna2 | 2561.957 | 2594.041 | 2577.999 |
| ENSMUSG00000039943 | protein_coding | Plcb4 | 1598.707 | 1612.961 | 1605.834 |
| ENSMUSG00000034968 | protein_coding | Lbx2 | 22.36329 | 23.26354 | 22.81341 |
| ENSMUSG00000057092 | protein_coding | Fxyd3 | 20.63898 | 21.61652 | 21.12775 |
| ENSMUSG00000060967 | protein_coding | Etd | 743.5501 | 754.0266 | 748.7884 |
| ENSMUSG00000057047 | protein_coding | 1700010B08Rik | 7.0944 | 6.579266 | 6.836833 |
| ENSMUSG00000067121 | pseudogene | Gm7027 | 57.5588 | 56.02043 | 56.78962 |
| ENSMUSG00000024639 | protein_coding | Gnaq | 2024.273 | 2006.935 | 2015.604 |
| ENSMUSG00000068823 | protein_coding | Csde1 | 13578.96 | 13691.35 | 13635.16 |
| ENSMUSG00000087050 | lincRNA | A030003K02Rik | 59.80999 | 61.37219 | 60.59109 |
| ENSMUSG00000080365 | snoRNA | SNORA79 | 12.34887 | 11.67301 | 12.01094 |

| | | | | | |
|---|---|---|---|---|---|
| ENSMUSG00000087507 | processed_transcrip | Gm16141 | 4.170809 | 3.814718 | 3.992763 |
| ENSMUSG00000085195 | lincRNA | 6430562O15Rik | 42.79853 | 44.22844 | 43.51348 |
| ENSMUSG00000001029 | protein_coding | Icam2 | 38.81841 | 40.02523 | 39.42182 |
| ENSMUSG00000035236 | protein_coding | Scai | 2388.319 | 2363.354 | 2375.836 |
| ENSMUSG00000059495 | protein_coding | Arhgef12 | 5357.249 | 5313.652 | 5335.451 |
| ENSMUSG00000004902 | protein_coding | Slc25a18 | 683.1208 | 691.865 | 687.4929 |
| ENSMUSG00000055210 | protein_coding | Foxd2 | 20.04985 | 19.20443 | 19.62714 |
| ENSMUSG00000082726 | pseudogene | Gm11424 | 1.917305 | 1.65059 | 1.783947 |
| ENSMUSG00000042233 | protein_coding | 2010015L04Rik | 84.34418 | 87.00589 | 85.67504 |
| ENSMUSG00000028719 | protein_coding | Cmpk1 | 2313.004 | 2295.675 | 2304.34 |
| ENSMUSG00000079728 | protein_coding | Dynlt1e | 13.58811 | 14.31215 | 13.95013 |
| ENSMUSG00000042286 | protein_coding | Stab1 | 574.3304 | 582.0165 | 578.1735 |
| ENSMUSG00000039660 | protein_coding | D2Wsu81e | 767.3243 | 759.1682 | 763.2463 |
| ENSMUSG00000041673 | protein_coding | Lrrc18 | 13.92305 | 13.19892 | 13.56098 |
| ENSMUSG00000025194 | protein_coding | Abcc2 | 25.8737 | 24.82145 | 25.34757 |
| ENSMUSG00000035953 | protein_coding | Tmem55b | 1223.591 | 1242.163 | 1232.877 |
| ENSMUSG00000042672 | protein_coding | Dcst1 | 35.84689 | 34.53724 | 35.19207 |
| ENSMUSG00000090986 | lincRNA | Gm17275 | 194.2221 | 197.9214 | 196.0718 |
| ENSMUSG00000019066 | protein_coding | Rab3d | 671.847 | 665.0469 | 668.4469 |
| ENSMUSG00000038246 | protein_coding | Fam50b | 8.411019 | 7.89397 | 8.152495 |
| ENSMUSG00000078909 | lincRNA | Gm17407 | 54.71907 | 53.30699 | 54.01303 |
| ENSMUSG00000002661 | protein_coding | Alkbh7 | 296.6033 | 291.5615 | 294.0824 |
| ENSMUSG00000082964 | pseudogene | Rpl13-ps5 | 1.937283 | 1.712931 | 1.825107 |
| ENSMUSG00000050347 | pseudogene | Gm9844 | 61.90621 | 63.39113 | 62.64867 |
| ENSMUSG00000091971 | protein_coding | Hspa1a | 354.4901 | 379.0698 | 366.7799 |
| ENSMUSG00000090120 | processed_transcrip | A730090N16Rik | 4.484719 | 4.132585 | 4.308652 |
| ENSMUSG00000029154 | protein_coding | Cwh43 | 1.943172 | 1.712931 | 1.828052 |
| ENSMUSG00000033949 | protein_coding | Trim36 | 214.1505 | 210.4132 | 212.2818 |
| ENSMUSG00000086299 | lincRNA | 4732463B04Rik | 5.165317 | 4.72291 | 4.944114 |
| ENSMUSG00000034154 | protein_coding | Ino80 | 2062.994 | 2045.125 | 2054.06 |
| ENSMUSG00000027953 | protein_coding | Slc50a1 | 418.0112 | 411.9294 | 414.9703 |
| ENSMUSG00000031537 | protein_coding | Ikbkb | 1205.463 | 1216.378 | 1210.92 |
| ENSMUSG00000048779 | protein_coding | P2ry6 | 104.6532 | 102.3287 | 103.491 |
| ENSMUSG00000024935 | protein_coding | Slc1a1 | 26.33118 | 25.22656 | 25.77887 |
| ENSMUSG00000033191 | protein_coding | Tie1 | 128.7786 | 126.4776 | 127.6281 |
| ENSMUSG00000085099 | pseudogene | Gm16288 | 1.951372 | 1.72194 | 1.836656 |
| ENSMUSG00000022195 | protein_coding | 6030458C11Rik | 1304.29 | 1293.016 | 1298.653 |
| ENSMUSG00000015305 | protein_coding | Sash1 | 1566.581 | 1548.676 | 1557.629 |
| ENSMUSG00000005268 | protein_coding | Prlr | 936.4639 | 923.3171 | 929.8905 |
| ENSMUSG00000039018 | protein_coding | Mtg1 | 416.5403 | 411.6191 | 414.0797 |
| ENSMUSG00000086285 | lincRNA | D630044L22Rik | 21.19449 | 22.23315 | 21.71382 |
| ENSMUSG00000025280 | protein_coding | Polr3a | 1589.969 | 1607.817 | 1598.893 |
| ENSMUSG00000032860 | protein_coding | P2ry2 | 512.0094 | 518.0717 | 515.0405 |
| ENSMUSG00000027356 | protein_coding | Fermt1 | 11.28183 | 10.60482 | 10.94333 |
| ENSMUSG00000031642 | protein_coding | Sh3rf1 | 674.3245 | 663.9067 | 669.1156 |
| ENSMUSG00000057710 | protein_coding | 9630041A04Rik | 1.629262 | 1.413083 | 1.521172 |
| ENSMUSG00000048502 | protein_coding | Duxbl | 12.51138 | 13.20829 | 12.85984 |
| ENSMUSG00000027822 | protein_coding | Slc33a1 | 408.1269 | 402.0498 | 405.0884 |
| ENSMUSG00000077639 | snoRNA | SNORA33 | 12.31607 | 13.00574 | 12.6609 |
| ENSMUSG00000083458 | pseudogene | Gm5510 | 9.070372 | 8.53799 | 8.804181 |
| ENSMUSG00000041891 | protein_coding | Lman1 | 3776.627 | 3740.24 | 3758.433 |
| ENSMUSG00000078088 | pseudogene | Gm7336 | 30.46532 | 27.27248 | 28.8689 |
| ENSMUSG00000065564 | miRNA | Mirlet7b | 4.233054 | 3.868412 | 4.050733 |
| ENSMUSG00000021470 | protein_coding | 0610007P08Rik | 1816.292 | 1799.834 | 1808.063 |
| ENSMUSG00000028269 | protein_coding | Gbp1 | 1.31304 | 1.086569 | 1.199805 |
| ENSMUSG00000060615 | protein_coding | Ang4 | 1.31304 | 1.086569 | 1.199805 |
| ENSMUSG00000059498 | protein_coding | Fcgr3 | 134.597 | 131.8644 | 133.2307 |
| ENSMUSG00000067038 | protein_coding | Rps12-ps3 | 740.4944 | 747.7449 | 744.1196 |

| | | | | | |
|---|---|---|---|---|---|
| ENSMUSG00000087074 | processed_transcrip | Gm14249 | 1.934972 | 1.695274 | 1.815123 |
| ENSMUSG00000085526 | lincRNA | Gm16083 | 8.065352 | 7.567818 | 7.816585 |
| ENSMUSG00000048895 | protein_coding | Cdk5r1 | 209.2576 | 212.8946 | 211.0761 |
| ENSMUSG00000040016 | protein_coding | Ptger3 | 9.371461 | 8.793515 | 9.082488 |
| ENSMUSG00000081611 | pseudogene | Gm13651 | 5.15585 | 4.740929 | 4.948389 |
| ENSMUSG00000058230 | protein_coding | Grlf1 | 4019.192 | 4065.55 | 4042.371 |
| ENSMUSG00000040550 | protein_coding | Otud6b | 1667.846 | 1686.227 | 1677.037 |
| ENSMUSG00000050565 | protein_coding | Tor1aip2 | 1199.787 | 1183.843 | 1191.815 |
| ENSMUSG00000048149 | pseudogene | Gm12663 | 360.6889 | 378.797 | 369.743 |
| ENSMUSG00000080503 | snoRNA | Snord88c | 10.35754 | 11.03552 | 10.69653 |
| ENSMUSG00000060470 | protein_coding | Gpr97 | 5.840026 | 5.394319 | 5.617173 |
| ENSMUSG00000009614 | protein_coding | Sardh | 376.8313 | 372.0076 | 374.4195 |
| ENSMUSG00000067642 | protein_coding | Gpr113 | 1.304841 | 1.086569 | 1.195705 |
| ENSMUSG00000068015 | protein_coding | Lrch1 | 918.7181 | 927.4721 | 923.0951 |
| ENSMUSG00000021929 | protein_coding | Kpna3 | 3521.572 | 3491.94 | 3506.756 |
| ENSMUSG00000026112 | protein_coding | 6330578E17Rik | 2883.125 | 2855.261 | 2869.193 |
| ENSMUSG00000082902 | pseudogene | Ccl19-ps1 | 1.264884 | 1.006571 | 1.135727 |
| ENSMUSG00000043340 | protein_coding | 6530409C15Rik | 54.97014 | 56.40421 | 55.68718 |
| ENSMUSG00000026936 | protein_coding | Lcn3 | 1.965461 | 1.712931 | 1.839196 |
| ENSMUSG00000070490 | protein_coding | Gm10293 | 1.937283 | 1.72194 | 1.829612 |
| ENSMUSG00000082990 | pseudogene | Gm14052 | 3.289012 | 2.861841 | 3.075427 |
| ENSMUSG00000091642 | pseudogene | Mocs3 | 1.923194 | 1.668247 | 1.79572 |
| ENSMUSG00000086930 | processed_transcrip | Gm14873 | 9.406571 | 8.864866 | 9.135718 |
| ENSMUSG00000020857 | protein_coding | Nme2 | 56.98666 | 55.45818 | 56.22242 |
| ENSMUSG00000090688 | protein_coding | Vmn2r12 | 47.57085 | 49.02958 | 48.30022 |
| ENSMUSG00000016757 | protein_coding | Ttll12 | 2185.067 | 2162.759 | 2173.913 |
| ENSMUSG00000081301 | pseudogene | Gm15951 | 4.184898 | 3.859402 | 4.02215 |
| ENSMUSG00000039201 | protein_coding | Tbc1d25 | 762.4946 | 770.633 | 766.5638 |
| ENSMUSG00000090613 | lincRNA | Gm17630 | 352.6834 | 357.1095 | 354.8964 |
| ENSMUSG00000013707 | protein_coding | Tnfaip8l2 | 36.80711 | 37.93245 | 37.36978 |
| ENSMUSG00000031641 | protein_coding | Cbr4 | 527.7012 | 521.9409 | 524.8211 |
| ENSMUSG00000070063 | snoRNA | Snora33 | 9.336126 | 8.731535 | 9.033831 |
| ENSMUSG00000020485 | protein_coding | Supt4h1 | 1398.813 | 1385.344 | 1392.079 |
| ENSMUSG00000029715 | protein_coding | Pop7 | 459.0098 | 464.5674 | 461.7886 |
| ENSMUSG00000029344 | protein_coding | Tpst2 | 587.8007 | 593.653 | 590.7269 |
| ENSMUSG00000048484 | protein_coding | Gm7461 | 19.69367 | 18.94963 | 19.32165 |
| ENSMUSG00000071532 | protein_coding | Gm10335 | 90.17639 | 91.85974 | 91.01806 |
| ENSMUSG00000033214 | protein_coding | Slitrk5 | 66.54151 | 68.13672 | 67.33911 |
| ENSMUSG00000065431 | miRNA | Mir186 | 47.25299 | 48.67997 | 47.96648 |
| ENSMUSG00000044206 | protein_coding | Vsig4 | 8.09122 | 8.716004 | 8.403612 |
| ENSMUSG00000032066 | protein_coding | Bco2 | 27.50378 | 26.49871 | 27.00124 |
| ENSMUSG00000092063 | lincRNA | Gm17242 | 1.929083 | 1.712931 | 1.821007 |
| ENSMUSG00000022431 | protein_coding | Ribc2 | 77.37696 | 75.58706 | 76.48201 |
| ENSMUSG00000032010 | protein_coding | Usp2 | 536.9105 | 530.1512 | 533.5309 |
| ENSMUSG00000036023 | protein_coding | Parp2 | 1083.843 | 1093.839 | 1088.841 |
| ENSMUSG00000092858 | miRNA | AL844147.1 | 1.953683 | 1.668608 | 1.811145 |
| ENSMUSG00000032624 | protein_coding | Eml4 | 3120.784 | 3094.4 | 3107.592 |
| ENSMUSG00000064427 | snoRNA | SNORA42 | 8.09942 | 8.627359 | 8.363389 |
| ENSMUSG00000038663 | protein_coding | Fsd2 | 3.899165 | 3.51487 | 3.707017 |
| ENSMUSG00000028373 | protein_coding | Astn2 | 11.71516 | 12.41541 | 12.06529 |
| ENSMUSG00000041360 | protein_coding | D19Bwg1357e | 2151.335 | 2133.107 | 2142.221 |
| ENSMUSG00000086220 | lincRNA | Gm16599 | 1.621062 | 1.422092 | 1.521577 |
| ENSMUSG00000050002 | protein_coding | 5133401N09Rik | 344.118 | 340.1318 | 342.1249 |
| ENSMUSG00000086206 | processed_transcrip | Gm13783 | 8.414373 | 9.078194 | 8.746283 |
| ENSMUSG00000002804 | protein_coding | Nudt14 | 385.6288 | 380.6023 | 383.1155 |
| ENSMUSG00000083734 | pseudogene | Gm16209 | 22.39482 | 23.40044 | 22.89763 |
| ENSMUSG00000090164 | protein_coding | BC035044 | 1.934972 | 1.712931 | 1.823952 |
| ENSMUSG00000028976 | protein_coding | Slc2a5 | 29.38471 | 30.41869 | 29.9017 |

| | | | | | |
|---|---|---|---|---|---|
| ENSMUSG00000089804 | processed_transcrip | Gm16136 | 1.920883 | 1.641581 | 1.781232 |
| ENSMUSG00000087444 | protein_coding | Gm5475 | 25.85454 | 24.77998 | 25.31726 |
| ENSMUSG00000049571 | protein_coding | 9330101J02Rik | 16.8195 | 16.14148 | 16.48049 |
| ENSMUSG00000085525 | processed_transcrip | Gm13166 | 7.09209 | 7.603131 | 7.34761 |
| ENSMUSG00000086056 | protein_coding | Gm4846 | 102.4375 | 104.9901 | 103.7138 |
| ENSMUSG00000026536 | protein_coding | Mnda | 5.815427 | 6.332388 | 6.073907 |
| ENSMUSG00000073421 | protein_coding | H2-Ab1 | 100.8815 | 104.3922 | 102.6369 |
| ENSMUSG00000024896 | protein_coding | Minpp1 | 2458.171 | 2440.503 | 2449.337 |
| ENSMUSG00000074219 | protein_coding | Gm10644 | 71.07564 | 73.32445 | 72.20005 |
| ENSMUSG00000040481 | protein_coding | Bptf | 4476.535 | 4438.465 | 4457.5 |
| ENSMUSG00000020922 | protein_coding | Lsm12 | 1881 | 1865.607 | 1873.304 |
| ENSMUSG00000057143 | protein_coding | Trim12c | 845.7701 | 854.5648 | 850.1674 |
| ENSMUSG00000091437 | protein_coding | C1galt1c1 | 4.829118 | 4.494774 | 4.661946 |
| ENSMUSG00000003970 | protein_coding | Rpl8 | 10471.48 | 10551.34 | 10511.41 |
| ENSMUSG00000067071 | protein_coding | Hes6 | 361.5291 | 365.8984 | 363.7137 |
| ENSMUSG00000009293 | protein_coding | Ube2g2 | 1425.485 | 1411.885 | 1418.685 |
| ENSMUSG00000072949 | protein_coding | Acot1 | 508.0381 | 513.9008 | 510.9695 |
| ENSMUSG00000077191 | snoRNA | Snord64 | 8.403862 | 8.909188 | 8.656525 |
| ENSMUSG00000024241 | protein_coding | Sos1 | 2808.268 | 2833.115 | 2820.692 |
| ENSMUSG00000046761 | protein_coding | Fam83h | 176.0879 | 172.9857 | 174.5368 |
| ENSMUSG00000079173 | protein_coding | Zan | 21.25607 | 20.46725 | 20.86166 |
| ENSMUSG00000007989 | protein_coding | Fzd3 | 1664.539 | 1683.55 | 1674.045 |
| ENSMUSG00000044786 | protein_coding | Zfp36 | 367.4318 | 362.0153 | 364.7235 |
| ENSMUSG00000028798 | protein_coding | Eif3i | 3674.695 | 3644.939 | 3659.817 |
| ENSMUSG00000078494 | processed_transcrip | Gm13201 | 8.124019 | 8.698709 | 8.411364 |
| ENSMUSG00000037795 | protein_coding | N4bp2 | 3093.052 | 3071.607 | 3082.33 |
| ENSMUSG00000072756 | protein_coding | Gm10414 | 1.606973 | 1.413083 | 1.510028 |
| ENSMUSG00000092083 | protein_coding | Kcnb2 | 8.039485 | 8.546999 | 8.293242 |
| ENSMUSG00000041530 | protein_coding | Eif2c1 | 1848.501 | 1865.358 | 1856.93 |
| ENSMUSG00000092364 | processed_transcrip | RP24-126J17.2 | 1.925505 | 1.686265 | 1.805885 |
| ENSMUSG00000045322 | protein_coding | Tlr9 | 9.376082 | 9.889093 | 9.632588 |
| ENSMUSG00000034820 | protein_coding | Cpsf7 | 3067.423 | 3042.523 | 3054.973 |
| ENSMUSG00000037358 | protein_coding | 4930578C19Rik | 8.376952 | 8.900179 | 8.638566 |
| ENSMUSG00000029771 | protein_coding | Irf5 | 148.5844 | 151.1629 | 149.8736 |
| ENSMUSG00000060680 | protein_coding | Gm8894 | 1.610551 | 1.341732 | 1.476142 |
| ENSMUSG00000030111 | protein_coding | A2m | 17.3815 | 16.67884 | 17.03017 |
| ENSMUSG00000049252 | protein_coding | Lrp1b | 1.592884 | 1.36876 | 1.480822 |
| ENSMUSG00000085823 | processed_transcrip | Gm13012 | 1.592884 | 1.36876 | 1.480822 |
| ENSMUSG00000076961 | miRNA | AC158749.1 | 5.517916 | 5.130146 | 5.324031 |
| ENSMUSG00000024026 | protein_coding | Glo1 | 3042.73 | 2874.641 | 2958.686 |
| ENSMUSG00000091034 | protein_coding | Gm17660 | 1.615173 | 1.422092 | 1.518632 |
| ENSMUSG00000042207 | protein_coding | Kdm5b | 10060.66 | 9977.869 | 10019.27 |
| ENSMUSG00000031851 | protein_coding | Ntpcr | 286.9556 | 283.4548 | 285.2052 |
| ENSMUSG00000057396 | protein_coding | Zfp759 | 280.4697 | 274.0805 | 277.2751 |
| ENSMUSG00000075227 | protein_coding | Znhit2-ps | 263.1292 | 259.8073 | 261.4683 |
| ENSMUSG00000040866 | protein_coding | Rsph6a | 5.185295 | 4.821288 | 5.003292 |
| ENSMUSG00000031766 | protein_coding | Slc12a3 | 20.14616 | 19.28479 | 19.71548 |
| ENSMUSG00000060402 | protein_coding | Chst8 | 81.2938 | 79.24322 | 80.26851 |
| ENSMUSG00000070287 | protein_coding | Tmem22 | 57.23922 | 58.61919 | 57.92921 |
| ENSMUSG00000027560 | protein_coding | Dok5 | 9.986236 | 9.419877 | 9.703056 |
| ENSMUSG00000031330 | protein_coding | Zcchc13 | 2.275793 | 2.021789 | 2.148791 |
| ENSMUSG00000015342 | protein_coding | Xk | 496.429 | 491.3327 | 493.8808 |
| ENSMUSG00000054667 | protein_coding | Irs4 | 50.89751 | 52.42661 | 51.66206 |
| ENSMUSG00000033917 | protein_coding | Gde1 | 1123.91 | 1115.168 | 1119.539 |
| ENSMUSG00000021565 | protein_coding | Slc6a19 | 2.261704 | 2.00377 | 2.132737 |
| ENSMUSG00000022462 | protein_coding | Slc38a2 | 6281.883 | 6337.902 | 6309.892 |
| ENSMUSG00000039477 | protein_coding | Tnrc18 | 2607.883 | 2578.427 | 2593.155 |
| ENSMUSG00000033307 | protein_coding | Mif | 2406.644 | 2424.824 | 2415.734 |

| | | | | | |
|---|---|---|---|---|---|
| ENSMUSG00000030934 | protein_coding | Oat | 5735.474 | 5776.414 | 5755.944 |
| ENSMUSG00000028687 | protein_coding | Mutyh | 286.3293 | 282.5416 | 284.4355 |
| ENSMUSG00000089953 | protein_coding | Gm757 | 7.133089 | 7.629797 | 7.381443 |
| ENSMUSG00000022122 | protein_coding | Ednrb | 183.0856 | 179.3931 | 181.2394 |
| ENSMUSG00000077100 | miRNA | AL606904.1 | 11.31821 | 11.97322 | 11.64572 |
| ENSMUSG00000032718 | protein_coding | Mansc1 | 167.7502 | 171.8064 | 169.7783 |
| ENSMUSG00000032384 | protein_coding | Csnk1g1 | 1943.693 | 1925.938 | 1934.816 |
| ENSMUSG00000032737 | protein_coding | Inppl1 | 3543.391 | 3568.869 | 3556.13 |
| ENSMUSG00000078903 | protein_coding | Gm14391 | 33.79712 | 32.64194 | 33.21953 |
| ENSMUSG00000039382 | protein_coding | Wdr45 | 448.8799 | 453.4889 | 451.1844 |
| ENSMUSG00000036751 | protein_coding | Cox6b1 | 1516.031 | 1532.912 | 1524.471 |
| ENSMUSG00000087681 | processed_transcrip | Gm13565 | 4.186165 | 3.859402 | 4.022784 |
| ENSMUSG00000058291 | protein_coding | Zfp68 | 1512.395 | 1492.518 | 1502.456 |
| ENSMUSG00000043807 | protein_coding | Ly6g5b | 12.62641 | 13.19027 | 12.90834 |
| ENSMUSG00000074071 | processed_transcrip | Fam169b | 35.50249 | 36.58963 | 36.04606 |
| ENSMUSG00000042474 | protein_coding | Faim3 | 7.096711 | 7.576104 | 7.336407 |
| ENSMUSG00000030180 | protein_coding | Kdm5a | 6717.867 | 6783.468 | 6750.668 |
| ENSMUSG00000043872 | protein_coding | Zmym1 | 874.0192 | 883.6375 | 878.8284 |
| ENSMUSG00000040396 | protein_coding | Abhd13 | 1536.114 | 1523.898 | 1530.006 |
| ENSMUSG00000042707 | protein_coding | Dnali1 | 13.9264 | 13.24397 | 13.58518 |
| ENSMUSG00000044827 | protein_coding | Tlr1 | 13.85953 | 13.2346 | 13.54706 |
| ENSMUSG00000014907 | protein_coding | Naf1 | 769.8449 | 782.2478 | 776.0464 |
| ENSMUSG00000074052 | lincRNA | BC048644 | 26.39321 | 25.54298 | 25.96809 |
| ENSMUSG00000038022 | protein_coding | Fam188b | 195.3695 | 192.3561 | 193.8628 |
| ENSMUSG00000029607 | protein_coding | Ankrd61 | 40.49225 | 41.6041 | 41.04818 |
| ENSMUSG00000065162 | snRNA | U6 | 1.923194 | 1.72194 | 1.822567 |
| ENSMUSG00000039367 | protein_coding | Sec24c | 2923.222 | 2945.071 | 2934.147 |
| ENSMUSG00000056267 | protein_coding | Cep70 | 811.0673 | 818.8723 | 814.9698 |
| ENSMUSG00000087436 | processed_transcrip | Gm16156 | 1.953683 | 1.72194 | 1.837811 |
| ENSMUSG00000092300 | polymorphic_pseuc | Cdk3-ps | 26.32112 | 27.43573 | 26.87843 |
| ENSMUSG00000024969 | protein_coding | Mark2 | 2508.571 | 2485.793 | 2497.182 |
| ENSMUSG00000085930 | lincRNA | 2310001H17Rik | 66.15909 | 64.24154 | 65.20032 |
| ENSMUSG00000015971 | protein_coding | Actr8 | 1008.657 | 1016.885 | 1012.771 |
| ENSMUSG00000039841 | protein_coding | Zfp800 | 1238.887 | 1226.391 | 1232.639 |
| ENSMUSG00000038173 | protein_coding | Enpp6 | 46.41823 | 45.11685 | 45.76754 |
| ENSMUSG00000039989 | protein_coding | Cbx4 | 1195.971 | 1205.973 | 1200.972 |
| ENSMUSG00000086240 | pseudogene | Gm7846 | 51.57684 | 52.85367 | 52.21525 |
| ENSMUSG00000072960 | protein_coding | BC065397 | 46.91969 | 49.37034 | 48.14502 |
| ENSMUSG00000070953 | protein_coding | Rabepk | 500.7481 | 495.4771 | 498.1126 |
| ENSMUSG00000046574 | protein_coding | Prr12 | 2838.57 | 2867.458 | 2853.014 |
| ENSMUSG00000037940 | protein_coding | Inpp4b | 37.24484 | 36.1215 | 36.68317 |
| ENSMUSG00000041889 | protein_coding | Shisa4 | 124.569 | 126.8151 | 125.692 |
| ENSMUSG00000027938 | protein_coding | Creb3l4 | 42.14738 | 40.94528 | 41.54633 |
| ENSMUSG00000022085 | protein_coding | Pebp4 | 9.327926 | 9.835761 | 9.581843 |
| ENSMUSG00000079642 | protein_coding | Gm6268 | 30.51191 | 29.44127 | 29.97659 |
| ENSMUSG00000056073 | protein_coding | Grik2 | 13.87131 | 13.18199 | 13.52665 |
| ENSMUSG00000025893 | protein_coding | Kbtbd3 | 216.2598 | 212.9644 | 214.6121 |
| ENSMUSG00000077380 | snoRNA | SNORA68 | 9.307948 | 9.826751 | 9.56735 |
| ENSMUSG00000025613 | protein_coding | Cct8 | 8216.362 | 8272.305 | 8244.334 |
| ENSMUSG00000085088 | lincRNA | 4931413K12Rik | 11.99038 | 11.43587 | 11.71313 |
| ENSMUSG00000073755 | protein_coding | 5730409E04Rik | 477.9734 | 484.069 | 481.0212 |
| ENSMUSG00000026932 | protein_coding | Nacc2 | 1650.91 | 1666.855 | 1658.883 |
| ENSMUSG00000087064 | processed_transcrip | Gm11721 | 13.30805 | 12.67057 | 12.98931 |
| ENSMUSG00000051518 | protein_coding | Rps19bp1 | 665.1663 | 671.1117 | 668.139 |
| ENSMUSG00000002781 | protein_coding | Tmem143 | 279.7549 | 275.9786 | 277.8667 |
| ENSMUSG00000021427 | protein_coding | Ssr1 | 7163.067 | 7117.028 | 7140.048 |
| ENSMUSG00000024887 | protein_coding | Asah2 | 420.8149 | 427.4604 | 424.1377 |
| ENSMUSG00000000340 | protein_coding | Dbt | 1058.684 | 1069.532 | 1064.108 |

| | | | | | |
|---|---|---|---|---|---|
| ENSMUSG00000030691 | protein_coding | Fchsd2 | 2125.222 | 2145.948 | 2135.585 |
| ENSMUSG00000030714 | protein_coding | Ccdc101 | 616.2621 | 607.3651 | 611.8136 |
| ENSMUSG00000091853 | lincRNA | Gm17702 | 21.28887 | 20.36996 | 20.82941 |
| ENSMUSG00000037649 | protein_coding | H2-DMa | 63.00567 | 64.87262 | 63.93914 |
| ENSMUSG00000029633 | pseudogene | Gm5578 | 854.4673 | 862.4135 | 858.4404 |
| ENSMUSG00000054594 | protein_coding | Oscar | 58.39013 | 56.91635 | 57.65324 |
| ENSMUSG00000020691 | protein_coding | Mettl2 | 1209.048 | 1218.351 | 1213.699 |
| ENSMUSG00000044811 | protein_coding | AF251705 | 41.34125 | 40.32507 | 40.83316 |
| ENSMUSG00000042564 | protein_coding | 4933432B09Rik | 71.99695 | 73.46942 | 72.73319 |
| ENSMUSG00000032174 | protein_coding | Icam5 | 20.02883 | 19.21059 | 19.61971 |
| ENSMUSG00000090877 | protein_coding | Hspa1b | 642.1616 | 649.3117 | 645.7366 |
| ENSMUSG00000035575 | protein_coding | Utp6 | 2478.615 | 2458.153 | 2468.384 |
| ENSMUSG00000087146 | processed_transcrip | Gm15205 | 57.09587 | 54.4731 | 55.78448 |
| ENSMUSG00000070777 | protein_coding | Ceacam20 | 10.05437 | 9.499876 | 9.777123 |
| ENSMUSG00000050377 | protein_coding | Il31ra | 39.24988 | 37.92059 | 38.58523 |
| ENSMUSG00000042595 | protein_coding | Fam199x | 1364.546 | 1348.545 | 1356.546 |
| ENSMUSG00000083798 | pseudogene | Gm14584 | 112.6613 | 106.2521 | 109.4567 |
| ENSMUSG00000038020 | protein_coding | Rapgefl1 | 386.6371 | 392.3591 | 389.4981 |
| ENSMUSG00000062373 | protein_coding | Tmem65 | 2179.411 | 2164.529 | 2171.97 |
| ENSMUSG00000038970 | protein_coding | Lmtk2 | 1910.554 | 1930.086 | 1920.32 |
| ENSMUSG00000069392 | protein_coding | Rpl17-ps3 | 154.8338 | 152.678 | 153.7559 |
| ENSMUSG00000041189 | protein_coding | Chrnb1 | 164.2044 | 166.6035 | 165.4039 |
| ENSMUSG00000076117 | miRNA | Mir680-2 | 2.267593 | 2.039445 | 2.153519 |
| ENSMUSG00000026641 | protein_coding | Usf1 | 2469.369 | 2452.587 | 2460.978 |
| ENSMUSG00000009092 | protein_coding | Derl3 | 552.01 | 562.7174 | 557.3637 |
| ENSMUSG00000056617 | protein_coding | 4931429L15Rik | 2.257082 | 2.00377 | 2.130426 |
| ENSMUSG00000051351 | protein_coding | Zfp46 | 1095.626 | 1080.532 | 1088.079 |
| ENSMUSG00000062488 | protein_coding | I830012O16Rik | 15.56051 | 14.91506 | 15.23778 |
| ENSMUSG00000084893 | pseudogene | Hba-ps4 | 3.248013 | 2.897516 | 3.072765 |
| ENSMUSG00000048218 | protein_coding | Amigo2 | 362.9889 | 367.6115 | 365.3002 |
| ENSMUSG00000031449 | protein_coding | Atp4b | 2.289882 | 2.048455 | 2.169168 |
| ENSMUSG00000029209 | protein_coding | Gnpda2 | 734.3382 | 742.9177 | 738.628 |
| ENSMUSG00000019577 | protein_coding | Pdk4 | 101.0268 | 102.6808 | 101.8538 |
| ENSMUSG00000074649 | lincRNA | BC029722 | 309.6186 | 313.3519 | 311.4853 |
| ENSMUSG00000052040 | protein_coding | Klf13 | 1878.808 | 1894.453 | 1886.631 |
| ENSMUSG00000030842 | protein_coding | 2400001E08Rik | 1701.625 | 1714.483 | 1708.054 |
| ENSMUSG00000048582 | protein_coding | Gja3 | 30.01864 | 29.11118 | 29.56491 |
| ENSMUSG00000030602 | protein_coding | Pak4 | 1514.276 | 1500.713 | 1507.494 |
| ENSMUSG00000033170 | protein_coding | Card10 | 651.9973 | 660.5068 | 656.2521 |
| ENSMUSG00000020516 | protein_coding | Rps6kb1 | 2024.952 | 2010.51 | 2017.731 |
| ENSMUSG00000029104 | protein_coding | Htt | 3850.408 | 3806.536 | 3828.472 |
| ENSMUSG00000024294 | protein_coding | Mib1 | 4604.555 | 4632.656 | 4618.606 |
| ENSMUSG00000085869 | processed_transcrip | Gm16079 | 5.204006 | 4.847954 | 5.02598 |
| ENSMUSG00000043329 | pseudogene | Gm8849 | 1.937283 | 1.748606 | 1.842945 |
| ENSMUSG00000057280 | protein_coding | Musk | 10.35292 | 10.83296 | 10.59294 |
| ENSMUSG00000028857 | protein_coding | Tmem222 | 1199.651 | 1209.119 | 1204.385 |
| ENSMUSG00000061462 | protein_coding | Obscn | 678.4236 | 653.5528 | 665.9882 |
| ENSMUSG00000026921 | protein_coding | Egfl7 | 291.1825 | 287.7387 | 289.4606 |
| ENSMUSG00000025423 | protein_coding | Pias2 | 4380.179 | 4349.632 | 4364.906 |
| ENSMUSG00000037071 | protein_coding | Scd1 | 221.4255 | 218.5255 | 219.9755 |
| ENSMUSG00000002803 | protein_coding | Btbd6 | 458.1378 | 464.4009 | 461.2694 |
| ENSMUSG00000040749 | protein_coding | Siah1b | 1421.4 | 1434.758 | 1428.079 |
| ENSMUSG00000039849 | protein_coding | Pcif1 | 1673.705 | 1686.246 | 1679.976 |
| ENSMUSG00000038215 | protein_coding | BC088983 | 573.8385 | 579.863 | 576.8507 |
| ENSMUSG00000074480 | protein_coding | Mex3a | 1907.364 | 1926.34 | 1916.852 |
| ENSMUSG00000003604 | protein_coding | Aven | 456.0162 | 451.7106 | 453.8634 |
| ENSMUSG00000027429 | protein_coding | Sec23b | 2269.739 | 2296.469 | 2283.104 |
| ENSMUSG00000027338 | protein_coding | Prnd | 190.4319 | 194.3538 | 192.3928 |

| | | | | | |
|---|---|---|---|---|---|
| ENSMUSG00000049507 | protein_coding | 4930429B21Rik | 6.148048 | 5.756508 | 5.952278 |
| ENSMUSG00000062275 | protein_coding | Fbxw24 | 1.920883 | 1.668608 | 1.794746 |
| ENSMUSG00000032128 | protein_coding | Robo3 | 11.34385 | 10.70828 | 11.02607 |
| ENSMUSG00000089982 | processed_transcrip | Gm7568 | 2.269904 | 1.986113 | 2.128008 |
| ENSMUSG00000026923 | protein_coding | Notch1 | 1987.97 | 2010.741 | 1999.356 |
| ENSMUSG00000029782 | protein_coding | Tmem209 | 1647.92 | 1661.843 | 1654.881 |
| ENSMUSG00000038248 | protein_coding | Sobp | 1410.57 | 1424.161 | 1417.366 |
| ENSMUSG00000071715 | protein_coding | Ncf4 | 32.05328 | 31.11531 | 31.5843 |
| ENSMUSG00000074895 | protein_coding | Eif4e1b | 25.93341 | 25.08598 | 25.5097 |
| ENSMUSG00000056832 | protein_coding | Ttc26 | 326.6255 | 333.5421 | 330.0838 |
| ENSMUSG00000038005 | protein_coding | 2700029M09Rik | 1326.912 | 1337.442 | 1332.177 |
| ENSMUSG00000054588 | protein_coding | Gbp10 | 17.20848 | 16.40022 | 16.80435 |
| ENSMUSG00000040797 | protein_coding | Iqsec3 | 13.87258 | 13.30595 | 13.58926 |
| ENSMUSG00000025215 | protein_coding | Tlx1 | 6.413802 | 6.843077 | 6.628439 |
| ENSMUSG00000043262 | protein_coding | Uevld | 870.4823 | 878.8988 | 874.6906 |
| ENSMUSG00000070345 | protein_coding | Hsf5 | 177.5401 | 172.7111 | 175.1256 |
| ENSMUSG00000047649 | protein_coding | Cd3eap | 480.4151 | 486.2278 | 483.3215 |
| ENSMUSG00000055333 | protein_coding | Fat2 | 2.259393 | 2.039445 | 2.149419 |
| ENSMUSG00000063801 | protein_coding | Ap3s2 | 1700.717 | 1712.905 | 1706.811 |
| ENSMUSG00000026810 | protein_coding | Dpm2 | 547.8996 | 553.084 | 550.4918 |
| ENSMUSG00000014592 | protein_coding | Camta1 | 1177.12 | 1163.059 | 1170.09 |
| ENSMUSG00000026358 | protein_coding | Rgs1 | 1.951372 | 1.739597 | 1.845485 |
| ENSMUSG00000075304 | protein_coding | Sp5 | 90.29313 | 87.74663 | 89.01988 |
| ENSMUSG00000093044 | snoRNA | AC159323.1 | 8.13453 | 7.647454 | 7.890992 |
| ENSMUSG00000081010 | pseudogene | Gm13880 | 4.840896 | 4.458737 | 4.649817 |
| ENSMUSG00000059336 | protein_coding | Slc14a1 | 7.090822 | 7.532142 | 7.311482 |
| ENSMUSG00000043065 | protein_coding | Spice1 | 1399.914 | 1387.307 | 1393.611 |
| ENSMUSG00000040242 | protein_coding | Fgfr1op2 | 3140.446 | 3116.127 | 3128.287 |
| ENSMUSG00000081855 | pseudogene | Rpl17-ps5 | 33.48298 | 32.49023 | 32.98661 |
| ENSMUSG00000056211 | protein_coding | R3hdm1 | 3347.668 | 3307.223 | 3327.445 |
| ENSMUSG00000085118 | processed_transcrip | Gm15774 | 6.44198 | 6.843077 | 6.642528 |
| ENSMUSG00000086105 | processed_transcrip | Gm11636 | 2.261704 | 2.039445 | 2.150575 |
| ENSMUSG00000087169 | lincRNA | C330002G04Rik | 8.068931 | 8.520694 | 8.294813 |
[truncated: 527,767 more chars]
